# ZIC2 induces pro-tumor macrophage polarization in nasopharyngeal carcinoma by activating the JUNB/MCSF axis

**DOI:** 10.1038/s41419-023-05983-x

**Published:** 2023-07-21

**Authors:** Qian Liu, Ting Yang, Yu Zhang, Ze-Dong Hu, Yan-Min Liu, Yi-Ling Luo, Shang-Xin Liu, Hua Zhang, Qian Zhong

**Affiliations:** 1 State Key Laboratory of Oncology in South China, Collaborative Innovation Center for Cancer Medicine, Guangdong Key Laboratory of Nasopharyngeal Carcinoma Diagnosis and Therapy, Sun Yat-sen University Cancer Center (SYSUCC), Guangzhou, China; 2 Department of Ultrasound Medicine, The Third Affiliated Hospital of Guangzhou Medical University, Guangzhou, China; 3 Department of Pathology, Sun Yat-sen University Cancer Center (SYSUCC), Guangzhou, China; 4 Department of Orthopedics, The First People’s Hospital of Anning, Kunming, China; 5 Department of Immunology, Zhongshan School of Medicine; Key Laboratory of Tropical Disease Control of Ministry of Education, Sun Yat-sen University, Guangzhou, China

**Keywords:** Cancer microenvironment, Prognostic markers

## Abstract

Nasopharyngeal carcinoma (NPC) is a common malignant epithelial tumor of the head and neck that often exhibits local recurrence and distant metastasis. The molecular mechanisms are understudied, and effective therapeutic targets are still lacking. In our study, we found that the transcription factor ZIC2 was highly expressed in NPC. Although ZIC family members play important roles in neural development and carcinogenesis, the specific mechanism and clinical significance of ZIC2 in the tumorigenesis and immune regulation of NPC remain elusive. Here, we first reported that high expression of ZIC2 triggered the secretion of MCSF in NPC cells, induced M2 polarization of tumor-associated macrophages (TAMs), and affected the secretion of TAM-related cytokines. Mechanistically, ChIP-seq and RNA-seq analyses identified JUNB as a downstream target of ZIC2. Furthermore, ZIC2 was significantly enriched in the promoter site of JUNB and activated JUNB promoter activity, as shown by ChIP-qPCR and luciferase assays. In addition, JUNB and MCSF participated in ZIC2-induced M2 TAMs polarization. Thus, blocking JUNB and MCSF could reverse ZIC2-mediated M2 TAMs polarization. Moreover, Kaplan-Meier survival analyses indicated that high expression of ZIC2, JUNB, and CD163 was positively associated with a poor prognosis in NPC. Overexpression of ZIC2 induced tumor growth in vivo, with the increase of JUNB, MCSF secretion, and CD163. In summary, our study implies that ZIC2 induces M2 TAM polarization, at least in part through regulation of JUNB/MCSF and that ZIC2, JUNB, and CD163 can be utilized as prognostic markers for NPC and as therapeutic targets for cancer immunotherapy.

## Background

Nasopharyngeal carcinoma (NPC) originates from the nasopharyngeal mucosal epithelium and often occurs in the pharyngeal recess. It is a common malignant epithelial tumor of the head and neck. The incidence of NPC is relatively high in northern Africa and southeastern Asia, especially in Guangdong Province, so NPC is also known as “Guangdong cancer” [1–3]. The morbidity and mortality in males are significantly higher than those in females [4]. At present, there are several treatments for NPC, including chemotherapy, biotherapy, surgical treatment, and radiotherapy, which is the most effective approach [5]. Although much progress in treatments has been achieved, local recurrence and distant metastasis are still the main reasons for unfavorable outcomes in NPC patients [6]. Therefore, it is of great significance to study the mechanisms of NPC development and identify novel molecular targets for the prognosis and treatment of NPC.

The zinc finger of the cerebellum (ZIC) family consists of five members (ZIC1–5), and these five human zinc finger proteins all contain a highly conserved domain composed of five tandem C_2_H_2_ zinc fingers. Furthermore, ZIC1, ZIC2, and ZIC3 have the same small domain of ZIC/Odd paired conserved (ZOC) motif at the N-terminus, which binds to their downstream target genes and regulates the transcriptional activity of these downstream genes [7–9]. At present, a large number of studies have reported that all ZIC family proteins play an important role in the development of the nervous system and function as transcription factors in regulating the transcriptional activity of downstream genes [10, 11]. Related evidence has shown that ZIC proteins regulate the expression of genes, not only as classic transcription factors that directly bind to DNA through the zinc finger domain but also through binding to other transcription factors to create a common factor [12]. The relationship between the ZIC protein family and tumors has been studied in recent years. The role of ZIC2 in cancer varies among different cancer types. Our previous study found that ZIC2 plays the role of a tumor suppressor gene in breast cancer by regulating its downstream target gene transcriptional activation factor 3 (STAT3) [13]. The transcriptional levels of ZIC1, ZIC2, and ZIC5 in meningiomas were found to be significantly higher than those in normal dura mater and could serve as new molecular markers for meningiomas [14]. A large number of studies have shown that ZIC2 is upregulated in renal cell carcinoma [15], prostate cancer [16], oral squamous cell carcinoma [17], and bladder cancer [18], in which high expression of ZIC2 is associated with a poor prognosis.

Tumor-associated macrophages (TAMs) derive from peripheral blood mononuclear cells (PBMCs), which can be further differentiated into macrophages after stimulation by various factors secreted by tumor cells and stromal cells, such as chemokines, cytokines, and growth factors [19]. TAMs are closely related to tumor proliferation, invasion, metastasis, and poor prognosis in the tumor microenvironment (TME) [20]. TAMs could be polarized into inflammation-promoting and antitumoral M1 phenotype (M1) and cancer-promoting M2-phenotype (M2), yet TAMs in vivo display more complex phenotypes that vary well beyond these denominations [21, 22]. M1-like phenotype macrophages can clear pathogens and participate in the inflammatory response and antitumor immunity [23]. M2 phenotype macrophages can exert an anti-inflammatory response and promote tumor formation [24]. Wang et al. reported that specifically neutralized CCL2 and CCL5 (BisCCL2/5i) mRNA nanoplatforms significantly induce the polarization of TAMs toward the antitumoral M1 phenotype and reduce immunosuppression in the tumor microenvironment. Combining BisCCL2/5i with PD-1 ligand inhibitor (PD-Li) achieves long-term survival in mouse models of primary liver cancer and liver metastasis of colorectal and pancreatic cancers [25].

In this study, we found that high expression of ZIC2 correlated with poor outcomes in NPC patients. ZIC2 regulated the expression of JUNB and MCSF, which in turn induced the M2 phenotype in TAMs. Our investigation provides new insights for potential cancer immunotherapy in NPC patients with high expression.

## Methods

### Patients and specimens

In total, 12 NPC specimens and 9 nontumorous nasopharyngeal tissue samples were collected for reverse transcription polymerase chain reaction (RT-PCR) analysis at the Sun Yat-sen University Cancer Center, Guangzhou, China. A total of 164 paraffin-embedded NPC specimens from patients who were first diagnosed between 2011 and 2013 at the Sun Yat-sen University Cancer Center were collected for immunohistochemical analysis. This study was conducted in accordance with the Helsinki Declaration, and all patients and healthy donors signed a consent form approved by the Research Ethics Committee of the Sun Yat-sen University Cancer Center.

### Cell culture and culture conditions

The NPC cell lines CNE1, SUNE2, CNE2, HNE1, HK1, SUNE1, C666-1, HNE1-EBV, CNE2-EBV, CNE2, HK1-EBV, and SUNE2, the human monocyte cell line THP1, and mouse colon adenocarcinoma cell line MC38 were cultured in RPMI-1640 medium (Invitrogen, Carlsbad CA, USA) supplemented with 10% fetal bovine serum (FBS, Invitrogen), and the normal nasopharyngeal epithelial cells (N01) and the nasopharyngeal immortalized epithelial cell lines (NPEC2-Bmi1, NPEC5-Bmi1, and NP69) were maintained in keratinocyte-SFM medium (Invitrogen). Human PBMCs were isolated from the blood of healthy donors. All the cell lines were cultured at 37 °C in a humidified 5% CO_2_ incubator at the Sun Yat-sen University Cancer Center. All cell lines were verified to be free of mycoplasma.

### Western blotting

Total protein was obtained with sodium dodecyl sulfate (SDS) sample buffer, and the protein was separated by 9% SDS-PAGE. Then, the protein was transferred to a PVDF membrane (Millipore, Burlington, MA). Membranes were blocked in 5% milk at room temperature for 1 h and subsequently incubated with an anti-ZIC2 antibody (ab150404, Abcam, Cambridge, UK) or anti-JUNB antibody (3753 S, CST, Danvers, USA) overnight at 4 °C. GAPDH (6004-1, Proteintech, Wuhan, China) and β-actin (66009-1-1g, Proteintech) served as internal controls. Species-matched secondary antibodies were then incubated with the PVDF membranes at room temperature for 1 h. Finally, enhanced chemiluminescence (Thermo, Waltham, MA) was used to visualize the antigen-antibody reactions.

### RNA extraction and real-time fluorescent quantitative PCR

Total RNA was extracted from various cells with TRIzol reagent (Invitrogen, Carlsbad, CA) according to the manufacturer’s instructions. mRNA expression levels were detected with iTaq Universal SYBR Green SuperMix (Bio-Rad, Hercules, CA) and a CFX96 real-time PCR detection system (Bio-Rad). With β-actin as the internal control, relative expression was calculated by the ${2^{-\Delta{\mathrm{Ct}}}}$ method. The primer sequences were as follows:

ZIC2-F: 5'-CACCTCCGATAAGCCCTATCT-3';

ZIC2-R: 5'-GGCGTGGACGACTCATAGC-3';

JUNB-F: 5'-AACAGCCCTTCTACCACGAC-3';

JUNB-R: 5'-CAGGCTCGGTTTCAGGAGTT-3';

NR4A1-F: 5'-GCTACGAAACTTGGGGGAGT-3';

NR4A1-R: 5'-CGGTGCTGGTGTCCCATATT-3';

TUBB2A-F: 5'-GAGGCCGAGAGCAACATGAA-3';

TUBB2A-R: 5'-AACAGAGGCAAAACTGAGCAC-3';

MCSF-F: 5'-GTGAGATTCCCGTACCCCAAG-3';

MCSF-R: 5'-TGCCTCTCATGGCCAGTTAC-3';

CD86-F: 5'-AGCACAGACACACGGATGAG-3';

CD86-R: 5'-AGAGGAGCAGCACCAGAGAG-3';

CD206-F: 5'-ACCTTCACAAGTATCCACACCATC-3';

CD206-R: 5'-CTTCATCACCACACAATCCT-3';

IL-13-F: 5'-CCTCATGGCGCTTTTGTTGAC-3';

IL-13-R: 5'-TCTGGTTCTGGGTGATGTTGA-3';

IFN-γ-F: 5'-TCTGCATCGTTTTGGGTTCTC-3';

IFN-γ-R: 5'-ATTTTTCTGTCACTCTCCTCTTTCC-3';

β-actin-F: 5'-GTGAAGGTGACAGCAGTCGGT-3';

β-actin-R: 5'-AAGTGGGGTGGCTTTTAGGAT-3'.

Mouse-ZIC2-F: 5'-CTTTCAGCACCATGCACGAG-3';

Mouse-ZIC2-R: 5'-CTGGAAAGGTTTCTCCCCTGT-3';

Mouse-JUNB-F: 5'-TTGATCGTCCCCAACAGCAA-3';

Mouse-JUNB-R: 5'-TGGGGAGTAACTGCTGAGGT-3';

Mouse-MCSF-F: 5'-AGTGCTCTAGCCGAGATGTG-3';

Mouse-MCSF-R: 5'-CCTCTATGCGAAGGGGAAGC-3';

Mouse-β-actin-F: 5'-TGGTTACAGGAAGTCCCTCAC-3'; and

Mouse-β-actin-R: 5'-ACAGAAGCAATGCTGTCACCTT-3'.

### Chromatin immunoprecipitation (ChIP) assay

C666-1 cells were fixed and cross-linked with 1% formaldehyde, collected with PBS, and resuspended in a lysis buffer. Genomic DNA was fragmented by a Covaris E220 ultrasonicator to approximately 200–500 bp, and then the supernatants were diluted and shaken overnight with an anti-ZIC2 antibody (ab150404, Abcam) at 4 °C. The next day, protein A/G beads were added and incubated at 4 °C for 1 h. After repeated washing of the immune complexes, the eluted DNA was uncrosslinked with 5 M NaCl in a 65 °C water bath for 4 h, and DNA was purified with the QIAquick PCR Purification Kit (QIAGEN, #28104, Hilden, Germany). Purified NDA was used for ChIP-seq or real-time PCR. The sequences of the primers used for ChIP-qPCR were as follows:

JUNB −1297 gDNA-F: 5'-CAACACCGTGTCGGCTCCTA-3';

JUNB −1297 gDNA-R: 5'-CACGCCCAGGTTCCTCTTCC-3';

JUNB −123 gDNA-F: 5'-CGTGGCCGCTGTTTACAAG-3';

JUNB −123 gDNA-R: 5'-TTTCCTGGCGTCGTTTCC-3';

JUNB +2163 gDNA-F: 5'-CCCATACAAGGACCGATTCTGC-3';

JUNB +2163 gDNA-R: 5'-GCGCCAGTGTCTTGAAGGTG-3';

JUNB +2676 gDNA-F: 5'-TGTCTGTTCCACTCACCCTA-3';

JUNB +2676 gDNA-R: 5'-CCTGCCAGTTTCCCCTCA-3';

JUNB +4740 gDNA-F: 5'-CAGAGTCCCTGCTGTGAG-3';

JUNB +4740 gDNA-R: 5'-CTGTAGGAGGAAATTGGG-3';

GAPDH-gDNA-F: 5'-CCCCACACACATGCACTTACC-3'; and

GAPDH-gDNA-R: 5'-CCTAGTCCCAGGGCTTTGATT-3'.

### Luciferase reporter assay

After reaching 30–40% confluence, 293 T cells were transfected with the truncation plasmid PGL3-JUNB (−1400/+250), PGL3-JUNB (−1400/−200), PGL3-JUNB (−1000/+250), PGL3-JUNB (−200/+250), PGL3-JUNB (+2000/+5000), PGL3-JUNB (+2000/+3000), PGL3-JUNB (+2000/+2400), PGL3-JUNB (+2400/+3000), or PGL3-JUNB (+3000/+5000), pBabe-vector or pBabe-ZIC2, and the TK-Renilla plasmid by Lipofectamine 3000 (Invitrogen) according to the manufacturer’s protocols. Then, 4–6 hours later, the normal medium was changed. At 24–48 h posttransfection, the cells were collected and analyzed for luciferase activity using the Dual-Glo Luciferase Assay System (Promega, #E2940, Madison, WI), and firefly luciferase activity was normalized to that of TK-Renilla luciferase according to the manufacturer’s instructions.

### siRNA interference experiment

When the density reached 50%, cells were transfected with siRNA and Lipofectamine RNAiMax (Invitrogen, #1795160) according to the manufacturer’s instructions. siRNA duplexes with the following sense and antisense sequences were used:

siRNA-ZIC2-1: AACTCCGGATTGCGTTCCT;

siRNA-ZIC2-2: TCTCCATGCCCACGTTCTT;

siRNA-JUNB-1: CTCTCTACACGACTACAAA;

siRNA-JUNB-2: AAACAGAAGGTCATGACCCAC; and

siRNA-MCSF: GATCCAGTGTGCTACCTTA.

All of the siRNAs were synthesized by RiboBio (Guangzhou, China), and negative control siRNA (siNC) was purchased from RiboBio. After transfection for 36–48 h, the cells were harvested for further experimentation.

### PBMC differentiation assay and coculture with tumor cells

Human PBMCs were isolated from donated peripheral blood by density gradient centrifugation. CD14^+^ cells were separated from healthy PBMCs using human CD14 microbeads (130-050-201, Miltenyi Biotec, Bergisch Gladbach, Germany) according to the manufacturer’s instructions. Briefly, after centrifugation, the layer of mononuclear cells was collected. Then the cells were incubated with anti-CD14 microbeads at 4 °C for 15 min. After washing, the CD14^+^ cells were isolated using a MACS cell isolation column (Miltenyi Biotec). The obtained monocytes were then seeded in 24-well-plated and cultured in RPMI 1640 medium supplemented with 10% FBS (HyClone) and 40 ng/ml macrophage colony-stimulating factor (MCSF; Novoprotein, catalog no. CB34) in 24-well plates with a humidified 5% CO_2_ incubator at 37 °C for 3–5 days. The culture medium was replaced every other day with a fresh medium containing MCSF. Matured macrophages were washed with PBS and cocultured with NPC cells which were seeded in upper transwell inserts (0.4-µm pore, 3470, Corning) for 3 days in 10% FBS RPMI 1640 medium without MCSF. Meantime, we continuously cultured CD14^+^ monocyte isolated from PBMC with 40 ng/ml MCSF in 24-well plates for a total of 10 days, which served as positive controls for M2-polarized macrophages. NPC cells (HK1-EBV and SUNE2) were treated with JUNB and MCSF siRNA for 24–48 h (RIBOBIO, Guangzhou, China) and JUNB inhibitor T5224 (HY-12270-10, MedChemExpress, China) for 2–3 days. Then the NPC cells were washed with PBS and cocultured with matured macrophages in Transwell apparatus with 10% FBS RPMI 1640 medium. MCSF inhibitor Pexidartinib (S7818-10, Selleck, USA) was added to the bottom wells of transwell plates to treat the macrophages cocultured with ZIC2-overexpressed tumor cells for 2–3 days.

### Cytokine array

A human inflammatory cytokine array was performed with the supernatants of NPC cell lines (HK1-EBV and C666-1) with ZIC2 knockdown according to the manufacturer’s protocol for the RayBio Human Cytokine Antibody Array (RayBiotech, catalog No. QAH-INF-3-1). The expression levels of M1- and M2-polarizing cytokines were detected by FACS and enzyme-linked immunosorbent assay (ELISA) kits. The procedures were performed according to the manufacturer’s protocols.

### Flow cytometric analysis

To analyze the expression of M1 or M2-phenotype markers and cytokines, the macrophages were stained with appropriate fluorophore-labeled antibodies according to the manufacturer’s instructions via fluorescence-activated cell sorting (FACS). Fluorophore-labeled antibodies against the following proteins, along with matched isotype controls, were purchased from BioLegend (San Diego, CA, USA): TNF-α (502912), TGF-β (349610), ARG-1 (17-3697-82), HLA-DR (307616), and CD163 (333606). All data were collected and analyzed using a CytExpert flow cytometer (BD Biosciences, USA).

### ELISA experiment

To detect the secretion of MCSF or the M1 or M2-phenotype related cytokines, the ELISAs were used with human IL-10 (430604, BioLegend, USA), human TNF-α (430204, BioLegend, USA), TGF-β (DY240-05, R&D, USA), human MCSF (CHE0042-96, 4Abio, China), and mouse MCSF (DY416-05, R&D, USA), mouse TGF-β (88-8350-88, Invitrogen, USA)and mouse IL-10 (431414, BioLegend, USA) kits. Briefly, a 96-well plate was coated with 100 µl diluted capture antibody overnight at 4 °C. Then, 200 µl per well blocking solution was incubated at room temperature for 2 hours. After washing the plate with PBST, a gradient diluted standard, and samples were added (100 µl per well) and incubated at room temperature for 2 h. Then, the plate was washed with PBST, and a diluted detection antibody (100 µl per well) was added and incubated at room temperature for 2 h. After washing the plate with PBST, 100 µl avidin–HRP was added and incubated for 20 min in the dark at room temperature. Then, the plate was washed with PBST, and the substrate TMB (100 µl) was added and incubated at room temperature for 20 min in the dark. A stop solution (50 µl) was then added to terminate the reaction, and the concentration of each sample was detected at 450 nm.

### Immunohistochemical analysis

All specimens were sectioned at a thickness of 4 µm, heated at 65 °C for 2 h, dewaxed with xylene, and rehydrated. Then, 3% H_2_O_2_ was used to block endogenous peroxidases for 10 min. EDTA was used for antigen retrieval. Subsequently, the sections were incubated with an anti-ZIC2 antibody (ab150404, Abcam) or anti-JUNB antibody (3753S, CST), anti-CD163 antibody (93498S, CST) overnight at 4 °C and with a secondary antibody at room temperature for 30 min. The sections were stained with diaminobenzidine (DAB) for 20 min, counterstained with Mayer’s hematoxylin, dehydrated, and mounted with a coverslip. All patient sections were scored by two experienced pathologists who were blinded toward the clinical parameters. The digital pathological system (HALO) was utilized to quantify the density of CD163-positive cells.

### Bioinformatic analysis

1. ChIP-seq: This approach compared sequencing data after ChIP with the Hg19 genome by BWA-MEM2 (version 2.0) and then converted and sorted the data and constructed an index with SAMtools (version 1.10). MACS2 (version 2.26) was used to find the enrichment peak region from the bam file of paired samples.
2. RNA-seq: Subread software (version 1.6.4) was used to compare the read length (reads) obtained by sequencing to the Hg19 genome and count and analyze the reads. After obtaining an expression matrix, DESeq2 was used for filtering and normalization (Normalization) to identify transcripts that were different among different samples according to a statistical model.

### Animal studies

All animal work was conducted in accordance with a protocol approved by the Institutional Animal Care and Use Committee at the Medical College of Sun Yat-sen University. Female 6- to 7-week-old C57BL/6 mice were randomly assigned to four groups, and 100 μl cell suspension mixture (5 × 10^5^) containing 75% MC38-Vector or MC38-ZIC2 cells and 25% Matrigel was subcutaneously injected. When the tumors could be palpated, we treated the mice with or without pexidartinib (an inhibitor of CSF-1R) (40 mg/kg each) via intragastric administration according to the manufacturer’s protocols. At the same time, tumor size was measured daily with calipers. When the mice were euthanized, the individual tumors were isolated and weighed.

### Data statistics and analysis

SPSS was used for statistical analysis. The chi-square test was performed to analyze the relationships between ZIC2 expression and clinical features, the Kaplan–Meier method was used to draw a cumulative survival distribution curve, and the log-rank test was used to compare differences among groups. A Cox proportional hazard regression model was used for univariate and multivariate regression analyses to determine the effects of specific prognostic factors on survival. GraphPad Prism 5.0 and 8.0 (GraphPad Software, San Diego, CA) were used to analyze experimental data. Comparisons between the two groups were analyzed by Student’s *t*-test. The data are presented as the mean SEM from at least three independent experiments. In all cases, a *p*-value less than 0.05 was considered statistically significant.

## Results

### ZIC2 is highly expressed in NPC

**Fig. 1:**
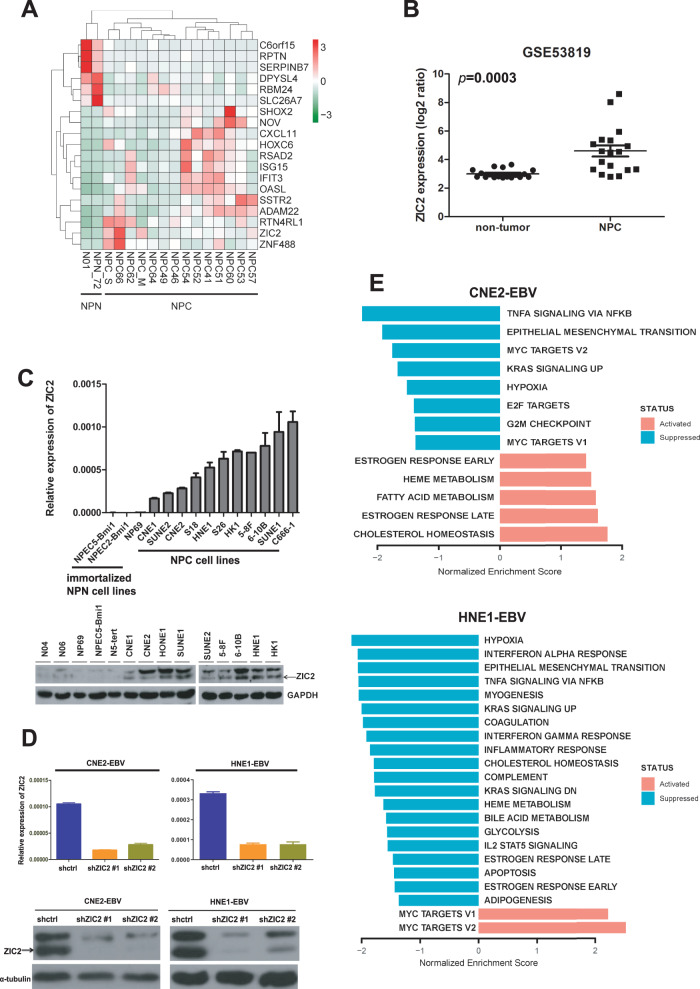
ZIC2 was highly expressed in nasopharyngeal carcinoma. **A** Gene expression profiles of NPC cell lines, normal nasopharyngeal epithelial cell lines, and NPC tissues. (N01 is a normal nasopharyngeal epithelial cell. NPN_72 is nontumorous nasopharyngeal tissue. NPC_S and NPC_M is primarycultured NPC cells. NPC_66, NPC_62, NPC_64, NPC_49, NPC_46, NPC_54, NPC_52, NPC_41, NPC_51, NPC_60, NPC_53, NPC_57 are NPC tissues). **B** The ZIC2 expression in the GEO database. **C** RT-PCR (upper panel) and Western blotting (lower panel) were used to detect the expression of ZIC2 in immortalized nasopharyngeal epithelial cells and NPC cells. GAPDH served as a loading control for western blotting, and β-actin served as an internal control for RT-PCR. (N04 and N06 are normal nasopharyngeal epithelial cells. NP69, N5-tert, NPEC5-Bmi1, and NPEC2-Bmi1 are immortalized nasopharyngeal epithelial cells. CNE1, CNE2, HONE1, SUNE1, SUNE2, 5–8F, 6–10B, HNE1, HK1, S18, S26, and C666-1 are NPC cells). **D** The expression levels of ZIC2 after shRNA knockdown in the nasopharyngeal carcinoma cell lines (CNE2-EBV and HNE1-EBV) were detected by both RT-PCR and western blotting. β-actin served as an internal control for RT-PCR, and α-tubulin served as the internal loading control for western blotting. shctrl (negative control shRNA). **E** GSEA analysis of RNA sequencing data for the nasopharyngeal carcinoma cell lines CNE2-EBV (upper panel) and HNE1-EBV (lower panel) (shZIC2 vs. shctrl).

To explore the specific regulatory molecular mechanism of the development of NPC, we performed RNA-seq analysis of NPC cell lines, normal nasopharyngeal epithelial cell lines, and NPC tissues. We found that ZIC2 was highly expressed in both NPC tissues and NPC cell lines (Fig. 1A, Supplemental Table 1). Similarly, the expression of ZIC2 was higher in NPC than in NPN (Non-neoplastic nasopharyngeal tissue) in the Gene Expression Omnibus (GEO) database (Fig. 1B). In addition, the mRNA and protein expression levels of ZIC2 were higher in NPC cell lines than in immortalized nasopharyngeal epithelial cells (Fig. 1C). C666-1 cell is the only EBV-positive cell line, and the other NPC cell lines lost EBV episomes during passages in vitro. Therefore, we reinfect these EBV-negative NPC cells with EBV to re-establish the EBV-positive NPC cell line. To explore the mechanism involving ZIC2 in the development of NPC, we established stable knockdown NPC cell lines with EBV (CNE2-EBV and HNE1-EBV) with two different shRNAs against ZIC2 (shZIC2 #1 and shZIC2 #2). In the previous validation results, we observed a higher amount of ZIC2 expression in the cell lines HNE1-EBV and CNE2-EBV. As shown in Fig. 1D, the expression of ZIC2 was significantly decreased at both the mRNA and protein levels in stable ZIC2-knockdown CNE2-EBV and HNE1-EBV cells. Then, we carried out RNA-seq analysis of these two ZIC2-knockdown cell lines (Supplemental Table 2) and found that there were 2642 co-downregulated genes and 675 co-upregulated genes in the two cell lines, which were enriched in TNF-α pathway, epithelial–mesenchymal transition (EMT), hypoxia and the KRAS signaling pathway by gene set enrichment analysis (GSEA) (Fig. 1E).

### High expression of ZIC2 in NPC cells could induce M2 phenotype polarization of TAMs

**Fig. 2:**
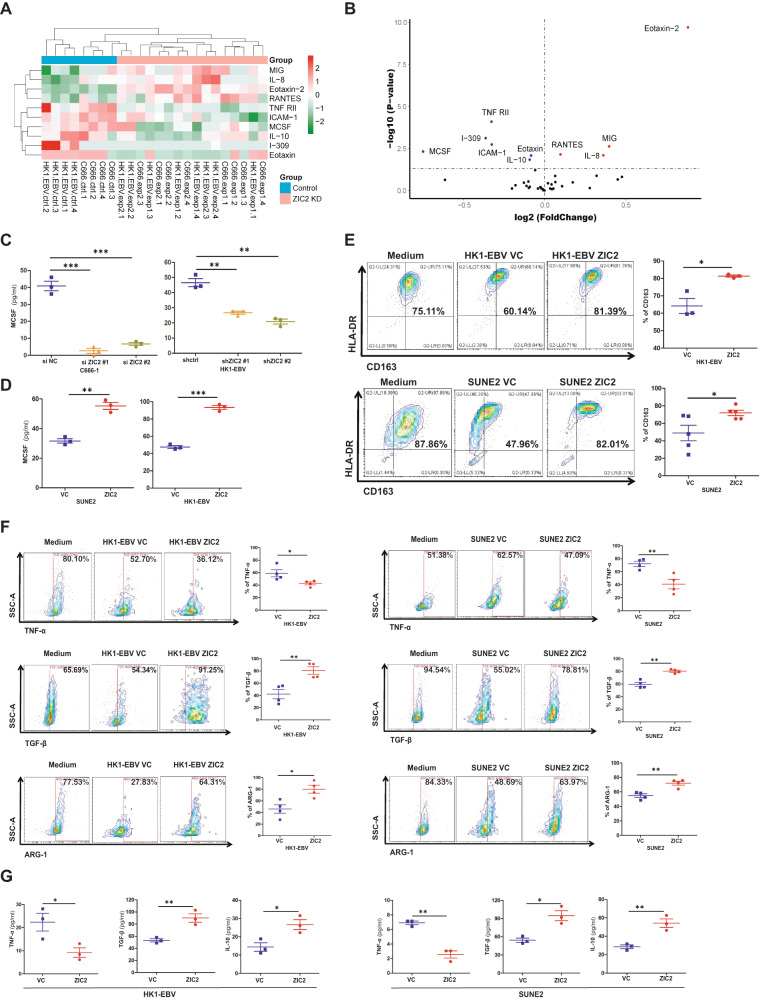
ZIC2 plays an important role in inducing M2 phenotype polarization of TAMs. **A** The Quantibody Human Cytokine Antibody Array was performed with the supernatants of the nasopharyngeal carcinoma cell lines C666-1 and HK1-EBV with ZIC2 knockdown. Cluster analysis showed the differential expression of cytokines after ZIC2 knockdown. **B** The volcano plot visualizes the cytokines according to their log2FC and −log10(*p*-value) after ZIC2 knockdown in NPC cell lines. **C** Relative secretion of MCSF in the culture supernatants of C666-1 and HK1-EBV cells after ZIC2 knockdown, detected by ELISA. siNC (Negative control siRNA), shctrl (Negative control shRNA). **D** Relative secretion of MCSF in the culture supernatants of SUNE2 and HK1-EBV cells overexpressing ZIC2 by ELISA. vc (Vector control). **E** PBMCs from healthy people were positively sorted with CD14 magnetic beads, stimulated with MCSF to differentiate the cells into macrophages, and then cocultured with HK1-EBV (upper panel) and SUNE2 cells (lower panel) overexpressing ZIC2 for 3–5 days. Representative plot (left panel) and quantification (right panel) of the expression of HLA-DR and CD163. **F** Representative image and quantification of the expression of the cytokines TNF-α, TGF-β, and related surface marker ARG-1 in macrophages cocultured with HK1-EBV (left panel) and SUNE2 cells (right panel) overexpressing ZIC2 for 3–5 days. **G** TNF-α, TGF-β, and IL-10 production were determined by ELISA analysis of the coculture supernatants of HK1-EBV (left panel) and SUNE2 cells (right panel) overexpressing ZIC2. All of the experiments were repeated at least three times, and statistical analyses were conducted with an unpaired Student’s *t*-test; *p < 0.05, **p < 0.01, and ***p < 0.001 vs. the corresponding control.

TNF-α is a well-known proinflammatory and anticancer cytokine in the TME [26] and is secreted mainly by M1 phenotype macrophages [23, 24, 27]. TNF-α secretion from monocytes strongly induces the expression of programmed death ligand 1 (PD-L1), and PD-L1-positive monocytes induce T cell dysfunction and reduce T cell proliferation [28]. Therefore, we performed Quantibody Human Cytokine Antibody Array analysis of the supernatants of NPC cell lines (HK1-EBV and C666-1) with ZIC2 knockdown. Cluster analysis showed that after ZIC2 was knocked down, the expression levels of TNF-α receptor 2 (TNF-RII), intercellular cell adhesion molecule-1 (ICAM-1), M-CSF, IL-10, I-309, and eotaxin were downregulated compared with those in the control group (Fig. 2A). The volcano plot shows that MCSF was the most significantly differentially expressed cytokine (Fig. 2B). Furthermore, we examined the high expression of MCSF in the supernatant of NPC cells by ELISA, and the results were consistent with the results of the cytokine array analysis. The expression level of MCSF was decreased after knocking down ZIC2 in NPC cells (Fig. 2C). Likewise, NPC cells (SUNE2 and HK1-EBV) overexpressing ZIC2 exhibited increased expression of MCSF (Fig. 2D).

MCSF, also known as colony-stimulating factor-1 (CSF-1), plays an important role in the proliferation, differentiation, and maintenance of monocyte activity. MCSF can act as a TAMs chemokine to promote tumor growth [29, 30]. Therefore, we aimed to explore the effects of ZIC2 on the differentiation of TAMs.

Macrophages differentiated from monocytes induced with MCSF were co-cultured with NPC cell lines with ZIC2 overexpression or knockdown for 3–5 days. Medium supplemented with MCSF served as a positive control for M2-like TAMs. The percentage of HLA-DR^+^CD163^+^ macrophages was increased after co-cultured with ZIC2-overexpressing HK1-EBV and SUNE2 cell lines (Fig. 2E) but was decreased after co-cultured with HNE1-EBV cells with stable knockdown of ZIC2 (Supplemental Fig. 1A). These results demonstrated that high expression of ZIC2 in tumor cells could increase the frequency of the HLA-DR^+^CD163^+^ phenotype in macrophages, suggesting that ZIC2 can induce macrophages to differentiate into the M2 phenotype.

Based on the above experimental results, we further explored the effects of NPC cells with ZIC2 overexpression on cytokines secreted and related surface markers by TAMs. The levels of TNF-α, an M1 phenotype cytokine secreted by macrophages, were decreased after co-cultured with ZIC2-overexpressing NPC cells, but the secretion of TGF-β, M2 phenotype cytokines and the expression of M2-related surface marker ARG-1 were increased (Fig. 2F). Opposite effects on the secretion of the M1 phenotype cytokine TNF-α and M2 phenotype cytokines TGF-β and related surface marker ARG-1 were observed after co-cultured with HNE1-EBV cells with ZIC2 knockdown (Supplemental Fig. 1B). Subsequently, similar results were observed for the secretion of TNF-α, IL-10, and TGF-β by ELISA in macrophages co-cultured with NPC cell lines (HK1-EBV and SUNE2) overexpressing ZIC2 (Fig. 2G).

Moreover, we found that overexpression of ZIC2 in NPC cells significantly increased the expression of the M2 phenotype cytokines IL-13 and related surface marker CD206 but decreased the expression of the M1 phenotype cytokines IFN-γ and related surface marker CD86 in THP-1 cells, as shown by RT-PCR (Supplemental Fig. 1C). Accordingly, these results consistently showed that ZIC2 could induce macrophages to secrete M2 phenotype cytokines and express related surface marker, but inhibit the secretion of M1 phenotype cytokines and the expression of related surface marker. Taken together, our data indicated that high expression of ZIC2 in NPC cells induced M2 phenotype polarization of TAMs and affected the secretion of TAMs-related cytokines and the expression of related surface markers.

### JUNB is a downstream target of ZIC2

**Fig. 3:**
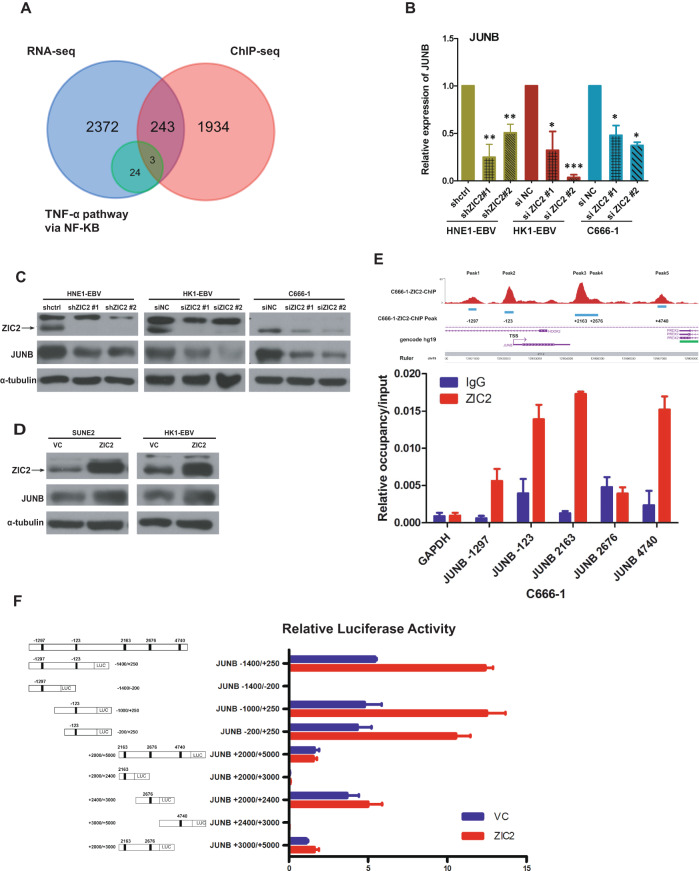
ZIC2 regulates the expression of JUNB. **A** Venn diagram analysis showing the downstream target genes of ZIC2 after gene overlap of ChIP-seq analysis results, RNA-seq analysis results, and the TNF-α/NF-κB signaling pathway. **B** RT-PCR detection of the expression of JUNB after ZIC2 knockdown in the NPC cell lines HNE1-EBV, HK1-EBV, and C666-1. β-actin served as the internal control. All of the experiments were repeated at least three times, and statistical analyses were conducted with an unpaired Student’s *t*-test; *p < 0.05, **p < 0.01, and ***p < 0.001 vs. the corresponding control. **C** The expression of JUNB in the NPC cell lines HNE1-EBV, HK1-EBV, and C666-1 after ZIC2 knockdown was detected by western blotting, and α-tubulin acted as the internal control. siNC (Negative control siRNA). **D** Western blotting was utilized to detect the expression of JUNB after overexpression of ZIC2 in the NPC cell lines SUNE2 and HK1-EBV. α-Tubulin acted as the internal control. vc (Vector control). **E** Bioinformatic analysis showing the promoter sites of JUNB (upper panel). ChIP-qPCR detected the relative occupancy of ZIC2 on the promoter of JUNB (lower panel). GAPDH gDNA amplification served as an internal control. **F** Schematic diagram of different length truncations of the promoter site of JUNB (left panel). Luciferase activity of nine JUNB promoter reporters in 293 T cells co-transfected with the pBabe empty vector or ZIC2 and normalized to Renilla luciferase activity (right panel).

ZIC2 is an important transcription factor that can regulate the expression of downstream target genes [12]. Therefore, we performed ChIP-seq to further screen and identify potential downstream target genes of ZIC2 in C666-1 cells. We found that 2179 genes were bound by ZIC2 with a peak score > 20 and a distance from the promoter position of −2000 to 1000 (Supplemental Table 3). Then, we overlapped these potential downstream genes with 27 genes enriched in the TNF-α pathway and identified NR4A1, TUBB2A, and JUNB as potential direct target genes of ZIC2 in the TNF-α pathway (Fig. 3A). Then, we detected the expression levels of NR4A1, TUBB2A, and JUNB in ZIC2-knockdown NPC cell lines by RT-PCR. Among these three genes, only JUNB was consistently reduced in several NPC cell lines after ZIC2 knockdown (Fig. 3B, Supplemental Fig. 2A).

Furthermore, the protein expression of JUNB was downregulated after knocking down ZIC2 (Fig. 3C), whereas it was enhanced by overexpression of ZIC2 (Fig. 3D, Supplemental Fig. 2B), indicating JUNB is potentially regulated by ZIC2.

According to our ChIP-seq analysis results, there are five binding sites in the promoter of JUNB bound by ZIC2 (Supplemental Table 4). Thus, we performed a ZIC2 ChIP assay with the C666-1 cell line and found that ZIC2 was significantly enriched in the promoter of JUNB, mainly at sites −123, −1297, 2163, and 4740, in the anti-ZIC2 antibody group compared with the IgG control group (Fig. 3E).

To determine the exact mechanisms underlying how ZIC2 binds to and regulates its downstream target gene JUNB, we constructed JUNB promoter luciferase reporter plasmids with the five binding sites in the JUNB promoter (−1400/+250, −1000/+250, −200/+250, +2000/+2400, +3000/+5000), as indicated in Fig. 3F. Compared to the vector control, ZIC2 significantly induced luciferase activity controlled by the promoter region (−1400/ + 250, −1000/+250, and −200/+250) and slightly induced luciferase activity controlled by the regions +2000/+2400 and +3000/+5000 (Fig. 3F), suggesting that ZIC2 might enhance JUNB promoter activity around the −123 binding site. Collectively, these data indicated that ZIC2 induced the transcription of JUNB by binding to its promoter.

### ZIC2 promotes the M2 phenotype polarization of TAMs through the regulation of JUNB in NPC cells

**Fig. 4:**
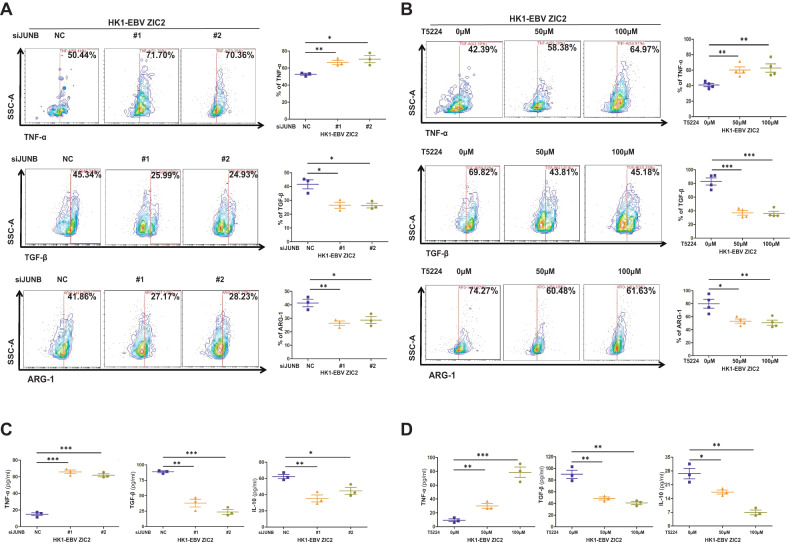
ZIC2 promotes the M2 phenotype polarization of TAMs through the regulation of JUNB in NPC cells. **A**, **B** Representative image (left panel) and quantification (right panel) of the expression of the cytokines TNF-α, TGF-β, and related surface marker ARG-1 in macrophages cocultured with HK1-EBV cells overexpressing ZIC2 treated with JUNB-specific siRNA (**A**) or JUNB inhibitor, T5224 (**B**). NC (Negative control siRNA). **C**, **D** TNF-α, TGF-β, and IL-10 production was determined by ELISA analysis of the coculture supernatants of HK1-EBV cells overexpressing ZIC2 treated with (**C**) or JUNB inhibitor, T5224 (**D**). NC (Negative control siRNA). All of the experiments were repeated at least three times, and statistical analyses were conducted with an unpaired Student’s *t*-test; *p < 0.05, **p < 0.01, and ***p < 0.001 vs. the corresponding control.

Given the above result that JUNB, downstream of the TNF-α pathway, was identified as a target gene of ZIC2, we speculated whether JUNB is involved in the M2 phenotype polarization of TAMs induced by ZIC2 in tumor cells. Therefore, we used JUNB-specific siRNA and the inhibitor T5224 to treat ZIC2-overexpressing HK1-EBV and SUNE2 cell lines and then co-cultured the treated cell lines with macrophages for 3–5 days. Treatment with the siRNA and inhibitor T5224 increased the expression of the M1 phenotype cytokine TNF-α but decreased the secretion of the M2 phenotype cytokines TGF-β and the expression of related surface marker ARG-1 in macrophages, as determined by flow cytometry (Fig. 4A, B, Supplemental Fig. 3A, B). Similar results were observed for the supernatant of macrophages co-cultured with NPC cells treated with the siRNA and inhibitor T5224 by ELISA (Fig. 4C, D, Supplemental Fig. 3C, D). These results showed that JUNB inhibition reversed the changes in TAMs-secreted cytokines triggered by ZIC2-overexpressing NPC cells, suggesting that ZIC2 promotes the M2 phenotype polarization of TAMs through the regulation of JUNB in NPC cells.

### ZIC2/JUNB/MCSF axis in NPC cells could promote M2 polarization of TAMs

**Fig. 5:**
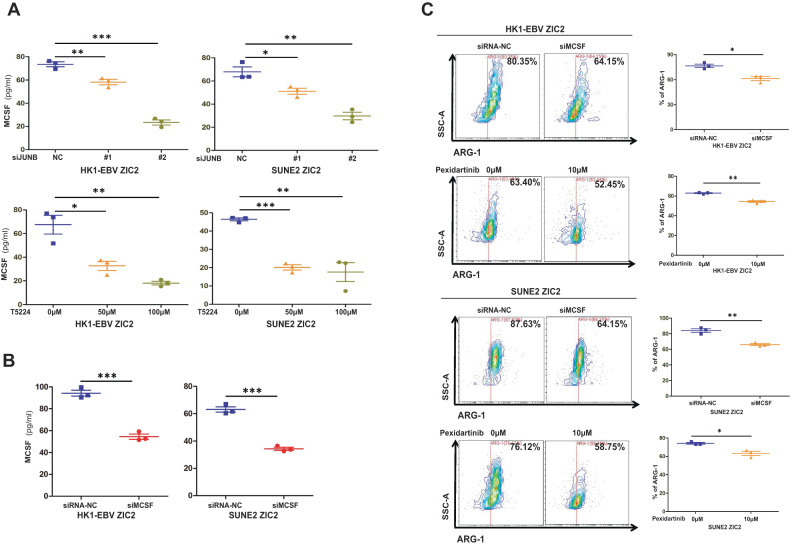
**A** ZIC2/JUNB/MCSF axis in NPC cells could promote M2 polarization of TAMs. **A** Detection of the relative secretion of MCSF vs. the corresponding control in NPC cells treated with JUNB-specific siRNA and an inhibitor by ELISA. NC (Negative control). **B** MCSF was determined by ELISA analysis of the supernatants of HK1-EBV and SUNE2 cells overexpressing ZIC2 treated with MCSF-specific siRNA. **C** Representative image (left panel) and quantification (right panel) of the expression of the related surface marker ARG-1 in macrophages cocultured with HK1-EBV and SUNE2 cells overexpressing ZIC2 transfected with MCSF-specific siRNA or treated with MCSFR inhibitor, Pexidartinib. All of the experiments were repeated at least three times, and statistical analyses were conducted with an unpaired Student’s t-test; *p < 0.05, **p < 0.01, and ***p < 0.001 vs. the corresponding control.

To investigate whether JUNB regulates the secretion of MCSF induced by ZIC2 overexpression, we detected the expression of MCSF in NPC cells treated with the JUNB-specific siRNA and inhibitor T5224 and found that the expression of MCSF was significantly decreased by ELISA (Fig. 5A), suggesting JUNB is involved in MCSF secretion in NPC cells.

To further explore the role of MCSF in ZIC2-induced M2 phenotype polarization of TAMs, we transfected ZIC2-overexpressed tumor cells with MCSF-specific siRNA, then cocultured them with macrophages for 3–5 days or added MCSF Receptor (MCSFR) inhibitor Pexidartinib to the bottom well of transwell plates to treat the macrophages cocultured with ZIC2-overexpressed tumor cells in order to abrogate MCSF/MCSFR axis (Fig. 5B, C). As shown in Fig. 5B, the expression of MCSF was significantly decreased after ZIC2-overexpressing cell lines were treated with the MCSF-specific siRNA. Under MCSF-siRNA or pexidartinib treatment, the expression of the M2-related surface marker ARG-1 was reduced (Fig. 5C), suggesting that interfering MCSF-MCSFR axis between the interactions of tumor cells and macrophages might inhibit the M2-like polarization of macrophages induced by ZIC2-overexpression tumor cells. Overall, ZIC2/JUNB/MCSF axis in NPC cells could promote M2 polarization of TAMs.

### High expression of ZIC2, JUNB, and CD163 is associated with a poor prognosis in NPC patients

**Fig. 6:**
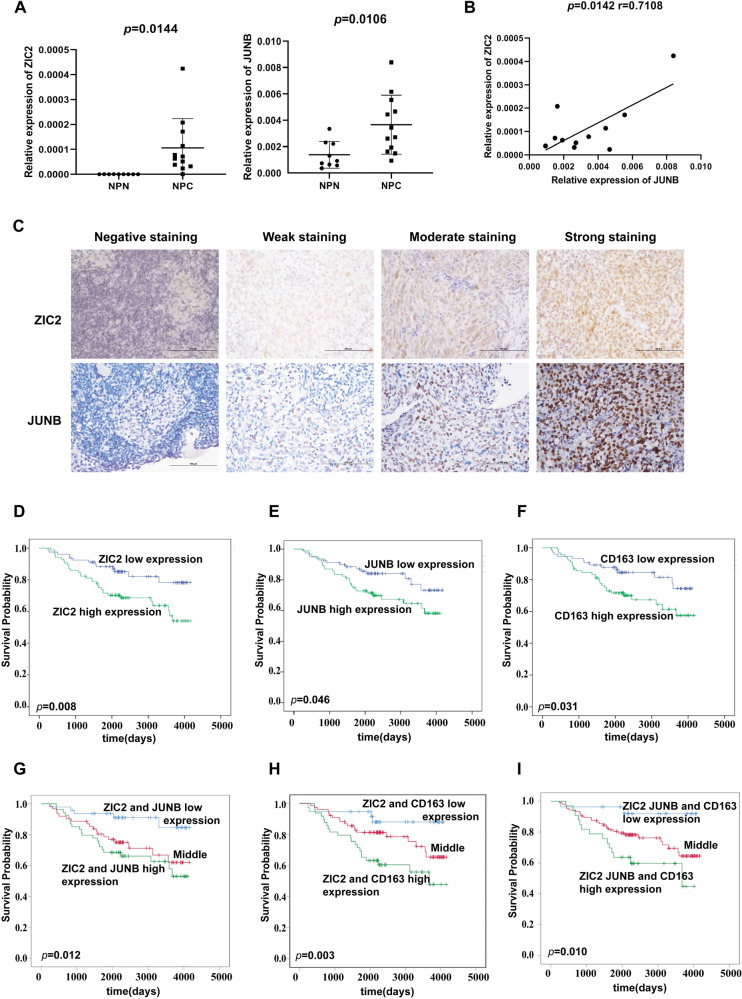
ZIC2 and JUNB are elevated in NPC tissue and are associated with a poor prognosis in NPC patients. **A** The expression levels of ZIC2 and JUNB in NPC tissues and unpaired nontumorous nasopharyngeal tissues were detected by RT-PCR, and β-actin served as an internal control. **B** Positive correlations were found between the expression of ZIC2 and JUNB in nasopharyngeal carcinoma. The statistical analysis was performed using Spearman’s correlation analysis and linear regression. **C** Representative images of immunohistochemical staining for ZIC2 and JUNB in NPC. **D**–**F** Overall survival curves of NPC patients with low and high expression of ZIC2 (**D**), JUNB (**E**), or CD163 (**F**) by Kaplan–Meier survival analysis (*p* = 0.008, *p* = 0.046, *p* = 0.031). **G** Overall survival curves of NPC patients with low and high expression of ZIC2 and JUNB (*p* = 0.012). **H** Overall survival curves of NPC patients with low and high expression of ZIC2 and CD163 (*p* = 0.003). **I** Overall survival curves of NPC patients with low and high expression of ZIC2, JUNB, and CD163 (*p* = 0.010).

To further verify the relationship between ZIC2 and JUNB in NPC, we detected the expression of ZIC2 and JUNB in both NPC tissues and unpaired nontumorous nasopharyngeal tissues by RT-PCR and immunohistochemistry (IHC). ZIC2 and JUNB were highly expressed in NPC tissues compared with nontumorous nasopharyngeal tissues (*p* = 0.0144, *p* = 0.0106) (Fig. 6A). In addition, the expression of ZIC2 was positively correlated with the expression of JUNB (r = 0.7108, *p* = 0.0142) (Fig. 6B). Representative immunohistochemical staining for ZIC2 and JUNB in NPC patients is shown in Fig. 6C, which shows that ZIC2 staining was mainly found in nuclei. High expression of ZIC2, JUNB, and CD163 was significantly related to a poor prognosis in NPC patients (*p* = 0.008, *p* = 0.046, *p* = 0.031, *p* = 0.005) (Fig. 6D–F). Moreover, the overall survival of NPC patients with high expression of both ZIC2 and JUNB or both ZIC2 and CD163 was significantly shorter than that of patients with low expression of both ZIC2 and JUNB or both ZIC2 and CD163 (*p* = 0.004, *p* = 0.013) (Fig. 6G–I).

To further explore the relationship between the expression of ZIC2 and the prognosis of NPC, we analyzed the clinical information of 164 NPC patients at the Sun Yat-sen University Cancer Center. High ZIC2 expression was related to tumor recurrence (*p* = 0.022) (Table 1). Additionally, a Cox proportional hazard model was used to examine the prognostic value of ZIC2, JUNB, and CD163 expression through univariate analysis (hazard ratio (HR): 2.342, 95% confidence interval (CI): 1.225–4.477, *p* = 0.010; HR: 1.897, 95% CI: 1.001–3.482, *p* = 0.050; HR: 1.987, 95% CI: 1.052–3.754, *p* = 0.034) and multivariate analysis of ZIC2 (HR: 2.019, 95% CI: 1.033–3.946, *p* = 0.040). The expression of ZIC2 was one of the independent prognostic factors for NPC (Table 2). In summary, the above results suggested that ZIC2, JUNB, and CD163 could be used as prognostic markers for NPC.

**Table 1:** Relationship between ZIC2 and clinicopathologic characteristics in Nasopharyngeal carcinoma patients.

Characteristics	ZIC2			*p*-Value
	All patients	Low	High	
*Gender*				0.435
Male	82	37	45	
Female	82	42	40	
*Age (years)*				0.504
≤44	89	45	44	
>44	75	34	41	
*TNM*				0.178
I + II	32	12	20	
III + IV	132	67	65	
*T*				0.238
T1 + 2	53	22	31	
T3 + 4	111	57	54	
*N*				0.986
N0-1	79	38	41	
N2-3	85	41	44	
*Metastasis*				0.374
No	139	69	70	
Yes	25	10	15	
*Recurrence*				**0.022**
No	155	78	77	
Yes	9	1	8	

Bold values mean the siginificance of *p*-Values.

**Table 2:** Univariate and multivariate Cox regression analysis for predictors of overall survival.

	HR	95% CI	*p*-Value
*Univariate analysis*			
Gender	1.017	0.554–1.866	0.957
Age(years)	1.490	0.823–2.698	0.188
Recurrence	4.843	2.248–10.435	**0.000**
T	1.762	0.869–3.571	0.116
N	2.517	1.336–4.743	**0.004**
Metastasis	8.816	4.654–17.081	**0.000**
TNM	4.530	1.397–14.692	**0.012**
ZIC2	2.342	1.225–4.477	**0.010**
JUNB	1.897	1.001–3.482	**0.050**
CD163	1.987	1.052–3.754	**0.034**
*Multivariate analysis*			
Recurrence	4.195	1.791–9.825	**0.001**
N	1.493	0.745–2.992	0.258
Metastasis	9.189	4.532–18.631	**0.000**
TNM	2.117	0.568–7.887	0.264
ZIC2	2.019	1.033–3.946	**0.040**
JUNB	1.494	0.782–2.855	0.225
CD163	1.162	0.600–2.251	0.656

Bold values mean the siginificance of *p*-Values.

Notes: Gender, male vs. female; Age, age ≤ 44 vs. 44; recurrence, No vs. Yes; T, T1 + 2 vs. T3 + 4; N, N0–1 vs. N2–3; Metastasis, No vs. Yes; TNM, I + II vs. III + IV; ZIC2, high expression vs. low expression.

*HR* hazard ratio, *CI* confidence interval.

### ZIC2 could induce M2 phenotype polarization of TAMs and affect the secretion of TAMs-related cytokines in vivo

**Fig. 7:**
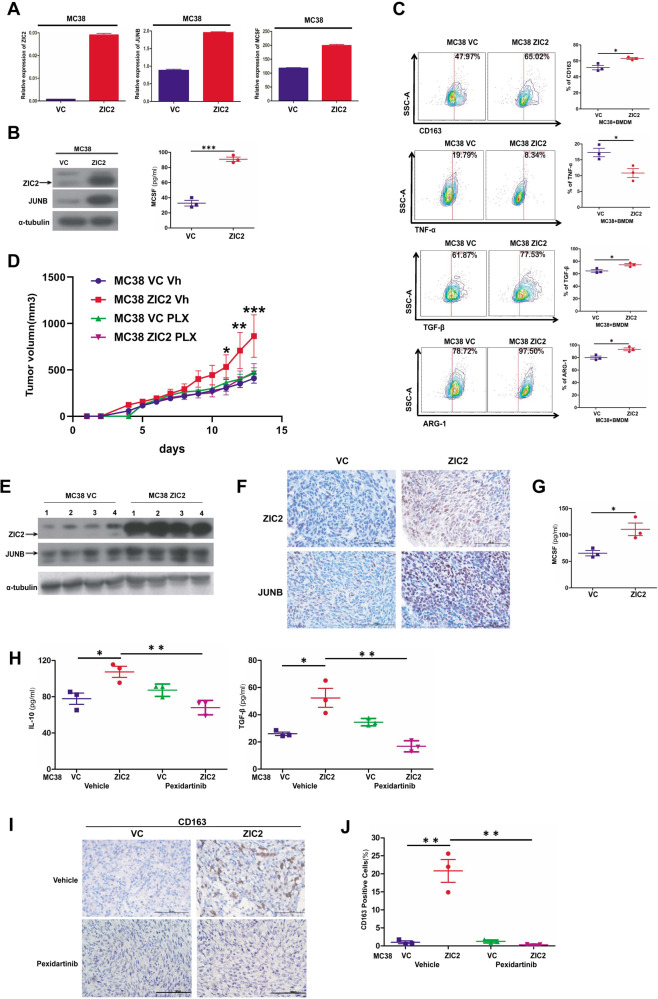
ZIC2 could induce tumorigenesis and affect the secretion of TAMs-related cytokines in vivo. **A**, **B** The expression of ZIC2, JUNB, and MCSF in the MC38 cell lines was detected by RT-PCR and western blotting. β-actin served as an internal control for RT-PCR, and α-tubulin served as the internal loading control for western blotting. VC (Vector control). **C** The expression of related surface markers of BMDM after co-culturing with ZIC2 or Vector-overexpressed MC38 cell lines. **D** The tumor growth curve of tumors formed by ZIC2 or Vector-overexpressed MC38 cell lines treated with pexidartinib or vehicle in C57BL/6 mice. **E** The expression of ZIC2 and JUNB in ZIC2-overexpressing xenograft tumors detected by western blotting. α-tubulin served as the internal loading control for western blotting. **F** Representative images of immunohistochemical staining for ZIC2 and JUNB in xenograft tumors. **G** MCSF production was determined by ELISA analysis of the xenograft tumors. **H** IL-10 and TGF-β production were determined by ELISA analysis of the xenograft tumors with pexidartinib or vehicle. **I** Representative images of immunohistochemical staining for CD163 in xenograft tumors with pexidartinib or vehicle. **J** The density of CD163-positive cells in xenograft tumors with pexidartinib or vehicle was calculated by HALO digital pathological platform. All of the experiments were repeated at least three times, and statistical analyses were conducted with an unpaired Student’s *t*-test; *p < 0.05, **p < 0.01, and ***p < 0.001 vs. the corresponding control.

Due to the lack of availability of NPC mouse immunocompetent models, we investigated the function of ZIC2 in vivo with MC38 immunocompetent models. We established MC38 cell lines with stable ZIC2 expression, and the expression levels of JUNB and MCSF were increased in ZIC2-overexpressing MC38 cells, as shown in Fig. 7A, B. Then, we co-cultured BMDM with MC38 cell lines with ZIC2 overexpression and detected the expression of related surface markers by FACS. The levels of M1 phenotype cytokine TNF-α were decreased after co-cultured with ZIC2-overexpressing, whereas the secretion of M2 phenotype cytokines TGF-β and the expression of M2-related surface markers ARG-1 and CD163 was increased (Fig. 7C). We subcutaneously injected ZIC2-overexpressing or vector-expressing MC38 cells into C57BL/6 mice, then treated them with pexidartinib (an inhibitor of CSF-1R) or vehicle. As shown in Fig. 7D, ZIC2 overexpression induced the tumor growth of MC38 xenografts in vivo, and the addition of pexidartinib abrogates this induction (Fig. 7D), suggesting that ZIC2 augmented NPC tumorigenesis in vivo through MCSF secretion. Furthermore, the expression levels of ZIC2 and JUNB were higher in ZIC2-overexpressing xenograft tumors than in vector xenografts by Western Blotting and IHC (Fig. 7E, F). The level of MCSF was higher in ZIC2-overexpressing xenograft tumors than in vector xenografts (Fig. 7G). Moreover, the expression of M2 cytokines IL-10 and TGF-β and the expression levels of CD163 was induced in the MC38 xenografts with ZIC2-overexpression, and the addition of pexidartinib inhibited this induction (Fig. 7H–J). Together, our results indicated that ZIC2 induced M2 phenotype polarization of TAMs by regulating JUNB and MCSF in vivo, contributing to tumor development.

## Discussion

An increasing amount of evidence has shown that ZIC2 plays an important role in many kinds of tumors. Recent studies have shown that high expression of ZIC2 promotes the proliferative and invasive abilities of NPC cells and is a prognostic marker for NPC [31, 32]. Nevertheless, the exact mechanisms involving ZIC2 in NPC development are not fully understood. Studies have shown that ZIC2 can promote stem cell transformation of lung adenocarcinoma by upregulating the expression of OCT4, which could be utilized as an indicator for monitoring the sensitivity and efficacy of treatment in patients with lung adenocarcinoma [33]. Moreover, ZIC2 regulates the expression of PAK4 by binding to the promoter of PAK4 to promote liver cancer progression [34]. Here, we first reported that ZIC2 is involved in immune regulation in NPC. Our RNA-seq analysis showed that the TNF-α pathway was significantly downregulated after ZIC2 knockdown (Fig. 1B). Additionally, MCSF was the most significantly differentially expressed cytokine in NPC cells after ZIC2 knockdown (Fig. 2A, B). The levels of the specific TAMs molecular markers HLA-DR and CD163 were increased after co-cultured with ZIC2-overexpressing NPC cells (Fig. 2E), accompanied by the induction of the secretion of M2 phenotype cytokines, such as TGF-β and related surface marker ARG-1, and a reduction in M1 phenotype cytokines, such as TNF-α (Fig. 2 and Supplemental Fig. 2), indicating an important role for ZIC2 in the polarization of macrophages in NPC.

It is worth noting that ZIC2 is a transcription factor that plays a regulatory role by binding to the DNA sequence of its downstream target genes [11]. Mechanistically, previous studies have shown that ZIC2 is highly expressed in clear cell renal cell carcinoma (ccRCC) and promotes the proliferation and migration of ccRCC by regulating the expression of the downstream target gene Runx2 and the ZIC2/Runx2/NOLC1 signaling axis [35]. Interestingly, in this study, through ChIP-seq analysis of ZIC2 and RNA-seq analysis, we found that JUNB was a downstream target gene of ZIC2 and that ZIC2 could significantly induce the promoter activity of JUNB at the −123 site (Fig. 3).

JUNB is one of the three members of the JUN protein family. JUNB has been reported to be a tumor-promoting factor in the processes of tumorigenesis and development [36–38]. Studies have shown that the combination of JUNB and miR-95t could act as a tumor promoter to promote proliferation and invasion in prostate cancer [39]. Latent membrane protein 1 (LMP1), an Epstein–Barr virus (EBV) latent gene, promotes the formation of dimers of c-Jun/JunB, which regulate the expression of p16 and cyclin D1 to affect the cell cycle, contributing to cell dysplasia and tumorigenesis in NPC [40]. In our study, inhibiting JUNB reduced the secretion of MCSF and the development of M2 phenotypes TAMs in macrophages cocultured with NPC cells overexpressing ZIC2 (Fig. 4), suggesting that ZIC2 directly binds to the JUNB promoter to induce JUNB expression and thus promotes MCSF secretion and M2 phenotype macrophage polarization in NPC.

TAMs are important immune cells in the TME [19, 20]. MCSF plays an important role in the proliferation and differentiation of mononuclear macrophages [31, 32]. TAMs are induced by GM-CSF secreted by tumor cells, while TAM-secreted C-C motif chemokine ligand 18 (CCL18) induces EMT in breast cancer cells, thus forming a positive feedback loop to promote lung and liver metastasis [41]. Additionally, disabled 2 mitogens responsive phosphoprotein (DAB2) is highly expressed in TAMs, which in turn accelerates tumor cell invasion through an integrin-ECM interaction [42]. We demonstrated that ZIC2 induced the secretion of MCSF in NPC cells to induce the M2 macrophage phenotype in vitro and in vivo. The application of the MCSF inhibitor pexidartinib, which is the first drug approved by the US Food and Drug Administration (FDA) for the treatment of symptomatic tenosynovial giant cell tumor (TGCT) patients, could reverse the M2 phenotype macrophage polarization induced by ZIC2 overexpression both in vitro and in vivo, suggesting that MCSF plays an important role in the interaction between NPC cells and TAMs.

To our knowledge, this is the first report on the role and potential mechanisms of ZIC2 in macrophage polarization in cancers. Due to the lack of availability of NPC mouse models, we subcutaneously injected ZIC2-overexpressing MC38 cells into C57BL/6 mice and served them as immunocompetent mouse tumor models to confirm that the function of ZIC2 in tumorigenesis is, at least in part, mediated through macrophage polarization. Although we couldn’t investigate the effects of TAMs on the development of NPC in vivo, the roles of TAMs in NPC have been studied in recent years. For example, Liao et al. reported that IL-6 secreted by both TAMs and NPC was suppressed by LPLUNC1 and could induce the proliferation of NPC cells [43]. In a previous study, we applied recombinant ISG15-treated macrophages mimic NPC-educated TAM to co-culture with NPC TILs and found ISG15-treated macrophages repressed the cytotoxicity of CD8^+^ cells [44], suggesting TAMs play a role in immune evasion in NPC. Taken together, TAMs might promote the development of NPC through tumor cell proliferation and immune suppression.

In summary, our results revealed that ZIC2 regulated its downstream target gene JUNB to induce M2 phenotype polarization of TAMs. The high expression levels of ZIC2 and JUNB were positively correlated and linked to a poor outcomes in NPC patients, implying that ZIC2, JUNB, and CD163 could be utilized as prognostic markers for NPC and as therapeutic targets for cancer immunotherapy.

## Supplementary information

supplemental figure 1

supplemental figure 2

supplemental figure 3

Supplemental table 1

Supplemental table 2

Supplemental table 3

Supplemental table 4

authors' ageements

checklist

Original Data File

## Data Availability

RNA-seq and ChIP-seq data are deposited to the National Genomics Data Center in China for public release upon publication under accession number HRA001547. Primary datasets have been generated and deposited in the Research Data Deposit (RDD) public platform (http://www.researchdata.org.cn), with the approval RDD number RDDB2023795754.
