# Supplementary figures and images for: ZIC2 induces pro-tumor macrophage polarization in nasopharyngeal carcinoma by activating the JUNB/MCSF axis
Source: Cell Death Dis. 2023 Jul 21;14(7):455. doi: 10.1038/s41419-023-05983-x (PMC10362010; doi:10.1038/s41419-023-05983-x)

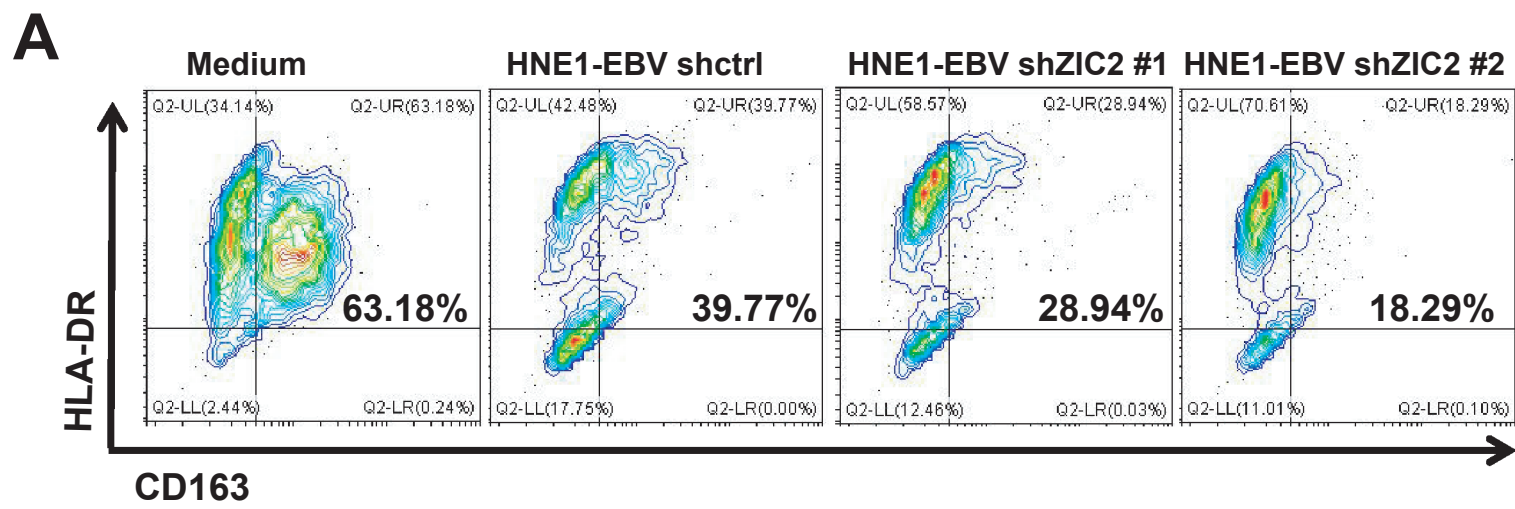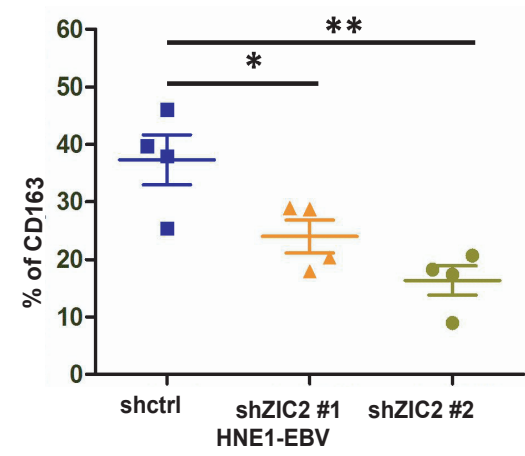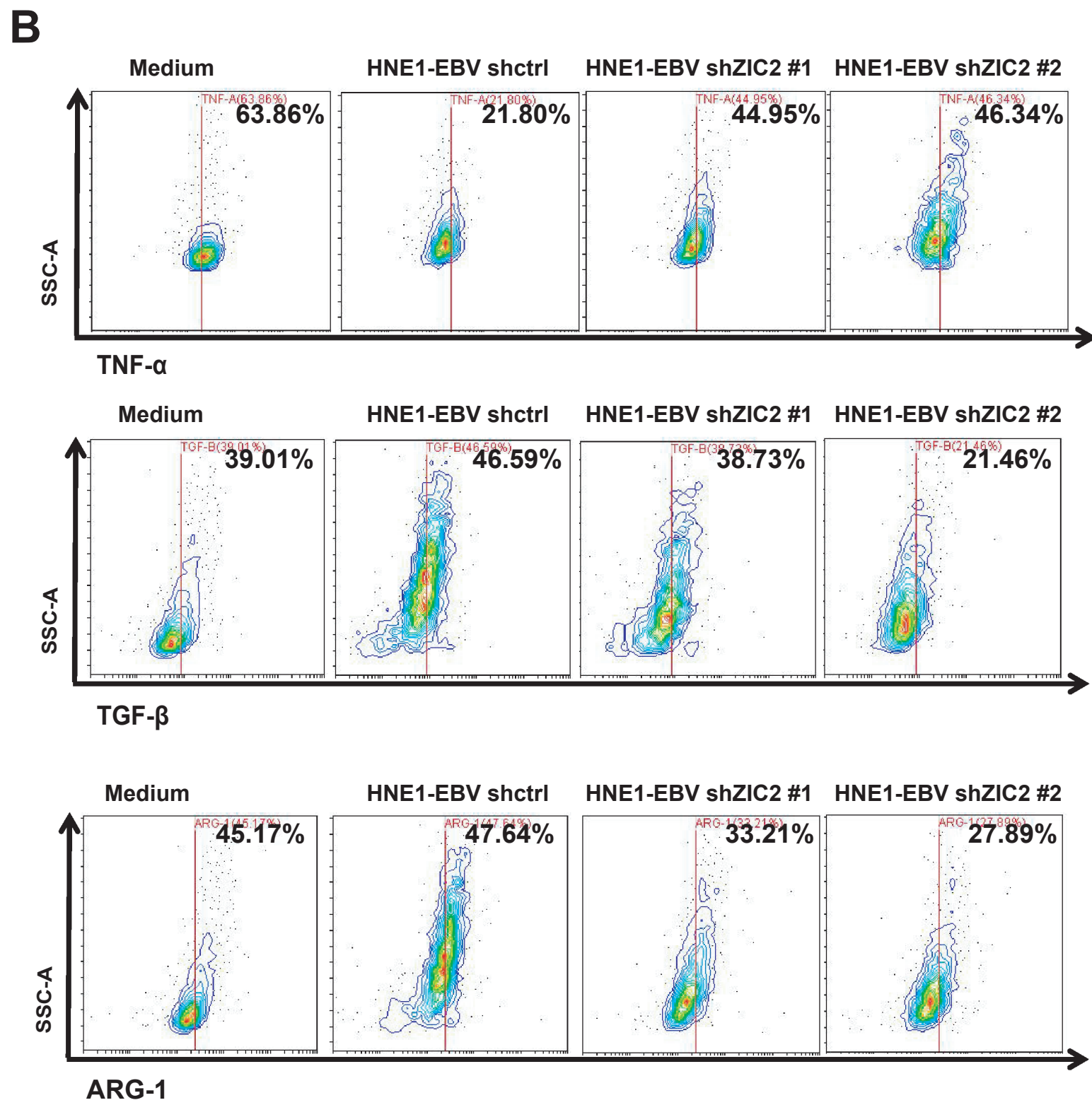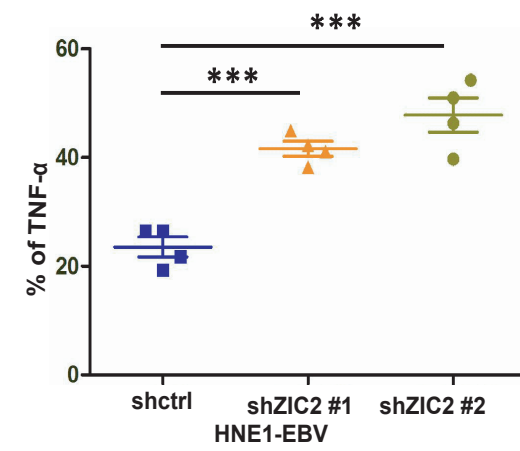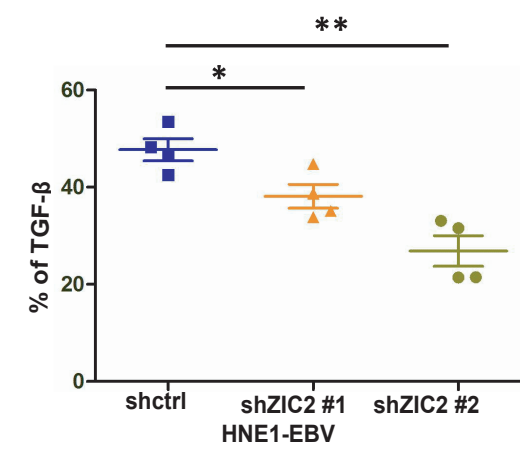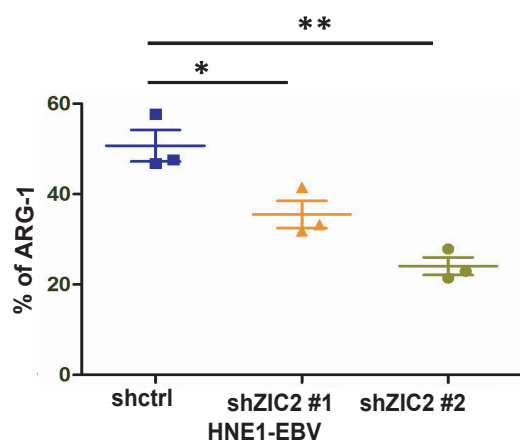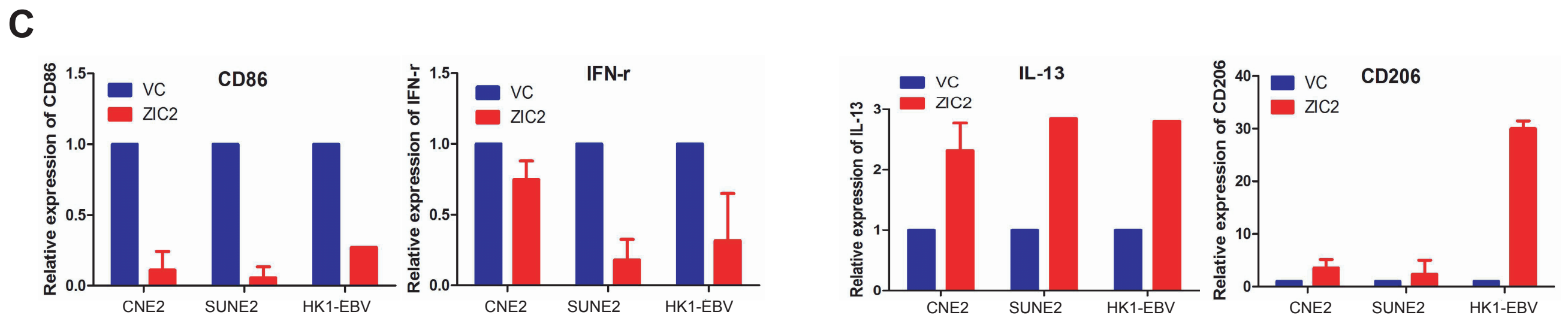

Supplement: Supplementary file 1 — supplemental figure 1 [file 41419_2023_5983_MOESM1_ESM.pdf]

**A**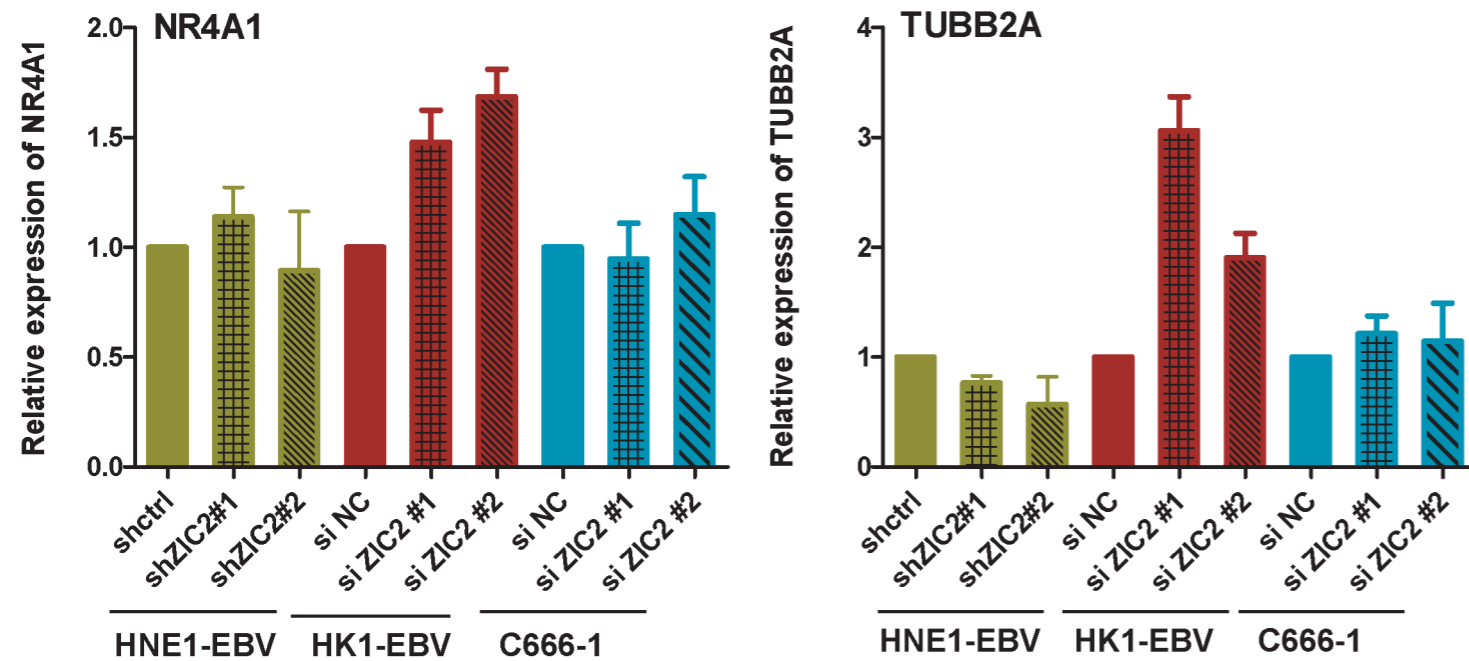**B**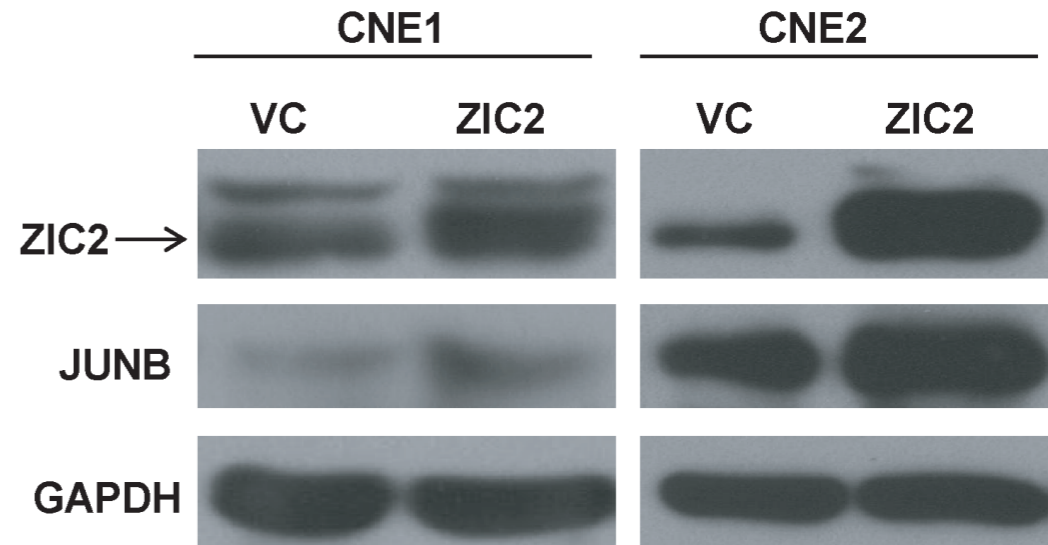

Supplement: Supplementary file 2 — supplemental figure 2 [file 41419_2023_5983_MOESM2_ESM.pdf]

**A**
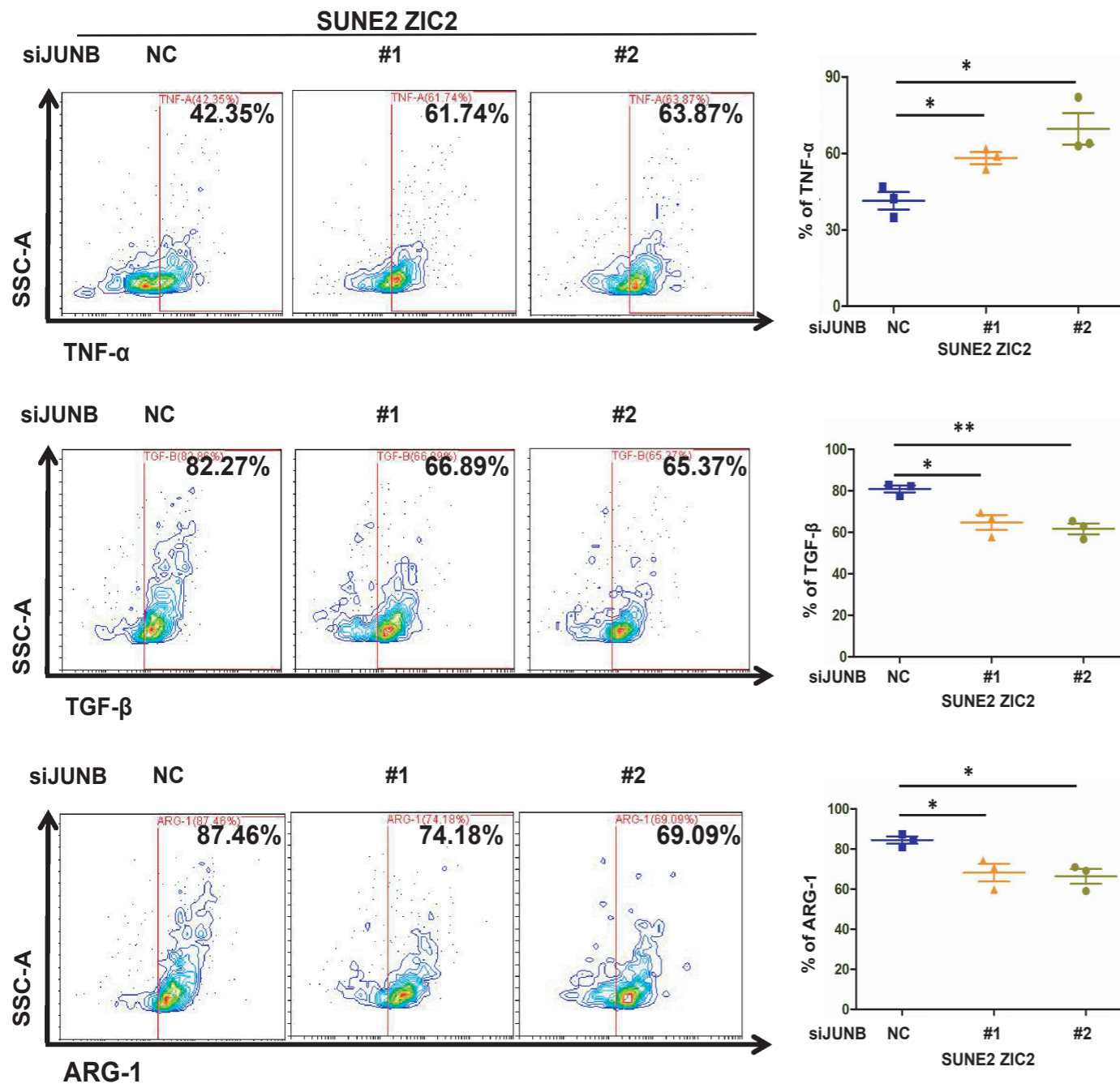
**B**
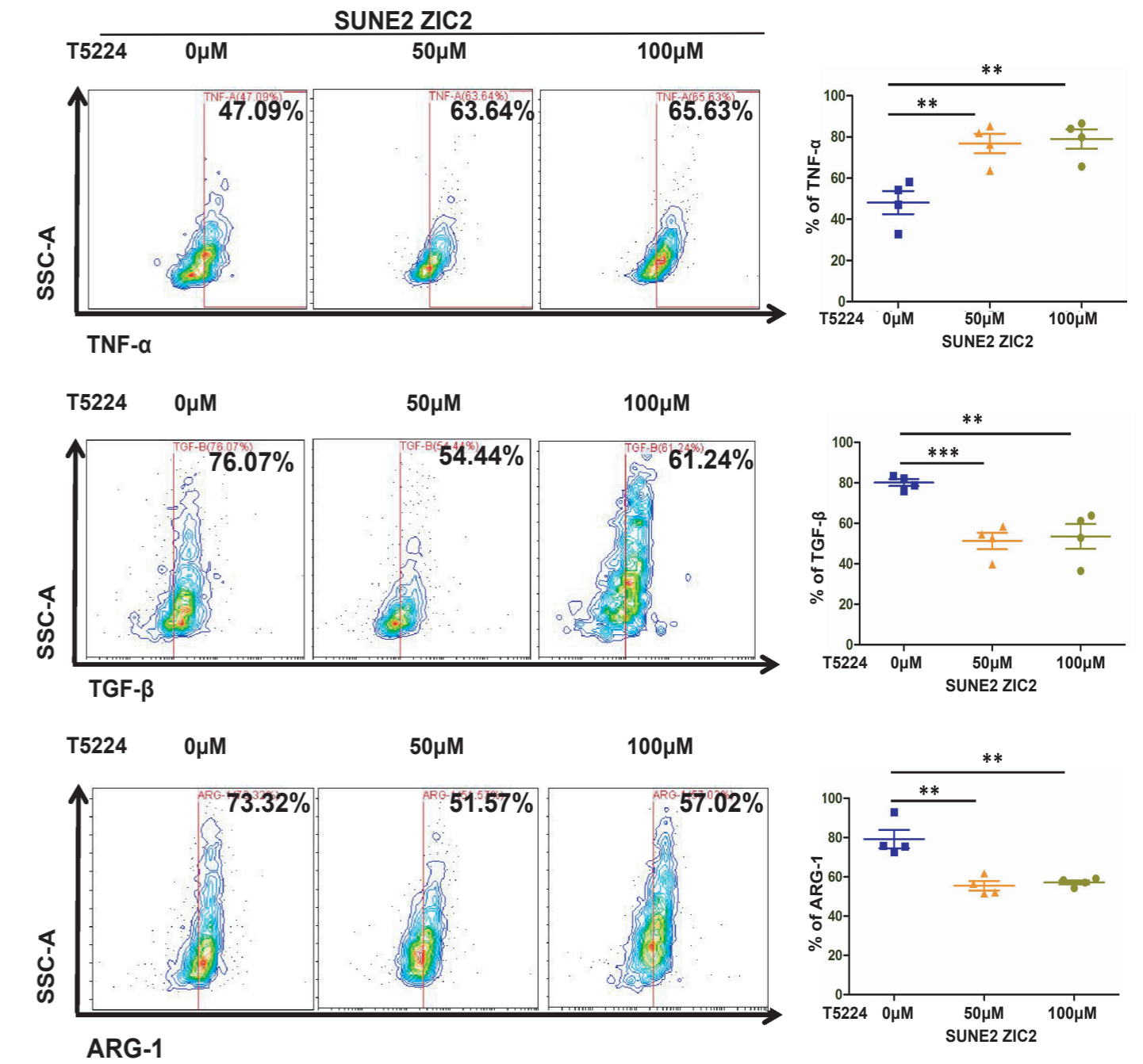
**C**
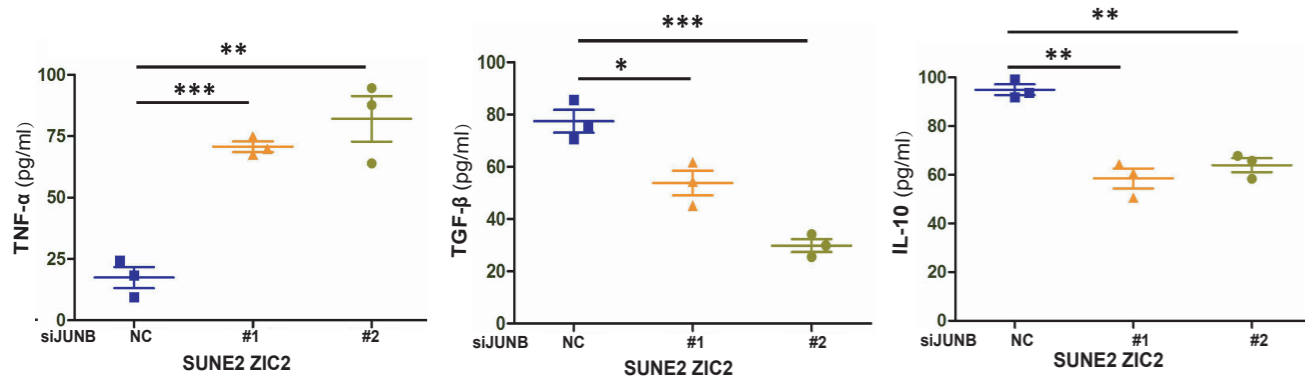
**D**
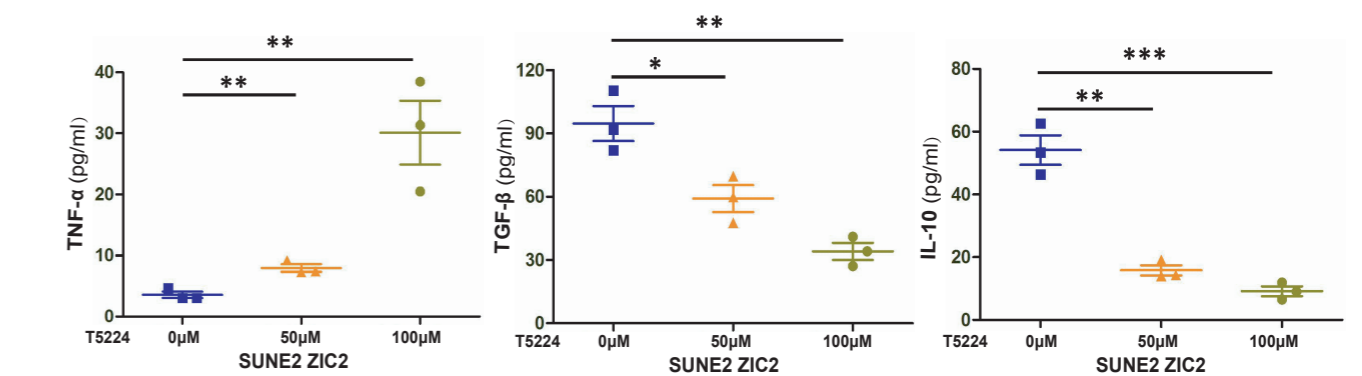

Supplement: Supplementary file 3 — supplemental figure 3 [file 41419_2023_5983_MOESM3_ESM.pdf]
