# Supplementary material for: ZIC2 induces pro-tumor macrophage polarization in nasopharyngeal carcinoma by activating the JUNB/MCSF axis
Source: Cell Death Dis. 2023 Jul 21;14(7):455. doi: 10.1038/s41419-023-05983-x (PMC10362010; doi:10.1038/s41419-023-05983-x)
Supplement: Supplementary file 4 — Supplemental table 1 [file 41419_2023_5983_MOESM4_ESM.pdf]

Supplemental table 1-NPC vs NPN mRNA-seq differential gene expression data

GeneID	Symbol	up/down	N01(rpkm)	NPN_72(rp	NPC_S(rpk	NPC_66(rp	NPC_62(rp	NPC_M(rpl	NPC_64(rp	NPC_49(rp	NPC_46(rp	NPC_54(rp	NPC_52(rp	NPC_41(rp	NPC_51(rp	NPC_60(rp	NPC_53(rp	NPC_57(rp
91543	RSAD2	up	0.081	3.231	11.572	11.261	64.34	0.841	14.776	19.831	18.95	107.446	36.357	107.412	83.311	51.041	42.551	8.437
3223	HOXC6	up	0.001	0.053	5.595	3.196	0.584	1.124	0.295	1.06	1.769	8.834	4.597	3.629	4.978	4.339	2.571	2.853
9636	ISG15	up	12.958	40.29	89.882	125.398	577.576	220.561	127.09	223.267	140.951	930.221	212.649	614.045	425.996	353.025	289.152	82.606
6373	CXCL11	up	1.084	2.106	13.037	9.077	23.907	2.539	31.287	16.658	48.998	53.73	137.414	96.644	132.548	62.425	28.578	25.256
3437	IFIT3	up	1.33	9.665	102.098	54.331	218.871	28.946	57.198	73.549	83.86	238.82	199.764	257.154	280.783	136.799	120.846	56.469
8638	OASL	up	0.134	4.864	15.13	36.611	56.826	50.596	14.631	23.357	24.581	74.684	73.006	82.915	78.924	58.501	50.613	23.637
7546	ZIC2	up	0.023	0.429	8.211	15.236	2.334	8.344	1.598	2.778	2.696	3.393	4.834	3.319	5.162	3.049	2.819	6.279
6474	SHOX2	up	0.038	0.102	1.921	0.932	0.861	0.241	0.322	0.398	0.338	1.579	1.484	0.541	0.583	5.543	0.776	1.11
4856	NOV	up	0.125	0.854	4.842	2.317	3.255	1.543	2.58	1.935	3.344	12.032	6.186	2.296	16.096	28.296	19.286	6.656
6752	SSTR2	up	0.014	0.573	0.749	21.939	2.04	0.121	2.701	2.264	2.5	9.528	3.344	19.023	21.903	9.334	45.341	38.363
146760	RTN4RL1	up	0.347	0.979	6.529	6.103	4.939	0.975	2.828	2.207	3.657	2.898	2.005	2.725	2.666	2.032	4.225	2.316
118738	ZNF488	up	0.745	0.266	10.375	16.226	3.824	3.913	3.13	1.855	3.931	1.411	0.92	2.578	7.336	0.832	2.26	3.238
53616	ADAM22	up	0.001	0.303	1.39	3.632	1.261	0.39	0.622	1.397	1.836	2.926	1.875	3.135	4.494	4.059	4.259	3.492
221662	RBM24	down	0.827	1.993	0.001	0.058	0.154	0.001	0.65	0.872	0.596	0.097	0.127	0.154	0.017	0.347	0.008	0.404
126638	RPTN	down	8.041	2.842	0.071	0.001	0.027	0.001	0.001	0.041	0.022	0.885	0.031	0.009	0.001	0.001	0.001	0.001
29113	C6orf15	down	2.096	0.82	0.176	0.001	0.001	0.001	0.207	0.001	0.001	0.03	0.032	0.029	0.001	0.001	0.001	0.001
10570	DPYSL4	down	0.808	1.099	0.001	0.182	0.157	0.014	0.476	0.091	0.219	0.176	0.149	0.321	0.023	0.247	0.056	0.228
115111	SLC26A7	down	0.321	1.625	0.038	0.008	0.009	0.007	0.036	0.084	0.124	0.013	0.056	0.013	0.024	0.054	0.015	0.019
8710	SERPINB7	down	41.682	14.606	0.961	0.451	4.499	0.11	1.285	1.678	1.347	1.757	0.049	1.435	0.037	0.664	0.384	0.633

km)
