# Supplementary material for: ZIC2 induces pro-tumor macrophage polarization in nasopharyngeal carcinoma by activating the JUNB/MCSF axis
Source: Cell Death Dis. 2023 Jul 21;14(7):455. doi: 10.1038/s41419-023-05983-x (PMC10362010; doi:10.1038/s41419-023-05983-x)
Supplement: Supplementary file 6 — Supplemental table 3 [file 41419_2023_5983_MOESM6_ESM.pdf]

Supplemental table 3 ZIC2 ChIP-seq data peak score

PeakID (cm	Chr	Start	End	Strand	Peak Score	Focus Ratio	Annotation	Detailed A	Distance to TSS	Nearest Pr	Entrez ID	Nearest Uni	Nearest Re	Nearest En	Gene Nam	Gene Alias	Gene Desc	Gene Type
lib-C666-l	chr3	1.42E+08	1.42E+08	+	62.30347	NA	promoter-	promoter-	19	NM_00128	54464	Hs.435103	NM_01900	ENSG0000	XRN1	1-Sep	5'-3' exoril	protein-co
lib-C666-l	chr4	1.2E+08	1.2E+08	+	813.4522	NA	promoter-	promoter-	-75	NR_03763	645513	Hs.677546	NR_03763	ENSG0000	LOC64551	-	septin 7 ps	pseudo
lib-C666-l	chr7	66309982	66309982	+	502.1209	NA	promoter-	promoter-	-169	NR_03393	729156	Hs.709878	NR_03393	ENSG0000	GTF2IRD1F	-	GTF2I repe	pseudo
lib-C666-l	chr19	36119930	36119930	+	499.4849	NA	promoter-	promoter-	12	NM_02432	79171	Hs.5086	NM_02432	ENSG0000	RBM42	-	RNA bindi	protein-co
lib-C666-l	chr3	10028555	10028555	+	468.7292	NA	promoter-	promoter-	-22	NR_10382	442075	Hs.690469	NM_001013730		EMC3-AS1	-	EMC3 antis	ncRNA
lib-C666-l	chr4	1.2E+08	1.2E+08	+	445.8763	NA	promoter-	promoter-	-26	NR_10998	729218	Hs.448518	NR_103825		LOC72921	-	uncharacte	pseudo
lib-C666-l	chr7	65958574	65958574	+	397.4668	NA	promoter-	promoter-	-45	NR_13457	1E+08	Hs.662276	NR_13457	ENSG0000	GS1-124K5	-	uncharacte	ncRNA
lib-C666-l	chr16	19079074	19079074	+	393.0909	NA	promoter-	promoter-	-61	NR_11938	1.03E+08	Hs.569608	NR_11937	ENSG0000	LOC10272	-	uncharacte	ncRNA
lib-C666-l	chr1	9489054	9489054	+	390.5467	NA	promoter-	promoter-	1	NR_10462	1.01E+08	Hs.131775	NR_104622		LOC10050	-	uncharacte	ncRNA
lib-C666-l	chr1	2.24E+08	2.24E+08	+	375.7795	NA	promoter-	promoter-	2	NR_13211	441124	Hs.520589	NR_132119		GTF2IP20	-	general tra	pseudo
lib-C666-l	chr11	60609520	60609520	+	366.1801	NA	promoter-	promoter-	91	NM_02409	79080	Hs.4253	NM_02409	ENSG0000	CCDC86	-	coiled-coil	protein-co
lib-C666-l	chr1	2.43E+08	2.43E+08	+	361.4463	NA	promoter-	promoter-	-59	NR_02940	731275	Hs.722350	NR_02940	ENSG0000	LINC01347	-	long interg	ncRNA
lib-C666-l	chr4	492961	492961	+	338.3255	NA	promoter-	promoter-	-1	NM_13347	170960	Hs.428360	NM_13347	ENSG0000	ZNF721	-	zinc finger	protein-co
lib-C666-l	chr4	1.53E+08	1.53E+08	+	329.087	NA	promoter-	promoter-	-63	NR_03379	54553	Hs.142307	NR_03379	ENSG0000	DKFZP434I	-	uncharacte	ncRNA
lib-C666-l	chr6	17707177	17707177	+	283.3171	NA	promoter-	promoter-	-97	NR_13461	1.05E+08	Hs.718703	NR_13461	ENSG0000	LOC10537	-	uncharacte	ncRNA
lib-C666-l	chr2	29093181	29093181	+	272.7369	NA	promoter-	promoter-	-6	NM_01791	55006	Hs.468026	NM_01791	ENSG0000	TRMT61B	-	tRNA meth	protein-co
lib-C666-l	chr1	714090	714090	+	265.5108	NA	promoter-	promoter-	-22	NR_03390	1E+08	Hs.527521	NR_033908		LOC10028	-	uncharacte	ncRNA
lib-C666-l	chr12	64616371	64616371	+	256.3782	NA	promoter-	promoter-	-295	NM_15244	144577	Hs.444671	NM_15244	ENSG0000	C12orf66	-	chromosor	protein-co
lib-C666-l	chr1	19536839	19536839	+	255.6728	NA	promoter-	promoter-	-41	NR_13511	1.02E+08	Hs.660534	NR_13511	ENSG0000	LOC10192	-	uncharacte	ncRNA
lib-C666-l	chr19	18043773	18043773	+	251.2096	NA	promoter-	promoter-	-51	NM_00113	115098	Hs.100043	NM_13844	ENSG0000	CCDC124	-	coiled-coil	protein-co
lib-C666-l	chr2	19548228	19548228	+	244.3756	NA	promoter-	promoter-	38	NR_03991	1.01E+08		NR_03991	ENSG0000	MIR4757	-	microRNA	ncRNA
lib-C666-l	chr17	38083899	38083899	+	226.3021	NA	promoter-	promoter-	158	NM_13928	94103	Hs.514151	NM_01647	ENSG0000	ORMDL3	-	ORMDL sp	protein-co
lib-C666-l	chr19	7553885	7553885	+	224.3673	NA	promoter-	promoter-	39	NM_08066	92960	Hs.515100	NM_08066	ENSG0000	PEX11G	-	peroxisom	protein-co
lib-C666-l	chr16	86588834	86588834	+	216.0169	NA	promoter-	promoter-	7	NM_00115	64779	Hs.343627	NM_02276	ENSG0000	MTHFSD	-	methenylte	protein-co
lib-C666-l	chr19	16683353	16683353	+	216.0169	NA	promoter-	promoter-	-160	NM_02488	79939	Hs.620596	NM_02488	ENSG0000	SLC35E1	-	solute carr	protein-co
lib-C666-l	chr7	65235854	65235854	+	211.379	NA	promoter-	promoter-	-57	NR_03837	441242	Hs.373941	NM_00101	ENSG0000	LOC44124	-	uncharacte	ncRNA
lib-C666-l	chr18	2655193	2655193	+	207.9531	NA	promoter-	promoter-	201	NR_03375	645158	Hs.712861	NR_03375	ENSG0000	CBX3P2	-	chromobo:	pseudo
lib-C666-l	chr19	44100538	44100538	+	202.3956	NA	promoter-	promoter-	-6	NM_02432	79177	Hs.11110	NM_02432	ENSG0000	ZNF576	-	zinc finger	protein-co
lib-C666-l	chr7	19748679	19748679	+	196.6477	NA	promoter-	promoter-	-19	NM_00100	221830	Hs.353035	NM_00100	ENSG0000	TWISTNB	-	TWIST neig	protein-co
lib-C666-l	chr3	1.19E+08	1.19E+08	+	192.3587	NA	promoter-	promoter-	26	NM_01826	55254	Hs.594171	NM_01826	ENSG0000	TMEM39A	-	transmemt	protein-co
lib-C666-l	chr3	44803175	44803175	+	186.1922	NA	promoter-	promoter-	-2	NM_02069	57456	Hs.744955	NM_02069	ENSG0000	KIAA1143	-	KIAA1143	protein-co
lib-C666-l	chr3	48488076	48488076	+	179.3365	NA	promoter-	promoter-	-38	NM_03216	84126	Hs.694840	NM_03216	ENSG0000	ATRIP	-	ATR intera	protein-co
lib-C666-l	chr20	306043	306043	+	176.7042	NA	promoter-	promoter-	-147	NR_10999	1.01E+08	Hs.659689	NR_109990		NRSN2-AS	-	NRSN2 ant	ncRNA
lib-C666-l	chr1	28052341	28052341	+	176.37	NA	promoter-	promoter-	-149	NM_00114	199870	Hs.469359	NM_15266	ENSG0000	FAM76A	-	family with	protein-co
lib-C666-l	chr3	1.96E+08	1.96E+08	+	175.7709	NA	promoter-	promoter-	83	NM_00134	348793	Hs.385865	NM_18262	ENSG0000	WDR53	-	WD repeat	protein-co
lib-C666-l	chr9	1.25E+08	1.25E+08	+	171.1925	NA	promoter-	promoter-	20	NR_02712	92400	Hs.415842	NM_03311	ENSG0000	RBM18	-	RNA bindi	protein-co
lib-C666-l	chr16	48644187	48644187	+	169.5727	NA	promoter-	promoter-	-67	NM_15302	9683	Hs.511839	NM_15302	ENSG0000	N4BP1	-	NEDD4 bir	protein-co
lib-C666-l	chr9	6015654	6015654	+	166.7792	NA	promoter-	promoter-	-14	NM_00124	26953	Hs.167496	NM_01241	ENSG0000	RANBP6	-	RAN bindi	protein-co
lib-C666-l	chr1	2.49E+08	2.49E+08	+	165.3986	NA	intron (NM	CpG	808	NM_02483	79884	Hs.521151	NM_02483	ENSG0000	ZNF672	-	zinc finger	protein-co
lib-C666-l	chr19	1240313	1240313	+	164.6524	NA	Intergenic	CpG	-1436	NM_00100	513	Hs.418668	NM_00168	ENSG0000	ATP5D	-	ATP syntha	protein-co
lib-C666-l	chr4	1.64E+08	1.64E+08	+	161.3808	NA	promoter-	promoter-	-42	NM_13838	92345	Hs.129095	NM_13838	ENSG0000	NAF1	-	nuclear ass	protein-co
lib-C666-l	chr3	1.86E+08	1.86E+08	+	161.019	NA	promoter-	promoter-	-5	NM_00128	55171	Hs.518469	NM_01813	ENSG0000	TBCCD1	-	TBCC dom	protein-co
lib-C666-l	chr7	1.25E+08	1.25E+08	+	160.6333	NA	promoter-	promoter-	41	NR_12571	401398	Hs.658551	NM_00102	ENSG0000	POT1-AS1	-	POT1 antis	ncRNA
lib-C666-l	chr18	77439687	77439687	+	156.7217	NA	promoter-	promoter-	58	NR_13664	284241		NR_13664	ENSG0000	LOC28424	-	uncharacte	ncRNA
lib-C666-l	chr7	6144358	6144358	+	145.2591	NA	promoter-	promoter-	-192	NM_03217	84132	Hs.31856	NM_03217	ENSG0000	USP42	-	ubiquitin s	protein-co
lib-C666-l	chr8	37605586	37605586	+	141.2457	NA	promoter-	promoter-	-22	NR_00367	728024	Hs.647135	NR_003671		LOC72802	-	chromosor	pseudo
lib-C666-l	chr16	30007399	30007399	+	139.3302	NA	promoter-	promoter-	18	NM_00360	8479	Hs.567370	NM_00360	ENSG0000	HIRIP3	-	HIRA inter	protein-co
lib-C666-l	chr11	66112607	66112607	+	138.2387	NA	promoter-	promoter-	-25	NM_01539	25855	Hs.100426	NM_01539	ENSG0000	BRMS1	-	breast canc	protein-co
lib-C666-l	chr22	19159185	19159185	+	137.2633	NA	promoter-	promoter-	-34	NR_10376	1.01E+08	Hs.572896	NR_103767		LINC01311	-	long interg	ncRNA
lib-C666-l	chr6	1.5E+08	1.5E+08	+	134.2487	NA	promoter-	promoter-	-63	NR_13459	1.05E+08	Hs.549678	NR_13459	ENSG0000	LOC10537	-	uncharacte	ncRNA
lib-C666-l	chr5	95297791	95297791	+	133.1091	NA	promoter-	promoter-	-16	NM_01208	22936	Hs.192221	NM_01208	ENSG0000	ELL2	-	elongation	protein-co
lib-C666-l	chr5	1.76E+08	1.76E+08	+	127.7119	NA	promoter-	promoter-	-512	NM_01490	22838	Hs.434888	NM_01490	ENSG0000	RNF44	-	ring finger	protein-co
lib-C666-l	chr21	44847231	44847231	+	125.5028	NA	promoter-	promoter-	-162	NM_00132	1.03E+08	Hs.282113	NM_00132	ENSG0000	LOC10272	-	serine/thre	protein-co
lib-C666-l	chr12	31882076	31882076	+	125.1886	NA	promoter-	promoter-	32	NR_10352	196394	Hs.585084	NM_00111	ENSG0000	AMN1	-	antagonist	protein-co
lib-C666-l	chr7	74508949	74508949	+	124.9432	NA	intron (NM	CpG	602	NM_00100	389524	Hs.647017	NM_00100	ENSG0000	GTF2IRD2E	-	GTF2I repe	protein-co
lib-C666-l	chr13	1.01E+08	1.01E+08	+	123.8327	NA	promoter-	promoter-	-63	NM_00107	84899	Hs.190983	NM_03281	ENSG0000	TMTC4	-	transmemt	protein-co
lib-C666-l	chr2	1.14E+08	1.14E+08	+	120.4604	NA	promoter-	promoter-	45	NM_17200	150472	Hs.531330	NM_17200	ENSG0000	CBWD2	-	COBW dor	protein-co
lib-C666-l	chr17	4607473	4607473	+	119.7632	NA	promoter-	promoter-	-52	NR_10348	1.02E+08	Hs.634966	NR_10348	ENSG0000	LOC10155	-	uncharacte	ncRNA
lib-C666-l	chr13	21872144	21872144	+	119.4814	NA	promoter-	promoter-	-120	NR_04646	650794	Hs.147563	NR_038939		MIPEPP3	-	mitochond	pseudo
lib-C666-l	chr1	70820664	70820664	+	119.4653	NA	promoter-	promoter-	171	NR_02740	11147	Hs.142245	NM_00707	ENSG0000	HHLA3	-	HERV-H L	protein-co
lib-C666-l	chr11	62607083	62607083	+	119.3373	NA	promoter-	promoter-	48	NM_00130	54663	Hs.654620	NM_01809	ENSG0000	WDR74	-	WD repeat	protein-co
lib-C666-l	chr11	65337863	65337863	+	119.0211	NA	promoter-	promoter-	21	NR_03892	254100	Hs.595927	NR_038923		SSSCA1-A	-	SSSCA1 an	ncRNA
lib-C666-l	chr19	56186457	56186457	+	116.1287	NA	promoter-	promoter-	-104	NM_01333	29924	Hs.279953	NM_01333	ENSG0000	EPN1	-	epsin 1	protein-co
lib-C666-l	chr15	71407757	71407757	+	115.2865	NA	promoter-	promoter-	82	NM_00110	196993	Hs.646533	NM_00110	ENSG0000	CT62	-	cancer/tes	protein-co
lib-C666-l	chr12	56862281	56862281	+	111.7598	NA	promoter-	promoter-	-20	NM_20734	283377	Hs.128676	NM_20734	ENSG0000	SPRYD4	-	SPRY dom	protein-co
lib-C666-l	chr3	33759692	33759692	+	110.4597	NA	promoter-	promoter-	13	NM_01509	23122	Hs.108614	NM_01509	ENSG0000	CLASP2	-	cytoplasmi	protein-co
lib-C666-l	chr9	88742510	88742510	+	110.0531	NA	promoter-	promoter-	55	NR_11099	1.02E+08	Hs.575644	NR_110996		LOC10192	-	uncharacte	ncRNA
lib-C666-l	chr19	9732019	9732019	+	109.4731	NA	promoter-	promoter-	-80	NM_00133	93134	Hs.720081	NM_15228	ENSG0000	ZNF561	-	zinc finger	protein-co
lib-C666-l	chr17	2615284	2615284	+	107.6347	NA	promoter-	promoter-	-319	NR_13641	1.05E+08	Hs.731739	NR_13641	ENSG0000	LOC10537	-	uncharacte	ncRNA
lib-C666-l	chr2	1.15E+08	1.15E+08	+	106.6573	NA	promoter-	promoter-	-43	NM_00133	80255	Hs.355655	NM_02518	ENSG0000	SLC35F5	-	solute carr	protein-co
lib-C666-l	chr16	67927136	67927136	+	106.6552	NA	promoter-	promoter-	-39	NM_00674	5681	Hs.513683	NM_00674	ENSG0000	PSKH1	-	protein ser	protein-co
lib-C666-l	chr14	68086496	68086496	+	105.6278	NA	promoter-	promoter-	-83	NM_00117	384	Hs.226007	NM_00117	ENSG0000	ARG2	-	arginase 2	protein-co
lib-C666-l	chr7	66119377	66119377	+	104.8272	NA	promoter-	promoter-	-129	NR_11003	1.01E+08	Hs.667408	NR_110037		LOC10099	-	uncharacte	ncRNA
lib-C666-l	chr9	34666087	34666087	+	101.7499	NA	promoter-	promoter-	-34	NR_13514	730098	Hs.459590	NM_00132	ENSG0000	LOC73009	-	uncharacte	protein-co
lib-C666-l	chr17	43325032	43325032	+	101.1509	NA	promoter-	promoter-	-260	NR_11032	1E+08	Hs.668927	NR_02443	ENSG0000	MAP3K14-	-	MAP3K14	ncRNA
lib-C666-l	chr19	47759820	47759820	+	100.0679	NA	promoter-	promoter-	89	NM_01560	26093	Hs.227782	NM_01560	ENSG0000	CCDC9	-	coiled-coil	protein-co
lib-C666-l	chr1	2.45E+08	2.45E+08	+	99.63164	NA	promoter-	promoter-	-168	NR_11190	1.02E+08	Hs.134857	NR_111907		LOC10192	-	uncharacte	ncRNA
lib-C666-l	chr11	1.12E+08	1.12E+08	+	99.40627	NA	promoter-	promoter-	28	NM_00133	64776	Hs.17546	NM_02276	ENSG0000	C11orf1	-	chromosor	protein-co
lib-C666-l	chr16	90039220	90039220	+	98.7459	NA	promoter-	promoter-	20	NM_14503	92806	Hs.513832	NM_14503	ENSG0000	CENPBD1	-	CENPB DN	protein-co
lib-C666-l	chr8	38243950	38243950	+	98.60414	NA	promoter-	promoter-	-9	NM_00128	137994	Hs.696457	NM_14465	ENSG0000	LETM2	-	leucine zip	protein-co
lib-C666-l	chr5	80597365	80597365	+	96.50365	NA	promoter-	promoter-	23	NR_03412	1E+08	Hs.655855	NR_03412	ENSG0000	CKMT2-AS	-	CKMT2 ant	ncRNA
lib-C666-l	chr19	57751995	57751995	+	95.9062	NA	promoter-	promoter-	-58	NM_00102	390980	Hs.22488	NM_00102	ENSG0000	ZNF805	-	zinc finger	protein-co
lib-C666-l	chr6	34664778	34664778	+	94.27481	NA	promoter-	promoter-	56	NR_13462	1.02E+08	Hs.643498	NR_13462	ENSG0000	LOC10192	-	uncharacte	ncRNA
lib-C666-l	chr19	45430487	45430487	+	93.50127	NA	intron (NR	intron (NR	427	NR_02841	342	Hs.110675	NR_02841	ENSG0000	APOC1P1	-	apolipopro	pseudo
lib-C666-l	chr11	2421606	2421606	+	93.50127	NA	promoter-	promoter-	-112	NM_00129	10078	Hs.523424	NM_00570	ENSG0000	TSSC4	-	tumor supp	protein-co
lib-C666-l	chr20	23338765	23338765	+	92.39822	NA	promoter-	promoter-	-1	NR_10988	1.01E+08	Hs.140295	NR_10988	ENSG0000	LINC01431	-	long interg	ncRNA
lib-C666-l	chr19	9785824	9785824	+	92.39822	NA	promoter-	promoter-	-48	NM_01765	54811	Hs.371107	NM_01765	ENSG0000	ZNF562	-	zinc finger	protein-co
lib-C666-l	chr2	1.1E+08	1.1E+08	+	91.79062	NA	promoter-	promoter-	-22	NM_00132	151011	Hs.469615	NM_14471	ENSG0000	10-Sep	-	septin 10	protein-co
lib-C666-l	chr16	2802610	2802610	+	85.88258	NA	promoter-	promoter-	-9	NR_02727	1E+08	Hs.311208	NR_02727	ENSG0000	SRRM2-AS	-	SRRM2 ant	ncRNA
lib-C666-l	chr3	1.57E+08	1.57E+08	+	85.88258	NA	promoter-	promoter-	-15	NM_00100	389170	Hs.478048	NM_00100	ENSG0000	LEKR1	-	leucine, glu	protein-co
lib-C666-l	chr1	1.2E+08	1.2E+08	+	85.64357	NA	promoter-	promoter-	34	NR_12597	1.02E+08	Hs.697821	NR_12597	ENSG0000	LOC10192	-	uncharacte	ncRNA
lib-C666-l	chr19	4247052	4247052	+	83.47607	NA	promoter-	promoter-	-15	NM_01807	55702	Hs.21811	NM_01807	ENSG0000	CCDC94	-	coiled-coil	protein-co
lib-C666-l	chr5	64859125	64859125	+	80.52238	NA	promoter-	promoter-	59	NM_00127	23398	Hs.121432	NM_01534	ENSG0000	PPWD1	-	peptidylpro	protein-co
lib-C666-l	chr1	1.45E+08	1.45E+08	+	78.77642	NA	promoter-	promoter-	-55	NR_12596	1.02E+08	Hs.735893	NR_12596	ENSG0000	LINC01719	-	long interg	ncRNA
lib-C666-l	chr8	42195983	42195983	+	78.63524	NA	promoter-	promoter-	10	NM_00269	5423	Hs.654484	NM_00269	ENSG0000	POLB	-	DNA polyr	protein-co
lib-C666-l	chr1	38156270	38156270	+	78.53495	NA	promoter-	promoter-	-3	NM_01785	54955	Hs.272673	NM_01785	ENSG0000	C1orf109	-	chromosor	protein-co
lib-C666-l	chr17	4046434	4046434	+	73.89001	NA	promoter-	promoter-	-28	NM_14461	124936	Hs.513871	NM_14461	ENSG0000	CYB5D2	-	cytochrom	protein-co
lib-C666-l	chr19	50637175	50637175	+	72.73944	NA	promoter-	promoter-	-54	NR_02423	1E+08	Hs.446545	NR_024231		SNAR-B1	-	small ILF3/	snRNA
lib-C666-l	chr22	24059563	24059563	+	72.31497	NA	promoter-	promoter-	47	NR_02444	91316	Hs.148656	NR_02444	ENSG0000	GUSBP11	-	glucuronid	pseudo
lib-C666-l	chr17	33895010	33895010	+	72.05244	NA	promoter-	promoter-	-123	NR_13547	1.05E+08		NR_135479		LINC02001	-	long interg	ncRNA
lib-C666-l	chr15	42264859	42264859	+	72.02663	NA	promoter-	promoter-	-102	NR_12033	1.02E+08	Hs.738043	NR_12033	ENSG0000	PLA2G4E-/	-	PLA2G4E a	ncRNA
lib-C666-l	chr4	1.2E+08	1.2E+08	+	70.69921	NA	promoter-	promoter-	3	NR_12593	1.02E+08	Hs.636184	NR_12593	ENSG0000	LOC10192	-	uncharacte	ncRNA
lib-C666-l	chr2	96676311	96676311	+	69.82498	NA	promoter-	promoter-	12	NR_00369	729234	Hs.720108	NR_00369	ENSG0000	FAHD2CP	-	fumarylace	pseudo
lib-C666-l	chr5	7869151	7869151	+	69.79861	NA	promoter-	promoter-	-1	NR_07360	79072	Hs.653162	NM_02409	ENSG0000	FASTKD3	-	FAST kinas	protein-co
lib-C666-l	chr9	93196025	93196025	+	69.18568	NA	promoter-	promoter-	-254	NR_10979	1.02E+08	Hs.598482	NR_10979	ENSG0000	LINC01508	-	long interg	ncRNA
lib-C666-l	chr15	83183054	83183054	+	69.07846	NA	promoter-	promoter-	-77	NR_03393	80154	Hs.454640	NR_025084		GOLGA2P1	-	golgin A2	pseudo
lib-C666-l	chr15	72766595	72766595	+	68.88256	NA	promoter-	promoter-	-15	NR_13567	1.05E+08	Hs.540241	NR_13567	ENSG0000	LOC10537	-	uncharacte	ncRNA
lib-C666-l	chr1	45956873	45956873	+	68.04845	NA	promoter-	promoter-	-33	NM_00717	10420	Hs.591499	NM_00717	ENSG0000	TESK2	-	testis-spec	protein-co
lib-C666-l	chr15	63481613	63481613	+	66.96093	NA	promoter-	promoter-	-115	NM_01653	51762	Hs.389733	NM_01653	ENSG0000	RAB8B	-	RAB8B, me	protein-co
lib-C666-l	chr19	59086778	59086778	+	65.54223	NA	promoter-	promoter-	12	NR_02605	65996	Hs.541177	NM_02393	ENSG0000	CENPBD1F	-	CENPB DN	pseudo
lib-C666-l	chr4	83822120	83822120	+	65.41075	NA	promoter-	promoter-	-51	NR_03407	1E+08	Hs.728916	NR_03407	ENSG0000	THAP9-AS	-	THAP9 ant	ncRNA
lib-C666-l	chr3	1.72E+08	1.72E+08	+	65.34157	NA	promoter-	promoter-	11	NM_00266	5337	Hs.382865	NM_00266	ENSG0000	PLD1	-	phospholip	protein-co
lib-C666-l	chr1	1.61E+08	1.61E+08	+	64.95611	NA	promoter-	promoter-	-26	NM_00118	4817	Hs.146406	NM_00560	ENSG0000	NIT1	-	nitrilase 1	protein-co
lib-C666-l	chr3	1.97E+08	1.97E+08	+	64.94322	NA	promoter-	promoter-	59	NM_15269	205564	Hs.240770	NM_15269	ENSG0000	SENP5	-	SUMO1/se	protein-co
lib-C666-l	chr6	10695068	10695068	+	64.17523	NA	promoter-	promoter-	-38	NR_02673	347744	Hs.61389	NM_00114	ENSG0000	C6orf52	-	chromosor	protein-co
lib-C666-l	chr17	78389037	78389037	+	63.68534	NA	promoter-	promoter-	-69	NR_02937	1E+08	Hs.657519	NR_02937	ENSG0000	LOC10029	-	uncharacte	ncRNA
lib-C666-l	chr19	50143641	50143641	+	63.30923	NA	promoter-	promoter-	-241	NM_00627	6237	Hs.515536	NM_00627	ENSG0000	RRAS	-	related RA	protein-co
lib-C666-l	chr4	77870929	77870929	+	62.77214	NA	promoter-	promoter-	62	NM_01824	55752	Hs.128199	NM_01824	ENSG0000	11-Sep	-	septin 11	protein-co
lib-C666-l	chr15	45021089	45021089	+	62.31611	NA	promoter-	promoter-	-97	NR_12537	1E+08	Hs.489254	NR_125376		LOC10041	-	ring finger	protein-co
lib-C666-l	chr6	15248932	15248932	+	59.09724	NA	promoter-	promoter-	-5	NR_12050	1.01E+08	Hs.664092	NR_12050	ENSG0000	JARID2-AS	-	JARID2 ant	ncRNA
lib-C666-l	chr2	1.32E+08	1.32E+08	+	57.14708	NA	promoter-	promoter-	268	NR_02692	150776	Hs.535622	NR_02692	ENSG0000	LOC15077	-	sphingomy	protein-co
lib-C666-l	chr16	67217839	67217839	+	56.63552	NA	promoter-	promoter-	44	NM_00104	653319	Hs.651201	NM_00104	ENSG0000	KIAA0895L	-	KIAA0895	protein-co
lib-C666-l	chrX	1.19E+08	1.19E+08	+	55.75688	NA	promoter-	promoter-	-8	NM_00117	63932	Hs.248572	NM_02210	ENSG0000	CXorf56	-	chromosor	protein-co
lib-C666-l	chr1	27339034	27339034	+	54.92945	NA	exon (NM_	exon (NM_	299	NM_05294	115572	Hs.632378	NM_05294	ENSG0000	FAM46B	-	family with	protein-co
lib-C666-l	chr9	1.32E+08	1.32E+08	+	54.91701	NA	non-codin	non-codin	108	NR_03895	1.01E+08	Hs.529860	NR_038955		LINC00963	-	long interg	ncRNA

lib-C666-l	chr2	1.36E+08	1.36E+08	+	54.30392	NA	promoter-	promoter-	-85	NR_03654	1E+08	Hs.729028	NR_036549		CCNT2-AS	-	CCNT2 ant	ncRNA
lib-C666-l	chr19	13906024	13906024	+	54.30392	NA	promoter-	promoter-	-250	NM_02307	65249	Hs.466015	NM_02307	ENSG0000	ZSWIM4	-	zinc finger	protein-co
lib-C666-l	chr7	1.07E+08	1.07E+08	+	54.19022	NA	promoter-	promoter-	-226	NM_00124	26959	Hs.162032	NM_01225	ENSG0000	HBP1	-	HMG-box	protein-co
lib-C666-l	chr10	1.35E+08	1.35E+08	+	53.15281	NA	promoter-	promoter-	18	NM_14580	118472	Hs.422113	NM_14580	ENSG0000	ZNF511	-	zinc finger	protein-co
lib-C666-l	chr19	13858654	13858654	+	52.99872	NA	promoter-	promoter-	-23	NM_03081	81576	Hs.24998	NM_03081	ENSG0000	CCDC130	-	coiled-coil	protein-co
lib-C666-l	chr17	7465212	7465212	+	52.74303	NA	promoter-	promoter-	-97	NR_03792	1.01E+08	Hs.129673	NR_037926		SENP3-EIF	-	SENP3-EIF	ncRNA
lib-C666-l	chr15	84899034	84899034	+	52.30908	NA	promoter-	promoter-	-114	NR_02700	388152	Hs.405809	NM_20342	ENSG0000	GOLGA2P7	-	golgin A2	pseudo
lib-C666-l	chr2	2.01E+08	2.01E+08	+	51.72884	NA	promoter-	promoter-	52	NM_15368	205327	Hs.154494	NM_15368	ENSG0000	C2orf69	-	chromosor	protein-co
lib-C666-l	chr1	2.12E+08	2.12E+08	+	51.38887	NA	promoter-	promoter-	-153	NR_12598	1.02E+08	Hs.632493	NR_12598	ENSG0000	LOC1019	-	uncharacte	ncRNA
lib-C666-l	chr10	99078920	99078920	+	51.38887	NA	promoter-	promoter-	-102	NM_00547	10023	Hs.126057	NM_00547	ENSG0000	FRAT1	-	FRAT1, WN	protein-co
lib-C666-l	chr8	64081042	64081042	+	51.11391	NA	promoter-	promoter-	-41	NR_10268	1.01E+08	Hs.163027	NR_10268	ENSG0000	YTHDF3-A	-	YTHDF3 ar	ncRNA
lib-C666-l	chr19	57901273	57901273	+	50.81684	NA	promoter-	promoter-	55	NM_00117	147694	Hs.126905	NM_15290	ENSG0000	ZNF548	-	zinc finger	protein-co
lib-C666-l	chr19	50610479	50610479	+	50.27643	NA	promoter-	promoter-	-88	NR_02421	1E+08	Hs.723094	NR_024214		SNAR-A3	-	small ILF3/	snRNA
lib-C666-l	chr1	1.11E+08	1.11E+08	+	50.11658	NA	promoter-	promoter-	-14	NR_10269	1.01E+08	Hs.559121	NR_102697		LAMTOR5-	-	LAMTOR5	ncRNA
lib-C666-l	chr3	1.58E+08	1.58E+08	+	49.83866	NA	promoter-	promoter-	-48	NR_10414	1.01E+08	Hs.369810	NR_10414	ENSG0000	LOC10099	-	uncharacte	ncRNA
lib-C666-l	chr19	12721705	12721705	+	49.31026	NA	promoter-	promoter-	-27	NM_15335	163049	Hs.720677	NM_15335	ENSG0000	ZNF791	-	zinc finger	protein-co
lib-C666-l	chr10	99094594	99094594	+	48.06386	NA	promoter-	promoter-	-136	NM_01208	23401	Hs.140720	NM_01208	ENSG0000	FRAT2	-	FRAT2, WN	protein-co
lib-C666-l	chr9	1.4E+08	1.4E+08	+	47.93446	NA	promoter-	promoter-	-109	NM_00100	441478	Hs.535075	NM_00100	ENSG0000	NRARP	-	NOTCH-re	protein-co
lib-C666-l	chr19	57862604	57862604	+	47.93181	NA	promoter-	promoter-	-38	NM_00129	57343	Hs.287374	NM_02065	ENSG0000	ZNF304	-	zinc finger	protein-co
lib-C666-l	chr7	66057345	66057345	+	47.37465	NA	promoter-	promoter-	49	NR_11197	493754	Hs.732409	NR_002933		GS1-124K5	-	RAB guani	pseudo
lib-C666-l	chr17	73267352	73267352	+	46.79055	NA	promoter-	promoter-	-28	NR_03652	1E+08	Hs.514470	NR_03652	ENSG0000	LOC10028	-	uncharacte	ncRNA
lib-C666-l	chrX	41135395	41135395	+	46.67128	NA	non-codin	non-codin	403	NR_13561	1.05E+08	Hs.546119	NR_13561	ENSG0000	LOC10537	-	uncharacte	ncRNA
lib-C666-l	chr2	1.98E+08	1.98E+08	+	46.35197	NA	promoter-	promoter-	-133	NM_00108	284992	Hs.132519	NM_17346	ENSG0000	CCDC150	-	coiled-coil	protein-co
lib-C666-l	chr2	1.28E+08	1.28E+08	+	46.32803	NA	promoter-	promoter-	-119	NM_01796	55677	Hs.469879	NM_01796	ENSG0000	IWS1	-	IWS1, SUP	protein-co
lib-C666-l	chr1	9242510	9242510	+	46.14268	NA	promoter-	promoter-	-113	NR_13274	1.07E+08	Hs.551837	NR_13274	ENSG0000	MIR34AHG	-	MIR34A hc	ncRNA
lib-C666-l	chr15	82798487	82798487	+	45.88523	NA	promoter-	promoter-	-88	NR_10349	80154	Hs.454640	NM_025084		GOLGA2P1	-	golgin A2	pseudo
lib-C666-l	chr10	8095645	8095645	+	45.81715	NA	promoter-	promoter-	-198	NR_02425	399717	Hs.158992	NM_20742	ENSG0000	GATA3-AS	-	GATA3 ant	ncRNA
lib-C666-l	chr19	48453514	48453514	+	45.79498	NA	promoter-	promoter-	-39	NR_02422	1E+08	Hs.467061	NR_024221		SNAR-C3	-	small ILF3/	snRNA
lib-C666-l	chr1	2.49E+08	2.49E+08	+	45.65358	NA	5' UTR (NM	5' UTR (NM	150	NM_02483	79894	Hs.521151	NM_02483	ENSG0000	ZNF672	-	zinc finger	protein-co
lib-C666-l	chr7	1.55E+08	1.55E+08	+	45.43086	NA	promoter-	promoter-	-614	NM_00142	2020	Hs.134989	NM_00142	ENSG0000	EN2	-	engrailed I	protein-co
lib-C666-l	chr17	45973415	45973415	+	45.42569	NA	promoter-	promoter-	-101	NM_00311	6668	Hs.514276	NM_00311	ENSG0000	SP2	-	Sp2 transc	protein-co
lib-C666-l	chr4	4249997	4249997	+	45.23903	NA	promoter-	promoter-	-28	NM_00129	85013	Hs.12845	NM_03292	ENSG0000	TMEM128	-	transmemt	protein-co
lib-C666-l	chr17	71162140	71162140	+	45.09843	NA	intron (NM	CpG	980	NM_00105	6752	Hs.514451	NM_00105	ENSG0000	SSTR2	-	somatosta	protein-co
lib-C666-l	chr2	87304199	87304199	+	45.09793	NA	promoter-	promoter-	-663	NR_02684	285074	Hs.424184	NM_001012626		LOC28507	-	anaphase	pseudo
lib-C666-l	chr1	70876804	70876804	+	45.09793	NA	promoter-	promoter-	-97	NM_00119	1491	Hs.19904	NM_00190	ENSG0000	CTH	-	cystathioni	protein-co
lib-C666-l	chr3	1.42E+08	1.42E+08	+	45.09793	NA	promoter-	promoter-	-39	NM_00103	256356	Hs.135904	NM_15277	ENSG0000	GK5	-	glycerol kir	protein-co
lib-C666-l	chr11	62554836	62554836	+	44.9307	NA	promoter-	promoter-	-38	NM_19933	374395	Hs.381134	NM_19933	ENSG0000	TMEM179E	-	transmemt	protein-co
lib-C666-l	chr17	40829321	40829321	+	44.5369	NA	promoter-	promoter-	-273	NM_02492	79990	Hs.632251	NM_02492	ENSG0000	PLEKHH3	-	pleckstrin	protein-co
lib-C666-l	chr7	97881607	97881607	+	44.31884	NA	promoter-	promoter-	-44	NM_01539	25851	Hs.592281	NM_01539	ENSG0000	TECPR1	-	tectonin b	protein-co
lib-C666-l	chr10	97850013	97850013	+	44.27569	NA	promoter-	promoter-	-21	NR_13432	728558	Hs.538374	NR_03844	ENSG0000	ENTPD1-A	-	ENTPD1 ar	ncRNA
lib-C666-l	chrX	1.34E+08	1.34E+08	+	43.81296	NA	promoter-	promoter-	-15	NM_00117	159091	Hs.269127	NM_13881	ENSG0000	FAM122C	-	family with	protein-co
lib-C666-l	chr2	97760595	97760595	+	43.3097	NA	promoter-	promoter-	24	NM_00132	151313	Hs.567723	NM_19933	ENSG0000	FAHD2B	-	fumarylace	protein-co
lib-C666-l	chr5	1.33E+08	1.33E+08	+	43.12596	NA	promoter-	promoter-	-18	NR_13424	1.05E+08	Hs.543907	NR_134247		LOC10537	-	uncharacte	ncRNA
lib-C666-l	chr4	1.07E+08	1.07E+08	+	42.49125	NA	promoter-	promoter-	-25	NM_02475	79807	Hs.161429	NM_02475	ENSG0000	GSTCD	-	glutathion	protein-co
lib-C666-l	chr20	35918049	35918049	+	42.39316	NA	promoter-	promoter-	-2	NM_02207	63905	Hs.6126	NM_02207	ENSG0000	MANBAL	-	mannosida	protein-co
lib-C666-l	chr15	40401122	40401122	+	42.39316	NA	promoter-	promoter-	-37	NM_00100	90427	Hs.591104	NM_03350	ENSG0000	BMF	-	Bcl2 modif	protein-co
lib-C666-l	chr19	49535117	49535117	+	42.35981	NA	promoter-	promoter-	-13	NM_03337	114336	Hs.567650	NM_03337	ENSG0000	CGB2	-	chorionic g	protein-co
lib-C666-l	chr11	63580859	63580859	+	42.35981	NA	promoter-	promoter-	13	NM_13847	144097	Hs.502793	NM_13847	ENSG0000	C11orf84	-	chromosor	protein-co
lib-C666-l	chr19	46009810	46009810	+	41.98367	NA	promoter-	promoter-	-878	NM_00337	7408	Hs.515469	NM_00337	ENSG0000	VASP	-	vasodilator	protein-co
lib-C666-l	chr1	85742257	85742257	+	41.94622	NA	non-codin	non-codin	216	NR_04548	646626	Hs.193516	NR_045484		LOC64662	-	uncharacte	ncRNA
lib-C666-l	chr5	1.41E+08	1.41E+08	+	41.83575	NA	promoter-	promoter-	-422	NR_03833	1.01E+08	Hs.655458	NR_03833	ENSG0000	LOC10050	-	uncharacte	ncRNA
lib-C666-l	chr1	33207250	33207250	+	41.31543	NA	promoter-	promoter-	-262	NM_02088	57648	Hs.591502	NM_02088	ENSG0000	KIAA1522	-	KIAA1522	protein-co
lib-C666-l	chr7	86781645	86781645	+	41.28836	NA	promoter-	promoter-	-12	NR_13625	1.02E+08	Hs.633960	NR_13625	ENSG0000	LOC10192	-	uncharacte	ncRNA
lib-C666-l	chr17	41116505	41116505	+	41.21722	NA	promoter-	promoter-	10	NM_00126	80755	Hs.317403	NM_00126	ENSG0000	AARSD1	-	alanyl-tRN	protein-co
lib-C666-l	chr16	3285233	3285233	+	41.17725	NA	promoter-	promoter-	-47	NM_00114	7752	Hs.632222	NM_00345	ENSG0000	ZNF200	-	zinc finger	protein-co
lib-C666-l	chr3	47324227	47324227	+	41.10975	NA	promoter-	promoter-	-103	NM_02501	23276	Hs.517946	NM_02501	ENSG0000	KLHL18	-	kelch like f	protein-co
lib-C666-l	chr6	1.7E+08	1.7E+08	+	40.86121	NA	promoter-	promoter-	85	NM_00131	387263	Hs.591375	NM_00102	ENSG0000	C6orf120	-	chromosor	protein-co
lib-C666-l	chr19	57831838	57831838	+	40.85015	NA	promoter-	promoter-	-27	NM_21359	125919	Hs.202544	NM_21359	ENSG0000	ZNF543	-	zinc finger	protein-co
lib-C666-l	chr22	38004587	38004587	+	40.85015	NA	promoter-	promoter-	106	NM_00117	26088	Hs.499158	NM_01336	ENSG0000	GGA1	-	golgi asso	protein-co
lib-C666-l	chr15	82944648	82944648	+	39.94134	NA	promoter-	promoter-	-98	NR_03393	80154	Hs.454640	NM_025084		GOLGA2P1	-	golgin A2	pseudo
lib-C666-l	chr2	2.19E+08	2.19E+08	+	39.94134	NA	promoter-	promoter-	-37	NR_12577	1.04E+08		NR_125777		CATIP-AS2	-	CATIP anti	ncRNA
lib-C666-l	chr19	50595922	50595922	+	39.78294	NA	promoter-	promoter-	-56	NR_02422	1E+08	Hs.723094	NR_024229		SNAR-A10	-	small ILF3/	snRNA
lib-C666-l	chr13	78272259	78272259	+	39.60187	NA	promoter-	promoter-	-8	NR_03743	1.01E+08		NR_03743	ENSG0000	MIR3665	-	microRNA	ncRNA
lib-C666-l	chr19	50621156	50621156	+	39.41852	NA	promoter-	promoter-	-59	NR_02422	1E+08	Hs.723094	NR_024226		SNAR-A9	-	small ILF3/	snRNA
lib-C666-l	chr11	47869945	47869945	+	39.32295	NA	exon (NM_	exon (NM_	151	NM_01523	23279	Hs.643526	NM_01523	ENSG0000	NUP160	-	nucleopori	protein-co
lib-C666-l	chr20	21283954	21283954	+	39.29799	NA	promoter-	promoter-	32	NM_01225	22803	Hs.255932	NM_01225	ENSG0000	XRN2	-	5'-3' exori	protein-co
lib-C666-l	chr19	38893717	38893717	+	39.19964	NA	promoter-	promoter-	-58	NM_17490	147965	Hs.355162	NM_17490	ENSG0000	FAM98C	-	family with	protein-co
lib-C666-l	chr10	22725876	22725876	+	38.6745	NA	non-codin	non-codin	982	NR_03653	1E+08	Hs.7444	NR_036533		LOC10049	-	uncharacte	ncRNA
lib-C666-l	chr10	75503871	75503871	+	38.60092	NA	promoter-	promoter-	-260	NM_19859	9632	Hs.81964	NM_00492	ENSG0000	SEC24C	-	SEC24 hon	protein-co
lib-C666-l	chr5	72252334	72252334	+	38.57241	NA	intron (NM	CpG	526	NM_00114	115548	Hs.165762	NM_13878	ENSG0000	FCHO2	-	FCH doma	protein-co
lib-C666-l	chr7	1.44E+08	1.44E+08	+	38.22284	NA	non-codin	non-codin	172	NR_12602	1.02E+08	Hs.591830	NR_126022		LOC10192	-	uncharacte	ncRNA
lib-C666-l	chr12	94954797	94954797	+	38.09782	NA	promoter-	promoter-	-768	NR_04988	1.01E+08		NR_04988	ENSG0000	MIR5700	-	microRNA	ncRNA
lib-C666-l	chr17	18528966	18528966	+	37.73319	NA	promoter-	promoter-	-36	NR_03664	284047	Hs.448012	NR_03664	ENSG0000	CCDC144B	-	coiled-coil	pseudo
lib-C666-l	chr14	36789883	36789883	+	37.4886	NA	promoter-	promoter-	-1	NM_00114	51562	Hs.368647	NM_01658	ENSG0000	MBIP	-	MAP3K12	protein-co
lib-C666-l	chr4	1.69E+08	1.69E+08	+	37.47171	NA	intron (NM	CpG	292	NM_01763	55601	Hs.591710	NM_01763	ENSG0000	DDX60	-	DExD/H-b	protein-co
lib-C666-l	chr9	3526750	3526750	+	37.41784	NA	promoter-	promoter-	-749	NM_13442	5991	Hs.136829	NM_00291	ENSG0000	RFX3	-	regulatory	protein-co
lib-C666-l	chr19	50528499	50528499	+	37.41784	NA	promoter-	promoter-	144	NM_00130	51231	Hs.443330	NM_01644	ENSG0000	VRK3	-	vaccinia re	protein-co
lib-C666-l	chr3	1.2E+08	1.2E+08	+	37.05378	NA	promoter-	promoter-	192	NM_17382	285282	Hs.444360	NM_17382	ENSG0000	RABL3	-	RAB, meml	protein-co
lib-C666-l	chr1	1.44E+08	1.44E+08	+	36.98516	NA	promoter-	promoter-	2	NR_11193	1E+08	Hs.585189	NR_033189		LOC10013	-	phosphodi	pseudo
lib-C666-l	chr19	58838636	58838636	+	36.85381	NA	promoter-	promoter-	-22	NR_14444	1.05E+08	Hs.659382	NR_144444		LOC10537	-	uncharacte	ncRNA
lib-C666-l	chrX	1.49E+08	1.49E+08	+	36.84144	NA	promoter-	promoter-	41	NR_02745	1E+08	Hs.496916	NR_02745	ENSG0000	LINC00894	-	long interg	ncRNA
lib-C666-l	chr12	89920055	89920055	+	36.70779	NA	promoter-	promoter-	-16	NM_00119	1.01E+08	Hs.25130	NM_00119	ENSG0000	POC1B-GA	-	POC1B-GA	protein-co
lib-C666-l	chr16	28722765	28722765	+	36.70747	NA	promoter-	promoter-	24	NM_00131	728689	Hs.567374	NM_00109	ENSG0000	EIF3CL	-	eukaryotic	protein-co
lib-C666-l	chr20	18774721	18774721	+	36.59122	NA	promoter-	promoter-	28	NR_02688	1E+08	Hs.598768	NR_026885		LOC10027	-	uncharacte	ncRNA
lib-C666-l	chrX	1.03E+08	1.03E+08	+	36.56424	NA	promoter-	promoter-	4	NR_13123	1.05E+08		NR_131236		LOC10537	-	uncharacte	ncRNA
lib-C666-l	chr19	39900210	39900210	+	36.11612	NA	TTS (NR_0	TTS (NR_0	108	NR_03975	1.01E+08		NR_03975	ENSG0000	MIR4530	-	microRNA	ncRNA
lib-C666-l	chr19	42901157	42901157	+	36.07033	NA	promoter-	promoter-	-123	NR_12604	1.02E+08	Hs.567935	NR_12604	ENSG0000	LOC10193	-	uncharacte	ncRNA
lib-C666-l	chr17	40706803	40706803	+	36.07033	NA	promoter-	promoter-	17	NR_14440	1.09E+08	Hs.655222	NR_144402		LOC10878	-	uncharacte	ncRNA
lib-C666-l	chr19	49540219	49540219	+	35.90556	NA	promoter-	promoter-	-28	NM_03337	114335	Hs.659014	NM_03337	ENSG0000	CGB1	-	chorionic g	protein-co
lib-C666-l	chr17	26662489	26662489	+	35.73373	NA	promoter-	promoter-	26	NM_00126	90410	Hs.705431	NM_17488	ENSG0000	IFT20	-	intraflagell	protein-co
lib-C666-l	chr4	1.1E+08	1.1E+08	+	35.47771	NA	promoter-	promoter-	-31	NR_02696	285456	Hs.683863	NM_18259	ENSG0000	RPL34-AS1	-	RPL34 anti	ncRNA
lib-C666-l	chr12	1.21E+08	1.21E+08	+	35.40551	NA	promoter-	promoter-	-11	NM_00128	64897	Hs.528671	NM_02289	ENSG0000	C12orf43	-	chromosor	protein-co
lib-C666-l	chr17	53342274	53342274	+	35.32367	NA	promoter-	promoter-	-47	NM_00212	3131	Hs.196952	NM_00212	ENSG0000	HLF	-	HLF, PAR b	protein-co
lib-C666-l	chr16	87799588	87799588	+	35.28811	NA	promoter-	promoter-	10	NM_00118	54758	Hs.123450	NM_01756	ENSG0000	KLHDC4	-	kelch dom	protein-co
lib-C666-l	chr13	1.14E+08	1.14E+08	+	35.28811	NA	promoter-	promoter-	22	NM_00127	8451	Hs.339735	NM_00358	ENSG0000	CUL4A	-	cullin 4A	protein-co
lib-C666-l	chr12	1.33E+08	1.33E+08	+	34.92958	NA	promoter-	promoter-	-302	NR_00329	347918	Hs.122115	NM_18261	ENSG0000	EP400NL	-	EP400 N-t	pseudo
lib-C666-l	chr17	4046922	4046922	+	34.83329	NA	promoter-	promoter-	7	NM_00125	124936	Hs.513871	NM_14461	ENSG0000	CYB5D2	-	cytochrom	protein-co
lib-C666-l	chr7	1.39E+08	1.39E+08	+	34.76791	NA	promoter-	promoter-	-379	NM_17356	254048	Hs.153458	NM_17356	ENSG0000	UBN2	-	ubinuclein	protein-co
lib-C666-l	chr11	95523030	95523030	+	34.62267	NA	promoter-	promoter-	-75	NM_00133	143684	Hs.288304	NM_14466	ENSG0000	FAM76B	-	family with	protein-co
lib-C666-l	chr10	1.34E+08	1.34E+08	+	34.53169	NA	promoter-	promoter-	21	NM_00114	80313	Hs.119897	NM_03062	ENSG0000	LRRC27	-	leucine ricl	protein-co
lib-C666-l	chr15	45571488	45571488	+	34.53169	NA	promoter-	promoter-	-68	NR_12033	1.02E+08		NR_12033	ENSG0000	LOC10192	-	uncharacte	ncRNA
lib-C666-l	chr8	1.25E+08	1.25E+08	+	34.19829	NA	promoter-	promoter-	-16	NM_00131	157769	Hs.459174	NM_14496	ENSG0000	FAM91A1	-	family with	protein-co
lib-C666-l	chr2	1.14E+08	1.14E+08	+	34.19829	NA	promoter-	promoter-	-63	NR_13071	150468	Hs.434250	NM_15251	ENSG0000	CKAP2L	-	cytoskelet	protein-co
lib-C666-l	chr11	1.18E+08	1.18E+08	+	34.0482	NA	promoter-	promoter-	42	NM_19827	196264	Hs.15396	NM_19827	ENSG0000	MPZL3	-	myelin pro	protein-co
lib-C666-l	chr15	76629100	76629100	+	33.43742	NA	promoter-	promoter-	35	NM_14580	64843	Hs.444677	NM_14580	ENSG0000	ISL2	-	ISL LIM ho	protein-co
lib-C666-l	chr19	1241693	1241693	+	33.11264	NA	promoter-	promoter-	-56	NM_00100	513	Hs.418668	NM_00168	ENSG0000	ATP5D	-	ATP syntha	protein-co
lib-C666-l	chr12	72148649	72148649	+	33.11264	NA	promoter-	promoter-	6	NM_01499	23011	Hs.524590	NM_01499	ENSG0000	RAB21	-	RAB21, me	protein-co
lib-C666-l	chr5	10761350	10761350	+	32.78343	NA	promoter-	promoter-	37	NM_00439	1611	Hs.75189	NM_00439	ENSG0000	DAP	-	death asso	protein-co
lib-C666-l	chr3	48130556	48130556	+	32.58685	NA	5' UTR (NM	5' UTR (NM	213	NM_03088	4134	Hs.517949	NM_00237	ENSG0000	MAP4	-	microtubul	protein-co
lib-C666-l	chr2	38265451	38265451	+	32.38963	NA	intron (NM	intron (NM	-1967	NR_10271	1.01E+08	Hs.591566	NR_102712		RMDN2-A	-	RMDN2 ar	ncRNA
lib-C666-l	chr19	1248729	1248729	+	32.38963	NA	intron (NM	CpG	177	NM_17740	55320	Hs.465529	NM_17740	ENSG0000	MIDN	-	midnolin	protein-co
lib-C666-l	chr5	41904234	41904234	+	32.29191	NA	promoter-	promoter-	-212	NM_17592	285636	Hs.29899	NM_17592	ENSG0000	C5orf51	-	chromosor	protein-co
lib-C666-l	chr14	96968648	96968648	+	32.29191	NA	promoter-	promoter-	15	NR_12212	730202	Hs.647565	NR_122121		LOC73020	-	uncharacte	ncRNA
lib-C666-l	chr4	1.2E+08	1.2E+08	+	32.11429	NA	promoter-	promoter-	-520	NR_12593	1.02E+08	Hs.570735	NR_125931		LOC10192	-	uncharacte	ncRNA
lib-C666-l	chr22	38054486	38054486	+	32.07197	NA	promoter-	promoter-	-102	NR_10995	1.02E+08		NR_10995	ENSG0000	LOC10192	-	uncharacte	ncRNA
lib-C666-l	chr16	23160877	23160877	+	32.04651	NA	promoter-	promoter-	-286	NM_02071	57478	Hs.183817	NM_02071	ENSG0000	USP31	-	ubiquitin s	protein-co
lib-C666-l	chr19	50615824	50615824	+	31.9103	NA	promoter-	promoter-	-79	NR_02421	1E+08	Hs.723094	NR_024214		SNAR-A3	-	small ILF3/	snRNA
lib-C666-l	chr14	65007134	65007134	+	31.49527	NA	promoter-	promoter-	-48	NR_11055	1.03E+08	Hs.667192	NR_11055	ENSG0000	LOC10272	-	uncharacte	ncRNA
lib-C666-l	chr4	1.2E+08	1.2E+08	+	31.45327	NA	promoter-	promoter-	-21	NR_10382	729218	Hs.448518	NR_103825		LOC72921	-	uncharacte	pseudo
lib-C666-l	chr3	49055872	49055872	+	31.10827	NA	exon (NM_	exon (NM_	169	NM_00100	55152	Hs.654815	NM_01811	ENSG0000	DALRD3	-	DALR antic	protein-co
lib-C666-l	chr3	62304633	62304633	+	31.0765	NA	promoter-	promoter-	-11	NR_03828	1.01E+08	Hs.656620	NR_03828	ENSG0000	PTPRG-AS	-	PTPRG ant	ncRNA
lib-C666-l	chr1	1334828	1334828	+	30.98434	NA	promoter-	promoter-	-82	NR_01543	148413	Hs.741357	NR_01543	ENSG0000	LOC14841	-	uncharacte	ncRNA
lib-C666-l	chr7	1.27E+08	1.27E+08	+	30.54026	NA	promoter-	promoter-	85	NM_00166	381	Hs.653202	NM_00166	ENSG0000	ARF5	-	ADP ribosy	protein-co
lib-C666-l	chr8	82754952	82754952	+	30.16874	NA	promoter-	promoter-	-431	NM_02213	64089	Hs.492121	NM_02213	ENSG0000	SNX16	-	sorting ne	protein-co
lib-C666-l	chr11	74660255	74660255	+	30.07415	NA	promoter-	promoter-	-23	NM_18296	143570	Hs.370145	NM_18296	ENSG0000	XRRA1	-	X-ray radi	protein-co
lib-C666-l	chr7	1.28E+08	1.28E+08	+	29.98795	NA	promoter-	promoter-	-29	NM_00119	813	Hs.7753	NM_00121	ENSG0000	CALU	-	calumenin	protein-co

lib-C666-l	chr2	33171937	33171937	+	29.93465	NA	promoter-	promoter-	-432	NM_20694	4052	Hs.619315	NM_00062	ENSG0000	LTBP1	-	latent trans	protein-co
lib-C666-l	chr15	72766173	72766173	+	29.93465	NA	promoter-	promoter-	407	NR_135678	1.05E+08	Hs.540241	NR_135678	ENSG0000	LOC105370	-	uncharacte	ncRNA
lib-C666-l	chr1	42801011	42801011	+	29.93465	NA	promoter-	promoter-	-108	NM_01494	22887	Hs.26023	NM_01494	ENSG0000	FOXJ3	-	forkhead b	protein-co
lib-C666-l	chr16	48645855	48645855	+	29.93113	NA	intron (NR	intron (NR	-1735	NM_15302	9683	Hs.511839	NM_15302	ENSG0000	N4BP1	-	NEDD4 bir	protein-co
lib-C666-l	chr15	23034395	23034395	+	29.41683	NA	promoter-	promoter-	32	NM_00118	81614	Hs.591003	NM_03092	ENSG0000	NIPA2	-	non imprir	protein-co
lib-C666-l	chr7	1.5E+08	1.5E+08	+	29.22024	NA	non-codin	non-codin	263	NR_027040	401431	Hs.556998	NM_00100	ENSG0000	ATP6V0E2	-	ATP6V0E2	ncRNA
lib-C666-l	chr19	10444099	10444099	+	29.21635	NA	TTS (NM_0	TTS (NM_0	215	NM_13345	125950	Hs.744952	NM_13345	ENSG0000	RAVER1	-	ribonucleo	protein-co
lib-C666-l	chr12	72057694	72057694	+	29.17165	NA	promoter-	promoter-	17	NM_03143	83591	Hs.245798	NM_03143	ENSG0000	THAP2	-	THAP dom	protein-co
lib-C666-l	chr16	74402191	74402191	+	29.115	NA	promoter-	promoter-	-38	NR_026950	283922	Hs.558726	NR_026950	ENSG0000	LOC283922	-	pyruvate d	pseudo
lib-C666-l	chr18	77711693	77711693	+	29.07066	NA	promoter-	promoter-	-40	NM_02507	80148	Hs.288284	NM_02507	ENSG0000	PQLC1	-	PQ loop re	protein-co
lib-C666-l	chr16	2059872	2059872	+	29.01149	NA	promoter-	promoter-	-50	NM_17816	90850	Hs.343828	NM_17816	ENSG0000	ZNF598	-	zinc finger	protein-co
lib-C666-l	chr2	1.91E+08	1.91E+08	+	28.88523	NA	promoter-	promoter-	48	NR_102429	1.01E+08	Hs.738558	NR_102429		OSGEPL1-	-	OSGEPL1 a	ncRNA
lib-C666-l	chr3	1.94E+08	1.94E+08	+	28.88523	NA	5' UTR (NM	5' UTR (NM	110	NM_01838	55341	Hs.744061	NM_01838	ENSG0000	LSG1	-	large 60S s	protein-co
lib-C666-l	chr15	41245550	41245550	+	28.88523	NA	5' UTR (NM	5' UTR (NM	203	NM_00114	79094	Hs.155569	NM_02411	ENSG0000	CHAC1	-	ChaC gluta	protein-co
lib-C666-l	chr16	3184864	3184864	+	28.8837	NA	promoter-	promoter-	19	NR_110900	1.01E+08	Hs.522143	NR_110900		ZNF213-AS	-	ZNF213 an	ncRNA
lib-C666-l	chr18	2655879	2655879	+	28.7732	NA	promoter-	promoter-	-7	NM_01529	23347	Hs.8118	NM_01529	ENSG0000	SMCHD1	-	structural r	protein-co
lib-C666-l	chr19	1275759	1275759	+	28.64047	NA	TTS (NM_0	TTS (NM_0	239	NM_01791	55009	Hs.591383	NM_01791	ENSG0000	C19orf24	-	chromosor	protein-co
lib-C666-l	chr10	94050867	94050867	+	28.64047	NA	promoter-	promoter-	8	NM_01491	22849	Hs.131683	NM_01491	ENSG0000	CPEB3	-	cytoplasmi	protein-co
lib-C666-l	chr1	1.6E+08	1.6E+08	+	28.42633	NA	exon (NM_	exon (NM_	436	NM_00113	284677	Hs.647718	NM_00113	ENSG0000	C1orf204	-	chromosor	protein-co
lib-C666-l	chr19	14247716	14247716	+	28.20039	NA	promoter-	promoter-	-248	NR_045214	1.01E+08	Hs.720437	NR_045214	ENSG0000	LOC100507	-	uncharacte	ncRNA
lib-C666-l	chr4	16228300	16228300	+	27.84631	NA	promoter-	promoter-	14	NR_027697	202020	Hs.445315	NR_027697	ENSG0000	TAPT1-AS1	-	TAPT1 anti	ncRNA
lib-C666-l	chr14	24028907	24028907	+	27.70954	NA	TTS (NM_0	TTS (NM_0	-1399	NR_110555	1.03E+08	Hs.569375	NR_110555	ENSG0000	LOC102724	-	uncharacte	ncRNA
lib-C666-l	chr19	36705638	36705638	+	27.69998	NA	promoter-	promoter-	-63	NM_15247	147929	Hs.596338	NM_15247	ENSG0000	ZNF565	-	zinc finger	protein-co
lib-C666-l	chr12	1.21E+08	1.21E+08	+	27.68428	NA	promoter-	promoter-	-25	NM_03231	84274	Hs.17250	NM_03231	ENSG0000	COQ5	-	coenzyme	protein-co
lib-C666-l	chr1	2.28E+08	2.28E+08	+	27.5466	NA	promoter-	promoter-	1	NM_00102	375	Hs.286221	NM_00165	ENSG0000	ARF1	-	ADP ribosy	protein-co
lib-C666-l	chr19	44529482	44529482	+	27.5466	NA	promoter-	promoter-	-12	NM_00112	7673	Hs.279840	NM_01336	ENSG0000	ZNF222	-	zinc finger	protein-co
lib-C666-l	chr19	50631875	50631875	+	27.42941	NA	promoter-	promoter-	-96	NR_024229	1E+08	Hs.723094	NR_024229		SNAR-A10	-	small ILF3/	snRNA
lib-C666-l	chr3	48701214	48701214	+	27.28371	NA	promoter-	promoter-	13	NR_111921	1.03E+08	Hs.663571	NR_111921	ENSG0000	CELSR3-AS	-	CELSR3 an	ncRNA
lib-C666-l	chr8	1.46E+08	1.46E+08	+	27.26713	NA	intron (NM	CpG	101	NM_02308	65265	Hs.169615	NM_02308	ENSG0000	C8orf33	-	chromosor	protein-co
lib-C666-l	chr9	84304799	84304799	+	27.26713	NA	promoter-	promoter-	171	NR_109772	1.02E+08	Hs.528628	NR_109772	ENSG0000	LOC101927	-	uncharacte	ncRNA
lib-C666-l	chr2	28114147	28114147	+	26.81569	NA	promoter-	promoter-	-166	NR_028308	1E+08	Hs.729719	NR_028308		BRE-AS1	-	BRE antiser	ncRNA
lib-C666-l	chr12	10902735	10902735	+	26.81569	NA	promoter-	promoter-	-98	NR_120463	1.02E+08	Hs.112950	NR_120463	ENSG0000	LOC101928	-	uncharacte	ncRNA
lib-C666-l	chr16	28415156	28415156	+	26.80209	NA	promoter-	promoter-	50	NM_00131	728689	Hs.567374	NM_00109	ENSG0000	EIF3CL	-	eukaryotic	protein-co
lib-C666-l	chr2	2.43E+08	2.43E+08	+	26.57948	NA	intron (NR	CpG	186	NR_110220	1.02E+08	Hs.130180	NR_110220	ENSG0000	LINC01237	-	long interg	ncRNA
lib-C666-l	chr4	775699	775699	+	26.54591	NA	promoter-	promoter-	-63	NR_036512	1E+08	Hs.731755	NR_036512	ENSG0000	LOC100129	-	uncharacte	ncRNA
lib-C666-l	chr4	76598755	76598755	+	26.52079	NA	promoter-	promoter-	-88	NM_20350	9908	Hs.303676	NM_01229	ENSG0000	G3BP2	-	G3BP stres	protein-co
lib-C666-l	chr7	1.52E+08	1.52E+08	+	26.46807	NA	non-codin	non-codin	302	NR_027387	1E+08	Hs.647112	NR_027387	ENSG0000	LINC01003	-	long interg	ncRNA
lib-C666-l	chr19	50380862	50380862	+	26.35145	NA	promoter-	promoter-	180	NM_00116	79735	Hs.631587	NM_02468	ENSG0000	TBC1D17	-	TBC1 dom	protein-co
lib-C666-l	chr11	82867960	82867960	+	25.96768	NA	promoter-	promoter-	-177	NM_01588	51585	Hs.128959	NM_01588	ENSG0000	PCF11	-	PCF11 clea	protein-co
lib-C666-l	chr2	1.09E+08	1.09E+08	+	25.68529	NA	promoter-	promoter-	169	NR_135290	644903		NR_03265	ENSG0000	GCC2-AS1	-	GCC2 antis	ncRNA
lib-C666-l	chr12	62860609	62860609	+	25.68529	NA	promoter-	promoter-	12	NM_01502	23041	Hs.389378	NM_01502	ENSG0000	MON2	-	MON2 hor	protein-co
lib-C666-l	chr16	50776287	50776287	+	25.61513	NA	promoter-	promoter-	71	NR_130462	1E+08		NR_130462	ENSG0000	MIR3181	-	microRNA	ncRNA
lib-C666-l	chr1	31191678	31191678	+	25.39487	NA	promoter-	promoter-	59	NR_034182	1E+08	Hs.659751	NR_034182	ENSG0000	MATN1-AS	-	MATN1 an	ncRNA
lib-C666-l	chr19	1249127	1249127	+	25.32183	NA	intron (NM	CpG	575	NM_17740	90007	Hs.465529	NM_17740	ENSG0000	MIDN	-	midnolin	protein-co
lib-C666-l	chr14	70234524	70234524	+	25.32183	NA	promoter-	promoter-	-94	NR_029378	1E+08	Hs.729250	NR_029378		LOC100289	-	uncharacte	ncRNA
lib-C666-l	chr16	699205	699205	+	25.32183	NA	promoter-	promoter-	108	NR_135177	1.05E+08		NR_135177		LOC105371	-	uncharacte	ncRNA
lib-C666-l	chr19	2840988	2840988	+	25.32183	NA	promoter-	promoter-	-445	NM_15279	148254	Hs.47712	NM_15279	ENSG0000	ZNF555	-	zinc finger	protein-co
lib-C666-l	chr19	48426994	48426994	+	24.97013	NA	promoter-	promoter-	-42	NR_024229	1E+08	Hs.723094	NR_024229		SNAR-A10	-	small ILF3/	snRNA
lib-C666-l	chr15	52472253	52472253	+	24.73139	NA	promoter-	promoter-	30	NR_102751	1E+08	Hs.569494	NR_102751		CERNA1	-	competing	ncRNA
lib-C666-l	chr9	6008363	6008363	+	24.43903	NA	promoter-	promoter-	-360	NM_00101	158358	Hs.535060	NM_00101	ENSG0000	KIAA2026	-	KIAA2026	protein-co
lib-C666-l	chr11	9781347	9781347	+	24.32656	NA	promoter-	promoter-	-267	NR_033972	440028	Hs.677541	NR_033972	ENSG0000	LOC440028	-	uncharacte	ncRNA
lib-C666-l	chr2	1.71E+08	1.71E+08	+	24.32656	NA	promoter-	promoter-	10	NM_00119	1.01E+08		NM_00119	ENSG0000	PHOSPHO	-	PHOSPHO	protein-co
lib-C666-l	chr7	36406822	36406822	+	24.28549	NA	promoter-	promoter-	20	NM_00119	23366	Hs.6224	NM_01531	ENSG0000	KIAA0895	-	KIAA0895	protein-co
lib-C666-l	chr8	1.46E+08	1.46E+08	+	24.25638	NA	promoter-	promoter-	-528	NR_134307	1.02E+08	Hs.529758	NR_134307		LOC101928	-	uncharacte	ncRNA
lib-C666-l	chr16	2732418	2732418	+	24.25638	NA	promoter-	promoter-	-77	NM_01899	54442	Hs.61960	NM_01899	ENSG0000	KCTD5	-	potassium	protein-co
lib-C666-l	chr1	1.48E+08	1.48E+08	+	24.25638	NA	promoter-	promoter-	6	NR_111930	1E+08	Hs.585189	NR_033189		LOC100132	-	phosphodi	pseudo
lib-C666-l	chr13	41707027	41707027	+	24.20134	NA	promoter-	promoter-	-37	NR_120423	1.02E+08	Hs.663088	NR_120423	ENSG0000	LOC101929	-	uncharacte	ncRNA
lib-C666-l	chr3	1.23E+08	1.23E+08	+	23.80446	NA	intron (NR	MER1B|DN	920	NR_121654	1.01E+08	Hs.556600	NR_038260	ENSG0000	MYLK-AS1	-	MYLK antis	ncRNA
lib-C666-l	chr2	1.14E+08	1.14E+08	+	23.73126	NA	promoter-	promoter-	-178	NR_024004	84771	Hs.712940	NR_024004		DDX11L2	-	DEAD/H-b	pseudo
lib-C666-l	chr12	1.34E+08	1.34E+08	+	23.60036	NA	promoter-	promoter-	-41	NM_00127	1.01E+08	Hs.614816	NM_00127	ENSG0000	ZNF891	-	zinc finger	protein-co
lib-C666-l	chr8	37620276	37620276	+	23.48385	NA	exon (NM_	exon (NM_	175	NM_00719	11212	Hs.304792	NM_00719	ENSG0000	PROSC	-	proline syr	protein-co
lib-C666-l	chr11	82782964	82782964	+	23.34793	NA	promoter-	promoter-	1	NM_00128	27314	Hs.40758	NM_01448	ENSG0000	RAB30	-	RAB30, me	protein-co
lib-C666-l	chr2	2.07E+08	2.07E+08	+	23.34793	NA	promoter-	promoter-	106	NM_02092	57683	Hs.110489	NM_02092	ENSG0000	ZDBF2	-	zinc finger	protein-co
lib-C666-l	chr12	56367722	56367722	+	23.34628	NA	promoter-	promoter-	25	NM_00125	5869	Hs.157659	NM_00286	ENSG0000	RAB5B	-	RAB5B, me	protein-co
lib-C666-l	chr19	50601300	50601300	+	23.242	NA	promoter-	promoter-	-97	NR_024227	1E+08	Hs.723094	NR_024227		SNAR-A6	-	small ILF3/	snRNA
lib-C666-l	chr5	1.5E+08	1.5E+08	+	23.1274	NA	promoter-	promoter-	-794	NM_00116	11346	Hs.435228	NM_00728	ENSG0000	SYNPO	-	synaptopo	protein-co
lib-C666-l	chr8	1.46E+08	1.46E+08	+	23.087	NA	promoter-	promoter-	-15	NM_17492	203054	Hs.283374	NM_17492	ENSG0000	ADCK5	-	aarF doma	protein-co
lib-C666-l	chr5	7306896	7306896	+	22.84581	NA	promoter-	promoter-	-69	NR_033906	442132	Hs.727388	NM_001034846		LOC442132	-	golgin A6	pseudo
lib-C666-l	chr11	18720289	18720289	+	22.82077	NA	promoter-	promoter-	7	NM_15334	144110	Hs.502100	NM_15334	ENSG0000	TMEM86A	-	transmemb	protein-co
lib-C666-l	chr19	50604321	50604321	+	22.7781	NA	promoter-	promoter-	-53	NR_024227	1E+08	Hs.723094	NR_024227		SNAR-A6	-	small ILF3/	snRNA
lib-C666-l	chr1	36023307	36023307	+	22.43333	NA	promoter-	promoter-	-86	NM_01428	23154	Hs.121870	NM_01428	ENSG0000	NCDN	-	neurochon	protein-co
lib-C666-l	chr19	50626514	50626514	+	22.31021	NA	promoter-	promoter-	-63	NR_024224	1E+08	Hs.723094	NR_024224		SNAR-A7	-	small ILF3/	snRNA
lib-C666-l	chr5	1.11E+08	1.11E+08	+	22.02847	NA	promoter-	promoter-	-74	NM_00130	134429	Hs.93842	NM_13916	ENSG0000	STARD4	-	StAR relate	protein-co
lib-C666-l	chr9	1.32E+08	1.32E+08	+	22.01132	NA	promoter-	promoter-	-72	NM_00443	2021	Hs.745161	NM_00443	ENSG0000	ENDOG	-	endonucle	protein-co
lib-C666-l	chr11	1.25E+08	1.25E+08	+	21.96185	NA	promoter-	promoter-	-44	NR_132372	1.05E+08	Hs.732083	NR_132372	ENSG0000	STT3A-AS1	-	STT3A anti	ncRNA
lib-C666-l	chr22	21271644	21271644	+	21.90047	NA	promoter-	promoter-	-70	NM_00520	1399	Hs.5613	NM_00520	ENSG0000	CRKL	-	CRK like pr	protein-co
lib-C666-l	chr9	1.03E+08	1.03E+08	+	21.87529	NA	promoter-	promoter-	-339	NR_109802	1.02E+08	Hs.637767	NR_109802	ENSG0000	LOC101928	-	uncharacte	ncRNA
lib-C666-l	chr18	34408904	34408904	+	21.87022	NA	promoter-	promoter-	-165	NR_136303	57536	Hs.4045	NM_02077	ENSG0000	KIAA1328	-	KIAA1328	protein-co
lib-C666-l	chr9	1.24E+08	1.24E+08	+	21.73991	NA	promoter-	promoter-	38	NR_024408	253039	Hs.594170	NR_024408		PSMD5-AS	-	PSMD5 an	ncRNA
lib-C666-l	chr14	35452170	35452170	+	21.73991	NA	promoter-	promoter-	66	NM_00313	6729	Hs.167535	NM_00313	ENSG0000	SRP54	-	signal reco	protein-co
lib-C666-l	chr19	50607390	50607390	+	21.61144	NA	promoter-	promoter-	-58	NR_024229	1E+08	Hs.723094	NR_024229		SNAR-A10	-	small ILF3/	snRNA
lib-C666-l	chr14	50359717	50359717	+	21.52202	NA	promoter-	promoter-	-19	NM_00166	382	Hs.525330	NM_00166	ENSG0000	ARF6	-	ADP ribosy	protein-co
lib-C666-l	chr17	63096873	63096873	+	21.35213	NA	promoter-	promoter-	-57	NR_110801	1.01E+08	Hs.729924	NR_110801		LOC100507	-	uncharacte	ncRNA
lib-C666-l	chr18	109300	109300	+	21.34108	NA	non-codin	non-codin	235	NR_033770	727758	Hs.585843	NR_033770	ENSG0000	ROCK1P1	-	Rho associ	pseudo
lib-C666-l	chr6	56708921	56708921	+	21.28624	NA	promoter-	promoter-	137	NR_125866	1.02E+08	Hs.650232	NR_125866		LOC101930	-	uncharacte	ncRNA
lib-C666-l	chr2	39005312	39005312	+	21.21068	NA	promoter-	promoter-	-15	NM_02477	79833	Hs.143818	NM_02477	ENSG0000	GEMIN6	-	gem nucle	protein-co
lib-C666-l	chr16	89284058	89284058	+	21.21068	NA	promoter-	promoter-	-53	NR_037705	197320	Hs.647385	NM_18253	ENSG0000	ZNF778	-	zinc finger	protein-co
lib-C666-l	chr5	1.49E+08	1.49E+08	+	20.94772	NA	promoter-	promoter-	-65	NM_00130	78991	Hs.644397	NM_02402	ENSG0000	PCYOX1L	-	prenylcyste	protein-co
lib-C666-l	chr11	66247732	66247732	+	20.90919	NA	promoter-	promoter-	-12	NR_120586	1.02E+08	Hs.523816	NR_120586	ENSG0000	LOC101928	-	uncharacte	ncRNA
lib-C666-l	chr5	94982519	94982519	+	20.90919	NA	promoter-	promoter-	38	NM_00113	317671	Hs.399758	NM_17336	ENSG0000	RFESD	-	Rieske Fe-	protein-co
lib-C666-l	chr17	17875893	17875893	+	20.87959	NA	promoter-	promoter-	-109	NM_00128	146691	Hs.462379	NM_14467	ENSG0000	TOM1L2	-	target of m	protein-co
lib-C666-l	chr19	45458268	45458268	+	20.84232	NA	promoter-	promoter-	18	NM_00128	1209	Hs.444441	NM_00129	ENSG0000	CLPTM1	-	CLPTM1, tr	protein-co
lib-C666-l	chr7	20369149	20369149	+	20.82108	NA	intron (NR	intron (NR	-1576	NM_00221	3696	Hs.592171	NM_00221	ENSG0000	ITGB8	-	integrin su	protein-co
lib-C666-l	chr7	1E+08	1E+08	+	20.82108	NA	promoter-	promoter-	0	NM_00527	2783	Hs.185172	NM_00527	ENSG0000	GNB2	-	G protein s	protein-co
lib-C666-l	chr8	80680302	80680302	+	20.7902	NA	promoter-	promoter-	-75	NR_125410	1.02E+08	Hs.144030	NR_125410	ENSG0000	LINC01607	-	long interg	ncRNA
lib-C666-l	chr1	40997227	40997227	+	20.72609	NA	promoter-	promoter-	-6	NM_15237	127396	Hs.524767	NM_15237	ENSG0000	ZNF684	-	zinc finger	protein-co
lib-C666-l	chr16	2517753	2517753	+	20.3278	NA	non-codin	non-codin	461	NR_135198	729652	Hs.638553	NR_135198	ENSG0000	LOC729652	-	uncharacte	ncRNA
lib-C666-l	chr11	1.08E+08	1.08E+08	+	20.19666	NA	promoter-	promoter-	-1	NM_00133	160140	Hs.653180	NM_15258	ENSG0000	C11orf65	-	chromosor	protein-co
lib-C666-l	chr14	23398869	23398869	+	20.19521	NA	promoter-	promoter-	51	NR_110002	1.02E+08	Hs.727321	NR_110002	ENSG0000	LOC101926	-	uncharacte	ncRNA
lib-C666-l	chr19	1249538	1249538	+	20.0808	NA	intron (NM	CpG	986	NM_17740	90007	Hs.465529	NM_17740	ENSG0000	MIDN	-	midnolin	protein-co
lib-C666-l	chr1	2.35E+08	2.35E+08	+	20.03192	NA	Intergenic	CpG	-1470	NM_18297	359948	Hs.350268	NM_18297	ENSG0000	IRF2BP2	-	interferon	protein-co
lib-C666-l	chr1	41157949	41157949	+	25.39084	NA	promoter-	promoter-	-16	NR_024567	1E+08	Hs.233458	NR_024567		NFYC-AS1	0808y08y	NFYC antis	ncRNA
lib-C666-l	chr7	1.06E+08	1.06E+08	+	29.98795	NA	promoter-	promoter-	80	NM_00574	10135	Hs.489615	NM_00574	ENSG0000	NAMPT	1110035O	nicotinami	protein-co
lib-C666-l	chr2	9771370	9771370	+	132.4598	NA	promoter-	promoter-	-186	NM_00682	10971	Hs.74405	NM_00682	ENSG0000	YWHAQ	14-3-3|1C	tyrosine 3-	protein-co
lib-C666-l	chr7	75988338	75988338	+	20.84232	NA	promoter-	promoter-	4	NM_01247	7532	Hs.744840	NM_01247	ENSG0000	YWHAG	14-3-3GAI	tyrosine 3-	protein-co
lib-C666-l	chr8	1.02E+08	1.02E+08	+	72.53808	NA	promoter-	promoter-	8	NM_14569	7534	Hs.492407	NM_00340	ENSG0000	YWHAZ	14-3-3-ze	tyrosine 3-	protein-co
lib-C666-l	chr22	42084966	42084966	+	22.13345	NA	promoter-	promoter-	-53	NM_00500	4809	Hs.182255	NM_00500	ENSG0000	SNU13	15.5K|FA-1	SNU13 hor	protein-co
lib-C666-l	chr12	1.21E+08	1.21E+08	+	303.5389	NA	promoter-	promoter-	-6	NM_17681	283459	Hs.706889	NM_17681	ENSG0000	GATC	15E1.2	glutamyl-t	protein-co
lib-C666-l	chr17	41322470	41322470	+	127.2685	NA	promoter-	promoter-	-18	NM_03186	4077	Hs.277721	NM_00589	ENSG0000	NBR1	1A1-3B|IAI	NBR1, auto	protein-co
lib-C666-l	chr2	30670089	30670089	+	72.05244	NA	promoter-	promoter-	-3	NM_00130	253558	Hs.468048	NM_18255	ENSG0000	LCLAT1	1AGPAT8|/	lysocardiol	protein-co
lib-C666-l	chr16	81041079	81041079	+	73.12756	NA	promoter-	promoter-	-577	NM_02018	56942	Hs.55028	NM_02018	ENSG0000	CMC2	2310061C1	C-X9-C mo	protein-co
lib-C666-l	chr15	73076122	73076122	+	484.9511	NA	promoter-	promoter-	4	NR_023318	83440	Hs.562587	NM_03128	ENSG0000	ADPGK	2610017G	ADP deper	protein-co
lib-C666-l	chr2	1.78E+08	1.78E+08	+	128.5813	NA	promoter-	promoter-	-135	NM_00133	220988	Hs.516539	NM_19424	ENSG0000	HNRNPA3	2610510D	heterogen	protein-co
lib-C666-l	chr1	59165806	59165806	+	25.39084	NA	promoter-	promoter-	-59	NM_00108	114803	Hs.744921	NM_00108	ENSG0000	MYSM1	2A-DUB|2/	Myb like, S	protein-co
lib-C666-l	chr4	1.13E+08	1.13E+08	+	43.79478	NA	promoter-	promoter-	-128	NM_01856	55435	Hs.435991	NM_01856	ENSG0000	AP1AR	2C18|C4or	adaptor re	protein-co
lib-C666-l	chr19	47987455	47987455	+	365.0136	NA	promoter-	promoter-	66	NR_111923	11133	Hs.25441	NM_00705	ENSG0000	KPTN	2E4|MRT41	kaptin, acti	protein-co
lib-C666-l	chr12	9102585	9102585	+	27.5466	NA	promoter-	promoter-	-55	NR_137427	10219	Hs.558446	NM_00581	ENSG0000	KLRG1	2F1|CLEC1	killer cell le	protein-co
lib-C666-l	chr4	2819849	2819849	+	41.21722	NA	promoter-	promoter-	-63	NM_00114	6452	Hs.167679	NM_00302	ENSG0000	SH3BP2	3BP-2|3BP	SH3 doma	protein-co
lib-C666-l	chr1	29213465	29213465	+	55.79842	NA	promoter-	promoter-	-138	NM_00116	2035	Hs.175437	NM_00443	ENSG0000	EPB41	4.1R|EL1|H	erythrocyte	protein-co
lib-C666-l	chr12	69754024	69754024	+	26.49922	NA	intron (NM	CpG	534	NM_00130	8089	Hs.4029	NM_00653	ENSG0000	YEATS4	4930573H	YEATS don	protein-co
lib-C666-l	chr10	18940550	18940550	+	28.64047	NA	promoter-	promoter-	16	NM_18254	221078	Hs.396175	NM_18254	ENSG0000	NSUN6	4933414E0	NOP2/Sun	protein-co
lib-C666-l	chr2	1.36E+08	1.36E+08	+	20.19521	NA	promoter-	promoter-	66	NM_00128	84083	Hs.658422	NM_03214	ENSG0000	ZRANB3	4933425L1	zinc finger	protein-co
lib-C666-l	chr10	72163847	72163847	+	26.54591	NA	promoter-	promoter-	-14	NM_00409	1979	Hs.621200	NM_00409	ENSG0000	EIF4EBP2	4EBP2|PHA	eukaryotic	protein-co
lib-C666-l	chr15	43785374	43785374	+	63.5671	NA	promoter-	promoter-	-20	NM_00114	7158	Hs.440968	NM_00565	ENSG0000	TP53BP1	53BP1|TDR	tumor prot	protein-co
lib-C666-l	chr16	12009955	12009955	+	109.0026	NA	promoter-	promoter-	-130	NM_00113	2935	Hs.528780	NM_00209	ENSG0000	GSPT1	551G9.2|E	G1 to S ph	protein-co
lib-C666-l	chr1	1.72E+08	1.72E+08	+	32.04651	NA	promoter-	promoter-	-112	NM_01495	51603	Hs.494705	NM_01495	ENSG0000	METTL13	5630401D	methyltran	protein-co

lib-C666-l	chr17	79670397	79670397	+	25.39487	NA	promoter-	promoter-	-3	NM_00294	6182	Hs.109059	NM_00294	ENSG0000	MRPL12	5c5-2|L12r	mitochond	protein-co
lib-C666-l	chr19	572509	572509	+	20.72609	NA	promoter-	promoter-	55	NM_19858	682	Hs.501293	NM_00172	ENSG0000	BSG	5F7|CD147	basigin (Ol	protein-co
lib-C666-l	chr10	1.34E+08	1.34E+08	+	48.61377	NA	promoter-	promoter-	-421	NM_00553	3632	Hs.523360	NM_00553	ENSG0000	INPP5A	5PTASE	inositol po	protein-co
lib-C666-l	chr1	1.53E+08	1.53E+08	+	92.73503	NA	promoter-	promoter-	-124	NM_00131	6279	Hs.416073	NM_00296	ENSG0000	S100A8	60B8AG|C/	S100 calciu	protein-co
lib-C666-l	chr6	31430854	31430854	+	27.84631	NA	promoter-	promoter-	-103	NR_04066	10866	Hs.691948	NR_04066	ENSG0000	HCP5	6S2650E|D	HLA comp	ncRNA
lib-C666-l	chrY	1660546	1660546	+	52.45363	NA	promoter-	promoter-	60	NR_02738	8227	Hs.522572	NM_00508	ENSG0000	AKAP17A	721P|AKAF	A-kinase a	protein-co
lib-C666-l	chr19	8373050	8373050	+	36.56565	NA	TTS (NR_1	TTS (NR_1	190	NM_01657	51293	Hs.558499	NM_01657	ENSG0000	CD320	8D6|8D6A|	CD320 mo	protein-co
lib-C666-l	chr4	39367962	39367962	+	115.2282	NA	promoter-	promoter-	39	NM_00120	5981	Hs.507475	NM_00291	ENSG0000	RFC1	A1|MHCBF	replication	protein-co
lib-C666-l	chr3	1.87E+08	1.87E+08	+	43.18268	NA	promoter-	promoter-	-56	NM_00291	5984	Hs.732098	NM_00291	ENSG0000	RFC4	A1|RFC37	replication	protein-co
lib-C666-l	chr17	39845623	39845623	+	153.3902	NA	intron (NM	CpG	496	NM_00580	10209	Hs.150580	NM_00580	ENSG0000	EIF1	A121|EIF-1	eukaryotic	protein-co
lib-C666-l	chr2	95831457	95831457	+	74.77618	NA	5' UTR (NN	5' UTR (NM	295	NM_00101	7549	Hs.590916	NM_02108	ENSG0000	ZNF2	A1-5|ZNF6	zinc finger	protein-co
lib-C666-l	chr5	1.79E+08	1.79E+08	+	22.66803	NA	intron (NM	intron (NM	-1735	NM_00390	8878	Hs.587290	NM_00390	ENSG0000	SQSTM1	A170|DMR	sequestos	protein-co
lib-C666-l	chr16	20911841	20911841	+	31.98049	NA	promoter-	promoter-	-147	NM_00112	57149	Hs.745012	NM_02042	ENSG0000	LYRM1	A211C6.1	LYR motif	protein-co
lib-C666-l	chr16	28834004	28834004	+	32.29191	NA	promoter-	promoter-	-365	NM_14841	11273	Hs.460499	NM_00724	ENSG0000	ATXN2L	A2D|A2LG|	ataxin 2 lik	protein-co
lib-C666-l	chr2	2.04E+08	2.04E+08	+	79.14428	NA	promoter-	promoter-	-294	NM_00111	65065	Hs.648846	NM_19894	ENSG0000	NBEAL1	A530083I0	neurobeac	protein-co
lib-C666-l	chr12	44200158	44200158	+	91.03855	NA	promoter-	promoter-	20	NM_00282	5756	Hs.189075	NM_00282	ENSG0000	TWF1	A6|PTK9	twinfilin ac	protein-co
lib-C666-l	chr7	99679513	99679513	+	22.02307	NA	promoter-	promoter-	-126	NM_00127	7551	Hs.435302	NM_01771	ENSG0000	ZNF3	A8-51|HF.1	zinc finger	protein-co
lib-C666-l	chr18	19180954	19180954	+	122.4882	NA	promoter-	promoter-	-261	NM_05291	114799	Hs.464733	NM_05291	ENSG0000	ESCO1	A930014I1	establishm	protein-co
lib-C666-l	chr11	12695883	12695883	+	117.5857	NA	promoter-	promoter-	-86	NM_02196	7003	Hs.655331	NM_02196	ENSG0000	TEAD1	AA|NTEF-1	TEA domai	protein-co
lib-C666-l	chr1	1407068	1407068	+	147.861	NA	promoter-	promoter-	-67	NM_03192	83858	Hs.729021	NM_03192	ENSG0000	ATAD3B	AAA-TOB3	ATPase far	protein-co
lib-C666-l	chr22	41253004	41253004	+	161.5162	NA	promoter-	promoter-	8	NM_00127	6767	Hs.712713	NM_00393	ENSG0000	ST13	AAG2|FAM	suppressio	protein-co
lib-C666-l	chr7	1.52E+08	1.52E+08	+	48.6123	NA	promoter-	promoter-	-134	NM_01620	51422	Hs.647072	NM_01620	ENSG0000	PRKAG2	AAKG|AAK	protein kin	protein-co
lib-C666-l	chr7	97736190	97736190	+	99.32263	NA	promoter-	promoter-	-7	NM_01491	22853	Hs.444179	NM_01491	ENSG0000	LMTK2	AATYK2|BR	lemur tyro	protein-co
lib-C666-l	chr1	2.28E+08	2.28E+08	+	284.3481	NA	promoter-	promoter-	-3	NM_18146	128308	Hs.63236	NM_18144	ENSG0000	MRPL55	AAVG5835	mitochond	protein-co
lib-C666-l	chr8	1.01E+08	1.01E+08	+	96.33944	NA	promoter-	promoter-	2	NM_00503	5440	Hs.351475	NM_00503	ENSG0000	POLR2K	ABC10-alp	RNA polyn	protein-co
lib-C666-l	chr6	32821825	32821825	+	41.43555	NA	promoter-	promoter-	-77	NM_00059	6890	Hs.352018	NM_00059	ENSG0000	TAP1	ABC17|ABC	transporte	protein-co
lib-C666-l	chr9	1.4E+08	1.4E+08	+	20.7902	NA	promoter-	promoter-	-16	NM_21253	20	Hs.421202	NM_00160	ENSG0000	ABCA2	ABC2	ATP bindin	protein-co
lib-C666-l	chr7	1.51E+08	1.51E+08	+	226.3797	NA	promoter-	promoter-	21	NM_00569	10061	Hs.654958	NM_00569	ENSG0000	ABCF2	ABC28|EST	ATP bindin	protein-co
lib-C666-l	chr7	40174398	40174398	+	22.01132	NA	promoter-	promoter-	-147	NM_13870	136647	Hs.654989	NM_13870	ENSG0000	MPLKIP	ABHS|C7or	M-phase s	protein-co
lib-C666-l	chr1	32110297	32110297	+	437.7954	NA	promoter-	promoter-	-79	NR_03368	553115	Hs.470417	NM_01239	ENSG0000	PEF1	ABP32|PEF	penta-EF-l	protein-co
lib-C666-l	chr1	32110650	32110650	+	198.453	NA	promoter-	promoter-	188	NM_01239	553115	Hs.470417	NM_01239	ENSG0000	PEF1	ABP32|PEF	penta-EF-l	protein-co
lib-C666-l	chr4	1.66E+08	1.66E+08	+	31.98049	NA	promoter-	promoter-	-67	NM_00724	11275	Hs.388668	NM_00724	ENSG0000	KLHL2	ABP-KELCI	kelch like f	protein-co
lib-C666-l	chr12	1.21E+08	1.21E+08	+	117.2161	NA	5' UTR (NN	5' UTR (NM	142	NM_00001	35	Hs.507076	NM_00001	ENSG0000	ACADS	ACAD3|SC	acyl-CoA c	protein-co
lib-C666-l	chr1	27719107	27719107	+	44.27569	NA	promoter-	promoter-	-41	NM_00528	2827	Hs.66542	NM_00528	ENSG0000	GPR3	ACCA	G protein-	protein-co
lib-C666-l	chr1	2.03E+08	2.03E+08	+	32.53922	NA	promoter-	promoter-	88	NM_01599	51094	Hs.5298	NM_01599	ENSG0000	ADIPOR1	ACDCR1|C	adiponecti	protein-co
lib-C666-l	chr12	1800118	1800118	+	111.7598	NA	promoter-	promoter-	-129	NM_02455	79602	Hs.371642	NM_02455	ENSG0000	ADIPOR2	ACDCR2|P	adiponecti	protein-co
lib-C666-l	chr7	1.57E+08	1.57E+08	+	128.1221	NA	promoter-	promoter-	-155	NM_02245	64327	Hs.209989	NM_02245	ENSG0000	LMBR1	ACHP|C7or	limb devel	protein-co
lib-C666-l	chr19	50354356	50354356	+	43.7029	NA	promoter-	promoter-	-21	NM_00130	53635	Hs.587979	NM_01743	ENSG0000	PTOV1	ACID2|PTC	prostate tu	protein-co
lib-C666-l	chr19	50354015	50354015	+	24.23745	NA	promoter-	promoter-	23	NM_00130	53635	Hs.587979	NM_01743	ENSG0000	PTOV1	ACID2|PTC	prostate tu	protein-co
lib-C666-l	chr22	41865098	41865098	+	119.4653	NA	promoter-	promoter-	-31	NM_00109	50	Hs.643610	NM_00109	ENSG0000	ACO2	ACONM|H	aconitase 2	protein-co
lib-C666-l	chr7	6388618	6388618	+	44.98312	NA	promoter-	promoter-	-28	NM_00103	84792	Hs.696146	NM_00103	ENSG0000	FAM220A	ACPIN1|C7	family with	protein-co
lib-C666-l	chr13	76112229	76112229	+	70.69921	NA	promoter-	promoter-	-221	NM_00128	170622	Hs.508266	NM_20349	ENSG0000	COMMD6	Acrg	COMM do	protein-co
lib-C666-l	chr4	57253676	57253676	+	361.7216	NA	promoter-	promoter-	-2	NM_00128	132949	Hs.104347	NM_18180	ENSG0000	AASDH	ACSF4|LYS	aminoadip	protein-co
lib-C666-l	chr17	79481845	79481845	+	37.42856	NA	Intergenic	CpG-1134	-1953	NM_00161	71	Hs.514581	NM_00161	ENSG0000	ACTG1	ACT|ACTG	actin gamr	protein-co
lib-C666-l	chr3	1.79E+08	1.79E+08	+	26.72399	NA	promoter-	promoter-	-30	NM_00430	86	Hs.435326	NM_00430	ENSG0000	ACTL6A	ACTL6|ARF	actin like 6	protein-co
lib-C666-l	chr9	1.32E+08	1.32E+08	+	36.16835	NA	promoter-	promoter-	31	NM_00128	28989	Hs.744027	NM_01406	ENSG0000	NTMT1	AD-003|C9	N-termina	protein-co
lib-C666-l	chr16	58664438	58664438	+	25.49921	NA	promoter-	promoter-	-648	NM_01628	23019	Hs.460923	NM_01628	ENSG0000	CNOT1	AD-005|CD	CCR4-NOT	protein-co
lib-C666-l	chr15	1.02E+08	1.02E+08	+	30.07415	NA	promoter-	promoter-	-21	NM_20347	55829	Hs.32148	NM_01844	ENSG0000	SELENOS	AD-015|AI	selenoprot	protein-co
lib-C666-l	chr13	45563648	45563648	+	153.9781	NA	promoter-	promoter-	-17	NM_00131	55425	Hs.731811	NM_01855	ENSG0000	GPALPP1	AD029|KIA	GPALPP m	protein-co
lib-C666-l	chr14	73603166	73603166	+	39.51209	NA	promoter-	promoter-	23	NM_00002	5663	Hs.3260	NM_00002	ENSG0000	PSEN1	AD3|FAD|P	presenilin 1	protein-co
lib-C666-l	chr2	1.09E+08	1.09E+08	+	312.781	NA	promoter-	promoter-	-27	NM_00626	5903	Hs.199561	NM_00626	ENSG0000	RANBP2	ADANE|AN	RAN bindi	protein-co
lib-C666-l	chr1	1.55E+08	1.55E+08	+	41.28836	NA	promoter-	promoter-	-77	NM_00102	103	Hs.12341	NM_00111	ENSG0000	ADAR	ADAR1|AG	adenosine	protein-co
lib-C666-l	chr19	41222801	41222801	+	28.88523	NA	promoter-	promoter-	-11	NM_02487	79934	Hs.130712	NM_02487	ENSG0000	COQ8B	ADCK4|NP	coenzyme	protein-co
lib-C666-l	chr4	95128792	95128792	+	56.28537	NA	promoter-	promoter-	33	NM_00112	56916	Hs.410406	NM_02015	ENSG0000	SMARCAD	ADERM|ET	SWI/SNF-r	protein-co
lib-C666-l	chr17	40540810	40540810	+	46.4068	NA	promoter-	promoter-	-297	NM_00315	6774	Hs.463059	NM_00315	ENSG0000	STAT3	ADMIO|AD	signal tran	protein-co
lib-C666-l	chr20	49548068	49548068	+	43.6853	NA	promoter-	promoter-	-318	NM_18144	23394	Hs.293736	NM_01533	ENSG0000	ADNP	ADNP1|HV	activity de	protein-co
lib-C666-l	chr6	1.11E+08	1.11E+08	+	30.98434	NA	promoter-	promoter-	-79	NR_10976	262	Hs.159118	NM_00163	ENSG0000	AMD1	ADOMETD	adenosylm	protein-co
lib-C666-l	chr22	44320005	44320005	+	21.51366	NA	intron (NM	CpG	386	NM_02522	80339	Hs.654800	NM_02522	ENSG0000	PNPLA3	ADPN|C22	patatin like	protein-co
lib-C666-l	chr14	20811773	20811773	+	80.31864	NA	promoter-	promoter-	0	NM_00548	10038	Hs.409412	NM_00548	ENSG0000	PARP2	ADPRT2|AI	poly(ADP-	protein-co
lib-C666-l	chr19	50270002	50270002	+	21.90047	NA	promoter-	promoter-	-178	NM_01420	160	Hs.467125	NM_01420	ENSG0000	AP2A1	ADTAA|AP	adaptor re	protein-co
lib-C666-l	chr17	33914283	33914283	+	373.6706	NA	promoter-	promoter-	1	NM_00128	163	Hs.514819	NM_00128	ENSG0000	AP2B1	ADTB2|AP	adaptor re	protein-co
lib-C666-l	chr16	71842901	71842901	+	149.53	NA	promoter-	promoter-	75	NM_00103	164	Hs.461253	NM_00112	ENSG0000	AP1G1	ADTG|CLA	adaptor re	protein-co
lib-C666-l	chr17	72869484	72869484	+	174.9811	NA	promoter-	promoter-	-328	NM_00411	2232	Hs.69745	NM_00411	ENSG0000	FDXR	ADXR	ferredoxin	protein-co
lib-C666-l	chr7	1.51E+08	1.51E+08	+	143.6353	NA	promoter-	promoter-	-27	NM_00119	6522	Hs.647069	NM_00304	ENSG0000	SLC4A2	AE2|BND3I	solute carr	protein-co
lib-C666-l	chr6	1.09E+08	1.09E+08	+	63.78558	NA	Intergenic	CpG	-1234	NM_20155	2309	Hs.220950	NM_00145	ENSG0000	FOXO3	AF6q21|FK	forkhead b	protein-co
lib-C666-l	chr9	99179711	99179711	+	65.27523	NA	intron (NM	CpG	958	NM_15369	195828	Hs.494557	NM_15369	ENSG0000	ZNF367	AFF29|CDC	zinc finger	protein-co
lib-C666-l	chr6	30181267	30181267	+	23.74671	NA	promoter-	promoter-	4	NM_00344	7726	Hs.485041	NM_00344	ENSG0000	TRIM26	AFP|RNF95	tripartite m	protein-co
lib-C666-l	chr19	11546396	11546396	+	22.23727	NA	promoter-	promoter-	-70	NM_00274	5589	Hs.610830	NM_00274	ENSG0000	PRKCSH	AGE-R2|G1	protein kin	protein-co
lib-C666-l	chr7	1.4E+08	1.4E+08	+	55.3054	NA	promoter-	promoter-	10	NM_00454	4708	Hs.655788	NM_00454	ENSG0000	NDUFB2	AGGG|CI-/	NADH:ubic	protein-co
lib-C666-l	chr9	1.32E+08	1.32E+08	+	84.30532	NA	promoter-	promoter-	-18	NM_01959	56262	Hs.643600	NM_01959	ENSG0000	LRRC8A	AGM5|LRR	leucine ricl	protein-co
lib-C666-l	chr19	1652845	1652845	+	204.2047	NA	promoter-	promoter-	-517	NM_00320	6929	Hs.371282	NM_00320	ENSG0000	TCF3	AGM8|E2A	transcripti	protein-co
lib-C666-l	chr19	10342299	10342299	+	29.83758	NA	promoter-	promoter-	-351	NM_00423	9294	Hs.655405	NM_00423	ENSG0000	S1PR2	AGR16|DFI	sphingosin	protein-co
lib-C666-l	chr13	51483951	51483951	+	82.44033	NA	5' UTR (NN	5' UTR (NM	137	NM_00114	79621	Hs.306291	NM_02457	ENSG0000	RNASEH2E	AGS2|DLEU	ribonuclea	protein-co
lib-C666-l	chr6	32163704	32163704	+	67.6042	NA	promoter-	promoter-	-404	NM_02210	63940	Hs.520046	NM_02210	ENSG0000	GPSM3	AGS4|C6or	G-protein	protein-co
lib-C666-l	chr6	1.36E+08	1.36E+08	+	50.46963	NA	promoter-	promoter-	-7	NM_00113	54806	Hs.386684	NM_01765	ENSG0000	AHI1	AHI-1|JBTS	Abelson he	protein-co
lib-C666-l	chr11	18343756	18343756	+	72.31497	NA	promoter-	promoter-	-35	NM_00721	11234	Hs.437599	NM_00721	ENSG0000	HPS5	AIBP63|BL(	HPS5, biog	protein-co
lib-C666-l	chr6	1.43E+08	1.43E+08	+	178.4308	NA	promoter-	promoter-	-254	NM_00128	51390	Hs.567501	NM_01610	ENSG0000	AIG1	AIG-1|dJ95	androgen	protein-co
lib-C666-l	chr20	43150747	43150747	+	36.56424	NA	promoter-	promoter-	10	NM_00681	10955	Hs.272168	NM_00681	ENSG0000	SERINC3	AIGP1|DIFF	serine inco	protein-co
lib-C666-l	chr16	31470705	31470705	+	46.59515	NA	5' UTR (NN	5' UTR (NM	388	NM_00110	79798	Hs.732945	NM_02474	ENSG0000	ARMC5	AIMAH2	armadillo r	protein-co
lib-C666-l	chr1	1310900	1310900	+	40.09574	NA	promoter-	promoter-	-82	NM_01790	54998	Hs.632515	NM_01790	ENSG0000	AURKAIP1	AIP|AKIP|M	aurora kina	protein-co
lib-C666-l	chr4	10118725	10118725	+	22.15977	NA	promoter-	promoter-	-152	NM_00511	9948	Hs.128548	NM_00511	ENSG0000	WDR1	AIP1|HEL-S	WD repeat	protein-co
lib-C666-l	chr5	14664825	14664825	+	22.23727	NA	promoter-	promoter-	71	NM_13834	90268	Hs.406335	NM_13834	ENSG0000	OTULIN	AIPDS|FAN	OTU deubi	protein-co
lib-C666-l	chr7	1199899	1199899	+	388.5561	NA	promoter-	promoter-	-44	NM_18249	90637	Hs.648111	NM_18249	ENSG0000	ZFAND2A	AIRAP	zinc finger	protein-co
lib-C666-l	chr2	2.2E+08	2.2E+08	+	43.79478	NA	promoter-	promoter-	-17	NM_13880	130617	Hs.534540	NM_13880	ENSG0000	ZFAND2B	AIRAPL	zinc finger	protein-co
lib-C666-l	chr19	15490652	15490652	+	36.97678	NA	promoter-	promoter-	-40	NM_00585	10270	Hs.594496	NM_00585	ENSG0000	AKAP8	AKAP 95|A	A-kinase a	protein-co
lib-C666-l	chr7	44646130	44646130	+	199.0501	NA	promoter-	promoter-	9	NM_00100	4967	Hs.488181	NM_00254	ENSG0000	OGDH	AKGDH|E1	oxoglutara	protein-co
lib-C666-l	chr1	1.47E+08	1.47E+08	+	53.02666	NA	intron (NM	CpG	301	NM_00125	9557	Hs.191164	NM_00428	ENSG0000	CHD1L	ALC1|CHD	chromodo	protein-co
lib-C666-l	chr16	30076923	30076923	+	22.85132	NA	promoter-	promoter-	-71	NM_00124	226	Hs.513490	NM_00003	ENSG0000	ALDOA	ALDA|GSD	aldolase, fr	protein-co
lib-C666-l	chr6	32812529	32812529	+	58.76075	NA	promoter-	promoter-	183	NM_00415	5696	Hs.180062	NM_00415	ENSG0000	PSMB8	ALDD|D6S	proteasom	protein-co
lib-C666-l	chr11	1.18E+08	1.18E+08	+	20.59244	NA	promoter-	promoter-	-192	NM_00593	4297	Hs.258855	NM_00593	ENSG0000	KMT2A	ALL-1|CXX	lysine metl	protein-co
lib-C666-l	chr11	34127105	34127105	+	119.4653	NA	promoter-	promoter-	-6	NM_02466	55226	Hs.577281	NM_02466	ENSG0000	NAT10	ALP|NET43	N-acetyltr	protein-co
lib-C666-l	chr1	1.15E+08	1.15E+08	+	116.1663	NA	TTS (NM_0	TTS (NM_0	105	NM_00252	4893	Hs.486502	NM_00252	ENSG0000	NRAS	ALPS4|CMI	neuroblast	protein-co
lib-C666-l	chr9	35072782	35072782	+	93.47076	NA	promoter-	promoter-	-43	NM_00712	7415	Hs.529782	NM_00712	ENSG0000	VCP	ALS14|CM	valosin cor	protein-co
lib-C666-l	chr12	54674373	54674373	+	21.84731	NA	promoter-	promoter-	-101	NM_03115	3178	Hs.546261	NM_00213	ENSG0000	HNRNPA1	ALS19|ALS	heterogen	protein-co
lib-C666-l	chr2	2.02E+08	2.02E+08	+	20.3278	NA	promoter-	promoter-	-1	NM_00113	60491	Hs.145284	NM_02182	ENSG0000	NIF3L1	ALS2CR1|C	NGG1 inte	protein-co
lib-C666-l	chr2	2.02E+08	2.02E+08	+	94.65162	NA	promoter-	promoter-	25	NM_01504	66008	Hs.152774	NM_01504	ENSG0000	TRAK2	ALS2CR3|C	trafficking	protein-co
lib-C666-l	chr2	2.03E+08	2.03E+08	+	81.64329	NA	5' UTR (NN	5' UTR (NM	212	NM_15238	65062	Hs.12319	NM_15238	ENSG0000	TMEM237	ALS2CR4|J	transmemt	protein-co
lib-C666-l	chr2	2.03E+08	2.03E+08	+	95.09922	NA	promoter-	promoter-	-109	NM_02091	57679	Hs.471096	NM_02091	ENSG0000	ALS2	ALS2CR6|A	ALS2, alsin	protein-co
lib-C666-l	chr2	2.03E+08	2.03E+08	+	21.23871	NA	intron (NM	CpG	376	NM_00113	57679	Hs.471096	NM_02091	ENSG0000	ALS2	ALS2CR6|A	ALS2, alsin	protein-co
lib-C666-l	chr9	1.35E+08	1.35E+08	+	47.23204	NA	promoter-	promoter-	-172	NM_01504	23064	Hs.460317	NM_01504	ENSG0000	SETX	ALS4|AOA2	senataxin	protein-co
lib-C666-l	chr9	27573835	27573835	+	137.9195	NA	promoter-	promoter-	29	NM_00125	203228	Hs.493639	NM_01832	ENSG0000	C9orf72	ALSFTD|DE	chromosor	protein-co
lib-C666-l	chr8	1.31E+08	1.31E+08	+	42.49125	NA	intron (NM	CpG	323	NM_01848	50807	Hs.655552	NM_01848	ENSG0000	ASAP1	AMAP1|CE	ArfGAP wit	protein-co
lib-C666-l	chr17	7218682	7218682	+	58.41369	NA	promoter-	promoter-	-24	NM_00448	2874	Hs.438219	NM_00448	ENSG0000	GPS2	AMF-1	G protein p	protein-co
lib-C666-l	chr12	49412595	49412595	+	29.41683	NA	promoter-	promoter-	34	NM_00120	5571	Hs.530862	NM_00273	ENSG0000	PRKAG1	AMPKG	protein kin	protein-co
lib-C666-l	chr13	60738153	60738153	+	51.02929	NA	promoter-	promoter-	-34	NM_00125	81624	Hs.283127	NM_03093	ENSG0000	DIAPH3	AN|AUNA1	diaphanou	protein-co
lib-C666-l	chr8	1.24E+08	1.24E+08	+	20.92054	NA	promoter-	promoter-	-80	NM_01410	29028	Hs.370834	NM_01410	ENSG0000	ATAD2	ANCCA|CT	ATPase far	protein-co
lib-C666-l	chr16	89557145	89557145	+	38.60092	NA	promoter-	promoter-	-176	NM_01327	29123	Hs.335003	NM_01327	ENSG0000	ANKRD11	ANCO-1|A	ankyrin rep	protein-co
lib-C666-l	chr3	1.57E+08	1.57E+08	+	137.9195	NA	5' UTR (NN	5' UTR (NM	529	NM_02030	57018	Hs.4859	NM_02030	ENSG0000	CCNL1	ANIA6A|BM	cyclin L1	protein-co
lib-C666-l	chr11	72145662	72145662	+	138.2052	NA	promoter-	promoter-	66	NM_00125	81570	Hs.523877	NM_03081	ENSG0000	CLPB	ANKCLB|H	ClpB homo	protein-co
lib-C666-l	chr20	18447787	18447787	+	81.67947	NA	promoter-	promoter-	42	NM_00109	55184	Hs.472225	NM_01815	ENSG0000	DZANK1	ANKRD64|	double zin	protein-co
lib-C666-l	chr6	34857034	34857034	+	41.21209	NA	promoter-	promoter-	-4	NM_01524	23294	Hs.656492	NM_01524	ENSG0000	ANKS1A	ANKS1	ankyrin rep	protein-co
lib-C666-l	chr19	50180111	50180111	+	39.42674	NA	promoter-	promoter-	-298	NM_00120	3276	Hs.20521	NM_00153	ENSG0000	PRMT1	ANM1|HCF	protein arg	protein-co
lib-C666-l	chr20	62612479	62612479	+	101.4314	NA	promoter-	promoter-	48	NM_01246	24148	Hs.31334	NM_01246	ENSG0000	PRPF6	ANT-1|AN	pre-mRNA	protein-co
lib-C666-l	chr10	46168306	46168306	+	26.82121	NA	promoter-	promoter-	-45	NM_00112	93550	Hs.89029	NM_17489	ENSG0000	ZFAND4	ANUBL1	zinc finger	protein-co
lib-C666-l	chr9	33001639	33001639	+	258.8831	NA	promoter-	promoter-	0	NM_00119	54840	Hs.20158	NM_01769	ENSG0000	APTX	AOA|AOA1	aprataxin	protein-co
lib-C666-l	chr9	97488926	97488926	+	132.2173	NA	promoter-	promoter-	-25	NM_03282	84909	Hs.434253	NM_03282	ENSG0000	C9orf3	AOPEP|AP	chromosor	protein-co
lib-C666-l	chr15	66993605	66993605	+	27.51092	NA	Intergenic	CpG	-1069	NR_02765	4091	Hs.153863	NM_00558	ENSG0000	SMAD6	AOVD2|Hs	SMAD fam	protein-co
lib-C666-l	chr19	18392605	18392605	+	55.1415	NA	promoter-	promoter-	-139	NM_00535	3727	Hs.2780	NM_00535	ENSG0000	JUND	AP-1	JunD proto	protein-co
lib-C666-l	chr19	12902187	12902187	+	27.69998	NA	promoter-	promoter-	-123	NM_00222	3726	Hs.25292	NM_00222	ENSG0000	JUNB	AP-1	JunB proto	protein-co
lib-C666-l	chr14	75745366	75745366	+	158.3171	NA	promoter-	promoter-	-115	NM_00525	2353	Hs.25647	NM_00525	ENSG0000	FOS	AP-1|C-FC	Fos proto-	protein-co
lib-C666-l	chr19	45971055	45971055	+	161.0245	NA	promoter-	promoter-	-198	NM_00673	2354	Hs.590958	NM_00673	ENSG0000	FOSB	AP-1|G0S3	FosB proto	protein-co
lib-C666-l	chr7	1.01E+08	1.01E+08	+	35.40551	NA	promoter-	promoter-	-67	NM_00128	1174	Hs.489365	NM_00128	ENSG0000	AP1S1	AP19|CLAF	adaptor re	protein-co
lib-C666-l	chr17	35969592	35969592	+	121.4374	NA	promoter-	promoter-	-106	NM_00116	11276	Hs.594647	NM_00724	ENSG0000	SYNRG	AP1GBP1|S	synergin g	protein-co
lib-C666-l	chr8	42010392	42010392	+	24.53984	NA	promoter-	promoter-	-72	NM_00680	10947	Hs.654529	NM_00680	ENSG0000	AP3M2	AP47B|CLA	adaptor re	protein-co
lib-C666-l	chr14	74353304	74353304	+	24.50625	NA	promoter-	promoter-	-14	NR_04025	57862	Hs.270869	NM_02118	ENSG0000	ZNF410	APA-1|APA	zinc finger	protein-co
lib-C666-l	chr1	15851236	15851236	+	40.12811	NA	promoter-	promoter-	49	NM_03299	842	Hs.329502	NM_00122	ENSG0000	CASP9	APAF-3|AF	caspase 9	protein-co

lib-C666-l	chr9	34329368	34329368	+	41.89289	NA	promoter-	promoter-	-136	NM_14717	318	Hs.493767	NM_00116	ENSG0000	NUDT2	APAH1	nudix hydr	protein-co
lib-C666-l	chr4	1.46E+08	1.46E+08	+	96.77992	NA	promoter-	promoter-	11	NM_00125	10393	Hs.480876	NM_01488	ENSG0000	ANAPC10	APC10|DO	anaphase	protein-co
lib-C666-l	chr17	79849533	79849533	+	51.11391	NA	promoter-	promoter-	-56	NM_00128	51529	Hs.534456	NM_01647	ENSG0000	ANAPC11	APC11|Apc	anaphase	protein-co
lib-C666-l	chr2	2.34E+08	2.34E+08	+	79.81274	NA	promoter-	promoter-	-11	NM_00119	55054	Hs.529322	NM_01797	ENSG0000	ATG16L1	APG16L|AT	autophagy	protein-co
lib-C666-l	chr5	1.32E+08	1.32E+08	+	51.89298	NA	promoter-	promoter-	-102	NM_00215	3308	Hs.90093	NM_00215	ENSG0000	HSPA4	APG-2|HEL	heat shock	protein-co
lib-C666-l	chr2	2.43E+08	2.43E+08	+	85.24104	NA	promoter-	promoter-	6	NM_17832	23192	Hs.283610	NM_01332	ENSG0000	ATG4B	APG4B|AU	autophagy	protein-co
lib-C666-l	chr6	1.07E+08	1.07E+08	+	92.67979	NA	promoter-	promoter-	12	NM_00128	9474	Hs.486063	NM_00484	ENSG0000	ATG5	APG5|APG	autophagy	protein-co
lib-C666-l	chr3	11314056	11314056	+	90.27715	NA	promoter-	promoter-	46	NM_00639	10533	Hs.38032	NM_00639	ENSG0000	ATG7	APG7-LIKE	autophagy	protein-co
lib-C666-l	chr12	10365522	10365522	+	64.54414	NA	promoter-	promoter-	33	NM_03141	23710	Hs.524250	NM_03141	ENSG0000	GABARAPL	APG8-LIKE	GABA type	protein-co
lib-C666-l	chr12	10365045	10365045	+	46.4068	NA	promoter-	promoter-	-444	NM_03141	23710	Hs.524250	NM_03141	ENSG0000	GABARAPL	APG8-LIKE	GABA type	protein-co
lib-C666-l	chr11	1.02E+08	1.02E+08	+	57.48363	NA	promoter-	promoter-	3	NM_00116	329	Hs.696238	NM_00116	ENSG0000	BIRC2	API1|HIAP2	baculoviral	protein-co
lib-C666-l	chr19	45418424	45418424	+	95.46208	NA	intron (NM	intron (NM	612	NM_00164	341	Hs.110675	NM_00164	ENSG0000	APOC1	Apo-CI|Ap	apolipopro	protein-co
lib-C666-l	chr10	1.12E+08	1.12E+08	+	34.02388	NA	promoter-	promoter-	144	NM_00132	7511	Hs.390623	NM_02038	ENSG0000	XPNPEP1	APP1|SAM	X-prolyl ar	protein-co
lib-C666-l	chr17	58603583	58603583	+	24.32656	NA	promoter-	promoter-	18	NM_00638	10513	Hs.84084	NM_00638	ENSG0000	APPBP2	APP-BP2|H	amyloid be	protein-co
lib-C666-l	chr18	57567001	57567001	+	26.26478	NA	promoter-	promoter-	-191	NM_02112	5366	Hs.96	NM_02112	ENSG0000	PMAIP1	APR|NOXA	phorbol-1:	protein-co
lib-C666-l	chr12	92539703	92539703	+	60.07874	NA	promoter-	promoter-	-30	NM_00173	694	Hs.255935	NM_00173	ENSG0000	BTG1	APRO2	BTG anti-p	protein-co
lib-C666-l	chr22	41843208	41843208	+	25.32183	NA	promoter-	promoter-	-181	NM_01627	10766	Hs.474978	NM_01627	ENSG0000	TOB2	APRO5|TO	transducer	protein-co
lib-C666-l	chr22	41844503	41844503	+	22.68206	NA	Intergenic	C-rich|Low	-1476	NM_01627	10766	Hs.474978	NM_01627	ENSG0000	TOB2	APRO5|TO	transducer	protein-co
lib-C666-l	chrX	40440075	40440075	+	28.21964	NA	promoter-	promoter-	-141	NM_00576	10159	Hs.495960	NM_00576	ENSG0000	ATP6AP2	APT6M8-9	ATPase H+	protein-co
lib-C666-l	chr4	75310808	75310808	+	30.542	NA	promoter-	promoter-	-7	NM_00165	374	Hs.270833	NM_00165	ENSG0000	AREG	AR|AREGB	amphiregu	protein-co
lib-C666-l	chr3	69101512	69101512	+	23.26437	NA	promoter-	promoter-	-28	NM_00711	7110	Hs.267632	NM_00711	ENSG0000	TMF1	ARA160|TM	TATA elem	protein-co
lib-C666-l	chr14	71067317	71067317	+	52.34435	NA	promoter-	promoter-	90	NM_00546	10001	Hs.497353	NM_00546	ENSG0000	MED6	ARC33|NY-	mediator c	protein-co
lib-C666-l	chr1	16482593	16482593	+	24.32656	NA	promoter-	promoter-	11	NM_00443	1969	Hs.171596	NM_00443	ENSG0000	EPHA2	ARCC2|CTI	EPH recept	protein-co
lib-C666-l	chr10	1.04E+08	1.04E+08	+	26.69347	NA	promoter-	promoter-	-39	NM_00419	8729	Hs.290243	NM_00419	ENSG0000	GBF1	ARF1GEF	golgi brefe	protein-co
lib-C666-l	chr3	57583215	57583215	+	108.2112	NA	promoter-	promoter-	0	NM_00166	378	Hs.652183	NM_00166	ENSG0000	ARF4	ARF2	ADP ribosy	protein-co
lib-C666-l	chr12	1.02E+08	1.02E+08	+	21.28624	NA	promoter-	promoter-	-166	NM_00130	400	Hs.372616	NM_00117	ENSG0000	ARL1	ARFL1	ADP ribosy	protein-co
lib-C666-l	chr1	8877821	8877821	+	26.39538	NA	promoter-	promoter-	-122	NM_01210	473	Hs.463041	NM_01210	ENSG0000	RERE	ARG|ARP|A	arginine-g	protein-co
lib-C666-l	chr17	57642943	57642943	+	206.5814	NA	promoter-	promoter-	57	NM_02461	79665	Hs.29403	NM_02461	ENSG0000	DHX40	ARG147|DI	DEAH-box	protein-co
lib-C666-l	chr1	36554570	36554570	+	38.83704	NA	TTS (NM_0	TTS (NM_0	117	NM_01782	54936	Hs.18021	NM_01782	ENSG0000	ADPRHL2	ARH3	ADP-ribos	protein-co
lib-C666-l	chr3	9291355	9291355	+	23.80446	NA	promoter-	promoter-	14	NM_00103	9901	Hs.654743	NM_01485	ENSG0000	SRGAP3	ARHGAP14	SLIT-ROBO	protein-co
lib-C666-l	chr3	48956267	48956267	+	58.30666	NA	promoter-	promoter-	14	NM_00131	10425	Hs.633601	NM_00632	ENSG0000	ARIH2	ARI2|TRIAD	ariadne RB	protein-co
lib-C666-l	chr8	32406015	32406015	+	25.42196	NA	5' UTR (NN	5' UTR (NN	287	NM_01395	3084	Hs.453951	NM_00449	ENSG0000	NRG1	ARIA|GGF|	neuregulin	protein-co
lib-C666-l	chr12	1.07E+08	1.07E+08	+	150.3604	NA	5' UTR (NN	5' UTR (NN	406	NM_01484	9891	Hs.524692	NM_01484	ENSG0000	NUAK1	ARK5	NUAK fam	protein-co
lib-C666-l	chr3	93698962	93698962	+	22.50982	NA	promoter-	promoter-	-21	NM_18289	200894	Hs.533086	NM_14499	ENSG0000	ARL13B	ARL2L1|JB1	ADP ribosy	protein-co
lib-C666-l	chr2	38604377	38604377	+	45.81715	NA	promoter-	promoter-	188	NM_00133	64225	Hs.594950	NM_02237	ENSG0000	ATL2	ARL3IP2|A	atlastin GT	protein-co
lib-C666-l	chr2	43823282	43823282	+	47.60628	NA	promoter-	promoter-	-97	NM_00134	63892	Hs.369592	NM_02206	ENSG0000	THADA	ARMC13|G	THADA, ar	protein-co
lib-C666-l	chr1	2.23E+08	2.23E+08	+	68.62961	NA	promoter-	promoter-	-17	NM_00132	375056	Hs.118474	NM_19855	ENSG0000	MIA3	ARNT|D320	MIA family	protein-co
lib-C666-l	chr15	52861281	52861281	+	25.80588	NA	promoter-	promoter-	48	NM_00662	10776	Hs.512908	NM_00662	ENSG0000	ARPP19	ARPP-16|A	cAMP regu	protein-co
lib-C666-l	chr1	1.51E+08	1.51E+08	+	89.57237	NA	promoter-	promoter-	-187	NM_20704	2029	Hs.632456	NM_00443	ENSG0000	ENSA	ARPP-19e	endosulfin	protein-co
lib-C666-l	chr1	1.51E+08	1.51E+08	+	76.14658	NA	promoter-	promoter-	12	NM_20704	2029	Hs.632456	NM_00443	ENSG0000	ENSA	ARPP-19e	endosulfin	protein-co
lib-C666-l	chrX	47518505	47518505	+	59.09724	NA	promoter-	promoter-	74	NM_00418	8409	Hs.172791	NM_00418	ENSG0000	UXT	ART-27|ST	ubiquitous	protein-co
lib-C666-l	chr7	1.4E+08	1.4E+08	+	47.52935	NA	non-codin	non-codin	653	NM_02275	64761	Hs.12646	NM_02275	ENSG0000	PARP12	ARTD12|M	poly(ADP-	protein-co
lib-C666-l	chr8	9413363	9413363	+	70.21218	NA	promoter-	promoter-	-82	NM_00374	8658	Hs.370267	NM_00374	ENSG0000	TNKS	ARTD5|PAI	tankyrase	protein-co
lib-C666-l	chr6	31325040	31325040	+	31.98049	NA	promoter-	promoter-	-18	NM_00551	3106	Hs.77961	NM_00551	ENSG0000	HLA-B	AS|B-4901	major histo	protein-co
lib-C666-l	chr7	65540685	65540685	+	236.6041	NA	promoter-	promoter-	-91	NM_00004	435	Hs.632015	NM_00004	ENSG0000	ASL	ASAL	argininosu	protein-co
lib-C666-l	chr2	2.39E+08	2.39E+08	+	24.28549	NA	promoter-	promoter-	-624	NM_00133	51665	Hs.516788	NM_01611	ENSG0000	ASB1	ASB-1	ankyrin rep	protein-co
lib-C666-l	chr6	1.01E+08	1.01E+08	+	34.77118	NA	promoter-	promoter-	17	NM_00128	10973	Hs.486031	NM_00682	ENSG0000	ASCC3	ASC1p200|	activating	protein-co
lib-C666-l	chr15	91537934	91537934	+	90.61259	NA	promoter-	promoter-	-53	NM_00398	9055	Hs.366401	NM_00398	ENSG0000	PRC1	ASE1	protein reg	protein-co
lib-C666-l	chr17	73401941	73401941	+	105.1162	NA	promoter-	promoter-	-151	NM_20350	2885	Hs.444356	NM_00208	ENSG0000	GRB2	ASH|EGFRI	growth fac	protein-co
lib-C666-l	chr18	55289065	55289065	+	35.0374	NA	5' UTR (NN	5' UTR (NN	112	NM_00453	4677	Hs.465224	NM_00453	ENSG0000	NARS	ASNRS|NA	asparaginy	protein-co
lib-C666-l	chr1	1.74E+08	1.74E+08	+	23.27622	NA	promoter-	promoter-	270	NM_01812	55157	Hs.647707	NM_01812	ENSG0000	DARS2	ASPRS|LBS	aspartyl-tR	protein-co
lib-C666-l	chr17	80231515	80231515	+	68.3168	NA	promoter-	promoter-	79	NM_00189	1453	Hs.631725	NM_00189	ENSG0000	CSNK1D	ASPS|CKId	casein kina	protein-co
lib-C666-l	chr10	1.01E+08	1.01E+08	+	28.68867	NA	promoter-	promoter-	-15	NM_00207	2805	Hs.500756	NM_00207	ENSG0000	GOT1	AST1|ASTQ	glutamic-o	protein-co
lib-C666-l	chr2	1.06E+08	1.06E+08	+	403.5553	NA	promoter-	promoter-	46	NM_02409	79074	Hs.549577	NM_02409	ENSG0000	C2orf49	asw	chromosor	protein-co
lib-C666-l	chr5	1.38E+08	1.38E+08	+	74.14931	NA	promoter-	promoter-	-164	NM_00196	1958	Hs.326035	NM_00196	ENSG0000	EGR1	AT225|G0S	early grow	protein-co
lib-C666-l	chr8	17658924	17658924	+	76.72826	NA	promoter-	promoter-	-498	NM_00100	57509	Hs.7946	NM_02074	ENSG0000	MTUS1	ATBP|ATIP	microtubul	protein-co
lib-C666-l	chr11	2466290	2466290	+	55.75688	NA	promoter-	promoter-	69	NM_00021	3784	Hs.95162	NM_00021	ENSG0000	KCNQ1	ATFB1|ATF	potassium	protein-co
lib-C666-l	chr5	37371189	37371189	+	107.4028	NA	promoter-	promoter-	39	NM_00127	9631	Hs.547696	NM_00429	ENSG0000	NUP155	ATFB15|N1	nucleopori	protein-co
lib-C666-l	chr11	72525397	72525397	+	26.46433	NA	promoter-	promoter-	-54	NM_00131	89849	Hs.653186	NM_03338	ENSG0000	ATG16L2	ATG16B|W	autophagy	protein-co
lib-C666-l	chr17	66453950	66453950	+	30.4351	NA	promoter-	promoter-	-285	NM_00132	55062	Hs.463964	NM_01798	ENSG0000	WIPI1	ATG18|ATC	WD repeat	protein-co
lib-C666-l	chr17	40976325	40976325	+	20.82108	NA	promoter-	promoter-	8	NM_00376	8678	Hs.716464	NM_00376	ENSG0000	BECN1	ATG6|VPS3	beclin 1	protein-co
lib-C666-l	chr16	87425823	87425823	+	66.77065	NA	promoter-	promoter-	22	NM_02281	81631	Hs.356061	NM_02281	ENSG0000	MAP1LC3E	ATG8F|LC3	microtubul	protein-co
lib-C666-l	chr20	5100657	5100657	+	33.11264	NA	promoter-	promoter-	-10	NM_18264	5111	Hs.147433	NM_00259	ENSG0000	PCNA	ATLD2	proliferatin	protein-co
lib-C666-l	chr18	43678145	43678145	+	28.64047	NA	exon (NM_	exon (NM_	174	NM_00100	498	Hs.298280	NM_00404	ENSG0000	ATP5A1	ATP5A|ATF	ATP syntha	protein-co
lib-C666-l	chr17	46969874	46969874	+	38.60092	NA	promoter-	promoter-	-274	NM_00517	516	Hs.80986	NM_00517	ENSG0000	ATP5G1	ATP5A|ATF	ATP syntha	protein-co
lib-C666-l	chr4	668212	668212	+	77.06716	NA	promoter-	promoter-	-85	NR_03374	521	Hs.85539	NM_00710	ENSG0000	ATP5I	ATP5K	ATP syntha	protein-co
lib-C666-l	chr16	2564270	2564270	+	143.2643	NA	intron (NM	CpG	399	NM_00169	527	Hs.389107	NM_00169	ENSG0000	ATP6V0C	ATP6C|ATF	ATPase H+	protein-co
lib-C666-l	chr18	76829173	76829173	+	195.8685	NA	promoter-	promoter-	-102	NM_19853	374868	Hs.465475	NM_19853	ENSG0000	ATP9B	ATPASEP|A	ATPase ph	protein-co
lib-C666-l	chr3	1.42E+08	1.42E+08	+	169.8697	NA	promoter-	promoter-	-488	NM_00167	483	Hs.477789	NM_00167	ENSG0000	ATP1B3	ATPB-3|CD	ATPase Na	protein-co
lib-C666-l	chr4	47487243	47487243	+	36.84144	NA	promoter-	promoter-	-167	NM_02045	57205	Hs.437241	NM_02045	ENSG0000	ATP10D	ATPVD	ATPase ph	protein-co
lib-C666-l	chr12	1.1E+08	1.1E+08	+	360.5904	NA	promoter-	promoter-	287	NR_03811	326625	Hs.12106	NM_05284	ENSG0000	MMAB	ATR|CFAP2	methylmal	protein-co
lib-C666-l	chr3	9975458	9975458	+	38.60092	NA	promoter-	promoter-	-66	NM_01551	78987	Hs.9383	NM_01551	ENSG0000	CRELD1	AVSD2|CIR	cysteine ric	protein-co
lib-C666-l	chr7	23571679	23571679	+	88.77095	NA	promoter-	promoter-	-19	NM_00128	29896	Hs.445652	NM_01329	ENSG0000	TRA2A	AWMS1|H	transforme	protein-co
lib-C666-l	chr15	80352399	80352399	+	111.1296	NA	promoter-	promoter-	-103	NM_00124	54469	Hs.596679	NM_01900	ENSG0000	ZFAND6	AWP1|ZA2	zinc finger	protein-co
lib-C666-l	chr15	80351994	80351994	+	38.30731	NA	promoter-	promoter-	84	NM_00124	54469	Hs.596679	NM_01900	ENSG0000	ZFAND6	AWP1|ZA2	zinc finger	protein-co
lib-C666-l	chr3	1.85E+08	1.85E+08	+	93.07718	NA	promoter-	promoter-	-8	NM_02162	59343	Hs.401388	NM_02162	ENSG0000	SENP2	AXAM2|SM	SUMO1/se	protein-co
lib-C666-l	chr17	79196835	79196835	+	26.93404	NA	promoter-	promoter-	-46	NM_00131	22994	Hs.514578	NM_01498	ENSG0000	CEP131	AZ1|AZI1|2	centrosom	protein-co
lib-C666-l	chr8	1.04E+08	1.04E+08	+	22.82077	NA	promoter-	promoter-	-34	NM_01587	51582	Hs.459106	NM_01587	ENSG0000	AZIN1	AZI|AZIA1|	antizyme ir	protein-co
lib-C666-l	chr7	1.23E+08	1.23E+08	+	26.49922	NA	promoter-	promoter-	48	NM_00500	4698	Hs.651219	NM_00500	ENSG0000	NDUFA5	B13|CI-13	NADH:ubic	protein-co
lib-C666-l	chr19	8386271	8386271	+	60.07874	NA	promoter-	promoter-	26	NM_00500	4701	Hs.333427	NM_00500	ENSG0000	NDUFA7	B14.5a|CI-	NADH:ubic	protein-co
lib-C666-l	chr9	32573169	32573169	+	63.78558	NA	promoter-	promoter-	13	NM_00249	4712	Hs.493668	NM_00249	ENSG0000	NDUFB6	B17|CI	NADH:ubic	protein-co
lib-C666-l	chr14	96671100	96671100	+	25.68529	NA	promoter-	promoter-	-35	NM_00062	624	Hs.654542	NM_00062	ENSG0000	BDKRB2	B2R|BK-2|E	bradykinin	protein-co
lib-C666-l	chr1	2.36E+08	2.36E+08	+	77.6848	NA	promoter-	promoter-	-136	NM_15249	148789	Hs.498143	NM_15249	ENSG0000	B3GALNT2	B3GalNAc-	beta-1,3-N	protein-co
lib-C666-l	chr11	17373379	17373379	+	30.04317	NA	promoter-	promoter-	70	NM_00120	374383	Hs.146274	NM_00100	ENSG0000	NCR3LG1	B7-H6|B7H	natural kill	protein-co
lib-C666-l	chr10	31320742	31320742	+	139.6843	NA	intron (NM	CpG	124	NM_00114	220929	Hs.660642	NM_18275	ENSG0000	ZNF438	bA330O11	zinc finger	protein-co
lib-C666-l	chr19	11071610	11071610	+	35.5104	NA	promoter-	promoter-	12	NM_00112	6597	Hs.327527	NM_00307	ENSG0000	SMARCA4	BAF190|BA	SWI/SNF r	protein-co
lib-C666-l	chr12	7079921	7079921	+	28.88523	NA	promoter-	promoter-	-5	NM_00114	11331	Hs.504620	NM_00114	ENSG0000	PHB2	BAP|BCAP	prohibitin	protein-co
lib-C666-l	chr17	6918120	6918120	+	34.3461	NA	promoter-	promoter-	64	NM_00114	124944	Hs.511801	NM_17489	ENSG0000	C17orf49	BAP18|HEF	chromosor	protein-co
lib-C666-l	chr7	1.07E+08	1.07E+08	+	29.74345	NA	promoter-	promoter-	241	NR_02783	55973	Hs.303787	NM_01884	ENSG0000	BCAP29	BAP29	B-cell rece	protein-co
lib-C666-l	chr7	1.38E+08	1.38E+08	+	36.70747	NA	promoter-	promoter-	-161	NM_00125	64764	Hs.490273	NM_19407	ENSG0000	CREB3L2	BBF2H7	cAMP resp	protein-co
lib-C666-l	chr11	64546550	64546550	+	25.68529	NA	promoter-	promoter-	-234	NM_00134	7536	Hs.502829	NM_00463	ENSG0000	SF1	BBP|D11S6	splicing fac	protein-co
lib-C666-l	chr17	56296608	56296608	+	27.62962	NA	promoter-	promoter-	58	NM_00132	54903	Hs.408843	NM_01777	ENSG0000	MKS1	BBS13|JBTS	Meckel syr	protein-co
lib-C666-l	chr9	26947359	26947359	+	219.4157	NA	promoter-	promoter-	49	NM_00109	80173	Hs.145402	NM_02510	ENSG0000	IFT74	BBS20|CCD	intraflagell	protein-co
lib-C666-l	chr2	27712682	27712682	+	37.48738	NA	promoter-	promoter-	-4	NM_01566	26160	Hs.127401	NM_01566	ENSG0000	IFT172	BBS20|NPH	intraflagell	protein-co
lib-C666-l	chr4	1.23E+08	1.23E+08	+	58.76075	NA	promoter-	promoter-	-9	NM_01819	55212	Hs.591694	NM_01819	ENSG0000	BBS7	BBS2L1	Bardet-Bie	protein-co
lib-C666-l	chr1	1.01E+08	1.01E+08	+	25.96768	NA	promoter-	promoter-	-76	NM_00191	1629	Hs.709187	NM_00191	ENSG0000	DBT	BCATE2|BC	dihydrolip	protein-co
lib-C666-l	chr5	70220924	70220924	+	28.8837	NA	5' UTR (NN	5' UTR (NN	156	NM_02287	6607	Hs.202179	NM_01741	ENSG0000	SMN2	BCD541|C-	survival of	protein-co
lib-C666-l	chr5	69345502	69345502	+	26.54591	NA	5' UTR (NN	5' UTR (NN	152	NM_02287	6607	Hs.202179	NM_01741	ENSG0000	SMN2	BCD541|C-	survival of	protein-co
lib-C666-l	chr7	1E+08	1E+08	+	24.50625	NA	promoter-	promoter-	-336	NM_01960	56257	Hs.178011	NM_01960	ENSG0000	MEPCE	BCDIN3	methylpho	protein-co
lib-C666-l	chr9	82186585	82186585	+	22.01132	NA	promoter-	promoter-	-103	NM_00128	7091	Hs.444213	NM_00700	ENSG0000	TLE4	BCE-1|BCE	transducin	protein-co
lib-C666-l	chr16	31119704	31119704	+	72.75481	NA	promoter-	promoter-	89	NM_00112	10295	Hs.513520	NM_00588	ENSG0000	BCKDK	BCKDKD|B	branched	protein-co
lib-C666-l	chr2	60781851	60781851	+	27.5466	NA	Intergenic	CpG	-1218	NM_13855	53335	Hs.370549	NM_01801	ENSG0000	BCL11A	BCL11A-L|	B-cell CLL/	protein-co
lib-C666-l	chr3	1.87E+08	1.87E+08	+	52.34435	NA	promoter-	promoter-	-32	NM_00170	604	Hs.478588	NM_00170	ENSG0000	BCL6	BCL5|BCL6	B-cell CLL/	protein-co
lib-C666-l	chr13	35516587	35516587	+	22.50982	NA	5' UTR (NN	5' UTR (NN	163	NM_01567	26960	Hs.491172	NM_01567	ENSG0000	NBEA	BCL8B|LYS	neurobeac	protein-co
lib-C666-l	chr1	33367086	33367086	+	32.78343	NA	promoter-	promoter-	-45	NM_00132	113452	Hs.534521	NM_03350	ENSG0000	TMEM54	BCLP|CAC-	transmeml	protein-co
lib-C666-l	chr22	18121475	18121475	+	23.05659	NA	exon (NM_	exon (NM_	125	NM_00127	23786	Hs.631672	NM_01536	ENSG0000	BCL2L13	BCL-RAME	BCL2 like 1	protein-co
lib-C666-l	chr14	23775967	23775967	+	89.36973	NA	promoter-	promoter-	-4	NM_00405	599	Hs.410026	NM_00405	ENSG0000	BCL2L2	BCL-W|BC	BCL2 like 2	protein-co
lib-C666-l	chr3	23987453	23987453	+	124.4789	NA	promoter-	promoter-	-159	NM_00114	9975	Hs.37288	NM_00512	ENSG0000	NR1D2	BD73|EAR-	nuclear rec	protein-co
lib-C666-l	chr4	89205884	89205884	+	88.70856	NA	promoter-	promoter-	99	NM_15254	152926	Hs.43744	NM_15254	ENSG0000	PPM1K	BDP|MSUD	protein ph	protein-co
lib-C666-l	chr7	1.39E+08	1.39E+08	+	33.97962	NA	promoter-	promoter-	-36	NM_00113	6916	Hs.520757	NM_00106	ENSG0000	TBXAS1	BDPLT14|C	thromboxa	protein-co
lib-C666-l	chr14	69446150	69446150	+	34.92812	NA	promoter-	promoter-	-67	NM_00113	87	Hs.235750	NM_00110	ENSG0000	ACTN1	BDPLT15	actinin alp	protein-co
lib-C666-l	chr13	1.09E+08	1.09E+08	+	41.89289	NA	promoter-	promoter-	120	NM_03285	84945	Hs.183528	NM_03285	ENSG0000	ABHD13	BEM46L1|C	abhydrolas	protein-co
lib-C666-l	chr13	1.12E+08	1.12E+08	+	83.757	NA	promoter-	promoter-	-27	NM_00133	8874	Hs.508738	NM_00389	ENSG0000	ARHGEF7	BETA-PIX|	Rho guanir	protein-co
lib-C666-l	chr17	35306207	35306207	+	35.28811	NA	promoter-	promoter-	32	NM_01213	26574	Hs.195740	NM_01213	ENSG0000	AATF	BFR2|CHE-	apoptosis	protein-co
lib-C666-l	chr7	65447256	65447256	+	52.42305	NA	promoter-	promoter-	45	NR_12053	2990	Hs.255230	NM_00018	ENSG0000	GUSB	BG|MPS7	glucuronid	protein-co
lib-C666-l	chr4	1.86E+08	1.86E+08	+	227.9757	NA	promoter-	promoter-	-60	NR_14431	55325	Hs.713548	NM_01835	ENSG0000	UFSP2	BHD|C4orf	UFM1 spec	protein-co
lib-C666-l	chr1	6480379	6480379	+	25.68529	NA	promoter-	promoter-	-400	NM_01908	54626	Hs.118727	NM_01908	ENSG0000	HES2	bHLHb40	hes family	protein-co
lib-C666-l	chr1	936272	936272	+	329.931	NA	promoter-	promoter-	-720	NM_02117	57801	Hs.154029	NM_02117	ENSG0000	HES4	bHLHb42	hes family	protein-co
lib-C666-l	chr1	937430	937430	+	26.52079	NA	Intergenic	Intergenic	-1878	NM_02117	57801	Hs.154029	NM_02117	ENSG0000	HES4	bHLHb42	hes family	protein-co
lib-C666-l	chr2	70141850	70141850	+	50.83079	NA	promoter-	promoter-	-323	NM_00120	4084	Hs.468908	NM_00235	ENSG0000	MXD1	BHLHC58|	MAX dime	protein-co
lib-C666-l	chr15	65822631	65822631	+	21.94284	NA	promoter-	promoter-	-196	NM_01639	51495	Hs.512973	NM_01639	ENSG0000	HACD3	B-IND1|BI	3-hydroxy	protein-co
lib-C666-l	chr9	1.28E+08	1.28E+08	+	53.38629	NA	promoter-	promoter-	-104	NM_00534	3309	Hs.743241	NM_00534	ENSG0000	HSPA5	BIP|GRP78	heat shock	protein-co
lib-C666-l	chr17	57785008	57785008	+	27.28371	NA	promoter-	promoter-	-49	NM_00101	51651	Hs.12677	NM_01607	ENSG0000	PTRH2	BIT1|CFAP	peptidyl-tR	protein-co
lib-C666-l	chr6	26458090	26458090	+	22.43333	NA	promoter-	promoter-	-42	NM_00704	11120	Hs.159028	NM_00704	ENSG0000	BTN2A1	BK14H9.1|	butyrophili	protein-co
lib-C666-l	chr14	75179860	75179860	+	53.15281	NA	promoter-	promoter-	10	NM_01596	51077	Hs.579828	NM_01596	ENSG0000	FCF1	Bka|C14orf	FCF1 rRNA	protein-co
lib-C666-l	chr6	97372643	97372643	+	26.72399	NA	non-codin	non-codin	147	NM_00132	114792	Hs.45056	NM_05290	ENSG0000	KLHL32	BKLHD5|KL	kelch like f	protein-co

lib-C666-l	chr3	1.49E+08	1.49E+08	+	26.52079	NA	promoter-	promoter-	99	NM_00130	84343	Hs.558314	NM_03238	ENSG0000	HPS3	BLOC2S1|S	HPS3, bioc	protein-co
lib-C666-l	chr10	1.04E+08	1.04E+08	+	52.3754	NA	promoter-	promoter-	5	NM_02474	79803	Hs.125133	NM_02474	ENSG0000	HPS6	BLOC2S3	HPS6, bioc	protein-co
lib-C666-l	chr22	26879835	26879835	+	498.8196	NA	promoter-	promoter-	-6	NR_07313	89781	Hs.474436	NM_02208	ENSG0000	HPS4	BLOC3S2|L	HPS4, bioc	protein-co
lib-C666-l	chr6	8064738	8064738	+	103.4946	NA	promoter-	promoter-	-91	NM_00119	63915	Hs.719272	NM_20128	ENSG0000	BLOC1S5	BLOS5|MU	biogenesis	protein-co
lib-C666-l	chr15	45879514	45879514	+	67.3806	NA	promoter-	promoter-	193	NR_13235	26258	Hs.7037	NM_01238	ENSG0000	BLOC1S6	BLOS6|HPS	biogenesis	protein-co
lib-C666-l	chr1	91487574	91487574	+	22.28596	NA	promoter-	promoter-	238	NM_20126	84146	Hs.173001	NM_01662	ENSG0000	ZNF644	BM-005|M	zinc finger	protein-co
lib-C666-l	chr18	48724352	48724352	+	25.39487	NA	promoter-	promoter-	-301	NM_01662	51320	Hs.465144	NM_01662	ENSG0000	MEX3C	BM-013|M	mex-3 RN/	protein-co
lib-C666-l	chr16	4897398	4897398	+	59.65469	NA	promoter-	promoter-	-9	NM_00132	84656	Hs.387255	NM_03256	ENSG0000	GLYR1	BM045|HIE	glyoxylate	protein-co
lib-C666-l	chr14	39901547	39901547	+	36.56565	NA	5' UTR (NM	5' UTR (NM	157	NM_20330	254170	Hs.146632	NM_20330	ENSG0000	FBXO33	BMND12|F	F-box prot	protein-co
lib-C666-l	chr10	75490035	75490035	+	25.3508	NA	promoter-	promoter-	237	NR_02659	729096	Hs.709171	NR_02659	ENSG0000	BMS1P4	BMS1LP4	BMS1, ribo	pseudo
lib-C666-l	chr1	9599466	9599466	+	92.67979	NA	promoter-	promoter-	-62	NM_03231	84275	Hs.568613	NM_03231	ENSG0000	SLC25A33	BMSC-MC	solute carr	protein-co
lib-C666-l	chr8	22102650	22102650	+	135.0517	NA	promoter-	promoter-	31	NM_00172	661	Hs.148342	NM_00172	ENSG0000	POLR3D	BN51T|RP(	RNA polym	protein-co
lib-C666-l	chr19	7600531	7600531	+	43.79478	NA	promoter-	promoter-	-58	NM_00116	10908	Hs.631863	NM_00670	ENSG0000	PNPLA6	BNHS|LNV	patatin like	protein-co
lib-C666-l	chr15	59981685	59981685	+	20.90919	NA	promoter-	promoter-	48	NM_00433	663	Hs.592515	NM_00433	ENSG0000	BNIP2	BNIP-2|NIP	BCL2 intera	protein-co
lib-C666-l	chr16	71879847	71879847	+	440.4756	NA	promoter-	promoter-	-47	NM_00113	342371	Hs.743239	NM_00113	ENSG0000	ATXN1L	BOAT|BOA	ataxin 1 lik	protein-co
lib-C666-l	chr19	18668656	18668656	+	43.37893	NA	promoter-	promoter-	84	NM_00117	79036	Hs.714548	NM_02406	ENSG0000	KXD1	BORCS4|C	KxDL motif	protein-co
lib-C666-l	chr17	48045843	48045843	+	26.54591	NA	promoter-	promoter-	-719	NM_13828	1748	Hs.591167	NM_00193	ENSG0000	DLX4	BP1|DLX7|[	distal-less	protein-co
lib-C666-l	chr12	1.13E+08	1.13E+08	+	174.441	NA	promoter-	promoter-	-12	NM_08060	5781	Hs.506852	NM_00283	ENSG0000	PTPN11	BPTP3|CFC	protein tyr	protein-co
lib-C666-l	chr3	9772803	9772803	+	25.39084	NA	promoter-	promoter-	-610	NM_00131	7862	Hs.1004	NM_00463	ENSG0000	BRPF1	BR140	bromodom	protein-co
lib-C666-l	chr12	82752201	82752201	+	297.279	NA	promoter-	promoter-	-2	NM_01416	29080	Hs.582627	NM_01416	ENSG0000	CCDC59	BR22|HSPC	coiled-coil	protein-co
lib-C666-l	chr7	1.41E+08	1.41E+08	+	214.5756	NA	promoter-	promoter-	-336	NM_00433	673	Hs.550061	NM_00433	ENSG0000	BRAF	B-RAF1|B-	B-Raf prot	protein-co
lib-C666-l	chr7	1.41E+08	1.41E+08	+	28.017	NA	intron (NM	CpG	360	NM_00433	673	Hs.550061	NM_00433	ENSG0000	BRAF	B-RAF1|B-	B-Raf prot	protein-co
lib-C666-l	chr15	60771335	60771335	+	68.23259	NA	promoter-	promoter-	24	NM_00127	79664	Hs.200943	NM_02461	ENSG0000	ICE2	BRCC1|NA	interactor	protein-co
lib-C666-l	chr13	32889558	32889558	+	40.85366	NA	promoter-	promoter-	-59	NM_00005	675	Hs.34012	NM_00005	ENSG0000	BRCA2	BRCC2|BR(	BRCA2, DN	protein-co
lib-C666-l	chr9	1.04E+08	1.04E+08	+	53.51636	NA	promoter-	promoter-	-145	NM_01959	56254	Hs.729085	NM_01959	ENSG0000	RNF20	BRE1|BRE1	ring finger	protein-co
lib-C666-l	chr16	30773603	30773603	+	25.90648	NA	promoter-	promoter-	-7	NM_00128	9810	Hs.65238	NM_01477	ENSG0000	RNF40	BRE1B|RBP	ring finger	protein-co
lib-C666-l	chr14	69260183	69260183	+	36.38376	NA	5' UTR (NM	5' UTR (NM	448	NM_00492	677	Hs.85155	NM_00492	ENSG0000	ZFP36L1	BRF1|Berg3	ZFP36 ring	protein-co
lib-C666-l	chr2	43453906	43453906	+	32.57434	NA	promoter-	promoter-	-161	NM_00688	678	Hs.503093	NM_00688	ENSG0000	ZFP36L2	BRF2|ERF-2	ZFP36 ring	protein-co
lib-C666-l	chr8	37707463	37707463	+	36.83792	NA	promoter-	promoter-	-32	NM_01831	55290	Hs.709301	NM_01831	ENSG0000	BRF2	BRFU|TFIII	BRF2, RNA	protein-co
lib-C666-l	chr14	36295436	36295436	+	66.35767	NA	promoter-	promoter-	-161	NM_03235	84312	Hs.525299	NM_03235	ENSG0000	BRMS1L	BRMS1	breast canc	protein-co
lib-C666-l	chr14	36295919	36295919	+	38.01809	NA	intron (NM	CpG	322	NM_03235	84312	Hs.525299	NM_03235	ENSG0000	BRMS1L	BRMS1	breast canc	protein-co
lib-C666-l	chrX	43514013	43514013	+	20.59244	NA	promoter-	promoter-	-142	NM_00024	4128	Hs.183109	NM_00024	ENSG0000	MAOA	BRNRS|MA	monoamin	protein-co
lib-C666-l	chr6	1.22E+08	1.22E+08	+	35.68805	NA	promoter-	promoter-	-238	NR_10445	221322	Hs.121396	NM_15273	ENSG0000	TBC1D32	BROMI|C6c	TBC1 dom	protein-co
lib-C666-l	chr2	2.19E+08	2.19E+08	+	45.10538	NA	promoter-	promoter-	18	NM_02257	25953	Hs.98475	NM_01548	ENSG0000	PNKD	BRP17|DYT	paroxysma	protein-co
lib-C666-l	chr2	97001511	97001511	+	23.12593	NA	promoter-	promoter-	32	NM_00128	23397	Hs.308045	NM_01534	ENSG0000	NCAPH	BRRN1|CAI	non-SMC	protein-co
lib-C666-l	chr20	24973423	24973423	+	55.66538	NA	promoter-	promoter-	2	NM_02053	57136	Hs.472330	NM_02053	ENSG0000	APMAP	BSCv|C20o	adipocyte	protein-co
lib-C666-l	chr4	1.19E+08	1.19E+08	+	38.6745	NA	promoter-	promoter-	-256	NM_00361	8492	Hs.445857	NM_00361	ENSG0000	PRSS12	BSSP-3|BS	protease, s	protein-co
lib-C666-l	chr6	26383245	26383245	+	35.0374	NA	promoter-	promoter-	-79	NM_00119	10385	Hs.373938	NM_00699	ENSG0000	BTN2A2	BT2.2|BTF2	butyrophili	protein-co
lib-C666-l	chr6	26365375	26365375	+	29.07066	NA	promoter-	promoter-	-12	NM_00119	11118	Hs.376046	NM_00704	ENSG0000	BTN3A2	BT3.2|BTF4	butyrophili	protein-co
lib-C666-l	chr16	3661657	3661657	+	25.75165	NA	promoter-	promoter-	-50	NM_03244	84464	Hs.143681	NM_03244	ENSG0000	SLX4	BTBD12|FA	SLX4 struct	protein-co
lib-C666-l	chr2	70056700	70056700	+	24.17107	NA	promoter-	promoter-	-38	NM_17843	64395	Hs.293971	NM_17843	ENSG0000	GMCL1	BTBD13|G(	germ cell-l	protein-co
lib-C666-l	chr17	47755454	47755454	+	24.41787	NA	promoter-	promoter-	71	NM_00100	8405	Hs.463382	NM_00356	ENSG0000	SPOP	BTBD32|TE	speckle typ	protein-co
lib-C666-l	chr14	45431238	45431238	+	22.57309	NA	promoter-	promoter-	-59	NM_01765	54813	Hs.550906	NM_01765	ENSG0000	KLHL28	BTBD5	kelch like f	protein-co
lib-C666-l	chr15	31618623	31618623	+	57.83654	NA	promoter-	promoter-	-435	NM_01599	51621	Hs.376443	NM_01599	ENSG0000	KLF13	BTEB3|FKLI	Kruppel lik	protein-co
lib-C666-l	chr6	26440694	26440694	+	20.59244	NA	promoter-	promoter-	-6	NM_00699	10384	Hs.167741	NM_00699	ENSG0000	BTN3A3	BTF3|BTN3	butyrophili	protein-co
lib-C666-l	chr13	45694580	45694580	+	40.85015	NA	promoter-	promoter-	-51	NM_00412	2963	Hs.654582	NM_00412	ENSG0000	GTF2F2	BTF4|RAP3	general tra	protein-co
lib-C666-l	chr19	6393416	6393416	+	28.30587	NA	promoter-	promoter-	-125	NM_00209	2962	Hs.68257	NM_00209	ENSG0000	GTF2F1	BTF4|RAP7	general tra	protein-co
lib-C666-l	chr17	38296665	38296665	+	36.97678	NA	5' UTR (NM	5' UTR (NM	158	NM_00735	22794	Hs.743287	NM_00735	ENSG0000	CASC3	BTZ|MLN5	cancer sus	protein-co
lib-C666-l	chr9	33076669	33076669	+	128.5813	NA	promoter-	promoter-	45	NM_01822	55234	Hs.655351	NM_01822	ENSG0000	SMU1	BWD|SMU	DNA replic	protein-co
lib-C666-l	chr19	10216978	10216978	+	34.19829	NA	promoter-	promoter-	79	NM_00134	56342	Hs.14468	NM_02023	ENSG0000	PPAN	BXDC3|SSF	peter pan	protein-co
lib-C666-l	chr6	41888931	41888931	+	96.67246	NA	promoter-	promoter-	-34	NM_00405	705	Hs.106880	NM_00405	ENSG0000	BYSL	BYSTIN	bystin like	protein-co
lib-C666-l	chr20	48807276	48807276	+	49.41553	NA	5' UTR (NM	5' UTR (NM	156	NM_00128	1051	Hs.517106	NM_00519	ENSG0000	CEBPB	C/EBP-bet	CCAAT/en	protein-co
lib-C666-l	chr18	11851385	11851385	+	114.7593	NA	promoter-	promoter-	-4	NM_02041	57132	Hs.656244	NM_02041	ENSG0000	CHMP1B	C10orf2|C1	charged m	protein-co
lib-C666-l	chr10	64564326	64564326	+	43.7015	NA	promoter-	promoter-	-190	NM_03280	84890	Hs.99821	NM_03280	ENSG0000	ADO	C10orf22	2-aminoet	protein-co
lib-C666-l	chr10	95256303	95256303	+	32.78343	NA	promoter-	promoter-	-66	NM_00112	55165	Hs.14559	NM_01813	ENSG0000	CEP55	C10orf3|C1	centrosom	protein-co
lib-C666-l	chr10	95255661	95255661	+	25.39487	NA	promoter-	promoter-	-708	NM_00112	55165	Hs.14559	NM_01813	ENSG0000	CEP55	C10orf3|C1	centrosom	protein-co
lib-C666-l	chr10	97453755	97453755	+	24.50625	NA	5' UTR (NM	5' UTR (NM	145	NM_01563	26123	Hs.438991	NM_01563	ENSG0000	TCTN3	C10orf61|J	tectonic fa	protein-co
lib-C666-l	chr10	1.06E+08	1.06E+08	+	33.60578	NA	promoter-	promoter-	27	NM_14524	119392	Hs.93667	NM_14524	ENSG0000	SFR1	C10orf78|N	SWI5 depe	protein-co
lib-C666-l	chr10	1.25E+08	1.25E+08	+	109.044	NA	exon (NM_	exon (NM_	515	NM_15333	118672	Hs.281004	NM_15333	ENSG0000	PSTK	C10orf89	phosphose	protein-co
lib-C666-l	chr7	1.02E+08	1.02E+08	+	218.6346	NA	promoter-	promoter-	-6	NM_02465	79706	Hs.722104	NM_02465	ENSG0000	PRKRIP1	C114|KRBC	PRKR inter	protein-co
lib-C666-l	chr11	8985959	8985959	+	75.60525	NA	promoter-	promoter-	-263	NR_07343	493900	Hs.591981	NR_07343	ENSG0000	TMEM9B-A	C11orf18	TMEM9B a	ncRNA
lib-C666-l	chr15	34394154	34394154	+	222.3334	NA	promoter-	promoter-	-101	NM_02015	56851	Hs.160565	NM_02015	ENSG0000	EMC7	C11orf3|C1	ER membr	protein-co
lib-C666-l	chr11	76155720	76155720	+	27.5466	NA	promoter-	promoter-	-349	NM_00130	56946	Hs.352588	NM_02019	ENSG0000	EMSY	C11orf30|C	EMSY, BRC	protein-co
lib-C666-l	chr11	64052238	64052238	+	24.25638	NA	promoter-	promoter-	-45	NM_00117	56834	Hs.523763	NM_02015	ENSG0000	GPR137	C11orf4|GF	G protein-	protein-co
lib-C666-l	chr11	64885281	64885281	+	23.80446	NA	promoter-	promoter-	-71	NM_01420	741	Hs.121025	NM_01420	ENSG0000	ZNHIT2	C11orf5|FC	zinc finger	protein-co
lib-C666-l	chr11	1593553	1593553	+	21.65402	NA	promoter-	promoter-	-403	NM_00442	1850	Hs.41688	NM_00442	ENSG0000	DUSP8	C11orf81|H	dual specif	protein-co
lib-C666-l	chr12	58165874	58165874	+	91.00045	NA	promoter-	promoter-	40	NM_02303	4234	Hs.42957	NM_00537	ENSG0000	METTL1	C12orf1|TR	methyltran	protein-co
lib-C666-l	chr12	66524598	66524598	+	24.32656	NA	promoter-	promoter-	-65	NM_03233	84298	Hs.504820	NM_03233	ENSG0000	LLPH	C12orf31|H	LLP homol	protein-co
lib-C666-l	chr12	4430289	4430289	+	24.50625	NA	promoter-	promoter-	-70	NM_02037	57103	Hs.504545	NM_02037	ENSG0000	TIGAR	C12orf5|FR	TP53 induc	protein-co
lib-C666-l	chr12	62996418	62996418	+	32.11429	NA	non-codin	non-codin	796	NR_12168	283416	Hs.375881	NM_17589	ENSG0000	LINC01465	C12orf61	long interg	ncRNA
lib-C666-l	chr12	50505603	50505603	+	88.49222	NA	promoter-	promoter-	-161	NM_00125	84987	Hs.388645	NM_03290	ENSG0000	COX14	C12orf62|F	COX14, cyt	protein-co
lib-C666-l	chr12	1.12E+08	1.12E+08	+	95.02626	NA	promoter-	promoter-	827	NM_00681	10961	Hs.75841	NM_00681	ENSG0000	ERP29	C12orf8|ER	endoplasm	protein-co
lib-C666-l	chr12	1.12E+08	1.12E+08	+	32.58685	NA	promoter-	promoter-	76	NM_00681	10961	Hs.75841	NM_00681	ENSG0000	ERP29	C12orf8|ER	endoplasm	protein-co
lib-C666-l	chr13	31191784	31191784	+	39.64852	NA	promoter-	promoter-	-32	NM_00132	10208	Hs.533831	NM_00580	ENSG0000	USPL1	C13orf22|[	ubiquitin s	protein-co
lib-C666-l	chr13	39612193	39612193	+	57.89614	NA	promoter-	promoter-	20	NM_02513	80209	Hs.318526	NM_02513	ENSG0000	PROSER1	C13orf23	proline anc	protein-co
lib-C666-l	chr13	53024939	53024939	+	30.08826	NA	promoter-	promoter-	-126	NM_00128	51028	Hs.109520	NM_01607	ENSG0000	VPS36	C13orf9|C(	vacuolar p	protein-co
lib-C666-l	chr14	45722613	45722613	+	56.64664	NA	promoter-	promoter-	-8	NM_01835	55320	Hs.732769	NM_01835	ENSG0000	MIS18BP1	C14orf106|	MIS18 binc	protein-co
lib-C666-l	chr14	23938789	23938789	+	44.91887	NA	promoter-	promoter-	-109	NM_00104	25983	Hs.9043	NM_01551	ENSG0000	NGDN	C14orf120|	neuroguidi	protein-co
lib-C666-l	chr14	24682746	24682746	+	185.0772	NA	TTS (NM_1	TTS (NM_1	290	NM_01416	29082	Hs.279761	NM_01416	ENSG0000	CHMP4A	C14orf123|	charged m	protein-co
lib-C666-l	chr14	32030603	32030603	+	140.9891	NA	promoter-	promoter-	12	NM_02515	80224	Hs.288981	NM_02515	ENSG0000	NUBPL	C14orf127|	nucleotide	protein-co
lib-C666-l	chr14	1.03E+08	1.03E+08	+	130.6388	NA	promoter-	promoter-	-363	NM_00126	55778	Hs.743359	NM_01833	ENSG0000	ZNF839	C14orf131	zinc finger	protein-co
lib-C666-l	chr14	1.03E+08	1.03E+08	+	66.80925	NA	promoter-	promoter-	-86	NM_01833	55778	Hs.743359	NM_01833	ENSG0000	ZNF839	C14orf131	zinc finger	protein-co
lib-C666-l	chr14	77924059	77924059	+	54.30392	NA	promoter-	promoter-	-76	NM_00119	63894	Hs.16157	NM_02206	ENSG0000	VIPAS39	C14orf133|	VPS33B int	protein-co
lib-C666-l	chr14	94492646	94492646	+	37.47379	NA	promoter-	promoter-	-78	NM_02311	78990	Hs.278815	NM_02311	ENSG0000	OTUB2	C14orf137|	OTU deubi	protein-co
lib-C666-l	chr14	50583326	50583326	+	155.4067	NA	promoter-	promoter-	-29	NM_00104	79609	Hs.558541	NM_02455	ENSG0000	VCPKMT	C14orf138|	valosin cor	protein-co
lib-C666-l	chr14	1.04E+08	1.04E+08	+	33.43742	NA	intron (NM	CpG	108	NM_15230	115708	Hs.525610	NM_15230	ENSG0000	TRMT61A	C14orf172|	tRNA meth	protein-co
lib-C666-l	chr14	76452142	76452142	+	53.07762	NA	promoter-	promoter-	46	NR_04566	112752	Hs.532626	NM_05287	ENSG0000	IFT43	C14orf179|	intraflagell	protein-co
lib-C666-l	chr14	35515334	35515334	+	66.67238	NA	promoter-	promoter-	-125	NM_00128	283635	Hs.446357	NM_17360	ENSG0000	FAM177A1	C14orf24	family with	protein-co
lib-C666-l	chr14	55518729	55518729	+	134.6364	NA	intron (NM	CpG	367	NM_14457	93487	Hs.594338	NM_14457	ENSG0000	MAPK1IP1L	C14orf32|M	mitogen-a	protein-co
lib-C666-l	chr14	1.04E+08	1.04E+08	+	25.39487	NA	intron (NR	intron (NR	507	NR_13803	1.05E+08	Hs.252904	NR_138038		LINC00677	C14orf74	long interg	ncRNA
lib-C666-l	chr14	21945338	21945338	+	40.85015	NA	promoter-	promoter-	3	NM_00130	9878	Hs.555910	NM_01482	ENSG0000	TOX4	C14orf92|K	TOX high r	protein-co
lib-C666-l	chr15	40861515	40861515	+	82.96959	NA	promoter-	promoter-	23	NM_15226	27079	Hs.173311	NM_15226	ENSG0000	RPUSD2	C15orf19|C	RNA pseuc	protein-co
lib-C666-l	chr15	42841044	42841044	+	102.4776	NA	promoter-	promoter-	33	NM_00132	55142	Hs.14347	NM_01809	ENSG0000	HAUS2	C15orf25|C	HAUS augr	protein-co
lib-C666-l	chr16	577587	577587	+	43.12596	NA	promoter-	promoter-	-180	NR_02412	64493	Hs.721553	NR_024121		LINC00235	C16orf10|L	long interg	ncRNA
lib-C666-l	chr16	765819	765819	+	50.68899	NA	exon (NM_	exon (NM_	646	NM_02404	79006	Hs.533772	NM_02404	ENSG0000	METRN	C16orf23|c	meteorin,	protein-co
lib-C666-l	chr16	771250	771250	+	27.94028	NA	5' UTR (NM	5' UTR (NM	108	NM_00127	65990	Hs.166244	NM_02393	ENSG0000	FAM173A	C16orf24	family with	protein-co
lib-C666-l	chr16	4588773	4588773	+	36.30428	NA	promoter-	promoter-	43	NM_01339	29965	Hs.654653	NM_01339	ENSG0000	CDIP1	C16orf5|CE	cell death i	protein-co
lib-C666-l	chr16	58035127	58035127	+	55.62774	NA	promoter-	promoter-	-150	NM_00119	79650	Hs.408702	NM_02459	ENSG0000	USB1	C16orf57|H	U6 snRNA	protein-co
lib-C666-l	chr16	58163462	58163462	+	83.41785	NA	promoter-	promoter-	-133	NR_13663	29105	Hs.532755	NM_01324	ENSG0000	CFAP20	C16orf80|E	cilia and fla	protein-co
lib-C666-l	chr16	88772864	88772864	+	336.5587	NA	promoter-	promoter-	-7	NM_00101	348180	Hs.592074	NM_00101	ENSG0000	CTU2	C16orf84|N	cytosolic th	protein-co
lib-C666-l	chr16	284809	284809	+	98.43693	NA	non-codin	non-codin	264	NM_03203	83986	Hs.513225	NM_03203	ENSG0000	FAM234A	C16orf9|ITI	family with	protein-co
lib-C666-l	chr17	685576	685576	+	363.2229	NA	promoter-	promoter-	-5	NM_01608	51031	Hs.279061	NM_01608	ENSG0000	GLOD4	C17orf25|C	glyoxalase	protein-co
lib-C666-l	chr17	37886770	37886770	+	53.07762	NA	promoter-	promoter-	46	NM_03233	84299	Hs.333526	NM_03233	ENSG0000	MIEN1	C17orf37|C	migration	protein-co
lib-C666-l	chr17	17942578	17942578	+	40.09574	NA	promoter-	promoter-	-33	NM_02405	79018	Hs.187422	NM_02405	ENSG0000	GID4	C17orf39|V	GID compl	protein-co
lib-C666-l	chr17	7155553	7155553	+	99.93166	NA	promoter-	promoter-	33	NM_20341	23587	Hs.417029	NM_01536	ENSG0000	ELP5	C17orf81|E	elongator	protein-co
lib-C666-l	chr18	5237987	5237987	+	64.69308	NA	promoter-	promoter-	41	NR_02684	147525	Hs.657197	NM_152471		LINC00526	C18orf18|H	long interg	ncRNA
lib-C666-l	chr18	60190176	60190176	+	21.28624	NA	promoter-	promoter-	-482	NM_01774	54877	Hs.114191	NM_01774	ENSG0000	ZCCHC2	C18orf49	zinc finger	protein-co
lib-C666-l	chr10	1.02E+08	1.02E+08	+	85.88258	NA	promoter-	promoter-	3	NM_00130	55280	Hs.215502	NM_01829	ENSG0000	CWF19L1	C19L1|SCA	CWF19-lik	protein-co
lib-C666-l	chr19	45394344	45394344	+	23.0291	NA	promoter-	promoter-	-133	NM_00611	10452	Hs.655909	NM_00611	ENSG0000	TOMM40	C19orf1|D1	translocase	protein-co
lib-C666-l	chr19	507399	507399	+	118.4713	NA	promoter-	promoter-	-98	NM_03351	91978	Hs.369613	NM_03351	ENSG0000	TPGS1	C19orf20|C	tubulin pol	protein-co
lib-C666-l	chr19	1885545	1885545	+	40.09574	NA	promoter-	promoter-	-27	NM_03121	81926	Hs.465542	NM_03121	ENSG0000	ABHD17A	C19orf27|F	abhydrolas	protein-co
lib-C666-l	chr19	50979544	50979544	+	346.466	NA	promoter-	promoter-	-190	NM_20653	284361	Hs.448941	NM_17506	ENSG0000	EMC10	C19orf63|H	ER membr	protein-co
lib-C666-l	chr19	47617070	47617070	+	62.69942	NA	promoter-	promoter-	-61	NM_01516	23211	Hs.104661	NM_01516	ENSG0000	ZC3H4	C19orf7	zinc finger	protein-co
lib-C666-l	chr19	14063088	14063088	+	24.32656	NA	promoter-	promoter-	-198	NM_13835	90379	Hs.443636	NM_13835	ENSG0000	DCAF15	C19orf72	DDB1 and	protein-co
lib-C666-l	chr19	46145685	46145685	+	29.01385	NA	non-codin	non-codin	933	NR_13072	1E+08	Hs.668611	NM_00124	ENSG0000	EML2-AS1	C19orf83	EML2 antis	ncRNA
lib-C666-l	chr1	39456905	39456905	+	71.09715	NA	promoter-	promoter-	-11	NM_02459	79647	Hs.293563	NM_02459	ENSG0000	AKIRIN1	C1orf108|S	akirin 1	protein-co
lib-C666-l	chr1	27648647	27648647	+	91.30406	NA	promoter-	promoter-	11	NR_03757	84065	Hs.469171	NM_03212	ENSG0000	TMEM222	C1orf160	transmemb	protein-co
lib-C666-l	chr1	20834624	20834624	+	64.98446	NA	promoter-	promoter-	50	NM_02454	79594	Hs.10101	NM_02454	ENSG0000	MUL1	C1orf166|C	mitochond	protein-co
lib-C666-l	chr1	27286851	27286851	+	28.64047	NA	promoter-	promoter-	50	NM_15236	126695	Hs.188881	NM_15236	ENSG0000	KDF1	C1orf172	keratinocyt	protein-co
lib-C666-l	chr1	23695388	23695388	+	33.38247	NA	promoter-	promoter-	-76	NR_03369	148898	Hs.61884	NM_13847	ENSG0000	ZNF436-AS	C1orf213	ZNF436 an	ncRNA
lib-C666-l	chr1	1.85E+08	1.85E+08	+	29.99407	NA	promoter-	promoter-	-92	NR_13511	80267	Hs.523811	NM_02519	ENSG0000	EDEM3	C1orf22	ER degrad	protein-co
lib-C666-l	chr1	2.03E+08	2.03E+08	+	161.3141	NA	promoter-	promoter-	-43	NR_13653	92703	Hs.497443	NM_13839	ENSG0000	TMEM183A	C1orf37	transmemb	protein-co
lib-C666-l	chr1	2.26E+08	2.26E+08	+	72.31497	NA	promoter-	promoter-	-10	NM_15260	163859	Hs.520192	NM_15260	ENSG0000	SDE2	C1orf55|dJ	SDE2 telon	protein-co
lib-C666-l	chr1	25664641	25664641	+	37.12814	NA	promoter-	promoter-	104	NR_13578	57035	Hs.259412	NM_02031	ENSG0000	RSRP1	C1orf63|NI	arginine ar	protein-co
lib-C666-l	chr1	1.57E+08	1.57E+08	+	307.9454	NA	promoter-	promoter-	391	NM_00114	51093	Hs.512597	NM_01599	ENSG0000	RRNAD1	C1orf66|C	ribosomal	protein-co
lib-C666-l	chr1	2.28E+08	2.28E+08	+	83.58523	NA	promoter-	promoter-	273	NM_00101	200205	Hs.237017	NM_00101	ENSG0000	IBA57	C1orf69|M	IBA57 hom	protein-co

lib-C666-l	chr1	2.12E+08	2.12E+08	+	60.02514	NA	promoter-	promoter-	-79	NR_037667	25896	Hs.369285	NM_01543	ENSG0000	INTS7	C1orf73|IN	integrator	protein-co
lib-C666-l	chr1	2144312	2144312	+	23.20869	NA	promoter-	promoter-	-153	NM_00128	199990	Hs.107101	NM_18253	ENSG0000	FAAP20	C1orf86|FP	Fanconi an	protein-co
lib-C666-l	chr1	1.73E+08	1.73E+08	+	21.94284	NA	promoter-	promoter-	-123	NM_01428	51430	Hs.204559	NM_01428	ENSG0000	SUCO	C1orf9|CH:	SUN doma	protein-co
lib-C666-l	chr20	34359867	34359867	+	55.96882	NA	promoter-	promoter-	-56	NM_01643	51230	Hs.517044	NM_01643	ENSG0000	PHF20	C20orf104|	PHD finger	protein-co
lib-C666-l	chr20	33292167	33292167	+	102.5183	NA	promoter-	promoter-	55	NM_02120	58476	Hs.516994	NM_02120	ENSG0000	TP53INP2	C20orf110|	tumor prot	protein-co
lib-C666-l	chr20	33292420	33292420	+	66.67216	NA	promoter-	promoter-	-126	NM_00132	58476	Hs.516994	NM_02120	ENSG0000	TP53INP2	C20orf110|	tumor prot	protein-co
lib-C666-l	chr20	43104502	43104502	+	63.78558	NA	promoter-	promoter-	-24	NM_02433	79183	Hs.283869	NM_02433	ENSG0000	TTPAL	C20orf121	alpha toco	protein-co
lib-C666-l	chr20	60982550	60982550	+	80.52238	NA	promoter-	promoter-	-211	NM_03121	81928	Hs.301040	NM_03121	ENSG0000	CABLES2	C20orf150|	Cdk5 and	protein-co
lib-C666-l	chr20	44420570	44420570	+	128.5813	NA	promoter-	promoter-	-6	NM_05295	116092	Hs.472852	NM_05295	ENSG0000	DNTTIP1	C20orf167|	deoxynucle	protein-co
lib-C666-l	chr20	32399008	32399008	+	215.9269	NA	promoter-	promoter-	-102	NM_17681	128866	Hs.472471	NM_17681	ENSG0000	CHMP4B	C20orf178|	charged m	protein-co
lib-C666-l	chr20	3801408	3801408	+	267.4425	NA	5' UTR (NM	intron (NM	237	NM_00120	55317	Hs.104806	NM_01834	ENSG0000	AP5S1	C20orf29	adaptor re	protein-co
lib-C666-l	chr20	5093744	5093744	+	168.3004	NA	promoter-	promoter-	-11	NM_00100	29058	Hs.472024	NM_01414	ENSG0000	TMEM230	C20orf30|H	transmemb	protein-co
lib-C666-l	chr20	60697350	60697350	+	25.49921	NA	promoter-	promoter-	-167	NM_14470	149986	Hs.105379	NM_14470	ENSG0000	LSM14B	C20orf40|F	LSM family	protein-co
lib-C666-l	chr20	55043643	55043643	+	100.1978	NA	promoter-	promoter-	2	NM_00128	51507	Hs.517134	NM_01640	ENSG0000	RTFDC1	C20orf43|C	replication	protein-co
lib-C666-l	chr20	30458449	30458449	+	30.07415	NA	promoter-	promoter-	18	NM_00132	128853	Hs.434108	NM_08061	ENSG0000	DUSP15	C20orf57|V	dual specif	protein-co
lib-C666-l	chr20	17949493	17949493	+	100.9143	NA	promoter-	promoter-	-41	NM_00131	92667	Hs.320823	NM_05286	ENSG0000	MGME1	C20orf72|D	mitochond	protein-co
lib-C666-l	chr20	42219523	42219523	+	36.56565	NA	5' UTR (NM	5' UTR (NM	270	NM_00132	51098	Hs.444332	NM_01600	ENSG0000	IFT52	C20orf9|C(	intraflagell	protein-co
lib-C666-l	chr21	30365326	30365326	+	72.37988	NA	promoter-	promoter-	-46	NM_00132	26046	Hs.288773	NM_01556	ENSG0000	LTN1	C21orf10|C	listerin E3	protein-co
lib-C666-l	chr21	40685693	40685693	+	307.9454	NA	promoter-	promoter-	19	NM_01896	54014	Hs.627139	NM_01896	ENSG0000	BRWD1	C21orf107|	bromodom	protein-co
lib-C666-l	chr21	43099300	43099300	+	25.49755	NA	promoter-	promoter-	-162	NR_024367	54090	Hs.570442	NR_024367		LINC00111	C21orf21|N	long interg	ncRNA
lib-C666-l	chr21	34852393	34852393	+	38.20619	NA	promoter-	promoter-	-77	NM_00613	757	Hs.433668	NM_00613	ENSG0000	TMEM50B	C21orf4|H(	transmemb	protein-co
lib-C666-l	chr21	47604517	47604517	+	51.32076	NA	promoter-	promoter-	-144	NM_03226	84221	Hs.381214	NM_03226	ENSG0000	SPATC1L	C21orf56	spermatog	protein-co
lib-C666-l	chr22	42342631	42342631	+	23.34628	NA	5' UTR (NM	5' UTR (NM	127	NM_00130	79019	Hs.208912	NM_02405	ENSG0000	CENPM	C22orf18|C	centromer	protein-co
lib-C666-l	chr22	47158465	47158465	+	158.3171	NA	promoter-	promoter-	-49	NM_00128	25771	Hs.435044	NM_01434	ENSG0000	TBC1D22A	C22orf4|HS	TBC1 dom	protein-co
lib-C666-l	chr22	47170021	47170021	+	66.08392	NA	5' UTR (NM	5' UTR (NM	197	NM_00128	25771	Hs.435044	NM_01434	ENSG0000	TBC1D22A	C22orf4|HS	TBC1 dom	protein-co
lib-C666-l	chr2	21022873	21022873	+	116.1534	NA	promoter-	promoter-	17	NM_00128	60526	Hs.187823	NM_02192	ENSG0000	LDAH	C2orf43|hL	lipid drople	protein-co
lib-C666-l	chr2	62115845	62115845	+	275.3644	NA	promoter-	promoter-	-13	NM_00132	150684	Hs.468702	NM_15251	ENSG0000	COMMD1	C2orf5|MU	copper me	protein-co
lib-C666-l	chr16	1359223	1359223	+	104.0705	NA	promoter-	promoter-	69	NM_19426	7329	Hs.302903	NM_00334	ENSG0000	UBE2I	C358B7.1|F	ubiquitin c	protein-co
lib-C666-l	chr1	2.03E+08	2.03E+08	+	41.67221	NA	promoter-	promoter-	8	NM_00130	59349	Hs.706793	NM_02163	ENSG0000	KLHL12	C3IP1|DKIF	kelch like f	protein-co
lib-C666-l	chr3	44379872	44379872	+	38.6745	NA	promoter-	promoter-	-72	NM_00128	285343	Hs.55131	NM_17382	ENSG0000	TCAIM	C3orf23|TC	T-cell activ	protein-co
lib-C666-l	chr3	99536631	99536631	+	92.15059	NA	promoter-	promoter-	-47	NM_03235	84319	Hs.581320	NM_03235	ENSG0000	CMSS1	C3orf26	cms1 ribos	protein-co
lib-C666-l	chr3	1.84E+08	1.84E+08	+	226.3886	NA	promoter-	promoter-	-53	NM_00117	131408	Hs.591307	NM_14463	ENSG0000	FAM131A	C3orf40|FL	family with	protein-co
lib-C666-l	chr3	1.91E+08	1.91E+08	+	41.5993	NA	promoter-	promoter-	93	NM_17490	152137	Hs.478682	NM_17490	ENSG0000	CCDC50	C3orf6|DFN	coiled-coil	protein-co
lib-C666-l	chr4	41992520	41992520	+	105.9344	NA	promoter-	promoter-	-3	NM_00634	10463	Hs.479634	NM_00634	ENSG0000	SLC30A9	C4orf1|GA(	solute carr	protein-co
lib-C666-l	chr4	1.14E+08	1.14E+08	+	137.0793	NA	promoter-	promoter-	-67	NM_01839	55345	Hs.380346	NM_01839	ENSG0000	ZGRF1	C4orf21	zinc finger	protein-co
lib-C666-l	chr4	8442498	8442498	+	446.2542	NA	promoter-	promoter-	-34	NM_15254	152992	Hs.566191	NM_02495	ENSG0000	TRMT44	C4orf23|M	tRNA meth	protein-co
lib-C666-l	chr4	20702037	20702037	+	72.05244	NA	promoter-	promoter-	1	NM_00125	133015	Hs.479298	NM_14504	ENSG0000	PACRGL	C4orf28	PARK2 cor	protein-co
lib-C666-l	chr5	93447382	93447382	+	31.27173	NA	promoter-	promoter-	22	NM_00116	83989	Hs.600086	NM_03204	ENSG0000	FAM172A	C5orf21	family with	protein-co
lib-C666-l	chr5	1.4E+08	1.4E+08	+	115.0328	NA	promoter-	promoter-	49	NM_03241	84418	Hs.529798	NM_03241	ENSG0000	CYSTM1	C5orf32|OI	cysteine ric	protein-co
lib-C666-l	chr5	1.38E+08	1.38E+08	+	142.8748	NA	promoter-	promoter-	-12	NM_00113	51307	Hs.54056	NM_01660	ENSG0000	FAM53C	C5orf6	family with	protein-co
lib-C666-l	chr6	1.39E+08	1.39E+08	+	20.72609	NA	intron (NM	CpG	859	NM_02124	58527	Hs.600861	NM_02124	ENSG0000	ABRACL	C6orf115|C	ABRA C-te	protein-co
lib-C666-l	chr6	30028967	30028967	+	52.45363	NA	promoter-	promoter-	-6	NR_026751	80862	Hs.653168	NR_026751	ENSG0000	ZNRD1ASP	C6orf12|HT	zinc ribbor	pseudo
lib-C666-l	chr6	30594646	30594646	+	20.59244	NA	promoter-	promoter-	-17	NM_00103	79969	Hs.654798	NM_02490	ENSG0000	ATAT1	C6orf134|N	alpha tubu	protein-co
lib-C666-l	chr6	43478130	43478130	+	35.0374	NA	promoter-	promoter-	-49	NM_00101	221424	Hs.445552	NM_00101	ENSG0000	LRRC73	C6orf154	leucine rich	protein-co
lib-C666-l	chr6	1.27E+08	1.27E+08	+	136.0045	NA	non-codin	non-codin	321	NM_00101	387103	Hs.486401	NM_00101	ENSG0000	CENPW	C6orf173|C	centromer	protein-co
lib-C666-l	chr6	1.09E+08	1.09E+08	+	53.6427	NA	promoter-	promoter-	296	NM_00127	285753	Hs.632616	NM_17383	ENSG0000	CEP57L1	C6orf182|b	centrosom	protein-co
lib-C666-l	chr6	37225474	37225474	+	32.78343	NA	promoter-	promoter-	-6	NR_130108	55633	Hs.731702	NM_01777	ENSG0000	TBC1D22B	C6orf197	TBC1 dom	protein-co
lib-C666-l	chr6	70506202	70506202	+	28.8334	NA	intron (NM	intron (NM	847	NM_01836	55788	Hs.271643	NM_01836	ENSG0000	LMBRD1	C6orf209|L	LMBR1 dor	protein-co
lib-C666-l	chr6	31774563	31774563	+	38.10056	NA	5' UTR (NM	5' UTR (NM	198	NM_02117	57819	Hs.103106	NM_02117	ENSG0000	LSM2	C6orf28|G7	LSM2 hom	protein-co
lib-C666-l	chr6	1.18E+08	1.18E+08	+	23.0291	NA	promoter-	promoter-	-44	NM_13845	116150	Hs.289008	NM_13845	ENSG0000	NUS1	C6orf68|CI	NUS1 dehy	protein-co
lib-C666-l	chr6	36409378	36409378	+	21.15243	NA	intron (NM	intron (NM	-1166	NM_17356	222658	Hs.188757	NM_17356	ENSG0000	KCTD20	C6orf69|dJ	potassium	protein-co
lib-C666-l	chr6	1.7E+08	1.7E+08	+	112.9303	NA	promoter-	promoter-	1	NM_01834	55780	Hs.47546	NM_01834	ENSG0000	ERMARD	C6orf70|PV	ER membra	protein-co
lib-C666-l	chr6	1.44E+08	1.44E+08	+	28.20039	NA	5' UTR (NM	5' UTR (NM	109	NM_03286	84946	Hs.185675	NM_03286	ENSG0000	LTV1	C6orf93|dJ	LTV1 ribos	protein-co
lib-C666-l	chr7	1.35E+08	1.35E+08	+	21.03795	NA	promoter-	promoter-	-167	NM_01513	23165	Hs.743250	NM_01513	ENSG0000	NUP205	C7orf14|NF	nucleopori	protein-co
lib-C666-l	chr7	56174247	56174247	+	29.7147	NA	promoter-	promoter-	76	NM_00132	51142	Hs.389996	NM_01613	ENSG0000	CHCHD2	C7orf17|M	coiled-coil	protein-co
lib-C666-l	chr7	916245	916245	+	27.40059	NA	promoter-	promoter-	54	NM_01594	51608	Hs.107387	NM_01594	ENSG0000	GET4	C7orf20|CE	golgi to ER	protein-co
lib-C666-l	chr7	86849074	86849074	+	52.00589	NA	5' UTR (NM	5' UTR (NM	240	NM_02431	79161	Hs.619593	NM_02431	ENSG0000	TMEM243	C7orf23|M	transmemb	protein-co
lib-C666-l	chr7	86849878	86849878	+	21.73991	NA	promoter-	promoter-	25	NM_00132	79161	Hs.619593	NM_02431	ENSG0000	TMEM243	C7orf23|M	transmemb	protein-co
lib-C666-l	chr7	23338942	23338942	+	36.59206	NA	promoter-	promoter-	2	NM_13844	115416	Hs.87385	NM_13844	ENSG0000	MALSU1	C7orf30|m	mitochond	protein-co
lib-C666-l	chr7	99149714	99149714	+	135.8552	NA	promoter-	promoter-	43	NM_14511	221786	Hs.596587	NM_14511	ENSG0000	FAM200A	C7orf38	family with	protein-co
lib-C666-l	chr7	45026343	45026343	+	91.53722	NA	promoter-	promoter-	-84	NR_003697	285958	Hs.25892	NR_003697	ENSG0000	SNHG15	C7orf40|Lir	small nucle	ncRNA
lib-C666-l	chr7	30174331	30174331	+	200.3882	NA	promoter-	promoter-	-221	NM_15279	222166	Hs.200100	NM_15279	ENSG0000	MTURN	C7orf41|Ell	maturin, ne	protein-co
lib-C666-l	chr7	1E+08	1E+08	+	27.88888	NA	promoter-	promoter-	-112	NM_00134	221908	Hs.413359	NM_14503	ENSG0000	PPP1R35	C7orf47	protein ph	protein-co
lib-C666-l	chr7	1E+08	1E+08	+	50.62957	NA	promoter-	promoter-	-579	NM_17356	222950	Hs.320086	NM_17356	ENSG0000	NYAP1	C7orf51	neuronal ty	protein-co
lib-C666-l	chr7	1.39E+08	1.39E+08	+	33.93039	NA	promoter-	promoter-	156	NR_073059	154791	Hs.718441	NM_19796	ENSG0000	FMC1	C7orf55|HS	formation	protein-co
lib-C666-l	chr7	1.39E+08	1.39E+08	+	31.02683	NA	promoter-	promoter-	-30	NM_19796	154791	Hs.718441	NM_19796	ENSG0000	FMC1	C7orf55|HS	formation	protein-co
lib-C666-l	chr7	92158039	92158039	+	27.5466	NA	promoter-	promoter-	-48	NM_03212	84060	Hs.21590	NM_03212	ENSG0000	RBM48	C7orf64|HS	RNA bindir	protein-co
lib-C666-l	chr7	1.28E+08	1.28E+08	+	44.9216	NA	promoter-	promoter-	-75	NM_01333	29923	Hs.706124	NM_01333	ENSG0000	HILPDA	C7orf68|HI	hypoxia inc	protein-co
lib-C666-l	chr8	90914071	90914071	+	34.92958	NA	promoter-	promoter-	-25	NM_00433	734	Hs.436445	NM_00433	ENSG0000	OSGIN2	C8orf1|hT4	oxidative s	protein-co
lib-C666-l	chr8	37594123	37594123	+	130.3773	NA	promoter-	promoter-	26	NM_00100	11160	Hs.705490	NM_00717	ENSG0000	ERLIN2	C8orf2|Erlir	ER lipid raf	protein-co
lib-C666-l	chr8	11059121	11059121	+	46.59515	NA	promoter-	promoter-	-246	NR_138153	286046	Hs.657083	NM_17368	ENSG0000	XKR6	C8orf21|C8	XK related	protein-co
lib-C666-l	chr8	1.26E+08	1.26E+08	+	85.88258	NA	promoter-	promoter-	0	NM_17368	286053	Hs.388297	NM_17368	ENSG0000	NSMCE2	C8orf36|M	NSE2/MMS	protein-co
lib-C666-l	chr8	42396787	42396787	+	82.1828	NA	promoter-	promoter-	22	NM_00113	114926	Hs.655320	NM_13843	ENSG0000	SMIM19	C8orf40	small integ	protein-co
lib-C666-l	chr8	1.18E+08	1.18E+08	+	20.19521	NA	promoter-	promoter-	-109	NM_03233	84294	Hs.86970	NM_03233	ENSG0000	UTP23	C8orf53	UTP23, sm	protein-co
lib-C666-l	chr8	11142552	11142552	+	23.80446	NA	exon (NM_	exon (NM_	552	NM_01545	66036	Hs.591395	NM_01545	ENSG0000	MTMR9	C8orf9|LIP-	myotubula	protein-co
lib-C666-l	chr9	1.3E+08	1.3E+08	+	89.57237	NA	promoter-	promoter-	-21	NM_00134	138428	Hs.653265	NM_00100	ENSG0000	PTRH1	C9orf115|P	peptidyl-tF	protein-co
lib-C666-l	chr9	1.31E+08	1.31E+08	+	23.34628	NA	promoter-	promoter-	15	NM_00104	375757	Hs.259594	NM_00104	ENSG0000	SWI5	C9orf119|S	SWI5 hom	protein-co
lib-C666-l	chr9	1.31E+08	1.31E+08	+	20.84232	NA	promoter-	promoter-	42	NM_00131	375757	Hs.259594	NM_00104	ENSG0000	SWI5	C9orf119|S	SWI5 hom	protein-co
lib-C666-l	chr9	35829279	35829279	+	21.90047	NA	promoter-	promoter-	57	NM_01644	51754	Hs.493808	NM_01644	ENSG0000	TMEM8B	C9orf127|N	transmemb	protein-co
lib-C666-l	chr9	1.01E+08	1.01E+08	+	61.38507	NA	intron (NM	CpG	294	NM_00133	51531	Hs.9196	NM_01648	ENSG0000	TRMO	C9orf156|H	tRNA meth	protein-co
lib-C666-l	chr9	77643422	77643422	+	65.54223	NA	promoter-	promoter-	-76	NM_15242	138199	Hs.567688	NM_15242	ENSG0000	CARNMT1	C9orf41|UF	carnosine I	protein-co
lib-C666-l	chr9	71394903	71394903	+	125.5274	NA	promoter-	promoter-	-19	NM_13833	116224	Hs.655572	NM_13833	ENSG0000	FAM122A	C9orf42	family with	protein-co
lib-C666-l	chr9	1.32E+08	1.32E+08	+	64.08802	NA	promoter-	promoter-	-40	NM_00633	10444	Hs.147950	NM_00633	ENSG0000	ZER1	C9orf60|ZY	zyg-11 rela	protein-co
lib-C666-l	chr9	1.15E+08	1.15E+08	+	73.41202	NA	promoter-	promoter-	18	NR_138054	58493	Hs.658575	NM_02121	ENSG0000	INIP	C9orf80|HS	INTS3 and	protein-co
lib-C666-l	chr9	26892789	26892789	+	60.75948	NA	promoter-	promoter-	37	NM_00116	79886	Hs.178357	NM_02482	ENSG0000	CAAP1	C9orf82|CA	caspase ac	protein-co
lib-C666-l	chr9	1.36E+08	1.36E+08	+	124.8021	NA	promoter-	promoter-	4	NM_00131	11092	Hs.62595	NM_01895	ENSG0000	SPACA9	C9orf9|Mas	sperm acro	protein-co
lib-C666-l	chrX	15693691	15693691	+	23.4136	NA	intron (NR_	CpG	652	NR_026551	340591	Hs.732244	NR_026551	ENSG0000	CA5BP1	CA5BL|CA5	carbonic a	pseudo
lib-C666-l	chr8	17103997	17103997	+	55.6757	NA	promoter-	promoter-	-30	NM_00132	29883	Hs.587465	NM_01335	ENSG0000	CNOT7	CAF-1|CAF	CCR4-NOT	protein-co
lib-C666-l	chr19	4402337	4402337	+	22.14682	NA	promoter-	promoter-	-323	NM_00548	10036	Hs.79018	NM_00548	ENSG0000	CHAF1A	CAF-1|CAF	chromatin	protein-co
lib-C666-l	chr12	57824857	57824857	+	106.0503	NA	promoter-	promoter-	-69	NM_00133	22864	Hs.443673	NM_01492	ENSG0000	R3HDM2	CAG6|PR01	R3H doma	protein-co
lib-C666-l	chr12	6798782	6798782	+	44.27569	NA	promoter-	promoter-	-44	NM_00113	171017	Hs.103315	NM_13347	ENSG0000	ZNF384	CAGH1|CA	zinc finger	protein-co
lib-C666-l	chr5	1.13E+08	1.13E+08	+	43.12596	NA	promoter-	promoter-	30	NM_00134	64848	Hs.231942	NM_02282	ENSG0000	YTHDC2	CAHL	YTH doma	protein-co
lib-C666-l	chr3	20227808	20227808	+	25.39084	NA	promoter-	promoter-	-83	NM_00119	151648	Hs.105153	NM_13848	ENSG0000	SGO1	CAID|NY-E	shugoshin	protein-co
lib-C666-l	chr9	1.34E+08	1.34E+08	+	318.6751	NA	promoter-	promoter-	6	NM_00508	8021	Hs.654530	NM_00508	ENSG0000	NUP214	CAIN|CAN	nucleopori	protein-co
lib-C666-l	chr5	86708758	86708758	+	98.87846	NA	promoter-	promoter-	-37	NM_00119	902	Hs.292524	NM_00123	ENSG0000	CCNH	CAK|CycH|	cyclin H	protein-co
lib-C666-l	chr7	27779727	27779727	+	30.97173	NA	promoter-	promoter-	13	NM_00107	8887	Hs.34576	NM_00602	ENSG0000	TAX1BP1	CALCOCO:	Tax1 bindi	protein-co
lib-C666-l	chr2	68479811	68479811	+	71.09715	NA	promoter-	promoter-	-160	NM_00094	5534	Hs.280604	NM_00094	ENSG0000	PPP3R1	CALNB1|CI	protein ph	protein-co
lib-C666-l	chr19	36631022	36631022	+	36.38376	NA	promoter-	promoter-	-7	NM_00130	826	Hs.515371	NM_00174	ENSG0000	CAPNS1	CALPAIN4|	calpain sm	protein-co
lib-C666-l	chr19	47104333	47104333	+	49.72588	NA	promoter-	promoter-	2	NM_00132	808	Hs.515487	NM_00518	ENSG0000	CALM3	CaM|CaMII	calmodulin	protein-co
lib-C666-l	chr2	47403888	47403888	+	94.79198	NA	promoter-	promoter-	-126	NM_00174	805	Hs.468442	NM_00174	ENSG0000	CALM2	CAMII|LQT	calmodulin	protein-co
lib-C666-l	chr17	3793564	3793564	+	171.1925	NA	intron (NM	intron (NM	473	NM_17220	84254	Hs.8417	NM_03229	ENSG0000	CAMKK1	CAMKKA	calcium/ca	protein-co
lib-C666-l	chr17	1619728	1619728	+	113.6233	NA	promoter-	promoter-	-89	NM_15234	124997	Hs.234572	NM_15234	ENSG0000	WDR81	CAMRQ2|F	WD repeat	protein-co
lib-C666-l	chr5	96271651	96271651	+	78.02469	NA	5' UTR (NM	5' UTR (NM	305	NM_00557	4012	Hs.527199	NM_00557	ENSG0000	LNPEP	CAP|IRAP|F	leucyl and	protein-co
lib-C666-l	chr5	96270943	96270943	+	39.64852	NA	promoter-	promoter-	-403	NM_00557	4012	Hs.527199	NM_00557	ENSG0000	LNPEP	CAP|IRAP|F	leucyl and	protein-co
lib-C666-l	chr4	1.3E+08	1.3E+08	+	65.54223	NA	promoter-	promoter-	2	NM_14464	132320	Hs.654690	NM_14464	ENSG0000	SCLT1	CAP-1A|CA	sodium ch	protein-co
lib-C666-l	chr20	34330363	34330363	+	32.04651	NA	promoter-	promoter-	-105	NR_040724	9584	Hs.282901	NM_00490	ENSG0000	RBM39	CAPER|CAF	RNA bindir	protein-co
lib-C666-l	chr14	23388154	23388154	+	541.5649	NA	promoter-	promoter-	242	NM_00107	55147	Hs.4997	NM_01810	ENSG0000	RBM23	CAPERbeta	RNA bindir	protein-co
lib-C666-l	chr7	1.58E+08	1.58E+08	+	22.66433	NA	promoter-	promoter-	-11	NM_00128	54892	Hs.18616	NM_01776	ENSG0000	NCAPG2	CAP-G2|CA	non-SMC	protein-co
lib-C666-l	chr22	50946654	50946654	+	176.0937	NA	promoter-	promoter-	9	NM_01455	29781	Hs.730607	NM_01455	ENSG0000	NCAPH2	CAPH2	non-SMC	protein-co
lib-C666-l	chr16	2771159	2771159	+	78.34339	NA	promoter-	promoter-	-607	NM_03194	83886	Hs.332878	NM_03194	ENSG0000	PRSS27	CAPH2|MP	protease, s	protein-co
lib-C666-l	chr1	1.54E+08	1.54E+08	+	45.65358	NA	promoter-	promoter-	-17	NM_00127	7170	Hs.535581	NM_15226	ENSG0000	TPM3	CAPM1|CF	tropomyos	protein-co
lib-C666-l	chr1	1.13E+08	1.13E+08	+	68.95776	NA	promoter-	promoter-	261	NM_00613	829	Hs.744974	NM_00613	ENSG0000	CAPZA1	CAPPA1|CA	capping ac	protein-co
lib-C666-l	chrX	41192401	41192401	+	50.76246	NA	promoter-	promoter-	-160	NM_00135	1654	Hs.728563	NM_00135	ENSG0000	DDX3X	CAP-Rf|DB	DEAD-box	protein-co
lib-C666-l	chr21	18885301	18885301	+	30.8056	NA	promoter-	promoter-	77	NM_00120	1525	Hs.627078	NM_00133	ENSG0000	CXADR	CAR|CAR4	coxsackie	protein-co
lib-C666-l	chr17	9479078	9479078	+	23.80446	NA	promoter-	promoter-	197	NR_033656	9482	Hs.431109	NM_00485	ENSG0000	STX8	CARB	syntaxin 8	protein-co
lib-C666-l	chr8	90770208	90770208	+	60.13322	NA	promoter-	promoter-	233	NM_00382	8767	Hs.103755	NM_00382	ENSG0000	RIPK2	CARD3|CA	receptor in	protein-co
lib-C666-l	chr17	33390797	33390797	+	23.5959	NA	promoter-	promoter-	-38	NM_00101	117584	Hs.13680	NM_05717	ENSG0000	RFFL	CARP-2|CA	ring finger	protein-co
lib-C666-l	chr2	1.7E+08	1.7E+08	+	137.7653	NA	promoter-	promoter-	33	NM_00479	9360	Hs.470544	NM_00479	ENSG0000	PPIG	CARS-Cyp	peptidylpro	protein-co
lib-C666-l	chr17	27071473	27071473	+	129.0074	NA	intron (NM	CpG	450	NM_00429	9618	Hs.8375	NM_00429	ENSG0000	TRAF4	CART1|MLI	TNF recept	protein-co
lib-C666-l	chr16	475529	475529	+	49.86005	NA	promoter-	promoter-	-139	NM_01470	9727	Hs.531642	NM_01470	ENSG0000	RAB11FIP3	CART1|Rab	RAB11 fam	protein-co
lib-C666-l	chr12	46384459	46384459	+	50.11658	NA	promoter-	promoter-	-58	NM_00471	9169	Hs.210367	NM_00471	ENSG0000	SCAF11	CASP11|SF	SR-related	protein-co
lib-C666-l	chr6	30524182	30524182	+	45.96589	NA	promoter-	promoter-	-304	NM_02526	80742	Hs.118354	NM_02526	ENSG0000	PRR3	CAT56	proline rich	protein-co
lib-C666-l	chr9	1E+08	1E+08	+	58.77799	NA	promoter-	promoter-	-186	NM_01429	23424	Hs.193842	NM_01429	ENSG0000	TDRD7	CATC4|PC1	tudor dom	protein-co
lib-C666-l	chr11	68671364	68671364	+	57.45638	NA	promoter-	promoter-	45	NM_00218	3508	Hs.503048	NM_00218	ENSG0000	IGHMBP2	CATF1|CM	immunoglo	protein-co
lib-C666-l	chr1	1.56E+08	1.56E+08	+	27.70954	NA	promoter-	promoter-	-54	NM_02038	57111	Hs.632469	NM_02038	ENSG0000	RAB25	CATX-8|RA	RAB25, me	protein-co
lib-C666-l	chr3	1.97E+08	1.97E+08	+	195.7658	NA	promoter-	promoter-	-10	NM_00736	22916	Hs.591671	NM_00736	ENSG0000	NCBP2	CBC2|CBP2	nuclear ca	protein-co

lib-C666-l	chr4	1.47E+08	1.47E+08	+	31.18315	NA	promoter-	promoter-	97	NM_17225	166785	Hs.452864	NM_17225	ENSG0000	MMAA	cblA	methylmal	protein-co
lib-C666-l	chr1	45965894	45965894	+	26.52079	NA	promoter-	promoter-	38	NM_01550	25974	Hs.13024	NM_01550	ENSG0000	MMACHC	cblC	methylmal	protein-co
lib-C666-l	chr16	3930852	3930852	+	22.23727	NA	promoter-	promoter-	-731	NM_00438	1387	Hs.459759	NM_00438	ENSG0000	CREBBP	CBP|KAT3A	CREB bind	protein-co
lib-C666-l	chr9	1E+08	1E+08	+	40.12713	NA	promoter-	promoter-	-72	NM_00248	4686	Hs.595669	NM_00248	ENSG0000	NCBP1	CBP80|NCI	nuclear cap	protein-co
lib-C666-l	chr9	1E+08	1E+08	+	23.05659	NA	promoter-	promoter-	-593	NM_00248	4686	Hs.595669	NM_00248	ENSG0000	NCBP1	CBP80|NCI	nuclear cap	protein-co
lib-C666-l	chr3	3168604	3168604	+	56.5598	NA	promoter-	promoter-	4	NM_18291	51095	Hs.732725	NM_01600	ENSG0000	TRNT1	CCA1|CGI-	tRNA nucle	protein-co
lib-C666-l	chr12	94853754	94853754	+	27.84631	NA	promoter-	promoter-	10	NM_01612	51134	Hs.279209	NM_01612	ENSG0000	CEP83	CCDC41|N	centrosom	protein-co
lib-C666-l	chr17	62502899	62502899	+	24.25638	NA	promoter-	promoter-	45	NR_13364	90799	Hs.569713	NM_13836	ENSG0000	CEP95	CCDC45	centrosom	protein-co
lib-C666-l	chr17	36981622	36981622	+	615.9252	NA	promoter-	promoter-	-19	NR_07342	54883	Hs.406223	NM_01774	ENSG0000	CWC25	CCDC49	CWC25 spl	protein-co
lib-C666-l	chr3	48481606	48481606	+	33.60578	NA	promoter-	promoter-	-42	NM_01593	51372	Hs.356440	NM_01593	ENSG0000	TMA7	CCDC72|H	translation	protein-co
lib-C666-l	chr4	1.23E+08	1.23E+08	+	105.0888	NA	promoter-	promoter-	45	NM_00123	890	Hs.58974	NM_00123	ENSG0000	CCNA2	CCN1|CCN	cyclin A2	protein-co
lib-C666-l	chr5	68462799	68462799	+	20.59244	NA	promoter-	promoter-	-38	NM_03196	891	Hs.23960	NM_03196	ENSG0000	CCNB1	CCNB	cyclin B1	protein-co
lib-C666-l	chr4	77997345	77997345	+	176.7042	NA	promoter-	promoter-	-220	NM_00683	10983	Hs.518827	NM_00683	ENSG0000	CCNI	CCNI1|CYC	cyclin I	protein-co
lib-C666-l	chr12	49110538	49110538	+	59.83187	NA	5' UTR (NM	5' UTR (NM	243	NM_00127	904	Hs.92308	NM_00124	ENSG0000	CCNT1	CCNT|CYC	cyclin T1	protein-co
lib-C666-l	chr12	49110870	49110870	+	25.3508	NA	promoter-	promoter-	-89	NM_00124	904	Hs.92308	NM_00124	ENSG0000	CCNT1	CCNT|CYC	cyclin T1	protein-co
lib-C666-l	chr9	88356914	88356914	+	25.49755	NA	promoter-	promoter-	30	NM_00128	23287	Hs.719980	NM_01523	ENSG0000	AGTPBP1	CCP1|NNA	ATP/GTP b	protein-co
lib-C666-l	chr9	90589613	90589613	+	36.70779	NA	promoter-	promoter-	82	NM_01211	23552	Hs.522274	NM_01211	ENSG0000	CDK20	CCRK|CDC	cyclin depe	protein-co
lib-C666-l	chr6	74230870	74230870	+	24.5284	NA	promoter-	promoter-	-115	NM_00140	1915	Hs.535192	NM_00140	ENSG0000	EEF1A1	CCS-3|CCS	eukaryotic	protein-co
lib-C666-l	chr16	1470813	1470813	+	242.0976	NA	promoter-	promoter-	-12	NM_00127	283951	Hs.58373	NM_00101	ENSG0000	C16orf91	CCSMST1|I	chromosor	protein-co
lib-C666-l	chr1	1.56E+08	1.56E+08	+	23.22307	NA	promoter-	promoter-	37	NM_00599	7203	Hs.491494	NM_00599	ENSG0000	CCT3	CCT-gamn	chaperonir	protein-co
lib-C666-l	chr7	56119349	56119349	+	27.28944	NA	promoter-	promoter-	-29	NM_00100	908	Hs.82916	NM_00176	ENSG0000	CCT6A	CCT-zeta|	chaperonir	protein-co
lib-C666-l	chr17	33288519	33288519	+	47.60628	NA	promoter-	promoter-	9	NM_00119	10693	Hs.73072	NM_00658	ENSG0000	CCT6B	CCT-zeta-	chaperonir	protein-co
lib-C666-l	chr13	1.14E+08	1.14E+08	+	24.18348	NA	promoter-	promoter-	-34	NM_00556	3916	Hs.494419	NM_00556	ENSG0000	LAMP1	CD107a|LA	lysosomal	protein-co
lib-C666-l	chr6	1.38E+08	1.38E+08	+	30.07415	NA	promoter-	promoter-	5	NM_00041	3459	Hs.520414	NM_00041	ENSG0000	IFNGR1	CD119|IFN	interferon	protein-co
lib-C666-l	chr15	89182042	89182042	+	37.31675	NA	promoter-	promoter-	68	NM_00130	3669	Hs.459265	NM_00220	ENSG0000	ISG20	CD25|HEM	interferon	protein-co
lib-C666-l	chr14	1.05E+08	1.05E+08	+	181.8953	NA	promoter-	promoter-	-17	NM_02170	10572	Hs.112058	NM_00642	ENSG0000	SIVA1	CD27BP|SI	SIVA1 apo	protein-co
lib-C666-l	chr10	33247193	33247193	+	35.7914	NA	promoter-	promoter-	100	NM_00221	3688	Hs.643813	NM_00221	ENSG0000	ITGB1	CD29|FNR	integrin su	protein-co
lib-C666-l	chr1	1.61E+08	1.61E+08	+	21.94284	NA	promoter-	promoter-	-51	NM_01694	50848	Hs.517293	NM_01694	ENSG0000	F11R	CD321|JAN	F11 recept	protein-co
lib-C666-l	chr17	37844450	37844450	+	46.69447	NA	promoter-	promoter-	113	NM_00128	2064	Hs.446352	NM_00444	ENSG0000	ERBB2	CD340|HEI	erb-b2 rec	protein-co
lib-C666-l	chr14	74486018	74486018	+	64.22771	NA	promoter-	promoter-	8	NM_00132	957	Hs.655070	NM_00124	ENSG0000	ENTPD5	CD39L4|N	ectonuclec	protein-co
lib-C666-l	chr3	1.96E+08	1.96E+08	+	112.7506	NA	promoter-	promoter-	-8	NM_00131	7037	Hs.529618	NM_00323	ENSG0000	TFRC	CD71|IMD	transferrin	protein-co
lib-C666-l	chrX	53111416	53111416	+	96.10687	NA	promoter-	promoter-	-126	NM_02211	64061	Hs.136164	NM_02211	ENSG0000	TSPYL2	CDA1|CIN	TSPY like 2	protein-co
lib-C666-l	chr6	29691136	29691136	+	26.54591	NA	promoter-	promoter-	19	NM_00109	3134	Hs.519972	NM_01895	ENSG0000	HLA-F	CDA12|HL	major histo	protein-co
lib-C666-l	chr12	29534188	29534188	+	23.087	NA	promoter-	promoter-	-45	NM_01657	51290	Hs.339453	NM_01657	ENSG0000	ERGIC2	CDA14|Erv	ERGIC and	protein-co
lib-C666-l	chr20	18488473	18488473	+	45.43086	NA	promoter-	promoter-	-134	NM_00117	10483	Hs.369373	NM_00636	ENSG0000	SEC23B	CDA-II|CD	Sec23 hom	protein-co
lib-C666-l	chr20	18488277	18488277	+	30.0396	NA	promoter-	promoter-	67	NM_00117	10483	Hs.369373	NM_00636	ENSG0000	SEC23B	CDA-II|CD	Sec23 hom	protein-co
lib-C666-l	chr7	35840530	35840530	+	23.087	NA	promoter-	promoter-	-66	NM_00101	989	Hs.191346	NM_00178	ENSG0000	7-Sep	CDC10|CD	septin 7	protein-co
lib-C666-l	chr10	38691854	38691854	+	69.18568	NA	promoter-	promoter-	-74	NR_02726	285961	Hs.406568	NR_027269		SEPT7P9	CDC10L|SE	septin 7 ps	pseudo
lib-C666-l	chr10	38692150	38692150	+	32.75483	NA	promoter-	promoter-	-370	NR_02726	285961	Hs.406568	NR_027269		SEPT7P9	CDC10L|SE	septin 7 ps	pseudo
lib-C666-l	chr9	99382151	99382151	+	134.7898	NA	promoter-	promoter-	-39	NM_03333	8555	Hs.40582	NM_00367	ENSG0000	CDC14B	CDC14B3|	cell divisio	protein-co
lib-C666-l	chr19	3506096	3506096	+	80.93561	NA	promoter-	promoter-	-199	NM_01626	51343	Hs.413133	NM_01626	ENSG0000	FZR1	CDC20C|C	fizzy/cell d	protein-co
lib-C666-l	chr1	1655854	1655854	+	180.0211	NA	promoter-	promoter-	5	NM_00178	984	Hs.651228	NM_00178	ENSG0000	CDK11B	CDC2L1|C	cyclin depe	protein-co
lib-C666-l	chr9	4679577	4679577	+	46.59515	NA	promoter-	promoter-	24	NM_01791	55664	Hs.666439	NM_01791	ENSG0000	CDC37L1	CDC37B|H	cell divisio	protein-co
lib-C666-l	chr1	22379156	22379156	+	68.38644	NA	promoter-	promoter-	36	NM_00103	998	Hs.467637	NM_00179	ENSG0000	CDC42	CDC42Hs|	cell divisio	protein-co
lib-C666-l	chr7	99698348	99698348	+	31.79085	NA	promoter-	promoter-	55	NM_18277	4176	Hs.438720	NM_00591	ENSG0000	MCM7	CDC47|MC	minichrom	protein-co
lib-C666-l	chr6	44355175	44355175	+	30.85659	NA	promoter-	promoter-	-76	NM_00125	988	Hs.485471	NM_00125	ENSG0000	CDC5L	CDC5|CDC	cell divisio	protein-co
lib-C666-l	chr2	2.43E+08	2.43E+08	+	32.29191	NA	promoter-	promoter-	89	NM_00132	1841	Hs.471873	NM_01214	ENSG0000	DTYMK	CDC8|PP3	deoxythym	protein-co
lib-C666-l	chr20	33265036	33265036	+	99.27254	NA	promoter-	promoter-	53	NM_08047	128869	Hs.253319	NM_08047	ENSG0000	PIGU	CDC91L1|C	phosphatic	protein-co
lib-C666-l	chr16	89989667	89989667	+	25.39487	NA	promoter-	promoter-	-20	NM_00608	10381	Hs.511743	NM_00608	ENSG0000	TUBB3	CDCBM|CD	tubulin bet	protein-co
lib-C666-l	chr6	3157845	3157845	+	29.07066	NA	promoter-	promoter-	-62	NM_00106	7280	Hs.654543	NM_00106	ENSG0000	TUBB2A	CDCBM5|T	tubulin bet	protein-co
lib-C666-l	chr22	19705913	19705913	+	41.5993	NA	promoter-	promoter-	-45	NM_00100	5413	Hs.728762	NM_00268	ENSG0000	5-Sep	CDCREL|C	septin 5	protein-co
lib-C666-l	chr3	53164464	53164464	+	76.14658	NA	promoter-	promoter-	6	NM_05285	91869	Hs.631910	NM_05285	ENSG0000	RFT1	CDG1N	RFT1 homo	protein-co
lib-C666-l	chr4	56212427	56212427	+	27.79032	NA	promoter-	promoter-	39	NM_02459	79644	Hs.39311	NM_02459	ENSG0000	SRD5A3	CDG1P|CD	steroid 5 a	protein-co
lib-C666-l	chrX	1.11E+08	1.11E+08	+	23.74671	NA	promoter-	promoter-	11	NM_00132	79868	Hs.443061	NM_01846	ENSG0000	ALG13	CDG1S|CX	ALG13, UD	protein-co
lib-C666-l	chr12	7282975	7282975	+	30.07415	NA	promoter-	promoter-	8	NM_01471	9746	Hs.535378	NM_01471	ENSG0000	CLSTN3	CDHR14|C	calsyntenir	protein-co
lib-C666-l	chrX	48334506	48334506	+	53.56082	NA	promoter-	promoter-	97	NM_17743	24140	Hs.23170	NM_01228	ENSG0000	FTSJ1	CDLIV|JM2	FtsJ RNA m	protein-co
lib-C666-l	chr7	1E+08	1E+08	+	27.84631	NA	promoter-	promoter-	-15	NM_00104	64598	Hs.521086	NM_02394	ENSG0000	MOSPD3	CDS3|NET	motile sper	protein-co
lib-C666-l	chr19	48949253	48949253	+	20.90919	NA	5' UTR (NM	5' UTR (NM	223	NM_03148	83743	Hs.400625	NM_03148	ENSG0000	GRWD1	CDW4|GR	glutamate	protein-co
lib-C666-l	chr1	7831439	7831439	+	43.37893	NA	5' UTR (NM	5' UTR (NM	110	NM_00478	9341	Hs.66708	NM_00478	ENSG0000	VAMP3	CEB	vesicle ass	protein-co
lib-C666-l	chr12	57914409	57914409	+	45.23877	NA	promoter-	promoter-	-109	NM_00408	1649	Hs.505777	NM_00408	ENSG0000	DDIT3	CEBPZ|CH	DNA dama	protein-co
lib-C666-l	chr6	32636299	32636299	+	38.21529	NA	Intergenic	Intergenic	-1833	NM_00212	3119	Hs.409934	NM_00212	ENSG0000	HLA-DQB1	CELIAC1|H	major histo	protein-co
lib-C666-l	chr4	1.04E+08	1.04E+08	+	67.49158	NA	promoter-	promoter-	-133	NM_00128	1062	Hs.75573	NM_00181	ENSG0000	CENPE	CENP-E|KI	centromer	protein-co
lib-C666-l	chr11	72433607	72433607	+	143.3197	NA	promoter-	promoter-	-204	NM_00113	116985	Hs.503165	NM_01524	ENSG0000	ARAP1	CENTD2	ArfGAP wit	protein-co
lib-C666-l	chr19	18682576	18682576	+	73.77308	NA	promoter-	promoter-	36	NM_00132	7311	Hs.5308	NM_00333	ENSG0000	UBA52	CEP52|HU	ubiquitin A	protein-co
lib-C666-l	chr17	7165988	7165988	+	45.24737	NA	5' UTR (NM	5' UTR (NM	276	NM_00118	1366	Hs.513915	NM_00130	ENSG0000	CLDN7	CEPTRL2|C	claudin 7	protein-co
lib-C666-l	chr9	1.4E+08	1.4E+08	+	92.98441	NA	promoter-	promoter-	-69	NM_00379	8721	Hs.174050	NM_00379	ENSG0000	EDF1	CFAP280|E	endothelia	protein-co
lib-C666-l	chr19	17530748	17530748	+	20.0808	NA	promoter-	promoter-	92	NM_13840	93343	Hs.515243	NM_13840	ENSG0000	MVB12A	CFBP|FAM	multivesicu	protein-co
lib-C666-l	chr15	66679082	66679082	+	167.6989	NA	promoter-	promoter-	-129	NM_00275	5604	Hs.145442	NM_00275	ENSG0000	MAP2K1	CFC3|MAP	mitogen-a	protein-co
lib-C666-l	chr2	2.09E+08	2.09E+08	+	36.59122	NA	promoter-	promoter-	-200	NM_15267	200576	Hs.744997	NM_01504	ENSG0000	PIKFYVE	CFD|FAB1|	phosphoin	protein-co
lib-C666-l	chr16	1832804	1832804	+	124.6706	NA	promoter-	promoter-	-120	NM_01222	10101	Hs.256549	NM_01222	ENSG0000	NUBP2	CFD1|NBP	nucleotide	protein-co
lib-C666-l	chrX	1.53E+08	1.53E+08	+	85.71884	NA	promoter-	promoter-	-585	NM_00533	3054	Hs.83634	NM_00533	ENSG0000	HCFC1	CFF|HCF|H	host cell fa	protein-co
lib-C666-l	chr12	69633341	69633341	+	37.64901	NA	promoter-	promoter-	24	NM_00130	11052	Hs.369606	NM_00700	ENSG0000	CPSF6	CFIM|CFIM	cleavage a	protein-co
lib-C666-l	chr7	23221586	23221586	+	168.2645	NA	5' UTR (NM	5' UTR (NM	140	NM_00734	11097	Hs.408241	NM_00734	ENSG0000	NUPL2	CG1|NLP-1	nucleopori	protein-co
lib-C666-l	chr1	51425987	51425987	+	130.7493	NA	promoter-	promoter-	-51	NM_00705	11124	Hs.530402	NM_00705	ENSG0000	FAF1	CGI-03|HF	Fas associa	protein-co
lib-C666-l	chr2	96068494	96068494	+	75.47253	NA	promoter-	promoter-	46	NM_01604	51011	Hs.531247	NM_01604	ENSG0000	FAHD2A	CGI-105	fumarylace	protein-co
lib-C666-l	chr2	24299277	24299277	+	61.35856	NA	promoter-	promoter-	37	NM_01604	51639	Hs.177861	NM_01604	ENSG0000	SF3B6	CGI-110|H	splicing fac	protein-co
lib-C666-l	chr1	51701961	51701961	+	34.12565	NA	promoter-	promoter-	16	NM_01437	26994	Hs.309641	NM_01437	ENSG0000	RNF11	CGI-123|SI	ring finger	protein-co
lib-C666-l	chr3	1.79E+08	1.79E+08	+	27.5466	NA	promoter-	promoter-	-42	NM_02040	57129	Hs.283734	NM_02040	ENSG0000	MRPL47	CGI-204|L	mitochond	protein-co
lib-C666-l	chr6	43027205	43027205	+	129.0765	NA	promoter-	promoter-	78	NM_01595	51069	Hs.55041	NM_01595	ENSG0000	MRPL2	CGI-22|MF	mitochond	protein-co
lib-C666-l	chr19	10362848	10362848	+	209.6795	NA	5' UTR (NM	5' UTR (NM	208	NM_01595	51073	Hs.279652	NM_01595	ENSG0000	MRPL4	CGI-28|L4	mitochond	protein-co
lib-C666-l	chr14	55738832	55738832	+	279.2322	NA	promoter-	promoter-	-40	NM_15223	55030	Hs.525348	NM_01794	ENSG0000	FBXO34	CGI-301|F	F-box prot	protein-co
lib-C666-l	chr14	55738048	55738048	+	144.0758	NA	promoter-	promoter-	27	NM_01794	55030	Hs.525348	NM_01794	ENSG0000	FBXO34	CGI-301|F	F-box prot	protein-co
lib-C666-l	chr11	72504946	72504946	+	25.10918	NA	promoter-	promoter-	-196	NM_00664	10809	Hs.188606	NM_00664	ENSG0000	STARD10	CGI-52|NY	StAR relate	protein-co
lib-C666-l	chr1	29557457	29557457	+	133.8809	NA	promoter-	promoter-	13	NM_00102	51102	Hs.183646	NM_01601	ENSG0000	MECR	CGI-63|FA	mitochond	protein-co
lib-C666-l	chr6	36954106	36954106	+	20.59244	NA	non-codin	non-codin	221	NR_13073	23787	Hs.485262	NM_01434	ENSG0000	MTCH1	CGI-64|PI	mitochond	protein-co
lib-C666-l	chr16	25123045	25123045	+	45.65358	NA	promoter-	promoter-	-2	NM_00103	51451	Hs.337730	NM_01630	ENSG0000	LCMT1	CGI-68|LC	leucine car	protein-co
lib-C666-l	chr8	1.34E+08	1.34E+08	+	142.8748	NA	promoter-	promoter-	-14	NM_01601	51105	Hs.304362	NM_01601	ENSG0000	PHF20L1	CGI-72|TD	PHD finger	protein-co
lib-C666-l	chrX	1.29E+08	1.29E+08	+	27.61921	NA	5' UTR (NM	5' UTR (NM	183	NM_00100	51114	Hs.193566	NM_01603	ENSG0000	ZDHHC9	CGI89|CX	zinc finger	protein-co
lib-C666-l	chr1	38478316	38478316	+	26.26353	NA	promoter-	promoter-	-68	NM_01603	51118	Hs.472038	NM_01603	ENSG0000	UTP11	CGI-94|CG	UTP11, sm	protein-co
lib-C666-l	chr14	52456209	52456209	+	77.40921	NA	promoter-	promoter-	-19	NM_01603	51637	Hs.534457	NM_01603	ENSG0000	C14orf166	CGI-99|CG	chromosor	protein-co
lib-C666-l	chr4	1.16E+08	1.16E+08	+	34.0482	NA	promoter-	promoter-	242	NM_00132	7368	Hs.144197	NM_00336	ENSG0000	UGT8	CGT|UGT4	UDP glyco	protein-co
lib-C666-l	chr20	61493502	61493502	+	25.39487	NA	promoter-	promoter-	-387	NM_00130	10732	Hs.126248	NM_00660	ENSG0000	TCFL5	CHA|E2BP	transcripti	protein-co
lib-C666-l	chr17	57697105	57697105	+	58.84504	NA	promoter-	promoter-	55	NM_00485	1213	Hs.491351	NM_00485	ENSG0000	CLTC	CHC|CHC1	clathrin he	protein-co
lib-C666-l	chr9	35657819	35657819	+	49.86005	NA	promoter-	promoter-	196	NR_00305	6023	Hs.587502	NR_00305	ENSG0000	RMRP	CHH|NME	RNA comp	ncRNA
lib-C666-l	chr11	67888952	67888952	+	34.02388	NA	promoter-	promoter-	-94	NM_21246	1119	Hs.77221	NM_00127	ENSG0000	CHKA	CHK|CK|CK	choline kin	protein-co
lib-C666-l	chr21	46961756	46961756	+	28.88196	NA	intron (NM	CpG	629	NM_00120	6573	Hs.84190	NM_00305	ENSG0000	SLC19A1	CHMD|FOI	solute carr	protein-co
lib-C666-l	chr9	1.03E+08	1.03E+08	+	166.1177	NA	promoter-	promoter-	-48	NM_17320	8013	Hs.279522	NM_00698	ENSG0000	NR4A3	CHN|CSMI	nuclear rec	protein-co
lib-C666-l	chr7	1.59E+08	1.59E+08	+	424.5518	NA	promoter-	promoter-	0	NM_02072	57488	Hs.490795	NM_02072	ENSG0000	ESYT2	CHR2SYT|E	extended s	protein-co
lib-C666-l	chr1	2.36E+08	2.36E+08	+	29.93113	NA	promoter-	promoter-	-181	NM_00008	1130	Hs.532411	NM_00008	ENSG0000	LYST	CHS|CHS1	lysosomal	protein-co
lib-C666-l	chr19	42759309	42759309	+	30.13508	NA	promoter-	promoter-	7	NM_00649	2077	Hs.655969	NM_00649	ENSG0000	ERF	CHYTS|CR	ETS2 repre	protein-co
lib-C666-l	chr21	44313260	44313260	+	61.37072	NA	promoter-	promoter-	-118	NM_00100	4731	Hs.473937	NM_02107	ENSG0000	NDUFV3	CI-10k|CI-	NADH:ubic	protein-co
lib-C666-l	chr18	9101920	9101920	+	32.75483	NA	promoter-	promoter-	-708	NM_02107	4729	Hs.464572	NM_02107	ENSG0000	NDUFV2	CI-24k	NADH:ubic	protein-co
lib-C666-l	chr2	2.41E+08	2.41E+08	+	233.4118	NA	promoter-	promoter-	-12	NM_00454	4705	Hs.277677	NM_00454	ENSG0000	NDUFA10	CI-42KD|C	NADH:ubic	protein-co
lib-C666-l	chr11	67374370	67374370	+	86.18742	NA	promoter-	promoter-	47	NM_00116	4723	Hs.7744	NM_00710	ENSG0000	NDUFV1	CI-51K|CI	NADH:ubic	protein-co
lib-C666-l	chr2	96931829	96931829	+	39.21146	NA	promoter-	promoter-	-55	NM_00480	9391	Hs.12109	NM_00480	ENSG0000	CIAO1	CIA1|WDR	cytosolic ir	protein-co
lib-C666-l	chr4	74904221	74904221	+	22.02847	NA	intron (NM	CpG-1988	269	NM_00209	2921	Hs.89690	NM_00209	ENSG0000	CXCL3	CINC-2b|G	C-X-C mo	protein-co
lib-C666-l	chr1	26758791	26758791	+	260.51	NA	promoter-	promoter-	18	NM_00124	79947	Hs.369385	NM_02488	ENSG0000	DHDDS	CIT|CPT|D	dehydrodc	protein-co
lib-C666-l	chr19	1940767	1940767	+	233.9893	NA	promoter-	promoter-	-394	NM_00131	1455	Hs.651905	NM_00131	ENSG0000	CSNK1G2	CK1g2	casein kina	protein-co
lib-C666-l	chr3	1.41E+08	1.41E+08	+	262.8556	NA	promoter-	promoter-	-44	NM_00120	9616	Hs.134623	NM_01424	ENSG0000	RNF7	CKBBP1|R	ring finger	protein-co
lib-C666-l	chr1	1.55E+08	1.55E+08	+	21.84731	NA	Intergenic	CpG	-1356	NR_04976	51043	Hs.729279	NM_00125	ENSG0000	ZBTB7B	CKROX|TH	zinc finger	protein-co
lib-C666-l	chr9	91925829	91925829	+	47.72539	NA	promoter-	promoter-	-281	NM_00182	1164	Hs.83758	NM_00182	ENSG0000	CKS2	CKSHS2	CDC28 pro	protein-co
lib-C666-l	chr5	1.72E+08	1.72E+08	+	47.69789	NA	promoter-	promoter-	-171	NM_00441	1843	Hs.171695	NM_00441	ENSG0000	DUSP1	CL100|HVI	dual specif	protein-co
lib-C666-l	chr7	1.55E+08	1.55E+08	+	111.5322	NA	promoter-	promoter-	-88	NM_00554	3638	Hs.520819	NM_00554	ENSG0000	INSIG1	CL6	insulin ind	protein-co
lib-C666-l	chr4	1.71E+08	1.71E+08	+	27.44508	NA	promoter-	promoter-	-28	NM_17387	1182	Hs.481186	NM_00182	ENSG0000	CLCN3	CLC3|CIC-	chloride vc	protein-co
lib-C666-l	chr17	79818886	79818886	+	38.6745	NA	promoter-	promoter-	-342	NM_00091	5034	Hs.464336	NM_00091	ENSG0000	P4HB	CLCRP1|D	prolyl 4-hy	protein-co
lib-C666-l	chr2	2.02E+08	2.02E+08	+	63.46463	NA	promoter-	promoter-	66	NR_02785	1195	Hs.433732	NM_00407	ENSG0000	CLK1	CLK|CLK/S	CDC like ki	protein-co
lib-C666-l	chr7	1.08E+08	1.08E+08	+	31.26176	NA	promoter-	promoter-	5	NM_00229	3912	Hs.650585	NM_00229	ENSG0000	LAMB1	CLM|LIS5	laminin sul	protein-co
lib-C666-l	chr7	66093680	66093680	+	24.44958	NA	promoter-	promoter-	-188	NM_00116	154881	Hs.546627	NM_15303	ENSG0000	KCTD7	CLN14|EP	potassium	protein-co
lib-C666-l	chr11	6640743	6640743	+	116.9047	NA	promoter-	promoter-	-51	NM_00039	1200	Hs.523454	NM_00039	ENSG0000	TPP1	CLN2|GIG1	tripeptidyl	protein-co
lib-C666-l	chr17	55038219	55038219	+	52.78568	NA	exon (NM_	exon (NM_	192	NM_00464	8161	Hs.532795	NM_00464	ENSG0000	COIL	CLN80|p8	coilin	protein-co
lib-C666-l	chr6	47445352	47445352	+	20.59244	NA	promoter-	promoter-	-173	NM_01212	23607	Hs.485518	NM_01212	ENSG0000	CD2AP	CMS	CD2 associ	protein-co
lib-C666-l	chr18	13726645	13726645	+	35.47771	NA	promoter-	promoter-	-14	NM_00130	8731	Hs.592347	NM_00379	ENSG0000	RNMT	CMT1|CMT	RNA guani	protein-co
lib-C666-l	chr1	10270508	10270508	+	37.56522	NA	promoter-	promoter-	-256	NM_18341	23095	Hs.97858	NM_01507	ENSG0000	KIF1B	CMT2|CMT	kinesin fan	protein-co
lib-C666-l	chr1	12040434	12040434	+	264.1204	NA	5' UTR (NM	5' UTR (NM	196	NM_00112	9927	Hs.376681	NM_01487	ENSG0000	MFN2	CMT2A|C	mitofusin 2	protein-co
lib-C666-l	chr7	30634419	30634419	+	40.85015	NA	promoter-	promoter-	68	NM_00131	2617	Hs.404321	NM_00204	ENSG0000	GARS	CMT2D|DS	glycyl-tRN	protein-co
lib-C666-l	chr9	1.3E+08	1.3E+08	+	66.96093	NA	promoter-	promoter-	15	NM_00100	90678	Hs.495188	NM_13836	ENSG0000	LRSAM1	CMT2P|RIF	leucine rich	protein-co

lib-C666-l	chr2	2.2E+08	2.2E+08	+	41.10975	NA	5' UTR (NM	5' UTR (NM	101	NM_00103	3300	Hs.77768	NM_00673	ENSG0000	DNAJB2	CMT2T|DS	DnaJ heat	protein-co
lib-C666-l	chr18	72163403	72163403	+	24.68378	NA	promoter-	promoter-	-97	NM_01823	55748	Hs.149185	NM_01823	ENSG0000	CNDP2	CN2|CPGL	CNDP dipe	protein-co
lib-C666-l	chr22	46692446	46692446	+	27.31171	NA	promoter-	promoter-	111	NR_02400	150384	Hs.348663	NR_02400	ENSG0000	GTSE1-AS	CN5H6.4|L	GTSE1 anti	ncRNA
lib-C666-l	chr10	13203494	13203494	+	128.8371	NA	promoter-	promoter-	-60	NM_01851	55388	Hs.198363	NM_01851	ENSG0000	MCM10	CNA43|DN	minichrom	protein-co
lib-C666-l	chr9	17579045	17579045	+	29.01385	NA	promoter-	promoter-	93	NM_00302	6456	Hs.75149	NM_00302	ENSG0000	SH3GL2	CNSA2|EEI	SH3 doma	protein-co
lib-C666-l	chr9	179085	179085	+	55.79842	NA	promoter-	promoter-	-10	NM_00114	55871	Hs.531330	NM_01849	ENSG0000	CBWD1	COBP	COBW dor	protein-co
lib-C666-l	chr3	37034942	37034942	+	48.61377	NA	promoter-	promoter-	101	NM_00125	4292	Hs.195364	NM_00024	ENSG0000	MLH1	COCA2|FC	mutL hom	protein-co
lib-C666-l	chr12	54121408	54121408	+	49.83866	NA	promoter-	promoter-	-101	NM_00114	57658	Hs.156667	NM_02089	ENSG0000	CALCOCO	Cocoa|PP1	calcium bir	protein-co
lib-C666-l	chr19	5720204	5720204	+	70.69921	NA	promoter-	promoter-	-28	NM_00127	9361	Hs.350265	NM_00479	ENSG0000	LONP1	CODASS|L	lon peptid	protein-co
lib-C666-l	chr13	1.03E+08	1.03E+08	+	95.85526	NA	promoter-	promoter-	-59	NM_00012	2073	Hs.258429	NM_00012	ENSG0000	ERCC5	COFS3|ER	ERCC excis	protein-co
lib-C666-l	chr18	20513267	20513267	+	33.11264	NA	promoter-	promoter-	-28	NM_00289	5932	Hs.546282	NM_00289	ENSG0000	RBBP8	COM1|CTI	RB binding	protein-co
lib-C666-l	chr2	2.38E+08	2.38E+08	+	246.3951	NA	5' UTR (NM	5' UTR (NM	422	NM_00671	10920	Hs.531713	NM_00671	ENSG0000	COPS8	COP9|CSN	COP9 sign	protein-co
lib-C666-l	chr10	64893009	64893009	+	36.04455	NA	promoter-	promoter-	2	NM_03075	29982	Hs.449628	NM_03075	ENSG0000	NRBF2	COPR|COP	nuclear rec	protein-co
lib-C666-l	chr11	63741978	63741978	+	41.28836	NA	promoter-	promoter-	-101	NM_00407	1351	Hs.743989	NM_00407	ENSG0000	COX8A	COX|COX8	cytochrom	protein-co
lib-C666-l	chr4	73935532	73935532	+	173.0244	NA	promoter-	promoter-	-56	NM_00130	285521	Hs.356697	NM_17382	ENSG0000	COX18	COX18HS	COX18, cy	protein-co
lib-C666-l	chr16	23568677	23568677	+	95.46208	NA	promoter-	promoter-	19	NR_00350	124454	Hs.620541	NM_00108	ENSG0000	EARS2	COXPD12|	glutamyl-t	protein-co
lib-C666-l	chr14	61447806	61447806	+	255.7837	NA	promoter-	promoter-	-9	NM_02081	57570	Hs.380159	NM_02081	ENSG0000	TRMT5	COXPD26|	tRNA meth	protein-co
lib-C666-l	chr13	1.11E+08	1.11E+08	+	94.41716	NA	exon (NM_	exon (NM_	151	NM_02453	79587	Hs.508725	NM_02453	ENSG0000	CARS2	COXPD27|	cysteinyl-t	protein-co
lib-C666-l	chr3	66271430	66271430	+	20.72954	NA	non-codin	non-codin	262	NR_02847	115286	Hs.379386	NM_17347	ENSG0000	SLC25A26	COXPD28|	solute carr	protein-co
lib-C666-l	chr22	36877597	36877597	+	145.0765	NA	promoter-	promoter-	90	NM_01247	25828	Hs.211929	NM_01247	ENSG0000	TXN2	COXPD29|	thioredoxi	protein-co
lib-C666-l	chr3	1.01E+08	1.01E+08	+	44.27569	NA	promoter-	promoter-	-25	NM_01781	54931	Hs.594839	NM_01781	ENSG0000	TRMT10C	COXPD30|	tRNA meth	protein-co
lib-C666-l	chr16	28857758	28857758	+	157.6147	NA	promoter-	promoter-	-29	NM_00332	7284	Hs.12084	NM_00332	ENSG0000	TUFM	COXPD4|E	Tu translat	protein-co
lib-C666-l	chr16	19535125	19535125	+	77.06716	NA	promoter-	promoter-	-54	NM_00132	9738	Hs.279912	NM_01471	ENSG0000	CCP110	CP110|Cep	centriolar	protein-co
lib-C666-l	chr14	1E+08	1E+08	+	64.98446	NA	promoter-	promoter-	-519	NM_00666	10858	Hs.25121	NM_00666	ENSG0000	CYP46A1	CP46|CYP4	cytochrom	protein-co
lib-C666-l	chr7	91764011	91764011	+	80.62132	NA	promoter-	promoter-	48	NM_00114	1595	Hs.417077	NM_00078	ENSG0000	CYP51A1	CP51|CYP5	cytochrom	protein-co
lib-C666-l	chrX	48659924	48659924	+	266.8418	NA	promoter-	promoter-	140	NM_00132	10013	Hs.6764	NM_00604	ENSG0000	HDAC6	CPBHM|HD	histone de	protein-co
lib-C666-l	chr4	15004021	15004021	+	61.35856	NA	promoter-	promoter-	-277	NM_00117	132864	Hs.656937	NM_18248	ENSG0000	CPEB2	CPE-BP2|C	cytoplasmi	protein-co
lib-C666-l	chr11	17229522	17229522	+	32.48059	NA	promoter-	promoter-	21	NM_00132	5286	Hs.175343	NM_00264	ENSG0000	PIK3C2A	CPK|PI3-K-	phosphatic	protein-co
lib-C666-l	chr7	45151322	45151322	+	137.8344	NA	promoter-	promoter-	24	NM_03090	9238	Hs.231411	NM_00474	ENSG0000	TBRG4	CPR2|FAST	transformi	protein-co
lib-C666-l	chr16	47007640	47007640	+	29.22024	NA	promoter-	promoter-	-15	NM_00588	10294	Hs.368078	NM_00588	ENSG0000	DNAJA2	CPR3|DJ3|	DnaJ heat	protein-co
lib-C666-l	chr14	99947694	99947694	+	121.9044	NA	promoter-	promoter-	-45	NM_00109	8812	Hs.510409	NM_00385	ENSG0000	CCNK	CPR4	cyclin K	protein-co
lib-C666-l	chr1	2.07E+08	2.07E+08	+	33.60578	NA	non-codin	non-codin	134	NM_00130	1604	Hs.126517	NM_00057	ENSG0000	CD55	CR|CROM|	CD55 mole	protein-co
lib-C666-l	chr12	42719941	42719941	+	71.09715	NA	promoter-	promoter-	-6	NM_00114	51535	Hs.444157	NM_01648	ENSG0000	PPHLN1	CR|HSPC2	periphilin 1	protein-co
lib-C666-l	chr9	1.35E+08	1.35E+08	+	96.94724	NA	promoter-	promoter-	-27	NM_00125	9442	Hs.374262	NM_00426	ENSG0000	MED27	CRAP34|C	mediator c	protein-co
lib-C666-l	chr2	2.08E+08	2.08E+08	+	22.82077	NA	non-codin	non-codin	154	NR_13547	1385	Hs.516646	NM_00437	ENSG0000	CREB1	CREB|CREE	cAMP resp	protein-co
lib-C666-l	chr2	61108659	61108659	+	45.65358	NA	promoter-	promoter-	29	NM_00290	5966	Hs.631886	NM_00290	ENSG0000	REL	C-Rel	REL proto-	protein-co
lib-C666-l	chr10	35484816	35484816	+	54.91701	NA	promoter-	promoter-	-14	NM_18272	1390	Hs.200250	NM_00188	ENSG0000	CREM	CREM-2|IC	cAMP resp	protein-co
lib-C666-l	chr10	35416023	35416023	+	37.48738	NA	promoter-	promoter-	254	NM_00126	1390	Hs.200250	NM_00188	ENSG0000	CREM	CREM-2|IC	cAMP resp	protein-co
lib-C666-l	chr1	2.04E+08	2.04E+08	+	42.50122	NA	promoter-	promoter-	-88	NM_03283	84919	Hs.304376	NM_03283	ENSG0000	PPP1R15B	CREP|MSS	protein ph	protein-co
lib-C666-l	chr10	1034338	1034338	+	39.64852	NA	promoter-	promoter-	-11	NM_01234	23560	Hs.215766	NM_01234	ENSG0000	GTPBP4	CRFG|NGB	GTP bindir	protein-co
lib-C666-l	chr17	1359601	1359601	+	36.5431	NA	promoter-	promoter-	-40	NM_01682	1398	Hs.461896	NM_00520	ENSG0000	CRK	CRKII|p38	CRK proto-	protein-co
lib-C666-l	chr1	1.56E+08	1.56E+08	+	32.22565	NA	promoter-	promoter-	-106	NR_13526	10485	Hs.380027	NM_00636	ENSG0000	C1orf61	CROC4	chromosor	protein-co
lib-C666-l	chr5	1345333	1345333	+	343.9041	NA	promoter-	promoter-	-148	NM_03078	81037	Hs.444673	NM_03078	ENSG0000	CLPTM1L	CRR9	CLPTM1 lik	protein-co
lib-C666-l	chr6	91297040	91297040	+	165.5633	NA	promoter-	promoter-	-20	NM_00318	6885	Hs.594838	NM_00318	ENSG0000	MAP3K7	CSCF|FMD	mitogen-a	protein-co
lib-C666-l	chr18	32621276	32621276	+	22.15977	NA	promoter-	promoter-	-48	NM_01426	10982	Hs.532824	NM_01426	ENSG0000	MAPRE2	CSCSC2|EB	microtubul	protein-co
lib-C666-l	chr16	11945474	11945474	+	24.54495	NA	promoter-	promoter-	-32	NM_01565	26156	Hs.401842	NM_01565	ENSG0000	RSL1D1	CSIG|L12|P	ribosomal	protein-co
lib-C666-l	chr17	17184613	17184613	+	26.35145	NA	promoter-	promoter-	4	NM_00131	8533	Hs.6076	NM_00365	ENSG0000	COPS3	CSN3|SGN	COP9 sign	protein-co
lib-C666-l	chr12	6833255	6833255	+	24.32656	NA	promoter-	promoter-	105	NM_00116	50813	Hs.530823	NM_01631	ENSG0000	COPS7A	CSN7|CSN	COP9 sign	protein-co
lib-C666-l	chr2	2.41E+08	2.41E+08	+	34.0482	NA	promoter-	promoter-	-71	NM_13833	150678	Hs.293884	NM_13833	ENSG0000	COPS9	CSNAP|MY	COP9 sign	protein-co
lib-C666-l	chr4	619136	619136	+	23.39665	NA	promoter-	promoter-	-227	NM_00114	5158	Hs.623810	NM_00028	ENSG0000	PDE6B	CSNB3|CS	phosphodi	protein-co
lib-C666-l	chr20	54967501	54967501	+	55.66538	NA	promoter-	promoter-	-73	NM_00132	1477	Hs.172865	NM_00132	ENSG0000	CSTF1	CstF-50|Cs	cleavage s	protein-co
lib-C666-l	chr10	53459609	53459609	+	24.25638	NA	promoter-	promoter-	-254	NM_01523	23283	Hs.591358	NM_01523	ENSG0000	CSTF2T	CstF-64T	cleavage s	protein-co
lib-C666-l	chr20	34203764	34203764	+	387.6584	NA	promoter-	promoter-	-42	NM_00311	6676	Hs.123159	NM_00311	ENSG0000	SPAG4	CT127|SU	sperm ass	protein-co
lib-C666-l	chr1	45205450	45205450	+	46.29081	NA	promoter-	promoter-	-40	NM_00129	11004	Hs.720061	NM_00684	ENSG0000	KIF2C	CT139|KN	kinesin fan	protein-co
lib-C666-l	chr9	1.28E+08	1.28E+08	+	99.63897	NA	promoter-	promoter-	33	NM_00148	2649	Hs.20131	NM_00148	ENSG0000	NR6A1	CT150|GC	nuclear rec	protein-co
lib-C666-l	chr6	28048916	28048916	+	42.40071	NA	5' UTR (NM	5' UTR (NM	434	NM_00344	7718	Hs.55481	NM_00344	ENSG0000	ZNF165	CT53|LD65	zinc finger	protein-co
lib-C666-l	chr17	49198390	49198390	+	62.69942	NA	promoter-	promoter-	-164	NM_00113	9043	Hs.463439	NM_00397	ENSG0000	SPAG9	CT89|HLC-	sperm ass	protein-co
lib-C666-l	chr14	1.04E+08	1.04E+08	+	54.91701	NA	promoter-	promoter-	-12	NM_00112	4140	Hs.35828	NM_00237	ENSG0000	MARK3	CTAK1|KP7	microtubul	protein-co
lib-C666-l	chr22	42017251	42017251	+	65.39317	NA	promoter-	promoter-	84	NM_00128	2547	Hs.292493	NM_00146	ENSG0000	XRCC6	CTC75|CT	X-ray repa	protein-co
lib-C666-l	chr15	72523683	72523683	+	25.39084	NA	5' UTR (NM	5' UTR (NM	282	NM_18247	5315	Hs.534770	NM_00265	ENSG0000	PKM	CTHBP|HE	pyruvate k	protein-co
lib-C666-l	chr3	41240839	41240839	+	22.02847	NA	promoter-	promoter-	-76	NM_00133	1499	Hs.476018	NM_00190	ENSG0000	CTNNB1	CTNNB|MF	catenin be	protein-co
lib-C666-l	chr10	1.04E+08	1.04E+08	+	184.2682	NA	promoter-	promoter-	-127	NM_00107	4791	Hs.73090	NM_00250	ENSG0000	NFKB2	CVID10|H2	nuclear fac	protein-co
lib-C666-l	chr10	1.04E+08	1.04E+08	+	54.43427	NA	promoter-	promoter-	-43	NM_00126	4791	Hs.73090	NM_00250	ENSG0000	NFKB2	CVID10|H2	nuclear fac	protein-co
lib-C666-l	chr5	1.5E+08	1.5E+08	+	36.38376	NA	promoter-	promoter-	-8	NM_01804	55696	Hs.713564	NM_01804	ENSG0000	RBM22	Cwc2|ZC3	RNA bindi	protein-co
lib-C666-l	chr11	3818881	3818881	+	153.7879	NA	promoter-	promoter-	-73	NM_00125	27315	Hs.133968	NM_01448	ENSG0000	PGAP2	CWH43-N	post-GPI a	protein-co
lib-C666-l	chr17	4643259	4643259	+	42.11224	NA	promoter-	promoter-	-36	NM_00110	58191	Hs.745037	NM_02205	ENSG0000	CXCL16	CXCLG16|	C-X-C mo	protein-co
lib-C666-l	chrX	1.53E+08	1.53E+08	+	64.17523	NA	5' UTR (NM	5' UTR (NM	141	NM_00349	8269	Hs.23119	NM_00349	ENSG0000	TMEM187	CXorf12|D	transmem	protein-co
lib-C666-l	chrX	1.53E+08	1.53E+08	+	42.40071	NA	promoter-	promoter-	-218	NM_00349	8269	Hs.23119	NM_00349	ENSG0000	TMEM187	CXorf12|D	transmem	protein-co
lib-C666-l	chrX	83757503	83757503	+	23.34793	NA	promoter-	promoter-	-16	NM_14465	139324	Hs.559546	NM_14465	ENSG0000	HDX	CXorf43|D	highly dive	protein-co
lib-C666-l	chr19	36208692	36208692	+	37.41784	NA	promoter-	promoter-	-229	NM_01472	9757	Hs.92236	NM_01472	ENSG0000	KMT2B	CXXC10|H	lysine meth	protein-co
lib-C666-l	chr16	30933879	30933879	+	55.01936	NA	promoter-	promoter-	-497	NM_00128	54620	Hs.152149	NM_01908	ENSG0000	FBXL19	CXXC11|F	F-box and	protein-co
lib-C666-l	chr18	47808035	47808035	+	60.82721	NA	TTS (NM_0	TTS (NM_0	102	NM_00120	4152	Hs.405610	NM_00238	ENSG0000	MBD1	CXXC3|PC	methyl-Cp	protein-co
lib-C666-l	chr11	61659400	61659400	+	30.8056	NA	promoter-	promoter-	-383	NM_02172	3995	Hs.21765	NM_02172	ENSG0000	FADS3	CYB5RP|LL	fatty acid c	protein-co
lib-C666-l	chr7	25165016	25165016	+	156.3776	NA	promoter-	promoter-	-36	NM_01894	54205	Hs.437060	NM_01894	ENSG0000	CYCS	CYC|HCS|T	cytochrom	protein-co
lib-C666-l	chr12	50419371	50419371	+	23.6003	NA	promoter-	promoter-	-31	NM_00132	29127	Hs.505469	NM_01327	ENSG0000	RACGAP1	CYK4|HsC	Rac GTPas	protein-co
lib-C666-l	chr5	1.77E+08	1.77E+08	+	49.31026	NA	intron (NM	intron (NM	331	NM_08088	1627	Hs.130316	NM_00439	ENSG0000	DBN1	D0S117E	drebrin 1	protein-co
lib-C666-l	chr10	70287274	70287274	+	85.64357	NA	promoter-	promoter-	6	NM_00132	8034	Hs.180408	NM_15270	ENSG0000	SLC25A16	D10S105E|	solute carr	protein-co
lib-C666-l	chr11	809836	809836	+	159.9151	NA	promoter-	promoter-	-100	NM_00100	6181	Hs.437594	NM_00100	ENSG0000	RPLP2	D11S2243	ribosomal	protein-co
lib-C666-l	chr11	809354	809354	+	25.40925	NA	promoter-	promoter-	-582	NM_00100	6181	Hs.437594	NM_00100	ENSG0000	RPLP2	D11S2243	ribosomal	protein-co
lib-C666-l	chr15	23378188	23378188	+	23.27622	NA	promoter-	promoter-	71	NR_00282	400322	Hs.458334	NR_00282	ENSG0000	HERC2P2	D15F37S3|	hect doma	pseudo
lib-C666-l	chr19	9695227	9695227	+	63.46463	NA	promoter-	promoter-	-18	NM_00130	7675	Hs.501537	NM_00100	ENSG0000	ZNF121	D19S204|Z	zinc finger	protein-co
lib-C666-l	chr21	45209397	45209397	+	266.4715	NA	promoter-	promoter-	-21	NM_00368	8568	Hs.110757	NM_00368	ENSG0000	RRP1	D21S2056	ribosomal	protein-co
lib-C666-l	chr3	10362836	10362836	+	75.07171	NA	promoter-	promoter-	36	NM_00127	6396	Hs.166924	NM_03067	ENSG0000	SEC13	D3S1231E|	SEC13 hon	protein-co
lib-C666-l	chr1	1.74E+08	1.74E+08	+	34.92958	NA	promoter-	promoter-	-37	NM_00134	84614	Hs.668497	NM_03252	ENSG0000	ZBTB37	D430004I0	zinc finger	protein-co
lib-C666-l	chr20	62496549	62496549	+	60.55943	NA	promoter-	promoter-	-32	NM_19936	7165	Hs.473296	NM_00328	ENSG0000	TPD52L2	D54|TPD5	tumor prot	protein-co
lib-C666-l	chr5	1.38E+08	1.38E+08	+	66.08471	NA	promoter-	promoter-	68	NM_00473	2107	Hs.483494	NM_00473	ENSG0000	ETF1	D5S1995|E	eukaryotic	protein-co
lib-C666-l	chr5	1.38E+08	1.38E+08	+	62.31611	NA	promoter-	promoter-	-361	NM_00129	2107	Hs.483494	NM_00473	ENSG0000	ETF1	D5S1995|E	eukaryotic	protein-co
lib-C666-l	chr6	32939750	32939750	+	22.23727	NA	5' UTR (NM	5' UTR (NM	378	NM_00119	6046	Hs.75243	NM_00510	ENSG0000	BRD2	D6S113E|F	bromodon	protein-co
lib-C666-l	chr6	31239939	31239939	+	27.26713	NA	promoter-	promoter-	-26	NM_00124	3107	Hs.656020	NM_00211	ENSG0000	HLA-C	D6S204|H	major histo	protein-co
lib-C666-l	chr6	32908884	32908884	+	60.38725	NA	promoter-	promoter-	-37	NM_00211	3109	Hs.351279	NM_00211	ENSG0000	HLA-DMB	D6S221E|R	major histo	protein-co
lib-C666-l	chr6	32920958	32920958	+	65.88593	NA	promoter-	promoter-	-59	NM_00612	3108	Hs.728759	NM_00612	ENSG0000	HLA-DMA	D6S222E|D	major histo	protein-co
lib-C666-l	chr6	35227247	35227247	+	42.78438	NA	promoter-	promoter-	-244	NM_00342	7629	Hs.388024	NM_00342	ENSG0000	ZNF76	D6S229E|Z	zinc finger	protein-co
lib-C666-l	chr6	31926789	31926789	+	98.40649	NA	promoter-	promoter-	75	NM_00290	7936	Hs.423935	NM_00290	ENSG0000	NELFE	D6S45|NEL	negative el	protein-co
lib-C666-l	chr9	86323065	86323065	+	149.53	NA	promoter-	promoter-	103	NM_01343	29979	Hs.9589	NM_01343	ENSG0000	UBQLN1	DA41|DSK	ubiquilin 1	protein-co
lib-C666-l	chr1	1.15E+08	1.15E+08	+	121.4374	NA	promoter-	promoter-	-122	NM_00587	10286	Hs.22960	NM_00587	ENSG0000	BCAS2	DAM1|SPF	BCAS2, pre	protein-co
lib-C666-l	chr10	1.06E+08	1.06E+08	+	21.94284	NA	promoter-	promoter-	-438	NM_00127	85450	Hs.523252	NM_03339	ENSG0000	ITPRIP	DANGER|K	inositol 1,4	protein-co
lib-C666-l	chr14	50053253	50053253	+	160.6232	NA	promoter-	promoter-	-119	NM_00103	6235	Hs.156367	NM_00103	ENSG0000	RPS29	DBA13|S2	ribosomal	protein-co
lib-C666-l	chr1	93297534	93297534	+	24.31114	NA	promoter-	promoter-	-60	NM_00096	6125	Hs.532359	NM_00096	ENSG0000	RPL5	DBA6|L5|M	ribosomal	protein-co
lib-C666-l	chr16	70333084	70333084	+	83.41785	NA	promoter-	promoter-	22	NM_00101	11269	Hs.221761	NM_00724	ENSG0000	DDX19B	DBP5|DDX	DEAD-box	protein-co
lib-C666-l	chr9	69262621	69262621	+	199.4978	NA	promoter-	promoter-	9	NM_00128	220869	Hs.355950	NM_00102	ENSG0000	CBWD5	DC36	COBW dor	protein-co
lib-C666-l	chr9	70856822	70856822	+	154.5329	NA	promoter-	promoter-	17	NM_00128	220869	Hs.355950	NM_00102	ENSG0000	CBWD5	DC36	COBW dor	protein-co
lib-C666-l	chr9	70490197	70490197	+	131.0062	NA	promoter-	promoter-	14	NR_10460	220869	Hs.355950	NM_00102	ENSG0000	CBWD5	DC36	COBW dor	protein-co
lib-C666-l	chr1	36930055	36930055	+	45.43086	NA	promoter-	promoter-	-15	NM_03128	64960	Hs.352839	NM_03128	ENSG0000	MRPS15	DC37|MPR	mitochond	protein-co
lib-C666-l	chrX	1.54E+08	1.54E+08	+	83.41785	NA	intron (NM	CpG	290	NM_02393	65991	Hs.356050	NM_02393	ENSG0000	FUNDC2	DC44|HCB	FUN14 dor	protein-co
lib-C666-l	chr1	1.11E+08	1.11E+08	+	50.12882	NA	promoter-	promoter-	-57	NM_00662	10768	Hs.743973	NM_00662	ENSG0000	AHCYL1	DCAL|IRBI	adenosylh	protein-co
lib-C666-l	chr12	57941067	57941067	+	21.94284	NA	promoter-	promoter-	47	NM_00126	10540	Hs.289123	NM_00640	ENSG0000	DCTN2	DCTN50|D	dynactin s	protein-co
lib-C666-l	chr8	48920963	48920963	+	99.26302	NA	promoter-	promoter-	-32	NM_00335	7336	Hs.491695	NM_00335	ENSG0000	UBE2V2	DDVIT1|D	ubiquitin c	protein-co
lib-C666-l	chr16	70380761	70380761	+	161.3141	NA	promoter-	promoter-	29	NM_01833	55308	Hs.656037	NM_01833	ENSG0000	DDX19A	DDX19-DI	DEAD-box	protein-co
lib-C666-l	chr17	7475997	7475997	+	107.7632	NA	promoter-	promoter-	-27	NM_00141	1973	Hs.129673	NM_00141	ENSG0000	EIF4A1	DDX2A|EIF	eukaryotic	protein-co
lib-C666-l	chr17	5372269	5372269	+	60.44312	NA	promoter-	promoter-	111	NM_02016	56919	Hs.250456	NM_02016	ENSG0000	DHX33	DDX33	DEAH-box	protein-co
lib-C666-l	chr3	1.54E+08	1.54E+08	+	155.9595	NA	promoter-	promoter-	-61	NM_00111	170506	Hs.446270	NM_02086	ENSG0000	DHX36	DDX36|G4	DEAH-box	protein-co
lib-C666-l	chr3	16306518	16306518	+	496.5448	NA	promoter-	promoter-	-22	NM_00104	285381	Hs.388087	NM_20683	ENSG0000	DPH3	DELGIP|DE	diphthami	protein-co
lib-C666-l	chr14	1.01E+08	1.01E+08	+	61.32821	NA	promoter-	promoter-	-113	NM_00340	7528	Hs.388927	NM_00340	ENSG0000	YY1	DELTA|INC	YY1 transc	protein-co
lib-C666-l	chr11	66139543	66139543	+	37.891	NA	promoter-	promoter-	-252	NR_12534	3177	Hs.569017	NM_00153	ENSG0000	SLC29A2	DER12|EN	solute carr	protein-co
lib-C666-l	chr11	64902096	64902096	+	25.39084	NA	promoter-	promoter-	-93	NM_17223	84447	Hs.75859	NM_03243	ENSG0000	SYVN1	DER3|HRD	synoviolin	protein-co
lib-C666-l	chr10	85899249	85899249	+	202.8664	NA	promoter-	promoter-	64	NM_01439	27069	Hs.352656	NM_01439	ENSG0000	GHITM	DERP2|HS	growth ho	protein-co
lib-C666-l	chr4	1.66E+08	1.66E+08	+	47.72539	NA	promoter-	promoter-	-102	NM_00101	6307	Hs.105269	NM_00674	ENSG0000	MSMO1	DESP4|ER	methylster	protein-co
lib-C666-l	chr14	73493964	73493964	+	28.94286	NA	promoter-	promoter-	-44	NM_02126	53349	Hs.335106	NM_02126	ENSG0000	ZFYVE1	DFCP1|PP	zinc finger	protein-co
lib-C666-l	chrX	40943985	40943985	+	29.74345	NA	promoter-	promoter-	-903	NM_00103	8239	Hs.77578	NM_00465	ENSG0000	USP9X	DFFRX|FAF	ubiquitin s	protein-co
lib-C666-l	chr11	67351198	67351198	+	23.20869	NA	5' UTR (NM	5' UTR (NM	132	NM_00085	2950	Hs.523836	NM_00085	ENSG0000	GSTP1	DFN7|FAE	glutathion	protein-co
lib-C666-l	chr6	76458888	76458888	+	24.99834	NA	promoter-	promoter-	-5	NM_00130	4646	Hs.149387	NM_00499	ENSG0000	MYO6	DFNA22|D	myosin VI	protein-co
lib-C666-l	chr12	1.23E+08	1.23E+08	+	39.83551	NA	promoter-	promoter-	-225	NM_00127	56616	Hs.169611	NM_01988	ENSG0000	DIABLO	DFNA64|S	diablo IAP-	protein-co
lib-C666-l	chr20	60813427	60813427	+	41.5993	NA	promoter-	promoter-	-114	NM_14449	9885	Hs.473254	NM_01483	ENSG0000	OSBPL2	DFNA67|D	oxysterol b	protein-co
lib-C666-l	chr3	1.22E+08	1.22E+08	+	33.78053	NA	promoter-	promoter-	86	NM_00119	286676	Hs.98484	NM_17592	ENSG0000	ILDR1	DFNB42|IL	immunogl	protein-co
lib-C666-l	chr22	19132266	19132266	+	86.40352	NA	promoter-	promoter-	-76	NR_13430	8220	Hs.517407	NM_02271	ENSG0000	DGCR14	DGCR13|D	DiGeorge	protein-co
lib-C666-l	chr3	1.14E+08	1.14E+08	+	40.13552	NA	promoter-	promoter-	-58	NM_00132	254887	Hs.21902	NM_17357	ENSG0000	ZDHHC23	DHHC-23|	zinc finger	protein-co

lib-C666-li	chr16	85045192	85045192	+	103.1159	NA	promoter-	promoter-	-51	NM_00114	55625	Hs.461610	NM_01774	ENSG0000	ZDHHC7	DHHC7|SE	zinc finger	protein-co
lib-C666-li	chr5	1.5E+08	1.5E+08	+	120.9748	NA	promoter-	promoter-	-26	NM_00102	972	Hs.436568	NM_00435	ENSG0000	CD74	DHLAG|HL	CD74 mole	protein-co
lib-C666-li	chr18	61034911	61034911	+	20.19666	NA	promoter-	promoter-	-405	NM_00203	2531	Hs.74050	NM_00203	ENSG0000	KDSR	DHSR|FVT1	3-ketodihy	protein-co
lib-C666-li	chr11	75526201	75526201	+	38.90989	NA	promoter-	promoter-	-11	NM_00336	7405	Hs.202470	NM_00336	ENSG0000	UVRAG	DHTX|VPS3	UV radiatic	protein-co
lib-C666-li	chr1	85527858	85527858	+	48.11517	NA	promoter-	promoter-	-123	NM_00128	126820	Hs.97933	NM_14517	ENSG0000	WDR63	DIC3|NYD-	WD repeat	protein-co
lib-C666-li	chr17	34901465	34901465	+	28.88523	NA	intron (NM	intron (NM	728	NM_02483	79893	Hs.514116	NM_02483	ENSG0000	GGNBP2	DIF-3|DIF3	gametoge	protein-co
lib-C666-li	chr17	34900902	34900902	+	20.72954	NA	5' UTR (NM	5' UTR (NM	165	NM_02483	79893	Hs.514116	NM_02483	ENSG0000	GGNBP2	DIF-3|DIF3	gametoge	protein-co
lib-C666-li	chr13	1.14E+08	1.14E+08	+	48.11517	NA	promoter-	promoter-	-54	NR_02658	7027	Hs.79353	NM_00711	ENSG0000	TFDP1	DILC|DP1|D	transcriptic	protein-co
lib-C666-li	chr1	8021660	8021660	+	79.67884	NA	promoter-	promoter-	-54	NM_00112	11315	Hs.419640	NM_00726	ENSG0000	PARK7	DJ-1|DJ1|H	Parkinsonis	protein-co
lib-C666-li	chr10	74114716	74114716	+	135.458	NA	exon (NM_	exon (NM_	191	NM_00100	54788	Hs.500013	NM_01762	ENSG0000	DNAJB12	DJ10	DnaJ heat	protein-co
lib-C666-li	chr1	1.75E+08	1.75E+08	+	24.25638	NA	promoter-	promoter-	-132	NR_03760	63931	Hs.654858	NM_02210	ENSG0000	MRPS14	DJ262D12.	mitochond	protein-co
lib-C666-li	chr6	38607879	38607879	+	244.3541	NA	promoter-	promoter-	45	NM_00109	114781	Hs.603858	NM_05289	ENSG0000	BTBD9	dJ322I12.1	BTB domai	protein-co
lib-C666-li	chr7	1.57E+08	1.57E+08	+	52.78568	NA	promoter-	promoter-	-57	NM_00549	10049	Hs.490745	NM_00549	ENSG0000	DNAJB6	DJ4|DnaJ|H	DnaJ heat	protein-co
lib-C666-li	chr22	39097076	39097076	+	426.7164	NA	promoter-	promoter-	-617	NM_01487	9929	Hs.3094	NM_01487	ENSG0000	JOSD1	dJ508I15.2	Josephin d	protein-co
lib-C666-li	chr22	43583719	43583719	+	26.19214	NA	promoter-	promoter-	-582	NM_01514	23170	Hs.517670	NM_01514	ENSG0000	TTLL12	dJ526I14.2	tubulin tyr	protein-co
lib-C666-li	chr1	70671347	70671347	+	243.4734	NA	promoter-	promoter-	14	NM_01776	55631	Hs.147836	NM_01776	ENSG0000	LRRC40	dJ677H15.	leucine ricl	protein-co
lib-C666-li	chr20	30697450	30697450	+	56.63552	NA	5' UTR (NM	5' UTR (NM	141	NM_01474	9777	Hs.654665	NM_01474	ENSG0000	TM9SF4	dJ836N17.	transmemk	protein-co
lib-C666-li	chr22	32058241	32058241	+	130.8321	NA	5' UTR (NM	5' UTR (NM	309	NM_00132	23761	Hs.420559	NM_01433	ENSG0000	PISD	DJ858B16|I	phosphatic	protein-co
lib-C666-li	chr14	24711873	24711873	+	27.44508	NA	promoter-	promoter-	7	NM_01246	26277	Hs.496191	NM_01246	ENSG0000	TINF2	DKCA3|TIN	TERF1 inte	protein-co
lib-C666-li	chr5	1.78E+08	1.78E+08	+	33.73372	NA	promoter-	promoter-	71	NM_01783	55651	Hs.744074	NM_01783	ENSG0000	NHP2	DKCB2|NH	NHP2 ribo	protein-co
lib-C666-li	chr2	1.1E+08	1.1E+08	+	21.28624	NA	promoter-	promoter-	-118	NM_02233	10913	Hs.171971	NM_02233	ENSG0000	EDAR	DL|ECTD10	ectodyspla	protein-co
lib-C666-li	chr7	1.08E+08	1.08E+08	+	78.00706	NA	promoter-	promoter-	2	NM_00128	1738	Hs.131711	NM_00010	ENSG0000	DLD	DLDD|DLD	dihydrolipo	protein-co
lib-C666-li	chr19	3971209	3971209	+	45.88523	NA	promoter-	promoter-	-88	NM_00134	1613	Hs.631844	NM_00134	ENSG0000	DAPK3	DLK|ZIP|ZIF	death asso	protein-co
lib-C666-li	chr11	68039497	68039497	+	29.41683	NA	promoter-	promoter-	-28	NM_02233	53838	Hs.303025	NM_02233	ENSG0000	C11orf24	DM4E3	chromosor	protein-co
lib-C666-li	chr9	36258496	36258496	+	23.27622	NA	promoter-	promoter-	0	NM_00119	10020	Hs.5920	NM_00547	ENSG0000	GNE	DMRV|GLC	glucosamir	protein-co
lib-C666-li	chr10	22292705	22292705	+	28.88196	NA	promoter-	promoter-	-55	NM_02236	64215	Hs.499000	NM_02236	ENSG0000	DNAJC1	DNAJL1|ER	DnaJ heat	protein-co
lib-C666-li	chr19	12992119	12992119	+	69.90313	NA	exon (NM_	exon (NM_	216	NM_00137	1777	Hs.118243	NM_00137	ENSG0000	DNASE2	DNASE2A|I	deoxyribor	protein-co
lib-C666-li	chr1	8378144	8378144	+	31.10827	NA	promoter-	promoter-	-1	NM_00108	50651	Hs.463036	NM_00108	ENSG0000	SLC45A1	DNB5	solute carr	protein-co
lib-C666-li	chr17	56160696	56160696	+	26.54911	NA	promoter-	promoter-	-84	NM_08067	140735	Hs.720595	NM_08067	ENSG0000	DYNLL2	DNCL1B|D	dynein ligh	protein-co
lib-C666-li	chr5	54603699	54603699	+	21.84731	NA	promoter-	promoter-	123	NM_01536	23517	Hs.274531	NM_01536	ENSG0000	SKIV2L2	Dob1|KIAA	Ski2 like RI	protein-co
lib-C666-li	chr11	67276086	67276086	+	72.82537	NA	promoter-	promoter-	113	NM_00585	10263	Hs.523835	NM_00585	ENSG0000	CDK2AP2	DOC-1R|p	cyclin depe	protein-co
lib-C666-li	chr19	9945855	9945855	+	21.28624	NA	promoter-	promoter-	-28	NM_00622	5300	Hs.465849	NM_00622	ENSG0000	PIN1	DOD|UBL5	peptidylpr	protein-co
lib-C666-li	chr6	31939953	31939953	+	234.3005	NA	promoter-	promoter-	79	NM_00551	1797	Hs.153299	NM_00551	ENSG0000	DXO	DOM3L|DC	decapping	protein-co
lib-C666-li	chr17	47022213	47022213	+	108.9712	NA	promoter-	promoter-	-9	NR_13367	11267	Hs.127249	NM_00724	ENSG0000	SNF8	Dot3|EAP3	SNF8, ESCI	protein-co
lib-C666-li	chr6	33041527	33041527	+	44.36932	NA	promoter-	promoter-	-73	NM_03355	3113	Hs.347270	NM_03355	ENSG0000	HLA-DPA1	DP(W3)|DF	major histc	protein-co
lib-C666-li	chr6	33043658	33043658	+	21.96185	NA	promoter-	promoter-	-45	NM_00212	3115	Hs.485130	NM_00212	ENSG0000	HLA-DPB1	DPB1|HLA-	major histc	protein-co
lib-C666-li	chr17	40713925	40713925	+	37.48154	NA	promoter-	promoter-	-167	NM_00104	80347	Hs.296422	NM_02523	ENSG0000	COASY	DPCK|NBIA	Coenzyme	protein-co
lib-C666-li	chr19	10527462	10527462	+	174.9811	NA	promoter-	promoter-	13	NM_00124	5141	Hs.89901	NM_00620	ENSG0000	PDE4A	DPDE2|PDI	phosphodi	protein-co
lib-C666-li	chr8	38126769	38126769	+	33.8063	NA	promoter-	promoter-	-31	NM_00110	84513	Hs.567619	NM_03248	ENSG0000	PLPP5	DPPL1|HTF	phospholip	protein-co
lib-C666-li	chr9	1.33E+08	1.33E+08	+	34.92958	NA	promoter-	promoter-	39	NR_13402	27348	Hs.252682	NM_01450	ENSG0000	TOR1B	DQ1	torsin fami	protein-co
lib-C666-li	chr6	32557675	32557675	+	56.44287	NA	promoter-	promoter-	-62	NM_00212	3123	Hs.534322	NM_00212	ENSG0000	HLA-DRB1	DRB1|HLA-	major histc	protein-co
lib-C666-li	chr11	67272031	67272031	+	23.0291	NA	intron (NM	CpG	812	NM_00491	9600	Hs.372295	NM_00491	ENSG0000	PITPNM1	DRES9|NIR	phosphatic	protein-co
lib-C666-li	chr1	38273863	38273863	+	21.21068	NA	promoter-	promoter-	2	NM_02464	79693	Hs.301564	NM_02464	ENSG0000	YRDC	DRIP3|IRIP|	yrdC N6-tl	protein-co
lib-C666-li	chr4	89618987	89618987	+	45.23877	NA	5' UTR (NM	5' UTR (NM	399	NM_15375	266812	Hs.12554	NM_15375	ENSG0000	NAP1L5	DRLM	nucleosom	protein-co
lib-C666-li	chr14	39644188	39644188	+	22.63618	NA	promoter-	promoter-	-199	NM_00268	5411	Hs.409965	NM_00268	ENSG0000	PNN	DRS|DRSP|	pinin, desn	protein-co
lib-C666-li	chr1	1284775	1284775	+	38.91483	NA	promoter-	promoter-	-283	NM_00133	1855	Hs.731450	NM_00442	ENSG0000	DVL1	DRS2|DVL|	dishevelled	protein-co
lib-C666-li	chr6	1.17E+08	1.17E+08	+	30.85659	NA	promoter-	promoter-	-16	NM_00132	29940	Hs.458358	NM_01335	ENSG0000	DSE	DS-epi1|D	dermatan	protein-co
lib-C666-li	chr11	57479858	57479858	+	37.12814	NA	promoter-	promoter-	-63	NM_15345	219541	Hs.43619	NM_15345	ENSG0000	MED19	DT2P1G7|L	mediator c	protein-co
lib-C666-li	chr17	76837073	76837073	+	266.4928	NA	promoter-	promoter-	-464	NM_00132	57602	Hs.464243	NM_02509	ENSG0000	USP36	DUB1	ubiquitin s	protein-co
lib-C666-li	chrX	48815601	48815601	+	24.18348	NA	promoter-	promoter-	47	NM_00113	55593	Hs.496098	NM_01760	ENSG0000	OTUD5	DUBA	OTU deubi	protein-co
lib-C666-li	chr10	23727892	23727892	+	24.23241	NA	promoter-	promoter-	-306	NM_00114	220213	Hs.499042	NM_00114	ENSG0000	OTUD1	DUBA7|OT	OTU deubi	protein-co
lib-C666-li	chr10	23729090	23729090	+	20.90919	NA	exon (NM_	exon (NM_	892	NM_00114	220213	Hs.499042	NM_00114	ENSG0000	OTUD1	DUBA7|OT	OTU deubi	protein-co
lib-C666-li	chr16	68057141	68057141	+	102.0361	NA	5' UTR (NM	5' UTR (NM	294	NM_01780	54920	Hs.534460	NM_01780	ENSG0000	DUS2	DUS2L|SM	dihydrouri	protein-co
lib-C666-li	chr19	5791272	5791272	+	92.67979	NA	promoter-	promoter-	-23	NM_02017	56931	Hs.284297	NM_02017	ENSG0000	DUS3L	DUS3	dihydrouri	protein-co
lib-C666-li	chr7	1.07E+08	1.07E+08	+	116.5974	NA	promoter-	promoter-	-1	NR_07300	11062	Hs.97627	NM_18158	ENSG0000	DUS4L	DUS4|PP35	dihydrouri	protein-co
lib-C666-li	chr10	1.12E+08	1.12E+08	+	21.84731	NA	promoter-	promoter-	-26	NM_00441	1847	Hs.2128	NM_00441	ENSG0000	DUSP5	DUSP|HVH	dual specif	protein-co
lib-C666-li	chr5	1.5E+08	1.5E+08	+	23.27622	NA	promoter-	promoter-	85	NM_00113	51164	Hs.675564	NM_01622	ENSG0000	DCTN4	DYN4|P62	dynactin su	protein-co
lib-C666-li	chr9	1.12E+08	1.12E+08	+	59.7967	NA	promoter-	promoter-	199	NM_00131	8518	Hs.494738	NM_00364	ENSG0000	IKBKAP	DYS|ELP1|F	inhibitor of	protein-co
lib-C666-li	chr14	55369682	55369682	+	126.6395	NA	promoter-	promoter-	-140	NM_00102	2643	Hs.86724	NM_00016	ENSG0000	GCH1	DYT14|DYT	GTP cycloh	protein-co
lib-C666-li	chr18	11857628	11857628	+	23.74671	NA	5' UTR (NM	5' UTR (NM	191	NM_00126	2774	Hs.136295	NM_00207	ENSG0000	GNAL	DYT25	G protein s	protein-co
lib-C666-li	chr8	42698606	42698606	+	59.74744	NA	promoter-	promoter-	-132	NM_01810	55145	Hs.7432	NM_01810	ENSG0000	THAP1	DYT6	THAP dom	protein-co
lib-C666-li	chr4	1.04E+08	1.04E+08	+	25.33067	NA	promoter-	promoter-	25	NM_18189	7323	Hs.518773	NM_00334	ENSG0000	UBE2D3	E2(17)KB3|	ubiquitin c	protein-co
lib-C666-li	chr7	1.3E+08	1.3E+08	+	133.1904	NA	promoter-	promoter-	14	NM_00120	7328	Hs.643548	NM_00334	ENSG0000	UBE2H	E2-20K|GID	ubiquitin c	protein-co
lib-C666-li	chr17	74449306	74449306	+	23.27622	NA	promoter-	promoter-	-18	NM_02206	63893	Hs.16130	NM_02206	ENSG0000	UBE2O	E2-230K	ubiquitin c	protein-co
lib-C666-li	chr19	55919316	55919316	+	42.49125	NA	promoter-	promoter-	9	NM_01450	27338	Hs.396393	NM_01450	ENSG0000	UBE2S	E2-EPF|E2E	ubiquitin c	protein-co
lib-C666-li	chr8	86089504	86089504	+	22.23727	NA	promoter-	promoter-	-115	NM_00195	1875	Hs.445758	NM_00195	ENSG0000	E2F5	E2F-5	E2F transcr	protein-co
lib-C666-li	chr11	1.18E+08	1.18E+08	+	31.27173	NA	promoter-	promoter-	-22	NM_00120	9354	Hs.75275	NM_00478	ENSG0000	UBE4A	E4|UBOX2|	ubiquitinat	protein-co
lib-C666-li	chr19	11616628	11616628	+	53.56082	NA	TTS (NM_0	TTS (NM_0	110	NM_13878	115950	Hs.465928	NM_13878	ENSG0000	ZNF653	E430039K0	zinc finger	protein-co
lib-C666-li	chr16	2273571	2273571	+	29.74345	NA	promoter-	promoter-	82	NM_00128	1877	Hs.513268	NM_00442	ENSG0000	E4F1	E4F	E4F transcr	protein-co
lib-C666-li	chr16	2318158	2318158	+	337.3493	NA	promoter-	promoter-	255	NR_10448	10921	Hs.355643	NM_00671	ENSG0000	RNPS1	E5.1	RNA bindir	protein-co
lib-C666-li	chr15	79165184	79165184	+	148.6877	NA	promoter-	promoter-	61	NM_20683	10933	Hs.374503	NM_00679	ENSG0000	MORF4L1	Eaf3|FWP0	mortality fa	protein-co
lib-C666-li	chr7	1.21E+08	1.21E+08	+	181.3347	NA	promoter-	promoter-	-113	NM_01907	54556	Hs.489811	NM_01907	ENSG0000	ING3	Eaf4|ING2|	inhibitor of	protein-co
lib-C666-li	chr7	1.21E+08	1.21E+08	+	38.71165	NA	intron (NM	CpG	373	NM_19826	54556	Hs.489811	NM_01907	ENSG0000	ING3	Eaf4|ING2|	inhibitor of	protein-co
lib-C666-li	chr3	45017722	45017722	+	87.45062	NA	promoter-	promoter-	-19	NM_01500	23016	Hs.719958	NM_01500	ENSG0000	EXOSC7	EAP1|RRP4	exosome c	protein-co
lib-C666-li	chr9	1.24E+08	1.24E+08	+	46.92278	NA	5' UTR (NM	5' UTR (NM	649	NM_00565	7185	Hs.531251	NM_00565	ENSG0000	TRAF1	EBI6|MGC:	TNF recept	protein-co
lib-C666-li	chr17	72744631	72744631	+	23.6003	NA	promoter-	promoter-	-120	NM_00425	9368	Hs.724482	NM_00425	ENSG0000	SLC9A3R1	EBP50|NHE	SLC9A3 re	protein-co
lib-C666-li	chr2	26467717	26467717	+	36.07033	NA	promoter-	promoter-	101	NM_00128	3032	Hs.515848	NM_00018	ENSG0000	HADHB	ECHB|MST	hydroxyac	protein-co
lib-C666-li	chr3	1.28E+08	1.28E+08	+	35.65661	NA	promoter-	promoter-	-233	NM_00131	8607	Hs.272822	NM_00370	ENSG0000	RUVBL1	ECP-54|EC	RuvB like A	protein-co
lib-C666-li	chr17	27224228	27224228	+	23.1274	NA	TTS (NM_1	TTS (NM_1	487	NM_00447	2319	Hs.514038	NM_00447	ENSG0000	FLOT2	ECS-1|ECS	flotillin 2	protein-co
lib-C666-li	chr3	5229369	5229369	+	29.08762	NA	promoter-	promoter-	10	NM_01467	9695	Hs.224616	NM_01467	ENSG0000	EDEM1	EDEM	ER degrad	protein-co
lib-C666-li	chr20	5591731	5591731	+	88.95569	NA	promoter-	promoter-	-59	NM_01959	56261	Hs.636359	NM_01959	ENSG0000	GPCPD1	EDI3|GDE5	glyceropho	protein-co
lib-C666-li	chrX	1.54E+08	1.54E+08	+	103.2754	NA	promoter-	promoter-	84	NM_00011	2010	Hs.522823	NM_00011	ENSG0000	EMD	EDMD|LEM	emerin	protein-co
lib-C666-li	chr7	30066581	30066581	+	32.22565	NA	promoter-	promoter-	-164	NR_04647	55033	Hs.390838	NM_01794	ENSG0000	FKBP14	EDSKMH|F	FK506 binc	protein-co
lib-C666-li	chr4	44680432	44680432	+	76.4537	NA	promoter-	promoter-	-1	NM_02192	60558	Hs.546419	NM_02192	ENSG0000	GUF1	EF-4|EF4|E	GUF1 hom	protein-co
lib-C666-li	chr19	14117684	14117684	+	399.1021	NA	promoter-	promoter-	-550	NM_00291	5989	Hs.655215	NM_00291	ENSG0000	RFX1	EFC|RFX	regulatory	protein-co
lib-C666-li	chr19	14117241	14117241	+	77.40723	NA	promoter-	promoter-	-107	NM_00291	5989	Hs.655215	NM_00291	ENSG0000	RFX1	EFC|RFX	regulatory	protein-co
lib-C666-li	chr16	11836789	11836789	+	23.83582	NA	promoter-	promoter-	-55	NM_00132	51061	Hs.313847	NM_01591	ENSG0000	TXNDC11	EFP1	thioredoxir	protein-co
lib-C666-li	chr10	94351538	94351538	+	33.11264	NA	Intergenic	CpG	-1287	NM_00452	3832	Hs.8878	NM_00452	ENSG0000	KIF11	EG5|HKSP|	kinesin far	protein-co
lib-C666-li	chr9	88556275	88556275	+	41.89289	NA	promoter-	promoter-	-169	NM_00132	60560	Hs.436098	NM_02192	ENSG0000	NAA35	EGAP|MAK	N(alpha)-a	protein-co
lib-C666-li	chr21	45432066	45432066	+	85.20388	NA	promoter-	promoter-	-140	NM_00327	7109	Hs.126221	NM_00327	ENSG0000	TRAPPC10	EHOC-1|EH	trafficking	protein-co
lib-C666-li	chr12	8234784	8234784	+	41.43555	NA	promoter-	promoter-	-23	NR_02426	25977	Hs.555927	NM_01550	ENSG0000	NECAP1	EIEE21	NECAP en	protein-co
lib-C666-li	chr1	2.31E+08	2.31E+08	+	49.05183	NA	promoter-	promoter-	-13	NM_02278	64801	Hs.275736	NM_02278	ENSG0000	ARV1	EIEE38	ARV1 hom	protein-co
lib-C666-li	chr20	32700151	32700151	+	59.93567	NA	promoter-	promoter-	11	NM_00390	8894	Hs.429180	NM_00390	ENSG0000	EIF2S2	EIF2|EIF2B|	eukaryotic	protein-co
lib-C666-li	chr12	1.24E+08	1.24E+08	+	382.1331	NA	promoter-	promoter-	8	NM_00141	1967	Hs.741273	NM_00141	ENSG0000	EIF2B1	EIF2B|EIF2E	eukaryotic	protein-co
lib-C666-li	chr1	36396733	36396733	+	20.87959	NA	promoter-	promoter-	50	NM_02485	192669	Hs.657659	NM_02485	ENSG0000	AGO3	EIF2C3	argonaute	protein-co
lib-C666-li	chr8	1.18E+08	1.18E+08	+	23.80446	NA	promoter-	promoter-	-45	NM_00375	8667	Hs.492599	NM_00375	ENSG0000	EIF3H	EIF3S3|eIF3	eukaryotic	protein-co
lib-C666-li	chrY	22737578	22737578	+	110.0531	NA	promoter-	promoter-	-19	NM_00127	9086	Hs.461178	NM_00468	ENSG0000	EIF1AY	eIF-4C	eukaryotic	protein-co
lib-C666-li	chr2	1.81E+08	1.81E+08	+	41.28836	NA	promoter-	promoter-	-180	NM_02094	57703	Hs.311363	NM_02094	ENSG0000	CWC22	EIF4GL|NC	CWC22 spl	protein-co
lib-C666-li	chr14	89258951	89258951	+	30.4351	NA	5' UTR (NM	5' UTR (NM	145	NM_18338	161436	Hs.325846	NM_18338	ENSG0000	EML5	EMAP-2	echinoderr	protein-co
lib-C666-li	chr4	1.07E+08	1.07E+08	+	41.72511	NA	promoter-	promoter-	-71	NM_00114	9255	Hs.591680	NM_00475	ENSG0000	AIMP1	EMAP2|EM	aminoacyl	protein-co
lib-C666-li	chr5	1.34E+08	1.34E+08	+	24.5284	NA	promoter-	promoter-	-4	NM_17067	6500	Hs.171626	NM_00693	ENSG0000	SKP1	EMC19|OC	S-phase ki	protein-co
lib-C666-li	chr10	98346810	98346810	+	30.07415	NA	promoter-	promoter-	-1	NM_02012	56889	Hs.500674	NM_02012	ENSG0000	TM9SF3	EP70-P-isc	transmemk	protein-co
lib-C666-li	chr19	4791479	4791479	+	156.7217	NA	promoter-	promoter-	-249	NM_01870	55527	Hs.515082	NM_01870	ENSG0000	FEM1A	EPRAP	fem-1 hon	protein-co
lib-C666-li	chr1	94344733	94344733	+	61.32821	NA	promoter-	promoter-	29	NM_01459	30836	Hs.85769	NM_01459	ENSG0000	DNTTIP2	ERBP|FCF2	deoxynucle	protein-co
lib-C666-li	chr14	76127526	76127526	+	85.99825	NA	promoter-	promoter-	12	NM_00717	11161	Hs.15106	NM_00717	ENSG0000	C14orf1	ERG28|NET	chromosor	protein-co
lib-C666-li	chr5	1.72E+08	1.72E+08	+	21.28624	NA	promoter-	promoter-	40	NM_00103	57222	Hs.509163	NM_02046	ENSG0000	ERGIC1	ERGIC-32|I	endoplasm	protein-co
lib-C666-li	chr15	52311356	52311356	+	20.19521	NA	promoter-	promoter-	-55	NM_00274	5597	Hs.411847	NM_00274	ENSG0000	MAPK6	ERK3|HsT1	mitogen-a	protein-co
lib-C666-li	chr3	1.86E+08	1.86E+08	+	22.01582	NA	promoter-	promoter-	-38	NM_00445	2119	Hs.43697	NM_00445	ENSG0000	ETV5	ERM	ETS variant	protein-co
lib-C666-li	chr1	1.45E+08	1.45E+08	+	48.04218	NA	promoter-	promoter-	-3	NM_00489	9554	Hs.632438	NM_00489	ENSG0000	SEC22B	ERS-24|SE	SEC22 hon	protein-co
lib-C666-li	chr9	1.36E+08	1.36E+08	+	26.82121	NA	promoter-	promoter-	-197	NM_00128	6836	Hs.512465	NM_03316	ENSG0000	SURF4	ERV29	surfeit 4	protein-co
lib-C666-li	chr15	65426194	65426194	+	40.51821	NA	promoter-	promoter-	-20	NM_00570	10081	Hs.458596	NM_00570	ENSG0000	PDCD7	ES18|HES1	programm	protein-co
lib-C666-li	chr11	65479628	65479628	+	97.78539	NA	5' UTR (NM	5' UTR (NM	155	NM_18270	10524	Hs.397010	NM_00638	ENSG0000	KAT5	ESA1|HTAT	lysine acet	protein-co
lib-C666-li	chr16	28962202	28962202	+	24.00146	NA	promoter-	promoter-	-116	NM_03281	84901	Hs.513470	NM_03281	ENSG0000	NFATC2IP	ESC2|NIP4	nuclear fac	protein-co
lib-C666-li	chr15	70392001	70392001	+	22.01132	NA	Intergenic	CpG-8039	-1745	NM_02090	7090	Hs.287362	NM_00507	ENSG0000	TLE3	ESG|ESG3|(	transducin	protein-co
lib-C666-li	chr17	74733951	74733951	+	44.06107	NA	promoter-	promoter-	368	NM_00124	79157	Hs.73965	NM_02431	ENSG0000	MFSD11	ET	major facili	protein-co
lib-C666-li	chr2	20850895	20850895	+	43.79266	NA	promoter-	promoter-	-31	NM_02246	64342	Hs.531785	NM_02246	ENSG0000	HS1BP3	ETM2|HS1-	HCLS1 binc	protein-co
lib-C666-li	chr14	31677086	31677086	+	35.40551	NA	promoter-	promoter-	-357	NM_01538	25831	Hs.708017	NM_01538	ENSG0000	HECTD1	EULIR	HECT dom	protein-co
lib-C666-li	chr17	76124985	76124985	+	50.11658	NA	promoter-	promoter-	-110	NM_00112	11322	Hs.632227	NM_00726	ENSG0000	TMC6	EV1|EVER1	transmemk	protein-co
lib-C666-li	chr12	51157677	51157677	+	24.60494	NA	promoter-	promoter-	-112	NM_00517	466	Hs.648565	NM_00517	ENSG0000	ATF1	EWS-ATF1	activating t	protein-co
lib-C666-li	chr13	21476940	21476940	+	54.30825	NA	promoter-	promoter-	-27	NM_02245	64328	Hs.507452	NM_02245	ENSG0000	XPO4	exp4	exportin 4	protein-co
lib-C666-li	chr8	1.19E+08	1.19E+08	+	26.46807	NA	promoter-	promoter-	-110	NM_00012	2131	Hs.492618	NM_00012	ENSG0000	EXT1	EXT|LGCR|I	exostosin g	protein-co
lib-C666-li	chr19	56652420	56652420	+	25.39084	NA	promoter-	promoter-	-115	NM_00125	55311	Hs.24545	NM_01833	ENSG0000	ZNF444	EZF-2|EZF2	zinc finger	protein-co
lib-C666-li	chr19	39881418	39881418	+	100.6476	NA	promoter-	promoter-	417	NM_00125	54623	Hs.466714	NM_01908	ENSG0000	PAF1	F23149_1|F	PAF1 hom	protein-co
lib-C666-li	chr19	39881876	39881876	+	26.80209	NA	promoter-	promoter-	-41	NM_01908	54623	Hs.466714	NM_01908	ENSG0000	PAF1	F23149_1|F	PAF1 hom	protein-co
lib-C666-li	chr7	55322665	55322665	+	23.60036	NA	intron (NR_	CpG	663	NR_11042	1.03E+08	Hs.590524	NR_110426		ELDR	FabI|LINC0	EGFR long	ncRNA
lib-C666-li	chr1	40723764	40723764	+	24.23241	NA	promoter-	promoter-	42	NM_00585	10269	Hs.132642	NM_00585	ENSG0000	ZMPSTE24	FACE-1|FA	zinc metall	protein-co

lib-C666-li	chr11	66610915	66610915	+	243.9554	NA	promoter-	promoter-	32	NM_00513	9986	Hs.654972	NM_00513	ENSG0000	RCE1	FACE2|RCE	Ras conver	protein-co
lib-C666-li	chr3	10068097	10068097	+	434.4169	NA	promoter-	promoter-	26	NM_00101	2177	Hs.208388	NM_03308	ENSG0000	FANCD2	FA-D2|FA4	Fanconi an	protein-co
lib-C666-li	chr10	1.02E+08	1.02E+08	+	40.99587	NA	promoter-	promoter-	100	NM_00506	6319	Hs.558396	NM_00506	ENSG0000	SCD	FADS5|MS	stearoyl-C	protein-co
lib-C666-li	chr11	22647424	22647424	+	23.34793	NA	promoter-	promoter-	-37	NM_02272	2188	Hs.632151	NM_02272	ENSG0000	FANCF	FAF	Fanconi an	protein-co
lib-C666-li	chr16	4665030	4665030	+	170.8208	NA	promoter-	promoter-	-103	NM_14525	124402	Hs.513313	NM_14525	ENSG0000	UBALD1	FAM100A|I	UBA like d	protein-co
lib-C666-li	chr16	4666293	4666293	+	91.98129	NA	Intergenic	CpG-8803	-1366	NM_14525	124402	Hs.513313	NM_14525	ENSG0000	UBALD1	FAM100A|I	UBA like d	protein-co
lib-C666-li	chr5	1.73E+08	1.73E+08	+	160.6333	NA	promoter-	promoter-	-97	NM_13836	91272	Hs.425091	NM_13836	ENSG0000	BOD1	FAM44B	biorientati	protein-co
lib-C666-li	chrX	14048080	14048080	+	55.46257	NA	promoter-	promoter-	-45	NM_00104	54960	Hs.592237	NM_01785	ENSG0000	GEMIN8	FAM51A1	gem nucle	protein-co
lib-C666-li	chr15	41047487	41047487	+	300.1926	NA	promoter-	promoter-	47	NM_00132	55177	Hs.511067	NM_01814	ENSG0000	RMDN3	FAM82A2|I	regulator c	protein-co
lib-C666-li	chr7	5632419	5632419	+	24.25638	NA	promoter-	promoter-	-17	NM_00308	6624	Hs.118400	NM_00308	ENSG0000	FSCN1	FAN1|HSN	fascin actir	protein-co
lib-C666-li	chr1	2.02E+08	2.02E+08	+	25.38683	NA	promoter-	promoter-	-133	NM_00131	29089	Hs.5199	NM_01417	ENSG0000	UBE2T	FANCT|HSI	ubiquitin c	protein-co
lib-C666-li	chr7	1.52E+08	1.52E+08	+	165.3579	NA	promoter-	promoter-	-109	NM_00543	7516	Hs.647093	NM_00543	ENSG0000	XRCC2	FANCU	X-ray repa	protein-co
lib-C666-li	chr1	11741188	11741188	+	61.18587	NA	promoter-	promoter-	83	NM_00634	10459	Hs.19400	NM_00634	ENSG0000	MAD2L2	FANCV|MA	MAD2 mit	protein-co
lib-C666-li	chr2	2.39E+08	2.39E+08	+	24.31114	NA	promoter-	promoter-	-323	NM_02281	8864	Hs.58756	NM_00389	ENSG0000	PER2	FASPS|FAS	period circ	protein-co
lib-C666-li	chr11	64889678	64889678	+	100.6476	NA	promoter-	promoter-	-6	NM_00199	2197	Hs.387208	NM_00199	ENSG0000	FAU	FAU1|Fub1	FAU, ubiqu	protein-co
lib-C666-li	chr19	54619125	54619125	+	121.0116	NA	promoter-	promoter-	-70	NM_01334	29844	Hs.590939	NM_01334	ENSG0000	TFPT	FB1|INO80	TCF3 fusio	protein-co
lib-C666-li	chr7	5553437	5553437	+	215.3851	NA	promoter-	promoter-	-8	NM_02496	80028	Hs.623974	NM_02496	ENSG0000	FBXL18	Fbl18	F-box and	protein-co
lib-C666-li	chr17	37558939	37558939	+	57.42312	NA	Intergenic	CpG-1053	-1030	NM_03287	84961	Hs.462946	NM_03287	ENSG0000	FBXL20	Fbl2|Fbl20	F-box and	protein-co
lib-C666-li	chr2	1.54E+08	1.54E+08	+	48.40399	NA	promoter-	promoter-	-105	NM_01789	55660	Hs.643580	NM_01789	ENSG0000	PRPF40A	FBP-11|FBI	pre-mRNA	protein-co
lib-C666-li	chr19	6424222	6424222	+	34.73338	NA	intron (NM	intron (NM	600	NM_00368	8570	Hs.727344	NM_00368	ENSG0000	KHSRP	FBP2|FUBP	KH-type sp	protein-co
lib-C666-li	chr19	6424950	6424950	+	28.88196	NA	promoter-	promoter-	-128	NM_00368	8570	Hs.727344	NM_00368	ENSG0000	KHSRP	FBP2|FUBP	KH-type sp	protein-co
lib-C666-li	chr16	30669541	30669541	+	33.76759	NA	Intergenic	CpG	-1299	NM_00110	64319	Hs.247186	NM_02245	ENSG0000	FBRS	FBS|FBS1	fibrosin	protein-co
lib-C666-li	chr9	1.24E+08	1.24E+08	+	62.55363	NA	promoter-	promoter-	52	NM_01216	26190	Hs.494985	NM_01216	ENSG0000	FBXW2	FBW2|Fwd	F-box and	protein-co
lib-C666-li	chr9	1.4E+08	1.4E+08	+	46.4068	NA	promoter-	promoter-	39	NM_01899	54461	Hs.522507	NM_01899	ENSG0000	FBXW5	Fbw5	F-box and	protein-co
lib-C666-li	chr19	12807489	12807489	+	101.7499	NA	promoter-	promoter-	-34	NM_03230	84261	Hs.515154	NM_03230	ENSG0000	FBXW9	Fbw9	F-box and	protein-co
lib-C666-li	chr7	1E+08	1E+08	+	23.1305	NA	promoter-	promoter-	2	NM_03350	26261	Hs.283764	NM_01217	ENSG0000	FBXO24	FBX24	F-box prot	protein-co
lib-C666-li	chr3	1.2E+08	1.2E+08	+	124.4398	NA	promoter-	promoter-	-59	NM_00551	2960	Hs.445272	NM_00551	ENSG0000	GTF2E1	FE|TF2E1|T	general tra	protein-co
lib-C666-li	chr5	1.4E+08	1.4E+08	+	24.41787	NA	promoter-	promoter-	4	NM_13317	10307	Hs.529449	NM_00605	ENSG0000	APBB3	FE65L2|SR	amyloid be	protein-co
lib-C666-li	chr17	8152607	8152607	+	184.7914	NA	promoter-	promoter-	11	NM_01239	5198	Hs.573976	NM_01239	ENSG0000	PFAS	FGAMS|FG	phosphori	protein-co
lib-C666-li	chr19	50400737	50400737	+	64.69308	NA	promoter-	promoter-	-590	NM_15289	259307	Hs.574492	NM_15289	ENSG0000	IL4I1	FIG1|LAAO	interleukin	protein-co
lib-C666-li	chrX	67718812	67718812	+	50.81684	NA	5' UTR (NM	5' UTR (NM	188	NM_00119	286451	Hs.82719	NM_17383	ENSG0000	YIPF6	FinGER6	Yip1 doma	protein-co
lib-C666-li	chr11	64008444	64008444	+	71.25437	NA	promoter-	promoter-	31	NM_00447	2286	Hs.227729	NM_00447	ENSG0000	FKBP2	FKBP-13|P	FK506 binc	protein-co
lib-C666-li	chr9	1.16E+08	1.16E+08	+	25.40925	NA	promoter-	promoter-	-47	NM_01525	23307	Hs.522351	NM_01525	ENSG0000	FKBP15	FKBP133|K	FK506 binc	protein-co
lib-C666-li	chr2	10184560	10184560	+	26.37215	NA	5' UTR (NM	5' UTR (NM	188	NM_00117	8462	Hs.12229	NM_00359	ENSG0000	KLF11	FKLF|FKLF1	Kruppel lik	protein-co
lib-C666-li	chr19	18303786	18303786	+	37.99633	NA	promoter-	promoter-	-254	NM_03268	84769	Hs.515254	NM_03268	ENSG0000	MPV17L2	FKSG24	MPV17 mit	protein-co
lib-C666-li	chr19	49977360	49977360	+	26.46433	NA	promoter-	promoter-	-106	NM_00127	2323	Hs.428	NM_00145	ENSG0000	FLT3LG	FL|FLT3L	fms relatec	protein-co
lib-C666-li	chr1	46049238	46049238	+	43.7015	NA	promoter-	promoter-	-422	NM_15229	4678	Hs.319334	NM_00248	ENSG0000	NASP	FLB7527|H	nuclear au	protein-co
lib-C666-li	chr14	91976886	91976886	+	117.5857	NA	promoter-	promoter-	-62	NM_00128	55671	Hs.533887	NM_01793	ENSG0000	PPP4R3A	FLFL1|KIAA	protein ph	protein-co
lib-C666-li	chr12	56615765	56615765	+	23.0291	NA	promoter-	promoter-	-12	NM_19435	10193	Hs.524502	NM_00578	ENSG0000	RNF41	FLRF|NRDF	ring finger	protein-co
lib-C666-li	chr14	24685285	24685285	+	47.23204	NA	promoter-	promoter-	-9	NM_13847	145553	Hs.220963	NM_13847	ENSG0000	MDP1	FN6PASE|M	magnesiun	protein-co
lib-C666-li	chr8	29939968	29939968	+	22.50982	NA	intron (NM	intron (NM	756	NM_01612	51669	Hs.521487	NM_01612	ENSG0000	SARAF	FOAP-7|HS	store-oper	protein-co
lib-C666-li	chr16	88729634	88729634	+	48.67325	NA	promoter-	promoter-	-77	NM_00246	4597	Hs.252457	NM_00246	ENSG0000	MVD	FP17780|M	mevalonat	protein-co
lib-C666-li	chr17	42264275	42264275	+	179.8454	NA	promoter-	promoter-	-44	NM_00133	79089	Hs.181391	NM_02410	ENSG0000	TMUB2	FP2653	transmemb	protein-co
lib-C666-li	chr7	74267302	74267302	+	107.244	NA	intron (NM	CpG	570	NM_00128	84163	Hs.647017	NM_17353	ENSG0000	GTF2IRD2	FP630|GTF	GTF2I repe	protein-co
lib-C666-li	chr11	1.26E+08	1.26E+08	+	148.6433	NA	promoter-	promoter-	-20	NM_01754	55572	Hs.317190	NM_01754	ENSG0000	FOXRED1	FP634|H17	FAD deper	protein-co
lib-C666-li	chr21	44527680	44527680	+	133.5783	NA	promoter-	promoter-	8	NM_00675	7307	Hs.365116	NM_00675	ENSG0000	U2AF1	FP793|RN|	U2 small n	protein-co
lib-C666-li	chr16	3333453	3333453	+	74.49267	NA	promoter-	promoter-	-34	NM_00574	10127	Hs.611475	NM_00574	ENSG0000	ZNF263	FPM315|Z	zinc finger	protein-co
lib-C666-li	chr1	1.55E+08	1.55E+08	+	85.95118	NA	promoter-	promoter-	24	NM_00200	2224	Hs.335918	NM_00200	ENSG0000	FDPS	FPPS|FPS|P	farnesyl di	protein-co
lib-C666-li	chr11	65668058	65668058	+	80.28929	NA	promoter-	promoter-	-61	NM_00130	8061	Hs.283565	NM_00543	ENSG0000	FOSL1	FRA|FRA1|	FOS like 1,	protein-co
lib-C666-li	chr3	1.29E+08	1.29E+08	+	49.86005	NA	promoter-	promoter-	-247	NM_00119	57461	Hs.512661	NM_02070	ENSG0000	ISY1	FSAP33	ISY1 splicir	protein-co
lib-C666-li	chr6	37400957	37400957	+	53.7204	NA	promoter-	promoter-	50	NM_01505	23070	Hs.520102	NM_01505	ENSG0000	CMTR1	FTSJD2|KIA	cap methy	protein-co
lib-C666-li	chr8	33330783	33330783	+	29.30929	NA	promoter-	promoter-	-119	NM_03266	84750	Hs.458713	NM_03266	ENSG0000	FUT10	FUCTX	fucosyltran	protein-co
lib-C666-li	chr22	41681566	41681566	+	25.49755	NA	5' UTR (NM	5' UTR (NM	455	NM_00127	5905	Hs.183800	NM_00288	ENSG0000	RANGAP1	Fug1|RAN(	Ran GTPas	protein-co
lib-C666-li	chr19	17337236	17337236	+	25.49755	NA	intron (NM	CpG	223	NM_02457	79629	Hs.422676	NM_02457	ENSG0000	OCEL1	FWP009|S	occludin/E	protein-co
lib-C666-li	chr16	30366738	30366738	+	40.31997	NA	promoter-	promoter-	-56	NM_00611	10421	Hs.202677	NM_00611	ENSG0000	CD2BP2	FWP010|LI	CD2 cytop	protein-co
lib-C666-li	chr11	6502632	6502632	+	95.85526	NA	promoter-	promoter-	-45	NM_01219	26515	Hs.54943	NM_01219	ENSG0000	TIMM10B	FXC1|TIM1	translocase	protein-co
lib-C666-li	chr17	56595428	56595428	+	20.90919	NA	promoter-	promoter-	-177	NM_00468	9110	Hs.514373	NM_00468	ENSG0000	MTMR4	FYVE-DSP	myotubula	protein-co
lib-C666-li	chr5	1.79E+08	1.79E+08	+	37.47379	NA	promoter-	promoter-	-1	NM_00128	55819	Hs.484363	NM_01843	ENSG0000	RNF130	G1RZFP|G(	ring finger	protein-co
lib-C666-li	chr5	54469061	54469061	+	22.28596	NA	promoter-	promoter-	-56	NM_00114	166979	Hs.669184	NM_15262	ENSG0000	CDC20B	G6VTS765	cell divisio	protein-co
lib-C666-li	chr6	31763815	31763815	+	66.08424	NA	promoter-	promoter-	-103	NM_00629	7407	Hs.520026	NM_00629	ENSG0000	VARS	G7A|VARS	valyl-tRNA	protein-co
lib-C666-li	chr1	1.51E+08	1.51E+08	+	38.21529	NA	promoter-	promoter-	13	NM_14461	126626	Hs.15671	NM_14461	ENSG0000	GABPB2	GABPB-2	GA binding	protein-co
lib-C666-li	chr19	49375577	49375577	+	41.60205	NA	promoter-	promoter-	-72	NM_01433	23645	Hs.631593	NM_01433	ENSG0000	PPP1R15A	GADD34	protein ph	protein-co
lib-C666-li	chr7	1.52E+08	1.52E+08	+	113.102	NA	promoter-	promoter-	-98	NM_02208	63917	Hs.647109	NM_02208	ENSG0000	GALNT11	GALNAC-T	polypeptid	protein-co
lib-C666-li	chr12	53693587	53693587	+	27.96363	NA	TTS (NM_1	TTS (NM_1	117	NM_02164	60314	Hs.655988	NM_02164	ENSG0000	C12orf10	Gamm1|M	chromosor	protein-co
lib-C666-li	chr16	81348496	81348496	+	151.1122	NA	promoter-	promoter-	-75	NM_02204	8139	Hs.112569	NM_02204	ENSG0000	GAN	GAN1|KLH	gigaxonin	protein-co
lib-C666-li	chr22	29702312	29702312	+	21.21068	NA	promoter-	promoter-	-673	NM_15223	10634	Hs.322852	NM_00647	ENSG0000	GAS2L1	GAR22	growth arr	protein-co
lib-C666-li	chr14	36278595	36278595	+	142.5945	NA	promoter-	promoter-	-66	NM_00134	253959	Hs.113150	NM_01499	ENSG0000	RALGAPA1	GARNL1|G	Ral GTPase	protein-co
lib-C666-li	chr9	6757734	6757734	+	25.3508	NA	promoter-	promoter-	93	NM_00114	23081	Hs.709425	NM_01506	ENSG0000	KDM4C	GASC1|JHD	lysine dem	protein-co
lib-C666-li	chr9	1.39E+08	1.39E+08	+	208.2466	NA	promoter-	promoter-	-62	NM_01617	10422	Hs.9194	NM_01617	ENSG0000	UBAC1	GBDR1|KP(	UBA doma	protein-co
lib-C666-li	chr2	1.09E+08	1.09E+08	+	28.16397	NA	non-codin	non-codin	125	NM_18145	9648	Hs.436505	NM_18145	ENSG0000	GCC2	GCC185|R	GRIP and c	protein-co
lib-C666-li	chr7	1.27E+08	1.27E+08	+	57.02325	NA	promoter-	promoter-	-16	NM_02452	79571	Hs.521168	NM_02452	ENSG0000	GCC1	GCC1P|GC	GRIP and c	protein-co
lib-C666-li	chr15	57998780	57998780	+	93.49621	NA	promoter-	promoter-	61	NM_01553	81488	Hs.437256	NM_01553	ENSG0000	POLR2M	GCOM1|G	RNA polyn	protein-co
lib-C666-li	chr22	50683468	50683468	+	315.4616	NA	promoter-	promoter-	-68	NM_02046	85378	Hs.336431	NM_02046	ENSG0000	TUBGCP6	GCP-6|GC	tubulin gar	protein-co
lib-C666-li	chr12	97301033	97301033	+	147.1243	NA	promoter-	promoter-	32	NM_00113	121441	Hs.270084	NM_15290	ENSG0000	NEDD1	GCP-WD|T	neural pre	protein-co
lib-C666-li	chr10	74927854	74927854	+	284.3587	NA	promoter-	promoter-	-1	NR_02420	11319	Hs.631822	NM_00726	ENSG0000	ECD	GCR2|HSG	ecdysonel	protein-co
lib-C666-li	chr3	57112762	57112762	+	21.0836	NA	intron (NM	CpG	574	NM_00112	50650	Hs.476402	NM_01955	ENSG0000	ARHGEF3	GEF3|STA3	Rho guani	protein-co
lib-C666-li	chr7	1.44E+08	1.44E+08	+	44.13626	NA	promoter-	promoter-	-103	NM_00543	7984	Hs.334	NM_00543	ENSG0000	ARHGEF5	GEF5|P60|T	Rho guani	protein-co
lib-C666-li	chr1	8086519	8086519	+	49.56857	NA	promoter-	promoter-	-126	NM_01894	54206	Hs.605445	NM_01894	ENSG0000	ERRFI1	GENE-33|M	ERBB recep	protein-co
lib-C666-li	chr12	52444934	52444934	+	57.02275	NA	promoter-	promoter-	-252	NM_00213	3164	Hs.524430	NM_00213	ENSG0000	NR4A1	GFRP1|HM	nuclear rec	protein-co
lib-C666-li	chr12	52445171	52445171	+	36.16835	NA	promoter-	promoter-	-15	NM_00213	3164	Hs.524430	NM_00213	ENSG0000	NR4A1	GFRP1|HM	nuclear rec	protein-co
lib-C666-li	chr12	52430795	52430795	+	28.57903	NA	promoter-	promoter-	-704	NM_00120	3164	Hs.524430	NM_00213	ENSG0000	NR4A1	GFRP1|HM	nuclear rec	protein-co
lib-C666-li	chr5	1.19E+08	1.19E+08	+	54.1948	NA	promoter-	promoter-	-491	NM_00128	25816	Hs.618488	NM_01435	ENSG0000	TNFAIP8	GG2-1|MD	TNF alpha	protein-co
lib-C666-li	chr22	24236466	24236466	+	30.07415	NA	promoter-	promoter-	-99	NM_00241	4282	Hs.407995	NM_00241	ENSG0000	MIF	GIF|GLIF|M	macropha	protein-co
lib-C666-li	chr1	1.75E+08	1.75E+08	+	127.9668	NA	promoter-	promoter-	45	NM_00100	27101	Hs.508524	NM_01441	ENSG0000	CACYBP	GIG5|PNA	calcyclin bi	protein-co
lib-C666-li	chr16	29465748	29465748	+	26.68159	NA	promoter-	promoter-	-74	NM_00101	548593	Hs.729791	NM_00101	ENSG0000	SLX1A	GIYD1	SLX1 hom	protein-co
lib-C666-li	chr17	61851128	61851128	+	57.48363	NA	promoter-	promoter-	-40	NM_02019	57003	Hs.202011	NM_02019	ENSG0000	CCDC47	GK001|MS	coiled-coil	protein-co
lib-C666-li	chr15	49462202	49462202	+	34.3461	NA	promoter-	promoter-	-11	NM_00128	2585	Hs.122006	NM_00204	ENSG0000	GALK2	GK2	galactokin	protein-co
lib-C666-li	chr14	24584036	24584036	+	58.5532	NA	promoter-	promoter-	50	NM_02523	80344	Hs.525251	NM_02523	ENSG0000	DCAF11	GL014|PRC	DDB1 and	protein-co
lib-C666-li	chr1	1.5E+08	1.5E+08	+	20.66352	NA	promoter-	promoter-	-122	NM_00352	8349	Hs.2178	NM_00352	ENSG0000	HIST2H2BE	GL105|H2E	histone clu	protein-co
lib-C666-li	chr11	62389665	62389665	+	37.64901	NA	promoter-	promoter-	-18	NM_00128	26229	Hs.502759	NM_01220	ENSG0000	B3GAT3	GLCATI|JD	beta-1,3-g	protein-co
lib-C666-li	chr16	1877078	1877078	+	40.6674	NA	promoter-	promoter-	117	NM_00532	3029	Hs.157394	NM_00532	ENSG0000	HAGH	GLO2|GLX	hydroxyac	protein-co
lib-C666-li	chr19	17666341	17666341	+	47.23204	NA	promoter-	promoter-	-62	NM_02465	79709	Hs.418795	NM_02465	ENSG0000	COLGALT1	GLT25D1	collagen b	protein-co
lib-C666-li	chr6	1.34E+08	1.34E+08	+	26.54591	NA	promoter-	promoter-	-4	NM_14517	154091	Hs.486508	NM_14517	ENSG0000	SLC2A12	GLUT12|GL	solute carr	protein-co
lib-C666-li	chr2	1.13E+08	1.13E+08	+	20.19521	NA	promoter-	promoter-	-47	NM_00541	6574	Hs.187946	NM_00541	ENSG0000	SLC20A1	GLVR1|Glv	solute carr	protein-co
lib-C666-li	chr2	2.41E+08	2.41E+08	+	23.74671	NA	promoter-	promoter-	-419	NM_00208	2817	Hs.328232	NM_00208	ENSG0000	GPC1	glypican	glypican 1	protein-co
lib-C666-li	chr18	46065880	46065880	+	22.5977	NA	intron (NM	CpG	453	NM_01477	9811	Hs.145230	NM_01477	ENSG0000	CTIF	Gm672|KIA	cap bindin	protein-co
lib-C666-li	chr6	2245921	2245921	+	58.76075	NA	promoter-	promoter-	-53	NM_00150	2762	Hs.144496	NM_00150	ENSG0000	GMDS	GMD|SDR3	GDP-manr	protein-co
lib-C666-li	chr1	2.28E+08	2.28E+08	+	145.4746	NA	promoter-	promoter-	-76	NM_00124	2987	Hs.376933	NM_00085	ENSG0000	GUK1	GMK	guanylate	protein-co
lib-C666-li	chr14	24701667	24701667	+	58.13765	NA	promoter-	promoter-	39	NM_00100	51292	Hs.368855	NM_01657	ENSG0000	GMPR2	GMPR 2	guanosine	protein-co
lib-C666-li	chr2	71295371	71295371	+	28.92533	NA	promoter-	promoter-	-37	NM_00133	55577	Hs.7036	NM_01756	ENSG0000	NAGK	GNK|HSA2	N-acetylgl	protein-co
lib-C666-li	chr9	1.28E+08	1.28E+08	+	60.89502	NA	promoter-	promoter-	13	NM_00207	2800	Hs.59504	NM_00207	ENSG0000	GOLGA1	golgin-97	golgin A1	protein-co
lib-C666-li	chr14	93260474	93260474	+	69.8176	NA	promoter-	promoter-	-102	NM_00511	9950	Hs.104320	NM_00511	ENSG0000	GOLGA5	GOLIM5|R	golgin A5	protein-co
lib-C666-li	chr1	32757568	32757568	+	31.81975	NA	promoter-	promoter-	-140	NM_00496	3065	Hs.88556	NM_00496	ENSG0000	HDAC1	GON-10|H	histone de	protein-co
lib-C666-li	chr5	32174456	32174456	+	28.8837	NA	promoter-	promoter-	-31	NM_02213	64083	Hs.408909	NM_02213	ENSG0000	GOLPH3	GOPP1|GP	golgi phos	protein-co
lib-C666-li	chr22	39101617	39101617	+	30.98434	NA	promoter-	promoter-	-190	NM_00428	9567	Hs.276925	NM_00428	ENSG0000	GTPBP1	GP-1|GP1|	GTP bindir	protein-co
lib-C666-li	chr17	28705907	28705907	+	24.25638	NA	promoter-	promoter-	-35	NM_00130	1362	Hs.446079	NM_00130	ENSG0000	CPD	GP180	carboxype	protein-co
lib-C666-li	chr3	1.98E+08	1.98E+08	+	25.49921	NA	promoter-	promoter-	-47	NM_03302	89782	Hs.518540	NM_03302	ENSG0000	LMLN	GP63|INV|I	leishmano	protein-co
lib-C666-li	chr10	1.14E+08	1.14E+08	+	432.3987	NA	promoter-	promoter-	7	NM_02091	57678	Hs.42586	NM_02091	ENSG0000	GPAM	GPAT|GPA	glycerol-3	protein-co
lib-C666-li	chrX	48980231	48980231	+	73.89001	NA	promoter-	promoter-	-80	NM_01569	27238	Hs.503666	NM_01569	ENSG0000	GPKOW	GPATC5|G	G-patch d	protein-co
lib-C666-li	chr5	56469523	56469523	+	62.69942	NA	promoter-	promoter-	-252	NM_00120	65056	Hs.444279	NM_02291	ENSG0000	GPBP1	GPBP|SSH	GC-rich pr	protein-co
lib-C666-li	chr3	1.7E+08	1.7E+08	+	20.19521	NA	promoter-	promoter-	-40	NM_01437	26996	Hs.231320	NM_01437	ENSG0000	GPR160	GPCR1|GP	G protein-	protein-co
lib-C666-li	chr1	1.46E+08	1.46E+08	+	41.28836	NA	promoter-	promoter-	58	NM_00109	653519	Hs.504320	NM_00109	ENSG0000	GPR89A	GPHR|GPR	G protein-	protein-co
lib-C666-li	chr1	1.47E+08	1.47E+08	+	36.99348	NA	promoter-	promoter-	-17	NM_01633	51463	Hs.504320	NM_01633	ENSG0000	GPR89B	GPHR|GPR	G protein-	protein-co
lib-C666-li	chrX	15353750	15353750	+	30.542	NA	promoter-	promoter-	-74	NM_00264	5277	Hs.137154	NM_00264	ENSG0000	PIGA	GPI3|MCA	phosphatic	protein-co
lib-C666-li	chr4	82393230	82393230	+	35.28811	NA	promoter-	promoter-	-148	NM_00130	153020	Hs.591696	NM_15254	ENSG0000	RASGEF1B	GPIG4	RasGEF do	protein-co
lib-C666-li	chr2	26568816	26568816	+	28.7791	NA	promoter-	promoter-	-106	NM_00132	165082	Hs.631878	NM_15383	ENSG0000	ADGRF3	GPR113|P	adhesion G	protein-co
lib-C666-li	chr3	1.27E+08	1.27E+08	+	50.86197	NA	promoter-	promoter-	-398	NR_07337	131601	Hs.6418	NM_01637	ENSG0000	TPRA1	GPR175|TM	transmemb	protein-co
lib-C666-li	chr7	55433987	55433987	+	262.548	NA	intron (NM	CpG	846	NM_01869	55915	Hs.595384	NM_01869	ENSG0000	LANCL2	GPR69B|TA	LanC like 2	protein-co
lib-C666-li	chr5	1.77E+08	1.77E+08	+	28.8837	NA	promoter-	promoter-	-227	NM_00100	2870	Hs.235116	NM_00208	ENSG0000	GRK6	GPRK6	G protein-	protein-co
lib-C666-li	chr10	27444221	27444221	+	59.83187	NA	promoter-	promoter-	468	NR_13546	84930	Hs.276905	NM_03284	ENSG0000	MASTL	GREATWA	microtubul	protein-co
lib-C666-li	chr9	95056002	95056002	+	20.19521	NA	promoter-	promoter-	36	NM_00216	3376	Hs.445403	NM_00216	ENSG0000	IARS	GRIDHH|IA	isoleucyl-t	protein-co
lib-C666-li	chr8	92082298	92082298	+	69.35086	NA	promoter-	promoter-	119	NR_11043	1.01E+08	Hs.153412	NR_11043	ENSG0000	OTUD6B-A	GS1-251I9	OTUD6B a	ncRNA
lib-C666-li	chr3	67704814	67704814	+	29.74345	NA	promoter-	promoter-	224	NM_00384	8801	Hs.644919	NM_00384	ENSG0000	SUCLG2	G-SCS|GB	succinate-	protein-co
lib-C666-li	chr11	18416059	18416059	+	53.15281	NA	non-codin	non-codin	123	NM_00116	3939	Hs.2795	NM_00556	ENSG0000	LDHA	GSD11|HE	lactate deh	protein-co
lib-C666-li	chr10	1.06E+08	1.06E+08	+	23.49208	NA	promoter-	promoter-	127	NM_18323	119391	Hs.107384	NM_18323	ENSG0000	GSTO2	GSTO 2-2|	glutathion	protein-co
lib-C666-li	chr4	74124898	74124898	+	82.96959	NA	promoter-	promoter-	-383	NM_19888	26057	Hs.601206	NM_01557	ENSG0000	ANKRD17	GTAR|MAS	ankyrin rep	protein-co

lib-C666-li	chr6	1.71E+08	1.71E+08	+	100.6476	NA	promoter-	promoter-	-14	NM_00117	6908	Hs.590872	NM_00319	ENSG0000	TBP	GTF2D|GTF	TATA-box	protein-co
lib-C666-li	chr20	60758088	60758088	+	110.1377	NA	promoter-	promoter-	7	NM_01566	26164	Hs.340636	NM_01566	ENSG0000	MTG2	GTPBP5|OI	mitochond	protein-co
lib-C666-li	chr10	70661004	70661004	+	34.25375	NA	promoter-	promoter-	-30	NM_02404	79009	Hs.522984	NM_02404	ENSG0000	DDX50	GU2|GUB|F	DExD-box	protein-co
lib-C666-li	chr10	70715978	70715978	+	28.88523	NA	promoter-	promoter-	99	NM_00472	9188	Hs.223141	NM_00472	ENSG0000	DDX21	GUA|GURD	DExD-box	protein-co
lib-C666-li	chr11	45944049	45944049	+	35.74596	NA	promoter-	promoter-	-178	NM_00130	120071	Hs.86543	NM_15231	ENSG0000	LARGE2	GYLTL1B|P	LARGE xylo	protein-co
lib-C666-li	chr20	30102224	30102224	+	30.98434	NA	promoter-	promoter-	11	NM_17858	81502	Hs.373741	NM_03078	ENSG0000	HM13	H13|IMP1|I	histocomp	protein-co
lib-C666-li	chr2	3605967	3605967	+	165.3986	NA	promoter-	promoter-	-6	NM_00128	246243	Hs.568006	NM_00293	ENSG0000	RNASEH1	H1RNA|PE	ribonuclea	protein-co
lib-C666-li	chr6	27832960	27832960	+	26.26478	NA	promoter-	promoter-	-147	NM_00351	8332	Hs.233568	NM_00351	ENSG0000	HIST1H2AL	H2A.i|H2A	histone clu	protein-co
lib-C666-li	chr7	44887908	44887908	+	34.83329	NA	promoter-	promoter-	-183	NM_20143	94239	Hs.488189	NM_01241	ENSG0000	H2AFV	H2A.Z-2|H	H2A histor	protein-co
lib-C666-li	chr6	27806077	27806077	+	26.26478	NA	promoter-	promoter-	40	NM_00351	8330	Hs.734717	NM_00351	ENSG0000	HIST1H2AH	H2A/d|H2A	histone clu	protein-co
lib-C666-li	chr6	27114716	27114716	+	20.59244	NA	promoter-	promoter-	-79	NM_00131	85236	Hs.437275	NM_08059	ENSG0000	HIST1H2BK	H2B/S|H2B	histone clu	protein-co
lib-C666-li	chr17	73775964	73775964	+	60.07874	NA	promoter-	promoter-	52	NM_00532	3021	Hs.180877	NM_00532	ENSG0000	H3F3B	H3.3B	H3 histone	protein-co
lib-C666-li	chr1	1.5E+08	1.5E+08	+	26.57948	NA	promoter-	promoter-	-89	NM_00100	333932	Hs.647745	NM_00100	ENSG0000	HIST2H3A	H3/n|H3/o	histone clu	protein-co
lib-C666-li	chr6	26204701	26204701	+	25.55305	NA	promoter-	promoter-	-172	NM_00354	8367	Hs.662174	NM_00354	ENSG0000	HIST1H4E	H4/j|H4FJ	histone clu	protein-co
lib-C666-li	chr9	2844278	2844278	+	97.29876	NA	promoter-	promoter-	-148	NM_01487	9933	Hs.493309	NM_01487	ENSG0000	PUM3	HA-8|HLA-	pumilio RN	protein-co
lib-C666-li	chr6	26597159	26597159	+	46.67128	NA	promoter-	promoter-	-12	NM_01337	29777	Hs.254406	NM_01337	ENSG0000	ABT1	hABT1	activator o	protein-co
lib-C666-li	chr4	1.09E+08	1.09E+08	+	41.28836	NA	promoter-	promoter-	92	NM_00118	3033	Hs.438289	NM_00532	ENSG0000	HADH	HAD|HADH	hydroxyacy	protein-co
lib-C666-li	chr16	75657208	75657208	+	69.78576	NA	promoter-	promoter-	13	NM_00132	23536	Hs.729312	NM_01209	ENSG0000	ADAT1	HADAT1	adenosine	protein-co
lib-C666-li	chr7	73097891	73097891	+	108.0891	NA	promoter-	promoter-	-7	NM_00120	114049	Hs.647063	NM_01752	ENSG0000	WBSCR22	HASJ4442|	Williams-B	protein-co
lib-C666-li	chr1	1447407	1447407	+	78.54099	NA	promoter-	promoter-	-116	NM_00117	55210	Hs.23413	NM_01818	ENSG0000	ATAD3A	HAYOS	ATPase far	protein-co
lib-C666-li	chrX	49125785	49125785	+	24.25638	NA	promoter-	promoter-	-521	NM_00118	89801	Hs.433652	NM_03321	ENSG0000	PPP1R3F	HB2E|LL0X	protein ph	protein-co
lib-C666-li	chr7	1.57E+08	1.57E+08	+	44.31884	NA	promoter-	promoter-	-50	NM_00551	3110	Hs.37035	NM_00551	ENSG0000	MNX1	HB9|HLXB9	motor neu	protein-co
lib-C666-li	chr9	1.4E+08	1.4E+08	+	74.35668	NA	promoter-	promoter-	15	NM_05304	94107	Hs.231029	NM_05304	ENSG0000	TMEM203	HBEBP1	transmeml	protein-co
lib-C666-li	chr3	52322004	52322004	+	68.04845	NA	TTS (NR_04	TTS (NR_04	168	NR_026701	132158	Hs.415312	NM_14526	ENSG0000	GLYCTK	HBEBP2|HE	glycerate k	protein-co
lib-C666-li	chr7	93633686	93633686	+	112.5251	NA	promoter-	promoter-	8	NM_00586	10282	Hs.489132	NM_00586	ENSG0000	BET1	HBET1	Bet1 golgi	protein-co
lib-C666-li	chr17	47865949	47865949	+	28.22301	NA	promoter-	promoter-	-32	NM_00134	11143	Hs.21907	NM_00706	ENSG0000	KAT7	HBO1|HBC	lysine acet	protein-co
lib-C666-li	chr4	1714230	1714230	+	56.02075	NA	5' UTR (NM	5' UTR (NM	238	NM_00130	7884	Hs.298345	NM_00652	ENSG0000	SLBP	HBP	stem-loop	protein-co
lib-C666-li	chr4	1714751	1714751	+	25.39084	NA	promoter-	promoter-	-283	NM_00130	7884	Hs.298345	NM_00652	ENSG0000	SLBP	HBP	stem-loop	protein-co
lib-C666-li	chr1	36107168	36107168	+	64.34737	NA	promoter-	promoter-	-16	NM_00119	5690	Hs.471441	NM_00279	ENSG0000	PSMB2	HC7-I	proteasom	protein-co
lib-C666-li	chr15	29562021	29562021	+	42.49125	NA	promoter-	promoter-	-1	NM_13870	56160	Hs.94011	NM_13870	ENSG0000	NSMCE3	HCA4|LICS	NSE3 hom	protein-co
lib-C666-li	chr16	4475614	4475614	+	21.81327	NA	promoter-	promoter-	-192	NM_00113	9093	Hs.459779	NM_00514	ENSG0000	DNAJA3	HCA57|TID	DnaJ heat	protein-co
lib-C666-li	chr9	1.33E+08	1.33E+08	+	72.59265	NA	promoter-	promoter-	-29	NM_01652	51759	Hs.278429	NM_01652	ENSG0000	C9orf78	HCA59|HSI	chromosor	protein-co
lib-C666-li	chr5	1.8E+08	1.8E+08	+	51.02929	NA	promoter-	promoter-	84	NR_04568(	1.01E+08	Hs.655606	NR_04568(	ENSG0000	HEIH	HCCAT2|LI	hepatocell	ncRNA
lib-C666-li	chr12	1.04E+08	1.04E+08	+	43.13729	NA	promoter-	promoter-	17	NM_01332	29915	Hs.506558	NM_01332	ENSG0000	HCFC2	HCF-2|HCF	host cell fa	protein-co
lib-C666-li	chr13	30424797	30424797	+	36.0188	NA	promoter-	promoter-	23	NM_00710	5412	Hs.145575	NM_00710	ENSG0000	UBL3	HCG-1|PN	ubiquitin li	protein-co
lib-C666-li	chr6	30294252	30294252	+	23.4136	NA	promoter-	promoter-	-341	NR_05201	414778	Hs.485041	NR_05201	ENSG0000	HCG17	HCG18|LIN	HLA comp	ncRNA
lib-C666-li	chr19	7694513	7694513	+	161.975	NA	promoter-	promoter-	-74	NM_02019	56949	Hs.9822	NM_02019	ENSG0000	XAB2	HCNP|HCR	XPA bindir	protein-co
lib-C666-li	chr8	66754790	66754790	+	126.2553	NA	promoter-	promoter-	-821	NM_00124	5150	Hs.527119	NM_00260	ENSG0000	PDE7A	HCP1|PDE7	phosphodi	protein-co
lib-C666-li	chr8	66754380	66754380	+	115.1238	NA	promoter-	promoter-	-411	NM_00124	5150	Hs.527119	NM_00260	ENSG0000	PDE7A	HCP1|PDE7	phosphodi	protein-co
lib-C666-li	chr8	17104364	17104364	+	341.5114	NA	promoter-	promoter-	-37	NM_00114	137492	Hs.343873	NM_15241	ENSG0000	VPS37A	HCRP1|PQ	VPS37A, ES	protein-co
lib-C666-li	chr6	1.39E+08	1.39E+08	+	320.639	NA	promoter-	promoter-	-133	NM_01621	51696	Hs.197644	NM_01621	ENSG0000	HECA	HDC|HDCL	hdc homol	protein-co
lib-C666-li	chr6	1.5E+08	1.5E+08	+	388.662	NA	promoter-	promoter-	-5	NM_13912	85313	Hs.744238	NM_13912	ENSG0000	PPIL4	HDCME13I	peptidylpr	protein-co
lib-C666-li	chr8	1.45E+08	1.45E+08	+	67.6042	NA	promoter-	promoter-	50	NM_00109	727957	Hs.443139	NM_03245	ENSG0000	MROH1	HEATR7A	maestro he	protein-co
lib-C666-li	chr7	26241058	26241058	+	43.37893	NA	promoter-	promoter-	227	NM_00727	11335	Hs.381189	NM_00727	ENSG0000	CBX3	HECH|HP1	chromobo:	protein-co
lib-C666-li	chr2	2.22E+08	2.22E+08	+	47.76595	NA	promoter-	promoter-	-18	NM_00130	2043	Hs.371218	NM_00443	ENSG0000	EPHA4	HEK8|SEK|T	EPH recept	protein-co
lib-C666-li	chr12	1.34E+08	1.34E+08	+	76.60542	NA	promoter-	promoter-	-10	NM_00133	7574	Hs.489608	NM_01959	ENSG0000	ZNF26	HEL-179|K	zinc finger	protein-co
lib-C666-li	chr8	30585276	30585276	+	25.65964	NA	exon (NM_	exon (NM_	210	NM_00063	2936	Hs.271510	NM_00063	ENSG0000	GSR	HEL-75|HE	glutathione	protein-co
lib-C666-li	chr6	53213821	53213821	+	105.3587	NA	promoter-	promoter-	-78	NM_00130	60481	Hs.520189	NM_02181	ENSG0000	ELOVL5	HELO1|SCA	ELOVL fatt	protein-co
lib-C666-li	chr19	16187210	16187210	+	78.53495	NA	promoter-	promoter-	75	NM_00329	7171	Hs.631618	NM_00329	ENSG0000	TPM4	HEL-S-108	tropomyos	protein-co
lib-C666-li	chr18	54306002	54306002	+	24.75602	NA	promoter-	promoter-	-82	NM_00478	9352	Hs.114412	NM_00478	ENSG0000	TXNL1	HEL-S-114	thioredoxir	protein-co
lib-C666-li	chr21	37506479	37506479	+	27.70954	NA	promoter-	promoter-	-784	NM_00123	874	Hs.154510	NM_00123	ENSG0000	CBR3	HEL-S-25|	carbonyl re	protein-co
lib-C666-li	chr17	40985261	40985261	+	203.47	NA	promoter-	promoter-	102	NM_00126	10197	Hs.152978	NM_00578	ENSG0000	PSME3	HEL-S-283	proteasom	protein-co
lib-C666-li	chr14	88851962	88851962	+	66.08471	NA	promoter-	promoter-	-26	NM_01841	55812	Hs.525518	NM_01841	ENSG0000	SPATA7	HEL-S-296	spermatog	protein-co
lib-C666-li	chr3	1.01E+08	1.01E+08	+	47.26464	NA	promoter-	promoter-	-71	NM_00098	6152	Hs.477028	NM_00098	ENSG0000	RPL24	HEL-S-310	ribosomal	protein-co
lib-C666-li	chr17	1466160	1466160	+	78.53495	NA	promoter-	promoter-	-50	NM_00622	5306	Hs.429819	NM_00622	ENSG0000	PITPNA	HEL-S-36|	phosphatic	protein-co
lib-C666-li	chr17	16284221	16284221	+	27.62962	NA	promoter-	promoter-	110	NM_01895	7314	Hs.356190	NM_01895	ENSG0000	UBB	HEL-S-50	ubiquitin B	protein-co
lib-C666-li	chrX	77359616	77359616	+	21.51366	NA	promoter-	promoter-	-50	NM_00029	5230	Hs.78771	NM_00029	ENSG0000	PGK1	HEL-S-68p	phosphogl	protein-co
lib-C666-li	chr2	28974608	28974608	+	21.99129	NA	promoter-	promoter-	-6	NM_20687	5500	Hs.702907	NM_00270	ENSG0000	PPP1CB	HEL-S-80p	protein ph	protein-co
lib-C666-li	chr4	1E+08	1E+08	+	66.96093	NA	promoter-	promoter-	-175	NM_15229	93587	Hs.380412	NM_15229	ENSG0000	TRMT10A	HEL-S-88|	tRNA meth	protein-co
lib-C666-li	chr3	1E+08	1E+08	+	36.70779	NA	promoter-	promoter-	97	NM_02020	56954	Hs.439152	NM_02020	ENSG0000	NIT2	HEL-S-8a	nitrilase fa	protein-co
lib-C666-li	chr19	38865203	38865203	+	21.73991	NA	promoter-	promoter-	13	NM_00281	5714	Hs.78466	NM_00281	ENSG0000	PSMD8	HEL-S-91r	proteasom	protein-co
lib-C666-li	chr5	1.45E+08	1.45E+08	+	58.46021	NA	promoter-	promoter-	100	NM_15255	153769	Hs.443728	NM_15255	ENSG0000	SH3RF2	HEPP1|PO	SH3 doma	protein-co
lib-C666-li	chr16	56965917	56965917	+	24.25638	NA	promoter-	promoter-	-85	NM_00127	9709	Hs.146393	NM_01468	ENSG0000	HERPUD1	HERP|Mif1|	homocyste	protein-co
lib-C666-li	chr1	2.42E+08	2.42E+08	+	57.02275	NA	promoter-	promoter-	-109	NM_13039	9156	Hs.498248	NM_00368	ENSG0000	EXO1	HEX1|hExo	exonucleas	protein-co
lib-C666-li	chr7	21467042	21467042	+	26.82121	NA	promoter-	promoter-	-610	NM_00132	6671	Hs.88013	NM_00311	ENSG0000	SP4	HF1B|SPR-	Sp4 transc	protein-co
lib-C666-li	chr17	73521127	73521127	+	55.62774	NA	promoter-	promoter-	-656	NM_00103	3993	Hs.514477	NM_00452	ENSG0000	LLGL2	HGL|Hugl-	LLGL2, scri	protein-co
lib-C666-li	chr8	42995514	42995514	+	42.90063	NA	promoter-	promoter-	-78	NM_15241	138050	Hs.600384	NM_15241	ENSG0000	HGSNAT	HGNAT|MI	heparan-a	protein-co
lib-C666-li	chr5	99871025	99871025	+	46.67128	NA	promoter-	promoter-	16	NM_19850	345757	Hs.356108	NM_19850	ENSG0000	FAM174A	HGS_RE408	family with	protein-co
lib-C666-li	chr1	32404074	32404074	+	34.19829	NA	promoter-	promoter-	-86	NM_08039	8073	Hs.470477	NM_00347	ENSG0000	PTP4A2	HH13|HH7	protein tyr	protein-co
lib-C666-li	chr9	1.4E+08	1.4E+08	+	78.99879	NA	promoter-	promoter-	-69	NM_00117	26012	Hs.455336	NM_01553	ENSG0000	NSMF	HH9|NELF	NMDA rec	protein-co
lib-C666-li	chr19	13056439	13056439	+	280.4812	NA	promoter-	promoter-	-189	NM_00505	5886	Hs.643267	NM_00505	ENSG0000	RAD23A	HHR23A|H	RAD23 hor	protein-co
lib-C666-li	chrX	1.19E+08	1.19E+08	+	32.0283	NA	promoter-	promoter-	-20	NM_00333	7319	Hs.379466	NM_00333	ENSG0000	UBE2A	HHR6A|MF	ubiquitin c	protein-co
lib-C666-li	chr19	45004798	45004798	+	95.85526	NA	promoter-	promoter-	-223	NM_00128	7733	Hs.22305	NM_01325	ENSG0000	ZNF180	HHZ168	zinc finger	protein-co
lib-C666-li	chr9	97136650	97136650	+	61.91626	NA	promoter-	promoter-	-183	NM_03255	84641	Hs.555996	NM_03255	ENSG0000	MFSD14B	HIATL1	major facil	protein-co
lib-C666-li	chr9	99776039	99776039	+	73.41202	NA	promoter-	promoter-	-177	NR_00289	84278	Hs.610084	NR_00289	ENSG0000	MFSD14C	HIATL2	major facil	pseudo
lib-C666-li	chrX	1.53E+08	1.53E+08	+	24.43903	NA	promoter-	promoter-	-9	NM_17486	3421	Hs.410197	NM_00413	ENSG0000	IDH3G	H-IDHG	isocitrate c	protein-co
lib-C666-li	chr19	40791078	40791078	+	53.56082	NA	intron (NM	CpG	224	NM_00124	208	Hs.631535	NM_00162	ENSG0000	AKT2	HIHGHH|P	AKT serine	protein-co
lib-C666-li	chr6	1.26E+08	1.26E+08	+	142.5108	NA	promoter-	promoter-	-12	NM_13857	135114	Hs.72325	NM_13857	ENSG0000	HINT3	HINT4	histidine tr	protein-co
lib-C666-li	chr3	1.49E+08	1.49E+08	+	52.49759	NA	promoter-	promoter-	-89	NM_13904	6596	Hs.3068	NM_00307	ENSG0000	HLTF	HIP116|HIF	helicase lik	protein-co
lib-C666-li	chr12	1.09E+08	1.09E+08	+	26.54591	NA	promoter-	promoter-	-147	NM_00707	11153	Hs.661891	NM_00707	ENSG0000	FICD	HIP13|HYP	FIC domair	protein-co
lib-C666-li	chr7	75368212	75368212	+	29.92316	NA	promoter-	promoter-	78	NM_00533	3092	Hs.329266	NM_00533	ENSG0000	HIP1	HIP-I|ILWE	huntingtin	protein-co
lib-C666-li	chr14	1.06E+08	1.06E+08	+	28.32976	NA	exon (NM_	exon (NM_	981	NM_00222	3714	Hs.433445	NM_00222	ENSG0000	JAG2	HJ2|SER2	jagged 2	protein-co
lib-C666-li	chr1	6640045	6640045	+	57.03545	NA	promoter-	promoter-	-6	NM_00534	3104	Hs.502330	NM_00534	ENSG0000	ZBTB48	HKR3|ZNF8	zinc finger	protein-co
lib-C666-li	chr6	30457202	30457202	+	30.542	NA	promoter-	promoter-	19	NM_00551	3133	Hs.650174	NM_00551	ENSG0000	HLA-E	HLA-6.2|Q	major histo	protein-co
lib-C666-li	chr6	32407560	32407560	+	25.80588	NA	promoter-	promoter-	-59	NM_01911	3122	Hs.520048	NM_01911	ENSG0000	HLA-DRA	HLA-DRA1	major histo	protein-co
lib-C666-li	chr1	1.81E+08	1.81E+08	+	55.90577	NA	5' UTR (NM	5' UTR (NM	502	NM_00119	3140	Hs.13500	NM_00153	ENSG0000	MR1	HLALS	major histo	protein-co
lib-C666-li	chr20	60962002	60962002	+	26.82121	NA	promoter-	promoter-	-119	NM_00102	6227	Hs.190968	NM_00102	ENSG0000	RPS21	HLDF|S21	ribosomal	protein-co
lib-C666-li	chr2	27603717	27603717	+	24.28549	NA	promoter-	promoter-	-106	NM_14463	130557	Hs.515872	NM_14463	ENSG0000	ZNF513	HMFT0656	zinc finger	protein-co
lib-C666-li	chr17	43210334	43210334	+	39.83551	NA	promoter-	promoter-	367	NM_00132	79777	Hs.110298	NM_02472	ENSG0000	ACBD4	HMFT0700	acyl-CoA k	protein-co
lib-C666-li	chr13	31040177	31040177	+	47.71827	NA	promoter-	promoter-	-75	NM_00212	3146	Hs.434102	NM_00212	ENSG0000	HMGB1	HMG-1|HM	high mobil	protein-co
lib-C666-li	chr21	40720681	40720681	+	63.61368	NA	intron (NM	CpG-1722	366	NM_00496	3150	Hs.356285	NM_00496	ENSG0000	HMGN1	HMG14	high mobil	protein-co
lib-C666-li	chr12	1.25E+08	1.25E+08	+	80.94106	NA	promoter-	promoter-	223	NM_02100	7316	Hs.520348	NM_02100	ENSG0000	UBC	HMG20	ubiquitin C	protein-co
lib-C666-li	chr12	1.25E+08	1.25E+08	+	23.05659	NA	intron (NM	CpG	876	NM_02100	7316	Hs.520348	NM_02100	ENSG0000	UBC	HMG20	ubiquitin C	protein-co
lib-C666-li	chr5	43313813	43313813	+	28.64047	NA	promoter-	promoter-	-199	NM_00132	3157	Hs.397729	NM_00213	ENSG0000	HMGCS1	HMGCS	3-hydroxy	protein-co
lib-C666-li	chr15	77713120	77713120	+	41.83575	NA	promoter-	promoter-	127	NM_00130	10363	Hs.69594	NM_01820	ENSG0000	HMG20A	HMGX1|HM	high mobil	protein-co
lib-C666-li	chr14	24664866	24664866	+	34.97044	NA	promoter-	promoter-	76	NM_00101	10548	Hs.91586	NM_00640	ENSG0000	TM9SF1	HMP70|MF	transmeml	protein-co
lib-C666-li	chr19	51506126	51506126	+	28.64047	NA	3' UTR (NM	3' UTR (NM	-1168	NM_14450	11202	Hs.104570	NM_00719	ENSG0000	KLK8	HNP|NP|NI	kallikrein re	protein-co
lib-C666-li	chr4	83350677	83350677	+	149.2554	NA	promoter-	promoter-	701	NM_03137	9987	Hs.527105	NM_00546	ENSG0000	HNRNPDL	HNRNP|HN	heterogen	protein-co
lib-C666-li	chr12	53845734	53845734	+	34.3461	NA	promoter-	promoter-	-152	NM_03198	5094	Hs.546271	NM_00501	ENSG0000	PCBP2	HNRNPE2|	poly(rC) bi	protein-co
lib-C666-li	chr5	1.37E+08	1.37E+08	+	41.10975	NA	promoter-	promoter-	-101	NM_00680	10949	Hs.96996	NM_00680	ENSG0000	HNRNPA0	HNRPA0	heterogen	protein-co
lib-C666-li	chr10	43904603	43904603	+	25.39487	NA	promoter-	promoter-	53	NM_00496	3185	Hs.808	NM_00496	ENSG0000	HNRNPF	HNRPF|OK	heterogen	protein-co
lib-C666-li	chr19	39340989	39340989	+	101.4314	NA	promoter-	promoter-	-372	NM_00153	3191	Hs.644906	NM_00153	ENSG0000	HNRNPL	HNRPL|P/C	heterogen	protein-co
lib-C666-li	chr1	2.3E+08	2.3E+08	+	125.5274	NA	promoter-	promoter-	-10	NM_01823	55746	Hs.12457	NM_01823	ENSG0000	NUP133	hNUP133	nucleopori	protein-co
lib-C666-li	chr5	78810453	78810453	+	202.629	NA	promoter-	promoter-	-794	NM_00427	9456	Hs.591761	NM_00427	ENSG0000	HOMER1	HOMER|H(	homer sca	protein-co
lib-C666-li	chr18	268060	268060	+	133.0043	NA	promoter-	promoter-	-1	NM_00513	9984	Hs.712543	NM_00513	ENSG0000	THOC1	HPR1|P84|I	THO comp	protein-co
lib-C666-li	chr16	791024	791024	+	25.9629	NA	promoter-	promoter-	14	NM_02249	64428	Hs.513247	NM_02249	ENSG0000	NARFL	HPRN|IOP1	nuclear pre	protein-co
lib-C666-li	chr9	1.16E+08	1.16E+08	+	123.0425	NA	promoter-	promoter-	-18	NM_00469	9128	Hs.744014	NM_00469	ENSG0000	PRPF4	HPRP4|HPI	pre-mRNA	protein-co
lib-C666-li	chr7	1E+08	1E+08	+	24.61613	NA	promoter-	promoter-	-297	NM_00607	3268	Hs.521083	NM_00607	ENSG0000	AGFG2	HRBL|RABI	ArfGAP wit	protein-co
lib-C666-li	chr8	1.25E+08	1.25E+08	+	317.9541	NA	promoter-	promoter-	-66	NM_00721	11236	Hs.744151	NM_00721	ENSG0000	RNF139	HRCA1|RC	ring finger	protein-co
lib-C666-li	chr13	45915385	45915385	+	230.7435	NA	promoter-	promoter-	34	NM_00128	7178	Hs.374596	NM_00329	ENSG0000	TPT1	HRF|TCTP|	tumor prot	protein-co
lib-C666-li	chr11	20408954	20408954	+	76.37573	NA	promoter-	promoter-	-122	NM_00114	10196	Hs.152337	NM_00578	ENSG0000	PRMT3	HRMT1L3	protein arg	protein-co
lib-C666-li	chr3	1.96E+08	1.96E+08	+	24.17107	NA	promoter-	promoter-	59	NM_15261	165918	Hs.250648	NM_15261	ENSG0000	RNF168	hRNF168	ring finger	protein-co
lib-C666-li	chr14	70233832	70233832	+	44.27569	NA	promoter-	promoter-	3	NM_00103	6430	Hs.632326	NM_00692	ENSG0000	SRSF5	HRS|SFRS5	serine and	protein-co
lib-C666-li	chr5	70751433	70751433	+	262.6899	NA	promoter-	promoter-	-9	NM_01842	55814	Hs.258272	NM_01842	ENSG0000	BDP1	HSA23852(	B double p	protein-co
lib-C666-li	chr3	53381686	53381686	+	20.82108	NA	promoter-	promoter-	-32	NM_00129	55802	Hs.476353	NM_01840	ENSG0000	DCP1A	HSA27598(	decapping	protein-co
lib-C666-li	chr4	1.42E+08	1.42E+08	+	69.8176	NA	promoter-	promoter-	6	NM_00129	27309	Hs.120766	NM_01448	ENSG0000	ZNF330	HSA6591|N	zinc finger	protein-co
lib-C666-li	chr9	94877707	94877707	+	327.2347	NA	promoter-	promoter-	49	NM_17832	10558	Hs.90458	NM_00641	ENSG0000	SPTLC1	HSAN1|HS	serine paln	protein-co
lib-C666-li	chr10	38645242	38645242	+	70.11733	NA	promoter-	promoter-	-66	NR_00308(	158160	Hs.652484	NM_18282	ENSG0000	HSD17B7P	HSD17B7|H	hydroxyste	pseudo
lib-C666-li	chr18	29671833	29671833	+	94.45538	NA	promoter-	promoter-	15	NM_01627	51444	Hs.302408	NM_01627	ENSG0000	RNF138	HSD-4|NA	ring finger	protein-co
lib-C666-li	chr6	33359315	33359315	+	26.18172	NA	promoter-	promoter-	2	NM_00226	3833	Hs.436912	NM_00226	ENSG0000	KIFC1	HSET|KNSL	kinesin far	protein-co
lib-C666-li	chr3	58291965	58291965	+	64.82358	NA	promoter-	promoter-	-7	NM_00704	11102	Hs.446320	NM_00704	ENSG0000	RPP14	HsHTD2|P1	ribonuclea	protein-co
lib-C666-li	chr3	1.5E+08	1.5E+08	+	25.39487	NA	5' UTR (NM	5' UTR (NM	198	NM_00506	6478	Hs.477959	NM_00506	ENSG0000	SIAH2	hSiah2	siah E3 ubi	protein-co
lib-C666-li	chr13	20207758	20207758	+	23.74671	NA	promoter-	promoter-	-30	NM_01752	54737	Hs.269654	NM_01752	ENSG0000	MPHOSPH	HSMPP8|T	M-phase p	protein-co
lib-C666-li	chr3	43328000	43328000	+	157.7011	NA	promoter-	promoter-	-4	NM_00133	54861	Hs.476052	NM_01771	ENSG0000	SNRK	HSNFRK	SNF relate	protein-co
lib-C666-li	chr16	3767622	3767622	+	144.2026	NA	promoter-	promoter-	-24	NM_00127	10131	Hs.30345	NM_01629	ENSG0000	TRAP1	HSP 75|HS	TNF recept	protein-co
lib-C666-li	chr13	31736847	31736847	+	27.70954	NA	promoter-	promoter-	-322	NM_00128	10808	Hs.743267	NM_00664	ENSG0000	HSPH1	HSP105|HS	heat shock	protein-co

lib-C666-l	chr10	14880086	14880086	+	66.1653	NA	promoter-	promoter-	-73	NM_00127	51182	Hs.534169	NM_01629	ENSG0000	HSPA14	HSP70-4|H	heat shock	protein-co
lib-C666-l	chr7	56019569	56019569	+	191.6877	NA	promoter-	promoter-	-42	NM_01596	51373	Hs.44298	NM_01596	ENSG0000	MRPS17	HSPC011|M	mitochond	protein-co
lib-C666-l	chr1	1.54E+08	1.54E+08	+	20.90919	NA	promoter-	promoter-	139	NM_00129	25912	Hs.287471	NM_01544	ENSG0000	C1orf43	HSPC012|N	chromosor	protein-co
lib-C666-l	chr3	53925995	53925995	+	197.3166	NA	promoter-	promoter-	47	NM_02123	58515	Hs.58471	NM_02123	ENSG0000	SELENOK	HSPC030|s	selenoprot	protein-co
lib-C666-l	chr16	11891253	11891253	+	52.3754	NA	promoter-	promoter-	-139	NM_01415	29066	Hs.371856	NM_01415	ENSG0000	ZC3H7A	HSPC055|Z	zinc finger	protein-co
lib-C666-l	chr16	58283855	58283855	+	102.4866	NA	promoter-	promoter-	15	NM_01415	29070	Hs.11614	NM_01415	ENSG0000	CCDC113	HSPC065	coiled-coil	protein-co
lib-C666-l	chr3	12598529	12598529	+	21.0836	NA	promoter-	promoter-	16	NM_00127	23609	Hs.591666	NM_01416	ENSG0000	MKRN2	HSPC070|R	makorin rir	protein-co
lib-C666-l	chr2	2.03E+08	2.03E+08	+	49.48694	NA	promoter-	promoter-	-1	NM_01593	51602	Hs.471104	NM_01593	ENSG0000	NOP58	HSPC120|N	NOP58 rib	protein-co
lib-C666-l	chr11	1.34E+08	1.34E+08	+	21.21068	NA	promoter-	promoter-	-24	NM_00103	29087	Hs.13645	NM_01417	ENSG0000	THYN1	HSPC144|M	thymocyte	protein-co
lib-C666-l	chr11	64085191	64085191	+	60.16793	NA	promoter-	promoter-	365	NM_00128	51504	Hs.333579	NM_01640	ENSG0000	TRMT112	HSPC152|H	tRNA meth	protein-co
lib-C666-l	chr16	67261016	67261016	+	190.8449	NA	promoter-	promoter-	0	NM_01418	29100	Hs.433203	NM_01418	ENSG0000	TMEM208	HSPC171	transmemt	protein-co
lib-C666-l	chr1	1510251	1510251	+	480.6659	NA	promoter-	promoter-	11	NM_01418	29101	Hs.30026	NM_01418	ENSG0000	SSU72	HSPC182|F	SSU72 hon	protein-co
lib-C666-l	chr5	1.46E+08	1.46E+08	+	54.91701	NA	promoter-	promoter-	35	NM_00131	51520	Hs.432674	NM_01646	ENSG0000	LARS	HSPC192|II	leucyl-tRN	protein-co
lib-C666-l	chr4	1.53E+08	1.53E+08	+	39.51209	NA	promoter-	promoter-	-33	NM_00456	5188	Hs.119316	NM_00456	ENSG0000	GATB	HSPC199|F	glutamyl-t	protein-co
lib-C666-l	chr1	31769759	31769759	+	32.48059	NA	promoter-	promoter-	-70	NM_00128	51538	Hs.524094	NM_01650	ENSG0000	ZCCHC17	HSPC251|F	zinc finger	protein-co
lib-C666-l	chr8	681308	681308	+	31.27173	NA	promoter-	promoter-	-69	NM_20733	157697	Hs.655310	NM_20733	ENSG0000	ERICH1	HSPC319	glutamate	protein-co
lib-C666-l	chr2	1.98E+08	1.98E+08	+	94.79198	NA	promoter-	promoter-	91	NM_00120	1.01E+08	Hs.633165	NM_00120	ENSG0000	HSPE1-MC	HSPE1-PH	HSPE1-MC	protein-co
lib-C666-l	chr19	49078151	49078151	+	68.00875	NA	promoter-	promoter-	-842	NM_00460	6820	Hs.369331	NM_00460	ENSG0000	SULT2B1	HSST2	sulfotransf	protein-co
lib-C666-l	chr18	33647552	33647552	+	41.21722	NA	promoter-	promoter-	5	NM_00130	55197	Hs.464912	NM_01817	ENSG0000	RPRD1A	HsT3101|P	regulation	protein-co
lib-C666-l	chr18	2571451	2571451	+	262.079	NA	promoter-	promoter-	51	NM_02284	64863	Hs.126888	NM_02284	ENSG0000	METTL4	HsT661	methyltran	protein-co
lib-C666-l	chr8	1.46E+08	1.46E+08	+	37.56522	NA	promoter-	promoter-	118	NM_00552	3297	Hs.530227	NM_00552	ENSG0000	HSF1	HSTF1	heat shock	protein-co
lib-C666-l	chr8	23386342	23386342	+	28.64047	NA	promoter-	promoter-	34	NM_01661	51312	Hs.596025	NM_01661	ENSG0000	SLC25A37	HT015|MFI	solute carr	protein-co
lib-C666-l	chr22	20104789	20104789	+	81.64329	NA	promoter-	promoter-	29	NM_00125	27037	Hs.713579	NM_02272	ENSG0000	TRMT2A	HTF9C	tRNA meth	protein-co
lib-C666-l	chr4	926129	926129	+	84.45766	NA	promoter-	promoter-	-46	NM_00129	84286	Hs.478936	NM_03232	ENSG0000	TMEM175	hTMEM175	transmemt	protein-co
lib-C666-l	chr3	1.86E+08	1.86E+08	+	42.99614	NA	promoter-	promoter-	33	NM_00124	6434	Hs.533122	NM_00459	ENSG0000	TRA2B	Htra2-beta	transforme	protein-co
lib-C666-l	chr3	52444042	52444042	+	217.739	NA	promoter-	promoter-	79	NM_00465	8314	Hs.106674	NM_00465	ENSG0000	BAP1	HUCEP-13	BRCA1 ass	protein-co
lib-C666-l	chr3	37217934	37217934	+	143.492	NA	promoter-	promoter-	-83	NM_00113	9209	Hs.740522	NM_00630	ENSG0000	LRRFIP2	HUFI-2	LRR bindin	protein-co
lib-C666-l	chr3	51428597	51428597	+	143.9767	NA	promoter-	promoter-	-102	NM_01328	29890	Hs.118738	NM_01328	ENSG0000	RBM15B	HUMAGCC	RNA bindir	protein-co
lib-C666-l	chr1	14026637	14026637	+	28.57903	NA	promoter-	promoter-	-98	NM_00113	7799	Hs.371823	NM_01223	ENSG0000	PRDM2	HUMHOXY	PR/SET doi	protein-co
lib-C666-l	chr15	50716557	50716557	+	87.64545	NA	promoter-	promoter-	-17	NM_00515	9101	Hs.443731	NM_00515	ENSG0000	USP8	HumORF8|	ubiquitin s	protein-co
lib-C666-l	chr1	1.55E+08	1.55E+08	+	114.9583	NA	promoter-	promoter-	-297	NM_00655	10654	Hs.30954	NM_00655	ENSG0000	PMVK	HUMPMKI|	phosphom	protein-co
lib-C666-l	chr5	1.4E+08	1.4E+08	+	25.3508	NA	promoter-	promoter-	-7	NR_02670	56664		NR_02670	ENSG0000	VTRNA1-1	HVG1|VAU	vault RNA	ncRNA
lib-C666-l	chr13	27825433	27825433	+	77.62498	NA	promoter-	promoter-	-259	NM_00098	6144	Hs.381123	NM_00098	ENSG0000	RPL21	HYPT12|L2	ribosomal	protein-co
lib-C666-l	chr19	8454862	8454862	+	156.0536	NA	promoter-	promoter-	-343	NM_00421	9230	Hs.558403	NM_00421	ENSG0000	RAB11B	H-YPT3	RAB11B, m	protein-co
lib-C666-l	chr12	1.34E+08	1.34E+08	+	88.33322	NA	promoter-	promoter-	32	NM_00116	10795	Hs.124047	NM_00341	ENSG0000	ZNF268	HZF3	zinc finger	protein-co
lib-C666-l	chr2	1.57E+08	1.57E+08	+	114.4773	NA	promoter-	promoter-	-24	NM_00618	4929	Hs.563344	NM_00618	ENSG0000	NR4A2	HZF-3|NO	nuclear rec	protein-co
lib-C666-l	chr2	1.57E+08	1.57E+08	+	25.68529	NA	Intergenic	CpG	-1978	NM_00618	4929	Hs.563344	NM_00618	ENSG0000	NR4A2	HZF-3|NO	nuclear rec	protein-co
lib-C666-l	chr19	57791802	57791802	+	73.77308	NA	promoter-	promoter-	-51	NM_00663	10794	Hs.99971	NM_00663	ENSG0000	ZNF460	HZF8|ZNF2	zinc finger	protein-co
lib-C666-l	chr20	20348464	20348464	+	21.28624	NA	promoter-	promoter-	-301	NM_00219	3642	Hs.89584	NM_00219	ENSG0000	INSM1	IA-1|IA1	INSM trans	protein-co
lib-C666-l	chrX	77151084	77151084	+	38.21529	NA	promoter-	promoter-	-19	NM_03212	84061	Hs.323562	NM_03212	ENSG0000	MAGT1	IAP|MRX95	magnesiun	protein-co
lib-C666-l	chr19	4909834	4909834	+	54.92945	NA	promoter-	promoter-	-271	NM_00129	29128	Hs.108106	NM_01328	ENSG0000	UHRF1	ICBP90|Np	ubiquitin li	protein-co
lib-C666-l	chr20	30192168	30192168	+	37.12814	NA	promoter-	promoter-	-918	NM_18135	3397	Hs.504609	NM_00216	ENSG0000	ID1	ID|bHLHb2	inhibitor of	protein-co
lib-C666-l	chr12	1.08E+08	1.08E+08	+	127.0525	NA	promoter-	promoter-	19	NM_00706	11137	Hs.506652	NM_00706	ENSG0000	PWP1	IEF-SSP-9	PWP1 hom	protein-co
lib-C666-l	chr3	50329974	50329974	+	25.68768	NA	promoter-	promoter-	52	NM_00676	7866	Hs.315177	NM_00676	ENSG0000	IFRD2	IFNRP|SKM	interferon	protein-co
lib-C666-l	chr3	39149131	39149131	+	54.01688	NA	promoter-	promoter-	-21	NM_00110	199223	Hs.443935	NM_14575	ENSG0000	TTC21A	IFT139A|ST	tetratricop	protein-co
lib-C666-l	chr22	22652085	22652085	+	27.16277	NA	promoter-	promoter-	-378	NR_02729	96610	Hs.449601	NM_08092	ENSG0000	BMS1P20	IGL|IGLV|IG	BMS1, ribo	pseudo
lib-C666-l	chr9	99212342	99212342	+	72.69891	NA	promoter-	promoter-	-95	NM_01428	22927	Hs.494567	NM_01428	ENSG0000	HABP4	IHABP-4|Ik	hyaluronar	protein-co
lib-C666-l	chr19	36390723	36390723	+	20.72954	NA	promoter-	promoter-	-84	NM_00132	84807	Hs.466531	NM_13923	ENSG0000	NFKBID	IkappaBNS	NFKB inhib	protein-co
lib-C666-l	chr19	39390324	39390324	+	69.79861	NA	promoter-	promoter-	-16	NM_00124	4793	Hs.9731	NM_00250	ENSG0000	NFKBIB	IKBB|TRIP9	NFKB inhib	protein-co
lib-C666-l	chr19	39390748	39390748	+	44.91887	NA	promoter-	promoter-	178	NM_00250	4793	Hs.9731	NM_00250	ENSG0000	NFKBIB	IKBB|TRIP9	NFKB inhib	protein-co
lib-C666-l	chr10	1.02E+08	1.02E+08	+	32.38963	NA	promoter-	promoter-	-13	NM_00132	1147	Hs.198998	NM_00127	ENSG0000	CHUK	IKBKA|IKK-	conserved	protein-co
lib-C666-l	chr8	42128773	42128773	+	62.11283	NA	promoter-	promoter-	-47	NM_00155	3551	Hs.597664	NM_00155	ENSG0000	IKBKB	IKK-beta|Ik	inhibitor of	protein-co
lib-C666-l	chr4	1.43E+08	1.43E+08	+	26.46433	NA	promoter-	promoter-	-145	NM_00058	3600	Hs.168132	NM_00058	ENSG0000	IL15	IL-15	interleukin	protein-co
lib-C666-l	chr13	21278322	21278322	+	44.8076	NA	exon (NM_	exon (NM_	840	NM_13828	53342	Hs.655142	NM_13828	ENSG0000	IL17D	IL-17D	interleukin	protein-co
lib-C666-l	chr12	56732613	56732613	+	36.59122	NA	promoter-	promoter-	-50	NM_01658	51561	Hs.98309	NM_01658	ENSG0000	IL23A	IL-23|IL-23	interleukin	protein-co
lib-C666-l	chr9	35732494	35732494	+	34.53169	NA	promoter-	promoter-	-102	NM_00628	7094	Hs.471014	NM_00628	ENSG0000	TLN1	ILWEQ|TLN	talin 1	protein-co
lib-C666-l	chr16	21531998	21531998	+	38.41137	NA	promoter-	promoter-	-233	NR_00259	387254	Hs.448808	NR_002594		SLC7A5P2	IMAA|MM/	solute carr	pseudo
lib-C666-l	chr17	45727173	45727173	+	21.99129	NA	promoter-	promoter-	-31	NM_00226	3837	Hs.532793	NM_00226	ENSG0000	KPNB1	IMB1|IPO1	karyopheri	protein-co
lib-C666-l	chr19	10491348	10491348	+	76.62533	NA	promoter-	promoter-	-100	NM_00333	7297	Hs.75516	NM_00333	ENSG0000	TYK2	IMD35|JTK	tyrosine kir	protein-co
lib-C666-l	chr15	45003631	45003631	+	123.3507	NA	promoter-	promoter-	-54	NM_00404	567	Hs.534255	NM_00404	ENSG0000	B2M	IMD43	beta-2-mi	protein-co
lib-C666-l	chr7	1.11E+08	1.11E+08	+	28.8837	NA	promoter-	promoter-	32	NM_00124	83943	Hs.655722	NM_03254	ENSG0000	IMMP2L	IMMP2L-IT	inner mito	protein-co
lib-C666-l	chr7	1.28E+08	1.28E+08	+	21.60686	NA	promoter-	promoter-	-359	NM_00130	3614	Hs.654401	NM_00088	ENSG0000	IMPDH1	IMPD|IMPD	inosine mc	protein-co
lib-C666-l	chr3	49066897	49066897	+	91.50885	NA	promoter-	promoter-	-22	NM_00088	3615	Hs.654400	NM_00088	ENSG0000	IMPDH2	IMPD2|IMF	inosine mc	protein-co
lib-C666-l	chr21	34100291	34100291	+	28.8334	NA	promoter-	promoter-	-41	NM_00116	8867	Hs.473632	NM_00389	ENSG0000	SYNJ1	INPP5G|PA	synaptojan	protein-co
lib-C666-l	chr2	1.19E+08	1.19E+08	+	21.46984	NA	promoter-	promoter-	-87	NM_00132	51141	Hs.7089	NM_01613	ENSG0000	INSIG2	INSIG-2	insulin indu	protein-co
lib-C666-l	chr10	6244847	6244847	+	23.05659	NA	promoter-	promoter-	7	NM_00132	5209	Hs.195471	NM_00456	ENSG0000	PFKFB3	IPFK2|PFK2	6-phospho	protein-co
lib-C666-l	chr9	1.03E+08	1.03E+08	+	23.12593	NA	promoter-	promoter-	4	NM_01774	54881	Hs.494648	NM_01774	ENSG0000	TEX10	Ipi1|bA208	testis expre	protein-co
lib-C666-l	chr19	984292	984292	+	187.0742	NA	promoter-	promoter-	-36	NM_02410	57418	Hs.325321	NM_02410	ENSG0000	WDR18	Ipi3|R3218	WD repeat	protein-co
lib-C666-l	chr7	1.08E+08	1.08E+08	+	49.69992	NA	promoter-	promoter-	61	NM_00125	50640	Hs.617340	NM_01572	ENSG0000	PNPLA8	IPLA2-2|IP	patatin like	protein-co
lib-C666-l	chr7	1.29E+08	1.29E+08	+	28.81777	NA	promoter-	promoter-	8	NR_03405	23534	Hs.193613	NM_01247	ENSG0000	TNPO3	IPO12|LGM	transportin	protein-co
lib-C666-l	chr6	1.6E+08	1.6E+08	+	41.94622	NA	promoter-	promoter-	-10	NM_00132	6648	Hs.487046	NM_00063	ENSG0000	SOD2	IPO-B|IPO	superoxide	protein-co
lib-C666-l	chr10	1095013	1095013	+	49.86005	NA	promoter-	promoter-	-190	NM_00131	3422	Hs.283652	NM_00450	ENSG0000	IDI1	IPP1|IPPI1	isopenteny	protein-co
lib-C666-l	chr14	24630333	24630333	+	284.3587	NA	promoter-	promoter-	-89	NM_00608	10379	Hs.1706	NM_00608	ENSG0000	IRF9	IRF-9|ISGF	interferon	protein-co
lib-C666-l	chr15	66084823	66084823	+	25.49755	NA	promoter-	promoter-	-192	NM_00114	10260	Hs.654567	NM_00584	ENSG0000	DENND4A	IRLB|MYCP	DENN dom	protein-co
lib-C666-l	chr16	54320784	54320784	+	28.88196	NA	promoter-	promoter-	-406	NM_02433	79191	Hs.499205	NM_02433	ENSG0000	IRX3	IRX-1|IRXB	iroquois ho	protein-co
lib-C666-l	chr12	6961804	6961804	+	38.10403	NA	promoter-	promoter-	513	NM_00348	8078	Hs.631661	NM_00348	ENSG0000	USP5	ISOT	ubiquitin s	protein-co
lib-C666-l	chr4	1.44E+08	1.44E+08	+	57.48363	NA	promoter-	promoter-	-303	NM_00360	8467	Hs.558422	NM_00360	ENSG0000	SMARCA5	ISWI|SNF2	SWI/SNF re	protein-co
lib-C666-l	chr4	1.44E+08	1.44E+08	+	31.26176	NA	non-codin	non-codin	345	NM_00360	8467	Hs.558422	NM_00360	ENSG0000	SMARCA5	ISWI|SNF2	SWI/SNF re	protein-co
lib-C666-l	chr19	49250327	49250327	+	20.83272	NA	promoter-	promoter-	-161	NM_00132	284359	Hs.400688	NM_18257	ENSG0000	IZUMO1	IZUMO|OB	izumo sper	protein-co
lib-C666-l	chr2	2.33E+08	2.33E+08	+	26.46807	NA	promoter-	promoter-	-106	NM_00260	5147	Hs.516808	NM_00260	ENSG0000	PDE6D	JBTS22|PD	phosphodi	protein-co
lib-C666-l	chr16	27561380	27561380	+	65.77034	NA	promoter-	promoter-	-88	NM_01520	23247	Hs.460459	NM_01520	ENSG0000	KIAA0556	JBTS26	KIAA0556	protein-co
lib-C666-l	chr2	1.11E+08	1.11E+08	+	110.485	NA	promoter-	promoter-	14	NM_20718	4867	Hs.280388	NM_00027	ENSG0000	NPHP1	JBTS4|NPH	nephrocyst	protein-co
lib-C666-l	chr1	2.06E+08	2.06E+08	+	117.9591	NA	promoter-	promoter-	-72	NM_02273	64710	Hs.213061	NM_02273	ENSG0000	NUCKS1	JC7|NUCKS	nuclear cas	protein-co
lib-C666-l	chr1	2.13E+08	2.13E+08	+	32.66768	NA	intron (NM	CpG	388	NM_01866	55509	Hs.62919	NM_01866	ENSG0000	BATF3	JDP1|JUNE	basic leuci	protein-co
lib-C666-l	chrX	54070491	54070491	+	25.49755	NA	promoter-	promoter-	116	NM_00118	23133	Hs.133352	NM_01510	ENSG0000	PHF8	JHDM1F|KI	PHD finger	protein-co
lib-C666-l	chrX	54070939	54070939	+	22.50982	NA	promoter-	promoter-	-332	NM_01510	23133	Hs.133352	NM_01510	ENSG0000	PHF8	JHDM1F|KI	PHD finger	protein-co
lib-C666-l	chr16	1756128	1756128	+	278.1266	NA	promoter-	promoter-	-56	NM_00131	23162	Hs.207763	NM_01513	ENSG0000	MAPK8IP3	JIP-3|JIP3|J	mitogen-a	protein-co
lib-C666-l	chr19	4968892	4968892	+	26.94522	NA	promoter-	promoter-	-232	NM_01501	23030	Hs.654816	NM_01501	ENSG0000	KDM4B	JMJD2B|TD	lysine dem	protein-co
lib-C666-l	chr10	49514670	49514670	+	39.15438	NA	promoter-	promoter-	-12	NR_13658	5599	Hs.138211	NM_00275	ENSG0000	MAPK8	JNK|JNK-4	mitogen-a	protein-co
lib-C666-l	chr7	6048868	6048868	+	23.70098	NA	promoter-	promoter-	-14	NM_00132	7965	Hs.301613	NM_00630	ENSG0000	AIMP2	JTV-1|JTV1	aminoacyl	protein-co
lib-C666-l	chr14	23451989	23451989	+	20.39357	NA	promoter-	promoter-	-138	NM_00128	84962	Hs.655832	NM_03287	ENSG0000	AJUBA	JUB	ajuba LIM	protein-co
lib-C666-l	chr19	59055569	59055569	+	37.99633	NA	promoter-	promoter-	-267	NM_00576	10155	Hs.467408	NM_00576	ENSG0000	TRIM28	KAP1|PPP1	tripartite m	protein-co
lib-C666-l	chr2	1.73E+08	1.73E+08	+	97.94548	NA	promoter-	promoter-	-3	NM_00364	8520	Hs.632532	NM_00364	ENSG0000	HAT1	KAT1	histone acc	protein-co
lib-C666-l	chr1	2.03E+08	2.03E+08	+	38.10403	NA	promoter-	promoter-	-53	NR_04632	1.01E+08	Hs.23459	NR_04632	ENSG0000	PCAT6	KDM5B-AS	prostate ca	ncRNA
lib-C666-l	chr2	68384977	68384977	+	24.99834	NA	promoter-	promoter-	15	NM_00132	56902	Hs.262858	NM_02014	ENSG0000	PNO1	KHRBP1|RF	partner of	protein-co
lib-C666-l	chr8	1.09E+08	1.09E+08	+	27.70954	NA	promoter-	promoter-	-6	NR_13803	9694	Hs.654351	NM_01467	ENSG0000	EMC2	KIAA0103|	ER membra	protein-co
lib-C666-l	chr21	45079420	45079420	+	147.9893	NA	promoter-	promoter-	-12	NM_01505	23076	Hs.565725	NM_01505	ENSG0000	RRP1B	KIAA0179|	ribosomal	protein-co
lib-C666-l	chr10	977733	977733	+	20.90919	NA	promoter-	promoter-	-88	NM_01515	23185	Hs.159069	NM_01515	ENSG0000	LARP4B	KIAA0217|	La ribonuc	protein-co
lib-C666-l	chr9	1.39E+08	1.39E+08	+	34.25375	NA	promoter-	promoter-	12	NM_01486	9919	Hs.522500	NM_01486	ENSG0000	SEC16A	KIAA0310|	SEC16 hon	protein-co
lib-C666-l	chr20	36661894	36661894	+	52.3754	NA	promoter-	promoter-	-24	NM_01465	9675	Hs.655481	NM_01465	ENSG0000	TTI1	KIAA0406|	TELO2 inte	protein-co
lib-C666-l	chr1	47184889	47184889	+	92.10336	NA	promoter-	promoter-	-153	NM_01477	9813	Hs.707688	NM_01477	ENSG0000	EFCAB14	KIAA0494	EF-hand ca	protein-co
lib-C666-l	chr10	99161055	99161055	+	29.21635	NA	promoter-	promoter-	72	NM_01517	23223	Hs.434251	NM_01517	ENSG0000	RRP12	KIAA0690	ribosomal	protein-co
lib-C666-l	chr6	96969654	96969654	+	24.99834	NA	promoter-	promoter-	-48	NM_01532	23376	Hs.149367	NM_01532	ENSG0000	UFL1	KIAA0776|	UFM1 spec	protein-co
lib-C666-l	chr19	36103616	36103616	+	370.0481	NA	promoter-	promoter-	-30	NM_01530	23354	Hs.7426	NM_01530	ENSG0000	HAUS5	KIAA0841|	HAUS augr	protein-co
lib-C666-l	chr13	46626898	46626898	+	35.72107	NA	promoter-	promoter-	15	NM_00133	23091	Hs.136102	NM_01507	ENSG0000	ZC3H13	KIAA0853	zinc finger	protein-co
lib-C666-l	chr5	5422496	5422496	+	146.9373	NA	promoter-	promoter-	-290	NM_01532	23379	Hs.449296	NM_01532	ENSG0000	ICE1	KIAA0947	interactor c	protein-co
lib-C666-l	chr15	52971641	52971641	+	24.43903	NA	promoter-	promoter-	-810	NR_10445	56204	Hs.152385	NM_01960	ENSG0000	FAM214A	KIAA1370	family with	protein-co
lib-C666-l	chr4	1340826	1340826	+	23.83582	NA	promoter-	promoter-	-228	NM_00131	57654	Hs.380475	NM_02089	ENSG0000	UVSSA	KIAA1530|	UV stimula	protein-co
lib-C666-l	chr4	1.75E+08	1.75E+08	+	46.71952	NA	promoter-	promoter-	75	NM_00104	80817	Hs.555989	NM_03063	ENSG0000	CEP44	KIAA1712|	centrosom	protein-co
lib-C666-l	chr8	95274628	95274628	+	65.54223	NA	promoter-	promoter-	-81	NM_18170	2669	Hs.654463	NM_00526	ENSG0000	GEM	KIR	GTP bindir	protein-co
lib-C666-l	chr14	1.04E+08	1.04E+08	+	82.10182	NA	promoter-	promoter-	-79	NM_18292	3831	Hs.20107	NM_00555	ENSG0000	KLC1	KLC|KNS2|	kinesin ligh	protein-co
lib-C666-l	chr19	55996526	55996526	+	50.83113	NA	promoter-	promoter-	-31	NM_02037	57106	Hs.31854	NM_02037	ENSG0000	NAT14	KLP1	N-acetyltra	protein-co
lib-C666-l	chr11	67195927	67195927	+	433.3791	NA	promoter-	promoter-	-8	NM_00395	6199	Hs.534345	NM_00395	ENSG0000	RPS6KB2	KLS|P70-b	ribosomal	protein-co
lib-C666-l	chr10	14920756	14920756	+	103.2754	NA	promoter-	promoter-	-26	NM_00119	79723	Hs.554883	NM_02467	ENSG0000	SUV39H2	KMT1B	suppressor	protein-co
lib-C666-l	chr4	1872727	1872727	+	36.59122	NA	promoter-	promoter-	-396	NM_00104	7468	Hs.113876	NM_00733	ENSG0000	NSD2	KMT3F|KM	nuclear rec	protein-co
lib-C666-l	chr8	38240620	38240620	+	36.04455	NA	promoter-	promoter-	-830	NM_02303	54904	Hs.434966	NM_01777	ENSG0000	NSD3	KMT3F|KM	nuclear rec	protein-co
lib-C666-l	chr15	66797542	66797542	+	30.13508	NA	promoter-	promoter-	121	NM_00128	55055	Hs.21331	NM_01797	ENSG0000	ZWILCH	KNTC1AP|	zwilch kine	protein-co
lib-C666-l	chr16	71496141	71496141	+	72.80621	NA	promoter-	promoter-	14	NM_00130	7571	Hs.656643	NM_14591	ENSG0000	ZNF23	KOX16|ZN	zinc finger	protein-co
lib-C666-l	chr10	38265649	38265649	+	59.88466	NA	promoter-	promoter-	-66	NR_13806	219749	Hs.499429	NM_14501	ENSG0000	ZNF25	KOX19|Zfp	zinc finger	protein-co
lib-C666-l	chr8	1.46E+08	1.46E+08	+	37.99392	NA	promoter-	promoter-	-11	NM_00128	80778	Hs.631854	NM_03058	ENSG0000	ZNF34	KOX32	zinc finger	protein-co
lib-C666-l	chr10	43932349	43932349	+	29.11329	NA	promoter-	promoter-	-225	NR_02669	642819	Hs.719657	NR_026693		ZNF487	KRBO1|ZN	zinc finger	pseudo
lib-C666-l	chr2	2.2E+08	2.2E+08	+	54.91701	NA	promoter-	promoter-	35	NM_00133	8576	Hs.153003	NM_00369	ENSG0000	STK16	KRCT|MPS	serine/thre	protein-co
lib-C666-l	chr19	50837555	50837555	+	262.3584	NA	promoter-	promoter-	-783	NR_11091	3748	Hs.467146	NM_00497	ENSG0000	KCNC3	KSHIIID|KV	potassium	protein-co
lib-C666-l	chr8	73449131	73449131	+	57.17088	NA	promoter-	promoter-	-495	NM_00477	9312	Hs.661102	NM_00477	ENSG0000	KCNB2	KV2.2	potassium	protein-co
lib-C666-l	chr8	73450068	73450068	+	37.41784	NA	5' UTR (NM	5' UTR (NM	442	NM_00477	9312	Hs.661102	NM_00477	ENSG0000	KCNB2	KV2.2	potassium	protein-co
lib-C666-l	chr19	49990767	49990767	+	36.59206	NA	promoter-	promoter-	-44	NR_07302	23521	Hs.523185	NM_01242	ENSG0000	RPL13A	L13A|TSTA	ribosomal	protein-co

lib-C666-l	chr11	1.17E+08	1.17E+08	+	20.59244	NA	promoter-	promoter-	-199	NM_00128	23387	Hs.167451	NM_02516	ENSG0000	SIK3	L19|QSK|SI	SIK family l	protein-co
lib-C666-l	chr17	37010106	37010106	+	64.0179	NA	promoter-	promoter-	-53	NM_00097	9349	Hs.406300	NM_00097	ENSG0000	RPL23	L23|rpL17	ribosomal	protein-co
lib-C666-l	chr17	27046770	27046770	+	30.07415	NA	promoter-	promoter-	-230	NM_00098	6147	Hs.419463	NM_00098	ENSG0000	RPL23A	L23A|MDA	ribosomal	protein-co
lib-C666-l	chr17	41150413	41150413	+	27.68428	NA	promoter-	promoter-	-33	NM_00098	6155	Hs.514196	NM_00098	ENSG0000	RPL27	L27	ribosomal	protein-co
lib-C666-l	chr2	1.02E+08	1.02E+08	+	33.4149	NA	promoter-	promoter-	-40	NM_00099	6160	Hs.469473	NM_00099	ENSG0000	RPL31	L31	ribosomal	protein-co
lib-C666-l	chr22	19419978	19419978	+	64.85778	NA	promoter-	promoter-	-49	NM_00131	64976	Hs.431307	NM_00377	ENSG0000	MRPL40	L40mt|MRI	mitochond	protein-co
lib-C666-l	chr12	56510316	56510316	+	34.92958	NA	promoter-	promoter-	-58	NM_02110	6171	Hs.112553	NM_02110	ENSG0000	RPL41	L41	ribosomal	protein-co
lib-C666-l	chr19	3762687	3762687	+	36.07033	NA	promoter-	promoter-	22	NM_17225	116541	Hs.356578	NM_17225	ENSG0000	MRPL54	L54mt|MRI	mitochond	protein-co
lib-C666-l	chr8	74206299	74206299	+	27.84631	NA	promoter-	promoter-	-430	NM_00097	6129	Hs.421257	NM_00097	ENSG0000	RPL7	L7|humL7-	ribosomal	protein-co
lib-C666-l	chr8	1.46E+08	1.46E+08	+	97.94548	NA	promoter-	promoter-	-11	NM_00097	6132	Hs.178551	NM_00097	ENSG0000	RPL8	L8	ribosomal	protein-co
lib-C666-l	chr4	39460601	39460601	+	279.8021	NA	promoter-	promoter-	-33	NM_00066	6133	Hs.412370	NM_00066	ENSG0000	RPL9	L9|NPC-A-	ribosomal	protein-co
lib-C666-l	chr5	6713215	6713215	+	20.19521	NA	Intergenic	CpG	-1503	NM_00117	11044	Hs.481542	NM_00699	ENSG0000	PAPD7	LAK-1|LAK	poly(A) RN	protein-co
lib-C666-l	chr9	1.36E+08	1.36E+08	+	20.19521	NA	promoter-	promoter-	-97	NM_00116	7248	Hs.370854	NM_00036	ENSG0000	TSC1	LAM|TSC	tuberous s	protein-co
lib-C666-l	chr1	1.5E+08	1.5E+08	+	27.96363	NA	promoter-	promoter-	-86	NM_00128	81611	Hs.656466	NM_03092	ENSG0000	ANP32E	LANP-L|LA	acidic nucl	protein-co
lib-C666-l	chr1	1.8E+08	1.8E+08	+	39.16209	NA	exon (NM_	exon (NM_	504	NM_00126	26092	Hs.496459	NM_01560	ENSG0000	TOR1AIP1	LAP1|LAP1	torsin 1A ir	protein-co
lib-C666-l	chr5	892826	892826	+	54.49234	NA	promoter-	promoter-	113	NM_02392	65980	Hs.449278	NM_02392	ENSG0000	BRD9	LAVS3040|	bromodon	protein-co
lib-C666-l	chr3	33482266	33482266	+	30.13508	NA	promoter-	promoter-	-369	NM_00112	7342	Hs.729120	NM_01451	ENSG0000	UBP1	LBP-1B|LB	upstream b	protein-co
lib-C666-l	chr12	51566916	51566916	+	137.2633	NA	promoter-	promoter-	10	NM_00565	7024	Hs.48849	NM_00565	ENSG0000	TFCP2	LBP1C|LSF	transcripti	protein-co
lib-C666-l	chr22	46731694	46731694	+	34.92958	NA	non-codin	non-codin	396	NR_10424	55687	Hs.439524	NM_01800	ENSG0000	TRMU	LCAL3|MT	tRNA 5-m	protein-co
lib-C666-l	chr5	1.76E+08	1.76E+08	+	49.72573	NA	promoter-	promoter-	14	NR_04572	1212	Hs.484241	NM_00183	ENSG0000	CLTB	LCB	clathrin lig	protein-co
lib-C666-l	chr5	74632919	74632919	+	48.61377	NA	promoter-	promoter-	-74	NM_00085	3156	Hs.628096	NM_00085	ENSG0000	HMGCR	LDLCQ3	3-hydroxy	protein-co
lib-C666-l	chr19	55629150	55629150	+	27.70954	NA	promoter-	promoter-	-182	NM_01760	54776	Hs.631579	NM_01760	ENSG0000	PPP1R12C	LENG3|MB	protein ph	protein-co
lib-C666-l	chr19	54695017	54695017	+	43.7015	NA	promoter-	promoter-	-48	NM_00107	79042	Hs.15580	NM_02407	ENSG0000	TSEN34	LENG5|PC	tRNA splic	protein-co
lib-C666-l	chr19	54694207	54694207	+	22.02307	NA	promoter-	promoter-	88	NM_00128	79042	Hs.15580	NM_02407	ENSG0000	TSEN34	LENG5|PC	tRNA splic	protein-co
lib-C666-l	chr19	49617481	49617481	+	72.82537	NA	promoter-	promoter-	-137	NM_00130	64130	Hs.221737	NM_02216	ENSG0000	LIN7B	LIN-7B|MA	lin-7 homo	protein-co
lib-C666-l	chr11	65189688	65189688	+	87.11781	NA	promoter-	promoter-	-581	NR_13101	283131	Hs.523789	NR_00280	ENSG0000	NEAT1	LINC00084	nuclear pa	ncRNA
lib-C666-l	chr12	90103474	90103474	+	61.94978	NA	non-codin	non-codin	742	NR_02813	338758	Hs.601766	NR_02813	ENSG0000	ATP2B1-A	LINC00936	ATP2B1 an	ncRNA
lib-C666-l	chr15	40615683	40615683	+	33.04877	NA	promoter-	promoter-	-574	NM_00130	1.01E+08	Hs.530791	NM_001301268		INAFM2	LINC00984	InaF motif	protein-co
lib-C666-l	chr11	70116735	70116735	+	25.33067	NA	promoter-	promoter-	-71	NM_17742	8500	Hs.530749	NM_00362	ENSG0000	PPFIA1	LIP.1|LIP1|L	PTPRF inte	protein-co
lib-C666-l	chr17	7835482	7835482	+	41.28836	NA	promoter-	promoter-	40	NM_00133	116840	Hs.348012	NM_05305	ENSG0000	CNTROB	LIP8|PP122	centrobin,	protein-co
lib-C666-l	chr7	1.22E+08	1.22E+08	+	124.4789	NA	promoter-	promoter-	-75	NM_00576	10157	Hs.156738	NM_00576	ENSG0000	AASS	LKR/SDH|L	aminoadip	protein-co
lib-C666-l	chrX	1.02E+08	1.02E+08	+	59.96872	NA	promoter-	promoter-	-13	NR_03898	1E+08	Hs.687084	NR_03898	ENSG0000	LINC00630	LL0XNC01-	long interg	ncRNA
lib-C666-l	chr14	23504386	23504386	+	23.242	NA	promoter-	promoter-	-32	NM_00114	5693	Hs.422990	NM_00279	ENSG0000	PSMB5	LMPX|MB1	proteasom	protein-co
lib-C666-l	chr1	40368009	40368009	+	117.9591	NA	promoter-	promoter-	-322	NM_00103	4610	Hs.437922	NM_00537	ENSG0000	MYCL	L-Myc|LM	v-myc avia	protein-co
lib-C666-l	chr11	27493753	27493753	+	23.22307	NA	exon (NM_	exon (NM_	475	NR_13116	1.05E+08	Hs.568999	NR_13116	ENSG0000	LOC10537	lnc-BBOX1	uncharacte	ncRNA
lib-C666-l	chr7	1.49E+08	1.49E+08	+	34.59844	NA	promoter-	promoter-	-86	NR_13010	1.03E+08	Hs.621982	NR_13010	ENSG0000	GHET1	lncRNA-G	gastric car	ncRNA
lib-C666-l	chr19	50379951	50379951	+	40.86121	NA	promoter-	promoter-	-445	NM_00127	84335	Hs.515542	NM_03237	ENSG0000	AKT1S1	Lobe|PRAS	AKT1 subs	protein-co
lib-C666-l	chr7	99933664	99933664	+	42.12788	NA	promoter-	promoter-	-24	NR_03657	1.02E+08	Hs.521075	NR_03656	ENSG0000	STAG3L5P	LOC10173	STAG3L5P	ncRNA
lib-C666-l	chr16	48278188	48278188	+	33.11264	NA	5' UTR (NN	5' UTR (NN	110	NM_03149	83752	Hs.295923	NM_03149	ENSG0000	LONP2	LONP|LON	lon peptid	protein-co
lib-C666-l	chr12	53894701	53894701	+	45.01834	NA	promoter-	promoter-	-4	NM_00417	6895	Hs.326	NM_00417	ENSG0000	TARBP2	LOQS|TRB	TARBP2, R	protein-co
lib-C666-l	chr19	290632	290632	+	35.17083	NA	intron (NM	CpG	537	NM_17754	8612	Hs.465506	NM_00371	ENSG0000	PLPP2	LPP2|PAP-	phospholip	protein-co
lib-C666-l	chr8	10697420	10697420	+	224.0763	NA	promoter-	promoter-	-11	NM_00128	54984	Hs.490991	NM_01788	ENSG0000	PINX1	LPTL|LPTS	PIN2/TERF	protein-co
lib-C666-l	chr7	1E+08	1E+08	+	25.49921	NA	intron (NM	intron (NM	947	NM_00231	4034	Hs.125742	NM_00231	ENSG0000	LRCH4	LRN|LRRN	leucine ric	protein-co
lib-C666-l	chr2	68290148	68290148	+	434.2848	NA	promoter-	promoter-	11	NM_17317	10438	Hs.602900	NM_00633	ENSG0000	C1D	LRP1|Rrp4	C1D nucle	protein-co
lib-C666-l	chr14	23340530	23340530	+	28.8837	NA	promoter-	promoter-	-396	NM_00132	26020	Hs.525232	NM_01404	ENSG0000	LRP10	LRP-10|LR	LDL recept	protein-co
lib-C666-l	chr4	1.86E+08	1.86E+08	+	134.6393	NA	promoter-	promoter-	-689	NM_18172	353322	Hs.508154	NM_18172	ENSG0000	ANKRD37	Lrp2bp	ankyrin rep	protein-co
lib-C666-l	chr12	7013900	7013900	+	21.0836	NA	promoter-	promoter-	3	NM_20165	10233	Hs.155586	NM_00699	ENSG0000	LRRC23	LRPB7	leucine ric	protein-co
lib-C666-l	chr8	1.46E+08	1.46E+08	+	113.4641	NA	promoter-	promoter-	-17	NM_00127	9684	Hs.459391	NM_01466	ENSG0000	LRRC14	LRRC14A	leucine ric	protein-co
lib-C666-l	chr21	45759306	45759306	+	157.3991	NA	promoter-	promoter-	-21	NM_00127	755	Hs.517331	NM_00492	ENSG0000	C21orf2	LRRC76|Y	chromosor	protein-co
lib-C666-l	chr15	74988368	74988368	+	53.07762	NA	promoter-	promoter-	18	NM_02508	80153	Hs.96852	NM_02508	ENSG0000	EDC3	LSM16|MR	enhancer c	protein-co
lib-C666-l	chr3	50362147	50362147	+	30.8056	NA	TTS (NM_0	TTS (NM_0	-1866	NM_03315	8692	Hs.76873	NM_00377	ENSG0000	HYAL2	LUCA2	hyaluronog	protein-co
lib-C666-l	chr19	6737745	6737745	+	126.4536	NA	promoter-	promoter-	-112	NM_00108	56927	Hs.167641	NM_02017	ENSG0000	GPR108	LUSTR2	G protein-	protein-co
lib-C666-l	chr1	23495362	23495362	+	20.7902	NA	promoter-	promoter-	-11	NM_03363	7798	Hs.257900	NM_03363	ENSG0000	LUZP1	LUZP	leucine zip	protein-co
lib-C666-l	chr19	50879747	50879747	+	43.12596	NA	promoter-	promoter-	67	NM_00125	7376	Hs.432976	NM_00712	ENSG0000	NR1H2	LXR-b|LXR	nuclear rec	protein-co
lib-C666-l	chr17	78075297	78075297	+	27.61921	NA	promoter-	promoter-	-28	NM_00107	2548	Hs.1437	NM_00015	ENSG0000	GAA	LYAG	glucosidas	protein-co
lib-C666-l	chr3	1.85E+08	1.85E+08	+	209.6724	NA	promoter-	promoter-	-34	NM_00124	9175	Hs.591306	NM_00472	ENSG0000	MAP3K13	LZK|MEKK	mitogen-a	protein-co
lib-C666-l	chr11	47429997	47429997	+	56.19669	NA	promoter-	promoter-	-49	NM_15226	91252	Hs.523664	NM_15226	ENSG0000	SLC39A13	LZT-Hs9	solute carr	protein-co
lib-C666-l	chr1	93544770	93544770	+	58.46021	NA	promoter-	promoter-	-22	NM_00116	22823	Hs.31016	NM_00735	ENSG0000	MTF2	M96|PCL2|	metal resp	protein-co
lib-C666-l	chr17	26646037	26646037	+	25.33067	NA	promoter-	promoter-	-84	NM_01457	27346	Hs.199695	NM_01457	ENSG0000	TMEM97	MAC30	transmemt	protein-co
lib-C666-l	chr1	25756751	25756751	+	26.37215	NA	promoter-	promoter-	-598	NM_00128	55219	Hs.189782	NM_01820	ENSG0000	TMEM57	MACOILIN	transmemt	protein-co
lib-C666-l	chr17	2304988	2304988	+	78.90324	NA	promoter-	promoter-	-730	NM_02031	4335	Hs.626579	NM_02031	ENSG0000	MNT	MAD6|MXI	MAX netw	protein-co
lib-C666-l	chr17	40719095	40719095	+	170.5978	NA	promoter-	promoter-	17	NM_19820	6945	Hs.383019	NM_17060	ENSG0000	MLX	MAD7|MXI	MLX, MAX	protein-co
lib-C666-l	chr1	53704199	53704199	+	294.1808	NA	promoter-	promoter-	83	NM_00237	4116	Hs.421576	NM_00237	ENSG0000	MAGOH	MAGOH1|	mago hom	protein-co
lib-C666-l	chr17	27503951	27503951	+	26.46433	NA	promoter-	promoter-	-181	NM_00134	399687	Hs.462590	NM_07847	ENSG0000	MYO18A	MAJN|MYS	myosin XV	protein-co
lib-C666-l	chr5	1.39E+08	1.39E+08	+	33.21417	NA	promoter-	promoter-	-402	NR_10273	1.01E+08	Hs.438871	NR_10273	ENSG0000	LINC01024	MA-linc1	long interg	ncRNA
lib-C666-l	chr5	1.39E+08	1.39E+08	+	25.39084	NA	non-codin	non-codin	254	NR_10274	1.01E+08	Hs.438871	NR_10273	ENSG0000	LINC01024	MA-linc1	long interg	ncRNA
lib-C666-l	chr5	1.79E+08	1.79E+08	+	21.41395	NA	promoter-	promoter-	-121	NM_01475	9794	Hs.631951	NM_01475	ENSG0000	MAML1	Mam-1|M	mastermin	protein-co
lib-C666-l	chr12	65563295	65563295	+	41.21722	NA	promoter-	promoter-	-56	NM_00116	23592	Hs.744150	NM_01431	ENSG0000	LEMD3	MAN1	LEM doma	protein-co
lib-C666-l	chr12	1.12E+08	1.12E+08	+	123.8325	NA	promoter-	promoter-	-210	NM_00366	8550	Hs.333120	NM_00366	ENSG0000	MAPKAPK5	MAPKAP-k	mitogen-a	protein-co
lib-C666-l	chr5	1.4E+08	1.4E+08	+	56.14594	NA	promoter-	promoter-	-164	NM_00119	54882	Hs.594084	NM_01774	ENSG0000	ANKHD1	MASK|MAS	ankyrin rep	protein-co
lib-C666-l	chr19	49224088	49224088	+	46.29081	NA	exon (NM_	exon (NM_	-1112	NM_00113	284358	Hs.191815	NM_18257	ENSG0000	MAMSTR	MASTR	MEF2 activ	protein-co
lib-C666-l	chr12	16035298	16035298	+	84.45766	NA	promoter-	promoter-	10	NM_00717	11171	Hs.743971	NM_00717	ENSG0000	STRAP	MAWD|PT	serine/thre	protein-co
lib-C666-l	chr8	97247879	97247879	+	21.21068	NA	promoter-	promoter-	-17	NM_00629	7381	Hs.131255	NM_00629	ENSG0000	UQCRB	MC3DN3|	ubiquinol-	protein-co
lib-C666-l	chr5	1.32E+08	1.32E+08	+	66.66461	NA	promoter-	promoter-	-11	NM_01440	27089	Hs.146602	NM_01440	ENSG0000	UQCRQ	MC3DN4|	ubiquinol-	protein-co
lib-C666-l	chrX	1.03E+08	1.03E+08	+	52.45363	NA	intron (NM	CpG	182	NM_00101	401612	Hs.660022	NM_00101	ENSG0000	SLC25A53	MCART6	solute carr	protein-co
lib-C666-l	chr4	1.29E+08	1.29E+08	+	71.09715	NA	promoter-	promoter-	46	NM_00119	10733	Hs.172052	NM_01426	ENSG0000	PLK4	MCCRP2|S	polo like ki	protein-co
lib-C666-l	chr4	1.29E+08	1.29E+08	+	29.74345	NA	promoter-	promoter-	118	NM_01426	10733	Hs.172052	NM_01426	ENSG0000	PLK4	MCCRP2|S	polo like ki	protein-co
lib-C666-l	chr6	49431032	49431032	+	49.56857	NA	promoter-	promoter-	9	NM_00025	4594	Hs.485527	NM_00025	ENSG0000	MUT	MCM	methylmal	protein-co
lib-C666-l	chr20	44600975	44600975	+	212.7112	NA	promoter-	promoter-	-142	NM_02209	63925	Hs.174193	NM_02209	ENSG0000	ZNF335	MCPH10|N	zinc finger	protein-co
lib-C666-l	chr1	47779945	47779945	+	42.35981	NA	promoter-	promoter-	-126	NM_00303	6491	Hs.525198	NM_00303	ENSG0000	STIL	MCPH7|SIL	SCL/TAL1 i	protein-co
lib-C666-l	chr17	80186862	80186862	+	24.32656	NA	promoter-	promoter-	-25	NM_00420	9123	Hs.500761	NM_00420	ENSG0000	SLC16A3	MCT 3|MC	solute carr	protein-co
lib-C666-l	chr17	984256	984256	+	28.64047	NA	intron (NM	intron (NM	-1870	NM_00128	29	Hs.159306	NM_00109	ENSG0000	ABR	MDB	active BCR	protein-co
lib-C666-l	chr3	49761443	49761443	+	77.89044	NA	promoter-	promoter-	-36	NM_01333	29925	Hs.567488	NM_01333	ENSG0000	GMPPB	MDDGA14	GDP-manr	protein-co
lib-C666-l	chr8	1.21E+08	1.21E+08	+	91.58624	NA	promoter-	promoter-	-93	NM_02204	27085	Hs.657656	NM_02204	ENSG0000	MTBP	MDM2BP	MDM2 bin	protein-co
lib-C666-l	chr12	2921885	2921885	+	46.4068	NA	promoter-	promoter-	97	NR_13074	55846	Hs.446098	NM_01846	ENSG0000	ITFG2	MDS028	integrin al	protein-co
lib-C666-l	chr17	17380342	17380342	+	102.2432	NA	promoter-	promoter-	42	NM_01801	55090	Hs.244595	NM_01801	ENSG0000	MED9	MED25	mediator c	protein-co
lib-C666-l	chr8	1.19E+08	1.19E+08	+	129.395	NA	promoter-	promoter-	22	NM_08065	90390	Hs.492612	NM_08065	ENSG0000	MED30	MED30S|T	mediator c	protein-co
lib-C666-l	chr19	40697285	40697285	+	44.79663	NA	promoter-	promoter-	-366	NM_00244	4294	Hs.466743	NM_00244	ENSG0000	MAP3K10	MEKK10|M	mitogen-a	protein-co
lib-C666-l	chr19	40696884	40696884	+	25.80588	NA	promoter-	promoter-	-767	NM_00244	4294	Hs.466743	NM_00244	ENSG0000	MAP3K10	MEKK10|M	mitogen-a	protein-co
lib-C666-l	chr19	16222582	16222582	+	65.27523	NA	promoter-	promoter-	92	NM_00537	4218	Hs.642874	NM_00537	ENSG0000	RAB8A	MEL|RAB8	RAB8A, me	protein-co
lib-C666-l	chr7	38217847	38217847	+	107.8209	NA	promoter-	promoter-	39	NM_03201	83930	Hs.309753	NM_03201	ENSG0000	STARD3NL	MENTHO	STARD3 N	protein-co
lib-C666-l	chr1	1.57E+08	1.57E+08	+	46.14268	NA	promoter-	promoter-	-100	NM_00524	2117	Hs.105636	NM_00524	ENSG0000	ETV3	METS|PE-1	ETS variant	protein-co
lib-C666-l	chr11	62572998	62572998	+	118.645	NA	promoter-	promoter-	-34	NM_00108	10482	Hs.523739	NM_00636	ENSG0000	NXF1	MEX67|TA	nuclear RN	protein-co
lib-C666-l	chr17	42976852	42976852	+	80.54002	NA	promoter-	promoter-	-116	NM_00125	9343	Hs.151787	NM_00424	ENSG0000	EFTUD2	MFDGA|M	elongation	protein-co
lib-C666-l	chr10	1.01E+08	1.01E+08	+	24.23241	NA	promoter-	promoter-	-87	NM_03121	81894	Hs.403790	NM_03121	ENSG0000	SLC25A28	MFRN2|MF	solute carr	protein-co
lib-C666-l	chr8	1.04E+08	1.04E+08	+	26.07785	NA	exon (NM_	exon (NM_	257	NM_03078	81034	Hs.532265	NM_03078	ENSG0000	SLC25A32	MFT|MFTC	solute carr	protein-co
lib-C666-l	chrX	48397778	48397778	+	57.02262	NA	promoter-	promoter-	-297	NM_00253	4943	Hs.694353	NM_00253	ENSG0000	TBC1D25	MG81|OAT	TBC1 dom	protein-co
lib-C666-l	chr19	46088272	46088272	+	133.6329	NA	promoter-	promoter-	-150	NM_00101	80207	Hs.466945	NM_02513	ENSG0000	OPA3	MGA3	OPA3, out	protein-co
lib-C666-l	chr6	34855844	34855844	+	58.46021	NA	promoter-	promoter-	4	NM_00127	6882	Hs.112444	NM_00564	ENSG0000	TAF11	MGC:1524	TATA-box	protein-co
lib-C666-l	chr9	1.4E+08	1.4E+08	+	20.76617	NA	promoter-	promoter-	-189	NM_02113	7186	Hs.522506	NM_02113	ENSG0000	TRAF2	MGC:4501	TNF recept	protein-co
lib-C666-l	chr6	1.34E+08	1.34E+08	+	91.58467	NA	promoter-	promoter-	-176	NM_00486	9519	Hs.486507	NM_00486	ENSG0000	TBPL1	MGC:8389	TATA-box	protein-co
lib-C666-l	chr12	10766201	10766201	+	282.1695	NA	promoter-	promoter-	7	NM_00131	55110	Hs.104650	NM_01804	ENSG0000	MAGOHB	MGN2|ma	mago hom	protein-co
lib-C666-l	chr19	5681012	5681012	+	90.07064	NA	promoter-	promoter-	-105	NM_00130	125988	Hs.356626	NM_20576	ENSG0000	C19orf70	MIC13|P11	chromosor	protein-co
lib-C666-l	chr18	61089774	61089774	+	65.54223	NA	promoter-	promoter-	-22	NM_00486	9525	Hs.126550	NM_00486	ENSG0000	VPS4B	MIG1|SKD	vacuolar p	protein-co
lib-C666-l	chr2	85132628	85132628	+	134.1589	NA	promoter-	promoter-	-135	NM_02110	9168	Hs.446574	NM_02110	ENSG0000	TMSB10	MIG12|TB	thymosin b	protein-co
lib-C666-l	chr7	1.33E+08	1.33E+08	+	30.13021	NA	non-codin	non-codin	129	NR_13367	54927	Hs.655010	NM_01781	ENSG0000	CHCHD3	MINOS3|M	coiled-coil	protein-co
lib-C666-l	chr19	40476985	40476985	+	20.90919	NA	promoter-	promoter-	73	NM_15300	5704	Hs.211594	NM_00650	ENSG0000	PSMC4	MIP224|RP	proteasom	protein-co
lib-C666-l	chr19	57874842	57874842	+	163.215	NA	promoter-	promoter-	-37	NR_00216	10597	Hs.446620	NR_00216	ENSG0000	TRAPPC2B	MIP-2A|SE	trafficking	pseudo
lib-C666-l	chr12	1.23E+08	1.23E+08	+	26.35145	NA	promoter-	promoter-	-40	NM_01493	22877	Hs.437153	NM_01493	ENSG0000	MLXIP	MIR|MON	MLX intera	protein-co
lib-C666-l	chr15	93447613	93447613	+	197.994	NA	promoter-	promoter-	-16	NR_03613	1E+08		NR_03613	ENSG0000	MIR3175	mir-3175	microRNA	ncRNA
lib-C666-l	chr16	2582081	2582081	+	24.31114	NA	promoter-	promoter-	-75	NR_03613	1E+08		NR_03613	ENSG0000	MIR3178	mir-3178	microRNA	ncRNA
lib-C666-l	chr2	64566297	64566297	+	61.67113	NA	Intergenic	Intergenic	-1596	NR_03963	1.01E+08		NR_03963	ENSG0000	MIR4433A	MIR4433|h	microRNA	ncRNA
lib-C666-l	chr3	25706535	25706535	+	147.861	NA	promoter-	promoter-	-105	NR_03964	1.01E+08		NR_03964	ENSG0000	MIR4442	mir-4442	microRNA	ncRNA
lib-C666-l	chr17	36860211	36860211	+	28.88523	NA	Intergenic	CpG	-1627	NR_03988	1.01E+08		NR_03988	ENSG0000	MIR4734	mir-4734	microRNA	ncRNA
lib-C666-l	chr17	1953739	1953739	+	115.7284	NA	promoter-	promoter-	-65	NR_02962	406994		NR_02962	ENSG0000	MIR212	MIRN212|	microRNA	ncRNA
lib-C666-l	chr3	44903310	44903310	+	36.38376	NA	promoter-	promoter-	-70	NR_03029	693149		NR_03029	ENSG0000	MIR564	MIRN564|	microRNA	ncRNA
lib-C666-l	chr11	13690152	13690152	+	27.5466	NA	promoter-	promoter-	-54	NM_03222	84188	Hs.501991	NM_03222	ENSG0000	FAR1	MLSTD2|P	fatty acyl-(	protein-co
lib-C666-l	chr16	58059466	58059466	+	29.01385	NA	promoter-	promoter-	-4	NM_00242	4324	Hs.80343	NM_00242	ENSG0000	MMP15	MMP-15|M	matrix met	protein-co
lib-C666-l	chr1	2.28E+08	2.28E+08	+	193.7784	NA	non-codin	non-codin	121	NM_02431	79169	Hs.445952	NM_02431	ENSG0000	C1orf35	MMTAG2	chromosor	protein-co
lib-C666-l	chr16	31128969	31128969	+	97.29876	NA	promoter-	promoter-	-16	NM_18295	84148	Hs.533803	NM_03218	ENSG0000	KAT8	MOF|MYS	lysine acet	protein-co
lib-C666-l	chrX	1.03E+08	1.03E+08	+	49.18845	NA	promoter-	promoter-	-18	NM_00114	9643	Hs.326387	NM_01228	ENSG0000	MORF4L2	MORFL2|M	mortality f	protein-co
lib-C666-l	chr10	93683528	93683528	+	56.28537	NA	promoter-	promoter-	-208	NM_00397	9044	Hs.500526	NM_00397	ENSG0000	BTAF1	MOT1|TAF	B-TFIID TA	protein-co
lib-C666-l	chr1	1.54E+08	1.54E+08	+	54.43427	NA	promoter-	promoter-	-52	NM_00103	6232	Hs.546291	NM_00103	ENSG0000	RPS27	MPS-1|MP	ribosomal	protein-co
lib-C666-l	chr16	67596204	67596204	+	93.22762	NA	promoter-	promoter-	-106	NM_00656	10664	Hs.368367	NM_00656	ENSG0000	CTCF	MRD21	CCCTC-bir	protein-co
lib-C666-l	chr1	27930699	27930699	+	25.50922	NA	promoter-	promoter-	-694	NM_00102	27245	Hs.469280	NM_00102	ENSG0000	AHDC1	MRD25	AT-hook D	protein-co
lib-C666-l	chr1	1.51E+08	1.51E+08	+	82.8533	NA	promoter-	promoter-	-92	NM_00119	23126	Hs.489873	NM_01510	ENSG0000	POGZ	MRD37|W	pogo trans	protein-co

lib-C666-l	chr1	1822582	1822582	+	57.48363	NA	promoter-	promoter-	-26	NM_00128	2782	Hs.430425	NM_00207	ENSG0000	GNB1	MRD42	G protein s	protein-co
lib-C666-l	chr2	1.19E+08	1.19E+08	+	68.38644	NA	promoter-	promoter-	-26	NM_00677	8886	Hs.744922	NM_00677	ENSG0000	DDX18	MrDb	DEAD-box	protein-co
lib-C666-l	chr7	1.35E+08	1.35E+08	+	93.75573	NA	promoter-	promoter-	-205	NM_00130	78996	Hs.643113	NM_02403	ENSG0000	C7orf49	MRI|MRI-2	chromosor	protein-co
lib-C666-l	chr9	1.04E+08	1.04E+08	+	32.11429	NA	promoter-	promoter-	-15	NM_01905	54534	Hs.288224	NM_01905	ENSG0000	MRPL50	MRP-L50	mitochond	protein-co
lib-C666-l	chr14	35591292	35591292	+	21.81327	NA	promoter-	promoter-	-235	NM_00125	9692	Hs.458487	NM_01467	ENSG0000	KIAA0391	MRPP3|PR(	KIAA0391	protein-co
lib-C666-l	chr1	859226	859226	+	42.35752	NA	Intergenic	CpG	-1895	NM_15248	148398	Hs.335293	NM_15248	ENSG0000	SAMD11	MRS	sterile alph	protein-co
lib-C666-l	chr17	55333680	55333680	+	31.76017	NA	promoter-	promoter-	-251	NM_00132	124540	Hs.658922	NM_13896	ENSG0000	MSI2	MSI2H	musashi R	protein-co
lib-C666-l	chr14	91527133	91527133	+	27.5466	NA	promoter-	promoter-	-140	NM_00132	9252	Hs.510225	NM_00475	ENSG0000	RPS6KA5	MSK1|MSP	ribosomal	protein-co
lib-C666-l	chr11	28129768	28129768	+	28.8837	NA	promoter-	promoter-	-22	NM_03121	81930	Hs.301052	NM_03121	ENSG0000	KIF18A	MS-KIF18A	kinesin fan	protein-co
lib-C666-l	chr20	16710633	16710633	+	48.59595	NA	promoter-	promoter-	24	NM_00309	6629	Hs.280378	NM_00309	ENSG0000	SNRPB2	Msl1|U2B''	small nucle	protein-co
lib-C666-l	chr3	52312755	52312755	+	27.96363	NA	promoter-	promoter-	-96	NM_02522	80335	Hs.194110	NM_02522	ENSG0000	WDR82	MST107|M	WD repeat	protein-co
lib-C666-l	chr4	37828184	37828184	+	26.47693	NA	promoter-	promoter-	-98	NM_01829	55276	Hs.23363	NM_01829	ENSG0000	PGM2	MSTP006	phosphogl	protein-co
lib-C666-l	chr16	56691749	56691749	+	24.99834	NA	promoter-	promoter-	-106	NM_00130	4494	Hs.513626	NM_00594	ENSG0000	MT1F	MT1	metallothic	protein-co
lib-C666-l	chr2	74153941	74153941	+	72.69891	NA	promoter-	promoter-	-12	NM_00131	1716	Hs.469022	NM_00192	ENSG0000	DGUOK	MTDPS3|N	deoxyguar	protein-co
lib-C666-l	chr1	38325293	38325293	+	48.22081	NA	promoter-	promoter-	-1	NM_00595	4520	Hs.471991	NM_00595	ENSG0000	MTF1	MTF-1|ZRF	metal regu	protein-co
lib-C666-l	chr13	48611862	48611862	+	46.29081	NA	non-codin	non-codin	159	NR_13668	55270	Hs.144407	NM_01828	ENSG0000	NUDT15	MTH2|NUD	nudix hydr	protein-co
lib-C666-l	chr8	21967199	21967199	+	396.068	NA	promoter-	promoter-	-267	NM_02481	79873	Hs.527101	NM_02481	ENSG0000	NUDT18	MTH3	nudix hydr	protein-co
lib-C666-l	chr19	41256559	41256559	+	41.94622	NA	promoter-	promoter-	-200	NM_00459	6626	Hs.466775	NM_00459	ENSG0000	SNRPA	Mud1|U1-A	small nucle	protein-co
lib-C666-l	chr1	1.09E+08	1.09E+08	+	97.8918	NA	promoter-	promoter-	-69	NM_00726	6814	Hs.530436	NM_00726	ENSG0000	STXBP3	MUNC18-3	syntaxin bi	protein-co
lib-C666-l	chr11	95523546	95523546	+	64.54414	NA	promoter-	promoter-	-79	NM_00124	9702	Hs.101014	NM_01467	ENSG0000	CEP57	MVA2|PIG	centrosom	protein-co
lib-C666-l	chr12	6772531	6772531	+	183.9704	NA	promoter-	promoter-	-223	NM_00112	51147	Hs.524210	NM_01616	ENSG0000	ING4	my036|p29	inhibitor of	protein-co
lib-C666-l	chr16	24550784	24550784	+	39.40619	NA	promoter-	promoter-	-124	NM_03262	5930	Hs.188553	NM_00691	ENSG0000	RBBP6	MY038|P2F	RB binding	protein-co
lib-C666-l	chr1	1.14E+08	1.14E+08	+	46.14268	NA	promoter-	promoter-	45	NM_15269	204851	Hs.532363	NM_15269	ENSG0000	HIPK1	Myak|Nbak	homeodor	protein-co
lib-C666-l	chr19	50935027	50935027	+	120.3606	NA	TTS (NM_0	TTS (NM_0	-1133	NM_00453	4606	Hs.85937	NM_00453	ENSG0000	MYBPC2	MYBPC|MY	myosin bin	protein-co
lib-C666-l	chr1	35524833	35524833	+	30.48199	NA	promoter-	promoter-	-554	NM_00128	79830	Hs.471243	NM_02477	ENSG0000	ZMYM1	MYM	zinc finger	protein-co
lib-C666-l	chr1	2.02E+08	2.02E+08	+	32.48059	NA	promoter-	promoter-	-157	NM_00248	4660	Hs.444403	NM_00248	ENSG0000	PPP1R12B	MYPT2|PP1	protein ph	protein-co
lib-C666-l	chr19	59084924	59084924	+	90.12853	NA	promoter-	promoter-	18	NM_19805	7593	Hs.399810	NM_00342	ENSG0000	MZF1	MZF-1|MZ	myeloid zi	protein-co
lib-C666-l	chr9	1.4E+08	1.4E+08	+	545.9794	NA	promoter-	promoter-	-8	NM_00373	8636	Hs.530314	NM_00373	ENSG0000	SSNA1	N14|NA-14	SS nuclear	protein-co
lib-C666-l	chr1	1.45E+08	1.45E+08	+	54.14909	NA	promoter-	promoter-	-84	NM_20345	388677	Hs.655156	NM_20345	ENSG0000	NOTCH2N	N2N	notch 2 N-	protein-co
lib-C666-l	chr7	1.18E+08	1.18E+08	+	24.23745	NA	5' UTR (NM	5' UTR (NM	186	NM_01620	51691	Hs.655046	NM_01620	ENSG0000	LSM8	NAA38	LSM8 hom	protein-co
lib-C666-l	chr9	34178978	34178978	+	23.27622	NA	promoter-	promoter-	-25	NM_01652	51271	Hs.268963	NM_01652	ENSG0000	UBAP1	NAG20|UA	ubiquitin a	protein-co
lib-C666-l	chr1	2344012	2344012	+	332.3961	NA	promoter-	promoter-	-2	NM_15381	5192	Hs.732228	NM_00261	ENSG0000	PEX10	NALD|PBD	peroxisom	protein-co
lib-C666-l	chr17	36507702	36507702	+	80.54002	NA	promoter-	promoter-	-305	NM_01459	30837	Hs.514132	NM_01459	ENSG0000	SOCS7	NAP4|NCK	suppressor	protein-co
lib-C666-l	chr11	63706244	63706244	+	97.88153	NA	promoter-	promoter-	-198	NM_02477	79829	Hs.523753	NM_02477	ENSG0000	NAA40	NAT11|PA	N(alpha)-a	protein-co
lib-C666-l	chr2	64751450	64751450	+	73.49606	NA	promoter-	promoter-	11	NM_20343	54812	Hs.655167	NM_01765	ENSG0000	AFTPH	Nbla10388	aftiphilin	protein-co
lib-C666-l	chr17	77813648	77813648	+	66.08424	NA	promoter-	promoter-	-435	NM_00365	8535	Hs.405046	NM_00365	ENSG0000	CBX4	NBP16|PC2	chromobo:	protein-co
lib-C666-l	chr13	77566223	77566223	+	55.60226	NA	exon (NM_	exon (NM_	164	NM_00649	1203	Hs.30213	NM_00649	ENSG0000	CLN5	NCL	ceroid-lipc	protein-co
lib-C666-l	chr16	2014999	2014999	+	116.8757	NA	promoter-	promoter-	2	NR_003142	735301	Hs.115329	NR_003142	ENSG0000	SNHG9	NCRNA000	small nucle	ncRNA
lib-C666-l	chr17	41277575	41277575	+	61.38507	NA	promoter-	promoter-	1	NR_13814	10230	Hs.373818	NM_00582	ENSG0000	NBR2	NCRNA001	neighbor o	ncRNA
lib-C666-l	chr7	39772982	39772982	+	139.3901	NA	promoter-	promoter-	-185	NR_026999	349114	Hs.414183	NM_198284		LINC00265	NCRNA002	long interg	ncRNA
lib-C666-l	chr12	27397037	27397037	+	145.6445	NA	promoter-	promoter-	-41	NM_01500	23012	Hs.184523	NM_01500	ENSG0000	STK38L	NDR2	serine/thre	protein-co
lib-C666-l	chr4	2010924	2010924	+	135.0517	NA	promoter-	promoter-	38	NM_00566	7469	Hs.21771	NM_00566	ENSG0000	NELFA	NELF-A|P/	negative el	protein-co
lib-C666-l	chr1	1.09E+08	1.09E+08	+	28.64047	NA	promoter-	promoter-	-46	NM_01806	55119	Hs.342307	NM_01806	ENSG0000	PRPF38B	NET1	pre-mRNA	protein-co
lib-C666-l	chr2	1.31E+08	1.31E+08	+	121.6727	NA	promoter-	promoter-	136	NM_01795	55627	Hs.516450	NM_01775	ENSG0000	SMPD4	NET13|NSN	sphingomy	protein-co
lib-C666-l	chr2	1.29E+08	1.29E+08	+	24.54495	NA	promoter-	promoter-	19	NM_00100	55339	Hs.554831	NM_01838	ENSG0000	WDR33	NET14|WD	WD repeat	protein-co
lib-C666-l	chr2	1.9E+08	1.9E+08	+	162.8322	NA	promoter-	promoter-	-353	NM_00130	84128	Hs.399984	NM_03216	ENSG0000	WDR75	NET16|UTF	WD repeat	protein-co
lib-C666-l	chr5	72861577	72861577	+	64.82358	NA	promoter-	promoter-	11	NM_00128	84135	Hs.406703	NM_03217	ENSG0000	UTP15	NET21	UTP15, sm	protein-co
lib-C666-l	chr12	1.33E+08	1.33E+08	+	488.6429	NA	promoter-	promoter-	-33	NM_02407	79050	Hs.558536	NM_02407	ENSG0000	NOC4L	NET49|NO	nucleolar c	protein-co
lib-C666-l	chr5	1.09E+08	1.09E+08	+	29.93113	NA	promoter-	promoter-	-174	NM_01481	9867	Hs.483036	NM_01481	ENSG0000	PJA2	Neurodap1	praja ring f	protein-co
lib-C666-l	chr9	33290417	33290417	+	260.868	NA	promoter-	promoter-	2	NM_14713	4799	Hs.413074	NM_00250	ENSG0000	NFX1	NFX2|TEG-	nuclear tra	protein-co
lib-C666-l	chr11	57228554	57228554	+	46.45276	NA	intron (NM	CpG-4141	544	NM_17857	349667	Hs.502618	NM_17857	ENSG0000	RTN4RL2	NGRH1|Ng	reticulon 4	protein-co
lib-C666-l	chr4	1.04E+08	1.04E+08	+	109.044	NA	promoter-	promoter-	30	NM_00110	150159	Hs.666728	NM_13917	ENSG0000	SLC9B1	NHA1|NHE	solute carr	protein-co
lib-C666-l	chr16	88636820	88636820	+	126.6171	NA	promoter-	promoter-	31	NM_00129	124245	Hs.93670	NM_14460	ENSG0000	ZC3H18	NHN1	zinc finger	protein-co
lib-C666-l	chr2	2.19E+08	2.19E+08	+	26.26478	NA	promoter-	promoter-	-25	NM_18264	58190	Hs.444468	NM_02119	ENSG0000	CTDSP1	NIF3|NLI-I	CTD small	protein-co
lib-C666-l	chr19	36036470	36036470	+	110.2966	NA	promoter-	promoter-	-32	NM_00124	10430	Hs.9234	NM_03263	ENSG0000	TMEM147	NIFIE14	transmemb	protein-co
lib-C666-l	chr9	95896953	95896953	+	28.8837	NA	promoter-	promoter-	-383	NM_00414	4814	Hs.494457	NM_00414	ENSG0000	NINJ1	NIN1|NINJ	ninjurin 1	protein-co
lib-C666-l	chr1	2.47E+08	2.47E+08	+	21.84731	NA	intron (NM	CpG	831	NM_03275	84838	Hs.168677	NM_03275	ENSG0000	ZNF496	NIZP1|ZFP	zinc finger	protein-co
lib-C666-l	chr10	28966307	28966307	+	42.39316	NA	promoter-	promoter-	-117	NM_01234	25805	Hs.533336	NM_01234	ENSG0000	BAMBI	NMA	BMP and a	protein-co
lib-C666-l	chr11	71791853	71791853	+	67.35306	NA	promoter-	promoter-	-114	NM_00128	4926	Hs.325978	NM_00618	ENSG0000	NUMA1	NMP-22|N	nuclear mi	protein-co
lib-C666-l	chr4	1.11E+08	1.11E+08	+	96.99037	NA	promoter-	promoter-	51	NM_03299	54433	Hs.69851	NM_01898	ENSG0000	GAR1	NOLA1	GAR1 ribor	protein-co
lib-C666-l	chr10	1.04E+08	1.04E+08	+	195.8685	NA	5' UTR (NM	5' UTR (NM	101	NM_00474	9221	Hs.523238	NM_00474	ENSG0000	NOLC1	NOPP130|I	nucleolar a	protein-co
lib-C666-l	chr3	1.29E+08	1.29E+08	+	335.7812	NA	promoter-	promoter-	62	NM_01404	28976	Hs.567482	NM_01404	ENSG0000	ACAD9	NPD002	acyl-CoA c	protein-co
lib-C666-l	chr17	38497697	38497697	+	36.16835	NA	promoter-	promoter-	-574	NM_00102	5914	Hs.654583	NM_00096	ENSG0000	RARA	NR1B1|RAI	retinoic aci	protein-co
lib-C666-l	chr1	52344640	52344640	+	91.86094	NA	promoter-	promoter-	-31	NM_00110	4898	Hs.584782	NM_00252	ENSG0000	NRDC	NRD1|hNR	nardilysin (	protein-co
lib-C666-l	chr7	1.12E+08	1.12E+08	+	33.00194	NA	promoter-	promoter-	-26	NM_02199	11179	Hs.655904	NM_02199	ENSG0000	ZNF277	NRIF4|ZNF	zinc finger	protein-co
lib-C666-l	chr3	99979799	99979799	+	20.82108	NA	5' UTR (NM	5' UTR (NM	138	NM_01830	55773	Hs.477003	NM_01830	ENSG0000	TBC1D23	NS4ATP1	TBC1 dom	protein-co
lib-C666-l	chr7	27702666	27702666	+	39.26979	NA	promoter-	promoter-	-46	NM_15274	11112	Hs.406758	NM_15274	ENSG0000	HIBADH	NS5ATP1	3-hydroxyi	protein-co
lib-C666-l	chr16	27280120	27280120	+	45.23903	NA	promoter-	promoter-	-7	NM_14508	197370	Hs.284295	NM_14508	ENSG0000	NSMCE1	NSE1	NSE1 hom	protein-co
lib-C666-l	chr19	6768015	6768015	+	99.9228	NA	promoter-	promoter-	-492	NM_00549	10045	Hs.439645	NM_00549	ENSG0000	SH2D3A	NSP1	SH2 doma	protein-co
lib-C666-l	chr1	94313656	94313656	+	50.2624	NA	promoter-	promoter-	-950	NM_00126	8412	Hs.36958	NM_00356	ENSG0000	BCAR3	NSP2|SH2I	breast canc	protein-co
lib-C666-l	chrX	24711910	24711910	+	99.63164	NA	promoter-	promoter-	-40	NM_00133	5422	Hs.567319	NM_01693	ENSG0000	POLA1	NSX|POLA	DNA polyn	protein-co
lib-C666-l	chr12	6862305	6862305	+	42.49125	NA	promoter-	promoter-	-223	NM_00543	8079	Hs.524214	NM_00543	ENSG0000	MLF2	NTN4	myeloid le	protein-co
lib-C666-l	chr22	26908419	26908419	+	36.59206	NA	promoter-	promoter-	53	NM_00134	24144	Hs.20225	NM_01214	ENSG0000	TFIP11	NTR1|STIP	tuftelin inte	protein-co
lib-C666-l	chr15	71055918	71055918	+	21.28624	NA	promoter-	promoter-	-68	NM_01800	55075	Hs.108049	NM_01800	ENSG0000	UACA	NUCLING	uveal auto	protein-co
lib-C666-l	chrX	1.29E+08	1.29E+08	+	26.37215	NA	promoter-	promoter-	-24	NM_00116	10813	Hs.458598	NM_00664	ENSG0000	UTP14A	NYCO16|SI	UTP14A sn	protein-co
lib-C666-l	chr9	1.39E+08	1.39E+08	+	27.61921	NA	promoter-	promoter-	-71	NM_00103	10807	Hs.94300	NM_00664	ENSG0000	SDCCAG3	NY-CO-3	serological	protein-co
lib-C666-l	chr13	49822035	49822035	+	122.4882	NA	promoter-	promoter-	-12	NR_036439	81602	Hs.388220	NM_03091	ENSG0000	CDADC1	NYD-SP15	cytidine an	protein-co
lib-C666-l	chr7	99647415	99647415	+	59.09209	NA	promoter-	promoter-	-2	NM_14591	7589	Hs.632294	NM_14591	ENSG0000	ZSCAN21	NY-REN-2	zinc finger	protein-co
lib-C666-l	chr4	1.71E+08	1.71E+08	+	40.09574	NA	promoter-	promoter-	54	NM_00119	4750	Hs.481181	NM_01222	ENSG0000	NEK1	NY-REN-5	NIMA relat	protein-co
lib-C666-l	chr17	58469704	58469704	+	23.0291	NA	promoter-	promoter-	-118	NM_03258	84669	Hs.132868	NM_03258	ENSG0000	USP32	NY-REN-6	ubiquitin s	protein-co
lib-C666-l	chrX	347741	347741	+	472.2298	NA	promoter-	promoter-	-51	NM_01323	28227	Hs.124942	NM_01323	ENSG0000	PPP2R3B	NYREN8|PI	protein ph	protein-co
lib-C666-l	chrY	297732	297732	+	382.1433	NA	promoter-	promoter-	-42	NM_01323	28227	Hs.124942	NM_01323	ENSG0000	PPP2R3B	NYREN8|PI	protein ph	protein-co
lib-C666-l	chr20	61273693	61273693	+	28.64047	NA	promoter-	promoter-	-104	NM_01635	28231	Hs.235782	NM_01635	ENSG0000	SLCO4A1	OATP-E|O	solute carr	protein-co
lib-C666-l	chr7	89975978	89975978	+	173.1897	NA	promoter-	promoter-	-1	NM_00104	85865	Hs.593547	NM_03310	ENSG0000	GTPBP10	ObgH2|UG	GTP bindir	protein-co
lib-C666-l	chr6	41755370	41755370	+	52.45363	NA	TTS (NM_0	TTS (NM_0	189	NM_00113	1E+08	Hs.731633	NM_00113	ENSG0000	TOMM6	OBTP|TOM	translocase	protein-co
lib-C666-l	chr1	1.86E+08	1.86E+08	+	154.2741	NA	promoter-	promoter-	19	NM_01784	54953	Hs.371210	NM_01784	ENSG0000	C1orf27	ODR4|TTG	chromosor	protein-co
lib-C666-l	chr7	1E+08	1E+08	+	58.59621	NA	promoter-	promoter-	-32	NM_00330	7205	Hs.534360	NM_00330	ENSG0000	TRIP6	OIP-1|OIP	thyroid hor	protein-co
lib-C666-l	chr12	98987410	98987410	+	176.7042	NA	promoter-	promoter-	7	NM_00263	5250	Hs.290404	NM_00263	ENSG0000	SLC25A3	OK/SW-cl.	solute carr	protein-co
lib-C666-l	chr16	71929273	71929273	+	22.15977	NA	promoter-	promoter-	-123	NM_00127	9798	Hs.232194	NM_01476	ENSG0000	IST1	OLC1	IST1, ESCR	protein-co
lib-C666-l	chr3	1.24E+08	1.24E+08	+	63.5671	NA	promoter-	promoter-	60	NR_033437	7372	Hs.2057	NM_00037	ENSG0000	UMPS	OPRT	uridine mc	protein-co
lib-C666-l	chr11	71934500	71934500	+	24.99834	NA	Intergenic	CpG	-1382	NM_00156	3636	Hs.523875	NM_00156	ENSG0000	INPPL1	OPSMD|SH	inositol po	protein-co
lib-C666-l	chr2	2.02E+08	2.02E+08	+	20.19521	NA	intron (NM	CpG	219	NR_033915	4999	Hs.444870	NM_00619	ENSG0000	ORC2	ORC2L	origin reco	protein-co
lib-C666-l	chr7	1.04E+08	1.04E+08	+	58.55077	NA	promoter-	promoter-	-5	NM_00255	5001	Hs.432948	NM_00255	ENSG0000	ORC5	ORC5L|OR	origin reco	protein-co
lib-C666-l	chr11	59383375	59383375	+	90.27715	NA	5' UTR (NM	5' UTR (NM	242	NM_00255	5007	Hs.597091	NM_00255	ENSG0000	OSBP	OSBP1	oxysterol b	protein-co
lib-C666-l	chr3	38206547	38206547	+	177.4851	NA	promoter-	promoter-	-479	NM_00510	9943	Hs.475970	NM_00510	ENSG0000	OXSR1	OSR1	oxidative s	protein-co
lib-C666-l	chr3	38206909	38206909	+	99.9228	NA	promoter-	promoter-	-117	NM_00510	9943	Hs.475970	NM_00510	ENSG0000	OXSR1	OSR1	oxidative s	protein-co
lib-C666-l	chr13	48877868	48877868	+	70.98327	NA	promoter-	promoter-	-15	NM_00032	5925	Hs.408528	NM_00032	ENSG0000	RB1	OSRC|PPP1	RB transcri	protein-co
lib-C666-l	chr3	1.28E+08	1.28E+08	+	40.09574	NA	promoter-	promoter-	-38	NM_00295	6184	Hs.518244	NM_00295	ENSG0000	RPN1	OST1|RBPH	ribophorin	protein-co
lib-C666-l	chr3	1.69E+08	1.69E+08	+	27.42941	NA	promoter-	promoter-	307	NR_033702	55892	Hs.507025	NM_01865	ENSG0000	MYNN	OSZF|SBBI	myoneurin	protein-co
lib-C666-l	chr16	88717948	88717948	+	24.99834	NA	promoter-	promoter-	-456	NM_00010	1535	Hs.513803	NM_00010	ENSG0000	CYBA	p22-PHOX	cytochrom	protein-co
lib-C666-l	chr12	1.24E+08	1.24E+08	+	32.48059	NA	promoter-	promoter-	-39	NM_00681	10959	Hs.733403	NM_00681	ENSG0000	TMED2	P24A|RNP2	transmemb	protein-co
lib-C666-l	chr19	10514393	10514393	+	130.7213	NA	promoter-	promoter-	-122	NM_00706	11140	Hs.160958	NM_00706	ENSG0000	CDC37	P50CDC37	cell divisio	protein-co
lib-C666-l	chr1	46598443	46598443	+	271.1168	NA	promoter-	promoter-	24	NM_00130	8503	Hs.655387	NM_00362	ENSG0000	PIK3R3	p55|p55-G	phosphoin	protein-co
lib-C666-l	chr17	38136779	38136779	+	155.3176	NA	promoter-	promoter-	-242	NM_00280	5709	Hs.12970	NM_00280	ENSG0000	PSMD3	P58|RPN3|	proteasom	protein-co
lib-C666-l	chr19	19496551	19496551	+	23.27622	NA	promoter-	promoter-	-91	NM_01766	54815	Hs.118964	NM_01766	ENSG0000	GATAD2A	p66alpha	GATA zinc	protein-co
lib-C666-l	chr3	39093531	39093531	+	61.48528	NA	promoter-	promoter-	54	NR_144399	57599	Hs.109778	NM_02083	ENSG0000	WDR48	P80|SPG60	WD repeat	protein-co
lib-C666-l	chr1	1.7E+08	1.7E+08	+	42.40071	NA	promoter-	promoter-	-272	NM_02042	57147	Hs.435560	NM_02042	ENSG0000	SCYL3	PACE-1|PA	SCY1 like p	protein-co
lib-C666-l	chr17	33905597	33905597	+	22.14682	NA	promoter-	promoter-	59	NM_00028	5193	Hs.591190	NM_00028	ENSG0000	PEX12	PAF-3|PBD	peroxisom	protein-co
lib-C666-l	chr1	89149908	89149908	+	97.42313	NA	promoter-	promoter-	81	NM_00132	5586	Hs.440833	NM_00625	ENSG0000	PKN2	PAK2|PRK2	protein kin	protein-co
lib-C666-l	chr11	77185455	77185455	+	282.6217	NA	promoter-	promoter-	-347	NM_00257	5058	Hs.435714	NM_00257	ENSG0000	PAK1	PAKalpha	p21 (RAC1	protein-co
lib-C666-l	chr11	62359130	62359130	+	36.38376	NA	promoter-	promoter-	-21	NM_02283	64852	Hs.728983	NM_02283	ENSG0000	TUT1	PAPD2|RB	terminal ur	protein-co
lib-C666-l	chr12	6873689	6873689	+	61.07141	NA	Intergenic	Intergenic	-1794	NM_00133	5763	Hs.504613	NM_00282	ENSG0000	PTMS	ParaT	parathymo	protein-co
lib-C666-l	chr1	63788159	63788159	+	47.79407	NA	promoter-	promoter-	-30	NR_121634	1.01E+08		NR_121634	ENSG0000	FOXD3-AS	pasFOXD3	FOXD3 ant	ncRNA
lib-C666-l	chr17	77771100	77771100	+	40.61611	NA	promoter-	promoter-	-185	NM_02064	57332	Hs.387258	NM_02064	ENSG0000	CBX8	PC3|RC1	chromobo:	protein-co
lib-C666-l	chr7	1.12E+08	1.12E+08	+	60.75948	NA	promoter-	promoter-	-45	NM_00155	3475	Hs.7879	NM_00155	ENSG0000	IFRD1	PC4|TIS7	interferon	protein-co
lib-C666-l	chr7	1.12E+08	1.12E+08	+	41.46989	NA	promoter-	promoter-	744	NR_120333	3475	Hs.7879	NM_00155	ENSG0000	IFRD1	PC4|TIS7	interferon	protein-co
lib-C666-l	chr11	65382869	65382869	+	29.92316	NA	promoter-	promoter-	-914	NM_03222	399909	Hs.380801	NM_03222	ENSG0000	PCNX3	PCNXL3	pecanex hc	protein-co
lib-C666-l	chr20	48532184	48532184	+	57.83654	NA	promoter-	promoter-	-104	NM_00603	9825	Hs.48513	NM_00603	ENSG0000	SPATA2	PD1|PPP1R	spermatog	protein-co
lib-C666-l	chr1	1.51E+08	1.51E+08	+	55.62774	NA	promoter-	promoter-	-12	NM_00130	57592	Hs.186756	NM_02083	ENSG0000	ZNF687	PDB6	zinc finger	protein-co
lib-C666-l	chrX	19362014	19362014	+	79.14428	NA	promoter-	promoter-	3	NM_00117	5160	Hs.530331	NM_00028	ENSG0000	PDHA1	PDHA|PDH	pyruvate d	protein-co
lib-C666-l	chr16	70148117	70148117	+	24.99834	NA	5' UTR (NM	5' UTR (NM	588	NM_00132	55066	Hs.744043	NM_01799	ENSG0000	PDPR	PDP3	pyruvate d	protein-co
lib-C666-l	chr1	47644937	47644937	+	103.278	NA	promoter-	promoter-	15	NR_047498	1.01E+08	Hs.435758	NR_047498	ENSG0000	LINC00853	PDZK1IP1-	long interg	ncRNA
lib-C666-l	chr10	1.19E+08	1.19E+08	+	56.19669	NA	promoter-	promoter-	-61	NM_17379	118987	Hs.501149	NM_17379	ENSG0000	PDZD8	PDZK8	PDZ doma	protein-co
lib-C666-l	chr16	67063290	67063290	+	20.72954	NA	5' UTR (NM	5' UTR (NM	240	NM_00175	865	Hs.460988	NM_00175	ENSG0000	CBFB	PEBP2B	core-bindi	protein-co
lib-C666-l	chr7	1.3E+08	1.3E+08	+	20.90919	NA	promoter-	promoter-	41	NM_00125	4232	Hs.270978	NM_00240	ENSG0000	MEST	PEG1	mesoderm	protein-co
lib-C666-l	chr2	88927179	88927179	+	24.18348	NA	promoter-	promoter-	-85	NM_00483	9451	Hs.591589	NM_00483	ENSG0000	EIF2AK3	PEK|PERK|	eukaryotic	protein-co

lib-C666-l	chr15	41186601	41186601	+	36.99348	NA	promoter-	promoter-	-27	NM_02085	57617	Hs.23876	NM_02085	ENSG0000	VPS18	PEP3	VPS18, CO	protein-co
lib-C666-l	chr17	8057644	8057644	+	24.25638	NA	Intergenic	Intergenic	-1891	NM_00261	5187	Hs.445534	NM_00261	ENSG0000	PER1	PER|RGUI	period circ	protein-co
lib-C666-l	chr17	63133415	63133415	+	27.70954	NA	promoter-	promoter-	-41	NM_00108	8787	Hs.664380	NM_00383	ENSG0000	RGS9	PERRS|RGS	regulator c	protein-co
lib-C666-l	chr22	30988079	30988079	+	109.565	NA	promoter-	promoter-	-152	NM_00124	23481	Hs.517543	NM_01430	ENSG0000	PES1	PES	pescadillo	protein-co
lib-C666-l	chr11	1.3E+08	1.3E+08	+	38.10056	NA	promoter-	promoter-	-365	NM_02022	56980	Hs.275086	NM_02022	ENSG0000	PRDM10	PFM7	PR/SET do	protein-co
lib-C666-l	chr1	6320174	6320174	+	82.17155	NA	intron (NM	CpG	861	NM_20737	387509	Hs.531581	NM_20737	ENSG0000	GPR153	PGR1	G protein-	protein-co
lib-C666-l	chr15	74908082	74908082	+	36.83792	NA	exon (NM_	exon (NM_	747	NM_00113	1198	Hs.511790	NM_00129	ENSG0000	CLK3	PHCLK3|PH	CDC like ki	protein-co
lib-C666-l	chr11	64014070	64014070	+	36.56565	NA	promoter-	promoter-	343	NM_13868	26472	Hs.523760	NM_13868	ENSG0000	PPP1R14B	PHI-1|PLCI	protein ph	protein-co
lib-C666-l	chr7	64254759	64254759	+	45.42569	NA	promoter-	promoter-	-7	NM_00127	7697	Hs.184080	NM_00652	ENSG0000	ZNF138	pHZ-32	zinc finger	protein-co
lib-C666-l	chr12	1.34E+08	1.34E+08	+	129.7455	NA	5' UTR (NN	5' UTR (NN	667	NM_00344	7699	Hs.181552	NM_00344	ENSG0000	ZNF140	pHZ-39	zinc finger	protein-co
lib-C666-l	chr12	1.34E+08	1.34E+08	+	65.45359	NA	promoter-	promoter-	-2	NM_00130	7699	Hs.181552	NM_00344	ENSG0000	ZNF140	pHZ-39	zinc finger	protein-co
lib-C666-l	chr4	25235862	25235862	+	21.69444	NA	exon (NM_	exon (NM_	209	NM_01832	55300	Hs.191701	NM_01832	ENSG0000	PI4K2B	PI4KIIB|PIK	phosphatic	protein-co
lib-C666-l	chr22	38453378	38453378	+	42.49125	NA	5' UTR (NN	5' UTR (NN	116	NM_01240	9463	Hs.180871	NM_01240	ENSG0000	PICK1	PICK|PRKC	protein int	protein-co
lib-C666-l	chr19	48248758	48248758	+	25.80588	NA	promoter-	promoter-	-35	NM_01571	29997	Hs.421907	NM_01571	ENSG0000	GLTSCR2	PICT-1|PIC	glioma tun	protein-co
lib-C666-l	chr15	34517244	34517244	+	50.38662	NA	promoter-	promoter-	46	NM_00128	51234	Hs.250905	NM_01645	ENSG0000	EMC4	PIG17|TME	ER membr	protein-co
lib-C666-l	chr22	50354283	50354283	+	66.77065	NA	5' UTR (NN	5' UTR (NN	140	NM_00100	415116	Hs.530381	NM_00100	ENSG0000	PIM3	pim-3	Pim-3 prot	protein-co
lib-C666-l	chr1	44820950	44820950	+	52.3754	NA	promoter-	promoter-	1	NM_00130	79033	Hs.731413	NM_02406	ENSG0000	ERI3	PINT1|PRN	ERI1 exorit	protein-co
lib-C666-l	chr16	67694761	67694761	+	61.32708	NA	promoter-	promoter-	-43	NM_02291	65057	Hs.78019	NM_02291	ENSG0000	ACD	PIP1|PTOP	ACD, shelt	protein-co
lib-C666-l	chr3	52188527	52188527	+	21.21068	NA	5' UTR (NN	5' UTR (NN	179	NM_00116	25886	Hs.476306	NM_01542	ENSG0000	POC1A	PIX2|SOFT	POC1 cent	protein-co
lib-C666-l	chr19	14228687	14228687	+	24.23241	NA	promoter-	promoter-	-128	NM_00273	5566	Hs.631630	NM_00273	ENSG0000	PRKACA	PKACA|PPI	protein kin	protein-co
lib-C666-l	chr7	23145332	23145332	+	119.6264	NA	promoter-	promoter-	-10	NR_04622	1.01E+08		NR_04622	ENSG0000	KLHL7-AS	PLATAK	KLHL7 anti	ncRNA
lib-C666-l	chr4	1.23E+08	1.23E+08	+	91.98129	NA	promoter-	promoter-	7	NM_00503	5393	Hs.91728	NM_00503	ENSG0000	EXOSC9	PM/Scl-75	exosome c	protein-co
lib-C666-l	chr1	1.56E+08	1.56E+08	+	23.12599	NA	TTS (NM_0	TTS (NM_0	249	NM_00119	1.01E+08	Hs.530479	NM_00119	ENSG0000	PMF1-BGL	PMF1	PMF1-BGL	protein-co
lib-C666-l	chr20	35234039	35234039	+	66.67216	NA	promoter-	promoter-	-98	NM_01884	55969	Hs.584985	NM_01884	ENSG0000	C20orf24	PNAS-11|F	chromosor	protein-co
lib-C666-l	chr17	42144053	42144053	+	36.59122	NA	5' UTR (NN	5' UTR (NN	934	NM_15234	124801	Hs.355570	NM_15234	ENSG0000	LSM12	PNAS-135	LSM12 hor	protein-co
lib-C666-l	chr3	1.41E+08	1.41E+08	+	47.52935	NA	promoter-	promoter-	-84	NM_01815	55186	Hs.144130	NM_01815	ENSG0000	SLC25A36	PNC2	solute carr	protein-co
lib-C666-l	chr1	6052568	6052568	+	80.54002	NA	promoter-	promoter-	-35	NM_01510	261734	Hs.462348	NM_01510	ENSG0000	NPHP4	POC10|SLS	nephrocyst	protein-co
lib-C666-l	chr1	35658860	35658860	+	60.13322	NA	promoter-	promoter-	-114	NR_13670	6421	Hs.355934	NM_00506	ENSG0000	SFPQ	POMP100|	splicing fac	protein-co
lib-C666-l	chr7	76256655	76256655	+	380.1244	NA	promoter-	promoter-	-35	NM_01223	22932	Hs.488877	NM_01223	ENSG0000	POMZP3	POM-ZP3|	POM121 a	protein-co
lib-C666-l	chr10	1.04E+08	1.04E+08	+	34.3461	NA	promoter-	promoter-	-18	NM_00699	10360	Hs.90691	NM_00699	ENSG0000	NPM3	PORMIN|T	nucleopho	protein-co
lib-C666-l	chr7	73153418	73153418	+	22.82077	NA	promoter-	promoter-	-221	NR_13562	83451	Hs.647045	NM_14891	ENSG0000	ABHD11	PP1226|WI	abhydrolas	protein-co
lib-C666-l	chr19	54960374	54960374	+	50.99376	NA	promoter-	promoter-	34	NM_05292	114823	Hs.502378	NM_05292	ENSG0000	LENG8	pp13842	leukocyte r	protein-co
lib-C666-l	chr5	1.34E+08	1.34E+08	+	81.64329	NA	TTS (NR_0	TTS (NR_0	124	NM_00271	5515	Hs.105818	NM_00271	ENSG0000	PPP2CA	PP2Ac|PP2	protein ph	protein-co
lib-C666-l	chr14	60715761	60715761	+	37.12814	NA	promoter-	promoter-	-205	NM_02100	5494	Hs.130036	NM_02100	ENSG0000	PPM1A	PP2C-ALPI	protein ph	protein-co
lib-C666-l	chr10	99447234	99447234	+	28.7791	NA	promoter-	promoter-	-219	NM_02173	60370	Hs.23918	NM_02173	ENSG0000	AVPI1	PP5395|VI	arginine va	protein-co
lib-C666-l	chr9	1.28E+08	1.28E+08	+	51.11391	NA	5' UTR (NN	5' UTR (NN	132	NM_00272	5537	Hs.744091	NM_00272	ENSG0000	PPP6C	PP6|PP6C	protein ph	protein-co
lib-C666-l	chr20	47835880	47835880	+	69.18568	NA	promoter-	promoter-	-48	NM_00131	6780	Hs.596704	NM_00460	ENSG0000	STAU1	PPP1R150|	staufen do	protein-co
lib-C666-l	chr17	1420034	1420034	+	30.85659	NA	promoter-	promoter-	148	NM_01653	51763	Hs.632238	NM_01653	ENSG0000	INPP5K	PPS|SKIP	inositol po	protein-co
lib-C666-l	chr2	10830091	10830091	+	102.4866	NA	promoter-	promoter-	22	NM_00126	79954	Hs.222494	NM_02489	ENSG0000	NOL10	PQBP5	nucleolar p	protein-co
lib-C666-l	chr1	1.63E+08	1.63E+08	+	57.48363	NA	promoter-	promoter-	-88	NM_00130	51478	Hs.492925	NM_01637	ENSG0000	HSD17B7	PRAP|SDR	hydroxyste	protein-co
lib-C666-l	chr21	44394680	44394680	+	88.35172	NA	promoter-	promoter-	60	NM_00457	5316	Hs.431043	NM_00457	ENSG0000	PKNOX1	PREP1|pko	PBX/knotte	protein-co
lib-C666-l	chr4	1.49E+08	1.49E+08	+	83.07521	NA	promoter-	promoter-	2	NM_13836	90826	Hs.591692	NM_13836	ENSG0000	PRMT9	PRMT10	protein arç	protein-co
lib-C666-l	chr19	10981973	10981973	+	21.28624	NA	promoter-	promoter-	-280	NM_19914	10498	Hs.323213	NM_19914	ENSG0000	CARM1	PRMT4	coactivato	protein-co
lib-C666-l	chr17	48474889	48474889	+	73.27174	NA	promoter-	promoter-	25	NM_01850	55379	Hs.370927	NM_01850	ENSG0000	LRRC59	PRO1855|p	leucine ricl	protein-co
lib-C666-l	chrX	16737652	16737652	+	95.73749	NA	promoter-	promoter-	-55	NM_03279	94056	Hs.744256	NM_03279	ENSG0000	SYAP1	PRO3113	synapse as	protein-co
lib-C666-l	chr13	52158063	52158063	+	43.12596	NA	promoter-	promoter-	-421	NM_05295	115825	Hs.46506	NM_05295	ENSG0000	WDFY2	PROF|WDF	WD repeat	protein-co
lib-C666-l	chr10	99496881	99496881	+	42.99614	NA	promoter-	promoter-	3	NM_00100	118813	Hs.744075	NM_14458	ENSG0000	ZFYVE27	PROTRUDI	zinc finger	protein-co
lib-C666-l	chr22	30752945	30752945	+	51.89298	NA	promoter-	promoter-	-9	NM_00587	10291	Hs.406277	NM_00587	ENSG0000	SF3A1	PRP21|PRP	splicing fac	protein-co
lib-C666-l	chr1	52870195	52870195	+	85.88258	NA	promoter-	promoter-	-24	NM_03286	84950	Hs.5301	NM_03228	ENSG0000	PRPF38A	PRP38A|Pr	pre-mRNA	protein-co
lib-C666-l	chr17	74722770	74722770	+	58.68336	NA	promoter-	promoter-	111	NM_01516	23210	Hs.514505	NM_01516	ENSG0000	JMJD6	PSR|PTDSR	arginine de	protein-co
lib-C666-l	chr19	2945401	2945401	+	69.90313	NA	promoter-	promoter-	-432	NM_02121	58492	Hs.38004	NM_02121	ENSG0000	ZNF77	pT1	zinc finger	protein-co
lib-C666-l	chr9	1.3E+08	1.3E+08	+	36.97678	NA	promoter-	promoter-	28	NM_00132	7633	Hs.522399	NM_00713	ENSG0000	ZNF79	pT7	zinc finger	protein-co
lib-C666-l	chr9	96793078	96793078	+	37.12814	NA	promoter-	promoter-	2	NM_17799	138639	Hs.437943	NM_15242	ENSG0000	PTPDC1	PTP9Q22	protein tyr	protein-co
lib-C666-l	chr11	1.12E+08	1.12E+08	+	20.19666	NA	exon (NM_	exon (NM_	157	NM_00031	5805	Hs.503860	NM_00031	ENSG0000	PTS	PTPS	6-pyruvoyl	protein-co
lib-C666-l	chr19	5340863	5340863	+	24.32656	NA	promoter-	promoter-	-49	NM_13085	5802	Hs.744928	NM_00285	ENSG0000	PTPRS	PTPSIGMA	protein tyr	protein-co
lib-C666-l	chr7	44925232	44925232	+	203.5799	NA	promoter-	promoter-	-248	NM_03322	5814	Hs.349150	NM_03322	ENSG0000	PURB	PURBETA	purine rich	protein-co
lib-C666-l	chr10	1.17E+08	1.17E+08	+	83.07521	NA	promoter-	promoter-	-9	NM_13916	142940	Hs.21187	NM_13916	ENSG0000	TRUB1	PUS4	TruB pseuc	protein-co
lib-C666-l	chr3	1.14E+08	1.14E+08	+	202.9887	NA	promoter-	promoter-	-35	NM_02463	79691	Hs.477162	NM_02463	ENSG0000	QTRT2	QTRTD1	queuine tR	protein-co
lib-C666-l	chr11	4116006	4116006	+	327.3639	NA	promoter-	promoter-	91	NM_00131	6240	Hs.445705	NM_00103	ENSG0000	RRM1	R1|RIR1|RR	ribonucleo	protein-co
lib-C666-l	chr19	19030404	19030404	+	221.5683	NA	promoter-	promoter-	-80	NM_01907	54555	Hs.143187	NM_01907	ENSG0000	DDX49	R27090_2	DEAD-box	protein-co
lib-C666-l	chr9	85677830	85677830	+	39.29799	NA	5' UTR (NN	5' UTR (NN	210	NM_15257	158158	Hs.129136	NM_15257	ENSG0000	RASEF	RAB45	RAS and El	protein-co
lib-C666-l	chr3	19988578	19988578	+	33.11719	NA	promoter-	promoter-	6	NM_00416	5868	Hs.475663	NM_00416	ENSG0000	RAB5A	RAB5	RAB5A, me	protein-co
lib-C666-l	chr11	73472352	73472352	+	27.96363	NA	promoter-	promoter-	-151	NM_00124	5870	Hs.503222	NM_00286	ENSG0000	RAB6A	RAB6	RAB6A, me	protein-co
lib-C666-l	chr12	72233567	72233567	+	25.39084	NA	promoter-	promoter-	80	NM_00114	64786	Hs.284630	NM_02277	ENSG0000	TBC1D15	RAB7-GAP	TBC1 dom	protein-co
lib-C666-l	chr7	66205589	66205589	+	57.08342	NA	promoter-	promoter-	-54	NM_01450	27342	Hs.530053	NM_01450	ENSG0000	RABGEF1	RABEX5|R/	RAB guani	protein-co
lib-C666-l	chr7	66147064	66147064	+	38.60092	NA	promoter-	promoter-	-14	NR_10467	27342	Hs.530053	NM_01450	ENSG0000	RABGEF1	RABEX5|R/	RAB guani	protein-co
lib-C666-l	chr1	2.29E+08	2.29E+08	+	79.67884	NA	promoter-	promoter-	-234	NM_00101	149603	Hs.356377	NM_00101	ENSG0000	RNF187	RACO-1|R/	ring finger	protein-co
lib-C666-l	chr16	66961093	66961093	+	86.72066	NA	Intergenic	Intergenic	-1654	NM_00416	6236	Hs.1027	NM_00416	ENSG0000	RRAD	RAD|RAD1	RRAD, Ras	protein-co
lib-C666-l	chr3	14220105	14220105	+	20.90919	NA	promoter-	promoter-	67	NM_00462	7508	Hs.475538	NM_00462	ENSG0000	XPC	RAD4|XP3	XPC compl	protein-co
lib-C666-l	chr11	506847	506847	+	139.6843	NA	promoter-	promoter-	-26	NM_20338	6050	Hs.530687	NM_00293	ENSG0000	RNH1	RAI|RNH	ribonuclea	protein-co
lib-C666-l	chr3	1.5E+08	1.5E+08	+	133.1091	NA	promoter-	promoter-	2	NM_01444	27230	Hs.518326	NM_01444	ENSG0000	SERP1	RAMP4	stress asso	protein-co
lib-C666-l	chr5	61708587	61708587	+	133.1904	NA	promoter-	promoter-	14	NM_01633	51194	Hs.482269	NM_01633	ENSG0000	IPO11	RanBP11	importin 1	protein-co
lib-C666-l	chr1	22110040	22110040	+	27.7824	NA	promoter-	promoter-	-352	NM_00103	84196	Hs.467524	NM_03223	ENSG0000	USP48	RAP1GA1|	ubiquitin s	protein-co
lib-C666-l	chr17	42385930	42385930	+	32.78343	NA	promoter-	promoter-	3	NM_00669	10900	Hs.500197	NM_00669	ENSG0000	RUNDC3A	RAP2IP|RP	RUN doma	protein-co
lib-C666-l	chr17	79633961	79633961	+	23.47534	NA	promoter-	promoter-	200	NM_19928	339230	Hs.744125	NM_19928	ENSG0000	CCDC137	RaRF	coiled-coil	protein-co
lib-C666-l	chr12	498756	498756	+	90.61259	NA	promoter-	promoter-	-135	NM_00104	5927	Hs.76272	NM_00505	ENSG0000	KDM5A	RBBP-2|RB	lysine dem	protein-co
lib-C666-l	chr2	25194967	25194967	+	63.68534	NA	promoter-	promoter-	-4	NM_00119	51277	Hs.434993	NM_01654	ENSG0000	DNAJC27	RBJ|RabJS	DnaJ heat	protein-co
lib-C666-l	chr6	90529495	90529495	+	106.0503	NA	promoter-	promoter-	18	NM_01461	23195	Hs.529948	NM_01461	ENSG0000	MDN1	Rea1	midasin A/	protein-co
lib-C666-l	chr15	78591975	78591975	+	94.67412	NA	promoter-	promoter-	93	NM_00130	80349	Hs.513055	NM_02523	ENSG0000	WDR61	REC14|SKI	WD repeat	protein-co
lib-C666-l	chr17	73663327	73663327	+	171.9321	NA	promoter-	promoter-	-58	NM_00100	9400	Hs.632229	NM_00425	ENSG0000	RECQL5	RECQ5	RecQ like I	protein-co
lib-C666-l	chr1	28241342	28241342	+	101.6547	NA	promoter-	promoter-	-34	NM_00129	6118	Hs.79411	NM_00294	ENSG0000	RPA2	REPA2|RP-	replication	protein-co
lib-C666-l	chr19	41283529	41283529	+	54.01688	NA	promoter-	promoter-	-595	NR_03779	1.01E+08	Hs.631539	NR_037791		RAB4B-EG	RERT-lncR	RAB4B-EG	ncRNA
lib-C666-l	chr13	34392207	34392207	+	38.60092	NA	promoter-	promoter-	1	NM_00291	5983	Hs.115474	NM_00291	ENSG0000	RFC3	RFC38	replication	protein-co
lib-C666-l	chr7	73668801	73668801	+	32.07197	NA	promoter-	promoter-	-13	NM_18147	5982	Hs.647062	NM_00291	ENSG0000	RFC2	RFC40	replication	protein-co
lib-C666-l	chr16	2205644	2205644	+	38.36071	NA	promoter-	promoter-	-155	NM_03227	84231	Hs.713632	NM_03227	ENSG0000	TRAF7	RFWD1|RN	TNF recept	protein-co
lib-C666-l	chr7	1.51E+08	1.51E+08	+	92.39822	NA	promoter-	promoter-	44	NM_00561	6009	Hs.283521	NM_00561	ENSG0000	RHEB	RHEB2	Ras homol	protein-co
lib-C666-l	chr10	49864423	49864423	+	53.51636	NA	promoter-	promoter-	-113	NR_04567	58504	Hs.655672	NM_02122	ENSG0000	ARHGAP22	RhoGAP2|F	Rho GTPas	protein-co
lib-C666-l	chr12	1.07E+08	1.07E+08	+	38.71165	NA	promoter-	promoter-	-39	NM_00133	55188	Hs.131306	NM_01815	ENSG0000	RIC8B	RIC8|hSyn	RIC8 guani	protein-co
lib-C666-l	chr6	33176263	33176263	+	32.0283	NA	promoter-	promoter-	-23	NM_00293	6015	Hs.631989	NM_00293	ENSG0000	RING1	RING1A|RN	ring finger	protein-co
lib-C666-l	chr3	51534047	51534047	+	116.3202	NA	promoter-	promoter-	-29	NM_01470	9730	Hs.716623	NM_01470	ENSG0000	DCAF1	RIP|VPRBP	DDB1 and	protein-co
lib-C666-l	chr19	29704147	29704147	+	33.78053	NA	promoter-	promoter-	-11	NM_00600	7386	Hs.743307	NM_00600	ENSG0000	UQCRFS1	RIP1|RIS1|F	ubiquinol-	protein-co
lib-C666-l	chr4	79860587	79860587	+	81.31076	NA	promoter-	promoter-	-5	NM_00104	152559	Hs.657312	NM_17745	ENSG0000	PAQR3	RKTG	progestin a	protein-co
lib-C666-l	chr5	1.81E+08	1.81E+08	+	55.66538	NA	promoter-	promoter-	-44	NM_00134	84851	Hs.458412	NM_03276	ENSG0000	TRIM52	RNF102	tripartite m	protein-co
lib-C666-l	chr16	4674693	4674693	+	38.20619	NA	promoter-	promoter-	-132	NM_00114	23295	Hs.526494	NM_01524	ENSG0000	MGRN1	RNF156	mahoguni	protein-co
lib-C666-l	chr6	30294999	30294999	+	39.64852	NA	promoter-	promoter-	-9	NM_17201	56658	Hs.413493	NM_02125	ENSG0000	TRIM39	RNF23|TFP	tripartite m	protein-co
lib-C666-l	chr3	9005142	9005142	+	236.5516	NA	promoter-	promoter-	17	NM_02016	56852	Hs.375684	NM_02016	ENSG0000	RAD18	RNF73	RAD18, E3	protein-co
lib-C666-l	chr1	1.48E+08	1.48E+08	+	57.45248	NA	promoter-	promoter-	-335	NR_10408	1.02E+08		NR_10408	ENSG0000	RNVU1-19	RNU1-126	RNA, varia	snRNA
lib-C666-l	chr9	1.37E+08	1.37E+08	+	58.41369	NA	promoter-	promoter-	-345	NR_02334	1E+08	Hs.545589	NR_02334	ENSG0000	RNU6ATAC	RNU6ATAC	RNA, U6at	snRNA
lib-C666-l	chr1	1.93E+08	1.93E+08	+	40.51821	NA	promoter-	promoter-	38	NM_00133	6738	Hs.288178	NM_00460	ENSG0000	TROVE2	RO60|ROR	TROVE dor	protein-co
lib-C666-l	chr20	2644856	2644856	+	119.6197	NA	promoter-	promoter-	13	NM_00133	3420	Hs.436405	NM_00689	ENSG0000	IDH3B	RP46	isocitrate c	protein-co
lib-C666-l	chr2	88991094	88991094	+	165.5633	NA	promoter-	promoter-	-82	NM_14456	22934	Hs.469264	NM_14456	ENSG0000	RPIA	RPI|RPIAD	ribose 5-p	protein-co
lib-C666-l	chr3	64009333	64009333	+	30.08826	NA	promoter-	promoter-	-51	NM_00127	9861	Hs.152536	NM_01481	ENSG0000	PSMD6	Rpn7|S10|S	proteasom	protein-co
lib-C666-l	chr19	30097183	30097183	+	301.0866	NA	promoter-	promoter-	13	NR_02736	10775	Hs.412870	NM_00662	ENSG0000	POP4	RPP29	POP4 hom	protein-co
lib-C666-l	chr6	1.29E+08	1.29E+08	+	20.19521	NA	promoter-	promoter-	-23	NM_00129	5796	Hs.155919	NM_00284	ENSG0000	PTPRK	R-PTP-kap	protein tyr	protein-co
lib-C666-l	chr2	2.28E+08	2.28E+08	+	20.3278	NA	promoter-	promoter-	100	NM_00116	84236	Hs.471514	NM_03227	ENSG0000	RHBDD1	RRP4	rhomboid	protein-co
lib-C666-l	chr7	77325598	77325598	+	23.14387	NA	promoter-	promoter-	-16	NR_13425	1.01E+08	Hs.72451	NR_03836	ENSG0000	APTR	RSBN1L-A	Alu-media	ncRNA
lib-C666-l	chr16	31519664	31519664	+	20.56906	NA	promoter-	promoter-	76	NM_02274	64755	Hs.9003	NM_02274	ENSG0000	C16orf58	RUS	chromosor	protein-co
lib-C666-l	chr19	49999601	49999601	+	33.47004	NA	promoter-	promoter-	-21	NM_00101	6205	Hs.433529	NM_00101	ENSG0000	RPS11	S11	ribosomal	protein-co
lib-C666-l	chr11	17099324	17099324	+	29.37119	NA	promoter-	promoter-	-104	NM_00101	6207	Hs.446588	NM_00101	ENSG0000	RPS13	S13	ribosomal	protein-co
lib-C666-l	chr11	75110485	75110485	+	38.21529	NA	promoter-	promoter-	-50	NM_00100	6188	Hs.546286	NM_00100	ENSG0000	RPS3	S3	ribosomal	protein-co
lib-C666-l	chr1	45241139	45241139	+	201.2371	NA	promoter-	promoter-	-107	NM_00101	6202	Hs.512675	NM_00101	ENSG0000	RPS8	S8	ribosomal	protein-co
lib-C666-l	chr19	54704650	54704650	+	41.28836	NA	promoter-	promoter-	-76	NR_13576	6203	Hs.467284	NM_00101	ENSG0000	RPS9	S9	ribosomal	protein-co
lib-C666-l	chr3	45730740	45730740	+	38.10403	NA	promoter-	promoter-	7	NM_00131	22908	Hs.156509	NM_01401	ENSG0000	SACM1L	SAC1	SAC1 supp	protein-co
lib-C666-l	chr17	74582303	74582303	+	36.04387	NA	promoter-	promoter-	-158	NM_00645	10610	Hs.592105	NM_00645	ENSG0000	ST6GALNA	SAITL1|SIA	ST6 N-ace	protein-co
lib-C666-l	chr3	49967334	49967334	+	25.3508	NA	5' UTR (NN	5' UTR (NN	111	NM_03235	84315	Hs.655014	NM_03235	ENSG0000	MON1A	SAND1	MON1 hor	protein-co
lib-C666-l	chr1	2.06E+08	2.06E+08	+	61.91626	NA	5' UTR (NN	5' UTR (NN	853	NM_00197	2005	Hs.497520	NM_00197	ENSG0000	ELK4	SAP1	ELK4, ETS t	protein-co
lib-C666-l	chr10	71930318	71930318	+	31.9103	NA	promoter-	promoter-	-33	NM_00114	56681	Hs.499960	NM_02015	ENSG0000	SAR1A	SAR1|SAR	secretion a	protein-co
lib-C666-l	chr19	39421507	39421507	+	27.7824	NA	promoter-	promoter-	29	NM_01782	54938	Hs.709416	NM_01782	ENSG0000	SARS2	SARS|SARS	seryl-tRNA	protein-co
lib-C666-l	chr12	58138410	58138410	+	257.3508	NA	promoter-	promoter-	-362	NM_00598	6302	Hs.632708	NM_00598	ENSG0000	TSPAN31	SAS	tetraspanir	protein-co
lib-C666-l	chr16	2022040	2022040	+	534.1061	NA	promoter-	promoter-	-24	NM_00645	10607	Hs.513267	NM_00645	ENSG0000	TBL3	SAZD|UTP	transducin	protein-co
lib-C666-l	chr19	51014794	51014794	+	413.1779	NA	promoter-	promoter-	-182	NM_00127	126119	Hs.467151	NM_13833	ENSG0000	JOSD2	SBBI54	Josephin d	protein-co
lib-C666-l	chr11	9482385	9482385	+	105.9638	NA	promoter-	promoter-	-127	NM_00128	7702	Hs.523471	NM_00344	ENSG0000	ZNF143	SBF|STAF|p	zinc finger	protein-co
lib-C666-l	chr9	91933438	91933438	+	114.9583	NA	promoter-	promoter-	50	NM_00128	79048	Hs.59804	NM_02407	ENSG0000	SECISBP2	SBP2	SECIS bind	protein-co
lib-C666-l	chr7	1.01E+08	1.01E+08	+	29.92316	NA	promoter-	promoter-	-64	NM_00337	7425	Hs.587325	NM_00337	ENSG0000	VGF	SCG7|SgVI	VGF nerve	protein-co
lib-C666-l	chr19	35168065	35168065	+	32.11429	NA	promoter-	promoter-	-129	NR_02762	643719	Hs.686754	NR_027620		SCGB1B2P	SCGB4A1P	secretoglo	pseudo
lib-C666-l	chr3	48514533	48514533	+	20.56906	NA	5' UTR (NN	5' UTR (NN	209	NM_00127	51246	Hs.414579	NM_01647	ENSG0000	SHISA5	SCOTIN	shisa famil	protein-co

lib-C666-l	chr16	4743584	4743584	+	24.03208	NA	promoter-	promoter-	-110	NM_03234	84309	Hs.513315	NM_03234	ENSG0000	NUDT16L1	SDOS	nudix hydr	protein-co
lib-C666-l	chr22	43411208	43411208	+	60.4974	NA	promoter-	promoter-	-24	NM_00118	11252	Hs.162877	NM_00722	ENSG0000	PACSIN2	SDPII	protein kin	protein-co
lib-C666-l	chr14	24769033	24769033	+	265.4836	NA	promoter-	promoter-	6	NM_00113	115817	Hs.348350	NM_13845	ENSG0000	DHRS1	SDR19C1	dehydroge	protein-co
lib-C666-l	chr13	52377866	52377866	+	164.7109	NA	intron (NM	CpG	432	NM_02470	79758	Hs.266728	NM_02470	ENSG0000	DHRS12	SDR40C1	dehydroge	protein-co
lib-C666-l	chr4	1.7E+08	1.7E+08	+	35.40551	NA	promoter-	promoter-	4	NM_03278	84869	Hs.659311	NM_03278	ENSG0000	CBR4	SDR45C1	carbonyl re	protein-co
lib-C666-l	chr19	55574692	55574692	+	25.49921	NA	promoter-	promoter-	-107	NR_02738	112724	Hs.327631	NM_13841	ENSG0000	RDH13	SDR7C3	retinol deh	protein-co
lib-C666-l	chr2	73053157	73053157	+	71.16089	NA	promoter-	promoter-	14	NM_00132	23233	Hs.303454	NM_01518	ENSG0000	EXOC6B	SEC15B|SE	exocyst co	protein-co
lib-C666-l	chr7	1.33E+08	1.33E+08	+	30.07198	NA	promoter-	promoter-	-82	NM_02180	60412	Hs.321273	NM_02180	ENSG0000	EXOC4	SEC8|SEC8	exocyst co	protein-co
lib-C666-l	chr11	62599505	62599505	+	62.69942	NA	promoter-	promoter-	58	NM_00133	6811	Hs.654602	NM_00316	ENSG0000	STX5	SED5|STX5	syntaxin 5	protein-co
lib-C666-l	chr7	45808663	45808663	+	356.2133	NA	promoter-	promoter-	-46	NR_02427	641977	Hs.723477	NR_02427	ENSG0000	SEPT7P2	SEPT13|SEI	septin 7 ps	pseudo
lib-C666-l	chr3	9438730	9438730	+	42.21183	NA	promoter-	promoter-	-423	NR_13278	440944	Hs.598958	NM_00101	ENSG0000	THUMPD3	SETD5-AS	THUMPD3	ncRNA
lib-C666-l	chr6	1.44E+08	1.44E+08	+	149.9894	NA	promoter-	promoter-	-33	NM_03128	83443	Hs.110695	NM_03128	ENSG0000	SF3B5	SF3b10|Ysf	splicing fac	protein-co
lib-C666-l	chr6	36562051	36562051	+	24.25638	NA	promoter-	promoter-	-39	NR_03661	6428	Hs.405144	NM_00301	ENSG0000	SRSF3	SFRS3|SRp	serine and	protein-co
lib-C666-l	chr19	2783369	2783369	+	30.98434	NA	promoter-	promoter-	-15	NM_00302	6449	Hs.203910	NM_00302	ENSG0000	SGTA	SGT|alphaS	small gluta	protein-co
lib-C666-l	chr11	10772592	10772592	+	33.95009	NA	promoter-	promoter-	58	NM_01463	9646	Hs.725151	NM_01463	ENSG0000	CTR9	SH2BP1|TS	CTR9 hom	protein-co
lib-C666-l	chr1	46806795	46806795	+	72.02663	NA	promoter-	promoter-	-55	NM_00125	387338	Hs.163424	NM_19904	ENSG0000	NSUN4	SHTAP	NOP2/Sun	protein-co
lib-C666-l	chr16	48419625	48419625	+	20.94772	NA	promoter-	promoter-	-396	NM_00303	6477	Hs.706828	NM_00303	ENSG0000	SIAH1	SIAH1A	siah E3 ubi	protein-co
lib-C666-l	chr8	74884579	74884579	+	52.95013	NA	promoter-	promoter-	-57	NM_00120	6921	Hs.533437	NM_00564	ENSG0000	ELOC	SIII|TCEB1	elongin C	protein-co
lib-C666-l	chr1	1.15E+08	1.15E+08	+	60.20627	NA	promoter-	promoter-	20	NR_04974	80143	Hs.709277	NM_02507	ENSG0000	SIKE1	SIKE	suppressor	protein-co
lib-C666-l	chr19	49122539	49122539	+	209.5932	NA	promoter-	promoter-	-9	NM_00124	56848	Hs.528006	NM_02012	ENSG0000	SPHK2	SK 2|SK-2|	sphingosin	protein-co
lib-C666-l	chr16	69345350	69345350	+	37.12814	NA	promoter-	promoter-	63	NM_01324	27183	Hs.128420	NM_01324	ENSG0000	VPS4A	SKD1|SKD1	vacuolar p	protein-co
lib-C666-l	chr8	41386539	41386539	+	105.1162	NA	promoter-	promoter-	-186	NM_03233	84296	Hs.656996	NM_03233	ENSG0000	GINS4	SLD5	GINS comp	protein-co
lib-C666-l	chr11	71159511	71159511	+	48.61959	NA	promoter-	promoter-	-34	NM_00136	1717	Hs.503134	NM_00136	ENSG0000	DHCR7	SLOS	7-dehydro	protein-co
lib-C666-l	chr15	74284687	74284687	+	105.6454	NA	promoter-	promoter-	2	NM_00125	9399	Hs.194816	NM_00480	ENSG0000	STOML1	SLP-1|STO	stomatin li	protein-co
lib-C666-l	chr9	95527304	95527304	+	48.61959	NA	promoter-	promoter-	-221	NM_00100	23299	Hs.436939	NM_01525	ENSG0000	BICD2	SMALED2|I	BICD carg	protein-co
lib-C666-l	chr19	39833015	39833015	+	36.16835	NA	promoter-	promoter-	-84	NM_00130	55095	Hs.612332	NM_01802	ENSG0000	SAMD4B	SMGB|Sma	sterile alph	protein-co
lib-C666-l	chr15	66790089	66790089	+	97.61671	NA	promoter-	promoter-	64	NM_00604	10302	Hs.30174	NM_00604	ENSG0000	SNAPC5	SNAP19	small nucle	protein-co
lib-C666-l	chr19	48018593	48018593	+	46.4068	NA	promoter-	promoter-	-78	NM_00382	8775	Hs.126938	NM_00382	ENSG0000	NAPA	SNAPA	NSF attach	protein-co
lib-C666-l	chr19	48437396	48437396	+	41.78738	NA	promoter-	promoter-	-42	NR_00443	1E+08	Hs.723085	NR_004436		SNAR-A2	SNAR-A53	small ILF3/	snRNA
lib-C666-l	chr19	48421675	48421675	+	28.36893	NA	promoter-	promoter-	-11	NR_00443	1E+08	Hs.723085	NR_004436		SNAR-A2	SNAR-A53	small ILF3/	snRNA
lib-C666-l	chr3	1.7E+08	1.7E+08	+	43.7015	NA	promoter-	promoter-	-36	NM_00541	6498	Hs.536655	NM_00541	ENSG0000	SKIL	SNO|SnoA	SKI like pro	protein-co
lib-C666-l	chr14	55493882	55493882	+	134.4805	NA	promoter-	promoter-	38	NM_08086	122809	Hs.744302	NM_08086	ENSG0000	SOCS4	SOCS7	suppressor	protein-co
lib-C666-l	chr1	24648374	24648374	+	41.10975	NA	intron (NM	CpG	-1156	NM_02118	57822	Hs.657920	NM_02118	ENSG0000	GRHL3	SOM|TFCP	grainyhea	protein-co
lib-C666-l	chr11	64851652	64851652	+	270.5995	NA	promoter-	promoter-	-37	NM_08066	113130	Hs.434886	NM_08066	ENSG0000	CDCA5	SORORIN	cell divisio	protein-co
lib-C666-l	chr18	23806339	23806339	+	51.11391	NA	promoter-	promoter-	-508	NM_00564	6875	Hs.369519	NM_00564	ENSG0000	TAF4B	SPGF13|TA	TATA-box	protein-co
lib-C666-l	chr17	74382077	74382077	+	48.20053	NA	exon (NM_	exon (NM_	788	NM_00114	8877	Hs.68061	NM_02197	ENSG0000	SPHK1	SPHK	sphingosin	protein-co
lib-C666-l	chr16	89895109	89895109	+	53.07762	NA	exon (NM_	exon (NM_	202	NM_03245	84501	Hs.461786	NM_03245	ENSG0000	SPIRE2	Spir-2	spire type	protein-co
lib-C666-l	chr16	30456794	30456794	+	23.6003	NA	exon (NM_	exon (NM_	502	NM_01224	22928	Hs.118725	NM_01224	ENSG0000	SEPHS2	SPS2|SPS2	selenopho	protein-co
lib-C666-l	chr11	18655986	18655986	+	108.8194	NA	promoter-	promoter-	34	NM_19428	144108	Hs.738650	NM_19428	ENSG0000	SPTY2D1	Spt2	SPT2 chror	protein-co
lib-C666-l	chr17	26989266	26989266	+	44.91887	NA	promoter-	promoter-	50	NM_00317	6830	Hs.250429	NM_00317	ENSG0000	SUPT6H	SPT6|SPT6	SPT6 hom	protein-co
lib-C666-l	chr5	1.4E+08	1.4E+08	+	25.61513	NA	promoter-	promoter-	-106	NR_04558	10011	Hs.653135	NM_00103	ENSG0000	SRA1	SRA|SRAP|	steroid rec	protein-co
lib-C666-l	chr19	50145193	50145193	+	66.80925	NA	promoter-	promoter-	-189	NM_02122	58506	Hs.103521	NM_02122	ENSG0000	SCAF1	SRA1	SR-related	protein-co
lib-C666-l	chr17	17739688	17739688	+	22.90968	NA	intron (NM	CpG	637	NM_00100	6720	Hs.592123	NM_00417	ENSG0000	SREBF1	SREBP-1c|	sterol regu	protein-co
lib-C666-l	chr22	42228965	42228965	+	32.07197	NA	promoter-	promoter-	-118	NM_00459	6721	Hs.443258	NM_00459	ENSG0000	SREBF2	SREBP-2|	sterol regu	protein-co
lib-C666-l	chr17	7531201	7531201	+	101.0015	NA	promoter-	promoter-	-7	NM_00132	112483	Hs.10846	NM_13349	ENSG0000	SAT2	SSAT2	spermidine	protein-co
lib-C666-l	chr7	1.17E+08	1.17E+08	+	64.34737	NA	promoter-	promoter-	-5	NR_00233	93653	Hs.597516	NR_00233	ENSG0000	ST7-AS1	ST7AS1|ST	ST7 antiser	ncRNA
lib-C666-l	chr20	56285088	56285088	+	20.90919	NA	promoter-	promoter-	-57	NM_02018	56937	Hs.517155	NM_02018	ENSG0000	PMEPA1	STAG1|TM	prostate tr	protein-co
lib-C666-l	chr7	72476510	72476510	+	91.93295	NA	promoter-	promoter-	-44	NR_04058	442578	Hs.632310	NM_001013739		STAG3L3	STAG3L1|S	stromal an	pseudo
lib-C666-l	chr7	74306784	74306784	+	32.56643	NA	promoter-	promoter-	-53	NR_04058	442582	Hs.632310	NM_001025202		STAG3L2	STAG3L1|S	stromal an	pseudo
lib-C666-l	chr7	74988377	74988377	+	80.42115	NA	promoter-	promoter-	-70	NR_04058	54441	Hs.632310	NM_018991		STAG3L1	STAG3L1P	stromal an	pseudo
lib-C666-l	chr7	66767563	66767563	+	51.38834	NA	promoter-	promoter-	-62	NR_04058	64940	Hs.632013	NM_02290	ENSG0000	STAG3L4	STAG3L4P	stromal an	pseudo
lib-C666-l	chr1	28099582	28099582	+	20.5987	NA	promoter-	promoter-	-112	NM_17742	23673	Hs.523855	NM_17742	ENSG0000	STX12	STX13|STX	syntaxin 12	protein-co
lib-C666-l	chr16	31044700	31044700	+	259.1773	NA	promoter-	promoter-	-112	NM_00460	6810	Hs.83734	NM_00460	ENSG0000	STX4	STX4A|p35	syntaxin 4	protein-co
lib-C666-l	chr4	54232252	54232252	+	121.6727	NA	promoter-	promoter-	-10	NM_15254	152579	Hs.302287	NM_15254	ENSG0000	SCFD2	STXBP1L1	sec1 family	protein-co
lib-C666-l	chr10	70939975	70939975	+	169.5727	NA	promoter-	promoter-	15	NM_00317	6832	Hs.106469	NM_00317	ENSG0000	SUPV3L1	SUV3	Suv3 like R	protein-co
lib-C666-l	chr19	55851088	55851088	+	46.59515	NA	promoter-	promoter-	-133	NM_03270	84787	Hs.590982	NM_03270	ENSG0000	KMT5C	SUV420H2	lysine meth	protein-co
lib-C666-l	chr17	25621065	25621065	+	44.1351	NA	promoter-	promoter-	-41	NM_01562	26118	Hs.446017	NM_01562	ENSG0000	WSB1	SWIP1|WSI	WD repeat	protein-co
lib-C666-l	chr17	15903000	15903000	+	231.5716	NA	promoter-	promoter-	6	NM_00104	125150	Hs.593985	NM_00104	ENSG0000	ZSWIM7	SWS1	zinc finger	protein-co
lib-C666-l	chr3	48282560	48282560	+	55.46257	NA	promoter-	promoter-	-36	NM_01608	51385	Hs.172602	NM_01608	ENSG0000	ZNF589	SZF1	zinc finger	protein-co
lib-C666-l	chr1	1.1E+08	1.1E+08	+	20.19521	NA	promoter-	promoter-	-30	NM_00564	6884	Hs.632426	NM_00564	ENSG0000	TAF13	TAF(II)18|T	TATA-box	protein-co
lib-C666-l	chr11	6633514	6633514	+	49.86005	NA	promoter-	promoter-	-39	NM_00628	6881	Hs.5158	NM_00628	ENSG0000	TAF10	TAF2A|TAF	TATA-box	protein-co
lib-C666-l	chr1	89990475	89990475	+	107.2762	NA	promoter-	promoter-	78	NM_00113	23507	Hs.482017	NM_01535	ENSG0000	LRRC8B	TA-LRRP|T	leucine rich	protein-co
lib-C666-l	chr16	20753126	20753126	+	195.8685	NA	5' UTR (NM	5' UTR (NM	160	NM_01773	55623	Hs.700004	NM_01773	ENSG0000	THUMPD1	Tan1	THUMP dc	protein-co
lib-C666-l	chr7	65670131	65670131	+	22.66433	NA	promoter-	promoter-	-128	NM_00359	8460	Hs.421194	NM_00359	ENSG0000	TPST1	TANGO13	tyrosylprot	protein-co
lib-C666-l	chr10	1.24E+08	1.24E+08	+	45.88523	NA	promoter-	promoter-	-16	NM_00133	59338	Hs.643512	NM_02162	ENSG0000	PLEKHA1	TAPP1	pleckstrin	protein-co
lib-C666-l	chrX	1.36E+08	1.36E+08	+	64.17523	NA	promoter-	promoter-	215	NM_00116	27336	Hs.204475	NM_01450	ENSG0000	HTATSF1	TAT-SF1|T	HIV-1 Tat	protein-co
lib-C666-l	chr13	1.14E+08	1.14E+08	+	34.78259	NA	intron (NM	intron (NM	608	NM_00128	79774	Hs.745043	NM_02471	ENSG0000	GRTP1	TBC1D6	growth hor	protein-co
lib-C666-l	chr16	81478279	81478279	+	24.99834	NA	promoter-	promoter-	-496	NM_19839	80790	Hs.594095	NM_03062	ENSG0000	CMIP	TCMIP	c-Maf indu	protein-co
lib-C666-l	chr6	1.23E+08	1.23E+08	+	34.3461	NA	promoter-	promoter-	17	NM_02075	57515	Hs.146668	NM_02075	ENSG0000	SERINC1	TDE1L|TDE	serine inco	protein-co
lib-C666-l	chr12	11802376	11802376	+	20.0808	NA	promoter-	promoter-	-412	NM_00198	2120	Hs.504765	NM_00198	ENSG0000	ETV6	TEL|TEL/AE	ETS variant	protein-co
lib-C666-l	chr7	1.16E+08	1.16E+08	+	161.3141	NA	promoter-	promoter-	-107	NM_01564	26136	Hs.592286	NM_01564	ENSG0000	TES	TESS|TESS-	testin LIM	protein-co
lib-C666-l	chr7	1.16E+08	1.16E+08	+	35.7914	NA	intron (NM	CpG	252	NM_01564	26136	Hs.592286	NM_01564	ENSG0000	TES	TESS|TESS-	testin LIM	protein-co
lib-C666-l	chr1	89357283	89357283	+	64.2674	NA	promoter-	promoter-	18	NM_00151	2959	Hs.481852	NM_00151	ENSG0000	GTF2B	TF2B|TFIIB	general tra	protein-co
lib-C666-l	chr2	27579733	27579733	+	77.28233	NA	promoter-	promoter-	168	NM_00152	2976	Hs.75782	NM_00152	ENSG0000	GTF3C2	TFIIIC-BET	general tra	protein-co
lib-C666-l	chr18	158593	158593	+	120.9748	NA	5' UTR (NM	5' UTR (NM	110	NM_00103	9097	Hs.464416	NM_00515	ENSG0000	USP14	TGT	ubiquitin s	protein-co
lib-C666-l	chr14	24025334	24025334	+	25.38683	NA	non-codin	non-codin	143	NM_00125	79178	Hs.655179	NM_02432	ENSG0000	THTPA	THTP|THTF	thiamine tr	protein-co
lib-C666-l	chr11	1.19E+08	1.19E+08	+	22.23727	NA	promoter-	promoter-	-23	NR_03416	1E+08	Hs.662365	NR_034160		USP2-AS1	THY1-AS1	USP2 antis	ncRNA
lib-C666-l	chr3	1.5E+08	1.5E+08	+	40.21248	NA	promoter-	promoter-	-78	NR_13013	9819	Hs.722470	NM_01477	ENSG0000	TSC22D2	TILZ4a|TIL	TSC22 dor	protein-co
lib-C666-l	chr19	2427814	2427814	+	115.2865	NA	promoter-	promoter-	100	NM_01245	26517	Hs.75056	NM_01245	ENSG0000	TIMM13	TIM13|TIM	translocase	protein-co
lib-C666-l	chr19	2427631	2427631	+	99.93166	NA	TTS (NM_0	TTS (NM_0	283	NM_01245	26517	Hs.75056	NM_01245	ENSG0000	TIMM13	TIM13|TIM	translocase	protein-co
lib-C666-l	chr19	8008634	8008634	+	29.74345	NA	promoter-	promoter-	74	NM_00635	10469	Hs.465784	NM_00635	ENSG0000	TIMM44	TIM44	translocase	protein-co
lib-C666-l	chr19	39971466	39971466	+	31.07983	NA	exon (NM_	exon (NM_	414	NM_00132	92609	Hs.590956	NM_00100	ENSG0000	TIMM50	TIM50|TIM	translocase	protein-co
lib-C666-l	chr1	1.68E+08	1.68E+08	+	36.90456	NA	promoter-	promoter-	-126	NM_00103	261726	Hs.209431	NM_15290	ENSG0000	TIPRL	TIP|TIP41|T	TOR signal	protein-co
lib-C666-l	chr12	1.17E+08	1.17E+08	+	39.72046	NA	promoter-	promoter-	-85	NM_00110	84900	Hs.437195	NM_03281	ENSG0000	RNFT2	TMEM118	ring finger	protein-co
lib-C666-l	chr16	75498637	75498637	+	20.94772	NA	promoter-	promoter-	-2	NM_14525	124491	Hs.487510	NM_14525	ENSG0000	TMEM170	TMEM170	transmemb	protein-co
lib-C666-l	chr2	2.2E+08	2.2E+08	+	198.6326	NA	promoter-	promoter-	-14	NM_00100	130612	Hs.446664	NM_00100	ENSG0000	TMEM198	TMEM198	transmemb	protein-co
lib-C666-l	chrX	52926252	52926252	+	125.5752	NA	promoter-	promoter-	-70	NM_00132	727866	Hs.6451	NM_00109	ENSG0000	FAM156B	TMEM29B	family with	protein-co
lib-C666-l	chrX	52987782	52987782	+	88.70856	NA	promoter-	promoter-	-67	NM_00132	727866	Hs.6451	NM_00109	ENSG0000	FAM156B	TMEM29B	family with	protein-co
lib-C666-l	chr2	2.33E+08	2.33E+08	+	27.68428	NA	Intergenic	CpG	-1760	NM_00282	5757	Hs.459927	NM_00282	ENSG0000	PTMA	TMSA	prothymos	protein-co
lib-C666-l	chr11	73087392	73087392	+	51.74843	NA	promoter-	promoter-	-13	NM_15222	84957	Hs.533720	NM_03287	ENSG0000	RELT	TNFRSF19	RELT tumo	protein-co
lib-C666-l	chr3	1.33E+08	1.33E+08	+	21.88699	NA	promoter-	promoter-	-59	NM_00702	11073	Hs.593379	NM_00702	ENSG0000	TOPBP1	TOP2BP1	topoisome	protein-co
lib-C666-l	chr13	1.03E+08	1.03E+08	+	76.37573	NA	promoter-	promoter-	-60	NM_00329	7174	Hs.432424	NM_00329	ENSG0000	TPP2	TPP-2|TPP	tripeptidyl	protein-co
lib-C666-l	chr5	1.59E+08	1.59E+08	+	129.173	NA	promoter-	promoter-	-43	NM_00331	7265	Hs.519718	NM_00331	ENSG0000	TTC1	TPR1	tetratricop	protein-co
lib-C666-l	chr1	36689890	36689890	+	82.1828	NA	promoter-	promoter-	-123	NM_00132	9967	Hs.744057	NM_00511	ENSG0000	THRAP3	TRAP150	thyroid ho	protein-co
lib-C666-l	chr3	1.56E+08	1.56E+08	+	25.68529	NA	promoter-	promoter-	-28	NM_00710	6747	Hs.518346	NM_00710	ENSG0000	SSR3	TRAPG	signal sequ	protein-co
lib-C666-l	chr9	1.13E+08	1.13E+08	+	26.26478	NA	intron (NM	CpG	977	NM_00332	7295	Hs.435136	NM_00332	ENSG0000	TXN	TRDX|TRX|	thioredoxir	protein-co
lib-C666-l	chr22	29166640	29166640	+	25.39487	NA	promoter-	promoter-	-80	NM_00107	7494	Hs.437638	NM_00508	ENSG0000	XBP1	TREB-5|TR	X-box binc	protein-co
lib-C666-l	chr19	13227546	13227546	+	66.27789	NA	promoter-	promoter-	17	NM_00113	55621	Hs.515169	NM_01772	ENSG0000	TRMT1	TRM1	tRNA meth	protein-co
lib-C666-l	chr8	1.25E+08	1.25E+08	+	41.84685	NA	promoter-	promoter-	17	NM_01795	55039	Hs.9925	NM_01795	ENSG0000	TRMT12	TRM12|TY	tRNA meth	protein-co
lib-C666-l	chr21	44299680	44299680	+	177.7008	NA	promoter-	promoter-	13	NM_00126	10785	Hs.248815	NM_01866	ENSG0000	WDR4	TRM82|TRI	WD repeat	protein-co
lib-C666-l	chr19	45681648	45681648	+	89.84392	NA	promoter-	promoter-	-147	NM_02410	79090	Hs.466929	NM_02410	ENSG0000	TRAPPC6A	TRS33	trafficking	protein-co
lib-C666-l	chr11	18548497	18548497	+	43.7015	NA	promoter-	promoter-	6	NM_00629	7251	Hs.523512	NM_00629	ENSG0000	TSG101	TSG10|VPS	tumor susc	protein-co
lib-C666-l	chr5	94891128	94891128	+	23.1274	NA	promoter-	promoter-	303	NM_19815	153642	Hs.585051	NM_19815	ENSG0000	ARSK	TSULF	arylsulfatas	protein-co
lib-C666-l	chr22	28315361	28315361	+	28.32976	NA	promoter-	promoter-	-3	NR_02696	284900	Hs.602319	NR_02696	ENSG0000	TTC28-AS	TTC28-AS|	TTC28 anti	ncRNA
lib-C666-l	chr6	34725249	34725249	+	75.47253	NA	non-codin	non-codin	378	NR_02947	6631	Hs.1063	NM_00309	ENSG0000	SNRPC	U1C|Yhc1	small nucle	protein-co
lib-C666-l	chr19	36236379	36236379	+	39.64852	NA	promoter-	promoter-	-36	NR_11017	199746	Hs.351558	NM_14498	ENSG0000	U2AF1L4	U2AF1-RS	U2 small n	protein-co
lib-C666-l	chr19	56165079	56165079	+	38.21529	NA	promoter-	promoter-	-337	NM_00101	11338	Hs.528007	NM_00727	ENSG0000	U2AF2	U2AF65	U2 small n	protein-co
lib-C666-l	chr19	1021710	1021710	+	132.3097	NA	promoter-	promoter-	189	NR_12573	1.04E+08		NR_12573	ENSG0000	RNU6-2	U6-2	RNA, U6 sr	snRNA
lib-C666-l	chr9	34049126	34049126	+	147.9688	NA	promoter-	promoter-	-179	NM_00128	55833	Hs.493739	NM_01844	ENSG0000	UBAP2	UBAP-2	ubiquitin a	protein-co
lib-C666-l	chr8	74791109	74791109	+	26.94522	NA	promoter-	promoter-	36	NM_00100	55284	Hs.128841	NM_01829	ENSG0000	UBE2W	UBC-16|U	ubiquitin c	protein-co
lib-C666-l	chr15	82647134	82647134	+	38.57241	NA	promoter-	promoter-	-220	NR_00484	1E+08	Hs.735222	NR_004847		UBE2Q2P2	UBE2Q2P3	ubiquitin c	pseudo
lib-C666-l	chr15	83023592	83023592	+	32.04651	NA	promoter-	promoter-	-249	NR_00484	1E+08	Hs.735222	NR_004847		UBE2Q2P2	UBE2Q2P3	ubiquitin c	pseudo
lib-C666-l	chr21	30397105	30397105	+	43.12596	NA	intron (NM	CpG	167	NM_00644	10600	Hs.99819	NM_00644	ENSG0000	USP16	UBP-M|UB	ubiquitin s	protein-co
lib-C666-l	chr4	4543757	4543757	+	42.49125	NA	promoter-	promoter-	36	NM_00134	53407	Hs.584913	NM_01693	ENSG0000	STX18	Ufe1	syntaxin 18	protein-co
lib-C666-l	chr17	47785503	47785503	+	116.9063	NA	promoter-	promoter-	-190	NM_00582	10237	Hs.154073	NM_00582	ENSG0000	SLC35B1	UGTREL1	solute carr	protein-co
lib-C666-l	chrX	47093063	47093063	+	21.21068	NA	intron (NM	CpG	749	NM_00465	8237	Hs.171501	NM_00465	ENSG0000	USP11	UHX1	ubiquitin s	protein-co
lib-C666-l	chr17	73781049	73781049	+	80.31881	NA	promoter-	promoter-	129	NR_03813	85451	Hs.584768	NM_00108	ENSG0000	UNK	UNKEMPT|	unkempt fa	protein-co
lib-C666-l	chr14	58862855	58862855	+	111.8992	NA	intron (NM	CpG	211	NM_20737	387990	Hs.592307	NM_20737	ENSG0000	TOMM20L	UNQ9438	translocase	protein-co
lib-C666-l	chr1	2.1E+08	2.1E+08	+	92.71857	NA	promoter-	promoter-	74	NM_15248	148304	Hs.497642	NM_15248	ENSG0000	C1orf74	URLC4	chromosor	protein-co
lib-C666-l	chr12	56727878	56727878	+	40.50761	NA	promoter-	promoter-	-41	NM_01487	9924	Hs.273397	NM_01487	ENSG0000	PAN2	USP52	PAN2 poly	protein-co
lib-C666-l	chr15	52122357	52122357	+	29.90332	NA	intron (NM	CpG	532	NM_01454	29766	Hs.4998	NM_01454	ENSG0000	TMOD3	UTMOD	tropomodu	protein-co
lib-C666-l	chr16	2198458	2198458	+	58.59621	NA	promoter-	promoter-	-193	NM_01435	25837	Hs.3797	NM_01435	ENSG0000	RAB26	V46133	RAB26, me	protein-co
lib-C666-l	chr16	2199042	2199042	+	25.39487	NA	intron (NM	CpG	391	NM_01435	25837	Hs.3797	NM_01435	ENSG0000	RAB26	V46133	RAB26, me	protein-co
lib-C666-l	chr9	1.37E+08	1.37E+08	+	85.64357	NA	promoter-	promoter-	-90	NM_00337	7410	Hs.369921	NM_00337	ENSG0000	VAV2	VAV-2	vav guanin	protein-co
lib-C666-l	chr17	41855745	41855745	+	48.61959	NA	intron (NM	CpG	623	NM_00409	1845	Hs.181046	NM_00409	ENSG0000	DUSP3	VHR	dual specif	protein-co
lib-C666-l	chr2	85788660	85788660	+	24.43903	NA	promoter-	promoter-	-3	NM_00082	2677	Hs.77719	NM_00082	ENSG0000	GGCX	VKCFD1	gamma-gl	protein-co

lib-C666-li	chr3	1.3E+08	1.3E+08	+	57.48363	NA	promoter-	promoter-	20	NM_01460	30849	Hs.149032	NM_01460	ENSG0000	PIK3R4	VPS15|p15	phosphoin	protein-co
lib-C666-li	chr2	74685314	74685314	+	22.81978	NA	promoter-	promoter-	-213	NM_01247	23559	Hs.516114	NM_01247	ENSG0000	WBP1	WBP-1	WW doma	protein-co
lib-C666-li	chr14	73393140	73393140	+	20.72609	NA	promoter-	promoter-	100	NM_18134	26094	Hs.331491	NM_01560	ENSG0000	DCAF4	WDR21|WI	DDB1 and	protein-co
lib-C666-li	chr11	9595083	9595083	+	32.2434	NA	promoter-	promoter-	-145	NM_00339	7465	Hs.249441	NM_00339	ENSG0000	WEE1	WEE1A|WE	WEE1 G2 c	protein-co
lib-C666-li	chrX	1.03E+08	1.03E+08	+	20.0808	NA	promoter-	promoter-	-23	NM_15333	90843	Hs.389734	NM_15333	ENSG0000	TCEAL8	WEX3	transcriptic	protein-co
lib-C666-li	chr5	78531902	78531902	+	25.38683	NA	promoter-	promoter-	-23	NM_15240	133746	Hs.482605	NM_15240	ENSG0000	JMY	WHAMM2	junction m	protein-co
lib-C666-li	chr7	1178006	1178006	+	171.6281	NA	promoter-	promoter-	-113	NM_03235	84310	Hs.653258	NM_03235	ENSG0000	C7orf50	YCR016W	chromosor	protein-co
lib-C666-li	chr7	1.34E+08	1.34E+08	+	40.50761	NA	promoter-	promoter-	46	NM_03282	84912	Hs.490181	NM_03282	ENSG0000	SLC35B4	YEA|YEA4	solute carr	protein-co
lib-C666-li	chr4	69215885	69215885	+	73.10241	NA	promoter-	promoter-	-50	NM_13337	91746	Hs.175955	NM_13337	ENSG0000	YTHDC1	YT521|YT5:	YTH doma	protein-co
lib-C666-li	chr9	74980239	74980239	+	70.91142	NA	promoter-	promoter-	-76	NM_00110	7763	Hs.406096	NM_00600	ENSG0000	ZFAND5	ZA20D2|ZF	zinc finger	protein-co
lib-C666-li	chr20	23342623	23342623	+	189.5765	NA	promoter-	promoter-	-146	NM_00131	64412	Hs.709462	NM_02248	ENSG0000	GZF1	ZBTB23|ZN	GDNF indu	protein-co
lib-C666-li	chr5	43121591	43121591	+	52.37	NA	promoter-	promoter-	28	NM_00133	7690	Hs.535804	NM_00343	ENSG0000	ZNF131	ZBTB35|pH	zinc finger	protein-co
lib-C666-li	chr12	56512201	56512201	+	29.08762	NA	promoter-	promoter-	-143	NM_00130	84872	Hs.632706	NM_03278	ENSG0000	ZC3H10	ZC3HDC1(	zinc finger	protein-co
lib-C666-li	chr8	1.45E+08	1.45E+08	+	390.5093	NA	promoter-	promoter-	-84	NM_01511	23144	Hs.521915	NM_01511	ENSG0000	ZC3H3	ZC3HDC3	zinc finger	protein-co
lib-C666-li	chr19	55987909	55987909	+	48.94471	NA	5' UTR (NM	5' UTR (NM	210	NM_03311	89887	Hs.525209	NM_03311	ENSG0000	ZNF628	ZEC|Zfp62{	zinc finger	protein-co
lib-C666-li	chr18	5296938	5296938	+	24.23241	NA	promoter-	promoter-	114	NM_00124	7541	Hs.592340	NM_00340	ENSG0000	ZBTB14	ZF5|ZFP-1(	zinc finger	protein-co
lib-C666-li	chr14	21572001	21572001	+	21.23871	NA	TTS (NM_0	TTS (NM_0	862	NM_00110	51222	Hs.250493	NM_01642	ENSG0000	ZNF219	ZFP219	zinc finger	protein-co
lib-C666-li	chr11	10562770	10562770	+	120.9748	NA	promoter-	promoter-	4	NM_01642	50862	Hs.44685	NM_01642	ENSG0000	RNF141	ZFP26|ZNF	ring finger	protein-co
lib-C666-li	chr8	1.46E+08	1.46E+08	+	27.84631	NA	promoter-	promoter-	-71	NM_00110	58500	Hs.532277	NM_02106	ENSG0000	ZNF250	ZFP647|ZN	zinc finger	protein-co
lib-C666-li	chr7	99102344	99102344	+	30.16874	NA	promoter-	promoter-	77	NM_00131	23660	Hs.110839	NM_01456	ENSG0000	ZKSCAN5	ZFP-95|ZFI	zinc finger	protein-co
lib-C666-li	chr14	69865125	69865125	+	58.68336	NA	promoter-	promoter-	29	NM_00125	55334	Hs.432690	NM_01837	ENSG0000	SLC39A9	ZIP-9|ZIP9	solute carr	protein-co
lib-C666-li	chr7	99097911	99097911	+	72.59265	NA	promoter-	promoter-	20	NM_03216	84124	Hs.386324	NM_03216	ENSG0000	ZNF394	ZKSCAN14	zinc finger	protein-co
lib-C666-li	chr16	4817213	4817213	+	25.49921	NA	promoter-	promoter-	6	NM_00130	26048	Hs.513316	NM_02164	ENSG0000	ZNF500	ZKSCAN18	zinc finger	protein-co
lib-C666-li	chr1	40626967	40626967	+	378.7383	NA	promoter-	promoter-	-74	NM_01242	6018	Hs.205627	NM_01242	ENSG0000	RLF	ZN-15L|ZN	rearrangec	protein-co
lib-C666-li	chr20	18268888	18268888	+	65.5106	NA	promoter-	promoter-	-39	NM_00128	7692	Hs.472221	NM_00343	ENSG0000	ZNF133	ZNF150|pH	zinc finger	protein-co
lib-C666-li	chr17	15602924	15602924	+	56.14594	NA	promoter-	promoter-	33	NM_00113	57335	Hs.585799	NM_02065	ENSG0000	ZNF286A	ZNF286	zinc finger	protein-co
lib-C666-li	chr21	43430365	43430365	+	308.3305	NA	5' UTR (NM	5' UTR (NM	131	NM_00109	49854	Hs.434947	NM_02072	ENSG0000	ZBTB21	ZNF295	zinc finger	protein-co
lib-C666-li	chr7	6617065	6617065	+	31.30805	NA	promoter-	promoter-	0	NM_01810	55146	Hs.5268	NM_01810	ENSG0000	ZDHHC4	ZNF374	zinc finger	protein-co
lib-C666-li	chr19	2900433	2900433	+	27.5466	NA	promoter-	promoter-	-463	NM_17348	126295	Hs.591378	NM_17348	ENSG0000	ZNF57	ZNF424	zinc finger	protein-co
lib-C666-li	chr19	56826096	56826096	+	28.8837	NA	promoter-	promoter-	-163	NM_00132	79149	Hs.177688	NM_02430	ENSG0000	ZSCAN5A	ZNF495|ZS	zinc finger	protein-co
lib-C666-li	chr18	77866954	77866954	+	33.04877	NA	promoter-	promoter-	39	NM_01491	22850	Hs.131915	NM_01491	ENSG0000	ADNP2	ZNF508	ADNP hom	protein-co
lib-C666-li	chr4	4291953	4291953	+	47.24198	NA	promoter-	promoter-	29	NM_14529	166793	Hs.419997	NM_14529	ENSG0000	ZBTB49	ZNF509	zinc finger	protein-co
lib-C666-li	chr16	30389221	30389221	+	35.90556	NA	promoter-	promoter-	-412	NM_00121	197407	Hs.513501	NM_15265	ENSG0000	ZNF48	ZNF553	zinc finger	protein-co
lib-C666-li	chr19	15560692	15560692	+	42.71758	NA	promoter-	promoter-	70	NM_02124	58525	Hs.442138	NM_02124	ENSG0000	WIZ	ZNF803	widely inte	protein-co
lib-C666-li	chr3	1.01E+08	1.01E+08	+	92.70942	NA	promoter-	promoter-	-70	NM_01441	27107	Hs.655286	NM_01441	ENSG0000	ZBTB11	ZNF-U692	zinc finger	protein-co
lib-C666-li	chr5	68389869	68389869	+	29.07066	NA	promoter-	promoter-	93	NM_02405	64924	Hs.631975	NM_02290	ENSG0000	SLC30A5	ZNT5|ZNTI	solute carr	protein-co
lib-C666-li	chr1	26496356	26496356	+	244.3541	NA	promoter-	promoter-	-32	NM_01587	51042	Hs.477273	NM_01587	ENSG0000	ZNF593	ZT86	zinc finger	protein-co
lib-C666-li	chr1	1550763	1550763	+	134.7347	NA	promoter-	promoter-	-32	NM_00117	142678	Hs.135805	NM_08087	ENSG0000	MIB2	ZZANK1|Z/	mindbomb	protein-co
