# Supplementary material for: ZIC2 induces pro-tumor macrophage polarization in nasopharyngeal carcinoma by activating the JUNB/MCSF axis
Source: Cell Death Dis. 2023 Jul 21;14(7):455. doi: 10.1038/s41419-023-05983-x (PMC10362010; doi:10.1038/s41419-023-05983-x)
Supplement: Supplementary file 7 — Supplemental table 4 [file 41419_2023_5983_MOESM7_ESM.pdf]

Supplemental table 4-ZIC2 ChIP-seq data

PeakID (cm	Chr	Start	End	Strand	Peak Score	Focus Ratio	Annotation	Detailed Annotation	Distance to TSS	Nearest Pr	Entrez ID	Nearest Ur	Nearest Re	Nearest En	Gene Nam	Gene Alias	Gene Desc	Gene Type
lib-C666-I	chr3	142166885	142166885	+	62.30347	NA	promoter-TSS (NM_001282859)	promoter-TSS (NM_001282859)	19	NM_00128	54464	Hs.435103	NM_01900	ENSG0000	XRN1	1-Sep	5'-3' exorit	protein-co
lib-C666-I	chr4	120375863	120375863	+	813.45215	NA	promoter-TSS (NR_135112)	promoter-TSS (NR_135112)	-75	NR_03763	645513	Hs.677546	NR_03763	ENSG0000	LOC64551	-	septin 7 ps	pseudo
lib-C666-I	chr7	66309982	66309982	+	502.12091	NA	promoter-TSS (NR_003934)	promoter-TSS (NR_003934)	-169	NR_00393	729156	Hs.709878	NR_00393	ENSG0000	GTF2IRD1F	-	GTF2I repe	pseudo
lib-C666-I	chr19	36119930	36119930	+	499.48486	NA	promoter-TSS (NM_001319113)	promoter-TSS (NM_001319113)	12	NM_02432	79171	Hs.5086	NM_02432	ENSG0000	RBM42	-	RNA bindir	protein-co
lib-C666-I	chr3	10028555	10028555	+	468.72922	NA	promoter-TSS (NR_103821)	promoter-TSS (NR_103821)	-22	NR_10382	442075	Hs.690469	NM_001013730		EMC3-AS1	-	EMC3 antis	ncRNA
lib-C666-I	chr4	119512873	119512873	+	445.87625	NA	promoter-TSS (NR_109983)	promoter-TSS (NR_109983)	-26	NR_10998	729218	Hs.448518	NR_103825		LOC72921	-	uncharacte	pseudo
lib-C666-I	chr7	75795903	75795903	+	428.02643	NA	Intergenic	CpG	-35308	NM_00129	222183	Hs.511025	NM_00103	ENSG0000	SRRM3	-	serine/argi	protein-co
lib-C666-I	chr7	65958574	65958574	+	397.46677	NA	promoter-TSS (NR_134573)	promoter-TSS (NR_134573)	-45	NR_13457	100289098	Hs.662276	NR_13457	ENSG0000	GS1-124K5	-	uncharacte	ncRNA
lib-C666-I	chr16	19079074	19079074	+	393.09094	NA	promoter-TSS (NR_119382)	promoter-TSS (NR_119382)	-61	NR_11938	102723385	Hs.569608	NR_11937	ENSG0000	LOC10272	-	uncharacte	ncRNA
lib-C666-I	chr1	9489054	9489054	+	390.54669	NA	promoter-TSS (NR_104622)	promoter-TSS (NR_104622)	1	NR_10462	100506022	Hs.131775	NR_104622		LOC10050	-	uncharacte	ncRNA
lib-C666-I	chr1	224180344	224180344	+	375.77948	NA	promoter-TSS (NR_132119)	promoter-TSS (NR_132119)	2	NR_13211	441124	Hs.520589	NR_132119		GTF2IP20	-	general tra	pseudo
lib-C666-I	chr11	60609520	60609520	+	366.18008	NA	promoter-TSS (NM_024098)	promoter-TSS (NM_024098)	91	NM_02409	79080	Hs.4253	NM_02409	ENSG0000	CCDC86	-	coiled-coil	protein-co
lib-C666-I	chr1	243265105	243265105	+	361.44632	NA	promoter-TSS (NR_029401)	promoter-TSS (NR_029401)	-59	NR_02940	731275	Hs.722350	NR_02940	ENSG0000	LINC01347	-	long interg	ncRNA
lib-C666-I	chr4	492961	492961	+	338.32547	NA	promoter-TSS (NR_144334)	promoter-TSS (NR_144334)	-1	NM_13347	170960	Hs.428360	NM_13347	ENSG0000	ZNF721	-	zinc finger	protein-co
lib-C666-I	chr4	153457353	153457353	+	329.08701	NA	promoter-TSS (NR_039658)	promoter-TSS (NR_039658)	-63	NR_03379	54553	Hs.142307	NR_03379	ENSG0000	DKFZP434I	-	uncharacte	ncRNA
lib-C666-I	chr6	17707177	17707177	+	283.31705	NA	promoter-TSS (NR_134618)	promoter-TSS (NR_134618)	-97	NR_13461	105374952	Hs.718703	NR_13461	ENSG0000	LOC10537	-	uncharacte	ncRNA
lib-C666-I	chr2	29093181	29093181	+	272.73685	NA	promoter-TSS (NM_017910)	promoter-TSS (NM_017910)	-6	NM_01791	55006	Hs.468026	NM_01791	ENSG0000	TRMT61B	-	tRNA meth	protein-co
lib-C666-I	chr1	714090	714090	+	265.5108	NA	promoter-TSS (NR_033908)	promoter-TSS (NR_033908)	-22	NR_03390	100288069	Hs.527521	NR_033908		LOC10028	-	uncharacte	ncRNA
lib-C666-I	chr12	64616371	64616371	+	256.3782	NA	promoter-TSS (NM_152440)	promoter-TSS (NM_152440)	-295	NM_15244	144577	Hs.444671	NM_15244	ENSG0000	C12orf66	-	chromosor	protein-co
lib-C666-I	chr1	19536839	19536839	+	255.67276	NA	promoter-TSS (NM_020765)	promoter-TSS (NM_020765)	-41	NR_13511	101927895	Hs.660534	NR_13511	ENSG0000	LOC10192	-	uncharacte	ncRNA
lib-C666-I	chr19	18043773	18043773	+	251.20961	NA	promoter-TSS (NM_001136203)	promoter-TSS (NM_001136203)	-51	NM_00113	115098	Hs.100043	NM_13844	ENSG0000	CCDC124	-	coiled-coil	protein-co
lib-C666-I	chr2	19548228	19548228	+	244.3756	NA	promoter-TSS (NR_039914)	promoter-TSS (NR_039914)	38	NR_03991	100616307		NR_03991	ENSG0000	MIR4757	-	microRNA	ncRNA
lib-C666-I	chr5	176881724	176881724	+	237.41806	NA	intron (NM_030567, intron 2 of 3)	CpG	6398	NM_00117	80758	Hs.534492	NM_03056	ENSG0000	PRR7	-	proline rich	protein-co
lib-C666-I	chr17	38083899	38083899	+	226.30214	NA	promoter-TSS (NM_001320803)	promoter-TSS (NM_001320803)	158	NM_13928	94103	Hs.514151	NM_01647	ENSG0000	ORMDL3	-	ORMDL sp	protein-co
lib-C666-I	chr19	7553885	7553885	+	224.36726	NA	promoter-TSS (NM_001270539)	promoter-TSS (NM_001270539)	39	NM_08066	92960	Hs.515100	NM_08066	ENSG0000	PEX11G	-	peroxisom	protein-co
lib-C666-I	chr19	51182818	51182818	+	224.36726	NA	intron (NM_016148, intron 20 of 22)	intron (NM_016148, intron 20 of 22	30116	NM_00119	342918	Hs.531650	NM_00119	ENSG0000	C19orf81	-	chromosor	protein-co
lib-C666-I	chr16	86588834	86588834	+	216.01685	NA	promoter-TSS (NM_001159380)	promoter-TSS (NM_001159380)	7	NM_00115	64779	Hs.343627	NM_02276	ENSG0000	MTHFSD	-	methenylte	protein-co
lib-C666-I	chr19	16683353	16683353	+	216.01685	NA	promoter-TSS (NM_024881)	promoter-TSS (NM_024881)	-160	NM_02488	79939	Hs.620596	NM_02488	ENSG0000	SLC35E1	-	solute carr	protein-co
lib-C666-I	chr7	65235854	65235854	+	211.379	NA	promoter-TSS (NR_038378)	promoter-TSS (NR_038378)	-57	NR_03837	441242	Hs.373941	NM_00101	ENSG0000	LOC44124	-	uncharacte	ncRNA
lib-C666-I	chr18	2655193	2655193	+	207.95309	NA	promoter-TSS (NM_015295)	promoter-TSS (NM_015295)	201	NR_03375	645158	Hs.712861	NR_03375	ENSG0000	CBX3P2	-	chromobo	pseudo
lib-C666-I	chr19	44100538	44100538	+	202.39555	NA	promoter-TSS (NM_024327)	promoter-TSS (NM_024327)	-6	NM_02432	79177	Hs.11110	NM_02432	ENSG0000	ZNF576	-	zinc finger	protein-co
lib-C666-I	chr7	19748679	19748679	+	196.64772	NA	promoter-TSS (NM_001002926)	promoter-TSS (NM_001002926)	-19	NM_00100	221830	Hs.353035	NM_00100	ENSG0000	TWISTNB	-	TWIST neig	protein-co
lib-C666-I	chr3	119182503	119182503	+	192.3587	NA	promoter-TSS (NM_018266)	promoter-TSS (NM_018266)	26	NM_01826	55254	Hs.594171	NM_01826	ENSG0000	TMEM39A	-	transmemt	protein-co
lib-C666-I	chr1	17066726	17066726	+	192.34555	NA	Intergenic	Intergenic	-5770	NM_00131	729587		NM_00131	ENSG0000	FAM231C	-	family with	protein-co
lib-C666-I	chr3	44803175	44803175	+	186.1922	NA	promoter-TSS (NM_001320334)	promoter-TSS (NM_001320334)	-2	NM_02069	57456	Hs.744955	NM_02069	ENSG0000	KIAA1143	-	KIAA1143	protein-co
lib-C666-I	chr3	48488076	48488076	+	179.33647	NA	promoter-TSS (NM_001271023)	promoter-TSS (NM_001271023)	-38	NM_03216	84126	Hs.694840	NM_03216	ENSG0000	ATRIP	-	ATR intera	protein-co
lib-C666-I	chr20	306043	306043	+	176.70418	NA	promoter-TSS (NM_006943)	promoter-TSS (NM_006943)	-147	NR_10999	100507459	Hs.659689	NR_109990		NRSN2-AS	-	NRSN2 ant	ncRNA
lib-C666-I	chr1	28052341	28052341	+	176.37	NA	promoter-TSS (NM_001143912)	promoter-TSS (NM_001143912)	-149	NM_00114	199870	Hs.469359	NM_15266	ENSG0000	FAM76A	-	family with	protein-co
lib-C666-I	chr3	196295462	196295462	+	175.7709	NA	promoter-TSS (NM_001105573)	promoter-TSS (NM_001105573)	83	NM_00134	348793	Hs.385865	NM_18262	ENSG0000	WDR53	-	WD repeat	protein-co
lib-C666-I	chr9	125027123	125027123	+	171.19254	NA	promoter-TSS (NM_033117)	promoter-TSS (NM_033117)	20	NR_02712	92400	Hs.415842	NM_03311	ENSG0000	RBM18	-	RNA bindir	protein-co
lib-C666-I	chr16	48644187	48644187	+	169.57268	NA	promoter-TSS (NM_153029)	promoter-TSS (NM_153029)	-67	NM_15302	9683	Hs.511839	NM_15302	ENSG0000	N4BP1	-	NEDD4 bir	protein-co
lib-C666-I	chr9	6015654	6015654	+	166.77919	NA	promoter-TSS (NM_001243202)	promoter-TSS (NM_001243202)	-14	NM_00124	26953	Hs.167496	NM_01241	ENSG0000	RANBP6	-	RAN bindir	protein-co
lib-C666-I	chr1	249133185	249133185	+	165.39861	NA	intron (NM_024836, intron 1 of 3)	CpG	808	NM_02483	79894	Hs.521151	NM_02483	ENSG0000	ZNF672	-	zinc finger	protein-co
lib-C666-I	chr1	27332738	27332738	+	165.29108	NA	exon (NM_052943, exon 2 of 2)	exon (NM_052943, exon 2 of 2)	6595	NM_05294	115572	Hs.632378	NM_05294	ENSG0000	FAM46B	-	family with	protein-co
lib-C666-I	chr19	1240313	1240313	+	164.65242	NA	Intergenic	CpG	-1436	NM_00100	513	Hs.418668	NM_00168	ENSG0000	ATP5D	-	ATP syntha	protein-co
lib-C666-I	chr4	164088115	164088115	+	161.38084	NA	promoter-TSS (NM_001128931)	promoter-TSS (NM_001128931)	-42	NM_13838	92345	Hs.129095	NM_13838	ENSG0000	NAF1	-	nuclear ass	protein-co
lib-C666-I	chr3	186288337	186288337	+	161.01898	NA	promoter-TSS (NM_001134415)	promoter-TSS (NM_001134415)	-5	NM_00128	55171	Hs.518469	NM_01813	ENSG0000	TBCCD1	-	TBCC dom	protein-co
lib-C666-I	chr7	124569968	124569968	+	160.63332	NA	promoter-TSS (NM_001042594)	promoter-TSS (NM_001042594)	41	NR_12571	401398	Hs.658551	NM_00102	ENSG0000	POT1-AS1	-	POT1 antis	ncRNA
lib-C666-I	chr18	77439687	77439687	+	156.72173	NA	promoter-TSS (NR_136643)	promoter-TSS (NR_136643)	58	NR_13664	284241		NR_13664	ENSG0000	LOC28424	-	uncharacte	ncRNA
lib-C666-I	chr1	182585442	182585442	+	153.96431	NA	non-coding (NR_036490, exon 1 of 1	non-coding (NR_036490, exon 1 of	1167	NR_03649	284648	Hs.253475	NR_03649	ENSG0000	LINC01686	-	long interg	ncRNA
lib-C666-I	chr1	27333204	27333204	+	147.1828	NA	exon (NM_052943, exon 2 of 2)	exon (NM_052943, exon 2 of 2)	6129	NM_05294	115572	Hs.632378	NM_05294	ENSG0000	FAM46B	-	family with	protein-co
lib-C666-I	chr7	6144358	6144358	+	145.25911	NA	promoter-TSS (NM_032172)	promoter-TSS (NM_032172)	-192	NM_03217	84132	Hs.31856	NM_03217	ENSG0000	USP42	-	ubiquitin s	protein-co
lib-C666-I	chr3	195361917	195361917	+	142.75381	NA	Intergenic	CpG	17113	NR_13618	105374297	Hs.655175	NR_136185		LOC10537	-	uncharacte	ncRNA
lib-C666-I	chr8	37605586	37605586	+	141.24567	NA	promoter-TSS (NR_003671)	promoter-TSS (NR_003671)	-22	NR_00367	728024	Hs.647135	NR_003671		LOC72802	-	chromosor	pseudo
lib-C666-I	chr16	30007399	30007399	+	139.33017	NA	promoter-TSS (NM_003609)	promoter-TSS (NM_003609)	18	NM_00360	8479	Hs.567370	NM_00360	ENSG0000	HIRIP3	-	HIRA intera	protein-co
lib-C666-I	chr11	66112607	66112607	+	138.23872	NA	promoter-TSS (NM_015399)	promoter-TSS (NM_015399)	-25	NM_01539	25855	Hs.100426	NM_01539	ENSG0000	BRMS1	-	breast canc	protein-co
lib-C666-I	chr22	19159185	19159185	+	137.26332	NA	promoter-TSS (NR_103767)	promoter-TSS (NR_103767)	-34	NR_10376	100652736	Hs.572896	NR_103767		LINC01311	-	long interg	ncRNA
lib-C666-I	chr6	150255586	150255586	+	134.24866	NA	promoter-TSS (NR_134598)	promoter-TSS (NR_134598)	-63	NR_13459	105378052	Hs.549678	NR_13459	ENSG0000	LOC10537	-	uncharacte	ncRNA
lib-C666-I	chr5	95297791	95297791	+	133.10905	NA	promoter-TSS (NM_012081)	promoter-TSS (NM_012081)	-16	NM_01208	22936	Hs.192221	NM_01208	ENSG0000	ELL2	-	elongation	protein-co
lib-C666-I	chr5	138061036	138061036	+	131.12344	NA	Intergenic	Intergenic	27962	NR_13424	105379194	Hs.445981	NR_13424	ENSG0000	LOC10537	-	uncharacte	ncRNA
lib-C666-I	chr4	8430248	8430248	+	130.83212	NA	intron (NM_003501, intron 1 of 17)	CpG	12204	NM_00350	8310	Hs.479122	NM_00350	ENSG0000	ACOX3	-	acyl-CoA c	protein-co
lib-C666-I	chr5	175964933	175964933	+	127.71187	NA	promoter-TSS (NM_014901)	promoter-TSS (NM_014901)	-512	NM_01490	22838	Hs.434888	NM_01490	ENSG0000	RNF44	-	ring finger	protein-co
lib-C666-I	chr21	44847231	44847231	+	125.50276	NA	promoter-TSS (NM_173354)	promoter-TSS (NM_173354)	-162	NM_00132	102724428	Hs.282113	NM_00132	ENSG0000	LOC10272	-	serine/thre	protein-co
lib-C666-I	chr12	31882076	31882076	+	125.18858	NA	promoter-TSS (NM_001113402)	promoter-TSS (NM_001113402)	32	NR_10352	196394	Hs.585084	NM_00111	ENSG0000	AMN1	-	antagonist	protein-co
lib-C666-I	chr7	74508949	74508949	+	124.94323	NA	intron (NM_173537, intron 1 of 15).2	CpG	602	NM_00100	389524	Hs.647017	NM_00100	ENSG0000	GTF2IRD2E	-	GTF2I repe	protein-co
lib-C666-I	chr13	101327252	101327252	+	123.83268	NA	promoter-TSS (NM_001079669)	promoter-TSS (NM_001079669)	-63	NM_00107	84899	Hs.190983	NM_03281	ENSG0000	TMTC4	-	transmemt	protein-co
lib-C666-I	chr2	114195313	114195313	+	120.46037	NA	promoter-TSS (NM_172003)	promoter-TSS (NM_172003)	45	NM_17200	150472	Hs.531330	NM_17200	ENSG0000	CBWD2	-	COBW dor	protein-co
lib-C666-I	chr17	4607473	4607473	+	119.76316	NA	promoter-TSS (NR_103482)	promoter-TSS (NR_103482)	-52	NR_10348	101559451	Hs.634966	NR_10348	ENSG0000	LOC10155	-	uncharacte	ncRNA
lib-C666-I	chr19	49164409	49164409	+	119.73698	NA	TTS (NM_145807)	TTS (NM_145807)	11929	NM_14580	126147	Hs.326217	NM_14580	ENSG0000	NTN5	-	netrin 5	protein-co
lib-C666-I	chr13	21872144	21872144	+	119.48137	NA	promoter-TSS (NR_038939)	promoter-TSS (NR_038939)	-120	NR_04646	650794	Hs.147563	NR_038939		MIPEPP3	-	mitochond	pseudo
lib-C666-I	chr1	70820664	70820664	+	119.46529	NA	promoter-TSS (NM_030816)	promoter-TSS (NM_030816)	171	NR_02740	11147	Hs.142245	NM_00707	ENSG0000	HHLA3	-	HERV-H L1	protein-co
lib-C666-I	chr11	62607083	62607083	+	119.33734	NA	promoter-TSS (NM_001307977)	promoter-TSS (NM_001307977)	48	NM_00130	54663	Hs.654620	NM_01809	ENSG0000	WDR74	-	WD repeat	protein-co
lib-C666-I	chr11	65337863	65337863	+	119.02105	NA	promoter-TSS (NR_038923)	promoter-TSS (NR_038923)	21	NR_03892	254100	Hs.595927	NR_038923		SSSCA1-A	-	SSSCA1 an	ncRNA
lib-C666-I	chr19	56186457	56186457	+	116.12865	NA	promoter-TSS (NM_001321263)	promoter-TSS (NM_001321263)	-104	NM_01333	29924	Hs.279953	NM_01333	ENSG0000	EPN1	-	epsin 1	protein-co
lib-C666-I	chr15	71407757	71407757	+	115.28649	NA	promoter-TSS (NM_001102658)	promoter-TSS (NM_001102658)	82	NM_00110	196993	Hs.646533	NM_00110	ENSG0000	CT62	-	cancer/test	protein-co
lib-C666-I	chr12	56862281	56862281	+	111.75975	NA	promoter-TSS (NM_207344)	promoter-TSS (NM_207344)	-20	NM_20734	283377	Hs.128676	NM_20734	ENSG0000	SPRYD4	-	SPRY dom	protein-co
lib-C666-I	chr3	33759692	33759692	+	110.45969	NA	promoter-TSS (NM_015097)	promoter-TSS (NM_015097)	13	NM_01509	23122	Hs.108614	NM_01509	ENSG0000	CLASP2	-	cytoplasmi	protein-co
lib-C666-I	chr9	88742510	88742510	+	110.05312	NA	promoter-TSS (NR_110996)	promoter-TSS (NR_110996)	55	NR_11099	101927623	Hs.575644	NR_110996		LOC10192	-	uncharacte	ncRNA
lib-C666-I	chr19	9732019	9732019	+	109.47309	NA	promoter-TSS (NM_152289)	promoter-TSS (NM_152289)	-80	NM_00133	93134	Hs.720081	NM_15228	ENSG0000	ZNF561	-	zinc finger	protein-co
lib-C666-I	chr17	2615284	2615284	+	107.63474	NA	promoter-TSS (NM_015229)	promoter-TSS (NM_015229)	-319	NR_13641	105371592	Hs.731739	NR_13641	ENSG0000	LOC10537	-	uncharacte	ncRNA
lib-C666-I	chr2	70369922	70369922	+	107.08224	NA	Intergenic	CpG	-17474	NR_02444	100133985	Hs.135528	NR_02444	ENSG0000	LINC01816	-	long interg	ncRNA
lib-C666-I	chr2	114514313	114514313	+	106.65726	NA	promoter-TSS (NM_001330314)	promoter-TSS (NM_001330314)	-43	NM_00133	80255	Hs.355655	NM_02518	ENSG0000	SLC35F5	-	solute carr	protein-co
lib-C666-I	chr16	67927136	67927136	+	106.65517	NA	promoter-TSS (NM_006742)	promoter-TSS (NM_006742)	-39	NM_00674	5681	Hs.513683	NM_00674	ENSG0000	PSKH1	-	protein ser	protein-co
lib-C666-I	chr1	156475241	156475241	+	106.05028	NA	Intergenic	CpG	-4607	NM_00592	4209	Hs.314327	NM_00592	ENSG0000	MEF2D	-	myocyte er	protein-co
lib-C666-I	chr14	68086496	68086496	+	105.62778	NA	promoter-TSS (NM_001172)	promoter-TSS (NM_001172)	-83	NM_00117	384	Hs.226007	NM_00117	ENSG0000	ARG2	-	arginase 2	protein-co
lib-C666-I	chr7	66119377	66119377	+	104.82721	NA	promoter-TSS (NR_110037)	promoter-TSS (NR_110037)	-129	NR_11003	100996437	Hs.667408	NR_110037		LOC10099	-	uncharacte	ncRNA
lib-C666-I	chr10	120183521	120183521	+	102.51401	NA	Intergenic	Intergenic	66901	NR_10804	100506126	Hs.69494	NR_10804	ENSG0000	LINC00867	-	long interg	ncRNA
lib-C666-I	chr9	34666087	34666087	+	101.74994	NA	promoter-TSS (NM_001320038)	promoter-TSS (NM_001320038)	-34	NR_13514	730098	Hs.459590	NM_00132	ENSG0000	LOC73009	-	uncharacte	protein-co
lib-C666-I	chr9	126759082	126759082	+	101.59872	NA	Intergenic	Intergenic	12593	NR_13512	100505588	Hs.547168	NR_13512	ENSG0000	LOC10050	-	uncharacte	ncRNA
lib-C666-I	chr17	43325032	43325032	+	101.15087	NA	promoter-TSS (NR_024434)	promoter-TSS (NR_024434)	-260	NR_11032	100133991	Hs.668927	NR_02443	ENSG0000	MAP3K14-	-	MAP3K14	ncRNA
lib-C666-I	chr19	47759820	47759820	+	100.06794	NA	promoter-TSS (NM_015603)	promoter-TSS (NM_015603)	89	NM_01560	26093	Hs.227782	NM_01560	ENSG0000	CCDC9	-	coiled-coil	protein-co
lib-C666-I	chr1	245134339	245134339	+	99.63164	NA	promoter-TSS (NR_111907)	promoter-TSS (NR_111907)	-168	NR_11190	101928068	Hs.134857	NR_111907		LOC10192	-	uncharacte	ncRNA
lib-C666-I	chr11	111749976	111749976	+	99.40627	NA	promoter-TSS (NM_022761)	promoter-TSS (NM_022761)	28	NM_00133	64776	Hs.17546	NM_02276	ENSG0000	C11orf1	-	chromosor	protein-co
lib-C666-I	chr22	42670248	42670248	+	99.34653	NA	non-coding (NR_036498, exon 2 of 2	non-coding (NR_036498, exon 2 of	4489	NR_03649	388906	Hs.730075	NR_03649	ENSG0000	OGFRP1	-	opioid gro	pseudo
lib-C666-I	chr1	153095528	153095528	+	98.7459	NA	Intergenic	L1PBa|LINE|L1	-9537	NM_00101	6705	Hs.490252	NM_00101	ENSG0000	SPRR2F	-	small proli	protein-co
lib-C666-I	chr19	51162297	51162297	+	98.7459	NA	TTS (NM_016148)	TTS (NM_016148)	9595	NM_00119	342918	Hs.531650	NM_00119	ENSG0000	C19orf81	-	chromosor	protein-co
lib-C666-I	chr16	90039220	90039220	+	98.7459	NA	promoter-TSS (NM_145039)	promoter-TSS (NM_145039)	20	NM_14503	92806	Hs.513832	NM_14503	ENSG0000	CENPBD1	-	CENPB DN	protein-co
lib-C666-I	chr8	38243950	38243950	+	98.60414	NA	promoter-TSS (NM_001286821)	promoter-TSS (NM_001286821)	-9	NM_00128	137994	Hs.696457	NM_14465	ENSG0000	LETM2	-	leucine zip	protein-co
lib-C666-I	chr5	80597365	80597365	+	96.50365	NA	promoter-TSS (NR_034121)	promoter-TSS (NR_034121)	23	NR_03412	100131067	Hs.655855	NR_03412	ENSG0000	CKMT2-AS	-	CKMT2 ant	ncRNA
lib-C666-I	chr1	148241142	148241142	+	96.50365	NA	intron (NM_001037501, intron 6 of 19	CpG	-38606	NM_00116	728945		NM_00116	ENSG0000	PPIAL4F	-	peptidylpro	protein-co
lib-C666-I	chr19	57751995	57751995	+	95.9062	NA	promoter-TSS (NM_001023563)	promoter-TSS (NM_001023563)	-58	NM_00102	390980	Hs.22488	NM_00102	ENSG0000	ZNF805	-	zinc finger	protein-co
lib-C666-I	chr10	43448000	43448000	+	95.73749	NA	Intergenic	Intergenic	28955	NR_12635	104266963	Hs.559152	NR_12635	ENSG0000	LINC01264	-	long interg	ncRNA
lib-C666-I	chr22	46431920	46431920	+	94.83107	NA	Intergenic	CpG	8828	NR_02703	100271722	Hs.528519	NR_027036		LINC00899	-	long interg	ncRNA
lib-C666-I	chr6	34664778	34664778	+	94.27481	NA	promoter-TSS (NR_134626)	promoter-TSS (NR_134626)	56	NR_13462	101929243	Hs.643498	NR_13462	ENSG0000	LOC10192	-	uncharacte	ncRNA
lib-C666-I	chr19	45430487	45430487	+	93.50127	NA	intron (NR_028413, intron 1 of 2)	intron (NR_028413, intron 1 of 2)	427	NR_02841	342	Hs.110675	NR_02841	ENSG0000	APOC1P1	-	apolipopro	pseudo
lib-C666-I	chr11	2421606	2421606	+	93.50127	NA	promoter-TSS (NM_001297658)	promoter-TSS (NM_001297658)	-112	NM_00129	10078	Hs.523424	NM_00570	ENSG0000	TSSC4	-	tumor supp	protein-co
lib-C666-I	chr3	5065180	5065180	+	93.3864	NA	Intergenic	Intergenic	-43534	NR_12591	100507582	Hs.434505	NR_03790	ENSG0000	BHLHE40-,	-	BHLHE40 a	ncRNA
lib-C666-I	chr5	1152483	1152483	+	93.07718	NA	Intergenic	Intergenic	26237	NR_10991	101928857	Hs.593732	NR_10991	ENSG0000	CTD-3080	-	uncharacte	ncRNA
lib-C666-I	chr20	23338765	23338765	+	92.39822	NA	promoter-TSS (NR_109884)	promoter-TSS (NR_109884)	-1	NR_10988	100505683	Hs.140295	NR_10988	ENSG0000	LINC01431	-	long interg	ncRNA
lib-C666-I	chr19	9785824	9785824	+	92.39822	NA	promoter-TSS (NM_001130031)	promoter-TSS (NM_001130031)	-48	NM_01765	54811	Hs.371107	NM_01765	ENSG0000	ZNF562	-	zinc finger	protein-co
lib-C666-I	chr2	110371805	110371805	+	91.79062	NA	promoter-TSS (NM_001321504)	promoter-TSS (NM_001321504)	-22	NM_00132	151011	Hs.469615	NM_14471	ENSG0000	10-Sep	-	septin 10	protein-co
lib-C666-I	chr10	102241420	102241420	+	91.58624	NA	intron (NM_003393, intron 4 of 5)	intron (NM_003393, intron 4 of 5)	18608	NM_00339	7479	Hs.421281	NM_00339	ENSG0000	WNT8B	-	Wnt family	protein-co
lib-C666-I	chr15	42349771	42349771	+	89.94785	NA	Intergenic	Intergenic	-6870	NM_00120	123745	Hs.668060	NM_00108	ENSG0000	PLA2G4E	-	phospholip	protein-co
lib-C666-I	chr1	17222240	17222240	+	86.65458	NA	Intergenic	CpG	-6079	NR_13505	105376805	Hs.465405	NR_13505	ENSG0000	LOC10537	-	uncharacte	ncRNA
lib-C666-I	chr16	2802610	2802610	+	85.88258	NA	promoter-TSS (NR_027275)	promoter-TSS (NR_027275)	-9	NR_02727	100128788	Hs.311208	NR_02727	ENSG0000	SRRM2-AS	-	SRRM2 ant	ncRNA
lib-C666-I	chr3	156544081	156544081	+	85.88258	NA	promoter-TSS (NM_001004316)	promoter-TSS (NM_001004316)	-15	NM_00100	389170	Hs.478048	NM_00100	ENSG0000	LEKR1	-	leucine, glu	protein-co
lib-C666-I	chr1	119683053	119683053	+	85.64357	NA	promoter-TSS (NR_125977)	promoter-TSS (NR_125977)	34	NR_12597	101929147	Hs.697821	NR_12597	ENSG0000	LOC10192	-	uncharacte	ncRNA
lib-C666-I	chr19	4247052	4247052	+	83.47607	NA	promoter-TSS (NM_018074)	promoter-TSS (NM_018074)	-15	NM_01807	55702	Hs.21811	NM_01807	ENSG0000	CCDC94	-	coiled-coil	protein-co
lib-C666-I	chr9	93763705	93763705	+	83.07521	NA	Intergenic	Intergenic	73709	NR_03391	100129316	Hs.458154	NR_033912		LOC10012	-	uncharacte	ncRNA
lib-C666-I	chr8	20233866	20233866	+	83.07521	NA	Intergenic	Intergenic	100582	NR_04750	100874051	Hs.385799	NR_04750	ENSG0000	LZTS1-AS1	-	LZTS1 anti	ncRNA
lib-C666-I	chr15	40361550	40361550	+	82.99603	NA	Intergenic	Intergenic	30038	NR_04006	100131089	Hs.386000	NR_04005	ENSG0000	SRP14-AS	-	SRP14 anti	ncRNA
lib-C666-I	chr16	25118457	25118457	+	82.92409	NA	intron (NR_120306, intron 1 of 2)	CpG	4419	NR_12030	102723510	Hs.569596	NR_120306		LCMT1-AS	-	LCMT1 ant	ncRNA
lib-C666-I	chr1	149224539	149224539	+	80.91374	NA	Intergenic	CpG	-62591	NR_11193	388692	Hs.271249	NM_001013644		LOC38869	-	uncharacte	ncRNA
lib-C666-I	chr22	47791500	47791500	+	80.82497	NA	Intergenic	Intergenic	50179	NR_14446	339685	Hs.434351	NR_038922		LOC33968	-	uncharacte	ncRNA
lib-C666-I	chr5	64859125	64859125	+	80.52238	NA	promoter-TSS (NM_001267038)	promoter-TSS (NM_001267038)	59	NM_00127	23398	Hs.121432	NM_01534	ENSG0000	PPWD1	-	peptidylpro	protein-co
lib-C666-I	chr8	102092996	102092996	+	78.98521	NA	Intergenic	CpG	28714	NR_03396	441374	Hs.652045	NR_03396	ENSG0000	FLJ42969	-	uncharacte	ncRNA
lib-C666-I	chr3	5028788	5028788	+	78.90324	NA	Intergenic	CpG	-7142	NR_12591	100507582	Hs.434505	NR_03790	ENSG0000	BHLHE40-,	-	BHLHE40 a	ncRNA
lib-C666-I	chr1	145382459	145382459	+	78.77642	NA	promoter-TSS (NR_125967)	promoter-TSS (NR_125967)	-55	NR_12596	101928979	Hs.735893	NR_12596	ENSG0000	LINC01719	-	long interg	ncRNA
lib-C666-I	chr8	42195983	42195983	+	78.63524	NA	promoter-TSS (NM_002690)	promoter-TSS (NM_002690)	10	NM_00269	5423	Hs.654484	NM_00269	ENSG0000	POLB	-	DNA polyn	protein-co
lib-C666-I	chr1	38156270	38156270	+	78.53495	NA	promoter-TSS (NM_001303030)	promoter-TSS (NM_001303030)	-3	NM_01785	54955	Hs.272673	NM_01785	ENSG0000	C1orf109	-	chromosor	protein-co
lib-C666-I	chr17	4046434	4046434	+	73.89001	NA	promoter-TSS (NM_144611)	promoter-TSS (NM_144611)	-28	NM_14461	124936	Hs.513871	NM_14461	ENSG0000	CYB5D2	-	cytochrom	protein-co
lib-C666-I	chr19	50637175	50637175	+	72.73944	NA	promoter-TSS (NR_024230)	promoter-TSS (NR_024230)	-54	NR_02423	100170224	Hs.446545	NR_024231		SNAR-B1	-	small ILF3/	snRNA
lib-C666-I	chr22	24059563	24059563	+	72.31497	NA	promoter-TSS (NR_024448)	promoter-TSS (NR_024448)	47	NR_02444	91316	Hs.148656	NR_02444	ENSG0000	GUSBP11	-	glucuronid	pseudo
lib-C666-I	chr17	33895010	33895010	+	72.05244	NA	promoter-TSS (NR_135479)	promoter-TSS (NR_135479)	-123	NR_13547	105371743		NR_135479		LINC02001	-	long interg	ncRNA
lib-C666-I	chr15	42264859	42264859	+	72.02663	NA	promoter-TSS (NR_120334)	promoter-TSS (NR_120334)	-102	NR_12033	101928388	Hs.738043	NR_12033	ENSG0000	PLA2G4E-/	-	PLA2G4E a	ncRNA
lib-C666-I	chr5	16934364	16934364	+	71.25437	NA	intron (NM_012334, intron 1 of 40)	intron (NM_012334, intron 1 of 40)	2021	NM_01233	4651	Hs.481720	NM_01233	ENSG0000	MYO10	-	myosin X	protein-co
lib-C666-I	chr4	119606493	119606493	+	70.69921	NA	promoter-TSS (NR_125930)	promoter-TSS (NR_125930)	3	NR_12593	101929741	Hs.636184	NR_12593	ENSG0000	LOC10192	-	uncharacte	ncRNA
lib-C666-I	chr22	25801382	25801382	+	70.00634	NA	Intergenic	CpG	-23838	NM_00113	91355	Hs.634058	NM_18249	ENSG0000	LRP5L	-	LDL recept	protein-co
lib-C666-I	chr2	96676311	96676311	+	69.82498	NA	promoter-TSS (NR_003698)	promoter-TSS (NR_003698)	12	NR_00369	729234	Hs.720108	NR_00369	ENSG0000	FAHD2CP	-	fumarylace	pseudo
lib-C666-I	chr5	7869151	7869151	+	69.79861	NA	promoter-TSS (NM_024091)	promoter-TSS (NM_024091)	-1	NR_07360	79072	Hs.653162	NM_02409	ENSG0000	FASTKD3	-	FAST kinas	protein-co
lib-C666-I	chr2	65058616	65058616	+	69.31723	NA	Intergenic	Intergenic	32149	NR_11022	101927438	Hs.580313	NR_11022	ENSG0000	LINC01800	-	long interg	ncRNA
lib-C666-I	chr9	93196025	93196025	+	69.18568	NA	promoter-TSS (NR_109795)	promoter-TSS (NR_109795)	-254	NR_10979	101927873	Hs.598482	NR_10979	ENSG0000	LINC01508	-	long interg	ncRNA
lib-C666-I	chr15	83183054	83183054	+	69.07846	NA	promoter-TSS (NR_026811).3	promoter-TSS (NR_026811).3	-77	NR_03393	80154	Hs.454640	NM_025084		GOLGA2P1	-	golgin A2	pseudo
lib-C666-I	chr15	72766595	72766595	+	68.88256	NA	promoter-TSS (NR_135678)	promoter-TSS (NR_135678)	-15	NR_13567	105370888	Hs.540241	NR_13567	ENSG0000	LOC10537	-	uncharacte	ncRNA
lib-C666-I	chr1	45956873	45956873	+	68.04845	NA	promoter-TSS (NM_007170)	promoter-TSS (NM_007170)	-33	NM_00717	10420	Hs.591499	NM_00717	ENSG0000	TESK2	-	testis-spec	protein-co
lib-C666-I	chr1	212732428	212732428	+	67.85292	NA	Intergenic	CpG	-6248	NM_00103	467	Hs.460	NM_00167	ENSG0000	ATF3	-	activating t	protein-co
lib-C666-I	chr2	102027525	102027525	+	67.6305	NA	intron (NM_001145664, intron 8 of 1	intron (NM_001145664, intron 8 of	-23560	NM_15383	200407	Hs.732289	NM_15383	ENSG0000	CREG2	-	cellular rep	protein-co
lib-C666-I	chr15	63481613	63481613	+	66.96093	NA	promoter-TSS (NM_016530)	promoter-TSS (NM_016530)	-115	NM_01653	51762	Hs.389733	NM_01653	ENSG0000	RAB8B	-	RAB8B, me	protein-co
lib-C666-I	chr2	5390828	5390828	+	66.42274	NA	Intergenic	LTR16E2|LTR|ERVL	440422	NR_11058	102723818	Hs.128107	NR_110580		LINC01248	-	long interg	ncRNA
lib-C666-I	chr19	59086778	59086778	+	65.54223	NA	promoter-TSS (NR_026052)	promoter-TSS (NR_026052)	12	NR_02605	65996	Hs.541177	NM_02393	ENSG0000	CENPBD1F	-	CENPB DN	pseudo
lib-C666-I	chr4	83822120	83822120	+	65.41075	NA	promoter-TSS (NM_001318120)	promoter-TSS (NM_001318120)	-51	NR_03407	100499177	Hs.728916	NR_03407	ENSG0000	THAP9-AS	-	THAP9 ant	ncRNA
lib-C666-I	chr3	170533335	170533335	+	65.34157	NA	Intergenic	MER1B|DNA|hAT-Charlie	54710	NR_13525	200916	Hs.380933	NM_00109	ENSG0000	RPL22L1	-	ribosomal	protein-co
lib-C666-I	chr3	171528273	171528273	+	65.34157	NA	promoter-TSS (NM_001130081)	promoter-TSS (NM_001130081)	11	NM_00266	5337	Hs.382865	NM_00266	ENSG0000	PLD1	-	phospholip	protein-co
lib-C666-I	chr1	161087836	161087836	+	64.95611	NA	promoter-TSS (NM_001185094)	promoter-TSS (NM_001185094)	-26	NM_00118	4817	Hs.146406	NM_00560	ENSG0000	NIT1	-	nitrilase 1	protein-co
lib-C666-I	chr3	196594786	196594786	+	64.94322	NA	promoter-TSS (NM_001308045)	promoter-TSS (NM_001308045)	59	NM_15269	205564	Hs.240770	NM_15269	ENSG0000	SENP5	-	SUMO1/se	protein-co
lib-C666-I	chr6	10695068	10695068	+	64.17523	NA	promoter-TSS (NR_026737)	promoter-TSS (NR_026737)	-38	NR_02673	347744	Hs.61389	NM_00114	ENSG0000	C6orf52	-	chromosor	protein-co

lib-C666-I	chr19	49285889	49285889	+	64.0179	NA	Intergenic	Intergenic	27073	NM_01911	26291	Hs.283015	NM_01911	ENSG0000	FGF21	-	fibroblast g	protein-co
lib-C666-I	chr17	78389037	78389037	+	63.68534	NA	promoter-TSS (NM_001164638)	promoter-TSS (NM_001164638)	-69	NR_02937(	100294362	Hs.657519	NR_02937(	ENSG0000	LOC10029	-	uncharacte	ncRNA
lib-C666-I	chr19	50143641	50143641	+	63.30923	NA	promoter-TSS (NM_006270)	promoter-TSS (NM_006270)	-241	NM_00627	6237	Hs.515536	NM_00627	ENSG0000	RRAS	-	related RA	protein-co
lib-C666-I	chr4	77870929	77870929	+	62.77214	NA	promoter-TSS (NM_001306147)	promoter-TSS (NM_001306147)	62	NM_01824	55752	Hs.128199	NM_01824	ENSG0000	11-Sep	-	septin 11	protein-co
lib-C666-I	chr17	1886241	1886241	+	62.69942	NA	intron (NM_178568, intron 1 of 1)	intron (NM_178568, intron 1 of 1)	-34769	NR_135642	105371485	Hs.655867	NR_135642	ENSG0000	LOC10537	-	uncharacte	ncRNA
lib-C666-I	chr15	45021089	45021089	+	62.31611	NA	promoter-TSS (NR_125376)	promoter-TSS (NR_125376)	-97	NR_12537(	100419583	Hs.489254	NR_125376		LOC10041	-	ring finger	pseudo
lib-C666-I	chr19	56143206	56143206	+	61.32821	NA	Intergenic	L2a|LINE|L2	-7265	NM_20337	147808	Hs.53996	NM_20337	ENSG0000	ZNF784	-	zinc finger	protein-co
lib-C666-I	chr9	139014896	139014896	+	60.4683	NA	Intergenic	Intergenic	-4165	NM_15283	90120	Hs.287411	NM_15283	ENSG0000	C9orf69	-	chromosor	protein-co
lib-C666-I	chr8	134864070	134864070	+	60.16496	NA	Intergenic	Helitron1Nb_Mam|RC|Helitron	-34669	NR_125424	101927822	Hs.129114	NR_125424	ENSG0000	LOC10192	-	uncharacte	ncRNA
lib-C666-I	chr6	15248932	15248932	+	59.09724	NA	promoter-TSS (NM_001267040)	promoter-TSS (NM_001267040)	-5	NR_120502	100506681	Hs.664092	NR_120502	ENSG0000	JARID2-AS	-	JARID2 ant	ncRNA
lib-C666-I	chr6	26474142	26474142	+	57.42312	NA	intron (NR_038992, intron 3 of 3)	intron (NR_038992, intron 3 of 3)	8595	NR_038992	285819	Hs.575357	NR_038992		LOC28581	-	uncharacte	ncRNA
lib-C666-I	chr2	132250654	132250654	+	57.14708	NA	promoter-TSS (NM_001085365)	promoter-TSS (NM_001085365)	268	NR_026922	150776	Hs.535622	NR_026922	ENSG0000	LOC15077	-	sphingomy	pseudo
lib-C666-I	chr16	67217839	67217839	+	56.63552	NA	promoter-TSS (NM_001040715)	promoter-TSS (NM_001040715)	44	NM_00104	653319	Hs.651201	NM_00104	ENSG0000	KIAA0895L	-	KIAA0895 l	protein-co
lib-C666-I	chrX	118699380	118699380	+	55.75688	NA	promoter-TSS (NM_001170570)	promoter-TSS (NM_001170570)	-8	NM_00117	63932	Hs.248572	NM_02210	ENSG0000	CXorf56	-	chromosor	protein-co
lib-C666-I	chr5	116102378	116102378	+	55.71593	NA	Intergenic	Intergenic	-4473	NR_104678	102467223	Hs.570916	NR_104678		LOC10246	-	uncharacte	ncRNA
lib-C666-I	chr17	58196672	58196672	+	55.62774	NA	Intergenic	Intergenic	-16392	NR_027408	653653	Hs.631741	NR_027408	ENSG0000	LOC65365	-	adaptor re	pseudo
lib-C666-I	chr1	54204991	54204991	+	55.62774	NA	Intergenic	CpG	-5114	NM_14719	148979	Hs.306691	NM_14719	ENSG0000	GLIS1	-	GLIS family	protein-co
lib-C666-I	chr7	150125960	150125960	+	55.1415	NA	Intergenic	Intergenic	-4782	NR_034033	285972	Hs.647110	NR_034033	ENSG0000	LINC00996	-	long interg	ncRNA
lib-C666-I	chr1	27339034	27339034	+	54.92945	NA	exon (NM_052943, exon 1 of 2)	exon (NM_052943, exon 1 of 2)	299	NM_05294	115572	Hs.632378	NM_05294	ENSG0000	FAM46B	-	family with	protein-co
lib-C666-I	chr9	132251047	132251047	+	54.91701	NA	non-coding (NR_038955, exon 1 of 5	non-coding (NR_038955, exon 1 of	108	NR_038955	100506190	Hs.529860	NR_038955		LINC00963	-	long interg	ncRNA
lib-C666-I	chr2	135676261	135676261	+	54.30392	NA	promoter-TSS (NR_037649)	promoter-TSS (NR_037649)	-85	NR_036549	100129961	Hs.729028	NR_036549		CCNT2-AS	-	CCNT2 ant	ncRNA
lib-C666-I	chr19	13906024	13906024	+	54.30392	NA	promoter-TSS (NM_023072)	promoter-TSS (NM_023072)	-250	NM_02307	65249	Hs.466015	NM_02307	ENSG0000	ZSWIM4	-	zinc finger	protein-co
lib-C666-I	chr7	106809180	106809180	+	54.19022	NA	promoter-TSS (NM_001244262)	promoter-TSS (NM_001244262)	-226	NM_00124	26959	Hs.162032	NM_01225	ENSG0000	HBP1	-	HMG-box	protein-co
lib-C666-I	chr1	17231469	17231469	+	54.10892	NA	Intergenic	CpG	-15308	NR_135059	105376805	Hs.465405	NR_13505(	ENSG0000	LOC10537	-	uncharacte	ncRNA
lib-C666-I	chr1	219748012	219748012	+	53.93396	NA	Intergenic	Intergenic	-17346	NR_135822	102723886	Hs.525963	NR_135822		LOC10272	-	uncharacte	ncRNA
lib-C666-I	chr1	78691013	78691013	+	53.56082	NA	Intergenic	ERVL-E-int|LTR|ERVL	-4270	NR_027310	149047	Hs.352665	NM_14470	ENSG0000	MGC27382	-	uncharacte	ncRNA
lib-C666-I	chr10	135122411	135122411	+	53.15281	NA	promoter-TSS (NM_145806)	promoter-TSS (NM_145806)	18	NM_14580	118472	Hs.422113	NM_14580	ENSG0000	ZNF511	-	zinc finger	protein-co
lib-C666-I	chr12	99849296	99849296	+	53.07762	NA	intron (NM_152788, intron 10 of 25)	L1PB1|LINE|L1	-192232	NM_15336	196472	Hs.591011	NM_15336	ENSG0000	FAM71C	-	family with	protein-co
lib-C666-I	chr19	13858654	13858654	+	52.99872	NA	promoter-TSS (NM_030818)	promoter-TSS (NM_030818)	-23	NM_03081	81576	Hs.24998	NM_03081	ENSG0000	CCDC130	-	coiled-coil	protein-co
lib-C666-I	chr19	49163223	49163223	+	52.78568	NA	intron (NR_004401, intron 1 of 4)	intron (NR_004401, intron 1 of 4)	13115	NM_14580	126147	Hs.326217	NM_14580	ENSG0000	NTN5	-	netrin 5	protein-co
lib-C666-I	chr17	7465212	7465212	+	52.74303	NA	promoter-TSS (NM_015670)	promoter-TSS (NM_015670)	-97	NR_03792(	100533955	Hs.129673	NR_037926		SENP3-EIF	-	SENP3-EIF	ncRNA
lib-C666-I	chr15	84899034	84899034	+	52.30908	NA	promoter-TSS (NR_027001)	promoter-TSS (NR_027001)	-114	NR_027001	388152	Hs.405809	NM_20342	ENSG0000	GOLGA2P7	-	golgin A2 p	pseudo
lib-C666-I	chr2	200776031	200776031	+	51.72884	NA	promoter-TSS (NM_153689)	promoter-TSS (NM_153689)	52	NM_15368	205327	Hs.154494	NM_15368	ENSG0000	C2orf69	-	chromosor	protein-co
lib-C666-I	chr1	212458516	212458516	+	51.38887	NA	promoter-TSS (NR_125984)	promoter-TSS (NR_125984)	-153	NR_125984	101929541	Hs.632493	NR_125982	ENSG0000	LOC10192	-	uncharacte	ncRNA
lib-C666-I	chr10	99078920	99078920	+	51.38887	NA	promoter-TSS (NM_005479)	promoter-TSS (NM_005479)	-102	NM_00547	10023	Hs.126057	NM_00547	ENSG0000	FRAT1	-	FRAT1, WN	protein-co
lib-C666-I	chr8	64081042	64081042	+	51.11391	NA	promoter-TSS (NR_102684)	promoter-TSS (NR_102684)	-41	NR_102684	101410533	Hs.163027	NR_102684	ENSG0000	YTHDF3-A	-	YTHDF3 ar	ncRNA
lib-C666-I	chr19	57901273	57901273	+	50.81684	NA	promoter-TSS (NM_001172773)	promoter-TSS (NM_001172773)	55	NM_00117	147694	Hs.126905	NM_15290	ENSG0000	ZNF548	-	zinc finger	protein-co
lib-C666-I	chr19	50610479	50610479	+	50.27643	NA	promoter-TSS (NR_024214).6	promoter-TSS (NR_024214).6	-88	NR_024214	100169951	Hs.723094	NR_024214		SNAR-A3	-	small ILF3/	snRNA
lib-C666-I	chr1	110950417	110950417	+	50.11658	NA	promoter-TSS (NR_102697)	promoter-TSS (NR_102697)	-14	NR_102697	101410535	Hs.559121	NR_102697		LAMTOR5-	-	LAMTOR5	ncRNA
lib-C666-I	chr3	158288903	158288903	+	49.83866	NA	promoter-TSS (NM_001195433)	promoter-TSS (NM_001195433)	-48	NR_104147	100996447	Hs.369810	NR_104147	ENSG0000	LOC10099	-	uncharacte	ncRNA
lib-C666-I	chr19	12721705	12721705	+	49.31026	NA	promoter-TSS (NM_020714)	promoter-TSS (NM_020714)	-27	NM_15335	163049	Hs.720677	NM_15335	ENSG0000	ZNF791	-	zinc finger	protein-co
lib-C666-I	chr10	99094594	99094594	+	48.06386	NA	promoter-TSS (NM_012083)	promoter-TSS (NM_012083)	-136	NM_01208	23401	Hs.140720	NM_01208	ENSG0000	FRAT2	-	FRAT2, WN	protein-co
lib-C666-I	chr9	140196812	140196812	+	47.93446	NA	promoter-TSS (NM_001004354)	promoter-TSS (NM_001004354)	-109	NM_00100	441478	Hs.535075	NM_00100	ENSG0000	NRARP	-	NOTCH-re	protein-co
lib-C666-I	chr19	57862604	57862604	+	47.93181	NA	promoter-TSS (NM_020657)	promoter-TSS (NM_020657)	-38	NM_00129	57343	Hs.287374	NM_02065	ENSG0000	ZNF304	-	zinc finger	protein-co
lib-C666-I	chr7	66057345	66057345	+	47.37465	NA	promoter-TSS (NR_111973)	promoter-TSS (NR_111973)	49	NR_111974	493754	Hs.732409	NR_002933		GS1-124K5	-	RAB guanii	pseudo
lib-C666-I	chr17	73267352	73267352	+	46.79055	NA	promoter-TSS (NM_001242498)	promoter-TSS (NM_001242498)	-28	NR_03652(	100287042	Hs.514470	NR_03652(	ENSG0000	LOC10028	-	uncharacte	ncRNA
lib-C666-I	chr16	67345837	67345837	+	46.69447	NA	intron (NM_001100915, intron 2 of 15	intron (NM_001100915, intron 2 of	14824	NM_00110	146212	Hs.299127	NM_00110	ENSG0000	KCTD19	-	potassium	protein-co
lib-C666-I	chrX	41135395	41135395	+	46.67128	NA	non-coding (NR_135616, exon 2 of 2	non-coding (NR_135616, exon 2 of	403	NR_13561(	105373185	Hs.546119	NR_13561(	ENSG0000	LOC10537	-	uncharacte	ncRNA
lib-C666-I	chr11	76802307	76802307	+	46.4068	NA	intron (NM_004055, intron 2 of 12)	intron (NM_004055, intron 2 of 12)	-11579	NM_00618	4975	Hs.673853	NM_00618	ENSG0000	OMP	-	olfactory m	protein-co
lib-C666-I	chr2	197504223	197504223	+	46.35197	NA	promoter-TSS (NM_001080539)	promoter-TSS (NM_001080539)	-133	NM_00108	284992	Hs.132519	NM_17346	ENSG0000	CCDC150	-	coiled-coil	protein-co
lib-C666-I	chr2	128284206	128284206	+	46.32803	NA	promoter-TSS (NM_017969)	promoter-TSS (NM_017969)	-119	NM_01796	55677	Hs.469879	NM_01796	ENSG0000	IWS1	-	IWS1, SUP	protein-co
lib-C666-I	chr1	9242510	9242510	+	46.14268	NA	promoter-TSS (NR_132742)	promoter-TSS (NR_132742)	-113	NR_132742	106614088	Hs.551837	NR_132742	ENSG0000	MIR34AHG	-	MIR34A hc	ncRNA
lib-C666-I	chr15	82798487	82798487	+	45.88523	NA	promoter-TSS (NR_026811)	promoter-TSS (NR_026811)	-88	NR_103496	80154	Hs.454640	NM_025084		GOLGA2P1	-	golgin A2 p	pseudo
lib-C666-I	chr10	8095645	8095645	+	45.81715	NA	promoter-TSS (NR_024256)	promoter-TSS (NR_024256)	-198	NR_024256	399717	Hs.158992	NM_20742	ENSG0000	GATA3-AS	-	GATA3 ant	ncRNA
lib-C666-I	chr19	48453514	48453514	+	45.79498	NA	promoter-TSS (NR_024221)	promoter-TSS (NR_024221)	-39	NR_024221	100170226	Hs.467061	NR_024221		SNAR-C3	-	small ILF3/	snRNA
lib-C666-I	chr1	249132527	249132527	+	45.65358	NA	5' UTR (NM_024836, exon 1 of 4)	5' UTR (NM_024836, exon 1 of 4)	150	NM_02483	79894	Hs.521151	NM_02483	ENSG0000	ZNF672	-	zinc finger	protein-co
lib-C666-I	chr7	155250210	155250210	+	45.43086	NA	promoter-TSS (NM_001427)	promoter-TSS (NM_001427)	-614	NM_00142	2020	Hs.134989	NM_00142	ENSG0000	EN2	-	engrailed h	protein-co
lib-C666-I	chr17	45973415	45973415	+	45.42569	NA	promoter-TSS (NM_003110)	promoter-TSS (NM_003110)	-101	NM_00311	6668	Hs.514276	NM_00311	ENSG0000	SP2	-	Sp2 transc	protein-co
lib-C666-I	chr4	4249997	4249997	+	45.23903	NA	promoter-TSS (NM_001297551)	promoter-TSS (NM_001297551)	-28	NM_00129	85013	Hs.12845	NM_03292	ENSG0000	TMEM128	-	transmemb	protein-co
lib-C666-I	chr3	121293543	121293543	+	45.23877	NA	intron (NM_001012659, intron 2 of 4)	MER1A|DNA|hAT-Charlie	6765	NM_00101	503582	Hs.224976	NM_00101	ENSG0000	ARGFX	-	arginine-fi	protein-co
lib-C666-I	chr17	71162140	71162140	+	45.09843	NA	intron (NM_001050, intron 1 of 1)	CpG	980	NM_00105	6752	Hs.514451	NM_00105	ENSG0000	SSTR2	-	somatostat	protein-co
lib-C666-I	chr2	87304199	87304199	+	45.09793	NA	promoter-TSS (NR_026846)	promoter-TSS (NR_026846)	-663	NR_026846	285074	Hs.424184	NM_001012626		LOC28507	-	anaphase p	pseudo
lib-C666-I	chr1	70876804	70876804	+	45.09793	NA	promoter-TSS (NM_001190463)	promoter-TSS (NM_001190463)	-97	NM_00119	1491	Hs.19904	NM_00190	ENSG0000	CTH	-	cystathioni	protein-co
lib-C666-I	chr11	62323780	62323780	+	45.09793	NA	Intergenic	Intergenic	3998	NR_037427	100500804		NR_037427		MIR3654	-	microRNA	ncRNA
lib-C666-I	chr3	141944488	141944488	+	45.09793	NA	promoter-TSS (NM_001039547)	promoter-TSS (NM_001039547)	-39	NM_00103	256356	Hs.135904	NM_15277	ENSG0000	GK5	-	glycerol kir	protein-co
lib-C666-I	chr11	62554836	62554836	+	44.9307	NA	promoter-TSS (NM_199337)	promoter-TSS (NM_199337)	-38	NM_19933	374395	Hs.381134	NM_19933	ENSG0000	TMEM179B	-	transmemb	protein-co
lib-C666-I	chr17	40829321	40829321	+	44.5369	NA	promoter-TSS (NR_073573)	promoter-TSS (NR_073573)	-273	NM_02492	79990	Hs.632251	NM_02492	ENSG0000	PLEKHH3	-	pleckstrin h	protein-co
lib-C666-I	chr20	12905956	12905956	+	44.36932	NA	intron (NR_109868, intron 4 of 11)	intron (NR_109868, intron 4 of 11)	11655	NR_109872	102606466	Hs.602196	NR_109872		LOC10260	-	uncharacte	ncRNA
lib-C666-I	chr7	97881607	97881607	+	44.31884	NA	promoter-TSS (NM_015395)	promoter-TSS (NM_015395)	-44	NM_01539	25851	Hs.592281	NM_01539	ENSG0000	TECPR1	-	tectonin be	protein-co
lib-C666-I	chr10	97850013	97850013	+	44.27569	NA	promoter-TSS (NR_038444)	promoter-TSS (NR_038444)	-21	NR_134320	728558	Hs.538374	NR_038444	ENSG0000	ENTPD1-A	-	ENTPD1 ar	ncRNA
lib-C666-I	chrX	133941190	133941190	+	43.81296	NA	promoter-TSS (NM_001170782)	promoter-TSS (NM_001170782)	-15	NM_00117	159091	Hs.269127	NM_13881	ENSG0000	FAM122C	-	family with	protein-co
lib-C666-I	chr2	97760595	97760595	+	43.3097	NA	promoter-TSS (NM_199336)	promoter-TSS (NM_199336)	24	NM_00132	151313	Hs.567723	NM_19933	ENSG0000	FAHD2B	-	fumarylace	protein-co
lib-C666-I	chr5	133304362	133304362	+	43.12596	NA	promoter-TSS (NM_020199)	promoter-TSS (NM_020199)	-18	NR_134247	105379183	Hs.543907	NR_134247		LOC10537	-	uncharacte	ncRNA
lib-C666-I	chr4	106629916	106629916	+	42.49125	NA	promoter-TSS (NM_024751)	promoter-TSS (NM_024751)	-25	NM_02475	79807	Hs.161429	NM_02475	ENSG0000	GSTCD	-	glutathione	protein-co
lib-C666-I	chr20	35918049	35918049	+	42.39316	NA	promoter-TSS (NM_001003897)	promoter-TSS (NM_001003897)	-2	NM_02207	63905	Hs.6126	NM_02207	ENSG0000	MANBAL	-	mannosida	protein-co
lib-C666-I	chr15	40401122	40401122	+	42.39316	NA	promoter-TSS (NM_001003943)	promoter-TSS (NM_001003943)	-37	NM_00100	90427	Hs.591104	NM_03350	ENSG0000	BMF	-	Bcl2 modif	protein-co
lib-C666-I	chr19	49535117	49535117	+	42.35981	NA	promoter-TSS (NR_024244)	promoter-TSS (NR_024244)	-13	NM_03337	114336	Hs.567650	NM_03337	ENSG0000	CGB2	-	chorionic g	protein-co
lib-C666-I	chr11	63580859	63580859	+	42.35981	NA	promoter-TSS (NM_138471)	promoter-TSS (NM_138471)	13	NM_13847	144097	Hs.502793	NM_13847	ENSG0000	C11orf84	-	chromosor	protein-co
lib-C666-I	chr6	170654166	170654166	+	42.11224	NA	intron (NM_032448, intron 4 of 10)	intron (NM_032448, intron 4 of 10)	14317	NR_039787	100616430		NR_039787	ENSG0000	MIR4644	-	microRNA	ncRNA
lib-C666-I	chr19	46009810	46009810	+	41.98367	NA	promoter-TSS (NM_003370)	promoter-TSS (NM_003370)	-878	NM_00337	7408	Hs.515469	NM_00337	ENSG0000	VASP	-	vasodilator	protein-co
lib-C666-I	chr3	9444686	9444686	+	41.98367	NA	intron (NM_001292043, intron 1 of 24	intron (NM_001292043, intron 1 of	5302	NM_00129	55209	Hs.288164	NM_01818	ENSG0000	SETD5	-	SET domai	protein-co
lib-C666-I	chr1	85742257	85742257	+	41.94622	NA	non-coding (NR_045484, exon 1 of 2	non-coding (NR_045484, exon 1 of	216	NR_045484	646626	Hs.193516	NR_045484		LOC64662	-	uncharacte	ncRNA
lib-C666-I	chr5	140937456	140937456	+	41.83575	NA	promoter-TSS (NR_038333)	promoter-TSS (NR_038333)	-422	NR_038333	100505658	Hs.655458	NR_038333	ENSG0000	LOC10050	-	uncharacte	ncRNA
lib-C666-I	chr1	33207250	33207250	+	41.31543	NA	promoter-TSS (NM_020888)	promoter-TSS (NM_020888)	-262	NM_02088	57648	Hs.591502	NM_02088	ENSG0000	KIAA1522	-	KIAA1522	protein-co
lib-C666-I	chr7	86781645	86781645	+	41.28836	NA	promoter-TSS (NM_021145)	promoter-TSS (NM_021145)	-12	NR_136252	101927420	Hs.633960	NR_136252	ENSG0000	LOC10192	-	uncharacte	ncRNA
lib-C666-I	chr17	41116505	41116505	+	41.21722	NA	promoter-TSS (NM_001261434)	promoter-TSS (NM_001261434)	10	NM_00126	80755	Hs.317403	NM_00126	ENSG0000	AARSD1	-	alanyl-tRN	protein-co
lib-C666-I	chr16	3285233	3285233	+	41.17725	NA	promoter-TSS (NM_001145447)	promoter-TSS (NM_001145447)	-47	NM_00114	7752	Hs.632222	NM_00345	ENSG0000	ZNF200	-	zinc finger	protein-co
lib-C666-I	chr3	47324227	47324227	+	41.10975	NA	promoter-TSS (NM_025010)	promoter-TSS (NM_025010)	-103	NM_02501	23276	Hs.517946	NM_02501	ENSG0000	KLHL18	-	kelch like f	protein-co
lib-C666-I	chr6	170102293	170102293	+	40.86121	NA	promoter-TSS (NM_001202550)	promoter-TSS (NM_001202550)	85	NM_00131	387263	Hs.591375	NM_00102	ENSG0000	C6orf120	-	chromosor	protein-co
lib-C666-I	chr19	57831838	57831838	+	40.85015	NA	promoter-TSS (NM_213598)	promoter-TSS (NM_213598)	-27	NM_21359	125919	Hs.202544	NM_21359	ENSG0000	ZNF543	-	zinc finger	protein-co
lib-C666-I	chr22	38004587	38004587	+	40.85015	NA	promoter-TSS (NM_001172688)	promoter-TSS (NM_001172688)	106	NM_00117	26088	Hs.499158	NM_01336	ENSG0000	GGA1	-	golgi assoc	protein-co
lib-C666-I	chr18	3004177	3004177	+	40.41586	NA	intron (NM_014646, intron 1 of 19)	MER11A|LTR|ERVK	7768	NM_01464	9663	Hs.132342	NM_01464	ENSG0000	LPIN2	-	lipin 2	protein-co
lib-C666-I	chr15	82944648	82944648	+	39.94134	NA	promoter-TSS (NR_026811).2	promoter-TSS (NR_026811).2	-98	NR_033936	80154	Hs.454640	NM_025084		GOLGA2P1	-	golgin A2 p	pseudo
lib-C666-I	chr2	219222726	219222726	+	39.94134	NA	promoter-TSS (NR_125777)	promoter-TSS (NR_125777)	-37	NR_125777	103689911		NR_125777		CATIP-AS2	-	CATIP antis	ncRNA
lib-C666-I	chr19	50595922	50595922	+	39.78294	NA	promoter-TSS (NR_024214).2	promoter-TSS (NR_024214).2	-56	NR_024229	100170216	Hs.723094	NR_024229		SNAR-A10	-	small ILF3/	snRNA
lib-C666-I	chr13	78272259	78272259	+	39.60187	NA	promoter-TSS (NR_037438)	promoter-TSS (NR_037438)	-8	NR_037438	100500861		NR_037438	ENSG0000	MIR3665	-	microRNA	ncRNA
lib-C666-I	chr19	50621156	50621156	+	39.41852	NA	promoter-TSS (NR_024214).8	promoter-TSS (NR_024214).8	-59	NR_024226	100169955	Hs.723094	NR_024226		SNAR-A9	-	small ILF3/	snRNA
lib-C666-I	chr11	47869945	47869945	+	39.32295	NA	exon (NM_001318399, exon 1 of 3)	exon (NM_001318399, exon 1 of 3)	151	NM_01523	23279	Hs.643526	NM_01523	ENSG0000	NUP160	-	nucleopori	protein-co
lib-C666-I	chr20	21283954	21283954	+	39.29799	NA	promoter-TSS (NM_012255)	promoter-TSS (NM_012255)	32	NM_01225	22803	Hs.255932	NM_01225	ENSG0000	XRN2	-	5'-3' exorit	protein-co
lib-C666-I	chr14	49030888	49030888	+	39.21146	NA	Intergenic	MER11B|LTR|ERVK	-766671	NR_039990	100506433	Hs.547419	NR_03999(	ENSG0000	LINC00648	-	long interg	ncRNA
lib-C666-I	chr19	38893717	38893717	+	39.19964	NA	promoter-TSS (NM_174905)	promoter-TSS (NM_174905)	-58	NM_17490	147965	Hs.355162	NM_17490	ENSG0000	FAM98C	-	family with	protein-co
lib-C666-I	chr9	138036561	138036561	+	38.90989	NA	Intergenic	Intergenic	-36894	NR_046107	401557	Hs.640138	NR_046107		LOC40155	-	uncharacte	ncRNA
lib-C666-I	chr10	22725876	22725876	+	38.6745	NA	non-coding (NR_036533, exon 1 of 1	non-coding (NR_036533, exon 1 of	982	NR_036533	100499489	Hs.7444	NR_036533		LOC10049	-	uncharacte	ncRNA
lib-C666-I	chr9	138114270	138114270	+	38.6731	NA	Intergenic	Intergenic	40815	NR_046107	401557	Hs.640138	NR_046107		LOC40155	-	uncharacte	ncRNA
lib-C666-I	chr10	75503871	75503871	+	38.60092	NA	promoter-TSS (NM_004922)	promoter-TSS (NM_004922)	-260	NM_19859	9632	Hs.81964	NM_00492	ENSG0000	SEC24C	-	SEC24 hon	protein-co
lib-C666-I	chr5	72252334	72252334	+	38.57241	NA	intron (NM_138782, intron 1 of 25)	CpG	526	NM_00114	115548	Hs.165762	NM_13878	ENSG0000	FCHO2	-	FCH doma	protein-co
lib-C666-I	chr15	99440355	99440355	+	38.30731	NA	intron (NM_000875, intron 4 of 20)	intron (NM_000875, intron 4 of 20)	108530	NM_00110	145814	Hs.592021	NM_00110	ENSG0000	PGPEP1L	-	pyroglutan	protein-co
lib-C666-I	chr7	143892586	143892586	+	38.22284	NA	non-coding (NR_126022, exon 1 of 5	non-coding (NR_126022, exon 1 of	172	NR_126022	101928605	Hs.591830	NR_126022		LOC10192	-	uncharacte	ncRNA
lib-C666-I	chr12	94954797	94954797	+	38.09782	NA	promoter-TSS (NR_049885)	promoter-TSS (NR_049885)	-768	NR_049885	100847031		NR_049885	ENSG0000	MIR5700	-	microRNA	ncRNA
lib-C666-I	chr17	18528966	18528966	+	37.73319	NA	promoter-TSS (NR_036647)	promoter-TSS (NR_036647)	-36	NR_036647	284047	Hs.448012	NR_036647	ENSG0000	CCDC144B	-	coiled-coil	pseudo
lib-C666-I	chr17	4693725	4693725	+	37.73319	NA	3' UTR (NM_001014985, exon 4 of 4)	3' UTR (NM_001014985, exon 4 of 4	1471	NM_00101	388323	Hs.131035	NM_00101	ENSG0000	GLTPD2	-	glycolipid t	protein-co
lib-C666-I	chr14	36789883	36789883	+	37.4886	NA	promoter-TSS (NM_001308110)	promoter-TSS (NM_001308110)	-1	NM_00114	51562	Hs.368647	NM_01658	ENSG0000	MBIP	-	MAP3K12 l	protein-co
lib-C666-I	chr11	13095368	13095368	+	37.48738	NA	Intergenic	Intergenic	64398	NM_00108	644943	Hs.134650	NM_00108	ENSG0000	RASSF10	-	Ras associa	protein-co
lib-C666-I	chr1	672476	672476	+	37.48738	NA	Intergenic	Intergenic	-6745	NR_028327	100133331	Hs.728864	NR_028327		LOC10013	-	uncharacte	ncRNA
lib-C666-I	chr4	169239666	169239666	+	37.47171	NA	intron (NM_017631, intron 1 of 37)	CpG	292	NM_01763	55601	Hs.591710	NM_01763	ENSG0000	DDX60	-	DExD/H-b	protein-co
lib-C666-I	chr9	3526750	3526750	+	37.41784	NA	promoter-TSS (NM_001282116)	promoter-TSS (NM_001282116)	-749	NM_13442	5991	Hs.136829	NM_00291	ENSG0000	RFX3	-	regulatory	protein-co
lib-C666-I	chr19	50528499	50528499	+	37.41784	NA	promoter-TSS (NM_001006656)	promoter-TSS (NM_001006656)	144	NM_00130	51231	Hs.443330	NM_01644	ENSG0000	VRK3	-	vaccinia re	protein-co
lib-C666-I	chr5	9562413	9562413	+	37.0874	NA	Intergenic	Intergenic	13474	NR_003689	100113384		NR_003689		SNORD123	-	small nucle	snoRNA
lib-C666-I	chr3	120461192	120461192	+	37.05378	NA	promoter-TSS (NM_005513)	promoter-TSS (NM_005513)	192	NM_17382	285282	Hs.444360	NM_17382	ENSG0000	RABL3	-	RAB, meml	protein-co
lib-C666-I	chr1	143687129	143687129	+	36.98516	NA	promoter-TSS (NR_111930)	promoter-TSS (NR_111930)	2	NR_111930	100132057	Hs.585189	NR_033189		LOC10013	-	phosphodi	pseudo
lib-C666-I	chr19	58838636	58838636	+	36.85381	NA	promoter-TSS (NR_144444)	promoter-TSS (NR_144444)	-22	NR_144444	105372480	Hs.659382	NR_144444		LOC10537	-	uncharacte	ncRNA
lib-C666-I	chrX	149106807	149106807	+	36.84144	NA	promoter-TSS (NR_027456)	promoter-TSS (NR_027456)	41	NR_027456	100272228	Hs.496916	NR_02745(	ENSG0000	LINC00894	-	long interg	ncRNA
lib-C666-I	chr12	89920055	89920055	+	36.70779	NA	promoter-TSS (NM_001199777)	promoter-TSS (NM_001199777)	-16	NM_00119	100528030	Hs.25130	NM_00119	ENSG0000	POC1B-GA	-	POC1B-GA	protein-co
lib-C666-I	chr16	28722765	28722765	+	36.70747	NA	promoter-TSS (NM_001286478).2	promoter-TSS (NM_001286478).2	24	NM_00131	728689	Hs.567374	NM_00109	ENSG0000	EIF3CL	-	eukaryotic	protein-co
lib-C666-I	chr20	18774721	18774721	+	36.59122	NA	promoter-TSS (NR_026884)	promoter-TSS (NR_026884)	28	NR_026885	100270804	Hs.598768	NR_026885		LOC10027	-	uncharacte	ncRNA
lib-C666-I	chrX	102809877	102809877	+	36.56424	NA	promoter-TSS (NR_131237)	promoter-TSS (NR_131237)	4	NR_131236	105373300		NR_131236		LOC10537	-	uncharacte	ncRNA
lib-C666-I	chr1	16209398	16209398	+	36.30428	NA	intron (NM_015001, intron 3 of 14)	intron (NM_015001, intron 3 of 14)	11754	NR_039973	100616427		NR_039973	ENSG0000	MIR5096	-	microRNA	ncRNA
lib-C666-I	chr15	29968347	29968347	+	36.16835	NA	intron (NR_135221, intron 1 of 1)	intron (NR_135221, intron 1 of 1)	1208	NR_135221	100130111	Hs.525720	NR_135221	ENSG0000	LOC10013	-	uncharacte	ncRNA
lib-C666-I	chr8	125265524	125265524	+	36.16835	NA	Intergenic	Intergenic	-5885	NR_125420	101927588	Hs.617072	NR_12542(	ENSG0000	LOC10192	-	uncharacte	ncRNA
lib-C666-I	chr10	129362413	129362413	+	36.11612	NA	Intergenic	Intergenic	14800	NM_00103	594857	Hs.643423	NM_00103	ENSG0000	NPS	-	neuropept	protein-co
lib-C666-I	chr19	39900210	39900210	+	36.11612	NA	TTS (NR_039755)	TTS (NR_039755)	108	NR_039755	100616163		NR_039755	ENSG0000	MIR4530	-	microRNA	ncRNA
lib-C666-I	chr19	42901157	42901157	+	36.07033	NA	promoter-TSS (NR_073180)	promoter-TSS (NR_073180)	-123	NR_126041	101930071	Hs.567935	NR_126041	ENSG0000	LOC10193	-	uncharacte	ncRNA
lib-C666-I	chr19	18701415	18701415	+	36.07033	NA	intron (NM_001100419, intron 3 of 4)	intron (NM_001100419, intron 3 of	1920	NM_00110	55049	Hs.655317	NM_01796	ENSG0000	C19orf60	-	chromosor	protein-co
lib-C666-I	chr17	40706803	40706803	+	36.07033	NA	promoter-TSS (NR_144402)	promoter-TSS (NR_144402)	17	NR_144402	108783654	Hs.655222	NR_144402		LOC10878	-	uncharacte	ncRNA
lib-C666-I	chr7	91808366	91808366	+	36.07033	NA	non-coding (NR_122109, exon 5 of 5	non-coding (NR_122109, exon 5 of	-13776	NM_00116	401387	Hs.671729	NM_00104	ENSG0000	LRRD1	-	leucine rich	protein-co
lib-C666-I	chr19	49540219	49540219	+	35.90556	NA	promoter-TSS (NR_004383)	promoter-TSS (NR_004383)	-28	NM_03337	114335	Hs.659014	NM_03337	ENSG0000	CGB1	-	chorionic g	protein-co
lib-C666-I	chr17	26662489	26662489	+	35.73373	NA	promoter-TSS (NM_001267774)	promoter-TSS (NM_001267774)	26	NM_00126	90410	Hs.705431	NM_17488	ENSG0000	IFT20	-	intraflagell	protein-co
lib-C666-I	chr15	93347629	93347629	+	35.48542	NA	Intergenic	MER63A|DNA|hAT-Blackjack	8915	NR_033769	728619	Hs.684129	NR_033769		ASB9P1	-	ankyrin rep	pseudo
lib-C666-I	chr4	109541644	109541644	+	35.47771	NA	promoter-TSS (NM_001319236)	promoter-TSS (NM_001319236)	-31	NR_026968	285456	Hs.683863	NM_18259	ENSG0000	RPL34-AS1	-	RPL34 anti	ncRNA
lib-C666-I	chr12	121454316	121454316	+	35.40551	NA	promoter-TSS (NM_001286191)	promoter-TSS (NM_001286191)	-11	NM_00128	64897	Hs.528671	NM_02289	ENSG0000	C12orf43	-	chromosor	protein-co
lib-C666-I	chr17	53342274	53342274	+	35.32367	NA	promoter-TSS (NM_002126)	promoter-TSS (NM_002126)	-47	NM_00212	3131	Hs.196952	NM_00212	ENSG0000	HLF	-	HLF, PAR b	protein-co
lib-C666-I	chr16	87799588	87799588	+	35.28811	NA	promoter-TSS (NM_001184854)	promoter-TSS (NM_001184854)	10	NM_00118	54758	Hs.123450	NM_01756	ENSG0000	KLHDC4	-	kelch dom	protein-co
lib-C666-I	chr13	113863030	113863030	+	35.28811	NA	promoter-TSS (NM_001320655)	promoter-TSS (NM_001320655)	22	NM_00127	8451	Hs.339735	NM_00358	ENSG0000	CUL4A	-	cullin 4A	protein-co
lib-C666-I	chr7	125848	125848	+	35.0374	NA	Intergenic	Intergenic	23590	NR_134325	102723672	Hs.605204	NR_134325		LOC10272	-	uncharacte	ncRNA
lib-C666-I	chr12	132568526	132568526	+	34.92958	NA	promoter-TSS (NR_003290)	promoter-TSS (NR_003290)	-302	NR_003290	347918	Hs.122115	NM_18261	ENSG0000	EP400NL	-	EP400 N-t	pseudo
lib-C666-I	chr16	11734530	11734530	+	34.84785	NA	Intergenic	Intergenic	-27759	NM_00349	8303	Hs.618526	NM_00349	ENSG0000	SNN	-	stannin	protein-co
lib-C666-I	chr17	4046922	4046922	+	34.83329	NA	promoter-TSS (NM_001254755)	promoter-TSS (NM_001254755)	7	NM_00125	124936	Hs.513871	NM_14461	ENSG0000	CYB5D2	-	cytochrom	protein-co
lib-C666-I	chr7	138915852	138915852	+	34.76791	NA	promoter-TSS (NM_173569)	promoter-TSS (NM_173569)	-379	NM_17356	254048	Hs.153458	NM_17356	ENSG0000	UBN2	-	ubinuclein	protein-co
lib-C666-I	chr5	17952049	17952049	+	34.62267	NA	Intergenic	L1PB1|LINE|L1	144666	NR_134286	646241	Hs.407197	NR_134286		LOC64624	-	uncharacte	ncRNA
lib-C666-I	chr11	95523030	95523030	+	34.62267	NA	promoter-TSS (NM_001243776)	promoter-TSS (NM_001243776)	-75	NM_00133	143684	Hs.288304	NM_14466	ENSG0000	FAM76B	-	family with	protein-co
lib-C666-I	chr10	134145635	134145635	+	34.53169	NA	promoter-TSS (NM_001318878)	promoter-TSS (NM_001318878)	21	NM_00114	80313	Hs.119897	NM_03062	ENSG0000	LRRC27	-	leucine rich	protein-co
lib-C666-I	chr15	45571488	45571488	+	34.53169	NA	promoter-TSS (NR_120335)	promoter-TSS (NR_120335)	-68	NR_120335	101928414		NR_120335	ENSG0000	LOC10192	-	uncharacte	ncRNA
lib-C666-I	chr1	28921512	28921512	+	34.3461	NA	TTS (NM_152304)	TTS (NM_152304)	2505	NM_00119	115273	Hs.652321	NM_15230	ENSG0000	RAB42	-	RAB42, me	protein-co
lib-C666-I	chr8	124780663	124780663	+	34.19829	NA	promoter-TSS (NM_001317918)	promoter-TSS (NM_001317918)	-16	NM_00131	157769	Hs.459174	NM_14496	ENSG0000	FAM91A1	-	family with	protein-co
lib-C666-I	chr2	113522317	113522317	+	34.19829	NA	promoter-TSS (NM_001304361)	promoter-TSS (NM_001304361)	-63	NR_130712	150468	Hs.434250	NM_15251	ENSG0000	CKAP2L	-	cytoskeletc	protein-co
lib-C666-I	chr10	121077143	121077143	+	34.12565	NA	intron (NM_005308, intron 1 of 15)	intron (NM_005308, intron 1 of 15)	-60341	NR_039829	100616398		NR_039829	ENSG0000	MIR4681	-	microRNA	ncRNA
lib-C666-I	chr11	118123041	118123041	+	34.0482	NA	promoter-TSS (NM_198275)	promoter-TSS (NM_198275)	42	NM_19827	196264	Hs.15396	NM_19827	ENSG0000	MPZL3	-	myelin pro	protein-co

lib-C666-l	chr8	49427165	49427165	+	34.04345	NA	Intergenic	CpG	-36962	NR_10500	101929268	Hs.683934	NR_10500	ENSG0000	LOC1019	-	uncharacte	ncRNA
lib-C666-l	chr6	30068684	30068684	+	33.95009	NA	Intergenic	CpG	-4333	NR_12647	104533120	Hs.548919	NR_12647	ENSG0000	TRIM31-A	-	TRIM31 an	ncRNA
lib-C666-l	chr16	87297187	87297187	+	33.43742	NA	Intergenic	Intergenic	28871	NR_10466	101928682	Hs.149004	NR_10466	ENSG0000	LOC1019	-	uncharacte	ncRNA
lib-C666-l	chr15	76629100	76629100	+	33.43742	NA	promoter-TSS (NM_145805)	promoter-TSS (NM_145805)	35	NM_14580	64843	Hs.444677	NM_14580	ENSG0000	ISL2	-	ISL LIM ho	protein-co
lib-C666-l	chr9	140205983	140205983	+	33.11719	NA	intron (NM_017820, intron 19 of 21)	intron (NM_017820, intron 19 of 21	-9280	NM_00100	441478	Hs.535075	NM_00100	ENSG0000	NRARP	-	NOTCH-re	protein-co
lib-C666-l	chr19	1241693	1241693	+	33.11264	NA	promoter-TSS (NM_001001975)	promoter-TSS (NM_001001975)	-56	NM_00100	513	Hs.418668	NM_00168	ENSG0000	ATP5D	-	ATP syntha	protein-co
lib-C666-l	chr12	72148649	72148649	+	33.11264	NA	promoter-TSS (NM_014999)	promoter-TSS (NM_014999)	6	NM_01499	23011	Hs.524590	NM_01499	ENSG0000	RAB21	-	RAB21, me	protein-co
lib-C666-l	chr5	172185241	172185241	+	33.11264	NA	5' UTR (NM_001287812, exon 2 of 4)	5' UTR (NM_001287812, exon 2 of 4	2735	NM_00128	101928093	Hs.646902	NM_001287812		LOC1019	-	uncharacte	protein-co
lib-C666-l	chr16	30645704	30645704	+	33.05473	NA	Intergenic	CpG	-16319	NM_00132	78994	Hs.293629	NM_02403	ENSG0000	PRR14	-	proline rich	protein-co
lib-C666-l	chr8	52783429	52783429	+	32.78343	NA	intron (NM_052937, intron 1 of 5)	intron (NM_052937, intron 1 of 5)	28317	NM_00128	115294	Hs.671268	NM_05293	ENSG0000	PCMTD1	-	protein-L-	protein-co
lib-C666-l	chr5	10761350	10761350	+	32.78343	NA	promoter-TSS (NM_001291963)	promoter-TSS (NM_001291963)	37	NM_00439	1611	Hs.75189	NM_00439	ENSG0000	DAP	-	death asso	protein-co
lib-C666-l	chr3	48130556	48130556	+	32.58685	NA	5' UTR (NM_002375, exon 1 of 19)	5' UTR (NM_002375, exon 1 of 19)	213	NM_03088	4134	Hs.517949	NM_00237	ENSG0000	MAP4	-	microtubul	protein-co
lib-C666-l	chr2	38265451	38265451	+	32.38963	NA	intron (NM_144713, intron 10 of 10)	intron (NM_144713, intron 10 of 10	-1967	NR_10271	101410544	Hs.591566	NR_102712		RMDN2-A	-	RMDN2 an	ncRNA
lib-C666-l	chr19	1248729	1248729	+	32.38963	NA	intron (NM_177401, intron 1 of 7)	CpG	177	NM_17740	90007	Hs.465529	NM_17740	ENSG0000	MIDN	-	midnolin	protein-co
lib-C666-l	chr5	41904234	41904234	+	32.29191	NA	promoter-TSS (NM_175921)	promoter-TSS (NM_175921)	-212	NM_17592	285636	Hs.29899	NM_17592	ENSG0000	C5orf51	-	chromosor	protein-co
lib-C666-l	chr14	96968648	96968648	+	32.29191	NA	promoter-TSS (NM_001252006)	promoter-TSS (NM_001252006)	15	NR_12212	730202	Hs.647565	NR_122121		LOC73020	-	uncharacte	ncRNA
lib-C666-l	chr4	120134319	120134319	+	32.11429	NA	promoter-TSS (NR_125931)	promoter-TSS (NR_125931)	-520	NR_12593	101929762	Hs.570735	NR_125931		LOC1019	-	uncharacte	ncRNA
lib-C666-l	chr22	38054486	38054486	+	32.07197	NA	promoter-TSS (NM_020315)	promoter-TSS (NM_020315)	-102	NR_10995	101927051		NR_10995	ENSG0000	LOC1019	-	uncharacte	ncRNA
lib-C666-l	chr16	23160877	23160877	+	32.04651	NA	promoter-TSS (NM_020718)	promoter-TSS (NM_020718)	-286	NM_02071	57478	Hs.183817	NM_02071	ENSG0000	USP31	-	ubiquitin s	protein-co
lib-C666-l	chr19	50615824	50615824	+	31.9103	NA	promoter-TSS (NR_024214).7	promoter-TSS (NR_024214).7	-79	NR_02421	100169951	Hs.723094	NR_024214		SNAR-A3	-	small ILF3/	snRNA
lib-C666-l	chr14	65007134	65007134	+	31.49527	NA	promoter-TSS (NR_110550)	promoter-TSS (NR_110550)	-48	NR_11055	102723809	Hs.667192	NR_11055	ENSG0000	LOC10272	-	uncharacte	ncRNA
lib-C666-l	chr4	119551063	119551063	+	31.45327	NA	promoter-TSS (NR_103825)	promoter-TSS (NR_103825)	-21	NR_10382	729218	Hs.448518	NR_103825		LOC72921	-	uncharacte	pseudo
lib-C666-l	chr17	25568748	25568748	+	31.45327	NA	Intergenic	Intergenic	52274	NR_03974	100616277		NR_03974	ENSG0000	MIR4522	-	microRNA	ncRNA
lib-C666-l	chr19	47178297	47178297	+	31.23797	NA	exon (NM_016457, exon 17 of 18)	exon (NM_016457, exon 17 of 18)	13562	NR_04004	100506068	Hs.434327	NR_04004	ENSG0000	DACT3-AS	-	DACT3 ant	ncRNA
lib-C666-l	chr3	49055872	49055872	+	31.10827	NA	exon (NM_001009996, exon 1 of 12)	exon (NM_001009996, exon 1 of 12	169	NM_00100	55152	Hs.654815	NM_01811	ENSG0000	DALRD3	-	DALR antic	protein-co
lib-C666-l	chr3	62304633	62304633	+	31.0765	NA	promoter-TSS (NR_038281)	promoter-TSS (NR_038281)	-11	NR_03828	100506994	Hs.656620	NR_03828	ENSG0000	PTPRG-AS	-	PTPRG ant	ncRNA
lib-C666-l	chr1	1334828	1334828	+	30.98434	NA	promoter-TSS (NR_015434)	promoter-TSS (NR_015434)	-82	NR_01543	148413	Hs.741357	NR_01543	ENSG0000	LOC14841	-	uncharacte	ncRNA
lib-C666-l	chr6	92009496	92009496	+	30.9373	NA	Intergenic	Intergenic	-221882	NR_03978	100616174		NR_03978	ENSG0000	MIR4643	-	microRNA	ncRNA
lib-C666-l	chr1	118166497	118166497	+	30.542	NA	exon (NM_017709, exon 2 of 2)	exon (NM_017709, exon 2 of 2)	17893	NM_01770	54855	Hs.356216	NM_01770	ENSG0000	FAM46C	-	family with	protein-co
lib-C666-l	chr7	127228491	127228491	+	30.54026	NA	promoter-TSS (NM_001662)	promoter-TSS (NM_001662)	85	NM_00166	381	Hs.653202	NM_00166	ENSG0000	ARF5	-	ADP ribosy	protein-co
lib-C666-l	chr3	177167231	177167231	+	30.4351	NA	intron (NR_047568, intron 1 of 3)	intron (NR_047568, intron 1 of 3)	7522	NR_04756	100505566	Hs.581170	NR_047568		LINC00578	-	long interg	ncRNA
lib-C666-l	chr12	90345189	90345189	+	30.4351	NA	Intergenic	MLT1I|LTR|ERVL-MaLR	3719	NR_13501	105369891	Hs.148998	NR_13501	ENSG0000	LOC10536	-	uncharacte	ncRNA
lib-C666-l	chr8	82754952	82754952	+	30.16874	NA	promoter-TSS (NM_152837)	promoter-TSS (NM_152837)	-431	NM_02213	64089	Hs.492121	NM_02213	ENSG0000	SNX16	-	sorting nex	protein-co
lib-C666-l	chr11	74660255	74660255	+	30.07415	NA	promoter-TSS (NM_001270380)	promoter-TSS (NM_001270380)	-23	NM_18296	143570	Hs.370145	NM_18296	ENSG0000	XRRA1	-	X-ray radia	protein-co
lib-C666-l	chr16	8618430	8618430	+	30.07415	NA	Intergenic	HAL1-2a_MD|LINE|L1	1577	NM_00129	283953	Hs.150849	NM_00114	ENSG0000	TMEM114	-	transmemb	protein-co
lib-C666-l	chr7	128379317	128379317	+	29.98795	NA	promoter-TSS (NM_001199672)	promoter-TSS (NM_001199672)	-29	NM_00119	813	Hs.7753	NM_00121	ENSG0000	CALU	-	calumenin	protein-co
lib-C666-l	chr2	33171937	33171937	+	29.93465	NA	promoter-TSS (NM_206943)	promoter-TSS (NM_206943)	-432	NM_20694	4052	Hs.619315	NM_00062	ENSG0000	LTBP1	-	latent trans	protein-co
lib-C666-l	chr15	72766173	72766173	+	29.93465	NA	promoter-TSS (NM_005744)	promoter-TSS (NM_005744)	407	NR_13567	105370888	Hs.540241	NR_13567	ENSG0000	LOC10537	-	uncharacte	ncRNA
lib-C666-l	chr1	42801011	42801011	+	29.93465	NA	promoter-TSS (NM_001198851)	promoter-TSS (NM_001198851)	-108	NM_01494	22887	Hs.26023	NM_01494	ENSG0000	FOXJ3	-	forkhead b	protein-co
lib-C666-l	chr16	48645855	48645855	+	29.93113	NA	intron (NR_039970, intron 4 of 4)	intron (NR_039970, intron 4 of 4)	-1735	NM_15302	9683	Hs.511839	NM_15302	ENSG0000	N4BP1	-	NEDD4 bir	protein-co
lib-C666-l	chr16	16952079	16952079	+	29.74345	NA	Intergenic	Intergenic	-276282	NR_13517	102723692	Hs.737758	NR_13517	ENSG0000	LOC10272	-	uncharacte	ncRNA
lib-C666-l	chr15	23034395	23034395	+	29.41683	NA	promoter-TSS (NM_001008860)	promoter-TSS (NM_001008860)	32	NM_00118	81614	Hs.591003	NM_03092	ENSG0000	NIPA2	-	non imprin	protein-co
lib-C666-l	chr7	149570688	149570688	+	29.22024	NA	non-coding (NR_110612, exon 1 of 5	non-coding (NR_110612, exon 1 of	263	NR_02704	401431	Hs.556998	NM_00100	ENSG0000	ATP6V0E2	-	ATP6V0E2	ncRNA
lib-C666-l	chr19	10444099	10444099	+	29.21635	NA	TTS (NM_001320608)	TTS (NM_001320608)	215	NM_13345	125950	Hs.744952	NM_13345	ENSG0000	RAVER1	-	ribonucleo	protein-co
lib-C666-l	chr12	72057694	72057694	+	29.17165	NA	promoter-TSS (NM_031435)	promoter-TSS (NM_031435)	17	NM_03143	83591	Hs.245798	NM_03143	ENSG0000	THAP2	-	THAP dom	protein-co
lib-C666-l	chr16	74402191	74402191	+	29.115	NA	promoter-TSS (NR_026950)	promoter-TSS (NR_026950)	-38	NR_02695	283922	Hs.558726	NR_02695	ENSG0000	LOC28392	-	pyruvate d	pseudo
lib-C666-l	chr18	77711693	77711693	+	29.07066	NA	promoter-TSS (NM_025078)	promoter-TSS (NM_025078)	-40	NM_02507	80148	Hs.288284	NM_02507	ENSG0000	PQLC1	-	PQ loop re	protein-co
lib-C666-l	chr1	16840980	16840980	+	29.03946	NA	Intergenic	CpG	21167	NR_13576	100132147	Hs.645945	NR_13576	ENSG0000	LINC01783	-	long interg	ncRNA
lib-C666-l	chr16	2059872	2059872	+	29.01149	NA	promoter-TSS (NM_178167)	promoter-TSS (NM_178167)	-50	NM_17816	90850	Hs.343828	NM_17816	ENSG0000	ZNF598	-	zinc finger	protein-co
lib-C666-l	chr12	2629821	2629821	+	28.97439	NA	intron (NM_001129830, intron 9 of 47	intron (NM_001129830, intron 9 of	151565	NR_04657	100874235	Hs.667644	NR_04657	ENSG0000	CACNA1C-	-	CACNA1C	ncRNA
lib-C666-l	chr2	190627554	190627554	+	28.88523	NA	promoter-TSS (NR_102429)	promoter-TSS (NR_102429)	48	NR_10242	101409258	Hs.738558	NR_102429		OSGEPL1-	-	OSGEPL1 a	ncRNA
lib-C666-l	chr3	194393096	194393096	+	28.88523	NA	5' UTR (NM_018385, exon 1 of 14)	5' UTR (NM_018385, exon 1 of 14)	110	NM_01838	55341	Hs.744061	NM_01838	ENSG0000	LSG1	-	large 60S s	protein-co
lib-C666-l	chr15	41245550	41245550	+	28.88523	NA	5' UTR (NM_001142776, exon 1 of 4)	5' UTR (NM_001142776, exon 1 of 4	203	NM_00114	79094	Hs.155569	NM_02411	ENSG0000	CHAC1	-	ChaC gluta	protein-co
lib-C666-l	chr6	170453422	170453422	+	28.88523	NA	Intergenic	Intergenic	-22444	NR_12587	102724511	Hs.550045	NR_125878		LOC10272	-	uncharacte	ncRNA
lib-C666-l	chr16	3184864	3184864	+	28.8837	NA	promoter-TSS (NM_004220)	promoter-TSS (NM_004220)	19	NR_11090	100507458	Hs.522143	NR_110900		ZNF213-A	-	ZNF213 an	ncRNA
lib-C666-l	chr1	235133155	235133155	+	28.8334	NA	Intergenic	Intergenic	-33409	NR_12594	101927851	Hs.520684	NR_12594	ENSG0000	LOC1019	-	uncharacte	ncRNA
lib-C666-l	chr9	116112927	116112927	+	28.8334	NA	intron (NM_017688, intron 1 of 5)	intron (NM_017688, intron 1 of 5)	1115	NM_00131	54836	Hs.614517	NM_01768	ENSG0000	BSPRY	-	B-box and	protein-co
lib-C666-l	chr18	2655879	2655879	+	28.7732	NA	promoter-TSS (NR_033754)	promoter-TSS (NR_033754)	-7	NM_01529	23347	Hs.8118	NM_01529	ENSG0000	SMCHD1	-	structural r	protein-co
lib-C666-l	chr19	1275759	1275759	+	28.64047	NA	TTS (NM_001300815)	TTS (NM_001300815)	239	NM_01791	55009	Hs.591383	NM_01791	ENSG0000	C19orf24	-	chromosor	protein-co
lib-C666-l	chr10	94050867	94050867	+	28.64047	NA	promoter-TSS (NM_017824)	promoter-TSS (NM_017824)	8	NM_01491	22849	Hs.131683	NM_01491	ENSG0000	CPEB3	-	cytoplasmi	protein-co
lib-C666-l	chr2	152220415	152220415	+	28.57903	NA	intron (NM_007115, intron 1 of 5)	intron (NM_007115, intron 1 of 5)	-4433	NR_03993	100616392		NR_03993	ENSG0000	MIR4773-1	-	microRNA	ncRNA
lib-C666-l	chr12	117581061	117581061	+	28.42633	NA	TTS (NM_033624)	TTS (NM_033624)	43789	NR_12046	101928244	Hs.742266	NR_120464		TESC-AS1	-	TESC antis	ncRNA
lib-C666-l	chr1	159824701	159824701	+	28.42633	NA	exon (NM_001134233, exon 2 of 6)	exon (NM_001134233, exon 2 of 6)	436	NM_00113	284677	Hs.647718	NM_00113	ENSG0000	C1orf204	-	chromosor	protein-co
lib-C666-l	chr22	41809943	41809943	+	28.22301	NA	Intergenic	CpG	32010	NM_00321	7008	Hs.181159	NM_00321	ENSG0000	TEF	-	TEF, PAR b	protein-co
lib-C666-l	chr19	14247716	14247716	+	28.20039	NA	promoter-TSS (NM_018154)	promoter-TSS (NM_018154)	-248	NR_04521	100507373	Hs.720437	NR_04521	ENSG0000	LOC10050	-	uncharacte	ncRNA
lib-C666-l	chr4	16228300	16228300	+	27.84631	NA	promoter-TSS (NM_153365)	promoter-TSS (NM_153365)	14	NR_02769	202020	Hs.445315	NM_15268	ENSG0000	TAPT1-AS	-	TAPT1 anti	ncRNA
lib-C666-l	chr11	67231523	67231523	+	27.7824	NA	TTS (NM_001078650)	TTS (NM_001078650)	5225	NM_00107	80194	Hs.288761	NM_02512	ENSG0000	TMEM134	-	transmemb	protein-co
lib-C666-l	chr14	24028907	24028907	+	27.70954	NA	TTS (NM_001256323)	TTS (NM_001256323)	-1399	NR_11055	102724814	Hs.569375	NR_11055	ENSG0000	LOC10272	-	uncharacte	ncRNA
lib-C666-l	chr1	205895080	205895080	+	27.70954	NA	intron (NM_134325, intron 12 of 21)	intron (NM_134325, intron 12 of 21	17508	NM_13432	115019	Hs.164073	NM_05293	ENSG0000	SLC26A9	-	solute carr	protein-co
lib-C666-l	chr15	85778062	85778062	+	27.70954	NA	Intergenic	CpG	-5579	NM_00131	100133220	Hs.454625	NM_001310153		GOLGA6L3	-	golgin A6 f	protein-co
lib-C666-l	chr6	89298854	89298854	+	27.70954	NA	Intergenic	Intergenic	-49766	NR_11086	101928936	Hs.533080	NR_110867		LOC1019	-	uncharacte	ncRNA
lib-C666-l	chr19	36705638	36705638	+	27.69998	NA	promoter-TSS (NM_152477)	promoter-TSS (NM_152477)	-63	NM_15247	147929	Hs.596338	NM_15247	ENSG0000	ZNF565	-	zinc finger	protein-co
lib-C666-l	chr12	120966989	120966989	+	27.68428	NA	promoter-TSS (NM_032314)	promoter-TSS (NM_032314)	-25	NM_03231	84274	Hs.17250	NM_03231	ENSG0000	COQ5	-	coenzyme	protein-co
lib-C666-l	chr1	228270362	228270362	+	27.5466	NA	promoter-TSS (NM_001658)	promoter-TSS (NM_001658)	1	NM_00102	375	Hs.286221	NM_00165	ENSG0000	ARF1	-	ADP ribosy	protein-co
lib-C666-l	chr19	44529482	44529482	+	27.5466	NA	promoter-TSS (NM_013360)	promoter-TSS (NM_013360)	-12	NM_00112	7673	Hs.279840	NM_01336	ENSG0000	ZNF222	-	zinc finger	protein-co
lib-C666-l	chr5	140943907	140943907	+	27.5466	NA	non-coding (NR_038333, exon 3 of 3	non-coding (NR_038333, exon 3 of	6029	NR_03833	100505658	Hs.655458	NR_03833	ENSG0000	LOC10050	-	uncharacte	ncRNA
lib-C666-l	chr19	50631875	50631875	+	27.42941	NA	promoter-TSS (NR_024214).10	promoter-TSS (NR_024214).10	-96	NR_02422	100170216	Hs.723094	NR_024229		SNAR-A10	-	small ILF3/	snRNA
lib-C666-l	chr3	48701214	48701214	+	27.28371	NA	promoter-TSS (NM_001407)	promoter-TSS (NM_001407)	13	NR_11192	102724368	Hs.663571	NR_11192	ENSG0000	CELSR3-AS	-	CELSR3 an	ncRNA
lib-C666-l	chr8	146277925	146277925	+	27.26713	NA	intron (NM_023080, intron 1 of 4)	CpG	101	NM_02308	65265	Hs.169615	NM_02308	ENSG0000	C8orf33	-	chromosor	protein-co
lib-C666-l	chr9	84304799	84304799	+	27.26713	NA	promoter-TSS (NM_001303103)	promoter-TSS (NM_001303103)	171	NR_10977	101927502	Hs.528628	NR_10977	ENSG0000	LOC1019	-	uncharacte	ncRNA
lib-C666-l	chr2	28114147	28114147	+	26.81569	NA	promoter-TSS (NM_001287580)	promoter-TSS (NM_001287580)	-166	NR_02830	100302650	Hs.729719	NR_028308		BRE-AS1	-	BRE antise	ncRNA
lib-C666-l	chr12	10902735	10902735	+	26.81569	NA	promoter-TSS (NR_120463)	promoter-TSS (NR_120463)	-98	NR_12046	101928162	Hs.112950	NR_12046	ENSG0000	LOC1019	-	uncharacte	ncRNA
lib-C666-l	chr16	28415156	28415156	+	26.80209	NA	promoter-TSS (NM_001286478)	promoter-TSS (NM_001286478)	50	NM_00131	728689	Hs.567374	NM_00109	ENSG0000	EIF3CL	-	eukaryotic	protein-co
lib-C666-l	chr2	242823700	242823700	+	26.57948	NA	intron (NR_110220, intron 1 of 3)	CpG	186	NR_11022	101927289	Hs.130180	NR_11022	ENSG0000	LINC01237	-	long interg	ncRNA
lib-C666-l	chr12	81640206	81640206	+	26.57948	NA	intron (NM_001330242, intron 13 of 1	L1MDa|LINE|L1	-32762	NR_12049	102724663	Hs.667264	NR_12049	ENSG0000	LOC10272	-	uncharacte	ncRNA
lib-C666-l	chr7	26429824	26429824	+	26.57948	NA	intron (NR_136272, intron 1 of 1)	intron (NR_136272, intron 1 of 1)	9214	NR_13627	105375304	Hs.735352	NR_13627	ENSG0000	LOC10537	-	uncharacte	ncRNA
lib-C666-l	chr4	775699	775699	+	26.54591	NA	promoter-TSS (NR_036511)	promoter-TSS (NR_036511)	-63	NR_03651	100129917	Hs.731755	NR_03651	ENSG0000	LOC10012	-	uncharacte	ncRNA
lib-C666-l	chr22	31451740	31451740	+	26.53025	NA	Intergenic	Intergenic	-25542	NM_13427	6525	Hs.149098	NM_00693	ENSG0000	SMTN	-	smoothelir	protein-co
lib-C666-l	chr4	76598755	76598755	+	26.52079	NA	promoter-TSS (NM_012297)	promoter-TSS (NM_012297)	-88	NM_20350	9908	Hs.303676	NM_01229	ENSG0000	G3BP2	-	G3BP stres	protein-co
lib-C666-l	chr17	7490147	7490147	+	26.4952	NA	exon (NM_001330073, exon 4 of 6)	exon (NM_001330073, exon 4 of 6)	-2750	NR_13640	100996842	Hs.720681	NM_00132	ENSG0000	LOC10099	-	uncharacte	protein-co
lib-C666-l	chr7	152161511	152161511	+	26.46807	NA	non-coding (NR_027387, exon 1 of 1	non-coding (NR_027387, exon 1 of	302	NR_02738	100128822	Hs.647112	NR_02738	ENSG0000	LINC01003	-	long interg	ncRNA
lib-C666-l	chr1	54590412	54590412	+	26.46433	NA	Intergenic	Intergenic	28267	NM_20154	200008	Hs.568555	NM_20154	ENSG0000	CDCP2	-	CUB doma	protein-co
lib-C666-l	chr19	50380862	50380862	+	26.35145	NA	promoter-TSS (NM_001098632)	promoter-TSS (NM_001098632)	180	NM_00116	79735	Hs.631587	NM_02468	ENSG0000	TBC1D17	-	TBC1 dom	protein-co
lib-C666-l	chr6	10383781	10383781	+	26.18172	NA	Intergenic	CpG	-28770	NR_03391	100130275	Hs.661431	NR_033910		TFAP2A-A	-	TFAP2A an	ncRNA
lib-C666-l	chr11	82867960	82867960	+	25.96768	NA	promoter-TSS (NM_015885)	promoter-TSS (NM_015885)	-177	NM_01588	51585	Hs.128959	NM_01588	ENSG0000	PCF11	-	PCF11 clea	protein-co
lib-C666-l	chr2	109150506	109150506	+	25.68529	NA	promoter-TSS (NM_001193483)	promoter-TSS (NM_001193483)	169	NR_13529	644903		NM_03265	ENSG0000	GCC2-AS1	-	GCC2 antis	ncRNA
lib-C666-l	chr12	62860609	62860609	+	25.68529	NA	promoter-TSS (NM_001278469)	promoter-TSS (NM_001278469)	12	NM_01502	23041	Hs.389378	NM_01502	ENSG0000	MON2	-	MON2 hor	protein-co
lib-C666-l	chr16	50776287	50776287	+	25.61513	NA	promoter-TSS (NR_130462)	promoter-TSS (NR_130462)	71	NR_13046	100422972		NR_13046	ENSG0000	MIR3181	-	microRNA	ncRNA
lib-C666-l	chr1	31125463	31125463	+	25.49921	NA	Intergenic	(TG)n|Simple_repeat|Simple_repeat	-66156	NR_03418	100129196	Hs.659751	NR_03418	ENSG0000	MATN1-A	-	MATN1 an	ncRNA
lib-C666-l	chr22	46361158	46361158	+	25.49755	NA	intron (NM_058238, intron 1 of 3)	L2c|LINE|L2	11850	NM_05823	7477	Hs.512714	NM_05823	ENSG0000	WNT7B	-	Wnt family	protein-co
lib-C666-l	chr1	31191678	31191678	+	25.39487	NA	promoter-TSS (NR_034182)	promoter-TSS (NR_034182)	59	NR_03418	100129196	Hs.659751	NR_03418	ENSG0000	MATN1-A	-	MATN1 an	ncRNA
lib-C666-l	chr5	141736764	141736764	+	25.39487	NA	intron (NR_120664, intron 3 of 3)	CpG	31906	NR_12066	101926941	Hs.658945	NR_12066	ENSG0000	LOC1019	-	uncharacte	ncRNA
lib-C666-l	chr19	1249127	1249127	+	25.32183	NA	intron (NM_177401, intron 1 of 7)	CpG	575	NM_17740	90007	Hs.465529	NM_17740	ENSG0000	MIDN	-	midnolin	protein-co
lib-C666-l	chr14	70234524	70234524	+	25.32183	NA	promoter-TSS (NR_029378)	promoter-TSS (NR_029378)	-94	NR_02937	100289511	Hs.729250	NR_029378		LOC10028	-	uncharacte	ncRNA
lib-C666-l	chr16	699205	699205	+	25.32183	NA	promoter-TSS (NM_145294)	promoter-TSS (NM_145294)	108	NR_13517	105371038		NR_135177		LOC10537	-	uncharacte	ncRNA
lib-C666-l	chr19	2840988	2840988	+	25.32183	NA	promoter-TSS (NM_001172775)	promoter-TSS (NM_001172775)	-445	NM_15279	148254	Hs.47712	NM_15279	ENSG0000	ZNF555	-	zinc finger	protein-co
lib-C666-l	chr19	48426994	48426994	+	24.97013	NA	promoter-TSS (NR_024214)	promoter-TSS (NR_024214)	-42	NR_02422	100170216	Hs.723094	NR_024229		SNAR-A10	-	small ILF3/	snRNA
lib-C666-l	chr15	52472253	52472253	+	24.73139	NA	promoter-TSS (NM_006578)	promoter-TSS (NM_006578)	30	NR_10275	100129973	Hs.569494	NR_102751		CERNA1	-	competing	ncRNA
lib-C666-l	chr10	99582226	99582226	+	24.69873	NA	Intergenic	Intergenic	27329	NR_10803	100505561	Hs.351904	NR_10803	ENSG0000	LINC00866	-	long interg	ncRNA
lib-C666-l	chr9	6008363	6008363	+	24.43903	NA	promoter-TSS (NM_001017969)	promoter-TSS (NM_001017969)	-360	NM_00101	158358	Hs.535060	NM_00101	ENSG0000	KIAA2026	-	KIAA2026	protein-co
lib-C666-l	chr19	39645418	39645418	+	24.43903	NA	intron (NM_001014834, intron 1 of 7)	intron (NM_001014834, intron 1 of	28998	NM_00588	10298	Hs.20447	NM_00588	ENSG0000	PAK4	-	p21 (RAC1	protein-co
lib-C666-l	chr11	9781347	9781347	+	24.32656	NA	promoter-TSS (NR_033972)	promoter-TSS (NR_033972)	-267	NR_03397	440028	Hs.677541	NR_03397	ENSG0000	LOC44002	-	uncharacte	ncRNA
lib-C666-l	chr2	170550951	170550951	+	24.32656	NA	promoter-TSS (NR_144936)	promoter-TSS (NR_144936)	10	NM_00119	100526832		NM_00119	ENSG0000	PHOSPHO	-	PHOSPHO	protein-co
lib-C666-l	chr7	36406822	36406822	+	24.28549	NA	promoter-TSS (NM_001199707)	promoter-TSS (NM_001199707)	20	NM_00119	23366	Hs.6224	NM_01531	ENSG0000	KIAA0895	-	KIAA0895	protein-co
lib-C666-l	chr8	145597176	145597176	+	24.25638	NA	promoter-TSS (NR_134307)	promoter-TSS (NR_134307)	-528	NR_13430	101928902	Hs.529758	NR_134307		LOC1019	-	uncharacte	ncRNA
lib-C666-l	chr16	2732418	2732418	+	24.25638	NA	promoter-TSS (NM_018992)	promoter-TSS (NM_018992)	-77	NM_01899	54442	Hs.61960	NM_01899	ENSG0000	KCTD5	-	potassium	protein-co
lib-C666-l	chr2	7273765	7273765	+	24.25638	NA	Intergenic	Intergenic	-55754	NR_11025	101929452		NR_11025	ENSG0000	LOC1019	-	uncharacte	ncRNA
lib-C666-l	chr1	147874642	147874642	+	24.25638	NA	promoter-TSS (NR_111930).2	promoter-TSS (NR_111930).2	6	NR_11193	100132057	Hs.585189	NR_033189		LOC10013	-	phosphodi	pseudo
lib-C666-l	chr19	13783630	13783630	+	24.25638	NA	Intergenic	AluSx|SINE|Alu	-58944	NM_00132	81576	Hs.24998	NM_03081	ENSG0000	CCDC130	-	coiled-coil	protein-co
lib-C666-l	chr17	2126298	2126298	+	24.2028	NA	intron (NM_017575, intron 10 of 18)	intron (NM_017575, intron 10 of 18	-9674	NR_11088	101927839	Hs.665648	NR_11088	ENSG0000	LOC1019	-	uncharacte	ncRNA
lib-C666-l	chr13	41707027	41707027	+	24.20134	NA	promoter-TSS (NM_152903)	promoter-TSS (NM_152903)	-37	NR_12042	101929140	Hs.663088	NR_12042	ENSG0000	LOC1019	-	uncharacte	ncRNA
lib-C666-l	chr1	20135987	20135987	+	24.0106	NA	Intergenic	MLT1N2|LTR|ERVL-MaLR	5784	NM_01906	54546	Hs.124835	NM_01906	ENSG0000	RNF186	-	ring finger	protein-co
lib-C666-l	chr3	123305280	123305280	+	23.80446	NA	intron (NR_121654, intron 2 of 3)	MER1B|DNA|hAT-Charlie	920	NR_12165	100506826	Hs.556600	NR_03826	ENSG0000	MYLK-AS1	-	MYLK antis	ncRNA
lib-C666-l	chr3	80820487	80820487	+	23.74671	NA	Intergenic	MLT1I|LTR|ERVL-MaLR	-222532	NR_13241	728290	Hs.563132	NR_13241	ENSG0000	LINC02027	-	long interg	ncRNA
lib-C666-l	chr2	114361472	114361472	+	23.73126	NA	promoter-TSS (NR_024004)	promoter-TSS (NR_024004)	-178	NR_02400	84771	Hs.712940	NR_024004		DDX11L2	-	DEAD/H-b	pseudo
lib-C666-l	chr12	133707100	133707100	+	23.60036	NA	promoter-TSS (NM_001277291)	promoter-TSS (NM_001277291)	-41	NM_00127	101060200	Hs.614816	NM_00127	ENSG0000	ZNF891	-	zinc finger	protein-co
lib-C666-l	chr8	37620276	37620276	+	23.48385	NA	exon (NM_007198, exon 1 of 8)	exon (NM_007198, exon 1 of 8)	175	NM_00719	11212	Hs.304792	NM_00719	ENSG0000	PROSC	-	proline syn	protein-co
lib-C666-l	chr11	82782964	82782964	+	23.34793	NA	promoter-TSS (NM_001286061)	promoter-TSS (NM_001286061)	1	NM_00128	27314	Hs.40758	NM_01448	ENSG0000	RAB30	-	RAB30, me	protein-co
lib-C666-l	chr2	207139471	207139471	+	23.34793	NA	promoter-TSS (NM_001285549)	promoter-TSS (NM_001285549)	106	NM_02092	57683	Hs.110489	NM_02092	ENSG0000	ZDBF2	-	zinc finger	protein-co
lib-C666-l	chr12	56367722	56367722	+	23.34628	NA	promoter-TSS (NM_002868)	promoter-TSS (NM_002868)	25	NM_00125	5869	Hs.157659	NM_00286	ENSG0000	RAB5B	-	RAB5B, me	protein-co
lib-C666-l	chr16	29623072	29623072	+	23.27622	NA	intron (NR_135317, intron 1 of 11)	intron (NR_135317, intron 1 of 11)	1966	NR_13531	440354	Hs.552700	NR_002473		SMG1P2	-	SMG1P2, n	pseudo
lib-C666-l	chr19	50601300	50601300	+	23.242	NA	promoter-TSS (NR_024214).3	promoter-TSS (NR_024214).3	-97	NR_02422	100169957	Hs.723094	NR_024227		SNAR-A6	-	small ILF3/	snRNA
lib-C666-l	chr5	149979848	149979848	+	23.1274	NA	promoter-TSS (NM_001166208)	promoter-TSS (NM_001166208)	-794	NM_00116	11346	Hs.435228	NM_00728	ENSG0000	SYNPO	-	synaptopo	protein-co
lib-C666-l	chr1	6661419	6661419	+	23.1274	NA	intron (NM_014851, intron 1 of 3)	intron (NM_014851, intron 1 of 3)	1539	NM_00132	9903	Hs.7764	NM_01485	ENSG0000	KLHL21	-	kelch like f	protein-co
lib-C666-l	chr8	145597689	145597689	+	23.087	NA	promoter-TSS (NM_174922)	promoter-TSS (NM_174922)	-15	NM_17492	203054	Hs.283374	NM_17492	ENSG0000	ADCK5	-	aarF doma	protein-co
lib-C666-l	chr5	142244451	142244451	+	23.05659	NA	intron (NR_046680, intron 3 of 5)	MIRb|SINE|MIR	4024	NR_04668	100874239	Hs.570896	NR_04668	ENSG0000	ARHGAP26	-	ARHGAP26	ncRNA
lib-C666-l	chr5	70585955	70585955	+	22.87742	NA	Intergenic	LTR12E|LTR|ERV1	-30833	NR_03396	100049076	Hs.665231	NR_033968		GUSBP9	-	glucuronid	pseudo
lib-C666-l	chr5	7306896	7306896	+	22.84581	NA	promoter-TSS (NR_033906)	promoter-TSS (NR_033906)	-69	NR_03390	442132	Hs.727388	NM_001034846		LOC44213	-	golgin A6 f	pseudo
lib-C666-l	chr11	18720289	18720289	+	22.82077	NA	promoter-TSS (NM_153347)	promoter-TSS (NM_153347)	7	NM_15334	144110	Hs.502100	NM_15334	ENSG0000	TMEM86A	-	transmemb	protein-co
lib-C666-l	chr19	50604321	50604321	+	22.7781	NA	promoter-TSS (NR_024214).4	promoter-TSS (NR_024214).4	-53	NR_02422	100169957	Hs.723094	NR_024227		SNAR-A6	-	small ILF3/	snRNA
lib-C666-l	chr1	34873054	34873054	+	22.66433	NA	Intergenic	Charlie1|DNA|hAT-Charlie	230501	NM_00113	84970	Hs.194610	NM_03288	ENSG0000	C1orf94	-	chromosor	protein-co
lib-C666-l	chr20	30135093	30135093	+	22.66433	NA	intron (NM_178580, intron 4 of 12)	CpG	25973	NR_04685	100874042	Hs.373741	NR_04685	ENSG0000	HM13-AS1	-	HM13 antis	ncRNA
lib-C666-l	chr12	116797045	116797045	+	22.50982	NA	Intergenic	Intergenic	69078	NR_03968	100616309		NR_03968	ENSG0000	MIR4472-2	-	microRNA	ncRNA
lib-C666-l	chr1	36023307	36023307	+	22.43333	NA	promoter-TSS (NM_001014839)	promoter-TSS (NM_001014839)	-86	NM_01428	23154	Hs.121870	NM_01428	ENSG0000	NCDN	-	neurochon	protein-co
lib-C666-l	chr19	50626514	50626514	+	22.31021	NA	promoter-TSS (NR_024214).9	promoter-TSS (NR_024214).9	-63	NR_02422	100169953	Hs.723094	NR_024224		SNAR-A7	-	small ILF3/	snRNA
lib-C666-l	chr2	41689467	41689467	+	22.23727	NA	Intergenic	MER1A|DNA|hAT-Charlie	-415228	NR_03399	388942	Hs.631883	NR_03399	ENSG0000	LINC01913	-	long interg	ncRNA
lib-C666-l	chr1	149215598	149215598	+	22.23727	NA	Intergenic	Intergenic	-71532	NR_11193	388692	Hs.271249	NM_001013644		LOC38869	-	uncharacte	ncRNA
lib-C666-l	chr11	60926225	60926225	+	22.14682	NA	intron (NM_017966, intron 1 of 4)	intron (NM_017966, intron 1 of 4)	2691	NM_01796	55048	Hs.523715	NM_01796	ENSG0000	VPS37C	-	VPS37C, E	protein-co
lib-C666-l	chr2	30440979	30440979	+	22.02847	NA	Intergenic	Intergenic	-13418	NM_03091	81606	Hs.567598	NM_03091	ENSG0000	LBH	-	limb bud a	protein-co
lib-C666-l	chr5	110848369	110848369	+	22.02847	NA	promoter-TSS (NM_139164)	promoter-TSS (NM_139164)	-74	NM_00130	134429	Hs.93842	NM_13916	ENSG0000	STARD4	-	StAR relate	protein-co
lib-C666-l	chr7	54841726	54841726	+	22.01437	NA	intron (NR_110040, intron 1 of 2)	intron (NR_110040, intron 1 of 2)	14720	NR_11004	100996654	Hs.127991	NR_110040		LOC10099	-	uncharacte	ncRNA
lib-C666-l	chr2	100799240	100799240	+	22.01132	NA	Intergenic	Intergenic	-25476	NR_10373	150577	Hs.434127	NM_00110	ENSG0000	LINC01104	-	long interg	ncRNA
lib-C666-l	chr9	131580707	131580707	+	22.01132	NA	promoter-TSS (NM_004435)	promoter-TSS (NM_004435)	-72	NM_00443	2021	Hs.745161	NM_00443	ENSG0000	ENDOG	-	endonucle	protein-co
lib-C666-l	chr5	76398591	76398591	+	21.99129	NA	intron (NR_024398, intron 2 of 3)	intron (NR_024398, intron 2 of 3)	-15520	NM_03236	84327	Hs.584988	NM_03236	ENSG0000	ZBED3	-	zinc finger	protein-co
lib-C666-l	chr3	99647017	99647017	+	21.99129	NA	intron (NM_001042459, intron 3 of 4)	intron (NM_001042459, intron 3 of	36225	NR_03748	100500859		NR_03748	ENSG0000	MIR3921	-	microRNA	ncRNA
lib-C666-l	chr16	86858553	86858553	+	21.99129	NA	Intergenic	Intergenic	102856	NR_13518	102724344	Hs.585769	NR_13518	ENSG0000	LOC10272	-	uncharacte	ncRNA
lib-C666-l	chr7	17478859	17478859	+	21.99129	NA	intron (NR_110015, intron 1 of 2)	THE1B|LTR|ERVL-MaLR	64318	NR_11001	102659288	Hs.559488	NR_110014		KCCAT333	-	renal clear	ncRNA

lib-C666-l	chr11	125462507	125462507	+	21.96185	NA	promoter-TSS (NM_001278503)	promoter-TSS (NM_001278503)	-44	NR_13237	105369550	Hs.732083	NR_13237	ENSG0000	STT3A-AS	-	STT3A anti	ncRNA
lib-C666-l	chr15	33162800	33162800	+	21.94284	NA	intron (NM_001103184, intron 12 of 1	intron (NM_001103184, intron 12 o	-151734	NR_10976	100131315	Hs.610545	NR_10976	ENSG0000	LOC10013	-	uncharacte	ncRNA
lib-C666-l	chr22	21271644	21271644	+	21.90047	NA	promoter-TSS (NM_005207)	promoter-TSS (NM_005207)	-70	NM_00520	1399	Hs.5613	NM_00520	ENSG0000	CRKL	-	CRK like pr	protein-co
lib-C666-l	chr9	102582510	102582510	+	21.87529	NA	promoter-TSS (NR_109802)	promoter-TSS (NR_109802)	-339	NR_10980	101928438	Hs.637767	NR_10980	ENSG0000	LOC10192	-	uncharacte	ncRNA
lib-C666-l	chr18	34408904	34408904	+	21.87022	NA	promoter-TSS (NM_001322327)	promoter-TSS (NM_001322327)	-165	NR_13630	57536	Hs.4045	NM_02077	ENSG0000	KIAA1328	-	KIAA1328	protein-co
lib-C666-l	chr9	132258365	132258365	+	21.84731	NA	intron (NR_038955, intron 2 of 4)	CpG	7426	NR_03895	100506190	Hs.529860	NR_038955		LINC00963	-	long interg	ncRNA
lib-C666-l	chr16	32630786	32630786	+	21.73991	NA	Intergenic	Intergenic	56644	NM_00133	102724127		NM_00133	ENSG0000	TP53TG3F	-	TP53 targe	protein-co
lib-C666-l	chr9	123605358	123605358	+	21.73991	NA	promoter-TSS (NR_024408)	promoter-TSS (NR_024408)	38	NR_02440	253039	Hs.594170	NR_024408		PSMD5-AS	-	PSMD5 ant	ncRNA
lib-C666-l	chr14	35452170	35452170	+	21.73991	NA	promoter-TSS (NM_001146282)	promoter-TSS (NM_001146282)	66	NM_00313	6729	Hs.167535	NM_00313	ENSG0000	SRP54	-	signal reco	protein-co
lib-C666-l	chr19	50607390	50607390	+	21.61144	NA	promoter-TSS (NR_024214).5	promoter-TSS (NR_024214).5	-58	NR_02422	100170216	Hs.723094	NR_024229		SNAR-A10	-	small ILF3/	snRNA
lib-C666-l	chr17	76642177	76642177	+	21.60686	NA	Intergenic	Intergenic	-14038	NR_11084	101928710	Hs.464224	NR_11084	ENSG0000	LINC02081	-	long interg	ncRNA
lib-C666-l	chr14	50359717	50359717	+	21.52202	NA	promoter-TSS (NM_001663)	promoter-TSS (NM_001663)	-19	NM_00166	382	Hs.525330	NM_00166	ENSG0000	ARF6	-	ADP ribosy	protein-co
lib-C666-l	chr10	42391647	42391647	+	21.52113	NA	Intergenic	HSATII|Satellite|Satellite	471846	NR_02438	441666	Hs.255729	NR_02438	ENSG0000	LOC44166	-	zinc finger	pseudo
lib-C666-l	chr17	63096873	63096873	+	21.35213	NA	promoter-TSS (NR_110801)	promoter-TSS (NR_110801)	-57	NR_11080	100507002	Hs.729924	NR_110801		LOC10050	-	uncharacte	ncRNA
lib-C666-l	chr18	109300	109300	+	21.34108	NA	non-coding (NR_033770, exon 1 of 5	non-coding (NR_033770, exon 1 of	235	NR_03377	727758	Hs.585843	NR_03377	ENSG0000	ROCK1P1	-	Rho associ	pseudo
lib-C666-l	chr4	174139996	174139996	+	21.33627	NA	intron (NM_017423, intron 1 of 11)	L2c|LINE|L2	-49193	NR_13424	101930370	Hs.563191	NR_13424	ENSG0000	LOC10193	-	uncharacte	ncRNA
lib-C666-l	chr14	100942997	100942997	+	21.31471	NA	intron (NM_024515, intron 3 of 6)	MIRb|SINE|MIR	91410	NM_00115	57596	Hs.211751	NM_02083	ENSG0000	BEGAIN	-	brain enric	protein-co
lib-C666-l	chr6	56708921	56708921	+	21.28624	NA	promoter-TSS (NM_183380)	promoter-TSS (NM_183380)	137	NR_12586	101930010	Hs.650232	NR_125866		LOC10193	-	uncharacte	ncRNA
lib-C666-l	chr2	39005312	39005312	+	21.21068	NA	promoter-TSS (NM_024775)	promoter-TSS (NM_024775)	-15	NM_02477	79833	Hs.143818	NM_02477	ENSG0000	GEMIN6	-	gem nucle	protein-co
lib-C666-l	chr3	5068369	5068369	+	21.21068	NA	Intergenic	CpG	-46723	NR_12591	100507582	Hs.434505	NR_03790	ENSG0000	BHLHE40-	-	BHLHE40 a	ncRNA
lib-C666-l	chr16	89284058	89284058	+	21.21068	NA	promoter-TSS (NM_001201407)	promoter-TSS (NM_001201407)	-53	NR_03770	197320	Hs.647385	NM_18253	ENSG0000	ZNF778	-	zinc finger	protein-co
lib-C666-l	chr7	113061938	113061938	+	21.21068	NA	Intergenic	Intergenic	-303301	NR_02441	401397	Hs.117929	NR_01544	ENSG0000	LINC00998	-	long interg	ncRNA
lib-C666-l	chr5	148737505	148737505	+	20.94772	NA	promoter-TSS (NM_001301054)	promoter-TSS (NM_001301054)	-65	NM_00130	78991	Hs.644397	NM_02402	ENSG0000	PCYOX1L	-	prenylcyste	protein-co
lib-C666-l	chr10	135462794	135462794	+	20.93221	NA	Intergenic	Intergenic	-22495	NM_00108	441581	Hs.690471	NM_00108	ENSG0000	FRG2B	-	FSHD regic	protein-co
lib-C666-l	chr17	47645380	47645380	+	20.90919	NA	intron (NR_103773, intron 1 of 2)	CpG	6046	NR_10377	100288866	Hs.720309	NR_10377	ENSG0000	LOC10028	-	uncharacte	ncRNA
lib-C666-l	chr11	66247732	66247732	+	20.90919	NA	promoter-TSS (NR_120586)	promoter-TSS (NR_120586)	-12	NR_12058	101928069	Hs.523816	NR_12058	ENSG0000	LOC10192	-	uncharacte	ncRNA
lib-C666-l	chr5	94982519	94982519	+	20.90919	NA	promoter-TSS (NM_001131066)	promoter-TSS (NM_001131066)	38	NM_00113	317671	Hs.399758	NM_17336	ENSG0000	RFESD	-	Rieske Fe-	protein-co
lib-C666-l	chr2	42158106	42158106	+	20.90919	NA	TTS (NR_110263)	TTS (NR_110263)	2757	NR_11026	101929723	Hs.369330	NR_11026	ENSG0000	LINC01914	-	long interg	ncRNA
lib-C666-l	chr17	17875893	17875893	+	20.87959	NA	promoter-TSS (NM_001033551)	promoter-TSS (NM_001033551)	-109	NM_00128	146691	Hs.462379	NM_14467	ENSG0000	TOM1L2	-	target of m	protein-co
lib-C666-l	chr2	65087039	65087039	+	20.84232	NA	intron (NR_110224, intron 1 of 4)	CpG	3726	NR_11022	101927438	Hs.580313	NR_11022	ENSG0000	LINC01800	-	long interg	ncRNA
lib-C666-l	chr19	45458268	45458268	+	20.84232	NA	promoter-TSS (NM_001282175)	promoter-TSS (NM_001282175)	18	NM_00128	1209	Hs.444441	NM_00129	ENSG0000	CLPTM1	-	CLPTM1, tr	protein-co
lib-C666-l	chr7	149478122	149478122	+	20.84232	NA	intron (NM_198455, intron 13 of 109)	intron (NM_198455, intron 13 of 10	4991	NM_19845	23145	Hs.632022	NM_19845	ENSG0000	SSPO	-	SCO-spon	protein-co
lib-C666-l	chr7	20369149	20369149	+	20.82108	NA	intron (NR_110120, intron 1 of 2)	intron (NR_110120, intron 1 of 2)	-1576	NM_00221	3696	Hs.592171	NM_00221	ENSG0000	ITGB8	-	integrin su	protein-co
lib-C666-l	chr7	100271363	100271363	+	20.82108	NA	promoter-TSS (NM_005273)	promoter-TSS (NM_005273)	0	NM_00527	2783	Hs.185172	NM_00527	ENSG0000	GNB2	-	G protein s	protein-co
lib-C666-l	chr8	80680302	80680302	+	20.7902	NA	promoter-TSS (NM_012258)	promoter-TSS (NM_012258)	-75	NR_12541	101927067	Hs.144030	NR_12541	ENSG0000	LINC01607	-	long interg	ncRNA
lib-C666-l	chr7	122319510	122319510	+	20.76617	NA	intron (NM_001009571, intron 2 of 27	MER11B|LTR|ERVK	19698	NM_13917	168433	Hs.126730	NM_13917	ENSG0000	RNF133	-	ring finger	protein-co
lib-C666-l	chr1	40997227	40997227	+	20.72609	NA	promoter-TSS (NM_152373)	promoter-TSS (NM_152373)	-6	NM_15237	127396	Hs.524767	NM_15237	ENSG0000	ZNF684	-	zinc finger	protein-co
lib-C666-l	chr12	32028435	32028435	+	20.72609	NA	Intergenic	Intergenic	11702	NR_13502	105369723	Hs.736954	NR_13502	ENSG0000	LOC10536	-	uncharacte	ncRNA
lib-C666-l	chr16	25061059	25061059	+	20.72609	NA	Intergenic	Tigger3b|DNA|TcMar-Tigger	-17149	NR_13519	283887	Hs.652665	NR_13519	ENSG0000	LOC28388	-	uncharacte	ncRNA
lib-C666-l	chr10	62896067	62896067	+	20.54946	NA	Intergenic	Intergenic	120034	NR_10805	100507058	Hs.145227	NR_10805	ENSG0000	LINC00845	-	long interg	ncRNA
lib-C666-l	chr16	2517753	2517753	+	20.3278	NA	non-coding (NR_135198, exon 1 of 1	non-coding (NR_135198, exon 1 of	461	NR_13519	729652	Hs.638553	NR_13519	ENSG0000	LOC72965	-	uncharacte	ncRNA
lib-C666-l	chr7	100048487	100048487	+	20.3278	NA	Intergenic	Intergenic	13407	NM_00100	402573	Hs.632306	NM_00100	ENSG0000	C7orf61	-	chromosor	protein-co
lib-C666-l	chr17	17817396	17817396	+	20.29774	NA	intron (NM_001288789, intron 1 of 12	intron (NM_001288789, intron 1 of	58388	NM_00108	146691	Hs.462379	NM_14467	ENSG0000	TOM1L2	-	target of m	protein-co
lib-C666-l	chr16	2977140	2977140	+	20.20244	NA	intron (NM_032296, intron 2 of 9)	intron (NM_032296, intron 2 of 9)	15160	NM_00130	84256	Hs.655321	NM_02091	ENSG0000	FLYWCH1	-	FLYWCH-t	protein-co
lib-C666-l	chr11	108338259	108338259	+	20.19666	NA	promoter-TSS (NM_152587)	promoter-TSS (NM_152587)	-1	NM_00133	160140	Hs.653180	NM_15258	ENSG0000	C11orf65	-	chromosor	protein-co
lib-C666-l	chr22	50329970	50329970	+	20.19521	NA	Intergenic	CpG	17692	NM_02432	79174	Hs.211282	NM_02432	ENSG0000	CRELD2	-	cysteine ric	protein-co
lib-C666-l	chr14	23398869	23398869	+	20.19521	NA	promoter-TSS (NM_001039619)	promoter-TSS (NM_001039619)	51	NR_11000	101926933	Hs.727321	NR_11000	ENSG0000	LOC10192	-	uncharacte	ncRNA
lib-C666-l	chr12	122125030	122125030	+	20.19521	NA	Intergenic	CpG	-17470	NM_17385	283385	Hs.434154	NM_17385	ENSG0000	MORN3	-	MORN rep	protein-co
lib-C666-l	chr19	1249538	1249538	+	20.0808	NA	intron (NM_177401, intron 1 of 7)	CpG	986	NM_17740	90007	Hs.465529	NM_17740	ENSG0000	MIDN	-	midnolin	protein-co
lib-C666-l	chr4	7541610	7541610	+	20.0808	NA	intron (NM_020777, intron 3 of 26)	intron (NM_020777, intron 3 of 26)	79855	NR_03623	100422826		NR_03623	ENSG0000	MIR4274	-	microRNA	ncRNA
lib-C666-l	chr22	48660767	48660767	+	20.0808	NA	Intergenic	Intergenic	-9409	NR_03617	100422916		NR_03617	ENSG0000	MIR3201	-	microRNA	ncRNA
lib-C666-l	chr1	218633430	218633430	+	20.03888	NA	intron (NR_031644, intron 2 of 6)	intron (NR_031644, intron 2 of 6)	17462	NR_12571	103611157	Hs.133379	NR_12571	ENSG0000	TGFB2-OT	-	TGFB2 ove	ncRNA
lib-C666-l	chr1	234746741	234746741	+	20.03192	NA	Intergenic	CpG	-1470	NM_18297	359948	Hs.350268	NM_18297	ENSG0000	IRF2BP2	-	interferon	protein-co
lib-C666-l	chr16	81110891	81110891	+	19.92425	NA	promoter-TSS (NM_001100873)	promoter-TSS (NM_001100873)	-19	NM_00110	123775	Hs.658684	NM_15233	ENSG0000	C16orf46	-	chromosor	protein-co
lib-C666-l	chr19	42784666	42784666	+	19.91222	NA	intron (NM_001304815, intron 2 of 20	CpG	-4151	NM_01512	23152	Hs.388236	NM_01512	ENSG0000	CIC	-	capicua tra	protein-co
lib-C666-l	chr3	181569129	181569129	+	19.83604	NA	Intergenic	Intergenic	-101023	NR_10414	100996490	Hs.606987	NR_10414	ENSG0000	LINC01206	-	long interg	ncRNA
lib-C666-l	chr3	101292914	101292914	+	19.82698	NA	promoter-TSS (NM_001320399)	promoter-TSS (NM_001320399)	-24	NR_13522	57092	Hs.732633	NM_02035	ENSG0000	PCNP	-	PEST prote	protein-co
lib-C666-l	chr2	24150075	24150075	+	19.82698	NA	promoter-TSS (NM_001242338)	promoter-TSS (NM_001242338)	-91	NM_01755	54454	Hs.467862	NM_01755	ENSG0000	ATAD2B	-	ATPase far	protein-co
lib-C666-l	chr11	49709957	49709957	+	19.82221	NA	intron (NR_027044, intron 2 of 6)	AluSq2|SINE|Alu	129877	NR_02704	440040	Hs.655746	NR_02704	ENSG0000	LOC44004	-	glutamate	pseudo
lib-C666-l	chr5	10551736	10551736	+	19.82221	NA	Intergenic	Intergenic	-12699	NM_00116	651746	Hs.26039	NM_00116	ENSG0000	ANKRD33E	-	ankyrin rep	protein-co
lib-C666-l	chr11	33664098	33664098	+	19.82221	NA	intron (NM_012194, intron 15 of 19)	LTR10F|LTR|ERV1	58188	NM_00116	100131378	Hs.709466	NM_00116	ENSG0000	C11orf91	-	chromosor	protein-co
lib-C666-l	chr20	37064036	37064036	+	19.75653	NA	promoter-TSS (NR_027241)	promoter-TSS (NR_027241)	-18	NR_02724	388796	Hs.400876	NR_01536	ENSG0000	SNHG17	-	small nucle	ncRNA
lib-C666-l	chr1	6684866	6684866	+	19.75653	NA	promoter-TSS (NM_001195753)	promoter-TSS (NM_001195753)	-59	NM_00119	90326	Hs.689006	NM_13835	ENSG0000	THAP3	-	THAP dom	protein-co
lib-C666-l	chr14	95757545	95757545	+	19.7012	NA	intron (NM_024734, intron 1 of 12)	intron (NM_024734, intron 1 of 12)	28700	NM_02473	79789	Hs.301478	NM_02473	ENSG0000	CLMN	-	calmin	protein-co
lib-C666-l	chr11	131840	131840	+	19.65763	NA	promoter-TSS (NR_028326)	promoter-TSS (NR_028326)	80	NR_02832	100133161	Hs.645398	NR_028326		LINC01001	-	long interg	ncRNA
lib-C666-l	chr20	30160983	30160983	+	19.65763	NA	promoter-TSS (NR_046853)	promoter-TSS (NR_046853)	83	NR_04685	100874042	Hs.373741	NR_04685	ENSG0000	HM13-AS1	-	HM13 antis	ncRNA
lib-C666-l	chr12	39300661	39300661	+	19.60174	NA	Intergenic	CpG	-1241	NM_15363	144402	Hs.40910	NM_15363	ENSG0000	CPNE8	-	copine 8	protein-co
lib-C666-l	chr19	46498565	46498565	+	19.57279	NA	promoter-TSS (NM_001267723)	promoter-TSS (NM_001267723)	-154	NM_00126	729440	Hs.515479	NM_00108	ENSG0000	CCDC61	-	coiled-coil	protein-co
lib-C666-l	chr7	72971628	72971628	+	19.54597	NA	promoter-TSS (NR_036682)	promoter-TSS (NR_036682)	-26	NR_03668	9275	Hs.647051	NM_00170	ENSG0000	BCL7B	-	BCL tumor	protein-co
lib-C666-l	chr10	42387262	42387262	+	19.42797	NA	Intergenic	HSATII|Satellite|Satellite	476231	NR_02438	441666	Hs.255729	NR_02438	ENSG0000	LOC44166	-	zinc finger	pseudo
lib-C666-l	chr7	139026479	139026479	+	19.24135	NA	intron (NM_197964, intron 1 of 1)	intron (NM_197964, intron 1 of 1)	601	NM_00124	100996928	Hs.731488	NM_00124	ENSG0000	C7orf55-LI	-	C7orf55-LI	protein-co
lib-C666-l	chr5	1634122	1634122	+	19.23598	NA	promoter-TSS (NR_003713)	promoter-TSS (NR_003713)	-2	NR_00371	728613	Hs.720393	NR_003713		LOC72861	-	programm	pseudo
lib-C666-l	chr15	67813413	67813413	+	19.2039	NA	promoter-TSS (NR_040055)	promoter-TSS (NR_040055)	-13	NR_04005	100506686	Hs.656245	NR_04005	ENSG0000	IQCH-AS1	-	IQCH antis	ncRNA
lib-C666-l	chr3	119042114	119042114	+	19.19377	NA	promoter-TSS (NR_046748)	promoter-TSS (NR_046748)	-507	NR_04674	100874246	Hs.607097	NR_04674	ENSG0000	ARHGAP31	-	ARHGAP31	ncRNA
lib-C666-l	chr12	127610696	127610696	+	19.19377	NA	Intergenic	L2a|LINE|L2	-65754	NR_10464	101927592	Hs.385663	NR_10464	ENSG0000	LOC10192	-	uncharacte	ncRNA
lib-C666-l	chr5	55419745	55419745	+	19.19377	NA	intron (NM_024669, intron 8 of 11)	intron (NM_024669, intron 8 of 11)	109441	NM_02466	79722	Hs.436214	NM_02466	ENSG0000	ANKRD55	-	ankyrin rep	protein-co
lib-C666-l	chr17	63100243	63100243	+	19.16794	NA	intron (NR_110801, intron 2 of 2)	intron (NR_110801, intron 2 of 2)	3313	NR_11080	100507002	Hs.729924	NR_110801		LOC10050	-	uncharacte	ncRNA
lib-C666-l	chr17	76311773	76311773	+	19.10251	NA	Intergenic	Intergenic	-37200	NR_07317	100996291	Hs.744493	NR_07317	ENSG0000	LINC01993	-	long interg	ncRNA
lib-C666-l	chr17	4890875	4890875	+	19.06818	NA	promoter-TSS (NM_001171168)	promoter-TSS (NM_001171168)	85	NM_01509	23125	Hs.632242	NM_01509	ENSG0000	CAMTA2	-	calmodulin	protein-co
lib-C666-l	chr19	9879336	9879336	+	19.01614	NA	promoter-TSS (NM_001077624)	promoter-TSS (NM_001077624)	74	NM_00107	162993	Hs.665717	NM_00107	ENSG0000	ZNF846	-	zinc finger	protein-co
lib-C666-l	chr17	58192915	58192915	+	19.01614	NA	Intergenic	Intergenic	-12635	NR_02740	653653	Hs.631741	NR_02740	ENSG0000	LOC65365	-	adaptor re	pseudo
lib-C666-l	chr19	47164629	47164629	+	19.01614	NA	promoter-TSS (NM_145056)	promoter-TSS (NM_145056)	-106	NR_04004	100506068	Hs.434327	NR_04004	ENSG0000	DACT3-AS	-	DACT3 ant	ncRNA
lib-C666-l	chr1	116915768	116915768	+	18.98417	NA	promoter-TSS (NM_000701)	promoter-TSS (NM_000701)	-27	NM_00070	476	Hs.371889	NM_00070	ENSG0000	ATP1A1	-	ATPase Na	protein-co
lib-C666-l	chr2	96676779	96676779	+	18.98417	NA	intron (NR_003698, intron 1 of 3)	intron (NR_003698, intron 1 of 3)	480	NR_00369	729234	Hs.720108	NR_00369	ENSG0000	FAHD2CP	-	fumarylace	pseudo
lib-C666-l	chr5	44808953	44808953	+	18.90251	NA	promoter-TSS (NR_109862)	promoter-TSS (NR_109862)	-58	NR_10986	100506674	Hs.575213	NR_109862		BRCAT54	-	breast canc	ncRNA
lib-C666-l	chr16	30583463	30583463	+	18.90009	NA	exon (NM_001024683, exon 1 of 3)	exon (NM_001024683, exon 1 of 3)	265	NM_00102	146542	Hs.301463	NM_14527	ENSG0000	ZNF688	-	zinc finger	protein-co
lib-C666-l	chr2	119067793	119067793	+	18.89222	NA	Intergenic	Intergenic	-123831	NR_14453	100506797	Hs.116176	NR_144530		LOC10050	-	uncharacte	ncRNA
lib-C666-l	chr8	27630182	27630182	+	18.89222	NA	promoter-TSS (NM_001304529)	promoter-TSS (NM_001304529)	-12	NM_00130	55246	Hs.445512	NM_01824	ENSG0000	CCDC25	-	coiled-coil	protein-co
lib-C666-l	chr5	164473131	164473131	+	18.89222	NA	Intergenic	L1PA5|LINE|L1	575846	NR_10506	102546299	Hs.437730	NR_10506	ENSG0000	LOC10254	-	uncharacte	ncRNA
lib-C666-l	chr9	132020736	132020736	+	18.89222	NA	Intergenic	CpG	-23959	NR_12158	101929331	Hs.522428	NR_12158	ENSG0000	LOC10192	-	uncharacte	ncRNA
lib-C666-l	chr10	93169844	93169844	+	18.82927	NA	promoter-TSS (NM_001284274)	promoter-TSS (NM_001284274)	-198	NM_00128	143279	Hs.596096	NM_17349	ENSG0000	HECTD2	-	HECT dom	protein-co
lib-C666-l	chr10	96162256	96162256	+	18.82927	NA	promoter-TSS (NM_015188)	promoter-TSS (NM_015188)	70	NM_01518	23232	Hs.500598	NM_01518	ENSG0000	TBC1D12	-	TBC1 dom	protein-co
lib-C666-l	chr5	142225859	142225859	+	18.74745	NA	intron (NM_015071, intron 1 of 22)	intron (NM_015071, intron 1 of 22)	22616	NR_04668	100874239	Hs.570896	NR_04668	ENSG0000	ARHGAP26	-	ARHGAP26	ncRNA
lib-C666-l	chr2	120460632	120460632	+	18.74745	NA	Intergenic	Intergenic	23889	NM_00110	80775	Hs.439991	NM_03057	ENSG0000	TMEM177	-	transmemb	protein-co
lib-C666-l	chr15	44222373	44222373	+	18.74745	NA	intron (NR_104455, intron 1 of 14)	intron (NR_104455, intron 1 of 14)	-52004	NR_00357	728758	Hs.658099	NR_003571		PIN4P1	-	peptidylpro	pseudo
lib-C666-l	chr19	2841520	2841520	+	18.74745	NA	promoter-TSS (NM_001172775)	promoter-TSS (NM_001172775)	87	NM_00117	148254	Hs.47712	NM_15279	ENSG0000	ZNF555	-	zinc finger	protein-co
lib-C666-l	chr20	39632330	39632330	+	18.74745	NA	intron (NM_001318197, intron 1 of 3)	intron (NM_001318197, intron 1 of	22929	NM_00131	100128988	Hs.636443	NM_001318196		LOC10012	-	uncharacte	protein-co
lib-C666-l	chr1	1051567	1051567	+	18.74745	NA	5' UTR (NM_017891, exon 1 of 10)	5' UTR (NM_017891, exon 1 of 10)	169	NM_00133	54991	Hs.235095	NM_01789	ENSG0000	C1orf159	-	chromosor	protein-co
lib-C666-l	chr11	33183167	33183167	+	18.74745	NA	promoter-TSS (NM_001326)	promoter-TSS (NM_001326)	-36	NR_03402	338739	Hs.423476	NR_03402	ENSG0000	CSTF3-AS1	-	CSTF3 anti	ncRNA
lib-C666-l	chr4	77069675	77069675	+	18.68431	NA	promoter-TSS (NM_001278603)	promoter-TSS (NM_001278603)	-7	NR_10378	53371	Hs.430435	NM_01742	ENSG0000	NUP54	-	nucleopori	protein-co
lib-C666-l	chr17	6916741	6916741	+	18.68431	NA	non-coding (NR_037715, exon 2 of 4	non-coding (NR_037715, exon 2 of	1005	NR_03771	440400	Hs.632232	NM_00100	ENSG0000	RNASEK	-	ribonuclea	protein-co
lib-C666-l	chr4	71705313	71705313	+	18.68431	NA	promoter-TSS (NM_001098477)	promoter-TSS (NM_001098477)	-93	NM_00109	2926	Hs.309763	NM_00209	ENSG0000	GRSF1	-	G-rich RN	protein-co
lib-C666-l	chr19	7894901	7894901	+	18.67864	NA	promoter-TSS (NM_145245)	promoter-TSS (NM_145245)	-260	NM_14524	115704	Hs.26870	NM_14524	ENSG0000	EVI5L	-	ecotropic v	protein-co
lib-C666-l	chr3	5058364	5058364	+	18.61761	NA	Intergenic	Intergenic	-36718	NR_12591	100507582	Hs.434505	NR_03790	ENSG0000	BHLHE40-	-	BHLHE40 a	ncRNA
lib-C666-l	chr22	44576748	44576748	+	18.60833	NA	promoter-TSS (NM_022141)	promoter-TSS (NM_022141)	-22	NM_02214	64098	Hs.658995	NM_02214	ENSG0000	PARVG	-	parvin gam	protein-co
lib-C666-l	chr1	212782010	212782010	+	18.49429	NA	promoter-TSS (NM_001040619)	promoter-TSS (NM_001040619)	40	NM_00120	467	Hs.460	NM_00167	ENSG0000	ATF3	-	activating t	protein-co
lib-C666-l	chr8	7812551	7812551	+	18.49429	NA	promoter-TSS (NR_027424)	promoter-TSS (NR_027424)	16	NR_02742	100132103	Hs.694680	NR_02742	ENSG0000	FAM66E	-	family with	ncRNA
lib-C666-l	chr1	221521362	221521362	+	18.47709	NA	Intergenic	ERVL-E-int|LTR|ERVL	-11724	NR_02423	400804	Hs.722626	NR_02423	ENSG0000	C1orf140	-	uncharacte	ncRNA
lib-C666-l	chr13	31444376	31444376	+	18.43022	NA	Intergenic	MIRc|SINE|MIR	-12323	NR_10381	440132	Hs.729493	NR_10381	ENSG0000	LINC00545	-	long interg	ncRNA
lib-C666-l	chr11	71640004	71640004	+	18.43022	NA	promoter-TSS (NR_029192)	promoter-TSS (NR_029192)	236	NR_02414	55298	Hs.368554	NM_01832	ENSG0000	RNF121	-	ring finger	protein-co
lib-C666-l	chr19	29081091	29081091	+	18.43022	NA	intron (NR_110759, intron 5 of 9)	intron (NR_110759, intron 5 of 9)	-16504	NR_13563	102724908		NR_13563	ENSG0000	LOC10272	-	uncharacte	ncRNA
lib-C666-l	chr15	90895687	90895687	+	18.43022	NA	intron (NM_001004309, intron 1 of 3)	CpG	210	NM_00100	342132	Hs.55307	NM_00100	ENSG0000	ZNF774	-	zinc finger	protein-co
lib-C666-l	chr15	75092574	75092574	+	18.43022	NA	intron (NM_001127190, intron 6 of 13	intron (NM_001127190, intron 6 of	-11476	NR_03973	100616183		NR_03973	ENSG0000	MIR4513	-	microRNA	ncRNA
lib-C666-l	chr16	31084988	31084988	+	18.41999	NA	promoter-TSS (NM_014699)	promoter-TSS (NM_014699)	-165	NM_00117	79759	Hs.102928	NM_02470	ENSG0000	ZNF668	-	zinc finger	protein-co
lib-C666-l	chr16	31084703	31084703	+	18.39104	NA	5' UTR (NM_001172668, exon 1 of 3)	5' UTR (NM_001172668, exon 1 of 3	120	NM_00117	79759	Hs.102928	NM_02470	ENSG0000	ZNF668	-	zinc finger	protein-co
lib-C666-l	chr19	58912764	58912764	+	18.20429	NA	Intergenic	CpG	5307	NM_00119	646862	Hs.728121	NM_00119	ENSG0000	RNF225	-	ring finger	protein-co
lib-C666-l	chr12	122150715	122150715	+	18.20429	NA	promoter-TSS (NM_001080825)	promoter-TSS (NM_001080825)	57	NM_00108	144404	Hs.644504	NM_00108	ENSG0000	TMEM120	-	transmemb	protein-co
lib-C666-l	chr7	44163261	44163261	+	18.20429	NA	promoter-TSS (NM_001127218)	promoter-TSS (NM_001127218)	-92	NM_00623	5425	Hs.306791	NM_00623	ENSG0000	POLD2	-	DNA polyn	protein-co
lib-C666-l	chr22	46409486	46409486	+	18.20429	NA	Intergenic	Intergenic	6990	NR_02724	730668	Hs.640071	NR_02724	ENSG0000	LOC73066	-	dynein hea	pseudo
lib-C666-l	chr19	40117367	40117367	+	18.20429	NA	Intergenic	MER1B|DNA|hAT-Charlie	-11958	NR_02687	100129935	Hs.659307	NR_02687	ENSG0000	LOC10012	-	lectin, gala	pseudo
lib-C666-l	chr3	194304588	194304588	+	18.17486	NA	promoter-TSS (NR_047573)	promoter-TSS (NR_047573)	-409	NR_04757	100507297	Hs.586059	NR_047573		TMEM44-A	-	TMEM44 a	ncRNA
lib-C666-l	chr7	35734798	35734798	+	18.1336	NA	promoter-TSS (NR_136255)	promoter-TSS (NR_136255)	-18	NR_13625	101930085	Hs.599851	NR_13625	ENSG0000	LOC10193	-	uncharacte	ncRNA
lib-C666-l	chr17	48357608	48357608	+	18.0551	NA	3' UTR (NM_001168215, exon 6 of 6)	3' UTR (NM_001168215, exon 6 of 6	5820	NM_15322	162461	Hs.224630	NM_15322	ENSG0000	TMEM92	-	transmemb	protein-co
lib-C666-l	chr3	182144264	182144264	+	17.90427	NA	Intergenic	L1PA2|LINE|L1	59886	NR_04010	401103	Hs.591304	NR_04010	ENSG0000	LINC01994	-	long interg	ncRNA
lib-C666-l	chr22	38003271	38003271	+	17.83432	NA	Intergenic	Intergenic	-1210	NM_01336	26088	Hs.499158	NM_01336	ENSG0000	GGA1	-	golgi assoc	protein-co
lib-C666-l	chr4	183065120	183065120	+	17.78771	NA	intron (NR_125905, intron 2 of 4)	CpG-20284	548	NR_02710	90768	Hs.175465	NR_027107		LOC90768	-	uncharacte	ncRNA
lib-C666-l	chr16	29911940	29911940	+	17.74278	NA	promoter-TSS (NM_181718)	promoter-TSS (NM_181718)	-207	NM_18171	253982	Hs.655761	NM_18171	ENSG0000	ASPHD1	-	aspartate b	protein-co
lib-C666-l	chr1	234975535	234975535	+	17.71746	NA	Intergenic	MER1A|DNA|hAT-Charlie	115746	NR_03885	100506810	Hs.586634	NR_03885	ENSG0000	LINC01132	-	long interg	ncRNA
lib-C666-l	chr5	17375713	17375713	+	17.71746	NA	Intergenic	Intergenic	11706	NR_03397	401177	Hs.508311	NR_03397	ENSG0000	LINC02111	-	long interg	ncRNA
lib-C666-l	chr8	122736834	122736834	+	17.68706	NA	Intergenic	Intergenic	-83204	NM_00532	3037	Hs.159226	NM_00532	ENSG0000	HAS2	-	hyaluronar	protein-co
lib-C666-l	chr17	1945310	1945310	+	17.68706	NA	promoter-TSS (NM_080822)	promoter-TSS (NM_080822)	33	NM_08082	124641	Hs.513856	NM_08082	ENSG0000	OVCA2	-	ovarian tur	protein-co
lib-C666-l	chr1	235099138	235099138	+	17.68706	NA	intron (NR_125945, intron 1 of 3)	intron (NR_125945, intron 1 of 3)	608	NR_12594	101927851	Hs.520684	NR_12594	ENSG0000	LOC10192	-	uncharacte	ncRNA
lib-C666-l	chr3	197944869	197944869	+	17.68706	NA	Intergenic	Intergenic	65632	NM_00114	728262	Hs.744675	NM_001145248		FAM157A	-	family with	protein-co
lib-C666-l	chr5	180611330	180611330	+	17.68706	NA	Intergenic	Intergenic	7578	NR_10803	102577426	Hs.277359	NR_10803	ENSG0000	LINC01962	-	long interg	ncRNA
lib-C666-l	chr13	111064694	111064694	+	17.68706	NA	intron (NM_001846, intron 4 of 47)	intron (NM_001846, intron 4 of 47)	50940	NM_00126	100129836	Hs.640035	NM_001267044		COL4A2-A	-	COL4A2 ar	protein-co
lib-C666-l	chr3	49044678	49044678	+	17.68706	NA	promoter-TSS (NM_001320547)	promoter-TSS (NM_001320547)	183	NM_00132	11180	Hs.654815	NM_01803	ENSG0000	WDR6	-	WD repeat	protein-co
lib-C666-l	chr16	2509427	2509427	+	17.68706	NA	promoter-TSS (NM_025108)	promoter-TSS (NM_025108)	-688	NM_02510	80178	Hs.534491	NM_02510	ENSG0000	C16orf59	-	chromosor	protein-co
lib-C666-l	chr14	105292759	105292759	+	17.68706	NA	Intergenic	Intergenic	5221	NR_02439	196872	Hs.352220	NR_02439	ENSG0000	LINC00638	-	long interg	ncRNA
lib-C666-l	chr15	93363672	93363672	+	17.68706	NA	Intergenic	Intergenic	24958	NR_03376	728619	Hs.684129	NR_033769		ASB9P1	-	ankyrin rep	pseudo
lib-C666-l	chr3	49591720	49591720	+	17.6458	NA	promoter-TSS (NR_038866)	promoter-TSS (NR_038866)	79	NR_03886	100132677	Hs.435651	NR_03886	ENSG0000	BSN-AS2	-	BSN antise	ncRNA
lib-C666-l	chr17	43128874	43128874	+	17.62684	NA	promoter-TSS (NM_001288655)	promoter-TSS (NM_001288655)	11	NM_00128	79877	Hs.463148	NM_02481	ENSG0000	DCAKD	-	dephosphc	protein-co
lib-C666-l	chr9	11407	11407	+	17.55871	NA	promoter-TSS (NR_051986)	promoter-TSS (NR_051986)	-580	NR_05198	100287596	Hs.719844	NR_051986		DDX11L5	-	DEAD/H-b	pseudo
lib-C666-l	chr2	118771008	118771008	+	17.48666	NA	intron (NM_019044, intron 1 of 23)	CpG	731	NM_01904	54520	Hs.107845	NM_01904	ENSG0000	CCDC93	-	coiled-coil	protein-co
lib-C666-l	chr19	47551986	47551986	+	17.48666	NA	promoter-TSS (NM_017854)	promoter-TSS (NM_017854)	-104	NM_01785	54958	Hs.105606	NM_01785	ENSG0000	TMEM160	-	transmemb	protein-co
lib-C666-l	chr9	37465553	37465553	+	17.48666	NA	promoter-TSS (NM_014872)	promoter-TSS (NM_014872)	-146	NM_01487	9925	Hs.161276	NM_01487	ENSG0000	ZBTB5	-	zinc finger	protein-co
lib-C666-l	chr2	8144603	8144603	+	17.41249	NA	Intergenic	Intergenic	-27658	NR_01540	339788	Hs.46864	NR_01540	ENSG0000	LINC00298	-	long interg	ncRNA
lib-C666-l	chr7	139226462	139226462	+	17.40556	NA	intron (NM_001080511, intron 3 of 4)	intron (NM_001080511, intron 3 of	17788	NM_00108	154790	Hs.57806	NM_00108	ENSG0000	CLEC2L	-	C-type lec	protein-co
lib-C666-l	chr19	45157803	45157803	+	17.37618	NA	promoter-TSS (NR_039756)	promoter-TSS (NR_039756)	-801	NR_03975	100616355		NR_03975	ENSG0000	MIR4531	-	microRNA	ncRNA
lib-C666-l	chr3	64963210	64963210	+	17.37618	NA	intron (NR_038264, intron 4 of 5)	MLT1K|LTR|ERVL-MaLR	-289845	NM_18292	56999	Hs.656071	NM_18292	ENSG0000	ADAMTS9	-	ADAM met	protein-co
lib-C666-l	chr1	85695587	85695587	+	17.37618	NA	Intergenic	Intergenic	-28859	NM_03218	84144	Hs.533853	NM_03218	ENSG0000	SYDE2	-	synapse de	protein-co
lib-C666-l	chr10	62895268	62895268	+	17.3027	NA	Intergenic	Intergenic	119235	NR_10805	100507058	Hs.145227	NR_10805	ENSG0000	LINC00845	-	long interg	ncRNA
lib-C666-l	chr8	124286718	124286718	+	17.3027	NA	promoter-TSS (NM_001204180)	promoter-TSS (NM_001204180)	9	NR_03787	11244	Hs.521800	NM_00722	ENSG0000	ZHX1	-	zinc finger	protein-co
lib-C666-l	chr11	124670349	124670349	+	17.27528	NA	promoter-TSS (NM_001308027)	promoter-TSS (NM_001308027)	25	NR_10386	100507283	Hs.564177	NR_10386	ENSG0000	LOC10050	-	uncharacte	ncRNA
lib-C666-l	chr12	19592518	19592518	+	17.27528	NA	promoter-TSS (NM_001267043)	promoter-TSS (NM_001267043)	-90	NM_00111	121536	Hs.126497	NM_15320	ENSG0000	AEBP2	-	AE binding	protein-co
lib-C666-l	chr11	10329733	10329733	+	17.25993	NA	promoter-TSS (NR_103765)	promoter-TSS (NR_103765)	-127	NR_10376	100130460	Hs.501890	NR_103765		CAND1.11	-	uncharacte	ncRNA
lib-C666-l	chr7	71101541	71101541	+	17.25993	NA	intron (NM_022479, intron 6 of 10)	L2a|LINE|L2	-328785	NR_03747	100500836		NR_03747	ENSG0000	MIR3914-1	-	microRNA	ncRNA
lib-C666-l	chr19	18527285	18527285	+	17.22931	NA	Intergenic	AluJb|SINE|Alu	-2861	NM_00100	170463	Hs.515259	NM_03262	ENSG0000	SSBP4	-	single strar	protein-co

lib-C666-l	chr6	165201034	165201034	+	17.22931	NA	Intergenic	L1PA5|LINE|L1	34518	NR_13192	105378111		NR_131926		MEAT6	-	melanoma	ncRNA
lib-C666-l	chr13	82090690	82090690	+	17.22931	NA	Intergenic	HUERS-P3b-int|LTR|ERV1	-289572	NR_04749	100861546	Hs.539621	NR_04749	ENSG0000	LINC00564	-	long interg	ncRNA
lib-C666-l	chr5	158606170	158606170	+	17.18456	NA	intron (NM_001199380, intron 4 of 1	intron (NM_001199380, intron 4 of	28486	NM_00119	153830	Hs.349306	NM_14472	ENSG0000	RNF145	-	ring finger	protein-co
lib-C666-l	chr10	42396071	42396071	+	17.14222	NA	Intergenic	HSATII|Satellite|Satellite	467422	NR_02438	441666	Hs.255729	NR_02438	ENSG0000	LOC44166	-	zinc finger	pseudo
lib-C666-l	chr3	169684535	169684535	+	17.13943	NA	promoter-TSS (NR_027622)	promoter-TSS (NR_027622)	-13	NR_02762	100128164	Hs.633070	NR_02440	ENSG0000	LOC10012	-	four and a	pseudo
lib-C666-l	chr7	73269930	73269930	+	17.13943	NA	Intergenic	Intergenic	-5559	NM_18250	135886	Hs.647026	NM_18250	ENSG0000	WBSCR28	-	Williams-B	protein-co
lib-C666-l	chr7	96634811	96634811	+	17.01151	NA	promoter-TSS (NM_005222)	promoter-TSS (NM_005222)	-479	NM_00522	1750	Hs.249196	NM_00522	ENSG0000	DLX6	-	distal-less	protein-co
lib-C666-l	chr9	135895066	135895066	+	16.96003	NA	promoter-TSS (NR_132980).3	promoter-TSS (NR_132980).3	-751	NR_13298	106635683		NR_132980		SNORD141	-	small nucle	snoRNA
lib-C666-l	chr19	12899522	12899522	+	16.9473	NA	Intergenic	Intergenic	1580	NR_04986	100847071		NR_04986	ENSG0000	MIR5684	-	microRNA	ncRNA
lib-C666-l	chr14	102829278	102829278	+	16.76091	NA	promoter-TSS (NM_001172631)	promoter-TSS (NM_001172631)	-25	NM_00132	51550	Hs.129634	NM_03263	ENSG0000	CINP	-	cyclin depe	protein-co
lib-C666-l	chr3	176586271	176586271	+	16.59065	NA	Intergenic	L1M1|LINE|L1	-51482	NR_11081	101928684	Hs.639352	NR_11081	ENSG0000	LINC01209	-	long interg	ncRNA
lib-C666-l	chr1	37899107	37899107	+	16.59065	NA	Intergenic	Intergenic	40937	NR_03884	728431	Hs.380738	NR_03884	ENSG0000	LINC01137	-	long interg	ncRNA
lib-C666-l	chrX	40506942	40506942	+	16.59065	NA	promoter-TSS (NM_144970)	promoter-TSS (NM_144970)	-7	NM_00133	159013	Hs.495961	NM_14497	ENSG0000	CXorf38	-	chromosor	protein-co
lib-C666-l	chr6	145431329	145431329	+	16.58224	NA	Intergenic	Intergenic	-624676	NR_03824	100507557	Hs.124537	NR_03824	ENSG0000	LOC10050	-	uncharacte	ncRNA
lib-C666-l	chr11	135258	135258	+	16.57122	NA	Intergenic	Intergenic	-3338	NR_02832	100133161	Hs.645398	NR_028326		LINC01001	-	long interg	ncRNA
lib-C666-l	chr18	22510679	22510679	+	16.5633	NA	Intergenic	Intergenic	204934	NR_13460	105372028	Hs.569901	NR_13460	ENSG0000	LOC10537	-	uncharacte	ncRNA
lib-C666-l	chr11	32916018	32916018	+	16.291	NA	intron (NM_001076786, intron 1 of 1	intron (NM_001076786, intron 1 of	1294	NM_00107	79832	Hs.369368	NM_02477	ENSG0000	QSER1	-	glutamine	protein-co
lib-C666-l	chr9	72726027	72726027	+	16.291	NA	intron (NM_001347990, intron 4 of 1	intron (NM_001347990, intron 4 of	64777	NR_04007	100507244	Hs.556816	NR_04007	ENSG0000	MAMDC2-	-	MAMDC2	ncRNA
lib-C666-l	chr12	110318235	110318235	+	16.264	NA	promoter-TSS (NM_016433)	promoter-TSS (NM_016433)	58	NM_01643	51228	Hs.381256	NM_01643	ENSG0000	GLTP	-	glycolipid t	protein-co
lib-C666-l	chr10	5591010	5591010	+	16.264	NA	Intergenic	Intergenic	-22801	NR_12049	100132159	Hs.239600	NR_120496		CALML3-A	-	CALML3 ar	ncRNA
lib-C666-l	chr14	100045529	100045529	+	16.264	NA	intron (NM_001144995, intron 1 of 5)	intron (NM_001144995, intron 1 of	25198	NM_00114	317762	Hs.709288	NM_00114	ENSG0000	CCDC85C	-	coiled-coil	protein-co
lib-C666-l	chr20	11248202	11248202	+	16.264	NA	non-coding (NR_038972, exon 2 of 2	non-coding (NR_038972, exon 2 of	5829	NR_03897	339593	Hs.434320	NR_038972		LOC33959	-	uncharacte	ncRNA
lib-C666-l	chr9	133424664	133424664	+	16.264	NA	Intergenic	MLT1J2|LTR|ERVL-MaLR	30217	NR_02744	100272217	Hs.601255	NR_027440		LOC10027	-	uncharacte	ncRNA
lib-C666-l	chr2	84331184	84331184	+	16.264	NA	Intergenic	L1P1|LINE|L1	-186622	NR_00366	388965	Hs.664098	NM_001013648		FUNDC2P2	-	FUN14 dor	pseudo
lib-C666-l	chr19	42746861	42746861	+	16.264	NA	promoter-TSS (NM_019884)	promoter-TSS (NM_019884)	-125	NM_01988	2931	Hs.466828	NM_01988	ENSG0000	GSK3A	-	glycogen s	protein-co
lib-C666-l	chr1	200638804	200638804	+	16.22497	NA	intron (NR_110787, intron 1 of 2)	CpG	169	NR_11078	101929224	Hs.639369	NR_11078	ENSG0000	LOC10192	-	uncharacte	ncRNA
lib-C666-l	chr10	72147398	72147398	+	16.20502	NA	Intergenic	Intergenic	-4992	NR_10346	55222	Hs.7778	NM_01820	ENSG0000	LRRC20	-	leucine rich	protein-co
lib-C666-l	chr5	43557358	43557358	+	16.19708	NA	promoter-TSS (NM_006451)	promoter-TSS (NM_006451)	-163	NM_00645	10605	Hs.482038	NM_00645	ENSG0000	PAIP1	-	poly(A) bin	protein-co
lib-C666-l	chr10	76995383	76995383	+	16.19708	NA	exon (NM_144589, exon 2 of 7)	exon (NM_144589, exon 2 of 7)	387	NM_14458	118881	Hs.355333	NM_14458	ENSG0000	COMTD1	-	catechol-C	protein-co
lib-C666-l	chr8	126010637	126010637	+	16.18257	NA	promoter-TSS (NM_003129)	promoter-TSS (NM_003129)	-83	NM_00312	6713	Hs.71465	NM_00312	ENSG0000	SQLE	-	squalene e	protein-co
lib-C666-l	chr11	68890313	68890313	+	16.14038	NA	Intergenic	Intergenic	39669	NR_03612	100422846		NR_03612	ENSG0000	MIR3164	-	microRNA	ncRNA
lib-C666-l	chr4	6919568	6919568	+	16.13885	NA	intron (NM_020773, intron 1 of 13)	intron (NM_020773, intron 1 of 13)	8073	NM_02077	57533	Hs.518611	NM_02077	ENSG0000	TBC1D14	-	TBC1 dom	protein-co
lib-C666-l	chr17	43107553	43107553	+	16.13885	NA	intron (NM_001321326, intron 3 of 4)	intron (NM_001321326, intron 3 of	21332	NM_00128	79877	Hs.463148	NM_02481	ENSG0000	DCAKD	-	dephosph	protein-co
lib-C666-l	chr2	8819050	8819050	+	15.92204	NA	TTS (NR_110153)	TTS (NR_110153)	2941	NR_11015	100506299	Hs.439031	NR_11015	ENSG0000	ID2-AS1	-	ID2 antiser	ncRNA
lib-C666-l	chr5	176830932	176830932	+	15.75122	NA	exon (NM_000505, exon 10 of 14)	exon (NM_000505, exon 10 of 14)	-3295	NM_00102	345456	Hs.130196	NM_00102	ENSG0000	PFN3	-	profilin 3	protein-co
lib-C666-l	chr2	196941729	196941729	+	15.75122	NA	Intergenic	Intergenic	-8193	NM_01889	56171	Hs.97403	NM_01889	ENSG0000	DNAH7	-	dynein axo	protein-co
lib-C666-l	chr16	29606953	29606953	+	15.74109	NA	promoter-TSS (NR_135305)	promoter-TSS (NR_135305)	-540	NR_13530	440354	Hs.552700	NR_002473		SMG1P2	-	SMG1P2, n	pseudo
lib-C666-l	chr21	35747970	35747970	+	15.60931	NA	intron (NM_058182, intron 1 of 3)	CpG	191	NM_00131	102723553	Hs.656195	NM_00131	ENSG0000	SMIM11B	-	small integ	protein-co
lib-C666-l	chr4	7044845	7044845	+	15.60931	NA	promoter-TSS (NM_153376)	promoter-TSS (NM_153376)	-117	NM_15337	257236	Hs.656757	NM_15337	ENSG0000	CCDC96	-	coiled-coil	protein-co
lib-C666-l	chr12	27167325	27167325	+	15.55328	NA	promoter-TSS (NM_016551)	promoter-TSS (NM_016551)	14	NM_01655	51768	Hs.438641	NM_01655	ENSG0000	TM7SF3	-	transmemb	protein-co
lib-C666-l	chr3	10050860	10050860	+	15.55328	NA	intron (NM_001008737, intron 2 of 3)	intron (NM_001008737, intron 2 of	1919	NM_00100	401052	Hs.662766	NM_001008737		LOC40105	-	uncharacte	protein-co
lib-C666-l	chr16	4784326	4784326	+	15.55328	NA	promoter-TSS (NM_001324130)	promoter-TSS (NM_001324130)	37	NM_13917	146562	Hs.602738	NM_13917	ENSG0000	C16orf71	-	chromosor	protein-co
lib-C666-l	chr4	77069022	77069022	+	15.55328	NA	intron (NR_103782, intron 1 of 11)	intron (NR_103782, intron 1 of 11)	646	NM_01742	53371	Hs.430435	NM_01742	ENSG0000	NUP54	-	nucleopori	protein-co
lib-C666-l	chr7	148032310	148032310	+	15.54425	NA	intron (NR_036093, intron 3 of 3)	intron (NR_036093, intron 3 of 3)	-255347	NM_14530	202865	Hs.352212	NM_14530	ENSG0000	C7orf33	-	chromosor	protein-co
lib-C666-l	chr9	35604101	35604101	+	15.54425	NA	Intergenic	CpG	-1180	NM_00628	7016	Hs.708096	NM_00628	ENSG0000	TESK1	-	testis-spec	protein-co
lib-C666-l	chr2	28549106	28549106	+	15.54425	NA	intron (NR_137438, intron 12 of 12)	intron (NR_137438, intron 12 of 12)	-15780	NR_03831	100505716	Hs.679092	NR_03831	ENSG0000	LOC10050	-	uncharacte	ncRNA
lib-C666-l	chr12	2629518	2629518	+	15.54425	NA	intron (NM_001129830, intron 9 of 4	intron (NM_001129830, intron 9 of	151868	NR_04657	100874235	Hs.667644	NR_04657	ENSG0000	CACNA1C-	-	CACNA1C	ncRNA
lib-C666-l	chr17	42277700	42277700	+	15.42216	NA	Intergenic	CpG	-2171	NM_00109	56970	Hs.512651	NM_02021	ENSG0000	ATXN7L3	-	ataxin 7 lik	protein-co
lib-C666-l	chr10	29910429	29910429	+	15.42216	NA	intron (NM_003174, intron 3 of 35)	intron (NM_003174, intron 3 of 35)	13472	NM_02173	6840	Hs.499209	NM_00317	ENSG0000	SVIL	-	supervillin	protein-co
lib-C666-l	chr1	87794122	87794122	+	15.3947	NA	promoter-TSS (NM_006769)	promoter-TSS (NM_006769)	-29	NM_00676	8543	Hs.436792	NM_00676	ENSG0000	LMO4	-	LIM domai	protein-co
lib-C666-l	chr7	149552527	149552527	+	15.33659	NA	intron (NM_001099220, intron 5 of 7)	LTR78|LTR|ERV1	17071	NM_00109	643641	Hs.731923	NM_00109	ENSG0000	ZNF862	-	zinc finger	protein-co
lib-C666-l	chr16	30661875	30661875	+	15.33659	NA	promoter-TSS (NM_024031)	promoter-TSS (NM_024031)	-148	NM_00132	78994	Hs.293629	NM_02403	ENSG0000	PRR14	-	proline rich	protein-co
lib-C666-l	chr19	54976027	54976027	+	15.31489	NA	TTS (NM_145057)	TTS (NM_145057)	-1133	NM_19898	94059	Hs.590976	NM_19898	ENSG0000	LENG9	-	leukocyte r	protein-co
lib-C666-l	chr2	219134994	219134994	+	15.31489	NA	promoter-TSS (NM_015488)	promoter-TSS (NM_015488)	-62	NM_00130	14	Hs.83347	NM_00108	ENSG0000	AAMP	-	angio asso	protein-co
lib-C666-l	chr7	131580440	131580440	+	15.31489	NA	Intergenic	Intergenic	-14539	NR_11083	101928782	Hs.571369	NR_11083	ENSG0000	LOC10192	-	uncharacte	ncRNA
lib-C666-l	chr17	9720647	9720647	+	15.30356	NA	intron (NM_001310219, intron 1 of 4)	intron (NM_001310219, intron 1 of	4741	NM_00131	644070		NM_00131	ENSG0000	GSG1L2	-	GSG1 like	protein-co
lib-C666-l	chr11	370273	370273	+	15.30356	NA	intron (NM_178537, intron 1 of 19)	intron (NM_178537, intron 1 of 19)	478	NM_17853	338707	Hs.148074	NM_17853	ENSG0000	B4GALNT4	-	beta-1,4-N	protein-co
lib-C666-l	chr9	34569576	34569576	+	15.30356	NA	intron (NM_001842, intron 1 of 8)	intron (NM_001842, intron 1 of 8)	1566	NR_02436	415056	Hs.734229	NR_024369		CNTFR-AS	-	CNTFR ant	ncRNA
lib-C666-l	chr22	19971958	19971958	+	15.22699	NA	intron (NM_001670, intron 3 of 19)	intron (NM_001670, intron 3 of 19)	20682	NR_03991	100616414		NR_03991	ENSG0000	MIR4761	-	microRNA	ncRNA
lib-C666-l	chr14	105957203	105957203	+	15.15463	NA	promoter-TSS (NM_001134875)	promoter-TSS (NM_001134875)	-383	NM_00113	283643	Hs.720306	NM_17360	ENSG0000	C14orf80	-	chromosor	protein-co
lib-C666-l	chr10	67330016	67330016	+	15.13929	NA	Intergenic	Intergenic	-1167	NR_12064	101928913	Hs.568857	NR_120647		LINC01515	-	long interg	ncRNA
lib-C666-l	chr5	60140368	60140368	+	15.13226	NA	promoter-TSS (NM_024930)	promoter-TSS (NM_024930)	-267	NM_00129	79993	Hs.274256	NM_02493	ENSG0000	ELOVL7	-	ELOVL fatt	protein-co
lib-C666-l	chr14	62680483	62680483	+	15.12452	NA	Intergenic	Intergenic	-73792	NR_10406	101954204	Hs.607093	NR_10406	ENSG0000	LINC00644	-	long interg	ncRNA
lib-C666-l	chr17	79360828	79360828	+	15.1031	NA	Intergenic	CpG	-1668	NM_00127	100130370	Hs.646594	NM_001272086		LOC10013	-	uncharacte	protein-co
lib-C666-l	chr18	19803856	19803856	+	15.1031	NA	Intergenic	Intergenic	54458	NM_00525	2627	Hs.514746	NM_00525	ENSG0000	GATA6	-	GATA bind	protein-co
lib-C666-l	chr1	52522167	52522167	+	15.1031	NA	promoter-TSS (NM_015913)	promoter-TSS (NM_015913)	310	NM_00124	91408	Hs.429839	NM_15226	ENSG0000	BTF3L4	-	basic trans	protein-co
lib-C666-l	chr19	42927973	42927973	+	15.09264	NA	promoter-TSS (NR_073179)	promoter-TSS (NR_073179)	66	NR_07317	100996307	Hs.466840	NR_07317	ENSG0000	LIPE-AS1	-	LIPE antise	ncRNA
lib-C666-l	chr7	562256	562256	+	15.06395	NA	non-coding (NR_033963, exon 1 of 3	non-coding (NR_033963, exon 1 of	2228	NR_03396	441307	Hs.521334	NR_03396	ENSG0000	HRAT92	-	heart tissu	ncRNA
lib-C666-l	chr1	23924958	23924958	+	15.01426	NA	Intergenic	Intergenic	-28866	NR_02704	259283	Hs.523369	NM_00134	ENSG0000	MDS2	-	myelodysp	protein-co
lib-C666-l	chr11	67056117	67056117	+	14.98892	NA	promoter-TSS (NR_030767)	promoter-TSS (NR_030767)	-645	NM_00134	338692	Hs.438673	NM_20735	ENSG0000	ANKRD13	-	ankyrin rep	protein-co
lib-C666-l	chr22	19435361	19435361	+	14.98502	NA	5' UTR (NM_001166242, exon 1 of 3)	5' UTR (NM_001166242, exon 1 of	394	NM_17379	128977	Hs.659565	NM_17379	ENSG0000	C22orf39	-	chromosor	protein-co
lib-C666-l	chr20	35202004	35202004	+	14.88921	NA	promoter-TSS (NR_109939)	promoter-TSS (NR_109939)	-6	NM_02180	60436	Hs.632264	NM_02180	ENSG0000	TGIF2	-	TGFB induc	protein-co
lib-C666-l	chr8	145733594	145733594	+	14.70559	NA	promoter-TSS (NM_138431)	promoter-TSS (NM_138431)	-825	NR_13012	113655	Hs.7678	NM_13843	ENSG0000	MFSD3	-	major facili	protein-co
lib-C666-l	chr11	74204207	74204207	+	14.70559	NA	promoter-TSS (NM_001319240)	promoter-TSS (NM_001319240)	-223	NM_00131	100287896	Hs.591971	NM_198519		LOC10028	-	uncharacte	protein-co
lib-C666-l	chr1	669009	669009	+	14.70559	NA	Intergenic	MER1A|DNA|hAT-Charlie	-3278	NR_02832	100133331	Hs.728864	NR_028327		LOC10013	-	uncharacte	ncRNA
lib-C666-l	chr17	47308183	47308183	+	14.59636	NA	promoter-TSS (NM_001143804)	promoter-TSS (NM_001143804)	-55	NM_17850	162466	Hs.405607	NM_17850	ENSG0000	PHOSPHO	-	phosphoet	protein-co
lib-C666-l	chr11	75479716	75479716	+	14.59636	NA	promoter-TSS (NR_046090)	promoter-TSS (NR_046090)	-24	NR_04609	283214	Hs.591972	NR_04609	ENSG0000	LOC28321	-	uncharacte	ncRNA
lib-C666-l	chr20	6572115	6572115	+	14.59636	NA	Intergenic	Intergenic	164736	NR_10995	101929244	Hs.542488	NR_10995	ENSG0000	CASC20	-	cancer sus	ncRNA
lib-C666-l	chr2	27294597	27294597	+	14.54258	NA	promoter-TSS (NM_001134693)	promoter-TSS (NM_001134693)	-30	NM_00113	100128731	Hs.502948	NM_00113	ENSG0000	OST4	-	oligosacch	protein-co
lib-C666-l	chr7	158396326	158396326	+	14.54258	NA	Intergenic	Intergenic	12018	NR_04989	100847032		NR_04989	ENSG0000	MIR5707	-	microRNA	ncRNA
lib-C666-l	chr3	14988953	14988953	+	14.54258	NA	promoter-TSS (NM_001291694)	promoter-TSS (NM_001291694)	59	NR_04625	100505641	Hs.517821	NR_04625	ENSG0000	FGD5-AS1	-	FGD5 antis	ncRNA
lib-C666-l	chr12	104351020	104351020	+	14.54258	NA	promoter-TSS (NM_001135570)	promoter-TSS (NM_001135570)	-27	NM_00113	728568	Hs.42547	NM_00113	ENSG0000	C12orf73	-	chromosor	protein-co
lib-C666-l	chr2	198318097	198318097	+	14.51965	NA	promoter-TSS (NM_001320819)	promoter-TSS (NM_001320819)	-50	NM_00132	80219	Hs.606592	NM_02514	ENSG0000	COQ10B	-	coenzyme	protein-co
lib-C666-l	chr2	26241124	26241124	+	14.45854	NA	Intergenic	Intergenic	-15605	NM_01613	10890	Hs.467960	NM_01613	ENSG0000	RAB10	-	RAB10, me	protein-co
lib-C666-l	chr19	55973111	55973111	+	14.45701	NA	promoter-TSS (NM_001136201)	promoter-TSS (NM_001136201)	-62	NM_02471	79763	Hs.467306	NM_02471	ENSG0000	ISOC2	-	isochorism	protein-co
lib-C666-l	chr19	18530078	18530078	+	14.45701	NA	promoter-TSS (NM_032627)	promoter-TSS (NM_032627)	-68	NM_00100	170463	Hs.515259	NM_03262	ENSG0000	SSBP4	-	single strar	protein-co
lib-C666-l	chr7	44240435	44240435	+	14.45701	NA	promoter-TSS (NM_006555)	promoter-TSS (NM_006555)	-143	NM_00655	10652	Hs.520794	NM_00655	ENSG0000	YKT6	-	YKT6 v-SN	protein-co
lib-C666-l	chr20	3389465	3389465	+	14.40921	NA	Intergenic	Intergenic	-1156	NM_00100	25943	Hs.516853	NM_00100	ENSG0000	C20orf194	-	chromosor	protein-co
lib-C666-l	chr2	70368467	70368467	+	14.37873	NA	Intergenic	MIRb|SINE|MIR	-16019	NR_02444	100133985	Hs.135528	NR_02444	ENSG0000	LINC01816	-	long interg	ncRNA
lib-C666-l	chr2	128459361	128459361	+	14.37873	NA	exon (NM_032740, exon 1 of 1)	exon (NM_032740, exon 1 of 1)	764	NM_03274	84826	Hs.345849	NM_03274	ENSG0000	SFT2D3	-	SFT2 dom	protein-co
lib-C666-l	chr15	41079935	41079935	+	14.37873	NA	intron (NM_018163, intron 1 of 10)	intron (NM_018163, intron 1 of 10)	17776	NM_00113	643338	Hs.631715	NM_00113	ENSG0000	C15orf62	-	chromosor	protein-co
lib-C666-l	chr10	3929282	3929282	+	14.37873	NA	Intergenic	Intergenic	53140	NR_13449	105376365	Hs.460114	NR_13449	ENSG0000	LOC10537	-	uncharacte	ncRNA
lib-C666-l	chr13	107001783	107001783	+	14.37873	NA	Intergenic	Intergenic	-27128	NR_03411	728192	Hs.559194	NR_034119		LINC00460	-	long interg	ncRNA
lib-C666-l	chr12	121367344	121367344	+	14.34866	NA	Intergenic	Intergenic	24846	NR_13121	105500240	Hs.742757	NR_131215		XLOC_009	-	uncharacte	ncRNA
lib-C666-l	chr17	819096	819096	+	14.34866	NA	intron (NM_022463, intron 1 of 7)	intron (NM_022463, intron 1 of 7)	6224	NR_13563	101927727	Hs.666198	NR_13563	ENSG0000	LOC10192	-	uncharacte	ncRNA
lib-C666-l	chr19	42747256	42747256	+	14.34866	NA	promoter-TSS (NM_019884)	promoter-TSS (NM_019884)	-520	NM_01988	2931	Hs.466828	NM_01988	ENSG0000	GSK3A	-	glycogen s	protein-co
lib-C666-l	chr17	53315819	53315819	+	14.34866	NA	Intergenic	CpG	-26502	NM_00212	3131	Hs.196952	NM_00212	ENSG0000	HLF	-	HLF, PAR b	protein-co
lib-C666-l	chr1	15527258	15527258	+	14.32599	NA	intron (NM_018022, intron 1 of 2)	intron (NM_018022, intron 1 of 2)	-29138	NM_00127	727684	Hs.667584	NM_001278501		C1orf195	-	chromosor	protein-co
lib-C666-l	chr22	20379352	20379352	+	14.3056	NA	exon (NM_001242313, exon 5 of 9)	exon (NM_001242313, exon 5 of 9)	1683	NM_00124	728229	Hs.376511	NM_00124	ENSG0000	TMEM191	-	transmemb	protein-co
lib-C666-l	chr14	79511933	79511933	+	14.3056	NA	intron (NM_001330195, intron 15 of	intron (NM_001330195, intron 15 o	-154392	NR_13515	105370586		NR_13515	ENSG0000	LOC10537	-	uncharacte	ncRNA
lib-C666-l	chr1	182584080	182584080	+	14.3056	NA	promoter-TSS (NR_036490)	promoter-TSS (NR_036490)	-195	NR_03649	284648	Hs.253475	NR_03649	ENSG0000	LINC01686	-	long interg	ncRNA
lib-C666-l	chr3	50297619	50297619	+	14.3056	NA	TTS (NM_001282619)	TTS (NM_001282619)	7184	NR_11070	101928931	Hs.587216	NR_11070	ENSG0000	SEMA3B-A	-	SEMA3B ar	ncRNA
lib-C666-l	chr19	46386185	46386185	+	14.27758	NA	TTS (NM_015649)	TTS (NM_015649)	3243	NM_01564	26145	Hs.515477	NM_01564	ENSG0000	IRF2BP1	-	interferon	protein-co
lib-C666-l	chr7	104653233	104653233	+	14.27758	NA	non-coding (NR_024586, exon 1 of 1	non-coding (NR_024586, exon 1 of	1355	NR_02458	100216545	Hs.631150	NR_024586		KMT2E-AS	-	KMT2E ant	ncRNA
lib-C666-l	chr4	26859237	26859237	+	14.26909	NA	Intergenic	CpG	2629	NR_13467	105374546	Hs.548859	NR_134673		LOC10537	-	uncharacte	ncRNA
lib-C666-l	chr22	18893841	18893841	+	14.26909	NA	5' UTR (NM_005675, exon 1 of 5)	5' UTR (NM_005675, exon 1 of 5)	105	NM_00567	8214	Hs.474185	NM_00567	ENSG0000	DGCR6	-	DiGeorge	protein-co
lib-C666-l	chr16	4012938	4012938	+	14.20852	NA	3' UTR (NM_001116, exon 11 of 11)	3' UTR (NM_001116, exon 11 of 11)	-12493	NR_12031	102724927	Hs.364739	NR_12031	ENSG0000	LOC10272	-	uncharacte	ncRNA
lib-C666-l	chr11	65150376	65150376	+	14.06854	NA	promoter-TSS (NM_001278250)	promoter-TSS (NM_001278250)	-231	NM_00107	283130	Hs.661604	NM_18255	ENSG0000	SLC25A45	-	solute carr	protein-co
lib-C666-l	chr22	20427781	20427781	+	14.04835	NA	intron (NR_136572, intron 3 of 5)	CpG	-29086	NR_00356	728233	Hs.592456	NR_00356	ENSG0000	PI4KAP1	-	phosphatic	pseudo
lib-C666-l	chr1	4052397	4052397	+	14.0317	NA	Intergenic	(ATGTG)n|Simple_repeat|Simple_rep	51725	NR_04006	728716	Hs.668359	NR_040065		LINC01346	-	long interg	ncRNA
lib-C666-l	chr22	41777514	41777514	+	14.02822	NA	promoter-TSS (NM_003216)	promoter-TSS (NM_003216)	-419	NM_00321	7008	Hs.181159	NM_00321	ENSG0000	TEF	-	TEF, PAR b	protein-co
lib-C666-l	chr16	19113900	19113900	+	14.02822	NA	Intergenic	Intergenic	-11354	NM_00103	162073	Hs.530899	NM_00103	ENSG0000	ITPRIPL2	-	inositol 1,4	protein-co
lib-C666-l	chr12	99418151	99418151	+	14.02822	NA	intron (NM_152788, intron 17 of 25)	L1ME5|LINE|L1	-68986	NR_11009	101928937	Hs.555499	NR_11009	ENSG0000	LOC10192	-	uncharacte	ncRNA
lib-C666-l	chr1	112938685	112938685	+	14.02822	NA	promoter-TSS (NM_018704)	promoter-TSS (NM_018704)	-115	NM_01870	55917	Hs.744100	NM_01870	ENSG0000	CTTNBP2N	-	CTTNBP2	protein-co
lib-C666-l	chr8	182441	182441	+	13.96005	NA	promoter-TSS (NR_003572)	promoter-TSS (NR_003572)	-19	NM_00104	169270	Hs.591388	NM_17353	ENSG0000	ZNF596	-	zinc finger	protein-co
lib-C666-l	chr2	73404594	73404594	+	13.91591	NA	Intergenic	CpG	-24792	NM_00113	344022	Hs.694384	NM_00113	ENSG0000	NOTO	-	notochord	protein-co
lib-C666-l	chr10	87427755	87427755	+	13.91591	NA	intron (NM_017551, intron 12 of 15)	intron (NM_017551, intron 12 of 15	90267	NR_03898	100507470	Hs.585483	NR_03898	ENSG0000	GRID1-AS	-	GRID1 anti	ncRNA
lib-C666-l	chr1	32671265	32671265	+	13.8811	NA	promoter-TSS (NM_001160042)	promoter-TSS (NM_001160042)	29	NM_01813	55721	Hs.274356	NM_01813	ENSG0000	IQCC	-	IQ motif cc	protein-co
lib-C666-l	chr18	46281658	46281658	+	13.8811	NA	intron (NM_014772, intron 7 of 11)	intron (NM_014772, intron 7 of 11)	84687	NR_03989	100616366		NR_03989	ENSG0000	MIR4743	-	microRNA	ncRNA
lib-C666-l	chr8	33424690	33424690	+	13.8811	NA	promoter-TSS (NM_024787)	promoter-TSS (NM_024787)	-44	NM_02478	79845	Hs.151237	NM_02478	ENSG0000	RNF122	-	ring finger	protein-co
lib-C666-l	chr10	127661946	127661946	+	13.85062	NA	promoter-TSS (NR_102707)	promoter-TSS (NR_102707)	-104	NR_10270	101410540	Hs.557889	NR_10270	ENSG0000	FANK1-AS	-	FANK1 ant	ncRNA
lib-C666-l	chr8	23104109	23104109	+	13.84327	NA	promoter-TSS (NM_001317899)	promoter-TSS (NM_001317899)	4	NM_00131	91782	Hs.5019	NM_15227	ENSG0000	CHMP7	-	charged m	protein-co
lib-C666-l	chr12	118407307	118407307	+	13.80771	NA	Intergenic	CpG	-1279	NM_17359	283455	Hs.375836	NM_17359	ENSG0000	KSR2	-	kinase sup	protein-co
lib-C666-l	chr4	37563645	37563645	+	13.80771	NA	intron (NM_001104629, intron 2 of 3)	MER82|DNA|TcMar-Tigger	-22210	NM_01830	55286	Hs.107527	NM_01830	ENSG0000	C4orf19	-	chromosor	protein-co
lib-C666-l	chr4	2420624	2420624	+	13.7499	NA	promoter-TSS (NM_001172656)	promoter-TSS (NM_001172656)	-48	NM_00119	402160	Hs.443116	NM_00119	ENSG0000	CFAP99	-	cilia and fla	protein-co
lib-C666-l	chr2	171571049	171571049	+	13.65283	NA	promoter-TSS (NR_027433)	promoter-TSS (NR_027433)	28	NR_02743	440925	Hs.668461	NM_00101	ENSG0000	LINC01124	-	long interg	ncRNA
lib-C666-l	chr8	67159847	67159847	+	13.65283	NA	Intergenic	MER11A|LTR|ERVK	55498	NR_03997	100505659	Hs.529729	NR_03997	ENSG0000	LINC00967	-	long interg	ncRNA
lib-C666-l	chr6	162339248	162339248	+	13.63441	NA	intron (NM_013987, intron 5 of 10)	intron (NM_013987, intron 5 of 10)	447239	NR_13459	105378098	Hs.691066	NR_134593		LOC10537	-	uncharacte	ncRNA
lib-C666-l	chr19	19174728	19174728	+	13.6111	NA	promoter-TSS (NM_001321544)	promoter-TSS (NM_001321544)	-42	NM_00132	284439	Hs.303669	NM_17852	ENSG0000	SLC25A42	-	solute carr	protein-co
lib-C666-l	chr17	26368639	26368639	+	13.59977	NA	Intergenic	CpG	-1049	NM_01623	51701	Hs.208759	NM_01623	ENSG0000	NLK	-	nemo like	protein-co
lib-C666-l	chr19	51897658	51897658	+	13.59977	NA	Intergenic	CpG	-3830	NM_00119	147646	Hs.148344	NM_00119	ENSG0000	C19orf84	-	chromosor	protein-co
lib-C666-l	chr3	183415715	183415715	+	13.59977	NA	5' UTR (NM_018023, exon 1 of 31)	5' UTR (NM_018023, exon 1 of 31)	109	NM_01802	55689	Hs.632575	NM_01802	ENSG0000	YEATS2	-	YEATS don	protein-co
lib-C666-l	chr2	157038916	157038916	+	13.59977	NA	intron (NR_110250, intron 1 of 3)	intron (NR_110250, intron 1 of 3)	72527	NR_11025	101929378	Hs.171192	NR_11024	ENSG0000	LINC01876	-	long interg	ncRNA
lib-C666-l	chr15	91498475	91498475	+	13.59267	NA	intron (NM_001017919, intron 1 of 7)	CpG	369	NM_00101	91433	Hs.655895	NM_03354	ENSG0000	RCCD1	-	RCC1 dom	protein-co
lib-C666-l	chr7	155137492	155137492	+	13.59254	NA	Intergenic	Intergenic	23136	NR_10354	338436	Hs.521326	NR_10354	ENSG0000	BLACE	-	B-cell acut	ncRNA
lib-C666-l	chr7	158769886	158769886	+	13.59254	NA	Intergenic	L1MC1|LINE|L1	-31159	NR_02439	154822	Hs.544783	NM_19834	ENSG0000	LINC00689	-	long interg	ncRNA
lib-C666-l	chr22	35695750	35695750	+	13.55025	NA	promoter-TSS (NM_001135732)	promoter-TSS (NM_001135732)	-47	NM_00113	10043	Hs.474705	NM_00548	ENSG0000	TOM1	-	target of m	protein-co
lib-C666-l	chr10	80826865	80826865	+	13.5289	NA	intron (NR_024431, intron 1 of 9)	intron (NR_024431, intron 1 of 9)	340	NR_02443	283050	Hs.309176	NR_01542	ENSG0000	ZMIZ1-AS	-	ZMIZ1 anti	ncRNA
lib-C666-l	chr2	97760258	97760258	+	13.46097	NA	intron (NM_001320848, intron 1 of 8)	CpG	361	NM_00132	151313	Hs.567723	NM_19933	ENSG0000	FAHD2B	-	fumarylace	protein-co
lib-C666-l	chr1	224401891	224401891	+	13.43408	NA	promoter-TSS (NR_110669)	promoter-TSS (NR_110669)	-910	NR_11066	101927143	Hs.638720	NR_11066	ENSG0000	LOC10192	-	uncharacte	ncRNA
lib-C666-l	chr4	39046837	39046837	+	13.43086	NA	intron (NM_001171654, intron 1 of 1	CpG	386	NM_00117	51088	Hs.272251	NM_01599	ENSG0000	KLHL5	-	kelch like f	protein-co
lib-C666-l	chr5	116099947	116099947	+	13.33918	NA	Intergenic	Intergenic	-2042	NR_10467	102467223	Hs.570916	NR_104678		LOC10246	-	uncharacte	ncRNA
lib-C666-l	chr1	1676541	1676541	+	13.33696	NA	intron (NM_001199787, intron 1 of 6)	intron (NM_001199787, intron 1 of	897	NM_18283	9906	Hs.655255	NM_18283	ENSG0000	SLC35E2	-	solute carr	protein-co
lib-C666-l	chr11	65149867	65149867	+	13.33696	NA	promoter-TSS (NM_001278250)	promoter-TSS (NM_001278250)	-10	NM_00127	283130	Hs.661604	NM_18255	ENSG0000	SLC25A45	-	solute carr	protein-co
lib-C666-l	chr3	46989678	46989678	+	13.33696	NA	intron (NM_144716, intron 2 of 7)	intron (NM_144716, intron 2 of 7)	28313	NR_10226	151903	Hs.631918	NM_14471	ENSG0000	CCDC12	-	coiled-coil	protein-co
lib-C666-l	chr6	94947129	94947129	+	13.20416	NA	Intergenic	Intergenic	530328	NR_01536	643432	Hs.509936	NR_015362		TSG1	-	tumor sup	ncRNA
lib-C666-l	chr11	43942457	43942457	+	13.20416	NA	promoter-TSS (NR_038907)	promoter-TSS (NR_038907)	37	NR_03890	100507300	Hs.664588	NR_03890	ENSG0000	ALKBH3-A	-	ALKBH3 ar	ncRNA
lib-C666-l	chr19	55943805	55943805	+	13.20416	NA	3' UTR (NM_001145176, exon 4 of 4)	3' UTR (NM_001145176, exon 4 of	10425	NM_00114	729956	Hs.6664	NM_17590	ENSG0000	SHISA7	-	shisa family	protein-co
lib-C666-l	chr3	129420208	129420208	+	13.20416	NA	intron (NM_001128224, intron 3 of 5)	intron (NM_001128224, intron 3 of	-12633	NR_03336	23023	Hs.477547	NM_01500	ENSG0000	TMCC1	-	transmemb	protein-co
lib-C666-l	chr7	41068801	41068801	+	13.20416	NA	Intergenic	Intergenic	-49264	NR_11083	101928744	Hs.639767	NR_11083	ENSG0000	LINC01450	-	long interg	ncRNA
lib-C666-l	chr4	56232967	56232967	+	13.16034	NA	non-coding (NR_037969, exon 4 of 4	non-coding (NR_037969, exon 4 of	18780	NR_03796	100506462	Hs.718667	NR_03796	ENSG0000	SRD5A3-A	-	SRD5A3 ar	ncRNA
lib-C666-l	chr17	65220379	65220379	+	13.15801	NA	intron (NM_001330447, intron 3 of 3	MLT1K|LTR|ERVL-MaLR	-20513	NR_13639	101928021	Hs.569824	NR_136398		LOC10192	-	uncharacte	ncRNA
lib-C666-l	chr2	7172033	7172033	+	13.14211	NA	intron (NM_014746, intron 8 of 8)	MER58A|DNA|hAT-Charlie	45978	NR_11025	101929452		NR_11025	ENSG0000	LOC10192	-	uncharacte	ncRNA
lib-C666-l	chr7	155284069	155284069	+	13.06669	NA	Intergenic	CpG	33245	NM_00142	2020	Hs.134989	NM_00142	ENSG0000	EN2	-	engrailed	protein-co
lib-C666-l	chr13	106796939	106796939	+	13.06669	NA	Intergenic	Eulor9C|DNA|DNA	-231972	NR_03411	728192	Hs.559194	NR_034119		LINC00460	-	long interg	ncRNA
lib-C666-l	chr10	111841923	111841923	+	13.03912	NA	intron (NM_016824, intron 1 of 14)	intron (NM_016824, intron 1 of 14)	-73784	NR_03894	100505933	Hs.604103	NR_038943		ADD3-AS1	-	ADD3 antis	ncRNA
lib-C666-l	chr17	66196997	66196997	+	12.99339	NA	TTS (NR_027283)	TTS (NR_027283)	2196	NR_02728	440461	Hs.661522	NR_027283		LOC44046	-	Rho GTPas	pseudo

lib-C666-l	chr5	116223041	116223041	+	12.97267	NA	Intergenic	Intergenic	-125136	NR_10467	102467223	Hs.570916	NR_104678		LOC10246	-	uncharacte	ncRNA
lib-C666-l	chr18	8375568	8375568	+	12.97267	NA	intron (NM_001105244, intron 24 of	intron (NM_001105244, intron 24 o	-8536	NR_02441	100192426	Hs.666655	NR_02441	ENSG0000	LOC10019	-	uncharacte	ncRNA
lib-C666-l	chr5	75599922	75599922	+	12.97267	NA	intron (NM_001297716, intron 12 of	LTR16B2|LTR|ERVL	-99158	NM_00663	10788	Hs.291030	NM_00663	ENSG0000	IQGAP2	-	IQ motif cc	protein-co
lib-C666-l	chr9	74384097	74384097	+	12.83248	NA	promoter-TSS (NM_001135820)	promoter-TSS (NM_001135820)	-297	NM_00113	23670	Hs.494146	NM_01339	ENSG0000	TMEM2	-	transmemb	protein-co
lib-C666-l	chr2	42795265	42795265	+	12.83248	NA	promoter-TSS (NM_001282756)	promoter-TSS (NM_001282756)	80	NM_00128	57504	Hs.435413	NM_02074	ENSG0000	MTA3	-	metastasis	protein-co
lib-C666-l	chr7	104624403	104624403	+	12.82883	NA	non-coding (NR_039981, exon 1 of 1	non-coding (NR_039981, exon 1 of	7209	NR_03998	100216546	Hs.369356	NR_039981		LINC01004	-	long interg	ncRNA
lib-C666-l	chr1	33241302	33241302	+	12.81406	NA	TTS (NM_020888)	TTS (NM_020888)	10067	NM_00119	57648	Hs.591502	NM_02088	ENSG0000	KIAA1522	-	KIAA1522	protein-co
lib-C666-l	chr10	89102762	89102762	+	12.81406	NA	promoter-TSS (NR_024397)	promoter-TSS (NR_024397)	269	NR_13529	439994	Hs.651188	NR_029408		LINC00863	-	long interg	ncRNA
lib-C666-l	chr17	55860905	55860905	+	12.78696	NA	Intergenic	Intergenic	-38215	NM_00128	101927581	Hs.540784	NM_00128	ENSG0000	CCDC182	-	coiled-coil	protein-co
lib-C666-l	chrX	131352386	131352386	+	12.78696	NA	promoter-TSS (NM_021183)	promoter-TSS (NM_021183)	-149	NR_11041	101928578	Hs.556853	NR_11041	ENSG0000	RAP2C-AS	-	RAP2C ant	ncRNA
lib-C666-l	chrX	149352987	149352987	+	12.78696	NA	Intergenic	Intergenic	-43252	NR_03174	100313839		NR_03174	ENSG0000	MIR2114	-	microRNA	ncRNA
lib-C666-l	chr8	41909401	41909401	+	12.73831	NA	non-coding (NR_136589, exon 1 of 2	non-coding (NR_136589, exon 1 of	133	NR_13658	105379393	Hs.742878	NR_136589		LOC10537	-	uncharacte	ncRNA
lib-C666-l	chr22	25390531	25390531	+	12.73831	NA	Intergenic	Intergenic	-33410	NM_00114	85379	Hs.419171	NM_00114	ENSG0000	KIAA1671	-	KIAA1671	protein-co
lib-C666-l	chr19	7933914	7933914	+	12.73831	NA	non-coding (NR_104345, exon 7 of 7	non-coding (NR_104345, exon 7 of	5412	NR_10434	80164	Hs.288540	NM_00108	ENSG0000	PRR36	-	proline rich	protein-co
lib-C666-l	chr15	30027505	30027505	+	12.7379	NA	intron (NM_001330239, intron 11 of	intron (NM_001330239, intron 11 o	60366	NR_13522	100130111	Hs.525720	NR_13522	ENSG0000	LOC10013	-	uncharacte	ncRNA
lib-C666-l	chr17	80164147	80164147	+	12.7379	NA	intron (NM_198082, intron 1 of 16)	intron (NM_198082, intron 1 of 16)	6542	NM_19808	284001	Hs.631724	NM_15267	ENSG0000	CCDC57	-	coiled-coil	protein-co
lib-C666-l	chr19	7069597	7069597	+	12.7379	NA	5' UTR (NM_001044387, exon 1 of 8)	5' UTR (NM_001044387, exon 1 of	126	NM_00104	79230	Hs.591380	NM_02434	ENSG0000	ZNF557	-	zinc finger	protein-co
lib-C666-l	chr6	40965403	40965403	+	12.72278	NA	intron (NR_110873, intron 1 of 5)	intron (NR_110873, intron 1 of 5)	25616	NR_11087	101929555	Hs.552555	NR_110873		LOC10192	-	uncharacte	ncRNA
lib-C666-l	chr15	72978569	72978569	+	12.70401	NA	promoter-TSS (NR_002780)	promoter-TSS (NR_002780)	49	NR_04556	585	Hs.208681	NM_03302	ENSG0000	BBS4	-	Bardet-Bie	protein-co
lib-C666-l	chr22	40421062	40421062	+	12.69461	NA	intron (NM_138435, intron 4 of 4)	intron (NM_138435, intron 4 of 4)	-7274	NR_03998	100130899	Hs.26192	NR_039988		LOC10013	-	uncharacte	ncRNA
lib-C666-l	chr1	15943847	15943847	+	12.6699	NA	promoter-TSS (NM_032341)	promoter-TSS (NM_032341)	-106	NM_03234	84301	Hs.718857	NM_03234	ENSG0000	DDI2	-	DNA dama	protein-co
lib-C666-l	chr22	21823145	21823145	+	12.62235	NA	intron (NM_001207052, intron 5 of 8)	CpG-17591	1686	NM_00120	645426	Hs.376511	NM_00120	ENSG0000	TMEM191	-	transmemb	protein-co
lib-C666-l	chr12	8464008	8464008	+	12.62235	NA	Intergenic	Intergenic	-68466	NR_02425	653113	Hs.661749	NR_024254		FAM86FP	-	family with	pseudo
lib-C666-l	chr7	112758686	112758686	+	12.62235	NA	promoter-TSS (NR_024412)	promoter-TSS (NR_024412)	-49	NR_02441	401397	Hs.117929	NR_01544	ENSG0000	LINC00998	-	long interg	ncRNA
lib-C666-l	chr3	193788771	193788771	+	12.62235	NA	promoter-TSS (NR_136179)	promoter-TSS (NR_136179)	-12	NR_13617	285389	Hs.157102	NR_13617	ENSG0000	LINC02028	-	long interg	ncRNA
lib-C666-l	chr3	156544673	156544673	+	12.62235	NA	5' UTR (NM_001193283, exon 1 of 5)	5' UTR (NM_001193283, exon 1 of	525	NM_00119	389170	Hs.478048	NM_00100	ENSG0000	LEKR1	-	leucine, glu	protein-co
lib-C666-l	chr3	177077814	177077814	+	12.62235	NA	Intergenic	Intergenic	65584	NR_04746	100820709	Hs.518409	NR_04746	ENSG0000	LINC00501	-	long interg	ncRNA
lib-C666-l	chr1	170561863	170561863	+	12.62235	NA	Intergenic	Intergenic	-60113	NR_12595	101928650	Hs.676662	NR_125958		LOC10192	-	uncharacte	ncRNA
lib-C666-l	chr20	48782742	48782742	+	12.62235	NA	Intergenic	Intergenic	-6365	NR_10994	101927541	Hs.570391	NR_10994	ENSG0000	LINC01273	-	long interg	ncRNA
lib-C666-l	chr4	144105835	144105835	+	12.5768	NA	promoter-TSS (NM_032557)	promoter-TSS (NM_032557)	179	NR_13620	105377623		NR_13620	ENSG0000	LOC10537	-	uncharacte	ncRNA
lib-C666-l	chr14	90422354	90422354	+	12.57335	NA	promoter-TSS (NM_001330205)	promoter-TSS (NM_001330205)	41	NM_00133	55775	Hs.209945	NM_01831	ENSG0000	TDP1	-	tyrosyl-DN	protein-co
lib-C666-l	chr3	8543388	8543388	+	12.55817	NA	promoter-TSS (NM_014583)	promoter-TSS (NM_014583)	-44	NR_03337	100288428	Hs.651576	NR_03337	ENSG0000	LMCD1-AS	-	LMCD1 an	ncRNA
lib-C666-l	chr2	219433146	219433146	+	12.55817	NA	promoter-TSS (NM_020935)	promoter-TSS (NM_020935)	-62	NM_02093	57695	Hs.166068	NM_02093	ENSG0000	USP37	-	ubiquitin s	protein-co
lib-C666-l	chr17	42092392	42092392	+	12.55817	NA	promoter-TSS (NM_032376)	promoter-TSS (NM_032376)	5	NM_03237	84336	Hs.514211	NM_03237	ENSG0000	TMEM101	-	transmemb	protein-co
lib-C666-l	chr2	109403207	109403207	+	12.55817	NA	promoter-TSS (NM_001303106)	promoter-TSS (NM_001303106)	1	NM_14497	165055	Hs.362702	NM_14497	ENSG0000	CCDC138	-	coiled-coil	protein-co
lib-C666-l	chr10	13141835	13141835	+	12.47847	NA	promoter-TSS (NM_001282658)	promoter-TSS (NM_001282658)	-62	NM_00128	83643	Hs.498720	NM_03145	ENSG0000	CCDC3	-	coiled-coil	protein-co
lib-C666-l	chr9	68726271	68726271	+	12.44501	NA	promoter-TSS (NR_034006).2	promoter-TSS (NR_034006).2	-195	NR_13630	102725126	Hs.231861	NR_136306		LOC10272	-	FSHD regic	pseudo
lib-C666-l	chr12	105065699	105065699	+	12.37529	NA	intron (NM_001173982, intron 2 of 2)	intron (NM_001173982, intron 2 of	80288	NR_03748	100500843		NR_03748	ENSG0000	MIR3922	-	microRNA	ncRNA
lib-C666-l	chr12	89511659	89511659	+	12.34228	NA	Intergenic	Intergenic	-98190	NR_03838	728084	Hs.132563	NR_03838	ENSG0000	LOC72808	-	uncharacte	ncRNA
lib-C666-l	chr19	1407211	1407211	+	12.34228	NA	promoter-TSS (NM_170711)	promoter-TSS (NM_170711)	-357	NM_17071	26528	Hs.222510	NM_01895	ENSG0000	DAZAP1	-	DAZ assoc	protein-co
lib-C666-l	chr15	28834508	28834508	+	12.26631	NA	Intergenic	CpG	-56365	NR_03335	100132565	Hs.510812	NM_00101	ENSG0000	GOLGA8F	-	golgin A8	pseudo
lib-C666-l	chr1	24543530	24543530	+	12.14922	NA	Intergenic	MIR|SINE|MIR	16800	NR_02708	284632	Hs.638906	NR_02708	ENSG0000	LOC28463	-	uncharacte	ncRNA
lib-C666-l	chr19	13842090	13842090	+	12.13776	NA	promoter-TSS (NM_001320561)	promoter-TSS (NM_001320561)	-484	NM_00132	81576	Hs.24998	NM_03081	ENSG0000	CCDC130	-	coiled-coil	protein-co
lib-C666-l	chr1	234735703	234735703	+	12.12723	NA	Intergenic	CpG	9568	NM_00107	359948	Hs.350268	NM_18297	ENSG0000	IRF2BP2	-	interferon	protein-co
lib-C666-l	chr18	38975902	38975902	+	12.12723	NA	Intergenic	Intergenic	124659	NR_00283	641516	Hs.116612	NR_00283	ENSG0000	KC6	-	keratocon	ncRNA
lib-C666-l	chr9	116173000	116173000	+	12.12723	NA	promoter-TSS (NM_001278255)	promoter-TSS (NM_001278255)	11	NM_00127	257169	Hs.632691	NM_15278	ENSG0000	C9orf43	-	chromosor	protein-co
lib-C666-l	chr3	64676821	64676821	+	12.12504	NA	intron (NR_038264, intron 1 of 5)	intron (NR_038264, intron 1 of 5)	-3456	NM_18292	56999	Hs.656071	NM_18292	ENSG0000	ADAMTS9	-	ADAM met	protein-co
lib-C666-l	chr1	93790355	93790355	+	12.12504	NA	intron (NR_034089, intron 7 of 8)	L3b|LINE|CR1	21013	NR_03408	100131564	Hs.732666	NR_034089		CCDC18-A	-	CCDC18 ar	ncRNA
lib-C666-l	chr5	69006857	69006857	+	12.12504	NA	promoter-TSS (NR_027386)	promoter-TSS (NR_027386)	-503	NR_02738	653188	Hs.631974	NR_02738	ENSG0000	GUSBP3	-	glucuronid	pseudo
lib-C666-l	chr22	19595166	19595166	+	12.12504	NA	Intergenic	Intergenic	-40804	NR_02438	150185	Hs.719752	NR_024381		LINC00895	-	long interg	ncRNA
lib-C666-l	chr15	23579524	23579524	+	12.12504	NA	promoter-TSS (NM_001271664).2	promoter-TSS (NM_001271664).2	-167	NM_00127	440243		NM_001271664		GOLGA6L2	-	golgin A6	protein-co
lib-C666-l	chrX	70288429	70288429	+	12.12504	NA	promoter-TSS (NM_013346)	promoter-TSS (NM_013346)	-131	NM_00125	29934	Hs.260750	NM_01334	ENSG0000	SNX12	-	sorting nex	protein-co
lib-C666-l	chr4	119810012	119810012	+	12.12504	NA	promoter-TSS (NM_001128934)	promoter-TSS (NM_001128934)	16	NM_13347	171024	Hs.655519	NM_13347	ENSG0000	SYNPO2	-	synaptopo	protein-co
lib-C666-l	chr5	105662363	105662363	+	12.12504	NA	Intergenic	THE1B|LTR|ERVL-MaLR	684352	NR_10467	102467213	Hs.570923	NR_10467	ENSG0000	LINC0195	-	long interg	ncRNA
lib-C666-l	chr8	11973451	11973451	+	12.10977	NA	TTS (NM_001039615)	TTS (NM_001039615)	160	NR_02742	100132923	Hs.546711	NR_027425		FAM66D	-	family with	ncRNA
lib-C666-l	chr5	174256751	174256751	+	12.02272	NA	Intergenic	MER77B|LTR|ERVL	78014	NR_03977	100616202		NR_03977	ENSG0000	MIR4634	-	microRNA	ncRNA
lib-C666-l	chr17	79650862	79650862	+	11.95031	NA	promoter-TSS (NM_001329609)	promoter-TSS (NM_001329609)	92	NR_13805	339231	Hs.658789	NM_00104	ENSG0000	ARL16	-	ADP ribosy	protein-co
lib-C666-l	chr10	82280843	82280843	+	11.93581	NA	3' UTR (NM_001128309, exon 6 of 6)	3' UTR (NM_001128309, exon 6 of	-10163	NR_12066	102723703	Hs.308615	NR_120662		LOC10272	-	uncharacte	ncRNA
lib-C666-l	chr4	106912346	106912346	+	11.93581	NA	Intergenic	Intergenic	31289	NR_12592	101929577	Hs.631131	NR_12592	ENSG0000	LOC10192	-	uncharacte	ncRNA
lib-C666-l	chr11	66233699	66233699	+	11.93581	NA	promoter-TSS (NM_001243135)	promoter-TSS (NM_001243135)	-99	NM_00124	246330	Hs.656875	NM_14506	ENSG0000	PELI3	-	pellino E3	protein-co
lib-C666-l	chr2	70529109	70529109	+	11.93581	NA	promoter-TSS (NM_001329752)	promoter-TSS (NM_001329752)	68	NM_00132	84908	Hs.516077	NM_03282	ENSG0000	FAM136A	-	family with	protein-co
lib-C666-l	chr1	994243	994243	+	11.93581	NA	Intergenic	Intergenic	15444	NM_00120	401934	Hs.568137	NM_00120	ENSG0000	RNF223	-	ring finger	protein-co
lib-C666-l	chr22	50983304	50983304	+	11.93581	NA	Intergenic	Intergenic	-3158	NM_13843	113730	Hs.137007	NM_13843	ENSG0000	KLHDC7B	-	kelch dom	protein-co
lib-C666-l	chr3	184495851	184495851	+	11.93581	NA	Intergenic	L2c|LINE|L2	-4908	NR_11004	101928992	Hs.507129	NR_11004	ENSG0000	LINC02069	-	long interg	ncRNA
lib-C666-l	chr19	58892229	58892229	+	11.90035	NA	intron (NR_049780, intron 1 of 2)	CpG	160	NR_04978	116412	Hs.222236	NM_13846	ENSG0000	ZNF837	-	zinc finger	protein-co
lib-C666-l	chr5	176728937	176728937	+	11.86993	NA	exon (NM_001031677, exon 7 of 8)	exon (NM_001031677, exon 7 of 8)	1808	NM_00103	53917	Hs.16258	NM_13078	ENSG0000	RAB24	-	RAB24, me	protein-co
lib-C666-l	chr8	82754420	82754420	+	11.86711	NA	5' UTR (NM_152837, exon 1 of 7)	5' UTR (NM_152837, exon 1 of 7)	101	NM_15283	64089	Hs.492121	NM_02213	ENSG0000	SNX16	-	sorting nex	protein-co
lib-C666-l	chr17	4463592	4463592	+	11.86603	NA	intron (NM_153338, intron 1 of 2)	intron (NM_153338, intron 1 of 2)	299	NM_00128	124975	Hs.130749	NM_15333	ENSG0000	GGT6	-	gamma-gl	protein-co
lib-C666-l	chr2	161135153	161135153	+	11.79284	NA	exon (NM_016836, exon 11 of 14)	exon (NM_016836, exon 11 of 14)	-6747	NR_10377	100505984	Hs.729532	NR_10377	ENSG0000	LOC10050	-	uncharacte	ncRNA
lib-C666-l	chr19	12893397	12893397	+	11.68542	NA	Intergenic	CpG	-4545	NR_04986	100847071		NR_04986	ENSG0000	MIR5684	-	microRNA	ncRNA
lib-C666-l	chr2	197097808	197097808	+	11.66438	NA	intron (NM_001304840, intron 19 of	intron (NM_001304840, intron 19 o	-26940	NR_11022	101927482	Hs.191841	NR_110225		LOC10192	-	uncharacte	ncRNA
lib-C666-l	chr16	2040460	2040460	+	11.66438	NA	intron (NM_004209, intron 1 of 3)	CpG	514	NM_00420	9143	Hs.435277	NM_00420	ENSG0000	SYNGR3	-	synaptogy	protein-co
lib-C666-l	chr1	118302944	118302944	+	11.66438	NA	Intergenic	Intergenic	154340	NM_01770	54855	Hs.356216	NM_01770	ENSG0000	FAM46C	-	family with	protein-co
lib-C666-l	chr1	187154706	187154706	+	11.66438	NA	intron (NR_126347, intron 1 of 3)	L1MB4|LINE|L1	92732	NR_12634	104169671		NR_126347		LINC01036	-	long interg	ncRNA
lib-C666-l	chr11	67559841	67559841	+	11.66012	NA	non-coding (NR_024249, exon 4 of 4	non-coding (NR_024249, exon 4 of	12966	NR_02424	645332	Hs.535094	NR_02424	ENSG0000	FAM86C2P	-	family with	pseudo
lib-C666-l	chr2	71454360	71454360	+	11.66012	NA	promoter-TSS (NM_020459)	promoter-TSS (NM_020459)	-127	NM_02045	400961	Hs.416735	NM_02045	ENSG0000	PAIP2B	-	poly(A) bin	protein-co
lib-C666-l	chr12	124086541	124086541	+	11.66012	NA	promoter-TSS (NM_020936)	promoter-TSS (NM_020936)	-83	NR_13510	57696	Hs.286173	NM_02093	ENSG0000	DDX55	-	DEAD-box	protein-co
lib-C666-l	chr12	117537331	117537331	+	11.66012	NA	promoter-TSS (NM_001168325)	promoter-TSS (NM_001168325)	59	NR_12046	101928244	Hs.742266	NR_120464		TESC-AS1	-	TESC antis	ncRNA
lib-C666-l	chr2	8821647	8821647	+	11.66012	NA	promoter-TSS (NM_002166)	promoter-TSS (NM_002166)	344	NR_11015	100506299	Hs.439031	NR_11015	ENSG0000	ID2-AS1	-	ID2 antiser	ncRNA
lib-C666-l	chr4	123653730	123653730	+	11.66012	NA	promoter-TSS (NM_001178007)	promoter-TSS (NM_001178007)	-117	NR_02404	729338	Hs.647968	NR_024041		CETN4P	-	centrin 4, p	pseudo
lib-C666-l	chr17	48556208	48556208	+	11.66012	NA	promoter-TSS (NM_018346)	promoter-TSS (NM_018346)	47	NM_01834	55316	Hs.8033	NM_01834	ENSG0000	RSAD1	-	radical S-a	protein-co
lib-C666-l	chr13	78582018	78582018	+	11.6497	NA	Intergenic	Intergenic	-5000	NR_04702	100874175	Hs.130364	NR_047028		LINC00446	-	long interg	ncRNA
lib-C666-l	chr8	74433043	74433043	+	11.6497	NA	intron (NM_001164383, intron 8 of 9)	MLT1L|LTR|ERVL-MaLR	100734	NR_03840	100128126	Hs.679921	NR_03840	ENSG0000	STAU2-AS	-	STAU2 ant	ncRNA
lib-C666-l	chr2	46727475	46727475	+	11.6497	NA	Intergenic	Intergenic	20771	NM_00114	388946	Hs.733348	NM_00114	ENSG0000	TMEM247	-	transmemb	protein-co
lib-C666-l	chr2	240323070	240323070	+	11.6145	NA	promoter-TSS (NR_135575)	promoter-TSS (NR_135575)	-60	NR_13557	101928111	Hs.534680	NR_13557	ENSG0000	LOC10192	-	uncharacte	ncRNA
lib-C666-l	chr15	44487585	44487585	+	11.57178	NA	promoter-TSS (NM_001286490)	promoter-TSS (NM_001286490)	-93	NM_00132	84978	Hs.578544	NM_03289	ENSG0000	FRMD5	-	FERM dom	protein-co
lib-C666-l	chrX	106809698	106809698	+	11.57178	NA	intron (NM_032428, intron 13 of 15)	intron (NM_032428, intron 13 of 15	-20647	NR_04675	100874122	Hs.600967	NR_04675	ENSG0000	FRMPD3-A	-	FRMPD3 a	ncRNA
lib-C666-l	chr5	66192181	66192181	+	11.57178	NA	intron (NM_015183, intron 1 of 27)	L1MD3|LINE|L1	-62517	NM_00129	375449	Hs.595458	NM_01518	ENSG0000	MAST4	-	microtubul	protein-co
lib-C666-l	chr5	33009007	33009007	+	11.57178	NA	Intergenic	Intergenic	61458	NR_03383	340113	Hs.434404	NR_03383	ENSG0000	LINC0212	-	long interg	ncRNA
lib-C666-l	chr4	136982137	136982137	+	11.57178	NA	Intergenic	Intergenic	-147302	NR_10376	100507528	Hs.518987	NR_10376	ENSG0000	LINC00613	-	long interg	ncRNA
lib-C666-l	chr11	124632708	124632708	+	11.57178	NA	promoter-TSS (NM_138961)	promoter-TSS (NM_138961)	381	NR_12057	101929340	Hs.173840	NR_12057	ENSG0000	LOC10192	-	uncharacte	ncRNA
lib-C666-l	chr1	48014073	48014073	+	11.57119	NA	Intergenic	Intergenic	-111084	NR_12635	102724077	Hs.159718	NR_126355		LINC01389	-	long interg	ncRNA
lib-C666-l	chr5	75519792	75519792	+	11.57119	NA	intron (NM_001297716, intron 4 of 12	MLT1A|LTR|ERVL-MaLR	140553	NM_01497	22987	Hs.646953	NM_01497	ENSG0000	SV2C	-	synaptic ve	protein-co
lib-C666-l	chr5	134361101	134361101	+	11.55724	NA	Intergenic	Intergenic	-7869	NM_00127	100996485		NM_001277348		C5orf66	-	chromosor	protein-co
lib-C666-l	chr1	105249621	105249621	+	11.55724	NA	Intergenic	Intergenic	633976	NR_03399	100129138	Hs.514487	NR_03399	ENSG0000	LOC10012	-	THAP dom	pseudo
lib-C666-l	chr20	3777280	3777280	+	11.5173	NA	exon (NM_004358, exon 1 of 16)	exon (NM_004358, exon 1 of 16)	894	NM_00128	994	Hs.153752	NM_00435	ENSG0000	CDC25B	-	cell divisior	protein-co
lib-C666-l	chr3	101395159	101395159	+	11.5173	NA	promoter-TSS (NR_024407)	promoter-TSS (NR_024407)	-115	NR_02440	100009676	Hs.743292	NR_02440	ENSG0000	ZBTB11-AS	-	ZBTB11 an	ncRNA
lib-C666-l	chr4	39529397	39529397	+	11.5173	NA	promoter-TSS (NM_001184701)	promoter-TSS (NM_001184701)	-62	NR_04767	100885776	Hs.640769	NR_04767	ENSG0000	UGDH-AS	-	UGDH anti	ncRNA
lib-C666-l	chr1	228263331	228263331	+	11.50397	NA	Intergenic	Intergenic	-7030	NM_00165	375	Hs.286221	NM_00165	ENSG0000	ARF1	-	ADP ribosy	protein-co
lib-C666-l	chr1	46943287	46943287	+	11.37021	NA	Intergenic	Intergenic	-27911	NR_12168	101929651	Hs.638707	NR_12168	ENSG0000	LINC01398	-	long interg	ncRNA
lib-C666-l	chr17	33601523	33601523	+	11.36743	NA	TTS (NM_001330183)	TTS (NM_001330183)	31437	NM_14497	162394	Hs.585792	NM_14497	ENSG0000	SLFN5	-	schlafen fa	protein-co
lib-C666-l	chr8	80813304	80813304	+	11.36743	NA	Intergenic	MER65B|LTR|ERV1	-29332	NR_11095	101927040	Hs.350876	NR_11095	ENSG0000	LOC10192	-	uncharacte	ncRNA
lib-C666-l	chr15	75165512	75165512	+	11.36743	NA	intron (NM_005697, intron 1 of 8)	CpG	208	NM_00132	10066	Hs.458917	NM_00569	ENSG0000	SCAMP2	-	secretory c	protein-co
lib-C666-l	chr13	27536204	27536204	+	11.36353	NA	Intergenic	Intergenic	-163466	NR_04654	100874070	Hs.524923	NR_046547		USP12-AS	-	USP12 anti	ncRNA
lib-C666-l	chr8	12459030	12459030	+	11.33964	NA	intron (NR_047662, intron 5 of 8)	CpG-25436	64090	NR_04766	729732	Hs.322761	NR_04766	ENSG0000	LOC72973	-	uncharacte	ncRNA
lib-C666-l	chr4	183786921	183786921	+	11.28944	NA	Intergenic	AluY|SINE|Alu	51709	NM_00101	1635	Hs.183850	NM_00192	ENSG0000	DCTD	-	dCMP dea	protein-co
lib-C666-l	chr7	28189512	28189512	+	11.23774	NA	intron (NM_175061, intron 1 of 4)	intron (NM_175061, intron 1 of 4)	-30564	NR_03409	100128081	Hs.434307	NR_034097		JAZF1-AS1	-	JAZF1 antis	ncRNA
lib-C666-l	chr14	23476350	23476350	+	11.22379	NA	intron (NM_001130706, intron 1 of 7)	CpG	3060	NM_02194	60686	Hs.255874	NM_02194	ENSG0000	C14orf93	-	chromosor	protein-co
lib-C666-l	chr19	7953517	7953517	+	11.22379	NA	intron (NM_001268285, intron 1 of 1)	CpG	127	NM_00126	80131	Hs.501511	NM_02506	ENSG0000	LRRC8E	-	leucine rich	protein-co
lib-C666-l	chr16	12642261	12642261	+	11.22379	NA	intron (NM_032167, intron 20 of 20)	intron (NM_032167, intron 20 of 20	-171917	NR_03986	100616195		NR_03986	ENSG0000	MIR4718	-	microRNA	ncRNA
lib-C666-l	chr1	87939233	87939233	+	11.22379	NA	Intergenic	Intergenic	-101895	NR_03832	100505768	Hs.125247	NR_03832	ENSG0000	LINC01364	-	long interg	ncRNA
lib-C666-l	chr2	54950804	54950804	+	11.20567	NA	Intergenic	CpG	-1345	NM_00103	400954	Hs.656692	NM_00103	ENSG0000	EML6	-	echinoderr	protein-co
lib-C666-l	chr16	3109408	3109408	+	11.13288	NA	promoter-TSS (NR_123723)	promoter-TSS (NR_123723)	-37	NR_12372	100507419	Hs.312579	NR_123723		MMP25-A	-	MMP25 an	ncRNA
lib-C666-l	chr5	34244832	34244832	+	11.13288	NA	Intergenic	LTR12E|LTR|ERV1	-120199	NR_03795	100534612	Hs.171929	NR_03795	ENSG0000	C1QTNF3-	-	C1QTNF3-	ncRNA
lib-C666-l	chr3	17081272	17081272	+	11.13288	NA	intron (NM_015184, intron 3 of 5)	intron (NM_015184, intron 3 of 5)	106584	NR_03746	100500913		NR_03746	ENSG0000	MIR3714	-	microRNA	ncRNA
lib-C666-l	chr1	41343611	41343611	+	11.13288	NA	Intergenic	Intergenic	-15593	NM_13346	163732	Hs.355820	NM_13346	ENSG0000	CITED4	-	Cbp/p300	protein-co
lib-C666-l	chr2	173420406	173420406	+	11.13288	NA	promoter-TSS (NM_001278549)	promoter-TSS (NM_001278549)	-291	NM_00261	5163	Hs.470633	NM_00261	ENSG0000	PDK1	-	pyruvate d	protein-co
lib-C666-l	chr5	74532809	74532809	+	11.13288	NA	promoter-TSS (NM_001164443)	promoter-TSS (NM_001164443)	-106	NM_00116	256006	Hs.482533	NM_00116	ENSG0000	ANKRD31	-	ankyrin rep	protein-co
lib-C666-l	chr19	51108143	51108143	+	11.11305	NA	promoter-TSS (NR_004384)	promoter-TSS (NR_004384)	-77	NR_00438	100126781	Hs.707968	NR_004384		SNAR-F	-	small ILF3/	snRNA
lib-C666-l	chr6	149354413	149354413	+	11.09374	NA	promoter-TSS (NR_134599)	promoter-TSS (NR_134599)	-616	NR_13459	105378047	Hs.659484	NR_134599		LOC10537	-	uncharacte	ncRNA
lib-C666-l	chr2	209676463	209676463	+	11.06679	NA	intron (NR_136588, intron 3 of 5)	L1PA2|LINE|L1	269096	NR_13658	101927960	Hs.159960	NR_136588		LOC10192	-	uncharacte	ncRNA
lib-C666-l	chr9	117160825	117160825	+	11.06679	NA	Intergenic	CpG	-4140	NM_00131	80709	Hs.494895	NM_03076	ENSG0000	AKNA	-	AT-hook t	protein-co
lib-C666-l	chr20	11210625	11210625	+	11.06679	NA	Intergenic	MLT1H1|LTR|ERVL-MaLR	43406	NR_03897	339593	Hs.434320	NR_038972		LOC33959	-	uncharacte	ncRNA
lib-C666-l	chr2	222917806	222917806	+	11.06679	NA	Intergenic	Intergenic	-245060	NM_15303	151278	Hs.350729	NM_15303	ENSG0000	CCDC140	-	coiled-coil	protein-co
lib-C666-l	chr15	39566250	39566250	+	11.06679	NA	Intergenic	Intergenic	23380	NM_00130	400360	Hs.376109	NM_207445		C15orf54	-	chromosor	ncRNA
lib-C666-l	chr1	41848453	41848453	+	11.06679	NA	3' UTR (NM_001291281, exon 3 of 3)	3' UTR (NM_001291281, exon 3 of	-7112	NR_13581	101929901		NR_13581	ENSG0000	LOC10192	-	uncharacte	ncRNA
lib-C666-l	chr12	11639626	11639626	+	11.06679	NA	Intergenic	Intergenic	-61338	NR_03389	338817	Hs.733066	NR_03389	ENSG0000	LINC01252	-	long interg	ncRNA
lib-C666-l	chr5	142237261	142237261	+	11.06076	NA	intron (NM_015071, intron 1 of 22)	intron (NM_015071, intron 1 of 22)	11214	NR_04668	100874239	Hs.570896	NR_04668	ENSG0000	ARHGAP26	-	ARHGAP2	ncRNA
lib-C666-l	chr7	90893704	90893704	+	11.0277	NA	promoter-TSS (NM_003505)	promoter-TSS (NM_003505)	-79	NM_00350	8321	Hs.94234	NM_00350	ENSG0000	FZD1	-	frizzled cla	protein-co
lib-C666-l	chr8	143712284	143712284	+	10.99112	NA	Intergenic	Intergenic	-7665	NR_13430	101928087	Hs.126101	NR_13430	ENSG0000	LOC10192	-	uncharacte	ncRNA
lib-C666-l	chr19	32896837	32896837	+	10.9422	NA	promoter-TSS (NR_046201)	promoter-TSS (NR_046201)	182	NM_00117	147991	Hs.194392	NM_20732	ENSG0000	DPY19L3	-	dpy-19 like	protein-co
lib-C666-l	chr2	197125064	197125064	+	10.92952	NA	non-coding (NR_110225, exon 2 of 3	non-coding (NR_110225, exon 2 of	316	NR_11022	101927482	Hs.191841	NR_110225		LOC10192	-	uncharacte	ncRNA
lib-C666-l	chr14	106004674	106004674	+	10.92275	NA	Intergenic	Intergenic	6482	NR_13520	105370697	Hs.148580	NR_135205		LOC10537	-	uncharacte	ncRNA
lib-C666-l	chr11	34460514	34460514	+	10.92275	NA	promoter-TSS (NM_001752)	promoter-TSS (NM_001752)	42	NM_00175	847	Hs.502302	NM_00175	ENSG0000	CAT	-	catalase	protein-co
lib-C666-l	chr1	167073001	167073001	+	10.92275	NA	intron (NM_001080426, intron 2 of 5)	intron (NM_001080426, intron 2 of	9674	NM_00108	92235	Hs.632462	NM_00108	ENSG0000	DUSP27	-	dual specif	protein-co
lib-C666-l	chr17	42215734	42215734	+	10.92275	NA	Intergenic	Intergenic	-3536	NM_00117	78995	Hs.437059	NM_02403	ENSG0000	C17orf53	-	chromosor	protein-co
lib-C666-l	chr17	18218529	18218529	+	10.92275	NA	promoter-TSS (NM_144775)	promoter-TSS (NM_144775)	-65	NM_14477	140775	Hs.592944	NM_14477	ENSG0000	SMCR8	-	Smith-Ma	protein-co
lib-C666-l	chr19	1407432	1407432	+	10.79304	NA	promoter-TSS (NM_170711)	promoter-TSS (NM_170711)	-136	NM_17071	26528	Hs.222510	NM_01895	ENSG0000	DAZAP1	-	DAZ assoc	protein-co
lib-C666-l	chr14	105282483	105282483	+	10.79304	NA	Intergenic	CpG	-5055	NR_02439	196872	Hs.352220	NR_02439	ENSG0000	LINC00638	-	long interg	ncRNA
lib-C666-l	chr4	78078219	78078219	+	10.79304	NA	promoter-TSS (NM_004354)	promoter-TSS (NM_004354)	-138	NM_00435	901	Hs.740456	NM_00435	ENSG0000	CCNG2	-	cyclin G2	protein-co
lib-C666-l	chr19	46390085	46390085	+	10.76537	NA	promoter-TSS (NM_015649)	promoter-TSS (NM_015649)	-657	NM_01564	26145	Hs.515477	NM_01564	ENSG0000	IRF2BP1	-	interferon	protein-co
lib-C666-l	chr4	8197300	8197300	+	10.76331	NA	Intergenic	Intergenic	-3671	NM_00131	54436	Hs.479116	NM_01898	ENSG0000	SH3TC1	-	SH3 doma	protein-co
lib-C666-l	chr15	27216282	27216282	+	10.73451	NA	promoter-TSS (NM_001270873)	promoter-TSS (NM_001270873)	-147	NM_03322	2567	Hs.569475	NM_03322	ENSG0000	GABRG3	-	gamma-ar	protein-co
lib-C666-l	chr17	73043203	73043203	+	10.73451	NA	promoter-TSS (NM_015353)	promoter-TSS (NM_015353)	-76	NR_11083	23510	Hs.514468	NM_01535	ENSG0000	KCTD2	-	potassium	protein-co
lib-C666-l	chr19	58919932	58919932	+	10.73451	NA	promoter-TSS (NM_001318002)	promoter-TSS (NM_001318002)	-109	NM_17354	201514	Hs.439551	NM_17354	ENSG0000	ZNF584	-	zinc finger	protein-co
lib-C666-l	chr9	138304587	138304587	+	10.73451	NA	Intergenic	CpG	59508	NR_03896	100506599	Hs.561897	NR_03896	ENSG0000	PPP1R26-A	-	PPP1R26 a	ncRNA
lib-C666-l	chr11	66313585	66313585	+	10.71733	NA	promoter-TSS (NM_001258371)	promoter-TSS (NM_001258371)	86	NM_20734	254359	Hs.659933	NM_20734	ENSG0000	ZDHHC24	-	zinc finger	protein-co
lib-C666-l	chr2	103752363	103752363	+	10.71733	NA	Intergenic	Intergenic	-151476	NR_13553	105373519		NR_13553	ENSG0000	LINC01935	-	long interg	ncRNA
lib-C666-l	chr6	139094863	139094863	+	10.71733	NA	promoter-TSS (NM_001242740)	promoter-TSS (NM_001242740)	-47	NM_00124	100507462	Hs.571054	NM_00124	ENSG0000	GVQW2	-	GVQW mo	protein-co
lib-C666-l	chr8	145980969	145980969	+	10.71733	NA	promoter-TSS (NM_138367)	promoter-TSS (NM_138367)	1	NM_13836	90987	Hs.534516	NM_13836	ENSG0000	ZNF251	-	zinc finger	protein-co
lib-C666-l	chr12	56109858	56109858	+	10.71733	NA	promoter-TSS (NR_037657)	promoter-TSS (NR_037657)	40	NR_03765	100528022	Hs.600940	NR_037658		BLOC1S1-	-	BLOC1S1-	ncRNA
lib-C666-l	chr10	103815992	103815992	+	10.71733	NA	promoter-TSS (NM_024541)	promoter-TSS (NM_024541)	-60	NM_02454	79591	Hs.16004	NM_02454	ENSG0000	C10orf76	-	chromosor	protein-co
lib-C666-l	chr3	182286196	182286196	+	10.71733	NA	Intergenic	Intergenic	-61284	NR_13494	105374244	Hs.130176	NR_13494	ENSG0000	LINC01995	-	long interg	ncRNA
lib-C666-l	chr10	17272537	17272537	+	10.71733	NA	promoter-TSS (NR_108061)	promoter-TSS (NR_108061)	-553	NR_10806	100507347	Hs.740502	NR_10806	ENSG0000	VIM-AS1	-	VIM antise	ncRNA
lib-C666-l	chr2	220852771	220852771	+	10.71733	NA	Intergenic	MLT1H2|LTR|ERVL-MaLR	-81485	NR_03622	100422959		NR_03622	ENSG0000	MIR4268	-	microRNA	ncRNA
lib-C666-l	chr7	148959122	148959122	+	10.70998	NA	promoter-TSS (NM_001195220)	promoter-TSS (NM_001195220)	-140	NM_00119	100289678	Hs.490512	NM_00119	ENSG0000	ZNF783	-	zinc finger	protein-co
lib-C666-l	chr1	222165794	222165794	+	10.68972	NA	Intergenic	MER57E1|LTR|ERV1	-151786	NR_12598	101929771	Hs.557001	NR_12598	ENSG0000	LINC01655	-	long interg	ncRNA

lib-C666-I	chr14	99370370	99370370	+	10.67459	NA	Intergenic	Intergenic	192420	NM_18256	283598	Hs.448754	NM_18256	ENSG0000	C14orf177	-	chromosor	protein-co
lib-C666-I	chr5	138088827	138088827	+	10.67459	NA	promoter-TSS (NM_001323986)	promoter-TSS (NM_001323986)	171	NR_13424	105379194	Hs.445981	NR_13424	ENSG0000	LOC10537	-	uncharacte	ncRNA
lib-C666-I	chr5	179105592	179105592	+	10.67459	NA	TTS (NM_001164444)	TTS (NM_001164444)	2383	NM_00116	646019	Hs.535767	NM_00116	ENSG0000	CBY3	-	chibby fam	protein-co
lib-C666-I	chr17	41438351	41438351	+	10.58189	NA	Intergenic	CpG	27915	NR_027412	100130581	Hs.546897	NR_02741	ENSG0000	LINC0091C	-	long interg	ncRNA
lib-C666-I	chr2	12072251	12072251	+	10.58189	NA	Intergenic	Intergenic	-74991	NR_11019	100506457	Hs.197824	NR_11019	ENSG0000	MIR3681H	-	MIR3681 h	ncRNA
lib-C666-I	chr1	112479958	112479958	+	10.58189	NA	intron (NM_004980, intron 2 of 7)	intron (NM_004980, intron 2 of 7)	27679	NR_04661	100873995	Hs.535274	NR_04661	ENSG0000	KCND3-AS	-	KCND3 ant	ncRNA
lib-C666-I	chr7	17411841	17411841	+	10.58189	NA	Intergenic	MLT1D|LTR|ERVL-MaLR	-2700	NR_11001	102659288	Hs.559488	NR_110014		KCCAT333	-	renal clear	ncRNA
lib-C666-I	chr19	44617504	44617504	+	10.58189	NA	promoter-TSS (NM_013362)	promoter-TSS (NM_013362)	-11	NM_00132	7768	Hs.279567	NM_01336	ENSG0000	ZNF225	-	zinc finger	protein-co
lib-C666-I	chr17	47841761	47841761	+	10.58189	NA	promoter-TSS (NM_030802)	promoter-TSS (NM_030802)	-243	NM_03080	81558	Hs.514308	NM_03080	ENSG0000	FAM117A	-	family with	protein-co
lib-C666-I	chr13	96328983	96328983	+	10.47352	NA	promoter-TSS (NM_006260)	promoter-TSS (NM_006260)	196	NR_13211	100289274	Hs.594844	NR_13211	ENSG0000	DNAJC3-A	-	DNAJC3 ar	ncRNA
lib-C666-I	chr8	22526588	22526588	+	10.4657	NA	promoter-TSS (NM_018688)	promoter-TSS (NM_018688)	73	NM_01868	55909	Hs.433722	NM_01868	ENSG0000	BIN3	-	bridging in	protein-co
lib-C666-I	chr5	34245209	34245209	+	10.40942	NA	Intergenic	LTR12E|LTR|ERV1	-120576	NR_03795	100534612	Hs.171929	NR_03795	ENSG0000	C1QTNF3-	-	C1QTNF3-	ncRNA
lib-C666-I	chr2	6055632	6055632	+	10.40942	NA	Intergenic	Intergenic	-17187	NR_02683	150622	Hs.560891	NR_026832		LINC01105	-	long interg	ncRNA
lib-C666-I	chr12	129696870	129696870	+	10.40942	NA	non-coding (NR_110058, exon 5 of 5	non-coding (NR_110058, exon 5 of	3724	NR_11005	101927735	Hs.569212	NR_11005	ENSG0000	LOC10192	-	uncharacte	ncRNA
lib-C666-I	chr5	10601785	10601785	+	10.40942	NA	intron (NM_001164440, intron 1 of 3)	intron (NM_001164440, intron 1 of	37350	NM_00116	651746	Hs.26039	NM_00116	ENSG0000	ANKRD33E	-	ankyrin rep	protein-co
lib-C666-I	chr8	143023891	143023891	+	10.40942	NA	Intergenic	Intergenic	55766	NR_13046	100616374		NR_13046	ENSG0000	MIR4539	-	microRNA	ncRNA
lib-C666-I	chr16	3019251	3019251	+	10.39031	NA	promoter-TSS (NM_001284511)	promoter-TSS (NM_001284511)	5	NM_15234	124222	Hs.351474	NM_15234	ENSG0000	PAQR4	-	progestin a	protein-co
lib-C666-I	chr3	156806854	156806854	+	10.38624	NA	promoter-TSS (NR_034008)	promoter-TSS (NR_034008)	-816	NR_03400	100498859	Hs.633655	NR_03400	ENSG0000	LINC00881	-	long interg	ncRNA
lib-C666-I	chr19	44952750	44952750	+	10.37767	NA	promoter-TSS (NM_014518)	promoter-TSS (NM_014518)	16	NM_00127	7772	Hs.709348	NM_01451	ENSG0000	ZNF229	-	zinc finger	protein-co
lib-C666-I	chr19	57946593	57946593	+	10.37767	NA	promoter-TSS (NM_001023561)	promoter-TSS (NM_001023561)	-100	NM_00132	388567	Hs.467358	NM_00102	ENSG0000	ZNF749	-	zinc finger	protein-co
lib-C666-I	chr11	75139515	75139515	+	10.37767	NA	exon (NM_001039548, exon 2 of 6)	exon (NM_001039548, exon 2 of 6)	2159	NM_00103	283212	Hs.292451	NM_17358	ENSG0000	KLHL35	-	kelch like f	protein-co
lib-C666-I	chr14	98179543	98179543	+	10.37767	NA	Intergenic	MIR|SINE|MIR	-26548	NR_03394	100129345	Hs.23632	NR_03394	ENSG0000	LOC10012	-	uncharacte	ncRNA
lib-C666-I	chr6	44020352	44020352	+	10.37767	NA	intron (NR_125864, intron 1 of 4)	LTR67B|LTR|ERVL	22037	NR_12586	101929705	Hs.670378	NR_12586	ENSG0000	LOC10192	-	uncharacte	ncRNA
lib-C666-I	chrX	101187095	101187095	+	10.37767	NA	promoter-TSS (NM_001282401)	promoter-TSS (NM_001282401)	-56	NM_00128	84460	Hs.496512	NM_00101	ENSG0000	ZMAT1	-	zinc finger	protein-co
lib-C666-I	chrX	39060808	39060808	+	10.37767	NA	Intergenic	Intergenic	125808	NR_03896	286442	Hs.638478	NR_03896	ENSG0000	LINC01281	-	long interg	ncRNA
lib-C666-I	chr8	95651763	95651763	+	10.32881	NA	promoter-TSS (NR_034034)	promoter-TSS (NR_034034)	-68	NR_03403	100288748	Hs.608210	NR_03403	ENSG0000	LOC10028	-	uncharacte	ncRNA
lib-C666-I	chr7	140884572	140884572	+	10.28419	NA	intron (NM_001195278, intron 1 of 3)	LTR88c|LTR|Gypsy?	110540	NM_00119	100507421	Hs.283851	NM_00119	ENSG0000	TMEM178E	-	transmemb	protein-co
lib-C666-I	chr9	132246257	132246257	+	10.27265	NA	Intergenic	Intergenic	-4682	NR_03895	100506190	Hs.529860	NR_038955		LINC00963	-	long interg	ncRNA
lib-C666-I	chr12	7000453	7000453	+	10.27265	NA	Intergenic	CpG	-1931	NR_13508	105369632	Hs.670096	NR_13508	ENSG0000	LOC10536	-	uncharacte	ncRNA
lib-C666-I	chr21	44937048	44937048	+	10.27265	NA	Intergenic	Intergenic	-37634	NR_13654	102724354	Hs.592161	NR_13654	ENSG0000	LINC01669	-	long interg	ncRNA
lib-C666-I	chr7	12250874	12250874	+	10.27265	NA	promoter-TSS (NM_001134232)	promoter-TSS (NM_001134232)	26	NM_01837	54664	Hs.396358	NM_01837	ENSG0000	TMEM106E	-	transmemb	protein-co
lib-C666-I	chr3	40494682	40494682	+	10.27265	NA	non-coding (NR_040100, exon 1 of 4	non-coding (NR_040100, exon 1 of	117	NR_04010	285266	Hs.619663	NR_04010	ENSG0000	ENTPD3-A	-	ENTPD3 ar	ncRNA
lib-C666-I	chr2	70369498	70369498	+	10.23572	NA	Intergenic	CpG	-17050	NR_02444	100133985	Hs.135528	NR_02444	ENSG0000	LINC01816	-	long interg	ncRNA
lib-C666-I	chr17	26368447	26368447	+	10.22271	NA	Intergenic	A-rich|Low_complexity|Low_comple	-1241	NM_01623	51701	Hs.208759	NM_01623	ENSG0000	NLK	-	nemo like l	protein-co
lib-C666-I	chr7	155281694	155281694	+	10.20361	NA	Intergenic	Intergenic	30870	NM_00142	2020	Hs.134989	NM_00142	ENSG0000	EN2	-	engrailed h	protein-co
lib-C666-I	chr8	132916386	132916386	+	10.20361	NA	promoter-TSS (NM_001323554)	promoter-TSS (NM_001323554)	51	NM_00132	23167	Hs.204564	NM_01513	ENSG0000	EFR3A	-	EFR3 homo	protein-co
lib-C666-I	chr7	71217322	71217322	+	10.1894	NA	Intergenic	Intergenic	-444566	NR_03747	100500836		NR_03747	ENSG0000	MIR3914-1	-	microRNA	ncRNA
lib-C666-I	chr19	41816234	41816234	+	10.16129	NA	promoter-TSS (NM_001346100)	promoter-TSS (NM_001346100)	-151	NM_00134	90324	Hs.437497	NM_05284	ENSG0000	CCDC97	-	coiled-coil	protein-co
lib-C666-I	chr2	44588845	44588845	+	10.16129	NA	promoter-TSS (NM_001171613)	promoter-TSS (NM_001171613)	-145	NM_00117	9581	Hs.444349	NM_00603	ENSG0000	PREPL	-	prolyl endo	protein-co
lib-C666-I	chr15	63449837	63449837	+	10.16129	NA	promoter-TSS (NM_015920)	promoter-TSS (NM_015920)	-96	NM_01592	51065	Hs.108957	NM_01592	ENSG0000	RPS27L	-	ribosomal	protein-co
lib-C666-I	chr3	35305118	35305118	+	10.07497	NA	intron (NR_110817, intron 2 of 3)	MLT1H1|LTR|ERVL-MaLR	130397	NR_11081	101928135	Hs.550715	NR_110817		LOC10192	-	uncharacte	ncRNA
lib-C666-I	chr19	50554323	50554323	+	10.03284	NA	non-coding (NR_027257, exon 1 of 3	non-coding (NR_027257, exon 1 of	477	NR_13809	400710	Hs.628968	NR_02725	ENSG0000	LOC40071	-	uncharacte	ncRNA
lib-C666-I	chr13	110790617	110790617	+	10.03284	NA	Intergenic	CpG	84985	NR_12638	104355146	Hs.508690	NR_12638	ENSG0000	LINC00396	-	long interg	ncRNA
lib-C666-I	chr19	2702960	2702960	+	10.03103	NA	promoter-TSS (NM_052847)	promoter-TSS (NM_052847)	-214	NM_05284	2788	Hs.515544	NM_05284	ENSG0000	GNG7	-	G protein s	protein-co
lib-C666-I	chr7	63767723	63767723	+	10.03103	NA	Intergenic	Intergenic	-6528	NM_00129	728927	Hs.386162	NM_00117	ENSG0000	ZNF736	-	zinc finger	protein-co
lib-C666-I	chr14	102975622	102975622	+	10.03103	NA	intron (NM_152326, intron 1 of 3)	CpG-7424	506	NM_15232	122416	Hs.432945	NM_15232	ENSG0000	ANKRD9	-	ankyrin rep	protein-co
lib-C666-I	chr11	19497138	19497138	+	10.03103	NA	intron (NM_001111018, intron 1 of 3	intron (NM_001111018, intron 1 of	31638	NR_04972	100874012	Hs.434602	NR_04972	ENSG0000	NAV2-AS5	-	NAV2 antis	ncRNA
lib-C666-I	chr19	49622352	49622352	+	10.03103	NA	promoter-TSS (NR_103842)	promoter-TSS (NR_103842)	45	NM_01811	55150	Hs.458310	NM_01811	ENSG0000	C19orf73	-	chromosor	protein-co
lib-C666-I	chr15	93206740	93206740	+	10.00688	NA	Intergenic	Intergenic	-7709	NM_20744	400451	Hs.27373	NM_20744	ENSG0000	FAM174B	-	family with	protein-co
lib-C666-I	chr1	214161270	214161270	+	9.97961	NA	promoter-TSS (NM_002763)	promoter-TSS (NM_002763)	-8	NM_00127	5629	Hs.741808	NM_00276	ENSG0000	PROX1	-	prospero h	protein-co
lib-C666-I	chr19	56146390	56146390	+	9.94769	NA	Intergenic	Intergenic	-6002	NM_20711	51157	Hs.631551	NM_01620	ENSG0000	ZNF580	-	zinc finger	protein-co
lib-C666-I	chr7	157294528	157294528	+	9.94769	NA	Intergenic	Intergenic	35603	NR_11015	101927914	Hs.623826	NR_11015	ENSG0000	LOC10192	-	uncharacte	ncRNA
lib-C666-I	chr14	23299182	23299182	+	9.94769	NA	promoter-TSS (NM_001318870)	promoter-TSS (NM_001318870)	90	NM_18130	122704	Hs.355935	NM_17833	ENSG0000	MRPL52	-	mitochond	protein-co
lib-C666-I	chr16	30886748	30886748	+	9.94769	NA	promoter-TSS (NR_039744)	promoter-TSS (NR_039744)	-33	NR_11094	101928736	Hs.303197	NR_11094	ENSG0000	MIR762HG	-	MIR762 ho	ncRNA
lib-C666-I	chr5	131132794	131132794	+	9.94079	NA	promoter-TSS (NM_133372)	promoter-TSS (NM_133372)	-38	NM_00134	96459	Hs.591273	NM_13337	ENSG0000	FNIP1	-	folliculin in	protein-co
lib-C666-I	chr1	149784029	149784029	+	9.94079	NA	promoter-TSS (NM_001161334)	promoter-TSS (NM_001161334)	-101	NM_00102	440689	Hs.632451	NM_00102	ENSG0000	HIST2H2BF	-	histone clu	protein-co
lib-C666-I	chr4	183666475	183666475	+	9.94079	NA	intron (NM_001080477, intron 19 of 2	intron (NM_001080477, intron 19 o	172155	NM_00101	1635	Hs.183850	NM_00192	ENSG0000	DCTD	-	dCMP dea	protein-co
lib-C666-I	chr5	76383480	76383480	+	9.93242	NA	promoter-TSS (NM_001329564)	promoter-TSS (NM_001329564)	-409	NM_03236	84327	Hs.584988	NM_03236	ENSG0000	ZBED3	-	zinc finger	protein-co
lib-C666-I	chr13	96144427	96144427	+	9.93242	NA	intron (NR_046533, intron 3 of 3)	L1PB1|LINE|L1	41737	NR_04653	100874194	Hs.569229	NR_04653	ENSG0000	CLDN10-A	-	CLDN10 ar	ncRNA
lib-C666-I	chr2	159001891	159001891	+	9.93242	NA	Intergenic	LTR10F|LTR|ERV1	-21271	NR_03885	554201	Hs.570262	NR_03885	ENSG0000	CCDC148-	-	CCDC148 a	ncRNA
lib-C666-I	chr4	38668941	38668941	+	9.93242	NA	intron (NM_016531, intron 1 of 5)	intron (NM_016531, intron 1 of 5)	-2692	NR_02680	79667	Hs.29725	NM_02461	ENSG0000	KLF3-AS1	-	KLF3 antise	ncRNA
lib-C666-I	chr2	28497891	28497891	+	9.79436	NA	intron (NR_137440, intron 10 of 13)	intron (NR_137440, intron 10 of 13)	35435	NR_03831	100505716	Hs.679092	NR_03831	ENSG0000	LOC10050	-	uncharacte	ncRNA
lib-C666-I	chr12	133101186	133101186	+	9.79436	NA	intron (NM_001142641, intron 2 of 16	intron (NM_001142641, intron 2 of	34029	NM_00114	57666	Hs.411138	NM_00114	ENSG0000	FBRSL1	-	fibrosin like	protein-co
lib-C666-I	chr6	170534922	170534922	+	9.79436	NA	Intergenic	Intergenic	36735	NR_00278	154449	Hs.447751	NR_00278	ENSG0000	LOC15444	-	uncharacte	ncRNA
lib-C666-I	chr3	125634872	125634872	+	9.79436	NA	promoter-TSS (NR_024251)	promoter-TSS (NR_024251)	-572	NR_02425	100125556	Hs.553949	NR_02425	ENSG0000	FAM86JP	-	family with	pseudo
lib-C666-I	chr1	28431732	28431732	+	9.79436	NA	Intergenic	Intergenic	-16584	NR_10421	2140	Hs.185774	NM_00199	ENSG0000	EYA3	-	EYA transc	protein-co
lib-C666-I	chr13	111468450	111468450	+	9.79436	NA	Intergenic	Intergenic	-3019	NR_13528	283486	Hs.447504	NR_13528	ENSG0000	LINC00567	-	long interg	ncRNA
lib-C666-I	chr7	128290749	128290749	+	9.75997	NA	intron (NR_024368, intron 3 of 4)	intron (NR_024368, intron 3 of 4)	9454	NR_02436	402483	Hs.722350	NM_001039682		LINC0100C	-	long interg	ncRNA
lib-C666-I	chr5	139125773	139125773	+	9.75997	NA	promoter-TSS (NR_105052)	promoter-TSS (NR_105052)	35	NR_10505	101929696	Hs.147408	NR_10505	ENSG0000	PSD2-AS1	-	PSD2 antis	ncRNA
lib-C666-I	chr7	121924870	121924870	+	9.75997	NA	Intergenic	L1PA2|LINE|L1	-18842	NR_03648	154860	Hs.664204	NR_03648	ENSG0000	FEZF1-AS1	-	FEZF1 antis	ncRNA
lib-C666-I	chr1	37940062	37940062	+	9.75997	NA	promoter-TSS (NR_038842)	promoter-TSS (NR_038842)	-18	NR_03884	728431	Hs.380738	NR_03884	ENSG0000	LINC01137	-	long interg	ncRNA
lib-C666-I	chr2	171572335	171572335	+	9.75997	NA	intron (NM_001003845, intron 1 of 1)	CpG	478	NM_00100	389058	Hs.368802	NM_00100	ENSG0000	SP5	-	Sp5 transc	protein-co
lib-C666-I	chr14	53196726	53196726	+	9.75396	NA	promoter-TSS (NM_145251)	promoter-TSS (NM_145251)	-157	NM_00113	6815	Hs.364980	NM_14525	ENSG0000	STYX	-	serine/thre	protein-co
lib-C666-I	chr7	3255644	3255644	+	9.75396	NA	Intergenic	Intergenic	-57438	NR_12602	100129603	Hs.652997	NR_126024		LOC10012	-	uncharacte	ncRNA
lib-C666-I	chr16	4009296	4009296	+	9.75396	NA	Intergenic	AluJb|SINE|Alu	-8851	NR_12031	102724927	Hs.364739	NR_12031	ENSG0000	LOC10272	-	uncharacte	ncRNA
lib-C666-I	chr7	140397054	140397054	+	9.73491	NA	promoter-TSS (NR_024454)	promoter-TSS (NR_024454)	-177	NR_02445	100134713	Hs.534141	NR_02445	ENSG0000	NDUFB2-A	-	NDUFB2 ar	ncRNA
lib-C666-I	chr9	35042192	35042192	+	9.7234	NA	promoter-TSS (NM_001040410)	promoter-TSS (NM_001040410)	-13	NM_20329	138724	Hs.148250	NM_20329	ENSG0000	C9orf131	-	chromosor	protein-co
lib-C666-I	chr17	37910780	37910780	+	9.7234	NA	Intergenic	CpG	14560	NM_00124	2886	Hs.86859	NM_00531	ENSG0000	GRB7	-	growth fac	protein-co
lib-C666-I	chr17	6711616	6711616	+	9.7234	NA	intron (NM_053285, intron 6 of 7)	L1PA4|LINE|L1	23444	NM_05328	83659	Hs.462108	NM_05328	ENSG0000	TEKT1	-	tektin 1	protein-co
lib-C666-I	chr16	11414131	11414131	+	9.7234	NA	Intergenic	MER1A|DNA|hAT-Charlie	24938	NR_13517	105371083	Hs.737495	NR_13517	ENSG0000	LOC10537	-	uncharacte	ncRNA
lib-C666-I	chr1	186979840	186979840	+	9.7234	NA	Intergenic	Intergenic	-82134	NR_12634	104169671		NR_126347		LINC01036	-	long interg	ncRNA
lib-C666-I	chr11	119205108	119205108	+	9.7234	NA	promoter-TSS (NM_032015)	promoter-TSS (NM_032015)	-102	NM_03201	79102	Hs.524084	NM_03201	ENSG0000	RNF26	-	ring finger	protein-co
lib-C666-I	chr15	42227249	42227249	+	9.615	NA	intron (NM_139265, intron 3 of 5)	L2b|LINE|L2	13634	NR_12033	101928363	Hs.680195	NR_120332		EHD4-AS1	-	EHD4 antis	ncRNA
lib-C666-I	chr2	159580545	159580545	+	9.56114	NA	intron (NR_135242, intron 1 of 3)	intron (NR_135242, intron 1 of 3)	10969	NR_13524	100129029	Hs.630724	NR_13524	ENSG0000	PKP4-AS1	-	PKP4 antis	ncRNA
lib-C666-I	chr1	180922981	180922981	+	9.53658	NA	Intergenic	CpG	40668	NM_02095	57710	Hs.734816	NM_02095	ENSG0000	KIAA1614	-	KIAA1614	protein-co
lib-C666-I	chr6	11537802	11537802	+	9.53658	NA	promoter-TSS (NM_001100829)	promoter-TSS (NM_001100829)	-658	NM_00110	100113407	Hs.146317	NM_00110	ENSG0000	TMEM170E	-	transmemb	protein-co
lib-C666-I	chr1	170560709	170560709	+	9.53658	NA	Intergenic	Intergenic	-58959	NR_12595	101928650	Hs.676662	NR_125958		LOC10192	-	uncharacte	ncRNA
lib-C666-I	chr5	473012	473012	+	9.53658	NA	promoter-TSS (NR_024158)	promoter-TSS (NR_024158)	68	NR_02415	25845	Hs.719132	NR_02415	ENSG0000	PP7080	-	uncharacte	ncRNA
lib-C666-I	chr15	100765001	100765001	+	9.4812	NA	intron (NM_139057, intron 7 of 21)	intron (NM_139057, intron 7 of 21)	117209	NM_13905	170691	Hs.513200	NM_13905	ENSG0000	ADAMTS1	-	ADAM met	protein-co
lib-C666-I	chr4	52709171	52709171	+	9.4812	NA	promoter-TSS (NM_001040402)	promoter-TSS (NM_001040402)	5	NM_00128	23142	Hs.605388	NM_01511	ENSG0000	DCUN1D4	-	defective ir	protein-co
lib-C666-I	chr7	107148342	107148342	+	9.4812	NA	intron (NM_181733, intron 6 of 20)	intron (NM_181733, intron 6 of 20)	37840	NM_00529	2845	Hs.657277	NM_00529	ENSG0000	GPR22	-	G protein-	protein-co
lib-C666-I	chr3	12779532	12779532	+	9.4812	NA	intron (NM_001284406, intron 7 of 11	intron (NM_001284406, intron 7 of	21276	NM_00128	55287	Hs.475502	NM_01830	ENSG0000	TMEM40	-	transmemb	protein-co
lib-C666-I	chr14	53647496	53647496	+	9.47655	NA	Intergenic	HERVIP10FH-int|LTR|ERV1	27424	NR_11006	101927620		NR_11006	ENSG0000	LOC10192	-	uncharacte	ncRNA
lib-C666-I	chr20	33865879	33865879	+	9.47655	NA	promoter-TSS (NR_102705)	promoter-TSS (NR_102705)	81	NR_10270	101410538	Hs.356273	NR_102705		MMP24-A	-	MMP24 an	ncRNA
lib-C666-I	chr4	10531885	10531885	+	9.45164	NA	intron (NM_052964, intron 12 of 18)	MIRc|SINE|MIR	-72853	NM_05304	85460	Hs.455089	NM_05304	ENSG0000	ZNF518B	-	zinc finger	protein-co
lib-C666-I	chr17	21454584	21454584	+	9.45164	NA	exon (NM_001113434, exon 1 of 2)	exon (NM_001113434, exon 1 of 2)	357	NM_00111	339263	Hs.514016	NM_00111	ENSG0000	C17orf51	-	chromosor	protein-co
lib-C666-I	chr1	85666701	85666701	+	9.45164	NA	promoter-TSS (NM_032184)	promoter-TSS (NM_032184)	27	NM_03218	84144	Hs.533853	NM_03218	ENSG0000	SYDE2	-	synapse de	protein-co
lib-C666-I	chr3	94648028	94648028	+	9.38975	NA	Intergenic	Intergenic	-9079	NR_01540	255025	Hs.130994	NR_01540	ENSG0000	LINC00879	-	long interg	ncRNA
lib-C666-I	chr7	81785662	81785662	+	9.3665	NA	intron (NM_000722, intron 4 of 38)	intron (NM_000722, intron 4 of 38)	147169	NR_11007	101927356	Hs.571348	NR_11007	ENSG0000	LOC10192	-	uncharacte	ncRNA
lib-C666-I	chr19	40128837	40128837	+	9.32825	NA	promoter-TSS (NR_026870)	promoter-TSS (NR_026870)	-488	NR_02687	100129935	Hs.659307	NR_02687	ENSG0000	LOC10012	-	lectin, gala	pseudo
lib-C666-I	chr10	3944775	3944775	+	9.29424	NA	Intergenic	Intergenic	68633	NR_13449	105376365	Hs.460114	NR_13449	ENSG0000	LOC10537	-	uncharacte	ncRNA
lib-C666-I	chr17	48350552	48350552	+	9.2832	NA	intron (NM_001168215, intron 1 of 5)	CpG	-1236	NM_15322	162461	Hs.224630	NM_15322	ENSG0000	TMEM92	-	transmemb	protein-co
lib-C666-I	chr17	20372668	20372668	+	9.21057	NA	Intergenic	L1ME3|LINE|L1	-1820	NM_00104	284194	Hs.462402	NM_00104	ENSG0000	LGALS9B	-	galectin 9E	protein-co
lib-C666-I	chrX	132088025	132088025	+	9.21057	NA	intron (NM_001077188, intron 3 of 5)	MIRb|SINE|MIR	7398	NM_00107	90161	Hs.385956	NM_14717	ENSG0000	HS6ST2	-	heparan su	protein-co
lib-C666-I	chr15	30763396	30763396	+	9.21057	NA	Intergenic	CpG	-56933	NM_00128	101059918		NM_00128	ENSG0000	GOLGA8R	-	golgin A8 f	protein-co
lib-C666-I	chr1	90823799	90823799	+	9.21057	NA	Intergenic	Intergenic	358995	NM_02006	343472	Hs.451956	NM_02006	ENSG0000	BARHL2	-	BarH like h	protein-co
lib-C666-I	chr3	184363216	184363216	+	9.21057	NA	Intergenic	L2c|LINE|L2	66620	NM_02214	64110	Hs.306123	NM_02214	ENSG0000	MAGEF1	-	MAGE fam	protein-co
lib-C666-I	chr19	23710798	23710798	+	9.21057	NA	Intergenic	LTR22A|LTR|ERVK	-111922	NR_12644	104472717	Hs.162105	NR_12644	ENSG0000	LINC01224	-	long interg	ncRNA
lib-C666-I	chrX	40730999	40730999	+	9.21057	NA	Intergenic	Intergenic	-38550	NR_02776	100132831	Hs.729923	NR_027761		LOC10013	-	A20-bindir	pseudo
lib-C666-I	chr10	87624965	87624965	+	9.21057	NA	intron (NM_017551, intron 6 of 15)	intron (NM_017551, intron 6 of 15)	287477	NR_03898	100507470	Hs.585483	NR_03898	ENSG0000	GRID1-AS	-	GRID1 anti	ncRNA
lib-C666-I	chr16	34768245	34768245	+	9.21057	NA	Intergenic	HSAT6|Satellite|Satellite	-26704	NR_03401	100130700	Hs.97712	NR_034018		TP53TG3H	-	TP53 targe	pseudo
lib-C666-I	chr3	135969425	135969425	+	9.20967	NA	intron (NM_000532, intron 1 of 14)	CpG	258	NM_00053	5096	Hs.63788	NM_00053	ENSG0000	PCCB	-	propionyl-	protein-co
lib-C666-I	chr17	44271465	44271465	+	9.20967	NA	intron (NR_034172, intron 1 of 1)	CpG	526	NR_03417	644246	Hs.644600	NR_03417	ENSG0000	KANSL1-A	-	KANSL1 ar	ncRNA
lib-C666-I	chr9	140500776	140500776	+	9.20967	NA	intron (NM_152285, intron 1 of 7)	CpG	684	NM_15228	92714	Hs.12999	NM_15228	ENSG0000	ARRDC1	-	arrestin do	protein-co
lib-C666-I	chr10	80734467	80734467	+	9.20967	NA	intron (NR_015429, intron 3 of 7)	intron (NR_015429, intron 3 of 7)	92738	NR_02443	283050	Hs.309176	NR_01542	ENSG0000	ZMIZ1-AS	-	ZMIZ1 anti	ncRNA
lib-C666-I	chr4	1858027	1858027	+	9.20633	NA	promoter-TSS (NM_012318)	promoter-TSS (NM_012318)	-53	NM_01231	3954	Hs.120165	NM_01231	ENSG0000	LETM1	-	leucine zip	protein-co
lib-C666-I	chr16	15188233	15188233	+	9.20633	NA	promoter-TSS (NM_001301064)	promoter-TSS (NM_001301064)	-35	NR_12543	100505915	Hs.678758	NR_12543	ENSG0000	LOC10050	-	uncharacte	ncRNA
lib-C666-I	chr13	109571764	109571764	+	9.14689	NA	intron (NM_001198950, intron 16 of 3	intron (NM_001198950, intron 16 o	247887	NR_04770	100885782	Hs.655942	NR_04770	ENSG0000	MYO16-AS	-	MYO16 an	ncRNA
lib-C666-I	chr22	39541022	39541022	+	9.14689	NA	intron (NM_175709, intron 2 of 5)	CpG	7633	NM_00134	23492	Hs.356416	NM_17570	ENSG0000	CBX7	-	chromobo	protein-co
lib-C666-I	chr9	97021563	97021563	+	9.10494	NA	promoter-TSS (NM_001301275)	promoter-TSS (NM_001301275)	15	NM_19432	169841	Hs.387623	NM_00344	ENSG0000	ZNF169	-	zinc finger	protein-co
lib-C666-I	chr5	55290942	55290942	+	9.10494	NA	promoter-TSS (NR_120480)	promoter-TSS (NR_120480)	-53	NR_10275	441072	Hs.482141	NR_10275	ENSG0000	FLJ31104	-	uncharacte	ncRNA
lib-C666-I	chr2	61061660	61061660	+	9.10494	NA	Intergenic	Intergenic	46789	NR_03398	400957	Hs.170358	NR_03398	ENSG0000	LINC01185	-	long interg	ncRNA
lib-C666-I	chr2	96657789	96657789	+	9.10494	NA	5' UTR (NM_001310154, exon 1 of 89	5' UTR (NM_001310154, exon 1 of 8	279	NM_00131	400986	Hs.646318	NM_00101	ENSG0000	ANKRD36C	-	ankyrin rep	protein-co
lib-C666-I	chr17	27947549	27947549	+	9.09067	NA	intron (NM_032854, intron 1 of 9)	intron (NM_032854, intron 1 of 9)	892	NM_03285	84940	Hs.143046	NM_03285	ENSG0000	CORO6	-	coronin 6	protein-co
lib-C666-I	chr19	19887413	19887413	+	9.04991	NA	promoter-TSS (NR_026956)	promoter-TSS (NR_026956)	-191	NR_02695	284440	Hs.665307	NR_026956		LINC00663	-	long interg	ncRNA
lib-C666-I	chr6	114012012	114012012	+	9.04008	NA	Intergenic	Intergenic	-40735	NR_12584	101927686	Hs.402044	NR_12584	ENSG0000	LOC10192	-	uncharacte	ncRNA
lib-C666-I	chr20	61300000	61300000	+	9.02258	NA	intron (NM_016354, intron 10 of 11)	intron (NM_016354, intron 10 of 11	-2027	NR_02447	100127888	Hs.671273	NR_02447	ENSG0000	SLCO4A1-	-	SLCO4A1 a	ncRNA
lib-C666-I	chr4	169401503	169401503	+	9.02258	NA	intron (NM_001345927, intron 1 of 37	CpG	162	NM_00101	91351	Hs.535011	NM_00101	ENSG0000	DDX60L	-	DEAD-box	protein-co
lib-C666-I	chr12	14956477	14956477	+	9.02258	NA	promoter-TSS (NM_175874)	promoter-TSS (NM_175874)	-29	NM_17587	144608	Hs.659951	NM_17587	ENSG0000	C12orf60	-	chromosor	protein-co
lib-C666-I	chr3	44754158	44754158	+	9.02258	NA	promoter-TSS (NM_001134440)	promoter-TSS (NM_001134440)	23	NM_00128	91392	Hs.224843	NM_03321	ENSG0000	ZNF502	-	zinc finger	protein-co
lib-C666-I	chr15	66649044	66649044	+	9.02258	NA	promoter-TSS (NM_001289986)	promoter-TSS (NM_001289986)	10	NM_00128	54962	Hs.744308	NM_01785	ENSG0000	TIPIN	-	TIMELESS i	protein-co
lib-C666-I	chr12	39436991	39436991	+	9.02258	NA	Intergenic	Intergenic	-137571	NM_15363	144402	Hs.40910	NM_15363	ENSG0000	CPNE8	-	copine 8	protein-co
lib-C666-I	chr2	179387871	179387871	+	9.02258	NA	promoter-TSS (NR_038271)	promoter-TSS (NR_038271)	-47	NR_03827	100506866	Hs.710694	NR_03827	ENSG0000	TTN-AS1	-	TTN antise	ncRNA
lib-C666-I	chr6	170341913	170341913	+	8.99157	NA	Intergenic	Intergenic	-133953	NR_12587	102724511	Hs.550045	NR_125878		LOC10272	-	uncharacte	ncRNA
lib-C666-I	chr16	21513642	21513642	+	8.97945	NA	promoter-TSS (NR_027155)	promoter-TSS (NR_027155)	-40	NR_02715	100271836	Hs.552700	NR_02715	ENSG0000	SMG1P3	-	SMG1P3, n	pseudo
lib-C666-I	chr14	53648377	53648377	+	8.97404	NA	Intergenic	LTR10F|LTR|ERV1	28305	NR_11006	101927620		NR_11006	ENSG0000	LOC10192	-	uncharacte	ncRNA
lib-C666-I	chr21	32898535	32898535	+	8.96177	NA	intron (NM_003253, intron 1 of 28)	intron (NM_003253, intron 1 of 28)	32755	NM_00325	7074	Hs.517228	NM_00325	ENSG0000	TIAM1	-	T-cell lymp	protein-co
lib-C666-I	chr12	109490401	109490401	+	8.96177	NA	promoter-TSS (NM_032663)	promoter-TSS (NM_032663)	26	NM_03266	84749	Hs.486434	NM_03266	ENSG0000	USP30	-	ubiquitin s	protein-co
lib-C666-I	chr15	72039986	72039986	+	8.96177	NA	intron (NM_001286429, intron 7 of 11	LTR44|LTR|ERV1	-56108	NR_12034	101929173	Hs.680593	NR_120346		THSD4-AS	-	THSD4 ant	ncRNA
lib-C666-I	chr17	1613527	1613527	+	8.96177	NA	exon (NM_001164407, exon 1 of 4)	exon (NM_001164407, exon 1 of 4)	135	NM_00116	727910	Hs.531005	NM_00116	ENSG0000	TLCD2	-	TLC domai	protein-co
lib-C666-I	chr3	123442650	123442650	+	8.96177	NA	intron (NM_053027, intron 12 of 32)	intron (NM_053027, intron 12 of 32	34159	NR_04662	100873940	Hs.667316	NR_04662	ENSG0000	MYLK-AS2	-	MYLK antis	ncRNA
lib-C666-I	chr22	42196559	42196559	+	8.96177	NA	promoter-TSS (NM_001304797)	promoter-TSS (NM_001304797)	-67	NM_02482	79879	Hs.474991	NM_02482	ENSG0000	CCDC134	-	coiled-coil	protein-co
lib-C666-I	chr16	4250388	4250388	+	8.95679	NA	intron (NM_001323667, intron 4 of 6)	intron (NM_001323667, intron 4 of	41693	NM_00132	6345	Hs.10041	NM_00109	ENSG0000	SRL	-	sarcalumer	protein-co
lib-C666-I	chr1	53686061	53686061	+	8.91001	NA	intron (NR_130897, intron 1 of 1)	CpG	250	NM_01788	54987	Hs.525391	NM_01788	ENSG0000	C1orf123	-	chromosor	protein-co
lib-C666-I	chr10	96162968	96162968	+	8.91001	NA	exon (NM_015188, exon 1 of 13)	exon (NM_015188, exon 1 of 13)	782	NM_01518	23232	Hs.500598	NM_01518	ENSG0000	TBC1D12	-	TBC1 dom	protein-co
lib-C666-I	chr5	89825454	89825454	+	8.89416	NA	promoter-TSS (NM_001286812)	promoter-TSS (NM_001286812)	-53	NM_00128	116068	Hs.136235	NM_19827	ENSG0000	LYSMD3	-	LysM dom	protein-co
lib-C666-I	chr19	58428044	58428044	+	8.89416	NA	promoter-TSS (NM_152475)	promoter-TSS (NM_152475)	-60	NM_00129	147687	Hs.567710	NM_15247	ENSG0000	ZNF417	-	zinc finger	protein-co
lib-C666-I	chr2	61108301	61108301	+	8.89416	NA	promoter-TSS (NM_002908)	promoter-TSS (NM_002908)	148	NR_03398	400957	Hs.170358	NR_03398	ENSG0000	LINC01185	-	long interg	ncRNA
lib-C666-I	chr17	35654	35654	+	8.89416	NA	intron (NR_104420, intron 1 of 1)	MER9a2|LTR|ERVK	1388	NR_10442	100506371		NR_104420		LINC02091	-	long interg	ncRNA
lib-C666-I	chr8	143484580	143484580	+	8.89416	NA	promoter-TSS (NM_145003)	promoter-TSS (NM_145003)	30	NM_00129	203062	Hs.370931	NM_14500	ENSG0000	TSNARE1	-	t-SNARE d	protein-co
lib-C666-I	chr10	30073460	30073460	+	8.89416	NA	Intergenic	Intergenic	-47530	NM_00132	6840	Hs.499209	NM_00317	ENSG0000	SVIL	-	supervillin	protein-co
lib-C666-I	chr16	86917443	86917443	+	8.89416	NA	Intergenic	Intergenic	161746	NR_13518	102724344	Hs.585769	NR_13518	ENSG0000	LOC10272	-	uncharacte	ncRNA
lib-C666-I	chr19	18314873	18314873	+	8.89416	NA	promoter-TSS (NM_002866)	promoter-TSS (NM_002866)	1	NM_00286	5864	Hs.27744	NM_00286	ENSG0000	RAB3A	-	RAB3A, me	protein-co
lib-C666-I	chr4	15683389	15683389	+	8.88643	NA	promoter-TSS (NM_001145191)	promoter-TSS (NM_001145191)	37	NM_00114	285550	Hs.399980	NM_00114	ENSG0000	FAM200B	-	family with	protein-co
lib-C666-I	chr9	36400475	36400475	+	8.85547	NA	promoter-TSS (NM_194329)	promoter-TSS (NM_194329)	-179	NM_02278	152006	Hs.333503	NM_02278	ENSG0000	RNF38	-	ring finger	protein-co
lib-C666-I	chr11	68934193	68934193	+	8.85547	NA	intron (NR_104161, intron 2 of 2)	intron (NR_104161, intron 2 of 2)	4837	NR_10416	338694	Hs.564235	NR_10416	ENSG0000	LOC33869	-	uncharacte	ncRNA
lib-C666-I	chr2	241588436	241588436	+	8.85547	NA	Intergenic	LTR45C|LTR|ERV1	23774	NM_00530	2859	Hs.112218	NM_00530	ENSG0000	GPR35	-	G protein-	protein-co

lib-C666-l	chr20	43595066	43595066	+	8.85547	NA	promoter-TSS (NR_038341)	promoter-TSS (NR_038341)	33	NR_03834	100505826	Hs.196065	NR_03834	ENSG0000	STK4-AS1	-	STK4 antis	ncRNA
lib-C666-l	chr20	3748529	3748529	+	8.85547	NA	promoter-TSS (NM_001039140)	promoter-TSS (NM_001039140)	-77	NM_00103	54976	Hs.274422	NM_01787	ENSG0000	C20orf27	-	chromosor	protein-co
lib-C666-l	chr14	75760916	75760916	+	8.85547	NA	promoter-TSS (NR_038421)	promoter-TSS (NR_038421)	-191	NR_03842	731223	Hs.534624	NR_038421		LINC01220	-	long interg	ncRNA
lib-C666-l	chr17	80674242	80674242	+	8.73012	NA	promoter-TSS (NR_046408)	promoter-TSS (NR_046408)	-113	NR_13640	101929552	Hs.375081	NR_13640	ENSG0000	LOC10192	-	uncharacte	ncRNA
lib-C666-l	chr9	102669121	102669121	+	8.73012	NA	promoter-TSS (NR_038853)	promoter-TSS (NR_038853)	206	NM_01791	55014	Hs.704031	NM_01791	ENSG0000	STX17	-	syntaxin 17	protein-co
lib-C666-l	chr12	5331380	5331380	+	8.73012	NA	Intergenic	Intergenic	20937	NR_12047	101929584	Hs.436529	NR_12047	ENSG0000	LOC10192	-	uncharacte	ncRNA
lib-C666-l	chr1	151254501	151254501	+	8.71138	NA	promoter-TSS (NR_135595)	promoter-TSS (NR_135595)	-96	NR_13559	100507670	Hs.225083	NR_13559	ENSG0000	LOC10050	-	uncharacte	ncRNA
lib-C666-l	chr19	12444565	12444565	+	8.71138	NA	promoter-TSS (NM_145276)	promoter-TSS (NM_145276)	-31	NM_14527	147837	Hs.663510	NM_14527	ENSG0000	ZNF563	-	zinc finger	protein-co
lib-C666-l	chr5	139124987	139124987	+	8.71138	NA	intron (NR_105053, intron 1 of 2)	intron (NR_105053, intron 1 of 2)	821	NR_10505	101929696	Hs.147408	NR_10505	ENSG0000	PSD2-AS1	-	PSD2 antis	ncRNA
lib-C666-l	chr17	40762273	40762273	+	8.71138	NA	promoter-TSS (NR_026697)	promoter-TSS (NR_026697)	-828	NR_02669	162427	Hs.632262	NM_17812	ENSG0000	FAM134C	-	family with	protein-co
lib-C666-l	chr8	74268737	74268737	+	8.71138	NA	promoter-TSS (NR_125388)	promoter-TSS (NR_125388)	-41	NR_12538	101926926	Hs.640090	NR_12538	ENSG0000	RDH10-AS	-	RDH10 ant	ncRNA
lib-C666-l	chr13	99852998	99852998	+	8.71138	NA	promoter-TSS (NR_036532)	promoter-TSS (NR_036532)	11	NR_03653	100289373	Hs.225914	NR_03653	ENSG0000	UBAC2-AS	-	UBAC2 ant	ncRNA
lib-C666-l	chr3	194207693	194207693	+	8.70719	NA	promoter-TSS (NR_033929)	promoter-TSS (NR_033929)	-176	NR_03392	401106	Hs.407087	NR_033929		LINC00884	-	long interg	ncRNA
lib-C666-l	chr11	356054	356054	+	8.65614	NA	Intergenic	CpG	-13741	NM_17853	338707	Hs.148074	NM_17853	ENSG0000	B4GALNT4	-	beta-1,4-N	protein-co
lib-C666-l	chr19	54663652	54663652	+	8.60974	NA	promoter-TSS (NM_024316)	promoter-TSS (NM_024316)	-171	NM_02431	79165	Hs.590974	NM_02431	ENSG0000	LENG1	-	leukocyte r	protein-co
lib-C666-l	chr16	12769817	12769817	+	8.59372	NA	intron (NM_001099455, intron 2 of 2)	intron (NM_001099455, intron 2 of	-44361	NR_03986	100616195		NR_03986	ENSG0000	MIR4718	-	microRNA	ncRNA
lib-C666-l	chr16	31083976	31083976	+	8.59372	NA	intron (NM_024706, intron 1 of 2)	intron (NM_024706, intron 1 of 2)	847	NM_00117	79759	Hs.102928	NM_02470	ENSG0000	ZNF668	-	zinc finger	protein-co
lib-C666-l	chr17	71161107	71161107	+	8.56376	NA	promoter-TSS (NM_001050)	promoter-TSS (NM_001050)	-53	NM_00105	6752	Hs.514451	NM_00105	ENSG0000	SSTR2	-	somatostat	protein-co
lib-C666-l	chr1	145395638	145395638	+	8.56376	NA	intron (NM_001039703, intron 68 of 8	intron (NM_001039703, intron 68 o	-13234	NR_12596	101928979	Hs.735893	NR_12596	ENSG0000	LINC01719	-	long interg	ncRNA
lib-C666-l	chr15	52029763	52029763	+	8.51971	NA	exon (NM_153374, exon 1 of 3)	exon (NM_153374, exon 1 of 3)	557	NM_15337	256586	Hs.603629	NM_15337	ENSG0000	LYSMD2	-	LysM dom	protein-co
lib-C666-l	chr17	6735151	6735151	+	8.50455	NA	promoter-TSS (NM_053285)	promoter-TSS (NM_053285)	-91	NM_05328	83659	Hs.462108	NM_05328	ENSG0000	TEKT1	-	tektin 1	protein-co
lib-C666-l	chr10	42385191	42385191	+	8.50435	NA	Intergenic	HSATII|Satellite|Satellite	478302	NR_02438	441666	Hs.255729	NR_02438	ENSG0000	LOC44166	-	zinc finger	pseudo
lib-C666-l	chr7	77427958	77427958	+	8.48019	NA	promoter-TSS (NM_032936)	promoter-TSS (NM_032936)	-151	NM_00112	57157	Hs.203965	NM_02043	ENSG0000	PHTF2	-	putative hc	protein-co
lib-C666-l	chr8	145159311	145159311	+	8.46313	NA	promoter-TSS (NR_038270)	promoter-TSS (NR_038270)	6	NM_03227	84232	Hs.19673	NM_03227	ENSG0000	MAF1	-	MAF1 hom	protein-co
lib-C666-l	chr17	41446581	41446581	+	8.42858	NA	TTS (NR_027412)	TTS (NR_027412)	19685	NR_02741	100130581	Hs.546897	NR_02741	ENSG0000	LINC0091	-	long interg	ncRNA
lib-C666-l	chr16	29466140	29466140	+	8.41666	NA	promoter-TSS (NM_001320628)	promoter-TSS (NM_001320628)	145	NM_00132	107282092	Hs.444600	NM_00132	ENSG0000	BOLA2-SM	-	BOLA2-SM	protein-co
lib-C666-l	chr3	5048541	5048541	+	8.40444	NA	Intergenic	Intergenic	-26895	NR_12591	100507582	Hs.434505	NR_03790	ENSG0000	BHLHE40-	-	BHLHE40 a	ncRNA
lib-C666-l	chr1	203259756	203259756	+	8.40444	NA	Intergenic	Intergenic	2928	NR_11709	100506775	Hs.443859	NR_11709	ENSG0000	LINC01353	-	long interg	ncRNA
lib-C666-l	chr7	582383	582383	+	8.40444	NA	Intergenic	Intergenic	-19212	NR_13238	101927000	Hs.551793	NR_13238	ENSG0000	LOC10192	-	uncharacte	ncRNA
lib-C666-l	chr12	108297567	108297567	+	8.40444	NA	promoter-TSS (NR_037629)	promoter-TSS (NR_037629)	-19	NR_03762	728739	Hs.741995	NR_037629		LOC72873	-	programm	pseudo
lib-C666-l	chr2	33295041	33295041	+	8.35712	NA	intron (NM_206943, intron 3 of 33)	intron (NM_206943, intron 3 of 33)	-64623	NM_00116	4052	Hs.619315	NM_00062	ENSG0000	LTBP1	-	latent trans	protein-co
lib-C666-l	chr16	2961885	2961885	+	8.35705	NA	promoter-TSS (NM_020912)	promoter-TSS (NM_020912)	-95	NM_02091	84256	Hs.655321	NM_02091	ENSG0000	FLYWCH1	-	FLYWCH-t	protein-co
lib-C666-l	chr1	169763885	169763885	+	8.33814	NA	promoter-TSS (NM_001320048)	promoter-TSS (NM_001320048)	14	NM_00132	55732	Hs.443551	NM_01818	ENSG0000	C1orf112	-	chromosor	protein-co
lib-C666-l	chr1	47973826	47973826	+	8.33814	NA	Intergenic	CpG	-70837	NR_12635	102724077	Hs.159718	NR_126355		LINC01389	-	long interg	ncRNA
lib-C666-l	chr3	122134884	122134884	+	8.33814	NA	promoter-TSS (NM_019069)	promoter-TSS (NM_019069)	-2	NM_01906	54554	Hs.567513	NM_01906	ENSG0000	WDR5B	-	WD repeat	protein-co
lib-C666-l	chr7	96187364	96187364	+	8.33814	NA	Intergenic	L1PA2|LINE|L1	-54529	NM_00120	401388	Hs.734935	NM_20750	ENSG0000	C7orf76	-	chromosor	protein-co
lib-C666-l	chr12	131617221	131617221	+	8.33814	NA	intron (NM_001330497, intron 22 of 2	intron (NM_001330497, intron 22 o	-32335	NR_02667	116437	Hs.675497	NR_02667	ENSG0000	LINC01257	-	long interg	ncRNA
lib-C666-l	chr16	57571171	57571171	+	8.33814	NA	promoter-TSS (NM_033212)	promoter-TSS (NM_033212)	-694	NM_03321	92922	Hs.644611	NM_03321	ENSG0000	CCDC102A	-	coiled-coil	protein-co
lib-C666-l	chr1	320557	320557	+	8.33814	NA	Intergenic	Intergenic	-3335	NR_02832	100133331	Hs.728864	NR_028327		LOC10013	-	uncharacte	ncRNA
lib-C666-l	chr16	29874943	29874943	+	8.31579	NA	promoter-TSS (NM_001286585)	promoter-TSS (NM_001286585)	-61	NR_02437	440356	Hs.448823	NR_01539	ENSG0000	CDIPT-AS1	-	CDIPT anti	ncRNA
lib-C666-l	chr1	214217964	214217964	+	8.30991	NA	Intergenic	Intergenic	56120	NM_00276	5629	Hs.741808	NM_00276	ENSG0000	PROX1	-	prospero h	protein-co
lib-C666-l	chr5	142562543	142562543	+	8.28735	NA	intron (NM_015071, intron 20 of 22)	intron (NM_015071, intron 20 of 22	-9522	NR_04681	100874372	Hs.666717	NR_04681	ENSG0000	ARHGAP26	-	ARHGAP26	ncRNA
lib-C666-l	chr9	37465917	37465917	+	8.28613	NA	promoter-TSS (NM_014872)	promoter-TSS (NM_014872)	-510	NM_01487	9925	Hs.161276	NM_01487	ENSG0000	ZBTB5	-	zinc finger	protein-co
lib-C666-l	chr19	40854500	40854500	+	8.28613	NA	promoter-TSS (NM_001256441)	promoter-TSS (NM_001256441)	-66	NM_00125	126526	Hs.631557	NM_17883	ENSG0000	C19orf47	-	chromosor	protein-co
lib-C666-l	chr2	119622866	119622866	+	8.28613	NA	Intergenic	Intergenic	-17107	NM_00142	2019	Hs.271977	NM_00142	ENSG0000	EN1	-	engrailed h	protein-co
lib-C666-l	chr20	1099127	1099127	+	8.28613	NA	promoter-TSS (NM_001323408)	promoter-TSS (NM_001323408)	-36	NR_13653	105372493		NR_13653	ENSG0000	LOC10537	-	uncharacte	ncRNA
lib-C666-l	chr19	16680963	16680963	+	8.28613	NA	intron (NM_024881, intron 2 of 5)	intron (NM_024881, intron 2 of 5)	2230	NM_02488	79939	Hs.620596	NM_02488	ENSG0000	SLC35E1	-	solute carr	protein-co
lib-C666-l	chr2	97198092	97198092	+	8.28613	NA	promoter-TSS (NR_135564)	promoter-TSS (NR_135564)	-49	NR_13556	105373496	Hs.570138	NR_13556	ENSG0000	LOC10537	-	uncharacte	ncRNA
lib-C666-l	chr7	81314461	81314461	+	8.28613	NA	intron (NR_126025, intron 1 of 10)	MLT1I|LTR|ERVL-MaLR	6261	NR_12602	100128317	Hs.571267	NR_12602	ENSG0000	LOC10012	-	uncharacte	ncRNA
lib-C666-l	chr10	5708634	5708634	+	8.28613	NA	promoter-TSS (NR_037164)	promoter-TSS (NR_037164)	-76	NR_03716	79754	Hs.445899	NM_02470	ENSG0000	ASB13	-	ankyrin rep	protein-co
lib-C666-l	chr16	4303996	4303996	+	8.28613	NA	promoter-TSS (NR_039999)	promoter-TSS (NR_039999)	-206	NR_03999	100507501	Hs.632979	NR_03999	ENSG0000	LINC01569	-	long interg	ncRNA
lib-C666-l	chr11	46867919	46867919	+	8.28333	NA	promoter-TSS (NM_014756)	promoter-TSS (NM_014756)	-43	NR_03890	100507401	Hs.675301	NR_03890	ENSG0000	LRP4-AS1	-	LRP4 antis	ncRNA
lib-C666-l	chr1	145396755	145396755	+	8.28333	NA	intron (NM_001039703, intron 68 of 8	intron (NM_001039703, intron 68 o	-14351	NR_12596	101928979	Hs.735893	NR_12596	ENSG0000	LINC01719	-	long interg	ncRNA
lib-C666-l	chr1	43424212	43424212	+	8.28333	NA	promoter-TSS (NR_033967)	promoter-TSS (NR_033967)	-508	NR_03396	440584	Hs.260928	NR_03396	ENSG0000	SLC2A1-A	-	SLC2A1 an	ncRNA
lib-C666-l	chr1	235110997	235110997	+	8.28333	NA	Intergenic	Intergenic	-11251	NR_12594	101927851	Hs.520684	NR_12594	ENSG0000	LOC10192	-	uncharacte	ncRNA
lib-C666-l	chr19	28284947	28284947	+	8.28333	NA	promoter-TSS (NR_027301)	promoter-TSS (NR_027301)	-99	NR_02730	148189	Hs.706547	NR_02730	ENSG0000	LINC00662	-	long interg	ncRNA
lib-C666-l	chr18	6729871	6729871	+	8.28216	NA	promoter-TSS (NR_134645)	promoter-TSS (NR_134645)	-10	NR_13464	101927168	Hs.242240	NR_134645		LOC10192	-	uncharacte	ncRNA
lib-C666-l	chr1	214276451	214276451	+	8.28216	NA	Intergenic	Intergenic	114607	NM_00276	5629	Hs.741808	NM_00276	ENSG0000	PROX1	-	prospero h	protein-co
lib-C666-l	chr2	152266283	152266283	+	8.28216	NA	promoter-TSS (NM_001177663)	promoter-TSS (NM_001177663)	-114	NM_01815	55183	Hs.536537	NM_01815	ENSG0000	RIF1	-	replication	protein-co
lib-C666-l	chr7	99063672	99063672	+	8.27411	NA	intron (NM_001198879, intron 1 of 8)	intron (NM_001198879, intron 1 of	152	NM_00119	100526740	Hs.632313	NM_00119	ENSG0000	ATP5J2-PT	-	ATP5J2-PT	protein-co
lib-C666-l	chr19	9247339	9247339	+	8.25582	NA	Intergenic	MER20|DNA|hAT-Charlie	-3717	NR_10243	57693	Hs.465829	NM_02093	ENSG0000	ZNF317	-	zinc finger	protein-co
lib-C666-l	chr19	2867298	2867298	+	8.20589	NA	promoter-TSS (NM_001300843)	promoter-TSS (NM_001300843)	-35	NM_02496	80032	Hs.287433	NM_02496	ENSG0000	ZNF556	-	zinc finger	protein-co
lib-C666-l	chr12	132863677	132863677	+	8.20589	NA	intron (NM_001122636, intron 1 of 1	intron (NM_001122636, intron 1 of	11700	NR_02456	100130238	Hs.536338	NR_024563		LOC10013	-	uncharacte	ncRNA
lib-C666-l	chrX	13687575	13687575	+	8.20589	NA	Intergenic	Intergenic	16265	NM_15263	170082	Hs.222855	NM_15263	ENSG0000	TCEANC	-	transcriptic	protein-co
lib-C666-l	chr9	12973255	12973255	+	8.20589	NA	promoter-TSS (NR_132976)	promoter-TSS (NR_132976)	-623	NR_13297	106635615		NR_132976		SNORD137	-	small nucle	snoRNA
lib-C666-l	chr8	12331052	12331052	+	8.11522	NA	intron (NR_040092, intron 4 of 5)	intron (NR_040092, intron 4 of 5)	36530	NR_04009	100506990	Hs.656893	NR_040091		LOC10050	-	uncharacte	ncRNA
lib-C666-l	chr20	52324105	52324105	+	8.08062	NA	Intergenic	(CA)n|Simple_repeat|Simple_repeat	99212	NR_13457	105372672	Hs.259350	NR_13457	ENSG0000	LOC10537	-	uncharacte	ncRNA
lib-C666-l	chr5	53942748	53942748	+	8.0787	NA	Intergenic	Intergenic	56404	NR_10465	102467080	Hs.637409	NR_104658		LOC10246	-	uncharacte	ncRNA
lib-C666-l	chr14	88277345	88277345	+	8.0787	NA	Intergenic	Intergenic	182270	NM_00120	2581	Hs.513439	NM_00015	ENSG0000	GALC	-	galactosylc	protein-co
lib-C666-l	chr5	66299881	66299881	+	8.0787	NA	promoter-TSS (NM_001290227)	promoter-TSS (NM_001290227)	101	NR_11190	101928794	Hs.672435	NR_111908		MAST4-AS	-	MAST4 ant	ncRNA
lib-C666-l	chr16	49118727	49118727	+	8.0787	NA	Intergenic	Intergenic	197015	NM_00435	869	Hs.458423	NM_00435	ENSG0000	CBLN1	-	cerebellin	protein-co
lib-C666-l	chr6	130511088	130511088	+	8.0787	NA	intron (NM_001258275, intron 7 of 1	L1MEb|LINE|L1	25942	NM_00127	154075	Hs.440508	NM_15255	ENSG0000	SAMD3	-	sterile alph	protein-co
lib-C666-l	chr19	57946847	57946847	+	8.0787	NA	5' UTR (NM_001023561, exon 1 of 3)	5' UTR (NM_001023561, exon 1 of	154	NM_00102	388567	Hs.467358	NM_00102	ENSG0000	ZNF749	-	zinc finger	protein-co
lib-C666-l	chr18	61962311	61962311	+	8.0787	NA	intron (NR_033881, intron 5 of 9)	intron (NR_033881, intron 5 of 9)	-35021	NR_03398	400654	Hs.434235	NR_03398	ENSG0000	LINC01538	-	long interg	ncRNA
lib-C666-l	chr10	43474720	43474720	+	8.0787	NA	intron (NR_126352, intron 2 of 2)	intron (NR_126352, intron 2 of 2)	2235	NR_12635	104266963	Hs.559152	NR_12635	ENSG0000	LINC01264	-	long interg	ncRNA
lib-C666-l	chr1	148177337	148177337	+	8.07793	NA	intron (NM_001037501, intron 6 of 1	intron (NM_001037501, intron 6 of	25199	NM_00116	645142	Hs.730589	NM_00116	ENSG0000	PPIAL4D	-	peptidylpr	protein-co
lib-C666-l	chr10	121302962	121302962	+	8.07793	NA	promoter-TSS (NM_001005339)	promoter-TSS (NM_001005339)	-740	NM_00100	6001	Hs.501200	NM_00292	ENSG0000	RGS10	-	regulator c	protein-co
lib-C666-l	chr6	10391669	10391669	+	8.03539	NA	Intergenic	Intergenic	-20882	NR_03391	100130275	Hs.661431	NR_033910		TFAP2A-A	-	TFAP2A an	ncRNA
lib-C666-l	chr3	159958767	159958767	+	8.03539	NA	Intergenic	LTR10F|LTR|ERV1	15344	NM_00116	401097	Hs.729755	NM_00116	ENSG0000	C3orf80	-	chromosor	protein-co
lib-C666-l	chr2	159312882	159312882	+	8.03539	NA	promoter-TSS (NM_003628)	promoter-TSS (NM_003628)	383	NM_00130	130940	Hs.668597	NM_13880	ENSG0000	CCDC148	-	coiled-coil	protein-co
lib-C666-l	chr11	111288644	111288644	+	8.03539	NA	non-coding (NR_046085, exon 1 of 4	non-coding (NR_046085, exon 1 of	267	NR_04608	100132078	Hs.672662	NR_04608	ENSG0000	LOC10013	-	uncharacte	ncRNA
lib-C666-l	chr4	187112555	187112555	+	8.03539	NA	promoter-TSS (NM_207352)	promoter-TSS (NM_207352)	89	NR_04626	651430	Hs.727796	NR_04626	ENSG0000	FLJ38576	-	uncharacte	ncRNA
lib-C666-l	chr7	29325181	29325181	+	8.03539	NA	intron (NM_001293072, intron 1 of 1	L3|LINE|CR1	-76595	NR_12052	102724484	Hs.638665	NR_120522		LOC10272	-	uncharacte	ncRNA
lib-C666-l	chr8	98450599	98450599	+	8.03539	NA	intron (NR_125390, intron 1 of 2)	intron (NR_125390, intron 1 of 2)	8126	NR_12539	101927066	Hs.732112	NR_125390		LOC10192	-	uncharacte	ncRNA
lib-C666-l	chr8	145754426	145754426	+	8.0262	NA	promoter-TSS (NM_001001795)	promoter-TSS (NM_001001795)	32	NM_00100	414919	Hs.105685	NM_00100	ENSG0000	C8orf82	-	chromosor	protein-co
lib-C666-l	chr11	36053841	36053841	+	8.01597	NA	intron (NM_001304264, intron 1 of 5)	intron (NM_001304264, intron 1 of	22193	NR_03976	100616311		NR_03976	ENSG0000	MIR3973	-	microRNA	ncRNA
lib-C666-l	chr4	102332699	102332699	+	8.0154	NA	Intergenic	L1PA2|LINE|L1	63765	NR_03387	90024	Hs.534513	NR_03387	ENSG0000	FLJ20021	-	uncharacte	ncRNA
lib-C666-l	chr6	3024257	3024257	+	8.0154	NA	promoter-TSS (NR_132750)	promoter-TSS (NR_132750)	748	NR_03388	401233	Hs.441039	NM_001013680		HTATSF1P	-	HIV-1 Tat	pseudo
lib-C666-l	chr3	65857194	65857194	+	8.0154	NA	intron (NM_015520, intron 1 of 24)	intron (NM_015520, intron 1 of 24)	-22297	NR_04657	100873983	Hs.663889	NR_04657	ENSG0000	MAGI1-AS	-	MAGI1 ant	ncRNA
lib-C666-l	chr12	66342773	66342773	+	8.0154	NA	intron (NM_001300918, intron 3 of 4)	intron (NM_001300918, intron 3 of	-66826	NM_00129	100129940	Hs.685856	NM_00129	ENSG0000	LOC10012	-	uncharacte	protein-co
lib-C666-l	chr2	101944466	101944466	+	8.0154	NA	Intergenic	Intergenic	18554	NR_04988	100847007		NR_04988	ENSG0000	MIR5696	-	microRNA	ncRNA
lib-C666-l	chr16	57850396	57850396	+	8.01457	NA	3' UTR (NM_001278081, exon 5 of 5)	3' UTR (NM_001278081, exon 5 of	5847	NM_00127	388282	Hs.434720	NM_001278081		LOC38828	-	uncharacte	protein-co
lib-C666-l	chr9	89356549	89356549	+	8.01457	NA	Intergenic	Intergenic	205555	NM_00204	2619	Hs.65029	NM_00204	ENSG0000	GAS1	-	growth arr	protein-co
lib-C666-l	chr11	60929048	60929048	+	8.01457	NA	promoter-TSS (NM_017966)	promoter-TSS (NM_017966)	-132	NM_01796	55048	Hs.523715	NM_01796	ENSG0000	VPS37C	-	VPS37C, E	protein-co
lib-C666-l	chr2	61292925	61292925	+	8.01457	NA	promoter-TSS (NM_001330435)	promoter-TSS (NM_001330435)	-80	NM_00112	84542	Hs.468653	NM_03250	ENSG0000	KIAA1841	-	KIAA1841	protein-co
lib-C666-l	chr4	80572094	80572094	+	8.01457	NA	Intergenic	Intergenic	158347	NR_03882	100506035	Hs.507664	NR_03882	ENSG0000	LINC00989	-	long interg	ncRNA
lib-C666-l	chr15	45492760	45492760	+	8.01457	NA	intron (NM_138356, intron 1 of 7)	intron (NM_138356, intron 1 of 7)	613	NM_13835	90525	Hs.310399	NM_13835	ENSG0000	SHF	-	Src homol	protein-co
lib-C666-l	chr4	183789053	183789053	+	8.01457	NA	Intergenic	Intergenic	49577	NM_00101	1635	Hs.183850	NM_00192	ENSG0000	DCTD	-	dCMP dea	protein-co
lib-C666-l	chr15	42565518	42565518	+	8.01457	NA	promoter-TSS (NM_001301409)	promoter-TSS (NM_001301409)	264	NM_00111	25963	Hs.511138	NM_01549	ENSG0000	TMEM87A	-	transmemb	protein-co
lib-C666-l	chr21	20230329	20230329	+	8.01457	NA	Intergenic	Intergenic	-98199	NR_10992	101927797	Hs.551743	NR_109925		MIR548XH	-	MIR548X h	ncRNA
lib-C666-l	chr8	74332339	74332339	+	8.01457	NA	promoter-TSS (NR_038406)	promoter-TSS (NR_038406)	30	NR_03840	100128126	Hs.679921	NR_03840	ENSG0000	STAU2-AS	-	STAU2 ant	ncRNA
lib-C666-l	chr1	675894	675894	+	7.97818	NA	Intergenic	Intergenic	-10163	NR_02832	100133331	Hs.728864	NR_028327		LOC10013	-	uncharacte	ncRNA
lib-C666-l	chr15	93425701	93425701	+	7.97818	NA	promoter-TSS (NR_037600)	promoter-TSS (NR_037600)	-372	NR_03760	100507217	Hs.622504	NR_037600		LINC01578	-	long interg	ncRNA
lib-C666-l	chr1	23158692	23158692	+	7.97818	NA	intron (NM_017449, intron 3 of 15)	MIRb|SINE|MIR	31027	NR_03621	100422914		NR_03621	ENSG0000	MIR4253	-	microRNA	ncRNA
lib-C666-l	chr16	2039947	2039947	+	7.97653	NA	promoter-TSS (NM_004209)	promoter-TSS (NM_004209)	1	NM_00420	9143	Hs.435277	NM_00420	ENSG0000	SYNGR3	-	synaptogy	protein-co
lib-C666-l	chr19	42773250	42773250	+	7.9573	NA	intron (NM_001304815, intron 1 of 2	CpG	561	NM_00130	23152	Hs.388236	NM_01512	ENSG0000	CIC	-	capicua tra	protein-co
lib-C666-l	chr1	12290125	12290125	+	7.9573	NA	promoter-TSS (NM_015378)	promoter-TSS (NM_015378)	29	NM_01537	55187	Hs.439381	NM_01537	ENSG0000	VPS13D	-	vacuolar p	protein-co
lib-C666-l	chr1	1677932	1677932	+	7.9573	NA	promoter-TSS (NM_001199787)	promoter-TSS (NM_001199787)	-494	NM_00119	9906	Hs.655255	NM_18283	ENSG0000	SLC35E2	-	solute carr	protein-co
lib-C666-l	chr4	148721301	148721301	+	7.9573	NA	intron (NM_024605, intron 1 of 22)	L1MDb|LINE|L1	17555	NR_03996	100616246		NR_03996	ENSG0000	MIR4799	-	microRNA	ncRNA
lib-C666-l	chr5	112196920	112196920	+	7.9573	NA	promoter-TSS (NM_001204196)	promoter-TSS (NM_001204196)	35	NM_00120	6728	Hs.637001	NM_00313	ENSG0000	SRP19	-	signal reco	protein-co
lib-C666-l	chr20	26190072	26190072	+	7.91164	NA	promoter-TSS (NR_040095)	promoter-TSS (NR_040095)	-203	NR_04009	284801	Hs.370699	NR_04009	ENSG0000	MIR663AH	-	MIR663A h	ncRNA
lib-C666-l	chr11	33713822	33713822	+	7.83708	NA	Intergenic	MER63B|DNA|hAT-Blackjack	8464	NM_00116	100131378	Hs.709466	NM_00116	ENSG0000	C11orf91	-	chromosor	protein-co
lib-C666-l	chr11	126462655	126462655	+	7.82019	NA	intron (NM_032531, intron 1 of 16)	intron (NM_032531, intron 1 of 16)	-60018	NR_12053	101929427	Hs.600341	NR_12053	ENSG0000	LOC10192	-	uncharacte	ncRNA
lib-C666-l	chr19	46379951	46379951	+	7.82019	NA	Intergenic	CpG	9477	NM_01564	26145	Hs.515477	NM_01564	ENSG0000	IRF2BP1	-	interferon	protein-co
lib-C666-l	chr2	106267795	106267795	+	7.76438	NA	Intergenic	MER1A|DNA|hAT-Charlie	-40779	NR_03889	285000	Hs.737917	NR_038891		LOC28500	-	uncharacte	ncRNA
lib-C666-l	chr4	40257414	40257414	+	7.76438	NA	Intergenic	MIRc|SINE|MIR	-51788	NR_12164	101060498	Hs.351492	NR_12164	ENSG0000	LOC10106	-	uncharacte	ncRNA
lib-C666-l	chr16	58549499	58549499	+	7.76438	NA	intron (NR_134583, intron 1 of 8)	CpG	116	NM_00116	79918	Hs.731691	NM_02486	ENSG0000	SETD6	-	SET domai	protein-co
lib-C666-l	chr9	125027744	125027744	+	7.76438	NA	promoter-TSS (NM_033117)	promoter-TSS (NM_033117)	-601	NM_03311	92400	Hs.415842	NM_03311	ENSG0000	RBM18	-	RNA bindi	protein-co
lib-C666-l	chr19	50167932	50167932	+	7.76438	NA	promoter-TSS (NR_104202)	promoter-TSS (NR_104202)	-467	NM_13863	83596	Hs.289052	NM_13863	ENSG0000	BCL2L12	-	BCL2 like 1	protein-co
lib-C666-l	chr3	56591159	56591159	+	7.76438	NA	promoter-TSS (NM_001012506)	promoter-TSS (NM_001012506)	-25	NM_00101	285331	Hs.476399	NM_00101	ENSG0000	CCDC66	-	coiled-coil	protein-co
lib-C666-l	chr1	212965155	212965155	+	7.76438	NA	promoter-TSS (NM_001042552)	promoter-TSS (NM_001042552)	-15	NM_00114	128387	Hs.530538	NM_00104	ENSG0000	TATDN3	-	TatD DNas	protein-co
lib-C666-l	chr19	51161257	51161257	+	7.76438	NA	exon (NM_001195076, exon 4 of 5)	exon (NM_001195076, exon 4 of 5)	8555	NM_00119	342918	Hs.531650	NM_00119	ENSG0000	C19orf81	-	chromosor	protein-co
lib-C666-l	chr15	45459239	45459239	+	7.76438	NA	TTS (NM_001301171)	TTS (NM_001301171)	14166	NM_00130	90525	Hs.310399	NM_13835	ENSG0000	SHF	-	Src homol	protein-co
lib-C666-l	chr12	53448189	53448189	+	7.75972	NA	promoter-TSS (NR_033854)	promoter-TSS (NR_033854)	33	NR_03385	283335	Hs.657060	NR_03385	ENSG0000	LOC28333	-	uncharacte	ncRNA
lib-C666-l	chr9	66553959	66553959	+	7.75972	NA	promoter-TSS (NR_122077)	promoter-TSS (NR_122077)	-48	NR_12207	403323	Hs.646340	NM_203449		LOC40332	-	uncharacte	ncRNA
lib-C666-l	chr11	10336384	10336384	+	7.7487	NA	intron (NR_103765, intron 1 of 3)	MER57B2|LTR|ERV1	6524	NR_10376	100130460	Hs.501890	NR_103765		CAND1.11	-	uncharacte	ncRNA
lib-C666-l	chr4	53027	53027	+	7.7024	NA	promoter-TSS (NM_001039127)	promoter-TSS (NM_001039127)	-152	NM_00128	152687	Hs.709469	NM_18252	ENSG0000	ZNF595	-	zinc finger	protein-co
lib-C666-l	chr2	64833742	64833742	+	7.6867	NA	promoter-TSS (NR_034023)	promoter-TSS (NR_034023)	-704	NR_03402	339807	Hs.434736	NR_03402	ENSG0000	LOC33980	-	uncharacte	ncRNA
lib-C666-l	chr8	12219551	12219551	+	7.6867	NA	promoter-TSS (NR_026789)	promoter-TSS (NR_026789)	23	NR_02678	100133172	Hs.721640	NR_026789		FAM66A	-	family with	ncRNA
lib-C666-l	chr9	30075624	30075624	+	7.67555	NA	Intergenic	Intergenic	332828	NR_04620	401497	Hs.680131	NR_046204		LINC01242	-	long interg	ncRNA
lib-C666-l	chr12	44152610	44152610	+	7.67555	NA	promoter-TSS (NM_031292)	promoter-TSS (NM_031292)	10	NM_00127	83448	Hs.445814	NM_03129	ENSG0000	PUS7L	-	pseudouric	protein-co
lib-C666-l	chr12	31911398	31911398	+	7.66023	NA	Intergenic	LTR10F|LTR|ERV1	-29290	NM_00127	196394	Hs.585084	NM_00111	ENSG0000	AMN1	-	antagonist	protein-co
lib-C666-l	chr14	100070777	100070777	+	7.60604	NA	promoter-TSS (NM_001144995)	promoter-TSS (NM_001144995)	-50	NM_00114	317762	Hs.709288	NM_00114	ENSG0000	CCDC85C	-	coiled-coil	protein-co
lib-C666-l	chr4	120331757	120331757	+	7.52748	NA	promoter-TSS (NR_037596)	promoter-TSS (NR_037596)	58	NR_03759	401149	Hs.448518	NR_037596		LINC01061	-	long interg	ncRNA
lib-C666-l	chr16	54520121	54520121	+	7.52748	NA	Intergenic	Intergenic	-115510	NR_13492	100996345	Hs.112680	NR_13492	ENSG0000	LINC0214	-	long interg	ncRNA
lib-C666-l	chr16	34449912	34449912	+	7.52748	NA	Intergenic	HSAT6|Satellite|Satellite	-45150	NR_00283	606551	Hs.646441	NR_002837		UBE2MP1	-	ubiquitin c	pseudo
lib-C666-l	chr1	27371438	27371438	+	7.52748	NA	Intergenic	Intergenic	-32105	NM_05294	115572	Hs.632378	NM_05294	ENSG0000	FAM46B	-	family with	protein-co
lib-C666-l	chr17	37894502	37894502	+	7.52748	NA	promoter-TSS (NM_001242442)	promoter-TSS (NM_001242442)	-74	NM_00103	2886	Hs.86859	NM_00531	ENSG0000	GRB7	-	growth fac	protein-co
lib-C666-l	chr19	14202118	14202118	+	7.52748	NA	promoter-TSS (NM_138352)	promoter-TSS (NM_138352)	-731	NM_13835	90378	Hs.140309	NM_13835	ENSG0000	SAMD1	-	sterile alph	protein-co
lib-C666-l	chr5	176831491	176831491	+	7.49651	NA	intron (NM_000505, intron 8 of 13)	CpG-21467	-3854	NM_00102	345456	Hs.130196	NM_00102	ENSG0000	PFN3	-	profilin 3	protein-co
lib-C666-l	chr8	8902839	8902839	+	7.49651	NA	Intergenic	Intergenic	-3116	NR_03980	100616350		NR_03980	ENSG0000	MIR4660	-	microRNA	ncRNA
lib-C666-l	chr15	75135481	75135481	+	7.49651	NA	promoter-TSS (NR_104309)	promoter-TSS (NR_104309)	71	NM_00128	25989	Hs.513034	NM_00109	ENSG0000	ULK3	-	unc-51 like	protein-co
lib-C666-l	chr4	6784326	6784326	+	7.49163	NA	promoter-TSS (NM_001100590)	promoter-TSS (NM_001100590)	-133	NM_00110	9778	Hs.79276	NM_01474	ENSG0000	KIAA0232	-	KIAA0232	protein-co
lib-C666-l	chr20	52680661	52680661	+	7.49163	NA	intron (NM_001316361, intron 1 of 9)	intron (NM_001316361, intron 1 of	4363	NR_03991	100616225		NR_03991	ENSG0000	MIR4756	-	microRNA	ncRNA
lib-C666-l	chrX	39612784	39612784	+	7.49163	NA	Intergenic	L2a|LINE|L2	92314	NR_03750	100500822		NR_03750	ENSG0000	MIR3937	-	microRNA	ncRNA
lib-C666-l	chr3	59035867	59035867	+	7.49163	NA	promoter-TSS (NM_198463)	promoter-TSS (NM_198463)	-152	NM_19846	200844	Hs.368434	NM_19846	ENSG0000	C3orf67	-	chromosor	protein-co
lib-C666-l	chr2	191208429	191208429	+	7.49163	NA	5' UTR (NM_001128928, exon 1 of 7)	5' UTR (NM_001128928, exon 1 of	233	NM_00112	3628	Hs.32309	NM_00219	ENSG0000	INPP1	-	inositol po	protein-co
lib-C666-l	chr12	93481378	93481378	+	7.49163	NA	intron (NR_040096, intron 3 of 4)	intron (NR_040096, intron 3 of 4)	-86764	NR_12049	102724933	Hs.663947	NR_12049	ENSG0000	LOC10272	-	uncharacte	ncRNA
lib-C666-l	chr6	145469761	145469761	+	7.4883	NA	Intergenic	LTR16A|LTR|ERVL	-586244	NR_03824	100507557	Hs.124537	NR_03824	ENSG0000	LOC10050	-	uncharacte	ncRNA
lib-C666-l	chr22	23864100	23864100	+	7.4883	NA	Intergenic	CpG	-34933	NR_11053	388882	Hs.542766	NR_11053	ENSG0000	LINC01658	-	long interg	ncRNA
lib-C666-l	chr16	67571843	67571843	+	7.43576	NA	intron (NM_001193524, intron 1 of 2	CpG	478	NM_00119	79567	Hs.152717	NM_02451	ENSG0000	FAM65A	-	family with	protein-co
lib-C666-l	chr10	52384307	52384307	+	7.43576	NA	promoter-TSS (NR_126411)	promoter-TSS (NR_126411)	-404	NR_12641	104355295	Hs.450493	NR_126411		SGMS1-AS	-	SGMS1 ant	ncRNA
lib-C666-l	chr19	1479534	1479534	+	7.43576	NA	promoter-TSS (NM_152482)	promoter-TSS (NM_152482)	-306	NM_15248	148223	Hs.532840	NM_15248	ENSG0000	C19orf25	-	chromosor	protein-co
lib-C666-l	chr4	78980793	78980793	+	7.42011	NA	intron (NM_001166133, intron 1 of 4	intron (NM_001166133, intron 1 of	2069	NM_00116	80144	Hs.369448	NM_02087	ENSG0000	FRAS1	-	Fraser extr	protein-co
lib-C666-l	chr17	62971874	62971874	+	7.37943	NA	promoter-TSS (NR_026903)	promoter-TSS (NR_026903)	-171	NR_02690	201283	Hs.396447	NM_15303	ENSG0000	AMZ2P1	-	archaelysir	pseudo
lib-C666-l	chr8	142085944	142085944	+	7.31982	NA	Intergenic	MIRb|SINE|MIR	-52776	NM_01495	22898	Hs.18166	NM_01495	ENSG0000	DENND3	-	DENN dom	protein-co
lib-C666-l	chr7	5128390	5128390	+	7.31982	NA	Intergenic	Intergenic	16699	NR_01534	389458	Hs.733005	NM_20339	ENSG0000	RBAKDN	-	RBAK dow	ncRNA

lib-C666-I	chr19	38908914	38908914	+	7.31982	NA	intron (NM_001146205, intron 7 of 1	CpG	8031	NM_00114	115727	Hs.130434	NM_05294	ENSG0000	RASGRP4	-	RAS guany	protein-co
lib-C666-I	chr4	185760257	185760257	+	7.31982	NA	Intergenic	Intergenic	7943	NR_03786	731424	Hs.427740	NR_037867		MIR3945H	-	MIR3945 h	ncRNA
lib-C666-I	chr5	103514925	103514925	+	7.31982	NA	Intergenic	HUERS-P3-int|LTR|ERV1	-616423	NM_00130	83594	Hs.434289	NM_03143	ENSG0000	NUDT12	-	nudix hydr	protein-co
lib-C666-I	chr17	6065730	6065730	+	7.31982	NA	Intergenic	LTR85a|LTR|Gypsy?	91796	NM_01525	23302	Hs.370166	NM_01525	ENSG0000	WSCD1	-	WSC doma	protein-co
lib-C666-I	chr19	12662251	12662251	+	7.26874	NA	5' UTR (NM_144976, exon 1 of 4)	5' UTR (NM_144976, exon 1 of 4)	105	NM_14497	163050	Hs.655615	NM_14497	ENSG0000	ZNF564	-	zinc finger	protein-co
lib-C666-I	chr5	142522251	142522251	+	7.23765	NA	intron (NM_015071, intron 19 of 22)	intron (NM_015071, intron 19 of 22	-49814	NR_04681	100874372	Hs.666717	NR_04681	ENSG0000	ARHGAP2	-	ARHGAP2	ncRNA
lib-C666-I	chr22	38610710	38610710	+	7.21092	NA	exon (NM_001161572, exon 3 of 3)	exon (NM_001161572, exon 3 of 3)	10017	NR_13277	106635525		NR_132774		SNORA92	-	small nucle	snoRNA
lib-C666-I	chr11	10879905	10879905	+	7.21092	NA	promoter-TSS (NM_021211)	promoter-TSS (NM_021211)	141	NR_03413	729013	Hs.726427	NR_03413	ENSG0000	ZBED5-AS	-	ZBED5 ant	ncRNA
lib-C666-I	chr9	132404515	132404515	+	7.21092	NA	promoter-TSS (NM_177999)	promoter-TSS (NM_177999)	-67	NM_01787	140459	Hs.125037	NM_01787	ENSG0000	ASB6	-	ankyrin rep	protein-co
lib-C666-I	chr16	30578733	30578733	+	7.21092	NA	Intergenic	Intergenic	4995	NM_00102	146542	Hs.301463	NM_14527	ENSG0000	ZNF688	-	zinc finger	protein-co
lib-C666-I	chr17	76414553	76414553	+	7.20016	NA	intron (NR_111989, intron 7 of 8)	L1ME4a|LINE|L1	39854	NM_02441	9489	Hs.654671	NM_02441	ENSG0000	PGS1	-	phosphatic	protein-co
lib-C666-I	chr8	12081653	12081653	+	7.17076	NA	Intergenic	HERVK-int|LTR|ERVK	-30029	NR_00349	85002	Hs.675799	NM_00108	ENSG0000	FAM86B1	-	family with	protein-co
lib-C666-I	chr8	145733919	145733919	+	7.15513	NA	promoter-TSS (NM_138431)	promoter-TSS (NM_138431)	-500	NR_13012	113655	Hs.7678	NM_13843	ENSG0000	MFSD3	-	major facili	protein-co
lib-C666-I	chr19	11708240	11708240	+	7.15513	NA	promoter-TSS (NM_001290083)	promoter-TSS (NM_001290083)	5	NM_00129	199692	Hs.526665	NM_14529	ENSG0000	ZNF627	-	zinc finger	protein-co
lib-C666-I	chr3	168595791	168595791	+	7.15277	NA	Intergenic	Intergenic	-23942	NR_10998	100507661	Hs.65918	NR_10998	ENSG0000	LINC02082	-	long interg	ncRNA
lib-C666-I	chr1	3713115	3713115	+	7.15277	NA	promoter-TSS (NM_020710)	promoter-TSS (NM_020710)	-47	NM_02071	57470	Hs.268488	NM_02071	ENSG0000	LRRC47	-	leucine rich	protein-co
lib-C666-I	chr10	72142468	72142468	+	7.15277	NA	promoter-TSS (NM_207119)	promoter-TSS (NM_207119)	-62	NR_10346	55222	Hs.7778	NM_01820	ENSG0000	LRRC20	-	leucine rich	protein-co
lib-C666-I	chr16	2723287	2723287	+	7.15277	NA	non-coding (NR_040023, exon 1 of 3	non-coding (NR_040023, exon 1 of	153	NR_04002	100507321	Hs.406976	NM_001012731		ERVK13-1	-	endogenou	ncRNA
lib-C666-I	chr2	73143399	73143399	+	7.15277	NA	Intergenic	CpG	-1205	NM_00409	2016	Hs.516090	NM_00409	ENSG0000	EMX1	-	empty spir	protein-co
lib-C666-I	chr3	49130511	49130511	+	7.15277	NA	intron (NM_017730, intron 1 of 10)	intron (NM_017730, intron 1 of 10)	554	NM_00132	54870	Hs.297389	NM_01773	ENSG0000	QRICH1	-	glutamine	protein-co
lib-C666-I	chr17	17991194	17991194	+	7.15277	NA	promoter-TSS (NM_001330144)	promoter-TSS (NM_001330144)	13	NM_00133	1819	Hs.78582	NM_00138	ENSG0000	DRG2	-	developme	protein-co
lib-C666-I	chr11	32914126	32914126	+	7.1502	NA	promoter-TSS (NM_001076786)	promoter-TSS (NM_001076786)	-598	NM_00107	79832	Hs.369368	NM_02477	ENSG0000	QSER1	-	glutamine	protein-co
lib-C666-I	chr4	28293689	28293689	+	7.1502	NA	Intergenic	L1MC1|LINE|L1	-527515	NR_03623	100422937		NR_03623	ENSG0000	MIR4275	-	microRNA	ncRNA
lib-C666-I	chr4	8196579	8196579	+	7.14523	NA	Intergenic	Intergenic	-4392	NM_00131	54436	Hs.479116	NM_01898	ENSG0000	SH3TC1	-	SH3 doma	protein-co
lib-C666-I	chr17	41132595	41132595	+	7.11942	NA	promoter-TSS (NM_025267)	promoter-TSS (NM_025267)	16	NM_17307	146923	Hs.632255	NM_17307	ENSG0000	RUNDC1	-	RUN doma	protein-co
lib-C666-I	chr22	40390756	40390756	+	7.11942	NA	promoter-TSS (NM_138435)	promoter-TSS (NM_138435)	-197	NM_13843	113828	Hs.197680	NM_13843	ENSG0000	FAM83F	-	family with	protein-co
lib-C666-I	chr1	3816902	3816902	+	7.11942	NA	promoter-TSS (NM_207356)	promoter-TSS (NM_207356)	-45	NM_20735	339448	Hs.103939	NM_20735	ENSG0000	C1orf174	-	chromosor	protein-co
lib-C666-I	chr12	77459446	77459446	+	7.11942	NA	promoter-TSS (NM_203394)	promoter-TSS (NM_203394)	-86	NM_20339	144455	Hs.416375	NM_20339	ENSG0000	E2F7	-	E2F transcr	protein-co
lib-C666-I	chr1	763076	763076	+	7.11942	NA	promoter-TSS (NR_047525)	promoter-TSS (NR_047525)	-102	NR_04752	643837	Hs.133183	NR_01536	ENSG0000	LINC01128	-	long interg	ncRNA
lib-C666-I	chr17	76374592	76374592	+	7.09685	NA	promoter-TSS (NM_024419)	promoter-TSS (NM_024419)	-107	NR_11060	9489	Hs.654671	NM_02441	ENSG0000	PGS1	-	phosphatic	protein-co
lib-C666-I	chr5	176884754	176884754	+	7.09685	NA	exon (NM_004395, exon 13 of 14)	exon (NM_004395, exon 13 of 14)	9428	NM_00117	80758	Hs.534492	NM_03056	ENSG0000	PRR7	-	proline rich	protein-co
lib-C666-I	chr1	112933509	112933509	+	7.09685	NA	Intergenic	Intergenic	-5291	NM_01870	55917	Hs.744100	NM_01870	ENSG0000	CTTNBP2N	-	CTTNBP2	protein-co
lib-C666-I	chr11	33655118	33655118	+	7.09685	NA	intron (NM_012194, intron 15 of 19)	intron (NM_012194, intron 15 of 19	67168	NM_00116	100131378	Hs.709466	NM_00116	ENSG0000	C11orf91	-	chromosor	protein-co
lib-C666-I	chr17	2240560	2240560	+	7.09685	NA	promoter-TSS (NM_001098509)	promoter-TSS (NM_001098509)	118	NM_01812	55720	Hs.388170	NM_01812	ENSG0000	TSR1	-	TSR1, ribos	protein-co
lib-C666-I	chr17	57500798	57500798	+	7.09685	NA	Intergenic	Intergenic	57354	NR_03988	100616204		NR_03988	ENSG0000	MIR4729	-	microRNA	ncRNA
lib-C666-I	chr7	35503067	35503067	+	7.09685	NA	Intergenic	L1M6|LINE|L1	149601	NR_03886	401324	Hs.377034	NR_038864		LOC40132	-	uncharacte	ncRNA
lib-C666-I	chr7	148982261	148982261	+	7.08977	NA	promoter-TSS (NR_036573)	promoter-TSS (NR_036573)	-111	NR_03657	155060	Hs.490512	NR_01535	ENSG0000	LOC15506	-	AI894139	pseudo
lib-C666-I	chr2	128785030	128785030	+	7.0832	NA	promoter-TSS (NM_024545)	promoter-TSS (NM_024545)	-161	NM_00114	79595	Hs.32995	NM_02454	ENSG0000	SAP130	-	Sin3A asso	protein-co
lib-C666-I	chr16	29477611	29477611	+	7.08136	NA	intron (NR_002557, intron 2 of 4)	CpG	1288	NR_00255	388242	Hs.662549	NR_00255	ENSG0000	LOC38824	-	SAGA com	pseudo
lib-C666-I	chr20	25677569	25677569	+	7.08136	NA	promoter-TSS (NM_015655)	promoter-TSS (NM_015655)	-29	NM_01565	26152	Hs.61881	NM_01565	ENSG0000	ZNF337	-	zinc finger	protein-co
lib-C666-I	chr19	10764608	10764608	+	7.08136	NA	promoter-TSS (NM_001137673)	promoter-TSS (NM_001137673)	-60	NR_02433	147727	Hs.631616	NR_02433	ENSG0000	ILF3-AS1	-	ILF3 antise	ncRNA
lib-C666-I	chr14	23770956	23770956	+	7.03995	NA	exon (NM_001276318, exon 2 of 5)	exon (NM_001276318, exon 2 of 5)	1101	NM_00127	90673	Hs.601513	NM_00127	ENSG0000	PPP1R3E	-	protein ph	protein-co
lib-C666-I	chr1	171220470	171220470	+	7.03089	NA	intron (NM_001282694, intron 1 of 7)	LTR22B|LTR|ERVK	2860	NM_00128	2326	Hs.1424	NM_00202	ENSG0000	FMO1	-	flavin conta	protein-co
lib-C666-I	chr8	145653946	145653946	+	7.03048	NA	promoter-TSS (NM_016208)	promoter-TSS (NM_016208)	0	NM_18305	51160	Hs.418175	NM_01620	ENSG0000	VPS28	-	VPS28, ES	protein-co
lib-C666-I	chr11	9336310	9336310	+	7.03048	NA	promoter-TSS (NM_015012)	promoter-TSS (NM_015012)	5	NM_00116	440026	Hs.594563	NM_01501	ENSG0000	TMEM41B	-	transmemb	protein-co
lib-C666-I	chr3	108800838	108800838	+	6.98764	NA	intron (NM_014429, intron 8 of 27)	L1MA4|LINE|L1	-19465	NR_14446	100506506	Hs.278908	NR_144461		MORC1-A	-	MORC1 an	ncRNA
lib-C666-I	chr12	105956219	105956219	+	6.98764	NA	Intergenic	Intergenic	-141762	NR_11010	101929110	Hs.145944	NR_11010	ENSG0000	CASC18	-	cancer sus	ncRNA
lib-C666-I	chr6	30710670	30710670	+	6.98764	NA	promoter-TSS (NM_001318875).2	promoter-TSS (NM_001318875).2	-42	NM_00580	10211	Hs.179986	NM_00580	ENSG0000	FLOT1	-	flotillin 1	protein-co
lib-C666-I	chrX	18721200	18721200	+	6.98764	NA	intron (NM_006240, intron 3 of 18)	intron (NM_006240, intron 3 of 18)	-10394	NR_04664	100874004		NR_046642		PPEF1-AS1	-	PPEF1 anti	ncRNA
lib-C666-I	chr12	41998843	41998843	+	6.98764	NA	Intergenic	MER102b|DNA|hAT-Charlie	-159148	NR_13504	101927038	Hs.98588	NR_13504	ENSG0000	LOC10192	-	uncharacte	ncRNA
lib-C666-I	chr6	42897080	42897080	+	6.98764	NA	non-coding (NR_134881, exon 1 of 8	non-coding (NR_134881, exon 1 of	212	NM_00131	107080644	Hs.144914	NM_001318856		CNPY3-GN	-	CNPY3-GN	protein-co
lib-C666-I	chr8	12293720	12293720	+	6.86398	NA	promoter-TSS (NR_040091)	promoter-TSS (NR_040091)	132	NM_00113	653333	Hs.458413	NM_00113	ENSG0000	FAM86B2	-	family with	protein-co
lib-C666-I	chr4	169142630	169142630	+	6.83635	NA	intron (NM_017631, intron 36 of 37)	intron (NM_017631, intron 36 of 37	97328	NM_01763	55601	Hs.591710	NM_01763	ENSG0000	DDX60	-	DExD/H-b	protein-co
lib-C666-I	chr3	16577590	16577590	+	6.82479	NA	promoter-TSS (NR_103843)	promoter-TSS (NR_103843)	-235	NR_10384	100996597	Hs.599432	NR_10384	ENSG0000	LINC00690	-	long interg	ncRNA
lib-C666-I	chr16	85160551	85160551	+	6.82479	NA	Intergenic	Intergenic	-10205	NR_03398	400548	Hs.592054	NR_03398	ENSG0000	LINC02139	-	long interg	ncRNA
lib-C666-I	chr9	6080483	6080483	+	6.82479	NA	Intergenic	Intergenic	-64843	NM_00124	26953	Hs.167496	NM_01241	ENSG0000	RANBP6	-	RAN bindi	protein-co
lib-C666-I	chr2	219773916	219773916	+	6.82479	NA	intron (NR_110238, intron 3 of 4)	CpG	21443	NR_11023	101928537	Hs.611129	NR_11023	ENSG0000	LINC01494	-	long interg	ncRNA
lib-C666-I	chr19	4343532	4343532	+	6.82479	NA	promoter-TSS (NM_001159846)	promoter-TSS (NM_001159846)	8	NM_03286	84954	Hs.321689	NM_03286	ENSG0000	MPND	-	MPN doma	protein-co
lib-C666-I	chr14	101053524	101053524	+	6.82479	NA	Intergenic	GC_rich|Low_complexity|Low_comp	-17393	NM_02083	57596	Hs.211751	NM_02083	ENSG0000	BEGAIN	-	brain enric	protein-co
lib-C666-I	chr16	19119399	19119399	+	6.82479	NA	Intergenic	AluSx|SINE|Alu	-5855	NM_00103	162073	Hs.530899	NM_00103	ENSG0000	ITPRIPL2	-	inositol 1,4	protein-co
lib-C666-I	chr11	62494688	62494688	+	6.82401	NA	promoter-TSS (NM_001318812)	promoter-TSS (NM_001318812)	168	NR_03794	100534595	Hs.533709	NR_03794	ENSG0000	HNRNPUL	-	HNRNPUL	ncRNA
lib-C666-I	chr17	71153786	71153786	+	6.81085	NA	Intergenic	Intergenic	-7374	NM_00105	6752	Hs.514451	NM_00105	ENSG0000	SSTR2	-	somatostat	protein-co
lib-C666-I	chr7	29603494	29603494	+	6.81085	NA	promoter-TSS (NM_175887)	promoter-TSS (NM_175887)	67	NM_17588	222171	Hs.91109	NM_17588	ENSG0000	PRR15	-	proline rich	protein-co
lib-C666-I	chr4	148848889	148848889	+	6.81085	NA	intron (NM_024605, intron 13 of 22)	intron (NM_024605, intron 13 of 22	145143	NR_03996	100616246		NR_03996	ENSG0000	MIR4799	-	microRNA	ncRNA
lib-C666-I	chr6	151389082	151389082	+	6.81085	NA	intron (NM_015440, intron 26 of 27)	intron (NM_015440, intron 26 of 27	160510	NR_12586	102723831	Hs.684425	NR_125868		LOC10272	-	uncharacte	ncRNA
lib-C666-I	chr10	77026262	77026262	+	6.79473	NA	Intergenic	Intergenic	-29879	NR_03822	253264	Hs.662111	NR_03822	ENSG0000	ZNF503-A	-	ZNF503 an	ncRNA
lib-C666-I	chr17	71694175	71694175	+	6.74226	NA	Intergenic	Intergenic	-39818	NM_00127	100134391	Hs.694655	NM_001278587		LOC10013	-	uncharacte	protein-co
lib-C666-I	chr17	65713907	65713907	+	6.71839	NA	promoter-TSS (NM_001303272)	promoter-TSS (NM_001303272)	-42	NM_00130	25926	Hs.463936	NM_01546	ENSG0000	NOL11	-	nucleolar p	protein-co
lib-C666-I	chr8	145734214	145734214	+	6.71839	NA	promoter-TSS (NM_138431)	promoter-TSS (NM_138431)	-205	NR_13012	113655	Hs.7678	NM_13843	ENSG0000	MFSD3	-	major facili	protein-co
lib-C666-I	chr9	43163041	43163041	+	6.71839	NA	Intergenic	Intergenic	-17557	NR_02747	642929	Hs.584288	NR_02747	ENSG0000	LOC64292	-	general tra	pseudo
lib-C666-I	chr2	218858000	218858000	+	6.71839	NA	TTS (NR_135524)	TTS (NR_135524)	14570	NR_13552	105373878	Hs.630709	NR_13552	ENSG0000	LOC10537	-	uncharacte	ncRNA
lib-C666-I	chr3	72677462	72677462	+	6.71839	NA	Intergenic	Intergenic	35938	NR_13553	105377162	Hs.123457	NR_135531		LOC10537	-	uncharacte	ncRNA
lib-C666-I	chr1	146643734	146643734	+	6.71839	NA	non-coding (NR_103870, exon 2 of 6	non-coding (NR_103870, exon 2 of	434	NM_00539	5565	Hs.50732	NM_00539	ENSG0000	PRKAB2	-	protein kin	protein-co
lib-C666-I	chr10	126313630	126313630	+	6.71839	NA	intron (NM_014661, intron 4 of 4)	intron (NM_014661, intron 4 of 4)	-78967	NR_12063	101927944	Hs.437448	NR_120630		FAM53B-A	-	FAM53B ar	ncRNA
lib-C666-I	chr1	10486141	10486141	+	6.71399	NA	Intergenic	LTR12C|LTR|ERV1	-4018	NM_00127	100526739	Hs.412311	NM_19854	ENSG0000	APITD1-C	-	APITD1-C	protein-co
lib-C666-I	chr11	568614	568614	+	6.70376	NA	promoter-TSS (NR_038262)	promoter-TSS (NR_038262)	-157	NR_03826	100506211	Hs.446388	NR_03826	ENSG0000	MIR210HG	-	MIR210 ho	ncRNA
lib-C666-I	chr4	7396549	7396549	+	6.64931	NA	intron (NM_020777, intron 1 of 26)	intron (NM_020777, intron 1 of 26)	40151	NM_00108	768239	Hs.662523	NM_00108	ENSG0000	PSAPL1	-	prosaposin	protein-co
lib-C666-I	chr1	665651	665651	+	6.63143	NA	promoter-TSS (NR_028327).2	promoter-TSS (NR_028327).2	80	NR_02832	100133331	Hs.728864	NR_028327		LOC10013	-	uncharacte	ncRNA
lib-C666-I	chr2	7172369	7172369	+	6.61453	NA	intron (NM_014746, intron 8 of 8)	CpG	45642	NR_11025	101929452		NR_11025	ENSG0000	LOC10192	-	uncharacte	ncRNA
lib-C666-I	chr2	97523920	97523920	+	6.61453	NA	promoter-TSS (NM_016466)	promoter-TSS (NM_016466)	-164	NM_01646	51239	Hs.709507	NM_01646	ENSG0000	ANKRD39	-	ankyrin rep	protein-co
lib-C666-I	chr2	133197502	133197502	+	6.61453	NA	intron (NM_001508, intron 1 of 1)	intron (NM_001508, intron 1 of 1)	23355	NM_00150	2863	Hs.432395	NM_00150	ENSG0000	GPR39	-	G protein-	protein-co
lib-C666-I	chr15	78286784	78286784	+	6.61453	NA	promoter-TSS (NR_026998)	promoter-TSS (NR_026998)	-217	NR_02699	91450	Hs.406766	NR_026998		LOC91450	-	uncharacte	ncRNA
lib-C666-I	chr1	10003442	10003442	+	6.61453	NA	promoter-TSS (NM_001316975)	promoter-TSS (NM_001316975)	43	NM_03236	84328	Hs.327252	NM_03236	ENSG0000	LZIC	-	leucine zip	protein-co
lib-C666-I	chr10	127408033	127408033	+	6.61453	NA	promoter-TSS (NM_001202438)	promoter-TSS (NM_001202438)	29	NR_03384	399821	Hs.535286	NR_03384	ENSG0000	FLJ37035	-	uncharacte	ncRNA
lib-C666-I	chr15	98503731	98503731	+	6.61453	NA	promoter-TSS (NM_183376)	promoter-TSS (NM_183376)	-202	NM_18337	91947	Hs.6093	NM_18337	ENSG0000	ARRDC4	-	arrestin do	protein-co
lib-C666-I	chr18	109032	109032	+	6.5798	NA	promoter-TSS (NR_033770)	promoter-TSS (NR_033770)	-33	NR_03377	727758	Hs.585843	NR_03377	ENSG0000	ROCK1P1	-	Rho associ	pseudo
lib-C666-I	chr12	125721839	125721839	+	6.56784	NA	Intergenic	Intergenic	-89323	NM_05290	114795	Hs.524838	NM_05290	ENSG0000	TMEM132	-	transmemb	protein-co
lib-C666-I	chr8	22722844	22722844	+	6.56784	NA	intron (NM_144962, intron 3 of 6)	CpG	-12641	NR_12543	101929237	Hs.677838	NR_12543	ENSG0000	LOC10192	-	uncharacte	ncRNA
lib-C666-I	chr5	121499701	121499701	+	6.56784	NA	intron (NM_001195535, intron 1 of 2)	intron (NM_001195535, intron 1 of	3830	NM_00119	100505841	Hs.733368	NM_001195535		LOC10050	-	zinc finger	protein-co
lib-C666-I	chr17	40021752	40021752	+	6.56784	NA	promoter-TSS (NM_018143)	promoter-TSS (NM_018143)	-68	NM_01814	55175	Hs.13268	NM_01814	ENSG0000	KLHL11	-	kelch like f	protein-co
lib-C666-I	chr1	232766547	232766547	+	6.55735	NA	Intergenic	Intergenic	111914	NR_11068	101927683	Hs.520568	NR_11068	ENSG0000	LINC01744	-	long interg	ncRNA
lib-C666-I	chr7	30387936	30387936	+	6.50091	NA	intron (NM_147128, intron 2 of 4)	intron (NM_147128, intron 2 of 4)	-21730	NR_00218	222161	Hs.112423	NR_002186		DKFZP586	-	uncharacte	pseudo
lib-C666-I	chr20	55141519	55141519	+	6.50091	NA	Intergenic	Intergenic	-11100	NR_13653	105372682	Hs.517135	NR_13653	ENSG0000	LINC01716	-	long interg	ncRNA
lib-C666-I	chr2	38709178	38709178	+	6.50091	NA	intron (NR_110259, intron 3 of 4)	intron (NR_110259, intron 3 of 4)	33704	NR_11025	101929596	Hs.537341	NR_110259		LOC10192	-	uncharacte	ncRNA
lib-C666-I	chr19	37178458	37178458	+	6.50091	NA	promoter-TSS (NR_110707)	promoter-TSS (NR_110707)	-56	NM_00132	163081	Hs.412517	NM_15260	ENSG0000	ZNF567	-	zinc finger	protein-co
lib-C666-I	chr1	28455732	28455732	+	6.49882	NA	Intergenic	Intergenic	-40584	NR_10421	2140	Hs.185774	NM_00199	ENSG0000	EYA3	-	EYA transc	protein-co
lib-C666-I	chr7	157667882	157667882	+	6.49644	NA	intron (NM_130843, intron 11 of 21)	intron (NM_130843, intron 11 of 21	20605	NR_03896	100506585	Hs.661265	NR_03896	ENSG0000	LOC10050	-	uncharacte	ncRNA
lib-C666-I	chr1	41826791	41826791	+	6.49644	NA	promoter-TSS (NM_001291281)	promoter-TSS (NM_001291281)	-812	NM_00129	100132074	Hs.559677	NM_001291281		FOXO6	-	forkhead b	protein-co
lib-C666-I	chr14	55337289	55337289	+	6.49644	NA	intron (NM_001024024, intron 1 of 6)	L1PA4|LINE|L1	7622	NR_03619	100422984		NR_03619	ENSG0000	MIR4308	-	microRNA	ncRNA
lib-C666-I	chr13	95309349	95309349	+	6.49644	NA	Intergenic	Tigger4|DNA|TcMar-Tigger	51391	NR_12041	101927248	Hs.626095	NR_120417		LOC10192	-	uncharacte	ncRNA
lib-C666-I	chr10	131909319	131909319	+	6.49644	NA	promoter-TSS (NR_034125)	promoter-TSS (NR_034125)	-238	NR_03412	387723	Hs.127394	NR_034125		LINC00959	-	long interg	ncRNA
lib-C666-I	chr17	75878891	75878891	+	6.49644	NA	non-coding (NR_028337, exon 2 of 2	non-coding (NR_028337, exon 2 of	1278	NR_02833	400624	Hs.514521	NM_00100	ENSG0000	LINC01973	-	long interg	ncRNA
lib-C666-I	chr11	65150080	65150080	+	6.49644	NA	promoter-TSS (NM_001278250)	promoter-TSS (NM_001278250)	65	NM_18255	283130	Hs.661604	NM_18255	ENSG0000	SLC25A45	-	solute carr	protein-co
lib-C666-I	chr1	227922989	227922989	+	6.49644	NA	promoter-TSS (NM_001323934)	promoter-TSS (NM_001323934)	123	NM_02300	65094	Hs.555974	NM_02300	ENSG0000	JMJD4	-	jumonji do	protein-co
lib-C666-I	chr1	112362905	112362905	+	6.49644	NA	intron (NM_004980, intron 2 of 7)	L2c|LINE|L2	36622	NR_04678	100874295	Hs.738190	NR_04678	ENSG0000	KCND3-IT	-	KCND3 int	ncRNA
lib-C666-I	chr7	68830963	68830963	+	6.49644	NA	Intergenic	Intergenic	231518	NR_10810	100507468	Hs.362492	NR_108105		LOC10050	-	uncharacte	ncRNA
lib-C666-I	chr17	74667869	74667869	+	6.49644	NA	promoter-TSS (NR_130916)	promoter-TSS (NR_130916)	-141	NR_13091	105274304	Hs.572495	NR_13091	ENSG0000	LOC10527	-	uncharacte	ncRNA
lib-C666-I	chr14	105452057	105452057	+	6.48676	NA	promoter-TSS (NM_174891)	promoter-TSS (NM_174891)	-559	NM_17489	122616	Hs.27183	NM_17489	ENSG0000	C14orf79	-	chromosor	protein-co
lib-C666-I	chr22	21356195	21356195	+	6.40179	NA	promoter-TSS (NR_027052)	promoter-TSS (NR_027052)	-16	NR_02705	439931	Hs.517430	NR_02705	ENSG0000	THAP7-AS	-	THAP7 ant	ncRNA
lib-C666-I	chr1	112421418	112421418	+	6.40179	NA	intron (NM_004980, intron 2 of 7)	MLT2B4|LTR|ERVL	-21891	NR_04678	100874295	Hs.738190	NR_04678	ENSG0000	KCND3-IT	-	KCND3 int	ncRNA
lib-C666-I	chr17	66201644	66201644	+	6.39985	NA	promoter-TSS (NM_001346471)	promoter-TSS (NM_001346471)	-626	NM_00134	51321	Hs.293560	NM_01662	ENSG0000	AMZ2	-	archaelysin	protein-co
lib-C666-I	chr7	50602820	50602820	+	6.38282	NA	intron (NR_033845, intron 1 of 4)	intron (NR_033845, intron 1 of 4)	3363	NR_03384	100129427	Hs.680434	NR_03384	ENSG0000	DDC-AS1	-	DDC antise	ncRNA
lib-C666-I	chr7	148959648	148959648	+	6.38282	NA	intron (NR_144366, intron 1 of 6)	CpG	386	NR_14436	100289678	Hs.490512	NM_00119	ENSG0000	ZNF783	-	zinc finger	protein-co
lib-C666-I	chr12	74296	74296	+	6.33582	NA	intron (NR_130745, intron 1 of 10)	CpG	571	NR_13074	100288778	Hs.459573	NR_13074	ENSG0000	LOC10028	-	WAS prote	pseudo
lib-C666-I	chr7	1609776	1609776	+	6.33432	NA	promoter-TSS (NM_032302)	promoter-TSS (NM_032302)	67	NR_02732	114796	Hs.436146	NR_02148	ENSG0000	PSMG3-AS	-	PSMG3 ant	ncRNA
lib-C666-I	chr10	99052171	99052171	+	6.33432	NA	intron (NR_037909, intron 1 of 14)	CpG	259	NM_00120	84986	Hs.80305	NM_03290	ENSG0000	ARHGAP1	-	Rho GTPas	protein-co
lib-C666-I	chr19	34269243	34269243	+	6.3317	NA	Intergenic	CpG	-18508	NM_00112	79047	Hs.221873	NM_02407	ENSG0000	KCTD15	-	potassium	protein-co
lib-C666-I	chr3	197941438	197941438	+	6.3317	NA	Intergenic	Intergenic	62201	NM_00114	728262	Hs.744675	NM_001145248		FAM157A	-	family with	protein-co
lib-C666-I	chr5	180617989	180617989	+	6.32351	NA	TTS (NR_108031)	TTS (NR_108031)	919	NR_10803	102577426	Hs.277359	NR_10803	ENSG0000	LINC01962	-	long interg	ncRNA
lib-C666-I	chr13	31444954	31444954	+	6.32351	NA	Intergenic	Intergenic	-11745	NR_10381	440132	Hs.729493	NR_10381	ENSG0000	LINC00545	-	long interg	ncRNA
lib-C666-I	chr7	3901744	3901744	+	6.32351	NA	intron (NM_152744, intron 5 of 44)	intron (NM_152744, intron 5 of 44)	-267573	NM_00107	221935	Hs.653013	NM_15274	ENSG0000	SDK1	-	sidekick ce	protein-co
lib-C666-I	chr3	196359440	196359440	+	6.32351	NA	promoter-TSS (NR_132101)	promoter-TSS (NR_132101)	18	NR_13210	101929769	Hs.518527	NR_13210	ENSG0000	LINC01063	-	long interg	ncRNA
lib-C666-I	chr19	42787986	42787986	+	6.32351	NA	promoter-TSS (NM_015125)	promoter-TSS (NM_015125)	-831	NM_01512	23152	Hs.388236	NM_01512	ENSG0000	CIC	-	capicua tra	protein-co
lib-C666-I	chr21	47393841	47393841	+	6.32351	NA	intron (NR_109931, intron 1 of 1)	CpG	957	NR_10993	101928796	Hs.130919	NR_109931		LOC10192	-	uncharacte	ncRNA
lib-C666-I	chr2	87303697	87303697	+	6.32351	NA	promoter-TSS (NR_026846)	promoter-TSS (NR_026846)	-161	NR_02684	285074	Hs.424184	NM_001012626		LOC28507	-	anaphase p	pseudo
lib-C666-I	chr2	133196437	133196437	+	6.32351	NA	intron (NM_001508, intron 1 of 1)	intron (NM_001508, intron 1 of 1)	22290	NM_00150	2863	Hs.432395	NM_00150	ENSG0000	GPR39	-	G protein-	protein-co
lib-C666-I	chr9	12955988	12955988	+	6.32351	NA	Intergenic	L1PBa|LINE|L1	16644	NR_13297	106635615		NR_132976		SNORD13	-	small nucle	snoRNA
lib-C666-I	chr4	14182943	14182943	+	6.32351	NA	Intergenic	Intergenic	69351	NR_03393	152742	Hs.135435	NR_03393	ENSG0000	LINC01085	-	long interg	ncRNA
lib-C666-I	chr10	131919834	131919834	+	6.32351	NA	Intergenic	Intergenic	-10753	NR_03412	387723	Hs.127394	NR_034125		LINC00959	-	long interg	ncRNA
lib-C666-I	chr12	95712030	95712030	+	6.32351	NA	Intergenic	Intergenic	8331	NR_03745	100500802		NR_03745	ENSG0000	MIR3685	-	microRNA	ncRNA
lib-C666-I	chr16	86918014	86918014	+	6.32351	NA	Intergenic	Intergenic	162317	NR_13518	102724344	Hs.585769	NR_13518	ENSG0000	LOC10272	-	uncharacte	ncRNA
lib-C666-I	chr4	14857850	14857850	+	6.32351	NA	intron (NR_126435, intron 2 of 6)	intron (NR_126435, intron 2 of 6)	31943	NR_12643	201853	Hs.551051	NR_12643	ENSG0000	LINC00504	-	long interg	ncRNA
lib-C666-I	chr17	19091433	19091433	+	6.32351	NA	Intergenic	U3|snRNA|snRNA	-25496	NM_00129	388436	Hs.677375	NM_00129	ENSG0000	LOC38843	-	uncharacte	protein-co
lib-C666-I	chr12	31886262	31886262	+	6.32351	NA	Intergenic	LTR8|LTR|ERV1	-4154	NM_00127	196394	Hs.585084	NM_00111	ENSG0000	AMN1	-	antagonist	protein-co
lib-C666-I	chr5	69696936	69696936	+	6.32351	NA	intron (NR_027386, intron 3 of 5).2	LTR12E|LTR|ERV1	-14261	NR_03341	653238	Hs.607501	NM_001098729		GTF2H2B	-	general tra	pseudo
lib-C666-I	chr11	78128157	78128157	+	6.32351	NA	intron (NM_080491, intron 1 of 9)	CpG	711	NM_08049	9846	Hs.429434	NM_01229	ENSG0000	GAB2	-	GRB2 asso	protein-co
lib-C666-I	chr9	99616843	99616843	+	6.32351	NA	promoter-TSS (NM_001001662)	promoter-TSS (NM_001001662)	-73	NM_00134	158431	Hs.30567	NM_00100	ENSG0000	ZNF782	-	zinc finger	protein-co
lib-C666-I	chr17	75954334	75954334	+	6.32351	NA	Intergenic	CpG	-45984	NM_00114	57690	Hs.406810	NM_01899	ENSG0000	TNRC6C	-	trinucleotic	protein-co
lib-C666-I	chr1	153606769	153606769	+	6.29902	NA	promoter-TSS (NM_001024210)	promoter-TSS (NM_001024210)	-201	NM_00102	6284	Hs.516505	NM_00597	ENSG0000	S100A13	-	S100 calciu	protein-co
lib-C666-I	chrX	39647190	39647190	+	6.29226	NA	Intergenic	Intergenic	126720	NR_03750	100500822		NR_03750	ENSG0000	MIR3937	-	microRNA	ncRNA
lib-C666-I	chr3	32726578	32726578	+	6.29226	NA	promoter-TSS (NR_046352)	promoter-TSS (NR_046352)	-59	NM_01544	25904	Hs.444851	NM_01544	ENSG0000	CNOT10	-	CCR4-NO	protein-co
lib-C666-I	chr11	9780421	9780421	+	6.29226	NA	intron (NR_036485, intron 1 of 2)	CpG	581	NR_03648	283104	Hs.632156	NR_03648	ENSG0000	SBF2-AS1	-	SBF2 antise	ncRNA
lib-C666-I	chr12	46121454	46121454	+	6.29226	NA	non-coding (NR_028408, exon 1 of 1	non-coding (NR_028408, exon 1 of	250	NR_02840	400027	Hs.597122	NR_028408		LINC00938	-	long interg	ncRNA
lib-C666-I	chr10	60027844	60027844	+	6.29226	NA	promoter-TSS (NM_152230)	promoter-TSS (NM_152230)	-150	NM_15223	253430	Hs.30280	NM_15223	ENSG0000	IPMK	-	inositol po	protein-co
lib-C666-I	chr17	74667290	74667290	+	6.29226	NA	promoter-TSS (NR_130916)	promoter-TSS (NR_130916)	-720	NR_13091	105274304	Hs.572495	NR_13091	ENSG0000	LOC10527	-	uncharacte	ncRNA
lib-C666-I	chr5	95297266	95297266	+	6.29226	NA	promoter-TSS (NR_130776)	promoter-TSS (NR_130776)	-439	NR_13077	101929710		NR_13077	ENSG0000	LOC10192	-	uncharacte	ncRNA
lib-C666-I	chr3	183947494	183947494	+	6.29226	NA	promoter-TSS (NM_138345)	promoter-TSS (NM_138345)	-723	NM_13834	90113	Hs.584997	NM_13834	ENSG0000	VWA5B2	-	von Willeb	protein-co
lib-C666-I	chr20	60951611	60951611	+	6.29226	NA	Intergenic	Intergenic	-9243	NM_00556	3911	Hs.473256	NM_00556	ENSG0000	LAMA5	-	laminin sul	protein-co
lib-C666-I	chr17	7227581	7227581	+	6.26612	NA	exon (NM_032442, exon 11 of 29)	exon (NM_032442, exon 11 of 29)	5057	NM_00100	84461	Hs.654794	NM_03244	ENSG0000	NEURL4	-	neuralized	protein-co
lib-C666-I	chr6	168648762	168648762	+	6.20473	NA	intron (NR_110870, intron 1 of 4)	intron (NR_110870, intron 1 of 4)	5144	NR_11087	101929420	Hs.571211	NR_11087	ENSG0000	LOC10192	-	uncharacte	ncRNA
lib-C666-I	chr16	30030162	30030162	+	6.20473	NA	Intergenic	L2c|LINE|L2	-4493	NM_00110	146378	Hs.651588	NM_00110	ENSG0000	C16orf92	-	chromosor	protein-co
lib-C666-I	chr2	8713763	8713763	+	6.20473	NA	intron (NR_110257, intron 4 of 5)	intron (NR_110257, intron 4 of 5)	10159	NR_11025	101929567	Hs.634706	NR_11025	ENSG0000	LINC01814	-	long interg	ncRNA
lib-C666-I	chr1	90098530	90098530	+	6.20473	NA	promoter-TSS (NR_033981)	promoter-TSS (NR_033981)	-77	NR_03398	400761	Hs.741594	NR_03398	ENSG0000	FLJ27354	-	uncharacte	ncRNA
lib-C666-I	chr12	110476978	110476978	+	6.20473	NA	3' UTR (NM_033121, exon 15 of 15)	3' UTR (NM_033121, exon 15 of 15)	28522	NM_20743	400073	Hs.44817	NM_20743	ENSG0000	C12orf76	-	chromosor	protein-co
lib-C666-I	chr8	56625703	56625703	+	6.20473	NA	Intergenic	Harlequin-int|LTR|ERV1	50836	NM_00128	137695	Hs.420076	NM_15241	ENSG0000	TMEM68	-	transmemb	protein-co
lib-C666-I	chr8	144373765	144373765	+	6.20473	NA	5' UTR (NM_030895, exon 1 of 3)	5' UTR (NM_030895, exon 1 of 3)	206	NM_03089	79943	Hs.512740	NM_03089	ENSG0000	ZNF696	-	zinc finger	protein-co
lib-C666-I	chr16	86789525	86789525	+	6.20473	NA	Intergenic	AluSq|SINE|Alu	33828	NR_13518	102724344	Hs.585769	NR_13518	ENSG0000	LOC10272	-	uncharacte	ncRNA

lib-C666-I	chrX	39473868	39473868	+	6.20473	NA	Intergenic	Intergenic	-46602	NR_03750	100500822		NR_03750	ENSG0000	MIR3937	-	microRNA	ncRNA
lib-C666-I	chr7	36331463	36331463	+	6.18732	NA	intron (NM_030636, intron 6 of 7)	intron (NM_030636, intron 6 of 7)	75319	NM_00119	23366	Hs.6224	NM_01531	ENSG0000	KIAA0895	-	KIAA0895	protein-co
lib-C666-I	chr2	63278008	63278008	+	6.18732	NA	promoter-TSS (NR_130153)	promoter-TSS (NR_130153)	71	NM_01456	5013	Hs.734360	NM_01456	ENSG0000	OTX1	-	orthodenti	protein-co
lib-C666-I	chr11	45001563	45001563	+	6.15313	NA	Intergenic	Intergenic	6110	NR_02668	221122	Hs.551656	NR_02668	ENSG0000	LOC22112	-	uncharacte	ncRNA
lib-C666-I	chr1	147807097	147807097	+	6.15313	NA	promoter-TSS (NR_049810)	promoter-TSS (NR_049810)	-419	NR_04981	100847044		NR_04981	ENSG0000	MIR5087	-	microRNA	ncRNA
lib-C666-I	chr15	39567563	39567563	+	6.15313	NA	Intergenic	Intergenic	24693	NM_00130	400360	Hs.376109	NM_207445		C15orf54	-	chromosor	ncRNA
lib-C666-I	chrX	116080809	116080809	+	6.15313	NA	Intergenic	L1PA2|LINE|L1	-128981	NM_00132	100126447	Hs.695847	NM_001321413		LOC10012	-	uncharacte	protein-co
lib-C666-I	chr17	7232829	7232829	+	6.15313	NA	promoter-TSS (NM_032442)	promoter-TSS (NM_032442)	-191	NM_03244	84461	Hs.654794	NM_03244	ENSG0000	NEURL4	-	neuralized	protein-co
lib-C666-I	chr2	219598523	219598523	+	6.1509	NA	intron (NM_014640, intron 2 of 19)	intron (NM_014640, intron 2 of 19)	22955	NM_01464	9654	Hs.471405	NM_01464	ENSG0000	TTLL4	-	tubulin tyr	protein-co
lib-C666-I	chr18	56279036	56279036	+	6.13111	NA	5' UTR (NM_052947, exon 2 of 13)	5' UTR (NM_052947, exon 2 of 13)	11184	NR_13296	106635543		NR_132963		SNORA10	-	small nucle	snoRNA
lib-C666-I	chr11	58910579	58910579	+	6.13111	NA	promoter-TSS (NR_110184)	promoter-TSS (NR_110184)	-298	NR_11018	101927204	Hs.659259	NR_11018	ENSG0000	LOC10192	-	uncharacte	ncRNA
lib-C666-I	chr1	221613470	221613470	+	6.13111	NA	Intergenic	Intergenic	-103832	NR_02423	400804	Hs.722626	NR_02423	ENSG0000	C1orf140	-	uncharacte	ncRNA
lib-C666-I	chr4	9104410	9104410	+	6.06312	NA	Intergenic	HERVE-int|LTR|ERV1	-107973	NM_00125	100287144	Hs.741130	NM_00125	ENSG0000	USP17L10	-	ubiquitin s	protein-co
lib-C666-I	chr5	118406653	118406653	+	6.01141	NA	promoter-TSS (NM_001290322)	promoter-TSS (NM_001290322)	-68	NR_13425	105379143	Hs.636285	NR_13424	ENSG0000	LOC10537	-	uncharacte	ncRNA
lib-C666-I	chr22	43659804	43659804	+	5.96761	NA	intron (NM_173050, intron 4 of 21)	intron (NM_173050, intron 4 of 21)	-12144	NR_13458	101927447	Hs.621227	NR_13458	ENSG0000	LOC10192	-	uncharacte	ncRNA
lib-C666-I	chr5	118324095	118324095	+	5.96761	NA	promoter-TSS (NM_001308081)	promoter-TSS (NM_001308081)	-180	NM_00130	285605	Hs.655891	NM_17366	ENSG0000	DTWD2	-	DTW doma	protein-co
lib-C666-I	chr4	87849168	87849168	+	5.96761	NA	intron (NR_038841, intron 2 of 2)	MIRb|SINE|MIR	6834	NR_03884	100506746	Hs.657766	NR_03884	ENSG0000	LOC10050	-	uncharacte	ncRNA
lib-C666-I	chr22	50449025	50449025	+	5.96422	NA	intron (NM_001001694, intron 1 of 14	CpG	2030	NM_00100	400935	Hs.526712	NM_00100	ENSG0000	IL17REL	-	interleukin	protein-co
lib-C666-I	chr8	17198719	17198719	+	5.96422	NA	intron (NM_004686, intron 6 of 13)	intron (NM_004686, intron 6 of 13)	72321	NM_00468	9108	Hs.625674	NM_00468	ENSG0000	MTMR7	-	myotubula	protein-co
lib-C666-I	chr19	36545275	36545275	+	5.96422	NA	promoter-TSS (NM_001083961)	promoter-TSS (NM_001083961)	389	NM_00133	199745	Hs.350209	NM_15265	ENSG0000	THAP8	-	THAP dom	protein-co
lib-C666-I	chr8	142163782	142163782	+	5.96422	NA	intron (NM_014957, intron 7 of 22)	intron (NM_014957, intron 7 of 22)	25062	NM_01495	22898	Hs.18166	NM_01495	ENSG0000	DENND3	-	DENN don	protein-co
lib-C666-I	chr1	32860200	32860200	+	5.96422	NA	promoter-TSS (NM_001143888)	promoter-TSS (NM_001143888)	-138	NM_00114	55108	Hs.353454	NM_01804	ENSG0000	BSDC1	-	BSD doma	protein-co
lib-C666-I	chr9	102581938	102581938	+	5.96422	NA	intron (NR_109802, intron 1 of 6)	CpG	233	NR_10980	101928438	Hs.637767	NR_10980	ENSG0000	LOC10192	-	uncharacte	ncRNA
lib-C666-I	chr16	30032917	30032917	+	5.96422	NA	Intergenic	CpG	-1738	NM_00110	146378	Hs.651588	NM_00110	ENSG0000	C16orf92	-	chromosor	protein-co
lib-C666-I	chr19	58326341	58326341	+	5.96422	NA	promoter-TSS (NM_024762)	promoter-TSS (NM_024762)	-60	NM_02476	79818	Hs.560727	NM_02476	ENSG0000	ZNF552	-	zinc finger	protein-co
lib-C666-I	chr6	52382517	52382517	+	5.92363	NA	intron (NM_012288, intron 2 of 10)	intron (NM_012288, intron 2 of 10)	59345	NM_01228	9697	Hs.520182	NM_01228	ENSG0000	TRAM2	-	translocati	protein-co
lib-C666-I	chr1	39957133	39957133	+	5.92363	NA	promoter-TSS (NM_181809)	promoter-TSS (NM_181809)	-185	NM_18180	353500	Hs.472497	NM_18180	ENSG0000	BMP8A	-	bone morp	protein-co
lib-C666-I	chr4	92188804	92188804	+	5.92363	NA	intron (NM_001145065, intron 10 of 1	intron (NM_001145065, intron 10 o	1009649	NR_12592	101929194	Hs.626389	NR_125920		LNCPRESS	-	lncRNA p5	ncRNA
lib-C666-I	chr6	95461837	95461837	+	5.92363	NA	Intergenic	MSTB1|LTR|ERVL-MaLR	563490	NR_10413	101927288	Hs.616643	NR_04750	ENSG0000	MANEA-A	-	MANEA an	ncRNA
lib-C666-I	chrX	3800160	3800160	+	5.92363	NA	Intergenic	CpG	-38225	NR_03403	389906	Hs.636199	NM_001045547		LOC38990	-	zinc finger	pseudo
lib-C666-I	chr5	154413829	154413829	+	5.9194	NA	Intergenic	L1PB1|LINE|L1	20514	NM_00109	285643	Hs.567824	NM_00109	ENSG0000	KIF4B	-	kinesin far	protein-co
lib-C666-I	chr2	58238021	58238021	+	5.9194	NA	intron (NM_001288838, intron 1 of 15	intron (NM_001288838, intron 1 of	-35756	NM_00113	7444	Hs.715298	NM_00629	ENSG0000	VRK2	-	vaccinia re	protein-co
lib-C666-I	chr11	393406	393406	+	5.9194	NA	promoter-TSS (NM_007183)	promoter-TSS (NM_007183)	-777	NM_00718	11187	Hs.534395	NM_00718	ENSG0000	PKP3	-	plakophilin	protein-co
lib-C666-I	chr2	102552418	102552418	+	5.9194	NA	Intergenic	L1P3|LINE|L1	-47509	NR_10379	100506328	Hs.516245	NR_103791		LINC01127	-	long interg	ncRNA
lib-C666-I	chr22	25516316	25516316	+	5.9194	NA	intron (NM_001145206, intron 4 of 10	intron (NM_001145206, intron 4 of	-7657	NR_03894	100128531	Hs.662126	NR_03894	ENSG0000	LOC10012	-	uncharacte	ncRNA
lib-C666-I	chr17	57440039	57440039	+	5.9194	NA	intron (NM_001005404, intron 2 of 4)	intron (NM_001005404, intron 2 of	-3405	NR_03988	100616204		NR_03988	ENSG0000	MIR4729	-	microRNA	ncRNA
lib-C666-I	chr2	20101679	20101679	+	5.9194	NA	promoter-TSS (NM_001008237)	promoter-TSS (NM_001008237)	68	NM_00100	130502	Hs.591547	NM_00100	ENSG0000	TTC32	-	tetratricop	protein-co
lib-C666-I	chr1	205512899	205512899	+	5.9194	NA	Intergenic	Intergenic	12864	NR_02708	284578	Hs.537020	NR_027086		LOC28457	-	uncharacte	ncRNA
lib-C666-I	chr2	151694819	151694819	+	5.89415	NA	Intergenic	L1PB1|LINE|L1	209408	NR_11024	101929282	Hs.126866	NR_11024	ENSG0000	LOC10192	-	uncharacte	ncRNA
lib-C666-I	chr15	65477894	65477894	+	5.89415	NA	promoter-TSS (NM_006660)	promoter-TSS (NM_006660)	-136	NM_00666	10845	Hs.113823	NM_00666	ENSG0000	CLPX	-	caseinolyti	protein-co
lib-C666-I	chr17	67571652	67571652	+	5.8798	NA	Intergenic	Intergenic	-18476	NR_10997	101928122	Hs.579342	NR_10997	ENSG0000	LINC01483	-	long interg	ncRNA
lib-C666-I	chr7	108210232	108210232	+	5.82086	NA	promoter-TSS (NM_012328)	promoter-TSS (NM_012328)	-20	NM_00113	168451	Hs.707689	NM_18252	ENSG0000	THAP5	-	THAP dom	protein-co
lib-C666-I	chr7	3255961	3255961	+	5.819	NA	Intergenic	Intergenic	-57755	NR_12602	100129603	Hs.652997	NR_126024		LOC10012	-	uncharacte	ncRNA
lib-C666-I	chr10	118502080	118502080	+	5.819	NA	promoter-TSS (NM_025015)	promoter-TSS (NM_025015)	5	NM_02501	259217	Hs.648448	NM_02501	ENSG0000	HSPA12A	-	heat shock	protein-co
lib-C666-I	chr5	178054102	178054102	+	5.819	NA	promoter-TSS (NM_020666)	promoter-TSS (NM_020666)	-48	NM_02066	57396	Hs.406557	NM_02066	ENSG0000	CLK4	-	CDC like ki	protein-co
lib-C666-I	chr15	81616699	81616699	+	5.819	NA	promoter-TSS (NR_120365)	promoter-TSS (NR_120365)	25	NR_12036	101929655	Hs.696654	NR_120365		TMC3-AS1	-	TMC3 antis	ncRNA
lib-C666-I	chr8	143807912	143807912	+	5.80117	NA	promoter-TSS (NM_016647)	promoter-TSS (NM_016647)	479	NR_03892	100288181	Hs.716823	NR_03892	ENSG0000	LOC10028	-	uncharacte	ncRNA
lib-C666-I	chr19	1248330	1248330	+	5.80117	NA	promoter-TSS (NM_177401)	promoter-TSS (NM_177401)	-222	NM_17740	90007	Hs.465529	NM_17740	ENSG0000	MIDN	-	midnolin	protein-co
lib-C666-I	chr9	138174002	138174002	+	5.80117	NA	Intergenic	Intergenic	-61093	NM_17352	157927	Hs.559511	NM_173520		C9orf62	-	chromosor	protein-co
lib-C666-I	chr1	999736	999736	+	5.80117	NA	Intergenic	CpG	9951	NM_00120	401934	Hs.568137	NM_00120	ENSG0000	RNF223	-	ring finger	protein-co
lib-C666-I	chr2	243030789	243030789	+	5.7614	NA	promoter-TSS (NR_130699)	promoter-TSS (NR_130699)	5	NR_13069	728323	Hs.380737	NR_024437		LINC01881	-	long interg	ncRNA
lib-C666-I	chr15	43621958	43621958	+	5.76059	NA	promoter-TSS (NM_001324364)	promoter-TSS (NM_001324364)	-591	NM_00132	161823	Hs.533913	NM_00101	ENSG0000	ADAL	-	adenosine	protein-co
lib-C666-I	chr1	43390036	43390036	+	5.74587	NA	Intergenic	CpG	-34684	NR_03396	440584	Hs.260928	NR_03396	ENSG0000	SLC2A1-AS	-	SLC2A1 an	ncRNA
lib-C666-I	chr18	3261944	3261944	+	5.74587	NA	promoter-TSS (NR_130143)	promoter-TSS (NR_130143)	-96	NR_13014	104968399	Hs.464472	NR_13014	ENSG0000	LOC10496	-	uncharacte	ncRNA
lib-C666-I	chr1	222013374	222013374	+	5.74587	NA	intron (NR_125989, intron 2 of 3)	intron (NR_125989, intron 2 of 3)	634	NR_12598	101929771	Hs.557001	NR_12598	ENSG0000	LINC01655	-	long interg	ncRNA
lib-C666-I	chr1	59369465	59369465	+	5.74587	NA	Intergenic	CpG	-116683	NR_11062	101926925		NR_110626		LINC01358	-	long interg	ncRNA
lib-C666-I	chr2	10633947	10633947	+	5.74587	NA	Intergenic	Intergenic	44093	NR_11059	101929715	Hs.634550	NR_110597		LOC10192	-	uncharacte	ncRNA
lib-C666-I	chr1	121484490	121484490	+	5.67774	NA	Intergenic	ALR/Alpha|Satellite|centr	223580	NR_00395	647121	Hs.697682	NR_00395	ENSG0000	EMBP1	-	embigin ps	pseudo
lib-C666-I	chr7	143078010	143078010	+	5.66964	NA	promoter-TSS (NM_003461)	promoter-TSS (NM_003461)	108	NM_00132	100507507	Hs.131743	NM_001322263		LOC10050	-	uncharacte	protein-co
lib-C666-I	chr22	42486928	42486928	+	5.66964	NA	promoter-TSS (NR_034118)	promoter-TSS (NR_034118)	-9	NR_03411	100132273	Hs.709677	NR_03411	ENSG0000	NDUFA6-A	-	NDUFA6 a	ncRNA
lib-C666-I	chr10	42599698	42599698	+	5.6574	NA	Intergenic	HSATII|Satellite|Satellite	263795	NR_02438	441666	Hs.255729	NR_02438	ENSG0000	LOC44166	-	zinc finger	pseudo
lib-C666-I	chr9	132259078	132259078	+	5.65398	NA	intron (NR_038955, intron 2 of 4)	CpG	8139	NR_03895	100506190	Hs.529860	NR_038955		LINC00963	-	long interg	ncRNA
lib-C666-I	chr13	110773021	110773021	+	5.65398	NA	Intergenic	Intergenic	67389	NR_12638	104355146	Hs.508690	NR_12638	ENSG0000	LINC00396	-	long interg	ncRNA
lib-C666-I	chr10	81918425	81918425	+	5.65398	NA	intron (NM_001278409, intron 14 of 1	intron (NM_001278409, intron 14 o	26167	NM_00101	219348	Hs.204947	NM_00101	ENSG0000	PLAC9	-	placenta sp	protein-co
lib-C666-I	chr1	110881259	110881259	+	5.64971	NA	promoter-TSS (NM_001201545)	promoter-TSS (NM_001201545)	534	NR_03659	440600	Hs.649375	NR_03659	ENSG0000	LOC44060	-	uncharacte	ncRNA
lib-C666-I	chr11	64491130	64491130	+	5.59206	NA	promoter-TSS (NM_138732)	promoter-TSS (NM_138732)	-470	NM_01508	9379	Hs.372938	NM_01508	ENSG0000	NRXN2	-	neurexin 2	protein-co
lib-C666-I	chr8	98366985	98366985	+	5.59206	NA	intron (NR_125390, intron 2 of 2)	intron (NR_125390, intron 2 of 2)	-76809	NM_03351	85453	Hs.173094	NM_03351	ENSG0000	TSPYL5	-	TSPY like 5	protein-co
lib-C666-I	chr16	67564326	67564326	+	5.57636	NA	intron (NM_001193523, intron 1 of 21	CpG	786	NM_00119	79567	Hs.152717	NM_02451	ENSG0000	FAM65A	-	family with	protein-co
lib-C666-I	chr9	89809581	89809581	+	5.57636	NA	Intergenic	Intergenic	46022	NM_00100	401535	Hs.657740	NM_001001709		C9orf170	-	chromosor	protein-co
lib-C666-I	chr8	74325319	74325319	+	5.53191	NA	Intergenic	Intergenic	-6990	NR_03840	100128126	Hs.679921	NR_03840	ENSG0000	STAU2-AS	-	STAU2 ant	ncRNA
lib-C666-I	chr10	35329910	35329910	+	5.51692	NA	intron (NM_001324375, intron 6 of 18	MER1B|DNA|hAT-Charlie	33348	NM_00119	8453	Hs.82919	NM_00359	ENSG0000	CUL2	-	cullin 2	protein-co
lib-C666-I	chr17	54910785	54910785	+	5.5166	NA	promoter-TSS (NM_003647)	promoter-TSS (NM_003647)	471	NM_00108	339210	Hs.658949	NM_00108	ENSG0000	C17orf67	-	chromosor	protein-co
lib-C666-I	chr4	163873630	163873630	+	5.5166	NA	Intergenic	Intergenic	141150	NR_03965	100616234		NR_03965	ENSG0000	MIR4454	-	microRNA	ncRNA
lib-C666-I	chr17	6915639	6915639	+	5.5166	NA	promoter-TSS (NR_037717)	promoter-TSS (NR_037717)	14	NR_04008	100506713	Hs.422967	NR_04008	ENSG0000	ALOX12-A	-	ALOX12 ar	ncRNA
lib-C666-I	chr5	180673288	180673288	+	5.5166	NA	promoter-TSS (NR_109909)	promoter-TSS (NR_109909)	-253	NR_10990	101928649	Hs.723307	NR_109909		CTC-338M	-	uncharacte	ncRNA
lib-C666-I	chr2	120010501	120010501	+	5.5166	NA	intron (NM_001008410, intron 3 of 4)	intron (NM_001008410, intron 3 of	-3854	NR_04672	100874111	Hs.704682	NR_04672	ENSG0000	STEAP3-AS	-	STEAP3 an	ncRNA
lib-C666-I	chr22	39002629	39002629	+	5.5166	NA	intron (NM_001013647, intron 12 of 1	intron (NM_001013647, intron 12 o	36124	NR_13462	105373031	Hs.144041	NR_134627		LOC10537	-	uncharacte	ncRNA
lib-C666-I	chr2	171626843	171626843	+	5.5166	NA	promoter-TSS (NR_110644)	promoter-TSS (NR_110644)	-349	NM_00129	285141	Hs.443729	NM_00128	ENSG0000	ERICH2	-	glutamate	protein-co
lib-C666-I	chr17	62223064	62223064	+	5.5166	NA	promoter-TSS (NR_004380)	promoter-TSS (NR_004380)	-266	NR_13227	105376843	Hs.405444	NR_13227	ENSG0000	SNHG25	-	small nucle	ncRNA
lib-C666-I	chr17	27920182	27920182	+	5.5098	NA	promoter-TSS (NM_152345)	promoter-TSS (NM_152345)	-345	NM_15234	124930	Hs.662164	NM_15234	ENSG0000	ANKRD13	-	ankyrin rep	protein-co
lib-C666-I	chr2	119998462	119998462	+	5.48804	NA	intron (NM_018234, intron 2 of 5)	intron (NM_018234, intron 2 of 5)	8185	NR_04672	100874111	Hs.704682	NR_04672	ENSG0000	STEAP3-AS	-	STEAP3 an	ncRNA
lib-C666-I	chr1	85667057	85667057	+	5.48804	NA	promoter-TSS (NM_032184)	promoter-TSS (NM_032184)	-329	NM_03218	84144	Hs.533853	NM_03218	ENSG0000	SYDE2	-	synapse de	protein-co
lib-C666-I	chr2	66662148	66662148	+	5.48804	NA	promoter-TSS (NM_002398)	promoter-TSS (NM_002398)	-384	NM_00239	4211	Hs.526754	NM_00239	ENSG0000	MEIS1	-	Meis home	protein-co
lib-C666-I	chr9	140210818	140210818	+	5.48804	NA	intron (NM_017820, intron 19 of 21)	CpG	-14115	NM_00100	441478	Hs.535075	NM_00100	ENSG0000	NRARP	-	NOTCH-re	protein-co
lib-C666-I	chr11	10831021	10831021	+	5.48804	NA	promoter-TSS (NM_001418)	promoter-TSS (NM_001418)	177	NR_12054	101928053	Hs.637773	NR_12054	ENSG0000	LOC10192	-	uncharacte	ncRNA
lib-C666-I	chr22	21996388	21996388	+	5.48804	NA	promoter-TSS (NM_022044)	promoter-TSS (NM_022044)	-154	NM_02204	23753	Hs.303116	NM_02204	ENSG0000	SDF2L1	-	stromal cel	protein-co
lib-C666-I	chr1	234859974	234859974	+	5.48804	NA	intron (NR_038856, intron 1 of 2)	intron (NR_038856, intron 1 of 2)	185	NR_03885	100506810	Hs.586634	NR_03885	ENSG0000	LINC01132	-	long interg	ncRNA
lib-C666-I	chr19	58816697	58816697	+	5.48804	NA	promoter-TSS (NR_144445)	promoter-TSS (NR_144445)	-43	NR_14444	105372481	Hs.435404	NR_14444	ENSG0000	ERVK3-1	-	endogenou	ncRNA
lib-C666-I	chr18	47721495	47721495	+	5.3823	NA	promoter-TSS (NM_001080467)	promoter-TSS (NM_001080467)	-44	NM_00108	4645	Hs.720076	NM_00108	ENSG0000	MYO5B	-	myosin VB	protein-co
lib-C666-I	chr7	155752781	155752781	+	5.3823	NA	Intergenic	Intergenic	-1132	NR_14462	389602	Hs.583071	NM_001291913		LOC38960	-	uncharacte	protein-co
lib-C666-I	chr15	94372909	94372909	+	5.37858	NA	Intergenic	Intergenic	-26880	NR_12032	101927153	Hs.371900	NR_120321		LOC10192	-	uncharacte	ncRNA
lib-C666-I	chr10	98208080	98208080	+	5.37858	NA	intron (NM_012465, intron 2 of 20)	intron (NM_012465, intron 2 of 20)	65603	NM_01246	7093	Hs.154296	NM_01246	ENSG0000	TLL2	-	tolloid like	protein-co
lib-C666-I	chr1	173446160	173446160	+	5.37858	NA	promoter-TSS (NM_004905)	promoter-TSS (NM_004905)	134	NR_03784	100506023	Hs.731284	NR_037845		LOC10050	-	uncharacte	ncRNA
lib-C666-I	chr11	33684036	33684036	+	5.3726	NA	intron (NM_012194, intron 19 of 19)	Tigger15a|DNA|TcMar-Tigger	38250	NM_00116	100131378	Hs.709466	NM_00116	ENSG0000	C11orf91	-	chromosor	protein-co
lib-C666-I	chr5	72509593	72509593	+	5.3726	NA	Intergenic	Intergenic	-11805	NR_13429	340090	Hs.434311	NR_134291		LOC34009	-	uncharacte	ncRNA
lib-C666-I	chr8	7106104	7106104	+	5.34948	NA	Intergenic	HERVE-int|LTR|ERV1	14910	NR_02700	349196	Hs.559040	NM_001025473		LINC00965	-	long interg	ncRNA
lib-C666-I	chr10	48342826	48342826	+	5.34142	NA	Intergenic	L1MB4|LINE|L1	-10629	NR_13450	107001062	Hs.576719	NR_134500		LOC10700	-	uncharacte	ncRNA
lib-C666-I	chr8	26240463	26240463	+	5.34142	NA	promoter-TSS (NM_004331)	promoter-TSS (NM_004331)	6	NR_14455	157489	Hs.586388	NR_144550		SDAD1P1	-	SDA1 dom	pseudo
lib-C666-I	chr7	100662098	100662098	+	5.34142	NA	3' UTR (NM_001164462, exon 12 of 1	3' UTR (NM_001164462, exon 12 of	-1209	NR_12052	102724094	Hs.571256	NR_12051	ENSG0000	LOC10272	-	uncharacte	ncRNA
lib-C666-I	chr15	28798589	28798589	+	5.34142	NA	Intergenic	Intergenic	-20446	NR_03335	100132565	Hs.510812	NM_00101	ENSG0000	GOLGA8F	-	golgin A8	pseudo
lib-C666-I	chr6	27721124	27721124	+	5.34142	NA	Intergenic	Intergenic	-8399	NR_03892	100131289	Hs.200573	NR_038929		LOC10013	-	uncharacte	ncRNA
lib-C666-I	chr10	32217567	32217567	+	5.34142	NA	intron (NM_018287, intron 1 of 19)	CpG	237	NM_01828	94134	Hs.499264	NM_01828	ENSG0000	ARHGAP12	-	Rho GTPas	protein-co
lib-C666-I	chr2	42068104	42068104	+	5.34142	NA	Intergenic	CpG	-36591	NR_03399	388942	Hs.631883	NR_03399	ENSG0000	LINC01913	-	long interg	ncRNA
lib-C666-I	chr15	90581764	90581764	+	5.34142	NA	intron (NM_198526, intron 1 of 4)	L1MC4|LINE|L1	31777	NR_03613	100422841		NR_03613	ENSG0000	MIR3174	-	microRNA	ncRNA
lib-C666-I	chr10	15035760	15035760	+	5.3293	NA	Intergenic	HERVE-int|LTR|ERV1	21197	NR_13449	105376430	Hs.615546	NR_13449	ENSG0000	LOC10537	-	uncharacte	ncRNA
lib-C666-I	chr2	96874210	96874210	+	5.3268	NA	promoter-TSS (NR_046322)	promoter-TSS (NR_046322)	56	NR_04632	285033	Hs.58648	NM_001037228		STARD7-A	-	STARD7 ar	ncRNA
lib-C666-I	chr11	1595939	1595939	+	5.3268	NA	intron (NR_021489, intron 1 of 1)	CpG	1968	NR_02148	338651	Hs.633229	NR_02148	ENSG0000	KRTAP5-A	-	KRTAP5-1/	ncRNA
lib-C666-I	chr16	28891275	28891275	+	5.30207	NA	promoter-TSS (NR_046287)	promoter-TSS (NR_046287)	-33	NR_04628	100289092	Hs.666631	NR_04628	ENSG0000	ATP2A1-A	-	ATP2A1 ar	ncRNA
lib-C666-I	chr3	4534919	4534919	+	5.30207	NA	promoter-TSS (NM_001168272)	promoter-TSS (NM_001168272)	-72	NR_10807	100996539	Hs.190365	NR_108075		ITPR1-AS1	-	ITPR1 antis	ncRNA
lib-C666-I	chr15	93352721	93352721	+	5.30207	NA	Intergenic	CpG	14007	NR_03376	728619	Hs.684129	NR_033769		ASB9P1	-	ankyrin rep	pseudo
lib-C666-I	chr15	99366607	99366607	+	5.25516	NA	intron (NM_000875, intron 2 of 20)	intron (NM_000875, intron 2 of 20)	38952	NR_03986	100616432		NR_03986	ENSG0000	MIR4714	-	microRNA	ncRNA
lib-C666-I	chr4	175699912	175699912	+	5.25516	NA	intron (NM_001042543, intron 2 of 8)	L1PA2|LINE|L1	50554	NM_00104	8001	Hs.413099	NM_00652	ENSG0000	GLRA3	-	glycine rec	protein-co
lib-C666-I	chr11	64490876	64490876	+	5.25516	NA	promoter-TSS (NM_138732)	promoter-TSS (NM_138732)	-216	NM_01508	9379	Hs.372938	NM_01508	ENSG0000	NRXN2	-	neurexin 2	protein-co
lib-C666-I	chr12	13254408	13254408	+	5.25516	NA	intron (NM_001206842, intron 1 of 5)	CpG	2222	NM_00120	83445	Hs.240053	NM_03128	ENSG0000	GSG1	-	germ cell a	protein-co
lib-C666-I	chr15	75493273	75493273	+	5.25516	NA	promoter-TSS (NM_015492)	promoter-TSS (NM_015492)	-948	NM_01549	56905	Hs.17936	NM_01549	ENSG0000	C15orf39	-	chromosor	protein-co
lib-C666-I	chr22	31608128	31608128	+	5.25516	NA	promoter-TSS (NM_005569)	promoter-TSS (NM_005569)	-122	NM_00556	3985	Hs.474596	NM_00556	ENSG0000	LIMK2	-	LIM domai	protein-co
lib-C666-I	chr8	27730411	27730411	+	5.25516	NA	intron (NM_173833, intron 8 of 8)	intron (NM_173833, intron 8 of 8)	13222	NR_03624	100422828		NR_03624	ENSG0000	MIR4287	-	microRNA	ncRNA
lib-C666-I	chr4	184718673	184718673	+	5.25516	NA	promoter-TSS (NR_132761)	promoter-TSS (NR_132761)	-502	NR_13276	56977	Hs.21958	NM_02022	ENSG0000	STOX2	-	storkhead	protein-co
lib-C666-I	chr3	186545341	186545341	+	5.25516	NA	Intergenic	CpG	-2031	NR_12540	102724699	Hs.518476	NR_12540	ENSG0000	LINC02043	-	long interg	ncRNA
lib-C666-I	chr12	122155190	122155190	+	5.25516	NA	intron (NM_001080825, intron 1 of 11	intron (NM_001080825, intron 1 of	4532	NM_00108	144404	Hs.644504	NM_00108	ENSG0000	TMEM120	-	transmemb	protein-co
lib-C666-I	chr15	72410324	72410324	+	5.25516	NA	promoter-TSS (NM_145204)	promoter-TSS (NM_145204)	116	NM_00690	4649	Hs.513000	NM_00690	ENSG0000	MYO9A	-	myosin IXA	protein-co
lib-C666-I	chr16	30709445	30709445	+	5.23679	NA	intron (NR_144637, intron 1 of 1)	CpG	365	NM_00125	730183	Hs.535456	NM_001256932		LOC73018	-	uncharacte	protein-co
lib-C666-I	chr1	16841304	16841304	+	5.23679	NA	Intergenic	Intergenic	20843	NR_13576	100132147	Hs.645945	NR_13576	ENSG0000	LINC01783	-	long interg	ncRNA
lib-C666-I	chr6	123774417	123774417	+	5.23609	NA	intron (NR_110844, intron 1 of 2)	L1PB1|LINE|L1	13668	NR_11084	101927990	Hs.660877	NR_110844		HRAT13	-	heart tissu	ncRNA
lib-C666-I	chr1	38259674	38259674	+	5.23609	NA	promoter-TSS (NM_001113482)	promoter-TSS (NM_001113482)	-100	NM_00111	149175	Hs.534562	NM_15249	ENSG0000	MANEAL	-	mannosida	protein-co
lib-C666-I	chr6	161106665	161106665	+	5.23609	NA	Intergenic	L1PA3|LINE|L1	-16560	NM_00116	5340	Hs.143436	NM_00030	ENSG0000	PLG	-	plasminog	protein-co
lib-C666-I	chr14	23352288	23352288	+	5.23609	NA	promoter-TSS (NM_173527)	promoter-TSS (NM_173527)	-144	NM_17352	161253	Hs.444911	NM_17352	ENSG0000	REM2	-	RRAD and	protein-co
lib-C666-I	chr15	59063517	59063517	+	5.23609	NA	promoter-TSS (NR_135474)	promoter-TSS (NR_135474)	-44	NM_00104	54629	Hs.591122	NM_01909	ENSG0000	FAM63B	-	family with	protein-co
lib-C666-I	chr5	76383103	76383103	+	5.23609	NA	promoter-TSS (NM_001329564)	promoter-TSS (NM_001329564)	-32	NM_03236	84327	Hs.584988	NM_03236	ENSG0000	ZBED3	-	zinc finger	protein-co
lib-C666-I	chr4	169224821	169224821	+	5.22168	NA	intron (NM_017631, intron 5 of 37)	Tigger3b|DNA|TcMar-Tigger	15137	NM_01763	55601	Hs.591710	NM_01763	ENSG0000	DDX60	-	DExD/H-b	protein-co
lib-C666-I	chr8	56634206	56634206	+	5.22168	NA	Intergenic	L1PB1|LINE|L1	42333	NM_00128	137695	Hs.420076	NM_15241	ENSG0000	TMEM68	-	transmemb	protein-co
lib-C666-I	chr19	47760263	47760263	+	5.22168	NA	intron (NM_015603, intron 1 of 11)	CpG	532	NM_01560	26093	Hs.227782	NM_01560	ENSG0000	CCDC9	-	coiled-coil	protein-co
lib-C666-I	chr11	356303	356303	+	5.2142	NA	Intergenic	CpG	-13492	NM_17853	338707	Hs.148074	NM_17853	ENSG0000	B4GALNT4	-	beta-1,4-N	protein-co
lib-C666-I	chr12	31902340	31902340	+	5.15932	NA	Intergenic	LTR8|LTR|ERV1	-20232	NM_00127	196394	Hs.585084	NM_00111	ENSG0000	AMN1	-	antagonist	protein-co
lib-C666-I	chr10	126849828	126849828	+	5.15932	NA	promoter-TSS (NM_001329)	promoter-TSS (NM_001329)	-198	NM_00108	1488	Hs.501345	NM_00132	ENSG0000	CTBP2	-	C-terminal	protein-co
lib-C666-I	chr6	168569345	168569345	+	5.15932	NA	Intergenic	Intergenic	57852	NR_13459	105378137	Hs.549668	NR_13459	ENSG0000	LOC10537	-	uncharacte	ncRNA
lib-C666-I	chr19	51842273	51842273	+	5.15932	NA	exon (NM_001163922, exon 5 of 10)	exon (NM_001163922, exon 5 of 10	3105	NM_00116	147645	Hs.293236	NM_00116	ENSG0000	VSIG10L	-	V-set and	protein-co
lib-C666-I	chr17	79303555	79303555	+	5.15932	NA	intron (NM_178520, intron 1 of 2)	intron (NM_178520, intron 1 of 2)	919	NM_17852	284186	Hs.364191	NM_17852	ENSG0000	TMEM105	-	transmemb	protein-co
lib-C666-I	chr1	42928974	42928974	+	5.15932	NA	intron (NM_001287511, intron 2 of 2)	CpG	6809	NM_02466	79717	Hs.706662	NM_02466	ENSG0000	PPCS	-	phosphopa	protein-co
lib-C666-I	chr16	27472063	27472063	+	5.15477	NA	3' UTR (NM_001520, exon 37 of 37)	3' UTR (NM_001520, exon 37 of 37)	-7349	NR_03715	283888	Hs.660935	NR_03715	ENSG0000	IL21R-AS1	-	IL21R antis	ncRNA
lib-C666-I	chr19	58963018	58963018	+	5.15278	NA	promoter-TSS (NM_207395)	promoter-TSS (NM_207395)	47	NM_20739	388569	Hs.186970	NM_20739	ENSG0000	ZNF324B	-	zinc finger	protein-co
lib-C666-I	chr5	151151174	151151174	+	5.15278	NA	promoter-TSS (NR_132346)	promoter-TSS (NR_132346)	-105	NR_13234	100652758	Hs.587054	NM_001278082		LOC10065	-	uncharacte	ncRNA
lib-C666-I	chr11	118401806	118401806	+	5.14484	NA	promoter-TSS (NR_120574)	promoter-TSS (NR_120574)	3	NM_00131	84866	Hs.564188	NM_03278	ENSG0000	TMEM25	-	transmemb	protein-co
lib-C666-I	chr19	58892615	58892615	+	5.12426	NA	promoter-TSS (NM_138466)	promoter-TSS (NM_138466)	-226	NM_13846	116412	Hs.222236	NM_13846	ENSG0000	ZNF837	-	zinc finger	protein-co
lib-C666-I	chr1	16767123	16767123	+	5.12426	NA	promoter-TSS (NM_001145277)	promoter-TSS (NM_001145277)	-44	NM_00114	55707	Hs.437385	NM_01809	ENSG0000	NECAP2	-	NECAP en	protein-co
lib-C666-I	chr12	53773658	53773658	+	5.12426	NA	promoter-TSS (NM_001251825)	promoter-TSS (NM_001251825)	-321	NM_00125	6667	Hs.620754	NM_00310	ENSG0000	SP1	-	Sp1 transc	protein-co
lib-C666-I	chr16	25043136	25043136	+	5.12426	NA	promoter-TSS (NR_038379)	promoter-TSS (NR_038379)	74	NR_03837	554206	Hs.552959	NR_03837	ENSG0000	LOC55420	-	leucine car	pseudo
lib-C666-I	chr6	150326721	150326721	+	5.12426	NA	promoter-TSS (NR_024045)	promoter-TSS (NR_024045)	-441	NR_02404	646024	Hs.560537	NR_02404	ENSG0000	RAET1K	-	retinoic aci	pseudo
lib-C666-I	chr9	100881605	100881605	+	5.12426	NA	promoter-TSS (NM_033219)	promoter-TSS (NM_033219)	30	NM_01478	9830	Hs.575631	NM_01478	ENSG0000	TRIM14	-	tripartite m	protein-co
lib-C666-I	chr8	132869135	132869135	+	5.12426	NA	Intergenic	MLT1G|LTR|ERVL-MaLR	-47200	NM_01513	23167	Hs.204564	NM_01513	ENSG0000	EFR3A	-	EFR3 homo	protein-co
lib-C666-I	chr19	4342927	4342927	+	5.10953	NA	promoter-TSS (NM_001159846)	promoter-TSS (NM_001159846)	-597	NM_00130	84954	Hs.321689	NM_03286	ENSG0000	MPND	-	MPN doma	protein-co
lib-C666-I	chr7	22603830	22603830	+	5.10953	NA	intron (NR_038393, intron 1 of 3)	intron (NR_038393, intron 1 of 3)	874	NR_03839	100506178	Hs.668122	NR_03839	ENSG0000	LOC10050	-	uncharacte	ncRNA
lib-C666-I	chr10	14424249	14424249	+	5.10953	NA	TTS (NR_036181)	TTS (NR_036181)	1027	NR_03618	100422843		NR_03618	ENSG0000	MIR4293	-	microRNA	ncRNA
lib-C666-I	chr20	60941358	60941358	+	5.10953	NA	intron (NM_005560, intron 1 of 79)	intron (NM_005560, intron 1 of 79)	1010	NM_00556	3911	Hs.473256	NM_00556	ENSG0000	LAMA5	-	laminin sul	protein-co
lib-C666-I	chr9	131549547	131549547	+	5.10953	NA	promoter-TSS (NM_018201)	promoter-TSS (NM_018201)	61	NM_01820	54662	Hs.3376	NM_01820	ENSG0000	TBC1D13	-	TBC1 doma	protein-co
lib-C666-I	chr19	27732147	27732147	+	5.06034	NA	Intergenic	ALR/Alpha|Satellite|centr	-552228	NR_11068	101927151	Hs.567934	NR_11068	ENSG0000	LOC10192	-	uncharacte	ncRNA
lib-C666-I	chr6	140784218	140784218	+	5.05119	NA	Intergenic	Intergenic	-220733	NR_03967	100616180		NR_03967	ENSG0000	MIR4465	-	microRNA	ncRNA

lib-C666-l	chr3	193547692	193547692	+	5.04826	NA	Intergenic	MIRb|SINE|MIR	164335	NR_02776	100128023	Hs.730112	NR_027764		DPPA2P3	-	developme	pseudo
lib-C666-l	chr5	50679117	50679117	+	5.04826	NA	promoter-TSS (NR_046243)	promoter-TSS (NR_046243)	49	NR_04624	642366	Hs.544139	NR_046243		LOC64236	-	uncharacte	ncRNA
lib-C666-l	chr11	66234853	66234853	+	5.04179	NA	intron (NM_001243136, intron 1 of 5)	CpG	517	NM_14506	246330	Hs.656875	NM_14506	ENSG0000	PELI3	-	pellino E3	protein-co
lib-C666-l	chr17	7819614	7819614	+	5.04179	NA	promoter-TSS (NR_024349)	promoter-TSS (NR_024349)	-349	NR_02434	284023	Hs.744470	NR_02434	ENSG0000	LOC28402	-	uncharacte	ncRNA
lib-C666-l	chr16	85784765	85784765	+	5.04179	NA	promoter-TSS (NM_206967)	promoter-TSS (NM_206967)	-76	NM_20696	404550	Hs.461655	NM_20696	ENSG0000	C16orf74	-	chromosor	protein-co
lib-C666-l	chr1	2457655	2457655	+	5.04179	NA	intron (NM_018216, intron 1 of 18)	CpG	412	NM_01821	55229	Hs.26156	NM_01821	ENSG0000	PANK4	-	pantothen	protein-co
lib-C666-l	chr16	80061254	80061254	+	5.04179	NA	Intergenic	Intergenic	232108	NR_10466	101928248	Hs.659802	NR_10466	ENSG0000	LINC01229	-	long interg	ncRNA
lib-C666-l	chr15	52030302	52030302	+	5.04179	NA	promoter-TSS (NM_153374)	promoter-TSS (NM_153374)	18	NM_15337	256586	Hs.603629	NM_15337	ENSG0000	LYSMD2	-	LysM dom	protein-co
lib-C666-l	chr5	89770423	89770423	+	5.04179	NA	promoter-TSS (NM_006467)	promoter-TSS (NM_006467)	162	NM_20340	153364	Hs.64004	NM_20340	ENSG0000	MBLAC2	-	metallo-be	protein-co
lib-C666-l	chr15	75494461	75494461	+	5.04179	NA	intron (NM_015492, intron 1 of 2)	CpG	240	NM_01549	56905	Hs.17936	NM_01549	ENSG0000	C15orf39	-	chromosor	protein-co
lib-C666-l	chr1	6266003	6266003	+	5.04179	NA	promoter-TSS (NR_125997)	promoter-TSS (NR_125997)	-163	NR_12599	102724450	Hs.669107	NR_12599	ENSG0000	LOC10272	-	uncharacte	ncRNA
lib-C666-l	chr4	77625767	77625767	+	5.04179	NA	intron (NM_020859, intron 2 of 10)	L2a|LINE|L2	129063	NR_03965	100616254		NR_03965	ENSG0000	MIR548AH	-	microRNA	ncRNA
lib-C666-l	chr9	132040831	132040831	+	4.99987	NA	Intergenic	Intergenic	-3864	NR_12158	101929331	Hs.522428	NR_12158	ENSG0000	LOC10192	-	uncharacte	ncRNA
lib-C666-l	chr11	1331142	1331142	+	4.98754	NA	promoter-TSS (NM_001318512)	promoter-TSS (NM_001318512)	204	NR_02940	255512	Hs.588291	NR_02940	ENSG0000	TOLLIP-AS	-	TOLLIP ant	ncRNA
lib-C666-l	chr2	64834335	64834335	+	4.98754	NA	promoter-TSS (NR_034023)	promoter-TSS (NR_034023)	-111	NR_03402	339807	Hs.434736	NR_03402	ENSG0000	LOC33980	-	uncharacte	ncRNA
lib-C666-l	chr9	66971247	66971247	+	4.97904	NA	intron (NR_135597, intron 4 of 4).3	ALR/Alpha|Satellite|centr	48280	NR_12157	101928381	Hs.708686	NR_121570		LOC10192	-	uncharacte	ncRNA
lib-C666-l	chr1	15853461	15853461	+	4.96104	NA	intron (NM_001287811, intron 1 of 1	CpG	153	NM_00128	23341	Hs.655410	NM_01529	ENSG0000	DNAJC16	-	DnaJ heat	protein-co
lib-C666-l	chr7	157793010	157793010	+	4.92693	NA	intron (NM_130843, intron 10 of 21)	intron (NM_130843, intron 10 of 21	145733	NR_03896	100506585	Hs.661265	NR_03896	ENSG0000	LOC10050	-	uncharacte	ncRNA
lib-C666-l	chr1	17222826	17222826	+	4.91694	NA	Intergenic	Intergenic	-6665	NR_13505	105376805	Hs.465405	NR_13505	ENSG0000	LOC10537	-	uncharacte	ncRNA
lib-C666-l	chr1	146976922	146976922	+	4.90117	NA	intron (NR_038423, intron 3 of 3)	intron (NR_038423, intron 3 of 3)	12777	NR_03842	100289211	Hs.323484	NR_03842	ENSG0000	LINC00624	-	long interg	ncRNA
lib-C666-l	chr6	20543441	20543441	+	4.90117	NA	intron (NM_017774, intron 2 of 15)	L1MD1|LINE|L1	8753	NM_01777	54901	Hs.657604	NM_01777	ENSG0000	CDKAL1	-	CDK5 regu	protein-co
lib-C666-l	chr18	11323487	11323487	+	4.90117	NA	Intergenic	Intergenic	-165082	NR_11077	101927433	Hs.331210	NR_11077	ENSG0000	LINC01255	-	long interg	ncRNA
lib-C666-l	chr11	126506732	126506732	+	4.90117	NA	intron (NM_032531, intron 1 of 16)	MIRb|SINE|MIR	-15941	NR_12053	101929427	Hs.600341	NR_12053	ENSG0000	LOC10192	-	uncharacte	ncRNA
lib-C666-l	chr6	41550941	41550941	+	4.90117	NA	intron (NM_001012426, intron 3 of 1	intron (NM_001012426, intron 3 of	-15520	NR_03978	100616178		NR_03978	ENSG0000	MIR4641	-	microRNA	ncRNA
lib-C666-l	chr6	5773129	5773129	+	4.90117	NA	Intergenic	Tigger3a|DNA|TcMar-Tigger	-77624	NR_11084	101927950	Hs.571136	NR_11084	ENSG0000	LOC10192	-	uncharacte	ncRNA
lib-C666-l	chrX	129906585	129906585	+	4.90117	NA	intron (NM_182314, intron 3 of 15)	intron (NM_182314, intron 3 of 15)	-30025	NR_13565	105373338	Hs.642023	NR_135655		LOC10537	-	uncharacte	ncRNA
lib-C666-l	chr7	51460906	51460906	+	4.90117	NA	Intergenic	Intergenic	-76348	NM_00134	23242	Hs.99141	NM_01519	ENSG0000	COBL	-	cordon-ble	protein-co
lib-C666-l	chr3	72041922	72041922	+	4.90117	NA	Intergenic	MER63A|DNA|hAT-Blackjack	107684	NR_10411	285286	Hs.559386	NR_10411	ENSG0000	LINC00877	-	long interg	ncRNA
lib-C666-l	chr6	138059468	138059468	+	4.90117	NA	intron (NR_121618, intron 1 of 2)	intron (NR_121618, intron 1 of 2)	706	NR_12161	100507406	Hs.556703	NR_12161	ENSG0000	LOC10050	-	uncharacte	ncRNA
lib-C666-l	chr6	29759743	29759743	+	4.90117	NA	promoter-TSS (NR_132323)	promoter-TSS (NR_132323)	60	NR_13232	554223	Hs.60856	NM_00120	ENSG0000	LOC55422	-	histocomp	pseudo
lib-C666-l	chr5	168527857	168527857	+	4.90117	NA	intron (NM_003062, intron 4 of 35)	intron (NM_003062, intron 4 of 35)	87625	NR_10989	728095	Hs.145601	NR_109897		LOC72809	-	uncharacte	ncRNA
lib-C666-l	chr11	119600084	119600084	+	4.90117	NA	promoter-TSS (NM_203285)	promoter-TSS (NM_203285)	-209	NR_12058	102724301	Hs.637640	NR_12058	ENSG0000	LOC10272	-	uncharacte	ncRNA
lib-C666-l	chr16	86935619	86935619	+	4.83287	NA	Intergenic	Intergenic	-155771	NR_12600	440390	Hs.513789	NR_12600	ENSG0000	LOC44039	-	uncharacte	ncRNA
lib-C666-l	chr13	111467698	111467698	+	4.82106	NA	Intergenic	Intergenic	-2267	NR_13528	283486	Hs.447504	NR_13528	ENSG0000	LINC00567	-	long interg	ncRNA
lib-C666-l	chr10	80827204	80827204	+	4.78638	NA	promoter-TSS (NR_015429)	promoter-TSS (NR_015429)	1	NR_02443	283050	Hs.309176	NR_01542	ENSG0000	ZMIZ1-AS	-	ZMIZ1 anti	ncRNA
lib-C666-l	chr10	80675154	80675154	+	4.74112	NA	Intergenic	Intergenic	152051	NR_02443	283050	Hs.309176	NR_01542	ENSG0000	ZMIZ1-AS	-	ZMIZ1 anti	ncRNA
lib-C666-l	chr4	44164824	44164824	+	4.74112	NA	Intergenic	L1M4|LINE|L1	145745	NR_13195	100506827	Hs.156197	NR_13195	ENSG0000	LVCAT1	-	liver cance	ncRNA
lib-C666-l	chr4	71870252	71870252	+	4.74112	NA	intron (NM_000788, intron 2 of 6)	L1PA7|LINE|L1	10987	NM_00078	1633	Hs.709	NM_00078	ENSG0000	DCK	-	deoxycytid	protein-co
lib-C666-l	chr19	32864756	32864756	+	4.73866	NA	intron (NM_001136156, intron 5 of 6)	Tigger6a|DNA|TcMar-Tigger	28242	NM_01491	22847	Hs.205392	NM_01491	ENSG0000	ZNF507	-	zinc finger	protein-co
lib-C666-l	chr6	163749140	163749140	+	4.73866	NA	Intergenic	LTR9|LTR|ERV1	-3635	NR_02839	285796	Hs.668693	NR_02839	ENSG0000	PACRG-AS	-	PACRG ant	ncRNA
lib-C666-l	chr2	20815653	20815653	+	4.73866	NA	Intergenic	Intergenic	-23345	NR_04683	100874343	Hs.591546	NR_04683	ENSG0000	HS1BP3-IT	-	HS1BP3 int	ncRNA
lib-C666-l	chr15	75550816	75550816	+	4.73866	NA	promoter-TSS (NM_001164404)	promoter-TSS (NM_001164404)	-83	NM_00116	653641	Hs.540230	NM_00116	ENSG0000	GOLGA6C	-	golgin A6	protein-co
lib-C666-l	chr1	235106313	235106313	+	4.73866	NA	Intergenic	CT-rich|Low_complexity|Low_compl	-6567	NR_12594	101927851	Hs.520684	NR_12594	ENSG0000	LOC10192	-	uncharacte	ncRNA
lib-C666-l	chr16	17299646	17299646	+	4.73866	NA	intron (NM_022166, intron 3 of 11)	intron (NM_022166, intron 3 of 11)	71285	NR_13517	102723692	Hs.737758	NR_13517	ENSG0000	LOC10272	-	uncharacte	ncRNA
lib-C666-l	chr19	16940139	16940139	+	4.73866	NA	promoter-TSS (NM_001297595)	promoter-TSS (NM_001297595)	-59	NM_01526	23309	Hs.13999	NM_01526	ENSG0000	SIN3B	-	SIN3 trans	protein-co
lib-C666-l	chr18	25090404	25090404	+	4.73866	NA	Intergenic	L1PA2|LINE|L1	-167784	NR_13458	105372038	Hs.722107	NR_13458	ENSG0000	LOC10537	-	uncharacte	ncRNA
lib-C666-l	chr10	34142427	34142427	+	4.73866	NA	Intergenic	L1PA4|LINE|L1	-80819	NR_03893	100505583	Hs.568804	NR_03893	ENSG0000	LINC00838	-	long interg	ncRNA
lib-C666-l	chr3	193590326	193590326	+	4.71278	NA	Intergenic	Intergenic	121701	NR_02776	100128023	Hs.730112	NR_027764		DPPA2P3	-	developme	pseudo
lib-C666-l	chr19	8943040	8943040	+	4.71278	NA	promoter-TSS (NM_144693)	promoter-TSS (NM_144693)	-60	NM_14469	148156	Hs.349444	NM_14469	ENSG0000	ZNF558	-	zinc finger	protein-co
lib-C666-l	chr16	23652705	23652705	+	4.67192	NA	promoter-TSS (NM_024675)	promoter-TSS (NM_024675)	18	NR_03757	84516	Hs.435941	NM_03248	ENSG0000	DCTN5	-	dynactin su	protein-co
lib-C666-l	chr3	32023003	32023003	+	4.64451	NA	promoter-TSS (NM_001137674)	promoter-TSS (NM_001137674)	-263	NM_00113	344787	Hs.585139	NM_00113	ENSG0000	ZNF860	-	zinc finger	protein-co
lib-C666-l	chr6	170534634	170534634	+	4.64451	NA	Intergenic	Intergenic	37023	NR_00278	154449	Hs.447751	NR_00278	ENSG0000	LOC15444	-	uncharacte	ncRNA
lib-C666-l	chr17	40828705	40828705	+	4.64451	NA	promoter-TSS (NR_073574)	promoter-TSS (NR_073574)	343	NR_07357	79990	Hs.632251	NM_02492	ENSG0000	PLEKHH3	-	pleckstrin	protein-co
lib-C666-l	chr19	11457145	11457145	+	4.61604	NA	promoter-TSS (NM_001321113)	promoter-TSS (NM_001321113)	-36	NM_00108	126075	Hs.631636	NM_00108	ENSG0000	CCDC159	-	coiled-coil	protein-co
lib-C666-l	chr11	51582714	51582714	+	4.56254	NA	Intergenic	ALR/Alpha|Satellite|centr	67432	NM_00100	119749	Hs.553564	NM_00100	ENSG0000	OR4C46	-	olfactory re	protein-co
lib-C666-l	chr3	177241990	177241990	+	4.56052	NA	intron (NR_047568, intron 1 of 3)	intron (NR_047568, intron 1 of 3)	82281	NR_04756	100505566	Hs.581170	NR_047568		LINC00578	-	long interg	ncRNA
lib-C666-l	chr8	67579681	67579681	+	4.56047	NA	promoter-TSS (NM_001204173)	promoter-TSS (NM_001204173)	-106	NM_01960	56260	Hs.661238	NM_01960	ENSG0000	C8orf44	-	chromosor	protein-co
lib-C666-l	chr2	47567118	47567118	+	4.56047	NA	intron (NR_110208, intron 1 of 4)	intron (NR_110208, intron 1 of 4)	5095	NR_11020	101927043	Hs.515976	NR_11020	ENSG0000	LOC10192	-	uncharacte	ncRNA
lib-C666-l	chr3	124605435	124605435	+	4.54787	NA	intron (NM_002213, intron 1 of 14)	CpG	717	NM_00221	3693	Hs.13155	NM_00221	ENSG0000	ITGB5	-	integrin su	protein-co
lib-C666-l	chr16	73398518	73398518	+	4.54787	NA	Intergenic	Intergenic	-22186	NR_03823	100506172	Hs.434230	NR_03823	ENSG0000	LINC01568	-	long interg	ncRNA
lib-C666-l	chr17	46184975	46184975	+	4.52748	NA	promoter-TSS (NM_013323)	promoter-TSS (NM_013323)	28	NM_01332	29916	Hs.15827	NM_01332	ENSG0000	SNX11	-	sorting nex	protein-co
lib-C666-l	chr11	61891429	61891429	+	4.52748	NA	promoter-TSS (NM_001040694)	promoter-TSS (NM_001040694)	-16	NM_02023	3619	Hs.142179	NM_02023	ENSG0000	INCENP	-	inner centr	protein-co
lib-C666-l	chr7	155201734	155201734	+	4.52748	NA	Intergenic	Intergenic	-41106	NR_10354	338436	Hs.521326	NR_10354	ENSG0000	BLACE	-	B-cell acut	ncRNA
lib-C666-l	chr7	151572614	151572614	+	4.52748	NA	intron (NM_016203, intron 1 of 15)	intron (NM_016203, intron 1 of 15)	-1513	NR_03892	100505483	Hs.649054	NR_038926		PRKAG2-A	-	PRKAG2 ar	ncRNA
lib-C666-l	chr14	105877677	105877677	+	4.52748	NA	intron (NM_001195082, intron 2 of 3)	CpG	8399	NR_12538	100507437	Hs.676548	NR_12538	ENSG0000	LOC10050	-	uncharacte	ncRNA
lib-C666-l	chr7	139877022	139877022	+	4.52748	NA	promoter-TSS (NM_030647)	promoter-TSS (NM_030647)	-39	NR_02445	100134229	Hs.634333	NR_02445	ENSG0000	JHDM1D-A	-	JHDM1D a	ncRNA
lib-C666-l	chr1	148176550	148176550	+	4.52748	NA	intron (NM_001037501, intron 6 of 1	CpG	25986	NM_00116	645142	Hs.730589	NM_00116	ENSG0000	PPIAL4D	-	peptidylpr	protein-co
lib-C666-l	chr9	47243509	47243509	+	4.52748	NA	Intergenic	Intergenic	399840	NR_12605	103908605	Hs.721139	NR_126050		LOC10390	-	uncharacte	ncRNA
lib-C666-l	chr7	151573983	151573983	+	4.52748	NA	promoter-TSS (NR_038926)	promoter-TSS (NR_038926)	-144	NR_03892	100505483	Hs.649054	NR_038926		PRKAG2-A	-	PRKAG2 ar	ncRNA
lib-C666-l	chr7	140882491	140882491	+	4.52748	NA	intron (NM_001195278, intron 1 of 3)	THE1B|LTR|ERVL-MaLR	108459	NM_00119	100507421	Hs.283851	NM_00119	ENSG0000	TMEM178	-	transmeml	protein-co
lib-C666-l	chr13	113864459	113864459	+	4.52748	NA	intron (NM_001008895, intron 2 of 1	CpG-6713	640	NM_00100	8451	Hs.339735	NM_00358	ENSG0000	CUL4A	-	cullin 4A	protein-co
lib-C666-l	chr11	63536612	63536612	+	4.52748	NA	promoter-TSS (NM_001144936)	promoter-TSS (NM_001144936)	-499	NM_00114	65998	Hs.191073	NM_00114	ENSG0000	C11orf95	-	chromosor	protein-co
lib-C666-l	chr7	20324615	20324615	+	4.51266	NA	Intergenic	Intergenic	-11716	NR_11011	101927769	Hs.256080	NR_11011	ENSG0000	LOC10192	-	uncharacte	ncRNA
lib-C666-l	chr4	184908890	184908890	+	4.51266	NA	intron (NM_020225, intron 1 of 3)	intron (NM_020225, intron 1 of 3)	82381	NM_02022	56977	Hs.21958	NM_02022	ENSG0000	STOX2	-	storkhead	protein-co
lib-C666-l	chr6	141647667	141647667	+	4.51266	NA	Intergenic	MER44D|DNA|TcMar-Tigger	642716	NR_03967	100616180		NR_03967	ENSG0000	MIR4465	-	microRNA	ncRNA
lib-C666-l	chr2	74756857	74756857	+	4.51266	NA	non-coding (NR_135769, exon 1 of 8	non-coding (NR_135769, exon 1 of	167	NM_18157	550	Hs.411480	NM_18157	ENSG0000	AUP1	-	ancient ub	protein-co
lib-C666-l	chr1	143913327	143913327	+	4.49043	NA	promoter-TSS (NM_001287385)	promoter-TSS (NM_001287385)	-167	NM_00128	554282		NM_00128	ENSG0000	FAM72C	-	family with	protein-co
lib-C666-l	chr11	77899809	77899809	+	4.49043	NA	promoter-TSS (NM_001029859)	promoter-TSS (NM_001029859)	-49	NM_02079	57558	Hs.531249	NM_02079	ENSG0000	USP35	-	ubiquitin s	protein-co
lib-C666-l	chr21	44375995	44375995	+	4.49043	NA	Intergenic	HERVE-int|LTR|ERV1	-4871	NR_04988	100847013		NR_04988	ENSG0000	MIR5692B	-	microRNA	ncRNA
lib-C666-l	chr17	77070988	77070988	+	4.46944	NA	promoter-TSS (NM_001042573)	promoter-TSS (NM_001042573)	-31	NM_00104	64772	Hs.29288	NM_02275	ENSG0000	ENGASE	-	endo-beta	protein-co
lib-C666-l	chr2	74669128	74669128	+	4.46944	NA	promoter-TSS (NM_001015055)	promoter-TSS (NM_001015055)	-68	NM_00101	6242	Hs.192854	NM_03304	ENSG0000	RTKN	-	rhotekin	protein-co
lib-C666-l	chr15	94774677	94774677	+	4.46944	NA	Intergenic	CpG	-66753	NM_00115	55784	Hs.33368	NM_01834	ENSG0000	MCTP2	-	multiple C	protein-co
lib-C666-l	chr3	101295607	101295607	+	4.46944	NA	non-coding (NR_135229, exon 2 of 5	non-coding (NR_135229, exon 2 of	2669	NR_13522	57092	Hs.732633	NM_02035	ENSG0000	PCNP	-	PEST prote	protein-co
lib-C666-l	chr12	30993944	30993944	+	4.46583	NA	Intergenic	MER66C|LTR|ERV1	45329	NR_04024	100287314	Hs.355210	NR_04024	ENSG0000	LINC00941	-	long interg	ncRNA
lib-C666-l	chr6	32121915	32121915	+	4.46583	NA	non-coding (NR_037861, exon 1 of 1	non-coding (NR_037861, exon 1 of	139	NR_03786	100532746	Hs.731888	NR_03786	ENSG0000	PPT2-EGFL	-	PPT2-EGFL	ncRNA
lib-C666-l	chr9	69240716	69240716	+	4.46583	NA	intron (NM_001085457, intron 6 of 1	intron (NM_001085457, intron 6 of	21877	NM_00108	644019	Hs.597638	NM_00108	ENSG0000	CBWD6	-	COBW dor	protein-co
lib-C666-l	chr19	38878904	38878904	+	4.46583	NA	promoter-TSS (NM_152657)	promoter-TSS (NM_152657)	-236	NM_15265	199720	Hs.447948	NM_15265	ENSG0000	GGN	-	gametoger	protein-co
lib-C666-l	chr2	35337862	35337862	+	4.39666	NA	Intergenic	L1PA3|LINE|L1	-358609	NR_03962	100616475		NR_03962	ENSG0000	MIR548AD	-	microRNA	ncRNA
lib-C666-l	chr9	94036698	94036698	+	4.39666	NA	intron (NM_001698, intron 6 of 9)	intron (NM_001698, intron 6 of 9)	87508	NM_00169	549	Hs.175905	NM_00169	ENSG0000	AUH	-	AU RNA bi	protein-co
lib-C666-l	chr19	48866631	48866631	+	4.39666	NA	exon (NM_001303538, exon 2 of 7)	exon (NM_001303538, exon 2 of 7)	863	NM_00130	55260	Hs.351335	NM_01827	ENSG0000	TMEM143	-	transmeml	protein-co
lib-C666-l	chr10	60656132	60656132	+	4.39666	NA	Intergenic	Intergenic	-103146	NR_10804	100507008	Hs.170953	NR_108046		LINC00844	-	long interg	ncRNA
lib-C666-l	chr2	8666053	8666053	+	4.39666	NA	Intergenic	Intergenic	57869	NR_11025	101929567	Hs.634706	NR_11025	ENSG0000	LINC01814	-	long interg	ncRNA
lib-C666-l	chr8	94457490	94457490	+	4.33814	NA	intron (NR_033858, intron 7 of 10)	intron (NR_033858, intron 7 of 10)	255171	NR_03385	642924	Hs.434882	NR_03385	ENSG0000	LINC00535	-	long interg	ncRNA
lib-C666-l	chr2	233853441	233853441	+	4.33814	NA	intron (NM_019850, intron 1 of 14)	L1MC4a|LINE|L1	-23883	NR_13632	101928881	Hs.570311	NR_13632	ENSG0000	LOC10192	-	uncharacte	ncRNA
lib-C666-l	chr3	143056404	143056404	+	4.30593	NA	intron (NM_173653, intron 14 of 15)	intron (NM_173653, intron 14 of 15	-4684	NR_04854	100885796	Hs.581618	NR_04854	ENSG0000	SLC9A9-A	-	SLC9A9 an	ncRNA
lib-C666-l	chr14	46361205	46361205	+	4.30593	NA	Intergenic	LTR18B|LTR|ERVL	-172157	NR_10270	100506412	Hs.207545	NR_10269	ENSG0000	LINC00871	-	long interg	ncRNA
lib-C666-l	chr15	101874407	101874407	+	4.30593	NA	promoter-TSS (NR_132373)	promoter-TSS (NR_132373)	-235	NR_13237	105371027		NR_13237	ENSG0000	PCSK6-AS	-	PCSK6 anti	ncRNA
lib-C666-l	chr12	110313457	110313457	+	4.30593	NA	intron (NM_016433, intron 1 of 4)	intron (NM_016433, intron 1 of 4)	4836	NM_01643	51228	Hs.381256	NM_01643	ENSG0000	GLTP	-	glycolipid	protein-co
lib-C666-l	chr3	142720182	142720182	+	4.27545	NA	promoter-TSS (NM_001320219)	promoter-TSS (NM_001320219)	127	NR_03403	100289361	Hs.599649	NR_03403	ENSG0000	LOC10028	-	uncharacte	ncRNA
lib-C666-l	chr1	2322756	2322756	+	4.27545	NA	promoter-TSS (NM_007033)	promoter-TSS (NM_007033)	434	NR_12536	79906	Hs.642701	NM_02484	ENSG0000	MORN1	-	MORN rep	protein-co
lib-C666-l	chr4	174291697	174291697	+	4.22173	NA	promoter-TSS (NM_003864)	promoter-TSS (NM_003864)	-396	NM_00386	8819	Hs.591715	NM_00386	ENSG0000	SAP30	-	Sin3A asso	protein-co
lib-C666-l	chr19	21580007	21580007	+	4.19683	NA	promoter-TSS (NM_175910)	promoter-TSS (NM_175910)	86	NM_17591	284443	Hs.656558	NM_14532	ENSG0000	ZNF493	-	zinc finger	protein-co
lib-C666-l	chr9	117481340	117481340	+	4.19683	NA	Intergenic	Intergenic	-36971	NR_14486	100505478	Hs.200101	NM_00119	ENSG0000	LOC10050	-	uncharacte	protein-co
lib-C666-l	chr10	87593021	87593021	+	4.19675	NA	intron (NM_017551, intron 8 of 15)	L1MD2|LINE|L1	255533	NR_03898	100507470	Hs.585483	NR_03898	ENSG0000	GRID1-AS	-	GRID1 anti	ncRNA
lib-C666-l	chr2	221484520	221484520	+	4.19675	NA	Intergenic	Intergenic	-713234	NR_03622	100422959		NR_03622	ENSG0000	MIR4268	-	microRNA	ncRNA
lib-C666-l	chr12	89492750	89492750	+	4.19675	NA	Intergenic	Intergenic	-79281	NR_03838	728084	Hs.132563	NR_03838	ENSG0000	LOC72808	-	uncharacte	ncRNA
lib-C666-l	chr2	85645726	85645726	+	4.16777	NA	promoter-TSS (NM_201594)	promoter-TSS (NM_201594)	-328	NM_20159	284948	Hs.209542	NM_19848	ENSG0000	SH2D6	-	SH2 doma	protein-co
lib-C666-l	chr10	92243864	92243864	+	4.16471	NA	intron (NR_110657, intron 2 of 6)	L1PB1|LINE|L1	56698	NR_11065	101926942	Hs.382666	NR_11065	ENSG0000	LOC10192	-	uncharacte	ncRNA
lib-C666-l	chr16	81745438	81745438	+	4.12121	NA	TTS (NM_198390)	TTS (NM_198390)	46479	NR_04511	100129617	Hs.657151	NR_045112		LOC10012	-	uncharacte	ncRNA
lib-C666-l	chr17	33570009	33570009	+	4.04484	NA	promoter-TSS (NM_144975)	promoter-TSS (NM_144975)	-46	NM_00133	162394	Hs.585792	NM_14497	ENSG0000	SLFN5	-	schlafen fa	protein-co
lib-C666-l	chr17	64948326	64948326	+	4.04037	NA	Intergenic	Intergenic	-12654	NM_01440	27092	Hs.514423	NM_01440	ENSG0000	CACNG4	-	calcium vo	protein-co
lib-C666-l	chr5	95066971	95066971	+	4.04037	NA	5' UTR (NM_014899, exon 1 of 12)	5' UTR (NM_014899, exon 1 of 12)	121	NM_01489	22836	Hs.445030	NM_01489	ENSG0000	RHOBTB3	-	Rho relate	protein-co
lib-C666-l	chr7	132003808	132003808	+	4.04037	NA	intron (NM_020911, intron 3 of 31)	intron (NM_020911, intron 3 of 31)	58188	NR_13456	101928807	Hs.552131	NR_134565		LOC10192	-	uncharacte	ncRNA
lib-C666-l	chr15	65176483	65176483	+	4.04037	NA	Intergenic	Intergenic	-27618	NM_18270	348094	Hs.207157	NM_18270	ENSG0000	ANKDD1A	-	ankyrin rep	protein-co
lib-C666-l	chr12	1906033	1906033	+	3.98736	NA	intron (NM_172364, intron 35 of 37)	CpG	-23400	NM_00103	654429	Hs.585579	NM_00103	ENSG0000	LRTM2	-	leucine rich	protein-co
lib-C666-l	chr5	98265230	98265230	+	3.98736	NA	non-coding (NR_036530, exon 1 of 1	non-coding (NR_036530, exon 1 of	392	NR_03653	100289230	Hs.552095	NR_036530		LOC10028	-	uncharacte	ncRNA
lib-C666-l	chr11	69795629	69795629	+	3.98718	NA	Intergenic	Intergenic	114401	NR_12053	101928443	Hs.313468	NR_12052	ENSG0000	LOC10192	-	uncharacte	ncRNA
lib-C666-l	chr8	12472051	12472051	+	3.98718	NA	intron (NR_047662, intron 2 of 8)	THE1B-int|LTR|ERVL-MaLR	51069	NR_04766	729732	Hs.322761	NR_04766	ENSG0000	LOC72973	-	uncharacte	ncRNA
lib-C666-l	chr15	42120507	42120507	+	3.98718	NA	TTS (NM_001128608)	TTS (NM_001128608)	224	NM_00111	100137047	Hs.198161	NM_00111	ENSG0000	JMJD7	-	jumonji do	protein-co
lib-C666-l	chr2	58237741	58237741	+	3.98718	NA	intron (NM_001288838, intron 1 of 1	intron (NM_001288838, intron 1 of	-36036	NM_00113	7444	Hs.715298	NM_00629	ENSG0000	VRK2	-	vaccinia re	protein-co
lib-C666-l	chr6	43968277	43968277	+	3.98718	NA	promoter-TSS (NM_001171992)	promoter-TSS (NM_001171992)	-60	NM_00117	221416	Hs.531926	NM_15324	ENSG0000	C6orf223	-	chromosor	protein-co
lib-C666-l	chr17	77979976	77979976	+	3.98718	NA	intron (NM_019020, intron 3 of 11)	intron (NM_019020, intron 3 of 11)	29681	NM_01902	125058	Hs.369819	NM_01902	ENSG0000	TBC1D16	-	TBC1 dom	protein-co
lib-C666-l	chr1	156554724	156554724	+	3.98718	NA	exon (NM_001105669, exon 6 of 10)	exon (NM_001105669, exon 6 of 10	3567	NM_00110	164118	Hs.447851	NM_00110	ENSG0000	TTC24	-	tetratricop	protein-co
lib-C666-l	chr8	120220916	120220916	+	3.98718	NA	intron (NM_052886, intron 2 of 4)	CpG-25965	306	NM_05288	114569	Hs.201083	NM_05288	ENSG0000	MAL2	-	mal, T-cell	protein-co
lib-C666-l	chr2	223184022	223184022	+	3.98718	NA	non-coding (NR_136638, exon 1 of 2	non-coding (NR_136638, exon 1 of	832	NR_13663	440934	Hs.732732	NR_13663	ENSG0000	LOC44093	-	uncharacte	ncRNA
lib-C666-l	chr3	155588383	155588383	+	3.98718	NA	promoter-TSS (NM_003875)	promoter-TSS (NM_003875)	58	NM_00387	8833	Hs.591314	NM_00387	ENSG0000	GMPS	-	guanine m	protein-co
lib-C666-l	chr10	32048863	32048863	+	3.98718	NA	Intergenic	CpG	148991	NM_00127	94134	Hs.499264	NM_01828	ENSG0000	ARHGAP12	-	Rho GTPas	protein-co
lib-C666-l	chr13	43837311	43837311	+	3.98718	NA	intron (NM_017993, intron 14 of 16)	intron (NM_017993, intron 14 of 16	150010	NR_04701	100874159	Hs.633241	NR_047013		LINC0040	-	long interg	ncRNA
lib-C666-l	chr16	90231990	90231990	+	3.98718	NA	intron (NR_126161, intron 3 of 3)	intron (NR_126161, intron 3 of 3)	63318	NR_12616	100996541	Hs.652849	NR_126161		FAM157C	-	family with	ncRNA
lib-C666-l	chr19	11908416	11908416	+	3.98718	NA	promoter-TSS (NM_152356)	promoter-TSS (NM_152356)	-975	NM_15235	126069	Hs.631634	NM_15235	ENSG0000	ZNF491	-	zinc finger	protein-co
lib-C666-l	chr1	9242977	9242977	+	3.96379	NA	promoter-TSS (NR_132742)	promoter-TSS (NR_132742)	-580	NR_13274	106614088	Hs.551837	NR_13274	ENSG0000	MIR34AHG	-	MIR34A hc	ncRNA
lib-C666-l	chr1	151319472	151319472	+	3.96379	NA	promoter-TSS (NR_131972)	promoter-TSS (NR_131972)	-14	NR_13197	101927886	Hs.634042	NR_131972		GBAT2	-	glioblaston	ncRNA
lib-C666-l	chr8	1693609	1693609	+	3.95418	NA	Intergenic	Intergenic	17891	NR_13430	101927752	Hs.356690	NR_13430	ENSG0000	LOC10192	-	uncharacte	ncRNA
lib-C666-l	chr12	31004495	31004495	+	3.94004	NA	Intergenic	LTR13_|LTR|ERVK	55880	NR_04024	100287314	Hs.355210	NR_04024	ENSG0000	LINC00941	-	long interg	ncRNA
lib-C666-l	chr10	126847418	126847418	+	3.93939	NA	5' UTR (NM_001321014, exon 1 of 11	5' UTR (NM_001321014, exon 1 of 1	269	NM_00132	1488	Hs.501345	NM_00132	ENSG0000	CTBP2	-	C-terminal	protein-co
lib-C666-l	chr15	50647023	50647023	+	3.93123	NA	promoter-TSS (NR_026891)	promoter-TSS (NR_026891)	53	NR_02689	55056	Hs.511316	NM_01797	ENSG0000	FLJ10038	-	uncharacte	ncRNA
lib-C666-l	chr4	32860849	32860849	+	3.92495	NA	Intergenic	MLT1F2|LTR|ERVL-MaLR	508189	NR_13467	101927363	Hs.559406	NR_13467	ENSG0000	LOC10192	-	uncharacte	ncRNA
lib-C666-l	chr1	74663940	74663940	+	3.92495	NA	promoter-TSS (NM_001199328)	promoter-TSS (NM_001199328)	44	NM_00111	100526835	Hs.480085	NM_00111	ENSG0000	FPGT-TNN	-	FPGT-TNN	protein-co
lib-C666-l	chrX	17755141	17755141	+	3.92495	NA	promoter-TSS (NM_006746)	promoter-TSS (NM_006746)	-428	NM_00103	6322	Hs.109655	NM_00674	ENSG0000	SCML1	-	sex comb	protein-co
lib-C666-l	chr22	49806999	49806999	+	3.92495	NA	Intergenic	Intergenic	130115	NR_03744	100500882		NR_03744	ENSG0000	MIR3667	-	microRNA	ncRNA
lib-C666-l	chr5	98356121	98356121	+	3.92495	NA	Intergenic	Intergenic	91283	NR_03653	100289230	Hs.552095	NR_036530		LOC10028	-	uncharacte	ncRNA
lib-C666-l	chr17	25708214	25708214	+	3.92495	NA	Intergenic	Intergenic	-36817	NR_03389	440419	Hs.385755	NR_03389	ENSG0000	TBC1D3P5	-	TBC1 dom	pseudo
lib-C666-l	chr7	148787861	148787861	+	3.89624	NA	promoter-TSS (NM_152411)	promoter-TSS (NM_152411)	8	NM_15241	136051	Hs.729572	NM_15241	ENSG0000	ZNF786	-	zinc finger	protein-co
lib-C666-l	chr1	212731873	212731873	+	3.89624	NA	Intergenic	CpG	-6803	NM_00103	467	Hs.460	NM_00167	ENSG0000	ATF3	-	activating	protein-co
lib-C666-l	chr16	30569685	30569685	+	3.89624	NA	promoter-TSS (NM_001172679)	promoter-TSS (NM_001172679)	-43	NM_03341	92595	Hs.132227	NM_03341	ENSG0000	ZNF764	-	zinc finger	protein-co
lib-C666-l	chr10	75532207	75532207	+	3.89624	NA	TTS (NM_004922)	TTS (NM_004922)	158	NM_00128	170384	Hs.588854	NM_17354	ENSG0000	FUT11	-	fucosyltran	protein-co
lib-C666-l	chr13	42535370	42535370	+	3.89624	NA	promoter-TSS (NM_015058)	promoter-TSS (NM_015058)	65	NR_03997	100507240	Hs.147766	NR_03997	ENSG0000	VWA8-AS	-	VWA8 anti	ncRNA
lib-C666-l	chr3	10289922	10289922	+	3.89624	NA	promoter-TSS (NM_014760)	promoter-TSS (NM_014760)	-255	NM_01476	9797	Hs.475401	NM_01476	ENSG0000	TATDN2	-	TatD DNas	protein-co
lib-C666-l	chr19	46010884	46010884	+	3.89034	NA	5' UTR (NM_003370, exon 1 of 13)	5' UTR (NM_003370, exon 1 of 13)	196	NM_00337	7408	Hs.515469	NM_00337	ENSG0000	VASP	-	vasodilator	protein-co
lib-C666-l	chr3	181930386	181930386	+	3.89034	NA	Intergenic	Intergenic	260234	NR_10414	100996490	Hs.606987	NR_10414	ENSG0000	LINC01206	-	long interg	ncRNA
lib-C666-l	chr6	3209494	3209494	+	3.89034	NA	Intergenic	Intergenic	-13493	NR_03829	100507194	Hs.678053	NR_03829	ENSG0000	LOC10050	-	uncharacte	ncRNA
lib-C666-l	chr20	25846738	25846738	+	3.8557	NA	Intergenic	SST1|Satellite|centr	-12081	NR_11000	101926935	Hs.737440	NR_110001		LOC10192	-	uncharacte	ncRNA
lib-C666-l	chr16	30346398	30346398	+	3.8557	NA	non-coding (NR_002453, exon 1 of 1	non-coding (NR_002453, exon 1 of	297	NR_00245	595101	Hs.654650	NR_00245	ENSG0000	SMG1P5	-	SMG1P5, n	pseudo
lib-C666-l	chr1	228645341	228645341	+	3.8557	NA	promoter-TSS (NM_175055)	promoter-TSS (NM_175055)	219	NM_03344	92815	Hs.26331	NM_03344	ENSG0000	HIST3H2A	-	histone clu	protein-co
lib-C666-l	chr3	184952330	184952330	+	3.8557	NA	intron (NM_001966, intron 3 of 6)	intron (NM_001966, intron 3 of 6)	18730	NR_10671	100847054		NR_10671	ENSG0000	MIR5588	-	microRNA	ncRNA
lib-C666-l	chr3	182076018	182076018	+	3.8557	NA	Intergenic	Intergenic	128132	NR_04010	401103	Hs.591304	NR_04010	ENSG0000	LINC01994	-	long interg	ncRNA
lib-C666-l	chr2	33171646	33171646	+	3.8557	NA	promoter-TSS (NM_206943)	promoter-TSS (NM_206943)	-723	NM_20694	4052	Hs.619315	NM_00062	ENSG0000	LTBP1	-	latent trans	protein-co

lib-C666-l	chr8	101428453	101428453	+	3.8557	NA	Intergenic	MER5A1|DNA|hAT-Charlie	33462	NR_03968	100616451		NR_03968	ENSG0000	MIR4471	-	microRNA	ncRNA
lib-C666-l	chr5	179105354	179105354	+	3.8557	NA	TTS (NM_001164444)	TTS (NM_001164444)	2621	NM_00116	646019	Hs.535767	NM_00116	ENSG0000	CBY3	-	chibby fam	protein-co
lib-C666-l	chr4	153857320	153857320	+	3.8557	NA	intron (NR_136614, intron 1 of 1)	CpG	669	NR_13661	729870	Hs.310357	NR_136614		LOC72987	-	uncharacte	ncRNA
lib-C666-l	chr6	41513611	41513611	+	3.8557	NA	promoter-TSS (NM_138457)	promoter-TSS (NM_138457)	370	NR_12641	101060264	Hs.571099	NR_12641	ENSG0000	FOXP4-AS	-	FOXP4 ant	ncRNA
lib-C666-l	chr1	247242148	247242148	+	3.8557	NA	promoter-TSS (NM_001204220)	promoter-TSS (NM_001204220)	-33	NR_03789	100533111	Hs.732305	NR_03789	ENSG0000	ZNF670-ZI	-	ZNF670-ZI	ncRNA
lib-C666-l	chr7	106810410	106810410	+	3.8557	NA	intron (NM_012257, intron 1 of 10)	intron (NM_012257, intron 1 of 10)	950	NM_01225	26959	Hs.162032	NM_01225	ENSG0000	HBP1	-	HMG-box	protein-co
lib-C666-l	chr2	177134185	177134185	+	3.8557	NA	promoter-TSS (NM_006554)	promoter-TSS (NM_006554)	62	NM_00131	10651	Hs.470728	NM_00655	ENSG0000	MTX2	-	metaxin 2	protein-co
lib-C666-l	chr8	145692269	145692269	+	3.84463	NA	intron (NM_145754, intron 2 of 16)	CpG-26246	549	NM_14575	90990	Hs.528713	NM_14575	ENSG0000	KIFC2	-	kinesin fam	protein-co
lib-C666-l	chr12	6560815	6560815	+	3.84463	NA	promoter-TSS (NR_015382)	promoter-TSS (NR_015382)	-132	NR_01538	678655	Hs.655224	NR_01538	ENSG0000	CD27-AS1	-	CD27 antis	ncRNA
lib-C666-l	chr1	85667293	85667293	+	3.84463	NA	promoter-TSS (NM_032184)	promoter-TSS (NM_032184)	-565	NM_03218	84144	Hs.533853	NM_03218	ENSG0000	SYDE2	-	synapse de	protein-co
lib-C666-l	chr2	70417972	70417972	+	3.84463	NA	intron (NM_017880, intron 1 of 9)	CpG	179	NM_01788	54980	Hs.720049	NM_01788	ENSG0000	C2orf42	-	chromosor	protein-co
lib-C666-l	chr1	20126510	20126510	+	3.82577	NA	promoter-TSS (NM_181719)	promoter-TSS (NM_181719)	-100	NM_18171	255104	Hs.656313	NM_18171	ENSG0000	TMCO4	-	transmeml	protein-co
lib-C666-l	chr12	122880042	122880042	+	3.82577	NA	promoter-TSS (NR_045382)	promoter-TSS (NR_045382)	-47	NR_04538	100507066	Hs.624253	NR_045382		CLIP1-AS1	-	CLIP1 antis	ncRNA
lib-C666-l	chr17	43096705	43096705	+	3.78778	NA	Intergenic	CpG	32180	NM_00128	79877	Hs.463148	NM_02481	ENSG0000	DCAKD	-	dephosph	protein-co
lib-C666-l	chr13	95752942	95752942	+	3.76263	NA	intron (NM_001105515, intron 20 of	intron (NM_001105515, intron 20 o	140647	NR_04748	100861544	Hs.48706	NR_04748	ENSG0000	LINC00557	-	long interg	ncRNA
lib-C666-l	chr1	16126849	16126849	+	3.7184	NA	Intergenic	Intergenic	7345	NM_00108	440567	Hs.568229	NM_00108	ENSG0000	UQCRHL	-	ubiquinol-	protein-co
lib-C666-l	chr2	64995406	64995406	+	3.62735	NA	Intergenic	CpG	95359	NR_11022	101927438	Hs.580313	NR_11022	ENSG0000	LINC0180C	-	long interg	ncRNA
lib-C666-l	chr10	82282768	82282768	+	3.62735	NA	TTS (NM_001128309)	TTS (NM_001128309)	-8238	NR_12066	102723703	Hs.308615	NR_120662		LOC10272	-	uncharacte	ncRNA
lib-C666-l	chr14	65722499	65722499	+	3.62735	NA	Intergenic	Intergenic	-33434	NR_10376	100128233	Hs.497323	NR_10376	ENSG0000	LOC10012	-	uncharacte	ncRNA
lib-C666-l	chr19	18026835	18026835	+	3.62735	NA	Intergenic	MER21C|LTR|ERVL	-16989	NM_00113	115098	Hs.100043	NM_13844	ENSG0000	CCDC124	-	coiled-coil	protein-co
lib-C666-l	chr19	42749020	42749020	+	3.61436	NA	Intergenic	CpG	-2284	NM_01988	2931	Hs.466828	NM_01988	ENSG0000	GSK3A	-	glycogen s	protein-co
lib-C666-l	chr14	59655419	59655419	+	3.59106	NA	promoter-TSS (NM_001270520)	promoter-TSS (NM_001270520)	38	NM_00127	23002	Hs.19156	NM_01499	ENSG0000	DAAM1	-	dishevellec	protein-co
lib-C666-l	chr8	144718176	144718176	+	3.59106	NA	promoter-TSS (NM_001261843)	promoter-TSS (NM_001261843)	-7	NM_00126	9831	Hs.43133	NM_01478	ENSG0000	ZNF623	-	zinc finger	protein-co
lib-C666-l	chr10	104210629	104210629	+	3.59106	NA	exon (NM_024886, exon 2 of 2)	exon (NM_024886, exon 2 of 2)	671	NM_02488	79946	Hs.225084	NM_024886		C10orf95	-	chromosor	protein-co
lib-C666-l	chr16	1052905	1052905	+	3.59106	NA	Intergenic	Intergenic	21097	NM_01458	30812	Hs.243678	NM_01458	ENSG0000	SOX8	-	SRY-box 8	protein-co
lib-C666-l	chr20	25604717	25604717	+	3.59106	NA	promoter-TSS (NR_126467)	promoter-TSS (NR_126467)	36	NR_12646	102724826	Hs.633621	NR_12646	ENSG0000	ZNF337-A	-	ZNF337 an	ncRNA
lib-C666-l	chr11	14866275	14866275	+	3.58839	NA	intron (NM_000922, intron 12 of 15)	L1PA3|LINE|L1	47476	NM_02451	120227	Hs.371427	NM_02451	ENSG0000	CYP2R1	-	cytochrom	protein-co
lib-C666-l	chr1	36023690	36023690	+	3.58839	NA	promoter-TSS (NM_024874)	promoter-TSS (NM_024874)	297	NM_00101	23154	Hs.121870	NM_01428	ENSG0000	NCDN	-	neurochon	protein-co
lib-C666-l	chr10	44212334	44212334	+	3.57421	NA	Intergenic	L1PA4|LINE|L1	61939	NR_13448	102724264	Hs.140667	NR_13448	ENSG0000	LOC10272	-	uncharacte	ncRNA
lib-C666-l	chr17	79319657	79319657	+	3.57421	NA	Intergenic	CpG	-15183	NM_17852	284186	Hs.364191	NM_17852	ENSG0000	TMEM105	-	transmeml	protein-co
lib-C666-l	chr18	22511445	22511445	+	3.57421	NA	Intergenic	Intergenic	205700	NR_13460	105372028	Hs.569901	NR_13460	ENSG0000	LOC10537	-	uncharacte	ncRNA
lib-C666-l	chr11	100607330	100607330	+	3.57421	NA	intron (NM_152432, intron 1 of 23)	intron (NM_152432, intron 1 of 23)	-48644	NR_13357	100128386	Hs.635125	NR_13357	ENSG0000	LOC10012	-	uncharacte	ncRNA
lib-C666-l	chr2	87557870	87557870	+	3.57421	NA	Intergenic	Intergenic	135961	NR_03992	100616165		NR_03992	ENSG0000	MIR4771-2	-	microRNA	ncRNA
lib-C666-l	chr22	25507081	25507081	+	3.57421	NA	intron (NR_038941, intron 2 of 4)	GA-rich|Low_complexity|Low_comp	1578	NR_03894	100128531	Hs.662126	NR_03894	ENSG0000	LOC10012	-	uncharacte	ncRNA
lib-C666-l	chr16	85336892	85336892	+	3.57421	NA	Intergenic	Intergenic	3039	NR_04981	100847022		NR_04981	ENSG0000	MIR5093	-	microRNA	ncRNA
lib-C666-l	chr1	64538406	64538406	+	3.57421	NA	intron (NM_001083592, intron 4 of 6)	intron (NM_001083592, intron 4 of	39482	NR_11066	101927034	Hs.680824	NR_11066	ENSG0000	ROR1-AS1	-	ROR1 antis	ncRNA
lib-C666-l	chr2	25896166	25896166	+	3.57421	NA	intron (NM_183361, intron 1 of 17)	CpG	350	NM_00125	1838	Hs.307720	NM_02190	ENSG0000	DTNB	-	dystrobrev	protein-co
lib-C666-l	chrX	15288040	15288040	+	3.57421	NA	5' UTR (NM_001168531, exon 2 of 8)	5' UTR (NM_001168531, exon 2 of 8	253	NM_00116	140462	Hs.19404	NM_02408	ENSG0000	ASB9	-	ankyrin rep	protein-co
lib-C666-l	chr3	97758353	97758353	+	3.57421	NA	Intergenic	Intergenic	-4205	NM_00110	200959	Hs.534578	NM_00110	ENSG0000	GABRR3	-	gamma-ar	protein-co
lib-C666-l	chrX	39621710	39621710	+	3.57421	NA	Intergenic	L1MB5|LINE|L1	101240	NR_03750	100500822		NR_03750	ENSG0000	MIR3937	-	microRNA	ncRNA
lib-C666-l	chr11	38111673	38111673	+	3.57421	NA	Intergenic	MER11A|LTR|ERVK	151522	NR_13511	105376633	Hs.135690	NR_13511	ENSG0000	LOC10537	-	uncharacte	ncRNA
lib-C666-l	chr2	119613568	119613568	+	3.57421	NA	Intergenic	CpG	-7809	NM_00142	2019	Hs.271977	NM_00142	ENSG0000	EN1	-	engrailed h	protein-co
lib-C666-l	chr7	76751634	76751634	+	3.54679	NA	promoter-TSS (NM_020879)	promoter-TSS (NM_020879)	-300	NM_02087	57639	Hs.113940	NM_02087	ENSG0000	CCDC146	-	coiled-coil	protein-co
lib-C666-l	chr5	17378894	17378894	+	3.47756	NA	TTS (NR_033975)	TTS (NR_033975)	8525	NR_03397	401177	Hs.508311	NR_03397	ENSG0000	LINC02111	-	long interg	ncRNA
lib-C666-l	chr19	47760451	47760451	+	3.41725	NA	intron (NM_015603, intron 1 of 11)	CpG	720	NM_01560	26093	Hs.227782	NM_01560	ENSG0000	CCDC9	-	coiled-coil	protein-co
lib-C666-l	chr15	43425964	43425964	+	3.38731	NA	non-coding (NR_144542, exon 1 of 1	non-coding (NR_144542, exon 1 of	242	NM_00134	80021	Hs.511175	NM_02495	ENSG0000	TMEM62	-	transmeml	protein-co
lib-C666-l	chr1	202995794	202995794	+	3.38731	NA	intron (NM_001304331, intron 1 of 2	CpG	145	NM_00130	8497	Hs.153648	NM_01505	ENSG0000	PPFIA4	-	PTPRF inte	protein-co
lib-C666-l	chr11	14913127	14913127	+	3.38731	NA	intron (NM_024514, intron 1 of 4)	CpG	624	NM_02451	120227	Hs.371427	NM_02451	ENSG0000	CYP2R1	-	cytochrom	protein-co
lib-C666-l	chr19	19702161	19702161	+	3.38731	NA	intron (NR_038198, intron 2 of 7)	intron (NR_038198, intron 2 of 7)	27564	NR_03819	80714	Hs.466257	NM_02524	ENSG0000	PBX4	-	PBX home	protein-co
lib-C666-l	chr7	108089288	108089288	+	3.38731	NA	intron (NM_005010, intron 1 of 27)	intron (NM_005010, intron 1 of 27)	7553	NM_00501	4897	Hs.21422	NM_00501	ENSG0000	NRCAM	-	neuronal c	protein-co
lib-C666-l	chr15	91498073	91498073	+	3.31563	NA	promoter-TSS (NM_001017919)	promoter-TSS (NM_001017919)	-33	NM_03354	91433	Hs.655895	NM_03354	ENSG0000	RCCD1	-	RCC1 dom	protein-co
lib-C666-l	chr1	145542728	145542728	+	3.31563	NA	3' UTR (NM_001303040, exon 27 of 2	3' UTR (NM_001303040, exon 27 of	-6481	NM_14469	148741	Hs.710624	NM_14469	ENSG0000	ANKRD35	-	ankyrin rep	protein-co
lib-C666-l	chr16	60141277	60141277	+	3.29276	NA	Intergenic	Intergenic	252020	NR_11091	101927580	Hs.552235	NR_11091	ENSG0000	LINC02141	-	long interg	ncRNA
lib-C666-l	chr5	180673778	180673778	+	3.28884	NA	non-coding (NR_109909, exon 1 of 3	non-coding (NR_109909, exon 1 of	237	NR_10990	101928649	Hs.723307	NR_109909		CTC-338M	-	uncharacte	ncRNA
lib-C666-l	chr1	52831090	52831090	+	3.28884	NA	intron (NM_032449, intron 1 of 23)	L2b|LINE|L2	787	NM_00133	200014	Hs.591451	NM_03244	ENSG0000	CC2D1B	-	coiled-coil	protein-co
lib-C666-l	chr2	87303430	87303430	+	3.27468	NA	non-coding (NR_026846, exon 1 of 1	non-coding (NR_026846, exon 1 of	106	NR_02684	285074	Hs.424184	NM_001012626		LOC28507	-	anaphase p	pseudo
lib-C666-l	chr10	44796613	44796613	+	3.27468	NA	Intergenic	Intergenic	8415	NM_00125	100130539	Hs.694626	NM_00125	ENSG0000	C10orf142	-	chromosor	protein-co
lib-C666-l	chr8	142138435	142138435	+	3.27468	NA	promoter-TSS (NM_014957)	promoter-TSS (NM_014957)	-285	NM_01495	22898	Hs.18166	NM_01495	ENSG0000	DENND3	-	DENN don	protein-co
lib-C666-l	chr14	102990983	102990983	+	3.27468	NA	Intergenic	Intergenic	-14855	NM_15232	122416	Hs.432945	NM_15232	ENSG0000	ANKRD9	-	ankyrin rep	protein-co
lib-C666-l	chr2	5527817	5527817	+	3.27468	NA	Intergenic	Intergenic	303433	NR_11058	102723818	Hs.128107	NR_110580		LINC01248	-	long interg	ncRNA
lib-C666-l	chr12	122751505	122751505	+	3.27468	NA	promoter-TSS (NM_022916)	promoter-TSS (NM_022916)	-437	NM_02291	65082	Hs.592009	NM_02291	ENSG0000	VPS33A	-	VPS33A, C	protein-co
lib-C666-l	chr16	2653371	2653371	+	3.27468	NA	promoter-TSS (NR_015441)	promoter-TSS (NR_015441)	-14	NR_01544	652276	Hs.720448	NR_01544	ENSG0000	LOC65227	-	potassium	pseudo
lib-C666-l	chr2	41817769	41817769	+	3.27468	NA	Intergenic	L1M2a1|LINE|L1	-286926	NR_03399	388942	Hs.631883	NR_03399	ENSG0000	LINC01913	-	long interg	ncRNA
lib-C666-l	chr5	112159185	112159185	+	3.27468	NA	intron (NM_001127510, intron 12 of	intron (NM_001127510, intron 12 o	-37700	NM_00120	6728	Hs.637001	NM_00313	ENSG0000	SRP19	-	signal reco	protein-co
lib-C666-l	chr12	53932150	53932150	+	3.27468	NA	intron (NM_001130060, intron 4 of 1	intron (NM_001130060, intron 4 of	24382	NR_04622	100652999	Hs.633602	NR_04622	ENSG0000	LOC10065	-	uncharacte	ncRNA
lib-C666-l	chr8	80803384	80803384	+	3.25853	NA	Intergenic	Intergenic	-19412	NR_11095	101927040	Hs.350876	NR_11095	ENSG0000	LOC10192	-	uncharacte	ncRNA
lib-C666-l	chr4	9154768	9154768	+	3.25502	NA	Intergenic	CpG	-57615	NM_00125	100287144	Hs.741130	NM_00125	ENSG0000	USP17L10	-	ubiquitin s	protein-co
lib-C666-l	chr21	46222466	46222466	+	3.25502	NA	promoter-TSS (NM_003343)	promoter-TSS (NM_003343)	310	NR_10992	101928689	Hs.116142	NR_109928		LINC01424	-	long interg	ncRNA
lib-C666-l	chr20	1373734	1373734	+	3.25502	NA	promoter-TSS (NM_054014)	promoter-TSS (NM_054014)	82	NR_03766	100528031	Hs.471933	NR_037661		FKBP1A-SI	-	FKBP1A-SI	ncRNA
lib-C666-l	chr18	22204619	22204619	+	3.25502	NA	Intergenic	L1PA2|LINE|L1	-3527	NR_04003	729950	Hs.585850	NR_04003	ENSG0000	LINC01915	-	long interg	ncRNA
lib-C666-l	chr21	32931428	32931428	+	3.23837	NA	promoter-TSS (NM_003253)	promoter-TSS (NM_003253)	-130	NM_00132	150051	Hs.708976	NM_001321023		LOC15005	-	uncharacte	protein-co
lib-C666-l	chr3	170588083	170588083	+	3.23837	NA	promoter-TSS (NR_135259)	promoter-TSS (NR_135259)	-38	NM_00109	200916	Hs.380933	NM_00109	ENSG0000	RPL22L1	-	ribosomal	protein-co
lib-C666-l	chr1	235123461	235123461	+	3.23837	NA	Intergenic	Intergenic	-23715	NR_12594	101927851	Hs.520684	NR_12594	ENSG0000	LOC10192	-	uncharacte	ncRNA
lib-C666-l	chr8	56685993	56685993	+	3.23837	NA	promoter-TSS (NM_001286661)	promoter-TSS (NM_001286661)	-27	NM_00128	137695	Hs.420076	NM_15241	ENSG0000	TMEM68	-	transmeml	protein-co
lib-C666-l	chr19	48867551	48867551	+	3.20885	NA	promoter-TSS (NM_001303538)	promoter-TSS (NM_001303538)	-57	NR_13031	55260	Hs.351335	NM_01827	ENSG0000	TMEM143	-	transmeml	protein-co
lib-C666-l	chr7	157902252	157902252	+	3.20885	NA	intron (NM_130843, intron 9 of 21)	intron (NM_130843, intron 9 of 21)	254975	NR_03896	100506585	Hs.661265	NR_03896	ENSG0000	LOC10050	-	uncharacte	ncRNA
lib-C666-l	chr2	127966174	127966174	+	3.20885	NA	Intergenic	L1PA8A|LINE|L1	-2831	NM_00100	339761	Hs.407639	NM_00100	ENSG0000	CYP27C1	-	cytochrom	protein-co
lib-C666-l	chr1	63988994	63988994	+	3.20885	NA	promoter-TSS (NM_032437)	promoter-TSS (NM_032437)	22	NM_03243	84455	Hs.652324	NM_03243	ENSG0000	EFCAB7	-	EF-hand c	protein-co
lib-C666-l	chr8	124287180	124287180	+	3.20885	NA	promoter-TSS (NM_001204180)	promoter-TSS (NM_001204180)	-453	NM_00120	100533106	Hs.707401	NM_00120	ENSG0000	ZHX1-C8o	-	ZHX1-C8o	protein-co
lib-C666-l	chr3	120068315	120068315	+	3.20885	NA	promoter-TSS (NM_001099678)	promoter-TSS (NM_001099678)	-129	NM_00109	116064	Hs.518084	NM_00109	ENSG0000	LRRC58	-	leucine ricl	protein-co
lib-C666-l	chr2	64995083	64995083	+	3.20885	NA	Intergenic	CpG	95682	NR_11022	101927438	Hs.580313	NR_11022	ENSG0000	LINC0180C	-	long interg	ncRNA
lib-C666-l	chr1	55181502	55181502	+	3.20885	NA	promoter-TSS (NM_001291333)	promoter-TSS (NM_001291333)	7	NM_00462	7268	Hs.412482	NM_00462	ENSG0000	TTC4	-	tetratricop	protein-co
lib-C666-l	chr9	132096312	132096312	+	3.20885	NA	Intergenic	Intergenic	-2890	NR_12068	100506119	Hs.624047	NR_12068	ENSG0000	LINC01503	-	long interg	ncRNA
lib-C666-l	chr1	148242055	148242055	+	3.17637	NA	intron (NM_001037501, intron 6 of 1	intron (NM_001037501, intron 6 of	-39519	NM_00116	728945		NM_00116	ENSG0000	PPIAL4F	-	peptidylpr	protein-co
lib-C666-l	chr7	5732702	5732702	+	3.13003	NA	intron (NM_207111, intron 13 of 16)	L1MC4a|LINE|L1	-12610	NR_04683	100874342	Hs.665728	NR_04683	ENSG0000	RNF216-IT	-	RNF216 int	ncRNA
lib-C666-l	chr10	3894615	3894615	+	3.13003	NA	Intergenic	Intergenic	18473	NR_13449	105376365	Hs.460114	NR_13449	ENSG0000	LOC10537	-	uncharacte	ncRNA
lib-C666-l	chr14	62217954	62217954	+	3.08629	NA	promoter-TSS (NR_144368)	promoter-TSS (NR_144368)	-139	NR_14436	105370526	Hs.661638	NR_144368		LOC10537	-	uncharacte	ncRNA
lib-C666-l	chr5	33162464	33162464	+	3.07144	NA	Intergenic	Intergenic	214915	NR_03383	340113	Hs.434404	NR_03383	ENSG0000	LINC0212C	-	long interg	ncRNA
lib-C666-l	chr6	41513862	41513862	+	3.07144	NA	promoter-TSS (NM_138457)	promoter-TSS (NM_138457)	119	NR_12641	101060264	Hs.571099	NR_12641	ENSG0000	FOXP4-AS	-	FOXP4 ant	ncRNA
lib-C666-l	chr3	15642964	15642964	+	3.07144	NA	promoter-TSS (NM_001281724)	promoter-TSS (NM_001281724)	105	NM_00128	686	Hs.444197	NM_00006	ENSG0000	BTD	-	biotinidase	protein-co
lib-C666-l	chr8	12546635	12546635	+	3.04651	NA	Intergenic	HERVE-int|LTR|ERV1	-23515	NR_04766	729732	Hs.322761	NR_04766	ENSG0000	LOC72973	-	uncharacte	ncRNA
lib-C666-l	chr9	89885517	89885517	+	3.02289	NA	Intergenic	Intergenic	121958	NM_00100	401535	Hs.657740	NM_001001709		C9orf170	-	chromosor	protein-co
lib-C666-l	chr1	50032792	50032792	+	2.99475	NA	intron (NR_136623, intron 2 of 12)	L1PA4|LINE|L1	-95035	NR_04683	100874313	Hs.551277	NR_04683	ENSG0000	AGBL4-IT1	-	AGBL4 intr	ncRNA
lib-C666-l	chr6	147232226	147232226	+	2.99475	NA	intron (NR_034115, intron 7 of 10)	intron (NR_034115, intron 7 of 10)	-107266	NR_00395	729176	Hs.486708	NR_003954		KATNBL1P	-	katanin re	pseudo
lib-C666-l	chr7	90032731	90032731	+	2.98619	NA	promoter-TSS (NM_001185073)	promoter-TSS (NM_001185073)	83	NM_00118	9069	Hs.731917	NM_01212	ENSG0000	CLDN12	-	claudin 12	protein-co
lib-C666-l	chr9	135895405	135895405	+	2.96591	NA	promoter-TSS (NR_132980).3	promoter-TSS (NR_132980).3	-412	NR_13298	106635683		NR_132980		SNORD14	-	small nucle	snoRNA
lib-C666-l	chr12	121363728	121363728	+	2.96041	NA	Intergenic	Intergenic	21230	NR_13121	105500240	Hs.742757	NR_131215		XLOC_009	-	uncharacte	ncRNA
lib-C666-l	chr4	39037782	39037782	+	2.96041	NA	Intergenic	Intergenic	-3741	NM_02494	80008	Hs.374147	NM_02494	ENSG0000	TMEM156	-	transmeml	protein-co
lib-C666-l	chr15	29963888	29963888	+	2.96041	NA	Intergenic	Intergenic	-3251	NR_13522	100130111	Hs.525720	NR_13522	ENSG0000	LOC10013	-	uncharacte	ncRNA
lib-C666-l	chr4	8646391	8646391	+	2.96041	NA	Intergenic	MLT1C|LTR|ERVL-MaLR	52004	NM_00365	8532	Hs.61995	NM_00365	ENSG0000	CPZ	-	carboxype	protein-co
lib-C666-l	chr1	10490599	10490599	+	2.93406	NA	promoter-TSS (NR_036462)	promoter-TSS (NR_036462)	-205	NM_00124	100526739	Hs.412311	NM_19854	ENSG0000	APITD1-C	-	APITD1-C	protein-co
lib-C666-l	chr14	64010169	64010169	+	2.92124	NA	promoter-TSS (NR_104104)	promoter-TSS (NR_104104)	-90	NR_10410	5529	Hs.334868	NM_00624	ENSG0000	PPP2R5E	-	protein ph	protein-co
lib-C666-l	chr2	225349917	225349917	+	2.91691	NA	intron (NM_003590, intron 13 of 15)	L1P3|LINE|L1	-83206	NM_02478	79843	Hs.147585	NM_02478	ENSG0000	FAM124B	-	family with	protein-co
lib-C666-l	chr15	61191740	61191740	+	2.91691	NA	intron (NM_134261, intron 1 of 10)	intron (NM_134261, intron 1 of 10)	217972	NR_12031	100996876	Hs.511626	NR_120318		RORA-AS2	-	RORA antis	ncRNA
lib-C666-l	chr12	105352550	105352550	+	2.91691	NA	Intergenic	Intergenic	-27548	NM_15231	121053	Hs.295563	NM_15231	ENSG0000	C12orf45	-	chromosor	protein-co
lib-C666-l	chr17	5095329	5095329	+	2.9164	NA	promoter-TSS (NR_034082)	promoter-TSS (NR_034082)	-50	NR_03408	100130950	Hs.668646	NR_03408	ENSG0000	LOC10013	-	uncharacte	ncRNA
lib-C666-l	chr5	178053563	178053563	+	2.9164	NA	intron (NM_020666, intron 1 of 12)	CpG	491	NM_02066	57396	Hs.406557	NM_02066	ENSG0000	CLK4	-	CDC like ki	protein-co
lib-C666-l	chr1	3817795	3817795	+	2.9164	NA	promoter-TSS (NM_207356)	promoter-TSS (NM_207356)	827	NR_02445	100133612	Hs.307652	NR_02445	ENSG0000	LINC01134	-	long interg	ncRNA
lib-C666-l	chr18	8609488	8609488	+	2.91431	NA	promoter-TSS (NM_001025300)	promoter-TSS (NM_001025300)	45	NM_00102	201475	Hs.270074	NM_00102	ENSG0000	RAB12	-	RAB12, me	protein-co
lib-C666-l	chr1	149223677	149223677	+	2.87341	NA	Intergenic	Intergenic	-63453	NR_11193	388692	Hs.271249	NM_001013644		LOC38869	-	uncharacte	ncRNA
lib-C666-l	chr7	155134025	155134025	+	2.84259	NA	Intergenic	Intergenic	26603	NR_10354	338436	Hs.521326	NR_10354	ENSG0000	BLACE	-	B-cell acut	ncRNA
lib-C666-l	chr4	22189090	22189090	+	2.84185	NA	Intergenic	Intergenic	152199	NR_03787	100505912	Hs.270471	NR_037877		LOC10050	-	uncharacte	ncRNA
lib-C666-l	chr1	221975575	221975575	+	2.80387	NA	Intergenic	Intergenic	38433	NR_12598	101929771	Hs.557001	NR_12598	ENSG0000	LINC01655	-	long interg	ncRNA
lib-C666-l	chr5	134986928	134986928	+	2.78587	NA	intron (NR_027127, intron 2 of 2)	LTR41|LTR|ERVL	2696	NR_02712	340074	Hs.434633	NR_02712	ENSG0000	LINC01959	-	long interg	ncRNA
lib-C666-l	chr2	8724098	8724098	+	2.78587	NA	promoter-TSS (NR_110257)	promoter-TSS (NR_110257)	-176	NR_11025	101929567	Hs.634706	NR_11025	ENSG0000	LINC01814	-	long interg	ncRNA
lib-C666-l	chr5	127270667	127270667	+	2.78587	NA	intron (NM_001317938, intron 6 of 6)	intron (NM_001317938, intron 6 of	148099	NR_01536	644873	Hs.340623	NR_01536	ENSG0000	LINC01184	-	long interg	ncRNA
lib-C666-l	chr8	90624191	90624191	+	2.78587	NA	Intergenic	Intergenic	145748	NR_12582	101929709	Hs.651324	NR_12582	ENSG0000	LOC10192	-	uncharacte	ncRNA
lib-C666-l	chr12	679200	679200	+	2.78587	NA	intron (NM_016533, intron 1 of 3)	CpG	16452	NR_13462	105369595	Hs.504422	NR_134624		LOC10536	-	uncharacte	ncRNA
lib-C666-l	chr15	86408540	86408540	+	2.78587	NA	Intergenic	Intergenic	39674	NR_04980	100847084		NR_04980	ENSG0000	MIR548AP	-	microRNA	ncRNA
lib-C666-l	chr17	66244090	66244090	+	2.77473	NA	promoter-TSS (NM_001289056)	promoter-TSS (NM_001289056)	-55	NM_00103	51321	Hs.293560	NM_01662	ENSG0000	AMZ2	-	archaelysir	protein-co
lib-C666-l	chr8	99129606	99129606	+	2.77473	NA	promoter-TSS (NM_005836)	promoter-TSS (NM_005836)	85	NM_00114	10940	Hs.252828	NM_01502	ENSG0000	POP1	-	POP1 hom	protein-co
lib-C666-l	chr2	74211006	74211006	+	2.77473	NA	Intergenic	Intergenic	-2440	NR_10402	100874048	Hs.600674	NR_10402	ENSG0000	DGUOK-A	-	DGUOK an	ncRNA
lib-C666-l	chrX	3733061	3733061	+	2.77473	NA	Intergenic	CpG	28874	NR_03403	389906	Hs.636199	NM_001045547		LOC38990	-	zinc finger	pseudo
lib-C666-l	chr12	29564949	29564949	+	2.77473	NA	intron (NR_073172, intron 1 of 1)	L1PA3|LINE|L1	22722	NR_07317	101055625	Hs.569158	NR_07317	ENSG0000	OVCH1-AS	-	OVCH1 an	ncRNA
lib-C666-l	chr10	46951670	46951670	+	2.77473	NA	non-coding (NR_134489, exon 1 of 2	non-coding (NR_134489, exon 1 of	198	NR_13448	102724593	Hs.663831	NR_134489		LOC10272	-	uncharacte	ncRNA
lib-C666-l	chr10	126718364	126718364	+	2.75718	NA	intron (NM_001083914, intron 3 of 1	intron (NM_001083914, intron 3 of	-1911	NM_02280	1488	Hs.501345	NM_00132	ENSG0000	CTBP2	-	C-terminal	protein-co
lib-C666-l	chr11	31893417	31893417	+	2.75718	NA	intron (NR_033971, intron 1 of 2)	intron (NR_033971, intron 1 of 2)	45303	NR_11709	103157000		NR_11709	ENSG0000	PAUPAR	-	PAX6 upst	ncRNA
lib-C666-l	chr4	14858228	14858228	+	2.70685	NA	intron (NR_126435, intron 2 of 6)	intron (NR_126435, intron 2 of 6)	31565	NR_12643	201853	Hs.551051	NR_12643	ENSG0000	LINC00504	-	long interg	ncRNA
lib-C666-l	chr2	216658930	216658930	+	2.65152	NA	intron (NR_037195, intron 1 of 9)	intron (NR_037195, intron 1 of 9)	49329	NR_03719	646324	Hs.655713	NR_03719	ENSG0000	LINC00607	-	long interg	ncRNA
lib-C666-l	chr10	134148911	134148911	+	2.65152	NA	intron (NM_001143757, intron 2 of 1	LTR71B|LTR|ERV1	-1700	NM_00130	80313	Hs.119897	NM_03062	ENSG0000	LRRC27	-	leucine ricl	protein-co
lib-C666-l	chr1	155247137	155247137	+	2.62544	NA	promoter-TSS (NM_020897)	promoter-TSS (NM_020897)	-81	NM_02089	57657	Hs.706960	NM_02089	ENSG0000	HCN3	-	hyperpolar	protein-co
lib-C666-l	chr17	9548635	9548635	+	2.62544	NA	promoter-TSS (NM_001267576)	promoter-TSS (NM_001267576)	-219	NM_00126	124739	Hs.709621	NM_15321	ENSG0000	USP43	-	ubiquitin s	protein-co
lib-C666-l	chr8	141129301	141129301	+	2.60101	NA	intron (NM_031466, intron 17 of 22)	intron (NM_031466, intron 17 of 22	-18667	NR_14443	359809	Hs.26814	NR_144431		PEG13	-	paternally	ncRNA
lib-C666-l	chr10	104210486	104210486	+	2.60101	NA	exon (NM_024886, exon 2 of 2)	exon (NM_024886, exon 2 of 2)	814	NM_02488	79946	Hs.225084	NM_024886		C10orf95	-	chromosor	protein-co
lib-C666-l	chr7	29603937	29603937	+	2.59723	NA	5' UTR (NM_175887, exon 1 of 2)	5' UTR (NM_175887, exon 1 of 2)	510	NM_17588	222171	Hs.91109	NM_17588	ENSG0000	PRR15	-	proline ricl	protein-co
lib-C666-l	chr4	14794915	14794915	+	2.59723	NA	intron (NR_126435, intron 2 of 6)	L1PBa1|LINE|L1	94878	NR_12643	201853	Hs.551051	NR_12643	ENSG0000	LINC00504	-	long interg	ncRNA
lib-C666-l	chr5	150017282	150017282	+	2.59723	NA	intron (NM_001166209, intron 2 of 2)	intron (NM_001166209, intron 2 of	-2925	NM_00728	11346	Hs.435228	NM_00728	ENSG0000	SYNPO	-	synaptopo	protein-co
lib-C666-l	chr7	64023525	64023525	+	2.59032	NA	promoter-TSS (NM_178558)	promoter-TSS (NM_178558)	-20	NM_00113	340252	Hs.520886	NM_17855	ENSG0000	ZNF680	-	zinc finger	protein-co
lib-C666-l	chr6	41471941	41471941	+	2.59032	NA	intron (NR_120347, intron 2 of 2)	intron (NR_120347, intron 2 of 2)	1759	NR_12034	103106903	Hs.563396	NR_12034	ENSG0000	LINC01276	-	long interg	ncRNA
lib-C666-l	chr7	44078915	44078915	+	2.59032	NA	intron (NR_072981, intron 1 of 2)	intron (NR_072981, intron 1 of 2)	267	NR_07298	255031	Hs.633978	NR_015401		LINC00957	-	long interg	ncRNA
lib-C666-l	chr10	60027045	60027045	+	2.59032	NA	intron (NM_152230, intron 1 of 5)	CpG	649	NM_15223	253430	Hs.30280	NM_15223	ENSG0000	IPMK	-	inositol po	protein-co
lib-C666-l	chr3	47206051	47206051	+	2.59032	NA	promoter-TSS (NM_014159)	promoter-TSS (NM_014159)	191	NR_03337	285352	Hs.570631	NR_033373		KIF9-AS1	-	KIF9 antise	ncRNA
lib-C666-l	chr3	122162711	122162711	+	2.58854	NA	TTS (NR_125405)	TTS (NR_125405)	27658	NR_12540	102723582	Hs.675056	NR_12540	ENSG0000	LOC10272	-	uncharacte	ncRNA
lib-C666-l	chr5	138032202	138032202	+	2.58854	NA	Intergenic	MIRb|SINE|MIR	56796	NR_13424	105379194	Hs.445981	NR_13424	ENSG0000	LOC10537	-	uncharacte	ncRNA
lib-C666-l	chr11	65121986	65121986	+	2.58799	NA	promoter-TSS (NM_145719)	promoter-TSS (NM_145719)	-296	NM_14571	220359	Hs.632121	NM_14571	ENSG0000	TIGD3	-	tigger tran	protein-co
lib-C666-l	chr8	144373392	144373392	+	2.58799	NA	promoter-TSS (NM_030895)	promoter-TSS (NM_030895)	-167	NM_03089	79943	Hs.512740	NM_03089	ENSG0000	ZNF696	-	zinc finger	protein-co
lib-C666-l	chr2	119640627	119640627	+	2.35408	NA	Intergenic	LTR41|LTR|ERVL	-34868	NM_00142	2019	Hs.271977	NM_00142	ENSG0000	EN1	-	engrailed h	protein-co
lib-C666-l	chr5	168006572	168006572	+	2.26435	NA	promoter-TSS (NM_024594)	promoter-TSS (NM_024594)	42	NM_02459	79646	Hs.388400	NM_02459	ENSG0000	PANK3	-	pantothen	protein-co
lib-C666-l	chr2	138880086	138880086	+	2.26435	NA	Intergenic	Intergenic	20632	NR_13552	105373635		NR_13552	ENSG0000	LINC01832	-	long interg	ncRNA
lib-C666-l	chr22	46360386	46360386	+	2.26435	NA	intron (NM_058238, intron 1 of 3)	intron (NM_058238, intron 1 of 3)	12622	NM_05823	7477	Hs.512714	NM_05823	ENSG0000	WNT7B	-	Wnt family	protein-co
lib-C666-l	chr19	58038679	58038679	+	2.24771	NA	promoter-TSS (NM_001199295)	promoter-TSS (NM_001199295)	-14	NM_15326	256051	Hs.744289	NM_15326	ENSG0000	ZNF549	-	zinc finger	protein-co
lib-C666-l	chr16	2932756	2932756	+	2.24771	NA	promoter-TSS (NM_001142499)	promoter-TSS (NM_001142499)	-440	NM_00114	114984	Hs.534525	NM_13843	ENSG0000	FLYWCH2	-	FLYWCH fa	protein-co
lib-C666-l	chr4	184425672	184425672	+	2.24771	NA	promoter-TSS (NM_001564)	promoter-TSS (NM_001564)	-4	NR_03399	389247	Hs.385787	NR_033995		LOC38924	-	uncharacte	ncRNA
lib-C666-l	chr15	42120359	42120359	+	2.24164	NA	promoter-TSS (NM_001198588)	promoter-TSS (NM_001198588)	76	NM_00111	100137047	Hs.198161	NM_00111	ENSG0000	JMJD7	-	jumonji do	protein-co
lib-C666-l	chr9	140193765	140193765	+	2.24164	NA	TTS (NM_001004354)	TTS (NM_001004354)	2938	NM_00100	441478	Hs.535075	NM_00100	ENSG0000	NRARP	-	NOTCH-re	protein-co
lib-C666-l	chr2	171627052	171627052	+	2.24164	NA	promoter-TSS (NR_110644)	promoter-TSS (NR_110644)	-140	NM_00129	285141	Hs.443729	NM_00128	ENSG0000	ERICH2	-	glutamate	protein-co
lib-C666-l	chr20	3748774	3748774	+	2.24164	NA	promoter-TSS (NM_001039140)	promoter-TSS (NM_001039140)	261	NR_04767	54976	Hs.274422	NM_01787	ENSG0000	C20orf27	-	chromosor	protein-co

lib-C666-l	chr20	3748758	3748758	+	2.24164	NA	promoter-TSS (NM_001039140)	promoter-TSS (NM_001039140)	277	NR_04767	54976	Hs.274422	NM_01787	ENSG0000	C20orf27	-	chromosor	protein-co
lib-C666-l	chr10	89102146	89102146	+	2.24066	NA	promoter-TSS (NR_029408)	promoter-TSS (NR_029408)	-22	NR_02940	439994	Hs.651188	NR_029408		LINC00863	-	long interg	ncRNA
lib-C666-l	chr2	60416502	60416502	+	2.24066	NA	Intergenic	L1PA3|LINE|L1	193669	NR_13299	106660609	Hs.565545	NR_13299	ENSG0000	MIR4432H	-	MIR4432 h	ncRNA
lib-C666-l	chr15	41836435	41836435	+	2.24066	NA	promoter-TSS (NM_015540)	promoter-TSS (NM_015540)	40	NM_01554	26015	Hs.371045	NM_01554	ENSG0000	RPAP1	-	RNA polym	protein-co
lib-C666-l	chr20	8581725	8581725	+	2.12162	NA	intron (NM_182734, intron 3 of 32)	L1PA2|LINE|L1	352374	NR_04680	100874337	Hs.662218	NR_046809		PLCB1-IT1	-	PLCB1 intro	ncRNA
lib-C666-l	chr3	123871259	123871259	+	2.12162	NA	intron (NM_001024660, intron 1 of 5	intron (NM_001024660, intron 1 of	19483	NR_04979	100847021		NR_04979	ENSG0000	MIR5002	-	microRNA	ncRNA
lib-C666-l	chr1	84464850	84464850	+	2.12162	NA	promoter-TSS (NM_024686)	promoter-TSS (NM_024686)	-17	NM_02468	79739	Hs.445826	NM_02468	ENSG0000	TTLL7	-	tubulin tyr	protein-co
lib-C666-l	chr3	23136713	23136713	+	2.12162	NA	Intergenic	Intergenic	107356	NR_13617	100505877	Hs.125904	NR_13617	ENSG0000	UBE2E2-A	-	UBE2E2 an	ncRNA
lib-C666-l	chr8	22735155	22735155	+	2.12162	NA	promoter-TSS (NR_125433)	promoter-TSS (NR_125433)	-330	NR_12543	101929237	Hs.677838	NR_12543	ENSG0000	LOC10192	-	uncharacte	ncRNA
lib-C666-l	chr3	48594498	48594498	+	2.11095	NA	promoter-TSS (NM_001317137)	promoter-TSS (NM_001317137)	-142	NM_00131	5210	Hs.476217	NM_00456	ENSG0000	PFKFB4	-	6-phospho	protein-co
lib-C666-l	chr1	110752934	110752934	+	2.10911	NA	promoter-TSS (NM_004978)	promoter-TSS (NM_004978)	-325	NR_04654	100873974	Hs.130515	NR_046546		KCNC4-AS	-	KCNC4 ant	ncRNA
lib-C666-l	chr20	32319936	32319936	+	2.10911	NA	intron (NR_104259, intron 1 of 13)	CpG	370	NR_10425	84905	Hs.516989	NM_03281	ENSG0000	ZNF341	-	zinc finger	protein-co
lib-C666-l	chr20	18548008	18548008	+	2.10911	NA	promoter-TSS (NR_015432)	promoter-TSS (NR_015432)	-65	NR_01543	388789	Hs.349092	NR_01543	ENSG0000	LINC00493	-	long interg	ncRNA
lib-C666-l	chr16	20817764	20817764	+	2.10911	NA	promoter-TSS (NM_080663)	promoter-TSS (NM_080663)	-3	NM_00114	81691	Hs.177926	NM_03094	ENSG0000	LOC81691	-	exonucleas	protein-co
lib-C666-l	chr6	99963412	99963412	+	2.10911	NA	promoter-TSS (NM_001080481)	promoter-TSS (NM_001080481)	-47	NR_14434	85015	Hs.143410	NM_03292	ENSG0000	USP45	-	ubiquitin s	protein-co
lib-C666-l	chr14	62004549	62004549	+	2.10911	NA	intron (NM_006255, intron 12 of 13)	intron (NM_006255, intron 12 of 13	32363	NR_11041	101927780	Hs.254789	NR_11041	ENSG0000	LOC10192	-	uncharacte	ncRNA
lib-C666-l	chr17	80170678	80170678	+	2.10911	NA	promoter-TSS (NM_198082)	promoter-TSS (NM_198082)	11	NM_19808	284001	Hs.631724	NM_15267	ENSG0000	CCDC57	-	coiled-coil	protein-co
lib-C666-l	chr19	36542595	36542595	+	2.10304	NA	TTS (NM_001347907)	TTS (NM_001347907)	3069	NM_00133	199745	Hs.350209	NM_15265	ENSG0000	THAP8	-	THAP dom	protein-co
lib-C666-l	chr2	51449201	51449201	+	2.0646	NA	intron (NR_135237, intron 2 of 10)	LTR43B|LTR|ERV1	189462	NR_13523	730100	Hs.640140	NR_13523	ENSG0000	LOC73010	-	uncharacte	ncRNA
lib-C666-l	chr1	247290032	247290032	+	2.05265	NA	intron (NM_001243740, intron 3 of 3)	CpG	-14313	NM_20740	388759	Hs.456511	NM_207401		C1orf229	-	chromosor	protein-co
lib-C666-l	chr10	28592233	28592233	+	2.05265	NA	promoter-TSS (NR_134517)	promoter-TSS (NR_134517)	-238	NM_00131	143098	Hs.499159	NM_17349	ENSG0000	MPP7	-	membrane	protein-co
lib-C666-l	chrX	15288383	15288383	+	2.05265	NA	promoter-TSS (NM_001168530)	promoter-TSS (NM_001168530)	-90	NM_02408	140462	Hs.19404	NM_02408	ENSG0000	ASB9	-	ankyrin rep	protein-co
lib-C666-l	chr5	150017174	150017174	+	2.02415	NA	intron (NM_001166209, intron 2 of 2)	intron (NM_001166209, intron 2 of	-3033	NM_00728	11346	Hs.435228	NM_00728	ENSG0000	SYNPO	-	synaptopo	protein-co
lib-C666-l	chr1	206138335	206138335	+	2.01461	NA	promoter-TSS (NM_001123168)	promoter-TSS (NM_001123168)	-105	NM_00128	554282		NM_00128	ENSG0000	FAM72C	-	family with	protein-co
lib-C666-l	chr11	14913846	14913846	+	2.00164	NA	promoter-TSS (NM_024514)	promoter-TSS (NM_024514)	-95	NM_02451	120227	Hs.371427	NM_02451	ENSG0000	CYP2R1	-	cytochrom	protein-co
lib-C666-l	chr13	111711353	111711353	+	2.00164	NA	Intergenic	L1PB1|LINE|L1	-36832	NR_12041	101927802	Hs.559193	NR_120415		LINC00368	-	long interg	ncRNA
lib-C666-l	chr5	140044344	140044344	+	2.00164	NA	promoter-TSS (NM_017706)	promoter-TSS (NM_017706)	-40	NM_01770	54853	Hs.286261	NM_01770	ENSG0000	WDR55	-	WD repeat	protein-co
lib-C666-l	chr4	7441540	7441540	+	2.00164	NA	intron (NM_020777, intron 2 of 26)	L1MC4|LINE|L1	-4840	NM_00108	768239	Hs.662523	NM_00108	ENSG0000	PSAPL1	-	prosaposin	protein-co
lib-C666-l	chr7	100303598	100303598	+	18.68977	NA	promoter-TSS (NM_005837)	promoter-TSS (NM_005837)	-78	NM_00583	10248	Hs.416994	NM_00583	ENSG0000	POP7	0610037N	POP7 hom	protein-co
lib-C666-l	chr1	41157949	41157949	+	25.39084	NA	promoter-TSS (NR_024567)	promoter-TSS (NR_024567)	-16	NR_02456	100130557	Hs.233458	NR_024567		NFYC-AS1	0808y08y	NFYC antis	ncRNA
lib-C666-l	chr10	88474071	88474071	+	3.20885	NA	intron (NM_001080114, intron 8 of 1	intron (NM_001080114, intron 8 of	-42325	NM_00432	657	Hs.524477	NM_00432	ENSG0000	BMPR1A	10q23del|	bone morp	protein-co
lib-C666-l	chr13	22178276	22178276	+	8.28333	NA	promoter-TSS (NM_152726)	promoter-TSS (NM_152726)	79	NM_15272	221154	Hs.412103	NM_15272	ENSG0000	MICU2	1110008L2	mitochond	protein-co
lib-C666-l	chr12	51441934	51441934	+	3.31563	NA	promoter-TSS (NM_015416)	promoter-TSS (NM_015416)	-148	NM_00124	25875	Hs.655272	NM_01541	ENSG0000	LETMD1	1110019O	LETM1 dor	protein-co
lib-C666-l	chr7	105925558	105925558	+	29.98795	NA	promoter-TSS (NM_005746)	promoter-TSS (NM_005746)	80	NM_00574	10135	Hs.489615	NM_00574	ENSG0000	NAMPT	1110035O	nicotinami	protein-co
lib-C666-l	chr7	106065625	106065625	+	25.75165	NA	Intergenic	Intergenic	-139987	NM_00574	10135	Hs.489615	NM_00574	ENSG0000	NAMPT	1110035O	nicotinami	protein-co
lib-C666-l	chr7	105924880	105924880	+	12.13776	NA	intron (NM_005746, intron 1 of 10)	CpG	758	NM_00574	10135	Hs.489615	NM_00574	ENSG0000	NAMPT	1110035O	nicotinami	protein-co
lib-C666-l	chr16	70488461	70488461	+	16.5633	NA	promoter-TSS (NM_145059)	promoter-TSS (NM_145059)	-37	NM_14505	197258	Hs.7907	NM_14505	ENSG0000	FUK	1110046B1	fucokinase	protein-co
lib-C666-l	chr1	154934338	154934338	+	7.11942	NA	promoter-TSS (NM_138300)	promoter-TSS (NM_138300)	-80	NM_13830	90780	Hs.533597	NM_13830	ENSG0000	PYGO2	1190004M	pygopus f	protein-co
lib-C666-l	chr19	5680607	5680607	+	19.88269	NA	promoter-TSS (NM_001267868)	promoter-TSS (NM_001267868)	-169	NM_19870	374875	Hs.631840	NM_19853	ENSG0000	HSD11B1L	11-DH3|11	hydroxyste	protein-co
lib-C666-l	chr3	71632876	71632876	+	8.89416	NA	promoter-TSS (NM_001244808)	promoter-TSS (NM_001244808)	28	NM_00124	27086	Hs.59368	NM_03268	ENSG0000	FOXP1	12CC4|HSF	forkhead b	protein-co
lib-C666-l	chr3	71150171	71150171	+	3.89034	NA	intron (NM_032682, intron 7 of 20)	intron (NM_032682, intron 7 of 20)	29921	NM_00124	27086	Hs.59368	NM_03268	ENSG0000	FOXP1	12CC4|HSF	forkhead b	protein-co
lib-C666-l	chr2	9771370	9771370	+	132.45979	NA	promoter-TSS (NM_006826)	promoter-TSS (NM_006826)	-186	NM_00682	10971	Hs.74405	NM_00682	ENSG0000	YWHAQ	14-3-3|1C	tyrosine 3-	protein-co
lib-C666-l	chr7	75988338	75988338	+	20.84232	NA	promoter-TSS (NM_012479)	promoter-TSS (NM_012479)	4	NM_01247	7532	Hs.744840	NM_01247	ENSG0000	YWHAG	14-3-3GAI	tyrosine 3-	protein-co
lib-C666-l	chr7	75988750	75988750	+	17.62684	NA	promoter-TSS (NM_012479)	promoter-TSS (NM_012479)	-408	NM_01247	7532	Hs.744840	NM_01247	ENSG0000	YWHAG	14-3-3GAI	tyrosine 3-	protein-co
lib-C666-l	chr8	101964349	101964349	+	72.53808	NA	promoter-TSS (NM_001135700)	promoter-TSS (NM_001135700)	8	NM_14569	7534	Hs.492407	NM_00340	ENSG0000	YWHAZ	14-3-3-ze	tyrosine 3-	protein-co
lib-C666-l	chr8	101912809	101912809	+	33.38247	NA	Intergenic	Intergenic	49990	NM_00113	7534	Hs.492407	NM_00340	ENSG0000	YWHAZ	14-3-3-ze	tyrosine 3-	protein-co
lib-C666-l	chr22	42078500	42078500	+	61.32821	NA	5' UTR (NM_005008, exon 2 of 4)	5' UTR (NM_005008, exon 2 of 4)	6413	NM_00100	4809	Hs.182255	NM_00500	ENSG0000	SNU13	15.5K|FA-1	SNU13 hor	protein-co
lib-C666-l	chr22	42084966	42084966	+	22.13345	NA	promoter-TSS (NM_005008)	promoter-TSS (NM_005008)	-53	NM_00500	4809	Hs.182255	NM_00500	ENSG0000	SNU13	15.5K|FA-1	SNU13 hor	protein-co
lib-C666-l	chr12	120884235	120884235	+	303.53885	NA	promoter-TSS (NM_176818)	promoter-TSS (NM_176818)	-6	NM_17681	283459	Hs.706889	NM_17681	ENSG0000	GATC	15E1.2	glutamyl-t	protein-co
lib-C666-l	chr1	24969401	24969401	+	13.06669	NA	promoter-TSS (NM_001303448)	promoter-TSS (NM_001303448)	-193	NM_00130	10250	Hs.18192	NM_00583	ENSG0000	SRRM1	160-KD|PC	serine and	protein-co
lib-C666-l	chr1	24969662	24969662	+	10.53493	NA	promoter-TSS (NM_001303448)	promoter-TSS (NM_001303448)	68	NM_00583	10250	Hs.18192	NM_00583	ENSG0000	SRRM1	160-KD|PC	serine and	protein-co
lib-C666-l	chr5	912721	912721	+	66.67216	NA	intron (NM_004237, intron 10 of 12)	CpG	19752	NM_00423	9319	Hs.436187	NM_00423	ENSG0000	TRIP13	16E1BP	thyroid hor	protein-co
lib-C666-l	chr17	27621308	27621308	+	12.99339	NA	promoter-TSS (NM_020772)	promoter-TSS (NM_020772)	-142	NM_02077	57532	Hs.462598	NM_02077	ENSG0000	NUFIP2	182-FIP|82	NUFIP2, FN	protein-co
lib-C666-l	chr11	318472	318472	+	14.02822	NA	Intergenic	CpG	2442	NR_04975	10410	Hs.374650	NM_02103	ENSG0000	IFITM3	1-8U|DSPA	interferon	protein-co
lib-C666-l	chr17	41322470	41322470	+	127.26852	NA	promoter-TSS (NR_110868)	promoter-TSS (NR_110868)	-18	NM_03186	4077	Hs.277721	NM_00589	ENSG0000	NBR1	1A1-3B|IAI	NBR1, autc	protein-co
lib-C666-l	chr17	41323151	41323151	+	4.52748	NA	promoter-TSS (NR_110868)	promoter-TSS (NR_110868)	164	NM_00589	4077	Hs.277721	NM_00589	ENSG0000	NBR1	1A1-3B|IAI	NBR1, autc	protein-co
lib-C666-l	chr8	41435775	41435775	+	16.6838	NA	promoter-TSS (NM_178819)	promoter-TSS (NM_178819)	68	NM_17881	137964	Hs.355753	NM_17881	ENSG0000	GPAT4	1-AGPAT 6	glycerol-3-	protein-co
lib-C666-l	chr6	32146010	32146010	+	9.21057	NA	promoter-TSS (NM_006913).4	promoter-TSS (NM_006913).4	-122	NM_03274	10554	Hs.409230	NM_00641	ENSG0000	AGPAT1	1-AGPAT1	1-acylglyce	protein-co
lib-C666-l	chr6	161695170	161695170	+	11.06679	NA	promoter-TSS (NM_020133)	promoter-TSS (NM_020133)	-63	NM_02013	56895	Hs.353175	NM_02013	ENSG0000	AGPAT4	1-AGPAT4	1-acylglyce	protein-co
lib-C666-l	chr8	6565832	6565832	+	3.76263	NA	promoter-TSS (NR_125386)	promoter-TSS (NR_125386)	-46	NM_01836	55326	Hs.624002	NM_01836	ENSG0000	AGPAT5	1AGPAT5|L	1-acylglyce	protein-co
lib-C666-l	chr2	30670089	30670089	+	72.05244	NA	promoter-TSS (NM_001002257)	promoter-TSS (NM_001002257)	-3	NM_00130	253558	Hs.468048	NM_18255	ENSG0000	LCLAT1	1AGPAT8|	lysocardiol	protein-co
lib-C666-l	chr13	50698552	50698552	+	34.25375	NA	intron (NR_109974, intron 2 of 6)	CpG	1125	NR_00261	8847	Hs.547964	NR_00261	ENSG0000	DLEU2	1B4|BCMS	deleted in	ncRNA
lib-C666-l	chr13	50698274	50698274	+	13.06669	NA	intron (NR_109974, intron 2 of 6)	CpG	1403	NR_00261	8847	Hs.547964	NR_00261	ENSG0000	DLEU2	1B4|BCMS	deleted in	ncRNA
lib-C666-l	chr13	50699960	50699960	+	10.71733	NA	promoter-TSS (NR_002612)	promoter-TSS (NR_002612)	-283	NR_00261	8847	Hs.547964	NR_00261	ENSG0000	DLEU2	1B4|BCMS	deleted in	ncRNA
lib-C666-l	chr1	192469896	192469896	+	2.62544	NA	Intergenic	L1PA2|LINE|L1	-74961	NM_00292	5996	Hs.75256	NM_00292	ENSG0000	RGS1	1R20|BL34	regulator c	protein-co
lib-C666-l	chr2	74734435	74734435	+	19.60174	NA	intron (NM_032673, intron 1 of 8)	CpG	386	NM_03267	84759	Hs.316750	NM_03267	ENSG0000	PCGF1	2010002K	polycomb	protein-co
lib-C666-l	chr19	15236307	15236307	+	15.74109	NA	intron (NM_006844, intron 1 of 15)	CpG	303	NM_00684	10994	Hs.78880	NM_00684	ENSG0000	ILVBL	209L8|AHA	ilvB acetola	protein-co
lib-C666-l	chr21	37560478	37560478	+	14.45701	NA	intron (NM_005128, intron 2 of 36)	intron (NM_005128, intron 2 of 36)	23639	NM_00512	9980	Hs.204575	NM_00512	ENSG0000	DOPEY2	21orf5|C21	dopey fam	protein-co
lib-C666-l	chr12	108956324	108956324	+	43.7029	NA	non-coding (NR_135127, exon 1 of 7	non-coding (NR_135127, exon 1 of	1085	NR_13512	23479	Hs.615131	NM_01430	ENSG0000	ISCU	2310020H	iron-sulfur	protein-co
lib-C666-l	chr16	47495099	47495099	+	10.71733	NA	promoter-TSS (NM_001305002)	promoter-TSS (NM_001305002)	86	NM_00130	81533	Hs.42217	NM_03079	ENSG0000	ITFG1	2310047C	integrin alp	protein-co
lib-C666-l	chr16	81041079	81041079	+	73.12756	NA	promoter-TSS (NM_020188)	promoter-TSS (NM_020188)	-577	NM_02018	56942	Hs.55028	NM_02018	ENSG0000	CMC2	2310061C1	C-X9-C m	protein-co
lib-C666-l	chr16	81040677	81040677	+	10.73451	NA	promoter-TSS (NM_020188)	promoter-TSS (NM_020188)	-175	NM_02018	56942	Hs.55028	NM_02018	ENSG0000	CMC2	2310061C1	C-X9-C m	protein-co
lib-C666-l	chr18	47814203	47814203	+	11.36743	NA	5' UTR (NM_014593, exon 1 of 15)	5' UTR (NM_014593, exon 1 of 15)	489	NM_00110	30827	Hs.180933	NM_01459	ENSG0000	CXXC1	2410002I1	CXXC finge	protein-co
lib-C666-l	chr17	5389929	5389929	+	14.3056	NA	promoter-TSS (NM_001304779)	promoter-TSS (NM_001304779)	235	NM_00125	79003	Hs.267194	NM_02403	ENSG0000	MIS12	2510025F0	MIS12, kin	protein-co
lib-C666-l	chr17	74099843	74099843	+	8.73012	NA	promoter-TSS (NM_001282313)	promoter-TSS (NM_001282313)	25	NM_00128	23265	Hs.514496	NM_01521	ENSG0000	EXOC7	2-5-3p|EX	exocyst co	protein-co
lib-C666-l	chr15	73076122	73076122	+	484.95114	NA	promoter-TSS (NM_031284)	promoter-TSS (NM_031284)	4	NR_02331	83440	Hs.562587	NM_03128	ENSG0000	ADPGK	2610017G	ADP deper	protein-co
lib-C666-l	chr2	178077287	178077287	+	128.58131	NA	promoter-TSS (NR_049831)	promoter-TSS (NR_049831)	-135	NM_00133	220988	Hs.516539	NM_19424	ENSG0000	HNRNPA3	2610510D	heterogen	protein-co
lib-C666-l	chr3	101443622	101443622	+	7.15277	NA	intron (NM_024548, intron 1 of 10)	CpG	185	NM_02454	79598	Hs.444135	NM_02454	ENSG0000	CEP97	2810403B	centrosom	protein-co
lib-C666-l	chr1	59165806	59165806	+	25.39084	NA	promoter-TSS (NM_001085487)	promoter-TSS (NM_001085487)	-59	NM_00108	114803	Hs.744921	NM_00108	ENSG0000	MYSM1	2A-DUB|2	Myb like, S	protein-co
lib-C666-l	chr16	7918036	7918036	+	9.10494	NA	Intergenic	MLT1E|LTR|ERVL-MaLR	535285	NM_14589	54715	Hs.459842	NM_01872	ENSG0000	RBFOX1	2BP1|A2BP	RNA bindir	protein-co
lib-C666-l	chr4	113152767	113152767	+	43.79478	NA	promoter-TSS (NM_018569)	promoter-TSS (NM_018569)	-128	NM_01856	55435	Hs.435991	NM_01856	ENSG0000	AP1AR	2C18|C4or	adaptor re	protein-co
lib-C666-l	chr2	198441472	198441472	+	7.0832	NA	intron (NM_144629, intron 8 of 8)	Tigger16a|DNA|TcMar-Tigger	60450	NM_00120	25843	Hs.633165	NM_01538	ENSG0000	MOB4	2C4D|CGI-	MOB famil	protein-co
lib-C666-l	chr2	198380706	198380706	+	6.82479	NA	promoter-TSS (NM_001100819)	promoter-TSS (NM_001100819)	-61	NM_01538	25843	Hs.633165	NM_01538	ENSG0000	MOB4	2C4D|CGI-	MOB famil	protein-co
lib-C666-l	chr7	130353621	130353621	+	2.46982	NA	promoter-TSS (NM_012133)	promoter-TSS (NM_012133)	-23	NM_00129	26958	Hs.6421	NM_01213	ENSG0000	COPG2	2-COP|gar	coatomer	protein-co
lib-C666-l	chr19	49436806	49436806	+	16.76091	NA	promoter-TSS (NM_014475)	promoter-TSS (NM_014475)	-133	NM_01447	27294	Hs.631555	NM_01447	ENSG0000	DHDH	2DD|HUM	dihydrodio	protein-co
lib-C666-l	chr19	47987455	47987455	+	365.01355	NA	promoter-TSS (NM_001291296)	promoter-TSS (NM_001291296)	66	NR_11192	11133	Hs.25441	NM_00705	ENSG0000	KPTN	2E4|MRT41	kaptin, acti	protein-co
lib-C666-l	chr12	9102585	9102585	+	27.5466	NA	promoter-TSS (NM_002355)	promoter-TSS (NM_002355)	-55	NR_13742	10219	Hs.558446	NM_00581	ENSG0000	KLRG1	2F1|CLEC1	killer cell le	protein-co
lib-C666-l	chr13	21714546	21714546	+	8.89416	NA	promoter-TSS (NM_005870)	promoter-TSS (NM_005870)	-107	NM_00587	10284	Hs.524899	NM_00587	ENSG0000	SAP18	2HOR0202	Sin3A asso	protein-co
lib-C666-l	chr6	149068787	149068787	+	4.12121	NA	exon (NM_005715, exon 1 of 8)	exon (NM_005715, exon 1 of 8)	516	NM_00571	10090	Hs.657370	NM_00571	ENSG0000	UST	2OST	uronyl 2-s	protein-co
lib-C666-l	chr3	49059071	49059071	+	12.13776	NA	promoter-TSS (NM_018114)	promoter-TSS (NM_018114)	-3	NM_19906	25915	Hs.31387	NM_19906	ENSG0000	NDUFAF3	2P1|C3orf6	NADH:ubic	protein-co
lib-C666-l	chr3	57541829	57541829	+	15.3763	NA	promoter-TSS (NM_001322177)	promoter-TSS (NM_001322177)	-145	NM_00132	201626	Hs.572993	NM_17796	ENSG0000	PDE12	2'-PDE|2-F	phosphodi	protein-co
lib-C666-l	chr9	131451311	131451311	+	10.71733	NA	promoter-TSS (NM_003011)	promoter-TSS (NM_003011)	-198	NM_00301	6418	Hs.436687	NM_00301	ENSG0000	SET	2PP2A|I2P	SET nuclea	protein-co
lib-C666-l	chr9	131452566	131452566	+	5.9099	NA	intron (NM_001248001, intron 1 of 7)	CpG	312	NM_00124	6418	Hs.436687	NM_00301	ENSG0000	SET	2PP2A|I2P	SET nuclea	protein-co
lib-C666-l	chr3	39448175	39448175	+	5.34948	NA	promoter-TSS (NM_002295)	promoter-TSS (NM_002295)	-5	NM_00229	3921	Hs.449909	NM_00229	ENSG0000	RPSA	37LRP|67L	ribosomal	protein-co
lib-C666-l	chr4	2819849	2819849	+	41.21722	NA	promoter-TSS (NM_003023)	promoter-TSS (NM_003023)	-63	NM_00114	6452	Hs.167679	NM_00302	ENSG0000	SH3BP2	3BP-2|3BP	SH3 doma	protein-co
lib-C666-l	chr4	2800661	2800661	+	18.39104	NA	intron (NM_001122681, intron 1 of 1	intron (NM_001122681, intron 1 of	5911	NM_00112	6452	Hs.167679	NM_00302	ENSG0000	SH3BP2	3BP-2|3BP	SH3 doma	protein-co
lib-C666-l	chr3	49977666	49977666	+	8.71138	NA	intron (NM_001167582, intron 1 of 1	CpG	189	NM_00577	10180	Hs.696735	NM_00577	ENSG0000	RBM6	3G2|DEF-3	RNA bindir	protein-co
lib-C666-l	chr2	62532355	62532355	+	25.33067	NA	Intergenic	Intergenic	89699	NM_00131	10678	Hs.173203	NM_00657	ENSG0000	B3GNT2	3-Gn-T1|3	UDP-GlcN	protein-co
lib-C666-l	chr2	62423049	62423049	+	6.56784	NA	promoter-TSS (NM_006577)	promoter-TSS (NM_006577)	-213	NM_00657	10678	Hs.173203	NM_00657	ENSG0000	B3GNT2	3-Gn-T1|3	UDP-GlcN	protein-co
lib-C666-l	chr17	81009515	81009515	+	5.48804	NA	intron (NM_001320742, intron 1 of 1	CpG	171	NR_13546	146712	Hs.607824	NM_00100	ENSG0000	B3GNTL1	3-Gn-T8|B	UDP-GlcN	protein-co
lib-C666-l	chr2	43037439	43037439	+	7.0832	NA	Intergenic	CpG	-17688	NM_01220	23498	Hs.368805	NM_01220	ENSG0000	HAAO	3-HAO|HA	3-hydroxy	protein-co
lib-C666-l	chr8	8860371	8860371	+	5.82086	NA	promoter-TSS (NM_153332)	promoter-TSS (NM_153332)	57	NM_15333	90459	Hs.20000	NM_15333	ENSG0000	ERI1	3'HEXO|HE	exoribonuc	protein-co
lib-C666-l	chr4	11430923	11430923	+	8.28216	NA	promoter-TSS (NM_005114)	promoter-TSS (NM_005114)	-386	NM_00511	9957	Hs.507348	NM_00511	ENSG0000	HS3ST1	3OST|3OS	heparan su	protein-co
lib-C666-l	chr16	25704004	25704004	+	3.93123	NA	exon (NM_006040, exon 1 of 2)	exon (NM_006040, exon 1 of 2)	657	NM_00604	9951	Hs.655275	NM_00604	ENSG0000	HS3ST4	3-OST-4|3	heparan su	protein-co
lib-C666-l	chr6	115036468	115036468	+	9.38975	NA	Intergenic	Intergenic	-652427	NM_15361	222537	Hs.645477	NM_15361	ENSG0000	HS3ST5	3-OST-5|3	heparan su	protein-co
lib-C666-l	chr16	1979818	1979818	+	29.74345	NA	Intergenic	CpG	-11494	NM_00100	64711	Hs.670144	NM_00100	ENSG0000	HS3ST6	3-OST-6|H	heparan su	protein-co
lib-C666-l	chr3	50649393	50649393	+	9.39802	NA	promoter-TSS (NM_145071)	promoter-TSS (NM_145071)	100	NM_00124	7867	Hs.234521	NM_00463	ENSG0000	MAPKAPK	3PK|MAPK	mitogen-a	protein-co
lib-C666-l	chr18	5630661	5630661	+	13.33918	NA	Intergenic	CpG-11574	-1671	NM_00128	23136	Hs.213394	NM_01230	ENSG0000	EPB41L3	4.1B|DAL-	erythrocyt	protein-co
lib-C666-l	chr6	131383873	131383873	+	3.8557	NA	intron (NM_001431, intron 1 of 19)	CpG	589	NM_00143	2037	Hs.486470	NM_00143	ENSG0000	EPB41L2	4.1-G|4.1G	erythrocyt	protein-co
lib-C666-l	chr1	29213465	29213465	+	55.79842	NA	promoter-TSS (NM_004437)	promoter-TSS (NM_004437)	-138	NM_00116	2035	Hs.175437	NM_00443	ENSG0000	EPB41	4.1R|EL1|H	erythrocyt	protein-co
lib-C666-l	chr1	8001085	8001085	+	3.20885	NA	intron (NM_001561, intron 1 of 8)	intron (NM_001561, intron 1 of 8)	2140	NM_00156	3604	Hs.86447	NM_00156	ENSG0000	TNFRSF9	4-1BB|CD1	TNF recept	protein-co
lib-C666-l	chr19	6536278	6536278	+	68.23259	NA	TTS (NM_003811)	TTS (NM_003811)	5268	NM_00381	8744	Hs.1524	NM_00381	ENSG0000	TNFSF9	4-1BB-L|C	tumor necr	protein-co
lib-C666-l	chr19	6531135	6531135	+	4.27545	NA	exon (NM_003811, exon 1 of 3)	exon (NM_003811, exon 1 of 3)	125	NM_00381	8744	Hs.1524	NM_00381	ENSG0000	TNFSF9	4-1BB-L|C	tumor necr	protein-co
lib-C666-l	chr17	73892648	73892648	+	2.00164	NA	exon (NM_001256124, exon 1 of 5)	exon (NM_001256124, exon 1 of 5)	436	NM_17354	201292	Hs.189823	NM_17354	ENSG0000	TRIM65	4732463G	tripartite m	protein-co
lib-C666-l	chr12	69754024	69754024	+	26.49922	NA	intron (NM_001300950, intron 1 of 4)	CpG	534	NM_00130	8089	Hs.4029	NM_00653	ENSG0000	YEATS4	4930573H	YEATS don	protein-co
lib-C666-l	chr10	18940550	18940550	+	28.64047	NA	promoter-TSS (NM_182543)	promoter-TSS (NM_182543)	16	NM_18254	221078	Hs.396175	NM_18254	ENSG0000	NSUN6	4933414E	NOP2/Sun	protein-co
lib-C666-l	chr13	21984742	21984742	+	6.20473	NA	intron (NR_104486, intron 4 of 14)	L1PA7|LINE|L1	48766	NM_15325	253832	Hs.564611	NM_15325	ENSG0000	ZDHHC20	4933421L1	zinc finger	protein-co
lib-C666-l	chr2	136288234	136288234	+	20.19521	NA	promoter-TSS (NM_001286568)	promoter-TSS (NM_001286568)	66	NM_00128	84083	Hs.658422	NM_03214	ENSG0000	ZRANB3	4933425L1	zinc finger	protein-co
lib-C666-l	chr10	72163847	72163847	+	26.54591	NA	promoter-TSS (NM_004096)	promoter-TSS (NM_004096)	-14	NM_00409	1979	Hs.621200	NM_00409	ENSG0000	EIF4EBP2	4EBP2|PHA	eukaryotic	protein-co
lib-C666-l	chr2	233415699	233415699	+	3.57421	NA	promoter-TSS (NM_145702)	promoter-TSS (NM_145702)	183	NM_00133	9470	Hs.292026	NM_00484	ENSG0000	EIF4E2	4E-LP|4EH	eukaryotic	protein-co
lib-C666-l	chr16	87901141	87901141	+	11.06679	NA	intron (NM_003486, intron 1 of 9)	intron (NM_003486, intron 1 of 9)	1964	NM_00348	8140	Hs.513797	NM_00348	ENSG0000	SLC7A5	4F2LC|CD9	solute carr	protein-co
lib-C666-l	chr15	44084433	44084433	+	13.0716	NA	promoter-TSS (NM_001199878)	promoter-TSS (NM_001199878)	-142	NM_00119	10169	Hs.424126	NM_00577	ENSG0000	SERF2	4F5REL|FA	small EDR	protein-co
lib-C666-l	chr11	44638721	44638721	+	9.02258	NA	intron (NM_002231, intron 7 of 9)	intron (NM_002231, intron 7 of 9)	51580	NM_00102	3732	Hs.527778	NM_00223	ENSG0000	CD82	4F9|C33|G	CD82 mole	protein-co
lib-C666-l	chr21	35014020	35014020	+	3.84463	NA	promoter-TSS (NM_003024)	promoter-TSS (NM_003024)	140	NM_14585	9946	Hs.352671	NM_14531	ENSG0000	CRYZL1	4P11|QOH	crystallin z	protein-co
lib-C666-l	chr17	48238954	48238954	+	5.04179	NA	Intergenic	CpG	-4392	NR_13555	6442	Hs.463412	NM_00002	ENSG0000	SGCA	50DAG|AD	sarcoglyca	protein-co
lib-C666-l	chr15	43785374	43785374	+	63.5671	NA	promoter-TSS (NM_001141979)	promoter-TSS (NM_001141979)	-20	NM_00114	7158	Hs.440968	NM_00565	ENSG0000	TP53BP1	53BP1|TDR	tumor prot	protein-co
lib-C666-l	chr1	224076016	224076016	+	61.07891	NA	Intergenic	Intergenic	-42342	NM_00542	7159	Hs.523968	NM_00542	ENSG0000	TP53BP2	53BP2|ASP	tumor prot	protein-co
lib-C666-l	chr1	224033592	224033592	+	2.65152	NA	promoter-TSS (NM_005426)	promoter-TSS (NM_005426)	82	NM_00103	7159	Hs.523968	NM_00542	ENSG0000	TP53BP2	53BP2|ASP	tumor prot	protein-co
lib-C666-l	chr11	66056626	66056626	+	13.59254	NA	promoter-TSS (NM_001300861)	promoter-TSS (NM_001300861)	12	NM_00130	10897	Hs.446445	NM_02047	ENSG0000	YIF1A	54TM|FinG	Yip1 intera	protein-co
lib-C666-l	chr16	12009955	12009955	+	109.00261	NA	promoter-TSS (NM_001130006)	promoter-TSS (NM_001130006)	-130	NM_00113	2935	Hs.528780	NM_00209	ENSG0000	GSPT1	551G9.2|E	G1 to S ph	protein-co
lib-C666-l	chr1	171750649	171750649	+	32.04651	NA	promoter-TSS (NM_015935)	promoter-TSS (NM_015935)	-112	NM_01495	51603	Hs.494705	NM_01495	ENSG0000	METTL13	5630401D	methyltran	protein-co
lib-C666-l	chr17	79670397	79670397	+	25.39487	NA	promoter-TSS (NM_002949)	promoter-TSS (NM_002949)	-3	NM_00294	6182	Hs.109059	NM_00294	ENSG0000	MRPL12	5c5-2|L12	mitochond	protein-co
lib-C666-l	chr19	572509	572509	+	20.72609	NA	promoter-TSS (NM_001322243)	promoter-TSS (NM_001322243)	55	NM_19858	682	Hs.501293	NM_00172	ENSG0000	BSG	5F7|CD147	basigin (O	protein-co
lib-C666-l	chr10	134350862	134350862	+	48.61377	NA	promoter-TSS (NM_005539)	promoter-TSS (NM_005539)	-421	NM_00553	3632	Hs.523360	NM_00553	ENSG0000	INPP5A	5PTASE	inositol po	protein-co
lib-C666-l	chr10	134355441	134355441	+	9.02258	NA	intron (NM_005539, intron 1 of 15)	intron (NM_005539, intron 1 of 15)	4158	NM_00553	3632	Hs.523360	NM_00553	ENSG0000	INPP5A	5PTASE	inositol po	protein-co
lib-C666-l	chr10	134388962	134388962	+	5.25516	NA	intron (NM_005539, intron 1 of 15)	intron (NM_005539, intron 1 of 15)	37679	NM_00553	3632	Hs.523360	NM_00553	ENSG0000	INPP5A	5PTASE	inositol po	protein-co
lib-C666-l	chr5	137688175	137688175	+	6.98764	NA	promoter-TSS (NM_016604)	promoter-TSS (NM_016604)	-110	NM_01660	51780	Hs.483486	NM_01660	ENSG0000	KDM3B	5qNCA|C5	lysine dem	protein-co
lib-C666-l	chr1	153395183	153395183	+	92.73503	NA	promoter-TSS (NM_001319198)	promoter-TSS (NM_001319198)	-124	NM_00131	6279	Hs.416073	NM_00296	ENSG0000	S100A8	60B8AG|C	S100 calciu	protein-co
lib-C666-l	chr1	153337974	153337974	+	2.96041	NA	Intergenic	Intergenic	7644	NM_00296	6280	Hs.112405	NM_00296	ENSG0000	S100A9	60B8AG|C	S100 calciu	protein-co
lib-C666-l	chr1	27986737	27986737	+	17.62684	NA	Intergenic	CpG	11987	NM_02287	2537	Hs.523847	NM_00203	ENSG0000	IFI6	6-16|FAM1	interferon	protein-co
lib-C666-l	chr16	18938050	18938050	+	11.20567	NA	promoter-TSS (NM_015092)	promoter-TSS (NM_015092)	-324	NM_01509	23049	Hs.460179	NM_01509	ENSG0000	SMG1	61E3.4|AT	SMG1, nor	protein-co
lib-C666-l	chr16	18939186	18939186	+	7.26874	NA	Intergenic	Intergenic	-1460	NM_01509	23049	Hs.460179	NM_01509	ENSG0000	SMG1	61E3.4|AT	SMG1, nor	protein-co
lib-C666-l	chr1	162025976	162025976	+	20.90919	NA	Intergenic	Intergenic	-13605	NM_00116	9722	Hs.731942	NM_01469	ENSG0000	NOS1AP	6330408P1	nitric oxide	protein-co
lib-C666-l	chr1	150241568	150241568	+	7.09685	NA	promoter-TSS (NM_016022)	promoter-TSS (NM_016022)	41	NR_04503	51107	Hs.108408	NM_01602	ENSG0000	APH1A	6530402N	aph-1 hon	protein-co
lib-C666-l	chr2	85829926	85829926	+	17.48666	NA	promoter-TSS (NR_033179)	promoter-TSS (NR_033179)	-39	NR_04634	10713	Hs.654745	NM_00659	ENSG0000	USP39	65K|CGI-2	ubiquitin s	protein-co
lib-C666-l	chr6	157041335	157041335	+	47.76595	NA	Intergenic	CpG	-57654	NM_00134	57492	Hs.291587	NM_01751	ENSG0000	ARID1B	6A3-5|BAF	AT-rich int	protein-co
lib-C666-l	chr6	157007962	157007962	+	3.57421	NA	Intergenic	Intergenic	-91027	NM_00134	57492	Hs.291587	NM_01751	ENSG0000	ARID1B	6A3-5|BAF	AT-rich int	protein-co
lib-C666-l	chr1	10459055	10459055	+	2.65152	NA	promoter-TSS (NM_001304451)	promoter-TSS (NM_001304451)	6	NM_00130	5226	Hs.464071	NM_00263	ENSG0000	PGD	6PGD	phosphogl	protein-co
lib-C666-l	chr6	31430854	31430854	+	27.84631	NA	promoter-TSS (NR_040662)	promoter-TSS (NR_040662)	-103	NR_04066	10866	Hs.691948	NR_04066	ENSG0000	HCP5	6S2650E|D	HLA comp	ncRNA
lib-C666-l	chrX	13753196	13753196	+	3.57421	NA	promoter-TSS (NM_001128835)	promoter-TSS (NM_001128835)	364	NM_00361	8481	Hs.6483	NM_00361	ENSG0000	OFD1	71-7A|CXc	OFD1, cent	protein-co
lib-C666-l	chrY	1660546	1660546	+	52.45363	NA	promoter-TSS (NM_005088).2	promoter-TSS (NM_005088).2	60	NR_02738	8227	Hs.522572	NM_00508	ENSG0000	AKAP17A	721P|AKAF	A-kinase a	protein-co
lib-C666-l	chrX	1710530	1710530	+	18.74745	NA	promoter-TSS (NM_005088)	promoter-TSS (NM_005088)	44	NM_00508	8227	Hs.522572	NM_00508	ENSG0000	AKAP17A	721P|AKAF	A-kinase a	protein-co
lib-C666-l	chr1	110091195	110091195	+	11.6145	NA	promoter-TSS (NM_006496)	promoter-TSS (NM_006496)	9	NM_00649	2773	Hs.73799	NM_00649	ENSG0000	GNAI3	87U6|ARC	G protein s	protein-co
lib-C666-l	chr19	8373050	8373050	+	36.56565	NA	TTS (NR_135539)	TTS (NR_135539)	190	NM_01657	51293	Hs.558499	NM_01657	ENSG0000	CD320	8D6|8D6A	CD320 mo	protein-co
lib-C666-l	chr11	311322	311322	+	9.02258	NA	Intergenic	CpG	-2669	NM_00364	8519	Hs.458414	NM_00364	ENSG0000	IFITM1	9-27|CD22	interferon	protein-co
lib-C666-l	chr12	69979187	69979187	+	17.22931	NA	promoter-TSS (NR_037475)	promoter-TSS (NR_037475)	-21	NM_00643	10576	Hs.189772	NM_00643	ENSG0000	CCT2	99D8.1|CC	chaperonir	protein-co
lib-C666-l	chr2	38978547	38978547	+	4.30593	NA	promoter-TSS (NM_001031684)	promoter-TSS (NM_001031684)	89	NM_00119	6432	Hs.309090	NM_00627	ENSG0000	SRSF7	9G8|AAG3|	serine and	protein-co

lib-C666-l	chr4	39367962	39367962	+	115.22815	NA	promoter-TSS (NM_001204747)	promoter-TSS (NM_001204747)	39	NM_00120	5981	Hs.507475	NM_00291	ENSG0000	RFC1	A1|MHCBF	replication	protein-co
lib-C666-l	chr3	186524346	186524346	+	43.18268	NA	promoter-TSS (NM_002916)	promoter-TSS (NM_002916)	-56	NM_00291	5984	Hs.732098	NM_00291	ENSG0000	RFC4	A1|RFC37	replication	protein-co
lib-C666-l	chr16	66864806	66864806	+	6.32351	NA	promoter-TSS (NM_001286500)	promoter-TSS (NM_001286500)	73	NM_00390	8883	Hs.460978	NM_00390	ENSG0000	NAE1	A-116A10.	NEDD8 act	protein-co
lib-C666-l	chr17	39845623	39845623	+	153.39015	NA	intron (NM_005801, intron 1 of 3)	CpG	496	NM_00580	10209	Hs.150580	NM_00580	ENSG0000	EIF1	A121|EIF-1	eukaryotic	protein-co
lib-C666-l	chr17	39845045	39845045	+	12.32267	NA	promoter-TSS (NM_005801)	promoter-TSS (NM_005801)	-82	NM_00580	10209	Hs.150580	NM_00580	ENSG0000	EIF1	A121|EIF-1	eukaryotic	protein-co
lib-C666-l	chr2	95831457	95831457	+	74.77618	NA	5' UTR (NM_001291605, exon 1 of 5)	5' UTR (NM_001291605, exon 1 of	295	NM_00101	7549	Hs.590916	NM_02108	ENSG0000	ZNF2	A1-5|ZNF	zinc finger	protein-co
lib-C666-l	chr5	179246107	179246107	+	22.66803	NA	intron (NM_001142298, intron 2 of 8)	intron (NM_001142298, intron 2 of	-1735	NM_00390	8878	Hs.587290	NM_00390	ENSG0000	SQSTM1	A170|DMR	sequestos	protein-co
lib-C666-l	chr2	86333136	86333136	+	7.21092	NA	promoter-TSS (NM_017952)	promoter-TSS (NM_017952)	142	NM_01542	25885	Hs.531818	NM_01542	ENSG0000	POLR1A	A190|AFD	RNA polym	protein-co
lib-C666-l	chrX	47050124	47050124	+	3.92495	NA	promoter-TSS (NM_153280)	promoter-TSS (NM_153280)	-75	NM_15328	7317	Hs.533273	NM_00333	ENSG0000	UBA1	A1S9|A1S9	ubiquitin li	protein-co
lib-C666-l	chr6	138159452	138159452	+	9.21057	NA	intron (NR_049793, intron 3 of 3)	L1PB1|LINE|L1	-28873	NM_00629	7128	Hs.211600	NM_00629	ENSG0000	TNFAIP3	A20|AISBL|	TNF alpha	protein-co
lib-C666-l	chr16	20911841	20911841	+	31.98049	NA	promoter-TSS (NR_126527)	promoter-TSS (NR_126527)	-147	NM_00112	57149	Hs.745012	NM_02042	ENSG0000	LYRM1	A211C6.1	LYR motif	protein-co
lib-C666-l	chr16	28834004	28834004	+	32.29191	NA	promoter-TSS (NM_148416)	promoter-TSS (NM_148416)	-365	NM_14841	11273	Hs.460499	NM_00724	ENSG0000	ATXN2L	A2D|A2LG|	ataxin 2 lik	protein-co
lib-C666-l	chr16	29801952	29801952	+	3.93532	NA	promoter-TSS (NM_001256269)	promoter-TSS (NM_001256269)	-82	NM_00731	3835	Hs.612151	NM_00731	ENSG0000	KIF22	A-328A3.2	kinesin fam	protein-co
lib-C666-l	chr16	12170039	12170039	+	5.96422	NA	intron (NM_032167, intron 10 of 20)	L1PA3|LINE|L1	99448	NM_03216	92017	Hs.458401	NM_03216	ENSG0000	SNX29	A-388D4.1	sorting ne	protein-co
lib-C666-l	chr22	39495708	39495708	+	3.23837	NA	intron (NM_001166002, intron 1 of 4)	intron (NM_001166002, intron 1 of	2479	NM_00116	164668	Hs.440515	NM_18177	ENSG0000	APOBEC3H	A3H|ARP-	apolipopro	protein-co
lib-C666-l	chr7	138437456	138437456	+	9.25113	NA	exon (NM_130841, exon 10 of 21)	exon (NM_130841, exon 10 of 21)	21326	NM_13084	50617	Hs.98967	NM_02063	ENSG0000	ATP6V0A4	A4|ATP6N	ATPase H+	protein-co
lib-C666-l	chr2	203879308	203879308	+	79.14428	NA	promoter-TSS (NM_001114132)	promoter-TSS (NM_001114132)	-294	NM_00111	65065	Hs.648846	NM_19894	ENSG0000	NBEAL1	A530083I0	neurobeac	protein-co
lib-C666-l	chr12	44200158	44200158	+	91.03855	NA	promoter-TSS (NR_073473)	promoter-TSS (NR_073473)	20	NM_00282	5756	Hs.189075	NM_00282	ENSG0000	TWF1	A6|PTK9	twinfilin ac	protein-co
lib-C666-l	chr7	99679513	99679513	+	22.02307	NA	promoter-TSS (NM_001318135)	promoter-TSS (NM_001318135)	-126	NM_00127	7551	Hs.435302	NM_01771	ENSG0000	ZNF3	A8-51|HF.	zinc finger	protein-co
lib-C666-l	chr7	99680225	99680225	+	2.59032	NA	promoter-TSS (NM_001318135)	promoter-TSS (NM_001318135)	-54	NM_00127	7551	Hs.435302	NM_01771	ENSG0000	ZNF3	A8-51|HF.	zinc finger	protein-co
lib-C666-l	chr18	19180954	19180954	+	122.48815	NA	promoter-TSS (NM_052911)	promoter-TSS (NM_052911)	-261	NM_05291	114799	Hs.464733	NM_05291	ENSG0000	ESCO1	A930014I1	establishm	protein-co
lib-C666-l	chr11	12695883	12695883	+	117.58568	NA	promoter-TSS (NM_021961)	promoter-TSS (NM_021961)	-86	NM_02196	7003	Hs.655331	NM_02196	ENSG0000	TEAD1	AA|NTEF-1	TEA domai	protein-co
lib-C666-l	chr11	12746272	12746272	+	17.13943	NA	intron (NM_021961, intron 2 of 12)	intron (NM_021961, intron 2 of 12)	50303	NM_02196	7003	Hs.655331	NM_02196	ENSG0000	TEAD1	AA|NTEF-1	TEA domai	protein-co
lib-C666-l	chr11	12768440	12768440	+	6.71839	NA	intron (NM_021961, intron 2 of 12)	intron (NM_021961, intron 2 of 12)	72471	NM_02196	7003	Hs.655331	NM_02196	ENSG0000	TEAD1	AA|NTEF-1	TEA domai	protein-co
lib-C666-l	chr12	53718729	53718729	+	44.98312	NA	Intergenic	CpG	-3317	NM_01566	8086	Hs.369144	NM_01566	ENSG0000	AAAS	AAA|AAAS	aladin WD	protein-co
lib-C666-l	chr21	27335749	27335749	+	2.78587	NA	intron (NM_001136131, intron 9 of 1	intron (NM_001136131, intron 9 of	176959	NM_00113	351	Hs.434980	NM_00048	ENSG0000	APP	AAA|ABET	amyloid be	protein-co
lib-C666-l	chr7	34975682	34975682	+	9.10494	NA	intron (NM_015283, intron 21 of 21)	intron (NM_015283, intron 21 of 21	-101739	NR_01535	404744	Hs.487951	NM_20728	ENSG0000	NPSR1-AS	AAA1	NPSR1 ant	ncRNA
lib-C666-l	chr1	1407068	1407068	+	147.86101	NA	promoter-TSS (NM_031921)	promoter-TSS (NM_031921)	-67	NM_03192	83858	Hs.729021	NM_03192	ENSG0000	ATAD3B	AAA-TOB	ATPase fan	protein-co
lib-C666-l	chr8	18067539	18067539	+	15.6921	NA	promoter-TSS (NM_000662)	promoter-TSS (NM_000662)	-73	NM_00116	9	Hs.591847	NM_00066	ENSG0000	NAT1	AAC1|MN	N-acetyltra	protein-co
lib-C666-l	chr8	18305805	18305805	+	13.8811	NA	Intergenic	Intergenic	57050	NM_00001	10	Hs.2	NM_00001	ENSG0000	NAT2	AAC2|NAT	N-acetyltra	protein-co
lib-C666-l	chrX	1511131	1511131	+	12.12504	NA	promoter-TSS (NM_001636)	promoter-TSS (NM_001636)	-92	NM_00163	293	Hs.350927	NM_00163	ENSG0000	SLC25A6	AAC3|ANT	solute carr	protein-co
lib-C666-l	chr14	95065512	95065512	+	13.14211	NA	Intergenic	Intergenic	-13202	NM_00108	12	Hs.534293	NM_00108	ENSG0000	SERPINA3	AACT|ACT|	serpin fam	protein-co
lib-C666-l	chr12	49454617	49454617	+	28.88523	NA	Intergenic	CpG	-5510	NM_00348	8085	Hs.731384	NM_00348	ENSG0000	KMT2D	AAD10|AL	lysine meth	protein-co
lib-C666-l	chr2	191659340	191659340	+	6.82479	NA	Intergenic	MLT1G3|LTR|ERVL-MaLR	-86207	NM_00125	2744	Hs.116448	NM_01490	ENSG0000	GLS	AAD20|GA	glutaminas	protein-co
lib-C666-l	chr2	191709239	191709239	+	6.79473	NA	Intergenic	ERV3-16A3_I-int|LTR|ERVL	-36308	NM_00125	2744	Hs.116448	NM_01490	ENSG0000	GLS	AAD20|GA	glutaminas	protein-co
lib-C666-l	chr3	172409413	172409413	+	5.65398	NA	intron (NM_020792, intron 1 of 4)	L2b|LINE|L2	19595	NM_00114	57552	Hs.444099	NM_02079	ENSG0000	NCEH1	AADACL1|	neutral ch	protein-co
lib-C666-l	chr7	50652814	50652814	+	13.20416	NA	Intergenic	Intergenic	-19660	NM_00108	1644	Hs.359698	NM_00079	ENSG0000	DDC	AADC	dopa deca	protein-co
lib-C666-l	chr7	50700468	50700468	+	12.12723	NA	intron (NM_001001549, intron 4 of 1	L1PB4|LINE|L1	-67314	NM_00108	1644	Hs.359698	NM_00079	ENSG0000	DDC	AADC	dopa deca	protein-co
lib-C666-l	chr17	8151548	8151548	+	10.40942	NA	promoter-TSS (NR_046431)	promoter-TSS (NR_046431)	-135	NR_04643	80169	Hs.156055	NM_02509	ENSG0000	CTC1	AAF-132|A	CST telom	protein-co
lib-C666-l	chr10	105668247	105668247	+	47.72539	NA	intron (NM_024928, intron 3 of 9)	intron (NM_024928, intron 3 of 9)	9798	NM_02492	79991	Hs.62314	NM_02492	ENSG0000	STN1	AAF-44|A	STN1, CST	protein-co
lib-C666-l	chr22	41253004	41253004	+	161.51622	NA	promoter-TSS (NM_001278589)	promoter-TSS (NM_001278589)	8	NM_00127	6767	Hs.712713	NM_00393	ENSG0000	ST13	AAG2|FAM	suppressio	protein-co
lib-C666-l	chr22	41252490	41252490	+	7.76438	NA	promoter-TSS (NM_001204827)	promoter-TSS (NM_001204827)	522	NM_00127	6767	Hs.712713	NM_00393	ENSG0000	ST13	AAG2|FAM	suppressio	protein-co
lib-C666-l	chr8	62491887	62491887	+	4.90117	NA	intron (NM_004318, intron 15 of 24)	intron (NM_004318, intron 15 of 24	110521	NM_03246	444	Hs.332422	NM_00431	ENSG0000	ASPH	AAH|BAH|	aspartate	protein-co
lib-C666-l	chr7	151574450	151574450	+	48.6123	NA	promoter-TSS (NM_016203)	promoter-TSS (NM_016203)	-134	NM_01620	51422	Hs.647072	NM_01620	ENSG0000	PRKAG2	AAKG|AAK	protein kin	protein-co
lib-C666-l	chr2	220363524	220363524	+	5.34142	NA	promoter-TSS (NM_013335)	promoter-TSS (NM_013335)	-63	NM_01333	29926	Hs.27059	NM_01333	ENSG0000	GMPPA	AAMR	GDP-manr	protein-co
lib-C666-l	chr3	72496384	72496384	+	12.14307	NA	promoter-TSS (NM_012234)	promoter-TSS (NM_012234)	-610	NM_01223	23429	Hs.7910	NM_012234		RYBP	AAP1|APAI	RING1 and	protein-co
lib-C666-l	chr3	72496615	72496615	+	9.7234	NA	promoter-TSS (NM_012234)	promoter-TSS (NM_012234)	-841	NM_01223	23429	Hs.7910	NM_012234		RYBP	AAP1|APAI	RING1 and	protein-co
lib-C666-l	chr3	49711440	49711440	+	8.73012	NA	promoter-TSS (NM_001640)	promoter-TSS (NM_001640)	5	NM_00164	327	Hs.517969	NM_00164	ENSG0000	APEH	AARE|ACP	acylamino	protein-co
lib-C666-l	chr7	140372981	140372981	+	3.89624	NA	promoter-TSS (NM_052853)	promoter-TSS (NM_052853)	28	NM_05285	90956	Hs.744886	NM_05285	ENSG0000	ADCK2	AARF	aarF doma	protein-co
lib-C666-l	chr3	123354266	123354266	+	51.60221	NA	intron (NM_053026, intron 28 of 32)	intron (NM_053026, intron 28 of 32	-14812	NM_05303	4638	Hs.477375	NM_00596	ENSG0000	MYLK	AAT7|KRP|	myosin ligh	protein-co
lib-C666-l	chr7	97736190	97736190	+	99.32263	NA	promoter-TSS (NM_014916)	promoter-TSS (NM_014916)	-7	NM_01491	22853	Hs.444179	NM_01491	ENSG0000	LMTK2	AATYK2|B	lemur tyro	protein-co
lib-C666-l	chr1	228297016	228297016	+	284.34808	NA	promoter-TSS (NM_181441)	promoter-TSS (NM_181441)	-3	NM_18146	128308	Hs.63236	NM_18144	ENSG0000	MRPL55	AAVG5835	mitochond	protein-co
lib-C666-l	chr1	148605402	148605402	+	36.56565	NA	intron (NR_104217, intron 2 of 17).2	CpG	44555	NM_17363	284565	Hs.523572	NM_17363	ENSG0000	NBPF15	AB14|AG3|	neuroblast	protein-co
lib-C666-l	chr1	148555935	148555935	+	2.96591	NA	intron (NR_104217, intron 2 of 17).2	CpG	-2253	NM_00117	284565	Hs.523572	NM_17363	ENSG0000	NBPF15	AB14|AG3|	neuroblast	protein-co
lib-C666-l	chr16	70719555	70719555	+	4.39666	NA	intron (NM_138383, intron 1 of 14)	CpG	399	NM_13838	92154	Hs.432387	NM_13838	ENSG0000	MTSS1L	ABBA|ABB	MTSS1L, I-	protein-co
lib-C666-l	chr16	70731076	70731076	+	2.78587	NA	exon (NM_018052, exon 16 of 19)	exon (NM_018052, exon 16 of 19)	-11122	NM_13838	92154	Hs.432387	NM_13838	ENSG0000	MTSS1L	ABBA|ABB	MTSS1L, I-	protein-co
lib-C666-l	chr2	220083566	220083566	+	8.56376	NA	TTS (NM_001077198)	TTS (NM_001077198)	146	NM_00568	10058	Hs.107911	NM_00568	ENSG0000	ABCB6	ABC|ABC1	ATP bindin	protein-co
lib-C666-l	chr1	94547665	94547665	+	2.10911	NA	intron (NM_000350, intron 7 of 49)	intron (NM_000350, intron 7 of 49)	39040	NM_00035	24	Hs.416707	NM_00035	ENSG0000	ABCA4	ABC10|AB	ATP bindin	protein-co
lib-C666-l	chr8	101162841	101162841	+	96.33944	NA	promoter-TSS (NM_005034)	promoter-TSS (NM_005034)	2	NM_00503	5440	Hs.351475	NM_00503	ENSG0000	POLR2K	ABC10-alp	RNA polym	protein-co
lib-C666-l	chr17	67323229	67323229	+	4.73866	NA	promoter-TSS (NM_172232)	promoter-TSS (NM_172232)	94	NM_17223	23461	Hs.731824	NM_01867	ENSG0000	ABCA5	ABC13|EST	ATP bindin	protein-co
lib-C666-l	chr2	169868877	169868877	+	6.56784	NA	intron (NM_003742, intron 5 of 27)	intron (NM_003742, intron 5 of 27)	18956	NM_00374	8647	Hs.658439	NM_00374	ENSG0000	ABCB11	ABC16|BR	ATP bindin	protein-co
lib-C666-l	chr6	32821825	32821825	+	41.43555	NA	promoter-TSS (NM_002800).2	promoter-TSS (NM_002800).2	-77	NM_00059	6890	Hs.352018	NM_00059	ENSG0000	TAP1	ABC17|AB	transporte	protein-co
lib-C666-l	chr9	139923390	139923390	+	20.7902	NA	promoter-TSS (NM_212533)	promoter-TSS (NM_212533)	-16	NM_21253	20	Hs.421202	NM_00160	ENSG0000	ABCA2	ABC2	ATP bindin	protein-co
lib-C666-l	chr6	30539189	30539189	+	5.5166	NA	promoter-TSS (NM_001025091)	promoter-TSS (NM_001025091)	19	NM_00102	23	Hs.655285	NM_00109	ENSG0000	ABCF1	ABC27|AB	ATP bindin	protein-co
lib-C666-l	chr7	150924439	150924439	+	226.37967	NA	promoter-TSS (NM_007189)	promoter-TSS (NM_007189)	21	NM_00569	10061	Hs.654958	NM_00569	ENSG0000	ABCF2	ABC28|EST	ATP bindin	protein-co
lib-C666-l	chr16	16119460	16119460	+	4.46944	NA	intron (NM_004996, intron 5 of 30)	intron (NM_004996, intron 5 of 30)	76026	NM_00499	4363	Hs.391464	NM_00499	ENSG0000	ABCC1	ABC29|AB	ATP bindin	protein-co
lib-C666-l	chr10	101537843	101537843	+	40.51821	NA	Intergenic	MER1B|DNA|hAT-Charlie	-4512	NM_00039	1244	Hs.368243	NM_00039	ENSG0000	ABCC2	ABC30|CM	ATP bindin	protein-co
lib-C666-l	chr10	101539812	101539812	+	11.77301	NA	Intergenic	L1MCa|LINE|L1	-2543	NM_00039	1244	Hs.368243	NM_00039	ENSG0000	ABCC2	ABC30|CM	ATP bindin	protein-co
lib-C666-l	chr21	43653837	43653837	+	8.59372	NA	intron (NM_207174, intron 2 of 14)	intron (NM_207174, intron 2 of 14)	13829	NM_20717	9619	Hs.124649	NM_00491	ENSG0000	ABCG1	ABC8|WHI	ATP bindin	protein-co
lib-C666-l	chr21	43639125	43639125	+	7.09685	NA	promoter-TSS (NM_004915)	promoter-TSS (NM_004915)	-142	NM_00491	9619	Hs.124649	NM_00491	ENSG0000	ABCG1	ABC8|WHI	ATP bindin	protein-co
lib-C666-l	chr21	43654912	43654912	+	6.50091	NA	intron (NM_207174, intron 2 of 14)	CpG	14904	NM_20717	9619	Hs.124649	NM_00491	ENSG0000	ABCG1	ABC8|WHI	ATP bindin	protein-co
lib-C666-l	chr16	2390884	2390884	+	7.26874	NA	promoter-TSS (NM_001089)	promoter-TSS (NM_001089)	-39	NR_00357	650655	Hs.376281	NR_00357	ENSG0000	ABCA17P	ABCA17	ATP bindin	pseudo
lib-C666-l	chr19	1040016	1040016	+	3.76263	NA	promoter-TSS (NM_019112)	promoter-TSS (NM_019112)	-86	NM_01911	10347	Hs.134514	NM_01911	ENSG0000	ABCA7	ABCA-SSN	ATP bindin	protein-co
lib-C666-l	chr16	57413610	57413610	+	7.7703	NA	exon (NM_002996, exon 2 of 3)	exon (NM_002996, exon 2 of 3)	7237	NM_00130	6376	Hs.531668	NM_00299	ENSG0000	CX3CL1	ABCD-3|C	C-X3-C m	protein-co
lib-C666-l	chr12	109531282	109531282	+	18.98417	NA	promoter-TSS (NM_001205179)	promoter-TSS (NM_001205179)	11	NM_00114	121642	Hs.374458	NM_00100	ENSG0000	ALKBH2	ABH2	alkB homo	protein-co
lib-C666-l	chr11	43902294	43902294	+	6.15313	NA	promoter-TSS (NM_139178)	promoter-TSS (NM_139178)	-63	NM_13917	221120	Hs.720708	NM_13917	ENSG0000	ALKBH3	ABH3|DEP	alkB homo	protein-co
lib-C666-l	chr14	23067187	23067187	+	7.49163	NA	promoter-TSS (NM_022060)	promoter-TSS (NM_022060)	40	NM_02206	63874	Hs.445665	NM_02206	ENSG0000	ABHD4	ABH4	abhydrolas	protein-co
lib-C666-l	chr17	18061550	18061550	+	21.96185	NA	intron (NM_016239, intron 50 of 64)	intron (NM_016239, intron 50 of 64	-25317	NM_01775	54890	Hs.744130	NM_01775	ENSG0000	ALKBH5	ABH5|OFO	alkB homo	protein-co
lib-C666-l	chr17	18087179	18087179	+	13.46097	NA	5' UTR (NM_017758, exon 1 of 4)	5' UTR (NM_017758, exon 1 of 4)	312	NM_01775	54890	Hs.744130	NM_01775	ENSG0000	ALKBH5	ABH5|OFO	alkB homo	protein-co
lib-C666-l	chr17	18086504	18086504	+	10.79304	NA	promoter-TSS (NM_017758)	promoter-TSS (NM_017758)	-363	NM_01775	54890	Hs.744130	NM_01775	ENSG0000	ALKBH5	ABH5|OFO	alkB homo	protein-co
lib-C666-l	chr19	36505199	36505199	+	8.96177	NA	promoter-TSS (NM_001297701)	promoter-TSS (NM_001297701)	-54	NM_00129	84964	Hs.71941	NM_03287	ENSG0000	ALKBH6	ABH6	alkB homo	protein-co
lib-C666-l	chr20	25371710	25371710	+	11.77301	NA	promoter-TSS (NM_015600)	promoter-TSS (NM_015600)	-92	NM_01560	26090	Hs.441550	NM_01560	ENSG0000	ABHD12	ABHD12A|	abhydrolas	protein-co
lib-C666-l	chr20	25371281	25371281	+	8.0154	NA	exon (NM_015600, exon 1 of 13)	exon (NM_015600, exon 1 of 13)	337	NM_00104	26090	Hs.441550	NM_01560	ENSG0000	ABHD12	ABHD12A|	abhydrolas	protein-co
lib-C666-l	chr7	40174398	40174398	+	22.01132	NA	promoter-TSS (NM_001193311)	promoter-TSS (NM_001193311)	-147	NM_13870	136647	Hs.654989	NM_13870	ENSG0000	MPLKIP	ABHS|C7or	M-phase s	protein-co
lib-C666-l	chr10	27070138	27070138	+	6.82479	NA	intron (NM_001012750, intron 2 of 1	HERVL40-int|LTR|ERVL	79878	NM_00117	10006	Hs.508148	NM_00547	ENSG0000	ABI1	ABI-1|ABL	abl interac	protein-co
lib-C666-l	chr5	150460689	150460689	+	14.45701	NA	promoter-TSS (NM_001258454)	promoter-TSS (NM_001258454)	-40	NM_00125	10318	Hs.355141	NM_00605	ENSG0000	TNIP1	ABIN-1|N	TNFAIP3 in	protein-co
lib-C666-l	chr4	2748059	2748059	+	4.6774	NA	intron (NM_001161527, intron 2 of 5)	intron (NM_001161527, intron 2 of	9693	NM_00116	79155	Hs.368551	NM_02430	ENSG0000	TNIP2	ABIN2|FLIP	TNFAIP3 in	protein-co
lib-C666-l	chr7	142583521	142583521	+	18.1336	NA	promoter-TSS (NM_018646)	promoter-TSS (NM_018646)	-31	NM_01864	55503	Hs.302740	NM_01427	ENSG0000	TRPV6	ABP/ZF|CA	transient r	protein-co
lib-C666-l	chr4	83812464	83812464	+	14.34866	NA	promoter-TSS (NM_001318119)	promoter-TSS (NM_001318119)	-31	NM_00107	22872	Hs.370024	NM_01493	ENSG0000	SEC31A	ABP125|A	SEC31 hon	protein-co
lib-C666-l	chr3	58007353	58007353	+	8.96177	NA	intron (NM_001164317, intron 1 of 4	intron (NM_001164317, intron 1 of	13226	NM_00145	2317	Hs.476448	NM_00145	ENSG0000	FLNB	ABP-278|A	filamin B	protein-co
lib-C666-l	chr3	57945817	57945817	+	5.819	NA	Intergenic	Intergenic	-48310	NM_00116	2317	Hs.476448	NM_00145	ENSG0000	FLNB	ABP-278|A	filamin B	protein-co
lib-C666-l	chrX	153571330	153571330	+	7.09685	NA	Intergenic	CpG	31676	NM_00145	2316	Hs.195464	NM_00145	ENSG0000	FLNA	ABP-280|A	filamin A	protein-co
lib-C666-l	chrX	153600073	153600073	+	6.98764	NA	intron (NM_001110556, intron 1 of 4	CpG-28471	2933	NM_00145	2316	Hs.195464	NM_00145	ENSG0000	FLNA	ABP-280|A	filamin A	protein-co
lib-C666-l	chr1	32110297	32110297	+	437.79544	NA	promoter-TSS (NR_033688)	promoter-TSS (NR_033688)	-79	NR_03368	553115	Hs.470417	NM_01239	ENSG0000	PEF1	ABP32|PEF	penta-EF-	protein-co
lib-C666-l	chr1	32110650	32110650	+	198.45299	NA	promoter-TSS (NR_033688)	promoter-TSS (NR_033688)	188	NM_01239	553115	Hs.470417	NM_01239	ENSG0000	PEF1	ABP32|PEF	penta-EF-	protein-co
lib-C666-l	chr1	32135348	32135348	+	7.21092	NA	intron (NM_001856, intron 49 of 70)	intron (NM_001856, intron 49 of 70	-24510	NR_03368	553115	Hs.470417	NM_01239	ENSG0000	PEF1	ABP32|PEF	penta-EF-	protein-co
lib-C666-l	chr4	166128703	166128703	+	31.98049	NA	promoter-TSS (NM_007246)	promoter-TSS (NM_007246)	-67	NM_00724	11275	Hs.388668	NM_00724	ENSG0000	KLHL2	ABP-KELC	kelch like f	protein-co
lib-C666-l	chr10	126490482	126490482	+	16.5633	NA	intron (NM_032182, intron 1 of 8)	CpG	128	NM_03218	23172	Hs.280695	NM_03218	ENSG0000	FAM175B	ABRO1|KIA	family with	protein-co
lib-C666-l	chr10	126559791	126559791	+	16.19708	NA	Intergenic	LTR16B1|LTR|ERVL	69437	NM_03218	23172	Hs.280695	NM_03218	ENSG0000	FAM175B	ABRO1|KIA	family with	protein-co
lib-C666-l	chr5	176944101	176944101	+	10.79304	NA	5' UTR (NM_001321732, exon 1 of 16	5' UTR (NM_001321732, exon 1 of	226	NM_00132	51428	Hs.484288	NM_01622	ENSG0000	DDX41	ABS|MPLP	DEAD-box	protein-co
lib-C666-l	chr11	34376553	34376553	+	27.64351	NA	intron (NM_145804, intron 1 of 16)	intron (NM_145804, intron 1 of 16)	3002	NM_14580	25841	Hs.23361	NM_14580	ENSG0000	ABTB2	ABTB2A|BT	ankyrin rep	protein-co
lib-C666-l	chr11	34296489	34296489	+	21.87022	NA	intron (NM_145804, intron 1 of 16)	intron (NM_145804, intron 1 of 16)	83066	NM_14580	25841	Hs.23361	NM_14580	ENSG0000	ABTB2	ABTB2A|BT	ankyrin rep	protein-co
lib-C666-l	chr11	34255781	34255781	+	18.74745	NA	intron (NM_145804, intron 1 of 16)	intron (NM_145804, intron 1 of 16)	123774	NM_14580	25841	Hs.23361	NM_14580	ENSG0000	ABTB2	ABTB2A|BT	ankyrin rep	protein-co
lib-C666-l	chr11	34379557	34379557	+	13.14211	NA	promoter-TSS (NM_145804)	promoter-TSS (NM_145804)	-2	NM_14580	25841	Hs.23361	NM_14580	ENSG0000	ABTB2	ABTB2A|BT	ankyrin rep	protein-co
lib-C666-l	chr11	34340095	34340095	+	9.7234	NA	intron (NM_145804, intron 1 of 16)	MIRb|SINE|MIR	39460	NM_14580	25841	Hs.23361	NM_14580	ENSG0000	ABTB2	ABTB2A|BT	ankyrin rep	protein-co
lib-C666-l	chr11	34416167	34416167	+	7.0832	NA	Intergenic	L1PB1|LINE|L1	-36612	NM_14580	25841	Hs.23361	NM_14580	ENSG0000	ABTB2	ABTB2A|BT	ankyrin rep	protein-co
lib-C666-l	chr8	17941692	17941692	+	7.42474	NA	promoter-TSS (NR_125430)	promoter-TSS (NR_125430)	187	NM_17792	427	Hs.527412	NM_00431	ENSG0000	ASAH1	AC|ACDas	N-acylsphi	protein-co
lib-C666-l	chr8	17942497	17942497	+	2.55248	NA	promoter-TSS (NM_177924)	promoter-TSS (NM_177924)	10	NM_00431	427	Hs.527412	NM_00431	ENSG0000	ASAH1	AC|ACDas	N-acylsphi	protein-co
lib-C666-l	chr8	132051311	132051311	+	4.46583	NA	intron (NM_001115, intron 1 of 17)	LTR78B|LTR|ERV1	1524	NM_00111	114	Hs.591859	NM_00111	ENSG0000	ADCY8	AC8|ADCY	adenylate	protein-co
lib-C666-l	chr14	103821295	103821295	+	20.90919	NA	Intergenic	ERV3-16A3_I-int|LTR|ERVL	17109	NR_00296	677811	Hs.658237	NR_00296	ENSG0000	SNORA28	ACA28	small nucle	snoRNA
lib-C666-l	chr12	121163683	121163683	+	117.21607	NA	5' UTR (NM_000017, exon 1 of 10)	5' UTR (NM_000017, exon 1 of 10)	142	NM_00001	35	Hs.507076	NM_00001	ENSG0000	ACADS	ACAD3|SC	acyl-CoA	protein-co
lib-C666-l	chr19	13001940	13001940	+	19.50061	NA	promoter-TSS (NM_013976)	promoter-TSS (NM_013976)	-3	NM_00015	2639	Hs.532699	NM_00015	ENSG0000	GCDH	ACAD5|GC	glutaryl-C	protein-co
lib-C666-l	chr17	7117941	7117941	+	17.71746	NA	intron (NR_135527, intron 3 of 20)	CpG	-2503	NM_00127	37	Hs.437178	NM_00001	ENSG0000	ACADVL	ACAD6|LC	acyl-CoA	protein-co
lib-C666-l	chr20	24998185	24998185	+	49.56857	NA	intron (NM_032501, intron 7 of 13)	intron (NM_032501, intron 7 of 13)	15157	NM_00125	84532	Hs.529353	NM_03250	ENSG0000	ACSS1	ACAS2L|A	acyl-CoA s	protein-co
lib-C666-l	chr20	25038753	25038753	+	15.31489	NA	promoter-TSS (NM_001252675)	promoter-TSS (NM_001252675)	65	NM_00125	84532	Hs.529353	NM_03250	ENSG0000	ACSS1	ACAS2L|A	acyl-CoA s	protein-co
lib-C666-l	chr20	25038050	25038050	+	12.73831	NA	intron (NM_032501, intron 1 of 13)	CpG	768	NM_00125	84532	Hs.529353	NM_03250	ENSG0000	ACSS1	ACAS2L|A	acyl-CoA s	protein-co
lib-C666-l	chrX	23761581	23761581	+	13.20416	NA	promoter-TSS (NM_001037171)	promoter-TSS (NM_001037171)	-174	NM_00103	23597	Hs.298885	NM_00103	ENSG0000	ACOT9	ACATE2|C	acyl-CoA t	protein-co
lib-C666-l	chr3	155572356	155572356	+	10.67459	NA	promoter-TSS (NM_001190992)	promoter-TSS (NM_001190992)	-108	NM_00473	9197	Hs.478031	NM_00473	ENSG0000	SLC33A1	ACATN|AT	solute carr	protein-co
lib-C666-l	chr10	43278432	43278432	+	13.48428	NA	intron (NM_014753, intron 1 of 22)	CpG	478	NM_01475	9790	Hs.10848	NM_01475	ENSG0000	BMS1	ACC|BMS1	BMS1, ribo	protein-co
lib-C666-l	chr15	79249473	79249473	+	9.93242	NA	Intergenic	LTR39|LTR|ERV1	-12033	NM_00439	1512	Hs.148641	NM_00439	ENSG0000	CTSH	ACC-4|AC	cathepsin	protein-co
lib-C666-l	chr1	27719107	27719107	+	44.27569	NA	promoter-TSS (NM_005281)	promoter-TSS (NM_005281)	-41	NM_00528	2827	Hs.66542	NM_00528	ENSG0000	GPR3	ACCA	G protein-	protein-co
lib-C666-l	chr15	34630281	34630281	+	10.03103	NA	promoter-TSS (NM_001042494)	promoter-TSS (NM_001042494)	-16	NM_00104	9990	Hs.510939	NM_00513	ENSG0000	SLC12A6	ACCPN|KC	solute carr	protein-co
lib-C666-l	chr1	202927436	202927436	+	32.53922	NA	promoter-TSS (NM_015999)	promoter-TSS (NM_015999)	88	NM_01599	51094	Hs.5298	NM_01599	ENSG0000	ADIPOR1	ACDCR1|C	adiponecti	protein-co
lib-C666-l	chr1	202926858	202926858	+	6.26151	NA	intron (NM_001290629, intron 1 of 8)	intron (NM_001290629, intron 1 of	254	NM_00129	51094	Hs.5298	NM_01599	ENSG0000	ADIPOR1	ACDCR1|C	adiponecti	protein-co
lib-C666-l	chr12	1800118	1800118	+	111.75975	NA	promoter-TSS (NM_024551)	promoter-TSS (NM_024551)	-129	NM_02455	79602	Hs.371642	NM_02455	ENSG0000	ADIPOR2	ACDCR2|P	adiponecti	protein-co
lib-C666-l	chr10	104678056	104678056	+	3.8557	NA	promoter-TSS (NM_199077)	promoter-TSS (NM_199077)	-19	NM_19907	54805	Hs.643509	NM_01764	ENSG0000	CNNM2	ACDP2|HC	cyclin and	protein-co
lib-C666-l	chr2	97430262	97430262	+	11.42528	NA	intron (NM_020184, intron 1 of 6)	intron (NM_020184, intron 1 of 6)	3623	NM_02018	26504	Hs.175043	NM_02018	ENSG0000	CNNM4	ACDP4	cyclin and	protein-co
lib-C666-l	chr14	35344138	35344138	+	13.0716	NA	intron (NM_013448, intron 1 of 26)	CpG-6938	715	NM_01344	11177	Hs.509140	NM_01344	ENSG0000	BAZ1A	ACF1|WAL	bromodom	protein-co
lib-C666-l	chr14	35343336	35343336	+	6.49644	NA	intron (NM_013448, intron 2 of 26)	CpG	1517	NM_01344	11177	Hs.509140	NM_01344	ENSG0000	BAZ1A	ACF1|WAL	bromodom	protein-co
lib-C666-l	chr14	35343811	35343811	+	3.59106	NA	5' UTR (NM_182648, exon 2 of 26)	5' UTR (NM_182648, exon 2 of 26)	1042	NM_01344	11177	Hs.509140	NM_01344	ENSG0000	BAZ1A	ACF1|WAL	bromodom	protein-co
lib-C666-l	chr6	33386118	33386118	+	12.72278	NA	promoter-TSS (NM_001014840)	promoter-TSS (NM_001014840)	-53	NM_00101	51596	Hs.520070	NM_01592	ENSG0000	CUTA	ACHAP|C6	cutA divale	protein-co
lib-C666-l	chr1	161736069	161736069	+	15.75122	NA	promoter-TSS (NM_007348)	promoter-TSS (NM_007348)	35	NM_00734	22926	Hs.492740	NM_00734	ENSG0000	ATF6	ACHM7|AT	activating	protein-co
lib-C666-l	chr1	161735005	161735005	+	10.0479	NA	Intergenic	Intergenic	-1029	NM_00734	22926	Hs.492740	NM_00734	ENSG0000	ATF6	ACHM7|AT	activating	protein-co
lib-C666-l	chr7	156686057	156686057	+	128.12206	NA	promoter-TSS (NM_022458)	promoter-TSS (NM_022458)	-155	NM_02245	64327	Hs.209989	NM_02245	ENSG0000	LMBR1	ACHP|C7o	limb devel	protein-co
lib-C666-l	chr2	175657476	175657476	+	15.33659	NA	Intergenic	Intergenic	-28276	NM_00103	1134	Hs.434479	NM_00007	ENSG0000	CHRNA1	ACHRA|AC	cholinergic	protein-co
lib-C666-l	chr19	50354356	50354356	+	43.7029	NA	promoter-TSS (NM_001305105)	promoter-TSS (NM_001305105)	-21	NM_00130	53635	Hs.587979	NM_01743	ENSG0000	PTOV1	ACID2|PTC	prostate tu	protein-co
lib-C666-l	chr19	50354015	50354015	+	24.23745	NA	promoter-TSS (NM_001305105)	promoter-TSS (NM_001305105)	23	NM_00130	53635	Hs.587979	NM_01743	ENSG0000	PTOV1	ACID2|PTC	prostate tu	protein-co
lib-C666-l	chr2	25142643	25142643	+	9.93242	NA	5' UTR (NM_001320613, exon 1 of 22	5' UTR (NM_001320613, exon 1 of	243	NM_00403	109	Hs.467898	NM_00403	ENSG0000	ADCY3	AC-III|AC3	adenylate	protein-co
lib-C666-l	chr3	195642042	195642042	+	16.42352	NA	Intergenic	Intergenic	-6162	NM_00578	10188	Hs.518513	NM_00578	ENSG0000	TNK2	ACK|ACK-	tyrosine ki	protein-co
lib-C666-l	chr17	40075330	40075330	+	12.41959	NA	promoter-TSS (NM_001303274)	promoter-TSS (NM_001303274)	-55	NM_00130	47	Hs.387567	NM_00109	ENSG0000	ACLY	ACL|ATPCL	ATP citrate	protein-co
lib-C666-l	chr7	42277316	42277316	+	6.49882	NA	promoter-TSS (NM_000168)	promoter-TSS (NM_000168)	-698	NM_00016	2737	Hs.21509	NM_00016	ENSG0000	GLI3	ACLS|GCP	GLI family	protein-co
lib-C666-l	chr22	29999455	29999455	+	17.25993	NA	promoter-TSS (NM_181830)	promoter-TSS (NM_181830)	-90	NM_18182	4771	Hs.187898	NM_00026	ENSG0000	NF2	ACN|BANF	neurofibro	protein-co
lib-C666-l	chr19	51339382	51339382	+	28.64047	NA	intron (NR_131203, intron 2 of 2)	CpG	-4603	NR_10227	55554	Hs.567535	NM_01750	ENSG0000	KLK15	ACO|HSRN	kallikrein r	protein-co
lib-C666-l	chr22	41865098	41865098	+	119.46529	NA	promoter-TSS (NM_001098)	promoter-TSS (NM_001098)	-31	NM_00109	50	Hs.643610	NM_00109	ENSG0000	ACO2	ACONM|H	aconitase 2	protein-co
lib-C666-l	chr9	32384793	32384793	+	10.92275	NA	intron (NM_002197, intron 1 of 20)	CpG	192	NM_00127	48	Hs.567229	NM_00219	ENSG0000	ACO1	ACONS|HE	aconitase 1	protein-co
lib-C666-l	chr16	23607693	23607693	+	9.7234	NA	promoter-TSS (NM_005003)	promoter-TSS (NM_005003)	-54	NM_00500	4706	Hs.189716	NM_00500	ENSG0000	NDUFAB1	ACP|FASN	NADH:ubic	protein-co
lib-C666-l	chr15	65282225	65282225	+	3.20885	NA	promoter-TSS (NM_001127889)	promoter-TSS (NM_001127889)	59	NM_00112	51324	Hs.242458	NM_01663	ENSG0000	SPG21	ACP33|BM	spastic par	protein-co
lib-C666-l	chr13	96329293	96329293	+	13.6111	NA	promoter-TSS (NR_132117)	promoter-TSS (NR_132117)	-100	NM_00626	5611	Hs.59214	NM_00626	ENSG0000	DNAJC3	ACPHD|ER	DnaJ heat	protein-co
lib-C666-l	chr7	6388618	6388618	+	44.98312	NA	promoter-TSS (NM_001037163)	promoter-TSS (NM_001037163)	-28	NM_00103	84792	Hs.696146	NM_00103	ENSG0000	FAM220A	ACPIN1|C7	family with	protein-co
lib-C666-l	chr3	140950689	140950689	+	2.58799	NA	promoter-TSS (NM_001037172)	promoter-TSS (NM_001037172)	22	NM_00128	92370	Hs.657887	NM_15228	ENSG0000	PXYLP1	ACPL2|HEL	2-phospho	protein-co
lib-C666-l	chr11	64085761	64085761	+	2.58854	NA	promoter-TSS (NM_001286082)	promoter-TSS (NM_001286082)	201	NM_18165	25824	Hs.502823	NM_01209	ENSG0000	PRDX5	ACR1|AOE	peroxiredo	protein-co
lib-C666-l	chr17	66508618	66508618	+	16.264	NA	promoter-TSS (NM_001276289)	promoter-TSS (NM_001276289)	98	NM_00127	5573	Hs.280342	NM_00273	ENSG0000	PRKAR1A	ACRDYS1|	protein kin	protein-co
lib-C666-l	chr5	58599212	58599212	+	5.14484	NA	intron (NM_001165899, intron 3 of 1	L1PB1|LINE|L1	-27267	NM_00119	5144	Hs.117545	NM_00620	ENSG0000	PDE4D	ACRDYS2|	phosphodi	protein-co
lib-C666-l	chr13	76112229	76112229	+	70.69921	NA	promoter-TSS (NM_001287393)	promoter-TSS (NM_001287393)	-221	NM_00128	170622	Hs.508266	NM_20349	ENSG0000	COMMD6	Acrg	COMM do	protein-co
lib-C666-l	chr17	4870368	4870368	+	4.61604	NA	TTS (NM_001171168)	TTS (NM_001171168)	764	NM_00489	9552	Hs.90436	NM_00489	ENSG0000	SPAG7	ACRP|FSA-	sperm asso	protein-co
lib-C666-l	chr2	223725692	223725692	+	8.89416	NA	promoter-TSS (NM_203372)	promoter-TSS (NM_203372)	-40	NM_00445	2181	Hs.655772	NM_00445	ENSG0000	ACSL3	ACS3|FACL	acyl-CoA s	protein-co
lib-C666-l	chrX	108976680	108976680	+	10.37767	NA	promoter-TSS (NM_004458)	promoter-TSS (NM_004458)	-48	NM_00131	2182	Hs.268785	NM_00445	ENSG0000	ACSL4	ACS4|FACL	acyl-CoA s	protein-co

lib-C666-l	chr12	125534309	125534309	+	11.48337	NA	Intergenic	CpG	-15604	NM_02392	65985	Hs.656073	NM_02392	ENSG0000	AACS	ACSF1|SUF	acetoacety	protein-co
lib-C666-l	chr4	57253676	57253676	+	361.72162	NA	promoter-TSS (NM_001286668)	promoter-TSS (NM_001286668)	-2	NM_00128	132949	Hs.104347	NM_18180	ENSG0000	AASDH	ACSF4|LYS	aminoadip	protein-co
lib-C666-l	chr1	153748545	153748545	+	6.94566	NA	exon (NM_001317929, exon 1 of 10)	exon (NM_001317929, exon 1 of 10	777	NM_02433	11000	Hs.438723	NM_02433	ENSG0000	SLC27A3	ACSVL3|FA	solute carr	protein-co
lib-C666-l	chr9	131102761	131102761	+	11.86993	NA	promoter-TSS (NM_005094)	promoter-TSS (NM_005094)	-78	NM_00509	10999	Hs.656699	NM_00509	ENSG0000	SLC27A4	ACSVL4|FA	solute carr	protein-co
lib-C666-l	chr17	79481845	79481845	+	37.42856	NA	Intergenic	CpG-11343	-1953	NM_00161	71	Hs.514581	NM_00161	ENSG0000	ACTG1	ACT|ACTG|	actin gamr	protein-co
lib-C666-l	chr17	34476460	34476460	+	46.07372	NA	Intergenic	Intergenic	45240	NM_00298	6351	Hs.75703	NM_00298	ENSG0000	CCL4	ACT2|AT74	C-C motif	protein-co
lib-C666-l	chr20	17550559	17550559	+	5.819	NA	promoter-TSS (NM_006870)	promoter-TSS (NM_006870)	-40	NM_00101	11034	Hs.304192	NM_00687	ENSG0000	DSTN	ACTDP|AD	destrin, act	protein-co
lib-C666-l	chr12	69229508	69229508	+	10.73451	NA	intron (NM_001145339, intron 6 of 8)	intron (NM_001145339, intron 6 of	26711	NM_00127	4193	Hs.484551	NM_00239	ENSG0000	MDM2	ACTFS|HDI	MDM2 prc	protein-co
lib-C666-l	chr19	39174563	39174563	+	40.29868	NA	intron (NM_001322033, intron 1 of 2(	intron (NM_001322033, intron 1 of	36307	NM_00132	81	Hs.270291	NM_00492	ENSG0000	ACTN4	ACTININ-4	actinin alpl	protein-co
lib-C666-l	chr19	39157907	39157907	+	8.03539	NA	intron (NM_001322033, intron 1 of 2(	intron (NM_001322033, intron 1 of	19651	NM_00132	81	Hs.270291	NM_00492	ENSG0000	ACTN4	ACTININ-4	actinin alpl	protein-co
lib-C666-l	chr3	179280638	179280638	+	26.72399	NA	promoter-TSS (NM_178042)	promoter-TSS (NM_178042)	-30	NM_00430	86	Hs.435326	NM_00430	ENSG0000	ACTL6A	ACTL6|ARF	actin like 6	protein-co
lib-C666-l	chr14	58666818	58666818	+	4.46944	NA	promoter-TSS (NM_018477)	promoter-TSS (NM_018477)	-15	NM_01847	55860	Hs.509451	NM_01847	ENSG0000	ACTR10	ACTR11|Ar	actin-relat	protein-co
lib-C666-l	chr3	52017318	52017318	+	5.12426	NA	promoter-TSS (NM_000666)	promoter-TSS (NM_000666)	18	NM_00066	95	Hs.334707	NM_00066	ENSG0000	ACY1	ACY-1|AC\	aminoacyl	protein-co
lib-C666-l	chr2	54198191	54198191	+	18.43022	NA	promoter-TSS (NM_001320587)	promoter-TSS (NM_001320587)	-49	NM_00132	98	Hs.516173	NM_13844	ENSG0000	ACYP2	ACYM|ACY	acylphospl	protein-co
lib-C666-l	chr2	54342832	54342832	+	3.7184	NA	5' UTR (NM_138448, exon 1 of 4)	5' UTR (NM_138448, exon 1 of 4)	422	NM_00132	98	Hs.516173	NM_13844	ENSG0000	ACYP2	ACYM|ACY	acylphospl	protein-co
lib-C666-l	chr9	132388463	132388463	+	36.16835	NA	promoter-TSS (NM_001286798)	promoter-TSS (NM_001286798)	31	NM_00128	28989	Hs.744027	NM_01406	ENSG0000	NTMT1	AD-003|C9	N-termina	protein-co
lib-C666-l	chr9	132319812	132319812	+	12.55817	NA	Intergenic	Intergenic	-51351	NM_00128	28989	Hs.744027	NM_01406	ENSG0000	NTMT1	AD-003|C9	N-termina	protein-co
lib-C666-l	chr16	58664438	58664438	+	25.49921	NA	promoter-TSS (NM_016284)	promoter-TSS (NM_016284)	-648	NM_01628	23019	Hs.460923	NM_01628	ENSG0000	CNOT1	AD-005|CD	CCR4-NOT	protein-co
lib-C666-l	chr16	58663876	58663876	+	8.59372	NA	promoter-TSS (NM_016284)	promoter-TSS (NM_016284)	-86	NM_01628	23019	Hs.460923	NM_01628	ENSG0000	CNOT1	AD-005|CD	CCR4-NOT	protein-co
lib-C666-l	chr16	53133797	53133797	+	5.64185	NA	intron (NM_025134, intron 1 of 38)	intron (NM_025134, intron 1 of 38)	44852	NM_02513	80205	Hs.59159	NM_01528	ENSG0000	CHD9	AD013|CHI	chromodo	protein-co
lib-C666-l	chr16	53164554	53164554	+	4.64451	NA	intron (NM_025134, intron 1 of 38)	CpG	-25284	NM_00130	80205	Hs.59159	NM_01528	ENSG0000	CHD9	AD013|CHI	chromodo	protein-co
lib-C666-l	chr15	101817746	101817746	+	30.07415	NA	promoter-TSS (NM_018445)	promoter-TSS (NM_018445)	-21	NM_20347	55829	Hs.32148	NM_01844	ENSG0000	SELENOS	AD-015|AD	selenoprot	protein-co
lib-C666-l	chr3	52739981	52739981	+	13.46097	NA	5' UTR (NM_001010983, exon 1 of 11	5' UTR (NM_001010983, exon 1 of 1	118	NM_01844	55830	Hs.297304	NM_01844	ENSG0000	GLT8D1	AD-017|M	glycosyltra	protein-co
lib-C666-l	chr1	109633458	109633458	+	8.28613	NA	promoter-TSS (NM_001322248)	promoter-TSS (NM_001322248)	55	NM_02014	56900	Hs.82933	NM_02014	ENSG0000	TMEM167B	AD-020|C1	transmemb	protein-co
lib-C666-l	chr17	73266640	73266640	+	18.89222	NA	promoter-TSS (NM_001242500)	promoter-TSS (NM_001242500)	-314	NM_00124	57409	Hs.325631	NM_02067	ENSG0000	MIF4GD	AD023|MIF	MIF4G dor	protein-co
lib-C666-l	chr13	45563648	45563648	+	153.97813	NA	promoter-TSS (NM_012345)	promoter-TSS (NM_012345)	-17	NM_00131	55425	Hs.731811	NM_01855	ENSG0000	GPALPP1	AD029|KIA	GPALPP m	protein-co
lib-C666-l	chr10	96122832	96122832	+	18.98417	NA	promoter-TSS (NM_022451)	promoter-TSS (NM_022451)	-99	NM_02245	64318	Hs.74899	NM_02245	ENSG0000	NOC3L	AD24|C10c	NOC3 like	protein-co
lib-C666-l	chr14	73603166	73603166	+	39.51209	NA	promoter-TSS (NM_000021)	promoter-TSS (NM_000021)	23	NM_00002	5663	Hs.3260	NM_00002	ENSG0000	PSEN1	AD3|FAD|P	presenilin	protein-co
lib-C666-l	chr2	101730332	101730332	+	23.4136	NA	intron (NR_138475, intron 1 of 18)	intron (NR_138475, intron 1 of 18)	37577	NM_00110	11138	Hs.442657	NM_00706	ENSG0000	TBC1D8	AD3|GRAN	TBC1 dom	protein-co
lib-C666-l	chr7	87563480	87563480	+	12.51077	NA	promoter-TSS (NM_001324417)	promoter-TSS (NM_001324417)	22	NM_00419	53616	Hs.256398	NM_00419	ENSG0000	ADAM22	ADAM 22|I	ADAM met	protein-co
lib-C666-l	chr7	87555964	87555964	+	5.72511	NA	Intergenic	L1PB1|LINE|L1	-7494	NM_00132	53616	Hs.256398	NM_00419	ENSG0000	ADAM22	ADAM 22|I	ADAM met	protein-co
lib-C666-l	chr8	24050689	24050689	+	7.15277	NA	Intergenic	MER1B|DNA|hAT-Charlie	-100864	NM_01426	10863	Hs.174030	NM_01426	ENSG0000	ADAM28	ADAM 28|I	ADAM met	protein-co
lib-C666-l	chr10	128062819	128062819	+	15.60931	NA	intron (NM_003474, intron 1 of 22)	L1ME3C|LINE|L1	14308	NM_00347	8038	Hs.594351	NM_00347	ENSG0000	ADAM12	ADAM12-(	ADAM met	protein-co
lib-C666-l	chr2	9696218	9696218	+	3.27468	NA	promoter-TSS (NM_003183)	promoter-TSS (NM_003183)	-269	NM_00318	6868	Hs.404914	NM_00318	ENSG0000	ADAM17	ADAM18|C	ADAM met	protein-co
lib-C666-l	chr2	9696017	9696017	+	3.20885	NA	promoter-TSS (NM_003183)	promoter-TSS (NM_003183)	-68	NM_00318	6868	Hs.404914	NM_00318	ENSG0000	ADAM17	ADAM18|C	ADAM met	protein-co
lib-C666-l	chr19	8675480	8675480	+	16.60407	NA	intron (NM_030957, intron 1 of 25)	CpG	140	NM_03095	81794	Hs.657508	NM_03095	ENSG0000	ADAMTS1(	ADAM-TS:	ADAM met	protein-co
lib-C666-l	chr2	109335910	109335910	+	312.78098	NA	promoter-TSS (NM_006267)	promoter-TSS (NM_006267)	-27	NM_00626	5903	Hs.199561	NM_00626	ENSG0000	RANBP2	ADANE|AN	RAN bindir	protein-co
lib-C666-l	chr1	154600533	154600533	+	41.28836	NA	promoter-TSS (NM_001025107)	promoter-TSS (NM_001025107)	-77	NM_00102	103	Hs.12341	NM_00111	ENSG0000	ADAR	ADAR1|AG	adenosine	protein-co
lib-C666-l	chr21	46494266	46494266	+	12.69461	NA	promoter-TSS (NM_001112)	promoter-TSS (NM_001112)	-227	NR_14448	104	Hs.474018	NM_00111	ENSG0000	ADARB1	ADAR2|DR	adenosine	protein-co
lib-C666-l	chr10	1758364	1758364	+	47.88828	NA	intron (NM_018702, intron 1 of 9)	intron (NM_018702, intron 1 of 9)	21306	NM_01870	105	Hs.586663	NM_01870	ENSG0000	ADARB2	ADAR3|REI	adenosine	protein-co
lib-C666-l	chr10	1779845	1779845	+	11.66012	NA	promoter-TSS (NM_018702)	promoter-TSS (NM_018702)	-175	NM_01870	105	Hs.586663	NM_01870	ENSG0000	ADARB2	ADAR3|REI	adenosine	protein-co
lib-C666-l	chr1	33593042	33593042	+	7.0832	NA	Intergenic	CpG	45263	NM_00130	113451	Hs.101807	NM_05299	ENSG0000	AZIN2	ADC|AZI2|	antizyme ir	protein-co
lib-C666-l	chr15	100106048	100106048	+	6.04359	NA	promoter-TSS (NM_005587)	promoter-TSS (NM_005587)	-85	NM_00558	4205	Hs.268675	NM_00558	ENSG0000	MEF2A	ADCAD1|R	myocyte er	protein-co
lib-C666-l	chr12	12420099	12420099	+	19.82221	NA	promoter-TSS (NM_002336)	promoter-TSS (NM_002336)	-288	NM_00233	4040	Hs.584775	NM_00233	ENSG0000	LRP6	ADCAD2|S	LDL recept	protein-co
lib-C666-l	chr3	63898248	63898248	+	13.84327	NA	intron (NM_000333, intron 2 of 12)	CpG	14173	NM_00117	6314	Hs.476595	NM_00033	ENSG0000	ATXN7	ADCAII|OP	ataxin 7	protein-co
lib-C666-l	chr3	63898025	63898025	+	5.04179	NA	intron (NM_000333, intron 2 of 12)	CpG	13950	NM_00117	6314	Hs.476595	NM_00033	ENSG0000	ATXN7	ADCAII|OP	ataxin 7	protein-co
lib-C666-l	chr19	41222801	41222801	+	28.88523	NA	promoter-TSS (NM_024876)	promoter-TSS (NM_024876)	-11	NM_02487	79934	Hs.130712	NM_02487	ENSG0000	COQ8B	ADCK4|NP	coenzyme	protein-co
lib-C666-l	chr10	111721516	111721516	+	51.32788	NA	intron (NR_038943, intron 2 of 4)	intron (NR_038943, intron 2 of 4)	-34592	NM_00132	120	Hs.501012	NM_00112	ENSG0000	ADD3	ADDL|CPS(	adducin 3	protein-co
lib-C666-l	chr4	95128792	95128792	+	56.28537	NA	promoter-TSS (NM_001128430)	promoter-TSS (NM_001128430)	33	NM_00112	56916	Hs.410406	NM_02015	ENSG0000	SMARCAD	ADERM|ET	SWI/SNF-r	protein-co
lib-C666-l	chr9	124048634	124048634	+	15.22999	NA	promoter-TSS (NM_001258030)	promoter-TSS (NM_001258030)	-224	NM_00125	2934	Hs.522373	NM_00017	ENSG0000	GSN	ADF|AGEL	gelsolin	protein-co
lib-C666-l	chr22	46941418	46941418	+	24.23241	NA	Intergenic	Intergenic	-8351	NM_01424	9620	Hs.252387	NM_01424	ENSG0000	CELSR1	ADGRC1|C	cadherin E	protein-co
lib-C666-l	chr8	67368599	67368599	+	11.6497	NA	intron (NM_144650, intron 10 of 13)	intron (NM_144650, intron 10 of 13	23881	NM_14465	137872	Hs.720023	NM_14465	ENSG0000	ADHFE1	ADH8|HMI	alcohol del	protein-co
lib-C666-l	chr1	85156141	85156141	+	5.64185	NA	promoter-TSS (NM_001166294)	promoter-TSS (NM_001166294)	10	NM_00116	117178	Hs.22587	NM_01402	ENSG0000	SSX2IP	ADIP|hMsc	SSX family	protein-co
lib-C666-l	chr19	860937	860937	+	2.62544	NA	exon (NM_001928, exon 3 of 5)	exon (NM_001928, exon 3 of 5)	1278	NM_00131	1675	Hs.155597	NM_00192	ENSG0000	CFD	ADIPSIN|A	compleme	protein-co
lib-C666-l	chr1	33502546	33502546	+	14.59636	NA	promoter-TSS (NM_001625)	promoter-TSS (NM_001625)	-34	NM_00131	204	Hs.470907	NM_00162	ENSG0000	AK2	ADK2	adenylate I	protein-co
lib-C666-l	chr5	126147737	126147737	+	8.94036	NA	intron (NM_005573, intron 5 of 10)	intron (NM_005573, intron 5 of 10)	34892	NM_00119	4001	Hs.89497	NM_00557	ENSG0000	LMNB1	ADLD|LMN	lamin B1	protein-co
lib-C666-l	chr5	126112669	126112669	+	8.59372	NA	promoter-TSS (NM_001198557)	promoter-TSS (NM_001198557)	-176	NM_00119	4001	Hs.89497	NM_00557	ENSG0000	LMNB1	ADLD|LMN	lamin B1	protein-co
lib-C666-l	chr5	126113369	126113369	+	7.37943	NA	exon (NM_005573, exon 1 of 11)	exon (NM_005573, exon 1 of 11)	524	NM_00119	4001	Hs.89497	NM_00557	ENSG0000	LMNB1	ADLD|LMN	lamin B1	protein-co
lib-C666-l	chr17	40540810	40540810	+	46.4068	NA	promoter-TSS (NM_213662)	promoter-TSS (NM_213662)	-297	NM_00315	6774	Hs.463059	NM_00315	ENSG0000	STAT3	ADMIO|AD	signal trans	protein-co
lib-C666-l	chr17	40539897	40539897	+	15.42216	NA	intron (NM_139276, intron 1 of 23)	CpG	508	NM_21366	6774	Hs.463059	NM_00315	ENSG0000	STAT3	ADMIO|AD	signal trans	protein-co
lib-C666-l	chr20	49548068	49548068	+	43.6853	NA	promoter-TSS (NM_001282531)	promoter-TSS (NM_001282531)	-318	NM_18144	23394	Hs.293736	NM_01533	ENSG0000	ADNP	ADNP1|HV	activity dep	protein-co
lib-C666-l	chr7	128991269	128991269	+	24.69873	NA	intron (NM_001130720, intron 1 of 1(	Charlie1|DNA|hAT-Charlie	-16695	NM_00113	23382	Hs.600789	NM_01532	ENSG0000	AHCYL2	ADOHCYA	adenosylh(	protein-co
lib-C666-l	chr7	129007832	129007832	+	4.38153	NA	promoter-TSS (NM_001130723)	promoter-TSS (NM_001130723)	-132	NM_00113	23382	Hs.600789	NM_01532	ENSG0000	AHCYL2	ADOHCYA	adenosylh(	protein-co
lib-C666-l	chr6	111195908	111195908	+	30.98434	NA	promoter-TSS (NR_109768)	promoter-TSS (NR_109768)	-79	NR_10976	262	Hs.159118	NM_00163	ENSG0000	AMD1	ADOMETD	adenosylm	protein-co
lib-C666-l	chr17	15824219	15824219	+	21.96185	NA	Intergenic	Intergenic	-24012	NM_00067	136	Hs.167046	NM_00067	ENSG0000	ADORA2B	ADORA2	adenosine	protein-co
lib-C666-l	chr1	27560954	27560954	+	13.91591	NA	promoter-TSS (NM_001276252)	promoter-TSS (NM_001276252)	-53	NM_00127	23038	Hs.469154	NM_01502	ENSG0000	WDTC1	ADP|DCAF	WD and te	protein-co
lib-C666-l	chr22	44320005	44320005	+	21.51366	NA	intron (NM_025225, intron 1 of 8)	CpG	386	NM_02522	80339	Hs.654800	NM_02522	ENSG0000	PNPLA3	ADPN|C22	patatin like	protein-co
lib-C666-l	chr1	226595756	226595756	+	17.97302	NA	promoter-TSS (NM_001618)	promoter-TSS (NM_001618)	45	NM_00161	142	Hs.177766	NM_00161	ENSG0000	PARP1	ADPRT|AD	poly(ADP-	protein-co
lib-C666-l	chr14	20811773	20811773	+	80.31864	NA	promoter-TSS (NM_005484)	promoter-TSS (NM_005484)	0	NM_00548	10038	Hs.409412	NM_00548	ENSG0000	PARP2	ADPRT2|AI	poly(ADP-	protein-co
lib-C666-l	chr2	32288498	32288498	+	12.55817	NA	promoter-TSS (NM_199436)	promoter-TSS (NM_199436)	-182	NM_01494	6683	Hs.468091	NM_01494	ENSG0000	SPAST	ADPSP|FSF	spastin	protein-co
lib-C666-l	chr5	159364797	159364797	+	24.19547	NA	intron (NM_000679, intron 1 of 1)	intron (NM_000679, intron 1 of 1)	21057	NM_00067	147	Hs.368632	NM_00067	ENSG0000	ADRA1B	ADRA1|ALI	adrenocep	protein-co
lib-C666-l	chr10	115786631	115786631	+	8.01457	NA	Intergenic	Intergenic	-17175	NM_00068	153	Hs.99913	NM_00068	ENSG0000	ADRB1	ADRB1R|B:	adrenocep	protein-co
lib-C666-l	chr22	26039798	26039798	+	3.48345	NA	intron (NM_005160, intron 2 of 20)	L2b|LINE|L2	78937	NM_00516	157	Hs.657494	NM_00516	ENSG0000	GRK3	ADRBK2|B/	G protein-	protein-co
lib-C666-l	chr6	165988411	165988411	+	10.03103	NA	intron (NM_001130690, intron 1 of 2:	intron (NM_001130690, intron 1 of	87177	NM_00666	10846	Hs.348762	NM_00666	ENSG0000	PDE10A	ADSD2|HS	phosphodi	protein-co
lib-C666-l	chr19	50270002	50270002	+	21.90047	NA	promoter-TSS (NM_130787)	promoter-TSS (NM_130787)	-178	NM_01420	160	Hs.467125	NM_01420	ENSG0000	AP2A1	ADTAA|AP	adaptor re	protein-co
lib-C666-l	chr22	29784595	29784595	+	7.49163	NA	promoter-TSS (NM_145730)	promoter-TSS (NM_145730)	-23	NM_00112	162	Hs.368794	NM_00112	ENSG0000	AP1B1	ADTB1|AP:	adaptor re	protein-co
lib-C666-l	chr17	33914283	33914283	+	373.67056	NA	promoter-TSS (NM_001282)	promoter-TSS (NM_001282)	1	NM_00128	163	Hs.514819	NM_00128	ENSG0000	AP2B1	ADTB2|AP:	adaptor re	protein-co
lib-C666-l	chr16	71842901	71842901	+	149.53001	NA	promoter-TSS (NM_001128)	promoter-TSS (NM_001128)	75	NM_00103	164	Hs.461253	NM_00112	ENSG0000	AP1G1	ADTG|CLA	adaptor re	protein-co
lib-C666-l	chr17	72869484	72869484	+	174.98105	NA	promoter-TSS (NM_001258012)	promoter-TSS (NM_001258012)	-328	NM_00411	2232	Hs.69745	NM_00411	ENSG0000	FDXR	ADXR	ferredoxin	protein-co
lib-C666-l	chr1	21736571	21736571	+	3.58839	NA	Intergenic	THE1B|LTR|ERVL-MaLR	-30012	NM_00125	84224	Hs.325422	NM_03226	ENSG0000	NBPF3	AE2	neuroblast	protein-co
lib-C666-l	chr7	150755260	150755260	+	143.63527	NA	promoter-TSS (NM_001199692)	promoter-TSS (NM_001199692)	-27	NM_00119	6522	Hs.647069	NM_00304	ENSG0000	SLC4A2	AE2|BND3I	solute carr	protein-co
lib-C666-l	chr2	220507286	220507286	+	14.75675	NA	TTS (NM_001326559)	TTS (NM_001326559)	14994	NR_04855	6508	Hs.1176	NM_00507	ENSG0000	SLC4A3	AE3|CAE3/	solute carr	protein-co
lib-C666-l	chr5	139743013	139743013	+	7.97818	NA	intron (NM_001258427, intron 7 of 2(	CpG	3226	NM_00125	83697	Hs.550313	NM_03146	ENSG0000	SLC4A9	AE4	solute carr	protein-co
lib-C666-l	chr14	93215051	93215051	+	8.89416	NA	promoter-TSS (NM_005606)	promoter-TSS (NM_005606)	-4	NM_00560	5641	Hs.18069	NM_00560	ENSG0000	LGMN	AEP|LGMN	legumain	protein-co
lib-C666-l	chr9	65718013	65718013	+	13.24366	NA	Intergenic	MIR3|SINE|MIR	-208403	NM_01566	26165	Hs.123004	NM_01566	ENSG0000	SPATA31A	AEP1|C9or	SPATA31 s	protein-co
lib-C666-l	chr21	42539816	42539816	+	9.02258	NA	promoter-TSS (NM_012105)	promoter-TSS (NM_012105)	88	NM_13899	25825	Hs.43047	NM_01210	ENSG0000	BACE2	AEPLC|ALP	beta-site A	protein-co
lib-C666-l	chr21	42540239	42540239	+	5.25516	NA	TTS (NR_036167)	TTS (NR_036167)	511	NM_13899	25825	Hs.43047	NM_01210	ENSG0000	BACE2	AEPLC|ALP	beta-site A	protein-co
lib-C666-l	chr21	34755605	34755605	+	8.27411	NA	Intergenic	Intergenic	-19597	NM_00553	3460	Hs.634632	NM_00553	ENSG0000	IFNGR2	AF-1|IFGR2	interferon	protein-co
lib-C666-l	chr10	21823166	21823166	+	14.98502	NA	promoter-TSS (NM_001195630)	promoter-TSS (NM_001195630)	65	NM_00464	8028	Hs.30385	NM_00464	ENSG0000	MLLT10	AF10	myeloid/ly	protein-co
lib-C666-l	chr15	40886139	40886139	+	10.67459	NA	promoter-TSS (NM_144508)	promoter-TSS (NM_144508)	-308	NM_14450	57082	Hs.181855	NM_14450	ENSG0000	KNL1	AF15Q14|(	kinetochor	protein-co
lib-C666-l	chr17	36862194	36862194	+	15.1031	NA	intron (NM_005937, intron 1 of 19)	intron (NM_005937, intron 1 of 19)	321	NM_00593	4302	Hs.91531	NM_00593	ENSG0000	MLLT6	AF17	MLLT6, PH	protein-co
lib-C666-l	chr17	75321311	75321311	+	6.49644	NA	intron (NM_006640, intron 1 of 10)	L1M4|LINE|L1	5714	NM_00664	10801	Hs.440932	NM_00664	ENSG0000	9-Sep	AF17q25|M	septin 9	protein-co
lib-C666-l	chr1	51887844	51887844	+	18.0551	NA	promoter-TSS (NM_001159969)	promoter-TSS (NM_001159969)	-51	NM_00115	2060	Hs.83722	NM_00198	ENSG0000	EPS15	AF-1P|AF1	epidermal	protein-co
lib-C666-l	chr4	87928239	87928239	+	9.53658	NA	promoter-TSS (NM_005935)	promoter-TSS (NM_005935)	86	NM_00593	4299	Hs.480190	NM_00593	ENSG0000	AFF1	AF4|MLLT2	AF4/FMR2	protein-co
lib-C666-l	chr5	132299510	132299510	+	5.12426	NA	promoter-TSS (NM_014423)	promoter-TSS (NM_014423)	-156	NM_01442	27125	Hs.519313	NM_01442	ENSG0000	AFF4	AF5Q31|CH	AF4/FMR2	protein-co
lib-C666-l	chr6	108879792	108879792	+	63.78558	NA	Intergenic	CpG	-1234	NM_20155	2309	Hs.220950	NM_00145	ENSG0000	FOXO3	AF6q21|FK	forkhead b	protein-co
lib-C666-l	chr9	124412588	124412588	+	53.02666	NA	intron (NM_032552, intron 1 of 16)	intron (NM_032552, intron 1 of 16)	83207	NM_03255	153090	Hs.522378	NM_03255	ENSG0000	DAB2IP	AF9Q34|AI	DAB2 inter	protein-co
lib-C666-l	chr9	124497589	124497589	+	29.21635	NA	intron (NM_032552, intron 3 of 16)	intron (NM_032552, intron 3 of 16)	-7475	NM_13870	153090	Hs.522378	NM_03255	ENSG0000	DAB2IP	AF9Q34|AI	DAB2 inter	protein-co
lib-C666-l	chr9	124650814	124650814	+	8.56376	NA	intron (NM_001139442, intron 6 of 8)	L2b|LINE|L2	145750	NM_13870	153090	Hs.522378	NM_03255	ENSG0000	DAB2IP	AF9Q34|AI	DAB2 inter	protein-co
lib-C666-l	chr9	124413884	124413884	+	6.13111	NA	intron (NM_032552, intron 1 of 16)	CpG	84503	NM_03255	153090	Hs.522378	NM_03255	ENSG0000	DAB2IP	AF9Q34|AI	DAB2 inter	protein-co
lib-C666-l	chr4	7973799	7973799	+	22.23727	NA	intron (NM_001130083, intron 20 of 2	intron (NM_001130083, intron 20 o	-32146	NM_00113	60312	Hs.529369	NM_02163	ENSG0000	AFAP1	AFAP|AFAF	actin filam	protein-co
lib-C666-l	chr2	85619748	85619748	+	8.73012	NA	TTS (NM_001135022)	TTS (NM_001135022)	17207	NM_00132	822	Hs.516155	NM_00174	ENSG0000	CAPG	AFCP|HEL-	capping ac	protein-co
lib-C666-l	chr1	149899664	149899664	+	8.28216	NA	TTS (NM_181873)	TTS (NM_181873)	480	NM_00585	10262	Hs.516160	NM_00585	ENSG0000	SF3B4	AFD1|Hsh4	splicing fac	protein-co
lib-C666-l	chr9	99179711	99179711	+	65.27523	NA	intron (NM_153695, intron 1 of 4)	CpG	958	NM_15369	195828	Hs.494557	NM_15369	ENSG0000	ZNF367	AFF29|CDC	zinc finger	protein-co
lib-C666-l	chr9	99180800	99180800	+	10.92275	NA	promoter-TSS (NM_153695)	promoter-TSS (NM_153695)	-131	NM_15369	195828	Hs.494557	NM_15369	ENSG0000	ZNF367	AFF29|CDC	zinc finger	protein-co
lib-C666-l	chr22	50765809	50765809	+	11.867	NA	promoter-TSS (NM_001001794)	promoter-TSS (NM_001001794)	-320	NM_00100	414918	Hs.530380	NM_00100	ENSG0000	DENND6B	AFI1B|FAM	DENN dor	protein-co
lib-C666-l	chr5	60628002	60628002	+	17.68706	NA	promoter-TSS (NM_020928)	promoter-TSS (NM_020928)	-98	NM_02092	57688	Hs.744939	NM_02092	ENSG0000	ZSWIM6	AFND	zinc finger	protein-co
lib-C666-l	chr5	60704621	60704621	+	4.90117	NA	intron (NM_020928, intron 1 of 13)	intron (NM_020928, intron 1 of 13)	76521	NM_02092	57688	Hs.744939	NM_02092	ENSG0000	ZSWIM6	AFND	zinc finger	protein-co
lib-C666-l	chr5	60626931	60626931	+	2.62544	NA	Intergenic	CpG	-1169	NM_02092	57688	Hs.744939	NM_02092	ENSG0000	ZSWIM6	AFND	zinc finger	protein-co
lib-C666-l	chr6	30181267	30181267	+	23.74671	NA	promoter-TSS (NM_003449)	promoter-TSS (NM_003449)	4	NM_00344	7726	Hs.485041	NM_00344	ENSG0000	TRIM26	AFP|RNF95	tripartite m	protein-co
lib-C666-l	chr8	123794612	123794612	+	5.97495	NA	intron (NM_014943, intron 1 of 3)	CpG-25975	711	NM_01494	22882	Hs.377090	NM_01494	ENSG0000	ZHX2	AFR1|RAF	zinc fingers	protein-co
lib-C666-l	chr8	123793840	123793840	+	4.04037	NA	promoter-TSS (NM_014943)	promoter-TSS (NM_014943)	-61	NM_01494	22882	Hs.377090	NM_01494	ENSG0000	ZHX2	AFR1|RAF	zinc fingers	protein-co
lib-C666-l	chr1	52521003	52521003	+	8.01457	NA	promoter-TSS (NM_152265)	promoter-TSS (NM_152265)	840	NR_04640	51060	Hs.476033	NM_01591	ENSG0000	TXNDC12	AG1|AGR1	thioredoxir	protein-co
lib-C666-l	chr2	236239900	236239900	+	6.3317	NA	Intergenic	L3|LINE|CR1	-162833	NM_00124	116987	Hs.435039	NM_01491	ENSG0000	AGAP1	AGAP-1|CI	ArfGAP wit	protein-co
lib-C666-l	chr7	150786013	150786013	+	7.59362	NA	intron (NM_001042535, intron 1 of 8)	L2|LINE|L2	2504	NM_00104	116988	Hs.647075	NM_03194	ENSG0000	AGAP3	AGAP-3|CI	ArfGAP wit	protein-co
lib-C666-l	chr2	112212130	112212130	+	13.74283	NA	intron (NR_024373, intron 1 of 1)	intron (NR_024373, intron 1 of 1)	40608	NR_02437	541471	Hs.560805	NR_01539	ENSG0000	MIR4435-2	AGD2|AK0(	MIR4435-2	ncRNA
lib-C666-l	chr1	20988013	20988013	+	3.27468	NA	promoter-TSS (NM_005216)	promoter-TSS (NM_005216)	24	NM_00521	1650	Hs.523145	NM_00521	ENSG0000	DDOST	AGER1|CD(	dolichyl-di	protein-co
lib-C666-l	chr19	11546396	11546396	+	22.23727	NA	promoter-TSS (NM_145045)	promoter-TSS (NM_145045)	-70	NM_00274	5589	Hs.610830	NM_00274	ENSG0000	PRKCSH	AGE-R2|G1	protein kin	protein-co
lib-C666-l	chr19	10207237	10207237	+	3.98718	NA	exon (NM_001321411, exon 2 of 6)	exon (NM_001321411, exon 2 of 6)	6096	NM_00132	83854	Hs.712850	NM_03191	ENSG0000	ANGPTL6	AGF|ARP5	angiopoiet	protein-co
lib-C666-l	chr7	140396491	140396491	+	55.3054	NA	promoter-TSS (NM_004546)	promoter-TSS (NM_004546)	10	NM_00454	4708	Hs.655788	NM_00454	ENSG0000	NDUFB2	AGGG|CI-/	NADH:ubiq	protein-co
lib-C666-l	chr9	131644373	131644373	+	84.30532	NA	promoter-TSS (NM_001127244)	promoter-TSS (NM_001127244)	-18	NM_01959	56262	Hs.643600	NM_01959	ENSG0000	LRRC8A	AGM5|LRR	leucine rich	protein-co
lib-C666-l	chr19	1652845	1652845	+	204.20467	NA	promoter-TSS (NM_003200)	promoter-TSS (NM_003200)	-517	NM_00320	6929	Hs.371282	NM_00320	ENSG0000	TCF3	AGM8|E2A	transcriptic	protein-co
lib-C666-l	chr1	170634168	170634168	+	6.82479	NA	intron (NM_006902, intron 1 of 4)	CpG	855	NM_02271	5396	Hs.283416	NM_00690	ENSG0000	PRRX1	AGOTC|PH	paired rela	protein-co
lib-C666-l	chr9	117050746	117050746	+	13.46097	NA	exon (NM_032888, exon 42 of 61)	exon (NM_032888, exon 42 of 61)	-34557	NM_00060	5004	Hs.522356	NM_00060	ENSG0000	ORM1	AGP-A|AG	orosomucc	protein-co
lib-C666-l	chr5	1522303	1522303	+	10.03284	NA	intron (NM_024830, intron 1 of 13)	intron (NM_024830, intron 1 of 13)	1789	NM_02483	79888	Hs.368853	NM_02483	ENSG0000	LPCAT1	AGPAT10|/	lysophospl	protein-co
lib-C666-l	chr19	10342299	10342299	+	29.83758	NA	promoter-TSS (NM_004230)	promoter-TSS (NM_004230)	-351	NM_00423	9294	Hs.655405	NM_00423	ENSG0000	S1PR2	AGR16|DFI	sphingosin	protein-co
lib-C666-l	chr19	10341774	10341774	+	4.30348	NA	TTS (NR_036206)	TTS (NR_036206)	174	NM_00423	9294	Hs.655405	NM_00423	ENSG0000	S1PR2	AGR16|DFI	sphingosin	protein-co
lib-C666-l	chr20	10654945	10654945	+	13.14211	NA	promoter-TSS (NM_000214)	promoter-TSS (NM_000214)	-251	NM_00021	182	Hs.224012	NM_00021	ENSG0000	JAG1	AGS|AGS1|	jagged 1	protein-co
lib-C666-l	chr17	17399833	17399833	+	7.21092	NA	promoter-TSS (NM_001199989)	promoter-TSS (NM_001199989)	-124	NM_01608	51655	Hs.25829	NM_01608	ENSG0000	RASD1	AGS1|DEXI	ras related	protein-co
lib-C666-l	chr13	51483951	51483951	+	82.44033	NA	5' UTR (NM_024570, exon 1 of 11)	5' UTR (NM_024570, exon 1 of 11)	137	NM_00114	79621	Hs.306291	NM_02457	ENSG0000	RNASEH2B	AGS2|DLEU	ribonuclea:	protein-co
lib-C666-l	chr1	120612251	120612251	+	14.51965	NA	promoter-TSS (NM_001200001)	promoter-TSS (NM_001200001)	66	NM_00120	4853	Hs.487360	NM_02440	ENSG0000	NOTCH2	AGS2|HJCY	notch 2	protein-co
lib-C666-l	chr11	65488159	65488159	+	11.07709	NA	exon (NM_032193, exon 1 of 4)	exon (NM_032193, exon 1 of 4)	250	NM_03219	84153	Hs.718438	NM_03219	ENSG0000	RNASEH2C	AGS3|AYP1	ribonuclea:	protein-co
lib-C666-l	chr6	32163704	32163704	+	67.6042	NA	promoter-TSS (NM_022107).4	promoter-TSS (NM_022107).4	-404	NM_02210	63940	Hs.520046	NM_02210	ENSG0000	GPSM3	AGS4|C6or	G-protein	protein-co
lib-C666-l	chr6	32164340	32164340	+	10.03103	NA	intron (NR_134950, intron 27 of 28).6	CpG	-1040	NM_02210	63940	Hs.520046	NM_02210	ENSG0000	GPSM3	AGS4|C6or	G-protein	protein-co
lib-C666-l	chr19	12917331	12917331	+	17.13943	NA	promoter-TSS (NM_006397)	promoter-TSS (NM_006397)	-97	NM_00639	10535	Hs.532851	NM_00639	ENSG0000	RNASEH2A	AGS4|JUNE	ribonuclea:	protein-co
lib-C666-l	chr4	178363408	178363408	+	10.19094	NA	exon (NM_000027, exon 1 of 9)	exon (NM_000027, exon 1 of 9)	249	NM_00117	175	Hs.207776	NM_00002	ENSG0000	AGA	AGU|ASRG	aspartylglu	protein-co
lib-C666-l	chr4	53578541	53578541	+	14.02822	NA	promoter-TSS (NR_039651)	promoter-TSS (NR_039651)	-20	NR_02403	57291	Hs.744077	NR_02403	ENSG0000	DANCR	AGU2|ANC	differentiat	ncRNA
lib-C666-l	chr1	162531277	162531277	+	14.70559	NA	promoter-TSS (NM_003115)	promoter-TSS (NM_003115)	-19	NM_00311	6675	Hs.492859	NM_00311	ENSG0000	UAP1	AGX|AGX1	UDP-N-ac	protein-co
lib-C666-l	chr4	109861951	109861951	+	19.23598	NA	intron (NM_001256074, intron 7 of 31	intron (NM_001256074, intron 7 of	-177716	NM_00114	64850	Hs.106576	NM_03127	ENSG0000	ETNPPL	AGXT2L1	ethanolam	protein-co
lib-C666-l	chr4	109861668	109861668	+	2.59723	NA	intron (NM_001256074, intron 8 of 31	intron (NM_001256074, intron 8 of	-177433	NM_00114	64850	Hs.106576	NM_03127	ENSG0000	ETNPPL	AGXT2L1	ethanolam	protein-co
lib-C666-l	chr3	185255449	185255449	+	48.67325	NA	intron (NM_139248, intron 1 of 9)	MIR3|SINE|MIR	14920	NM_13924	200879	Hs.68864	NM_13924	ENSG0000	LIPH	AH|ARWH2	lipase H	protein-co
lib-C666-l	chr2	61404254	61404254	+	7.49163	NA	promoter-TSS (NM_001321303)	promoter-TSS (NM_001321303)	-299	NM_00132	130872	Hs.655602	NM_15239	ENSG0000	AHSA2	AHA1|Hch1	activator o	protein-co
lib-C666-l	chr5	318630	318630	+	10.37767	NA	intron (NM_020731, intron 1 of 11)	intron (NM_020731, intron 1 of 11)	14339	NM_02073	57491	Hs.50823	NM_02073	ENSG0000	AHRR	AHH|AHHF	aryl-hydro	protein-co
lib-C666-l	chr6	135818910	135818910	+	50.46963	NA	promoter-TSS (NM_001134832)	promoter-TSS (NM_001134832)	-7	NM_00113	54806	Hs.386684	NM_01765	ENSG0000	AHI1	AHI-1|JBTS	Abelson he	protein-co
lib-C666-l	chr12	51419694	51419694	+	19.82221	NA	5' UTR (NM_001174129, exon 1 of 16	5' UTR (NM_001174129, exon 1 of 1	230	NM_00117	4891	Hs.505545	NM_00061	ENSG0000	SLC11A2	AHMIO1|D	solute carr	protein-co
lib-C666-l	chr2	119976265	119976265	+	24.32656	NA	Intergenic	Intergenic	-5119	NM_00100	55240	Hs.647822	NM_01823	ENSG0000	STEAP3	AHMIO2|S	STEAP3 me	protein-co
lib-C666-l	chr11	62314097	62314097	+	4.51266	NA	promoter-TSS (NM_001346446)	promoter-TSS (NM_001346446)	235	NM_00162	79026	Hs.502756	NM_00162	ENSG0000	AHNAK	AHNAKRS|	AHNAK nu	protein-co
lib-C666-l	chr20	57445982	57445982	+	29.90332	NA	intron (NR_126334, intron 3 of 4)	intron (NR_126334, intron 3 of 4)	-17486	NR_13227	2778	Hs.125898	NM_00051	ENSG0000	GNAS	AHO|C20o	GNAS com	protein-co
lib-C666-l	chr1	207925368	207925368	+	12.13776	NA	promoter-TSS (NM_172351)	promoter-TSS (NM_172351)	-15	NM_17235	4179	Hs.510402	NM_00238	ENSG0000	CD46	AHUS2|MC	CD46 mole	protein-co
lib-C666-l	chr1	209822696	209822696	+	10.79304	NA	intron (NM_001017402, intron 2 of 2:	intron (NM_001017402, intron 2 of	1983	NM_00101	3914	Hs.497636	NM_00022	ENSG0000	LAMB3	AI1A|BM6(	laminin sut	protein-co
lib-C666-l	chr1	209826583	209826583	+	8.95679	NA	promoter-TSS (NM_000228)	promoter-TSS (NM_000228)	-763	NM_00022	3914	Hs.497636	NM_00022	ENSG0000	LAMB3	AI1A|BM6(	laminin sut	protein-co
lib-C666-l	chr1	209825876	209825876	+	4.52748	NA	promoter-TSS (NM_000228)	promoter-TSS (NM_000228)	-56	NM_00022	3914	Hs.497636	NM_00022	ENSG0000	LAMB3	AI1A|BM6(	laminin sut	protein-co
lib-C666-l	chr4	71450299	71450299	+	2.31621	NA	Intergenic	Intergenic	-7676	NM_01651	258	Hs.272396	NM_01651	ENSG0000	AMBN	AI1F	ameloblast	protein-co
lib-C666-l	chr2	161061289	161061289	+	7.21092	NA	Intergenic	Intergenic	-4465	NM_00088	3694	Hs.470399	NM_00088	ENSG0000	ITGB6	AI1H	integrin su	protein-co
lib-C666-l	chr8	144815932	144815932	+	3.58499	NA	promoter-TSS (NR_039810)	promoter-TSS (NR_039810)	-18	NM_19848	286077	Hs.67776	NM_19848	ENSG0000	FAM83H	AI3	family with	protein-co
lib-C666-l	chr11	18343756	18343756	+	72.31497	NA	promoter-TSS (NM_001142307)	promoter-TSS (NM_001142307)	-35	NM_00721	11234	Hs.437599	NM_00721	ENSG0000	HPS5	AIBP63|BL(	HPS5, biog	protein-co
lib-C666-l	chr2	100106629	100106629	+	6.82479	NA	promoter-TSS (NM_001037872)	promoter-TSS (NM_001037872)	-111	NR_13565	51455	Hs.443077	NM_01631	ENSG0000	REV1	AIBP80|RE\	REV1, DNA	protein-co

lib-C666-l	chr2	100106091	100106091	+	3.17541	NA	intron (NR_135653, intron 1 of 21)	CpG	427	NR_135652	51455	Hs.443077	NM_01631	ENSG0000	REV1	AIBP80|REV	REV1, DNA	protein-co
lib-C666-l	chr6	143381725	143381725	+	178.43076	NA	promoter-TSS (NM_016108)	promoter-TSS (NM_016108)	-254	NM_00128	51390	Hs.567501	NM_01610	ENSG0000	AIG1	AIG-1|dJ95	androgen i	protein-co
lib-C666-l	chr6	11713314	11713314	+	36.56424	NA	TTS (NM_001143948)	TTS (NM_001143948)	65966	NM_00114	84830	Hs.126409	NM_03274	ENSG0000	ADTRP	AIG1L|C6o	androgen (	protein-co
lib-C666-l	chr6	11806984	11806984	+	23.74671	NA	Intergenic	Intergenic	-27704	NM_03274	84830	Hs.126409	NM_03274	ENSG0000	ADTRP	AIG1L|C6o	androgen (	protein-co
lib-C666-l	chr20	43150747	43150747	+	36.56424	NA	promoter-TSS (NM_198941)	promoter-TSS (NM_198941)	10	NM_00681	10955	Hs.272168	NM_00681	ENSG0000	SERINC3	AIGP1|DIFF	serine inco	protein-co
lib-C666-l	chr17	8113968	8113968	+	19.82221	NA	promoter-TSS (NR_132731)	promoter-TSS (NR_132731)	-24	NM_00128	9212	Hs.442658	NM_00421	ENSG0000	AURKB	AIK2|AIM-1	aurora kina	protein-co
lib-C666-l	chr16	31470705	31470705	+	46.59515	NA	5' UTR (NM_001105247, exon 1 of 6)	5' UTR (NM_001105247, exon 1 of 6	388	NM_00110	79798	Hs.732945	NM_02474	ENSG0000	ARMC5	AIMAH2	armadillo r	protein-co
lib-C666-l	chr16	31471486	31471486	+	25.39084	NA	intron (NM_024742, intron 1 of 3)	intron (NM_024742, intron 1 of 3)	1169	NM_00110	79798	Hs.732945	NM_02474	ENSG0000	ARMC5	AIMAH2	armadillo r	protein-co
lib-C666-l	chr17	38020513	38020513	+	3.20885	NA	promoter-TSS (NM_001284514)	promoter-TSS (NM_001284514)	-72	NM_00128	22806	Hs.371680	NM_01248	ENSG0000	IKZF3	AIO|AIOLC	IKAROS far	protein-co
lib-C666-l	chr1	1310900	1310900	+	40.09574	NA	promoter-TSS (NM_001127229)	promoter-TSS (NM_001127229)	-82	NM_01790	54998	Hs.632515	NM_01790	ENSG0000	AURKAIP1	AIP|AKIP|M	aurora kina	protein-co
lib-C666-l	chr11	102188188	102188188	+	8.0154	NA	promoter-TSS (NM_001165)	promoter-TSS (NM_001165)	7	NM_00116	330	Hs.127799	NM_00116	ENSG0000	BIRC3	AIP1|API2|(	baculoviral	protein-co
lib-C666-l	chr16	18813102	18813102	+	17.13943	NA	promoter-TSS (NM_001313858)	promoter-TSS (NM_001313858)	-102	NM_01516	23204	Hs.634882	NM_01516	ENSG0000	ARL6IP1	AIP1|ARL6I	ADP ribosy	protein-co
lib-C666-l	chr4	10118725	10118725	+	22.15977	NA	promoter-TSS (NM_005112)	promoter-TSS (NM_005112)	-152	NM_00511	9948	Hs.128548	NM_00511	ENSG0000	WDR1	AIP1|HEL-S	WD repeat	protein-co
lib-C666-l	chr8	87317833	87317833	+	14.34866	NA	Intergenic	Intergenic	-37161	NM_00701	11059	Hs.655189	NM_00701	ENSG0000	WWP1	AIP5|Tiul1|	WW doma	protein-co
lib-C666-l	chr2	153574918	153574918	+	19.88269	NA	promoter-TSS (NM_017892)	promoter-TSS (NM_017892)	511	NR_02452	151188	Hs.643580	NM_15252	ENSG0000	ARL6IP6	AIP-6|AIP6	ADP ribosy	protein-co
lib-C666-l	chr5	14664825	14664825	+	22.23727	NA	promoter-TSS (NM_138348)	promoter-TSS (NM_138348)	71	NM_13834	90268	Hs.406335	NM_13834	ENSG0000	OTULIN	AIPDS|FAN	OTU deubi	protein-co
lib-C666-l	chr9	37120572	37120572	+	11.07709	NA	promoter-TSS (NM_001289120)	promoter-TSS (NM_001289120)	-23	NM_00128	84186	Hs.654700	NM_03222	ENSG0000	ZCCHC7	AIR1|HSPC	zinc finger	protein-co
lib-C666-l	chr7	1199899	1199899	+	388.55606	NA	promoter-TSS (NR_110069)	promoter-TSS (NR_110069)	-44	NM_18249	90637	Hs.648111	NM_18249	ENSG0000	ZFAND2A	AIRAP	zinc finger	protein-co
lib-C666-l	chr2	220071489	220071489	+	43.79478	NA	promoter-TSS (NM_001270998)	promoter-TSS (NM_001270998)	-17	NM_13880	130617	Hs.534540	NM_13880	ENSG0000	ZFAND2B	AIRAPL	zinc finger	protein-co
lib-C666-l	chr21	34915239	34915239	+	18.82927	NA	promoter-TSS (NM_001291412)	promoter-TSS (NM_001291412)	-41	NM_00113	2618	Hs.473648	NM_00081	ENSG0000	GART	AIRS|GARS	phosphoril	protein-co
lib-C666-l	chr8	135724889	135724889	+	11.06679	NA	intron (NM_001289394, intron 1 of 1	CpG	403	NM_00117	57623	Hs.446172	NM_02086	ENSG0000	ZFAT	AITD3|ZFA	zinc finger	protein-co
lib-C666-l	chr8	135725301	135725301	+	7.21092	NA	promoter-TSS (NM_020863)	promoter-TSS (NM_020863)	-9	NM_00128	57623	Hs.446172	NM_02086	ENSG0000	ZFAT	AITD3|ZFA	zinc finger	protein-co
lib-C666-l	chr10	75936632	75936632	+	5.82086	NA	exon (NM_001202449, exon 1 of 10)	exon (NM_001202449, exon 1 of 10	385	NM_00120	132	Hs.656586	NM_00112	ENSG0000	ADK	AK	adenosine	protein-co
lib-C666-l	chr9	4741294	4741294	+	6.50091	NA	promoter-TSS (NM_016282)	promoter-TSS (NM_016282)	15	NM_01628	50808	Hs.732022	NM_01628	ENSG0000	AK3	AK3L1|AK6	adenylate l	protein-co
lib-C666-l	chr9	4740959	4740959	+	4.73866	NA	exon (NM_016282, exon 1 of 5)	exon (NM_016282, exon 1 of 5)	350	NM_01628	50808	Hs.732022	NM_01628	ENSG0000	AK3	AK3L1|AK6	adenylate l	protein-co
lib-C666-l	chr19	15490652	15490652	+	36.97678	NA	promoter-TSS (NM_005858)	promoter-TSS (NM_005858)	-40	NM_00585	10270	Hs.594496	NM_00585	ENSG0000	AKAP8	AKAP 95|A	A-kinase a	protein-co
lib-C666-l	chr17	55162552	55162552	+	15.31489	NA	promoter-TSS (NM_003488)	promoter-TSS (NM_003488)	-1	NM_00348	8165	Hs.463506	NM_00348	ENSG0000	AKAP1	AKAP|AKAI	A-kinase a	protein-co
lib-C666-l	chr17	55162915	55162915	+	4.51266	NA	promoter-TSS (NM_001242903)	promoter-TSS (NM_001242903)	-163	NM_00124	8165	Hs.463506	NM_00348	ENSG0000	AKAP1	AKAP|AKAI	A-kinase a	protein-co
lib-C666-l	chr9	112428225	112428225	+	14.0317	NA	intron (NM_001037293, intron 1 of 6)	L1PA8|LINE|L1	25157	NM_00103	114299	Hs.591908	NM_05301	ENSG0000	PALM2	AKAP2	paralemmi	protein-co
lib-C666-l	chr7	91570356	91570356	+	7.67555	NA	5' UTR (NM_147185, exon 1 of 50)	5' UTR (NM_147185, exon 1 of 50)	167	NM_14718	10142	Hs.651221	NM_00575	ENSG0000	AKAP9	AKAP-9|AK	A-kinase a	protein-co
lib-C666-l	chr7	91570032	91570032	+	4.73866	NA	promoter-TSS (NM_147185)	promoter-TSS (NM_147185)	-157	NM_00575	10142	Hs.651221	NM_00575	ENSG0000	AKAP9	AKAP-9|AK	A-kinase a	protein-co
lib-C666-l	chr7	44646130	44646130	+	199.05013	NA	promoter-TSS (NM_001165036)	promoter-TSS (NM_001165036)	9	NM_00100	4967	Hs.488181	NM_00254	ENSG0000	OGDH	AKGDH|E1	oxoglutara	protein-co
lib-C666-l	chr9	116163617	116163617	+	7.0832	NA	promoter-TSS (NM_000031)	promoter-TSS (NM_000031)	1	NM_00100	210	Hs.1227	NM_00003	ENSG0000	ALAD	ALADH|PB	aminolevul	protein-co
lib-C666-l	chr1	146714592	146714592	+	53.02666	NA	intron (NM_001256338, intron 1 of 1	CpG	301	NM_00125	9557	Hs.191164	NM_00428	ENSG0000	CHD1L	ALC1|CHD	chromodo	protein-co
lib-C666-l	chr6	150263111	150263111	+	17.13943	NA	promoter-TSS (NM_025217)	promoter-TSS (NM_025217)	37	NM_02521	80328	Hs.656778	NM_02521	ENSG0000	ULBP2	ALCAN-alp	UL16 bindi	protein-co
lib-C666-l	chr16	30076923	30076923	+	22.85132	NA	promoter-TSS (NM_001243177)	promoter-TSS (NM_001243177)	-71	NM_00124	226	Hs.513490	NM_00003	ENSG0000	ALDOA	ALDA|GSD	aldolase, fr	protein-co
lib-C666-l	chr16	30075637	30075637	+	8.11643	NA	promoter-TSS (NM_184043)	promoter-TSS (NM_184043)	37	NM_18404	226	Hs.513490	NM_00003	ENSG0000	ALDOA	ALDA|GSD	aldolase, fr	protein-co
lib-C666-l	chr16	30076574	30076574	+	3.08629	NA	promoter-TSS (NM_001243177)	promoter-TSS (NM_001243177)	-420	NM_00124	226	Hs.513490	NM_00003	ENSG0000	ALDOA	ALDA|GSD	aldolase, fr	protein-co
lib-C666-l	chr6	32812529	32812529	+	58.76075	NA	promoter-TSS (NM_148919).8	promoter-TSS (NM_148919).8	183	NM_00415	5696	Hs.180062	NM_00415	ENSG0000	PSMB8	ALDD|D6S	proteasom	protein-co
lib-C666-l	chr15	101300280	101300280	+	2.59723	NA	Intergenic	Intergenic	-119617	NM_00069	220	Hs.459538	NM_00069	ENSG0000	ALDH1A3	ALDH1A6|	aldehyde c	protein-co
lib-C666-l	chr12	34175268	34175268	+	6.82479	NA	promoter-TSS (NM_032834)	promoter-TSS (NM_032834)	52	NM_03283	84920	Hs.102971	NM_03283	ENSG0000	ALG10	ALG10A|DI	ALG10, alp	protein-co
lib-C666-l	chr12	34286343	34286343	+	4.19675	NA	Intergenic	L1PA4|LINE|L1	111127	NM_03283	84920	Hs.102971	NM_03283	ENSG0000	ALG10	ALG10A|DI	ALG10, alp	protein-co
lib-C666-l	chr22	23524936	23524936	+	8.91001	NA	intron (NM_021574, intron 1 of 21)	intron (NM_021574, intron 1 of 21)	2384	NM_00432	613	Hs.517461	NM_00432	ENSG0000	BCR	ALL|BCR1|(	BCR, RhoG	protein-co
lib-C666-l	chr11	118307013	118307013	+	20.59244	NA	promoter-TSS (NM_005933)	promoter-TSS (NM_005933)	-192	NM_00593	4297	Hs.258855	NM_00593	ENSG0000	KMT2A	ALL-1|CXX	lysine meth	protein-co
lib-C666-l	chr17	6782792	6782792	+	18.78674	NA	intron (NR_002710, intron 2 of 13)	HERVK9-int|LTR|ERVK	-14304	NR_12045	245	Hs.378695	NR_00271	ENSG0000	ALOX12P2	ALOX12E	arachidona	pseudo
lib-C666-l	chr4	186480858	186480858	+	29.01385	NA	Intergenic	L1PB1|LINE|L1	-24146	NM_00125	27295	Hs.701364	NM_01447	ENSG0000	PDLIM3	ALP	PDZ and LI	protein-co
lib-C666-l	chr4	186456619	186456619	+	7.1502	NA	promoter-TSS (NM_001114107)	promoter-TSS (NM_001114107)	93	NM_00125	27295	Hs.701364	NM_01447	ENSG0000	PDLIM3	ALP	PDZ and LI	protein-co
lib-C666-l	chr11	34127105	34127105	+	119.46529	NA	promoter-TSS (NM_001144030)	promoter-TSS (NM_001144030)	-6	NM_02466	55226	Hs.577281	NM_02466	ENSG0000	NAT10	ALP|NET43	N-acetyltra	protein-co
lib-C666-l	chr11	34139462	34139462	+	9.79436	NA	intron (NM_024662, intron 6 of 28)	intron (NM_024662, intron 6 of 28)	12351	NM_00114	55226	Hs.577281	NM_02466	ENSG0000	NAT10	ALP|NET43	N-acetyltra	protein-co
lib-C666-l	chr2	233193076	233193076	+	14.3056	NA	intron (NR_046476, intron 14 of 20)	intron (NR_046476, intron 14 of 20)	-50168	NM_00163	250	Hs.284255	NM_00163	ENSG0000	ALPP	ALP|PALP|F	alkaline ph	protein-co
lib-C666-l	chr7	129251488	129251488	+	8.71138	NA	promoter-TSS (NM_001293163)	promoter-TSS (NM_001293163)	-55	NM_00129	4899	Hs.654363	NM_00501	ENSG0000	NRF1	ALPHA-PA	nuclear res	protein-co
lib-C666-l	chr7	129251918	129251918	+	6.06312	NA	intron (NM_005011, intron 1 of 10)	CpG	375	NM_00501	4899	Hs.654363	NM_00501	ENSG0000	NRF1	ALPHA-PA	nuclear res	protein-co
lib-C666-l	chr6	41701802	41701802	+	2.05265	NA	intron (NM_007162, intron 1 of 9)	intron (NM_007162, intron 1 of 9)	337	NM_00127	7942	Hs.485360	NM_00716	ENSG0000	TFEB	ALPHATFE	transcriptic	protein-co
lib-C666-l	chr1	115259410	115259410	+	116.16629	NA	TTS (NM_007158)	TTS (NM_007158)	105	NM_00252	4893	Hs.486502	NM_00252	ENSG0000	NRAS	ALPS4|CMI	neuroblast	protein-co
lib-C666-l	chr16	2033820	2033820	+	3.56279	NA	promoter-TSS (NM_005262)	promoter-TSS (NM_005262)	-330	NM_00526	2671	Hs.27184	NM_00526	ENSG0000	GFER	ALR|ERV1|I	growth fac	protein-co
lib-C666-l	chr9	35072782	35072782	+	93.47076	NA	promoter-TSS (NM_007126)	promoter-TSS (NM_007126)	-43	NM_00712	7415	Hs.529782	NM_00712	ENSG0000	VCP	ALS14|CM	valosin cor	protein-co
lib-C666-l	chr12	54674373	54674373	+	21.84731	NA	promoter-TSS (NM_012117)	promoter-TSS (NM_012117)	-101	NM_03115	3178	Hs.546261	NM_00213	ENSG0000	HNRNPA1	ALS19|ALS	heterogen	protein-co
lib-C666-l	chr2	201754049	201754049	+	20.3278	NA	promoter-TSS (NM_021824)	promoter-TSS (NM_021824)	-1	NM_00113	60491	Hs.145284	NM_02182	ENSG0000	NIF3L1	ALS2CR1|C	NGG1 inte	protein-co
lib-C666-l	chr2	203499573	203499573	+	15.60931	NA	promoter-TSS (NM_173511)	promoter-TSS (NM_173511)	-328	NM_17351	150864	Hs.471130	NM_17351	ENSG0000	FAM117B	ALS2CR13	family with	protein-co
lib-C666-l	chr2	203736148	203736148	+	5.15932	NA	promoter-TSS (NM_001288623)	promoter-TSS (NM_001288623)	47	NM_00128	130026	Hs.516629	NM_13846	ENSG0000	ICA1L	ALS2CR14|	islet cell au	protein-co
lib-C666-l	chr2	205356566	205356566	+	12.62235	NA	Intergenic	L3|LINE|CR1	-53950	NM_15252	117583	Hs.657382	NM_05717	ENSG0000	PARD3B	ALS2CR19|	par-3 fami	protein-co
lib-C666-l	chr2	202316294	202316294	+	94.65162	NA	promoter-TSS (NM_001206864)	promoter-TSS (NM_001206864)	25	NM_01504	66008	Hs.152774	NM_01504	ENSG0000	TRAK2	ALS2CR3|C	trafficking	protein-co
lib-C666-l	chr2	202507455	202507455	+	81.64329	NA	5' UTR (NM_152388, exon 1 of 12)	5' UTR (NM_152388, exon 1 of 12)	212	NM_15238	65062	Hs.12319	NM_15238	ENSG0000	TMEM237	ALS2CR4|J	transmemb	protein-co
lib-C666-l	chr2	202508110	202508110	+	3.59106	NA	promoter-TSS (NM_152388)	promoter-TSS (NM_152388)	142	NM_00104	65062	Hs.12319	NM_15238	ENSG0000	TMEM237	ALS2CR4|J	transmemb	protein-co
lib-C666-l	chr2	202646004	202646004	+	95.09922	NA	promoter-TSS (NM_020919)	promoter-TSS (NM_020919)	-109	NM_02091	57679	Hs.471096	NM_02091	ENSG0000	ALS2	ALS2CR6|A	ALS2, alsin	protein-co
lib-C666-l	chr2	202645519	202645519	+	21.23871	NA	intron (NM_001135745, intron 1 of 3)	CpG	376	NM_00113	57679	Hs.471096	NM_02091	ENSG0000	ALS2	ALS2CR6|A	ALS2, alsin	protein-co
lib-C666-l	chr2	202747085	202747085	+	44.18462	NA	intron (NM_001261435, intron 12 of 1	intron (NM_001261435, intron 12 o	75783	NM_00126	65061	Hs.348711	NM_13915	ENSG0000	CDK15	ALS2CR7|P	cyclin depe	protein-co
lib-C666-l	chr9	135230544	135230544	+	47.23204	NA	promoter-TSS (NM_015046)	promoter-TSS (NM_015046)	-172	NM_01504	23064	Hs.460317	NM_01504	ENSG0000	SETX	ALS4|AOA2	senataxin	protein-co
lib-C666-l	chr15	44955291	44955291	+	8.85547	NA	intron (NM_001160227, intron 1 of 3	CpG	585	NM_02513	80208	Hs.656271	NM_02513	ENSG0000	SPG11	ALS5|CMT2	spastic par	protein-co
lib-C666-l	chr16	31191376	31191376	+	5.10953	NA	promoter-TSS (NM_004960)	promoter-TSS (NM_004960)	-55	NM_00496	2521	Hs.46894	NM_00496	ENSG0000	FUS	ALS6|ETM4	FUS RNA b	protein-co
lib-C666-l	chr20	56964649	56964649	+	7.52748	NA	intron (NR_036633, intron 1 of 3)	CpG	474	NR_03663	9217	Hs.182625	NM_00473	ENSG0000	VAPB	ALS8|VAM	VAMP assc	protein-co
lib-C666-l	chr20	56964218	56964218	+	5.12426	NA	promoter-TSS (NM_001195677)	promoter-TSS (NM_001195677)	43	NR_03663	9217	Hs.182625	NM_00473	ENSG0000	VAPB	ALS8|VAM	VAMP assc	protein-co
lib-C666-l	chr9	27573835	27573835	+	137.91954	NA	promoter-TSS (NM_018325)	promoter-TSS (NM_018325)	29	NM_00125	203228	Hs.493639	NM_01832	ENSG0000	C9orf72	ALSFTD|DE	chromosor	protein-co
lib-C666-l	chr15	50979061	50979061	+	4.30593	NA	promoter-TSS (NM_001301212)	promoter-TSS (NM_001301212)	-49	NM_00130	54822	Hs.512894	NM_01767	ENSG0000	TRPM7	ALSPDC|CI	transient re	protein-co
lib-C666-l	chr16	46918088	46918088	+	5.06913	NA	promoter-TSS (NM_133443)	promoter-TSS (NM_133443)	-204	NM_13344	84706	Hs.460693	NM_13344	ENSG0000	GPT2	ALT2|GPT 2	glutamic--	protein-co
lib-C666-l	chrY	2270011	2270011	+	8.20589	NA	intron (NM_145177, intron 3 of 6).2	intron (NM_145177, intron 3 of 6).2	98569	NM_00117	9189	Hs.131452	NM_00472	ENSG0000	ZBED1	ALTE|DREF	zinc finger	protein-co
lib-C666-l	chr8	21988712	21988712	+	11.42226	NA	promoter-TSS (NM_005144)	promoter-TSS (NM_005144)	-147	NM_00514	55806	Hs.272367	NM_00514	ENSG0000	HR	ALUNC|AU	HR, lysine (	protein-co
lib-C666-l	chr8	21988010	21988010	+	3.2873	NA	5' UTR (NM_005144, exon 1 of 19)	5' UTR (NM_005144, exon 1 of 19)	555	NM_01841	55806	Hs.272367	NM_00514	ENSG0000	HR	ALUNC|AU	HR, lysine (	protein-co
lib-C666-l	chr1	225908427	225908427	+	10.08274	NA	Intergenic	ERV3-16A3_I-int|LTR|ERVL	-57088	NM_00113	6726	Hs.511425	NM_00313	ENSG0000	SRP9	ALURBP	signal reco	protein-co
lib-C666-l	chr16	75584436	75584436	+	15.75122	NA	intron (NR_074083, intron 2 of 6)	LTR50|LTR|ERVL	5748	NR_07408	79583	Hs.156784	NM_00107	ENSG0000	TMEM231	ALYE870|JE	transmemb	protein-co
lib-C666-l	chr16	75590202	75590202	+	3.20885	NA	promoter-TSS (NM_001077416)	promoter-TSS (NM_001077416)	-18	NM_00107	79583	Hs.156784	NM_00107	ENSG0000	TMEM231	ALYE870|JE	transmemb	protein-co
lib-C666-l	chr12	14339094	14339094	+	5.92363	NA	Intergenic	L2a|LINE|L2	-179472	NM_00128	55729	Hs.504856	NM_01817	ENSG0000	ATF7IP	AM|ATF-IP	activating t	protein-co
lib-C666-l	chr10	116080784	116080784	+	44.91887	NA	intron (NM_001001936, intron 5 of 1	intron (NM_001001936, intron 5 of	81411	NM_00132	340706	Hs.197741	NM_19849	ENSG0000	VWA2	AMACO|C(	von Willeb	protein-co
lib-C666-l	chr5	109025066	109025066	+	9.10494	NA	promoter-TSS (NM_002372)	promoter-TSS (NM_002372)	-1	NM_00237	4124	Hs.432822	NM_00237	ENSG0000	MAN2A1	AMan II|G(	mannosida	protein-co
lib-C666-l	chr8	131455583	131455583	+	42.49125	NA	intron (NM_001247996, intron 1 of 3	CpG	323	NM_01848	50807	Hs.655552	NM_01848	ENSG0000	ASAP1	AMAP1|CE	ArfGAP wit	protein-co
lib-C666-l	chr8	131440361	131440361	+	13.10988	NA	intron (NM_001247996, intron 1 of 3	intron (NM_001247996, intron 1 of	15545	NM_01848	50807	Hs.655552	NM_01848	ENSG0000	ASAP1	AMAP1|CE	ArfGAP wit	protein-co
lib-C666-l	chr2	9346537	9346537	+	11.05669	NA	promoter-TSS (NM_003887)	promoter-TSS (NM_003887)	-357	NM_00388	8853	Hs.555902	NM_00388	ENSG0000	ASAP2	AMAP2|CE	ArfGAP wit	protein-co
lib-C666-l	chr2	9346920	9346920	+	6.93532	NA	promoter-TSS (NM_003887)	promoter-TSS (NM_003887)	26	NM_00113	8853	Hs.555902	NM_00388	ENSG0000	ASAP2	AMAP2|CE	ArfGAP wit	protein-co
lib-C666-l	chr2	9311259	9311259	+	2.56114	NA	Intergenic	LTR8|LTR|ERV1	-35635	NM_00388	8853	Hs.555902	NM_00388	ENSG0000	ASAP2	AMAP2|CE	ArfGAP wit	protein-co
lib-C666-l	chrX	57313110	57313110	+	8.73012	NA	promoter-TSS (NM_174912)	promoter-TSS (NM_174912)	0	NM_17491	158584	Hs.496205	NM_17491	ENSG0000	FAAH2	AMDD	fatty acid a	protein-co
lib-C666-l	chr16	67464744	67464744	+	9.77951	NA	promoter-TSS (NM_000196)	promoter-TSS (NM_000196)	-292	NM_00019	3291	Hs.1376	NM_00019	ENSG0000	HSD11B2	AME|AME1	hydroxyste	protein-co
lib-C666-l	chr17	7218682	7218682	+	58.41369	NA	promoter-TSS (NM_004489)	promoter-TSS (NM_004489)	-24	NM_00448	2874	Hs.438219	NM_00448	ENSG0000	GPS2	AMF-1	G protein p	protein-co
lib-C666-l	chr10	71867293	71867293	+	7.08136	NA	intron (NM_018649, intron 7 of 8)	intron (NM_018649, intron 7 of 8)	25397	NM_03279	84883	Hs.650680	NM_03279	ENSG0000	AIFM2	AMID|PRG	apoptosis i	protein-co
lib-C666-l	chr21	36217111	36217111	+	24.18348	NA	intron (NM_001001890, intron 3 of 5)	intron (NM_001001890, intron 3 of	43876	NM_00100	861	Hs.149261	NM_00175	ENSG0000	RUNX1	AML1|AML	runt relate	protein-co
lib-C666-l	chr21	36262351	36262351	+	11.6145	NA	intron (NM_001754, intron 3 of 8)	CpG	-1364	NM_00112	861	Hs.149261	NM_00175	ENSG0000	RUNX1	AML1|AML	runt relate	protein-co
lib-C666-l	chr21	36262672	36262672	+	9.93242	NA	intron (NM_001754, intron 3 of 8)	CpG	-1685	NM_00112	861	Hs.149261	NM_00175	ENSG0000	RUNX1	AML1|AML	runt relate	protein-co
lib-C666-l	chr21	36628871	36628871	+	7.15277	NA	Intergenic	Intergenic	-207276	NM_00175	861	Hs.149261	NM_00175	ENSG0000	RUNX1	AML1|AML	runt relate	protein-co
lib-C666-l	chr21	36261271	36261271	+	4.73866	NA	promoter-TSS (NM_001122607)	promoter-TSS (NM_001122607)	-284	NM_00112	861	Hs.149261	NM_00175	ENSG0000	RUNX1	AML1|AML	runt relate	protein-co
lib-C666-l	chr1	25256877	25256877	+	16.35923	NA	promoter-TSS (NM_004350)	promoter-TSS (NM_004350)	-107	NM_00435	864	Hs.170019	NM_00435	ENSG0000	RUNX3	AML2|CBF/	runt relate	protein-co
lib-C666-l	chr10	12110794	12110794	+	7.9573	NA	promoter-TSS (NM_018706)	promoter-TSS (NM_018706)	-122	NM_01870	55526	Hs.104980	NM_01870	ENSG0000	DHTKD1	AMOXAD|(	dehydroge	protein-co
lib-C666-l	chr2	127822495	127822495	+	8.28333	NA	intron (NM_139351, intron 6 of 12)	intron (NM_139351, intron 6 of 12)	17146	NM_00132	274	Hs.193163	NM_00430	ENSG0000	BIN1	AMPH2|AN	bridging in	protein-co
lib-C666-l	chr2	127864913	127864913	+	3.8557	NA	promoter-TSS (NM_139348)	promoter-TSS (NM_139348)	-10	NM_13934	274	Hs.193163	NM_00430	ENSG0000	BIN1	AMPH2|AN	bridging in	protein-co
lib-C666-l	chr12	120105540	120105540	+	6.29226	NA	promoter-TSS (NM_006253)	promoter-TSS (NM_006253)	-221	NM_00625	5564	Hs.741184	NM_00625	ENSG0000	PRKAB1	AMPK|HAN	protein kin	protein-co
lib-C666-l	chr12	49412595	49412595	+	29.41683	NA	promoter-TSS (NM_001206710)	promoter-TSS (NM_001206710)	34	NM_00120	5571	Hs.530862	NM_00273	ENSG0000	PRKAG1	AMPKG	protein kin	protein-co
lib-C666-l	chr2	74056107	74056107	+	19.92425	NA	promoter-TSS (NM_006463)	promoter-TSS (NM_006463)	-7	NM_00646	10617	Hs.469018	NM_00646	ENSG0000	STAMBP	AMSH|MIC	STAM binc	protein-co
lib-C666-l	chr9	137967145	137967145	+	7.49163	NA	promoter-TSS (NM_006334)	promoter-TSS (NM_006334)	42	NM_00633	10439	Hs.522484	NM_00633	ENSG0000	OLFM1	AMY|NOE1	olfactomec	protein-co
lib-C666-l	chr11	31531334	31531334	+	5.12426	NA	promoter-TSS (NM_001304274)	promoter-TSS (NM_001304274)	58	NM_00128	26610	Hs.175534	NM_01904	ENSG0000	ELP4	AN|AN2|C1	elongator a	protein-co
lib-C666-l	chr13	60738153	60738153	+	51.02929	NA	promoter-TSS (NM_001258366)	promoter-TSS (NM_001258366)	-34	NM_00125	81624	Hs.283127	NM_03093	ENSG0000	DIAPH3	AN|AUNA1	diaphanou	protein-co
lib-C666-l	chr13	60737511	60737511	+	7.1502	NA	intron (NM_001042517, intron 1 of 2	intron (NM_001042517, intron 1 of	608	NM_00125	81624	Hs.283127	NM_03093	ENSG0000	DIAPH3	AN|AUNA1	diaphanou	protein-co
lib-C666-l	chr17	61627698	61627698	+	17.68706	NA	promoter-TSS (NR_073585)	promoter-TSS (NR_073585)	-98	NM_00582	10238	Hs.410596	NM_00582	ENSG0000	DCAF7	AN11|HAN	DDB1 and	protein-co
lib-C666-l	chr11	35684642	35684642	+	12.26631	NA	5' UTR (NM_017583, exon 1 of 5)	5' UTR (NM_017583, exon 1 of 5)	342	NM_01758	54765	Hs.192103	NM_01758	ENSG0000	TRIM44	AN3|DIPB|I	tripartite m	protein-co
lib-C666-l	chr21	18983972	18983972	+	14.45701	NA	intron (NM_006806, intron 1 of 4)	intron (NM_006806, intron 1 of 4)	1296	NM_00680	10950	Hs.473420	NM_00680	ENSG0000	BTG3	ANA|APRC	BTG anti-p	protein-co
lib-C666-l	chr17	45197497	45197497	+	6.20473	NA	3' UTR (NM_001293091, exon 18 of 1	3' UTR (NM_001293091, exon 18 of	69181	NM_00129	996	Hs.463295	NM_00125	ENSG0000	CDC27	ANAPC3|A	cell divisior	protein-co
lib-C666-l	chr5	137549135	137549135	+	11.66438	NA	promoter-TSS (NM_004661)	promoter-TSS (NM_004661)	-103	NM_00466	8697	Hs.73625	NM_00466	ENSG0000	CDC23	ANAPC8|A	cell divisior	protein-co
lib-C666-l	chr3	179041335	179041335	+	6.32351	NA	promoter-TSS (NM_016331)	promoter-TSS (NM_016331)	-216	NM_01633	51193	Hs.632578	NM_01633	ENSG0000	ZNF639	ANC-2H01	zinc finger	protein-co
lib-C666-l	chr3	179040746	179040746	+	6.32351	NA	promoter-TSS (NM_001303425)	promoter-TSS (NM_001303425)	-33	NM_00130	51193	Hs.632578	NM_01633	ENSG0000	ZNF639	ANC-2H01	zinc finger	protein-co
lib-C666-l	chr8	124408785	124408785	+	20.92054	NA	promoter-TSS (NM_014109)	promoter-TSS (NM_014109)	-80	NM_01410	29028	Hs.370834	NM_01410	ENSG0000	ATAD2	ANCCA|CT	ATPase far	protein-co
lib-C666-l	chr16	89557145	89557145	+	38.60092	NA	promoter-TSS (NM_001256182)	promoter-TSS (NM_001256182)	-176	NM_01327	29123	Hs.335003	NM_01327	ENSG0000	ANKRD11	ANCO-1|A	ankyrin rep	protein-co
lib-C666-l	chr5	133927157	133927157	+	15.30356	NA	Intergenic	Intergenic	41376	NM_01610	51128	Hs.432984	NM_01610	ENSG0000	SAR1B	ANDD|CM	secretion a	protein-co
lib-C666-l	chr7	127983913	127983913	+	16.58224	NA	promoter-TSS (NM_018077)	promoter-TSS (NM_018077)	49	NM_00116	55131	Hs.274263	NM_01807	ENSG0000	RBM28	ANES	RNA bindir	protein-co
lib-C666-l	chr11	64863723	64863723	+	9.93242	NA	exon (NM_013265, exon 1 of 10)	exon (NM_013265, exon 1 of 10)	136	NR_07351	738	Hs.277517	NM_01326	ENSG0000	VPS51	ANG2|ANG	VPS51, GA	protein-co
lib-C666-l	chr1	178816900	178816900	+	4.74112	NA	intron (NM_152663, intron 8 of 19)	intron (NM_152663, intron 8 of 19)	23315	NM_00467	9068	Hs.591474	NM_00467	ENSG0000	ANGPTL1	ANG3|ANG	angiopoiet	protein-co
lib-C666-l	chr1	11229542	11229542	+	10.92275	NA	intron (NM_004958, intron 28 of 57)	MIRc|SINE|MIR	-19804	NM_02114	10218	Hs.146559	NM_02114	ENSG0000	ANGPTL7	AngX|CDT(	angiopoiet	protein-co
lib-C666-l	chr12	26985974	26985974	+	3.89034	NA	5' UTR (NM_002223, exon 1 of 57)	5' UTR (NM_002223, exon 1 of 57)	157	NM_00222	3709	Hs.512235	NM_00222	ENSG0000	ITPR2	ANHD|CFA	inositol 1,4	protein-co
lib-C666-l	chr3	156878020	156878020	+	137.91954	NA	5' UTR (NM_001308185, exon 1 of 11	5' UTR (NM_001308185, exon 1 of 1	529	NM_02030	57018	Hs.4859	NM_02030	ENSG0000	CCNL1	ANIA6A|BN	cyclin L1	protein-co
lib-C666-l	chr3	156892895	156892895	+	66.51361	NA	Intergenic	CpG	-14346	NM_00130	57018	Hs.4859	NM_02030	ENSG0000	CCNL1	ANIA6A|BN	cyclin L1	protein-co
lib-C666-l	chr3	156877546	156877546	+	5.37858	NA	intron (NM_020307, intron 1 of 10)	CpG-19080	1003	NM_02030	57018	Hs.4859	NM_02030	ENSG0000	CCNL1	ANIA6A|BN	cyclin L1	protein-co
lib-C666-l	chr5	14880340	14880340	+	3.98718	NA	Intergenic	Intergenic	-8453	NM_05402	56172	Hs.156727	NM_05402	ENSG0000	ANKH	ANK|CCAL	ANKH inor	protein-co
lib-C666-l	chr5	14872379	14872379	+	2.78587	NA	promoter-TSS (NM_054027)	promoter-TSS (NM_054027)	-492	NM_05402	56172	Hs.156727	NM_05402	ENSG0000	ANKH	ANK|CCAL	ANKH inor	protein-co
lib-C666-l	chr8	41676363	41676363	+	12.73831	NA	intron (NM_001142446, intron 1 of 4	MER61E|LTR|ERV1	-21223	NM_02047	286	Hs.654438	NM_00003	ENSG0000	ANK1	ANK|SPH1|	ankyrin 1	protein-co
lib-C666-l	chr8	41559458	41559458	+	8.94859	NA	intron (NM_000037, intron 21 of 42)	CpG	-36654	NM_02047	286	Hs.654438	NM_00003	ENSG0000	ANK1	ANK|SPH1|	ankyrin 1	protein-co
lib-C666-l	chr8	41649838	41649838	+	4.96104	NA	intron (NM_001142446, intron 1 of 4	intron (NM_001142446, intron 1 of	5302	NM_00003	286	Hs.654438	NM_00003	ENSG0000	ANK1	ANK|SPH1|	ankyrin 1	protein-co
lib-C666-l	chr4	113985570	113985570	+	20.83272	NA	intron (NM_001148, intron 1 of 45)	intron (NM_001148, intron 1 of 45)	14785	NM_02097	287	Hs.620557	NM_00114	ENSG0000	ANK2	ANK-2|LQ	ankyrin 2	protein-co
lib-C666-l	chr11	72145662	72145662	+	138.2052	NA	promoter-TSS (NM_001258392)	promoter-TSS (NM_001258392)	66	NM_00125	81570	Hs.523877	NM_03081	ENSG0000	CLPB	ANKCLB|H	ClpB homc	protein-co
lib-C666-l	chr11	72125433	72125433	+	6.13111	NA	intron (NM_001258394, intron 2 of 1	intron (NM_001258394, intron 2 of	20295	NM_00125	81570	Hs.523877	NM_03081	ENSG0000	CLPB	ANKCLB|H	ClpB homc	protein-co
lib-C666-l	chr17	4151477	4151477	+	10.71733	NA	intron (NR_047571, intron 1 of 23)	MER1B|DNA|hAT-Charlie	15665	NM_00125	51479	Hs.696087	NM_01637	ENSG0000	ANKFY1	ANKHZN|B	ankyrin rep	protein-co
lib-C666-l	chr21	43187234	43187234	+	19.82221	NA	promoter-TSS (NM_020639)	promoter-TSS (NM_020639)	15	NM_02063	54101	Hs.517310	NM_02063	ENSG0000	RIPK4	ANKK2|AN	receptor in	protein-co
lib-C666-l	chr12	110437251	110437251	+	3.98718	NA	promoter-TSS (NM_033121)	promoter-TSS (NM_033121)	16	NM_03312	88455	Hs.528703	NM_03312	ENSG0000	ANKRD13A	ANKRD13|	ankyrin rep	protein-co
lib-C666-l	chr19	11308426	11308426	+	13.55025	NA	promoter-TSS (NM_001136191)	promoter-TSS (NM_001136191)	-183	NM_00113	25959	Hs.284208	NM_01549	ENSG0000	KANK2	ANKRD25|	KN motif a	protein-co
lib-C666-l	chr20	37454775	37454775	+	7.31982	NA	intron (NM_001172735, intron 1 of 9)	intron (NM_001172735, intron 1 of	20439	NM_01556	26051	Hs.45719	NM_01556	ENSG0000	PPP1R16B	ANKRD4|T	protein ph	protein-co
lib-C666-l	chr20	18447787	18447787	+	81.67947	NA	promoter-TSS (NM_001099407)	promoter-TSS (NM_001099407)	42	NM_00109	55184	Hs.472225	NM_01815	ENSG0000	DZANK1	ANKRD64|	double zin	protein-co
lib-C666-l	chr8	101522449	101522449	+	17.71746	NA	3' UTR (NM_001270379, exon 6 of 6)	3' UTR (NM_001270379, exon 6 of 6	49490	NM_00127	157567	Hs.530199	NM_19840	ENSG0000	ANKRD46	ANK-S|GEI	ankyrin rep	protein-co
lib-C666-l	chr8	101514808	101514808	+	15.1031	NA	Intergenic	Intergenic	57131	NM_00127	157567	Hs.530199	NM_19840	ENSG0000	ANKRD46	ANK-S|GEI	ankyrin rep	protein-co
lib-C666-l	chr8	101572040	101572040	+	9.94079	NA	promoter-TSS (NM_001270377)	promoter-TSS (NM_001270377)	-26	NM_00127	157567	Hs.530199	NM_19840	ENSG0000	ANKRD46	ANK-S|GEI	ankyrin rep	protein-co
lib-C666-l	chr6	34857034	34857034	+	41.21209	NA	promoter-TSS (NM_015245)	promoter-TSS (NM_015245)	-4	NM_01524	23294	Hs.656492	NM_01524	ENSG0000	ANKS1A	ANKS1	ankyrin rep	protein-co
lib-C666-l	chr8	72873905	72873905	+	65.69007	NA	intron (NR_033652, intron 2 of 4)	intron (NR_033652, intron 2 of 4)	113914	NM_00733	8989	Hs.716816	NM_00733	ENSG0000	TRPA1	ANKTM1|F	transient re	protein-co
lib-C666-l	chr8	73056862	73056862	+	7.67555	NA	Intergenic	Intergenic	-69043	NM_00733	8989	Hs.716816	NM_00733	ENSG0000	TRPA1	ANKTM1|F	transient re	protein-co
lib-C666-l	chr10	62113690	62113690	+	15.42216	NA	intron (NM_001204403, intron 2 of 4	intron (NM_001204403, intron 2 of	36052	NM_00132	288	Hs.499725	NM_00114	ENSG0000	ANK3	ANKYRIN-	ankyrin 3	protein-co
lib-C666-l	chr10	62414706	62414706	+	8.51971	NA	intron (NM_001204403, intron 1 of 4	Tigger3b|DNA|TcMar-Tigger	78578	NM_00120	288	Hs.499725	NM_00114	ENSG0000	ANK3	ANKYRIN-	ankyrin 3	protein-co
lib-C666-l	chr19	50180111	50180111	+	39.42674	NA	promoter-TSS (NM_001207042)	promoter-TSS (NM_001207042)	-298	NM_00120	3276	Hs.20521	NM_00153	ENSG0000	PRMT1	ANM1|HCF	protein arg	protein-co
lib-C666-l	chrX	40029025	40029025	+	11.57178	NA	intron (NM_001123384, intron 1 of 1	CpG	7557	NM_00112	54880	Hs.659681	NM_01774	ENSG0000	BCOR	ANOP2|M/	BCL6 corep	protein-co
lib-C666-l	chrX	40013062	40013062	+	5.92363	NA	intron (NM_001123384, intron 1 of 1	CpG	23520	NM_00112	54880	Hs.659681	NM_01774	ENSG0000	BCOR	ANOP2|M/	BCL6 corep	protein-co
lib-C666-l	chrX	39960124	39960124	+	2.99475	NA	intron (NM_001123384, intron 1 of 1	CpG-27857	-3405	NM_00112	54880	Hs.659681	NM_01774	ENSG0000	BCOR	ANOP2|M/	BCL6 corep	protein-co
lib-C666-l	chr3	181430846	181430846	+	16.13885	NA	exon (NM_003106, exon 1 of 1)	exon (NM_003106, exon 1 of 1)	1134	NM_00310	6657	Hs.518438	NM_00310	ENSG0000	SOX2	ANOP3|M(	SRY-box 2	protein-co
lib-C666-l	chr3	181429344	181429344	+	7.49163	NA	promoter-TSS (NM_003106)	promoter-TSS (NM_003106)	-368	NM_00310	6657	Hs.518438	NM_00310	ENSG0000	SOX2	ANOP3|M(	SRY-box 2	protein-co
lib-C666-l	chr3	181500281	181500281	+	4.04037	NA	Intergenic	HAL1|LINE|L1	70569	NM_00310	6657	Hs.518438	NM_00310	ENSG0000	SOX2	ANOP3|M(	SRY-box 2	protein-co
lib-C666-l	chr9	21994812	21994812	+	3.98718	NA	promoter-TSS (NR_047536)	promoter-TSS (NR_047536)	22	NR_04753	100048912	Hs.493614	NR_00352	ENSG0000	CDKN2B-A	ANRIL|CDK	CDKN2B a	ncRNA

lib-C666-l	chr20	62612479	62612479	+	101.43139	NA	promoter-TSS (NM_012469)	promoter-TSS (NM_012469)	48	NM_01246	24148	Hs.31334	NM_01246	ENSG0000	PRPF6	ANT-1|AN	pre-mRNA	protein-co
lib-C666-l	chr10	46168306	46168306	+	26.82121	NA	promoter-TSS (NM_001282905)	promoter-TSS (NM_001282905)	-45	NM_00112	93550	Hs.89029	NM_17489	ENSG0000	ZFAND4	ANUBL1	zinc finger	protein-co
lib-C666-l	chr10	81965340	81965340	+	2.00164	NA	promoter-TSS (NM_145869)	promoter-TSS (NM_145869)	-12	NM_00127	311	Hs.530291	NM_00115	ENSG0000	ANXA11	ANX11|CA	annexin A1	protein-co
lib-C666-l	chr4	122589613	122589613	+	35.32367	NA	3' UTR (NM_001154, exon 13 of 13)	3' UTR (NM_001154, exon 13 of 13)	28534	NM_00115	308	Hs.480653	NM_00115	ENSG0000	ANXA5	ANX5|ENX	annexin A5	protein-co
lib-C666-l	chr4	122618324	122618324	+	9.7234	NA	promoter-TSS (NM_001154)	promoter-TSS (NM_001154)	-177	NM_00115	308	Hs.480653	NM_00115	ENSG0000	ANXA5	ANX5|ENX	annexin A5	protein-co
lib-C666-l	chr10	75173683	75173683	+	8.56376	NA	intron (NM_004034, intron 1 of 13)	CpG	160	NM_00132	310	Hs.631827	NM_00115	ENSG0000	ANXA7	ANX7|SNX	annexin A7	protein-co
lib-C666-l	chr10	47171782	47171782	+	23.27622	NA	intron (NM_001271702, intron 1 of 1	intron (NM_001271702, intron 1 of	2361	NM_00104	653145	Hs.535306	NM_00104	ENSG0000	ANXA8	ANX8|CH1	annexin A8	protein-co
lib-C666-l	chr10	48257554	48257554	+	15.31489	NA	intron (NM_001271703, intron 1 of 9)	intron (NM_001271703, intron 1 of	2350	NM_00104	653145	Hs.535306	NM_00104	ENSG0000	ANXA8	ANX8|CH1	annexin A8	protein-co
lib-C666-l	chr9	33001639	33001639	+	258.88312	NA	promoter-TSS (NM_001195250)	promoter-TSS (NM_001195250)	0	NM_00119	54840	Hs.20158	NM_01769	ENSG0000	APTX	AOA|AOA1	aprataxin	protein-co
lib-C666-l	chr19	50372884	50372884	+	18.47709	NA	3' UTR (NM_001098632, exon 5 of 5)	3' UTR (NM_001098632, exon 5 of 5	-2062	NM_00725	11284	Hs.78016	NM_00725	ENSG0000	PNKP	AOA4|EIEE	polynucleo	protein-co
lib-C666-l	chr19	50370712	50370712	+	17.89429	NA	intron (NM_007254, intron 1 of 16)	CpG	110	NM_00725	11284	Hs.78016	NM_00725	ENSG0000	PNKP	AOA4|EIEE	polynucleo	protein-co
lib-C666-l	chr19	50372542	50372542	+	3.62735	NA	3' UTR (NM_001098632, exon 5 of 5)	3' UTR (NM_001098632, exon 5 of 5	-1720	NM_00725	11284	Hs.78016	NM_00725	ENSG0000	PNKP	AOA4|EIEE	polynucleo	protein-co
lib-C666-l	chr9	97488926	97488926	+	132.21727	NA	promoter-TSS (NM_001193331)	promoter-TSS (NM_001193331)	-25	NM_03282	84909	Hs.434253	NM_03282	ENSG0000	C9orf3	AOPEP|AP	chromosor	protein-co
lib-C666-l	chr4	26321404	26321404	+	7.37943	NA	promoter-TSS (NM_005349)	promoter-TSS (NM_005349)	72	NM_00534	3516	Hs.479396	NM_00534	ENSG0000	RBPJ	AOS3|CBF1	recombina	protein-co
lib-C666-l	chr4	26348009	26348009	+	5.5166	NA	intron (NM_015874, intron 1 of 10)	LTR9|LTR|ERV1	25580	NM_20328	3516	Hs.479396	NM_00534	ENSG0000	RBPJ	AOS3|CBF1	recombina	protein-co
lib-C666-l	chr9	139434755	139434755	+	5.96422	NA	intron (NM_017617, intron 2 of 33)	L2b|LINE|L2	5483	NM_01761	4851	Hs.495473	NM_01761	ENSG0000	NOTCH1	AOS5|AOV	notch 1	protein-co
lib-C666-l	chr15	41220915	41220915	+	11.6145	NA	promoter-TSS (NM_019074)	promoter-TSS (NM_019074)	-616	NM_01907	54567	Hs.511076	NM_01907	ENSG0000	DLL4	AOS6|hdel	delta like c	protein-co
lib-C666-l	chr15	66993605	66993605	+	27.51092	NA	Intergenic	CpG	-1069	NR_02765	4091	Hs.153863	NM_00558	ENSG0000	SMAD6	AOVD2|Hs	SMAD fam	protein-co
lib-C666-l	chr15	67058260	67058260	+	25.68529	NA	intron (NR_027654, intron 4 of 4)	intron (NR_027654, intron 4 of 4)	63586	NM_00558	4091	Hs.153863	NM_00558	ENSG0000	SMAD6	AOVD2|Hs	SMAD fam	protein-co
lib-C666-l	chr15	66993312	66993312	+	10.03103	NA	Intergenic	GC_rich|Low_complexity|Low_comp	-1362	NR_02765	4091	Hs.153863	NM_00558	ENSG0000	SMAD6	AOVD2|Hs	SMAD fam	protein-co
lib-C666-l	chr15	66994579	66994579	+	7.15725	NA	promoter-TSS (NM_005585)	promoter-TSS (NM_005585)	-95	NR_02765	4091	Hs.153863	NM_00558	ENSG0000	SMAD6	AOVD2|Hs	SMAD fam	protein-co
lib-C666-l	chr2	201577954	201577954	+	31.23797	NA	non-coding (NR_037886, exon 3 of 3	non-coding (NR_037886, exon 3 of	17508	NR_13501	107161151	Hs.148269	NR_135011		AOX3P-AC	AOX3/AOX	AOX3P-AC	pseudo
lib-C666-l	chr2	201508759	201508759	+	5.25516	NA	intron (NM_001159, intron 25 of 34)	L1PB1|LINE|L1	-51687	NR_13501	107161151	Hs.148269	NR_135011		AOX3P-AC	AOX3/AOX	AOX3P-AC	pseudo
lib-C666-l	chr19	18392605	18392605	+	55.1415	NA	promoter-TSS (NM_001286968)	promoter-TSS (NM_001286968)	-139	NM_00535	3727	Hs.2780	NM_00535	ENSG0000	JUND	AP-1	JunD proto	protein-co
lib-C666-l	chr19	12904473	12904473	+	35.40551	NA	TTS (NM_002229)	TTS (NM_002229)	2163	NM_00222	3726	Hs.25292	NM_00222	ENSG0000	JUNB	AP-1	JunB proto	protein-co
lib-C666-l	chr19	12902187	12902187	+	27.69998	NA	promoter-TSS (NM_002229)	promoter-TSS (NM_002229)	-123	NM_00222	3726	Hs.25292	NM_00222	ENSG0000	JUNB	AP-1	JunB proto	protein-co
lib-C666-l	chr19	12907050	12907050	+	11.86993	NA	TTS (NM_005809)	TTS (NM_005809)	4740	NM_00222	3726	Hs.25292	NM_00222	ENSG0000	JUNB	AP-1	JunB proto	protein-co
lib-C666-l	chr19	18392214	18392214	+	7.75972	NA	promoter-TSS (NR_036155)	promoter-TSS (NR_036155)	252	NM_00128	3727	Hs.2780	NM_00535	ENSG0000	JUND	AP-1	JunD proto	protein-co
lib-C666-l	chr19	12901013	12901013	+	4.52748	NA	Intergenic	CpG	-1297	NM_00222	3726	Hs.25292	NM_00222	ENSG0000	JUNB	AP-1	JunB proto	protein-co
lib-C666-l	chr19	12904986	12904986	+	2.84146	NA	TTS (NM_002229)	TTS (NM_002229)	2676	NM_00222	3726	Hs.25292	NM_00222	ENSG0000	JUNB	AP-1	JunB proto	protein-co
lib-C666-l	chr1	59235156	59235156	+	20.0808	NA	Intergenic	Intergenic	14629	NM_00222	3725	Hs.696684	NM_00222	ENSG0000	JUN	AP-1|AP1|(	Jun proto-	protein-co
lib-C666-l	chr1	59249914	59249914	+	8.89416	NA	promoter-TSS (NR_034014)	promoter-TSS (NR_034014)	-129	NM_00222	3725	Hs.696684	NM_00222	ENSG0000	JUN	AP-1|AP1|(	Jun proto-	protein-co
lib-C666-l	chr14	75745366	75745366	+	158.31706	NA	promoter-TSS (NM_005252)	promoter-TSS (NM_005252)	-115	NM_00525	2353	Hs.25647	NM_00525	ENSG0000	FOS	AP-1|C-FC	Fos proto-	protein-co
lib-C666-l	chr14	75725843	75725843	+	46.32803	NA	Intergenic	CpG	-19638	NM_00525	2353	Hs.25647	NM_00525	ENSG0000	FOS	AP-1|C-FC	Fos proto-	protein-co
lib-C666-l	chr19	45971055	45971055	+	161.02449	NA	promoter-TSS (NM_001114171)	promoter-TSS (NM_001114171)	-198	NM_00673	2354	Hs.590958	NM_00673	ENSG0000	FOSB	AP-1|G0S3	FosB proto	protein-co
lib-C666-l	chr19	45972535	45972535	+	52.30908	NA	intron (NM_006732, intron 1 of 3)	CpG	1282	NM_00111	2354	Hs.590958	NM_00673	ENSG0000	FOSB	AP-1|G0S3	FosB proto	protein-co
lib-C666-l	chr19	45959179	45959179	+	8.89416	NA	Intergenic	Intergenic	-12074	NM_00673	2354	Hs.590958	NM_00673	ENSG0000	FOSB	AP-1|G0S3	FosB proto	protein-co
lib-C666-l	chr17	43568184	43568184	+	7.08136	NA	promoter-TSS (NM_014798)	promoter-TSS (NM_014798)	-38	NR_02778	9842	Hs.514242	NM_01479	ENSG0000	PLEKHM1	AP162|B2|(	pleckstrin h	protein-co
lib-C666-l	chr19	47352722	47352722	+	4.74112	NA	intron (NM_004069, intron 1 of 4)	intron (NM_004069, intron 1 of 4)	825	NM_00130	1175	Hs.119591	NM_00406	ENSG0000	AP2S1	AP17|CLAF	adaptor re	protein-co
lib-C666-l	chr7	100797619	100797619	+	35.40551	NA	promoter-TSS (NM_001283)	promoter-TSS (NM_001283)	-67	NM_00128	1174	Hs.489365	NM_00128	ENSG0000	AP1S1	AP19|CLAF	adaptor re	protein-co
lib-C666-l	chr17	35969592	35969592	+	121.43741	NA	promoter-TSS (NM_001163547)	promoter-TSS (NM_001163547)	-106	NM_00116	11276	Hs.594647	NM_00724	ENSG0000	SYNRG	AP1GBP1|S	synergin g	protein-co
lib-C666-l	chr20	55204178	55204178	+	16.63918	NA	promoter-TSS (NM_003222)	promoter-TSS (NM_003222)	-180	NM_00322	7022	Hs.473152	NM_00322	ENSG0000	TFAP2C	AP2-GAMI	transcriptic	protein-co
lib-C666-l	chr13	74469257	74469257	+	12.04446	NA	intron (NM_007249, intron 3 of 7)	intron (NM_007249, intron 3 of 7)	238809	NM_00724	11278	Hs.373857	NM_00724	ENSG0000	KLF12	AP-2rep|A	Kruppel lik	protein-co
lib-C666-l	chr16	4323413	4323413	+	5.23609	NA	promoter-TSS (NM_003223)	promoter-TSS (NM_003223)	-412	NM_00322	7023	Hs.513305	NM_00322	ENSG0000	TFAP4	AP-4|bHLH	transcriptic	protein-co
lib-C666-l	chr16	4323222	4323222	+	3.20885	NA	promoter-TSS (NM_003223)	promoter-TSS (NM_003223)	-221	NM_00322	7023	Hs.513305	NM_00322	ENSG0000	TFAP4	AP-4|bHLH	transcriptic	protein-co
lib-C666-l	chr16	4323875	4323875	+	2.00164	NA	promoter-TSS (NM_003223)	promoter-TSS (NM_003223)	-874	NM_00322	7023	Hs.513305	NM_00322	ENSG0000	TFAP4	AP-4|bHLH	transcriptic	protein-co
lib-C666-l	chr7	150065120	150065120	+	10.39983	NA	promoter-TSS (NM_001099695)	promoter-TSS (NM_001099695)	-759	NM_00109	29803	Hs.647086	NM_01340	ENSG0000	REPIN1	AP4|RIP60|	replication	protein-co
lib-C666-l	chr8	42010392	42010392	+	24.53984	NA	promoter-TSS (NM_001134296)	promoter-TSS (NM_001134296)	-72	NM_00680	10947	Hs.654529	NM_00680	ENSG0000	AP3M2	AP47B|CLA	adaptor re	protein-co
lib-C666-l	chr8	41997691	41997691	+	11.67529	NA	Intergenic	CpG	-12773	NM_00680	10947	Hs.654529	NM_00680	ENSG0000	AP3M2	AP47B|CLA	adaptor re	protein-co
lib-C666-l	chr14	74353304	74353304	+	24.50625	NA	promoter-TSS (NM_001242924)	promoter-TSS (NM_001242924)	-14	NR_04025	57862	Hs.270869	NM_02118	ENSG0000	ZNF410	APA-1|AP/	zinc finger	protein-co
lib-C666-l	chr1	15851236	15851236	+	40.12811	NA	promoter-TSS (NR_102732)	promoter-TSS (NR_102732)	49	NM_03299	842	Hs.329502	NM_00122	ENSG0000	CASP9	APAF-3|AF	caspase 9	protein-co
lib-C666-l	chr1	15850905	15850905	+	13.72417	NA	promoter-TSS (NR_102732)	promoter-TSS (NR_102732)	35	NR_10273	842	Hs.329502	NM_00122	ENSG0000	CASP9	APAF-3|AF	caspase 9	protein-co
lib-C666-l	chr1	15850476	15850476	+	5.31153	NA	intron (NM_032996, intron 1 of 8)	intron (NM_032996, intron 1 of 8)	464	NR_10273	842	Hs.329502	NM_00122	ENSG0000	CASP9	APAF-3|AF	caspase 9	protein-co
lib-C666-l	chr9	34329368	34329368	+	41.89289	NA	promoter-TSS (NM_147173)	promoter-TSS (NM_147173)	-136	NM_14717	318	Hs.493767	NM_00116	ENSG0000	NUDT2	APAH1	nudix hydr	protein-co
lib-C666-l	chr3	81810387	81810387	+	6.56784	NA	intron (NM_000158, intron 1 of 15)	CpG	563	NM_00015	2632	Hs.436062	NM_00015	ENSG0000	GBE1	APBD|GBE|	1,4-alpha-	protein-co
lib-C666-l	chr2	128145518	128145518	+	98.43693	NA	Intergenic	CpG	-30478	NM_00031	5624	Hs.224698	NM_00031	ENSG0000	PROC	APC|PC|PR	protein C,	protein-co
lib-C666-l	chr2	128165753	128165753	+	33.43742	NA	Intergenic	G-rich|Low_complexity|Low_comple	-10243	NM_00031	5624	Hs.224698	NM_00031	ENSG0000	PROC	APC|PC|PR	protein C,	protein-co
lib-C666-l	chr2	128177522	128177522	+	10.03103	NA	exon (NM_000312, exon 2 of 9)	exon (NM_000312, exon 2 of 9)	1526	NM_00031	5624	Hs.224698	NM_00031	ENSG0000	PROC	APC|PC|PR	protein C,	protein-co
lib-C666-l	chr4	146019360	146019360	+	96.77992	NA	promoter-TSS (NM_001318367)	promoter-TSS (NM_001318367)	11	NM_00125	10393	Hs.480876	NM_01488	ENSG0000	ANAPC10	APC10|DO	anaphase p	protein-co
lib-C666-l	chr17	79849533	79849533	+	51.11391	NA	promoter-TSS (NM_005782)	promoter-TSS (NM_005782)	-56	NM_00128	51529	Hs.534456	NM_01647	ENSG0000	ANAPC11	APC11|Apc	anaphase p	protein-co
lib-C666-l	chr11	71823832	71823832	+	12.55817	NA	promoter-TSS (NM_001278488)	promoter-TSS (NM_001278488)	-6	NM_00133	25906	Hs.38044	NM_01404	ENSG0000	ANAPC15	APC15|C11	anaphase p	protein-co
lib-C666-l	chr11	71823483	71823483	+	4.52748	NA	promoter-TSS (NM_001278491)	promoter-TSS (NM_001278491)	-139	NM_00127	25906	Hs.38044	NM_01404	ENSG0000	ANAPC15	APC15|C11	anaphase p	protein-co
lib-C666-l	chr19	6460373	6460373	+	12.62235	NA	promoter-TSS (NM_024103)	promoter-TSS (NM_024103)	-592	NM_02410	79085	Hs.356231	NM_02410	ENSG0000	SLC25A23	APC2|MCS	solute carr	protein-co
lib-C666-l	chr12	121790343	121790343	+	8.01457	NA	promoter-TSS (NM_016237)	promoter-TSS (NM_016237)	-78	NM_00133	51433	Hs.7101	NM_01623	ENSG0000	ANAPC5	APC5	anaphase p	protein-co
lib-C666-l	chr19	1445449	1445449	+	41.98367	NA	Intergenic	CpG	-4699	NM_00588	10297	Hs.446376	NM_00588	ENSG0000	APC2	APCL|SOT(	APC2, WN	protein-co
lib-C666-l	chr14	20923404	20923404	+	3.65763	NA	promoter-TSS (NM_017807)	promoter-TSS (NM_017807)	114	NM_00164	328	Hs.73722	NM_00164	ENSG0000	APEX1	APE|APE1|/	apurinic/ap	protein-co
lib-C666-l	chr2	220341152	220341152	+	8.89416	NA	intron (NM_005876, intron 18 of 40)	MER102b|DNA|hAT-Charlie	15618	NM_00117	10290	Hs.21639	NM_00587	ENSG0000	SPEG	APEG-1|AF	SPEG comp	protein-co
lib-C666-l	chr2	68694683	68694683	+	17.22931	NA	promoter-TSS (NM_001024680)	promoter-TSS (NM_001024680)	-8	NM_17354	200558	Hs.720369	NM_17354	ENSG0000	APLF	APFL|C2orl	aprataxin a	protein-co
lib-C666-l	chr6	142644747	142644747	+	7.15277	NA	intron (NM_001032394, intron 2 of 2	Tigger3a|DNA|TcMar-Tigger	21691	NM_00103	57211	Hs.743302	NM_02045	ENSG0000	ADGRG6	APG1|DRE(	adhesion G	protein-co
lib-C666-l	chr5	115193795	115193795	+	11.07709	NA	intron (NM_001002924, intron 2 of 6)	HAL1|LINE|L1	-16247	NR_07360	9140	Hs.264482	NM_00470	ENSG0000	ATG12	APG12|AP(	autophagy	protein-co
lib-C666-l	chr2	234160206	234160206	+	79.81274	NA	promoter-TSS (NM_198890)	promoter-TSS (NM_198890)	-11	NM_00119	55054	Hs.529322	NM_01797	ENSG0000	ATG16L1	APG16L|AT	autophagy	protein-co
lib-C666-l	chr5	132387560	132387560	+	51.89298	NA	promoter-TSS (NM_002154)	promoter-TSS (NM_002154)	-102	NM_00215	3308	Hs.90093	NM_00215	ENSG0000	HSPA4	APG-2|HEL	heat shock	protein-co
lib-C666-l	chr3	112280651	112280651	+	8.40444	NA	promoter-TSS (NM_017945)	promoter-TSS (NM_017945)	159	NM_02248	64422	Hs.477126	NM_02248	ENSG0000	ATG3	APG3|APG	autophagy	protein-co
lib-C666-l	chr3	112280042	112280042	+	8.28613	NA	promoter-TSS (NM_017945)	promoter-TSS (NM_017945)	768	NM_02248	64422	Hs.477126	NM_02248	ENSG0000	ATG3	APG3|APG	autophagy	protein-co
lib-C666-l	chr2	242577033	242577033	+	85.24104	NA	promoter-TSS (NM_178326)	promoter-TSS (NM_178326)	6	NM_17832	23192	Hs.283610	NM_01332	ENSG0000	ATG4B	APG4B|AU	autophagy	protein-co
lib-C666-l	chr1	63249673	63249673	+	8.01457	NA	promoter-TSS (NM_032852)	promoter-TSS (NM_032852)	-104	NM_03285	84938	Hs.7353	NM_03285	ENSG0000	ATG4C	APG4-C|AI	autophagy	protein-co
lib-C666-l	chr6	106773683	106773683	+	92.67979	NA	promoter-TSS (NM_001286111)	promoter-TSS (NM_001286111)	12	NM_00128	9474	Hs.486063	NM_00484	ENSG0000	ATG5	APG5|APG	autophagy	protein-co
lib-C666-l	chr6	106808413	106808413	+	9.79436	NA	Intergenic	Intergenic	-34718	NM_00484	9474	Hs.486063	NM_00484	ENSG0000	ATG5	APG5|APG	autophagy	protein-co
lib-C666-l	chr3	11314056	11314056	+	90.27715	NA	promoter-TSS (NM_001144912)	promoter-TSS (NM_001144912)	46	NM_00639	10533	Hs.38032	NM_00639	ENSG0000	ATG7	APG7-LIKE	autophagy	protein-co
lib-C666-l	chr12	10365522	10365522	+	64.54414	NA	promoter-TSS (NM_031412)	promoter-TSS (NM_031412)	33	NM_03141	23710	Hs.524250	NM_03141	ENSG0000	GABARAPL	APG8-LIKE	GABA type	protein-co
lib-C666-l	chr12	10365045	10365045	+	46.4068	NA	promoter-TSS (NM_031412)	promoter-TSS (NM_031412)	-444	NM_03141	23710	Hs.524250	NM_03141	ENSG0000	GABARAPL	APG8-LIKE	GABA type	protein-co
lib-C666-l	chr11	102217916	102217916	+	57.48363	NA	promoter-TSS (NM_001256166)	promoter-TSS (NM_001256166)	3	NM_00116	329	Hs.696238	NM_00116	ENSG0000	BIRC2	API1|HIAP2	baculoviral	protein-co
lib-C666-l	chr11	102216849	102216849	+	4.77753	NA	Intergenic	Intergenic	-1064	NM_00125	329	Hs.696238	NM_00116	ENSG0000	BIRC2	API1|HIAP2	baculoviral	protein-co
lib-C666-l	chrX	122984101	122984101	+	18.64332	NA	Intergenic	LTR10F|LTR|ERV1	-9561	NM_00120	331	Hs.356076	NM_00116	ENSG0000	XIAP	API3|BIRC4	X-linked in	protein-co
lib-C666-l	chr17	76210319	76210319	+	6.32351	NA	promoter-TSS (NM_001168)	promoter-TSS (NM_001168)	42	NM_00101	332	Hs.744872	NM_00116	ENSG0000	BIRC5	API4|EPR-1	baculoviral	protein-co
lib-C666-l	chr16	81764095	81764095	+	19.59432	NA	Intergenic	Intergenic	-48768	NM_00266	5336	Hs.372303	NM_00266	ENSG0000	PLCG2	APLAID|FC	phospholip	protein-co
lib-C666-l	chrX	77154871	77154871	+	12.78696	NA	promoter-TSS (NM_001866)	promoter-TSS (NM_001866)	-90	NM_00186	1349	Hs.522699	NM_00186	ENSG0000	COX7B	APLCC|LSD	cytochrom	protein-co
lib-C666-l	chr11	129939865	129939865	+	3.92495	NA	promoter-TSS (NM_001243299)	promoter-TSS (NM_001243299)	149	NM_00132	334	Hs.370247	NM_00164	ENSG0000	APLP2	APLP-2|AP	amyloid be	protein-co
lib-C666-l	chr18	45688436	45688436	+	18.64332	NA	intron (NM_001318841, intron 3 of 4)	CpG	-24756	NM_00103	201501	Hs.515388	NM_00103	ENSG0000	ZBTB7C	APM-1|AP	zinc finger	protein-co
lib-C666-l	chr18	45936650	45936650	+	6.82479	NA	promoter-TSS (NM_001318841)	promoter-TSS (NM_001318841)	-857	NM_00131	201501	Hs.515388	NM_00103	ENSG0000	ZBTB7C	APM-1|AP	zinc finger	protein-co
lib-C666-l	chr18	45936108	45936108	+	6.15313	NA	promoter-TSS (NM_001318841)	promoter-TSS (NM_001318841)	-315	NM_00131	201501	Hs.515388	NM_00103	ENSG0000	ZBTB7C	APM-1|AP	zinc finger	protein-co
lib-C666-l	chr1	27481925	27481925	+	9.73491	NA	promoter-TSS (NR_046474)	promoter-TSS (NR_046474)	-304	NM_00304	6548	Hs.469116	NM_00304	ENSG0000	SLC9A1	APNH|LIKN	solute carr	protein-co
lib-C666-l	chr1	27426119	27426119	+	4.46583	NA	non-coding (NR_046474, exon 11 of	non-coding (NR_046474, exon 11 c	55502	NR_04647	6548	Hs.469116	NM_00304	ENSG0000	SLC9A1	APNH|LIKN	solute carr	protein-co
lib-C666-l	chr3	172325486	172325486	+	8.01457	NA	Intergenic	MIRb|SINE|MIR	-84189	NR_03399	8743	Hs.478275	NM_00381	ENSG0000	TNFSF10	APO2L|Apo	tumor necr	protein-co
lib-C666-l	chr3	172310115	172310115	+	6.15313	NA	Intergenic	L1PREC2|LINE|L1	-68818	NR_03399	8743	Hs.478275	NM_00381	ENSG0000	TNFSF10	APO2L|Apo	tumor necr	protein-co
lib-C666-l	chr1	6531729	6531729	+	6.13111	NA	intron (NM_198681, intron 12 of 21)	CpG-337	-5474	NM_00103	8718	Hs.462529	NM_00379	ENSG0000	TNFRSF25	APO-3|DD	TNF recept	protein-co
lib-C666-l	chr6	160941262	160941262	+	13.20416	NA	Intergenic	Intergenic	-9106	NR_02809	80350	Hs.654503	NM_02449	ENSG0000	LPAL2	APOA2|AP	lipoprotein	pseudo
lib-C666-l	chr19	45418424	45418424	+	95.46208	NA	intron (NM_001321065, intron 2 of 3)	intron (NM_001321065, intron 2 of	612	NM_00164	341	Hs.110675	NM_00164	ENSG0000	APOC1	Apo-CI|Ap	apolipopro	protein-co
lib-C666-l	chr1	53792204	53792204	+	2.59032	NA	intron (NM_001018054, intron 2 of 1	CpG	1617	NM_00101	7804	Hs.280387	NM_00463	ENSG0000	LRP8	APOER2|H	LDL recept	protein-co
lib-C666-l	chr22	36520107	36520107	+	21.21068	NA	Intergenic	Intergenic	36870	NM_01434	80833	Hs.474737	NM_01434	ENSG0000	APOL3	APOLIII|CG	apolipopro	protein-co
lib-C666-l	chr1	114447897	114447897	+	16.264	NA	promoter-TSS (NM_001319946)	promoter-TSS (NM_001319946)	-18	NM_02283	64858	Hs.591412	NM_02283	ENSG0000	DCLRE1B	APOLLO|SI	DNA cross	protein-co
lib-C666-l	chr2	32582168	32582168	+	17.27528	NA	promoter-TSS (NM_016252)	promoter-TSS (NM_016252)	72	NM_01625	57448	Hs.150107	NM_01625	ENSG0000	BIRC6	APOLLON|	baculoviral	protein-co
lib-C666-l	chr22	36082624	36082624	+	6.32351	NA	Intergenic	Intergenic	-31295	NM_03064	80831	Hs.326561	NM_03064	ENSG0000	APOL5	APOL-V|AI	apolipopro	protein-co
lib-C666-l	chr10	111683167	111683167	+	34.02388	NA	promoter-TSS (NM_001324136)	promoter-TSS (NM_001324136)	144	NM_00132	7511	Hs.390623	NM_02038	ENSG0000	XPNPEP1	APP1|SAM	X-prolyl ar	protein-co
lib-C666-l	chr17	58603583	58603583	+	24.32656	NA	promoter-TSS (NM_001282476)	promoter-TSS (NM_001282476)	18	NM_00638	10513	Hs.84084	NM_00638	ENSG0000	APPBP2	APP-BP2|H	amyloid be	protein-co
lib-C666-l	chr5	115276903	115276903	+	3.38731	NA	Intergenic	Intergenic	-21248	NM_17380	206338	Hs.98288	NM_17380	ENSG0000	LVRN	APQ|AQPE	laeverin	protein-co
lib-C666-l	chr18	57567001	57567001	+	26.26478	NA	promoter-TSS (NM_021127)	promoter-TSS (NM_021127)	-191	NM_02112	5366	Hs.96	NM_02112	ENSG0000	PMAIP1	APR|NOXA	phorbol-1	protein-co
lib-C666-l	chr18	57515427	57515427	+	8.03539	NA	Intergenic	Intergenic	-51765	NM_02112	5366	Hs.96	NM_02112	ENSG0000	PMAIP1	APR|NOXA	phorbol-1	protein-co
lib-C666-l	chr9	100745250	100745250	+	2.59032	NA	promoter-TSS (NM_006401)	promoter-TSS (NM_006401)	-239	NM_00640	10541	Hs.730654	NM_00640	ENSG0000	ANP32B	APRIL|PHA	acidic nucl	protein-co
lib-C666-l	chr12	92539703	92539703	+	60.07874	NA	promoter-TSS (NR_135037)	promoter-TSS (NR_135037)	-30	NM_00173	694	Hs.255935	NM_00173	ENSG0000	BTG1	APRO2	BTG anti-p	protein-co
lib-C666-l	chr17	48943823	48943823	+	18.91821	NA	promoter-TSS (NR_038458)	promoter-TSS (NR_038458)	-108	NM_00124	10140	Hs.703321	NM_00574	ENSG0000	TOB1	APRO5|AP	transducer	protein-co
lib-C666-l	chr17	48944923	48944923	+	12.55817	NA	non-coding (NR_038458, exon 2 of 2	non-coding (NR_038458, exon 2 of	416	NM_00124	10140	Hs.703321	NM_00574	ENSG0000	TOB1	APRO5|AP	transducer	protein-co
lib-C666-l	chr17	49027849	49027849	+	3.8557	NA	Intergenic	Intergenic	-82510	NM_00124	10140	Hs.703321	NM_00574	ENSG0000	TOB1	APRO5|AP	transducer	protein-co
lib-C666-l	chr22	41843208	41843208	+	25.32183	NA	promoter-TSS (NM_016272)	promoter-TSS (NM_016272)	-181	NM_01627	10766	Hs.474978	NM_01627	ENSG0000	TOB2	APRO5|TO	transducer	protein-co
lib-C666-l	chr22	41844503	41844503	+	22.68206	NA	Intergenic	C-rich|Low_complexity|Low_comple	-1476	NM_01627	10766	Hs.474978	NM_01627	ENSG0000	TOB2	APRO5|TO	transducer	protein-co
lib-C666-l	chr7	101880035	101880035	+	41.03913	NA	intron (NM_001202543, intron 22 of 2	intron (NM_001202543, intron 22 o	-48318	NM_02097	10603	Hs.489448	NM_02097	ENSG0000	SH2B2	APS	SH2B adap	protein-co
lib-C666-l	chr7	101768708	101768708	+	14.98892	NA	intron (NM_181500, intron 8 of 22)	intron (NM_181500, intron 8 of 22)	-159645	NM_02097	10603	Hs.489448	NM_02097	ENSG0000	SH2B2	APS	SH2B adap	protein-co
lib-C666-l	chr19	51347743	51347743	+	17.71746	NA	intron (NR_131203, intron 2 of 2)	L1PBa|LINE|L1	-10428	NM_00103	354	Hs.171995	NM_00164	ENSG0000	KLK3	APS|KLK2A	kallikrein re	protein-co
lib-C666-l	chrX	51239802	51239802	+	10.37767	NA	promoter-TSS (NM_018159)	promoter-TSS (NM_018159)	-343	NM_01815	55190	Hs.200016	NM_01815	ENSG0000	NUDT11	APS1|ASP1	nudix hydr	protein-co
lib-C666-l	chrX	40331760	40331760	+	28.8334	NA	Intergenic	ERV3-16A3_LTR|LTR|ERVL	-108456	NM_00576	10159	Hs.495960	NM_00576	ENSG0000	ATP6AP2	APT6M8-9	ATPase H+	protein-co
lib-C666-l	chrX	40440075	40440075	+	28.21964	NA	promoter-TSS (NM_005765)	promoter-TSS (NM_005765)	-141	NM_00576	10159	Hs.495960	NM_00576	ENSG0000	ATP6AP2	APT6M8-9	ATPase H+	protein-co
lib-C666-l	chrX	40430076	40430076	+	3.92495	NA	Intergenic	Intergenic	-10140	NM_00576	10159	Hs.495960	NM_00576	ENSG0000	ATP6AP2	APT6M8-9	ATPase H+	protein-co
lib-C666-l	chr14	75594053	75594053	+	5.72511	NA	promoter-TSS (NM_001329237)	promoter-TSS (NM_001329237)	-270	NM_00132	91754	Hs.7200	NM_03311	ENSG0000	NEK9	APUG|LCC	NIMA relat	protein-co
lib-C666-l	chr9	33415733	33415733	+	8.28613	NA	Intergenic	Intergenic	-13053	NR_13451	364	Hs.455323	NM_00117	ENSG0000	AQP7	AQP7L|AQ	aquaporin	protein-co
lib-C666-l	chr7	30899697	30899697	+	9.75396	NA	intron (NM_032222, intron 13 of 17)	intron (NM_032222, intron 13 of 17	-51612	NM_19809	358	Hs.76152	NM_00038	ENSG0000	AQP1	AQP-CHIP	aquaporin	protein-co
lib-C666-l	chr4	75310808	75310808	+	30.542	NA	promoter-TSS (NM_001657)	promoter-TSS (NM_001657)	-7	NM_00165	374	Hs.270833	NM_00165	ENSG0000	AREG	AR|AREGB|	amphiregu	protein-co
lib-C666-l	chr4	75480590	75480590	+	16.13885	NA	promoter-TSS (NM_001657).2	promoter-TSS (NM_001657).2	-1	NM_00165	374	Hs.270833	NM_00165	ENSG0000	AREG	AR|AREGB|	amphiregu	protein-co
lib-C666-l	chr4	75311126	75311126	+	10.03103	NA	intron (NM_001657, intron 1 of 5)	CpG	311	NM_00165	374	Hs.270833	NM_00165	ENSG0000	AREG	AR|AREGB|	amphiregu	protein-co
lib-C666-l	chr22	42710104	42710104	+	53.7204	NA	Intergenic	L2a|LINE|L2	-30171	NM_00565	6942	Hs.475018	NM_00565	ENSG0000	TCF20	AR1|SPBP|	transcriptic	protein-co
lib-C666-l	chr22	42689141	42689141	+	29.34323	NA	Intergenic	Intergenic	-9208	NM_00565	6942	Hs.475018	NM_00565	ENSG0000	TCF20	AR1|SPBP|	transcriptic	protein-co
lib-C666-l	chr22	42690142	42690142	+	15.74109	NA	Intergenic	Intergenic	-10209	NM_00565	6942	Hs.475018	NM_00565	ENSG0000	TCF20	AR1|SPBP|	transcriptic	protein-co
lib-C666-l	chr6	1671601	1671601	+	14.0317	NA	intron (NM_001500, intron 9 of 10)	intron (NM_001500, intron 9 of 10)	60920	NM_00145	2296	Hs.348883	NM_00145	ENSG0000	FOXC1	ARA|FKHL7	forkhead b	protein-co
lib-C666-l	chr11	65729063	65729063	+	2.59723	NA	promoter-TSS (NM_005146)	promoter-TSS (NM_005146)	-97	NM_00514	9092	Hs.502883	NM_00514	ENSG0000	SART1	Ara1|HOM	squamous	protein-co
lib-C666-l	chr3	69101512	69101512	+	23.26437	NA	promoter-TSS (NM_007114)	promoter-TSS (NM_007114)	-28	NM_00711	7110	Hs.267632	NM_00711	ENSG0000	TMF1	ARA160|TM	TATA elem	protein-co
lib-C666-l	chr5	141348656	141348656	+	2.00164	NA	promoter-TSS (NM_183401)	promoter-TSS (NM_183401)	-56	NM_18340	9604	Hs.483616	NM_00429	ENSG0000	RNF14	ARA54|HFE	ring finger	protein-co
lib-C666-l	chr16	31484289	31484289	+	7.21092	NA	promoter-TSS (NM_015927)	promoter-TSS (NM_015927)	-237	NM_01592	7041	Hs.513530	NM_01592	ENSG0000	TGFB1I1	ARA55|HIC	transformi	protein-co
lib-C666-l	chr11	67250585	67250585	+	5.48804	NA	promoter-TSS (NM_003977)	promoter-TSS (NM_003977)	85	NM_00130	9049	Hs.412433	NM_00397	ENSG0000	AIP	ARA9|FKBF	aryl hydroc	protein-co
lib-C666-l	chrX	47420811	47420811	+	3.58839	NA	intron (NM_001256196, intron 1 of 1	CpG	312	NM_00125	369	Hs.446641	NM_00165	ENSG0000	ARAF	A-RAF|ARA	A-Raf prot	protein-co
lib-C666-l	chrX	47382879	47382879	+	3.27468	NA	Intergenic	CpG	-37620	NM_00165	369	Hs.446641	NM_00165	ENSG0000	ARAF	A-RAF|ARA	A-Raf prot	protein-co
lib-C666-l	chr11	46402300	46402300	+	11.66438	NA	promoter-TSS (NM_002391)	promoter-TSS (NM_002391)	-34	NM_00127	4192	Hs.82045	NM_00239	ENSG0000	MDK	ARAP|MK|N	midkine (n	protein-co
lib-C666-l	chr8	145550539	145550539	+	5.15932	NA	promoter-TSS (NM_012079)	promoter-TSS (NM_012079)	43	NM_01207	8694	Hs.521954	NM_01207	ENSG0000	DGAT1	ARAT|ARG	diacylglyce	protein-co
lib-C666-l	chr16	67212296	67212296	+	14.17553	NA	exon (NM_001040715, exon 5 of 7)	exon (NM_001040715, exon 5 of 7)	4540	NM_00118	8996	Hs.513667	NM_00394	ENSG0000	NOL3	ARC|FCM|N	nucleolar p	protein-co
lib-C666-l	chr9	127631357	127631357	+	11.5173	NA	5' UTR (NM_030978, exon 3 of 6)	5' UTR (NM_030978, exon 3 of 6)	6960	NM_03097	81873	Hs.132499	NM_03097	ENSG0000	ARPC5L	ARC16-2	actin relate	protein-co
lib-C666-l	chr9	127624391	127624391	+	5.23609	NA	promoter-TSS (NM_007209)	promoter-TSS (NM_007209)	-6	NM_03097	81873	Hs.132499	NM_03097	ENSG0000	ARPC5L	ARC16-2	actin relate	protein-co
lib-C666-l	chr3	9834205	9834205	+	10.79304	NA	promoter-TSS (NM_001198793)	promoter-TSS (NM_001198793)	26	NM_00102	10093	Hs.323342	NM_00571	ENSG0000	ARPC4	ARC20|P2C	actin relate	protein-co
lib-C666-l	chr17	60143177	60143177	+	5.5166	NA	promoter-TSS (NM_005121)	promoter-TSS (NM_005121)	-534	NM_00512	9969	Hs.282678	NM_00512	ENSG0000	MED13	ARC250|DI	mediator c	protein-co
lib-C666-l	chr14	71067317	71067317	+	52.34435	NA	promoter-TSS (NM_001284209)	promoter-TSS (NM_001284209)	90	NM_00546	10001	Hs.497353	NM_00546	ENSG0000	MED6	ARC33|NY-	mediator c	protein-co
lib-C666-l	chr2	219081774	219081774	+	5.9194	NA	promoter-TSS (NM_152862)	promoter-TSS (NM_152862)	-43	NM_15286	10109	Hs.529303	NM_00573	ENSG0000	ARPC2	ARC34|PN/	actin relate	protein-co
lib-C666-l	chr7	98923335	98923335	+	5.5166	NA	promoter-TSS (NM_001190996)	promoter-TSS (NM_001190996)	-161	NM_00119	10552	Hs.124126	NM_00640	ENSG0000	ARPC1A	Arc40|HEL-	actin relate	protein-co
lib-C666-l	chr7	98923660	98923660	+	2.58799	NA	intron (NM_006409, intron 1 of 9)	CpG	164	NM_00640	10552	Hs.124126	NM_00640	ENSG0000	ARPC1A	Arc40|HEL-	actin relate	protein-co
lib-C666-l	chr1	16491001	16491001	+	90.27715	NA	Intergenic	MIR|SINE|MIR	-8397	NM_00132	1969	Hs.171596	NM_00443	ENSG0000	EPHA2	ARCC2|CTI	EPH recept	protein-co
lib-C666-l	chr1	16482593	16482593	+	24.32656	NA	promoter-TSS (NM_004431)	promoter-TSS (NM_004431)	11	NM_00443	1969	Hs.171596	NM_00443	ENSG0000	EPHA2	ARCC2|CTI	EPH recept	protein-co
lib-C666-l	chr1	16482098	16482098	+	8.96177	NA	intron (NM_004431, intron 1 of 16)	CpG	506	NM_00443	1969	Hs.171596	NM_00443	ENSG0000	EPHA2	ARCC2|CTI	EPH recept	protein-co
lib-C666-l	chr1	33077895	33077895	+	116.93324	NA	Intergenic	Intergenic	38296	NM_00130	339487	Hs.655921	NM_17854	ENSG0000	ZBTB8OS	ARCH|ARC	zinc finger	protein-co
lib-C666-l	chr19	15610034	15610034	+	10.67459	NA	Intergenic	Intergenic	-9302	NM_17348	126410	Hs.156452	NM_17348	ENSG0000	CYP4F22	ARCI5|INLI	cytochrom	protein-co
lib-C666-l	chr19	15610438	15610438	+	3.27468	NA	Intergenic	Intergenic	-8898	NM_17348	126410	Hs.156452	NM_17348	ENSG0000	CYP4F22	ARCI5|INLI	cytochrom	protein-co
lib-C666-l	chr19	41104429	41104429	+	4.83287	NA	intron (NM_001042544, intron 1 of 3	intron (NM_001042544, intron 1 of	1288	NM_00104	8425	Hs.466766	NM_00357	ENSG0000	LTBP4	ARCL1C|LT	latent trans	protein-co

lib-C666-l	chr19	41129848	41129848	+	3.27468	NA	exon (NM_003573, exon 30 of 34)	exon (NM_003573, exon 30 of 34)	22571	NM_00104	8425	Hs.466766	NM_00357	ENSG0000	LTBP4	ARCL1C|LT	latent trans	protein-co
lib-C666-l	chr20	9050058	9050058	+	15.81359	NA	intron (NM_001172646, intron 1 of 37	intron (NM_001172646, intron 1 of	357	NM_00117	5332	Hs.472101	NM_00093	ENSG0000	PLCB4	ARCND2|P	phospholip	protein-co
lib-C666-l	chr20	9002479	9002479	+	10.16129	NA	Intergenic	Intergenic	-47222	NM_00117	5332	Hs.472101	NM_00093	ENSG0000	PLCB4	ARCND2|P	phospholip	protein-co
lib-C666-l	chr6	12261934	12261934	+	6.98764	NA	Intergenic	L2|LINE|L2	-28595	NM_00116	1906	Hs.511899	NM_00195	ENSG0000	EDN1	ARCND3|E	endothelin	protein-co
lib-C666-l	chr8	66546518	66546518	+	9.55098	NA	promoter-TSS (NM_001286702)	promoter-TSS (NM_001286702)	-66	NM_00128	55156	Hs.269542	NM_01812	ENSG0000	ARMC1	Arcp	armadillo r	protein-co
lib-C666-l	chr10	31892538	31892538	+	8.03539	NA	Intergenic	CpG	281988	NM_00132	6935	Hs.124503	NM_03075	ENSG0000	ZEB1	AREB6|BZF	zinc finger	protein-co
lib-C666-l	chr1	249168389	249168389	+	36.91569	NA	Intergenic	Intergenic	-15074	NM_00119	55657	Hs.377705	NM_01786	ENSG0000	ZNF692	AREBP|Zfp	zinc finger	protein-co
lib-C666-l	chr1	249168017	249168017	+	16.291	NA	Intergenic	Intergenic	-14702	NM_00119	55657	Hs.377705	NM_01786	ENSG0000	ZNF692	AREBP|Zfp	zinc finger	protein-co
lib-C666-l	chr20	61904169	61904169	+	5.12426	NA	promoter-TSS (NM_001281484)	promoter-TSS (NM_001281484)	32	NM_01820	55738	Hs.25584	NM_01820	ENSG0000	ARFGAP1	ARF1GAP|H	ADP ribosy	protein-co
lib-C666-l	chr10	104005216	104005216	+	26.69347	NA	promoter-TSS (NM_004193)	promoter-TSS (NM_004193)	-39	NM_00419	8729	Hs.290243	NM_00419	ENSG0000	GBF1	ARF1GEF	golgi brefe	protein-co
lib-C666-l	chr17	44439232	44439232	+	16.96353	NA	promoter-TSS (NM_016632)	promoter-TSS (NM_016632)	-69	NM_01663	51326	Hs.559259	NM_01663	ENSG0000	ARL17A	ARF1P2|AR	ADP ribosy	protein-co
lib-C666-l	chr3	57583215	57583215	+	108.21124	NA	promoter-TSS (NM_001660)	promoter-TSS (NM_001660)	0	NM_00166	378	Hs.652183	NM_00166	ENSG0000	ARF4	ARF2	ADP ribosy	protein-co
lib-C666-l	chr3	57568651	57568651	+	13.20416	NA	intron (NM_001660, intron 3 of 5)	MER20|DNA|hAT-Charlie	14564	NM_00166	378	Hs.652183	NM_00166	ENSG0000	ARF4	ARF2	ADP ribosy	protein-co
lib-C666-l	chr17	41476313	41476313	+	12.73831	NA	promoter-TSS (NM_001661)	promoter-TSS (NM_001661)	-40	NM_00166	379	Hs.183153	NM_00166	ENSG0000	ARL4D	ARF4L|ARL	ADP ribosy	protein-co
lib-C666-l	chr3	160395596	160395596	+	8.15043	NA	exon (NM_025047, exon 1 of 1)	exon (NM_025047, exon 1 of 1)	648	NM_02504	80117	Hs.287702	NM_02504	ENSG0000	ARL14	ARF7	ADP ribosy	protein-co
lib-C666-l	chrX	53741705	53741705	+	5.92363	NA	Intergenic	MLT1N2|LTR|ERVL-MaLR	-28031	NM_03140	10075	Hs.136905	NM_03140	ENSG0000	HUWE1	ARF-BP1|H	HECT, UBA	protein-co
lib-C666-l	chrX	53711036	53711036	+	3.92495	NA	intron (NM_031407, intron 2 of 83)	CpG	2638	NM_03140	10075	Hs.136905	NM_03140	ENSG0000	HUWE1	ARF-BP1|H	HECT, UBA	protein-co
lib-C666-l	chr22	43253205	43253205	+	13.59254	NA	5' UTR (NM_014570, exon 1 of 16)	5' UTR (NM_014570, exon 1 of 16)	203	NM_01457	26286	Hs.685225	NM_01457	ENSG0000	ARFGAP3	ARFGAP1	ADP ribosy	protein-co
lib-C666-l	chr8	68255977	68255977	+	4.71278	NA	promoter-TSS (NR_136224)	promoter-TSS (NR_136224)	-65	NM_00642	10565	Hs.656902	NM_00642	ENSG0000	ARFGEF1	ARFGEP1|E	ADP ribosy	protein-co
lib-C666-l	chr3	13064953	13064953	+	8.89416	NA	intron (NM_001134382, intron 1 of 14	intron (NM_001134382, intron 1 of	-36417	NM_00133	9922	Hs.475506	NM_01486	ENSG0000	IQSEC1	ARF-GEP1(	IQ motif ar	protein-co
lib-C666-l	chr3	13153199	13153199	+	3.98736	NA	Intergenic	MLT1A|LTR|ERVL-MaLR	-38582	NM_00113	9922	Hs.475506	NM_01486	ENSG0000	IQSEC1	ARF-GEP1(	IQ motif ar	protein-co
lib-C666-l	chr12	101801764	101801764	+	21.28624	NA	promoter-TSS (NM_001301068)	promoter-TSS (NM_001301068)	-166	NM_00130	400	Hs.372616	NM_00117	ENSG0000	ARL1	ARFL1	ADP ribosy	protein-co
lib-C666-l	chr11	64781857	64781857	+	17.68706	NA	intron (NM_001199745, intron 1 of 3)	CpG	272	NM_00166	402	Hs.502836	NM_00166	ENSG0000	ARL2	ARFL2	ADP ribosy	protein-co
lib-C666-l	chr11	64781439	64781439	+	5.3823	NA	promoter-TSS (NR_037650)	promoter-TSS (NR_037650)	-146	NM_00119	402	Hs.502836	NM_00166	ENSG0000	ARL2	ARFL2	ADP ribosy	protein-co
lib-C666-l	chr1	8877821	8877821	+	26.39538	NA	promoter-TSS (NM_001042681)	promoter-TSS (NM_001042681)	-122	NM_01210	473	Hs.463041	NM_01210	ENSG0000	RERE	ARG|ARP|A	arginine-g	protein-co
lib-C666-l	chr1	8763477	8763477	+	8.96177	NA	intron (NM_001042681, intron 1 of 22	CpG	114222	NM_00104	473	Hs.463041	NM_01210	ENSG0000	RERE	ARG|ARP|A	arginine-g	protein-co
lib-C666-l	chr1	32042041	32042041	+	3.98718	NA	promoter-TSS (NM_001204414)	promoter-TSS (NM_001204414)	-45	NM_00120	64129	Hs.199368	NM_02216	ENSG0000	TINAGL1	ARG1|LCN	tubulointer	protein-co
lib-C666-l	chr19	39109789	39109789	+	11.23774	NA	promoter-TSS (NM_001308393)	promoter-TSS (NM_001308393)	75	NM_01323	27335	Hs.314359	NM_01323	ENSG0000	EIF3K	ARG134|EI	eukaryotic	protein-co
lib-C666-l	chr17	57642943	57642943	+	206.58141	NA	promoter-TSS (NM_001166301)	promoter-TSS (NM_001166301)	57	NM_02461	79665	Hs.29403	NM_02461	ENSG0000	DHX40	ARG147|DI	DEAH-box	protein-co
lib-C666-l	chr12	29903859	29903859	+	26.54591	NA	intron (NM_175861, intron 5 of 17)	MER57E1|LTR|ERV1	32884	NM_00119	83857	Hs.401954	NM_17586	ENSG0000	TMTC1	ARG99|OLI	transmemb	protein-co
lib-C666-l	chr17	76250617	76250617	+	5.15932	NA	Intergenic	CpG	23226	NM_00120	283999	Hs.632228	NM_00114	ENSG0000	TMEM235	ARGM1	transmemb	protein-co
lib-C666-l	chr14	94812651	94812651	+	5.14484	NA	Intergenic	Intergenic	20388	NM_00622	390502	Hs.525557	NM_00622	ENSG0000	SERPINA2	ARGS|ATR|	serpin fam	protein-co
lib-C666-l	chr3	119301172	119301172	+	14.02822	NA	exon (NM_001125, exon 3 of 5)	exon (NM_001125, exon 3 of 5)	2892	NM_00112	141	Hs.99884	NM_00112	ENSG0000	ADPRH	ARH1	ADP-ribos	protein-co
lib-C666-l	chr1	36554570	36554570	+	38.83704	NA	TTS (NM_014466)	TTS (NM_014466)	117	NM_01782	54936	Hs.18021	NM_01782	ENSG0000	ADPRHL2	ARH3	ADP-ribos	protein-co
lib-C666-l	chr2	20646528	20646528	+	7.03995	NA	promoter-TSS (NM_004040)	promoter-TSS (NM_004040)	-304	NM_00404	388	Hs.502876	NM_00404	ENSG0000	RHOB	ARH6|ARH	ras homolo	protein-co
lib-C666-l	chr2	20638475	20638475	+	5.12426	NA	Intergenic	Intergenic	-8357	NM_00404	388	Hs.502876	NM_00404	ENSG0000	RHOB	ARH6|ARH	ras homolo	protein-co
lib-C666-l	chr1	113249729	113249729	+	5.5166	NA	promoter-TSS (NM_001042679)	promoter-TSS (NM_001042679)	-51	NM_00104	389	Hs.502659	NM_17574	ENSG0000	RHOC	ARH9|ARH	ras homolo	protein-co
lib-C666-l	chr12	63360572	63360572	+	6.32351	NA	Intergenic	LTR10F|LTR|ERV1	-31907	NM_02070	57460	Hs.435479	NM_02070	ENSG0000	PPM1H	ARHCL1|N	protein ph	protein-co
lib-C666-l	chr2	151342739	151342739	+	4.73866	NA	intron (NM_005168, intron 3 of 5)	CpG	1470	NM_00516	390	Hs.6838	NM_00516	ENSG0000	RND3	ARHE|Rho8	Rho family	protein-co
lib-C666-l	chr2	151343092	151343092	+	3.57421	NA	intron (NM_005168, intron 3 of 5)	CpG	1117	NM_00516	390	Hs.6838	NM_00516	ENSG0000	RND3	ARHE|Rho8	Rho family	protein-co
lib-C666-l	chr11	3862694	3862694	+	6.71399	NA	promoter-TSS (NM_001665)	promoter-TSS (NM_001665)	-481	NM_00166	391	Hs.501728	NM_00166	ENSG0000	RHOG	ARHG	ras homolo	protein-co
lib-C666-l	chr3	9291355	9291355	+	23.80446	NA	promoter-TSS (NM_014850)	promoter-TSS (NM_014850)	14	NM_00103	9901	Hs.654743	NM_01485	ENSG0000	SRGAP3	ARHGAP14	SLIT-ROBO	protein-co
lib-C666-l	chr1	206531322	206531322	+	7.76438	NA	intron (NM_001170637, intron 1 of 19	intron (NM_001170637, intron 1 of	15122	NM_00130	23380	Hs.497575	NM_01532	ENSG0000	SRGAP2	ARHGAP34	SLIT-ROBO	protein-co
lib-C666-l	chr13	34116737	34116737	+	16.14038	NA	intron (NM_001243476, intron 3 of 17	CpG	134235	NM_00124	90627	Hs.156551	NM_05285	ENSG0000	STARD13	ARHGAP37	StAR relate	protein-co
lib-C666-l	chr13	34116948	34116948	+	9.10494	NA	intron (NM_001243476, intron 3 of 17	CpG	134024	NM_00124	90627	Hs.156551	NM_05285	ENSG0000	STARD13	ARHGAP37	StAR relate	protein-co
lib-C666-l	chr13	34253429	34253429	+	7.76438	NA	Intergenic	Intergenic	-2457	NM_00124	90627	Hs.156551	NM_05285	ENSG0000	STARD13	ARHGAP37	StAR relate	protein-co
lib-C666-l	chr8	12990778	12990778	+	13.59254	NA	promoter-TSS (NM_006094)	promoter-TSS (NM_006094)	31	NM_00609	10395	Hs.134296	NM_00609	ENSG0000	DLC1	ARHGAP7|	DLC1 Rho	protein-co
lib-C666-l	chr13	113623463	113623463	+	16.20502	NA	promoter-TSS (NR_034002)	promoter-TSS (NR_034002)	-72	NM_00111	23263	Hs.170422	NM_02497	ENSG0000	MCF2L	ARHGEF14	MCF.2 cell	protein-co
lib-C666-l	chr13	113683920	113683920	+	8.28613	NA	intron (NM_024979, intron 3 of 29)	intron (NM_024979, intron 3 of 29)	27892	NM_00132	23263	Hs.170422	NM_02497	ENSG0000	MCF2L	ARHGEF14	MCF.2 cell	protein-co
lib-C666-l	chrX	138840612	138840612	+	4.90117	NA	intron (NM_173694, intron 22 of 29)	MER1B|DNA|hAT-Charlie	-50231	NM_00117	4168	Hs.387262	NM_00536	ENSG0000	MCF2	ARHGEF21	MCF.2 cell	protein-co
lib-C666-l	chr3	183089198	183089198	+	3.20885	NA	intron (NM_015078, intron 3 of 29)	MER4A1_|LTR|ERV1	56859	NM_01507	23101	Hs.208267	NM_01507	ENSG0000	MCF2L2	ARHGEF22	MCF.2 cell	protein-co
lib-C666-l	chr5	14264915	14264915	+	5.12426	NA	intron (NM_007118, intron 1 of 56)	intron (NM_007118, intron 1 of 56)	121104	NM_00711	7204	Hs.130031	NM_00711	ENSG0000	TRIO	ARHGEF23	trio Rho gu	protein-co
lib-C666-l	chr5	14212504	14212504	+	4.73866	NA	intron (NM_007118, intron 1 of 56)	intron (NM_007118, intron 1 of 56)	68693	NM_00711	7204	Hs.130031	NM_00711	ENSG0000	TRIO	ARHGEF23	trio Rho gu	protein-co
lib-C666-l	chr5	14143135	14143135	+	2.65152	NA	promoter-TSS (NR_134469)	promoter-TSS (NR_134469)	-676	NR_13446	7204	Hs.130031	NM_00711	ENSG0000	TRIO	ARHGEF23	trio Rho gu	protein-co
lib-C666-l	chr5	14143098	14143098	+	2.59723	NA	promoter-TSS (NR_134469)	promoter-TSS (NR_134469)	-713	NR_13446	7204	Hs.130031	NM_00711	ENSG0000	TRIO	ARHGEF23	trio Rho gu	protein-co
lib-C666-l	chr3	124311621	124311621	+	6.32351	NA	intron (NM_001322993, intron 1 of 26	intron (NM_001322993, intron 1 of	8115	NM_00706	8997	Hs.8004	NM_00394	ENSG0000	KALRN	ARHGEF24	kalirin, Rhc	protein-co
lib-C666-l	chr13	24567968	24567968	+	10.32881	NA	intron (NM_001286792, intron 2 of 14	intron (NM_001286792, intron 2 of	14203	NM_00128	221178	Hs.434298	NM_15302	ENSG0000	SPATA13	ARHGEF29	spermatog	protein-co
lib-C666-l	chr10	101769960	101769960	+	10.17139	NA	promoter-TSS (NM_015221)	promoter-TSS (NM_015221)	-250	NM_01522	23268	Hs.500771	NM_01522	ENSG0000	DNMBP	ARHGEF36	dynamin b	protein-co
lib-C666-l	chr6	151042082	151042082	+	12.81406	NA	promoter-TSS (NM_001329806)	promoter-TSS (NM_001329806)	13	NM_00132	57480	Hs.189781	NM_00102	ENSG0000	PLEKHG1	ARHGEF41	pleckstrin h	protein-co
lib-C666-l	chr6	150889341	150889341	+	11.15301	NA	Intergenic	Intergenic	-31660	NR_13813	57480	Hs.189781	NM_00102	ENSG0000	PLEKHG1	ARHGEF41	pleckstrin h	protein-co
lib-C666-l	chr19	39903356	39903356	+	13.91591	NA	promoter-TSS (NM_022835)	promoter-TSS (NM_022835)	-394	NM_02283	64857	Hs.631574	NM_02283	ENSG0000	PLEKHG2	ARHGEF42	pleckstrin h	protein-co
lib-C666-l	chr19	39903016	39903016	+	7.37943	NA	promoter-TSS (NM_022835)	promoter-TSS (NM_022835)	-734	NM_02283	64857	Hs.631574	NM_02283	ENSG0000	PLEKHG2	ARHGEF42	pleckstrin h	protein-co
lib-C666-l	chr14	63690759	63690759	+	8.89416	NA	intron (NM_020663, intron 1 of 4)	intron (NM_020663, intron 1 of 4)	19657	NM_02066	57381	Hs.656339	NM_02066	ENSG0000	RHOJ	ARHJ|RASL	ras homolo	protein-co
lib-C666-l	chr12	49259901	49259901	+	5.23609	NA	promoter-TSS (NM_014470)	promoter-TSS (NM_014470)	-248	NM_01447	27289	Hs.124940	NM_01447	ENSG0000	RND1	ARHS|RHO	Rho family	protein-co
lib-C666-l	chr16	717934	717934	+	5.23609	NA	promoter-TSS (NM_138769)	promoter-TSS (NM_138769)	-149	NM_13876	89941	Hs.513242	NM_13876	ENSG0000	RHOT2	ARHT2|C16	ras homolo	protein-co
lib-C666-l	chr1	228978156	228978156	+	18.27857	NA	Intergenic	Intergenic	107332	NM_02120	58480	Hs.647774	NM_02120	ENSG0000	RHOU	ARHU|CDC	ras homolo	protein-co
lib-C666-l	chr15	41154451	41154451	+	14.70559	NA	Intergenic	Intergenic	12036	NM_13363	171177	Hs.447901	NM_13363	ENSG0000	RHOV	ARHV|CHP	ras homolo	protein-co
lib-C666-l	chr15	41166491	41166491	+	11.66012	NA	promoter-TSS (NM_133639)	promoter-TSS (NM_133639)	-4	NM_13363	171177	Hs.447901	NM_13363	ENSG0000	RHOV	ARHV|CHP	ras homolo	protein-co
lib-C666-l	chr15	41166123	41166123	+	7.26874	NA	intron (NM_133639, intron 1 of 2)	CpG-7778	364	NM_13363	171177	Hs.447901	NM_13363	ENSG0000	RHOV	ARHV|CHP	ras homolo	protein-co
lib-C666-l	chr3	48956267	48956267	+	58.30666	NA	promoter-TSS (NM_001317334)	promoter-TSS (NM_001317334)	14	NM_00131	10425	Hs.633601	NM_00632	ENSG0000	ARIH2	ARI2|TRIAD	ariadne RB	protein-co
lib-C666-l	chr8	32406015	32406015	+	25.42196	NA	5' UTR (NM_013960, exon 1 of 13)	5' UTR (NM_013960, exon 1 of 13)	287	NM_01395	3084	Hs.453951	NM_00449	ENSG0000	NRG1	ARIA|GGF|(	neuregulin	protein-co
lib-C666-l	chr18	44497426	44497426	+	9.7234	NA	promoter-TSS (NM_001324046)	promoter-TSS (NM_001324046)	69	NM_00132	9063	Hs.57769	NM_00467	ENSG0000	PIAS2	ARIP3|DIP|	protein inh	protein-co
lib-C666-l	chr6	163148917	163148917	+	14.34866	NA	promoter-TSS (NM_013988)	promoter-TSS (NM_013988)	-83	NM_01398	5071	Hs.132954	NM_00456	ENSG0000	PARK2	AR-JP|LPRS	parkin RBR	protein-co
lib-C666-l	chr12	106533405	106533405	+	150.36041	NA	5' UTR (NM_014840, exon 1 of 7)	5' UTR (NM_014840, exon 1 of 7)	406	NM_01484	9891	Hs.524692	NM_01484	ENSG0000	NUAK1	ARK5	NUAK fam	protein-co
lib-C666-l	chr12	106530107	106530107	+	15.31489	NA	intron (NM_014840, intron 1 of 6)	intron (NM_014840, intron 1 of 6)	3704	NM_01484	9891	Hs.524692	NM_01484	ENSG0000	NUAK1	ARK5	NUAK fam	protein-co
lib-C666-l	chr18	43754003	43754003	+	7.11942	NA	promoter-TSS (NM_001008239)	promoter-TSS (NM_001008239)	15	NM_00100	147339	Hs.116486	NM_14505	ENSG0000	C18orf25	ARKL1|RNF	chromosor	protein-co
lib-C666-l	chr18	43830495	43830495	+	3.62735	NA	intron (NM_145055, intron 3 of 4)	L2b|LINE|L2	76507	NM_00100	147339	Hs.116486	NM_14505	ENSG0000	C18orf25	ARKL1|RNF	chromosor	protein-co
lib-C666-l	chr18	43987422	43987422	+	8.01457	NA	intron (NM_001256758, intron 1 of 5)	intron (NM_001256758, intron 1 of	73235	NR_04635	494470	Hs.501114	NM_15247	ENSG0000	RNF165	ARKL2|RNF	ring finger	protein-co
lib-C666-l	chr18	44005111	44005111	+	5.12426	NA	intron (NM_001256758, intron 1 of 5)	intron (NM_001256758, intron 1 of	90924	NR_04635	494470	Hs.501114	NM_15247	ENSG0000	RNF165	ARKL2|RNF	ring finger	protein-co
lib-C666-l	chr18	43913953	43913953	+	4.74112	NA	promoter-TSS (NR_046357)	promoter-TSS (NR_046357)	-234	NM_00125	494470	Hs.501114	NM_15247	ENSG0000	RNF165	ARKL2|RNF	ring finger	protein-co
lib-C666-l	chr18	43987708	43987708	+	4.73866	NA	intron (NM_001256758, intron 1 of 5)	intron (NM_001256758, intron 1 of	73521	NR_04635	494470	Hs.501114	NM_15247	ENSG0000	RNF165	ARKL2|RNF	ring finger	protein-co
lib-C666-l	chr18	43913703	43913703	+	3.62735	NA	promoter-TSS (NR_046357)	promoter-TSS (NR_046357)	-484	NM_00125	494470	Hs.501114	NM_15247	ENSG0000	RNF165	ARKL2|RNF	ring finger	protein-co
lib-C666-l	chr3	5163974	5163974	+	5.23679	NA	promoter-TSS (NM_018184)	promoter-TSS (NM_018184)	44	NM_01818	55207	Hs.250009	NM_01818	ENSG0000	ARL8B	ARL10C|Gi	ADP ribosy	protein-co
lib-C666-l	chr17	44656642	44656642	+	15.60931	NA	intron (NM_001330240, intron 1 of 3)	CpG	436	NM_00133	100506084	Hs.559259	NM_00103	ENSG0000	ARL17B	ARL17|ARL	ADP ribosy	protein-co
lib-C666-l	chr17	44438675	44438675	+	5.74587	NA	intron (NM_001039083, intron 1 of 3)	CpG	459	NM_00103	100506084	Hs.559259	NM_00103	ENSG0000	ARL17B	ARL17|ARL	ADP ribosy	protein-co
lib-C666-l	chr20	62339342	62339342	+	2.60101	NA	promoter-TSS (NM_181485)	promoter-TSS (NM_181485)	23	NM_00126	10139	Hs.389277	NM_00322	ENSG0000	ARFRP1	ARL18|ARF	ADP ribosy	protein-co
lib-C666-l	chr3	93698962	93698962	+	22.50982	NA	promoter-TSS (NM_001321328)	promoter-TSS (NM_001321328)	-21	NM_18289	200894	Hs.533086	NM_14499	ENSG0000	ARL13B	ARL2L1|JBT	ADP ribosy	protein-co
lib-C666-l	chr2	38604377	38604377	+	45.81715	NA	promoter-TSS (NM_001308076)	promoter-TSS (NM_001308076)	188	NM_00133	64225	Hs.594950	NM_02237	ENSG0000	ATL2	ARL3IP2|AI	atlastin GT	protein-co
lib-C666-l	chr2	38623367	38623367	+	11.66203	NA	Intergenic	Intergenic	-17641	NM_00133	64225	Hs.594950	NM_02237	ENSG0000	ATL2	ARL3IP2|AI	atlastin GT	protein-co
lib-C666-l	chr7	12879580	12879580	+	21.90047	NA	Intergenic	L1PA7|LINE|L1	152669	NM_00119	10124	Hs.245540	NM_00573	ENSG0000	ARL4A	ARL4	ADP ribosy	protein-co
lib-C666-l	chr7	12726218	12726218	+	14.7189	NA	promoter-TSS (NM_005738)	promoter-TSS (NM_005738)	-234	NM_00103	10124	Hs.245540	NM_00573	ENSG0000	ARL4A	ARL4	ADP ribosy	protein-co
lib-C666-l	chr7	13194994	13194994	+	8.03539	NA	Intergenic	Intergenic	468083	NM_00119	10124	Hs.245540	NM_00573	ENSG0000	ARL4A	ARL4	ADP ribosy	protein-co
lib-C666-l	chr10	18948175	18948175	+	9.94079	NA	promoter-TSS (NM_178815)	promoter-TSS (NM_178815)	-138	NM_17881	221079	Hs.25362	NM_17881	ENSG0000	ARL5B	ARL8	ADP ribosy	protein-co
lib-C666-l	chr20	60877890	60877890	+	14.37873	NA	promoter-TSS (NM_001281438)	promoter-TSS (NM_001281438)	-62	NM_00128	11047	Hs.90107	NM_00700	ENSG0000	ADRM1	ARM-1|AR	adhesion r	protein-co
lib-C666-l	chr2	43823282	43823282	+	47.60628	NA	promoter-TSS (NR_073394)	promoter-TSS (NR_073394)	-97	NM_00134	63892	Hs.369592	NM_02206	ENSG0000	THADA	ARMC13|G	THADA, ar	protein-co
lib-C666-l	chr20	23618310	23618310	+	6.82479	NA	exon (NM_000099, exon 1 of 3)	exon (NM_000099, exon 1 of 3)	375	NM_00128	1471	Hs.304682	NM_00009	ENSG0000	CST3	ARMD11|H	cystatin C	protein-co
lib-C666-l	chr3	51422596	51422596	+	18.20429	NA	promoter-TSS (NM_006010)	promoter-TSS (NM_006010)	-72	NM_00601	7873	Hs.436446	NM_00601	ENSG0000	MANF	ARMET|AR	mesenceph	protein-co
lib-C666-l	chr10	14854413	14854413	+	6.15313	NA	Intergenic	MER51A|LTR|ERV1	25570	NM_00102	441549	Hs.559067	NM_00102	ENSG0000	CDNF	ARMETL1	cerebral dc	protein-co
lib-C666-l	chr19	48994399	48994399	+	15.60931	NA	exon (NM_001080434, exon 15 of 16)	exon (NM_001080434, exon 15 of 1	21934	NM_00422	9266	Hs.144011	NM_00422	ENSG0000	CYTH2	ARNO|CTS	cytohesin 2	protein-co
lib-C666-l	chr19	48972423	48972423	+	13.06669	NA	promoter-TSS (NM_004228)	promoter-TSS (NM_004228)	-42	NM_01745	9266	Hs.144011	NM_00422	ENSG0000	CYTH2	ARNO|CTS	cytohesin 2	protein-co
lib-C666-l	chr1	222817628	222817628	+	68.62961	NA	promoter-TSS (NM_001300867)	promoter-TSS (NM_001300867)	-17	NM_00132	375056	Hs.118474	NM_19855	ENSG0000	MIA3	ARNT|D32(	MIA family	protein-co
lib-C666-l	chr12	102513984	102513984	+	9.79436	NA	promoter-TSS (NM_001319996)	promoter-TSS (NM_001319996)	35	NM_00131	55010	Hs.330663	NM_01791	ENSG0000	PARPBP	AROM|C12	PARP1 bin	protein-co
lib-C666-l	chr22	39928790	39928790	+	4.52748	NA	promoter-TSS (NM_194326)	promoter-TSS (NM_194326)	70	NM_19432	91582	Hs.526933	NM_19432	ENSG0000	RPS19BP1	AROS|S19E	ribosomal	protein-co
lib-C666-l	chr19	19249311	19249311	+	9.79436	NA	promoter-TSS (NM_001256766)	promoter-TSS (NM_001256766)	-1	NM_01781	54929	Hs.631629	NM_01781	ENSG0000	TMEM161A	AROS-29	transmemb	protein-co
lib-C666-l	chr4	111826030	111826030	+	21.28624	NA	Intergenic	Intergenic	-262751	NM_15342	5308	Hs.643588	NM_00032	ENSG0000	PITX2	ARP1|Brx1|	paired like	protein-co
lib-C666-l	chr15	96596601	96596601	+	38.20619	NA	Intergenic	Intergenic	-272556	NM_00114	7026	Hs.347991	NM_02100	ENSG0000	NR2F2	ARP1|CHTI	nuclear rec	protein-co
lib-C666-l	chr15	96900181	96900181	+	19.8879	NA	Intergenic	CpG	23612	NM_00114	7026	Hs.347991	NM_02100	ENSG0000	NR2F2	ARP1|CHTI	nuclear rec	protein-co
lib-C666-l	chr7	152456938	152456938	+	8.48019	NA	non-coding (NR_073001, exon 1 of 1	non-coding (NR_073001, exon 1 of	104	NM_02044	57180	Hs.647117	NM_02044	ENSG0000	ACTR3B	ARP11|ARF	ARP3 actin	protein-co
lib-C666-l	chr2	98280619	98280619	+	13.46097	NA	promoter-TSS (NM_005735)	promoter-TSS (NM_005735)	-58	NM_00573	10120	Hs.98791	NM_00573	ENSG0000	ACTR1B	ARP1B|CTF	ARP1 actin	protein-co
lib-C666-l	chr9	129893656	129893656	+	7.03892	NA	intron (NM_001190728, intron 7 of 16	intron (NM_001190728, intron 7 of	-8612	NM_01209	23452	Hs.653262	NM_01209	ENSG0000	ANGPTL2	ARP2|HARI	angiopoiet	protein-co
lib-C666-l	chr20	37376976	37376976	+	5.12426	NA	promoter-TSS (NM_024855)	promoter-TSS (NM_024855)	-121	NM_02485	79913	Hs.371585	NM_02485	ENSG0000	ACTR5	Arp5|INO8	ARP5 actin	protein-co
lib-C666-l	chr12	100594653	100594653	+	6.15313	NA	promoter-TSS (NR_048568)	promoter-TSS (NR_048568)	98	NR_04856	64431	Hs.115088	NM_02249	ENSG0000	ACTR6	ARP6|CDA	ARP6 actin	protein-co
lib-C666-l	chr16	70835173	70835173	+	6.15313	NA	promoter-TSS (NM_018052)	promoter-TSS (NM_018052)	-112	NM_01805	55697	Hs.445061	NM_01805	ENSG0000	VAC14	ArPIKfyve|S	Vac14, PIK	protein-co
lib-C666-l	chr15	52861281	52861281	+	25.80588	NA	promoter-TSS (NM_001306196)	promoter-TSS (NM_001306196)	48	NM_00662	10776	Hs.512908	NM_00662	ENSG0000	ARPP19	ARPP-16|A	cAMP regu	protein-co
lib-C666-l	chr15	52857781	52857781	+	8.0154	NA	intron (NM_001306196, intron 1 of 3)	intron (NM_001306196, intron 1 of	3248	NM_00133	10776	Hs.512908	NM_00662	ENSG0000	ARPP19	ARPP-16|A	cAMP regu	protein-co
lib-C666-l	chr1	150601796	150601796	+	89.57237	NA	promoter-TSS (NM_207045)	promoter-TSS (NM_207045)	-187	NM_20704	2029	Hs.632456	NM_00443	ENSG0000	ENSA	ARPP-19e	endosulfin	protein-co
lib-C666-l	chr1	150602086	150602086	+	76.14658	NA	promoter-TSS (NM_207045)	promoter-TSS (NM_207045)	12	NM_20704	2029	Hs.632456	NM_00443	ENSG0000	ENSA	ARPP-19e	endosulfin	protein-co
lib-C666-l	chr7	100472769	100472769	+	8.28613	NA	promoter-TSS (NM_001128854)	promoter-TSS (NM_001128854)	68	NM_00112	51593	Hs.111801	NM_01590	ENSG0000	SRRT	ARS2|ASR2	serrate, RN	protein-co
lib-C666-l	chrX	47518505	47518505	+	59.09724	NA	promoter-TSS (NR_045560)	promoter-TSS (NR_045560)	74	NM_00418	8409	Hs.172791	NM_00418	ENSG0000	UXT	ART-27|ST	ubiquitous	protein-co
lib-C666-l	chr3	107241747	107241747	+	12.97267	NA	promoter-TSS (NM_001142568)	promoter-TSS (NM_001142568)	-36	NM_00114	56987	Hs.124366	NM_02023	ENSG0000	BBX	ARTC1|HBI	BBX, HMG-	protein-co
lib-C666-l	chr12	4140619	4140619	+	9.93242	NA	Intergenic	CpG	-158005	NM_00128	57097	Hs.657268	NM_02036	ENSG0000	PARP11	ARTD11|C1	poly(ADP-	protein-co
lib-C666-l	chr12	3982551	3982551	+	4.83287	NA	promoter-TSS (NM_001286522)	promoter-TSS (NM_001286522)	63	NR_10446	57097	Hs.657268	NM_02036	ENSG0000	PARP11	ARTD11|C1	poly(ADP-	protein-co
lib-C666-l	chr7	139762868	139762868	+	47.52935	NA	non-coding (NR_130117, exon 1 of 1	non-coding (NR_130117, exon 1 of	653	NM_02275	64761	Hs.12646	NM_02275	ENSG0000	PARP12	ARTD12|M	poly(ADP-	protein-co
lib-C666-l	chr3	156392332	156392332	+	3.84463	NA	promoter-TSS (NM_001184717)	promoter-TSS (NM_001184717)	127	NM_01550	25976	Hs.744050	NM_01550	ENSG0000	TIPARP	ARTD14|PA	TCDD indu	protein-co
lib-C666-l	chr15	65593092	65593092	+	8.28216	NA	Intergenic	LTR12C|LTR|ERV1	-13911	NM_00131	54956	Hs.30634	NM_01785	ENSG0000	PARP16	ARTD15|C1	poly(ADP-	protein-co
lib-C666-l	chr5	49963748	49963748	+	16.96353	NA	intron (NM_001331028, intron 1 of 25	CpG	368	NM_00133	79668	Hs.369581	NM_02461	ENSG0000	PARP8	ARTD16|pA	poly(ADP-	protein-co
lib-C666-l	chr5	49962246	49962246	+	10.07497	NA	promoter-TSS (NM_024615)	promoter-TSS (NM_024615)	513	NM_00117	79668	Hs.369581	NM_02461	ENSG0000	PARP8	ARTD16|pA	poly(ADP-	protein-co
lib-C666-l	chr15	72564975	72564975	+	13.46097	NA	promoter-TSS (NR_136606)	promoter-TSS (NR_136606)	-59	NM_00132	56965	Hs.270244	NM_02021	ENSG0000	PARP6	ARTD17|PA	poly(ADP-	protein-co
lib-C666-l	chr15	72564562	72564562	+	5.5166	NA	intron (NM_001323516, intron 1 of 23	CpG	354	NM_00132	56965	Hs.270244	NM_02021	ENSG0000	PARP6	ARTD17|PA	poly(ADP-	protein-co
lib-C666-l	chr8	9413363	9413363	+	70.21218	NA	promoter-TSS (NM_003747)	promoter-TSS (NM_003747)	-82	NM_00374	8658	Hs.370267	NM_00374	ENSG0000	TNKS	ARTD5|PAI	tankyrase	protein-co
lib-C666-l	chr10	93558184	93558184	+	15.1031	NA	promoter-TSS (NR_108070)	promoter-TSS (NR_108070)	33	NM_02523	80351	Hs.329327	NM_02523	ENSG0000	TNKS2	ARTD6|PAI	tankyrase 2	protein-co
lib-C666-l	chrX	106871588	106871588	+	5.92363	NA	promoter-TSS (NM_002764)	promoter-TSS (NM_002764)	-66	NM_00276	5631	Hs.56	NM_00276	ENSG0000	PRPS1	ARTS|CMT	phosphorit	protein-co
lib-C666-l	chr14	76334336	76334336	+	6.79473	NA	intron (NM_015072, intron 29 of 31)	intron (NM_015072, intron 29 of 31	114029	NM_00323	7043	Hs.592317	NM_00323	ENSG0000	TGFB3	ARVD|ARV	transformi	protein-co
lib-C666-l	chr18	28683619	28683619	+	7.37943	NA	intron (NR_110785, intron 2 of 3)	intron (NR_110785, intron 2 of 3)	-1224	NM_00494	1824	Hs.95612	NM_00494	ENSG0000	DSC2	ARVD11|CI	desmocolli	protein-co
lib-C666-l	chr17	39943117	39943117	+	4.52748	NA	promoter-TSS (NM_002230)	promoter-TSS (NM_002230)	-153	NM_00223	3728	Hs.514174	NM_00223	ENSG0000	JUP	ARVD12|C	junction pl	protein-co
lib-C666-l	chr12	33109489	33109489	+	10.4657	NA	Intergenic	L1PA4|LINE|L1	-59709	NM_00457	5318	Hs.164384	NM_00457	ENSG0000	PKP2	ARVD9	plakophilin	protein-co
lib-C666-l	chr6	31325040	31325040	+	31.98049	NA	promoter-TSS (NM_005514).6	promoter-TSS (NM_005514).6	-18	NM_00551	3106	Hs.77961	NM_00551	ENSG0000	HLA-B	AS|B-4901	major histo	protein-co
lib-C666-l	chr20	20693471	20693471	+	10.32881	NA	promoter-TSS (NM_020343)	promoter-TSS (NM_020343)	-205	NM_02034	57186	Hs.472285	NM_02034	ENSG0000	RALGAPA2	AS250|C20	Ral GTPase	protein-co
lib-C666-l	chr10	52499910	52499910	+	4.39666	NA	intron (NM_001321960, intron 1 of 6)	CpG	462	NM_00132	653308	Hs.659820	NM_00107	ENSG0000	ASAH2B	ASAH2C|A	N-acylsphi	protein-co
lib-C666-l	chr7	65540685	65540685	+	236.60414	NA	promoter-TSS (NM_000048)	promoter-TSS (NM_000048)	-91	NM_00004	435	Hs.632015	NM_00004	ENSG0000	ASL	ASAL	argininosu	protein-co
lib-C666-l	chr8	131260025	131260025	+	4.19675	NA	intron (NM_001247996, intron 4 of 30	intron (NM_001247996, intron 4 of	48754	NR_00276	29065	Hs.639318	NR_002765		ASAP1-IT1	ASAP1-IT|	ASAP1 intr	ncRNA
lib-C666-l	chr5	78223637	78223637	+	26.18172	NA	intron (NM_198709, intron 5 of 7)	L1MEa|LINE|L1	58129	NM_19870	411	Hs.149103	NM_00004	ENSG0000	ARSB	ASB|G4S|N	arylsulfata	protein-co
lib-C666-l	chr2	239334945	239334945	+	24.28549	NA	promoter-TSS (NM_001330196)	promoter-TSS (NM_001330196)	-624	NM_00133	51665	Hs.516788	NM_01611	ENSG0000	ASB1	ASB-1	ankyrin rep	protein-co
lib-C666-l	chr1	76358530	76358530	+	19.57279	NA	intron (NM_002440, intron 17 of 19)	Charlie4a|DNA|hAT-Charlie	39586	NR_02654	127247	Hs.125423	NM_08086	ENSG0000	ASB17	Asb-17	ankyrin rep	protein-co
lib-C666-l	chr19	1021110	1021110	+	7.84109	NA	promoter-TSS (NM_033420)	promoter-TSS (NM_033420)	31	NM_03342	91304	Hs.515003	NM_03342	ENSG0000	TMEM259	ASBABP1|(	transmemb	protein-co
lib-C666-l	chr22	39715740	39715740	+	19.18403	NA	promoter-TSS (NR_002439)	promoter-TSS (NR_002439)	-70	NM_00096	6122	Hs.119598	NM_00096	ENSG0000	RPL3	ASC-1|L3|T	ribosomal	protein-co
lib-C666-l	chr22	30207605	30207605	+	6.64931	NA	intron (NM_001242906, intron 7 of 18	intron (NM_001242906, intron 7 of	26688	NM_03220	84164	Hs.731754	NM_03220	ENSG0000	ASCC2	ASC1p100|	activating s	protein-co
lib-C666-l	chr22	30217451	30217451	+	5.23609	NA	intron (NM_001242906, intron 4 of 18	MIRb|SINE|MIR	16842	NM_03220	84164	Hs.731754	NM_03220	ENSG0000	ASCC2	ASC1p100|	activating s	protein-co
lib-C666-l	chr6	101329231	101329231	+	34.77118	NA	promoter-TSS (NM_006828)	promoter-TSS (NM_006828)	17	NM_00128	10973	Hs.486031	NM_00682	ENSG0000	ASCC3	ASC1p200|	activating s	protein-co
lib-C666-l	chr2	96971123	96971123	+	8.28216	NA	intron (NM_014014, intron 1 of 44)	intron (NM_014014, intron 1 of 44)	184	NM_01401	23020	Hs.246112	NM_01401	ENSG0000	SNRNP200	ASCC3L1|B	small nucle	protein-co
lib-C666-l	chr20	4667449	4667449	+	9.79436	NA	intron (NM_001080123, intron 1 of 1)	CpG	292	NM_00108	5621	Hs.472010	NM_00031	ENSG0000	PRNP	ASCR|AltPr	prion prote	protein-co
lib-C666-l	chr20	4573284	4573284	+	3.20885	NA	Intergenic	CpG	-93513	NM_00127	5621	Hs.472010	NM_00031	ENSG0000	PRNP	ASCR|AltPr	prion prote	protein-co
lib-C666-l	chr2	65216414	65216414	+	8.0154	NA	promoter-TSS (NM_003038)	promoter-TSS (NM_003038)	-81	NM_00303	6509	Hs.654352	NM_00303	ENSG0000	SLC1A4	ASCT1|SAT	solute carr	protein-co
lib-C666-l	chrX	2847613	2847613	+	2.99475	NA	promoter-TSS (NM_009589)	promoter-TSS (NM_009589)	-197	NM_00958	414	Hs.528631	NM_00166	ENSG0000	ARSD	ASD	arylsulfata	protein-co
lib-C666-l	chr6	139697152	139697152	+	4.52748	NA	Intergenic	Intergenic	-1365	NM_00607	10370	Hs.82071	NM_00607	ENSG0000	CITED2	ASD8|MRG	Cbp/p300	protein-co
lib-C666-l	chr15	91537934	91537934	+	90.61259	NA	promoter-TSS (NM_003981)	promoter-TSS (NM_003981)	-53	NM_00398	9055	Hs.366401	NM_00398	ENSG0000	PRC1	ASE1	protein reg	protein-co
lib-C666-l	chr19	45908921	45908921	+	9.14689	NA	promoter-TSS (NM_012099)	promoter-TSS (NM_012099)	-546	NM_00129	10849	Hs.710495	NM_01209	ENSG0000	CD3EAP	ASE-1|ASE	CD3e mole	protein-co

lib-C666-l	chr17	56084800	56084800	+	11.80692	NA	promoter-TSS (NM_001078166)	promoter-TSS (NM_001078166)	-93	NR_03404	6426	Hs.68714	NM_00692	ENSG0000	SRSF1	ASF|SF2|SF	serine and	protein-co
lib-C666-l	chrX	146993379	146993379	+	4.90117	NA	promoter-TSS (NR_024499)	promoter-TSS (NR_024499)	-44	NR_02450	100126270	Hs.561999	NM_02449	ENSG0000	FMR1-AS1	ASFMR1|FI	FMR1 antis	ncRNA
lib-C666-l	chr17	7036250	7036250	+	6.20473	NA	Intergenic	Intergenic	-18120	NM_08091	433	Hs.654440	NM_00118	ENSG0000	ASGR2	ASGP-R2|A	asialoglyco	protein-co
lib-C666-l	chr17	73401941	73401941	+	105.11623	NA	promoter-TSS (NM_203506)	promoter-TSS (NM_203506)	-151	NM_20350	2885	Hs.444356	NM_00208	ENSG0000	GRB2	ASH|EGFRE	growth fac	protein-co
lib-C666-l	chr17	73390061	73390061	+	15.75122	NA	intron (NM_002086, intron 1 of 5)	intron (NM_002086, intron 1 of 5)	11728	NM_00208	2885	Hs.444356	NM_00208	ENSG0000	GRB2	ASH|EGFRE	growth fac	protein-co
lib-C666-l	chr1	155532872	155532872	+	7.0832	NA	promoter-TSS (NM_018489)	promoter-TSS (NM_018489)	-548	NM_01848	55870	Hs.491060	NM_01848	ENSG0000	ASH1L	ASH1|ASH1	ASH1 like h	protein-co
lib-C666-l	chr8	37962978	37962978	+	14.31593	NA	promoter-TSS (NM_001282272)	promoter-TSS (NM_001282272)	-33	NM_00128	9070	Hs.521530	NM_00467	ENSG0000	ASH2L	ASH2|ASH2	ASH2 like h	protein-co
lib-C666-l	chr11	2292358	2292358	+	9.10494	NA	promoter-TSS (NM_005170)	promoter-TSS (NM_005170)	-176	NM_00517	430	Hs.152475	NM_00517	ENSG0000	ASCL2	ASH2|HASI	achaete-sc	protein-co
lib-C666-l	chr11	2278839	2278839	+	3.20885	NA	Intergenic	Intergenic	13343	NM_00517	430	Hs.152475	NM_00517	ENSG0000	ASCL2	ASH2|HASI	achaete-sc	protein-co
lib-C666-l	chr10	102289640	102289640	+	9.79436	NA	promoter-TSS (NM_005004)	promoter-TSS (NM_005004)	40	NM_00128	4714	Hs.523215	NM_00500	ENSG0000	NDUFB8	ASHI|CI-AS	NADH:ubic	protein-co
lib-C666-l	chr10	35047545	35047545	+	7.0832	NA	intron (NM_001184785, intron 1 of 2	intron (NM_001184785, intron 1 of	56708	NM_00118	56288	Hs.131489	NM_01961	ENSG0000	PARD3	ASIP|Baz|P	par-3 fami	protein-co
lib-C666-l	chr6	137113644	137113644	+	13.91591	NA	promoter-TSS (NM_005923)	promoter-TSS (NM_005923)	12	NM_00592	4217	Hs.186486	NM_00592	ENSG0000	MAP3K5	ASK1|MAP	mitogen-a	protein-co
lib-C666-l	chr1	27695928	27695928	+	17.62684	NA	exon (NM_003665, exon 8 of 8)	exon (NM_003665, exon 8 of 8)	-2571	NM_00467	9064	Hs.194694	NM_00467	ENSG0000	MAP3K6	ASK2|MAP	mitogen-a	protein-co
lib-C666-l	chr1	28908477	28908477	+	5.96422	NA	promoter-TSS (NR_024127)	promoter-TSS (NR_024127)	-111	NR_02412	85028	Hs.632377	NM_05304	ENSG0000	SNHG12	ASLNC040	small nucle	ncRNA
lib-C666-l	chr11	6411400	6411400	+	7.49163	NA	promoter-TSS (NM_000543)	promoter-TSS (NM_000543)	-244	NR_13450	6609	Hs.498173	NM_00054	ENSG0000	SMPD1	ASM|ASM/	sphingomy	protein-co
lib-C666-l	chr6	123110979	123110979	+	2.26435	NA	intron (NM_001286138, intron 1 of 6)	intron (NM_001286138, intron 1 of	785	NM_00128	10924	Hs.486357	NM_00671	ENSG0000	SMPDL3A	ASM3A|AS	sphingomy	protein-co
lib-C666-l	chrY	1730219	1730219	+	155.64427	NA	Intergenic	Intergenic	46193	NM_00117	438	Hs.522572	NM_00404	ENSG0000	ASMT	ASMTY|HIC	acetylserot	protein-co
lib-C666-l	chrX	1780216	1780216	+	85.95118	NA	Intergenic	Intergenic	46190	NM_00117	438	Hs.522572	NM_00404	ENSG0000	ASMT	ASMTY|HIC	acetylserot	protein-co
lib-C666-l	chr18	55289065	55289065	+	35.0374	NA	5' UTR (NM_004539, exon 1 of 14)	5' UTR (NM_004539, exon 1 of 14)	112	NM_00453	4677	Hs.465224	NM_00453	ENSG0000	NARS	ASNRS|NA	asparaginy	protein-co
lib-C666-l	chr7	97501829	97501829	+	14.70559	NA	promoter-TSS (NM_001178075)	promoter-TSS (NM_001178075)	25	NM_18335	440	Hs.489207	NM_00167	ENSG0000	ASNS	ASNSD|TS	asparagine	protein-co
lib-C666-l	chr1	197116003	197116003	+	3.07144	NA	promoter-TSS (NM_001206846)	promoter-TSS (NM_001206846)	-179	NM_01813	259266	Hs.121028	NM_01813	ENSG0000	ASPM	ASP|Calmb	abnormal s	protein-co
lib-C666-l	chr17	79935644	79935644	+	11.90035	NA	intron (NM_001330528, intron 1 of 1	CpG	218	NM_00125	79058	Hs.298351	NM_02408	ENSG0000	ASPSCR1	ASPCR1|AS	ASPSCR1, U	protein-co
lib-C666-l	chr1	173794067	173794067	+	23.27622	NA	promoter-TSS (NM_001171182)	promoter-TSS (NM_001171182)	270	NM_01812	55157	Hs.647707	NM_01812	ENSG0000	DARS2	ASPRS|LBS	aspartyl-tR	protein-co
lib-C666-l	chr17	80231515	80231515	+	68.3168	NA	promoter-TSS (NM_001893)	promoter-TSS (NM_001893)	79	NM_00189	1453	Hs.631725	NM_00189	ENSG0000	CSNK1D	ASPS|CKId	casein kina	protein-co
lib-C666-l	chr4	48833126	48833126	+	12.10977	NA	promoter-TSS (NM_001079840)	promoter-TSS (NM_001079840)	66	NM_01783	54940	Hs.518750	NM_01783	ENSG0000	OCIAD1	ASRIJ|OCIA	OCIA dom	protein-co
lib-C666-l	chr10	101190545	101190545	+	28.68867	NA	promoter-TSS (NM_002079)	promoter-TSS (NM_002079)	-15	NM_00207	2805	Hs.500756	NM_00207	ENSG0000	GOT1	AST1|ASTQ	glutamic-o	protein-co
lib-C666-l	chr2	105953862	105953862	+	403.55533	NA	promoter-TSS (NM_024093)	promoter-TSS (NM_024093)	46	NM_02409	79074	Hs.549577	NM_02409	ENSG0000	C2orf49	asw	chromosor	protein-co
lib-C666-l	chr2	55277743	55277743	+	6.81085	NA	promoter-TSS (NM_153828)	promoter-TSS (NM_153828)	-9	NM_00132	57142	Hs.637850	NM_00700	ENSG0000	RTN4	ASY|NI220	reticulon 4	protein-co
lib-C666-l	chr5	137801017	137801017	+	74.14931	NA	promoter-TSS (NM_001964)	promoter-TSS (NM_001964)	-164	NM_00196	1958	Hs.326035	NM_00196	ENSG0000	EGR1	AT225|G0S	early grow	protein-co
lib-C666-l	chr5	137799851	137799851	+	5.819	NA	Intergenic	CpG	-1330	NM_00196	1958	Hs.326035	NM_00196	ENSG0000	EGR1	AT225|G0S	early grow	protein-co
lib-C666-l	chr10	64576295	64576295	+	13.14211	NA	promoter-TSS (NM_001321037)	promoter-TSS (NM_001321037)	-155	NM_00039	1959	Hs.1395	NM_00039	ENSG0000	EGR2	AT591|CMT	early grow	protein-co
lib-C666-l	chr12	46766587	46766587	+	9.79436	NA	promoter-TSS (NM_001307936)	promoter-TSS (NM_001307936)	58	NM_00130	54407	Hs.221847	NM_01897	ENSG0000	SLC38A2	ATA2|PRO	solute carr	protein-co
lib-C666-l	chr1	168572895	168572895	+	18.64332	NA	Intergenic	LTR88a|LTR|Gypsy?	27184	NM_00299	6375	Hs.546295	NM_00299	ENSG0000	XCL1	ATAC|LPTN	X-C motif	protein-co
lib-C666-l	chr1	160313021	160313021	+	9.79436	NA	promoter-TSS (NM_001290184)	promoter-TSS (NM_001290184)	-42	NM_00129	23385	Hs.517249	NM_01533	ENSG0000	NCSTN	ATAG1874	nicastrin	protein-co
lib-C666-l	chr1	2480254	2480254	+	4.71278	NA	Intergenic	AluY|SINE|Alu	-7550	NM_00129	8764	Hs.512898	NM_00382	ENSG0000	TNFRSF14	ATAR|CD27	TNF recept	protein-co
lib-C666-l	chr16	73087698	73087698	+	8.33814	NA	intron (NM_001164766, intron 1 of 8)	intron (NM_001164766, intron 1 of	4836	NM_00116	463	Hs.598297	NM_00688	ENSG0000	ZFHX3	ATBF1|ATB	zinc finger	protein-co
lib-C666-l	chr16	73104712	73104712	+	5.4536	NA	Intergenic	Intergenic	-12178	NM_00116	463	Hs.598297	NM_00688	ENSG0000	ZFHX3	ATBF1|ATB	zinc finger	protein-co
lib-C666-l	chr8	17658924	17658924	+	76.72826	NA	promoter-TSS (NM_001001925)	promoter-TSS (NM_001001925)	-498	NM_00100	57509	Hs.7946	NM_02074	ENSG0000	MTUS1	ATBP|ATIP|	microtubul	protein-co
lib-C666-l	chr11	102980000	102980000	+	5.92363	NA	promoter-TSS (NM_001080463)	promoter-TSS (NM_001080463)	-160	NM_00137	79659	Hs.503721	NM_00137	ENSG0000	DYNC2H1	ATD3|DHC	dynein cyt	protein-co
lib-C666-l	chr2	166810659	166810659	+	19.19377	NA	promoter-TSS (NM_024753)	promoter-TSS (NM_024753)	-311	NM_02475	79809	Hs.310672	NM_02475	ENSG0000	TTC21B	ATD4|IFT1	tetratricop	protein-co
lib-C666-l	chr2	166810317	166810317	+	15.3763	NA	promoter-TSS (NM_024753)	promoter-TSS (NM_024753)	31	NM_02475	79809	Hs.310672	NM_02475	ENSG0000	TTC21B	ATD4|IFT1	tetratricop	protein-co
lib-C666-l	chr5	37696697	37696697	+	4.74112	NA	intron (NM_001345998, intron 10 of 1	intron (NM_001345998, intron 10 o	138227	NM_00127	2668	Hs.248114	NM_00051	ENSG0000	GDNF	ATF|ATF1|A	glial cell de	protein-co
lib-C666-l	chr10	75668957	75668957	+	8.65614	NA	TTS (NM_001001791)	TTS (NM_001001791)	-1902	NM_00131	5328	Hs.77274	NM_00265	ENSG0000	PLAU	ATF|BDPLT	plasminog	protein-co
lib-C666-l	chr11	2466290	2466290	+	55.75688	NA	promoter-TSS (NM_000218)	promoter-TSS (NM_000218)	69	NM_00021	3784	Hs.95162	NM_00021	ENSG0000	KCNQ1	ATFB1|ATF	potassium	protein-co
lib-C666-l	chr5	37371189	37371189	+	107.40278	NA	promoter-TSS (NM_001278312)	promoter-TSS (NM_001278312)	39	NM_00127	9631	Hs.547696	NM_00429	ENSG0000	NUP155	ATFB15|N1	nucleopori	protein-co
lib-C666-l	chr12	132380756	132380756	+	5.23609	NA	intron (NM_003565, intron 3 of 27)	CpG	1477	NM_00356	8408	Hs.47061	NM_00356	ENSG0000	ULK1	ATG1|ATG	unc-51 like	protein-co
lib-C666-l	chr11	72525397	72525397	+	26.46433	NA	promoter-TSS (NM_001318766)	promoter-TSS (NM_001318766)	-54	NM_00131	89849	Hs.653186	NM_03338	ENSG0000	ATG16L2	ATG16B|W	autophagy	protein-co
lib-C666-l	chr17	66453950	66453950	+	30.4351	NA	promoter-TSS (NM_017983)	promoter-TSS (NM_017983)	-285	NM_00132	55062	Hs.463964	NM_01798	ENSG0000	WIPI1	ATG18|AT	WD repeat	protein-co
lib-C666-l	chr17	40976325	40976325	+	20.82108	NA	promoter-TSS (NM_001313998)	promoter-TSS (NM_001313998)	8	NM_00376	8678	Hs.716464	NM_00376	ENSG0000	BECN1	ATG6|VPS	beclin 1	protein-co
lib-C666-l	chr16	75600202	75600202	+	3.38731	NA	promoter-TSS (NM_007285)	promoter-TSS (NM_007285)	-47	NM_00728	11345	Hs.461379	NM_00728	ENSG0000	GABARAPL	ATG8|ATG	GABA type	protein-co
lib-C666-l	chr17	7146264	7146264	+	4.73866	NA	promoter-TSS (NM_007278)	promoter-TSS (NM_007278)	-511	NM_00727	11337	Hs.647421	NM_00727	ENSG0000	GABARAP	ATG8A|GA	GABA type	protein-co
lib-C666-l	chr17	7145699	7145699	+	4.71278	NA	promoter-TSS (NM_007278)	promoter-TSS (NM_007278)	54	NM_00727	11337	Hs.647421	NM_00727	ENSG0000	GABARAP	ATG8A|GA	GABA type	protein-co
lib-C666-l	chr16	87425823	87425823	+	66.77065	NA	promoter-TSS (NM_001282683)	promoter-TSS (NM_001282683)	22	NM_02281	81631	Hs.356061	NM_02281	ENSG0000	MAP1LC3B	ATG8F|LC3	microtubul	protein-co
lib-C666-l	chr1	242153527	242153527	+	8.56376	NA	Intergenic	MLT1G|LTR|ERVL-MaLR	8858	NM_00100	440738	Hs.534971	NM_00100	ENSG0000	MAP1LC3C	ATG8J|LC3	microtubul	protein-co
lib-C666-l	chr14	99827490	99827490	+	48.59595	NA	Intergenic	Intergenic	-89440	NM_00128	64919	Hs.709690	NM_02289	ENSG0000	BCL11B	ATL1|ATL1	B-cell CLL/	protein-co
lib-C666-l	chr20	5100657	5100657	+	33.11264	NA	promoter-TSS (NM_182649)	promoter-TSS (NM_182649)	-10	NM_18264	5111	Hs.147433	NM_00259	ENSG0000	PCNA	ATLD2	proliferatin	protein-co
lib-C666-l	chr1	47134095	47134095	+	7.0832	NA	promoter-TSS (NM_001042546)	promoter-TSS (NM_001042546)	4	NM_02274	64756	Hs.100874	NM_02274	ENSG0000	ATPAF1	ATP11|ATP	ATP syntha	protein-co
lib-C666-l	chr19	19774540	19774540	+	8.01457	NA	promoter-TSS (NM_020410)	promoter-TSS (NM_020410)	-37	NM_02041	57130	Hs.501794	NM_02041	ENSG0000	ATP13A1	ATP13A|CC	ATPase 13	protein-co
lib-C666-l	chr11	117692979	117692979	+	7.31982	NA	intron (NM_001243598, intron 7 of 9)	intron (NM_001243598, intron 7 of	2480	NM_00168	486	Hs.731865	NM_00168	ENSG0000	FXYD2	ATP1G1|HC	FXYD dom	protein-co
lib-C666-l	chr1	203632569	203632569	+	12.55817	NA	intron (NM_001684, intron 1 of 20)	intron (NM_001684, intron 1 of 20)	36654	NM_00100	493	Hs.343522	NM_00168	ENSG0000	ATP2B4	ATP2B2|MX	ATPase pla	protein-co
lib-C666-l	chr1	203626692	203626692	+	10.73451	NA	intron (NM_001684, intron 1 of 20)	intron (NM_001684, intron 1 of 20)	30777	NM_00100	493	Hs.343522	NM_00168	ENSG0000	ATP2B4	ATP2B2|MX	ATPase pla	protein-co
lib-C666-l	chr18	43678145	43678145	+	28.64047	NA	exon (NM_004046, exon 1 of 12)	exon (NM_004046, exon 1 of 12)	174	NM_00100	498	Hs.298280	NM_00404	ENSG0000	ATP5A1	ATP5A|ATF	ATP syntha	protein-co
lib-C666-l	chr17	46969874	46969874	+	38.60092	NA	promoter-TSS (NM_005175)	promoter-TSS (NM_005175)	-274	NM_00517	516	Hs.80986	NM_00517	ENSG0000	ATP5G1	ATP5A|ATF	ATP syntha	protein-co
lib-C666-l	chr4	668212	668212	+	77.06716	NA	promoter-TSS (NM_007100)	promoter-TSS (NM_007100)	-85	NR_03374	521	Hs.85539	NM_00710	ENSG0000	ATP5I	ATP5K	ATP syntha	protein-co
lib-C666-l	chr3	113465703	113465703	+	19.65763	NA	promoter-TSS (NM_001308445)	promoter-TSS (NM_001308445)	-163	NM_00169	523	Hs.477155	NM_00169	ENSG0000	ATP6V1A	ATP6A1|AT	ATPase H+	protein-co
lib-C666-l	chr16	2564270	2564270	+	143.2643	NA	intron (NM_001694, intron 1 of 2)	CpG	399	NM_00169	527	Hs.389107	NM_00169	ENSG0000	ATP6V0C	ATP6C|ATF	ATPase H+	protein-co
lib-C666-l	chr16	2563849	2563849	+	17.13943	NA	promoter-TSS (NM_001694)	promoter-TSS (NM_001694)	-22	NM_00169	527	Hs.389107	NM_00169	ENSG0000	ATP6V0C	ATP6C|ATF	ATPase H+	protein-co
lib-C666-l	chr16	67515128	67515128	+	15.60931	NA	promoter-TSS (NM_004691)	promoter-TSS (NM_004691)	-39	NM_00469	9114	Hs.106876	NM_00469	ENSG0000	ATP6V0D1	ATP6D|ATF	ATPase H+	protein-co
lib-C666-l	chr22	18111617	18111617	+	13.14211	NA	promoter-TSS (NM_001696)	promoter-TSS (NM_001696)	-29	NM_00169	529	Hs.517338	NM_00169	ENSG0000	ATP6V1E1	ATP6E|ATP	ATPase H+	protein-co
lib-C666-l	chr2	46769490	46769490	+	8.96177	NA	promoter-TSS (NM_012249)	promoter-TSS (NM_012249)	206	NM_00131	90423	Hs.659656	NM_08065	ENSG0000	ATP6V1E2	ATP6E1|AT	ATPase H+	protein-co
lib-C666-l	chr1	44440570	44440570	+	14.31268	NA	5' UTR (NM_004047, exon 1 of 8)	5' UTR (NM_004047, exon 1 of 8)	250	NM_00404	533	Hs.596514	NM_00404	ENSG0000	ATP6V0B	ATP6F|HAT	ATPase H+	protein-co
lib-C666-l	chr4	42659251	42659251	+	5.5166	NA	promoter-TSS (NM_001105529)	promoter-TSS (NM_001105529)	-129	NM_00110	10396	Hs.435052	NM_00609	ENSG0000	ATP8A1	ATPASEII|A	ATPase ph	protein-co
lib-C666-l	chr18	76829173	76829173	+	195.86848	NA	promoter-TSS (NM_001306085)	promoter-TSS (NM_001306085)	-102	NM_19853	374868	Hs.465475	NM_19853	ENSG0000	ATP9B	ATPASEP|A	ATPase ph	protein-co
lib-C666-l	chr3	141594946	141594946	+	169.86967	NA	promoter-TSS (NM_001679)	promoter-TSS (NM_001679)	-488	NM_00167	483	Hs.477789	NM_00167	ENSG0000	ATP1B3	ATPB-3|CD	ATPase Na	protein-co
lib-C666-l	chr1	27216772	27216772	+	4.73866	NA	promoter-TSS (NM_018066)	promoter-TSS (NM_018066)	97	NM_01806	54707	Hs.14333	NM_01806	ENSG0000	GPN2	ATPBD1B	GPN-loop	protein-co
lib-C666-l	chr19	51611851	51611851	+	4.71278	NA	promoter-TSS (NM_145232)	promoter-TSS (NM_145232)	-204	NM_14523	90353	Hs.148425	NM_14523	ENSG0000	CTU1	ATPBD3|N	cytosolic th	protein-co
lib-C666-l	chr15	35838452	35838452	+	13.59977	NA	promoter-TSS (NR_038251)	promoter-TSS (NR_038251)	-48	NM_08065	89978	Hs.107196	NM_08065	ENSG0000	DPH6	ATPBD4	diphthamir	protein-co
lib-C666-l	chr16	89787407	89787407	+	6.29226	NA	promoter-TSS (NR_110122)	promoter-TSS (NR_110122)	-13	NM_00491	9605	Hs.164410	NM_00491	ENSG0000	VPS9D1	ATP-BL|C1	VPS9 dom	protein-co
lib-C666-l	chr1	28573880	28573880	+	8.01457	NA	Intergenic	CpG	11278	NM_17819	93974	Hs.744914	NM_01631	ENSG0000	ATPIF1	ATPI|ATPIP	ATPase inh	protein-co
lib-C666-l	chr18	55470518	55470518	+	3.29276	NA	promoter-TSS (NM_005603)	promoter-TSS (NM_005603)	-191	NM_00560	5205	Hs.216623	NM_00560	ENSG0000	ATP8B1	ATPIC|BRIC	ATPase ph	protein-co
lib-C666-l	chr18	55487352	55487352	+	2.78587	NA	Intergenic	Intergenic	-17025	NM_00560	5205	Hs.216623	NM_00560	ENSG0000	ATP8B1	ATPIC|BRIC	ATPase ph	protein-co
lib-C666-l	chr1	154301819	154301819	+	5.96422	NA	intron (NM_020452, intron 2 of 27)	intron (NM_020452, intron 2 of 27)	1543	NM_02045	57198	Hs.435700	NM_02045	ENSG0000	ATP8B2	ATPID	ATPase ph	protein-co
lib-C666-l	chr3	182511071	182511071	+	16.14038	NA	promoter-TSS (NM_014616)	promoter-TSS (NM_014616)	-220	NM_01461	23200	Hs.478429	NM_01461	ENSG0000	ATP11B	ATPIF|ATPI	ATPase ph	protein-co
lib-C666-l	chr3	182458944	182458944	+	9.28096	NA	Intergenic	Intergenic	-52347	NM_01461	23200	Hs.478429	NM_01461	ENSG0000	ATP11B	ATPIF|ATPI	ATPase ph	protein-co
lib-C666-l	chr20	50384158	50384158	+	6.32351	NA	intron (NM_006045, intron 1 of 27)	CpG	792	NM_00604	10079	Hs.649234	NM_00604	ENSG0000	ATP9A	ATPIIA	ATPase ph	protein-co
lib-C666-l	chr20	50384357	50384357	+	4.39666	NA	intron (NM_006045, intron 1 of 27)	CpG	593	NM_00604	10079	Hs.649234	NM_00604	ENSG0000	ATP9A	ATPIIA	ATPase ph	protein-co
lib-C666-l	chr12	57039903	57039903	+	19.60174	NA	promoter-TSS (NM_001686)	promoter-TSS (NM_001686)	-51	NM_00168	506	Hs.406510	NM_00168	ENSG0000	ATP5B	ATPMB|AT	ATP syntha	protein-co
lib-C666-l	chr21	35174167	35174167	+	13.46097	NA	intron (NM_001331012, intron 19 of 2	intron (NM_001331012, intron 19 o	113991	NM_00169	539	Hs.409140	NM_00169	ENSG0000	ATP5O	ATPO|HMC	ATP syntha	protein-co
lib-C666-l	chr21	35288126	35288126	+	3.62735	NA	promoter-TSS (NM_001697)	promoter-TSS (NM_001697)	32	NM_00169	539	Hs.409140	NM_00169	ENSG0000	ATP5O	ATPO|HMC	ATP syntha	protein-co
lib-C666-l	chr21	45719687	45719687	+	6.20473	NA	promoter-TSS (NM_001002021)	promoter-TSS (NM_001002021)	-230	NM_00262	5211	Hs.255093	NM_00262	ENSG0000	PFKL	ATP-PFK|P	phosphofru	protein-co
lib-C666-l	chr4	108607099	108607099	+	23.73126	NA	intron (NM_005443, intron 4 of 11)	intron (NM_005443, intron 4 of 11)	34320	NM_00544	9061	Hs.368610	NM_00544	ENSG0000	PAPSS1	ATPSK1|PA	3'-phosph	protein-co
lib-C666-l	chr4	47487243	47487243	+	36.84144	NA	promoter-TSS (NM_020453)	promoter-TSS (NM_020453)	-167	NM_02045	57205	Hs.437241	NM_02045	ENSG0000	ATP10D	ATPVD	ATPase ph	protein-co
lib-C666-l	chr12	110011071	110011071	+	360.59042	NA	promoter-TSS (NM_000431)	promoter-TSS (NM_000431)	287	NR_03811	326625	Hs.12106	NM_05284	ENSG0000	MMAB	ATR|CFAP2	methylmal	protein-co
lib-C666-l	chrX	77041828	77041828	+	6.32351	NA	promoter-TSS (NM_000489)	promoter-TSS (NM_000489)	-73	NM_00048	546	Hs.533526	NM_00048	ENSG0000	ATRX	ATR2|JMS|I	ATRX, chrc	protein-co
lib-C666-l	chr13	30169956	30169956	+	2.78587	NA	promoter-TSS (NM_003045)	promoter-TSS (NM_003045)	-131	NM_00304	6541	Hs.14846	NM_00304	ENSG0000	SLC7A1	ATRC1|CAT	solute carr	protein-co
lib-C666-l	chr8	90996822	90996822	+	11.51321	NA	promoter-TSS (NM_002485)	promoter-TSS (NM_002485)	77	NM_00248	4683	Hs.492208	NM_00248	ENSG0000	NBN	AT-V1|AT-	nibrin	protein-co
lib-C666-l	chr5	151109331	151109331	+	12.62235	NA	Intergenic	MER11B|LTR|ERVK	28879	NM_00404	475	Hs.125213	NM_00404	ENSG0000	ATOX1	ATX1|HAH	antioxidant	protein-co
lib-C666-l	chr7	105305468	105305468	+	116.66657	NA	intron (NM_001318229, intron 3 of 9)	intron (NM_001318229, intron 3 of	14141	NM_13849	222255	Hs.745056	NM_02072	ENSG0000	ATXN7L1	ATXN7L4	ataxin 7 lik	protein-co
lib-C666-l	chr7	105315238	105315238	+	4.52748	NA	intron (NM_020725, intron 3 of 11)	intron (NM_020725, intron 3 of 11)	4371	NM_13849	222255	Hs.745056	NM_02072	ENSG0000	ATXN7L1	ATXN7L4	ataxin 7 lik	protein-co
lib-C666-l	chr4	83295381	83295381	+	5.72511	NA	promoter-TSS (NM_002138)	promoter-TSS (NM_002138)	-232	NM_03136	3184	Hs.480073	NM_00213	ENSG0000	HNRNPD	AUF1|AUF1	heterogen	protein-co
lib-C666-l	chr9	86595090	86595090	+	5.80117	NA	promoter-TSS (NM_001318187)	promoter-TSS (NM_001318187)	96	NM_00131	3190	Hs.522257	NM_00214	ENSG0000	HNRNPK	AUKS|CSBF	heterogen	protein-co
lib-C666-l	chr9	86595342	86595342	+	3.23837	NA	promoter-TSS (NM_001318187)	promoter-TSS (NM_001318187)	-156	NM_00131	3190	Hs.522257	NM_00214	ENSG0000	HNRNPK	AUKS|CSBF	heterogen	protein-co
lib-C666-l	chr16	2265142	2265142	+	15.60931	NA	promoter-TSS (NM_001042371)	promoter-TSS (NM_001042371)	-320	NM_00104	283871	Hs.442634	NM_00104	ENSG0000	PGP	AUM|G3PP	phosphogl	protein-co
lib-C666-l	chr2	26726231	26726231	+	22.01132	NA	intron (NM_001287489, intron 6 of 4	CpG	-25321	NM_19432	9381	Hs.91608	NM_00480	ENSG0000	OTOF	AUNB1|DF	otoferlin	protein-co
lib-C666-l	chr2	26726626	26726626	+	3.25502	NA	intron (NM_001287489, intron 6 of 4	CpG	-25716	NM_19432	9381	Hs.91608	NM_00480	ENSG0000	OTOF	AUNB1|DF	otoferlin	protein-co
lib-C666-l	chr14	21905404	21905404	+	2.59032	NA	promoter-TSS (NM_020920)	promoter-TSS (NM_020920)	53	NM_02092	57680	Hs.530698	NM_02092	ENSG0000	CHD8	AUTS18|HE	chromodo	protein-co
lib-C666-l	chr4	99851205	99851205	+	12.62235	NA	promoter-TSS (NM_001130678)	promoter-TSS (NM_001130678)	583	NM_00196	1977	Hs.13211	NM_00196	ENSG0000	EIF4E	AUTS19|CE	eukaryotic	protein-co
lib-C666-l	chr7	116312222	116312222	+	15.31489	NA	promoter-TSS (NM_001324402)	promoter-TSS (NM_001324402)	-191	NM_00132	4233	Hs.132966	NM_00024	ENSG0000	MET	AUTS9|DFN	MET proto	protein-co
lib-C666-l	chrX	153617552	153617552	+	16.63918	NA	Intergenic	CpG	-8854	NM_00601	6134	Hs.534404	NM_00601	ENSG0000	RPL10	AUTSX5|D	ribosomal	protein-co
lib-C666-l	chrX	153618586	153618586	+	9.21057	NA	Intergenic	CpG	-7820	NM_00601	6134	Hs.534404	NM_00601	ENSG0000	RPL10	AUTSX5|D	ribosomal	protein-co
lib-C666-l	chrX	153626571	153626571	+	7.76438	NA	promoter-TSS (NM_001303624)	promoter-TSS (NM_001303624)	-142	NM_00130	6134	Hs.534404	NM_00601	ENSG0000	RPL10	AUTSX5|D	ribosomal	protein-co
lib-C666-l	chr3	9975458	9975458	+	38.60092	NA	promoter-TSS (NM_015513)	promoter-TSS (NM_015513)	-66	NM_01551	78987	Hs.9383	NM_01551	ENSG0000	CRELD1	AVSD2|CIR	cysteine ric	protein-co
lib-C666-l	chr3	9974711	9974711	+	9.45164	NA	promoter-TSS (NM_015513)	promoter-TSS (NM_015513)	-813	NM_01551	78987	Hs.9383	NM_01551	ENSG0000	CRELD1	AVSD2|CIR	cysteine ric	protein-co
lib-C666-l	chr6	122170863	122170863	+	206.28981	NA	Intergenic	Intergenic	414140	NM_00016	2697	Hs.74471	NM_00016	ENSG0000	GJA1	AVSD3|CM	gap junctic	protein-co
lib-C666-l	chr6	122161252	122161252	+	22.50982	NA	Intergenic	Intergenic	404529	NM_00016	2697	Hs.74471	NM_00016	ENSG0000	GJA1	AVSD3|CM	gap junctic	protein-co
lib-C666-l	chr6	121825542	121825542	+	5.04179	NA	Intergenic	Intergenic	68819	NM_00016	2697	Hs.74471	NM_00016	ENSG0000	GJA1	AVSD3|CM	gap junctic	protein-co
lib-C666-l	chr6	122160182	122160182	+	2.91431	NA	Intergenic	Intergenic	403459	NM_00016	2697	Hs.74471	NM_00016	ENSG0000	GJA1	AVSD3|CM	gap junctic	protein-co
lib-C666-l	chr7	23571679	23571679	+	88.77095	NA	promoter-TSS (NM_001282758)	promoter-TSS (NM_001282758)	-19	NM_00128	29896	Hs.445652	NM_01329	ENSG0000	TRA2A	AWMS1|HS	transforme	protein-co
lib-C666-l	chr15	80352399	80352399	+	111.12958	NA	promoter-TSS (NM_001242914)	promoter-TSS (NM_001242914)	-103	NM_00124	54469	Hs.596679	NM_01900	ENSG0000	ZFAND6	AWP1|ZA2	zinc finger	protein-co
lib-C666-l	chr15	80351994	80351994	+	38.30731	NA	promoter-TSS (NM_001242911)	promoter-TSS (NM_001242911)	84	NM_00124	54469	Hs.596679	NM_01900	ENSG0000	ZFAND6	AWP1|ZA2	zinc finger	protein-co
lib-C666-l	chr3	185304023	185304023	+	93.07718	NA	promoter-TSS (NM_021627)	promoter-TSS (NM_021627)	-8	NM_02162	59343	Hs.401388	NM_02162	ENSG0000	SENP2	AXAM2|SM	SUMO1/se	protein-co
lib-C666-l	chr1	213031550	213031550	+	8.28613	NA	promoter-TSS (NR_027285)	promoter-TSS (NR_027285)	-47	NM_01405	28982	Hs.7055	NM_01405	ENSG0000	FLVCR1	AXPC1|FLV	feline leuk	protein-co
lib-C666-l	chr3	39194420	39194420	+	8.71138	NA	intron (NM_001320560, intron 1 of 4)	CpG	682	NM_03302	64651	Hs.370950	NM_03302	ENSG0000	CSRNP1	AXUD1|CS	cysteine ar	protein-co
lib-C666-l	chr3	39195071	39195071	+	3.84463	NA	promoter-TSS (NM_033027)	promoter-TSS (NM_033027)	31	NM_03302	64651	Hs.370950	NM_03302	ENSG0000	CSRNP1	AXUD1|CS	cysteine ar	protein-co
lib-C666-l	chr16	79634653	79634653	+	9.10494	NA	promoter-TSS (NM_005360)	promoter-TSS (NM_005360)	-31	NM_00536	4094	Hs.134859	NM_00536	ENSG0000	MAF	AYGRP|CC	MAF bZIP	protein-co
lib-C666-l	chr17	79196835	79196835	+	26.93404	NA	promoter-TSS (NM_001009811)	promoter-TSS (NM_001009811)	-46	NM_00131	22994	Hs.514578	NM_01498	ENSG0000	CEP131	AZ1|AZI1|Z	centrosom	protein-co
lib-C666-l	chr8	103876462	103876462	+	22.82077	NA	promoter-TSS (NM_015878)	promoter-TSS (NM_015878)	-34	NM_01587	51582	Hs.459106	NM_01587	ENSG0000	AZIN1	AZI|AZIA1|	antizyme ir	protein-co
lib-C666-l	chr8	103819129	103819129	+	6.94566	NA	Intergenic	CpG	57299	NM_00130	51582	Hs.459106	NM_01587	ENSG0000	AZIN1	AZI|AZIA1|	antizyme ir	protein-co
lib-C666-l	chr8	103820940	103820940	+	3.98718	NA	Intergenic	Intergenic	55488	NM_00130	51582	Hs.459106	NM_01587	ENSG0000	AZIN1	AZI|AZIA1|	antizyme ir	protein-co
lib-C666-l	chr8	103781598	103781598	+	3.07144	NA	Intergenic	Intergenic	94830	NM_00130	51582	Hs.459106	NM_01587	ENSG0000	AZIN1	AZI|AZIA1|	antizyme ir	protein-co
lib-C666-l	chr19	2269414	2269414	+	15.42216	NA	promoter-TSS (NM_001301020)	promoter-TSS (NM_001301020)	-71	NM_00130	4946	Hs.446427	NM_00415	ENSG0000	OAZ1	AZI|OAZ	ornithine d	protein-co
lib-C666-l	chr2	173868104	173868104	+	10.03103	NA	intron (NM_001100397, intron 14 of 2	intron (NM_001100397, intron 14 o	-72461	NM_13364	51776	Hs.444451	NM_01665	ENSG0000	MAP3K20	AZK|MLK7|	mitogen-a	protein-co
lib-C666-l	chr10	123778817	123778817	+	9.45164	NA	intron (NM_206862, intron 1 of 22)	intron (NM_206862, intron 1 of 22)	30128	NM_20686	10579	Hs.501252	NM_00699	ENSG0000	TACC2	AZU-1|EC1	transformir	protein-co
lib-C666-l	chr10	123779177	123779177	+	3.96379	NA	intron (NM_206862, intron 1 of 22)	intron (NM_206862, intron 1 of 22)	30488	NM_20686	10579	Hs.501252	NM_00699	ENSG0000	TACC2	AZU-1|EC1	transformir	protein-co
lib-C666-l	chr7	33534320	33534320	+	10.40942	NA	intron (NM_001033604, intron 18 of 2	L2c|LINE|L2	365168	NM_19842	27241	Hs.372360	NM_01445	ENSG0000	BBS9	B1|C18|D1|	Bardet-Bie	protein-co
lib-C666-l	chr7	123197910	123197910	+	26.49922	NA	promoter-TSS (NM_001282422)	promoter-TSS (NM_001282422)	48	NM_00500	4698	Hs.651219	NM_00500	ENSG0000	NDUFA5	B13|CI-13K	NADH:ubic	protein-co
lib-C666-l	chr19	8386271	8386271	+	60.07874	NA	promoter-TSS (NR_135539)	promoter-TSS (NR_135539)	26	NM_00500	4701	Hs.333427	NM_00500	ENSG0000	NDUFA7	B14.5a|CI-	NADH:ubic	protein-co
lib-C666-l	chr3	120341547	120341547	+	3.76263	NA	Intergenic	HERVIP10FH-int|LTR|ERV1	26419	NM_00454	4710	Hs.304613	NM_00454	ENSG0000	NDUFB4	B15|CI-B15	NADH:ubic	protein-co
lib-C666-l	chr21	34960983	34960983	+	15.1031	NA	promoter-TSS (NM_017613)	promoter-TSS (NM_017613)	31	NM_01761	29980	Hs.436341	NM_01761	ENSG0000	DONSON	B17|C21orf	downstrea	protein-co
lib-C666-l	chr9	32573169	32573169	+	63.78558	NA	promoter-TSS (NM_001199987)	promoter-TSS (NM_001199987)	13	NM_00249	4712	Hs.493668	NM_00249	ENSG0000	NDUFB6	B17|CI	NADH:ubic	protein-co
lib-C666-l	chr21	33784051	33784051	+	17.27528	NA	promoter-TSS (NR_135467)	promoter-TSS (NR_135467)	51	NM_00132	59271	Hs.208358	NM_05818	ENSG0000	EVA1C	B18|B19|C2	eva-1 hom	protein-co
lib-C666-l	chr21	33784618	33784618	+	12.97267	NA	promoter-TSS (NR_135467)	promoter-TSS (NR_135467)	-71	NR_13546	59271	Hs.208358	NM_05818	ENSG0000	EVA1C	B18|B19|C2	eva-1 hom	protein-co
lib-C666-l	chr21	33785112	33785112	+	4.19675	NA	non-coding (NR_135467, exon 1 of 8	non-coding (NR_135467, exon 1 of	367	NM_05818	59271	Hs.208358	NM_05818	ENSG0000	EVA1C	B18|B19|C2	eva-1 hom	protein-co
lib-C666-l	chr19	14682903	14682903	+	3.62735	NA	promoter-TSS (NM_004146)	promoter-TSS (NM_004146)	-14	NM_00414	4713	Hs.532853	NM_00414	ENSG0000	NDUFB7	B18|CI-B18	NADH:ubic	protein-co
lib-C666-l	chr14	96671100	96671100	+	25.68529	NA	promoter-TSS (NM_000623)	promoter-TSS (NM_000623)	-35	NM_00062	624	Hs.654542	NM_00062	ENSG0000	BDKRB2	B2R|BK-2|E	bradykinin	protein-co
lib-C666-l	chr14	96653753	96653753	+	17.37618	NA	Intergenic	Intergenic	-17382	NM_00062	624	Hs.654542	NM_00062	ENSG0000	BDKRB2	B2R|BK-2|E	bradykinin	protein-co
lib-C666-l	chr1	183204135	183204135	+	22.50982	NA	intron (NM_018891, intron 15 of 21)	intron (NM_018891, intron 15 of 21	48961	NM_00556	3918	Hs.591484	NM_00556	ENSG0000	LAMC2	B2T|BM60(	laminin sub	protein-co
lib-C666-l	chr1	183129269	183129269	+	6.81085	NA	Intergenic	Intergenic	-25905	NM_01889	3918	Hs.591484	NM_00556	ENSG0000	LAMC2	B2T|BM60(	laminin sub	protein-co
lib-C666-l	chr1	235667917	235667917	+	77.6848	NA	promoter-TSS (NM_152490)	promoter-TSS (NM_152490)	-136	NM_15249	148789	Hs.498143	NM_15249	ENSG0000	B3GALNT2	B3GalNAc-	beta-1,3-N	protein-co
lib-C666-l	chr19	17889579	17889579	+	27.28371	NA	exon (NM_001161357, exon 20 of 29)	exon (NM_001161357, exon 20 of 2	-16340	NM_01425	10331	Hs.69009	NM_01425	ENSG0000	B3GNT3	B3GAL-T8|	UDP-GlcN	protein-co
lib-C666-l	chr13	31774883	31774883	+	5.04826	NA	intron (NM_194318, intron 1 of 14)	CpG	771	NM_19431	145173	Hs.13205	NM_19431	ENSG0000	B3GLCT	B3GALTL|B	beta 3-glu	protein-co
lib-C666-l	chr3	183003823	183003823	+	44.52938	NA	intron (NM_015078, intron 14 of 29)	intron (NM_015078, intron 14 of 29	32791	NM_03204	84002	Hs.718506	NM_03204	ENSG0000	B3GNT5	B3GN-T5|E	UDP-GlcN	protein-co
lib-C666-l	chr3	182970843	182970843	+	5.25516	NA	promoter-TSS (NM_032047)	promoter-TSS (NM_032047)	-189	NM_03204	84002	Hs.718506	NM_03204	ENSG0000	B3GNT5	B3GN-T5|E	UDP-GlcN	protein-co
lib-C666-l	chr6	89791516	89791516	+	34.3461	NA	intron (NM_006813, intron 1 of 1)	CpG	1087	NM_00681	10957	Hs.75969	NM_00681	ENSG0000	PNRC1	B4-2|PNAS	proline rich	protein-co
lib-C666-l	chr3	115503391	115503391	+	17.3027	NA	Intergenic	CpG	161240	NM_00204	2596	Hs.134974	NM_00204	ENSG0000	GAP43	B-50|PP46	growth ass	protein-co
lib-C666-l	chr20	42086479	42086479	+	13.89614	NA	promoter-TSS (NR_034009)	promoter-TSS (NR_034009)	-25	NM_00627	6431	Hs.684950	NM_00627	ENSG0000	SRSF6	B52|HEL-S	serine and	protein-co
lib-C666-l	chr17	4736614	4736614	+	12.83248	NA	promoter-TSS (NM_001321236)	promoter-TSS (NM_001321236)	-21	NM_17066	50488	Hs.443417	NM_01571	ENSG0000	MINK1	B55|MAP4I	misshapen	protein-co

lib-C666-l	chr8	26149027	26149027	+	9.21057	NA	promoter-TSS (NM_002717)	promoter-TSS (NM_002717)	20	NM_00271	5520	Hs.146339	NM_00271	ENSG0000	PPP2R2A	B55A|B55A	protein ph	protein-co
lib-C666-l	chr11	64692075	64692075	+	6.71839	NA	promoter-TSS (NM_006244)	promoter-TSS (NM_006244)	-68	NM_00624	5526	Hs.75199	NM_00624	ENSG0000	PPP2R5B	B56B|PR61	protein ph	protein-co
lib-C666-l	chr14	102276644	102276644	+	14.54258	NA	intron (NM_178586, intron 1 of 12)	intron (NM_178586, intron 1 of 12)	504	NM_00271	5527	Hs.368264	NM_00271	ENSG0000	PPP2R5C	B56G|PR61	protein ph	protein-co
lib-C666-l	chr14	102276151	102276151	+	13.59254	NA	promoter-TSS (NM_178587)	promoter-TSS (NM_178587)	11	NM_00271	5527	Hs.368264	NM_00271	ENSG0000	PPP2R5C	B56G|PR61	protein ph	protein-co
lib-C666-l	chr22	43045325	43045325	+	11.13288	NA	promoter-TSS (NM_000398)	promoter-TSS (NM_000398)	80	NM_00039	1727	Hs.561064	NM_00039	ENSG0000	CYB5R3	B5R|DIA1	cytochrom	protein-co
lib-C666-l	chr1	201280745	201280745	+	7.9573	NA	intron (NM_001005337, intron 2 of 1	intron (NM_001005337, intron 2 of	28165	NM_00029	5317	Hs.497350	NM_00029	ENSG0000	PKP1	B6P	plakophilin	protein-co
lib-C666-l	chr1	201280058	201280058	+	6.01141	NA	intron (NM_001005337, intron 2 of 1	intron (NM_001005337, intron 2 of	27478	NM_00029	5317	Hs.497350	NM_00029	ENSG0000	PKP1	B6P	plakophilin	protein-co
lib-C666-l	chr10	73533323	73533323	+	4.49043	NA	promoter-TSS (NM_022153)	promoter-TSS (NM_022153)	14	NM_02215	64115	Hs.47382	NM_02215	ENSG0000	VSIR	B7-H5|B7H	V-set imm	protein-co
lib-C666-l	chr11	17373379	17373379	+	30.04317	NA	promoter-TSS (NM_001202439)	promoter-TSS (NM_001202439)	70	NM_00120	374383	Hs.146274	NM_00100	ENSG0000	NCR3LG1	B7-H6|B7H	natural kill	protein-co
lib-C666-l	chr17	19265816	19265816	+	7.08136	NA	intron (NM_001243475, intron 1 of 6)	CpG	235	NM_00133	27077	Hs.309467	NM_01568	ENSG0000	B9D1	B9|EPPB9|J	B9 domain	protein-co
lib-C666-l	chr14	103605174	103605174	+	7.21092	NA	Intergenic	CpG	12510	NM_00629	7127	Hs.525607	NM_00629	ENSG0000	TNFAIP2	B94|EXOC3	TNF alpha	protein-co
lib-C666-l	chr10	102792114	102792114	+	24.32656	NA	5' UTR (NM_030971, exon 2 of 12)	5' UTR (NM_030971, exon 2 of 12)	1118	NM_03097	81855	Hs.283844	NM_03097	ENSG0000	SFXN3	BA108L7.2	sideroflexir	protein-co
lib-C666-l	chr10	102791213	102791213	+	6.94566	NA	promoter-TSS (NM_001195263)	promoter-TSS (NM_001195263)	217	NM_03097	81855	Hs.283844	NM_03097	ENSG0000	SFXN3	BA108L7.2	sideroflexir	protein-co
lib-C666-l	chr9	70146024	70146024	+	6.81085	NA	Intergenic	Intergenic	32791	NM_00112	653427	Hs.714683	NM_00112	ENSG0000	FOXD4L5	bA15J10.2	forkhead b	protein-co
lib-C666-l	chr10	38146615	38146615	+	14.98892	NA	promoter-TSS (NM_001267606)	promoter-TSS (NM_001267606)	-51	NM_02104	57209	Hs.528423	NM_02104	ENSG0000	ZNF248	bA162G10	zinc finger	protein-co
lib-C666-l	chr10	105836697	105836697	+	13.59254	NA	intron (NM_000494, intron 4 of 55)	intron (NM_000494, intron 4 of 55)	8941	NM_00049	1308	Hs.117938	NM_00049	ENSG0000	COL17A1	BA16H23.2	collagen ty	protein-co
lib-C666-l	chr9	114393581	114393581	+	13.46097	NA	promoter-TSS (NM_001015882)	promoter-TSS (NM_001015882)	-51	NR_03714	548645	Hs.534196	NM_00101	ENSG0000	DNAJC25	bA16L21.2	DnaJ heat	protein-co
lib-C666-l	chr9	131938206	131938206	+	20.72609	NA	3' UTR (NM_203434, exon 1 of 1)	3' UTR (NM_203434, exon 1 of 1)	2334	NM_20343	389792	Hs.529857	NM_20343	ENSG0000	IER5L	bA247A12	immediate	protein-co
lib-C666-l	chr9	131936217	131936217	+	9.94079	NA	Intergenic	Intergenic	4323	NM_20343	389792	Hs.529857	NM_20343	ENSG0000	IER5L	bA247A12	immediate	protein-co
lib-C666-l	chr20	14813596	14813596	+	8.01457	NA	intron (NM_080676, intron 5 of 16)	LTR16E1|LTR|ERVL	96568	NR_11031	100379174	Hs.407533	NR_03784	ENSG0000	MACROD2	BA318C17.	MACROD2	ncRNA
lib-C666-l	chr10	31320742	31320742	+	139.68425	NA	intron (NM_182755, intron 1 of 7)	CpG	124	NM_00114	220929	Hs.660642	NM_18275	ENSG0000	ZNF438	bA330O11	zinc finger	protein-co
lib-C666-l	chr10	31388788	31388788	+	9.47655	NA	Intergenic	Intergenic	-67922	NM_18275	220929	Hs.660642	NM_18275	ENSG0000	ZNF438	bA330O11	zinc finger	protein-co
lib-C666-l	chr9	130007526	130007526	+	66.82613	NA	Intergenic	CpG	-18415	NM_00128	84253	Hs.29304	NM_03229	ENSG0000	GARNL3	bA356B19.	GTPase act	protein-co
lib-C666-l	chr20	44997321	44997321	+	3.8557	NA	intron (NM_182764, intron 20 of 20)	intron (NM_182764, intron 20 of 20	-4224	NM_00128	51006	Hs.593344	NM_01594	ENSG0000	SLC35C2	BA394O2.1	solute carr	protein-co
lib-C666-l	chr9	38068056	38068056	+	8.28613	NA	exon (NM_003028, exon 1 of 6)	exon (NM_003028, exon 1 of 6)	1154	NM_00302	6461	Hs.521482	NM_00302	ENSG0000	SHB	bA3J10.2	SH2 doma	protein-co
lib-C666-l	chr9	38069741	38069741	+	7.15277	NA	promoter-TSS (NM_003028)	promoter-TSS (NM_003028)	-531	NM_00302	6461	Hs.521482	NM_00302	ENSG0000	SHB	bA3J10.2	SH2 doma	protein-co
lib-C666-l	chr9	38047938	38047938	+	4.87548	NA	intron (NM_003028, intron 1 of 5)	intron (NM_003028, intron 1 of 5)	21272	NM_00302	6461	Hs.521482	NM_00302	ENSG0000	SHB	bA3J10.2	SH2 doma	protein-co
lib-C666-l	chr9	38227356	38227356	+	2.2973	NA	Intergenic	Intergenic	-158146	NM_00302	6461	Hs.521482	NM_00302	ENSG0000	SHB	bA3J10.2	SH2 doma	protein-co
lib-C666-l	chr10	75692294	75692294	+	6.61453	NA	Intergenic	Intergenic	-9759	NM_00100	414236	Hs.585453	NM_00100	ENSG0000	C10orf55	bA417O11	chromosor	protein-co
lib-C666-l	chr13	37573633	37573633	+	17.22931	NA	promoter-TSS (NM_001142364)	promoter-TSS (NM_001142364)	-129	NM_00114	29880	Hs.507769	NM_01333	ENSG0000	ALG5	bA421P11.	ALG5, dolic	protein-co
lib-C666-l	chr9	112956770	112956770	+	12.12504	NA	Intergenic	MER4C|LTR|ERV1	13643	NM_00101	401546	Hs.125608	NM_00101	ENSG0000	C9orf152	bA470J20.2	chromosor	protein-co
lib-C666-l	chr10	29228937	29228937	+	8.71456	NA	Intergenic	Intergenic	93600	NM_00127	283080		NM_001278522		C10orf126	bA492M23	chromosor	protein-co
lib-C666-l	chr6	146285559	146285559	+	7.15277	NA	promoter-TSS (NM_001042683)	promoter-TSS (NM_001042683)	-326	NM_17308	257218	Hs.723297	NM_17308	ENSG0000	SHPRH	bA545I5.2	SNF2 histo	protein-co
lib-C666-l	chr22	41347604	41347604	+	2.93406	NA	intron (NM_014248, intron 1 of 4)	CpG	253	NM_01424	9978	Hs.474949	NM_01424	ENSG0000	RBX1	BA554C12.	ring-box 1	protein-co
lib-C666-l	chr10	97667415	97667415	+	13.46097	NA	promoter-TSS (NM_001130446)	promoter-TSS (NM_001130446)	-307	NM_00113	100127889	Hs.719076	NM_00109	ENSG0000	C10orf131	bA690P14.	chromosor	protein-co
lib-C666-l	chr6	42105246	42105246	+	3.92495	NA	intron (NM_001164446, intron 1 of 4)	intron (NM_001164446, intron 1 of	5469	NM_00116	647024	Hs.444277	NM_00116	ENSG0000	C6orf132	bA7K24.2	chromosor	protein-co
lib-C666-l	chr15	50647423	50647423	+	3.25502	NA	promoter-TSS (NR_026891)	promoter-TSS (NR_026891)	182	NM_00204	2553	Hs.654350	NM_00204	ENSG0000	GABPB1	BABPB2|E4	GA binding	protein-co
lib-C666-l	chr17	59940975	59940975	+	3.27468	NA	promoter-TSS (NM_032043)	promoter-TSS (NM_032043)	-55	NM_03204	83990	Hs.128903	NM_03204	ENSG0000	BRIP1	BACH1|FAI	BRCA1 inte	protein-co
lib-C666-l	chr19	11071610	11071610	+	35.5104	NA	promoter-TSS (NM_001128844)	promoter-TSS (NM_001128844)	12	NM_00112	6597	Hs.327527	NM_00307	ENSG0000	SMARCA4	BAF190|BA	SWI/SNF re	protein-co
lib-C666-l	chr19	11057911	11057911	+	19.60174	NA	Intergenic	Intergenic	-13687	NM_00307	6597	Hs.327527	NM_00307	ENSG0000	SMARCA4	BAF190|BA	SWI/SNF re	protein-co
lib-C666-l	chr9	2178296	2178296	+	7.21092	NA	intron (NM_003070, intron 29 of 33)	intron (NM_003070, intron 29 of 33	19840	NM_00128	6595	Hs.298990	NM_00307	ENSG0000	SMARCA2	BAF190|BR	SWI/SNF re	protein-co
lib-C666-l	chr9	2015154	2015154	+	7.0832	NA	promoter-TSS (NM_139045)	promoter-TSS (NM_139045)	-65	NM_00307	6595	Hs.298990	NM_00307	ENSG0000	SMARCA2	BAF190|BR	SWI/SNF re	protein-co
lib-C666-l	chr12	46122722	46122722	+	5.04179	NA	promoter-TSS (NM_152641)	promoter-TSS (NM_152641)	-770	NM_15264	196528	Hs.317304	NM_15264	ENSG0000	ARID2	BAF200|p2	AT-rich int	protein-co
lib-C666-l	chr19	38714910	38714910	+	6.29226	NA	promoter-TSS (NM_001135155)	promoter-TSS (NM_001135155)	-20	NM_00113	8193	Hs.631576	NM_00464	ENSG0000	DPF1	BAF45b|NE	double PH	protein-co
lib-C666-l	chr17	38804375	38804375	+	16.291	NA	promoter-TSS (NM_003079)	promoter-TSS (NM_003079)	-272	NM_00307	6605	Hs.743978	NM_00307	ENSG0000	SMARCE1	BAF57|CSS	SWI/SNF re	protein-co
lib-C666-l	chr7	150945968	150945968	+	10.73451	NA	promoter-TSS (NM_001003801)	promoter-TSS (NM_001003801)	-219	NM_00100	6604	Hs.647067	NM_00307	ENSG0000	SMARCD3	BAF60C|CR	SWI/SNF re	protein-co
lib-C666-l	chr14	104029123	104029123	+	7.49163	NA	promoter-TSS (NM_001302654)	promoter-TSS (NM_001302654)	28	NM_00101	9529	Hs.5443	NM_00487	ENSG0000	BAG5	BAG-5	BCL2 assoc	protein-co
lib-C666-l	chr14	104028831	104028831	+	5.23609	NA	promoter-TSS (NM_001302654)	promoter-TSS (NM_001302654)	-176	NM_00487	9529	Hs.5443	NM_00487	ENSG0000	BAG5	BAG-5	BCL2 assoc	protein-co
lib-C666-l	chr6	31620082	31620082	+	3.89034	NA	promoter-TSS (NR_045828).4	promoter-TSS (NR_045828).4	88	NM_00109	7917	Hs.440900	NM_00463	ENSG0000	BAG6	BAG-6|BA1	BCL2 assoc	protein-co
lib-C666-l	chr2	111925247	111925247	+	25.80569	NA	3' UTR (NM_001204106, exon 4 of 4)	3' UTR (NM_001204106, exon 4 of 4	46756	NM_00120	10018	Hs.469658	NM_00653	ENSG0000	BCL2L11	BAM|BIM|E	BCL2 like 1	protein-co
lib-C666-l	chr2	111954154	111954154	+	7.4883	NA	non-coding (NR_136162, exon 5 of 5	non-coding (NR_136162, exon 5 of	75663	NM_00120	10018	Hs.469658	NM_00653	ENSG0000	BCL2L11	BAM|BIM|E	BCL2 like 1	protein-co
lib-C666-l	chr10	112328002	112328002	+	8.59372	NA	intron (NM_005445, intron 1 of 28)	intron (NM_005445, intron 1 of 28)	553	NM_00544	9126	Hs.24485	NM_00544	ENSG0000	SMC3	BAM|BMH|	structural r	protein-co
lib-C666-l	chr12	7079921	7079921	+	28.88523	NA	promoter-TSS (NM_001267700)	promoter-TSS (NM_001267700)	-5	NM_00114	11331	Hs.504620	NM_00114	ENSG0000	PHB2	BAP|BCAP3	prohibitin 2	protein-co
lib-C666-l	chr5	138546524	138546524	+	20.87959	NA	Intergenic	Charlie3|DNA|hAT-Charlie	-12459	NM_00103	64374	Hs.483521	NM_02246	ENSG0000	SIL1	BAP|MSS|L	SIL1 nucle	protein-co
lib-C666-l	chr7	74072906	74072906	+	9.55098	NA	intron (NM_001163636, intron 1 of 3	CpG	915	NM_03300	2969	Hs.647041	NM_00151	ENSG0000	GTF2I	BAP135|BT	general tra	protein-co
lib-C666-l	chr7	74071827	74071827	+	6.0202	NA	promoter-TSS (NM_001280800)	promoter-TSS (NM_001280800)	-164	NM_00151	2969	Hs.647041	NM_00151	ENSG0000	GTF2I	BAP135|BT	general tra	protein-co
lib-C666-l	chr17	6918120	6918120	+	34.3461	NA	promoter-TSS (NM_174893)	promoter-TSS (NM_174893)	64	NM_00114	124944	Hs.511801	NM_17489	ENSG0000	C17orf49	BAP18|HEF	chromosor	protein-co
lib-C666-l	chr17	6918469	6918469	+	10.9422	NA	TTS (NR_037716)	TTS (NR_037716)	413	NM_00114	124944	Hs.511801	NM_17489	ENSG0000	C17orf49	BAP18|HEF	chromosor	protein-co
lib-C666-l	chr17	6918678	6918678	+	9.4812	NA	TTS (NR_037716)	TTS (NR_037716)	622	NM_00114	124944	Hs.511801	NM_17489	ENSG0000	C17orf49	BAP18|HEF	chromosor	protein-co
lib-C666-l	chr1	236767833	236767833	+	4.56047	NA	promoter-TSS (NM_018072)	promoter-TSS (NM_018072)	8	NM_01807	55127	Hs.708114	NM_01807	ENSG0000	HEATR1	BAP28|UTF	HEAT repe	protein-co
lib-C666-l	chr7	107220663	107220663	+	29.74345	NA	promoter-TSS (NM_001008405)	promoter-TSS (NM_001008405)	241	NR_02783	55973	Hs.303787	NM_01884	ENSG0000	BCAP29	BAP29	B-cell rece	protein-co
lib-C666-l	chr16	14728087	14728087	+	2.60101	NA	intron (NM_001330500, intron 1 of 5)	intron (NM_001330500, intron 1 of	1419	NM_00133	51283	Hs.435556	NM_01656	ENSG0000	BFAR	BAR|RNF47	bifunctiona	protein-co
lib-C666-l	chr1	226496875	226496875	+	3.8557	NA	exon (NM_173083, exon 1 of 15)	exon (NM_173083, exon 1 of 15)	329	NM_00127	286826	Hs.120817	NM_17308	ENSG0000	LIN9	BARA|BARI	lin-9 DREA	protein-co
lib-C666-l	chr4	1242208	1242208	+	5.04826	NA	intron (NM_001012614, intron 1 of 9)	CpG	700	NM_00132	1487	Hs.208597	NM_00132	ENSG0000	CTBP1	BARS	C-terminal	protein-co
lib-C666-l	chr16	57294643	57294643	+	7.66023	NA	intron (NM_015993, intron 2 of 3)	intron (NM_015993, intron 2 of 3)	15605	NM_01210	23568	Hs.632873	NM_01210	ENSG0000	ARL2BP	BART|BAR1	ADP ribosy	protein-co
lib-C666-l	chr16	57278773	57278773	+	5.5166	NA	promoter-TSS (NM_012106)	promoter-TSS (NM_012106)	-265	NM_01210	23568	Hs.632873	NM_01210	ENSG0000	ARL2BP	BART|BAR1	ADP ribosy	protein-co
lib-C666-l	chr19	14517121	14517121	+	6.56784	NA	intron (NM_078481, intron 14 of 19)	CpG	13074	NM_00580	10212	Hs.311609	NM_00580	ENSG0000	DDX39A	BAT1|BAT1	DExD-box	protein-co
lib-C666-l	chr19	14530238	14530238	+	5.74587	NA	promoter-TSS (NM_005804)	promoter-TSS (NM_005804)	-43	NR_04636	10212	Hs.311609	NM_00580	ENSG0000	DDX39A	BAT1|BAT1	DExD-box	protein-co
lib-C666-l	chr6	31587832	31587832	+	10.40942	NA	promoter-TSS (NM_004638).7	promoter-TSS (NM_004638).7	-618	NM_00463	7916	Hs.123239	NM_00463	ENSG0000	PRRC2A	BAT2|D6S5	proline rich	protein-co
lib-C666-l	chr6	31588280	31588280	+	7.0832	NA	promoter-TSS (NM_004638).7	promoter-TSS (NM_004638).7	-170	NM_00463	7916	Hs.123239	NM_00463	ENSG0000	PRRC2A	BAT2|D6S5	proline rich	protein-co
lib-C666-l	chr1	171454650	171454650	+	11.90035	NA	promoter-TSS (NM_015172)	promoter-TSS (NM_015172)	-16	NM_01517	23215	Hs.494614	NM_01517	ENSG0000	PRRC2C	BAT2-iso|E	proline rich	protein-co
lib-C666-l	chr9	134248872	134248872	+	19.82916	NA	Intergenic	CpG	-56605	NM_01331	84726	Hs.743955	NM_01331	ENSG0000	PRRC2B	BAT2L|BAT	proline rich	protein-co
lib-C666-l	chr6	31671205	31671205	+	7.15277	NA	promoter-TSS (NM_001177515).2	promoter-TSS (NM_001177515).2	-68	NM_02116	7920	Hs.388188	NM_02116	ENSG0000	ABHD16A	BAT5|D6S8	abhydrolas	protein-co
lib-C666-l	chr6	31870152	31870152	+	8.33814	NA	promoter-TSS (NM_181842)	promoter-TSS (NM_181842)	-383	NM_18184	221527	Hs.234027	NM_18184	ENSG0000	ZBTB12	Bat9|C6orf	zinc finger	protein-co
lib-C666-l	chr17	6927075	6927075	+	8.89416	NA	exon (NM_181844, exon 2 of 9)	exon (NM_181844, exon 2 of 9)	706	NM_18184	255877	Hs.22575	NM_18184	ENSG0000	BCL6B	BAZF|ZBTB	B-cell CLL/	protein-co
lib-C666-l	chr16	89627008	89627008	+	18.98417	NA	promoter-TSS (NM_001243130)	promoter-TSS (NM_001243130)	-57	NM_00124	6137	Hs.410817	NM_00097	ENSG0000	RPL13	BBC1|D16S	ribosomal	protein-co
lib-C666-l	chr10	123413814	123413814	+	11.66971	NA	Intergenic	Intergenic	-55842	NM_00114	2263	Hs.533683	NM_00014	ENSG0000	FGFR2	BBDS|BEK|I	fibroblast g	protein-co
lib-C666-l	chr10	123084387	123084387	+	10.79304	NA	Intergenic	MER1B|DNA|hAT-Charlie	206441	NM_00132	2263	Hs.533683	NM_00014	ENSG0000	FGFR2	BBDS|BEK|I	fibroblast g	protein-co
lib-C666-l	chr7	137687008	137687008	+	36.70747	NA	promoter-TSS (NM_001253775)	promoter-TSS (NM_001253775)	-161	NM_00125	64764	Hs.490273	NM_19407	ENSG0000	CREB3L2	BBF2H7	cAMP resp	protein-co
lib-C666-l	chr7	137714911	137714911	+	13.41844	NA	Intergenic	MER4C|LTR|ERV1	-28064	NM_00125	64764	Hs.490273	NM_19407	ENSG0000	CREB3L2	BBF2H7	cAMP resp	protein-co
lib-C666-l	chr7	137686410	137686410	+	7.97818	NA	exon (NM_001253775, exon 1 of 4)	exon (NM_001253775, exon 1 of 4)	437	NM_19407	64764	Hs.490273	NM_19407	ENSG0000	CREB3L2	BBF2H7	cAMP resp	protein-co
lib-C666-l	chr7	137710484	137710484	+	4.46944	NA	Intergenic	HUERS-P1-int|LTR|ERV1	-23637	NM_00125	64764	Hs.490273	NM_19407	ENSG0000	CREB3L2	BBF2H7	cAMP resp	protein-co
lib-C666-l	chr2	73298657	73298657	+	4.71278	NA	promoter-TSS (NM_001330403)	promoter-TSS (NM_001330403)	-198	NM_00133	94097	Hs.368171	NM_14457	ENSG0000	SFXN5	BBG-TCC	sideroflexir	protein-co
lib-C666-l	chr10	112678963	112678963	+	8.28613	NA	promoter-TSS (NM_001269039)	promoter-TSS (NM_001269039)	161	NM_00119	92482	Hs.232543	NM_00119	ENSG0000	BBIP1	BBIP10|BBS	BBSome in	protein-co
lib-C666-l	chr5	92918433	92918433	+	10.67506	NA	promoter-TSS (NM_005654)	promoter-TSS (NM_005654)	-610	NM_00565	7025	Hs.519445	NM_00565	ENSG0000	NR2F1	BBOAS|BBS	nuclear rec	protein-co
lib-C666-l	chr11	64546550	64546550	+	25.68529	NA	promoter-TSS (NM_004630)	promoter-TSS (NM_004630)	-234	NM_00134	7536	Hs.502829	NM_00463	ENSG0000	SF1	BBP|D11S6	splicing fac	protein-co
lib-C666-l	chr11	64546330	64546330	+	11.48337	NA	promoter-TSS (NM_004630)	promoter-TSS (NM_004630)	-14	NM_00134	7536	Hs.502829	NM_00463	ENSG0000	SF1	BBP|D11S6	splicing fac	protein-co
lib-C666-l	chr16	56554075	56554075	+	18.89222	NA	promoter-TSS (NM_031885)	promoter-TSS (NM_031885)	-67	NM_03188	583	Hs.333738	NM_03188	ENSG0000	BBS2	BBS|RP74	Bardet-Bie	protein-co
lib-C666-l	chr17	56296608	56296608	+	27.62962	NA	promoter-TSS (NM_001321268)	promoter-TSS (NM_001321268)	58	NM_00132	54903	Hs.408843	NM_01777	ENSG0000	MKS1	BBS13|JBTS	Meckel syn	protein-co
lib-C666-l	chr2	63815940	63815940	+	16.96353	NA	promoter-TSS (NM_001199112)	promoter-TSS (NM_001199112)	-73	NM_01591	51057	Hs.414952	NM_01591	ENSG0000	WDPCP	BBS15|C2o	WD repeat	protein-co
lib-C666-l	chr22	37172388	37172388	+	10.16129	NA	promoter-TSS (NM_006860)	promoter-TSS (NM_006860)	-211	NR_03353	11020	Hs.415172	NM_00686	ENSG0000	IFT27	BBS19|RAB	intraflagell	protein-co
lib-C666-l	chr9	26947359	26947359	+	219.41573	NA	promoter-TSS (NM_001099222)	promoter-TSS (NM_001099222)	49	NM_00109	80173	Hs.145402	NM_02510	ENSG0000	IFT74	BBS20|CCD	intraflagell	protein-co
lib-C666-l	chr2	27712682	27712682	+	37.48738	NA	promoter-TSS (NM_015662)	promoter-TSS (NM_015662)	-4	NM_01566	26160	Hs.127401	NM_01566	ENSG0000	IFT172	BBS20|NPH	intraflagell	protein-co
lib-C666-l	chr4	122791661	122791661	+	58.76075	NA	promoter-TSS (NM_176824)	promoter-TSS (NM_176824)	-9	NM_01819	55212	Hs.591694	NM_01819	ENSG0000	BBS7	BBS2L1	Bardet-Bie	protein-co
lib-C666-l	chr20	10415086	10415086	+	10.73451	NA	promoter-TSS (NR_072977)	promoter-TSS (NR_072977)	-199	NM_17078	8195	Hs.472119	NM_01884	ENSG0000	MKKS	BBS6|HMC	McKusick-	protein-co
lib-C666-l	chr20	36156347	36156347	+	7.09685	NA	promoter-TSS (NM_001167822)	promoter-TSS (NM_001167822)	-14	NM_00116	10904	Hs.472651	NM_00669	ENSG0000	BLCAP	BC10	bladder ca	protein-co
lib-C666-l	chr19	59066469	59066469	+	14.81115	NA	promoter-TSS (NM_014453)	promoter-TSS (NM_014453)	22	NM_01445	27243	Hs.12107	NM_01445	ENSG0000	CHMP2A	BC-2|BC2|(	charged m	protein-co
lib-C666-l	chr19	20163121	20163121	+	25.3508	NA	Intergenic	Intergenic	-12844	NM_03319	91120	Hs.306298	NM_03319	ENSG0000	ZNF682	BC39498_3	zinc finger	protein-co
lib-C666-l	chr19	20150157	20150157	+	10.37767	NA	promoter-TSS (NM_001077349)	promoter-TSS (NM_001077349)	-93	NM_00107	91120	Hs.306298	NM_03319	ENSG0000	ZNF682	BC39498_3	zinc finger	protein-co
lib-C666-l	chr19	49314368	49314368	+	5.5166	NA	promoter-TSS (NM_001190)	promoter-TSS (NM_001190)	-48	NM_00119	587	Hs.512670	NM_00119	ENSG0000	BCAT2	BCAM|BCA	branched c	protein-co
lib-C666-l	chr10	98510295	98510295	+	11.07709	NA	Intergenic	CpG	-30016	NM_15230	118788	Hs.310456	NM_15230	ENSG0000	PIK3AP1	BCAP	phosphoin	protein-co
lib-C666-l	chr1	100715485	100715485	+	25.96768	NA	promoter-TSS (NM_001918)	promoter-TSS (NM_001918)	-76	NM_00191	1629	Hs.709187	NM_00191	ENSG0000	DBT	BCATE2|BC	dihydrolip	protein-co
lib-C666-l	chr4	187112819	187112819	+	10.73451	NA	promoter-TSS (NR_046264)	promoter-TSS (NR_046264)	145	NM_20735	285440	Hs.587231	NM_20735	ENSG0000	CYP4V2	BCD|CYP4	cytochrom	protein-co
lib-C666-l	chr5	70220924	70220924	+	28.8837	NA	5' UTR (NM_022875, exon 1 of 8)	5' UTR (NM_022875, exon 1 of 8)	156	NM_02287	6607	Hs.202179	NM_01741	ENSG0000	SMN2	BCD541|C-	survival of	protein-co
lib-C666-l	chr5	69345502	69345502	+	26.54591	NA	5' UTR (NM_017411, exon 1 of 9)	5' UTR (NM_017411, exon 1 of 9)	152	NM_02287	6607	Hs.202179	NM_01741	ENSG0000	SMN2	BCD541|C-	survival of	protein-co
lib-C666-l	chr7	100026918	100026918	+	24.50625	NA	promoter-TSS (NM_017984)	promoter-TSS (NM_017984)	-336	NM_01960	56257	Hs.178011	NM_01960	ENSG0000	MEPCE	BCDIN3	methylpho	protein-co
lib-C666-l	chr7	100026336	100026336	+	10.30802	NA	promoter-TSS (NM_017984)	promoter-TSS (NM_017984)	-77	NM_00119	56257	Hs.178011	NM_01960	ENSG0000	MEPCE	BCDIN3	methylpho	protein-co
lib-C666-l	chr7	100027863	100027863	+	5.74587	NA	exon (NM_019606, exon 1 of 4)	exon (NM_019606, exon 1 of 4)	609	NM_01960	56257	Hs.178011	NM_01960	ENSG0000	MEPCE	BCDIN3	methylpho	protein-co
lib-C666-l	chr16	81271977	81271977	+	7.11942	NA	promoter-TSS (NM_017429)	promoter-TSS (NM_017429)	-319	NM_01742	53630	Hs.212172	NM_01742	ENSG0000	BCO1	BCDO|BCD	beta-carot	protein-co
lib-C666-l	chr9	82186585	82186585	+	22.01132	NA	promoter-TSS (NM_001282748)	promoter-TSS (NM_001282748)	-103	NM_00128	7091	Hs.444213	NM_00700	ENSG0000	TLE4	BCE-1|BCE	transducin	protein-co
lib-C666-l	chr9	81977356	81977356	+	11.57178	NA	Intergenic	Intergenic	-209332	NM_00128	7091	Hs.444213	NM_00700	ENSG0000	TLE4	BCE-1|BCE	transducin	protein-co
lib-C666-l	chr9	82239261	82239261	+	3.98718	NA	intron (NM_001282749, intron 5 of 1	intron (NM_001282749, intron 5 of	52573	NR_10423	7091	Hs.444213	NM_00700	ENSG0000	TLE4	BCE-1|BCE	transducin	protein-co
lib-C666-l	chr14	103988626	103988626	+	14.64638	NA	intron (NM_001823, intron 2 of 7)	CpG-7449	570	NM_00182	1152	Hs.173724	NM_00182	ENSG0000	CKB	B-CK|BCK|(	creatine kir	protein-co
lib-C666-l	chr14	103987160	103987160	+	10.27265	NA	intron (NM_001823, intron 5 of 7)	CpG	2036	NM_00182	1152	Hs.173724	NM_00182	ENSG0000	CKB	B-CK|BCK|(	creatine kir	protein-co
lib-C666-l	chr14	103988045	103988045	+	6.15313	NA	intron (NM_001823, intron 4 of 7)	CpG-7449	1151	NM_00182	1152	Hs.173724	NM_00182	ENSG0000	CKB	B-CK|BCK|(	creatine kir	protein-co
lib-C666-l	chr6	80816324	80816324	+	8.01457	NA	promoter-TSS (NM_000056)	promoter-TSS (NM_000056)	-3	NM_18305	594	Hs.654441	NM_00005	ENSG0000	BCKDHB	BCKDE1B|E	branched c	protein-co
lib-C666-l	chr16	31119704	31119704	+	72.75481	NA	promoter-TSS (NM_001122957)	promoter-TSS (NM_001122957)	89	NM_00112	10295	Hs.513520	NM_00588	ENSG0000	BCKDK	BCKDKD|BI	branched c	protein-co
lib-C666-l	chr2	60781851	60781851	+	27.5466	NA	Intergenic	CpG	-1218	NM_13855	53335	Hs.370549	NM_01801	ENSG0000	BCL11A	BCL11A-L|	B-cell CLL/	protein-co
lib-C666-l	chr1	150551636	150551636	+	3.8557	NA	exon (NM_182763, exon 1 of 2)	exon (NM_182763, exon 1 of 2)	578	NM_00119	4170	Hs.632486	NM_02196	ENSG0000	MCL1	BCL2L3|EA	BCL2 famil	protein-co
lib-C666-l	chr19	49458135	49458135	+	5.65398	NA	promoter-TSS (NM_138764)	promoter-TSS (NM_138764)	18	NM_00129	581	Hs.624291	NM_00432	ENSG0000	BAX	BCL2L4	BCL2 assoc	protein-co
lib-C666-l	chr2	242483508	242483508	+	29.50576	NA	TTS (NR_033346)	TTS (NR_033346)	-14638	NM_03251	666	Hs.293753	NM_03251	ENSG0000	BOK	BCL2L9|BC	BOK, BCL2	protein-co
lib-C666-l	chr19	45256723	45256723	+	12.73831	NA	intron (NM_005178, intron 2 of 8)	intron (NM_005178, intron 2 of 8)	4745	NM_00517	602	Hs.31210	NM_00517	ENSG0000	BCL3	BCL4|D19S	B-cell CLL/	protein-co
lib-C666-l	chr19	45242276	45242276	+	9.06551	NA	Intergenic	Intergenic	-9702	NM_00517	602	Hs.31210	NM_00517	ENSG0000	BCL3	BCL4|D19S	B-cell CLL/	protein-co
lib-C666-l	chr19	45251903	45251903	+	4.73866	NA	promoter-TSS (NM_005178)	promoter-TSS (NM_005178)	-75	NM_00517	602	Hs.31210	NM_00517	ENSG0000	BCL3	BCL4|D19S	B-cell CLL/	protein-co
lib-C666-l	chr3	187463545	187463545	+	52.34435	NA	promoter-TSS (NM_001706)	promoter-TSS (NM_001706)	-32	NM_00170	604	Hs.478588	NM_00170	ENSG0000	BCL6	BCL5|BCL6	B-cell CLL/	protein-co
lib-C666-l	chr13	35516587	35516587	+	22.50982	NA	5' UTR (NM_015678, exon 1 of 58)	5' UTR (NM_015678, exon 1 of 58)	163	NM_01567	26960	Hs.491172	NM_01567	ENSG0000	NBEA	BCL8B|LYS	neurobeac	protein-co
lib-C666-l	chr11	118783877	118783877	+	9.02258	NA	Intergenic	Intergenic	-2264	NM_18255	283149	Hs.414740	NM_18255	ENSG0000	BCL9L	BCL9-2|DL	B-cell CLL/	protein-co
lib-C666-l	chr11	118799114	118799114	+	2.2973	NA	Intergenic	Intergenic	-17501	NM_18255	283149	Hs.414740	NM_18255	ENSG0000	BCL9L	BCL9-2|DL	B-cell CLL/	protein-co
lib-C666-l	chr11	118783097	118783097	+	2.2973	NA	Intergenic	Intergenic	-1484	NM_18255	283149	Hs.414740	NM_18255	ENSG0000	BCL9L	BCL9-2|DL	B-cell CLL/	protein-co
lib-C666-l	chr1	33367086	33367086	+	32.78343	NA	promoter-TSS (NM_033504)	promoter-TSS (NM_033504)	-45	NM_00132	113452	Hs.534521	NM_03350	ENSG0000	TMEM54	BCLP|CAC-	transmemb	protein-co
lib-C666-l	chr22	18121475	18121475	+	23.05659	NA	exon (NM_001270727, exon 1 of 5)	exon (NM_001270727, exon 1 of 5)	125	NM_00127	23786	Hs.631672	NM_01536	ENSG0000	BCL2L13	BCL-RAME	BCL2 like 1	protein-co
lib-C666-l	chr14	23775967	23775967	+	89.36973	NA	promoter-TSS (NM_001199839)	promoter-TSS (NM_001199839)	-4	NM_00405	599	Hs.410026	NM_00405	ENSG0000	BCL2L2	BCL-W|BC	BCL2 like 2	protein-co
lib-C666-l	chr20	30311539	30311539	+	15.08005	NA	promoter-TSS (NM_001317919)	promoter-TSS (NM_001317919)	263	NM_00131	598	Hs.516966	NM_00119	ENSG0000	BCL2L1	BCL-XL/S|E	BCL2 like 1	protein-co
lib-C666-l	chr8	127570698	127570698	+	5.59206	NA	promoter-TSS (NM_174911)	promoter-TSS (NM_174911)	13	NM_17491	157638	Hs.741352	NM_17491	ENSG0000	FAM84B	BCMP101|I	family with	protein-co
lib-C666-l	chr8	127570226	127570226	+	3.38731	NA	intron (NM_174911, intron 1 of 1)	CpG	485	NM_17491	157638	Hs.741352	NM_17491	ENSG0000	FAM84B	BCMP101|I	family with	protein-co
lib-C666-l	chr9	98273417	98273417	+	3.04651	NA	intron (NM_001083603, intron 1 of 2	GC_rich|Low_complexity|Low_comp	-2586	NM_00026	5727	Hs.494538	NM_00026	ENSG0000	PTCH1	BCNS|HPE	patched 1	protein-co
lib-C666-l	chr9	98273156	98273156	+	2.92124	NA	intron (NM_001083603, intron 1 of 2	CpG	-2325	NM_00026	5727	Hs.494538	NM_00026	ENSG0000	PTCH1	BCNS|HPE	patched 1	protein-co
lib-C666-l	chr9	98270123	98270123	+	2.59723	NA	promoter-TSS (NM_001083607)	promoter-TSS (NM_001083607)	199	NM_00108	5727	Hs.494538	NM_00026	ENSG0000	PTCH1	BCNS|HPE	patched 1	protein-co
lib-C666-l	chr16	75467384	75467384	+	11.07709	NA	promoter-TSS (NM_006324)	promoter-TSS (NM_006324)	3	NM_00632	10428	Hs.461361	NM_00632	ENSG0000	CFDP1	BCNT|BUC	craniofacia	protein-co
lib-C666-l	chr17	26972314	26972314	+	10.56916	NA	promoter-TSS (NM_014680)	promoter-TSS (NM_014680)	-137	NM_00132	9703	Hs.151761	NM_01468	ENSG0000	KIAA0100	BCOX|BCO	KIAA0100	protein-co
lib-C666-l	chr14	69620024	69620024	+	14.98502	NA	promoter-TSS (NM_001284208)	promoter-TSS (NM_001284208)	-110	NM_00128	8816	Hs.509780	NM_00386	ENSG0000	DCAF5	BCRG2|BCI	DDB1 and	protein-co
lib-C666-l	chr10	88720344	88720344	+	46.45163	NA	intron (NM_001330120, intron 5 of 6)	intron (NM_001330120, intron 5 of	2056	NM_00308	6623	Hs.349470	NM_00308	ENSG0000	SNCG	BCSG1|SR	synuclein g	protein-co
lib-C666-l	chr10	88632840	88632840	+	3.57421	NA	intron (NM_004329, intron 2 of 12)	intron (NM_004329, intron 2 of 12)	-82718	NM_00133	6623	Hs.349470	NM_00308	ENSG0000	SNCG	BCSG1|SR	synuclein g	protein-co
lib-C666-l	chr21	35402613	35402613	+	11.36743	NA	Intergenic	MLT1J2|LTR|ERVL-MaLR	-43210	NM_00693	6526	Hs.302742	NM_00693	ENSG0000	SLC5A3	BCW2|SMI	solute carr	protein-co
lib-C666-l	chr3	23987453	23987453	+	124.47887	NA	promoter-TSS (NM_001145425)	promoter-TSS (NM_001145425)	-159	NM_00114	9975	Hs.37288	NM_00512	ENSG0000	NR1D2	BD73|EAR-	nuclear rec	protein-co
lib-C666-l	chr10	33798419	33798419	+	11.93581	NA	Intergenic	Intergenic	-174586	NM_00124	8829	Hs.131704	NM_00387	ENSG0000	NRP1	BDCA4|CD	neuropilin	protein-co
lib-C666-l	chr16	87526598	87526598	+	8.28613	NA	Intergenic	Intergenic	-1138	NM_01514	23174	Hs.156231	NM_01514	ENSG0000	ZCCHC14	BDG-29|BI	zinc finger	protein-co
lib-C666-l	chr3	197281796	197281796	+	13.46097	NA	intron (NM_203314, intron 1 of 7)	CpG	1062	NM_00405	622	Hs.274539	NM_00405	ENSG0000	BDH1	BDH|SDR9	3-hydroxyl	protein-co
lib-C666-l	chr9	21832326	21832326	+	83.98973	NA	intron (NM_002451, intron 4 of 7)	intron (NM_002451, intron 4 of 7)	29691	NM_00245	4507	Hs.193268	NM_00245	ENSG0000	MTAP	BDMF|DMS	methylthio	protein-co
lib-C666-l	chr15	74838640	74838640	+	7.15277	NA	intron (NM_006465, intron 2 of 8)	intron (NM_006465, intron 2 of 8)	5122	NM_00646	10620	Hs.647642	NM_00646	ENSG0000	ARID3B	BDP|DRIL2	AT-rich int	protein-co
lib-C666-l	chr4	89205884	89205884	+	88.70856	NA	promoter-TSS (NM_152542)	promoter-TSS (NM_152542)	99	NM_15254	152926	Hs.43744	NM_15254	ENSG0000	PPM1K	BDP|MSUD	protein ph	protein-co
lib-C666-l	chr14	105714776	105714776	+	16.36809	NA	promoter-TSS (NM_033271)	promoter-TSS (NM_033271)	-103	NM_03327	90135	Hs.7367	NM_03327	ENSG0000	BTBD6	BDPL	BTB domai	protein-co
lib-C666-l	chr19	55517520	55517520	+	73.41202	NA	Intergenic	Intergenic	32112	NM_01636	51206	Hs.661752	NM_01636	ENSG0000	GP6	BDPLT11|G	glycoprote	protein-co
lib-C666-l	chr7	139478011	139478011	+	33.97962	NA	promoter-TSS (NM_022740)	promoter-TSS (NM_022740)	-36	NM_00113	6916	Hs.520757	NM_00106	ENSG0000	TBXAS1	BDPLT14|C	thromboxa	protein-co
lib-C666-l	chr14	69446150	69446150	+	34.92812	NA	promoter-TSS (NM_001130004)	promoter-TSS (NM_001130004)	-67	NM_00113	87	Hs.235750	NM_00110	ENSG0000	ACTN1	BDPLT15	actinin alpl	protein-co
lib-C666-l	chr22	36761248	36761248	+	12.14922	NA	intron (NM_002473, intron 1 of 40)	intron (NM_002473, intron 1 of 40)	22864	NM_00247	4627	Hs.474751	NM_00247	ENSG0000	MYH9	BDPLT6|DF	myosin hea	protein-co
lib-C666-l	chr22	36782835	36782835	+	3.58839	NA	intron (NM_002473, intron 1 of 40)	intron (NM_002473, intron 1 of 40)	1277	NM_00247	4627	Hs.474751	NM_00247	ENSG0000	MYH9	BDPLT6|DF	myosin hea	protein-co

lib-C666-l	chr2	120770493	120770493	+	17.48666	NA	promoter-TSS (NM_020909)	promoter-TSS (NM_020909)	-111	NM_00133	57669	Hs.369232	NM_02090	ENSG0000	EPB41L5	BE37|YMO	erythrocyte	protein-co
lib-C666-l	chr13	108870883	108870883	+	41.89289	NA	promoter-TSS (NM_001098268)	promoter-TSS (NM_001098268)	120	NM_03285	84945	Hs.183528	NM_03285	ENSG0000	ABHD13	BEM46L1|C	abhydrolas	protein-co
lib-C666-l	chr16	87984603	87984603	+	3.89624	NA	promoter-TSS (NM_001173541)	promoter-TSS (NM_001173541)	-435	NM_00117	54971	Hs.461705	NM_01786	ENSG0000	BANP	BEND1|SM	BTG3 assoc	protein-co
lib-C666-l	chr16	23347379	23347379	+	4.73866	NA	intron (NM_000336, intron 1 of 12)	intron (NM_000336, intron 1 of 12)	33788	NM_00033	6338	Hs.414614	NM_00033	ENSG0000	SCNN1B	BESC1|ENa	sodium cha	protein-co
lib-C666-l	chr12	6479978	6479978	+	8.28613	NA	intron (NM_001159576, intron 1 of 1:	intron (NM_001159576, intron 1 of	4412	NM_00115	6337	Hs.591047	NM_00103	ENSG0000	SCNN1A	BESC2|ENa	sodium cha	protein-co
lib-C666-l	chr16	23193891	23193891	+	11.86993	NA	promoter-TSS (NM_001039)	promoter-TSS (NM_001039)	-149	NM_00103	6340	Hs.371727	NM_00103	ENSG0000	SCNN1G	BESC3|ENa	sodium cha	protein-co
lib-C666-l	chr1	36615115	36615115	+	5.96422	NA	promoter-TSS (NR_073098)	promoter-TSS (NR_073098)	0	NM_00127	27095	Hs.523131	NM_01440	ENSG0000	TRAPPC3	BET3	trafficking	protein-co
lib-C666-l	chr9	140135491	140135491	+	6.20473	NA	promoter-TSS (NM_006088)	promoter-TSS (NM_006088)	-220	NM_00608	10383	Hs.433615	NM_00608	ENSG0000	TUBB4B	Beta2|TUBI	tubulin bet	protein-co
lib-C666-l	chr2	232276821	232276821	+	8.85547	NA	Intergenic	CpG	16486	NM_14523	93010	Hs.299329	NM_14523	ENSG0000	B3GNT7	beta3GnT7|	UDP-GlcNA	protein-co
lib-C666-l	chr1	161147515	161147515	+	6.15313	NA	5' UTR (NM_001199873, exon 1 of 8)	5' UTR (NM_001199873, exon 1 of 8	243	NM_00377	8703	Hs.321231	NM_00377	ENSG0000	B4GALT3	beta4Gal-1	beta-1,4-g	protein-co
lib-C666-l	chr3	139108545	139108545	+	7.49163	NA	promoter-TSS (NM_004766)	promoter-TSS (NM_004766)	-23	NM_00476	9276	Hs.681616	NM_00476	ENSG0000	COPB2	beta'-COP	coatomer p	protein-co
lib-C666-l	chr5	72794302	72794302	+	7.9573	NA	promoter-TSS (NM_001037637)	promoter-TSS (NM_001037637)	52	NM_00120	689	Hs.591768	NM_00120	ENSG0000	BTF3	BETA-NAC	basic trans	protein-co
lib-C666-l	chr13	111805967	111805967	+	83.757	NA	promoter-TSS (NM_001320851)	promoter-TSS (NM_001320851)	-27	NM_00133	8874	Hs.508738	NM_00389	ENSG0000	ARHGEF7	BETA-PIX|	Rho guanin	protein-co
lib-C666-l	chr10	103113665	103113665	+	15.22999	NA	promoter-TSS (NM_001256856)	promoter-TSS (NM_001256856)	-125	NM_00125	8945	Hs.643802	NM_00393	ENSG0000	BTRC	BETA-TRCI	beta-trans	protein-co
lib-C666-l	chrX	102659769	102659769	+	16.59065	NA	Intergenic	Intergenic	27660	NM_01438	27018	Hs.448588	NM_01438	ENSG0000	BEX3	Bex|DXS69	brain expre	protein-co
lib-C666-l	chrX	102565925	102565925	+	5.92363	NA	promoter-TSS (NM_001168399)	promoter-TSS (NM_001168399)	13	NM_00116	84707	Hs.398989	NM_03262	ENSG0000	BEX2	BEX1|DJ79	brain expre	protein-co
lib-C666-l	chr8	47171568	47171568	+	20.82108	NA	Intergenic	GSAT|Satellite|centr	-580940	NR_027012	497634	Hs.406982	NR_02701	ENSG0000	LINC00293	BEYLA|NCF	long interg	ncRNA
lib-C666-l	chr1	54954707	54954707	+	4.19675	NA	Intergenic	CpG	-59100	NM_14716	26027	Hs.745173	NM_01554	ENSG0000	ACOT11	BFIT|STARD	acyl-CoA t	protein-co
lib-C666-l	chr8	133359584	133359584	+	18.64332	NA	intron (NM_004519, intron 1 of 14)	(ATG)n|Simple_repeat|Simple_repea	99925	NM_00120	3786	Hs.374023	NM_00451	ENSG0000	KCNQ3	BFNC2|EBN	potassium	protein-co
lib-C666-l	chr17	35306207	35306207	+	35.28811	NA	promoter-TSS (NM_012138)	promoter-TSS (NM_012138)	32	NM_01213	26574	Hs.195740	NM_01213	ENSG0000	AATF	BFR2|CHE-	apoptosis a	protein-co
lib-C666-l	chr7	65447256	65447256	+	52.42305	NA	promoter-TSS (NM_000181)	promoter-TSS (NM_000181)	45	NR_12053	2990	Hs.255230	NM_00018	ENSG0000	GUSB	BG|MPS7	glucuronid	protein-co
lib-C666-l	chr1	92351687	92351687	+	7.15277	NA	non-coding (NR_036634, exon 1 of 1	non-coding (NR_036634, exon 1 of	149	NM_00324	7049	Hs.482390	NM_00324	ENSG0000	TGFBR3	BGCAN|be	transformi	protein-co
lib-C666-l	chr4	151936931	151936931	+	5.66964	NA	promoter-TSS (NM_001199282)	promoter-TSS (NM_001199282)	-282	NM_00672	987	Hs.480938	NM_00672	ENSG0000	LRBA	BGL|CDC4L	LPS respor	protein-co
lib-C666-l	chr19	6205568	6205568	+	3.31563	NA	Intergenic	AluSz|SINE|Alu	69924	NM_00128	81616	Hs.465720	NM_03092	ENSG0000	ACSBG2	BGR|BRGL|	acyl-CoA s	protein-co
lib-C666-l	chr17	28619122	28619122	+	9.94769	NA	promoter-TSS (NM_000386)	promoter-TSS (NM_000386)	62	NM_00038	642	Hs.371914	NM_00038	ENSG0000	BLMH	BH|BMH	bleomycin	protein-co
lib-C666-l	chr17	28618584	28618584	+	9.55098	NA	intron (NM_000386, intron 1 of 11)	CpG	600	NM_00038	642	Hs.371914	NM_00038	ENSG0000	BLMH	BH|BMH	bleomycin	protein-co
lib-C666-l	chr17	28618210	28618210	+	3.96379	NA	intron (NM_000386, intron 2 of 11)	intron (NM_000386, intron 2 of 11)	974	NM_00038	642	Hs.371914	NM_00038	ENSG0000	BLMH	BH|BMH	bleomycin	protein-co
lib-C666-l	chr14	100339305	100339305	+	30.48199	NA	intron (NM_004434, intron 3 of 21)	intron (NM_004434, intron 3 of 21)	79560	NM_00443	2009	Hs.12451	NM_00443	ENSG0000	EML1	BH|ELP79|	echinoderr	protein-co
lib-C666-l	chr22	45460644	45460644	+	10.32881	NA	Intergenic	Intergenic	-54835	NM_00113	112885	Hs.254097	NM_13841	ENSG0000	PHF21B	BHC80L|PH	PHD finger	protein-co
lib-C666-l	chr22	45349464	45349464	+	8.96177	NA	intron (NM_001284296, intron 2 of 1:	intron (NM_001284296, intron 2 of	55410	NM_00128	112885	Hs.254097	NM_13841	ENSG0000	PHF21B	BHC80L|PH	PHD finger	protein-co
lib-C666-l	chr4	186347199	186347199	+	227.97565	NA	promoter-TSS (NR_028085)	promoter-TSS (NR_028085)	-60	NR_14431	55325	Hs.713548	NM_01835	ENSG0000	UFSP2	BHD|C4orf	UFM1 spec	protein-co
lib-C666-l	chr17	17140862	17140862	+	3.20885	NA	promoter-TSS (NM_144997)	promoter-TSS (NM_144997)	-360	NM_14460	201163	Hs.31652	NM_14460	ENSG0000	FLCN	BHD|FLCL	folliculin	protein-co
lib-C666-l	chr19	13204803	13204803	+	95.73749	NA	intron (NM_001271043, intron 10 of 1	intron (NM_001271043, intron 10 o	9171	NM_00558	4066	Hs.46446	NM_00558	ENSG0000	LYL1	bHLHa18	LYL1, basic	protein-co
lib-C666-l	chr3	5020459	5020459	+	7.8041	NA	promoter-TSS (NM_003670)	promoter-TSS (NM_003670)	-638	NM_00367	8553	Hs.744856	NM_00367	ENSG0000	BHLHE40	BHLHB2|C	basic helix	protein-co
lib-C666-l	chr12	26267466	26267466	+	26.72399	NA	Intergenic	CpG	10537	NM_03076	79365	Hs.177841	NM_03076	ENSG0000	BHLHE41	BHLHB3|D	basic helix	protein-co
lib-C666-l	chr1	6480379	6480379	+	25.68529	NA	promoter-TSS (NM_019089)	promoter-TSS (NM_019089)	-400	NM_01908	54626	Hs.118727	NM_01908	ENSG0000	HES2	bHLHb40	hes family	protein-co
lib-C666-l	chr1	936272	936272	+	329.931	NA	promoter-TSS (NM_021170)	promoter-TSS (NM_021170)	-720	NM_02117	57801	Hs.154029	NM_02117	ENSG0000	HES4	bHLHb42	hes family	protein-co
lib-C666-l	chr1	937430	937430	+	26.52079	NA	Intergenic	Intergenic	-1878	NM_02117	57801	Hs.154029	NM_02117	ENSG0000	HES4	bHLHb42	hes family	protein-co
lib-C666-l	chr1	935624	935624	+	9.75997	NA	promoter-TSS (NM_021170)	promoter-TSS (NM_021170)	-72	NM_02117	57801	Hs.154029	NM_02117	ENSG0000	HES4	bHLHb42	hes family	protein-co
lib-C666-l	chr1	934830	934830	+	3.34207	NA	intron (NM_001142467, intron 2 of 2)	CpG	722	NM_00114	57801	Hs.154029	NM_02117	ENSG0000	HES4	bHLHb42	hes family	protein-co
lib-C666-l	chr21	34451346	34451346	+	12.55817	NA	Intergenic	Intergenic	8896	NM_13898	116448	Hs.56663	NM_13898	ENSG0000	OLIG1	BHLHB6|B	oligodendr	protein-co
lib-C666-l	chr2	70141850	70141850	+	50.83079	NA	promoter-TSS (NM_001202514)	promoter-TSS (NM_001202514)	-323	NM_00120	4084	Hs.468908	NM_00235	ENSG0000	MXD1	BHLHC58|	MAX dime	protein-co
lib-C666-l	chr14	65569289	65569289	+	8.96177	NA	promoter-TSS (NM_002382)	promoter-TSS (NM_002382)	-27	NM_14511	4149	Hs.285354	NM_00238	ENSG0000	MAX	bHLHd4	MYC assoc	protein-co
lib-C666-l	chr7	17246013	17246013	+	23.02293	NA	Intergenic	Intergenic	-92263	NM_00162	196	Hs.171189	NM_00162	ENSG0000	AHR	bHLHe76	aryl hydroc	protein-co
lib-C666-l	chr7	17338044	17338044	+	9.53658	NA	promoter-TSS (NM_001621)	promoter-TSS (NM_001621)	-232	NM_00162	196	Hs.171189	NM_00162	ENSG0000	AHR	bHLHe76	aryl hydroc	protein-co
lib-C666-l	chr17	1171929	1171929	+	5.04179	NA	Intergenic	CpG	-1929	NM_00116	727857	Hs.723790	NM_00116	ENSG0000	BHLHA9	BHLHF42|C	basic helix	protein-co
lib-C666-l	chr4	30210613	30210613	+	11.20643	NA	Intergenic	AluSz|SINE|Alu	-511417	NM_03245	5099	Hs.479439	NM_00258	ENSG0000	PCDH7	BH-Pcdh|B	protocadhe	protein-co
lib-C666-l	chr12	120450228	120450228	+	11.6497	NA	intron (NM_207311, intron 2 of 8)	intron (NM_207311, intron 2 of 8)	22580	NM_20731	92558	Hs.369763	NM_20731	ENSG0000	BICDL1	BICDR-1|B	BICD family	protein-co
lib-C666-l	chr20	47538158	47538158	+	2.60101	NA	promoter-TSS (NM_006420)	promoter-TSS (NM_006420)	-117	NM_00642	10564	Hs.62578	NM_00642	ENSG0000	ARFGEF2	BIG2|PVNH	ADP ribos	protein-co
lib-C666-l	chr4	79697131	79697131	+	10.73451	NA	promoter-TSS (NM_017593)	promoter-TSS (NM_017593)	-401	NM_19889	55589	Hs.146551	NM_01759	ENSG0000	BMP2K	BIKE|HRIHF	BMP2 indu	protein-co
lib-C666-l	chr9	95857938	95857938	+	7.84231	NA	promoter-TSS (NR_134468)	promoter-TSS (NR_134468)	-467	NR_13446	84270	Hs.434213	NM_03231	ENSG0000	CARD19	BinCARD|C	caspase re	protein-co
lib-C666-l	chr15	65822631	65822631	+	21.94284	NA	promoter-TSS (NM_016395)	promoter-TSS (NM_016395)	-196	NM_01639	51495	Hs.512973	NM_01639	ENSG0000	HACD3	B-IND1|BI	3-hydroxya	protein-co
lib-C666-l	chr10	32735029	32735029	+	9.02258	NA	promoter-TSS (NR_120660)	promoter-TSS (NR_120660)	19	NR_10982	79741	Hs.585464	NM_02468	ENSG0000	CCDC7	BIOT2|Bio	coiled-coil	protein-co
lib-C666-l	chr9	128003770	128003770	+	53.38629	NA	promoter-TSS (NM_005347)	promoter-TSS (NM_005347)	-104	NM_00534	3309	Hs.743241	NM_00534	ENSG0000	HSPA5	BIP|GRP78	heat shock	protein-co
lib-C666-l	chr21	39235139	39235139	+	10.03103	NA	intron (NM_002240, intron 1 of 3)	intron (NM_002240, intron 1 of 3)	53602	NM_00224	3763	Hs.626242	NM_00224	ENSG0000	KCNJ6	BIR1|GIRK-	potassium	protein-co
lib-C666-l	chr17	57785008	57785008	+	27.28371	NA	promoter-TSS (NM_001015509)	promoter-TSS (NM_001015509)	-49	NM_00101	51651	Hs.12677	NM_01607	ENSG0000	PTRH2	BIT1|CFAP	peptidyl-tR	protein-co
lib-C666-l	chr3	138313173	138313173	+	9.87814	NA	promoter-TSS (NM_001288964)	promoter-TSS (NM_001288964)	52	NM_00128	11190	Hs.531962	NM_02449	ENSG0000	CEP70	BITE	centrosom	protein-co
lib-C666-l	chr20	33113844	33113844	+	3.7184	NA	intron (NM_001281728, intron 2 of 4)	intron (NM_001281728, intron 2 of	9655	NM_00128	83658	Hs.593920	NM_01418	ENSG0000	DYNLRB1	BITH|BLP|C	dynein ligh	protein-co
lib-C666-l	chr6	26458090	26458090	+	22.43333	NA	promoter-TSS (NM_001197234)	promoter-TSS (NM_001197234)	-42	NM_00704	11120	Hs.159028	NM_00704	ENSG0000	BTN2A1	BK14H9.1|	butyrophil	protein-co
lib-C666-l	chr14	75179860	75179860	+	53.15281	NA	promoter-TSS (NM_015962)	promoter-TSS (NM_015962)	10	NM_01596	51077	Hs.579828	NM_01596	ENSG0000	FCF1	Bka|C14orf	FCF1 rRNA	protein-co
lib-C666-l	chr7	32931124	32931124	+	13.15801	NA	intron (NM_015483, intron 1 of 3)	CpG	344	NM_01548	25948	Hs.372541	NM_01548	ENSG0000	KBTBD2	BKLHD1	kelch repe	protein-co
lib-C666-l	chr6	97372643	97372643	+	26.72399	NA	non-coding (NR_136580, exon 1 of 1	non-coding (NR_136580, exon 1 of	147	NM_00132	114792	Hs.45056	NM_05290	ENSG0000	KLHL32	BKLHD5|KI	kelch like f	protein-co
lib-C666-l	chr10	79291565	79291565	+	14.27758	NA	intron (NM_001322832, intron 1 of 27	MIR3|SINE|MIR	106012	NM_00127	3778	Hs.144795	NM_00224	ENSG0000	KCNMA1	BKTM|KCa	potassium	protein-co
lib-C666-l	chr10	79110548	79110548	+	8.96177	NA	promoter-TSS (NM_001322838)	promoter-TSS (NM_001322838)	-29	NM_00132	3778	Hs.144795	NM_00224	ENSG0000	KCNMA1	BKTM|KCa	potassium	protein-co
lib-C666-l	chr16	11439272	11439272	+	6.06312	NA	promoter-TSS (NM_152308)	promoter-TSS (NM_152308)	-23	NR_13075	116028	Hs.347524	NM_15230	ENSG0000	RMI2	BLAP18|C1	RecQ med	protein-co
lib-C666-l	chr16	11473567	11473567	+	2.24771	NA	Intergenic	L1MB2|LINE|L1	34272	NM_15230	116028	Hs.347524	NM_15230	ENSG0000	RMI2	BLAP18|C1	RecQ med	protein-co
lib-C666-l	chr11	65680437	65680437	+	5.74587	NA	Intergenic	Intergenic	6094	NM_00113	83638	Hs.433573	NM_03145	ENSG0000	C11orf68	BLES03|P5	chromosor	protein-co
lib-C666-l	chr6	106208035	106208035	+	8.01457	NA	Intergenic	Intergenic	-326160	NM_00119	639	Hs.436023	NM_00119	ENSG0000	PRDM1	BLIMP1|PR	PR/SET do	protein-co
lib-C666-l	chr1	153631176	153631176	+	4.38153	NA	promoter-TSS (NM_012437)	promoter-TSS (NM_012437)	46	NR_05201	23557	Hs.32018	NM_01243	ENSG0000	SNAPIN	BLOC1S7|S	SNAP asso	protein-co
lib-C666-l	chr3	148847470	148847470	+	26.52079	NA	promoter-TSS (NM_001308258)	promoter-TSS (NM_001308258)	99	NM_00130	84343	Hs.558314	NM_03238	ENSG0000	HPS3	BLOC2S1|S	HPS3, biog	protein-co
lib-C666-l	chr10	103825129	103825129	+	52.3754	NA	promoter-TSS (NM_024747)	promoter-TSS (NM_024747)	5	NM_02474	79803	Hs.125133	NM_02474	ENSG0000	HPS6	BLOC2S3	HPS6, biog	protein-co
lib-C666-l	chr22	26879835	26879835	+	498.81958	NA	promoter-TSS (NR_073135)	promoter-TSS (NR_073135)	-6	NR_07313	89781	Hs.474436	NM_00308	ENSG0000	HPS4	BLOC2S2|L	HPS4, biog	protein-co
lib-C666-l	chr1	155904286	155904286	+	14.5394	NA	promoter-TSS (NM_014949)	promoter-TSS (NM_014949)	-53	NM_01494	22889	Hs.24656	NM_01494	ENSG0000	KIAA0907	BLOM7|SN	KIAA0907	protein-co
lib-C666-l	chr10	102061028	102061028	+	5.92363	NA	intron (NM_016112, intron 2 of 15)	MER11B|LTR|ERVK	-14559	NM_00128	282991	Hs.34906	NM_17380	ENSG0000	BLOC1S2	BLOS2|BO	biogenesis	protein-co
lib-C666-l	chr6	8064738	8064738	+	103.49458	NA	promoter-TSS (NM_001199322)	promoter-TSS (NM_001199322)	-91	NM_00119	63915	Hs.719272	NM_20128	ENSG0000	BLOC1S5	BLOS5|MU	biogenesis	protein-co
lib-C666-l	chr15	45879514	45879514	+	67.3806	NA	promoter-TSS (NM_001311256)	promoter-TSS (NM_001311256)	193	NR_13235	26258	Hs.7037	NM_01238	ENSG0000	BLOC1S6	BLOS6|HP	biogenesis	protein-co
lib-C666-l	chr8	38854113	38854113	+	11.54352	NA	promoter-TSS (NM_031940)	promoter-TSS (NM_031940)	-72	NM_03194	83877	Hs.7471	NM_03194	ENSG0000	TM2D2	BLP1	TM2 doma	protein-co
lib-C666-l	chr8	38843136	38843136	+	3.28837	NA	intron (NM_153692, intron 8 of 8)	intron (NM_153692, intron 8 of 8)	10905	NM_07847	83877	Hs.7471	NM_03194	ENSG0000	TM2D2	BLP1	TM2 doma	protein-co
lib-C666-l	chr15	102156530	102156530	+	10.92275	NA	Intergenic	Intergenic	36064	NM_07847	80213	Hs.288912	NM_02514	ENSG0000	TM2D3	BLP2	TM2 doma	protein-co
lib-C666-l	chr15	102120106	102120106	+	7.15277	NA	Intergenic	MLT1K|LTR|ERVL-MaLR	72488	NM_07847	80213	Hs.288912	NM_02514	ENSG0000	TM2D3	BLP2	TM2 doma	protein-co
lib-C666-l	chr13	38923844	38923844	+	6.32351	NA	promoter-TSS (NM_001286706)	promoter-TSS (NM_001286706)	-64	NM_00128	51569	Hs.643655	NM_01661	ENSG0000	UFM1	BM-002|C	ubiquitin f	protein-co
lib-C666-l	chr1	91487574	91487574	+	22.28596	NA	promoter-TSS (NM_016620)	promoter-TSS (NM_016620)	238	NM_20126	84146	Hs.173001	NM_01662	ENSG0000	ZNF644	BM-005|M	zinc finger	protein-co
lib-C666-l	chr8	131028771	131028771	+	8.28613	NA	non-coding (NR_046361, exon 1 of 1	non-coding (NR_046361, exon 1 of	126	NR_04636	51571	Hs.126941	NM_01662	ENSG0000	FAM49B	BM-009|L1	family with	protein-co
lib-C666-l	chr3	186501163	186501163	+	17.68706	NA	promoter-TSS (NM_001967)	promoter-TSS (NM_001967)	-198	NM_00196	1974	Hs.518475	NM_00196	ENSG0000	EIF4A2	BM-010|D	eukaryotic	protein-co
lib-C666-l	chr3	157828051	157828051	+	3.27468	NA	intron (NM_001271838, intron 1 of 9)	CpG	210	NM_00127	51319	Hs.591283	NM_01662	ENSG0000	RSRC1	BM-011|S	arginine ar	protein-co
lib-C666-l	chr18	48724352	48724352	+	25.39487	NA	promoter-TSS (NM_016626)	promoter-TSS (NM_016626)	-301	NM_01662	51320	Hs.465144	NM_01662	ENSG0000	MEX3C	BM-013|M	mex-3 RN	protein-co
lib-C666-l	chr10	28821555	28821555	+	6.61453	NA	promoter-TSS (NM_100486)	promoter-TSS (NM_100486)	38	NM_10048	51322	Hs.743224	NM_01662	ENSG0000	WAC	BM-016|D	WW doma	protein-co
lib-C666-l	chr6	47277643	47277643	+	4.90117	NA	promoter-TSS (NM_014452)	promoter-TSS (NM_014452)	40	NM_01445	27242	Hs.443577	NM_01445	ENSG0000	TNFRSF21	BM-018|C	TNF recept	protein-co
lib-C666-l	chr4	78783304	78783304	+	9.93242	NA	promoter-TSS (NM_020236)	promoter-TSS (NM_020236)	-501	NM_02023	65008	Hs.532019	NM_02023	ENSG0000	MRPL1	BM022|L1	mitochond	protein-co
lib-C666-l	chr13	25497106	25497106	+	13.59254	NA	promoter-TSS (NR_047594)	promoter-TSS (NR_047594)	-79	NR_04759	55835	Hs.513379	NM_01845	ENSG0000	CENPJ	BM032|CE	centromer	protein-co
lib-C666-l	chr14	35025230	35025230	+	35.71493	NA	Intergenic	CpG	-16287	NM_01845	55837	Hs.433269	NM_01845	ENSG0000	EAPP	BM036|C1	E2F associ	protein-co
lib-C666-l	chr14	35016267	35016267	+	25.24625	NA	Intergenic	CpG	-7324	NM_01845	55837	Hs.433269	NM_01845	ENSG0000	EAPP	BM036|C1	E2F associ	protein-co
lib-C666-l	chr14	35008861	35008861	+	10.47352	NA	promoter-TSS (NM_018453)	promoter-TSS (NM_018453)	82	NM_00131	55837	Hs.433269	NM_01845	ENSG0000	EAPP	BM036|C1	E2F associ	protein-co
lib-C666-l	chr14	35024838	35024838	+	4.96122	NA	Intergenic	Intergenic	-15895	NM_01845	55837	Hs.433269	NM_01845	ENSG0000	EAPP	BM036|C1	E2F associ	protein-co
lib-C666-l	chr3	121553978	121553978	+	14.02237	NA	promoter-TSS (NM_001319107)	promoter-TSS (NM_001319107)	-49	NM_00132	55840	Hs.477325	NM_01845	ENSG0000	EAF2	BM040|TR	ELL associ	protein-co
lib-C666-l	chr16	4897398	4897398	+	59.65469	NA	promoter-TSS (NM_001288656)	promoter-TSS (NM_001288656)	-9	NM_00132	84656	Hs.387255	NM_03256	ENSG0000	GLYR1	BM045|HII	glyoxylate	protein-co
lib-C666-l	chr3	127317198	127317198	+	12.99339	NA	promoter-TSS (NR_073375)	promoter-TSS (NR_073375)	-2	NR_07337	4171	Hs.477481	NM_00452	ENSG0000	MCM2	BM28|CCN	minichrom	protein-co
lib-C666-l	chr11	74082839	74082839	+	7.15277	NA	exon (NM_173582, exon 3 of 14)	exon (NM_173582, exon 3 of 14)	26671	NM_17358	283209	Hs.26612	NM_17358	ENSG0000	PGM2L1	BM32A|PM	phosphogl	protein-co
lib-C666-l	chr18	19749149	19749149	+	4.46583	NA	promoter-TSS (NR_102763)	promoter-TSS (NR_102763)	-220	NR_10276	100128893	Hs.514745	NR_10276	ENSG0000	GATA6-AS	BM742401	GATA6 ant	ncRNA
lib-C666-l	chr11	13299076	13299076	+	2.58799	NA	promoter-TSS (NM_001178)	promoter-TSS (NM_001178)	-198	NM_00103	406	Hs.65734	NM_00117	ENSG0000	ARNTL	BMAL1|BM	aryl hydroc	protein-co
lib-C666-l	chr12	27485971	27485971	+	4.38153	NA	5' UTR (NM_001248002, exon 1 of 16	5' UTR (NM_001248002, exon 1 of 1	184	NM_02018	56938	Hs.445447	NM_02018	ENSG0000	ARNTL2	BMAL2|CL	aryl hydroc	protein-co
lib-C666-l	chr11	73694429	73694429	+	3.23837	NA	promoter-TSS (NM_003355)	promoter-TSS (NM_003355)	-540	NM_00335	7351	Hs.80658	NM_00335	ENSG0000	UCP2	BMIQ4|SL	uncouplin	protein-co
lib-C666-l	chr18	77748650	77748650	+	19.8879	NA	promoter-TSS (NM_001303471)	promoter-TSS (NM_001303471)	-58	NM_00130	10907	Hs.465498	NM_00670	ENSG0000	TXNL4A	BMKS|DIB1	thioredoxir	protein-co
lib-C666-l	chr11	68110407	68110407	+	32.48059	NA	intron (NM_001291902, intron 1 of 22	intron (NM_001291902, intron 1 of	30330	NM_00233	4041	Hs.6347	NM_00233	ENSG0000	LRP5	BMND1|EV	LDL recept	protein-co
lib-C666-l	chr11	68080836	68080836	+	3.58839	NA	intron (NM_001291902, intron 1 of 22	CpG	759	NM_00233	4041	Hs.6347	NM_00233	ENSG0000	LRP5	BMND1|EV	LDL recept	protein-co
lib-C666-l	chr14	39901547	39901547	+	36.56565	NA	5' UTR (NM_203301, exon 1 of 4)	5' UTR (NM_203301, exon 1 of 4)	157	NM_20330	254170	Hs.146632	NM_20330	ENSG0000	FBXO33	BMND12|F	F-box prot	protein-co
lib-C666-l	chr10	47243239	47243239	+	12.83248	NA	intron (NR_024495, intron 1 of 8),2	CpG	272	NR_02449	642826	Hs.463017	NR_024495		BMS1P6	BMS1LP2|E	BMS1, ribo	pseudo
lib-C666-l	chr10	48186112	48186112	+	9.10494	NA	intron (NR_024495, intron 1 of 8)	CpG	273	NR_02449	642826	Hs.463017	NR_024495		BMS1P6	BMS1LP2|E	BMS1, ribo	pseudo
lib-C666-l	chr10	75490035	75490035	+	25.3508	NA	promoter-TSS (NR_048575)	promoter-TSS (NR_048575)	237	NR_02659	729096	Hs.709171	NR_02659	ENSG0000	BMS1P4	BMS1LP4	BMS1, ribo	pseudo
lib-C666-l	chr1	9599466	9599466	+	92.67979	NA	promoter-TSS (NM_032315)	promoter-TSS (NM_032315)	-62	NM_03231	84275	Hs.568613	NM_03231	ENSG0000	SLC25A33	BMSC-MC	solute carr	protein-co
lib-C666-l	chr20	42286171	42286171	+	166.00273	NA	Intergenic	CpG	-9488	NM_00127	4605	Hs.179718	NM_00246	ENSG0000	MYBL2	B-MYB|BM	MYB proto	protein-co
lib-C666-l	chr8	22102650	22102650	+	135.05168	NA	promoter-TSS (NM_001722)	promoter-TSS (NM_001722)	31	NM_00172	661	Hs.148342	NM_00172	ENSG0000	POLR3D	BN51T|RP	RNA polym	protein-co
lib-C666-l	chr19	7600531	7600531	+	43.79478	NA	promoter-TSS (NM_001166114)	promoter-TSS (NM_001166114)	-58	NM_00116	10908	Hs.631863	NM_00670	ENSG0000	PNPLA6	BNHS|LNV	patatin lik	protein-co
lib-C666-l	chr19	7616300	7616300	+	4.58897	NA	exon (NM_001166112, exon 20 of 34)	exon (NM_001166112, exon 20 of 3	15711	NM_00116	10908	Hs.631863	NM_00670	ENSG0000	PNPLA6	BNHS|LNV	patatin lik	protein-co
lib-C666-l	chr15	59981685	59981685	+	20.90919	NA	promoter-TSS (NM_001320674)	promoter-TSS (NM_001320674)	48	NM_00433	663	Hs.592515	NM_00433	ENSG0000	BNIP2	BNIP-2|NI	BCL2 inter	protein-co
lib-C666-l	chr19	49939753	49939753	+	21.88699	NA	intron (NM_020309, intron 2 of 11)	CpG	5055	NM_02030	57030	Hs.375616	NM_02030	ENSG0000	SLC17A7	BNPI|VGL	solute carr	protein-co
lib-C666-l	chr16	71879847	71879847	+	440.47562	NA	promoter-TSS (NM_001137675)	promoter-TSS (NM_001137675)	-47	NM_00113	342371	Hs.743239	NM_00113	ENSG0000	ATXN1L	BOAT|BOA	ataxin 1 lik	protein-co
lib-C666-l	chr15	81282008	81282008	+	9.53658	NA	non-coding (NR_126327, exon 1 of 5	non-coding (NR_126327, exon 1 of	211	NM_01515	23184	Hs.578450	NM_01515	ENSG0000	MESDC2	BOCA|MES	mesoderm	protein-co
lib-C666-l	chr4	13629396	13629396	+	11.66012	NA	promoter-TSS (NM_148894)	promoter-TSS (NM_148894)	-68	NM_14889	259282	Hs.744935	NM_14889	ENSG0000	BOD1L1	BOD1L|FA	biorientati	protein-co
lib-C666-l	chr20	18477817	18477817	+	10.4657	NA	promoter-TSS (NM_006606)	promoter-TSS (NM_006606)	70	NM_00660	10741	Hs.69330	NM_00660	ENSG0000	RBBP9	BOG|RBBP	RB binding	protein-co
lib-C666-l	chr16	30205436	30205436	+	11.20036	NA	promoter-TSS (NR_037608)	promoter-TSS (NR_037608)	191	NM_00103	654483	Hs.444600	NM_00103	ENSG0000	BOLA2B	BOLA2	bolA family	protein-co
lib-C666-l	chr8	102517102	102517102	+	19.60174	NA	intron (NM_001330593, intron 1 of 15	intron (NM_001330593, intron 1 of	12434	NM_02491	79977	Hs.661088	NM_02491	ENSG0000	GRHL2	BOM|DFNA	grainyhead	protein-co
lib-C666-l	chr8	102551241	102551241	+	13.91591	NA	intron (NM_001330593, intron 1 of 15	intron (NM_001330593, intron 1 of	46573	NM_02491	79977	Hs.661088	NM_02491	ENSG0000	GRHL2	BOM|DFNA	grainyhead	protein-co
lib-C666-l	chr8	102504896	102504896	+	5.73967	NA	5' UTR (NM_024915, exon 1 of 16)	5' UTR (NM_024915, exon 1 of 16)	228	NM_02491	79977	Hs.661088	NM_02491	ENSG0000	GRHL2	BOM|DFNA	grainyhead	protein-co
lib-C666-l	chr1	38158060	38158060	+	9.55098	NA	promoter-TSS (NM_001256875)	promoter-TSS (NM_001256875)	-13	NM_01810	55143	Hs.524571	NM_01810	ENSG0000	CDCA8	BOR|BORE	cell divisior	protein-co
lib-C666-l	chr19	46273812	46273812	+	2.58854	NA	exon (NM_001081562, exon 15 of 15)	exon (NM_001081562, exon 15 of 1	-1315	NM_17587	147912	Hs.43314	NM_17587	ENSG0000	SIX5	BOR2|DMA	SIX homeo	protein-co
lib-C666-l	chr19	18668656	18668656	+	43.37893	NA	promoter-TSS (NM_001171948)	promoter-TSS (NM_001171948)	84	NM_00117	79036	Hs.714548	NM_02406	ENSG0000	KXD1	BORCS4|C	KxDL motif	protein-co
lib-C666-l	chr17	71313198	71313198	+	32.04651	NA	Intergenic	Intergenic	-5055	NM_01212	23580	Hs.3903	NM_01212	ENSG0000	CDC42EP4	BORG4|CE	CDC42 effe	protein-co
lib-C666-l	chr17	71338505	71338505	+	6.96291	NA	intron (NM_001144952, intron 44 of 4	intron (NM_001144952, intron 44 o	-30362	NM_01212	23580	Hs.3903	NM_01212	ENSG0000	CDC42EP4	BORG4|CE	CDC42 effe	protein-co
lib-C666-l	chr17	71308264	71308264	+	3.76263	NA	promoter-TSS (NM_012121)	promoter-TSS (NM_012121)	-121	NM_01212	23580	Hs.3903	NM_01212	ENSG0000	CDC42EP4	BORG4|CE	CDC42 effe	protein-co
lib-C666-l	chr22	37957904	37957904	+	7.97818	NA	intron (NM_152243, intron 1 of 2)	CpG	1433	NM_15224	11135	Hs.225356	NM_15224	ENSG0000	CDC42EP1	BORG5|CE	CDC42 effe	protein-co
lib-C666-l	chr1	145507524	145507524	+	15.55328	NA	promoter-TSS (NM_005105)	promoter-TSS (NM_005105)	-33	NM_00510	9939	Hs.591455	NM_00510	ENSG0000	RBM8A	BOV-1A|B	RNA bindir	protein-co
lib-C666-l	chr17	48045843	48045843	+	26.54591	NA	promoter-TSS (NM_138281)	promoter-TSS (NM_138281)	-719	NM_13828	1748	Hs.591167	NM_00193	ENSG0000	DLX4	BP1|DLX7|	distal-less	protein-co
lib-C666-l	chr17	48046506	48046506	+	3.89624	NA	promoter-TSS (NM_138281)	promoter-TSS (NM_138281)	-56	NM_13828	1748	Hs.591167	NM_00193	ENSG0000	DLX4	BP1|DLX7|	distal-less	protein-co
lib-C666-l	chr7	46193749	46193749	+	10.9422	NA	Intergenic	Intergenic	-232878	NM_00059	3486	Hs.450230	NM_00059	ENSG0000	IGFBP3	BP-53|IBP3	insulin like	protein-co
lib-C666-l	chr1	43147959	43147959	+	8.28333	NA	promoter-TSS (NM_004559)	promoter-TSS (NM_004559)	53	NM_00455	4904	Hs.473583	NM_00455	ENSG0000	YBX1	BP-8|CBF-:	Y-box binc	protein-co
lib-C666-l	chr22	43548299	43548299	+	6.81085	NA	intron (NM_001256530, intron 1 of 3)	intron (NM_001256530, intron 1 of	358	NM_00125	706	Hs.202	NM_00071	ENSG0000	TSPO	BPBS|BZRP	translocat	protein-co
lib-C666-l	chr12	112856690	112856690	+	174.44098	NA	promoter-TSS (NM_001320141)	promoter-TSS (NM_001320141)	-12	NM_08060	5781	Hs.506852	NM_00283	ENSG0000	PTPN11	BPTP3|CFC	protein tyr	protein-co
lib-C666-l	chr3	9772803	9772803	+	25.39084	NA	promoter-TSS (NM_001319050)	promoter-TSS (NM_001319050)	-610	NM_00131	7862	Hs.1004	NM_00463	ENSG0000	BRPF1	BR140	bromodon	protein-co
lib-C666-l	chr3	9773296	9773296	+	16.43544	NA	promoter-TSS (NM_001319050)	promoter-TSS (NM_001319050)	-117	NM_00131	7862	Hs.1004	NM_00463	ENSG0000	BRPF1	BR140	bromodon	protein-co
lib-C666-l	chr12	82752201	82752201	+	297.27899	NA	promoter-TSS (NM_001319675)	promoter-TSS (NM_001319675)	-2	NM_01416	29080	Hs.582627	NM_01416	ENSG0000	CCDC59	BR22|HSPC	coiled-coil	protein-co
lib-C666-l	chr7	140624900	140624900	+	214.57555	NA	promoter-TSS (NM_004333)	promoter-TSS (NM_004333)	-336	NM_00433	673	Hs.550061	NM_00433	ENSG0000	BRAF	B-RAF1|B-	B-Raf prot	protein-co
lib-C666-l	chr7	140624204	140624204	+	28.017	NA	intron (NM_004333, intron 1 of 17)	CpG	360	NM_00433	673	Hs.550061	NM_00433	ENSG0000	BRAF	B-RAF1|B-	B-Raf prot	protein-co
lib-C666-l	chr7	140511246	140511246	+	23.73126	NA	intron (NM_004333, intron 3 of 17)	LTR55|LTR|ERV	113318	NM_00433	673	Hs.550061	NM_00433	ENSG0000	BRAF	B-RAF1|B-	B-Raf prot	protein-co
lib-C666-l	chr10	125765779	125765779	+	31.99681	NA	Intergenic	Intergenic	86161	NM_01486	51363	Hs.287537	NM_01486	ENSG0000	CHST15	BRAG|GAL	carbohydra	protein-co
lib-C666-l	chr15	60771335	60771335	+	68.23259	NA	promoter-TSS (NM_001018089)	promoter-TSS (NM_001018089)	24	NM_00127	79664	Hs.200943	NM_02461	ENSG0000	ICE2	BRCC1|NA	interactor	protein-co
lib-C666-l	chr13	32889558	32889558	+	40.85366	NA	promoter-TSS (NM_000059)	promoter-TSS (NM_000059)	-59	NM_00005	675	Hs.34012	NM_00005	ENSG0000	BRCA2	BRCC2|BR	BRCA2, DN	protein-co
lib-C666-l	chr13	32890280	32890280	+	5.96422	NA	intron (NM_000059, intron 1 of 26)	MER3|DNA|hAT-Charlie	663	NM_00005	675	Hs.34012	NM_00005	ENSG0000	BRCA2	BRCC2|BR	BRCA2, DN	protein-co
lib-C666-l	chr5	59996005	59996005	+	18.0551	NA	promoter-TSS (NM_001145208)	promoter-TSS (NM_001145208)	-12	NM_00114	55789	Hs.482233	NM_01836	ENSG0000	DEPDC1B	BRCC3|XT	DEP doma	protein-co
lib-C666-l	chr15	40987354	40987354	+	7.6867	NA	promoter-TSS (NR_040058)	promoter-TSS (NR_040058)	-24	NM_00116	5888	Hs.631709	NM_00287	ENSG0000	RAD51	BRCC5|FA	RAD51 rec	protein-co
lib-C666-l	chr9	104295986	104295986	+	53.51636	NA	promoter-TSS (NM_019592)	promoter-TSS (NM_019592)	-145	NM_01959	56254	Hs.729085	NM_01959	ENSG0000	RNF20	BRE1|BRE1	ring finger	protein-co
lib-C666-l	chr16	30773603	30773603	+	25.90648	NA	promoter-TSS (NM_001286572)	promoter-TSS (NM_001286572)	-7	NM_00128	9810	Hs.65238	NM_01477	ENSG0000	RNF40	BRE1B|RB	ring finger	protein-co
lib-C666-l	chr14	69260183	69260183	+	36.38376	NA	5' UTR (NM_001244698, exon 1 of 3)	5' UTR (NM_001244698, exon 1 of 3	448	NM_00492	677	Hs.85155	NM_00492	ENSG0000	ZFP36L1	BRF1|Berg	ZFP36 ring	protein-co
lib-C666-l	chr14	69263108	69263108	+	10.79304	NA	promoter-TSS (NM_001244701)	promoter-TSS (NM_001244701)	-148	NM_00124	677	Hs.85155	NM_00492	ENSG0000	ZFP36L1	BRF1|Berg	ZFP36 ring	protein-co
lib-C666-l	chr14	69262579	69262579	+	8.71138	NA	intron (NM_001244701, intron 1 of 2)	CpG	381	NM_00124	677	Hs.85155	NM_00492	ENSG0000	ZFP36L1	BRF1|Berg	ZFP36 ring	protein-co
lib-C666-l	chr1	17099343	17099343	+	11.92838	NA	Intergenic	Intergenic	-8368	NM_00127	11223	Hs.349110	NM_001271733		MST1L	BRF-1|D1F	macrophag	protein-co
lib-C666-l	chr2	43453906	43453906	+	32.57434	NA	promoter-TSS (NM_006887)	promoter-TSS (NM_006887)	-161	NM_00688	676	Hs.503093	NM_00688	ENSG0000	ZFP36L2	BRF2|ERF-2	ZFP36 ring	protein-co

lib-C666-l	chr2	43395987	43395987	+	9.15272	NA	Intergenic	Intergenic	57758	NM_00688	678	Hs.503093	NM_00688	ENSG0000	ZFP36L2	BRF2|ERF-:	ZFP36 ring	protein-co
lib-C666-l	chr8	37707463	37707463	+	36.83792	NA	promoter-TSS (NM_018310)	promoter-TSS (NM_018310)	-32	NM_01831	55290	Hs.709301	NM_01831	ENSG0000	BRF2	BRFU|TFIIIE	BRF2, RNA	protein-co
lib-C666-l	chr12	125498693	125498693	+	4.6552	NA	intron (NM_080626, intron 2 of 2)	intron (NM_080626, intron 2 of 2)	13276	NR_11037!	102659353	Hs.596464	NR_11037!	ENSG0000	THRIL	BRI3BP-AS	TNF and H	ncRNA
lib-C666-l	chr19	925176	925176	+	8.28333	NA	promoter-TSS (NM_005224)	promoter-TSS (NM_005224)	-861	NM_00522	1820	Hs.501296	NM_00522	ENSG0000	ARID3A	BRIGHT|DF	AT-rich int	protein-co
lib-C666-l	chr3	144812576	144812576	+	9.79436	NA	Intergenic	MER65B|LTR|ERV1	1066706	NM_18294	5352	Hs.477866	NM_00093	ENSG0000	PLOD2	BRKS2|LH2	procollage	protein-co
lib-C666-l	chr14	36295436	36295436	+	66.35767	NA	promoter-TSS (NM_032352)	promoter-TSS (NM_032352)	-161	NM_03235	84312	Hs.525299	NM_03235	ENSG0000	BRMS1L	BRMS1	breast canc	protein-co
lib-C666-l	chr14	36295919	36295919	+	38.01809	NA	intron (NM_032352, intron 1 of 9)	CpG	322	NM_03235	84312	Hs.525299	NM_03235	ENSG0000	BRMS1L	BRMS1	breast canc	protein-co
lib-C666-l	chrX	43514013	43514013	+	20.59244	NA	promoter-TSS (NM_001270458)	promoter-TSS (NM_001270458)	-142	NM_00024	4128	Hs.183109	NM_00024	ENSG0000	MAOA	BRNRS|MA	monoamin	protein-co
lib-C666-l	chrX	43411113	43411113	+	15.30356	NA	Intergenic	LTR16B1|LTR|ERVL	-103042	NM_00024	4128	Hs.183109	NM_00024	ENSG0000	MAOA	BRNRS|MA	monoamin	protein-co
lib-C666-l	chr6	121655884	121655884	+	35.68805	NA	promoter-TSS (NR_104452)	promoter-TSS (NR_104452)	-238	NR_10445	221322	Hs.121396	NM_15273	ENSG0000	TBC1D32	BROMI|C6	TBC1 dom	protein-co
lib-C666-l	chr17	56769924	56769924	+	9.94769	NA	promoter-TSS (NM_001201457)	promoter-TSS (NM_001201457)	-10	NR_10387	5889	Hs.412587	NM_00287	ENSG0000	RAD51C	BROVCA3|	RAD51 par	protein-co
lib-C666-l	chr17	33446726	33446726	+	4.04037	NA	non-coding (NR_037712, exon 1 of 8	non-coding (NR_037712, exon 1 of	162	NM_00287	5892	Hs.631757	NM_00287	ENSG0000	RAD51D	BROVCA4|	RAD51 par	protein-co
lib-C666-l	chr8	145448134	145448134	+	52.49759	NA	Intergenic	CpG	10254	NM_01645	51236	Hs.300224	NM_01645	ENSG0000	HGH1	BRP16|BRP	HGH1 hom	protein-co
lib-C666-l	chr2	219187920	219187920	+	45.10538	NA	promoter-TSS (NM_022572)	promoter-TSS (NM_022572)	18	NM_02257	25953	Hs.98475	NM_01548	ENSG0000	PNKD	BRP17|DYT	paroxysma	protein-co
lib-C666-l	chr6	166796584	166796584	+	8.85547	NA	promoter-TSS (NM_001270879)	promoter-TSS (NM_001270879)	-83	NM_01609	51660	Hs.172755	NM_01609	ENSG0000	MPC1	BRP44L|CG	mitochond	protein-co
lib-C666-l	chr2	97001511	97001511	+	23.12593	NA	promoter-TSS (NM_015341)	promoter-TSS (NM_015341)	32	NM_00128	23397	Hs.308045	NM_01534	ENSG0000	NCAPH	BRRN1|CA	non-SMC	protein-co
lib-C666-l	chr2	97008062	97008062	+	6.56784	NA	intron (NM_001281710, intron 3 of 17	intron (NM_001281710, intron 3 of	6583	NM_00128	23397	Hs.308045	NM_01534	ENSG0000	NCAPH	BRRN1|CA	non-SMC	protein-co
lib-C666-l	chr16	50775940	50775940	+	10.40942	NA	promoter-TSS (NM_001042355)	promoter-TSS (NM_001042355)	-21	NM_01524	1540	Hs.578973	NM_01524	ENSG0000	CYLD	BRSS|CDM	CYLD lysin	protein-co
lib-C666-l	chr11	47574767	47574767	+	10.71733	NA	promoter-TSS (NM_006560)	promoter-TSS (NM_006560)	25	NM_00656	10658	Hs.595333	NM_00656	ENSG0000	CELF1	BRUNOL2|	CUGBP, Ela	protein-co
lib-C666-l	chr19	3275253	3275253	+	9.20967	NA	intron (NM_021938, intron 3 of 12)	intron (NM_021938, intron 3 of 12)	49770	NR_03334	60680	Hs.655747	NM_02193	ENSG0000	CELF5	BRUNOL-5	CUGBP, Ela	protein-co
lib-C666-l	chr19	3275757	3275757	+	9.02258	NA	intron (NM_021938, intron 3 of 12)	CpG	50274	NR_03334	60680	Hs.655747	NM_02193	ENSG0000	CELF5	BRUNOL-5	CUGBP, Ela	protein-co
lib-C666-l	chr15	72612640	72612640	+	8.51971	NA	promoter-TSS (NM_052840)	promoter-TSS (NM_052840)	-115	NM_05284	60677	Hs.348342	NM_05284	ENSG0000	CELF6	BRUNOL6	CUGBP, Ela	protein-co
lib-C666-l	chr13	27103761	27103761	+	38.21529	NA	Intergenic	Intergenic	-28079	NM_00664	10810	Hs.618732	NM_00664	ENSG0000	WASF3	Brush-1|SC	WAS prote	protein-co
lib-C666-l	chr11	2954804	2954804	+	11.66438	NA	Intergenic	Intergenic	-4154	NM_00331	7262	Hs.154036	NM_00331	ENSG0000	PHLDA2	BRW1C|BW	pleckstrin	protein-co
lib-C666-l	chr11	2954521	2954521	+	4.64451	NA	Intergenic	Intergenic	-3871	NM_00331	7262	Hs.154036	NM_00331	ENSG0000	PHLDA2	BRW1C|BW	pleckstrin	protein-co
lib-C666-l	chr7	5598723	5598723	+	18.17486	NA	Intergenic	Intergenic	-28491	NM_00110	60	Hs.520640	NM_00110	ENSG0000	ACTB	BRWS1|PS	actin beta	protein-co
lib-C666-l	chr7	5569455	5569455	+	5.96422	NA	intron (NM_001101, intron 1 of 5)	CpG-23985	777	NM_00110	60	Hs.520640	NM_00110	ENSG0000	ACTB	BRWS1|PS	actin beta	protein-co
lib-C666-l	chr7	5570239	5570239	+	2.00164	NA	promoter-TSS (NM_001101)	promoter-TSS (NM_001101)	-7	NM_00110	60	Hs.520640	NM_00110	ENSG0000	ACTB	BRWS1|PS	actin beta	protein-co
lib-C666-l	chr2	111334778	111334778	+	16.19708	NA	promoter-TSS (NM_005054).2	promoter-TSS (NM_005054).2	-100	NM_00505	84220	Hs.469630	NM_00505	ENSG0000	RGPD5	BS-63|BS6	RANBP2-li	protein-co
lib-C666-l	chr2	110551874	110551874	+	14.54258	NA	promoter-TSS (NM_005054)	promoter-TSS (NM_005054)	-92	NM_00505	84220	Hs.469630	NM_00505	ENSG0000	RGPD5	BS-63|BS6	RANBP2-li	protein-co
lib-C666-l	chr22	40924117	40924117	+	36.70779	NA	intron (NM_001282662, intron 3 of 14	intron (NM_001282662, intron 3 of	5767	NM_00131	57591	Hs.654688	NM_02083	ENSG0000	MKL1	BSAC|MAL	megakaryc	protein-co
lib-C666-l	chr22	41048146	41048146	+	10.27265	NA	Intergenic	Intergenic	-15423	NM_02083	57591	Hs.654688	NM_02083	ENSG0000	MKL1	BSAC|MAL	megakaryc	protein-co
lib-C666-l	chr22	41032866	41032866	+	5.74587	NA	promoter-TSS (NM_001282661)	promoter-TSS (NM_001282661)	-143	NM_02083	57591	Hs.654688	NM_02083	ENSG0000	MKL1	BSAC|MAL	megakaryc	protein-co
lib-C666-l	chr20	24973423	24973423	+	55.66538	NA	promoter-TSS (NM_020531)	promoter-TSS (NM_020531)	2	NM_02053	57136	Hs.472330	NM_02053	ENSG0000	APMAP	BSCv|C20o	adipocyte	protein-co
lib-C666-l	chr11	67140911	67140911	+	3.7491	NA	intron (NM_001166212, intron 1 of 2)	CpG	295	NM_01324	23529	Hs.502977	NM_01324	ENSG0000	CLCF1	BSF-3|BSF	cardiotrop	protein-co
lib-C666-l	chr16	29911341	29911341	+	2.26435	NA	promoter-TSS (NM_201575)	promoter-TSS (NM_201575)	-756	NM_20157	26470	Hs.6314	NM_01241	ENSG0000	SEZ6L2	BSRPA|PSK	seizure rela	protein-co
lib-C666-l	chr4	119274178	119274178	+	38.6745	NA	promoter-TSS (NM_003619)	promoter-TSS (NM_003619)	-256	NM_00361	8492	Hs.445857	NM_00361	ENSG0000	PRSS12	BSSP-3|BS	protease, s	protein-co
lib-C666-l	chr16	2918685	2918685	+	28.16397	NA	Intergenic	Intergenic	-10514	NM_02211	64063	Hs.459709	NM_02211	ENSG0000	PRSS22	BSSP-4|SP	protease, s	protein-co
lib-C666-l	chr16	2908725	2908725	+	11.48337	NA	promoter-TSS (NM_022119)	promoter-TSS (NM_022119)	-554	NM_02211	64063	Hs.459709	NM_02211	ENSG0000	PRSS22	BSSP-4|SP	protease, s	protein-co
lib-C666-l	chr13	110914001	110914001	+	8.89416	NA	intron (NM_001303110, intron 1 of 24	intron (NM_001303110, intron 1 of	45503	NM_00130	1282	Hs.17441	NM_00184	ENSG0000	COL4A1	BSVD|RAT	collagen ty	protein-co
lib-C666-l	chr6	26383245	26383245	+	35.0374	NA	promoter-TSS (NM_001197238)	promoter-TSS (NM_001197238)	-79	NM_00119	10385	Hs.373938	NM_00699	ENSG0000	BTN2A2	BT2.2|BTF2	butyrophili	protein-co
lib-C666-l	chr6	26402469	26402469	+	15.42216	NA	promoter-TSS (NM_007048)	promoter-TSS (NM_007048)	4	NM_00114	11119	Hs.191510	NM_00704	ENSG0000	BTN3A1	BT3.1|BTF5	butyrophili	protein-co
lib-C666-l	chr6	26365375	26365375	+	29.07066	NA	promoter-TSS (NM_001197249)	promoter-TSS (NM_001197249)	-12	NM_00119	11118	Hs.376046	NM_00704	ENSG0000	BTN3A2	BT3.2|BTF4	butyrophili	protein-co
lib-C666-l	chr6	26341921	26341921	+	10.37767	NA	Intergenic	Intergenic	-23466	NM_00119	11118	Hs.376046	NM_00704	ENSG0000	BTN3A2	BT3.2|BTF4	butyrophili	protein-co
lib-C666-l	chr16	3661657	3661657	+	25.75165	NA	promoter-TSS (NM_032444)	promoter-TSS (NM_032444)	-50	NM_03244	84464	Hs.143681	NM_03244	ENSG0000	SLX4	BTBD12|FA	SLX4 struct	protein-co
lib-C666-l	chr2	70056700	70056700	+	24.17107	NA	promoter-TSS (NM_178439)	promoter-TSS (NM_178439)	-38	NM_17843	64395	Hs.293971	NM_17843	ENSG0000	GMCL1	BTBD13|G	germ cell-l	protein-co
lib-C666-l	chr6	90927892	90927892	+	15.4157	NA	intron (NM_001170794, intron 1 of 6)	intron (NM_001170794, intron 1 of	78735	NM_02181	60468	Hs.269764	NM_02181	ENSG0000	BACH2	BTBD25	BTB domai	protein-co
lib-C666-l	chr6	82970190	82970190	+	10.73451	NA	Intergenic	Intergenic	-12719	NM_00130	25998	Hs.306425	NM_01552	ENSG0000	IBTK	BTBD26|BT	inhibitor of	protein-co
lib-C666-l	chr6	82811373	82811373	+	3.8557	NA	Intergenic	L1PA2|LINE|L1	146098	NM_01552	25998	Hs.306425	NM_01552	ENSG0000	IBTK	BTBD26|BT	inhibitor of	protein-co
lib-C666-l	chr17	47755454	47755454	+	24.41787	NA	promoter-TSS (NM_003563)	promoter-TSS (NM_003563)	71	NM_00100	8405	Hs.463382	NM_00356	ENSG0000	SPOP	BTBD32|TE	speckle typ	protein-co
lib-C666-l	chr14	45431238	45431238	+	22.57309	NA	promoter-TSS (NM_001308112)	promoter-TSS (NM_001308112)	-59	NM_01765	54813	Hs.550906	NM_01765	ENSG0000	KLHL28	BTBD5	kelch like f	protein-co
lib-C666-l	chr7	20838214	20838214	+	11.66438	NA	Intergenic	CpG	-11706	NM_18270	221833	Hs.195922	NM_18270	ENSG0000	SP8	BTD	Sp8 transc	protein-co
lib-C666-l	chr7	20826578	20826578	+	8.73012	NA	promoter-TSS (NM_182700)	promoter-TSS (NM_182700)	-70	NM_18270	221833	Hs.195922	NM_18270	ENSG0000	SP8	BTD	Sp8 transc	protein-co
lib-C666-l	chr9	73029263	73029263	+	19.82221	NA	5' UTR (NM_001206, exon 1 of 2)	5' UTR (NM_001206, exon 1 of 2)	310	NM_00120	687	Hs.150557	NM_00120	ENSG0000	KLF9	BTEB|BTEB	Kruppel lik	protein-co
lib-C666-l	chr9	73028920	73028920	+	14.37873	NA	5' UTR (NM_001206, exon 1 of 2)	5' UTR (NM_001206, exon 1 of 2)	653	NM_00120	687	Hs.150557	NM_00120	ENSG0000	KLF9	BTEB|BTEB	Kruppel lik	protein-co
lib-C666-l	chr9	73033278	73033278	+	7.21092	NA	Intergenic	MER5A|DNA|hAT-Charlie	-3705	NM_00120	687	Hs.150557	NM_00120	ENSG0000	KLF9	BTEB|BTEB	Kruppel lik	protein-co
lib-C666-l	chr9	73028517	73028517	+	5.51524	NA	5' UTR (NM_001206, exon 1 of 2)	5' UTR (NM_001206, exon 1 of 2)	1056	NM_00120	687	Hs.150557	NM_00120	ENSG0000	KLF9	BTEB|BTEB	Kruppel lik	protein-co
lib-C666-l	chr13	73633077	73633077	+	6.50091	NA	5' UTR (NM_001730, exon 1 of 4)	5' UTR (NM_001730, exon 1 of 4)	147	NM_00173	688	Hs.508234	NM_00173	ENSG0000	KLF5	BTEB2|CKL	Kruppel lik	protein-co
lib-C666-l	chr15	31618623	31618623	+	57.83654	NA	promoter-TSS (NM_001302461)	promoter-TSS (NM_001302461)	-435	NM_01599	51621	Hs.376443	NM_01599	ENSG0000	KLF13	BTEB3|FKL	Kruppel lik	protein-co
lib-C666-l	chr15	31692285	31692285	+	10.9422	NA	intron (NM_001302461, intron 1 of 1)	intron (NM_001302461, intron 1 of	73227	NM_00130	51621	Hs.376443	NM_01599	ENSG0000	KLF13	BTEB3|FKL	Kruppel lik	protein-co
lib-C666-l	chr15	31619436	31619436	+	2.00164	NA	exon (NM_001302461, exon 1 of 2)	exon (NM_001302461, exon 1 of 2)	378	NM_00130	51621	Hs.376443	NM_01599	ENSG0000	KLF13	BTEB3|FKL	Kruppel lik	protein-co
lib-C666-l	chr6	136611011	136611011	+	12.55817	NA	promoter-TSS (NM_001301038)	promoter-TSS (NM_001301038)	-22	NM_00107	9774	Hs.486542	NM_01473	ENSG0000	BCLAF1	BTF|bK211	BCL2 assoc	protein-co
lib-C666-l	chr6	26440694	26440694	+	20.59244	NA	promoter-TSS (NM_001242803)	promoter-TSS (NM_001242803)	-6	NM_00699	10384	Hs.167741	NM_00699	ENSG0000	BTN3A3	BTF3|BTN3	butyrophili	protein-co
lib-C666-l	chr13	77492205	77492205	+	2.24164	NA	Intergenic	L1PBa1|LINE|L1	-10380	NR_02698	690	Hs.567241	NR_026983		BTF3P11	BTF3L1|HU	basic trans	pseudo
lib-C666-l	chr13	45694580	45694580	+	40.85015	NA	promoter-TSS (NM_004128)	promoter-TSS (NM_004128)	-51	NM_00412	2963	Hs.654582	NM_00412	ENSG0000	GTF2F2	BTF4|RAP3	general tra	protein-co
lib-C666-l	chr19	6393416	6393416	+	28.30587	NA	promoter-TSS (NM_002096)	promoter-TSS (NM_002096)	-125	NM_00209	2962	Hs.68257	NM_00209	ENSG0000	GTF2F1	BTF4|RAP7	general tra	protein-co
lib-C666-l	chr19	6393793	6393793	+	3.93123	NA	promoter-TSS (NM_002096)	promoter-TSS (NM_002096)	-502	NM_00209	2962	Hs.68257	NM_00209	ENSG0000	GTF2F1	BTF4|RAP7	general tra	protein-co
lib-C666-l	chr21	47415937	47415937	+	3.31562	NA	intron (NM_001848, intron 20 of 34)	intron (NM_001848, intron 20 of 34	14274	NM_00184	1291	Hs.474053	NM_00184	ENSG0000	COL6A1	BTHLM1|O	collagen ty	protein-co
lib-C666-l	chr6	26421573	26421573	+	12.55817	NA	promoter-TSS (NR_027795)	promoter-TSS (NR_027795)	-46	NR_02779	54718	Hs.729655	NM_02401	ENSG0000	BTN2A3P	BTN2.3|BT	butyrophili	pseudo
lib-C666-l	chr6	29617932	29617932	+	33.69979	NA	Intergenic	CpG	-6826	NM_20681	4340	Hs.141308	NM_00243	ENSG0000	MOG	BTN6|BTN	myelin olig	protein-co
lib-C666-l	chr20	3219823	3219823	+	5.48804	NA	promoter-TSS (NM_001174089)	promoter-TSS (NM_001174089)	13	NR_13500	83959	Hs.105607	NM_03203	ENSG0000	SLC4A11	BTR1|CDP	solute carr	protein-co
lib-C666-l	chr17	38296665	38296665	+	36.97678	NA	5' UTR (NM_007359, exon 1 of 14)	5' UTR (NM_007359, exon 1 of 14)	158	NM_00735	22794	Hs.743287	NM_00735	ENSG0000	CASC3	BTZ|MLN5	cancer sus	protein-co
lib-C666-l	chr16	20687403	20687403	+	9.7234	NA	intron (NR_134918, intron 3 of 10)	HERV15-int|LTR|ERV1	15175	NM_05295	116285	Hs.306812	NM_05295	ENSG0000	ACSM1	BUCS1|MA	acyl-CoA s	protein-co
lib-C666-l	chr5	121297517	121297517	+	10.70998	NA	promoter-TSS (NM_152546)	promoter-TSS (NM_152546)	-139	NM_15254	153443	Hs.107622	NM_15254	ENSG0000	SRFBP1	BUD22|P4	serum resp	protein-co
lib-C666-l	chr9	33076669	33076669	+	128.58131	NA	promoter-TSS (NM_018225)	promoter-TSS (NM_018225)	45	NM_01822	55234	Hs.655351	NM_01822	ENSG0000	SMU1	BWD|SMU	DNA replic	protein-co
lib-C666-l	chr6	111303266	111303266	+	16.58224	NA	promoter-TSS (NM_001289111)	promoter-TSS (NM_001289111)	46	NM_00128	84154	Hs.372265	NM_03219	ENSG0000	RPF2	BXDC1|bA	ribosome p	protein-co
lib-C666-l	chr19	10216978	10216978	+	34.19829	NA	promoter-TSS (NM_020230)	promoter-TSS (NM_020230)	79	NM_00134	56342	Hs.14468	NM_02023	ENSG0000	PPAN	BXDC3|SSF	peter pan l	protein-co
lib-C666-l	chr12	133287262	133287262	+	12.00566	NA	promoter-TSS (NM_138575)	promoter-TSS (NM_138575)	-131	NM_00117	192111	Hs.102558	NM_13857	ENSG0000	PGAM5	BXLBV68	PGAM fam	protein-co
lib-C666-l	chr6	41888931	41888931	+	96.67246	NA	promoter-TSS (NM_004053)	promoter-TSS (NM_004053)	-34	NM_00405	705	Hs.106880	NM_00405	ENSG0000	BYSL	BYSTIN	bystin like	protein-co
lib-C666-l	chr2	201677192	201677192	+	9.94769	NA	promoter-TSS (NR_110275)	promoter-TSS (NR_110275)	136	NM_00120	9689	Hs.355983	NM_01467	ENSG0000	BZW1	BZAP45|N	basic leuci	protein-co
lib-C666-l	chr19	33793568	33793568	+	9.47655	NA	promoter-TSS (NM_001285829)	promoter-TSS (NM_001285829)	-98	NM_00128	1050	Hs.76171	NM_00436	ENSG0000	CEBPA	C/EBP-alp	CCAAT/en	protein-co
lib-C666-l	chr20	48807276	48807276	+	49.41553	NA	5' UTR (NM_001285879, exon 1 of 1)	5' UTR (NM_001285879, exon 1 of 1	156	NM_00128	1051	Hs.517106	NM_00519	ENSG0000	CEBPB	C/EBP-bet	CCAAT/en	protein-co
lib-C666-l	chr20	48807751	48807751	+	3.38731	NA	exon (NM_001285878, exon 1 of 1)	exon (NM_001285878, exon 1 of 1)	631	NM_00128	1051	Hs.517106	NM_00519	ENSG0000	CEBPB	C/EBP-bet	CCAAT/en	protein-co
lib-C666-l	chr8	48650887	48650887	+	14.18438	NA	promoter-TSS (NM_005195)	promoter-TSS (NM_005195)	-161	NM_00519	1052	Hs.440829	NM_00519	ENSG0000	CEBPD	C/EBP-del	CCAAT/en	protein-co
lib-C666-l	chr8	48678448	48678448	+	13.59254	NA	Intergenic	MER11B|LTR|ERVK	-27722	NM_00519	1052	Hs.440829	NM_00519	ENSG0000	CEBPD	C/EBP-del	CCAAT/en	protein-co
lib-C666-l	chr8	48644158	48644158	+	11.48337	NA	intron (NR_104581, intron 16 of 16)	intron (NR_104581, intron 16 of 16)	6568	NM_00519	1052	Hs.440829	NM_00519	ENSG0000	CEBPD	C/EBP-del	CCAAT/en	protein-co
lib-C666-l	chr8	48650614	48650614	+	8.91001	NA	exon (NM_005195, exon 1 of 1)	exon (NM_005195, exon 1 of 1)	112	NM_00519	1052	Hs.440829	NM_00519	ENSG0000	CEBPD	C/EBP-del	CCAAT/en	protein-co
lib-C666-l	chr14	21737825	21737825	+	10.06445	NA	promoter-TSS (NM_031314)	promoter-TSS (NM_031314)	-187	NM_00450	3183	Hs.508848	NM_00450	ENSG0000	HNRNPC	C1|C2|HNF	heterogen	protein-co
lib-C666-l	chr12	7047250	7047250	+	35.73373	NA	intron (NM_001007026, intron 6 of 9)	CpG-5080	-5351	NM_00130	113246	Hs.405913	NM_13842	ENSG0000	C12orf57	C10|GRCC	chromosor	protein-co
lib-C666-l	chr10	80063150	80063150	+	3.04651	NA	Intergenic	CpG	36065	NR_07344	414243	Hs.55047	NR_07344	ENSG0000	LINC00595	C10orf101	long interg	ncRNA
lib-C666-l	chr10	115934044	115934044	+	13.00105	NA	promoter-TSS (NM_018017)	promoter-TSS (NM_018017)	-65	NM_00132	55088	Hs.159066	NM_01801	ENSG0000	CCDC186	C10orf118	coiled-coil	protein-co
lib-C666-l	chr10	134717901	134717901	+	5.74587	NA	intron (NM_001200049, intron 22 of 5	intron (NM_001200049, intron 22 o	38188	NM_00120	54777	Hs.298038	NM_01760	ENSG0000	CFAP46	C10orf123	cilia and fla	protein-co
lib-C666-l	chr10	96943533	96943533	+	7.97818	NA	Intergenic	CpG	-10424	NM_20732	142827	Hs.134229	NM_20732	ENSG0000	ACSM6	C10orf129	acyl-CoA s	protein-co
lib-C666-l	chr10	99621802	99621802	+	6.32351	NA	intron (NM_001010917, intron 2 of 4)	intron (NM_001010917, intron 2 of	11807	NM_00101	401647	Hs.567524	NM_00101	ENSG0000	GOLGA7B	C10orf132	golgin A7	protein-co
lib-C666-l	chr10	99629357	99629357	+	3.25502	NA	3' UTR (NM_001010917, exon 5 of 5)	3' UTR (NM_001010917, exon 5 of 5	19362	NM_00101	401647	Hs.567524	NM_00101	ENSG0000	GOLGA7B	C10orf132	golgin A7	protein-co
lib-C666-l	chr10	5734865	5734865	+	5.9194	NA	intron (NM_001321783, intron 1 of 21	intron (NM_001321783, intron 1 of	8064	NM_00132	54906	Hs.610717	NM_01778	ENSG0000	FAM208B	C10orf18|	family with	protein-co
lib-C666-l	chr18	11851385	11851385	+	114.75929	NA	promoter-TSS (NM_020412)	promoter-TSS (NM_020412)	-4	NM_02041	57132	Hs.656244	NM_02041	ENSG0000	CHMP1B	C10orf2|C1	charged m	protein-co
lib-C666-l	chr10	64564326	64564326	+	43.7015	NA	promoter-TSS (NM_032804)	promoter-TSS (NM_032804)	-190	NM_03280	84890	Hs.99821	NM_03280	ENSG0000	ADO	C10orf22	2-aminoet	protein-co
lib-C666-l	chr10	104503598	104503598	+	12.55817	NA	promoter-TSS (NM_001083913)	promoter-TSS (NM_001083913)	-129	NM_00108	54838	Hs.500897	NM_01778	ENSG0000	WBP1L	C10orf26|	WW doma	protein-co
lib-C666-l	chr10	104499171	104499171	+	9.20967	NA	TTS (NM_178858)	TTS (NM_178858)	-4556	NM_00108	54838	Hs.500897	NM_01778	ENSG0000	WBP1L	C10orf26|	WW doma	protein-co
lib-C666-l	chr10	104563234	104563234	+	6.50091	NA	intron (NM_017787, intron 2 of 3)	intron (NM_017787, intron 2 of 3)	27346	NM_01778	54838	Hs.500897	NM_01778	ENSG0000	WBP1L	C10orf26|	WW doma	protein-co
lib-C666-l	chr10	99894386	99894386	+	17.97302	NA	promoter-TSS (NM_014472)	promoter-TSS (NM_014472)	5	NM_00125	27291	Hs.419800	NM_01447	ENSG0000	R3HCC1L	C10orf28|	R3H doma	protein-co
lib-C666-l	chr10	95256303	95256303	+	32.78343	NA	promoter-TSS (NM_001127182)	promoter-TSS (NM_001127182)	-66	NM_00112	55165	Hs.14559	NM_01813	ENSG0000	CEP55	C10orf3|C	centrosom	protein-co
lib-C666-l	chr10	95255661	95255661	+	25.39487	NA	promoter-TSS (NM_001127182)	promoter-TSS (NM_001127182)	-708	NM_00112	55165	Hs.14559	NM_01813	ENSG0000	CEP55	C10orf3|C	centrosom	protein-co
lib-C666-l	chr10	13482610	13482610	+	3.20885	NA	TTS (NM_001100912)	TTS (NM_001100912)	62366	NM_00110	222389	Hs.498740	NM_15275	ENSG0000	BEND7	C10orf30	BEN doma	protein-co
lib-C666-l	chr10	104614215	104614215	+	9.02258	NA	intron (NM_001136200, intron 1 of 4)	CpG	248	NM_00113	119032	Hs.34492	NM_14459	ENSG0000	BORCS7	C10orf32	BLOC-1 re	protein-co
lib-C666-l	chr10	15412434	15412434	+	7.97818	NA	intron (NM_001010924, intron 1 of 7)	CpG	624	NM_00101	221061	Hs.66762	NM_00101	ENSG0000	FAM171A1	C10orf38	family with	protein-co
lib-C666-l	chr10	14602451	14602451	+	14.72053	NA	intron (NM_001320736, intron 1 of 4)	intron (NM_001320736, intron 1 of	-4272	NM_00132	83641	Hs.446315	NM_03145	ENSG0000	FAM107B	C10orf45|	family with	protein-co
lib-C666-l	chr10	14647453	14647453	+	6.56784	NA	intron (NM_001282695, intron 2 of 5)	intron (NM_001282695, intron 2 of	-1036	NM_00128	83641	Hs.446315	NM_03145	ENSG0000	FAM107B	C10orf45|	family with	protein-co
lib-C666-l	chr10	120514670	120514670	+	14.37873	NA	promoter-TSS (NM_153810)	promoter-TSS (NM_153810)	88	NM_15381	143384	Hs.420024	NM_15381	ENSG0000	CACUL1	C10orf46|	CDK2 asso	protein-co
lib-C666-l	chr10	120514153	120514153	+	2.58854	NA	exon (NM_153810, exon 1 of 9)	exon (NM_153810, exon 1 of 9)	605	NM_15381	143384	Hs.420024	NM_15381	ENSG0000	CACUL1	C10orf46|	CDK2 asso	protein-co
lib-C666-l	chr10	11865385	11865385	+	8.28613	NA	promoter-TSS (NM_153256)	promoter-TSS (NM_153256)	-12	NM_15325	254427	Hs.435775	NM_15325	ENSG0000	PROSER2	C10orf47	proline and	protein-co
lib-C666-l	chr10	97453755	97453755	+	24.50625	NA	5' UTR (NM_001143973, exon 1 of 10	5' UTR (NM_001143973, exon 1 of 1	145	NM_01563	26123	Hs.438991	NM_01563	ENSG0000	TCTN3	C10orf61|	tectonic fa	protein-co
lib-C666-l	chr10	25350938	25350938	+	10.03103	NA	intron (NM_001270383, intron 1 of 5)	intron (NM_001270383, intron 1 of	270	NM_00127	219670	Hs.534486	NM_14501	ENSG0000	ENKUR	C10orf63|	enkurin, TF	protein-co
lib-C666-l	chr10	25351501	25351501	+	3.84463	NA	promoter-TSS (NM_001270383)	promoter-TSS (NM_001270383)	-293	NM_00127	219670	Hs.534486	NM_14501	ENSG0000	ENKUR	C10orf63|	enkurin, TF	protein-co
lib-C666-l	chr10	105881843	105881843	+	33.60578	NA	promoter-TSS (NM_145247)	promoter-TSS (NM_145247)	27	NM_14524	119392	Hs.93667	NM_14524	ENSG0000	SFR1	C10orf78|	SWI5 depe	protein-co
lib-C666-l	chr10	106151259	106151259	+	21.28624	NA	intron (NM_001008723, intron 9 of 17	intron (NM_001008723, intron 9 of	37737	NM_00100	159686	Hs.253576	NM_00100	ENSG0000	CFAP58	C10orf80|	cilia and fla	protein-co
lib-C666-l	chr10	120101913	120101913	+	6.82479	NA	promoter-TSS (NM_001134672)	promoter-TSS (NM_001134672)	-74	NM_00113	63877	Hs.105575	NM_02206	ENSG0000	FAM204A	C10orf84|	family with	protein-co
lib-C666-l	chr10	123734505	123734505	+	5.65398	NA	exon (NM_001167865, exon 1 of 11)	exon (NM_001167865, exon 1 of 11	238	NM_00116	54780	Hs.258798	NM_01761	ENSG0000	NSMCE4A	C10orf86|	NSE4 hom	protein-co
lib-C666-l	chr10	124046624	124046624	+	21.90047	NA	intron (NM_001318189, intron 5 of 15	intron (NM_001318189, intron 5 of	15813	NM_00131	118663	Hs.422466	NM_14458	ENSG0000	BTBD16	C10orf87	BTB domai	protein-co
lib-C666-l	chr10	124740071	124740071	+	109.04404	NA	exon (NM_153336, exon 1 of 7)	exon (NM_153336, exon 1 of 7)	515	NM_15333	118672	Hs.281004	NM_15333	ENSG0000	PSTK	C10orf89	phosphose	protein-co
lib-C666-l	chr10	35625486	35625486	+	6.13111	NA	promoter-TSS (NM_001282852)	promoter-TSS (NM_001282852)	-316	NM_00128	219771	Hs.14745	NM_14501	ENSG0000	CCNY	C10orf9|C	cyclin Y	protein-co
lib-C666-l	chr10	15981753	15981753	+	8.20589	NA	Intergenic	THE1B|LTR|ERVL-MaLR	-79221	NM_02494	80013	Hs.158870	NM_02494	ENSG0000	FAM188A	C10orf97|	family with	protein-co
lib-C666-l	chr16	103700	103700	+	11.90035	NA	promoter-TSS (NM_016310)	promoter-TSS (NM_016310)	-68	NM_01631	51728	Hs.632223	NM_01631	ENSG0000	POLR3K	C11|C11-R	RNA polym	protein-co
lib-C666-l	chr7	102036798	102036798	+	218.63457	NA	promoter-TSS (NM_024653)	promoter-TSS (NM_024653)	-6	NM_02465	79706	Hs.722104	NM_02465	ENSG0000	PRKRIP1	C114|KRB	PRKR inter	protein-co
lib-C666-l	chr11	61463615	61463615	+	7.49163	NA	intron (NM_006133, intron 1 of 19)	intron (NM_006133, intron 1 of 19)	15710	NM_00613	747	Hs.241564	NM_00613	ENSG0000	DAGLA	C11orf11|	diacylglyce	protein-co
lib-C666-l	chr11	8985959	8985959	+	75.60525	NA	promoter-TSS (NR_073431)	promoter-TSS (NR_073431)	-263	NR_07343	493900	Hs.591981	NR_07343	ENSG0000	TMEM9B-A	C11orf18	TMEM9B a	ncRNA
lib-C666-l	chr11	68227807	68227807	+	6.15313	NA	promoter-TSS (NM_001164164)	promoter-TSS (NM_001164164)	-379	NM_00116	55291	Hs.503022	NM_01831	ENSG0000	PPP6R3	C11orf23|	protein ph	protein-co
lib-C666-l	chr15	34394154	34394154	+	222.33336	NA	promoter-TSS (NM_020154)	promoter-TSS (NM_020154)	-101	NM_02015	56851	Hs.160565	NM_02015	ENSG0000	EMC7	C11orf3|C1	ER membr	protein-co
lib-C666-l	chr11	76155720	76155720	+	27.5466	NA	promoter-TSS (NM_020193)	promoter-TSS (NM_020193)	-349	NM_00130	56946	Hs.352588	NM_02019	ENSG0000	EMSY	C11orf30|	EMSY, BRC	protein-co
lib-C666-l	chr11	121322883	121322883	+	2.12162	NA	promoter-TSS (NM_003105)	promoter-TSS (NM_003105)	-29	NM_00310	6653	Hs.368592	NM_00310	ENSG0000	SORL1	C11orf32|	sortilin rela	protein-co
lib-C666-l	chr11	560666	560666	+	2.59032	NA	promoter-TSS (NM_001143993)	promoter-TSS (NM_001143993)	113	NM_17357	256329	Hs.669395	NM_17357	ENSG0000	LMNTD2	C11orf35	lamin tail d	protein-co
lib-C666-l	chr11	131479359	131479359	+	3.8557	NA	intron (NM_001048209, intron 1 of 7)	intron (NM_001048209, intron 1 of	54091	NR_12615	101929637	Hs.730330	NM_20743	ENSG0000	NTM-AS1	C11orf39	NTM antise	ncRNA
lib-C666-l	chr11	64052238	64052238	+	24.25638	NA	promoter-TSS (NM_004322)	promoter-TSS (NM_004322)	-45	NM_00117	56834	Hs.523763	NM_02015	ENSG0000	GPR137	C11orf4|G	G protein-	protein-co
lib-C666-l	chr11	64038698	64038698	+	7.76438	NA	intron (NM_032989, intron 3 of 3)	(CA)n|Simple_repeat|Simple_repeat	-13113	NM_00117	56834	Hs.523763	NM_02015	ENSG0000	GPR137	C11orf4|G	G protein-	protein-co
lib-C666-l	chr11	64053004	64053004	+	3.27468	NA	promoter-TSS (NM_020155)	promoter-TSS (NM_020155)	-578	NM_00117	56834	Hs.523763	NM_02015	ENSG0000	GPR137	C11orf4|G	G protein-	protein-co
lib-C666-l	chr11	64054067	64054067	+	2.78587	NA	exon (NM_020155, exon 1 of 7)	exon (NM_020155, exon 1 of 7)	485	NM_02015	56834	Hs.523763	NM_02015	ENSG0000	GPR137	C11orf4|G	G protein-	protein-co
lib-C666-l	chr11	62439505	62439505	+	3.96379	NA	promoter-TSS (NM_024099)	promoter-TSS (NM_024099)	-264	NM_02409	79081	Hs.9061	NM_02409	ENSG0000	LBHD1	C11orf48	LBH doma	protein-co
lib-C666-l	chr11	64885281	64885281	+	23.80446	NA	promoter-TSS (NM_014205)	promoter-TSS (NM_014205)	-71	NM_01420	741	Hs.121025	NM_01420	ENSG0000	ZNHIT2	C11orf5|F	zinc finger	protein-co
lib-C666-l	chr11	64884936	64884936	+	3.20885	NA	exon (NM_014205, exon 1 of 1)	exon (NM_014205, exon 1 of 1)	274	NM_01420	741	Hs.121025	NM_01420	ENSG0000	ZNHIT2	C11orf5|F	zinc finger	protein-co
lib-C666-l	chr11	6255980	6255980	+	5.3268	NA	promoter-TSS (NM_032127)	promoter-TSS (NM_032127)	-39	NM_03212	84067	Hs.501793	NM_03212	ENSG0000	FAM160A2	C11orf56|	family with	protein-co
lib-C666-l	chr11	71814249	71814249	+	5.3823	NA	exon (NM_017907, exon 1 of 5)	exon (NM_017907, exon 1 of 5)	184	NM_01790	55004	Hs.731528	NM_01790	ENSG0000	LAMTOR1	C11orf59|	late endos	protein-co
lib-C666-l	chr11	1593553	1593553	+	21.65402	NA	promoter-TSS (NR_021489)	promoter-TSS (NR_021489)	-403	NM_00442	1850	Hs.41688	NM_00442	ENSG0000	DUSP8	C11orf81|	dual specif	protein-co
lib-C666-l	chr11	1568378	1568378	+	2.59723	NA	Intergenic	CpG	24772	NM_00442	1850	Hs.41688	NM_00442	ENSG0000	DUSP8	C11orf81|	dual specif	protein-co
lib-C666-l	chr12	106751516	106751516	+	3.8557	NA	promoter-TSS (NM_018082)	promoter-TSS (NM_018082)	80	NM_01808	55703	Hs.62696	NM_01808	ENSG0000	POLR3B	C128|HLD	RNA polym	protein-co
lib-C666-l	chr12	58165874	58165874	+	91.00045	NA	promoter-TSS (NM_015433)	promoter-TSS (NM_015433)	40	NM_02303	4234	Hs.42957	NM_00537	ENSG0000	METTL1	C12orf1|T	methyltran	protein-co
lib-C666-l	chr12	107350648	107350648	+	3.27468	NA	promoter-TSS (NM_001319662)	promoter-TSS (NM_001319662)	85	NM_00131	90488	Hs.257664	NM_15226	ENSG0000	TMEM263	C12orf23	transmemb	protein-co
lib-C666-l	chr12	121399587	121399587	+	3.98718	NA	Intergenic	Intergenic	10508	NR_02434	283460	Hs.612351	NR_02434	ENSG0000	HNF1A-AS	C12orf27|	HNF1A ant	ncRNA
lib-C666-l	chr12	112546389	112546389	+	10.9095	NA	intron (NM_024953, intron 1 of 23)	CpG	246	NM_02495	80018	Hs.530941	NM_02495	ENSG0000	NAA25	C12orf30|	N(alpha)-a	protein-co
lib-C666-l	chr12	66524598	66524598	+	24.32656	NA	promoter-TSS (NM_032338)	promoter-TSS (NM_032338)	-65	NM_03233	84298	Hs.504820	NM_03233	ENSG0000	LLPH	C12orf31|	LLP homol	protein-co
lib-C666-l	chr12	110153196	110153196	+	89.80472	NA	intron (NM_032829, intron 1 of 2)	intron (NM_032829, intron 1 of 2)	1009	NM_03282	84915	Hs.661785	NM_03282	ENSG0000	FAM222A	C12orf34	family with	protein-co
lib-C666-l	chr12	110151511	110151511	+	10.73451	NA	promoter-TSS (NM_032829)	promoter-TSS (NM_032829)	-676	NM_03282	84915	Hs.661785	NM_03282	ENSG0000	FAM222A	C12orf34	family with	protein-co
lib-C666-l	chr12	52463656	52463656	+	13.98985	NA	promoter-TSS (NM_001098673)	promoter-TSS (NM_001098673)	-102	NM_02193	60673	Hs.9911	NM_02193	ENSG0000	ATG101	C12orf44	autophagy	protein-co
lib-C666-l	chr12	4430289	4430289	+	24.50625	NA	promoter-TSS (NM_020375)	promoter-TSS (NM_020375)	-70	NM_02037	57103	Hs.504545	NM_02037	ENSG0000	TIGAR	C12orf5|F	TP53 induc	protein-co
lib-C666-l	chr12	62996418	62996418	+	32.11429	NA	non-coding (NR_121682, exon 1 of 1	non-coding (NR_121682, exon 1 of	796	NR_12168	283416	Hs.375881	NM_17589	ENSG0000	LINC01465	C12orf61	long interg	ncRNA
lib-C666-l	chr12	62996903	62996903	+	8.01457	NA	promoter-TSS (NR_029661)	promoter-TSS (NR_029661)	311	NR_12168	283416	Hs.375881	NM_17589	ENSG0000	LINC01465	C12orf61	long interg	ncRNA
lib-C666-l	chr12	50505603	50505603	+	88.49222	NA	promoter-TSS (NM_001257133)	promoter-TSS (NM_001257133)	-161	NM_00125	84987	Hs.388645	NM_03290	ENSG0000	COX14	C12orf62|	COX14, cyt	protein-co

lib-C666-l	chr12	31835296	31835296	+	31.45327	NA	intron (NR_103522, intron 7 of 8)	LTR55|LTR|ERV	22687	NM_00113	254013	Hs.740628	NM_17380	ENSG0000	ETFBKMT	C12orf72|E	electron tr	protein-co
lib-C666-l	chr12	31812249	31812249	+	9.75396	NA	promoter-TSS (NM_001135863)	promoter-TSS (NM_001135863)	128	NM_17380	254013	Hs.740628	NM_17380	ENSG0000	ETFBKMT	C12orf72|E	electron tr	protein-co
lib-C666-l	chr12	112451979	112451979	+	95.02626	NA	promoter-TSS (NM_001294314)	promoter-TSS (NM_001294314)	827	NM_00681	10961	Hs.75841	NM_00681	ENSG0000	ERP29	C12orf8|ER	endoplasm	protein-co
lib-C666-l	chr12	112451228	112451228	+	32.58685	NA	promoter-TSS (NM_001294314)	promoter-TSS (NM_001294314)	76	NM_00681	10961	Hs.75841	NM_00681	ENSG0000	ERP29	C12orf8|ER	endoplasm	protein-co
lib-C666-l	chr13	114145227	114145227	+	5.65398	NA	promoter-TSS (NM_001014283)	promoter-TSS (NM_001014283)	-81	NM_01790	55002	Hs.317593	NM_01790	ENSG0000	TMCO3	C13orf11	transmemb	protein-co
lib-C666-l	chr13	29160903	29160903	+	36.70779	NA	Intergenic	Intergenic	-72238	NM_01593	51371	Hs.268742	NM_01593	ENSG0000	POMP	C13orf12|H	proteasom	protein-co
lib-C666-l	chr13	31191784	31191784	+	39.64852	NA	promoter-TSS (NM_001313893)	promoter-TSS (NM_001313893)	-32	NM_00132	10208	Hs.533831	NM_00580	ENSG0000	USPL1	C13orf22|D	ubiquitin s	protein-co
lib-C666-l	chr13	39612193	39612193	+	57.89614	NA	promoter-TSS (NM_025138)	promoter-TSS (NM_025138)	20	NM_02513	80209	Hs.318526	NM_02513	ENSG0000	PROSER1	C13orf23	proline anc	protein-co
lib-C666-l	chr13	92001336	92001336	+	3.98718	NA	intron (NR_027350, intron 1 of 1)	CpG	1262	NR_02734	407975	Hs.24115	NR_02734	ENSG0000	MIR17HG	C13orf25|F	miR-17-92	ncRNA
lib-C666-l	chr13	21750795	21750795	+	6.49644	NA	promoter-TSS (NM_001166017)	promoter-TSS (NM_001166017)	-54	NM_00116	221150	Hs.88523	NM_14506	ENSG0000	SKA3	C13orf3|RA	spindle anc	protein-co
lib-C666-l	chr13	43294770	43294770	+	8.89416	NA	Intergenic	Intergenic	-60916	NM_00131	144809	Hs.16970	NM_18250	ENSG0000	FAM216B	C13orf30	family with	protein-co
lib-C666-l	chr13	53024939	53024939	+	30.08826	NA	promoter-TSS (NM_001282169)	promoter-TSS (NM_001282169)	-126	NM_00128	51028	Hs.109520	NM_01607	ENSG0000	VPS36	C13orf9|CC	vacuolar pr	protein-co
lib-C666-l	chr14	57046272	57046272	+	14.09262	NA	promoter-TSS (NM_017799)	promoter-TSS (NM_017799)	-239	NM_01779	54916	Hs.497253	NM_01779	ENSG0000	TMEM260	C14orf101	transmemb	protein-co
lib-C666-l	chr14	96829694	96829694	+	17.62684	NA	promoter-TSS (NM_001271905)	promoter-TSS (NM_001271905)	44	NM_01803	55102	Hs.168241	NM_01803	ENSG0000	ATG2B	C14orf103	autophagy	protein-co
lib-C666-l	chr14	50100450	50100450	+	24.32656	NA	exon (NM_018139, exon 1 of 3)	exon (NM_018139, exon 1 of 3)	1498	NM_01813	55172	Hs.231761	NM_01813	ENSG0000	DNAAF2	C14orf104	dynein axo	protein-co
lib-C666-l	chr14	45722613	45722613	+	56.64664	NA	promoter-TSS (NM_018353)	promoter-TSS (NM_018353)	-8	NM_01835	55320	Hs.732769	NM_01835	ENSG0000	MIS18BP1	C14orf106	MIS18 binc	protein-co
lib-C666-l	chr14	76618206	76618206	+	10.32881	NA	promoter-TSS (NM_001322028)	promoter-TSS (NM_001322028)	-22	NM_01792	55668	Hs.410231	NM_01792	ENSG0000	GPATCH2L	C14orf118	G-patch do	protein-co
lib-C666-l	chr14	23938789	23938789	+	44.91887	NA	promoter-TSS (NM_001042635)	promoter-TSS (NM_001042635)	-109	NM_00104	25983	Hs.9043	NM_01551	ENSG0000	NGDN	C14orf120	neuroguidi	protein-co
lib-C666-l	chr14	24682746	24682746	+	185.07722	NA	TTS (NM_138476)	TTS (NM_138476)	290	NM_01416	29082	Hs.279761	NM_01416	ENSG0000	CHMP4A	C14orf123	charged m	protein-co
lib-C666-l	chr14	24912089	24912089	+	8.85547	NA	promoter-TSS (NM_001308075)	promoter-TSS (NM_001308075)	22	NM_00129	56948	Hs.643552	NM_02019	ENSG0000	SDR39U1	C14orf124	short chain	protein-co
lib-C666-l	chr14	31889841	31889841	+	6.71839	NA	promoter-TSS (NR_110045)	promoter-TSS (NR_110045)	-53	NM_01547	25938	Hs.744979	NM_01547	ENSG0000	HEATR5A	C14orf125	HEAT repe	protein-co
lib-C666-l	chr14	31926657	31926657	+	4.49043	NA	promoter-TSS (NM_080664)	promoter-TSS (NM_080664)	23	NM_08066	112487	Hs.116014	NM_08066	ENSG0000	DTD2	C14orf126	D-tyrosyl-t	protein-co
lib-C666-l	chr14	32030603	32030603	+	140.98909	NA	promoter-TSS (NM_025152)	promoter-TSS (NM_025152)	12	NM_02515	80224	Hs.288981	NM_02515	ENSG0000	NUBPL	C14orf127	nucleotide	protein-co
lib-C666-l	chr14	93673381	93673381	+	5.74587	NA	promoter-TSS (NM_032490)	promoter-TSS (NM_032490)	-20	NR_03815	55148	Hs.648806	NM_17574	ENSG0000	UBR7	C14orf130	ubiquitin p	protein-co
lib-C666-l	chr14	102783351	102783351	+	130.63884	NA	promoter-TSS (NM_001267827)	promoter-TSS (NM_001267827)	-363	NM_00126	55778	Hs.743359	NM_01833	ENSG0000	ZNF839	C14orf131	zinc finger	protein-co
lib-C666-l	chr14	102786002	102786002	+	66.80925	NA	promoter-TSS (NM_018335)	promoter-TSS (NM_018335)	-86	NM_01833	55778	Hs.743359	NM_01833	ENSG0000	ZNF839	C14orf131	zinc finger	protein-co
lib-C666-l	chr14	77924059	77924059	+	54.30392	NA	promoter-TSS (NM_012111)	promoter-TSS (NM_012111)	-76	NM_00119	63894	Hs.16157	NM_02206	ENSG0000	VIPAS39	C14orf133	VPS33B int	protein-co
lib-C666-l	chr14	60558684	60558684	+	7.6867	NA	promoter-TSS (NM_022495)	promoter-TSS (NM_022495)	55	NM_02249	64430	Hs.509499	NM_02249	ENSG0000	PCNX4	C14orf135	pecanex hc	protein-co
lib-C666-l	chr14	94492646	94492646	+	37.47379	NA	promoter-TSS (NM_023112)	promoter-TSS (NM_023112)	-78	NM_02311	78990	Hs.278815	NM_02311	ENSG0000	OTUB2	C14orf137	OTU deubi	protein-co
lib-C666-l	chr14	50583326	50583326	+	155.40666	NA	promoter-TSS (NM_001040662)	promoter-TSS (NM_001040662)	-29	NM_00104	79609	Hs.558541	NM_02455	ENSG0000	VCPKMT	C14orf138	valosin cor	protein-co
lib-C666-l	chr14	95861571	95861571	+	7.31982	NA	Intergenic	Intergenic	14856	NR_02677	161176	Hs.41502	NM_02463	ENSG0000	SYNE3	C14orf139	spectrin re	protein-co
lib-C666-l	chr14	34931613	34931613	+	8.50275	NA	promoter-TSS (NM_138288)	promoter-TSS (NM_138288)	-145	NM_13828	171546	Hs.740577	NM_13828	ENSG0000	SPTSSA	C14orf147	serine paln	protein-co
lib-C666-l	chr14	59950686	59950686	+	10.38624	NA	promoter-TSS (NM_001098625)	promoter-TSS (NM_001098625)	387	NM_14458	112849	Hs.729061	NM_14458	ENSG0000	L3HYPDH	C14orf149	trans-L-3-	protein-co
lib-C666-l	chr14	59951021	59951021	+	3.84463	NA	promoter-TSS (NM_001098625)	promoter-TSS (NM_001098625)	52	NM_14458	112849	Hs.729061	NM_14458	ENSG0000	L3HYPDH	C14orf149	trans-L-3-	protein-co
lib-C666-l	chr14	64108763	64108763	+	15.54425	NA	promoter-TSS (NM_001008726)	promoter-TSS (NM_001008726)	-122	NM_00100	112840	Hs.655666	NM_08066	ENSG0000	WDR89	C14orf150	WD repeat	protein-co
lib-C666-l	chr14	99946699	99946699	+	2.78587	NA	intron (NM_199123, intron 1 of 8)	CpG	527	NM_03223	84193	Hs.510407	NM_03223	ENSG0000	SETD3	C14orf154	SET domai	protein-co
lib-C666-l	chr14	31091498	31091498	+	17.97302	NA	promoter-TSS (NM_001283031)	promoter-TSS (NM_001283031)	-27	NM_00125	23256	Hs.369168	NM_01610	ENSG0000	SCFD1	C14orf163	sec1 family	protein-co
lib-C666-l	chr14	73958229	73958229	+	17.62684	NA	exon (NM_024644, exon 2 of 2)	exon (NM_024644, exon 2 of 2)	590	NM_02464	79697	Hs.509916	NM_02464	ENSG0000	RIOX1	C14orf169	ribosomal	protein-co
lib-C666-l	chr14	103995617	103995617	+	33.43742	NA	intron (NM_152307, intron 1 of 3)	CpG	108	NM_15230	115708	Hs.525610	NM_15230	ENSG0000	TRMT61A	C14orf172	tRNA meth	protein-co
lib-C666-l	chr14	76452142	76452142	+	53.07762	NA	promoter-TSS (NM_001255995)	promoter-TSS (NM_001255995)	46	NR_04566	112752	Hs.532626	NM_05287	ENSG0000	IFT43	C14orf179	intraflagell	protein-co
lib-C666-l	chr14	50470225	50470225	+	11.66012	NA	non-coding (NR_126496, exon 1 of 5	non-coding (NR_126496, exon 1 of	3012	NR_12649	283551	Hs.292580	NM_00101	ENSG0000	LINC01588	C14orf182	long interg	ncRNA
lib-C666-l	chr14	35515334	35515334	+	66.67238	NA	promoter-TSS (NM_173607)	promoter-TSS (NM_173607)	-125	NM_00128	283635	Hs.446357	NM_17360	ENSG0000	FAM177A1	C14orf24	family with	protein-co
lib-C666-l	chr14	38065167	38065167	+	19.59432	NA	promoter-TSS (NM_004496)	promoter-TSS (NM_004496)	763	NM_00131	319089	Hs.659936	NM_00100	ENSG0000	TTC6	C14orf25|N	tetratricop	protein-co
lib-C666-l	chr14	52118791	52118791	+	6.82401	NA	promoter-TSS (NR_037676)	promoter-TSS (NR_037676)	215	NM_15233	122786	Hs.434914	NM_15233	ENSG0000	FRMD6	C14orf31|E	FERM dom	protein-co
lib-C666-l	chr14	55518729	55518729	+	134.63637	NA	intron (NM_144578, intron 1 of 3)	CpG	367	NM_14457	93487	Hs.594338	NM_14457	ENSG0000	MAPK1IP1	C14orf32|M	mitogen-a	protein-co
lib-C666-l	chr14	57810362	57810362	+	6.71839	NA	Intergenic	L1PA3|LINE|L1	-46909	NM_00101	122830	Hs.165465	NM_00101	ENSG0000	NAA30	C14orf35|N	N(alpha)-a	protein-co
lib-C666-l	chr14	77500352	77500352	+	68.63446	NA	Intergenic	Intergenic	-5310	NM_02449	64207	Hs.179260	NM_02449	ENSG0000	IRF2BPL	C14orf4|EA	interferon	protein-co
lib-C666-l	chr14	77495034	77495034	+	16.6838	NA	promoter-TSS (NM_024496)	promoter-TSS (NM_024496)	8	NM_02449	64207	Hs.179260	NM_02449	ENSG0000	IRF2BPL	C14orf4|EA	interferon	protein-co
lib-C666-l	chr14	77494579	77494579	+	3.84463	NA	5' UTR (NM_024496, exon 1 of 1)	5' UTR (NM_024496, exon 1 of 1)	463	NM_02449	64207	Hs.179260	NM_02449	ENSG0000	IRF2BPL	C14orf4|EA	interferon	protein-co
lib-C666-l	chr14	73925397	73925397	+	15.22999	NA	promoter-TSS (NM_001320114)	promoter-TSS (NM_001320114)	-111	NM_00132	8650	Hs.525443	NM_00374	ENSG0000	NUMB	C14orf41|S	NUMB, enc	protein-co
lib-C666-l	chr14	64970382	64970382	+	8.89416	NA	promoter-TSS (NM_001123329)	promoter-TSS (NM_001123329)	181	NM_00697	7597	Hs.654571	NM_00697	ENSG0000	ZBTB25	C14orf51|K	zinc finger	protein-co
lib-C666-l	chr14	65346724	65346724	+	13.20416	NA	Intergenic	CpG	-34355	NM_14516	91612	Hs.731537	NM_14516	ENSG0000	CHURC1	C14orf52|N	churchill do	protein-co
lib-C666-l	chr14	66479820	66479820	+	5.70312	NA	Intergenic	Intergenic	-473269	NR_02433	440184	Hs.412138	NM_00100	ENSG0000	LINC00238	C14orf53|N	long interg	ncRNA
lib-C666-l	chr14	78471820	78471820	+	20.87959	NA	Intergenic	Intergenic	-164896	NM_00133	9369	Hs.368307	NM_00479	ENSG0000	NRXN3	C14orf60	neurexin 3	protein-co
lib-C666-l	chr14	78494171	78494171	+	13.20416	NA	Intergenic	MIRc|SINE|MIR	-142545	NM_00133	9369	Hs.368307	NM_00479	ENSG0000	NRXN3	C14orf60	neurexin 3	protein-co
lib-C666-l	chr14	96001169	96001169	+	11.86993	NA	promoter-TSS (NR_001459)	promoter-TSS (NR_001459)	40	NR_00313	283596	Hs.448753	NR_001459		SNHG10	C14orf62|L	small nucle	ncRNA
lib-C666-l	chr14	100659359	100659359	+	6.06312	NA	Intergenic	CpG	-33347	NM_20691	123099	Hs.159643	NM_20691	ENSG0000	DEGS2	C14orf66|D	delta 4-de	protein-co
lib-C666-l	chr14	100842734	100842734	+	9.55098	NA	promoter-TSS (NM_213646)	promoter-TSS (NM_213646)	-21	NM_00116	79446	Hs.497600	NM_02451	ENSG0000	WDR25	C14orf67	WD repeat	protein-co
lib-C666-l	chr14	102197906	102197906	+	5.96422	NA	intron (NR_026774, intron 2 of 3)	intron (NR_026774, intron 2 of 3)	1132	NR_02677	145200	Hs.150840	NR_02677	ENSG0000	LINC00239	C14orf72|N	long interg	ncRNA
lib-C666-l	chr14	103575349	103575349	+	9.79436	NA	intron (NM_001077594, intron 10 of 1	intron (NM_001077594, intron 10 o	8868	NM_00107	91828	Hs.37712	NM_00107	ENSG0000	EXOC3L4	C14orf73	exocyst co	protein-co
lib-C666-l	chr14	103588739	103588739	+	25.39487	NA	intron (NR_138038, intron 1 of 1)	intron (NR_138038, intron 1 of 1)	507	NR_13803	105370683	Hs.252904	NR_138038		LINC00677	C14orf74	long interg	ncRNA
lib-C666-l	chr14	103589531	103589531	+	6.29902	NA	promoter-TSS (NR_138038)	promoter-TSS (NR_138038)	-285	NR_13803	105370683	Hs.252904	NR_138038		LINC00677	C14orf74	long interg	ncRNA
lib-C666-l	chr14	103578891	103578891	+	5.23609	NA	Intergenic	Intergenic	10355	NR_13803	105370683	Hs.252904	NR_138038		LINC00677	C14orf74	long interg	ncRNA
lib-C666-l	chr14	105442695	105442695	+	147.86101	NA	intron (NM_138420, intron 1 of 6)	intron (NM_138420, intron 1 of 6)	1999	NM_13842	113146	Hs.441783	NM_13842	ENSG0000	AHNAK2	C14orf78	AHNAK nu	protein-co
lib-C666-l	chr14	96562294	96562294	+	6.64452	NA	Intergenic	Intergenic	56633	NM_00128	56967	Hs.6434	NM_02021	ENSG0000	C14orf132	C14orf88	chromosor	protein-co
lib-C666-l	chr14	20929811	20929811	+	11.48337	NA	promoter-TSS (NM_001100814)	promoter-TSS (NM_001100814)	-40	NM_00110	90809	Hs.7001	NM_14456	ENSG0000	TMEM55B	C14orf9	transmemb	protein-co
lib-C666-l	chr14	21945338	21945338	+	40.85015	NA	promoter-TSS (NM_001163380)	promoter-TSS (NM_001163380)	3	NM_00130	9878	Hs.555910	NM_01482	ENSG0000	TOX4	C14orf92|K	TOX high r	protein-co
lib-C666-l	chr14	23426312	23426312	+	6.32351	NA	promoter-TSS (NM_017815)	promoter-TSS (NM_017815)	39	NM_01781	54930	Hs.442782	NM_01781	ENSG0000	HAUS4	C14orf94	HAUS augr	protein-co
lib-C666-l	chr14	106805119	106805119	+	11.90035	NA	Intergenic	Intergenic	60850	NR_03337	338004	Hs.569411	NR_033375		LINC00226	C14orf97|N	long interg	ncRNA
lib-C666-l	chr4	84074036	84074036	+	16.63918	NA	Intergenic	Intergenic	-38125	NM_00113	51316	Hs.546392	NM_01661	ENSG0000	PLAC8	C15|DGIC|F	placenta sp	protein-co
lib-C666-l	chr15	63340893	63340893	+	13.48428	NA	exon (NM_001301289, exon 1 of 8)	exon (NM_001301289, exon 1 of 8)	257	NM_00130	7168	Hs.133892	NM_00036	ENSG0000	TPM1	C15orf13|C	tropomyos	protein-co
lib-C666-l	chr15	63340495	63340495	+	7.60409	NA	promoter-TSS (NM_001301289)	promoter-TSS (NM_001301289)	-23	NM_00133	7168	Hs.133892	NM_00036	ENSG0000	TPM1	C15orf13|C	tropomyos	protein-co
lib-C666-l	chr15	55489018	55489018	+	13.33696	NA	exon (NM_016304, exon 1 of 6)	exon (NM_016304, exon 1 of 6)	213	NM_01630	51187	Hs.274772	NM_01630	ENSG0000	RSL24D1	C15orf15|H	ribosomal	protein-co
lib-C666-l	chr15	32162965	32162965	+	19.82221	NA	promoter-TSS (NM_130901)	promoter-TSS (NM_130901)	-90	NM_00132	161725	Hs.355236	NM_13090	ENSG0000	OTUD7A	C15orf16|C	OTU deubi	protein-co
lib-C666-l	chr15	40861515	40861515	+	82.96959	NA	promoter-TSS (NM_001286407)	promoter-TSS (NM_001286407)	23	NM_15226	27079	Hs.173311	NM_15226	ENSG0000	RPUSD2	C15orf19|C	RNA pseuc	protein-co
lib-C666-l	chr15	42841044	42841044	+	102.47761	NA	promoter-TSS (NM_001130447)	promoter-TSS (NM_001130447)	33	NM_00132	55142	Hs.14347	NM_01809	ENSG0000	HAUS2	C15orf25|C	HAUS augr	protein-co
lib-C666-l	chr15	49170207	49170207	+	7.09685	NA	promoter-TSS (NM_014335)	promoter-TSS (NM_014335)	-83	NM_01433	23741	Hs.255973	NM_01433	ENSG0000	EID1	C15orf3|CF	EP300 inte	protein-co
lib-C666-l	chr15	90118470	90118470	+	3.27468	NA	promoter-TSS (NM_152259)	promoter-TSS (NM_152259)	-348	NM_15225	90381	Hs.441708	NM_15225	ENSG0000	TICRR	C15orf42|S	TOPBP1 in	protein-co
lib-C666-l	chr15	90041642	90041642	+	10.79304	NA	Intergenic	L1M5|LINE|L1	-1798	NR_11026	51458	Hs.459284	NM_01632	ENSG0000	RHCG	C15orf6|PD	Rh family (	protein-co
lib-C666-l	chr16	577587	577587	+	43.12596	NA	promoter-TSS (NM_005632)	promoter-TSS (NM_005632)	-180	NR_02412	64493	Hs.721553	NR_024121		LINC00235	C16orf10|L	long interg	ncRNA
lib-C666-l	chr16	691836	691836	+	10.79304	NA	promoter-TSS (NM_138418)	promoter-TSS (NM_138418)	32	NM_00133	84331	Hs.706105	NM_03237	ENSG0000	MCRIP2	C16orf14|F	MAPK regu	protein-co
lib-C666-l	chr16	734446	734446	+	10.2663	NA	promoter-TSS (NM_001323918)	promoter-TSS (NM_001323918)	0	NM_00132	339123	Hs.533771	NM_00100	ENSG0000	JMJD8	C16orf20|F	jumonji do	protein-co
lib-C666-l	chr16	745726	745726	+	5.23609	NA	exon (NM_153350, exon 3 of 6)	exon (NM_153350, exon 3 of 6)	-5302	NM_03225	84219	Hs.459632	NM_03225	ENSG0000	WDR24	C16orf21|J	WD repeat	protein-co
lib-C666-l	chr16	765819	765819	+	50.68899	NA	exon (NM_024042, exon 2 of 4)	exon (NM_024042, exon 2 of 4)	646	NM_02404	79006	Hs.533772	NM_02404	ENSG0000	METRN	C16orf23|c	meteorin, g	protein-co
lib-C666-l	chr16	771250	771250	+	27.94028	NA	5' UTR (NM_001271285, exon 1 of 4)	5' UTR (NM_001271285, exon 1 of 4	108	NM_00127	65990	Hs.166244	NM_02393	ENSG0000	FAM173A	C16orf24	family with	protein-co
lib-C666-l	chr16	1728132	1728132	+	12.73831	NA	promoter-TSS (NM_144570)	promoter-TSS (NM_144570)	-146	NM_14457	90861	Hs.513261	NM_14457	ENSG0000	HN1L	C16orf34|L	hematolog	protein-co
lib-C666-l	chr16	838649	838649	+	4.46944	NA	promoter-TSS (NM_001324086)	promoter-TSS (NM_001324086)	27	NM_02209	63922	Hs.153850	NM_02209	ENSG0000	CHTF18	C16orf41|C	chromosor	protein-co
lib-C666-l	chr16	57481388	57481388	+	10.79304	NA	promoter-TSS (NM_001308358)	promoter-TSS (NM_001308358)	51	NM_02031	57017	Hs.513632	NM_02031	ENSG0000	COQ9	C16orf49|C	coenzyme	protein-co
lib-C666-l	chr16	4588773	4588773	+	36.30428	NA	promoter-TSS (NM_001199054)	promoter-TSS (NM_001199054)	43	NM_01339	29965	Hs.654653	NM_01339	ENSG0000	CDIP1	C16orf5|CD	cell death i	protein-co
lib-C666-l	chr16	29827583	29827583	+	3.64888	NA	promoter-TSS (NM_024516)	promoter-TSS (NM_024516)	55	NM_02451	79447	Hs.702841	NM_02451	ENSG0000	PAGR1	C16orf53|G	PAXIP1 ass	protein-co
lib-C666-l	chr16	58035127	58035127	+	55.62774	NA	promoter-TSS (NM_001204911)	promoter-TSS (NM_001204911)	-150	NM_00119	79650	Hs.408702	NM_02459	ENSG0000	USB1	C16orf57|H	U6 snRNA	protein-co
lib-C666-l	chr16	67143752	67143752	+	10.40942	NA	promoter-TSS (NM_001320542)	promoter-TSS (NM_001320542)	-44	NM_00132	80262	Hs.513666	NM_02518	ENSG0000	C16orf70	C16orf6|LI	chromosor	protein-co
lib-C666-l	chr16	89766746	89766746	+	57.89614	NA	intron (NM_152339, intron 2 of 2)	intron (NM_152339, intron 2 of 2)	1375	NM_15233	124044	Hs.374556	NM_15233	ENSG0000	SPATA2L	C16orf76|t	spermatog	protein-co
lib-C666-l	chr16	89768125	89768125	+	5.65398	NA	promoter-TSS (NM_152339)	promoter-TSS (NM_152339)	-4	NM_15233	124044	Hs.374556	NM_15233	ENSG0000	SPATA2L	C16orf76|t	spermatog	protein-co
lib-C666-l	chr16	58163462	58163462	+	83.41785	NA	promoter-TSS (NR_136632)	promoter-TSS (NR_136632)	-133	NR_13663	29105	Hs.532755	NM_01324	ENSG0000	CFAP20	C16orf80|E	cilia and fla	protein-co
lib-C666-l	chr16	88772864	88772864	+	336.55869	NA	promoter-TSS (NM_001171815)	promoter-TSS (NM_001171815)	-7	NM_00101	348180	Hs.592074	NM_00101	ENSG0000	CTU2	C16orf84|N	cytosolic th	protein-co
lib-C666-l	chr16	284809	284809	+	98.43693	NA	non-coding (NR_104317, exon 1 of 1	non-coding (NR_104317, exon 1 of	264	NM_03203	83986	Hs.513225	NM_03203	ENSG0000	FAM234A	C16orf9|ITI	family with	protein-co
lib-C666-l	chr17	15549010	15549010	+	44.27569	NA	intron (NM_006470, intron 4 of 8)	intron (NM_006470, intron 4 of 8)	-25992	NM_00128	374286	Hs.548021	NM_00638	ENSG0000	CDRT1	C17ORF1|C	CMT1A du	protein-co
lib-C666-l	chr17	15546319	15546319	+	11.86993	NA	intron (NM_006470, intron 4 of 8)	intron (NM_006470, intron 4 of 8)	-23301	NM_00128	374286	Hs.548021	NM_00638	ENSG0000	CDRT1	C17ORF1|C	CMT1A du	protein-co
lib-C666-l	chr17	80376521	80376521	+	14.75675	NA	promoter-TSS (NR_033265)	promoter-TSS (NR_033265)	-8	NM_02464	79701	Hs.187505	NM_02464	ENSG0000	OGFOD3	C17orf101	2-oxoglut	protein-co
lib-C666-l	chr17	2952242	2952242	+	23.087	NA	Intergenic	CpG	14659	NM_01456	8386	Hs.553508	NM_01456	ENSG0000	OR1D5	C17orf2|OI	olfactory re	protein-co
lib-C666-l	chr17	685576	685576	+	363.22293	NA	promoter-TSS (NM_016080)	promoter-TSS (NM_016080)	-5	NM_01608	51031	Hs.279061	NM_01608	ENSG0000	GLOD4	C17orf25|G	glyoxalase	protein-co
lib-C666-l	chr17	71088813	71088813	+	8.85547	NA	promoter-TSS (NM_139177)	promoter-TSS (NM_139177)	40	NM_00115	201266	Hs.221127	NM_13917	ENSG0000	SLC39A11	C17orf26|Z	solute carr	protein-co
lib-C666-l	chr17	72256445	72256445	+	30.98434	NA	3' UTR (NM_052869, exon 8 of 8)	3' UTR (NM_052869, exon 8 of 8)	11940	NM_05286	94015	Hs.27935	NM_03264	ENSG0000	TTYH2	C17orf29	tweety fam	protein-co
lib-C666-l	chr17	21117836	21117836	+	9.75396	NA	promoter-TSS (NM_003876)	promoter-TSS (NM_003876)	72	NR_02454	8834	Hs.592945	NM_00387	ENSG0000	TMEM11	C17orf35|F	transmemb	protein-co
lib-C666-l	chr17	21117713	21117713	+	5.819	NA	non-coding (NR_024547, exon 1 of 3	non-coding (NR_024547, exon 1 of	195	NR_02454	8834	Hs.592945	NM_00387	ENSG0000	TMEM11	C17orf35|F	transmemb	protein-co
lib-C666-l	chr17	37886770	37886770	+	53.07762	NA	promoter-TSS (NM_032339)	promoter-TSS (NM_032339)	46	NM_03233	84299	Hs.333526	NM_03233	ENSG0000	MIEN1	C17orf37|C	migration a	protein-co
lib-C666-l	chr17	8788476	8788476	+	11.57178	NA	intron (NM_014308, intron 14 of 18)	intron (NM_014308, intron 14 of 18	-17482	NR_11086	146850	Hs.255809	NM_00101	ENSG0000	PIK3R6	C17orf38|H	phosphoin	protein-co
lib-C666-l	chr17	17942578	17942578	+	40.09574	NA	promoter-TSS (NM_145691)	promoter-TSS (NM_145691)	-33	NM_02405	79018	Hs.187422	NM_02405	ENSG0000	GID4	C17orf39|V	GID compl	protein-co
lib-C666-l	chr17	30228734	30228734	+	7.76438	NA	promoter-TSS (NM_018428)	promoter-TSS (NM_018428)	-5	NM_01842	55813	Hs.589862	NM_01842	ENSG0000	UTP6	C17orf40|H	UTP6, sma	protein-co
lib-C666-l	chr17	29233306	29233306	+	5.10953	NA	promoter-TSS (NM_024683)	promoter-TSS (NM_024683)	-20	NM_02468	79736	Hs.317340	NM_02468	ENSG0000	TEFM	C17orf42	transcriptic	protein-co
lib-C666-l	chr17	8126408	8126408	+	5.10953	NA	non-coding (NR_026951, exon 2 of 3	non-coding (NR_026951, exon 2 of	953	NR_02695	284029	Hs.121692	NM_17362	ENSG0000	LINC00324	C17orf44|N	long interg	ncRNA
lib-C666-l	chr17	71870615	71870615	+	31.921	NA	Intergenic	Intergenic	-45939	NR_02714	283982	Hs.464079	NM_182564		LINC00469	C17orf54	long interg	ncRNA
lib-C666-l	chr17	8093324	8093324	+	2.92124	NA	exon (NM_017622, exon 1 of 1)	exon (NM_017622, exon 1 of 1)	240	NM_01762	54785	Hs.129563	NM_01762	ENSG0000	BORCS6	C17orf59|F	BLOC-1 re	protein-co
lib-C666-l	chr17	7308244	7308244	+	6.15313	NA	promoter-TSS (NM_152766)	promoter-TSS (NM_152766)	-794	NM_15276	254863	Hs.730789	NM_15276	ENSG0000	TMEM256	C17orf61	transmemb	protein-co
lib-C666-l	chr17	27139613	27139613	+	6.82479	NA	promoter-TSS (NM_001288634)	promoter-TSS (NM_001288634)	-160	NM_00128	55731	Hs.564533	NM_01818	ENSG0000	FAM222B	C17orf63	family with	protein-co
lib-C666-l	chr17	79519404	79519404	+	10.86393	NA	promoter-TSS (NR_033338)	promoter-TSS (NR_033338)	25	NR_03333	80233	Hs.313905	NM_02516	ENSG0000	FAAP100	C17orf70	Fanconi an	protein-co
lib-C666-l	chr17	7155553	7155553	+	99.93166	NA	promoter-TSS (NM_001143775)	promoter-TSS (NM_001143775)	33	NM_20341	23587	Hs.417029	NM_01536	ENSG0000	ELP5	C17orf81|D	elongator a	protein-co
lib-C666-l	chr17	36831342	36831342	+	4.73866	NA	promoter-TSS (NM_001130677)	promoter-TSS (NM_001130677)	-155	NM_00113	100170841	Hs.445574	NM_00113	ENSG0000	EPOP	C17orf96|F	elongin BC	protein-co
lib-C666-l	chr18	13218092	13218092	+	5.92363	NA	promoter-TSS (NM_181482)	promoter-TSS (NM_181482)	-637	NM_18148	753	Hs.731853	NM_00433	ENSG0000	LDLRAD4	C18orf1	low density	protein-co
lib-C666-l	chr18	29952584	29952584	+	7.42565	NA	intron (NM_022751, intron 2 of 5)	intron (NM_022751, intron 2 of 5)	97863	NM_00124	64762	Hs.444314	NM_02275	ENSG0000	GAREM1	C18orf11|F	GRB2 asso	protein-co
lib-C666-l	chr18	5237987	5237987	+	64.69308	NA	promoter-TSS (NR_026849)	promoter-TSS (NR_026849)	41	NR_02684	147525	Hs.657197	NM_152471		LINC00526	C18orf18|H	long interg	ncRNA
lib-C666-l	chr18	33077941	33077941	+	3.25502	NA	promoter-TSS (NM_001308064)	promoter-TSS (NM_001308064)	14	NM_00109	125476	Hs.464903	NM_19428	ENSG0000	INO80C	C18orf37|I	INO80 con	protein-co
lib-C666-l	chr18	65434309	65434309	+	4.39666	NA	intron (NR_033921, intron 1 of 4)	Tigger3b|DNA|TcMar-Tigger	-250342	NM_03216	92126	Hs.124673	NM_03216	ENSG0000	DSEL	C18orf4	dermatan s	protein-co
lib-C666-l	chr18	60190176	60190176	+	21.28624	NA	promoter-TSS (NM_017742)	promoter-TSS (NM_017742)	-482	NM_01774	54877	Hs.114191	NM_01774	ENSG0000	ZCCHC2	C18orf49	zinc finger	protein-co
lib-C666-l	chr18	658190	658190	+	8.01457	NA	exon (NM_001012716, exon 1 of 2)	exon (NM_001012716, exon 1 of 2)	150	NM_00101	494514	Hs.274959	NM_001012716		TYMSOS	C18orf56	TYMS oppo	protein-co
lib-C666-l	chr18	73120950	73120950	+	10.37767	NA	TTS (NM_001037331)	TTS (NM_001037331)	18708	NM_00130	284274	Hs.666744	NM_00103	ENSG0000	SMIM21	C18orf62	small integ	protein-co
lib-C666-l	chr10	102027435	102027435	+	85.88258	NA	promoter-TSS (NM_001303404)	promoter-TSS (NM_001303404)	3	NM_00130	55280	Hs.215502	NM_01829	ENSG0000	CWF19L1	C19L1|SCA	CWF19-like	protein-co
lib-C666-l	chr19	45394344	45394344	+	23.0291	NA	promoter-TSS (NM_006114)	promoter-TSS (NM_006114)	-133	NM_00611	10452	Hs.655909	NM_00611	ENSG0000	TOMM40	C19orf1|D1	translocase	protein-co
lib-C666-l	chr19	45393860	45393860	+	10.9422	NA	promoter-TSS (NM_006114)	promoter-TSS (NM_006114)	-617	NM_00611	10452	Hs.655909	NM_00611	ENSG0000	TOMM40	C19orf1|D1	translocase	protein-co
lib-C666-l	chr19	4670368	4670368	+	11.06679	NA	promoter-TSS (NM_019107)	promoter-TSS (NM_019107)	47	NM_01910	56005	Hs.465645	NM_01910	ENSG0000	MYDGF	C19orf10|E	myeloid de	protein-co
lib-C666-l	chr19	36561319	36561319	+	4.30593	NA	intron (NM_173636, intron 7 of 31)	AluSx1|SINE|Alu	15536	NM_00108	284403	Hs.116244	NM_17363	ENSG0000	WDR62	C19orf14|N	WD repeat	protein-co
lib-C666-l	chr19	18594161	18594161	+	7.49163	NA	intron (NM_006532, intron 1 of 11)	intron (NM_006532, intron 1 of 11)	38776	NM_00653	8178	Hs.515260	NM_00653	ENSG0000	ELL	C19orf17|E	elongation	protein-co
lib-C666-l	chr19	18633185	18633185	+	3.23837	NA	promoter-TSS (NM_006532)	promoter-TSS (NM_006532)	-248	NM_00653	8178	Hs.515260	NM_00653	ENSG0000	ELL	C19orf17|E	elongation	protein-co
lib-C666-l	chr19	30364509	30364509	+	11.66012	NA	Intergenic	CpG	-50042	NM_00125	8725	Hs.466391	NM_00379	ENSG0000	URI1	C19orf2|NI	URI1, prefo	protein-co
lib-C666-l	chr19	507399	507399	+	118.47128	NA	promoter-TSS (NM_033513)	promoter-TSS (NM_033513)	-98	NM_03351	91978	Hs.369613	NM_03351	ENSG0000	TPGS1	C19orf20|G	tubulin pol	protein-co
lib-C666-l	chr19	913200	913200	+	3.58839	NA	promoter-TSS (NM_138774)	promoter-TSS (NM_138774)	25	NM_13877	91300	Hs.557655	NM_13877	ENSG0000	R3HDM4	C19orf22	R3H doma	protein-co
lib-C666-l	chr19	1885545	1885545	+	40.09574	NA	promoter-TSS (NM_001130111)	promoter-TSS (NM_001130111)	-27	NM_03121	81926	Hs.465542	NM_03121	ENSG0000	ABHD17A	C19orf27|F	abhydrolas	protein-co
lib-C666-l	chr19	3557845	3557845	+	5.72511	NA	promoter-TSS (NM_001287529)	promoter-TSS (NM_001287529)	-263	NM_00128	126321	Hs.656901	NM_02173	ENSG0000	MFSD12	C19orf28|F	major facili	protein-co
lib-C666-l	chr19	3626951	3626951	+	14.37873	NA	promoter-TSS (NM_001080543)	promoter-TSS (NM_001080543)	-138	NM_00108	58509	Hs.128425	NM_02123	ENSG0000	CACTIN	C19orf29|N	cactin, splic	protein-co
lib-C666-l	chr19	14607196	14607196	+	4.96104	NA	promoter-TSS (NM_202469)	promoter-TSS (NM_202469)	-235	NM_20249	10755	Hs.655012	NM_00571	ENSG0000	GIPC1	C19orf3|GI	GIPC PDZ (	protein-co
lib-C666-l	chr19	14606986	14606986	+	2.78587	NA	promoter-TSS (NM_202469)	promoter-TSS (NM_202469)	-25	NM_20249	10755	Hs.655012	NM_00571	ENSG0000	GIPC1	C19orf3|GI	GIPC PDZ (	protein-co
lib-C666-l	chr19	1491174	1491174	+	6.49644	NA	promoter-TSS (NM_017573)	promoter-TSS (NM_017573)	170	NM_00132	92840	Hs.76277	NM_13839	ENSG0000	REEP6	C19orf32|D	receptor ac	protein-co
lib-C666-l	chr19	11485426	11485426	+	10.27265	NA	promoter-TSS (NM_175871)	promoter-TSS (NM_175871)	43	NM_17587	126074	Hs.631619	NM_17587	ENSG0000	SWSAP1	C19orf39|S	SWIM-type	protein-co
lib-C666-l	chr19	33496523	33496523	+	17.71746	NA	intron (NM_033103, intron 7 of 14)	intron (NM_033103, intron 7 of 14)	33400	NM_15226	91442	Hs.579899	NM_15226	ENSG0000	FAAP24	C19orf40	Fanconi an	protein-co
lib-C666-l	chr19	33463087	33463087	+	10.03103	NA	promoter-TSS (NM_001300978)	promoter-TSS (NM_001300978)	-36	NM_00130	91442	Hs.579899	NM_15226	ENSG0000	FAAP24	C19orf40	Fanconi an	protein-co
lib-C666-l	chr19	16770894	16770894	+	10.27265	NA	promoter-TSS (NM_001300925)	promoter-TSS (NM_001300925)	74	NM_02410	79086	Hs.356467	NM_02410	ENSG0000	SMIM7	C19orf42	small integ	protein-co
lib-C666-l	chr19	55678143	55678143	+	6.3317	NA	promoter-TSS (NM_001256716)	promoter-TSS (NM_001256716)	-53	NM_00125	352909	Hs.351582	NM_17883	ENSG0000	DNAAF3	C19orf51|C	dynein axo	protein-co
lib-C666-l	chr19	12780411	12780411	+	5.23609	NA	promoter-TSS (NM_016145)	promoter-TSS (NM_016145)	54	NM_01614	51398	Hs.108969	NM_01614	ENSG0000	WDR83OS	C19orf56|F	WD repeat	protein-co
lib-C666-l	chr19	17420862	17420862	+	9.17935	NA	intron (NM_024050, intron 1 of 4)	CpG	525	NM_02405	79016	Hs.466154	NM_02405	ENSG0000	DDA1	C19orf58|F	DET1 and I	protein-co
lib-C666-l	chr19	17420335	17420335	+	7.95564	NA	promoter-TSS (NM_024050)	promoter-TSS (NM_024050)	-2	NM_02405	79016	Hs.466154	NM_02405	ENSG0000	DDA1	C19orf58|F	DET1 and I	protein-co
lib-C666-l	chr19	44258843	44258843	+	3.59106	NA	5' UTR (NM_019108, exon 1 of 14)	5' UTR (NM_019108, exon 1 of 14)	345	NM_01910	56006	Hs.466875	NM_01910	ENSG0000	SMG9	C19orf61|F	SMG9, nor	protein-co
lib-C666-l	chr19	17377980	17377980	+	4.52748	NA	promoter-TSS (NM_014173)	promoter-TSS (NM_014173)	-205	NM_00128	29086	Hs.190722	NM_01417	ENSG0000	BABAM1	C19orf62|H	BRISC and	protein-co
lib-C666-l	chr19	50979544	50979544	+	346.46603	NA	promoter-TSS (NM_175063)	promoter-TSS (NM_175063)	-190	NM_20653	284361	Hs.448941	NM_17506	ENSG0000	EMC10	C19orf63|H	ER membra	protein-co
lib-C666-l	chr19	47617070	47617070	+	62.69942	NA	promoter-TSS (NM_015168)	promoter-TSS (NM_015168)	-61	NM_01516	23211	Hs.104661	NM_01516	ENSG0000	ZC3H4	C19orf7	zinc finger	protein-co
lib-C666-l	chr19	14063088	14063088	+	24.32656	NA	promoter-TSS (NM_138353)	promoter-TSS (NM_138353)	-198	NM_13835	90379	Hs.443636	NM_13835	ENSG0000	DCAF15	C19orf72	DDB1 and	protein-co
lib-C666-l	chr19	46145685	46145685	+	29.01385	NA	non-coding (NR_130728, exon 3 of 3	non-coding (NR_130728, exon 3 of	933	NR_13072	100287177	Hs.668611	NM_00124	ENSG0000	EML2-AS1	C19orf83	EML2 antis	ncRNA

lib-C666-I	chr19	46145297	46145297	+	5.819	NA	non-coding (NR_130728, exon 2 of 3	non-coding (NR_130728, exon 2 of	545	NR_13072	100287177	Hs.668611	NM_00124	ENSG0000	EML2-AS1	C19orf83	EML2 antis	ncRNA
lib-C666-I	chr7	7222147	7222147	+	11.13288	NA	promoter-TSS (NM_020156)	promoter-TSS (NM_020156)	-99	NM_02015	56913	Hs.239666	NM_02015	ENSG0000	C1GALT1	C1GALT|T-	core 1 synt	protein-co
lib-C666-I	chr1	36916119	36916119	+	3.98718	NA	promoter-TSS (NM_145047)	promoter-TSS (NM_145047)	-33	NM_20683	127700	Hs.202207	NM_14504	ENSG0000	OSCP1	C1orf102|N	organic so	protein-co
lib-C666-I	chr1	36916395	36916395	+	3.59106	NA	promoter-TSS (NM_145047)	promoter-TSS (NM_145047)	-309	NM_20683	127700	Hs.202207	NM_14504	ENSG0000	OSCP1	C1orf102|N	organic so	protein-co
lib-C666-I	chr1	111506731	111506731	+	17.22931	NA	promoter-TSS (NM_018372)	promoter-TSS (NM_018372)	-165	NM_00100	55791	Hs.25245	NM_01837	ENSG0000	LRIF1	C1orf103|R	ligand dep	protein-co
lib-C666-I	chr1	39456905	39456905	+	71.09715	NA	promoter-TSS (NM_024595)	promoter-TSS (NM_024595)	-11	NM_02459	79647	Hs.293563	NM_02459	ENSG0000	AKIRIN1	C1orf108|S	akirin 1	protein-co
lib-C666-I	chr1	36765834	36765834	+	21.84731	NA	intron (NM_005119, intron 9 of 11)	intron (NM_005119, intron 9 of 11)	-6160	NM_00116	79729	Hs.524496	NM_02467	ENSG0000	SH3D21	C1orf113	SH3 doma	protein-co
lib-C666-I	chr1	179335054	179335054	+	11.6145	NA	promoter-TSS (NR_073544)	promoter-TSS (NR_073544)	-53	NR_07354	126859	Hs.658505	NM_14469	ENSG0000	AXDND1	C1orf125	axonemal (	protein-co
lib-C666-I	chr1	15451017	15451017	+	49.00246	NA	intron (NR_027136, intron 1 of 4)	intron (NR_027136, intron 1 of 4)	27943	NR_02713	200197	Hs.531424	NM_18253	ENSG0000	TMEM51-A	C1orf126	TMEM51 a	ncRNA
lib-C666-I	chr1	24104832	24104832	+	18.74745	NA	promoter-TSS (NM_020362)	promoter-TSS (NM_020362)	-44	NM_02036	57095	Hs.31819	NM_02036	ENSG0000	PITHD1	C1orf128|H	PITH doma	protein-co
lib-C666-I	chr1	24889314	24889314	+	11.06679	NA	intron (NM_001010980, intron 1 of 3)	intron (NM_001010980, intron 1 of	6747	NM_00101	400746	Hs.200253	NM_00101	ENSG0000	NCMAP	C1orf130|N	non-comp	protein-co
lib-C666-I	chr1	16694067	16694067	+	8.28333	NA	intron (NR_073500, intron 1 of 1)	CpG	542	NM_00111	26099	Hs.252967	NM_00111	ENSG0000	SZRD1	C1orf144	SUZ RNA b	protein-co
lib-C666-I	chr1	16693627	16693627	+	7.49163	NA	non-coding (NR_073500, exon 1 of 2	non-coding (NR_073500, exon 1 of	102	NM_00111	26099	Hs.252967	NM_00111	ENSG0000	SZRD1	C1orf144	SUZ RNA b	protein-co
lib-C666-I	chr1	228352061	228352061	+	7.09685	NA	non-coding (NR_103539, exon 2 of 2	non-coding (NR_103539, exon 2 of	488	NR_10353	574432	Hs.568467	NR_10353	ENSG0000	IBA57-AS1	C1orf148	IBA57 antis	ncRNA
lib-C666-I	chr1	38000147	38000147	+	5.25516	NA	TTS (NM_024700)	TTS (NM_024700)	-19727	NR_07309	64769	Hs.17118	NM_02275	ENSG0000	MEAF6	C1orf149|C	MYST/Esa1	protein-co
lib-C666-I	chr1	37980318	37980318	+	2.9164	NA	non-coding (NR_073092, exon 1 of 9	non-coding (NR_073092, exon 1 of	102	NM_02275	64769	Hs.17118	NM_02275	ENSG0000	MEAF6	C1orf149|C	MYST/Esa1	protein-co
lib-C666-I	chr1	144533799	144533799	+	50.79384	NA	intron (NM_001278267, intron 52 of 1	CpG	78920	NR_00324	767846	Hs.657186	NR_00324	ENSG0000	PFN1P2	C1orf152|C	profilin 1 p	pseudo
lib-C666-I	chr1	144491837	144491837	+	26.26478	NA	intron (NM_001278267, intron 52 of 1	CpG	120882	NR_00324	767846	Hs.657186	NR_00324	ENSG0000	PFN1P2	C1orf152|C	profilin 1 p	pseudo
lib-C666-I	chr1	144520151	144520151	+	18.99577	NA	intron (NM_001278267, intron 52 of 1	CpG	92568	NR_00324	767846	Hs.657186	NR_00324	ENSG0000	PFN1P2	C1orf152|C	profilin 1 p	pseudo
lib-C666-I	chr1	173793695	173793695	+	11.13288	NA	promoter-TSS (NM_001171182)	promoter-TSS (NM_001171182)	82	NM_00112	91687	Hs.531856	NM_03331	ENSG0000	CENPL	C1orf155|C	centromere	protein-co
lib-C666-I	chr1	183441790	183441790	+	5.9194	NA	promoter-TSS (NR_040063)	promoter-TSS (NR_040063)	284	NM_00117	9887	Hs.591463	NM_01483	ENSG0000	SMG7	C1orf16|ES	SMG7, nor	protein-co
lib-C666-I	chr1	27648647	27648647	+	91.30406	NA	promoter-TSS (NR_037576)	promoter-TSS (NR_037576)	11	NR_03757	84065	Hs.469171	NM_03212	ENSG0000	TMEM222	C1orf160	transmemb	protein-co
lib-C666-I	chr1	116681773	116681773	+	4.74112	NA	Intergenic	Intergenic	27397	NM_15236	126868	Hs.376194	NM_15236	ENSG0000	MAB21L3	C1orf161	mab-21 lik	protein-co
lib-C666-I	chr1	20834624	20834624	+	64.98446	NA	promoter-TSS (NM_024544)	promoter-TSS (NM_024544)	50	NM_02454	79594	Hs.10101	NM_02454	ENSG0000	MUL1	C1orf166|G	mitochond	protein-co
lib-C666-I	chr1	183912316	183912316	+	9.02258	NA	intron (NM_001303421, intron 10 of 1	intron (NM_001303421, intron 10 o	94588	NM_01510	23127	Hs.387995	NM_01510	ENSG0000	COLGALT2	C1orf17|GL	collagen be	protein-co
lib-C666-I	chr1	27286851	27286851	+	28.64047	NA	promoter-TSS (NM_152365)	promoter-TSS (NM_152365)	50	NM_15236	126695	Hs.188881	NM_15236	ENSG0000	KDF1	C1orf172	keratinocyt	protein-co
lib-C666-I	chr1	112281617	112281617	+	5.5166	NA	promoter-TSS (NR_038952)	promoter-TSS (NR_038952)	429	NM_01909	55924	Hs.193406	NM_01909	ENSG0000	FAM212B	C1orf183	family with	protein-co
lib-C666-I	chr1	170115285	170115285	+	16.19708	NA	promoter-TSS (NM_001136107)	promoter-TSS (NM_001136107)	97	NM_00113	149281	Hs.553612	NM_00113	ENSG0000	METTL11B	C1orf184|H	methyltran	protein-co
lib-C666-I	chr1	35325451	35325451	+	6.15313	NA	promoter-TSS (NM_001164824)	promoter-TSS (NM_001164824)	-34	NM_00132	113444	Hs.27160	NM_13842	ENSG0000	SMIM12	C1orf212	small integ	protein-co
lib-C666-I	chr1	23695388	23695388	+	33.38247	NA	promoter-TSS (NR_033690)	promoter-TSS (NR_033690)	-76	NR_03369	148898	Hs.61884	NM_13847	ENSG0000	ZNF436-A	C1orf213	ZNF436 an	ncRNA
lib-C666-I	chr1	184724133	184724133	+	29.99407	NA	promoter-TSS (NM_001319960)	promoter-TSS (NM_001319960)	-92	NR_13511	80267	Hs.523811	NM_02519	ENSG0000	EDEM3	C1orf22	ER degrad	protein-co
lib-C666-I	chr1	193090909	193090909	+	8.89416	NA	promoter-TSS (NM_024529)	promoter-TSS (NM_024529)	-179	NM_02452	79577	Hs.378996	NM_02452	ENSG0000	CDC73	C1orf28|FI	cell divisio	protein-co
lib-C666-I	chr1	110778776	110778776	+	2.58854	NA	Intergenic	Intergenic	25440	NR_03643	3749	Hs.153521	NM_00497	ENSG0000	KCNC4	C1orf30|HI	potassium	protein-co
lib-C666-I	chr1	211688777	211688777	+	88.95569	NA	Intergenic	CpG	-22518	NM_18305	343035	Hs.632495	NM_18305	ENSG0000	RD3	C1orf36|LC	retinal deg	protein-co
lib-C666-I	chr1	211665098	211665098	+	5.74041	NA	intron (NM_183059, intron 1 of 2)	intron (NM_183059, intron 1 of 2)	1161	NM_00116	343035	Hs.632495	NM_18305	ENSG0000	RD3	C1orf36|LC	retinal deg	protein-co
lib-C666-I	chr1	202976452	202976452	+	161.31409	NA	promoter-TSS (NR_036557)	promoter-TSS (NR_036557)	-43	NR_13653	92703	Hs.497443	NM_13839	ENSG0000	TMEM183A	C1orf37	transmemb	protein-co
lib-C666-I	chr1	201865392	201865392	+	4.74112	NA	TTS (NM_012134)	TTS (NM_012134)	7587	NM_19814	149345	Hs.632471	NM_19814	ENSG0000	SHISA4	C1orf40|TN	shisa family	protein-co
lib-C666-I	chr1	54411090	54411090	+	11.66012	NA	promoter-TSS (NM_052940)	promoter-TSS (NM_052940)	406	NR_13363	51668	Hs.525462	NM_01612	ENSG0000	HSPB11	C1orf41|HS	heat shock	protein-co
lib-C666-I	chr1	226187076	226187076	+	72.31497	NA	promoter-TSS (NM_152608)	promoter-TSS (NM_152608)	-10	NM_15260	163859	Hs.520192	NM_15260	ENSG0000	SDE2	C1orf55|dJ	SDE2 telon	protein-co
lib-C666-I	chr1	222886023	222886023	+	9.48188	NA	promoter-TSS (NM_022831)	promoter-TSS (NM_022831)	128	NM_14469	148362	Hs.534965	NM_14469	ENSG0000	BROX	C1orf58	BRO1 dom	protein-co
lib-C666-I	chr1	25664641	25664641	+	37.12814	NA	promoter-TSS (NM_014313)	promoter-TSS (NM_014313)	104	NR_13578	57035	Hs.259412	NM_02031	ENSG0000	RSRP1	C1orf63|NF	arginine ar	protein-co
lib-C666-I	chr1	25594329	25594329	+	9.4812	NA	promoter-TSS (NR_135777)	promoter-TSS (NR_135777)	87	NR_13577	57035	Hs.259412	NM_02031	ENSG0000	RSRP1	C1orf63|NF	arginine ar	protein-co
lib-C666-I	chr1	25573759	25573759	+	6.15313	NA	intron (NR_135788, intron 1 of 3)	CpG	251	NR_13577	57035	Hs.259412	NM_02031	ENSG0000	RSRP1	C1orf63|NF	arginine ar	protein-co
lib-C666-I	chr1	156698654	156698654	+	307.94543	NA	promoter-TSS (NM_001303095)	promoter-TSS (NM_001303095)	391	NM_00114	51093	Hs.512597	NM_01599	ENSG0000	RRNAD1	C1orf66|C(	ribosomal	protein-co
lib-C666-I	chr1	228353782	228353782	+	83.58523	NA	promoter-TSS (NR_103540)	promoter-TSS (NR_103540)	273	NM_00101	200205	Hs.237017	NM_00101	ENSG0000	IBA57	C1orf69|M	IBA57 hom	protein-co
lib-C666-I	chr1	228361875	228361875	+	15.42216	NA	promoter-TSS (NM_001310327)	promoter-TSS (NM_001310327)	-376	NM_00131	200205	Hs.237017	NM_00101	ENSG0000	IBA57	C1orf69|M	IBA57 hom	protein-co
lib-C666-I	chr1	228354276	228354276	+	2.00164	NA	intron (NM_001010867, intron 1 of 2)	intron (NM_001010867, intron 1 of	767	NM_00101	200205	Hs.237017	NM_00101	ENSG0000	IBA57	C1orf69|M	IBA57 hom	protein-co
lib-C666-I	chr1	212209081	212209081	+	60.02514	NA	promoter-TSS (NM_001199809)	promoter-TSS (NM_001199809)	-79	NR_03766	25896	Hs.369285	NM_01543	ENSG0000	INTS7	C1orf73|IN	integrator	protein-co
lib-C666-I	chr1	153606479	153606479	+	18.82927	NA	promoter-TSS (NM_001206612)	promoter-TSS (NM_001206612)	21	NM_00120	26097	Hs.611057	NM_01560	ENSG0000	CHTOP	C1orf77|FL	chromatin	protein-co
lib-C666-I	chr1	54519067	54519067	+	5.96422	NA	promoter-TSS (NR_039942)	promoter-TSS (NR_039942)	44	NM_00130	9528	Hs.118947	NM_00487	ENSG0000	TMEM59	C1orf8|DCI	transmemb	protein-co
lib-C666-I	chr1	43855540	43855540	+	17.22931	NA	promoter-TSS (NM_001001653)	promoter-TSS (NM_001001653)	-16	NM_01528	23334	Hs.643560	NM_01528	ENSG0000	SZT2	C1orf84|EI	seizure thr	protein-co
lib-C666-I	chr1	2144312	2144312	+	23.20869	NA	promoter-TSS (NM_001282670)	promoter-TSS (NM_001282670)	-153	NM_00128	199990	Hs.107101	NM_18253	ENSG0000	FAAP20	C1orf86|FP	Fanconi an	protein-co
lib-C666-I	chr1	16563077	16563077	+	16.264	NA	intron (NM_030907, intron 1 of 4)	intron (NM_030907, intron 1 of 4)	582	NM_03090	79363	Hs.546430	NM_03090	ENSG0000	RSG1	C1orf89	REM2 and	protein-co
lib-C666-I	chr1	172502135	172502135	+	21.94284	NA	promoter-TSS (NM_014283)	promoter-TSS (NM_014283)	-123	NM_01428	51430	Hs.204559	NM_01428	ENSG0000	SUCO	C1orf9|CH	SUN doma	protein-co
lib-C666-I	chr1	172501670	172501670	+	3.31563	NA	promoter-TSS (NM_014283)	promoter-TSS (NM_014283)	181	NM_01622	51430	Hs.204559	NM_01428	ENSG0000	SUCO	C1orf9|CH	SUN doma	protein-co
lib-C666-I	chr1	229478162	229478162	+	7.52748	NA	exon (NM_145257, exon 2 of 4)	exon (NM_145257, exon 2 of 4)	526	NM_14525	126731	Hs.585011	NM_14525	ENSG0000	CCSAP	C1orf96|CS	centriole, c	protein-co
lib-C666-I	chr1	211556205	211556205	+	10.92275	NA	non-coding (NR_026761, exon 1 of 6	non-coding (NR_026761, exon 1 of	108	NR_02676	84791	Hs.523932	NM_032705		LINC00467	C1orf97	long interg	ncRNA
lib-C666-I	chr17	43045646	43045646	+	8.28613	NA	promoter-TSS (NM_006688)	promoter-TSS (NM_006688)	-2	NM_00668	10882	Hs.134012	NM_00668	ENSG0000	C1QL1	C1QRF|C1(	compleme	protein-co
lib-C666-I	chr2	119884425	119884425	+	6.09173	NA	Intergenic	Intergenic	32046	NM_18252	165257	Hs.433493	NM_18252	ENSG0000	C1QL2	C1QTNF10	compleme	protein-co
lib-C666-I	chr20	34359867	34359867	+	55.96882	NA	promoter-TSS (NM_016436)	promoter-TSS (NM_016436)	-56	NM_01643	51230	Hs.517044	NM_01643	ENSG0000	PHF20	C20orf104	PHD finger	protein-co
lib-C666-I	chr20	33292167	33292167	+	102.51831	NA	promoter-TSS (NM_001329430)	promoter-TSS (NM_001329430)	55	NM_02120	58476	Hs.516994	NM_02120	ENSG0000	TP53INP2	C20orf110	tumor prot	protein-co
lib-C666-I	chr20	33292420	33292420	+	66.67216	NA	promoter-TSS (NM_001329431)	promoter-TSS (NM_001329431)	-126	NM_00132	58476	Hs.516994	NM_02120	ENSG0000	TP53INP2	C20orf110	tumor prot	protein-co
lib-C666-I	chr20	33297188	33297188	+	5.23609	NA	exon (NM_001329430, exon 3 of 4)	exon (NM_001329430, exon 3 of 4)	4642	NM_00132	58476	Hs.516994	NM_02120	ENSG0000	TP53INP2	C20orf110	tumor prot	protein-co
lib-C666-I	chr20	43104502	43104502	+	63.78558	NA	promoter-TSS (NM_024331)	promoter-TSS (NM_024331)	-24	NM_02433	79183	Hs.283869	NM_02433	ENSG0000	TTPAL	C20orf121	alpha toco	protein-co
lib-C666-I	chr20	34164170	34164170	+	2.01461	NA	non-coding (NR_119376, exon 35 of	non-coding (NR_119376, exon 35 c	31314	NR_11937	80307	Hs.72222	NR_00144	ENSG0000	FER1L4	C20orf124	fer-1 like f	pseudo
lib-C666-I	chr20	30539788	30539788	+	8.40444	NA	promoter-TSS (NM_030815)	promoter-TSS (NM_030815)	95	NM_03081	81572	Hs.435755	NM_03081	ENSG0000	PDRG1	C20orf126	p53 and D	protein-co
lib-C666-I	chr20	40104989	40104989	+	16.5633	NA	intron (NM_032221, intron 16 of 36)	intron (NM_032221, intron 16 of 36	-109491	NM_05284	90187	Hs.726525	NM_05284	ENSG0000	EMILIN3	C20orf130	elastin mic	protein-co
lib-C666-I	chr20	62152023	62152023	+	7.15277	NA	promoter-TSS (NM_024299)	promoter-TSS (NM_024299)	-110	NM_02429	79144	Hs.79625	NM_02429	ENSG0000	PPDPF	C20orf149	pancreatic	protein-co
lib-C666-I	chr20	37678794	37678794	+	9.79436	NA	Intergenic	CpG	87813	NM_00119	60625	Hs.444520	NM_02193	ENSG0000	DHX35	C20orf15|D	DEAH-box	protein-co
lib-C666-I	chr20	60982550	60982550	+	80.52238	NA	promoter-TSS (NM_031215)	promoter-TSS (NM_031215)	-211	NM_03121	81928	Hs.301040	NM_03121	ENSG0000	CABLES2	C20orf150	Cdk5 and /	protein-co
lib-C666-I	chr20	5986676	5986676	+	5.66964	NA	promoter-TSS (NR_110101)	promoter-TSS (NR_110101)	-43	NR_13661	54675	Hs.743956	NM_01909	ENSG0000	CRLS1	C20orf155	cardiolipin	protein-co
lib-C666-I	chr20	4116311	4116311	+	14.34866	NA	Intergenic	Intergenic	-13115	NM_17584	54498	Hs.433337	NM_17583	ENSG0000	SMOX	C20orf16|P	spermine c	protein-co
lib-C666-I	chr20	44519958	44519958	+	4.73866	NA	promoter-TSS (NM_080749)	promoter-TSS (NM_080749)	-32	NM_00127	140825	Hs.517094	NM_08074	ENSG0000	NEURL2	C20orf163	neuralized	protein-co
lib-C666-I	chr20	44486152	44486152	+	2.62544	NA	promoter-TSS (NR_037628)	promoter-TSS (NR_037628)	-68	NR_03762	140831	Hs.292135	NM_08075	ENSG0000	ZSWIM3	C20orf164	zinc finger	protein-co
lib-C666-I	chr20	44420570	44420570	+	128.58131	NA	promoter-TSS (NM_080614)	promoter-TSS (NM_080614)	-6	NM_05295	116092	Hs.472852	NM_05295	ENSG0000	DNTTIP1	C20orf167	deoxynucle	protein-co
lib-C666-I	chr20	43988646	43988646	+	6.29226	NA	Intergenic	Intergenic	-1931	NM_00119	90196	Hs.655055	NM_03354	ENSG0000	SYS1	C20orf169	SYS1, golg	protein-co
lib-C666-I	chr20	43989958	43989958	+	3.96379	NA	promoter-TSS (NM_001197129)	promoter-TSS (NM_001197129)	-619	NM_00119	90196	Hs.655055	NM_03354	ENSG0000	SYS1	C20orf169	SYS1, golg	protein-co
lib-C666-I	chr20	35402155	35402155	+	4.6774	NA	promoter-TSS (NM_001145316)	promoter-TSS (NM_001145316)	-1	NM_02491	79980	Hs.632268	NM_02491	ENSG0000	DSN1	C20orf172	DSN1 hom	protein-co
lib-C666-I	chr20	57798021	57798021	+	14.45701	NA	intron (NM_178457, intron 3 of 4)	intron (NM_178457, intron 3 of 4)	31946	NM_17845	128611	Hs.473204	NM_17845	ENSG0000	ZNF831	C20orf174	zinc finger	protein-co
lib-C666-I	chr20	49306499	49306499	+	12.12504	NA	intron (NM_001290268, intron 1 of 2	intron (NM_001290268, intron 1 of	1568	NM_00129	140876	Hs.372578	NM_08082	ENSG0000	FAM65C	C20orf175	family with	protein-co
lib-C666-I	chr20	49255058	49255058	+	4.38153	NA	intron (NM_001290268, intron 1 of 2	intron (NM_001290268, intron 1 of	-1603	NM_08082	140876	Hs.372578	NM_08082	ENSG0000	FAM65C	C20orf175	family with	protein-co
lib-C666-I	chr20	32399008	32399008	+	215.92688	NA	promoter-TSS (NR_110623)	promoter-TSS (NR_110623)	-102	NM_17681	128866	Hs.472471	NM_17681	ENSG0000	CHMP4B	C20orf178	charged m	protein-co
lib-C666-I	chr20	400366	400366	+	73.53196	NA	exon (NM_001323958, exon 5 of 10)	exon (NM_001323958, exon 5 of 10	11672	NM_00132	10616	Hs.29546	NM_00646	ENSG0000	RBCK1	C20orf18|H	RANBP2-ty	protein-co
lib-C666-I	chr20	401604	401604	+	10.48892	NA	exon (NM_006462, exon 6 of 11)	exon (NM_006462, exon 6 of 11)	12910	NM_00132	10616	Hs.29546	NM_00646	ENSG0000	RBCK1	C20orf18|H	RANBP2-ty	protein-co
lib-C666-I	chr20	33680724	33680724	+	6.39985	NA	promoter-TSS (NM_199368)	promoter-TSS (NM_199368)	-106	NM_19936	26133	Hs.168073	NM_01563	ENSG0000	TRPC4AP	C20orf188	transient re	protein-co
lib-C666-I	chr20	3026856	3026856	+	9.20967	NA	TTS (NM_178332)	TTS (NM_178332)	181	NM_03081	64949	Hs.18946	NM_03081	ENSG0000	MRPS26	C20orf193	mitochond	protein-co
lib-C666-I	chr20	61425540	61425540	+	17.22931	NA	Intergenic	MIRc|SINE|MIR	-2217	NM_01827	55257	Hs.443260	NM_01827	ENSG0000	MRGBP	C20orf20|E	MRG doma	protein-co
lib-C666-I	chr20	61425977	61425977	+	12.97267	NA	Intergenic	Intergenic	-1780	NM_01827	55257	Hs.443260	NM_01827	ENSG0000	MRGBP	C20orf20|E	MRG doma	protein-co
lib-C666-I	chr20	61856378	61856378	+	22.23727	NA	Intergenic	Intergenic	-8840	NM_01779	54915	Hs.744852	NM_01779	ENSG0000	YTHDF1	C20orf21	YTH N6-m	protein-co
lib-C666-I	chr20	20033225	20033225	+	9.14689	NA	promoter-TSS (NM_001278628)	promoter-TSS (NM_001278628)	67	NM_01558	26074	Hs.729168	NM_01558	ENSG0000	CFAP61	C20orf26|C	cilia and fla	protein-co
lib-C666-I	chr20	3801408	3801408	+	267.44254	NA	5' UTR (NM_001204446, exon 1 of 3)	intron (NM_001204447, intron 1 of	237	NM_00120	55317	Hs.104806	NM_01834	ENSG0000	AP5S1	C20orf29	adaptor re	protein-co
lib-C666-I	chr20	5093744	5093744	+	168.30037	NA	promoter-TSS (NM_014145)	promoter-TSS (NM_014145)	-11	NM_00100	29058	Hs.472024	NM_01414	ENSG0000	TMEM230	C20orf30|H	transmemb	protein-co
lib-C666-I	chr20	5053873	5053873	+	6.32351	NA	intron (NM_001330987, intron 3 of 3)	intron (NM_001330987, intron 3 of	39860	NM_00133	29058	Hs.472024	NM_01414	ENSG0000	TMEM230	C20orf30|H	transmemb	protein-co
lib-C666-I	chr20	62886972	62886972	+	3.89034	NA	promoter-TSS (NM_018257)	promoter-TSS (NM_018257)	-76	NM_00110	55251	Hs.744845	NM_01825	ENSG0000	PCMTD2	C20orf36	protein-L-	protein-co
lib-C666-I	chr20	12990573	12990573	+	5.23609	NA	intron (NM_018327, intron 1 of 11)	intron (NM_018327, intron 1 of 11)	946	NM_01832	55304	Hs.425023	NM_01832	ENSG0000	SPTLC3	C20orf38|L	serine paln	protein-co
lib-C666-I	chr20	60697350	60697350	+	25.49921	NA	promoter-TSS (NM_144703)	promoter-TSS (NM_144703)	-167	NM_14470	149986	Hs.105379	NM_14470	ENSG0000	LSM14B	C20orf40|F	LSM family	protein-co
lib-C666-I	chr20	60696947	60696947	+	7.11942	NA	promoter-TSS (NM_144703)	promoter-TSS (NM_144703)	-570	NM_14470	149986	Hs.105379	NM_14470	ENSG0000	LSM14B	C20orf40|F	LSM family	protein-co
lib-C666-I	chr20	55043643	55043643	+	100.19778	NA	promoter-TSS (NM_001283035)	promoter-TSS (NM_001283035)	2	NM_00128	51507	Hs.517134	NM_01640	ENSG0000	RTFDC1	C20orf43|C	replication	protein-co
lib-C666-I	chr20	13975990	13975990	+	8.01457	NA	promoter-TSS (NR_073207)	promoter-TSS (NR_073207)	-11	NR_07320	80343	Hs.590879	NM_02522	ENSG0000	SEL1L2	C20orf50	SEL1L2 ER/	protein-co
lib-C666-I	chr20	821933	821933	+	10.40942	NA	promoter-TSS (NM_001289147)	promoter-TSS (NM_001289147)	15	NM_00128	83541	Hs.574822	NM_03142	ENSG0000	FAM110A	C20orf55|F	family with	protein-co
lib-C666-I	chr20	822931	822931	+	8.28333	NA	intron (NM_001289147, intron 1 of 1)	intron (NM_001289147, intron 1 of	1013	NM_00128	83541	Hs.574822	NM_03142	ENSG0000	FAM110A	C20orf55|F	family with	protein-co
lib-C666-I	chr20	30458449	30458449	+	30.07415	NA	promoter-TSS (NM_001320479)	promoter-TSS (NM_001320479)	18	NM_00132	128853	Hs.434108	NM_08061	ENSG0000	DUSP15	C20orf57|V	dual specif	protein-co
lib-C666-I	chr20	30483074	30483074	+	29.06486	NA	intron (NR_134519, intron 4 of 17)	MER65B|LTR|ERV1	-24527	NM_08061	128853	Hs.434108	NM_08061	ENSG0000	DUSP15	C20orf57|V	dual specif	protein-co
lib-C666-I	chr20	44563076	44563076	+	8.89416	NA	promoter-TSS (NM_022104)	promoter-TSS (NM_022104)	-241	NM_02210	63935	Hs.472856	NM_02210	ENSG0000	PCIF1	C20orf67|P	PDX1 C-te	protein-co
lib-C666-I	chr20	17949493	17949493	+	100.9143	NA	promoter-TSS (NM_052865)	promoter-TSS (NM_052865)	-41	NM_00131	92667	Hs.320823	NM_05286	ENSG0000	MGME1	C20orf72|D	mitochond	protein-co
lib-C666-I	chr20	18568776	18568776	+	6.04359	NA	intron (NM_080820, intron 1 of 5)	CpG	239	NM_08082	92675	Hs.659442	NM_08082	ENSG0000	DTD1	C20orf88|D	D-tyrosyl-	protein-co
lib-C666-I	chr20	42219523	42219523	+	36.56565	NA	5' UTR (NM_001323580, exon 1 of 14	5' UTR (NM_001323580, exon 1 of 1	270	NM_00132	51098	Hs.444332	NM_01600	ENSG0000	IFT52	C20orf9|C(	intraflagell	protein-co
lib-C666-I	chr20	354269	354269	+	73.89001	NA	Intergenic	MIRb|SINE|MIR	-7004	NM_02115	57761	Hs.516826	NM_02115	ENSG0000	TRIB3	C20orf97|N	tribbles ps	protein-co
lib-C666-I	chr20	328380	328380	+	10.92952	NA	promoter-TSS (NM_001323685)	promoter-TSS (NM_001323685)	-169	NM_00132	80023	Hs.416024	NM_02495	ENSG0000	NRSN2	C20orf98|c	neurensin	protein-co
lib-C666-I	chr20	278129	278129	+	4.56047	NA	promoter-TSS (NM_033089)	promoter-TSS (NM_033089)	-75	NM_03308	85364	Hs.28608	NM_03308	ENSG0000	ZCCHC3	C20orf99	zinc finger	protein-co
lib-C666-I	chr3	176915632	176915632	+	14.18215	NA	promoter-TSS (NM_024665)	promoter-TSS (NM_024665)	-518	NM_02466	79718	Hs.714201	NM_02466	ENSG0000	TBL1XR1	C21|DC42|	transducin	protein-co
lib-C666-I	chr21	30365326	30365326	+	72.37988	NA	promoter-TSS (NM_015565)	promoter-TSS (NM_015565)	-46	NM_00132	26046	Hs.288773	NM_01556	ENSG0000	LTN1	C21orf10|C	listerin E3 u	protein-co
lib-C666-I	chr21	30375055	30375055	+	8.28613	NA	Intergenic	CpG	-9775	NM_00132	26046	Hs.288773	NM_01556	ENSG0000	LTN1	C21orf10|C	listerin E3 u	protein-co
lib-C666-I	chr21	47878708	47878708	+	12.13776	NA	promoter-TSS (NM_206891)	promoter-TSS (NM_206891)	-154	NM_01515	23181	Hs.189585	NM_01515	ENSG0000	DIP2A	C21orf106	disco inter	protein-co
lib-C666-I	chr21	40685693	40685693	+	307.94543	NA	promoter-TSS (NM_018963)	promoter-TSS (NM_018963)	19	NM_01896	54014	Hs.627139	NM_01896	ENSG0000	BRWD1	C21orf107	bromodom	protein-co
lib-C666-I	chr21	30446020	30446020	+	7.21092	NA	promoter-TSS (NM_006585)	promoter-TSS (NM_006585)	98	NM_00128	10694	Hs.125113	NM_00658	ENSG0000	CCT8	C21orf112	chaperonir	protein-co
lib-C666-I	chr21	45175138	45175138	+	33.60578	NA	intron (NM_001331030, intron 8 of 9)	intron (NM_001331030, intron 8 of	13873	NM_00133	8566	Hs.284491	NM_00368	ENSG0000	PDXK	C21orf124	pyridoxal (	protein-co
lib-C666-I	chr21	45139153	45139153	+	8.40444	NA	5' UTR (NM_003681, exon 1 of 11)	5' UTR (NM_003681, exon 1 of 11)	175	NM_00368	8566	Hs.284491	NM_00368	ENSG0000	PDXK	C21orf124	pyridoxal (	protein-co
lib-C666-I	chr21	44866609	44866609	+	7.49163	NA	Intergenic	Intergenic	-3295	NR_02696	284836	Hs.146127	NM_19430	ENSG0000	LINC00319	C21orf125	long interg	ncRNA
lib-C666-I	chr21	44866244	44866244	+	3.8557	NA	Intergenic	Intergenic	-3660	NR_02696	284836	Hs.146127	NM_19430	ENSG0000	LINC00319	C21orf125	long interg	ncRNA
lib-C666-I	chr21	19191866	19191866	+	5.37858	NA	promoter-TSS (NM_001100420)	promoter-TSS (NM_001100420)	-163	NM_00110	54149	Hs.293811	NM_01744	ENSG0000	C21orf91	C21orf14|C	chromosor	protein-co
lib-C666-I	chr21	43099300	43099300	+	25.49755	NA	promoter-TSS (NR_024367)	promoter-TSS (NR_024367)	-162	NR_02436	54090	Hs.570442	NR_024367		LINC00111	C21orf21|N	long interg	ncRNA
lib-C666-I	chr21	34852393	34852393	+	38.20619	NA	promoter-TSS (NR_040016)	promoter-TSS (NR_040016)	-77	NM_00613	757	Hs.433668	NM_00613	ENSG0000	TMEM50B	C21orf4|H(	transmemb	protein-co
lib-C666-I	chr21	34851713	34851713	+	3.23837	NA	intron (NM_006134, intron 1 of 6)	CpG	603	NR_04001	757	Hs.433668	NM_00613	ENSG0000	TMEM50B	C21orf4|H(	transmemb	protein-co
lib-C666-I	chr21	34863898	34863898	+	9.04991	NA	promoter-TSS (NM_017833)	promoter-TSS (NM_017833)	-108	NM_01783	54943	Hs.458308	NM_01783	ENSG0000	DNAJC28	C21orf55|C	DnaJ heat	protein-co
lib-C666-I	chr21	47604517	47604517	+	51.32076	NA	promoter-TSS (NM_001142854)	promoter-TSS (NM_001142854)	-144	NM_03226	84221	Hs.381214	NM_03226	ENSG0000	SPATC1L	C21orf56	spermatog	protein-co
lib-C666-I	chr21	47621329	47621329	+	5.37858	NA	intron (NM_001001438, intron 17 of 2	intron (NM_001001438, intron 17 o	-16956	NM_03226	84221	Hs.381214	NM_03226	ENSG0000	SPATC1L	C21orf56	spermatog	protein-co
lib-C666-I	chr21	47706307	47706307	+	8.89416	NA	promoter-TSS (NM_003906)	promoter-TSS (NM_003906)	63	NM_00131	54059	Hs.474066	NM_05818	ENSG0000	YBEY	C21orf57	ybeY meta	protein-co
lib-C666-I	chr21	46360301	46360301	+	3.62735	NA	promoter-TSS (NR_027128)	promoter-TSS (NR_027128)	389	NM_00131	85395	Hs.410830	NM_05819	ENSG0000	FAM207A	C21orf70|F	family with	protein-co
lib-C666-I	chr21	46707834	46707834	+	10.03103	NA	promoter-TSS (NM_015227)	promoter-TSS (NM_015227)	-23	NR_00485	23275	Hs.592164	NM_01522	ENSG0000	POFUT2	C21orf80|F	protein O-	protein-co
lib-C666-I	chr21	44897992	44897992	+	10.16129	NA	non-coding (NR_026863, exon 1 of 4	non-coding (NR_026863, exon 1 of	111	NR_02686	114038	Hs.592161	NM_15375	ENSG0000	LINC00313	C21orf84|C	long interg	ncRNA
lib-C666-I	chr22	24181439	24181439	+	5.37858	NA	promoter-TSS (NM_001135751)	promoter-TSS (NM_001135751)	-240	NM_00100	91319	Hs.593679	NM_19844	ENSG0000	DERL3	C22orf14|I	derlin 3	protein-co
lib-C666-I	chr22	42342631	42342631	+	23.34628	NA	5' UTR (NM_001304370, exon 1 of 5)	5' UTR (NM_001304370, exon 1 of 5	127	NM_00130	79019	Hs.208912	NM_02405	ENSG0000	CENPM	C22orf18|C	centromere	protein-co
lib-C666-I	chr22	20008512	20008512	+	9.79436	NA	promoter-TSS (NM_001322175)	promoter-TSS (NM_001322175)	-80	NM_00132	128989	Hs.474233	NM_15290	ENSG0000	TANGO2	C22orf25|N	transport a	protein-co
lib-C666-I	chr22	20004420	20004420	+	6.39985	NA	promoter-TSS (NM_001322174)	promoter-TSS (NM_001322174)	-103	NM_00132	128989	Hs.474233	NM_15290	ENSG0000	TANGO2	C22orf25|N	transport a	protein-co
lib-C666-I	chr22	46465869	46465869	+	27.61921	NA	Intergenic	Intergenic	-15845	NM_01828	55267	Hs.567529	NM_01828	ENSG0000	PRR34	C22orf26	proline rich	protein-co
lib-C666-I	chr22	42475988	42475988	+	4.29847	NA	TTS (NM_001002034)	TTS (NM_001002034)	293	NM_03331	91689	Hs.306083	NM_03331	ENSG0000	SMDT1	C22orf32|D	single-pas	protein-co
lib-C666-I	chr22	42475634	42475634	+	3.76263	NA	promoter-TSS (NM_033318)	promoter-TSS (NM_033318)	-61	NM_03331	91689	Hs.306083	NM_03331	ENSG0000	SMDT1	C22orf32|D	single-pas	protein-co
lib-C666-I	chr22	27041365	27041365	+	4.19683	NA	Intergenic	Intergenic	-12081	NR_00349	440823	Hs.517502	NR_00349	ENSG0000	MIAT	C22orf35|G	myocardia	ncRNA
lib-C666-I	chr22	18264482	18264482	+	7.69468	NA	Intergenic	Intergenic	4426	NR_10371	200298	Hs.517397	NR_10371	ENSG0000	LINC00528	C22orf37	long interg	ncRNA
lib-C666-I	chr22	47158465	47158465	+	158.31706	NA	promoter-TSS (NM_014346)	promoter-TSS (NM_014346)	-49	NM_00128	25771	Hs.435044	NM_01434	ENSG0000	TBC1D22A	C22orf4|HS	TBC1 dom	protein-co
lib-C666-I	chr22	47170021	47170021	+	66.08392	NA	5' UTR (NM_001284305, exon 1 of 13	5' UTR (NM_001284305, exon 1 of 1	197	NM_00128	25771	Hs.435044	NM_01434	ENSG0000	TBC1D22A	C22orf4|HS	TBC1 dom	protein-co
lib-C666-I	chr22	47150187	47150187	+	18.20429	NA	Intergenic	Intergenic	-8327	NM_00128	25771	Hs.435044	NM_01434	ENSG0000	TBC1D22A	C22orf4|HS	TBC1 dom	protein-co
lib-C666-I	chr22	51001393	51001393	+	3.23837	NA	promoter-TSS (NM_001123225)	promoter-TSS (NM_001123225)	-12	NM_00112	644186	Hs.535660	NM_00112	ENSG0000	SYCE3	C22orf41|T	synaptone	protein-co
lib-C666-I	chr22	43485265	43485265	+	10.27265	NA	intron (NM_012263, intron 1 of 10)	CpG	169	NR_02777	25809	Hs.660298	NM_01226	ENSG0000	TTLL1	C22orf7|HS	tubulin tyr	protein-co
lib-C666-I	chr22	45608429	45608429	+	8.71138	NA	promoter-TSS (NM_015264)	promoter-TSS (NM_015264)	-82	NM_01526	23313	Hs.592207	NM_01526	ENSG0000	KIAA0930	C22orf9|LS	KIAA0930	protein-co
lib-C666-I	chr2	232329352	232329352	+	5.15932	NA	promoter-TSS (NM_005381)	promoter-TSS (NM_005381)	-147	NM_00538	4691	Hs.79110	NM_00538	ENSG0000	NCL	C23	nucleolin	protein-co
lib-C666-I	chr9	79073997	79073997	+	7.76438	NA	promoter-TSS (NM_001490)	promoter-TSS (NM_001490)	-71	NM_00149	2650	Hs.521568	NM_00149	ENSG0000	GCNT1	C2GNT|C2(	glucosamir	protein-co
lib-C666-I	chr5	74333177	74333177	+	16.85814	NA	Intergenic	Intergenic	-6453	NM_01659	51301	Hs.272404	NM_01659	ENSG0000	GCNT4	C2GNT3	glucosamir	protein-co
lib-C666-I	chr5	74262656	74262656	+	7.76438	NA	Intergenic	LTR36|LTR|ERV1	64068	NM_01659	51301	Hs.272404	NM_01659	ENSG0000	GCNT4	C2GNT3	glucosamir	protein-co
lib-C666-I	chr5	74262907	74262907	+	3.43712	NA	Intergenic	LTR36|LTR|ERV1	63817	NM_01659	51301	Hs.272404	NM_01659	ENSG0000	GCNT4	C2GNT3	glucosamir	protein-co
lib-C666-I	chr1	244211173	244211173	+	9.93242	NA	TTS (NR_033883)	TTS (NR_033883)	-1068	NM_00127	10472	Hs.69997	NM_00635	ENSG0000	ZBTB18	C2H2-171|	zinc finger	protein-co
lib-C666-I	chr1	244326965	244326965	+	7.09685	NA	Intergenic	Intergenic	110458	NM_00635	10472	Hs.69997	NM_00635	ENSG0000	ZBTB18	C2H2-171|	zinc finger	protein-co
lib-C666-I	chr19	58144596	58144596	+	10.37767	NA	promoter-TSS (NM_001322306)	promoter-TSS (NM_001322306)	61	NM_00132	10520	Hs.590977	NM_00638	ENSG0000	ZNF211	C2H2-25|C	zinc finger	protein-co
lib-C666-I	chr9	130548509	130548509	+	4.39666	NA	TTS (NR_039767)	TTS (NR_039767)	204	NM_00126	1025	Hs.150423	NM_00126	ENSG0000	CDK9	C-2k|CDC2	cyclin depe	protein-co
lib-C666-I	chr2	198492941	198492941	+	8.0154	NA	intron (NM_144629, intron 5 of 8)	intron (NM_144629, intron 5 of 8)	47643	NM_14462	130132	Hs.591615	NM_14462	ENSG0000	RFTN2	C2orf11|Ra	raftlin fami	protein-co
lib-C666-I	chr2	42396370	42396370	+	17.41249	NA	promoter-TSS (NM_019063)	promoter-TSS (NM_019063)	-108	NM_01906	27436	Hs.713173	NM_01906	ENSG0000	EML4	C2orf2|ELP	echinoderr	protein-co

lib-C666-l	chr2	42360312	42360312	+	6.39985	NA	Intergenic	Intergenic	-36166	NM_01906	27436	Hs.713173	NM_01906	ENSG0000	EML4	C2orf2|ELP	echinoderr	protein-co
lib-C666-l	chr2	42359735	42359735	+	6.29226	NA	Intergenic	Intergenic	-36743	NM_01906	27436	Hs.713173	NM_01906	ENSG0000	EML4	C2orf2|ELP	echinoderr	protein-co
lib-C666-l	chr2	208634493	208634493	+	6.32351	NA	promoter-TSS (NM_003468)	promoter-TSS (NM_003468)	-350	NM_00346	7855	Hs.17631	NM_00346	ENSG0000	FZD5	C2orf31|HF	frizzled cla	protein-co
lib-C666-l	chr2	32236025	32236025	+	12.12504	NA	promoter-TSS (NR_126034)	promoter-TSS (NR_126034)	96	NR_12603	51072	Hs.444969	NM_01595	ENSG0000	MEMO1	C2orf4|CGI	mediator c	protein-co
lib-C666-l	chr2	32205959	32205959	+	10.1894	NA	intron (NM_001301852, intron 1 of 7)	intron (NM_001301852, intron 1 of	29774	NM_00113	51072	Hs.444969	NM_01595	ENSG0000	MEMO1	C2orf4|CGI	mediator c	protein-co
lib-C666-l	chr2	21022873	21022873	+	116.1534	NA	promoter-TSS (NM_001282719)	promoter-TSS (NM_001282719)	17	NM_00128	60526	Hs.187823	NM_02192	ENSG0000	LDAH	C2orf43|hL	lipid drople	protein-co
lib-C666-l	chr2	8363357	8363357	+	28.88196	NA	intron (NR_034135, intron 7 of 8)	intron (NR_034135, intron 7 of 8)	105192	NR_03413	339789	Hs.565619	NM_20735	ENSG0000	LINC00299	C2orf46|N(	long interg	ncRNA
lib-C666-l	chr2	62115845	62115845	+	275.36435	NA	promoter-TSS (NM_001256721)	promoter-TSS (NM_001256721)	-13	NM_00132	150684	Hs.468702	NM_15251	ENSG0000	COMMD1	C2orf5|MU	copper me	protein-co
lib-C666-l	chr2	232379024	232379024	+	17.62684	NA	promoter-TSS (NR_024079)	promoter-TSS (NR_024079)	26	NR_02407	151477	Hs.375211	NM_17351	ENSG0000	LINC00471	C2orf52	long interg	ncRNA
lib-C666-l	chr2	37458790	37458790	+	14.81115	NA	promoter-TSS (NM_144736)	promoter-TSS (NM_144736)	16	NM_00108	55471	Hs.433466	NM_01860	ENSG0000	NDUFAF7	C2orf56|M	NADH:ubic	protein-co
lib-C666-l	chr2	232469578	232469578	+	25.61513	NA	Intergenic	Intergenic	12003	NM_15261	165100	Hs.98104	NM_15261	ENSG0000	TEX44	C2orf57	testis expre	protein-co
lib-C666-l	chr2	232478992	232478992	+	4.67061	NA	Intergenic	CpG	21417	NM_15261	165100	Hs.98104	NM_15261	ENSG0000	TEX44	C2orf57	testis expre	protein-co
lib-C666-l	chr2	38374240	38374240	+	17.78771	NA	intron (NR_027252, intron 2 of 2)	L2a|LINE|L2	15993	NR_02725	285154	Hs.353894	NM_17365	ENSG0000	CYP1B1-A	C2orf58	CYP1B1 an	ncRNA
lib-C666-l	chr2	87795862	87795862	+	7.03048	NA	intron (NR_024204, intron 3 of 3)	intron (NR_024204, intron 3 of 3)	40888	NR_02420	112597	Hs.652166	NM_05287	ENSG0000	CYTOR	C2orf59|LI	cytoskeleto	ncRNA
lib-C666-l	chr2	74405701	74405701	+	5.37858	NA	promoter-TSS (NM_001317111)	promoter-TSS (NM_001317111)	-260	NM_00131	55233	Hs.602092	NM_01822	ENSG0000	MOB1A	C2orf6|MA	MOB kinas	protein-co
lib-C666-l	chr2	74406006	74406006	+	2.05265	NA	promoter-TSS (NM_001317111)	promoter-TSS (NM_001317111)	88	NM_00131	55233	Hs.602092	NM_01822	ENSG0000	MOB1A	C2orf6|MA	MOB kinas	protein-co
lib-C666-l	chr2	73460456	73460456	+	3.98736	NA	promoter-TSS (NR_029403)	promoter-TSS (NR_029403)	-44	NM_03231	84279	Hs.61268	NM_03231	ENSG0000	PRADC1	C2orf7|PA	protease a	protein-co
lib-C666-l	chr2	113342089	113342089	+	5.5166	NA	promoter-TSS (NM_001304354)	promoter-TSS (NM_001304354)	75	NM_03230	84269	Hs.375707	NM_03230	ENSG0000	CHCHD5	C2orf9|MI(	coiled-coil	protein-co
lib-C666-l	chr9	116337119	116337119	+	22.82077	NA	intron (NM_134427, intron 1 of 4)	intron (NM_134427, intron 1 of 4)	-6038	NM_00128	5998	Hs.494875	NM_01779	ENSG0000	RGS3	C2PA|RGP	regulator c	protein-co
lib-C666-l	chr1	204463786	204463786	+	2.65152	NA	Intergenic	CpG	-4312	NM_00264	5287	Hs.497487	NM_00264	ENSG0000	PIK3C2B	C2-PI3K	phosphatic	protein-co
lib-C666-l	chr16	1359223	1359223	+	104.07054	NA	promoter-TSS (NM_003345)	promoter-TSS (NM_003345)	69	NM_19426	7329	Hs.302903	NM_00334	ENSG0000	UBE2I	C358B7.1|F	ubiquitin c	protein-co
lib-C666-l	chr21	28168544	28168544	+	11.55724	NA	Intergenic	Intergenic	49184	NM_00698	9510	Hs.643357	NM_00698	ENSG0000	ADAMTS1	C3-C5|ME	ADAM met	protein-co
lib-C666-l	chr12	7125852	7125852	+	3.98718	NA	promoter-TSS (NM_005768)	promoter-TSS (NM_005768)	-10	NM_00576	10162	Hs.655248	NM_00576	ENSG0000	LPCAT3	C3F|LPCAT	lysophospl	protein-co
lib-C666-l	chr9	134631164	134631164	+	6.15313	NA	Intergenic	Intergenic	-15833	NM_00130	2889	Hs.127897	NM_00531	ENSG0000	RAPGEF1	C3G|GRF2	Rap guanir	protein-co
lib-C666-l	chr1	202896392	202896392	+	41.67221	NA	promoter-TSS (NM_001303109)	promoter-TSS (NM_001303109)	8	NM_00130	59349	Hs.706793	NM_02163	ENSG0000	KLHL12	C3IP1|DKIF	kelch like f	protein-co
lib-C666-l	chr3	119217266	119217266	+	9.20633	NA	promoter-TSS (NM_016589)	promoter-TSS (NM_016589)	-102	NM_01658	51300	Hs.477287	NM_01658	ENSG0000	TIMMDC1	C3orf1	translocase	protein-co
lib-C666-l	chr3	10157255	10157255	+	16.23236	NA	promoter-TSS (NM_018462)	promoter-TSS (NM_018462)	-78	NM_01846	55845	Hs.649307	NM_01846	ENSG0000	BRK1	C3orf10|HS	BRICK1, SC	protein-co
lib-C666-l	chr3	190303719	190303719	+	15.42216	NA	intron (NM_001167931, intron 3 of 11	intron (NM_001167931, intron 3 of	71879	NM_00116	3556	Hs.478673	NM_00218	ENSG0000	IL1RAP	C3orf13|IL-	interleukin	protein-co
lib-C666-l	chr3	14693157	14693157	+	12.55817	NA	promoter-TSS (NM_016474)	promoter-TSS (NM_016474)	-96	NR_13552	51244	Hs.517820	NM_01647	ENSG0000	CCDC174	C3orf19|HS	coiled-coil	protein-co
lib-C666-l	chr3	194991795	194991795	+	5.04826	NA	promoter-TSS (NM_152531)	promoter-TSS (NM_152531)	100	NM_15253	152002	Hs.478741	NM_15253	ENSG0000	XXYLT1	C3orf21	xyloside xy	protein-co
lib-C666-l	chr3	44379872	44379872	+	38.6745	NA	promoter-TSS (NM_001282914)	promoter-TSS (NM_001282914)	-72	NM_00128	285343	Hs.55131	NM_17382	ENSG0000	TCAIM	C3orf23|TC	T-cell activ	protein-co
lib-C666-l	chr3	99536631	99536631	+	92.15059	NA	promoter-TSS (NM_032359)	promoter-TSS (NM_032359)	-47	NM_03235	84319	Hs.581320	NM_03235	ENSG0000	CMSS1	C3orf26	cms1 ribos	protein-co
lib-C666-l	chr3	122102989	122102989	+	8.01457	NA	promoter-TSS (NM_001017928)	promoter-TSS (NM_001017928)	-34	NM_01436	26355	Hs.584881	NM_01436	ENSG0000	FAM162A	C3orf28|E2	family with	protein-co
lib-C666-l	chr3	8723903	8723903	+	3.93123	NA	Intergenic	HERVE-int|LTR|ERV1	-30139	NM_00125	51066	Hs.736935	NM_01593	ENSG0000	SSUH2	C3orf32|SS	ssu-2 hom	protein-co
lib-C666-l	chr3	128997646	128997646	+	16.19708	NA	promoter-TSS (NM_020187)	promoter-TSS (NM_020187)	-38	NM_02018	56941	Hs.458320	NM_02018	ENSG0000	HMCES	C3orf37|D(	5-hydroxy	protein-co
lib-C666-l	chr3	98241760	98241760	+	8.71138	NA	5' UTR (NM_019895, exon 1 of 6)	5' UTR (NM_019895, exon 1 of 6)	150	NM_00104	56650	Hs.531371	NM_01989	ENSG0000	CLDND1	C3orf4|GEI	claudin do	protein-co
lib-C666-l	chr3	184053664	184053664	+	226.38863	NA	promoter-TSS (NM_001171093)	promoter-TSS (NM_001171093)	-53	NM_00117	131408	Hs.591307	NM_14463	ENSG0000	FAM131A	C3orf40|FL	family with	protein-co
lib-C666-l	chr3	139063038	139063038	+	6.15313	NA	exon (NM_020191, exon 1 of 8)	exon (NM_020191, exon 1 of 8)	177	NM_02019	56945	Hs.745001	NM_02019	ENSG0000	MRPS22	C3orf5|CO	mitochond	protein-co
lib-C666-l	chr3	191046967	191046967	+	41.5993	NA	promoter-TSS (NM_174908)	promoter-TSS (NM_174908)	93	NM_17490	152137	Hs.478682	NM_17490	ENSG0000	CCDC50	C3orf6|DFI	coiled-coil	protein-co
lib-C666-l	chr3	56717390	56717390	+	16.19847	NA	promoter-TSS (NM_001112736)	promoter-TSS (NM_001112736)	-255	NM_00111	23272	Hs.116877	NM_01522	ENSG0000	FAM208A	C3orf63|RA	family with	protein-co
lib-C666-l	chr3	185406730	185406730	+	10.67459	NA	intron (NR_138486, intron 6 of 15)	intron (NR_138486, intron 6 of 15)	-24310	NR_12632	646600	Hs.647949	NR_02731	ENSG0000	IGF2BP2-A	C3orf65	IGF2BP2 ar	ncRNA
lib-C666-l	chr3	128746045	128746045	+	8.48019	NA	intron (NM_024768, intron 2 of 7)	CpG	25573	NM_02476	79825	Hs.134807	NM_02476	ENSG0000	EFCC1	C3orf73|C(	EF-hand a	protein-co
lib-C666-l	chr3	47554986	47554986	+	5.96422	NA	intron (NM_001031703, intron 1 of 6)	CpG	213	NM_00103	54859	Hs.311100	NM_01771	ENSG0000	ELP6	C3orf75|TN	elongator a	protein-co
lib-C666-l	chr3	119187923	119187923	+	18.68431	NA	exon (NM_152305, exon 1 of 11)	exon (NM_152305, exon 1 of 11)	138	NR_02426	56983	Hs.231750	NM_15230	ENSG0000	POGLUT1	C3orf9|CLF	protein O-	protein-co
lib-C666-l	chr19	43979597	43979597	+	63.5671	NA	exon (NM_198850, exon 16 of 16)	exon (NM_198850, exon 16 of 16)	-9766	NM_01440	27076	Hs.631594	NM_01440	ENSG0000	LYPD3	C4.4A	LY6/PLAUF	protein-co
lib-C666-l	chr19	43969907	43969907	+	11.22379	NA	promoter-TSS (NM_014400)	promoter-TSS (NM_014400)	-76	NM_01440	27076	Hs.631594	NM_01440	ENSG0000	LYPD3	C4.4A	LY6/PLAUF	protein-co
lib-C666-l	chr9	123342518	123342518	+	16.0143	NA	promoter-TSS (NR_073557)	promoter-TSS (NR_073557)	-70	NM_01824	55755	Hs.269560	NM_01824	ENSG0000	CDK5RAP2	C48|Cep21	CDK5 regu	protein-co
lib-C666-l	chr4	41992520	41992520	+	105.93439	NA	promoter-TSS (NM_006345)	promoter-TSS (NM_006345)	-3	NM_00634	10463	Hs.479634	NM_00634	ENSG0000	SLC30A9	C4orf1|GA(	solute carr	protein-co
lib-C666-l	chr4	2939119	2939119	+	13.91591	NA	TTS (NM_001291978)	TTS (NM_001291978)	1846	NR_01545	317648	Hs.398178	NR_01545	ENSG0000	NOP14-AS	C4orf10|RE	NOP14 ant	ncRNA
lib-C666-l	chr4	147443563	147443563	+	14.59636	NA	promoter-TSS (NR_133924)	promoter-TSS (NR_133924)	-440	NM_03212	84068	Hs.659209	NM_03212	ENSG0000	SLC10A7	C4orf13|P7	solute carr	protein-co
lib-C666-l	chr4	113558218	113558218	+	137.0793	NA	promoter-TSS (NM_015454)	promoter-TSS (NM_015454)	-67	NM_01839	55345	Hs.380346	NM_01839	ENSG0000	ZGRF1	C4orf21	zinc finger	protein-co
lib-C666-l	chr4	8442498	8442498	+	446.25415	NA	promoter-TSS (NM_152544)	promoter-TSS (NM_152544)	-34	NM_15254	152992	Hs.566191	NM_02495	ENSG0000	TRMT44	C4orf23|M	tRNA meth	protein-co
lib-C666-l	chr4	170678904	170678904	+	10.79304	NA	intron (NM_017867, intron 1 of 7)	intron (NM_017867, intron 1 of 7)	189	NM_01786	54969	Hs.406756	NM_01786	ENSG0000	HPF1	C4orf27	histone PA	protein-co
lib-C666-l	chr4	20702037	20702037	+	72.05244	NA	promoter-TSS (NM_001317849)	promoter-TSS (NM_001317849)	1	NM_00125	133015	Hs.479298	NM_14504	ENSG0000	PACRGL	C4orf28	PARK2 cor	protein-co
lib-C666-l	chr4	17812406	17812406	+	4.30593	NA	promoter-TSS (NR_073124)	promoter-TSS (NR_073124)	-25	NM_00134	54876	Hs.614787	NM_01774	ENSG0000	DCAF16	C4orf30	DDB1 and	protein-co
lib-C666-l	chr4	39640627	39640627	+	19.82221	NA	promoter-TSS (NM_001317896)	promoter-TSS (NM_001317896)	-9	NM_17492	201895	Hs.205952	NM_17492	ENSG0000	SMIM14	C4orf34	small integ	protein-co
lib-C666-l	chr4	184644168	184644168	+	7.26874	NA	Intergenic	CpG	63748	NM_02194	60684	Hs.443240	NM_02194	ENSG0000	TRAPPC11	C4orf41|FC	trafficking	protein-co
lib-C666-l	chr4	1243218	1243218	+	6.32351	NA	promoter-TSS (NM_001328)	promoter-TSS (NM_001328)	-10	NR_03333	92070	Hs.561314	NM_052861		CTBP1-AS	C4orf42|CT	CTBP1 anti	ncRNA
lib-C666-l	chr4	26024303	26024303	+	2.59723	NA	Intergenic	Intergenic	108489	NM_00114	389203	Hs.479386	NM_00114	ENSG0000	SMIM20	C4orf52|M	small integ	protein-co
lib-C666-l	chr4	2965193	2965193	+	19.75653	NA	promoter-TSS (NM_005307)	promoter-TSS (NM_005307)	40	NM_00129	8602	Hs.627133	NM_00370	ENSG0000	NOP14	C4orf9|NO	NOP14 nu	protein-co
lib-C666-l	chr17	46048328	46048328	+	6.94566	NA	promoter-TSS (NM_001278217)	promoter-TSS (NM_001278217)	6	NM_00127	80279	Hs.20157	NM_02519	ENSG0000	CDK5RAP3	C53|HSF-2	CDK5 regu	protein-co
lib-C666-l	chr5	88578856	88578856	+	5.25516	NA	intron (NR_136219, intron 7 of 9)	intron (NR_136219, intron 7 of 9)	-378934	NM_00113	4208	Hs.649965	NM_00239	ENSG0000	MEF2C	C5DELq14.	myocyte e	protein-co
lib-C666-l	chr5	96470596	96470596	+	36.11612	NA	intron (NM_153234, intron 1 of 5)	intron (NM_153234, intron 1 of 5)	7924	NM_15323	167410	Hs.656702	NM_15323	ENSG0000	LIX1	C5orf11|Lft	limb and C	protein-co
lib-C666-l	chr5	111093351	111093351	+	10.58189	NA	promoter-TSS (NM_004772)	promoter-TSS (NM_004772)	48	NM_00114	9315	Hs.36053	NM_00477	ENSG0000	NREP	C5orf13|D4	neuronal r	protein-co
lib-C666-l	chr5	141016481	141016481	+	7.49163	NA	promoter-TSS (NM_001130029)	promoter-TSS (NM_001130029)	-36	NM_17382	285613	Hs.120094	NM_17382	ENSG0000	RELL2	C5orf16	RELT like 2	protein-co
lib-C666-l	chr5	112257315	112257315	+	14.45701	NA	intron (NM_005669, intron 1 of 4)	CpG	716	NM_00566	7905	Hs.429608	NM_00566	ENSG0000	REEP5	C5orf18|D5	receptor a	protein-co
lib-C666-l	chr5	112257967	112257967	+	3.8557	NA	promoter-TSS (NM_005669)	promoter-TSS (NM_005669)	64	NM_00566	7905	Hs.429608	NM_00566	ENSG0000	REEP5	C5orf18|D5	receptor a	protein-co
lib-C666-l	chr5	137785352	137785352	+	3.89034	NA	Intergenic	Intergenic	10662	NR_07344	51308	Hs.416090	NM_01660	ENSG0000	REEP2	C5orf19|SG	receptor a	protein-co
lib-C666-l	chr5	93447382	93447382	+	31.27173	NA	promoter-TSS (NM_001163418)	promoter-TSS (NM_001163418)	22	NM_00116	83989	Hs.600086	NM_03204	ENSG0000	FAM172A	C5orf21	family with	protein-co
lib-C666-l	chr5	111496444	111496444	+	2.59723	NA	promoter-TSS (NR_002922)	promoter-TSS (NR_002922)	221	NR_01537	114915	Hs.12082	NM_053000		EPB41L4A-	C5orf26|N(	EPB41L4A	ncRNA
lib-C666-l	chr5	43484029	43484029	+	11.66012	NA	promoter-TSS (NM_022483)	promoter-TSS (NM_022483)	-37	NM_02248	64417	Hs.732093	NM_02248	ENSG0000	TMEM267	C5orf28	transmemb	protein-co
lib-C666-l	chr5	130506580	130506580	+	11.23774	NA	promoter-TSS (NM_001293735)	promoter-TSS (NM_001293735)	-27	NR_12165	90624	Hs.115467	NM_18170	ENSG0000	LYRM7	C5orf31|M	LYR motif	protein-co
lib-C666-l	chr5	139554702	139554702	+	115.03284	NA	promoter-TSS (NM_032412)	promoter-TSS (NM_032412)	49	NM_03241	84418	Hs.529798	NM_03241	ENSG0000	CYSTM1	C5orf32|OI	cysteine ric	protein-co
lib-C666-l	chr5	36242291	36242291	+	3.8557	NA	promoter-TSS (NM_001287340)	promoter-TSS (NM_001287340)	90	NM_00128	133686	Hs.81907	NM_15301	ENSG0000	NADK2	C5orf33|DI	NAD kinas	protein-co
lib-C666-l	chr5	171184989	171184989	+	13.20416	NA	Intergenic	MLT1K|LTR|ERVL-MaLR	-27887	NM_00128	644994	Hs.591740	NM_001289970		SMIM23	C5orf50	small integ	protein-co
lib-C666-l	chr5	137673212	137673212	+	142.87477	NA	promoter-TSS (NM_016605)	promoter-TSS (NM_016605)	-12	NM_00113	51307	Hs.54056	NM_01660	ENSG0000	FAM53C	C5orf6	family with	protein-co
lib-C666-l	chr20	60718592	60718592	+	16.63918	NA	promoter-TSS (NM_001301778)	promoter-TSS (NM_001301778)	-78	NM_00279	5688	Hs.233952	NM_00279	ENSG0000	PSMA7	C6|HEL-S-	proteasom	protein-co
lib-C666-l	chr6	139350678	139350678	+	20.72609	NA	intron (NM_021243, intron 1 of 2)	CpG	859	NM_02124	58527	Hs.600861	NM_02124	ENSG0000	ABRACL	C6orf115|C	ABRA C-te	protein-co
lib-C666-l	chr6	30028967	30028967	+	52.45363	NA	promoter-TSS (NM_001278786).3	promoter-TSS (NM_001278786).3	-6	NR_02675	80862	Hs.653168	NR_02675	ENSG0000	ZNRD1ASI	C6orf12|H1	zinc ribbor	pseudo
lib-C666-l	chr6	168226592	168226592	+	2.00045	NA	non-coding (NR_027906, exon 1 of 2	non-coding (NR_027906, exon 1 of	884	NR_02790	653483	Hs.520556	NM_00104	ENSG0000	AFDN-AS1	C6orf124|H	AFDN antis	ncRNA
lib-C666-l	chr6	30594646	30594646	+	20.59244	NA	promoter-TSS (NM_001318762)	promoter-TSS (NM_001318762)	-17	NM_00103	79969	Hs.654798	NM_02490	ENSG0000	ATAT1	C6orf134|N	alpha tubu	protein-co
lib-C666-l	chr6	54563863	54563863	+	4.90117	NA	Intergenic	Intergenic	-147706	NM_00101	222584	Hs.152423	NM_00101	ENSG0000	FAM83B	C6orf143	family with	protein-co
lib-C666-l	chr6	43478130	43478130	+	35.0374	NA	promoter-TSS (NM_001012974)	promoter-TSS (NM_001012974)	-49	NM_00101	221424	Hs.445552	NM_00101	ENSG0000	LRRC73	C6orf154	leucine ricl	protein-co
lib-C666-l	chr6	72130437	72130437	+	2.45439	NA	promoter-TSS (NR_026807)	promoter-TSS (NR_026807)	11	NR_12161	79940	Hs.368337	NR_026807		LINC00472	C6orf155	long interg	ncRNA
lib-C666-l	chr6	86388670	86388670	+	2.58799	NA	promoter-TSS (NR_003038)	promoter-TSS (NR_003038)	-219	NR_00303	387066	Hs.292457	NR_00303	ENSG0000	SNHG5	C6orf160|L	small nucle	ncRNA
lib-C666-l	chr6	88146378	88146378	+	14.0317	NA	intron (NM_001031743, intron 11 of 1	intron (NM_001031743, intron 11 o	28688	NM_00103	154313	Hs.82921	NM_17882	ENSG0000	CFAP206	C6orf165|c	cilia and fla	protein-co
lib-C666-l	chr6	88442079	88442079	+	5.5166	NA	Intergenic	Intergenic	-30094	NM_01806	55122	Hs.485915	NM_01806	ENSG0000	AKIRIN2	C6orf166|F	akirin 2	protein-co
lib-C666-l	chr6	126661256	126661256	+	136.00446	NA	non-coding (NR_104462, exon 1 of 4	non-coding (NR_104462, exon 1 of	321	NM_00101	387103	Hs.486401	NM_00101	ENSG0000	CENPW	C6orf173|C	centromer	protein-co
lib-C666-l	chr6	126589452	126589452	+	10.9422	NA	Intergenic	Intergenic	-71483	NM_00128	387103	Hs.486401	NM_00101	ENSG0000	CENPW	C6orf173|C	centromer	protein-co
lib-C666-l	chr6	126574732	126574732	+	6.9373	NA	Intergenic	Tigger3b|DNA|TcMar-Tigger	-86203	NM_00128	387103	Hs.486401	NM_00101	ENSG0000	CENPW	C6orf173|C	centromer	protein-co
lib-C666-l	chr6	31125928	31125928	+	2.78587	NA	promoter-TSS (NM_001105564)	promoter-TSS (NM_001105564)	87	NM_01905	54535	Hs.485075	NM_01905	ENSG0000	CCHCR1	C6orf18|H(	coiled-coil	protein-co
lib-C666-l	chr6	109010701	109010701	+	7.20016	NA	Intergenic	Intergenic	-62156	NR_03337	387111	Hs.520310	NR_03337	ENSG0000	LINC00222	C6orf181|N	long interg	ncRNA
lib-C666-l	chr6	109103626	109103626	+	5.04179	NA	Intergenic	Intergenic	30769	NR_03337	387111	Hs.520310	NR_03337	ENSG0000	LINC00222	C6orf181|N	long interg	ncRNA
lib-C666-l	chr6	109416652	109416652	+	53.6427	NA	promoter-TSS (NM_014454)	promoter-TSS (NM_014454)	296	NM_00127	285753	Hs.632616	NM_17383	ENSG0000	CEP57L1	C6orf182|b	centrosom	protein-co
lib-C666-l	chr6	37225474	37225474	+	32.78343	NA	promoter-TSS (NR_130108)	promoter-TSS (NR_130108)	-6	NR_13010	55633	Hs.731702	NM_01777	ENSG0000	TBC1D22B	C6orf197	TBC1 dom	protein-co
lib-C666-l	chr6	116421923	116421923	+	5.66964	NA	promoter-TSS (NM_152729)	promoter-TSS (NM_152729)	-76	NM_15272	221294	Hs.520341	NM_15272	ENSG0000	NT5DC1	C6orf200|L	5'-nucleoti	protein-co
lib-C666-l	chr6	70506202	70506202	+	28.8334	NA	intron (NM_018368, intron 1 of 15)	intron (NM_018368, intron 1 of 15)	847	NM_01836	55788	Hs.271643	NM_01836	ENSG0000	LMBRD1	C6orf209|L	LMBR1 do	protein-co
lib-C666-l	chr6	123275122	123275122	+	8.89416	NA	Intergenic	L1PA14|LINE|L1	-41994	NM_00101	134829	Hs.486361	NM_00101	ENSG0000	CLVS2	C6orf212|C	clavesin 2	protein-co
lib-C666-l	chr6	135908219	135908219	+	8.51971	NA	intron (NR_026805, intron 1 of 3)	intron (NR_026805, intron 1 of 3)	89280	NR_02680	100131814	Hs.734007	NR_02680	ENSG0000	LINC00271	C6orf217|N	long interg	ncRNA
lib-C666-l	chr6	11094101	11094101	+	14.59636	NA	promoter-TSS (NM_001135575)	promoter-TSS (NM_001135575)	-165	NM_00113	221710	Hs.656600	NM_00113	ENSG0000	SMIM13	C6orf228	small integ	protein-co
lib-C666-l	chr6	31774563	31774563	+	38.10056	NA	5' UTR (NM_021177, exon 1 of 5).5	5' UTR (NM_021177, exon 1 of 5).5	198	NM_02117	57819	Hs.103106	NM_02117	ENSG0000	LSM2	C6orf28|G7	LSM2 hom	protein-co
lib-C666-l	chr6	82161640	82161640	+	7.7703	NA	Intergenic	Intergenic	300788	NM_01763	55603	Hs.10784	NM_01763	ENSG0000	FAM46A	C6orf37|XT	family with	protein-co
lib-C666-l	chr6	82463125	82463125	+	6.29226	NA	promoter-TSS (NM_017633)	promoter-TSS (NM_017633)	-697	NM_01763	55603	Hs.10784	NM_01763	ENSG0000	FAM46A	C6orf37|XT	family with	protein-co
lib-C666-l	chr6	161549846	161549846	+	20.90919	NA	Intergenic	Intergenic	33168	NR_02427	79992	Hs.664873	NM_02492	ENSG0000	AGPAT4-I1	C6orf59|N(	AGPAT4 in	ncRNA
lib-C666-l	chr6	56819984	56819984	+	12.78696	NA	promoter-TSS (NM_001144769)	promoter-TSS (NM_001144769)	211	NM_15273	221336	Hs.582993	NM_15273	ENSG0000	BEND6	C6orf65	BEN doma	protein-co
lib-C666-l	chr6	117996573	117996573	+	23.0291	NA	promoter-TSS (NM_138459)	promoter-TSS (NM_138459)	-44	NM_13845	116150	Hs.289008	NM_13845	ENSG0000	NUS1	C6orf68|CI	NUS1 deh	protein-co
lib-C666-l	chr6	36409378	36409378	+	21.15243	NA	intron (NM_152990, intron 1 of 4)	intron (NM_152990, intron 1 of 4)	-1166	NM_17356	222658	Hs.188757	NM_17356	ENSG0000	KCTD20	C6orf69|dJ	potassium	protein-co
lib-C666-l	chr6	170151719	170151719	+	112.93029	NA	promoter-TSS (NM_001278531)	promoter-TSS (NM_001278531)	1	NM_01834	55780	Hs.47546	NM_01834	ENSG0000	ERMARD	C6orf70|P\	ER membr	protein-co
lib-C666-l	chr6	150740432	150740432	+	27.54452	NA	Intergenic	Intergenic	50404	NM_00131	389434	Hs.310225	NM_20339	ENSG0000	IYD	C6orf71|DI	iodotyrosir	protein-co
lib-C666-l	chr6	150724725	150724725	+	6.32351	NA	non-coding (NR_134655, exon 6 of 6	non-coding (NR_134655, exon 6 of	34697	NM_00131	389434	Hs.310225	NM_20339	ENSG0000	IYD	C6orf71|DI	iodotyrosir	protein-co
lib-C666-l	chr6	166756084	166756084	+	3.98718	NA	promoter-TSS (NM_145169)	promoter-TSS (NM_145169)	10	NR_13011	113402	Hs.487143	NM_14516	ENSG0000	SFT2D1	C6orf83|pF	SFT2 doma	protein-co
lib-C666-l	chr6	144164519	144164519	+	28.20039	NA	5' UTR (NM_001329953, exon 1 of 11	5' UTR (NM_001329953, exon 1 of 1	109	NM_03286	84946	Hs.185675	NM_03286	ENSG0000	LTV1	C6orf93|dJ	LTV1 ribos	protein-co
lib-C666-l	chr6	149818073	149818073	+	8.96177	NA	Intergenic	L1MEd|LINE|L1	-11925	NM_20736	340152	Hs.632618	NM_20736	ENSG0000	ZC3H12D	C6orf95|M	zinc finger	protein-co
lib-C666-l	chr3	142876963	142876963	+	4.5708	NA	Intergenic	MIRb|SINE|MIR	38345	NM_00426	9435	Hs.8786	NM_00426	ENSG0000	CHST2	C6ST|GST-	carbohydra	protein-co
lib-C666-l	chr6	111804371	111804371	+	18.89222	NA	promoter-TSS (NR_034111)	promoter-TSS (NR_034111)	-304	NR_03411	643749	Hs.486228	NR_03410	ENSG0000	TRAF3IP2-	C6UAS|C6	TRAF3IP2 a	ncRNA
lib-C666-l	chr6	111804709	111804709	+	5.5166	NA	promoter-TSS (NR_034111)	promoter-TSS (NR_034111)	34	NR_03410	643749	Hs.486228	NR_03410	ENSG0000	TRAF3IP2-	C6UAS|C6	TRAF3IP2 a	ncRNA
lib-C666-l	chr3	52720024	52720024	+	10.71733	NA	promoter-TSS (NM_018313)	promoter-TSS (NM_018313)	88	NM_01436	26354	Hs.313544	NM_01436	ENSG0000	GNL3	C77032|E2	G protein r	protein-co
lib-C666-l	chr7	73173290	73173290	+	12.71281	NA	Intergenic	Intergenic	11310	NM_00130	1365	Hs.647023	NM_00130	ENSG0000	CLDN3	C7orf1|CPE	claudin 3	protein-co
lib-C666-l	chr7	73207132	73207132	+	5.74587	NA	Intergenic	MER21C|LTR|ERVL	-22532	NM_00130	1365	Hs.647023	NM_00130	ENSG0000	CLDN3	C7orf1|CPE	claudin 3	protein-co
lib-C666-l	chr7	135242495	135242495	+	21.03795	NA	promoter-TSS (NM_015135)	promoter-TSS (NM_015135)	-167	NM_01513	23165	Hs.743250	NM_01513	ENSG0000	NUP205	C7orf14|NI	nucleopori	protein-co
lib-C666-l	chr7	56174247	56174247	+	29.7147	NA	promoter-TSS (NM_001320327)	promoter-TSS (NM_001320327)	76	NM_00132	51142	Hs.389996	NM_01613	ENSG0000	CHCHD2	C7orf17|M	coiled-coil	protein-co
lib-C666-l	chr7	56173415	56173415	+	6.39985	NA	intron (NM_016139, intron 1 of 3)	AluJr|SINE|Alu	908	NM_00132	51142	Hs.389996	NM_01613	ENSG0000	CHCHD2	C7orf17|M	coiled-coil	protein-co
lib-C666-l	chr7	102066508	102066508	+	11.95031	NA	non-coding (NR_038967, exon 9 of 9	non-coding (NR_038967, exon 9 of	-7469	NM_00112	80228	Hs.363308	NM_03283	ENSG0000	ORAI2	C7orf19|CE	ORAI calci	protein-co
lib-C666-l	chr7	916245	916245	+	27.40059	NA	promoter-TSS (NM_015949)	promoter-TSS (NM_015949)	54	NM_01594	51608	Hs.107387	NM_01594	ENSG0000	GET4	C7orf20|CE	golgi to ER	protein-co
lib-C666-l	chr7	150780788	150780788	+	4.61604	NA	promoter-TSS (NM_031434)	promoter-TSS (NM_031434)	-168	NM_00113	83590	Hs.726215	NM_03143	ENSG0000	TMUB1	C7orf21|DU	transmemb	protein-co
lib-C666-l	chr7	150780562	150780562	+	3.23837	NA	promoter-TSS (NM_031434)	promoter-TSS (NM_031434)	58	NM_00113	83590	Hs.726215	NM_03143	ENSG0000	TMUB1	C7orf21|DU	transmemb	protein-co
lib-C666-l	chr7	86849074	86849074	+	52.00589	NA	5' UTR (NM_024315, exon 1 of 4)	5' UTR (NM_024315, exon 1 of 4)	240	NM_02431	79161	Hs.619593	NM_02431	ENSG0000	TMEM243	C7orf23|M	transmemb	protein-co
lib-C666-l	chr7	86849878	86849878	+	21.73991	NA	promoter-TSS (NR_138030)	promoter-TSS (NR_138030)	25	NM_00132	79161	Hs.619593	NM_02431	ENSG0000	TMEM243	C7orf23|M	transmemb	protein-co
lib-C666-l	chr7	5938273	5938273	+	3.20885	NA	promoter-TSS (NM_015622)	promoter-TSS (NM_015622)	-68	NM_01562	51622	Hs.530000	NM_01562	ENSG0000	CCZ1	C7orf28A|(	CCZ1 hom	protein-co
lib-C666-l	chr7	6970529	6970529	+	8.73012	NA	Intergenic	Intergenic	-104603	NM_19809	221960	Hs.567779	NM_19809	ENSG0000	CCZ1B	C7orf28B|H	CCZ1 hom	protein-co
lib-C666-l	chr7	156742347	156742347	+	11.867	NA	promoter-TSS (NM_138400)	promoter-TSS (NM_138400)	-70	NM_13840	64434	Hs.15825	NM_13840	ENSG0000	NOM1	C7orf3|PPF	nucleolar p	protein-co
lib-C666-l	chr7	23338942	23338942	+	36.59206	NA	promoter-TSS (NM_138446)	promoter-TSS (NM_138446)	2	NM_13844	115416	Hs.87385	NM_13844	ENSG0000	MALSU1	C7orf30|m	mitochond	protein-co
lib-C666-l	chr7	99149714	99149714	+	135.85521	NA	promoter-TSS (NM_145111)	promoter-TSS (NM_145111)	43	NM_14511	221786	Hs.596587	NM_14511	ENSG0000	FAM200A	C7orf38	family with	protein-co
lib-C666-l	chr7	156347364	156347364	+	25.49921	NA	intron (NR_103858, intron 1 of 1)	L1ME1|LINE|L1	14179	NR_02411	64433	Hs.404170	NR_02411	ENSG0000	LINC00244	C7orf4|NC	long interg	ncRNA
lib-C666-l	chr7	45026343	45026343	+	91.53722	NA	promoter-TSS (NR_003697)	promoter-TSS (NR_003697)	-84	NR_00369	285958	Hs.25892	NR_00369	ENSG0000	SNHG15	C7orf40|Lir	small nucle	ncRNA
lib-C666-l	chr7	30174331	30174331	+	200.38818	NA	promoter-TSS (NM_152793)	promoter-TSS (NM_152793)	-221	NM_15279	222166	Hs.200100	NM_15279	ENSG0000	MTURN	C7orf41|Ell	maturin, n	protein-co
lib-C666-l	chr7	66386091	66386091	+	12.71281	NA	promoter-TSS (NM_017994)	promoter-TSS (NM_017994)	-112	NM_01799	55069	Hs.488478	NM_01799	ENSG0000	TMEM248	C7orf42	transmemb	protein-co
lib-C666-l	chr7	100034208	100034208	+	27.88888	NA	promoter-TSS (NM_001346938)	promoter-TSS (NM_001346938)	-112	NM_00134	221908	Hs.413359	NM_14503	ENSG0000	PPP1R35	C7orf47	protein ph	protein-co
lib-C666-l	chr7	1608809	1608809	+	15.75122	NA	promoter-TSS (NR_021487)	promoter-TSS (NR_021487)	820	NM_03230	84262	Hs.446311	NM_03230	ENSG0000	PSMG3	C7orf48|PA	proteasom	protein-co
lib-C666-l	chr7	100080971	100080971	+	50.62957	NA	promoter-TSS (NM_173564)	promoter-TSS (NM_173564)	-579	NM_17356	222950	Hs.320086	NM_17356	ENSG0000	NYAP1	C7orf51	neuronal t	protein-co
lib-C666-l	chr7	139025352	139025352	+	33.93039	NA	promoter-TSS (NM_001244584)	promoter-TSS (NM_001244584)	156	NR_07305	154791	Hs.718441	NM_19796	ENSG0000	FMC1	C7orf55|HS	formation	protein-co
lib-C666-l	chr7	139025848	139025848	+	31.02683	NA	promoter-TSS (NM_001244584)	promoter-TSS (NM_001244584)	-30	NM_19796	154791	Hs.718441	NM_19796	ENSG0000	FMC1	C7orf55|HS	formation	protein-co
lib-C666-l	chr7	99746454	99746454	+	15.22999	NA	promoter-TSS (NM_001008395)	promoter-TSS (NM_001008395)	-68	NM_00100	389541	Hs.406520	NM_00100	ENSG0000	LAMTOR4	C7orf59	late endos	protein-co
lib-C666-l	chr7	112580313	112580313	+	8.94859	NA	promoter-TSS (NM_152556)	promoter-TSS (NM_152556)	-381	NM_15255	154743	Hs.489734	NM_15255	ENSG0000	BMT2	C7orf60	base meth	protein-co
lib-C666-l	chr7	92158039	92158039	+	27.5466	NA	promoter-TSS (NM_000466)	promoter-TSS (NM_000466)	-48	NM_03212	84060	Hs.21590	NM_03212	ENSG0000	RBM48	C7orf64|HS	RNA bindi	protein-co
lib-C666-l	chr7	128095809	128095809	+	44.9216	NA	promoter-TSS (NM_001098786)	promoter-TSS (NM_001098786)	-75	NM_01333	29923	Hs.706124	NM_01333	ENSG0000	HILPDA	C7orf68|HI	hypoxia in	protein-co
lib-C666-l	chr7	106301079	106301079	+	13.55025	NA	exon (NM_175884, exon 1 of 1)	exon (NM_175884, exon 1 of 1)	555	NM_17588	168455	Hs.29692	NM_17588	ENSG0000	CCDC71L	C7orf74	coiled-coil	protein-co
lib-C666-l	chr7	106300753	106300753	+	4.52748	NA	exon (NM_175884, exon 1 of 1)	exon (NM_175884, exon 1 of 1)	881	NM_17588	168455	Hs.29692	NM_17588	ENSG0000	CCDC71L	C7orf74	coiled-coil	protein-co
lib-C666-l	chr8	126441968	126441968	+	8.03225	NA	promoter-TSS (NM_025195)	promoter-TSS (NM_025195)	-595	NM_02519	10221	Hs.444947	NM_02519	ENSG0000	TRIB1	C8FW|GIG-	tribbles ps	protein-co
lib-C666-l	chr8	126286237	126286237	+	3.89034	NA	intron (NM_173685, intron 5 of 7)	intron (NM_173685, intron 5 of 7)	-156326	NM_02519	10221	Hs.444947	NM_02519	ENSG0000	TRIB1	C8FW|GIG-	tribbles ps	protein-co
lib-C666-l	chr8	90914071	90914071	+	34.92958	NA	promoter-TSS (NM_004337)	promoter-TSS (NM_004337)	-25	NM_00433	734	Hs.436445	NM_00433	ENSG0000	OSGIN2	C8orf1|hT4	oxidative s	protein-co
lib-C666-l	chr8	90914838	90914838	+	3.98736	NA	promoter-TSS (NM_001126111)	promoter-TSS (NM_001126111)	76	NM_00112	734	Hs.436445	NM_00433	ENSG0000	OSGIN2	C8orf1|hT4	oxidative s	protein-co
lib-C666-l	chr8	11272787	11272787	+	11.90035	NA	intron (NR_026814, intron 3 of 6)	intron (NR_026814, intron 3 of 6)	46876	NR_02681	83656	Hs.583896	NM_05401	ENSG0000	FAM167A-	C8orf12	FAM167A	ncRNA
lib-C666-l	chr8	11265471	11265471	+	2.96591	NA	intron (NR_026814, intron 3 of 6)	L1PA4|LINE|L1	39560	NR_02681	83656	Hs.583896	NM_05401	ENSG0000	FAM167A-	C8orf12	FAM167A	ncRNA
lib-C666-l	chr8	11324203	11324203	+	9.45948	NA	promoter-TSS (NM_053279)	promoter-TSS (NM_053279)	73	NM_05327	83648	Hs.124299	NM_05327	ENSG0000	FAM167A	C8orf13|D8	family with	protein-co
lib-C666-l	chr8	37594123	37594123	+	130.37727	NA	promoter-TSS (NM_001003791)	promoter-TSS (NM_001003791)	26	NM_00100	11160	Hs.705490	NM_00717	ENSG0000	ERLIN2	C8orf2|Erli	ER lipid raf	protein-co
lib-C666-l	chr8	21998873	21998873	+	11.07714	NA	intron (NM_025232, intron 1 of 7)	CpG	591	NM_00131	80346	Hs.289063	NM_02523	ENSG0000	REEP4	C8orf20|PF	receptor a	protein-co

lib-C666-I	chr8	11059121	11059121	+	46.59515	NA	promoter-TSS (NM_173683)	promoter-TSS (NM_173683)	-246	NR_13815	286046	Hs.657083	NM_17368	ENSG0000	XKR6	C8orf21|C	XK related	protein-co
lib-C666-I	chr8	135844612	135844612	+	9.10494	NA	Intergenic	CpG	-5700	NR_12602	552853	Hs.575612	NR_12602	ENSG0000	NCRNA002	C8orf24	non-prote	ncRNA
lib-C666-I	chr8	126104083	126104083	+	85.88258	NA	promoter-TSS (NM_173685)	promoter-TSS (NM_173685)	0	NM_17368	286053	Hs.388297	NM_17368	ENSG0000	NSMCE2	C8orf36|M	NSE2/MMS	protein-co
lib-C666-I	chr8	96037237	96037237	+	8.48019	NA	promoter-TSS (NM_152416)	promoter-TSS (NM_152416)	23	NM_15241	137682	Hs.729144	NM_15241	ENSG0000	NDUFAF6	C8orf38	NADH:ubic	protein-co
lib-C666-I	chr8	42396787	42396787	+	82.1828	NA	promoter-TSS (NM_001257181)	promoter-TSS (NM_001257181)	22	NM_00113	114926	Hs.655320	NM_13843	ENSG0000	SMIM19	C8orf40	small integ	protein-co
lib-C666-I	chr8	33370757	33370757	+	14.7189	NA	promoter-TSS (NM_025115)	promoter-TSS (NM_025115)	-47	NM_00133	80185	Hs.77135	NM_02511	ENSG0000	TTI2	C8orf41|M	TELO2 inte	protein-co
lib-C666-I	chr8	28558891	28558891	+	10.37767	NA	promoter-TSS (NM_001440)	promoter-TSS (NM_001440)	90	NR_12602	101929402	Hs.667366	NR_12602	ENSG0000	EXTL3-AS1	C8orf50	EXTL3 anti	ncRNA
lib-C666-I	chr8	28479846	28479846	+	8.0787	NA	Intergenic	CpG	79135	NR_12602	101929402	Hs.667366	NR_12602	ENSG0000	EXTL3-AS1	C8orf50	EXTL3 anti	ncRNA
lib-C666-I	chr8	95835625	95835625	+	3.20885	NA	non-coding (NR_073444, exon 1 of 2	non-coding (NR_073444, exon 1 of	107	NR_07344	55656	Hs.727669	NM_01786	ENSG0000	INTS8	C8orf52|IN	integrator	protein-co
lib-C666-I	chr8	117778633	117778633	+	20.19521	NA	promoter-TSS (NM_032334)	promoter-TSS (NM_032334)	-109	NM_03233	84294	Hs.86970	NM_03233	ENSG0000	UTP23	C8orf53	UTP23, sm	protein-co
lib-C666-I	chr8	143808531	143808531	+	10.47352	NA	promoter-TSS (NR_038925)	promoter-TSS (NR_038925)	-90	NM_01664	51337	Hs.368402	NM_01664	ENSG0000	THEM6	C8orf55|D	thioesteras	protein-co
lib-C666-I	chr8	29595133	29595133	+	11.06679	NA	intron (NR_026765, intron 1 of 3)	intron (NR_026765, intron 1 of 3)	10492	NR_02676	619351	Hs.566911	NR_02676	ENSG0000	LINC00589	C8orf75	long interg	ncRNA
lib-C666-I	chr8	29499579	29499579	+	5.34142	NA	Intergenic	Intergenic	106046	NR_02676	619351	Hs.566911	NR_02676	ENSG0000	LINC00589	C8orf75	long interg	ncRNA
lib-C666-I	chr8	125212451	125212451	+	2.59032	NA	intron (NR_125420, intron 2 of 2)	intron (NR_125420, intron 2 of 2)	-28688	NR_10354	157376	Hs.662360	NM_18252	ENSG0000	FER1L6-AS	C8orf78	FER1L6 ant	ncRNA
lib-C666-I	chr8	11142552	11142552	+	23.80446	NA	exon (NM_015458, exon 1 of 10)	exon (NM_015458, exon 1 of 10)	552	NM_01545	66036	Hs.591395	NM_01545	ENSG0000	MTMR9	C8orf9|LIP	myotubula	protein-co
lib-C666-I	chr8	11141903	11141903	+	5.23679	NA	promoter-TSS (NM_015458)	promoter-TSS (NM_015458)	-97	NM_01545	66036	Hs.591395	NM_01545	ENSG0000	MTMR9	C8orf9|LIP	myotubula	protein-co
lib-C666-I	chr8	11138466	11138466	+	4.96122	NA	Intergenic	MER51A|LTR|ERV1	-3534	NM_01545	66036	Hs.591395	NM_01545	ENSG0000	MTMR9	C8orf9|LIP	myotubula	protein-co
lib-C666-I	chr9	37592584	37592584	+	3.20885	NA	promoter-TSS (NM_001001790)	promoter-TSS (NM_001001790)	52	NM_00113	401505	Hs.130774	NM_00100	ENSG0000	TOMM5	C9orf105|T	translocase	protein-co
lib-C666-I	chr9	96213876	96213876	+	7.01081	NA	promoter-TSS (NM_001322224)	promoter-TSS (NM_001322224)	10	NR_13623	158293	Hs.350364	NM_19884	ENSG0000	FAM120AC	C9orf10OS	family with	protein-co
lib-C666-I	chr9	131591998	131591998	+	14.3056	NA	intron (NM_016390, intron 1 of 11)	CpG	102	NM_01639	51490	Hs.224137	NM_01639	ENSG0000	SPOUT1	C9orf114|C	SPOUT dor	protein-co
lib-C666-I	chr9	130477976	130477976	+	89.57237	NA	promoter-TSS (NM_001317037)	promoter-TSS (NM_001317037)	-21	NM_00134	138428	Hs.653265	NM_00100	ENSG0000	PTRH1	C9orf115|P	peptidyl-tF	protein-co
lib-C666-I	chr9	131038440	131038440	+	23.34628	NA	promoter-TSS (NM_004486)	promoter-TSS (NM_004486)	15	NM_00104	375757	Hs.259594	NM_00104	ENSG0000	SWI5	C9orf119|S	SWI5 hom	protein-co
lib-C666-I	chr9	131037700	131037700	+	20.84232	NA	promoter-TSS (NM_001318092)	promoter-TSS (NM_001318092)	42	NM_00131	375757	Hs.259594	NM_00104	ENSG0000	SWI5	C9orf119|S	SWI5 hom	protein-co
lib-C666-I	chr9	95432505	95432505	+	14.02822	NA	promoter-TSS (NM_022755)	promoter-TSS (NM_022755)	42	NM_02275	64768	Hs.459896	NM_02275	ENSG0000	IPPK	C9orf12|IN	inositol-pe	protein-co
lib-C666-I	chr9	127908647	127908647	+	9.79436	NA	TTS (NM_001123355)	TTS (NM_001123355)	-2809	NM_17369	286205	Hs.59504	NM_17369	ENSG0000	SCAI	C9orf126|N	suppressor	protein-co
lib-C666-I	chr9	35829279	35829279	+	21.90047	NA	promoter-TSS (NM_016446)	promoter-TSS (NM_016446)	57	NM_01644	51754	Hs.493808	NM_01644	ENSG0000	TMEM8B	C9orf127|N	transmemb	protein-co
lib-C666-I	chr9	98638250	98638250	+	4.46944	NA	promoter-TSS (NR_023390)	promoter-TSS (NR_023390)	9	NR_02338	100128782	Hs.494549	NR_02338	ENSG0000	LINC00476	C9orf130|N	long interg	ncRNA
lib-C666-I	chr9	98639072	98639072	+	2.78587	NA	promoter-TSS (NR_023390)	promoter-TSS (NR_023390)	-813	NR_02339	100128782	Hs.494549	NR_02338	ENSG0000	LINC00476	C9orf130|N	long interg	ncRNA
lib-C666-I	chr9	130730284	130730284	+	4.92623	NA	intron (NM_001035254, intron 1 of 1	FLAM_A|SINE|Alu	12528	NM_00103	399665	Hs.535972	NM_20330	ENSG0000	FAM102A	C9orf132|E	family with	protein-co
lib-C666-I	chr9	32550889	32550889	+	7.49163	NA	promoter-TSS (NR_033991)	promoter-TSS (NR_033991)	-253	NR_03399	100129250	Hs.664395	NR_03399	ENSG0000	TOPORS-A	C9orf133	TOPORS a	ncRNA
lib-C666-I	chr9	130890057	130890057	+	17.50692	NA	promoter-TSS (NR_024425)	promoter-TSS (NR_024425)	410	NM_00125	80142	Hs.495219	NM_02507	ENSG0000	PTGES2	C9orf15|G	prostaglan	protein-co
lib-C666-I	chr9	100684558	100684558	+	61.38507	NA	intron (NM_001330725, intron 1 of 5)	CpG	294	NM_00133	51531	Hs.9196	NM_01648	ENSG0000	TRMO	C9orf156|H	tRNA meth	protein-co
lib-C666-I	chr9	36136636	36136636	+	6.15313	NA	promoter-TSS (NM_001287014)	promoter-TSS (NM_001287014)	103	NR_10463	152007	Hs.493819	NM_02234	ENSG0000	GLIPR2	C9orf19|G	GLI pathog	protein-co
lib-C666-I	chr9	36149369	36149369	+	6.15313	NA	intron (NM_001287010, intron 3 of 3)	intron (NM_001287010, intron 3 of	12587	NM_00128	152007	Hs.493819	NM_02234	ENSG0000	GLIPR2	C9orf19|G	GLI pathog	protein-co
lib-C666-I	chr9	36136337	36136337	+	4.30593	NA	promoter-TSS (NR_104639)	promoter-TSS (NR_104639)	-196	NR_10464	152007	Hs.493819	NM_02234	ENSG0000	GLIPR2	C9orf19|G	GLI pathog	protein-co
lib-C666-I	chr9	95088100	95088100	+	6.50091	NA	promoter-TSS (NM_001330722)	promoter-TSS (NM_001330722)	-224	NR_04610	55035	Hs.442199	NM_01794	ENSG0000	NOL8	C9orf34|N	nucleolar p	protein-co
lib-C666-I	chr9	140513194	140513194	+	5.56385	NA	promoter-TSS (NR_122036)	promoter-TSS (NR_122036)	153	NR_12203	85026	Hs.522520	NM_03293	ENSG0000	ARRDC1-A	C9orf37	ARRDC1 a	ncRNA
lib-C666-I	chr9	77643422	77643422	+	65.54223	NA	promoter-TSS (NM_152420)	promoter-TSS (NM_152420)	-76	NM_15242	138199	Hs.567688	NM_15242	ENSG0000	CARNMT1	C9orf41|U	carnosine I	protein-co
lib-C666-I	chr9	71394903	71394903	+	125.52737	NA	promoter-TSS (NM_138333)	promoter-TSS (NM_138333)	-19	NM_13833	116224	Hs.655572	NM_13833	ENSG0000	FAM122A	C9orf42	family with	protein-co
lib-C666-I	chr9	19230872	19230872	+	12.12723	NA	intron (NM_001330640, intron 1 of 3	CpG	109	NR_07320	55667	Hs.249591	NM_01792	ENSG0000	DENND4C	C9orf55|C	DENN dor	protein-co
lib-C666-I	chr9	131534253	131534253	+	64.08802	NA	promoter-TSS (NM_006336)	promoter-TSS (NM_006336)	-40	NM_00633	10444	Hs.147950	NM_00633	ENSG0000	ZER1	C9orf60|Z	zyg-11 rel	protein-co
lib-C666-I	chr9	72092029	72092029	+	23.80446	NA	intron (NM_001163, intron 2 of 12)	L1PA2|LINE|L1	147788	NM_00481	9413	Hs.118003	NM_00481	ENSG0000	FAM189A2	C9orf61|X1	family with	protein-co
lib-C666-I	chr9	140095107	140095107	+	3.98718	NA	promoter-TSS (NM_001128228)	promoter-TSS (NM_001128228)	56	NM_00112	286262	Hs.323445	NM_17369	ENSG0000	TPRN	C9orf75|D	taperin	protein-co
lib-C666-I	chr9	115480411	115480411	+	73.41202	NA	promoter-TSS (NM_001329588)	promoter-TSS (NM_001329588)	18	NR_13805	58493	Hs.658575	NM_02121	ENSG0000	INIP	C9orf80|H	INTS3 and	protein-co
lib-C666-I	chr9	26892789	26892789	+	60.75948	NA	promoter-TSS (NM_001167575)	promoter-TSS (NM_001167575)	37	NM_00116	79886	Hs.178357	NM_02482	ENSG0000	CAAP1	C9orf82|C	caspase ac	protein-co
lib-C666-I	chr9	130352371	130352371	+	7.31982	NA	Intergenic	Intergenic	-11085	NM_00103	64855	Hs.522401	NM_02283	ENSG0000	FAM129B	C9orf88|M	family with	protein-co
lib-C666-I	chr9	135753711	135753711	+	124.80209	NA	promoter-TSS (NM_001316898)	promoter-TSS (NM_001316898)	4	NM_00131	11092	Hs.62595	NM_01895	ENSG0000	SPACA9	C9orf9|Ma	sperm acro	protein-co
lib-C666-I	chr9	117374008	117374008	+	19.88269	NA	intron (NM_153045, intron 1 of 8)	CpG	302	NM_15304	203197	Hs.522357	NM_15304	ENSG0000	TMEM268	C9orf91	transmemb	protein-co
lib-C666-I	chr9	15553249	15553249	+	5.9194	NA	5' UTR (NM_001348002, exon 1 of 27	5' UTR (NM_001348002, exon 1 of 2	244	NM_17355	203238	Hs.17267	NM_17355	ENSG0000	CCDC171	C9orf93|b	coiled-coil	protein-co
lib-C666-I	chr5	145826741	145826741	+	4.46944	NA	promoter-TSS (NM_006706)	promoter-TSS (NM_006706)	-132	NM_00670	10915	Hs.443465	NM_00670	ENSG0000	TCERG1	CA150|TAF	transcriptic	protein-co
lib-C666-I	chr16	87959125	87959125	+	19.23598	NA	intron (NM_001739, intron 2 of 6)	MER68|LTR|ERVL	10987	NM_00173	763	Hs.177446	NM_00173	ENSG0000	CA5A	CA5|CA5A	carbonic a	protein-co
lib-C666-I	chrX	15693691	15693691	+	23.4136	NA	intron (NR_026551, intron 1 of 4)	CpG	652	NR_02655	340591	Hs.732244	NR_02655	ENSG0000	CA5BP1	CA5BL|CA5	carbonic a	pseudo
lib-C666-I	chrX	15693468	15693468	+	18.20429	NA	intron (NR_026551, intron 1 of 4)	CpG	429	NR_02655	340591	Hs.732244	NR_02655	ENSG0000	CA5BP1	CA5BL|CA5	carbonic a	pseudo
lib-C666-I	chr17	37793538	37793538	+	8.0154	NA	TTS (NM_032192)	TTS (NM_032192)	205	NM_00680	10948	Hs.728838	NM_00680	ENSG0000	STARD3	CAB1|MLN	StAR relate	protein-co
lib-C666-I	chr12	49208680	49208680	+	4.83287	NA	promoter-TSS (NM_001206916)	promoter-TSS (NM_001206916)	465	NM_00120	784	Hs.250712	NM_00072	ENSG0000	CACNB3	CAB3|CAC	calcium vo	protein-co
lib-C666-I	chr12	49208776	49208776	+	2.66659	NA	promoter-TSS (NM_001206916)	promoter-TSS (NM_001206916)	561	NM_00120	784	Hs.250712	NM_00072	ENSG0000	CACNB3	CAB3|CAC	calcium vo	protein-co
lib-C666-I	chr1	1167464	1167464	+	5.3823	NA	promoter-TSS (NM_016176)	promoter-TSS (NM_016176)	-17	NM_01617	51150	Hs.42806	NM_01617	ENSG0000	SDF4	Cab45|SDF	stromal cel	protein-co
lib-C666-I	chr18	21718961	21718961	+	10.79304	NA	promoter-TSS (NM_012189)	promoter-TSS (NM_012189)	41	NM_13864	26256	Hs.511983	NM_01218	ENSG0000	CABYR	CABYRa|C	calcium bir	protein-co
lib-C666-I	chr5	80724856	80724856	+	9.94079	NA	intron (NM_012446, intron 15 of 16)	intron (NM_012446, intron 15 of 16	-34868	NM_13076	134526	Hs.591756	NM_13076	ENSG0000	ACOT12	CACH-1|C	acyl-CoA t	protein-co
lib-C666-I	chr12	2244798	2244798	+	5.04826	NA	intron (NM_001129830, intron 3 of 4	L1PA2|LINE|L1	82347	NM_00116	775	Hs.118262	NM_00071	ENSG0000	CACNA1C	CACH2|CA	calcium vo	protein-co
lib-C666-I	chr3	53578716	53578716	+	2.62544	NA	intron (NM_001128840, intron 3 of 4	intron (NM_001128840, intron 3 of	49640	NM_00072	776	Hs.476358	NM_00072	ENSG0000	CACNA1D	CACH3|CA	calcium vo	protein-co
lib-C666-I	chr7	82073538	82073538	+	8.89416	NA	promoter-TSS (NM_001302890)	promoter-TSS (NM_001302890)	-416	NM_00130	781	Hs.282151	NM_00072	ENSG0000	CACNA2D	CACNA2|C	calcium vo	protein-co
lib-C666-I	chr10	18695286	18695286	+	4.90117	NA	intron (NM_001330060, intron 2 of 1	MER1A|DNA|hAT-Charlie	5773	NM_20157	783	Hs.59093	NM_00072	ENSG0000	CACNB2	CACNLB2|	calcium vo	protein-co
lib-C666-I	chr20	60012179	60012179	+	5.80117	NA	intron (NM_001794, intron 2 of 15)	intron (NM_001794, intron 2 of 15)	-62298	NM_00125	1002	Hs.473231	NM_00179	ENSG0000	CDH4	CAD4|R-C	cadherin 4	protein-co
lib-C666-I	chr20	60017456	60017456	+	3.20885	NA	intron (NM_001794, intron 2 of 15)	intron (NM_001794, intron 2 of 15)	-57021	NM_00125	1002	Hs.473231	NM_00179	ENSG0000	CDH4	CAD4|R-C	cadherin 4	protein-co
lib-C666-I	chr19	15306583	15306583	+	4.73866	NA	intron (NM_000435, intron 2 of 32)	CpG	5209	NM_00043	4854	Hs.8546	NM_00043	ENSG0000	NOTCH3	CADASIL|C	notch 3	protein-co
lib-C666-I	chr3	20081575	20081575	+	10.4657	NA	promoter-TSS (NM_003884)	promoter-TSS (NM_003884)	51	NM_00388	8850	Hs.533055	NM_00388	ENSG0000	KAT2B	CAF|P/CAF	lysine acet	protein-co
lib-C666-I	chr8	17103997	17103997	+	55.6757	NA	promoter-TSS (NM_001322089)	promoter-TSS (NM_001322089)	-30	NM_00132	29883	Hs.587465	NM_01335	ENSG0000	CNOT7	CAF-1|CAF	CCR4-NOT	protein-co
lib-C666-I	chr19	4402337	4402337	+	22.14682	NA	promoter-TSS (NM_005483)	promoter-TSS (NM_005483)	-323	NM_00548	10036	Hs.79018	NM_00548	ENSG0000	CHAF1A	CAF-1|CAF	chromatin	protein-co
lib-C666-I	chr21	37757692	37757692	+	9.7234	NA	promoter-TSS (NM_005441)	promoter-TSS (NM_005441)	3	NM_00544	8208	Hs.75238	NM_00544	ENSG0000	CHAF1B	CAF-1|CAF	chromatin	protein-co
lib-C666-I	chr6	42896734	42896734	+	17.90427	NA	promoter-TSS (NM_001318856)	promoter-TSS (NM_001318856)	-134	NR_13488	10695	Hs.414099	NM_00658	ENSG0000	CNPY3	CAG4A|ER	canopy FG	protein-co
lib-C666-I	chr12	57824857	57824857	+	106.05028	NA	promoter-TSS (NM_014925)	promoter-TSS (NM_014925)	-69	NM_00133	22864	Hs.443673	NM_01492	ENSG0000	R3HDM2	CAG6|PR0	R3H doma	protein-co
lib-C666-I	chr12	6798782	6798782	+	44.27569	NA	promoter-TSS (NM_133476)	promoter-TSS (NM_133476)	-44	NM_00113	171017	Hs.103315	NM_13347	ENSG0000	ZNF384	CAGH1|CA	zinc finger	protein-co
lib-C666-I	chr16	24740849	24740849	+	14.70559	NA	promoter-TSS (NM_014494)	promoter-TSS (NM_014494)	-200	NM_00133	27327	Hs.655057	NM_01449	ENSG0000	TNRC6A	CAGH26|G	trinucleotic	protein-co
lib-C666-I	chr12	132434509	132434509	+	8.28216	NA	promoter-TSS (NM_015409)	promoter-TSS (NM_015409)	44	NM_01540	57634	Hs.723478	NM_01540	ENSG0000	EP400	CAGH32|P	E1A bindin	protein-co
lib-C666-I	chr7	113725928	113725928	+	10.16129	NA	promoter-TSS (NR_033766)	promoter-TSS (NR_033766)	-437	NR_03376	93986	Hs.282787	NM_01449	ENSG0000	FOXP2	CAGH44|S	forkhead b	protein-co
lib-C666-I	chr3	40498768	40498768	+	10.03103	NA	promoter-TSS (NM_003973)	promoter-TSS (NM_003973)	-15	NM_00397	9045	Hs.446522	NM_00397	ENSG0000	RPL14	CAG-ISL-7	ribosomal	protein-co
lib-C666-I	chr7	5492807	5492807	+	5.92363	NA	Intergenic	Intergenic	-29630	NM_00108	84629	Hs.520638	NM_00101	ENSG0000	TNRC18	CAGL79|T	trinucleotic	protein-co
lib-C666-I	chr1	1840337	1840337	+	23.11805	NA	Intergenic	CpG	-5929	NM_00133	163688	Hs.85902	NM_13870	ENSG0000	CALML6	CAGLP	calmodulin	protein-co
lib-C666-I	chr7	5821259	5821259	+	3.31563	NA	5' UTR (NM_207116, exon 1 of 17)	5' UTR (NM_207116, exon 1 of 17)	102	NM_20711	54476	Hs.487458	NM_01901	ENSG0000	RNF216	CAHH|TRIA	ring finger	protein-co
lib-C666-I	chr5	112849389	112849389	+	43.12596	NA	promoter-TSS (NM_001345975)	promoter-TSS (NM_001345975)	30	NM_00134	64848	Hs.231942	NM_02282	ENSG0000	YTHDC2	CAHL	YTH doma	protein-co
lib-C666-I	chr1	29063296	29063296	+	6.71839	NA	promoter-TSS (NM_001172828)	promoter-TSS (NM_001172828)	-156	NM_00117	51441	Hs.532286	NM_01625	ENSG0000	YTHDF2	CAHL|HGR	YTH N6-m	protein-co
lib-C666-I	chr3	20227808	20227808	+	25.39084	NA	promoter-TSS (NM_001012410)	promoter-TSS (NM_001012410)	-83	NM_00119	151648	Hs.105153	NM_13848	ENSG0000	SGO1	CAID|NY-E	shugoshin	protein-co
lib-C666-I	chr9	134000954	134000954	+	318.67514	NA	promoter-TSS (NM_001318324)	promoter-TSS (NM_001318324)	6	NM_00508	8021	Hs.654530	NM_00508	ENSG0000	NUP214	CAIN|CAN	nucleopori	protein-co
lib-C666-I	chr9	134103711	134103711	+	6.71839	NA	exon (NM_001318324, exon 33 of 36)	exon (NM_001318324, exon 33 of 3	38209	NM_00131	8021	Hs.654530	NM_00508	ENSG0000	NUP214	CAIN|CAN	nucleopori	protein-co
lib-C666-I	chr22	24407640	24407640	+	12.55817	NA	promoter-TSS (NM_001201429)	promoter-TSS (NM_001201429)	-125	NM_01229	23523	Hs.517478	NM_01229	ENSG0000	CABIN1	CAIN|KB-3	calcineurin	protein-co
lib-C666-I	chr22	38576330	38576330	+	10.16134	NA	intron (NM_001199562, intron 1 of 1	intron (NM_001199562, intron 1 of	1431	NM_00100	8398	Hs.170479	NM_00356	ENSG0000	PLA2G6	Cal-PLA2|	phospholip	protein-co
lib-C666-I	chr5	86708758	86708758	+	98.87846	NA	promoter-TSS (NM_001239)	promoter-TSS (NM_001239)	-37	NM_00119	902	Hs.292524	NM_00123	ENSG0000	CCNH	CAK|CycH|	cyclin H	protein-co
lib-C666-I	chr1	205180760	205180760	+	14.54258	NA	promoter-TSS (NM_199462)	promoter-TSS (NM_199462)	-33	NM_19946	25778	Hs.6874	NM_01537	ENSG0000	DSTYK	CAKUT1|D	dual serine	protein-co
lib-C666-I	chr6	117923502	117923502	+	4.83287	NA	5' UTR (NM_020399, exon 1 of 9)	5' UTR (NM_020399, exon 1 of 9)	203	NM_02039	57120	Hs.191539	NM_02039	ENSG0000	GOPC	CAL|FIG|G	golgi asso	protein-co
lib-C666-I	chr7	27779727	27779727	+	30.97173	NA	promoter-TSS (NM_001206902)	promoter-TSS (NM_001206902)	13	NM_00107	8887	Hs.34576	NM_00602	ENSG0000	TAX1BP1	CALCOCO	Tax1 bindi	protein-co
lib-C666-I	chr3	154604456	154604456	+	10.99112	NA	Intergenic	Intergenic	-192980	NM_00090	4311	Hs.307734	NM_00090	ENSG0000	MME	CALLA|CD	membrane	protein-co
lib-C666-I	chr11	85780303	85780303	+	19.19377	NA	promoter-TSS (NM_007166)	promoter-TSS (NM_007166)	-164	NM_00120	8301	Hs.163893	NM_00716	ENSG0000	PICALM	CALM|CLT	phosphatic	protein-co
lib-C666-I	chr14	90849735	90849735	+	65.80097	NA	Intergenic	CpG	-13592	NM_00688	801	Hs.282410	NM_00688	ENSG0000	CALM1	CALML2|C	calmodulin	protein-co
lib-C666-I	chr14	90849135	90849135	+	9.02258	NA	Intergenic	CpG	-14192	NM_00688	801	Hs.282410	NM_00688	ENSG0000	CALM1	CALML2|C	calmodulin	protein-co
lib-C666-I	chr14	90863274	90863274	+	5.3823	NA	promoter-TSS (NM_006888)	promoter-TSS (NM_006888)	-53	NM_00688	801	Hs.282410	NM_00688	ENSG0000	CALM1	CALML2|C	calmodulin	protein-co
lib-C666-I	chr22	30129694	30129694	+	7.1502	NA	intron (NM_001003692, intron 5 of 5)	MIRc|SINE|MIR	13350	NM_18252	164633	Hs.643608	NM_18252	ENSG0000	CABP7	CALN2	calcium bir	protein-co
lib-C666-I	chr8	22298912	22298912	+	5.96422	NA	5' UTR (NM_001243974, exon 1 of 15	5' UTR (NM_001243974, exon 1 of 1	429	NM_00124	5533	Hs.731683	NM_00560	ENSG0000	PPP3CC	CALNA3|C	protein ph	protein-co
lib-C666-I	chr2	68479811	68479811	+	71.09715	NA	promoter-TSS (NM_000945)	promoter-TSS (NM_000945)	-160	NM_00094	5534	Hs.280604	NM_00094	ENSG0000	PPP3R1	CALNB1|C	protein ph	protein-co
lib-C666-I	chr19	36631022	36631022	+	36.38376	NA	promoter-TSS (NM_001302632)	promoter-TSS (NM_001302632)	-7	NM_00130	826	Hs.515371	NM_00174	ENSG0000	CAPNS1	CALPAIN4|	calpain sm	protein-co
lib-C666-I	chr3	15247623	15247623	+	6.20473	NA	promoter-TSS (NR_027927)	promoter-TSS (NR_027927)	-110	NM_01429	23473	Hs.595234	NM_01429	ENSG0000	CAPN7	CALPAIN7|	calpain 7	protein-co
lib-C666-I	chr19	47104333	47104333	+	49.72588	NA	promoter-TSS (NM_001329926)	promoter-TSS (NM_001329926)	2	NM_00132	808	Hs.515487	NM_00518	ENSG0000	CALM3	CaM|CaMI	calmodulin	protein-co
lib-C666-I	chr7	44309110	44309110	+	4.90117	NA	intron (NM_172079, intron 2 of 19)	intron (NM_172079, intron 2 of 19)	56120	NM_17208	816	Hs.351887	NM_00122	ENSG0000	CAMK2B	CAM2|CAM	calcium/ca	protein-co
lib-C666-I	chr17	43505705	43505705	+	15.31489	NA	intron (NM_001282290, intron 1 of 1	MIRb|SINE|MIR	1958	NM_00128	201176	Hs.205326	NM_17491	ENSG0000	ARHGAP27	CAMGAP1	Rho GTPas	protein-co
lib-C666-I	chr17	43463042	43463042	+	12.41959	NA	Intergenic	Intergenic	39970	NM_19928	201176	Hs.205326	NM_17491	ENSG0000	ARHGAP27	CAMGAP1	Rho GTPas	protein-co
lib-C666-I	chr17	43449142	43449142	+	7.21092	NA	Intergenic	CpG	53870	NM_19928	201176	Hs.205326	NM_17491	ENSG0000	ARHGAP27	CAMGAP1	Rho GTPas	protein-co
lib-C666-I	chr17	43509149	43509149	+	4.19675	NA	intron (NM_174919, intron 2 of 3)	CpG-10725	1133	NM_17491	201176	Hs.205326	NM_17491	ENSG0000	ARHGAP27	CAMGAP1	Rho GTPas	protein-co
lib-C666-I	chr2	47403888	47403888	+	94.79198	NA	promoter-TSS (NM_001305624)	promoter-TSS (NM_001305624)	-126	NM_00174	805	Hs.468442	NM_00174	ENSG0000	CALM2	CAMII|LQT	calmodulin	protein-co
lib-C666-I	chr5	110560197	110560197	+	7.15277	NA	promoter-TSS (NM_001323377)	promoter-TSS (NM_001323377)	-43	NM_00132	814	Hs.438801	NM_00174	ENSG0000	CAMK4	CaMK IV|C	calcium/ca	protein-co
lib-C666-I	chr4	114683872	114683872	+	9.4812	NA	promoter-TSS (NM_001321589)	promoter-TSS (NM_001321589)	-789	NM_17212	817	Hs.144114	NM_00122	ENSG0000	CAMK2D	CAMKD	calcium/ca	protein-co
lib-C666-I	chr4	114506103	114506103	+	6.56784	NA	intron (NM_001321576, intron 4 of 1	intron (NM_001321576, intron 4 of	176980	NM_17211	817	Hs.144114	NM_00122	ENSG0000	CAMK2D	CAMKD	calcium/ca	protein-co
lib-C666-I	chr3	183979049	183979049	+	6.3317	NA	exon (NM_033259, exon 1 of 2)	exon (NM_033259, exon 1 of 2)	202	NM_03325	94032	Hs.585003	NM_03325	ENSG0000	CAMK2N2	CAM-KIIN|	calcium/ca	protein-co
lib-C666-I	chr17	3793564	3793564	+	171.19254	NA	intron (NM_172207, intron 1 of 15)	intron (NM_172207, intron 1 of 15)	473	NM_17220	84254	Hs.8417	NM_03229	ENSG0000	CAMKK1	CAMKKA	calcium/ca	protein-co
lib-C666-I	chr17	3791957	3791957	+	16.389	NA	intron (NM_172207, intron 1 of 15)	CpG	2080	NM_17220	84254	Hs.8417	NM_03229	ENSG0000	CAMKK1	CAMKKA	calcium/ca	protein-co
lib-C666-I	chr22	22296994	22296994	+	13.06669	NA	intron (NM_014634, intron 2 of 7)	intron (NM_014634, intron 2 of 7)	10256	NM_01463	9647	Hs.112728	NM_01463	ENSG0000	PPM1F	CAMKP|Ca	protein ph	protein-co
lib-C666-I	chr5	134074130	134074130	+	8.89416	NA	promoter-TSS (NM_001745)	promoter-TSS (NM_001745)	-40	NM_00174	819	Hs.529846	NM_00174	ENSG0000	CAMLG	CAML|GET	calcium m	protein-co
lib-C666-I	chr17	1619728	1619728	+	113.62334	NA	promoter-TSS (NR_028502)	promoter-TSS (NR_028502)	-89	NM_15234	124997	Hs.234572	NM_15234	ENSG0000	WDR81	CAMRQ2|F	WD repeat	protein-co
lib-C666-I	chr17	1627904	1627904	+	3.38731	NA	promoter-TSS (NM_001163811)	promoter-TSS (NM_001163811)	70	NM_00116	124997	Hs.234572	NM_15234	ENSG0000	WDR81	CAMRQ2|F	WD repeat	protein-co
lib-C666-I	chr17	1620901	1620901	+	2.92124	NA	intron (NM_001163673, intron 1 of 9)	intron (NM_001163673, intron 1 of	1084	NM_00116	124997	Hs.234572	NM_15234	ENSG0000	WDR81	CAMRQ2|F	WD repeat	protein-co
lib-C666-I	chr2	191879019	191879019	+	7.97818	NA	promoter-TSS (NM_139266)	promoter-TSS (NM_139266)	-43	NM_13926	6772	Hs.642990	NM_00731	ENSG0000	STAT1	CANDF7|I	signal tran	protein-co
lib-C666-I	chr1	6845114	6845114	+	17.22931	NA	promoter-TSS (NM_015215)	promoter-TSS (NM_015215)	-270	NM_01521	23261	Hs.397705	NM_01521	ENSG0000	CAMTA1	CANPMR	calmodulin	protein-co
lib-C666-I	chr1	7023559	7023559	+	12.00555	NA	intron (NM_015215, intron 3 of 22)	intron (NM_015215, intron 3 of 22)	178175	NM_00124	23261	Hs.397705	NM_01521	ENSG0000	CAMTA1	CANPMR	calmodulin	protein-co
lib-C666-I	chr1	6844473	6844473	+	3.57421	NA	promoter-TSS (NM_015215)	promoter-TSS (NM_015215)	-911	NM_01521	23261	Hs.397705	NM_01521	ENSG0000	CAMTA1	CANPMR	calmodulin	protein-co
lib-C666-I	chr10	97205922	97205922	+	8.96177	NA	intron (NM_015385, intron 2 of 24)	intron (NM_015385, intron 2 of 24)	-4985	NM_00643	10580	Hs.38621	NM_00643	ENSG0000	SORBS1	CAP|FLAF2	sorbin and	protein-co
lib-C666-I	chr10	97235891	97235891	+	5.96761	NA	intron (NM_015385, intron 2 of 24)	intron (NM_015385, intron 2 of 24)	-34954	NM_00643	10580	Hs.38621	NM_00643	ENSG0000	SORBS1	CAP|FLAF2	sorbin and	protein-co
lib-C666-I	chr19	15443465	15443465	+	3.38731	NA	promoter-TSS (NM_001330384)	promoter-TSS (NM_001330384)	-111	NM_00133	23476	Hs.187763	NM_01429	ENSG0000	BRD4	CAP|HUNK	bromodon	protein-co
lib-C666-I	chr5	96271651	96271651	+	78.02469	NA	5' UTR (NM_005575, exon 1 of 18)	5' UTR (NM_005575, exon 1 of 18)	305	NM_00557	4012	Hs.527199	NM_00557	ENSG0000	LNPEP	CAP|IRAP|	leucyl and	protein-co
lib-C666-I	chr5	96270943	96270943	+	39.64852	NA	promoter-TSS (NM_005575)	promoter-TSS (NM_005575)	-403	NM_00557	4012	Hs.527199	NM_00557	ENSG0000	LNPEP	CAP|IRAP|	leucyl and	protein-co
lib-C666-I	chr5	96270445	96270445	+	2.24164	NA	promoter-TSS (NM_005575)	promoter-TSS (NM_005575)	-901	NM_00557	4012	Hs.527199	NM_00557	ENSG0000	LNPEP	CAP|IRAP|	leucyl and	protein-co
lib-C666-I	chr14	103274017	103274017	+	28.8837	NA	intron (NM_001199427, intron 1 of 7)	intron (NM_001199427, intron 1 of	30201	NM_00330	7187	Hs.510528	NM_00330	ENSG0000	TRAF3	CAP-1|CAF	TNF recept	protein-co
lib-C666-I	chr4	130014760	130014760	+	65.54223	NA	promoter-TSS (NM_144643)	promoter-TSS (NM_144643)	2	NM_14464	132320	Hs.654690	NM_14464	ENSG0000	SCLT1	CAP-1A|C	sodium ch	protein-co
lib-C666-I	chr6	89723339	89723339	+	7.49163	NA	Intergenic	LTR84a|LTR|ERVL	-49991	NM_00128	8732	Hs.567378	NM_00380	ENSG0000	RNGTT	CAP1A|HC	RNA guan	protein-co
lib-C666-I	chr18	61637787	61637787	+	5.92363	NA	intron (NM_198833, intron 1 of 6)	CpG	524	NM_00264	5271	Hs.368077	NM_00264	ENSG0000	SERPINB8	CAP2|PI8|P	serpin fam	protein-co
lib-C666-I	chr5	17186667	17186667	+	7.67555	NA	intron (NR_027253, intron 1 of 2)	intron (NR_027253, intron 1 of 2)	-30265	NM_00127	10409	Hs.201641	NM_00631	ENSG0000	BASP1	CAP-23|C	brain abun	protein-co
lib-C666-I	chr5	17146049	17146049	+	5.14484	NA	intron (NR_027253, intron 2 of 2)	intron (NR_027253, intron 2 of 2)	-70883	NM_00127	10409	Hs.201641	NM_00631	ENSG0000	BASP1	CAP-23|C	brain abun	protein-co
lib-C666-I	chr5	17097286	17097286	+	4.90117	NA	Intergenic	Intergenic	-119646	NM_00127	10409	Hs.201641	NM_00631	ENSG0000	BASP1	CAP-23|C	brain abun	protein-co
lib-C666-I	chr8	134309677	134309677	+	7.08929	NA	promoter-TSS (NM_001258432)	promoter-TSS (NM_001258432)	-130	NM_00609	10397	Hs.372914	NM_00609	ENSG0000	NDRG1	CAP43|CM	N-myc do	protein-co
lib-C666-I	chr3	160117061	160117061	+	18.56491	NA	promoter-TSS (NM_001288753)	promoter-TSS (NM_001288753)	-31	NM_00128	10051	Hs.58992	NM_00549	ENSG0000	SMC4	CAP-C|CA	structural r	protein-co
lib-C666-I	chr20	34330363	34330363	+	32.04651	NA	promoter-TSS (NM_001242599)	promoter-TSS (NM_001242599)	-105	NR_04072	9584	Hs.282901	NM_00490	ENSG0000	RBM39	CAPER|CA	RNA bindi	protein-co
lib-C666-I	chr14	23388154	23388154	+	541.56488	NA	promoter-TSS (NR_120599)	promoter-TSS (NR_120599)	242	NM_00107	55147	Hs.4997	NM_01810	ENSG0000	RBM23	CAPERbeta	RNA bindi	protein-co
lib-C666-I	chr7	158497533	158497533	+	22.66433	NA	promoter-TSS (NM_001281932)	promoter-TSS (NM_001281932)	-11	NM_00128	54892	Hs.18616	NM_01776	ENSG0000	NCAPG2	CAP-G2|C	non-SMC	protein-co
lib-C666-I	chr22	50946654	50946654	+	176.09369	NA	promoter-TSS (NM_152299)	promoter-TSS (NM_152299)	9	NM_01455	29781	Hs.730607	NM_01455	ENSG0000	NCAPH2	CAPH2	non-SMC	protein-co
lib-C666-I	chr16	2771159	2771159	+	78.34339	NA	promoter-TSS (NM_001318395)	promoter-TSS (NM_001318395)	-607	NM_03194	83886	Hs.332878	NM_03194	ENSG0000	PRSS27	CAPH2|MP	protease, s	protein-co
lib-C666-I	chr1	154155742	154155742	+	45.65358	NA	promoter-TSS (NR_103461)	promoter-TSS (NR_103461)	-17	NM_00127	7170	Hs.535581	NM_15226	ENSG0000	TPM3	CAPM1|CF	tropomyos	protein-co
lib-C666-I	chr1	113162336	113162336	+	68.95776	NA	promoter-TSS (NM_001308264)	promoter-TSS (NM_001308264)	261	NM_00613	829	Hs.744974	NM_00613	ENSG0000	CAPZA1	CAPPA1|C	capping ac	protein-co
lib-C666-I	chr7	116421246	116421246	+	8.85547	NA	intron (NM_001127500, intron 17 of 2	MER74A|LTR|ERVL	-81317	NM_00613	830	Hs.446123	NM_00613	ENSG0000	CAPZA2	CAPPA2|C	capping ac	protein-co
lib-C666-I	chr7	116420955	116420955	+	4.38153	NA	intron (NM_001127500, intron 17 of 2	MER74A|LTR|ERVL	-81608	NM_00613	830	Hs.446123	NM_00613	ENSG0000	CAPZA2	CAPPA2|C	capping ac	protein-co
lib-C666-I	chrX	41192401	41192401	+	50.76246	NA	promoter-TSS (NR_126093)	promoter-TSS (NR_126093)	-160	NM_00135	1654	Hs.728563	NM_00135	ENSG0000	DDX3X	CAP-Rf|DB	DEAD-box	protein-co
lib-C666-I	chrX	41193361	41193361	+	9.79436	NA	promoter-TSS (NR_126094)	promoter-TSS (NR_126094)	-54	NR_12609	1654	Hs.728563	NM_00135	ENSG0000	DDX3X	CAP-Rf|DB	DEAD-box	protein-co
lib-C666-I	chrX	41193847	41193847	+	3.92495	NA	intron (NM_001193416, intron 1 of 1	CpG	432	NM_00119	1654	Hs.728563	NM_00135	ENSG0000	DDX3X	CAP-Rf|DB	DEAD-box	protein-co
lib-C666-I	chr21	18885301	18885301	+	30.8056	NA	promoter-TSS (NM_001207064)	promoter-TSS (NM_001207064)	77	NM_00120	1525	Hs.627078	NM_00133	ENSG0000	CXADR	CAR|CAR4	coxsackie	protein-co
lib-C666-I	chr21	18885770	18885770	+	3.7184	NA	intron (NM_001207063, intron 1 of 4)	CpG	546	NM_00120	1525	Hs.627078	NM_00133	ENSG0000	CXADR	CAR|CAR4	coxsackie	protein-co
lib-C666-I	chr17	9479078	9479078	+	23.80446	NA	promoter-TSS (NM_145054)	promoter-TSS (NM_145054)	197	NR_03365	9482	Hs.431109	NM_00485	ENSG0000	STX8	CARB	syntaxin 8	protein-co
lib-C666-I	chr8	90770208	90770208	+	60.13322	NA	promoter-TSS (NR_125822)	promoter-TSS (NR_125822)	233	NM_00382	8767	Hs.103755	NM_00382	ENSG0000	RIPK2	CARD3|CA	receptor in	protein-co
lib-C666-I	chr17	5560671	5560671	+	5.9194	NA	Intergenic	Intergenic	-72839	NM_03300	22861	Hs.652273	NM_01492	ENSG0000	NLRP1	CARD7|CI	NLR family	protein-co
lib-C666-I	chr20	3827551	3827551	+	4.38153	NA	intron (NR_037921, intron 1 of 5)	CpG	105	NM_02074	57506	Hs.570362	NM_02074	ENSG0000	MAVS	CARDIF|IPS	mitochond	protein-co
lib-C666-I	chr4	184365699	184365699	+	6.39985	NA	promoter-TSS (NM_017632)	promoter-TSS (NM_017632)	-45	NM_00131	55602	Hs.592508	NM_01763	ENSG0000	CDKN2AIP	CARF	CDKN2A ir	protein-co

lib-C666-l	chr17	3539685	3539685	+	4.46944	NA	promoter-TSS (NM_004937)	promoter-TSS (NM_004937)	-69	NM_01327	23729	Hs.579217	NM_01327	ENSG0000	SHPK	CARKL|SH	sedoheptu	protein-co
lib-C666-l	chr8	128746432	128746432	+	5.9194	NA	promoter-TSS (NR_117102)	promoter-TSS (NR_117102)	-219	NR_11710	100270680	Hs.333720	NR_11710	ENSG0000	CASC11	CARLo-7|L	cancer sus	ncRNA
lib-C666-l	chr12	121837859	121837859	+	9.3976	NA	promoter-TSS (NM_001256858)	promoter-TSS (NM_001256858)	-27	NM_02512	80196	Hs.292804	NM_02512	ENSG0000	RNF34	CARP-1|C	ring finger	protein-co
lib-C666-l	chr17	33390797	33390797	+	23.5959	NA	promoter-TSS (NM_001017368)	promoter-TSS (NM_001017368)	-38	NM_00101	117584	Hs.13680	NM_05717	ENSG0000	RFFL	CARP-2|C	ring finger	protein-co
lib-C666-l	chr17	33415207	33415207	+	7.15277	NA	intron (NR_037713, intron 1 of 6)	intron (NR_037713, intron 1 of 6)	1141	NR_03771	117584	Hs.13680	NM_05717	ENSG0000	RFFL	CARP-2|C	ring finger	protein-co
lib-C666-l	chr17	33415996	33415996	+	5.34142	NA	intron (NR_037713, intron 1 of 6)	intron (NR_037713, intron 1 of 6)	352	NR_03771	117584	Hs.13680	NM_05717	ENSG0000	RFFL	CARP-2|C	ring finger	protein-co
lib-C666-l	chr11	3117155	3117155	+	21.87022	NA	intron (NM_145638, intron 13 of 20)	L1MC5|LINE|L1	-38474	NM_13927	833	Hs.274873	NM_00175	ENSG0000	CARS	CARS1|CY	cysteinyl-t	protein-co
lib-C666-l	chr2	170440883	170440883	+	137.76534	NA	promoter-TSS (NM_004792)	promoter-TSS (NM_004792)	33	NM_00479	9360	Hs.470544	NM_00479	ENSG0000	PPIG	CARS-Cyp	peptidylpr	protein-co
lib-C666-l	chr17	27071473	27071473	+	129.00739	NA	intron (NM_004295, intron 1 of 6)	CpG	450	NM_00429	9618	Hs.8375	NM_00429	ENSG0000	TRAF4	CART1|ML	TNF recept	protein-co
lib-C666-l	chr17	27071008	27071008	+	11.93581	NA	promoter-TSS (NM_004295)	promoter-TSS (NM_004295)	-15	NM_00429	9618	Hs.8375	NM_00429	ENSG0000	TRAF4	CART1|ML	TNF recept	protein-co
lib-C666-l	chr17	27070384	27070384	+	8.59372	NA	promoter-TSS (NM_004295)	promoter-TSS (NM_004295)	-639	NM_00429	9618	Hs.8375	NM_00429	ENSG0000	TRAF4	CART1|ML	TNF recept	protein-co
lib-C666-l	chr16	475529	475529	+	49.86005	NA	promoter-TSS (NM_014700)	promoter-TSS (NM_014700)	-139	NM_01470	9727	Hs.531642	NM_01470	ENSG0000	RAB11FIP3	CART1|Ra	RAB11 fam	protein-co
lib-C666-l	chr16	75339564	75339564	+	21.16833	NA	intron (NM_006324, intron 5 of 6)	MIR|SINE|MIR	-37613	NM_00117	9564	Hs.479747	NM_01456	ENSG0000	BCAR1	CAS|CAS1|	BCAR1, Ca	protein-co
lib-C666-l	chr16	75285337	75285337	+	13.33918	NA	intron (NM_001170715, intron 1 of 6)	CpG	189	NM_00117	9564	Hs.479747	NM_01456	ENSG0000	BCAR1	CAS|CAS1|	BCAR1, Ca	protein-co
lib-C666-l	chr16	75284992	75284992	+	9.20633	NA	intron (NM_001170715, intron 1 of 6)	CpG	534	NM_00117	9564	Hs.479747	NM_01456	ENSG0000	BCAR1	CAS|CAS1|	BCAR1, Ca	protein-co
lib-C666-l	chr20	47663428	47663428	+	7.15277	NA	promoter-TSS (NR_110624)	promoter-TSS (NR_110624)	645	NM_00131	1434	Hs.90073	NM_00131	ENSG0000	CSE1L	CAS|CSE1|	chromosor	protein-co
lib-C666-l	chr11	57566598	57566598	+	4.73866	NA	intron (NM_001085461, intron 6 of 1	intron (NM_001085461, intron 6 of	37364	NM_00108	1500	Hs.166011	NM_00133	ENSG0000	CTNND1	CAS|CTNN	catenin de	protein-co
lib-C666-l	chr1	10700308	10700308	+	24.19547	NA	intron (NM_001079843, intron 20 of	intron (NM_001079843, intron 20 o	156425	NM_00107	54897	Hs.439894	NM_01776	ENSG0000	CASZ1	CAS11|CST	castor zinc	protein-co
lib-C666-l	chr1	10839541	10839541	+	13.20416	NA	intron (NM_017766, intron 1 of 15)	intron (NM_017766, intron 1 of 15)	17192	NM_00107	54897	Hs.439894	NM_01776	ENSG0000	CASZ1	CAS11|CST	castor zinc	protein-co
lib-C666-l	chr1	10864362	10864362	+	8.0787	NA	Intergenic	Intergenic	-7629	NM_01776	54897	Hs.439894	NM_01776	ENSG0000	CASZ1	CAS11|CST	castor zinc	protein-co
lib-C666-l	chr1	10864612	10864612	+	2.12162	NA	Intergenic	Intergenic	-7879	NM_01776	54897	Hs.439894	NM_01776	ENSG0000	CASZ1	CAS11|CST	castor zinc	protein-co
lib-C666-l	chr7	101526434	101526434	+	19.60174	NA	intron (NM_181552, intron 1 of 23)	intron (NM_181552, intron 1 of 23)	65552	NM_18155	1523	Hs.191482	NM_00191	ENSG0000	CUX1	CASP|CDP|	cut like ho	protein-co
lib-C666-l	chr7	101458776	101458776	+	18.98417	NA	promoter-TSS (NM_001913)	promoter-TSS (NM_001913)	-408	NM_00120	1523	Hs.191482	NM_00191	ENSG0000	CUX1	CASP|CDP|	cut like ho	protein-co
lib-C666-l	chr7	101457636	101457636	+	17.68706	NA	Intergenic	CpG	-1548	NM_00120	1523	Hs.191482	NM_00191	ENSG0000	CUX1	CASP|CDP|	cut like ho	protein-co
lib-C666-l	chr7	101460845	101460845	+	9.94079	NA	promoter-TSS (NM_181552)	promoter-TSS (NM_181552)	-37	NM_18155	1523	Hs.191482	NM_00191	ENSG0000	CUX1	CASP|CDP|	cut like ho	protein-co
lib-C666-l	chr7	101500311	101500311	+	6.4187	NA	intron (NM_181552, intron 1 of 23)	intron (NM_181552, intron 1 of 23)	39429	NM_18155	1523	Hs.191482	NM_00191	ENSG0000	CUX1	CASP|CDP|	cut like ho	protein-co
lib-C666-l	chr7	101456875	101456875	+	5.80117	NA	Intergenic	Intergenic	-2309	NM_00120	1523	Hs.191482	NM_00191	ENSG0000	CUX1	CASP|CDP|	cut like ho	protein-co
lib-C666-l	chr7	101460579	101460579	+	3.25502	NA	promoter-TSS (NM_181552)	promoter-TSS (NM_181552)	-303	NM_18155	1523	Hs.191482	NM_00191	ENSG0000	CUX1	CASP|CDP|	cut like ho	protein-co
lib-C666-l	chr12	46384459	46384459	+	50.11658	NA	promoter-TSS (NM_004719)	promoter-TSS (NM_004719)	-58	NM_00471	9169	Hs.210367	NM_00471	ENSG0000	SCAF11	CASP11|SF	SR-related	protein-co
lib-C666-l	chr16	3208872	3208872	+	8.91001	NA	Intergenic	CpG	14580	NR_13232	197350	Hs.434132	NR_13232	ENSG0000	CASP16P	CASP16	caspase 16	pseudo
lib-C666-l	chr9	39014750	39014750	+	10.03103	NA	Intergenic	MIR3|SINE|MIR	273550	NM_03365	79937	Hs.128474	NM_03365	ENSG0000	CNTNAP3	CASPR3|C	contactin a	protein-co
lib-C666-l	chr12	1100257	1100257	+	19.92425	NA	promoter-TSS (NR_027949)	promoter-TSS (NR_027949)	-117	NR_02794	23085	Hs.601216	NM_17803	ENSG0000	ERC1	Cast2|ELKS	ELKS/RAB6	protein-co
lib-C666-l	chr22	30685610	30685610	+	9.20633	NA	promoter-TSS (NM_001037666)	promoter-TSS (NM_001037666)	6	NM_00103	652968	Hs.444950	NM_00103	ENSG0000	GATSL3	CASTOR1	GATS prot	protein-co
lib-C666-l	chr7	74838231	74838231	+	16.59065	NA	intron (NM_001145064, intron 1 of 8)	intron (NM_001145064, intron 1 of	29278	NM_00114	729438	Hs.594823	NM_00114	ENSG0000	GATSL2	CASTOR2|	GATS prot	protein-co
lib-C666-l	chr16	19098055	19098055	+	4.46944	NA	Intergenic	CpG	18816	NM_00119	10229	Hs.157113	NM_01613	ENSG0000	COQ7	CAT5|CLK-	coenzyme	protein-co
lib-C666-l	chr6	30524182	30524182	+	45.96589	NA	promoter-TSS (NM_001077497)	promoter-TSS (NM_001077497)	-304	NM_02526	80742	Hs.118354	NM_02526	ENSG0000	PRR3	CAT56	proline ric	protein-co
lib-C666-l	chr6	30524825	30524825	+	10.79304	NA	5' UTR (NM_005275, exon 1 of 12).6	5' UTR (NM_005275, exon 1 of 12).	339	NM_00107	80742	Hs.118354	NM_02526	ENSG0000	PRR3	CAT56	proline ric	protein-co
lib-C666-l	chr9	100174116	100174116	+	58.77799	NA	promoter-TSS (NM_001302884)	promoter-TSS (NM_001302884)	-186	NM_01429	23424	Hs.193842	NM_01429	ENSG0000	TDRD7	CATC4|PC	tudor dom	protein-co
lib-C666-l	chr11	68671364	68671364	+	57.45638	NA	promoter-TSS (NM_181514)	promoter-TSS (NM_181514)	45	NM_00218	3508	Hs.503048	NM_00218	ENSG0000	IGHMBP2	CATF1|CM	immunogl	protein-co
lib-C666-l	chr19	50221183	50221183	+	5.04179	NA	Intergenic	AluY|SINE|Alu	26818	NM_00119	126129	Hs.112195	NM_15235	ENSG0000	CPT1C	CATL1|CPT	carnitine p	protein-co
lib-C666-l	chr1	156030886	156030886	+	27.70954	NA	promoter-TSS (NM_020387)	promoter-TSS (NM_020387)	-54	NM_02038	57111	Hs.632469	NM_02038	ENSG0000	RAB25	CATX-8|R	RAB25, me	protein-co
lib-C666-l	chr9	103502470	103502470	+	6.89274	NA	Intergenic	L1PBa|LINE|L1	162109	NM_00101	347273	Hs.99004	NM_00101	ENSG0000	MURC	CAVIN4|ca	muscle rel	protein-co
lib-C666-l	chr8	86158339	86158339	+	8.0154	NA	intron (NM_198584, intron 1 of 6)	intron (NM_198584, intron 1 of 6)	623	NM_19858	377677	Hs.127189	NM_19858	ENSG0000	CA13	CAXIII	carbonic a	protein-co
lib-C666-l	chr16	31007924	31007924	+	28.88196	NA	intron (NM_052874, intron 7 of 9)	intron (NM_052874, intron 7 of 9)	11405	NM_02519	80270	Hs.460618	NM_02519	ENSG0000	HSD3B7	CBAS1|PFI	hydroxy-d	protein-co
lib-C666-l	chr3	196669474	196669474	+	195.76578	NA	promoter-TSS (NM_007362)	promoter-TSS (NM_007362)	-10	NM_00736	22916	Hs.591671	NM_00736	ENSG0000	NCBP2	CBC2|CBP	nuclear ca	protein-co
lib-C666-l	chr1	41157448	41157448	+	5.10953	NA	promoter-TSS (NM_001142590)	promoter-TSS (NM_001142590)	206	NM_00114	4802	Hs.713051	NM_01422	ENSG0000	NFYC	CBF-C|CBF	nuclear tra	protein-co
lib-C666-l	chr4	146540637	146540637	+	31.18315	NA	promoter-TSS (NM_172250)	promoter-TSS (NM_172250)	97	NM_17225	166785	Hs.452864	NM_17225	ENSG0000	MMAA	cblA	methylmal	protein-co
lib-C666-l	chr1	45965894	45965894	+	26.52079	NA	promoter-TSS (NM_015506)	promoter-TSS (NM_015506)	38	NM_01550	25974	Hs.13024	NM_01550	ENSG0000	MMACHC	cblC	methylmal	protein-co
lib-C666-l	chr16	3930852	3930852	+	22.23727	NA	promoter-TSS (NM_004380)	promoter-TSS (NM_004380)	-731	NM_00438	1387	Hs.459759	NM_00438	ENSG0000	CREBBP	CBP|KAT3	CREB bindi	protein-co
lib-C666-l	chr14	55596131	55596131	+	3.93123	NA	intron (NM_002306, intron 1 of 5)	CpG	196	NM_00117	3958	Hs.531081	NM_00230	ENSG0000	LGALS3	CBP35|GA	galectin 3	protein-co
lib-C666-l	chr9	100395633	100395633	+	40.12713	NA	promoter-TSS (NM_002486)	promoter-TSS (NM_002486)	-72	NM_00248	4686	Hs.595669	NM_00248	ENSG0000	NCBP1	CBP80|NC	nuclear ca	protein-co
lib-C666-l	chr9	100395112	100395112	+	23.05659	NA	promoter-TSS (NM_002486)	promoter-TSS (NM_002486)	-593	NM_00248	4686	Hs.595669	NM_00248	ENSG0000	NCBP1	CBP80|NC	nuclear ca	protein-co
lib-C666-l	chr2	98612668	98612668	+	10.20361	NA	promoter-TSS (NM_015348)	promoter-TSS (NM_015348)	-314	NM_01534	23505	Hs.469376	NM_01534	ENSG0000	TMEM131	CC28|PRO	transmem	protein-co
lib-C666-l	chr11	20385280	20385280	+	8.56376	NA	promoter-TSS (NM_006410)	promoter-TSS (NM_006410)	-9	NM_00109	10553	Hs.90753	NM_00641	ENSG0000	HTATIP2	CC3|SDR4	HIV-1 Tat i	protein-co
lib-C666-l	chr5	128018172	128018172	+	28.21964	NA	Intergenic	Intergenic	-144437	NM_00199	2201	Hs.519294	NM_00199	ENSG0000	FBN2	CCA|DA9|E	fibrillin 2	protein-co
lib-C666-l	chr3	3168604	3168604	+	56.5598	NA	promoter-TSS (NM_001302946)	promoter-TSS (NM_001302946)	4	NM_18291	51095	Hs.732725	NM_01600	ENSG0000	TRNT1	CCA1|CGI-	tRNA nucle	protein-co
lib-C666-l	chr10	13986164	13986164	+	8.89416	NA	intron (NM_018027, intron 2 of 24)	MER1A|DNA|hAT-Charlie	28205	NM_00131	55691	Hs.330463	NM_01802	ENSG0000	FRMD4A	CCAFCA|F	FERM dom	protein-co
lib-C666-l	chr10	13732561	13732561	+	6.56784	NA	intron (NM_018027, intron 15 of 24)	intron (NM_018027, intron 15 of 24	17006	NM_00131	55691	Hs.330463	NM_01802	ENSG0000	FRMD4A	CCAFCA|F	FERM dom	protein-co
lib-C666-l	chr7	100450058	100450058	+	5.15278	NA	promoter-TSS (NM_001267814)	promoter-TSS (NM_001267814)	-283	NM_02024	56996	Hs.521087	NM_02024	ENSG0000	SLC12A9	CCC6|CIP1	solute carr	protein-co
lib-C666-l	chr5	122796120	122796120	+	12.69461	NA	Intergenic	L2a|LINE|L2	-36834	NM_15322	153241	Hs.483209	NM_15322	ENSG0000	CEP120	CCDC100|	centrosom	protein-co
lib-C666-l	chr5	122614022	122614022	+	8.33814	NA	Intergenic	L2b|LINE|L2	145264	NM_00116	153241	Hs.483209	NM_15322	ENSG0000	CEP120	CCDC100|	centrosom	protein-co
lib-C666-l	chr5	122759465	122759465	+	7.23273	NA	promoter-TSS (NM_001166226)	promoter-TSS (NM_001166226)	-179	NM_15322	153241	Hs.483209	NM_15322	ENSG0000	CEP120	CCDC100|	centrosom	protein-co
lib-C666-l	chr5	122759138	122759138	+	5.04179	NA	intron (NM_153223, intron 1 of 20)	CpG	148	NM_00116	153241	Hs.483209	NM_15322	ENSG0000	CEP120	CCDC100|	centrosom	protein-co
lib-C666-l	chr16	28565228	28565228	+	9.4812	NA	promoter-TSS (NM_138414)	promoter-TSS (NM_138414)	-21	NM_13841	112869	Hs.655476	NM_13841	ENSG0000	SGF29	CCDC101|	SAGA com	protein-co
lib-C666-l	chr2	48667782	48667782	+	12.72278	NA	promoter-TSS (NM_001135629)	promoter-TSS (NM_001135629)	-126	NR_02418	129285	Hs.654619	NM_15299	ENSG0000	PPP1R21	CCDC128|	protein ph	protein-co
lib-C666-l	chr7	92861652	92861652	+	2.64885	NA	promoter-TSS (NM_024553)	promoter-TSS (NM_024553)	-1	NM_02455	55610	Hs.202424	NM_01766	ENSG0000	VPS50	CCDC132|	VPS50, EA	protein-co
lib-C666-l	chr17	20224439	20224439	+	14.17553	NA	promoter-TSS (NR_023380)	promoter-TSS (NR_023380)	-48	NR_02338	348254	Hs.462475	NR_023380		CCDC144	CCDC144C	coiled-coil	pseudo
lib-C666-l	chr5	138739556	138739556	+	10.27265	NA	intron (NM_194296, intron 1 of 5)	CpG	220	NM_19429	202051	Hs.373612	NM_19429	ENSG0000	SPATA24	CCDC161|	spermatog	protein-co
lib-C666-l	chr18	8840547	8840547	+	23.20869	NA	Intergenic	Intergenic	123178	NM_01521	23255	Hs.707920	NM_01521	ENSG0000	MTCL1	CCDC165|	microtubul	protein-co
lib-C666-l	chr1	159870407	159870407	+	10.73451	NA	promoter-TSS (NR_130464)	promoter-TSS (NR_130464)	-501	NM_01233	25790	Hs.647705	NM_01233	ENSG0000	CFAP45	CCDC19|N	cilia and fl	protein-co
lib-C666-l	chr1	26560563	26560563	+	7.21092	NA	promoter-TSS (NM_001281517)	promoter-TSS (NM_001281517)	-81	NM_02277	64793	Hs.63795	NM_02277	ENSG0000	CEP85	CCDC21	centrosom	protein-co
lib-C666-l	chr15	40857273	40857273	+	10.71733	NA	promoter-TSS (NM_001289132)	promoter-TSS (NM_001289132)	-17	NM_00128	90416	Hs.654661	NM_05284	ENSG0000	C15orf57	CCDC32	chromosor	protein-co
lib-C666-l	chr12	94853754	94853754	+	27.84631	NA	promoter-TSS (NR_027035)	promoter-TSS (NR_027035)	10	NM_01612	51134	Hs.279209	NM_01612	ENSG0000	CEP83	CCDC41|N	centrosom	protein-co
lib-C666-l	chr17	62502899	62502899	+	24.25638	NA	promoter-TSS (NM_001320597)	promoter-TSS (NM_001320597)	45	NR_13364	90799	Hs.569713	NM_13836	ENSG0000	CEP95	CCDC45	centrosom	protein-co
lib-C666-l	chr17	36981622	36981622	+	615.92517	NA	promoter-TSS (NR_039880)	promoter-TSS (NR_039880)	-19	NR_07342	54883	Hs.406223	NM_01774	ENSG0000	CWC25	CCDC49	CWC25 spl	protein-co
lib-C666-l	chr12	102455944	102455944	+	17.68706	NA	promoter-TSS (NM_016053)	promoter-TSS (NM_016053)	-42	NM_01605	51019	Hs.405692	NM_01605	ENSG0000	WASHC3	CCDC53|C	WASH com	protein-co
lib-C666-l	chr17	28443834	28443834	+	19.51538	NA	promoter-TSS (NM_001261467)	promoter-TSS (NM_001261467)	9	NM_03214	84081	Hs.462663	NM_03214	ENSG0000	NSRP1	CCDC55|H	nuclear sp	protein-co
lib-C666-l	chr3	48481606	48481606	+	33.60578	NA	promoter-TSS (NM_001256964)	promoter-TSS (NM_001256964)	-42	NM_01593	51372	Hs.356440	NM_01593	ENSG0000	TMA7	CCDC72|H	translation	protein-co
lib-C666-l	chr1	100598750	100598750	+	6.29226	NA	promoter-TSS (NM_019083)	promoter-TSS (NM_019083)	44	NM_01908	54482	Hs.729095	NM_01908	ENSG0000	TRMT13	CCDC76	tRNA meth	protein-co
lib-C666-l	chr18	77480199	77480199	+	10.37767	NA	intron (NM_048368, intron 10 of 11)	intron (NM_048368, intron 10 of 11	38769	NM_00120	9150	Hs.465490	NM_00471	ENSG0000	CTDP1	CCFDN|FC	CTD phos	protein-co
lib-C666-l	chr3	167452703	167452703	+	6.81085	NA	promoter-TSS (NM_007217)	promoter-TSS (NM_007217)	-52	NM_14585	11235	Hs.478150	NM_00721	ENSG0000	PDCD10	CCM3|TFA	programm	protein-co
lib-C666-l	chr20	2451521	2451521	+	12.13776	NA	promoter-TSS (NM_003091)	promoter-TSS (NM_003091)	-22	NM_00309	6628	Hs.83753	NM_00309	ENSG0000	SNRPB	CCMS|COD	small nucle	protein-co
lib-C666-l	chr4	122745111	122745111	+	105.08881	NA	promoter-TSS (NM_001237)	promoter-TSS (NM_001237)	45	NM_00123	890	Hs.58974	NM_00123	ENSG0000	CCNA2	CCN1|CCN	cyclin A2	protein-co
lib-C666-l	chr8	134247204	134247204	+	3.58839	NA	Intergenic	Intergenic	43922	NR_03794	8840	Hs.492974	NM_00388	ENSG0000	WISP1	CCN4|WIS	WNT1 indu	protein-co
lib-C666-l	chr6	112364966	112364966	+	9.21057	NA	Intergenic	Intergenic	-10312	NR_12535	8838	Hs.558428	NM_00388	ENSG0000	WISP3	CCN6|LIBC	WNT1 indu	protein-co
lib-C666-l	chr5	68462799	68462799	+	20.59244	NA	promoter-TSS (NM_031966)	promoter-TSS (NM_031966)	-38	NM_03196	891	Hs.23960	NM_03196	ENSG0000	CCNB1	CCNB	cyclin B1	protein-co
lib-C666-l	chr5	162864505	162864505	+	4.73866	NA	promoter-TSS (NM_199246)	promoter-TSS (NM_199246)	-72	NM_19924	900	Hs.79101	NM_00406	ENSG0000	CCNG1	CCNG	cyclin G1	protein-co
lib-C666-l	chr4	77997345	77997345	+	176.70418	NA	promoter-TSS (NM_006835)	promoter-TSS (NM_006835)	-220	NM_00683	10983	Hs.518827	NM_00683	ENSG0000	CCNI	CCNI1|CYC	cyclin I	protein-co
lib-C666-l	chr12	49110538	49110538	+	59.83187	NA	5' UTR (NM_001277842, exon 1 of 8)	5' UTR (NM_001277842, exon 1 of	243	NM_00127	904	Hs.92308	NM_00124	ENSG0000	CCNT1	CCNT|CYC	cyclin T1	protein-co
lib-C666-l	chr12	49110870	49110870	+	25.3508	NA	promoter-TSS (NM_001277842)	promoter-TSS (NM_001277842)	-89	NM_00124	904	Hs.92308	NM_00124	ENSG0000	CCNT1	CCNT|CYC	cyclin T1	protein-co
lib-C666-l	chr5	54529667	54529667	+	8.521	NA	promoter-TSS (NR_125348)	promoter-TSS (NR_125348)	-122	NM_02114	10309	Hs.3041	NM_02114	ENSG0000	CCNO	CCNU|CILD	cyclin O	protein-co
lib-C666-l	chr19	38943554	38943554	+	5.25516	NA	exon (NM_000540, exon 13 of 106)	exon (NM_000540, exon 13 of 106)	19214	NM_00054	6261	Hs.466664	NM_00054	ENSG0000	RYR1	CCO|MHS|	ryanodine	protein-co
lib-C666-l	chr9	88356914	88356914	+	25.49755	NA	promoter-TSS (NM_001286717)	promoter-TSS (NM_001286717)	30	NM_00128	23287	Hs.719980	NM_01523	ENSG0000	AGTPBP1	CCP1|NNA	ATP/GTP b	protein-co
lib-C666-l	chr9	88358554	88358554	+	7.75972	NA	Intergenic	Intergenic	-1610	NM_00128	23287	Hs.719980	NM_01523	ENSG0000	AGTPBP1	CCP1|NNA	ATP/GTP b	protein-co
lib-C666-l	chr9	88356388	88356388	+	5.48804	NA	intron (NM_001330701, intron 1 of 2	CpG	556	NM_00128	23287	Hs.719980	NM_01523	ENSG0000	AGTPBP1	CCP1|NNA	ATP/GTP b	protein-co
lib-C666-l	chr7	134671383	134671383	+	7.0832	NA	non-coding (NR_144293, exon 1 of 1	non-coding (NR_144293, exon 1 of	124	NM_00134	340351	Hs.648616	NM_17856	ENSG0000	AGBL3	CCP3	ATP/GTP b	protein-co
lib-C666-l	chr6	170615589	170615589	+	13.85062	NA	promoter-TSS (NM_001286381)	promoter-TSS (NM_001286381)	-221	NM_00128	84498	Hs.369522	NM_03244	ENSG0000	FAM120B	CCPG|KIA	family with	protein-co
lib-C666-l	chr6	170615938	170615938	+	5.04179	NA	intron (NM_001286381, intron 1 of 8)	CpG	128	NM_03244	84498	Hs.369522	NM_03244	ENSG0000	FAM120B	CCPG|KIA	family with	protein-co
lib-C666-l	chr5	179868809	179868809	+	49.50627	NA	Intergenic	Intergenic	-52590	NM_00130	57472	Hs.608380	NM_01545	ENSG0000	CNOT6	CCR4|Ccr4	CCR4-NO	protein-co
lib-C666-l	chr5	179921314	179921314	+	6.39985	NA	promoter-TSS (NM_001303241)	promoter-TSS (NM_001303241)	-85	NM_00130	57472	Hs.608380	NM_01545	ENSG0000	CNOT6	CCR4|Ccr4	CCR4-NO	protein-co
lib-C666-l	chr14	77279211	77279211	+	9.47655	NA	promoter-TSS (NM_015305)	promoter-TSS (NM_015305)	72	NM_01530	23357	Hs.497448	NM_01530	ENSG0000	ANGEL1	Ccr4e|KIA	angel hom	protein-co
lib-C666-l	chr4	139936772	139936772	+	16.264	NA	promoter-TSS (NM_012118)	promoter-TSS (NM_012118)	-141	NM_01211	25819	Hs.639842	NM_01211	ENSG0000	NOCT	CCR4L|CC	nocturnin	protein-co
lib-C666-l	chr9	90589613	90589613	+	36.70779	NA	promoter-TSS (NM_001170639)	promoter-TSS (NM_001170639)	82	NM_01211	23552	Hs.522274	NM_01211	ENSG0000	CDK20	CCRK|CDC	cyclin dep	protein-co
lib-C666-l	chr6	74290202	74290202	+	36.56424	NA	Intergenic	Intergenic	-59447	NM_00140	1915	Hs.535192	NM_00140	ENSG0000	EEF1A1	CCS-3|CC	eukaryotic	protein-co
lib-C666-l	chr6	74230870	74230870	+	24.5284	NA	promoter-TSS (NM_001402)	promoter-TSS (NM_001402)	-115	NM_00140	1915	Hs.535192	NM_00140	ENSG0000	EEF1A1	CCS-3|CC	eukaryotic	protein-co
lib-C666-l	chr16	1470813	1470813	+	242.09764	NA	promoter-TSS (NM_001272051)	promoter-TSS (NM_001272051)	-12	NM_00127	283951	Hs.58373	NM_00101	ENSG0000	C16orf91	CCSMST1|	chromosor	protein-co
lib-C666-l	chr7	64541733	64541733	+	137.89944	NA	Intergenic	CpG	43001	NR_03341	643180	Hs.510847	NR_03341	ENSG0000	CCT6P3	CCT6-3P	chaperoni	pseudo
lib-C666-l	chr7	64498850	64498850	+	11.05669	NA	non-coding (NR_033416, exon 1 of 1	non-coding (NR_033416, exon 1 of	118	NR_03341	643180	Hs.510847	NR_03341	ENSG0000	CCT6P3	CCT6-3P	chaperoni	pseudo
lib-C666-l	chr1	156308169	156308169	+	23.22307	NA	promoter-TSS (NM_001304817)	promoter-TSS (NM_001304817)	37	NM_00599	7203	Hs.491494	NM_00599	ENSG0000	CCT3	CCT-gam	chaperoni	protein-co
lib-C666-l	chr7	56119349	56119349	+	27.28944	NA	promoter-TSS (NM_004577)	promoter-TSS (NM_004577)	-29	NM_00100	908	Hs.82916	NM_00176	ENSG0000	CCT6A	CCT-zeta|	chaperoni	protein-co
lib-C666-l	chr17	33288519	33288519	+	47.60628	NA	promoter-TSS (NM_001193529)	promoter-TSS (NM_001193529)	9	NM_00119	10693	Hs.73072	NM_00658	ENSG0000	CCT6B	CCT-zeta-	chaperoni	protein-co
lib-C666-l	chr5	68665551	68665551	+	10.37767	NA	promoter-TSS (NM_133342)	promoter-TSS (NM_133342)	-7	NM_13334	5884	Hs.16184	NM_00287	ENSG0000	RAD17	CCYC|HRA	RAD17 che	protein-co
lib-C666-l	chr13	113951435	113951435	+	24.18348	NA	promoter-TSS (NM_005561)	promoter-TSS (NM_005561)	-34	NM_00556	3916	Hs.494419	NM_00556	ENSG0000	LAMP1	CD107a|L	lysosomal	protein-co
lib-C666-l	chr13	113968458	113968458	+	3.38731	NA	intron (NM_005561, intron 4 of 8)	intron (NM_005561, intron 4 of 8)	16989	NM_00556	3916	Hs.494419	NM_00556	ENSG0000	LAMP1	CD107a|L	lysosomal	protein-co
lib-C666-l	chr15	74723135	74723135	+	16.13885	NA	intron (NM_001146029, intron 1 of 1	intron (NM_001146029, intron 1 of	2910	NM_00114	8482	Hs.24640	NM_00361	ENSG0000	SEMA7A	CD108|CD	semaphori	protein-co
lib-C666-l	chr19	45349653	45349653	+	10.71733	NA	5' UTR (NM_002856, exon 1 of 6)	5' UTR (NM_002856, exon 1 of 6)	260	NM_00104	5819	Hs.655455	NM_00285	ENSG0000	NECTIN2	CD112|HV	nectin cell	protein-co
lib-C666-l	chr6	137540562	137540562	+	30.07415	NA	promoter-TSS (NM_000416)	promoter-TSS (NM_000416)	5	NM_00041	3459	Hs.520414	NM_00041	ENSG0000	IFNGR1	CD119|IFN	interferon	protein-co
lib-C666-l	chr22	37557807	37557807	+	4.52748	NA	intron (NM_001346222, intron 1 of 9)	intron (NM_001346222, intron 1 of	-11777	NM_00087	3560	Hs.474787	NM_00087	ENSG0000	IL2RB	CD122|IL1	interleukin	protein-co
lib-C666-l	chr1	154378356	154378356	+	10.79304	NA	intron (NM_181359, intron 1 of 8)	CpG	687	NM_00120	3570	Hs.135087	NM_00056	ENSG0000	IL6R	CD126|IL-	interleukin	protein-co
lib-C666-l	chr22	37288559	37288559	+	6.71839	NA	Intergenic	Intergenic	-21116	NM_00039	1439	Hs.592192	NM_00039	ENSG0000	CSF2RB	CD131|CD	colony stin	protein-co
lib-C666-l	chr1	95100639	95100639	+	34.02388	NA	Intergenic	Intergenic	-93226	NM_00199	2152	Hs.62192	NM_00199	ENSG0000	F3	CD142|TF|	coagulatio	protein-co
lib-C666-l	chr11	48002324	48002324	+	4.98959	NA	5' UTR (NM_001098503, exon 1 of 9)	5' UTR (NM_001098503, exon 1 of	214	NM_00109	5795	Hs.318547	NM_00284	ENSG0000	PTPRJ	CD148|DE	protein tyr	protein-co
lib-C666-l	chr9	117670630	117670630	+	11.93581	NA	intron (NM_001252290, intron 2 of 4)	intron (NM_001252290, intron 2 of	22245	NM_00124	944	Hs.494901	NM_00124	ENSG0000	TNFSF8	CD153|CD	tumor nec	protein-co
lib-C666-l	chr19	45147273	45147273	+	13.77909	NA	5' UTR (NM_006505, exon 1 of 8)	5' UTR (NM_006505, exon 1 of 8)	175	NM_00650	5817	Hs.171844	NM_00650	ENSG0000	PVR	CD155|HV	poliovirus	protein-co
lib-C666-l	chr1	161507258	161507258	+	8.43855	NA	Intergenic	MER11A|LTR|ERVK	12635	NM_00112	2214	Hs.372679	NM_00056	ENSG0000	FCGR3A	CD16|CD1	Fc fragmer	protein-co
lib-C666-l	chr12	109011330	109011330	+	106.19211	NA	Intergenic	Intergenic	14524	NM_00120	6404	Hs.591014	NM_00300	ENSG0000	SELPLG	CD162|CL	selectin P	protein-co
lib-C666-l	chr3	105086798	105086798	+	27.79032	NA	intron (NM_001243280, intron 1 of 1	intron (NM_001243280, intron 1 of	1241	NM_00124	214	Hs.591293	NM_00162	ENSG0000	ALCAM	CD166|ME	activated l	protein-co
lib-C666-l	chr3	105087220	105087220	+	3.27468	NA	intron (NM_001243280, intron 1 of 1	intron (NM_001243280, intron 1 of	1663	NM_00124	214	Hs.591293	NM_00162	ENSG0000	ALCAM	CD166|ME	activated l	protein-co
lib-C666-l	chr2	137067757	137067757	+	11.66438	NA	Intergenic	Intergenic	-192032	NM_00346	7852	Hs.593413	NM_00346	ENSG0000	CXCR4	CD184|D2	C-X-C mo	protein-co
lib-C666-l	chr2	136874577	136874577	+	3.98718	NA	promoter-TSS (NM_001008540)	promoter-TSS (NM_001008540)	-764	NM_00100	7852	Hs.593413	NM_00346	ENSG0000	CXCR4	CD184|D2	C-X-C mo	protein-co
lib-C666-l	chr2	160752193	160752193	+	8.43277	NA	intron (NM_001198759, intron 2 of 3	intron (NM_001198759, intron 2 of	9074	NM_00234	4065	Hs.153563	NM_00234	ENSG0000	LY75	CD205|CLE	lymphocyt	protein-co
lib-C666-l	chr2	160761126	160761126	+	3.93123	NA	exon (NM_001198760, exon 1 of 38)	exon (NM_001198760, exon 1 of 38	141	NM_00234	4065	Hs.153563	NM_00234	ENSG0000	LY75	CD205|CLE	lymphocyt	protein-co
lib-C666-l	chr3	182877336	182877336	+	3.27468	NA	intron (NM_014398, intron 1 of 5)	intron (NM_014398, intron 1 of 5)	3331	NM_01439	27074	Hs.518448	NM_01439	ENSG0000	LAMP3	CD208|DC	lysosomal	protein-co
lib-C666-l	chr19	18215372	18215372	+	115.06194	NA	intron (NM_015016, intron 1 of 26)	intron (NM_015016, intron 1 of 26)	-5746	NM_00129	3594	Hs.567294	NM_00553	ENSG0000	IL12RB1	CD212|IL-	interleukin	protein-co
lib-C666-l	chr10	6019627	6019627	+	9.20967	NA	promoter-TSS (NM_172200)	promoter-TSS (NM_172200)	-53	NM_00125	3601	Hs.445124	NM_00218	ENSG0000	IL15RA	CD215	interleukin	protein-co
lib-C666-l	chr19	7198117	7198117	+	2.05918	NA	intron (NM_001079817, intron 2 of 2	CpG	96196	NM_00107	3643	Hs.465744	NM_00020	ENSG0000	INSR	CD220|HH	insulin rec	protein-co
lib-C666-l	chr15	99190783	99190783	+	4.51266	NA	promoter-TSS (NM_001291858)	promoter-TSS (NM_001291858)	-985	NM_00129	3480	Hs.643120	NM_00087	ENSG0000	IGF1R	CD221|IGF	insulin like	protein-co
lib-C666-l	chr3	196760062	196760062	+	7.0832	NA	Intergenic	Intergenic	-3375	NM_00592	4241	Hs.184727	NM_00592	ENSG0000	MELTF	CD228|MA	melanotra	protein-co
lib-C666-l	chr3	196760749	196760749	+	6.04359	NA	Intergenic	Intergenic	-4062	NM_00592	4241	Hs.184727	NM_00592	ENSG0000	MELTF	CD228|MA	melanotra	protein-co
lib-C666-l	chr2	127415442	127415442	+	4.90117	NA	intron (NM_016815, intron 1 of 2)	intron (NM_016815, intron 1 of 2)	1931	NM_01681	2995	Hs.59138	NM_00210	ENSG0000	GYPC	CD236|CD	glycophori	protein-co
lib-C666-l	chr15	89182042	89182042	+	37.31675	NA	promoter-TSS (NM_001303237)	promoter-TSS (NM_001303237)	68	NM_00130	3669	Hs.459265	NM_00220	ENSG0000	ISG20	CD25|HEM	interferon	protein-co
lib-C666-l	chr17	16894849	16894849	+	29.07066	NA	Intergenic	Intergenic	-19447	NM_01245	23495	Hs.158341	NM_01245	ENSG0000	TNFRSF13	CD267|CV	TNF recept	protein-co
lib-C666-l	chr14	105219453	105219453	+	181.89525	NA	promoter-TSS (NM_021709)	promoter-TSS (NM_021709)	-17	NM_02170	10572	Hs.112058	NM_00642	ENSG0000	SIVA1	CD27BP|S	SIVA1 apo	protein-co
lib-C666-l	chr4	38859518	38859518	+	7.15277	NA	Intergenic	Intergenic	-1080	NM_00606	10333	Hs.575090	NM_00606	ENSG0000	TLR6	CD286	toll like rec	protein-co
lib-C666-l	chr10	33247193	33247193	+	35.7914	NA	promoter-TSS (NM_133376)	promoter-TSS (NM_133376)	100	NM_00221	3688	Hs.643813	NM_00221	ENSG0000	ITGB1	CD29|FNR	integrin su	protein-co
lib-C666-l	chr10	33246786	33246786	+	4.04037	NA	promoter-TSS (NM_133376)	promoter-TSS (NM_133376)	36	NM_13337	3688	Hs.643813	NM_00221	ENSG0000	ITGB1	CD29|FNR	integrin su	protein-co
lib-C666-l	chr17	72510354	72510354	+	11.55724	NA	Intergenic	(ATGTG)n|Simple_repeat|Simple_rep	17259	NM_17489	124599	Hs.313343	NM_17489	ENSG0000	CD300LB	CD300b|C	CD300 mo	protein-co
lib-C666-l	chr17	72510826	72510826	+	9.04008	NA	Intergenic	(ATGTG)n|Simple_repeat|Simple_rep	16787	NM_17489	124599	Hs.313343	NM_17489	ENSG0000	CD300LB	CD300b|C	CD300 mo	protein-co
lib-C666-l	chr17	72565792	72565792	+	23.20869	NA	Intergenic	Intergenic	-2310	NM_00132	100130520		NM_001324073		LOC10013	CD300H|C	CD300c m	protein-co
lib-C666-l	chr4	56050880	56050880	+	3.92495	NA	Intergenic	Intergenic	-59118	NM_00225	3791	Hs.479756	NM_00225	ENSG0000	KDR	CD309|FLK	kinase inse	protein-co
lib-C666-l	chr1	117396549	117396549	+	4.90117	NA	Intergenic	Intergenic	-55996	NM_02044	5738	Hs.418093	NM_02044	ENSG0000	PTGFRN	CD315|CD	prostaglan	protein-co
lib-C666-l	chr1	160069273	160069273	+	4.19675	NA	promoter-TSS (NM_001320247)	promoter-TSS (NM_001320247)	-655	NM_00120	93185	Hs.332012	NM_05286	ENSG0000	IGSF8	CD316|CD	immunogl	protein-co
lib-C666-l	chr1	160991184	160991184	+	21.94284	NA	promoter-TSS (NM_016946)	promoter-TSS (NM_016946)	-51	NM_01694	50848	Hs.517293	NM_01694	ENSG0000	F11R	CD321|JAM	F11 recept	protein-co
lib-C666-l	chr1	160983377	160983377	+	3.90449	NA	intron (NM_016946, intron 1 of 9)	intron (NM_016946, intron 1 of 9)	7756	NM_01694	50848	Hs.517293	NM_01694	ENSG0000	F11R	CD321|JAM	F11 recept	protein-co
lib-C666-l	chr5	176514113	176514113	+	6.98764	NA	intron (NM_213647, intron 1 of 17)	CpG	240	NM_00201	2264	Hs.165950	NM_00201	ENSG0000	FGFR4	CD334|JTK	fibroblast	protein-co
lib-C666-l	chr17	37844450	37844450	+	46.69447	NA	promoter-TSS (NM_001291726)	promoter-TSS (NM_001291726)	113	NM_00128	2064	Hs.446352	NM_00444	ENSG0000	ERBB2	CD340|HE	erb-b2 rec	protein-co
lib-C666-l	chr17	37859848	37859848	+	8.01457	NA	intron (NR_110535, intron 1 of 26)	intron (NR_110535, intron 1 of 26)	3617	NM_00128	2064	Hs.446352	NM_00444	ENSG0000	ERBB2	CD340|HE	erb-b2 rec	protein-co

lib-C666-l	chr7	72850721	72850721	+	7.03995	NA	TTS (NM_003508)	TTS (NM_003508)	2612	NM_00350	8326	Hs.647029	NM_00350	ENSG0000	FZD9	CD349|FZD	frizzled cla	protein-co
lib-C666-l	chr7	72848158	72848158	+	4.96104	NA	promoter-TSS (NM_003508)	promoter-TSS (NM_003508)	49	NM_00350	8326	Hs.647029	NM_00350	ENSG0000	FZD9	CD349|FZD	frizzled cla	protein-co
lib-C666-l	chr8	97507352	97507352	+	5.23609	NA	intron (NM_002998, intron 1 of 4)	CpG	1470	NM_00299	6383	Hs.1501	NM_00299	ENSG0000	SDC2	CD362|HSI	syndecan 2	protein-co
lib-C666-l	chr12	125348337	125348337	+	15.27523	NA	5' UTR (NM_005505, exon 1 of 13)	5' UTR (NM_005505, exon 1 of 13)	182	NM_00108	949	Hs.731377	NM_00550	ENSG0000	SCARB1	CD36L1|CL	scavenger	protein-co
lib-C666-l	chr20	25176376	25176376	+	19.7012	NA	promoter-TSS (NM_001322388)	promoter-TSS (NM_001322388)	70	NM_00132	955	Hs.500375	NM_00124	ENSG0000	ENTPD6	CD39L2|IL-	ectonuclec	protein-co
lib-C666-l	chr14	74486018	74486018	+	64.22771	NA	promoter-TSS (NM_025057)	promoter-TSS (NM_025057)	8	NM_00132	957	Hs.655070	NM_00124	ENSG0000	ENTPD5	CD39L4|NT	ectonuclec	protein-co
lib-C666-l	chr2	173135416	173135416	+	4.39666	NA	Intergenic	L1PA6|LINE|L1	-156666	NM_00131	3655	Hs.133397	NM_00021	ENSG0000	ITGA6	CD49f|ITG/	integrin su	protein-co
lib-C666-l	chr1	149764536	149764536	+	2.26435	NA	TTS (NM_000566)	TTS (NM_000566)	10286	NM_00056	2209	Hs.77424	NM_00056	ENSG0000	FCGR1A	CD64|CD6	Fc fragmer	protein-co
lib-C666-l	chr1	120925834	120925834	+	3.25502	NA	TTS (NM_001004340)	TTS (NM_001004340)	10110	NR_04521	2210	Hs.534956	NM_00100	ENSG0000	FCGR1B	CD64b|IGF	Fc fragmer	protein-co
lib-C666-l	chr3	195809040	195809040	+	112.75057	NA	promoter-TSS (NM_003234)	promoter-TSS (NM_003234)	-8	NM_00131	7037	Hs.529618	NM_00323	ENSG0000	TFRC	CD71|IMD	transferrin	protein-co
lib-C666-l	chr2	87035279	87035279	+	4.83287	NA	5' UTR (NM_001145873, exon 1 of 9)	5' UTR (NM_001145873, exon 1 of 9	240	NM_00114	925	Hs.85258	NM_00176	ENSG0000	CD8A	CD8|Leu2|I	CD8a mole	protein-co
lib-C666-l	chr19	55166597	55166597	+	6.15313	NA	Intergenic	Intergenic	-7674	NM_00127	11006	Hs.67846	NM_00684	ENSG0000	LILRB4	CD85K|ILT	leukocyte i	protein-co
lib-C666-l	chr19	44179667	44179667	+	21.99129	NA	Intergenic	Intergenic	-5169	NM_00265	5329	Hs.466871	NM_00265	ENSG0000	PLAUR	CD87|U-P/	plasminog	protein-co
lib-C666-l	chr15	43029346	43029346	+	14.75675	NA	promoter-TSS (NM_138477)	promoter-TSS (NM_138477)	71	NM_13847	146059	Hs.599232	NM_13847	ENSG0000	CDAN1	CDA1|CDA	codanin 1	protein-co
lib-C666-l	chrX	53111416	53111416	+	96.10687	NA	promoter-TSS (NM_022117)	promoter-TSS (NM_022117)	-126	NM_02211	64061	Hs.136164	NM_02211	ENSG0000	TSPYL2	CDA1|CIN/	TSPY like 2	protein-co
lib-C666-l	chr6	29691136	29691136	+	26.54591	NA	promoter-TSS (NM_018950).5	promoter-TSS (NM_018950).5	19	NM_00109	3134	Hs.519972	NM_01895	ENSG0000	HLA-F	CDA12|HL/	major histc	protein-co
lib-C666-l	chr12	29534188	29534188	+	23.087	NA	promoter-TSS (NM_016570)	promoter-TSS (NM_016570)	-45	NM_01657	51290	Hs.339453	NM_01657	ENSG0000	ERGIC2	CDA14|Erv	ERGIC and	protein-co
lib-C666-l	chr20	18488473	18488473	+	45.43086	NA	promoter-TSS (NM_001172745)	promoter-TSS (NM_001172745)	-134	NM_00117	10483	Hs.369373	NM_00636	ENSG0000	SEC23B	CDA-II|CD.	Sec23 hom	protein-co
lib-C666-l	chr20	18488277	18488277	+	30.0396	NA	promoter-TSS (NM_006363)	promoter-TSS (NM_006363)	67	NM_00117	10483	Hs.369373	NM_00636	ENSG0000	SEC23B	CDA-II|CD.	Sec23 hom	protein-co
lib-C666-l	chr20	18485470	18485470	+	8.51971	NA	Intergenic	CpG	-2718	NM_00636	10483	Hs.369373	NM_00636	ENSG0000	SEC23B	CDA-II|CD.	Sec23 hom	protein-co
lib-C666-l	chr7	35840530	35840530	+	23.087	NA	promoter-TSS (NM_001242956)	promoter-TSS (NM_001242956)	-66	NM_00101	989	Hs.191346	NM_00178	ENSG0000	7-Sep	CDC10|CD	septin 7	protein-co
lib-C666-l	chr10	38691854	38691854	+	69.18568	NA	promoter-TSS (NR_027269)	promoter-TSS (NR_027269)	-74	NR_02726	285961	Hs.406568	NR_027269		SEPT7P9	CDC10L|SE	septin 7 ps	pseudo
lib-C666-l	chr10	38692150	38692150	+	32.75483	NA	promoter-TSS (NR_027269)	promoter-TSS (NR_027269)	-370	NR_02726	285961	Hs.406568	NR_027269		SEPT7P9	CDC10L|SE	septin 7 ps	pseudo
lib-C666-l	chr7	48677188	48677188	+	6.98764	NA	intron (NM_152701, intron 59 of 61)	intron (NM_152701, intron 59 of 61	-286969	NR_00359	168448	Hs.567757	NM_152627		CDC14C	CDC14B2|(	cell divisior	pseudo
lib-C666-l	chr9	99382151	99382151	+	134.78979	NA	promoter-TSS (NM_033331)	promoter-TSS (NM_033331)	-39	NM_03333	8555	Hs.40582	NM_00367	ENSG0000	CDC14B	CDC14B3|(	cell divisior	protein-co
lib-C666-l	chr9	99329243	99329243	+	3.8557	NA	promoter-TSS (NM_001077181)	promoter-TSS (NM_001077181)	-40	NM_00107	8555	Hs.40582	NM_00367	ENSG0000	CDC14B	CDC14B3|(	cell divisior	protein-co
lib-C666-l	chr19	50896228	50896228	+	12.62235	NA	intron (NR_046402, intron 1 of 25)	L1MC4a|LINE|L1	-5881	NM_00130	5424	Hs.279413	NM_00269	ENSG0000	POLD1	CDC2|CRC	DNA polyn	protein-co
lib-C666-l	chr1	43815076	43815076	+	4.38153	NA	intron (NM_005373, intron 10 of 11)	CpG-1000	-9550	NM_00125	991	Hs.524947	NM_00125	ENSG0000	CDC20	CDC20A|b	cell divisior	protein-co
lib-C666-l	chr19	3506096	3506096	+	80.93561	NA	promoter-TSS (NM_016263)	promoter-TSS (NM_016263)	-199	NM_01626	51343	Hs.413133	NM_01626	ENSG0000	FZR1	CDC20C|C	fizzy/cell d	protein-co
lib-C666-l	chr8	48873406	48873406	+	11.20567	NA	promoter-TSS (NM_006904)	promoter-TSS (NM_006904)	643	NM_00591	4173	Hs.460184	NM_00591	ENSG0000	MCM4	CDC21|CD	minichrom	protein-co
lib-C666-l	chr3	48229958	48229958	+	4.61604	NA	promoter-TSS (NM_001789)	promoter-TSS (NM_001789)	-157	NM_00178	993	Hs.437705	NM_00178	ENSG0000	CDC25A	CDC25A2	cell divisior	protein-co
lib-C666-l	chr1	1655854	1655854	+	180.02109	NA	promoter-TSS (NM_033487)	promoter-TSS (NM_033487)	5	NM_00178	984	Hs.651228	NM_00178	ENSG0000	CDK11B	CDC2L1|C	cyclin depe	protein-co
lib-C666-l	chr6	111137181	111137181	+	10.70998	NA	promoter-TSS (NM_001300963)	promoter-TSS (NM_001300963)	-93	NM_00130	23097	Hs.744895	NM_01507	ENSG0000	CDK19	CDC2L6|C	cyclin depe	protein-co
lib-C666-l	chr9	33816902	33816902	+	6.29226	NA	promoter-TSS (NM_017811)	promoter-TSS (NM_017811)	-280	NM_01781	54926	Hs.643648	NM_01781	ENSG0000	UBE2R2	CDC34B|E2	ubiquitin c	protein-co
lib-C666-l	chr12	70636648	70636648	+	10.71733	NA	promoter-TSS (NM_001199302)	promoter-TSS (NM_001199302)	-126	NM_01451	4848	Hs.133350	NM_01451	ENSG0000	CNOT2	CDC36|HSI	CCR4-NOT	protein-co
lib-C666-l	chr9	4679577	4679577	+	46.59515	NA	promoter-TSS (NM_017913)	promoter-TSS (NM_017913)	24	NM_01791	55664	Hs.666439	NM_01791	ENSG0000	CDC37L1	CDC37B|H.	cell divisior	protein-co
lib-C666-l	chr11	46722160	46722160	+	2.26435	NA	promoter-TSS (NM_024741)	promoter-TSS (NM_024741)	55	NM_00430	392	Hs.138860	NM_00430	ENSG0000	ARHGAP1	CDC42GAF	Rho GTPas	protein-co
lib-C666-l	chr1	22379156	22379156	+	68.38644	NA	promoter-TSS (NM_001791)	promoter-TSS (NM_001791)	36	NM_00103	998	Hs.467637	NM_00179	ENSG0000	CDC42	CDC42Hs|(	cell divisior	protein-co
lib-C666-l	chr22	19467134	19467134	+	13.33918	NA	promoter-TSS (NM_001178010)	promoter-TSS (NM_001178010)	-215	NM_00117	8318	Hs.474217	NM_00350	ENSG0000	CDC45	CDC45L|C	cell divisior	protein-co
lib-C666-l	chr7	99698348	99698348	+	31.79085	NA	promoter-TSS (NM_182776)	promoter-TSS (NM_182776)	55	NM_18277	4176	Hs.438720	NM_00591	ENSG0000	MCM7	CDC47|MC	minichrom	protein-co
lib-C666-l	chr6	44355175	44355175	+	30.85659	NA	promoter-TSS (NM_001253)	promoter-TSS (NM_001253)	-76	NM_00125	988	Hs.485471	NM_00125	ENSG0000	CDC5L	CDC5|CDC	cell divisior	protein-co
lib-C666-l	chr2	242626294	242626294	+	32.29191	NA	promoter-TSS (NM_001165031)	promoter-TSS (NM_001165031)	89	NM_00132	1841	Hs.471873	NM_01214	ENSG0000	DTYMK	CDC8|PP3	deoxythym	protein-co
lib-C666-l	chr20	33265036	33265036	+	99.27254	NA	promoter-TSS (NM_080476)	promoter-TSS (NM_080476)	53	NM_08047	128869	Hs.253319	NM_08047	ENSG0000	PIGU	CDC91L1|(	phosphatic	protein-co
lib-C666-l	chr16	89989667	89989667	+	25.39487	NA	promoter-TSS (NM_006086)	promoter-TSS (NM_006086)	-20	NM_00608	10381	Hs.511743	NM_00608	ENSG0000	TUBB3	CDCBM|C	tubulin bet	protein-co
lib-C666-l	chr5	61601849	61601849	+	8.0787	NA	promoter-TSS (NM_004520)	promoter-TSS (NM_004520)	-140	NM_00124	3796	Hs.558351	NM_00452	ENSG0000	KIF2A	CDCBM3|I	kinesin far	protein-co
lib-C666-l	chr6	3157845	3157845	+	29.07066	NA	promoter-TSS (NM_001069)	promoter-TSS (NM_001069)	-62	NM_00106	7280	Hs.654543	NM_00106	ENSG0000	TUBB2A	CDCBM5|T	tubulin bet	protein-co
lib-C666-l	chr6	30689153	30689153	+	2.12162	NA	promoter-TSS (NM_001293212).7	promoter-TSS (NM_001293212).7	-173	NM_00129	203068	Hs.636480	NM_17801	ENSG0000	TUBB	CDCBM6|C	tubulin bet	protein-co
lib-C666-l	chr1	156076234	156076234	+	13.33696	NA	intron (NM_001282625, intron 3 of 12	intron (NM_001282625, intron 3 of	-8227	NM_00557	4000	Hs.594444	NM_00557	ENSG0000	LMNA	CDCD1|CD	lamin A/C	protein-co
lib-C666-l	chr1	156093647	156093647	+	8.28333	NA	intron (NM_001282625, intron 4 of 12	intron (NM_001282625, intron 4 of	-2261	NM_00128	4000	Hs.594444	NM_00557	ENSG0000	LMNA	CDCD1|CD	lamin A/C	protein-co
lib-C666-l	chr22	19705913	19705913	+	41.5993	NA	promoter-TSS (NR_037612)	promoter-TSS (NR_037612)	-45	NM_00100	5413	Hs.728762	NM_00268	ENSG0000	5-Sep	CDCREL|C	septin 5	protein-co
lib-C666-l	chr13	98795233	98795233	+	10.03103	NA	promoter-TSS (NM_001001715)	promoter-TSS (NM_001001715)	-119	NM_00100	10160	Hs.403917	NM_00576	ENSG0000	FARP1	CDEP|FARF	FERM, ARH	protein-co
lib-C666-l	chr22	30592310	30592310	+	20.7902	NA	Intergenic	Intergenic	50530	NM_00125	3976	Hs.2250	NM_00230	ENSG0000	LIF	CDF|DIA|H	leukemia ir	protein-co
lib-C666-l	chr22	30648774	30648774	+	15.1031	NA	Intergenic	Intergenic	-5934	NM_00230	3976	Hs.2250	NM_00230	ENSG0000	LIF	CDF|DIA|H	leukemia ir	protein-co
lib-C666-l	chr22	30592758	30592758	+	3.89034	NA	Intergenic	Intergenic	50082	NM_00125	3976	Hs.2250	NM_00230	ENSG0000	LIF	CDF|DIA|H	leukemia ir	protein-co
lib-C666-l	chr15	75182405	75182405	+	5.80117	NA	promoter-TSS (NM_002435)	promoter-TSS (NM_002435)	53	NM_00128	4351	Hs.75694	NM_00243	ENSG0000	MPI	CDG1B|PM	mannose p	protein-co
lib-C666-l	chr16	5134912	5134912	+	8.89416	NA	3' UTR (NM_019109, exon 13 of 13)	3' UTR (NM_019109, exon 13 of 13)	12663	NM_00133	56052	Hs.592086	NM_01910	ENSG0000	ALG1	CDG1K|HM	ALG1, chitc	protein-co
lib-C666-l	chr16	5121861	5121861	+	4.71278	NA	promoter-TSS (NM_019109)	promoter-TSS (NM_019109)	51	NM_01910	56052	Hs.592086	NM_01910	ENSG0000	ALG1	CDG1K|HM	ALG1, chitc	protein-co
lib-C666-l	chr3	53164464	53164464	+	76.14658	NA	promoter-TSS (NM_052859)	promoter-TSS (NM_052859)	6	NM_05285	91869	Hs.631910	NM_05285	ENSG0000	RFT1	CDG1N	RFT1 homc	protein-co
lib-C666-l	chr4	56212427	56212427	+	27.79032	NA	promoter-TSS (NM_024592)	promoter-TSS (NM_024592)	39	NM_02459	79644	Hs.39311	NM_02459	ENSG0000	SRD5A3	CDG1P|CD	steroid 5 a	protein-co
lib-C666-l	chr4	56212750	56212750	+	5.23609	NA	intron (NM_024592, intron 1 of 4)	CpG	362	NM_02459	79644	Hs.39311	NM_02459	ENSG0000	SRD5A3	CDG1P|CD	steroid 5 a	protein-co
lib-C666-l	chr4	56212985	56212985	+	2.59723	NA	intron (NM_024592, intron 1 of 4)	CpG	597	NM_02459	79644	Hs.39311	NM_02459	ENSG0000	SRD5A3	CDG1P|CD	steroid 5 a	protein-co
lib-C666-l	chr13	52586374	52586374	+	8.56376	NA	promoter-TSS (NM_001243182)	promoter-TSS (NM_001243182)	-149	NR_03657	440138	Hs.512963	NM_00100	ENSG0000	ALG11	CDG1P|GT	ALG11, alp	protein-co
lib-C666-l	chrX	110924409	110924409	+	23.74671	NA	promoter-TSS (NM_001324291)	promoter-TSS (NM_001324291)	11	NM_00132	79868	Hs.443061	NM_01846	ENSG0000	ALG13	CDG1S|CX	ALG13, UD	protein-co
lib-C666-l	chr3	31514435	31514435	+	14.98502	NA	Intergenic	THE1B|LTR|ERVL-MaLR	-59558	NM_17886	201595	Hs.475812	NM_17886	ENSG0000	STT3B	CDG1X|SIN	STT3B, cata	protein-co
lib-C666-l	chr3	31573919	31573919	+	8.01457	NA	promoter-TSS (NM_178862)	promoter-TSS (NM_178862)	-74	NM_17886	201595	Hs.475812	NM_17886	ENSG0000	STT3B	CDG1X|SIN	STT3B, cata	protein-co
lib-C666-l	chr3	31573710	31573710	+	3.25502	NA	promoter-TSS (NM_178862)	promoter-TSS (NM_178862)	-283	NM_17886	201595	Hs.475812	NM_17886	ENSG0000	STT3B	CDG1X|SIN	STT3B, cata	protein-co
lib-C666-l	chr16	23435043	23435043	+	15.66421	NA	intron (NM_153603, intron 7 of 16)	intron (NM_153603, intron 7 of 16)	29469	NM_15360	91949	Hs.185807	NM_15360	ENSG0000	COG7	CDG2E	componen	protein-co
lib-C666-l	chr6	88182506	88182506	+	10.03284	NA	promoter-TSS (NM_001168398)	promoter-TSS (NM_001168398)	-137	NM_00116	10559	Hs.423163	NM_00641	ENSG0000	SLC35A1	CDG2F|CM	solute carr	protein-co
lib-C666-l	chr4	56262041	56262041	+	18.39104	NA	promoter-TSS (NM_018475)	promoter-TSS (NM_018475)	-39	NR_07307	55858	Hs.479766	NM_01847	ENSG0000	TMEM165	CDG2K|FT2	transmeml	protein-co
lib-C666-l	chr4	56335167	56335167	+	3.92495	NA	intron (NM_004898, intron 8 of 22)	Tigger3b|DNA|TcMar-Tigger	73087	NM_01847	55858	Hs.479766	NM_01847	ENSG0000	TMEM165	CDG2K|FT2	transmeml	protein-co
lib-C666-l	chrX	48768976	48768976	+	9.4812	NA	5' UTR (NM_001282647, exon 1 of 5)	5' UTR (NM_001282647, exon 1 of 5	259	NM_00104	7355	Hs.21899	NM_00566	ENSG0000	SLC35A2	CDG2M|C	solute carr	protein-co
lib-C666-l	chr20	49575163	49575163	+	15.31489	NA	promoter-TSS (NM_003859)	promoter-TSS (NM_003859)	-62	NR_13364	8813	Hs.654951	NM_00385	ENSG0000	DPM1	CDGIE|MPI	dolichyl-pl	protein-co
lib-C666-l	chr17	7487068	7487068	+	2.59723	NA	promoter-TSS (NM_001322800)	promoter-TSS (NM_001322800)	103	NR_02460	9526	Hs.246381	NM_00487	ENSG0000	MPDU1	CDGIF|HBE	mannose-l	protein-co
lib-C666-l	chr5	19881095	19881095	+	25.96768	NA	intron (NM_001291957, intron 1 of 12	intron (NM_001291957, intron 1 of	5286	NM_00129	1016	Hs.317632	NM_00493	ENSG0000	CDH18	CDH14|CD	cadherin 1	protein-co
lib-C666-l	chr7	105650101	105650101	+	26.52079	NA	intron (NM_152750, intron 8 of 18)	CpG	46444	NM_15275	222256	Hs.150120	NM_15275	ENSG0000	CDHR3	CDH28	cadherin re	protein-co
lib-C666-l	chr4	187648004	187648004	+	95.13348	NA	Intergenic	CpG-20331	-3017	NM_00524	2195	Hs.481371	NM_00524	ENSG0000	FAT1	CDHF7|CD	FAT atypica	protein-co
lib-C666-l	chr4	187644968	187644968	+	3.13003	NA	promoter-TSS (NM_005245)	promoter-TSS (NM_005245)	19	NM_00524	2195	Hs.481371	NM_00524	ENSG0000	FAT1	CDHF7|CD	FAT atypica	protein-co
lib-C666-l	chr5	150997697	150997697	+	3.99607	NA	Intergenic	Intergenic	-49192	NM_00144	2196	Hs.591255	NM_00144	ENSG0000	FAT2	CDHF8|CD	FAT atypica	protein-co
lib-C666-l	chr12	7282975	7282975	+	30.07415	NA	promoter-TSS (NM_014718)	promoter-TSS (NM_014718)	8	NM_01471	9746	Hs.535378	NM_01471	ENSG0000	CLSTN3	CDHR14|C	calsyntenir	protein-co
lib-C666-l	chr14	54863604	54863604	+	12.26631	NA	promoter-TSS (NM_005192)	promoter-TSS (NM_005192)	18	NM_00133	1033	Hs.84113	NM_00519	ENSG0000	CDKN3	CDI1|CIP2|	cyclin depe	protein-co
lib-C666-l	chr9	22052859	22052859	+	2.00164	NA	intron (NR_120536, intron 3 of 3)	intron (NR_120536, intron 3 of 3)	-43547	NM_00493	1030	Hs.72901	NM_00493	ENSG0000	CDKN2B	CDK4I|INK	cyclin depe	protein-co
lib-C666-l	chr17	30810599	30810599	+	13.85062	NA	TTS (NM_001270482)	TTS (NM_001270482)	-3506	NM_00388	8851	Hs.500015	NM_00388	ENSG0000	CDK5R1	CDK5P35|(	cyclin depe	protein-co
lib-C666-l	chrX	48334506	48334506	+	53.56082	NA	promoter-TSS (NM_001282157)	promoter-TSS (NM_001282157)	97	NM_17743	24140	Hs.23170	NM_01228	ENSG0000	FTSJ1	CDLIV|JM2	FtsJ RNA m	protein-co
lib-C666-l	chr5	36877495	36877495	+	3.98718	NA	promoter-TSS (NR_046262)	promoter-TSS (NR_046262)	634	NM_01538	25836	Hs.481927	NM_01538	ENSG0000	NIPBL	CDLS|CDLS	NIPBL, coh	protein-co
lib-C666-l	chr7	134529209	134529209	+	10.03103	NA	intron (NM_033157, intron 2 of 14)	intron (NM_033157, intron 2 of 14)	-46942	NM_03314	800	Hs.490203	NM_00434	ENSG0000	CALD1	CDM|H-CA	caldesmon	protein-co
lib-C666-l	chr12	22697517	22697517	+	7.26874	NA	promoter-TSS (NM_001286177)	promoter-TSS (NM_001286177)	-37	NM_00128	9847	Hs.271014	NM_01480	ENSG0000	C2CD5	CDP138|KI	C2 calcium	protein-co
lib-C666-l	chr4	85503923	85503923	+	19.57279	NA	promoter-TSS (NM_001263)	promoter-TSS (NM_001263)	-134	NM_00126	1040	Hs.654899	NM_00126	ENSG0000	CDS1	CDS	CDP-diacy	protein-co
lib-C666-l	chr7	100209710	100209710	+	27.84631	NA	promoter-TSS (NM_023948)	promoter-TSS (NM_023948)	-15	NM_00104	64598	Hs.521086	NM_02394	ENSG0000	MOSPD3	CDS3|NET	motile sper	protein-co
lib-C666-l	chr1	212208645	212208645	+	3.20885	NA	promoter-TSS (NM_001286229)	promoter-TSS (NM_001286229)	-250	NM_00128	51514	Hs.656473	NM_01644	ENSG0000	DTL	CDT2|DCA	denticleles	protein-co
lib-C666-l	chr12	110562236	110562236	+	14.3056	NA	promoter-TSS (NM_031473)	promoter-TSS (NM_031473)	96	NR_14495	28981	Hs.528382	NM_01405	ENSG0000	IFT81	CDV-1|CD	intraflagell	protein-co
lib-C666-l	chr21	34641631	34641631	+	6.82479	NA	intron (NM_000628, intron 2 of 6)	intron (NM_000628, intron 2 of 6)	2966	NM_00062	3588	Hs.654593	NM_00062	ENSG0000	IL10RB	CDW210B|	interleukin	protein-co
lib-C666-l	chr19	48949253	48949253	+	20.90919	NA	5' UTR (NM_031485, exon 1 of 7)	5' UTR (NM_031485, exon 1 of 7)	223	NM_03148	83743	Hs.400625	NM_03148	ENSG0000	GRWD1	CDW4|GRV	glutamate	protein-co
lib-C666-l	chr4	47916603	47916603	+	3.96379	NA	promoter-TSS (NM_152995)	promoter-TSS (NM_152995)	-29	NM_00127	152518	Hs.646325	NM_15299	ENSG0000	NFXL1	CDZFP|HO	nuclear tra	protein-co
lib-C666-l	chr19	45225570	45225570	+	8.71138	NA	Intergenic	Intergenic	23149	NM_00103	388551	Hs.456381	NM_00103	ENSG0000	CEACAM16	CEAL2|DFN	carcinoeml	protein-co
lib-C666-l	chr1	7764696	7764696	+	101.26558	NA	intron (NM_015215, intron 11 of 22)	CpG	-66633	NM_00478	9341	Hs.66708	NM_00478	ENSG0000	VAMP3	CEB	vesicle ass	protein-co
lib-C666-l	chr1	7831439	7831439	+	43.37893	NA	5' UTR (NM_004781, exon 1 of 5)	5' UTR (NM_004781, exon 1 of 5)	110	NM_00478	9341	Hs.66708	NM_00478	ENSG0000	VAMP3	CEB	vesicle ass	protein-co
lib-C666-l	chr1	7729723	7729723	+	9.79436	NA	intron (NM_015215, intron 9 of 22)	intron (NM_015215, intron 9 of 22)	-101606	NM_00478	9341	Hs.66708	NM_00478	ENSG0000	VAMP3	CEB	vesicle ass	protein-co
lib-C666-l	chr1	7462153	7462153	+	6.82479	NA	intron (NM_015215, intron 5 of 22)	intron (NM_015215, intron 5 of 22)	-369176	NM_00478	9341	Hs.66708	NM_00478	ENSG0000	VAMP3	CEB	vesicle ass	protein-co
lib-C666-l	chr1	7531143	7531143	+	3.98718	NA	intron (NM_015215, intron 6 of 22)	intron (NM_015215, intron 6 of 22)	-300186	NM_00478	9341	Hs.66708	NM_00478	ENSG0000	VAMP3	CEB	vesicle ass	protein-co
lib-C666-l	chr12	57914409	57914409	+	45.23877	NA	promoter-TSS (NM_004083)	promoter-TSS (NM_004083)	-109	NM_00408	1649	Hs.505777	NM_00408	ENSG0000	DDIT3	CEBPZ|CH	DNA dama	protein-co
lib-C666-l	chr22	17387083	17387083	+	11.5173	NA	Intergenic	Intergenic	78719	NR_00360	27437	Hs.143955	NR_00360	ENSG0000	HSFY1P1	CECR8|HSF	heat shock	pseudo
lib-C666-l	chr19	41859990	41859990	+	13.91591	NA	promoter-TSS (NM_000660)	promoter-TSS (NM_000660)	-202	NM_00066	7040	Hs.645227	NM_00066	ENSG0000	TGFB1	CED|DPD1	transformi	protein-co
lib-C666-l	chr22	21213222	21213222	+	13.59254	NA	promoter-TSS (NM_058004)	promoter-TSS (NM_058004)	-70	NM_00478	9342	Hs.108002	NM_00478	ENSG0000	SNAP29	CEDNIK|SN	synaptoso	protein-co
lib-C666-l	chr9	131183572	131183572	+	3.38731	NA	intron (NM_016174, intron 1 of 12)	intron (NM_016174, intron 1 of 12)	813	NM_01617	51148	Hs.495230	NM_01617	ENSG0000	CERCAM	CEECAM1|	cerebral er	protein-co
lib-C666-l	chr6	35150054	35150054	+	6.32351	NA	Intergenic	CpG	-31785	NM_15275	222663	Hs.12923	NM_15275	ENSG0000	SCUBE3	CEGF3	signal pept	protein-co
lib-C666-l	chr8	27351681	27351681	+	3.25502	NA	intron (NM_001256483, intron 1 of 17	HERV9-int|LTR|ERV1	2996	NM_00125	2053	Hs.212088	NM_00197	ENSG0000	EPHX2	CEH|SEH	epoxide hy	protein-co
lib-C666-l	chr6	32605115	32605115	+	11.57178	NA	promoter-TSS (NM_002122)	promoter-TSS (NM_002122)	-68	NM_00212	3117	Hs.387679	NM_00212	ENSG0000	HLA-DQA1	CELIAC1|D	major histc	protein-co
lib-C666-l	chr6	32636299	32636299	+	38.21529	NA	Intergenic	Intergenic	-1833	NM_00212	3119	Hs.409934	NM_00212	ENSG0000	HLA-DQB1	CELIAC1|H	major histc	protein-co
lib-C666-l	chr6	32634519	32634519	+	15.42216	NA	promoter-TSS (NM_002123)	promoter-TSS (NM_002123)	-53	NM_00212	3119	Hs.409934	NM_00212	ENSG0000	HLA-DQB1	CELIAC1|H	major histc	protein-co
lib-C666-l	chr22	50965224	50965224	+	5.12426	NA	promoter-TSS (NM_001169110)	promoter-TSS (NM_001169110)	-356	NM_00116	9997	Hs.180903	NM_00513	ENSG0000	SCO2	CEMCOX1	SCO2, cytc	protein-co
lib-C666-l	chr22	50964984	50964984	+	3.89624	NA	promoter-TSS (NM_005138)	promoter-TSS (NM_005138)	-116	NM_00116	9997	Hs.180903	NM_00513	ENSG0000	SCO2	CEMCOX1	SCO2, cytc	protein-co
lib-C666-l	chr1	224301819	224301819	+	16.63918	NA	promoter-TSS (NM_001136115)	promoter-TSS (NM_001136115)	30	NM_00113	23219	Hs.64691	NM_01517	ENSG0000	FBXO28	CENP-30|F	F-box prot	protein-co
lib-C666-l	chr20	4850331	4850331	+	2.99475	NA	intron (NM_203327, intron 12 of 16)	intron (NM_203327, intron 12 of 16	-46040	NM_01473	9770	Hs.631504	NM_01473	ENSG0000	RASSF2	CENP-34|F	Ras associa	protein-co
lib-C666-l	chr4	185655416	185655416	+	11.6145	NA	promoter-TSS (NR_104593)	promoter-TSS (NR_104593)	-130	NM_02462	79682	Hs.575032	NM_02462	ENSG0000	CENPU	CENP50|C	centromere	protein-co
lib-C666-l	chr2	27008748	27008748	+	11.36743	NA	promoter-TSS (NM_001809)	promoter-TSS (NM_001809)	-134	NM_00180	1058	Hs.1594	NM_00180	ENSG0000	CENPA	CENP-A|C	centromere	protein-co
lib-C666-l	chr4	68410841	68410841	+	9.89412	NA	intron (NM_001812, intron 1 of 18)	intron (NM_001812, intron 1 of 18)	415	NM_00181	1060	Hs.479867	NM_00181	ENSG0000	CENPC	CENP-C|C	centromere	protein-co
lib-C666-l	chr4	104119699	104119699	+	67.49158	NA	promoter-TSS (NM_001813)	promoter-TSS (NM_001813)	-133	NM_00128	1062	Hs.75573	NM_00181	ENSG0000	CENPE	CENP-E|KI	centromere	protein-co
lib-C666-l	chrX	100353037	100353037	+	12.78696	NA	promoter-TSS (NM_001318521)	promoter-TSS (NM_001318521)	-134	NM_00131	2491	Hs.348920	NM_00673	ENSG0000	CENPI	CENP-I|FSI	centromere	protein-co
lib-C666-l	chr17	79961652	79961652	+	17.13943	NA	intron (NM_001330528, intron 6 of 14	CpG	19133	NM_00127	201254	Hs.37616	NM_14499	ENSG0000	CENPX	CENP-X|D	centromere	protein-co
lib-C666-l	chr7	941661	941661	+	10.86006	NA	intron (NM_001284308, intron 6 of 10	intron (NM_001284308, intron 6 of	18865	NM_00128	11033	Hs.602573	NM_00686	ENSG0000	ADAP1	CENTA1|G	ArfGAP wit	protein-co
lib-C666-l	chr7	953996	953996	+	4.38153	NA	intron (NM_001284308, intron 4 of 10	intron (NM_001284308, intron 4 of	6530	NM_00128	11033	Hs.602573	NM_00686	ENSG0000	ADAP1	CENTA1|G	ArfGAP wit	protein-co
lib-C666-l	chr3	195163965	195163965	+	3.98718	NA	promoter-TSS (NM_012287)	promoter-TSS (NM_012287)	-148	NM_01228	23527	Hs.593373	NM_01228	ENSG0000	ACAP2	CENTB2|C	ArfGAP wit	protein-co
lib-C666-l	chr1	1243504	1243504	+	17.22931	NA	promoter-TSS (NM_030649)	promoter-TSS (NM_030649)	-235	NM_03064	116983	Hs.535257	NM_03064	ENSG0000	ACAP3	CENTB5	ArfGAP wit	protein-co
lib-C666-l	chr11	72433607	72433607	+	143.31969	NA	promoter-TSS (NM_015242)	promoter-TSS (NM_015242)	-204	NM_00113	116985	Hs.503165	NM_01524	ENSG0000	ARAP1	CENTD2	ArfGAP wit	protein-co
lib-C666-l	chr11	72429360	72429360	+	9.4812	NA	intron (NM_001040118, intron 3 of 34	intron (NM_001040118, intron 3 of	4043	NM_01524	116985	Hs.503165	NM_01524	ENSG0000	ARAP1	CENTD2	ArfGAP wit	protein-co
lib-C666-l	chr4	56814738	56814738	+	2.58854	NA	promoter-TSS (NM_025009)	promoter-TSS (NM_025009)	-236	NM_02500	9662	Hs.518767	NM_01464	ENSG0000	CEP135	CEP4|KIAA	centrosom	protein-co
lib-C666-l	chr2	99757528	99757528	+	7.97818	NA	promoter-TSS (NM_001317992)	promoter-TSS (NM_001317992)	509	NM_18291	80705	Hs.120267	NM_02524	ENSG0000	TSGA10	CEP4L|CT7	testis speci	protein-co
lib-C666-l	chr19	18682576	18682576	+	73.77308	NA	promoter-TSS (NM_001321017)	promoter-TSS (NM_001321017)	36	NM_00132	7311	Hs.5308	NM_00333	ENSG0000	UBA52	CEP52|HUE	ubiquitin A	protein-co
lib-C666-l	chr7	1116055	1116055	+	23.12593	NA	intron (NM_032350, intron 2 of 4)	intron (NM_032350, intron 2 of 4)	-10388	NM_00103	2852	Hs.20961	NM_00150	ENSG0000	GPER1	CEPR|CMK	G protein-	protein-co
lib-C666-l	chr7	1115775	1115775	+	10.79304	NA	intron (NM_032350, intron 2 of 4)	intron (NM_032350, intron 2 of 4)	-10668	NM_00103	2852	Hs.20961	NM_00150	ENSG0000	GPER1	CEPR|CMK	G protein-	protein-co
lib-C666-l	chr17	7165988	7165988	+	45.24737	NA	5' UTR (NM_001307, exon 1 of 4)	5' UTR (NM_001307, exon 1 of 4)	276	NM_00118	1366	Hs.513915	NM_00130	ENSG0000	CLDN7	CEPTRL2|C	claudin 7	protein-co
lib-C666-l	chr5	74807738	74807738	+	7.76438	NA	promoter-TSS (NM_005713)	promoter-TSS (NM_005713)	68	NM_00113	10087	Hs.270437	NM_00571	ENSG0000	COL4A3BP	CERT|CERT	collagen ty	protein-co
lib-C666-l	chr19	13030182	13030182	+	16.63918	NA	promoter-TSS (NM_001105578)	promoter-TSS (NM_001105578)	-96	NM_00110	256126	Hs.655173	NM_00110	ENSG0000	SYCE2	CESC1	synaptone	protein-co
lib-C666-l	chr5	139043996	139043996	+	22.40513	NA	intron (NM_001317207, intron 1 of 2)	intron (NM_001317207, intron 1 of	4856	NM_00131	51523	Hs.189119	NM_01646	ENSG0000	CXXC5	CF5|HSPC1	CXXC finge	protein-co
lib-C666-l	chr5	139047851	139047851	+	4.96122	NA	intron (NM_001317203, intron 2 of 3)	intron (NM_001317203, intron 2 of	-7174	NM_00131	51523	Hs.189119	NM_01646	ENSG0000	CXXC5	CF5|HSPC1	CXXC finge	protein-co
lib-C666-l	chr6	30034999	30034999	+	3.8557	NA	promoter-TSS (NM_021959).2	promoter-TSS (NM_021959).2	67	NM_02195	6992	Hs.82887	NM_02195	ENSG0000	PPP1R11	CFAP255|I	protein ph	protein-co
lib-C666-l	chr9	139760857	139760857	+	92.98441	NA	promoter-TSS (NM_153200)	promoter-TSS (NM_153200)	-69	NM_00379	8721	Hs.174050	NM_00379	ENSG0000	EDF1	CFAP280|E	endothelia	protein-co
lib-C666-l	chr19	17530748	17530748	+	20.0808	NA	promoter-TSS (NM_138401)	promoter-TSS (NM_138401)	92	NM_13840	93343	Hs.515243	NM_13840	ENSG0000	MVB12A	CFBP|FAM	multivesicu	protein-co
lib-C666-l	chr15	66679082	66679082	+	167.6989	NA	promoter-TSS (NM_002755)	promoter-TSS (NM_002755)	-129	NM_00275	5604	Hs.145442	NM_00275	ENSG0000	MAP2K1	CFC3|MAP	mitogen-a	protein-co
lib-C666-l	chr2	209130791	209130791	+	36.59122	NA	promoter-TSS (NM_152671)	promoter-TSS (NM_152671)	-200	NM_15267	200576	Hs.744997	NM_01504	ENSG0000	PIKFYVE	CFD|FAB1|I	phosphoin	protein-co
lib-C666-l	chr2	209159606	209159606	+	11.36743	NA	intron (NM_001178000, intron 7 of 11	L1MCa|LINE|L1	28615	NM_00117	200576	Hs.744997	NM_01504	ENSG0000	PIKFYVE	CFD|FAB1|I	phosphoin	protein-co
lib-C666-l	chr16	1832804	1832804	+	124.67059	NA	promoter-TSS (NR_104318)	promoter-TSS (NR_104318)	-120	NM_01222	10101	Hs.256549	NM_01222	ENSG0000	NUBP2	CFD1|NBP	nucleotide	protein-co
lib-C666-l	chrX	153237404	153237404	+	85.71884	NA	promoter-TSS (NM_005334)	promoter-TSS (NM_005334)	-585	NM_00533	3054	Hs.83634	NM_00533	ENSG0000	HCFC1	CFF|HCF|H	host cell fa	protein-co
lib-C666-l	chr12	69633341	69633341	+	37.64901	NA	promoter-TSS (NM_001300947)	promoter-TSS (NM_001300947)	24	NM_00130	11052	Hs.369606	NM_00700	ENSG0000	CPSF6	CFIM|CFIM	cleavage a	protein-co
lib-C666-l	chr11	61197467	61197467	+	13.06669	NA	promoter-TSS (NM_024811)	promoter-TSS (NM_024811)	-3	NM_00113	79869	Hs.718984	NM_02481	ENSG0000	CPSF7	CFIm59	cleavage a	protein-co
lib-C666-l	chr9	112117866	112117866	+	16.14038	NA	Intergenic	Intergenic	-34622	NM_01911	54566	Hs.591901	NM_01842	ENSG0000	EPB41L4B	CG1|EHM2	erythrocyte	protein-co
lib-C666-l	chr9	112083419	112083419	+	14.37873	NA	promoter-TSS (NM_019114)	promoter-TSS (NM_019114)	-175	NM_01911	54566	Hs.591901	NM_01842	ENSG0000	EPB41L4B	CG1|EHM2	erythrocyte	protein-co
lib-C666-l	chr7	23221586	23221586	+	168.26447	NA	5' UTR (NM_007342, exon 1 of 7)	5' UTR (NM_007342, exon 1 of 7)	140	NM_00734	11097	Hs.408241	NM_00734	ENSG0000	NUPL2	CG1|NLP-1	nucleopori	protein-co
lib-C666-l	chr7	100858132	100858132	+	4.73866	NA	intron (NM_001084, intron 6 of 18)	MIRb|SINE|MIR	-2853	NM_00634	10467	Hs.211079	NM_00634	ENSG0000	ZNHIT1	CG1I|ZNFN	zinc finger	protein-co
lib-C666-l	chr19	36611124	36611124	+	9.4812	NA	intron (NM_001300971, intron 2 of 5)	L2a|LINE|L2	4394	NM_00130	1155	Hs.31053	NM_00128	ENSG0000	TBCB	CG22|CKAI	tubulin folc	protein-co
lib-C666-l	chr19	36618632	36618632	+	5.64971	NA	Intergenic	Intergenic	11902	NM_00130	1155	Hs.31053	NM_00128	ENSG0000	TBCB	CG22|CKAI	tubulin folc	protein-co
lib-C666-l	chr10	43745441	43745441	+	15.22699	NA	intron (NM_145313, intron 1 of 12)	intron (NM_145313, intron 1 of 12)	16926	NM_14531	221002	Hs.125293	NM_14531	ENSG0000	RASGEF1A	CG4853	RasGEF do	protein-co
lib-C666-l	chr9	37954231	37954231	+	8.56376	NA	intron (NM_003028, intron 4 of 5)	intron (NM_003028, intron 4 of 5)	-49881	NR_02487	92014	Hs.634120	NM_03341	ENSG0000	SLC25A51	CG7943|M	solute carr	protein-co
lib-C666-l	chr9	37903883	37903883	+	5.66964	NA	intron (NR_024872, intron 1 of 4)	CpG	467	NM_03341	92014	Hs.634120	NM_03341	ENSG0000	SLC25A51	CG7943|M	solute carr	protein-co
lib-C666-l	chr9	37904166	37904166	+	4.98754	NA	non-coding (NR_024872, exon 1 of 5	non-coding (NR_024872, exon 1 of	184	NM_03341	92014	Hs.634120	NM_03341	ENSG0000	SLC25A51	CG7943|M	solute carr	protein-co
lib-C666-l	chr14	93390787	93390787	+	12.12504	NA	intron (NM_001275, intron 2 of 7)	intron (NM_001275, intron 2 of 7)	1342	NM_00127	1113	Hs.150793	NM_00127	ENSG0000	CHGA	CGA	chromogra	protein-co
lib-C666-l	chr19	49521082	49521082	+	5.9194	NA	promoter-TSS (NM_000894)	promoter-TSS (NM_000894)	-735	NM_00089	3972	Hs.154704	NM_00089	ENSG0000	LHB	CGB4|HH2	luteinizing	protein-co

lib-C666-l	chr19	49521391	49521391	+	5.23609	NA	TTS (NR_073552)	TTS (NR_073552)	-1044	NM_00089	3972	Hs.154704	NM_00089	ENSG0000	LHB	CGB4|HH2	luteinizing	protein-co
lib-C666-l	chr4	120550139	120550139	+	11.66012	NA	promoter-TSS (NM_001083)	promoter-TSS (NM_001083)	-158	NM_00108	8654	Hs.647971	NM_00108	ENSG0000	PDE5A	CGB-PDE|(	phosphodi	protein-co
lib-C666-l	chr22	37269801	37269801	+	7.95564	NA	intron (NM_000631, intron 7 of 9)	MER20|DNA|hAT-Charlie	12771	NM_00063	4689	Hs.474781	NM_00063	ENSG0000	NCF4	CGD3|NCF	neutrophil	protein-co
lib-C666-l	chr3	88108156	88108156	+	9.20967	NA	promoter-TSS (NM_003663)	promoter-TSS (NM_003663)	-10	NM_00100	8545	Hs.444818	NM_00366	ENSG0000	CGGBP1	CGGBP|p2(	CGG triplet	protein-co
lib-C666-l	chr1	51425987	51425987	+	130.74927	NA	promoter-TSS (NM_007051)	promoter-TSS (NM_007051)	-51	NM_00705	11124	Hs.530402	NM_00705	ENSG0000	FAF1	CGI-03|HF.	Fas associa	protein-co
lib-C666-l	chr12	32908952	32908952	+	2.05265	NA	promoter-TSS (NM_001040436)	promoter-TSS (NM_001040436)	-65	NM_00104	51067	Hs.505231	NM_01593	ENSG0000	YARS2	CGI-04|ML	tyrosyl-tRN	protein-co
lib-C666-l	chr20	44044734	44044734	+	3.93123	NA	promoter-TSS (NM_001184728)	promoter-TSS (NM_001184728)	27	NR_04769	51604	Hs.437388	NM_01593	ENSG0000	PIGT	CGI-06|MC	phosphatic	protein-co
lib-C666-l	chr3	160939104	160939104	+	12.12723	NA	promoter-TSS (NM_001320227)	promoter-TSS (NM_001320227)	26	NM_00132	51068	Hs.598836	NM_01593	ENSG0000	NMD3	CGI-07	NMD3 ribo	protein-co
lib-C666-l	chr20	5931249	5931249	+	11.77301	NA	promoter-TSS (NM_015939)	promoter-TSS (NM_015939)	-45	NM_00128	51605	Hs.128791	NM_01593	ENSG0000	TRMT6	CGI-09|GC	tRNA meth	protein-co
lib-C666-l	chr14	74416989	74416989	+	7.77438	NA	promoter-TSS (NM_182476)	promoter-TSS (NM_182476)	34	NM_18247	51004	Hs.131555	NM_18247	ENSG0000	COQ6	CGI-10|CG	coenzyme	protein-co
lib-C666-l	chr1	93646159	93646159	+	7.43067	NA	promoter-TSS (NM_001167830)	promoter-TSS (NM_001167830)	87	NM_00116	50999	Hs.482873	NM_01604	ENSG0000	TMED5	CGI-100|p	transmemb	protein-co
lib-C666-l	chr9	37784883	37784883	+	6.49644	NA	exon (NM_016042, exon 1 of 4)	exon (NM_016042, exon 1 of 4)	206	NM_00100	51010	Hs.602571	NM_01604	ENSG0000	EXOSC3	CGI-102|P	exosome c	protein-co
lib-C666-l	chr11	118889246	118889246	+	15.30356	NA	promoter-TSS (NM_001028)	promoter-TSS (NM_001028)	67	NM_00131	51399	Hs.132760	NM_01614	ENSG0000	TRAPPC4	CGI-104|H	trafficking	protein-co
lib-C666-l	chr2	96068494	96068494	+	75.47253	NA	promoter-TSS (NM_016044)	promoter-TSS (NM_016044)	46	NM_01604	51011	Hs.531247	NM_01604	ENSG0000	FAHD2A	CGI-105	fumarylace	protein-co
lib-C666-l	chr5	176730792	176730792	+	10.16129	NA	promoter-TSS (NM_001271828)	promoter-TSS (NM_001271828)	29	NM_01323	27166	Hs.744904	NM_01323	ENSG0000	PRELID1	CGI-106|PI	PRELI dom	protein-co
lib-C666-l	chr5	114961863	114961863	+	10.40942	NA	promoter-TSS (NM_181836)	promoter-TSS (NM_181836)	13	NM_18183	51014	Hs.642817	NM_18183	ENSG0000	TMED7	CGI-109|p	transmemb	protein-co
lib-C666-l	chr8	54755628	54755628	+	5.04179	NA	promoter-TSS (NM_213620)	promoter-TSS (NM_213620)	-26	NM_21362	51606	Hs.491737	NM_01594	ENSG0000	ATP6V1H	CGI-11|MS	ATPase H+	protein-co
lib-C666-l	chr2	24299277	24299277	+	61.35856	NA	promoter-TSS (NM_001291328)	promoter-TSS (NM_001291328)	37	NM_01604	51639	Hs.177861	NM_01604	ENSG0000	SF3B6	CGI-110|H	splicing fac	protein-co
lib-C666-l	chr11	73490397	73490397	+	9.14689	NA	Intergenic	Intergenic	-8520	NM_01605	51642	Hs.503239	NM_01605	ENSG0000	MRPL48	CGI-118|H	mitochond	protein-co
lib-C666-l	chr11	73490788	73490788	+	5.80117	NA	Intergenic	CpG	-8129	NM_01605	51642	Hs.503239	NM_01605	ENSG0000	MRPL48	CGI-118|H	mitochond	protein-co
lib-C666-l	chr12	54718892	54718892	+	7.97818	NA	promoter-TSS (NR_073424)	promoter-TSS (NR_073424)	18	NM_00127	22818	Hs.505652	NM_01605	ENSG0000	COPZ1	CGI-120|C	coatomer p	protein-co
lib-C666-l	chr1	51701961	51701961	+	34.12565	NA	promoter-TSS (NM_014372)	promoter-TSS (NM_014372)	16	NM_01437	26994	Hs.309641	NM_01437	ENSG0000	RNF11	CGI-123|SI	ring finger	protein-co
lib-C666-l	chr2	30369339	30369339	+	15.42216	NA	promoter-TSS (NM_016061)	promoter-TSS (NM_016061)	-411	NM_00112	51646	Hs.515890	NM_01606	ENSG0000	YPEL5	CGI-127	yippee like	protein-co
lib-C666-l	chr17	55927487	55927487	+	13.14211	NA	promoter-TSS (NM_016070)	promoter-TSS (NM_016070)	-54	NM_01607	51649	Hs.5836	NM_01607	ENSG0000	MRPS23	CGI-138|H	mitochond	protein-co
lib-C666-l	chr7	140714706	140714706	+	6.38282	NA	promoter-TSS (NM_016071)	promoter-TSS (NM_016071)	75	NM_05303	51650	Hs.416207	NM_01607	ENSG0000	MRPS33	CGI-139|M	mitochond	protein-co
lib-C666-l	chr12	21654654	21654654	+	5.10953	NA	promoter-TSS (NM_032941)	promoter-TSS (NM_032941)	-45	NM_01607	51026	Hs.62275	NM_01607	ENSG0000	GOLT1B	CGI-141|G	golgi trans	protein-co
lib-C666-l	chr1	149871347	149871347	+	19.18403	NA	intron (NM_001321026, intron 1 of 1)	CpG	212	NM_00132	51027	Hs.13880	NM_01607	ENSG0000	BOLA1	CGI-143	bolA family	protein-co
lib-C666-l	chr2	86790313	86790313	+	8.73558	NA	intron (NM_001198954, intron 3 of 7)	CpG	307	NM_01607	51652	Hs.591582	NM_01607	ENSG0000	CHMP3	CGI-149|N	charged m	protein-co
lib-C666-l	chr3	179322476	179322476	+	27.5466	NA	promoter-TSS (NM_001199957)	promoter-TSS (NM_001199957)	-42	NM_02040	57129	Hs.283734	NM_02040	ENSG0000	MRPL47	CGI-204|L	mitochond	protein-co
lib-C666-l	chr6	43027205	43027205	+	129.07648	NA	promoter-TSS (NM_001300848)	promoter-TSS (NM_001300848)	78	NM_01595	51069	Hs.55041	NM_01595	ENSG0000	MRPL2	CGI-22|MF	mitochond	protein-co
lib-C666-l	chr6	116892294	116892294	+	4.30593	NA	promoter-TSS (NM_015952)	promoter-TSS (NM_015952)	-236	NM_01610	51389	Hs.532164	NM_01595	ENSG0000	RWDD1	CGI-24|PT	RWD doma	protein-co
lib-C666-l	chr19	10362848	10362848	+	209.67952	NA	5' UTR (NM_015956, exon 1 of 9)	5' UTR (NM_015956, exon 1 of 9)	208	NM_01595	51073	Hs.279652	NM_01595	ENSG0000	MRPL4	CGI-28|L4r	mitochond	protein-co
lib-C666-l	chr14	55738832	55738832	+	279.23224	NA	promoter-TSS (NM_152231)	promoter-TSS (NM_152231)	-40	NM_15223	55030	Hs.525348	NM_01794	ENSG0000	FBXO34	CGI-301|Fb	F-box prot	protein-co
lib-C666-l	chr14	55738048	55738048	+	144.07582	NA	promoter-TSS (NM_017943)	promoter-TSS (NM_017943)	27	NM_01794	55030	Hs.525348	NM_01794	ENSG0000	FBXO34	CGI-301|Fb	F-box prot	protein-co
lib-C666-l	chr10	101491892	101491892	+	4.38153	NA	promoter-TSS (NM_015960)	promoter-TSS (NM_015960)	-66	NM_01596	51076	Hs.16606	NM_01596	ENSG0000	CUTC	CGI-32	cutC coppe	protein-co
lib-C666-l	chr2	69664640	69664640	+	2.42134	NA	non-coding (NR_045631, exon 1 of 7	non-coding (NR_045631, exon 1 of	120	NM_00100	27247	Hs.430439	NM_01570	ENSG0000	NFU1	CGI-33|HIF	NFU1 iron-	protein-co
lib-C666-l	chr16	69373412	69373412	+	7.09685	NA	promoter-TSS (NM_001199434)	promoter-TSS (NM_001199434)	-3	NM_01610	51388	Hs.585728	NM_01610	ENSG0000	NIP7	CGI-37|HS	NIP7, nucle	protein-co
lib-C666-l	chr11	117049449	117049449	+	7.49163	NA	promoter-TSS (NM_001040455)	promoter-TSS (NM_001040455)	-490	NM_00104	51092	Hs.712144	NM_01599	ENSG0000	SIDT2	CGI-40	SID1 transr	protein-co
lib-C666-l	chr15	45926983	45926983	+	5.6488	NA	promoter-TSS (NM_021199)	promoter-TSS (NM_021199)	45	NM_02119	58472	Hs.511251	NM_02119	ENSG0000	SQRDL	CGI-44|PR	sulfide qui	protein-co
lib-C666-l	chr11	72504946	72504946	+	25.10918	NA	promoter-TSS (NM_006645)	promoter-TSS (NM_006645)	-196	NM_00664	10809	Hs.188606	NM_00664	ENSG0000	STARD10	CGI-52|NY	StAR relate	protein-co
lib-C666-l	chr1	67896716	67896716	+	2.00164	NA	promoter-TSS (NM_015640)	promoter-TSS (NM_015640)	-593	NM_01564	26135	Hs.530412	NM_01564	ENSG0000	SERBP1	CGI-55|CH	SERPINE1	protein-co
lib-C666-l	chr7	139044586	139044586	+	6.4187	NA	promoter-TSS (NM_001270643)	promoter-TSS (NM_001270643)	-6	NM_01601	51631	Hs.731488	NM_01601	ENSG0000	LUC7L2	CGI-59|CG	LUC7 like 2	protein-co
lib-C666-l	chr2	44001293	44001293	+	15.31489	NA	intron (NM_001193464, intron 1 of 1	intron (NM_001193464, intron 1 of	115	NM_00119	51626	Hs.371597	NM_01552	ENSG0000	DYNC2LI1	CGI-60|D2	dynein cyt	protein-co
lib-C666-l	chr1	29557457	29557457	+	133.88091	NA	promoter-TSS (NM_001024732)	promoter-TSS (NM_001024732)	13	NM_00102	51102	Hs.183646	NM_01601	ENSG0000	MECR	CGI-63|FA	mitochond	protein-co
lib-C666-l	chr6	36954106	36954106	+	20.59244	NA	non-coding (NR_130739, exon 1 of 1	non-coding (NR_130739, exon 1 of	221	NR_13073	23787	Hs.485262	NM_01434	ENSG0000	MTCH1	CGI-64|PI	mitochond	protein-co
lib-C666-l	chr2	231577519	231577519	+	5.98967	NA	promoter-TSS (NM_016289)	promoter-TSS (NM_016289)	-38	NM_01628	51719	Hs.603930	NM_01628	ENSG0000	CAB39	CGI-66|MC	calcium bir	protein-co
lib-C666-l	chr16	25123045	25123045	+	45.65358	NA	promoter-TSS (NR_120306)	promoter-TSS (NR_120306)	-2	NM_00103	51451	Hs.337730	NM_01630	ENSG0000	LCMT1	CGI-68|LC	leucine car	protein-co
lib-C666-l	chr17	42404390	42404390	+	12.7393	NA	Intergenic	Intergenic	-2152	NM_00132	51629	Hs.744880	NM_01601	ENSG0000	SLC25A39	CGI-69|CG	solute carr	protein-co
lib-C666-l	chr8	133787590	133787590	+	142.87477	NA	promoter-TSS (NM_198513)	promoter-TSS (NM_198513)	-14	NM_01601	51105	Hs.304362	NM_01601	ENSG0000	PHF20L1	CGI-72|TD	PHD finger	protein-co
lib-C666-l	chr16	21610816	21610816	+	12.13776	NA	promoter-TSS (NR_110930)	promoter-TSS (NR_110930)	19	NM_00107	51108	Hs.279583	NM_01602	ENSG0000	METTL9	CGI-81|DR	methyltran	protein-co
lib-C666-l	chr11	67981312	67981312	+	10.79304	NA	promoter-TSS (NM_001300907)	promoter-TSS (NM_001300907)	-73	NM_00130	51111	Hs.632120	NM_01602	ENSG0000	KMT5B	CGI-85|CG	lysine meth	protein-co
lib-C666-l	chr1	43833795	43833795	+	11.66012	NA	promoter-TSS (NM_001256399)	promoter-TSS (NM_001256399)	-50	NM_00125	64834	Hs.25597	NM_02282	ENSG0000	ELOVL1	CGI-88|Ssc	ELOVL fatt	protein-co
lib-C666-l	chrX	128977378	128977378	+	27.61921	NA	5' UTR (NM_001008222, exon 1 of 10	5' UTR (NM_001008222, exon 1 of 1	183	NM_00100	51114	Hs.193566	NM_01603	ENSG0000	ZDHHC9	CGI89|CXo	zinc finger	protein-co
lib-C666-l	chrX	128977888	128977888	+	10.37767	NA	promoter-TSS (NM_016032)	promoter-TSS (NM_016032)	22	NM_01603	51114	Hs.193566	NM_01603	ENSG0000	ZDHHC9	CGI89|CXo	zinc finger	protein-co
lib-C666-l	chr17	21030231	21030231	+	13.46097	NA	promoter-TSS (NM_015510)	promoter-TSS (NM_015510)	-10	NM_01551	25979	Hs.386989	NM_01551	ENSG0000	DHRS7B	CGI-93|SD	dehydroge	protein-co
lib-C666-l	chr1	38478316	38478316	+	26.26353	NA	promoter-TSS (NM_016037)	promoter-TSS (NM_016037)	-68	NM_01603	51118	Hs.472038	NM_01603	ENSG0000	UTP11	CGI-94|CG	UTP11, sm	protein-co
lib-C666-l	chr7	66460628	66460628	+	13.46097	NA	promoter-TSS (NM_016038)	promoter-TSS (NM_016038)	-40	NM_01603	51119	Hs.110445	NM_01603	ENSG0000	SBDS	CGI-97|SD	SBDS ribos	protein-co
lib-C666-l	chr6	119216379	119216379	+	5.34142	NA	intron (NM_014034, intron 1 of 3)	CpG	1138	NM_01403	25842	Hs.292316	NM_01403	ENSG0000	ASF1A	CGI-98|CI	anti-silenci	protein-co
lib-C666-l	chr14	52456209	52456209	+	77.40921	NA	promoter-TSS (NM_016039)	promoter-TSS (NM_016039)	-19	NM_01603	51637	Hs.534457	NM_01603	ENSG0000	C14orf166	CGI-99|CG	chromosor	protein-co
lib-C666-l	chr14	54976472	54976472	+	12.6699	NA	promoter-TSS (NM_006568)	promoter-TSS (NM_006568)	-115	NM_00656	10668	Hs.59106	NM_00656	ENSG0000	CGRRF1	CGR19|RN	cell growth	protein-co
lib-C666-l	chr14	54976831	54976831	+	8.30991	NA	intron (NM_006568, intron 1 of 5)	CpG	244	NM_00656	10668	Hs.59106	NM_00656	ENSG0000	CGRRF1	CGR19|RN	cell growth	protein-co
lib-C666-l	chr7	65580418	65580418	+	17.48788	NA	intron (NM_001040648, intron 1 of 2)	intron (NM_001040648, intron 1 of	613	NR_02454	27297	Hs.300684	NM_01447	ENSG0000	CRCP	CGRP-RCP	CGRP rece	protein-co
lib-C666-l	chr11	126338113	126338113	+	32.75483	NA	intron (NM_032531, intron 5 of 16)	intron (NM_032531, intron 5 of 16)	62036	NM_00125	6484	Hs.591947	NM_00627	ENSG0000	ST3GAL4	CGS23|NA	ST3 beta-g	protein-co
lib-C666-l	chr4	115519800	115519800	+	34.0482	NA	promoter-TSS (NM_001322114)	promoter-TSS (NM_001322114)	242	NM_00132	7368	Hs.144197	NM_00336	ENSG0000	UGT8	CGT|UGT4	UDP glyco	protein-co
lib-C666-l	chr20	61493502	61493502	+	25.39487	NA	promoter-TSS (NM_001301726)	promoter-TSS (NM_001301726)	-387	NM_00130	10732	Hs.126248	NM_00660	ENSG0000	TCFL5	CHA|E2BP-	transcriptic	protein-co
lib-C666-l	chr20	61492697	61492697	+	7.9573	NA	exon (NM_001301726, exon 1 of 6)	exon (NM_001301726, exon 1 of 6)	418	NM_00660	10732	Hs.126248	NM_00660	ENSG0000	TCFL5	CHA|E2BP-	transcriptic	protein-co
lib-C666-l	chr22	50557231	50557231	+	10.58189	NA	intron (NM_001164105, intron 9 of 2	LTR45C|LTR|ERV1	-28253	NM_00116	54456	Hs.62880	NM_01899	ENSG0000	MOV10L1	CHAMP|DJ	Mov10 RIS	protein-co
lib-C666-l	chr8	141521526	141521526	+	3.98718	NA	promoter-TSS (NR_040712)	promoter-TSS (NR_040712)	129	NM_01744	54108	Hs.279704	NM_01744	ENSG0000	CHRAC1	CHARC1|C	chromatin	protein-co
lib-C666-l	chr17	57697105	57697105	+	58.84504	NA	promoter-TSS (NM_004859)	promoter-TSS (NM_004859)	55	NM_00485	1213	Hs.491351	NM_00485	ENSG0000	CLTC	CHC|CHC1	clathrin he	protein-co
lib-C666-l	chr11	73587930	73587930	+	3.96379	NA	promoter-TSS (NM_001267803)	promoter-TSS (NM_001267803)	-40	NM_01656	51287	Hs.475387	NM_01656	ENSG0000	COA4	CHCHD8|C	cytochrom	protein-co
lib-C666-l	chr13	47222492	47222492	+	34.78259	NA	intron (NM_001164211, intron 1 of 1	MER1A|DNA|hAT-Charlie	95196	NM_00116	23143	Hs.507971	NM_01511	ENSG0000	LRCH1	CHDC1|NP	leucine rich	protein-co
lib-C666-l	chr6	126070417	126070417	+	2.26435	NA	promoter-TSS (NM_012259)	promoter-TSS (NM_012259)	-315	NM_01225	23493	Hs.144287	NM_01225	ENSG0000	HEY2	CHF1|GRID	hes related	protein-co
lib-C666-l	chr9	35657819	35657819	+	49.86005	NA	promoter-TSS (NM_001195200)	promoter-TSS (NM_001195200)	196	NR_00305	6023	Hs.587502	NR_00305	ENSG0000	RMRP	CHH|NME1	RNA comp	ncRNA
lib-C666-l	chr10	43857510	43857510	+	4.46583	NA	Intergenic	CpG	-9582	NM_00118	53828	Hs.130497	NM_17316	ENSG0000	FXYD4	CHIF	FXYD dom	protein-co
lib-C666-l	chr16	730283	730283	+	11.36743	NA	promoter-TSS (NR_136337)	promoter-TSS (NR_136337)	172	NM_00129	10273	Hs.592081	NM_00586	ENSG0000	STUB1	CHIP|HSPA	STIP1 hom	protein-co
lib-C666-l	chr11	67888952	67888952	+	34.02388	NA	promoter-TSS (NM_212469)	promoter-TSS (NM_212469)	-94	NM_21246	1119	Hs.77221	NM_00127	ENSG0000	CHKA	CHK|CK|CK	choline kin	protein-co
lib-C666-l	chr21	46961756	46961756	+	28.88196	NA	intron (NM_194255, intron 1 of 5)	CpG	629	NM_00120	6573	Hs.84190	NM_00305	ENSG0000	SLC19A1	CHMD|FOL	solute carr	protein-co
lib-C666-l	chr21	47065900	47065900	+	24.75602	NA	Intergenic	Intergenic	-103515	NM_19425	6573	Hs.84190	NM_00305	ENSG0000	SLC19A1	CHMD|FOL	solute carr	protein-co
lib-C666-l	chr9	102584089	102584089	+	166.11772	NA	promoter-TSS (NM_173200)	promoter-TSS (NM_173200)	-48	NM_17320	8013	Hs.279522	NM_00698	ENSG0000	NR4A3	CHN|CSMF	nuclear rec	protein-co
lib-C666-l	chr5	172629030	172629030	+	7.52748	NA	Intergenic	Intergenic	33285	NM_00116	1482	Hs.54473	NM_00438	ENSG0000	NKX2-5	CHNG5|CS	NK2 home	protein-co
lib-C666-l	chr15	69706590	69706590	+	8.71138	NA	promoter-TSS (NM_001281301)	promoter-TSS (NM_001281301)	5	NM_00485	9493	Hs.270845	NM_00485	ENSG0000	KIF23	CHO1|KNS	kinesin fan	protein-co
lib-C666-l	chr15	41523371	41523371	+	8.28613	NA	promoter-TSS (NM_001286441)	promoter-TSS (NM_001286441)	-66	NM_00723	11261	Hs.406234	NM_00723	ENSG0000	CHP1	CHP|SLC9A	calcineurin	protein-co
lib-C666-l	chr8	66556935	66556935	+	4.52748	NA	promoter-TSS (NM_001145839)	promoter-TSS (NM_001145839)	47	NM_01463	9650	Hs.584788	NM_01463	ENSG0000	MTFR1	CHPPR|FAI	mitochond	protein-co
lib-C666-l	chr7	158622319	158622319	+	424.55182	NA	promoter-TSS (NM_020728)	promoter-TSS (NM_020728)	0	NM_02072	57488	Hs.490795	NM_02072	ENSG0000	ESYT2	CHR2SYT|E	extended s	protein-co
lib-C666-l	chr7	158620309	158620309	+	58.41369	NA	intron (NM_020728, intron 1 of 21)	intron (NM_020728, intron 1 of 21)	2010	NM_02072	57488	Hs.490795	NM_02072	ENSG0000	ESYT2	CHR2SYT|E	extended s	protein-co
lib-C666-l	chr7	73050886	73050886	+	68.52034	NA	Intergenic	Intergenic	-12013	NR_13454	51085	Hs.647055	NM_03295	ENSG0000	MLXIPL	CHREBP|M	MLX intera	protein-co
lib-C666-l	chr7	73027295	73027295	+	22.70376	NA	intron (NM_032951, intron 2 of 16)	L2c|LINE|L2	11575	NM_03295	51085	Hs.647055	NM_03295	ENSG0000	MLXIPL	CHREBP|M	MLX intera	protein-co
lib-C666-l	chr8	145682194	145682194	+	124.42715	NA	intron (NM_138496, intron 2 of 4)	intron (NM_138496, intron 2 of 4)	6040	NM_00133	50626	Hs.459379	NM_03268	ENSG0000	CYHR1	CHRP	cysteine ar	protein-co
lib-C666-l	chr1	236030408	236030408	+	29.93113	NA	promoter-TSS (NM_000081)	promoter-TSS (NM_000081)	-181	NM_00008	1130	Hs.532411	NM_00008	ENSG0000	LYST	CHS|CHS1	lysosomal	protein-co
lib-C666-l	chr8	100025423	100025423	+	4.71278	NA	promoter-TSS (NM_181661)	promoter-TSS (NM_181661)	-71	NR_04758	157680	Hs.191540	NM_01524	ENSG0000	VPS13B	CHS1|COH	vacuolar p	protein-co
lib-C666-l	chr15	101792222	101792222	+	5.819	NA	promoter-TSS (NM_014918)	promoter-TSS (NM_014918)	-85	NM_01491	22856	Hs.110488	NM_01491	ENSG0000	CHSY1	CHSY|CSS1	chondroitir	protein-co
lib-C666-l	chr2	220395053	220395053	+	6.3317	NA	intron (NM_018674, intron 1 of 9)	intron (NM_018674, intron 1 of 9)	12766	NM_00119	79586	Hs.516711	NM_02453	ENSG0000	CHPF	CHSY2|CSS	chondroitir	protein-co
lib-C666-l	chr2	220406573	220406573	+	4.56047	NA	exon (NM_001195731, exon 2 of 4)	exon (NM_001195731, exon 2 of 4)	1246	NM_00119	79586	Hs.516711	NM_02453	ENSG0000	CHPF	CHSY2|CSS	chondroitir	protein-co
lib-C666-l	chr6	149638493	149638493	+	8.73012	NA	promoter-TSS (NM_001292034)	promoter-TSS (NM_001292034)	-570	NM_00129	23118	Hs.269775	NM_01509	ENSG0000	TAB2	CHTD2|MA	TGF-beta a	protein-co
lib-C666-l	chr6	149554507	149554507	+	7.09685	NA	intron (NM_001292035, intron 2 of 6)	intron (NM_001292035, intron 2 of	15447	NR_12586	23118	Hs.269775	NM_01509	ENSG0000	TAB2	CHTD2|MA	TGF-beta a	protein-co
lib-C666-l	chr6	149492411	149492411	+	2.26435	NA	Intergenic	Intergenic	-46649	NM_00129	23118	Hs.269775	NM_01509	ENSG0000	TAB2	CHTD2|MA	TGF-beta a	protein-co
lib-C666-l	chr19	42759309	42759309	+	30.13508	NA	promoter-TSS (NM_006494)	promoter-TSS (NM_006494)	7	NM_00649	2077	Hs.655969	NM_00649	ENSG0000	ERF	CHYTS|CRS	ETS2 repre	protein-co
lib-C666-l	chr21	44313260	44313260	+	61.37072	NA	promoter-TSS (NM_001001503)	promoter-TSS (NM_001001503)	-118	NM_00100	4731	Hs.473937	NM_02107	ENSG0000	NDUFV3	CI-10k|CI-	NADH:ubic	protein-co
lib-C666-l	chr19	1383599	1383599	+	15.22699	NA	promoter-TSS (NM_024407)	promoter-TSS (NM_024407)	-284	NM_02440	374291	Hs.211914	NM_02440	ENSG0000	NDUFS7	CI-20|CI-2	NADH:ubic	protein-co
lib-C666-l	chr18	9101920	9101920	+	32.75483	NA	promoter-TSS (NM_021074)	promoter-TSS (NM_021074)	-708	NM_02107	4729	Hs.464572	NM_02107	ENSG0000	NDUFV2	CI-24k	NADH:ubic	protein-co
lib-C666-l	chr18	9102405	9102405	+	4.52748	NA	promoter-TSS (NM_021074)	promoter-TSS (NM_021074)	-223	NM_02107	4729	Hs.464572	NM_02107	ENSG0000	NDUFV2	CI-24k	NADH:ubic	protein-co
lib-C666-l	chr11	47600526	47600526	+	5.48804	NA	promoter-TSS (NM_004551)	promoter-TSS (NM_004551)	-36	NM_00455	4722	Hs.502528	NM_00455	ENSG0000	NDUFS3	CI-30	NADH:ubic	protein-co
lib-C666-l	chr2	240964831	240964831	+	233.41176	NA	promoter-TSS (NM_001322020)	promoter-TSS (NM_001322020)	-12	NM_00454	4705	Hs.277677	NM_00454	ENSG0000	NDUFA10	CI-42KD|C	NADH:ubic	protein-co
lib-C666-l	chr1	161172034	161172034	+	11.96837	NA	promoter-TSS (NM_001166159)	promoter-TSS (NM_001166159)	97	NM_00116	4720	Hs.173611	NM_00455	ENSG0000	NDUFS2	CI-49	NADH:ubic	protein-co
lib-C666-l	chr11	67374370	67374370	+	86.18742	NA	promoter-TSS (NR_130935)	promoter-TSS (NR_130935)	47	NM_00116	4723	Hs.7744	NM_00710	ENSG0000	NDUFV1	CI-51K|CI5	NADH:ubic	protein-co
lib-C666-l	chr2	96931829	96931829	+	39.21146	NA	promoter-TSS (NM_001193304)	promoter-TSS (NM_001193304)	-55	NM_00480	9391	Hs.12109	NM_00480	ENSG0000	CIAO1	CIA1|WDR	cytosolic ir	protein-co
lib-C666-l	chr15	64386449	64386449	+	18.74745	NA	promoter-TSS (NM_001289108)	promoter-TSS (NM_001289108)	-242	NM_00128	84191	Hs.439548	NM_03223	ENSG0000	FAM96A	CIA2A	family with	protein-co
lib-C666-l	chr15	90777254	90777254	+	17.48666	NA	promoter-TSS (NM_001013657)	promoter-TSS (NM_001013657)	25	NM_00127	10519	Hs.715556	NM_00638	ENSG0000	CIB1	CIB|CIBP|KI	calcium an	protein-co
lib-C666-l	chr15	90809089	90809089	+	13.14211	NA	promoter-TSS (NR_102428)	promoter-TSS (NR_102428)	-98	NR_10242	10519	Hs.715556	NM_00638	ENSG0000	CIB1	CIB|CIBP|KI	calcium an	protein-co
lib-C666-l	chr8	120845030	120845030	+	16.96353	NA	promoter-TSS (NM_003184)	promoter-TSS (NM_003184)	44	NM_00318	6873	Hs.122752	NM_00318	ENSG0000	TAF2	CIF150|MR	TATA-box	protein-co
lib-C666-l	chr2	216301112	216301112	+	13.46097	NA	promoter-TSS (NM_002026)	promoter-TSS (NM_002026)	-319	NM_00130	2335	Hs.203717	NM_00202	ENSG0000	FN1	CIG|ED-B|F	fibronectin	protein-co
lib-C666-l	chr2	216275700	216275700	+	4.19675	NA	intron (NM_001306132, intron 13 of	intron (NM_001306132, intron 13 o	25091	NM_21247	2335	Hs.203717	NM_00202	ENSG0000	FN1	CIG|ED-B|F	fibronectin	protein-co
lib-C666-l	chr10	103990328	103990328	+	114.95827	NA	TTS (NM_152310)	TTS (NM_152310)	4293	NM_15231	83401	Hs.302130	NM_15231	ENSG0000	ELOVL3	CIG-30|CI	ELOVL fatt	protein-co
lib-C666-l	chr6	116937390	116937390	+	8.85547	NA	promoter-TSS (NM_001010892)	promoter-TSS (NM_001010892)	-252	NM_00116	345895	Hs.160380	NM_00101	ENSG0000	RSPH4A	CILD11|RS	radial spok	protein-co
lib-C666-l	chr17	78010048	78010048	+	3.9966	NA	promoter-TSS (NM_019020)	promoter-TSS (NM_019020)	-383	NM_00133	55036	Hs.202542	NM_01795	ENSG0000	CCDC40	CILD15|FA	coiled-coil	protein-co
lib-C666-l	chr7	766280	766280	+	5.76059	NA	promoter-TSS (NR_075098)	promoter-TSS (NR_075098)	-58	NM_01780	54919	Hs.535896	NM_01780	ENSG0000	DNAAF5	CILD18|HE	dynein axo	protein-co
lib-C666-l	chr8	101225283	101225283	+	15.42216	NA	intron (NM_172218, intron 10 of 18)	CpG	54720	NM_00311	6674	Hs.591866	NM_00311	ENSG0000	SPAG1	CILD28|CT	sperm assc	protein-co
lib-C666-l	chr5	14036189	14036189	+	7.09685	NA	Intergenic	Intergenic	-91600	NM_00136	1767	Hs.212360	NM_00136	ENSG0000	DNAH5	CILD3|DNA	dynein axo	protein-co
lib-C666-l	chr4	74904221	74904221	+	22.02847	NA	intron (NM_002090, intron 1 of 3)	CpG-19880	269	NM_00209	2921	Hs.89690	NM_00209	ENSG0000	CXCL3	CINC-2b|G	C-X-C mo	protein-co
lib-C666-l	chr9	5628982	5628982	+	4.51266	NA	promoter-TSS (NM_001135920)	promoter-TSS (NM_001135920)	-137	NM_00120	57589	Hs.211520	NM_02082	ENSG0000	RIC1	CIP150|KIA	RIC1 homo	protein-co
lib-C666-l	chr3	108308506	108308506	+	3.98718	NA	promoter-TSS (NM_020890)	promoter-TSS (NM_020890)	-15	NM_02089	57650	Hs.591308	NM_02089	ENSG0000	KIAA1524	CIP2A|p90	KIAA1524	protein-co
lib-C666-l	chr9	117264179	117264179	+	13.72417	NA	intron (NM_015404, intron 1 of 11)	intron (NM_015404, intron 1 of 11)	1316	NM_00108	25861	Hs.93836	NM_01540	ENSG0000	WHRN	CIP98|DFN	whirlin	protein-co
lib-C666-l	chr9	117267720	117267720	+	4.39666	NA	promoter-TSS (NM_001173425)	promoter-TSS (NM_001173425)	16	NM_00117	25861	Hs.93836	NM_01540	ENSG0000	WHRN	CIP98|DFN	whirlin	protein-co
lib-C666-l	chr1	212662037	212662037	+	21.60686	NA	Intergenic	Intergenic	55808	NR_02659	29937	Hs.461787	NM_01334	ENSG0000	NENF	CIR2|SCIRP	neudesin n	protein-co
lib-C666-l	chr1	212606137	212606137	+	3.20885	NA	promoter-TSS (NM_013349)	promoter-TSS (NM_013349)	-92	NM_01334	29937	Hs.461787	NM_01334	ENSG0000	NENF	CIR2|SCIRP	neudesin n	protein-co
lib-C666-l	chr6	12008721	12008721	+	31.1618	NA	Intergenic	Intergenic	-4003	NM_00211	3096	Hs.567284	NM_00211	ENSG0000	HIVEP1	CIRIP|CRYE	human imr	protein-co
lib-C666-l	chr19	14318126	14318126	+	9.94079	NA	Intergenic	CpG-13106	-1129	NM_01492	22859	Hs.94229	NM_01492	ENSG0000	ADGRL1	CIRL1|CL1|	adhesion G	protein-co
lib-C666-l	chr19	1260979	1260979	+	18.56491	NA	Intergenic	CpG	-8286	NM_00130	1153	Hs.618145	NM_00128	ENSG0000	CIRBP	CIRP	cold induci	protein-co
lib-C666-l	chr16	11277273	11277273	+	17.89429	NA	Intergenic	Intergenic	72766	NM_00374	8651	Hs.50640	NM_00374	ENSG0000	SOCS1	CIS1|CISH1	suppressor	protein-co
lib-C666-l	chr16	11285809	11285809	+	15.48898	NA	Intergenic	L2a|LINE|L2	64230	NM_00374	8651	Hs.50640	NM_00374	ENSG0000	SOCS1	CIS1|CISH1	suppressor	protein-co
lib-C666-l	chr16	11230644	11230644	+	9.14689	NA	intron (NM_015226, intron 21 of 22)	intron (NM_015226, intron 21 of 22	119395	NM_00374	8651	Hs.50640	NM_00374	ENSG0000	SOCS1	CIS1|CISH1	suppressor	protein-co
lib-C666-l	chr16	11295893	11295893	+	6.48676	NA	Intergenic	Intergenic	54146	NM_00374	8651	Hs.50640	NM_00374	ENSG0000	SOCS1	CIS1|CISH1	suppressor	protein-co
lib-C666-l	chr16	11296606	11296606	+	5.6488	NA	Intergenic	Intergenic	53433	NM_00374	8651	Hs.50640	NM_00374	ENSG0000	SOCS1	CIS1|CISH1	suppressor	protein-co
lib-C666-l	chr2	46926120	46926120	+	9.53658	NA	promoter-TSS (NM_014011)	promoter-TSS (NM_014011)	21	NM_01401	9655	Hs.468426	NM_01401	ENSG0000	SOCS5	CIS6|CISH6	suppressor	protein-co
lib-C666-l	chr8	67687533	67687533	+	13.20416	NA	5' UTR (NM_170709, exon 1 of 16)	5' UTR (NM_170709, exon 1 of 16)	117	NM_01325	23678	Hs.613417	NM_01325	ENSG0000	SGK3	CISK|SGK2|	serum/gluc	protein-co
lib-C666-l	chr1	26758791	26758791	+	260.51001	NA	promoter-TSS (NM_001243564)	promoter-TSS (NM_001243564)	18	NM_00124	79947	Hs.369385	NM_02488	ENSG0000	DHDDS	CIT|CPT|DS	dehydrodo	protein-co
lib-C666-l	chr12	120315266	120315266	+	9.20967	NA	promoter-TSS (NM_007174)	promoter-TSS (NM_007174)	-171	NM_00120	11113	Hs.119594	NM_00717	ENSG0000	CIT	CITK|CRIK|	citron rho-	protein-co
lib-C666-l	chr5	148931144	148931144	+	12.41572	NA	promoter-TSS (NM_001892)	promoter-TSS (NM_001892)	-29	NM_00102	1452	Hs.529862	NM_00189	ENSG0000	CSNK1A1	CK1|CK1a|	casein kina	protein-co
lib-C666-l	chr17	39749150	39749150	+	11.06679	NA	Intergenic	MER1B|DNA|hAT-Charlie	-6003	NM_00052	3861	Hs.654380	NM_00052	ENSG0000	KRT14	CK14|EBS3	keratin 14	protein-co
lib-C666-l	chr12	53342650	53342650	+	5.3823	NA	promoter-TSS (NM_199187)	promoter-TSS (NM_199187)	-5	NM_19918	3875	Hs.406013	NM_00022	ENSG0000	KRT18	CK-18|CYK	keratin 18	protein-co
lib-C666-l	chr19	1940767	1940767	+	233.98932	NA	promoter-TSS (NM_001319)	promoter-TSS (NM_001319)	-394	NM_00131	1455	Hs.651905	NM_00131	ENSG0000	CSNK1G2	CK1g2	casein kina	protein-co
lib-C666-l	chr17	232600	232600	+	8.33814	NA	Intergenic	Intergenic	-27518	NM_00101	400566	Hs.499607	NM_00101	ENSG0000	C17orf97	CK20|LIAT1	chromosor	protein-co
lib-C666-l	chr6	31633715	31633715	+	10.37767	NA	promoter-TSS (NM_001199240).3	promoter-TSS (NM_001199240).3	58	NM_00132	1460	Hs.73527	NM_00132	ENSG0000	CSNK2B	CK2B|CK2N	casein kina	protein-co
lib-C666-l	chr12	53049747	53049747	+	14.17553	NA	Intergenic	Intergenic	-3788	NM_00042	3849	Hs.707	NM_00042	ENSG0000	KRT2	CK-2e|K2e	keratin 2	protein-co
lib-C666-l	chr12	52637977	52637977	+	5.80117	NA	intron (NM_005556, intron 6 of 8)	intron (NM_005556, intron 6 of 8)	11023	NM_00555	3855	Hs.411501	NM_00555	ENSG0000	KRT7	CK7|K2C7|	keratin 7	protein-co
lib-C666-l	chr12	52615857	52615857	+	3.28884	NA	intron (NR_027358, intron 4 of 4)	intron (NR_027358, intron 4 of 4)	-11097	NM_00555	3855	Hs.411501	NM_00555	ENSG0000	KRT7	CK7|K2C7|	keratin 7	protein-co
lib-C666-l	chr12	53268221	53268221	+	13.14211	NA	Intergenic	CpG	-25443	NM_17335	196374	Hs.665267	NM_17335	ENSG0000	KRT78	CK-78|K5B	keratin 78	protein-co
lib-C666-l	chr19	8163846	8163846	+	8.60785	NA	intron (NM_001321431, intron 41 of	L1ME5|LINE|L1	46200	NM_00120	6370	Hs.310511	NM_00562	ENSG0000	CCL25	Ckb15|SCY	C-C motif	protein-co
lib-C666-l	chr3	141457007	141457007	+	262.85562	NA	promoter-TSS (NM_183237)	promoter-TSS (NM_183237)	-44	NM_00120	9616	Hs.134623	NM_01424	ENSG0000	RNF7	CKBBP1|RC	ring finger	protein-co
lib-C666-l	chr19	13067926	13067926	+	2.10911	NA	exon (NM_052850, exon 1 of 2)	exon (NM_052850, exon 1 of 2)	142	NM_05285	90480	Hs.515164	NM_05285	ENSG0000	GADD45GI	CKBBP2|CK	GADD45G	protein-co
lib-C666-l	chr22	38732233	38732233	+	5.64185	NA	intron (NM_001289912, intron 5 of 1	GC_rich|Low_complexity|Low_comp	-18144	NM_00189	1454	Hs.474833	NM_00189	ENSG0000	CSNK1E	CKIepsilon	casein kina	protein-co
lib-C666-l	chr5	122847382	122847382	+	3.27468	NA	promoter-TSS (NM_001270573)	promoter-TSS (NM_001270573)	-411	NM_00127	1456	Hs.129206	NM_00438	ENSG0000	CSNK1G3	CKI-gamm	casein kina	protein-co
lib-C666-l	chr5	122847966	122847966	+	2.59723	NA	5' UTR (NM_001270573, exon 1 of 12	5' UTR (NM_001270573, exon 1 of 1	173	NM_00127	1456	Hs.129206	NM_00438	ENSG0000	CSNK1G3	CKI-gamm	casein kina	protein-co
lib-C666-l	chr3	32463848	32463848	+	45.19756	NA	intron (NM_138410, intron 1 of 4)	intron (NM_138410, intron 1 of 4)	30685	NM_18147	112616	Hs.440494	NM_13841	ENSG0000	CMTM7	CKLFSF7	CKLF like M	protein-co
lib-C666-l	chr10	12375911	12375911	+	16.13885	NA	Intergenic	Intergenic	-15631	NM_15349	57118	Hs.600547	NM_02039	ENSG0000	CAMK1D	CKLiK|CaM	calcium/ca	protein-co
lib-C666-l	chr19	45829460	45829460	+	29.98795	NA	Intergenic	Intergenic	-3225	NM_00182	1158	Hs.334347	NM_00182	ENSG0000	CKM	CKMM|M-	creatine kir	protein-co
lib-C666-l	chr15	43982351	43982351	+	12.83248	NA	Intergenic	CpG	-2633	NM_00132	548596	Hs.654988	NM_00101	ENSG0000	CKMT1A	CKMT1|U-	creatine kir	protein-co

lib-C666-l	chr12	25539194	25539194	+	21.73991	NA	Intergenic	CpG	-135329	NM_00498	3845	Hs.505033	NM_00498	ENSG0000	KRAS	C-K-RAS|C	KRAS prot	protein-co
lib-C666-l	chr1	154973750	154973750	+	21.84731	NA	Intergenic	CpG	-1356	NR_04976	51043	Hs.729279	NM_00125	ENSG0000	ZBTB7B	CKROX|TH	zinc finger	protein-co
lib-C666-l	chr1	154974596	154974596	+	7.23765	NA	promoter-TSS (NM_001252406)	promoter-TSS (NM_001252406)	-510	NR_04976	51043	Hs.729279	NM_00125	ENSG0000	ZBTB7B	CKROX|TH	zinc finger	protein-co
lib-C666-l	chr1	154974145	154974145	+	6.56784	NA	promoter-TSS (NM_001252406)	promoter-TSS (NM_001252406)	-961	NR_04976	51043	Hs.729279	NM_00125	ENSG0000	ZBTB7B	CKROX|TH	zinc finger	protein-co
lib-C666-l	chr1	154990149	154990149	+	6.20473	NA	TTS (NM_144622)	TTS (NM_144622)	15043	NR_04620	51043	Hs.729279	NM_00125	ENSG0000	ZBTB7B	CKROX|TH	zinc finger	protein-co
lib-C666-l	chr9	91925829	91925829	+	47.72539	NA	promoter-TSS (NM_001827)	promoter-TSS (NM_001827)	-281	NM_00182	1164	Hs.83758	NM_00182	ENSG0000	CKS2	CKSHS2	CDC28 pro	protein-co
lib-C666-l	chr13	36497687	36497687	+	21.73075	NA	intron (NM_001330071, intron 4 of 1	intron (NM_001330071, intron 4 of	-67689	NM_00119	9201	Hs.507755	NM_00473	ENSG0000	DCLK1	CL1|CLICK	doublecort	protein-co
lib-C666-l	chr13	36628398	36628398	+	10.4657	NA	intron (NM_001330071, intron 3 of 1	LTR40a|LTR|ERVL	77116	NM_00133	9201	Hs.507755	NM_00473	ENSG0000	DCLK1	CL1|CLICK	doublecort	protein-co
lib-C666-l	chr13	36501985	36501985	+	7.15513	NA	intron (NM_001330071, intron 4 of 1	MER21C|LTR|ERVL	-71987	NM_00119	9201	Hs.507755	NM_00473	ENSG0000	DCLK1	CL1|CLICK	doublecort	protein-co
lib-C666-l	chr5	172198374	172198374	+	47.69789	NA	promoter-TSS (NM_004417)	promoter-TSS (NM_004417)	-171	NM_00441	1843	Hs.171695	NM_00441	ENSG0000	DUSP1	CL100|HVH	dual specif	protein-co
lib-C666-l	chr5	172192551	172192551	+	23.11805	NA	Intergenic	Intergenic	5652	NM_00441	1843	Hs.171695	NM_00441	ENSG0000	DUSP1	CL100|HVH	dual specif	protein-co
lib-C666-l	chr5	172204580	172204580	+	15.55328	NA	Intergenic	Intergenic	-6377	NM_00441	1843	Hs.171695	NM_00441	ENSG0000	DUSP1	CL100|HVH	dual specif	protein-co
lib-C666-l	chr5	172199374	172199374	+	12.33851	NA	Intergenic	CpG-21390	-1171	NM_00441	1843	Hs.171695	NM_00441	ENSG0000	DUSP1	CL100|HVH	dual specif	protein-co
lib-C666-l	chr4	151000644	151000644	+	111.38619	NA	intron (NM_001040260, intron 1 of 1	CpG	1218	NM_00104	166614	Hs.591683	NM_15261	ENSG0000	DCLK2	CL2|CLICK-	doublecort	protein-co
lib-C666-l	chr7	155089394	155089394	+	111.53218	NA	promoter-TSS (NM_001346590)	promoter-TSS (NM_001346590)	-88	NM_00554	3638	Hs.520819	NM_00554	ENSG0000	INSIG1	CL6	insulin ind	protein-co
lib-C666-l	chr7	154998349	154998349	+	63.56729	NA	Intergenic	MSTB|LTR|ERVL-MaLR	-91133	NM_00554	3638	Hs.520819	NM_00554	ENSG0000	INSIG1	CL6	insulin ind	protein-co
lib-C666-l	chr7	154997325	154997325	+	30.85659	NA	Intergenic	MIR|SINE|MIR	-92157	NM_00554	3638	Hs.520819	NM_00554	ENSG0000	INSIG1	CL6	insulin ind	protein-co
lib-C666-l	chr4	84205878	84205878	+	18.1336	NA	exon (NM_015697, exon 1 of 7)	exon (NM_015697, exon 1 of 7)	189	NM_01569	27235	Hs.144304	NM_01569	ENSG0000	COQ2	CL640|COC	coenzyme	protein-co
lib-C666-l	chr16	30194828	30194828	+	17.78771	NA	promoter-TSS (NM_001193333)	promoter-TSS (NM_001193333)	97	NM_00707	11151	Hs.415067	NM_00707	ENSG0000	CORO1A	CLABP|CLI	coronin 1A	protein-co
lib-C666-l	chr19	45542365	45542365	+	5.65398	NA	promoter-TSS (NM_007056)	promoter-TSS (NM_007056)	67	NM_00705	11129	Hs.466917	NM_00705	ENSG0000	CLASRP	CLASP|SFR	CLK4 asso	protein-co
lib-C666-l	chr1	31297499	31297499	+	6.61453	NA	Intergenic	Intergenic	-66816	NM_00676	7805	Hs.371021	NM_00676	ENSG0000	LAPTM5	CLAST6	lysosomal	protein-co
lib-C666-l	chr12	9840095	9840095	+	9.20967	NA	intron (NM_001004419, intron 2 of 5)	MER1A|DNA|hAT-Charlie	17791	NM_01326	29121	Hs.268326	NM_01326	ENSG0000	CLEC2D	CLAX|LLT1	C-type lec	protein-co
lib-C666-l	chr4	170541644	170541644	+	27.44508	NA	promoter-TSS (NM_001243372)	promoter-TSS (NM_001243372)	-28	NM_17387	1182	Hs.481186	NM_00182	ENSG0000	CLCN3	CLC3|ClC-	chloride vc	protein-co
lib-C666-l	chr4	170581333	170581333	+	16.14038	NA	5' UTR (NM_001243374, exon 1 of 12	5' UTR (NM_001243374, exon 1 of 1	120	NM_00124	1182	Hs.481186	NM_00182	ENSG0000	CLCN3	CLC3|ClC-	chloride vc	protein-co
lib-C666-l	chr1	11866147	11866147	+	17.13943	NA	promoter-TSS (NR_046428)	promoter-TSS (NR_046428)	-6	NM_00125	1185	Hs.193043	NM_00128	ENSG0000	CLCN6	CLC-6	chloride vc	protein-co
lib-C666-l	chr16	1525062	1525062	+	5.819	NA	promoter-TSS (NM_001287)	promoter-TSS (NM_001287)	23	NM_00128	1186	Hs.459649	NM_00128	ENSG0000	CLCN7	CLC-7|CLC	chloride vc	protein-co
lib-C666-l	chr3	98690205	98690205	+	7.23765	NA	Intergenic	Intergenic	-69672	NM_08092	131566	Hs.203691	NM_08092	ENSG0000	DCBLD2	CLCP1|ESD	discoidin, (	protein-co
lib-C666-l	chr17	79818886	79818886	+	38.6745	NA	promoter-TSS (NM_000918)	promoter-TSS (NM_000918)	-342	NM_00091	5034	Hs.464336	NM_00091	ENSG0000	P4HB	CLCRP1|DS	prolyl 4-hy	protein-co
lib-C666-l	chr4	119757733	119757733	+	5.96422	NA	promoter-TSS (NM_001318066)	promoter-TSS (NM_001318066)	-382	NM_01482	9871	Hs.189641	NM_01482	ENSG0000	SEC24D	CLCRP2	SEC24 hon	protein-co
lib-C666-l	chr8	86019456	86019456	+	15.60931	NA	5' UTR (NM_033402, exon 1 of 19)	5' UTR (NM_033402, exon 1 of 19)	133	NM_03340	85444	Hs.193115	NM_03340	ENSG0000	LRRCC1	CLERC|SAF	leucine rich	protein-co
lib-C666-l	chr16	55478736	55478736	+	16.5633	NA	Intergenic	Intergenic	-34006	NM_00130	4313	Hs.513617	NM_00453	ENSG0000	MMP2	CLG4|CLG4	matrix met	protein-co
lib-C666-l	chr1	209750336	209750336	+	5.96761	NA	Intergenic	Intergenic	-6709	NM_02043	57172	Hs.199068	NM_02043	ENSG0000	CAMK1G	CLICK3|CLI	calcium/ca	protein-co
lib-C666-l	chr1	26438294	26438294	+	11.66012	NA	promoter-TSS (NM_001243533)	promoter-TSS (NM_001243533)	26	NM_00124	149420	Hs.468801	NM_15283	ENSG0000	PDIK1L	CLIK1L|STK	PDLIM1 int	protein-co
lib-C666-l	chr10	97087301	97087301	+	34.25375	NA	intron (NM_001034956, intron 24 of 2	intron (NM_001034956, intron 24 o	-36396	NM_02099	9124	Hs.368525	NM_02099	ENSG0000	PDLIM1	CLIM1|CLP	PDZ and LI	protein-co
lib-C666-l	chr12	106641625	106641625	+	2.05265	NA	promoter-TSS (NM_006825)	promoter-TSS (NM_006825)	88	NM_00682	10970	Hs.74368	NM_00682	ENSG0000	CKAP4	CLIMP-63|	cytoskelet	protein-co
lib-C666-l	chr5	157286176	157286176	+	18.0551	NA	promoter-TSS (NM_001195555)	promoter-TSS (NM_001195555)	7	NM_00119	9685	Hs.644000	NM_01466	ENSG0000	CLINT1	CLINT|ENT	clathrin int	protein-co
lib-C666-l	chr7	73699182	73699182	+	133.49304	NA	Intergenic	Intergenic	-4623	NM_03242	7461	Hs.647018	NM_00338	ENSG0000	CLIP2	CLIP|CLIP-	CAP-Gly d	protein-co
lib-C666-l	chr7	73694125	73694125	+	38.90989	NA	Intergenic	Intergenic	-9680	NM_03242	7461	Hs.647018	NM_00338	ENSG0000	CLIP2	CLIP|CLIP-	CAP-Gly d	protein-co
lib-C666-l	chr21	43835331	43835331	+	25.61513	NA	intron (NM_018961, intron 5 of 14)	intron (NM_018961, intron 5 of 14)	11360	NM_00100	53347	Hs.473912	NM_01896	ENSG0000	UBASH3A	CLIP4|STS-	ubiquitin a	protein-co
lib-C666-l	chr15	68852341	68852341	+	7.11942	NA	intron (NM_001324014, intron 2 of 1	ERV3-16A3_LTR|LTR|ERVL	-18967	NM_00609	10391	Hs.551213	NM_00609	ENSG0000	CORO2B	CLIPINC	coronin 2B	protein-co
lib-C666-l	chr15	68870180	68870180	+	3.8557	NA	intron (NM_001324014, intron 2 of 1	L2c|LINE|L2	-1128	NM_00609	10391	Hs.551213	NM_00609	ENSG0000	CORO2B	CLIPINC	coronin 2B	protein-co
lib-C666-l	chr2	201729401	201729401	+	63.46463	NA	promoter-TSS (NM_004071)	promoter-TSS (NM_004071)	66	NR_02785	1195	Hs.433732	NM_00407	ENSG0000	CLK1	CLK|CLK/S	CDC like ki	protein-co
lib-C666-l	chr2	201728999	201728999	+	6.32351	NA	5' UTR (NM_001162407, exon 1 of 13	5' UTR (NM_001162407, exon 1 of 1	285	NM_00116	1195	Hs.433732	NM_00407	ENSG0000	CLK1	CLK|CLK/S	CDC like ki	protein-co
lib-C666-l	chr7	107643799	107643799	+	31.26176	NA	promoter-TSS (NM_002291)	promoter-TSS (NM_002291)	5	NM_00229	3912	Hs.650585	NM_00229	ENSG0000	LAMB1	CLM|LIS5	laminin sul	protein-co
lib-C666-l	chr7	107635837	107635837	+	18.61761	NA	intron (NM_002291, intron 4 of 33)	intron (NM_002291, intron 4 of 33)	7967	NM_00229	3912	Hs.650585	NM_00229	ENSG0000	LAMB1	CLM|LIS5	laminin sul	protein-co
lib-C666-l	chr17	72450363	72450363	+	8.01457	NA	Intergenic	CpG	-12149	NM_00133	11314	Hs.9688	NM_00726	ENSG0000	CD300A	CLM-8|CM	CD300a m	protein-co
lib-C666-l	chr1	17356813	17356813	+	12.12504	NA	intron (NM_003000, intron 3 of 7)	AluJb|SINE|Alu	-18346	NM_00114	23400	Hs.128866	NM_02208	ENSG0000	ATP13A2	CLN12|HSA	ATPase 13A	protein-co
lib-C666-l	chr7	66093680	66093680	+	24.44958	NA	promoter-TSS (NM_001167961)	promoter-TSS (NM_001167961)	-188	NM_00116	154881	Hs.546627	NM_15303	ENSG0000	KCTD7	CLN14|EPN	potassium	protein-co
lib-C666-l	chr11	6640743	6640743	+	116.90468	NA	promoter-TSS (NM_000391)	promoter-TSS (NM_000391)	-51	NM_00039	1200	Hs.523454	NM_00039	ENSG0000	TPP1	CLN2|GIG1	tripeptidyl	protein-co
lib-C666-l	chr20	62526362	62526362	+	5.23609	NA	promoter-TSS (NM_025219)	promoter-TSS (NM_025219)	-93	NM_02521	80331	Hs.164419	NM_02521	ENSG0000	DNAJC5	CLN4|CLN4	DnaJ heat	protein-co
lib-C666-l	chr17	55038219	55038219	+	52.78568	NA	exon (NM_004645, exon 1 of 7)	exon (NM_004645, exon 1 of 7)	192	NM_00464	8161	Hs.532795	NM_00464	ENSG0000	COIL	CLN80|p80	coilin	protein-co
lib-C666-l	chr2	44223082	44223082	+	10.67459	NA	promoter-TSS (NM_133259)	promoter-TSS (NM_133259)	62	NM_13325	10128	Hs.368084	NM_13325	ENSG0000	LRPPRC	CLONE-23	leucine rich	protein-co
lib-C666-l	chr19	18111941	18111941	+	16.96353	NA	promoter-TSS (NM_001025604)	promoter-TSS (NM_001025604)	0	NM_00102	27106	Hs.515249	NM_01568	ENSG0000	ARRDC2	CLONE249	arrestin do	protein-co
lib-C666-l	chr17	43225693	43225693	+	12.81406	NA	5' UTR (NM_006460, exon 1 of 1)	5' UTR (NM_006460, exon 1 of 1)	1009	NM_00646	10614	Hs.586945	NM_00646	ENSG0000	HEXIM1	CLP1|EDG1	hexamethy	protein-co
lib-C666-l	chr17	43229482	43229482	+	12.55817	NA	TTS (NM_006460)	TTS (NM_006460)	4798	NM_00646	10614	Hs.586945	NM_00646	ENSG0000	HEXIM1	CLP1|EDG1	hexamethy	protein-co
lib-C666-l	chr17	43226065	43226065	+	5.5098	NA	5' UTR (NM_006460, exon 1 of 1)	5' UTR (NM_006460, exon 1 of 1)	1381	NM_00646	10614	Hs.586945	NM_00646	ENSG0000	HEXIM1	CLP1|EDG1	hexamethy	protein-co
lib-C666-l	chr16	57023582	57023582	+	3.89034	NA	intron (NM_001330552, intron 1 of 4	CpG	185	NM_00133	84166	Hs.528836	NM_03220	ENSG0000	NLRC5	CLR16.1|N	NLR family	protein-co
lib-C666-l	chrX	41301500	41301500	+	2.12162	NA	Intergenic	CpG	-5213	NM_02256	60506	Hs.302019	NM_02256	ENSG0000	NYX	CLRP|CSNE	nyctalopin	protein-co
lib-C666-l	chr10	5544157	5544157	+	18.74745	NA	Intergenic	Intergenic	-2624	NM_01742	51806	Hs.180142	NM_01742	ENSG0000	CALML5	CLSP	calmodulin	protein-co
lib-C666-l	chr9	80645723	80645723	+	8.73012	NA	intron (NM_002072, intron 1 of 6)	intron (NM_002072, intron 1 of 6)	642	NM_00207	2776	Hs.269782	NM_00207	ENSG0000	GNAQ	CMC1|G-A	G protein s	protein-co
lib-C666-l	chr1	109419792	109419792	+	5.5166	NA	5' UTR (NM_013296, exon 1 of 15)	5' UTR (NM_013296, exon 1 of 15)	189	NM_00132	29899	Hs.584901	NM_01329	ENSG0000	GPSM2	CMCS|DFN	G-protein	protein-co
lib-C666-l	chr10	69913457	69913457	+	30.48199	NA	intron (NR_045663, intron 6 of 17)	intron (NR_045663, intron 6 of 17)	44267	NM_03257	84665	Hs.55205	NM_03257	ENSG0000	MYPN	CMD1DD|(	myopallad	protein-co
lib-C666-l	chr3	12705735	12705735	+	7.08136	NA	promoter-TSS (NM_002880)	promoter-TSS (NM_002880)	-35	NM_00288	5894	Hs.159130	NM_00288	ENSG0000	RAF1	CMD1NN|(	Raf-1 prot	protein-co
lib-C666-l	chr3	12705410	12705410	+	3.59106	NA	5' UTR (NM_002880, exon 1 of 17)	5' UTR (NM_002880, exon 1 of 17)	290	NM_00288	5894	Hs.159130	NM_00288	ENSG0000	RAF1	CMD1NN|(	Raf-1 prot	protein-co
lib-C666-l	chr9	108320392	108320392	+	11.06679	NA	promoter-TSS (NM_001198963)	promoter-TSS (NM_001198963)	-19	NM_00107	2218	Hs.55777	NM_00673	ENSG0000	FKTN	CMD1X|FC	fukutin	protein-co
lib-C666-l	chr7	92077686	92077686	+	6.39985	NA	intron (NR_052016, intron 1 of 5)	intron (NR_052016, intron 1 of 5)	924	NM_02116	57798	Hs.21145	NM_02116	ENSG0000	GATAD1	CMD2B|OD	GATA zinc	protein-co
lib-C666-l	chr10	46090339	46090339	+	10.71733	NA	promoter-TSS (NM_145021)	promoter-TSS (NM_145021)	15	NM_14502	220972	Hs.499489	NM_14502	ENSG0000	8-Mar	CMIR|MAR	membrane	protein-co
lib-C666-l	chr2	237509795	237509795	+	6.29226	NA	Intergenic	Intergenic	31415	NM_02031	57007	Hs.471751	NM_02031	ENSG0000	ACKR3	CMKOR1|C	atypical ch	protein-co
lib-C666-l	chr12	58146344	58146344	+	5.04826	NA	promoter-TSS (NM_000075)	promoter-TSS (NM_000075)	-114	NM_00007	1019	Hs.95577	NM_00007	ENSG0000	CDK4	CMM3|PSK	cyclin depe	protein-co
lib-C666-l	chr16	89984402	89984402	+	13.52135	NA	5' UTR (NM_002386, exon 1 of 1)	5' UTR (NM_002386, exon 1 of 1)	115	NM_00238	4157	Hs.513829	NM_00238	ENSG0000	MC1R	CMM5|MS	melanocor	protein-co
lib-C666-l	chr14	104181948	104181948	+	7.26874	NA	promoter-TSS (NM_001100118)	promoter-TSS (NM_001100118)	-125	NM_00543	7517	Hs.592325	NM_00543	ENSG0000	XRCC3	CMM6	X-ray repa	protein-co
lib-C666-l	chr6	47445352	47445352	+	20.59244	NA	promoter-TSS (NM_012120)	promoter-TSS (NM_012120)	-173	NM_01212	23607	Hs.485518	NM_01212	ENSG0000	CD2AP	CMS	CD2 associ	protein-co
lib-C666-l	chr2	69614469	69614469	+	4.49043	NA	promoter-TSS (NM_001244710)	promoter-TSS (NM_001244710)	-83	NM_00205	2673	Hs.580300	NM_00205	ENSG0000	GFPT1	CMS12|CM	glutamine-	protein-co
lib-C666-l	chr1	95538364	95538364	+	10.9422	NA	exon (NM_001305242, exon 1 of 5)	exon (NM_001305242, exon 1 of 5)	143	NR_13103	199857	Hs.408927	NM_14498	ENSG0000	ALG14	CMS15	ALG14, UD	protein-co
lib-C666-l	chr10	71529345	71529345	+	75.60525	NA	Intergenic	Intergenic	-32299	NM_00113	1305	Hs.695934	NM_00520	ENSG0000	COL13A1	CMS19|CO	collagen ty	protein-co
lib-C666-l	chr10	71561519	71561519	+	8.73012	NA	promoter-TSS (NM_080802)	promoter-TSS (NM_080802)	-125	NM_00113	1305	Hs.695934	NM_00520	ENSG0000	COL13A1	CMS19|CO	collagen ty	protein-co
lib-C666-l	chr1	202614332	202614332	+	10.03103	NA	intron (NM_177402, intron 1 of 8)	intron (NM_177402, intron 1 of 8)	-1751	NM_00113	127833	Hs.25422	NM_17740	ENSG0000	SYT2	CMS7|MYS	synaptotag	protein-co
lib-C666-l	chr1	968524	968524	+	19.8879	NA	intron (NM_198576, intron 2 of 35)	CpG	13021	NM_00130	375790	Hs.273330	NM_19857	ENSG0000	AGRN	CMS8|CMS	agrin	protein-co
lib-C666-l	chr1	955037	955037	+	14.06854	NA	promoter-TSS (NM_001305275)	promoter-TSS (NM_001305275)	-466	NM_19857	375790	Hs.273330	NM_19857	ENSG0000	AGRN	CMS8|CMS	agrin	protein-co
lib-C666-l	chr1	956391	956391	+	2.80387	NA	intron (NM_198576, intron 1 of 35)	intron (NM_198576, intron 1 of 35)	888	NM_00130	375790	Hs.273330	NM_19857	ENSG0000	AGRN	CMS8|CMS	agrin	protein-co
lib-C666-l	chr9	113507307	113507307	+	20.84232	NA	intron (NM_005592, intron 6 of 13)	intron (NM_005592, intron 6 of 13)	76256	NM_00116	4593	Hs.521653	NM_00559	ENSG0000	MUSK	CMS9|FAD	muscle ass	protein-co
lib-C666-l	chr18	13726645	13726645	+	35.47771	NA	promoter-TSS (NM_001098801)	promoter-TSS (NM_001098801)	-14	NM_00130	8731	Hs.592347	NM_00379	ENSG0000	RNMT	CMT1|CMT	RNA guani	protein-co
lib-C666-l	chr6	43597136	43597136	+	13.91591	NA	promoter-TSS (NM_019096)	promoter-TSS (NM_019096)	-143	NM_00100	9587	Hs.122346	NM_01462	ENSG0000	MAD2L1BP	CMT2	MAD2L1 b	protein-co
lib-C666-l	chr1	10270508	10270508	+	37.56522	NA	promoter-TSS (NM_183416)	promoter-TSS (NM_183416)	-256	NM_18341	23095	Hs.97858	NM_01507	ENSG0000	KIF1B	CMT2|CMT	kinesin fam	protein-co
lib-C666-l	chr1	12040434	12040434	+	264.12036	NA	5' UTR (NM_001127660, exon 1 of 18	5' UTR (NM_001127660, exon 1 of 1	196	NM_00112	9927	Hs.376681	NM_01487	ENSG0000	MFN2	CMT2A|CM	mitofusin 2	protein-co
lib-C666-l	chr22	29866559	29866559	+	26.26478	NA	Intergenic	CpG	-9622	NM_02107	4744	Hs.198760	NM_02107	ENSG0000	NEFH	CMT2CC|N	neurofilam	protein-co
lib-C666-l	chr7	30634419	30634419	+	40.85015	NA	promoter-TSS (NM_001316772)	promoter-TSS (NM_001316772)	68	NM_00131	2617	Hs.404321	NM_00204	ENSG0000	GARS	CMT2D|DS	glycyl-tRN	protein-co
lib-C666-l	chr7	75948191	75948191	+	14.37873	NA	Intergenic	Intergenic	16316	NM_00154	3315	Hs.520973	NM_00154	ENSG0000	HSPB1	CMT2F|HEI	heat shock	protein-co
lib-C666-l	chr7	75924148	75924148	+	12.73831	NA	Intergenic	Intergenic	-7727	NM_00154	3315	Hs.520973	NM_00154	ENSG0000	HSPB1	CMT2F|HEI	heat shock	protein-co
lib-C666-l	chr19	10828464	10828464	+	15.22699	NA	promoter-TSS (NM_001190716)	promoter-TSS (NM_001190716)	-265	NM_00494	1785	Hs.211463	NM_00494	ENSG0000	DNM2	CMT2M|CN	dynamin 2	protein-co
lib-C666-l	chr16	70323423	70323423	+	12.12235	NA	promoter-TSS (NM_001257174)	promoter-TSS (NM_001257174)	-11	NM_00160	16	Hs.315137	NM_00160	ENSG0000	AARS	CMT2N|EIE	alanyl-tRN	protein-co
lib-C666-l	chr14	102414442	102414442	+	195.76578	NA	Intergenic	CpG	-16423	NM_00137	1778	Hs.614080	NM_00137	ENSG0000	DYNC1H1	CMT2O|DH	dynein cyt	protein-co
lib-C666-l	chr9	130213780	130213780	+	66.96093	NA	promoter-TSS (NM_000976)	promoter-TSS (NM_000976)	15	NM_00100	90678	Hs.495188	NM_13836	ENSG0000	LRSAM1	CMT2P|RIF	leucine rich	protein-co
lib-C666-l	chr4	154073463	154073463	+	13.91591	NA	promoter-TSS (NM_001130067)	promoter-TSS (NM_001130067)	-807	NM_00113	23321	Hs.435711	NM_01527	ENSG0000	TRIM2	CMT2R|RN	tripartite m	protein-co
lib-C666-l	chr4	154028050	154028050	+	6.56784	NA	Intergenic	L1PB2|LINE|L1	-46220	NM_00113	23321	Hs.435711	NM_01527	ENSG0000	TRIM2	CMT2R|RN	tripartite m	protein-co
lib-C666-l	chr4	154074563	154074563	+	3.8557	NA	intron (NM_001130067, intron 1 of 1	CpG	293	NM_00113	23321	Hs.435711	NM_01527	ENSG0000	TRIM2	CMT2R|RN	tripartite m	protein-co
lib-C666-l	chr2	220144141	220144141	+	41.10975	NA	5' UTR (NM_001039550, exon 1 of 10	5' UTR (NM_001039550, exon 1 of 1	101	NM_00103	3300	Hs.77768	NM_00673	ENSG0000	DNAJB2	CMT2T|DS	DnaJ heat	protein-co
lib-C666-l	chr17	40688150	40688150	+	2.59032	NA	5' UTR (NM_000263, exon 1 of 6)	5' UTR (NM_000263, exon 1 of 6)	199	NM_00026	4669	Hs.50727	NM_00026	ENSG0000	NAGLU	CMT2V|MF	N-acetyl-a	protein-co
lib-C666-l	chr11	10315202	10315202	+	6.20473	NA	intron (NM_030962, intron 1 of 39)	intron (NM_030962, intron 1 of 39)	552	NM_03096	81846	Hs.577252	NM_03096	ENSG0000	SBF2	CMT4B2|D	SET bindin	protein-co
lib-C666-l	chr12	32552700	32552700	+	7.75972	NA	Intergenic	CpG	-86206	NM_00130	121512	Hs.117835	NM_13924	ENSG0000	FGD4	CMT4H|FR.	FYVE, Rho(	protein-co
lib-C666-l	chr1	33283676	33283676	+	8.96177	NA	promoter-TSS (NM_003680)	promoter-TSS (NM_003680)	-43	NM_00368	8565	Hs.213264	NM_00368	ENSG0000	YARS	CMTDIC|TY	tyrosyl-tRN	protein-co
lib-C666-l	chrX	24483303	24483303	+	10.03103	NA	promoter-TSS (NM_005391)	promoter-TSS (NM_005391)	-41	NM_00114	5165	Hs.296031	NM_00539	ENSG0000	PDK3	CMTX6|GS	pyruvate d	protein-co
lib-C666-l	chr3	39248134	39248134	+	26.81569	NA	Intergenic	LTR78|LTR|ERV1	-14047	NM_00119	165904	Hs.447868	NM_19429	ENSG0000	XIRP1	CMYA1|Xir	xin actin bi	protein-co
lib-C666-l	chr1	144936872	144936872	+	32.84146	NA	intron (NR_144517, intron 7 of 42)	intron (NR_144517, intron 7 of 42)	-4507	NM_00100	9659	Hs.584841	NM_01464	ENSG0000	PDE4DIP	CMYA2|MN	phosphodi	protein-co
lib-C666-l	chr1	144932370	144932370	+	15.97188	NA	promoter-TSS (NM_001002811)	promoter-TSS (NM_001002811)	-5	NM_00100	9659	Hs.584841	NM_01464	ENSG0000	PDE4DIP	CMYA2|MN	phosphodi	protein-co
lib-C666-l	chr6	135502140	135502140	+	2.59723	NA	promoter-TSS (NM_001161659)	promoter-TSS (NM_001161659)	-313	NM_00116	4602	Hs.606320	NM_00537	ENSG0000	MYB	Cmyb|c-my	MYB proto	protein-co
lib-C666-l	chr18	72163403	72163403	+	24.68378	NA	promoter-TSS (NM_018235)	promoter-TSS (NM_018235)	-97	NM_01823	55748	Hs.149185	NM_01823	ENSG0000	CNDP2	CN2|CPGL|	CNDP dipe	protein-co
lib-C666-l	chr22	46692446	46692446	+	27.31171	NA	promoter-TSS (NM_016426)	promoter-TSS (NM_016426)	111	NR_02400	150384	Hs.348663	NR_02400	ENSG0000	GTSE1-AS	CN5H6.4|L	GTSE1 anti	ncRNA
lib-C666-l	chr10	13203494	13203494	+	128.83711	NA	promoter-TSS (NM_018518)	promoter-TSS (NM_018518)	-60	NM_01851	55388	Hs.198363	NM_01851	ENSG0000	MCM10	CNA43|DN	minichrom	protein-co
lib-C666-l	chr22	42835916	42835916	+	8.28613	NA	Intergenic	Intergenic	-7515	NM_14591	150372	Hs.436677	NM_14591	ENSG0000	NFAM1	CNAIP	NFAT activ	protein-co
lib-C666-l	chr3	128902818	128902818	+	5.96422	NA	promoter-TSS (NM_003418)	promoter-TSS (NM_003418)	-8	NM_00112	7555	Hs.518249	NM_00341	ENSG0000	CNBP	CNBP1|DM	CCHC-type	protein-co
lib-C666-l	chrX	73563940	73563940	+	48.11517	NA	Intergenic	LTR55|LTR|ERV	39915	NM_20330	389874	Hs.157231	NM_20330	ENSG0000	ZCCHC13	CNBP2|ZN	zinc finger	protein-co
lib-C666-l	chr1	224622583	224622583	+	10.4657	NA	promoter-TSS (NM_001322302)	promoter-TSS (NM_001322302)	-14	NR_13629	149111	Hs.731723	NM_15249	ENSG0000	CNIH3	CNIH-3	cornichon	protein-co
lib-C666-l	chr20	49407411	49407411	+	5.5166	NA	Intergenic	Intergenic	-4020	NM_19879	55653	Hs.381178	NM_01784	ENSG0000	BCAS4	CNOL	breast carc	protein-co
lib-C666-l	chr13	44373507	44373507	+	10.73451	NA	Intergenic	MIRc|SINE|MIR	-12391	NM_00134	55068	Hs.128258	NM_01799	ENSG0000	ENOX1	CNOX|PIG	ecto-NOX	protein-co
lib-C666-l	chr13	44360238	44360238	+	5.25516	NA	non-coding (NR_145132, exon 2 of 2	non-coding (NR_145132, exon 2 of	878	NM_00134	55068	Hs.128258	NM_01799	ENSG0000	ENOX1	CNOX|PIG	ecto-NOX	protein-co
lib-C666-l	chr2	232791175	232791175	+	10.06764	NA	promoter-TSS (NM_024409)	promoter-TSS (NM_024409)	-62	NM_02440	4880	Hs.247916	NM_02440	ENSG0000	NPPC	CNP|CNP2	natriuretic	protein-co
lib-C666-l	chr4	160725944	160725944	+	11.66438	NA	Intergenic	L1PA3|LINE|L1	536946	NM_01424	9693	Hs.744884	NM_01424	ENSG0000	RAPGEF2	CNrasGEF|	Rap guanir	protein-co
lib-C666-l	chr4	161240429	161240429	+	11.06679	NA	Intergenic	ERVL-B4-int|LTR|ERVL	1051431	NM_01424	9693	Hs.744884	NM_01424	ENSG0000	RAPGEF2	CNrasGEF|	Rap guanir	protein-co
lib-C666-l	chr9	17579045	17579045	+	29.01385	NA	promoter-TSS (NM_003026)	promoter-TSS (NM_003026)	93	NM_00302	6456	Hs.75149	NM_00302	ENSG0000	SH3GL2	CNSA2|EEN	SH3 doma	protein-co
lib-C666-l	chr9	17863371	17863371	+	8.0154	NA	Intergenic	Intergenic	284419	NM_00302	6456	Hs.75149	NM_00302	ENSG0000	SH3GL2	CNSA2|EEN	SH3 doma	protein-co
lib-C666-l	chr15	85458475	85458475	+	8.33814	NA	intron (NM_001321722, intron 9 of 1	intron (NM_001321722, intron 9 of	30583	NM_20165	9154	Hs.459187	NM_00421	ENSG0000	SLC28A1	CNT1|HCN	solute carr	protein-co
lib-C666-l	chr9	86955188	86955188	+	17.68706	NA	intron (NM_001199633, intron 1 of 1	intron (NM_001199633, intron 1 of	506	NM_00119	64078	Hs.535966	NM_02212	ENSG0000	SLC28A3	CNT3	solute carr	protein-co
lib-C666-l	chr5	179125815	179125815	+	17.3027	NA	promoter-TSS (NM_001746)	promoter-TSS (NM_001746)	-115	NM_00102	821	Hs.567968	NM_00174	ENSG0000	CANX	CNX|IP90|F	calnexin	protein-co
lib-C666-l	chr5	179125438	179125438	+	4.71278	NA	promoter-TSS (NM_001746)	promoter-TSS (NM_001746)	-492	NM_00102	821	Hs.567968	NM_00174	ENSG0000	CANX	CNX|IP90|F	calnexin	protein-co
lib-C666-l	chr11	66383995	66383995	+	3.61436	NA	promoter-TSS (NM_001198837)	promoter-TSS (NM_001198837)	-58	NM_00119	10432	Hs.533712	NM_00632	ENSG0000	RBM14	COAA|PSP	RNA bindir	protein-co
lib-C666-l	chr1	147931256	147931256	+	30.08538	NA	intron (NM_001037501, intron 18 of 1	CpG	24163	NM_00114	653505	Hs.573713	NM_00114	ENSG0000	PPIAL4A	COAS-2|C(	peptidylpr	protein-co
lib-C666-l	chr1	147932288	147932288	+	5.96422	NA	intron (NM_001037501, intron 18 of 1	CpG	23131	NM_00114	653505	Hs.573713	NM_00114	ENSG0000	PPIAL4A	COAS-2|C(	peptidylpr	protein-co
lib-C666-l	chr11	66384398	66384398	+	14.5394	NA	exon (NM_001198837, exon 1 of 2)	exon (NM_001198837, exon 1 of 2)	345	NM_00119	100526737	Hs.523822	NM_00119	ENSG0000	RBM14-RB	COAZ	RBM14-RB	protein-co
lib-C666-l	chr11	101981797	101981797	+	8.0787	NA	exon (NM_001130145, exon 1 of 9)	exon (NM_001130145, exon 1 of 9)	605	NM_00128	10413	Hs.503692	NM_00610	ENSG0000	YAP1	COB1|YAP|	Yes associa	protein-co
lib-C666-l	chr9	179085	179085	+	55.79842	NA	promoter-TSS (NM_001145355)	promoter-TSS (NM_001145355)	-10	NM_00114	55871	Hs.531330	NM_01849	ENSG0000	CBWD1	COBP	COBW dor	protein-co
lib-C666-l	chr3	37034942	37034942	+	48.61377	NA	promoter-TSS (NM_001167617)	promoter-TSS (NM_001167617)	101	NM_00125	4292	Hs.195364	NM_00024	ENSG0000	MLH1	COCA2|FC	mutL hom	protein-co
lib-C666-l	chr12	54121408	54121408	+	49.83866	NA	promoter-TSS (NM_020898)	promoter-TSS (NM_020898)	-101	NM_00114	57658	Hs.156667	NM_02089	ENSG0000	CALCOCO	Cocoa|PP1	calcium bir	protein-co
lib-C666-l	chr19	5720204	5720204	+	70.69921	NA	promoter-TSS (NM_004793)	promoter-TSS (NM_004793)	-28	NM_00127	9361	Hs.350265	NM_00479	ENSG0000	LONP1	CODASS|L	lon peptida	protein-co
lib-C666-l	chr5	158374451	158374451	+	9.10494	NA	intron (NM_182708, intron 5 of 14)	L1PA7|LINE|L1	152319	NM_00129	1879	Hs.573143	NM_02400	ENSG0000	EBF1	COE1|EBF|(	early B-cel	protein-co
lib-C666-l	chr13	103498132	103498132	+	95.85526	NA	promoter-TSS (NM_000123)	promoter-TSS (NM_000123)	-59	NM_00012	2073	Hs.258429	NM_00012	ENSG0000	ERCC5	COFS3|ERC	ERCC excis	protein-co
lib-C666-l	chr19	45927158	45927158	+	5.34142	NA	promoter-TSS (NM_001166049)	promoter-TSS (NM_001166049)	19	NM_00116	2067	Hs.435981	NM_00198	ENSG0000	ERCC1	COFS4|RAD	ERCC excis	protein-co
lib-C666-l	chrX	62975041	62975041	+	13.20416	NA	promoter-TSS (NM_015185)	promoter-TSS (NM_015185)	-10	NM_01518	23229	Hs.54697	NM_01518	ENSG0000	ARHGEF9	COLLYBIST	Cdc42 gua	protein-co
lib-C666-l	chr18	20513267	20513267	+	33.11264	NA	promoter-TSS (NM_002894)	promoter-TSS (NM_002894)	-28	NM_00289	5932	Hs.546282	NM_00289	ENSG0000	RBBP8	COM1|CTII	RB binding	protein-co
lib-C666-l	chr18	20493950	20493950	+	9.93242	NA	Intergenic	Intergenic	-19345	NM_00289	5932	Hs.546282	NM_00289	ENSG0000	RBBP8	COM1|CTII	RB binding	protein-co
lib-C666-l	chr12	31257157	31257157	+	5.23679	NA	3' UTR (NM_004399, exon 26 of 26)	3' UTR (NM_004399, exon 26 of 26)	-30376	NR_03892	100506660	Hs.586774	NR_03892	ENSG0000	DDX11-AS	CONCR	DDX11 ant	ncRNA
lib-C666-l	chr2	237994506	237994506	+	246.39511	NA	5' UTR (NM_006710, exon 1 of 8)	5' UTR (NM_006710, exon 1 of 8)	422	NM_00671	10920	Hs.531713	NM_00671	ENSG0000	COPS8	COP9|CSN	COP9 signa	protein-co
lib-C666-l	chr2	238037012	238037012	+	28.81777	NA	Intergenic	Intergenic	42928	NM_00671	10920	Hs.531713	NM_00671	ENSG0000	COPS8	COP9|CSN	COP9 signa	protein-co
lib-C666-l	chr11	14521406	14521406	+	5.5166	NA	promoter-TSS (NM_016451)	promoter-TSS (NM_016451)	-2	NM_01645	1315	Hs.339278	NM_01645	ENSG0000	COPB1	COPB	coatomer	protein-co
lib-C666-l	chr20	34252911	34252911	+	5.76059	NA	promoter-TSS (NM_001198863)	promoter-TSS (NM_001198863)	-33	NM_15292	8904	Hs.246413	NM_00391	ENSG0000	CPNE1	COPN1|CP	copine 1	protein-co
lib-C666-l	chr10	64893009	64893009	+	36.04455	NA	promoter-TSS (NM_001282405)	promoter-TSS (NM_001282405)	2	NM_03075	29982	Hs.449628	NM_03075	ENSG0000	NRBF2	COPR|COP	nuclear rec	protein-co
lib-C666-l	chr17	80009867	80009867	+	7.1502	NA	promoter-TSS (NM_002917)	promoter-TSS (NM_002917)	126	NM_00132	2873	Hs.268530	NM_00412	ENSG0000	GPS1	COPS1|CSN	G protein	protein-co
lib-C666-l	chr4	13486159	13486159	+	19.75653	NA	promoter-TSS (NM_001017979)	promoter-TSS (NM_001017979)	-170	NM_00115	9364	Hs.656060	NM_00424	ENSG0000	RAB28	CORD18	RAB28, me	protein-co
lib-C666-l	chr4	13331805	13331805	+	7.09685	NA	Intergenic	Intergenic	154184	NM_00424	9364	Hs.656060	NM_00424	ENSG0000	RAB28	CORD18	RAB28, me	protein-co
lib-C666-l	chr14	76178370	76178370	+	5.72511	NA	intron (NM_015072, intron 9 of 31)	intron (NM_015072, intron 9 of 31)	50819	NM_01507	23093	Hs.709609	NM_01507	ENSG0000	TTLL5	CORD19|K	tubulin tyr	protein-co
lib-C666-l	chr14	103058592	103058592	+	12.23699	NA	promoter-TSS (NM_015156)	promoter-TSS (NM_015156)	-404	NM_01515	23186	Hs.510521	NM_01515	ENSG0000	RCOR1	COREST|R(	REST corep	protein-co
lib-C666-l	chr1	9291868	9291868	+	8.40444	NA	Intergenic	MIRc|SINE|MIR	-2995	NM_00428	9563	Hs.463511	NM_00428	ENSG0000	H6PD	CORTRD1|	hexose-6-	protein-co
lib-C666-l	chr1	9292041	9292041	+	3.84463	NA	Intergenic	Intergenic	-2822	NM_00428	9563	Hs.463511	NM_00428	ENSG0000	H6PD	CORTRD1|	hexose-6-	protein-co
lib-C666-l	chr22	24093325	24093325	+	8.56376	NA	promoter-TSS (NM_021916)	promoter-TSS (NM_021916)	-46	NM_02191	7621	Hs.382874	NM_02191	ENSG0000	ZNF70	Cos17	zinc finger	protein-co
lib-C666-l	chr22	20748427	20748427	+	2.59032	NA	promoter-TSS (NM_001256523)	promoter-TSS (NM_001256523)	22	NM_00125	7625	Hs.517418	NM_00342	ENSG0000	ZNF74	COS52|ZFF	zinc finger	protein-co
lib-C666-l	chr16	85833847	85833847	+	5.96422	NA	promoter-TSS (NM_006067)	promoter-TSS (NM_006067)	543	NM_00131	1327	Hs.433419	NM_00186	ENSG0000	COX4I1	COX IV-1|K	cytochrom	protein-co
lib-C666-l	chr11	63741978	63741978	+	41.28836	NA	promoter-TSS (NM_004074)	promoter-TSS (NM_004074)	-101	NM_00407	1351	Hs.743989	NM_00407	ENSG0000	COX8A	COX|COX8	cytochrom	protein-co

lib-C666-l	chr15	75230399	75230399	+	7.03995	NA	promoter-TSS (NM_004255)	promoter-TSS (NM_004255)	96	NM_00425	9377	Hs.401903	NM_00425	ENSG0000	COX5A	COX|COX-	cytochrom	protein-co
lib-C666-l	chr17	13972798	13972798	+	11.66438	NA	promoter-TSS (NM_001303)	promoter-TSS (NM_001303)	-23	NR_04971	100874058	Hs.720411	NR_04971	ENSG0000	COX10-AS	COX10-AS	COX10 ant	ncRNA
lib-C666-l	chr4	73935532	73935532	+	173.02444	NA	promoter-TSS (NM_173827)	promoter-TSS (NM_173827)	-56	NM_00130	285521	Hs.356697	NM_17382	ENSG0000	COX18	COX18HS	COX18, cyt	protein-co
lib-C666-l	chr1	186649526	186649526	+	2.24771	NA	promoter-TSS (NR_125801)	promoter-TSS (NR_125801)	33	NM_00096	5743	Hs.196384	NM_00096	ENSG0000	PTGS2	COX-2|CO	prostaglan	protein-co
lib-C666-l	chr8	57124370	57124370	+	16.63918	NA	promoter-TSS (NM_002655)	promoter-TSS (NM_002655)	173	NM_00101	79145	Hs.436913	NM_02430	ENSG0000	CHCHD7	COX23	coiled-coil	protein-co
lib-C666-l	chr16	31441898	31441898	+	5.23609	NA	Intergenic	Intergenic	-2149	NM_00520	1339	Hs.250760	NM_00520	ENSG0000	COX6A2	COX6AH|C	cytochrom	protein-co
lib-C666-l	chr19	36661715	36661715	+	5.12426	NA	Intergenic	Intergenic	-17944	NM_00186	1346	Hs.421621	NM_00186	ENSG0000	COX7A1	COX7A|CC	cytochrom	protein-co
lib-C666-l	chr16	23568677	23568677	+	95.46208	NA	promoter-TSS (NM_019116)	promoter-TSS (NM_019116)	19	NR_00350	124454	Hs.620541	NM_00108	ENSG0000	EARS2	COXPD12|	glutamyl-t	protein-co
lib-C666-l	chr17	12921306	12921306	+	9.10494	NA	promoter-TSS (NM_001165962)	promoter-TSS (NM_001165962)	75	NM_17371	60528	Hs.434232	NM_01812	ENSG0000	ELAC2	COXPD17|	elaC ribon	protein-co
lib-C666-l	chr6	30882138	30882138	+	11.07709	NA	promoter-TSS (NM_001167734)	promoter-TSS (NM_001167734)	30	NM_00116	57176	Hs.597526	NM_02044	ENSG0000	VARS2	COXPD20|	valyl-tRNA	protein-co
lib-C666-l	chr1	150459807	150459807	+	7.11942	NA	promoter-TSS (NR_073514)	promoter-TSS (NR_073514)	-33	NM_00127	80222	Hs.288974	NM_02515	ENSG0000	TARS2	COXPD21|	threonyl-t	protein-co
lib-C666-l	chr19	17448298	17448298	+	11.86993	NA	promoter-TSS (NM_001128855)	promoter-TSS (NM_001128855)	-28	NM_03262	84705	Hs.334885	NM_03262	ENSG0000	GTPBP3	COXPD23|	GTP bindin	protein-co
lib-C666-l	chr19	17447344	17447344	+	3.18342	NA	promoter-TSS (NM_001128855)	promoter-TSS (NM_001128855)	-982	NM_03262	84705	Hs.334885	NM_03262	ENSG0000	GTPBP3	COXPD23|	GTP bindin	protein-co
lib-C666-l	chr2	198570218	198570218	+	6.32351	NA	exon (NM_138395, exon 1 of 1)	exon (NM_138395, exon 1 of 1)	190	NM_13839	92935	Hs.744330	NM_13839	ENSG0000	MARS2	COXPD25|	methionyl-	protein-co
lib-C666-l	chr14	61447806	61447806	+	255.78366	NA	promoter-TSS (NM_153811)	promoter-TSS (NM_153811)	-9	NM_02081	57570	Hs.380159	NM_02081	ENSG0000	TRMT5	COXPD26|	tRNA meth	protein-co
lib-C666-l	chr13	111358376	111358376	+	94.41716	NA	exon (NM_024537, exon 1 of 15)	exon (NM_024537, exon 1 of 15)	151	NM_02453	79587	Hs.508725	NM_02453	ENSG0000	CARS2	COXPD27|	cysteinyl-t	protein-co
lib-C666-l	chr3	66271430	66271430	+	20.72954	NA	non-coding (NR_028475, exon 1 of 1	non-coding (NR_028475, exon 1 of	262	NR_02847	115286	Hs.379386	NM_17347	ENSG0000	SLC25A26	COXPD28|	solute carr	protein-co
lib-C666-l	chr22	36877597	36877597	+	145.07654	NA	promoter-TSS (NM_012473)	promoter-TSS (NM_012473)	90	NM_01247	25828	Hs.211929	NM_01247	ENSG0000	TXN2	COXPD29|	thioredoxi	protein-co
lib-C666-l	chr22	36851263	36851263	+	6.49644	NA	Intergenic	CpG	26424	NM_01247	25828	Hs.211929	NM_01247	ENSG0000	TXN2	COXPD29|	thioredoxi	protein-co
lib-C666-l	chr22	36858557	36858557	+	3.23837	NA	Intergenic	Intergenic	19130	NM_01247	25828	Hs.211929	NM_01247	ENSG0000	TXN2	COXPD29|	thioredoxi	protein-co
lib-C666-l	chr3	101280655	101280655	+	44.27569	NA	promoter-TSS (NM_017819)	promoter-TSS (NM_017819)	-25	NM_01781	54931	Hs.594839	NM_01781	ENSG0000	TRMT10C	COXPD30|	tRNA meth	protein-co
lib-C666-l	chr16	28857758	28857758	+	157.61473	NA	promoter-TSS (NM_003321)	promoter-TSS (NM_003321)	-29	NM_00332	7284	Hs.12084	NM_00332	ENSG0000	TUFM	COXPD4|E	Tu translat	protein-co
lib-C666-l	chr3	131221771	131221771	+	17.68706	NA	promoter-TSS (NM_007208)	promoter-TSS (NM_007208)	89	NM_00720	11222	Hs.205163	NM_00720	ENSG0000	MRPL3	COXPD9|M	mitochond	protein-co
lib-C666-l	chr20	35724542	35724542	+	12.62235	NA	promoter-TSS (NM_002895)	promoter-TSS (NM_002895)	76	NM_00132	5933	Hs.207745	NM_00289	ENSG0000	RBL1	CP107|PRB	RB transcri	protein-co
lib-C666-l	chr16	19535125	19535125	+	77.06716	NA	promoter-TSS (NM_001199022)	promoter-TSS (NM_001199022)	-54	NM_00132	9738	Hs.279912	NM_01471	ENSG0000	CCP110	CP110|Cep	centriolar c	protein-co
lib-C666-l	chr2	38285456	38285456	+	7.50042	NA	intron (NM_144713, intron 10 of 10)	intron (NM_144713, intron 10 of 10	17867	NM_00010	1545	Hs.154654	NM_00010	ENSG0000	CYP1B1	CP1B|CYPI	cytochrom	protein-co
lib-C666-l	chr2	38323495	38323495	+	4.27545	NA	Intergenic	Intergenic	-20172	NM_00010	1545	Hs.154654	NM_00010	ENSG0000	CYP1B1	CP1B|CYPI	cytochrom	protein-co
lib-C666-l	chr7	87849549	87849549	+	13.77909	NA	promoter-TSS (NM_003130)	promoter-TSS (NM_003130)	-150	NM_00125	6717	Hs.489040	NM_00313	ENSG0000	SRI	CP-22|CP2	sorcin	protein-co
lib-C666-l	chr16	2576322	2576322	+	5.59206	NA	intron (NM_001330449, intron 3 of 1	HAL1|LINE|L1	5087	NM_00104	752014	Hs.433499	NM_00104	ENSG0000	CEMP1	CP-23|CP2	cementum	protein-co
lib-C666-l	chr10	94968981	94968981	+	23.80446	NA	Intergenic	Intergenic	135334	NM_00078	1592	Hs.150595	NM_00078	ENSG0000	CYP26A1	CP26|CYP2	cytochrom	protein-co
lib-C666-l	chr2	219650075	219650075	+	10.36806	NA	intron (NM_000784, intron 1 of 8)	MER1A|DNA|hAT-Charlie	3603	NM_00078	1593	Hs.516700	NM_00078	ENSG0000	CYP27A1	CP27|CTX|	cytochrom	protein-co
lib-C666-l	chr14	100150236	100150236	+	64.98446	NA	promoter-TSS (NM_006668)	promoter-TSS (NM_006668)	-519	NM_00666	10858	Hs.25121	NM_00666	ENSG0000	CYP46A1	CP46|CYP4	cytochrom	protein-co
lib-C666-l	chr7	91764011	91764011	+	80.62132	NA	promoter-TSS (NM_001146152)	promoter-TSS (NM_001146152)	48	NM_00114	1595	Hs.417077	NM_00078	ENSG0000	CYP51A1	CP51|CYP5	cytochrom	protein-co
lib-C666-l	chr19	41506424	41506424	+	51.38887	NA	intron (NM_000767, intron 1 of 8)	intron (NM_000767, intron 1 of 8)	9220	NM_00076	1555	Hs.1360	NM_00076	ENSG0000	CYP2B6	CPB6|CYP2	cytochrom	protein-co
lib-C666-l	chrX	48659924	48659924	+	266.84183	NA	promoter-TSS (NM_001321226)	promoter-TSS (NM_001321226)	140	NM_00132	10013	Hs.6764	NM_00604	ENSG0000	HDAC6	CPBHM|HD	histone de	protein-co
lib-C666-l	chr10	135337275	135337275	+	11.77013	NA	Intergenic	Intergenic	-3592	NM_00077	1571	Hs.12907	NM_00077	ENSG0000	CYP2E1	CPE1|CYP2	cytochrom	protein-co
lib-C666-l	chr4	15004021	15004021	+	61.35856	NA	promoter-TSS (NM_182485)	promoter-TSS (NM_182485)	-277	NM_00117	132864	Hs.656937	NM_18248	ENSG0000	CPEB2	CPE-BP2|C	cytoplasmi	protein-co
lib-C666-l	chr5	173315263	173315263	+	5.25516	NA	promoter-TSS (NM_001308189)	promoter-TSS (NM_001308189)	-68	NM_03062	80315	Hs.127126	NM_03062	ENSG0000	CPEB4	CPE-BP4|h	cytoplasmi	protein-co
lib-C666-l	chr7	73246783	73246783	+	9.97417	NA	3' UTR (NM_001305, exon 1 of 1)	3' UTR (NM_001305, exon 1 of 1)	1590	NM_00130	1364	Hs.647036	NM_00130	ENSG0000	CLDN4	CPE-R|CPE	claudin 4	protein-co
lib-C666-l	chr7	73242201	73242201	+	5.23609	NA	Intergenic	Intergenic	-2992	NM_00130	1364	Hs.647036	NM_00130	ENSG0000	CLDN4	CPE-R|CPE	claudin 4	protein-co
lib-C666-l	chr7	73224244	73224244	+	2.58799	NA	Intergenic	Intergenic	-20949	NM_00130	1364	Hs.647036	NM_00130	ENSG0000	CLDN4	CPE-R|CPE	claudin 4	protein-co
lib-C666-l	chr3	87553353	87553353	+	26.18172	NA	Intergenic	Intergenic	-227616	NM_00112	5449	Hs.591654	NM_00030	ENSG0000	POU1F1	CPHD1|GH	POU class	protein-co
lib-C666-l	chr3	87382248	87382248	+	9.53658	NA	Intergenic	Intergenic	-56511	NM_00112	5449	Hs.591654	NM_00030	ENSG0000	POU1F1	CPHD1|GH	POU class	protein-co
lib-C666-l	chr9	139062436	139062436	+	27.70954	NA	Intergenic	Intergenic	32583	NM_01456	8022	Hs.148427	NM_01456	ENSG0000	LHX3	CPHD3|LIM	LIM home	protein-co
lib-C666-l	chr6	150465323	150465323	+	16.14038	NA	intron (NM_030949, intron 1 of 3)	intron (NM_030949, intron 1 of 3)	1135	NM_03094	81706	Hs.486798	NM_03094	ENSG0000	PPP1R14C	CPI17-like|	protein ph	protein-co
lib-C666-l	chr21	45195927	45195927	+	15.01861	NA	intron (NM_000100, intron 1 of 2)	CpG	329	NM_00010	1476	Hs.695	NM_00010	ENSG0000	CSTB	CPI-B|CST	cystatin B	protein-co
lib-C666-l	chr11	17229522	17229522	+	32.48059	NA	promoter-TSS (NM_001321378)	promoter-TSS (NM_001321378)	21	NM_00132	5286	Hs.175343	NM_00264	ENSG0000	PIK3C2A	CPK|PI3-K-	phosphati	protein-co
lib-C666-l	chr19	48625397	48625397	+	5.89415	NA	intron (NR_135501, intron 21 of 25)	intron (NR_135501, intron 21 of 25)	-11288	NM_00115	8605	Hs.631562	NM_00370	ENSG0000	PLA2G4C	CPLA2-ga	phospholi	protein-co
lib-C666-l	chr11	88070916	88070916	+	12.78696	NA	promoter-TSS (NM_001814)	promoter-TSS (NM_001814)	39	NM_14817	1075	Hs.128065	NM_00181	ENSG0000	CTSC	CPPI|DPP-	cathepsin	protein-co
lib-C666-l	chr7	75544371	75544371	+	17.78771	NA	promoter-TSS (NM_000941)	promoter-TSS (NM_000941)	-49	NM_00094	5447	Hs.354056	NM_00094	ENSG0000	POR	CPR|CYPO	cytochrom	protein-co
lib-C666-l	chr7	45151322	45151322	+	137.83443	NA	promoter-TSS (NM_001261834)	promoter-TSS (NM_001261834)	24	NM_03090	9238	Hs.231411	NM_00474	ENSG0000	TBRG4	CPR2|FAST	transformi	protein-co
lib-C666-l	chr16	47007640	47007640	+	29.22024	NA	promoter-TSS (NM_005880)	promoter-TSS (NM_005880)	-15	NM_00588	10294	Hs.368078	NM_00588	ENSG0000	DNAJA2	CPR3|DJ3|D	DnaJ heat	protein-co
lib-C666-l	chr14	99947694	99947694	+	121.90443	NA	promoter-TSS (NM_032233)	promoter-TSS (NM_032233)	-45	NM_00109	8812	Hs.510409	NM_00385	ENSG0000	CCNK	CPR4	cyclin K	protein-co
lib-C666-l	chr15	55700531	55700531	+	7.03048	NA	promoter-TSS (NM_001198784)	promoter-TSS (NM_001198784)	43	NM_00474	9236	Hs.285051	NM_00474	ENSG0000	CCPG1	CPR8	cell cycle p	protein-co
lib-C666-l	chr7	99699129	99699129	+	12.62235	NA	promoter-TSS (NM_182776)	promoter-TSS (NM_182776)	-1	NM_00472	9179	Hs.632317	NM_00472	ENSG0000	AP4M1	CPSQ3|MU	adaptor re	protein-co
lib-C666-l	chr15	51200722	51200722	+	8.03539	NA	promoter-TSS (NM_001252127)	promoter-TSS (NM_001252127)	-147	NM_00734	23431	Hs.413366	NM_00734	ENSG0000	AP4E1	CPSQ4|SP	adaptor re	protein-co
lib-C666-l	chr15	51200999	51200999	+	7.0832	NA	exon (NM_007347, exon 1 of 21)	exon (NM_007347, exon 1 of 21)	130	NM_00125	23431	Hs.413366	NM_00734	ENSG0000	AP4E1	CPSQ4|SP	adaptor re	protein-co
lib-C666-l	chr10	125617652	125617652	+	13.14211	NA	intron (NM_198148, intron 3 of 13)	Tigger9a|DNA|TcMar-Tigger	33848	NM_19814	119587	Hs.656887	NM_19814	ENSG0000	CPXM2	CPX2|UNQ	carboxype	protein-co
lib-C666-l	chr1	207494951	207494951	+	33.60578	NA	non-coding (NR_125349, exon 1 of 1	non-coding (NR_125349, exon 1 of	134	NM_00130	1604	Hs.126517	NM_00057	ENSG0000	CD55	CR|CROM|	CD55 mole	protein-co
lib-C666-l	chr12	42719941	42719941	+	71.09715	NA	promoter-TSS (NM_033114)	promoter-TSS (NM_033114)	-6	NM_00114	51535	Hs.444157	NM_01648	ENSG0000	PPHLN1	CR|HSPC2	periphilin 1	protein-co
lib-C666-l	chr14	24411985	24411985	+	18.20429	NA	intron (NR_023922, intron 1 of 1)	L1PA7|LINE|L1	-10959	NM_00128	10901	Hs.528385	NM_02100	ENSG0000	DHRS4	CR|NRDR|F	dehydroge	protein-co
lib-C666-l	chr14	24422892	24422892	+	6.01141	NA	promoter-TSS (NM_001282988)	promoter-TSS (NM_001282988)	-52	NM_00128	10901	Hs.528385	NM_02100	ENSG0000	DHRS4	CR|NRDR|F	dehydroge	protein-co
lib-C666-l	chr16	3185447	3185447	+	3.25502	NA	promoter-TSS (NR_110900)	promoter-TSS (NR_110900)	313	NM_00113	7760	Hs.115284	NM_00422	ENSG0000	ZNF213	CR53|ZKSC	zinc finger	protein-co
lib-C666-l	chr9	92219817	92219817	+	7.76377	NA	promoter-TSS (NM_006705)	promoter-TSS (NM_006705)	-110	NM_00670	10912	Hs.9701	NM_00670	ENSG0000	GADD45G	CR6|DDIT2	growth arr	protein-co
lib-C666-l	chr12	122064405	122064405	+	6.48676	NA	promoter-TSS (NM_032790)	promoter-TSS (NM_032790)	-50	NM_03279	84876	Hs.55148	NM_032790		ORAI1	CRACM1|I	ORAI calci	protein-co
lib-C666-l	chr15	89722445	89722445	+	7.52748	NA	intron (NM_152924, intron 6 of 10)	intron (NM_152924, intron 6 of 10)	42477	NM_00032	6017	Hs.1933	NM_00032	ENSG0000	RLBP1	CRALBP	retinaldeh	protein-co
lib-C666-l	chr9	134955301	134955301	+	96.94724	NA	promoter-TSS (NM_001253881)	promoter-TSS (NM_001253881)	-27	NM_00125	9442	Hs.374262	NM_00426	ENSG0000	MED27	CRAP34|C	mediator c	protein-co
lib-C666-l	chr12	133264074	133264074	+	12.62235	NA	promoter-TSS (NM_006231)	promoter-TSS (NM_006231)	36	NM_00623	5426	Hs.524871	NM_00623	ENSG0000	POLE	CRCS12|FI	DNA polyn	protein-co
lib-C666-l	chr18	46474930	46474930	+	15.60931	NA	intron (NM_005904, intron 1 of 3)	intron (NM_005904, intron 1 of 3)	773	NM_00119	4092	Hs.465087	NM_00590	ENSG0000	SMAD7	CRCS3|MA	SMAD fam	protein-co
lib-C666-l	chr18	46476264	46476264	+	8.89416	NA	promoter-TSS (NM_001190822)	promoter-TSS (NM_001190822)	-561	NM_00119	4092	Hs.465087	NM_00590	ENSG0000	SMAD7	CRCS3|MA	SMAD fam	protein-co
lib-C666-l	chr18	46476607	46476607	+	7.0832	NA	promoter-TSS (NM_001190822)	promoter-TSS (NM_001190822)	474	NM_00119	4092	Hs.465087	NM_00590	ENSG0000	SMAD7	CRCS3|MA	SMAD fam	protein-co
lib-C666-l	chr18	46502570	46502570	+	6.81085	NA	Intergenic	CpG	-25489	NM_00590	4092	Hs.465087	NM_00590	ENSG0000	SMAD7	CRCS3|MA	SMAD fam	protein-co
lib-C666-l	chr18	46475879	46475879	+	4.39666	NA	promoter-TSS (NM_001190822)	promoter-TSS (NM_001190822)	-176	NM_00119	4092	Hs.465087	NM_00590	ENSG0000	SMAD7	CRCS3|MA	SMAD fam	protein-co
lib-C666-l	chr18	46359516	46359516	+	2.78587	NA	intron (NM_014772, intron 10 of 11)	intron (NM_014772, intron 10 of 11	109661	NM_00119	4092	Hs.465087	NM_00590	ENSG0000	SMAD7	CRCS3|MA	SMAD fam	protein-co
lib-C666-l	chr17	47109637	47109637	+	15.42216	NA	intron (NM_001160423, intron 5 of 1	intron (NM_001160423, intron 5 of	34863	NM_00654	10642	Hs.144936	NM_00654	ENSG0000	IGF2BP1	CRD-BP|C	insulin like	protein-co
lib-C666-l	chr2	208394770	208394770	+	22.82077	NA	non-coding (NR_135473, exon 1 of 1	non-coding (NR_135473, exon 1 of	154	NR_13547	1385	Hs.516646	NM_00437	ENSG0000	CREB1	CREB|CREE	cAMP resp	protein-co
lib-C666-l	chr22	39916375	39916375	+	10.40942	NA	promoter-TSS (NM_001675)	promoter-TSS (NM_001675)	-189	NM_00167	468	Hs.496487	NM_00167	ENSG0000	ATF4	CREB-2|CR	activating t	protein-co
lib-C666-l	chr7	28651692	28651692	+	24.25638	NA	intron (NM_182899, intron 4 of 9)	intron (NM_182899, intron 4 of 9)	-73906	NM_00101	9586	Hs.437075	NM_00490	ENSG0000	CREB5	CRE-BPA|C	cAMP resp	protein-co
lib-C666-l	chr7	28459563	28459563	+	19.65763	NA	intron (NM_182898, intron 1 of 10)	intron (NM_182898, intron 1 of 10)	7419	NM_18289	9586	Hs.437075	NM_00490	ENSG0000	CREB5	CRE-BPA|C	cAMP resp	protein-co
lib-C666-l	chr7	28448986	28448986	+	8.35705	NA	intron (NM_182899, intron 1 of 9)	CpG	-3158	NM_18289	9586	Hs.437075	NM_00490	ENSG0000	CREB5	CRE-BPA|C	cAMP resp	protein-co
lib-C666-l	chr1	167522786	167522786	+	10.67459	NA	exon (NM_003851, exon 1 of 4)	exon (NM_003851, exon 1 of 4)	270	NM_00385	8804	Hs.5710	NM_00385	ENSG0000	CREG1	CREG	cellular rep	protein-co
lib-C666-l	chr2	61108659	61108659	+	45.65358	NA	promoter-TSS (NR_033980)	promoter-TSS (NR_033980)	29	NM_00290	5966	Hs.631886	NM_00290	ENSG0000	REL	C-Rel	REL proto-	protein-co
lib-C666-l	chr10	35484816	35484816	+	54.91701	NA	promoter-TSS (NM_182723)	promoter-TSS (NM_182723)	-14	NM_18272	1390	Hs.200250	NM_00188	ENSG0000	CREM	CREM-2|I	cAMP resp	protein-co
lib-C666-l	chr10	35416023	35416023	+	37.48738	NA	promoter-TSS (NM_181571)	promoter-TSS (NM_181571)	254	NM_00126	1390	Hs.200250	NM_00188	ENSG0000	CREM	CREM-2|I	cAMP resp	protein-co
lib-C666-l	chr10	35484041	35484041	+	13.46097	NA	promoter-TSS (NM_001267568)	promoter-TSS (NM_001267568)	-14	NM_18271	1390	Hs.200250	NM_00188	ENSG0000	CREM	CREM-2|I	cAMP resp	protein-co
lib-C666-l	chr1	204381033	204381033	+	42.50122	NA	promoter-TSS (NM_032833)	promoter-TSS (NM_032833)	-88	NM_03283	84919	Hs.304376	NM_03283	ENSG0000	PPP1R15B	CREP|MSS	protein ph	protein-co
lib-C666-l	chr6	137493547	137493547	+	3.92495	NA	intron (NM_181310, intron 1 of 4)	intron (NM_181310, intron 1 of 4)	1238	NM_18131	116379	Hs.126891	NM_05296	ENSG0000	IL22RA2	CRF2-10|C	interleukin	protein-co
lib-C666-l	chr6	137402017	137402017	+	3.92495	NA	Intergenic	MER61A|LTR|ERV1	-35700	NM_01443	53832	Hs.445868	NM_01443	ENSG0000	IL20RA	CRF2-8|IL-	interleukin	protein-co
lib-C666-l	chr10	1034338	1034338	+	39.64852	NA	promoter-TSS (NM_012341)	promoter-TSS (NM_012341)	-11	NM_01234	23560	Hs.215766	NM_01234	ENSG0000	GTPBP4	CRFG|NGB	GTP bindin	protein-co
lib-C666-l	chr8	61564663	61564663	+	41.46989	NA	Intergenic	CpG	-26661	NM_01778	55636	Hs.20395	NM_01778	ENSG0000	CHD7	CRG|HH5|I	chromodo	protein-co
lib-C666-l	chr8	61590986	61590986	+	2.05265	NA	promoter-TSS (NM_017780)	promoter-TSS (NM_017780)	-338	NM_01778	55636	Hs.20395	NM_01778	ENSG0000	CHD7	CRG|HH5|I	chromodo	protein-co
lib-C666-l	chr8	145911390	145911390	+	5.819	NA	promoter-TSS (NM_001308208)	promoter-TSS (NM_001308208)	-193	NM_00130	80728	Hs.148988	NM_02525	ENSG0000	ARHGAP3	CrGAP|Vils	Rho GTPas	protein-co
lib-C666-l	chr16	8962507	8962507	+	13.59977	NA	promoter-TSS (NM_001278261)	promoter-TSS (NM_001278261)	69	NM_00127	23589	Hs.632184	NM_01431	ENSG0000	CARHSP1	CRHSP-24	calcium re	protein-co
lib-C666-l	chr16	85649532	85649532	+	27.5466	NA	intron (NM_001278184, intron 1 of 1	intron (NM_001278184, intron 1 of	2690	NM_00127	23199	Hs.461647	NM_01461	ENSG0000	GSE1	CRHSP24|	Gse1 coile	protein-co
lib-C666-l	chr16	85587143	85587143	+	17.68706	NA	Intergenic	CpG	-57886	NM_00113	23199	Hs.461647	NM_01461	ENSG0000	GSE1	CRHSP24|	Gse1 coile	protein-co
lib-C666-l	chr16	85557452	85557452	+	11.66438	NA	Intergenic	Intergenic	-87577	NM_00113	23199	Hs.461647	NM_01461	ENSG0000	GSE1	CRHSP24|	Gse1 coile	protein-co
lib-C666-l	chr16	85558677	85558677	+	11.48337	NA	Intergenic	Intergenic	-86352	NM_00113	23199	Hs.461647	NM_01461	ENSG0000	GSE1	CRHSP24|	Gse1 coile	protein-co
lib-C666-l	chr16	85648818	85648818	+	11.06679	NA	intron (NM_001278184, intron 1 of 1	CpG-9602	1976	NM_00127	23199	Hs.461647	NM_01461	ENSG0000	GSE1	CRHSP24|	Gse1 coile	protein-co
lib-C666-l	chr16	85646738	85646738	+	10.13529	NA	promoter-TSS (NM_001278184)	promoter-TSS (NM_001278184)	-104	NM_01461	23199	Hs.461647	NM_01461	ENSG0000	GSE1	CRHSP24|	Gse1 coile	protein-co
lib-C666-l	chr16	85590858	85590858	+	6.16704	NA	Intergenic	Intergenic	-54171	NM_00113	23199	Hs.461647	NM_01461	ENSG0000	GSE1	CRHSP24|	Gse1 coile	protein-co
lib-C666-l	chr16	85612566	85612566	+	2.96041	NA	Intergenic	L2b|LINE|L2	-32463	NM_00113	23199	Hs.461647	NM_01461	ENSG0000	GSE1	CRHSP24|	Gse1 coile	protein-co
lib-C666-l	chr8	144897452	144897452	+	6.04359	NA	promoter-TSS (NM_182706)	promoter-TSS (NM_182706)	97	NM_18270	23513	Hs.436329	NM_01535	ENSG0000	SCRIB	CRIB1|SCR	scribbled p	protein-co
lib-C666-l	chr8	144897843	144897843	+	4.38153	NA	promoter-TSS (NM_182706)	promoter-TSS (NM_182706)	-294	NM_01535	23513	Hs.436329	NM_01535	ENSG0000	SCRIB	CRIB1|SCR	scribbled p	protein-co
lib-C666-l	chr14	105940711	105940711	+	6.98764	NA	promoter-TSS (NR_073083)	promoter-TSS (NR_073083)	-401	NR_07308	1397	Hs.534309	NM_00131	ENSG0000	CRIP2	CRIP|CRP2	cysteine ric	protein-co
lib-C666-l	chr14	105940339	105940339	+	4.90117	NA	promoter-TSS (NR_073083)	promoter-TSS (NR_073083)	-773	NR_07308	1397	Hs.534309	NM_00131	ENSG0000	CRIP2	CRIP|CRP2	cysteine ric	protein-co
lib-C666-l	chr17	1359601	1359601	+	36.5431	NA	promoter-TSS (NM_005206)	promoter-TSS (NM_005206)	-40	NM_01682	1398	Hs.461896	NM_00520	ENSG0000	CRK	CRKII|p38	CRK proto-	protein-co
lib-C666-l	chr5	55199388	55199388	+	28.88523	NA	intron (NM_001242639, intron 9 of 1	intron (NM_001242639, intron 9 of	50238	NM_00124	133396	Hs.55378	NM_13901	ENSG0000	IL31RA	CRL|CRL3|	interleukin	protein-co
lib-C666-l	chr4	71554100	71554100	+	8.73558	NA	promoter-TSS (NM_020368)	promoter-TSS (NM_020368)	-96	NM_02036	57050	Hs.322901	NM_02036	ENSG0000	UTP3	CRL1|CRLZ	UTP3, sma	protein-co
lib-C666-l	chr2	61825730	61825730	+	21.21068	NA	Intergenic	LTR10F|LTR|ERV1	-60312	NM_00340	7514	Hs.370770	NM_00340	ENSG0000	XPO1	CRM1|emb	exportin 1	protein-co
lib-C666-l	chr2	61765608	61765608	+	9.14689	NA	promoter-TSS (NM_003400)	promoter-TSS (NM_003400)	-190	NM_00340	7514	Hs.370770	NM_00340	ENSG0000	XPO1	CRM1|emb	exportin 1	protein-co
lib-C666-l	chr2	61765881	61765881	+	7.21092	NA	promoter-TSS (NM_003400)	promoter-TSS (NM_003400)	-463	NM_00340	7514	Hs.370770	NM_00340	ENSG0000	XPO1	CRM1|emb	exportin 1	protein-co
lib-C666-l	chr8	26486327	26486327	+	5.25516	NA	intron (NM_001386, intron 7 of 13)	intron (NM_001386, intron 7 of 13)	50406	NM_00124	1808	Hs.593187	NM_00138	ENSG0000	DPYSL2	CRMP-2|C	dihydropyr	protein-co
lib-C666-l	chr10	133972259	133972259	+	6.15313	NA	intron (NM_001323090, intron 19 of 2	intron (NM_001323090, intron 19 o	-28155	NM_00642	10570	Hs.100058	NM_00642	ENSG0000	DPYSL4	CRMP3|DR	dihydropyr	protein-co
lib-C666-l	chr16	54963176	54963176	+	14.02822	NA	promoter-TSS (NM_001308963)	promoter-TSS (NM_001308963)	-75	NR_03410	643911	Hs.237396	NM_001308963		CRNDE	CRNDEP|L	colorectal	protein-co
lib-C666-l	chr1	156391664	156391664	+	32.22565	NA	promoter-TSS (NR_135268)	promoter-TSS (NR_135268)	-106	NR_13526	10485	Hs.380027	NM_00636	ENSG0000	C1orf61	CROC4	chromoso	protein-co
lib-C666-l	chr1	156426776	156426776	+	12.97267	NA	Intergenic	CpG	-27436	NR_13526	10485	Hs.380027	NM_00636	ENSG0000	C1orf61	CROC4	chromoso	protein-co
lib-C666-l	chr1	156427188	156427188	+	8.56376	NA	Intergenic	CpG	-27848	NR_13526	10485	Hs.380027	NM_00636	ENSG0000	C1orf61	CROC4	chromoso	protein-co
lib-C666-l	chr1	201475869	201475869	+	8.33814	NA	promoter-TSS (NM_001193571)	promoter-TSS (NM_001193571)	98	NM_00119	1465	Hs.108080	NM_00407	ENSG0000	CSRP1	CRP|CRP1|	cysteine ar	protein-co
lib-C666-l	chr1	201476406	201476406	+	8.28613	NA	promoter-TSS (NM_001193571)	promoter-TSS (NM_001193571)	-19	NM_00407	1465	Hs.108080	NM_00407	ENSG0000	CSRP1	CRP|CRP1|	cysteine ar	protein-co
lib-C666-l	chr12	77273270	77273270	+	2.24164	NA	promoter-TSS (NM_001300965)	promoter-TSS (NM_001300965)	-450	NM_00132	1466	Hs.530904	NM_00132	ENSG0000	CSRP2	CRP2|LMO	cysteine ar	protein-co
lib-C666-l	chr5	137911207	137911207	+	8.59372	NA	5' UTR (NM_004134, exon 1 of 17)	5' UTR (NM_004134, exon 1 of 17)	111	NM_00413	3313	Hs.184233	NM_00413	ENSG0000	HSPA9	CRP40|CS	heat shock	protein-co
lib-C666-l	chr3	37284720	37284720	+	3.84463	NA	promoter-TSS (NM_001172713)	promoter-TSS (NM_001172713)	38	NM_00117	2803	Hs.344151	NM_00207	ENSG0000	GOLGA4	CRPF46|G	golgin A4	protein-co
lib-C666-l	chr5	1345333	1345333	+	343.90414	NA	promoter-TSS (NM_030782)	promoter-TSS (NM_030782)	-148	NM_03078	81037	Hs.444673	NM_03078	ENSG0000	CLPTM1L	CRR9	CLPTM1 lik	protein-co
lib-C666-l	chr15	57179491	57179491	+	12.55817	NA	non-coding (NR_015419, exon 4 of 4	non-coding (NR_015419, exon 4 of	-30797	NM_00132	6938	Hs.511504	NM_00320	ENSG0000	TCF12	CRS3|HEB|	transcripti	protein-co
lib-C666-l	chrX	40595189	40595189	+	18.89222	NA	promoter-TSS (NM_004229)	promoter-TSS (NM_004229)	-385	NM_00422	9282	Hs.407604	NM_00422	ENSG0000	MED14	CRSP150|C	mediator c	protein-co
lib-C666-l	chr19	16739142	16739142	+	8.85547	NA	promoter-TSS (NM_004831)	promoter-TSS (NM_004831)	-127	NM_00483	9441	Hs.715040	NM_00483	ENSG0000	MED26	CRSP7|CRS	mediator c	protein-co
lib-C666-l	chr1	26680728	26680728	+	7.52748	NA	promoter-TSS (NM_001039775)	promoter-TSS (NM_001039775)	-107	NM_00103	55057	Hs.128738	NM_01797	ENSG0000	AIM1L	CRYBG2	absent in n	protein-co
lib-C666-l	chr6	91297040	91297040	+	165.56329	NA	promoter-TSS (NM_145332)	promoter-TSS (NM_145332)	-20	NM_00318	6885	Hs.594838	NM_00318	ENSG0000	MAP3K7	CSCF|FMD	mitogen-a	protein-co
lib-C666-l	chr6	91212721	91212721	+	17.3027	NA	Intergenic	Intergenic	84299	NM_14533	6885	Hs.594838	NM_00318	ENSG0000	MAP3K7	CSCF|FMD	mitogen-a	protein-co
lib-C666-l	chr18	32621276	32621276	+	22.15977	NA	promoter-TSS (NR_046177)	promoter-TSS (NR_046177)	-48	NM_01426	10982	Hs.532824	NM_01426	ENSG0000	MAPRE2	CSCSC2|EB	microtubul	protein-co
lib-C666-l	chr12	10874659	10874659	+	10.16129	NA	intron (NM_001145426, intron 1 of 8)	intron (NM_001145426, intron 1 of	1294	NM_00114	8531	Hs.221889	NM_00365	ENSG0000	YBX3	CSDA|CSD.	Y-box binc	protein-co
lib-C666-l	chr2	95999629	95999629	+	8.28613	NA	intron (NM_013434, intron 2 of 8)	intron (NM_013434, intron 2 of 8)	-13139	NM_00103	30818	Hs.437376	NM_01343	ENSG0000	KCNIP3	CSEN|DRE	potassium	protein-co
lib-C666-l	chr2	95999838	95999838	+	5.66964	NA	intron (NM_013434, intron 2 of 8)	intron (NM_013434, intron 2 of 8)	-12930	NM_00103	30818	Hs.437376	NM_01343	ENSG0000	KCNIP3	CSEN|DRE	potassium	protein-co
lib-C666-l	chr8	19535983	19535983	+	107.6729	NA	intron (NR_024040, intron 1 of 9)	L3|LINE|CR1	4111	NM_01837	55790	Hs.613729	NM_01837	ENSG0000	CSGALNAC	CSGalNAc	chondroiti	protein-co
lib-C666-l	chr8	19536490	19536490	+	29.90333	NA	intron (NR_024040, intron 1 of 9)	MIRb|SINE|MIR	3604	NM_01837	55790	Hs.613729	NM_01837	ENSG0000	CSGALNAC	CSGalNAc	chondroiti	protein-co
lib-C666-l	chr8	19537241	19537241	+	10.23386	NA	intron (NR_024040, intron 1 of 9)	intron (NR_024040, intron 1 of 9)	2853	NM_01837	55790	Hs.613729	NM_01837	ENSG0000	CSGALNAC	CSGalNAc	chondroiti	protein-co
lib-C666-l	chr16	11945474	11945474	+	24.54495	NA	promoter-TSS (NM_015659)	promoter-TSS (NM_015659)	-32	NM_01565	26156	Hs.401842	NM_01565	ENSG0000	RSL1D1	CSIG|L12|P	ribosomal	protein-co
lib-C666-l	chr17	17184613	17184613	+	26.35145	NA	promoter-TSS (NM_001199125)	promoter-TSS (NM_001199125)	4	NM_00131	8533	Hs.6076	NM_00365	ENSG0000	COPS3	CSN3|SGN	COP9 sign	protein-co
lib-C666-l	chr17	17184118	17184118	+	5.819	NA	promoter-TSS (NM_001199125)	promoter-TSS (NM_001199125)	-106	NM_00119	8533	Hs.6076	NM_00365	ENSG0000	COPS3	CSN3|SGN	COP9 sign	protein-co
lib-C666-l	chr4	83956045	83956045	+	4.61604	NA	promoter-TSS (NM_001258006)	promoter-TSS (NM_001258006)	-194	NM_00133	51138	Hs.190384	NM_01612	ENSG0000	COPS4	CSN4|SGN	COP9 sign	protein-co
lib-C666-l	chr12	6833255	6833255	+	24.32656	NA	promoter-TSS (NM_001164093)	promoter-TSS (NM_001164093)	105	NM_00116	50813	Hs.530823	NM_01631	ENSG0000	COPS7A	CSN7|CSN	COP9 sign	protein-co
lib-C666-l	chr2	241075835	241075835	+	34.0482	NA	promoter-TSS (NM_138336)	promoter-TSS (NM_138336)	-71	NM_13833	150678	Hs.293884	NM_13833	ENSG0000	COPS9	CSNAP|MY	COP9 sign	protein-co
lib-C666-l	chr17	36497412	36497412	+	29.74082	NA	intron (NM_001004334, intron 1 of 1	intron (NM_001004334, intron 1 of	2281	NM_00100	440435	Hs.462915	NM_00100	ENSG0000	GPR179	CSNB1E|G	G protein-	protein-co
lib-C666-l	chr4	619136	619136	+	23.39665	NA	promoter-TSS (NM_000283)	promoter-TSS (NM_000283)	-227	NM_00114	5158	Hs.623810	NM_00028	ENSG0000	PDE6B	CSNB3|CS	phosphodi	protein-co
lib-C666-l	chr3	129253616	129253616	+	8.12619	NA	3' UTR (NM_000539, exon 5 of 5)	3' UTR (NM_000539, exon 5 of 5)	6134	NM_00053	6010	Hs.247565	NM_00053	ENSG0000	RHO	CSNBAD1|	rhodopsin	protein-co
lib-C666-l	chr8	66886193	66886193	+	40.92836	NA	Intergenic	MLT1A1-int|LTR|ERVL-MaLR	-47598	NM_03310	85479	Hs.491885	NM_03310	ENSG0000	DNAJC5B	CSP-beta	DnaJ heat	protein-co
lib-C666-l	chr19	19325981	19325981	+	8.0787	NA	intron (NM_004386, intron 1 of 14)	intron (NM_004386, intron 1 of 14)	3199	NM_00438	1463	Hs.169047	NM_00438	ENSG0000	NCAN	CSPG3	neurocan	protein-co
lib-C666-l	chr8	67976535	67976535	+	14.45701	NA	promoter-TSS (NM_024790)	promoter-TSS (NM_024790)	-68	NM_02479	79848	Hs.370147	NM_02479	ENSG0000	CSPP1	CSPP|JBTS	centrosom	protein-co
lib-C666-l	chr20	18118563	18118563	+	3.98718	NA	promoter-TSS (NM_001164811)	promoter-TSS (NM_001164811)	64	NM_00116	100303755	Hs.488051	NM_00116	ENSG0000	PET117	CSRP2BP	PET117 ho	protein-co
lib-C666-l	chr20	54967501	54967501	+	55.66538	NA	promoter-TSS (NM_001324)	promoter-TSS (NM_001324)	-73	NM_00132	1477	Hs.172865	NM_00132	ENSG0000	CSTF1	CstF-50|Cs	cleavage st	protein-co
lib-C666-l	chr10	53459609	53459609	+	24.25638	NA	promoter-TSS (NM_015235)	promoter-TSS (NM_015235)	-254	NM_01523	23283	Hs.591358	NM_01523	ENSG0000	CSTF2T	CstF-64T	cleavage st	protein-co
lib-C666-l	chr16	12897753	12897753	+	3.20885	NA	promoter-TSS (NM_001099455)	promoter-TSS (NM_001099455)	-9	NM_01834	55313	Hs.460002	NM_01834	ENSG0000	CPPED1	CSTP1	calcineurin	protein-co
lib-C666-l	chr10	101298388	101298388	+	10.9422	NA	Intergenic	Intergenic	5698	NM_14528	159296	Hs.243272	NM_14528	ENSG0000	NKX2-3	CSX3|NK2.	NK2 home	protein-co
lib-C666-l	chr16	30907766	30907766	+	2.58799	NA	promoter-TSS (NM_001142544)	promoter-TSS (NM_001142544)	-162	NM_00114	1489	Hs.483811	NM_00133	ENSG0000	CTF1	CT-1|CT1	cardiotrop	protein-co
lib-C666-l	chr15	23692530	23692530	+	12.12504	NA	promoter-TSS (NM_001304388)	promoter-TSS (NM_001304388)	-140	NM_00130	283685		NM_18256	ENSG0000	GOLGA6L2	CT105	golgin A6	protein-co
lib-C666-l	chr17	56709473	56709473	+	58.77799	NA	intron (NM_198393, intron 2 of 32)	intron (NM_198393, intron 2 of 32)	59943	NM_00120	56155	Hs.390221	NM_03127	ENSG0000	TEX14	CT113	testis expre	protein-co
lib-C666-l	chr17	56736884	56736884	+	16.76091	NA	intron (NM_198393, intron 1 of 32)	CpG	32532	NM_00120	56155	Hs.390221	NM_03127	ENSG0000	TEX14	CT113	testis expre	protein-co

lib-C666-l	chr20	34203764	34203764	+	387.65842	NA	promoter-TSS (NM_001317931)	promoter-TSS (NM_001317931)	-42	NM_00311	6676	Hs.123159	NM_00311	ENSG0000	SPAG4	CT127|SUN	sperm assc	protein-co
lib-C666-l	chr11	133724642	133724642	+	5.92363	NA	Intergenic	L1PB1|LINE|L1	-9209	NM_17492	219938	Hs.97541	NM_17492	ENSG0000	SPATA19	CT132|SPA	spermatog	protein-co
lib-C666-l	chr9	131218326	131218326	+	16.96353	NA	promoter-TSS (NM_001242354)	promoter-TSS (NM_001242354)	41	NM_15343	4957	Hs.129055	NM_00254	ENSG0000	ODF2	CT134|ODI	outer dens	protein-co
lib-C666-l	chr9	131218995	131218995	+	8.28216	NA	promoter-TSS (NM_153437)	promoter-TSS (NM_153437)	-191	NM_15343	4957	Hs.129055	NM_00254	ENSG0000	ODF2	CT134|ODI	outer dens	protein-co
lib-C666-l	chr1	45205450	45205450	+	46.29081	NA	promoter-TSS (NM_001297655)	promoter-TSS (NM_001297655)	-40	NM_00129	11004	Hs.720061	NM_00684	ENSG0000	KIF2C	CT139|KNS	kinesin fan	protein-co
lib-C666-l	chr10	22629242	22629242	+	18.43022	NA	Intergenic	Intergenic	-5132	NM_00125	9576	Hs.655170	NM_01244	ENSG0000	SPAG6	CT141|Rep	sperm assc	protein-co
lib-C666-l	chr10	22629937	22629937	+	3.27468	NA	Intergenic	CpG	-4437	NM_00125	9576	Hs.655170	NM_01244	ENSG0000	SPAG6	CT141|Rep	sperm assc	protein-co
lib-C666-l	chr9	127533556	127533556	+	99.63897	NA	promoter-TSS (NM_001489)	promoter-TSS (NM_001489)	33	NM_00148	2649	Hs.20131	NM_00148	ENSG0000	NR6A1	CT150|GCN	nuclear rec	protein-co
lib-C666-l	chr6	110813951	110813951	+	4.52748	NA	Intergenic	Intergenic	-16107	NM_03312	85413	Hs.520319	NM_03312	ENSG0000	SLC22A16	CT2|FLIPT2	solute carr	protein-co
lib-C666-l	chr6	7389706	7389706	+	11.57178	NA	promoter-TSS (NM_031480)	promoter-TSS (NM_031480)	236	NM_00117	285782	Hs.721486	NM_17574	ENSG0000	CAGE1	CT3|CT95|(	cancer anti	protein-co
lib-C666-l	chrX	31088100	31088100	+	4.90117	NA	Intergenic	Intergenic	2070	NM_03189	53940	Hs.333125	NM_03189	ENSG0000	FTHL17	CT38	ferritin hea	protein-co
lib-C666-l	chr6	28048916	28048916	+	42.40071	NA	5' UTR (NM_003447, exon 1 of 4)	5' UTR (NM_003447, exon 1 of 4)	434	NM_00344	7718	Hs.55481	NM_00344	ENSG0000	ZNF165	CT53|LD65	zinc finger	protein-co
lib-C666-l	chr1	18188296	18188296	+	5.92363	NA	Intergenic	Intergenic	106488	NM_03081	81569	Hs.2149	NM_03081	ENSG0000	ACTL8	CT57	actin like 8	protein-co
lib-C666-l	chr21	43916516	43916516	+	13.06669	NA	promoter-TSS (NR_135518)	promoter-TSS (NR_135518)	-52	NM_00128	89765	Hs.661069	NM_08086	ENSG0000	RSPH1	CT79|RSP4	radial spok	protein-co
lib-C666-l	chr21	43916084	43916084	+	3.23837	NA	promoter-TSS (NR_135518)	promoter-TSS (NR_135518)	380	NM_08086	89765	Hs.661069	NM_08086	ENSG0000	RSPH1	CT79|RSP4	radial spok	protein-co
lib-C666-l	chr8	27695475	27695475	+	3.92495	NA	5' UTR (NM_001278945, exon 1 of 8)	5' UTR (NM_001278945, exon 1 of 8	137	NM_01849	55872	Hs.104741	NM_01849	ENSG0000	PBK	CT84|HEL1	PDZ bindir	protein-co
lib-C666-l	chr17	49198390	49198390	+	62.69942	NA	promoter-TSS (NM_003971)	promoter-TSS (NM_003971)	-164	NM_00113	9043	Hs.463439	NM_00397	ENSG0000	SPAG9	CT89|HLC-	sperm assc	protein-co
lib-C666-l	chr17	49199363	49199363	+	7.9119	NA	Intergenic	Intergenic	-1137	NM_00113	9043	Hs.463439	NM_00397	ENSG0000	SPAG9	CT89|HLC-	sperm assc	protein-co
lib-C666-l	chr6	80714278	80714278	+	11.66012	NA	promoter-TSS (NM_001166691)	promoter-TSS (NM_001166691)	-44	NM_00331	7272	Hs.169840	NM_00331	ENSG0000	TTK	CT96|ESK|	TTK proteir	protein-co
lib-C666-l	chr14	103851689	103851689	+	54.91701	NA	promoter-TSS (NM_001128918)	promoter-TSS (NM_001128918)	-12	NM_00112	4140	Hs.35828	NM_00237	ENSG0000	MARK3	CTAK1|KP7	microtubul	protein-co
lib-C666-l	chr22	42017251	42017251	+	65.39317	NA	promoter-TSS (NM_001288978)	promoter-TSS (NM_001288978)	84	NM_00128	2547	Hs.292493	NM_00146	ENSG0000	XRCC6	CTC75|CTC	X-ray repa	protein-co
lib-C666-l	chr5	38789841	38789841	+	25.49755	NA	intron (NR_109951, intron 2 of 3)	MLT1L|LTR|ERVL-MaLR	56090	NR_10995	101929768	Hs.546623	NR_109951		OSMR-AS:	CTD-2127	OSMR anti	ncRNA
lib-C666-l	chr5	9431797	9431797	+	5.48804	NA	intron (NM_003966, intron 2 of 22)	intron (NM_003966, intron 2 of 22)	-79648	NR_10993	101929338	Hs.675861	NR_10993	ENSG0000	SEMA5A-A	CTD-2201	SEMA5A ar	ncRNA
lib-C666-l	chr9	14353685	14353685	+	8.51971	NA	intron (NM_001190738, intron 1 of 8)	intron (NM_001190738, intron 1 of	-39640	NM_00119	4781	Hs.644095	NM_00559	ENSG0000	NFIB	CTF|HMG(	nuclear fac	protein-co
lib-C666-l	chr9	14251465	14251465	+	4.19675	NA	intron (NM_001190737, intron 2 of 10	intron (NM_001190737, intron 2 of	62580	NM_00559	4781	Hs.644095	NM_00559	ENSG0000	NFIB	CTF|HMG(	nuclear fac	protein-co
lib-C666-l	chr1	61542927	61542927	+	3.12293	NA	promoter-TSS (NM_001145511)	promoter-TSS (NM_001145511)	-19	NM_00114	4774	Hs.740757	NM_00559	ENSG0000	NFIA	CTF|NF-I/	nuclear fac	protein-co
lib-C666-l	chr16	69166476	69166476	+	15.01426	NA	promoter-TSS (NM_032830)	promoter-TSS (NM_032830)	17	NR_03322	54921	Hs.85962	NM_01780	ENSG0000	CHTF8	CTF8|DERF	chromosor	protein-co
lib-C666-l	chr22	37593772	37593772	+	7.15277	NA	Intergenic	Intergenic	-9442	NM_03191	114904	Hs.22011	NM_03191	ENSG0000	C1QTNF6	CTFP6|CTR	C1q and tu	protein-co
lib-C666-l	chr12	125093832	125093832	+	72.00107	NA	Intergenic	Intergenic	-41822	NM_00120	9612	Hs.137510	NM_00631	ENSG0000	NCOR2	CTG26|N-(	nuclear rec	protein-co
lib-C666-l	chr16	67876081	67876081	+	10.58189	NA	promoter-TSS (NM_020457)	promoter-TSS (NM_020457)	-132	NM_02045	57215	Hs.632200	NM_02045	ENSG0000	THAP11	CTG-B43a|	THAP dom	protein-co
lib-C666-l	chr15	72523683	72523683	+	25.39084	NA	5' UTR (NM_001206796, exon 1 of 12	5' UTR (NM_001206796, exon 1 of 1	282	NM_18247	5315	Hs.534770	NM_00265	ENSG0000	PKM	CTHBP|HEI	pyruvate k	protein-co
lib-C666-l	chr15	72520972	72520972	+	12.57335	NA	intron (NM_001206798, intron 1 of 10	intron (NM_001206798, intron 1 of	1091	NM_00120	5315	Hs.534770	NM_00265	ENSG0000	PKM	CTHBP|HEI	pyruvate k	protein-co
lib-C666-l	chr19	10736104	10736104	+	7.21092	NA	promoter-TSS (NM_020428)	promoter-TSS (NM_020428)	-67	NM_02042	57153	Hs.534560	NM_02042	ENSG0000	SLC44A2	CTL2|PP12	solute carr	protein-co
lib-C666-l	chr2	86947306	86947306	+	13.91591	NA	promoter-TSS (NM_022780)	promoter-TSS (NM_022780)	-108	NM_02278	64795	Hs.75277	NM_02278	ENSG0000	RMND5A	CTLH|GID2	required fc	protein-co
lib-C666-l	chr3	41240839	41240839	+	22.02847	NA	promoter-TSS (NM_001904)	promoter-TSS (NM_001904)	-76	NM_00133	1499	Hs.476018	NM_00190	ENSG0000	CTNNB1	CTNNB|MF	catenin be	protein-co
lib-C666-l	chr3	41200208	41200208	+	11.07709	NA	Intergenic	Intergenic	-40707	NM_00133	1499	Hs.476018	NM_00190	ENSG0000	CTNNB1	CTNNB|MF	catenin be	protein-co
lib-C666-l	chr22	19166316	19166316	+	17.47044	NA	promoter-TSS (NM_001256534)	promoter-TSS (NM_001256534)	60	NR_04629	6576	Hs.111024	NM_00598	ENSG0000	SLC25A1	CTP|D2L2A	solute carr	protein-co
lib-C666-l	chr22	19166679	19166679	+	3.58839	NA	promoter-TSS (NM_001256534)	promoter-TSS (NM_001256534)	-303	NM_00598	6576	Hs.111024	NM_00598	ENSG0000	SLC25A1	CTP|D2L2A	solute carr	protein-co
lib-C666-l	chr1	41418261	41418261	+	8.28613	NA	Intergenic	Intergenic	-26710	NM_00190	1503	Hs.473087	NM_00190	ENSG0000	CTPS1	CTPS|IMD2	CTP synthe	protein-co
lib-C666-l	chrX	17395588	17395588	+	22.50982	NA	intron (NM_001291867, intron 1 of 8)	CpG	2045	NM_00129	4810	Hs.201623	NM_19827	ENSG0000	NHS	CTRCT40|	NHS actin	protein-co
lib-C666-l	chr19	38423903	38423903	+	8.71138	NA	intron (NM_015073, intron 1 of 21)	L2a|LINE|L2	26042	NM_01507	23094	Hs.655502	NM_01507	ENSG0000	SIPA1L3	CTRCT45|	signal indu	protein-co
lib-C666-l	chr19	38423128	38423128	+	6.15313	NA	intron (NM_015073, intron 1 of 21)	intron (NM_015073, intron 1 of 21)	25267	NM_01507	23094	Hs.655502	NM_01507	ENSG0000	SIPA1L3	CTRCT45|	signal indu	protein-co
lib-C666-l	chr17	77034751	77034751	+	16.10678	NA	intron (NM_198593, intron 1 of 3)	intron (NM_198593, intron 1 of 3)	4370	NM_19859	114897	Hs.201398	NM_03096	ENSG0000	C1QTNF1	CTRP1|GIP	C1q and tu	protein-co
lib-C666-l	chr4	157215251	157215251	+	5.5166	NA	Intergenic	Intergenic	-340203	NM_00133	1519	Hs.75262	NM_00133	ENSG0000	CTSO	CTSO1	cathepsin (	protein-co
lib-C666-l	chr11	65819775	65819775	+	6.33432	NA	promoter-TSS (NM_006842)	promoter-TSS (NM_006842)	-41	NM_00684	10992	Hs.406423	NM_00684	ENSG0000	SF3B2	Cus1|SAP1	splicing fac	protein-co
lib-C666-l	chr12	100660909	100660909	+	9.10494	NA	promoter-TSS (NR_135073)	promoter-TSS (NR_135073)	-9	NM_00131	55681	Hs.506481	NM_01798	ENSG0000	SCYL2	CVAK104	SCY1 like 2	protein-co
lib-C666-l	chrX	153707749	153707749	+	3.92495	NA	promoter-TSS (NM_006014)	promoter-TSS (NM_006014)	-153	NM_00601	8270	Hs.444619	NM_00601	ENSG0000	LAGE3	CVG5|DXS\	L antigen f	protein-co
lib-C666-l	chr10	104154208	104154208	+	184.26823	NA	promoter-TSS (NM_001077494)	promoter-TSS (NM_001077494)	-127	NM_00107	4791	Hs.73090	NM_00250	ENSG0000	NFKB2	CVID10|H2	nuclear fac	protein-co
lib-C666-l	chr10	104155310	104155310	+	54.43427	NA	promoter-TSS (NM_001322934)	promoter-TSS (NM_001322934)	-43	NM_00126	4791	Hs.73090	NM_00250	ENSG0000	NFKB2	CVID10|H2	nuclear fac	protein-co
lib-C666-l	chr6	159239413	159239413	+	4.80341	NA	promoter-TSS (NM_003379)	promoter-TSS (NM_003379)	-73	NM_00337	7430	Hs.487027	NM_00337	ENSG0000	EZR	CVIL|CVL|	ezrin	protein-co
lib-C666-l	chr7	99006473	99006473	+	18.35738	NA	promoter-TSS (NM_003910)	promoter-TSS (NM_003910)	-128	NM_00391	8896	Hs.380233	NM_00391	ENSG0000	BUD31	Cwc14|EDC	BUD31 hor	protein-co
lib-C666-l	chr5	150080677	150080677	+	36.38376	NA	promoter-TSS (NM_018047)	promoter-TSS (NM_018047)	-8	NM_01804	55696	Hs.713564	NM_01804	ENSG0000	RBM22	Cwc2|ZC3H	RNA bindir	protein-co
lib-C666-l	chr11	3818881	3818881	+	153.78793	NA	promoter-TSS (NM_001256235)	promoter-TSS (NM_001256235)	-73	NM_00125	27315	Hs.133968	NM_01448	ENSG0000	PGAP2	CWH43-N	post-GPI a	protein-co
lib-C666-l	chr1	17380583	17380583	+	7.67555	NA	promoter-TSS (NM_003000)	promoter-TSS (NM_003000)	82	NM_00300	6390	Hs.465924	NM_00300	ENSG0000	SDHB	CWS2|IP|P	succinate (	protein-co
lib-C666-l	chr10	89622004	89622004	+	3.8557	NA	exon (NM_001126049, exon 1 of 1)	exon (NM_001126049, exon 1 of 1)	1190	NM_00112	100144748	Hs.559820	NM_00112	ENSG0000	KLLN	CWS4|KILL	killin, p53-	protein-co
lib-C666-l	chr1	35220980	35220980	+	7.15277	NA	intron (NM_005268, intron 1 of 1)	intron (NM_005268, intron 1 of 1)	332	NM_00526	2709	Hs.198249	NM_00526	ENSG0000	GJB5	CX31.1	gap junctic	protein-co
lib-C666-l	chr17	38519084	38519084	+	2.23837	NA	3' UTR (NM_152219, exon 1 of 1)	3' UTR (NM_152219, exon 1 of 1)	1861	NM_15221	125111	Hs.444663	NM_15221	ENSG0000	GJD3	CX31.9|Cx3	gap junctic	protein-co
lib-C666-l	chr4	83934269	83934269	+	16.07623	NA	promoter-TSS (NM_001115007)	promoter-TSS (NM_001115007)	-175	NM_00111	132660	Hs.96952	NM_19428	ENSG0000	LIN54	CXCDC1|(	lin-54 DRE	protein-co
lib-C666-l	chr17	4643259	4643259	+	42.11224	NA	promoter-TSS (NM_001267822)	promoter-TSS (NM_001267822)	-36	NM_00110	58191	Hs.745037	NM_02205	ENSG0000	CXCL16	CXCLG16|S	C-X-C mo	protein-co
lib-C666-l	chrX	153238132	153238132	+	64.17523	NA	5' UTR (NM_003492, exon 1 of 2)	5' UTR (NM_003492, exon 1 of 2)	141	NM_00349	8269	Hs.23119	NM_00349	ENSG0000	TMEM187	CXorf12|D	transment	protein-co
lib-C666-l	chrX	153237773	153237773	+	42.40071	NA	promoter-TSS (NM_005334)	promoter-TSS (NM_005334)	-218	NM_00349	8269	Hs.23119	NM_00349	ENSG0000	TMEM187	CXorf12|D	transment	protein-co
lib-C666-l	chrX	16804584	16804584	+	4.74112	NA	promoter-TSS (NM_001168683)	promoter-TSS (NM_001168683)	29	NM_00116	55787	Hs.555961	NM_01836	ENSG0000	TXLNG	CXorf15|EL	taxilin gam	protein-co
lib-C666-l	chrX	122911800	122911800	+	29.07066	NA	Intergenic	Intergenic	-44896	NM_00108	57187	Hs.149991	NM_02044	ENSG0000	THOC2	CXorf3|MR	THO comp	protein-co
lib-C666-l	chrX	148622416	148622416	+	8.03539	NA	promoter-TSS (NM_001324277)	promoter-TSS (NM_001324277)	-103	NM_00132	91966	Hs.458216	NM_17812	ENSG0000	CXorf40A	CXorf40|E	chromosor	protein-co
lib-C666-l	chrX	149096027	149096027	+	4.39666	NA	3' UTR (NM_001171909, exon 5 of 5)	3' UTR (NM_001171909, exon 5 of 5	7175	NM_00117	91966	Hs.458216	NM_13312	ENSG0000	CXorf40A	CXorf40|E	chromosor	protein-co
lib-C666-l	chrX	83757503	83757503	+	23.34793	NA	promoter-TSS (NM_001177478)	promoter-TSS (NM_001177478)	-16	NM_14465	139324	Hs.559546	NM_14465	ENSG0000	HDX	CXorf43|D	highly dive	protein-co
lib-C666-l	chr19	36208692	36208692	+	37.41784	NA	promoter-TSS (NM_014727)	promoter-TSS (NM_014727)	-229	NM_01472	9757	Hs.92236	NM_01472	ENSG0000	KMT2B	CXXC10|H	lysine metl	protein-co
lib-C666-l	chr16	30933879	30933879	+	55.01936	NA	promoter-TSS (NM_001282351)	promoter-TSS (NM_001282351)	-497	NM_00128	54620	Hs.152149	NM_01908	ENSG0000	FBXL19	CXXC11|Ft	F-box and	protein-co
lib-C666-l	chr16	30934359	30934359	+	5.3823	NA	promoter-TSS (NM_001282351)	promoter-TSS (NM_001282351)	-17	NM_00128	54620	Hs.152149	NM_01908	ENSG0000	FBXL19	CXXC11|Ft	F-box and	protein-co
lib-C666-l	chr18	47808035	47808035	+	60.82721	NA	TTS (NM_014593)	TTS (NM_014593)	102	NM_00120	4152	Hs.405610	NM_00238	ENSG0000	MBD1	CXXC3|PC	methyl-Cp	protein-co
lib-C666-l	chr10	70314991	70314991	+	14.59636	NA	Intergenic	AluSx|SINE|Alu	-5126	NM_03062	80312	Hs.258855	NM_03062	ENSG0000	TET1	CXXC6|LC)	tet methyl	protein-co
lib-C666-l	chr18	71959520	71959520	+	2.1162	NA	promoter-TSS (NM_001190807)	promoter-TSS (NM_001190807)	-269	NM_00191	1528	Hs.465413	NM_00191	ENSG0000	CYB5A	CYB5|MCB	cytochrom	protein-co
lib-C666-l	chr1	161284183	161284183	+	16.39562	NA	promoter-TSS (NM_001278172)	promoter-TSS (NM_001278172)	17	NM_00127	6391	Hs.444472	NM_00300	ENSG0000	SDHC	CYB560|CY	succinate (	protein-co
lib-C666-l	chr17	61523807	61523807	+	16.47235	NA	promoter-TSS (NM_001915)	promoter-TSS (NM_001915)	-85	NM_00191	1534	Hs.355264	NM_00191	ENSG0000	CYB561	CYB561A1	cytochrom	protein-co
lib-C666-l	chr17	61524514	61524514	+	6.15313	NA	promoter-TSS (NM_001915)	promoter-TSS (NM_001915)	-792	NM_00191	1534	Hs.355264	NM_00191	ENSG0000	CYB561	CYB561A1	cytochrom	protein-co
lib-C666-l	chr11	61659400	61659400	+	30.8056	NA	promoter-TSS (NM_021727)	promoter-TSS (NM_021727)	-383	NM_02172	3995	Hs.21765	NM_02172	ENSG0000	FADS3	CYB5RP|LL	fatty acid c	protein-co
lib-C666-l	chr7	25165016	25165016	+	156.37758	NA	promoter-TSS (NM_018947)	promoter-TSS (NM_018947)	-36	NM_01894	54205	Hs.437060	NM_01894	ENSG0000	CYCS	CYC|HCS|T	cytochrom	protein-co
lib-C666-l	chr22	22020538	22020538	+	10.79304	NA	intron (NM_148176, intron 1 of 20)	CpG	265	NM_00131	23759	Hs.438587	NM_01433	ENSG0000	PPIL2	CYC4|CYPE	peptidylprc	protein-co
lib-C666-l	chr12	50419371	50419371	+	23.6003	NA	promoter-TSS (NM_001320000)	promoter-TSS (NM_001320000)	-31	NM_00132	29127	Hs.505469	NM_01327	ENSG0000	RACGAP1	CYK4|HsCY	Rac GTPas	protein-co
lib-C666-l	chr2	72372164	72372164	+	23.27622	NA	intron (NM_019885, intron 1 of 5)	CpG	2827	NM_00127	56603	Hs.91546	NM_01988	ENSG0000	CYP26B1	CYP26A2|P	cytochrom	protein-co
lib-C666-l	chr19	41426984	41426984	+	88.51586	NA	Intergenic	Intergenic	-3186	NR_00127	1556	Hs.529117	NR_00127	ENSG0000	CYP2B7P	CYP2B|CYF	cytochrom	pseudo
lib-C666-l	chr10	81008299	81008299	+	20.90919	NA	intron (NM_020338, intron 7 of 24)	intron (NM_020338, intron 7 of 24)	-98921	NM_00572	10105	Hs.381072	NM_00572	ENSG0000	PPIF	CYP3|CyP-	peptidylprc	protein-co
lib-C666-l	chr10	81091730	81091730	+	7.0832	NA	Intergenic	Intergenic	-15490	NM_00572	10105	Hs.381072	NM_00572	ENSG0000	PPIF	CYP3|CyP-	peptidylprc	protein-co
lib-C666-l	chr10	81107952	81107952	+	3.98736	NA	intron (NM_005729, intron 1 of 5)	intron (NM_005729, intron 1 of 5)	732	NM_00572	10105	Hs.381072	NM_00572	ENSG0000	PPIF	CYP3|CyP-	peptidylprc	protein-co
lib-C666-l	chr10	81107158	81107158	+	3.27468	NA	promoter-TSS (NM_005729)	promoter-TSS (NM_005729)	-62	NM_00572	10105	Hs.381072	NM_00572	ENSG0000	PPIF	CYP3|CyP-	peptidylprc	protein-co
lib-C666-l	chr7	44836210	44836210	+	10.03284	NA	promoter-TSS (NM_001300981)	promoter-TSS (NM_001300981)	-25	NM_02113	5478	Hs.356331	NM_02113	ENSG0000	PPIA	CYPA|CYPH	peptidylprc	protein-co
lib-C666-l	chr19	41689431	41689431	+	21.0836	NA	Intergenic	Intergenic	-9681	NM_03062	29785	Hs.98370	NM_03062	ENSG0000	CYP2S1	CYPIIS1	cytochrom	protein-co
lib-C666-l	chr2	204103651	204103651	+	9.47655	NA	5' UTR (NM_177538, exon 1 of 13)	5' UTR (NM_177538, exon 1 of 13)	487	NM_17753	57404	Hs.446065	NM_02067	ENSG0000	CYP20A1	CYP-M	cytochrom	protein-co
lib-C666-l	chr2	204104009	204104009	+	8.96177	NA	intron (NM_177538, intron 1 of 12)	CpG	845	NM_17753	57404	Hs.446065	NM_02067	ENSG0000	CYP20A1	CYP-M	cytochrom	protein-co
lib-C666-l	chr2	204055761	204055761	+	3.82577	NA	intron (NM_001114132, intron 45 of 5	intron (NM_001114132, intron 45 o	-47403	NM_17753	57404	Hs.446065	NM_02067	ENSG0000	CYP20A1	CYP-M	cytochrom	protein-co
lib-C666-l	chr15	64455366	64455366	+	19.57279	NA	promoter-TSS (NM_000942)	promoter-TSS (NM_000942)	-12	NM_00094	5479	Hs.434937	NM_00094	ENSG0000	PPIB	CYP-S1|CY	peptidylprc	protein-co
lib-C666-l	chr15	41576095	41576095	+	6.49644	NA	promoter-TSS (NR_026757)	promoter-TSS (NR_026757)	-106	NR_02675	729082	Hs.380164	NR_026757		OIP5-AS1	cyrano	OIP5 antise	ncRNA
lib-C666-l	chr5	176899540	176899540	+	49.31026	NA	intron (NM_080881, intron 1 of 14)	intron (NM_080881, intron 1 of 14)	331	NM_08088	1627	Hs.130316	NM_00439	ENSG0000	DBN1	D0S117E	drebrin 1	protein-co
lib-C666-l	chr10	70287274	70287274	+	85.64357	NA	promoter-TSS (NM_001324312)	promoter-TSS (NM_001324312)	6	NM_00132	8034	Hs.180408	NM_15270	ENSG0000	SLC25A16	D10S105E|	solute carr	protein-co
lib-C666-l	chr10	61667581	61667581	+	2.56932	NA	Intergenic	Intergenic	-1167	NM_00543	8030	Hs.591360	NM_00543	ENSG0000	CCDC6	D10S170|	coiled-coil	protein-co
lib-C666-l	chr11	809836	809836	+	159.9151	NA	promoter-TSS (NM_001004)	promoter-TSS (NM_001004)	-100	NM_00100	6181	Hs.437594	NM_00100	ENSG0000	RPLP2	D11S2243I	ribosomal	protein-co
lib-C666-l	chr11	809354	809354	+	25.40925	NA	promoter-TSS (NM_001004)	promoter-TSS (NM_001004)	-582	NM_00100	6181	Hs.437594	NM_00100	ENSG0000	RPLP2	D11S2243I	ribosomal	protein-co
lib-C666-l	chr11	3876303	3876303	+	8.71138	NA	promoter-TSS (NM_001277962)	promoter-TSS (NM_001277962)	-630	NM_00315	6786	Hs.501735	NM_00315	ENSG0000	STIM1	D11S4896I	stromal int	protein-co
lib-C666-l	chr12	56360503	56360503	+	7.15277	NA	promoter-TSS (NM_001200053)	promoter-TSS (NM_001200053)	-7	NM_00692	6490	Hs.95972	NM_00692	ENSG0000	PMEL	D12S53E|N	premelano	protein-co
lib-C666-l	chr15	28567266	28567266	+	3.84463	NA	promoter-TSS (NM_004667)	promoter-TSS (NM_004667)	32	NM_00466	8924	Hs.434890	NM_00466	ENSG0000	HERC2	D15F37S1|	HECT and	protein-co
lib-C666-l	chr15	23378188	23378188	+	23.27622	NA	promoter-TSS (NR_002824)	promoter-TSS (NR_002824)	71	NR_00282	400322	Hs.458334	NR_00282	ENSG0000	HERC2P2	D15F37S3|	hect doma	pseudo
lib-C666-l	chr15	20711551	20711551	+	8.90036	NA	promoter-TSS (NR_036432)	promoter-TSS (NR_036432)	-118	NR_03643	283755	Hs.608918	NM_00102	ENSG0000	HERC2P3	D15F37S4	hect doma	pseudo
lib-C666-l	chr15	20711203	20711203	+	4.73866	NA	intron (NR_036432, intron 1 of 25)	intron (NR_036432, intron 1 of 25)	230	NR_03643	283755	Hs.608918	NM_00102	ENSG0000	HERC2P3	D15F37S4	hect doma	pseudo
lib-C666-l	chr19	9695227	9695227	+	63.46463	NA	promoter-TSS (NM_001308269)	promoter-TSS (NM_001308269)	-18	NM_00130	7675	Hs.501537	NM_00100	ENSG0000	ZNF121	D19S204|Z	zinc finger	protein-co
lib-C666-l	chr1	115300753	115300753	+	3.8557	NA	promoter-TSS (NM_007158)	promoter-TSS (NM_007158)	-82	NM_00124	7812	Hs.69855	NM_00715	ENSG0000	CSDE1	D1S155E|L	cold shock	protein-co
lib-C666-l	chr21	45209397	45209397	+	266.4715	NA	promoter-TSS (NM_003683)	promoter-TSS (NM_003683)	-21	NM_00368	8568	Hs.110757	NM_00368	ENSG0000	RRP1	D21S2056I	ribosomal	protein-co
lib-C666-l	chr22	32446913	32446913	+	13.20416	NA	intron (NM_000343, intron 2 of 14)	intron (NM_000343, intron 2 of 14)	7894	NM_00034	6523	Hs.1964	NM_00034	ENSG0000	SLC5A1	D22S675|N	solute carr	protein-co
lib-C666-l	chr15	90603078	90603078	+	38.57241	NA	intron (NM_198526, intron 1 of 4)	intron (NM_198526, intron 1 of 4)	40775	NM_00128	3418	Hs.596461	NM_00216	ENSG0000	IDH2	D2HGA2|I	isocitrate c	protein-co
lib-C666-l	chr11	113871592	113871592	+	27.5466	NA	intron (NM_001290809, intron 4 of 7)	intron (NM_001290809, intron 4 of	26031	NM_00129	1814	Hs.121478	NM_00079	ENSG0000	DRD3	D3DR|ETM	dopamine	protein-co
lib-C666-l	chr3	10362836	10362836	+	75.07171	NA	promoter-TSS (NM_183352)	promoter-TSS (NM_183352)	36	NM_00127	6396	Hs.166924	NM_00067	ENSG0000	SEC13	D3S1231E|	SEC13 hon	protein-co
lib-C666-l	chr1	173837183	173837183	+	34.92958	NA	promoter-TSS (NR_002579)	promoter-TSS (NR_002579)	37	NM_00134	84614	Hs.668497	NM_03252	ENSG0000	ZBTB37	D430004I0	zinc finger	protein-co
lib-C666-l	chr4	1722901	1722901	+	15.33659	NA	promoter-TSS (NM_006342)	promoter-TSS (NM_006342)	183	NM_13838	92305	Hs.518562	NM_13838	ENSG0000	TMEM129	D4S2561E	transment	protein-co
lib-C666-l	chr8	81083810	81083810	+	7.67555	NA	promoter-TSS (NR_105036)	promoter-TSS (NR_105036)	84	NM_00102	7163	Hs.368433	NM_00507	ENSG0000	TPD52	D52|N8L|P	tumor prot	protein-co
lib-C666-l	chr20	62496549	62496549	+	60.55943	NA	promoter-TSS (NM_001243895)	promoter-TSS (NM_001243895)	-32	NM_19936	7165	Hs.473296	NM_00328	ENSG0000	TPD52L2	D54|TPD54	tumor prot	protein-co
lib-C666-l	chr11	61584479	61584479	+	6.15313	NA	promoter-TSS (NM_013402)	promoter-TSS (NM_013402)	50	NM_01340	3992	Hs.503546	NM_01340	ENSG0000	FADS1	D5D|FADS	fatty acid c	protein-co
lib-C666-l	chr5	137878921	137878921	+	66.08471	NA	promoter-TSS (NM_004730)	promoter-TSS (NM_004730)	68	NM_00473	2107	Hs.483494	NM_00473	ENSG0000	ETF1	D5S1995E|	eukaryotic	protein-co
lib-C666-l	chr5	137879350	137879350	+	62.31611	NA	promoter-TSS (NM_004730)	promoter-TSS (NM_004730)	-361	NM_00129	2107	Hs.483494	NM_00473	ENSG0000	ETF1	D5S1995E|	eukaryotic	protein-co
lib-C666-l	chr6	32939750	32939750	+	22.23727	NA	5' UTR (NM_001291986, exon 2 of 14	5' UTR (NM_001291986, exon 2 of 1	378	NM_00119	6046	Hs.75243	NM_00510	ENSG0000	BRD2	D6S113E|F	bromodon	protein-co
lib-C666-l	chr6	44214749	44214749	+	12.78696	NA	promoter-TSS (NM_007355)	promoter-TSS (NM_007355)	54	NM_00127	3326	Hs.509736	NM_00735	ENSG0000	HSP90AB1	D6S182|HS	heat shock	protein-co
lib-C666-l	chr6	44214243	44214243	+	6.98764	NA	promoter-TSS (NM_007355)	promoter-TSS (NM_007355)	340	NM_00127	3326	Hs.509736	NM_00735	ENSG0000	HSP90AB1	D6S182|HS	heat shock	protein-co
lib-C666-l	chr6	31239939	31239939	+	27.26713	NA	promoter-TSS (NM_002117).7	promoter-TSS (NM_002117).7	-26	NM_00124	3107	Hs.656020	NM_00211	ENSG0000	HLA-C	D6S204|HL	major histc	protein-co
lib-C666-l	chr6	33239843	33239843	+	16.59065	NA	promoter-TSS (NM_001289176).3	promoter-TSS (NM_001289176).3	-9	NM_02255	6222	Hs.448854	NM_02255	ENSG0000	RPS18	D6S218E|H	ribosomal	protein-co
lib-C666-l	chr6	32908884	32908884	+	60.38725	NA	promoter-TSS (NM_002118)	promoter-TSS (NM_002118)	-37	NM_00211	3109	Hs.351279	NM_00211	ENSG0000	HLA-DMB	D6S221E|R	major histc	protein-co
lib-C666-l	chr6	32920958	32920958	+	65.88593	NA	promoter-TSS (NM_006120)	promoter-TSS (NM_006120)	-59	NM_00612	3108	Hs.728759	NM_00612	ENSG0000	HLA-DMA	D6S222E|D	major histc	protein-co
lib-C666-l	chr6	35227247	35227247	+	42.78438	NA	promoter-TSS (NM_001292032)	promoter-TSS (NM_001292032)	-244	NM_00342	7629	Hs.388024	NM_00342	ENSG0000	ZNF76	D6S229E|Z	zinc finger	protein-co
lib-C666-l	chr6	35075601	35075601	+	3.92495	NA	Intergenic	AluSc8|SINE|Alu	33586	NM_00126	6954	Hs.435371	NM_01867	ENSG0000	TCP11	D6S230E|F	t-complex	protein-co
lib-C666-l	chr6	3849396	3849396	+	6.98764	NA	promoter-TSS (NM_012135)	promoter-TSS (NM_012135)	-204	NM_01213	26240	Hs.140944	NM_01213	ENSG0000	FAM50B	D6S2654E|	family with	protein-co
lib-C666-l	chr6	31926789	31926789	+	98.40649	NA	promoter-TSS (NM_002904).4	promoter-TSS (NM_002904).4	75	NM_00290	7936	Hs.423935	NM_00290	ENSG0000	NELFE	D6S45|NEL	negative el	protein-co
lib-C666-l	chr9	72121344	72121344	+	4.70431	NA	intron (NM_001163, intron 2 of 12)	L1PA2|LINE|L1	165931	NM_00116	320	Hs.171939	NM_00116	ENSG0000	APBA1	D9S411E|L	amyloid be	protein-co
lib-C666-l	chr9	100274448	100274448	+	7.67555	NA	intron (NM_003275, intron 1 of 9)	L1ME3|LINE|L1	10527	NM_00327	7111	Hs.404289	NM_00327	ENSG0000	TMOD1	D9S57E|ET	tropomodi	protein-co
lib-C666-l	chr9	86323065	86323065	+	149.53001	NA	promoter-TSS (NR_135840)	promoter-TSS (NR_135840)	103	NM_01343	29979	Hs.9589	NM_01343	ENSG0000	UBQLN1	DA41|DSK2	ubiquilin 1	protein-co
lib-C666-l	chr19	49140835	49140835	+	3.23837	NA	promoter-TSS (NM_001352)	promoter-TSS (NM_001352)	-28	NM_00135	1628	Hs.414480	NM_00135	ENSG0000	DBP	DABP	D-box binc	protein-co
lib-C666-l	chr2	25430249	25430249	+	8.03539	NA	intron (NR_132378, intron 1 of 1)	intron (NR_132378, intron 1 of 1)	3067	NR_13237	106144568	Hs.580474	NR_13237	ENSG0000	LINC01381	DADOT	long interg	ncRNA
lib-C666-l	chr12	26427835	26427835	+	15.31489	NA	Intergenic	Intergenic	79329	NM_00508	8082	Hs.183428	NM_00508	ENSG0000	SSPN	DAGA5|KR	sarcospan	protein-co
lib-C666-l	chr12	26392815	26392815	+	6.71839	NA	Intergenic	Intergenic	44309	NM_00508	8082	Hs.183428	NM_00508	ENSG0000	SSPN	DAGA5|KR	sarcospan	protein-co
lib-C666-l	chr7	14460626	14460626	+	9.94079	NA	intron (NM_145695, intron 20 of 23)	MER51C|LTR|ERV1	420449	NM_14569	1607	Hs.567255	NM_00408	ENSG0000	DGKB	DAGK2|DG	diacylglyce	protein-co
lib-C666-l	chr1	115124387	115124387	+	121.43741	NA	promoter-TSS (NM_005872)	promoter-TSS (NM_005872)	-122	NM_00587	10286	Hs.22960	NM_00587	ENSG0000	BCAS2	DAM1|SPF	BCAS2, pre	protein-co
lib-C666-l	chrX	75555504	75555504	+	2.99475	NA	Intergenic	Intergenic	-92542	NM_02093	57692	Hs.8453	NM_02093	ENSG0000	MAGEE1	DAMAGE|F	MAGE fam	protein-co
lib-C666-l	chr16	14724096	14724096	+	13.02989	NA	promoter-TSS (NM_001134477)	promoter-TSS (NM_001134477)	32	NM_00113	5073	Hs.253197	NM_00258	ENSG0000	PARN	DAN|DKCE	poly(A)-sp	protein-co
lib-C666-l	chr19	16653453	16653453	+	5.9194	NA	promoter-TSS (NM_006387)	promoter-TSS (NM_006387)	-190	NM_00638	10523	Hs.740364	NM_00638	ENSG0000	CHERP	DAN16|SC	calcium ho	protein-co
lib-C666-l	chr19	16653117	16653117	+	5.5166	NA	intron (NM_006387, intron 1 of 16)	CpG-13182	146	NM_00638	10523	Hs.740364	NM_00638	ENSG0000	CHERP	DAN16|SC	calcium ho	protein-co
lib-C666-l	chr10	106089188	106089188	+	21.94284	NA	promoter-TSS (NM_001272012)	promoter-TSS (NM_001272012)	-438	NM_00127	85450	Hs.523252	NM_03339	ENSG0000	ITPRIP	DANGER|K	inositol 1,4	protein-co
lib-C666-l	chr11	63997834	63997834	+	11.86993	NA	promoter-TSS (NM_001307981)	promoter-TSS (NM_001307981)	-70	NM_00130	3338	Hs.172847	NM_00552	ENSG0000	DNAJC4	DANJC4|H	DnaJ heat	protein-co
lib-C666-l	chr2	203103314	203103314	+	5.82086	NA	promoter-TSS (NM_003352)	promoter-TSS (NM_003352)	-8	NM_00100	7341	Hs.81424	NM_00335	ENSG0000	SUMO1	DAP1|GMF	small ubiq	protein-co
lib-C666-l	chr20	35089832	35089832	+	12.14307	NA	promoter-TSS (NM_183006)	promoter-TSS (NM_183006)	14	NM_00104	22839	Hs.249600	NM_01490	ENSG0000	DLGAP4	DAP-4|DA	DLG associ	protein-co
lib-C666-l	chr20	35110285	35110285	+	10.16129	NA	intron (NM_183006, intron 1 of 6)	intron (NM_183006, intron 1 of 6)	20099	NM_18300	22839	Hs.249600	NM_01490	ENSG0000	DLGAP4	DAP-4|DA	DLG associ	protein-co
lib-C666-l	chr20	35096231	35096231	+	7.97818	NA	intron (NM_183006, intron 1 of 6)	MIRb|SINE|MIR	6045	NM_18300	22839	Hs.249600	NM_01490	ENSG0000	DLGAP4	DAP-4|DA	DLG associ	protein-co
lib-C666-l	chr20	35094054	35094054	+	6.32351	NA	intron (NM_183006, intron 1 of 6)	intron (NM_183006, intron 1 of 6)	3868	NM_18300	22839	Hs.249600	NM_01490	ENSG0000	DLGAP4	DAP-4|DA	DLG associ	protein-co
lib-C666-l	chr9	90185889	90185889	+	15.42216	NA	intron (NM_001288729, intron 2 of 25	intron (NM_001288729, intron 2 of	72439	NM_00128	1612	Hs.380277	NM_00493	ENSG0000	DAPK1	DAPK	death asso	protein-co
lib-C666-l	chr14	91880072	91880072	+	7.37943	NA	intron (NM_001080414, intron 2 of 25	intron (NM_001080414, intron 2 of	4116	NM_00108	440193	Hs.525536	NM_00108	ENSG0000	CCDC88C	DAPLE|HKI	coiled-coil	protein-co
lib-C666-l	chr14	91833780	91833780	+	4.38153	NA	intron (NM_001080414, intron 3 of 25	intron (NM_001080414, intron 3 of	50408	NM_00108	440193	Hs.525536	NM_00108	ENSG0000	CCDC88C	DAPLE|HKI	coiled-coil	protein-co
lib-C666-l	chr17	37774110	37774110	+	19.59432	NA	Intergenic	CpG	-9067	NM_03219	84152	Hs.286192	NM_03219	ENSG0000	PPP1R1B	DARPP-32	protein ph	protein-co
lib-C666-l	chr12	56435763	56435763	+	14.98502	NA	promoter-TSS (NM_001029)	promoter-TSS (NM_001029)	77	NM_00102	6231	Hs.447562	NM_00102	ENSG0000	RPS26	DBA10|S26	ribosomal	protein-co
lib-C666-l	chr17	8286572	8286572	+	4.83287	NA	promoter-TSS (NM_000987)	promoter-TSS (NM_000987)	-7	NM_00098	6154	Hs.482144	NM_00098	ENSG0000	RPL26	DBA11|L26	ribosomal	protein-co
lib-C666-l	chr14	50053253	50053253	+	160.62315	NA	promoter-TSS (NM_001032)	promoter-TSS (NM_001032)	-119	NM_00103	6235	Hs.156367	NM_00103	ENSG0000	RPS29	DBA13|S29	ribosomal	protein-co
lib-C666-l	chr10	79793498	79793498	+	6.50091	NA	promoter-TSS (NM_001026)	promoter-TSS (NM_001026)	-20	NM_00114	6229	Hs.280130	NM_00102	ENSG0000	RPS24	DBA3|S24	ribosomal	protein-co
lib-C666-l	chr1	93297534	93297534	+	24.31114	NA	promoter-TSS (NM_000969)	promoter-TSS (NM_000969)	-60	NM_00096	6125	Hs.532359	NM_00096	ENSG0000	RPL5	DBA6|L5|M	ribosomal	protein-co

lib-C666-l	chr8	22462160	22462160	+	3.84463	NA	promoter-TSS (NR_033902)	promoter-TSS (NR_033902)	15	NR_033902	57805	Hs.744848	NM_02117	ENSG0000	CCAR2	DBC-1|DB(	cell cycle a	protein-co
lib-C666-l	chr4	9693348	9693348	+	2.99475	NA	Intergenic	CpG	-89910	NM_00079	1816	Hs.380681	NM_00079	ENSG0000	DRD5	DBDR|DRD	dopamine	protein-co
lib-C666-l	chr13	52027237	52027237	+	8.73012	NA	promoter-TSS (NM_001039937)	promoter-TSS (NM_001039937)	38	NM_01214	26512	Hs.439440	NM_01214	ENSG0000	INTS6	DBI-1|DDX	integrator	protein-co
lib-C666-l	chr5	118788339	118788339	+	6.29226	NA	5' UTR (NM_001292028, exon 1 of 24	5' UTR (NM_001292028, exon 1 of 2	147	NM_00129	3295	Hs.406861	NM_00041	ENSG0000	HSD17B4	DBP|MFE-2	hydroxyste	protein-co
lib-C666-l	chr16	70333084	70333084	+	83.41785	NA	promoter-TSS (NM_007242)	promoter-TSS (NM_007242)	22	NM_00101	11269	Hs.221761	NM_00724	ENSG0000	DDX19B	DBP5|DDX	DEAD-box	protein-co
lib-C666-l	chr16	17930975	17930975	+	7.43576	NA	Intergenic	MLT1I|LTR|ERVL-MaLR	-366237	NM_02216	64131	Hs.22907	NM_02216	ENSG0000	XYLT1	DBQD2|PX	xylosyltran	protein-co
lib-C666-l	chr16	17871628	17871628	+	3.84463	NA	Intergenic	Intergenic	-306890	NM_02216	64131	Hs.22907	NM_02216	ENSG0000	XYLT1	DBQD2|PX	xylosyltran	protein-co
lib-C666-l	chr12	110939528	110939528	+	7.0832	NA	promoter-TSS (NM_001286531)	promoter-TSS (NM_001286531)	417	NM_00128	51699	Hs.600114	NM_01622	ENSG0000	VPS29	DC15|DC7|	VPS29, retr	protein-co
lib-C666-l	chr4	109571576	109571576	+	10.67459	NA	promoter-TSS (NM_001267817)	promoter-TSS (NM_001267817)	-165	NM_00126	58505	Hs.445803	NM_02122	ENSG0000	OSTC	DC2	oligosacch	protein-co
lib-C666-l	chrX	23801195	23801195	+	15.30356	NA	promoter-TSS (NM_002970)	promoter-TSS (NM_002970)	-80	NR_02778	6303	Hs.28491	NM_00297	ENSG0000	SAT1	DC21|KFSD	spermidine	protein-co
lib-C666-l	chrX	23825610	23825610	+	6.32351	NA	Intergenic	Intergenic	24335	NM_00297	6303	Hs.28491	NM_00297	ENSG0000	SAT1	DC21|KFSD	spermidine	protein-co
lib-C666-l	chr9	69262621	69262621	+	199.49777	NA	promoter-TSS (NM_001286836)	promoter-TSS (NM_001286836)	9	NM_00128	220869	Hs.355950	NM_00102	ENSG0000	CBWD5	DC36	COBW dor	protein-co
lib-C666-l	chr9	70856822	70856822	+	154.53293	NA	promoter-TSS (NM_001286836).2	promoter-TSS (NM_001286836).2	17	NM_00128	220869	Hs.355950	NM_00102	ENSG0000	CBWD5	DC36	COBW dor	protein-co
lib-C666-l	chr9	70490197	70490197	+	131.0062	NA	promoter-TSS (NM_001286835)	promoter-TSS (NM_001286835)	14	NR_10460	220869	Hs.355950	NM_00102	ENSG0000	CBWD5	DC36	COBW dor	protein-co
lib-C666-l	chr1	36930055	36930055	+	45.43086	NA	promoter-TSS (NM_031280)	promoter-TSS (NM_031280)	-15	NM_03128	64960	Hs.352839	NM_03128	ENSG0000	MRPS15	DC37|MPR	mitochond	protein-co
lib-C666-l	chrX	154255354	154255354	+	83.41785	NA	intron (NM_023934, intron 1 of 4)	CpG	290	NM_02393	65991	Hs.356050	NM_02393	ENSG0000	FUNDC2	DC44|HCB	FUN14 dor	protein-co
lib-C666-l	chr5	68513808	68513808	+	6.98764	NA	intron (NM_033281, intron 1 of 3)	CpG	235	NM_03328	92259	Hs.631971	NM_03328	ENSG0000	MRPS36	DC47|MRP	mitochond	protein-co
lib-C666-l	chr8	110346474	110346474	+	3.89624	NA	promoter-TSS (NM_032869)	promoter-TSS (NM_032869)	-78	NM_00119	56943	Hs.492555	NM_02018	ENSG0000	ENY2	DC6|Sus1|e	ENY2, tran	protein-co
lib-C666-l	chr11	46615574	46615574	+	4.51266	NA	promoter-TSS (NM_001267782)	promoter-TSS (NM_001267782)	45	NM_00126	55626	Hs.654644	NM_01774	ENSG0000	AMBRA1	DCAF3|WD	autophagy	protein-co
lib-C666-l	chr1	110527330	110527330	+	50.12882	NA	promoter-TSS (NM_006621)	promoter-TSS (NM_006621)	-57	NM_00662	10768	Hs.743973	NM_00662	ENSG0000	AHCYL1	DCAL|IRBI	adenosylho	protein-co
lib-C666-l	chr16	2301549	2301549	+	14.26909	NA	promoter-TSS (NM_001178029)	promoter-TSS (NM_001178029)	53	NM_00117	1632	Hs.403436	NM_00191	ENSG0000	ECI1	DCI	enoyl-CoA	protein-co
lib-C666-l	chr20	62314051	62314051	+	19.75653	NA	intron (NM_001283010, intron 13 of	intron (NM_001283010, intron 13 o	-13953	NM_00382	8771	Hs.434878	NM_00382	ENSG0000	TNFRSF6B	DCR3|DJ58	TNF recept	protein-co
lib-C666-l	chr20	62330173	62330173	+	19.57279	NA	TTS (NM_003823)	TTS (NM_003823)	2169	NM_00382	8771	Hs.434878	NM_00382	ENSG0000	TNFRSF6B	DCR3|DJ58	TNF recept	protein-co
lib-C666-l	chr20	62330567	62330567	+	11.77301	NA	TTS (NM_003823)	TTS (NM_003823)	2563	NM_00382	8771	Hs.434878	NM_00382	ENSG0000	TNFRSF6B	DCR3|DJ58	TNF recept	protein-co
lib-C666-l	chr21	38640095	38640095	+	3.23837	NA	promoter-TSS (NM_006052)	promoter-TSS (NM_006052)	-262	NM_00605	10311	Hs.369488	NM_00605	ENSG0000	DSCR3	DCRA|DSC	DSCR3 arre	protein-co
lib-C666-l	chr21	38639778	38639778	+	2.60101	NA	promoter-TSS (NM_006052)	promoter-TSS (NM_006052)	55	NM_00605	10311	Hs.369488	NM_00605	ENSG0000	DSCR3	DCRA|DSC	DSCR3 arre	protein-co
lib-C666-l	chr21	38445728	38445728	+	4.04484	NA	promoter-TSS (NM_001320480)	promoter-TSS (NM_001320480)	244	NM_00133	7267	Hs.368214	NM_00331	ENSG0000	TTC3	DCRR1|RN	tetratricop	protein-co
lib-C666-l	chr10	82213697	82213697	+	9.20967	NA	promoter-TSS (NM_001128309)	promoter-TSS (NM_001128309)	-341	NM_00112	81619	Hs.718943	NM_03092	ENSG0000	TSPAN14	DC-TM4F2	tetraspanir	protein-co
lib-C666-l	chr9	34620551	34620551	+	17.48666	NA	promoter-TSS (NM_001281425)	promoter-TSS (NM_001281425)	-31	NM_00128	11258	Hs.511768	NM_00723	ENSG0000	DCTN3	DCTN-22|[	dynactin su	protein-co
lib-C666-l	chr12	57941067	57941067	+	21.94284	NA	promoter-TSS (NM_006400)	promoter-TSS (NM_006400)	47	NM_00126	10540	Hs.289123	NM_00640	ENSG0000	DCTN2	DCTN50|D	dynactin su	protein-co
lib-C666-l	chr6	7505813	7505813	+	14.3056	NA	Intergenic	Intergenic	-35995	NM_00441	1832	Hs.519873	NM_00441	ENSG0000	DSP	DCWHKTA	desmoplak	protein-co
lib-C666-l	chr11	47235944	47235944	+	17.3027	NA	promoter-TSS (NM_001300734)	promoter-TSS (NM_001300734)	-549	NM_00010	1643	Hs.700338	NM_00010	ENSG0000	DDB2	DDBB|UV-	damage sp	protein-co
lib-C666-l	chr1	68166596	68166596	+	16.23236	NA	TTS (NM_018841)	TTS (NM_018841)	15736	NM_00192	1647	Hs.80409	NM_00192	ENSG0000	GADD45A	DDIT1|GAD	growth arr	protein-co
lib-C666-l	chr1	68215398	68215398	+	7.97818	NA	intron (NM_018841, intron 2 of 3)	intron (NM_018841, intron 2 of 3)	64538	NM_00192	1647	Hs.80409	NM_00192	ENSG0000	GADD45A	DDIT1|GAD	growth arr	protein-co
lib-C666-l	chr8	48920963	48920963	+	99.26302	NA	promoter-TSS (NM_003350)	promoter-TSS (NM_003350)	-32	NM_00335	7336	Hs.491695	NM_00335	ENSG0000	UBE2V2	DDVIT1|DD	ubiquitin c	protein-co
lib-C666-l	chr16	61018	61018	+	9.04991	NA	promoter-TSS (NR_045117)	promoter-TSS (NR_045117)	-537	NR_04511	100287029	Hs.592089	NR_04511	ENSG0000	DDX11L10	DDX11L1|D	DEAD/H-b	pseudo
lib-C666-l	chr16	70380761	70380761	+	161.31409	NA	promoter-TSS (NM_001320525)	promoter-TSS (NM_001320525)	29	NM_01833	55308	Hs.656037	NM_01833	ENSG0000	DDX19A	DDX19-DD	DEAD-box	protein-co
lib-C666-l	chr7	44613438	44613438	+	3.27468	NA	exon (NM_001257189, exon 1 of 13)	exon (NM_001257189, exon 1 of 13	699	NM_01908	54606	Hs.654762	NM_01908	ENSG0000	DDX56	DDX21|DD	DEAD-box	protein-co
lib-C666-l	chr17	7475997	7475997	+	107.76324	NA	promoter-TSS (NM_001416)	promoter-TSS (NM_001416)	-27	NM_00141	1973	Hs.129673	NM_00141	ENSG0000	EIF4A1	DDX2A|EIF	eukaryotic	protein-co
lib-C666-l	chr3	47844524	47844524	+	12.73831	NA	non-coding (NR_075079, exon 1 of 4	non-coding (NR_075079, exon 1 of	125	NM_13861	22907	Hs.517948	NM_01496	ENSG0000	DHX30	DDX30|RE1	DExH-box	protein-co
lib-C666-l	chr17	5372269	5372269	+	60.44312	NA	promoter-TSS (NR_135644)	promoter-TSS (NR_135644)	111	NM_02016	56919	Hs.250456	NM_02016	ENSG0000	DHX33	DDX33	DEAH-box	protein-co
lib-C666-l	chr19	47854974	47854974	+	5.23609	NA	intron (NM_014681, intron 1 of 16)	MLT1K|LTR|ERVL-MaLR	2436	NM_01468	9704	Hs.151706	NM_01468	ENSG0000	DHX34	DDX34|HR	DExH-box	protein-co
lib-C666-l	chr3	154042347	154042347	+	155.95952	NA	promoter-TSS (NM_020865)	promoter-TSS (NM_020865)	-61	NM_00111	170506	Hs.446270	NM_02086	ENSG0000	DHX36	DDX36|G4	DEAH-box	protein-co
lib-C666-l	chr16	72127674	72127674	+	16.42352	NA	promoter-TSS (NM_017853)	promoter-TSS (NM_017853)	59	NM_01400	9785	Hs.151218	NM_01400	ENSG0000	DHX38	DDX38|PR	DEAH-box	protein-co
lib-C666-l	chr17	78120983	78120983	+	4.49043	NA	promoter-TSS (NM_014740)	promoter-TSS (NM_014740)	-1	NM_01474	9775	Hs.389649	NM_01474	ENSG0000	EIF4A3	DDX48|MU	eukaryotic	protein-co
lib-C666-l	chr2	39103019	39103019	+	3.8557	NA	promoter-TSS (NM_001145450)	promoter-TSS (NM_001145450)	56	NM_00132	90957	Hs.468226	NM_19896	ENSG0000	DHX57	DDX57	DExH-box	protein-co
lib-C666-l	chr4	153186441	153186441	+	4.52748	NA	Intergenic	Intergenic	-72366	NR_10427	102191832	Hs.712524	NR_10427	ENSG0000	FBXW7-AS	DEAR|DEsp	FBXW7 ant	pseudo
lib-C666-l	chr2	85185638	85185638	+	7.49163	NA	Intergenic	Intergenic	-12593	NM_02012	56888	Hs.654968	NM_02012	ENSG0000	KCMF1	DEBT91|FI	potassium	protein-co
lib-C666-l	chr17	26926081	26926081	+	2.42134	NA	promoter-TSS (NM_006461)	promoter-TSS (NM_006461)	-25	NM_00646	10615	Hs.514033	NM_00646	ENSG0000	SPAG5	DEEPEST|M	sperm asso	protein-co
lib-C666-l	chr8	6777030	6777030	+	8.73012	NA	Intergenic	Intergenic	6568	NM_00192	1671	Hs.711	NM_00192	ENSG0000	DEFA6	DEF6|HD-6	defensin al	protein-co
lib-C666-l	chr5	83731963	83731963	+	3.98718	NA	Intergenic	MER11A|LTR|ERVK	-51278	NM_00127	10085	Hs.482730	NM_00571	ENSG0000	EDIL3	DEL1	EGF like re	protein-co
lib-C666-l	chr5	141303283	141303283	+	13.0716	NA	promoter-TSS (NM_001142603)	promoter-TSS (NM_001142603)	-102	NM_00114	9812	Hs.210532	NM_01477	ENSG0000	KIAA0141	DELE	KIAA0141	protein-co
lib-C666-l	chr3	16306518	16306518	+	496.54483	NA	promoter-TSS (NM_001047434)	promoter-TSS (NM_001047434)	-22	NM_00104	285381	Hs.388087	NM_20683	ENSG0000	DPH3	DELGIP|DE	diphthamic	protein-co
lib-C666-l	chr14	100704989	100704989	+	61.32821	NA	promoter-TSS (NM_003403)	promoter-TSS (NM_003403)	-113	NM_00340	7528	Hs.388927	NM_00340	ENSG0000	YY1	DELTA|INC	YY1 transc	protein-co
lib-C666-l	chr17	4699452	4699452	+	7.15277	NA	promoter-TSS (NM_002798)	promoter-TSS (NM_002798)	13	NM_00127	5694	Hs.77060	NM_00279	ENSG0000	PSMB6	DELTA|LMI	proteasom	protein-co
lib-C666-l	chr8	120885945	120885945	+	5.04826	NA	promoter-TSS (NM_001283012)	promoter-TSS (NM_001283012)	50	NM_00128	64798	Hs.112981	NM_02278	ENSG0000	DEPTOR	DEP.6|DEP	DEP doma	protein-co
lib-C666-l	chr10	45471181	45471181	+	30.77943	NA	TTS (NM_007021)	TTS (NM_007021)	3149	NM_00702	11067	Hs.93675	NM_00702	ENSG0000	C10orf10	DEPP|FIG|F	chromosor	protein-co
lib-C666-l	chr10	45470534	45470534	+	8.56376	NA	intron (NM_032023, intron 3 of 10)	GC_rich|Low_complexity|Low_comp	3796	NM_00702	11067	Hs.93675	NM_00702	ENSG0000	C10orf10	DEPP|FIG|F	chromosor	protein-co
lib-C666-l	chr8	124054541	124054541	+	9.79436	NA	5' UTR (NM_001134671, exon 1 of 8)	5' UTR (NM_001134671, exon 1 of	122	NM_00133	79139	Hs.241576	NM_02429	ENSG0000	DERL1	DER-1|DEF	derlin 1	protein-co
lib-C666-l	chr11	66139543	66139543	+	37.891	NA	promoter-TSS (NM_001532)	promoter-TSS (NM_001532)	-252	NR_12534	3177	Hs.569017	NM_00153	ENSG0000	SLC29A2	DER12|EN1	solute carr	protein-co
lib-C666-l	chr11	64902096	64902096	+	25.39084	NA	promoter-TSS (NM_032431)	promoter-TSS (NM_032431)	-93	NM_17223	84447	Hs.75859	NM_03243	ENSG0000	SYVN1	DER3|HRD	synoviolin	protein-co
lib-C666-l	chr10	85899249	85899249	+	202.86639	NA	promoter-TSS (NM_014394)	promoter-TSS (NM_014394)	64	NM_01439	27069	Hs.352656	NM_01439	ENSG0000	GHITM	DERP2|HSF	growth hor	protein-co
lib-C666-l	chr10	85878517	85878517	+	15.60931	NA	Intergenic	LTR10F|LTR|ERV1	-20668	NM_01439	27069	Hs.352656	NM_01439	ENSG0000	GHITM	DERP2|HSF	growth hor	protein-co
lib-C666-l	chr4	166248716	166248716	+	47.72539	NA	promoter-TSS (NM_006745)	promoter-TSS (NM_006745)	-102	NM_00101	6307	Hs.105269	NM_00674	ENSG0000	MSMO1	DESP4|ERG	methylster	protein-co
lib-C666-l	chr10	63575487	63575487	+	17.71746	NA	Intergenic	Charlie20a|DNA|hAT-Charlie	-85526	NM_03219	84159	Hs.535297	NM_03219	ENSG0000	ARID5B	DESRT|MR	AT-rich int	protein-co
lib-C666-l	chr10	63582880	63582880	+	17.71746	NA	Intergenic	Intergenic	-78133	NM_03219	84159	Hs.535297	NM_03219	ENSG0000	ARID5B	DESRT|MR	AT-rich int	protein-co
lib-C666-l	chr14	73493964	73493964	+	28.94286	NA	promoter-TSS (NM_001281734)	promoter-TSS (NM_001281734)	-44	NM_02126	53349	Hs.335106	NM_02126	ENSG0000	ZFYVE1	DFCP1|PPF	zinc finger	protein-co
lib-C666-l	chrX	40943985	40943985	+	29.74345	NA	promoter-TSS (NM_001039590)	promoter-TSS (NM_001039590)	-903	NM_00103	8239	Hs.77578	NM_00465	ENSG0000	USP9X	DFFRX|FAF	ubiquitin s	protein-co
lib-C666-l	chr11	67351198	67351198	+	23.20869	NA	5' UTR (NM_000852, exon 1 of 7)	5' UTR (NM_000852, exon 1 of 7)	132	NM_00085	2950	Hs.523836	NM_00085	ENSG0000	GSTP1	DFN7|FAEE	glutathione	protein-co
lib-C666-l	chr5	140998746	140998746	+	3.20885	NA	promoter-TSS (NM_005219)	promoter-TSS (NM_005219)	-124	NM_00131	1729	Hs.529451	NM_00521	ENSG0000	DIAPH1	DFNA1|DIA	diaphanou	protein-co
lib-C666-l	chr6	33140441	33140441	+	2.05265	NA	intron (NM_080679, intron 35 of 62).6	intron (NM_080679, intron 35 of 62	19804	NM_00116	1302	Hs.390171	NM_08067	ENSG0000	COL11A2	DFNA13|D	collagen ty	protein-co
lib-C666-l	chr6	76458888	76458888	+	24.99834	NA	promoter-TSS (NM_001300899)	promoter-TSS (NM_001300899)	-5	NM_00130	4646	Hs.149387	NM_00499	ENSG0000	MYO6	DFNA22|D	myosin VI	protein-co
lib-C666-l	chr6	76457191	76457191	+	6.98764	NA	Intergenic	L2c|LINE|L2	-1702	NM_00130	4646	Hs.149387	NM_00499	ENSG0000	MYO6	DFNA22|D	myosin VI	protein-co
lib-C666-l	chr9	75142316	75142316	+	6.15313	NA	intron (NM_138691, intron 1 of 23)	intron (NM_138691, intron 1 of 23)	5599	NM_13869	117531	Hs.670211	NM_13869	ENSG0000	TMC1	DFNA36|D	transmemb	protein-co
lib-C666-l	chr19	50703440	50703440	+	105.49278	NA	Intergenic	Intergenic	-3445	NM_00107	79784	Hs.467142	NM_02472	ENSG0000	MYH14	DFNA4|DF	myosin hea	protein-co
lib-C666-l	chr19	50708643	50708643	+	30.24892	NA	intron (NM_001077186, intron 1 of 4	CpG	1758	NM_02472	79784	Hs.467142	NM_02472	ENSG0000	MYH14	DFNA4|DF	myosin hea	protein-co
lib-C666-l	chr19	50706861	50706861	+	12.34228	NA	promoter-TSS (NM_001145809)	promoter-TSS (NM_001145809)	-24	NM_00107	79784	Hs.467142	NM_02472	ENSG0000	MYH14	DFNA4|DF	myosin hea	protein-co
lib-C666-l	chr12	122710622	122710622	+	39.83551	NA	promoter-TSS (NR_103497)	promoter-TSS (NR_103497)	-225	NM_00127	56616	Hs.169611	NM_01988	ENSG0000	DIABLO	DFNA64|SI	diablo IAP-	protein-co
lib-C666-l	chr16	2524086	2524086	+	4.52748	NA	TTS (NM_006181)	TTS (NM_006181)	-1061	NM_00119	57465	Hs.353087	NM_02070	ENSG0000	TBC1D24	DFNA65|D	TBC1 dom	protein-co
lib-C666-l	chr16	2524668	2524668	+	2.00164	NA	promoter-TSS (NM_001199107)	promoter-TSS (NM_001199107)	-479	NM_00119	57465	Hs.353087	NM_02070	ENSG0000	TBC1D24	DFNA65|D	TBC1 dom	protein-co
lib-C666-l	chr6	109703536	109703536	+	16.264	NA	promoter-TSS (NM_001346500)	promoter-TSS (NM_001346500)	479	NM_00114	8763	Hs.520313	NM_00601	ENSG0000	CD164	DFNA66|M	CD164 mo	protein-co
lib-C666-l	chr20	60813427	60813427	+	41.5993	NA	promoter-TSS (NM_001278649)	promoter-TSS (NM_001278649)	-114	NM_14449	9885	Hs.473254	NM_01483	ENSG0000	OSBPL2	DFNA67|D	oxysterol b	protein-co
lib-C666-l	chr1	100828649	100828649	+	12.97267	NA	intron (NM_033312, intron 2 of 14)	LTR9|LTR|ERV1	10626	NM_03331	8556	Hs.127411	NM_00367	ENSG0000	CDC14A	DFNB105|c	cell divisior	protein-co
lib-C666-l	chr22	38143016	38143016	+	3.07144	NA	intron (NM_007032, intron 1 of 13)	intron (NM_007032, intron 1 of 13)	775	NM_13863	11078	Hs.533030	NM_00703	ENSG0000	TRIOBP	DFNB28|HI	TRIO and F	protein-co
lib-C666-l	chr21	37881577	37881577	+	5.9099	NA	intron (NM_001146077, intron 2 of 2)	intron (NM_001146077, intron 2 of	-29189	NM_00114	23562	Hs.660278	NM_01213	ENSG0000	CLDN14	DFNB29	claudin 14	protein-co
lib-C666-l	chr14	76843610	76843610	+	7.15277	NA	intron (NM_004452, intron 1 of 10)	CpG	5920	NM_00445	2103	Hs.435845	NM_00445	ENSG0000	ESRRB	DFNB35|EF	estrogen re	protein-co
lib-C666-l	chr3	121741041	121741041	+	33.78053	NA	promoter-TSS (NM_175924)	promoter-TSS (NM_175924)	86	NM_00119	286676	Hs.98484	NM_17592	ENSG0000	ILDR1	DFNB42|ILI	immunoglo	protein-co
lib-C666-l	chr5	131629630	131629630	+	11.55573	NA	promoter-TSS (NM_003059)	promoter-TSS (NM_003059)	-515	NM_00305	6583	Hs.310591	NM_00305	ENSG0000	SLC22A4	DFNB60|O	solute carr	protein-co
lib-C666-l	chr7	103047438	103047438	+	7.21092	NA	intron (NR_135801, intron 8 of 17)	MER119|DNA|hAT-Charlie	36220	NR_12044	375611	Hs.585146	NM_19899	ENSG0000	SLC26A5	DFNB61|PF	solute carr	protein-co
lib-C666-l	chr18	44116618	44116618	+	10.16129	NA	intron (NM_144612, intron 26 of 39)	intron (NM_144612, intron 26 of 39	-17569	NM_00114	125336	Hs.345877	NM_14461	ENSG0000	LOXHD1	DFNB77|LH	lipoxygena	protein-co
lib-C666-l	chr11	78285883	78285883	+	18.20429	NA	promoter-TSS (NM_001243251)	promoter-TSS (NM_001243251)	26	NM_00124	79731	Hs.503389	NM_02467	ENSG0000	NARS2	DFNB94|SL	asparaginy	protein-co
lib-C666-l	chr9	15511072	15511072	+	3.8557	NA	promoter-TSS (NM_001317898)	promoter-TSS (NM_001317898)	-55	NM_00131	11168	Hs.658434	NM_02114	ENSG0000	PSIP1	DFS70|LED	PC4 and SF	protein-co
lib-C666-l	chr4	129209321	129209321	+	8.73558	NA	5' UTR (NM_006320, exon 1 of 3)	5' UTR (NM_006320, exon 1 of 3)	663	NM_00632	10424	Hs.507910	NM_00632	ENSG0000	PGRMC2	DG6|PMBP	progesterc	protein-co
lib-C666-l	chr22	19132266	19132266	+	86.40352	NA	promoter-TSS (NM_022719)	promoter-TSS (NM_022719)	-76	NR_13430	8220	Hs.517407	NM_02271	ENSG0000	DGCR14	DGCR13|D	DiGeorge s	protein-co
lib-C666-l	chr13	42614479	42614479	+	9.93242	NA	intron (NM_001204504, intron 1 of 2	CpG	307	NM_00120	160851	Hs.630380	NM_15291	ENSG0000	DGKH	DGKeta	diacylglyce	protein-co
lib-C666-l	chr7	137459710	137459710	+	8.01457	NA	intron (NM_001321708, intron 1 of 3	intron (NM_001321708, intron 1 of	71368	NM_00132	9162	Hs.242947	NM_00471	ENSG0000	DGKI	DGK-IOTA	diacylglyce	protein-co
lib-C666-l	chr8	11660098	11660098	+	5.29271	NA	promoter-TSS (NM_001287745)	promoter-TSS (NM_001287745)	-22	NM_00446	2222	Hs.593928	NM_00446	ENSG0000	FDFT1	DGPT|ERG	farnesyl-di	protein-co
lib-C666-l	chr22	19109938	19109938	+	3.20885	NA	promoter-TSS (NM_001184781)	promoter-TSS (NM_001184781)	29	NM_00118	9993	Hs.517357	NM_00513	ENSG0000	DGCR2	DGS-C|IDD	DiGeorge s	protein-co
lib-C666-l	chrX	132860653	132860653	+	6.98764	NA	intron (NM_001164617, intron 3 of 8)	intron (NM_001164617, intron 3 of	259020	NM_00116	2719	Hs.644108	NM_00448	ENSG0000	GPC3	DGSX|GTR	glypican 3	protein-co
lib-C666-l	chr19	17186414	17186414	+	16.23236	NA	promoter-TSS (NM_004145)	promoter-TSS (NM_004145)	-71	NM_00101	93323	Hs.404088	NM_03341	ENSG0000	HAUS8	DGT4|HICE	HAUS augr	protein-co
lib-C666-l	chr9	19103251	19103251	+	4.04037	NA	promoter-TSS (NM_001270890)	promoter-TSS (NM_001270890)	-311	NM_01764	54801	Hs.533468	NM_01764	ENSG0000	HAUS6	Dgt6|FAM2	HAUS augr	protein-co
lib-C666-l	chr1	225615890	225615890	+	5.12426	NA	promoter-TSS (NM_002296)	promoter-TSS (NM_002296)	-75	NM_00229	3930	Hs.435166	NM_00229	ENSG0000	LBR	DHCR14B|I	lamin B rec	protein-co
lib-C666-l	chr1	27160178	27160178	+	13.55025	NA	intron (NM_032283, intron 2 of 7)	intron (NM_032283, intron 2 of 7)	6977	NM_03228	84243	Hs.523710	NM_03228	ENSG0000	ZDHHC18	DHHC-18|	zinc finger	protein-co
lib-C666-l	chr8	17013857	17013857	+	9.87814	NA	promoter-TSS (NM_016353)	promoter-TSS (NM_016353)	21	NM_01635	51201	Hs.728241	NM_01635	ENSG0000	ZDHHC2	DHHC2|ZN	zinc finger	protein-co
lib-C666-l	chr8	17014856	17014856	+	3.55617	NA	intron (NM_016353, intron 1 of 12)	intron (NM_016353, intron 1 of 12)	1020	NM_01635	51201	Hs.728241	NM_01635	ENSG0000	ZDHHC2	DHHC2|ZN	zinc finger	protein-co
lib-C666-l	chr3	113666690	113666690	+	40.13552	NA	promoter-TSS (NM_001320466)	promoter-TSS (NM_001320466)	-58	NM_00132	254887	Hs.21902	NM_17357	ENSG0000	ZDHHC23	DHHC-23|	zinc finger	protein-co
lib-C666-l	chr11	57435314	57435314	+	2.78666	NA	promoter-TSS (NM_015457)	promoter-TSS (NM_015457)	-160	NM_01545	25921	Hs.27239	NM_01545	ENSG0000	ZDHHC5	DHHC5|ZN	zinc finger	protein-co
lib-C666-l	chr16	85045192	85045192	+	103.11586	NA	promoter-TSS (NM_001145548)	promoter-TSS (NM_001145548)	-51	NM_00114	55625	Hs.461610	NM_01774	ENSG0000	ZDHHC7	DHHC7|SE	zinc finger	protein-co
lib-C666-l	chr5	149792525	149792525	+	120.97476	NA	promoter-TSS (NM_004355)	promoter-TSS (NM_004355)	-26	NM_00102	972	Hs.436568	NM_00435	ENSG0000	CD74	DHLAG|HL	CD74 mole	protein-co
lib-C666-l	chr5	149791052	149791052	+	30.07415	NA	intron (NM_001025158, intron 1 of 5)	intron (NM_001025158, intron 1 of	1447	NM_00102	972	Hs.436568	NM_00435	ENSG0000	CD74	DHLAG|HL	CD74 mole	protein-co
lib-C666-l	chr5	149790279	149790279	+	21.73991	NA	intron (NM_001025158, intron 1 of 5)	intron (NM_001025158, intron 1 of	2220	NM_00102	972	Hs.436568	NM_00435	ENSG0000	CD74	DHLAG|HL	CD74 mole	protein-co
lib-C666-l	chr5	149789501	149789501	+	12.62235	NA	intron (NM_001025158, intron 1 of 5)	intron (NM_001025158, intron 1 of	2998	NM_00102	972	Hs.436568	NM_00435	ENSG0000	CD74	DHLAG|HL	CD74 mole	protein-co
lib-C666-l	chr5	53686071	53686071	+	6.82479	NA	intron (NR_126379, intron 1 of 4)	intron (NR_126379, intron 1 of 4)	-65360	NM_00630	8988	Hs.41707	NM_00630	ENSG0000	HSPB3	DHMN2C|I	heat shock	protein-co
lib-C666-l	chr16	72042518	72042518	+	11.03618	NA	promoter-TSS (NM_001361)	promoter-TSS (NM_001361)	-125	NM_00136	1723	Hs.654427	NM_00136	ENSG0000	DHODH	DHOdehas	dihydroorc	protein-co
lib-C666-l	chr4	17504646	17504646	+	10.73451	NA	intron (NM_001306140, intron 2 of 5)	L1MC4|LINE|L1	9211	NM_00130	5860	Hs.75438	NM_00032	ENSG0000	QDPR	DHPR|PKU	quinoid dih	protein-co
lib-C666-l	chr4	17513761	17513761	+	3.9966	NA	promoter-TSS (NM_000320)	promoter-TSS (NM_000320)	96	NM_00130	5860	Hs.75438	NM_00032	ENSG0000	QDPR	DHPR|PKU	quinoid dih	protein-co
lib-C666-l	chr18	61034911	61034911	+	20.19666	NA	promoter-TSS (NM_002035)	promoter-TSS (NM_002035)	-405	NM_00203	2531	Hs.74050	NM_00203	ENSG0000	KDSR	DHSR|FVT1	3-ketodihy	protein-co
lib-C666-l	chr11	75526201	75526201	+	38.90989	NA	promoter-TSS (NM_003369)	promoter-TSS (NM_003369)	-11	NM_00336	7405	Hs.202470	NM_00336	ENSG0000	UVRAG	DHTX|VPS3	UV radiatic	protein-co
lib-C666-l	chr17	58104606	58104606	+	40.21248	NA	Intergenic	Intergenic	-8193	NR_00292	653645	Hs.448884	NR_00292	ENSG0000	TBC1D3P1	DHX40P|D	TBC1D3P1	pseudo
lib-C666-l	chr17	58108373	58108373	+	13.52963	NA	Intergenic	MER103C|DNA|hAT-Charlie	-11960	NR_00292	653645	Hs.448884	NR_00292	ENSG0000	TBC1D3P1	DHX40P|D	TBC1D3P1	pseudo
lib-C666-l	chr3	143690161	143690161	+	3.7184	NA	promoter-TSS (NM_173552)	promoter-TSS (NM_173552)	-479	NM_17355	205428	Hs.288954	NM_17355	ENSG0000	C3orf58	DIA1|GoPr	chromosor	protein-co
lib-C666-l	chr19	38754798	38754798	+	7.97818	NA	promoter-TSS (NM_001166103)	promoter-TSS (NM_001166103)	-300	NM_02110	10653	Hs.31439	NM_02110	ENSG0000	SPINT2	DIAR3|HAI	serine pep	protein-co
lib-C666-l	chr19	38755282	38755282	+	4.71278	NA	5' UTR (NM_001166103, exon 1 of 6)	5' UTR (NM_001166103, exon 1 of 6	184	NM_00116	10653	Hs.31439	NM_02110	ENSG0000	SPINT2	DIAR3|HAI	serine pep	protein-co
lib-C666-l	chr2	47597160	47597160	+	14.3056	NA	intron (NM_002354, intron 1 of 8)	CpG	873	NM_00235	4072	Hs.542050	NM_00235	ENSG0000	EPCAM	DIAR5|EGP	epithelial c	protein-co
lib-C666-l	chr2	47596611	47596611	+	6.40179	NA	5' UTR (NM_002354, exon 1 of 9)	5' UTR (NM_002354, exon 1 of 9)	324	NM_00235	4072	Hs.542050	NM_00235	ENSG0000	EPCAM	DIAR5|EGP	epithelial c	protein-co
lib-C666-l	chr12	14818587	14818587	+	19.23598	NA	intron (NM_004963, intron 10 of 26)	L2b|LINE|L2	30932	NM_00496	2984	Hs.524278	NM_00496	ENSG0000	GUCY2C	DIAR6|GC-	guanylate	protein-co
lib-C666-l	chr17	79679022	79679022	+	9.75396	NA	promoter-TSS (NM_001270888)	promoter-TSS (NM_001270888)	-244	NM_00127	1468	Hs.548187	NM_01214	ENSG0000	SLC25A10	DIC	solute carr	protein-co
lib-C666-l	chr17	79702646	79702646	+	3.23837	NA	Intergenic	Intergenic	23380	NM_01214	1468	Hs.548187	NM_01214	ENSG0000	SLC25A10	DIC	solute carr	protein-co
lib-C666-l	chr1	85527858	85527858	+	48.11517	NA	promoter-TSS (NM_001288563)	promoter-TSS (NM_001288563)	-123	NM_00128	126820	Hs.97933	NM_14517	ENSG0000	WDR63	DIC3|NYD-	WD repeat	protein-co
lib-C666-l	chr9	131419049	131419049	+	5.96422	NA	promoter-TSS (NM_052844)	promoter-TSS (NM_052844)	80	NM_05284	89891	Hs.495240	NM_05284	ENSG0000	WDR34	DIC5|FAP1	WD repeat	protein-co
lib-C666-l	chr2	172543930	172543930	+	11.06679	NA	promoter-TSS (NM_001320882)	promoter-TSS (NM_001320882)	11	NM_00127	1781	Hs.546250	NM_00137	ENSG0000	DYNC1I2	DIC74|DNC	dynein cyto	protein-co
lib-C666-l	chr2	172544304	172544304	+	3.7184	NA	5' UTR (NM_001320884, exon 1 of 16	5' UTR (NM_001320884, exon 1 of 1	122	NM_00127	1781	Hs.546250	NM_00137	ENSG0000	DYNC1I2	DIC74|DNC	dynein cyto	protein-co
lib-C666-l	chr6	31546124	31546124	+	5.14484	NA	TTS (NM_000594).4	TTS (NM_000594).4	2780	NM_00059	7124	Hs.241570	NM_00059	ENSG0000	TNF	DIF|TNF-al	tumor necr	protein-co
lib-C666-l	chr17	34901465	34901465	+	28.88523	NA	intron (NM_024835, intron 1 of 13)	intron (NM_024835, intron 1 of 13)	728	NM_02483	79893	Hs.514116	NM_02483	ENSG0000	GGNBP2	DIF-3|DIF3	gametoger	protein-co
lib-C666-l	chr17	34900902	34900902	+	20.72954	NA	5' UTR (NM_024835, exon 1 of 14)	5' UTR (NM_024835, exon 1 of 14)	165	NM_02483	79893	Hs.514116	NM_02483	ENSG0000	GGNBP2	DIF-3|DIF3	gametoger	protein-co
lib-C666-l	chr17	34900512	34900512	+	2.65152	NA	promoter-TSS (NM_024835)	promoter-TSS (NM_024835)	-225	NM_02483	79893	Hs.514116	NM_02483	ENSG0000	GGNBP2	DIF-3|DIF3	gametoger	protein-co
lib-C666-l	chr2	242255557	242255557	+	8.73012	NA	promoter-TSS (NM_001321030)	promoter-TSS (NM_001321030)	-36	NM_00132	4735	Hs.721234	NM_00440	ENSG0000	2-Sep	DIFF6|NED	septin 2	protein-co
lib-C666-l	chr10	74033602	74033602	+	7.09685	NA	promoter-TSS (NM_019058)	promoter-TSS (NM_019058)	-75	NM_01905	54541	Hs.744875	NM_01905	ENSG0000	DDIT4	Dig2|REDD	DNA dama	protein-co
lib-C666-l	chr10	74016910	74016910	+	5.96687	NA	Intergenic	Intergenic	-16767	NM_01905	54541	Hs.744875	NM_01905	ENSG0000	DDIT4	Dig2|REDD	DNA dama	protein-co
lib-C666-l	chr10	74024532	74024532	+	3.7184	NA	Intergenic	Intergenic	-9145	NM_01905	54541	Hs.744875	NM_01905	ENSG0000	DDIT4	Dig2|REDD	DNA dama	protein-co
lib-C666-l	chr13	114238949	114238949	+	48.11517	NA	promoter-TSS (NR_026580)	promoter-TSS (NR_026580)	-54	NR_02658	7027	Hs.79353	NM_00711	ENSG0000	TFDP1	DILC|DP1|D	transcriptic	protein-co
lib-C666-l	chr22	47057518	47057518	+	10.47352	NA	intron (NM_015124, intron 5 of 18)	intron (NM_015124, intron 5 of 18)	41219	NM_01512	23151	Hs.475150	NM_01512	ENSG0000	GRAMD4	DIP|dA59H	GRAM don	protein-co
lib-C666-l	chr22	46980567	46980567	+	6.15313	NA	Intergenic	Intergenic	-35732	NM_01512	23151	Hs.475150	NM_01512	ENSG0000	GRAMD4	DIP|dA59H	GRAM don	protein-co
lib-C666-l	chr11	65658121	65658121	+	9.10494	NA	5' UTR (NM_006848, exon 1 of 1)	5' UTR (NM_006848, exon 1 of 1)	246	NM_00684	11007	Hs.66713	NM_00684	ENSG0000	CCDC85B	DIPA	coiled-coil	protein-co
lib-C666-l	chr6	32098147	32098147	+	3.8557	NA	promoter-TSS (NM_022110)	promoter-TSS (NM_022110)	-80	NM_02211	63943	Hs.520042	NM_02211	ENSG0000	FKBPL	DIR1|FKBP	FK506 binc	protein-co
lib-C666-l	chr1	8021660	8021660	+	79.67884	NA	promoter-TSS (NM_001123377)	promoter-TSS (NM_001123377)	-54	NM_00112	11315	Hs.419640	NM_00726	ENSG0000	PARK7	DJ-1|DJ1|F	Parkinsonis	protein-co
lib-C666-l	chr1	8014362	8014362	+	39.72046	NA	Intergenic	CpG	-7352	NM_00112	11315	Hs.419640	NM_00726	ENSG0000	PARK7	DJ-1|DJ1|F	Parkinsonis	protein-co
lib-C666-l	chr10	74114716	74114716	+	135.45795	NA	exon (NM_001002762, exon 1 of 8)	exon (NM_001002762, exon 1 of 8)	191	NM_00100	54788	Hs.500013	NM_01762	ENSG0000	DNAJB12	DJ10	DnaJ heat	protein-co
lib-C666-l	chr17	40169136	40169136	+	14.54258	NA	promoter-TSS (NM_001144766)	promoter-TSS (NM_001144766)	47	NM_00114	7266	Hs.500156	NM_00331	ENSG0000	DNAJC7	DJ11|DJC7	DnaJ heat	protein-co
lib-C666-l	chr17	40169706	40169706	+	2.00164	NA	promoter-TSS (NM_001144766)	promoter-TSS (NM_001144766)	9	NR_02943	7266	Hs.500156	NM_00331	ENSG0000	DNAJC7	DJ11|DJC7	DnaJ heat	protein-co
lib-C666-l	chr6	28864170	28864170	+	9.93242	NA	promoter-TSS (NR_104117).6	promoter-TSS (NR_104117).6	-137	NR_10411	414760	Hs.211005	NR_10411	ENSG0000	HCG14	dJ111M5.4	HLA comp	ncRNA
lib-C666-l	chr1	17042060	17042060	+	58.9546	NA	intron (NR_026567, intron 1 of 10)	intron (NR_026567, intron 1 of 10)	4592	NR_02656	284729	Hs.548239	NR_02656	ENSG0000	ESPNP	dJ1182A14	espin pseu	pseudo
lib-C666-l	chr20	3140536	3140536	+	19.01614	NA	promoter-TSS (NM_001267584)	promoter-TSS (NM_001267584)	-4	NM_02182	60493	Hs.741168	NM_02182	ENSG0000	FASTKD5	dJ1187M1	FAST kinas	protein-co
lib-C666-l	chr1	6761994	6761994	+	8.89416	NA	promoter-TSS (NM_018198)	promoter-TSS (NM_018198)	-28	NM_01819	55735	Hs.462640	NM_01819	ENSG0000	DNAJC11	dJ126A5.1	DnaJ heat	protein-co
lib-C666-l	chr9	33025193	33025193	+	18.1336	NA	promoter-TSS (NM_001314039)	promoter-TSS (NM_001314039)	-8	NM_00153	3301	Hs.445203	NM_00153	ENSG0000	DNAJA1	DJ-2|DjA1|	DnaJ heat	protein-co
lib-C666-l	chr9	33044361	33044361	+	13.46097	NA	3' UTR (NM_018225, exon 12 of 12)	3' UTR (NM_018225, exon 12 of 12)	19160	NM_00131	3301	Hs.445203	NM_00153	ENSG0000	DNAJA1	DJ-2|DjA1|	DnaJ heat	protein-co
lib-C666-l	chr1	174992723	174992723	+	24.25638	NA	promoter-TSS (NM_022100)	promoter-TSS (NM_022100)	-132	NR_03760	63931	Hs.654858	NM_02210	ENSG0000	MRPS14	DJ262D12.	mitochond	protein-co

lib-C666-l	chr6	38607879	38607879	+	244.35405	NA	promoter-TSS (NM_052893)	promoter-TSS (NM_052893)	45	NM_00109	114781	Hs.603858	NM_05289	ENSG0000	BTBD9	dJ322I12.1	BTB domai	protein-co
lib-C666-l	chr22	29168802	29168802	+	8.28333	NA	promoter-TSS (NM_001284265)	promoter-TSS (NM_001284265)	140	NM_00128	150275	Hs.406460	NM_17351	ENSG0000	CCDC117	dJ366L4.1	coiled-coil	protein-co
lib-C666-l	chr7	157089997	157089997	+	118.24409	NA	Intergenic	Intergenic	-39713	NM_00549	10049	Hs.490745	NM_00549	ENSG0000	DNAJB6	DJ4|DnaJ|H	DnaJ heat	protein-co
lib-C666-l	chr7	157129653	157129653	+	52.78568	NA	promoter-TSS (NM_005494)	promoter-TSS (NM_005494)	-57	NM_00549	10049	Hs.490745	NM_00549	ENSG0000	DNAJB6	DJ4|DnaJ|H	DnaJ heat	protein-co
lib-C666-l	chr1	24742185	24742185	+	5.04179	NA	promoter-TSS (NM_001199012)	promoter-TSS (NM_001199012)	-55	NM_00132	57185	Hs.523442	NM_02044	ENSG0000	NIPAL3	DJ462O23.	NIPA like d	protein-co
lib-C666-l	chr6	42847412	42847412	+	5.5166	NA	promoter-TSS (NR_134563)	promoter-TSS (NR_134563)	58	NR_13456	285855	Hs.520133	NM_19848	ENSG0000	RPL7L1	dJ475N16.	ribosomal	protein-co
lib-C666-l	chr6	116574739	116574739	+	9.94769	NA	promoter-TSS (NR_136520)	promoter-TSS (NR_136520)	522	NM_02164	23270	Hs.284141	NM_02164	ENSG0000	TSPYL4	dJ486I3.2	TSPY like 4	protein-co
lib-C666-l	chr22	39097076	39097076	+	426.71643	NA	promoter-TSS (NM_014876)	promoter-TSS (NM_014876)	-617	NM_01487	9929	Hs.3094	NM_01487	ENSG0000	JOSD1	dJ508I15.2	Josephin d	protein-co
lib-C666-l	chr22	43583719	43583719	+	26.19214	NA	promoter-TSS (NM_015140)	promoter-TSS (NM_015140)	-582	NM_01514	23170	Hs.517670	NM_01514	ENSG0000	TTLL12	dJ526I14.2	tubulin tyr	protein-co
lib-C666-l	chr1	70671347	70671347	+	243.47342	NA	promoter-TSS (NM_017768)	promoter-TSS (NM_017768)	14	NM_01776	55631	Hs.147836	NM_01776	ENSG0000	LRRC40	dJ677H15.	leucine ric	protein-co
lib-C666-l	chr20	11871553	11871553	+	5.4536	NA	exon (NM_181443, exon 1 of 5)	exon (NM_181443, exon 1 of 5)	182	NM_00128	22903	Hs.709366	NM_01496	ENSG0000	BTBD3	dJ742J24.1	BTB domai	protein-co
lib-C666-l	chr20	30697450	30697450	+	56.63552	NA	5' UTR (NM_014742, exon 1 of 18)	5' UTR (NM_014742, exon 1 of 18)	141	NM_01474	9777	Hs.654665	NM_01474	ENSG0000	TM9SF4	dJ836N17.	transmemb	protein-co
lib-C666-l	chr22	32058241	32058241	+	130.83212	NA	5' UTR (NM_001326411, exon 1 of 8)	5' UTR (NM_001326411, exon 1 of 8	309	NM_00132	23761	Hs.420559	NM_01433	ENSG0000	PISD	DJ858B16|	phosphatic	protein-co
lib-C666-l	chr22	32042723	32042723	+	6.82479	NA	intron (NM_001326416, intron 3 of 1(	intron (NM_001326416, intron 3 of	-8234	NM_17802	23761	Hs.420559	NM_01433	ENSG0000	PISD	DJ858B16|	phosphatic	protein-co
lib-C666-l	chr11	33061328	33061328	+	6.82401	NA	promoter-TSS (NM_001145541)	promoter-TSS (NM_001145541)	-233	NM_00114	55346	Hs.655341	NM_01839	ENSG0000	TCP11L1	dJ85M6.3	t-complex	protein-co
lib-C666-l	chrX	107334818	107334818	+	3.57421	NA	promoter-TSS (NM_001321287)	promoter-TSS (NM_001321287)	56	NM_00281	5716	Hs.522752	NM_00281	ENSG0000	PSMD10	dJ889N15.	proteasom	protein-co
lib-C666-l	chr6	117803674	117803674	+	3.98718	NA	promoter-TSS (NM_173674)	promoter-TSS (NM_173674)	-93	NM_17367	285761	Hs.658304	NM_17367	ENSG0000	DCBLD1	dJ94G16.1	discoidin,	protein-co
lib-C666-l	chr1	86861762	86861762	+	11.48337	NA	5' UTR (NM_001184765, exon 1 of 17	5' UTR (NM_001184765, exon 1 of 1	263	NM_00118	57489	Hs.149360	NM_02072	ENSG0000	ODF2L	dJ977L11.1	outer dens	protein-co
lib-C666-l	chr14	24711873	24711873	+	27.44508	NA	promoter-TSS (NM_001099274)	promoter-TSS (NM_001099274)	7	NM_01246	26277	Hs.496191	NM_01246	ENSG0000	TINF2	DKCA3|TIN	TERF1 inte	protein-co
lib-C666-l	chr5	177580890	177580890	+	33.73372	NA	promoter-TSS (NM_001034833)	promoter-TSS (NM_001034833)	71	NM_01783	55651	Hs.744074	NM_01783	ENSG0000	NHP2	DKCB2|NH	NHP2 ribo	protein-co
lib-C666-l	chr17	7589347	7589347	+	8.0154	NA	promoter-TSS (NM_001143990)	promoter-TSS (NM_001143990)	-42	NM_00114	55135	Hs.408312	NM_01808	ENSG0000	WRAP53	DKCB3|TC	WD repeat	protein-co
lib-C666-l	chr1	183440494	183440494	+	7.15277	NA	non-coding (NR_040063, exon 1 of 5	non-coding (NR_040063, exon 1 of	623	NR_04006	284649	Hs.127384	NR_04006	ENSG0000	SMG7-AS1	DKFZP564	SMG7 anti	ncRNA
lib-C666-l	chr1	183441108	183441108	+	2.25647	NA	promoter-TSS (NR_040063)	promoter-TSS (NR_040063)	9	NR_04006	284649	Hs.127384	NR_04006	ENSG0000	SMG7-AS1	DKFZP564	SMG7 anti	ncRNA
lib-C666-l	chr2	109605946	109605946	+	21.28624	NA	promoter-TSS (NM_022336)	promoter-TSS (NM_022336)	-118	NM_02233	10913	Hs.171971	NM_02233	ENSG0000	EDAR	DL|ECTD1	ectodyspla	protein-co
lib-C666-l	chr3	32612635	32612635	+	9.11849	NA	promoter-TSS (NM_016141)	promoter-TSS (NM_016141)	-219	NM_00132	51143	Hs.529495	NM_01614	ENSG0000	DYNC1LI1	DLC-A|DN	dynein cyt	protein-co
lib-C666-l	chr3	32612224	32612224	+	3.20885	NA	exon (NM_016141, exon 1 of 13)	exon (NM_016141, exon 1 of 13)	142	NM_01614	51143	Hs.529495	NM_01614	ENSG0000	DYNC1LI1	DLC-A|DN	dynein cyt	protein-co
lib-C666-l	chr16	75097130	75097130	+	8.03539	NA	intron (NM_032268, intron 1 of 4)	intron (NM_032268, intron 1 of 4)	53540	NM_19443	197257	Hs.380929	NM_15348	ENSG0000	LDHD	DLD	lactate del	protein-co
lib-C666-l	chr7	107531554	107531554	+	78.00706	NA	promoter-TSS (NM_001289752)	promoter-TSS (NM_001289752)	2	NM_00128	1738	Hs.131711	NM_00010	ENSG0000	DLD	DLDD|DLD	dihydrolip	protein-co
lib-C666-l	chr17	41984959	41984959	+	14.3056	NA	5' UTR (NM_001278372, exon 1 of 14	5' UTR (NM_001278372, exon 1 of 1	351	NM_00537	4355	Hs.514208	NM_00537	ENSG0000	MPP2	DLG2	membrane	protein-co
lib-C666-l	chr17	41910206	41910206	+	6.82479	NA	intron (NM_001330233, intron 1 of 19	CpG-10661	341	NR_03562	4356	Hs.396566	NM_00193	ENSG0000	MPP3	DLG3	membrane	protein-co
lib-C666-l	chr17	41917344	41917344	+	3.59271	NA	Intergenic	Intergenic	-6782	NM_00133	4356	Hs.396566	NM_00193	ENSG0000	MPP3	DLG3	membrane	protein-co
lib-C666-l	chr14	55658471	55658471	+	6.68451	NA	promoter-TSS (NM_014750)	promoter-TSS (NM_014750)	-75	NM_00114	9787	Hs.77695	NM_01475	ENSG0000	DLGAP5	DLG7|HUR	DLG associ	protein-co
lib-C666-l	chr19	3971209	3971209	+	45.88523	NA	promoter-TSS (NM_001348)	promoter-TSS (NM_001348)	-88	NM_00134	1613	Hs.631844	NM_00134	ENSG0000	DAPK3	DLK|ZIP|ZI	death asso	protein-co
lib-C666-l	chr11	111896059	111896059	+	3.89034	NA	5' UTR (NM_001931, exon 1 of 14)	5' UTR (NM_001931, exon 1 of 14)	521	NM_00193	1737	Hs.335551	NM_00193	ENSG0000	DLAT	DLTA|PDC-	dihydrolip	protein-co
lib-C666-l	chr14	75348750	75348750	+	3.98718	NA	intron (NR_045209, intron 1 of 13)	CpG	156	NM_00193	1743	Hs.525459	NM_00193	ENSG0000	DLST	DLTS	dihydrolip	protein-co
lib-C666-l	chr11	68039497	68039497	+	29.41683	NA	promoter-TSS (NM_022338)	promoter-TSS (NM_022338)	-28	NM_02233	53838	Hs.303025	NM_02233	ENSG0000	C11orf24	DM4E3	chromosor	protein-co
lib-C666-l	chr19	42976231	42976231	+	6.09173	NA	intron (NR_073179, intron 1 of 1)	intron (NR_073179, intron 1 of 1)	-29031	NM_19847	284340	Hs.445586	NM_19847	ENSG0000	CXCL17	DMC|Dcip1	C-X-C mo	protein-co
lib-C666-l	chr18	46986998	46986998	+	5.15932	NA	promoter-TSS (NM_017653)	promoter-TSS (NM_017653)	81	NM_01765	54808	Hs.162996	NM_01765	ENSG0000	DYM	DMC|SMC	dymeclin	protein-co
lib-C666-l	chr22	38965701	38965701	+	17.30443	NA	promoter-TSS (NR_134627)	promoter-TSS (NR_134627)	500	NM_00706	11144	Hs.339396	NM_00706	ENSG0000	DMC1	DMC1H|LII	DNA meio	protein-co
lib-C666-l	chr7	86782131	86782131	+	18.1956	NA	promoter-TSS (NR_136252)	promoter-TSS (NR_136252)	261	NM_02114	9988	Hs.196129	NM_02114	ENSG0000	DMTF1	DMP1|DM	cyclin D bi	protein-co
lib-C666-l	chr7	192167	192167	+	7.08136	NA	promoter-TSS (NM_020223)	promoter-TSS (NM_020223)	-802	NM_02022	56975	Hs.134742	NM_02022	ENSG0000	FAM20C	DMP-4|DN	FAM20C, g	protein-co
lib-C666-l	chr9	36258496	36258496	+	23.27622	NA	promoter-TSS (NM_001190384)	promoter-TSS (NM_001190384)	0	NM_00119	10020	Hs.5920	NM_00547	ENSG0000	GNE	DMRV|GLC	glucosami	protein-co
lib-C666-l	chr17	76554615	76554615	+	67.6305	NA	intron (NM_173628, intron 13 of 80)	intron (NM_173628, intron 13 of 80	18861	NM_17362	8632	Hs.375975	NM_00372	ENSG0000	DNAH17	DNAHL1|D	dynein axo	protein-co
lib-C666-l	chr17	76529289	76529289	+	25.39487	NA	intron (NM_173628, intron 19 of 80)	intron (NM_173628, intron 19 of 80	44187	NM_17362	8632	Hs.375975	NM_00372	ENSG0000	DNAH17	DNAHL1|D	dynein axo	protein-co
lib-C666-l	chr12	56223490	56223490	+	14.45701	NA	promoter-TSS (NR_036479)	promoter-TSS (NR_036479)	-70	NM_03236	85406	Hs.709320	NM_03236	ENSG0000	DNAJC14	DNAJ|DRIP	DnaJ heat	protein-co
lib-C666-l	chr12	56223829	56223829	+	4.52748	NA	promoter-TSS (NM_032364)	promoter-TSS (NM_032364)	-409	NM_03236	85406	Hs.709320	NM_03236	ENSG0000	DNAJC14	DNAJ|DRIP	DnaJ heat	protein-co
lib-C666-l	chr22	29137927	29137927	+	5.819	NA	promoter-TSS (NM_001318315)	promoter-TSS (NM_001318315)	-93	NM_00131	150274	Hs.632780	NM_17200	ENSG0000	HSCB	DNAJC20|I	HscB mitoc	protein-co
lib-C666-l	chr13	43632697	43632697	+	9.10494	NA	intron (NM_013238, intron 1 of 5)	L1PB1|LINE|L1	35335	NM_01323	29103	Hs.438830	NM_01323	ENSG0000	DNAJC15	DNAJD1|H	DnaJ heat	protein-co
lib-C666-l	chr10	22292705	22292705	+	28.88196	NA	promoter-TSS (NM_022365)	promoter-TSS (NM_022365)	-55	NM_02236	64215	Hs.499000	NM_02236	ENSG0000	DNAJC1	DNAJL1|EF	DnaJ heat	protein-co
lib-C666-l	chr8	48872711	48872711	+	7.77438	NA	promoter-TSS (NM_006904)	promoter-TSS (NM_006904)	32	NM_00690	5591	Hs.491682	NM_00690	ENSG0000	PRKDC	DNA-PKcs	protein kin	protein-co
lib-C666-l	chr19	12992119	12992119	+	69.90313	NA	exon (NM_001375, exon 1 of 6)	exon (NM_001375, exon 1 of 6)	216	NM_00137	1777	Hs.118243	NM_00137	ENSG0000	DNASE2	DNASE2A|	deoxyribor	protein-co
lib-C666-l	chr1	8378144	8378144	+	31.10827	NA	promoter-TSS (NM_001080397)	promoter-TSS (NM_001080397)	-1	NM_00108	50651	Hs.463036	NM_00108	ENSG0000	SLC45A1	DNB5	solute carr	protein-co
lib-C666-l	chr17	73285618	73285618	+	2.59032	NA	promoter-TSS (NM_021734)	promoter-TSS (NM_021734)	-88	NM_02173	60386	Hs.514470	NM_02173	ENSG0000	SLC25A19	DNC|MCPI	solute carr	protein-co
lib-C666-l	chr17	56160696	56160696	+	26.54911	NA	promoter-TSS (NR_110809)	promoter-TSS (NR_110809)	-84	NM_08067	140735	Hs.720595	NM_08067	ENSG0000	DYNLL2	DNCL1B|D	dynein ligh	protein-co
lib-C666-l	chr16	66785405	66785405	+	2.2973	NA	promoter-TSS (NR_133569)	promoter-TSS (NR_133569)	10	NM_00128	1783	Hs.369068	NM_00614	ENSG0000	DYNC1LI2	DNCLI2|LI(	dynein cyt	protein-co
lib-C666-l	chr2	84743577	84743577	+	3.38731	NA	promoter-TSS (NM_001370)	promoter-TSS (NM_001370)	1768	NM_00137	1768	Hs.249972	NM_00137	ENSG0000	DNAH6	DNHL1|Dn	dynein axo	protein-co
lib-C666-l	chr2	25585809	25585809	+	9.94079	NA	Intergenic	Intergenic	-20350	NM_17562	1788	Hs.515840	NM_02255	ENSG0000	DNMT3A	DNMT3A2	DNA meth	protein-co
lib-C666-l	chr17	73127612	73127612	+	2.00164	NA	intron (NM_001252377, intron 1 of 3)	CpG	278	NR_04551	30833	Hs.67201	NM_01459	ENSG0000	NT5C	DNT|DNT1	5', 3'-nucle	protein-co
lib-C666-l	chr4	1581326	1581326	+	8.28216	NA	Intergenic	CpG	104392	NM_00101	152877	Hs.143314	NM_00101	ENSG0000	FAM53A	DNTNP	family with	protein-co
lib-C666-l	chr5	10354074	10354074	+	17.37618	NA	intron (NM_001270661, intron 1 of 2	CpG	323	NM_00127	10299	Hs.432862	NM_00588	ENSG0000	6-Mar	DOA10|MA	membrane	protein-co
lib-C666-l	chr5	10353739	10353739	+	9.94079	NA	promoter-TSS (NM_001270660)	promoter-TSS (NM_001270660)	-12	NM_00127	10299	Hs.432862	NM_00588	ENSG0000	6-Mar	DOA10|MA	membrane	protein-co
lib-C666-l	chr6	32784844	32784844	+	14.59636	NA	promoter-TSS (NM_002120).2	promoter-TSS (NM_002120).2	-19	NM_00212	3112	Hs.1802	NM_00212	ENSG0000	HLA-DOB	DOB	major histo	protein-co
lib-C666-l	chr6	32795068	32795068	+	8.03539	NA	3' UTR (NM_001290043, exon 12 of 1	3' UTR (NM_001290043, exon 12 of	-10243	NM_00212	3112	Hs.1802	NM_00212	ENSG0000	HLA-DOB	DOB	major histo	protein-co
lib-C666-l	chr5	54603699	54603699	+	21.84731	NA	promoter-TSS (NM_001345965)	promoter-TSS (NM_001345965)	123	NM_01536	23517	Hs.274531	NM_01536	ENSG0000	SKIV2L2	Dob1|KIAA	Ski2 like R	protein-co
lib-C666-l	chr12	123755904	123755904	+	2.0646	NA	promoter-TSS (NR_073008)	promoter-TSS (NR_073008)	-255	NR_03004	8099	Hs.433201	NM_00464	ENSG0000	CDK2AP1	DOC1|DO	cyclin dep	protein-co
lib-C666-l	chr11	67276086	67276086	+	72.82537	NA	promoter-TSS (NR_073484)	promoter-TSS (NR_073484)	113	NM_00585	10263	Hs.523835	NM_00585	ENSG0000	CDK2AP2	DOC-1R|p	cyclin dep	protein-co
lib-C666-l	chr11	67275545	67275545	+	4.52748	NA	non-coding (NR_073484, exon 1 of 3	non-coding (NR_073484, exon 1 of	113	NM_00127	10263	Hs.523835	NM_00585	ENSG0000	CDK2AP2	DOC-1R|p	cyclin dep	protein-co
lib-C666-l	chr17	13648	13648	+	13.72417	NA	intron (NM_003585, intron 3 of 5)	intron (NM_003585, intron 3 of 5)	17773	NM_00358	8447	Hs.551705	NM_00358	ENSG0000	DOC2B	DOC2BL	double C2	protein-co
lib-C666-l	chr11	79651484	79651484	+	11.06679	NA	Intergenic	L2b|LINE|L2	-499789	NM_00109	26011	Hs.213087	NM_00109	ENSG0000	TENM4	Doc4|ETM	teneurin tr	protein-co
lib-C666-l	chr11	79468529	79468529	+	6.74226	NA	Intergenic	L2c|LINE|L2	-316834	NM_00109	26011	Hs.213087	NM_00109	ENSG0000	TENM4	Doc4|ETM	teneurin tr	protein-co
lib-C666-l	chr10	128594006	128594006	+	7.4883	NA	promoter-TSS (NM_001290223)	promoter-TSS (NM_001290223)	28	NM_00129	1793	Hs.159195	NM_00138	ENSG0000	DOCK1	DOCK180|	dedicator c	protein-co
lib-C666-l	chr19	9945855	9945855	+	21.28624	NA	promoter-TSS (NR_038830)	promoter-TSS (NR_038830)	-28	NM_00622	5300	Hs.465849	NM_00622	ENSG0000	PIN1	DOD|UBL5	peptidylpr	protein-co
lib-C666-l	chr6	31939953	31939953	+	234.30052	NA	promoter-TSS (NM_005510)	promoter-TSS (NM_005510)	79	NM_00551	1797	Hs.153299	NM_00551	ENSG0000	DXO	DOM3L|DO	decapping	protein-co
lib-C666-l	chr5	139335536	139335536	+	7.11942	NA	intron (NM_013983, intron 1 of 10)	MIRc|SINE|MIR	87348	NM_01398	9542	Hs.408515	NM_00488	ENSG0000	NRG2	DON1|HRG	neuregulin	protein-co
lib-C666-l	chr19	2163830	2163830	+	11.13288	NA	promoter-TSS (NM_032482)	promoter-TSS (NM_032482)	-318	NM_03248	84444	Hs.713641	NM_03248	ENSG0000	DOT1L	DOT1|KMT	DOT1 like	protein-co
lib-C666-l	chr17	47022213	47022213	+	108.97115	NA	promoter-TSS (NM_001317192)	promoter-TSS (NM_001317192)	-9	NR_13367	11267	Hs.127249	NM_00724	ENSG0000	SNF8	Dot3|EAP3	SNF8, ESCI	protein-co
lib-C666-l	chr6	33041527	33041527	+	44.36932	NA	promoter-TSS (NM_033554).5	promoter-TSS (NM_033554).5	-73	NM_03355	3113	Hs.347270	NM_03355	ENSG0000	HLA-DPA1	DP(W3)|DF	major histo	protein-co
lib-C666-l	chr1	112298235	112298235	+	13.59254	NA	promoter-TSS (NR_125963)	promoter-TSS (NR_125963)	45	NM_00720	11218	Hs.591405	NM_00720	ENSG0000	DDX20	DP103|GEM	DEAD-box	protein-co
lib-C666-l	chr13	114868434	114868434	+	4.52748	NA	promoter-TSS (NM_001178138)	promoter-TSS (NM_001178138)	-48	NM_00117	7029	Hs.379018	NM_00628	ENSG0000	TFDP2	DP2	transcripti	protein-co
lib-C666-l	chr6	33080144	33080144	+	10.37767	NA	promoter-TSS (NR_001435)	promoter-TSS (NR_001435)	-149	NR_00143	3116	Hs.665450	NR_00143	ENSG0000	HLA-DPB2	DP2B|DPB2	major histo	pseudo
lib-C666-l	chr12	117257290	117257290	+	183.31183	NA	intron (NM_032814, intron 7 of 10)	CpG	61942	NR_07318	8739	Hs.87247	NM_00380	ENSG0000	HRK	DP5|HARA	harakiri, BC	protein-co
lib-C666-l	chr19	4723923	4723923	+	9.75997	NA	promoter-TSS (NM_139159)	promoter-TSS (NM_139159)	-68	NM_13915	91039	Hs.515081	NM_13915	ENSG0000	DPP9	DP9|DPLP9	dipeptidyl	protein-co
lib-C666-l	chr19	4720251	4720251	+	3.37191	NA	intron (NM_139159, intron 2 of 21)	intron (NM_139159, intron 2 of 21)	3604	NM_13915	91039	Hs.515081	NM_13915	ENSG0000	DPP9	DP9|DPLP9	dipeptidyl	protein-co
lib-C666-l	chr6	33043658	33043658	+	21.96185	NA	promoter-TSS (NM_002121).4	promoter-TSS (NM_002121).4	-45	NM_00212	3115	Hs.485130	NM_00212	ENSG0000	HLA-DPB1	DPB1|HLA-	major histo	protein-co
lib-C666-l	chr17	40713925	40713925	+	37.48154	NA	promoter-TSS (NM_001042529)	promoter-TSS (NM_001042529)	-167	NM_00104	80347	Hs.296422	NM_02523	ENSG0000	COASY	DPCK|NBIA	Coenzyme	protein-co
lib-C666-l	chr19	18342009	18342009	+	4.51266	NA	intron (NM_001330172, intron 2 of 15	intron (NM_001330172, intron 2 of	3180	NM_00133	5143	Hs.132584	NM_00092	ENSG0000	PDE4C	DPDE1	phosphodi	protein-co
lib-C666-l	chr19	10527462	10527462	+	174.98105	NA	promoter-TSS (NM_001243121)	promoter-TSS (NM_001243121)	13	NM_00124	5141	Hs.89901	NM_00620	ENSG0000	PDE4A	DPDE2|PDI	phosphodi	protein-co
lib-C666-l	chr19	10585847	10585847	+	10.58189	NA	Intergenic	Intergenic	22214	NM_00620	5141	Hs.89901	NM_00620	ENSG0000	PDE4A	DPDE2|PDI	phosphodi	protein-co
lib-C666-l	chr1	66748139	66748139	+	7.15725	NA	intron (NM_001037340, intron 5 of 14	intron (NM_001037340, intron 5 of	-49652	NM_00103	5142	Hs.198072	NM_00260	ENSG0000	PDE4B	DPDE4|PDI	phosphodi	protein-co
lib-C666-l	chr1	66815969	66815969	+	6.15313	NA	intron (NM_001037339, intron 1 of 9)	intron (NM_001037339, intron 1 of	-4092	NM_00129	5142	Hs.198072	NM_00260	ENSG0000	PDE4B	DPDE4|PDI	phosphodi	protein-co
lib-C666-l	chr1	233749648	233749648	+	11.93581	NA	promoter-TSS (NM_002245)	promoter-TSS (NM_002245)	-102	NM_00224	3775	Hs.208544	NM_00224	ENSG0000	KCNK1	DPK|HOHO	potassium	protein-co
lib-C666-l	chr12	118684138	118684138	+	12.55817	NA	intron (NM_001346493, intron 3 of 22	intron (NM_001346493, intron 3 of	-55771	NM_00134	51347	Hs.644420	NM_01628	ENSG0000	TAOK3	DPK|JIK|MA	TAO kinas	protein-co
lib-C666-l	chr9	140009330	140009330	+	7.15513	NA	promoter-TSS (NM_013379)	promoter-TSS (NM_013379)	-135	NM_01337	29952	Hs.37916	NM_01337	ENSG0000	DPP7	DPP2|DPPI	dipeptidyl	protein-co
lib-C666-l	chr8	38126769	38126769	+	33.8063	NA	promoter-TSS (NM_001102559)	promoter-TSS (NM_001102559)	-31	NM_00110	56994	Hs.567619	NM_03248	ENSG0000	PLPP5	DPPL1|HTF	phosphatic	protein-co
lib-C666-l	chr20	3451612	3451612	+	7.09685	NA	promoter-TSS (NM_001207047)	promoter-TSS (NM_001207047)	-53	NM_00120	8455	Hs.276252	NM_01207	ENSG0000	ATRN	DPPT-L|M(	attractin	protein-co
lib-C666-l	chr3	114866887	114866887	+	9.20633	NA	promoter-TSS (NM_015642)	promoter-TSS (NM_015642)	-760	NM_01564	26137	Hs.202577	NM_01564	ENSG0000	ZBTB20	DPZF|HOF	zinc finger	protein-co
lib-C666-l	chr9	132565461	132565461	+	34.92958	NA	promoter-TSS (NM_001317894)	promoter-TSS (NM_001317894)	39	NR_13402	27348	Hs.252682	NM_01450	ENSG0000	TOR1B	DQ1	torsin fami	protein-co
lib-C666-l	chr9	132586503	132586503	+	3.57421	NA	promoter-TSS (NM_000113)	promoter-TSS (NM_000113)	-62	NM_00011	1861	Hs.534312	NM_00011	ENSG0000	TOR1A	DQ2|DYT1	torsin fami	protein-co
lib-C666-l	chr14	70078051	70078051	+	8.03539	NA	promoter-TSS (NM_014734)	promoter-TSS (NM_014734)	-259	NM_01473	9766	Hs.440025	NM_01473	ENSG0000	SUSD6	DRAGO|KI	sushi dom	protein-co
lib-C666-l	chr12	102255618	102255618	+	47.62605	NA	Intergenic	MER1A|DNA|hAT-Charlie	-15487	NM_01837	55332	Hs.525634	NM_01837	ENSG0000	DRAM1	DRAM	DNA dama	protein-co
lib-C666-l	chr12	102271287	102271287	+	3.23837	NA	5' UTR (NM_018370, exon 1 of 7)	5' UTR (NM_018370, exon 1 of 7)	182	NM_01837	55332	Hs.525634	NM_01837	ENSG0000	DRAM1	DRAM	DNA dama	protein-co
lib-C666-l	chr6	32557675	32557675	+	56.44287	NA	promoter-TSS (NM_002124)	promoter-TSS (NM_002124)	-62	NM_00212	3123	Hs.534322	NM_00212	ENSG0000	HLA-DRB1	DRB1|HLA-	major histo	protein-co
lib-C666-l	chr6	32577772	32577772	+	53.97609	NA	Intergenic	Intergenic	-20159	NM_00212	3123	Hs.534322	NM_00212	ENSG0000	HLA-DRB1	DRB1|HLA-	major histo	protein-co
lib-C666-l	chr6	32572430	32572430	+	30.542	NA	Intergenic	Intergenic	-14817	NM_00212	3123	Hs.534322	NM_00212	ENSG0000	HLA-DRB1	DRB1|HLA-	major histo	protein-co
lib-C666-l	chr19	55598589	55598589	+	2.96041	NA	intron (NM_133180, intron 18 of 19)	CpG-14396	6829	NM_01772	54869	Hs.438862	NM_01772	ENSG0000	EPS8L1	DRC3|EPS8	EPS8 like 1	protein-co
lib-C666-l	chr7	23053134	23053134	+	8.01457	NA	intron (NM_032581, intron 1 of 10)	CpG	636	NM_03258	84668	Hs.85603	NM_03258	ENSG0000	FAM126A	DRCTNNB1	family with	protein-co
lib-C666-l	chr10	135143538	135143538	+	5.96422	NA	intron (NM_015722, intron 1 of 5)	CpG	6937	NM_01572	50632	Hs.148680	NM_01572	ENSG0000	CALY	DRD1IP|N!	calcyon ne	protein-co
lib-C666-l	chr3	183353289	183353289	+	14.37873	NA	promoter-TSS (NM_017644)	promoter-TSS (NM_017644)	-122	NM_01764	54800	Hs.407709	NM_01764	ENSG0000	KLHL24	DRE1|KRIP	kelch like f	protein-co
lib-C666-l	chr1	150980893	150980893	+	15.31489	NA	promoter-TSS (NM_021222)	promoter-TSS (NM_021222)	26	NR_13013	58497	Hs.78524	NM_02122	ENSG0000	PRUNE1	DRES-17|D	prune exo	protein-co
lib-C666-l	chr11	67272031	67272031	+	23.0291	NA	intron (NM_001130848, intron 1 of 23	CpG	812	NM_00491	9600	Hs.372295	NM_00491	ENSG0000	PITPNM1	DRES9|NIR	phosphatic	protein-co
lib-C666-l	chr11	67272741	67272741	+	8.71138	NA	5' UTR (NM_004910, exon 1 of 24)	5' UTR (NM_004910, exon 1 of 24)	102	NM_00491	9600	Hs.372295	NM_00491	ENSG0000	PITPNM1	DRES9|NIR	phosphatic	protein-co
lib-C666-l	chr2	71127564	71127564	+	16.59065	NA	promoter-TSS (NM_012476)	promoter-TSS (NM_012476)	-156	NM_01247	25806	Hs.249170	NM_01247	ENSG0000	VAX2	DRES93	ventral ant	protein-co
lib-C666-l	chr10	50603628	50603628	+	11.07709	NA	intron (NM_001276451, intron 1 of 6)	CpG-2821	434	NM_00127	644168	Hs.534530	NM_00108	ENSG0000	DRGX	DRG11|PR	dorsal root	protein-co
lib-C666-l	chr1	56880592	56880592	+	16.63918	NA	Intergenic	Intergenic	164665	NM_00371	8613	Hs.405156	NM_00371	ENSG0000	PLPP3	Dri42|LPP3	phospholip	protein-co
lib-C666-l	chr1	56798033	56798033	+	6.1054	NA	Intergenic	Intergenic	247224	NM_00371	8613	Hs.405156	NM_00371	ENSG0000	PLPP3	Dri42|LPP3	phospholip	protein-co
lib-C666-l	chr2	225713472	225713472	+	8.07187	NA	intron (NM_014689, intron 19 of 55)	intron (NM_014689, intron 19 of 55	98310	NM_01468	55619	Hs.465978	NM_01468	ENSG0000	DOCK10	DRIP2|Nbl	dedicator c	protein-co
lib-C666-l	chr15	38273863	38273863	+	21.21068	NA	promoter-TSS (NM_024640)	promoter-TSS (NM_024640)	2	NM_02464	79693	Hs.301564	NM_02464	ENSG0000	YRDC	DRIP3|IRIP	yrdC N6-th	protein-co
lib-C666-l	chr4	89618987	89618987	+	45.23877	NA	5' UTR (NM_153757, exon 1 of 1)	5' UTR (NM_153757, exon 1 of 1)	399	NM_15375	266812	Hs.12554	NM_15375	ENSG0000	NAP1L5	DRLM	nucleosom	protein-co
lib-C666-l	chr12	123237209	123237209	+	8.51971	NA	promoter-TSS (NM_003677)	promoter-TSS (NM_003677)	-159	NM_00367	8562	Hs.22393	NM_00367	ENSG0000	DENR	DRP|DRP1	density reg	protein-co
lib-C666-l	chr14	39644188	39644188	+	22.63618	NA	promoter-TSS (NM_002687)	promoter-TSS (NM_002687)	-199	NM_00268	5411	Hs.409965	NM_00268	ENSG0000	PNN	DRS|DRSP	pinin, desn	protein-co
lib-C666-l	chr1	1284775	1284775	+	38.91483	NA	promoter-TSS (NM_004421)	promoter-TSS (NM_004421)	-283	NM_00133	1855	Hs.731450	NM_00442	ENSG0000	DVL1	DRS2|DVL	dishevellec	protein-co
lib-C666-l	chr1	1285171	1285171	+	10.52288	NA	promoter-TSS (NM_004421)	promoter-TSS (NM_004421)	-679	NM_00133	1855	Hs.731450	NM_00442	ENSG0000	DVL1	DRS2|DVL	dishevellec	protein-co
lib-C666-l	chr17	73008758	73008758	+	6.15313	NA	promoter-TSS (NM_001303265)	promoter-TSS (NM_001303265)	-1	NM_00154	3396	Hs.407955	NM_00154	ENSG0000	MRPL58	DS-1|DS1|	mitochond	protein-co
lib-C666-l	chr17	73009050	73009050	+	5.96422	NA	intron (NM_001545, intron 1 of 5)	CpG	291	NM_00154	3396	Hs.407955	NM_00154	ENSG0000	MRPL58	DS-1|DS1|	mitochond	protein-co
lib-C666-l	chr18	47216867	47216867	+	5.65398	NA	Intergenic	L1MC4a|LINE|L1	123384	NM_00611	10449	Hs.200136	NM_00611	ENSG0000	ACAA2	DSAEC	acetyl-CoA	protein-co
lib-C666-l	chr18	47252706	47252706	+	5.04826	NA	Intergenic	Intergenic	87545	NM_00611	10449	Hs.200136	NM_00611	ENSG0000	ACAA2	DSAEC	acetyl-CoA	protein-co
lib-C666-l	chr12	108955117	108955117	+	17.37618	NA	promoter-TSS (NM_001301141)	promoter-TSS (NM_001301141)	48	NM_01470	9733	Hs.584842	NM_01470	ENSG0000	SART3	DSAP1|P1(	squamous	protein-co
lib-C666-l	chr6	116575299	116575299	+	30.85659	NA	promoter-TSS (NR_136520)	promoter-TSS (NR_136520)	-16	NM_00132	29940	Hs.458358	NM_01335	ENSG0000	DSE	DS-epi1|D	dermatan s	protein-co
lib-C666-l	chr6	116601166	116601166	+	5.819	NA	promoter-TSS (NR_136521)	promoter-TSS (NR_136521)	-60	NR_13652	29940	Hs.458358	NM_01335	ENSG0000	DSE	DS-epi1|D	dermatan s	protein-co
lib-C666-l	chr6	116692137	116692137	+	4.85425	NA	TTS (NR_136521)	TTS (NR_136521)	104	NM_00132	29940	Hs.458358	NM_01335	ENSG0000	DSE	DS-epi1|D	dermatan s	protein-co
lib-C666-l	chr17	1196881	1196881	+	107.40278	NA	intron (NM_172367, intron 1 of 2)	HAL1-2a_MD|LINE|L1	13924	NM_17236	286753	Hs.146059	NM_17236	ENSG0000	TUSC5	DSPB1|IFIT	tumor sup	protein-co
lib-C666-l	chr7	96339034	96339034	+	9.47655	NA	exon (NM_006304, exon 1 of 3)	exon (NM_006304, exon 1 of 3)	169	NM_00630	7979	Hs.489201	NM_00630	ENSG0000	SEM1	DSS1|ECD|	SEM1, 26S	protein-co
lib-C666-l	chr12	76425439	76425439	+	3.59106	NA	exon (NM_007350, exon 1 of 2)	exon (NM_007350, exon 1 of 2)	117	NM_00735	22822	Hs.602085	NM_00735	ENSG0000	PHLDA1	DT1P1B11|	pleckstrin	protein-co
lib-C666-l	chr11	57479858	57479858	+	37.12814	NA	promoter-TSS (NM_001317078)	promoter-TSS (NM_001317078)	-63	NM_15345	219541	Hs.43619	NM_15345	ENSG0000	MED19	DT2P1G7|L	mediator c	protein-co
lib-C666-l	chr6	35454152	35454152	+	16.36809	NA	intron (NM_003214, intron 2 of 12)	CpG	10709	NM_00321	7005	Hs.485205	NM_00321	ENSG0000	TEAD3	DTEF-1|ET	TEA domai	protein-co
lib-C666-l	chr17	76837073	76837073	+	266.49277	NA	promoter-TSS (NM_001321291)	promoter-TSS (NM_001321291)	-464	NM_00132	57602	Hs.464243	NM_02509	ENSG0000	USP36	DUB1	ubiquitin s	protein-co
lib-C666-l	chr4	9382966	9382966	+	6.61453	NA	Intergenic	CpG	13366	NR_02727	391622	Hs.722658	NR_02727	ENSG0000	USP17L6P	DUB1|USP	ubiquitin s	pseudo
lib-C666-l	chrX	48815601	48815601	+	24.18348	NA	promoter-TSS (NM_001136157)	promoter-TSS (NM_001136157)	-47	NM_00113	55593	Hs.496098	NM_01760	ENSG0000	OTUD5	DUBA	OTU deubi	protein-co
lib-C666-l	chr4	146101980	146101980	+	4.73866	NA	Intergenic	Intergenic	-1148	NM_00110	54726	Hs.270851	NM_01749	ENSG0000	OTUD4	DUBA6|HII	OTU deubi	protein-co
lib-C666-l	chr10	23727892	23727892	+	24.23241	NA	promoter-TSS (NM_001145373)	promoter-TSS (NM_001145373)	-306	NM_00114	220213	Hs.499042	NM_00114	ENSG0000	OTUD1	DUBA7|OT	OTU deubi	protein-co
lib-C666-l	chr10	23729090	23729090	+	20.90919	NA	exon (NM_001145373, exon 1 of 1)	exon (NM_001145373, exon 1 of 1)	892	NM_00114	220213	Hs.499042	NM_00114	ENSG0000	OTUD1	DUBA7|OT	OTU deubi	protein-co
lib-C666-l	chr6	136571563	136571563	+	18.20429	NA	promoter-TSS (NM_001099286)	promoter-TSS (NM_001099286)	-90	NM_13841	113115	Hs.121536	NM_13841	ENSG0000	MTFR2	DUFD1|FA	mitochond	protein-co
lib-C666-l	chr6	136547470	136547470	+	5.07147	NA	Intergenic	Intergenic	24003	NM_00109	113115	Hs.121536	NM_13841	ENSG0000	MTFR2	DUFD1|FA	mitochond	protein-co
lib-C666-l	chr16	68057141	68057141	+	102.03614	NA	5' UTR (NM_001271762, exon 1 of 16	5' UTR (NM_001271762, exon 1 of 1	294	NM_01780	54920	Hs.534460	NM_01780	ENSG0000	DUS2	DUS2L|SM	dihydrouri	protein-co
lib-C666-l	chr19	5791272	5791272	+	92.67979	NA	promoter-TSS (NM_001161619)	promoter-TSS (NM_001161619)	-23	NM_02017	56931	Hs.284297	NM_02017	ENSG0000	DUS3L	DUS3	dihydrouri	protein-co
lib-C666-l	chr19	5804308	5804308	+	5.04179	NA	Intergenic	CpG	-13059	NM_02017	56931	Hs.284297	NM_02017	ENSG0000	DUS3L	DUS3	dihydrouri	protein-co
lib-C666-l	chr7	107204401	107204401	+	116.5974	NA	promoter-TSS (NM_181581)	promoter-TSS (NM_181581)	-1	NR_07300	11062	Hs.97627	NM_18158	ENSG0000	DUS4L	DUS4|PP35	dihydrouri	protein-co
lib-C666-l	chr10	112257599	112257599	+	21.84731	NA	promoter-TSS (NM_004419)	promoter-TSS (NM_004419)	-26	NM_00441	1847	Hs.2128	NM_00441	ENSG0000	DUSP5	DUSP|HVH	dual specif	protein-co
lib-C666-l	chr10	112174481	112174481	+	11.66012	NA	Intergenic	Intergenic	-83144	NM_00441	1847	Hs.2128	NM_00441	ENSG0000	DUSP5	DUSP|HVH	dual specif	protein-co
lib-C666-l	chr7	75677324	75677324	+	4.72928	NA	promoter-TSS (NM_005918)	promoter-TSS (NM_005918)	-3	NM_00131	51657	Hs.11615	NM_01608	ENSG0000	STYXL1	DUSP24|M	serine/thre	protein-co
lib-C666-l	chr1	159750590	159750590	+	7.15513	NA	promoter-TSS (NM_001319658)	promoter-TSS (NM_001319658)	-146	NM_01782	54935	Hs.425801	NM_01782	ENSG0000	DUSP23	DUSP25|D	dual specif	protein-co
lib-C666-l	chr2	241499764	241499764	+	8.01457	NA	5' UTR (NM_001033575, exon 1 of 3)	5' UTR (NM_001033575, exon 1 of 3	293	NM_00103	285193	Hs.369297	NM_00103	ENSG0000	DUSP28	DUSP26|V	dual specif	protein-co
lib-C666-l	chr15	48695234	48695234	+	13.59977	NA	Intergenic	Intergenic	70869	NM_00194	1854	Hs.527980	NM_00194	ENSG0000	DUT	dUTPase	deoxyuridi	protein-co
lib-C666-l	chr15	48650801	48650801	+	4.74112	NA	Intergenic	Intergenic	26436	NM_00194	1854	Hs.527980	NM_00194	ENSG0000	DUT	dUTPase	deoxyuridi	protein-co
lib-C666-l	chr15	48624252	48624252	+	2.24164	NA	promoter-TSS (NM_001948)	promoter-TSS (NM_001948)	-113	NM_00194	1854	Hs.527980	NM_00194	ENSG0000	DUT	dUTPase	deoxyuridi	protein-co
lib-C666-l	chr5	135468566	135468566	+	7.0832	NA	promoter-TSS (NM_005903)	promoter-TSS (NM_005903)	32	NM_00100	4090	Hs.167700	NM_00590	ENSG0000	SMAD5	DWFC|JV5	SMAD fam	protein-co
lib-C666-l	chrX	153714934	153714934	+	9.29081	NA	promoter-TSS (NM_014235)	promoter-TSS (NM_014235)	75	NM_01423	8266	Hs.76480	NM_01423	ENSG0000	UBL4A	DX254E|D)	ubiquitin li	protein-co

lib-C666-l	chrX	53254511	53254511	+	15.01861	NA	promoter-TSS (NM_001146702)	promoter-TSS (NM_001146702)	93	NM_00114	8242	Hs.631768	NM_00418	ENSG0000	KDM5C	DXS1272E|	lysine dem	protein-co
lib-C666-l	chrX	7895510	7895510	+	18.47709	NA	promoter-TSS (NM_004650)	promoter-TSS (NM_004650)	-35	NM_00465	8228	Hs.264	NM_00465	ENSG0000	PNPLA4	DXS1283E|	patatin like	protein-co
lib-C666-l	chrX	70474151	70474151	+	2.05265	NA	promoter-TSS (NM_201599)	promoter-TSS (NM_201599)	-113	NM_20159	9203	Hs.522684	NM_00509	ENSG0000	ZMYM3	DXS6673E|	zinc finger	protein-co
lib-C666-l	chr5	150138572	150138572	+	23.27622	NA	promoter-TSS (NM_001135644)	promoter-TSS (NM_001135644)	85	NM_00113	51164	Hs.675564	NM_01622	ENSG0000	DCTN4	DYN4|P62	dynactin su	protein-co
lib-C666-l	chr5	150138283	150138283	+	2.65152	NA	promoter-TSS (NM_001135644)	promoter-TSS (NM_001135644)	-201	NM_00113	51164	Hs.675564	NM_01622	ENSG0000	DCTN4	DYN4|P62	dynactin su	protein-co
lib-C666-l	chr21	38936857	38936857	+	14.18438	NA	Intergenic	Intergenic	144256	NM_13043	1859	Hs.368240	NM_00139	ENSG0000	DYRK1A	DYRK|DYRI	dual specif	protein-co
lib-C666-l	chr21	38944083	38944083	+	3.23837	NA	Intergenic	L1M4|LINE|L1	151482	NM_13043	1859	Hs.368240	NM_00139	ENSG0000	DYRK1A	DYRK|DYRI	dual specif	protein-co
lib-C666-l	chr1	206808840	206808840	+	13.33696	NA	promoter-TSS (NM_003582)	promoter-TSS (NM_003582)	-41	NM_00100	8444	Hs.164267	NM_00358	ENSG0000	DYRK3	DYRK5|RED	dual specif	protein-co
lib-C666-l	chr11	33398218	33398218	+	15.01861	NA	Intergenic	CpG	118341	NM_00127	10114	Hs.201918	NM_00573	ENSG0000	HIPK3	DYRK6|FIS	homeodor	protein-co
lib-C666-l	chr11	33397685	33397685	+	8.28266	NA	Intergenic	CpG	117808	NM_00127	10114	Hs.201918	NM_00573	ENSG0000	HIPK3	DYRK6|FIS	homeodor	protein-co
lib-C666-l	chr9	111696413	111696413	+	59.7967	NA	promoter-TSS (NM_017832)	promoter-TSS (NM_017832)	199	NM_00131	8518	Hs.494738	NM_00364	ENSG0000	IKBKAP	DYS|ELP1|F	inhibitor of	protein-co
lib-C666-l	chr17	79798607	79798607	+	8.96177	NA	Intergenic	Intergenic	-5681	NM_00100	116729	Hs.454720	NM_00100	ENSG0000	PPP1R27	DYSFIP1	protein ph	protein-co
lib-C666-l	chr14	55369682	55369682	+	126.63946	NA	promoter-TSS (NM_000161)	promoter-TSS (NM_000161)	-140	NM_00102	2643	Hs.86724	NM_00016	ENSG0000	GCH1	DYT14|DYT	GTP cycloh	protein-co
lib-C666-l	chr14	55368808	55368808	+	5.30207	NA	intron (NM_001024024, intron 1 of 6)	CpG	734	NM_00102	2643	Hs.86724	NM_00016	ENSG0000	GCH1	DYT14|DYT	GTP cycloh	protein-co
lib-C666-l	chr2	179315448	179315448	+	7.21092	NA	promoter-TSS (NM_001139517)	promoter-TSS (NM_001139517)	36	NM_00113	8575	Hs.570274	NM_00369	ENSG0000	PRKRA	DYT16|HSD	protein act	protein-co
lib-C666-l	chr18	11857628	11857628	+	23.74671	NA	5' UTR (NM_001261444, exon 1 of 8)	5' UTR (NM_001261444, exon 1 of	191	NM_00126	2774	Hs.136295	NM_00207	ENSG0000	GNAL	DYT25	G protein s	protein-co
lib-C666-l	chr8	42698606	42698606	+	59.74744	NA	promoter-TSS (NM_018105)	promoter-TSS (NM_018105)	-132	NM_01810	55145	Hs.7432	NM_01810	ENSG0000	THAP1	DYT6	THAP dom	protein-co
lib-C666-l	chr8	42697940	42697940	+	3.31563	NA	intron (NM_018105, intron 1 of 2)	CpG	534	NM_19900	55145	Hs.7432	NM_01810	ENSG0000	THAP1	DYT6	THAP dom	protein-co
lib-C666-l	chr19	41770129	41770129	+	16.27126	NA	promoter-TSS (NM_001321208)	promoter-TSS (NM_001321208)	50	NM_00704	11100	Hs.155218	NM_00704	ENSG0000	HNRNPUL	E1B-AP5|E	heterogen	protein-co
lib-C666-l	chr5	138940763	138940763	+	4.52748	NA	promoter-TSS (NM_181838)	promoter-TSS (NM_181838)	12	NM_00333	7322	Hs.108332	NM_00333	ENSG0000	UBE2D2	E2(17)KB2|	ubiquitin c	protein-co
lib-C666-l	chr4	103790025	103790025	+	25.33067	NA	promoter-TSS (NM_181890)	promoter-TSS (NM_181890)	25	NM_18189	7323	Hs.518773	NM_00334	ENSG0000	UBE2D3	E2(17)KB3|	ubiquitin c	protein-co
lib-C666-l	chr4	103748714	103748714	+	8.56376	NA	promoter-TSS (NM_181887)	promoter-TSS (NM_181887)	-4	NM_18188	7323	Hs.518773	NM_00334	ENSG0000	UBE2D3	E2(17)KB3|	ubiquitin c	protein-co
lib-C666-l	chr4	103748146	103748146	+	8.07963	NA	5' UTR (NM_181892, exon 1 of 8)	5' UTR (NM_181892, exon 1 of 8)	194	NM_18189	7323	Hs.518773	NM_00334	ENSG0000	UBE2D3	E2(17)KB3|	ubiquitin c	protein-co
lib-C666-l	chr4	103749407	103749407	+	3.20885	NA	promoter-TSS (NM_181887)	promoter-TSS (NM_181887)	-70	NM_18188	7323	Hs.518773	NM_00334	ENSG0000	UBE2D3	E2(17)KB3|	ubiquitin c	protein-co
lib-C666-l	chr17	4233356	4233356	+	30.13508	NA	intron (NM_003342, intron 1 of 5)	intron (NM_003342, intron 1 of 5)	36613	NM_00334	7326	Hs.741319	NM_00334	ENSG0000	UBE2G1	E217K|UBC	ubiquitin c	protein-co
lib-C666-l	chr7	129592769	129592769	+	133.19038	NA	promoter-TSS (NM_003344)	promoter-TSS (NM_003344)	14	NM_00120	7328	Hs.643548	NM_00334	ENSG0000	UBE2H	E2-20K|GID	ubiquitin c	protein-co
lib-C666-l	chr17	74449306	74449306	+	23.27622	NA	promoter-TSS (NM_022066)	promoter-TSS (NM_022066)	-18	NM_02206	63893	Hs.16130	NM_02206	ENSG0000	UBE2O	E2-230K	ubiquitin c	protein-co
lib-C666-l	chr19	531406	531406	+	14.98892	NA	promoter-TSS (NM_004359)	promoter-TSS (NM_004359)	-327	NM_00435	997	Hs.514997	NM_00435	ENSG0000	CDC34	E2-CDC34	cell divisior	protein-co
lib-C666-l	chr19	55919316	55919316	+	42.49125	NA	promoter-TSS (NM_014501)	promoter-TSS (NM_014501)	9	NM_01450	27338	Hs.396393	NM_01450	ENSG0000	UBE2S	E2-EPF|E2E	ubiquitin c	protein-co
lib-C666-l	chr20	32274234	32274234	+	17.68706	NA	promoter-TSS (NM_005225)	promoter-TSS (NM_005225)	-24	NM_00522	1869	Hs.654393	NM_00522	ENSG0000	E2F1	E2F-1|RBA	E2F transcr	protein-co
lib-C666-l	chr1	23866317	23866317	+	104.06357	NA	Intergenic	MIR3|SINE|MIR	-8605	NM_00409	1870	Hs.194333	NM_00409	ENSG0000	E2F2	E2F-2	E2F transcr	protein-co
lib-C666-l	chr16	67225990	67225990	+	3.7491	NA	promoter-TSS (NM_001950)	promoter-TSS (NM_001950)	-78	NM_00195	1874	Hs.108371	NM_00195	ENSG0000	E2F4	E2F-4	E2F transcr	protein-co
lib-C666-l	chr8	86089504	86089504	+	22.23727	NA	promoter-TSS (NR_134311)	promoter-TSS (NR_134311)	-115	NM_00195	1875	Hs.445758	NM_00195	ENSG0000	E2F5	E2F-5	E2F transcr	protein-co
lib-C666-l	chr8	86090143	86090143	+	6.29226	NA	promoter-TSS (NR_134311)	promoter-TSS (NR_134311)	524	NM_00108	1875	Hs.445758	NM_00195	ENSG0000	E2F5	E2F-5	E2F transcr	protein-co
lib-C666-l	chr11	76499498	76499498	+	20.84232	NA	intron (NM_001318478, intron 1 of 1)	intron (NM_001318478, intron 1 of	-3298	NM_00131	25987	Hs.8361	NM_01551	ENSG0000	TSKU	E2IG4|LRR(	tsukushi, sr	protein-co
lib-C666-l	chr11	76494244	76494244	+	12.47847	NA	promoter-TSS (NR_120561)	promoter-TSS (NR_120561)	-41	NM_01551	25987	Hs.8361	NM_01551	ENSG0000	TSKU	E2IG4|LRR(	tsukushi, sr	protein-co
lib-C666-l	chr11	76499885	76499885	+	6.81085	NA	intron (NM_001318478, intron 1 of 1)	intron (NM_001318478, intron 1 of	-2911	NM_00131	25987	Hs.8361	NM_01551	ENSG0000	TSKU	E2IG4|LRR(	tsukushi, sr	protein-co
lib-C666-l	chr11	118230274	118230274	+	31.27173	NA	promoter-TSS (NM_001204077)	promoter-TSS (NM_001204077)	-22	NM_00120	9354	Hs.75275	NM_00478	ENSG0000	UBE4A	E4|UBOX2|	ubiquitinat	protein-co
lib-C666-l	chr19	11616628	11616628	+	53.56082	NA	TTS (NM_001243204)	TTS (NM_001243204)	110	NM_13878	115950	Hs.465928	NM_13878	ENSG0000	ZNF653	E430039KC	zinc finger	protein-co
lib-C666-l	chr22	46067805	46067805	+	4.52748	NA	5' UTR (NM_001167621, exon 1 of 11	5' UTR (NM_001167621, exon 1 of 1	127	NM_01323	25814	Hs.475125	NM_01323	ENSG0000	ATXN10	E46L|HUM	ataxin 10	protein-co
lib-C666-l	chr8	143869962	143869962	+	2.59032	NA	Intergenic	Intergenic	-1954	NM_00369	8581	Hs.415762	NM_00369	ENSG0000	LY6D	E48|Ly-6D	lymphocyt	protein-co
lib-C666-l	chr16	2273571	2273571	+	29.74345	NA	promoter-TSS (NM_004424)	promoter-TSS (NM_004424)	82	NM_00128	1877	Hs.513268	NM_00442	ENSG0000	E4F1	E4F	E4F transcr	protein-co
lib-C666-l	chr21	27107282	27107282	+	5.819	NA	promoter-TSS (NM_001197297)	promoter-TSS (NM_001197297)	24	NM_00119	2551	Hs.473470	NM_00204	ENSG0000	GABPA	E4TF1-60|E	GA binding	protein-co
lib-C666-l	chr16	2318158	2318158	+	337.34933	NA	promoter-TSS (NR_132988)	promoter-TSS (NR_132988)	255	NR_10448	10921	Hs.355643	NM_00671	ENSG0000	RNPS1	E5.1	RNA bindir	protein-co
lib-C666-l	chr15	77223989	77223989	+	15.33659	NA	promoter-TSS (NM_001271837)	promoter-TSS (NM_001271837)	27	NM_00290	5955	Hs.79088	NM_00290	ENSG0000	RCN2	E6BP|ERC-	reticulocalb	protein-co
lib-C666-l	chr5	36724095	36724095	+	4.73866	NA	Intergenic	Intergenic	117638	NM_00128	6507	Hs.481918	NM_00417	ENSG0000	SLC1A3	EA6|EAAT1	solute carr	protein-co
lib-C666-l	chr6	101674764	101674764	+	21.87022	NA	Intergenic	HERVE-int|LTR|ERV1	-172097	NM_17576	2898	Hs.98262	NM_02195	ENSG0000	GRIK2	EAA4|GLR6	glutamate	protein-co
lib-C666-l	chr11	35358990	35358990	+	10.71733	NA	intron (NM_001195728, intron 1 of 11	intron (NM_001195728, intron 1 of	82115	NM_00417	6506	Hs.502338	NM_00417	ENSG0000	SLC1A2	EAAT2|EIEE	solute carr	protein-co
lib-C666-l	chr11	35326060	35326060	+	3.99113	NA	intron (NM_001252652, intron 6 of 11	intron (NM_001252652, intron 6 of	115045	NM_00417	6506	Hs.502338	NM_00417	ENSG0000	SLC1A2	EAAT2|EIEE	solute carr	protein-co
lib-C666-l	chr15	79165184	79165184	+	148.6877	NA	promoter-TSS (NM_001265605)	promoter-TSS (NM_001265605)	61	NM_20683	10933	Hs.374503	NM_00679	ENSG0000	MORF4L1	Eaf3|FWP0	mortality fa	protein-co
lib-C666-l	chr7	120590704	120590704	+	181.33469	NA	promoter-TSS (NM_019071)	promoter-TSS (NM_019071)	-113	NM_01907	54556	Hs.489811	NM_01907	ENSG0000	ING3	Eaf4|ING2|	inhibitor of	protein-co
lib-C666-l	chr7	120591190	120591190	+	38.71165	NA	intron (NM_198267, intron 1 of 4)	CpG	373	NM_19826	54556	Hs.489811	NM_01907	ENSG0000	ING3	Eaf4|ING2|	inhibitor of	protein-co
lib-C666-l	chr3	45017722	45017722	+	87.45062	NA	promoter-TSS (NM_016598)	promoter-TSS (NM_016598)	-19	NM_01500	23016	Hs.719958	NM_01500	ENSG0000	EXOSC7	EAP1|RRP4	exosome c	protein-co
lib-C666-l	chr1	220219954	220219954	+	11.07709	NA	promoter-TSS (NM_004446)	promoter-TSS (NM_004446)	46	NM_00444	2058	Hs.497788	NM_00444	ENSG0000	EPRS	EARS|GLUF	glutamyl-p	protein-co
lib-C666-l	chr20	31407635	31407635	+	13.20416	NA	promoter-TSS (NM_012325)	promoter-TSS (NM_012325)	-64	NM_01232	22919	Hs.472437	NM_01232	ENSG0000	MAPRE1	EB1	microtubul	protein-co
lib-C666-l	chr19	48903671	48903671	+	8.28613	NA	intron (NM_000836, intron 2 of 12)	intron (NM_000836, intron 2 of 12)	5539	NM_00083	2906	Hs.445015	NM_00083	ENSG0000	GRIN2D	EB11|EIEE4	glutamate	protein-co
lib-C666-l	chr2	27193577	27193577	+	4.39666	NA	5' UTR (NM_012326, exon 1 of 7)	5' UTR (NM_012326, exon 1 of 7)	338	NM_01232	22924	Hs.515860	NM_01232	ENSG0000	MAPRE3	EB3|EBF3|E	microtubul	protein-co
lib-C666-l	chr8	110551801	110551801	+	8.01457	NA	promoter-TSS (NM_004215)	promoter-TSS (NM_004215)	-128	NM_19812	9166	Hs.409368	NM_00421	ENSG0000	EBAG9	EB9|PDAF	estrogen re	protein-co
lib-C666-l	chr3	48633016	48633016	+	16.13885	NA	promoter-TSS (NM_000094)	promoter-TSS (NM_000094)	-423	NM_00009	1294	Hs.476218	NM_00009	ENSG0000	COL7A1	EBD1|EBD(	collagen ty	protein-co
lib-C666-l	chrX	9432991	9432991	+	10.58189	NA	promoter-TSS (NM_005647)	promoter-TSS (NM_005647)	-210	NM_00564	6907	Hs.495656	NM_00564	ENSG0000	TBL1X	EBI|SMAP5	transducin	protein-co
lib-C666-l	chr9	123688524	123688524	+	46.92278	NA	5' UTR (NM_005658, exon 2 of 8)	5' UTR (NM_005658, exon 2 of 8)	649	NM_00565	7185	Hs.531251	NM_00565	ENSG0000	TRAF1	EBI6|MGC:	TNF recept	protein-co
lib-C666-l	chr10	22518268	22518268	+	5.9194	NA	Intergenic	CpG	-19356	NM_00119	340900	Hs.450519	NM_00119	ENSG0000	EBLN1	EBLN-1	endogenou	protein-co
lib-C666-l	chr9	37079936	37079936	+	5.98967	NA	promoter-TSS (NR_036592)	promoter-TSS (NR_036592)	43	NR_03659	100506710	Hs.446271	NR_03659	ENSG0000	EBLN3P	EBLN3	endogenou	pseudo
lib-C666-l	chr12	56497953	56497953	+	13.33696	NA	promoter-TSS (NM_006191)	promoter-TSS (NM_006191)	-150	NM_00619	5036	Hs.524498	NM_00619	ENSG0000	PA2G4	EBP1|HG4-	proliferatio	protein-co
lib-C666-l	chr17	72744631	72744631	+	23.6003	NA	promoter-TSS (NM_004252)	promoter-TSS (NM_004252)	-120	NM_00425	9368	Hs.724482	NM_00425	ENSG0000	SLC9A3R1	EBP50|NHE	SLC9A3 reg	protein-co
lib-C666-l	chr8	145017613	145017613	+	14.70772	NA	promoter-TSS (NM_201383)	promoter-TSS (NM_201383)	513	NM_20138	5339	Hs.434248	NM_00044	ENSG0000	PLEC	EBS1|EBSM	plectin	protein-co
lib-C666-l	chr8	145026921	145026921	+	12.11076	NA	intron (NM_000445, intron 2 of 32)	intron (NM_000445, intron 2 of 32)	1167	NM_20137	5339	Hs.434248	NM_00044	ENSG0000	PLEC	EBS1|EBSM	plectin	protein-co
lib-C666-l	chr8	145014383	145014383	+	8.0154	NA	promoter-TSS (NM_201384)	promoter-TSS (NM_201384)	-625	NM_20138	5339	Hs.434248	NM_00044	ENSG0000	PLEC	EBS1|EBSM	plectin	protein-co
lib-C666-l	chr8	145051417	145051417	+	4.51266	NA	promoter-TSS (NM_000445)	promoter-TSS (NM_000445)	-504	NM_00044	5339	Hs.434248	NM_00044	ENSG0000	PLEC	EBS1|EBSM	plectin	protein-co
lib-C666-l	chr8	145026575	145026575	+	3.98718	NA	intron (NM_000445, intron 2 of 32)	intron (NM_000445, intron 2 of 32)	1513	NM_20137	5339	Hs.434248	NM_00044	ENSG0000	PLEC	EBS1|EBSM	plectin	protein-co
lib-C666-l	chr8	145010893	145010893	+	3.98718	NA	intron (NM_000445, intron 6 of 32)	intron (NM_000445, intron 6 of 32)	2865	NM_20138	5339	Hs.434248	NM_00044	ENSG0000	PLEC	EBS1|EBSM	plectin	protein-co
lib-C666-l	chr1	21682593	21682593	+	6.82479	NA	Intergenic	Intergenic	-10559	NM_00111	1889	Hs.195080	NM_00139	ENSG0000	ECE1	ECE	endothelin	protein-co
lib-C666-l	chr22	50968510	50968510	+	4.61604	NA	promoter-TSS (NM_001113755)	promoter-TSS (NM_001113755)	4	NM_00125	1890	Hs.180903	NM_00195	ENSG0000	TYMP	ECGF|ECGF	thymidine	protein-co
lib-C666-l	chr19	33572177	33572177	+	3.28884	NA	intron (NM_018025, intron 1 of 19)	CpG	391	NM_01802	55094	Hs.466436	NM_01802	ENSG0000	GPATCH1	ECGP|GPAT	G-patch do	protein-co
lib-C666-l	chr2	26467717	26467717	+	36.07033	NA	promoter-TSS (NM_000182)	promoter-TSS (NM_000182)	101	NM_00128	3032	Hs.515848	NM_00018	ENSG0000	HADHB	ECHB|MST	hydroxyac	protein-co
lib-C666-l	chr9	114245495	114245495	+	6.15313	NA	intron (NM_001080398, intron 3 of 5	CpG	1530	NM_00108	23392	Hs.368255	NM_00108	ENSG0000	KIAA0368	ECM29	KIAA0368	protein-co
lib-C666-l	chr9	114246522	114246522	+	3.27468	NA	exon (NM_001080398, exon 3 of 51)	exon (NM_001080398, exon 3 of 51	503	NM_00108	23392	Hs.368255	NM_00108	ENSG0000	KIAA0368	ECM29	KIAA0368	protein-co
lib-C666-l	chr3	127842963	127842963	+	35.65661	NA	promoter-TSS (NM_001319084)	promoter-TSS (NM_001319084)	-233	NM_00131	8607	Hs.272822	NM_00370	ENSG0000	RUVBL1	ECP-54|EC	RuvB like A	protein-co
lib-C666-l	chr12	71203860	71203860	+	12.62235	NA	intron (NM_002849, intron 2 of 13)	intron (NM_002849, intron 2 of 13)	-21150	NM_00120	5801	Hs.506076	NM_00284	ENSG0000	PTPRR	EC-PTP|PC	protein tyr	protein-co
lib-C666-l	chr17	27224228	27224228	+	23.1274	NA	TTS (NM_144683)	TTS (NM_144683)	487	NM_00447	2319	Hs.514038	NM_00447	ENSG0000	FLOT2	ECS-1|ECS	flotillin 2	protein-co
lib-C666-l	chr4	4861197	4861197	+	11.86993	NA	promoter-TSS (NM_002448)	promoter-TSS (NM_002448)	-195	NM_00244	4487	Hs.424414	NM_00244	ENSG0000	MSX1	ECTD3|HO	msh home	protein-co
lib-C666-l	chr12	54261704	54261704	+	5.18033	NA	Intergenic	Intergenic	-70872	NM_01741	3229	Hs.118608	NM_01741	ENSG0000	HOXC13	ECTD9|HO	homeobox	protein-co
lib-C666-l	chr1	115016005	115016005	+	12.13776	NA	intron (NM_033020, intron 1 of 18)	LTR10F|LTR|ERV1	37776	NM_03302	51592	Hs.26837	NM_01590	ENSG0000	TRIM33	ECTO|PTC7	tripartite m	protein-co
lib-C666-l	chr1	114889297	114889297	+	5.23609	NA	Intergenic	Intergenic	164484	NM_03302	51592	Hs.26837	NM_01590	ENSG0000	TRIM33	ECTO|PTC7	tripartite m	protein-co
lib-C666-l	chr1	115054138	115054138	+	3.20885	NA	promoter-TSS (NM_015906)	promoter-TSS (NM_015906)	-357	NM_01590	51592	Hs.26837	NM_01590	ENSG0000	TRIM33	ECTO|PTC7	tripartite m	protein-co
lib-C666-l	chr2	46283553	46283553	+	12.71554	NA	intron (NM_005400, intron 10 of 14)	intron (NM_005400, intron 10 of 14	-240988	NM_00143	2034	Hs.468410	NM_00143	ENSG0000	EPAS1	ECYT4|HIF2	endothelia	protein-co
lib-C666-l	chr2	46325125	46325125	+	11.89365	NA	intron (NM_005400, intron 11 of 14)	intron (NM_005400, intron 11 of 14	-199416	NM_00143	2034	Hs.468410	NM_00143	ENSG0000	EPAS1	ECYT4|HIF2	endothelia	protein-co
lib-C666-l	chr3	5229369	5229369	+	29.08762	NA	promoter-TSS (NM_014674)	promoter-TSS (NM_014674)	10	NM_01467	9695	Hs.224616	NM_01467	ENSG0000	EDEM1	EDEM	ER degrada	protein-co
lib-C666-l	chr9	113800112	113800112	+	2.12162	NA	intron (NM_057159, intron 1 of 4)	CpG	253	NM_05715	1902	Hs.126667	NM_00140	ENSG0000	LPAR1	EDG2|GPR2	lysophosph	protein-co
lib-C666-l	chr9	99102085	99102085	+	8.94036	NA	intron (NR_104627, intron 8 of 12)	intron (NR_104627, intron 8 of 12)	-37651	NM_00019	3293	Hs.477	NM_00019	ENSG0000	HSD17B3	EDH17B3|S	hydroxyste	protein-co
lib-C666-l	chr20	5591731	5591731	+	88.95569	NA	promoter-TSS (NM_019593)	promoter-TSS (NM_019593)	-59	NM_01959	56261	Hs.636359	NM_01959	ENSG0000	GPCPD1	EDI3|GDE5	glyceropho	protein-co
lib-C666-l	chr18	47139631	47139631	+	15.47905	NA	Intergenic	Intergenic	51230	NM_00130	9388	Hs.465102	NM_00603	ENSG0000	LIPG	EDL|EL|PRC	lipase G, er	protein-co
lib-C666-l	chrX	153607681	153607681	+	103.27538	NA	promoter-TSS (NM_000117)	promoter-TSS (NM_000117)	84	NM_00011	2010	Hs.522823	NM_00011	ENSG0000	EMD	EDMD|LEM	emerin	protein-co
lib-C666-l	chr14	64320296	64320296	+	3.23837	NA	intron (NM_182914, intron 1 of 115)	CpG	613	NM_18291	23224	Hs.745014	NM_01518	ENSG0000	SYNE2	EDMD5|NU	spectrin re	protein-co
lib-C666-l	chr9	137533582	137533582	+	6.29226	NA	promoter-TSS (NM_000093)	promoter-TSS (NM_000093)	-69	NM_00009	1289	Hs.210283	NM_00009	ENSG0000	COL5A1	EDSC	collagen ty	protein-co
lib-C666-l	chr9	137533810	137533810	+	2.59032	NA	5' UTR (NM_000093, exon 1 of 66)	5' UTR (NM_000093, exon 1 of 66)	159	NM_00127	1289	Hs.210283	NM_00009	ENSG0000	COL5A1	EDSC	collagen ty	protein-co
lib-C666-l	chr17	48277226	48277226	+	43.79478	NA	exon (NM_000088, exon 2 of 51)	exon (NM_000088, exon 2 of 51)	1774	NM_00008	1277	Hs.172928	NM_00008	ENSG0000	COL1A1	EDSC|OI1|(	collagen ty	protein-co
lib-C666-l	chr7	30066581	30066581	+	32.22565	NA	promoter-TSS (NM_017946)	promoter-TSS (NM_017946)	-164	NR_04647	55033	Hs.390838	NM_01794	ENSG0000	FKBP14	EDSKMH|F	FK506 binc	protein-co
lib-C666-l	chr1	161053427	161053427	+	33.60578	NA	intron (NM_030916, intron 1 of 8)	intron (NM_030916, intron 1 of 8)	5958	NM_03091	81607	Hs.492490	NM_03091	ENSG0000	NECTIN4	EDSS1|LNII	nectin cell	protein-co
lib-C666-l	chr2	207024426	207024426	+	13.53348	NA	promoter-TSS (NM_001199981)	promoter-TSS (NM_001199981)	108	NM_00195	1933	Hs.421608	NM_00195	ENSG0000	EEF1B2	EEF1B|EEF1	eukaryotic	protein-co
lib-C666-l	chr19	3985493	3985493	+	18.20429	NA	promoter-TSS (NM_001961)	promoter-TSS (NM_001961)	-32	NM_00196	1938	Hs.515070	NM_00196	ENSG0000	EEF2	EEF-2|EF-2	eukaryotic	protein-co
lib-C666-l	chr6	135375945	135375945	+	16.23236	NA	promoter-TSS (NM_001145207)	promoter-TSS (NM_001145207)	91	NM_00114	10767	Hs.378532	NM_00662	ENSG0000	HBS1L	EF-1a|ERFS	HBS1 like t	protein-co
lib-C666-l	chr8	144679759	144679759	+	11.95031	NA	promoter-TSS (NM_032862)	promoter-TSS (NM_032862)	86	NM_00113	1936	Hs.333388	NM_00196	ENSG0000	EEF1D	EF-1D|EF1I	eukaryotic	protein-co
lib-C666-l	chr4	44680432	44680432	+	76.4537	NA	promoter-TSS (NM_001345869)	promoter-TSS (NM_001345869)	-1	NM_02192	60558	Hs.546419	NM_02192	ENSG0000	GUF1	EF-4|EF4|E	GUF1 hom	protein-co
lib-C666-l	chr10	104181064	104181064	+	18.74745	NA	intron (NM_024326, intron 1 of 3)	CpG-3230	-1373	NM_00127	5662	Hs.154658	NM_00277	ENSG0000	PSD	EFA6|EFA6	pleckstrin a	protein-co
lib-C666-l	chr2	113961733	113961733	+	15.60931	NA	Intergenic	Intergenic	30173	NM_01245	23550	Hs.516306	NM_01245	ENSG0000	PSD4	EFA6B|TIC	pleckstrin a	protein-co
lib-C666-l	chr2	113915273	113915273	+	3.07144	NA	Intergenic	CpG	-16287	NM_01245	23550	Hs.516306	NM_01245	ENSG0000	PSD4	EFA6B|TIC	pleckstrin a	protein-co
lib-C666-l	chr19	14117684	14117684	+	399.10214	NA	promoter-TSS (NM_002918)	promoter-TSS (NM_002918)	-550	NM_00291	5989	Hs.655215	NM_00291	ENSG0000	RFX1	EFC|RFX	regulatory	protein-co
lib-C666-l	chr19	14117241	14117241	+	77.40723	NA	promoter-TSS (NM_002918)	promoter-TSS (NM_002918)	-107	NM_00291	5989	Hs.655215	NM_00291	ENSG0000	RFX1	EFC|RFX	regulatory	protein-co
lib-C666-l	chr8	16884507	16884507	+	4.56047	NA	promoter-TSS (NM_181723)	promoter-TSS (NM_181723)	-240	NM_18172	286097	Hs.403594	NM_18172	ENSG0000	MICU3	EFHA2	mitochond	protein-co
lib-C666-l	chr16	11836789	11836789	+	23.83582	NA	promoter-TSS (NM_001324022)	promoter-TSS (NM_001324022)	-55	NM_00132	51061	Hs.313847	NM_01591	ENSG0000	TXNDC11	EFP1	thioredoxir	protein-co
lib-C666-l	chr12	58176526	58176526	+	16.19708	NA	promoter-TSS (NM_001172695)	promoter-TSS (NM_001172695)	-2	NM_00117	10102	Hs.632704	NM_00572	ENSG0000	TSFM	EFTS|EFTSM	Ts translati	protein-co
lib-C666-l	chr15	82555105	82555105	+	9.47655	NA	promoter-TSS (NM_024580)	promoter-TSS (NM_024580)	-1	NM_00104	79631	Hs.459114	NM_02458	ENSG0000	EFL1	EFTUD1|FA	elongation	protein-co
lib-C666-l	chr10	94351538	94351538	+	33.11264	NA	Intergenic	CpG	-1287	NM_00452	3832	Hs.8878	NM_00452	ENSG0000	KIF11	EG5|HKSP|	kinesin fam	protein-co
lib-C666-l	chr10	94353203	94353203	+	9.10494	NA	exon (NM_004523, exon 1 of 22)	exon (NM_004523, exon 1 of 22)	378	NM_00452	3832	Hs.8878	NM_00452	ENSG0000	KIF11	EG5|HKSP|	kinesin fam	protein-co
lib-C666-l	chr10	94352724	94352724	+	2.00164	NA	promoter-TSS (NM_004523)	promoter-TSS (NM_004523)	-101	NM_00452	3832	Hs.8878	NM_00452	ENSG0000	KIF11	EG5|HKSP|	kinesin fam	protein-co
lib-C666-l	chr9	88556275	88556275	+	41.89289	NA	promoter-TSS (NM_001321882)	promoter-TSS (NM_001321882)	-169	NM_00132	60560	Hs.436098	NM_02192	ENSG0000	NAA35	EGAP|MAK	N(alpha)-a	protein-co
lib-C666-l	chr4	106831038	106831038	+	14.98502	NA	intron (NM_001184693, intron 2 of 11	intron (NM_001184693, intron 2 of	14441	NM_00118	255743	Hs.518921	NM_19827	ENSG0000	NPNT	EGFL6L|PO	nephronec	protein-co
lib-C666-l	chr4	100868007	100868007	+	2.59723	NA	promoter-TSS (NM_001031723)	promoter-TSS (NM_001031723)	-124	NM_00127	79982	Hs.577426	NM_02492	ENSG0000	DNAJB14	EGNR9427	DnaJ heat s	protein-co
lib-C666-l	chr3	4818965	4818965	+	11.20567	NA	exon (NM_001168272, exon 47 of 61)	exon (NM_001168272, exon 47 of 6	-25691	NR_00442	100126791	Hs.663059	NR_00442	ENSG0000	EGOT	EGO|NCRN	eosinophil	ncRNA
lib-C666-l	chr3	4792852	4792852	+	3.78771	NA	non-coding (NR_004428, exon 1 of 2	non-coding (NR_004428, exon 1 of	422	NR_00442	100126791	Hs.663059	NR_00442	ENSG0000	EGOT	EGO|NCRN	eosinophil	ncRNA
lib-C666-l	chr21	45432066	45432066	+	85.20388	NA	promoter-TSS (NM_003274)	promoter-TSS (NM_003274)	-140	NM_00327	7109	Hs.126221	NM_00327	ENSG0000	TRAPPC10	EHOC-1|EH	trafficking	protein-co
lib-C666-l	chr21	45527144	45527144	+	17.22931	NA	promoter-TSS (NM_005049)	promoter-TSS (NM_005049)	-64	NM_00504	5822	Hs.449076	NM_00504	ENSG0000	PWP2	EHOC-17|F	PWP2 peri	protein-co
lib-C666-l	chr20	32077677	32077677	+	7.57121	NA	promoter-TSS (NM_001032999)	promoter-TSS (NM_001032999)	-251	NM_00103	9139	Hs.153934	NM_00509	ENSG0000	CBFA2T2	EHT|MTGR	CBFA2/RUI	protein-co
lib-C666-l	chr19	40023385	40023385	+	3.98718	NA	exon (NM_152361, exon 1 of 1)	exon (NM_152361, exon 1 of 1)	122	NM_15236	126272	Hs.135181	NM_15236	ENSG0000	EID2B	EID-2B|EID	EP300 inte	protein-co
lib-C666-l	chr1	44173245	44173245	+	7.15277	NA	promoter-TSS (NM_001270462)	promoter-TSS (NM_001270462)	41	NM_00127	6487	Hs.597915	NM_00627	ENSG0000	ST3GAL3	EIEE15|MR	ST3 beta-g	protein-co
lib-C666-l	chr12	8234784	8234784	+	41.43555	NA	promoter-TSS (NR_024260)	promoter-TSS (NR_024260)	-23	NR_02426	25977	Hs.555927	NM_01550	ENSG0000	NECAP1	EIEE21	NECAP enc	protein-co
lib-C666-l	chr1	111154748	111154748	+	8.01457	NA	intron (NM_001204269, intron 1 of 4)	intron (NM_001204269, intron 1 of	-5773	NM_00497	3737	Hs.248139	NM_00497	ENSG0000	KCNA2	EIEE32|HBK	potassium	protein-co
lib-C666-l	chr20	44650407	44650407	+	5.34142	NA	promoter-TSS (NM_001134771)	promoter-TSS (NM_001134771)	78	NM_00113	57468	Hs.21413	NM_02070	ENSG0000	SLC12A5	EIEE34|EIG	solute carr	protein-co
lib-C666-l	chr1	231114782	231114782	+	49.05183	NA	promoter-TSS (NM_001122835)	promoter-TSS (NM_001122835)	-13	NM_02278	64801	Hs.275736	NM_02278	ENSG0000	ARV1	EIEE38	ARV1 hom	protein-co
lib-C666-l	chrX	20160044	20160044	+	12.97267	NA	promoter-TSS (NM_001412)	promoter-TSS (NM_001412)	-78	NM_00141	1964	Hs.522590	NM_00141	ENSG0000	EIF1AX	EIF1A|EIF1	eukaryotic	protein-co
lib-C666-l	chr20	32700151	32700151	+	59.93567	NA	promoter-TSS (NM_001316364)	promoter-TSS (NM_001316364)	11	NM_00390	8894	Hs.429180	NM_00390	ENSG0000	EIF2S2	EIF2|EIF2B|	eukaryotic	protein-co
lib-C666-l	chr20	32699725	32699725	+	9.93242	NA	intron (NM_003908, intron 1 of 8)	CpG	437	NM_00390	8894	Hs.429180	NM_00390	ENSG0000	EIF2S2	EIF2|EIF2B|	eukaryotic	protein-co
lib-C666-l	chrX	24072399	24072399	+	12.12504	NA	promoter-TSS (NM_001415)	promoter-TSS (NM_001415)	-666	NM_00141	1968	Hs.539684	NM_00141	ENSG0000	EIF2S3	EIF2|EIF2G|	eukaryotic	protein-co
lib-C666-l	chr2	27593318	27593318	+	7.21092	NA	promoter-TSS (NM_001267060)	promoter-TSS (NM_001267060)	6	NM_00103	8890	Hs.169474	NM_01563	ENSG0000	EIF2B4	EIF-2B|EIF2	eukaryotic	protein-co
lib-C666-l	chr12	124118315	124118315	+	382.13306	NA	promoter-TSS (NM_001414)	promoter-TSS (NM_001414)	8	NM_00141	1967	Hs.741273	NM_00141	ENSG0000	EIF2B1	EIF2B|EIF2E	eukaryotic	protein-co
lib-C666-l	chr1	45452361	45452361	+	5.10953	NA	promoter-TSS (NM_001261418)	promoter-TSS (NM_001261418)	33	NM_00126	8891	Hs.533549	NM_02036	ENSG0000	EIF2B3	EIF-2B|EIF2	eukaryotic	protein-co
lib-C666-l	chr1	36348726	36348726	+	17.1806	NA	promoter-TSS (NM_001317122)	promoter-TSS (NM_001317122)	-70	NM_01219	26523	Hs.22867	NM_01219	ENSG0000	AGO1	EIF2C|EIF2(	argonaute	protein-co
lib-C666-l	chr8	141801079	141801079	+	21.18233	NA	intron (NM_001316342, intron 11 of 3	intron (NM_001316342, intron 11 o	-155433	NM_00116	27161	Hs.743313	NM_01215	ENSG0000	AGO2	EIF2C2|Q1(	argonaute	protein-co
lib-C666-l	chr8	141644486	141644486	+	5.819	NA	intron (NM_001164623, intron 1 of 17	CpG	1160	NM_01215	27161	Hs.743313	NM_01215	ENSG0000	AGO2	EIF2C2|Q1(	argonaute	protein-co
lib-C666-l	chr1	36396733	36396733	+	20.87959	NA	promoter-TSS (NM_177422)	promoter-TSS (NM_177422)	50	NM_02485	192669	Hs.657659	NM_02485	ENSG0000	AGO3	EIF2C3	argonaute	protein-co
lib-C666-l	chr10	120840991	120840991	+	9.47655	NA	promoter-TSS (NM_003750)	promoter-TSS (NM_003750)	-657	NM_00375	8661	Hs.523299	NM_00375	ENSG0000	EIF3A	EIF3|EIF3S1	eukaryotic	protein-co
lib-C666-l	chr10	120840412	120840412	+	8.35705	NA	promoter-TSS (NM_003750)	promoter-TSS (NM_003750)	-78	NM_00375	8661	Hs.523299	NM_00375	ENSG0000	EIF3A	EIF3|EIF3S1	eukaryotic	protein-co
lib-C666-l	chr7	2394456	2394456	+	3.8557	NA	promoter-TSS (NM_003751)	promoter-TSS (NM_003751)	-18	NM_00375	8662	Hs.371001	NM_00375	ENSG0000	EIF3B	EIF3-ETA|E	eukaryotic	protein-co
lib-C666-l	chr7	2393811	2393811	+	2.24771	NA	promoter-TSS (NM_003751)	promoter-TSS (NM_003751)	-663	NM_00375	8662	Hs.371001	NM_00375	ENSG0000	EIF3B	EIF3-ETA|E	eukaryotic	protein-co
lib-C666-l	chr15	44829339	44829339	+	4.52748	NA	promoter-TSS (NM_003758)	promoter-TSS (NM_003758)	73	NM_00128	8669	Hs.404056	NM_00375	ENSG0000	EIF3J	EIF3S1|eIF3	eukaryotic	protein-co
lib-C666-l	chr8	117768107	117768107	+	23.80446	NA	promoter-TSS (NM_003756)	promoter-TSS (NM_003756)	-45	NM_00375	8667	Hs.492599	NM_00375	ENSG0000	EIF3H	EIF3S3|eIF3	eukaryotic	protein-co
lib-C666-l	chr11	8008763	8008763	+	11.13288	NA	promoter-TSS (NM_003754)	promoter-TSS (NM_003754)	-104	NM_00375	8665	Hs.516023	NM_00375	ENSG0000	EIF3F	EIF3S5|eIF3	eukaryotic	protein-co
lib-C666-l	chr22	36925327	36925327	+	13.59254	NA	promoter-TSS (NM_003753)	promoter-TSS (NM_003753)	-50	NM_00375	8664	Hs.55682	NM_00375	ENSG0000	EIF3D	EIF3S7|eIF3	eukaryotic	protein-co
lib-C666-l	chrY	22737578	22737578	+	110.05312	NA	promoter-TSS (NM_001278612)	promoter-TSS (NM_001278612)	-19	NM_00127	9086	Hs.461178	NM_00468	ENSG0000	EIF1AY	eIF-4C	eukaryotic	protein-co
lib-C666-l	chr2	180871960	180871960	+	41.28836	NA	promoter-TSS (NM_020943)	promoter-TSS (NM_020943)	-180	NM_02094	57703	Hs.311363	NM_02094	ENSG0000	CWC22	EIF4GL|NC	CWC22 spl	protein-co
lib-C666-l	chr14	103800460	103800460	+	3.8557	NA	promoter-TSS (NM_183004)	promoter-TSS (NM_183004)	121	NM_00196	1983	Hs.433702	NM_00196	ENSG0000	EIF5	EIF-5|EIF-5	eukaryotic	protein-co
lib-C666-l	chr3	170626538	170626538	+	7.97818	NA	promoter-TSS (NM_020390)	promoter-TSS (NM_020390)	-112	NM_02039	56648	Hs.164144	NM_02039	ENSG0000	EIF5A2	EIF-5A2|eII	eukaryotic	protein-co
lib-C666-l	chr2	3381612	3381612	+	19.38944	NA	promoter-TSS (NM_003310)	promoter-TSS (NM_003310)	91	NM_00331	7260	Hs.502770	NM_00331	ENSG0000	TSSC1	EIPR1	tumor sup	protein-co
lib-C666-l	chr19	41304908	41304908	+	13.33696	NA	promoter-TSS (NM_080732)	promoter-TSS (NM_080732)	-140	NM_08073	112398	Hs.515417	NM_01755	ENSG0000	EGLN2	EIT6|HIF-P	egl-9 fami	protein-co
lib-C666-l	chr17	47974429	47974429	+	5.5166	NA	Intergenic	Intergenic	-49050	NM_00107	255061	Hs.652130	NM_17068	ENSG0000	TAC4	EK|HK-1|H	tachykinin	protein-co
lib-C666-l	chr3	184283819	184283819	+	4.46664	NA	intron (NM_004443, intron 1 of 15)	intron (NM_004443, intron 1 of 15)	4232	NM_00444	2049	Hs.2913	NM_00444	ENSG0000	EPHB3	EK2|ETK2|H	EPH recept	protein-co

lib-C666-l	chr3	89993787	89993787	+	8.0787	NA	Intergenic	L1PA3|LINE|L1	837113	NM_18264	2042	Hs.123642	NM_00523	ENSG0000	EPHA3	EK4|ETK|ET	EPH recept	protein-co
lib-C666-l	chr12	22778098	22778098	+	10.92952	NA	promoter-TSS (NR_135031)	promoter-TSS (NR_135031)	22	NM_00103	55500	Hs.29464	NM_01863	ENSG0000	ETNK1	EKI|EKI 1|El	ethanolam	protein-co
lib-C666-l	chr1	204121250	204121250	+	8.89416	NA	promoter-TSS (NM_001297760)	promoter-TSS (NM_001297760)	60	NM_00129	55224	Hs.497469	NM_01820	ENSG0000	ETNK2	EKI2|HMFT	ethanolam	protein-co
lib-C666-l	chr14	65279345	65279345	+	9.615	NA	intron (NM_000347, intron 1 of 30)	intron (NM_000347, intron 1 of 30)	10521	NM_00102	6710	Hs.417303	NM_00034	ENSG0000	SPTB	EL3|HS2|H	spectrin be	protein-co
lib-C666-l	chr14	102552935	102552935	+	8.89416	NA	intron (NM_001017963, intron 2 of 1	CpG	577	NM_00534	3320	Hs.525600	NM_00534	ENSG0000	HSP90AA1	EL52|HEL-	heat shock	protein-co
lib-C666-l	chr19	8070811	8070811	+	11.07709	NA	promoter-TSS (NM_001419)	promoter-TSS (NM_001419)	-282	NM_00141	1994	Hs.184492	NM_00141	ENSG0000	ELAVL1	ELAV1|HUI	ELAV like F	protein-co
lib-C666-l	chr3	134515328	134515328	+	16.63918	NA	intron (NM_004441, intron 1 of 15)	CpG	1229	NM_00444	2047	Hs.116092	NM_00444	ENSG0000	EPHB1	ELK|EPHT2	EPH recept	protein-co
lib-C666-l	chr3	134514348	134514348	+	7.15513	NA	5' UTR (NM_004441, exon 1 of 16)	5' UTR (NM_004441, exon 1 of 16)	249	NM_00444	2047	Hs.116092	NM_00444	ENSG0000	EPHB1	ELK|EPHT2	EPH recept	protein-co
lib-C666-l	chr19	1848702	1848702	+	8.17853	NA	promoter-TSS (NM_020695)	promoter-TSS (NM_020695)	-250	NM_02069	57455	Hs.192477	NM_02069	ENSG0000	REXO1	ELOABP1|E	RNA exonu	protein-co
lib-C666-l	chr1	79350987	79350987	+	31.23797	NA	Intergenic	Intergenic	121508	NM_02215	64123	Hs.132314	NM_02215	ENSG0000	ADGRL4	ELTD1|ETL|	adhesion G	protein-co
lib-C666-l	chr1	247095572	247095572	+	6.20473	NA	promoter-TSS (NM_015446)	promoter-TSS (NM_015446)	-292	NR_13658	25909	Hs.300887	NM_01544	ENSG0000	AHCTF1	ELYS|MST1	AT-hook c	protein-co
lib-C666-l	chr15	76603960	76603960	+	8.59372	NA	promoter-TSS (NM_000126)	promoter-TSS (NM_000126)	-150	NM_00012	2108	Hs.39925	NM_00012	ENSG0000	ETFA	EMA|GA2|N	electron tra	protein-co
lib-C666-l	chr6	136871519	136871519	+	5.25516	NA	exon (NM_003980, exon 1 of 18)	exon (NM_003980, exon 1 of 18)	438	NM_00398	9053	Hs.486548	NM_00398	ENSG0000	MAP7	E-MAP-11	microtubul	protein-co
lib-C666-l	chr6	136871758	136871758	+	2.05265	NA	5' UTR (NM_003980, exon 1 of 18)	5' UTR (NM_003980, exon 1 of 18)	199	NM_00398	9053	Hs.486548	NM_00398	ENSG0000	MAP7	E-MAP-11	microtubul	protein-co
lib-C666-l	chr14	89258951	89258951	+	30.4351	NA	5' UTR (NM_183387, exon 1 of 43)	5' UTR (NM_183387, exon 1 of 43)	145	NM_18338	161436	Hs.325846	NM_18338	ENSG0000	EML5	EMAP-2	echinoderr	protein-co
lib-C666-l	chr14	89259426	89259426	+	9.7234	NA	promoter-TSS (NM_183387)	promoter-TSS (NM_183387)	-330	NM_18338	161436	Hs.325846	NM_18338	ENSG0000	EML5	EMAP-2	echinoderr	protein-co
lib-C666-l	chr4	107237593	107237593	+	41.72511	NA	promoter-TSS (NM_001142416)	promoter-TSS (NM_001142416)	-71	NM_00114	9255	Hs.591680	NM_00475	ENSG0000	AIMP1	EMAP2|EM	aminoacyl	protein-co
lib-C666-l	chr5	133512728	133512728	+	24.5284	NA	promoter-TSS (NM_170679)	promoter-TSS (NM_170679)	-4	NM_17067	6500	Hs.171626	NM_00693	ENSG0000	SKP1	EMC19|OC	S-phase ki	protein-co
lib-C666-l	chrX	135055906	135055906	+	13.59977	NA	5' UTR (NM_001330000, exon 2 of 5)	5' UTR (NM_001330000, exon 2 of 5	316	NM_17347	93380	Hs.110702	NM_17347	ENSG0000	MMGT1	EMC5|TME	membrane	protein-co
lib-C666-l	chr4	1283566	1283566	+	17.40556	NA	promoter-TSS (NR_123716)	promoter-TSS (NR_123716)	-73	NR_12371	10296	Hs.139896	NM_00588	ENSG0000	MAEA	EMLP|EMP	macrophag	protein-co
lib-C666-l	chr5	149829426	149829426	+	3.23837	NA	promoter-TSS (NM_005617)	promoter-TSS (NM_005617)	-107	NM_00102	6208	Hs.744846	NM_00561	ENSG0000	RPS14	EMTB|S14	ribosomal	protein-co
lib-C666-l	chr10	119412869	119412869	+	23.74671	NA	Intergenic	Intergenic	-108290	NR_00279	196047	Hs.312592	NR_00279	ENSG0000	EMX2OS	EMX2-AS1	EMX2 opp	ncRNA
lib-C666-l	chr11	118938375	118938375	+	4.90117	NA	promoter-TSS (NM_001290185)	promoter-TSS (NM_001290185)	-88	NM_00129	55823	Hs.234282	NM_02172	ENSG0000	VPS11	END1|HLD	VPS11, CO	protein-co
lib-C666-l	chr1	156024715	156024715	+	13.77909	NA	exon (NM_001145264, exon 1 of 3)	exon (NM_001145264, exon 1 of 3)	198	NM_01401	28956	Hs.632483	NM_01401	ENSG0000	LAMTOR2	ENDAP|HS	late endos	protein-co
lib-C666-l	chr6	96025684	96025684	+	10.73451	NA	promoter-TSS (NR_104136)	promoter-TSS (NR_104136)	311	NM_02464	79694	Hs.734632	NM_02464	ENSG0000	MANEA	ENDO|hEn	mannosida	protein-co
lib-C666-l	chr5	54177390	54177390	+	4.46583	NA	Intergenic	Intergenic	104024	NM_00703	11082	Hs.129944	NM_00703	ENSG0000	ESM1	endocan	endothelia	protein-co
lib-C666-l	chr19	6280037	6280037	+	19.75261	NA	promoter-TSS (NM_005934)	promoter-TSS (NM_005934)	-78	NM_00593	4298	Hs.10095	NM_00593	ENSG0000	MLLT1	ENL|LTG19	MLLT1, sup	protein-co
lib-C666-l	chr7	148581447	148581447	+	2.46982	NA	promoter-TSS (NM_004456)	promoter-TSS (NM_004456)	-6	NM_15299	2146	Hs.444082	NM_00445	ENSG0000	EZH2	ENX-1|EN	enhancer c	protein-co
lib-C666-l	chr17	8339306	8339306	+	14.02822	NA	5' UTR (NM_001330129, exon 1 of 8)	5' UTR (NM_001330129, exon 1 of 8	136	NM_00102	81565	Hs.372123	NM_03080	ENSG0000	NDEL1	EOPA|MIT	nudE neur	protein-co
lib-C666-l	chr5	40486865	40486865	+	28.21964	NA	Intergenic	Intergenic	-193167	NM_00095	5734	Hs.199248	NM_00095	ENSG0000	PTGER4	EP4|EP4R	prostaglan	protein-co
lib-C666-l	chr5	40411340	40411340	+	12.26631	NA	Intergenic	Intergenic	-268692	NM_00095	5734	Hs.199248	NM_00095	ENSG0000	PTGER4	EP4|EP4R	prostaglan	protein-co
lib-C666-l	chr5	40446924	40446924	+	8.51971	NA	Intergenic	THE1D|LTR|ERVL-MaLR	-233108	NM_00095	5734	Hs.199248	NM_00095	ENSG0000	PTGER4	EP4|EP4R	prostaglan	protein-co
lib-C666-l	chr13	103451431	103451431	+	15.33659	NA	promoter-TSS (NM_024089)	promoter-TSS (NM_024089)	-27	NM_00131	79070	Hs.408629	NM_02408	ENSG0000	KDELC1	EP58|ERp5	KDEL motif	protein-co
lib-C666-l	chr10	98346810	98346810	+	30.07415	NA	promoter-TSS (NM_020123)	promoter-TSS (NM_020123)	-1	NM_02012	56889	Hs.500674	NM_02012	ENSG0000	TM9SF3	EP70-P-isc	transmemb	protein-co
lib-C666-l	chr1	226005016	226005016	+	13.71937	NA	intron (NM_001291163, intron 1 of 8)	intron (NM_001291163, intron 1 of	7180	NM_00129	2052	Hs.89649	NM_00012	ENSG0000	EPHX1	EPHX|EPO	epoxide hy	protein-co
lib-C666-l	chr17	34122414	34122414	+	10.79304	NA	non-coding (NR_111988, exon 1 of 9	non-coding (NR_111988, exon 1 of	297	NM_03295	79148	Hs.380710	NM_02430	ENSG0000	MMP28	EPILYSIN|M	matrix met	protein-co
lib-C666-l	chr12	131249263	131249263	+	34.12565	NA	Intergenic	HAL1-2a_MD|LINE|L1	74556	NM_19435	2054	Hs.437585	NM_00198	ENSG0000	STX2	EPIM|EPM|	syntaxin 2	protein-co
lib-C666-l	chr12	131249911	131249911	+	16.59065	NA	Intergenic	HAL1-2a_MD|LINE|L1	73908	NM_19435	2054	Hs.437585	NM_00198	ENSG0000	STX2	EPIM|EPM|	syntaxin 2	protein-co
lib-C666-l	chr12	131248860	131248860	+	12.69461	NA	Intergenic	HAL1-2a_MD|LINE|L1	74959	NM_19435	2054	Hs.437585	NM_00198	ENSG0000	STX2	EPIM|EPM|	syntaxin 2	protein-co
lib-C666-l	chr16	10215930	10215930	+	6.81085	NA	intron (NM_000833, intron 3 of 13)	intron (NM_000833, intron 3 of 13)	59994	NM_00113	2903	Hs.411472	NM_00083	ENSG0000	GRIN2A	EPND|FESD	glutamate	protein-co
lib-C666-l	chr19	11492438	11492438	+	14.70559	NA	non-coding (NR_033663, exon 4 of 8	non-coding (NR_033663, exon 4 of	2580	NM_00012	2057	Hs.631624	NM_00012	ENSG0000	EPOR	EPO-R	erythropoi	protein-co
lib-C666-l	chr19	11491928	11491928	+	7.08136	NA	intron (NM_000121, intron 4 of 7)	CpG-12947	3090	NM_00012	2057	Hs.631624	NM_00012	ENSG0000	EPOR	EPO-R	erythropoi	protein-co
lib-C666-l	chr1	201977348	201977348	+	5.25516	NA	Intergenic	Intergenic	-2342	NM_00443	1999	Hs.603657	NM_00443	ENSG0000	ELF3	EPR-1|ERT	E74 like ET	protein-co
lib-C666-l	chr19	4791479	4791479	+	156.72173	NA	promoter-TSS (NM_018708)	promoter-TSS (NM_018708)	-249	NM_01870	55527	Hs.515082	NM_01870	ENSG0000	FEM1A	EPRAP	fem-1 hon	protein-co
lib-C666-l	chr15	44038723	44038723	+	10.92275	NA	promoter-TSS (NR_002318)	promoter-TSS (NR_002318)	133	NM_00531	2923	Hs.591095	NM_00531	ENSG0000	PDIA3	ER60|ERp5	protein dis	protein-co
lib-C666-l	chr7	14031082	14031082	+	4.52748	NA	promoter-TSS (NM_001163149)	promoter-TSS (NM_001163149)	-32	NM_00116	2115	Hs.22634	NM_00495	ENSG0000	ETV1	ER81	ETS variant	protein-co
lib-C666-l	chr6	126235119	126235119	+	11.06679	NA	intron (NM_001122842, intron 10 of 1	intron (NM_001122842, intron 10 o	-5251	NM_00119	135112	Hs.171426	NM_18178	ENSG0000	NCOA7	ERAP140|E	nuclear rec	protein-co
lib-C666-l	chr14	64805833	64805833	+	9.7234	NA	promoter-TSS (NR_073496)	promoter-TSS (NR_073496)	-565	NR_07349	2100	Hs.660607	NM_00143	ENSG0000	ESR2	ER-BETA|E	estrogen re	protein-co
lib-C666-l	chr1	94344733	94344733	+	61.32821	NA	promoter-TSS (NM_014597)	promoter-TSS (NM_014597)	29	NM_01459	30836	Hs.85769	NM_01459	ENSG0000	DNTTIP2	ERBP|FCF2	deoxynucle	protein-co
lib-C666-l	chr16	14051882	14051882	+	7.03048	NA	Intergenic	Intergenic	37868	NM_00523	2072	Hs.567265	NM_00523	ENSG0000	ERCC4	ERCC11|FA	ERCC excis	protein-co
lib-C666-l	chr2	183580831	183580831	+	8.85547	NA	promoter-TSS (NM_001271581)	promoter-TSS (NM_001271581)	63	NR_07336	54431	Hs.516632	NM_01898	ENSG0000	DNAJC10	ERdj5|JPDI	DnaJ heat s	protein-co
lib-C666-l	chr2	183507241	183507241	+	5.74587	NA	Intergenic	MLT1K|LTR|ERVL-MaLR	-73527	NR_07336	54431	Hs.516632	NM_01898	ENSG0000	DNAJC10	ERdj5|JPDI	DnaJ heat s	protein-co
lib-C666-l	chr7	150676141	150676141	+	7.15545	NA	promoter-TSS (NM_000238)	promoter-TSS (NM_000238)	-739	NM_00023	3757	Hs.647099	NM_00023	ENSG0000	KCNH2	ERG-1|ERG	potassium	protein-co
lib-C666-l	chr7	150672053	150672053	+	5.96422	NA	intron (NM_172056, intron 1 of 8)	CpG	3349	NM_17205	3757	Hs.647099	NM_00023	ENSG0000	KCNH2	ERG-1|ERG	potassium	protein-co
lib-C666-l	chr14	76127526	76127526	+	85.99825	NA	promoter-TSS (NM_015072)	promoter-TSS (NM_015072)	12	NM_00717	11161	Hs.15106	NM_00717	ENSG0000	C14orf1	ERG28|NET	chromosor	protein-co
lib-C666-l	chr11	121163429	121163429	+	6.13111	NA	promoter-TSS (NM_006918)	promoter-TSS (NM_006918)	41	NM_00691	6309	Hs.287749	NM_00691	ENSG0000	SC5D	ERG3|S5DE	sterol-C5-	protein-co
lib-C666-l	chr5	172261263	172261263	+	21.28624	NA	promoter-TSS (NM_001031711)	promoter-TSS (NM_001031711)	40	NM_00103	57222	Hs.509163	NM_02046	ENSG0000	ERGIC1	ERGIC-32|I	endoplasm	protein-co
lib-C666-l	chr2	134193064	134193064	+	25.39084	NA	intron (NM_207481, intron 3 of 17)	intron (NM_207481, intron 3 of 17)	132967	NM_20748	344148	Hs.537329	NM_20736	ENSG0000	NCKAP5	ERIH1|ERIH	NCK assoc	protein-co
lib-C666-l	chr4	103811181	103811181	+	22.50982	NA	3' UTR (NM_001008388, exon 3 of 3)	3' UTR (NM_001008388, exon 3 of 3	21046	NM_00100	493856	Hs.444955	NM_00100	ENSG0000	CISD2	ERIS|Miner	CDGSH iro	protein-co
lib-C666-l	chr22	22222205	22222205	+	4.73866	NA	promoter-TSS (NM_138957)	promoter-TSS (NM_138957)	-235	NM_00274	5594	Hs.431850	NM_00274	ENSG0000	MAPK1	ERK|ERK-2	mitogen-a	protein-co
lib-C666-l	chr15	52311356	52311356	+	20.19521	NA	promoter-TSS (NM_002748)	promoter-TSS (NM_002748)	-55	NM_00274	5597	Hs.411847	NM_00274	ENSG0000	MAPK6	ERK3|HsT1	mitogen-a	protein-co
lib-C666-l	chr12	58087806	58087806	+	10.62959	NA	promoter-TSS (NM_001261422)	promoter-TSS (NM_001261422)	68	NM_00126	10956	Hs.527861	NM_00681	ENSG0000	OS9	ERLEC2|OS	OS9, endo	protein-co
lib-C666-l	chr3	185826939	185826939	+	22.01582	NA	promoter-TSS (NM_004454)	promoter-TSS (NM_004454)	-38	NM_00445	2119	Hs.43697	NM_00445	ENSG0000	ETV5	ERM	ETS variant	protein-co
lib-C666-l	chr14	53167682	53167682	+	7.15513	NA	Intergenic	Intergenic	-5033	NM_01458	30001	Hs.525339	NM_01458	ENSG0000	ERO1A	ERO1-L|ER	endoplasm	protein-co
lib-C666-l	chr12	96652490	96652490	+	179.01491	NA	intron (NM_001303511, intron 1 of 2)	intron (NM_001303511, intron 1 of	3252	NM_00130	2004	Hs.46523	NM_00523	ENSG0000	ELK3	ERP|NET|S	ELK3, ETS t	protein-co
lib-C666-l	chr12	96710023	96710023	+	3.27468	NA	intron (NM_002595, intron 3 of 16)	L1PA2|LINE|L1	60785	NM_00130	2004	Hs.46523	NM_00523	ENSG0000	ELK3	ERP|NET|S	ELK3, ETS t	protein-co
lib-C666-l	chr12	96709844	96709844	+	2.59032	NA	intron (NM_002595, intron 3 of 16)	L1PA2|LINE|L1	60606	NM_00130	2004	Hs.46523	NM_00523	ENSG0000	ELK3	ERP|NET|S	ELK3, ETS t	protein-co
lib-C666-l	chr12	96617941	96617941	+	2.12162	NA	intron (NM_005230, intron 2 of 4)	intron (NM_005230, intron 2 of 4)	29781	NM_00523	2004	Hs.46523	NM_00523	ENSG0000	ELK3	ERP|NET|S	ELK3, ETS t	protein-co
lib-C666-l	chr2	10952950	10952950	+	9.10494	NA	promoter-TSS (NM_001282707)	promoter-TSS (NM_001282707)	15	NM_00128	10130	Hs.212102	NM_00574	ENSG0000	PDIA6	ERP5|P5|T	protein dis	protein-co
lib-C666-l	chr7	148725526	148725526	+	2.92124	NA	5' UTR (NM_004911, exon 1 of 10)	5' UTR (NM_004911, exon 1 of 10)	256	NM_00491	9601	Hs.93659	NM_00491	ENSG0000	PDIA4	ERP70|ERP	protein dis	protein-co
lib-C666-l	chr14	53019294	53019294	+	5.82086	NA	promoter-TSS (NM_001160047)	promoter-TSS (NM_001160047)	7	NM_02078	57544	Hs.532609	NM_02078	ENSG0000	TXNDC16	ERp90|KIA	thioredoxir	protein-co
lib-C666-l	chr5	149110565	149110565	+	15.60931	NA	intron (NM_133263, intron 1 of 11)	CpG	750	NM_00117	133522	Hs.483816	NM_13326	ENSG0000	PPARGC1B	ERRL1|PER	PPARG coa	protein-co
lib-C666-l	chr1	145096404	145096404	+	48.04218	NA	promoter-TSS (NM_004892)	promoter-TSS (NM_004892)	-3	NM_00489	9554	Hs.632438	NM_00489	ENSG0000	SEC22B	ERS-24|SE	SEC22 hon	protein-co
lib-C666-l	chr9	136245017	136245017	+	26.82121	NA	promoter-TSS (NM_001280788)	promoter-TSS (NM_001280788)	-197	NM_00128	6836	Hs.512465	NM_03316	ENSG0000	SURF4	ERV29	surfeit 4	protein-co
lib-C666-l	chr9	136242984	136242984	+	11.77301	NA	promoter-TSS (NR_103997)	promoter-TSS (NR_103997)	53	NM_00128	6836	Hs.512465	NM_03316	ENSG0000	SURF4	ERV29	surfeit 4	protein-co
lib-C666-l	chr21	45592769	45592769	+	14.43224	NA	Intergenic	Intergenic	39275	NM_00464	8209	Hs.413482	NM_00464	ENSG0000	C21orf33	ES1|GT335	chromosor	protein-co
lib-C666-l	chr15	65426194	65426194	+	40.51821	NA	promoter-TSS (NM_005707)	promoter-TSS (NM_005707)	-20	NM_00570	10081	Hs.458596	NM_00570	ENSG0000	PDCD7	ES18|HES1	programm	protein-co
lib-C666-l	chr7	94938334	94938334	+	6.29226	NA	intron (NM_000446, intron 5 of 8)	intron (NM_000446, intron 5 of 8)	15550	NM_00044	5444	Hs.370995	NM_00044	ENSG0000	PON1	ESA|MVCD	paraoxona	protein-co
lib-C666-l	chr11	65479628	65479628	+	97.78539	NA	5' UTR (NM_006388, exon 1 of 14)	5' UTR (NM_006388, exon 1 of 14)	155	NM_18270	10524	Hs.397010	NM_00638	ENSG0000	KAT5	ESA1|HTA1	lysine acet	protein-co
lib-C666-l	chr16	28962202	28962202	+	24.00146	NA	promoter-TSS (NM_032815)	promoter-TSS (NM_032815)	-116	NM_03281	84901	Hs.513470	NM_03281	ENSG0000	NFATC2IP	ESC2|NIP4	nuclear fac	protein-co
lib-C666-l	chr16	28956912	28956912	+	5.2142	NA	Intergenic	Intergenic	-5406	NM_03281	84901	Hs.513470	NM_03281	ENSG0000	NFATC2IP	ESC2|NIP4	nuclear fac	protein-co
lib-C666-l	chr8	76236897	76236897	+	17.37618	NA	Intergenic	L1PA5|LINE|L1	-45773	NR_10384	101805492	Hs.571424	NR_103848		CASC9	ESCCAL-1|	cancer sus	ncRNA
lib-C666-l	chr11	34706953	34706953	+	2.59032	NA	Intergenic	Intergenic	52942	NM_00120	26298	Hs.653859	NM_01215	ENSG0000	EHF	ESE3|ESE3	ETS homol	protein-co
lib-C666-l	chr1	150898772	150898772	+	6.71839	NA	promoter-TSS (NM_001243491)	promoter-TSS (NM_001243491)	-43	NM_00124	9869	Hs.643565	NM_01243	ENSG0000	SETDB1	ESET|H3-K	SET domai	protein-co
lib-C666-l	chr9	83832197	83832197	+	3.59106	NA	Intergenic	L1PA4|LINE|L1	472253	NM_00130	7088	Hs.197320	NM_00507	ENSG0000	TLE1	ESG|ESG1|	transducin	protein-co
lib-C666-l	chr9	84304388	84304388	+	3.02289	NA	promoter-TSS (NR_109772)	promoter-TSS (NR_109772)	62	NM_00130	7088	Hs.197320	NM_00507	ENSG0000	TLE1	ESG|ESG1|	transducin	protein-co
lib-C666-l	chr19	3035720	3035720	+	33.78053	NA	intron (NM_001144761, intron 2 of 1	CpG	-6108	NM_00114	7089	Hs.332173	NM_00326	ENSG0000	TLE2	ESG|ESG2|	transducin	protein-co
lib-C666-l	chr15	70433181	70433181	+	36.07033	NA	Intergenic	Intergenic	-42925	NM_02090	7090	Hs.287362	NM_00507	ENSG0000	TLE3	ESG|ESG3|	transducin	protein-co
lib-C666-l	chr15	70392001	70392001	+	22.01132	NA	Intergenic	CpG-8039	-1745	NM_02090	7090	Hs.287362	NM_00507	ENSG0000	TLE3	ESG|ESG3|	transducin	protein-co
lib-C666-l	chr15	70489138	70489138	+	18.1336	NA	Intergenic	Intergenic	-98882	NM_02090	7090	Hs.287362	NM_00507	ENSG0000	TLE3	ESG|ESG3|	transducin	protein-co
lib-C666-l	chr15	70383043	70383043	+	4.39666	NA	intron (NM_001105192, intron 4 of 1	intron (NM_001105192, intron 4 of	4081	NM_00128	7090	Hs.287362	NM_00507	ENSG0000	TLE3	ESG|ESG3|	transducin	protein-co
lib-C666-l	chr12	56552126	56552126	+	17.37618	NA	promoter-TSS (NM_021019)	promoter-TSS (NM_021019)	81	NM_02101	4637	Hs.632717	NM_02101	ENSG0000	MYL6	ESMLC|LC1	myosin ligh	protein-co
lib-C666-l	chr12	53662036	53662036	+	4.49043	NA	promoter-TSS (NM_012291)	promoter-TSS (NM_012291)	-47	NM_01229	9700	Hs.153479	NM_01229	ENSG0000	ESPL1	ESP1|SEPA	extra spind	protein-co
lib-C666-l	chr3	107941347	107941347	+	14.70559	NA	promoter-TSS (NM_018010)	promoter-TSS (NM_018010)	70	NM_01801	55081	Hs.412196	NM_01801	ENSG0000	IFT57	ESRRBL1|H	intraflagell	protein-co
lib-C666-l	chr6	43395151	43395151	+	9.79436	NA	promoter-TSS (NM_001198934)	promoter-TSS (NM_001198934)	-141	NM_00119	89845	Hs.55879	NM_03345	ENSG0000	ABCC10	EST182763	ATP bindin	protein-co
lib-C666-l	chr3	183903911	183903911	+	5.819	NA	promoter-TSS (NM_018358)	promoter-TSS (NM_018358)	48	NM_01835	55324	Hs.361323	NM_01835	ENSG0000	ABCF3	EST201864	ATP bindin	protein-co
lib-C666-l	chr1	229694848	229694848	+	6.71839	NA	promoter-TSS (NM_012089)	promoter-TSS (NM_012089)	-406	NM_01208	23456	Hs.17614	NM_01208	ENSG0000	ABCB10	EST20237|I	ATP bindin	protein-co
lib-C666-l	chr7	150725328	150725328	+	13.59254	NA	promoter-TSS (NM_001282293)	promoter-TSS (NM_001282293)	-181	NM_00128	11194	Hs.647118	NM_00718	ENSG0000	ABCB8	EST328128	ATP bindin	protein-co
lib-C666-l	chr7	150725657	150725657	+	6.39985	NA	exon (NM_001282293, exon 1 of 15)	exon (NM_001282293, exon 1 of 15	148	NM_00718	11194	Hs.647118	NM_00718	ENSG0000	ABCB8	EST328128	ATP bindin	protein-co
lib-C666-l	chr17	74733951	74733951	+	44.06107	NA	promoter-TSS (NM_001195427)	promoter-TSS (NM_001195427)	368	NM_00124	79157	Hs.73965	NM_02431	ENSG0000	MFSD11	ET	major facili	protein-co
lib-C666-l	chr17	74733591	74733591	+	3.84463	NA	promoter-TSS (NM_001242533)	promoter-TSS (NM_001242533)	8	NM_00124	79157	Hs.73965	NM_02431	ENSG0000	MFSD11	ET	major facili	protein-co
lib-C666-l	chr1	42128505	42128505	+	88.70856	NA	intron (NM_001127714, intron 1 of 7)	CpG	-178151	NM_00195	1907	Hs.1407	NM_00195	ENSG0000	EDN2	ET-2|ET2|P	endothelin	protein-co
lib-C666-l	chr1	41898552	41898552	+	77.28233	NA	Intergenic	Intergenic	51802	NM_00130	1907	Hs.1407	NM_00195	ENSG0000	EDN2	ET-2|ET2|P	endothelin	protein-co
lib-C666-l	chr1	42133148	42133148	+	7.9573	NA	intron (NM_001127714, intron 1 of 7)	intron (NM_001127714, intron 1 of	-182794	NM_00195	1907	Hs.1407	NM_00195	ENSG0000	EDN2	ET-2|ET2|P	endothelin	protein-co
lib-C666-l	chr1	41961970	41961970	+	5.15932	NA	Intergenic	CpG	-11616	NM_00195	1907	Hs.1407	NM_00195	ENSG0000	EDN2	ET-2|ET2|P	endothelin	protein-co
lib-C666-l	chr5	175866105	175866105	+	10.71733	NA	Intergenic	Intergenic	-9251	NM_01461	23197	Hs.484242	NM_01461	ENSG0000	FAF2	ETEA|UBXD	Fas associa	protein-co
lib-C666-l	chr5	175875363	175875363	+	9.4812	NA	promoter-TSS (NM_014613)	promoter-TSS (NM_014613)	7	NM_01461	23197	Hs.484242	NM_01461	ENSG0000	FAF2	ETEA|UBXD	Fas associa	protein-co
lib-C666-l	chr2	20850895	20850895	+	43.79266	NA	promoter-TSS (NM_022460)	promoter-TSS (NM_022460)	-31	NM_02246	64342	Hs.531785	NM_02246	ENSG0000	HS1BP3	ETM2|HS1-	HCLS1 bin	protein-co
lib-C666-l	chr19	13274005	13274005	+	60.44312	NA	Intergenic	CpG	12723	NM_00490	9592	Hs.501629	NM_00490	ENSG0000	IER2	ETR101	immediate	protein-co
lib-C666-l	chr19	13263793	13263793	+	31.58745	NA	5' UTR (NM_004907, exon 2 of 2)	5' UTR (NM_004907, exon 2 of 2)	2511	NM_00490	9592	Hs.501629	NM_00490	ENSG0000	IER2	ETR101	immediate	protein-co
lib-C666-l	chr19	13261258	13261258	+	19.65763	NA	promoter-TSS (NM_001271611)	promoter-TSS (NM_001271611)	-24	NM_00490	9592	Hs.501629	NM_00490	ENSG0000	IER2	ETR101	immediate	protein-co
lib-C666-l	chr19	13262751	13262751	+	18.47709	NA	intron (NM_004907, intron 1 of 1)	CpG	1469	NM_00490	9592	Hs.501629	NM_00490	ENSG0000	IER2	ETR101	immediate	protein-co
lib-C666-l	chr19	13262440	13262440	+	10.71733	NA	intron (NM_004907, intron 1 of 1)	CpG	1158	NM_00490	9592	Hs.501629	NM_00490	ENSG0000	IER2	ETR101	immediate	protein-co
lib-C666-l	chr19	13265966	13265966	+	2.00164	NA	TTS (NM_004907)	TTS (NM_004907)	4684	NM_00490	9592	Hs.501629	NM_00490	ENSG0000	IER2	ETR101	immediate	protein-co
lib-C666-l	chr11	128337859	128337859	+	11.57178	NA	intron (NM_001330451, intron 5 of 6)	intron (NM_001330451, intron 5 of	54346	NM_00116	2113	Hs.369438	NM_00523	ENSG0000	ETS1	ETS-1|EWS	ETS proto-	protein-co
lib-C666-l	chr21	40177161	40177161	+	7.0832	NA	promoter-TSS (NM_001256295)	promoter-TSS (NM_001256295)	-70	NM_00125	2114	Hs.644231	NM_00523	ENSG0000	ETS2	ETS2IT1	ETS proto-	protein-co
lib-C666-l	chr4	140099135	140099135	+	9.20633	NA	promoter-TSS (NM_001331036)	promoter-TSS (NM_001331036)	-763	NM_00133	1998	Hs.634040	NM_00687	ENSG0000	ELF2	EU32|NERF	E74 like ET	protein-co
lib-C666-l	chr4	140004166	140004166	+	4.74112	NA	intron (NM_001331036, intron 4 of 9)	CpG	1402	NM_00687	1998	Hs.634040	NM_00687	ENSG0000	ELF2	EU32|NERF	E74 like ET	protein-co
lib-C666-l	chr4	140098813	140098813	+	4.52748	NA	promoter-TSS (NM_001331036)	promoter-TSS (NM_001331036)	-441	NM_00133	1998	Hs.634040	NM_00687	ENSG0000	ELF2	EU32|NERF	E74 like ET	protein-co
lib-C666-l	chr14	31677086	31677086	+	35.40551	NA	promoter-TSS (NM_015382)	promoter-TSS (NM_015382)	-357	NM_01538	25831	Hs.708017	NM_01538	ENSG0000	HECTD1	EULIR	HECT dom	protein-co
lib-C666-l	chr20	18038492	18038492	+	7.09685	NA	promoter-TSS (NM_021220)	promoter-TSS (NM_021220)	34	NM_02122	58495	Hs.661013	NM_02122	ENSG0000	OVOL2	EUROIMA	ovo like zir	protein-co
lib-C666-l	chr20	18007878	18007878	+	5.9194	NA	intron (NM_001303462, intron 2 of 2)	intron (NM_001303462, intron 2 of	30648	NM_02122	58495	Hs.661013	NM_02122	ENSG0000	OVOL2	EUROIMA	ovo like zir	protein-co
lib-C666-l	chr17	76124985	76124985	+	50.11658	NA	promoter-TSS (NM_001321185)	promoter-TSS (NM_001321185)	-110	NM_00112	11322	Hs.632227	NM_00726	ENSG0000	TMC6	EV1|EVER1	transmemb	protein-co
lib-C666-l	chr22	38330073	38330073	+	57.47389	NA	intron (NM_033386, intron 13 of 15)	intron (NM_033386, intron 13 of 15	19581	NM_03256	84645	Hs.517612	NM_03256	ENSG0000	C22orf23	EVG1|dJ10	chromosor	protein-co
lib-C666-l	chr6	21588286	21588286	+	8.73012	NA	Intergenic	CpG	-5686	NM_00310	6659	Hs.643910	NM_00310	ENSG0000	SOX4	EVI16	SRY-box 4	protein-co
lib-C666-l	chr6	21587924	21587924	+	7.15277	NA	Intergenic	CpG	-6048	NM_00310	6659	Hs.643910	NM_00310	ENSG0000	SOX4	EVI16	SRY-box 4	protein-co
lib-C666-l	chr2	131862852	131862852	+	13.8811	NA	5' UTR (NM_001100623, exon 1 of 8)	5' UTR (NM_001100623, exon 1 of 8	432	NM_00126	55041	Hs.469944	NM_01795	ENSG0000	PLEKHB2	EVT2	pleckstrin h	protein-co
lib-C666-l	chr12	51157677	51157677	+	24.60494	NA	promoter-TSS (NM_005171)	promoter-TSS (NM_005171)	-112	NM_00517	466	Hs.648565	NM_00517	ENSG0000	ATF1	EWS-ATF1	activating t	protein-co
lib-C666-l	chr12	51158054	51158054	+	11.57119	NA	5' UTR (NM_005171, exon 1 of 7)	5' UTR (NM_005171, exon 1 of 7)	265	NM_00517	466	Hs.648565	NM_00517	ENSG0000	ATF1	EWS-ATF1	activating t	protein-co
lib-C666-l	chr12	51158419	51158419	+	4.52748	NA	intron (NM_005171, intron 1 of 6)	CpG	630	NM_00517	466	Hs.648565	NM_00517	ENSG0000	ATF1	EWS-ATF1	activating t	protein-co
lib-C666-l	chr12	54753052	54753052	+	9.87982	NA	intron (NR_120487, intron 1 of 3)	intron (NR_120487, intron 1 of 3)	5218	NM_02037	53831	Hs.306199	NM_02037	ENSG0000	GPR84	EX33|GPCF	G protein-	protein-co
lib-C666-l	chr15	41522987	41522987	+	7.21092	NA	promoter-TSS (NM_001286441)	promoter-TSS (NM_001286441)	-32	NM_15259	161829	Hs.307999	NM_15259	ENSG0000	EXD1	EXDL1	exonucleas	protein-co
lib-C666-l	chr15	89440808	89440808	+	11.93581	NA	Intergenic	Intergenic	-1951	NM_17823	145864	Hs.447530	NM_17823	ENSG0000	HAPLN3	EXLD1|HsT	hyaluronar	protein-co
lib-C666-l	chr13	21476940	21476940	+	54.30825	NA	promoter-TSS (NM_022459)	promoter-TSS (NM_022459)	-27	NM_02245	64328	Hs.507452	NM_02245	ENSG0000	XPO4	exp4	exportin 4	protein-co
lib-C666-l	chr16	28223510	28223510	+	2.10911	NA	promoter-TSS (NM_015171)	promoter-TSS (NM_015171)	-271	NM_01517	23214	Hs.460468	NM_01517	ENSG0000	XPO6	EXP6|RANE	exportin 6	protein-co
lib-C666-l	chr8	21777121	21777121	+	19.19377	NA	promoter-TSS (NM_015024)	promoter-TSS (NM_015024)	-59	NM_01502	23039	Hs.172685	NM_01502	ENSG0000	XPO7	EXP7|RANE	exportin 7	protein-co
lib-C666-l	chr19	1354873	1354873	+	11.66012	NA	promoter-TSS (NM_032853)	promoter-TSS (NM_032853)	-103	NM_03285	84939	Hs.515016	NM_03285	ENSG0000	MUM1	EXPAND1|I	melanoma	protein-co
lib-C666-l	chr19	1355228	1355228	+	3.27468	NA	intron (NM_032853, intron 1 of 13)	CpG	252	NM_03285	84939	Hs.515016	NM_03285	ENSG0000	MUM1	EXPAND1|I	melanoma	protein-co
lib-C666-l	chr8	119124168	119124168	+	26.46807	NA	promoter-TSS (NM_000127)	promoter-TSS (NM_000127)	-110	NM_00012	2131	Hs.492618	NM_00012	ENSG0000	EXT1	EXT|LGCR|I	exostosin g	protein-co
lib-C666-l	chr1	101360405	101360405	+	6.32351	NA	non-coding (NR_048570, exon 1 of 6	non-coding (NR_048570, exon 1 of	330	NM_00143	2135	Hs.357637	NM_00143	ENSG0000	EXTL2	EXTR2	exostosin l	protein-co
lib-C666-l	chr12	120639065	120639065	+	14.83525	NA	promoter-TSS (NM_053275)	promoter-TSS (NM_053275)	-29	NR_03892	100506649	Hs.569205	NR_03892	ENSG0000	PXN-AS1	EyeLinc4	PXN antise	ncRNA
lib-C666-l	chr9	110267294	110267294	+	7.0832	NA	Intergenic	Intergenic	-15293	NM_00131	9314	Hs.376206	NM_00423	ENSG0000	KLF4	EZF|GKLF	Kruppel lik	protein-co
lib-C666-l	chr9	110252409	110252409	+	5.04179	NA	promoter-TSS (NM_001314052)	promoter-TSS (NM_001314052)	-408	NM_00131	9314	Hs.376206	NM_00423	ENSG0000	KLF4	EZF|GKLF	Kruppel lik	protein-co
lib-C666-l	chr19	56652420	56652420	+	25.39084	NA	promoter-TSS (NM_001253792)	promoter-TSS (NM_001253792)	-115	NM_00125	55311	Hs.24545	NM_01833	ENSG0000	ZNF444	EZF-2|EZF	zinc finger	protein-co
lib-C666-l	chr19	39881418	39881418	+	100.64755	NA	promoter-TSS (NM_001317770)	promoter-TSS (NM_001317770)	417	NM_00125	54623	Hs.466714	NM_01908	ENSG0000	PAF1	F23149_1|	PAF1 hom	protein-co
lib-C666-l	chr19	39881876	39881876	+	26.80209	NA	promoter-TSS (NR_046384)	promoter-TSS (NR_046384)	-41	NM_01908	54623	Hs.466714	NM_01908	ENSG0000	PAF1	F23149_1|	PAF1 hom	protein-co
lib-C666-l	chr17	8838722	8838722	+	10.71733	NA	intron (NM_001251853, intron 1 of 1	intron (NM_001251853, intron 1 of	-22888	NM_01430	23533	Hs.278901	NM_01430	ENSG0000	PIK3R5	F730038I1	phosphoin	protein-co
lib-C666-l	chr12	6172532	6172532	+	5.25516	NA	intron (NM_000552, intron 12 of 51)	intron (NM_000552, intron 12 of 51	61309	NM_00055	7450	Hs.440848	NM_00055	ENSG0000	VWF	F8VWF|VW	von Willeb	protein-co
lib-C666-l	chr12	6187757	6187757	+	4.49043	NA	intron (NM_000552, intron 6 of 51)	intron (NM_000552, intron 6 of 51)	46084	NM_00055	7450	Hs.440848	NM_00055	ENSG0000	VWF	F8VWF|VW	von Willeb	protein-co
lib-C666-l	chr16	89883184	89883184	+	4.52748	NA	promoter-TSS (NM_001286167)	promoter-TSS (NM_001286167)	-119	NM_00128	2175	Hs.744083	NM_00013	ENSG0000	FANCA	FA|FA-H|F	Fanconi an	protein-co
lib-C666-l	chr9	98079994	98079994	+	10.71733	NA	promoter-TSS (NM_001243744)	promoter-TSS (NM_001243744)	-3	NM_00013	2176	Hs.494529	NM_00013	ENSG0000	FANCC	FA3|FAC|F	Fanconi an	protein-co
lib-C666-l	chr16	74794480	74794480	+	8.71138	NA	intron (NM_024306, intron 1 of 6)	intron (NM_024306, intron 1 of 6)	14249	NM_02430	79152	Hs.461329	NM_02430	ENSG0000	FA2H	FAAH|FAH	fatty acid 2	protein-co
lib-C666-l	chr16	74808814	74808814	+	5.96761	NA	promoter-TSS (NM_024306)	promoter-TSS (NM_024306)	-85	NM_02430	79152	Hs.461329	NM_02430	ENSG0000	FA2H	FAAH|FAH	fatty acid 2	protein-co
lib-C666-l	chr16	74777898	74777898	+	5.96422	NA	intron (NM_024306, intron 1 of 6)	intron (NM_024306, intron 1 of 6)	30831	NM_02430	79152	Hs.461329	NM_02430	ENSG0000	FA2H	FAAH|FAH	fatty acid 2	protein-co
lib-C666-l	chr14	45605001	45605001	+	8.91001	NA	promoter-TSS (NM_001308134)	promoter-TSS (NM_001308134)	-141	NM_00130	57697	Hs.509229	NM_02093	ENSG0000	FANCM	FAAP250|K	Fanconi an	protein-co
lib-C666-l	chr2	58468622	58468622	+	10.73451	NA	promoter-TSS (NM_018062)	promoter-TSS (NM_018062)	-107	NM_00111	55120	Hs.631890	NM_01806	ENSG0000	FANCL	FAAP43|PH	Fanconi an	protein-co
lib-C666-l	chr6	35310246	35310246	+	16.59065	NA	promoter-TSS (NM_006238)	promoter-TSS (NM_006238)	-89	NM_17743	5467	Hs.696032	NM_00623	ENSG0000	PPARD	FAAR|NR1	peroxisom	protein-co

lib-C666-l	chr7	55322665	55322665	+	23.60036	NA	intron (NR_110426, intron 2 of 3)	CpG	663	NR_11042	102725541	Hs.590524	NR_110426		ELDR	Fabl|LINC0	EGFR long	ncRNA
lib-C666-l	chr7	152133902	152133902	+	15.33659	NA	promoter-TSS (NM_170606)	promoter-TSS (NM_170606)	-78	NR_00293	220832	Hs.647108	NR_00293	ENSG0000	FABP5P3	FABP5L3	fatty acid b	pseudo
lib-C666-l	chr4	111115081	111115081	+	10.03103	NA	intron (NM_001130721, intron 2 of 4)	intron (NM_001130721, intron 2 of	4690	NM_00113	79071	Hs.412939	NM_02409	ENSG0000	ELOVL6	FACE|FAE|L	ELOVL fatt	protein-co
lib-C666-l	chr1	40723764	40723764	+	24.23241	NA	promoter-TSS (NM_005857)	promoter-TSS (NM_005857)	42	NM_00585	10269	Hs.132642	NM_00585	ENSG0000	ZMPSTE24	FACE-1|FA	zinc metall	protein-co
lib-C666-l	chr11	66610915	66610915	+	243.95541	NA	promoter-TSS (NM_005133)	promoter-TSS (NM_005133)	32	NM_00513	9986	Hs.654972	NM_00513	ENSG0000	RCE1	FACE2|RCE	Ras conver	protein-co
lib-C666-l	chr1	154960777	154960777	+	4.7223	NA	exon (NM_001184892, exon 3 of 3)	exon (NM_001184892, exon 3 of 3)	5007	NM_20139	80308	Hs.118666	NM_02520	ENSG0000	FLAD1	FAD1|FADS	flavin aden	protein-co
lib-C666-l	chr3	171757340	171757340	+	18.0551	NA	promoter-TSS (NM_022763)	promoter-TSS (NM_022763)	-78	NM_02276	64778	Hs.744888	NM_02276	ENSG0000	FNDC3B	FAD104|PR	fibronectin	protein-co
lib-C666-l	chr3	10068097	10068097	+	434.4169	NA	promoter-TSS (NM_033084)	promoter-TSS (NM_033084)	26	NM_00101	2177	Hs.208388	NM_03308	ENSG0000	FANCD2	FA-D2|FA4	Fanconi an	protein-co
lib-C666-l	chr10	102106872	102106872	+	40.99587	NA	promoter-TSS (NM_005063)	promoter-TSS (NM_005063)	100	NM_00506	6319	Hs.558396	NM_00506	ENSG0000	SCD	FADS5|MS	stearoyl-C	protein-co
lib-C666-l	chr11	22647424	22647424	+	23.34793	NA	promoter-TSS (NM_022725)	promoter-TSS (NM_022725)	-37	NM_02272	2188	Hs.632151	NM_02272	ENSG0000	FANCF	FAF	Fanconi an	protein-co
lib-C666-l	chr11	22647034	22647034	+	4.04037	NA	exon (NM_022725, exon 1 of 1)	exon (NM_022725, exon 1 of 1)	353	NM_02272	2188	Hs.632151	NM_02272	ENSG0000	FANCF	FAF	Fanconi an	protein-co
lib-C666-l	chr3	138327573	138327573	+	7.49163	NA	promoter-TSS (NM_001033030)	promoter-TSS (NM_001033030)	31	NM_00103	55179	Hs.173438	NM_01814	ENSG0000	FAIM	FAIM1	Fas apopto	protein-co
lib-C666-l	chr1	207083038	207083038	+	13.33918	NA	intron (NM_005449, intron 6 of 7)	CpG-2116	12197	NM_00119	9214	Hs.58831	NM_00544	ENSG0000	FCMR	FAIM3|TOS	Fc fragmen	protein-co
lib-C666-l	chr16	4665030	4665030	+	170.82079	NA	promoter-TSS (NM_145253)	promoter-TSS (NM_145253)	-103	NM_14525	124402	Hs.513313	NM_14525	ENSG0000	UBALD1	FAM100A|I	UBA like d	protein-co
lib-C666-l	chr16	4666293	4666293	+	91.98129	NA	Intergenic	CpG-8803	-1366	NM_14525	124402	Hs.513313	NM_14525	ENSG0000	UBALD1	FAM100A|I	UBA like d	protein-co
lib-C666-l	chr17	74261210	74261210	+	11.66012	NA	promoter-TSS (NM_182565)	promoter-TSS (NM_182565)	-76	NM_18256	283991	Hs.744981	NM_18256	ENSG0000	UBALD2	FAM100B	UBA like d	protein-co
lib-C666-l	chr7	143599135	143599135	+	3.8557	NA	promoter-TSS (NM_014719)	promoter-TSS (NM_014719)	37	NM_01471	9747	Hs.735149	NM_01471	ENSG0000	TCAF1	FAM115A	TRPM8 cha	protein-co
lib-C666-l	chr2	208490173	208490173	+	19.82698	NA	promoter-TSS (NM_001330136)	promoter-TSS (NM_001330136)	-71	NM_00133	151194	Hs.664764	NM_14528	ENSG0000	METTL21A	FAM119A|I	methyltran	protein-co
lib-C666-l	chr2	120980780	120980780	+	4.52748	NA	5' UTR (NM_024121, exon 1 of 1)	5' UTR (NM_024121, exon 1 of 1)	204	NM_02412	79134	Hs.376722	NM_02412	ENSG0000	TMEM185B	FAM11B	transmemb	protein-co
lib-C666-l	chrX	63425727	63425727	+	4.90117	NA	promoter-TSS (NM_152424)	promoter-TSS (NM_152424)	-103	NM_15242	139285	Hs.314225	NM_15242	ENSG0000	AMER1	FAM123B|(	APC memb	protein-co
lib-C666-l	chr2	130939760	130939760	+	2.42134	NA	promoter-TSS (NM_017951)	promoter-TSS (NM_017951)	265	NM_02502	80097	Hs.469925	NM_02502	ENSG0000	MZT2B	FAM128B|I	mitotic spi	protein-co
lib-C666-l	chr16	10936933	10936933	+	12.62235	NA	Intergenic	Intergenic	-24282	NR_13490	780776	Hs.371576	NM_00107	ENSG0000	TVP23A	FAM18A|YI	trans-golg	protein-co
lib-C666-l	chr16	10915103	10915103	+	3.2667	NA	Intergenic	Intergenic	-2452	NR_13490	780776	Hs.371576	NM_00107	ENSG0000	TVP23A	FAM18A|YI	trans-golg	protein-co
lib-C666-l	chr10	86287384	86287384	+	5.34142	NA	Intergenic	HERV17-int|LTR|ERV1	102446	NM_00128	54462	Hs.461988	NM_01899	ENSG0000	CCSER2	FAM190B|(	coiled-coil	protein-co
lib-C666-l	chr1	16993724	16993724	+	182.3006	NA	Intergenic	Intergenic	-5774	NM_00128	729574		NM_00128	ENSG0000	FAM231A	FAM231C	family with	protein-co
lib-C666-l	chr1	16992912	16992912	+	4.45654	NA	Intergenic	Intergenic	-6586	NM_00128	729574		NM_00128	ENSG0000	FAM231A	FAM231C	family with	protein-co
lib-C666-l	chr10	49197534	49197534	+	22.01132	NA	Intergenic	Intergenic	10291	NM_00113	100133093	Hs.723078	NM_00113	ENSG0000	FAM25G	FAM25A|F	family with	protein-co
lib-C666-l	chr10	49196157	49196157	+	3.92495	NA	Intergenic	Intergenic	11668	NM_00113	100133093	Hs.723078	NM_00113	ENSG0000	FAM25G	FAM25A|F	family with	protein-co
lib-C666-l	chr9	45729200	45729200	+	31.30805	NA	Intergenic	SST1|Satellite|centr	-387743	NR_10371	100289124	Hs.567050	NM_001024608		FAM27E2	FAM27E1	family with	ncRNA
lib-C666-l	chr19	6476311	6476311	+	7.11942	NA	intron (NM_024898, intron 10 of 22)	intron (NM_024898, intron 10 of 22	5508	NM_00129	79958	Hs.236449	NM_02489	ENSG0000	DENND1C	FAM31C	DENN dom	protein-co
lib-C666-l	chr17	57232771	57232771	+	4.52748	NA	promoter-TSS (NM_018304)	promoter-TSS (NM_018304)	29	NM_00133	348235	Hs.463607	NM_18262	ENSG0000	SKA2	FAM33A	spindle an	protein-co
lib-C666-l	chr10	88855126	88855126	+	9.79436	NA	promoter-TSS (NM_001318905)	promoter-TSS (NM_001318905)	173	NM_01905	54537	Hs.500419	NM_01905	ENSG0000	FAM35A	FAM35A1|I	family with	protein-co
lib-C666-l	chr10	88855381	88855381	+	2.78587	NA	promoter-TSS (NM_001318905)	promoter-TSS (NM_001318905)	428	NM_01905	54537	Hs.500419	NM_01905	ENSG0000	FAM35A	FAM35A1|I	family with	protein-co
lib-C666-l	chr10	47396050	47396050	+	10.71733	NA	intron (NR_027634, intron 2 of 6)	CpG	16330	NR_02763	439965	Hs.283521	NR_027634		FAM35DP	FAM35B2	family with	pseudo
lib-C666-l	chr15	102501723	102501723	+	9.75684	NA	promoter-TSS (NR_036051)	promoter-TSS (NR_036051)	707	NR_00365	374666	Hs.459573	NM_199163		WASH3P	FAM39DP	WAS prote	pseudo
lib-C666-l	chr1	29195	29195	+	10.92275	NA	intron (NR_024540, intron 1 of 10)	CpG	175	NR_02454	653635	Hs.446466	NR_024540		WASH7P	FAM39F|W	WAS prote	pseudo
lib-C666-l	chr1	110577421	110577421	+	3.64267	NA	exon (NM_033088, exon 1 of 21)	exon (NM_033088, exon 1 of 21)	198	NM_03308	85369	Hs.584996	NM_03308	ENSG0000	STRIP1	FAM40A|F	striatin inte	protein-co
lib-C666-l	chr7	129074297	129074297	+	6.74574	NA	promoter-TSS (NM_020704)	promoter-TSS (NM_020704)	23	NM_00113	57464	Hs.489988	NM_02070	ENSG0000	STRIP2	FAM40B|F	striatin inte	protein-co
lib-C666-l	chr5	173043763	173043763	+	160.63332	NA	promoter-TSS (NM_001159651)	promoter-TSS (NM_001159651)	-97	NM_13836	91272	Hs.425091	NM_13836	ENSG0000	BOD1	FAM44B	biorientati	protein-co
lib-C666-l	chrX	14048080	14048080	+	55.46257	NA	promoter-TSS (NM_017856)	promoter-TSS (NM_017856)	-45	NM_00104	54960	Hs.592237	NM_01785	ENSG0000	GEMIN8	FAM51A1	gem nucle	protein-co
lib-C666-l	chr1	26137456	26137456	+	136.28183	NA	intron (NM_206926, intron 6 of 11)	intron (NM_206926, intron 6 of 11)	-8941	NM_00109	56181	Hs.149305	NM_01955	ENSG0000	MTFR1L	FAM54B|H	mitochond	protein-co
lib-C666-l	chr12	56521789	56521789	+	14.45701	NA	promoter-TSS (NM_015292)	promoter-TSS (NM_015292)	-197	NM_00118	23344	Hs.632729	NM_01529	ENSG0000	ESYT1	FAM62A|M	extended s	protein-co
lib-C666-l	chr9	84614800	84614800	+	5.5166	NA	Intergenic	L1MB4|LINE|L1	11113	NM_00100	389763	Hs.209545	NM_00100	ENSG0000	SPATA31D	FAM75D1	SPATA31 s	protein-co
lib-C666-l	chr1	31643772	31643772	+	15.22999	NA	Intergenic	Intergenic	68962	NM_02452	79570	Hs.470259	NM_02452	ENSG0000	NKAIN1	FAM77C	Sodium/pc	protein-co
lib-C666-l	chr1	31628428	31628428	+	13.33883	NA	Intergenic	CpG	84306	NM_02452	79570	Hs.470259	NM_02452	ENSG0000	NKAIN1	FAM77C	Sodium/pc	protein-co
lib-C666-l	chr8	63442945	63442945	+	6.3317	NA	intron (NR_130764, intron 1 of 6)	intron (NR_130764, intron 1 of 6)	281444	NM_00130	286183	Hs.654662	NM_17368	ENSG0000	NKAIN3	FAM77D	Sodium/pc	protein-co
lib-C666-l	chr1	3541454	3541454	+	8.28216	NA	promoter-TSS (NM_182752)	promoter-TSS (NM_182752)	-102	NM_18275	127262	Hs.20529	NM_18275	ENSG0000	TPRG1L	FAM79A|h	tumor prot	protein-co
lib-C666-l	chr3	42003783	42003783	+	14.02822	NA	non-coding (NR_136342, exon 1 of 3	non-coding (NR_136342, exon 1 of	149	NR_13634	54986	Hs.656192	NM_01788	ENSG0000	ULK4	FAM7C1|R	unc-51 like	protein-co
lib-C666-l	chr3	42055098	42055098	+	3.27468	NA	Intergenic	CpG	-51166	NM_00132	54986	Hs.656192	NM_01788	ENSG0000	ULK4	FAM7C1|R	unc-51 like	protein-co
lib-C666-l	chr15	41047487	41047487	+	300.1926	NA	promoter-TSS (NM_001323894)	promoter-TSS (NM_001323894)	47	NM_00132	55177	Hs.511067	NM_01814	ENSG0000	RMDN3	FAM82A2|I	regulator c	protein-co
lib-C666-l	chr16	5148180	5148180	+	6.49644	NA	promoter-TSS (NM_001289029)	promoter-TSS (NM_001289029)	-359	NM_20159	196483	Hs.406461	NM_20140	ENSG0000	EEF2KMT	FAM86A|SI	eukaryotic	protein-co
lib-C666-l	chr8	7468912	7468912	+	11.00418	NA	Intergenic	CpG	-36992	NR_04634	441317	Hs.715411	NM_001136572		FAM90A7P	FAM90A7	putative pr	pseudo
lib-C666-l	chr8	7448450	7448450	+	7.69829	NA	Intergenic	HERVE-int|LTR|ERV1	-16530	NR_04634	441317	Hs.715411	NM_001136572		FAM90A7P	FAM90A7	putative pr	pseudo
lib-C666-l	chr8	7448793	7448793	+	4.78788	NA	Intergenic	HERVE-int|LTR|ERV1	-16873	NR_04634	441317	Hs.715411	NM_001136572		FAM90A7P	FAM90A7	putative pr	pseudo
lib-C666-l	chr4	183899772	183899772	+	3.84463	NA	Intergenic	L1PBa1|LINE|L1	-59046	NR_00361	403315	Hs.652187	NR_00361	ENSG0000	FAM92A1P	FAM92A3	family with	pseudo
lib-C666-l	chr7	5632419	5632419	+	24.25638	NA	promoter-TSS (NM_003088)	promoter-TSS (NM_003088)	-17	NM_00308	6624	Hs.118400	NM_00308	ENSG0000	FSCN1	FAN1|HSN	fascin actir	protein-co
lib-C666-l	chr1	202311227	202311227	+	25.38683	NA	promoter-TSS (NM_014176)	promoter-TSS (NM_014176)	-133	NM_00131	29089	Hs.5199	NM_01417	ENSG0000	UBE2T	FANCT|HSI	ubiquitin c	protein-co
lib-C666-l	chr7	152373359	152373359	+	165.35788	NA	promoter-TSS (NM_005431)	promoter-TSS (NM_005431)	-109	NM_00543	7516	Hs.647093	NM_00543	ENSG0000	XRCC2	FANCU	X-ray repa	protein-co
lib-C666-l	chr7	152406955	152406955	+	35.90556	NA	Intergenic	Intergenic	-33705	NM_00543	7516	Hs.647093	NM_00543	ENSG0000	XRCC2	FANCU	X-ray repa	protein-co
lib-C666-l	chr7	152272167	152272167	+	18.36277	NA	Intergenic	Intergenic	101083	NM_00543	7516	Hs.647093	NM_00543	ENSG0000	XRCC2	FANCU	X-ray repa	protein-co
lib-C666-l	chr1	11741188	11741188	+	61.18587	NA	promoter-TSS (NM_006341)	promoter-TSS (NM_006341)	83	NM_00634	10459	Hs.19400	NM_00634	ENSG0000	MAD2L2	FANCV|MA	MAD2 mit	protein-co
lib-C666-l	chr1	92764647	92764647	+	4.52748	NA	promoter-TSS (NM_001319683)	promoter-TSS (NM_001319683)	-81	NR_13508	11146	Hs.49105	NM_00707	ENSG0000	GLMN	FAP|FAP48	glomulin, F	protein-co
lib-C666-l	chr7	158649507	158649507	+	4.30593	NA	intron (NM_018051, intron 1 of 24)	CpG	238	NM_01805	55112	Hs.389945	NM_01805	ENSG0000	WDR60	FAP163|SR	WD repeat	protein-co
lib-C666-l	chr16	2097877	2097877	+	11.0277	NA	promoter-TSS (NM_001318829)	promoter-TSS (NM_001318829)	-7	NM_00252	4913	Hs.66196	NM_00252	ENSG0000	NTHL1	FAP3|NTH	nth like DN	protein-co
lib-C666-l	chr16	2093972	2093972	+	4.96104	NA	intron (NM_001318193, intron 2 of 4)	intron (NM_001318193, intron 2 of	3898	NM_00131	4913	Hs.66196	NM_00252	ENSG0000	NTHL1	FAP3|NTH	nth like DN	protein-co
lib-C666-l	chr2	179345082	179345082	+	6.82479	NA	promoter-TSS (NM_019091)	promoter-TSS (NM_019091)	-117	NM_01909	65977	Hs.41086	NM_01909	ENSG0000	PLEKHA3	FAPP1	pleckstrin h	protein-co
lib-C666-l	chr7	30067873	30067873	+	7.7703	NA	promoter-TSS (NM_001197026)	promoter-TSS (NM_001197026)	-104	NM_00119	84725	Hs.233495	NM_03263	ENSG0000	PLEKHA8	FAPP2	pleckstrin h	protein-co
lib-C666-l	chr7	30068117	30068117	+	2.59032	NA	5' UTR (NM_001197026, exon 1 of 14	5' UTR (NM_001197026, exon 1 of 1	140	NM_00119	84725	Hs.233495	NM_03263	ENSG0000	PLEKHA8	FAPP2	pleckstrin h	protein-co
lib-C666-l	chr22	43525256	43525256	+	5.80117	NA	exon (NM_001197, exon 5 of 5)	exon (NM_001197, exon 5 of 5)	14147	NR_04642	27349	Hs.349111	NM_01450	ENSG0000	MCAT	FASN2C|M	malonyl-C	protein-co
lib-C666-l	chr3	25831565	25831565	+	5.23609	NA	promoter-TSS (NM_001145294)	promoter-TSS (NM_001145294)	2	NM_00114	54995	Hs.55781	NM_01789	ENSG0000	OXSM	FASN2D|K	3-oxoacyl-	protein-co
lib-C666-l	chr2	239197530	239197530	+	24.31114	NA	promoter-TSS (NM_022817)	promoter-TSS (NM_022817)	-323	NM_02281	8864	Hs.58756	NM_00389	ENSG0000	PER2	FASPS|FAS	period circ	protein-co
lib-C666-l	chr1	7844178	7844178	+	13.77909	NA	promoter-TSS (NM_001289861)	promoter-TSS (NM_001289861)	-311	NM_01683	8863	Hs.162200	NM_01683	ENSG0000	PER3	FASPS3|GI	period circ	protein-co
lib-C666-l	chr7	150778041	150778041	+	7.50041	NA	promoter-TSS (NM_001258461)	promoter-TSS (NM_001258461)	-71	NM_03301	10922	Hs.647094	NM_00671	ENSG0000	FASTK	FAST	Fas activat	protein-co
lib-C666-l	chr11	64889678	64889678	+	100.64755	NA	promoter-TSS (NM_001997)	promoter-TSS (NM_001997)	-6	NM_00199	2197	Hs.387208	NM_00199	ENSG0000	FAU	FAU1|Fub1	FAU, ubiqu	protein-co
lib-C666-l	chr19	36193389	36193389	+	9.10494	NA	Intergenic	CpG	-2000	NM_01438	27033	Hs.99430	NM_01438	ENSG0000	ZBTB32	FAXF|FAZF	zinc finger	protein-co
lib-C666-l	chr19	54619125	54619125	+	121.01157	NA	promoter-TSS (NM_001321792)	promoter-TSS (NM_001321792)	-70	NM_01334	29844	Hs.590939	NM_01334	ENSG0000	TFPT	FB1|INO80	TCF3 fusio	protein-co
lib-C666-l	chr11	33795651	33795651	+	6.40797	NA	promoter-TSS (NR_120550)	promoter-TSS (NR_120550)	420	NM_01217	26273	Hs.406787	NM_01217	ENSG0000	FBXO3	FBA|FBX3	F-box prot	protein-co
lib-C666-l	chr1	11727713	11727713	+	11.6145	NA	intron (NM_018438, intron 1 of 5)	intron (NM_018438, intron 1 of 5)	3563	NM_01843	26270	Hs.464419	NM_01843	ENSG0000	FBXO6	FBG2|FBS2	F-box prot	protein-co
lib-C666-l	chr1	11724158	11724158	+	6.91525	NA	promoter-TSS (NM_018438)	promoter-TSS (NM_018438)	8	NM_01843	26270	Hs.464419	NM_01843	ENSG0000	FBXO6	FBG2|FBS2	F-box prot	protein-co
lib-C666-l	chr10	5932016	5932016	+	13.8811	NA	promoter-TSS (NM_001009941)	promoter-TSS (NM_001009941)	-150	NM_17815	84893	Hs.498543	NM_03280	ENSG0000	FBXO18	FBH1|Fbx1	F-box prot	protein-co
lib-C666-l	chr19	4067580	4067580	+	11.06679	NA	promoter-TSS (NM_015898)	promoter-TSS (NM_015898)	-637	NM_01589	51341	Hs.591384	NM_01589	ENSG0000	ZBTB7A	FBI-1|FBI1|	zinc finger	protein-co
lib-C666-l	chr19	9929561	9929561	+	2.42134	NA	5' UTR (NM_001316936, exon 1 of 2)	5' UTR (NM_001316936, exon 1 of	218	NM_00131	54850	Hs.12439	NM_01770	ENSG0000	FBXL12	Fbl12	F-box and	protein-co
lib-C666-l	chr12	1703200	1703200	+	5.64185	NA	exon (NM_152441, exon 1 of 2)	exon (NM_152441, exon 1 of 2)	131	NM_15244	144699	Hs.367956	NM_15244	ENSG0000	FBXL14	Fbl14	F-box and	protein-co
lib-C666-l	chr7	5553437	5553437	+	215.3851	NA	promoter-TSS (NR_126168)	promoter-TSS (NR_126168)	-8	NM_02496	80028	Hs.623974	NM_02496	ENSG0000	FBXL18	Fbl18	F-box and	protein-co
lib-C666-l	chr17	37558939	37558939	+	57.42312	NA	Intergenic	CpG-10535	-1030	NM_03287	84961	Hs.462946	NM_03287	ENSG0000	FBXL20	Fbl2|Fbl20	F-box and	protein-co
lib-C666-l	chr17	37557886	37557886	+	8.28333	NA	promoter-TSS (NM_032875)	promoter-TSS (NM_032875)	23	NM_00118	84961	Hs.462946	NM_03287	ENSG0000	FBXL20	Fbl2|Fbl20	F-box and	protein-co
lib-C666-l	chr13	77601098	77601098	+	5.25516	NA	5' UTR (NM_012158, exon 1 of 5)	5' UTR (NM_012158, exon 1 of 5)	233	NM_01215	26224	Hs.508284	NM_01215	ENSG0000	FBXL3	FBL3|FBL3	F-box and	protein-co
lib-C666-l	chr4	15656660	15656660	+	4.52748	NA	intron (NR_036464, intron 1 of 11)	CpG	375	NM_01216	26234	Hs.643433	NM_01216	ENSG0000	FBXL5	FBL4|FBL5|	F-box and	protein-co
lib-C666-l	chr8	145582183	145582183	+	9.10494	NA	promoter-TSS (NM_012162)	promoter-TSS (NM_012162)	0	NM_01216	26233	Hs.12271	NM_01216	ENSG0000	FBXL6	FBL6|FBL6	F-box and	protein-co
lib-C666-l	chr5	15500305	15500305	+	3.07144	NA	promoter-TSS (NM_012304)	promoter-TSS (NM_012304)	0	NM_01230	23194	Hs.433057	NM_01230	ENSG0000	FBXL7	FBL6|FBL7	F-box and	protein-co
lib-C666-l	chr9	97431979	97431979	+	7.7487	NA	Intergenic	AluY|SINE|Alu	-29448	NM_00112	2203	Hs.494496	NM_00050	ENSG0000	FBP1	FBP	fructose-b	protein-co
lib-C666-l	chr9	123968947	123968947	+	5.04179	NA	Intergenic	Intergenic	-4582	NM_01632	51552	Hs.371563	NM_01632	ENSG0000	RAB14	FBP|RAB-1	RAB14, me	protein-co
lib-C666-l	chr2	153574080	153574080	+	48.40399	NA	promoter-TSS (NM_152522)	promoter-TSS (NM_152522)	-105	NM_01789	55660	Hs.643580	NM_01789	ENSG0000	PRPF40A	FBP-11|FBI	pre-mRNA	protein-co
lib-C666-l	chr9	132772882	132772882	+	5.96761	NA	intron (NM_015033, intron 1 of 16)	MLT1L|LTR|ERVL-MaLR	32591	NM_01503	23048	Hs.189409	NM_01503	ENSG0000	FNBP1	FBP17	formin bin	protein-co
lib-C666-l	chr19	6424222	6424222	+	34.73338	NA	intron (NM_003685, intron 1 of 19)	intron (NM_003685, intron 1 of 19)	600	NM_00368	8570	Hs.727344	NM_00368	ENSG0000	KHSRP	FBP2|FUBP	KH-type sp	protein-co
lib-C666-l	chr19	6424950	6424950	+	28.88196	NA	promoter-TSS (NM_003685)	promoter-TSS (NM_003685)	-128	NM_00368	8570	Hs.727344	NM_00368	ENSG0000	KHSRP	FBP2|FUBP	KH-type sp	protein-co
lib-C666-l	chr19	6425430	6425430	+	9.79436	NA	promoter-TSS (NM_003685)	promoter-TSS (NM_003685)	-608	NM_00368	8570	Hs.727344	NM_00368	ENSG0000	KHSRP	FBP2|FUBP	KH-type sp	protein-co
lib-C666-l	chr13	41635639	41635639	+	6.82479	NA	promoter-TSS (NM_007187)	promoter-TSS (NM_007187)	-58	NM_00718	11193	Hs.411300	NM_00718	ENSG0000	WBP4	FBP21	WW doma	protein-co
lib-C666-l	chr13	41636075	41636075	+	5.9099	NA	intron (NM_007187, intron 1 of 9)	CpG-6388	378	NM_00718	11193	Hs.411300	NM_00718	ENSG0000	WBP4	FBP21	WW doma	protein-co
lib-C666-l	chr13	41634517	41634517	+	3.98718	NA	Intergenic	CpG	-1180	NM_00718	11193	Hs.411300	NM_00718	ENSG0000	WBP4	FBP21	WW doma	protein-co
lib-C666-l	chr16	30669541	30669541	+	33.76759	NA	Intergenic	CpG	-1299	NM_00110	64319	Hs.247186	NM_02245	ENSG0000	FBRS	FBS|FBS1	fibrosin	protein-co
lib-C666-l	chr16	30670607	30670607	+	2.26435	NA	promoter-TSS (NM_001105079)	promoter-TSS (NM_001105079)	-233	NM_00110	64319	Hs.247186	NM_02245	ENSG0000	FBRS	FBS|FBS1	fibrosin	protein-co
lib-C666-l	chr9	123555688	123555688	+	62.55363	NA	promoter-TSS (NM_012164)	promoter-TSS (NM_012164)	52	NM_01216	26190	Hs.494985	NM_01216	ENSG0000	FBXW2	FBW2|Fwd	F-box and	protein-co
lib-C666-l	chr22	23634886	23634886	+	3.48675	NA	intron (NM_021574, intron 15 of 21)	intron (NM_021574, intron 15 of 21	29932	NR_03340	26226	Hs.729589	NR_03340	ENSG0000	FBXW4P1	FBW3|FBX	F-box and	pseudo
lib-C666-l	chr9	139839167	139839167	+	46.4068	NA	promoter-TSS (NM_000606)	promoter-TSS (NM_000606)	39	NM_01899	54461	Hs.522507	NM_01899	ENSG0000	FBXW5	Fbw5	F-box and	protein-co
lib-C666-l	chr19	12807489	12807489	+	101.74994	NA	promoter-TSS (NM_032301)	promoter-TSS (NM_032301)	-34	NM_03230	84261	Hs.515154	NM_03230	ENSG0000	FBXW9	Fbw9	F-box and	protein-co
lib-C666-l	chr22	32870924	32870924	+	5.9194	NA	promoter-TSS (NM_001033024)	promoter-TSS (NM_001033024)	217	NM_01217	25793	Hs.5912	NM_01217	ENSG0000	FBXO7	FBX|FBX07	F-box prot	protein-co
lib-C666-l	chr16	2479206	2479206	+	6.0459	NA	promoter-TSS (NM_001323538)	promoter-TSS (NM_001323538)	-186	NM_00132	899	Hs.1973	NM_00176	ENSG0000	CCNF	FBX1|FBXC	cyclin F	protein-co
lib-C666-l	chr16	87422144	87422144	+	12.73831	NA	intron (NM_001282683, intron 2 of 9)	intron (NM_001282683, intron 2 of	3569	NM_00128	79791	Hs.567582	NM_02473	ENSG0000	FBXO31	FBX14|FBX	F-box prot	protein-co
lib-C666-l	chr15	76196285	76196285	+	18.89222	NA	promoter-TSS (NM_012170)	promoter-TSS (NM_012170)	85	NM_14718	26263	Hs.591115	NM_01217	ENSG0000	FBXO22	FBX22|FIST	F-box prot	protein-co
lib-C666-l	chr7	100183958	100183958	+	23.1305	NA	promoter-TSS (NM_001289934)	promoter-TSS (NM_001289934)	2	NM_03350	26261	Hs.283764	NM_01217	ENSG0000	FBXO24	FBX24	F-box prot	protein-co
lib-C666-l	chr8	356995	356995	+	9.06551	NA	5' UTR (NM_012173, exon 1 of 9)	5' UTR (NM_012173, exon 1 of 9)	187	NM_01217	26260	Hs.438454	NM_01217	ENSG0000	FBXO25	FBX25	F-box prot	protein-co
lib-C666-l	chr2	230787712	230787712	+	4.40211	NA	promoter-TSS (NM_001284216)	promoter-TSS (NM_001284216)	505	NM_17489	130888	Hs.140666	NM_17489	ENSG0000	FBXO36	Fbx36	F-box prot	protein-co
lib-C666-l	chr5	147763490	147763490	+	6.71839	NA	promoter-TSS (NR_105057)	promoter-TSS (NR_105057)	-8	NM_03079	81545	Hs.483772	NM_03079	ENSG0000	FBXO38	Fbx38|HMI	F-box prot	protein-co
lib-C666-l	chr1	16679014	16679014	+	19.82221	NA	promoter-TSS (NM_018994)	promoter-TSS (NM_018994)	-28	NM_01899	54455	Hs.522384	NM_01899	ENSG0000	FBXO42	Fbx42|JFK	F-box prot	protein-co
lib-C666-l	chr5	107718007	107718007	+	5.6488	NA	promoter-TSS (NM_001163315)	promoter-TSS (NM_001163315)	-208	NM_00116	64839	Hs.657225	NM_02282	ENSG0000	FBXL17	FBXO13|Fb	F-box and	protein-co
lib-C666-l	chr19	39684809	39684809	+	17.51603	NA	Intergenic	Intergenic	-2795	NM_00100	342897	Hs.726934	NM_00100	ENSG0000	NCCRP1	FBXO50|N	non-specif	protein-co
lib-C666-l	chr19	39685908	39685908	+	5.5166	NA	Intergenic	MIR|SINE|MIR	-1696	NM_00100	342897	Hs.726934	NM_00100	ENSG0000	NCCRP1	FBXO50|N	non-specif	protein-co
lib-C666-l	chr3	142297506	142297506	+	2.92124	NA	exon (NM_001184, exon 1 of 47)	exon (NM_001184, exon 1 of 47)	162	NM_00118	545	Hs.271791	NM_00118	ENSG0000	ATR	FCTCS|FRP	ATR serine	protein-co
lib-C666-l	chr3	120461499	120461499	+	124.4398	NA	promoter-TSS (NM_005513)	promoter-TSS (NM_005513)	-59	NM_00551	2960	Hs.445272	NM_00551	ENSG0000	GTF2E1	FE|TF2E1|T	general tra	protein-co
lib-C666-l	chr4	41218669	41218669	+	13.59254	NA	Intergenic	CpG	-2034	NM_00430	323	Hs.479602	NM_00430	ENSG0000	APBB2	FE65L|FE65	amyloid be	protein-co
lib-C666-l	chr4	40858973	40858973	+	3.58839	NA	intron (NM_001166051, intron 1 of 6)	CpG	199	NM_00116	323	Hs.479602	NM_00430	ENSG0000	APBB2	FE65L|FE65	amyloid be	protein-co
lib-C666-l	chr5	139944185	139944185	+	24.41787	NA	promoter-TSS (NM_133173)	promoter-TSS (NM_133173)	4	NM_13317	10307	Hs.529449	NM_00605	ENSG0000	APBB3	FE65L2|SR	amyloid be	protein-co
lib-C666-l	chr5	90228131	90228131	+	13.20416	NA	intron (NR_003149, intron 83 of 89)	intron (NR_003149, intron 83 of 89)	373514	NM_03211	84059	Hs.591777	NM_03211	ENSG0000	ADGRV1	FEB4|GPR9	adhesion G	protein-co
lib-C666-l	chr11	61560374	61560374	+	8.28613	NA	promoter-TSS (NM_014206)	promoter-TSS (NM_014206)	265	NM_00411	2237	Hs.409065	NM_00411	ENSG0000	FEN1	FEN-1|MF1	flap structu	protein-co
lib-C666-l	chr2	71785870	71785870	+	24.28549	NA	intron (NM_001130978, intron 22 of 5	intron (NM_001130978, intron 22 o	92038	NM_00113	8291	Hs.252180	NM_00349	ENSG0000	DYSF	FER1L1|LGI	dysferlin	protein-co
lib-C666-l	chr2	72012223	72012223	+	5.23609	NA	Intergenic	Intergenic	318391	NM_00113	8291	Hs.252180	NM_00349	ENSG0000	DYSF	FER1L1|LGI	dysferlin	protein-co
lib-C666-l	chr10	95231068	95231068	+	11.07709	NA	intron (NM_013451, intron 1 of 53)	(T)n|Simple_repeat|Simple_repeat	11006	NM_13333	26509	Hs.602086	NM_01345	ENSG0000	MYOF	FER1L3	myoferlin	protein-co
lib-C666-l	chr10	95093510	95093510	+	6.49644	NA	intron (NM_013451, intron 42 of 53)	intron (NM_013451, intron 42 of 53	148564	NM_13333	26509	Hs.602086	NM_01345	ENSG0000	MYOF	FER1L3	myoferlin	protein-co
lib-C666-l	chr19	55791495	55791495	+	9.14689	NA	promoter-TSS (NM_001297600)	promoter-TSS (NM_001297600)	256	NM_01226	23640	Hs.53066	NM_01226	ENSG0000	HSPBP1	FES1	HSPA (Hsp	protein-co
lib-C666-l	chr12	71833588	71833588	+	9.79436	NA	promoter-TSS (NM_001277227)	promoter-TSS (NM_001277227)	38	NM_00127	8549	Hs.658889	NM_00366	ENSG0000	LGR5	FEX|GPR49	leucine rich	protein-co
lib-C666-l	chr3	50388408	50388408	+	13.48428	NA	promoter-TSS (NM_006545)	promoter-TSS (NM_006545)	78	NM_00654	10641	Hs.437083	NM_00654	ENSG0000	NPRL2	FFEVF2|NP	NPR2-like,	protein-co
lib-C666-l	chr17	8152607	8152607	+	184.79135	NA	promoter-TSS (NM_012393)	promoter-TSS (NM_012393)	11	NM_01239	5198	Hs.573976	NM_01239	ENSG0000	PFAS	FGAMS|FG	phosphorib	protein-co
lib-C666-l	chr13	22244212	22244212	+	9.94079	NA	Intergenic	CpG	-1003	NM_00201	2254	Hs.111	NM_00201	ENSG0000	FGF9	FGF-9|GAF	fibroblast g	protein-co
lib-C666-l	chr11	65656017	65656017	+	12.58342	NA	promoter-TSS (NM_198897)	promoter-TSS (NM_198897)	-7	NM_19889	9158	Hs.7768	NM_00421	ENSG0000	FIBP	FGFIBP|FIB	FGF1 intrac	protein-co
lib-C666-l	chr7	44224273	44224273	+	26.53025	NA	intron (NM_000162, intron 1 of 9)	intron (NM_000162, intron 1 of 9)	4749	NM_00016	2645	Hs.1270	NM_00016	ENSG0000	GCK	FGQTL3|GI	glucokinas	protein-co
lib-C666-l	chr19	11200175	11200175	+	3.8557	NA	5' UTR (NM_001195803, exon 1 of 16	5' UTR (NM_001195803, exon 1 of 1	137	NM_00119	3949	Hs.213289	NM_00052	ENSG0000	LDLR	FH|FHC|LD	low density	protein-co
lib-C666-l	chr11	61735160	61735160	+	9.45164	NA	promoter-TSS (NM_002032)	promoter-TSS (NM_002032)	-28	NM_00203	2495	Hs.524910	NM_00203	ENSG0000	FTH1	FHC|FTH|F	ferritin hea	protein-co
lib-C666-l	chr11	61793293	61793293	+	8.40444	NA	Intergenic	Intergenic	-58161	NM_00203	2495	Hs.524910	NM_00203	ENSG0000	FTH1	FHC|FTH|F	ferritin hea	protein-co
lib-C666-l	chr6	144471392	144471392	+	14.47615	NA	promoter-TSS (NM_003764)	promoter-TSS (NM_003764)	-262	NM_00376	8676	Hs.118958	NM_00376	ENSG0000	STX11	FHL4|HLH4	syntaxin 11	protein-co
lib-C666-l	chr19	7701982	7701982	+	11.07709	NA	promoter-TSS (NR_073560)	promoter-TSS (NR_073560)	-5	NR_07356	6813	Hs.515104	NM_00694	ENSG0000	STXBP2	FHL5|Hunc	syntaxin bi	protein-co
lib-C666-l	chr2	153191562	153191562	+	3.8557	NA	promoter-TSS (NM_052905)	promoter-TSS (NM_052905)	-189	NM_05290	114793	Hs.654630	NM_05290	ENSG0000	FMNL2	FHOD2	formin like	protein-co
lib-C666-l	chr16	67280174	67280174	+	7.51944	NA	intron (NM_001318202, intron 1 of 2	CpG	1251	NM_01324	29109	Hs.95231	NM_01324	ENSG0000	FHOD1	FHOS	formin hor	protein-co
lib-C666-l	chr18	33827278	33827278	+	6.98764	NA	intron (NM_017947, intron 9 of 14)	L1PB1|LINE|L1	-50381	NM_00128	80206	Hs.436636	NM_02513	ENSG0000	FHOD3	FHOS2|For	formin hor	protein-co
lib-C666-l	chr12	8179131	8179131	+	13.46097	NA	Intergenic	Intergenic	-6228	NM_01841	55810	Hs.120844	NM_01841	ENSG0000	FOXJ2	FHX	forkhead b	protein-co
lib-C666-l	chr19	40337028	40337028	+	8.89416	NA	promoter-TSS (NM_001436)	promoter-TSS (NM_001436)	26	NM_00143	2091	Hs.299002	NM_00143	ENSG0000	FBL	FIB|FLRN|R	fibrillarin	protein-co
lib-C666-l	chr19	50400737	50400737	+	64.69308	NA	promoter-TSS (NM_152899)	promoter-TSS (NM_152899)	-590	NM_15289	259307	Hs.574492	NM_15289	ENSG0000	IL4I1	FIG1|LAAO	interleukin	protein-co
lib-C666-l	chr19	50401230	50401230	+	7.49163	NA	intron (NR_047577, intron 4 of 10)	intron (NR_047577, intron 4 of 10)	-1083	NM_15289	259307	Hs.574492	NM_15289	ENSG0000	IL4I1	FIG1|LAAO	interleukin	protein-co
lib-C666-l	chr19	36422511	36422511	+	12.10977	NA	Intergenic	CpG	-5511	NM_02450	79414	Hs.143792	NM_02450	ENSG0000	LRFN3	FIGLER1|S	leucine rich	protein-co
lib-C666-l	chr3	3708645	3708645	+	33.48615	NA	Intergenic	Intergenic	-132470	NM_00132	57633	Hs.163244	NM_02087	ENSG0000	LRRN1	FIGLER3|NI	leucine rich	protein-co
lib-C666-l	chr3	4004776	4004776	+	17.62684	NA	Intergenic	L1HS|LINE|L1	163661	NM_00132	57633	Hs.163244	NM_02087	ENSG0000	LRRN1	FIGLER3|NI	leucine rich	protein-co
lib-C666-l	chr3	3657424	3657424	+	6.97783	NA	Intergenic	Intergenic	-183691	NM_00132	57633	Hs.163244	NM_02087	ENSG0000	LRRN1	FIGLER3|NI	leucine rich	protein-co
lib-C666-l	chr3	3840870	3840870	+	6.48676	NA	promoter-TSS (NM_001324189)	promoter-TSS (NM_001324189)	-245	NM_00132	57633	Hs.163244	NM_02087	ENSG0000	LRRN1	FIGLER3|NI	leucine rich	protein-co
lib-C666-l	chr1	204621187	204621187	+	4.90117	NA	intron (NM_201630, intron 1 of 1)	intron (NM_201630, intron 1 of 1)	33294	NM_00633	10446	Hs.26312	NM_00633	ENSG0000	LRRN2	FIGLER7|G	leucine rich	protein-co
lib-C666-l	chr13	20534311	20534311	+	4.19675	NA	intron (NM_001190965, intron 2 of 2	intron (NM_001190965, intron 2 of	1337	NM_00119	7750	Hs.507433	NM_00345	ENSG0000	ZMYM2	FIM|MYM|F	zinc finger	protein-co
lib-C666-l	chr19	11039307	11039307	+	5.9194	NA	promoter-TSS (NM_138358)	promoter-TSS (NM_138358)	36	NM_00132	78992	Hs.164026	NM_02402	ENSG0000	YIPF2	FinGER2	Yip1 doma	protein-co
lib-C666-l	chr5	143550086	143550086	+	3.7184	NA	promoter-TSS (NM_020768)	promoter-TSS (NM_020768)	192	NM_03079	81555	Hs.372050	NM_03079	ENSG0000	YIPF5	FinGER5|SE	Yip1 doma	protein-co

lib-C666-l	chrX	67718812	67718812	+	50.81684	NA	5' UTR (NM_001195214, exon 1 of 6)	5' UTR (NM_001195214, exon 1 of 6	188	NM_00119	286451	Hs.82719	NM_17383	ENSG0000	YIPF6	FinGER6	Yip1 doma	protein-co
lib-C666-l	chr8	144911534	144911534	+	5.72511	NA	promoter-TSS (NM_001271098)	promoter-TSS (NM_001271098)	22	NM_00127	22827	Hs.521924	NM_01428	ENSG0000	PUF60	FIR|RoBPI|S	poly(U) bin	protein-co
lib-C666-l	chr10	105615147	105615147	+	6.71839	NA	promoter-TSS (NM_014631)	promoter-TSS (NM_014631)	17	NM_01463	9644	Hs.678727	NM_01463	ENSG0000	SH3PXD2A	FISH|SH3M	SH3 and P.	protein-co
lib-C666-l	chr10	105630306	105630306	+	4.58897	NA	Intergenic	Intergenic	-15142	NM_01463	9644	Hs.678727	NM_01463	ENSG0000	SH3PXD2A	FISH|SH3M	SH3 and P.	protein-co
lib-C666-l	chr20	1408623	1408623	+	33.11264	NA	Intergenic	L1MD2|LINE|L1	-34807	NM_00119	2280	Hs.471933	NM_00080	ENSG0000	FKBP1A	FKBP-12|FI	FK506 binc	protein-co
lib-C666-l	chr20	1408474	1408474	+	23.27622	NA	Intergenic	L1MD2|LINE|L1	-34658	NM_00119	2280	Hs.471933	NM_00080	ENSG0000	FKBP1A	FKBP-12|FI	FK506 binc	protein-co
lib-C666-l	chr11	64008444	64008444	+	71.25437	NA	promoter-TSS (NM_001135208)	promoter-TSS (NM_001135208)	31	NM_00447	2286	Hs.227729	NM_00447	ENSG0000	FKBP2	FKBP-13|P	FK506 binc	protein-co
lib-C666-l	chr9	115983688	115983688	+	25.40925	NA	promoter-TSS (NM_015258)	promoter-TSS (NM_015258)	-47	NM_01525	23307	Hs.522351	NM_01525	ENSG0000	FKBP15	FKBP133|K	FK506 binc	protein-co
lib-C666-l	chr2	179343289	179343289	+	17.22931	NA	promoter-TSS (NM_181342)	promoter-TSS (NM_181342)	66	NM_00113	51661	Hs.410378	NM_01610	ENSG0000	FKBP7	FKBP23|PP	FK506 binc	protein-co
lib-C666-l	chr14	45603804	45603804	+	13.8811	NA	promoter-TSS (NM_002013)	promoter-TSS (NM_002013)	-72	NM_00201	2287	Hs.509226	NM_00201	ENSG0000	FKBP3	FKBP-25|FI	FK506 binc	protein-co
lib-C666-l	chr17	76195138	76195138	+	15.33659	NA	intron (NM_001145526, intron 2 of 1(	intron (NM_001145526, intron 2 of	11740	NM_00114	125061	Hs.558614	NM_00101	ENSG0000	AFMID	FKF|KF|KFA	arylforman	protein-co
lib-C666-l	chr16	86609468	86609468	+	9.10494	NA	Intergenic	CpG	-2647	NM_00525	2300	Hs.533830	NM_00525	ENSG0000	FOXL1	FKH6|FKHL	forkhead b	protein-co
lib-C666-l	chr9	100565856	100565856	+	48.94471	NA	Intergenic	CpG	-49681	NM_00447	2304	Hs.159234	NM_00447	ENSG0000	FOXE1	FKHL15|FC	forkhead b	protein-co
lib-C666-l	chr9	100572167	100572167	+	10.71733	NA	Intergenic	Intergenic	-43370	NM_00447	2304	Hs.159234	NM_00447	ENSG0000	FOXE1	FKHL15|FC	forkhead b	protein-co
lib-C666-l	chr9	100616339	100616339	+	7.08136	NA	exon (NM_004473, exon 1 of 1)	exon (NM_004473, exon 1 of 1)	802	NM_00447	2304	Hs.159234	NM_00447	ENSG0000	FOXE1	FKHL15|FC	forkhead b	protein-co
lib-C666-l	chr17	26828029	26828029	+	12.55817	NA	Intergenic	Intergenic	-22930	NM_00359	8456	Hs.58611	NM_00359	ENSG0000	FOXN1	FKHL20|RC	forkhead b	protein-co
lib-C666-l	chr2	10184560	10184560	+	26.37215	NA	5' UTR (NM_001177718, exon 1 of 4)	5' UTR (NM_001177718, exon 1 of 4	188	NM_00117	8462	Hs.12229	NM_00359	ENSG0000	KLF11	FKLF|FKLF1	Kruppel lik	protein-co
lib-C666-l	chr2	10184125	10184125	+	10.58189	NA	promoter-TSS (NM_001177718)	promoter-TSS (NM_001177718)	169	NM_00117	8462	Hs.12229	NM_00359	ENSG0000	KLF11	FKLF|FKLF1	Kruppel lik	protein-co
lib-C666-l	chr13	29293057	29293057	+	6.98764	NA	promoter-TSS (NM_001135919)	promoter-TSS (NM_001135919)	93	NM_00113	283537	Hs.117167	NM_18178	ENSG0000	SLC46A3	FKSG16	solute carr	protein-co
lib-C666-l	chr8	102354083	102354083	+	19.70589	NA	Intergenic	Intergenic	-27038	NR_00218	83955	Hs.567608	NM_03203	ENSG0000	NACAP1	FKSG17	nascent pc	pseudo
lib-C666-l	chr9	86433005	86433005	+	5.64185	NA	promoter-TSS (NM_001135953)	promoter-TSS (NM_001135953)	-253	NM_00113	80318	Hs.522255	NM_02521	ENSG0000	GKAP1	FKSG21|GK	G kinase ar	protein-co
lib-C666-l	chr19	18303786	18303786	+	37.99633	NA	promoter-TSS (NM_032683)	promoter-TSS (NM_032683)	-254	NM_03268	84769	Hs.515254	NM_03268	ENSG0000	MPV17L2	FKSG24	MPV17 mit	protein-co
lib-C666-l	chr16	89940695	89940695	+	8.96177	NA	intron (NM_014972, intron 1 of 17)	CpG	701	NM_01497	22980	Hs.415342	NM_01497	ENSG0000	TCF25	FKSG26|Hu	transcriptic	protein-co
lib-C666-l	chr2	92325130	92325130	+	12.00741	NA	Intergenic	ALR/Alpha|Satellite|centr	195971	NR_02771	440888	Hs.730239	NM_001032412		ACTR3BP2	FKSG73	ACTR3B ps	pseudo
lib-C666-l	chr19	49977360	49977360	+	26.46433	NA	promoter-TSS (NM_001278638)	promoter-TSS (NM_001278638)	-106	NM_00127	2323	Hs.428	NM_00145	ENSG0000	FLT3LG	FL|FLT3L	fms relatec	protein-co
lib-C666-l	chr1	170043944	170043944	+	7.52748	NA	promoter-TSS (NM_014970)	promoter-TSS (NM_014970)	-65	NM_00120	22920	Hs.433442	NM_01497	ENSG0000	KIFAP3	FLA3|KAP-	kinesin ass	protein-co
lib-C666-l	chr19	42721071	42721071	+	5.5098	NA	exon (NM_001270614, exon 2 of 5)	exon (NM_001270614, exon 2 of 5)	873	NR_07305	162989	Hs.515432	NM_13332	ENSG0000	DEDD2	FLAME-3|F	death effec	protein-co
lib-C666-l	chr19	42721973	42721973	+	4.27545	NA	promoter-TSS (NR_073048)	promoter-TSS (NR_073048)	-29	NR_07304	162989	Hs.515432	NM_13332	ENSG0000	DEDD2	FLAME-3|F	death effec	protein-co
lib-C666-l	chr2	238600925	238600925	+	5.51692	NA	5' UTR (NM_001137552, exon 1 of 11	5' UTR (NM_001137552, exon 1 of 1	118	NM_00113	9208	Hs.471779	NM_00473	ENSG0000	LRRFIP1	FLAP-1|FL/	LRR bindin	protein-co
lib-C666-l	chr1	185285470	185285470	+	5.96422	NA	intron (NM_006469, intron 1 of 14)	CpG	991	NM_00646	10625	Hs.497183	NM_00646	ENSG0000	IVNS1ABP	FLARA3|HS	influenza v	protein-co
lib-C666-l	chr1	185286706	185286706	+	3.8432	NA	promoter-TSS (NM_006469)	promoter-TSS (NM_006469)	-245	NM_00646	10625	Hs.497183	NM_00646	ENSG0000	IVNS1ABP	FLARA3|HS	influenza v	protein-co
lib-C666-l	chr1	46049238	46049238	+	43.7015	NA	promoter-TSS (NM_001195193)	promoter-TSS (NM_001195193)	-422	NM_15229	4678	Hs.319334	NM_00248	ENSG0000	NASP	FLB7527|H	nuclear aut	protein-co
lib-C666-l	chr14	91976886	91976886	+	117.58568	NA	promoter-TSS (NM_001284281)	promoter-TSS (NM_001284281)	-62	NM_00128	55671	Hs.533887	NM_01793	ENSG0000	PPP4R3A	FLFL1|KIAA	protein ph	protein-co
lib-C666-l	chr14	92040545	92040545	+	40.85015	NA	Intergenic	CpG	-63721	NM_00128	55671	Hs.533887	NM_01793	ENSG0000	PPP4R3A	FLFL1|KIAA	protein ph	protein-co
lib-C666-l	chr2	55845038	55845038	+	13.14211	NA	promoter-TSS (NM_001282850)	promoter-TSS (NM_001282850)	-178	NM_00128	57223	Hs.516182	NM_02046	ENSG0000	PPP4R3B	FLFL2|PP4F	protein ph	protein-co
lib-C666-l	chr22	45098932	45098932	+	5.96761	NA	intron (NM_001017528, intron 2 of 8)	intron (NM_001017528, intron 2 of	854	NM_18133	55615	Hs.102336	NM_01536	ENSG0000	PRR5	FLJ20185k|	proline rich	protein-co
lib-C666-l	chr12	56615765	56615765	+	23.0291	NA	promoter-TSS (NM_005785)	promoter-TSS (NM_005785)	-12	NM_19435	10193	Hs.524502	NM_00578	ENSG0000	RNF41	FLRF|NRDF	ring finger	protein-co
lib-C666-l	chr19	680405	680405	+	14.88921	NA	exon (NM_005860, exon 3 of 5)	exon (NM_005860, exon 3 of 5)	4016	NM_00586	10272	Hs.529038	NM_00586	ENSG0000	FSTL3	FLRG|FSRP	follistatin li	protein-co
lib-C666-l	chr1	29562983	29562983	+	11.20567	NA	promoter-TSS (NM_001195001)	promoter-TSS (NM_001195001)	-45	NM_13317	10076	Hs.19718	NM_00570	ENSG0000	PTPRU	FMI|PCP-2	protein tyr	protein-co
lib-C666-l	chr15	33191607	33191607	+	8.01457	NA	intron (NM_001103184, intron 10 of 1	intron (NM_001103184, intron 10 o	168626	NM_00110	342184	Hs.657649	NM_19850	ENSG0000	FMN1	FMN|LD	formin 1	protein-co
lib-C666-l	chr15	33487031	33487031	+	5.5166	NA	promoter-TSS (NM_001277314)	promoter-TSS (NM_001277314)	-97	NM_00127	342184	Hs.657649	NM_19850	ENSG0000	FMN1	FMN|LD	formin 1	protein-co
lib-C666-l	chr1	171283191	171283191	+	10.79304	NA	promoter-TSS (NM_002022)	promoter-TSS (NM_002022)	-131	NM_00202	2329	Hs.386502	NM_00202	ENSG0000	FMO4	FMO2	flavin conta	protein-co
lib-C666-l	chr7	102789658	102789658	+	19.38538	NA	promoter-TSS (NM_001122838)	promoter-TSS (NM_001122838)	-89	NM_19899	222236	Hs.324271	NM_19899	ENSG0000	NAPEPLD	FMP30|NA	N-acyl pho	protein-co
lib-C666-l	chr17	7518518	7518518	+	11.66012	NA	promoter-TSS (NM_004860)	promoter-TSS (NM_004860)	-303	NM_00486	9513	Hs.52788	NM_00486	ENSG0000	FXR2	FMR1L2|FX	FMR1 auto	protein-co
lib-C666-l	chr14	24685285	24685285	+	47.23204	NA	promoter-TSS (NM_138476)	promoter-TSS (NM_138476)	-9	NM_13847	145553	Hs.220963	NM_13847	ENSG0000	MDP1	FN6PASE|N	magnesium	protein-co
lib-C666-l	chr1	149832829	149832829	+	8.85547	NA	promoter-TSS (NM_003548)	promoter-TSS (NM_003548)	-104	NM_00354	8370	Hs.55468	NM_00354	ENSG0000	HIST2H4A	FO108|H4|	histone clu	protein-co
lib-C666-l	chr8	29939968	29939968	+	22.50982	NA	intron (NM_016127, intron 1 of 5)	intron (NM_016127, intron 1 of 5)	756	NM_01612	51669	Hs.521487	NM_01612	ENSG0000	SARAF	FOAP-7|HS	store-oper	protein-co
lib-C666-l	chr8	29940623	29940623	+	13.46097	NA	5' UTR (NM_016127, exon 1 of 6)	5' UTR (NM_016127, exon 1 of 6)	101	NM_01612	51669	Hs.521487	NM_01612	ENSG0000	SARAF	FOAP-7|HS	store-oper	protein-co
lib-C666-l	chr10	88281620	88281620	+	6.32351	NA	promoter-TSS (NM_001318328)	promoter-TSS (NM_001318328)	-79	NM_01504	23063	Hs.203099	NM_01504	ENSG0000	WAPL	FOE|KIAA0	WAPL cohe	protein-co
lib-C666-l	chr6	167412801	167412801	+	11.06679	NA	promoter-TSS (NM_194429)	promoter-TSS (NM_194429)	-4	NM_00127	11116	Hs.487175	NM_00704	ENSG0000	FGFR1OP	FOP	FGFR1 onc	protein-co
lib-C666-l	chr22	36461892	36461892	+	7.11942	NA	Intergenic	CpG	-37307	NM_00108	23543	Hs.282998	NM_01430	ENSG0000	RBFOX2	FOX2|Fox-	RNA bindir	protein-co
lib-C666-l	chr9	42678668	42678668	+	10.13529	NA	Intergenic	Intergenic	-39398	NM_19924	349334	Hs.712520	NM_19924	ENSG0000	FOXD4L4	FOXD4L2|F	forkhead b	protein-co
lib-C666-l	chr16	88729634	88729634	+	48.67325	NA	promoter-TSS (NR_024402)	promoter-TSS (NR_024402)	-77	NM_00246	4597	Hs.252457	NM_00246	ENSG0000	MVD	FP17780|M	mevalonat	protein-co
lib-C666-l	chr1	159915773	159915773	+	5.15932	NA	promoter-TSS (NM_020789)	promoter-TSS (NM_020789)	-387	NM_00113	57549	Hs.591472	NM_02078	ENSG0000	IGSF9	FP18798|IC	immunoglo	protein-co
lib-C666-l	chr17	42264275	42264275	+	179.84544	NA	promoter-TSS (NR_049729)	promoter-TSS (NR_049729)	-44	NM_00133	79089	Hs.181391	NM_02410	ENSG0000	TMUB2	FP2653	transmemb	protein-co
lib-C666-l	chr7	74267302	74267302	+	107.24402	NA	intron (NM_001281447, intron 1 of 2)	CpG	570	NM_00128	84163	Hs.647017	NM_17353	ENSG0000	GTF2IRD2	FP630|GTF	GTF2I repe	protein-co
lib-C666-l	chr11	126138915	126138915	+	148.64328	NA	promoter-TSS (NM_017547)	promoter-TSS (NM_017547)	-20	NM_01754	55572	Hs.317190	NM_01754	ENSG0000	FOXRED1	FP634|H17	FAD deper	protein-co
lib-C666-l	chr21	44527680	44527680	+	133.57831	NA	promoter-TSS (NM_001320651)	promoter-TSS (NM_001320651)	8	NM_00675	7307	Hs.365116	NM_00675	ENSG0000	U2AF1	FP793|RN|I	U2 small n	protein-co
lib-C666-l	chr19	42580139	42580139	+	19.65763	NA	promoter-TSS (NM_022752)	promoter-TSS (NM_022752)	-151	NM_02275	64763	Hs.13323	NM_02275	ENSG0000	ZNF574	FP972	zinc finger	protein-co
lib-C666-l	chr16	3333453	3333453	+	74.49267	NA	promoter-TSS (NM_005741)	promoter-TSS (NM_005741)	-34	NM_00574	10127	Hs.611475	NM_00574	ENSG0000	ZNF263	FPM315|ZI	zinc finger	protein-co
lib-C666-l	chr16	3332931	3332931	+	11.90035	NA	promoter-TSS (NM_005741)	promoter-TSS (NM_005741)	-556	NM_00574	10127	Hs.611475	NM_00574	ENSG0000	ZNF263	FPM315|ZI	zinc finger	protein-co
lib-C666-l	chr1	155278563	155278563	+	85.95118	NA	promoter-TSS (NM_001135822)	promoter-TSS (NM_001135822)	24	NM_00200	2224	Hs.335918	NM_00200	ENSG0000	FDPS	FPPS|FPS|P	farnesyl dip	protein-co
lib-C666-l	chr11	65668058	65668058	+	80.28929	NA	promoter-TSS (NM_005438)	promoter-TSS (NM_005438)	-61	NM_00130	8061	Hs.283565	NM_00543	ENSG0000	FOSL1	FRA|FRA1|f	FOS like 1,	protein-co
lib-C666-l	chr2	28615544	28615544	+	14.31405	NA	promoter-TSS (NM_005253)	promoter-TSS (NM_005253)	-235	NM_00525	2355	Hs.220971	NM_00525	ENSG0000	FOSL2	FRA2	FOS like 2,	protein-co
lib-C666-l	chr1	11322766	11322766	+	3.98718	NA	promoter-TSS (NM_004958)	promoter-TSS (NM_004958)	-158	NM_00495	2475	Hs.338207	NM_00495	ENSG0000	MTOR	FRAP|FRAF	mechanisti	protein-co
lib-C666-l	chr4	154994770	154994770	+	8.73012	NA	Intergenic	MLT1D|LTR|ERVL-MaLR	-284542	NM_00301	6423	Hs.481022	NM_00301	ENSG0000	SFRP2	FRP-2|SAR	secreted fr	protein-co
lib-C666-l	chr4	123073457	123073457	+	85.77416	NA	Intergenic	CpG	-18301	NM_01531	84162	Hs.408142	NM_01531	ENSG0000	KIAA1109	FSA|Tweek	KIAA1109	protein-co
lib-C666-l	chr3	128880320	128880320	+	49.86005	NA	promoter-TSS (NM_001204890)	promoter-TSS (NM_001204890)	-247	NM_00119	57461	Hs.512661	NM_02070	ENSG0000	ISY1	FSAP33	ISY1 splicir	protein-co
lib-C666-l	chr7	36429329	36429329	+	7.11942	NA	promoter-TSS (NM_001284301)	promoter-TSS (NM_001284301)	-83	NM_01868	54443	Hs.62180	NM_01868	ENSG0000	ANLN	FSGS8|Scra	anillin actir	protein-co
lib-C666-l	chr4	74783347	74783347	+	13.20416	NA	Intergenic	MER1B|DNA|hAT-Charlie	48238	NR_04603	2919	Hs.789	NM_00151	ENSG0000	CXCL1	FSP|GRO1|	C-X-C mo	protein-co
lib-C666-l	chr15	23096828	23096828	+	33.69979	NA	intron (NR_040057, intron 4 of 4)	intron (NR_040057, intron 4 of 4)	-9985	NM_00114	123606	Hs.511797	NM_14459	ENSG0000	NIPA1	FSP3|SPG6	non imprin	protein-co
lib-C666-l	chr2	24713695	24713695	+	8.96177	NA	Intergenic	MIR3|SINE|MIR	-93651	NM_00374	8648	Hs.596314	NM_00374	ENSG0000	NCOA1	F-SRC-1|K	nuclear rec	protein-co
lib-C666-l	chr16	53537420	53537420	+	14.85625	NA	promoter-TSS (NM_001308325)	promoter-TSS (NM_001308325)	-204	NM_00130	64400	Hs.380897	NM_02247	ENSG0000	AKTIP	FT1|FTS	AKT intera	protein-co
lib-C666-l	chr16	53536799	53536799	+	10.92275	NA	intron (NM_022476, intron 1 of 9)	CpG	417	NM_02247	64400	Hs.380897	NM_02247	ENSG0000	AKTIP	FT1|FTS	AKT intera	protein-co
lib-C666-l	chr17	43394241	43394241	+	4.38153	NA	intron (NM_003954, intron 1 of 15)	CpG	189	NM_00395	9020	Hs.404183	NM_00395	ENSG0000	MAP3K14	FTDCR1B|H	mitogen-a	protein-co
lib-C666-l	chr4	152020573	152020573	+	10.27265	NA	promoter-TSS (NM_001006)	promoter-TSS (NM_001006)	-152	NM_00126	6189	Hs.356572	NM_00100	ENSG0000	RPS3A	FTE1|MFTL	ribosomal	protein-co
lib-C666-l	chr6	151134531	151134531	+	30.87451	NA	intron (NM_001329800, intron 10 of 1	intron (NM_001329800, intron 10 o	-52284	NM_01544	25902	Hs.591343	NM_01544	ENSG0000	MTHFD1L	FTHFSDC1	methylene	protein-co
lib-C666-l	chr6	151135292	151135292	+	6.15313	NA	intron (NM_001329800, intron 10 of 1	intron (NM_001329800, intron 10 o	-51523	NM_01544	25902	Hs.591343	NM_01544	ENSG0000	MTHFD1L	FTHFSDC1	methylene	protein-co
lib-C666-l	chr6	151186793	151186793	+	3.31563	NA	promoter-TSS (NM_001242768)	promoter-TSS (NM_001242768)	-22	NM_01544	25902	Hs.591343	NM_01544	ENSG0000	MTHFD1L	FTHFSDC1	methylene	protein-co
lib-C666-l	chrX	100663212	100663212	+	10.27265	NA	promoter-TSS (NM_019597)	promoter-TSS (NM_019597)	91	NM_00103	3188	Hs.432485	NM_01959	ENSG0000	HNRNPH2	FTP3|HNRF	heterogen	protein-co
lib-C666-l	chr6	37400957	37400957	+	53.7204	NA	promoter-TSS (NM_015050)	promoter-TSS (NM_015050)	50	NM_01505	23070	Hs.520102	NM_01505	ENSG0000	CMTR1	FTSJD2|KIA	cap methy	protein-co
lib-C666-l	chr8	33101011	33101011	+	63.75269	NA	Intergenic	Intergenic	229653	NM_03266	84750	Hs.458713	NM_03266	ENSG0000	FUT10	FUCTX	fucosyltran	protein-co
lib-C666-l	chr8	33330783	33330783	+	29.30929	NA	promoter-TSS (NM_032664)	promoter-TSS (NM_032664)	-119	NM_03266	84750	Hs.458713	NM_03266	ENSG0000	FUT10	FUCTX	fucosyltran	protein-co
lib-C666-l	chr22	41681566	41681566	+	25.49755	NA	5' UTR (NM_001278651, exon 1 of 17	5' UTR (NM_001278651, exon 1 of 1	455	NM_00127	5905	Hs.183800	NM_00288	ENSG0000	RANGAP1	Fug1|RAN(	Ran GTPas	protein-co
lib-C666-l	chr22	41682340	41682340	+	8.27411	NA	promoter-TSS (NM_001278651)	promoter-TSS (NM_001278651)	-85	NM_00288	5905	Hs.183800	NM_00288	ENSG0000	RANGAP1	Fug1|RAN(	Ran GTPas	protein-co
lib-C666-l	chr22	41684564	41684564	+	6.63143	NA	intron (NM_001317930, intron 1 of 15	intron (NM_001317930, intron 1 of	-2309	NM_00288	5905	Hs.183800	NM_00288	ENSG0000	RANGAP1	Fug1|RAN(	Ran GTPas	protein-co
lib-C666-l	chr22	41681954	41681954	+	3.20885	NA	promoter-TSS (NM_001278651)	promoter-TSS (NM_001278651)	67	NM_00127	5905	Hs.183800	NM_00288	ENSG0000	RANGAP1	Fug1|RAN(	Ran GTPas	protein-co
lib-C666-l	chrX	74493847	74493847	+	11.57178	NA	promoter-TSS (NM_001307944)	promoter-TSS (NM_001307944)	-47	NM_00130	139596	Hs.91612	NM_14505	ENSG0000	UPRT	FUR1|UPP	uracil phos	protein-co
lib-C666-l	chr3	50336874	50336874	+	13.77909	NA	promoter-TSS (NM_001200018)	promoter-TSS (NM_001200018)	-22	NM_00120	24142	Hs.129910	NM_01219	ENSG0000	NAT6	FUS-2|FUS	N-acetyltra	protein-co
lib-C666-l	chr3	50336463	50336463	+	3.8557	NA	promoter-TSS (NM_001200018)	promoter-TSS (NM_001200018)	-143	NM_00120	24142	Hs.129910	NM_01219	ENSG0000	NAT6	FUS-2|FUS	N-acetyltra	protein-co
lib-C666-l	chr1	24306530	24306530	+	10.03284	NA	intron (NR_034035, intron 1 of 6)	CpG	423	NM_00130	10772	Hs.3530	NM_00662	ENSG0000	SRSF10	FUSIP1|FUS	serine and	protein-co
lib-C666-l	chr19	17337236	17337236	+	25.49755	NA	intron (NM_024578, intron 1 of 5)	CpG	223	NM_02457	79629	Hs.422676	NM_02457	ENSG0000	OCEL1	FWP009|S8	occludin/E	protein-co
lib-C666-l	chr16	30366738	30366738	+	40.31997	NA	promoter-TSS (NM_001243646)	promoter-TSS (NM_001243646)	-56	NM_00611	10421	Hs.202677	NM_00611	ENSG0000	CD2BP2	FWP010|LI	CD2 cytop	protein-co
lib-C666-l	chr8	144699983	144699983	+	9.02258	NA	promoter-TSS (NM_001317783)	promoter-TSS (NM_001317783)	-251	NM_00331	7264	Hs.404119	NM_00331	ENSG0000	TSTA3	FX|P35B|SD	tissue spec	protein-co
lib-C666-l	chr11	6502632	6502632	+	95.85526	NA	promoter-TSS (NM_012402)	promoter-TSS (NM_012402)	-45	NM_01219	26515	Hs.54943	NM_01219	ENSG0000	TIMM10B	FXC1|TIM1	translocase	protein-co
lib-C666-l	chr9	5833227	5833227	+	9.79436	NA	promoter-TSS (NM_024896)	promoter-TSS (NM_024896)	-146	NM_02489	79956	Hs.591078	NM_02489	ENSG0000	ERMP1	FXNA|KIAA	endoplasm	protein-co
lib-C666-l	chr3	180630575	180630575	+	2.02415	NA	intron (NM_001013438, intron 1 of 15	CpG	341	NM_00101	8087	Hs.478407	NM_00508	ENSG0000	FXR1	FXR1P	FMR1 auto	protein-co
lib-C666-l	chr14	68283373	68283373	+	5.15477	NA	promoter-TSS (NM_015346)	promoter-TSS (NM_015346)	-67	NM_01534	23503	Hs.98041	NM_01534	ENSG0000	ZFYVE26	FYVE-CEN	zinc finger	protein-co
lib-C666-l	chr17	56595428	56595428	+	20.90919	NA	promoter-TSS (NM_004687)	promoter-TSS (NM_004687)	-177	NM_00468	9110	Hs.514373	NM_00468	ENSG0000	MTMR4	FYVE-DSP2	myotubula	protein-co
lib-C666-l	chr19	39894695	39894695	+	12.70401	NA	Intergenic	CpG	-2792	NM_00340	7538	Hs.534052	NM_00340	ENSG0000	ZFP36	G0S24|GO	ZFP36 ring	protein-co
lib-C666-l	chr19	39893620	39893620	+	8.28613	NA	Intergenic	Intergenic	-3867	NM_00340	7538	Hs.534052	NM_00340	ENSG0000	ZFP36	G0S24|GO	ZFP36 ring	protein-co
lib-C666-l	chr1	192778041	192778041	+	8.56376	NA	promoter-TSS (NM_002923)	promoter-TSS (NM_002923)	-128	NM_00292	5997	Hs.78944	NM_00292	ENSG0000	RGS2	G0S8	regulator c	protein-co
lib-C666-l	chr5	179499119	179499119	+	37.47379	NA	promoter-TSS (NM_001280801)	promoter-TSS (NM_001280801)	-1	NM_00128	55819	Hs.484363	NM_01843	ENSG0000	RNF130	G1RZFP|G(	ring finger	protein-co
lib-C666-l	chr5	179563739	179563739	+	14.59636	NA	intron (NM_175062, intron 3 of 13)	CpG-21553	-64621	NM_00128	55819	Hs.484363	NM_01843	ENSG0000	RNF130	G1RZFP|G(	ring finger	protein-co
lib-C666-l	chr14	105512198	105512198	+	9.02258	NA	Intergenic	CpG	19689	NM_00127	29933	Hs.532504	NM_01334	ENSG0000	GPR132	G2A	G protein-	protein-co
lib-C666-l	chr11	62414125	62414125	+	5.23609	NA	promoter-TSS (NM_001278193)	promoter-TSS (NM_001278193)	73	NM_00127	23193	Hs.595071	NM_01461	ENSG0000	GANAB	G2AN|GIIA	glucosidas	protein-co
lib-C666-l	chr8	11594094	11594094	+	9.20633	NA	intron (NM_002052, intron 2 of 6)	intron (NM_002052, intron 2 of 6)	-24671	NR_10355	606553	Hs.545529	NM_00103	ENSG0000	C8orf49	G4DM	chromosor	ncRNA
lib-C666-l	chrX	153775370	153775370	+	3.29276	NA	promoter-TSS (NM_000402)	promoter-TSS (NM_000402)	-137	NM_00040	2539	Hs.461047	NM_00040	ENSG0000	G6PD	G6PD1	glucose-6-	protein-co
lib-C666-l	chr11	118901721	118901721	+	5.14484	NA	promoter-TSS (NM_001164277)	promoter-TSS (NM_001164277)	-105	NM_00116	2542	Hs.719203	NM_00146	ENSG0000	SLC37A4	G6PT1|G6F	solute carr	protein-co
lib-C666-l	chr5	54469061	54469061	+	22.28596	NA	promoter-TSS (NR_031572)	promoter-TSS (NR_031572)	-56	NM_00114	166979	Hs.669184	NM_15262	ENSG0000	CDC20B	G6VTS765	cell divisior	protein-co
lib-C666-l	chr5	54472478	54472478	+	17.37618	NA	Intergenic	Intergenic	-3473	NM_00114	166979	Hs.669184	NM_15262	ENSG0000	CDC20B	G6VTS765	cell divisior	protein-co
lib-C666-l	chr6	31707870	31707870	+	3.57421	NA	5' UTR (NM_002441, exon 1 of 25).3	5' UTR (NM_002441, exon 1 of 25).3	145	NM_17216	4439	Hs.647011	NM_00244	ENSG0000	MSH5	G7|MUTSH	mutS hom	protein-co
lib-C666-l	chr6	31763815	31763815	+	66.08424	NA	promoter-TSS (NM_006295).7	promoter-TSS (NM_006295).7	-103	NM_00629	7407	Hs.520026	NM_00629	ENSG0000	VARS	G7A|VARS	valyl-tRNA	protein-co
lib-C666-l	chr12	56882530	56882530	+	5.45221	NA	promoter-TSS (NM_001280796)	promoter-TSS (NM_001280796)	-332	NM_01326	27165	Hs.212606	NM_01326	ENSG0000	GLS2	GA|GLS|LG	glutaminas	protein-co
lib-C666-l	chr8	38578182	38578182	+	94.04023	NA	Intergenic	LTR12C|LTR|ERV1	-7522	NM_00133	6867	Hs.279245	NM_00628	ENSG0000	TACC1	Ga55	transformir	protein-co
lib-C666-l	chr8	38599602	38599602	+	39.6673	NA	intron (NM_001330521, intron 1 of 12	intron (NM_001330521, intron 1 of	13898	NM_00114	6867	Hs.279245	NM_00628	ENSG0000	TACC1	Ga55	transformir	protein-co
lib-C666-l	chr1	151043093	151043093	+	38.21529	NA	promoter-TSS (NM_001323913)	promoter-TSS (NM_001323913)	13	NM_14461	126626	Hs.15671	NM_14461	ENSG0000	GABPB2	GABPB-2	GA binding	protein-co
lib-C666-l	chr10	126068086	126068086	+	23.60036	NA	Intergenic	Intergenic	39459	NM_00132	4942	Hs.523332	NM_00027	ENSG0000	OAT	GACR|HO(	ornithine a	protein-co
lib-C666-l	chr10	126107807	126107807	+	11.66438	NA	promoter-TSS (NM_001322971)	promoter-TSS (NM_001322971)	-262	NM_00132	4942	Hs.523332	NM_00027	ENSG0000	OAT	GACR|HO(	ornithine a	protein-co
lib-C666-l	chr10	126067702	126067702	+	10.92952	NA	Intergenic	Intergenic	39843	NM_00132	4942	Hs.523332	NM_00027	ENSG0000	OAT	GACR|HO(	ornithine a	protein-co
lib-C666-l	chr19	49375577	49375577	+	41.60205	NA	promoter-TSS (NM_014330)	promoter-TSS (NM_014330)	-72	NM_01433	23645	Hs.631593	NM_01433	ENSG0000	PPP1R15A	GADD34	protein ph	protein-co
lib-C666-l	chr19	49378642	49378642	+	3.85762	NA	intron (NM_014330, intron 2 of 2)	intron (NM_014330, intron 2 of 2)	2993	NM_01433	23645	Hs.631593	NM_01433	ENSG0000	PPP1R15A	GADD34	protein ph	protein-co
lib-C666-l	chr19	2476776	2476776	+	8.01457	NA	intron (NM_015675, intron 2 of 3)	CpG-12408	653	NM_01567	4616	Hs.110571	NM_01567	ENSG0000	GADD45B	GADD45BE	growth arr	protein-co
lib-C666-l	chr8	29120432	29120432	+	9.21057	NA	intron (NM_015254, intron 1 of 39)	CpG	178	NM_01525	23303	Hs.444767	NM_01525	ENSG0000	KIF13B	GAKIN	kinesin far	protein-co
lib-C666-l	chr19	39290330	39290330	+	2.05265	NA	Intergenic	L1ME2z|LINE|L1	10480	NM_00104	653499	Hs.558355	NM_00104	ENSG0000	LGALS7B	GAL7|Gal-1	galectin 7E	protein-co
lib-C666-l	chr5	153569791	153569791	+	5.25516	NA	promoter-TSS (NM_198321)	promoter-TSS (NM_198321)	-504	NM_19832	55568	Hs.631797	NM_01754	ENSG0000	GALNT10	GALNACT1	polypeptid	protein-co
lib-C666-l	chr7	151722661	151722661	+	113.10201	NA	promoter-TSS (NM_001304514)	promoter-TSS (NM_001304514)	-98	NM_02208	63917	Hs.647109	NM_02208	ENSG0000	GALNT11	GALNAC-1	polypeptid	protein-co
lib-C666-l	chr7	151728046	151728046	+	4.27545	NA	intron (NM_001304514, intron 1 of 1(	HERV9-int|LTR|ERV1	5287	NM_00130	63917	Hs.647109	NM_02208	ENSG0000	GALNT11	GALNAC-1	polypeptid	protein-co
lib-C666-l	chr3	16143089	16143089	+	4.38153	NA	Intergenic	LTR10F|LTR|ERV1	-72740	NM_00131	117248	Hs.411308	NM_05411	ENSG0000	GALNT15	GALNACT1	polypeptid	protein-co
lib-C666-l	chr7	70683543	70683543	+	10.06764	NA	intron (NM_022479, intron 1 of 10)	MER1A|DNA|hAT-Charlie	86020	NM_02247	64409	Hs.488591	NM_02247	ENSG0000	WBSCR17	GALNACT1	Williams-B	protein-co
lib-C666-l	chr7	70172878	70172878	+	7.42011	NA	intron (NM_015570, intron 6 of 18)	intron (NM_015570, intron 6 of 18)	-424645	NM_02247	64409	Hs.488591	NM_02247	ENSG0000	WBSCR17	GALNACT1	Williams-B	protein-co
lib-C666-l	chr2	166650734	166650734	+	10.9422	NA	promoter-TSS (NM_004482)	promoter-TSS (NM_004482)	69	NM_00448	2591	Hs.170986	NM_00448	ENSG0000	GALNT3	GalNAc-T3	polypeptid	protein-co
lib-C666-l	chr12	132674318	132674318	+	13.77909	NA	Intergenic	CpG	16255	NM_02180	50614	Hs.301062	NM_02180	ENSG0000	GALNT9	GALNAC-1	polypeptid	protein-co
lib-C666-l	chr12	53693587	53693587	+	27.96363	NA	TTS (NM_145897)	TTS (NM_145897)	117	NM_02164	60314	Hs.655988	NM_02164	ENSG0000	C12orf10	Gamm1|M	chromosor	protein-co
lib-C666-l	chr16	81348496	81348496	+	151.1122	NA	promoter-TSS (NM_022041)	promoter-TSS (NM_022041)	-75	NM_02204	8139	Hs.112569	NM_02204	ENSG0000	GAN	GAN1|KLH	gigaxonin	protein-co
lib-C666-l	chr13	114830971	114830971	+	72.44688	NA	intron (NM_001320821, intron 2 of 25	intron (NM_001320821, intron 2 of	12483	NM_00132	22821	Hs.593075	NM_00736	ENSG0000	RASA3	GAP1IP4BF	RAS p21 p	protein-co
lib-C666-l	chr3	141206851	141206851	+	4.46583	NA	intron (NM_001303245, intron 1 of 23	intron (NM_001303245, intron 1 of	962	NM_00130	5922	Hs.98445	NM_00650	ENSG0000	RASA2	GAP1M	RAS p21 p	protein-co
lib-C666-l	chr22	29702312	29702312	+	21.21068	NA	promoter-TSS (NM_001278730)	promoter-TSS (NM_001278730)	-673	NM_15223	10634	Hs.322852	NM_00647	ENSG0000	GAS2L1	GAR22	growth arr	protein-co
lib-C666-l	chr14	36278595	36278595	+	142.59454	NA	promoter-TSS (NR_104269)	promoter-TSS (NR_104269)	-66	NM_00134	253959	Hs.113150	NM_01499	ENSG0000	RALGAPA1	GARNL1|G	Ral GTPase	protein-co
lib-C666-l	chr9	6757734	6757734	+	25.3508	NA	promoter-TSS (NM_001304341)	promoter-TSS (NM_001304341)	93	NM_00114	23081	Hs.709425	NM_01506	ENSG0000	KDM4C	GASC1|JHD	lysine dem	protein-co
lib-C666-l	chr3	10890163	10890163	+	13.84327	NA	intron (NM_014229, intron 5 of 13)	intron (NM_014229, intron 5 of 13)	32278	NM_01422	6538	Hs.655078	NM_01422	ENSG0000	SLC6A11	GAT-3|GA	solute carr	protein-co
lib-C666-l	chr6	107077563	107077563	+	8.96177	NA	promoter-TSS (NM_032730)	promoter-TSS (NM_032730)	122	NM_01829	55278	Hs.406917	NM_01829	ENSG0000	QRSL1	GatA	glutaminyl	protein-co
lib-C666-l	chr20	61077098	61077098	+	4.46583	NA	Intergenic	THE1C|LTR|ERVL-MaLR	-26072	NM_08047	140628	Hs.352250	NM_08047	ENSG0000	GATA5	GATAS|bB3	GATA bind	protein-co
lib-C666-l	chr1	155214567	155214567	+	6.49644	NA	promoter-TSS (NM_001171811)	promoter-TSS (NM_001171811)	86	NM_00117	2629	Hs.282997	NM_00015	ENSG0000	GBA	GBA1|GCB	glucosylcer	protein-co
lib-C666-l	chr2	44125622	44125622	+	34.19829	NA	intron (NM_133259, intron 34 of 37)	intron (NM_133259, intron 34 of 37	59519	NM_02243	64241	Hs.413931	NM_02243	ENSG0000	ABCG8	GBD4|STSL	ATP bindin	protein-co
lib-C666-l	chr9	138853288	138853288	+	208.24658	NA	promoter-TSS (NM_016172)	promoter-TSS (NM_016172)	-62	NM_01617	10422	Hs.9194	NM_01617	ENSG0000	UBAC1	GBDR1|KP(	UBA doma	protein-co
lib-C666-l	chr16	2255843	2255843	+	9.47171	NA	5' UTR (NM_001199174, exon 1 of 9)	5' UTR (NM_001199174, exon 1 of 9	111	NM_00119	64223	Hs.29203	NM_02237	ENSG0000	MLST8	GBL|Gbeta	MTOR assc	protein-co
lib-C666-l	chr16	2255586	2255586	+	3.44441	NA	promoter-TSS (NM_001199174)	promoter-TSS (NM_001199174)	-146	NM_00119	64223	Hs.29203	NM_02237	ENSG0000	MLST8	GBL|Gbeta	MTOR assc	protein-co
lib-C666-l	chr1	89727804	89727804	+	8.8228	NA	intron (NM_001134486, intron 10 of 1	MIR|SINE|MIR	10740	NM_05294	115362	Hs.513726	NM_05294	ENSG0000	GBP5	GBP-5	guanylate	protein-co
lib-C666-l	chr3	40351099	40351099	+	7.09685	NA	promoter-TSS (NR_033965)	promoter-TSS (NR_033965)	-74	NM_00587	10289	Hs.315230	NM_00587	ENSG0000	EIF1B	GC20	eukaryotic	protein-co
lib-C666-l	chr2	109065702	109065702	+	28.16397	NA	non-coding (NR_028063, exon 1 of 2	non-coding (NR_028063, exon 1 of	125	NM_18145	9648	Hs.436505	NM_18145	ENSG0000	GCC2	GCC185|R/	GRIP and c	protein-co
lib-C666-l	chr7	127225670	127225670	+	57.02325	NA	promoter-TSS (NM_024523)	promoter-TSS (NM_024523)	-16	NM_02452	79571	Hs.521168	NM_02452	ENSG0000	GCC1	GCC1P|GC	GRIP and c	protein-co
lib-C666-l	chr9	132836039	132836039	+	9.94769	NA	intron (NM_020960, intron 1 of 17)	AluSz|SINE|Alu	20054	NM_02096	57720	Hs.512461	NM_02096	ENSG0000	GPR107	GCDRP|LU	G protein-	protein-co
lib-C666-l	chr9	132835358	132835358	+	8.73012	NA	intron (NM_020960, intron 1 of 17)	L1ME3A|LINE|L1	19373	NM_02096	57720	Hs.512461	NM_02096	ENSG0000	GPR107	GCDRP|LU	G protein-	protein-co
lib-C666-l	chr9	132834963	132834963	+	7.83708	NA	intron (NM_020960, intron 1 of 17)	intron (NM_020960, intron 1 of 17)	18978	NM_02096	57720	Hs.512461	NM_02096	ENSG0000	GPR107	GCDRP|LU	G protein-	protein-co
lib-C666-l	chr9	6681751	6681751	+	14.54258	NA	Intergenic	CpG	-36059	NM_00017	2731	Hs.584238	NM_00017	ENSG0000	GLDC	GCE|GCSP|	glycine dec	protein-co
lib-C666-l	chr9	6681313	6681313	+	2.24066	NA	Intergenic	CpG	-35621	NM_00017	2731	Hs.584238	NM_00017	ENSG0000	GLDC	GCE|GCSP|	glycine dec	protein-co

lib-C666-l	chr16	81130114	81130114	+	8.03539	NA	promoter-TSS (NM_004483)	promoter-TSS (NM_004483)	-134	NM_00448	2653	Hs.546256	NM_00448	ENSG0000	GCSH	GCE|NKH	glycine clea	protein-co
lib-C666-l	chr16	81129740	81129740	+	5.37858	NA	exon (NM_004483, exon 1 of 5)	exon (NM_004483, exon 1 of 5)	240	NR_03324	2653	Hs.546256	NM_00448	ENSG0000	GCSH	GCE|NKH	glycine clea	protein-co
lib-C666-l	chr14	50999411	50999411	+	11.07709	NA	promoter-TSS (NM_006575)	promoter-TSS (NM_006575)	-35	NM_19879	11183	Hs.130491	NM_00657	ENSG0000	MAP4K5	GCKR|KHS|	mitogen-a	protein-co
lib-C666-l	chr14	50998916	50998916	+	6.48676	NA	promoter-TSS (NM_001127713)	promoter-TSS (NM_001127713)	460	NM_00657	11183	Hs.130491	NM_00657	ENSG0000	MAP4K5	GCKR|KHS|	mitogen-a	protein-co
lib-C666-l	chr12	120632640	120632640	+	3.59106	NA	promoter-TSS (NM_006836)	promoter-TSS (NM_006836)	-127	NM_00683	10985	Hs.298716	NM_00683	ENSG0000	GCN1	GCN1L|GC	GCN1, eIF2	protein-co
lib-C666-l	chr15	57998780	57998780	+	93.49621	NA	promoter-TSS (NM_001018102)	promoter-TSS (NM_001018102)	61	NM_01553	81488	Hs.437256	NM_01553	ENSG0000	POLR2M	GCOM1|GI	RNA polym	protein-co
lib-C666-l	chr8	41347899	41347899	+	2.59032	NA	promoter-TSS (NM_001174124)	promoter-TSS (NM_001174124)	-182	NM_01609	51125	Hs.654773	NM_01609	ENSG0000	GOLGA7	GCP16|GO	golgin A7	protein-co
lib-C666-l	chr12	133405604	133405604	+	19.82221	NA	promoter-TSS (NM_005895)	promoter-TSS (NM_005895)	-178	NM_00117	2802	Hs.507333	NM_00589	ENSG0000	GOLGA3	GCP170|M	golgin A3	protein-co
lib-C666-l	chr15	22833616	22833616	+	2.91691	NA	exon (NM_001102610, exon 1 of 22)	exon (NM_001102610, exon 1 of 22	221	NM_05290	114791	Hs.533898	NM_05290	ENSG0000	TUBGCP5	GCP5	tubulin gar	protein-co
lib-C666-l	chr22	50683468	50683468	+	315.46158	NA	promoter-TSS (NM_020461)	promoter-TSS (NM_020461)	-68	NM_02046	85378	Hs.336431	NM_02046	ENSG0000	TUBGCP6	GCP-6|GCI	tubulin gar	protein-co
lib-C666-l	chr1	226374418	226374418	+	5.96761	NA	promoter-TSS (NM_022735)	promoter-TSS (NM_022735)	5	NM_02273	64746	Hs.520207	NM_02273	ENSG0000	ACBD3	GCP60|GO	acyl-CoA b	protein-co
lib-C666-l	chr14	20922826	20922826	+	11.0277	NA	promoter-TSS (NM_001244249)	promoter-TSS (NM_001244249)	441	NM_01780	55644	Hs.525196	NM_01780	ENSG0000	OSGEP	GCPL1|KAE	O-sialoglyc	protein-co
lib-C666-l	chr12	97301033	97301033	+	147.12431	NA	promoter-TSS (NM_152905)	promoter-TSS (NM_152905)	32	NM_00113	121441	Hs.270084	NM_15290	ENSG0000	NEDD1	GCP-WD|T	neural prec	protein-co
lib-C666-l	chr10	74927854	74927854	+	284.35867	NA	promoter-TSS (NM_173348)	promoter-TSS (NM_173348)	-1	NR_02420	11319	Hs.631822	NM_00726	ENSG0000	ECD	GCR2|HSG	ecdysonele	protein-co
lib-C666-l	chr13	51654700	51654700	+	4.04037	NA	Intergenic	L1PA6|LINE|L1	-14407	NR_00392	2974	Hs.411573	NM_00412	ENSG0000	GUCY1B2	GC-SB2	guanylate c	pseudo
lib-C666-l	chr15	91478246	91478246	+	3.8557	NA	5' UTR (NM_001323621, exon 1 of 20	5' UTR (NM_001323621, exon 1 of 2	130	NM_01867	55898	Hs.389461	NM_01797	ENSG0000	UNC45A	GC-UNC45	unc-45 my	protein-co
lib-C666-l	chr17	32579732	32579732	+	20.19521	NA	Intergenic	Intergenic	-2564	NM_00298	6347	Hs.303649	NM_00298	ENSG0000	CCL2	GDCF-2|HC	C-C motif	protein-co
lib-C666-l	chr1	100315648	100315648	+	7.15277	NA	promoter-TSS (NM_000642)	promoter-TSS (NM_000642)	8	NM_00064	178	Hs.904	NM_00002	ENSG0000	AGL	GDE	amylo-alph	protein-co
lib-C666-l	chr11	75203364	75203364	+	15.33659	NA	intron (NM_030792, intron 1 of 16)	intron (NM_030792, intron 1 of 16)	33235	NM_03079	81544	Hs.503297	NM_03079	ENSG0000	GDPD5	GDE2|PP16	glycerophc	protein-co
lib-C666-l	chr11	77011288	77011288	+	9.38975	NA	Intergenic	LTR10F|LTR|ERV1	-12825	NM_18283	220032	Hs.249795	NM_18283	ENSG0000	GDPD4	GDE6	glycerophc	protein-co
lib-C666-l	chr17	79831859	79831859	+	3.20885	NA	Intergenic	L1M4|LINE|L1	-2577	NM_00118	396	Hs.159161	NM_00430	ENSG0000	ARHGDIA	GDIA1|HEL	Rho GDP c	protein-co
lib-C666-l	chr8	21461776	21461776	+	22.82077	NA	Intergenic	ERV3-16A3_I-int|LTR|ERVL	184570	NM_00116	2675	Hs.441202	NM_00149	ENSG0000	GFRA2	GDNFRB|N	GDNF fam	protein-co
lib-C666-l	chr4	99181884	99181884	+	8.01457	NA	promoter-TSS (NM_001100426)	promoter-TSS (NM_001100426)	-643	NM_00110	5910	Hs.132858	NM_02115	ENSG0000	RAP1GDS1	GDS1|Smg	Rap1 GTPa	protein-co
lib-C666-l	chr1	3371079	3371079	+	3.84463	NA	promoter-TSS (NM_014448)	promoter-TSS (NM_014448)	-68	NM_01444	27237	Hs.87435	NM_01444	ENSG0000	ARHGEF16	GEF16|NBF	Rho guanir	protein-co
lib-C666-l	chr3	57112762	57112762	+	21.0836	NA	intron (NM_001128615, intron 1 of 12	CpG	574	NM_00112	50650	Hs.476402	NM_01955	ENSG0000	ARHGEF3	GEF3|STA3	Rho guanir	protein-co
lib-C666-l	chr7	144052386	144052386	+	44.13626	NA	promoter-TSS (NM_005435)	promoter-TSS (NM_005435)	-103	NM_00543	7984	Hs.334	NM_00543	ENSG0000	ARHGEF5	GEF5|P60|T	Rho guanir	protein-co
lib-C666-l	chr6	24775257	24775257	+	3.89034	NA	promoter-TSS (NM_001251989)	promoter-TSS (NM_001251989)	98	NM_00125	51053	Hs.234896	NM_01589	ENSG0000	GMNN	Gem|MGO	geminin, D	protein-co
lib-C666-l	chr3	190580599	190580599	+	11.66012	NA	promoter-TSS (NM_001146686)	promoter-TSS (NM_001146686)	-134	NM_00114	647309	Hs.518490	NM_00114	ENSG0000	GMNC	GEMC1	geminin cc	protein-co
lib-C666-l	chr5	154317804	154317804	+	5.25516	NA	promoter-TSS (NM_015465)	promoter-TSS (NM_015465)	-28	NM_00125	25929	Hs.483921	NM_01546	ENSG0000	GEMIN5	GEMIN-5	gem nucle	protein-co
lib-C666-l	chr1	8086519	8086519	+	49.56857	NA	promoter-TSS (NM_018948)	promoter-TSS (NM_018948)	-126	NM_01894	54206	Hs.605445	NM_01894	ENSG0000	ERRFI1	GENE-33|N	ERBB recep	protein-co
lib-C666-l	chr1	8157718	8157718	+	13.20416	NA	Intergenic	Intergenic	-71325	NM_01894	54206	Hs.605445	NM_01894	ENSG0000	ERRFI1	GENE-33|N	ERBB recep	protein-co
lib-C666-l	chr1	155146726	155146726	+	8.22036	NA	promoter-TSS (NR_046329)	promoter-TSS (NR_046329)	463	NM_00128	80128	Hs.287735	NM_02505	ENSG0000	TRIM46	GENEY|TRI	tripartite m	protein-co
lib-C666-l	chr10	104402078	104402078	+	16.63918	NA	Intergenic	CpG	-2174	NR_14432	81603	Hs.336810	NM_03091	ENSG0000	TRIM8	GERP|RNF2	tripartite m	protein-co
lib-C666-l	chr10	104370392	104370392	+	9.63603	NA	intron (NM_016169, intron 8 of 11)	L3|LINE|CR1	-33860	NR_14432	81603	Hs.336810	NM_03091	ENSG0000	TRIM8	GERP|RNF2	tripartite m	protein-co
lib-C666-l	chr10	104405326	104405326	+	4.98682	NA	intron (NM_001345950, intron 1 of 4)	UCON4|Unknown|Unknown	1074	NM_00134	81603	Hs.336810	NM_03091	ENSG0000	TRIM8	GERP|RNF2	tripartite m	protein-co
lib-C666-l	chr2	39351530	39351530	+	27.44508	NA	Intergenic	CpG	-3926	NM_00563	6654	Hs.709893	NM_00563	ENSG0000	SOS1	GF1|GGF1|	SOS Ras/R	protein-co
lib-C666-l	chr12	52444934	52444934	+	57.02275	NA	promoter-TSS (NM_002135)	promoter-TSS (NM_002135)	-252	NM_00213	3164	Hs.524430	NM_00213	ENSG0000	NR4A1	GFRP1|HM	nuclear rec	protein-co
lib-C666-l	chr12	52445171	52445171	+	36.16835	NA	promoter-TSS (NM_002135)	promoter-TSS (NM_002135)	-15	NM_00213	3164	Hs.524430	NM_00213	ENSG0000	NR4A1	GFRP1|HM	nuclear rec	protein-co
lib-C666-l	chr12	52430795	52430795	+	28.57903	NA	promoter-TSS (NM_001202234)	promoter-TSS (NM_001202234)	-704	NM_00120	3164	Hs.524430	NM_00213	ENSG0000	NR4A1	GFRP1|HM	nuclear rec	protein-co
lib-C666-l	chr12	52426894	52426894	+	20.72954	NA	intron (NM_001202233, intron 1 of 7)	CpG	-4605	NM_00120	3164	Hs.524430	NM_00213	ENSG0000	NR4A1	GFRP1|HM	nuclear rec	protein-co
lib-C666-l	chr12	52419624	52419624	+	15.33659	NA	intron (NM_001202233, intron 1 of 7)	intron (NM_001202233, intron 1 of	3008	NM_00120	3164	Hs.524430	NM_00213	ENSG0000	NR4A1	GFRP1|HM	nuclear rec	protein-co
lib-C666-l	chr12	52414562	52414562	+	2.58854	NA	Intergenic	Intergenic	-2054	NM_00120	3164	Hs.524430	NM_00213	ENSG0000	NR4A1	GFRP1|HM	nuclear rec	protein-co
lib-C666-l	chr5	118603896	118603896	+	54.1948	NA	promoter-TSS (NM_001286817)	promoter-TSS (NM_001286817)	-491	NM_00128	25816	Hs.618488	NM_01435	ENSG0000	TNFAIP8	GG2-1|MD	TNF alpha	protein-co
lib-C666-l	chr5	118660136	118660136	+	3.23837	NA	intron (NM_001286815, intron 2 of 2)	intron (NM_001286815, intron 2 of	-8734	NM_00128	25816	Hs.618488	NM_01435	ENSG0000	TNFAIP8	GG2-1|MD	TNF alpha	protein-co
lib-C666-l	chr17	47269430	47269430	+	6.1054	NA	Intergenic	CpG	17313	NM_03149	2793	Hs.181781	NM_03149	ENSG0000	GNGT2	G-GAMMA	G protein s	protein-co
lib-C666-l	chr8	63951310	63951310	+	19.60174	NA	exon (NM_003878, exon 1 of 9)	exon (NM_003878, exon 1 of 9)	300	NM_00387	8836	Hs.78619	NM_00387	ENSG0000	GGH	GH	gamma-gl	protein-co
lib-C666-l	chr7	30978534	30978534	+	8.96177	NA	Intergenic	Intergenic	-25102	NM_00082	2692	Hs.767	NM_00082	ENSG0000	GHRHR	GHRFR|GR	growth hor	protein-co
lib-C666-l	chr5	177557821	177557821	+	14.17553	NA	promoter-TSS (NM_001288794)	promoter-TSS (NM_001288794)	-141	NM_00128	64777	Hs.27222	NM_02276	ENSG0000	RMND5B	GID2|GID2	required fc	protein-co
lib-C666-l	chr2	47168041	47168041	+	3.23837	NA	promoter-TSS (NM_001288951)	promoter-TSS (NM_001288951)	-272	NM_02045	57217	Hs.370603	NM_02045	ENSG0000	TTC7A	GIDID|MIN	tetratricop	protein-co
lib-C666-l	chr22	24236466	24236466	+	30.07415	NA	promoter-TSS (NM_002415)	promoter-TSS (NM_002415)	-99	NM_00241	4282	Hs.407995	NM_00241	ENSG0000	MIF	GIF|GLIF|M	macrophag	protein-co
lib-C666-l	chr22	24237200	24237200	+	9.7234	NA	non-coding (NR_038911, exon 3 of 3	non-coding (NR_038911, exon 3 of	635	NM_00241	4282	Hs.407995	NM_00241	ENSG0000	MIF	GIF|GLIF|M	macrophag	protein-co
lib-C666-l	chr11	70005959	70005959	+	32.29191	NA	intron (NM_018043, intron 16 of 25)	intron (NM_018043, intron 16 of 25	-43310	NM_00382	8772	Hs.86131	NM_00382	ENSG0000	FADD	GIG3|MOR	Fas associa	protein-co
lib-C666-l	chr1	174968616	174968616	+	127.96682	NA	promoter-TSS (NM_014412)	promoter-TSS (NM_014412)	45	NM_00100	27101	Hs.508524	NM_01441	ENSG0000	CACYBP	GIG5|PNAS	calcyclin bi	protein-co
lib-C666-l	chr2	8825976	8825976	+	43.03704	NA	Intergenic	CpG	3863	NM_00216	3398	Hs.180919	NM_00216	ENSG0000	ID2	GIG8|ID2A|	inhibitor of	protein-co
lib-C666-l	chr2	8822329	8822329	+	10.16134	NA	promoter-TSS (NR_110154)	promoter-TSS (NR_110154)	216	NM_00216	3398	Hs.180919	NM_00216	ENSG0000	ID2	GIG8|ID2A|	inhibitor of	protein-co
lib-C666-l	chr3	167813692	167813692	+	4.52748	NA	promoter-TSS (NM_001308155)	promoter-TSS (NM_001308155)	21	NM_01449	27333	Hs.143600	NM_01449	ENSG0000	GOLIM4	GIMPC|GO	golgi integ	protein-co
lib-C666-l	chr5	102455895	102455895	+	3.98718	NA	promoter-TSS (NM_001317954)	promoter-TSS (NM_001317954)	-53	NM_00131	54826	Hs.24088	NM_01767	ENSG0000	GIN1	GIN-1|TGII	gypsy retrc	protein-co
lib-C666-l	chr7	6746465	6746465	+	3.1901	NA	5' UTR (NM_006956, exon 1 of 6)	5' UTR (NM_006956, exon 1 of 6)	101	NM_00695	7559	Hs.431471	NM_00695	ENSG0000	ZNF12	GIOT-3|HZ	zinc finger	protein-co
lib-C666-l	chr3	50263940	50263940	+	6.50091	NA	promoter-TSS (NR_106714)	promoter-TSS (NR_106714)	-180	NM_00128	2771	Hs.77269	NM_00207	ENSG0000	GNAI2	GIP|GNAI2	G protein s	protein-co
lib-C666-l	chr3	50280763	50280763	+	2.0708	NA	intron (NM_001282618, intron 1 of 7)	intron (NM_001282618, intron 1 of	-3563	NM_00116	2771	Hs.77269	NM_00207	ENSG0000	GNAI2	GIP|GNAI2	G protein s	protein-co
lib-C666-l	chr6	108395968	108395968	+	19.75653	NA	promoter-TSS (NM_014028)	promoter-TSS (NM_014028)	-27	NM_01402	28962	Hs.226780	NM_01402	ENSG0000	OSTM1	GIPN|GL|H	osteopetro	protein-co
lib-C666-l	chr2	156299706	156299706	+	7.52748	NA	Intergenic	MLT1J|LTR|ERVL-MaLR	744613	NM_00126	3760	Hs.591606	NM_00223	ENSG0000	KCNJ3	GIRK1|KGA	potassium	protein-co
lib-C666-l	chr16	29465748	29465748	+	26.68159	NA	promoter-TSS (NM_001320628)	promoter-TSS (NM_001320628)	-74	NM_00101	548593	Hs.729791	NM_00101	ENSG0000	SLX1A	GIYD1	SLX1 homo	protein-co
lib-C666-l	chr16	30205075	30205075	+	12.18875	NA	promoter-TSS (NR_037608)	promoter-TSS (NR_037608)	-89	NM_00101	548593	Hs.729791	NM_00101	ENSG0000	SLX1A	GIYD1	SLX1 homo	protein-co
lib-C666-l	chr17	61851128	61851128	+	57.48363	NA	promoter-TSS (NM_020198)	promoter-TSS (NM_020198)	-40	NM_02019	57003	Hs.202011	NM_02019	ENSG0000	CCDC47	GK001|MS	coiled-coil	protein-co
lib-C666-l	chrX	30671320	30671320	+	10.37767	NA	promoter-TSS (NM_203391)	promoter-TSS (NM_203391)	-156	NM_00016	2710	Hs.1466	NM_00016	ENSG0000	GK	GK1|GKD	glycerol kir	protein-co
lib-C666-l	chr15	49462202	49462202	+	34.3461	NA	promoter-TSS (NM_001289031)	promoter-TSS (NM_001289031)	-11	NM_00128	2585	Hs.122006	NM_00204	ENSG0000	GALK2	GK2	galactokina	protein-co
lib-C666-l	chr11	65307871	65307871	+	10.0479	NA	intron (NM_001164266, intron 22 of 2	CpG-4341	15323	NM_02068	57410	Hs.238839	NM_02068	ENSG0000	SCYL1	GKLP|HT01	SCY1 like p	protein-co
lib-C666-l	chr3	9932575	9932575	+	3.25502	NA	intron (NM_032492, intron 1 of 1)	CpG	304	NM_03249	84522	Hs.24054	NM_03249	ENSG0000	JAGN1	GL009|SCN	jagunal ho	protein-co
lib-C666-l	chr14	24584036	24584036	+	58.5532	NA	promoter-TSS (NM_181357)	promoter-TSS (NM_181357)	50	NM_02523	80344	Hs.525251	NM_02523	ENSG0000	DCAF11	GL014|PRC	DDB1 and	protein-co
lib-C666-l	chr1	149858354	149858354	+	20.66352	NA	promoter-TSS (NM_003517)	promoter-TSS (NM_003517)	-122	NM_00352	8349	Hs.2178	NM_00352	ENSG0000	HIST2H2BE	GL105|H2E	histone clu	protein-co
lib-C666-l	chr10	73620233	73620233	+	7.15277	NA	Intergenic	Intergenic	-9101	NM_00104	5660	Hs.523004	NM_00277	ENSG0000	PSAP	GLBA|SAP1	prosaposin	protein-co
lib-C666-l	chr5	110428003	110428003	+	16.20502	NA	exon (NM_139281, exon 1 of 23)	exon (NM_139281, exon 1 of 23)	133	NM_13928	134430	Hs.533237	NM_13928	ENSG0000	WDR36	GLC1G|TA-	WD repeat	protein-co
lib-C666-l	chr11	62389665	62389665	+	37.64901	NA	promoter-TSS (NM_001288721)	promoter-TSS (NM_001288721)	-18	NM_00128	26229	Hs.502759	NM_01220	ENSG0000	B3GAT3	GLCATI|JDS	beta-1,3-g	protein-co
lib-C666-l	chr6	71549739	71549739	+	7.52748	NA	intron (NM_001281440, intron 7 of 10	intron (NM_001281440, intron 7 of	117049	NM_08074	135152	Hs.713609	NM_08074	ENSG0000	B3GAT2	GLCATS	beta-1,3-g	protein-co
lib-C666-l	chr1	94375105	94375105	+	7.49163	NA	promoter-TSS (NM_001308253)	promoter-TSS (NM_001308253)	49	NM_00206	2730	Hs.315562	NM_00206	ENSG0000	GCLM	GLCLR	glutamate-	protein-co
lib-C666-l	chr12	57855204	57855204	+	28.88523	NA	intron (NM_001160045, intron 1 of 9)	intron (NM_001160045, intron 1 of	1270	NM_00116	2735	Hs.632702	NM_00526	ENSG0000	GLI1	GLI	GLI family z	protein-co
lib-C666-l	chr12	57853130	57853130	+	15.42216	NA	promoter-TSS (NM_001160045)	promoter-TSS (NM_001160045)	-788	NM_00116	2735	Hs.632702	NM_00526	ENSG0000	GLI1	GLI	GLI family z	protein-co
lib-C666-l	chr2	39664189	39664189	+	2.59032	NA	promoter-TSS (NR_037875)	promoter-TSS (NR_037875)	264	NM_00127	8491	Hs.655750	NM_00361	ENSG0000	MAP4K3	GLK|MAPK	mitogen-a	protein-co
lib-C666-l	chr1	182360468	182360468	+	5.5166	NA	promoter-TSS (NM_001033056)	promoter-TSS (NM_001033056)	71	NM_00103	2752	Hs.132016	NM_00206	ENSG0000	GLUL	GLNS|GS|P	glutamate-	protein-co
lib-C666-l	chr16	1877078	1877078	+	40.6674	NA	promoter-TSS (NM_031208)	promoter-TSS (NM_031208)	117	NM_00532	3029	Hs.157394	NM_00532	ENSG0000	HAGH	GLO2|GLX2	hydroxyacy	protein-co
lib-C666-l	chr16	1876237	1876237	+	8.91001	NA	promoter-TSS (NM_031208)	promoter-TSS (NM_031208)	958	NM_00532	3029	Hs.157394	NM_00532	ENSG0000	HAGH	GLO2|GLX2	hydroxyacy	protein-co
lib-C666-l	chr15	74375000	74375000	+	11.6145	NA	promoter-TSS (NM_001038640)	promoter-TSS (NM_001038640)	-109	NM_00103	342096	Hs.546408	NM_00103	ENSG0000	GOLGA6A	GLP|GOLG	golgin A6 f	protein-co
lib-C666-l	chr10	132099762	132099762	+	20.59244	NA	Intergenic	Intergenic	165089	NM_00132	10539	Hs.42644	NM_00654	ENSG0000	GLRX3	GLRX4|GR	glutaredox	protein-co
lib-C666-l	chr2	200324242	200324242	+	10.40942	NA	intron (NM_001172517, intron 2 of 11	CpG-15850	-1423	NR_13496	23314	Hs.516617	NM_01526	ENSG0000	SATB2	GLSS	SATB home	protein-co
lib-C666-l	chr19	17666341	17666341	+	47.23204	NA	promoter-TSS (NM_024656)	promoter-TSS (NM_024656)	-62	NM_02465	79709	Hs.418795	NM_02465	ENSG0000	COLGALT1	GLT25D1	collagen be	protein-co
lib-C666-l	chr19	17717079	17717079	+	12.83248	NA	exon (NM_001080421, exon 42 of 42)	exon (NM_001080421, exon 42 of 4	50676	NM_02465	79709	Hs.418795	NM_02465	ENSG0000	COLGALT1	GLT25D1	collagen be	protein-co
lib-C666-l	chr11	105590272	105590272	+	20.59244	NA	intron (NM_001077243, intron 3 of 16	intron (NM_001077243, intron 3 of	108861	NM_00111	2893	Hs.503743	NM_00082	ENSG0000	GRIA4	GLUR4|GLU	glutamate	protein-co
lib-C666-l	chrX	122474728	122474728	+	16.59065	NA	intron (NM_007325, intron 4 of 15)	intron (NM_007325, intron 4 of 15)	156632	NM_00125	2892	Hs.377070	NM_00082	ENSG0000	GRIA3	GLUR-C|GI	glutamate	protein-co
lib-C666-l	chr6	134373793	134373793	+	26.54591	NA	promoter-TSS (NM_145176)	promoter-TSS (NM_145176)	-4	NM_14517	154091	Hs.486508	NM_14517	ENSG0000	SLC2A12	GLUT12|GL	solute carr	protein-co
lib-C666-l	chr3	170695990	170695990	+	6.13111	NA	Intergenic	Intergenic	48778	NM_00034	6514	Hs.167584	NM_00034	ENSG0000	SLC2A2	GLUT2	solute carr	protein-co
lib-C666-l	chr9	136344361	136344361	+	5.80117	NA	promoter-TSS (NM_001145099)	promoter-TSS (NM_001145099)	-85	NM_00114	11182	Hs.244378	NM_01758	ENSG0000	SLC2A6	GLUT6|GLU	solute carr	protein-co
lib-C666-l	chr9	130150830	130150830	+	14.3056	NA	intron (NM_032293, intron 25 of 27)	intron (NM_032293, intron 25 of 27	-8587	NM_00127	29988	Hs.179522	NM_01458	ENSG0000	SLC2A8	GLUT8|GLU	solute carr	protein-co
lib-C666-l	chr9	130158866	130158866	+	9.93242	NA	promoter-TSS (NM_001271711)	promoter-TSS (NM_001271711)	-551	NM_00127	29988	Hs.179522	NM_01458	ENSG0000	SLC2A8	GLUT8|GLU	solute carr	protein-co
lib-C666-l	chr2	113403387	113403387	+	20.19521	NA	promoter-TSS (NM_005415)	promoter-TSS (NM_005415)	-47	NM_00541	6574	Hs.187946	NM_00541	ENSG0000	SLC20A1	GLVR1|Glv	solute carr	protein-co
lib-C666-l	chr8	42334290	42334290	+	7.03232	NA	intron (NM_006749, intron 1 of 10)	Tigger8|DNA|TcMar-Tigger	24688	NM_00125	6575	Hs.653173	NM_00674	ENSG0000	SLC20A2	GLVR-2|GL	solute carr	protein-co
lib-C666-l	chr9	37409478	37409478	+	14.81317	NA	Intergenic	Intergenic	-13229	NM_01220	9380	Hs.155742	NM_01220	ENSG0000	GRHPR	GLXR|GLYD	glyoxylate	protein-co
lib-C666-l	chr2	241374696	241374696	+	23.74671	NA	promoter-TSS (NM_002081)	promoter-TSS (NM_002081)	-419	NM_00208	2817	Hs.328232	NM_00208	ENSG0000	GPC1	glypican	glypican 1	protein-co
lib-C666-l	chr1	44495850	44495850	+	10.03103	NA	intron (NM_001024845, intron 1 of 13	CpG	945	NM_00132	6536	Hs.442590	NM_00693	ENSG0000	SLC6A9	GLYT1	solute carr	protein-co
lib-C666-l	chr18	46065880	46065880	+	22.5977	NA	intron (NM_014772, intron 1 of 11)	CpG	453	NM_01477	9811	Hs.145230	NM_01477	ENSG0000	CTIF	Gm672|KIA	cap bindin	protein-co
lib-C666-l	chr18	46072903	46072903	+	8.89416	NA	intron (NM_001142397, intron 2 of 12	L1MEd|LINE|L1	7476	NM_01477	9811	Hs.145230	NM_01477	ENSG0000	CTIF	Gm672|KIA	cap bindin	protein-co
lib-C666-l	chr6	2245921	2245921	+	58.76075	NA	promoter-TSS (NR_046229)	promoter-TSS (NR_046229)	-53	NM_00150	2762	Hs.144496	NM_00150	ENSG0000	GMDS	GMD|SDR3	GDP-manr	protein-co
lib-C666-l	chr14	54939275	54939275	+	24.41787	NA	Intergenic	Intergenic	16469	NM_00412	2764	Hs.151413	NM_00412	ENSG0000	GMFB	GMF	glia matura	protein-co
lib-C666-l	chr2	99225078	99225078	+	14.37873	NA	promoter-TSS (NM_001008215)	promoter-TSS (NM_001008215)	67	NM_00133	25972	Hs.13370	NM_01404	ENSG0000	UNC50	GMH1|HSD	unc-50 inn	protein-co
lib-C666-l	chr1	228327709	228327709	+	145.47461	NA	promoter-TSS (NM_000858)	promoter-TSS (NM_000858)	-76	NM_00124	2987	Hs.376933	NM_00085	ENSG0000	GUK1	GMK	guanylate	protein-co
lib-C666-l	chr5	177019108	177019108	+	7.67555	NA	promoter-TSS (NM_017510)	promoter-TSS (NM_017510)	-41	NM_01751	54732	Hs.279929	NM_01751	ENSG0000	TMED9	GMP25|HS	transmemb	protein-co
lib-C666-l	chr14	24701667	24701667	+	58.13765	NA	promoter-TSS (NM_006156)	promoter-TSS (NM_006156)	39	NM_00100	51292	Hs.368855	NM_01657	ENSG0000	GMPR2	GMPR 2	guanosine	protein-co
lib-C666-l	chr5	180671294	180671294	+	2.31621	NA	promoter-TSS (NM_006098)	promoter-TSS (NM_006098)	-388	NM_00609	10399	Hs.5662	NM_00609	ENSG0000	RACK1	GNB2L1|Gr	receptor fc	protein-co
lib-C666-l	chr2	71295371	71295371	+	28.92533	NA	promoter-TSS (NM_017567)	promoter-TSS (NM_017567)	-37	NM_00133	55577	Hs.7036	NM_01756	ENSG0000	NAGK	GNK|HSA2	N-acetylgl	protein-co
lib-C666-l	chr2	71295910	71295910	+	19.19377	NA	intron (NM_001330425, intron 1 of 8)	CpG	502	NM_00133	55577	Hs.7036	NM_01756	ENSG0000	NAGK	GNK|HSA2	N-acetylgl	protein-co
lib-C666-l	chr12	104324047	104324047	+	13.46097	NA	promoter-TSS (NR_037425)	promoter-TSS (NR_037425)	-58	NR_02724	253724	Hs.506549	NR_02724	ENSG0000	TTC41P	GNN|GNN	tetratricop	pseudo
lib-C666-l	chr12	104323211	104323211	+	11.06679	NA	promoter-TSS (NR_037425)	promoter-TSS (NR_037425)	778	NR_02724	253724	Hs.506549	NR_02724	ENSG0000	TTC41P	GNN|GNN	tetratricop	pseudo
lib-C666-l	chr12	104319276	104319276	+	11.06679	NA	intron (NR_027249, intron 1 of 15)	MLT1F|LTR|ERVL-MaLR	4713	NR_02724	253724	Hs.506549	NR_02724	ENSG0000	TTC41P	GNN|GNN	tetratricop	pseudo
lib-C666-l	chr22	39858663	39858663	+	62.02255	NA	intron (NM_002409, intron 1 of 1)	intron (NM_002409, intron 1 of 1)	5338	NM_00240	4248	Hs.276808	NM_00240	ENSG0000	MGAT3	GNT-III|GN	mannosyl (	protein-co
lib-C666-l	chr12	87106388	87106388	+	10.92275	NA	intron (NM_013244, intron 1 of 6)	intron (NM_013244, intron 1 of 6)	126293	NM_01324	25834	Hs.589093	NM_01324	ENSG0000	MGAT4C	GNTIVH|H	MGAT4 far	protein-co
lib-C666-l	chr2	135219314	135219314	+	29.98795	NA	intron (NM_030923, intron 6 of 7)	intron (NM_030923, intron 6 of 7)	207484	NM_00241	4249	Hs.4988	NM_00241	ENSG0000	MGAT5	GNT-V|GN	mannosyl (	protein-co
lib-C666-l	chr1	170501468	170501468	+	10.40942	NA	intron (NM_152281, intron 1 of 4)	intron (NM_152281, intron 1 of 4)	205	NM_15228	92344	Hs.183702	NM_15228	ENSG0000	GORAB	GO|NTKLB	golgin, RAI	protein-co
lib-C666-l	chr15	72946913	72946913	+	17.62684	NA	promoter-TSS (NM_018652)	promoter-TSS (NM_018652)	-125	NM_01865	55889	Hs.546408	NM_01865	ENSG0000	GOLGA6B	GOLGA|GC	golgin A6 f	protein-co
lib-C666-l	chr12	100557717	100557717	+	8.48019	NA	intron (NR_036632, intron 2 of 9)	intron (NR_036632, intron 2 of 9)	9404	NR_02426	55592	Hs.524660	NM_01760	ENSG0000	GOLGA2P5	GOLGA2B|	golgin A2 p	pseudo
lib-C666-l	chr15	23414260	23414260	+	9.48322	NA	intron (NM_001271664, intron 9 of 11	intron (NM_001271664, intron 9 of	-20810	NR_03335	390535	Hs.454647	NM_001012423		GOLGA8EF	GOLGA8E	golgin A8 f	pseudo
lib-C666-l	chr15	23452619	23452619	+	4.39666	NA	intron (NM_001271664, intron 9 of 11	AluSp|SINE|Alu	17549	NR_03335	390535	Hs.454647	NM_001012423		GOLGA8EF	GOLGA8E	golgin A8 f	pseudo
lib-C666-l	chr9	127703373	127703373	+	60.89502	NA	promoter-TSS (NM_002077)	promoter-TSS (NM_002077)	13	NM_00207	2800	Hs.59504	NM_00207	ENSG0000	GOLGA1	golgin-97	golgin A1	protein-co
lib-C666-l	chr14	93260474	93260474	+	69.8176	NA	promoter-TSS (NM_005113)	promoter-TSS (NM_005113)	-102	NM_00511	9950	Hs.104320	NM_00511	ENSG0000	GOLGA5	GOLIM5|RI	golgin A5	protein-co
lib-C666-l	chr2	171785619	171785619	+	3.98718	NA	5' UTR (NM_015530, exon 1 of 10)	5' UTR (NM_015530, exon 1 of 10)	583	NM_01553	26003	Hs.431317	NM_01553	ENSG0000	GORASP2	GOLPH6|G	golgi reass	protein-co
lib-C666-l	chr1	32757568	32757568	+	31.81975	NA	promoter-TSS (NM_004964)	promoter-TSS (NM_004964)	-140	NM_00496	3065	Hs.88556	NM_00496	ENSG0000	HDAC1	GON-10|H	histone de	protein-co
lib-C666-l	chr1	155827074	155827074	+	12.12504	NA	promoter-TSS (NM_032292)	promoter-TSS (NM_032292)	12	NM_00128	54856	Hs.656361	NM_03229	ENSG0000	GON4L	GON-4|GC	gon-4 like	protein-co
lib-C666-l	chr19	49935010	49935010	+	9.79436	NA	intron (NM_020309, intron 9 of 11)	intron (NM_020309, intron 9 of 11)	5322	NM_00119	100507003	Hs.553831	NM_00119	ENSG0000	GFY	Goofy	golgi assoc	protein-co
lib-C666-l	chr16	11059656	11059656	+	11.90035	NA	intron (NM_015226, intron 3 of 22)	intron (NM_015226, intron 3 of 22)	21311	NM_00124	23274	Hs.35490	NM_01522	ENSG0000	CLEC16A	Gop-1|KIA	C-type lec	protein-co
lib-C666-l	chr5	32174456	32174456	+	28.8837	NA	promoter-TSS (NM_022130)	promoter-TSS (NM_022130)	-31	NM_02213	64083	Hs.408909	NM_02213	ENSG0000	GOLPH3	GOPP1|GP	golgi phos	protein-co
lib-C666-l	chr5	32173788	32173788	+	14.3056	NA	intron (NM_022130, intron 1 of 3)	CpG	637	NM_02213	64083	Hs.408909	NM_02213	ENSG0000	GOLPH3	GOPP1|GP	golgi phos	protein-co
lib-C666-l	chr5	32174060	32174060	+	7.09685	NA	exon (NM_022130, exon 1 of 4)	exon (NM_022130, exon 1 of 4)	365	NM_02213	64083	Hs.408909	NM_02213	ENSG0000	GOLPH3	GOPP1|GP	golgi phos	protein-co
lib-C666-l	chr22	39101617	39101617	+	30.98434	NA	promoter-TSS (NM_004286)	promoter-TSS (NM_004286)	-190	NM_00428	9567	Hs.276925	NM_00428	ENSG0000	GTPBP1	GP-1|GP1|	GTP bindin	protein-co
lib-C666-l	chr3	52866195	52866195	+	3.92495	NA	TTS (NM_001198974)	TTS (NM_001198974)	-1478	NM_00221	3700	Hs.709406	NM_00221	ENSG0000	ITIH4	GP120|H4F	inter-alpha	protein-co
lib-C666-l	chr9	87374397	87374397	+	9.02258	NA	intron (NM_001018064, intron 11 of 1	intron (NM_001018064, intron 11 o	89105	NM_00101	4915	Hs.494312	NM_00618	ENSG0000	NTRK2	GP145-Trk	neurotropl	protein-co
lib-C666-l	chr9	87555271	87555271	+	8.01457	NA	intron (NM_001018064, intron 13 of 1	intron (NM_001018064, intron 13 o	269979	NM_00101	4915	Hs.494312	NM_00618	ENSG0000	NTRK2	GP145-Trk	neurotropl	protein-co
lib-C666-l	chr9	87283300	87283300	+	4.73866	NA	promoter-TSS (NM_006180)	promoter-TSS (NM_006180)	-73	NM_00618	4915	Hs.494312	NM_00618	ENSG0000	NTRK2	GP145-Trk	neurotropl	protein-co
lib-C666-l	chr17	28705907	28705907	+	24.25638	NA	promoter-TSS (NM_001304)	promoter-TSS (NM_001304)	-35	NM_00130	1362	Hs.446079	NM_00130	ENSG0000	CPD	GP180	carboxype	protein-co
lib-C666-l	chr7	131305611	131305611	+	52.3754	NA	Intergenic	Intergenic	-64235	NM_00539	5420	Hs.744213	NM_00539	ENSG0000	PODXL	Gp200|PC|	podocalyxi	protein-co
lib-C666-l	chr7	131198409	131198409	+	8.85547	NA	intron (NM_001018111, intron 1 of 8)	intron (NM_001018111, intron 1 of	42967	NM_00101	5420	Hs.744213	NM_00539	ENSG0000	PODXL	Gp200|PC|	podocalyxi	protein-co
lib-C666-l	chr3	197687024	197687024	+	25.49921	NA	promoter-TSS (NM_033029)	promoter-TSS (NM_033029)	-47	NM_03302	89782	Hs.518540	NM_03302	ENSG0000	LMLN	GP63|INV|l	leishmanol	protein-co
lib-C666-l	chr16	56459505	56459505	+	3.57421	NA	promoter-TSS (NM_001144)	promoter-TSS (NM_001144)	-55	NM_00114	267	Hs.295137	NM_00114	ENSG0000	AMFR	GP78|RNF4	autocrine r	protein-co
lib-C666-l	chr1	109940672	109940672	+	5.5166	NA	promoter-TSS (NM_002959)	promoter-TSS (NM_002959)	-105	NM_00295	6272	Hs.485195	NM_00295	ENSG0000	SORT1	Gp95|LDLC	sortilin 1	protein-co
lib-C666-l	chr10	113943530	113943530	+	432.39868	NA	promoter-TSS (NM_020918)	promoter-TSS (NM_020918)	7	NM_02091	57678	Hs.42586	NM_02091	ENSG0000	GPAM	GPAT|GPA	glycerol-3-	protein-co
lib-C666-l	chr10	113977372	113977372	+	8.89416	NA	Intergenic	MIRc|SINE|MIR	-33835	NM_00124	57678	Hs.42586	NM_02091	ENSG0000	GPAM	GPAT|GPA	glycerol-3-	protein-co
lib-C666-l	chrX	48980231	48980231	+	73.89001	NA	promoter-TSS (NM_015698)	promoter-TSS (NM_015698)	-80	NM_01569	27238	Hs.503666	NM_01569	ENSG0000	GPKOW	GPATC5|GI	G-patch do	protein-co
lib-C666-l	chr20	62339499	62339499	+	2.59032	NA	promoter-TSS (NM_001267547)	promoter-TSS (NM_001267547)	119	NM_18148	84619	Hs.590868	NM_03252	ENSG0000	ZGPAT	GPATC6|GI	zinc finger	protein-co
lib-C666-l	chr20	25229143	25229143	+	4.39666	NA	intron (NM_002862, intron 1 of 19)	CpG	437	NM_00286	5834	Hs.368157	NM_00286	ENSG0000	PYGB	GPBB	glycogen p	protein-co
lib-C666-l	chr5	56469523	56469523	+	62.69942	NA	promoter-TSS (NM_001203246)	promoter-TSS (NM_001203246)	-252	NM_00120	65056	Hs.444279	NM_02291	ENSG0000	GPBP1	GPBP|SSH	GC-rich pr	protein-co
lib-C666-l	chr3	169755695	169755695	+	20.19521	NA	promoter-TSS (NM_014373)	promoter-TSS (NM_014373)	-40	NM_01437	26996	Hs.231320	NM_01437	ENSG0000	GPR160	GPCR1|GP	G protein-	protein-co
lib-C666-l	chr19	33865059	33865059	+	9.20967	NA	promoter-TSS (NM_001252296)	promoter-TSS (NM_001252296)	-371	NM_00125	1054	Hs.429666	NM_00180	ENSG0000	CEBPG	GPE1BP|IG	CCAAT/en	protein-co
lib-C666-l	chr19	33864555	33864555	+	4.73866	NA	promoter-TSS (NM_001252296)	promoter-TSS (NM_001252296)	-20	NM_00180	1054	Hs.429666	NM_00180	ENSG0000	CEBPG	GPE1BP|IG	CCAAT/en	protein-co
lib-C666-l	chr1	145827045	145827045	+	41.28836	NA	promoter-TSS (NM_001097612)	promoter-TSS (NM_001097612)	58	NM_00109	653519	Hs.504320	NM_00109	ENSG0000	GPR89A	GPHR|GPR	G protein-	protein-co
lib-C666-l	chr1	147400489	147400489	+	36.99348	NA	promoter-TSS (NM_016334)	promoter-TSS (NM_016334)	-17	NM_01633	51463	Hs.504320	NM_01633	ENSG0000	GPR89B	GPHR|GPR	G protein-	protein-co
lib-C666-l	chrX	15353750	15353750	+	30.542	NA	promoter-TSS (NM_020473)	promoter-TSS (NM_020473)	-74	NM_00264	5277	Hs.137154	NM_00264	ENSG0000	PIGA	GPI3|MCAH	phosphatic	protein-co
lib-C666-l	chrX	15354329	15354329	+	6.98764	NA	promoter-TSS (NM_020473)	promoter-TSS (NM_020473)	-653	NM_00264	5277	Hs.137154	NM_00264	ENSG0000	PIGA	GPI3|MCAH	phosphatic	protein-co
lib-C666-l	chr11	34073817	34073817	+	18.89222	NA	intron (NM_005898, intron 1 of 18)	CpG	587	NM_20336	4076	Hs.471818	NM_00589	ENSG0000	CAPRIN1	GPIAP1|GP	cell cycle a	protein-co
lib-C666-l	chr11	34073115	34073115	+	14.88921	NA	promoter-TSS (NM_203364)	promoter-TSS (NM_203364)	-115	NM_00589	4076	Hs.471818	NM_00589	ENSG0000	CAPRIN1	GPIAP1|GP	cell cycle a	protein-co

lib-C666-l	chr4	82393230	82393230	+	35.28811	NA	promoter-TSS (NM_152545)	promoter-TSS (NM_152545)	-148	NM_00130	153020	Hs.591696	NM_15254	ENSG0000	RASGEF1B	GPIG4	RasGEF do	protein-co
lib-C666-l	chr4	82504598	82504598	+	31.42602	NA	Intergenic	MER2B|DNA|TcMar-Tigger	-111516	NM_00130	153020	Hs.591696	NM_15254	ENSG0000	RASGEF1B	GPIG4	RasGEF do	protein-co
lib-C666-l	chr4	82471307	82471307	+	9.79436	NA	Intergenic	Intergenic	-78225	NM_00130	153020	Hs.591696	NM_15254	ENSG0000	RASGEF1B	GPIG4	RasGEF do	protein-co
lib-C666-l	chr4	82505226	82505226	+	2.89057	NA	Intergenic	Intergenic	-112144	NM_00130	153020	Hs.591696	NM_15254	ENSG0000	RASGEF1B	GPIG4	RasGEF do	protein-co
lib-C666-l	chr15	55611245	55611245	+	10.70998	NA	5' UTR (NM_004855, exon 1 of 12)	5' UTR (NM_004855, exon 1 of 12)	112	NM_00485	9488	Hs.612814	NM_00485	ENSG0000	PIGB	GPI-MT-III	phosphatic	protein-co
lib-C666-l	chr3	196696779	196696779	+	3.89624	NA	Intergenic	CpG-19308	-1037	NM_02516	80235	Hs.518403	NM_02516	ENSG0000	PIGZ	GPI-MT-IV	phosphatic	protein-co
lib-C666-l	chr12	123198299	123198299	+	9.20967	NA	Intergenic	Intergenic	3140	NM_00601	8843	Hs.458425	NM_00601	ENSG0000	HCAR3	GPR109B|H	hydroxycar	protein-co
lib-C666-l	chr12	123201521	123201521	+	5.96422	NA	promoter-TSS (NM_006018)	promoter-TSS (NM_006018)	-82	NM_00601	8843	Hs.458425	NM_00601	ENSG0000	HCAR3	GPR109B|H	hydroxycar	protein-co
lib-C666-l	chr2	26568816	26568816	+	28.7791	NA	promoter-TSS (NM_001321975)	promoter-TSS (NM_001321975)	-106	NM_00132	165082	Hs.631878	NM_15383	ENSG0000	ADGRF3	GPR113|PC	adhesion G	protein-co
lib-C666-l	chr8	37653948	37653948	+	7.9428	NA	promoter-TSS (NM_032777)	promoter-TSS (NM_032777)	-453	NM_03277	25960	Hs.274136	NM_03277	ENSG0000	ADGRA2	GPR124|TE	adhesion G	protein-co
lib-C666-l	chr8	37654763	37654763	+	4.04484	NA	5' UTR (NM_032777, exon 1 of 19)	5' UTR (NM_032777, exon 1 of 19)	362	NM_03277	25960	Hs.274136	NM_03277	ENSG0000	ADGRA2	GPR124|TE	adhesion G	protein-co
lib-C666-l	chr17	80325884	80325884	+	15.3763	NA	Intergenic	LTR71B|LTR|ERV1	-6269	NM_01894	2837	Hs.192720	NM_01894	ENSG0000	UTS2R	GPR14|UR-	urotensin 2	protein-co
lib-C666-l	chr3	127310000	127310000	+	50.86197	NA	promoter-TSS (NR_073377)	promoter-TSS (NR_073377)	-398	NR_07337	131601	Hs.6418	NM_01637	ENSG0000	TPRA1	GPR175|TM	transmemb	protein-co
lib-C666-l	chr3	127310936	127310936	+	5.25516	NA	Intergenic	Intergenic	-1334	NR_07337	131601	Hs.6418	NM_01637	ENSG0000	TPRA1	GPR175|TM	transmemb	protein-co
lib-C666-l	chr6	158981111	158981111	+	102.93473	NA	intron (NM_020823, intron 1 of 16)	CpG	23643	NM_02082	57583	Hs.99145	NM_02082	ENSG0000	TMEM181	GPR178|KI	transmemb	protein-co
lib-C666-l	chr6	158981597	158981597	+	27.70954	NA	intron (NM_020823, intron 1 of 16)	CpG	24129	NM_02082	57583	Hs.99145	NM_02082	ENSG0000	TMEM181	GPR178|KI	transmemb	protein-co
lib-C666-l	chr22	41079189	41079189	+	10.73451	NA	TTS (NM_005297)	TTS (NM_005297)	4007	NM_00529	2847	Hs.248122	NM_00529	ENSG0000	MCHR1	GPR24|MC	melanin co	protein-co
lib-C666-l	chr7	55433987	55433987	+	262.548	NA	intron (NM_018697, intron 1 of 8)	CpG	846	NM_01869	55915	Hs.595384	NM_01869	ENSG0000	LANCL2	GPR69B|TA	LanC like 2	protein-co
lib-C666-l	chr7	55433349	55433349	+	13.46097	NA	5' UTR (NM_018697, exon 1 of 9)	5' UTR (NM_018697, exon 1 of 9)	208	NM_01869	55915	Hs.595384	NM_01869	ENSG0000	LANCL2	GPR69B|TA	LanC like 2	protein-co
lib-C666-l	chr20	62735997	62735997	+	4.90117	NA	Intergenic	Intergenic	2187	NM_00528	2832	Hs.248118	NM_00528	ENSG0000	NPBWR2	GPR8	neuropept	protein-co
lib-C666-l	chr11	88663990	88663990	+	16.59065	NA	intron (NM_000842, intron 2 of 8)	AluSq2|SINE|Alu	117250	NM_00114	2915	Hs.147361	NM_00084	ENSG0000	GRM5	GPRC1E|M	glutamate	protein-co
lib-C666-l	chr10	120966484	120966484	+	8.03539	NA	promoter-TSS (NM_005308)	promoter-TSS (NM_005308)	-713	NM_00530	2869	Hs.524625	NM_00530	ENSG0000	GRK5	GPRK5	G protein-	protein-co
lib-C666-l	chr5	176853460	176853460	+	28.8837	NA	promoter-TSS (NM_002082)	promoter-TSS (NM_002082)	-227	NM_00100	2870	Hs.235116	NM_00208	ENSG0000	GRK6	GPRK6	G protein-	protein-co
lib-C666-l	chr19	2061585	2061585	+	15.01861	NA	Intergenic	CpG	-10342	NM_01757	2872	Hs.515032	NM_01757	ENSG0000	MKNK2	GPRK7|MN	MAP kinas	protein-co
lib-C666-l	chr19	14640377	14640377	+	3.08629	NA	promoter-TSS (NR_030369)	promoter-TSS (NR_030369)	-2	NM_13850	9524	Hs.515642	NM_00486	ENSG0000	TECR	GPSN2|MR	trans-2,3-	protein-co
lib-C666-l	chr19	1103912	1103912	+	10.03284	NA	promoter-TSS (NM_002085)	promoter-TSS (NM_002085)	-13	NM_00208	2879	Hs.433951	NM_00208	ENSG0000	GPX4	GPx-4|GSH	glutathione	protein-co
lib-C666-l	chr19	1113570	1113570	+	8.03539	NA	exon (NM_001100122, exon 16 of 29)	exon (NM_001100122, exon 16 of 2	9093	NM_00103	2879	Hs.433951	NM_00208	ENSG0000	GPX4	GPx-4|GSH	glutathione	protein-co
lib-C666-l	chr19	1104767	1104767	+	4.39666	NA	exon (NM_001039848, exon 1 of 7)	exon (NM_001039848, exon 1 of 7)	290	NM_00103	2879	Hs.433951	NM_00208	ENSG0000	GPX4	GPx-4|GSH	glutathione	protein-co
lib-C666-l	chr5	142149926	142149926	+	10.40942	NA	promoter-TSS (NM_001135608)	promoter-TSS (NM_001135608)	-366	NM_01507	23092	Hs.654668	NM_01507	ENSG0000	ARHGAP26	GRAF|GRA	Rho GTPas	protein-co
lib-C666-l	chr4	148653021	148653021	+	6.56447	NA	promoter-TSS (NM_024605)	promoter-TSS (NM_024605)	-432	NM_02460	79658	Hs.368631	NM_02460	ENSG0000	ARHGAP10	GRAF2|PS-	Rho GTPas	protein-co
lib-C666-l	chr4	148653747	148653747	+	3.98718	NA	intron (NM_024605, intron 1 of 22)	CpG	294	NM_02460	79658	Hs.368631	NM_02460	ENSG0000	ARHGAP10	GRAF2|PS-	Rho GTPas	protein-co
lib-C666-l	chr17	73852122	73852122	+	9.10494	NA	promoter-TSS (NM_012478)	promoter-TSS (NM_012478)	-229	NM_00133	23558	Hs.514489	NM_01247	ENSG0000	WBP2	GRAMD6|W	WW doma	protein-co
lib-C666-l	chr17	73851540	73851540	+	6.50091	NA	promoter-TSS (NM_012478)	promoter-TSS (NM_012478)	-39	NM_01247	23558	Hs.514489	NM_01247	ENSG0000	WBP2	GRAMD6|W	WW doma	protein-co
lib-C666-l	chrX	106045955	106045955	+	4.90117	NA	promoter-TSS (NM_198881)	promoter-TSS (NM_198881)	36	NM_19888	54885	Hs.351798	NM_01775	ENSG0000	TBC1D8B	GRAMD8B	TBC1 dom	protein-co
lib-C666-l	chr4	141677982	141677982	+	7.75972	NA	promoter-TSS (NM_015130)	promoter-TSS (NM_015130)	-511	NM_01513	23158	Hs.480819	NM_01513	ENSG0000	TBC1D9	GRAMD9|N	TBC1 dom	protein-co
lib-C666-l	chr2	106361725	106361725	+	5.64185	NA	intron (NM_001004722, intron 1 of 3)	CpG	205	NM_00358	8440	Hs.529244	NM_00358	ENSG0000	NCK2	GRB4|NCK	NCK adapt	protein-co
lib-C666-l	chr7	50889268	50889268	+	28.56445	NA	Intergenic	Intergenic	-28109	NM_00100	2887	Hs.164060	NM_00531	ENSG0000	GRB10	GRB-IR|Gr	growth fac	protein-co
lib-C666-l	chr7	50860590	50860590	+	7.40317	NA	intron (NM_001001555, intron 1 of 1	intron (NM_001001555, intron 1 of	569	NM_00100	2887	Hs.164060	NM_00531	ENSG0000	GRB10	GRB-IR|Gr	growth fac	protein-co
lib-C666-l	chr12	6934958	6934958	+	10.37767	NA	intron (NM_019858, intron 3 of 4)	intron (NM_019858, intron 3 of 4)	-2580	NM_01426	10536	Hs.631655	NM_01426	ENSG0000	P3H3	GRCB|HSU	prolyl 3-hy	protein-co
lib-C666-l	chr12	6960596	6960596	+	8.59372	NA	promoter-TSS (NM_003481)	promoter-TSS (NM_003481)	498	NR_13853	83461	Hs.524216	NM_03129	ENSG0000	CDCA3	GRCC8|TO	cell divisior	protein-co
lib-C666-l	chr6	108582495	108582495	+	3.98718	NA	promoter-TSS (NM_152827)	promoter-TSS (NM_152827)	-31	NM_00130	8724	Hs.12102	NM_00379	ENSG0000	SNX3	Grd19|MC	sorting ne	protein-co
lib-C666-l	chr10	27444221	27444221	+	59.83187	NA	promoter-TSS (NM_014263)	promoter-TSS (NM_014263)	468	NR_13546	84930	Hs.276905	NM_03284	ENSG0000	MASTL	GREATWA	microtubul	protein-co
lib-C666-l	chr19	2962179	2962179	+	100.08857	NA	Intergenic	L1ME1|LINE|L1	-15352	NR_13861	79816	Hs.334507	NM_02476	ENSG0000	TLE6	GRG6|PREI	transducin	protein-co
lib-C666-l	chr9	95056002	95056002	+	20.19521	NA	promoter-TSS (NR_073446)	promoter-TSS (NR_073446)	36	NM_00216	3376	Hs.445403	NM_00216	ENSG0000	IARS	GRIDHH|IA	isoleucyl-t	protein-co
lib-C666-l	chr12	104680482	104680482	+	9.94769	NA	promoter-TSS (NM_001261445)	promoter-TSS (NM_001261445)	22	NM_18274	7296	Hs.654922	NM_00333	ENSG0000	TXNRD1	GRIM-12|T	thioredoxir	protein-co
lib-C666-l	chr4	90359495	90359495	+	74.14931	NA	Intergenic	Intergenic	-130334	NM_19828	285513	Hs.100912	NM_19828	ENSG0000	GPRIN3	GRIN3	GPRIN fam	protein-co
lib-C666-l	chr15	57884298	57884298	+	18.43022	NA	exon (NM_001018100, exon 1 of 13)	exon (NM_001018100, exon 1 of 13	196	NM_00101	145781	Hs.437256	NM_00101	ENSG0000	GCOM1	GRINL1A|G	GRINL1A c	protein-co
lib-C666-l	chr1	40318048	40318048	+	14.11985	NA	intron (NR_132410, intron 1 of 6)	intron (NR_132410, intron 1 of 6)	31135	NR_13240	54802	Hs.356554	NM_01764	ENSG0000	TRIT1	GRO1|IPPT	tRNA isope	protein-co
lib-C666-l	chr1	40311424	40311424	+	6.49644	NA	intron (NR_132413, intron 1 of 3)	intron (NR_132413, intron 1 of 3)	37759	NR_13240	54802	Hs.356554	NM_01764	ENSG0000	TRIT1	GRO1|IPPT	tRNA isope	protein-co
lib-C666-l	chr11	118928040	118928040	+	10.37767	NA	promoter-TSS (NM_006389)	promoter-TSS (NM_006389)	-83	NM_00113	10525	Hs.277704	NM_00638	ENSG0000	HYOU1	GRP-170|C	hypoxia up	protein-co
lib-C666-l	chr4	7077715	7077715	+	4.46583	NA	Intergenic	Intergenic	-7778	NM_02519	80273	Hs.443723	NM_02519	ENSG0000	GRPEL1	GrpE|HMG	GrpE like 1	protein-co
lib-C666-l	chr4	7069814	7069814	+	3.62735	NA	5' UTR (NM_025196, exon 1 of 4)	5' UTR (NM_025196, exon 1 of 4)	123	NM_02519	80273	Hs.443723	NM_02519	ENSG0000	GRPEL1	GrpE|HMG	GrpE like 1	protein-co
lib-C666-l	chr17	8076962	8076962	+	4.37202	NA	3' UTR (NM_032354, exon 5 of 5)	3' UTR (NM_032354, exon 5 of 5)	2752	NM_03235	84314	Hs.513933	NM_03235	ENSG0000	TMEM107	GRVS638|F	transmemb	protein-co
lib-C666-l	chr6	86353064	86353064	+	6.15313	NA	promoter-TSS (NM_001253771)	promoter-TSS (NM_001253771)	-21	NM_00115	10492	Hs.571177	NM_00637	ENSG0000	SYNCRIP	GRY-RBP|C	synaptotag	protein-co
lib-C666-l	chr8	92082298	92082298	+	69.35086	NA	promoter-TSS (NM_001286745)	promoter-TSS (NM_001286745)	119	NR_11043	100506365	Hs.153412	NR_11043	ENSG0000	OTUD6B-A	GS1-251I9	OTUD6B a	ncRNA
lib-C666-l	chr16	857874	857874	+	196.80406	NA	intron (NM_001013638, intron 2 of 2)	CpG-8474	2431	NM_00101	388199	Hs.528461	NM_00101	ENSG0000	PRR25	gs64	proline rich	protein-co
lib-C666-l	chr3	67704814	67704814	+	29.74345	NA	promoter-TSS (NR_109993)	promoter-TSS (NR_109993)	224	NM_00384	8801	Hs.644919	NM_00384	ENSG0000	SUCLG2	G-SCS|GBE	succinate-	protein-co
lib-C666-l	chr11	18416059	18416059	+	53.15281	NA	non-coding (NR_028500, exon 1 of 7	non-coding (NR_028500, exon 1 of	123	NM_00116	3939	Hs.2795	NM_00556	ENSG0000	LDHA	GSD11|HEI	lactate deh	protein-co
lib-C666-l	chr14	51422301	51422301	+	21.94284	NA	Intergenic	LTR88b|LTR|Gypsy?	-11053	NM_00116	5836	Hs.282417	NM_00286	ENSG0000	PYGL	GSD6	glycogen p	protein-co
lib-C666-l	chr20	33543453	33543453	+	2.58799	NA	promoter-TSS (NM_020884)	promoter-TSS (NM_020884)	-15	NM_00132	2937	Hs.82327	NM_00017	ENSG0000	GSS	GSHS|HEL-	glutathione	protein-co
lib-C666-l	chr7	142960663	142960663	+	7.26874	NA	exon (NM_001143681, exon 1 of 7)	exon (NM_001143681, exon 1 of 7)	141	NM_01591	373156	Hs.390667	NM_01591	ENSG0000	GSTK1	GST|GST 1	glutathione	protein-co
lib-C666-l	chr4	140651773	140651773	+	7.76438	NA	exon (NM_018717, exon 4 of 6)	exon (NM_018717, exon 4 of 6)	64851	NM_00120	4258	Hs.81874	NM_00241	ENSG0000	MGST2	GST2|MGS	microsoma	protein-co
lib-C666-l	chr4	140735689	140735689	+	2.59723	NA	intron (NM_018717, intron 3 of 5)	intron (NM_018717, intron 3 of 5)	148767	NM_00120	4258	Hs.81874	NM_00241	ENSG0000	MGST2	GST2|MGS	microsoma	protein-co
lib-C666-l	chr1	110187555	110187555	+	6.20473	NA	Intergenic	Intergenic	-11143	NM_14714	2948	Hs.348387	NM_00085	ENSG0000	GSTM4	GSTM4-4|(	glutathione	protein-co
lib-C666-l	chr10	106028758	106028758	+	23.49208	NA	promoter-TSS (NR_039702)	promoter-TSS (NR_039702)	127	NM_18323	119391	Hs.107384	NM_18323	ENSG0000	GSTO2	GSTO 2-2|	glutathione	protein-co
lib-C666-l	chr19	49496635	49496635	+	9.14689	NA	promoter-TSS (NR_135578)	promoter-TSS (NR_135578)	-25	NR_02776	2997	Hs.386225	NM_00210	ENSG0000	GYS1	GSY|GYS	glycogen s	protein-co
lib-C666-l	chr1	154531407	154531407	+	10.79304	NA	promoter-TSS (NM_017582)	promoter-TSS (NM_017582)	-287	NM_01758	55585	Hs.607928	NM_01758	ENSG0000	UBE2Q1	GTAP|NICE	ubiquitin c	protein-co
lib-C666-l	chr4	74124898	74124898	+	82.96959	NA	promoter-TSS (NM_015574)	promoter-TSS (NM_015574)	-383	NM_19888	26057	Hs.601206	NM_01557	ENSG0000	ANKRD17	GTAR|MAS	ankyrin rep	protein-co
lib-C666-l	chr4	74124250	74124250	+	8.71138	NA	exon (NM_015574, exon 1 of 34)	exon (NM_015574, exon 1 of 34)	265	NM_01557	26057	Hs.601206	NM_01557	ENSG0000	ANKRD17	GTAR|MAS	ankyrin rep	protein-co
lib-C666-l	chr2	48009992	48009992	+	10.40942	NA	promoter-TSS (NM_001281493)	promoter-TSS (NM_001281493)	-229	NM_00128	2956	Hs.445052	NM_00017	ENSG0000	MSH6	GTBP|GTM	mutS hom	protein-co
lib-C666-l	chr2	48010299	48010299	+	3.8557	NA	promoter-TSS (NM_001281494)	promoter-TSS (NM_001281494)	78	NM_00017	2956	Hs.445052	NM_00017	ENSG0000	MSH6	GTBP|GTM	mutS hom	protein-co
lib-C666-l	chr6	170863407	170863407	+	100.64755	NA	promoter-TSS (NM_003194)	promoter-TSS (NM_003194)	-14	NM_00117	6908	Hs.590872	NM_00319	ENSG0000	TBP	GTF2D|GTF	TATA-box	protein-co
lib-C666-l	chr8	54935038	54935038	+	3.59106	NA	promoter-TSS (NM_201437)	promoter-TSS (NM_201437)	-22	NR_10990	6917	Hs.491745	NM_00675	ENSG0000	TCEA1	GTF2S|SII|T	transcriptic	protein-co
lib-C666-l	chr20	60758088	60758088	+	110.13766	NA	promoter-TSS (NM_015666)	promoter-TSS (NM_015666)	7	NM_01566	26164	Hs.340636	NM_01566	ENSG0000	MTG2	GTPBP5|O	mitochond	protein-co
lib-C666-l	chr10	76586359	76586359	+	4.52748	NA	5' UTR (NM_001256468, exon 1 of 18	5' UTR (NM_001256468, exon 1 of 1	188	NM_01233	23522	Hs.35758	NM_01233	ENSG0000	KAT6B	GTPTS|MO	lysine acet	protein-co
lib-C666-l	chr2	96874587	96874587	+	13.46097	NA	promoter-TSS (NM_020151)	promoter-TSS (NM_020151)	-14	NM_02015	56910	Hs.469331	NM_02015	ENSG0000	STARD7	GTT1	StAR relate	protein-co
lib-C666-l	chr10	70661004	70661004	+	34.25375	NA	promoter-TSS (NM_024045)	promoter-TSS (NM_024045)	-30	NM_02404	79009	Hs.522984	NM_02404	ENSG0000	DDX50	GU2|GUB|F	DExD-box	protein-co
lib-C666-l	chr10	70631187	70631187	+	10.03103	NA	intron (NM_001130161, intron 1 of 4)	intron (NM_001130161, intron 1 of	-29847	NM_02404	79009	Hs.522984	NM_02404	ENSG0000	DDX50	GU2|GUB|F	DExD-box	protein-co
lib-C666-l	chr10	70715978	70715978	+	28.88523	NA	promoter-TSS (NM_001256910)	promoter-TSS (NM_001256910)	99	NM_00472	9188	Hs.223141	NM_00472	ENSG0000	DDX21	GUA|GURD	DExD-box	protein-co
lib-C666-l	chr20	43514462	43514462	+	3.53586	NA	5' UTR (NM_139323, exon 1 of 6)	5' UTR (NM_139323, exon 1 of 6)	222	NM_13932	7529	Hs.643544	NM_00340	ENSG0000	YWHAB	GW128|HE	tyrosine 3-	protein-co
lib-C666-l	chr1	178995734	178995734	+	2.59032	NA	intron (NM_001324311, intron 1 of 7)	CpG	475	NM_00132	9917	Hs.5737	NM_01486	ENSG0000	FAM20B	gxk1	FAM20B, g	protein-co
lib-C666-l	chrY	13467429	13467429	+	24.08985	NA	Intergenic	BSR/Beta|Satellite|Satellite	1065960	NR_03366	352887	Hs.546221	NR_03366	ENSG0000	GYG2P1	GYG2P	glycogenin	pseudo
lib-C666-l	chrY	13484333	13484333	+	22.89944	NA	Intergenic	BSR/Beta|Satellite|Satellite	1049056	NR_03366	352887	Hs.546221	NR_03366	ENSG0000	GYG2P1	GYG2P	glycogenin	pseudo
lib-C666-l	chrY	13465048	13465048	+	18.38442	NA	Intergenic	BSR/Beta|Satellite|Satellite	1068341	NR_03366	352887	Hs.546221	NR_03366	ENSG0000	GYG2P1	GYG2P	glycogenin	pseudo
lib-C666-l	chrY	13481678	13481678	+	10.92023	NA	Intergenic	BSR/Beta|Satellite|Satellite	1051711	NR_03366	352887	Hs.546221	NR_03366	ENSG0000	GYG2P1	GYG2P	glycogenin	pseudo
lib-C666-l	chrY	13482102	13482102	+	8.58379	NA	Intergenic	BSR/Beta|Satellite|Satellite	1051287	NR_03366	352887	Hs.546221	NR_03366	ENSG0000	GYG2P1	GYG2P	glycogenin	pseudo
lib-C666-l	chrY	13473004	13473004	+	7.6466	NA	Intergenic	BSR/Beta|Satellite|Satellite	1060385	NR_03366	352887	Hs.546221	NR_03366	ENSG0000	GYG2P1	GYG2P	glycogenin	pseudo
lib-C666-l	chrY	13466793	13466793	+	5.42166	NA	Intergenic	BSR/Beta|Satellite|Satellite	1066596	NR_03366	352887	Hs.546221	NR_03366	ENSG0000	GYG2P1	GYG2P	glycogenin	pseudo
lib-C666-l	chrY	13472732	13472732	+	5.22892	NA	Intergenic	BSR/Beta|Satellite|Satellite	1060657	NR_03366	352887	Hs.546221	NR_03366	ENSG0000	GYG2P1	GYG2P	glycogenin	pseudo
lib-C666-l	chrY	13466470	13466470	+	2.17341	NA	Intergenic	BSR/Beta|Satellite|Satellite	1066919	NR_03366	352887	Hs.546221	NR_03366	ENSG0000	GYG2P1	GYG2P	glycogenin	pseudo
lib-C666-l	chr11	45944049	45944049	+	35.74596	NA	promoter-TSS (NM_001300721)	promoter-TSS (NM_001300721)	-178	NM_00130	120071	Hs.86543	NM_15231	ENSG0000	LARGE2	GYLTL1B|P	LARGE xylo	protein-co
lib-C666-l	chr1	150039353	150039353	+	7.11942	NA	promoter-TSS (NM_001279353)	promoter-TSS (NM_001279353)	3	NM_00725	11311	Hs.443750	NM_00725	ENSG0000	VPS45	H1|H1VPS4	vacuolar pr	protein-co
lib-C666-l	chrX	151999453	151999453	+	3.20885	NA	promoter-TSS (NM_004344)	promoter-TSS (NM_004344)	-58	NM_00112	50814	Hs.57698	NM_01592	ENSG0000	NSDHL	H105E3|SD	NAD(P) de	protein-co
lib-C666-l	chr20	30102224	30102224	+	30.98434	NA	promoter-TSS (NM_178581)	promoter-TSS (NM_178581)	11	NM_17858	81502	Hs.373741	NM_03078	ENSG0000	HM13	H13|IMP1|I	histocomp	protein-co
lib-C666-l	chr2	3605967	3605967	+	165.39861	NA	promoter-TSS (NM_001286837)	promoter-TSS (NM_001286837)	-6	NM_00128	246243	Hs.568006	NM_00293	ENSG0000	RNASEH1	H1RNA|PE	ribonuclea	protein-co
lib-C666-l	chr14	20811194	20811194	+	4.49043	NA	promoter-TSS (NM_005484)	promoter-TSS (NM_005484)	376	NR_00231	85495	Hs.613102	NR_00231	ENSG0000	RPPH1	H1RNA|RP	ribonuclea	ncRNA
lib-C666-l	chr6	27832960	27832960	+	26.26478	NA	promoter-TSS (NM_003511)	promoter-TSS (NM_003511)	-147	NM_00351	8332	Hs.233568	NM_00351	ENSG0000	HIST1H2AI	H2A.i|H2A	histone clu	protein-co
lib-C666-l	chr4	100871548	100871548	+	10.03103	NA	promoter-TSS (NM_002106)	promoter-TSS (NM_002106)	-36	NM_00210	3015	Hs.119192	NM_00210	ENSG0000	H2AFZ	H2A.Z-1|H	H2A histor	protein-co
lib-C666-l	chr7	44887908	44887908	+	34.83329	NA	promoter-TSS (NM_012412)	promoter-TSS (NM_012412)	-183	NM_20143	94239	Hs.488189	NM_01241	ENSG0000	H2AFV	H2A.Z-2|H	H2A histor	protein-co
lib-C666-l	chr7	44887600	44887600	+	3.20885	NA	5' UTR (NM_012412, exon 1 of 5)	5' UTR (NM_012412, exon 1 of 5)	125	NM_20151	94239	Hs.488189	NM_01241	ENSG0000	H2AFV	H2A.Z-2|H	H2A histor	protein-co
lib-C666-l	chr6	27806077	27806077	+	26.26478	NA	promoter-TSS (NM_003520)	promoter-TSS (NM_003520)	40	NM_00351	8330	Hs.734717	NM_00351	ENSG0000	HIST1H2AH	H2A/d|H2A	histone clu	protein-co
lib-C666-l	chr1	149859643	149859643	+	8.07793	NA	promoter-TSS (NM_175065)	promoter-TSS (NM_175065)	-177	NM_17506	317772	Hs.664173	NM_17506	ENSG0000	HIST2H2AB	H2AB	histone clu	protein-co
lib-C666-l	chr6	26123352	26123352	+	15.30356	NA	TTS (NM_003526)	TTS (NM_003526)	780	NM_00352	8347	Hs.658713	NM_00352	ENSG0000	HIST1H2BC	H2B.1|H2B	histone clu	protein-co
lib-C666-l	chr6	26158292	26158292	+	9.21057	NA	promoter-TSS (NM_138720)	promoter-TSS (NM_138720)	-57	NM_13872	3017	Hs.591797	NM_02106	ENSG0000	HIST1H2BD	H2B.1B|H2	histone clu	protein-co
lib-C666-l	chr6	27870634	27870634	+	8.0787	NA	Intergenic	Intergenic	9431	NM_00352	8348	Hs.484991	NM_00352	ENSG0000	HIST1H2BO	H2B.2|H2B	histone clu	protein-co
lib-C666-l	chr6	27863079	27863079	+	4.39666	NA	Intergenic	Intergenic	1876	NM_00352	8348	Hs.484991	NM_00352	ENSG0000	HIST1H2BO	H2B.2|H2B	histone clu	protein-co
lib-C666-l	chr6	27861153	27861153	+	2.59723	NA	promoter-TSS (NM_003527)	promoter-TSS (NM_003527)	-50	NM_00352	8348	Hs.484991	NM_00352	ENSG0000	HIST1H2BO	H2B.2|H2B	histone clu	protein-co
lib-C666-l	chr6	27782728	27782728	+	8.03539	NA	promoter-TSS (NM_003521)	promoter-TSS (NM_003521)	-94	NM_00352	8342	Hs.182432	NM_00352	ENSG0000	HIST1H2BM	H2B/e|H2B	histone clu	protein-co
lib-C666-l	chr6	27100683	27100683	+	6.98764	NA	promoter-TSS (NM_021064)	promoter-TSS (NM_021064)	-108	NM_02105	8970	Hs.591807	NM_02105	ENSG0000	HIST1H2BJ	H2B/r|H2B	histone clu	protein-co
lib-C666-l	chr6	27114716	27114716	+	20.59244	NA	promoter-TSS (NM_001312653)	promoter-TSS (NM_001312653)	-79	NM_00131	85236	Hs.437275	NM_08059	ENSG0000	HIST1H2BK	H2B/S|H2B	histone clu	protein-co
lib-C666-l	chr1	228645708	228645708	+	12.99339	NA	promoter-TSS (NM_175055)	promoter-TSS (NM_175055)	-100	NM_17505	128312	Hs.376691	NM_17505	ENSG0000	HIST3H2BB	H2Bb	histone clu	protein-co
lib-C666-l	chr17	73775964	73775964	+	60.07874	NA	promoter-TSS (NM_005324)	promoter-TSS (NM_005324)	52	NM_00532	3021	Hs.180877	NM_00532	ENSG0000	H3F3B	H3.3B	H3 histone	protein-co
lib-C666-l	chr6	26020570	26020570	+	15.13226	NA	promoter-TSS (NM_003529)	promoter-TSS (NM_003529)	-148	NM_00352	8350	Hs.546315	NM_00352	ENSG0000	HIST1H3A	H3/A|H3FA	histone clu	protein-co
lib-C666-l	chr6	26032465	26032465	+	16.59065	NA	promoter-TSS (NM_003537)	promoter-TSS (NM_003537)	-177	NM_00353	8358	Hs.533292	NM_00353	ENSG0000	HIST1H3B	H3/l|H3FL	histone clu	protein-co
lib-C666-l	chr1	149812854	149812854	+	26.57948	NA	promoter-TSS (NM_001005464).2	promoter-TSS (NM_001005464).2	-89	NM_00100	333932	Hs.647745	NM_00100	ENSG0000	HIST2H3A	H3/n|H3/o	histone clu	protein-co
lib-C666-l	chr1	149824088	149824088	+	18.82927	NA	promoter-TSS (NM_001005464)	promoter-TSS (NM_001005464)	-93	NM_00100	333932	Hs.647745	NM_00100	ENSG0000	HIST2H3A	H3/n|H3/o	histone clu	protein-co
lib-C666-l	chr6	26285883	26285883	+	15.30356	NA	promoter-TSS (NM_003543)	promoter-TSS (NM_003543)	-156	NM_00354	8365	Hs.591790	NM_00354	ENSG0000	HIST1H4H	H4/h|H4FH	histone clu	protein-co
lib-C666-l	chr6	26026577	26026577	+	16.5633	NA	TTS (NM_003544)	TTS (NM_003544)	903	NM_00354	8366	Hs.143080	NM_00354	ENSG0000	HIST1H4B	H4/I|H4FI	histone clu	protein-co
lib-C666-l	chr6	26204701	26204701	+	25.55305	NA	promoter-TSS (NM_003545)	promoter-TSS (NM_003545)	-172	NM_00354	8367	Hs.662174	NM_00354	ENSG0000	HIST1H4E	H4/j|H4FJ	histone clu	protein-co
lib-C666-l	chr1	149803836	149803836	+	5.13457	NA	promoter-TSS (NM_003548).2	promoter-TSS (NM_003548).2	-385	NM_00103	554313	Hs.55468	NM_00103	ENSG0000	HIST2H4B	H4/o	histone clu	protein-co
lib-C666-l	chr3	133293404	133293404	+	7.11942	NA	5' UTR (NM_001134423, exon 1 of 5)	5' UTR (NM_001134423, exon 1 of 5	126	NM_00128	55573	Hs.518265	NM_01754	ENSG0000	CDV3	H41	CDV3 hom	protein-co
lib-C666-l	chr4	8763543	8763543	+	4.27545	NA	Intergenic	Intergenic	110000	NM_00130	3166	Hs.104134	NM_01894	ENSG0000	HMX1	H6|NKX5-3	H6 family h	protein-co
lib-C666-l	chr15	44580915	44580915	+	2.45439	NA	promoter-TSS (NM_177974)	promoter-TSS (NM_177974)	6	NM_17797	113201	Hs.512867	NM_13842	ENSG0000	CASC4	H63	cancer sus	protein-co
lib-C666-l	chr10	112632514	112632514	+	6.15313	NA	intron (NM_145341, intron 1 of 12)	intron (NM_145341, intron 1 of 12)	961	NM_14534	27250	Hs.711490	NM_01445	ENSG0000	PDCD4	H731	programm	protein-co
lib-C666-l	chr19	1082703	1082703	+	5.96422	NA	intron (NM_001321232, intron 19 of 2	CpG	6070	NM_00128	23526	Hs.465521	NM_01229	ENSG0000	ARHGAP45	HA-1|HLA-	Rho GTPas	protein-co
lib-C666-l	chr1	159894503	159894503	+	5.23609	NA	promoter-TSS (NM_001277223)	promoter-TSS (NM_001277223)	-162	NM_00127	8407	Hs.517168	NM_00356	ENSG0000	TAGLN2	HA1756	transgelin	protein-co
lib-C666-l	chr17	39653124	39653124	+	129.17302	NA	Intergenic	Intergenic	-7008	NM_00377	8689	Hs.248189	NM_00377	ENSG0000	KRT36	HA6|KRTH	keratin 36	protein-co
lib-C666-l	chr9	2844278	2844278	+	97.29876	NA	promoter-TSS (NM_014878)	promoter-TSS (NM_014878)	-148	NM_01487	9933	Hs.493309	NM_01487	ENSG0000	PUM3	HA-8|HLA-	pumilio RN	protein-co
lib-C666-l	chr19	15529956	15529956	+	9.14689	NA	promoter-TSS (NM_001291478)	promoter-TSS (NM_001291478)	-24	NM_01437	26993	Hs.399800	NM_01437	ENSG0000	AKAP8L	HA95|HAP	A-kinase a	protein-co
lib-C666-l	chr6	26597159	26597159	+	46.67128	NA	promoter-TSS (NM_013375)	promoter-TSS (NM_013375)	-12	NM_01337	29777	Hs.254406	NM_01337	ENSG0000	ABT1	hABT1	activator o	protein-co
lib-C666-l	chr4	108910962	108910962	+	41.28836	NA	promoter-TSS (NM_001184705)	promoter-TSS (NM_001184705)	92	NM_00118	3033	Hs.438289	NM_00532	ENSG0000	HADH	HAD|HADH	hydroxyacy	protein-co
lib-C666-l	chr16	75657208	75657208	+	69.78576	NA	promoter-TSS (NM_001324444)	promoter-TSS (NM_001324444)	13	NM_00132	23536	Hs.729312	NM_01209	ENSG0000	ADAT1	HADAT1	adenosine	protein-co
lib-C666-l	chr3	25490755	25490755	+	28.21964	NA	intron (NR_110893, intron 1 of 6)	intron (NR_110893, intron 1 of 6)	-9046	NM_00129	5915	Hs.543218	NM_00096	ENSG0000	RARB	HAP|MCOF	retinoic aci	protein-co
lib-C666-l	chr3	24871934	24871934	+	24.5284	NA	intron (NM_001290216, intron 1 of 1	(TG)n|Simple_repeat|Simple_repeat	1120	NM_00129	5915	Hs.543218	NM_00096	ENSG0000	RARB	HAP|MCOF	retinoic aci	protein-co
lib-C666-l	chr3	24870786	24870786	+	9.75396	NA	promoter-TSS (NM_001290216)	promoter-TSS (NM_001290216)	-28	NM_00129	5915	Hs.543218	NM_00096	ENSG0000	RARB	HAP|MCOF	retinoic aci	protein-co
lib-C666-l	chr5	114505766	114505766	+	4.73866	NA	promoter-TSS (NM_001300759)	promoter-TSS (NM_001300759)	-106	NM_00130	55521	Hs.519514	NM_01870	ENSG0000	TRIM36	HAPRIN|RE	tripartite m	protein-co
lib-C666-l	chr2	217277054	217277054	+	18.98417	NA	promoter-TSS (NM_001127207)	promoter-TSS (NM_001127207)	-83	NM_01414	50485	Hs.516674	NM_01414	ENSG0000	SMARCAL1	HARP|HHA	SWI/SNF re	protein-co
lib-C666-l	chr5	140071132	140071132	+	5.5166	NA	5' UTR (NM_001258040, exon 1 of 12	5' UTR (NM_001258040, exon 1 of 1	121	NM_01220	23438	Hs.432560	NM_01220	ENSG0000	HARS2	HARSL|HAI	histidyl-tR	protein-co
lib-C666-l	chr1	48938053	48938053	+	12.78696	NA	promoter-TSS (NM_001286238)	promoter-TSS (NM_001286238)	-173	NM_01907	54558	Hs.538103	NM_01907	ENSG0000	SPATA6	HASH|SRF-	spermatog	protein-co
lib-C666-l	chr7	73097891	73097891	+	108.08909	NA	promoter-TSS (NM_017528)	promoter-TSS (NM_017528)	-7	NM_00120	114049	Hs.647063	NM_01752	ENSG0000	WBSCR22	HASJ4442|	Williams-B	protein-co
lib-C666-l	chr16	3507807	3507807	+	5.12426	NA	promoter-TSS (NM_001317098)	promoter-TSS (NM_001317098)	-178	NM_00131	79903	Hs.513296	NM_02484	ENSG0000	NAA60	HAT4|NAT	N(alpha)-a	protein-co
lib-C666-l	chr1	1447407	1447407	+	78.54099	NA	promoter-TSS (NM_001170535)	promoter-TSS (NM_001170535)	-116	NM_00117	55210	Hs.23413	NM_01818	ENSG0000	ATAD3A	HAYOS	ATPase fan	protein-co
lib-C666-l	chr5	143204832	143204832	+	12.83248	NA	Intergenic	Intergenic	13106	NM_02118	57824	Hs.158320	NM_02118	ENSG0000	HMHB1	HB-1|HB-1	histocomp	protein-co
lib-C666-l	chrX	49125785	49125785	+	24.25638	NA	promoter-TSS (NM_033215)	promoter-TSS (NM_033215)	-521	NM_00118	89801	Hs.433652	NM_03321	ENSG0000	PPP1R3F	HB2E|LL0X	protein ph	protein-co
lib-C666-l	chrX	49126165	49126165	+	8.56376	NA	promoter-TSS (NM_033215)	promoter-TSS (NM_033215)	-141	NM_00118	89801	Hs.433652	NM_03321	ENSG0000	PPP1R3F	HB2E|LL0X	protein ph	protein-co
lib-C666-l	chr10	70883713	70883713	+	7.31982	NA	promoter-TSS (NM_004896)	promoter-TSS (NM_004896)	-196	NM_00131	9559	Hs.499925	NM_00489	ENSG0000	VPS26A	HB58|Hbet	VPS26, retr	protein-co
lib-C666-l	chr12	52674267	52674267	+	4.30593	NA	intron (NM_001320198, intron 2 of 1	intron (NM_001320198, intron 2 of	5838	NM_00132	3892	Hs.278658	NM_00228	ENSG0000	KRT86	HB6|Hb1|K	keratin 86	protein-co
lib-C666-l	chr7	156803397	156803397	+	44.31884	NA	promoter-TSS (NR_038835)	promoter-TSS (NR_038835)	-50	NM_00551	3110	Hs.37035	NM_00551	ENSG0000	MNX1	HB9|HLXB9	motor neu	protein-co
lib-C666-l	chr7	156802995	156802995	+	5.30207	NA	promoter-TSS (NM_001165255)	promoter-TSS (NM_001165255)	352	NM_00551	3110	Hs.37035	NM_00551	ENSG0000	MNX1	HB9|HLXB9	motor neu	protein-co
lib-C666-l	chrY	2558589	2558589	+	11.57178	NA	promoter-TSS (NM_001321369)	promoter-TSS (NM_001321369)	-585	NM_00112	4267	Hs.653349	NM_00241	ENSG0000	CD99	HBA71|MI(	CD99 mole	protein-co
lib-C666-l	chrX	2608614	2608614	+	10.03103	NA	promoter-TSS (NM_001321370)	promoter-TSS (NM_001321370)	-560	NM_00132	4267	Hs.653349	NM_00241	ENSG0000	CD99	HBA71|MI(	CD99 mole	protein-co
lib-C666-l	chrX	2609380	2609380	+	5.92363	NA	non-coding (NR_135623, exon 1 of 1	non-coding (NR_135623, exon 1 of	206	NR_13562	4267	Hs.653349	NM_00241	ENSG0000	CD99	HBA71|MI(	CD99 mole	protein-co
lib-C666-l	chrY	2559628	2559628	+	3.57421	NA	intron (NM_001122898, intron 1 of 8)	CpG	454	NM_00241	4267	Hs.653349	NM_00241	ENSG0000	CD99	HBA71|MI(	CD99 mole	protein-co
lib-C666-l	chr16	220132	220132	+	13.84327	NA	Intergenic	Intergenic	-2714	NM_00051	3040	Hs.449630	NM_00051	ENSG0000	HBA2	HBA-T2|HE	hemoglobi	protein-co
lib-C666-l	chr16	224450	224450	+	8.89416	NA	TTS (NM_000517)	TTS (NM_000517)	1604	NM_00051	3040	Hs.449630	NM_00051	ENSG0000	HBA2	HBA-T2|HE	hemoglobi	protein-co
lib-C666-l	chr9	140100075	140100075	+	74.35668	NA	promoter-TSS (NM_001144028)	promoter-TSS (NM_001144028)	15	NM_05304	94107	Hs.231029	NM_05304	ENSG0000	TMEM203	HBEBP1	transmemb	protein-co
lib-C666-l	chr3	52322004	52322004	+	68.04845	NA	TTS (NR_046719)	TTS (NR_046719)	168	NR_02670	132158	Hs.415312	NM_14526	ENSG0000	GLYCTK	HBEBP2|HE	glycerate k	protein-co

lib-C666-I	chr7	93633686	93633686	+	112.52512	NA	promoter-TSS (NM_001317739)	promoter-TSS (NM_001317739)	8	NM_00586	10282	Hs.489132	NM_00586	ENSG0000	BET1	HBET1	Bet1 golgi	protein-co
lib-C666-I	chr7	22893968	22893968	+	5.23679	NA	Intergenic	CpG	-2264	NR_00307	692210		NR_00307	ENSG0000	SNORD93	HBII-336	small nucle	snoRNA
lib-C666-I	chr9	88897741	88897741	+	17.71746	NA	promoter-TSS (NM_030940)	promoter-TSS (NM_030940)	-251	NM_03094	81689	Hs.449291	NM_03094	ENSG0000	ISCA1	HBLD2|ISA	iron-sulfur	protein-co
lib-C666-I	chr9	88897264	88897264	+	17.48666	NA	intron (NM_030940, intron 1 of 3)	CpG	226	NM_03094	81689	Hs.449291	NM_03094	ENSG0000	ISCA1	HBLD2|ISA	iron-sulfur	protein-co
lib-C666-I	chr17	47865949	47865949	+	28.22301	NA	promoter-TSS (NM_001199158)	promoter-TSS (NM_001199158)	-32	NM_00134	11143	Hs.21907	NM_00706	ENSG0000	KAT7	HBO1|HBC	lysine acet	protein-co
lib-C666-I	chr17	47866600	47866600	+	2.10911	NA	intron (NM_001346706, intron 1 of 1	CpG	619	NM_00706	11143	Hs.21907	NM_00706	ENSG0000	KAT7	HBO1|HBC	lysine acet	protein-co
lib-C666-I	chr4	1714230	1714230	+	56.02075	NA	5' UTR (NM_006527, exon 1 of 8)	5' UTR (NM_006527, exon 1 of 8)	238	NM_00130	7884	Hs.298345	NM_00652	ENSG0000	SLBP	HBP	stem-loop	protein-co
lib-C666-I	chr4	1714751	1714751	+	25.39084	NA	promoter-TSS (NM_006527)	promoter-TSS (NM_006527)	-283	NM_00130	7884	Hs.298345	NM_00652	ENSG0000	SLBP	HBP	stem-loop	protein-co
lib-C666-I	chr2	242254674	242254674	+	17.05103	NA	promoter-TSS (NM_001321035)	promoter-TSS (NM_001321035)	33	NM_00132	3069	Hs.471851	NM_00533	ENSG0000	HDLBP	HBP|PRO2	high densit	protein-co
lib-C666-I	chr2	242255230	242255230	+	5.5166	NA	promoter-TSS (NM_001321035)	promoter-TSS (NM_001321035)	27	NM_00533	3069	Hs.471851	NM_00533	ENSG0000	HDLBP	HBP|PRO2	high densit	protein-co
lib-C666-I	chr2	242212360	242212360	+	3.7184	NA	promoter-TSS (NM_001320967)	promoter-TSS (NM_001320967)	-59	NM_00124	3069	Hs.471851	NM_00533	ENSG0000	HDLBP	HBP|PRO2	high densit	protein-co
lib-C666-I	chr3	47205303	47205303	+	19.19377	NA	promoter-TSS (NR_033373)	promoter-TSS (NR_033373)	164	NM_01415	29072	Hs.517941	NM_01227	ENSG0000	SETD2	HBP231|HI	SET domai	protein-co
lib-C666-I	chr16	231449	231449	+	10.03103	NA	TTS (NM_005331)	TTS (NM_005331)	1116	NM_00533	3049	Hs.247921	NM_00533	ENSG0000	HBQ1	HBQ	hemoglobi	protein-co
lib-C666-I	chr7	43966378	43966378	+	11.07709	NA	promoter-TSS (NM_001077664)	promoter-TSS (NM_001077664)	343	NM_01598	51619	Hs.19196	NM_01598	ENSG0000	UBE2D4	HBUCE1	ubiquitin c	protein-co
lib-C666-I	chr11	77531979	77531979	+	6.29676	NA	promoter-TSS (NM_016578)	promoter-TSS (NM_016578)	-99	NM_01657	51773	Hs.420229	NM_01657	ENSG0000	RSF1	HBXAP|RSF	remodeling	protein-co
lib-C666-I	chr11	14542008	14542008	+	5.15932	NA	promoter-TSS (NM_001143937)	promoter-TSS (NM_001143937)	-17	NM_00114	5682	Hs.102798	NM_00278	ENSG0000	PSMA1	HC2|HEL-S	proteasom	protein-co
lib-C666-I	chr6	170862367	170862367	+	15.60931	NA	promoter-TSS (NM_002793)	promoter-TSS (NM_002793)	50	NM_00279	5689	Hs.352768	NM_00279	ENSG0000	PSMB1	HC5|PMSB	proteasom	protein-co
lib-C666-I	chr1	36107168	36107168	+	64.34737	NA	promoter-TSS (NM_001199779)	promoter-TSS (NM_001199779)	-16	NM_00119	5690	Hs.471441	NM_00279	ENSG0000	PSMB2	HC7-I	proteasom	protein-co
lib-C666-I	chr14	58711677	58711677	+	2.03963	NA	intron (NR_038123, intron 1 of 9)	CpG	154	NM_00278	5684	Hs.558799	NM_00278	ENSG0000	PSMA3	HC8|PSC3	proteasom	protein-co
lib-C666-I	chr14	58711799	58711799	+	2.03963	NA	intron (NR_038123, intron 1 of 9)	CpG	276	NM_00278	5684	Hs.558799	NM_00278	ENSG0000	PSMA3	HC8|PSC3	proteasom	protein-co
lib-C666-I	chr15	29562021	29562021	+	42.49125	NA	promoter-TSS (NM_138704)	promoter-TSS (NM_138704)	-1	NM_13870	56160	Hs.94011	NM_13870	ENSG0000	NSMCE3	HCA4|LICS	NSE3 hom	protein-co
lib-C666-I	chr16	4475614	4475614	+	21.81327	NA	promoter-TSS (NM_001135110)	promoter-TSS (NM_001135110)	-192	NM_00113	9093	Hs.459779	NM_00514	ENSG0000	DNAJA3	HCA57|TID	DnaJ heat	protein-co
lib-C666-I	chr9	132597601	132597601	+	72.59265	NA	promoter-TSS (NM_006676)	promoter-TSS (NM_006676)	-29	NM_01652	51759	Hs.278429	NM_01652	ENSG0000	C9orf78	HCA59|HSI	chromosor	protein-co
lib-C666-I	chr15	89015719	89015719	+	6.3317	NA	3' UTR (NM_001321976, exon 3 of 3)	3' UTR (NM_001321976, exon 3 of 3	5035	NM_00132	64963	Hs.111286	NM_02283	ENSG0000	MRPS11	HCC-2|MR	mitochond	protein-co
lib-C666-I	chr15	89010814	89010814	+	4.61604	NA	promoter-TSS (NM_022163)	promoter-TSS (NM_022163)	130	NM_00132	64963	Hs.111286	NM_02283	ENSG0000	MRPS11	HCC-2|MR	mitochond	protein-co
lib-C666-I	chr5	180258534	180258534	+	51.02929	NA	promoter-TSS (NR_045680)	promoter-TSS (NR_045680)	84	NR_04568	100859930	Hs.655606	NR_04568	ENSG0000	HEIH	HCCAT2|LI	hepatocell	ncRNA
lib-C666-I	chr15	80237094	80237094	+	9.58102	NA	Intergenic	Intergenic	-20998	NR_03765	400410	Hs.729127	NM_00110	ENSG0000	ST20	HCCS-1	suppressor	protein-co
lib-C666-I	chr17	618224	618224	+	15.22699	NA	promoter-TSS (NM_018289)	promoter-TSS (NM_018289)	-128	NM_01828	55275	Hs.461819	NM_01828	ENSG0000	VPS53	HCCS1|PCI	VPS53, GA	protein-co
lib-C666-I	chr16	3451098	3451098	+	14.47615	NA	promoter-TSS (NM_001324340)	promoter-TSS (NM_001324340)	-33	NM_00132	54925	Hs.592078	NM_01781	ENSG0000	ZSCAN32	HCCS-5|ZN	zinc finger	protein-co
lib-C666-I	chr12	104458253	104458253	+	43.13729	NA	promoter-TSS (NM_001316967)	promoter-TSS (NM_001316967)	17	NM_01332	29915	Hs.506558	NM_01332	ENSG0000	HCFC2	HCF-2|HCI	host cell fa	protein-co
lib-C666-I	chr2	74237422	74237422	+	7.7703	NA	intron (NM_001287491, intron 2 of 1	intron (NM_001287491, intron 2 of	23891	NM_00128	200424	Hs.516107	NM_00128	ENSG0000	TET3	hCG_4073	tet methyl	protein-co
lib-C666-I	chr13	30424797	30424797	+	36.0188	NA	promoter-TSS (NM_007106)	promoter-TSS (NM_007106)	23	NM_00710	5412	Hs.145575	NM_00710	ENSG0000	UBL3	HCG-1|PN	ubiquitin li	protein-co
lib-C666-I	chr6	30294252	30294252	+	23.4136	NA	promoter-TSS (NR_052012).6	promoter-TSS (NR_052012).6	-341	NR_05201	414778	Hs.485041	NR_05201	ENSG0000	HCG17	HCG18|LIN	HLA comp	ncRNA
lib-C666-I	chr14	50234846	50234846	+	11.07714	NA	promoter-TSS (NM_014315)	promoter-TSS (NM_014315)	59	NM_01431	23588	Hs.509264	NM_01431	ENSG0000	KLHDC2	HCLP-1|H	kelch doma	protein-co
lib-C666-I	chr14	50234379	50234379	+	6.35487	NA	promoter-TSS (NM_014315)	promoter-TSS (NM_014315)	-408	NM_01431	23588	Hs.509264	NM_01431	ENSG0000	KLHDC2	HCLP-1|H	kelch doma	protein-co
lib-C666-I	chr11	65259464	65259464	+	25.39487	NA	Intergenic	Intergenic	-5745	NR_14456	378938	Hs.621695	NR_002819		MALAT1	HCN|LINC	metastasis	ncRNA
lib-C666-I	chr11	65239695	65239695	+	19.30132	NA	Intergenic	Intergenic	-25514	NR_14456	378938	Hs.621695	NR_002819		MALAT1	HCN|LINC	metastasis	ncRNA
lib-C666-I	chr11	65265006	65265006	+	8.22136	NA	promoter-TSS (NR_002819)	promoter-TSS (NR_002819)	-203	NR_14456	378938	Hs.621695	NR_002819		MALAT1	HCN|LINC	metastasis	ncRNA
lib-C666-I	chr12	118573801	118573801	+	6.49644	NA	5' UTR (NM_002567, exon 1 of 4)	5' UTR (NM_002567, exon 1 of 4)	112	NM_00256	5037	Hs.433863	NM_00256	ENSG0000	PEBP1	HCNP|HCN	phosphatic	protein-co
lib-C666-I	chr19	7694513	7694513	+	161.97499	NA	promoter-TSS (NM_020196)	promoter-TSS (NM_020196)	-74	NM_02019	56949	Hs.9822	NM_02019	ENSG0000	XAB2	HCNP|HCR	XPA bindin	protein-co
lib-C666-I	chr8	66754790	66754790	+	126.2553	NA	promoter-TSS (NM_001242318)	promoter-TSS (NM_001242318)	-821	NM_00124	5150	Hs.527119	NM_00260	ENSG0000	PDE7A	HCP1|PDE	phosphodi	protein-co
lib-C666-I	chr8	66754380	66754380	+	115.12381	NA	promoter-TSS (NM_001242318)	promoter-TSS (NM_001242318)	-411	NM_00124	5150	Hs.527119	NM_00260	ENSG0000	PDE7A	HCP1|PDE	phosphodi	protein-co
lib-C666-I	chr2	85838958	85838958	+	6.29226	NA	promoter-TSS (NM_001256727)	promoter-TSS (NM_001256727)	221	NM_00101	388969	Hs.516159	NM_00101	ENSG0000	C2orf68	HCRCN81	chromosor	protein-co
lib-C666-I	chr8	17104364	17104364	+	341.51138	NA	promoter-TSS (NM_013354)	promoter-TSS (NM_013354)	-37	NM_00114	137492	Hs.343873	NM_15241	ENSG0000	VPS37A	HCRP1|PQ	VPS37A, E	protein-co
lib-C666-I	chr11	45868859	45868859	+	12.83248	NA	promoter-TSS (NM_021117)	promoter-TSS (NM_021117)	-98	NM_02111	1408	Hs.532491	NM_02111	ENSG0000	CRY2	HCRY2|PHI	cryptochro	protein-co
lib-C666-I	chr11	45864876	45864876	+	2.24164	NA	Intergenic	Intergenic	-3793	NM_00112	1408	Hs.532491	NM_02111	ENSG0000	CRY2	HCRY2|PHI	cryptochro	protein-co
lib-C666-I	chr21	38362458	38362458	+	13.33918	NA	promoter-TSS (NM_000411)	promoter-TSS (NM_000411)	87	NM_00041	3141	Hs.371350	NM_00041	ENSG0000	HLCS	HCS	holocarbo	protein-co
lib-C666-I	chr3	13521691	13521691	+	11.6145	NA	promoter-TSS (NM_001136041)	promoter-TSS (NM_001136041)	-24	NM_02482	79885	Hs.744132	NM_02482	ENSG0000	HDAC11	HD11	histone de	protein-co
lib-C666-I	chr6	139456116	139456116	+	320.63895	NA	promoter-TSS (NM_016217)	promoter-TSS (NM_016217)	-133	NM_01621	51696	Hs.197644	NM_01621	ENSG0000	HECA	HDC|HDCL	hdc homol	protein-co
lib-C666-I	chr6	149867243	149867243	+	388.66202	NA	promoter-TSS (NM_139126)	promoter-TSS (NM_139126)	-5	NM_13912	85313	Hs.744238	NM_13912	ENSG0000	PPIL4	HDCME13	peptidylpr	protein-co
lib-C666-I	chr10	126150431	126150431	+	9.47655	NA	promoter-TSS (NM_022126)	promoter-TSS (NM_022126)	90	NM_00131	64077	Hs.527748	NM_02212	ENSG0000	LHPP	HDHD2B	phospholy	protein-co
lib-C666-I	chr10	75007058	75007058	+	7.6867	NA	promoter-TSS (NM_015190)	promoter-TSS (NM_015190)	31	NM_01519	23234	Hs.408577	NM_01519	ENSG0000	DNAJC9	HDJC9|JDD	DnaJ heat	protein-co
lib-C666-I	chr15	58624447	58624447	+	10.32881	NA	Intergenic	Intergenic	-99728	NM_00023	3990	Hs.654472	NM_00023	ENSG0000	LIPC	HDLCQ12|	lipase C, h	protein-co
lib-C666-I	chr1	204485675	204485675	+	17.13943	NA	intron (NM_001278516, intron 1 of 9)	CpG	168	NM_00120	4194	Hs.497492	NM_00239	ENSG0000	MDM4	HDMX|MD	MDM4, p5	protein-co
lib-C666-I	chr3	47422124	47422124	+	17.97302	NA	promoter-TSS (NM_015466)	promoter-TSS (NM_015466)	-348	NM_01546	25930	Hs.25524	NM_01546	ENSG0000	PTPN23	HD-PTP|HI	protein tyr	protein-co
lib-C666-I	chr10	8137582	8137582	+	8.6111	NA	Intergenic	Intergenic	40915	NM_00205	2625	Hs.524134	NM_00205	ENSG0000	GATA3	HDR|HDRS	GATA bind	protein-co
lib-C666-I	chr17	11900883	11900883	+	3.20885	NA	promoter-TSS (NM_001303281)	promoter-TSS (NM_001303281)	-56	NM_00130	7566	Hs.370473	NM_14468	ENSG0000	ZNF18	HDSG1|KO	zinc finger	protein-co
lib-C666-I	chr8	42249055	42249055	+	6.62035	NA	promoter-TSS (NM_005662)	promoter-TSS (NM_005662)	-224	NM_00113	7419	Hs.699301	NM_00566	ENSG0000	VDAC3	HD-VDAC	voltage de	protein-co
lib-C666-I	chr11	57425150	57425150	+	10.47352	NA	promoter-TSS (NM_006831)	promoter-TSS (NM_006831)	-66	NM_00114	10978	Hs.523687	NM_00683	ENSG0000	CLP1	HEAB|hClp	cleavage a	protein-co
lib-C666-I	chr8	145202969	145202969	+	67.6042	NA	promoter-TSS (NM_001099280)	promoter-TSS (NM_001099280)	50	NM_00109	727957	Hs.443139	NM_03245	ENSG0000	MROH1	HEATR7A	maestro he	protein-co
lib-C666-I	chr2	178128452	178128452	+	8.89416	NA	5' UTR (NM_001313904, exon 1 of 5)	5' UTR (NM_001313904, exon 1 of 5	407	NM_00131	4780	Hs.744006	NM_00616	ENSG0000	NFE2L2	HEBP1|NRI	nuclear fac	protein-co
lib-C666-I	chr2	178129667	178129667	+	7.26874	NA	promoter-TSS (NM_001313904)	promoter-TSS (NM_001313904)	192	NM_00131	4780	Hs.744006	NM_00616	ENSG0000	NFE2L2	HEBP1|NRI	nuclear fac	protein-co
lib-C666-I	chr7	26241058	26241058	+	43.37893	NA	promoter-TSS (NM_031243)	promoter-TSS (NM_031243)	227	NM_00727	11335	Hs.381189	NM_00727	ENSG0000	CBX3	HECH|HP1	chromobo	protein-co
lib-C666-I	chr7	156931411	156931411	+	9.6047	NA	promoter-TSS (NM_014671)	promoter-TSS (NM_014671)	-244	NM_01467	9690	Hs.118351	NM_01467	ENSG0000	UBE3C	HECTH2	ubiquitin p	protein-co
lib-C666-I	chr11	85904643	85904643	+	23.74671	NA	Intergenic	Intergenic	-50783	NM_00130	8726	Hs.503510	NM_00379	ENSG0000	EED	HEED|WAI	embryonic	protein-co
lib-C666-I	chr3	124774957	124774957	+	10.67313	NA	promoter-TSS (NM_020733)	promoter-TSS (NM_020733)	-155	NM_02073	57493	Hs.477420	NM_02073	ENSG0000	HEG1	HEG|MST1	heart deve	protein-co
lib-C666-I	chr1	23881643	23881643	+	9.79436	NA	Intergenic	CpG	4642	NM_00216	3399	Hs.76884	NM_00216	ENSG0000	ID3	HEIR-1|bH	inhibitor o	protein-co
lib-C666-I	chr1	23888977	23888977	+	4.49043	NA	Intergenic	Intergenic	-2692	NM_00216	3399	Hs.76884	NM_00216	ENSG0000	ID3	HEIR-1|bH	inhibitor o	protein-co
lib-C666-I	chr3	194033534	194033534	+	9.55098	NA	Intergenic	CpG	-2941	NR_02448	100131551	Hs.128066	NR_02448	ENSG0000	LINC00887	HEIRCC	long interg	ncRNA
lib-C666-I	chr2	222437096	222437096	+	47.76595	NA	promoter-TSS (NM_001304537)	promoter-TSS (NM_001304537)	-18	NM_00130	2043	Hs.371218	NM_00443	ENSG0000	EPHA4	HEK8|SEK|	EPH recept	protein-co
lib-C666-I	chr2	222376221	222376221	+	16.50555	NA	intron (NM_001304536, intron 4 of 1	MER57A1|LTR|ERV1	60857	NM_00443	2043	Hs.371218	NM_00443	ENSG0000	EPHA4	HEK8|SEK|	EPH recept	protein-co
lib-C666-I	chr4	90022955	90022955	+	77.62498	NA	Intergenic	MER1B|DNA|hAT-Charlie	-11013	NM_14571	166815	Hs.58924	NM_14571	ENSG0000	TIGD2	HEL106	tigger tran	protein-co
lib-C666-I	chr12	133562924	133562924	+	76.60542	NA	promoter-TSS (NM_001256279)	promoter-TSS (NM_001256279)	-10	NM_00133	7574	Hs.489608	NM_01959	ENSG0000	ZNF26	HEL-179|K	zinc finger	protein-co
lib-C666-I	chr7	64838705	64838705	+	8.59372	NA	promoter-TSS (NM_001287532)	promoter-TSS (NM_001287532)	-7	NM_15262	168374	Hs.9521	NM_00713	ENSG0000	ZNF92	HEL-203|H	zinc finger	protein-co
lib-C666-I	chrX	64926335	64926335	+	8.01457	NA	intron (NM_002444, intron 1 of 12)	intron (NM_002444, intron 1 of 12)	38824	NM_00244	4478	Hs.87752	NM_00244	ENSG0000	MSN	HEL70|IMD	moesin	protein-co
lib-C666-I	chr8	30580618	30580618	+	54.28952	NA	intron (NM_000637, intron 1 of 12)	intron (NM_000637, intron 1 of 12)	4868	NM_00063	2936	Hs.271510	NM_00063	ENSG0000	GSR	HEL-75|HE	glutathione	protein-co
lib-C666-I	chr8	30560652	30560652	+	27.67001	NA	exon (NM_001195104, exon 5 of 11)	exon (NM_001195104, exon 5 of 11	24834	NM_00063	2936	Hs.271510	NM_00063	ENSG0000	GSR	HEL-75|HE	glutathione	protein-co
lib-C666-I	chr8	30585276	30585276	+	25.65964	NA	exon (NM_001195104, exon 1 of 11)	exon (NM_001195104, exon 1 of 11	210	NM_00063	2936	Hs.271510	NM_00063	ENSG0000	GSR	HEL-75|HE	glutathione	protein-co
lib-C666-I	chr6	53213821	53213821	+	105.35873	NA	promoter-TSS (NM_001301856)	promoter-TSS (NM_001301856)	-78	NM_00130	60481	Hs.520189	NM_02181	ENSG0000	ELOVL5	HELO1|SC	ELOVL fatt	protein-co
lib-C666-I	chr6	53224321	53224321	+	8.96177	NA	Intergenic	CpG	-10344	NM_00124	60481	Hs.520189	NM_02181	ENSG0000	ELOVL5	HELO1|SC	ELOVL fatt	protein-co
lib-C666-I	chr9	100807802	100807802	+	6.49644	NA	Intergenic	MER57B1|LTR|ERV1	-11157	NM_01894	54187	Hs.522310	NM_01894	ENSG0000	NANS	HEL-S-10	N-acetylne	protein-co
lib-C666-I	chr9	100848530	100848530	+	6.32351	NA	3' UTR (NM_014788, exon 6 of 6)	3' UTR (NM_014788, exon 6 of 6)	29571	NM_01894	54187	Hs.522310	NM_01894	ENSG0000	NANS	HEL-S-10	N-acetylne	protein-co
lib-C666-I	chr4	17578758	17578758	+	16.63918	NA	promoter-TSS (NM_015907)	promoter-TSS (NM_015907)	-169	NM_01590	51056	Hs.570791	NM_01590	ENSG0000	LAP3	HEL-S-10	leucine am	protein-co
lib-C666-I	chr19	16187210	16187210	+	78.53495	NA	promoter-TSS (NM_003290)	promoter-TSS (NM_003290)	75	NM_00329	7171	Hs.631618	NM_00329	ENSG0000	TPM4	HEL-S-10	tropomyos	protein-co
lib-C666-I	chr11	17281737	17281737	+	12.12723	NA	Intergenic	Intergenic	-16528	NM_00133	4925	Hs.654599	NM_00501	ENSG0000	NUCB2	HEL-S-10	nucleobinc	protein-co
lib-C666-I	chr11	17281268	17281268	+	10.71733	NA	Intergenic	MER102b|DNA|hAT-Charlie	-16997	NM_00133	4925	Hs.654599	NM_00501	ENSG0000	NUCB2	HEL-S-10	nucleobinc	protein-co
lib-C666-I	chr18	54306002	54306002	+	24.75602	NA	promoter-TSS (NM_004786)	promoter-TSS (NM_004786)	-82	NM_00478	9352	Hs.114412	NM_00478	ENSG0000	TXNL1	HEL-S-114	thioredoxir	protein-co
lib-C666-I	chr19	11646991	11646991	+	47.76595	NA	Intergenic	Intergenic	-2541	NM_00130	1264	Hs.465929	NM_00129	ENSG0000	CNN1	HEL-S-14|	calponin 1	protein-co
lib-C666-I	chr21	37506479	37506479	+	27.70954	NA	promoter-TSS (NM_001236)	promoter-TSS (NM_001236)	-784	NM_00123	874	Hs.154510	NM_00123	ENSG0000	CBR3	HEL-S-25|	carbonyl re	protein-co
lib-C666-I	chr21	37507500	37507500	+	8.28613	NA	exon (NM_001236, exon 1 of 3)	exon (NM_001236, exon 1 of 3)	237	NM_00123	874	Hs.154510	NM_00123	ENSG0000	CBR3	HEL-S-25|	carbonyl re	protein-co
lib-C666-I	chr21	37507934	37507934	+	8.28216	NA	intron (NM_001236, intron 1 of 2)	CpG	671	NM_00123	874	Hs.154510	NM_00123	ENSG0000	CBR3	HEL-S-25|	carbonyl re	protein-co
lib-C666-I	chr17	40985261	40985261	+	203.47003	NA	promoter-TSS (NM_001330229)	promoter-TSS (NM_001330229)	102	NM_00126	10197	Hs.152978	NM_00578	ENSG0000	PSME3	HEL-S-28	proteasom	protein-co
lib-C666-I	chr14	88851962	88851962	+	66.08471	NA	promoter-TSS (NM_001040428)	promoter-TSS (NM_001040428)	-26	NM_01841	55812	Hs.525518	NM_01841	ENSG0000	SPATA7	HEL-S-29	spermatog	protein-co
lib-C666-I	chr1	26606514	26606514	+	11.13288	NA	5' UTR (NM_031286, exon 1 of 3)	5' UTR (NM_031286, exon 1 of 3)	301	NM_03128	83442	Hs.109051	NM_03128	ENSG0000	SH3BGRL3	HEL-S-297	SH3 doma	protein-co
lib-C666-I	chr17	46019040	46019040	+	10.79304	NA	promoter-TSS (NR_103856)	promoter-TSS (NR_103856)	151	NM_01812	55163	Hs.631742	NM_01812	ENSG0000	PNPO	HEL-S-302	pyridoxam	protein-co
lib-C666-I	chrX	24043695	24043695	+	12.78696	NA	intron (NM_030624, intron 1 of 3)	CpG-27799	1608	NM_03062	80311	Hs.495854	NM_03062	ENSG0000	KLHL15	HEL-S-30	kelch like f	protein-co
lib-C666-I	chrX	24044292	24044292	+	11.57178	NA	intron (NM_030624, intron 1 of 3)	intron (NM_030624, intron 1 of 3)	1011	NM_03062	80311	Hs.495854	NM_03062	ENSG0000	KLHL15	HEL-S-30	kelch like f	protein-co
lib-C666-I	chr3	101405634	101405634	+	47.26464	NA	promoter-TSS (NM_000986)	promoter-TSS (NM_000986)	-71	NM_00098	6152	Hs.477028	NM_00098	ENSG0000	RPL24	HEL-S-31	ribosomal	protein-co
lib-C666-I	chr17	1466160	1466160	+	78.53495	NA	promoter-TSS (NM_006224)	promoter-TSS (NM_006224)	-50	NM_00622	5306	Hs.429819	NM_00622	ENSG0000	PITPNA	HEL-S-36|	phosphatic	protein-co
lib-C666-I	chr3	53304193	53304193	+	12.64827	NA	Intergenic	Intergenic	-14063	NM_00106	7086	Hs.89643	NM_00106	ENSG0000	TKT	HEL-S-48|	transketola	protein-co
lib-C666-I	chr17	16284221	16284221	+	27.62962	NA	promoter-TSS (NM_001281718)	promoter-TSS (NM_001281718)	110	NM_01895	7314	Hs.356190	NM_01895	ENSG0000	UBB	HEL-S-50	ubiquitin B	protein-co
lib-C666-I	chr10	32428397	32428397	+	10.67459	NA	Intergenic	Intergenic	-83026	NM_00452	3799	Hs.327736	NM_00452	ENSG0000	KIF5B	HEL-S-61|	kinesin fam	protein-co
lib-C666-I	chr10	71992366	71992366	+	4.52748	NA	intron (NM_021129, intron 1 of 10)	intron (NM_021129, intron 1 of 10)	824	NM_02112	5464	Hs.437403	NM_02112	ENSG0000	PPA1	HEL-S-66	pyrophosp	protein-co
lib-C666-I	chr10	71982406	71982406	+	4.04037	NA	intron (NM_021129, intron 2 of 10)	intron (NM_021129, intron 2 of 10)	10784	NM_02112	5464	Hs.437403	NM_02112	ENSG0000	PPA1	HEL-S-66	pyrophosp	protein-co
lib-C666-I	chrX	77359616	77359616	+	21.51366	NA	promoter-TSS (NM_000291)	promoter-TSS (NM_000291)	-50	NM_00029	5230	Hs.78771	NM_00029	ENSG0000	PGK1	HEL-S-68	phosphogl	protein-co
lib-C666-I	chr17	34271906	34271906	+	9.29081	NA	Intergenic	Intergenic	-1192	NM_00119	57151	Hs.97477	NM_02042	ENSG0000	LYZL6	HEL-S-6a|	lysozyme li	protein-co
lib-C666-I	chr12	93835890	93835890	+	10.27265	NA	5' UTR (NM_003348, exon 1 of 4)	5' UTR (NM_003348, exon 1 of 4)	136	NM_00334	7334	Hs.524630	NM_00334	ENSG0000	UBE2N	HEL-S-71|	ubiquitin c	protein-co
lib-C666-I	chr2	28974608	28974608	+	21.99129	NA	promoter-TSS (NM_002709)	promoter-TSS (NM_002709)	-6	NM_20687	5500	Hs.702907	NM_00270	ENSG0000	PPP1CB	HEL-S-80	protein ph	protein-co
lib-C666-I	chr2	28939430	28939430	+	7.08136	NA	Intergenic	Intergenic	-35184	NM_20687	5500	Hs.702907	NM_00270	ENSG0000	PPP1CB	HEL-S-80	protein ph	protein-co
lib-C666-I	chr4	100485000	100485000	+	66.96093	NA	promoter-TSS (NM_001300785)	promoter-TSS (NM_001300785)	-175	NM_15229	93587	Hs.380412	NM_15229	ENSG0000	TRMT10A	HEL-S-88|	tRNA meth	protein-co
lib-C666-I	chr3	100053659	100053659	+	36.70779	NA	promoter-TSS (NM_020202)	promoter-TSS (NM_020202)	97	NM_02020	56954	Hs.439152	NM_02020	ENSG0000	NIT2	HEL-S-8a	nitrilase fa	protein-co
lib-C666-I	chr19	38865203	38865203	+	21.73991	NA	promoter-TSS (NM_002812)	promoter-TSS (NM_002812)	13	NM_00281	5714	Hs.78466	NM_00281	ENSG0000	PSMD8	HEL-S-91	proteasom	protein-co
lib-C666-I	chr11	63953337	63953337	+	5.72511	NA	promoter-TSS (NM_006819)	promoter-TSS (NM_006819)	-250	NM_00128	10963	Hs.337295	NM_00681	ENSG0000	STIP1	HEL-S-94	stress indu	protein-co
lib-C666-I	chr15	45316090	45316090	+	12.13776	NA	intron (NR_034039, intron 1 of 8)	intron (NR_034039, intron 1 of 8)	788	NM_00310	6652	Hs.878	NM_00310	ENSG0000	SORD	HEL-S-95	sorbitol de	protein-co
lib-C666-I	chr14	24110050	24110050	+	8.01457	NA	intron (NM_005794, intron 4 of 8)	intron (NM_005794, intron 4 of 8)	4477	NM_18290	10202	Hs.272499	NM_00579	ENSG0000	DHRS2	HEP27|SDI	dehydroge	protein-co
lib-C666-I	chr14	105487489	105487489	+	6.71839	NA	promoter-TSS (NM_017955)	promoter-TSS (NM_017955)	-64	NM_14570	55038	Hs.34045	NM_01795	ENSG0000	CDCA4	HEPP|SEI-	cell divisior	protein-co
lib-C666-I	chr5	145316226	145316226	+	58.46021	NA	promoter-TSS (NM_152550)	promoter-TSS (NM_152550)	100	NM_15255	153769	Hs.443728	NM_15255	ENSG0000	SH3RF2	HEPP1|PO	SH3 doma	protein-co
lib-C666-I	chr16	56965917	56965917	+	24.25638	NA	promoter-TSS (NM_001010989)	promoter-TSS (NM_001010989)	-85	NM_00127	9709	Hs.146393	NM_01468	ENSG0000	HERPUD1	HERP|Mif1	homocyste	protein-co
lib-C666-I	chr3	193830411	193830411	+	17.27528	NA	Intergenic	Intergenic	-23520	NM_00552	3280	Hs.250666	NM_00552	ENSG0000	HES1	HES-1|HHI	hes family	protein-co
lib-C666-I	chr3	193852852	193852852	+	10.92275	NA	Intergenic	(CA)n|Simple_repeat|Simple_repeat	-1079	NM_00552	3280	Hs.250666	NM_00552	ENSG0000	HES1	HES-1|HHI	hes family	protein-co
lib-C666-I	chr3	193853831	193853831	+	3.89624	NA	promoter-TSS (NM_005524)	promoter-TSS (NM_005524)	-100	NM_00552	3280	Hs.250666	NM_00552	ENSG0000	HES1	HES-1|HHI	hes family	protein-co
lib-C666-I	chr3	54469033	54469033	+	19.7012	NA	intron (NM_018398, intron 4 of 37)	LTR69|LTR|ERVL	204851	NR_02712	790952	Hs.720658	NR_02712	ENSG0000	ESRG	HESRG	embryonic	ncRNA
lib-C666-I	chr3	54584762	54584762	+	4.46583	NA	intron (NM_018398, intron 5 of 37)	intron (NM_018398, intron 5 of 37)	89122	NR_02712	790952	Hs.720658	NR_02712	ENSG0000	ESRG	HESRG	embryonic	ncRNA
lib-C666-I	chr1	242011384	242011384	+	57.02275	NA	promoter-TSS (NM_001319224)	promoter-TSS (NM_001319224)	-109	NM_13039	9156	Hs.498248	NM_00368	ENSG0000	EXO1	HEX1|hExo	exonucleas	protein-co
lib-C666-I	chr7	21467042	21467042	+	26.82121	NA	promoter-TSS (NR_137166)	promoter-TSS (NR_137166)	-610	NM_00132	6671	Hs.88013	NM_00311	ENSG0000	SP4	HF1B|SPR-	Sp4 transc	protein-co
lib-C666-I	chr6	1311756	1311756	+	15.30356	NA	promoter-TSS (NM_033260)	promoter-TSS (NM_033260)	-919	NM_03326	94234	Hs.591352	NM_03326	ENSG0000	FOXQ1	HFH1	forkhead b	protein-co
lib-C666-I	chr6	1311410	1311410	+	4.90117	NA	Intergenic	CpG	-1265	NM_03326	94234	Hs.591352	NM_03326	ENSG0000	FOXQ1	HFH1	forkhead b	protein-co
lib-C666-I	chr11	3498285	3498285	+	6.20473	NA	Intergenic	HERVE-int|LTR|ERV1	-85140	NR_04499	10821	Hs.637689	NR_04499	ENSG0000	OR7E12P	HG1058|O	olfactory re	pseudo
lib-C666-I	chr22	31686814	31686814	+	11.55573	NA	intron (NM_001135911, intron 3 of 4)	CpG	1706	NM_05288	113791	Hs.26670	NM_05288	ENSG0000	PIK3IP1	HGFL|hHG	phosphoin	protein-co
lib-C666-I	chr17	73521127	73521127	+	55.62774	NA	promoter-TSS (NM_001015002)	promoter-TSS (NM_001015002)	-656	NM_00103	3993	Hs.514477	NM_00452	ENSG0000	LLGL2	HGL|HugI-	LLGL2, scri	protein-co
lib-C666-I	chr8	42995514	42995514	+	42.90063	NA	promoter-TSS (NM_152419)	promoter-TSS (NM_152419)	-78	NM_15241	138050	Hs.600384	NM_15241	ENSG0000	HGSNAT	HGNAT|MI	heparan-a	protein-co
lib-C666-I	chr8	42995994	42995994	+	5.12426	NA	intron (NM_152419, intron 1 of 17)	CpG	402	NM_15241	138050	Hs.600384	NM_15241	ENSG0000	HGSNAT	HGNAT|MI	heparan-a	protein-co
lib-C666-I	chrX	133594491	133594491	+	8.03539	NA	intron (NM_000194, intron 1 of 8)	CpG	316	NM_00019	3251	Hs.412707	NM_00019	ENSG0000	HPRT1	HGPRT|HP	hypoxanth	protein-co
lib-C666-I	chr5	99871025	99871025	+	46.67128	NA	promoter-TSS (NM_198507)	promoter-TSS (NM_198507)	16	NM_19850	345757	Hs.356108	NM_19850	ENSG0000	FAM174A	HGS_RE40	family with	protein-co
lib-C666-I	chr1	32404074	32404074	+	34.19829	NA	promoter-TSS (NM_080391)	promoter-TSS (NM_080391)	-86	NM_08039	8073	Hs.470477	NM_00347	ENSG0000	PTP4A2	HH13|HH7	protein tyr	protein-co
lib-C666-I	chr2	129079977	129079977	+	34.59844	NA	Intergenic	CpG	-3806	NM_00480	9394	Hs.512841	NM_00480	ENSG0000	HS6ST1	HH15|HS6	heparan su	protein-co
lib-C666-I	chr12	89619338	89619338	+	5.80117	NA	Intergenic	Intergenic	127298	NM_00194	1848	Hs.298654	NM_00194	ENSG0000	DUSP6	HH19|MKP	dual specif	protein-co
lib-C666-I	chr12	89619836	89619836	+	5.04179	NA	Intergenic	Intergenic	126800	NM_00194	1848	Hs.298654	NM_00194	ENSG0000	DUSP6	HH19|MKP	dual specif	protein-co
lib-C666-I	chr12	89747265	89747265	+	4.73866	NA	promoter-TSS (NM_001946)	promoter-TSS (NM_001946)	-629	NM_02265	1848	Hs.298654	NM_00194	ENSG0000	DUSP6	HH19|MKP	dual specif	protein-co
lib-C666-I	chr6	64282304	64282304	+	14.45701	NA	5' UTR (NM_003463, exon 1 of 6)	5' UTR (NM_003463, exon 1 of 6)	387	NM_00346	7803	Hs.227777	NM_00346	ENSG0000	PTP4A1	HH72|PRL-	protein tyr	protein-co
lib-C666-I	chr6	64281720	64281720	+	4.90117	NA	promoter-TSS (NM_003463)	promoter-TSS (NM_003463)	-197	NM_00346	7803	Hs.227777	NM_00346	ENSG0000	PTP4A1	HH72|PRL-	protein tyr	protein-co
lib-C666-I	chr9	140353855	140353855	+	78.99879	NA	promoter-TSS (NM_001130969)	promoter-TSS (NM_001130969)	-69	NM_00117	26012	Hs.455336	NM_01553	ENSG0000	NSMF	HH9|NELF	NMDA rec	protein-co
lib-C666-I	chr10	25138694	25138694	+	10.4657	NA	3' UTR (NM_001282786, exon 8 of 8)	3' UTR (NM_001282786, exon 8 of 8	102879	NM_02020	56952	Hs.405619	NM_02020	ENSG0000	PRTFDC1	HHGP	phosphori	protein-co
lib-C666-I	chr19	13056439	13056439	+	280.48123	NA	promoter-TSS (NM_005053)	promoter-TSS (NM_005053)	-189	NM_00505	5886	Hs.643267	NM_00505	ENSG0000	RAD23A	HHR23A|H	RAD23 hor	protein-co
lib-C666-I	chr9	110006340	110006340	+	8.89416	NA	Intergenic	Intergenic	-39177	NM_00287	5887	Hs.521640	NM_00287	ENSG0000	RAD23B	HHR23B|H	RAD23 hor	protein-co
lib-C666-I	chr9	110124340	110124340	+	5.96422	NA	Intergenic	L2c|LINE|L2	77290	NM_00124	5887	Hs.521640	NM_00287	ENSG0000	RAD23B	HHR23B|H	RAD23 hor	protein-co
lib-C666-I	chrX	118708410	118708410	+	32.0283	NA	promoter-TSS (NM_181762)	promoter-TSS (NM_181762)	-20	NM_00333	7319	Hs.379466	NM_00333	ENSG0000	UBE2A	HHR6A|MI	ubiquitin c	protein-co
lib-C666-I	chr7	48019204	48019204	+	13.20416	NA	promoter-TSS (NM_004507)	promoter-TSS (NM_004507)	18	NR_03791	3364	Hs.152983	NM_00450	ENSG0000	HUS1	hHUS1	HUS1 chec	protein-co
lib-C666-I	chrX	47863524	47863524	+	12.69461	NA	promoter-TSS (NM_001178099)	promoter-TSS (NM_001178099)	-130	NM_00117	7569	Hs.189690	NM_00696	ENSG0000	ZNF182	HHZ150|K	zinc finger	protein-co
lib-C666-I	chr19	45004798	45004798	+	95.85526	NA	promoter-TSS (NM_001291633)	promoter-TSS (NM_001291633)	-223	NM_00128	7733	Hs.22305	NM_01325	ENSG0000	ZNF180	HHZ168	zinc finger	protein-co
lib-C666-I	chr1	100503658	100503658	+	12.99339	NA	promoter-TSS (NM_033055)	promoter-TSS (NM_033055)	-131	NM_03305	64645	Hs.124156	NM_03305	ENSG0000	MFSD14A	HIAT1	major facili	protein-co
lib-C666-I	chr1	100504309	100504309	+	5.3823	NA	intron (NM_033055, intron 1 of 11)	CpG	520	NM_03305	64645	Hs.124156	NM_03305	ENSG0000	MFSD14A	HIAT1	major facili	protein-co
lib-C666-I	chr9	97136650	97136650	+	61.91626	NA	promoter-TSS (NM_032558)	promoter-TSS (NM_032558)	-183	NM_03255	84641	Hs.555996	NM_03255	ENSG0000	MFSD14B	HIATL1	major facili	protein-co
lib-C666-I	chr9	99776039	99776039	+	73.41202	NA	promoter-TSS (NR_002894)	promoter-TSS (NR_002894)	-177	NR_00289	84278	Hs.610084	NR_00289	ENSG0000	MFSD14C	HIATL2	major facili	pseudo
lib-C666-I	chr2	191184634	191184634	+	12.57335	NA	5' UTR (NM_014362, exon 1 of 14)	5' UTR (NM_014362, exon 1 of 14)	137	NM_19804	26275	Hs.656685	NM_01436	ENSG0000	HIBCH	HIBYLCOA	3-hydroxyi	protein-co
lib-C666-I	chrX	153059987	153059987	+	24.43903	NA	promoter-TSS (NM_004135)	promoter-TSS (NM_004135)	-9	NM_17486	3421	Hs.410197	NM_00413	ENSG0000	IDH3G	H-IDHG	isocitrate d	protein-co
lib-C666-I	chr19	46806860	46806860	+	10.03103	NA	promoter-TSS (NM_152794)	promoter-TSS (NM_152794)	4	NM_15279	64344	Hs.420830	NM_02246	ENSG0000	HIF3A	HIF-3A|HIF	hypoxia in	protein-co

lib-C666-l	chr19	46807378	46807378	+	9.02258	NA	intron (NM_152796, intron 2 of 11)	CpG-13989	522	NM_15279	64344	Hs.420830	NM_02246	ENSG0000	HIF3A	HIF-3A|HIF	hypoxia inc	protein-co
lib-C666-l	chr19	46768799	46768799	+	5.5166	NA	Intergenic	AluJb|SINE|Alu	-31504	NM_15279	64344	Hs.420830	NM_02246	ENSG0000	HIF3A	HIF-3A|HIF	hypoxia inc	protein-co
lib-C666-l	chr14	34547511	34547511	+	54.91701	NA	Intergenic	LTR16D1|LTR|ERVL	-127227	NM_02207	112399	Hs.135507	NM_02207	ENSG0000	EGLN3	HIFP4H3|H	egl-9 fami	protein-co
lib-C666-l	chr14	34420306	34420306	+	3.95418	NA	promoter-TSS (NM_001308103)	promoter-TSS (NM_001308103)	-22	NM_02207	112399	Hs.135507	NM_02207	ENSG0000	EGLN3	HIFP4H3|H	egl-9 fami	protein-co
lib-C666-l	chr14	34420098	34420098	+	3.25502	NA	promoter-TSS (NM_001308103)	promoter-TSS (NM_001308103)	-66	NM_00130	112399	Hs.135507	NM_02207	ENSG0000	EGLN3	HIFP4H3|H	egl-9 fami	protein-co
lib-C666-l	chr19	40791078	40791078	+	53.56082	NA	intron (NM_001243027, intron 1 of 13	CpG	224	NM_00124	208	Hs.631535	NM_00162	ENSG0000	AKT2	HIHGHH|P	AKT serine	protein-co
lib-C666-l	chr19	40791477	40791477	+	10.92275	NA	promoter-TSS (NM_001626)	promoter-TSS (NM_001626)	-175	NM_00162	208	Hs.631535	NM_00162	ENSG0000	AKT2	HIHGHH|P	AKT serine	protein-co
lib-C666-l	chr17	70515228	70515228	+	6.1509	NA	intron (NR_137281, intron 1 of 4)	intron (NR_137281, intron 1 of 4)	73715	NR_03648	100499467	Hs.157726	NR_036488		LINC00673	HI-LNC75|	long interg	ncRNA
lib-C666-l	chr5	130311193	130311193	+	8.28216	NA	Intergenic	Intergenic	189848	NR_02461	3094	Hs.483305	NM_00534	ENSG0000	HINT1	HINT|NMA	histidine tr	protein-co
lib-C666-l	chr5	130274310	130274310	+	7.49163	NA	Intergenic	Intergenic	226731	NR_02461	3094	Hs.483305	NM_00534	ENSG0000	HINT1	HINT|NMA	histidine tr	protein-co
lib-C666-l	chr5	130233363	130233363	+	2.00164	NA	Intergenic	L1PA3|LINE|L1	267678	NR_02461	3094	Hs.483305	NM_00534	ENSG0000	HINT1	HINT|NMA	histidine tr	protein-co
lib-C666-l	chr6	126277849	126277849	+	142.51076	NA	promoter-TSS (NM_138571)	promoter-TSS (NM_138571)	-12	NM_13857	135114	Hs.72325	NM_13857	ENSG0000	HINT3	HINT4	histidine tr	protein-co
lib-C666-l	chr6	126278344	126278344	+	3.20885	NA	intron (NM_138571, intron 1 of 4)	CpG	483	NM_13857	135114	Hs.72325	NM_13857	ENSG0000	HINT3	HINT4	histidine tr	protein-co
lib-C666-l	chr3	148804430	148804430	+	52.49759	NA	promoter-TSS (NM_139048)	promoter-TSS (NM_139048)	-89	NM_13904	6596	Hs.3068	NM_00307	ENSG0000	HLTF	HIP116|HIF	helicase lik	protein-co
lib-C666-l	chr12	123333496	123333496	+	16.19847	NA	intron (NM_001303099, intron 4 of 17	intron (NM_001303099, intron 4 of	13511	NM_00130	9026	Hs.524815	NM_00395	ENSG0000	HIP1R	HIP12|HIP3	huntingtin	protein-co
lib-C666-l	chr12	123326309	123326309	+	8.0154	NA	intron (NM_003959, intron 1 of 31)	intron (NM_003959, intron 1 of 31)	6324	NM_00130	9026	Hs.524815	NM_00395	ENSG0000	HIP1R	HIP12|HIP3	huntingtin	protein-co
lib-C666-l	chr12	108908904	108908904	+	26.54591	NA	promoter-TSS (NM_007076)	promoter-TSS (NM_007076)	-147	NM_00707	11153	Hs.661891	NM_00707	ENSG0000	FICD	HIP13|HYP	FIC domair	protein-co
lib-C666-l	chr11	19138682	19138682	+	4.39666	NA	promoter-TSS (NM_019028)	promoter-TSS (NM_019028)	-10	NM_01902	54503	Hs.188569	NM_01902	ENSG0000	ZDHHC13	HIP14L|HIF	zinc finger	protein-co
lib-C666-l	chr7	75368212	75368212	+	29.92316	NA	promoter-TSS (NM_001243198)	promoter-TSS (NM_001243198)	78	NM_00533	3092	Hs.329266	NM_00533	ENSG0000	HIP1	HIP-I|ILWE	huntingtin	protein-co
lib-C666-l	chr7	75368726	75368726	+	12.73831	NA	promoter-TSS (NM_001243198)	promoter-TSS (NM_001243198)	-436	NM_00124	3092	Hs.329266	NM_00533	ENSG0000	HIP1	HIP-I|ILWE	huntingtin	protein-co
lib-C666-l	chr2	227656530	227656530	+	5.65398	NA	intron (NM_005544, intron 1 of 1)	CpG	6976	NM_00554	3667	Hs.471508	NM_00554	ENSG0000	IRS1	HIRS-1	insulin rece	protein-co
lib-C666-l	chr4	70885240	70885240	+	3.7184	NA	Intergenic	L1HS|LINE|L1	-8890	NM_00020	3347	Hs.654442	NM_00020	ENSG0000	HTN3	HIS2|HTN2	histatin 3	protein-co
lib-C666-l	chr15	43882551	43882551	+	7.84109	NA	promoter-TSS (NM_001190214)	promoter-TSS (NM_001190214)	-100	NM_00113	9677	Hs.156814	NM_01465	ENSG0000	PPIP5K1	HISPPD2A|	diphospho	protein-co
lib-C666-l	chr19	12511956	12511956	+	4.71278	NA	promoter-TSS (NM_001322497)	promoter-TSS (NM_001322497)	132	NM_00108	90576	Hs.546637	NM_20338	ENSG0000	ZNF799	HIT-40|ZN	zinc finger	protein-co
lib-C666-l	chr6	143206676	143206676	+	21.21068	NA	intron (NM_006734, intron 1 of 9)	intron (NM_006734, intron 1 of 9)	59662	NM_00673	3097	Hs.510172	NM_00673	ENSG0000	HIVEP2	HIV-EP2|M	human imr	protein-co
lib-C666-l	chr6	143267600	143267600	+	10.32881	NA	Intergenic	CpG-22589	-1262	NM_00673	3097	Hs.510172	NM_00673	ENSG0000	HIVEP2	HIV-EP2|M	human imr	protein-co
lib-C666-l	chr6	143265856	143265856	+	5.26803	NA	intron (NM_006734, intron 1 of 9)	CpG	482	NM_00673	3097	Hs.510172	NM_00673	ENSG0000	HIVEP2	HIV-EP2|M	human imr	protein-co
lib-C666-l	chr14	105634180	105634180	+	28.32976	NA	exon (NM_002226, exon 2 of 26)	exon (NM_002226, exon 2 of 26)	981	NM_00222	3714	Hs.433445	NM_00222	ENSG0000	JAG2	HJ2|SER2	jagged 2	protein-co
lib-C666-l	chr19	12886560	12886560	+	6.93532	NA	promoter-TSS (NM_001100176)	promoter-TSS (NM_001100176)	-126	NM_01331	29911	Hs.30792	NM_01331	ENSG0000	HOOK2	HK2	hook micro	protein-co
lib-C666-l	chr8	42752316	42752316	+	9.04991	NA	promoter-TSS (NR_039679)	promoter-TSS (NR_039679)	283	NM_03241	84376	Hs.162852	NM_03241	ENSG0000	HOOK3	HK3	hook micro	protein-co
lib-C666-l	chr1	6640045	6640045	+	57.03545	NA	promoter-TSS (NM_001278648)	promoter-TSS (NM_001278648)	-6	NM_00534	3104	Hs.502330	NM_00534	ENSG0000	ZBTB48	HKR3|ZNF	zinc finger	protein-co
lib-C666-l	chr22	41601314	41601314	+	16.5633	NA	promoter-TSS (NM_031488)	promoter-TSS (NM_031488)	1	NM_03148	83746	Hs.517641	NM_03148	ENSG0000	L3MBTL2	H-l(3)mbt-	L3MBTL2 p	protein-co
lib-C666-l	chr6	30457202	30457202	+	30.542	NA	promoter-TSS (NM_005516).6	promoter-TSS (NM_005516).6	19	NM_00551	3133	Hs.650174	NM_00551	ENSG0000	HLA-E	HLA-6.2|Q	major histc	protein-co
lib-C666-l	chr6	30227245	30227245	+	8.28613	NA	promoter-TSS (NR_027822)	promoter-TSS (NR_027822)	-94	NR_02782	3139	Hs.656020	NR_02782	ENSG0000	HLA-L	HLA-92|HL	major histc	pseudo
lib-C666-l	chr6	32997387	32997387	+	20.0808	NA	Intergenic	Intergenic	-19998	NM_00211	3111	Hs.631991	NM_00211	ENSG0000	HLA-DOA	HLA-DNA|	major histc	protein-co
lib-C666-l	chr6	32977449	32977449	+	8.89416	NA	promoter-TSS (NM_002119).5	promoter-TSS (NM_002119).5	-60	NM_00211	3111	Hs.631991	NM_00211	ENSG0000	HLA-DOA	HLA-DNA|	major histc	protein-co
lib-C666-l	chr6	32441453	32441453	+	50.81684	NA	Intergenic	Intergenic	33834	NM_01911	3122	Hs.520048	NM_01911	ENSG0000	HLA-DRA	HLA-DRA1	major histc	protein-co
lib-C666-l	chr6	32407560	32407560	+	25.80588	NA	promoter-TSS (NM_019111).7	promoter-TSS (NM_019111).7	-59	NM_01911	3122	Hs.520048	NM_01911	ENSG0000	HLA-DRA	HLA-DRA1	major histc	protein-co
lib-C666-l	chr6	32405429	32405429	+	25.55305	NA	Intergenic	Intergenic	-2190	NM_01911	3122	Hs.520048	NM_01911	ENSG0000	HLA-DRA	HLA-DRA1	major histc	protein-co
lib-C666-l	chr1	181003063	181003063	+	55.90577	NA	5' UTR (NM_001531, exon 2 of 7)	5' UTR (NM_001531, exon 2 of 7)	502	NM_00119	3140	Hs.13500	NM_00153	ENSG0000	MR1	HLALS	major histc	protein-co
lib-C666-l	chr1	226112006	226112006	+	13.49465	NA	promoter-TSS (NM_001271681)	promoter-TSS (NM_001271681)	34	NM_00127	29920	Hs.654718	NM_01332	ENSG0000	PYCR2	HLD10|P5C	pyrroline-5	protein-co
lib-C666-l	chr20	60962002	60962002	+	26.82121	NA	promoter-TSS (NM_001024)	promoter-TSS (NM_001024)	-119	NM_00102	6227	Hs.190968	NM_00102	ENSG0000	RPS21	HLDF|S21	ribosomal	protein-co
lib-C666-l	chr2	17720293	17720293	+	5.9194	NA	Intergenic	CpG	-1514	NM_00338	7447	Hs.444212	NM_00338	ENSG0000	VSNL1	HLP3|HPCA	visinin like	protein-co
lib-C666-l	chr17	79884674	79884674	+	13.7534	NA	intron (NM_002359, intron 1 of 2)	intron (NM_002359, intron 1 of 2)	913	NM_00235	4097	Hs.252229	NM_00235	ENSG0000	MAFG	hMAF	MAF bZIP t	protein-co
lib-C666-l	chr2	145123089	145123089	+	15.42216	NA	Intergenic	Intergenic	-32988	NM_00128	79712	Hs.44780	NM_02465	ENSG0000	GTDC1	Hmat-Xa|n	glycosyltra	protein-co
lib-C666-l	chr2	145090126	145090126	+	8.73012	NA	promoter-TSS (NM_001164629)	promoter-TSS (NM_001164629)	-25	NM_00128	79712	Hs.44780	NM_02465	ENSG0000	GTDC1	Hmat-Xa|n	glycosyltra	protein-co
lib-C666-l	chr2	230272147	230272147	+	4.19683	NA	intron (NM_139072, intron 9 of 12)	intron (NM_139072, intron 9 of 12)	-136090	NM_00133	55022	Hs.745038	NM_01793	ENSG0000	PID1	HMFN2073	phosphoty	protein-co
lib-C666-l	chr2	27603717	27603717	+	24.28549	NA	promoter-TSS (NM_001201459)	promoter-TSS (NM_001201459)	-106	NM_14463	130557	Hs.515872	NM_14463	ENSG0000	ZNF513	HMFT0656	zinc finger	protein-co
lib-C666-l	chr17	43210334	43210334	+	39.83551	NA	promoter-TSS (NM_133373)	promoter-TSS (NM_133373)	367	NM_00132	79777	Hs.110298	NM_02472	ENSG0000	ACBD4	HMFT0700	acyl-CoA b	protein-co
lib-C666-l	chr13	31040177	31040177	+	47.71827	NA	promoter-TSS (NM_002128)	promoter-TSS (NM_002128)	-75	NM_00212	3146	Hs.434102	NM_00212	ENSG0000	HMGB1	HMG-1|HN	high mobil	protein-co
lib-C666-l	chr21	40720681	40720681	+	63.61368	NA	intron (NM_004965, intron 1 of 5)	CpG-17220	366	NM_00496	3150	Hs.356285	NM_00496	ENSG0000	HMGN1	HMG14	high mobil	protein-co
lib-C666-l	chr1	26827424	26827424	+	2.59032	NA	Intergenic	CpG	28522	NM_00551	3151	Hs.181163	NM_00551	ENSG0000	HMGN2	HMG17	high mobil	protein-co
lib-C666-l	chr1	26827075	26827075	+	2.10911	NA	Intergenic	CpG	28173	NM_00551	3151	Hs.181163	NM_00551	ENSG0000	HMGN2	HMG17	high mobil	protein-co
lib-C666-l	chr6	26537837	26537837	+	5.14484	NA	promoter-TSS (NM_006353)	promoter-TSS (NM_006353)	-735	NM_00635	10473	Hs.236774	NM_00635	ENSG0000	HMGN4	HMG17L3|	high mobil	protein-co
lib-C666-l	chr12	125399364	125399364	+	80.94106	NA	promoter-TSS (NR_049820)	promoter-TSS (NR_049820)	223	NM_02100	7316	Hs.520348	NM_02100	ENSG0000	UBC	HMG20	ubiquitin C	protein-co
lib-C666-l	chr12	125398711	125398711	+	23.05659	NA	intron (NM_021009, intron 1 of 1)	CpG	876	NM_02100	7316	Hs.520348	NM_02100	ENSG0000	UBC	HMG20	ubiquitin C	protein-co
lib-C666-l	chr12	125389183	125389183	+	4.19675	NA	Intergenic	Intergenic	10404	NM_02100	7316	Hs.520348	NM_02100	ENSG0000	UBC	HMG20	ubiquitin C	protein-co
lib-C666-l	chr5	43313813	43313813	+	28.64047	NA	promoter-TSS (NM_001324223)	promoter-TSS (NM_001324223)	-199	NM_00132	3157	Hs.397729	NM_00213	ENSG0000	HMGCS1	HMGCS	3-hydroxy	protein-co
lib-C666-l	chr15	77713120	77713120	+	41.83575	NA	promoter-TSS (NM_024776)	promoter-TSS (NM_024776)	127	NM_00130	10363	Hs.69594	NM_01820	ENSG0000	HMG20A	HMGX1|HN	high mobil	protein-co
lib-C666-l	chr16	74734530	74734530	+	5.64185	NA	5' UTR (NM_152649, exon 1 of 11)	5' UTR (NM_152649, exon 1 of 11)	516	NM_00114	197259	Hs.119878	NM_15264	ENSG0000	MLKL	hMLKL	mixed linea	protein-co
lib-C666-l	chr3	9791483	9791483	+	2.10911	NA	promoter-TSS (NM_016829)	promoter-TSS (NM_016829)	-145	NM_01682	4968	Hs.380271	NM_00254	ENSG0000	OGG1	HMMH|HC	8-oxoguar	protein-co
lib-C666-l	chr8	22224753	22224753	+	13.7499	NA	promoter-TSS (NM_001128431)	promoter-TSS (NM_001128431)	-9	NM_01535	23516	Hs.491232	NM_01535	ENSG0000	SLC39A14	HMNDYT2	solute carr	protein-co
lib-C666-l	chr2	86422580	86422580	+	6.39985	NA	5' UTR (NM_001100169, exon 1 of 15	5' UTR (NM_001100169, exon 1 of 1	313	NM_00683	10989	Hs.148559	NM_00683	ENSG0000	IMMT	HMP|MINC	inner mem	protein-co
lib-C666-l	chr2	86422202	86422202	+	2.60101	NA	intron (NM_006839, intron 1 of 14)	CpG	691	NM_00683	10989	Hs.148559	NM_00683	ENSG0000	IMMT	HMP|MINC	inner mem	protein-co
lib-C666-l	chr14	24664866	24664866	+	34.97044	NA	promoter-TSS (NM_001289006)	promoter-TSS (NM_001289006)	76	NM_00101	10548	Hs.91586	NM_00640	ENSG0000	TM9SF1	HMP70|MF	transmemb	protein-co
lib-C666-l	chr5	110074924	110074924	+	2.64885	NA	exon (NM_138773, exon 1 of 8)	exon (NM_138773, exon 1 of 8)	251	NM_00130	91137	Hs.75639	NM_13877	ENSG0000	SLC25A46	HMSN6B	solute carr	protein-co
lib-C666-l	chr1	151371896	151371896	+	3.27468	NA	promoter-TSS (NM_002796)	promoter-TSS (NM_002796)	-145	NM_00279	5692	Hs.89545	NM_00279	ENSG0000	PSMB4	HN3|HsN3	proteasom	protein-co
lib-C666-l	chr10	37967911	37967911	+	6.81085	NA	Intergenic	Intergenic	-76052	NM_00119	100288485	Hs.727092	NM_00119	ENSG0000	MTRNR2L7	HN7	MT-RNR2-	protein-co
lib-C666-l	chr20	44485899	44485899	+	2.59723	NA	promoter-TSS (NR_037628)	promoter-TSS (NR_037628)	149	NM_00546	10005	Hs.444776	NM_00546	ENSG0000	ACOT8	HNAACTE|	acyl-CoA t	protein-co
lib-C666-l	chr14	38052990	38052990	+	5.72511	NA	Intergenic	CpG	11335	NM_00449	3169	Hs.163484	NM_00449	ENSG0000	FOXA1	HNF3A|TCI	forkhead b	protein-co
lib-C666-l	chr17	79917564	79917564	+	3.20885	NA	exon (NM_178493, exon 2 of 11)	exon (NM_178493, exon 2 of 11)	1493	NM_17849	147111	Hs.106137	NM_17849	ENSG0000	NOTUM	hNOTUM	NOTUM, p	protein-co
lib-C666-l	chr19	51506126	51506126	+	28.64047	NA	3' UTR (NM_012315, exon 5 of 5)	3' UTR (NM_012315, exon 5 of 5)	-1168	NM_14450	11202	Hs.104570	NM_00719	ENSG0000	KLK8	HNP|NP|NI	kallikrein re	protein-co
lib-C666-l	chr2	190648941	190648941	+	13.59254	NA	non-coding (NR_110332, exon 1 of 1	non-coding (NR_110332, exon 1 of	130	NM_00132	5378	Hs.111749	NM_00053	ENSG0000	PMS1	HNPCC3|N	PMS1 hom	protein-co
lib-C666-l	chr4	83350677	83350677	+	149.25539	NA	promoter-TSS (NM_001292017)	promoter-TSS (NM_001292017)	701	NM_03137	9987	Hs.527105	NM_00546	ENSG0000	HNRNPDL	HNRNP|HN	heterogen	protein-co
lib-C666-l	chr4	83351282	83351282	+	2.24066	NA	promoter-TSS (NR_003249)	promoter-TSS (NR_003249)	96	NM_03137	9987	Hs.527105	NM_00546	ENSG0000	HNRNPDL	HNRNP|HN	heterogen	protein-co
lib-C666-l	chr7	26240455	26240455	+	19.01614	NA	promoter-TSS (NM_031243)	promoter-TSS (NM_031243)	-42	NM_00213	3181	Hs.487774	NM_00213	ENSG0000	HNRNPA2I	HNRNPA2|	heterogen	protein-co
lib-C666-l	chr12	53845734	53845734	+	34.3461	NA	promoter-TSS (NM_001128912)	promoter-TSS (NM_001128912)	-152	NM_03198	5094	Hs.546271	NM_00501	ENSG0000	PCBP2	HNRNPE2|	poly(rC) bi	protein-co
lib-C666-l	chr12	53846497	53846497	+	3.70361	NA	intron (NM_001128911, intron 1 of 14	CpG	611	NM_00112	5094	Hs.546271	NM_00501	ENSG0000	PCBP2	HNRNPE2|	poly(rC) bi	protein-co
lib-C666-l	chr19	797233	797233	+	13.48428	NA	promoter-TSS (NM_031990)	promoter-TSS (NM_031990)	-159	NM_03199	5725	Hs.172550	NM_00281	ENSG0000	PTBP1	HNRNP-I|H	polypyrimi	protein-co
lib-C666-l	chr1	245026553	245026553	+	23.74671	NA	intron (NM_004501, intron 1 of 13)	CpG	1274	NM_00450	3192	Hs.106212	NM_00450	ENSG0000	HNRNPU	HNRNPU-/	heterogen	protein-co
lib-C666-l	chr1	245027902	245027902	+	9.02258	NA	promoter-TSS (NM_004501)	promoter-TSS (NM_004501)	-75	NM_03184	3192	Hs.106212	NM_00450	ENSG0000	HNRNPU	HNRNPU-/	heterogen	protein-co
lib-C666-l	chr5	137090140	137090140	+	41.10975	NA	promoter-TSS (NM_006805)	promoter-TSS (NM_006805)	-101	NM_00680	10949	Hs.96996	NM_00680	ENSG0000	HNRNPA0	HNRPA0	heterogen	protein-co
lib-C666-l	chr20	32581569	32581569	+	10.71733	NA	5' UTR (NM_016732, exon 1 of 10)	5' UTR (NM_016732, exon 1 of 10)	111	NM_00736	22913	Hs.136947	NM_00736	ENSG0000	RALY	HNRPCL2|I	RALY heter	protein-co
lib-C666-l	chr10	43904603	43904603	+	25.39487	NA	promoter-TSS (NM_004966)	promoter-TSS (NM_004966)	53	NM_00496	3185	Hs.808	NM_00496	ENSG0000	HNRNPF	HNRPF|OK	heterogen	protein-co
lib-C666-l	chr10	43892161	43892161	+	8.76935	NA	5' UTR (NM_001098208, exon 1 of 3)	5' UTR (NM_001098208, exon 1 of	118	NM_00109	3185	Hs.808	NM_00496	ENSG0000	HNRNPF	HNRPF|OK	heterogen	protein-co
lib-C666-l	chr10	43916492	43916492	+	7.9573	NA	Intergenic	Intergenic	-11796	NM_00109	3185	Hs.808	NM_00496	ENSG0000	HNRNPF	HNRPF|OK	heterogen	protein-co
lib-C666-l	chr10	43903652	43903652	+	7.0832	NA	promoter-TSS (NM_001098206)	promoter-TSS (NM_001098206)	-353	NM_00109	3185	Hs.808	NM_00496	ENSG0000	HNRNPF	HNRPF|OK	heterogen	protein-co
lib-C666-l	chr10	43892764	43892764	+	6.82479	NA	promoter-TSS (NM_001098208)	promoter-TSS (NM_001098208)	30	NM_00109	3185	Hs.808	NM_00496	ENSG0000	HNRNPF	HNRPF|OK	heterogen	protein-co
lib-C666-l	chr5	179050749	179050749	+	7.9573	NA	promoter-TSS (NM_005520)	promoter-TSS (NM_005520)	-27	NM_00552	3187	Hs.604001	NM_00552	ENSG0000	HNRNPH1	HNRPH|HN	heterogen	protein-co
lib-C666-l	chr19	39340989	39340989	+	101.43139	NA	promoter-TSS (NM_001533)	promoter-TSS (NM_001533)	-372	NM_00153	3191	Hs.644906	NM_00153	ENSG0000	HNRNPL	HNRPL|P/C	heterogen	protein-co
lib-C666-l	chr19	39342593	39342593	+	14.54258	NA	intron (NM_001005335, intron 1 of 12	CpG-13673	386	NM_00100	3191	Hs.644906	NM_00153	ENSG0000	HNRNPL	HNRPL|P/C	heterogen	protein-co
lib-C666-l	chr2	38830182	38830182	+	8.28613	NA	promoter-TSS (NM_001142650)	promoter-TSS (NM_001142650)	-4	NM_13839	92906	Hs.445497	NM_13839	ENSG0000	HNRNPLL	HNRPLL|SR	heterogen	protein-co
lib-C666-l	chr1	23670950	23670950	+	14.3056	NA	promoter-TSS (NM_001102397)	promoter-TSS (NM_001102397)	-93	NM_00110	10236	Hs.373763	NM_00582	ENSG0000	HNRNPR	HNRPR|hn	heterogen	protein-co
lib-C666-l	chr1	27248192	27248192	+	9.75396	NA	promoter-TSS (NM_006600)	promoter-TSS (NM_006600)	-21	NM_00660	10726	Hs.263812	NM_00660	ENSG0000	NUDC	HNUDC|M	nuclear dis	protein-co
lib-C666-l	chr1	229644098	229644098	+	125.52737	NA	promoter-TSS (NM_018230)	promoter-TSS (NM_018230)	-10	NM_01823	55746	Hs.12457	NM_01823	ENSG0000	NUP133	hNUP133	nucleopori	protein-co
lib-C666-l	chr16	4526369	4526369	+	7.08136	NA	promoter-TSS (NM_001127204)	promoter-TSS (NM_001127204)	28	NM_00128	3163	Hs.284279	NM_00213	ENSG0000	HMOX2	HO-2	heme oxyg	protein-co
lib-C666-l	chr5	78810453	78810453	+	202.62904	NA	promoter-TSS (NM_004272)	promoter-TSS (NM_004272)	-794	NM_00427	9456	Hs.591761	NM_00427	ENSG0000	HOMER1	HOMER|H	homer sca	protein-co
lib-C666-l	chr16	15737124	15737124	+	12.41959	NA	promoter-TSS (NM_001184998)	promoter-TSS (NM_001184998)	0	NM_00114	54820	Hs.655378	NM_01766	ENSG0000	NDE1	HOM-TES-	nudE neur	protein-co
lib-C666-l	chr12	114679832	114679832	+	4.39666	NA	Intergenic	MLT1K|LTR|ERVL-MaLR	164136	NM_18148	6910	Hs.381715	NM_00019	ENSG0000	TBX5	HOS	T-box 5	protein-co
lib-C666-l	chr7	27169821	27169821	+	14.54258	NA	exon (NM_002141, exon 1 of 2)	exon (NM_002141, exon 1 of 2)	578	NM_00214	3201	Hs.533357	NM_00214	ENSG0000	HOXA4	HOX1|HOX	homeobox	protein-co
lib-C666-l	chr17	46703988	46703988	+	15.39922	NA	promoter-TSS (NM_024017)	promoter-TSS (NM_024017)	-153	NM_02401	3219	Hs.463350	NM_02401	ENSG0000	HOXB9	HOX-2.5|H	homeobox	protein-co
lib-C666-l	chr12	54378829	54378829	+	13.74283	NA	promoter-TSS (NM_017409)	promoter-TSS (NM_017409)	-117	NM_01740	3226	Hs.44276	NM_01740	ENSG0000	HOXC10	HOX3I	homeobox	protein-co
lib-C666-l	chr5	108063794	108063794	+	19.65763	NA	non-coding (NR_046368, exon 1 of 1	non-coding (NR_046368, exon 1 of	168	NR_04636	100652853	Hs.133009	NR_04636	ENSG0000	LINC01023	HP07349	long interg	ncRNA
lib-C666-l	chr12	56709770	56709770	+	8.96177	NA	TTS (NM_001127460)	TTS (NM_001127460)	358	NM_00119	10330	Hs.8752	NM_01425	ENSG0000	CNPY2	HP10390|N	canopy FG	protein-co
lib-C666-l	chr1	21113516	21113516	+	3.20885	NA	promoter-TSS (NM_016287)	promoter-TSS (NM_016287)	-335	NM_01628	50809	Hs.142442	NM_01628	ENSG0000	HP1BP3	HP1-BP74|	heterochro	protein-co
lib-C666-l	chr7	73130167	73130167	+	6.61453	NA	intron (NM_001165903, intron 1 of 9)	intron (NM_001165903, intron 1 of	3850	NM_00116	6804	Hs.647024	NM_00460	ENSG0000	STX1A	HPC-1|P35	syntaxin 1A	protein-co
lib-C666-l	chr2	62932751	62932751	+	3.8557	NA	promoter-TSS (NM_015252)	promoter-TSS (NM_015252)	-250	NM_00114	23301	Hs.271667	NM_01525	ENSG0000	EHBP1	HPC12|NA	EH domain	protein-co
lib-C666-l	chr12	133613952	133613952	+	7.15277	NA	promoter-TSS (NR_110091)	promoter-TSS (NR_110091)	80	NM_00128	7637	Hs.445019	NM_00342	ENSG0000	ZNF84	HPF2	zinc finger	protein-co
lib-C666-l	chr19	57922501	57922501	+	5.12426	NA	promoter-TSS (NM_006959)	promoter-TSS (NM_006959)	-14	NM_00133	7565	Hs.729403	NM_00695	ENSG0000	ZNF17	HPF3|KOX	zinc finger	protein-co
lib-C666-l	chr19	23578345	23578345	+	8.20589	NA	promoter-TSS (NM_001300951)	promoter-TSS (NM_001300951)	17	NM_00130	7644	Hs.58834	NM_00343	ENSG0000	ZNF91	HPF7|HTF1	zinc finger	protein-co
lib-C666-l	chr16	3074329	3074329	+	4.12121	NA	promoter-TSS (NM_001002017)	promoter-TSS (NM_001002017)	-42	NM_00128	54985	Hs.423103	NM_01788	ENSG0000	HCFC1R1	HPIP	host cell fa	protein-co
lib-C666-l	chr9	36487891	36487891	+	15.01426	NA	Intergenic	CpG	-84968	NM_00125	9833	Hs.184339	NM_01479	ENSG0000	MELK	HPK38	maternal e	protein-co
lib-C666-l	chr9	35096506	35096506	+	6.32351	NA	promoter-TSS (NM_032634)	promoter-TSS (NM_032634)	40	NM_03263	84720	Hs.522099	NM_03263	ENSG0000	PIGO	HPMRS2	phosphatic	protein-co
lib-C666-l	chr18	268060	268060	+	133.00432	NA	promoter-TSS (NM_005131)	promoter-TSS (NM_005131)	-1	NM_00513	9984	Hs.712543	NM_00513	ENSG0000	THOC1	HPR1|P84|I	THO comp	protein-co
lib-C666-l	chr5	35317210	35317210	+	11.66012	NA	Intergenic	Intergenic	-86519	NM_00094	5618	Hs.368587	NM_00094	ENSG0000	PRLR	HPRL|MFA	prolactin re	protein-co
lib-C666-l	chr16	791024	791024	+	25.9629	NA	promoter-TSS (NM_022493)	promoter-TSS (NM_022493)	14	NM_02249	64428	Hs.513247	NM_02249	ENSG0000	NARFL	HPRN|IOP1	nuclear pre	protein-co
lib-C666-l	chr9	116037896	116037896	+	123.04253	NA	promoter-TSS (NM_001244926)	promoter-TSS (NM_001244926)	-18	NM_00469	9128	Hs.744014	NM_00469	ENSG0000	PRPF4	HPRP4|HPI	pre-mRNA	protein-co
lib-C666-l	chr17	1588187	1588187	+	10.27265	NA	promoter-TSS (NM_006445)	promoter-TSS (NM_006445)	-11	NM_00644	10594	Hs.181368	NM_00644	ENSG0000	PRPF8	HPRP8|PRF	pre-mRNA	protein-co
lib-C666-l	chr5	34915305	34915305	+	7.15277	NA	promoter-TSS (NM_018321)	promoter-TSS (NM_018321)	426	NR_02659	5810	Hs.38114	NM_00285	ENSG0000	RAD1	HRAD1|RE	RAD1 chec	protein-co
lib-C666-l	chr12	120524989	120524989	+	26.46433	NA	intron (NM_207311, intron 7 of 8)	CpG	29654	NM_00686	11021	Hs.524788	NM_00686	ENSG0000	RAB35	H-ray|RAB	RAB35, me	protein-co
lib-C666-l	chr7	100136537	100136537	+	24.61613	NA	promoter-TSS (NM_006076)	promoter-TSS (NM_006076)	-297	NM_00607	3268	Hs.521083	NM_00607	ENSG0000	AGFG2	HRBL|RABI	ArfGAP wit	protein-co
lib-C666-l	chr8	125486942	125486942	+	317.95407	NA	promoter-TSS (NM_007218)	promoter-TSS (NM_007218)	-66	NM_00721	11236	Hs.744151	NM_00721	ENSG0000	RNF139	HRCA1|RC	ring finger	protein-co
lib-C666-l	chr13	45915385	45915385	+	230.74353	NA	promoter-TSS (NM_001286273)	promoter-TSS (NM_001286273)	34	NM_00128	7178	Hs.374596	NM_00329	ENSG0000	TPT1	HRF|TCTP|t	tumor prot	protein-co
lib-C666-l	chr10	83574819	83574819	+	18.20429	NA	Intergenic	L1MD2|LINE|L1	-60251	NM_00101	10718	Hs.125119	NM_00101	ENSG0000	NRG3	HRG3|pro-	neuregulin	protein-co
lib-C666-l	chr11	108535682	108535682	+	14.3056	NA	promoter-TSS (NM_004398)	promoter-TSS (NM_004398)	-70	NM_00439	1662	Hs.591931	NM_00439	ENSG0000	DDX10	HRH-J8	DEAD-box	protein-co
lib-C666-l	chr21	48055022	48055022	+	8.56376	NA	promoter-TSS (NM_001242865)	promoter-TSS (NM_001242865)	-485	NM_20696	3275	Hs.154163	NM_00153	ENSG0000	PRMT2	HRMT1L1	protein arg	protein-co
lib-C666-l	chr11	20408954	20408954	+	76.37573	NA	promoter-TSS (NM_001145166)	promoter-TSS (NM_001145166)	-122	NM_00114	10196	Hs.152337	NM_00578	ENSG0000	PRMT3	HRMT1L3	protein arg	protein-co
lib-C666-l	chr12	3420302	3420302	+	43.81296	NA	Intergenic	Intergenic	-70213	NM_00125	56341	Hs.504530	NM_01985	ENSG0000	PRMT8	HRMT1L3|I	protein arg	protein-co
lib-C666-l	chr12	3573895	3573895	+	6.83635	NA	intron (NM_001256536, intron 1 of 9)	MER101B|LTR|ERV1	-26469	NM_01985	56341	Hs.504530	NM_01985	ENSG0000	PRMT8	HRMT1L3|I	protein arg	protein-co
lib-C666-l	chr3	196230580	196230580	+	24.17107	NA	promoter-TSS (NM_152617)	promoter-TSS (NM_152617)	59	NM_15261	165918	Hs.250648	NM_15261	ENSG0000	RNF168	hRNF168	ring finger	protein-co
lib-C666-l	chr7	102312101	102312101	+	16.27126	NA	promoter-TSS (NM_032959)	promoter-TSS (NM_032959)	75	NM_03295	246721	Hs.610255	NM_03295	ENSG0000	POLR2J2	HRPB11B|F	RNA polym	protein-co
lib-C666-l	chr14	70233832	70233832	+	44.27569	NA	promoter-TSS (NM_001320214)	promoter-TSS (NM_001320214)	3	NM_00103	6430	Hs.632326	NM_00692	ENSG0000	SRSF5	HRS|SFRS5	serine and	protein-co
lib-C666-l	chr8	99129390	99129390	+	3.93123	NA	promoter-TSS (NM_005836)	promoter-TSS (NM_005836)	28	NM_00583	10247	Hs.18426	NM_00583	ENSG0000	RIDA	HRSP12|P1	reactive int	protein-co
lib-C666-l	chr15	89645190	89645190	+	11.90035	NA	intron (NM_007011, intron 2 of 14)	intron (NM_007011, intron 2 of 14)	13809	NM_00701	11057	Hs.122337	NM_00701	ENSG0000	ABHD2	HS1-2|LAB	abhydrolas	protein-co
lib-C666-l	chr15	89631504	89631504	+	7.37684	NA	5' UTR (NM_007011, exon 1 of 15)	5' UTR (NM_007011, exon 1 of 15)	123	NM_00701	11057	Hs.122337	NM_00701	ENSG0000	ABHD2	HS1-2|LAB	abhydrolas	protein-co
lib-C666-l	chr5	70751433	70751433	+	262.68985	NA	promoter-TSS (NM_018429)	promoter-TSS (NM_018429)	-9	NM_01842	55814	Hs.258272	NM_01842	ENSG0000	BDP1	HSA23852	B double p	protein-co
lib-C666-l	chr3	54156342	54156342	+	9.58102	NA	promoter-TSS (NM_018398)	promoter-TSS (NM_018398)	-351	NM_01839	55799	Hs.656687	NM_01839	ENSG0000	CACNA2D3	HSA27226	calcium vo	protein-co
lib-C666-l	chr3	53381686	53381686	+	20.82108	NA	promoter-TSS (NM_001290204)	promoter-TSS (NM_001290204)	-32	NM_00129	55802	Hs.476353	NM_01840	ENSG0000	DCP1A	HSA27598	decapping	protein-co
lib-C666-l	chr4	142142047	142142047	+	69.8176	NA	promoter-TSS (NM_001292002)	promoter-TSS (NM_001292002)	6	NM_00129	27309	Hs.120766	NM_01448	ENSG0000	ZNF330	HSA6591|N	zinc finger	protein-co
lib-C666-l	chr4	167947256	167947256	+	10.79304	NA	intron (NM_001204354, intron 2 of 9)	Tigger3a|DNA|TcMar-Tigger	208321	NM_00120	50859	Hs.481133	NM_01695	ENSG0000	SPOCK3	HSAJ1454|	SPARC/ost	protein-co
lib-C666-l	chr1	26947280	26947280	+	23.47534	NA	Intergenic	CpG	66247	NR_03174	100302190		NR_03174	ENSG0000	MIR1976	hsa-mir-1	microRNA	ncRNA
lib-C666-l	chr1	26946858	26946858	+	8.22136	NA	Intergenic	CpG	65825	NR_03174	100302190		NR_03174	ENSG0000	MIR1976	hsa-mir-1	microRNA	ncRNA
lib-C666-l	chr8	75610554	75610554	+	2.99475	NA	intron (NR_033830, intron 2 of 5)	L1PB1|LINE|L1	-7374	NR_03174	100302260		NR_03174	ENSG0000	MIR2052	hsa-mir-2	microRNA	ncRNA
lib-C666-l	chr10	6205833	6205833	+	10.03103	NA	intron (NM_001282630, intron 1 of 14	CpG	-11608	NR_03969	100628560		NR_03969	ENSG0000	MIR3155B	hsa-mir-3	microRNA	ncRNA
lib-C666-l	chr21	9827291	9827291	+	8.50855	NA	Intergenic	SSU-rRNA_Hsa|rRNA|rRNA	1088	NR_12871	103504728		NR_12871	ENSG0000	MIR3687-2	hsa-mir-3	microRNA	ncRNA
lib-C666-l	chr3	50265028	50265028	+	6.32351	NA	TTS (NR_106714)	TTS (NR_106714)	160	NR_10671	102464817		NR_10671	ENSG0000	MIR5787	hsa-mir-5	microRNA	ncRNA
lib-C666-l	chr21	45029651	45029651	+	14.3056	NA	TTS (NR_106718)	TTS (NR_106718)	219	NR_10671	102464825		NR_10671	ENSG0000	MIR6070	hsa-mir-6	microRNA	ncRNA
lib-C666-l	chr2	85999695	85999695	+	16.61004	NA	intron (NM_032827, intron 2 of 2)	intron (NM_032827, intron 2 of 2)	11105	NR_10671	102466516		NR_10671	ENSG0000	MIR6071	hsa-mir-6	microRNA	ncRNA
lib-C666-l	chr17	62773269	62773269	+	7.67555	NA	TTS (NR_027487)	TTS (NR_027487)	-3608	NR_10672	102464831		NR_10672	ENSG0000	MIR6080	hsa-mir-6	microRNA	ncRNA
lib-C666-l	chr1	20963640	20963640	+	8.01457	NA	intron (NM_032409, intron 1 of 7)	intron (NM_032409, intron 1 of 7)	3468	NR_10673	102464833		NR_10673	ENSG0000	MIR6084	hsa-mir-6	microRNA	ncRNA
lib-C666-l	chr15	62682679	62682679	+	59.08633	NA	Intergenic	CpG	47451	NR_10673	102464834		NR_10673	ENSG0000	MIR6085	hsa-mir-6	microRNA	ncRNA
lib-C666-l	chr15	62750051	62750051	+	11.57119	NA	Intergenic	Intergenic	114823	NR_10673	102464834		NR_10673	ENSG0000	MIR6085	hsa-mir-6	microRNA	ncRNA
lib-C666-l	chr19	45946804	45946804	+	42.99614	NA	Intergenic	CpG	6892	NR_10673	102464836		NR_10673	ENSG0000	MIR6088	hsa-mir-6	microRNA	ncRNA
lib-C666-l	chr17	47601272	47601272	+	8.28333	NA	intron (NR_103773, intron 1 of 2)	intron (NR_103773, intron 1 of 2)	13093	NR_10675	102465141		NR_10675	ENSG0000	MIR6165	hsa-mir-6	microRNA	ncRNA
lib-C666-l	chr16	15670900	15670900	+	9.75997	NA	intron (NM_001142469, intron 3 of 5)	LTR9|LTR|ERV1	34052	NR_10676	102465252		NR_10676	ENSG0000	MIR6506	hsa-mir-6	microRNA	ncRNA
lib-C666-l	chr7	134881114	134881114	+	9.14689	NA	intron (NM_014149, intron 7 of 14)	intron (NM_014149, intron 7 of 14)	10716	NR_10676	102465254		NR_10676	ENSG0000	MIR6509	hsa-mir-6	microRNA	ncRNA
lib-C666-l	chr11	118530463	118530463	+	48.11517	NA	exon (NM_001301065, exon 10 of 14)	exon (NM_001301065, exon 10 of 1	15745	NR_10677	102466719		NR_10677	ENSG0000	MIR6716	hsa-mir-6	microRNA	ncRNA

lib-C666-l	chr19	40315431	40315431	+	3.8231	NA	TTS (NM_006484)	TTS (NM_006484)	5011	NR_10677	102465974		NR_10677	ENSG0000	MIR6719	hsa-mir-6	microRNA	ncRNA
lib-C666-l	chr1	12605175	12605175	+	26.37215	NA	Intergenic	Intergenic	33876	NR_10678	102466722		NR_10678	ENSG0000	MIR6730	hsa-mir-6	microRNA	ncRNA
lib-C666-l	chr1	12609966	12609966	+	17.37618	NA	Intergenic	Intergenic	29085	NR_10678	102466722		NR_10678	ENSG0000	MIR6730	hsa-mir-6	microRNA	ncRNA
lib-C666-l	chr1	228532646	228532646	+	12.78696	NA	intron (NM_001271223, intron 86 of 1	intron (NM_001271223, intron 86 o	52164	NR_10680	102465444		NR_10680	ENSG0000	MIR6742	hsa-mir-6	microRNA	ncRNA
lib-C666-l	chr1	228560841	228560841	+	4.83287	NA	intron (NM_001271223, intron 105 of	intron (NM_001271223, intron 105	23969	NR_10680	102465444		NR_10680	ENSG0000	MIR6742	hsa-mir-6	microRNA	ncRNA
lib-C666-l	chr11	1283919	1283919	+	27.5466	NA	TTS (NM_002458)	TTS (NM_002458)	6084	NR_10680	102466725		NR_10680	ENSG0000	MIR6744	hsa-mir-6	microRNA	ncRNA
lib-C666-l	chr11	1281347	1281347	+	5.37858	NA	intron (NM_002458, intron 46 of 48)	intron (NM_002458, intron 46 of 48	3512	NR_10680	102466725		NR_10680	ENSG0000	MIR6744	hsa-mir-6	microRNA	ncRNA
lib-C666-l	chr11	71192585	71192585	+	3.8557	NA	intron (NM_018161, intron 12 of 20)	intron (NM_018161, intron 12 of 20	8036	NR_10681	102466728		NR_10681	ENSG0000	MIR6754	hsa-mir-6	microRNA	ncRNA
lib-C666-l	chr16	4713839	4713839	+	27.68428	NA	intron (NM_001142291, intron 5 of 1	intron (NM_001142291, intron 5 of	-7480	NR_10682	102466731		NR_10682	ENSG0000	MIR6769A	hsa-mir-6	microRNA	ncRNA
lib-C666-l	chr16	86072563	86072563	+	2.96041	NA	Intergenic	Intergenic	120610	NR_10683	102466732		NR_10683	ENSG0000	MIR6774	hsa-mir-6	microRNA	ncRNA
lib-C666-l	chr19	6736492	6736492	+	14.17553	NA	TTS (NR_106849)	TTS (NR_106849)	297	NR_10684	102465474		NR_10684	ENSG0000	MIR6791	hsa-mir-6	microRNA	ncRNA
lib-C666-l	chr2	218718580	218718580	+	3.98718	NA	intron (NM_001308023, intron 15 of 2	intron (NM_001308023, intron 15 o	46771	NR_10686	102465485		NR_10686	ENSG0000	MIR6809	hsa-mir-6	microRNA	ncRNA
lib-C666-l	chr22	36727613	36727613	+	10.92275	NA	intron (NM_002473, intron 3 of 40)	intron (NM_002473, intron 3 of 40)	-44660	NR_10687	102465491		NR_10687	ENSG0000	MIR6819	hsa-mir-6	microRNA	ncRNA
lib-C666-l	chr22	36682557	36682557	+	10.03103	NA	TTS (NR_106877)	TTS (NR_106877)	396	NR_10687	102465491		NR_10687	ENSG0000	MIR6819	hsa-mir-6	microRNA	ncRNA
lib-C666-l	chr22	50364544	50364544	+	24.25638	NA	Intergenic	Intergenic	8030	NR_10687	102465493		NR_10687	ENSG0000	MIR6821	hsa-mir-6	microRNA	ncRNA
lib-C666-l	chr3	134031445	134031445	+	7.15277	NA	Intergenic	Intergenic	55259	NR_10688	102466744		NR_10688	ENSG0000	MIR6827	hsa-mir-6	microRNA	ncRNA
lib-C666-l	chr3	195603813	195603813	+	10.79304	NA	intron (NM_001010938, intron 9 of 14	intron (NM_001010938, intron 9 of	5453	NR_10688	102465977		NR_10688	ENSG0000	MIR6829	hsa-mir-6	microRNA	ncRNA
lib-C666-l	chr9	132648104	132648104	+	64.77209	NA	Intergenic	CpG	16220	NR_10691	102466750		NR_10691	ENSG0000	MIR6855	hsa-mir-6	microRNA	ncRNA
lib-C666-l	chr9	132643929	132643929	+	39.72046	NA	3' UTR (NM_006676, exon 25 of 25)	3' UTR (NM_006676, exon 25 of 25)	12045	NR_10691	102466750		NR_10691	ENSG0000	MIR6855	hsa-mir-6	microRNA	ncRNA
lib-C666-l	chr9	132643495	132643495	+	4.29847	NA	3' UTR (NM_006676, exon 25 of 25)	3' UTR (NM_006676, exon 25 of 25)	11611	NR_10691	102466750		NR_10691	ENSG0000	MIR6855	hsa-mir-6	microRNA	ncRNA
lib-C666-l	chr12	112587607	112587607	+	6.50091	NA	exon (NM_006700, exon 9 of 12)	exon (NM_006700, exon 9 of 12)	13518	NR_10692	102465519		NR_10692	ENSG0000	MIR6861	hsa-mir-6	microRNA	ncRNA
lib-C666-l	chr17	8042531	8042531	+	8.07963	NA	Intergenic	Intergenic	5858	NR_10694	102465532		NR_10694	ENSG0000	MIR6883	hsa-mir-6	microRNA	ncRNA
lib-C666-l	chr17	8042211	8042211	+	4.27545	NA	Intergenic	tRNA-Ser-TCG|tRNA|tRNA	6178	NR_10694	102465532		NR_10694	ENSG0000	MIR6883	hsa-mir-6	microRNA	ncRNA
lib-C666-l	chr12	121881205	121881205	+	9.93242	NA	TTS (NR_106957)	TTS (NR_106957)	950	NR_10695	102465665		NR_10695	ENSG0000	MIR7107	hsa-mir-7	microRNA	ncRNA
lib-C666-l	chr12	121905450	121905450	+	2.75718	NA	intron (NM_032590, intron 12 of 22)	intron (NM_032590, intron 12 of 22	-23295	NR_10695	102465665		NR_10695	ENSG0000	MIR7107	hsa-mir-7	microRNA	ncRNA
lib-C666-l	chr8	2075676	2075676	+	269.56012	NA	intron (NM_003970, intron 31 of 36)	CpG	51007	NR_10698	102465695		NR_10698	ENSG0000	MIR7160	hsa-mir-7	microRNA	ncRNA
lib-C666-l	chr16	81555834	81555834	+	24.99834	NA	intron (NM_030629, intron 1 of 20)	intron (NM_030629, intron 1 of 20)	-11673	NR_10700	102465840		NR_10700	ENSG0000	MIR7854	hsa-mir-7	microRNA	ncRNA
lib-C666-l	chr15	37324621	37324621	+	16.19847	NA	intron (NM_170677, intron 8 of 12)	intron (NM_170677, intron 8 of 12)	-59519	NR_10703	102466875		NR_10703	ENSG0000	MIR8063	hsa-mir-8	microRNA	ncRNA
lib-C666-l	chr14	106083118	106083118	+	2.12162	NA	Intergenic	Intergenic	-4335	NR_10705	102466889		NR_10705	ENSG0000	MIR8071-2	hsa-mir-8	microRNA	ncRNA
lib-C666-l	chr13	111024357	111024357	+	3.44441	NA	intron (NM_001846, intron 4 of 47)	intron (NM_001846, intron 4 of 47)	31052	NR_10704	102465872		NR_10704	ENSG0000	MIR8073	hsa-mir-8	microRNA	ncRNA
lib-C666-l	chr13	44842241	44842241	+	3.82577	NA	Intergenic	Intergenic	-71905	NR_10704	102465876		NR_10704	ENSG0000	MIR8079	hsa-mir-8	microRNA	ncRNA
lib-C666-l	chr2	80182677	80182677	+	9.89412	NA	intron (NM_001282597, intron 7 of 18	intron (NM_001282597, intron 7 of	-88968	NR_10704	102465877		NR_10704	ENSG0000	MIR8080	hsa-mir-8	microRNA	ncRNA
lib-C666-l	chr4	114367325	114367325	+	12.62235	NA	Intergenic	Intergenic	293887	NR_10704	102465878		NR_10704	ENSG0000	MIR8082	hsa-mir-8	microRNA	ncRNA
lib-C666-l	chr9	94877707	94877707	+	327.23465	NA	promoter-TSS (NM_178324)	promoter-TSS (NM_178324)	49	NM_17832	10558	Hs.90458	NM_00641	ENSG0000	SPTLC1	HSAN1|HS	serine paln	protein-co
lib-C666-l	chr12	860515	860515	+	4.78638	NA	Intergenic	Intergenic	-1574	NM_00118	65125	Hs.744906	NM_01482	ENSG0000	WNK1	HSAN2|HS	WNK lysine	protein-co
lib-C666-l	chr9	34989573	34989573	+	8.94859	NA	promoter-TSS (NR_109756)	promoter-TSS (NR_109756)	-65	NM_00113	25822	Hs.237506	NM_01226	ENSG0000	DNAJB5	Hsc40	DnaJ heat	protein-co
lib-C666-l	chr9	35002099	35002099	+	5.66433	NA	Intergenic	Intergenic	11832	NM_00113	25822	Hs.237506	NM_01226	ENSG0000	DNAJB5	Hsc40	DnaJ heat	protein-co
lib-C666-l	chr9	34992597	34992597	+	3.59106	NA	intron (NM_001135005, intron 1 of 3)	intron (NM_001135005, intron 1 of	2330	NM_00113	25822	Hs.237506	NM_01226	ENSG0000	DNAJB5	Hsc40	DnaJ heat	protein-co
lib-C666-l	chr16	4524645	4524645	+	9.94079	NA	promoter-TSS (NM_001127206)	promoter-TSS (NM_001127206)	6	NM_00130	57407	Hs.288969	NM_02067	ENSG0000	NMRAL1	HSCARG|S	NmrA like	protein-co
lib-C666-l	chr10	38645242	38645242	+	70.11733	NA	promoter-TSS (NR_003086)	promoter-TSS (NR_003086)	-66	NR_00308	158160	Hs.652484	NM_18282	ENSG0000	HSD17B7P	HSD17B7|H	hydroxyste	pseudo
lib-C666-l	chr18	29671833	29671833	+	94.45538	NA	promoter-TSS (NM_016271)	promoter-TSS (NM_016271)	15	NM_01627	51444	Hs.302408	NM_01627	ENSG0000	RNF138	HSD-4|NA	ring finger	protein-co
lib-C666-l	chr15	100890499	100890499	+	4.98754	NA	promoter-TSS (NR_028140)	promoter-TSS (NR_028140)	-61	NR_02814	388182	Hs.136982	NM_20738	ENSG0000	SPATA41	HSD47	spermatog	ncRNA
lib-C666-l	chr12	28343412	28343412	+	5.80117	NA	promoter-TSS (NM_001330367)	promoter-TSS (NM_001330367)	52	NM_01831	55297	Hs.653125	NM_01831	ENSG0000	CCDC91	HSD8|p56	coiled-coil	protein-co
lib-C666-l	chr12	28343559	28343559	+	4.52748	NA	5' UTR (NM_001330367, exon 1 of 12	5' UTR (NM_001330367, exon 1 of 1	199	NM_01831	55297	Hs.653125	NM_01831	ENSG0000	CCDC91	HSD8|p56	coiled-coil	protein-co
lib-C666-l	chr1	26324690	26324690	+	13.14211	NA	promoter-TSS (NM_000437)	promoter-TSS (NM_000437)	-42	NM_00043	5051	Hs.477083	NM_00043	ENSG0000	PAFAH2	HSD-PLA2	platelet act	protein-co
lib-C666-l	chr14	57735643	57735643	+	2.64233	NA	promoter-TSS (NM_006544)	promoter-TSS (NM_006544)	-26	NM_00654	10640	Hs.743987	NM_00654	ENSG0000	EXOC5	HSEC10|PR	exocyst co	protein-co
lib-C666-l	chr6	33359315	33359315	+	26.18172	NA	promoter-TSS (NM_002263)	promoter-TSS (NM_002263)	2	NM_00226	3833	Hs.436912	NM_00226	ENSG0000	KIFC1	HSET|KNSL	kinesin fam	protein-co
lib-C666-l	chr3	58291965	58291965	+	64.82358	NA	promoter-TSS (NM_001098783)	promoter-TSS (NM_001098783)	-7	NM_00704	11102	Hs.446320	NM_00704	ENSG0000	RPP14	HsHTD2|P1	ribonuclea	protein-co
lib-C666-l	chr3	150481065	150481065	+	25.39487	NA	5' UTR (NM_005067, exon 1 of 2)	5' UTR (NM_005067, exon 1 of 2)	198	NM_00506	6478	Hs.477959	NM_00506	ENSG0000	SIAH2	hSiah2	siah E3 ubi	protein-co
lib-C666-l	chr1	214454607	214454607	+	8.56376	NA	promoter-TSS (NM_020197)	promoter-TSS (NM_020197)	42	NM_02019	56950	Hs.66170	NM_02019	ENSG0000	SMYD2	HSKM-B|K	SET and M	protein-co
lib-C666-l	chr4	120988033	120988033	+	7.7703	NA	promoter-TSS (NM_002358)	promoter-TSS (NM_002358)	-20	NM_00235	4085	Hs.591697	NM_00235	ENSG0000	MAD2L1	HSMAD2|N	MAD2 mit	protein-co
lib-C666-l	chr12	53646057	53646057	+	15.01861	NA	intron (NM_032889, intron 1 of 1)	CpG	181	NM_03288	84975	Hs.654660	NM_03288	ENSG0000	MFSD5	hsMOT2	major facili	protein-co
lib-C666-l	chr13	20207758	20207758	+	23.74671	NA	promoter-TSS (NM_017520)	promoter-TSS (NM_017520)	-30	NM_01752	54737	Hs.269654	NM_01752	ENSG0000	MPHOSPH	HSMPP8|T	M-phase p	protein-co
lib-C666-l	chr14	78083114	78083114	+	6.3317	NA	promoter-TSS (NM_004863)	promoter-TSS (NM_004863)	-4	NM_00486	9517	Hs.435661	NM_00486	ENSG0000	SPTLC2	HSN1C|LCI	serine paln	protein-co
lib-C666-l	chr3	43328000	43328000	+	157.70113	NA	promoter-TSS (NM_017719)	promoter-TSS (NM_017719)	-4	NM_00133	54861	Hs.476052	NM_01771	ENSG0000	SNRK	HSNFRK	SNF relate	protein-co
lib-C666-l	chr16	3767622	3767622	+	144.20264	NA	promoter-TSS (NM_016292)	promoter-TSS (NM_016292)	-24	NM_00127	10131	Hs.30345	NM_01629	ENSG0000	TRAP1	HSP 75|HS	TNF recept	protein-co
lib-C666-l	chr13	31736847	31736847	+	27.70954	NA	promoter-TSS (NM_001286503)	promoter-TSS (NM_001286503)	-322	NM_00128	10808	Hs.743267	NM_00664	ENSG0000	HSPH1	HSP105|HS	heat shock	protein-co
lib-C666-l	chr10	14880086	14880086	+	66.1653	NA	promoter-TSS (NM_001029954)	promoter-TSS (NM_001029954)	-73	NM_00127	51182	Hs.534169	NM_01629	ENSG0000	HSPA14	HSP70-4|H	heat shock	protein-co
lib-C666-l	chr1	161582334	161582334	+	9.20633	NA	Intergenic	CpG	6485	NR_02415	3311	Hs.662180	NR_024151		HSPA7	HSP70B	heat shock	pseudo
lib-C666-l	chr8	80942382	80942382	+	8.28333	NA	exon (NM_014018, exon 1 of 3)	exon (NM_014018, exon 1 of 3)	124	NM_01401	28957	Hs.521124	NM_01401	ENSG0000	MRPS28	HSPC007|N	mitochond	protein-co
lib-C666-l	chr7	56019569	56019569	+	191.68773	NA	promoter-TSS (NM_015969)	promoter-TSS (NM_015969)	-42	NM_01596	51373	Hs.44298	NM_01596	ENSG0000	MRPS17	HSPC011|N	mitochond	protein-co
lib-C666-l	chr1	154193134	154193134	+	20.90919	NA	promoter-TSS (NM_014847)	promoter-TSS (NM_014847)	139	NM_00129	25912	Hs.287471	NM_01544	ENSG0000	C1orf43	HSPC012|N	chromosor	protein-co
lib-C666-l	chr19	13885287	13885287	+	4.38153	NA	promoter-TSS (NM_014047)	promoter-TSS (NM_014047)	30	NM_01404	28974	Hs.231616	NM_01404	ENSG0000	C19orf53	HSPC023|L	chromosor	protein-co
lib-C666-l	chr7	16718674	16718674	+	15.3763	NA	intron (NR_027624, intron 3 of 10)	MER11A|LTR|ERVK	32915	NR_02762	28969	Hs.487635	NM_01403	ENSG0000	BZW2	HSPC028|N	basic leuci	protein-co
lib-C666-l	chr3	53925995	53925995	+	197.31662	NA	promoter-TSS (NM_021237)	promoter-TSS (NM_021237)	47	NM_02123	58515	Hs.58471	NM_02123	ENSG0000	SELENOK	HSPC030|H	selenoprot	protein-co
lib-C666-l	chr3	53961719	53961719	+	6.13111	NA	Intergenic	L1PA2|LINE|L1	-35677	NM_02123	58515	Hs.58471	NM_02123	ENSG0000	SELENOK	HSPC030|H	selenoprot	protein-co
lib-C666-l	chr11	47685083	47685083	+	10.63893	NA	intron (NM_024783, intron 17 of 18)	intron (NM_024783, intron 17 of 18	-20877	NM_00131	23788	Hs.269944	NM_01434	ENSG0000	MTCH2	HSPC032|N	mitochond	protein-co
lib-C666-l	chr8	102218093	102218093	+	11.90035	NA	promoter-TSS (NM_001042510)	promoter-TSS (NM_001042510)	-133	NM_01609	51123	Hs.374485	NM_01609	ENSG0000	ZNF706	HSPC038|F	zinc finger	protein-co
lib-C666-l	chr8	102217654	102217654	+	4.98754	NA	intron (NM_001267708, intron 1 of 5)	CpG	306	NM_00126	51123	Hs.374485	NM_01609	ENSG0000	ZNF706	HSPC038|F	zinc finger	protein-co
lib-C666-l	chr3	149470378	149470378	+	17.3027	NA	promoter-TSS (NM_016094)	promoter-TSS (NM_016094)	-92	NM_01609	51122	Hs.432729	NM_01609	ENSG0000	COMMD2	HSPC042	COMM do	protein-co
lib-C666-l	chr7	134896309	134896309	+	10.67459	NA	promoter-TSS (NM_014149)	promoter-TSS (NM_014149)	7	NM_01414	29062	Hs.459858	NM_01414	ENSG0000	WDR91	HSPC049|S	WD repeat	protein-co
lib-C666-l	chr13	20437917	20437917	+	4.52748	NA	promoter-TSS (NM_001142684)	promoter-TSS (NM_001142684)	-141	NM_00103	9205	Hs.530988	NM_01424	ENSG0000	ZMYM5	HSPC050|N	zinc finger	protein-co
lib-C666-l	chr22	30163294	30163294	+	3.23837	NA	promoter-TSS (NM_019103)	promoter-TSS (NM_019103)	-64	NM_01338	29796	Hs.284292	NM_01338	ENSG0000	UQCR10	HSPC051|H	ubiquinol-	protein-co
lib-C666-l	chr16	11891253	11891253	+	52.3754	NA	promoter-TSS (NM_014153)	promoter-TSS (NM_014153)	-139	NM_01415	29066	Hs.371856	NM_01415	ENSG0000	ZC3H7A	HSPC055|Z	zinc finger	protein-co
lib-C666-l	chr16	58283855	58283855	+	102.48656	NA	promoter-TSS (NM_001142302)	promoter-TSS (NM_001142302)	15	NM_01415	29070	Hs.11614	NM_01415	ENSG0000	CCDC113	HSPC065	coiled-coil	protein-co
lib-C666-l	chr3	12598529	12598529	+	21.0836	NA	promoter-TSS (NM_014160)	promoter-TSS (NM_014160)	16	NM_00127	23609	Hs.591666	NM_01416	ENSG0000	MKRN2	HSPC070|R	makorin rir	protein-co
lib-C666-l	chr6	160211409	160211409	+	13.33696	NA	promoter-TSS (NM_030752)	promoter-TSS (NM_030752)	-69	NM_01416	29074	Hs.416998	NM_01416	ENSG0000	MRPL18	HSPC071|L	mitochond	protein-co
lib-C666-l	chr2	203130438	203130438	+	49.48694	NA	promoter-TSS (NM_015934)	promoter-TSS (NM_015934)	-1	NM_01593	51602	Hs.471104	NM_01593	ENSG0000	NOP58	HSPC120|N	NOP58 rib	protein-co
lib-C666-l	chr4	106395168	106395168	+	16.19847	NA	promoter-TSS (NM_006903)	promoter-TSS (NM_006903)	59	NM_17686	27068	Hs.654957	NM_00690	ENSG0000	PPA2	HSPC124|S	pyrophosp	protein-co
lib-C666-l	chr3	112710129	112710129	+	12.81406	NA	exon (NM_014170, exon 1 of 6)	exon (NM_014170, exon 1 of 6)	329	NM_01417	29083	Hs.127496	NM_01417	ENSG0000	GTPBP8	HSPC135	GTP bindin	protein-co
lib-C666-l	chr11	134123284	134123284	+	21.21068	NA	promoter-TSS (NM_001037304)	promoter-TSS (NM_001037304)	-24	NM_00103	29087	Hs.13645	NM_01417	ENSG0000	THYN1	HSPC144|N	thymocyte	protein-co
lib-C666-l	chr11	64085191	64085191	+	60.16793	NA	promoter-TSS (NM_181651)	promoter-TSS (NM_181651)	365	NM_00128	51504	Hs.333579	NM_01640	ENSG0000	TRMT112	HSPC152|H	tRNA meth	protein-co
lib-C666-l	chr14	25519249	25519249	+	7.05018	NA	promoter-TSS (NM_014178)	promoter-TSS (NM_014178)	-154	NM_01417	29091	Hs.508958	NM_01417	ENSG0000	STXBP6	HSPC156|a	syntaxin bi	protein-co
lib-C666-l	chr14	25393963	25393963	+	6.29226	NA	intron (NM_001304477, intron 2 of 5)	L2c|LINE|L2	124243	NM_00130	29091	Hs.508958	NM_01417	ENSG0000	STXBP6	HSPC156|a	syntaxin bi	protein-co
lib-C666-l	chr16	67261016	67261016	+	190.84485	NA	promoter-TSS (NM_012163)	promoter-TSS (NM_012163)	0	NM_01418	29100	Hs.433203	NM_01418	ENSG0000	TMEM208	HSPC171	transmemb	protein-co
lib-C666-l	chr1	1510251	1510251	+	480.66586	NA	promoter-TSS (NM_014188)	promoter-TSS (NM_014188)	11	NM_01418	29101	Hs.30026	NM_01418	ENSG0000	SSU72	HSPC182|F	SSU72 hon	protein-co
lib-C666-l	chr19	47220438	47220438	+	5.96687	NA	promoter-TSS (NM_016457)	promoter-TSS (NM_016457)	-54	NM_00107	25865	Hs.466987	NM_01645	ENSG0000	PRKD2	HSPC187|F	protein kin	protein-co
lib-C666-l	chr5	145562315	145562315	+	54.91701	NA	promoter-TSS (NM_001317964)	promoter-TSS (NM_001317964)	35	NM_00131	51520	Hs.432674	NM_01646	ENSG0000	LARS	HSPC192|I	leucyl-tRN	protein-co
lib-C666-l	chr15	67399687	67399687	+	9.94079	NA	intron (NM_005902, intron 1 of 8)	intron (NM_005902, intron 1 of 8)	-18367	NM_00114	4088	Hs.727986	NM_00590	ENSG0000	SMAD3	HSPC193|H	SMAD fam	protein-co
lib-C666-l	chr15	67442941	67442941	+	4.39666	NA	intron (NM_001145103, intron 1 of 8)	intron (NM_001145103, intron 1 of	12582	NM_00114	4088	Hs.727986	NM_00590	ENSG0000	SMAD3	HSPC193|H	SMAD fam	protein-co
lib-C666-l	chr4	152682208	152682208	+	39.51209	NA	promoter-TSS (NM_004564)	promoter-TSS (NM_004564)	-33	NM_00456	5188	Hs.119316	NM_00456	ENSG0000	GATB	HSPC199|F	glutamyl-t	protein-co
lib-C666-l	chr4	152621824	152621824	+	9.79436	NA	intron (NM_004564, intron 9 of 12)	intron (NM_004564, intron 9 of 12)	60351	NM_00456	5188	Hs.119316	NM_00456	ENSG0000	GATB	HSPC199|F	glutamyl-t	protein-co
lib-C666-l	chr7	129648995	129648995	+	7.9573	NA	Intergenic	Intergenic	42296	NM_01647	51530	Hs.194157	NM_01647	ENSG0000	ZC3HC1	HSPC216|N	zinc finger	protein-co
lib-C666-l	chr6	107349538	107349538	+	9.14689	NA	5' UTR (NM_001142468, exon 1 of 4)	5' UTR (NM_001142468, exon 1 of 4	162	NM_00114	51250	Hs.486084	NM_01648	ENSG0000	C6orf203	HSPC230|F	chromosor	protein-co
lib-C666-l	chr6	107388585	107388585	+	4.92623	NA	3' UTR (NM_001080450, exon 5 of 5)	3' UTR (NM_001080450, exon 5 of 5	39209	NM_00114	51250	Hs.486084	NM_01648	ENSG0000	C6orf203	HSPC230|F	chromosor	protein-co
lib-C666-l	chr2	85822748	85822748	+	5.23609	NA	promoter-TSS (NM_016494)	promoter-TSS (NM_016494)	-89	NM_01649	51255	Hs.356187	NM_01649	ENSG0000	RNF181	HSPC238	ring finger	protein-co
lib-C666-l	chr19	8478160	8478160	+	10.79304	NA	promoter-TSS (NM_001005415)	promoter-TSS (NM_001005415)	-27	NM_00100	51257	Hs.631861	NM_01649	ENSG0000	2-Mar	HSPC240|N	membrane	protein-co
lib-C666-l	chr19	8488839	8488839	+	2.05265	NA	intron (NM_016496, intron 3 of 5)	AluJo|SINE|Alu	10652	NM_01649	51257	Hs.631861	NM_01649	ENSG0000	2-Mar	HSPC240|N	membrane	protein-co
lib-C666-l	chr22	30831862	30831862	+	55.79842	NA	Intergenic	Intergenic	10251	NM_00100	51537	Hs.713636	NM_01649	ENSG0000	MTFP1	HSPC242|N	mitochond	protein-co
lib-C666-l	chr22	30820091	30820091	+	13.0716	NA	3' UTR (NM_012429, exon 12 of 12)	3' UTR (NM_012429, exon 12 of 12)	-1520	NM_01649	51537	Hs.713636	NM_01649	ENSG0000	MTFP1	HSPC242|N	mitochond	protein-co
lib-C666-l	chr1	31769759	31769759	+	32.48059	NA	promoter-TSS (NM_004814)	promoter-TSS (NM_004814)	-70	NM_00128	51538	Hs.524094	NM_01650	ENSG0000	ZCCHC17	HSPC251|F	zinc finger	protein-co
lib-C666-l	chr16	1995665	1995665	+	26.46433	NA	intron (NM_005061, intron 8 of 9)	intron (NM_005061, intron 8 of 9)	-2338	NM_01633	51734	Hs.655346	NM_01633	ENSG0000	MSRB1	HSPC270|S	methionine	protein-co
lib-C666-l	chr16	1993075	1993075	+	6.49644	NA	intron (NM_016332, intron 1 of 3)	CpG	252	NM_01633	51734	Hs.655346	NM_01633	ENSG0000	MSRB1	HSPC270|S	methionine	protein-co
lib-C666-l	chr7	150020411	150020411	+	6.0459	NA	5' UTR (NM_001142928, exon 1 of 3)	5' UTR (NM_001142928, exon 1 of 3	115	NM_00114	65999	Hs.647119	NM_02394	ENSG0000	LRRC61	HSPC295	leucine rich	protein-co
lib-C666-l	chr17	4634661	4634661	+	5.80117	NA	promoter-TSS (NM_001001683)	promoter-TSS (NM_001001683)	-42	NM_00130	400569	Hs.513885	NM_00100	ENSG0000	MED11	HSPC296	mediator c	protein-co
lib-C666-l	chr8	681308	681308	+	31.27173	NA	promoter-TSS (NM_001303100)	promoter-TSS (NM_001303100)	-69	NM_20733	157697	Hs.655310	NM_20733	ENSG0000	ERICH1	HSPC319	glutamate	protein-co
lib-C666-l	chr8	636802	636802	+	6.06312	NA	intron (NM_207332, intron 3 of 5)	intron (NM_207332, intron 3 of 5)	44437	NM_00130	157697	Hs.655310	NM_20733	ENSG0000	ERICH1	HSPC319	glutamate	protein-co
lib-C666-l	chr8	637692	637692	+	2.64233	NA	intron (NM_207332, intron 3 of 5)	CpG	43547	NM_00130	157697	Hs.655310	NM_20733	ENSG0000	ERICH1	HSPC319	glutamate	protein-co
lib-C666-l	chr2	198364812	198364812	+	94.79198	NA	promoter-TSS (NM_002157)	promoter-TSS (NM_002157)	91	NM_00120	100529241	Hs.633165	NM_00120	ENSG0000	HSPE1-MC	HSPE1-PH	HSPE1-MC	protein-co
lib-C666-l	chr22	18921519	18921519	+	4.74112	NA	intron (NM_001195226, intron 1 of 13	intron (NM_001195226, intron 1 of	2445	NM_00119	5625	Hs.517352	NM_01633	ENSG0000	PRODH	HSPOX2|P	proline del	protein-co
lib-C666-l	chr16	77224742	77224742	+	18.68431	NA	promoter-TSS (NM_001286639)	promoter-TSS (NM_001286639)	-74	NM_00128	22879	Hs.513743	NM_01494	ENSG0000	MON1B	HSRG1|SAI	MON1 hor	protein-co
lib-C666-l	chr19	49078151	49078151	+	68.00875	NA	promoter-TSS (NM_004605)	promoter-TSS (NM_004605)	-842	NM_00460	6820	Hs.369331	NM_00460	ENSG0000	SULT2B1	HSST2	sulfotransf	protein-co
lib-C666-l	chr10	75571518	75571518	+	4.73866	NA	promoter-TSS (NM_003635)	promoter-TSS (NM_003635)	71	NM_00363	8509	Hs.654758	NM_00363	ENSG0000	NDST2	HSST2|NST	N-deacety	protein-co
lib-C666-l	chr1	155107770	155107770	+	6.50091	NA	promoter-TSS (NM_001287590)	promoter-TSS (NM_001287590)	-50	NM_00128	55974	Hs.292154	NM_01884	ENSG0000	SLC50A1	HsSWEET1	solute carr	protein-co
lib-C666-l	chr18	6414967	6414967	+	18.20429	NA	promoter-TSS (NM_173464)	promoter-TSS (NM_173464)	-49	NM_00133	91133	Hs.128279	NM_17346	ENSG0000	L3MBTL4	HsT1031	l(3)mbt-lik	protein-co
lib-C666-l	chr15	23959646	23959646	+	15.42216	NA	Intergenic	L1PA3|LINE|L1	-27196	NM_00248	4692	Hs.50130	NM_00248	ENSG0000	NDN	HsT16328|	necdin, MA	protein-co
lib-C666-l	chr15	59397293	59397293	+	5.65398	NA	promoter-TSS (NM_004701)	promoter-TSS (NM_004701)	9	NM_00470	9133	Hs.194698	NM_00470	ENSG0000	CCNB2	HsT17299	cyclin B2	protein-co
lib-C666-l	chr15	80166725	80166725	+	6.32351	NA	intron (NR_037654, intron 2 of 2)	L1MEf|LINE|L1	22614	NR_03765	10588	Hs.194294	NM_00644	ENSG0000	MTHFS	HsT19268	methenylte	protein-co
lib-C666-l	chr15	80189197	80189197	+	4.38153	NA	non-coding (NR_037654, exon 1 of 3	non-coding (NR_037654, exon 1 of	142	NR_03765	10588	Hs.194294	NM_00644	ENSG0000	MTHFS	HsT19268	methenylte	protein-co
lib-C666-l	chr15	80043127	80043127	+	2.60101	NA	Intergenic	L1PA3|LINE|L1	146212	NR_03765	10588	Hs.194294	NM_00644	ENSG0000	MTHFS	HsT19268	methenylte	protein-co
lib-C666-l	chr15	85524586	85524586	+	14.70559	NA	promoter-TSS (NM_173454)	promoter-TSS (NM_173454)	-619	NM_17345	5151	Hs.9333	NM_00260	ENSG0000	PDE8A	HsT19550	phosphodi	protein-co
lib-C666-l	chr15	85523762	85523762	+	5.96761	NA	promoter-TSS (NM_001243137)	promoter-TSS (NM_001243137)	18	NM_00124	5151	Hs.9333	NM_00260	ENSG0000	PDE8A	HsT19550	phosphodi	protein-co
lib-C666-l	chr15	85524896	85524896	+	3.25502	NA	promoter-TSS (NM_173454)	promoter-TSS (NM_173454)	-309	NM_17345	5151	Hs.9333	NM_00260	ENSG0000	PDE8A	HsT19550	phosphodi	protein-co
lib-C666-l	chr18	33647552	33647552	+	41.21722	NA	promoter-TSS (NM_001303411)	promoter-TSS (NM_001303411)	5	NM_00130	55197	Hs.464912	NM_01817	ENSG0000	RPRD1A	HsT3101|P	regulation	protein-co
lib-C666-l	chr18	2571451	2571451	+	262.07895	NA	promoter-TSS (NM_001308401)	promoter-TSS (NM_001308401)	51	NM_02284	64863	Hs.126888	NM_02284	ENSG0000	METTL4	HsT661	methyltran	protein-co
lib-C666-l	chr8	145515369	145515369	+	37.56522	NA	promoter-TSS (NM_015201)	promoter-TSS (NM_015201)	118	NM_00552	3297	Hs.530227	NM_00552	ENSG0000	HSF1	HSTF1	heat shock	protein-co
lib-C666-l	chr8	145516270	145516270	+	6.32351	NA	intron (NM_005526, intron 1 of 12)	intron (NM_005526, intron 1 of 12)	1019	NM_00552	3297	Hs.530227	NM_00552	ENSG0000	HSF1	HSTF1	heat shock	protein-co
lib-C666-l	chr4	153701039	153701039	+	8.71138	NA	promoter-TSS (NM_001287433)	promoter-TSS (NM_001287433)	-50	NM_00128	27236	Hs.416089	NM_01444	ENSG0000	ARFIP1	HSU52521	ADP ribosy	protein-co
lib-C666-l	chr16	22202782	22202782	+	10.9422	NA	Intergenic	CpG	-14810	NM_01330	29904	Hs.498892	NM_01330	ENSG0000	EEF2K	HSU93850	eukaryotic	protein-co
lib-C666-l	chr16	22217472	22217472	+	5.15278	NA	promoter-TSS (NM_013302)	promoter-TSS (NM_013302)	-120	NM_01330	29904	Hs.498892	NM_01330	ENSG0000	EEF2K	HSU93850	eukaryotic	protein-co
lib-C666-l	chr19	11849965	11849965	+	7.75972	NA	promoter-TSS (NM_001080493)	promoter-TSS (NM_001080493)	-141	NM_01750	55552	Hs.142167	NM_01750	ENSG0000	ZNF823	HSZFP36	zinc finger	protein-co
lib-C666-l	chr2	187350843	187350843	+	3.20885	NA	promoter-TSS (NM_018471)	promoter-TSS (NM_018471)	-42	NM_01847	55854	Hs.741180	NM_01847	ENSG0000	ZC3H15	HT010|LER	zinc finger	protein-co
lib-C666-l	chr8	23386342	23386342	+	28.64047	NA	promoter-TSS (NM_016612)	promoter-TSS (NM_016612)	34	NM_01661	51312	Hs.596025	NM_01661	ENSG0000	SLC25A37	HT015|MFI	solute carr	protein-co
lib-C666-l	chr5	115910462	115910462	+	5.83894	NA	5' UTR (NM_020796, exon 1 of 19)	5' UTR (NM_020796, exon 1 of 19)	160	NM_02079	57556	Hs.156967	NM_02068	ENSG0000	SEMA6A	HT018|SEM	semaphori	protein-co
lib-C666-l	chr12	104359491	104359491	+	3.20885	NA	promoter-TSS (NM_003211)	promoter-TSS (NM_003211)	-102	NM_00321	6996	Hs.584809	NM_00321	ENSG0000	TDG	hTDG	thymine DI	protein-co
lib-C666-l	chr22	20104789	20104789	+	81.64329	NA	promoter-TSS (NM_022727)	promoter-TSS (NM_022727)	29	NM_00125	27037	Hs.713579	NM_02272	ENSG0000	TRMT2A	HTF9C	tRNA meth	protein-co
lib-C666-l	chr7	100401338	100401338	+	22.66433	NA	intron (NM_004444, intron 16 of 16)	intron (NM_004444, intron 16 of 16	23805	NM_00444	2050	Hs.437008	NM_00444	ENSG0000	EPHB4	HTK|MYK1	EPH recept	protein-co
lib-C666-l	chr2	48341588	48341588	+	24.20134	NA	Intergenic	Intergenic	-200207	NM_00215	3344	Hs.468478	NM_00215	ENSG0000	FOXN2	HTLF	forkhead b	protein-co
lib-C666-l	chr2	48542014	48542014	+	8.33814	NA	intron (NM_002158, intron 1 of 6)	CpG	219	NM_00215	3344	Hs.468478	NM_00215	ENSG0000	FOXN2	HTLF	forkhead b	protein-co
lib-C666-l	chr4	926129	926129	+	84.45766	NA	promoter-TSS (NM_001318134)	promoter-TSS (NM_001318134)	-46	NM_00129	84286	Hs.478936	NM_03232	ENSG0000	TMEM175	hTMEM17	transmemb	protein-co
lib-C666-l	chr4	919898	919898	+	8.73012	NA	intron (NM_005255, intron 1 of 27)	intron (NM_005255, intron 1 of 27)	-6277	NM_00129	84286	Hs.478936	NM_03232	ENSG0000	TMEM175	hTMEM17	transmemb	protein-co
lib-C666-l	chr3	185655891	185655891	+	42.99614	NA	promoter-TSS (NM_001243879)	promoter-TSS (NM_001243879)	33	NM_00124	6434	Hs.533122	NM_00459	ENSG0000	TRA2B	Htra2-beta	transforme	protein-co
lib-C666-l	chr1	26827661	26827661	+	3.98718	NA	Intergenic	Intergenic	-28588	NM_00295	6195	Hs.149957	NM_00295	ENSG0000	RPS6KA1	HU-1|MAP	ribosomal	protein-co
lib-C666-l	chr1	26856311	26856311	+	3.28884	NA	promoter-TSS (NM_002953)	promoter-TSS (NM_002953)	62	NM_00295	6195	Hs.149957	NM_00295	ENSG0000	RPS6KA1	HU-1|MAP	ribosomal	protein-co
lib-C666-l	chr6	167054093	167054093	+	2.59723	NA	intron (NM_001006932, intron 2 of 21	intron (NM_001006932, intron 2 of	-13328	NM_02113	6196	Hs.655277	NM_02113	ENSG0000	RPS6KA2	HU-2|MAP	ribosomal	protein-co
lib-C666-l	chr19	11593497	11593497	+	4.19675	NA	TTS (NM_138783)	TTS (NM_138783)	-1694	NM_00142	1995	Hs.1701	NM_00142	ENSG0000	ELAVL3	HUC|HUCL	ELAV like F	protein-co
lib-C666-l	chr1	205196999	205196999	+	6.50091	NA	promoter-TSS (NM_014858)	promoter-TSS (NM_014858)	-39	NM_01485	9911	Hs.6360	NM_01485	ENSG0000	TMCC2	HUCEP11	transmemb	protein-co
lib-C666-l	chr3	52444042	52444042	+	217.73895	NA	promoter-TSS (NM_001321127)	promoter-TSS (NM_001321127)	79	NM_00465	8314	Hs.106674	NM_00465	ENSG0000	BAP1	HUCEP-13	BRCA1 ass	protein-co
lib-C666-l	chr3	37217934	37217934	+	143.492	NA	promoter-TSS (NM_001134369)	promoter-TSS (NM_001134369)	-83	NM_00113	9209	Hs.740522	NM_00630	ENSG0000	LRRFIP2	HUFI-2	LRR bindin	protein-co
lib-C666-l	chr13	96705056	96705056	+	18.20429	NA	intron (NM_020121, intron 1 of 38)	intron (NM_020121, intron 1 of 38)	680	NM_02012	55757	Hs.193226	NM_02012	ENSG0000	UGGT2	HUGT2|UG	UDP-gluco	protein-co
lib-C666-l	chr13	96705505	96705505	+	11.20567	NA	exon (NM_020121, exon 1 of 39)	exon (NM_020121, exon 1 of 39)	231	NM_02012	55757	Hs.193226	NM_02012	ENSG0000	UGGT2	HUGT2|UG	UDP-gluco	protein-co
lib-C666-l	chr3	127513339	127513339	+	2.62544	NA	intron (NM_001003794, intron 2 of 7)	intron (NM_001003794, intron 2 of	28386	NM_00728	11343	Hs.277035	NM_00728	ENSG0000	MGLL	HU-K5|HU	monoglyce	protein-co
lib-C666-l	chr3	51428597	51428597	+	143.97672	NA	promoter-TSS (NM_013286)	promoter-TSS (NM_013286)	-102	NM_01328	29890	Hs.118738	NM_01328	ENSG0000	RBM15B	HUMAGCG	RNA bindir	protein-co
lib-C666-l	chr1	38061546	38061546	+	7.9699	NA	promoter-TSS (NM_001323623)	promoter-TSS (NM_001323623)	40	NM_01328	29889	Hs.75528	NM_01328	ENSG0000	GNL2	HUMAUAN	G protein r	protein-co
lib-C666-l	chr1	14026637	14026637	+	28.57903	NA	promoter-TSS (NM_001135610)	promoter-TSS (NM_001135610)	-98	NM_00113	7799	Hs.371823	NM_01223	ENSG0000	PRDM2	HUMHOXY	PR/SET do	protein-co
lib-C666-l	chr1	13973290	13973290	+	6.71839	NA	Intergenic	Intergenic	-53445	NM_00113	7799	Hs.371823	NM_01223	ENSG0000	PRDM2	HUMHOXY	PR/SET do	protein-co
lib-C666-l	chr1	14027050	14027050	+	6.15313	NA	intron (NM_001135610, intron 1 of 7)	CpG	315	NM_00113	7799	Hs.371823	NM_01223	ENSG0000	PRDM2	HUMHOXY	PR/SET do	protein-co

lib-C666-I	chr16	30382314	30382314	+	11.867	NA	promoter-TSS (NM_015527)	promoter-TSS (NM_015527)	59	NM_00132	29895	Hs.50889	NM_01329	ENSG0000	MYLPF	HUMMLC2	myosin ligl	protein-co
lib-C666-I	chr16	30381955	30381955	+	2.10911	NA	promoter-TSS (NM_015527)	promoter-TSS (NM_015527)	-300	NM_00132	29895	Hs.50889	NM_01329	ENSG0000	MYLPF	HUMMLC2	myosin ligl	protein-co
lib-C666-I	chr6	84140867	84140867	+	2.10304	NA	promoter-TSS (NM_002395)	promoter-TSS (NM_002395)	71	NM_00239	4199	Hs.21160	NM_00239	ENSG0000	ME1	HUMNDM	malic enzy	protein-co
lib-C666-I	chr2	20251759	20251759	+	18.15328	NA	promoter-TSS (NM_014713)	promoter-TSS (NM_014713)	30	NM_01471	9741	Hs.467807	NM_01471	ENSG0000	LAPTM4A	HUMORF1	lysosomal	protein-co
lib-C666-I	chr15	50716557	50716557	+	87.64545	NA	promoter-TSS (NM_001128610)	promoter-TSS (NM_001128610)	-17	NM_00515	9101	Hs.443731	NM_00515	ENSG0000	USP8	HumORF8|	ubiquitin s	protein-co
lib-C666-I	chr1	154909781	154909781	+	114.95827	NA	promoter-TSS (NM_006556)	promoter-TSS (NM_006556)	-297	NM_00655	10654	Hs.30954	NM_00655	ENSG0000	PMVK	HUMPMKI|	phosphom	protein-co
lib-C666-I	chr1	154909152	154909152	+	11.22379	NA	promoter-TSS (NM_001323011)	promoter-TSS (NM_001323011)	-214	NM_00132	10654	Hs.30954	NM_00655	ENSG0000	PMVK	HUMPMKI|	phosphom	protein-co
lib-C666-I	chr19	18954100	18954100	+	17.3027	NA	intron (NM_001297549, intron 1 of 23	intron (NM_001297549, intron 1 of	11356	NM_00291	5976	Hs.515266	NM_00291	ENSG0000	UPF1	HUPF1|NO	UPF1, RNA	protein-co
lib-C666-I	chr19	18942727	18942727	+	2.65152	NA	promoter-TSS (NM_002911)	promoter-TSS (NM_002911)	-17	NM_00129	5976	Hs.515266	NM_00291	ENSG0000	UPF1	HUPF1|NO	UPF1, RNA	protein-co
lib-C666-I	chrX	118987163	118987163	+	12.69461	NA	promoter-TSS (NM_023010)	promoter-TSS (NM_023010)	-172	NM_08063	65109	Hs.103832	NM_02301	ENSG0000	UPF3B	HUPF3B|M	UPF3 regu	protein-co
lib-C666-I	chr20	34287348	34287348	+	9.79436	NA	promoter-TSS (NM_021100)	promoter-TSS (NM_021100)	-61	NR_03757(	9054	Hs.194692	NM_02110	ENSG0000	NFS1	HUSSY-08	NFS1, cyst	protein-co
lib-C666-I	chr18	43313728	43313728	+	2.99475	NA	intron (NM_001146037, intron 3 of 8)	MER1B|DNA|hAT-Charlie	6810	NM_00114	6563	Hs.101307	NM_01586	ENSG0000	SLC14A1	HUT11|Hs1	solute carr	protein-co
lib-C666-I	chr5	140090854	140090854	+	25.3508	NA	promoter-TSS (NR_026703)	promoter-TSS (NR_026703)	-7	NR_02670	56664		NR_02670	ENSG0000	VTRNA1-1	HVG1|VAU	vault RNA	ncRNA
lib-C666-I	chr8	29208600	29208600	+	3.92495	NA	promoter-TSS (NM_001394)	promoter-TSS (NM_001394)	-333	NM_00139	1846	Hs.417962	NM_00139	ENSG0000	DUSP4	HVH2|MKF	dual specif	protein-co
lib-C666-I	chr20	2821014	2821014	+	3.27468	NA	promoter-TSS (NM_080413)	promoter-TSS (NM_080413)	-329	NM_08041	64601	Hs.269577	NM_02257	ENSG0000	VPS16	hVPS16	VPS16, CO	protein-co
lib-C666-I	chrY	21906600	21906600	+	2.12162	NA	5' UTR (NM_001146705, exon 1 of 28	5' UTR (NM_001146705, exon 1 of 2	225	NM_00465	8284	Hs.80358	NM_00465	ENSG0000	KDM5D	HY|HYA|JA	lysine dem	protein-co
lib-C666-I	chr12	50017096	50017096	+	5.5166	NA	promoter-TSS (NM_012272)	promoter-TSS (NM_012272)	-101	NM_00103	25766	Hs.706827	NM_01227	ENSG0000	PRPF40B	HYPC	pre-mRNA	protein-co
lib-C666-I	chr13	27825433	27825433	+	77.62498	NA	promoter-TSS (NM_000982)	promoter-TSS (NM_000982)	-259	NM_00098	6144	Hs.381123	NM_00098	ENSG0000	RPL21	HYPT12|L2	ribosomal	protein-co
lib-C666-I	chr19	8454862	8454862	+	156.05363	NA	promoter-TSS (NR_049795)	promoter-TSS (NR_049795)	-343	NM_00421	9230	Hs.558403	NM_00421	ENSG0000	RAB11B	H-YPT3	RAB11B, m	protein-co
lib-C666-I	chr19	9546214	9546214	+	17.68706	NA	promoter-TSS (NM_001271314)	promoter-TSS (NM_001271314)	40	NM_00663	10781	Hs.656185	NM_00663	ENSG0000	ZNF266	HZF1	zinc finger	protein-co
lib-C666-I	chr19	19779756	19779756	+	2.24164	NA	5' UTR (NM_033204, exon 1 of 4)	5' UTR (NM_033204, exon 1 of 4)	159	NM_03320	94039	Hs.63187	NM_03320	ENSG0000	ZNF101	HZF12	zinc finger	protein-co
lib-C666-I	chr1	247335081	247335081	+	5.04179	NA	intron (NM_003431, intron 1 of 3)	CpG	238	NM_00129	7678	Hs.421238	NM_00343	ENSG0000	ZNF124	HZF-16|HZ	zinc finger	protein-co
lib-C666-I	chr12	133758092	133758092	+	88.33322	NA	promoter-TSS (NM_001165883)	promoter-TSS (NM_001165883)	32	NM_00116	10795	Hs.124047	NM_00341	ENSG0000	ZNF268	HZF3	zinc finger	protein-co
lib-C666-I	chr2	157189311	157189311	+	114.47734	NA	promoter-TSS (NM_006186)	promoter-TSS (NM_006186)	-24	NM_00618	4929	Hs.563344	NM_00618	ENSG0000	NR4A2	HZF-3|NO	nuclear rec	protein-co
lib-C666-I	chr2	157191265	157191265	+	25.68529	NA	Intergenic	CpG	-1978	NM_00618	4929	Hs.563344	NM_00618	ENSG0000	NR4A2	HZF-3|NO	nuclear rec	protein-co
lib-C666-I	chr2	157199091	157199091	+	24.20134	NA	Intergenic	CpG	-9804	NM_00618	4929	Hs.563344	NM_00618	ENSG0000	NR4A2	HZF-3|NO	nuclear rec	protein-co
lib-C666-I	chr19	57791802	57791802	+	73.77308	NA	promoter-TSS (NR_136529)	promoter-TSS (NR_136529)	-51	NM_00663	10794	Hs.99971	NM_00663	ENSG0000	ZNF460	HZF8|ZNF2	zinc finger	protein-co
lib-C666-I	chr20	20393669	20393669	+	52.95013	NA	intron (NM_020343, intron 37 of 39)	intron (NM_020343, intron 37 of 39	44904	NM_00219	3642	Hs.89584	NM_00219	ENSG0000	INSM1	IA-1|IA1	INSM trans	protein-co
lib-C666-I	chr20	20348464	20348464	+	21.28624	NA	promoter-TSS (NM_002196)	promoter-TSS (NM_002196)	-301	NM_00219	3642	Hs.89584	NM_00219	ENSG0000	INSM1	IA-1|IA1	INSM trans	protein-co
lib-C666-I	chr3	107706013	107706013	+	10.79304	NA	Intergenic	Intergenic	103922	NM_19879	961	Hs.446414	NM_00177	ENSG0000	CD47	IAP|MER6|(	CD47 mole	protein-co
lib-C666-I	chrX	77151084	77151084	+	38.21529	NA	promoter-TSS (NM_032121)	promoter-TSS (NM_032121)	-19	NM_03212	84061	Hs.323562	NM_03212	ENSG0000	MAGT1	IAP|MRX95	magnesiun	protein-co
lib-C666-I	chr19	45909811	45909811	+	3.8557	NA	promoter-TSS (NM_001142502)	promoter-TSS (NM_001142502)	-204	NM_00114	10848	Hs.466937	NM_00666	ENSG0000	PPP1R13L	IASPP|NKIF	protein ph	protein-co
lib-C666-I	chr22	39638920	39638920	+	8.73012	NA	intron (NM_002608, intron 1 of 6)	CpG	-2006	NM_03301	5155	Hs.1976	NM_00260	ENSG0000	PDGFB	IBGC5|PDG	platelet de	protein-co
lib-C666-I	chr8	141474943	141474943	+	95.30233	NA	Intergenic	CpG	-6265	NM_03146	83696	Hs.654911	NM_03146	ENSG0000	TRAPPC9	IBP|IKBKBB	trafficking	protein-co
lib-C666-I	chr8	141474442	141474442	+	77.94225	NA	Intergenic	CpG	-5764	NM_03146	83696	Hs.654911	NM_03146	ENSG0000	TRAPPC9	IBP|IKBKBB	trafficking	protein-co
lib-C666-I	chr15	35262020	35262020	+	9.53658	NA	promoter-TSS (NM_014691)	promoter-TSS (NM_014691)	-25	NM_01469	9716	Hs.510958	NM_01469	ENSG0000	AQR	IBP160|fSA	aquarius in	protein-co
lib-C666-I	chr2	217526458	217526458	+	28.7732	NA	intron (NM_000597, intron 3 of 4)	MIR|SINE|MIR	2117	NM_00131	3485	Hs.438102	NM_00059	ENSG0000	IGFBP2	IBP2|IGF-B	insulin like	protein-co
lib-C666-I	chr19	50433194	50433194	+	5.96422	NA	promoter-TSS (NM_001290746)	promoter-TSS (NM_001290746)	-206	NM_01655	23636	Hs.574492	NM_01234	ENSG0000	NUP62	IBSN|SNDI	nucleopori	protein-co
lib-C666-I	chr7	8330711	8330711	+	71.14091	NA	intron (NR_125740, intron 3 of 5)	intron (NR_125740, intron 3 of 5)	-28469	NM_00127	3382	Hs.487561	NM_00496	ENSG0000	ICA1	ICA69|ICAp	islet cell au	protein-co
lib-C666-I	chr7	8302249	8302249	+	18.49429	NA	promoter-TSS (NM_001136020)	promoter-TSS (NM_001136020)	-7	NM_00127	3382	Hs.487561	NM_00496	ENSG0000	ICA1	ICA69|ICAp	islet cell au	protein-co
lib-C666-I	chr7	8301731	8301731	+	3.23837	NA	promoter-TSS (NM_022307)	promoter-TSS (NM_022307)	-49	NM_02230	3382	Hs.487561	NM_00496	ENSG0000	ICA1	ICA69|ICAp	islet cell au	protein-co
lib-C666-I	chr1	9946344	9946344	+	8.88643	NA	intron (NM_020248, intron 1 of 5)	MER20|DNA|hAT-Charlie	23972	NM_02024	56998	Hs.463759	NM_02024	ENSG0000	CTNNBIP1	ICAT	catenin bet	protein-co
lib-C666-I	chr19	4909834	4909834	+	54.92945	NA	promoter-TSS (NM_001290051)	promoter-TSS (NM_001290051)	-271	NM_00129	29128	Hs.108106	NM_01328	ENSG0000	UHRF1	ICBP90|Np	ubiquitin li	protein-co
lib-C666-I	chr2	174455270	174455270	+	7.15725	NA	Intergenic	Intergenic	235709	NM_14581	83879	Hs.470654	NM_03194	ENSG0000	CDCA7	ICF3|JPO1	cell divisior	protein-co
lib-C666-I	chr2	174219464	174219464	+	4.52748	NA	promoter-TSS (NM_031942)	promoter-TSS (NM_031942)	-97	NM_03194	83879	Hs.470654	NM_03194	ENSG0000	CDCA7	ICF3|JPO1	cell divisior	protein-co
lib-C666-I	chr2	174220158	174220158	+	3.98718	NA	intron (NM_145810, intron 1 of 8)	CpG	597	NM_14581	83879	Hs.470654	NM_03194	ENSG0000	CDCA7	ICF3|JPO1	cell divisior	protein-co
lib-C666-I	chr2	174397821	174397821	+	2.64885	NA	Intergenic	L1PA3|LINE|L1	178260	NM_14581	83879	Hs.470654	NM_03194	ENSG0000	CDCA7	ICF3|JPO1	cell divisior	protein-co
lib-C666-I	chr20	30192168	30192168	+	37.12814	NA	promoter-TSS (NM_181353)	promoter-TSS (NM_181353)	-918	NM_18135	3397	Hs.504609	NM_00216	ENSG0000	ID1	ID|bHLHb2	inhibitor ol	protein-co
lib-C666-I	chr20	30190870	30190870	+	14.75675	NA	Intergenic	Intergenic	-2216	NM_18135	3397	Hs.504609	NM_00216	ENSG0000	ID1	ID|bHLHb2	inhibitor ol	protein-co
lib-C666-I	chr5	54515067	54515067	+	2.96041	NA	TTS (NM_001190787)	TTS (NM_001190787)	8076	NM_00119	345643	Hs.394578	NM_00119	ENSG0000	MCIDAS	IDAS|MCI|N	multiciliate	protein-co
lib-C666-I	chr12	111843271	111843271	+	17.37618	NA	promoter-TSS (NM_005475)	promoter-TSS (NM_005475)	-481	NM_00547	10019	Hs.506784	NM_00547	ENSG0000	SH2B3	IDDM20|LN	SH2B adap	protein-co
lib-C666-I	chr6	16129114	16129114	+	6.98764	NA	promoter-TSS (NM_013262)	promoter-TSS (NM_013262)	-203	NM_01326	29116	Hs.484738	NM_01326	ENSG0000	MYLIP	IDOL|MIR	myosin reg	protein-co
lib-C666-I	chr12	108079528	108079528	+	127.05254	NA	promoter-TSS (NM_001317963)	promoter-TSS (NM_001317963)	19	NM_00706	11137	Hs.506652	NM_00706	ENSG0000	PWP1	IEF-SSP-95	PWP1 hom	protein-co
lib-C666-I	chr12	25801724	25801724	+	3.92495	NA	promoter-TSS (NM_001145727)	promoter-TSS (NM_001145727)	-228	NM_00114	160492	Hs.44647	NM_15259	ENSG0000	LMNTD1	IFLTD1|LM	lamin tail d	protein-co
lib-C666-I	chr21	34576361	34576361	+	7.37943	NA	Intergenic	Intergenic	-25839	NM_20758	3455	Hs.708195	NM_00087	ENSG0000	IFNAR2	IFN-R|IFN-	interferon a	protein-co
lib-C666-I	chr21	34572549	34572549	+	4.51266	NA	Intergenic	MLT1B|LTR|ERVL-MaLR	-29651	NM_20758	3455	Hs.708195	NM_00087	ENSG0000	IFNAR2	IFN-R|IFN-	interferon a	protein-co
lib-C666-I	chr3	50329974	50329974	+	25.68768	NA	promoter-TSS (NM_006764)	promoter-TSS (NM_006764)	52	NM_00676	7866	Hs.315177	NM_00676	ENSG0000	IFRD2	IFNRP|SKM	interferon	protein-co
lib-C666-I	chr17	41158807	41158807	+	7.67555	NA	promoter-TSS (NM_005533)	promoter-TSS (NM_005533)	65	NM_00133	3430	Hs.632258	NM_00553	ENSG0000	IFI35	IFP35	interferon	protein-co
lib-C666-I	chr3	187130404	187130404	+	17.71746	NA	Intergenic	L1PA2|LINE|L1	44236	NM_02214	64108	Hs.43388	NM_02214	ENSG0000	RTP4	IFRG28|Z3(	receptor tr	protein-co
lib-C666-I	chr3	39149131	39149131	+	54.01688	NA	promoter-TSS (NM_001105513)	promoter-TSS (NM_001105513)	-21	NM_00110	199223	Hs.443935	NM_14575	ENSG0000	TTC21A	IFT139A|ST	tetratricop	protein-co
lib-C666-I	chr13	80915516	80915516	+	8.01457	NA	promoter-TSS (NM_005842)	promoter-TSS (NM_005842)	-266	NM_00584	10253	Hs.18676	NM_00584	ENSG0000	SPRY2	IGAN3|hSP	sprouty RT	protein-co
lib-C666-I	chr16	29973402	29973402	+	19.75653	NA	promoter-TSS (NM_001083613)	promoter-TSS (NM_001083613)	51	NM_19428	124446	Hs.460574	NM_19428	ENSG0000	TMEM219	IGFBP-3R	transmemb	protein-co
lib-C666-I	chr22	22652085	22652085	+	27.16277	NA	promoter-TSS (NR_027293)	promoter-TSS (NR_027293)	-378	NR_02729	96610	Hs.449601	NM_08092	ENSG0000	BMS1P20	IGL|IGLV|IG	BMS1, ribo	pseudo
lib-C666-I	chr16	75551321	75551321	+	2.65152	NA	Intergenic	CpG	17747	NM_02453	23563	Hs.745424	NM_01212	ENSG0000	CHST5	I-GlcNAc-(	carbohydra	protein-co
lib-C666-I	chr11	132952833	132952833	+	5.92363	NA	intron (NM_001012393, intron 1 of 7)	CpG	-139170	NM_00131	4978	Hs.4817	NM_00254	ENSG0000	OPCML	IGLON1|OI	opioid binc	protein-co
lib-C666-I	chr9	99212342	99212342	+	72.69891	NA	promoter-TSS (NM_014282)	promoter-TSS (NM_014282)	-95	NM_01428	22927	Hs.494567	NM_01428	ENSG0000	HABP4	IHABP-4|IH	hyaluronar	protein-co
lib-C666-I	chr12	117777069	117777069	+	5.39586	NA	intron (NM_001204218, intron 1 of 29	intron (NM_001204218, intron 1 of	22538	NM_00120	4842	Hs.654410	NM_00062	ENSG0000	NOS1	IHPS1|N-N	nitric oxide	protein-co
lib-C666-I	chr11	67723119	67723119	+	11.11305	NA	Intergenic	CpG	48476	NM_03093	81622	Hs.502989	NM_03093	ENSG0000	UNC93B1	IIAE1|UNC9	unc-93 ho	protein-co
lib-C666-I	chr11	67771763	67771763	+	2.05265	NA	promoter-TSS (NM_030930)	promoter-TSS (NM_030930)	-168	NM_03093	81622	Hs.502989	NM_03093	ENSG0000	UNC93B1	IIAE1|UNC9	unc-93 ho	protein-co
lib-C666-I	chr19	50169056	50169056	+	7.49163	NA	promoter-TSS (NM_001197122)	promoter-TSS (NM_001197122)	76	NM_00119	3661	Hs.75254	NM_00157	ENSG0000	IRF3	IIAE7	interferon	protein-co
lib-C666-I	chr1	12079688	12079688	+	4.27545	NA	intron (NM_021933, intron 1 of 9)	CpG	389	NM_02193	60672	Hs.8595	NM_02193	ENSG0000	MIIP	IIP45	migration a	protein-co
lib-C666-I	chr19	36390723	36390723	+	20.72954	NA	promoter-TSS (NM_001321831)	promoter-TSS (NM_001321831)	-84	NM_00132	84807	Hs.466531	NM_13923	ENSG0000	NFKBID	IkappaBNS	NFKB inhib	protein-co
lib-C666-I	chr14	35874118	35874118	+	12.73831	NA	promoter-TSS (NM_020529)	promoter-TSS (NM_020529)	-158	NM_02052	4792	Hs.81328	NM_02052	ENSG0000	NFKBIA	IKBA|MAD-	NFKB inhib	protein-co
lib-C666-I	chr14	35845190	35845190	+	6.50091	NA	Intergenic	Intergenic	28770	NM_02052	4792	Hs.81328	NM_02052	ENSG0000	NFKBIA	IKBA|MAD-	NFKB inhib	protein-co
lib-C666-I	chr14	35873059	35873059	+	5.80816	NA	intron (NM_020529, intron 1 of 5)	CpG	901	NM_02052	4792	Hs.81328	NM_02052	ENSG0000	NFKBIA	IKBA|MAD-	NFKB inhib	protein-co
lib-C666-I	chr19	39390324	39390324	+	69.79861	NA	promoter-TSS (NM_001243116)	promoter-TSS (NM_001243116)	-16	NM_00124	4793	Hs.9731	NM_00250	ENSG0000	NFKBIB	IKBB|TRIP9	NFKB inhib	protein-co
lib-C666-I	chr19	39390748	39390748	+	44.91887	NA	promoter-TSS (NM_030593)	promoter-TSS (NM_030593)	178	NM_00250	4793	Hs.9731	NM_00250	ENSG0000	NFKBIB	IKBB|TRIP9	NFKB inhib	protein-co
lib-C666-I	chr10	101989380	101989380	+	32.38963	NA	promoter-TSS (NM_001278)	promoter-TSS (NM_001278)	-13	NM_00132	1147	Hs.198998	NM_00127	ENSG0000	CHUK	IKBKA|IKK-	conserved	protein-co
lib-C666-I	chr8	145669768	145669768	+	6.71839	NA	promoter-TSS (NM_013432)	promoter-TSS (NM_013432)	44	NM_01343	4796	Hs.675285	NM_01343	ENSG0000	TONSL	IKBR|NFKB	tonsoku-lik	protein-co
lib-C666-I	chr12	99038292	99038292	+	12.54595	NA	promoter-TSS (NM_181869)	promoter-TSS (NM_181869)	537	NM_20161	121457	Hs.252543	NM_15368	ENSG0000	IKBIP	IKIP	IKBKB inter	protein-co
lib-C666-I	chr8	42128773	42128773	+	62.11283	NA	promoter-TSS (NR_040009)	promoter-TSS (NR_040009)	-47	NM_00155	3551	Hs.597664	NM_00155	ENSG0000	IKBKB	IKK-beta|Ik	inhibitor ol	protein-co
lib-C666-I	chr1	32645578	32645578	+	7.15277	NA	intron (NM_175852, intron 1 of 10)	CpG	233	NM_17585	200081	Hs.17987	NM_17585	ENSG0000	TXLNA	IL14|TXLN	taxilin alph	protein-co
lib-C666-I	chr4	142557604	142557604	+	26.46433	NA	promoter-TSS (NM_172175)	promoter-TSS (NM_172175)	-145	NM_00058	3600	Hs.168132	NM_00058	ENSG0000	IL15	IL-15	interleukin	protein-co
lib-C666-I	chr4	142558147	142558147	+	10.38266	NA	non-coding (NR_037840, exon 1 of 8	non-coding (NR_037840, exon 1 of	398	NM_17217	3600	Hs.168132	NM_00058	ENSG0000	IL15	IL-15	interleukin	protein-co
lib-C666-I	chr13	21278322	21278322	+	44.8076	NA	exon (NM_138284, exon 2 of 3)	exon (NM_138284, exon 2 of 3)	840	NM_13828	53342	Hs.655142	NM_13828	ENSG0000	IL17D	IL-17D	interleukin	protein-co
lib-C666-I	chr11	407561	407561	+	12.72278	NA	exon (NM_001135054, exon 6 of 10)	exon (NM_001135054, exon 6 of 10	7438	NM_00113	59307	Hs.501624	NM_02180	ENSG0000	SIGIRR	IL-1R8|TIR8	single Ig ar	protein-co
lib-C666-I	chr19	10947281	10947281	+	11.48337	NA	promoter-TSS (NR_104015)	promoter-TSS (NR_104015)	-298	NM_00685	11018	Hs.211463	NM_00685	ENSG0000	TMED1	IL1RL1LG|II	transmemb	protein-co
lib-C666-I	chr2	102844246	102844246	+	5.25516	NA	intron (NM_003854, intron 9 of 11)	intron (NM_003854, intron 9 of 11)	40813	NM_00385	8808	Hs.659863	NM_00385	ENSG0000	IL1RL2	IL-1Rrp2|IL	interleukin	protein-co
lib-C666-I	chr12	56732613	56732613	+	36.59122	NA	promoter-TSS (NM_016584)	promoter-TSS (NM_016584)	-50	NM_01658	51561	Hs.98309	NM_01658	ENSG0000	IL23A	IL-23|IL-23	interleukin	protein-co
lib-C666-I	chr16	28519037	28519037	+	18.17142	NA	promoter-TSS (NM_145659)	promoter-TSS (NM_145659)	-882	NM_14565	246778	Hs.528111	NM_14565	ENSG0000	IL27	IL-27|IL-27	interleukin	protein-co
lib-C666-I	chr19	4229316	4229316	+	16.76091	NA	promoter-TSS (NM_005755)	promoter-TSS (NM_005755)	-224	NM_00575	10148	Hs.501452	NM_00575	ENSG0000	EBI3	IL-27B|IL27	Epstein-Ba	protein-co
lib-C666-I	chr16	3115457	3115457	+	7.08929	NA	promoter-TSS (NM_001308078)	promoter-TSS (NM_001308078)	144	NM_00101	9235	Hs.943	NM_00422	ENSG0000	IL32	IL-32alpha	interleukin	protein-co
lib-C666-I	chr19	35738897	35738897	+	2.00164	NA	promoter-TSS (NM_001260490)	promoter-TSS (NM_001260490)	-662	NM_00126	51599	Hs.466507	NM_01592	ENSG0000	LSR	ILDR3|LISC	lipolysis sti	protein-co
lib-C666-I	chr17	80477438	80477438	+	15.55328	NA	promoter-TSS (NM_004514)	promoter-TSS (NM_004514)	-156	NM_00451	3607	Hs.591140	NM_00451	ENSG0000	FOXK2	ILF|ILF-1|IL	forkhead b	protein-co
lib-C666-I	chr9	35732494	35732494	+	34.53169	NA	promoter-TSS (NR_106912)	promoter-TSS (NR_106912)	-102	NM_00628	7094	Hs.471014	NM_00628	ENSG0000	TLN1	ILWEQ|TLN	talin 1	protein-co
lib-C666-I	chr16	21531998	21531998	+	38.41137	NA	promoter-TSS (NR_002594)	promoter-TSS (NR_002594)	-233	NR_00259	387254	Hs.448808	NR_002594		SLC7A5P2	IMAA|MMA	solute carr	pseudo
lib-C666-I	chr16	21529779	21529779	+	32.50085	NA	non-coding (NR_002594, exon 1 of 1	non-coding (NR_002594, exon 1 of	1986	NR_00259	387254	Hs.448808	NR_002594		SLC7A5P2	IMAA|MMA	solute carr	pseudo
lib-C666-I	chr16	21531661	21531661	+	19.06818	NA	non-coding (NR_002594, exon 1 of 1	non-coding (NR_002594, exon 1 of	104	NR_00259	387254	Hs.448808	NR_002594		SLC7A5P2	IMAA|MMA	solute carr	pseudo
lib-C666-I	chr16	21526979	21526979	+	17.21813	NA	Intergenic	LTR22B|LTR|ERVK	4786	NR_00259	387254	Hs.448808	NR_002594		SLC7A5P2	IMAA|MMA	solute carr	pseudo
lib-C666-I	chr11	16760151	16760151	+	10.70998	NA	promoter-TSS (NM_014267)	promoter-TSS (NM_014267)	3	NM_01426	10944	Hs.368226	NM_01426	ENSG0000	C11orf58	IMAGE145	chromosor	protein-co
lib-C666-I	chr17	45727173	45727173	+	21.99129	NA	promoter-TSS (NM_002265)	promoter-TSS (NM_002265)	-31	NM_00226	3837	Hs.532793	NM_00226	ENSG0000	KPNB1	IMB1|IPO1	karyopheri	protein-co
lib-C666-I	chr17	45726709	45726709	+	2.59723	NA	promoter-TSS (NM_002265)	promoter-TSS (NM_002265)	-495	NM_00226	3837	Hs.532793	NM_00226	ENSG0000	KPNB1	IMB1|IPO1	karyopheri	protein-co
lib-C666-I	chr1	27816780	27816780	+	7.26874	NA	promoter-TSS (NM_001201404)	promoter-TSS (NM_001201404)	-82	NM_00120	10163	Hs.469244	NM_00699	ENSG0000	WASF2	IMD2|SCAF	WAS prote	protein-co
lib-C666-I	chrX	48534677	48534677	+	5.92363	NA	Intergenic	CpG	-7509	NM_00037	7454	Hs.2157	NM_00037	ENSG0000	WAS	IMD2|SCN)	Wiskott-Al	protein-co
lib-C666-I	chr19	10491348	10491348	+	76.62533	NA	promoter-TSS (NM_003331)	promoter-TSS (NM_003331)	-100	NM_00333	7297	Hs.75516	NM_00333	ENSG0000	TYK2	IMD35|JTK	tyrosine kir	protein-co
lib-C666-I	chr11	615587	615587	+	2.05265	NA	TTS (NM_001171968)	TTS (NM_001171968)	141	NM_00403	3665	Hs.166120	NM_00157	ENSG0000	IRF7	IMD39|IRF-	interferon	protein-co
lib-C666-I	chr15	45003631	45003631	+	123.35068	NA	promoter-TSS (NM_004048)	promoter-TSS (NM_004048)	-54	NM_00404	567	Hs.534255	NM_00404	ENSG0000	B2M	IMD43	beta-2-mi	protein-co
lib-C666-I	chr14	21979557	21979557	+	15.22267	NA	promoter-TSS (NM_019852)	promoter-TSS (NM_019852)	-40	NM_01985	56339	Hs.168799	NM_01985	ENSG0000	METTL3	IME4|M6A|	methyltran	protein-co
lib-C666-I	chr7	111202541	111202541	+	28.8837	NA	promoter-TSS (NM_032549)	promoter-TSS (NM_032549)	32	NM_00124	83943	Hs.655722	NM_03254	ENSG0000	IMMP2L	IMMP2L-IT	inner mitoc	protein-co
lib-C666-I	chr13	41345305	41345305	+	8.01457	NA	promoter-TSS (NM_005830)	promoter-TSS (NM_005830)	42	NM_00583	10240	Hs.596607	NM_00583	ENSG0000	MRPS31	IMOGN38|	mitochond	protein-co
lib-C666-I	chr1	44412261	44412261	+	7.03048	NA	promoter-TSS (NM_014652)	promoter-TSS (NM_014652)	-217	NM_01465	9670	Hs.158497	NM_01465	ENSG0000	IPO13	IMP13|KAP	importin 1;	protein-co
lib-C666-I	chr14	24658127	24658127	+	5.23609	NA	promoter-TSS (NR_051979)	promoter-TSS (NR_051979)	-3	NR_05197	79711	Hs.411865	NM_02465	ENSG0000	IPO4	Imp4	importin 4	protein-co
lib-C666-I	chr11	9386091	9386091	+	38.90989	NA	Intergenic	CpG	-20078	NM_00639	10527	Hs.744911	NM_00639	ENSG0000	IPO7	Imp7|RANB	importin 7	protein-co
lib-C666-I	chr7	128050400	128050400	+	21.60686	NA	promoter-TSS (NM_000883)	promoter-TSS (NM_000883)	-359	NM_00130	3614	Hs.654401	NM_00088	ENSG0000	IMPDH1	IMPD|IMPD	inosine mo	protein-co
lib-C666-I	chr3	49066897	49066897	+	91.50885	NA	promoter-TSS (NM_000884)	promoter-TSS (NM_000884)	-22	NM_00088	3615	Hs.654400	NM_00088	ENSG0000	IMPDH2	IMPD2|IMP	inosine mo	protein-co
lib-C666-I	chr4	184427374	184427374	+	10.76537	NA	5' UTR (NM_001291959, exon 1 of 2)	5' UTR (NM_001291959, exon 1 of 2	139	NM_00129	3622	Hs.107153	NM_00156	ENSG0000	ING2	ING1L|p33	inhibitor ol	protein-co
lib-C666-I	chr1	51443987	51443987	+	42.99614	NA	Intergenic	CpG	8345	NM_07862	1031	Hs.525324	NM_00126	ENSG0000	CDKN2C	INK4C|p18	cyclin depe	protein-co
lib-C666-I	chr19	10679782	10679782	+	12.26631	NA	promoter-TSS (NM_079421)	promoter-TSS (NM_079421)	-127	NM_07942	1032	Hs.435051	NM_00180	ENSG0000	CDKN2D	INK4D|p19	cyclin depe	protein-co
lib-C666-I	chr15	41408645	41408645	+	2.38795	NA	promoter-TSS (NR_104038)	promoter-TSS (NR_104038)	-201	NR_10403	54617	Hs.292949	NM_01755	ENSG0000	INO80	INO80A|IN	INO80 com	protein-co
lib-C666-I	chr11	129780557	129780557	+	9.79436	NA	intron (NM_199439, intron 13 of 15)	intron (NM_199439, intron 13 of 15	-15067	NM_00114	4798	Hs.530539	NM_00616	ENSG0000	NFRKB	INO80G	nuclear fac	protein-co
lib-C666-I	chr2	99061785	99061785	+	9.10494	NA	intron (NM_001134225, intron 1 of 24	CpG	464	NM_00156	3631	Hs.469386	NM_00156	ENSG0000	INPP4A	INPP4|TVA	inositol po	protein-co
lib-C666-I	chr22	31518959	31518959	+	9.94769	NA	promoter-TSS (NM_001284286)	promoter-TSS (NM_001284286)	50	NM_00100	27124	Hs.517549	NM_01442	ENSG0000	INPP5J	INPP5|PIB5	inositol po	protein-co
lib-C666-I	chr21	34100291	34100291	+	28.8334	NA	promoter-TSS (NR_038880)	promoter-TSS (NR_038880)	-41	NM_00116	8867	Hs.473632	NM_00389	ENSG0000	SYNJ1	INPP5G|PA	synaptojan	protein-co
lib-C666-I	chr6	158460341	158460341	+	14.35303	NA	intron (NM_003898, intron 4 of 26)	intron (NM_003898, intron 4 of 26)	22261	NM_00117	8871	Hs.434494	NM_00389	ENSG0000	SYNJ2	INPP5H	synaptojan	protein-co
lib-C666-I	chr6	158447829	158447829	+	12.12504	NA	intron (NM_003898, intron 2 of 26)	intron (NM_003898, intron 2 of 26)	9749	NM_00117	8871	Hs.434494	NM_00389	ENSG0000	SYNJ2	INPP5H	synaptojan	protein-co
lib-C666-I	chr19	10613686	10613686	+	17.48666	NA	promoter-TSS (NM_012289)	promoter-TSS (NM_012289)	-205	NM_01228	9817	Hs.465870	NM_01228	ENSG0000	KEAP1	INrf2|KLHL	kelch like E	protein-co
lib-C666-I	chr2	118845907	118845907	+	21.46984	NA	promoter-TSS (NM_001321333)	promoter-TSS (NM_001321333)	-87	NM_00132	51141	Hs.7089	NM_01613	ENSG0000	INSIG2	INSIG-2	insulin indu	protein-co
lib-C666-I	chr7	1544171	1544171	+	4.04484	NA	promoter-TSS (NM_001080453)	promoter-TSS (NM_001080453)	-153	NM_00108	26173	Hs.532188	NM_00108	ENSG0000	INTS1	INT1|NET2	integrator	protein-co
lib-C666-I	chr11	77705901	77705901	+	10.92275	NA	promoter-TSS (NM_033547)	promoter-TSS (NM_033547)	-184	NM_03354	92105	Hs.533723	NM_03354	ENSG0000	INTS4	INT4|MST0	integrator	protein-co
lib-C666-I	chr17	80416278	80416278	+	6.15313	NA	promoter-TSS (NM_001083608)	promoter-TSS (NM_001083608)	218	NM_00103	26502	Hs.256526	NM_01233	ENSG0000	NARF	IOP2	nuclear pre	protein-co
lib-C666-I	chr14	35801079	35801079	+	8.30991	NA	Intergenic	Intergenic	39556	NM_00279	5687	Hs.446260	NM_00279	ENSG0000	PSMA6	IOTA|PROS	proteasom	protein-co
lib-C666-I	chr14	35801410	35801410	+	8.11522	NA	Intergenic	Intergenic	39887	NM_00279	5687	Hs.446260	NM_00279	ENSG0000	PSMA6	IOTA|PROS	proteasom	protein-co
lib-C666-I	chr14	35801691	35801691	+	7.49163	NA	Intergenic	Intergenic	40168	NM_00279	5687	Hs.446260	NM_00279	ENSG0000	PSMA6	IOTA|PROS	proteasom	protein-co
lib-C666-I	chr14	35806115	35806115	+	5.74587	NA	Intergenic	Intergenic	44592	NM_00279	5687	Hs.446260	NM_00279	ENSG0000	PSMA6	IOTA|PROS	proteasom	protein-co
lib-C666-I	chr1	226921396	226921396	+	13.84327	NA	intron (NM_002221, intron 2 of 7)	intron (NM_002221, intron 2 of 7)	5480	NM_00222	3707	Hs.528087	NM_00222	ENSG0000	ITPKB	IP3-3KB|IP3	inositol-tris	protein-co
lib-C666-I	chr1	205648952	205648952	+	12.62235	NA	intron (NM_033102, intron 1 of 4)	intron (NM_033102, intron 1 of 4)	678	NM_03310	85414	Hs.278695	NM_03310	ENSG0000	SLC45A3	IPCA-2|IPC	solute carr	protein-co
lib-C666-I	chr1	205628198	205628198	+	7.06733	NA	3' UTR (NM_033102, exon 5 of 5)	3' UTR (NM_033102, exon 5 of 5)	21432	NM_03310	85414	Hs.278695	NM_03310	ENSG0000	SLC45A3	IPCA-2|IPC	solute carr	protein-co
lib-C666-I	chr11	61348950	61348950	+	7.0832	NA	promoter-TSS (NM_001300773)	promoter-TSS (NM_001300773)	-606	NM_00130	9066	Hs.131188	NM_00420	ENSG0000	SYT7	IPCA-7|IPC	synaptotag	protein-co
lib-C666-I	chr11	61334933	61334933	+	4.90117	NA	intron (NM_001252065, intron 1 of 9)	intron (NM_001252065, intron 1 of	13411	NM_00420	9066	Hs.131188	NM_00420	ENSG0000	SYT7	IPCA-7|IPC	synaptotag	protein-co
lib-C666-I	chr10	6244847	6244847	+	23.05659	NA	promoter-TSS (NM_001314063)	promoter-TSS (NM_001314063)	7	NM_00132	5209	Hs.195471	NM_00456	ENSG0000	PFKFB3	IPFK2|PFK2	6-phospho	protein-co
lib-C666-I	chr10	6186768	6186768	+	10.27265	NA	promoter-TSS (NM_001145443)	promoter-TSS (NM_001145443)	-73	NM_00114	5209	Hs.195471	NM_00456	ENSG0000	PFKFB3	IPFK2|PFK2	6-phospho	protein-co
lib-C666-I	chr10	6245353	6245353	+	8.71138	NA	intron (NM_004566, intron 1 of 14)	CpG-2555	513	NM_00132	5209	Hs.195471	NM_00456	ENSG0000	PFKFB3	IPFK2|PFK2	6-phospho	protein-co
lib-C666-I	chr9	103115255	103115255	+	23.12593	NA	promoter-TSS (NM_001161584)	promoter-TSS (NM_001161584)	4	NM_01774	54881	Hs.494648	NM_01774	ENSG0000	TEX10	Ipi1|bA208	testis expre	protein-co
lib-C666-I	chr19	984292	984292	+	187.07423	NA	promoter-TSS (NM_024100)	promoter-TSS (NM_024100)	-36	NM_02410	57418	Hs.325321	NM_02410	ENSG0000	WDR18	Ipi3|R3218	WD repeat	protein-co
lib-C666-I	chr3	48343022	48343022	+	9.94769	NA	promoter-TSS (NM_001308430)	promoter-TSS (NM_001308430)	-121	NM_00130	10201	Hs.465558	NM_00579	ENSG0000	NME6	IPIA-ALPH	NME/NM2	protein-co
lib-C666-I	chr7	108166701	108166701	+	49.69992	NA	promoter-TSS (NM_015723)	promoter-TSS (NM_015723)	61	NM_00125	50640	Hs.617340	NM_01572	ENSG0000	PNPLA8	IPLA2-2|IP	patatin like	protein-co
lib-C666-I	chr7	128695219	128695219	+	28.81777	NA	promoter-TSS (NR_034053)	promoter-TSS (NR_034053)	8	NR_03405	23534	Hs.193613	NM_01247	ENSG0000	TNPO3	IPO12|LGM	transportin	protein-co
lib-C666-I	chr19	12833348	12833348	+	3.34207	NA	promoter-TSS (NM_013433)	promoter-TSS (NM_013433)	114	NM_00113	30000	Hs.416049	NM_01343	ENSG0000	TNPO2	IPO3|KPNB	transportin	protein-co
lib-C666-I	chr17	66032359	66032359	+	17.13943	NA	5' UTR (NM_001320611, exon 1 of 11	5' UTR (NM_001320611, exon 1 of 1	283	NM_00132	3838	Hs.594238	NM_00226	ENSG0000	KPNA2	IPOA1|QIP	karyopheri	protein-co
lib-C666-I	chr17	66016163	66016163	+	8.89416	NA	Intergenic	CpG	-15555	NM_00226	3838	Hs.594238	NM_00226	ENSG0000	KPNA2	IPOA1|QIP	karyopheri	protein-co
lib-C666-I	chr3	122233561	122233561	+	5.11732	NA	intron (NM_002264, intron 1 of 13)	CpG	225	NM_00226	3836	Hs.161008	NM_00226	ENSG0000	KPNA1	IPOA5|NPI	karyopheri	protein-co
lib-C666-I	chr6	160147986	160147986	+	41.94622	NA	promoter-TSS (NM_001270533)	promoter-TSS (NM_001270533)	-10	NM_00132	6648	Hs.487046	NM_00063	ENSG0000	SOD2	IPO-B|IPOI	superoxide	protein-co
lib-C666-I	chr10	1095013	1095013	+	49.86005	NA	promoter-TSS (NM_001317956)	promoter-TSS (NM_001317956)	-190	NM_00131	3422	Hs.283652	NM_00450	ENSG0000	IDI1	IPP1|IPPI1	isopenteny	protein-co
lib-C666-I	chr2	182895877	182895877	+	7.97818	NA	intron (NM_001080545, intron 2 of 4)	L1PA2|LINE|L1	45326	NM_00126	151242	Hs.10941	NM_00108	ENSG0000	PPP1R1C	IPP5	protein ph	protein-co
lib-C666-I	chr10	1082371	1082371	+	5.37858	NA	intron (NR_027709, intron 3 of 4)	L2b|LINE|L2	-10572	NM_03326	91734	Hs.9270	NM_03326	ENSG0000	IDI2	IPPI2	isopenteny	protein-co
lib-C666-I	chr3	10240526	10240526	+	14.5394	NA	intron (NM_001570, intron 2 of 12)	intron (NM_001570, intron 2 of 12)	33963	NM_00157	3656	Hs.449207	NM_00157	ENSG0000	IRAK2	IRAK-2	interleukin	protein-co
lib-C666-I	chr19	45504569	45504569	+	12.62235	NA	promoter-TSS (NM_006509)	promoter-TSS (NM_006509)	-138	NM_00650	5971	Hs.654402	NM_00650	ENSG0000	RELB	I-REL|IREL|	RELB protc	protein-co

lib-C666-l	chr5	131826649	131826649	+	4.67192	NA	promoter-TSS (NM_002198)	promoter-TSS (NM_002198)	-184	NM_00219	3659	Hs.436061	NM_00219	ENSG0000	IRF1	IRF-1|MAR	interferon	protein-co
lib-C666-l	chr4	185395986	185395986	+	4.39666	NA	promoter-TSS (NM_002199)	promoter-TSS (NM_002199)	-260	NM_00219	3660	Hs.654566	NM_00219	ENSG0000	IRF2	IRF-2	interferon	protein-co
lib-C666-l	chr14	24630333	24630333	+	284.35867	NA	promoter-TSS (NM_006084)	promoter-TSS (NM_006084)	-89	NM_00608	10379	Hs.1706	NM_00608	ENSG0000	IRF9	IRF-9|ISGF:	interferon	protein-co
lib-C666-l	chr15	66084823	66084823	+	25.49755	NA	promoter-TSS (NM_001144823)	promoter-TSS (NM_001144823)	-192	NM_00114	10260	Hs.654567	NM_00584	ENSG0000	DENND4A	IRLB|MYCP	DENN don	protein-co
lib-C666-l	chr13	110485480	110485480	+	5.23609	NA	Intergenic	L1PA8A|LINE|L1	-46566	NM_00374	8660	Hs.442344	NM_00374	ENSG0000	IRS2	IRS-2	insulin rece	protein-co
lib-C666-l	chr7	98048433	98048433	+	64.08802	NA	Intergenic	Intergenic	-18006	NM_01884	55971	Hs.656063	NM_01884	ENSG0000	BAIAP2L1	IRTKS	BAI1 assoc	protein-co
lib-C666-l	chr16	54320784	54320784	+	28.88196	NA	promoter-TSS (NM_024336)	promoter-TSS (NM_024336)	-406	NM_02433	79191	Hs.499205	NM_02433	ENSG0000	IRX3	IRX-1|IRXB	iroquois hc	protein-co
lib-C666-l	chr16	55358641	55358641	+	14.54258	NA	5' UTR (NM_024335, exon 1 of 6)	5' UTR (NM_024335, exon 1 of 6)	170	NM_02433	79190	Hs.369907	NM_02433	ENSG0000	IRX6	IRX-3|IRX7	iroquois hc	protein-co
lib-C666-l	chr16	55331269	55331269	+	3.57421	NA	Intergenic	MLT1K|LTR|ERVL-MaLR	-27202	NM_02433	79190	Hs.369907	NM_02433	ENSG0000	IRX6	IRX-3|IRX7	iroquois hc	protein-co
lib-C666-l	chr11	47198522	47198522	+	13.15801	NA	TTS (NM_001184975)	TTS (NM_001184975)	154	NM_03238	84364	Hs.436204	NM_03238	ENSG0000	ARFGAP2	IRZ|NBLA1	ADP ribosy	protein-co
lib-C666-l	chrX	48432530	48432530	+	19.82221	NA	promoter-TSS (NM_006743)	promoter-TSS (NM_006743)	-211	NM_00674	5935	Hs.301404	NM_00674	ENSG0000	RBM3	IS1-RNPL|F	RNA bindir	protein-co
lib-C666-l	chr12	6961804	6961804	+	38.10403	NA	promoter-TSS (NM_001297603)	promoter-TSS (NM_001297603)	513	NM_00348	8078	Hs.631661	NM_00348	ENSG0000	USP5	ISOT	ubiquitin s	protein-co
lib-C666-l	chr12	6961198	6961198	+	2.59032	NA	promoter-TSS (NM_003481)	promoter-TSS (NM_003481)	-87	NM_00109	8078	Hs.631661	NM_00348	ENSG0000	USP5	ISOT	ubiquitin s	protein-co
lib-C666-l	chr4	144434313	144434313	+	57.48363	NA	promoter-TSS (NM_003601)	promoter-TSS (NM_003601)	-303	NM_00360	8467	Hs.558422	NM_00360	ENSG0000	SMARCA5	ISWI|SNF2I	SWI/SNF re	protein-co
lib-C666-l	chr4	144434961	144434961	+	31.26176	NA	non-coding (NR_104027, exon 2 of 2	non-coding (NR_104027, exon 2 of	345	NM_00360	8467	Hs.558422	NM_00360	ENSG0000	SMARCA5	ISWI|SNF2I	SWI/SNF re	protein-co
lib-C666-l	chrX	118740454	118740454	+	8.03539	NA	promoter-TSS (NM_001173487)	promoter-TSS (NM_001173487)	-608	NM_01754	55922	Hs.437084	NM_01754	ENSG0000	NKRF	ITBA4|NRF	NFKB repre	protein-co
lib-C666-l	chr10	7583517	7583517	+	5.23609	NA	Intergenic	Intergenic	78119	NM_03281	80760	Hs.498586	NM_03056	ENSG0000	ITIH5	ITI-HC5|PP	inter-alpha	protein-co
lib-C666-l	chr13	95254099	95254099	+	5.12426	NA	promoter-TSS (NM_180989)	promoter-TSS (NM_180989)	-5	NM_18098	160897	Hs.439363	NM_18098	ENSG0000	GPR180	ITR	G protein-	protein-co
lib-C666-l	chr2	162023589	162023589	+	4.74112	NA	intron (NM_001199135, intron 1 of 7)	intron (NM_001199135, intron 1 of	6650	NM_00119	10010	Hs.132257	NM_00418	ENSG0000	TANK	I-TRAF|ITR.	TRAF famil	protein-co
lib-C666-l	chr2	161993613	161993613	+	4.73866	NA	intron (NM_004180, intron 1 of 7)	intron (NM_004180, intron 1 of 7)	147	NM_00418	10010	Hs.132257	NM_00418	ENSG0000	TANK	I-TRAF|ITR.	TRAF famil	protein-co
lib-C666-l	chr14	93582124	93582124	+	2.89057	NA	5' UTR (NM_014216, exon 1 of 11)	5' UTR (NM_014216, exon 1 of 11)	140	NM_01421	3705	Hs.308122	NM_01421	ENSG0000	ITPK1	ITRPK1	inositol-tet	protein-co
lib-C666-l	chr19	49250327	49250327	+	20.83272	NA	promoter-TSS (NM_001321864)	promoter-TSS (NM_001321864)	-161	NM_00132	284359	Hs.400688	NM_18257	ENSG0000	IZUMO1	IZUMO|OB	izumo sper	protein-co
lib-C666-l	chr5	133860353	133860353	+	5.92363	NA	promoter-TSS (NM_001289984)	promoter-TSS (NM_001289984)	287	NM_00130	23338	Hs.732155	NM_01528	ENSG0000	JADE2	JADE-2|PH	jade family	protein-co
lib-C666-l	chr5	133860912	133860912	+	5.25516	NA	promoter-TSS (NM_001289984)	promoter-TSS (NM_001289984)	-428	NM_00128	23338	Hs.732155	NM_01528	ENSG0000	JADE2	JADE-2|PH	jade family	protein-co
lib-C666-l	chr15	43398388	43398388	+	19.38944	NA	promoter-TSS (NM_174916)	promoter-TSS (NM_174916)	-102	NM_17491	197131	Hs.591121	NM_17491	ENSG0000	UBR1	JBS	ubiquitin p	protein-co
lib-C666-l	chr2	232646143	232646143	+	26.46807	NA	promoter-TSS (NM_001291018)	promoter-TSS (NM_001291018)	-106	NM_00260	5147	Hs.516808	NM_00260	ENSG0000	PDE6D	JBTS22|PD	phosphodi	protein-co
lib-C666-l	chr14	58922632	58922632	+	3.20885	NA	intron (NM_001329947, intron 9 of 2	L1M4|LINE|L1	16233	NM_00124	9786	Hs.232532	NM_01474	ENSG0000	KIAA0586	JBTS23|SR1	KIAA0586	protein-co
lib-C666-l	chr16	27561380	27561380	+	65.77034	NA	promoter-TSS (NM_001520)	promoter-TSS (NM_001520)	-88	NM_01520	23247	Hs.460459	NM_01520	ENSG0000	KIAA0556	JBTS26	KIAA0556	protein-co
lib-C666-l	chr2	110962625	110962625	+	110.48502	NA	promoter-TSS (NM_000272)	promoter-TSS (NM_000272)	14	NM_20718	4867	Hs.280388	NM_00027	ENSG0000	NPHP1	JBTS4|NPH	nephrocyst	protein-co
lib-C666-l	chr1	205719444	205719444	+	117.95905	NA	promoter-TSS (NM_022731)	promoter-TSS (NM_022731)	-72	NM_02273	64710	Hs.213061	NM_02273	ENSG0000	NUCKS1	JC7|NUCKS	nuclear cas	protein-co
lib-C666-l	chr17	27055646	27055646	+	15.55328	NA	promoter-TSS (NM_178170)	promoter-TSS (NM_178170)	-186	NM_17817	284086	Hs.448468	NM_17817	ENSG0000	NEK8	JCK|NEK12	NIMA relat	protein-co
lib-C666-l	chr17	27054989	27054989	+	9.14689	NA	promoter-TSS (NM_178170)	promoter-TSS (NM_178170)	-843	NM_17817	284086	Hs.448468	NM_17817	ENSG0000	NEK8	JCK|NEK12	NIMA relat	protein-co
lib-C666-l	chr1	212872939	212872939	+	32.66768	NA	intron (NM_018664, intron 1 of 2)	CpG	388	NM_01866	55509	Hs.62919	NM_01866	ENSG0000	BATF3	JDP1|JUNE	basic leucir	protein-co
lib-C666-l	chr1	212872629	212872629	+	7.09685	NA	intron (NM_018664, intron 1 of 2)	CpG	698	NM_01866	55509	Hs.62919	NM_01866	ENSG0000	BATF3	JDP1|JUNE	basic leucir	protein-co
lib-C666-l	chr11	94501396	94501396	+	6.32351	NA	promoter-TSS (NM_130847)	promoter-TSS (NM_130847)	-112	NM_00130	154810	Hs.503594	NM_13084	ENSG0000	AMOTL1	JEAP	angiomotir	protein-co
lib-C666-l	chrX	54070491	54070491	+	25.49755	NA	promoter-TSS (NM_001184898)	promoter-TSS (NM_001184898)	116	NM_00118	23133	Hs.133352	NM_01510	ENSG0000	PHF8	JHDM1F|KI	PHD finger	protein-co
lib-C666-l	chrX	54070939	54070939	+	22.50982	NA	promoter-TSS (NM_015107)	promoter-TSS (NM_015107)	-332	NM_01510	23133	Hs.133352	NM_01510	ENSG0000	PHF8	JHDM1F|KI	PHD finger	protein-co
lib-C666-l	chr2	86668106	86668106	+	6.94566	NA	promoter-TSS (NM_018433)	promoter-TSS (NM_018433)	-165	NM_01843	55818	Hs.557425	NM_01843	ENSG0000	KDM3A	JHDM2A|JH	lysine dem	protein-co
lib-C666-l	chr1	44115658	44115658	+	6.32351	NA	promoter-TSS (NM_014663)	promoter-TSS (NM_014663)	-139	NM_01466	9682	Hs.155983	NM_01466	ENSG0000	KDM4A	JHDM3A|JI	lysine dem	protein-co
lib-C666-l	chr16	1756128	1756128	+	278.12662	NA	promoter-TSS (NM_015133)	promoter-TSS (NM_015133)	-56	NM_00131	23162	Hs.207763	NM_01513	ENSG0000	MAPK8IP3	JIP-3|JIP3|J	mitogen-a	protein-co
lib-C666-l	chr5	16617116	16617116	+	18.43022	NA	promoter-TSS (NM_001034850)	promoter-TSS (NM_001034850)	51	NM_00103	54463	Hs.481704	NM_01900	ENSG0000	FAM134B	JK-1|JK1	family with	protein-co
lib-C666-l	chr3	97629560	97629560	+	3.89624	NA	intron (NM_153605, intron 15 of 21)	L1PA3|LINE|L1	61735	NM_15318	84864	Hs.570562	NM_03277	ENSG0000	RIOX2	JMJD10|MI	ribosomal	protein-co
lib-C666-l	chr3	97691301	97691301	+	3.07144	NA	promoter-TSS (NM_032778)	promoter-TSS (NM_032778)	-6	NM_00126	84864	Hs.570562	NM_03277	ENSG0000	RIOX2	JMJD10|MI	ribosomal	protein-co
lib-C666-l	chr19	4968892	4968892	+	26.94522	NA	promoter-TSS (NM_015015)	promoter-TSS (NM_015015)	-232	NM_01501	23030	Hs.654816	NM_01501	ENSG0000	KDM4B	JMJD2B|TD	lysine dem	protein-co
lib-C666-l	chr17	7747462	7747462	+	42.40071	NA	intron (NM_001080424, intron 1 of 21	CpG	4227	NM_00108	23135	Hs.223678	NM_00108	ENSG0000	KDM6B	JMJD3	lysine dem	protein-co
lib-C666-l	chr17	7738945	7738945	+	28.81777	NA	Intergenic	CpG	-4290	NM_00108	23135	Hs.223678	NM_00108	ENSG0000	KDM6B	JMJD3	lysine dem	protein-co
lib-C666-l	chr17	7740211	7740211	+	9.10494	NA	Intergenic	Intergenic	-3024	NM_00108	23135	Hs.223678	NM_00108	ENSG0000	KDM6B	JMJD3	lysine dem	protein-co
lib-C666-l	chr17	7748193	7748193	+	2.65152	NA	intron (NM_001080424, intron 1 of 21	CpG	4958	NM_00108	23135	Hs.223678	NM_00108	ENSG0000	KDM6B	JMJD3	lysine dem	protein-co
lib-C666-l	chr17	7746057	7746057	+	2.59723	NA	intron (NM_001080424, intron 1 of 21	CpG	2822	NM_00108	23135	Hs.223678	NM_00108	ENSG0000	KDM6B	JMJD3	lysine dem	protein-co
lib-C666-l	chr16	27237890	27237890	+	17.3027	NA	intron (NM_145080, intron 6 of 7)	intron (NM_145080, intron 6 of 7)	22594	NM_00114	79831	Hs.145717	NM_02477	ENSG0000	KDM8	JMJD5	lysine dem	protein-co
lib-C666-l	chr16	27214855	27214855	+	15.96918	NA	promoter-TSS (NM_024773)	promoter-TSS (NM_024773)	48	NM_02477	79831	Hs.145717	NM_02477	ENSG0000	KDM8	JMJD5	lysine dem	protein-co
lib-C666-l	chr16	27228749	27228749	+	7.21092	NA	intron (NM_001145348, intron 5 of 7)	intron (NM_001145348, intron 5 of	13453	NM_00114	79831	Hs.145717	NM_02477	ENSG0000	KDM8	JMJD5	lysine dem	protein-co
lib-C666-l	chr10	49542048	49542048	+	39.42674	NA	intron (NM_139049, intron 3 of 13)	intron (NM_139049, intron 3 of 13)	27366	NM_00132	5599	Hs.138211	NM_00275	ENSG0000	MAPK8	JNK|JNK-4	mitogen-a	protein-co
lib-C666-l	chr10	49514670	49514670	+	39.15438	NA	promoter-TSS (NM_001278547)	promoter-TSS (NM_001278547)	-12	NR_13658	5599	Hs.138211	NM_00275	ENSG0000	MAPK8	JNK|JNK-4	mitogen-a	protein-co
lib-C666-l	chr10	49572160	49572160	+	7.76438	NA	intron (NM_139049, intron 3 of 13)	intron (NM_139049, intron 3 of 13)	-37495	NM_00127	5599	Hs.138211	NM_00275	ENSG0000	MAPK8	JNK|JNK-4	mitogen-a	protein-co
lib-C666-l	chr10	49540512	49540512	+	5.74587	NA	intron (NM_139049, intron 3 of 13)	HERVP71A-int|LTR|ERV1	25830	NM_00132	5599	Hs.138211	NM_00275	ENSG0000	MAPK8	JNK|JNK-4	mitogen-a	protein-co
lib-C666-l	chr5	179720778	179720778	+	3.23837	NA	Intergenic	Intergenic	-1707	NM_00275	5601	Hs.484371	NM_00275	ENSG0000	MAPK9	JNK-55|JN	mitogen-a	protein-co
lib-C666-l	chr15	42066674	42066674	+	7.49163	NA	promoter-TSS (NM_001128608)	promoter-TSS (NM_001128608)	42	NR_04976	23005	Hs.513661	NM_01499	ENSG0000	MAPKBP1	JNKBP-1|JI	mitogen-a	protein-co
lib-C666-l	chr19	2256568	2256568	+	11.43915	NA	promoter-TSS (NM_144616)	promoter-TSS (NM_144616)	-146	NM_14461	126306	Hs.330742	NM_14461	ENSG0000	JSRP1	JP-45|JP45	junctional	protein-co
lib-C666-l	chr7	1458267	1458267	+	129.74547	NA	Intergenic	Intergenic	40842	NM_18292	79778	Hs.376617	NM_02472	ENSG0000	MICALL2	JRAB|MICA	MICAL like	protein-co
lib-C666-l	chr7	1408736	1408736	+	13.40382	NA	Intergenic	CpG	90373	NM_18292	79778	Hs.376617	NM_02472	ENSG0000	MICALL2	JRAB|MICA	MICAL like	protein-co
lib-C666-l	chr7	1452602	1452602	+	11.03618	NA	Intergenic	CpG	46507	NM_18292	79778	Hs.376617	NM_02472	ENSG0000	MICALL2	JRAB|MICA	MICAL like	protein-co
lib-C666-l	chr9	4984838	4984838	+	6.32351	NA	promoter-TSS (NM_004972)	promoter-TSS (NM_004972)	-248	NM_00132	3717	Hs.656213	NM_00497	ENSG0000	JAK2	JTK10|THC	Janus kinas	protein-co
lib-C666-l	chr7	6048868	6048868	+	23.70098	NA	promoter-TSS (NM_000535)	promoter-TSS (NM_000535)	-14	NM_00132	7965	Hs.301613	NM_00630	ENSG0000	AIMP2	JTV-1|JTV1	aminoacyl	protein-co
lib-C666-l	chr14	23451989	23451989	+	20.39357	NA	promoter-TSS (NM_032876)	promoter-TSS (NM_032876)	-138	NM_00128	84962	Hs.655832	NM_03287	ENSG0000	AJUBA	JUB	ajuba LIM	protein-co
lib-C666-l	chr18	45457586	45457586	+	3.89624	NA	promoter-TSS (NM_005901)	promoter-TSS (NM_005901)	-69	NM_00100	4087	Hs.12253	NM_00590	ENSG0000	SMAD2	JV18|JV18-	SMAD fam	protein-co
lib-C666-l	chr18	45456713	45456713	+	2.00164	NA	intron (NM_001135937, intron 1 of 9)	CpG	257	NM_00590	4087	Hs.12253	NM_00590	ENSG0000	SMAD2	JV18|JV18-	SMAD fam	protein-co
lib-C666-l	chr17	80291343	80291343	+	8.96177	NA	intron (NM_003004, intron 1 of 4)	CpG	578	NM_00300	6398	Hs.558009	NM_00300	ENSG0000	SECTM1	K12	secreted ar	protein-co
lib-C666-l	chr8	140718151	140718151	+	11.22379	NA	Intergenic	CpG-26022	-2852	NR_10421	51305	Hs.493037	NM_00128	ENSG0000	KCNK9	K2p9.1|KT3	potassium	protein-co
lib-C666-l	chrX	44732254	44732254	+	10.71733	NA	promoter-TSS (NM_021140)	promoter-TSS (NM_021140)	-167	NM_00129	7403	Hs.522616	NM_02114	ENSG0000	KDM6A	KABUK2|U1	lysine dem	protein-co
lib-C666-l	chr17	7387329	7387329	+	3.7184	NA	promoter-TSS (NM_000937)	promoter-TSS (NM_000937)	239	NM_02089	57659	Hs.35096	NM_02089	ENSG0000	ZBTB4	KAISO-L1|:	zinc finger	protein-co
lib-C666-l	chr19	50862279	50862279	+	16.76091	NA	exon (NM_004851, exon 8 of 9)	exon (NM_004851, exon 8 of 9)	6808	NM_00485	9476	Hs.512843	NM_00485	ENSG0000	NAPSA	KAP|Kdap|I	napsin A a:	protein-co
lib-C666-l	chr19	59055569	59055569	+	37.99633	NA	promoter-TSS (NM_005762)	promoter-TSS (NM_005762)	-267	NM_00576	10155	Hs.467408	NM_00576	ENSG0000	TRIM28	KAP1|PPP1	tripartite m	protein-co
lib-C666-l	chr17	39362720	39362720	+	4.39666	NA	Intergenic	L1PA2|LINE|L1	16581	NM_00119	728318	Hs.727478	NM_00119	ENSG0000	KRTAP9-1	KAP9.1|KR1	keratin ass	protein-co
lib-C666-l	chr17	39369050	39369050	+	5.31153	NA	Intergenic	Intergenic	-13850	NM_03196	83899	Hs.307013	NM_03196	ENSG0000	KRTAP9-2	KAP9.2|KR1	keratin ass	protein-co
lib-C666-l	chr11	43702185	43702185	+	16.39562	NA	promoter-TSS (NM_016142)	promoter-TSS (NM_016142)	42	NM_01614	51144	Hs.132513	NM_01614	ENSG0000	HSD17B12	KAR|SDR12	hydroxyste	protein-co
lib-C666-l	chr19	49907268	49907268	+	8.01457	NA	intron (NM_144688, intron 9 of 19)	intron (NM_144688, intron 9 of 19)	15793	NM_14468	147872	Hs.662085	NM_14468	ENSG0000	CCDC155	KASH5	coiled-coil	protein-co
lib-C666-l	chr2	172778932	172778932	+	97.94548	NA	promoter-TSS (NM_003642)	promoter-TSS (NM_003642)	-3	NM_00364	8520	Hs.632532	NM_00364	ENSG0000	HAT1	KAT1	histone ace	protein-co
lib-C666-l	chr9	135545149	135545149	+	3.84463	NA	promoter-TSS (NM_012204)	promoter-TSS (NM_012204)	-273	NM_01220	9329	Hs.22302	NM_01220	ENSG0000	GTF3C4	KAT12|TF3	general tra	protein-co
lib-C666-l	chr4	56412342	56412342	+	5.25516	NA	promoter-TSS (NM_001267843)	promoter-TSS (NM_001267843)	-243	NM_00126	9575	Hs.436975	NM_00489	ENSG0000	CLOCK	KAT13D|bH	clock circa	protein-co
lib-C666-l	chr1	14944493	14944493	+	10.27265	NA	intron (NM_201628, intron 1 of 14)	intron (NM_201628, intron 1 of 14)	19280	NM_01520	23254	Hs.368823	NM_01520	ENSG0000	KAZN	KAZ	kazrin, peri	protein-co
lib-C666-l	chr1	14843908	14843908	+	9.03945	NA	Intergenic	MLT1K|LTR|ERVL-MaLR	-81305	NM_20162	23254	Hs.368823	NM_01520	ENSG0000	KAZN	KAZ	kazrin, peri	protein-co
lib-C666-l	chr1	15354823	15354823	+	7.06733	NA	intron (NM_001017999, intron 2 of 7)	intron (NM_001017999, intron 2 of	82408	NM_00101	23254	Hs.368823	NM_01520	ENSG0000	KAZN	KAZ	kazrin, peri	protein-co
lib-C666-l	chr12	52575780	52575780	+	8.73012	NA	intron (NM_001081492, intron 2 of 8)	intron (NM_001081492, intron 2 of	10004	NM_18250	144501	Hs.140978	NM_18250	ENSG0000	KRT80	KB20	keratin 80	protein-co
lib-C666-l	chr12	52585886	52585886	+	5.32392	NA	promoter-TSS (NM_182507)	promoter-TSS (NM_182507)	-102	NM_00108	144501	Hs.140978	NM_18250	ENSG0000	KRT80	KB20	keratin 80	protein-co
lib-C666-l	chr22	38203915	38203915	+	4.73866	NA	promoter-TSS (NM_014291)	promoter-TSS (NM_014291)	3	NM_01429	23464	Hs.54609	NM_01429	ENSG0000	GCAT	KBL	glycine C-a	protein-co
lib-C666-l	chr1	42421340	42421340	+	9.79436	NA	intron (NR_038261, intron 1 of 3)	intron (NR_038261, intron 1 of 3)	-36844	NR_03826	59269	Hs.403972	NM_02450	ENSG0000	HIVEP3	KBP-1|KBP	human imr	protein-co
lib-C666-l	chr1	42468042	42468042	+	5.25516	NA	intron (NR_038261, intron 1 of 3)	intron (NR_038261, intron 1 of 3)	33554	NR_03826	59269	Hs.403972	NM_02450	ENSG0000	HIVEP3	KBP-1|KBP	human imr	protein-co
lib-C666-l	chr2	23608929	23608929	+	15.55328	NA	intron (NM_052920, intron 1 of 13)	CpG	631	NM_05292	114818	Hs.130593	NM_02506	ENSG0000	KLHL29	KBTBD9	kelch like f:	protein-co
lib-C666-l	chr19	18063512	18063512	+	3.27468	NA	intron (NM_002248, intron 1 of 10)	intron (NM_002248, intron 1 of 10)	1401	NM_00224	3780	Hs.158173	NM_00224	ENSG0000	KCNN1	KCa2.1|SK1	potassium	protein-co
lib-C666-l	chr5	113275759	113275759	+	9.7234	NA	Intergenic	Intergenic	-422257	NM_02161	3781	Hs.98280	NM_02161	ENSG0000	KCNN2	KCa2.2|SK2	potassium	protein-co
lib-C666-l	chr1	154839712	154839712	+	2.58854	NA	intron (NM_002249, intron 1 of 7)	intron (NM_002249, intron 1 of 7)	3042	NM_00120	3782	Hs.490765	NM_00224	ENSG0000	KCNN3	KCa2.3|SK3	potassium	protein-co
lib-C666-l	chr11	77899555	77899555	+	8.85547	NA	promoter-TSS (NM_020798)	promoter-TSS (NM_020798)	120	NM_00102	283219	Hs.528439	NM_00102	ENSG0000	KCTD21	KCASH2	potassium	protein-co
lib-C666-l	chr3	58457222	58457222	+	10.16129	NA	Intergenic	Intergenic	-20601	NM_00112	200845	Hs.13982	NM_15333	ENSG0000	KCTD6	KCASH3	potassium	protein-co
lib-C666-l	chr3	58477930	58477930	+	4.46583	NA	intron (NM_001128214, intron 1 of 2)	CpG	107	NM_00112	200845	Hs.13982	NM_15333	ENSG0000	KCTD6	KCASH3	potassium	protein-co
lib-C666-l	chr5	1140635	1140635	+	5.6488	NA	Intergenic	Intergenic	-28463	NM_00659	10723	Hs.172613	NM_00659	ENSG0000	SLC12A7	KCC4	solute carr	protein-co
lib-C666-l	chr3	177545469	177545469	+	7.84231	NA	intron (NR_110826, intron 1 of 2)	intron (NR_110826, intron 1 of 2)	10816	NR_11082	102724550	Hs.570526	NR_11082	ENSG0000	LINC02015	KCCAT211	long interg	ncRNA
lib-C666-l	chr3	177382872	177382872	+	3.27468	NA	intron (NR_047568, intron 3 of 3)	L1PA4|LINE|L1	-151781	NR_11082	102724550	Hs.570526	NR_11082	ENSG0000	LINC02015	KCCAT211	long interg	ncRNA
lib-C666-l	chr10	103590594	103590594	+	6.82401	NA	intron (NM_014591, intron 2 of 9)	CpG	9017	NM_17319	30819	Hs.97044	NM_01459	ENSG0000	KCNIP2	KCHIP2	potassium	protein-co
lib-C666-l	chr11	2721292	2721292	+	9.21057	NA	promoter-TSS (NR_002728)	promoter-TSS (NR_002728)	-64	NR_00272	10984	Hs.604823	NR_00272	ENSG0000	KCNQ1OT	KCNQ1-AS	KCNQ1 op	ncRNA
lib-C666-l	chr11	2721918	2721918	+	3.92495	NA	promoter-TSS (NR_002728)	promoter-TSS (NR_002728)	-690	NR_00272	10984	Hs.604823	NR_00272	ENSG0000	KCNQ1OT	KCNQ1-AS	KCNQ1 op	ncRNA
lib-C666-l	chr6	138409604	138409604	+	6.15313	NA	TTS (NM_022121)	TTS (NM_022121)	19056	NM_02212	64065	Hs.201446	NM_02212	ENSG0000	PERP	KCP1|KRTC	PERP, TP53	protein-co
lib-C666-l	chr10	65282318	65282318	+	12.47485	NA	promoter-TSS (NM_001318154)	promoter-TSS (NM_001318154)	-483	NM_00131	221037	Hs.413416	NM_00424	ENSG0000	JMJD1C	KDM3C|TR	jumonji do	protein-co
lib-C666-l	chr10	65467405	65467405	+	2.78587	NA	Intergenic	Intergenic	-185570	NM_00131	221037	Hs.413416	NM_00424	ENSG0000	JMJD1C	KDM3C|TR	jumonji do	protein-co
lib-C666-l	chr1	202780021	202780021	+	38.10403	NA	promoter-TSS (NR_046326)	promoter-TSS (NR_046326)	-53	NR_04632	100506696	Hs.23459	NR_04632	ENSG0000	PCAT6	KDM5B-AS	prostate ca	ncRNA
lib-C666-l	chr21	47743962	47743962	+	12.12504	NA	promoter-TSS (NM_001286477)	promoter-TSS (NM_001286477)	-74	NM_00603	5116	Hs.474069	NM_00603	ENSG0000	PCNT	KEN|MOPD	pericentrin	protein-co
lib-C666-l	chr21	47744236	47744236	+	8.73012	NA	promoter-TSS (NM_001286477)	promoter-TSS (NM_001286477)	200	NM_00603	5116	Hs.474069	NM_00603	ENSG0000	PCNT	KEN|MOPD	pericentrin	protein-co
lib-C666-l	chr22	26165685	26165685	+	8.03539	NA	intron (NM_001318245, intron 4 of 4	intron (NM_001318245, intron 4 of	27574	NM_00131	84700	Hs.417959	NM_03260	ENSG0000	MYO18B	KFS4	myosin XV	protein-co
lib-C666-l	chrX	132549897	132549897	+	6.98764	NA	promoter-TSS (NM_001448)	promoter-TSS (NM_001448)	-692	NM_00144	2239	Hs.58367	NM_00144	ENSG0000	GPC4	K-glypican	glypican 4	protein-co
lib-C666-l	chrX	132579249	132579249	+	5.92363	NA	Intergenic	MIRb|SINE|MIR	-30044	NM_00144	2239	Hs.58367	NM_00144	ENSG0000	GPC4	K-glypican	glypican 4	protein-co
lib-C666-l	chr2	68384977	68384977	+	24.99834	NA	promoter-TSS (NM_138458)	promoter-TSS (NM_138458)	15	NM_00132	56902	Hs.262858	NM_02014	ENSG0000	PNO1	KHRBP1|RF	partner of	protein-co
lib-C666-l	chr2	68417497	68417497	+	2.05265	NA	intron (NM_000945, intron 2 of 5)	AluSq2|SINE|Alu	32535	NM_00132	56902	Hs.262858	NM_02014	ENSG0000	PNO1	KHRBP1|RF	partner of	protein-co
lib-C666-l	chr5	130970978	130970978	+	5.72511	NA	promoter-TSS (NM_001164386)	promoter-TSS (NM_001164386)	-49	NM_01634	51735	Hs.483329	NM_01634	ENSG0000	RAPGEF6	KIA001LB|F	Rap guanir	protein-co
lib-C666-l	chr15	64673815	64673815	+	8.03539	NA	promoter-TSS (NM_014736)	promoter-TSS (NM_014736)	-106	NM_00102	9768	Hs.81892	NM_01473	ENSG0000	PCLAF	KIAA0101|I	PCNA clam	protein-co
lib-C666-l	chr8	109455806	109455806	+	27.70954	NA	promoter-TSS (NM_001329494)	promoter-TSS (NM_001329494)	-6	NR_13803	9694	Hs.654351	NM_01467	ENSG0000	EMC2	KIAA0103|1	ER membra	protein-co
lib-C666-l	chr1	229761929	229761929	+	15.01861	NA	promoter-TSS (NM_014777)	promoter-TSS (NM_014777)	-34	NM_00131	9816	Hs.533628	NM_01477	ENSG0000	URB2	KIAA0133|I	URB2 ribos	protein-co
lib-C666-l	chr8	48173454	48173454	+	4.96104	NA	promoter-TSS (NM_001080394)	promoter-TSS (NM_001080394)	-16	NR_10458	23514	Hs.381058	NM_00108	ENSG0000	SPIDR	KIAA0146	scaffolding	protein-co
lib-C666-l	chr12	121124701	121124701	+	5.12426	NA	promoter-TSS (NM_001303628)	promoter-TSS (NM_001303628)	-226	NM_00130	9761	Hs.744910	NM_01473	ENSG0000	MLEC	KIAA0152	malectin	protein-co
lib-C666-l	chr12	121124374	121124374	+	3.23837	NA	promoter-TSS (NM_001303628)	promoter-TSS (NM_001303628)	-553	NM_00130	9761	Hs.744910	NM_01473	ENSG0000	MLEC	KIAA0152	malectin	protein-co
lib-C666-l	chr9	131709944	131709944	+	6.94566	NA	promoter-TSS (NM_014908)	promoter-TSS (NM_014908)	-28	NM_01535	23511	Hs.308340	NM_01535	ENSG0000	NUP188	KIAA0169|I	nucleopori	protein-co
lib-C666-l	chr21	45079420	45079420	+	147.98929	NA	promoter-TSS (NM_007031)	promoter-TSS (NM_007031)	-12	NM_01505	23076	Hs.565725	NM_01505	ENSG0000	RRP1B	KIAA0179|I	ribosomal	protein-co
lib-C666-l	chr10	977733	977733	+	20.90919	NA	promoter-TSS (NM_015155)	promoter-TSS (NM_015155)	-88	NM_01515	23185	Hs.159069	NM_01515	ENSG0000	LARP4B	KIAA0217|I	La ribonuc	protein-co
lib-C666-l	chr11	73309393	73309393	+	16.60407	NA	promoter-TSS (NM_001286051)	promoter-TSS (NM_001286051)	-159	NM_00128	23201	Hs.475334	NM_01515	ENSG0000	FAM168A	KIAA0280|1	family with	protein-co
lib-C666-l	chr9	139377495	139377495	+	34.25375	NA	promoter-TSS (NM_014866)	promoter-TSS (NM_014866)	12	NM_01486	9919	Hs.522500	NM_01486	ENSG0000	SEC16A	KIAA0310|S	SEC16 hon	protein-co
lib-C666-l	chr14	24883290	24883290	+	5.48804	NA	intron (NM_025081, intron 8 of 8)	intron (NM_025081, intron 8 of 8)	-15202	NM_00129	23351	Hs.713590	NM_01529	ENSG0000	KHNYN	KIAA0323	KH and NY	protein-co
lib-C666-l	chr20	36661894	36661894	+	52.3754	NA	promoter-TSS (NM_021215)	promoter-TSS (NM_021215)	-24	NM_01465	9675	Hs.655481	NM_01465	ENSG0000	TTI1	KIAA0406|s	TELO2 inte	protein-co
lib-C666-l	chr11	6624901	6624901	+	4.52748	NA	promoter-TSS (NM_015324)	promoter-TSS (NM_015324)	-21	NM_01532	23378	Hs.652255	NM_01532	ENSG0000	RRP8	KIAA0409|I	ribosomal	protein-co
lib-C666-l	chr7	4815183	4815183	+	17.13943	NA	promoter-TSS (NM_014855)	promoter-TSS (NM_014855)	-79	NM_01485	9907	Hs.558440	NM_01485	ENSG0000	AP5Z1	KIAA0415|S	adaptor re	protein-co
lib-C666-l	chr1	153917211	153917211	+	30.48199	NA	intron (NM_014856, intron 1 of 27)	intron (NM_014856, intron 1 of 27)	1943	NM_01485	9909	Hs.744878	NM_01485	ENSG0000	DENND4B	KIAA0476	DENN don	protein-co
lib-C666-l	chr1	153917680	153917680	+	7.08136	NA	intron (NM_014856, intron 1 of 27)	intron (NM_014856, intron 1 of 27)	1474	NM_01485	9909	Hs.744878	NM_01485	ENSG0000	DENND4B	KIAA0476	DENN don	protein-co
lib-C666-l	chr1	47184889	47184889	+	92.10336	NA	promoter-TSS (NM_014774)	promoter-TSS (NM_014774)	-153	NM_01477	9813	Hs.707688	NM_01477	ENSG0000	EFCAB14	KIAA0494	EF-hand ca	protein-co
lib-C666-l	chr2	65251287	65251287	+	9.14689	NA	TTS (NM_001193493)	TTS (NM_001193493)	-32208	NM_00131	23177	Hs.709257	NM_01514	ENSG0000	CEP68	KIAA0582	centrosom	protein-co
lib-C666-l	chr2	65276638	65276638	+	7.97818	NA	Intergenic	LTR45B|LTR|ERV1	-6857	NM_00131	23177	Hs.709257	NM_01514	ENSG0000	CEP68	KIAA0582	centrosom	protein-co
lib-C666-l	chr11	46638886	46638886	+	10.40942	NA	promoter-TSS (NM_173811)	promoter-TSS (NM_173811)	60	NM_00134	9776	Hs.127403	NM_01474	ENSG0000	ATG13	KIAA0652|I	autophagy	protein-co
lib-C666-l	chr22	50781549	50781549	+	9.22467	NA	promoter-TSS (NM_001242898)	promoter-TSS (NM_001242898)	-197	NM_00124	9701	Hs.449098	NM_01467	ENSG0000	PPP6R2	KIAA0685|I	protein ph	protein-co
lib-C666-l	chr10	99161055	99161055	+	29.21635	NA	promoter-TSS (NM_001145114)	promoter-TSS (NM_001145114)	72	NM_01517	23223	Hs.434251	NM_01517	ENSG0000	RRP12	KIAA0690	ribosomal	protein-co
lib-C666-l	chr6	96969654	96969654	+	24.99834	NA	promoter-TSS (NM_015323)	promoter-TSS (NM_015323)	-48	NM_01532	23376	Hs.149367	NM_01532	ENSG0000	UFL1	KIAA0776|I	UFM1 spec	protein-co
lib-C666-l	chr1	226063301	226063301	+	15.33659	NA	intron (NM_014698, intron 3 of 24)	intron (NM_014698, intron 3 of 24)	7119	NM_01469	9725	Hs.119387	NM_01469	ENSG0000	TMEM63A	KIAA0792	transmemb	protein-co
lib-C666-l	chr1	226070089	226070089	+	2.00164	NA	5' UTR (NM_014698, exon 1 of 25)	5' UTR (NM_014698, exon 1 of 25)	331	NM_01469	9725	Hs.119387	NM_01469	ENSG0000	TMEM63A	KIAA0792	transmemb	protein-co
lib-C666-l	chr19	36103616	36103616	+	370.0481	NA	promoter-TSS (NM_015302)	promoter-TSS (NM_015302)	-30	NM_01530	23354	Hs.7426	NM_01530	ENSG0000	HAUS5	KIAA0841|(	HAUS augr	protein-co
lib-C666-l	chr13	46626898	46626898	+	35.72107	NA	promoter-TSS (NR_046227)	promoter-TSS (NR_046227)	15	NM_00133	23091	Hs.136102	NM_01507	ENSG0000	ZC3H13	KIAA0853	zinc finger	protein-co
lib-C666-l	chr5	5422496	5422496	+	146.93733	NA	promoter-TSS (NM_015325)	promoter-TSS (NM_015325)	-290	NM_01532	23379	Hs.449296	NM_01532	ENSG0000	ICE1	KIAA0947	interactor (	protein-co
lib-C666-l	chr19	1177933	1177933	+	13.59267	NA	Intergenic	Intergenic	-3651	NM_01496	22904	Hs.408708	NM_01496	ENSG0000	SBNO2	KIAA0963|S	strawberry	protein-co
lib-C666-l	chr15	31195889	31195889	+	18.43022	NA	promoter-TSS (NM_001146096)	promoter-TSS (NM_001146096)	-166	NM_00114	22909	Hs.584863	NM_01496	ENSG0000	FAN1	KIAA1018|I	FANCD2 ai	protein-co
lib-C666-l	chr12	105501624	105501624	+	2.26435	NA	promoter-TSS (NR_121211)	promoter-TSS (NR_121211)	132	NM_01527	23325	Hs.12144	NM_01527	ENSG0000	WASHC4	KIAA1033|I	WASH con	protein-co
lib-C666-l	chr19	55770731	55770731	+	8.28333	NA	promoter-TSS (NM_014931)	promoter-TSS (NM_014931)	-693	NM_01493	22870	Hs.515610	NM_01493	ENSG0000	PPP6R1	KIAA1115|I	protein ph	protein-co
lib-C666-l	chr19	55770504	55770504	+	5.5166	NA	promoter-TSS (NM_014931)	promoter-TSS (NM_014931)	-466	NM_01493	22870	Hs.515610	NM_01493	ENSG0000	PPP6R1	KIAA1115|I	protein ph	protein-co
lib-C666-l	chr19	50102974	50102974	+	2.45439	NA	exon (NM_020719, exon 5 of 14)	exon (NM_020719, exon 5 of 14)	8074	NM_02071	57479	Hs.590971	NM_02071	ENSG0000	PRR12	KIAA1205	proline rich	protein-co
lib-C666-l	chr20	37101318	37101318	+	8.28333	NA	promoter-TSS (NM_001282917)	promoter-TSS (NM_001282917)	-131	NM_00128	57148	Hs.598850	NM_02033	ENSG0000	RALGAPB	KIAA1219|I	Ral GTPase	protein-co
lib-C666-l	chr15	52971641	52971641	+	24.43903	NA	promoter-TSS (NM_019600)	promoter-TSS (NM_019600)	-810	NR_10445	56204	Hs.152385	NM_01960	ENSG0000	FAM214A	KIAA1370	family with	protein-co
lib-C666-l	chr14	93799705	93799705	+	11.95031	NA	promoter-TSS (NM_001002860)	promoter-TSS (NM_001002860)	140	NR_14439	57578	Hs.126561	NM_02081	ENSG0000	UNC79	KIAA1409	unc-79 ho	protein-co
lib-C666-l	chr6	71122898	71122898	+	6.98764	NA	promoter-TSS (NM_001331001)	promoter-TSS (NM_001331001)	-209	NM_00133	57579	Hs.211700	NM_02081	ENSG0000	FAM135A	KIAA1411	family with	protein-co
lib-C666-l	chr14	23755509	23755509	+	11.66012	NA	promoter-TSS (NM_020834)	promoter-TSS (NM_020834)	-200	NM_02083	57594	Hs.632332	NM_02083	ENSG0000	HOMEZ	KIAA1443	homeobox	protein-co
lib-C666-l	chr4	48343543	48343543	+	4.73866	NA	promoter-TSS (NM_020846)	promoter-TSS (NM_020846)	-70	NM_02084	57606	Hs.479677	NM_02084	ENSG0000	SLAIN2	KIAA1458	SLAIN mot	protein-co
lib-C666-l	chr4	1340826	1340826	+	23.83582	NA	promoter-TSS (NM_001317935)	promoter-TSS (NM_001317935)	-228	NM_00131	57654	Hs.380475	NM_02089	ENSG0000	UVSSA	KIAA1530|U	UV stimula	protein-co
lib-C666-l	chr19	35491174	35491174	+	12.36452	NA	promoter-TSS (NM_001320035)	promoter-TSS (NM_001320035)	-50	NM_02089	57655	Hs.515351	NM_02089	ENSG0000	GRAMD1A	KIAA1533	GRAM don	protein-co
lib-C666-l	chr4	106068019	106068019	+	17.62684	NA	5' UTR (NM_001127208, exon 1 of 11	5' UTR (NM_001127208, exon 1 of 1	177	NM_00112	54790	Hs.367639	NM_01762	ENSG0000	TET2	KIAA1546|I	tet methylo	protein-co

lib-C666-I	chr2	98206124	98206124	+	6.32351	NA	exon (NM_025190, exon 1 of 44)	exon (NM_025190, exon 1 of 44)	304	NM_02519	57730	Hs.532921	NM_02097	ENSG0000	ANKRD36B	KIAA1641	ankyrin rep	protein-co
lib-C666-I	chr4	175204903	175204903	+	46.71952	NA	promoter-TSS (NM_001145314)	promoter-TSS (NM_001145314)	75	NM_00104	80817	Hs.555989	NM_03063	ENSG0000	CEP44	KIAA1712|I	centrosom	protein-co
lib-C666-I	chr14	77564556	77564556	+	9.02258	NA	promoter-TSS (NM_033426)	promoter-TSS (NM_033426)	-22	NM_03342	85457	Hs.709066	NM_03342	ENSG0000	CIPC	KIAA1737	CLOCK inte	protein-co
lib-C666-I	chr14	77554857	77554857	+	7.97818	NA	Intergenic	Intergenic	-9721	NM_03342	85457	Hs.709066	NM_03342	ENSG0000	CIPC	KIAA1737	CLOCK inte	protein-co
lib-C666-I	chr6	111580399	111580399	+	3.20885	NA	promoter-TSS (NM_153369)	promoter-TSS (NM_153369)	-83	NM_15336	91749	Hs.400572	NM_15336	ENSG0000	MFSD4B	KIAA1919|I	major facili	protein-co
lib-C666-I	chr3	113415660	113415660	+	11.57119	NA	promoter-TSS (NM_001009899)	promoter-TSS (NM_001009899)	-167	NM_00100	205717	Hs.632570	NM_00100	ENSG0000	USF3	KIAA2018	upstream t	protein-co
lib-C666-I	chr12	4381210	4381210	+	11.06679	NA	intron (NR_125790, intron 1 of 1)	CpG	-1692	NM_00175	894	Hs.376071	NM_00175	ENSG0000	CCND2	KIAK0002|I	cyclin D2	protein-co
lib-C666-I	chr12	4262800	4262800	+	4.68875	NA	Intergenic	Intergenic	-120102	NM_00175	894	Hs.376071	NM_00175	ENSG0000	CCND2	KIAK0002|I	cyclin D2	protein-co
lib-C666-I	chr15	42867520	42867520	+	7.15277	NA	promoter-TSS (NM_020759)	promoter-TSS (NM_020759)	-337	NM_02075	57519	Hs.122061	NM_02075	ENSG0000	STARD9	KIF16A	StAR relate	protein-co
lib-C666-I	chr1	21044554	21044554	+	5.96422	NA	promoter-TSS (NM_001122819)	promoter-TSS (NM_001122819)	-44	NM_02081	57576	Hs.130411	NM_02081	ENSG0000	KIF17	KIF17B|KIF	kinesin fam	protein-co
lib-C666-I	chr1	21023181	21023181	+	2.59032	NA	intron (NM_020816, intron 6 of 14)	CpG	21329	NM_00128	57576	Hs.130411	NM_02081	ENSG0000	KIF17	KIF17B|KIF	kinesin fam	protein-co
lib-C666-I	chr8	95274628	95274628	+	65.54223	NA	promoter-TSS (NM_005261)	promoter-TSS (NM_005261)	-81	NM_18170	2669	Hs.654463	NM_00526	ENSG0000	GEM	KIR	GTP bindin	protein-co
lib-C666-I	chr17	21360601	21360601	+	39.72046	NA	Intergenic	Intergenic	52153	NM_00119	100134444	Hs.200629	NM_00119	ENSG0000	KCNJ18	KIR2.6|TTP	potassium	protein-co
lib-C666-I	chr17	21361214	21361214	+	4.39666	NA	Intergenic	Intergenic	52766	NM_00119	100134444	Hs.200629	NM_00119	ENSG0000	KCNJ18	KIR2.6|TTP	potassium	protein-co
lib-C666-I	chr1	116184424	116184424	+	10.58189	NA	promoter-TSS (NM_001172411)	promoter-TSS (NM_001172411)	-150	NM_13895	81839	Hs.515130	NM_13895	ENSG0000	VANGL1	KITENIN|LF	VANGL pla	protein-co
lib-C666-I	chr14	104095446	104095446	+	82.10182	NA	promoter-TSS (NM_005552)	promoter-TSS (NM_005552)	-79	NM_18292	3831	Hs.20107	NM_00555	ENSG0000	KLC1	KLC|KNS2|I	kinesin ligh	protein-co
lib-C666-I	chr19	45844038	45844038	+	13.8811	NA	promoter-TSS (NM_177417)	promoter-TSS (NM_177417)	40	NM_17741	147700	Hs.298079	NM_14527	ENSG0000	KLC3	KLC2|KLC2	kinesin ligh	protein-co
lib-C666-I	chr19	55996526	55996526	+	50.83113	NA	promoter-TSS (NM_020378)	promoter-TSS (NM_020378)	-31	NM_02037	57106	Hs.31854	NM_02037	ENSG0000	NAT14	KLP1	N-acetyltra	protein-co
lib-C666-I	chr11	67195927	67195927	+	433.37909	NA	promoter-TSS (NM_003952)	promoter-TSS (NM_003952)	-8	NM_00395	6199	Hs.534345	NM_00395	ENSG0000	RPS6KB2	KLS|P70-b	ribosomal	protein-co
lib-C666-I	chr13	45992621	45992621	+	7.15277	NA	promoter-TSS (NM_001286807)	promoter-TSS (NM_001286807)	-31	NM_00128	253512	Hs.591230	NM_00101	ENSG0000	SLC25A30	KMCP1	solute carr	protein-co
lib-C666-I	chr10	14920756	14920756	+	103.27538	NA	promoter-TSS (NM_001193424)	promoter-TSS (NM_001193424)	-26	NM_00119	79723	Hs.554883	NM_02467	ENSG0000	SUV39H2	KMT1B	suppressor	protein-co
lib-C666-I	chr16	30969139	30969139	+	13.46097	NA	5' UTR (NM_014712, exon 1 of 19)	5' UTR (NM_014712, exon 1 of 19)	524	NM_01471	9739	Hs.297483	NM_01471	ENSG0000	SETD1A	KMT2F|Set	SET domai	protein-co
lib-C666-I	chr16	30968853	30968853	+	2.64885	NA	5' UTR (NM_014712, exon 1 of 19)	5' UTR (NM_014712, exon 1 of 19)	238	NM_01471	9739	Hs.297483	NM_01471	ENSG0000	SETD1A	KMT2F|Set	SET domai	protein-co
lib-C666-I	chr4	1872727	1872727	+	36.59122	NA	promoter-TSS (NM_001042424)	promoter-TSS (NM_001042424)	-396	NM_00104	7468	Hs.113876	NM_00733	ENSG0000	NSD2	KMT3F|KM	nuclear rec	protein-co
lib-C666-I	chr8	38240620	38240620	+	36.04455	NA	promoter-TSS (NM_023034)	promoter-TSS (NM_023034)	-830	NM_02303	54904	Hs.434966	NM_01777	ENSG0000	NSD3	KMT3F|KM	nuclear rec	protein-co
lib-C666-I	chr4	140476540	140476540	+	12.12723	NA	intron (NR_131339, intron 1 of 3)	CpG	1383	NR_13133	80854	Hs.480792	NM_03064	ENSG0000	SETD7	KMT7|SET7	SET domai	protein-co
lib-C666-I	chr15	66797542	66797542	+	30.13508	NA	promoter-TSS (NM_000968)	promoter-TSS (NM_000968)	121	NM_00128	55055	Hs.21331	NM_01797	ENSG0000	ZWILCH	KNTC1AP|I	zwilch kine	protein-co
lib-C666-I	chr16	71518542	71518542	+	26.94522	NA	5' UTR (NM_006961, exon 2 of 6)	5' UTR (NM_006961, exon 2 of 6)	4712	NM_00696	7567	Hs.660076	NM_00696	ENSG0000	ZNF19	KOX12	zinc finger	protein-co
lib-C666-I	chr16	71496141	71496141	+	72.80621	NA	promoter-TSS (NM_145911)	promoter-TSS (NM_145911)	14	NM_00130	7571	Hs.656643	NM_14591	ENSG0000	ZNF23	KOX16|ZN	zinc finger	protein-co
lib-C666-I	chr16	71446570	71446570	+	9.7234	NA	Intergenic	Intergenic	49556	NM_14591	7571	Hs.656643	NM_14591	ENSG0000	ZNF23	KOX16|ZN	zinc finger	protein-co
lib-C666-I	chr18	32924166	32924166	+	3.62961	NA	intron (NM_001308123, intron 1 of 3)	CpG	265	NM_00696	7572	Hs.514802	NM_00696	ENSG0000	ZNF24	KOX17|RS(	zinc finger	protein-co
lib-C666-I	chr18	32923964	32923964	+	2.62544	NA	intron (NM_001308123, intron 1 of 3)	CpG	467	NM_00696	7572	Hs.514802	NM_00696	ENSG0000	ZNF24	KOX17|RS(	zinc finger	protein-co
lib-C666-I	chr7	99613158	99613158	+	15.09264	NA	promoter-TSS (NM_001346581)	promoter-TSS (NM_001346581)	-17	NR_14447	7586	Hs.615360	NM_00343	ENSG0000	ZKSCAN1	KOX18|PH	zinc finger	protein-co
lib-C666-I	chr10	38265649	38265649	+	59.88466	NA	promoter-TSS (NM_145011)	promoter-TSS (NM_145011)	-66	NR_13806	219749	Hs.499429	NM_14501	ENSG0000	ZNF25	KOX19|Zfp	zinc finger	protein-co
lib-C666-I	chr10	38299591	38299591	+	19.92425	NA	promoter-TSS (NM_006974)	promoter-TSS (NM_006974)	13	NM_00697	7581	Hs.435774	NM_00695	ENSG0000	ZNF33A	KOX2|KOX	zinc finger	protein-co
lib-C666-I	chr1	33896605	33896605	+	5.9194	NA	Intergenic	CpG	-41627	NM_14523	7579	Hs.442705	NM_14523	ENSG0000	ZSCAN20	KOX29|ZFF	zinc finger	protein-co
lib-C666-I	chr8	146012741	146012741	+	37.99392	NA	promoter-TSS (NM_001286769)	promoter-TSS (NM_001286769)	-11	NM_00128	80778	Hs.631854	NM_03058	ENSG0000	ZNF34	KOX32	zinc finger	protein-co
lib-C666-I	chr8	146011503	146011503	+	7.26874	NA	intron (NM_030580, intron 1 of 5)	AluY|SINE|Alu	1227	NM_00128	80778	Hs.631854	NM_03058	ENSG0000	ZNF34	KOX32	zinc finger	protein-co
lib-C666-I	chr11	73358603	73358603	+	11.6145	NA	promoter-TSS (NM_001130034)	promoter-TSS (NM_001130034)	9	NM_00113	58473	Hs.445489	NM_02120	ENSG0000	PLEKHB1	KPL1|PHR1	pleckstrin I	protein-co
lib-C666-I	chr13	21635732	21635732	+	9.4812	NA	promoter-TSS (NM_014572)	promoter-TSS (NM_014572)	-10	NM_01457	26524	Hs.78960	NM_01457	ENSG0000	LATS2	KPM	large tumc	protein-co
lib-C666-I	chr10	43932349	43932349	+	29.11329	NA	promoter-TSS (NR_026693)	promoter-TSS (NR_026693)	-225	NR_02669	642819	Hs.719657	NR_026693		ZNF487	KRBO1|ZN	zinc finger	pseudo
lib-C666-I	chr10	43951094	43951094	+	16.43544	NA	intron (NR_026693, intron 1 of 3)	CpG	18520	NR_02669	642819	Hs.719657	NR_026693		ZNF487	KRBO1|ZN	zinc finger	pseudo
lib-C666-I	chr2	220110212	220110212	+	54.91701	NA	promoter-TSS (NM_001286423)	promoter-TSS (NM_001286423)	35	NM_00133	8576	Hs.153003	NM_00369	ENSG0000	STK16	KRCT|MPSI	serine/thre	protein-co
lib-C666-I	chr13	98085677	98085677	+	17.68706	NA	promoter-TSS (NM_021033)	promoter-TSS (NM_021033)	-798	NM_02103	5911	Hs.508480	NM_02103	ENSG0000	RAP2A	K-REV|KRE	RAP2A, me	protein-co
lib-C666-I	chr13	98085421	98085421	+	14.34866	NA	Intergenic	CpG	-1054	NM_02103	5911	Hs.508480	NM_02103	ENSG0000	RAP2A	K-REV|KRE	RAP2A, me	protein-co
lib-C666-I	chr16	3006698	3006698	+	8.71138	NA	Intergenic	Intergenic	-7519	NM_00125	79412	Hs.661128	NM_02450	ENSG0000	KREMEN2	KRM2	kringle cor	protein-co
lib-C666-I	chr16	3013921	3013921	+	2.98619	NA	promoter-TSS (NM_001253725)	promoter-TSS (NM_001253725)	-296	NM_00125	79412	Hs.661128	NM_02450	ENSG0000	KREMEN2	KRM2	kringle cor	protein-co
lib-C666-I	chr11	71300599	71300599	+	3.22338	NA	Intergenic	HERVE-int|LTR|ERV1	-6678	NM_00100	440051	Hs.679617	NM_00100	ENSG0000	KRTAP5-1	KRTAP5-5|	keratin ass	protein-co
lib-C666-I	chr6	27440212	27440212	+	5.25516	NA	non-coding (NR_144687, exon 2 of 9	non-coding (NR_144687, exon 2 of	465	NM_00131	7738	Hs.158174	NM_00714	ENSG0000	ZNF184	kr-ZNF3	zinc finger	protein-co
lib-C666-I	chr6	27440803	27440803	+	4.52748	NA	promoter-TSS (NM_001318893)	promoter-TSS (NM_001318893)	94	NM_00131	7738	Hs.158174	NM_00714	ENSG0000	ZNF184	kr-ZNF3	zinc finger	protein-co
lib-C666-I	chr19	50837555	50837555	+	262.35843	NA	promoter-TSS (NR_110912)	promoter-TSS (NR_110912)	-783	NR_11091	3748	Hs.467146	NM_00497	ENSG0000	KCNC3	KSHIIID|KV	potassium	protein-co
lib-C666-I	chr19	50836832	50836832	+	10.9422	NA	promoter-TSS (NR_110912)	promoter-TSS (NR_110912)	-60	NR_11091	3748	Hs.467146	NM_00497	ENSG0000	KCNC3	KSHIIID|KV	potassium	protein-co
lib-C666-I	chr19	50819403	50819403	+	4.19683	NA	intron (NR_110912, intron 2 of 3)	intron (NR_110912, intron 2 of 3)	13231	NM_00497	3748	Hs.467146	NM_00497	ENSG0000	KCNC3	KSHIIID|KV	potassium	protein-co
lib-C666-I	chr17	25833863	25833863	+	8.01457	NA	intron (NM_014238, intron 2 of 20)	intron (NM_014238, intron 2 of 20)	34827	NM_01423	8844	Hs.133534	NM_01423	ENSG0000	KSR1	KSR|RSU2	kinase sup	protein-co
lib-C666-I	chr17	25799436	25799436	+	5.80117	NA	intron (NM_014238, intron 1 of 20)	intron (NM_014238, intron 1 of 20)	400	NM_01423	8844	Hs.133534	NM_01423	ENSG0000	KSR1	KSR|RSU2	kinase sup	protein-co
lib-C666-I	chr16	24875257	24875257	+	2.00164	NA	intron (NM_001258412, intron 4 of 14	L1M4|LINE|L1	17705	NM_05294	115584	Hs.164118	NM_05294	ENSG0000	SLC5A11	KST1|RKST	solute carr	protein-co
lib-C666-I	chr20	48770382	48770382	+	6.49644	NA	promoter-TSS (NM_199203)	promoter-TSS (NM_199203)	-47	NM_19912	387521	Hs.744839	NM_19912	ENSG0000	TMEM189	KUA	transmemb	protein-co
lib-C666-I	chr8	73449131	73449131	+	57.17088	NA	promoter-TSS (NM_004770)	promoter-TSS (NM_004770)	-495	NM_00477	9312	Hs.661102	NM_00477	ENSG0000	KCNB2	KV2.2	potassium	protein-co
lib-C666-I	chr8	73450068	73450068	+	37.41784	NA	5' UTR (NM_004770, exon 1 of 3)	5' UTR (NM_004770, exon 1 of 3)	442	NM_00477	9312	Hs.661102	NM_00477	ENSG0000	KCNB2	KV2.2	potassium	protein-co
lib-C666-I	chr2	18198842	18198842	+	32.78343	NA	Intergenic	L1PA2|LINE|L1	138918	NM_00225	3790	Hs.414489	NM_00225	ENSG0000	KCNS3	KV9.3	potassium	protein-co
lib-C666-I	chr2	18364552	18364552	+	5.64185	NA	Intergenic	Intergenic	304628	NM_00225	3790	Hs.414489	NM_00225	ENSG0000	KCNS3	KV9.3	potassium	protein-co
lib-C666-I	chr2	143631157	143631157	+	24.17107	NA	Intergenic	Intergenic	-4038	NM_00393	8942	Hs.470126	NM_00393	ENSG0000	KYNU	KYNUU	kynurenina	protein-co
lib-C666-I	chr19	49990767	49990767	+	36.59206	NA	promoter-TSS (NR_073024)	promoter-TSS (NR_073024)	-44	NR_07302	23521	Hs.523185	NM_01242	ENSG0000	RPL13A	L13A|TSTA	ribosomal	protein-co
lib-C666-I	chr11	116969330	116969330	+	20.59244	NA	promoter-TSS (NM_001281748)	promoter-TSS (NM_001281748)	-199	NM_00128	23387	Hs.167451	NM_02516	ENSG0000	SIK3	L19|QSK|SI	SIK family I	protein-co
lib-C666-I	chr12	27677028	27677028	+	3.27468	NA	promoter-TSS (NM_177444)	promoter-TSS (NM_177444)	-17	NM_00119	8496	Hs.172445	NM_00362	ENSG0000	PPFIBP1	L2|SGT2|hS	PPFIA bind	protein-co
lib-C666-I	chr17	37010106	37010106	+	64.0179	NA	promoter-TSS (NR_002576)	promoter-TSS (NR_002576)	-53	NM_00097	9349	Hs.406300	NM_00097	ENSG0000	RPL23	L23|rpL17	ribosomal	protein-co
lib-C666-I	chr17	27046770	27046770	+	30.07415	NA	promoter-TSS (NM_000984)	promoter-TSS (NM_000984)	-230	NM_00098	6147	Hs.419463	NM_00098	ENSG0000	RPL23A	L23A|MDA	ribosomal	protein-co
lib-C666-I	chr1	156711082	156711082	+	2.59032	NA	promoter-TSS (NM_145729)	promoter-TSS (NM_145729)	-159	NM_02454	79590	Hs.418233	NM_02454	ENSG0000	MRPL24	L24mt|MRI	mitochond	protein-co
lib-C666-I	chr17	41150413	41150413	+	27.68428	NA	promoter-TSS (NM_000988)	promoter-TSS (NM_000988)	-33	NM_00098	6155	Hs.514196	NM_00098	ENSG0000	RPL27	L27	ribosomal	protein-co
lib-C666-I	chr11	8704256	8704256	+	3.8557	NA	5' UTR (NM_000990, exon 1 of 5)	5' UTR (NM_000990, exon 1 of 5)	261	NM_00099	6157	Hs.523463	NM_00099	ENSG0000	RPL27A	L27A	ribosomal	protein-co
lib-C666-I	chr19	55897201	55897201	+	12.55817	NA	promoter-TSS (NM_001136134)	promoter-TSS (NM_001136134)	-99	NM_00113	6158	Hs.652114	NM_00099	ENSG0000	RPL28	L28	ribosomal	protein-co
lib-C666-I	chr2	99797670	99797670	+	4.73866	NA	promoter-TSS (NM_001320417)	promoter-TSS (NM_001320417)	128	NM_14521	51263	Hs.346736	NM_01650	ENSG0000	MRPL30	L28MT|L30	mitochond	protein-co
lib-C666-I	chr17	43239035	43239035	+	12.55817	NA	promoter-TSS (NM_001303440)	promoter-TSS (NM_001303440)	-177	NM_00130	124790	Hs.56382	NM_14460	ENSG0000	HEXIM2	L3	hexamethy	protein-co
lib-C666-I	chr2	101618651	101618651	+	33.4149	NA	promoter-TSS (NM_000993)	promoter-TSS (NM_000993)	-40	NM_00099	6160	Hs.469473	NM_00099	ENSG0000	RPL31	L31	ribosomal	protein-co
lib-C666-I	chr3	12883075	12883075	+	5.9194	NA	promoter-TSS (NM_000994)	promoter-TSS (NM_000994)	6	NM_00100	6161	Hs.265174	NM_00099	ENSG0000	RPL32	L32|PP993	ribosomal	protein-co
lib-C666-I	chr19	17416365	17416365	+	6.71839	NA	promoter-TSS (NM_023937)	promoter-TSS (NM_023937)	-112	NM_02393	64981	Hs.515242	NM_02393	ENSG0000	MRPL34	L34mt	mitochond	protein-co
lib-C666-I	chr19	5687302	5687302	+	21.0836	NA	exon (NM_001267868, exon 7 of 9)	exon (NM_001267868, exon 7 of 9)	-2970	NM_03364	25873	Hs.408018	NM_01541	ENSG0000	RPL36	L36	ribosomal	protein-co
lib-C666-I	chr22	19419978	19419978	+	64.85778	NA	promoter-TSS (NM_001318152)	promoter-TSS (NM_001318152)	-49	NM_00131	64976	Hs.431307	NM_00377	ENSG0000	MRPL40	L40mt|MRI	mitochond	protein-co
lib-C666-I	chr12	56510316	56510316	+	34.92958	NA	promoter-TSS (NM_021104)	promoter-TSS (NM_021104)	-58	NM_02110	6171	Hs.112553	NM_02110	ENSG0000	RPL41	L41	ribosomal	protein-co
lib-C666-I	chr19	3762687	3762687	+	36.07033	NA	promoter-TSS (NM_172251)	promoter-TSS (NM_172251)	22	NM_17225	116541	Hs.356578	NM_17225	ENSG0000	MRPL54	L54mt|MRI	mitochond	protein-co
lib-C666-I	chr8	74206299	74206299	+	27.84631	NA	promoter-TSS (NM_000971)	promoter-TSS (NM_000971)	-430	NM_00097	6129	Hs.421257	NM_00097	ENSG0000	RPL7	L7|humL7-	ribosomal	protein-co
lib-C666-I	chr8	146017842	146017842	+	97.94548	NA	promoter-TSS (NR_106909)	promoter-TSS (NR_106909)	-11	NM_00097	6132	Hs.178551	NM_00097	ENSG0000	RPL8	L8	ribosomal	protein-co
lib-C666-I	chr4	39460601	39460601	+	279.80209	NA	promoter-TSS (NM_000661)	promoter-TSS (NM_000661)	-33	NM_00066	6133	Hs.412370	NM_00066	ENSG0000	RPL9	L9|NPC-A-	ribosomal	protein-co
lib-C666-I	chr16	88279313	88279313	+	2.94047	NA	Intergenic	Intergenic	51426	NR_11094	101928880	Hs.634835	NR_11094	ENSG0000	LOC10192	LA16c-444	uncharacte	ncRNA
lib-C666-I	chr1	201361208	201361208	+	13.59977	NA	intron (NM_005558, intron 1 of 9)	intron (NM_005558, intron 1 of 9)	7461	NM_00555	3898	Hs.519035	NM_00555	ENSG0000	LAD1	LadA	ladinin 1	protein-co
lib-C666-I	chr1	201368807	201368807	+	10.27265	NA	promoter-TSS (NM_005558)	promoter-TSS (NM_005558)	-138	NM_00555	3898	Hs.519035	NM_00555	ENSG0000	LAD1	LadA	ladinin 1	protein-co
lib-C666-I	chr2	100496817	100496817	+	8.20589	NA	intron (NM_001025108, intron 6 of 23	intron (NM_001025108, intron 6 of	225228	NM_00102	3899	Hs.444414	NM_00228	ENSG0000	AFF3	LAF4|MLLT	AF4/FMR2	protein-co
lib-C666-I	chr5	6713215	6713215	+	20.19521	NA	Intergenic	CpG	-1503	NM_00117	11044	Hs.481542	NM_00699	ENSG0000	PAPD7	LAK-1|LAK	poly(A) RN	protein-co
lib-C666-I	chr8	23315203	23315203	+	16.5633	NA	promoter-TSS (NM_001128930)	promoter-TSS (NM_001128930)	41	NM_00112	9583	Hs.444389	NM_00490	ENSG0000	ENTPD4	LALP70|LA	ectonuclec	protein-co
lib-C666-I	chr9	135820117	135820117	+	20.19521	NA	promoter-TSS (NR_049862)	promoter-TSS (NR_049862)	-97	NM_00116	7248	Hs.370854	NM_00036	ENSG0000	TSC1	LAM|TSC	tuberous s	protein-co
lib-C666-I	chr12	56123438	56123438	+	8.56376	NA	promoter-TSS (NM_001267698)	promoter-TSS (NM_001267698)	19	NM_00125	967	Hs.445570	NM_00178	ENSG0000	CD63	LAMP-3|M	CD63 mole	protein-co
lib-C666-I	chr12	56123046	56123046	+	8.01457	NA	promoter-TSS (NM_001257392)	promoter-TSS (NM_001257392)	-124	NM_00178	967	Hs.445570	NM_00178	ENSG0000	CD63	LAMP-3|M	CD63 mole	protein-co
lib-C666-I	chr6	53664467	53664467	+	4.73866	NA	intron (NM_018214, intron 1 of 13)	intron (NM_018214, intron 1 of 13)	4689	NM_01821	55227	Hs.606493	NM_01821	ENSG0000	LRRC1	LANO|dJ52	leucine rich	protein-co
lib-C666-I	chr6	53659455	53659455	+	4.46583	NA	promoter-TSS (NM_018214)	promoter-TSS (NM_018214)	-323	NM_01821	55227	Hs.606493	NM_01821	ENSG0000	LRRC1	LANO|dJ52	leucine rich	protein-co
lib-C666-I	chr1	150208590	150208590	+	27.96363	NA	promoter-TSS (NM_001136478)	promoter-TSS (NM_001136478)	-86	NM_00128	81611	Hs.656466	NM_03092	ENSG0000	ANP32E	LANP-L|LA	acidic nucl	protein-co
lib-C666-I	chr11	47270360	47270360	+	3.27468	NA	promoter-TSS (NM_001302489)	promoter-TSS (NM_001302489)	38	NM_00130	53	Hs.532492	NM_00161	ENSG0000	ACP2	LAP	acid phosp	protein-co
lib-C666-I	chr1	179851681	179851681	+	39.16209	NA	exon (NM_001267578, exon 1 of 10)	exon (NM_001267578, exon 1 of 10	504	NM_00126	26092	Hs.496459	NM_01560	ENSG0000	TOR1AIP1	LAP1|LAP1	torsin 1A ir	protein-co
lib-C666-I	chr8	98787974	98787974	+	19.60174	NA	exon (NM_018407, exon 1 of 7)	exon (NM_018407, exon 1 of 7)	165	NM_01840	55353	Hs.492314	NM_01840	ENSG0000	LAPTM4B	LAPTM4be	lysosomal	protein-co
lib-C666-I	chr11	120302982	120302982	+	2.12162	NA	intron (NM_001301084, intron 11 of 4	intron (NM_001301084, intron 11 o	46979	NM_00130	23365	Hs.24598	NM_01531	ENSG0000	ARHGEF12	LARG|PRO	Rho guanir	protein-co
lib-C666-I	chr11	66406011	66406011	+	9.47655	NA	promoter-TSS (NM_002896)	promoter-TSS (NM_002896)	-77	NM_00289	5936	Hs.523822	NM_00289	ENSG0000	RBM4	LARK|RBM	RNA bindir	protein-co
lib-C666-I	chr11	66418705	66418705	+	2.59723	NA	intron (NM_001198844, intron 2 of 2)	intron (NM_001198844, intron 2 of	12617	NM_00119	5936	Hs.523822	NM_00289	ENSG0000	RBM4	LARK|RBM	RNA bindir	protein-co
lib-C666-I	chr5	154092195	154092195	+	14.45701	NA	promoter-TSS (NM_015315)	promoter-TSS (NM_015315)	-267	NM_01531	23367	Hs.292078	NM_01531	ENSG0000	LARP1	LARP	La ribonuc	protein-co
lib-C666-I	chr4	128982347	128982347	+	12.70401	NA	promoter-TSS (NM_001278604)	promoter-TSS (NM_001278604)	-74	NM_03223	55132	Hs.657067	NM_01807	ENSG0000	LARP1B	LARP2	La ribonuc	protein-co
lib-C666-I	chrX	64754763	64754763	+	14.0317	NA	promoter-TSS (NM_031206)	promoter-TSS (NM_031206)	-77	NM_03120	81887	Hs.522675	NM_03120	ENSG0000	LAS1L	Las1-like|V	LAS1 like, r	protein-co
lib-C666-I	chr17	37025770	37025770	+	6.71839	NA	promoter-TSS (NM_001271608)	promoter-TSS (NM_001271608)	-342	NM_00127	3927	Hs.741156	NM_00614	ENSG0000	LASP1	Lasp-1|ML	LIM and SI	protein-co
lib-C666-I	chr19	8274454	8274454	+	9.02258	NA	intron (NM_024552, intron 1 of 11)	CpG	237	NM_02455	79603	Hs.515111	NM_02455	ENSG0000	CERS4	LASS4|Trh1	ceramide s	protein-co
lib-C666-I	chr19	8258921	8258921	+	5.96761	NA	Intergenic	Intergenic	-15296	NM_02455	79603	Hs.515111	NM_02455	ENSG0000	CERS4	LASS4|Trh1	ceramide s	protein-co
lib-C666-I	chr16	68298354	68298354	+	6.49644	NA	promoter-TSS (NM_001076785)	promoter-TSS (NM_001076785)	-65	NM_00398	9057	Hs.679580	NM_00398	ENSG0000	SLC7A6	LAT-2|LAT	solute carr	protein-co
lib-C666-I	chr16	68298655	68298655	+	2.62544	NA	intron (NM_003983, intron 1 of 10)	CpG	236	NM_00107	9057	Hs.679580	NM_00398	ENSG0000	SLC7A6	LAT-2|LAT	solute carr	protein-co
lib-C666-I	chr11	57261075	57261075	+	13.59977	NA	intron (NM_001198810, intron 8 of 14	intron (NM_001198810, intron 8 of	21283	NM_00119	8501	Hs.591952	NM_00362	ENSG0000	SLC43A1	LAT3|PB39	solute carr	protein-co
lib-C666-I	chr17	1532470	1532470	+	3.23837	NA	promoter-TSS (NM_001284498)	promoter-TSS (NM_001284498)	-290	NM_15234	124935	Hs.160550	NM_15234	ENSG0000	SLC43A2	LAT4	solute carr	protein-co
lib-C666-I	chr5	892826	892826	+	54.49234	NA	promoter-TSS (NM_001166260)	promoter-TSS (NM_001166260)	113	NM_02392	65980	Hs.449278	NM_02392	ENSG0000	BRD9	LAVS3040|	bromodon	protein-co
lib-C666-I	chr13	53029290	53029290	+	5.23609	NA	promoter-TSS (NM_001286687)	promoter-TSS (NM_001286687)	-205	NM_00109	26586	Hs.444028	NM_01820	ENSG0000	CKAP2	LB1|TMAP|	cytoskeletc	protein-co
lib-C666-I	chr6	34213115	34213115	+	6.98764	NA	3' UTR (NM_145903, exon 5 of 5)	3' UTR (NM_145903, exon 5 of 5)	3770	NM_00100	221491	Hs.381300	NM_17850	ENSG0000	C6orf1	LBH	chromosor	protein-co
lib-C666-I	chr3	33482266	33482266	+	30.13508	NA	promoter-TSS (NM_001128160)	promoter-TSS (NM_001128160)	-369	NM_00112	7342	Hs.729120	NM_01451	ENSG0000	UBP1	LBP-1B|LBI	upstream I	protein-co
lib-C666-I	chr12	51566916	51566916	+	137.26332	NA	promoter-TSS (NM_001173452)	promoter-TSS (NM_001173452)	10	NM_00565	7024	Hs.48849	NM_00565	ENSG0000	TFCP2	LBP1C|LSF|	transcriptic	protein-co
lib-C666-I	chr2	10131027	10131027	+	14.02237	NA	intron (NM_198182, intron 10 of 15)	intron (NM_198182, intron 10 of 15	39235	NM_19818	29841	Hs.418493	NM_19818	ENSG0000	GRHL1	LBP32|MGI	grainyhead	protein-co
lib-C666-I	chr2	10091931	10091931	+	11.06679	NA	5' UTR (NM_198182, exon 1 of 16)	5' UTR (NM_198182, exon 1 of 16)	139	NM_19818	29841	Hs.418493	NM_19818	ENSG0000	GRHL1	LBP32|MGI	grainyhead	protein-co
lib-C666-I	chr14	68171334	68171334	+	7.0832	NA	intron (NM_152443, intron 1 of 8)	intron (NM_152443, intron 1 of 8)	2731	NM_15244	145226	Hs.415322	NM_15244	ENSG0000	RDH12	LCA13|RP5	retinol deh	protein-co
lib-C666-I	chr22	46731694	46731694	+	34.92958	NA	non-coding (NR_104240, exon 1 of 1	non-coding (NR_104240, exon 1 of	396	NR_10424	55687	Hs.439524	NM_01800	ENSG0000	TRMU	LCAL3|MT(	tRNA 5-me	protein-co
lib-C666-I	chr2	216483923	216483923	+	4.6254	NA	intron (NR_037195, intron 8 of 9)	intron (NR_037195, intron 8 of 9)	-98843	NR_13238	105373869	Hs.152812	NR_132383		LINC01614	LCAL4	long interg	ncRNA
lib-C666-I	chr17	70657817	70657817	+	73.41202	NA	intron (NM_001159770, intron 7 of 9)	intron (NM_001159770, intron 7 of	-21206	NR_03387	400619	Hs.447555	NR_03387	ENSG0000	LINC00511	LCAL5|onc	long interg	ncRNA
lib-C666-I	chr5	175843556	175843556	+	49.72573	NA	promoter-TSS (NR_045724)	promoter-TSS (NR_045724)	14	NR_04572	1212	Hs.484241	NM_00183	ENSG0000	CLTB	LCB	clathrin ligl	protein-co
lib-C666-I	chr3	134093933	134093933	+	13.89614	NA	promoter-TSS (NM_016201)	promoter-TSS (NM_016201)	326	NM_00127	51421	Hs.426312	NM_01620	ENSG0000	AMOTL2	LCCP	angiomotir	protein-co
lib-C666-I	chr3	134092643	134092643	+	13.57551	NA	intron (NM_001278683, intron 1 of 9)	CpG	784	NM_01620	51421	Hs.426312	NM_01620	ENSG0000	AMOTL2	LCCP	angiomotir	protein-co
lib-C666-I	chr3	134093147	134093147	+	2.13257	NA	intron (NM_001278683, intron 1 of 9)	CpG	280	NM_01620	51421	Hs.426312	NM_01620	ENSG0000	AMOTL2	LCCP	angiomotir	protein-co
lib-C666-I	chr19	3700509	3700509	+	7.0832	NA	promoter-TSS (NM_001300849)	promoter-TSS (NM_001300849)	-19	NM_00130	23396	Hs.282177	NM_01239	ENSG0000	PIP5K1C	LCCS3|PIP5	phosphatic	protein-co
lib-C666-I	chr12	102002783	102002783	+	3.20885	NA	intron (NM_001254722, intron 1 of 27	intron (NM_001254722, intron 1 of	14074	NM_00125	4604	Hs.654589	NM_00246	ENSG0000	MYBPC1	LCCS4|MYI	myosin bin	protein-co
lib-C666-I	chr14	105271830	105271830	+	9.02258	NA	TTS (NM_001137601)	TTS (NM_001137601)	4312	NM_00113	100128927	Hs.170853	NM_00113	ENSG0000	ZBTB42	LCCS6|ZNF	zinc finger	protein-co
lib-C666-I	chr17	46125568	46125568	+	11.66012	NA	promoter-TSS (NM_003204)	promoter-TSS (NM_003204)	-99	NM_00133	4779	Hs.514284	NM_00320	ENSG0000	NFE2L1	LCR-F1|NR	nuclear fac	protein-co
lib-C666-I	chr5	74632919	74632919	+	48.61377	NA	promoter-TSS (NM_000859)	promoter-TSS (NM_000859)	-74	NM_00085	3156	Hs.628096	NM_00085	ENSG0000	HMGCR	LDLCQ3	3-hydroxy	protein-co
lib-C666-I	chr5	74621397	74621397	+	9.02258	NA	Intergenic	L1M4|LINE|L1	-11596	NM_00085	3156	Hs.628096	NM_00085	ENSG0000	HMGCR	LDLCQ3	3-hydroxy	protein-co
lib-C666-I	chrX	134555981	134555981	+	2.99475	NA	non-coding (NR_024359, exon 1 of 2	non-coding (NR_024359, exon 1 of	123	NR_02435	399668	Hs.374414	NM_20330	ENSG0000	SMIM10L2	LED|LINC0	small integ	ncRNA
lib-C666-I	chr4	109089753	109089753	+	8.89416	NA	5' UTR (NM_016269, exon 1 of 12)	5' UTR (NM_016269, exon 1 of 12)	359	NM_00113	51176	Hs.743478	NM_01626	ENSG0000	LEF1	LEF-1|TCF1	lymphoid e	protein-co
lib-C666-I	chr4	109090174	109090174	+	4.04037	NA	promoter-TSS (NM_016269)	promoter-TSS (NM_016269)	-62	NM_01626	51176	Hs.743478	NM_01626	ENSG0000	LEF1	LEF-1|TCF1	lymphoid e	protein-co
lib-C666-I	chr4	23790249	23790249	+	12.58342	NA	Intergenic	Intergenic	92666	NM_00133	10891	Hs.527078	NM_01326	ENSG0000	PPARGC1A	LEM6|PGC	PPARG coa	protein-co
lib-C666-I	chr19	54641186	54641186	+	5.96422	NA	promoter-TSS (NM_014516)	promoter-TSS (NM_014516)	-250	NM_01451	4849	Hs.343571	NM_01451	ENSG0000	CNOT3	LENG2|NO	CCR4-NOT	protein-co
lib-C666-I	chr19	55629150	55629150	+	27.70954	NA	promoter-TSS (NM_017607)	promoter-TSS (NM_017607)	-182	NM_01760	54776	Hs.631579	NM_01760	ENSG0000	PPP1R12C	LENG3|MB	protein ph	protein-co
lib-C666-I	chr19	54695017	54695017	+	43.7015	NA	promoter-TSS (NM_001077446)	promoter-TSS (NM_001077446)	-48	NM_00107	79042	Hs.15580	NM_02407	ENSG0000	TSEN34	LENG5|PCI	tRNA splici	protein-co
lib-C666-I	chr19	54694207	54694207	+	22.02307	NA	promoter-TSS (NM_001282333)	promoter-TSS (NM_001282333)	88	NM_00128	79042	Hs.15580	NM_02407	ENSG0000	TSEN34	LENG5|PCI	tRNA splici	protein-co
lib-C666-I	chr19	54694399	54694399	+	11.62669	NA	promoter-TSS (NM_001282332)	promoter-TSS (NM_001282332)	-183	NM_00128	79042	Hs.15580	NM_02407	ENSG0000	TSEN34	LENG5|PCI	tRNA splici	protein-co
lib-C666-I	chr7	127899831	127899831	+	2.98619	NA	Intergenic	LTR8|LTR|ERV1	18500	NM_00023	3952	Hs.194236	NM_00023	ENSG0000	LEP	LEPD|OB|C	leptin	protein-co
lib-C666-I	chr1	65885660	65885660	+	15.33659	NA	promoter-TSS (NM_002303)	promoter-TSS (NM_002303)	-471	NM_01752	54741	Hs.23581	NM_01752	ENSG0000	LEPROT	LEPR|OB-R	leptin rece	protein-co
lib-C666-I	chr9	96929056	96929056	+	76.26201	NA	Intergenic	CpG	-9183	NR_02947	406881		NR_02947	ENSG0000	MIRLET7A1	LET7A1|MI	microRNA	ncRNA
lib-C666-I	chr9	96928408	96928408	+	16.13885	NA	Intergenic	CpG	-9831	NR_02947	406881		NR_02947	ENSG0000	MIRLET7A1	LET7A1|MI	microRNA	ncRNA
lib-C666-I	chr19	52193711	52193711	+	7.0832	NA	intron (NM_001316994, intron 1 of 8)	intron (NM_001316994, intron 1 of	1024	NM_00131	147650	Hs.467174	NM_20732	ENSG0000	SPACA6	LET7EH|LIN	sperm acrc	protein-co
lib-C666-I	chr19	49468751	49468751	+	5.819	NA	5' UTR (NM_000146, exon 1 of 4)	5' UTR (NM_000146, exon 1 of 4)	185	NM_00014	2512	Hs.433670	NM_00014	ENSG0000	FTL	LFTD|NBIA	ferritin ligh	protein-co
lib-C666-I	chr8	61429934	61429934	+	12.62235	NA	intron (NM_001242644, intron 1 of 6)	CpG	465	NM_00286	5862	Hs.369017	NM_00286	ENSG0000	RAB2A	LHX|RAB2	RAB2A, me	protein-co
lib-C666-I	chr3	66479295	66479295	+	10.40942	NA	intron (NM_015541, intron 3 of 18)	intron (NM_015541, intron 3 of 18)	71550	NM_01554	26018	Hs.518055	NM_01554	ENSG0000	LRIG1	LIG-1|LIG1	leucine rich	protein-co
lib-C666-I	chr17	33307755	33307755	+	14.26909	NA	intron (NM_013975, intron 1 of 19)	CpG	238	NM_00231	3980	Hs.100299	NM_00231	ENSG0000	LIG3	LIG2	DNA ligase	protein-co
lib-C666-I	chr7	73509087	73509087	+	6.15313	NA	intron (NM_001204426, intron 1 of 14	intron (NM_001204426, intron 1 of	1601	NM_00120	3984	Hs.647035	NM_00231	ENSG0000	LIMK1	LIMK|LIMK	LIM domai	protein-co
lib-C666-I	chr12	49504708	49504708	+	8.28613	NA	promoter-TSS (NM_001300751)	promoter-TSS (NM_001300751)	-25	NM_01811	55716	Hs.272838	NM_01811	ENSG0000	LMBR1L	LIMR	limb devel	protein-co
lib-C666-I	chr12	122237481	122237481	+	4.73866	NA	intron (NR_002809, intron 5 of 6)	intron (NR_002809, intron 5 of 6)	3909	NR_00280	338799	Hs.524804	NR_00280	ENSG0000	LINC01089	LIMT	long interg	ncRNA
lib-C666-I	chr12	122238214	122238214	+	2.59032	NA	intron (NR_002809, intron 4 of 6)	CpG	3176	NR_00280	338799	Hs.524804	NR_00280	ENSG0000	LINC01089	LIMT	long interg	ncRNA
lib-C666-I	chr3	32823059	32823059	+	3.58839	NA	Intergenic	CpG	-36451	NM_00103	131405	Hs.113170	NM_00103	ENSG0000	TRIM71	LIN-41|LIN	tripartite m	protein-co
lib-C666-I	chr19	49617481	49617481	+	72.82537	NA	promoter-TSS (NM_022165)	promoter-TSS (NM_022165)	-137	NM_00130	64130	Hs.221737	NM_02216	ENSG0000	LIN7B	LIN-7B|MA	lin-7 homc	protein-co
lib-C666-I	chr11	27528237	27528237	+	7.08136	NA	promoter-TSS (NR_002832)	promoter-TSS (NR_002832)	89	NM_01836	55327	Hs.91393	NM_01836	ENSG0000	LIN7C	LIN-7-C|LI	lin-7 homc	protein-co

lib-C666-l	chr4	119199861	119199861	+	11.90035	NA	promoter-TSS (NR_003584)	promoter-TSS (NR_003584)	-56	NR_00358	100093630	Hs.535762	NR_00358	ENSG0000	SNHG8	LINC0006	small nucle	ncRNA
lib-C666-l	chr22	31365160	31365160	+	3.59106	NA	promoter-TSS (NM_001303256)	promoter-TSS (NM_001303256)	-37	NR_11049	55000	Hs.554829	NR_002323		TUG1	LINC0008	taurine up-	ncRNA
lib-C666-l	chr11	65189688	65189688	+	87.11781	NA	promoter-TSS (NR_028272)	promoter-TSS (NR_028272)	-581	NR_13101	283131	Hs.523789	NR_00280	ENSG0000	NEAT1	LINC00084	nuclear pa	ncRNA
lib-C666-l	chr11	65190159	65190159	+	6.85244	NA	promoter-TSS (NR_028272)	promoter-TSS (NR_028272)	-110	NR_13101	283131	Hs.523789	NR_00280	ENSG0000	NEAT1	LINC00084	nuclear pa	ncRNA
lib-C666-l	chr7	86974847	86974847	+	13.33918	NA	promoter-TSS (NM_001243745)	promoter-TSS (NM_001243745)	-39	NR_01538	11257	Hs.274329	NM_00723	ENSG0000	TP53TG1	LINC00096	TP53 targe	ncRNA
lib-C666-l	chr15	69384872	69384872	+	40.99806	NA	intron (NR_026949, intron 3 of 3)	L1ME3A|LINE|L1	11682	NR_02694	283673	Hs.177927	NM_17361	ENSG0000	EWSAT1	LINC00277	Ewing sarc	ncRNA
lib-C666-l	chr6	163835103	163835103	+	3.57421	NA	promoter-TSS (NM_006775)	promoter-TSS (NM_006775)	-121	NR_03759	100526820	Hs.644159	NR_03759	ENSG0000	CAHM	LINC00468	colon ader	ncRNA
lib-C666-l	chr20	48655293	48655293	+	17.71746	NA	Intergenic	Intergenic	2873	NR_05197	100887755	Hs.195705	NR_051976		TRERNA1	LINC00651	translation	ncRNA
lib-C666-l	chr20	34638680	34638680	+	5.23609	NA	non-coding (NR_027451, exon 1 of 1	non-coding (NR_027451, exon 1 of	202	NR_02745	647979	Hs.743232	NR_02745	ENSG0000	NORAD	LINC00657	non-codin	ncRNA
lib-C666-l	chr1	143743644	143743644	+	13.8811	NA	intron (NR_027468, intron 1 of 4)	CpG	875	NR_02746	388685	Hs.713362	NM_20740	ENSG0000	LINC01138	LINC00875	long interg	ncRNA
lib-C666-l	chr1	143744677	143744677	+	12.71281	NA	promoter-TSS (NR_104014)	promoter-TSS (NR_104014)	-158	NR_10401	388685	Hs.713362	NM_20740	ENSG0000	LINC01138	LINC00875	long interg	ncRNA
lib-C666-l	chr12	90103474	90103474	+	61.94978	NA	non-coding (NR_028138, exon 1 of 1	non-coding (NR_028138, exon 1 of	742	NR_02813	338758	Hs.601766	NR_02813	ENSG0000	ATP2B1-A	LINC00936	ATP2B1 an	ncRNA
lib-C666-l	chr12	90163141	90163141	+	12.62235	NA	Intergenic	ERVL-B4-int|LTR|ERVL	60409	NR_02813	338758	Hs.601766	NR_02813	ENSG0000	ATP2B1-A	LINC00936	ATP2B1 an	ncRNA
lib-C666-l	chr12	90102873	90102873	+	2.94047	NA	non-coding (NR_028138, exon 1 of 1	non-coding (NR_028138, exon 1 of	141	NR_02813	338758	Hs.601766	NR_02813	ENSG0000	ATP2B1-A	LINC00936	ATP2B1 an	ncRNA
lib-C666-l	chr15	40615683	40615683	+	33.04877	NA	promoter-TSS (NM_001301268)	promoter-TSS (NM_001301268)	-574	NM_00130	100505573	Hs.530791	NM_001301268		INAFM2	LINC00984	InaF motif	protein-co
lib-C666-l	chr19	52207671	52207671	+	7.21092	NA	exon (NM_001316972, exon 8 of 9)	exon (NM_001316972, exon 8 of 9)	-10962	NR_10810	102238594	Hs.640185	NR_10810	ENSG0000	SPACA6P-	LINC01129	SPACA6P a	ncRNA
lib-C666-l	chr19	52205720	52205720	+	4.30593	NA	intron (NR_024330, intron 2 of 7)	intron (NR_024330, intron 2 of 7)	-9011	NR_10810	102238594	Hs.640185	NR_10810	ENSG0000	SPACA6P-	LINC01129	SPACA6P a	ncRNA
lib-C666-l	chr8	129665719	129665719	+	3.27468	NA	Intergenic	L3|LINE|CR1	-88794	NR_12167	101927774	Hs.628730	NR_121672		LINC00824	LINC01263	long interg	ncRNA
lib-C666-l	chr13	50931954	50931954	+	7.43576	NA	intron (NR_109974, intron 2 of 6)	intron (NR_109974, intron 2 of 6)	169631	NR_12575	103689915	Hs.722230	NR_12575	ENSG0000	DLEU1-AS	LINC01308	DLEU1 ant	ncRNA
lib-C666-l	chr14	77371428	77371428	+	24.41787	NA	Intergenic	MIRb|SINE|MIR	-54553	NR_13391	105370578	Hs.574184	NR_13391	ENSG0000	LINC01629	linc-KIAA1	long interg	ncRNA
lib-C666-l	chr14	77428266	77428266	+	23.087	NA	intron (NR_133913, intron 1 of 2)	intron (NR_133913, intron 1 of 2)	2285	NR_13391	105370578	Hs.574184	NR_13391	ENSG0000	LINC01629	linc-KIAA1	long interg	ncRNA
lib-C666-l	chr14	77420119	77420119	+	4.98754	NA	Intergenic	Intergenic	-5862	NR_13391	105370578	Hs.574184	NR_13391	ENSG0000	LINC01629	linc-KIAA1	long interg	ncRNA
lib-C666-l	chr14	77422986	77422986	+	3.66964	NA	Intergenic	CpG	-2995	NR_13391	105370578	Hs.574184	NR_13391	ENSG0000	LINC01629	linc-KIAA1	long interg	ncRNA
lib-C666-l	chr2	97151905	97151905	+	12.34228	NA	Intergenic	Intergenic	19217	NM_00128	93082	Hs.149219	NM_00108	ENSG0000	NEURL3	LINCR	neuralized	protein-co
lib-C666-l	chr1	238415904	238415904	+	17.62684	NA	Intergenic	LTR55|LTR|ERV	233413	NR_01540	339535	Hs.532047	NR_01540	ENSG0000	LINC01139	LINKA	long interg	ncRNA
lib-C666-l	chr17	38336518	38336518	+	18.99577	NA	intron (NM_016339, intron 1 of 14)	intron (NM_016339, intron 1 of 14)	2276	NM_01633	51195	Hs.632254	NM_01633	ENSG0000	RAPGEFL1	Link-GEFII	Rap guanir	protein-co
lib-C666-l	chr11	70116735	70116735	+	25.33067	NA	promoter-TSS (NM_177423)	promoter-TSS (NM_177423)	-71	NM_17742	8500	Hs.530749	NM_00362	ENSG0000	PPFIA1	LIP.1|LIP1|L	PTPRF inte	protein-co
lib-C666-l	chr2	220462655	220462655	+	11.90035	NA	promoter-TSS (NM_052902)	promoter-TSS (NM_052902)	82	NM_05290	114790	Hs.22410	NM_05290	ENSG0000	STK11IP	LIP1|LKB1I	serine/thre	protein-co
lib-C666-l	chr17	7835482	7835482	+	41.28836	NA	promoter-TSS (NR_030697)	promoter-TSS (NR_030697)	40	NM_00133	116840	Hs.348012	NM_05305	ENSG0000	CNTROB	LIP8|PP122	centrobin,	protein-co
lib-C666-l	chr11	62009690	62009690	+	16.5633	NA	promoter-TSS (NM_006551)	promoter-TSS (NM_006551)	-34	NM_00655	10647	Hs.204096	NM_00655	ENSG0000	SCGB1D2	LIPB|LPHB|	secretoglo	protein-co
lib-C666-l	chr2	99771365	99771365	+	11.867	NA	promoter-TSS (NM_025244)	promoter-TSS (NM_025244)	-53	NM_14519	51601	Hs.719172	NM_01592	ENSG0000	LIPT1	LIPT1D	lipoyltransf	protein-co
lib-C666-l	chr17	2496909	2496909	+	19.19377	NA	promoter-TSS (NM_000430)	promoter-TSS (NM_000430)	-14	NM_00043	5048	Hs.77318	NM_00043	ENSG0000	PAFAH1B1	LIS1|LIS2|M	platelet act	protein-co
lib-C666-l	chr18	33709357	33709357	+	12.62235	NA	promoter-TSS (NM_012319)	promoter-TSS (NM_012319)	0	NM_00109	25800	Hs.79136	NM_01231	ENSG0000	SLC39A6	LIV-1|ZIP6	solute carr	protein-co
lib-C666-l	chr22	47134488	47134488	+	14.52676	NA	promoter-TSS (NM_022766)	promoter-TSS (NM_022766)	-336	NM_02276	64781	Hs.200668	NM_02276	ENSG0000	CERK	LK4|dA59H	ceramide k	protein-co
lib-C666-l	chr19	1205682	1205682	+	7.15277	NA	promoter-TSS (NM_000455)	promoter-TSS (NM_000455)	-116	NM_00045	6794	Hs.515005	NM_00045	ENSG0000	STK11	LKB1|PJS|h	serine/thre	protein-co
lib-C666-l	chr7	121784419	121784419	+	124.47887	NA	promoter-TSS (NM_005763)	promoter-TSS (NM_005763)	-75	NM_00576	10157	Hs.156738	NM_00576	ENSG0000	AASS	LKR/SDH|L	aminoadip	protein-co
lib-C666-l	chrX	102024082	102024082	+	59.96872	NA	promoter-TSS (NR_038988)	promoter-TSS (NR_038988)	-13	NR_03898	100287765	Hs.687084	NR_03898	ENSG0000	LINC00630	LL0XNC01-	long interg	ncRNA
lib-C666-l	chr3	111578362	111578362	+	4.71278	NA	promoter-TSS (NM_001134439)	promoter-TSS (NM_001134439)	-206	NM_00113	90102	Hs.603252	NM_14575	ENSG0000	PHLDB2	LL5b|LL5be	pleckstrin h	protein-co
lib-C666-l	chr5	54830768	54830768	+	10.16129	NA	promoter-TSS (NR_001575)	promoter-TSS (NR_001575)	138	NM_17689	8611	Hs.696231	NM_00371	ENSG0000	PLPP1	LLP1a|LPP1	phospholip	protein-co
lib-C666-l	chrX	49047720	49047720	+	11.93581	NA	intron (NM_003179, intron 6 of 6)	intron (NM_003179, intron 6 of 6)	-4867	NM_00615	4007	Hs.732712	NM_00615	ENSG0000	PRICKLE3	LMO6	prickle plar	protein-co
lib-C666-l	chr14	23504386	23504386	+	23.242	NA	promoter-TSS (NM_001144932)	promoter-TSS (NM_001144932)	-32	NM_00114	5693	Hs.422990	NM_00279	ENSG0000	PSMB5	LMPX|MB1	proteasom	protein-co
lib-C666-l	chr19	48996992	48996992	+	6.56784	NA	exon (NM_001080434, exon 13 of 16)	exon (NM_001080434, exon 13 of 1	19454	NM_00108	114783	Hs.207426	NM_00108	ENSG0000	LMTK3	LMR3|PPP1	lemur tyros	protein-co
lib-C666-l	chr1	40368009	40368009	+	117.95905	NA	promoter-TSS (NM_001033081)	promoter-TSS (NM_001033081)	-322	NM_00103	4610	Hs.437922	NM_00537	ENSG0000	MYCL	L-Myc|LMY	v-myc avia	protein-co
lib-C666-l	chr10	13344130	13344130	+	50.38662	NA	Intergenic	Intergenic	-2000	NM_00621	5264	Hs.498732	NM_00621	ENSG0000	PHYH	LN1|LNAP1	phytanoyl-	protein-co
lib-C666-l	chr11	27493753	27493753	+	23.22307	NA	exon (NM_001346432, exon 1 of 17)	exon (NM_001346432, exon 1 of 17	475	NR_13116	105376671	Hs.568999	NR_13116	ENSG0000	LOC10537	lnc-BBOX1	uncharacte	ncRNA
lib-C666-l	chr11	27451559	27451559	+	6.82479	NA	intron (NM_018490, intron 1 of 17)	intron (NM_018490, intron 1 of 17)	-41719	NR_13117	105376671	Hs.568999	NR_13116	ENSG0000	LOC10537	lnc-BBOX1	uncharacte	ncRNA
lib-C666-l	chr7	148684533	148684533	+	34.59844	NA	promoter-TSS (NR_130107)	promoter-TSS (NR_130107)	-86	NR_13010	102723099	Hs.621982	NR_13010	ENSG0000	GHET1	lncRNA-GH	gastric carc	ncRNA
lib-C666-l	chr12	74902867	74902867	+	77.06716	NA	Intergenic	L1PA3|LINE|L1	-28684	NM_00113	552889	Hs.744849	NM_00113	ENSG0000	ATXN7L3B	lnc-SCA7	ataxin 7 lik	protein-co
lib-C666-l	chr19	50379951	50379951	+	40.86121	NA	promoter-TSS (NM_024682)	promoter-TSS (NM_024682)	-445	NM_00127	84335	Hs.515542	NM_03237	ENSG0000	AKT1S1	Lobe|PRAS	AKT1 subst	protein-co
lib-C666-l	chr7	99933664	99933664	+	42.12788	NA	promoter-TSS (NR_036569)	promoter-TSS (NR_036569)	-24	NR_03657	101752399	Hs.521075	NR_03656	ENSG0000	STAG3L5P-	LOC10173	STAG3L5P-	ncRNA
lib-C666-l	chr2	241525895	241525895	+	11.93581	NA	promoter-TSS (NR_023085)	promoter-TSS (NR_023085)	-162	NR_10379	101752400	Hs.585987	NR_10379	ENSG0000	CAPN10-A	locus959	CAPN10 ar	ncRNA
lib-C666-l	chr5	171615571	171615571	+	2.24066	NA	promoter-TSS (NM_005990)	promoter-TSS (NM_005990)	-225	NM_00599	6793	Hs.744005	NM_00599	ENSG0000	STK10	LOK|PRO2	serine/thre	protein-co
lib-C666-l	chr16	48278188	48278188	+	33.11264	NA	5' UTR (NM_001300948, exon 1 of 14	5' UTR (NM_001300948, exon 1 of 1	110	NM_03149	83752	Hs.295923	NM_03149	ENSG0000	LONP2	LONP|LON	lon peptida	protein-co
lib-C666-l	chr12	53894701	53894701	+	45.01834	NA	promoter-TSS (NM_134324)	promoter-TSS (NM_134324)	-4	NM_00417	6895	Hs.326	NM_00417	ENSG0000	TARBP2	LOQS|TRBI	TARBP2, RI	protein-co
lib-C666-l	chr10	105726973	105726973	+	2.59032	NA	promoter-TSS (NM_014720)	promoter-TSS (NM_014720)	30	NM_01472	9748	Hs.591922	NM_01472	ENSG0000	SLK	LOSK|STK2	STE20 like	protein-co
lib-C666-l	chr6	144384960	144384960	+	7.76438	NA	intron (NM_001289043, intron 1 of 6)	CpG	776	NM_00128	5325	Hs.743225	NM_00265	ENSG0000	PLAGL1	LOT1|ZAC|	PLAG1 like	protein-co
lib-C666-l	chr4	25162034	25162034	+	4.52748	NA	promoter-TSS (NR_037934)	promoter-TSS (NR_037934)	170	NM_01695	51091	Hs.253305	NM_01695	ENSG0000	SEPSECS	LP|PCH2D|	Sep (O-ph	protein-co
lib-C666-l	chr9	136890592	136890592	+	18.90251	NA	promoter-TSS (NR_015427)	promoter-TSS (NR_015427)	31	NR_01542	266655	Hs.654869	NR_00280	ENSG0000	LINC00094	LP2477|NC	long interg	ncRNA
lib-C666-l	chr22	50628912	50628912	+	5.5166	NA	promoter-TSS (NM_001320488)	promoter-TSS (NM_001320488)	-67	NM_00132	80305	Hs.517731	NM_02520	ENSG0000	TRABD	LP6054|PP	TraB doma	protein-co
lib-C666-l	chr16	15068721	15068721	+	18.44668	NA	promoter-TSS (NM_001285445)	promoter-TSS (NM_001285445)	61	NM_00128	23042	Hs.370781	NM_01502	ENSG0000	PDXDC1	LP8165	pyridoxal c	protein-co
lib-C666-l	chr21	45377447	45377447	+	15.13226	NA	intron (NM_001037553, intron 1 of 8)	intron (NM_001037553, intron 1 of	32168	NM_00103	56894	Hs.248785	NM_02013	ENSG0000	AGPAT3	LPAAT-GA	1-acylglyce	protein-co
lib-C666-l	chr21	45285568	45285568	+	5.96422	NA	intron (NM_020132, intron 1 of 9)	GC_rich|Low_complexity|Low_comp	452	NM_02013	56894	Hs.248785	NM_02013	ENSG0000	AGPAT3	LPAAT-GA	1-acylglyce	protein-co
lib-C666-l	chr11	117103232	117103232	+	8.0787	NA	promoter-TSS (NM_207343)	promoter-TSS (NM_207343)	9	NM_00471	9159	Hs.648612	NM_00471	ENSG0000	PCSK7	LPC|PC7|P(	proprotein	protein-co
lib-C666-l	chr10	79609117	79609117	+	8.73012	NA	intron (NM_004747, intron 5 of 31)	intron (NM_004747, intron 5 of 31)	77231	NM_00474	9231	Hs.652690	NM_00474	ENSG0000	DLG5	LP-DLG|P-	discs large	protein-co
lib-C666-l	chr6	20212632	20212632	+	2.12162	NA	promoter-TSS (NM_001080480)	promoter-TSS (NM_001080480)	63	NM_00108	154141	Hs.377830	NM_00108	ENSG0000	MBOAT1	LPEAT1|LP	membrane	protein-co
lib-C666-l	chr19	290632	290632	+	35.17083	NA	intron (NM_177526, intron 1 of 5)	CpG	537	NM_17754	8612	Hs.465506	NM_00371	ENSG0000	PLPP2	LPP2|PAP-	phospholip	protein-co
lib-C666-l	chr19	290322	290322	+	15.56873	NA	intron (NM_177526, intron 1 of 5)	MIR|SINE|MIR	847	NM_17754	8612	Hs.465506	NM_00371	ENSG0000	PLPP2	LPP2|PAP-	phospholip	protein-co
lib-C666-l	chr19	291240	291240	+	4.28252	NA	promoter-TSS (NM_177543)	promoter-TSS (NM_177543)	-71	NM_17754	8612	Hs.465506	NM_00371	ENSG0000	PLPP2	LPP2|PAP-	phospholip	protein-co
lib-C666-l	chr1	209979525	209979525	+	18.98417	NA	promoter-TSS (NM_006147)	promoter-TSS (NM_006147)	-5	NM_00120	3664	Hs.591415	NM_00614	ENSG0000	IRF6	LPS|OFC6|	interferon	protein-co
lib-C666-l	chr8	10697420	10697420	+	224.07634	NA	promoter-TSS (NM_001284356)	promoter-TSS (NM_001284356)	-11	NM_00128	54984	Hs.490991	NM_01788	ENSG0000	PINX1	LPTL|LPTS	PIN2/TERF	protein-co
lib-C666-l	chr11	805303	805303	+	5.819	NA	promoter-TSS (NM_145886)	promoter-TSS (NM_145886)	-53	NM_14588	55367	Hs.592290	NM_01849	ENSG0000	PIDD1	LRDD|PIDD	p53-induc	protein-co
lib-C666-l	chr7	100182864	100182864	+	25.49921	NA	intron (NM_002319, intron 1 of 17)	intron (NM_002319, intron 1 of 17)	947	NM_00231	4034	Hs.125742	NM_00231	ENSG0000	LRCH4	LRN|LRRN	leucine rich	protein-co
lib-C666-l	chr2	68290148	68290148	+	434.28476	NA	promoter-TSS (NM_006333)	promoter-TSS (NM_006333)	11	NM_17317	10438	Hs.602900	NM_00633	ENSG0000	C1D	LRP1|Rrp47	C1D nuclea	protein-co
lib-C666-l	chr14	23340530	23340530	+	28.8837	NA	promoter-TSS (NM_014045)	promoter-TSS (NM_014045)	-396	NM_00132	26020	Hs.525232	NM_01404	ENSG0000	LRP10	LRP-10|LRI	LDL recept	protein-co
lib-C666-l	chr11	63933938	63933938	+	3.89624	NA	promoter-TSS (NM_014067)	promoter-TSS (NM_014067)	-353	NM_01406	28992	Hs.602898	NM_01406	ENSG0000	MACROD1	LRP16	MACRO dc	protein-co
lib-C666-l	chr4	186317151	186317151	+	134.63931	NA	promoter-TSS (NM_181726)	promoter-TSS (NM_181726)	-689	NM_18172	353322	Hs.508154	NM_18172	ENSG0000	ANKRD37	Lrp2bp	ankyrin rep	protein-co
lib-C666-l	chr12	7013900	7013900	+	21.0836	NA	promoter-TSS (NM_001135217)	promoter-TSS (NM_001135217)	3	NM_20165	10233	Hs.155586	NM_00699	ENSG0000	LRRC23	LRPB7	leucine rich	protein-co
lib-C666-l	chr8	145743332	145743332	+	113.46414	NA	promoter-TSS (NM_004260)	promoter-TSS (NM_004260)	-17	NM_00127	9684	Hs.459391	NM_01466	ENSG0000	LRRC14	LRRC14A	leucine rich	protein-co
lib-C666-l	chr8	145747839	145747839	+	103.28344	NA	TTS (NM_001024678)	TTS (NM_001024678)	4490	NM_01466	9684	Hs.459391	NM_01466	ENSG0000	LRRC14	LRRC14A	leucine rich	protein-co
lib-C666-l	chr8	145752445	145752445	+	12.69461	NA	promoter-TSS (NM_001024678)	promoter-TSS (NM_001024678)	-29	NM_00102	441381	Hs.740859	NM_00102	ENSG0000	LRRC24	LRRC14OS	leucine rich	protein-co
lib-C666-l	chr17	62915667	62915667	+	18.43022	NA	promoter-TSS (NM_001303255)	promoter-TSS (NM_001303255)	-69	NM_00130	374819	Hs.551962	NM_19934	ENSG0000	LRRC37A3	LRRC37|LR	leucine rich	protein-co
lib-C666-l	chr22	37825262	37825262	+	5.25516	NA	Intergenic	Intergenic	-1757	NR_11051	114794	Hs.344170	NM_05290	ENSG0000	ELFN2	LRRC62|PP	extracellula	protein-co
lib-C666-l	chr8	8729913	8729913	+	16.23236	NA	intron (NM_004225, intron 1 of 2)	intron (NM_004225, intron 1 of 2)	21218	NM_00422	9258	Hs.379414	NM_00422	ENSG0000	MFHAS1	LRRC65|M	malignant	protein-co
lib-C666-l	chr8	8663815	8663815	+	7.9428	NA	intron (NM_004225, intron 1 of 2)	intron (NM_004225, intron 1 of 2)	87316	NM_00422	9258	Hs.379414	NM_00422	ENSG0000	MFHAS1	LRRC65|M	malignant	protein-co
lib-C666-l	chr19	45596286	45596286	+	9.4812	NA	promoter-TSS (NM_019121)	promoter-TSS (NM_019121)	-145	NM_01912	284352	Hs.285363	NM_01912	ENSG0000	PPP1R37	LRRC68	protein ph	protein-co
lib-C666-l	chr21	45759306	45759306	+	157.39911	NA	promoter-TSS (NM_001271440)	promoter-TSS (NM_001271440)	-21	NM_00127	755	Hs.517331	NM_00492	ENSG0000	C21orf2	LRRC76|YF	chromosor	protein-co
lib-C666-l	chr9	130981082	130981082	+	6.82479	NA	intron (NM_001005336, intron 3 of 22	CpG	-14420	NM_00113	25792	Hs.212395	NM_01212	ENSG0000	CIZ1	LSFR1|NP9	CDKN1A ir	protein-co
lib-C666-l	chr9	131843203	131843203	+	8.28613	NA	promoter-TSS (NM_020438)	promoter-TSS (NM_020438)	-180	NR_07298	57171	Hs.21701	NM_02043	ENSG0000	DOLPP1	LSFR2	dolichyldip	protein-co
lib-C666-l	chr15	74988368	74988368	+	53.07762	NA	promoter-TSS (NM_001142443)	promoter-TSS (NM_001142443)	18	NM_02508	80153	Hs.96852	NM_02508	ENSG0000	EDC3	LSM16|MR	enhancer c	protein-co
lib-C666-l	chr2	234881792	234881792	+	4.71278	NA	intron (NM_024080, intron 17 of 25)	intron (NM_024080, intron 17 of 25	55749	NM_02408	79054	Hs.366053	NM_02408	ENSG0000	TRPM8	LTRPC6|TR	transient re	protein-co
lib-C666-l	chr16	279495	279495	+	11.62669	NA	promoter-TSS (NM_201412)	promoter-TSS (NM_201412)	-14	NM_00133	55692	Hs.16803	NM_01803	ENSG0000	LUC7L	LUC7B1|Lu	LUC7 like	protein-co
lib-C666-l	chr3	50362147	50362147	+	30.8056	NA	TTS (NM_007275)	TTS (NM_007275)	-1866	NM_03315	8692	Hs.76873	NM_00377	ENSG0000	HYAL2	LUCA2	hyalurono	protein-co
lib-C666-l	chr3	50358783	50358783	+	6.56784	NA	intron (NM_003773, intron 1 of 3)	CpG	1498	NM_00377	8692	Hs.76873	NM_00377	ENSG0000	HYAL2	LUCA2	hyalurono	protein-co
lib-C666-l	chr9	35727143	35727143	+	11.66971	NA	intron (NM_006289, intron 1 of 56)	intron (NM_006289, intron 1 of 56)	-5174	NM_00636	10488	Hs.522110	NM_00636	ENSG0000	CREB3	LUMAN|LZ	cAMP resp	protein-co
lib-C666-l	chr9	32552549	32552549	+	5.05119	NA	promoter-TSS (NM_001195622)	promoter-TSS (NM_001195622)	77	NM_00119	10210	Hs.589962	NM_00580	ENSG0000	TOPORS	LUN|P53BF	TOP1 bind	protein-co
lib-C666-l	chr19	6737745	6737745	+	126.45357	NA	promoter-TSS (NR_106849)	promoter-TSS (NR_106849)	-112	NM_00108	56927	Hs.167641	NM_02017	ENSG0000	GPR108	LUSTR2	G protein-	protein-co
lib-C666-l	chr1	23495362	23495362	+	20.7902	NA	promoter-TSS (NM_001142546)	promoter-TSS (NM_001142546)	-11	NM_03363	7798	Hs.257900	NM_03363	ENSG0000	LUZP1	LUZP	leucine zip	protein-co
lib-C666-l	chr11	47279070	47279070	+	4.74112	NA	promoter-TSS (NM_001130101)	promoter-TSS (NM_001130101)	-398	NM_00113	10062	Hs.438863	NM_00569	ENSG0000	NR1H3	LXR-a|LXR	nuclear rec	protein-co
lib-C666-l	chr19	50879747	50879747	+	43.12596	NA	promoter-TSS (NM_001256647)	promoter-TSS (NM_001256647)	67	NM_00125	7376	Hs.432976	NM_00712	ENSG0000	NR1H2	LXR-b|LXR	nuclear rec	protein-co
lib-C666-l	chr6	6811681	6811681	+	14.59636	NA	Intergenic	Intergenic	-188622	NR_02697	285780	Hs.655737	NM_17367	ENSG0000	LY86-AS1	LY86-AS|L	LY86 antise	ncRNA
lib-C666-l	chr6	6820952	6820952	+	5.25516	NA	Intergenic	Intergenic	-197893	NR_02697	285780	Hs.655737	NM_17367	ENSG0000	LY86-AS1	LY86-AS|L	LY86 antise	ncRNA
lib-C666-l	chr17	78075297	78075297	+	27.61921	NA	promoter-TSS (NR_134848)	promoter-TSS (NR_134848)	-28	NM_00107	2548	Hs.1437	NM_00015	ENSG0000	GAA	LYAG	glucosidas	protein-co
lib-C666-l	chr17	61819061	61819061	+	10.38624	NA	intron (NM_153335, intron 1 of 8)	CpG	269	NM_15333	92335	Hs.514402	NM_15333	ENSG0000	STRADA	LYK5|NY-B	STE20-rela	protein-co
lib-C666-l	chr17	61820142	61820142	+	3.07144	NA	promoter-TSS (NM_001165969)	promoter-TSS (NM_001165969)	-812	NM_00100	92335	Hs.514402	NM_15333	ENSG0000	STRADA	LYK5|NY-B	STE20-rela	protein-co
lib-C666-l	chr2	133372842	133372842	+	11.66012	NA	intron (NM_001508, intron 1 of 1)	intron (NM_001508, intron 1 of 1)	55101	NM_00132	116372	Hs.432395	NM_14458	ENSG0000	LYPD1	LYPDC1|PH	LY6/PLAUF	protein-co
lib-C666-l	chr19	36486011	36486011	+	15.31489	NA	promoter-TSS (NM_001042631)	promoter-TSS (NM_001042631)	-79	NM_00104	644096	Hs.356460	NM_00104	ENSG0000	SDHAF1	LYRM8	succinate c	protein-co
lib-C666-l	chr3	185000695	185000695	+	209.67239	NA	promoter-TSS (NM_001242314)	promoter-TSS (NM_001242314)	-34	NM_00124	9175	Hs.591306	NM_00472	ENSG0000	MAP3K13	LZK|MEKK1	mitogen-a	protein-co
lib-C666-l	chr3	185080864	185080864	+	12.62235	NA	promoter-TSS (NM_004721)	promoter-TSS (NM_004721)	28	NM_00472	9175	Hs.591306	NM_00472	ENSG0000	MAP3K13	LZK|MEKK1	mitogen-a	protein-co
lib-C666-l	chr11	47429997	47429997	+	56.19669	NA	promoter-TSS (NM_152264)	promoter-TSS (NM_152264)	-49	NM_15226	91252	Hs.523664	NM_15226	ENSG0000	SLC39A13	LZT-Hs9	solute carr	protein-co
lib-C666-l	chr5	177541274	177541274	+	8.01457	NA	intron (NM_015111, intron 1 of 4)	intron (NM_015111, intron 1 of 4)	718	NM_01511	23138	Hs.101761	NM_01511	ENSG0000	N4BP3	LZTS4	NEDD4 bir	protein-co
lib-C666-l	chr12	80328454	80328454	+	12.7379	NA	intron (NM_001244990, intron 2 of 24	CpG	277	NM_00124	4659	Hs.49582	NM_00248	ENSG0000	PPP1R12A	M130|MBS	protein ph	protein-co
lib-C666-l	chr19	4861297	4861297	+	81.48571	NA	intron (NM_001164189, intron 2 of 7)	intron (NM_001164189, intron 2 of	6483	NM_00581	10226	Hs.140452	NM_00581	ENSG0000	PLIN3	M6PRBP1|I	perilipin 3	protein-co
lib-C666-l	chr1	93544770	93544770	+	58.46021	NA	promoter-TSS (NM_001164392)	promoter-TSS (NM_001164392)	-22	NM_00116	22823	Hs.31016	NM_00735	ENSG0000	MTF2	M96|PCL2|	metal resp	protein-co
lib-C666-l	chr17	26646037	26646037	+	25.33067	NA	promoter-TSS (NM_014573)	promoter-TSS (NM_014573)	-84	NM_01457	27346	Hs.199695	NM_01457	ENSG0000	TMEM97	MAC30	transmemb	protein-co
lib-C666-l	chr17	26653227	26653227	+	7.0832	NA	intron (NM_014573, intron 2 of 2)	intron (NM_014573, intron 2 of 2)	7106	NM_01457	27346	Hs.199695	NM_01457	ENSG0000	TMEM97	MAC30	transmemb	protein-co
lib-C666-l	chr19	489036	489036	+	6.61453	NA	Intergenic	CpG	-7454	NM_13076	8174	Hs.102598	NM_13076	ENSG0000	MADCAM1	MACAM1	mucosal va	protein-co
lib-C666-l	chr6	130031452	130031452	+	5.96422	NA	promoter-TSS (NM_033515)	promoter-TSS (NM_033515)	-82	NM_03351	93663	Hs.486458	NM_03351	ENSG0000	ARHGAP18	MacGAP|SI	Rho GTPas	protein-co
lib-C666-l	chr1	25756751	25756751	+	26.37215	NA	promoter-TSS (NM_001282564)	promoter-TSS (NM_001282564)	-598	NM_00128	55219	Hs.189782	NM_01820	ENSG0000	TMEM57	MACOILIN	transmemb	protein-co
lib-C666-l	chr1	25799221	25799221	+	17.71746	NA	intron (NM_018202, intron 6 of 10)	intron (NM_018202, intron 6 of 10)	41872	NM_01820	55219	Hs.189782	NM_01820	ENSG0000	TMEM57	MACOILIN	transmemb	protein-co
lib-C666-l	chr1	25757269	25757269	+	7.52748	NA	promoter-TSS (NM_001282564)	promoter-TSS (NM_001282564)	-80	NM_00128	55219	Hs.189782	NM_01820	ENSG0000	TMEM57	MACOILIN	transmemb	protein-co
lib-C666-l	chr1	118472246	118472246	+	3.44441	NA	promoter-TSS (NM_001135589)	promoter-TSS (NM_001135589)	56	NM_01768	54834	Hs.310809	NM_01768	ENSG0000	GDAP2	MACROD3	ganglioside	protein-co
lib-C666-l	chr20	19673597	19673597	+	6.15313	NA	intron (NM_020689, intron 12 of 16)	intron (NM_020689, intron 12 of 16	-193568	NM_00124	54453	Hs.472270	NM_01899	ENSG0000	RIN2	MACS|RAS	Ras and Ra	protein-co
lib-C666-l	chr20	19738522	19738522	+	3.59106	NA	Intergenic	CpG	-128643	NM_00124	54453	Hs.472270	NM_01899	ENSG0000	RIN2	MACS|RAS	Ras and Ra	protein-co
lib-C666-l	chr10	111971068	111971068	+	126.06001	NA	intron (NM_001008541, intron 1 of 4)	intron (NM_001008541, intron 1 of	1079	NM_00100	4601	Hs.602078	NM_00596	ENSG0000	MXI1	MAD2|MXI	MAX intera	protein-co
lib-C666-l	chr10	111986003	111986003	+	6.49644	NA	exon (NM_005962, exon 1 of 6)	exon (NM_005962, exon 1 of 6)	241	NM_00596	4601	Hs.602078	NM_00596	ENSG0000	MXI1	MAD2|MXI	MAX intera	protein-co
lib-C666-l	chr4	2264291	2264291	+	9.45164	NA	promoter-TSS (NM_006454)	promoter-TSS (NM_006454)	-552	NM_00645	10608	Hs.655020	NM_00645	ENSG0000	MXD4	MAD4|MST	MAX dime	protein-co
lib-C666-l	chr17	2304988	2304988	+	78.90324	NA	promoter-TSS (NM_020310)	promoter-TSS (NM_020310)	-730	NM_02031	4335	Hs.626579	NM_02031	ENSG0000	MNT	MAD6|MXI	MAX netw	protein-co
lib-C666-l	chr17	2303079	2303079	+	37.41784	NA	intron (NM_020310, intron 1 of 5)	CpG	1179	NM_02031	4335	Hs.626579	NM_02031	ENSG0000	MNT	MAD6|MXI	MAX netw	protein-co
lib-C666-l	chr17	2300205	2300205	+	25.49875	NA	intron (NM_020310, intron 1 of 5)	intron (NM_020310, intron 1 of 5)	4053	NM_02031	4335	Hs.626579	NM_02031	ENSG0000	MNT	MAD6|MXI	MAX netw	protein-co
lib-C666-l	chr17	2297121	2297121	+	9.10494	NA	intron (NM_020310, intron 4 of 5)	CpG	7137	NM_02031	4335	Hs.626579	NM_02031	ENSG0000	MNT	MAD6|MXI	MAX netw	protein-co
lib-C666-l	chr17	2304456	2304456	+	6.29226	NA	promoter-TSS (NM_020310)	promoter-TSS (NM_020310)	-198	NM_02031	4335	Hs.626579	NM_02031	ENSG0000	MNT	MAD6|MXI	MAX netw	protein-co
lib-C666-l	chr17	40719095	40719095	+	170.59778	NA	promoter-TSS (NM_198204)	promoter-TSS (NM_198204)	17	NM_19820	6945	Hs.383019	NM_17060	ENSG0000	MLX	MAD7|MXI	MLX, MAX	protein-co
lib-C666-l	chr12	57480851	57480851	+	6.82479	NA	Intergenic	CpG	-1826	NM_00133	4665	Hs.159223	NM_00596	ENSG0000	NAB2	MADER	NGFI-A bir	protein-co
lib-C666-l	chr21	9964548	9964548	+	8.53649	NA	intron (NR_038329, intron 2 of 2)	MER57A1|LTR|ERV1	4046	NR_03832	100132288	Hs.487562	NM_17494	ENSG0000	TEKT4P2	MAFIPL|TE	tektin 4 ps	pseudo
lib-C666-l	chr6	154927841	154927841	+	7.76438	NA	Intergenic	Intergenic	-96088	NM_17351	154043	Hs.16064	NM_17351	ENSG0000	CNKSR3	MAGI1	CNKSR fan	protein-co
lib-C666-l	chr1	113932992	113932992	+	3.25502	NA	promoter-TSS (NM_001142782)	promoter-TSS (NM_001142782)	-483	NM_15290	260425	Hs.486189	NM_02096	ENSG0000	MAGI3	MAGI-3|dJ	membrane	protein-co
lib-C666-l	chr1	53704199	53704199	+	294.18082	NA	promoter-TSS (NM_002370)	promoter-TSS (NM_002370)	83	NM_00237	4116	Hs.421576	NM_00237	ENSG0000	MAGOH	MAGOH1|I	mago hom	protein-co
lib-C666-l	chr17	27503951	27503951	+	26.46433	NA	promoter-TSS (NM_001346767)	promoter-TSS (NM_001346767)	-181	NM_00134	399687	Hs.462590	NM_07847	ENSG0000	MYO18A	MAJN|MYS	myosin XV	protein-co
lib-C666-l	chr8	33342530	33342530	+	4.12121	NA	promoter-TSS (NM_032509)	promoter-TSS (NM_032509)	-155	NM_03250	84549	Hs.583805	NM_03250	ENSG0000	MAK16	MAK16L|RI	MAK16 ho	protein-co
lib-C666-l	chr5	139487999	139487999	+	33.21417	NA	promoter-TSS (NR_102741)	promoter-TSS (NR_102741)	-402	NR_10273	100505636	Hs.438871	NR_10273	ENSG0000	LINC01024	MA-linc1	long interg	ncRNA
lib-C666-l	chr5	139487343	139487343	+	25.39084	NA	non-coding (NR_102741, exon 1 of 4	non-coding (NR_102741, exon 1 of	254	NR_10274	100505636	Hs.438871	NR_10273	ENSG0000	LINC01024	MA-linc1	long interg	ncRNA
lib-C666-l	chr5	179159730	179159730	+	21.41395	NA	promoter-TSS (NM_014757)	promoter-TSS (NM_014757)	-121	NM_01475	9794	Hs.631951	NM_01475	ENSG0000	MAML1	Mam-1|Ma	mastermin	protein-co
lib-C666-l	chr12	65563295	65563295	+	41.21722	NA	promoter-TSS (NM_001167614)	promoter-TSS (NM_001167614)	-56	NM_00116	23592	Hs.744150	NM_01431	ENSG0000	LEMD3	MAN1	LEM doma	protein-co
lib-C666-l	chr1	117909779	117909779	+	2.62544	NA	promoter-TSS (NM_006699)	promoter-TSS (NM_006699)	-306	NM_00669	10905	Hs.435938	NM_00669	ENSG0000	MAN1A2	MAN1B	mannosida	protein-co
lib-C666-l	chr15	75660458	75660458	+	5.23609	NA	exon (NM_001256495, exon 2 of 26)	exon (NM_001256495, exon 2 of 26	510	NM_00125	4123	Hs.26232	NM_00671	ENSG0000	MAN2C1	MAN6A8|M	mannosida	protein-co
lib-C666-l	chr4	103658495	103658495	+	111.87783	NA	intron (NM_005908, intron 1 of 16)	L1PB1|LINE|L1	23656	NM_00590	4126	Hs.480415	NM_00590	ENSG0000	MANBA	MANB1	mannosida	protein-co
lib-C666-l	chr2	131850970	131850970	+	5.12426	NA	promoter-TSS (NM_001321748)	promoter-TSS (NM_001321748)	63	NM_00132	130074	Hs.534679	NM_00100	ENSG0000	FAM168B	MANI	family with	protein-co
lib-C666-l	chr14	93651209	93651209	+	15.60931	NA	promoter-TSS (NM_022151)	promoter-TSS (NM_022151)	40	NM_02215	64112	Hs.24719	NM_02215	ENSG0000	MOAP1	MAP-1|PN	modulator	protein-co
lib-C666-l	chr16	29984936	29984936	+	3.96379	NA	promoter-TSS (NM_004783)	promoter-TSS (NM_004783)	-252	NM_00478	9344	Hs.291623	NM_00478	ENSG0000	TAOK2	MAP3K17|I	TAO kinase	protein-co
lib-C666-l	chrX	30907628	30907628	+	3.92495	NA	promoter-TSS (NM_152787)	promoter-TSS (NM_152787)	-117	NM_15278	257397	Hs.188256	NM_15278	ENSG0000	TAB3	MAP3K7IP	TGF-beta a	protein-co
lib-C666-l	chr6	36098075	36098075	+	2.99475	NA	promoter-TSS (NM_002754)	promoter-TSS (NM_002754)	-186	NM_00275	5603	Hs.178695	NM_00275	ENSG0000	MAPK13	MAPK 13|N	mitogen-a	protein-co
lib-C666-l	chr12	112279822	112279822	+	123.83248	NA	promoter-TSS (NM_003668)	promoter-TSS (NM_003668)	-210	NM_00366	8550	Hs.333120	NM_00366	ENSG0000	MAPKAPK5	MAPKAP-K	mitogen-a	protein-co
lib-C666-l	chr17	21191354	21191354	+	18.43022	NA	promoter-TSS (NM_002756)	promoter-TSS (NM_002756)	6	NM_00275	5606	Hs.514012	NM_00275	ENSG0000	MAP2K3	MAPKK3|M	mitogen-a	protein-co
lib-C666-l	chr17	21220190	21220190	+	9.88517	NA	Intergenic	CpG	28842	NM_00275	5606	Hs.514012	NM_00275	ENSG0000	MAP2K3	MAPKK3|M	mitogen-a	protein-co
lib-C666-l	chr17	21183594	21183594	+	6.20473	NA	Intergenic	Intergenic	-4374	NM_14510	5606	Hs.514012	NM_00275	ENSG0000	MAP2K3	MAPKK3|M	mitogen-a	protein-co
lib-C666-l	chr17	67449214	67449214	+	8.0154	NA	intron (NM_002758, intron 1 of 11)	intron (NM_002758, intron 1 of 11)	38376	NM_00275	5608	Hs.49329	NM_00275	ENSG0000	MAP2K6	MAPKK6|M	mitogen-a	protein-co
lib-C666-l	chr5	56111701	56111701	+	2.59723	NA	exon (NM_005921, exon 1 of 20)	exon (NM_005921, exon 1 of 20)	801	NM_00592	4214	Hs.653654	NM_00592	ENSG0000	MAP3K1	MAPKKK1|	mitogen-a	protein-co

lib-C666-I	chr17	61699582	61699582	+	7.0832	NA	promoter-TSS (NM_203351)	promoter-TSS (NM_203351)	-203	NM_00133	4215	Hs.29282	NM_00240	ENSG0000	MAP3K3	MAPKKK3|	mitogen-a	protein-co
lib-C666-I	chr22	44894249	44894249	+	7.1502	NA	promoter-TSS (NM_032287)	promoter-TSS (NM_032287)	-244	NM_03228	84247	Hs.744991	NM_03228	ENSG0000	LDOC1L	Mar6|Mart	leucine zip	protein-co
lib-C666-I	chr5	126366401	126366401	+	7.6867	NA	promoter-TSS (NM_178450)	promoter-TSS (NM_178450)	99	NM_17845	115123	Hs.132441	NM_17845	ENSG0000	3-Mar	MARCH-III	membrane	protein-co
lib-C666-I	chr16	71645120	71645120	+	9.53658	NA	Intergenic	Intergenic	-14936	NM_05285	91862	Hs.513706	NM_05285	ENSG0000	MARVELD3	MARVD3|M	MARVEL d	protein-co
lib-C666-I	chr1	235292188	235292188	+	6.32351	NA	promoter-TSS (NR_002956)	promoter-TSS (NR_002956)	68	NM_01476	9804	Hs.533192	NM_01476	ENSG0000	TOMM20	MAS20|MO	translocase	protein-co
lib-C666-I	chr5	139781235	139781235	+	56.14594	NA	promoter-TSS (NM_001197030)	promoter-TSS (NM_001197030)	-164	NM_00119	54882	Hs.594084	NM_01774	ENSG0000	ANKHD1	MASK|MAS	ankyrin rep	protein-co
lib-C666-I	chr4	88423913	88423913	+	2.05265	NA	intron (NM_004684, intron 1 of 10)	HAL1|LINE|L1	26742	NM_00468	8404	Hs.62886	NM_00468	ENSG0000	SPARCL1	MAST 9|MA	SPARC like	protein-co
lib-C666-I	chr1	46269387	46269387	+	2.62544	NA	5' UTR (NM_001324320, exon 1 of 30	5' UTR (NM_001324320, exon 1 of 3	102	NM_01511	23139	Hs.319481	NM_01511	ENSG0000	MAST2	MAST205|M	microtubul	protein-co
lib-C666-I	chr19	49224088	49224088	+	46.29081	NA	exon (NM_017805, exon 12 of 12)	exon (NM_017805, exon 12 of 12)	-1112	NM_00113	284358	Hs.191815	NM_18257	ENSG0000	MAMSTR	MASTR	MEF2 activ	protein-co
lib-C666-I	chr19	49217600	49217600	+	6.01141	NA	intron (NM_001297753, intron 4 of 6)	CpG	2614	NM_18257	284358	Hs.191815	NM_18257	ENSG0000	MAMSTR	MASTR	MEF2 activ	protein-co
lib-C666-I	chr19	49229942	49229942	+	3.20885	NA	intron (NM_017805, intron 8 of 11)	intron (NM_017805, intron 8 of 11)	-6966	NM_00113	284358	Hs.191815	NM_18257	ENSG0000	MAMSTR	MASTR	MEF2 activ	protein-co
lib-C666-I	chr2	85766327	85766327	+	13.46097	NA	promoter-TSS (NR_038942)	promoter-TSS (NR_038942)	226	NM_00591	4144	Hs.516157	NM_00591	ENSG0000	MAT2A	MATA2|MA	methionine	protein-co
lib-C666-I	chr17	19411309	19411309	+	5.96687	NA	Intergenic	CpG	-25858	NM_01824	55244	Hs.232054	NM_01824	ENSG0000	SLC47A1	MATE1	solute carr	protein-co
lib-C666-I	chr12	16035298	16035298	+	84.45766	NA	promoter-TSS (NM_007178)	promoter-TSS (NM_007178)	10	NM_00717	11171	Hs.743971	NM_00717	ENSG0000	STRAP	MAWD|PT-	serine/thre	protein-co
lib-C666-I	chr16	68027373	68027373	+	6.82479	NA	intron (NM_022355, intron 1 of 10)	CpG	821	NM_00132	64174	Hs.372633	NM_02235	ENSG0000	DPEP2	MBD2	dipeptidas	protein-co
lib-C666-I	chr13	97973543	97973543	+	57.17088	NA	intron (NM_001306070, intron 2 of 7)	MamRep38|DNA|hAT	98993	NM_14477	10150	Hs.657347	NM_14477	ENSG0000	MBNL2	MBLL|MBL	muscleblin	protein-co
lib-C666-I	chr13	97910269	97910269	+	26.46807	NA	intron (NM_001306070, intron 1 of 7)	intron (NM_001306070, intron 1 of	35719	NM_14477	10150	Hs.657347	NM_14477	ENSG0000	MBNL2	MBLL|MBL	muscleblin	protein-co
lib-C666-I	chr13	97975239	97975239	+	13.59254	NA	intron (NM_001306070, intron 2 of 7)	MamRep38|DNA|hAT	100689	NM_14477	10150	Hs.657347	NM_14477	ENSG0000	MBNL2	MBLL|MBL	muscleblin	protein-co
lib-C666-I	chr13	97874538	97874538	+	10.16129	NA	promoter-TSS (NM_207304)	promoter-TSS (NM_207304)	-5	NM_00130	10150	Hs.657347	NM_14477	ENSG0000	MBNL2	MBLL|MBL	muscleblin	protein-co
lib-C666-I	chr13	97907350	97907350	+	9.20967	NA	intron (NM_001306070, intron 1 of 7)	intron (NM_001306070, intron 1 of	32800	NM_14477	10150	Hs.657347	NM_14477	ENSG0000	MBNL2	MBLL|MBL	muscleblin	protein-co
lib-C666-I	chr13	97960380	97960380	+	2.51791	NA	intron (NM_001306070, intron 2 of 7)	intron (NM_001306070, intron 2 of	85830	NM_14477	10150	Hs.657347	NM_14477	ENSG0000	MBNL2	MBLL|MBL	muscleblin	protein-co
lib-C666-I	chr10	105111010	105111010	+	8.73012	NA	promoter-TSS (NM_001011663)	promoter-TSS (NM_001011663)	-119	NM_03215	84108	Hs.335808	NM_03215	ENSG0000	PCGF6	MBLR|RNF	polycomb	protein-co
lib-C666-I	chr8	97247879	97247879	+	21.21068	NA	promoter-TSS (NM_001254752)	promoter-TSS (NM_001254752)	-17	NM_00629	7381	Hs.131255	NM_00629	ENSG0000	UQCRB	MC3DN3|C	ubiquinol-	protein-co
lib-C666-I	chr5	132202308	132202308	+	66.66461	NA	promoter-TSS (NM_001288827)	promoter-TSS (NM_001288827)	-11	NM_01440	27089	Hs.146602	NM_01440	ENSG0000	UQCRQ	MC3DN4|C	ubiquinol-	protein-co
lib-C666-I	chr16	21964572	21964572	+	3.59271	NA	5' UTR (NM_003366, exon 1 of 14)	5' UTR (NM_003366, exon 1 of 14)	187	NM_00336	7385	Hs.528803	NM_00336	ENSG0000	UQCRC2	MC3DN5|C	ubiquinol-	protein-co
lib-C666-I	chrX	103401526	103401526	+	52.45363	NA	intron (NM_001012755, intron 1 of 1)	CpG	182	NM_00101	401612	Hs.660022	NM_00101	ENSG0000	SLC25A53	MCART6	solute carr	protein-co
lib-C666-I	chr5	112482882	112482882	+	4.74112	NA	intron (NM_001085377, intron 4 of 1	intron (NM_001085377, intron 4 of	147730	NM_00238	4163	Hs.593171	NM_00238	ENSG0000	MCC	MCC1	mutated in	protein-co
lib-C666-I	chr5	70829640	70829640	+	11.57119	NA	intron (NM_018429, intron 26 of 38)	L1MB7|LINE|L1	-53475	NM_02213	64087	Hs.604789	NM_02213	ENSG0000	MCCC2	MCCB	methylcrot	protein-co
lib-C666-I	chr3	182817420	182817420	+	4.39666	NA	promoter-TSS (NM_001293273)	promoter-TSS (NM_001293273)	-45	NM_02016	56922	Hs.47649	NM_02016	ENSG0000	MCCC1	MCC-B|MC	methylcrot	protein-co
lib-C666-I	chr4	128802445	128802445	+	71.09715	NA	promoter-TSS (NM_001190801)	promoter-TSS (NM_001190801)	46	NM_00119	10733	Hs.172052	NM_01426	ENSG0000	PLK4	MCCRP2|S.	polo like ki	protein-co
lib-C666-I	chr4	128802134	128802134	+	29.74345	NA	promoter-TSS (NM_001190801)	promoter-TSS (NM_001190801)	118	NM_01426	10733	Hs.172052	NM_01426	ENSG0000	PLK4	MCCRP2|S.	polo like ki	protein-co
lib-C666-I	chr7	87505736	87505736	+	7.66979	NA	promoter-TSS (NM_018843)	promoter-TSS (NM_018843)	-44	NM_01884	55972	Hs.208414	NM_01884	ENSG0000	SLC25A40	MCFP	solute carr	protein-co
lib-C666-I	chr2	136634022	136634022	+	12.13776	NA	promoter-TSS (NM_005915)	promoter-TSS (NM_005915)	25	NM_00591	4175	Hs.444118	NM_00591	ENSG0000	MCM6	MCG40308	minichrom	protein-co
lib-C666-I	chr4	110624416	110624416	+	9.94079	NA	intron (NM_001226, intron 1 of 6)	CpG	213	NM_03299	839	Hs.654616	NM_00122	ENSG0000	CASP6	MCH2	caspase 6	protein-co
lib-C666-I	chr1	109505999	109505999	+	8.91001	NA	5' UTR (NM_015127, exon 1 of 12)	5' UTR (NM_015127, exon 1 of 12)	122	NM_01512	23155	Hs.658489	NM_01512	ENSG0000	CLCC1	MCLC	chloride ch	protein-co
lib-C666-I	chr6	49431032	49431032	+	49.56857	NA	promoter-TSS (NM_000255)	promoter-TSS (NM_000255)	9	NM_00025	4594	Hs.485527	NM_00025	ENSG0000	MUT	MCM	methylmal	protein-co
lib-C666-I	chr6	43138947	43138947	+	8.0787	NA	promoter-TSS (NM_003131)	promoter-TSS (NM_003131)	-86	NM_00313	6722	Hs.520140	NM_00313	ENSG0000	SRF	MCM1	serum resp	protein-co
lib-C666-I	chr15	74494273	74494273	+	5.11556	NA	intron (NM_001142619, intron 2 of 1	intron (NM_001142619, intron 2 of	947	NM_00119	64220	Hs.24553	NM_02236	ENSG0000	STRA6	MCOPCB8	stimulated	protein-co
lib-C666-I	chr16	1322017	1322017	+	4.73866	NA	Intergenic	Intergenic	15744	NM_01221	23430	Hs.677789	NM_01221	ENSG0000	TPSD1	MCP7-LIKE	tryptase de	protein-co
lib-C666-I	chr20	44600975	44600975	+	212.7112	NA	promoter-TSS (NM_022095)	promoter-TSS (NM_022095)	-142	NM_02209	63925	Hs.174193	NM_02209	ENSG0000	ZNF335	MCPH10|N	zinc finger	protein-co
lib-C666-I	chr7	92393226	92393226	+	19.18403	NA	intron (NM_001145306, intron 3 of 7)	intron (NM_001145306, intron 3 of	70005	NM_00125	1021	Hs.119882	NM_00125	ENSG0000	CDK6	MCPH12|P	cyclin depe	protein-co
lib-C666-I	chr1	100598523	100598523	+	4.64451	NA	promoter-TSS (NM_019083)	promoter-TSS (NM_019083)	128	NM_19429	163786	Hs.591447	NM_19429	ENSG0000	SASS6	MCPH14|S.	SAS-6 cent	protein-co
lib-C666-I	chr1	47779945	47779945	+	42.35981	NA	promoter-TSS (NM_001282939)	promoter-TSS (NM_001282939)	-126	NM_00303	6491	Hs.525198	NM_00303	ENSG0000	STIL	MCPH7|SIL	SCL/TAL1 i	protein-co
lib-C666-I	chr1	47780421	47780421	+	3.98718	NA	promoter-TSS (NM_001282939)	promoter-TSS (NM_001282939)	-602	NM_00303	6491	Hs.525198	NM_00303	ENSG0000	STIL	MCPH7|SIL	SCL/TAL1 i	protein-co
lib-C666-I	chr9	130860600	130860600	+	14.37873	NA	promoter-TSS (NM_052901)	promoter-TSS (NM_052901)	-161	NR_04976	114789	Hs.5476	NM_05290	ENSG0000	SLC25A25	MCSC|PCS	solute carr	protein-co
lib-C666-I	chr9	130830347	130830347	+	13.55025	NA	promoter-TSS (NM_197956)	promoter-TSS (NM_197956)	-132	NM_00100	114789	Hs.5476	NM_05290	ENSG0000	SLC25A25	MCSC|PCS	solute carr	protein-co
lib-C666-I	chr9	130856831	130856831	+	9.02258	NA	intron (NM_001006642, intron 1 of 9)	intron (NM_001006642, intron 1 of	3088	NM_00126	114789	Hs.5476	NM_05290	ENSG0000	SLC25A25	MCSC|PCS	solute carr	protein-co
lib-C666-I	chr17	80186862	80186862	+	24.32656	NA	promoter-TSS (NM_004207)	promoter-TSS (NM_004207)	-25	NM_00420	9123	Hs.500761	NM_00420	ENSG0000	SLC16A3	MCT 3|MC	solute carr	protein-co
lib-C666-I	chr17	66286758	66286758	+	15.1031	NA	promoter-TSS (NM_001267727)	promoter-TSS (NM_001267727)	647	NM_00469	9120	Hs.42645	NM_00469	ENSG0000	SLC16A6	MCT6|MCT	solute carr	protein-co
lib-C666-I	chr8	59465740	59465740	+	6.49644	NA	promoter-TSS (NM_005625)	promoter-TSS (NM_005625)	12	NM_00562	6386	Hs.200804	NM_00562	ENSG0000	SDCBP	MDA-9|MD	syndecan b	protein-co
lib-C666-I	chr17	1018475	1018475	+	38.25723	NA	intron (NM_001322841, intron 2 of 2	intron (NM_001322841, intron 2 of	-6135	NM_00109	29	Hs.159306	NM_00109	ENSG0000	ABR	MDB	active BCR	protein-co
lib-C666-I	chr17	984256	984256	+	28.64047	NA	intron (NM_001322841, intron 5 of 2	intron (NM_001322841, intron 5 of	-1870	NM_00128	29	Hs.159306	NM_00109	ENSG0000	ABR	MDB	active BCR	protein-co
lib-C666-I	chr17	1132327	1132327	+	9.93242	NA	exon (NM_001322841, exon 1 of 23)	exon (NM_001322841, exon 1 of 23	647	NM_00132	29	Hs.159306	NM_00109	ENSG0000	ABR	MDB	active BCR	protein-co
lib-C666-I	chr17	1131881	1131881	+	4.92967	NA	intron (NM_001322842, intron 1 of 1)	CpG	1093	NM_00132	29	Hs.159306	NM_00109	ENSG0000	ABR	MDB	active BCR	protein-co
lib-C666-I	chr17	1014668	1014668	+	3.59106	NA	intron (NM_001322841, intron 2 of 2	intron (NM_001322841, intron 2 of	-2328	NM_00109	29	Hs.159306	NM_00109	ENSG0000	ABR	MDB	active BCR	protein-co
lib-C666-I	chr17	42856672	42856672	+	30.13508	NA	intron (NM_001318933, intron 26 of 2	CpG	20125	NM_00131	4185	Hs.6088	NM_00239	ENSG0000	ADAM11	MDC	ADAM met	protein-co
lib-C666-I	chr17	42852971	42852971	+	4.6254	NA	exon (NM_002390, exon 17 of 27)	exon (NM_002390, exon 17 of 27)	16424	NM_00131	4185	Hs.6088	NM_00239	ENSG0000	ADAM11	MDC	ADAM met	protein-co
lib-C666-I	chr6	30675219	30675219	+	27.61921	NA	exon (NM_014641, exon 9 of 15)	exon (NM_014641, exon 9 of 15)	4375	NR_13364	106478956	Hs.708954	NR_13364	ENSG0000	MDC1-AS	MDC1-AS	MDC1 anti	ncRNA
lib-C666-I	chr3	49761443	49761443	+	77.89044	NA	promoter-TSS (NM_013334)	promoter-TSS (NM_013334)	-36	NM_01333	29925	Hs.567488	NM_01333	ENSG0000	GMPPB	MDDGA14	GDP-manr	protein-co
lib-C666-I	chr8	121457545	121457545	+	91.58624	NA	promoter-TSS (NM_022045)	promoter-TSS (NM_022045)	-93	NM_02204	27085	Hs.657656	NM_02204	ENSG0000	MTBP	MDM2BP	MDM2 bin	protein-co
lib-C666-I	chr15	89878199	89878199	+	10.16129	NA	promoter-TSS (NM_002693)	promoter-TSS (NM_002693)	-173	NM_00112	5428	Hs.706868	NM_00269	ENSG0000	POLG	MDP1|MIR	DNA polyn	protein-co
lib-C666-I	chr15	50158637	50158637	+	6.72475	NA	non-coding (NR_073598, exon 27 of	non-coding (NR_073598, exon 27 c	245411	NM_02023	56986	Hs.127432	NM_02023	ENSG0000	DTWD1	MDS009	DTW doma	protein-co
lib-C666-I	chr11	82997468	82997468	+	6.98764	NA	promoter-TSS (NM_001286116)	promoter-TSS (NM_001286116)	-18	NM_00128	60492	Hs.368866	NM_02182	ENSG0000	CCDC90B	MDS011|M	coiled-coil	protein-co
lib-C666-I	chr11	82996957	82996957	+	6.98764	NA	exon (NM_021825, exon 1 of 9)	exon (NM_021825, exon 1 of 9)	493	NM_00128	60492	Hs.368866	NM_02182	ENSG0000	CCDC90B	MDS011|M	coiled-coil	protein-co
lib-C666-I	chr1	150266260	150266260	+	14.75675	NA	promoter-TSS (NM_031901)	promoter-TSS (NM_031901)	-2	NM_01899	54460	Hs.405880	NM_01899	ENSG0000	MRPS21	MDS016|M	mitochond	protein-co
lib-C666-I	chr15	75400679	75400679	+	6.29226	NA	Intergenic	Looper|DNA|PiggyBac	65069	NM_00130	60490	Hs.458922	NM_02182	ENSG0000	PPCDC	MDS018|PI	phosphopa	protein-co
lib-C666-I	chr12	2921885	2921885	+	46.4068	NA	promoter-TSS (NM_018463)	promoter-TSS (NM_018463)	97	NR_13074	55846	Hs.446098	NM_01846	ENSG0000	ITFG2	MDS028	integrin alp	protein-co
lib-C666-I	chr10	103577915	103577915	+	14.43224	NA	promoter-TSS (NR_045118)	promoter-TSS (NR_045118)	307	NM_00114	10724	Hs.500842	NM_01221	ENSG0000	MGEA5	MEA5|NCC	meningiom	protein-co
lib-C666-I	chr10	103578328	103578328	+	7.23273	NA	promoter-TSS (NM_001142434)	promoter-TSS (NM_001142434)	-106	NM_01221	10724	Hs.500842	NM_01221	ENSG0000	MGEA5	MEA5|NCC	meningiom	protein-co
lib-C666-I	chrX	150565584	150565584	+	8.20589	NA	promoter-TSS (NM_001017980)	promoter-TSS (NM_001017980)	-73	NM_00101	203547	Hs.58633	NM_00101	ENSG0000	VMA21	MEAX|XME	VMA21 vac	protein-co
lib-C666-I	chr3	129159124	129159124	+	7.11942	NA	promoter-TSS (NM_003925)	promoter-TSS (NM_003925)	-102	NM_00127	8930	Hs.35947	NM_00392	ENSG0000	MBD4	MED1	methyl-Cp	protein-co
lib-C666-I	chr9	136213733	136213733	+	18.89222	NA	intron (NM_181491, intron 1 of 3)	intron (NM_181491, intron 1 of 3)	1278	NM_18149	6837	Hs.78354	NM_00675	ENSG0000	MED22	MED24|SU	mediator c	protein-co
lib-C666-I	chr17	17380342	17380342	+	102.24316	NA	promoter-TSS (NM_018019)	promoter-TSS (NM_018019)	42	NM_01801	55090	Hs.244595	NM_01801	ENSG0000	MED9	MED25	mediator c	protein-co
lib-C666-I	chr8	118532974	118532974	+	129.39502	NA	promoter-TSS (NM_001282986)	promoter-TSS (NM_001282986)	22	NM_08065	90390	Hs.492612	NM_08065	ENSG0000	MED30	MED30S|TF	mediator c	protein-co
lib-C666-I	chr19	19303260	19303260	+	2.00164	NA	promoter-TSS (NM_001278728)	promoter-TSS (NM_001278728)	140	NM_00114	729991	Hs.153629	NM_00114	ENSG0000	BORCS8	MEF2BNB	BLOC-1 re	protein-co
lib-C666-I	chr15	75871657	75871657	+	8.71138	NA	promoter-TSS (NM_002833)	promoter-TSS (NM_002833)	-25	NM_00283	5780	Hs.445775	NM_00283	ENSG0000	PTPN9	MEG2|PTPI	protein tyr	protein-co
lib-C666-I	chr1	186538092	186538092	+	2.0646	NA	Intergenic	Intergenic	-107852	NM_00259	5132	Hs.654381	NM_00259	ENSG0000	PDC	MEKA|PHD	phosducin	protein-co
lib-C666-I	chr19	40697285	40697285	+	44.79663	NA	promoter-TSS (NM_002446)	promoter-TSS (NM_002446)	-366	NM_00244	4294	Hs.466743	NM_00244	ENSG0000	MAP3K10	MEKK10|M	mitogen-a	protein-co
lib-C666-I	chr19	40696884	40696884	+	25.80588	NA	promoter-TSS (NM_002446)	promoter-TSS (NM_002446)	-767	NM_00244	4294	Hs.466743	NM_00244	ENSG0000	MAP3K10	MEKK10|M	mitogen-a	protein-co
lib-C666-I	chr11	65381810	65381810	+	19.19377	NA	promoter-TSS (NM_002419)	promoter-TSS (NM_002419)	-90	NM_00241	4296	Hs.502872	NM_00241	ENSG0000	MAP3K11	MEKK11|M	mitogen-a	protein-co
lib-C666-I	chr14	71276358	71276358	+	3.93123	NA	promoter-TSS (NM_001284230)	promoter-TSS (NM_001284230)	-470	NM_03314	4293	Hs.445496	NM_03314	ENSG0000	MAP3K9	MEKK9|ML	mitogen-a	protein-co
lib-C666-I	chr19	16222582	16222582	+	65.27523	NA	promoter-TSS (NM_005370)	promoter-TSS (NM_005370)	92	NM_00537	4218	Hs.642874	NM_00537	ENSG0000	RAB8A	MEL|RAB8	RAB8A, me	protein-co
lib-C666-I	chr19	16217292	16217292	+	18.68431	NA	Intergenic	Intergenic	-5198	NM_00537	4218	Hs.642874	NM_00537	ENSG0000	RAB8A	MEL|RAB8	RAB8A, me	protein-co
lib-C666-I	chr1	223254736	223254736	+	54.49234	NA	Intergenic	Intergenic	61888	NM_00326	7100	Hs.604542	NM_00326	ENSG0000	TLR5	MELIOS|SL	toll like rec	protein-co
lib-C666-I	chr1	223363762	223363762	+	43.7015	NA	Intergenic	Intergenic	-47138	NM_00326	7100	Hs.604542	NM_00326	ENSG0000	TLR5	MELIOS|SL	toll like rec	protein-co
lib-C666-I	chr1	223316470	223316470	+	7.11942	NA	intron (NM_003268, intron 1 of 5)	CpG	154	NM_00326	7100	Hs.604542	NM_00326	ENSG0000	TLR5	MELIOS|SL	toll like rec	protein-co
lib-C666-I	chr16	46723254	46723254	+	2.65152	NA	promoter-TSS (NM_014321)	promoter-TSS (NM_014321)	-110	NM_01820	55737	Hs.454528	NM_01820	ENSG0000	VPS35	MEM3|PAR	VPS35, retr	protein-co
lib-C666-I	chr7	38217847	38217847	+	107.82085	NA	promoter-TSS (NM_032016)	promoter-TSS (NM_032016)	39	NM_03201	83930	Hs.309753	NM_03201	ENSG0000	STARD3NL	MENTHO	STARD3 N	protein-co
lib-C666-I	chr7	128114424	128114424	+	11.66438	NA	Intergenic	Intergenic	-2359	NM_01839	55798	Hs.433213	NM_01839	ENSG0000	METTL2B	METL|MET	methyltran	protein-co
lib-C666-I	chr7	128116774	128116774	+	4.49043	NA	promoter-TSS (NM_018396)	promoter-TSS (NM_018396)	-9	NM_01839	55798	Hs.433213	NM_01839	ENSG0000	METTL2B	METL|MET	methyltran	protein-co
lib-C666-I	chr1	157108277	157108277	+	46.14268	NA	promoter-TSS (NM_005240)	promoter-TSS (NM_005240)	-100	NM_00524	2117	Hs.105636	NM_00524	ENSG0000	ETV3	METS|PE-1	ETS variant	protein-co
lib-C666-I	chr17	60498906	60498906	+	10.79304	NA	Intergenic	Intergenic	-2340	NM_18172	339175	Hs.381204	NM_18172	ENSG0000	METTL2A	METTL2	methyltran	protein-co
lib-C666-I	chr2	187636772	187636772	+	87.45062	NA	Intergenic	L1PBa|LINE|L1	77125	NM_18252	151112	Hs.375054	NM_18252	ENSG0000	ZSWIM2	MEX|ZZZ2	zinc finger	protein-co
lib-C666-I	chr15	82339281	82339281	+	6.29902	NA	promoter-TSS (NM_032246)	promoter-TSS (NM_032246)	-797	NM_03224	84206	Hs.104744	NM_03224	ENSG0000	MEX3B	MEX-3B|RK	mex-3 RNA	protein-co
lib-C666-I	chr15	82090615	82090615	+	3.28884	NA	Intergenic	L1PA2|LINE|L1	247869	NM_03224	84206	Hs.104744	NM_03224	ENSG0000	MEX3B	MEX-3B|RK	mex-3 RNA	protein-co
lib-C666-I	chr11	62572998	62572998	+	118.64497	NA	promoter-TSS (NM_006362)	promoter-TSS (NM_006362)	-34	NM_00108	10482	Hs.523739	NM_00636	ENSG0000	NXF1	MEX67|TAP	nuclear RN	protein-co
lib-C666-I	chr17	42976852	42976852	+	80.54002	NA	promoter-TSS (NM_001258353)	promoter-TSS (NM_001258353)	-116	NM_00125	9343	Hs.151787	NM_00424	ENSG0000	EFTUD2	MFDGA|MI	elongation	protein-co
lib-C666-I	chr3	196729962	196729962	+	10.58189	NA	3' UTR (NM_005929, exon 16 of 16)	3' UTR (NM_005929, exon 16 of 16)	185	NR_03828	100507057	Hs.666451	NR_03828	ENSG0000	MELTF-AS	MFI2-AS1	MELTF ant	ncRNA
lib-C666-I	chr10	101380308	101380308	+	24.23241	NA	promoter-TSS (NM_031212)	promoter-TSS (NM_031212)	-87	NM_03121	81894	Hs.403790	NM_03121	ENSG0000	SLC25A28	MFRN2|MF	solute carr	protein-co
lib-C666-I	chr10	101380630	101380630	+	5.15278	NA	promoter-TSS (NM_031212)	promoter-TSS (NM_031212)	-409	NM_03121	81894	Hs.403790	NM_03121	ENSG0000	SLC25A28	MFRN2|MF	solute carr	protein-co
lib-C666-I	chr8	104427306	104427306	+	26.07785	NA	exon (NM_015420, exon 1 of 11)	exon (NM_015420, exon 1 of 11)	257	NM_03078	81034	Hs.532265	NM_03078	ENSG0000	SLC25A32	MFT|MFTC	solute carr	protein-co
lib-C666-I	chr11	1260254	1260254	+	23.74671	NA	exon (NM_002458, exon 26 of 49)	exon (NM_002458, exon 26 of 49)	15959	NM_00245	727897	Hs.523395	NM_00245	ENSG0000	MUC5B	MG1|MUC	mucin 5B,	protein-co
lib-C666-I	chrX	48397778	48397778	+	57.02262	NA	promoter-TSS (NM_002536)	promoter-TSS (NM_002536)	-297	NM_00253	4943	Hs.694353	NM_00253	ENSG0000	TBC1D25	MG81|OAT	TBC1 doma	protein-co
lib-C666-I	chr19	46088272	46088272	+	133.63286	NA	promoter-TSS (NM_025136)	promoter-TSS (NM_025136)	-150	NM_00101	80207	Hs.466945	NM_02513	ENSG0000	OPA3	MGA3	OPA3, oute	protein-co
lib-C666-I	chr6	34855844	34855844	+	58.46021	NA	promoter-TSS (NM_005643)	promoter-TSS (NM_005643)	4	NM_00127	6882	Hs.112444	NM_00564	ENSG0000	TAF11	MGC:1524	TATA-box	protein-co
lib-C666-I	chr16	84220701	84220701	+	6.20473	NA	promoter-TSS (NM_001243156)	promoter-TSS (NM_001243156)	-25	NM_00124	9013	Hs.153022	NM_00567	ENSG0000	TAF1C	MGC:3997	TATA-box	protein-co
lib-C666-I	chr9	139780776	139780776	+	20.76617	NA	promoter-TSS (NM_021138)	promoter-TSS (NM_021138)	-189	NM_02113	7186	Hs.522506	NM_02113	ENSG0000	TRAF2	MGC:4501	TNF recept	protein-co
lib-C666-I	chr22	35937313	35937313	+	5.5166	NA	promoter-TSS (NM_014310)	promoter-TSS (NM_014310)	-39	NM_01431	23551	Hs.474711	NM_01431	ENSG0000	RASD2	MGC:4834	RASD fami	protein-co
lib-C666-I	chr22	35936392	35936392	+	4.39666	NA	promoter-TSS (NM_014310)	promoter-TSS (NM_014310)	-960	NM_01431	23551	Hs.474711	NM_01431	ENSG0000	RASD2	MGC:4834	RASD fami	protein-co
lib-C666-I	chr6	134274125	134274125	+	91.58467	NA	promoter-TSS (NM_004865)	promoter-TSS (NM_004865)	-176	NM_00486	9519	Hs.486507	NM_00486	ENSG0000	TBPL1	MGC:8389	TATA-box	protein-co
lib-C666-I	chr2	9983683	9983683	+	5.12426	NA	exon (NM_005680, exon 1 of 15)	exon (NM_005680, exon 1 of 15)	112	NM_00131	9014	Hs.584833	NM_00568	ENSG0000	TAF1B	MGC:9349	TATA-box	protein-co
lib-C666-I	chr6	13328802	13328802	+	5.92363	NA	promoter-TSS (NM_001318809)	promoter-TSS (NM_001318809)	13	NM_00125	51256	Hs.484678	NM_01649	ENSG0000	TBC1D7	MGCPH|PI	TBC1 doma	protein-co
lib-C666-I	chr22	28114447	28114447	+	29.74082	NA	Intergenic	Intergenic	83039	NM_00243	4330	Hs.268515	NM_00243	ENSG0000	MN1	MGCR|MG	MN1 proto	protein-co
lib-C666-I	chr3	184089557	184089557	+	57.36837	NA	TTS (NM_001289998)	TTS (NM_001289998)	6439	NM_00117	7066	Hs.1166	NM_00046	ENSG0000	THPO	MGDF|MK	thrombopo	protein-co
lib-C666-I	chr17	40440260	40440260	+	9.75396	NA	5' UTR (NM_003152, exon 2 of 20)	5' UTR (NM_003152, exon 2 of 20)	186	NM_00128	6776	Hs.437058	NM_00315	ENSG0000	STAT5A	MGF|STAT	signal trans	protein-co
lib-C666-I	chr17	40441072	40441072	+	4.51266	NA	intron (NM_001288720, intron 1 of 1	intron (NM_001288720, intron 1 of	998	NM_00128	6776	Hs.437058	NM_00315	ENSG0000	STAT5A	MGF|STAT	signal trans	protein-co
lib-C666-I	chr12	10766201	10766201	+	282.16949	NA	promoter-TSS (NM_001300739)	promoter-TSS (NM_001300739)	7	NM_00131	55110	Hs.104650	NM_01804	ENSG0000	MAGOHB	MGN2|mag	mago hom	protein-co
lib-C666-I	chr9	132539560	132539560	+	20.19521	NA	Intergenic	Intergenic	-24216	NM_00487	9536	Hs.146688	NM_00487	ENSG0000	PTGES	MGST-IV|M	prostaglan	protein-co
lib-C666-I	chr19	5681012	5681012	+	90.07064	NA	promoter-TSS (NM_001308240)	promoter-TSS (NM_001308240)	-105	NM_00130	125988	Hs.356626	NM_20576	ENSG0000	C19orf70	MIC13|P11	chromosor	protein-co
lib-C666-I	chr22	18330376	18330376	+	60.44312	NA	intron (NM_001122731, intron 18 of 1	intron (NM_001122731, intron 18 o	59276	NM_00112	57553	Hs.528024	NM_01524	ENSG0000	MICAL3	MICAL-3	microtubul	protein-co
lib-C666-I	chr22	18329331	18329331	+	13.59977	NA	intron (NM_001122731, intron 18 of 1	intron (NM_001122731, intron 18 o	60321	NM_00112	57553	Hs.528024	NM_01524	ENSG0000	MICAL3	MICAL-3	microtubul	protein-co
lib-C666-I	chr22	38302256	38302256	+	3.59106	NA	5' UTR (NM_033386, exon 1 of 16)	5' UTR (NM_033386, exon 1 of 16)	101	NM_03338	85377	Hs.517610	NM_03338	ENSG0000	MICALL1	MICAL-L1|	MICAL like	protein-co
lib-C666-I	chr17	18163711	18163711	+	15.31489	NA	promoter-TSS (NM_001144900)	promoter-TSS (NM_001144900)	-137	NM_00114	125170	Hs.655555	NM_13916	ENSG0000	MIEF2	MID49|SM	mitochond	protein-co
lib-C666-I	chr1	1590542	1590542	+	298.30591	NA	intron (NM_033490, intron 5 of 18)	CpG	22383	NR_00294	8511	Hs.671760	NR_00294	ENSG0000	MMP23A	MIFR|MIFR	matrix met	pseudo
lib-C666-I	chr18	61089774	61089774	+	65.54223	NA	promoter-TSS (NM_004869)	promoter-TSS (NM_004869)	-22	NM_00486	9525	Hs.126550	NM_00486	ENSG0000	VPS4B	MIG1|SKD1	vacuolar p	protein-co
lib-C666-I	chr2	85132628	85132628	+	134.15894	NA	promoter-TSS (NM_021103)	promoter-TSS (NM_021103)	-135	NM_02110	9168	Hs.446574	NM_02110	ENSG0000	TMSB10	MIG12|TB1	thymosin b	protein-co
lib-C666-I	chr2	85153228	85153228	+	19.38538	NA	Intergenic	Intergenic	20465	NM_02110	9168	Hs.446574	NM_02110	ENSG0000	TMSB10	MIG12|TB1	thymosin b	protein-co
lib-C666-I	chr3	16518502	16518502	+	21.84731	NA	intron (NM_015150, intron 2 of 9)	intron (NM_015150, intron 2 of 9)	36720	NM_01515	23180	Hs.98910	NM_01515	ENSG0000	RFTN1	MIG2|PIB1	raftlin, lipic	protein-co
lib-C666-I	chr3	16490306	16490306	+	16.19708	NA	intron (NM_015150, intron 2 of 9)	intron (NM_015150, intron 2 of 9)	64916	NM_01515	23180	Hs.98910	NM_01515	ENSG0000	RFTN1	MIG2|PIB1	raftlin, lipic	protein-co
lib-C666-I	chr17	36886427	36886427	+	2.59032	NA	promoter-TSS (NM_001136498)	promoter-TSS (NM_001136498)	-83	NM_00113	284106	Hs.713595	NM_00113	ENSG0000	CISD3	Miner2	CDGSH iro	protein-co
lib-C666-I	chr7	132766789	132766789	+	30.13021	NA	non-coding (NR_133671, exon 1 of 1	non-coding (NR_133671, exon 1 of	129	NR_13367	54927	Hs.655010	NM_01781	ENSG0000	CHCHD3	MINOS3|M	coiled-coil	protein-co
lib-C666-I	chr7	7606598	7606598	+	5.34142	NA	promoter-TSS (NM_019005)	promoter-TSS (NM_019005)	-18	NM_01900	54468	Hs.520215	NM_01900	ENSG0000	MIOS	MIO|Sea4	meiosis reg	protein-co
lib-C666-I	chr7	7606416	7606416	+	4.73866	NA	promoter-TSS (NM_019005)	promoter-TSS (NM_019005)	-200	NM_01900	54468	Hs.520215	NM_01900	ENSG0000	MIOS	MIO|Sea4	meiosis reg	protein-co
lib-C666-I	chr19	40476985	40476985	+	20.90919	NA	promoter-TSS (NM_006503)	promoter-TSS (NM_006503)	73	NM_15300	5704	Hs.211594	NM_00650	ENSG0000	PSMC4	MIP224|RP	proteasom	protein-co
lib-C666-I	chr19	57874842	57874842	+	163.21504	NA	promoter-TSS (NR_002166)	promoter-TSS (NR_002166)	-37	NR_00216	10597	Hs.446620	NR_00216	ENSG0000	TRAPPC2B	MIP-2A|SE	trafficking	pseudo
lib-C666-I	chr12	122516594	122516594	+	26.35145	NA	promoter-TSS (NM_014938)	promoter-TSS (NM_014938)	-40	NM_01493	22877	Hs.437153	NM_01493	ENSG0000	MLXIP	MIR|MOND	MLX intera	protein-co
lib-C666-I	chr14	104583406	104583406	+	2.59032	NA	promoter-TSS (NR_029620)	promoter-TSS (NR_029620)	-336	NR_02962	406986		NR_02962	ENSG0000	MIR203A	MIR203|MI	microRNA	ncRNA
lib-C666-I	chr9	131012643	131012643	+	10.27265	NA	intron (NM_001005336, intron 20 of 2	CpG	-5334	NR_03611	100422893		NR_03611	ENSG0000	MIR3154	mir-3154	microRNA	ncRNA
lib-C666-I	chr10	45655878	45655878	+	4.19675	NA	intron (NR_033891, intron 2 of 7)	MER44D|DNA|TcMar-Tigger	-3584	NR_03611	100422988		NR_03611	ENSG0000	MIR3156-1	mir-3156-	microRNA	ncRNA
lib-C666-I	chr15	93447613	93447613	+	197.99403	NA	promoter-TSS (NR_036136)	promoter-TSS (NR_036136)	-16	NR_03613	100422995		NR_03613	ENSG0000	MIR3175	mir-3175	microRNA	ncRNA
lib-C666-I	chr16	2582081	2582081	+	24.31114	NA	promoter-TSS (NM_001048212)	promoter-TSS (NM_001048212)	-75	NR_03613	100422974		NR_03613	ENSG0000	MIR3178	mir-3178	microRNA	ncRNA
lib-C666-I	chr16	16403746	16403746	+	3.89624	NA	promoter-TSS (NR_036141)	promoter-TSS (NR_036141)	10	NR_03614	100422956		NR_03614	ENSG0000	MIR3180-2	mir-3180-	microRNA	ncRNA
lib-C666-I	chr16	2186111	2186111	+	3.93123	NA	promoter-TSS (NR_037467)	promoter-TSS (NR_037467)	19	NR_03746	100500916		NR_03746	ENSG0000	MIR3180-5	mir-3180-	microRNA	ncRNA
lib-C666-I	chr17	79397033	79397033	+	63.9862	NA	intron (NM_001291324, intron 1 of 2	intron (NM_001291324, intron 1 of	21181	NR_03615	100422944		NR_03615	ENSG0000	MIR3186	mir-3186	microRNA	ncRNA
lib-C666-I	chr19	18403104	18403104	+	27.44508	NA	Intergenic	CpG	10217	NR_03615	100422833		NR_03615	ENSG0000	MIR3188	mir-3188	microRNA	ncRNA
lib-C666-I	chr20	49983099	49983099	+	13.20416	NA	Intergenic	Intergenic	86415	NR_03616	100422889		NR_03616	ENSG0000	MIR3194	mir-3194	microRNA	ncRNA
lib-C666-I	chr20	49982810	49982810	+	10.03103	NA	Intergenic	Intergenic	86704	NR_03616	100422889		NR_03616	ENSG0000	MIR3194	mir-3194	microRNA	ncRNA
lib-C666-I	chr20	50063870	50063870	+	4.39666	NA	intron (NM_001258294, intron 6 of 1	intron (NM_001258294, intron 6 of	5644	NR_03616	100422889		NR_03616	ENSG0000	MIR3194	mir-3194	microRNA	ncRNA
lib-C666-I	chr14	104584277	104584277	+	8.01457	NA	promoter-TSS (NR_039859)	promoter-TSS (NR_039859)	-437	NR_03985	100616173		NR_03985	ENSG0000	MIR203B	MIR3545|h	microRNA	ncRNA
lib-C666-I	chr17	72755036	72755036	+	7.67555	NA	intron (NM_004252, intron 1 of 5)	intron (NM_004252, intron 1 of 5)	10284	NR_03740	100500847		NR_03740	ENSG0000	MIR3615	mir-3615	microRNA	ncRNA
lib-C666-I	chr16	14403526	14403526	+	6.32351	NA	TTS (NR_029854)	TTS (NR_029854)	384	NR_02985	100126355		NR_02985	ENSG0000	MIR365A	MIR365-1|	microRNA	ncRNA
lib-C666-I	chr5	89380306	89380306	+	9.93242	NA	Intergenic	L1MA5A|LINE|L1	-67769	NR_03743	100500825		NR_03743	ENSG0000	MIR3660	mir-3660	microRNA	ncRNA
lib-C666-I	chr5	89318682	89318682	+	7.0832	NA	Intergenic	Intergenic	-6145	NR_03743	100500825		NR_03743	ENSG0000	MIR3660	mir-3660	microRNA	ncRNA
lib-C666-I	chr11	70797312	70797312	+	16.59065	NA	intron (NM_012309, intron 7 of 22)	intron (NM_012309, intron 7 of 22)	-78839	NR_03743	100500844		NR_03743	ENSG0000	MIR3664	mir-3664	microRNA	ncRNA
lib-C666-I	chr4	160024137	160024137	+	21.0836	NA	Intergenic	CpG	-25820	NR_03996	100616303		NR_03996	ENSG0000	MIR3688-2	mir-3688-	microRNA	ncRNA
lib-C666-I	chr8	94886323	94886323	+	12.12504	NA	Intergenic	MER41B|LTR|ERV1	42024	NR_03960	100616169		NR_03960	ENSG0000	MIR378D2	mir-378d-	microRNA	ncRNA
lib-C666-I	chr1	247374234	247374234	+	29.74082	NA	Intergenic	CpG	-8872	NR_03748	100500849		NR_037480		MIR3916	mir-3916	microRNA	ncRNA

lib-C666-l	chr2	64566297	64566297	+	61.67113	NA	Intergenic	Intergenic	-1596	NR_03963	100616265		NR_03963	ENSG0000	MIR4433A	MIR4433|h	microRNA	ncRNA
lib-C666-l	chr2	87849293	87849293	+	3.98718	NA	Intergenic	Intergenic	-79981	NR_03963	100616499		NR_03963	ENSG0000	MIR4435-1	mir-4435-	microRNA	ncRNA
lib-C666-l	chr2	112045909	112045909	+	6.15313	NA	intron (NR_136165, intron 2 of 3)	intron (NR_136165, intron 2 of 3)	32754	NR_03963	100616341		NR_03963	ENSG0000	MIR4435-2	mir-4435-	microRNA	ncRNA
lib-C666-l	chr3	25706535	25706535	+	147.86101	NA	promoter-TSS (NM_001068)	promoter-TSS (NM_001068)	-105	NR_03964	100616477		NR_03964	ENSG0000	MIR4442	mir-4442	microRNA	ncRNA
lib-C666-l	chr5	8578079	8578079	+	29.90332	NA	Intergenic	L1MB8|LINE|L1	117041	NR_03966	100616142		NR_03966	ENSG0000	MIR4458	mir-4458	microRNA	ncRNA
lib-C666-l	chr6	157342704	157342704	+	7.37943	NA	intron (NM_020732, intron 5 of 19)	CpG	-241839	NR_03967	100616154		NR_03967	ENSG0000	MIR4466	mir-4466	microRNA	ncRNA
lib-C666-l	chr8	42751575	42751575	+	14.7189	NA	promoter-TSS (NR_039679)	promoter-TSS (NR_039679)	-157	NR_03967	100616115		NR_03967	ENSG0000	MIR4469	mir-4469	microRNA	ncRNA
lib-C666-l	chr8	62637577	62637577	+	16.59065	NA	Intergenic	MER1B|DNA|hAT-Charlie	10230	NR_03968	100616484		NR_03968	ENSG0000	MIR4470	mir-4470	microRNA	ncRNA
lib-C666-l	chr8	62627301	62627301	+	3.95418	NA	promoter-TSS (NM_001164754)	promoter-TSS (NM_001164754)	-46	NR_03968	100616484		NR_03968	ENSG0000	MIR4470	mir-4470	microRNA	ncRNA
lib-C666-l	chr9	36924201	36924201	+	12.26631	NA	intron (NM_001280556, intron 5 of 8)	intron (NM_001280556, intron 5 of	-30673	NR_03968	100616456		NR_03968	ENSG0000	MIR4476	mir-4476	microRNA	ncRNA
lib-C666-l	chr9	36903597	36903597	+	4.73866	NA	intron (NR_103999, intron 6 of 8)	intron (NR_103999, intron 6 of 8)	-10069	NR_03968	100616456		NR_03968	ENSG0000	MIR4476	mir-4476	microRNA	ncRNA
lib-C666-l	chr20	56475470	56475470	+	7.52748	NA	Intergenic	LTR33A_|LTR|ERVL	5020	NR_03975	100616353		NR_03975	ENSG0000	MIR4532	mir-4532	microRNA	ncRNA
lib-C666-l	chr20	56592771	56592771	+	7.11942	NA	Intergenic	Intergenic	122321	NR_03975	100616353		NR_03975	ENSG0000	MIR4532	mir-4532	microRNA	ncRNA
lib-C666-l	chr20	56566853	56566853	+	3.27468	NA	Intergenic	Intergenic	96403	NR_03975	100616353		NR_03975	ENSG0000	MIR4532	mir-4532	microRNA	ncRNA
lib-C666-l	chr5	180649565	180649565	+	12.62235	NA	promoter-TSS (NM_201627)	promoter-TSS (NM_201627)	68	NR_03978	100616342		NR_03978	ENSG0000	MIR4638	mir-4638	microRNA	ncRNA
lib-C666-l	chr9	114724011	114724011	+	20.7902	NA	Intergenic	L2b|LINE|L2	29631	NR_03981	100616114		NR_03981	ENSG0000	MIR4668	mir-4668	microRNA	ncRNA
lib-C666-l	chr14	65823238	65823238	+	2.59032	NA	Intergenic	Intergenic	-21337	NR_03985	100616176		NR_03985	ENSG0000	MIR4708	mir-4708	microRNA	ncRNA
lib-C666-l	chr17	36860211	36860211	+	28.88523	NA	Intergenic	CpG	-1627	NR_03988	100616203		NR_03988	ENSG0000	MIR4734	mir-4734	microRNA	ncRNA
lib-C666-l	chr19	58898192	58898192	+	3.96379	NA	promoter-TSS (NM_001009)	promoter-TSS (NM_001009)	33	NR_03991	100616168		NR_03991	ENSG0000	MIR4754	mir-4754	microRNA	ncRNA
lib-C666-l	chr2	240867200	240867200	+	5.96422	NA	intron (NR_136158, intron 7 of 10)	intron (NR_136158, intron 7 of 10)	15311	NR_03994	100616417		NR_03994	ENSG0000	MIR4786	mir-4786	microRNA	ncRNA
lib-C666-l	chr8	130994196	130994196	+	6.20473	NA	intron (NR_046360, intron 1 of 14)	L2b|LINE|L2	26503	NR_04982	100847051		NR_04982	ENSG0000	MIR5194	mir-5194	microRNA	ncRNA
lib-C666-l	chr2	228331162	228331162	+	11.6497	NA	Intergenic	Intergenic	-5686	NR_04988	100847081		NR_049888		MIR5703	mir-5703	microRNA	ncRNA
lib-C666-l	chr1	143673517	143673517	+	15.30356	NA	TTS (NR_106967)	TTS (NR_106967)	596	NR_10696	102466225		NR_10696	ENSG0000	MIR6077	MIR6077-1	microRNA	ncRNA
lib-C666-l	chr1	147861043	147861043	+	12.69461	NA	TTS (NR_106967).2	TTS (NR_106967).2	609	NR_10696	102466225		NR_10696	ENSG0000	MIR6077	MIR6077-1	microRNA	ncRNA
lib-C666-l	chr8	128992867	128992867	+	5.74587	NA	intron (NR_003367, intron 3 of 8)	intron (NR_003367, intron 3 of 8)	19988	NR_03161	100302161		NR_03161	ENSG0000	MIR1205	MIRN1205	microRNA	ncRNA
lib-C666-l	chr8	129000123	129000123	+	15.55328	NA	intron (NR_003367, intron 4 of 8)	intron (NR_003367, intron 4 of 8)	-21021	NR_03161	100302170		NR_03161	ENSG0000	MIR1206	MIRN1206	microRNA	ncRNA
lib-C666-l	chr8	129089339	129089339	+	42.49125	NA	intron (NR_003367, intron 6 of 8)	intron (NR_003367, intron 6 of 8)	27941	NR_03161	100302175		NR_03161	ENSG0000	MIR1207	MIRN1207	microRNA	ncRNA
lib-C666-l	chr8	129047511	129047511	+	8.89416	NA	intron (NR_003367, intron 5 of 8)	intron (NR_003367, intron 5 of 8)	-13887	NR_03161	100302175		NR_03161	ENSG0000	MIR1207	MIRN1207	microRNA	ncRNA
lib-C666-l	chr8	129061013	129061013	+	8.0154	NA	promoter-TSS (NR_031612)	promoter-TSS (NR_031612)	-385	NR_03161	100302175		NR_03161	ENSG0000	MIR1207	MIRN1207	microRNA	ncRNA
lib-C666-l	chr8	129189060	129189060	+	8.28613	NA	Intergenic	Intergenic	26698	NR_03161	100302281		NR_03161	ENSG0000	MIR1208	MIRN1208	microRNA	ncRNA
lib-C666-l	chr5	179223398	179223398	+	2.24164	NA	3' UTR (NM_145867, exon 5 of 5)	3' UTR (NM_145867, exon 5 of 5)	1948	NR_03159	100302156		NR_03159	ENSG0000	MIR1229	MIRN1229	microRNA	ncRNA
lib-C666-l	chr1	21349196	21349196	+	6.72475	NA	intron (NM_001198803, intron 4 of 1	intron (NM_001198803, intron 4 of	-34271	NR_03165	100302155		NR_03165	ENSG0000	MIR1256	MIRN1256	microRNA	ncRNA
lib-C666-l	chr22	41488034	41488034	+	5.74041	NA	promoter-TSS (NM_001429)	promoter-TSS (NM_001429)	-483	NR_03169	100302237		NR_03169	ENSG0000	MIR1281	MIRN1281	microRNA	ncRNA
lib-C666-l	chr5	132628187	132628187	+	7.15277	NA	intron (NM_015082, intron 6 of 15)	intron (NM_015082, intron 6 of 15)	135211	NR_03162	100302134		NR_03162	ENSG0000	MIR1289-2	MIRN1289	microRNA	ncRNA
lib-C666-l	chr5	132650231	132650231	+	3.7184	NA	intron (NM_015082, intron 5 of 15)	intron (NM_015082, intron 5 of 15)	113167	NR_03162	100302134		NR_03162	ENSG0000	MIR1289-2	MIRN1289	microRNA	ncRNA
lib-C666-l	chr20	2633302	2633302	+	2.00164	NA	promoter-TSS (NR_031699)	promoter-TSS (NR_031699)	-121	NR_03169	100302138		NR_03169	ENSG0000	MIR1292	MIRN1292	microRNA	ncRNA
lib-C666-l	chr2	208199403	208199403	+	11.36743	NA	Intergenic	Intergenic	-65255	NR_03163	100302130		NR_03163	ENSG0000	MIR1302-4	MIRN1302	microRNA	ncRNA
lib-C666-l	chr2	208202768	208202768	+	9.73491	NA	Intergenic	Intergenic	-68620	NR_03163	100302130		NR_03163	ENSG0000	MIR1302-4	MIRN1302	microRNA	ncRNA
lib-C666-l	chr2	208153530	208153530	+	8.73012	NA	Intergenic	Intergenic	-19382	NR_03163	100302130		NR_03163	ENSG0000	MIR1302-4	MIRN1302	microRNA	ncRNA
lib-C666-l	chr2	208154269	208154269	+	3.8557	NA	Intergenic	Intergenic	-20121	NR_03163	100302130		NR_03163	ENSG0000	MIR1302-4	MIRN1302	microRNA	ncRNA
lib-C666-l	chr15	45748924	45748924	+	20.81433	NA	Intergenic	Intergenic	23676	NR_03059	100126311		NR_03059	ENSG0000	MIR147B	MIRN147B	microRNA	ncRNA
lib-C666-l	chr7	25896743	25896743	+	19.01614	NA	Intergenic	CpG	92863	NR_02959	406940		NR_02959	ENSG0000	MIR148A	MIRN148|	microRNA	ncRNA
lib-C666-l	chr7	25902032	25902032	+	16.63918	NA	Intergenic	Intergenic	87574	NR_02959	406940		NR_02959	ENSG0000	MIR148A	MIRN148|	microRNA	ncRNA
lib-C666-l	chr7	25894818	25894818	+	11.86993	NA	Intergenic	Intergenic	94788	NR_02959	406940		NR_02959	ENSG0000	MIR148A	MIRN148|	microRNA	ncRNA
lib-C666-l	chr1	235994560	235994560	+	18.43022	NA	intron (NM_000081, intron 2 of 52)	MLT1M|LTR|ERVL-MaLR	21800	NR_03171	100302139		NR_03171	ENSG0000	MIR1537	MIRN1537	microRNA	ncRNA
lib-C666-l	chr18	47013713	47013713	+	13.46097	NA	promoter-TSS (NR_031720)	promoter-TSS (NR_031720)	-30	NR_03172	100302257		NR_03172	ENSG0000	MIR1539	MIRN1539	microRNA	ncRNA
lib-C666-l	chr7	129419859	129419859	+	9.94769	NA	Intergenic	CpG	-5005	NR_02961	406959		NR_02961	ENSG0000	MIR183	MIRN183|r	microRNA	ncRNA
lib-C666-l	chr7	129421734	129421734	+	8.91001	NA	Intergenic	CpG	-6880	NR_02961	406959		NR_02961	ENSG0000	MIR183	MIRN183|r	microRNA	ncRNA
lib-C666-l	chr7	129420321	129420321	+	3.8557	NA	Intergenic	CpG	-5467	NR_02961	406959		NR_02961	ENSG0000	MIR183	MIRN183|r	microRNA	ncRNA
lib-C666-l	chr11	61582840	61582840	+	5.66433	NA	promoter-TSS (NM_001281501)	promoter-TSS (NM_001281501)	-128	NR_03172	100302263		NR_03172	ENSG0000	MIR1908	MIRN1908	microRNA	ncRNA
lib-C666-l	chr10	21653600	21653600	+	9.93242	NA	Intergenic	LTR88b|LTR|Gypsy?	131970	NR_03173	100302129		NR_03173	ENSG0000	MIR1915	MIRN1915	microRNA	ncRNA
lib-C666-l	chr17	46708812	46708812	+	2.77473	NA	Intergenic	Intergenic	1109	NR_02958	406972		NR_02958	ENSG0000	MIR196A1	MIRN196-	microRNA	ncRNA
lib-C666-l	chr1	1098223	1098223	+	15.33659	NA	Intergenic	CpG	-4261	NR_02963	406984		NR_02963	ENSG0000	MIR200B	MIRN200B	microRNA	ncRNA
lib-C666-l	chr1	1098672	1098672	+	4.27545	NA	Intergenic	CpG	-3812	NR_02963	406984		NR_02963	ENSG0000	MIR200B	MIRN200B	microRNA	ncRNA
lib-C666-l	chr17	57915126	57915126	+	6.81085	NA	intron (NM_001329398, intron 10 of 1	intron (NM_001329398, intron 10 o	-3501	NR_02949	406991	Hs.444569	NR_02949	ENSG0000	MIR21	MIRN21|hs	microRNA	ncRNA
lib-C666-l	chr17	57923819	57923819	+	6.76094	NA	Intergenic	Intergenic	5192	NR_02949	406991	Hs.444569	NR_02949	ENSG0000	MIR21	MIRN21|hs	microRNA	ncRNA
lib-C666-l	chr17	1953739	1953739	+	115.72837	NA	promoter-TSS (NR_029674)	promoter-TSS (NR_029674)	-65	NR_02962	406994		NR_02962	ENSG0000	MIR212	MIRN212|r	microRNA	ncRNA
lib-C666-l	chr5	168249905	168249905	+	7.37943	NA	intron (NM_003062, intron 7 of 35)	MamRep564|Unknown|Unknown	-54645	NR_02963	407001		NR_02963	ENSG0000	MIR218-2	MIRN218-	microRNA	ncRNA
lib-C666-l	chr19	13958060	13958060	+	39.94134	NA	Intergenic	Intergenic	-10587	NR_02949	407010		NR_02949	ENSG0000	MIR23A	MIRN23A|	microRNA	ncRNA
lib-C666-l	chr19	13957560	13957560	+	17.68706	NA	Intergenic	Intergenic	-10087	NR_02949	407010		NR_02949	ENSG0000	MIR23A	MIRN23A|	microRNA	ncRNA
lib-C666-l	chr19	13954539	13954539	+	7.75972	NA	Intergenic	Intergenic	-7066	NR_02949	407010		NR_02949	ENSG0000	MIR23A	MIRN23A|	microRNA	ncRNA
lib-C666-l	chr19	13951583	13951583	+	3.28884	NA	Intergenic	Intergenic	-4110	NR_02949	407010		NR_02949	ENSG0000	MIR23A	MIRN23A|	microRNA	ncRNA
lib-C666-l	chr22	22006115	22006115	+	5.5166	NA	Intergenic	CpG	-1155	NR_03062	100126318		NR_03062	ENSG0000	MIR301B	MIRN301B	microRNA	ncRNA
lib-C666-l	chr6	72117416	72117416	+	30.542	NA	TTS (NR_026807)	TTS (NR_026807)	-4092	NR_02950	407029		NR_02950	ENSG0000	MIR30A	MIRN30A|r	microRNA	ncRNA
lib-C666-l	chr19	46135976	46135976	+	23.1274	NA	intron (NM_012155, intron 6 of 18)	intron (NM_012155, intron 6 of 18)	6369	NR_02988	442902		NR_02988	ENSG0000	MIR330	MIRN330|	microRNA	ncRNA
lib-C666-l	chr19	46133160	46133160	+	9.20967	NA	intron (NM_012155, intron 7 of 18)	intron (NM_012155, intron 7 of 18)	9185	NR_02988	442902		NR_02988	ENSG0000	MIR330	MIRN330|	microRNA	ncRNA
lib-C666-l	chr10	88014474	88014474	+	8.01457	NA	intron (NM_017551, intron 2 of 15)	intron (NM_017551, intron 2 of 15)	10071	NR_02990	442911		NR_02990	ENSG0000	MIR346	MIRN346|	microRNA	ncRNA
lib-C666-l	chr3	99721850	99721850	+	5.23609	NA	intron (NM_001042459, intron 1 of 4)	intron (NM_001042459, intron 1 of	-4791	NR_03166	100313938		NR_03166	ENSG0000	MIR548G	MIRN548G	microRNA	ncRNA
lib-C666-l	chr3	168235519	168235519	+	14.3056	NA	intron (NR_021485, intron 3 of 15)	intron (NR_021485, intron 3 of 15)	-34123	NR_03029	693136		NR_03029	ENSG0000	MIR551B	MIRN551B	microRNA	ncRNA
lib-C666-l	chr3	44903310	44903310	+	36.38376	NA	promoter-TSS (NR_030290)	promoter-TSS (NR_030290)	-70	NR_03029	693149		NR_03029	ENSG0000	MIR564	MIRN564|	microRNA	ncRNA
lib-C666-l	chr4	166398011	166398011	+	12.12504	NA	intron (NM_001873, intron 3 of 8)	intron (NM_001873, intron 3 of 8)	90617	NR_03030	693163		NR_03030	ENSG0000	MIR578	MIRN578|	microRNA	ncRNA
lib-C666-l	chr4	166424670	166424670	+	11.6145	NA	Intergenic	Intergenic	117276	NR_03030	693163		NR_03030	ENSG0000	MIR578	MIRN578|	microRNA	ncRNA
lib-C666-l	chr4	166361632	166361632	+	3.57421	NA	intron (NM_001873, intron 1 of 8)	intron (NM_001873, intron 1 of 8)	54238	NR_03030	693163		NR_03030	ENSG0000	MIR578	MIRN578|	microRNA	ncRNA
lib-C666-l	chr6	107194747	107194747	+	12.12723	NA	intron (NR_033557, intron 1 of 6)	intron (NR_033557, intron 1 of 6)	-37253	NR_03031	693172		NR_03031	ENSG0000	MIR587	MIRN587|	microRNA	ncRNA
lib-C666-l	chr6	107194413	107194413	+	4.83287	NA	intron (NR_033557, intron 1 of 6)	intron (NR_033557, intron 1 of 6)	-37587	NR_03031	693172		NR_03031	ENSG0000	MIR587	MIRN587|	microRNA	ncRNA
lib-C666-l	chr7	127775961	127775961	+	32.48059	NA	Intergenic	CpG	54048	NR_03032	693178		NR_03032	ENSG0000	MIR593	MIRN593|	microRNA	ncRNA
lib-C666-l	chr7	158224883	158224883	+	38.71165	NA	intron (NM_130842, intron 1 of 21)	intron (NM_130842, intron 1 of 21)	100622	NR_03032	693180		NR_03032	ENSG0000	MIR595	MIRN595|	microRNA	ncRNA
lib-C666-l	chr7	158225505	158225505	+	3.20885	NA	intron (NM_130842, intron 1 of 21)	LTR69|LTR|ERVL	100000	NR_03032	693180		NR_03032	ENSG0000	MIR595	MIRN595|	microRNA	ncRNA
lib-C666-l	chr12	12902550	12902550	+	11.06679	NA	intron (NM_001130415, intron 1 of 1)	MER51A|LTR|ERV1	-15033	NR_03034	693198		NR_03034	ENSG0000	MIR613	MIRN613|	microRNA	ncRNA
lib-C666-l	chr17	64726905	64726905	+	63.56729	NA	intron (NM_002737, intron 8 of 16)	MER41B|LTR|ERV1	-56285	NR_03036	693219		NR_03036	ENSG0000	MIR634	MIRN634|	microRNA	ncRNA
lib-C666-l	chr22	18470796	18470796	+	6.3317	NA	intron (NM_015241, intron 1 of 31)	intron (NM_015241, intron 1 of 31)	-7069	NR_03037	693233		NR_03037	ENSG0000	MIR648	MIRN648|	microRNA	ncRNA
lib-C666-l	chr17	79093630	79093630	+	228.60843	NA	intron (NM_001080395, intron 12 of 1	CpG-11309	5543	NR_03039	724027		NR_03039	ENSG0000	MIR657	MIRN657|	microRNA	ncRNA
lib-C666-l	chr22	38240598	38240598	+	6.49644	NA	promoter-TSS (NM_138797)	promoter-TSS (NM_138797)	-220	NR_03039	724028		NR_03039	ENSG0000	MIR658	MIRN658|	microRNA	ncRNA
lib-C666-l	chr11	78913212	78913212	+	6.98764	NA	intron (NM_001098816, intron 3 of 3	intron (NM_001098816, intron 3 of	199941	NR_03059	100126333		NR_03059	ENSG0000	MIR708	MIRN708|	microRNA	ncRNA
lib-C666-l	chr3	197381785	197381785	+	129.90346	NA	Intergenic	CpG	19662	NR_03062	100126321		NR_03062	ENSG0000	MIR922	MIRN922|	microRNA	ncRNA
lib-C666-l	chr1	155164599	155164599	+	8.01457	NA	promoter-TSS (NR_030281)	promoter-TSS (NR_030281)	-369	NR_03028	693235		NR_03028	ENSG0000	MIR92B	MIRN92B|	microRNA	ncRNA
lib-C666-l	chr4	8031697	8031697	+	44.40054	NA	intron (NM_001130083, intron 10 of 2	intron (NM_001130083, intron 10 o	-24589	NR_02951	407052		NR_02951	ENSG0000	MIR95	MIRN95|hs	microRNA	ncRNA
lib-C666-l	chr4	7979855	7979855	+	27.79032	NA	intron (NM_001130083, intron 20 of 2	intron (NM_001130083, intron 20 o	27253	NR_02951	407052		NR_02951	ENSG0000	MIR95	MIRN95|hs	microRNA	ncRNA
lib-C666-l	chr10	80962526	80962526	+	15.06395	NA	intron (NM_020338, intron 5 of 24)	intron (NM_020338, intron 5 of 24)	133734	NM_02033	57178	Hs.193118	NM_02033	ENSG0000	ZMIZ1	MIZ|RAI17|	zinc finger	protein-co
lib-C666-l	chr9	140485119	140485119	+	9.94079	NA	promoter-TSS (NM_138462)	promoter-TSS (NM_138462)	-182	NM_13846	116225	Hs.128096	NM_13846	ENSG0000	ZMYND19	MIZIP	zinc finger	protein-co
lib-C666-l	chr17	35848655	35848655	+	11.5173	NA	Intergenic	Intergenic	-1296	NM_00702	11072	Hs.91448	NM_00702	ENSG0000	DUSP14	MKP-L|MK	dual specif	protein-co
lib-C666-l	chr3	52091421	52091421	+	4.52748	NA	promoter-TSS (NM_001947)	promoter-TSS (NM_001947)	-960	NM_00194	1849	Hs.591664	NM_00194	ENSG0000	DUSP7	MKPX|PYS	dual specif	protein-co
lib-C666-l	chr12	88150028	88150028	+	34.54802	NA	Intergenic	Intergenic	28460	NR_03341	400058	Hs.572212	NR_033410		MKRN9P	MKRN5|M	makorin rir	pseudo
lib-C666-l	chr1	200589927	200589927	+	13.77909	NA	promoter-TSS (NM_014875)	promoter-TSS (NM_014875)	-65	NM_01487	9928	Hs.3104	NM_01487	ENSG0000	KIF14	MKS12	kinesin fan	protein-co
lib-C666-l	chr22	51059896	51059896	+	6.50091	NA	Intergenic	L1ME3|LINE|L1	6705	NM_00108	410	Hs.88251	NM_00048	ENSG0000	ARSA	MLD	arylsulfatas	protein-co
lib-C666-l	chr17	10023247	10023247	+	45.23877	NA	intron (NM_201433, intron 1 of 13)	intron (NM_201433, intron 1 of 13)	78621	NM_20143	8522	Hs.462214	NM_00364	ENSG0000	GAS7	MLL/GAS7	growth arr	protein-co
lib-C666-l	chr4	18023339	18023339	+	6.40179	NA	non-coding (NR_136669, exon 1 of 5	non-coding (NR_136669, exon 1 of	159	NR_13666	254251	Hs.446201	NM_15368	ENSG0000	LCORL	MLR1	ligand dep	protein-co
lib-C666-l	chr11	13690152	13690152	+	27.5466	NA	promoter-TSS (NM_032228)	promoter-TSS (NM_032228)	-54	NM_03222	84188	Hs.501991	NM_03222	ENSG0000	FAR1	MLSTD2|P	fatty acyl-(	protein-co
lib-C666-l	chr11	13690554	13690554	+	15.01861	NA	intron (NM_032228, intron 1 of 11)	CpG	348	NM_03222	84188	Hs.501991	NM_03222	ENSG0000	FAR1	MLSTD2|P	fatty acyl-(	protein-co
lib-C666-l	chr10	114206826	114206826	+	17.40556	NA	promoter-TSS (NR_134521)	promoter-TSS (NR_134521)	80	NM_00131	143187	Hs.194554	NM_14520	ENSG0000	VTI1A	MMDS3|M	vesicle trar	protein-co
lib-C666-l	chr17	1391524	1391524	+	17.13943	NA	intron (NM_001080779, intron 1 of 3	CpG	-2473	NM_00108	4641	Hs.286226	NM_03337	ENSG0000	MYO1C	MMI-beta|	myosin IC	protein-co
lib-C666-l	chr17	1388949	1388949	+	8.8556	NA	intron (NM_001080779, intron 1 of 3	CpG	102	NM_00108	4641	Hs.286226	NM_03337	ENSG0000	MYO1C	MMI-beta|	myosin IC	protein-co
lib-C666-l	chr16	58059466	58059466	+	29.01385	NA	promoter-TSS (NM_002428)	promoter-TSS (NM_002428)	-4	NM_00242	4324	Hs.80343	NM_00242	ENSG0000	MMP15	MMP-15|M	matrix met	protein-co
lib-C666-l	chr11	102417362	102417362	+	3.89034	NA	Intergenic	Intergenic	-15878	NM_00242	4316	Hs.2256	NM_00242	ENSG0000	MMP7	MMP-7|M	matrix met	protein-co
lib-C666-l	chr2	191273579	191273579	+	3.8557	NA	intron (NM_017694, intron 1 of 7)	CpG	498	NM_01769	54842	Hs.418581	NM_01769	ENSG0000	MFSD6	MMR2|hM	major facil	protein-co
lib-C666-l	chr14	74551324	74551324	+	13.8811	NA	promoter-TSS (NM_005589)	promoter-TSS (NM_005589)	-128	NM_00127	4329	Hs.293970	NM_00558	ENSG0000	ALDH6A1	MMSADH/	aldehyde c	protein-co
lib-C666-l	chr1	228291042	228291042	+	193.77844	NA	non-coding (NR_130975, exon 1 of 9	non-coding (NR_130975, exon 1 of	121	NM_02431	79169	Hs.445952	NM_02431	ENSG0000	C1orf35	MMTAG2	chromosor	protein-co
lib-C666-l	chr1	228289857	228289857	+	7.0832	NA	exon (NM_024319, exon 6 of 8)	exon (NM_024319, exon 6 of 8)	1306	NM_02431	79169	Hs.445952	NM_02431	ENSG0000	C1orf35	MMTAG2	chromosor	protein-co
lib-C666-l	chr3	146262486	146262486	+	13.0716	NA	5' UTR (NM_021105, exon 1 of 9)	5' UTR (NM_021105, exon 1 of 9)	142	NM_02110	5359	Hs.130759	NM_02110	ENSG0000	PLSCR1	MMTRA1B	phospholip	protein-co
lib-C666-l	chr13	50018235	50018235	+	3.31563	NA	promoter-TSS (NM_001320727)	promoter-TSS (NM_001320727)	27	NM_00128	81617	Hs.87159	NM_03092	ENSG0000	CAB39L	MO25-BE	calcium bir	protein-co
lib-C666-l	chr13	95953826	95953826	+	4.28252	NA	promoter-TSS (NM_001301830)	promoter-TSS (NM_001301830)	-126	NM_00110	10257	Hs.508423	NM_00584	ENSG0000	ABCC4	MOAT-B|	ATP bindin	protein-co
lib-C666-l	chr10	52339896	52339896	+	4.19683	NA	intron (NM_147156, intron 2 of 10)	HAL1|LINE|L1	43841	NM_14715	259230	Hs.654698	NM_14715	ENSG0000	SGMS1	MOB|MOB	sphingomy	protein-co
lib-C666-l	chr19	2096430	2096430	+	5.12426	NA	promoter-TSS (NM_130807)	promoter-TSS (NM_130807)	-161	NM_13080	126308	Hs.744938	NM_13080	ENSG0000	MOB3A	MOB-LAK|	MOB kinas	protein-co
lib-C666-l	chr16	31128969	31128969	+	97.29876	NA	promoter-TSS (NM_032188)	promoter-TSS (NM_032188)	-16	NM_18295	84148	Hs.533803	NM_03218	ENSG0000	KAT8	MOF|MYS	lysine acet	protein-co
lib-C666-l	chr3	58318652	58318652	+	15.3763	NA	promoter-TSS (NM_001289095)	promoter-TSS (NM_001289095)	35	NM_00128	54899	Hs.190544	NM_01777	ENSG0000	PXK	MONaKA	PX domain	protein-co
lib-C666-l	chr2	122247031	122247031	+	34.0482	NA	intron (NM_001142274, intron 8 of 3	intron (NM_001142274, intron 8 of	-41425	NR_02334	100151683	Hs.689638	NR_02334	ENSG0000	RNU4ATA(	MOPD1|RF	RNA, U4at	snRNA
lib-C666-l	chrX	102941764	102941764	+	49.18845	NA	promoter-TSS (NM_001142418)	promoter-TSS (NM_001142418)	-18	NM_00114	9643	Hs.326387	NM_01228	ENSG0000	MORF4L2	MORFL2|M	mortality f	protein-co
lib-C666-l	chr10	93683528	93683528	+	56.28537	NA	promoter-TSS (NM_003972)	promoter-TSS (NM_003972)	-208	NM_00397	9044	Hs.500526	NM_00397	ENSG0000	BTAF1	MOT1|TAF	B-TFIID TA	protein-co
lib-C666-l	chr10	93817335	93817335	+	9.7234	NA	intron (NM_014912, intron 9 of 9)	L1MA1|LINE|L1	133599	NM_00397	9044	Hs.500526	NM_00397	ENSG0000	BTAF1	MOT1|TAF	B-TFIID TA	protein-co
lib-C666-l	chr16	74330614	74330614	+	8.28333	NA	promoter-TSS (NM_002811)	promoter-TSS (NM_002811)	-59	NM_00281	5713	Hs.440604	NM_00281	ENSG0000	PSMD7	MOV34|P4	proteasom	protein-co
lib-C666-l	chr2	183989092	183989092	+	7.21092	NA	promoter-TSS (NM_001287585)	promoter-TSS (NM_001287585)	9	NM_13828	129401	Hs.180591	NM_13828	ENSG0000	NUP35	MP-44|MP	nucleopori	protein-co
lib-C666-l	chr11	59051806	59051806	+	3.92495	NA	Intergenic	Intergenic	-71312	NM_00103	219972	Hs.744920	NM_00103	ENSG0000	MPEG1	MPG1|MP	macrophag	protein-co
lib-C666-l	chr16	82143118	82143118	+	8.00643	NA	Intergenic	THE1B|LTR|ERVL-MaLR	60711	NM_00579	10200	Hs.344400	NM_00579	ENSG0000	MPHOSPH	MPP|MPP-	M-phase p	protein-co
lib-C666-l	chr16	82203868	82203868	+	7.09685	NA	promoter-TSS (NM_005792)	promoter-TSS (NM_005792)	-39	NM_00579	10200	Hs.344400	NM_00579	ENSG0000	MPHOSPH	MPP|MPP-	M-phase p	protein-co
lib-C666-l	chr1	244013928	244013928	+	13.2252	NA	Intergenic	CpG	-7042	NM_00120	10000	Hs.498292	NM_00546	ENSG0000	AKT3	MPPH|MP	AKT serine	protein-co
lib-C666-l	chr15	69591345	69591345	+	15.06395	NA	promoter-TSS (NM_017705)	promoter-TSS (NM_017705)	51	NM_01770	54852	Hs.591096	NM_01770	ENSG0000	PAQR5	MPRG	progestin a	protein-co
lib-C666-l	chr1	153963187	153963187	+	54.43427	NA	promoter-TSS (NM_001030)	promoter-TSS (NM_001030)	-52	NM_00103	6232	Hs.546291	NM_00103	ENSG0000	RPS27	MPS-1|MP	ribosomal	protein-co
lib-C666-l	chrX	148586880	148586880	+	16.59065	NA	promoter-TSS (NM_000202)	promoter-TSS (NM_000202)	4	NM_00116	3423	Hs.6795	NM_00020	ENSG0000	IDS	MPS2|SIDS	iduronate :	protein-co
lib-C666-l	chr1	167711870	167711870	+	9.79436	NA	intron (NM_003953, intron 1 of 5)	L1PB|LINE|L1	20683	NM_00114	9019	Hs.493919	NM_00395	ENSG0000	MPZL1	MPZL1b|P	myelin pro	protein-co
lib-C666-l	chr1	167691124	167691124	+	2.26435	NA	promoter-TSS (NM_024569)	promoter-TSS (NM_024569)	-63	NM_02456	9019	Hs.493919	NM_00395	ENSG0000	MPZL1	MPZL1b|P	myelin pro	protein-co
lib-C666-l	chr22	37056027	37056027	+	10.92275	NA	intron (NM_006078, intron 1 of 3)	intron (NM_006078, intron 1 of 3)	42663	NM_00607	10369	Hs.146766	NM_00607	ENSG0000	CACNG2	MRD10	calcium vo	protein-co
lib-C666-l	chr16	67596204	67596204	+	93.22762	NA	promoter-TSS (NM_001191022)	promoter-TSS (NM_001191022)	-106	NM_00656	10664	Hs.368367	NM_00656	ENSG0000	CTCF	MRD21	CCCTC-bir	protein-co
lib-C666-l	chr1	27930699	27930699	+	25.50922	NA	promoter-TSS (NM_001029882)	promoter-TSS (NM_001029882)	-694	NM_00102	27245	Hs.469280	NM_00102	ENSG0000	AHDC1	MRD25	AT-hook D	protein-co
lib-C666-l	chr1	27927261	27927261	+	5.34142	NA	intron (NM_001029882, intron 1 of 6)	intron (NM_001029882, intron 1 of	2744	NM_00102	27245	Hs.469280	NM_00102	ENSG0000	AHDC1	MRD25	AT-hook D	protein-co
lib-C666-l	chr2	5938411	5938411	+	20.7902	NA	Intergenic	HAL1|LINE|L1	105612	NM_00310	6664	Hs.432638	NM_00310	ENSG0000	SOX11	MRD27	SRY-box 1	protein-co
lib-C666-l	chr19	52692880	52692880	+	4.73866	NA	promoter-TSS (NM_014225)	promoter-TSS (NM_014225)	-175	NM_01422	5518	Hs.467192	NM_01422	ENSG0000	PPP2R1A	MRD36|PP	protein ph	protein-co
lib-C666-l	chr1	151432033	151432033	+	82.8533	NA	promoter-TSS (NM_001194937)	promoter-TSS (NM_001194937)	-92	NM_00119	23126	Hs.489873	NM_01510	ENSG0000	POGZ	MRD37|W	pogo trans	protein-co
lib-C666-l	chr2	2577824	2577824	+	14.0317	NA	Intergenic	HAL1-2a_MD|LINE|L1	-242739	NM_00130	23040	Hs.434418	NM_01502	ENSG0000	MYT1L	MRD39|NZ	myelin trar	protein-co
lib-C666-l	chr1	1822582	1822582	+	57.48363	NA	promoter-TSS (NM_001282538)	promoter-TSS (NM_001282538)	-26	NM_00128	2782	Hs.430425	NM_00207	ENSG0000	GNB1	MRD42	G protein s	protein-co
lib-C666-l	chr2	118572229	118572229	+	68.38644	NA	promoter-TSS (NM_006773)	promoter-TSS (NM_006773)	-26	NM_00677	8886	Hs.744922	NM_00677	ENSG0000	DDX18	MrDb	DEAD-box	protein-co
lib-C666-l	chr17	65418115	65418115	+	4.46583	NA	intron (NM_181671, intron 1 of 9)	intron (NM_181671, intron 1 of 9)	44718	NM_01241	26207	Hs.591185	NM_01241	ENSG0000	PITPNC1	M-RDGB-I	phosphatic	protein-co
lib-C666-l	chr12	116774640	116774640	+	14.70559	NA	Intergenic	Intergenic	-59649	NM_01533	23389	Hs.603766	NM_01533	ENSG0000	MED13L	MRFACD|P	mediator c	protein-co
lib-C666-l	chr7	134855783	134855783	+	93.75573	NA	promoter-TSS (NM_001305629)	promoter-TSS (NM_001305629)	-205	NM_00130	78996	Hs.643113	NM_02403	ENSG0000	C7orf49	MRI|MRI-2	chromosor	protein-co
lib-C666-l	chr7	134873629	134873629	+	9.75997	NA	intron (NM_014149, intron 12 of 14)	MER5A|DNA|hAT-Charlie	-18051	NM_00130	78996	Hs.643113	NM_02403	ENSG0000	C7orf49	MRI|MRI-2	chromosor	protein-co
lib-C666-l	chr17	16913550	16913550	+	10.63893	NA	Intergenic	Intergenic	-32524	NM_01513	23164	Hs.462341	NM_01513	ENSG0000	MPRIP	M-RIP|MR	myosin ph	protein-co
lib-C666-l	chr9	104160934	104160934	+	32.11429	NA	promoter-TSS (NR_103480)	promoter-TSS (NR_103480)	-15	NM_01905	54534	Hs.288224	NM_01905	ENSG0000	MRPL50	MRP-L50	mitochond	protein-co
lib-C666-l	chr14	35591292	35591292	+	21.81327	NA	promoter-TSS (NM_001256678)	promoter-TSS (NM_001256678)	-235	NM_00125	9692	Hs.458487	NM_01467	ENSG0000	KIAA0391	MRPP3|PR	KIAA0391	protein-co
lib-C666-l	chr14	35629403	35629403	+	3.19102	NA	intron (NM_001256681, intron 2 of 5)	intron (NM_001256681, intron 2 of	37645	NM_00125	9692	Hs.458487	NM_01467	ENSG0000	KIAA0391	MRPP3|PR	KIAA0391	protein-co
lib-C666-l	chr17	73257853	73257853	+	3.8557	NA	promoter-TSS (NM_014001)	promoter-TSS (NM_014001)	104	NM_01597	51081	Hs.71787	NM_01597	ENSG0000	MRPS7	MRP-S|MR	mitochond	protein-co
lib-C666-l	chr5	71604316	71604316	+	12.55817	NA	intron (NM_015084, intron 2 of 10)	CpG	11768	NM_00128	23107	Hs.482491	NM_01508	ENSG0000	MRPS27	MRP-S27|	mitochond	protein-co
lib-C666-l	chr2	95787573	95787573	+	4.51266	NA	promoter-TSS (NM_001321995)	promoter-TSS (NM_001321995)	199	NM_00132	64969	Hs.655259	NM_03190	ENSG0000	MRPS5	MRP-S5|S	mitochond	protein-co
lib-C666-l	chr1	859226	859226	+	42.35752	NA	Intergenic	CpG	-1895	NM_15248	148398	Hs.335293	NM_15248	ENSG0000	SAMD11	MRS	sterile alph	protein-co
lib-C666-l	chr1	877272	877272	+	20.72954	NA	intron (NM_152486, intron 8 of 13)	CpG-18	16151	NM_15248	148398	Hs.335293	NM_15248	ENSG0000	SAMD11	MRS	sterile alph	protein-co
lib-C666-l	chr3	3221347	3221347	+	3.93123	NA	promoter-TSS (NM_001173482)	promoter-TSS (NM_001173482)	54	NM_00117	51185	Hs.18925	NM_01630	ENSG0000	CRBN	MRT2|MRT	cereblon	protein-co
lib-C666-l	chr12	94071255	94071255	+	16.63918	NA	promoter-TSS (NM_001320099)	promoter-TSS (NM_001320099)	108	NM_00132	8738	Hs.38533	NM_00380	ENSG0000	CRADD	MRT34|RA	CASP2 anc	protein-co
lib-C666-l	chr12	94019066	94019066	+	2.96041	NA	Intergenic	Intergenic	-52081	NR_13514	8738	Hs.38533	NM_00380	ENSG0000	CRADD	MRT34|RA	CASP2 anc	protein-co
lib-C666-l	chr14	89097176	89097176	+	10.06764	NA	intron (NM_183387, intron 32 of 42)	MLT1L|LTR|ERVL-MaLR	36446	NM_20766	79882	Hs.684046	NM_02482	ENSG0000	ZC3H14	MRT56|MS	zinc finger	protein-co

lib-C666-l	chrX	69653871	69653871	+	10.03103	NA	TTS (NM_017711)	TTS (NM_017711)	-10834	NM_02112	1741	Hs.721586	NM_02073	ENSG0000	DLG3	MRX|MRXS	discs large	protein-co
lib-C666-l	chrX	48755118	48755118	+	8.01457	NA	promoter-TSS (NM_001167990)	promoter-TSS (NM_001167990)	-77	NM_14449	10084	Hs.534384	NM_00571	ENSG0000	PQBP1	MRX2|MRX	polyglutam	protein-co
lib-C666-l	chrX	73834539	73834539	+	14.3056	NA	promoter-TSS (NM_016120)	promoter-TSS (NM_016120)	-78	NM_18335	51132	Hs.653288	NM_01612	ENSG0000	RLIM	MRX61|NY	ring finger	protein-co
lib-C666-l	chrX	70503512	70503512	+	8.0787	NA	5' UTR (NM_007363, exon 1 of 12)	5' UTR (NM_007363, exon 1 of 12)	470	NM_00114	4841	Hs.533282	NM_00736	ENSG0000	NONO	MRXS34|N	non-POU (	protein-co
lib-C666-l	chr2	97535881	97535881	+	8.28333	NA	promoter-TSS (NM_017789)	promoter-TSS (NM_017789)	-146	NM_01778	54910	Hs.516220	NM_01778	ENSG0000	SEMA4C	M-SEMA-I	semaphori	protein-co
lib-C666-l	chr2	97547453	97547453	+	6.94566	NA	intron (NM_016490, intron 2 of 4)	intron (NM_016490, intron 2 of 4)	-11718	NM_01778	54910	Hs.516220	NM_01778	ENSG0000	SEMA4C	M-SEMA-I	semaphori	protein-co
lib-C666-l	chr2	97540446	97540446	+	5.23679	NA	Intergenic	Intergenic	-4711	NM_01778	54910	Hs.516220	NM_01778	ENSG0000	SEMA4C	M-SEMA-I	semaphori	protein-co
lib-C666-l	chr7	83057477	83057477	+	40.21248	NA	intron (NM_001178129, intron 4 of 1(	intron (NM_001178129, intron 4 of	213270	NM_00117	9723	Hs.528721	NM_01243	ENSG0000	SEMA3E	M-SEMAH	semaphori	protein-co
lib-C666-l	chr17	55333680	55333680	+	31.76017	NA	promoter-TSS (NM_001322251)	promoter-TSS (NM_001322251)	-251	NM_00132	124540	Hs.658922	NM_13896	ENSG0000	MSI2	MSI2H	musashi RI	protein-co
lib-C666-l	chr21	44829316	44829316	+	8.01457	NA	Intergenic	L1ME1|LINE|L1	17696	NM_17335	150094	Hs.282113	NM_17335	ENSG0000	SIK1	MSK|SIK|SI	salt induci	protein-co
lib-C666-l	chr14	91527133	91527133	+	27.5466	NA	promoter-TSS (NM_001322238)	promoter-TSS (NM_001322238)	-140	NM_00132	9252	Hs.510225	NM_00475	ENSG0000	RPS6KA5	MSK1|MSP	ribosomal	protein-co
lib-C666-l	chr11	28129768	28129768	+	28.8837	NA	promoter-TSS (NM_031217)	promoter-TSS (NM_031217)	-22	NM_03121	81930	Hs.301052	NM_03121	ENSG0000	KIF18A	MS-KIF18A	kinesin fan	protein-co
lib-C666-l	chr20	16710633	16710633	+	48.59595	NA	promoter-TSS (NM_003092)	promoter-TSS (NM_003092)	24	NM_00309	6629	Hs.280378	NM_00309	ENSG0000	SNRPB2	Msl1|U2B''	small nucle	protein-co
lib-C666-l	chr3	135914783	135914783	+	2.9164	NA	promoter-TSS (NM_018133)	promoter-TSS (NM_018133)	-95	NM_01813	55167	Hs.18631	NM_01813	ENSG0000	MSL2	MSL-2|MS	male-spec	protein-co
lib-C666-l	chrX	11777699	11777699	+	5.25516	NA	promoter-TSS (NM_006800)	promoter-TSS (NM_006800)	-36	NM_00680	10943	Hs.655288	NM_00680	ENSG0000	MSL3	MSL3L1	male-spec	protein-co
lib-C666-l	chr18	67873042	67873042	+	8.0787	NA	promoter-TSS (NM_001318520)	promoter-TSS (NM_001318520)	-80	NM_17363	25914	Hs.654809	NM_17363	ENSG0000	RTTN	MSSP	rotatin	protein-co
lib-C666-l	chr1	36863586	36863586	+	12.55817	NA	promoter-TSS (NM_032881)	promoter-TSS (NM_032881)	-26	NM_03288	84967	Hs.603131	NM_03288	ENSG0000	LSM10	MST074|M	LSM10, U7	protein-co
lib-C666-l	chr1	163291605	163291605	+	10.92275	NA	promoter-TSS (NM_031423)	promoter-TSS (NM_031423)	-24	NR_04563(	8490	Hs.24950	NM_00361	ENSG0000	RGS5	MST092|M	regulator c	protein-co
lib-C666-l	chr5	159546556	159546556	+	13.59254	NA	promoter-TSS (NM_001267035)	promoter-TSS (NM_001267035)	-104	NM_05292	114825	Hs.744960	NM_05292	ENSG0000	PWWP2A	MST101	PWWP dor	protein-co
lib-C666-l	chr2	32390735	32390735	+	3.59106	NA	promoter-TSS (NM_001330476)	promoter-TSS (NM_001330476)	-175	NM_00133	55676	Hs.23248	NM_01796	ENSG0000	SLC30A6	MST103|M	solute carr	protein-co
lib-C666-l	chr12	93323355	93323355	+	19.82221	NA	promoter-TSS (NM_003566)	promoter-TSS (NM_003566)	-248	NM_00356	8411	Hs.567367	NM_00356	ENSG0000	EEA1	MST105|M	early endo	protein-co
lib-C666-l	chr3	52312755	52312755	+	27.96363	NA	promoter-TSS (NM_025222)	promoter-TSS (NM_025222)	-96	NM_02522	80335	Hs.194110	NM_02522	ENSG0000	WDR82	MST107|M	WD repeat	protein-co
lib-C666-l	chr11	134201951	134201951	+	6.98764	NA	5' UTR (NM_138342, exon 1 of 20)	5' UTR (NM_138342, exon 1 of 20)	183	NM_13834	89944	Hs.436178	NM_13834	ENSG0000	GLB1L2	MST114|M	galactosida	protein-co
lib-C666-l	chr12	76953501	76953501	+	14.45701	NA	promoter-TSS (NM_001319653)	promoter-TSS (NM_001319653)	88	NM_02084	114882	Hs.430849	NM_02084	ENSG0000	OSBPL8	MST120|M	oxysterol b	protein-co
lib-C666-l	chr2	233489045	233489045	+	9.10494	NA	intron (NM_001243252, intron 1 of 3)	intron (NM_001243252, intron 1 of	-9162	NM_02520	80303	Hs.516769	NM_02520	ENSG0000	EFHD1	MST133|M	EF-hand d	protein-co
lib-C666-l	chr4	37828184	37828184	+	26.47693	NA	promoter-TSS (NM_018290)	promoter-TSS (NM_018290)	-98	NM_01829	55276	Hs.23363	NM_01829	ENSG0000	PGM2	MSTP006	phosphogl	protein-co
lib-C666-l	chr10	121596509	121596509	+	14.72053	NA	exon (NM_001256378, exon 13 of 16)	exon (NM_001256378, exon 13 of 1	17740	NM_00124	22876	Hs.369755	NM_01493	ENSG0000	INPP5F	MSTP007|	inositol po	protein-co
lib-C666-l	chr4	186130649	186130649	+	39.60187	NA	intron (NM_001317781, intron 1 of 18	CpG	5258	NM_00131	83891	Hs.369091	NM_03195	ENSG0000	SNX25	MSTP043|	sorting nex	protein-co
lib-C666-l	chr8	95565838	95565838	+	11.90035	NA	promoter-TSS (NM_015496)	promoter-TSS (NM_015496)	-92	NM_01549	25962	Hs.202238	NM_01549	ENSG0000	KIAA1429	MSTP054|f	KIAA1429	protein-co
lib-C666-l	chr16	56691749	56691749	+	24.99834	NA	promoter-TSS (NM_001301272)	promoter-TSS (NM_001301272)	-106	NM_00130	4494	Hs.513626	NM_00594	ENSG0000	MT1F	MT1	metallothic	protein-co
lib-C666-l	chr1	237176569	237176569	+	10.37767	NA	Intergenic	Intergenic	-8851	NM_00127	645745	Hs.632513	NM_00103	ENSG0000	MT1HL1	MT1P2	metallothic	protein-co
lib-C666-l	chr18	8705160	8705160	+	4.74112	NA	intron (NR_120598, intron 1 of 1)	CpG	2459	NR_12059(	100287082	Hs.439434	NR_12059(	ENSG0000	GACAT2	MTCL1-AS	gastric can	ncRNA
lib-C666-l	chr2	74153941	74153941	+	72.69891	NA	promoter-TSS (NM_001318863)	promoter-TSS (NM_001318863)	-12	NM_00131	1716	Hs.469022	NM_00192	ENSG0000	DGUOK	MTDPS3|N	deoxyguar	protein-co
lib-C666-l	chr7	91510110	91510110	+	9.62701	NA	promoter-TSS (NM_001301134)	promoter-TSS (NM_001301134)	-76	NM_00130	7978	Hs.532216	NM_00698	ENSG0000	MTERF1	MTERF	mitochond	protein-co
lib-C666-l	chr2	242003266	242003266	+	4.46583	NA	intron (NM_001080437, intron 18 of 3	intron (NM_001080437, intron 18 o	38481	NR_02805	130916	Hs.159556	NM_18250	ENSG0000	MTERF4	MTERFD2	mitochond	protein-co
lib-C666-l	chr1	38325293	38325293	+	48.22081	NA	promoter-TSS (NM_005955)	promoter-TSS (NM_005955)	-1	NM_00595	4520	Hs.471991	NM_00595	ENSG0000	MTF1	MTF-1|ZRF	metal regu	protein-co
lib-C666-l	chr9	123638651	123638651	+	7.67555	NA	intron (NM_001286840, intron 1 of 14	intron (NM_001286840, intron 1 of	955	NM_00128	26147	Hs.460124	NM_01565	ENSG0000	PHF19	MTF2L1|PC	PHD finger	protein-co
lib-C666-l	chr6	33378153	33378153	+	14.3056	NA	promoter-TSS (NM_024165).2	promoter-TSS (NM_024165).2	-620	NR_02769	5252	Hs.166204	NM_00263	ENSG0000	PHF1	MTF2L2|PC	PHD finger	protein-co
lib-C666-l	chr13	48611862	48611862	+	46.29081	NA	non-coding (NR_136687, exon 1 of 5	non-coding (NR_136687, exon 1 of	159	NR_13668	55270	Hs.144407	NM_01828	ENSG0000	NUDT15	MTH2|NUD	nudix hydr	protein-co
lib-C666-l	chr13	48612190	48612190	+	13.8811	NA	intron (NM_018283, intron 1 of 2)	CpG	487	NR_13668	55270	Hs.144407	NM_01828	ENSG0000	NUDT15	MTH2|NUD	nudix hydr	protein-co
lib-C666-l	chr8	21967199	21967199	+	396.06802	NA	promoter-TSS (NM_024815)	promoter-TSS (NM_024815)	-267	NM_02481	79873	Hs.527101	NM_02481	ENSG0000	NUDT18	MTH3	nudix hydr	protein-co
lib-C666-l	chr3	10334573	10334573	+	4.61604	NA	promoter-TSS (NR_126505)	promoter-TSS (NR_126505)	58	NM_00113	51738	Hs.590080	NM_01636	ENSG0000	GHRL	MTLRP	ghrelin and	protein-co
lib-C666-l	chr1	55240766	55240766	+	2.75718	NA	Intergenic	Intergenic	-10540	NM_15226	25973	Hs.380169	NM_15226	ENSG0000	PARS2	MT-PRORS	prolyl-tRN	protein-co
lib-C666-l	chr20	23331044	23331044	+	16.14038	NA	promoter-TSS (NM_013248)	promoter-TSS (NM_013248)	-329	NM_01324	29107	Hs.516933	NM_01324	ENSG0000	NXT1	MTR2|P15	nuclear tra	protein-co
lib-C666-l	chr7	141438163	141438163	+	10.73967	NA	promoter-TSS (NM_003143)	promoter-TSS (NM_003143)	42	NM_00125	6742	Hs.490394	NM_00314	ENSG0000	SSBP1	Mt-SSB|SC	single strar	protein-co
lib-C666-l	chr1	155178655	155178655	+	3.20885	NA	promoter-TSS (NR_045553)	promoter-TSS (NR_045553)	165	NM_19888	4580	Hs.490874	NM_00245	ENSG0000	MTX1	MTX|MTXN	metaxin 1	protein-co
lib-C666-l	chr12	40867963	40867963	+	6.15313	NA	intron (NM_173600, intron 52 of 173)	intron (NM_173600, intron 52 of 17	80767	NM_17360	283463	Hs.632712	NM_173600		MUC19	MUC-19	mucin 19, (	protein-co
lib-C666-l	chr19	41256559	41256559	+	41.94622	NA	promoter-TSS (NM_198476)	promoter-TSS (NM_198476)	-200	NM_00459	6626	Hs.466775	NM_00459	ENSG0000	SNRPA	Mud1|U1-A	small nucle	protein-co
lib-C666-l	chr9	35162163	35162163	+	8.0154	NA	5' UTR (NM_006377, exon 1 of 39)	5' UTR (NM_006377, exon 1 of 39)	174	NM_00637	10497	Hs.493791	NM_00637	ENSG0000	UNC13B	MUNC13|L	unc-13 ho	protein-co
lib-C666-l	chr9	130374325	130374325	+	9.45194	NA	promoter-TSS (NM_003165)	promoter-TSS (NM_003165)	-161	NM_00316	6812	Hs.288229	NM_00316	ENSG0000	STXBP1	MUNC18-1	syntaxin bi	protein-co
lib-C666-l	chr1	109289216	109289216	+	97.8918	NA	promoter-TSS (NM_007269)	promoter-TSS (NM_007269)	-69	NM_00726	6814	Hs.530436	NM_00726	ENSG0000	STXBP3	MUNC18-3	syntaxin bi	protein-co
lib-C666-l	chr9	140317597	140317597	+	5.72511	NA	promoter-TSS (NM_006647)	promoter-TSS (NM_006647)	117	NR_10459	54932	Hs.495553	NM_01782	ENSG0000	EXD3	mut-7	exonucleas	protein-co
lib-C666-l	chr11	95523546	95523546	+	64.54414	NA	promoter-TSS (NM_001243776)	promoter-TSS (NM_001243776)	-79	NM_00124	9702	Hs.101014	NM_01467	ENSG0000	CEP57	MVA2|PIG8	centrosom	protein-co
lib-C666-l	chr6	43777268	43777268	+	4.73866	NA	Intergenic	L2b|LINE|L2	37546	NM_00128	7422	Hs.73793	NM_00337	ENSG0000	VEGFA	MVCD1|VE	vascular er	protein-co
lib-C666-l	chr12	6772531	6772531	+	183.97044	NA	promoter-TSS (NM_001127582)	promoter-TSS (NM_001127582)	-223	NM_00112	51147	Hs.524210	NM_01616	ENSG0000	ING4	my036|p29	inhibitor of	protein-co
lib-C666-l	chr16	24550784	24550784	+	39.40619	NA	promoter-TSS (NM_018703)	promoter-TSS (NM_018703)	-124	NM_03262	5930	Hs.188553	NM_00691	ENSG0000	RBBP6	MY038|P2P	RB binding	protein-co
lib-C666-l	chr7	156433147	156433147	+	16.76091	NA	promoter-TSS (NM_001308273)	promoter-TSS (NM_001308273)	201	NR_02686	129790	Hs.647014	NM_03262	ENSG0000	C7orf13	MY040	chromosor	ncRNA
lib-C666-l	chr1	114472041	114472041	+	46.14268	NA	promoter-TSS (NM_152696)	promoter-TSS (NM_152696)	45	NM_15269	204851	Hs.532363	NM_15269	ENSG0000	HIPK1	Myak|Nbak	homeodor	protein-co
lib-C666-l	chr19	50935027	50935027	+	120.36063	NA	TTS (NM_001243998)	TTS (NM_001243998)	-1133	NM_00453	4606	Hs.85937	NM_00453	ENSG0000	MYBPC2	MYBPC|MY	myosin bin	protein-co
lib-C666-l	chr3	38183678	38183678	+	14.54258	NA	3' UTR (NM_001172567, exon 5 of 5)	3' UTR (NM_001172567, exon 5 of 5	3709	NM_00117	4615	Hs.82116	NM_00246	ENSG0000	MYD88	MYD88D	myeloid di	protein-co
lib-C666-l	chr1	203031758	203031758	+	31.41263	NA	intron (NM_001304331, intron 24 of 2	intron (NM_001304331, intron 24 o	23408	NM_00247	4656	Hs.2830	NM_00247	ENSG0000	MYOG	MYF4|bHLH	myogenin	protein-co
lib-C666-l	chr1	45805835	45805835	+	8.85547	NA	promoter-TSS (NM_001048174)	promoter-TSS (NM_001048174)	-47	NM_00129	4595	Hs.271353	NM_01222	ENSG0000	MUTYH	MYH	mutY DNA	protein-co
lib-C666-l	chr16	11036379	11036379	+	4.96104	NA	promoter-TSS (NM_014015)	promoter-TSS (NM_014015)	-122	NM_01401	28955	Hs.592051	NM_01401	ENSG0000	DEXI	MYLE	Dexi homo	protein-co
lib-C666-l	chr1	35524833	35524833	+	30.48199	NA	promoter-TSS (NM_001289088)	promoter-TSS (NM_001289088)	-554	NM_00128	79830	Hs.471243	NM_02477	ENSG0000	ZMYM1	MYM	zinc finger	protein-co
lib-C666-l	chr1	35523787	35523787	+	7.37943	NA	Intergenic	L1P1|LINE|L1	-1600	NM_00128	79830	Hs.471243	NM_02477	ENSG0000	ZMYM1	MYM	zinc finger	protein-co
lib-C666-l	chr12	6431137	6431137	+	5.5166	NA	intron (NM_001144856, intron 13 of 1	MIRb|SINE|MIR	9320	NM_00114	55200	Hs.631660	NM_01817	ENSG0000	PLEKHG6	MyoGEF	pleckstrin I	protein-co
lib-C666-l	chr5	131563477	131563477	+	2.13399	NA	promoter-TSS (NM_001017973)	promoter-TSS (NM_001017973)	79	NM_00114	8974	Hs.519568	NM_00419	ENSG0000	P4HA2	MYP25	prolyl 4-hy	protein-co
lib-C666-l	chr1	202317673	202317673	+	32.48059	NA	promoter-TSS (NM_001167858)	promoter-TSS (NM_001167858)	-157	NM_00248	4660	Hs.444403	NM_00248	ENSG0000	PPP1R12B	MYPT2|PP1	protein ph	protein-co
lib-C666-l	chr19	59084924	59084924	+	90.12853	NA	promoter-TSS (NM_003422)	promoter-TSS (NM_003422)	18	NM_19805	7593	Hs.399810	NM_00342	ENSG0000	MZF1	MZF-1|MZ	myeloid zir	protein-co
lib-C666-l	chr16	1662100	1662100	+	16.43544	NA	promoter-TSS (NM_014714)	promoter-TSS (NM_014714)	9	NM_01471	9742	Hs.389438	NM_01471	ENSG0000	IFT140	MZSDS|SR	intraflagell	protein-co
lib-C666-l	chr9	140083046	140083046	+	545.97943	NA	promoter-TSS (NM_013366)	promoter-TSS (NM_013366)	-8	NM_00373	8636	Hs.530314	NM_00373	ENSG0000	SSNA1	N14|NA-14	SS nuclear	protein-co
lib-C666-l	chr6	150285001	150285001	+	7.9428	NA	promoter-TSS (NM_001317089)	promoter-TSS (NM_001317089)	-109	NM_02521	80329	Hs.653255	NM_02521	ENSG0000	ULBP1	N2DL-1|NI	UL16 bindi	protein-co
lib-C666-l	chr1	145209029	145209029	+	54.14909	NA	promoter-TSS (NM_203458)	promoter-TSS (NM_203458)	-84	NM_20345	388677	Hs.655156	NM_20345	ENSG0000	NOTCH2N	N2N	notch 2 N-	protein-co
lib-C666-l	chr1	145209881	145209881	+	6.38282	NA	intron (NM_203458, intron 1 of 4)	CpG	768	NM_20345	388677	Hs.655156	NM_20345	ENSG0000	NOTCH2N	N2N	notch 2 N-	protein-co
lib-C666-l	chr7	117824272	117824272	+	24.23745	NA	5' UTR (NM_016200, exon 1 of 4)	5' UTR (NM_016200, exon 1 of 4)	186	NM_01620	51691	Hs.655046	NM_01620	ENSG0000	LSM8	NAA38	LSM8 hom	protein-co
lib-C666-l	chr7	117749881	117749881	+	6.15313	NA	Intergenic	Tigger3a|DNA|TcMar-Tigger	-74205	NM_01620	51691	Hs.655046	NM_01620	ENSG0000	LSM8	NAA38	LSM8 hom	protein-co
lib-C666-l	chr11	842692	842692	+	6.48676	NA	promoter-TSS (NM_003271)	promoter-TSS (NM_003271)	-132	NM_00102	7106	Hs.654836	NM_00327	ENSG0000	TSPAN4	NAG-2|NA	tetraspanir	protein-co
lib-C666-l	chr9	34178978	34178978	+	23.27622	NA	promoter-TSS (NM_016525)	promoter-TSS (NM_016525)	-25	NM_01652	51271	Hs.268963	NM_01652	ENSG0000	UBAP1	NAG20|UA	ubiquitin a	protein-co
lib-C666-l	chr1	2344012	2344012	+	332.39606	NA	promoter-TSS (NM_002617)	promoter-TSS (NM_002617)	-2	NM_15381	5192	Hs.732228	NM_00261	ENSG0000	PEX10	NALD|PBD	peroxisom	protein-co
lib-C666-l	chr6	31830847	31830847	+	17.71746	NA	promoter-TSS (NM_000434).4	promoter-TSS (NM_000434).4	-138	NM_00043	4758	Hs.520037	NM_00043	ENSG0000	NEU1	NANH|NEU	neuraminic	protein-co
lib-C666-l	chr19	13991107	13991107	+	9.20967	NA	intron (NM_001098622, intron 1 of 1)	CpG	3044	NM_00109	342977	Hs.127982	NM_00109	ENSG0000	NANOS3	NANOS1L|	nanos C2H	protein-co
lib-C666-l	chr17	36507702	36507702	+	80.54002	NA	promoter-TSS (NM_014598)	promoter-TSS (NM_014598)	-305	NM_01459	30837	Hs.514132	NM_01459	ENSG0000	SOCS7	NAP4|NCK	suppressor	protein-co
lib-C666-l	chr15	83349312	83349312	+	9.53658	NA	exon (NM_004644, exon 8 of 26)	exon (NM_004644, exon 8 of 26)	29348	NM_00464	8120	Hs.199593	NM_00464	ENSG0000	AP3B2	NAPTB	adaptor re	protein-co
lib-C666-l	chr13	41885235	41885235	+	17.37618	NA	promoter-TSS (NM_001110798)	promoter-TSS (NM_001110798)	-106	NM_00111	79612	Hs.512914	NM_01852	ENSG0000	NAA16	NARG1L	N(alpha)-a	protein-co
lib-C666-l	chr7	122794442	122794442	+	32.11429	NA	intron (NM_001324400, intron 7 of 17	intron (NM_001324400, intron 7 of	45583	NM_00132	6561	Hs.489849	NM_02244	ENSG0000	SLC13A1	NAS1|NaSi	solute carr	protein-co
lib-C666-l	chr7	122830311	122830311	+	3.23837	NA	intron (NM_022444, intron 1 of 14)	LTR69|LTR|ERVL	9714	NM_00132	6561	Hs.489849	NM_02244	ENSG0000	SLC13A1	NAS1|NaSi	solute carr	protein-co
lib-C666-l	chr11	63706244	63706244	+	97.88153	NA	promoter-TSS (NM_001300800)	promoter-TSS (NM_001300800)	-198	NM_02477	79829	Hs.523753	NM_02477	ENSG0000	NAA40	NAT11|PAT	N(alpha)-a	protein-co
lib-C666-l	chr20	19998054	19998054	+	2.00164	NA	promoter-TSS (NM_181527)	promoter-TSS (NM_181527)	120	NM_18152	51126	Hs.368783	NM_01610	ENSG0000	NAA20	NAT3|NAT	N(alpha)-a	protein-co
lib-C666-l	chr3	893084	893084	+	11.95031	NA	Intergenic	HERVE-int|LTR|ERV1	-241258	NM_00128	27255	Hs.387300	NM_01446	ENSG0000	CNTN6	NB3	contactin 6	protein-co
lib-C666-l	chr3	27575557	27575557	+	8.01457	NA	Intergenic	Intergenic	-49646	NM_00132	9497	Hs.250072	NM_00361	ENSG0000	SLC4A7	NBC2|NBC	solute carr	protein-co
lib-C666-l	chr3	27526022	27526022	+	7.97818	NA	promoter-TSS (NM_001321108)	promoter-TSS (NM_001321108)	-111	NM_00132	9497	Hs.250072	NM_00361	ENSG0000	SLC4A7	NBC2|NBC	solute carr	protein-co
lib-C666-l	chr6	24721532	24721532	+	30.542	NA	Intergenic	CpG	-2129	NM_03093	81688	Hs.744857	NM_03093	ENSG0000	C6orf62	Nbla00237	chromosor	protein-co
lib-C666-l	chr2	64751450	64751450	+	73.49606	NA	promoter-TSS (NR_136167)	promoter-TSS (NR_136167)	11	NM_20343	54812	Hs.655167	NM_01765	ENSG0000	AFTPH	Nbla10388	aftiphilin	protein-co
lib-C666-l	chr4	41712049	41712049	+	17.30443	NA	Intergenic	Intergenic	38938	NM_00392	8929	Hs.87202	NM_00392	ENSG0000	PHOX2B	NBLST2|NE	paired like	protein-co
lib-C666-l	chr17	77813648	77813648	+	66.08424	NA	promoter-TSS (NM_003655)	promoter-TSS (NM_003655)	-435	NM_00365	8535	Hs.405046	NM_00365	ENSG0000	CBX4	NBP16|PC2	chromobo:	protein-co
lib-C666-l	chr20	4981756	4981756	+	11.05669	NA	intron (NM_203327, intron 1 of 16)	intron (NM_203327, intron 1 of 16)	389	NM_00511	9962	Hs.516866	NM_00511	ENSG0000	SLC23A2	NBTL1|SLC	solute carr	protein-co
lib-C666-l	chr11	65686961	65686961	+	12.43796	NA	promoter-TSS (NM_001135635)	promoter-TSS (NM_001135635)	233	NM_00644	10589	Hs.356742	NM_00644	ENSG0000	DRAP1	NC2-alpha	DR1 associ	protein-co
lib-C666-l	chr6	84569390	84569390	+	13.59977	NA	promoter-TSS (NM_016230)	promoter-TSS (NM_016230)	20	NM_01623	51167	Hs.5741	NM_01623	ENSG0000	CYB5R4	NCB5OR|cl	cytochrom	protein-co
lib-C666-l	chr7	74180945	74180945	+	10.40378	NA	Intergenic	Intergenic	-7364	NM_00026	653361	Hs.647047	NM_00026	ENSG0000	NCF1	NCF1A|NC	neutrophil	protein-co
lib-C666-l	chr7	74203336	74203336	+	2.38795	NA	intron (NM_000265, intron 10 of 10)	CpG-24511	15027	NM_00026	653361	Hs.647047	NM_00026	ENSG0000	NCF1	NCF1A|NC	neutrophil	protein-co
lib-C666-l	chr7	72627297	72627297	+	9.85986	NA	Intergenic	Intergenic	-7377	NR_00318	654816	Hs.655201	NR_00318	ENSG0000	NCF1B	NCF-1B|SH	neutrophil	pseudo
lib-C666-l	chr2	219824378	219824378	+	3.27468	NA	promoter-TSS (NM_003936)	promoter-TSS (NM_003936)	28	NM_00393	8941	Hs.158460	NM_00393	ENSG0000	CDK5R2	NCK5AI|P3	cyclin depe	protein-co
lib-C666-l	chr13	77566223	77566223	+	55.60226	NA	exon (NM_006493, exon 1 of 4)	exon (NM_006493, exon 1 of 4)	164	NM_00649	1203	Hs.30213	NM_00649	ENSG0000	CLN5	NCL	ceroid-lipc	protein-co
lib-C666-l	chr9	139622664	139622664	+	16.23236	NA	promoter-TSS (NR_024542)	promoter-TSS (NR_024542)	-28	NR_00367	84973	Hs.495480	NR_00367	ENSG0000	SNHG7	NCRNA00(	small nucle	ncRNA
lib-C666-l	chr16	2014999	2014999	+	116.87568	NA	promoter-TSS (NM_002952)	promoter-TSS (NM_002952)	2	NR_00314	735301	Hs.115329	NR_00314	ENSG0000	SNHG9	NCRNA00(	small nucle	ncRNA
lib-C666-l	chr16	30934662	30934662	+	7.49163	NA	promoter-TSS (NR_024348)	promoter-TSS (NR_024348)	-72	NR_02434	283932	Hs.711535	NM_17590	ENSG0000	FBXL19-AS	NCRNA00(	FBXL19 an	ncRNA
lib-C666-l	chr1	762785	762785	+	19.57279	NA	promoter-TSS (NR_047526)	promoter-TSS (NR_047526)	117	NR_02432	79854	Hs.351445	NM_02479	ENSG0000	LINC00115	NCRNA001	long interg	ncRNA
lib-C666-l	chr9	136523902	136523902	+	18.89222	NA	3' UTR (NM_000787, exon 12 of 12)	3' UTR (NM_000787, exon 12 of 12)	-1372	NR_10273	138948	Hs.223858	NR_00278	ENSG0000	DBH-AS1	NCRNA001	DBH antise	ncRNA
lib-C666-l	chrY	7872450	7872450	+	16.59065	NA	Intergenic	BSR/Beta|Satellite|Satellite	199485	NR_00155	83867	Hs.667616	NR_00155	ENSG0000	TTTY12	NCRNA001	testis-spec	ncRNA
lib-C666-l	chr20	62670028	62670028	+	9.10494	NA	non-coding (NR_027686, exon 4 of 4	non-coding (NR_027686, exon 4 of	4331	NR_02768	284739	Hs.97840	NM_20734	ENSG0000	LINC00176	NCRNA001	long interg	protein-co
lib-C666-l	chr17	41277575	41277575	+	61.38507	NA	promoter-TSS (NR_003108)	promoter-TSS (NR_003108)	1	NR_13814	10230	Hs.373818	NM_00582	ENSG0000	NBR2	NCRNA001	neighbor c	ncRNA
lib-C666-l	chr7	39772982	39772982	+	139.39011	NA	promoter-TSS (NR_026999)	promoter-TSS (NR_026999)	-185	NR_02699	349114	Hs.414183	NM_198284		LINC00265	NCRNA002	long interg	ncRNA
lib-C666-l	chr7	39830762	39830762	+	8.0787	NA	intron (NR_026999, intron 10 of 11)	intron (NR_026999, intron 10 of 11)	57595	NR_02699	349114	Hs.414183	NM_198284		LINC00265	NCRNA002	long interg	ncRNA
lib-C666-l	chr7	39773873	39773873	+	8.01457	NA	intron (NR_026999, intron 1 of 11)	LTR24|LTR|ERV1	706	NR_02699	349114	Hs.414183	NM_198284		LINC00265	NCRNA002	long interg	ncRNA
lib-C666-l	chr16	33963137	33963137	+	3.0357	NA	promoter-TSS (NR_038368)	promoter-TSS (NR_038368)	-634	NR_03836	649159	Hs.532675	NM_00104	ENSG0000	LINC00273	NCRNA002	long interg	ncRNA
lib-C666-l	chr2	64431470	64431470	+	4.52748	NA	non-coding (NR_033837, exon 1 of 6	non-coding (NR_033837, exon 1 of	1149	NR_03383	150992	Hs.272318	NR_03383	ENSG0000	LINC00309	NCRNA003	long interg	ncRNA
lib-C666-l	chr19	47950739	47950739	+	2.45439	NA	intron (NM_015063, intron 4 of 9)	intron (NM_015063, intron 4 of 9)	24568	NM_01506	6543	Hs.172884	NM_01506	ENSG0000	SLC8A2	NCX2	solute carr	protein-co
lib-C666-l	chr14	70655861	70655861	+	5.04179	NA	promoter-TSS (NM_182932)	promoter-TSS (NM_182932)	-74	NM_18293	6547	Hs.337696	NM_03326	ENSG0000	SLC8A3	NCX3	solute carr	protein-co
lib-C666-l	chr17	46908353	46908353	+	16.58224	NA	promoter-TSS (NM_001261390)	promoter-TSS (NM_001261390)	3	NM_00126	10241	Hs.514920	NM_00583	ENSG0000	CALCOCO1	NDP52	calcium bir	protein-co
lib-C666-l	chr12	27397037	27397037	+	145.64447	NA	promoter-TSS (NM_015000)	promoter-TSS (NM_015000)	-41	NM_01500	23012	Hs.184523	NM_01500	ENSG0000	STK38L	NDR2	serine/thre	protein-co
lib-C666-l	chr18	55711978	55711978	+	5.04179	NA	intron (NM_001144967, intron 1 of 3(	CpG	368	NM_00124	23327	Hs.185677	NM_01527	ENSG0000	NEDD4L	NEDD4-2|I	neural pre(	protein-co
lib-C666-l	chr7	43116111	43116111	+	5.5166	NA	Intergenic	Intergenic	-36087	NM_01505	23072	Hs.164453	NM_01505	ENSG0000	HECW1	NEDL1	HECT, C2 a	protein-co
lib-C666-l	chr12	49682292	49682292	+	6.81003	NA	Intergenic	Intergenic	-6617	NM_00626	5630	Hs.37044	NM_00626	ENSG0000	PRPH	NEF4|PRPH	peripherin	protein-co
lib-C666-l	chr4	2010924	2010924	+	135.05168	NA	promoter-TSS (NM_005663)	promoter-TSS (NM_005663)	38	NM_00566	7469	Hs.21771	NM_00566	ENSG0000	NELFA	NELF-A|P/(	negative el	protein-co
lib-C666-l	chr14	35192276	35192276	+	22.81978	NA	Intergenic	MER119|DNA|hAT-Charlie	-8247	NR_02813	1073	Hs.180141	NM_02191	ENSG0000	CFL2	NEM7	cofilin 2	protein-co
lib-C666-l	chr1	155294960	155294960	+	17.78771	NA	non-coding (NR_103478, exon 2 of 8	non-coding (NR_103478, exon 2 of	774	NR_10347	23623	Hs.226499	NM_01432	ENSG0000	RUSC1	NESCA	RUN and S	protein-co
lib-C666-l	chr1	109234886	109234886	+	28.64047	NA	promoter-TSS (NM_018061)	promoter-TSS (NM_018061)	-46	NM_01806	55119	Hs.342307	NM_01806	ENSG0000	PRPF38B	NET1	pre-mRNA	protein-co
lib-C666-l	chr2	29117462	29117462	+	5.6488	NA	promoter-TSS (NM_015131)	promoter-TSS (NM_015131)	-47	NM_01513	23160	Hs.622982	NM_01513	ENSG0000	WDR43	NET12|UTF	WD repeat	protein-co
lib-C666-l	chr2	130939194	130939194	+	121.67265	NA	promoter-TSS (NM_001330282)	promoter-TSS (NM_001330282)	136	NM_01795	55627	Hs.516450	NM_01775	ENSG0000	SMPD4	NET13|NSM	sphingomy	protein-co
lib-C666-l	chr2	128568742	128568742	+	24.54495	NA	promoter-TSS (NM_018383)	promoter-TSS (NM_018383)	19	NM_00100	55339	Hs.554831	NM_01838	ENSG0000	WDR33	NET14|WD	WD repeat	protein-co
lib-C666-l	chr2	190305806	190305806	+	162.83224	NA	promoter-TSS (NM_001303096)	promoter-TSS (NM_001303096)	-353	NM_00130	84128	Hs.399984	NM_03216	ENSG0000	WDR75	NET16|UTF	WD repeat	protein-co
lib-C666-l	chr4	40631987	40631987	+	5.2142	NA	promoter-TSS (NM_001098634)	promoter-TSS (NM_001098634)	-104	NM_00109	54502	Hs.518727	NM_01902	ENSG0000	RBM47	NET18	RNA bindir	protein-co
lib-C666-l	chr4	40631555	40631555	+	3.23837	NA	5' UTR (NM_001098634, exon 1 of 7)	5' UTR (NM_001098634, exon 1 of 7	328	NM_00109	54502	Hs.518727	NM_01902	ENSG0000	RBM47	NET18	RNA bindir	protein-co
lib-C666-l	chr1	70687018	70687018	+	8.96177	NA	intron (NM_001190987, intron 1 of 12	intron (NM_001190987, intron 1 of	15653	NM_00119	9295	Hs.479693	NM_00476	ENSG0000	SRSF11	NET2|SFRS	serine and	protein-co
lib-C666-l	chr5	72861577	72861577	+	64.82358	NA	promoter-TSS (NM_023039)	promoter-TSS (NM_023039)	11	NM_00128	84135	Hs.406703	NM_03217	ENSG0000	UTP15	NET21	UTP15, sm	protein-co
lib-C666-l	chr7	44788308	44788308	+	10.17139	NA	promoter-TSS (NM_031449)	promoter-TSS (NM_031449)	143	NM_00130	83637	Hs.77978	NM_03144	ENSG0000	ZMIZ2	NET27|TRA	zinc finger	protein-co
lib-C666-l	chr7	75620518	75620518	+	11.66438	NA	intron (NM_001317803, intron 3 of 11	intron (NM_001317803, intron 3 of	3474	NM_03192	83862	Hs.488835	NM_03192	ENSG0000	TMEM120A	NET29|TMI	transmemb	protein-co
lib-C666-l	chr9	34376884	34376884	+	2.26435	NA	promoter-TSS (NM_020702)	promoter-TSS (NM_020702)	10	NM_02070	57462	Hs.522083	NM_02070	ENSG0000	KIAA1161	NET37	KIAA1161	protein-co
lib-C666-l	chr4	99579966	99579966	+	8.03539	NA	promoter-TSS (NM_005723)	promoter-TSS (NM_005723)	-154	NM_00572	10098	Hs.118118	NM_00572	ENSG0000	TSPAN5	NET-4|NET	tetraspanir	protein-co
lib-C666-l	chr4	99582972	99582972	+	6.15313	NA	Intergenic	L2c|LINE|L2	-3160	NM_00572	10098	Hs.118118	NM_00572	ENSG0000	TSPAN5	NET-4|NET	tetraspanir	protein-co
lib-C666-l	chr10	71905905	71905905	+	4.46944	NA	exon (NM_001040273, exon 1 of 2)	exon (NM_001040273, exon 1 of 2)	591	NM_17355	219743	Hs.533655	NM_17355	ENSG0000	TYSND1	NET41	trypsin dor	protein-co
lib-C666-l	chr10	71906379	71906379	+	3.31563	NA	non-coding (NR_073581, exon 1 of 4	non-coding (NR_073581, exon 1 of	117	NM_17355	219743	Hs.533655	NM_17355	ENSG0000	TYSND1	NET41	trypsin dor	protein-co
lib-C666-l	chr12	132628939	132628939	+	488.64294	NA	promoter-TSS (NM_175066)	promoter-TSS (NM_175066)	-33	NM_02407	79050	Hs.558536	NM_02407	ENSG0000	NOC4L	NET49|NO	nucleolar c	protein-co
lib-C666-l	chr12	3317232	3317232	+	35.47771	NA	intron (NM_006675, intron 3 of 8)	MIRb|SINE|MIR	130711	NM_00116	10867	Hs.504517	NM_00667	ENSG0000	TSPAN9	NET-5|NET	tetraspanir	protein-co
lib-C666-l	chr19	3185397	3185397	+	3.96379	NA	promoter-TSS (NM_001321463)	promoter-TSS (NM_001321463)	-478	NM_02017	56926	Hs.657032	NM_02017	ENSG0000	NCLN	NET59	nicalin	protein-co
lib-C666-l	chr7	16793066	16793066	+	7.6867	NA	promoter-TSS (NM_014399)	promoter-TSS (NM_014399)	-285	NM_01439	27075	Hs.364544	NM_01439	ENSG0000	TSPAN13	NET-6|NET	tetraspanir	protein-co
lib-C666-l	chr5	171995650	171995650	+	12.12504	NA	Intergenic	L2b|LINE|L2	-72619	NM_00130	54492	Hs.91521	NM_00114	ENSG0000	NEURL1B	NEURL3|hN	neuralized	protein-co
lib-C666-l	chr5	108745849	108745849	+	29.93113	NA	promoter-TSS (NM_014819)	promoter-TSS (NM_014819)	-174	NM_01481	9867	Hs.483036	NM_01481	ENSG0000	PJA2	Neurodap1	praja ring f	protein-co
lib-C666-l	chr6	157802055	157802055	+	9.94079	NA	promoter-TSS (NM_024630)	promoter-TSS (NM_024630)	-502	NM_02463	79683	Hs.596214	NM_02463	ENSG0000	ZDHHC14	NEW1CP	zinc finger	protein-co
lib-C666-l	chr1	204776565	204776565	+	36.84144	NA	Intergenic	Intergenic	-21217	NM_00100	23114	Hs.13349	NM_01509	ENSG0000	NFASC	NF|NRCAM	neurofasci	protein-co
lib-C666-l	chr1	153643641	153643641	+	12.34228	NA	promoter-TSS (NM_001267809)	promoter-TSS (NM_001267809)	-137	NM_00451	3608	Hs.75117	NM_00451	ENSG0000	ILF2	NF45|PRO:	interleukin	protein-co
lib-C666-l	chr1	153644442	153644442	+	3.57421	NA	promoter-TSS (NM_001267809)	promoter-TSS (NM_001267809)	-938	NM_00451	3608	Hs.75117	NM_00451	ENSG0000	ILF2	NF45|PRO:	interleukin	protein-co
lib-C666-l	chr14	24837052	24837052	+	12.13776	NA	promoter-TSS (NM_004554)	promoter-TSS (NM_004554)	-174	NM_00455	4776	Hs.77810	NM_00455	ENSG0000	NFATC4	NF-AT3|NF	nuclear fac	protein-co
lib-C666-l	chr14	24835999	24835999	+	10.27265	NA	promoter-TSS (NM_001136022)	promoter-TSS (NM_001136022)	-118	NM_00113	4776	Hs.77810	NM_00455	ENSG0000	NFATC4	NF-AT3|NF	nuclear fac	protein-co
lib-C666-l	chr14	24836631	24836631	+	5.66964	NA	promoter-TSS (NM_004554)	promoter-TSS (NM_004554)	514	NM_00132	4776	Hs.77810	NM_00455	ENSG0000	NFATC4	NF-AT3|NF	nuclear fac	protein-co
lib-C666-l	chr16	68119114	68119114	+	17.37618	NA	promoter-TSS (NM_004555)	promoter-TSS (NM_004555)	-155	NM_00455	4775	Hs.436585	NM_00455	ENSG0000	NFATC3	NFAT4|NFA	nuclear fac	protein-co
lib-C666-l	chr18	77180386	77180386	+	10.37767	NA	intron (NM_172389, intron 2 of 9)	CpG	20112	NM_17238	4772	Hs.534074	NM_00616	ENSG0000	NFATC1	NF-ATC|NI	nuclear fac	protein-co

lib-C666-I	chr18	77180752	77180752	+	4.46583	NA	intron (NM_172389, intron 2 of 9)	CpG	20478	NM_17238	4772	Hs.534074	NM_00616	ENSG0000	NFATC1	NF-ATC|NI	nuclear fac	protein-co
lib-C666-I	chr6	30685221	30685221	+	4.90117	NA	5' UTR (NM_014641, exon 1 of 15)	5' UTR (NM_014641, exon 1 of 15)	237	NM_01464	9656	Hs.653495	NM_01464	ENSG0000	MDC1	NFBD1	mediator c	protein-co
lib-C666-I	chr7	1560402	1560402	+	8.48019	NA	Intergenic	MLT1G3|LTR|ERVL-MaLR	-9966	NM_00236	7975	Hs.520612	NM_00236	ENSG0000	MAFK	NFE2U|P18	MAF bZIP t	protein-co
lib-C666-I	chr11	65430462	65430462	+	11.07709	NA	promoter-TSS (NM_001243984)	promoter-TSS (NM_001243984)	-19	NM_00124	5970	Hs.502875	NM_02197	ENSG0000	RELA	NFKB3|p65	RELA protc	protein-co
lib-C666-I	chr17	29421874	29421874	+	9.02258	NA	promoter-TSS (NM_000267)	promoter-TSS (NM_000267)	-71	NM_00026	4763	Hs.113577	NM_00026	ENSG0000	NF1	NFNS|VRN	neurofibro	protein-co
lib-C666-I	chr9	33290417	33290417	+	260.86801	NA	promoter-TSS (NM_147134)	promoter-TSS (NM_147134)	2	NM_14713	4799	Hs.413074	NM_00250	ENSG0000	NFX1	NFX2|TEG-	nuclear tra	protein-co
lib-C666-I	chr1	178062903	178062903	+	7.15277	NA	promoter-TSS (NM_170692)	promoter-TSS (NM_170692)	39	NM_17069	9462	Hs.496139	NM_00484	ENSG0000	RASAL2	NGAP	RAS protei	protein-co
lib-C666-I	chr11	57228554	57228554	+	46.45276	NA	intron (NM_178570, intron 1 of 2)	CpG-4141	544	NM_17857	349667	Hs.502618	NM_17857	ENSG0000	RTN4RL2	NGRH1|Ng	reticulon 4	protein-co
lib-C666-I	chr11	57228182	57228182	+	3.07144	NA	5' UTR (NM_178570, exon 1 of 3)	5' UTR (NM_178570, exon 1 of 3)	172	NM_17857	349667	Hs.502618	NM_17857	ENSG0000	RTN4RL2	NGRH1|Ng	reticulon 4	protein-co
lib-C666-I	chr17	1927231	1927231	+	10.9095	NA	intron (NM_178568, intron 1 of 1)	CpG	1408	NM_17856	146760	Hs.22917	NM_17856	ENSG0000	RTN4RL1	NGRH2|Ng	reticulon 4	protein-co
lib-C666-I	chr4	103940866	103940866	+	109.04404	NA	promoter-TSS (NM_001100874)	promoter-TSS (NM_001100874)	30	NM_00110	150159	Hs.666728	NM_13917	ENSG0000	SLC9B1	NHA1|NHE	solute carr	protein-co
lib-C666-I	chr2	103274515	103274515	+	14.17553	NA	intron (NM_003048, intron 2 of 11)	intron (NM_003048, intron 2 of 11)	38522	NM_00304	6549	Hs.250083	NM_00304	ENSG0000	SLC9A2	NHE2	solute carr	protein-co
lib-C666-I	chr20	48388984	48388984	+	14.17553	NA	Intergenic	Intergenic	-40266	NR_048539	23315	Hs.444202	NM_01526	ENSG0000	SLC9A8	NHE-8|NH	solute carr	protein-co
lib-C666-I	chr16	88636820	88636820	+	126.61713	NA	promoter-TSS (NM_001294340)	promoter-TSS (NM_001294340)	31	NM_00129	124245	Hs.93670	NM_14460	ENSG0000	ZC3H18	NHN1	zinc finger	protein-co
lib-C666-I	chr11	124492792	124492792	+	16.59065	NA	promoter-TSS (NM_032811)	promoter-TSS (NM_032811)	50	NR_016021	84897	Hs.436410	NM_03281	ENSG0000	TBRG1	NIAM|TB-5	transformir	protein-co
lib-C666-I	chr2	219264453	219264453	+	26.26478	NA	promoter-TSS (NM_021198)	promoter-TSS (NM_021198)	-25	NM_18264	58190	Hs.444468	NM_02119	ENSG0000	CTDSP1	NIF3|NLI-II	CTD small	protein-co
lib-C666-I	chr2	219265096	219265096	+	10.03103	NA	intron (NM_182642, intron 1 of 6)	CpG-15947	618	NM_02119	58190	Hs.444468	NM_02119	ENSG0000	CTDSP1	NIF3|NLI-II	CTD small	protein-co
lib-C666-I	chr2	219260956	219260956	+	3.98718	NA	3' UTR (NM_000578, exon 15 of 15)	3' UTR (NM_000578, exon 15 of 15)	-2105	NM_00120	58190	Hs.444468	NM_02119	ENSG0000	CTDSP1	NIF3|NLI-II	CTD small	protein-co
lib-C666-I	chr19	36036470	36036470	+	110.29659	NA	promoter-TSS (NM_032635)	promoter-TSS (NM_032635)	-32	NM_00124	10430	Hs.9234	NM_03263	ENSG0000	TMEM147	NIFIE14	transmemb	protein-co
lib-C666-I	chr1	41134634	41134634	+	17.27528	NA	Intergenic	Intergenic	-3310	NM_01474	9783	Hs.654808	NM_01474	ENSG0000	RIMS3	NIM3|RIM3	regulating	protein-co
lib-C666-I	chr9	95896953	95896953	+	28.8837	NA	promoter-TSS (NM_004148)	promoter-TSS (NM_004148)	-383	NM_00414	4814	Hs.494457	NM_00414	ENSG0000	NINJ1	NIN1|NINJ	ninjurin 1	protein-co
lib-C666-I	chr16	75034858	75034858	+	2.58854	NA	intron (NM_032268, intron 1 of 4)	intron (NM_032268, intron 1 of 4)	1943	NM_03226	84937	Hs.427284	NM_03226	ENSG0000	ZNRF1	NIN283	zinc and rii	protein-co
lib-C666-I	chr5	172602726	172602726	+	4.83287	NA	Intergenic	Intergenic	31281	NM_00120	662	Hs.145726	NM_00120	ENSG0000	BNIP1	NIP1|SEC2(	BCL2 intera	protein-co
lib-C666-I	chr5	172571941	172571941	+	4.52748	NA	intron (NM_013980, intron 1 of 5)	intron (NM_013980, intron 1 of 5)	496	NM_00120	662	Hs.145726	NM_00120	ENSG0000	BNIP1	NIP1|SEC2(	BCL2 intera	protein-co
lib-C666-I	chr10	133795541	133795541	+	3.98718	NA	promoter-TSS (NM_004052)	promoter-TSS (NM_004052)	-24	NM_00405	664	Hs.144873	NM_00405	ENSG0000	BNIP3	NIP3	BCL2 intera	protein-co
lib-C666-I	chr5	36744741	36744741	+	10.27265	NA	Intergenic	Intergenic	132055	NR_046262	646719	Hs.355684	NR_046262		NIPBL-AS1	NIPBL-AS	NIPBL antis	ncRNA
lib-C666-I	chr5	36745496	36745496	+	9.22467	NA	Intergenic	CpG	131300	NR_046262	646719	Hs.355684	NR_046262		NIPBL-AS1	NIPBL-AS	NIPBL antis	ncRNA
lib-C666-I	chr5	36744370	36744370	+	3.59106	NA	Intergenic	Intergenic	132426	NR_046262	646719	Hs.355684	NR_046262		NIPBL-AS1	NIPBL-AS	NIPBL antis	ncRNA
lib-C666-I	chr7	56032340	56032340	+	11.55573	NA	promoter-TSS (NM_001202469)	promoter-TSS (NM_001202469)	70	NM_00148	2631	Hs.591069	NM_00148	ENSG0000	GBAS	NIPSNAP2	glioblaston	protein-co
lib-C666-I	chr1	247494411	247494411	+	21.84731	NA	intron (NM_001329733, intron 2 of 9)	CpG	831	NM_03275	84838	Hs.168677	NM_03275	ENSG0000	ZNF496	NIZP1|ZFP4	zinc finger	protein-co
lib-C666-I	chr16	4357828	4357828	+	22.98772	NA	Intergenic	CpG	-6934	NM_00131	84662	Hs.592087	NM_03257	ENSG0000	GLIS2	NKL|NPHP	GLIS family	protein-co
lib-C666-I	chr16	4365189	4365189	+	5.50154	NA	5' UTR (NM_001318918, exon 1 of 8)	5' UTR (NM_001318918, exon 1 of 8	427	NM_00131	84662	Hs.592087	NM_03257	ENSG0000	GLIS2	NKL|NPHP	GLIS family	protein-co
lib-C666-I	chr16	4359850	4359850	+	5.3823	NA	Intergenic	Intergenic	-4912	NM_00131	84662	Hs.592087	NM_03257	ENSG0000	GLIS2	NKL|NPHP	GLIS family	protein-co
lib-C666-I	chr16	4365459	4365459	+	4.73866	NA	intron (NM_001318918, intron 1 of 7)	CpG	697	NM_00131	84662	Hs.592087	NM_03257	ENSG0000	GLIS2	NKL|NPHP	GLIS family	protein-co
lib-C666-I	chr4	85420688	85420688	+	4.73866	NA	Intergenic	CpG-19950	-1301	NM_00616	4825	Hs.546270	NM_00616	ENSG0000	NKX6-1	NKX6.1|NK	NK6 home	protein-co
lib-C666-I	chr3	173113279	173113279	+	4.19683	NA	Intergenic	CpG	-2959	NM_01493	22871	Hs.478289	NM_01493	ENSG0000	NLGN1	NL1	neuroligin	protein-co
lib-C666-I	chr17	33469292	33469292	+	7.08929	NA	promoter-TSS (NM_001014445)	promoter-TSS (NM_001014445)	-22	NM_00101	54475	Hs.85570	NM_01809	ENSG0000	NLE1	Nle	notchless h	protein-co
lib-C666-I	chr20	25524374	25524374	+	9.79436	NA	intron (NM_025176, intron 1 of 23)	MER51A|LTR|ERV1	41793	NM_02517	22981	Hs.631508	NM_02517	ENSG0000	NINL	NLP	ninein like	protein-co
lib-C666-I	chr8	37553299	37553299	+	3.61436	NA	promoter-TSS (NM_025069)	promoter-TSS (NM_025069)	30	NM_02506	80139	Hs.288042	NM_02506	ENSG0000	ZNF703	NLZ1|ZEPP	zinc finger	protein-co
lib-C666-I	chr17	49230879	49230879	+	12.55817	NA	promoter-TSS (NM_198175)	promoter-TSS (NM_198175)	-18	NR_037149	654364	Hs.463456	NM_00101	ENSG0000	NME1-NM	NM23-LV|	NME1-NM	protein-co
lib-C666-I	chr10	28966307	28966307	+	42.39316	NA	promoter-TSS (NM_012342)	promoter-TSS (NM_012342)	-117	NM_01234	25805	Hs.533336	NM_01234	ENSG0000	BAMBI	NMA	BMP and a	protein-co
lib-C666-I	chr2	74425543	74425543	+	5.76059	NA	promoter-TSS (NM_006636)	promoter-TSS (NM_006636)	-147	NR_027405	10797	Hs.469030	NM_00663	ENSG0000	MTHFD2	NMDMC	methylenet	protein-co
lib-C666-I	chr17	8534709	8534709	+	13.8811	NA	promoter-TSS (NM_001256012)	promoter-TSS (NM_001256012)	-630	NM_00125	4628	Hs.16355	NM_00596	ENSG0000	MYH10	NMMHC-I	myosin hea	protein-co
lib-C666-I	chr17	8534358	8534358	+	7.21092	NA	promoter-TSS (NM_001256012)	promoter-TSS (NM_001256012)	-279	NM_00125	4628	Hs.16355	NM_00596	ENSG0000	MYH10	NMMHC-I	myosin hea	protein-co
lib-C666-I	chr11	71791853	71791853	+	67.35306	NA	promoter-TSS (NM_001286561)	promoter-TSS (NM_001286561)	-114	NM_00128	4926	Hs.325978	NM_00618	ENSG0000	NUMA1	NMP-22|N	nuclear mit	protein-co
lib-C666-I	chr7	2797997	2797997	+	12.73831	NA	intron (NM_007353, intron 2 of 3)	Charlie1|DNA|hAT-Charlie	4777	NM_00128	2768	Hs.487341	NM_00735	ENSG0000	GNA12	NNX3|RMF	G protein s	protein-co
lib-C666-I	chr7	2884056	2884056	+	3.31563	NA	promoter-TSS (NM_001293092)	promoter-TSS (NM_001293092)	-97	NM_00735	2768	Hs.487341	NM_00735	ENSG0000	GNA12	NNX3|RMF	G protein s	protein-co
lib-C666-I	chr7	2884302	2884302	+	3.25502	NA	promoter-TSS (NM_001293092)	promoter-TSS (NM_001293092)	-343	NM_00735	2768	Hs.487341	NM_00735	ENSG0000	GNA12	NNX3|RMF	G protein s	protein-co
lib-C666-I	chr19	10044869	10044869	+	2.05265	NA	intron (NM_058164, intron 1 of 5)	intron (NM_058164, intron 1 of 5)	2359	NM_05816	93145	Hs.169743	NM_05816	ENSG0000	OLFM2	NOE2|NOE	olfactomec	protein-co
lib-C666-I	chr8	37749479	37749479	+	14.45701	NA	intron (NM_001002814, intron 1 of 5)	intron (NM_001002814, intron 1 of	7536	NM_00100	80223	Hs.191179	NM_02515	ENSG0000	RAB11FIP1	NOEL1A|R(	RAB11 fam	protein-co
lib-C666-I	chr7	72395577	72395577	+	388.40335	NA	intron (NM_001257190, intron 4 of 1	CpG	29725	NR_033323	260294	Hs.510927	NM_14893	ENSG0000	NSUN5P2	NOL1R2|N	NOP2/Sun	pseudo
lib-C666-I	chr7	72425382	72425382	+	14.54258	NA	promoter-TSS (NR_033323)	promoter-TSS (NR_033323)	-80	NR_033323	260294	Hs.510927	NM_14893	ENSG0000	NSUN5P2	NOL1R2|N	NOP2/Sun	pseudo
lib-C666-I	chr4	110736717	110736717	+	96.99037	NA	promoter-TSS (NM_018983)	promoter-TSS (NM_018983)	51	NM_03299	54433	Hs.69851	NM_01898	ENSG0000	GAR1	NOLA1	GAR1 ribor	protein-co
lib-C666-I	chr22	38082461	38082461	+	7.75972	NA	exon (NM_024313, exon 1 of 6)	exon (NM_024313, exon 1 of 6)	117	NM_02431	79159	Hs.632778	NM_02431	ENSG0000	NOL12	Nop25|dJ3	nucleolar p	protein-co
lib-C666-I	chr10	103912034	103912034	+	195.86848	NA	5' UTR (NM_001284388, exon 1 of 13	5' UTR (NM_001284388, exon 1 of 1	101	NM_00474	9221	Hs.523238	NM_00474	ENSG0000	NOLC1	NOPP130|I	nucleolar a	protein-co
lib-C666-I	chr5	34737038	34737038	+	34.25375	NA	intron (NM_001145525, intron 4 of 1	MER1B|DNA|hAT-Charlie	49374	NM_00114	26064	Hs.431400	NM_01557	ENSG0000	RAI14	NORPEG|R	retinoic aci	protein-co
lib-C666-I	chr5	34626284	34626284	+	21.21068	NA	Intergenic	Tigger3b|DNA|TcMar-Tigger	-30149	NM_00114	26064	Hs.431400	NM_01557	ENSG0000	RAI14	NORPEG|R	retinoic aci	protein-co
lib-C666-I	chr5	34656415	34656415	+	6.29226	NA	promoter-TSS (NM_001145522)	promoter-TSS (NM_001145522)	-18	NM_00114	26064	Hs.431400	NM_01557	ENSG0000	RAI14	NORPEG|R	retinoic aci	protein-co
lib-C666-I	chr5	34585874	34585874	+	4.52748	NA	Intergenic	Intergenic	-70559	NM_00114	26064	Hs.431400	NM_01557	ENSG0000	RAI14	NORPEG|R	retinoic aci	protein-co
lib-C666-I	chr4	48018789	48018789	+	4.73866	NA	promoter-TSS (NM_207330)	promoter-TSS (NM_207330)	0	NM_20733	152519	Hs.134190	NM_20733	ENSG0000	NIPAL1	NPAL1	NIPA like d	protein-co
lib-C666-I	chr18	21166850	21166850	+	6.20473	NA	promoter-TSS (NM_000271)	promoter-TSS (NM_000271)	-269	NM_00027	4864	Hs.464779	NM_00027	ENSG0000	NPC1	NPC	NPC intrac	protein-co
lib-C666-I	chr18	21166646	21166646	+	3.27468	NA	promoter-TSS (NM_000271)	promoter-TSS (NM_000271)	-65	NM_00027	4864	Hs.464779	NM_00027	ENSG0000	NPC1	NPC	NPC intrac	protein-co
lib-C666-I	chr3	128598395	128598395	+	335.78116	NA	promoter-TSS (NM_014049)	promoter-TSS (NM_014049)	62	NM_01404	28976	Hs.567482	NM_01404	ENSG0000	ACAD9	NPD002	acyl-CoA c	protein-co
lib-C666-I	chr10	95753481	95753481	+	5.9194	NA	promoter-TSS (NM_001288989)	promoter-TSS (NM_001288989)	-265	NM_00128	51196	Hs.655033	NM_01634	ENSG0000	PLCE1	NPHS3|PL(	phospholip	protein-co
lib-C666-I	chr1	151284255	151284255	+	10.58189	NA	intron (NM_002651, intron 3 of 12)	intron (NM_002651, intron 3 of 12)	15936	NM_00119	5298	Hs.632465	NM_00265	ENSG0000	PI4KB	NPIK|PI4K-	phosphatic	protein-co
lib-C666-I	chr16	70100063	70100063	+	7.0832	NA	promoter-TSS (NR_003610)	promoter-TSS (NR_003610)	-212	NR_003610	283970	Hs.513695	NM_19913	ENSG0000	PDXDC2P	NPIP|PDXD	pyridoxal c	pseudo
lib-C666-I	chr4	185129798	185129798	+	4.98754	NA	intron (NM_153343, intron 1 of 7)	L1PA3|LINE|L1	9316	NM_15334	133121	Hs.297814	NM_15334	ENSG0000	ENPP6	NPP6	ectonuclec	protein-co
lib-C666-I	chr17	38497697	38497697	+	36.16835	NA	promoter-TSS (NM_001024809)	promoter-TSS (NM_001024809)	-574	NM_00102	5914	Hs.654583	NM_00096	ENSG0000	RARA	NR1B1|RAI	retinoic aci	protein-co
lib-C666-I	chr17	38468027	38468027	+	5.96422	NA	intron (NM_000964, intron 1 of 8)	intron (NM_000964, intron 1 of 8)	2604	NM_00096	5914	Hs.654583	NM_00096	ENSG0000	RARA	NR1B1|RAI	retinoic aci	protein-co
lib-C666-I	chr12	53629967	53629967	+	3.27468	NA	Intergenic	Intergenic	-3927	NM_00096	5916	Hs.1497	NM_00096	ENSG0000	RARG	NR1B3|RAI	retinoic aci	protein-co
lib-C666-I	chr15	60885111	60885111	+	3.57421	NA	promoter-TSS (NM_134262)	promoter-TSS (NM_134262)	-404	NM_13426	6095	Hs.560343	NM_00294	ENSG0000	RORA	NR1F1|ROI	RAR relate	protein-co
lib-C666-I	chr12	48290730	48290730	+	23.27622	NA	intron (NM_000376, intron 1 of 9)	intron (NM_000376, intron 1 of 9)	8084	NM_00101	7421	Hs.524368	NM_00037	ENSG0000	VDR	NR1I1|PPP	vitamin D (	protein-co
lib-C666-I	chr9	137208882	137208882	+	20.90919	NA	Intergenic	L3|LINE|CR1	-9427	NM_00295	6256	Hs.590886	NM_00295	ENSG0000	RXRA	NR2B1	retinoid X r	protein-co
lib-C666-I	chr9	137209648	137209648	+	12.62235	NA	Intergenic	Intergenic	-8661	NM_00295	6256	Hs.590886	NM_00295	ENSG0000	RXRA	NR2B1	retinoid X r	protein-co
lib-C666-I	chr9	137210187	137210187	+	8.48019	NA	Intergenic	Intergenic	-8122	NM_00295	6256	Hs.590886	NM_00295	ENSG0000	RXRA	NR2B1	retinoid X r	protein-co
lib-C666-I	chr9	137210422	137210422	+	5.23609	NA	Intergenic	Intergenic	-7887	NM_00295	6256	Hs.590886	NM_00295	ENSG0000	RXRA	NR2B1	retinoid X r	protein-co
lib-C666-I	chr9	137249145	137249145	+	4.92693	NA	intron (NM_002957, intron 1 of 9)	intron (NM_002957, intron 1 of 9)	-21996	NM_00129	6256	Hs.590886	NM_00295	ENSG0000	RXRA	NR2B1	retinoid X r	protein-co
lib-C666-I	chr9	137208325	137208325	+	4.51266	NA	Intergenic	Intergenic	-9984	NM_00295	6256	Hs.590886	NM_00295	ENSG0000	RXRA	NR2B1	retinoid X r	protein-co
lib-C666-I	chr1	52344640	52344640	+	91.86094	NA	promoter-TSS (NM_001242361)	promoter-TSS (NM_001242361)	-31	NM_00110	4898	Hs.584782	NM_00252	ENSG0000	NRDC	NRD1|hNR	nardilysin (	protein-co
lib-C666-I	chr7	26195489	26195489	+	14.75675	NA	intron (NM_004289, intron 1 of 3)	MIRb|SINE|MIR	3642	NM_00428	9603	Hs.404741	NM_00428	ENSG0000	NFE2L3	NRF3	nuclear fac	protein-co
lib-C666-I	chr7	26192005	26192005	+	2.98619	NA	5' UTR (NM_004289, exon 1 of 4)	5' UTR (NM_004289, exon 1 of 4)	158	NM_00428	9603	Hs.404741	NM_00428	ENSG0000	NFE2L3	NRF3	nuclear fac	protein-co
lib-C666-I	chr7	111846617	111846617	+	33.00194	NA	promoter-TSS (NM_021994)	promoter-TSS (NM_021994)	-26	NM_02199	11179	Hs.655904	NM_02199	ENSG0000	ZNF277	NRIF4|ZNF	zinc finger	protein-co
lib-C666-I	chr6	6009316	6009316	+	4.90117	NA	Intergenic	Intergenic	-1478	NM_00127	51299	Hs.103291	NM_01658	ENSG0000	NRN1	NRN|dJ38(	neuritin 1	protein-co
lib-C666-I	chr12	45342691	45342691	+	4.46583	NA	Intergenic	Intergenic	-34980	NM_00114	4753	Hs.505326	NM_00615	ENSG0000	NELL2	NRP2	neural EGF	protein-co
lib-C666-I	chr4	57775154	57775154	+	2.00164	NA	promoter-TSS (NM_001193508)	promoter-TSS (NM_001193508)	75	NM_00119	5978	Hs.307836	NM_00561	ENSG0000	REST	NRSF|WT6	RE1 silenci	protein-co
lib-C666-I	chr17	860437	860437	+	11.42528	NA	intron (NM_022463, intron 1 of 7)	intron (NM_022463, intron 1 of 7)	22561	NM_02246	64359	Hs.527989	NM_02246	ENSG0000	NXN	NRX|TRG-4	nucleoredc	protein-co
lib-C666-I	chr5	125731242	125731242	+	7.76438	NA	intron (NM_001146319, intron 1 of 1	intron (NM_001146319, intron 1 of	-27627	NM_00114	65983	Hs.363558	NM_02392	ENSG0000	GRAMD3	NS3TP2	GRAM don	protein-co
lib-C666-I	chr3	99979799	99979799	+	20.82108	NA	5' UTR (NM_018309, exon 1 of 18)	5' UTR (NM_018309, exon 1 of 18)	138	NM_01830	55773	Hs.477003	NM_01830	ENSG0000	TBC1D23	NS4ATP1	TBC1 doma	protein-co
lib-C666-I	chr7	27702666	27702666	+	39.26979	NA	promoter-TSS (NM_152740)	promoter-TSS (NM_152740)	-46	NM_15274	11112	Hs.406758	NM_15274	ENSG0000	HIBADH	NS5ATP1	3-hydroxyi	protein-co
lib-C666-I	chr11	120081869	120081869	+	9.21057	NA	5' UTR (NM_178507, exon 1 of 4)	5' UTR (NM_178507, exon 1 of 4)	122	NM_17850	220323	Hs.445081	NM_17850	ENSG0000	OAF	NS5ATP13	out at first	protein-co
lib-C666-I	chr16	27280120	27280120	+	45.23903	NA	promoter-TSS (NM_145080)	promoter-TSS (NM_145080)	-7	NM_14508	197370	Hs.284295	NM_14508	ENSG0000	NSMCE1	NSE1	NSE1 hom	protein-co
lib-C666-I	chr17	44449982	44449982	+	10.27265	NA	promoter-TSS (NR_033799)	promoter-TSS (NR_033799)	-197	NR_033799	728806	Hs.735097	NR_033799		NSFP1	NSFP	N-ethylma	pseudo
lib-C666-I	chr16	68482741	68482741	+	2.78587	NA	promoter-TSS (NM_018667)	promoter-TSS (NM_018667)	-332	NM_01866	55512	Hs.368421	NM_01866	ENSG0000	SMPD3	NSMASE2	sphingomy	protein-co
lib-C666-I	chr19	6768015	6768015	+	99.9228	NA	promoter-TSS (NM_005490)	promoter-TSS (NM_005490)	-492	NM_00549	10045	Hs.439645	NM_00549	ENSG0000	SH2D3A	NSP1	SH2 doma	protein-co
lib-C666-I	chr1	94313656	94313656	+	50.2624	NA	promoter-TSS (NM_001261408)	promoter-TSS (NM_001261408)	-950	NM_00126	8412	Hs.36958	NM_00356	ENSG0000	BCAR3	NSP2|SH2D	breast canc	protein-co
lib-C666-I	chr1	94220146	94220146	+	13.20416	NA	intron (NM_001261408, intron 2 of 1	Tigger3b|DNA|TcMar-Tigger	-72752	NM_00356	8412	Hs.36958	NM_00356	ENSG0000	BCAR3	NSP2|SH2D	breast canc	protein-co
lib-C666-I	chr1	94171620	94171620	+	9.93242	NA	intron (NM_001261408, intron 3 of 1	intron (NM_001261408, intron 3 of	-24226	NM_00356	8412	Hs.36958	NM_00356	ENSG0000	BCAR3	NSP2|SH2D	breast canc	protein-co
lib-C666-I	chr7	75072071	75072071	+	428.0585	NA	intron (NM_001099415, intron 3 of 1	CpG	32466	NR_104013	155400	Hs.436034	NM_00103	ENSG0000	NSUN5P1	NSUN5B|W	NOP2/Sun	pseudo
lib-C666-I	chr7	75039497	75039497	+	8.48019	NA	promoter-TSS (NR_033322)	promoter-TSS (NR_033322)	-108	NR_033322	155400	Hs.436034	NM_00103	ENSG0000	NSUN5P1	NSUN5B|W	NOP2/Sun	pseudo
lib-C666-I	chrX	24711910	24711910	+	99.63164	NA	promoter-TSS (NM_001330360)	promoter-TSS (NM_001330360)	-40	NM_00133	5422	Hs.567319	NM_01693	ENSG0000	POLA1	NSX|POLA	DNA polyn	protein-co
lib-C666-I	chr12	6862305	6862305	+	42.49125	NA	promoter-TSS (NM_005439)	promoter-TSS (NM_005439)	-223	NM_00543	8079	Hs.524214	NM_00543	ENSG0000	MLF2	NTN4	myeloid leu	protein-co
lib-C666-I	chr12	6852547	6852547	+	6.15313	NA	Intergenic	THE1B|LTR|ERVL-MaLR	9535	NM_00543	8079	Hs.524214	NM_00543	ENSG0000	MLF2	NTN4	myeloid leu	protein-co
lib-C666-I	chr20	61334979	61334979	+	22.14682	NA	Intergenic	Intergenic	-5210	NM_00253	4923	Hs.590869	NM_00253	ENSG0000	NTSR1	NTR	neurotensi	protein-co
lib-C666-I	chr22	26908419	26908419	+	36.59206	NA	promoter-TSS (NM_001008697)	promoter-TSS (NM_001008697)	53	NM_00134	24144	Hs.20225	NM_01214	ENSG0000	TFIP11	NTR1|STIP|	tuftelin inte	protein-co
lib-C666-I	chr15	71055918	71055918	+	21.28624	NA	promoter-TSS (NM_018003)	promoter-TSS (NM_018003)	-68	NM_01800	55075	Hs.108049	NM_01800	ENSG0000	UACA	NUCLING	uveal auto	protein-co
lib-C666-I	chr7	44530442	44530442	+	3.20885	NA	promoter-TSS (NM_015332)	promoter-TSS (NM_015332)	-57	NM_01533	23386	Hs.488171	NM_01533	ENSG0000	NUDCD3	NudCL	NudC dom	protein-co
lib-C666-I	chr4	88343780	88343780	+	5.9194	NA	promoter-TSS (NM_001248011)	promoter-TSS (NM_001248011)	31	NM_19803	53343	Hs.149500	NM_02404	ENSG0000	NUDT9	NUDT10	nudix hydr	protein-co
lib-C666-I	chr13	45492388	45492388	+	7.75972	NA	Intergenic	Intergenic	71225	NM_01234	26747	Hs.525006	NM_01234	ENSG0000	NUFIP1	NUFIP|bA5	NUFIP1, FM	protein-co
lib-C666-I	chr12	57637589	57637589	+	7.16095	NA	non-coding (NR_104422, exon 8 of 8	non-coding (NR_104422, exon 8 of	-3114	NM_02014	56901	Hs.75069	NM_02014	ENSG0000	NDUFA4L2	NUOMS	NDUFA4, r	protein-co
lib-C666-I	chr7	56264065	56264065	+	8.51971	NA	Intergenic	MER11A|LTR|ERVK	-79975	NM_00114	389493	Hs.177948	NM_00114	ENSG0000	NUPR2	NUPR1L	nuclear prc	protein-co
lib-C666-I	chr1	33116724	33116724	+	7.67555	NA	promoter-TSS (NM_001308141)	promoter-TSS (NM_001308141)	-25	NM_00561	5928	Hs.16003	NM_00561	ENSG0000	RBBP4	NURF55|RE	RB binding	protein-co
lib-C666-I	chr7	123389223	123389223	+	7.08136	NA	promoter-TSS (NM_003941)	promoter-TSS (NM_003941)	-98	NM_00394	8976	Hs.143728	NM_00394	ENSG0000	WASL	N-WASP|N	Wiskott-Al	protein-co
lib-C666-I	chr11	72853589	72853589	+	9.93242	NA	promoter-TSS (NM_014824)	promoter-TSS (NM_014824)	-446	NM_01482	9873	Hs.744959	NM_01482	ENSG0000	FCHSD2	NWK|SH3N	FCH and d	protein-co
lib-C666-I	chr11	72859003	72859003	+	9.47655	NA	Intergenic	Intergenic	-5860	NM_01482	9873	Hs.744959	NM_01482	ENSG0000	FCHSD2	NWK|SH3N	FCH and d	protein-co
lib-C666-I	chr10	37014709	37014709	+	86.51872	NA	Intergenic	Intergenic	-400076	NM_05299	91074	Hs.373787	NM_05299	ENSG0000	ANKRD30A	NY-BR-1	ankyrin rep	protein-co
lib-C666-I	chr10	37435493	37435493	+	16.5633	NA	intron (NM_052997, intron 8 of 35)	LTR22B|LTR|ERVK	20708	NM_05299	91074	Hs.373787	NM_05299	ENSG0000	ANKRD30A	NY-BR-1	ankyrin rep	protein-co
lib-C666-I	chr14	50329639	50329639	+	250.74324	NA	Intergenic	CpG	-9718	NM_00471	9147	Hs.655964	NM_00471	ENSG0000	NEMF	NY-CO-1|S	nuclear exp	protein-co
lib-C666-I	chr14	50319814	50319814	+	15.22267	NA	5' UTR (NM_001301732, exon 1 of 32	5' UTR (NM_001301732, exon 1 of 3	107	NM_00130	9147	Hs.655964	NM_00471	ENSG0000	NEMF	NY-CO-1|S	nuclear exp	protein-co
lib-C666-I	chr5	64331547	64331547	+	8.89416	NA	Intergenic	CpG	266802	NM_00131	10283	Hs.371372	NM_00586	ENSG0000	CWC27	NY-CO-10	CWC27 spl	protein-co
lib-C666-I	chrX	129040073	129040073	+	26.37215	NA	promoter-TSS (NM_001166221)	promoter-TSS (NM_001166221)	-24	NM_00116	10813	Hs.458598	NM_00664	ENSG0000	UTP14A	NYCO16|SI	UTP14A sn	protein-co
lib-C666-I	chr9	139305125	139305125	+	27.61921	NA	promoter-TSS (NM_001039707)	promoter-TSS (NM_001039707)	-71	NM_00103	10807	Hs.94300	NM_00664	ENSG0000	SDCCAG3	NY-CO-3	serological	protein-co
lib-C666-I	chr9	95921995	95921995	+	9.20967	NA	Intergenic	L2b|LINE|L2	-25217	NM_00128	65268	Hs.654856	NM_00664	ENSG0000	WNK2	NY-CO-43	WNK lysine	protein-co
lib-C666-I	chr13	49822035	49822035	+	122.48815	NA	promoter-TSS (NM_001193478)	promoter-TSS (NM_001193478)	-12	NR_036439	81602	Hs.388220	NM_03091	ENSG0000	CDADC1	NYD-SP15	cytidine an	protein-co
lib-C666-I	chr15	67547107	67547107	+	7.49163	NA	promoter-TSS (NM_001271886)	promoter-TSS (NM_001271886)	-31	NM_00132	64799	Hs.657894	NM_02278	ENSG0000	IQCH	NYDSP5	IQ motif cc	protein-co
lib-C666-I	chr7	99647415	99647415	+	59.09209	NA	promoter-TSS (NM_145914)	promoter-TSS (NM_145914)	-2	NM_14591	7589	Hs.632294	NM_14591	ENSG0000	ZSCAN21	NY-REN-2	zinc finger	protein-co
lib-C666-I	chr5	123991742	123991742	+	25.39084	NA	intron (NM_020747, intron 2 of 8)	intron (NM_020747, intron 2 of 8)	89063	NM_02074	57507	Hs.266616	NM_02074	ENSG0000	ZNF608	NY-REN-3	zinc finger	protein-co
lib-C666-I	chr1	215805059	215805059	+	14.3056	NA	intron (NM_206933, intron 70 of 71)	intron (NM_206933, intron 70 of 71	64337	NM_01612	51133	Hs.335139	NM_01612	ENSG0000	KCTD3	NY-REN-4	potassium	protein-co
lib-C666-I	chr1	215740920	215740920	+	9.10494	NA	5' UTR (NM_001319294, exon 1 of 18	5' UTR (NM_001319294, exon 1 of 1	198	NM_01612	51133	Hs.335139	NM_01612	ENSG0000	KCTD3	NY-REN-4	potassium	protein-co
lib-C666-I	chr4	170533724	170533724	+	40.09574	NA	promoter-TSS (NM_012224)	promoter-TSS (NM_012224)	54	NM_00119	4750	Hs.481181	NM_01222	ENSG0000	NEK1	NY-REN-5	NIMA relat	protein-co
lib-C666-I	chr17	58469704	58469704	+	23.0291	NA	promoter-TSS (NM_032582)	promoter-TSS (NM_032582)	-118	NM_03258	84669	Hs.132868	NM_03258	ENSG0000	USP32	NY-REN-6	ubiquitin s	protein-co
lib-C666-I	chr17	58469284	58469284	+	17.38993	NA	exon (NM_032582, exon 1 of 34)	exon (NM_032582, exon 1 of 34)	302	NM_03258	84669	Hs.132868	NM_03258	ENSG0000	USP32	NY-REN-6	ubiquitin s	protein-co
lib-C666-I	chrX	347741	347741	+	472.2298	NA	promoter-TSS (NM_013239)	promoter-TSS (NM_013239)	-51	NM_01323	28227	Hs.124942	NM_01323	ENSG0000	PPP2R3B	NYREN8|PI	protein ph	protein-co
lib-C666-I	chrY	297732	297732	+	382.14325	NA	promoter-TSS (NM_013239).2	promoter-TSS (NM_013239).2	-42	NM_01323	28227	Hs.124942	NM_01323	ENSG0000	PPP2R3B	NYREN8|PI	protein ph	protein-co
lib-C666-I	chr11	32812843	32812843	+	4.39666	NA	intron (NM_001008391, intron 1 of 17	intron (NM_001008391, intron 1 of	3361	NM_00100	493860	Hs.706808	NM_00100	ENSG0000	CCDC73	NY-SAR-7	coiled-coil	protein-co
lib-C666-I	chr11	46259828	46259828	+	14.17553	NA	Intergenic	CpG	-39361	NM_05285	90993	Hs.405961	NM_05285	ENSG0000	CREB3L1	OASIS	cAMP resp	protein-co
lib-C666-I	chr11	64334204	64334204	+	14.59636	NA	intron (NM_001307985, intron 6 of 7)	intron (NM_001307985, intron 6 of	11131	NM_00130	55867	Hs.220844	NM_01848	ENSG0000	SLC22A11	OAT4|hOA	solute carr	protein-co
lib-C666-I	chr20	61273693	61273693	+	28.64047	NA	promoter-TSS (NM_016354)	promoter-TSS (NM_016354)	-104	NM_01635	28231	Hs.235782	NM_01635	ENSG0000	SLCO4A1	OATP-E|O.	solute carr	protein-co
lib-C666-I	chr8	70746846	70746846	+	6.13111	NA	intron (NM_030958, intron 1 of 9)	CpG	453	NM_03095	81796	Hs.152460	NM_03095	ENSG0000	SLCO5A1	OATP-J|O/	solute carr	protein-co
lib-C666-I	chr2	192542747	192542747	+	18.43022	NA	promoter-TSS (NR_045622)	promoter-TSS (NR_045622)	-51	NR_045622	64859	Hs.591610	NM_02283	ENSG0000	NABP1	OBFC2A|S(	nucleic aci	protein-co
lib-C666-I	chr2	192543361	192543361	+	5.64185	NA	non-coding (NR_045623, exon 1 of 7	non-coding (NR_045623, exon 1 of	499	NR_045623	64859	Hs.591610	NM_02283	ENSG0000	NABP1	OBFC2A|S(	nucleic aci	protein-co
lib-C666-I	chr12	56618065	56618065	+	12.26631	NA	promoter-TSS (NM_024068)	promoter-TSS (NM_024068)	-60	NM_02406	79035	Hs.240170	NM_02406	ENSG0000	NABP2	OBFC2B|S(	nucleic aci	protein-co
lib-C666-I	chr12	56618293	56618293	+	11.07709	NA	intron (NM_024068, intron 1 of 6)	CpG	168	NM_02406	79035	Hs.240170	NM_02406	ENSG0000	NABP2	OBFC2B|S(	nucleic aci	protein-co
lib-C666-I	chr7	89975978	89975978	+	173.18965	NA	promoter-TSS (NM_033107)	promoter-TSS (NM_033107)	-1	NM_00104	85865	Hs.593547	NM_03310	ENSG0000	GTPBP10	ObgH2|UG	GTP bindin	protein-co
lib-C666-I	chr11	3186510	3186510	+	8.01597	NA	promoter-TSS (NM_020896)	promoter-TSS (NM_020896)	72	NM_14563	114879	Hs.436166	NM_02089	ENSG0000	OSBPL5	OBPH1|OR	oxysterol b	protein-co
lib-C666-I	chr6	41755370	41755370	+	52.45363	NA	TTS (NM_013397)	TTS (NM_013397)	189	NM_00113	100188893	Hs.731633	NM_00113	ENSG0000	TOMM6	OBTP|TOM	translocase	protein-co
lib-C666-I	chr11	69064704	69064704	+	23.20869	NA	TTS (NM_001293296)	TTS (NM_001293296)	3099	NM_00129	26579	Hs.523848	NM_13876	ENSG0000	MYEOV	OCIM	myeloma c	protein-co
lib-C666-I	chr1	167189876	167189876	+	10.27265	NA	promoter-TSS (NM_001198786)	promoter-TSS (NM_001198786)	-190	NM_00119	5451	Hs.283402	NM_00269	ENSG0000	POU2F1	OCT1|OTF:	POU class	protein-co
lib-C666-I	chr1	167189497	167189497	+	8.17853	NA	promoter-TSS (NM_001198786)	promoter-TSS (NM_001198786)	-569	NM_00119	5451	Hs.283402	NM_00269	ENSG0000	POU2F1	OCT1|OTF:	POU class	protein-co
lib-C666-I	chr1	38512762	38512762	+	14.3056	NA	promoter-TSS (NM_002699)	promoter-TSS (NM_002699)	-312	NM_00269	5453	Hs.1837	NM_00269	ENSG0000	POU3F1	OCT6|OTF(	POU class	protein-co
lib-C666-I	chr8	144465873	144465873	+	8.0787	NA	3' UTR (NM_052924, exon 15 of 15)	3' UTR (NM_052924, exon 15 of 15)	14848	NM_05292	114822	Hs.521912	NM_05292	ENSG0000	RHPN1	ODF5|RHC	rhophilin R	protein-co
lib-C666-I	chr8	144450936	144450936	+	6.49644	NA	promoter-TSS (NM_052924)	promoter-TSS (NM_052924)	-89	NM_05292	114822	Hs.521912	NM_05292	ENSG0000	RHPN1	ODF5|RHC	rhophilin R	protein-co
lib-C666-I	chr1	186344909	186344909	+	154.27405	NA	promoter-TSS (NM_001164245)	promoter-TSS (NM_001164245)	19	NM_01784	54953	Hs.371210	NM_01784	ENSG0000	C1orf27	ODR4|TTG	chromosor	protein-co
lib-C666-I	chr18	48405409	48405409	+	7.75972	NA	promoter-TSS (NM_001168335)	promoter-TSS (NM_001168335)	-23	NM_00239	4200	Hs.233119	NM_00239	ENSG0000	ME2	ODS1	malic enzy	protein-co
lib-C666-I	chr11	47395689	47395689	+	7.08136	NA	intron (NM_001080547, intron 2 of 4)	intron (NM_001080547, intron 2 of	4438	NM_00312	6688	Hs.502511	NM_00312	ENSG0000	SPI1	OF|PU.1|SF	Spi-1 protc	protein-co
lib-C666-I	chr4	88813120	88813120	+	3.27468	NA	Intergenic	Intergenic	57265	NM_00129	56955	Hs.676357	NM_02020	ENSG0000	MEPE	OF45	matrix extr	protein-co
lib-C666-I	chr4	88813951	88813951	+	2.59032	NA	Intergenic	Intergenic	58096	NM_00129	56955	Hs.676357	NM_02020	ENSG0000	MEPE	OF45	matrix extr	protein-co
lib-C666-I	chr12	53739231	53739231	+	11.57119	NA	promoter-TSS (NM_001300837)	promoter-TSS (NM_001300837)	-654	NM_00130	121340	Hs.209402	NM_15286	ENSG0000	SP7	OI11|OI12|	Sp7 transcr	protein-co

lib-C666-l	chr8	22036569	22036569	+	22.87742	NA	intron (NR_033404, intron 6 of 15)	intron (NR_033404, intron 6 of 15)	13916	NM_00119	649	Hs.1274	NM_00119	ENSG0000	BMP1	OI13|PCOL	bone morp	protein-co
lib-C666-l	chr7	100464918	100464918	+	58.59621	NA	promoter-TSS (NR_106935)	promoter-TSS (NR_106935)	-32	NM_00330	7205	Hs.534360	NM_00330	ENSG0000	TRIP6	OIP-1|OIP	thyroid hor	protein-co
lib-C666-l	chr12	98987410	98987410	+	176.70418	NA	promoter-TSS (NM_005888)	promoter-TSS (NM_005888)	7	NM_00263	5250	Hs.290404	NM_00263	ENSG0000	SLC25A3	OK/SW-cl.	solute carr	protein-co
lib-C666-l	chr16	71929273	71929273	+	22.15977	NA	promoter-TSS (NM_001270975)	promoter-TSS (NM_001270975)	-123	NM_00127	9798	Hs.232194	NM_01476	ENSG0000	IST1	OLC1	IST1, ESCR	protein-co
lib-C666-l	chr2	219761248	219761248	+	6.4187	NA	Intergenic	Intergenic	15993	NM_02521	80326	Hs.121540	NM_02521	ENSG0000	WNT10A	OODD|SSP	Wnt family	protein-co
lib-C666-l	chr2	219745155	219745155	+	4.39666	NA	promoter-TSS (NM_025216)	promoter-TSS (NM_025216)	-100	NM_02521	80326	Hs.121540	NM_02521	ENSG0000	WNT10A	OODD|SSP	Wnt family	protein-co
lib-C666-l	chr11	85359018	85359018	+	9.21057	NA	promoter-TSS (NM_001244735)	promoter-TSS (NM_001244735)	55	NM_03227	84233	Hs.533725	NM_03227	ENSG0000	TMEM126	OPA7	transmem	protein-co
lib-C666-l	chr14	23790424	23790424	+	19.91222	NA	5' UTR (NM_004643, exon 1 of 7)	5' UTR (NM_004643, exon 1 of 7)	1027	NM_00464	8106	Hs.707712	NM_00464	ENSG0000	PABPN1	OPMD|PAB	poly(A) bin	protein-co
lib-C666-l	chr3	124449273	124449273	+	63.5671	NA	promoter-TSS (NR_033434)	promoter-TSS (NR_033434)	60	NR_03343	7372	Hs.2057	NM_00037	ENSG0000	UMPS	OPRT	uridine mo	protein-co
lib-C666-l	chr11	71934500	71934500	+	24.99834	NA	Intergenic	CpG	-1382	NM_00156	3636	Hs.523875	NM_00156	ENSG0000	INPPL1	OPSMD|SH	inositol po	protein-co
lib-C666-l	chr7	26331803	26331803	+	2.31621	NA	promoter-TSS (NM_001318199)	promoter-TSS (NM_001318199)	288	NM_01332	29887	Hs.741316	NM_01332	ENSG0000	SNX10	OPTB8	sorting nex	protein-co
lib-C666-l	chr12	56018064	56018064	+	9.56114	NA	Intergenic	Intergenic	-12612	NM_20689	121130	Hs.554551	NM_20689	ENSG0000	OR10P1	OR10P1P|(	olfactory re	protein-co
lib-C666-l	chr11	59318242	59318242	+	3.58839	NA	Intergenic	Intergenic	35856	NM_00100	390199	Hs.553757	NM_00100	ENSG0000	OR4D9	OR11-253	olfactory re	protein-co
lib-C666-l	chr19	9166303	9166303	+	16.37572	NA	Intergenic	Intergenic	-37618	NM_00100	125963	Hs.553577	NM_00100	ENSG0000	OR1M1	OR19-5|OI	olfactory re	protein-co
lib-C666-l	chr19	9186457	9186457	+	16.264	NA	Intergenic	Intergenic	-17464	NM_00100	125963	Hs.553577	NM_00100	ENSG0000	OR1M1	OR19-5|OI	olfactory re	protein-co
lib-C666-l	chr19	9165698	9165698	+	11.06679	NA	Intergenic	Intergenic	-38223	NM_00100	125963	Hs.553577	NM_00100	ENSG0000	OR1M1	OR19-5|OI	olfactory re	protein-co
lib-C666-l	chr7	143991508	143991508	+	26.61344	NA	intron (NR_126022, intron 3 of 4)	CpG	-4958	NR_00215	401428	Hs.490448	NR_00215	ENSG0000	OR2A20P	OR2A20	olfactory re	pseudo
lib-C666-l	chr7	143991081	143991081	+	26.37099	NA	intron (NR_126022, intron 3 of 4)	intron (NR_126022, intron 3 of 4)	-5385	NR_00215	401428	Hs.490448	NR_00215	ENSG0000	OR2A20P	OR2A20	olfactory re	pseudo
lib-C666-l	chr5	180588308	180588308	+	30.07415	NA	Intergenic	Intergenic	6365	NM_20688	285659	Hs.553681	NM_20688	ENSG0000	OR2V2	OR2V3|OS	olfactory re	protein-co
lib-C666-l	chr5	180587615	180587615	+	26.4506	NA	Intergenic	Intergenic	5672	NM_20688	285659	Hs.553681	NM_20688	ENSG0000	OR2V2	OR2V3|OS	olfactory re	protein-co
lib-C666-l	chr2	201828205	201828205	+	20.19521	NA	intron (NM_006190, intron 1 of 17)	CpG	219	NR_03391	4999	Hs.444870	NM_00619	ENSG0000	ORC2	ORC2L	origin reco	protein-co
lib-C666-l	chr7	103848500	103848500	+	58.55077	NA	promoter-TSS (NM_002553)	promoter-TSS (NM_002553)	-5	NM_00255	5001	Hs.432948	NM_00255	ENSG0000	ORC5	ORC5L|OR	origin reco	protein-co
lib-C666-l	chr9	136933548	136933548	+	5.5166	NA	promoter-TSS (NM_007371)	promoter-TSS (NM_007371)	-407	NM_00737	8019	Hs.522472	NM_00737	ENSG0000	BRD3	ORFX|RINC	bromodom	protein-co
lib-C666-l	chr7	25020004	25020004	+	9.3976	NA	promoter-TSS (NM_015550)	promoter-TSS (NM_015550)	-173	NM_01555	26031	Hs.520259	NM_01555	ENSG0000	OSBPL3	ORP-3|OR	oxysterol b	protein-co
lib-C666-l	chr17	45887963	45887963	+	5.96422	NA	exon (NM_145798, exon 18 of 23)	exon (NM_145798, exon 18 of 23)	11184	NM_14579	114881	Hs.463320	NM_01773	ENSG0000	OSBPL7	ORP7	oxysterol b	protein-co
lib-C666-l	chr1	52195257	52195257	+	3.98718	NA	promoter-TSS (NM_148904)	promoter-TSS (NM_148904)	-229	NM_14890	114883	Hs.21938	NM_02458	ENSG0000	OSBPL9	ORP-9|OR	oxysterol b	protein-co
lib-C666-l	chr12	58259145	58259145	+	2.31621	NA	Intergenic	CpG	-18398	NM_00573	10106	Hs.524530	NM_00573	ENSG0000	CTDSP2	OS4|PSR2|	CTD small	protein-co
lib-C666-l	chr11	59383375	59383375	+	90.27715	NA	5' UTR (NM_002556, exon 1 of 14)	5' UTR (NM_002556, exon 1 of 14)	242	NM_00255	5007	Hs.597091	NM_00255	ENSG0000	OSBP	OSBP1	oxysterol b	protein-co
lib-C666-l	chr3	38206547	38206547	+	177.48514	NA	promoter-TSS (NM_005109)	promoter-TSS (NM_005109)	-479	NM_00510	9943	Hs.475970	NM_00510	ENSG0000	OXSR1	OSR1	oxidative s	protein-co
lib-C666-l	chr3	38206909	38206909	+	99.9228	NA	promoter-TSS (NM_005109)	promoter-TSS (NM_005109)	-117	NM_00510	9943	Hs.475970	NM_00510	ENSG0000	OXSR1	OSR1	oxidative s	protein-co
lib-C666-l	chr13	48877868	48877868	+	70.98327	NA	promoter-TSS (NR_046414)	promoter-TSS (NR_046414)	-15	NM_00032	5925	Hs.408528	NM_00032	ENSG0000	RB1	OSRC|PPP	RB transcri	protein-co
lib-C666-l	chr3	128369757	128369757	+	40.09574	NA	promoter-TSS (NM_002950)	promoter-TSS (NM_002950)	-38	NM_00295	6184	Hs.518244	NM_00295	ENSG0000	RPN1	OST1|RBPH	ribophorin	protein-co
lib-C666-l	chr3	128399799	128399799	+	33.11264	NA	Intergenic	CpG	-30080	NM_00295	6184	Hs.518244	NM_00295	ENSG0000	RPN1	OST1|RBPH	ribophorin	protein-co
lib-C666-l	chr14	23025401	23025401	+	28.16397	NA	Intergenic	Intergenic	32742	NM_00134	1603	Hs.82890	NM_00134	ENSG0000	DAD1	OST2	defender a	protein-co
lib-C666-l	chr14	22852247	22852247	+	6.82401	NA	Intergenic	Intergenic	205896	NM_00134	1603	Hs.82890	NM_00134	ENSG0000	DAD1	OST2	defender a	protein-co
lib-C666-l	chr14	22782747	22782747	+	6.71839	NA	Intergenic	Intergenic	275396	NM_00134	1603	Hs.82890	NM_00134	ENSG0000	DAD1	OST2	defender a	protein-co
lib-C666-l	chr3	169491160	169491160	+	27.42941	NA	promoter-TSS (NM_001185119)	promoter-TSS (NM_001185119)	307	NR_03370	55892	Hs.507025	NM_01865	ENSG0000	MYNN	OSZF|SBBI	myoneurin	protein-co
lib-C666-l	chr2	241191017	241191017	+	5.04179	NA	Intergenic	Intergenic	-110944	NM_14896	150677	Hs.148586	NM_14896	ENSG0000	OTOS	OTOSP	otospiralin	protein-co
lib-C666-l	chr1	110881908	110881908	+	10.79304	NA	promoter-TSS (NR_036595)	promoter-TSS (NR_036595)	-37	NM_02276	64783	Hs.435947	NM_02276	ENSG0000	RBM15	OTT|OTT1|	RNA bindi	protein-co
lib-C666-l	chr14	57463670	57463670	+	4.67192	NA	Intergenic	Intergenic	183769	NR_02938	100309464	Hs.637962	NR_02938	ENSG0000	OTX2-AS1	OTX2OS1	OTX2 antis	ncRNA
lib-C666-l	chr5	41870544	41870544	+	7.49163	NA	5' UTR (NM_000436, exon 1 of 17)	5' UTR (NM_000436, exon 1 of 17)	247	NM_00043	5019	Hs.278277	NM_00043	ENSG0000	OXCT1	OXCT|SCO	3-oxoacid	protein-co
lib-C666-l	chr2	159313349	159313349	+	6.56784	NA	promoter-TSS (NM_003628)	promoter-TSS (NM_003628)	-43	NM_00130	8502	Hs.407580	NM_00362	ENSG0000	PKP4	p0071	plakophilin	protein-co
lib-C666-l	chr3	138553488	138553488	+	4.52748	NA	Intergenic	CpG	-75287	NM_00621	5291	Hs.239818	NM_00621	ENSG0000	PIK3CB	P110BETA|	phosphatic	protein-co
lib-C666-l	chr1	226309375	226309375	+	3.31563	NA	Intergenic	CpG	58947	NR_00231	440926	Hs.533624	NR_002315		H3F3AP4	p13	H3 histone	pseudo
lib-C666-l	chr2	136002832	136002832	+	4.74112	NA	intron (NM_032143, intron 12 of 20)	intron (NM_032143, intron 12 of 20	192997	NM_00117	22930	Hs.306327	NM_01223	ENSG0000	RAB3GAP1	P130|RAB3	RAB3 GTPa	protein-co
lib-C666-l	chr15	22977381	22977381	+	14.02822	NA	intron (NM_001033028, intron 7 of 1	MER77B|LTR|ERVL	21626	NM_00103	23191	Hs.26704	NM_01460	ENSG0000	CYFIP1	P140SRA-	cytoplasmi	protein-co
lib-C666-l	chr5	32585633	32585633	+	7.11942	NA	promoter-TSS (NM_006713)	promoter-TSS (NM_006713)	28	NM_00671	10923	Hs.229641	NM_00671	ENSG0000	SUB1	P15|PC4|p	SUB1 hom	protein-co
lib-C666-l	chrX	108811928	108811928	+	11.57178	NA	Intergenic	Intergenic	31581	NM_00124	55916	Hs.25010	NM_01869	ENSG0000	NXT2	P15-2	nuclear tra	protein-co
lib-C666-l	chrX	108812456	108812456	+	11.57178	NA	Intergenic	Intergenic	32109	NM_00124	55916	Hs.25010	NM_01869	ENSG0000	NXT2	P15-2	nuclear tra	protein-co
lib-C666-l	chrX	108813382	108813382	+	9.21057	NA	Intergenic	Intergenic	33035	NM_00124	55916	Hs.25010	NM_01869	ENSG0000	NXT2	P15-2	nuclear tra	protein-co
lib-C666-l	chr17	4458738	4458738	+	2.25647	NA	promoter-TSS (NM_014520)	promoter-TSS (NM_014520)	-57	NM_00110	10514	Hs.22824	NM_01452	ENSG0000	MYBBP1A	P160|PAP2	MYB bindi	protein-co
lib-C666-l	chr1	120838578	120838578	+	2.59032	NA	promoter-TSS (NM_001320149)	promoter-TSS (NM_001320149)	-427	NM_00110	653820	Hs.339665	NM_00110	ENSG0000	FAM72B	p17	family with	protein-co
lib-C666-l	chr1	120838438	120838438	+	2.38795	NA	promoter-TSS (NM_001320149)	promoter-TSS (NM_001320149)	-567	NM_00110	653820	Hs.339665	NM_00110	ENSG0000	FAM72B	p17	family with	protein-co
lib-C666-l	chr16	88717948	88717948	+	24.99834	NA	promoter-TSS (NM_000101)	promoter-TSS (NM_000101)	-456	NM_00010	1535	Hs.513803	NM_00010	ENSG0000	CYBA	p22-PHOX	cytochrom	protein-co
lib-C666-l	chr12	57082050	57082050	+	14.02822	NA	promoter-TSS (NR_104219)	promoter-TSS (NR_104219)	142	NM_00128	10728	Hs.50425	NM_00660	ENSG0000	PTGES3	P23|TEBP|c	prostaglan	protein-co
lib-C666-l	chr12	124069037	124069037	+	32.48059	NA	promoter-TSS (NR_110049)	promoter-TSS (NR_110049)	-39	NM_00681	10959	Hs.733403	NM_00681	ENSG0000	TMED2	P24A|RNP2	transmemb	protein-co
lib-C666-l	chr13	111368079	111368079	+	9.3976	NA	exon (NM_005537, exon 1 of 2)	intron (NM_001267728, intron 1 of	295	NM_00126	3621	Hs.46700	NM_00553	ENSG0000	ING1	p24ING1c|	inhibitor of	protein-co
lib-C666-l	chr13	111365386	111365386	+	5.10953	NA	promoter-TSS (NM_198219)	promoter-TSS (NM_198219)	-224	NM_19821	3621	Hs.46700	NM_00553	ENSG0000	ING1	p24ING1c|	inhibitor of	protein-co
lib-C666-l	chr1	28655327	28655327	+	16.19847	NA	promoter-TSS (NM_001127350)	promoter-TSS (NM_001127350)	-186	NM_01763	54797	Hs.479911	NM_01763	ENSG0000	MED18	p28b	mediator c	protein-co
lib-C666-l	chr2	242641498	242641498	+	9.20967	NA	promoter-TSS (NM_001330162)	promoter-TSS (NM_001330162)	64	NM_03232	84289	Hs.529172	NM_03232	ENSG0000	ING5	p28ING5	inhibitor of	protein-co
lib-C666-l	chr12	121648065	121648065	+	9.14689	NA	exon (NM_002560, exon 1 of 12)	exon (NM_002560, exon 1 of 12)	401	NM_00126	5025	Hs.321709	NM_00256	ENSG0000	P2RX4	P2X4|P2X4	purinergic	protein-co
lib-C666-l	chr12	121667775	121667775	+	7.09685	NA	intron (NR_046373, intron 7 of 10)	MLT1C|LTR|ERVL-MaLR	20111	NM_00126	5025	Hs.321709	NM_00256	ENSG0000	P2RX4	P2X4|P2X4	purinergic	protein-co
lib-C666-l	chr11	73000563	73000563	+	6.82401	NA	intron (NM_001277208, intron 1 of 1)	intron (NM_001277208, intron 1 of	17376	NM_00127	5031	Hs.16362	NM_00415	ENSG0000	P2RY6	P2Y6	pyrimidine	protein-co
lib-C666-l	chr7	76147727	76147727	+	8.48019	NA	intron (NM_182684, intron 6 of 6)	intron (NM_182684, intron 6 of 6)	7987	NM_18268	105375355	Hs.488861	NM_03057	ENSG0000	UPK3B	P35|UP3B|	uroplakin 3	protein-co
lib-C666-l	chr7	76149866	76149866	+	7.72386	NA	intron (NM_182684, intron 6 of 6)	L2|LINE|L2	10126	NM_18268	105375355	Hs.488861	NM_03057	ENSG0000	UPK3B	P35|UP3B|	uroplakin 3	protein-co
lib-C666-l	chr8	59323902	59323902	+	12.72278	NA	promoter-TSS (NM_001077619)	promoter-TSS (NM_001077619)	79	NM_00133	137886	Hs.155572	NM_00107	ENSG0000	UBXN2B	p37	UBX doma	protein-co
lib-C666-l	chr22	50721048	50721048	+	19.23598	NA	intron (NM_012401, intron 18 of 36)	CpG-18126	-12226	NM_00275	5600	Hs.57732	NM_00275	ENSG0000	MAPK11	P38B|P38B	mitogen-a	protein-co
lib-C666-l	chr16	2029345	2029345	+	16.6838	NA	TTS (NM_006453)	TTS (NM_006453)	2205	NM_17216	124056	Hs.191762	NM_14460	ENSG0000	NOXO1	P41NOX|P	NADPH ox	protein-co
lib-C666-l	chr16	2031717	2031717	+	2.92124	NA	promoter-TSS (NM_001267721)	promoter-TSS (NM_001267721)	-167	NM_00126	124056	Hs.191762	NM_14460	ENSG0000	NOXO1	P41NOX|P	NADPH ox	protein-co
lib-C666-l	chr20	1447513	1447513	+	3.59106	NA	non-coding (NR_038164, exon 1 of 8	non-coding (NR_038164, exon 1 of	824	NM_01614	55968	Hs.12865	NM_01614	ENSG0000	NSFL1C	P47|UBX1|	NSFL1 cofa	protein-co
lib-C666-l	chr19	10514393	10514393	+	130.72133	NA	promoter-TSS (NR_031592)	promoter-TSS (NR_031592)	-122	NM_00706	11140	Hs.160958	NM_00706	ENSG0000	CDC37	P50CDC37	cell divisio	protein-co
lib-C666-l	chr19	10514972	10514972	+	8.27411	NA	promoter-TSS (NR_031592)	promoter-TSS (NR_031592)	-701	NM_00706	11140	Hs.160958	NM_00706	ENSG0000	CDC37	P50CDC37	cell divisio	protein-co
lib-C666-l	chr7	148844619	148844619	+	8.2273	NA	promoter-TSS (NM_170686)	promoter-TSS (NM_170686)	59	NM_17068	57541	Hs.632032	NM_02078	ENSG0000	ZNF398	P51|P71|ZE	zinc finger	protein-co
lib-C666-l	chr7	148845280	148845280	+	3.98736	NA	intron (NM_170686, intron 1 of 5)	CpG	720	NM_17068	57541	Hs.632032	NM_02078	ENSG0000	ZNF398	P51|P71|ZE	zinc finger	protein-co
lib-C666-l	chr16	32707669	32707669	+	32.11429	NA	Intergenic	HSAT6|Satellite|Satellite	-20219	NR_11088	24150	Hs.592038	NM_01536	ENSG0000	TP53TG3	P53TG3|TP	TP53 targe	protein-co
lib-C666-l	chr1	46598443	46598443	+	271.11682	NA	promoter-TSS (NM_001328650)	promoter-TSS (NM_001328650)	24	NM_00130	8503	Hs.655387	NM_00362	ENSG0000	PIK3R3	p55|p55-G	phosphoin	protein-co
lib-C666-l	chr1	46640223	46640223	+	12.62235	NA	promoter-TSS (NM_005727)	promoter-TSS (NM_005727)	350	NM_00132	8503	Hs.655387	NM_00362	ENSG0000	PIK3R3	p55|p55-G	phosphoin	protein-co
lib-C666-l	chr1	46637715	46637715	+	2.55621	NA	intron (NM_001303427, intron 1 of 9)	intron (NM_001303427, intron 1 of	2858	NM_00132	8503	Hs.655387	NM_00362	ENSG0000	PIK3R3	p55|p55-G	phosphoin	protein-co
lib-C666-l	chr17	38136779	38136779	+	155.31761	NA	promoter-TSS (NM_002809)	promoter-TSS (NM_002809)	-242	NM_00280	5709	Hs.12970	NM_00280	ENSG0000	PSMD3	P58|RPN3|	proteasom	protein-co
lib-C666-l	chr11	74303582	74303582	+	3.07144	NA	promoter-TSS (NM_006591)	promoter-TSS (NM_006591)	7	NM_00659	10714	Hs.82502	NM_00659	ENSG0000	POLD3	P66|P68|PF	DNA polyn	protein-co
lib-C666-l	chr19	19496551	19496551	+	23.27622	NA	promoter-TSS (NM_017660)	promoter-TSS (NM_017660)	-91	NM_01766	54815	Hs.118964	NM_01766	ENSG0000	GATAD2A	p66alpha	GATA zinc	protein-co
lib-C666-l	chr19	19516258	19516258	+	3.7184	NA	intron (NM_017660, intron 1 of 11)	CpG	19616	NM_01766	54815	Hs.118964	NM_01766	ENSG0000	GATAD2A	p66alpha	GATA zinc	protein-co
lib-C666-l	chr22	38901693	38901693	+	5.83894	NA	intron (NM_006386, intron 1 of 12)	CpG	652	NM_00109	10521	Hs.528305	NM_00638	ENSG0000	DDX17	P72|RH70	DEAD-box	protein-co
lib-C666-l	chr9	93564437	93564437	+	17.37618	NA	intron (NM_001174168, intron 1 of 1	CpG	228	NM_00117	6850	Hs.371720	NM_00317	ENSG0000	SYK	p72-Syk	spleen ass	protein-co
lib-C666-l	chr1	3590739	3590739	+	7.08929	NA	intron (NM_005427, intron 1 of 13)	intron (NM_005427, intron 1 of 13)	-16497	NM_00112	7161	Hs.192132	NM_00542	ENSG0000	TP73	P73	tumor prot	protein-co
lib-C666-l	chr1	3568996	3568996	+	6.15313	NA	promoter-TSS (NM_001204184)	promoter-TSS (NM_001204184)	-133	NM_00120	7161	Hs.192132	NM_00542	ENSG0000	TP73	P73	tumor prot	protein-co
lib-C666-l	chr1	3569534	3569534	+	2.78587	NA	intron (NM_005427, intron 1 of 13)	CpG-281	405	NM_00120	7161	Hs.192132	NM_00542	ENSG0000	TP73	P73	tumor prot	protein-co
lib-C666-l	chr1	3568735	3568735	+	2.05265	NA	promoter-TSS (NM_001204184)	promoter-TSS (NM_001204184)	-394	NM_00120	7161	Hs.192132	NM_00542	ENSG0000	TP73	P73	tumor prot	protein-co
lib-C666-l	chr3	39093531	39093531	+	61.48528	NA	promoter-TSS (NM_001346226)	promoter-TSS (NM_001346226)	54	NR_14439	57599	Hs.109778	NM_02083	ENSG0000	WDR48	P80|SPG60	WD repeat	protein-co
lib-C666-l	chr6	109330853	109330853	+	16.76091	NA	promoter-TSS (NM_001199933)	promoter-TSS (NM_001199933)	-95	NM_00119	27244	Hs.591336	NM_01445	ENSG0000	SESN1	PA26|SEST	sestrin 1	protein-co
lib-C666-l	chr8	101734417	101734417	+	18.74745	NA	promoter-TSS (NM_002568)	promoter-TSS (NM_002568)	-102	NM_00256	26986	Hs.387804	NM_00256	ENSG0000	PABPC1	PAB1|PABF	poly(A) bin	protein-co
lib-C666-l	chr8	101734852	101734852	+	18.0551	NA	promoter-TSS (NM_002568)	promoter-TSS (NM_002568)	-537	NM_00256	26986	Hs.387804	NM_00256	ENSG0000	PABPC1	PAB1|PABF	poly(A) bin	protein-co
lib-C666-l	chr8	101842896	101842896	+	6.15313	NA	Intergenic	MER4D|LTR|ERV1	-108581	NM_00256	26986	Hs.387804	NM_00256	ENSG0000	PABPC1	PAB1|PABF	poly(A) bin	protein-co
lib-C666-l	chr2	146445380	146445380	+	13.91591	NA	Intergenic	Intergenic	-899245	NR_02690	728773	Hs.334462	NR_026904		PABPC1P2	PABP2|PAE	poly(A) bin	pseudo
lib-C666-l	chr13	25670955	25670955	+	3.92495	NA	exon (NM_030979, exon 1 of 1)	exon (NM_030979, exon 1 of 1)	679	NM_03097	5042	Hs.458280	NM_03097	ENSG0000	PABPC3	PABP3|PAE	poly(A) bin	protein-co
lib-C666-l	chr13	25671822	25671822	+	2.99475	NA	exon (NM_030979, exon 1 of 1)	exon (NM_030979, exon 1 of 1)	1546	NM_03097	5042	Hs.458280	NM_03097	ENSG0000	PABPC3	PABP3|PAE	poly(A) bin	protein-co
lib-C666-l	chr1	169863372	169863372	+	42.40071	NA	promoter-TSS (NM_020423)	promoter-TSS (NM_020423)	-272	NM_02042	57147	Hs.435560	NM_02042	ENSG0000	SCYL3	PACE-1|PA	SCY1 like p	protein-co
lib-C666-l	chr1	169860547	169860547	+	4.19675	NA	intron (NM_181093, intron 1 of 13)	MLT1M|LTR|ERVL-MaLR	2553	NM_18109	57147	Hs.435560	NM_02042	ENSG0000	SCYL3	PACE-1|PA	SCY1 like p	protein-co
lib-C666-l	chr1	169860092	169860092	+	3.84463	NA	intron (NM_181093, intron 1 of 13)	MLT1M|LTR|ERVL-MaLR	3008	NM_18109	57147	Hs.435560	NM_02042	ENSG0000	SCYL3	PACE-1|PA	SCY1 like p	protein-co
lib-C666-l	chr17	33905597	33905597	+	22.14682	NA	promoter-TSS (NM_000286)	promoter-TSS (NM_000286)	59	NM_00028	5193	Hs.591190	NM_00028	ENSG0000	PEX12	PAF-3|PBD	peroxisom	protein-co
lib-C666-l	chr7	98476296	98476296	+	15.22999	NA	intron (NM_003496, intron 1 of 70)	CpG	183	NM_00124	8295	Hs.203952	NM_00349	ENSG0000	TRRAP	PAF350/40	transforma	protein-co
lib-C666-l	chr7	98476670	98476670	+	3.61436	NA	intron (NM_003496, intron 1 of 70)	CpG	557	NM_00124	8295	Hs.203952	NM_00349	ENSG0000	TRRAP	PAF350/40	transforma	protein-co
lib-C666-l	chr9	37485876	37485876	+	9.4812	NA	promoter-TSS (NM_001282766)	promoter-TSS (NM_001282766)	-56	NM_00128	64425	Hs.591087	NM_02249	ENSG0000	POLR1E	PAF53|PRA	RNA polym	protein-co
lib-C666-l	chr3	178789387	178789387	+	2.10911	NA	5' UTR (NM_022470, exon 1 of 6)	5' UTR (NM_022470, exon 1 of 6)	269	NM_02247	64393	Hs.371609	NM_02247	ENSG0000	ZMAT3	PAG608|W	zinc finger	protein-co
lib-C666-l	chr7	100751813	100751813	+	12.80342	NA	Intergenic	Intergenic	-18557	NM_00060	5054	Hs.414795	NM_00060	ENSG0000	SERPINE1	PAI|PAI-1|	serpin fam	protein-co
lib-C666-l	chr7	100781857	100781857	+	8.11522	NA	3' UTR (NM_000602, exon 9 of 9)	3' UTR (NM_000602, exon 9 of 9)	11487	NM_00060	5054	Hs.414795	NM_00060	ENSG0000	SERPINE1	PAI|PAI-1|	serpin fam	protein-co
lib-C666-l	chr5	138677493	138677493	+	9.94769	NA	promoter-TSS (NM_016480)	promoter-TSS (NM_016480)	-28	NR_10983	51247	Hs.396644	NM_01648	ENSG0000	PAIP2	PAIP-2|PAI	poly(A) bin	protein-co
lib-C666-l	chr1	89149908	89149908	+	97.42313	NA	promoter-TSS (NM_001320708)	promoter-TSS (NM_001320708)	81	NM_00132	5586	Hs.440833	NM_00625	ENSG0000	PKN2	PAK2|PRK2	protein kin	protein-co
lib-C666-l	chr3	196516858	196516858	+	104.53465	NA	intron (NM_002577, intron 2 of 14)	MER1B|DNA|hAT-Charlie	50130	NM_00257	5062	Hs.518530	NM_00257	ENSG0000	PAK2	PAK65|PAK	p21 (RAC1	protein-co
lib-C666-l	chr11	77185455	77185455	+	282.62173	NA	promoter-TSS (NM_002576)	promoter-TSS (NM_002576)	-347	NM_00257	5058	Hs.435714	NM_00257	ENSG0000	PAK1	PAKalpha	p21 (RAC1	protein-co
lib-C666-l	chr16	46655299	46655299	+	18.20429	NA	promoter-TSS (NR_136738)	promoter-TSS (NR_136738)	12	NM_02474	79801	Hs.123253	NM_02474	ENSG0000	SHCBP1	PAL	SHC bindi	protein-co
lib-C666-l	chr5	102089696	102089696	+	5.48804	NA	promoter-TSS (NM_001319943)	promoter-TSS (NM_001319943)	-791	NM_00131	5066	Hs.369430	NM_00091	ENSG0000	PAM	PAL|PHM	peptidylgly	protein-co
lib-C666-l	chr14	67708440	67708440	+	11.66012	NA	intron (NM_022474, intron 1 of 14)	CpG	428	NM_00125	64398	Hs.652312	NM_02247	ENSG0000	MPP5	PALS1	membrane	protein-co
lib-C666-l	chr2	18741916	18741916	+	17.27528	NA	promoter-TSS (NM_020905)	promoter-TSS (NM_020905)	43	NM_02090	57665	Hs.120319	NM_02090	ENSG0000	RDH14	PAN2|SDR	retinol deh	protein-co
lib-C666-l	chr2	11886887	11886887	+	19.35617	NA	intron (NM_145693, intron 1 of 19)	CpG	165	NM_14569	23175	Hs.467740	NM_14569	ENSG0000	LPIN1	PAP1	lipin 1	protein-co
lib-C666-l	chr2	11884401	11884401	+	8.03539	NA	intron (NM_001261429, intron 2 of 9)	intron (NM_001261429, intron 2 of	-2321	NM_14569	23175	Hs.467740	NM_14569	ENSG0000	LPIN1	PAP1	lipin 1	protein-co
lib-C666-l	chr2	11893657	11893657	+	6.71839	NA	intron (NM_145693, intron 1 of 19)	intron (NM_145693, intron 1 of 19)	6935	NM_14569	23175	Hs.467740	NM_14569	ENSG0000	LPIN1	PAP1	lipin 1	protein-co
lib-C666-l	chr7	33148955	33148955	+	16.19708	NA	promoter-TSS (NM_203288)	promoter-TSS (NM_203288)	47	NM_20328	6100	Hs.326805	NM_20328	ENSG0000	RP9	PAP-1|PAF	retinitis pig	protein-co
lib-C666-l	chr17	18759594	18759594	+	12.12723	NA	promoter-TSS (NM_001243942)	promoter-TSS (NM_001243942)	-18	NM_00124	5636	Hs.632236	NM_00276	ENSG0000	PRPSAP2	PAP41	phosphori	protein-co
lib-C666-l	chr11	62359130	62359130	+	36.38376	NA	promoter-TSS (NM_022830)	promoter-TSS (NM_022830)	-21	NM_02283	64852	Hs.728983	NM_02283	ENSG0000	TUT1	PAPD2|RBI	terminal ur	protein-co
lib-C666-l	chr9	88969040	88969040	+	19.82221	NA	intron (NM_001185059, intron 1 of 2	CpG	325	NM_00133	79670	Hs.597057	NM_02461	ENSG0000	ZCCHC6	PAPD6|TU	zinc finger	protein-co
lib-C666-l	chr9	89029122	89029122	+	13.33696	NA	Intergenic	Intergenic	-59720	NM_02461	79670	Hs.597057	NM_02461	ENSG0000	ZCCHC6	PAPD6|TU	zinc finger	protein-co
lib-C666-l	chr9	88969366	88969366	+	11.66438	NA	promoter-TSS (NM_001185074)	promoter-TSS (NM_001185074)	-1	NM_00133	79670	Hs.597057	NM_02461	ENSG0000	ZCCHC6	PAPD6|TU	zinc finger	protein-co
lib-C666-l	chr9	88998284	88998284	+	3.27468	NA	Intergenic	L1PA4|LINE|L1	-28882	NM_02461	79670	Hs.597057	NM_02461	ENSG0000	ZCCHC6	PAPD6|TU	zinc finger	protein-co
lib-C666-l	chr19	39581663	39581663	+	14.59636	NA	intron (NM_001004318, intron 2 of 1	L1MC4a|LINE|L1	6718	NM_00100	390928	Hs.448934	NM_00100	ENSG0000	ACP7	PAPL|PAPL	acid phosp	protein-co
lib-C666-l	chr14	53620016	53620016	+	6.29226	NA	promoter-TSS (NM_030637)	promoter-TSS (NM_030637)	30	NM_03063	80821	Hs.125525	NM_03063	ENSG0000	DDHD1	PA-PLA1|P	DDHD don	protein-co
lib-C666-l	chr1	11120231	11120231	+	7.09685	NA	promoter-TSS (NM_003132)	promoter-TSS (NM_003132)	-140	NM_00313	6723	Hs.76244	NM_00313	ENSG0000	SRM	PAPT|SPDS	spermidine	protein-co
lib-C666-l	chr20	49347907	49347907	+	10.38266	NA	promoter-TSS (NM_032521)	promoter-TSS (NM_032521)	-174	NM_03252	84612	Hs.589848	NM_03252	ENSG0000	PARD6B	PAR6B	par-6 fami	protein-co
lib-C666-l	chr20	49378432	49378432	+	9.3665	NA	Intergenic	Intergenic	30351	NM_03252	84612	Hs.589848	NM_03252	ENSG0000	PARD6B	PAR6B	par-6 fami	protein-co
lib-C666-l	chr12	6873689	6873689	+	61.07141	NA	Intergenic	Intergenic	-1794	NM_00133	5763	Hs.504613	NM_00282	ENSG0000	PTMS	ParaT	parathymo	protein-co
lib-C666-l	chr7	149194748	149194748	+	12.57335	NA	5' UTR (NM_152557, exon 1 of 7)	5' UTR (NM_152557, exon 1 of 7)	150	NM_00116	155061	Hs.24643	NM_15255	ENSG0000	ZNF746	PARIS	zinc finger	protein-co
lib-C666-l	chr2	85765818	85765818	+	6.20473	NA	promoter-TSS (NM_005911)	promoter-TSS (NM_005911)	191	NR_03894	100630918	Hs.66049	NR_038942		PARTICL	PARTICLE	promoter (	ncRNA
lib-C666-l	chr1	63788159	63788159	+	47.79407	NA	promoter-TSS (NR_121634)	promoter-TSS (NR_121634)	-30	NR_12163	100996301		NR_12163	ENSG0000	FOXD3-AS	pasFOXD3	FOXD3 ant	ncRNA
lib-C666-l	chr2	242088904	242088904	+	11.77301	NA	promoter-TSS (NM_015148)	promoter-TSS (NM_015148)	15	NM_01514	23178	Hs.397891	NM_01514	ENSG0000	PASK	PASKIN|ST	PAS doma	protein-co
lib-C666-l	chr19	48226450	48226450	+	10.03284	NA	intron (NM_014601, intron 3 of 5)	L2b|LINE|L2	9849	NM_01460	30846	Hs.744963	NM_01460	ENSG0000	EHD2	PAST2	EH domain	protein-co
lib-C666-l	chr15	44975428	44975428	+	4.71278	NA	Intergenic	L1PA7|LINE|L1	-6342	NM_00133	197135	Hs.11594	NM_00114	ENSG0000	PATL2	Pat1a|hPat	PAT1 hom	protein-co
lib-C666-l	chr11	59437001	59437001	+	14.59636	NA	promoter-TSS (NM_152716)	promoter-TSS (NM_152716)	-490	NM_15271	219988	Hs.591960	NM_15271	ENSG0000	PATL1	Pat1b|hPat	PAT1 hom	protein-co
lib-C666-l	chr5	150667896	150667896	+	18.43022	NA	intron (NR_181774, intron 5 of 9)	L3|LINE|CR1	15438	NR_18177	285641	Hs.720893	NM_18177	ENSG0000	SLC36A3	PAT3|TRAM	solute carr	protein-co
lib-C666-l	chr7	154756906	154756906	+	12.62235	NA	intron (NM_007349, intron 8 of 20)	MLT1F1|LTR|ERVL-MaLR	36679	NR_02447	100132707	Hs.586358	NR_02447	ENSG0000	PAXIP1-AS	PAXIP1 ant	ncRNA	
lib-C666-l	chr6	143771902	143771902	+	3.8557	NA	promoter-TSS (NM_003630)	promoter-TSS (NM_003630)	-16	NM_00363	8504	Hs.7277	NM_00363	ENSG0000	PEX3	PBD10A|TF	peroxisom	protein-co
lib-C666-l	chr11	118955647	118955647	+	15.42216	NA	promoter-TSS (NM_000190)	promoter-TSS (NM_000190)	60	NM_00019	3145	Hs.82609	NM_00019	ENSG0000	HMBS	PBG-D|PBC	hydroxyme	protein-co
lib-C666-l	chr17	77771100	77771100	+	40.61611	NA	promoter-TSS (NM_020649)	promoter-TSS (NM_020649)	-185	NM_02064	57332	Hs.387258	NM_02064	ENSG0000	CBX8	PC3|RC1	chromobo	protein-co
lib-C666-l	chr17	77770837	77770837	+	19.59555	NA	promoter-TSS (NM_020649)	promoter-TSS (NM_020649)	78	NM_02064	57332	Hs.387258	NM_02064	ENSG0000	CBX8	PC3|RC1	chromobo	protein-co
lib-C666-l	chr17	77775219	77775219	+	16.23236	NA	Intergenic	CpG-11232	-4304	NM_02064	57332	Hs.387258	NM_02064	ENSG0000	CBX8	PC3|RC1	chromobo	protein-co
lib-C666-l	chr7	112090438	112090438	+	60.75948	NA	promoter-TSS (NR_120333)	promoter-TSS (NR_120333)	-45	NM_00155	3475	Hs.7879	NM_00155	ENSG0000	IFRD1	PC4|TIS7	interferon	protein-co
lib-C666-l	chr7	112091307	112091307	+	41.46989	NA	promoter-TSS (NM_001197079)	promoter-TSS (NM_001197079)	744	NR_12033	3475	Hs.7879	NM_00155	ENSG0000	IFRD1	PC4|TIS7	interferon	protein-co
lib-C666-l	chr5	141258449	141258449	+	8.71138	NA	promoter-TSS (NM_032420)	promoter-TSS (NM_032420)	-474	NM_03242	5097	Hs.79769	NM_00258	ENSG0000	PCDH1	PC42|PCDH	protocadh	protein-co
lib-C666-l	chr8	127837119	127837119	+	4.52748	NA	Intergenic	Intergenic	-188280	NR_04526	100750225	Hs.571530	NR_04526	ENSG0000	PCAT1	PCA1|PCAT	prostate ca	ncRNA
lib-C666-l	chr11	66717778	66717778	+	36.98516	NA	intron (NM_001040716, intron 3 of 2	intron (NM_001040716, intron 3 of	8069	NM_00092	5091	Hs.89890	NM_00092	ENSG0000	PC	PCB	pyruvate c	protein-co
lib-C666-l	chr11	66726021	66726021	+	4.98754	NA	promoter-TSS (NM_000920)	promoter-TSS (NM_000920)	-174	NM_00104	5091	Hs.89890	NM_00092	ENSG0000	PC	PCB	pyruvate c	protein-co
lib-C666-l	chr5	140384077	140384077	+	10.70998	NA	intron (NM_031860, intron 3 of 3)	intron (NM_031860, intron 3 of 3)	37725	NM_03188	56134	Hs.199343	NM_01889	ENSG0000	PCDHAC2	PCDH-ALF	protocadh	protein-co
lib-C666-l	chr5	140420907	140420907	+	12.35626	NA	Intergenic	Intergenic	-10054	NM_01334	29930	Hs.278950	NM_01334	ENSG0000	PCDHB1	PCDH-BET	protocadh	protein-co
lib-C666-l	chr3	12525924	12525924	+	2.26435	NA	promoter-TSS (NM_001145393)	promoter-TSS (NM_001145393)	-7	NR_13560	80746	Hs.335550	NM_02526	ENSG0000	TSEN2	PCH2B|SEN	tRNA splic	protein-co
lib-C666-l	chr20	5100250	5100250	+	6.15313	NA	promoter-TSS (NR_028370)	promoter-TSS (NR_028370)	18	NR_02837	100302739	Hs.744934	NR_028370		PCNA-AS1	PCNA-AS|	PCNA antis	ncRNA
lib-C666-l	chr14	71374059	71374059	+	7.09685	NA	promoter-TSS (NM_001308160)	promoter-TSS (NM_001308160)	-63	NM_01498	22990	Hs.446559	NM_01498	ENSG0000	PCNX1	PCNX|PCN	pecanex h	protein-co
lib-C666-l	chr11	65382869	65382869	+	29.92316	NA	promoter-TSS (NM_032223)	promoter-TSS (NM_032223)	-914	NM_03222	399909	Hs.380801	NM_03222	ENSG0000	PCNXL3	PCNXL3	pecanex h	protein-co
lib-C666-l	chr13	24463594	24463594	+	5.74587	NA	promoter-TSS (NM_001135816)	promoter-TSS (NM_001135816)	2	NM_00113	542767	Hs.642624	NM_001014442		C1QTNF9E	PCOTH	C1QTNF9E	protein-co

lib-C666-I	chr3	142585902	142585902	+	6.15313	NA	intron (NM_013363, intron 2 of 8)	LTR9B|LTR|ERV1	22143	NM_01336	26577	Hs.8944	NM_01336	ENSG0000	PCOLCE2	PCPE2	procollage	protein-co
lib-C666-I	chr3	142547748	142547748	+	4.52604	NA	intron (NM_013363, intron 6 of 8)	intron (NM_013363, intron 6 of 8)	60297	NM_01336	26577	Hs.8944	NM_01336	ENSG0000	PCOLCE2	PCPE2	procollage	protein-co
lib-C666-I	chr1	205498312	205498312	+	8.0154	NA	intron (NM_212503, intron 11 of 15)	CpG-2099	24628	NM_21250	5129	Hs.445402	NM_00259	ENSG0000	CDK18	PCTAIRE|P(	cyclin depe	protein-co
lib-C666-I	chr20	48484213	48484213	+	57.97734	NA	intron (NR_048540, intron 8 of 11)	intron (NR_048540, intron 8 of 11)	46063	NM_00113	9825	Hs.48513	NM_00603	ENSG0000	SPATA2	PD1|PPP1R	spermatog	protein-co
lib-C666-I	chr20	48532184	48532184	+	57.83654	NA	promoter-TSS (NM_006038)	promoter-TSS (NM_006038)	-104	NM_00603	9825	Hs.48513	NM_00603	ENSG0000	SPATA2	PD1|PPP1R	spermatog	protein-co
lib-C666-I	chr1	151254019	151254019	+	55.62774	NA	promoter-TSS (NM_001304763)	promoter-TSS (NM_001304763)	-12	NM_00130	57592	Hs.186756	NM_02083	ENSG0000	ZNF687	PDB6	zinc finger	protein-co
lib-C666-I	chr7	558573	558573	+	4.79659	NA	intron (NM_002607, intron 1 of 6)	CpG-23715	908	NM_03302	5154	Hs.535898	NM_00260	ENSG0000	PDGFA	PDGF-A|PI	platelet de	protein-co
lib-C666-I	chr8	95003892	95003892	+	74.14931	NA	Intergenic	Intergenic	74613	NM_00116	54704	Hs.22265	NM_01844	ENSG0000	PDP1	PDH|PDP|F	pyruvate d	protein-co
lib-C666-I	chr8	94928965	94928965	+	7.49163	NA	promoter-TSS (NR_039602)	promoter-TSS (NR_039602)	-118	NM_01844	54704	Hs.22265	NM_01844	ENSG0000	PDP1	PDH|PDP|F	pyruvate d	protein-co
lib-C666-I	chrX	19362014	19362014	+	79.14428	NA	promoter-TSS (NM_001173456)	promoter-TSS (NM_001173456)	3	NM_00117	5160	Hs.530331	NM_00028	ENSG0000	PDHA1	PDHA|PDH	pyruvate d	protein-co
lib-C666-I	chr20	62205804	62205804	+	13.05292	NA	promoter-TSS (NM_001037335)	promoter-TSS (NM_001037335)	-212	NM_00103	85441	Hs.517180	NM_03340	ENSG0000	HELZ2	PDIP-1|PRI	helicase wi	protein-co
lib-C666-I	chr17	26684650	26684650	+	16.14038	NA	promoter-TSS (NM_001290145)	promoter-TSS (NM_001290145)	-38	NM_01558	26073	Hs.241543	NM_01558	ENSG0000	POLDIP2	PDIP38|PO	DNA polyn	protein-co
lib-C666-I	chr3	122785833	122785833	+	8.07793	NA	promoter-TSS (NR_028444)	promoter-TSS (NR_028444)	-23	NM_00681	10954	Hs.477352	NM_00681	ENSG0000	PDIA5	PDIR	protein dis	protein-co
lib-C666-I	chr9	4662418	4662418	+	12.12723	NA	exon (NM_203453, exon 1 of 1)	exon (NM_203453, exon 1 of 1)	124	NM_20345	403313	Hs.107510	NM_20345	ENSG0000	PLPP6	PDP1|PPAF	phospholip	protein-co
lib-C666-I	chr16	70148117	70148117	+	24.99834	NA	5' UTR (NM_017990, exon 1 of 19)	5' UTR (NM_017990, exon 1 of 19)	588	NM_00132	55066	Hs.744043	NM_01799	ENSG0000	PDPR	PDP3	pyruvate d	protein-co
lib-C666-I	chr16	2009441	2009441	+	12.73831	NA	promoter-TSS (NM_004548)	promoter-TSS (NM_004548)	-76	NM_00454	4716	Hs.513266	NM_00454	ENSG0000	NDUFB10	PDSW	NADH:ubic	protein-co
lib-C666-I	chr1	47644937	47644937	+	103.27804	NA	promoter-TSS (NR_047498)	promoter-TSS (NR_047498)	15	NR_04749	100874253	Hs.435758	NR_04749	ENSG0000	LINC00853	PDZK1IP1-	long interg	ncRNA
lib-C666-I	chr10	119135039	119135039	+	56.19669	NA	promoter-TSS (NM_173791)	promoter-TSS (NM_173791)	-61	NM_17379	118987	Hs.501149	NM_17379	ENSG0000	PDZD8	PDZK8	PDZ doma	protein-co
lib-C666-I	chr13	28194483	28194483	+	4.38153	NA	promoter-TSS (NM_001206559)	promoter-TSS (NM_001206559)	237	NM_15337	222484	Hs.132359	NM_15337	ENSG0000	LNX2	PDZRN1	ligand of n	protein-co
lib-C666-I	chr17	17495110	17495110	+	10.33571	NA	promoter-TSS (NM_148172)	promoter-TSS (NM_148172)	-93	NM_00126	10400	Hs.714193	NM_00716	ENSG0000	PEMT	PEAMT|PEI	phosphatic	protein-co
lib-C666-I	chr17	80709849	80709849	+	19.7012	NA	promoter-TSS (NM_005993)	promoter-TSS (NM_005993)	-91	NM_00599	6904	Hs.464391	NM_00599	ENSG0000	TBCD	PEBAT|SSD	tubulin folc	protein-co
lib-C666-I	chr16	67063290	67063290	+	20.72954	NA	5' UTR (NM_022845, exon 1 of 6)	5' UTR (NM_022845, exon 1 of 6)	240	NM_00175	865	Hs.460988	NM_00175	ENSG0000	CBFB	PEBP2B	core-bindi	protein-co
lib-C666-I	chr7	130126057	130126057	+	20.90919	NA	promoter-TSS (NM_177524)	promoter-TSS (NM_177524)	41	NM_00125	4232	Hs.270978	NM_00240	ENSG0000	MEST	PEG1	mesoderm	protein-co
lib-C666-I	chr10	124768350	124768350	+	3.96379	NA	promoter-TSS (NR_073475)	promoter-TSS (NR_073475)	16	NR_07347	64376	Hs.501289	NM_00127	ENSG0000	IKZF5	PEGASUS|Z	IKAROS far	protein-co
lib-C666-I	chr2	88927179	88927179	+	24.18348	NA	promoter-TSS (NR_135540)	promoter-TSS (NR_135540)	-85	NM_00483	9451	Hs.591589	NM_00483	ENSG0000	EIF2AK3	PEK|PERK|V	eukaryotic	protein-co
lib-C666-I	chr2	88899806	88899806	+	11.06679	NA	intron (NM_004836, intron 2 of 16)	intron (NM_004836, intron 2 of 16)	24641	NM_00131	9451	Hs.591589	NM_00483	ENSG0000	EIF2AK3	PEK|PERK|V	eukaryotic	protein-co
lib-C666-I	chr15	41186601	41186601	+	36.99348	NA	promoter-TSS (NM_020857)	promoter-TSS (NM_020857)	-27	NM_02085	57617	Hs.23876	NM_02085	ENSG0000	VPS18	PEP3	VPS18, CO	protein-co
lib-C666-I	chr11	134095551	134095551	+	5.5166	NA	intron (NM_052875, intron 1 of 5)	intron (NM_052875, intron 1 of 5)	1052	NM_05287	112936	Hs.334684	NM_05287	ENSG0000	VPS26B	Pep8b	VPS26, retr	protein-co
lib-C666-I	chr17	8059957	8059957	+	41.89289	NA	Intergenic	CpG	-4204	NM_00261	5187	Hs.445534	NM_00261	ENSG0000	PER1	PER|RIGUI|	period circ	protein-co
lib-C666-I	chr17	8057644	8057644	+	24.25638	NA	Intergenic	Intergenic	-1891	NM_00261	5187	Hs.445534	NM_00261	ENSG0000	PER1	PER|RIGUI|	period circ	protein-co
lib-C666-I	chr17	63198402	63198402	+	34.92958	NA	intron (NM_003835, intron 14 of 18)	intron (NM_003835, intron 14 of 18	64946	NM_00116	8787	Hs.664380	NM_00383	ENSG0000	RGS9	PERRS|RGS	regulator c	protein-co
lib-C666-I	chr17	63133415	63133415	+	27.70954	NA	promoter-TSS (NM_001165933)	promoter-TSS (NM_001165933)	-41	NM_00108	8787	Hs.664380	NM_00383	ENSG0000	RGS9	PERRS|RGS	regulator c	protein-co
lib-C666-I	chr22	30988079	30988079	+	109.56496	NA	promoter-TSS (NM_014303)	promoter-TSS (NM_014303)	-152	NM_00124	23481	Hs.517543	NM_01430	ENSG0000	PES1	PES	pescadillo	protein-co
lib-C666-I	chr1	214725192	214725192	+	10.92275	NA	promoter-TSS (NM_005401)	promoter-TSS (NM_005401)	-168	NM_00540	5784	Hs.193557	NM_00540	ENSG0000	PTPN14	PEZ|PTP36	protein tyr	protein-co
lib-C666-I	chr1	214725453	214725453	+	2.96041	NA	promoter-TSS (NM_005401)	promoter-TSS (NM_005401)	-429	NM_00540	5784	Hs.193557	NM_00540	ENSG0000	PTPN14	PEZ|PTP36	protein tyr	protein-co
lib-C666-I	chr17	27279516	27279516	+	5.819	NA	Intergenic	CpG	-1008	NM_00103	57649	Hs.444173	NM_02088	ENSG0000	PHF12	PF1	PHD finger	protein-co
lib-C666-I	chr2	214149365	214149365	+	9.09067	NA	promoter-TSS (NM_001242575)	promoter-TSS (NM_001242575)	262	NM_02453	79582	Hs.743365	NM_02453	ENSG0000	SPAG16	PF20|WDR	sperm assc	protein-co
lib-C666-I	chr2	214149043	214149043	+	5.96687	NA	promoter-TSS (NM_001025436)	promoter-TSS (NM_001025436)	-60	NM_00102	79582	Hs.743365	NM_02453	ENSG0000	SPAG16	PF20|WDR	sperm assc	protein-co
lib-C666-I	chr3	46935417	46935417	+	8.03539	NA	exon (NM_000316, exon 4 of 16)	exon (NM_000316, exon 4 of 16)	11678	NM_00118	5745	Hs.1019	NM_00031	ENSG0000	PTH1R	PFE|PTHR|F	parathyroi	protein-co
lib-C666-I	chr1	207226595	207226595	+	2.31621	NA	promoter-TSS (NM_006212)	promoter-TSS (NM_006212)	-25	NM_00621	5208	Hs.282702	NM_00621	ENSG0000	PFKFB2	PFK-2/FBP	6-phospho	protein-co
lib-C666-I	chr12	108154987	108154987	+	12.26631	NA	promoter-TSS (NM_012406)	promoter-TSS (NM_012406)	-73	NM_01240	11108	Hs.506655	NM_01240	ENSG0000	PRDM4	PFM1	PR/SET do	protein-co
lib-C666-I	chr11	129873095	129873095	+	38.10056	NA	promoter-TSS (NM_199437)	promoter-TSS (NM_199437)	-365	NM_02022	56980	Hs.275086	NM_02022	ENSG0000	PRDM10	PFM7	PR/SET do	protein-co
lib-C666-I	chr11	45201824	45201824	+	85.77059	NA	intron (NM_001256696, intron 1 of 6)	CpG	32930	NM_00125	56981	Hs.178715	NM_02022	ENSG0000	PRDM11	PFM8	PR/SET do	protein-co
lib-C666-I	chr11	45168824	45168824	+	19.75653	NA	promoter-TSS (NM_001256695)	promoter-TSS (NM_001256695)	-70	NM_00125	56981	Hs.178715	NM_02022	ENSG0000	PRDM11	PFM8	PR/SET do	protein-co
lib-C666-I	chr11	45202433	45202433	+	7.76438	NA	intron (NM_001256696, intron 1 of 6)	CpG	33539	NM_00125	56981	Hs.178715	NM_02022	ENSG0000	PRDM11	PFM8	PR/SET do	protein-co
lib-C666-I	chr1	6320174	6320174	+	82.17155	NA	intron (NM_207370, intron 1 of 5)	CpG	861	NM_20737	387509	Hs.531581	NM_20737	ENSG0000	GPR153	PGR1	G protein-	protein-co
lib-C666-I	chr1	6320666	6320666	+	3.27468	NA	intron (NM_207370, intron 1 of 5)	CpG	369	NM_20737	387509	Hs.531581	NM_20737	ENSG0000	GPR153	PGR1	G protein-	protein-co
lib-C666-I	chr15	74908082	74908082	+	36.83792	NA	exon (NM_001130028, exon 1 of 13)	exon (NM_001130028, exon 1 of 13	747	NM_00113	1198	Hs.511790	NM_00129	ENSG0000	CLK3	PHCLK3|PH	CDC like ki	protein-co
lib-C666-I	chr4	129733235	129733235	+	12.97267	NA	intron (NM_001287443, intron 1 of 10	CpG	236	NM_00128	79960	Hs.12420	NM_02490	ENSG0000	JADE1	PHF17	jade family	protein-co
lib-C666-I	chr4	129731288	129731288	+	8.80021	NA	5' UTR (NM_001287439, exon 1 of 11	5' UTR (NM_001287439, exon 1 of 1	214	NM_00128	79960	Hs.12420	NM_02490	ENSG0000	JADE1	PHF17	jade family	protein-co
lib-C666-I	chr4	129733604	129733604	+	2.05265	NA	intron (NM_001287443, intron 1 of 10	CpG	605	NM_00128	79960	Hs.12420	NM_02490	ENSG0000	JADE1	PHF17	jade family	protein-co
lib-C666-I	chr1	6673655	6673655	+	8.89416	NA	promoter-TSS (NM_153812)	promoter-TSS (NM_153812)	-101	NM_15381	148479	Hs.516079	NM_15381	ENSG0000	PHF13	PHF5|SPOC	PHD finger	protein-co
lib-C666-I	chr11	64014070	64014070	+	36.56565	NA	promoter-TSS (NR_135086)	promoter-TSS (NR_135086)	343	NM_13868	26472	Hs.523760	NM_13868	ENSG0000	PPP1R14B	PHI-1|PLCI	protein ph	protein-co
lib-C666-I	chr12	107487332	107487332	+	7.21092	NA	5' UTR (NM_004075, exon 1 of 13)	5' UTR (NM_004075, exon 1 of 13)	303	NM_00407	1407	Hs.151573	NM_00407	ENSG0000	CRY1	PHLL1	cryptochro	protein-co
lib-C666-I	chr18	60382389	60382389	+	11.66012	NA	promoter-TSS (NM_194449)	promoter-TSS (NM_194449)	-283	NM_19444	23239	Hs.465337	NM_19444	ENSG0000	PHLPP1	PHLPP|PLE	PH domair	protein-co
lib-C666-I	chr16	71757258	71757258	+	4.52748	NA	intron (NM_015020, intron 1 of 18)	CpG	1346	NM_01502	23035	Hs.709458	NM_01502	ENSG0000	PHLPP2	PHLPPL|PP	PH domair	protein-co
lib-C666-I	chr7	64254759	64254759	+	45.42569	NA	promoter-TSS (NM_001160183)	promoter-TSS (NM_001160183)	-7	NM_00127	7697	Hs.184080	NM_00652	ENSG0000	ZNF138	pHZ-32	zinc finger	protein-co
lib-C666-I	chr12	133657456	133657456	+	129.74547	NA	5' UTR (NM_001300776, exon 1 of 4)	5' UTR (NM_001300776, exon 1 of 4	667	NM_00344	7699	Hs.181552	NM_00344	ENSG0000	ZNF140	pHZ-39	zinc finger	protein-co
lib-C666-I	chr12	133656787	133656787	+	65.45359	NA	promoter-TSS (NM_001300776)	promoter-TSS (NM_001300776)	-2	NM_00130	7699	Hs.181552	NM_00344	ENSG0000	ZNF140	pHZ-39	zinc finger	protein-co
lib-C666-I	chr19	44488211	44488211	+	8.96177	NA	promoter-TSS (NM_001260486)	promoter-TSS (NM_001260486)	-111	NM_19808	7711	Hs.279567	NM_00344	ENSG0000	ZNF155	pHZ-96	zinc finger	protein-co
lib-C666-I	chr22	21212898	21212898	+	7.97818	NA	promoter-TSS (NM_004782)	promoter-TSS (NM_004782)	202	NM_05800	5297	Hs.529438	NM_00265	ENSG0000	PI4KA	PI4K-ALPH	phosphatic	protein-co
lib-C666-I	chr4	25235862	25235862	+	21.69444	NA	exon (NM_018323, exon 1 of 10)	exon (NM_018323, exon 1 of 10)	209	NM_01832	55300	Hs.191701	NM_01832	ENSG0000	PI4K2B	PI4KIIB|PIK	phosphatic	protein-co
lib-C666-I	chr17	36956274	36956274	+	5.51692	NA	promoter-TSS (NM_003559)	promoter-TSS (NM_003559)	-116	NM_00355	8396	Hs.260603	NM_00355	ENSG0000	PIP4K2B	PI5P4KB|PI	phosphatic	protein-co
lib-C666-I	chr20	52532287	52532287	+	8.27411	NA	Intergenic	Intergenic	-40039	NR_00218	391257	Hs.449977	NR_002189		SUMO1P1	PIC1L|UBL2	SUMO1 ps	pseudo
lib-C666-I	chr22	38453378	38453378	+	42.49125	NA	5' UTR (NM_012407, exon 1 of 13)	5' UTR (NM_012407, exon 1 of 13)	116	NM_01240	9463	Hs.180871	NM_01240	ENSG0000	PICK1	PICK|PRKC	protein inte	protein-co
lib-C666-I	chr19	48248758	48248758	+	25.80588	NA	promoter-TSS (NM_015710)	promoter-TSS (NM_015710)	-35	NM_01571	29997	Hs.421907	NM_01571	ENSG0000	GLTSCR2	PICT-1|PIC	glioma tun	protein-co
lib-C666-I	chr11	44972136	44972136	+	3.7184	NA	promoter-TSS (NM_001258320)	promoter-TSS (NM_001258320)	319	NM_00131	9537	Hs.554791	NM_00603	ENSG0000	TP53I11	PIG11	tumor prot	protein-co
lib-C666-I	chr15	34517244	34517244	+	50.38662	NA	promoter-TSS (NM_001286420)	promoter-TSS (NM_001286420)	46	NM_00128	51234	Hs.250905	NM_01645	ENSG0000	EMC4	PIG17|TME	ER membra	protein-co
lib-C666-I	chr11	443793	443793	+	9.48923	NA	Intergenic	Intergenic	-1782	NM_00134	338440	Hs.501622	NM_00101	ENSG0000	ANO9	PIG5|TMEN	anoctamin	protein-co
lib-C666-I	chr16	11702453	11702453	+	10.65273	NA	Intergenic	L1MB8|LINE|L1	-21131	NR_02432	9516	Hs.459940	NM_00486	ENSG0000	LITAF	PIG7|SIMPI	lipopolysac	protein-co
lib-C666-I	chr16	11706233	11706233	+	5.12175	NA	Intergenic	Intergenic	-24911	NR_02432	9516	Hs.459940	NM_00486	ENSG0000	LITAF	PIG7|SIMPI	lipopolysac	protein-co
lib-C666-I	chr16	11702143	11702143	+	2.42134	NA	Intergenic	Intergenic	-20821	NR_02432	9516	Hs.459940	NM_00486	ENSG0000	LITAF	PIG7|SIMPI	lipopolysac	protein-co
lib-C666-I	chr3	196439382	196439382	+	4.73866	NA	promoter-TSS (NM_032898)	promoter-TSS (NM_032898)	137	NM_00116	54965	Hs.223296	NM_01786	ENSG0000	PIGX	PIG-X	phosphatic	protein-co
lib-C666-I	chr22	50354283	50354283	+	66.77065	NA	5' UTR (NM_001001852, exon 1 of 6)	5' UTR (NM_001001852, exon 1 of 6	140	NM_00100	415116	Hs.530381	NM_00100	ENSG0000	PIM3	pim-3	Pim-3 prot	protein-co
lib-C666-I	chr22	50338924	50338924	+	9.7234	NA	Intergenic	Intergenic	-15219	NM_00100	415116	Hs.530381	NM_00100	ENSG0000	PIM3	pim-3	Pim-3 prot	protein-co
lib-C666-I	chr6	150070954	150070954	+	3.12293	NA	exon (NM_001252053, exon 1 of 7)	exon (NM_001252053, exon 1 of 7)	123	NM_00125	5110	Hs.279257	NM_00538	ENSG0000	PCMT1	PIMT	protein-L-	protein-co
lib-C666-I	chr1	44820950	44820950	+	52.3754	NA	promoter-TSS (NM_001301700)	promoter-TSS (NM_001301700)	1	NM_00130	79033	Hs.731413	NM_02406	ENSG0000	ERI3	PINT1|PRN	ERI1 exorit	protein-co
lib-C666-I	chr7	77045872	77045872	+	8.61897	NA	promoter-TSS (NM_017439)	promoter-TSS (NM_017439)	-155	NM_01743	54103	Hs.186649	NM_01743	ENSG0000	GSAP	PION	gamma-se	protein-co
lib-C666-I	chr16	67694761	67694761	+	61.32708	NA	promoter-TSS (NM_016948)	promoter-TSS (NM_016948)	-43	NM_02291	65057	Hs.78019	NM_02291	ENSG0000	ACD	PIP1|PTOP	ACD, shelt	protein-co
lib-C666-I	chr6	154614187	154614187	+	6.50091	NA	intron (NM_001130700, intron 1 of 11	intron (NM_001130700, intron 1 of	37028	NM_00113	26034	Hs.146100	NM_01555	ENSG0000	IPCEF1	PIP3-E	interaction	protein-co
lib-C666-I	chr12	57984840	57984840	+	3.93123	NA	promoter-TSS (NM_001146258)	promoter-TSS (NM_001146258)	-102	NM_02477	79837	Hs.745011	NM_02477	ENSG0000	PIP4K2C	PIP5K2C	phosphatic	protein-co
lib-C666-I	chr9	130693093	130693093	+	5.25516	NA	promoter-TSS (NM_001135219)	promoter-TSS (NM_001135219)	-17	NM_00113	138429	Hs.734928	NM_17349	ENSG0000	PIP5KL1	PIPKH|bA2	phosphatic	protein-co
lib-C666-I	chr22	31933082	31933082	+	8.85547	NA	intron (NM_014775, intron 4 of 31)	MLT1J|LTR|ERVL-MaLR	40957	NM_00125	9814	Hs.62209	NM_01477	ENSG0000	SFI1	PISD|PPP1I	SFI1 centri	protein-co
lib-C666-I	chr3	15901379	15901379	+	5.9194	NA	promoter-TSS (NM_015199)	promoter-TSS (NM_015199)	-326	NM_01519	23243	Hs.335239	NM_01519	ENSG0000	ANKRD28	PITK|PPP1F	ankyrin rep	protein-co
lib-C666-I	chr3	52188527	52188527	+	21.21068	NA	5' UTR (NM_001161580, exon 1 of 10	5' UTR (NM_001161580, exon 1 of 1	179	NM_00116	25886	Hs.476306	NM_01542	ENSG0000	POC1A	PIX2|SOFT|	POC1 cent	protein-co
lib-C666-I	chr19	14228687	14228687	+	24.23241	NA	promoter-TSS (NM_002730)	promoter-TSS (NM_002730)	-128	NM_00273	5566	Hs.631630	NM_00273	ENSG0000	PRKACA	PKACA|PPI	protein kin	protein-co
lib-C666-I	chr2	45871001	45871001	+	19.75653	NA	Intergenic	CpG	-8042	NM_00540	5581	Hs.580351	NM_00540	ENSG0000	PRKCE	PKCE|nPKC	protein kin	protein-co
lib-C666-I	chr2	46163642	46163642	+	16.63918	NA	intron (NM_005400, intron 2 of 14)	intron (NM_005400, intron 2 of 14)	284599	NM_00540	5581	Hs.580351	NM_00540	ENSG0000	PRKCE	PKCE|nPKC	protein kin	protein-co
lib-C666-I	chr2	45878245	45878245	+	16.5633	NA	promoter-TSS (NM_005400)	promoter-TSS (NM_005400)	-798	NM_00540	5581	Hs.580351	NM_00540	ENSG0000	PRKCE	PKCE|nPKC	protein kin	protein-co
lib-C666-I	chr2	45877277	45877277	+	2.59723	NA	Intergenic	CpG	-1766	NM_00540	5581	Hs.580351	NM_00540	ENSG0000	PRKCE	PKCE|nPKC	protein kin	protein-co
lib-C666-I	chr14	61788749	61788749	+	10.6239	NA	5' UTR (NM_006255, exon 1 of 14)	5' UTR (NM_006255, exon 1 of 14)	588	NM_00625	5583	Hs.333907	NM_00625	ENSG0000	PRKCH	PKC-L|PKC	protein kin	protein-co
lib-C666-I	chr14	61789280	61789280	+	6.32351	NA	intron (NM_006255, intron 1 of 13)	CpG	1119	NM_00625	5583	Hs.333907	NM_00625	ENSG0000	PRKCH	PKC-L|PKC	protein kin	protein-co
lib-C666-I	chr1	2064719	2064719	+	9.10494	NA	intron (NM_001033582, intron 1 of 14	CpG	28564	NM_00103	5590	Hs.496255	NM_00274	ENSG0000	PRKCZ	PKC-ZETA|	protein kin	protein-co
lib-C666-I	chr2	217138763	217138763	+	12.57335	NA	intron (NM_020814, intron 3 of 3)	LTR87|LTR|ERVL?	57151	NR_03770	150967	Hs.56876	NM_00119	ENSG0000	LINC01963	PKI55	long interg	ncRNA
lib-C666-I	chr2	172117350	172117350	+	10.73451	NA	Intergenic	MER4C|LTR|ERV1	-29526	NM_00113	9874	Hs.744917	NM_01229	ENSG0000	TLK1	PKU-beta	tousled like	protein-co
lib-C666-I	chr7	135347268	135347268	+	19.91222	NA	promoter-TSS (NM_001130929)	promoter-TSS (NM_001130929)	47	NM_00113	647087	Hs.200022	NM_00113	ENSG0000	C7orf73	PL-5283	chromosor	protein-co
lib-C666-I	chr1	186732547	186732547	+	8.73012	NA	Intergenic	Intergenic	-65485	NM_02442	5321	Hs.497200	NM_02442	ENSG0000	PLA2G4A	PLA2G4|cP	phospholip	protein-co
lib-C666-I	chr1	20424863	20424863	+	27.44508	NA	Intergenic	Intergenic	21196	NM_00127	26279	Hs.189507	NM_01240	ENSG0000	PLA2G2D	PLA2IID|SP	phospholip	protein-co
lib-C666-I	chr7	23145332	23145332	+	119.6264	NA	promoter-TSS (NM_018846)	promoter-TSS (NM_018846)	-10	NR_04622	100775104		NR_04622	ENSG0000	KLHL7-AS1	PLATAK	KLHL7 anti	ncRNA
lib-C666-I	chr2	43864393	43864393	+	8.01457	NA	promoter-TSS (NM_172069)	promoter-TSS (NM_172069)	-46	NM_17206	130271	Hs.164162	NM_17206	ENSG0000	PLEKHH2	PLEKHH1L	pleckstrin h	protein-co
lib-C666-I	chr17	62833270	62833270	+	16.6838	NA	promoter-TSS (NR_024386)	promoter-TSS (NR_024386)	32	NR_02438	440456	Hs.646585	NR_024386		PLEKHM1P	PLEKHM1P	pleckstrin h	pseudo
lib-C666-I	chr16	23690117	23690117	+	10.84249	NA	promoter-TSS (NM_005030)	promoter-TSS (NM_005030)	24	NM_00503	5347	Hs.592049	NM_00503	ENSG0000	PLK1	PLK|STPK1	polo like ki	protein-co
lib-C666-I	chr4	122722479	122722479	+	91.98129	NA	promoter-TSS (NM_005033)	promoter-TSS (NM_005033)	7	NM_00503	5393	Hs.91728	NM_00503	ENSG0000	EXOSC9	PM/Scl-75	exosome c	protein-co
lib-C666-I	chr11	73882273	73882273	+	6.82401	NA	promoter-TSS (NM_015531)	promoter-TSS (NM_015531)	165	NM_01614	51400	Hs.503251	NM_01614	ENSG0000	PPME1	PME-1	protein ph	protein-co
lib-C666-I	chr1	156183028	156183028	+	23.12599	NA	TTS (NM_001286184)	TTS (NM_001286184)	249	NM_00119	100527963	Hs.530479	NM_00119	ENSG0000	PMF1-BGL	PMF1	PMF1-BGL	protein-co
lib-C666-I	chr16	57318515	57318515	+	11.13288	NA	promoter-TSS (NM_015993)	promoter-TSS (NM_015993)	69	NM_01599	51090	Hs.632215	NM_01599	ENSG0000	PLLP	PMLP|TM4	plasmolipi	protein-co
lib-C666-I	chr16	57351099	57351099	+	7.09685	NA	Intergenic	Intergenic	-32515	NM_01599	51090	Hs.632215	NM_01599	ENSG0000	PLLP	PMLP|TM4	plasmolipi	protein-co
lib-C666-I	chr20	32308180	32308180	+	17.48666	NA	promoter-TSS (NM_007238)	promoter-TSS (NM_007238)	-44	NM_18339	11264	Hs.654857	NM_00723	ENSG0000	PXMP4	PMP24	peroxisom	protein-co
lib-C666-I	chr11	86748712	86748712	+	19.23598	NA	promoter-TSS (NM_001168724)	promoter-TSS (NM_001168724)	-174	NR_03314	65084	Hs.188591	NM_02291	ENSG0000	TMEM135	PMP52	transmemt	protein-co
lib-C666-I	chr8	52630853	52630853	+	15.1031	NA	intron (NM_144651, intron 1 of 22)	intron (NM_144651, intron 1 of 22)	91152	NM_14465	137902	Hs.444882	NM_14465	ENSG0000	PXDNL	PMR1|PRM	peroxidasi	protein-co
lib-C666-I	chr7	76637551	76637551	+	6.52841	NA	intron (NR_023383, intron 6 of 10)	intron (NR_023383, intron 6 of 10)	27412	NR_02338	441263	Hs.675888	NR_02338	ENSG0000	DTX2P1-U	PMS2L11	DTX2P1-U	pseudo
lib-C666-I	chr16	15150078	15150078	+	8.56376	NA	promoter-TSS (NM_001270767)	promoter-TSS (NM_001270767)	-142	NM_00127	123803	Hs.592045	NM_17347	ENSG0000	NTAN1	PNAA|PNA	N-termina	protein-co
lib-C666-I	chr20	35234039	35234039	+	66.67216	NA	promoter-TSS (NM_001199534)	promoter-TSS (NM_001199534)	-98	NM_01884	55969	Hs.584985	NM_01884	ENSG0000	C20orf24	PNAS-11|F	chromosor	protein-co
lib-C666-I	chr20	35220673	35220673	+	14.3056	NA	3' UTR (NM_021809, exon 3 of 3)	3' UTR (NM_021809, exon 3 of 3)	-13464	NM_01884	55969	Hs.584985	NM_01884	ENSG0000	C20orf24	PNAS-11|F	chromosor	protein-co
lib-C666-I	chr17	42144053	42144053	+	36.59122	NA	5' UTR (NM_152344, exon 2 of 6)	5' UTR (NM_152344, exon 2 of 6)	934	NM_15234	124801	Hs.355570	NM_15234	ENSG0000	LSM12	PNAS-135	LSM12 hor	protein-co
lib-C666-I	chr8	71520635	71520635	+	10.92275	NA	promoter-TSS (NR_038881)	promoter-TSS (NR_038881)	59	NM_00131	23471	Hs.491988	NM_01429	ENSG0000	TRAM1	PNAS8|TRA	translocati	protein-co
lib-C666-I	chr3	140660578	140660578	+	47.52935	NA	promoter-TSS (NM_018155)	promoter-TSS (NM_018155)	-84	NM_01815	55186	Hs.144130	NM_01815	ENSG0000	SLC25A36	PNC2	solute carr	protein-co
lib-C666-I	chr1	6052568	6052568	+	80.54002	NA	promoter-TSS (NR_111987)	promoter-TSS (NR_111987)	-35	NM_01510	261734	Hs.462348	NM_01510	ENSG0000	NPHP4	POC10|SLS	nephrocys	protein-co
lib-C666-I	chrX	84784601	84784601	+	4.90117	NA	Intergenic	L1PA3|LINE|L1	-149853	NM_02492	79983	Hs.267038	NM_02492	ENSG0000	POF1B	POF|POF2E	premature	protein-co
lib-C666-I	chr4	57844913	57844913	+	5.15932	NA	5' UTR (NM_000938, exon 1 of 25)	5' UTR (NM_000938, exon 1 of 25)	107	NM_00130	5431	Hs.602757	NM_00093	ENSG0000	POLR2B	POL2RB|RF	RNA polyn	protein-co
lib-C666-I	chr17	7388059	7388059	+	4.30348	NA	promoter-TSS (NM_020899)	promoter-TSS (NM_020899)	361	NM_00093	5430	Hs.270017	NM_00093	ENSG0000	POLR2A	POLR2|POI	RNA polyn	protein-co
lib-C666-I	chr7	102233253	102233253	+	2.59723	NA	intron (NM_001079877, intron 15 of 1	intron (NM_001079877, intron 15 o	-20185	NM_00109	548644	Hs.610255	NM_00101	ENSG0000	POLR2J3	POLR2J2|R	RNA polyn	protein-co
lib-C666-I	chr1	201617070	201617070	+	5.39586	NA	promoter-TSS (NM_020443)	promoter-TSS (NM_020443)	-380	NM_02044	89796	Hs.585374	NM_02044	ENSG0000	NAV1	POMFIL3|S	neuron nav	protein-co
lib-C666-I	chr1	35658860	35658860	+	60.13322	NA	promoter-TSS (NR_136703)	promoter-TSS (NR_136703)	-114	NR_13670	6421	Hs.355934	NM_00506	ENSG0000	SFPQ	POMP100|	splicing fac	protein-co
lib-C666-I	chr1	35658115	35658115	+	3.59106	NA	non-coding (NR_136703, exon 1 of 1	non-coding (NR_136703, exon 1 of	628	NM_00506	6421	Hs.355934	NM_00506	ENSG0000	SFPQ	POMP100|	splicing fac	protein-co
lib-C666-I	chr7	76256655	76256655	+	380.12436	NA	promoter-TSS (NM_152992)	promoter-TSS (NM_152992)	-35	NM_01223	22932	Hs.488877	NM_01223	ENSG0000	POMZP3	POM-ZP3|	POM121 a	protein-co
lib-C666-I	chr3	119357859	119357859	+	6.44411	NA	Intergenic	Intergenic	21578	NM_00130	64091	Hs.534383	NM_02213	ENSG0000	POPDC2	POP2	popeye do	protein-co
lib-C666-I	chr10	76970297	76970297	+	18.89222	NA	intron (NM_001184823, intron 1 of 9)	CpG	385	NM_00132	7417	Hs.355927	NM_00337	ENSG0000	VDAC2	POR	voltage de	protein-co
lib-C666-I	chr10	103543188	103543188	+	34.3461	NA	promoter-TSS (NM_006993)	promoter-TSS (NM_006993)	-18	NM_00699	10360	Hs.90691	NM_00699	ENSG0000	NPM3	PORMIN|T	nucleopho	protein-co
lib-C666-I	chr4	170192229	170192229	+	3.93123	NA	promoter-TSS (NM_020870)	promoter-TSS (NM_020870)	20	NM_02087	57630	Hs.301804	NM_02087	ENSG0000	SH3RF1	POSH|RNF	SH3 doma	protein-co
lib-C666-I	chr13	78634999	78634999	+	6.64452	NA	intron (NR_047001, intron 1 of 5)	L1PBa1|LINE|L1	6009	NR_04700	100874222	Hs.493062	NR_04700	ENSG0000	RNF219-A	POU4F1-A	RNF219 an	ncRNA
lib-C666-I	chr7	73157476	73157476	+	79.18531	NA	Intergenic	Intergenic	-4279	NR_13562	83451	Hs.647045	NM_14891	ENSG0000	ABHD11	PP1226|WI	abhydrolas	protein-co
lib-C666-I	chr7	73153418	73153418	+	22.82077	NA	promoter-TSS (NM_001301058)	promoter-TSS (NM_001301058)	-221	NR_13562	83451	Hs.647045	NM_14891	ENSG0000	ABHD11	PP1226|WI	abhydrolas	protein-co
lib-C666-I	chr7	73156120	73156120	+	9.4812	NA	Intergenic	Intergenic	-2923	NR_13562	83451	Hs.647045	NM_14891	ENSG0000	ABHD11	PP1226|WI	abhydrolas	protein-co
lib-C666-I	chr1	26361932	26361932	+	16.96353	NA	3' UTR (NM_004455, exon 11 of 11)	3' UTR (NM_004455, exon 11 of 11)	10697	NM_03251	7780	Hs.143545	NM_03251	ENSG0000	SLC30A2	PP12488|T	solute carr	protein-co
lib-C666-I	chr19	54960374	54960374	+	50.99376	NA	promoter-TSS (NR_126418)	promoter-TSS (NR_126418)	34	NM_05292	114823	Hs.502378	NM_05292	ENSG0000	LENG8	pp13842	leukocyte r	protein-co
lib-C666-I	chr8	30670270	30670270	+	18.85495	NA	promoter-TSS (NM_001009552)	promoter-TSS (NM_001009552)	82	NM_00100	5516	Hs.491440	NM_00415	ENSG0000	PPP2CB	PP2Abeta|I	protein ph	protein-co
lib-C666-I	chr5	133561826	133561826	+	81.64329	NA	TTS (NR_037434)	TTS (NR_037434)	124	NM_00271	5515	Hs.105818	NM_00271	ENSG0000	PPP2CA	PP2Ac|PP2	protein ph	protein-co
lib-C666-I	chr14	60715761	60715761	+	37.12814	NA	promoter-TSS (NM_021003)	promoter-TSS (NM_021003)	-205	NM_02100	5494	Hs.130036	NM_02100	ENSG0000	PPM1A	PP2C-ALPI	protein ph	protein-co
lib-C666-I	chr2	44395598	44395598	+	19.54889	NA	promoter-TSS (NM_002706)	promoter-TSS (NM_002706)	-344	NM_17796	5495	Hs.416769	NM_00270	ENSG0000	PPM1B	PP2C-beta	protein ph	protein-co
lib-C666-I	chr2	44314547	44314547	+	9.20633	NA	Intergenic	CpG	-81395	NM_17796	5495	Hs.416769	NM_00270	ENSG0000	PPM1B	PP2C-beta	protein ph	protein-co
lib-C666-I	chr17	58677494	58677494	+	15.54425	NA	promoter-TSS (NM_003620)	promoter-TSS (NM_003620)	-50	NM_00362	8493	Hs.286073	NM_00362	ENSG0000	PPM1D	PP2C-DEL	protein ph	protein-co
lib-C666-I	chr2	27632531	27632531	+	8.28216	NA	promoter-TSS (NM_177983)	promoter-TSS (NM_177983)	19	NM_17798	5496	Hs.643951	NM_17798	ENSG0000	PPM1G	PP2CG|PP2	protein ph	protein-co
lib-C666-I	chr12	48840371	48840371	+	7.76438	NA	Intergenic	Intergenic	-26077	NM_01240	23519	Hs.532658	NM_01240	ENSG0000	ANP32D	PP32R2	acidic nucl	protein-co
lib-C666-I	chr10	99447234	99447234	+	28.7791	NA	promoter-TSS (NM_021732)	promoter-TSS (NM_021732)	-219	NM_02173	60370	Hs.23918	NM_02173	ENSG0000	AVPI1	PP5395|VIF	arginine va	protein-co
lib-C666-I	chr9	127952086	127952086	+	51.11391	NA	5' UTR (NM_001123355, exon 1 of 8)	5' UTR (NM_001123355, exon 1 of 8	132	NM_00272	5537	Hs.744091	NM_00272	ENSG0000	PPP6C	PP6|PP6C	protein ph	protein-co
lib-C666-I	chr9	131790380	131790380	+	11.36743	NA	exon (NM_020145, exon 1 of 11)	exon (NM_020145, exon 1 of 11)	252	NM_02014	56904	Hs.460238	NM_02014	ENSG0000	SH3GLB2	PP6569|PP	SH3 doma	protein-co
lib-C666-I	chr21	42932731	42932731	+	6.50091	NA	Intergenic	MER5A|DNA|hAT-Charlie	-52646	NM_00565	7113	Hs.439309	NM_00565	ENSG0000	TMPRSS2	PP9284|PR	transmemt	protein-co
lib-C666-I	chr15	43622757	43622757	+	13.91591	NA	promoter-TSS (NM_014793)	promoter-TSS (NM_014793)	63	NM_01479	9836	Hs.200596	NM_01479	ENSG0000	LCMT2	PPM2|TYW	leucine car	protein-co
lib-C666-I	chr1	161136167	161136167	+	2.58799	NA	promoter-TSS (NM_000309)	promoter-TSS (NM_000309)	-14	NM_00112	5498	Hs.517373	NM_00030	ENSG0000	PPOX	PPO|V290I	protoporpl	protein-co
lib-C666-I	chr1	28696184	28696184	+	9.20633	NA	promoter-TSS (NM_001048183)	promoter-TSS (NM_001048183)	91	NM_00104	65979	Hs.225641	NM_02392	ENSG0000	PHACTR4	PPP1R124	phosphata	protein-co
lib-C666-I	chr1	28696762	28696762	+	7.49163	NA	intron (NM_001048183, intron 1 of 13	intron (NM_001048183, intron 1 of	669	NM_00104	65979	Hs.225641	NM_02392	ENSG0000	PHACTR4	PPP1R124	phosphata	protein-co
lib-C666-I	chr20	47835880	47835880	+	69.18568	NA	promoter-TSS (NM_001319135)	promoter-TSS (NM_001319135)	-48	NM_00131	6780	Hs.596704	NM_00460	ENSG0000	STAU1	PPP1R150|	staufen do	protein-co
lib-C666-I	chr8	19266198	19266198	+	9.94079	NA	exon (NM_001130518, exon 9 of 10)	exon (NM_001130518, exon 9 of 10	94711	NM_00117	63898	Hs.303208	NM_02207	ENSG0000	SH2D4A	PPP1R38|S	SH2 doma	protein-co
lib-C666-I	chr8	19171911	19171911	+	9.45164	NA	intron (NM_001174159, intron 1 of 9)	CpG	424	NM_00117	63898	Hs.303208	NM_02207	ENSG0000	SH2D4A	PPP1R38|S	SH2 doma	protein-co
lib-C666-I	chr11	83255321	83255321	+	5.92363	NA	intron (NM_001142702, intron 2 of 10	MER68|LTR|ERVL	138147	NM_00114	1740	Hs.367656	NM_00136	ENSG0000	DLG2	PPP1R58|P	discs large	protein-co
lib-C666-I	chr11	82904725	82904725	+	9.21057	NA	promoter-TSS (NM_001300976)	promoter-TSS (NM_001300976)	-56	NM_00130	338699	Hs.503438	NM_18260	ENSG0000	ANKRD42	PPP1R79|S	ankyrin rep	protein-co

lib-C666-l	chr2	37193740	37193740	+	15.55328	NA	promoter-TSS (NM_003162)	promoter-TSS (NM_003162)	-125	NM_00316	6801	Hs.127486	NM_00316	ENSG0000	STRN	PPP2R6A|S	striatin	protein-co
lib-C666-l	chr17	1420034	1420034	+	30.85659	NA	promoter-TSS (NR_028514)	promoter-TSS (NR_028514)	148	NM_01653	51763	Hs.632238	NM_01653	ENSG0000	INPP5K	PPS|SKIP	inositol po	protein-co
lib-C666-l	chr16	30087255	30087255	+	18.43022	NA	promoter-TSS (NM_001303507)	promoter-TSS (NM_001303507)	-42	NM_00130	5531	Hs.534338	NM_00272	ENSG0000	PPP4C	PP-X|PP4|F	protein ph	protein-co
lib-C666-l	chr2	10830091	10830091	+	102.48656	NA	promoter-TSS (NM_001261392)	promoter-TSS (NM_001261392)	22	NM_00126	79954	Hs.222494	NM_02489	ENSG0000	NOL10	PQBP5	nucleolar p	protein-co
lib-C666-l	chr6	4018928	4018928	+	10.03103	NA	Intergenic	CpG	-2641	NM_00391	8899	Hs.159014	NM_00391	ENSG0000	PRPF4B	PR4H|PRP4	pre-mRNA	protein-co
lib-C666-l	chr19	42463536	42463536	+	7.7703	NA	promoter-TSS (NM_006423)	promoter-TSS (NM_006423)	-8	NM_00642	10567	Hs.11417	NM_00642	ENSG0000	RABAC1	PRA1|PRAF	Rab accept	protein-co
lib-C666-l	chr7	26904426	26904426	+	12.26631	NA	promoter-TSS (NM_003930)	promoter-TSS (NM_003930)	-64	NM_00393	8935	Hs.200770	NM_00393	ENSG0000	SKAP2	PRAP|RA7(	src kinase a	protein-co
lib-C666-l	chr1	162760404	162760404	+	57.48363	NA	promoter-TSS (NM_001304513)	promoter-TSS (NM_001304513)	-88	NM_00130	51478	Hs.492925	NM_01637	ENSG0000	HSD17B7	PRAP|SDR3	hydroxyste	protein-co
lib-C666-l	chr1	182558205	182558205	+	11.66438	NA	intron (NM_021133, intron 1 of 6)	intron (NM_021133, intron 1 of 6)	189	NM_02113	6041	Hs.518545	NM_02113	ENSG0000	RNASEL	PRCA1|RNS	ribonuclea	protein-co
lib-C666-l	chr17	75136754	75136754	+	15.60931	NA	promoter-TSS (NM_003003)	promoter-TSS (NM_003003)	-251	NM_00300	6397	Hs.464184	NM_00300	ENSG0000	SEC14L1	PRELID4A|S	SEC14 like	protein-co
lib-C666-l	chr17	75137641	75137641	+	10.19094	NA	intron (NM_001143999, intron 1 of 16	CpG	636	NM_00114	6397	Hs.464184	NM_00300	ENSG0000	SEC14L1	PRELID4A|S	SEC14 like	protein-co
lib-C666-l	chr17	75084716	75084716	+	9.47655	NA	promoter-TSS (NR_106997)	promoter-TSS (NR_106997)	-9	NM_00120	6397	Hs.464184	NM_00300	ENSG0000	SEC14L1	PRELID4A|S	SEC14 like	protein-co
lib-C666-l	chr21	44394680	44394680	+	88.35172	NA	promoter-TSS (NM_001320694)	promoter-TSS (NM_001320694)	60	NM_00457	5316	Hs.431043	NM_00457	ENSG0000	PKNOX1	PREP1|pko	PBX/knotte	protein-co
lib-C666-l	chr18	72782909	72782909	+	2.12162	NA	Intergenic	Intergenic	134559	NM_00130	284273	Hs.465433	NM_17590	ENSG0000	ZADH2	PRG-3	zinc bindin	protein-co
lib-C666-l	chr11	32851398	32851398	+	5.48804	NA	promoter-TSS (NM_024081)	promoter-TSS (NM_024081)	-83	NM_02408	79056	Hs.471695	NM_02408	ENSG0000	PRRG4	PRGP4|TM	proline rich	protein-co
lib-C666-l	chr14	94154746	94154746	+	11.80692	NA	intron (NM_001346218, intron 45 of 5	intron (NM_001346218, intron 45 o	100020	NM_17801	145270	Hs.432401	NM_17800	ENSG0000	PRIMA1	PRIMA	proline rich	protein-co
lib-C666-l	chr7	106684900	106684900	+	13.89614	NA	promoter-TSS (NM_002736)	promoter-TSS (NM_002736)	-278	NM_00273	5577	Hs.433068	NM_00273	ENSG0000	PRKAR2B	PRKAR2|RI	protein kin	protein-co
lib-C666-l	chr20	46012884	46012884	+	35.29357	NA	Intergenic	Intergenic	-27251	NM_00128	23613	Hs.446240	NM_01240	ENSG0000	ZMYND8	PRKCBP1|F	zinc finger	protein-co
lib-C666-l	chr4	82074896	82074896	+	6.82479	NA	intron (NM_006259, intron 6 of 18)	intron (NM_006259, intron 6 of 18)	415	NM_00128	5593	Hs.232044	NM_00625	ENSG0000	PRKG2	PRKGR2|cG	protein kin	protein-co
lib-C666-l	chr4	148605379	148605379	+	83.07521	NA	promoter-TSS (NM_001304458)	promoter-TSS (NM_001304458)	2	NM_13836	90826	Hs.591692	NM_13836	ENSG0000	PRMT9	PRMT10	protein arg	protein-co
lib-C666-l	chr19	10981973	10981973	+	21.28624	NA	promoter-TSS (NM_199141)	promoter-TSS (NM_199141)	-280	NM_19914	10498	Hs.323213	NM_19914	ENSG0000	CARM1	PRMT4	coactivator	protein-co
lib-C666-l	chr16	9185384	9185384	+	18.76627	NA	promoter-TSS (NM_014117)	promoter-TSS (NM_014117)	-153	NM_01411	29035	Hs.221497	NM_01411	ENSG0000	C16orf72	PRO0149	chromosor	protein-co
lib-C666-l	chr16	9218029	9218029	+	9.94079	NA	Intergenic	Intergenic	32492	NM_01411	29035	Hs.221497	NM_01411	ENSG0000	C16orf72	PRO0149	chromosor	protein-co
lib-C666-l	chr16	9184975	9184975	+	5.34142	NA	promoter-TSS (NM_014117)	promoter-TSS (NM_014117)	-562	NM_01411	29035	Hs.221497	NM_01411	ENSG0000	C16orf72	PRO0149	chromosor	protein-co
lib-C666-l	chr5	122181096	122181096	+	2.65152	NA	promoter-TSS (NM_014035)	promoter-TSS (NM_014035)	-64	NM_01403	28966	Hs.483200	NM_01403	ENSG0000	SNX24	PRO1284|S	sorting nex	protein-co
lib-C666-l	chr17	48474889	48474889	+	73.27174	NA	promoter-TSS (NM_018509)	promoter-TSS (NM_018509)	25	NM_01850	55379	Hs.370927	NM_01850	ENSG0000	LRRC59	PRO1855|p	leucine rich	protein-co
lib-C666-l	chr2	24583430	24583430	+	13.33696	NA	promoter-TSS (NM_147152)	promoter-TSS (NM_147152)	-33	NM_14715	50618	Hs.432562	NM_00627	ENSG0000	ITSN2	PRO2015|S	intersectin	protein-co
lib-C666-l	chr1	223432184	223432184	+	8.85547	NA	intron (NM_001037175, intron 5 of 5)	intron (NM_001037175, intron 5 of	105360	NM_01798	55061	Hs.497841	NM_01798	ENSG0000	SUSD4	PRO222	sushi doma	protein-co
lib-C666-l	chr3	128444957	128444957	+	15.3763	NA	promoter-TSS (NM_004637)	promoter-TSS (NM_004637)	-22	NM_00463	7879	Hs.684374	NM_00463	ENSG0000	RAB7A	PRO2706|F	RAB7A, me	protein-co
lib-C666-l	chrX	16737652	16737652	+	95.73749	NA	promoter-TSS (NM_032796)	promoter-TSS (NM_032796)	-55	NM_03279	94056	Hs.744256	NM_03279	ENSG0000	SYAP1	PRO3113	synapse as	protein-co
lib-C666-l	chr9	127574920	127574920	+	11.13288	NA	3' UTR (NM_182487, exon 8 of 8)	3' UTR (NM_182487, exon 8 of 8)	11767	NM_00128	169611	Hs.357004	NM_18248	ENSG0000	OLFML2A	PRO34319	olfactomec	protein-co
lib-C666-l	chr2	228271191	228271191	+	8.8556	NA	Intergenic	Intergenic	-27169	NM_02479	79853	Hs.156652	NM_02479	ENSG0000	TM4SF20	PRO994|SL	transmemb	protein-co
lib-C666-l	chr13	52158063	52158063	+	43.12596	NA	promoter-TSS (NM_052950)	promoter-TSS (NM_052950)	-421	NM_05295	115825	Hs.46506	NM_05295	ENSG0000	WDFY2	PROF|WDF	WD repeat	protein-co
lib-C666-l	chr13	52158537	52158537	+	3.04651	NA	promoter-TSS (NM_052950)	promoter-TSS (NM_052950)	53	NM_05295	115825	Hs.46506	NM_05295	ENSG0000	WDFY2	PROF|WDF	WD repeat	protein-co
lib-C666-l	chr11	36406924	36406924	+	6.64931	NA	intron (NM_024841, intron 2 of 9)	intron (NM_024841, intron 2 of 9)	9389	NM_00116	79899	Hs.19987	NM_02484	ENSG0000	PRR5L	PROTOR2	proline rich	protein-co
lib-C666-l	chr3	129721923	129721923	+	7.49163	NA	Intergenic	CpG	28687	NM_00711	7200	Hs.182231	NM_00711	ENSG0000	TRH	Pro-TRH|T	thyrotropir	protein-co
lib-C666-l	chr10	99496881	99496881	+	42.99614	NA	promoter-TSS (NM_001174122)	promoter-TSS (NM_001174122)	3	NM_00100	118813	Hs.744075	NM_14458	ENSG0000	ZFYVE27	PROTRUDI	zinc finger	protein-co
lib-C666-l	chr10	13628912	13628912	+	2.45439	NA	promoter-TSS (NM_003675)	promoter-TSS (NM_003675)	-27	NM_00367	8559	Hs.660649	NM_00367	ENSG0000	PRPF18	PRP18|hPr	pre-mRNA	protein-co
lib-C666-l	chr22	30752945	30752945	+	51.89298	NA	promoter-TSS (NM_005877)	promoter-TSS (NM_005877)	-9	NM_00587	10291	Hs.406277	NM_00587	ENSG0000	SF3A1	PRP21|PRP	splicing fac	protein-co
lib-C666-l	chr1	52870195	52870195	+	85.88258	NA	promoter-TSS (NM_001190818)	promoter-TSS (NM_001190818)	-24	NM_03286	84950	Hs.5301	NM_03228	ENSG0000	PRPF38A	PRP38A|Pr	pre-mRNA	protein-co
lib-C666-l	chr1	38455923	38455923	+	6.56784	NA	promoter-TSS (NM_006802)	promoter-TSS (NM_006802)	-141	NM_00132	10946	Hs.77897	NM_00680	ENSG0000	SF3A3	PRP9|PRPF	splicing fac	protein-co
lib-C666-l	chr12	49246038	49246038	+	9.06551	NA	promoter-TSS (NM_004818)	promoter-TSS (NM_004818)	-81	NM_00481	9416	Hs.130098	NM_00481	ENSG0000	DDX23	PRPF28|SN	DEAD-box	protein-co
lib-C666-l	chr5	134094334	134094334	+	3.29276	NA	promoter-TSS (NM_001300860)	promoter-TSS (NM_001300860)	-90	NM_01482	9879	Hs.406549	NM_01482	ENSG0000	DDX46	PRPF5|Prp5	DEAD-box	protein-co
lib-C666-l	chr19	47776870	47776870	+	10.06764	NA	promoter-TSS (NR_037675)	promoter-TSS (NR_037675)	-789	NR_037675	255783	Hs.128690	NM_17851	ENSG0000	INAFM1	PRR24	InaF motif	protein-co
lib-C666-l	chr7	155495576	155495576	+	60.44312	NA	intron (NM_053043, intron 6 of 17)	intron (NM_053043, intron 6 of 17)	58373	NM_05304	155435	Hs.591815	NM_05304	ENSG0000	RBM33	PRR8	RNA bindir	protein-co
lib-C666-l	chr12	123874194	123874194	+	4.92623	NA	intron (NR_136910, intron 2 of 8)	CpG-5949	-1628	NM_00132	387893	Hs.443735	NM_02038	ENSG0000	KMT5A	PR-Set7|SE	lysine meth	protein-co
lib-C666-l	chr1	109969138	109969138	+	4.64451	NA	promoter-TSS (NM_001199772)	promoter-TSS (NM_001199772)	-30	NM_00119	5686	Hs.485246	NM_00279	ENSG0000	PSMA5	PSC5|ZETA	proteasom	protein-co
lib-C666-l	chr17	7108593	7108593	+	12.73831	NA	promoter-TSS (NM_001321076)	promoter-TSS (NM_001321076)	-106	NM_00132	1742	Hs.463928	NM_00136	ENSG0000	DLG4	PSD95|SAF	discs large	protein-co
lib-C666-l	chr20	25388287	25388287	+	4.46583	NA	promoter-TSS (NM_021067)	promoter-TSS (NM_021067)	-32	NR_13457	9837	Hs.658464	NM_02106	ENSG0000	GINS1	PSF1	GINS comp	protein-co
lib-C666-l	chr16	58426825	58426825	+	8.0154	NA	intron (NM_001126130, intron 1 of 1)	CpG	527	NM_00112	64785	Hs.47125	NM_02277	ENSG0000	GINS3	PSF3	GINS comp	protein-co
lib-C666-l	chr16	28874845	28874845	+	5.23609	NA	promoter-TSS (NM_001145795)	promoter-TSS (NM_001145795)	-233	NM_00130	25970	Hs.15744	NM_01550	ENSG0000	SH2B1	PSM|SH2B	SH2B adap	protein-co
lib-C666-l	chr16	28875174	28875174	+	3.20885	NA	promoter-TSS (NM_001145795)	promoter-TSS (NM_001145795)	96	NM_00114	25970	Hs.15744	NM_01550	ENSG0000	SH2B1	PSM|SH2B	SH2B adap	protein-co
lib-C666-l	chr10	115614265	115614265	+	2.96041	NA	promoter-TSS (NM_198514)	promoter-TSS (NM_198514)	-102	NM_00127	9937	Hs.1560	NM_01488	ENSG0000	DCLRE1A	PSO2|SNM	DNA cross	protein-co
lib-C666-l	chr20	48552845	48552845	+	6.61453	NA	promoter-TSS (NM_018683)	promoter-TSS (NM_018683)	-69	NM_01868	55905	Hs.144949	NM_01868	ENSG0000	RNF114	PSORS12|Z	ring finger	protein-co
lib-C666-l	chr2	85906679	85906679	+	2.78587	NA	Intergenic	Intergenic	-10815	NM_19884	6439	Hs.512690	NM_00054	ENSG0000	SFTPB	PSP-B|SFTI	surfactant	protein-co
lib-C666-l	chr17	74722770	74722770	+	58.68336	NA	promoter-TSS (NM_001302705)	promoter-TSS (NM_001302705)	111	NM_01516	23210	Hs.514505	NM_01516	ENSG0000	JMJD6	PSR|PTDSR	arginine de	protein-co
lib-C666-l	chr17	74722204	74722204	+	7.37943	NA	promoter-TSS (NM_001302705)	promoter-TSS (NM_001302705)	677	NM_01516	23210	Hs.514505	NM_01516	ENSG0000	JMJD6	PSR|PTDSR	arginine de	protein-co
lib-C666-l	chr11	450254	450254	+	3.44441	NA	promoter-TSS (NM_001329544)	promoter-TSS (NM_001329544)	-26	NR_13804	81490	Hs.12851	NM_03078	ENSG0000	PTDSS2	PSS2	phosphatic	protein-co
lib-C666-l	chr19	2945401	2945401	+	69.90313	NA	promoter-TSS (NM_021217)	promoter-TSS (NM_021217)	-432	NM_02121	58492	Hs.38004	NM_02121	ENSG0000	ZNF77	pT1	zinc finger	protein-co
lib-C666-l	chr9	130186681	130186681	+	36.97678	NA	promoter-TSS (NM_001286698)	promoter-TSS (NM_001286698)	28	NM_00132	7633	Hs.522399	NM_00713	ENSG0000	ZNF79	pT7	zinc finger	protein-co
lib-C666-l	chr1	97144994	97144994	+	7.9573	NA	Intergenic	Intergenic	-42167	NM_00130	58155	Hs.596061	NM_02119	ENSG0000	PTBP2	PTBLP|brPT	polypyrimi	protein-co
lib-C666-l	chr1	45297361	45297361	+	5.25516	NA	intron (NM_003738, intron 5 of 21)	intron (NM_003738, intron 5 of 21)	11255	NM_00116	8643	Hs.591497	NM_00373	ENSG0000	PTCH2	PTC2	patched 2	protein-co
lib-C666-l	chr8	17780149	17780149	+	9.06551	NA	promoter-TSS (NM_001315507)	promoter-TSS (NM_001315507)	-217	NM_00619	5108	Hs.491148	NM_00619	ENSG0000	PCM1	PTC4|RET/I	pericentrio	protein-co
lib-C666-l	chr9	15422744	15422744	+	10.79304	NA	promoter-TSS (NM_001039697)	promoter-TSS (NM_001039697)	-38	NM_00103	6619	Hs.546299	NM_00308	ENSG0000	SNAPC3	PTFbeta|SN	small nucle	protein-co
lib-C666-l	chr2	209223936	209223936	+	3.27468	NA	promoter-TSS (NM_001309516)	promoter-TSS (NM_001309516)	-633	NM_00130	5746	Hs.570296	NM_00504	ENSG0000	PTH2R	PTHR2	parathyroic	protein-co
lib-C666-l	chr20	49057468	49057468	+	6.20473	NA	Intergenic	Plat_L3|LINE|CR1	-69390	NM_00282	5770	Hs.417549	NM_00282	ENSG0000	PTPN1	PTP1B	protein tyr	protein-co
lib-C666-l	chr9	96793078	96793078	+	37.12814	NA	promoter-TSS (NM_177995)	promoter-TSS (NM_177995)	2	NM_17799	138639	Hs.437943	NM_15242	ENSG0000	PTPDC1	PTP9Q22	protein tyr	protein-co
lib-C666-l	chr9	112262285	112262285	+	17.62684	NA	Intergenic	CpG	-1692	NM_00114	5774	Hs.436429	NM_00282	ENSG0000	PTPN3	PTP-H1|PT	protein tyr	protein-co
lib-C666-l	chr9	112260488	112260488	+	2.75718	NA	intron (NM_001145368, intron 1 of 24	intron (NM_001145368, intron 1 of	105	NM_00282	5774	Hs.436429	NM_00282	ENSG0000	PTPN3	PTP-H1|PT	protein tyr	protein-co
lib-C666-l	chr7	77166238	77166238	+	5.96761	NA	promoter-TSS (NM_002835)	promoter-TSS (NM_002835)	-535	NM_00283	5782	Hs.61812	NM_00283	ENSG0000	PTPN12	PTP-PEST|I	protein tyr	protein-co
lib-C666-l	chr18	7830599	7830599	+	14.3056	NA	intron (NM_001105244, intron 2 of 32	intron (NM_001105244, intron 2 of	263285	NM_00284	5797	Hs.49774	NM_00284	ENSG0000	PTPRM	PTPRL1|R-	protein tyr	protein-co
lib-C666-l	chr18	7567341	7567341	+	9.29081	NA	promoter-TSS (NM_002845)	promoter-TSS (NM_002845)	27	NM_00284	5797	Hs.49774	NM_00284	ENSG0000	PTPRM	PTPRL1|R-	protein tyr	protein-co
lib-C666-l	chr11	112097245	112097245	+	20.19666	NA	exon (NM_000317, exon 1 of 6)	exon (NM_000317, exon 1 of 6)	157	NM_00031	5805	Hs.503860	NM_00031	ENSG0000	PTS	PTPS	6-pyruvoy	protein-co
lib-C666-l	chr19	5339787	5339787	+	57.42312	NA	intron (NM_130853, intron 1 of 28)	CpG	1027	NM_00285	5802	Hs.744928	NM_00285	ENSG0000	PTPRS	PTPSIGMA	protein tyr	protein-co
lib-C666-l	chr19	5340863	5340863	+	24.32656	NA	promoter-TSS (NM_002850)	promoter-TSS (NM_002850)	-49	NM_13085	5802	Hs.744928	NM_00285	ENSG0000	PTPRS	PTPSIGMA	protein tyr	protein-co
lib-C666-l	chr12	58119786	58119786	+	4.19675	NA	promoter-TSS (NR_027032)	promoter-TSS (NR_027032)	-237	NR_02703	100130776	Hs.656080	NR_02703	ENSG0000	AGAP2-AS	PUNISHER	AGAP2 ant	ncRNA
lib-C666-l	chr7	44925232	44925232	+	203.5799	NA	promoter-TSS (NM_033224)	promoter-TSS (NM_033224)	-248	NM_03322	5814	Hs.349150	NM_03322	ENSG0000	PURB	PURBETA	purine rich	protein-co
lib-C666-l	chr8	30891446	30891446	+	18.0551	NA	promoter-TSS (NM_001323311)	promoter-TSS (NM_001323311)	-215	NM_00101	29942	Hs.373778	NM_01335	ENSG0000	PURG	PURG-A|PU	purine rich	protein-co
lib-C666-l	chr10	116697943	116697943	+	83.07521	NA	promoter-TSS (NM_139169)	promoter-TSS (NM_139169)	-9	NM_13916	142940	Hs.21187	NM_13916	ENSG0000	TRUB1	PUS4	TruB pseuc	protein-co
lib-C666-l	chr10	134221595	134221595	+	53.7204	NA	intron (NM_138499, intron 2 of 2)	intron (NM_138499, intron 2 of 2)	10893	NM_13849	170394	Hs.527751	NM_13849	ENSG0000	PWWP2B	PWWP2|bA	PWWP dor	protein-co
lib-C666-l	chr10	134201449	134201449	+	18.43022	NA	Intergenic	Intergenic	-9253	NM_00109	170394	Hs.527751	NM_13849	ENSG0000	PWWP2B	PWWP2|bA	PWWP dor	protein-co
lib-C666-l	chr1	180137309	180137309	+	11.66012	NA	intron (NM_001004128, intron 2 of 12	intron (NM_001004128, intron 2 of	13341	NM_00100	5768	Hs.744925	NM_00282	ENSG0000	QSOX1	Q6|QSCN6	quiescin su	protein-co
lib-C666-l	chr3	113775547	113775547	+	202.98869	NA	promoter-TSS (NM_001256835)	promoter-TSS (NM_001256835)	-35	NM_02463	79691	Hs.477162	NM_02463	ENSG0000	QTRT2	QTRTD1	queuine tR	protein-co
lib-C666-l	chr11	4116006	4116006	+	327.36392	NA	promoter-TSS (NM_001033)	promoter-TSS (NM_001033)	91	NM_00131	6240	Hs.445705	NM_00103	ENSG0000	RRM1	R1|RIR1|RR	ribonucleo	protein-co
lib-C666-l	chr19	19030404	19030404	+	221.5683	NA	promoter-TSS (NM_001330469)	promoter-TSS (NM_001330469)	-80	NM_01907	54555	Hs.143187	NM_01907	ENSG0000	DDX49	R27090_2	DEAD-box	protein-co
lib-C666-l	chr17	74561480	74561480	+	42.16684	NA	TTS (NR_038111)	TTS (NR_038111)	3765	NR_00439	677848		NR_00439	ENSG0000	SNORD1A	R38A|snR3	small nucle	snoRNA
lib-C666-l	chr14	68286765	68286765	+	17.13943	NA	intron (NM_001321809, intron 1 of 11	intron (NM_001321809, intron 1 of	225	NM_00132	5890	Hs.172587	NM_00287	ENSG0000	RAD51B	R51H2|RAD	RAD51 par	protein-co
lib-C666-l	chr2	65357538	65357538	+	7.49163	NA	promoter-TSS (NM_015543)	promoter-TSS (NM_015543)	-103	NM_00416	5861	Hs.310645	NM_00416	ENSG0000	RAB1A	RAB1|YPT1	RAB1A, me	protein-co
lib-C666-l	chr10	119806367	119806367	+	4.61604	NA	promoter-TSS (NR_026941)	promoter-TSS (NR_026941)	28	NM_00133	22841	Hs.173656	NM_01490	ENSG0000	RAB11FIP2	Rab11-FIP2	RAB11 fam	protein-co
lib-C666-l	chr1	220445852	220445852	+	12.83248	NA	promoter-TSS (NM_012414)	promoter-TSS (NM_012414)	-9	NM_01241	25782	Hs.654849	NM_01241	ENSG0000	RAB3GAP2	RAB3-GAP	RAB3 GTPa	protein-co
lib-C666-l	chr9	85677830	85677830	+	39.29799	NA	5' UTR (NM_152573, exon 1 of 17)	5' UTR (NM_152573, exon 1 of 17)	213	NM_15257	158158	Hs.129136	NM_15257	ENSG0000	RASEF	RAB45	RAS and EI	protein-co
lib-C666-l	chr9	85677049	85677049	+	8.01457	NA	intron (NM_152573, intron 1 of 16)	CpG	994	NM_15257	158158	Hs.129136	NM_15257	ENSG0000	RASEF	RAB45	RAS and EI	protein-co
lib-C666-l	chr3	19988578	19988578	+	33.11719	NA	promoter-TSS (NM_001330688)	promoter-TSS (NM_001330688)	6	NM_00416	5868	Hs.475663	NM_00416	ENSG0000	RAB5A	RAB5	RAB5A, me	protein-co
lib-C666-l	chr3	19989010	19989010	+	16.96353	NA	promoter-TSS (NM_001330688)	promoter-TSS (NM_001330688)	438	NM_00416	5868	Hs.475663	NM_00416	ENSG0000	RAB5A	RAB5	RAB5A, me	protein-co
lib-C666-l	chr11	73472352	73472352	+	27.96363	NA	promoter-TSS (NM_002869)	promoter-TSS (NM_002869)	-151	NM_00124	5870	Hs.503222	NM_00286	ENSG0000	RAB6A	RAB6	RAB6A, me	protein-co
lib-C666-l	chr12	72233567	72233567	+	25.39084	NA	promoter-TSS (NR_027449)	promoter-TSS (NR_027449)	80	NM_00114	64786	Hs.284630	NM_02277	ENSG0000	TBC1D15	RAB7-GAP	TBC1 dom	protein-co
lib-C666-l	chr5	104553897	104553897	+	9.29081	NA	Intergenic	Eulor2A|DNA?|DNA?	118722	NR_00003	9366	Hs.158296	NR_00003	ENSG0000	RAB9BP1	RAB9P1	RAB9B, me	pseudo
lib-C666-l	chr3	15140655	15140655	+	15.08005	NA	promoter-TSS (NM_001302378)	promoter-TSS (NM_001302378)	9	NM_00130	64145	Hs.475565	NM_02234	ENSG0000	RBSN	Rabenosyn	rabenosyn	protein-co
lib-C666-l	chr7	66205589	66205589	+	57.08342	NA	promoter-TSS (NM_001287062)	promoter-TSS (NM_001287062)	-54	NM_01450	27342	Hs.530053	NM_01450	ENSG0000	RABGEF1	RABEX5|RA	RAB guani	protein-co
lib-C666-l	chr7	66147064	66147064	+	38.60092	NA	promoter-TSS (NR_104676)	promoter-TSS (NR_104676)	-14	NR_10467	27342	Hs.530053	NM_01450	ENSG0000	RABGEF1	RABEX5|RA	RAB guani	protein-co
lib-C666-l	chr1	228674834	228674834	+	79.67884	NA	promoter-TSS (NM_001010858)	promoter-TSS (NM_001010858)	-234	NM_00101	149603	Hs.356377	NM_00101	ENSG0000	RNF187	RACO-1|R	ring finger	protein-co
lib-C666-l	chr1	228674445	228674445	+	4.52748	NA	promoter-TSS (NM_001010858)	promoter-TSS (NM_001010858)	-623	NM_00101	149603	Hs.356377	NM_00101	ENSG0000	RNF187	RACO-1|R	ring finger	protein-co
lib-C666-l	chr16	66961093	66961093	+	86.72066	NA	Intergenic	Intergenic	-1654	NM_00416	6236	Hs.1027	NM_00416	ENSG0000	RRAD	RAD|RAD1	RRAD, Ras	protein-co
lib-C666-l	chr6	43543848	43543848	+	2.62544	NA	promoter-TSS (NM_020750)	promoter-TSS (NM_020750)	-30	NM_00129	5429	Hs.655467	NM_00650	ENSG0000	POLH	RAD30|RAI	DNA polyn	protein-co
lib-C666-l	chr3	14220105	14220105	+	20.90919	NA	promoter-TSS (NM_004628)	promoter-TSS (NM_004628)	67	NM_00462	7508	Hs.475538	NM_00462	ENSG0000	XPC	RAD4|XP3|	XPC compl	protein-co
lib-C666-l	chr11	67159272	67159272	+	8.89416	NA	promoter-TSS (NM_004584)	promoter-TSS (NM_004584)	-151	NM_00458	5883	Hs.655354	NM_00458	ENSG0000	RAD9A	RAD9	RAD9 chec	protein-co
lib-C666-l	chr6	150185036	150185036	+	8.28216	NA	exon (NM_032832, exon 1 of 7)	exon (NM_032832, exon 1 of 7)	402	NR_04512	100652739	Hs.642734	NR_04512	ENSG0000	RAET1E-AS	RAET1G-A	RAET1E an	ncRNA
lib-C666-l	chr11	506847	506847	+	139.68425	NA	promoter-TSS (NM_002939)	promoter-TSS (NM_002939)	-26	NM_20338	6050	Hs.530687	NM_00293	ENSG0000	RNH1	RAI|RNH	ribonuclea	protein-co
lib-C666-l	chr8	21946640	21946640	+	15.07075	NA	promoter-TSS (NM_022749)	promoter-TSS (NM_022749)	-74	NM_02274	64760	Hs.491223	NM_02274	ENSG0000	FAM160B2	RAI16	family with	protein-co
lib-C666-l	chr8	21947129	21947129	+	2.42134	NA	intron (NM_022749, intron 1 of 16)	CpG	415	NM_02274	64760	Hs.491223	NM_02274	ENSG0000	FAM160B2	RAI16	family with	protein-co
lib-C666-l	chr16	19864635	19864635	+	5.74587	NA	intron (NR_130968, intron 8 of 8)	intron (NR_130968, intron 8 of 8)	31598	NM_01623	51704	Hs.148685	NM_01623	ENSG0000	GPRC5B	RAIG-2|RA	G protein-	protein-co
lib-C666-l	chr7	39662946	39662946	+	2.59723	NA	promoter-TSS (NM_005402)	promoter-TSS (NM_005402)	-206	NM_00540	5898	Hs.6906	NM_00540	ENSG0000	RALA	RAL	RAS like pr	protein-co
lib-C666-l	chr9	129677104	129677104	+	8.0154	NA	promoter-TSS (NM_001322320)	promoter-TSS (NM_001322320)	51	NM_00119	9649	Hs.432842	NM_01463	ENSG0000	RALGPS1	RALGEF2|R	Ral GEF wit	protein-co
lib-C666-l	chr9	129748257	129748257	+	7.43576	NA	intron (NM_001190728, intron 3 of 16	intron (NM_001190728, intron 3 of	23753	NM_00119	9649	Hs.432842	NM_01463	ENSG0000	RALGPS1	RALGEF2|R	Ral GEF wit	protein-co
lib-C666-l	chr15	49218188	49218188	+	2.77473	NA	intron (NM_203349, intron 1 of 11)	L2a|LINE|L2	37453	NM_20334	399694	Hs.642615	NM_20334	ENSG0000	SHC4	RaLP|SHCD	SHC adapt	protein-co
lib-C666-l	chr3	150264426	150264426	+	133.10905	NA	promoter-TSS (NM_014445)	promoter-TSS (NM_014445)	2	NM_01444	27230	Hs.518326	NM_01444	ENSG0000	SERP1	RAMP4	stress asso	protein-co
lib-C666-l	chr5	61708587	61708587	+	133.19038	NA	promoter-TSS (NM_016338)	promoter-TSS (NM_016338)	14	NM_01633	51194	Hs.482269	NM_01633	ENSG0000	IPO11	RanBP11	importin 1	protein-co
lib-C666-l	chr1	22110040	22110040	+	27.7824	NA	promoter-TSS (NM_001032730)	promoter-TSS (NM_001032730)	-352	NM_00103	84196	Hs.467524	NM_03223	ENSG0000	USP48	RAP1GA1|U	ubiquitin s	protein-co
lib-C666-l	chr1	22109714	22109714	+	14.26909	NA	promoter-TSS (NM_001032730)	promoter-TSS (NM_001032730)	-26	NM_00103	84196	Hs.467524	NM_03223	ENSG0000	USP48	RAP1GA1|U	ubiquitin s	protein-co
lib-C666-l	chr1	22108896	22108896	+	7.43576	NA	intron (NM_001330394, intron 1 of 25	intron (NM_001330394, intron 1 of	792	NM_03223	84196	Hs.467524	NM_03223	ENSG0000	USP48	RAP1GA1|U	ubiquitin s	protein-co
lib-C666-l	chr17	42385930	42385930	+	32.78343	NA	promoter-TSS (NR_110802)	promoter-TSS (NR_110802)	3	NM_00669	10900	Hs.500197	NM_00669	ENSG0000	RUNDC3A	RAP2IP|RPI	RUN doma	protein-co
lib-C666-l	chr5	176433492	176433492	+	3.27468	NA	promoter-TSS (NM_016290)	promoter-TSS (NM_016290)	-49	NM_01629	51720	Hs.232721	NM_01629	ENSG0000	UIMC1	RAP80|X2H	ubiquitin ir	protein-co
lib-C666-l	chr17	80656650	80656650	+	11.6497	NA	promoter-TSS (NM_006822)	promoter-TSS (NM_006822)	-52	NM_00682	10966	Hs.484068	NM_00682	ENSG0000	RAB40B	RAR|SEC4L	RAB40B, m	protein-co
lib-C666-l	chr17	79633961	79633961	+	23.47534	NA	promoter-TSS (NM_001304999)	promoter-TSS (NM_001304999)	200	NM_19928	339230	Hs.744125	NM_19928	ENSG0000	CCDC137	RaRF	coiled-coil	protein-co
lib-C666-l	chr16	658293	658293	+	27.5466	NA	intron (NM_021168, intron 1 of 5)	THE1C|LTR|ERVL-MaLR	18120	NM_02116	57799	Hs.459630	NM_02116	ENSG0000	RAB40C	RARL|RASL	RAB40C, m	protein-co
lib-C666-l	chr16	639765	639765	+	8.521	NA	promoter-TSS (NM_001172664)	promoter-TSS (NM_001172664)	96	NM_00117	57799	Hs.459630	NM_02116	ENSG0000	RAB40C	RARL|RASL	RAB40C, m	protein-co
lib-C666-l	chr16	639445	639445	+	4.95623	NA	promoter-TSS (NM_001172663)	promoter-TSS (NM_001172663)	88	NM_00117	57799	Hs.459630	NM_02116	ENSG0000	RAB40C	RARL|RASL	RAB40C, m	protein-co
lib-C666-l	chr12	65004724	65004724	+	6.15313	NA	intron (NR_040718, intron 1 of 3)	CpG	431	NR_04071	283349	Hs.643605	NM_17816	ENSG0000	RASSF3	RASSF5	Ras associa	protein-co
lib-C666-l	chr12	65004310	65004310	+	5.65398	NA	promoter-TSS (NM_178169)	promoter-TSS (NM_178169)	17	NR_04071	283349	Hs.643605	NM_17816	ENSG0000	RASSF3	RASSF5	Ras associa	protein-co
lib-C666-l	chr2	264187	264187	+	12.62235	NA	promoter-TSS (NR_024080)	promoter-TSS (NR_024080)	-90	NM_00115	26751	Hs.515951	NM_01567	ENSG0000	SH3YL1	RAY	SH3 and S	protein-co
lib-C666-l	chr2	264789	264789	+	4.19675	NA	promoter-TSS (NR_024080)	promoter-TSS (NR_024080)	-46	NR_10422	26751	Hs.515951	NM_01567	ENSG0000	SH3YL1	RAY	SH3 and S	protein-co
lib-C666-l	chr14	58766327	58766327	+	4.10929	NA	intron (NM_023001, intron 1 of 22)	CpG	1105	NM_02300	5926	Hs.161000	NM_00289	ENSG0000	ARID4A	RBBP-1|RB	AT-rich int	protein-co
lib-C666-l	chr12	498756	498756	+	90.61259	NA	promoter-TSS (NM_001042603)	promoter-TSS (NM_001042603)	-135	NM_00104	5927	Hs.76272	NM_00505	ENSG0000	KDM5A	RBBP-2|RB	lysine dem	protein-co
lib-C666-l	chr2	25194967	25194967	+	63.68534	NA	promoter-TSS (NM_001198559)	promoter-TSS (NM_001198559)	-4	NM_00119	51277	Hs.434993	NM_01654	ENSG0000	DNAJC27	RBJ|RabJS	DnaJ heat	protein-co
lib-C666-l	chr11	66445429	66445429	+	11.53603	NA	promoter-TSS (NM_031492)	promoter-TSS (NM_031492)	-37	NM_03149	83759	Hs.618713	NM_03149	ENSG0000	RBM4B	RBM30|RBI	RNA bindir	protein-co
lib-C666-l	chr8	95653173	95653173	+	4.52748	NA	promoter-TSS (NM_001122827)	promoter-TSS (NM_001122827)	-191	NM_00112	54845	Hs.487471	NM_01769	ENSG0000	ESRP1	RBM35A|R	epithelial s	protein-co
lib-C666-l	chr16	68270100	68270100	+	9.75997	NA	promoter-TSS (NM_024939)	promoter-TSS (NM_024939)	36	NM_02493	80004	Hs.436585	NM_02493	ENSG0000	ESRP2	RBM35B	epithelial s	protein-co
lib-C666-l	chr20	43930817	43930817	+	13.59977	NA	intron (NM_003833, intron 4 of 10)	LTR85a|LTR|Gypsy?	-4666	NM_00128	11317	Hs.248217	NM_01427	ENSG0000	RBPJL	RBPL|RBPS	recombina	protein-co
lib-C666-l	chr1	205091443	205091443	+	3.96379	NA	promoter-TSS (NM_001193273)	promoter-TSS (NM_001193273)	-293	NM_00119	5929	Hs.519230	NM_00505	ENSG0000	RBBP5	RBQ3|SWD	RB binding	protein-co
lib-C666-l	chr19	40950595	40950595	+	18.0551	NA	promoter-TSS (NM_203344)	promoter-TSS (NM_203344)	-313	NM_20334	29946	Hs.515412	NM_01336	ENSG0000	SERTAD3	RBT1	SERTA don	protein-co
lib-C666-l	chr19	40950279	40950279	+	15.31489	NA	promoter-TSS (NM_203344)	promoter-TSS (NM_203344)	3	NM_20334	29946	Hs.515412	NM_01336	ENSG0000	SERTAD3	RBT1	SERTA don	protein-co
lib-C666-l	chr19	40949138	40949138	+	8.89416	NA	promoter-TSS (NM_013368)	promoter-TSS (NM_013368)	-633	NM_01336	29946	Hs.515412	NM_01336	ENSG0000	SERTAD3	RBT1	SERTA don	protein-co
lib-C666-l	chr11	8364881	8364881	+	5.23679	NA	Intergenic	Intergenic	-74699	NM_00127	4004	Hs.654426	NM_00231	ENSG0000	LMO1	RBTN1|RH(	LIM domai	protein-co
lib-C666-l	chr19	44079813	44079813	+	19.88269	NA	promoter-TSS (NM_006297)	promoter-TSS (NM_006297)	-83	NM_00629	7515	Hs.98493	NM_00629	ENSG0000	XRCC1	RCC	X-ray repa	protein-co
lib-C666-l	chr3	122513728	122513728	+	14.45701	NA	promoter-TSS (NM_032839)	promoter-TSS (NM_032839)	-173	NM_03283	84925	Hs.477346	NM_03283	ENSG0000	DIRC2	RCC4	disrupted i	protein-co
lib-C666-l	chr1	156737113	156737113	+	11.66012	NA	promoter-TSS (NM_005973)	promoter-TSS (NM_005973)	-161	NM_00597	5546	Hs.516948	NM_00597	ENSG0000	PRCC	RCCP1|TPR	papillary re	protein-co
lib-C666-l	chr1	156737517	156737517	+	10.58189	NA	5' UTR (NM_005973, exon 1 of 7)	5' UTR (NM_005973, exon 1 of 7)	243	NM_00597	5546	Hs.516948	NM_00597	ENSG0000	PRCC	RCCP1|TPR	papillary re	protein-co
lib-C666-l	chrX	48901183	48901183	+	9.02258	NA	promoter-TSS (NM_001282142)	promoter-TSS (NM_001282142)	-140	NM_00128	7030	Hs.730740	NM_00652	ENSG0000	TFE3	RCCP2|RC(	transcriptic	protein-co
lib-C666-l	chr4	159593365	159593365	+	9.94769	NA	promoter-TSS (NM_001008393)	promoter-TSS (NM_001008393)	42	NR_07723	201725	Hs.380920	NM_00100	ENSG0000	C4orf46	RCDG1	chromosor	protein-co
lib-C666-l	chr6	90529495	90529495	+	106.05028	NA	promoter-TSS (NM_014611)	promoter-TSS (NM_014611)	18	NM_01461	23195	Hs.529948	NM_01461	ENSG0000	MDN1	Rea1	midasin AA	protein-co
lib-C666-l	chr15	78591975	78591975	+	94.67412	NA	promoter-TSS (NM_001303247)	promoter-TSS (NM_001303247)	93	NM_00130	80349	Hs.513055	NM_02523	ENSG0000	WDR61	REC14|SKI8	WD repeat	protein-co
lib-C666-l	chr17	73663327	73663327	+	171.93208	NA	promoter-TSS (NM_004259)	promoter-TSS (NM_004259)	-58	NM_00100	9400	Hs.632229	NM_00425	ENSG0000	RECQL5	RECQ5	RecQ like h	protein-co
lib-C666-l	chr1	28241342	28241342	+	101.65468	NA	promoter-TSS (NM_001297558)	promoter-TSS (NM_001297558)	-34	NM_00129	6118	Hs.79411	NM_00294	ENSG0000	RPA2	REPA2|RP-	replication	protein-co
lib-C666-l	chr2	154400554	154400554	+	5.92363	NA	Intergenic	Intergenic	-65232	NM_01984	56475	Hs.100890	NM_01984	ENSG0000	RPRM	REPRIMO	reprimo, TI	protein-co
lib-C666-l	chr19	41283529	41283529	+	54.01688	NA	promoter-TSS (NM_016154)	promoter-TSS (NM_016154)	-595	NR_03779	100529264	Hs.631539	NR_037791		RAB4B-EG	RERT-lncR	RAB4B-EG	ncRNA
lib-C666-l	chr4	2538094	2538094	+	34.12565	NA	Intergenic	CpG	66914	NM_00118	6047	Hs.66394	NM_00293	ENSG0000	RNF4	RES4-26|SI	ring finger	protein-co
lib-C666-l	chr4	2470730	2470730	+	7.15513	NA	promoter-TSS (NM_001185010)	promoter-TSS (NM_001185010)	-65	NM_00293	6047	Hs.66394	NM_00293	ENSG0000	RNF4	RES4-26|SI	ring finger	protein-co

lib-C666-l	chr4	2538980	2538980	+	3.58499	NA	Intergenic	CpG	67800	NM_00118	6047	Hs.66394	NM_00293	ENSG0000	RNF4	RES4-26|SI	ring finger	protein-co
lib-C666-l	chr12	118432657	118432657	+	3.57421	NA	Intergenic	L2b|LINE|L2	-21809	NM_00113	5985	Hs.731908	NM_00737	ENSG0000	RFC5	RFC36	replication	protein-co
lib-C666-l	chr13	34392207	34392207	+	38.60092	NA	promoter-TSS (NM_002915)	promoter-TSS (NM_002915)	1	NM_00291	5983	Hs.115474	NM_00291	ENSG0000	RFC3	RFC38	replication	protein-co
lib-C666-l	chr7	73668801	73668801	+	32.07197	NA	promoter-TSS (NM_001278791)	promoter-TSS (NM_001278791)	-13	NM_18147	5982	Hs.647062	NM_00291	ENSG0000	RFC2	RFC40	replication	protein-co
lib-C666-l	chr16	2205644	2205644	+	38.36071	NA	promoter-TSS (NR_132114)	promoter-TSS (NR_132114)	-155	NM_03227	84231	Hs.713632	NM_03227	ENSG0000	TRAF7	RFWD1|RN	TNF recept	protein-co
lib-C666-l	chr15	56535770	56535770	+	17.32164	NA	promoter-TSS (NM_022841)	promoter-TSS (NM_022841)	-287	NM_02284	64864	Hs.745089	NM_02284	ENSG0000	RFX7	RFXDC2	regulatory	protein-co
lib-C666-l	chr7	109923395	109923395	+	7.51944	NA	Intergenic	Intergenic	-323125	NR_00302	442720	Hs.530096	NR_00302	ENSG0000	EIF3IP1	RG208K23	eukaryotic	pseudo
lib-C666-l	chr9	136024979	136024979	+	16.46929	NA	promoter-TSS (NM_001042368)	promoter-TSS (NM_001042368)	-372	NM_00104	5900	Hs.106185	NM_00626	ENSG0000	RALGDS	RGDS|RGF|	ral guanine	protein-co
lib-C666-l	chr9	136019922	136019922	+	2.9164	NA	intron (NM_001042368, intron 1 of 1	CpG	4685	NM_00104	5900	Hs.106185	NM_00626	ENSG0000	RALGDS	RGDS|RGF|	ral guanine	protein-co
lib-C666-l	chr1	183605271	183605271	+	6.15313	NA	promoter-TSS (NM_015149)	promoter-TSS (NM_015149)	89	NM_00129	23179	Hs.497148	NM_01514	ENSG0000	RGL1	RGL	ral guanine	protein-co
lib-C666-l	chr5	72921775	72921775	+	3.94004	NA	promoter-TSS (NM_001080479)	promoter-TSS (NM_001080479)	-208	NM_00117	64283	Hs.482521	NM_00108	ENSG0000	ARHGEF28	RGNEF|RIP	Rho guanir	protein-co
lib-C666-l	chr2	107153729	107153729	+	8.28613	NA	Intergenic	Intergenic	-68928	NM_00114	653489	Hs.726367	NM_00114	ENSG0000	RGPD3	RGP3	RANBP2-li	protein-co
lib-C666-l	chr2	107141084	107141084	+	2.62544	NA	Intergenic	Intergenic	-56283	NM_00114	653489	Hs.726367	NM_00114	ENSG0000	RGPD3	RGP3	RANBP2-li	protein-co
lib-C666-l	chr7	17980208	17980208	+	10.03103	NA	promoter-TSS (NM_015132)	promoter-TSS (NM_015132)	-77	NM_01513	23161	Hs.487648	NM_01513	ENSG0000	SNX13	RGS-PX1	sorting nex	protein-co
lib-C666-l	chr6	86303628	86303628	+	14.59636	NA	5' UTR (NM_153816, exon 1 of 29)	5' UTR (NM_153816, exon 1 of 29)	246	NM_15381	57231	Hs.485871	NM_02046	ENSG0000	SNX14	RGS-PX2|S	sorting nex	protein-co
lib-C666-l	chr17	74494445	74494445	+	5.2142	NA	intron (NM_024599, intron 1 of 18)	intron (NM_024599, intron 1 of 18)	3064	NM_02459	79651	Hs.464157	NM_02459	ENSG0000	RHBDF2	RHBDL5|RI	rhomboid	protein-co
lib-C666-l	chr7	151216966	151216966	+	92.39822	NA	promoter-TSS (NM_005614)	promoter-TSS (NM_005614)	44	NM_00561	6009	Hs.283521	NM_00561	ENSG0000	RHEB	RHEB2	Ras homol	protein-co
lib-C666-l	chr12	49463796	49463796	+	15.60931	NA	promoter-TSS (NM_001303126)	promoter-TSS (NM_001303126)	12	NM_00130	121268	Hs.159013	NM_14459	ENSG0000	RHEBL1	RHEBL1c	Ras homol	protein-co
lib-C666-l	chr10	49864423	49864423	+	53.51636	NA	promoter-TSS (NM_001347736)	promoter-TSS (NM_001347736)	-113	NR_04567	58504	Hs.655672	NM_02122	ENSG0000	ARHGAP22	RhoGAP2|F	Rho GTPas	protein-co
lib-C666-l	chr20	35807491	35807491	+	8.71138	NA	promoter-TSS (NM_002951)	promoter-TSS (NM_002951)	42	NM_00132	6185	Hs.370895	NM_00295	ENSG0000	RPN2	RIBIIR|RPN	ribophorin	protein-co
lib-C666-l	chr11	207643	207643	+	8.48019	NA	promoter-TSS (NM_016526)	promoter-TSS (NM_016526)	132	NM_00128	60626	Hs.592292	NM_02193	ENSG0000	RIC8A	RIC8	RIC8 guani	protein-co
lib-C666-l	chr12	107168295	107168295	+	38.71165	NA	promoter-TSS (NM_001330145)	promoter-TSS (NM_001330145)	-39	NM_00133	55188	Hs.131306	NM_01815	ENSG0000	RIC8B	RIC8|hSyn	RIC8 guani	protein-co
lib-C666-l	chr19	1444099	1444099	+	9.02258	NA	Intergenic	Intergenic	5675	NM_00130	6209	Hs.406683	NM_00101	ENSG0000	RPS15	RIG|S15	ribosomal	protein-co
lib-C666-l	chr19	1438375	1438375	+	7.08136	NA	promoter-TSS (NM_001018)	promoter-TSS (NM_001018)	12	NM_00101	6209	Hs.406683	NM_00101	ENSG0000	RPS15	RIG|S15	ribosomal	protein-co
lib-C666-l	chr8	144105564	144105564	+	19.50061	NA	Intergenic	Intergenic	5662	NM_00234	4061	Hs.521903	NM_00234	ENSG0000	LY6E	RIG-E|RIGE	lymphocyt	protein-co
lib-C666-l	chr8	144100085	144100085	+	4.73866	NA	promoter-TSS (NR_026913)	promoter-TSS (NR_026913)	183	NM_00234	4061	Hs.521903	NM_00234	ENSG0000	LY6E	RIG-E|RIGE	lymphocyt	protein-co
lib-C666-l	chr5	131603928	131603928	+	6.29226	NA	intron (NM_003687, intron 3 of 6)	intron (NM_003687, intron 3 of 6)	10577	NM_00368	8572	Hs.424312	NM_00368	ENSG0000	PDLIM4	RIL	PDZ and LI	protein-co
lib-C666-l	chr5	131604500	131604500	+	5.13457	NA	intron (NM_003687, intron 3 of 6)	intron (NM_003687, intron 3 of 6)	11149	NM_00368	8572	Hs.424312	NM_00368	ENSG0000	PDLIM4	RIL	PDZ and LI	protein-co
lib-C666-l	chr6	33176263	33176263	+	32.0283	NA	promoter-TSS (NM_002931).5	promoter-TSS (NM_002931).5	-23	NM_00293	6015	Hs.631989	NM_00293	ENSG0000	RING1	RING1A|RI	ring finger	protein-co
lib-C666-l	chr5	96528966	96528966	+	43.03704	NA	Intergenic	L1PB1|LINE|L1	-9961	NM_01834	55781	Hs.27021	NM_01834	ENSG0000	RIOK2	RIO2	RIO kinase	protein-co
lib-C666-l	chr6	3068900	3068900	+	4.19683	NA	intron (NM_003804, intron 1 of 10)	CpG	4778	NM_00380	8737	Hs.519842	NM_00380	ENSG0000	RIPK1	RIP|RIP-1|F	receptor in	protein-co
lib-C666-l	chr3	51534047	51534047	+	116.32015	NA	promoter-TSS (NM_001171904)	promoter-TSS (NM_001171904)	-29	NM_01470	9730	Hs.716623	NM_01470	ENSG0000	DCAF1	RIP|VPRBP	DDB1 and	protein-co
lib-C666-l	chr19	29704147	29704147	+	33.78053	NA	promoter-TSS (NM_006003)	promoter-TSS (NM_006003)	-11	NM_00600	7386	Hs.743307	NM_00600	ENSG0000	UQCRFS1	RIP1|RIS1|F	ubiquinol-	protein-co
lib-C666-l	chr18	9475374	9475374	+	14.26909	NA	promoter-TSS (NM_006788)	promoter-TSS (NM_006788)	-156	NM_00678	10928	Hs.528993	NM_00678	ENSG0000	RALBP1	RIP1|RLIP1	ralA bindin	protein-co
lib-C666-l	chr8	144513813	144513813	+	16.19708	NA	Intergenic	CpG	-1211	NM_20158	389692	Hs.521914	NM_20158	ENSG0000	MAFA	RIPE3b1|hN	MAF bZIP t	protein-co
lib-C666-l	chr19	54041162	54041162	+	6.98764	NA	promoter-TSS (NM_001079906)	promoter-TSS (NM_001079906)	-171	NM_00131	55422	Hs.185674	NM_01855	ENSG0000	ZNF331	RITA|ZNF3	zinc finger	protein-co
lib-C666-l	chr19	54041430	54041430	+	6.13111	NA	promoter-TSS (NM_001079906)	promoter-TSS (NM_001079906)	97	NM_00107	55422	Hs.185674	NM_01855	ENSG0000	ZNF331	RITA|ZNF3	zinc finger	protein-co
lib-C666-l	chr19	54024152	54024152	+	5.5166	NA	promoter-TSS (NM_018555)	promoter-TSS (NM_018555)	-25	NM_00131	55422	Hs.185674	NM_01855	ENSG0000	ZNF331	RITA|ZNF3	zinc finger	protein-co
lib-C666-l	chr4	79860587	79860587	+	81.31076	NA	promoter-TSS (NM_001040202)	promoter-TSS (NM_001040202)	-5	NM_00104	152559	Hs.657312	NM_17745	ENSG0000	PAQR3	RKTG	progestin a	protein-co
lib-C666-l	chr5	180688163	180688163	+	55.66538	NA	promoter-TSS (NR_102759)	promoter-TSS (NR_102759)	-44	NM_00134	84851	Hs.458412	NM_03276	ENSG0000	TRIM52	RNF102	tripartite m	protein-co
lib-C666-l	chr16	4674693	4674693	+	38.20619	NA	promoter-TSS (NM_015246)	promoter-TSS (NM_015246)	-132	NM_00114	23295	Hs.526494	NM_01524	ENSG0000	MGRN1	RNF156	mahogunir	protein-co
lib-C666-l	chr10	92922403	92922403	+	9.2055	NA	promoter-TSS (NR_046435)	promoter-TSS (NR_046435)	-366	NR_04643	84333	Hs.500512	NM_03237	ENSG0000	PCGF5	RNF159	polycomb	protein-co
lib-C666-l	chr8	101322516	101322516	+	7.26874	NA	promoter-TSS (NM_183419)	promoter-TSS (NM_183419)	-122	NM_18341	25897	Hs.292882	NM_01543	ENSG0000	RNF19A	RNF19	ring finger	protein-co
lib-C666-l	chr8	12611691	12611691	+	54.10892	NA	intron (NR_138256, intron 1 of 10)	CpG	1260	NR_13825	91694	Hs.180178	NM_15227	ENSG0000	LONRF1	RNF191	LON pepti	protein-co
lib-C666-l	chr8	12613214	12613214	+	5.99089	NA	promoter-TSS (NM_001329976)	promoter-TSS (NM_001329976)	-263	NR_13825	91694	Hs.180178	NM_15227	ENSG0000	LONRF1	RNF191	LON pepti	protein-co
lib-C666-l	chr8	12612889	12612889	+	3.20885	NA	promoter-TSS (NM_001329976)	promoter-TSS (NM_001329976)	62	NR_13825	91694	Hs.180178	NM_15227	ENSG0000	LONRF1	RNF191	LON pepti	protein-co
lib-C666-l	chr16	74700885	74700885	+	14.3056	NA	promoter-TSS (NM_018124)	promoter-TSS (NM_018124)	-106	NM_01812	55159	Hs.567525	NM_01812	ENSG0000	RFWD3	RNF201	ring finger	protein-co
lib-C666-l	chr3	49894030	49894030	+	13.46097	NA	promoter-TSS (NM_005879)	promoter-TSS (NM_005879)	-38	NM_00587	10293	Hs.517972	NM_00587	ENSG0000	TRAIP	RNF206|SC	TRAF intera	protein-co
lib-C666-l	chr6	113134640	113134640	+	16.20502	NA	Intergenic	L1PA2|LINE|L1	466108	NM_00101	442247	Hs.448264	NM_00101	ENSG0000	RFPL4B	RNF211	ret finger p	protein-co
lib-C666-l	chr7	5013874	5013874	+	14.3056	NA	intron (NR_023385, intron 1 of 4)	CpG	258	NR_01544	441191	Hs.520636	NR_01544	ENSG0000	RNF216P1	RNF216L|h	ring finger	pseudo
lib-C666-l	chr6	30294999	30294999	+	39.64852	NA	promoter-TSS (NR_024052).5	promoter-TSS (NR_024052).5	-9	NM_17201	56658	Hs.413493	NM_02125	ENSG0000	TRIM39	RNF23|TFP	tripartite m	protein-co
lib-C666-l	chr7	76097485	76097485	+	6.39985	NA	intron (NM_001102594, intron 2 of 1	intron (NM_001102594, intron 2 of	6513	NM_00110	113878	Hs.187058	NM_02089	ENSG0000	DTX2	RNF58	deltex E3 u	protein-co
lib-C666-l	chr7	140178894	140178894	+	7.15277	NA	intron (NM_001291663, intron 1 of 6)	CpG	475	NM_00114	23608	Hs.744883	NM_01344	ENSG0000	MKRN1	RNF61	makorin rir	protein-co
lib-C666-l	chr3	9005142	9005142	+	236.55156	NA	promoter-TSS (NM_020165)	promoter-TSS (NM_020165)	17	NM_02016	56852	Hs.375684	NM_02016	ENSG0000	RAD18	RNF73	RAD18, E3	protein-co
lib-C666-l	chr11	5706447	5706447	+	7.76438	NA	promoter-TSS (NM_033034)	promoter-TSS (NM_033034)	-108	NM_03303	85363	Hs.125300	NM_03303	ENSG0000	TRIM5	RNF88|TRII	tripartite m	protein-co
lib-C666-l	chr6	30131112	30131112	+	3.92495	NA	5' UTR (NM_033229, exon 1 of 7).7	5' UTR (NM_033229, exon 1 of 7).7	129	NM_03322	89870	Hs.591789	NM_03322	ENSG0000	TRIM15	RNF93|ZNI	tripartite m	protein-co
lib-C666-l	chr1	117664526	117664526	+	10.71733	NA	promoter-TSS (NM_025188)	promoter-TSS (NM_025188)	-115	NM_02518	80263	Hs.301526	NM_02518	ENSG0000	TRIM45	RNF99	tripartite m	protein-co
lib-C666-l	chr19	49588538	49588538	+	5.30207	NA	5' UTR (NM_003089, exon 1 of 10)	5' UTR (NM_003089, exon 1 of 10)	141	NM_00308	6625	Hs.467097	NM_00308	ENSG0000	SNRNP70	RNPU1Z|RI	small nucle	protein-co
lib-C666-l	chr10	11653804	11653804	+	9.10494	NA	promoter-TSS (NM_014688)	promoter-TSS (NM_014688)	-16	NM_01468	9712	Hs.498661	NM_01468	ENSG0000	USP6NL	RNTRE|TRE	USP6 N-te	protein-co
lib-C666-l	chr1	149605767	149605767	+	12.12504	NA	promoter-TSS (NR_125392)	promoter-TSS (NR_125392)	-150	NR_12539	101954268		NR_125392		RNVU1-20	RNU1-110	RNA, varia	snRNA
lib-C666-l	chr1	147993811	147993811	+	57.45248	NA	promoter-TSS (NR_104086)	promoter-TSS (NR_104086)	-335	NR_10408	101954277		NR_10408	ENSG0000	RNVU1-19	RNU1-126	RNA, varia	snRNA
lib-C666-l	chr1	149514598	149514598	+	16.59065	NA	promoter-TSS (NR_104086).2	promoter-TSS (NR_104086).2	-342	NR_10408	101954277		NR_10408	ENSG0000	RNVU1-19	RNU1-126	RNA, varia	snRNA
lib-C666-l	chr14	71708027	71708027	+	17.3027	NA	Intergenic	Intergenic	-157027	NR_00127	319139		NR_00127	ENSG0000	SNORD56	RNU56B	small nucle	snoRNA
lib-C666-l	chr14	71791730	71791730	+	7.43576	NA	Intergenic	Intergenic	-73324	NR_00127	319139		NR_00127	ENSG0000	SNORD56	RNU56B	small nucle	snoRNA
lib-C666-l	chr14	71788211	71788211	+	5.819	NA	Intergenic	CpG	-76843	NR_00127	319139		NR_00127	ENSG0000	SNORD56	RNU56B	small nucle	snoRNA
lib-C666-l	chr9	137030031	137030031	+	58.41369	NA	promoter-TSS (NR_023344)	promoter-TSS (NR_023344)	-345	NR_02334	100151684	Hs.545589	NR_02334	ENSG0000	RNU6ATA	RNU6ATA	RNA, U6at	snRNA
lib-C666-l	chr22	39688709	39688709	+	13.91591	NA	Intergenic	MIRb|SINE|MIR	21207	NR_00002	116938		NR_00002	ENSG0000	SNORD83	RNU83B|U	small nucle	snoRNA
lib-C666-l	chr1	193028590	193028590	+	40.51821	NA	promoter-TSS (NM_001199261)	promoter-TSS (NM_001199261)	38	NM_00133	6738	Hs.288178	NM_00460	ENSG0000	TROVE2	RO60|ROR	TROVE do	protein-co
lib-C666-l	chr2	11488500	11488500	+	12.57335	NA	Intergenic	Intergenic	-2967	NM_00132	9475	Hs.681743	NM_00485	ENSG0000	ROCK2	ROCK-II	Rho associ	protein-co
lib-C666-l	chr2	11485170	11485170	+	10.71733	NA	promoter-TSS (NM_004850)	promoter-TSS (NM_004850)	363	NM_00132	9475	Hs.681743	NM_00485	ENSG0000	ROCK2	ROCK-II	Rho associ	protein-co
lib-C666-l	chr1	17240656	17240656	+	3.59106	NA	Intergenic	CpG	-7789	NM_01467	9696	Hs.309403	NM_01467	ENSG0000	CROCC	ROLT|TAX1	ciliary root	protein-co
lib-C666-l	chr11	62379914	62379914	+	2.65152	NA	promoter-TSS (NM_000327)	promoter-TSS (NM_000327)	-299	NM_00032	6094	Hs.281564	NM_00032	ENSG0000	ROM1	ROM|ROSI	retinal out	protein-co
lib-C666-l	chr1	114355156	114355156	+	6.32351	NA	promoter-TSS (NM_018364)	promoter-TSS (NM_018364)	-58	NR_13089	54665	Hs.486285	NM_01836	ENSG0000	RSBN1	ROSBIN	round sper	protein-co
lib-C666-l	chr19	33183186	33183186	+	3.98718	NA	exon (NM_001105570, exon 1 of 3)	exon (NM_001105570, exon 1 of 3)	319	NM_00110	390916	Hs.203961	NM_00110	ENSG0000	NUDT19	RP2	nudix hydr	protein-co
lib-C666-l	chr2	61991404	61991404	+	65.39317	NA	Intergenic	CpG	89874	NR_03771	84140	Hs.440466	NM_03218	ENSG0000	FAM161A	RP28	family with	protein-co
lib-C666-l	chrX	37706869	37706869	+	10.03103	NA	promoter-TSS (NM_006520)	promoter-TSS (NM_006520)	20	NM_00652	6990	Hs.446392	NM_00652	ENSG0000	DYNLT3	RP3|TCTE1	dynein ligh	protein-co
lib-C666-l	chr20	2644856	2644856	+	119.61965	NA	promoter-TSS (NM_174855)	promoter-TSS (NM_174855)	13	NM_00133	3420	Hs.436405	NM_00689	ENSG0000	IDH3B	RP46	isocitrate d	protein-co
lib-C666-l	chr6	170996311	170996311	+	6.74574	NA	Intergenic	Intergenic	-102531	NM_14478	5134	Hs.367900	NM_00259	ENSG0000	PDCD2	RP8|ZMYN	programm	protein-co
lib-C666-l	chr6	170893656	170893656	+	3.23837	NA	promoter-TSS (NM_001199462)	promoter-TSS (NM_001199462)	86	NM_00119	5134	Hs.367900	NM_00259	ENSG0000	PDCD2	RP8|ZMYN	programm	protein-co
lib-C666-l	chr2	113299600	113299600	+	4.52748	NA	promoter-TSS (NM_001282772)	promoter-TSS (NM_001282772)	-13	NM_00128	84172	Hs.745127	NM_01901	ENSG0000	POLR1B	RPA135|RP	RNA polym	protein-co
lib-C666-l	chr7	7680201	7680201	+	14.45701	NA	promoter-TSS (NM_001302350)	promoter-TSS (NM_001302350)	-66	NM_00130	729852	Hs.652371	NM_00130	ENSG0000	UMAD1	RPA3-AS1	UBAP1-MV	protein-co
lib-C666-l	chr7	7680603	7680603	+	10.16129	NA	5' UTR (NM_002947, exon 5 of 8)	5' UTR (NM_002947, exon 5 of 8)	336	NM_00130	729852	Hs.652371	NM_00130	ENSG0000	UMAD1	RPA3-AS1	UBAP1-MV	protein-co
lib-C666-l	chr7	44059052	44059052	+	2.9164	NA	promoter-TSS (NR_003655)	promoter-TSS (NR_003655)	-259	NR_00365	84820	Hs.657028	NM_03273	ENSG0000	POLR2J4	RPB11-phi	RNA polym	pseudo
lib-C666-l	chr22	41940556	41940556	+	16.14038	NA	promoter-TSS (NM_001018050)	promoter-TSS (NM_001018050)	54	NM_00128	171568	Hs.712617	NM_13833	ENSG0000	POLR3H	RPC22.9|RI	RNA polym	protein-co
lib-C666-l	chr1	145455381	145455381	+	18.61761	NA	TTS (NM_032305)	TTS (NM_032305)	15027	NM_03230	84265	Hs.591456	NM_03230	ENSG0000	POLR3GL	RPC32HOI	RNA polym	protein-co
lib-C666-l	chr1	145470447	145470447	+	3.92495	NA	promoter-TSS (NM_001039888)	promoter-TSS (NM_001039888)	-39	NM_00133	84265	Hs.591456	NM_03230	ENSG0000	POLR3GL	RPC32HOI	RNA polym	protein-co
lib-C666-l	chr16	22309724	22309724	+	13.6111	NA	intron (NM_001258035, intron 1 of 1	intron (NM_001258035, intron 1 of	1028	NM_00125	55718	Hs.460298	NM_01811	ENSG0000	POLR3E	RPC5|SIN	RNA polym	protein-co
lib-C666-l	chr16	22308619	22308619	+	2.42134	NA	promoter-TSS (NM_001258033)	promoter-TSS (NM_001258033)	-77	NM_00125	55718	Hs.460298	NM_01811	ENSG0000	POLR3E	RPC5|SIN	RNA polym	protein-co
lib-C666-l	chr2	88991094	88991094	+	165.56329	NA	promoter-TSS (NM_144563)	promoter-TSS (NM_144563)	-82	NM_14456	22934	Hs.469264	NM_14456	ENSG0000	RPIA	RPI|RPIAD	ribose 5-p	protein-co
lib-C666-l	chr14	56209138	56209138	+	13.59254	NA	Intergenic	Intergenic	-23825	NR_00484	645683	Hs.663461	NR_00484	ENSG0000	RPL13AP3	RPL13A_11	ribosomal	pseudo
lib-C666-l	chr2	114378918	114378918	+	5.5166	NA	TTS (NR_024530)	TTS (NR_024530)	5797	NR_02452	118433	Hs.595056	NR_000029		RPL23AP7	RPL23AL1|	ribosomal	pseudo
lib-C666-l	chr5	172332108	172332108	+	7.49163	NA	intron (NM_001031711, intron 3 of 9)	intron (NM_001031711, intron 3 of	-53624	NM_00131	51121	Hs.546390	NM_01609	ENSG0000	RPL26L1	RPL26P1	ribosomal	protein-co
lib-C666-l	chr12	122326629	122326629	+	5.96422	NA	promoter-TSS (NM_001261400)	promoter-TSS (NM_001261400)	-8	NM_00126	5715	Hs.131151	NM_00281	ENSG0000	PSMD9	Rpn4|p27	proteasom	protein-co
lib-C666-l	chr3	64009333	64009333	+	30.08826	NA	promoter-TSS (NM_001271781)	promoter-TSS (NM_001271781)	-51	NM_00127	9861	Hs.152536	NM_01481	ENSG0000	PSMD6	Rpn7|S10|S	proteasom	protein-co
lib-C666-l	chr19	30097183	30097183	+	301.08664	NA	promoter-TSS (NM_006627)	promoter-TSS (NM_006627)	13	NR_02736	10775	Hs.412870	NM_00662	ENSG0000	POP4	RPP29	POP4 hom	protein-co
lib-C666-l	chr19	24010321	24010321	+	4.19675	NA	non-coding (NR_003662, exon 4 of 4	non-coding (NR_003662, exon 4 of	64505	NR_00366	388524	Hs.560655	NM_001005472		RPSAP58	RPSA_30_1	ribosomal	pseudo
lib-C666-l	chr6	128841842	128841842	+	20.19521	NA	promoter-TSS (NM_002844)	promoter-TSS (NM_002844)	-23	NM_00129	5796	Hs.155919	NM_00284	ENSG0000	PTPRK	R-PTP-kap	protein tyr	protein-co
lib-C666-l	chr9	136201225	136201225	+	10.71733	NA	intron (NM_006753, intron 2 of 4)	intron (NM_006753, intron 2 of 4)	1822	NR_10387	6838	Hs.274430	NM_00675	ENSG0000	SURF6	RRP14	surfeit 6	protein-co
lib-C666-l	chr9	136203076	136203076	+	7.69829	NA	promoter-TSS (NM_001278942)	promoter-TSS (NM_001278942)	-29	NM_00127	6838	Hs.274430	NM_00675	ENSG0000	SURF6	RRP14	surfeit 6	protein-co
lib-C666-l	chr2	227700771	227700771	+	20.3278	NA	promoter-TSS (NM_001167608)	promoter-TSS (NM_001167608)	100	NM_00116	84236	Hs.471514	NM_03227	ENSG0000	RHBDD1	RRP4	rhomboid	protein-co
lib-C666-l	chr8	145133419	145133419	+	5.04826	NA	promoter-TSS (NM_019037)	promoter-TSS (NM_019037)	-103	NM_01903	54512	Hs.632041	NM_01903	ENSG0000	EXOSC4	RRP41|RRP	exosome c	protein-co
lib-C666-l	chr7	77325598	77325598	+	23.14387	NA	promoter-TSS (NR_134251)	promoter-TSS (NR_134251)	-16	NR_13425	100505854	Hs.72451	NR_03836	ENSG0000	APTR	RSBN1L-A	Alu-media	ncRNA
lib-C666-l	chr18	712500	712500	+	4.46944	NA	promoter-TSS (NM_202758)	promoter-TSS (NM_202758)	17	NM_20275	55556	Hs.658550	NM_01751	ENSG0000	ENOSF1	RTS|TYMSA	enolase su	protein-co
lib-C666-l	chr16	29302356	29302356	+	5.48804	NA	Intergenic	Intergenic	-11252	NR_00293	440352	Hs.658149	NR_002939		SNX29P2	RUNDC2C	sorting nex	pseudo
lib-C666-l	chr16	31519664	31519664	+	20.56906	NA	promoter-TSS (NM_022744)	promoter-TSS (NM_022744)	76	NM_02274	64755	Hs.9003	NM_02274	ENSG0000	C16orf58	RUS	chromosor	protein-co
lib-C666-l	chr17	2241304	2241304	+	9.02258	NA	promoter-TSS (NM_018128)	promoter-TSS (NM_018128)	496	NM_00109	9905	Hs.513861	NM_01485	ENSG0000	SGSM2	RUTBC1	small G prc	protein-co
lib-C666-l	chr22	25202207	25202207	+	2.96041	NA	promoter-TSS (NM_001039948)	promoter-TSS (NM_001039948)	19	NM_00103	129049	Hs.474397	NM_13345	ENSG0000	SGSM1	RUTBC2	small G prc	protein-co
lib-C666-l	chr6	83903032	83903032	+	6.82401	NA	promoter-TSS (NM_033411)	promoter-TSS (NM_033411)	0	NM_00132	112611	Hs.423336	NM_03341	ENSG0000	RWDD2A	RWDD2|dJ	RWD doma	protein-co
lib-C666-l	chr19	10196851	10196851	+	6.32351	NA	promoter-TSS (NM_001308277)	promoter-TSS (NM_001308277)	56	NM_01838	55337	Hs.655613	NM_01838	ENSG0000	C19orf66	RyDEN	chromosor	protein-co
lib-C666-l	chr1	153522021	153522021	+	18.85495	NA	promoter-TSS (NM_002960)	promoter-TSS (NM_002960)	-287	NM_00296	6274	Hs.557609	NM_00296	ENSG0000	S100A3	S100E	S100 calciu	protein-co
lib-C666-l	chr1	33283009	33283009	+	15.60931	NA	promoter-TSS (NM_022753)	promoter-TSS (NM_022753)	-34	NM_00125	64766	Hs.440880	NM_02275	ENSG0000	S100PBP	S100PBPR	S100P binc	protein-co
lib-C666-l	chr19	49999601	49999601	+	33.47004	NA	promoter-TSS (NM_001015)	promoter-TSS (NM_001015)	-21	NM_00101	6205	Hs.433529	NM_00101	ENSG0000	RPS11	S11	ribosomal	protein-co
lib-C666-l	chr6	133135441	133135441	+	6.39985	NA	promoter-TSS (NM_001016)	promoter-TSS (NM_001016)	-267	NM_00101	6206	Hs.546289	NM_00101	ENSG0000	RPS12	S12	ribosomal	protein-co
lib-C666-l	chr11	17099324	17099324	+	29.37119	NA	promoter-TSS (NM_001017)	promoter-TSS (NM_001017)	-104	NM_00101	6207	Hs.446588	NM_00101	ENSG0000	RPS13	S13	ribosomal	protein-co
lib-C666-l	chr19	39921582	39921582	+	28.42633	NA	Intergenic	MER6B|DNA|TcMar-Tigger	5078	NM_00132	6217	Hs.397609	NM_00102	ENSG0000	RPS16	S16	ribosomal	protein-co
lib-C666-l	chr19	39926721	39926721	+	9.94079	NA	promoter-TSS (NM_001321111)	promoter-TSS (NM_001321111)	-61	NM_00102	6217	Hs.397609	NM_00102	ENSG0000	RPS16	S16	ribosomal	protein-co
lib-C666-l	chr11	75110485	75110485	+	38.21529	NA	promoter-TSS (NM_001005)	promoter-TSS (NM_001005)	-50	NM_00100	6188	Hs.546286	NM_00100	ENSG0000	RPS3	S3	ribosomal	protein-co
lib-C666-l	chr19	58898547	58898547	+	8.71138	NA	promoter-TSS (NM_001009)	promoter-TSS (NM_001009)	-89	NM_00100	6193	Hs.378103	NM_00100	ENSG0000	RPS5	S5	ribosomal	protein-co
lib-C666-l	chr19	56015311	56015311	+	197.29065	NA	intron (NM_001144950, intron 12 of 1	CpG	15441	NM_00119	284297	Hs.554182	NM_18257	ENSG0000	SSC5D	S5D-SRCR	scavenger	protein-co
lib-C666-l	chr9	19380327	19380327	+	11.66012	NA	promoter-TSS (NM_001010)	promoter-TSS (NM_001010)	-92	NM_00101	6194	Hs.408073	NM_00101	ENSG0000	RPS6	S6	ribosomal	protein-co
lib-C666-l	chr1	45241139	45241139	+	201.23711	NA	promoter-TSS (NR_000015)	promoter-TSS (NR_000015)	-107	NM_00101	6202	Hs.512675	NM_00101	ENSG0000	RPS8	S8	ribosomal	protein-co
lib-C666-l	chr19	54704650	54704650	+	41.28836	NA	promoter-TSS (NM_001321702)	promoter-TSS (NM_001321702)	-76	NR_13576	6203	Hs.467284	NM_00101	ENSG0000	RPS9	S9	ribosomal	protein-co
lib-C666-l	chr3	136471212	136471212	+	4.19675	NA	promoter-TSS (NM_005862)	promoter-TSS (NM_005862)	33	NM_00586	10274	Hs.412586	NM_00586	ENSG0000	STAG1	SA1|SCC3A	stromal an	protein-co
lib-C666-l	chr3	15419820	15419820	+	7.15277	NA	Intergenic	MER66C|LTR|ERV1	-36919	NM_00101	9467	Hs.257761	NM_00484	ENSG0000	SH3BP5	SAB|SH3BF	SH3 doma	protein-co
lib-C666-l	chr3	45730740	45730740	+	38.10403	NA	promoter-TSS (NM_001319072)	promoter-TSS (NM_001319072)	7	NM_00131	22908	Hs.156509	NM_01401	ENSG0000	SACM1L	SAC1	SAC1 supp	protein-co
lib-C666-l	chr20	32890856	32890856	+	18.43022	NA	promoter-TSS (NM_001322086)	promoter-TSS (NM_001322086)	-44	NM_00132	191	Hs.388004	NM_00068	ENSG0000	AHCY	SAHH|adol	adenosylh	protein-co
lib-C666-l	chr17	74582303	74582303	+	36.04387	NA	promoter-TSS (NM_006456)	promoter-TSS (NM_006456)	-158	NM_00645	10610	Hs.592105	NM_00645	ENSG0000	ST6GALNA	SAITL1|SIA	ST6 N-ace	protein-co
lib-C666-l	chr1	32452964	32452964	+	6.15313	NA	Intergenic	Intergenic	-26331	NR_07349	10657	Hs.445893	NM_00655	ENSG0000	KHDRBS1	Sam68|p62	KH RNA bi	protein-co
lib-C666-l	chr1	32479708	32479708	+	2.78587	NA	non-coding (NR_073499, exon 1 of 1	non-coding (NR_073499, exon 1 of	413	NM_00127	10657	Hs.445893	NM_00655	ENSG0000	KHDRBS1	Sam68|p62	KH RNA bi	protein-co
lib-C666-l	chr14	55032794	55032794	+	6.74574	NA	Intergenic	CpG	-1536	NM_01558	23034	Hs.98259	NM_01558	ENSG0000	SAMD4A	SAMD4|SM	sterile alph	protein-co
lib-C666-l	chr14	55033573	55033573	+	4.96104	NA	promoter-TSS (NM_001161576)	promoter-TSS (NM_001161576)	-757	NM_01558	23034	Hs.98259	NM_01558	ENSG0000	SAMD4A	SAMD4|SM	sterile alph	protein-co
lib-C666-l	chr14	55032411	55032411	+	4.46944	NA	Intergenic	CpG	-1919	NM_01558	23034	Hs.98259	NM_01558	ENSG0000	SAMD4A	SAMD4|SM	sterile alph	protein-co
lib-C666-l	chr3	49967334	49967334	+	25.3508	NA	5' UTR (NM_001142501, exon 1 of 5)	5' UTR (NM_001142501, exon 1 of 5	111	NM_03235	84315	Hs.655014	NM_03235	ENSG0000	MON1A	SAND1	MON1 hor	protein-co
lib-C666-l	chr1	205601147	205601147	+	61.91626	NA	5' UTR (NM_021795, exon 1 of 3)	5' UTR (NM_021795, exon 1 of 3)	853	NM_00197	2005	Hs.497520	NM_00197	ENSG0000	ELK4	SAP1	ELK4, ETS t	protein-co
lib-C666-l	chr1	205600632	205600632	+	3.84463	NA	intron (NM_001973, intron 1 of 4)	CpG	1368	NM_00197	2005	Hs.497520	NM_00197	ENSG0000	ELK4	SAP1	ELK4, ETS t	protein-co
lib-C666-l	chr10	71930318	71930318	+	31.9103	NA	promoter-TSS (NM_001142648)	promoter-TSS (NM_001142648)	-33	NM_00114	56681	Hs.499960	NM_02015	ENSG0000	SAR1A	SAR1|SARA	secretion a	protein-co
lib-C666-l	chr10	99552008	99552008	+	30.07415	NA	Intergenic	Intergenic	-20252	NM_00301	6425	Hs.279565	NM_00301	ENSG0000	SFRP5	SARP3	secreted fr	protein-co
lib-C666-l	chr19	39421507	39421507	+	27.7824	NA	promoter-TSS (NM_017827)	promoter-TSS (NM_017827)	29	NM_01782	54938	Hs.709416	NM_01782	ENSG0000	SARS2	SARS|SARS	seryl-tRNA	protein-co
lib-C666-l	chr12	58138410	58138410	+	257.3508	NA	promoter-TSS (NM_005981)	promoter-TSS (NM_005981)	-362	NM_00598	6302	Hs.632708	NM_00598	ENSG0000	TSPAN31	SAS	tetraspanir	protein-co
lib-C666-l	chr2	86116212	86116212	+	18.74745	NA	promoter-TSS (NM_003896)	promoter-TSS (NM_003896)	-55	NM_00389	8869	Hs.415117	NM_00389	ENSG0000	ST3GAL5	SATI|SIAT9	ST3 beta-g	protein-co
lib-C666-l	chr16	2022040	2022040	+	534.10608	NA	promoter-TSS (NM_006453)	promoter-TSS (NM_006453)	-24	NM_00645	10607	Hs.513267	NM_00645	ENSG0000	TBL3	SAZD|UTP1	transducin	protein-co
lib-C666-l	chr19	54370885	54370885	+	7.7487	NA	promoter-TSS (NM_138373)	promoter-TSS (NM_138373)	-234	NM_00102	91663	Hs.380906	NM_13837	ENSG0000	MYADM	SB135	myeloid as	protein-co
lib-C666-l	chr19	54372936	54372936	+	3.94004	NA	5' UTR (NM_001290189, exon 1 of 3)	5' UTR (NM_001290189, exon 1 of 3	291	NM_00102	91663	Hs.380906	NM_13837	ENSG0000	MYADM	SB135	myeloid as	protein-co
lib-C666-l	chr1	181096397	181096397	+	18.82927	NA	Intergenic	Intergenic	38759	NM_01654	51278	Hs.15725	NM_01654	ENSG0000	IER5	SBBI48	immediate	protein-co
lib-C666-l	chr1	181086976	181086976	+	11.6497	NA	Intergenic	Intergenic	29338	NM_01654	51278	Hs.15725	NM_01654	ENSG0000	IER5	SBBI48	immediate	protein-co
lib-C666-l	chr1	181059143	181059143	+	5.10953	NA	3' UTR (NM_016545, exon 1 of 1)	3' UTR (NM_016545, exon 1 of 1)	1505	NM_01654	51278	Hs.15725	NM_01654	ENSG0000	IER5	SBBI48	immediate	protein-co
lib-C666-l	chr1	181058765	181058765	+	4.83287	NA	exon (NM_016545, exon 1 of 1)	exon (NM_016545, exon 1 of 1)	1127	NM_01654	51278	Hs.15725	NM_01654	ENSG0000	IER5	SBBI48	immediate	protein-co
lib-C666-l	chr19	51014794	51014794	+	413.17792	NA	promoter-TSS (NM_001270686)	promoter-TSS (NM_001270686)	-182	NM_00127	126119	Hs.467151	NM_13833	ENSG0000	JOSD2	SBBI54	Josephin d	protein-co
lib-C666-l	chr12	55286090	55286090	+	6.15313	NA	Intergenic	Intergenic	37791	NM_05817	118430	Hs.348419	NM_05817	ENSG0000	MUCL1	SBEM	mucin like	protein-co
lib-C666-l	chr11	9482385	9482385	+	105.96375	NA	promoter-TSS (NR_036539)	promoter-TSS (NR_036539)	-127	NM_00128	7702	Hs.523471	NM_00344	ENSG0000	ZNF143	SBF|STAF|p	zinc finger	protein-co
lib-C666-l	chr16	68362772	68362772	+	9.2055	NA	intron (NM_001290018, intron 5 of 1	intron (NM_001290018, intron 5 of	17755	NM_00129	54496	Hs.640229	NM_01902	ENSG0000	PRMT7	SBIDDS	protein arg	protein-co
lib-C666-l	chr9	91933438	91933438	+	114.95827	NA	promoter-TSS (NM_001282690)	promoter-TSS (NM_001282690)	50	NM_00128	79048	Hs.59804	NM_02407	ENSG0000	SECISBP2	SBP2	SECIS bind	protein-co
lib-C666-l	chr15	43213118	43213118	+	3.65763	NA	promoter-TSS (NM_173500)	promoter-TSS (NM_173500)	-111	NM_17350	146057	Hs.646511	NM_17350	ENSG0000	TTBK2	SCA11|TTB	tau tubulin	protein-co
lib-C666-l	chr5	90576577	90576577	+	28.80463	NA	Intergenic	CpG	33642	NR_10354	100505994	Hs.628363	NR_10354	ENSG0000	LUCAT1	SCAL1	lung cance	ncRNA
lib-C666-l	chr5	90354499	90354499	+	15.42216	NA	intron (NR_003149, intron 85 of 89)	intron (NR_003149, intron 85 of 89)	255720	NR_10354	100505994	Hs.628363	NR_10354	ENSG0000	LUCAT1	SCAL1	lung cance	ncRNA
lib-C666-l	chr5	90458768	90458768	+	8.03539	NA	intron (NR_003149, intron 89 of 89)	intron (NR_003149, intron 89 of 89)	151451	NR_10354	100505994	Hs.628363	NR_10354	ENSG0000	LUCAT1	SCAL1	lung cance	ncRNA
lib-C666-l	chr5	90515217	90515217	+	3.38731	NA	Intergenic	Intergenic	95002	NR_10354	100505994	Hs.628363	NR_10354	ENSG0000	LUCAT1	SCAL1	lung cance	ncRNA

lib-C666-l	chr5	90607944	90607944	+	2.59723	NA	intron (NR_103549, intron 2 of 2)	intron (NR_103549, intron 2 of 2)	2275	NR_10354	100505994	Hs.628363	NR_10354	ENSG0000	LUCAT1	SCAL1	lung cance	ncRNA
lib-C666-l	chr8	22423587	22423587	+	15.66421	NA	intron (NM_001018003, intron 1 of 9)	CpG	408	NM_00101	10174	Hs.528572	NM_00577	ENSG0000	SORBS3	SCAM-1|S	sorbin and	protein-co
lib-C666-l	chr8	22424789	22424789	+	8.81145	NA	intron (NM_001018003, intron 5 of 9)	intron (NM_001018003, intron 5 of	1610	NM_00101	10174	Hs.528572	NM_00577	ENSG0000	SORBS3	SCAM-1|S	sorbin and	protein-co
lib-C666-l	chr15	85174626	85174626	+	13.20416	NA	promoter-TSS (NR_003654)	promoter-TSS (NR_003654)	-65	NR_00485	54581	Hs.658114	NM_022050		SCAND2P	SCAND2	SCAN dom	pseudo
lib-C666-l	chr3	43749800	43749800	+	12.23699	NA	intron (NM_016006, intron 3 of 6)	intron (NM_016006, intron 3 of 6)	-16714	NM_00134	55129	Hs.656657	NM_01807	ENSG0000	ANO10	SCAR10|TM	anoctamin	protein-co
lib-C666-l	chr11	102962960	102962960	+	11.57178	NA	promoter-TSS (NM_001318739)	promoter-TSS (NM_001318739)	-16	NM_03229	84259	Hs.503716	NM_03229	ENSG0000	DCUN1D5	SCCRO5	defective ir	protein-co
lib-C666-l	chr7	2559052	2559052	+	5.92363	NA	promoter-TSS (NM_001040168)	promoter-TSS (NM_001040168)	-427	NM_00104	3955	Hs.159142	NM_00230	ENSG0000	LFNG	SCDO3	LFNG O-fu	protein-co
lib-C666-l	chr16	30103376	30103376	+	8.11522	NA	promoter-TSS (NM_004608)	promoter-TSS (NM_004608)	-171	NM_00460	6911	Hs.198301	NM_00460	ENSG0000	TBX6	SCDO5	T-box 6	protein-co
lib-C666-l	chr20	5825192	5825192	+	8.96177	NA	intron (NM_001303478, intron 3 of 3)	intron (NM_001303478, intron 3 of	-66782	NM_00181	1114	Hs.516874	NM_00181	ENSG0000	CHGB	SCG1	chromogra	protein-co
lib-C666-l	chr7	100808916	100808916	+	29.92316	NA	promoter-TSS (NM_003378)	promoter-TSS (NM_003378)	-64	NM_00337	7425	Hs.587325	NM_00337	ENSG0000	VGF	SCG7|SgVI	VGF nerve	protein-co
lib-C666-l	chr19	35168065	35168065	+	32.11429	NA	promoter-TSS (NR_027620)	promoter-TSS (NR_027620)	-129	NR_02762	643719	Hs.686754	NR_027620		SCGB1B2P	SCGB4A1P	secretoglo	pseudo
lib-C666-l	chr14	51297850	51297850	+	7.9573	NA	promoter-TSS (NM_020921)	promoter-TSS (NM_020921)	-11	NM_18294	51199	Hs.310429	NM_01635	ENSG0000	NIN	SCKL7	ninein	protein-co
lib-C666-l	chr2	238969507	238969507	+	19.06818	NA	promoter-TSS (NM_016510)	promoter-TSS (NM_016510)	-58	NM_01651	51540	Hs.731909	NM_01651	ENSG0000	SCLY	SCL|hSCL	selenocyst	protein-co
lib-C666-l	chr1	41708041	41708041	+	10.4657	NA	promoter-TSS (NM_001172220)	promoter-TSS (NM_001172220)	-226	NM_00117	22955	Hs.571874	NM_01223	ENSG0000	SCMH1	Scml3	sex comb c	protein-co
lib-C666-l	chr3	48514533	48514533	+	20.56906	NA	5' UTR (NM_001272082, exon 1 of 4)	5' UTR (NM_001272082, exon 1 of 4	209	NM_00127	51246	Hs.414579	NM_01647	ENSG0000	SHISA5	SCOTIN	shisa family	protein-co
lib-C666-l	chr3	195681528	195681528	+	8.28735	NA	Intergenic	CpG	35622	NR_00326	255812	Hs.566872	NR_00326	ENSG0000	SDHAP1	SDHAL1|SD	succinate c	pseudo
lib-C666-l	chr16	4743584	4743584	+	24.03208	NA	promoter-TSS (NM_001193452)	promoter-TSS (NM_001193452)	-110	NM_03234	84309	Hs.513315	NM_03234	ENSG0000	NUDT16L1	SDOS	nudix hydr	protein-co
lib-C666-l	chr6	158184553	158184553	+	11.86993	NA	Intergenic	Intergenic	-59650	NM_01622	51429	Hs.191213	NM_01622	ENSG0000	SNX9	SDP1|SH3P	sorting nex	protein-co
lib-C666-l	chr22	43411208	43411208	+	60.4974	NA	promoter-TSS (NM_001184970)	promoter-TSS (NM_001184970)	-24	NM_00118	11252	Hs.162877	NM_00722	ENSG0000	PACSIN2	SDPII	protein kin	protein-co
lib-C666-l	chr22	43387677	43387677	+	9.79436	NA	intron (NM_001184970, intron 1 of 10	intron (NM_001184970, intron 1 of	23507	NM_00118	11252	Hs.162877	NM_00722	ENSG0000	PACSIN2	SDPII	protein kin	protein-co
lib-C666-l	chr14	24769033	24769033	+	265.48364	NA	promoter-TSS (NM_138452)	promoter-TSS (NM_138452)	6	NM_00113	115817	Hs.348350	NM_13845	ENSG0000	DHRS1	SDR19C1	dehydroge	protein-co
lib-C666-l	chr1	24127359	24127359	+	18.43022	NA	promoter-TSS (NM_000403)	promoter-TSS (NM_000403)	-65	NM_00100	2582	Hs.632380	NM_00040	ENSG0000	GALE	SDR1E1	UDP-galac	protein-co
lib-C666-l	chr2	73114601	73114601	+	2.31621	NA	promoter-TSS (NM_003124)	promoter-TSS (NM_003124)	89	NM_00312	6697	Hs.301540	NM_00312	ENSG0000	SPR	SDR38C1	sepiapterin	protein-co
lib-C666-l	chr13	52377866	52377866	+	164.71092	NA	intron (NM_001031719, intron 1 of 7)	CpG	432	NM_02470	79758	Hs.266728	NM_02470	ENSG0000	DHRS12	SDR40C1	dehydroge	protein-co
lib-C666-l	chr13	52338474	52338474	+	7.37943	NA	Intergenic	L2b|LINE|L2	39824	NM_02470	79758	Hs.266728	NM_02470	ENSG0000	DHRS12	SDR40C1	dehydroge	protein-co
lib-C666-l	chr13	52391078	52391078	+	5.5166	NA	non-coding (NR_027047, exon 5 of 5	non-coding (NR_027047, exon 5 of	-12780	NM_00127	79758	Hs.266728	NM_02470	ENSG0000	DHRS12	SDR40C1	dehydroge	protein-co
lib-C666-l	chr4	169931464	169931464	+	35.40551	NA	promoter-TSS (NM_032783)	promoter-TSS (NM_032783)	4	NM_03278	84869	Hs.659311	NM_03278	ENSG0000	CBR4	SDR45C1	carbonyl re	protein-co
lib-C666-l	chr2	106760211	106760211	+	14.27758	NA	intron (NR_045607, intron 5 of 13)	MLT1E2|LTR|ERVL-MaLR	-4874	NM_00125	80146	Hs.469561	NM_02507	ENSG0000	UXS1	SDR6E1|UC	UDP-gluc	protein-co
lib-C666-l	chr19	55574692	55574692	+	25.49921	NA	promoter-TSS (NR_027382)	promoter-TSS (NR_027382)	-107	NR_02738	112724	Hs.327631	NM_13841	ENSG0000	RDH13	SDR7C3	retinol deh	protein-co
lib-C666-l	chr19	49142352	49142352	+	23.0291	NA	intron (NR_136241, intron 7 of 8)	intron (NR_136241, intron 7 of 8)	1056	NR_00440	653677	Hs.443160	NR_00440	ENSG0000	SEC1P	SEC1	secretory b	pseudo
lib-C666-l	chr2	27357551	27357551	+	7.60409	NA	promoter-TSS (NM_001330484)	promoter-TSS (NM_001330484)	-8	NM_01338	10113	Hs.279784	NM_01338	ENSG0000	PREB	SEC12	prolactin re	protein-co
lib-C666-l	chr2	73053157	73053157	+	71.16089	NA	promoter-TSS (NM_001321730)	promoter-TSS (NM_001321730)	14	NM_00132	23233	Hs.303454	NM_01518	ENSG0000	EXOC6B	SEC15B|SE	exocyst co	protein-co
lib-C666-l	chr3	122920760	122920760	+	18.0551	NA	promoter-TSS (NM_012430)	promoter-TSS (NM_012430)	-14	NM_01243	26984	Hs.477361	NM_01243	ENSG0000	SEC22A	SEC22L2	SEC22 hon	protein-co
lib-C666-l	chr3	42632256	42632256	+	40.50761	NA	intron (NM_016305, intron 1 of 3)	CpG	-8736	NM_00420	9117	Hs.445892	NM_00420	ENSG0000	SEC22C	SEC22L3	SEC22 hon	protein-co
lib-C666-l	chr4	110354839	110354839	+	12.99339	NA	promoter-TSS (NM_001042734)	promoter-TSS (NM_001042734)	-32	NM_00131	10427	Hs.292472	NM_00632	ENSG0000	SEC24B	SEC24	SEC24 hon	protein-co
lib-C666-l	chr13	46038936	46038936	+	7.75972	NA	promoter-TSS (NM_031431)	promoter-TSS (NM_031431)	-94	NM_03143	83548	Hs.507948	NM_03143	ENSG0000	COG3	SEC34	componen	protein-co
lib-C666-l	chr6	693046	693046	+	12.78696	NA	promoter-TSS (NM_018303)	promoter-TSS (NM_018303)	95	NR_07306	55770	Hs.484412	NM_01830	ENSG0000	EXOC2	SEC5|SEC5	exocyst co	protein-co
lib-C666-l	chr6	692645	692645	+	6.98764	NA	intron (NR_073064, intron 1 of 29)	CpG	496	NR_07306	55770	Hs.484412	NM_01830	ENSG0000	EXOC2	SEC5|SEC5	exocyst co	protein-co
lib-C666-l	chr7	132937741	132937741	+	30.07198	NA	promoter-TSS (NM_021807)	promoter-TSS (NM_021807)	-82	NM_02180	60412	Hs.321273	NM_02180	ENSG0000	EXOC4	SEC8|SEC8	exocyst co	protein-co
lib-C666-l	chr11	62599505	62599505	+	62.69942	NA	promoter-TSS (NR_135084)	promoter-TSS (NR_135084)	58	NM_00133	6811	Hs.654602	NM_00316	ENSG0000	STX5	SED5|STX5	syntaxin 5	protein-co
lib-C666-l	chr19	40931906	40931906	+	5.3823	NA	promoter-TSS (NM_013376)	promoter-TSS (NM_013376)	26	NM_01337	29950	Hs.269898	NM_01337	ENSG0000	SERTAD1	SEI1|TRIP-I	SERTA don	protein-co
lib-C666-l	chr2	64872380	64872380	+	101.17616	NA	intron (NM_014755, intron 1 of 1)	intron (NM_014755, intron 1 of 1)	8666	NM_01475	9792	Hs.591569	NM_01475	ENSG0000	SERTAD2	Sei-2|TRIP-	SERTA don	protein-co
lib-C666-l	chr2	64955193	64955193	+	31.21486	NA	Intergenic	Intergenic	-74147	NM_01475	9792	Hs.591569	NM_01475	ENSG0000	SERTAD2	Sei-2|TRIP-	SERTA don	protein-co
lib-C666-l	chr10	13390543	13390543	+	9.02258	NA	promoter-TSS (NM_012247)	promoter-TSS (NM_012247)	-245	NM_01224	22929	Hs.124027	NM_01224	ENSG0000	SEPHS1	SELD|SPS|S	selenopho	protein-co
lib-C666-l	chr22	50639308	50639308	+	13.91591	NA	promoter-TSS (NM_031454)	promoter-TSS (NM_031454)	-100	NM_03145	83642	Hs.365405	NM_03145	ENSG0000	SELENOO	SELO	selenoprot	protein-co
lib-C666-l	chr3	122730100	122730100	+	3.76263	NA	intron (NR_046079, intron 1 of 22)	intron (NR_046079, intron 1 of 22)	16559	NM_00103	54437	Hs.210870	NM_01898	ENSG0000	SEMA5B	SEMAG|Se	semaphori	protein-co
lib-C666-l	chr19	4584841	4584841	+	31.98049	NA	Intergenic	CpG	-25070	NM_03210	10501	Hs.465642	NM_02024	ENSG0000	SEMA6B	SEM-SEMA	semaphori	protein-co
lib-C666-l	chr7	45808663	45808663	+	356.21329	NA	promoter-TSS (NR_024271)	promoter-TSS (NR_024271)	-46	NR_02427	641977	Hs.723477	NR_02427	ENSG0000	SEPT7P2	SEPT13|SE	septin 7 ps	pseudo
lib-C666-l	chr7	45846605	45846605	+	9.21057	NA	Intergenic	Intergenic	-37988	NR_02427	641977	Hs.723477	NR_02427	ENSG0000	SEPT7P2	SEPT13|SE	septin 7 ps	pseudo
lib-C666-l	chr19	48281840	48281840	+	5.04826	NA	promoter-TSS (NM_003009)	promoter-TSS (NM_003009)	-2	NM_00300	6415	Hs.631549	NM_00300	ENSG0000	SELENOW	SEPW1|sel	selenoprot	protein-co
lib-C666-l	chr19	48282077	48282077	+	4.51266	NA	intron (NM_003009, intron 1 of 5)	CpG	235	NM_00300	6415	Hs.631549	NM_00300	ENSG0000	SELENOW	SEPW1|sel	selenoprot	protein-co
lib-C666-l	chr14	95147780	95147780	+	13.59977	NA	Intergenic	MLT1K|LTR|ERVL-MaLR	40718	NR_01534	388007	Hs.527795	NR_01534	ENSG0000	SERPINA13	SERPINA13	serpin fam	pseudo
lib-C666-l	chr11	94987637	94987637	+	12.72278	NA	Intergenic	Intergenic	-21932	NM_00127	143686	Hs.120633	NM_14466	ENSG0000	SESN3	SEST3	sestrin 3	protein-co
lib-C666-l	chr3	9438730	9438730	+	42.21183	NA	promoter-TSS (NR_132781)	promoter-TSS (NR_132781)	-423	NR_13278	440944	Hs.598958	NM_00101	ENSG0000	THUMPD3	SETD5-AS	THUMPD3	ncRNA
lib-C666-l	chr6	144416787	144416787	+	149.98938	NA	promoter-TSS (NM_031287)	promoter-TSS (NM_031287)	-33	NM_03128	83443	Hs.110695	NM_03128	ENSG0000	SF3B5	SF3b10|Ysf	splicing fac	protein-co
lib-C666-l	chr5	65493846	65493846	+	10.37767	NA	Intergenic	L1M1|LINE|L1	53183	NM_13916	140890	Hs.49367	NM_13916	ENSG0000	SREK1	SFRS12|SR	splicing reg	protein-co
lib-C666-l	chr12	123465267	123465267	+	3.25502	NA	TTS (NM_001304833)	TTS (NM_001304833)	660	NM_00127	51329	Hs.103561	NM_01663	ENSG0000	ARL6IP4	SFRS20|SR	ADP ribosy	protein-co
lib-C666-l	chr6	36562051	36562051	+	24.25638	NA	promoter-TSS (NM_003017)	promoter-TSS (NM_003017)	-39	NR_03661	6428	Hs.405144	NM_00301	ENSG0000	SRSF3	SFRS3|SRp	serine and	protein-co
lib-C666-l	chr1	29508506	29508506	+	4.73866	NA	5' UTR (NM_005626, exon 1 of 6)	5' UTR (NM_005626, exon 1 of 6)	131	NM_00562	6429	Hs.469970	NM_00562	ENSG0000	SRSF4	SFRS4|SRP	serine and	protein-co
lib-C666-l	chr12	120907519	120907519	+	12.0509	NA	promoter-TSS (NM_003769)	promoter-TSS (NM_003769)	39	NM_00376	8683	Hs.706889	NM_00376	ENSG0000	SRSF9	SFRS9|SRp	serine and	protein-co
lib-C666-l	chr6	134684714	134684714	+	15.60931	NA	Intergenic	Intergenic	-45518	NM_00114	6446	Hs.510078	NM_00562	ENSG0000	SGK1	SGK	serum/gluc	protein-co
lib-C666-l	chr6	134496111	134496111	+	12.12504	NA	promoter-TSS (NM_005627)	promoter-TSS (NM_005627)	-77	NM_00562	6446	Hs.510078	NM_00562	ENSG0000	SGK1	SGK	serum/gluc	protein-co
lib-C666-l	chr19	56028474	56028474	+	140.66429	NA	intron (NM_001144950, intron 13 of 1	CpG	19961	NM_00110	646643	Hs.532676	NM_00110	ENSG0000	SBK2	SGK069	SH3 doma	protein-co
lib-C666-l	chr2	201390844	201390844	+	7.49163	NA	promoter-TSS (NM_152524)	promoter-TSS (NM_152524)	-21	NM_00116	151246	Hs.655182	NM_15252	ENSG0000	SGO2	SGOL2|TRI	shugoshin	protein-co
lib-C666-l	chr1	2187496	2187496	+	106.05028	NA	intron (NM_003036, intron 1 of 6)	intron (NM_003036, intron 1 of 6)	27362	NM_00303	6497	Hs.656507	NM_00303	ENSG0000	SKI	SGS|SKV	SKI proto-c	protein-co
lib-C666-l	chr1	2208448	2208448	+	8.0787	NA	intron (NM_003036, intron 1 of 6)	intron (NM_003036, intron 1 of 6)	48314	NM_00303	6497	Hs.656507	NM_00303	ENSG0000	SKI	SGS|SKV	SKI proto-c	protein-co
lib-C666-l	chr1	2158954	2158954	+	4.73866	NA	Intergenic	CpG	-1180	NM_00303	6497	Hs.656507	NM_00303	ENSG0000	SKI	SGS|SKV	SKI proto-c	protein-co
lib-C666-l	chr19	2783369	2783369	+	30.98434	NA	promoter-TSS (NM_003021)	promoter-TSS (NM_003021)	-15	NM_00302	6449	Hs.203910	NM_00302	ENSG0000	SGTA	SGT|alpha	small gluta	protein-co
lib-C666-l	chr13	53226484	53226484	+	7.31982	NA	promoter-TSS (NM_001130912)	promoter-TSS (NM_001130912)	-342	NM_00670	10910	Hs.281902	NM_00670	ENSG0000	SUGT1	SGT1	SGT1 hom	protein-co
lib-C666-l	chr13	53226910	53226910	+	4.74112	NA	promoter-TSS (NM_001130912)	promoter-TSS (NM_001130912)	84	NM_00132	10910	Hs.281902	NM_00670	ENSG0000	SUGT1	SGT1	SGT1 hom	protein-co
lib-C666-l	chr11	10772592	10772592	+	33.95009	NA	promoter-TSS (NM_014633)	promoter-TSS (NM_014633)	58	NM_01463	9646	Hs.725151	NM_01463	ENSG0000	CTR9	SH2BP1|TS	CTR9 hom	protein-co
lib-C666-l	chr15	42783417	42783417	+	13.35078	NA	promoter-TSS (NM_001284307)	promoter-TSS (NM_001284307)	-22	NM_00128	64397	Hs.511143	NM_02247	ENSG0000	ZNF106	SH3BP3|ZF	zinc finger	protein-co
lib-C666-l	chr5	53813527	53813527	+	13.8811	NA	promoter-TSS (NM_052870)	promoter-TSS (NM_052870)	-62	NM_00114	112574	Hs.432755	NM_05287	ENSG0000	SNX18	SH3PX2|SH	sorting nex	protein-co
lib-C666-l	chr7	74595129	74595129	+	8.73558	NA	Intergenic	Intergenic	-7327	NR_00318	654817	Hs.647047	NR_00318	ENSG0000	NCF1C	SH3PXD1C	neutrophil	pseudo
lib-C666-l	chr11	68816352	68816352	+	9.75396	NA	promoter-TSS (NM_139075)	promoter-TSS (NM_139075)	2	NM_13907	219931	Hs.131851	NM_13907	ENSG0000	TPCN2	SHEP10|TP	two pore s	protein-co
lib-C666-l	chr1	36851575	36851575	+	14.32762	NA	promoter-TSS (NM_001282546)	promoter-TSS (NM_001282546)	-47	NM_03201	83931	Hs.471768	NM_03201	ENSG0000	STK40	SHIK|SgK4	serine/thre	protein-co
lib-C666-l	chr1	46806795	46806795	+	72.02663	NA	promoter-TSS (NM_001256128)	promoter-TSS (NM_001256128)	-55	NM_00125	387338	Hs.163424	NM_19904	ENSG0000	NSUN4	SHTAP	NOP2/Sun	protein-co
lib-C666-l	chr16	48419625	48419625	+	20.94772	NA	promoter-TSS (NM_003031)	promoter-TSS (NM_003031)	-396	NM_00303	6477	Hs.706828	NM_00303	ENSG0000	SIAH1	SIAH1A	siah E3 ubi	protein-co
lib-C666-l	chr3	39425033	39425033	+	7.9428	NA	5' UTR (NM_017875, exon 1 of 7)	5' UTR (NM_017875, exon 1 of 7)	218	NM_01787	54977	Hs.369615	NM_01787	ENSG0000	SLC25A38	SIDBA2	solute carr	protein-co
lib-C666-l	chr3	39425887	39425887	+	7.67555	NA	intron (NM_017875, intron 1 of 6)	CpG	1072	NM_01787	54977	Hs.369615	NM_01787	ENSG0000	SLC25A38	SIDBA2	solute carr	protein-co
lib-C666-l	chr3	39425280	39425280	+	2.10911	NA	exon (NM_017875, exon 1 of 7)	exon (NM_017875, exon 1 of 7)	465	NM_01787	54977	Hs.369615	NM_01787	ENSG0000	SLC25A38	SIDBA2	solute carr	protein-co
lib-C666-l	chr15	63793890	63793890	+	5.65398	NA	Intergenic	Intergenic	-2820	NM_00125	9960	Hs.458499	NM_00653	ENSG0000	USP3	SIH003|UB	ubiquitin s	protein-co
lib-C666-l	chr1	24070426	24070426	+	4.19675	NA	intron (NM_003198, intron 1 of 10)	CpG	570	NM_00319	6924	Hs.15535	NM_00319	ENSG0000	ELOA	SIII|SIII p11	elongin A	protein-co
lib-C666-l	chr8	74884579	74884579	+	52.95013	NA	promoter-TSS (NM_005648)	promoter-TSS (NM_005648)	-57	NM_00120	6921	Hs.533437	NM_00564	ENSG0000	ELOC	SIII|TCEB1	elongin C	protein-co
lib-C666-l	chr16	2827395	2827395	+	7.21981	NA	promoter-TSS (NM_007108)	promoter-TSS (NM_007108)	-98	NM_00710	6923	Hs.172772	NM_00710	ENSG0000	ELOB	SIII|TCEB2	elongin B	protein-co
lib-C666-l	chr1	115323288	115323288	+	60.20627	NA	promoter-TSS (NR_049742)	promoter-TSS (NR_049742)	20	NR_04974	80143	Hs.709277	NM_02507	ENSG0000	SIKE1	SIKE	suppressor	protein-co
lib-C666-l	chr19	45582447	45582447	+	2.17032	NA	promoter-TSS (NM_001319055)	promoter-TSS (NM_001319055)	-6	NM_00131	79760	Hs.466919	NM_02470	ENSG0000	GEMIN7	SIP3	gem nucle	protein-co
lib-C666-l	chr12	120729941	120729941	+	55.66538	NA	Intergenic	Intergenic	-10183	NM_01224	23409	Hs.50861	NM_01224	ENSG0000	SIRT4	SIR2L4	sirtuin 4	protein-co
lib-C666-l	chr12	120731104	120731104	+	4.83287	NA	Intergenic	Intergenic	-9020	NM_01224	23409	Hs.50861	NM_01224	ENSG0000	SIRT4	SIR2L4	sirtuin 4	protein-co
lib-C666-l	chr19	4182518	4182518	+	3.8557	NA	promoter-TSS (NM_001193285)	promoter-TSS (NM_001193285)	83	NM_00132	51548	Hs.423756	NM_01653	ENSG0000	SIRT6	SIR2L6	sirtuin 6	protein-co
lib-C666-l	chr19	4182348	4182348	+	2.65152	NA	promoter-TSS (NM_001321064)	promoter-TSS (NM_001321064)	-45	NM_00132	51548	Hs.423756	NM_01653	ENSG0000	SIRT6	SIR2L6	sirtuin 6	protein-co
lib-C666-l	chr20	1306371	1306371	+	13.59977	NA	non-coding (NR_040047, exon 1 of 3	non-coding (NR_040047, exon 1 of	142	NM_00119	27111	Hs.657015	NM_01568	ENSG0000	SDCBP2	SITAC|SITA	syndecan b	protein-co
lib-C666-l	chr19	11639821	11639821	+	5.04179	NA	promoter-TSS (NM_001243204)	promoter-TSS (NM_001243204)	-141	NM_00124	51295	Hs.515146	NM_01658	ENSG0000	ECSIT	SITPEC	ECSIT signa	protein-co
lib-C666-l	chr19	49122539	49122539	+	209.59317	NA	promoter-TSS (NM_020126)	promoter-TSS (NM_020126)	-9	NM_00124	56848	Hs.528006	NM_02012	ENSG0000	SPHK2	SK 2|SK-2|	sphingosin	protein-co
lib-C666-l	chr16	69345350	69345350	+	37.12814	NA	promoter-TSS (NM_013245)	promoter-TSS (NM_013245)	63	NM_01324	27183	Hs.128420	NM_01324	ENSG0000	VPS4A	SKD1|SKD1	vacuolar p	protein-co
lib-C666-l	chr17	44667910	44667910	+	6.71839	NA	promoter-TSS (NR_033799).2	promoter-TSS (NR_033799).2	-125	NM_00617	4905	Hs.431279	NM_00617	ENSG0000	NSF	SKD2	N-ethylma	protein-co
lib-C666-l	chr1	16011360	16011360	+	5.04179	NA	intron (NM_015164, intron 1 of 19)	CpG	533	NM_01516	23207	Hs.646775	NM_01516	ENSG0000	PLEKHM2	SKIP	pleckstrin h	protein-co
lib-C666-l	chr10	24772808	24772808	+	5.5166	NA	intron (NM_001282768, intron 6 of 17	intron (NM_001282768, intron 6 of	17374	NM_00128	56243	Hs.445885	NM_01959	ENSG0000	KIAA1217	SKT	KIAA1217	protein-co
lib-C666-l	chr11	102629196	102629196	+	5.92363	NA	Intergenic	Intergenic	22163	NM_00242	4319	Hs.2258	NM_00242	ENSG0000	MMP10	SL-2|STMY	matrix met	protein-co
lib-C666-l	chr5	74199328	74199328	+	8.28333	NA	Intergenic	MER74A|LTR|ERVL	-36665	NM_01556	26049	Hs.91662	NM_01556	ENSG0000	FAM169A	SLAP75	family with	protein-co
lib-C666-l	chr1	1623232	1623232	+	14.98892	NA	intron (NM_001290264, intron 1 of 9)	intron (NM_001290264, intron 1 of	1011	NM_00129	728661	Hs.655255	NM_00111	ENSG0000	SLC35E2B	SLC35E2	solute carr	protein-co
lib-C666-l	chr1	1624187	1624187	+	12.4773	NA	promoter-TSS (NM_001290264)	promoter-TSS (NM_001290264)	56	NM_00129	728661	Hs.655255	NM_00111	ENSG0000	SLC35E2B	SLC35E2	solute carr	protein-co
lib-C666-l	chr1	1624904	1624904	+	6.71839	NA	promoter-TSS (NM_001290264)	promoter-TSS (NM_001290264)	-661	NM_00111	728661	Hs.655255	NM_00111	ENSG0000	SLC35E2B	SLC35E2	solute carr	protein-co
lib-C666-l	chr3	136580974	136580974	+	14.18438	NA	promoter-TSS (NR_110175)	promoter-TSS (NR_110175)	-69	NR_11017	101927597	Hs.559587	NR_110175		NCK1-AS1	SLC35G2-A	NCK1 antis	ncRNA
lib-C666-l	chr3	125803087	125803087	+	3.76263	NA	promoter-TSS (NM_001008486)	promoter-TSS (NM_001008486)	47	NM_00100	54946	Hs.573007	NM_01783	ENSG0000	SLC41A3	SLC41A1-L	solute carr	protein-co
lib-C666-l	chr8	41386539	41386539	+	105.11623	NA	promoter-TSS (NM_032336)	promoter-TSS (NM_032336)	-186	NM_03233	84296	Hs.656996	NM_03233	ENSG0000	GINS4	SLD5	GINS comp	protein-co
lib-C666-l	chr7	128578289	128578289	+	10.47352	NA	promoter-TSS (NM_032643)	promoter-TSS (NM_032643)	18	NM_03264	3663	Hs.521181	NM_03264	ENSG0000	IRF5	SLEB10	interferon	protein-co
lib-C666-l	chr7	129710168	129710168	+	19.01614	NA	promoter-TSS (NM_014997)	promoter-TSS (NM_014997)	-181	NM_01499	23008	Hs.520710	NM_01499	ENSG0000	KLHDC10	slim	kelch dom	protein-co
lib-C666-l	chr11	71159511	71159511	+	48.61959	NA	promoter-TSS (NM_001360)	promoter-TSS (NM_001360)	-34	NM_00136	1717	Hs.503134	NM_00136	ENSG0000	DHCR7	SLOS	7-dehydro	protein-co
lib-C666-l	chr15	74284687	74284687	+	105.64542	NA	promoter-TSS (NM_004809)	promoter-TSS (NM_004809)	2	NM_00125	9399	Hs.194816	NM_00480	ENSG0000	STOML1	SLP-1|STO	stomatin li	protein-co
lib-C666-l	chr6	159125514	159125514	+	5.25516	NA	intron (NM_001318745, intron 3 of 14	intron (NM_001318745, intron 3 of	41079	NM_00131	94120	Hs.436977	NM_00100	ENSG0000	SYTL3	SLP3	synaptotag	protein-co
lib-C666-l	chr8	49838580	49838580	+	6.32351	NA	Intergenic	Intergenic	-4581	NM_00306	6591	Hs.360174	NM_00306	ENSG0000	SNAI2	SLUG|SLUG	snail family	protein-co
lib-C666-l	chr9	95527304	95527304	+	48.61959	NA	promoter-TSS (NM_001003800)	promoter-TSS (NM_001003800)	-221	NM_00100	23299	Hs.436939	NM_01525	ENSG0000	BICD2	SMALED2|I	BICD cargo	protein-co
lib-C666-l	chr1	40840309	40840309	+	11.13288	NA	promoter-TSS (NM_001198978)	promoter-TSS (NM_001198978)	4	NM_00119	64744	Hs.15200	NM_02273	ENSG0000	SMAP2	SMAP1L	small ArfG	protein-co
lib-C666-l	chr4	140375064	140375064	+	2.9164	NA	5' UTR (NM_031296, exon 1 of 2)	5' UTR (NM_031296, exon 1 of 2)	103	NM_03129	83452	Hs.591679	NM_03129	ENSG0000	RAB33B	SMC2	RAB33B, m	protein-co
lib-C666-l	chr9	72873839	72873839	+	15.31489	NA	promoter-TSS (NR_039990)	promoter-TSS (NR_039990)	-39	NM_01511	23137	Hs.534189	NM_01511	ENSG0000	SMC5	SMC5L1	structural r	protein-co
lib-C666-l	chr9	72874305	72874305	+	4.46583	NA	promoter-TSS (NR_039990)	promoter-TSS (NR_039990)	427	NM_01511	23137	Hs.534189	NM_01511	ENSG0000	SMC5	SMC5L1	structural r	protein-co
lib-C666-l	chr17	17585843	17585843	+	19.65763	NA	intron (NM_030665, intron 1 of 5)	CpG	1056	NM_03066	10743	Hs.655395	NM_03066	ENSG0000	RAI1	SMCR|SMS	retinoic aci	protein-co
lib-C666-l	chr17	17586413	17586413	+	13.0716	NA	intron (NM_030665, intron 1 of 5)	intron (NM_030665, intron 1 of 5)	1626	NM_03066	10743	Hs.655395	NM_03066	ENSG0000	RAI1	SMCR|SMS	retinoic aci	protein-co
lib-C666-l	chr19	46195523	46195523	+	7.03048	NA	promoter-TSS (NM_001163377)	promoter-TSS (NM_001163377)	51	NM_00459	6633	Hs.515472	NM_00459	ENSG0000	SNRPD2	SMD2|SNR	small nucle	protein-co
lib-C666-l	chr2	70520905	70520905	+	9.20633	NA	promoter-TSS (NM_001317165)	promoter-TSS (NM_001317165)	-2	NM_00131	6637	Hs.465167	NM_00309	ENSG0000	SNRPG	SMG|Sm-G	small nucle	protein-co
lib-C666-l	chr19	39833015	39833015	+	36.16835	NA	promoter-TSS (NM_001303614)	promoter-TSS (NM_001303614)	-84	NM_00130	55095	Hs.612332	NM_01802	ENSG0000	SAMD4B	SMGB|Sma	sterile alph	protein-co
lib-C666-l	chr10	112064809	112064809	+	13.20416	NA	promoter-TSS (NM_005871)	promoter-TSS (NM_005871)	-102	NM_00587	10285	Hs.632093	NM_00587	ENSG0000	SMNDC1	SMNR|SPF	survival mo	protein-co
lib-C666-l	chr10	112064276	112064276	+	8.89416	NA	intron (NM_005871, intron 1 of 5)	CpG	431	NM_00587	10285	Hs.632093	NM_00587	ENSG0000	SMNDC1	SMNR|SPF	survival mo	protein-co
lib-C666-l	chr10	112155767	112155767	+	7.43576	NA	Intergenic	Intergenic	-91060	NM_00587	10285	Hs.632093	NM_00587	ENSG0000	SMNDC1	SMNR|SPF	survival mo	protein-co
lib-C666-l	chr10	112085006	112085006	+	5.80117	NA	Intergenic	Intergenic	-20299	NM_00587	10285	Hs.632093	NM_00587	ENSG0000	SMNDC1	SMNR|SPF	survival mo	protein-co
lib-C666-l	chr4	108746071	108746071	+	8.56376	NA	intron (NM_001136258, intron 1 of 6)	CpG	350	NM_00113	166929	Hs.595423	NM_15262	ENSG0000	SGMS2	SMS2	sphingomy	protein-co
lib-C666-l	chr2	27435135	27435135	+	12.12723	NA	promoter-TSS (NM_021095)	promoter-TSS (NM_021095)	40	NR_02832	8884	Hs.435735	NM_02109	ENSG0000	SLC5A6	SMVT	solute carr	protein-co
lib-C666-l	chr15	66790089	66790089	+	97.61671	NA	promoter-TSS (NM_001329615)	promoter-TSS (NM_001329615)	64	NM_00604	10302	Hs.30174	NM_00604	ENSG0000	SNAPC5	SNAP19	small nucle	protein-co
lib-C666-l	chr19	48018593	48018593	+	46.4068	NA	promoter-TSS (NR_038457)	promoter-TSS (NR_038457)	-78	NM_00382	8775	Hs.126938	NM_00382	ENSG0000	NAPA	SNAPA	NSF attach	protein-co
lib-C666-l	chr3	190629388	190629388	+	4.46583	NA	Intergenic	Intergenic	33669	NR_02434	100170222	Hs.717310	NR_024343		SNAR-I	snaR-3	small ILF3/	snRNA
lib-C666-l	chr19	48437396	48437396	+	41.78738	NA	promoter-TSS (NR_004435).2	promoter-TSS (NR_004435).2	-42	NR_00443	100126799	Hs.723085	NR_004436		SNAR-A2	SNAR-A53	small ILF3/	snRNA
lib-C666-l	chr19	48421675	48421675	+	28.36893	NA	promoter-TSS (NR_004435)	promoter-TSS (NR_004435)	-11	NR_00443	100126799	Hs.723085	NR_004436		SNAR-A2	SNAR-A53	small ILF3/	snRNA
lib-C666-l	chr8	82644618	82644618	+	3.25502	NA	promoter-TSS (NM_152284)	promoter-TSS (NM_152284)	-70	NM_15228	92421	Hs.183861	NM_15228	ENSG0000	CHMP4C	SNF7-3|Sh	charged m	protein-co
lib-C666-l	chr3	170075437	170075437	+	43.7015	NA	promoter-TSS (NM_005414)	promoter-TSS (NM_005414)	-36	NM_00541	6498	Hs.536655	NM_00541	ENSG0000	SKIL	SNO|SnoA	SKI like pro	protein-co
lib-C666-l	chr3	170074776	170074776	+	6.3317	NA	promoter-TSS (NM_005414)	promoter-TSS (NM_005414)	-697	NM_00541	6498	Hs.536655	NM_00541	ENSG0000	SKIL	SNO|SnoA	SKI like pro	protein-co
lib-C666-l	chr22	30162985	30162985	+	4.71278	NA	promoter-TSS (NM_019103)	promoter-TSS (NM_019103)	15	NM_00131	55954	Hs.713647	NM_01910	ENSG0000	ZMAT5	SNRNP20|U	zinc finger	protein-co
lib-C666-l	chr14	55493882	55493882	+	134.48051	NA	promoter-TSS (NM_080867)	promoter-TSS (NM_080867)	38	NM_08086	122809	Hs.744302	NM_08086	ENSG0000	SOCS4	SOCS7	suppressor	protein-co
lib-C666-l	chr2	242448309	242448309	+	16.19847	NA	promoter-TSS (NM_001271979)	promoter-TSS (NM_001271979)	-199	NM_00637	10494	Hs.516807	NM_00637	ENSG0000	STK25	SOK1|YSK1	serine/thre	protein-co
lib-C666-l	chr2	180104208	180104208	+	6.3317	NA	intron (NM_178123, intron 1 of 17)	intron (NM_178123, intron 1 of 17)	25142	NM_17812	91404	Hs.30977	NM_17812	ENSG0000	SESTD1	SOLO	SEC14 and	protein-co
lib-C666-l	chr1	24648374	24648374	+	41.10975	NA	intron (NM_001195010, intron 1 of 15	CpG	-1156	NM_02118	57822	Hs.657920	NM_02118	ENSG0000	GRHL3	SOM|TFCP	grainyhead	protein-co
lib-C666-l	chr1	24648767	24648767	+	15.31489	NA	promoter-TSS (NM_021180)	promoter-TSS (NM_021180)	-763	NM_02118	57822	Hs.657920	NM_02118	ENSG0000	GRHL3	SOM|TFCP	grainyhead	protein-co
lib-C666-l	chr1	24610627	24610627	+	9.80074	NA	Intergenic	Intergenic	-35185	NM_19817	57822	Hs.657920	NM_02118	ENSG0000	GRHL3	SOM|TFCP	grainyhead	protein-co
lib-C666-l	chr11	64851652	64851652	+	270.59946	NA	promoter-TSS (NM_080668)	promoter-TSS (NM_080668)	-37	NM_08066	113130	Hs.434886	NM_08066	ENSG0000	CDCA5	SORORIN	cell divisior	protein-co
lib-C666-l	chr20	307018	307018	+	6.48676	NA	exon (NM_006943, exon 1 of 1)	exon (NM_006943, exon 1 of 1)	803	NM_00694	6666	Hs.43627	NM_00694	ENSG0000	SOX12	SOX22	SRY-box 1	protein-co
lib-C666-l	chr3	137483058	137483058	+	9.79436	NA	promoter-TSS (NM_004189)	promoter-TSS (NM_004189)	-76	NM_00418	8403	Hs.248184	NM_00418	ENSG0000	SOX14	SOX28	SRY-box 1	protein-co
lib-C666-l	chr1	46152872	46152872	+	15.75122	NA	promoter-TSS (NM_016486)	promoter-TSS (NM_016486)	-570	NM_02163	60313	Hs.238432	NM_02163	ENSG0000	GPBP1L1	SP192	GC-rich pr	protein-co
lib-C666-l	chr12	63814189	63814189	+	38.10056	NA	Intergenic	LTR55|LTR|ERV	248165	NM_17381	283417	Hs.533644	NM_17381	ENSG0000	DPY19L2	SPATA34|S	dpy-19 like	protein-co
lib-C666-l	chr12	6579918	6579918	+	7.52748	NA	non-coding (NR_123717, exon 1 of 4	non-coding (NR_123717, exon 1 of	147	NM_00129	6843	Hs.20021	NM_01423	ENSG0000	VAMP1	SPAX1|SYB	vesicle ass	protein-co
lib-C666-l	chr12	6580202	6580202	+	4.38153	NA	promoter-TSS (NR_123717)	promoter-TSS (NR_123717)	-137	NR_12371	6843	Hs.20021	NM_01423	ENSG0000	VAMP1	SPAX1|SYB	vesicle ass	protein-co
lib-C666-l	chr4	177116574	177116574	+	7.0832	NA	exon (NM_144644, exon 1 of 6)	exon (NM_144644, exon 1 of 6)	248	NM_14464	132851	Hs.481235	NM_14464	ENSG0000	SPATA4	SPEF1B|TS	spermatog	protein-co
lib-C666-l	chr5	32444974	32444974	+	8.56376	NA	promoter-TSS (NM_016107)	promoter-TSS (NM_016107)	-107	NM_01610	51663	Hs.435231	NM_01610	ENSG0000	ZFR	SPG71|ZFR	zinc finger	protein-co
lib-C666-l	chr18	23806339	23806339	+	51.11391	NA	promoter-TSS (NM_001293725)	promoter-TSS (NM_001293725)	-508	NM_00564	6875	Hs.369519	NM_00564	ENSG0000	TAF4B	SPGF13|TA	TATA-box	protein-co
lib-C666-l	chr17	74382077	74382077	+	48.20053	NA	exon (NM_021972, exon 3 of 6)	exon (NM_021972, exon 3 of 6)	788	NM_00114	8877	Hs.68061	NM_02197	ENSG0000	SPHK1	SPHK	sphingosin	protein-co
lib-C666-l	chr19	50923079	50923079	+	6.38282	NA	intron (NM_003121, intron 1 of 5)	intron (NM_003121, intron 1 of 5)	884	NM_00312	6689	Hs.437905	NM_00312	ENSG0000	SPIB	SPI-B	Spi-B trans	protein-co
lib-C666-l	chr19	50920452	50920452	+	5.03541	NA	exon (NM_001308632, exon 25 of 26)	exon (NM_001308632, exon 25 of 2	-1743	NM_00124	6689	Hs.437905	NM_00312	ENSG0000	SPIB	SPI-B	Spi-B trans	protein-co
lib-C666-l	chr19	50920835	50920835	+	3.41241	NA	intron (NR_046402, intron 25 of 25)	intron (NR_046402, intron 25 of 25)	-1360	NM_00124	6689	Hs.437905	NM_00312	ENSG0000	SPIB	SPI-B	Spi-B trans	protein-co

lib-C666-l	chr16	89895109	89895109	+	53.07762	NA	exon (NM_032451, exon 1 of 15)	exon (NM_032451, exon 1 of 15)	202	NM_03245	84501	Hs.461786	NM_03245	ENSG0000	SPIRE2	Spir-2	spire type a	protein-co
lib-C666-l	chr19	46365862	46365862	+	7.15277	NA	intron (NM_004819, intron 1 of 26)	L2b|LINE|L2	686	NM_00481	8189	Hs.515475	NM_00481	ENSG0000	SYMPK	SPK|SYM	symplekin	protein-co
lib-C666-l	chr6	105307679	105307679	+	5.5166	NA	promoter-TSS (NM_001321083)	promoter-TSS (NM_001321083)	115	NM_00132	57531	Hs.434340	NM_02077	ENSG0000	HACE1	SPPRS	HECT dom	protein-co
lib-C666-l	chr2	65659752	65659752	+	18.56491	NA	promoter-TSS (NM_181784)	promoter-TSS (NM_181784)	-96	NM_18178	200734	Hs.59332	NM_18178	ENSG0000	SPRED2	Spred-2	sprouty rel	protein-co
lib-C666-l	chr2	65758274	65758274	+	11.06679	NA	Intergenic	MER1B|DNA|hAT-Charlie	-98618	NM_18178	200734	Hs.59332	NM_18178	ENSG0000	SPRED2	Spred-2	sprouty rel	protein-co
lib-C666-l	chr2	65662997	65662997	+	8.85547	NA	Intergenic	CpG	-3341	NM_18178	200734	Hs.59332	NM_18178	ENSG0000	SPRED2	Spred-2	sprouty rel	protein-co
lib-C666-l	chrY	58989861	58989861	+	23.23604	NA	Intergenic	BSR/Beta|Satellite|Satellite	-110542	NM_00584	10251	Hs.381912	NM_00584	ENSG0000	SPRY3	spry-3	sprouty RT	protein-co
lib-C666-l	chrY	58985199	58985199	+	8.55484	NA	Intergenic	BSR/Beta|Satellite|Satellite	-115204	NM_00584	10251	Hs.381912	NM_00584	ENSG0000	SPRY3	spry-3	sprouty RT	protein-co
lib-C666-l	chr16	30456794	30456794	+	23.6003	NA	exon (NM_012248, exon 1 of 1)	exon (NM_012248, exon 1 of 1)	502	NM_01224	22928	Hs.118725	NM_01224	ENSG0000	SEPHS2	SPS2|SPS2l	selenopho	protein-co
lib-C666-l	chr16	30457275	30457275	+	7.49163	NA	promoter-TSS (NM_012248)	promoter-TSS (NM_012248)	21	NM_01224	22928	Hs.118725	NM_01224	ENSG0000	SEPHS2	SPS2|SPS2l	selenopho	protein-co
lib-C666-l	chr11	18655986	18655986	+	108.81936	NA	promoter-TSS (NM_194285)	promoter-TSS (NM_194285)	34	NM_19428	144108	Hs.738650	NM_19428	ENSG0000	SPTY2D1	Spt2	SPT2 chror	protein-co
lib-C666-l	chr17	26989266	26989266	+	44.91887	NA	promoter-TSS (NM_003170)	promoter-TSS (NM_003170)	50	NM_00317	6830	Hs.250429	NM_00317	ENSG0000	SUPT6H	SPT6|SPT6	SPT6 hom	protein-co
lib-C666-l	chr2	27886668	27886668	+	5.48804	NA	promoter-TSS (NM_001282729)	promoter-TSS (NM_001282729)	39	NM_00128	9913	Hs.6232	NM_01486	ENSG0000	SUPT7L	SPT7L|STA	SPT7-like S	protein-co
lib-C666-l	chr5	139937147	139937147	+	25.61513	NA	promoter-TSS (NR_045587)	promoter-TSS (NR_045587)	-106	NR_04558	10011	Hs.653135	NM_00103	ENSG0000	SRA1	SRA|SRAP|	steroid rec	protein-co
lib-C666-l	chr19	50145193	50145193	+	66.80925	NA	promoter-TSS (NM_021228)	promoter-TSS (NM_021228)	-189	NM_02122	58506	Hs.103521	NM_02122	ENSG0000	SCAF1	SRA1	SR-related	protein-co
lib-C666-l	chr12	27175478	27175478	+	8.89416	NA	promoter-TSS (NM_001271811)	promoter-TSS (NM_001271811)	23	NM_00127	9412	Hs.286145	NM_00426	ENSG0000	MED21	SRB7|SURE	mediator c	protein-co
lib-C666-l	chr1	27977371	27977371	+	25.32183	NA	Intergenic	LTR16E1|LTR|ERVL	-15644	NM_00524	2268	Hs.1422	NM_00524	ENSG0000	FGR	SRC2|c-fgr	FGR proto-	protein-co
lib-C666-l	chr17	17743544	17743544	+	36.56565	NA	Intergenic	CpG	-3219	NM_00417	6720	Hs.592123	NM_00417	ENSG0000	SREBF1	SREBP-1c|	sterol regu	protein-co
lib-C666-l	chr17	17753518	17753518	+	29.50576	NA	intron (NM_001288788, intron 10 of 1	intron (NM_001288788, intron 10 o	-13193	NM_00417	6720	Hs.592123	NM_00417	ENSG0000	SREBF1	SREBP-1c|	sterol regu	protein-co
lib-C666-l	chr17	17739688	17739688	+	22.90968	NA	intron (NM_001005291, intron 1 of 1	CpG	637	NM_00100	6720	Hs.592123	NM_00417	ENSG0000	SREBF1	SREBP-1c|	sterol regu	protein-co
lib-C666-l	chr17	17788484	17788484	+	5.61899	NA	intron (NM_001033551, intron 3 of 1	intron (NM_001033551, intron 3 of	-48159	NM_00417	6720	Hs.592123	NM_00417	ENSG0000	SREBF1	SREBP-1c|	sterol regu	protein-co
lib-C666-l	chr22	42228965	42228965	+	32.07197	NA	promoter-TSS (NR_103834)	promoter-TSS (NR_103834)	-118	NM_00459	6721	Hs.443258	NM_00459	ENSG0000	SREBF2	SREBP-2|b	sterol regu	protein-co
lib-C666-l	chr17	1546916	1546916	+	9.7234	NA	non-coding (NR_028075, exon 4 of 1	non-coding (NR_028075, exon 4 of	2167	NM_00369	8578	Hs.647430	NM_00369	ENSG0000	SCARF1	SREC|SREC	scavenger	protein-co
lib-C666-l	chr17	1546287	1546287	+	8.51971	NA	intron (NM_003693, intron 4 of 10)	AluSx|SINE|Alu	2796	NM_00369	8578	Hs.647430	NM_00369	ENSG0000	SCARF1	SREC|SREC	scavenger	protein-co
lib-C666-l	chr3	57102976	57102976	+	5.5166	NA	intron (NM_181727, intron 1 of 1)	AluSg7|SINE|Alu	8507	NM_18172	353324	Hs.129794	NM_18172	ENSG0000	SPATA12	SRG5	spermatog	protein-co
lib-C666-l	chr17	7531201	7531201	+	101.00147	NA	promoter-TSS (NM_001289113)	promoter-TSS (NM_001289113)	-7	NM_00132	112483	Hs.10846	NM_13349	ENSG0000	SAT2	SSAT2	spermidine	protein-co
lib-C666-l	chr1	9353684	9353684	+	5.25516	NA	intron (NM_025106, intron 1 of 2)	GC_rich|Low_complexity|Low_comp	743	NM_02510	80176	Hs.8261	NM_02510	ENSG0000	SPSB1	SSB-1|SSB	spIA/ryano	protein-co
lib-C666-l	chr3	140819124	140819124	+	31.27173	NA	intron (NM_080862, intron 2 of 2)	MLT1C|LTR|ERVL-MaLR	48880	NM_08086	92369	Hs.655112	NM_08086	ENSG0000	SPSB4	SSB-4|SSB	spIA/ryano	protein-co
lib-C666-l	chr17	28071827	28071827	+	11.6145	NA	intron (NM_001282130, intron 2 of 7)	intron (NM_001282130, intron 2 of	16623	NM_00128	85464	Hs.654754	NM_03338	ENSG0000	SSH2	SSH-2|SSH	slingshot p	protein-co
lib-C666-l	chr17	28257890	28257890	+	5.65398	NA	promoter-TSS (NM_033389)	promoter-TSS (NM_033389)	-644	NM_00128	85464	Hs.654754	NM_03338	ENSG0000	SSH2	SSH-2|SSH	slingshot p	protein-co
lib-C666-l	chr7	54826938	54826938	+	3.84463	NA	promoter-TSS (NM_001012456)	promoter-TSS (NM_001012456)	1	NM_00101	23480	Hs.488282	NM_01430	ENSG0000	SEC61G	SSS1	Sec61 tran	protein-co
lib-C666-l	chr15	73655349	73655349	+	2.24164	NA	intron (NM_005477, intron 1 of 7)	intron (NM_005477, intron 1 of 7)	6256	NM_00547	10021	Hs.86941	NM_00547	ENSG0000	HCN4	SSS2	hyperpolar	protein-co
lib-C666-l	chr8	105601519	105601519	+	7.15277	NA	promoter-TSS (NM_001135703)	promoter-TSS (NM_001135703)	-267	NM_01343	29967	Hs.600630	NM_01343	ENSG0000	LRP12	ST7	LDL recept	protein-co
lib-C666-l	chr7	116594393	116594393	+	64.34737	NA	promoter-TSS (NR_002330)	promoter-TSS (NR_002330)	-5	NR_00233	93653	Hs.597516	NR_00233	ENSG0000	ST7-AS1	ST7AS1|ST	ST7 antiser	ncRNA
lib-C666-l	chr20	56293661	56293661	+	78.65518	NA	Intergenic	CpG	-7069	NM_19917	56937	Hs.517155	NM_02018	ENSG0000	PMEPA1	STAG1|TM	prostate tr	protein-co
lib-C666-l	chr20	56285088	56285088	+	20.90919	NA	promoter-TSS (NR_131157)	promoter-TSS (NR_131157)	-57	NM_02018	56937	Hs.517155	NM_02018	ENSG0000	PMEPA1	STAG1|TM	prostate tr	protein-co
lib-C666-l	chr20	56347720	56347720	+	16.264	NA	Intergenic	Intergenic	-61128	NM_19917	56937	Hs.517155	NM_02018	ENSG0000	PMEPA1	STAG1|TM	prostate tr	protein-co
lib-C666-l	chr20	56286721	56286721	+	15.86559	NA	promoter-TSS (NM_199171)	promoter-TSS (NM_199171)	-129	NM_19917	56937	Hs.517155	NM_02018	ENSG0000	PMEPA1	STAG1|TM	prostate tr	protein-co
lib-C666-l	chr7	72476510	72476510	+	91.93295	NA	promoter-TSS (NR_040583)	promoter-TSS (NR_040583)	-44	NR_04058	442578	Hs.632310	NM_001013739		STAG3L3	STAG3L1|	stromal an	pseudo
lib-C666-l	chr7	74306784	74306784	+	32.56643	NA	promoter-TSS (NR_040584)	promoter-TSS (NR_040584)	-53	NR_04058	442582	Hs.632310	NM_001025202		STAG3L2	STAG3L1|	stromal an	pseudo
lib-C666-l	chr7	74988377	74988377	+	80.42115	NA	promoter-TSS (NR_027776).3	promoter-TSS (NR_027776).3	-70	NR_04058	54441	Hs.632310	NM_018991		STAG3L1	STAG3L1P	stromal an	pseudo
lib-C666-l	chr7	66767563	66767563	+	51.39834	NA	promoter-TSS (NR_046297)	promoter-TSS (NR_046297)	-62	NR_04058	64940	Hs.632013	NM_02290	ENSG0000	STAG3L4	STAG3L4P	stromal an	pseudo
lib-C666-l	chr17	40428006	40428006	+	12.7379	NA	intron (NM_012448, intron 1 of 18)	CpG	418	NM_01244	6777	Hs.595276	NM_01244	ENSG0000	STAT5B	STAT5	signal tran	protein-co
lib-C666-l	chr5	172755575	172755575	+	4.7846	NA	5' UTR (NM_003714, exon 1 of 4)	5' UTR (NM_003714, exon 1 of 4)	931	NM_00371	8614	Hs.233160	NM_00371	ENSG0000	STC2	STC-2|STC	stanniocalc	protein-co
lib-C666-l	chr21	15755545	15755545	+	19.38538	NA	promoter-TSS (NM_006948)	promoter-TSS (NM_006948)	-36	NM_00694	6782	Hs.352341	NM_00694	ENSG0000	HSPA13	STCH	heat shock	protein-co
lib-C666-l	chr3	52931586	52931586	+	5.23609	NA	promoter-TSS (NM_001198974)	promoter-TSS (NM_001198974)	11	NM_19856	375346	Hs.744189	NM_19856	ENSG0000	TMEM110	STIMATE	transmemb	protein-co
lib-C666-l	chr1	28099582	28099582	+	20.5987	NA	promoter-TSS (NM_177424)	promoter-TSS (NM_177424)	-112	NM_17742	23673	Hs.523855	NM_17742	ENSG0000	STX12	STX13|STX	syntaxin 12	protein-co
lib-C666-l	chr16	31044700	31044700	+	259.17731	NA	promoter-TSS (NM_004604)	promoter-TSS (NM_004604)	-112	NM_00460	6810	Hs.83734	NM_00460	ENSG0000	STX4	STX4A|p35	syntaxin 4	protein-co
lib-C666-l	chr4	54232252	54232252	+	121.67265	NA	promoter-TSS (NM_152540)	promoter-TSS (NM_152540)	-10	NM_15254	152579	Hs.302287	NM_15254	ENSG0000	SCFD2	STXBP1L1	sec1 family	protein-co
lib-C666-l	chr10	70939975	70939975	+	169.57268	NA	promoter-TSS (NM_001301683)	promoter-TSS (NM_001301683)	15	NM_00132	6832	Hs.106469	NM_00317	ENSG0000	SUPV3L1	SUV3	Suv3 like R	protein-co
lib-C666-l	chr10	70939443	70939443	+	5.76059	NA	promoter-TSS (NM_001301683)	promoter-TSS (NM_001301683)	-517	NM_00132	6832	Hs.106469	NM_00317	ENSG0000	SUPV3L1	SUV3	Suv3 like R	protein-co
lib-C666-l	chr19	55851088	55851088	+	46.59515	NA	promoter-TSS (NM_032701)	promoter-TSS (NM_032701)	-133	NM_03270	84787	Hs.590982	NM_03270	ENSG0000	KMT5C	SUV420H2	lysine meth	protein-co
lib-C666-l	chr17	29035874	29035874	+	8.71138	NA	promoter-TSS (NR_024187)	promoter-TSS (NR_024187)	-752	NR_14439	440423	Hs.628886	NR_024187		SUZ12P1	SUZ12P	SUZ12 poly	pseudo
lib-C666-l	chr17	29035429	29035429	+	5.5166	NA	Intergenic	CpG	-1197	NR_14439	440423	Hs.628886	NR_024187		SUZ12P1	SUZ12P	SUZ12 poly	pseudo
lib-C666-l	chr17	25621065	25621065	+	44.1351	NA	promoter-TSS (NR_039748)	promoter-TSS (NR_039748)	-41	NM_01562	26118	Hs.446017	NM_01562	ENSG0000	WSB1	SWIP1|WSI	WD repeat	protein-co
lib-C666-l	chr17	25680840	25680840	+	15.52737	NA	Intergenic	CpG	59734	NM_13426	26118	Hs.446017	NM_01562	ENSG0000	WSB1	SWIP1|WSI	WD repeat	protein-co
lib-C666-l	chr17	25659738	25659738	+	11.25496	NA	Intergenic	Intergenic	38632	NM_13426	26118	Hs.446017	NM_01562	ENSG0000	WSB1	SWIP1|WSI	WD repeat	protein-co
lib-C666-l	chr17	15903000	15903000	+	231.57161	NA	promoter-TSS (NM_001042698)	promoter-TSS (NM_001042698)	6	NM_00104	125150	Hs.593985	NM_00104	ENSG0000	ZSWIM7	SWS1	zinc finger	protein-co
lib-C666-l	chr1	15656686	15656686	+	27.5466	NA	intron (NM_052929, intron 14 of 30)	L1MC4a|LINE|L1	-79705	NM_02432	79180	Hs.465374	NM_02432	ENSG0000	EFHD2	SWS1	EF-hand d	protein-co
lib-C666-l	chr17	15952636	15952636	+	5.96422	NA	intron (NM_006311, intron 40 of 45)	intron (NM_006311, intron 40 of 45	-49630	NM_00104	125150	Hs.593985	NM_00104	ENSG0000	ZSWIM7	SWS1	zinc finger	protein-co
lib-C666-l	chr1	15736286	15736286	+	4.04064	NA	promoter-TSS (NM_024329)	promoter-TSS (NM_024329)	-105	NM_02432	79180	Hs.465374	NM_02432	ENSG0000	EFHD2	SWS1	EF-hand d	protein-co
lib-C666-l	chrX	155111456	155111456	+	9.21057	NA	intron (NR_033714, intron 1 of 6)	intron (NR_033714, intron 1 of 6)	513	NM_00114	6845	Hs.24167	NM_00563	ENSG0000	VAMP7	SYBL1|TI-V	vesicle ass	protein-co
lib-C666-l	chrY	59213960	59213960	+	8.0787	NA	promoter-TSS (NR_033715).2	promoter-TSS (NR_033715).2	11	NR_03371	6845	Hs.24167	NM_00563	ENSG0000	VAMP7	SYBL1|TI-V	vesicle ass	protein-co
lib-C666-l	chrY	59214415	59214415	+	8.0787	NA	intron (NM_001145149, intron 1 of 6)	intron (NM_001145149, intron 1 of	466	NR_03371	6845	Hs.24167	NM_00563	ENSG0000	VAMP7	SYBL1|TI-V	vesicle ass	protein-co
lib-C666-l	chr1	109102514	109102514	+	5.25516	NA	promoter-TSS (NM_001010883)	promoter-TSS (NM_001010883)	-457	NM_00101	284611	Hs.200230	NM_00101	ENSG0000	FAM102B	SYM-3B	family with	protein-co
lib-C666-l	chr1	109102698	109102698	+	5.04179	NA	promoter-TSS (NM_001010883)	promoter-TSS (NM_001010883)	-273	NM_00101	284611	Hs.200230	NM_00101	ENSG0000	FAM102B	SYM-3B	family with	protein-co
lib-C666-l	chrX	47479382	47479382	+	13.20416	NA	promoter-TSS (NM_133499)	promoter-TSS (NM_133499)	-126	NM_00695	6853	Hs.225936	NM_00695	ENSG0000	SYN1	SYN1a|SYN	synapsin I	protein-co
lib-C666-l	chr20	43977298	43977298	+	10.73451	NA	promoter-TSS (NM_002999)	promoter-TSS (NM_002999)	-234	NM_00299	6385	Hs.632267	NM_00299	ENSG0000	SDC4	SYND4	syndecan 4	protein-co
lib-C666-l	chr5	121572130	121572130	+	13.8811	NA	Intergenic	Intergenic	-75256	NR_13176	9627	Hs.426463	NM_00546	ENSG0000	SNCAIP	SYPH1|Sph	synuclein a	protein-co
lib-C666-l	chr3	48282560	48282560	+	55.46257	NA	promoter-TSS (NM_016089)	promoter-TSS (NM_016089)	-36	NM_01608	51385	Hs.172602	NM_01608	ENSG0000	ZNF589	SZF1	zinc finger	protein-co
lib-C666-l	chr7	143131989	143131989	+	39.14433	NA	intron (NR_033897, intron 2 of 4)	intron (NR_033897, intron 2 of 4)	-8557	NM_17743	338398	Hs.688222	NM_17743	ENSG0000	TAS2R60	T2R56|T2R	taste 2 rec	protein-co
lib-C666-l	chr9	111342482	111342482	+	12.12504	NA	Intergenic	Intergenic	275793	NM_00668	10880	Hs.534390	NM_00668	ENSG0000	ACTL7B	Tact1	actin like 7	protein-co
lib-C666-l	chr1	109618654	109618654	+	20.19521	NA	promoter-TSS (NM_005645)	promoter-TSS (NM_005645)	-30	NM_00564	6884	Hs.632426	NM_00564	ENSG0000	TAF13	TAF(II)18|T	TATA-box	protein-co
lib-C666-l	chr9	32704499	32704499	+	6.44764	NA	Intergenic	Intergenic	-68832	NM_15380	138474	Hs.591086	NM_15380	ENSG0000	TAF1L	TAF(II)210|	TATA-box	protein-co
lib-C666-l	chr11	6633514	6633514	+	49.86005	NA	promoter-TSS (NM_006284)	promoter-TSS (NM_006284)	-39	NM_00628	6881	Hs.5158	NM_00628	ENSG0000	TAF10	TAF2A|TAF	TATA-box	protein-co
lib-C666-l	chr20	60663698	60663698	+	11.66012	NA	Intergenic	Tigger3b|DNA|TcMar-Tigger	-22832	NM_00318	6874	Hs.18857	NM_00318	ENSG0000	TAF4	TAF2C|TAF	TATA-box	protein-co
lib-C666-l	chr5	140700459	140700459	+	9.10494	NA	promoter-TSS (NM_005642)	promoter-TSS (NM_005642)	-108	NM_00564	6879	Hs.438838	NM_00564	ENSG0000	TAF7	TAF2F|TAF	TATA-box	protein-co
lib-C666-l	chr13	24153672	24153672	+	9.20967	NA	intron (NM_018647, intron 1 of 8)	CpG	173	NM_01864	55504	Hs.149168	NM_01864	ENSG0000	TNFRSF19	TAJ|TAJ-al	TNF recept	protein-co
lib-C666-l	chr1	89990475	89990475	+	107.27621	NA	promoter-TSS (NM_001134476)	promoter-TSS (NM_001134476)	78	NM_00113	23507	Hs.482017	NM_01535	ENSG0000	LRRC8B	TA-LRRP|T	leucine ric	protein-co
lib-C666-l	chr16	20753126	20753126	+	195.86848	NA	5' UTR (NM_001304550, exon 1 of 5)	5' UTR (NM_001304550, exon 1 of 5	160	NM_01773	55623	Hs.700004	NM_01773	ENSG0000	THUMPD1	Tan1	THUMP do	protein-co
lib-C666-l	chr7	65670131	65670131	+	22.66433	NA	promoter-TSS (NM_003596)	promoter-TSS (NM_003596)	-128	NM_00359	8460	Hs.421194	NM_00359	ENSG0000	TPST1	TANGO13a	tyrosylprot	protein-co
lib-C666-l	chr22	26986088	26986088	+	12.34228	NA	promoter-TSS (NM_003595)	promoter-TSS (NM_003595)	1	NM_00359	8459	Hs.632768	NM_00359	ENSG0000	TPST2	TANGO13b	tyrosylprot	protein-co
lib-C666-l	chr10	124096106	124096106	+	79.83466	NA	exon (NM_144587, exon 15 of 16)	exon (NM_144587, exon 15 of 16)	-37988	NM_00133	59338	Hs.643512	NM_02162	ENSG0000	PLEKHA1	TAPP1	pleckstrin	protein-co
lib-C666-l	chr10	124134078	124134078	+	45.88523	NA	promoter-TSS (NM_001001974)	promoter-TSS (NM_001001974)	-16	NM_00133	59338	Hs.643512	NM_02162	ENSG0000	PLEKHA1	TAPP1	pleckstrin	protein-co
lib-C666-l	chr10	124134744	124134744	+	2.94047	NA	intron (NM_001001974, intron 1 of 1	CpG	650	NM_00100	59338	Hs.643512	NM_02162	ENSG0000	PLEKHA1	TAPP1	pleckstrin	protein-co
lib-C666-l	chr10	124134507	124134507	+	2.24771	NA	intron (NM_001001974, intron 1 of 1	CpG	413	NM_00100	59338	Hs.643512	NM_02162	ENSG0000	PLEKHA1	TAPP1	pleckstrin	protein-co
lib-C666-l	chr12	49716934	49716934	+	11.22379	NA	promoter-TSS (NM_005480)	promoter-TSS (NM_005480)	-37	NM_00548	10024	Hs.524399	NM_00548	ENSG0000	TROAP	TASTIN	trophinin a	protein-co
lib-C666-l	chrX	135579453	135579453	+	64.17523	NA	promoter-TSS (NM_014500)	promoter-TSS (NM_014500)	215	NM_00116	27336	Hs.204475	NM_01450	ENSG0000	HTATSF1	TAT-SF1|T	HIV-1 Tat s	protein-co
lib-C666-l	chr3	14481273	14481273	+	6.15313	NA	intron (NM_001134367, intron 10 of 2	intron (NM_001134367, intron 10 o	37197	NR_10350	6533	Hs.529488	NM_00304	ENSG0000	SLC6A6	TAUT	solute carr	protein-co
lib-C666-l	chr4	38320648	38320648	+	29.98945	NA	Intergenic	Intergenic	199073	NM_00125	23216	Hs.176503	NM_01517	ENSG0000	TBC1D1	TBC|TBC1	TBC1 dom	protein-co
lib-C666-l	chr4	38190392	38190392	+	2.59032	NA	Intergenic	FordPrefect|DNA|hAT-Tip100	68817	NM_00125	23216	Hs.176503	NM_01517	ENSG0000	TBC1D1	TBC|TBC1	TBC1 dom	protein-co
lib-C666-l	chr13	114017855	114017855	+	34.78259	NA	intron (NM_024719, intron 2 of 7)	intron (NM_024719, intron 2 of 7)	608	NM_00128	79774	Hs.745043	NM_02471	ENSG0000	GRTP1	TBC1D6	growth hor	protein-co
lib-C666-l	chr11	47448052	47448052	+	3.27468	NA	promoter-TSS (NM_002804)	promoter-TSS (NM_002804)	-28	NM_00280	5702	Hs.250758	NM_00280	ENSG0000	PSMC3	TBP1	proteasom	protein-co
lib-C666-l	chr3	27722895	27722895	+	34.54802	NA	Intergenic	Intergenic	41311	NM_00127	8320	Hs.591663	NM_00544	ENSG0000	EOMES	TBR2	eomesode	protein-co
lib-C666-l	chr3	27664577	27664577	+	6.81003	NA	Intergenic	Intergenic	99629	NM_00127	8320	Hs.591663	NM_00544	ENSG0000	EOMES	TBR2	eomesode	protein-co
lib-C666-l	chr1	168256653	168256653	+	9.77951	NA	intron (NM_005149, intron 1 of 7)	L2a|LINE|L2	6375	NM_00514	9095	Hs.716656	NM_00514	ENSG0000	TBX19	TBS19|TPI	T-box 19	protein-co
lib-C666-l	chr12	115685887	115685887	+	6.38027	NA	Intergenic	Intergenic	-563918	NM_00599	6926	Hs.744016	NM_00599	ENSG0000	TBX3	TBX3-ISO|	T-box 3	protein-co
lib-C666-l	chr12	115821599	115821599	+	5.80117	NA	Intergenic	Intergenic	-699630	NM_00599	6926	Hs.744016	NM_00599	ENSG0000	TBX3	TBX3-ISO|	T-box 3	protein-co
lib-C666-l	chr19	23869222	23869222	+	8.51971	NA	intron (NM_138330, intron 1 of 3)	AluSq2|SINE|Alu	795	NM_13833	171392	Hs.264345	NM_13833	ENSG0000	ZNF675	TBZF|TIZ	zinc finger	protein-co
lib-C666-l	chr8	40016647	40016647	+	11.66438	NA	Intergenic	MIR3|SINE|MIR	5660	NM_02013	56892	Hs.591849	NM_02013	ENSG0000	C8orf4	TC-1|TC1	chromosor	protein-co
lib-C666-l	chr8	40010945	40010945	+	9.96422	NA	promoter-TSS (NM_020130)	promoter-TSS (NM_020130)	-42	NM_02013	56892	Hs.591849	NM_02013	ENSG0000	C8orf4	TC-1|TC1	chromosor	protein-co
lib-C666-l	chr8	39912464	39912464	+	7.7703	NA	Intergenic	Intergenic	-98523	NM_02013	56892	Hs.591849	NM_02013	ENSG0000	C8orf4	TC-1|TC1	chromosor	protein-co
lib-C666-l	chr8	39976688	39976688	+	4.49043	NA	Intergenic	Intergenic	-34299	NM_02013	56892	Hs.591849	NM_02013	ENSG0000	C8orf4	TC-1|TC1	chromosor	protein-co
lib-C666-l	chr1	169455315	169455315	+	16.14038	NA	promoter-TSS (NM_006996)	promoter-TSS (NM_006996)	-107	NM_00131	10560	Hs.30246	NM_00699	ENSG0000	SLC19A2	TC1|THMD	solute carr	protein-co
lib-C666-l	chr10	121356667	121356667	+	6.82479	NA	promoter-TSS (NM_001323969)	promoter-TSS (NM_001323969)	-126	NM_00132	7073	Hs.501203	NM_00325	ENSG0000	TIAL1	TCBP|TIAR	TIA1 cytot	protein-co
lib-C666-l	chr5	174969412	174969412	+	24.80022	NA	Intergenic	Intergenic	63911	NM_00132	94081	Hs.369440	NM_02275	ENSG0000	SFXN1	TCC	sideroflexir	protein-co
lib-C666-l	chr16	81478279	81478279	+	24.99834	NA	promoter-TSS (NM_198390)	promoter-TSS (NM_198390)	-496	NM_19839	80790	Hs.594095	NM_03062	ENSG0000	CMIP	TCMIP	c-Maf indu	protein-co
lib-C666-l	chr16	81478002	81478002	+	10.03103	NA	promoter-TSS (NM_198390)	promoter-TSS (NM_198390)	-773	NM_19839	80790	Hs.594095	NM_03062	ENSG0000	CMIP	TCMIP	c-Maf indu	protein-co
lib-C666-l	chr1	193467377	193467377	+	78.53495	NA	Intergenic	Intergenic	193502	NR_12578	101929184	Hs.146728	NR_12578	ENSG0000	LINC01031	TCONS_00	long interg	ncRNA
lib-C666-l	chr10	130010359	130010359	+	22.43333	NA	Intergenic	Intergenic	-73855	NR_12061	101927381	Hs.523342	NR_12061	ENSG0000	LINC01163	TCONS_00	long interg	ncRNA
lib-C666-l	chr10	109488833	109488833	+	6.98764	NA	Intergenic	Intergenic	340218	NR_12576	103695365	Hs.648991	NR_12576	ENSG0000	LINC01435	TCONS_00	long interg	ncRNA
lib-C666-l	chr13	100150921	100150921	+	10.38624	NA	intron (NR_110731, intron 1 of 4)	CpG	639	NR_11073	102725509	Hs.648968	NR_110731		LINC01232	TCONS_00	long interg	ncRNA
lib-C666-l	chr13	100085084	100085084	+	4.73866	NA	Intergenic	L2b|LINE|L2	66476	NR_11073	102725509	Hs.648968	NR_110731		LINC01232	TCONS_00	long interg	ncRNA
lib-C666-l	chr13	100089123	100089123	+	2.62544	NA	Intergenic	Intergenic	62437	NR_11073	102725509	Hs.648968	NR_110731		LINC01232	TCONS_00	long interg	ncRNA
lib-C666-l	chr13	81553370	81553370	+	3.92495	NA	Intergenic	Intergenic	-39156	NR_12577	103724386	Hs.539622	NR_125770		LINC00377	TCONS_00	long interg	ncRNA
lib-C666-l	chr16	86176505	86176505	+	45.38641	NA	Intergenic	Intergenic	-53282	NR_10385	100506542	Hs.111902	NR_10385	ENSG0000	LINC01082	TCONS_00	long interg	ncRNA
lib-C666-l	chr21	44772652	44772652	+	19.50061	NA	Intergenic	Intergenic	9577	NR_13190	101928399	Hs.517319	NR_13190	ENSG0000	LINC01679	TCONS_00	long interg	ncRNA
lib-C666-l	chr21	44796659	44796659	+	19.19377	NA	Intergenic	Intergenic	-14430	NR_13190	101928399	Hs.517319	NR_13190	ENSG0000	LINC01679	TCONS_00	long interg	ncRNA
lib-C666-l	chr4	190064656	190064656	+	4.90117	NA	Intergenic	LTR16E2|LTR|ERVL	-516104	NR_12167	101928971	Hs.519164	NR_12167	ENSG0000	LINC01262	TCONS_l2	long interg	ncRNA
lib-C666-l	chr7	153142048	153142048	+	8.51971	NA	Intergenic	Intergenic	-32729	NR_12577	103724390	Hs.614280	NR_12577	ENSG0000	LINC01287	TCONS_l2	long interg	ncRNA
lib-C666-l	chr1	17738308	17738308	+	8.96177	NA	intron (NM_001136204, intron 10 of 1	intron (NM_001136204, intron 10 o	26751	NM_00113	55920	Hs.380857	NM_01871	ENSG0000	RCC2	TD-60	regulator c	protein-co
lib-C666-l	chr1	17764654	17764654	+	5.09942	NA	intron (NM_001136204, intron 1 of 1	CpG	405	NM_00113	55920	Hs.380857	NM_01871	ENSG0000	RCC2	TD-60	regulator c	protein-co
lib-C666-l	chr1	17739036	17739036	+	3.59106	NA	intron (NM_001136204, intron 9 of 1	intron (NM_001136204, intron 9 of	26023	NM_00113	55920	Hs.380857	NM_01871	ENSG0000	RCC2	TD-60	regulator c	protein-co
lib-C666-l	chr6	122793009	122793009	+	34.3461	NA	promoter-TSS (NM_001270394)	promoter-TSS (NM_001270394)	17	NM_00127	57515	Hs.146668	NM_02075	ENSG0000	SERINC1	TDE1L|TDE	serine inco	protein-co
lib-C666-l	chr7	127291885	127291885	+	7.66979	NA	promoter-TSS (NM_014390)	promoter-TSS (NM_014390)	-158	NM_01439	27044	Hs.122523	NM_01439	ENSG0000	SND1	TDRD11|T	staphyloco	protein-co
lib-C666-l	chr1	151763168	151763168	+	8.89416	NA	promoter-TSS (NM_006862)	promoter-TSS (NM_006862)	-158	NM_00108	11022	Hs.144439	NM_00686	ENSG0000	TDRKH	TDRD2	tudor and	protein-co
lib-C666-l	chr12	11802376	11802376	+	20.8008	NA	promoter-TSS (NM_001987)	promoter-TSS (NM_001987)	-412	NM_00198	2120	Hs.504765	NM_00198	ENSG0000	ETV6	TEL|TEL/AE	ETS variant	protein-co
lib-C666-l	chr12	11802067	11802067	+	11.15301	NA	promoter-TSS (NM_001987)	promoter-TSS (NM_001987)	-721	NM_00198	2120	Hs.504765	NM_00198	ENSG0000	ETV6	TEL|TEL/AE	ETS variant	protein-co
lib-C666-l	chr12	11881302	11881302	+	8.28613	NA	intron (NM_001987, intron 1 of 7)	MER1B|DNA|hAT-Charlie	78514	NM_00198	2120	Hs.504765	NM_00198	ENSG0000	ETV6	TEL|TEL/AE	ETS variant	protein-co
lib-C666-l	chr12	11958831	11958831	+	6.15313	NA	intron (NM_001987, intron 2 of 7)	intron (NM_001987, intron 2 of 7)	156043	NM_00198	2120	Hs.504765	NM_00198	ENSG0000	ETV6	TEL|TEL/AE	ETS variant	protein-co
lib-C666-l	chr17	37295468	37295468	+	19.82221	NA	intron (NM_020405, intron 2 of 13)	intron (NM_020405, intron 2 of 13)	12434	NM_02040	57125	Hs.125036	NM_02040	ENSG0000	PLXDC1	TEM3|TEM	plexin dom	protein-co
lib-C666-l	chr7	47615809	47615809	+	15.01861	NA	intron (NM_022748, intron 1 of 30)	intron (NM_022748, intron 1 of 30)	5933	NM_02274	64759	Hs.520814	NM_02274	ENSG0000	TNS3	TEM6|TENS	tensin 3	protein-co
lib-C666-l	chr7	47267642	47267642	+	4.39666	NA	Intergenic	Intergenic	354100	NM_02274	64759	Hs.520814	NM_02274	ENSG0000	TNS3	TEM6|TENS	tensin 3	protein-co
lib-C666-l	chr7	115850440	115850440	+	161.31409	NA	promoter-TSS (NM_015641)	promoter-TSS (NM_015641)	-107	NM_01564	26136	Hs.592286	NM_01564	ENSG0000	TES	TESS|TESS-	testin LIM	protein-co
lib-C666-l	chr7	115850799	115850799	+	35.7914	NA	intron (NM_015641, intron 1 of 6)	CpG	252	NM_01564	26136	Hs.592286	NM_01564	ENSG0000	TES	TESS|TESS-	testin LIM	protein-co
lib-C666-l	chr10	73857646	73857646	+	15.8174	NA	intron (NR_045564, intron 9 of 9)	intron (NR_045564, intron 9 of 9)	-8856	NM_00113	9806	Hs.523009	NM_01476	ENSG0000	SPOCK2	testican-2	SPARC/ost	protein-co
lib-C666-l	chr10	73825320	73825320	+	11.66012	NA	intron (NM_014767, intron 9 of 11)	Tigger3a|DNA|TcMar-Tigger	23211	NM_00124	9806	Hs.523009	NM_01476	ENSG0000	SPOCK2	testican-2	SPARC/ost	protein-co
lib-C666-l	chr4	2936645	2936645	+	7.7703	NA	promoter-TSS (NM_001120)	promoter-TSS (NM_001120)	-59	NM_00114	10227	Hs.632581	NM_00112	ENSG0000	MFSD10	TETRAN|TE	major facil	protein-co
lib-C666-l	chr17	900261	900261	+	7.03995	NA	5' UTR (NM_013337, exon 1 of 4)	5' UTR (NM_013337, exon 1 of 4)	128	NM_01333	29928	Hs.745034	NM_01333	ENSG0000	TIMM22	TEX4|TIM2	translocase	protein-co
lib-C666-l	chr17	899919	899919	+	4.29847	NA	promoter-TSS (NM_013337)	promoter-TSS (NM_013337)	-214	NM_01333	29928	Hs.745034	NM_01333	ENSG0000	TIMM22	TEX4|TIM2	translocase	protein-co
lib-C666-l	chr14	81685748	81685748	+	8.35705	NA	intron (NM_201595, intron 1 of 8)	intron (NM_201595, intron 1 of 8)	1548	NM_01585	2957	Hs.592334	NM_01585	ENSG0000	GTF2A1	TF2A1|TFI	general tra	protein-co
lib-C666-l	chr14	81687611	81687611	+	6.72475	NA	promoter-TSS (NM_001278940)	promoter-TSS (NM_001278940)	-36	NM_20159	2957	Hs.592334	NM_01585	ENSG0000	GTF2A1	TF2A1|TFI	general tra	protein-co
lib-C666-l	chr1	89357283	89357283	+	64.2674	NA	promoter-TSS (NM_001514)	promoter-TSS (NM_001514)	18	NM_00151	2959	Hs.481852	NM_00151	ENSG0000	GTF2B	TF2B|TFIIB	general tra	protein-co
lib-C666-l	chr9	135906261	135906261	+	8.28613	NA	promoter-TSS (NR_135119)	promoter-TSS (NR_135119)	199	NM_01208	9328	Hs.495417	NM_01208	ENSG0000	GTF3C5	TFIIIC63|T	general tra	protein-co
lib-C666-l	chr2	27579733	27579733	+	77.28233	NA	promoter-TSS (NM_001318909)	promoter-TSS (NM_001318909)	168	NM_00152	2976	Hs.75782	NM_00152	ENSG0000	GTF3C2	TFIIIC-BET	general tra	protein-co
lib-C666-l	chr20	62694425	62694425	+	11.66438	NA	5' UTR (NM_003195, exon 1 of 10)	5' UTR (NM_003195, exon 1 of 10)	415	NM_00319	6919	Hs.505004	NM_00319	ENSG0000	TCEA2	TFIIS	transcriptio	protein-co
lib-C666-l	chr20	62699794	62699794	+	5.3726	NA	intron (NM_003195, intron 4 of 9)	intron (NM_003195, intron 4 of 9)	5784	NM_00319	6919	Hs.505004	NM_00319	ENSG0000	TCEA2	TFIIS	transcriptio	protein-co
lib-C666-l	chr20	62688674	62688674	+	4.04064	NA	5' UTR (NM_198723, exon 1 of 11)	5' UTR (NM_198723, exon 1 of 11)	235	NM_19872	6919	Hs.505004	NM_00319	ENSG0000	TCEA2	TFIIS	transcriptio	protein-co
lib-C666-l	chr1	23778618	23778618	+	4.46944	NA	intron (NM_017707, intron 4 of 24)	intron (NM_017707, intron 4 of 24)	-27346	NM_00319	6920	Hs.446354	NM_00319	ENSG0000	TCEA3	TFIIS|TFIIS.	transcriptio	protein-co
lib-C666-l	chr2	85555377	85555377	+	12.83248	NA	promoter-TSS (NM_001206841)	promoter-TSS (NM_001206841)	42	NM_00120	10618	Hs.593382	NM_00646	ENSG0000	TGOLN2	TGN38|TG	trans-golg	protein-co
lib-C666-l	chr3	44925470	44925470	+	13.71937	NA	intron (NM_003241, intron 1 of 13)	intron (NM_003241, intron 1 of 13)	9372	NM_00324	7047	Hs.438265	NM_00324	ENSG0000	TGM4	TGP|hTGP	transglutar	protein-co
lib-C666-l	chr18	158593	158593	+	120.97476	NA	5' UTR (NM_005151, exon 1 of 16)	5' UTR (NM_005151, exon 1 of 16)	110	NM_00103	9097	Hs.464416	NM_00515	ENSG0000	USP14	TGT	ubiquitin s	protein-co
lib-C666-l	chr7	32869134	32869134	+	7.0832	NA	intron (NR_132360, intron 2 of 5)	intron (NR_132360, intron 2 of 5)	3220	NR_13236	107724668	Hs.636806	NR_132360		DPY19L1P1	THC10725	DPY19L1 p	pseudo
lib-C666-l	chr5	33441042	33441042	+	3.59106	NA	non-coding (NR_047676, exon 1 of 2	non-coding (NR_047676, exon 1 of	160	NM_00476	6897	Hs.481860	NM_15229	ENSG0000	TARS	ThrRS	threonyl-tF	protein-co
lib-C666-l	chr19	1507984	1507984	+	26.54591	NA	intron (NM_213604, intron 7 of 11)	CpG	5204	NM_21360	339366	Hs.371674	NM_21360	ENSG0000	ADAMTSL5	THSD6	ADAMTS li	protein-co
lib-C666-l	chr14	24025334	24025334	+	25.38683	NA	non-coding (NR_023314, exon 1 of 2	non-coding (NR_023314, exon 1 of	143	NM_00125	79178	Hs.655179	NM_02432	ENSG0000	THTPA	THTP|THTF	thiamine tr	protein-co
lib-C666-l	chr11	119252465	119252465	+	22.23727	NA	promoter-TSS (NM_001243759)	promoter-TSS (NM_001243759)	-23	NR_03416	100499227	Hs.662365	NR_034160		USP2-AS1	THY1-AS1	USP2 antis	ncRNA

lib-C666-l	chr1	201438375	201438375	+	5.23609	NA	promoter-TSS (NR_073080)	promoter-TSS (NR_073080)	-76	NR_07308(	23612	Hs.153944	NM_01239	ENSG0000	PHLDA3	TIH1	pleckstrin h	protein-co
lib-C666-l	chr1	48509436	48509436	+	30.85659	NA	Intergenic	Intergenic	-46874	NM_00119	388630	Hs.61504	NM_00119	ENSG0000	TRABD2B	TIKI2	TraB doma	protein-co
lib-C666-l	chr1	48505156	48505156	+	10.79304	NA	Intergenic	Intergenic	-42594	NM_00119	388630	Hs.61504	NM_00119	ENSG0000	TRABD2B	TIKI2	TraB doma	protein-co
lib-C666-l	chr1	48400027	48400027	+	2.78587	NA	intron (NM_001194986, intron 2 of 6)	intron (NM_001194986, intron 2 of	62535	NM_00119	388630	Hs.61504	NM_00119	ENSG0000	TRABD2B	TIKI2	TraB doma	protein-co
lib-C666-l	chr3	150126044	150126044	+	40.21248	NA	promoter-TSS (NR_130136)	promoter-TSS (NR_130136)	-78	NR_13013(	9819	Hs.722470	NM_01477	ENSG0000	TSC22D2	TILZ4a|TILZ	TSC22 don	protein-co
lib-C666-l	chr3	150126367	150126367	+	9.14689	NA	non-coding (NR_130136, exon 1 of 5	non-coding (NR_130136, exon 1 of	245	NM_01477	9819	Hs.722470	NM_01477	ENSG0000	TSC22D2	TILZ4a|TILZ	TSC22 don	protein-co
lib-C666-l	chr11	57298486	57298486	+	3.89034	NA	promoter-TSS (NM_012456)	promoter-TSS (NM_012456)	-254	NM_01245	26519	Hs.235750	NM_01245	ENSG0000	TIMM10	TIM10|TIM	translocase	protein-co
lib-C666-l	chr19	2427814	2427814	+	115.28649	NA	promoter-TSS (NM_012458)	promoter-TSS (NM_012458)	100	NM_01245	26517	Hs.75056	NM_01245	ENSG0000	TIMM13	TIM13|TIM	translocase	protein-co
lib-C666-l	chr19	2427631	2427631	+	99.93166	NA	TTS (NM_032737)	TTS (NM_032737)	283	NM_01245	26517	Hs.75056	NM_01245	ENSG0000	TIMM13	TIM13|TIM	translocase	protein-co
lib-C666-l	chr19	8008634	8008634	+	29.74345	NA	promoter-TSS (NM_006351)	promoter-TSS (NM_006351)	74	NM_00635	10469	Hs.465784	NM_00635	ENSG0000	TIMM44	TIM44	translocase	protein-co
lib-C666-l	chr19	39971466	39971466	+	31.07983	NA	exon (NM_001001563, exon 1 of 11)	exon (NM_001001563, exon 1 of 11	414	NM_00132	92609	Hs.590956	NM_00100	ENSG0000	TIMM50	TIM50|TIM	translocase	protein-co
lib-C666-l	chr14	58894314	58894314	+	4.11503	NA	promoter-TSS (NR_130751)	promoter-TSS (NR_130751)	18	NM_01246	26520	Hs.440525	NM_01246	ENSG0000	TIMM9	TIM9|TIM9	translocase	protein-co
lib-C666-l	chr2	172291077	172291077	+	4.30593	NA	promoter-TSS (NM_001321154)	promoter-TSS (NM_001321154)	236	NM_00132	79828	Hs.135146	NM_02477	ENSG0000	METTL8	TIP	methyltran	protein-co
lib-C666-l	chr1	168147957	168147957	+	36.90456	NA	promoter-TSS (NM_152902)	promoter-TSS (NM_152902)	-126	NM_00103	261726	Hs.209431	NM_15290	ENSG0000	TIPRL	TIP|TIP41|T	TOR signal	protein-co
lib-C666-l	chr12	67663084	67663084	+	6.40179	NA	promoter-TSS (NM_018448)	promoter-TSS (NM_018448)	23	NM_00132	55832	Hs.546407	NM_01844	ENSG0000	CAND1	TIP120|TIP	cullin asso	protein-co
lib-C666-l	chr3	12851550	12851550	+	6.40797	NA	intron (NM_001162499, intron 4 of 1	CpG	13379	NM_00116	23066	Hs.343664	NM_01229	ENSG0000	CAND2	TIP120B|Tp	cullin asso	protein-co
lib-C666-l	chr19	56632796	56632796	+	9.79436	NA	promoter-TSS (NM_001002836)	promoter-TSS (NM_001002836)	-54	NM_00100	126208	Hs.397153	NM_00100	ENSG0000	ZNF787	TIP20	zinc finger	protein-co
lib-C666-l	chr12	57030742	57030742	+	8.85547	NA	promoter-TSS (NM_013449)	promoter-TSS (NM_013449)	-579	NM_01344	11176	Hs.314263	NM_01344	ENSG0000	BAZ2A	TIP5|WALp	bromodom	protein-co
lib-C666-l	chr12	57024229	57024229	+	8.28735	NA	promoter-TSS (NM_001300905)	promoter-TSS (NM_001300905)	-114	NM_00130	11176	Hs.314263	NM_01344	ENSG0000	BAZ2A	TIP5|WALp	bromodom	protein-co
lib-C666-l	chr16	30609729	30609729	+	6.72475	NA	Intergenic	LTR40c|LTR|ERVL	11420	NR_10416(	115509	Hs.454685	NM_13844	ENSG0000	ZNF689	TIPUH1	zinc finger	protein-co
lib-C666-l	chr9	117592980	117592980	+	6.71839	NA	Intergenic	Intergenic	-24572	NM_00511	9966	Hs.23349	NM_00511	ENSG0000	TNFSF15	TL1|TL1A|T	tumor necr	protein-co
lib-C666-l	chr5	90676131	90676131	+	7.0832	NA	promoter-TSS (NM_001329671)	promoter-TSS (NM_001329671)	-17	NM_00132	57561	Hs.24684	NM_02080	ENSG0000	ARRDC3	TLIMP	arrestin do	protein-co
lib-C666-l	chr5	90721046	90721046	+	4.90117	NA	Intergenic	LTR16C|LTR|ERVL	-41856	NM_00132	57561	Hs.24684	NM_02080	ENSG0000	ARRDC3	TLIMP	arrestin do	protein-co
lib-C666-l	chr15	77363494	77363494	+	5.65398	NA	promoter-TSS (NM_001168412)	promoter-TSS (NM_001168412)	76	NM_00572	10099	Hs.744863	NM_00572	ENSG0000	TSPAN3	TM4-A|TM	tetraspanir	protein-co
lib-C666-l	chr12	117176011	117176011	+	39.72046	NA	promoter-TSS (NM_024738)	promoter-TSS (NM_024738)	-85	NM_00110	84900	Hs.437195	NM_03281	ENSG0000	RNFT2	TMEM118	ring finger	protein-co
lib-C666-l	chr4	154354357	154354357	+	19.75653	NA	Intergenic	Intergenic	-33141	NM_01519	23240	Hs.732450	NM_01519	ENSG0000	KIAA0922	TMEM131L	KIAA0922	protein-co
lib-C666-l	chr16	75498637	75498637	+	20.94772	NA	promoter-TSS (NM_001304997)	promoter-TSS (NM_001304997)	-2	NM_14525	124491	Hs.487510	NM_14525	ENSG0000	TMEM170A	TMEM170	transmemk	protein-co
lib-C666-l	chr22	21057810	21057810	+	6.50091	NA	non-coding (NR_026815, exon 4 of 8	non-coding (NR_026815, exon 4 of	2408	NR_02681!	84222	Hs.546454	NM_03226	ENSG0000	TMEM191A	TMEM191A	transmemk	pseudo
lib-C666-l	chr2	220408714	220408714	+	198.6326	NA	promoter-TSS (NM_001005209)	promoter-TSS (NM_001005209)	-14	NM_00100	130612	Hs.446664	NM_00100	ENSG0000	TMEM198	TMEM198A	transmemk	protein-co
lib-C666-l	chrX	52926252	52926252	+	125.57522	NA	promoter-TSS (NM_001321179)	promoter-TSS (NM_001321179)	-70	NM_00132	727866	Hs.6451	NM_00109	ENSG0000	FAM156B	TMEM29B	family with	protein-co
lib-C666-l	chrX	52987782	52987782	+	88.70856	NA	promoter-TSS (NM_001321179).2	promoter-TSS (NM_001321179).2	-67	NM_00132	727866	Hs.6451	NM_00109	ENSG0000	FAM156B	TMEM29B	family with	protein-co
lib-C666-l	chrX	52964407	52964407	+	11.57178	NA	Intergenic	CpG	21546	NM_00109	727866	Hs.6451	NM_00109	ENSG0000	FAM156B	TMEM29B	family with	protein-co
lib-C666-l	chr19	35558195	35558195	+	8.03539	NA	TTS (NM_002151)	TTS (NM_002151)	26785	NM_00215	3249	Hs.182385	NM_00215	ENSG0000	HPN	TMPRSS1	hepsin	protein-co
lib-C666-l	chr2	232571475	232571475	+	27.68428	NA	Intergenic	CpG	-1760	NM_00282	5757	Hs.459927	NM_00282	ENSG0000	PTMA	TMSA	prothymos	protein-co
lib-C666-l	chr2	232573075	232573075	+	10.67459	NA	promoter-TSS (NM_001099285)	promoter-TSS (NM_001099285)	-160	NM_00282	5757	Hs.459927	NM_00282	ENSG0000	PTMA	TMSA	prothymos	protein-co
lib-C666-l	chr11	73087392	73087392	+	51.74843	NA	promoter-TSS (NM_152222)	promoter-TSS (NM_152222)	-13	NM_15222	84957	Hs.533720	NM_03287	ENSG0000	RELT	TNFRSF19L	RELT tumo	protein-co
lib-C666-l	chr17	38574282	38574282	+	2.10911	NA	promoter-TSS (NM_001067)	promoter-TSS (NM_001067)	-80	NM_00106	7153	Hs.156346	NM_00106	ENSG0000	TOP2A	TOP2|TP2A	topoisome	protein-co
lib-C666-l	chr3	133380796	133380796	+	21.88699	NA	promoter-TSS (NM_007027)	promoter-TSS (NM_007027)	-59	NM_00702	11073	Hs.593379	NM_00702	ENSG0000	TOPBP1	TOP2BP1	topoisome	protein-co
lib-C666-l	chr20	39657372	39657372	+	11.07709	NA	promoter-TSS (NM_003286)	promoter-TSS (NM_003286)	-86	NM_00328	7150	Hs.472737	NM_00328	ENSG0000	TOP1	TOPI	topoisome	protein-co
lib-C666-l	chr20	39637142	39637142	+	4.39666	NA	intron (NM_001318196, intron 2 of 2)	L2b|LINE|L2	-20316	NM_00328	7150	Hs.472737	NM_00328	ENSG0000	TOP1	TOPI	topoisome	protein-co
lib-C666-l	chr3	25634160	25634160	+	4.19683	NA	intron (NR_110893, intron 5 of 6)	intron (NR_110893, intron 5 of 6)	71703	NM_00106	7155	Hs.475733	NM_00106	ENSG0000	TOP2B	TOPIIB|top	topoisome	protein-co
lib-C666-l	chr9	130497765	130497765	+	6.15313	NA	promoter-TSS (NM_130459)	promoter-TSS (NM_130459)	-137	NM_00125	27433	Hs.444106	NM_13045	ENSG0000	TOR2A	TORP1	torsin fami	protein-co
lib-C666-l	chr16	32244444	32244444	+	29.50576	NA	Intergenic	HSAT6|Satellite|Satellite	-20201	NR_11089(	729264	Hs.513537	NM_00124	ENSG0000	TP53TG3D	TP53TG3|T	TP53 targe	protein-co
lib-C666-l	chr8	42068216	42068216	+	45.38224	NA	Intergenic	MER1A|DNA|hAT-Charlie	-2974	NM_03301	5327	Hs.491582	NM_00093	ENSG0000	PLAT	T-PA|TPA	plasminog	protein-co
lib-C666-l	chr8	42065355	42065355	+	7.03995	NA	promoter-TSS (NM_033011)	promoter-TSS (NM_033011)	-113	NM_03301	5327	Hs.491582	NM_00093	ENSG0000	PLAT	T-PA|TPA	plasminog	protein-co
lib-C666-l	chr11	113167414	113167414	+	2.99475	NA	Intergenic	L1PB1|LINE|L1	-17837	NM_01786	54970	Hs.288772	NM_01786	ENSG0000	TTC12	TPARM	tetratricop	protein-co
lib-C666-l	chr12	113683817	113683817	+	16.291	NA	intron (NM_001301214, intron 1 of 2	intron (NM_001301214, intron 1 of	1751	NM_00130	53373	Hs.524763	NM_01790	ENSG0000	TPCN1	TPC1	two pore s	protein-co
lib-C666-l	chr13	103249226	103249226	+	76.37573	NA	promoter-TSS (NM_003291)	promoter-TSS (NM_003291)	-60	NM_00329	7174	Hs.432424	NM_00329	ENSG0000	TPP2	TPP-2|TPP	tripeptidyl	protein-co
lib-C666-l	chr5	159436064	159436064	+	129.17302	NA	promoter-TSS (NM_001282500)	promoter-TSS (NM_001282500)	-43	NM_00331	7265	Hs.519718	NM_00331	ENSG0000	TTC1	TPR1	tetratricop	protein-co
lib-C666-l	chr12	95524096	95524096	+	15.3763	NA	intron (NM_018351, intron 8 of 20)	intron (NM_018351, intron 8 of 20)	-56692	NM_00103	7181	Hs.108301	NM_00329	ENSG0000	NR2C1	TR2	nuclear rec	protein-co
lib-C666-l	chr12	95467425	95467425	+	7.52748	NA	promoter-TSS (NM_001127362)	promoter-TSS (NM_001127362)	-21	NM_00103	7181	Hs.108301	NM_00329	ENSG0000	NR2C1	TR2	nuclear rec	protein-co
lib-C666-l	chr12	95466614	95466614	+	2.58854	NA	intron (NM_001032287, intron 1 of 1	intron (NM_001032287, intron 1 of	790	NM_00112	7181	Hs.108301	NM_00329	ENSG0000	NR2C1	TR2	nuclear rec	protein-co
lib-C666-l	chr10	126605353	126605353	+	65.34157	NA	Intergenic	CpG	-25339	NM_01758	54764	Hs.595158	NM_01758	ENSG0000	ZRANB1	TRABID	zinc finger	protein-co
lib-C666-l	chr1	36689890	36689890	+	82.1828	NA	promoter-TSS (NM_001321473)	promoter-TSS (NM_001321473)	-123	NM_00132	9967	Hs.744057	NM_00511	ENSG0000	THRAP3	TRAP150	thyroid hor	protein-co
lib-C666-l	chr3	156273017	156273017	+	25.68529	NA	promoter-TSS (NM_001308204)	promoter-TSS (NM_001308204)	-28	NM_00710	6747	Hs.518346	NM_00710	ENSG0000	SSR3	TRAPG	signal sequ	protein-co
lib-C666-l	chr1	231664369	231664369	+	8.71138	NA	promoter-TSS (NM_005999)	promoter-TSS (NM_005999)	-30	NM_00599	7257	Hs.13318	NM_00599	ENSG0000	TSNAX	TRAX	translin ass	protein-co
lib-C666-l	chr16	69430175	69430175	+	16.59065	NA	Intergenic	Intergenic	-10284	NM_00565	7014	Hs.63335	NM_00565	ENSG0000	TERF2	TRBF2|TRF	telomeric r	protein-co
lib-C666-l	chr16	69419864	69419864	+	9.10494	NA	promoter-TSS (NM_005652)	promoter-TSS (NM_005652)	27	NM_00565	7014	Hs.63335	NM_00565	ENSG0000	TERF2	TRBF2|TRF	telomeric r	protein-co
lib-C666-l	chr9	113017943	113017943	+	26.26478	NA	intron (NM_001244938, intron 1 of 3)	CpG	977	NM_00332	7295	Hs.435136	NM_00332	ENSG0000	TXN	TRDX|TRX|	thioredoxir	protein-co
lib-C666-l	chr9	113018934	113018934	+	4.74112	NA	promoter-TSS (NM_003329)	promoter-TSS (NM_003329)	-14	NM_00124	7295	Hs.435136	NM_00332	ENSG0000	TXN	TRDX|TRX|	thioredoxir	protein-co
lib-C666-l	chr22	29196640	29196640	+	25.39487	NA	promoter-TSS (NM_005080)	promoter-TSS (NM_005080)	-80	NM_00107	7494	Hs.437638	NM_00508	ENSG0000	XBP1	TREB-5|TR	X-box binc	protein-co
lib-C666-l	chr16	50186913	50186913	+	9.38975	NA	promoter-TSS (NM_001040284)	promoter-TSS (NM_001040284)	84	NM_00104	64282	Hs.514342	NM_02244	ENSG0000	PAPD5	TRF4-2	poly(A) RN	protein-co
lib-C666-l	chr17	18622642	18622642	+	36.90456	NA	Intergenic	Intergenic	-2760	NM_00103	147166	Hs.164324	NM_00103	ENSG0000	TRIM16L	TRIM70	tripartite m	protein-co
lib-C666-l	chr2	230786851	230786851	+	7.43576	NA	promoter-TSS (NM_001284216)	promoter-TSS (NM_001284216)	-126	NM_00423	9320	Hs.572642	NM_00423	ENSG0000	TRIP12	TRIP-12|UI	thyroid hor	protein-co
lib-C666-l	chr19	13227546	13227546	+	66.27789	NA	promoter-TSS (NM_017722)	promoter-TSS (NM_017722)	17	NM_00113	55621	Hs.515169	NM_01772	ENSG0000	TRMT1	TRM1	tRNA meth	protein-co
lib-C666-l	chr8	125463065	125463065	+	41.84685	NA	promoter-TSS (NM_017956)	promoter-TSS (NM_017956)	17	NM_01795	55039	Hs.9925	NM_01795	ENSG0000	TRMT12	TRM12|TYW	tRNA meth	protein-co
lib-C666-l	chr21	44299680	44299680	+	177.70084	NA	promoter-TSS (NM_033661)	promoter-TSS (NM_033661)	13	NM_00126	10785	Hs.248815	NM_01866	ENSG0000	WDR4	TRM82|TRI	WD repeat	protein-co
lib-C666-l	chr19	45681648	45681648	+	89.84392	NA	promoter-TSS (NM_001270891)	promoter-TSS (NM_001270891)	-147	NM_02410	79090	Hs.466929	NM_02410	ENSG0000	TRAPPC6A	TRS33	trafficking	protein-co
lib-C666-l	chr11	18548497	18548497	+	43.7015	NA	promoter-TSS (NM_006292)	promoter-TSS (NM_006292)	6	NM_00629	7251	Hs.523512	NM_00629	ENSG0000	TSG101	TSG10|VPS	tumor susc	protein-co
lib-C666-l	chr9	25678383	25678383	+	4.64451	NA	5' UTR (NM_001004125, exon 1 of 1)	5' UTR (NM_001004125, exon 1 of 1	473	NM_00100	286319	Hs.26268	NM_00100	ENSG0000	TUSC1	TSG-9|TSG	tumor supp	protein-co
lib-C666-l	chr6	169796313	169796313	+	14.0317	NA	Intergenic	Intergenic	-142104	NM_00324	7058	Hs.371147	NM_00324	ENSG0000	THBS2	TSP2	thrombosp	protein-co
lib-C666-l	chr11	44747979	44747979	+	8.01597	NA	Intergenic	Intergenic	-37997	NM_13078	90139	Hs.385634	NM_13078	ENSG0000	TSPAN18	TSPAN	tetraspanir	protein-co
lib-C666-l	chr5	94891128	94891128	+	23.1274	NA	promoter-TSS (NM_014639)	promoter-TSS (NM_014639)	303	NM_19815	153642	Hs.585051	NM_19815	ENSG0000	ARSK	TSULF	arylsulfatas	protein-co
lib-C666-l	chr22	28315361	28315361	+	28.32976	NA	promoter-TSS (NM_001284277)	promoter-TSS (NM_001284277)	-3	NR_02696	284900	Hs.602319	NR_02696	ENSG0000	TTC28-AS1	TTC28-AS|	TTC28 anti	ncRNA
lib-C666-l	chr14	91294559	91294559	+	6.56784	NA	Intergenic	Intergenic	-11798	NM_00101	145567	Hs.655697	NM_00101	ENSG0000	TTC7B	TTC7L1|c14	tetratricop	protein-co
lib-C666-l	chr14	71132188	71132188	+	9.64253	NA	intron (NM_015351, intron 1 of 2)	intron (NM_015351, intron 1 of 2)	23684	NM_01535	23508	Hs.79170	NM_01535	ENSG0000	TTC9	TTC9A	tetratricop	protein-co
lib-C666-l	chr3	111805305	111805305	+	16.43544	NA	exon (NM_024616, exon 1 of 6)	exon (NM_024616, exon 1 of 6)	123	NM_00117	79669	Hs.434247	NM_02461	ENSG0000	C3orf52	TTMP	chromosor	protein-co
lib-C666-l	chr3	111816388	111816388	+	7.37943	NA	intron (NM_001171747, intron 2 of 3)	intron (NM_001171747, intron 2 of	11206	NM_00117	79669	Hs.434247	NM_02461	ENSG0000	C3orf52	TTMP	chromosor	protein-co
lib-C666-l	chrY	10103511	10103511	+	2.78587	NA	Intergenic	ALR/Alpha|Satellite|centr	355104	NR_00359	100101121	Hs.522846	NR_00359	ENSG0000	TTTY23B	TTTY23	testis-spec	ncRNA
lib-C666-l	chr12	104274443	104274443	+	26.46807	NA	intron (NR_027249, intron 9 of 15)	intron (NR_027249, intron 9 of 15)	-39468	NM_00103	51559	Hs.48428	NM_01657	ENSG0000	NT5DC3	TU12B1-TY	5'-nucleoti	protein-co
lib-C666-l	chr12	104163110	104163110	+	8.28216	NA	Intergenic	Intergenic	71865	NM_00103	51559	Hs.48428	NM_01657	ENSG0000	NT5DC3	TU12B1-TY	5'-nucleoti	protein-co
lib-C666-l	chr12	3021234	3021234	+	15.31489	NA	intron (NM_001160408, intron 2 of 1	intron (NM_001160408, intron 2 of	21201	NM_00116	7289	Hs.655333	NM_00332	ENSG0000	TULP3	TUBL3	tubby like	protein-co
lib-C666-l	chr1	179560697	179560697	+	3.8557	NA	promoter-TSS (NM_001199091)	promoter-TSS (NM_001199091)	-51	NM_00119	163589	Hs.197354	NM_17353	ENSG0000	TDRD5	TUDOR3	tudor dom	protein-co
lib-C666-l	chr7	130845022	130845022	+	33.38247	NA	intron (NM_001145354, intron 2 of 1	L1MB7|LINE|L1	50167	NM_00114	4289	Hs.44693	NM_01325	ENSG0000	MKLN1	TWA2	muskelin 1	protein-co
lib-C666-l	chr16	67840883	67840883	+	15.3763	NA	promoter-TSS (NM_001320238)	promoter-TSS (NM_001320238)	102	NM_00128	55815	Hs.632212	NM_01843	ENSG0000	TSNAXIP1	TXI1	translin ass	protein-co
lib-C666-l	chr5	172444224	172444224	+	9.7234	NA	intron (NM_003945, intron 2 of 3)	intron (NM_003945, intron 2 of 3)	-3505	NR_00298	677841	Hs.692720	NR_00298	ENSG0000	SNORA74B	U19-2	small nucle	snoRNA
lib-C666-l	chr6	34725249	34725249	+	75.47253	NA	non-coding (NR_029472, exon 1 of 5	non-coding (NR_029472, exon 1 of	378	NR_02947	6631	Hs.1063	NM_00309	ENSG0000	SNRPC	U1C|Yhc1	small nucle	protein-co
lib-C666-l	chr19	36236379	36236379	+	39.64852	NA	promoter-TSS (NM_144987)	promoter-TSS (NM_144987)	-36	NR_11017	199746	Hs.351558	NM_14498	ENSG0000	U2AF1L4	U2AF1-RS	U2 small n	protein-co
lib-C666-l	chr19	36235943	36235943	+	13.53348	NA	promoter-TSS (NM_001281532)	promoter-TSS (NM_001281532)	400	NR_11017	199746	Hs.351558	NM_14498	ENSG0000	U2AF1L4	U2AF1-RS	U2 small n	protein-co
lib-C666-l	chr19	56165079	56165079	+	38.21529	NA	promoter-TSS (NM_007279)	promoter-TSS (NM_007279)	-337	NM_00101	11338	Hs.528007	NM_00727	ENSG0000	U2AF2	U2AF65	U2 small n	protein-co
lib-C666-l	chr19	56166797	56166797	+	9.20967	NA	intron (NM_001012478, intron 1 of 1	CpG-14459	1381	NM_00727	11338	Hs.528007	NM_00727	ENSG0000	U2AF2	U2AF65	U2 small n	protein-co
lib-C666-l	chr19	1021710	1021710	+	132.30969	NA	promoter-TSS (NM_033420)	promoter-TSS (NM_033420)	189	NR_12573	103625684		NR_12573	ENSG0000	RNU6-2	U6-2	RNA, U6 sr	snRNA
lib-C666-l	chr18	47438030	47438030	+	18.90251	NA	intron (NM_001080467, intron 18 of 3	LTR22B|LTR|ERVK	97637	NR_00300	677769		NR_00300	ENSG0000	SCARNA17	U91	small Cajal	ncRNA
lib-C666-l	chr18	47461789	47461789	+	5.15932	NA	intron (NM_001080467, intron 16 of 3	MER3|DNA|hAT-Charlie	121396	NR_00300	677769		NR_00300	ENSG0000	SCARNA17	U91	small Cajal	ncRNA
[truncated: 43,760 more chars]
