# Supplementary material for: ZIC2 induces pro-tumor macrophage polarization in nasopharyngeal carcinoma by activating the JUNB/MCSF axis
Source: Cell Death Dis. 2023 Jul 21;14(7):455. doi: 10.1038/s41419-023-05983-x (PMC10362010; doi:10.1038/s41419-023-05983-x)

Dr. Qian Liu's agreement:


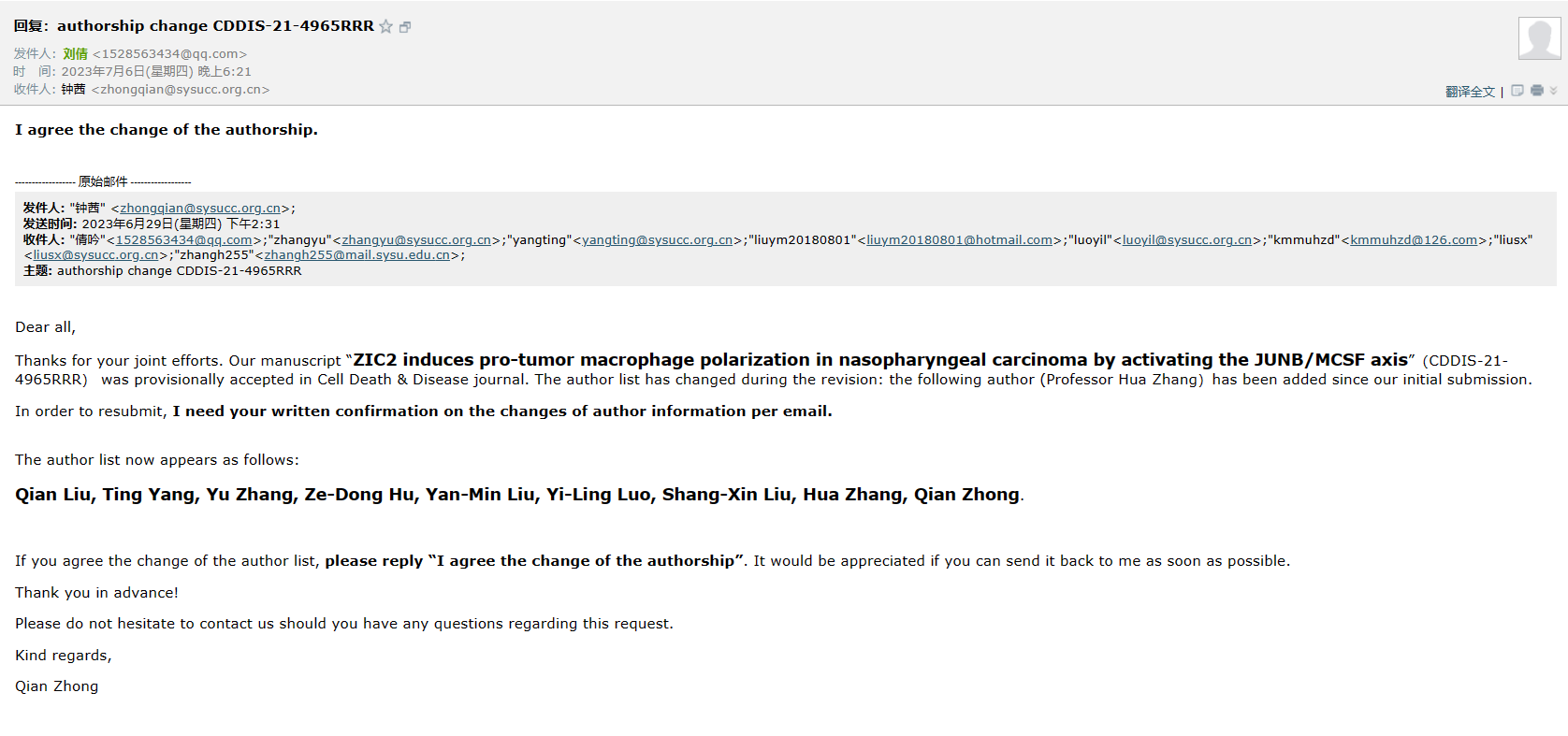


Dr. Ting Yang's agreement:


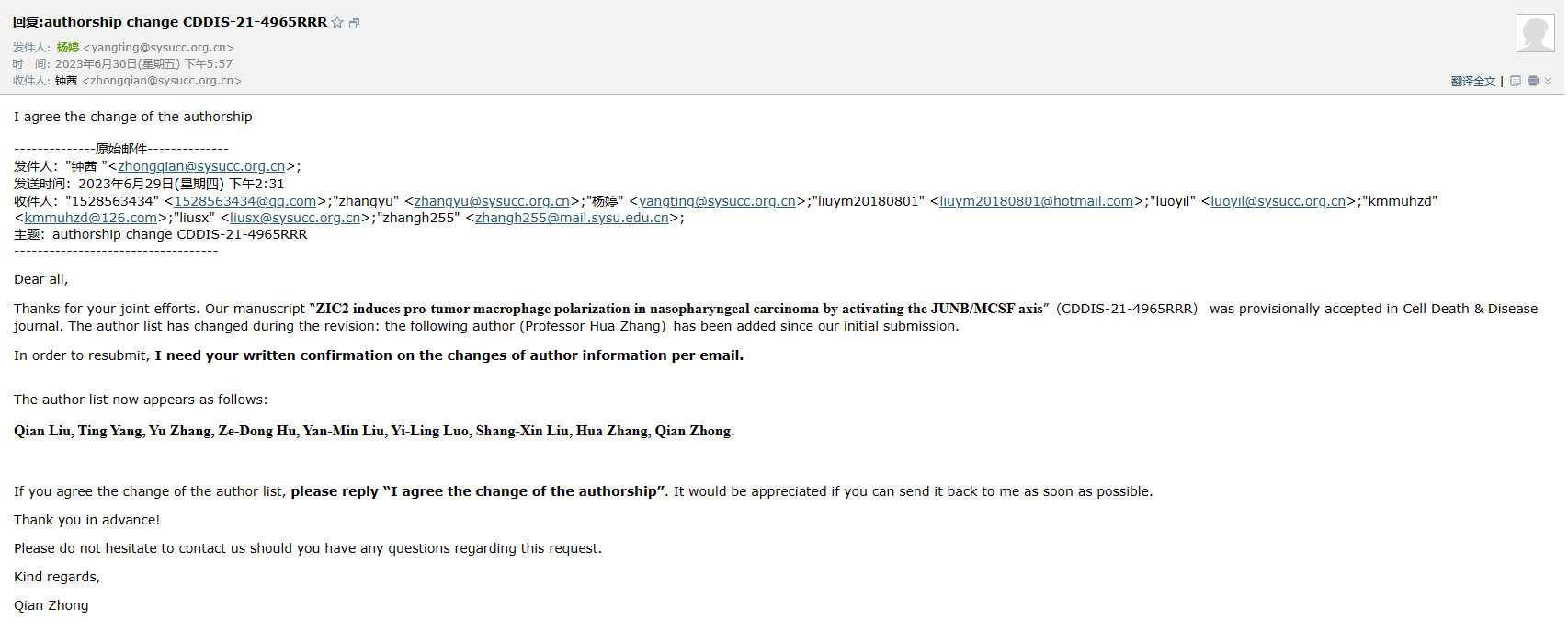


Dr. Yu Zhang's agreement:


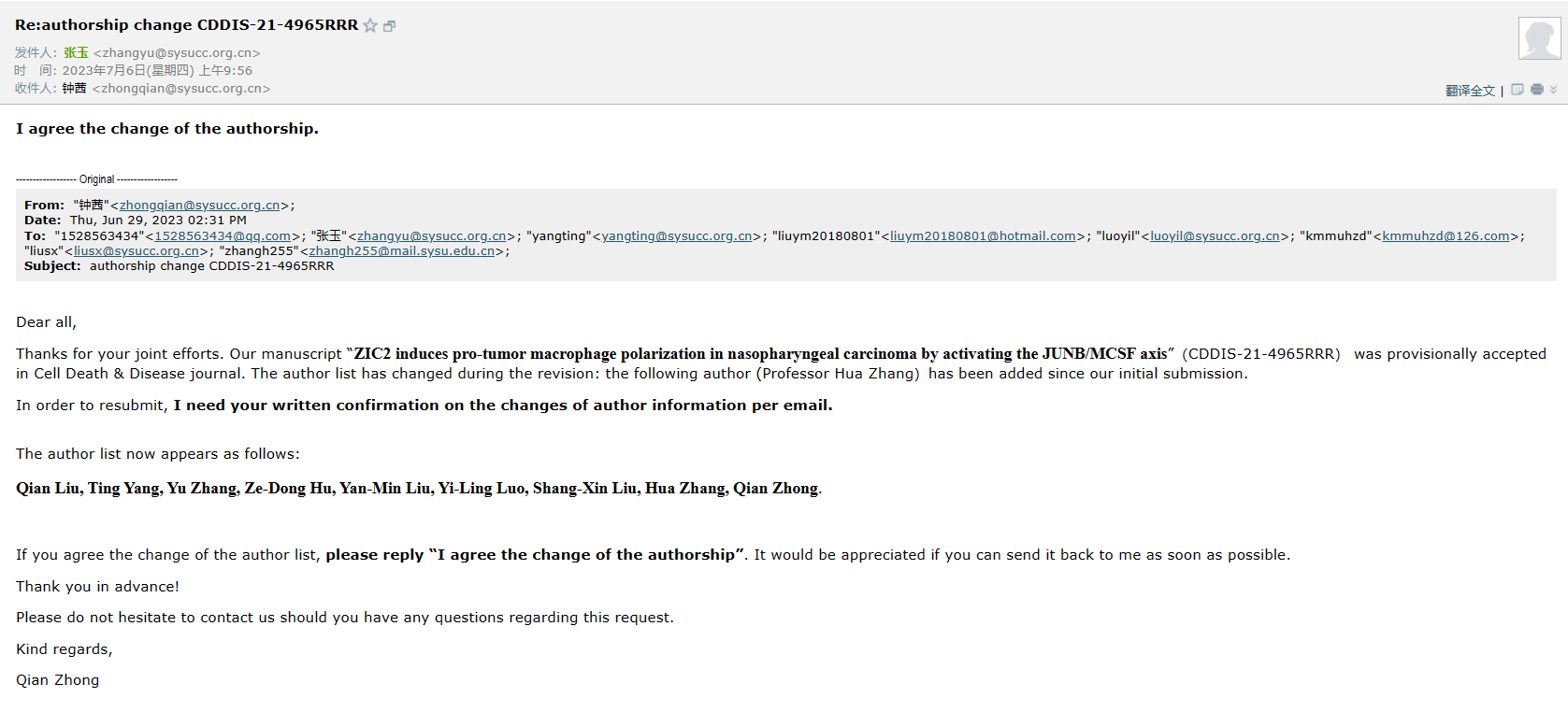


Dr. Ze-dong Hu's agreement:


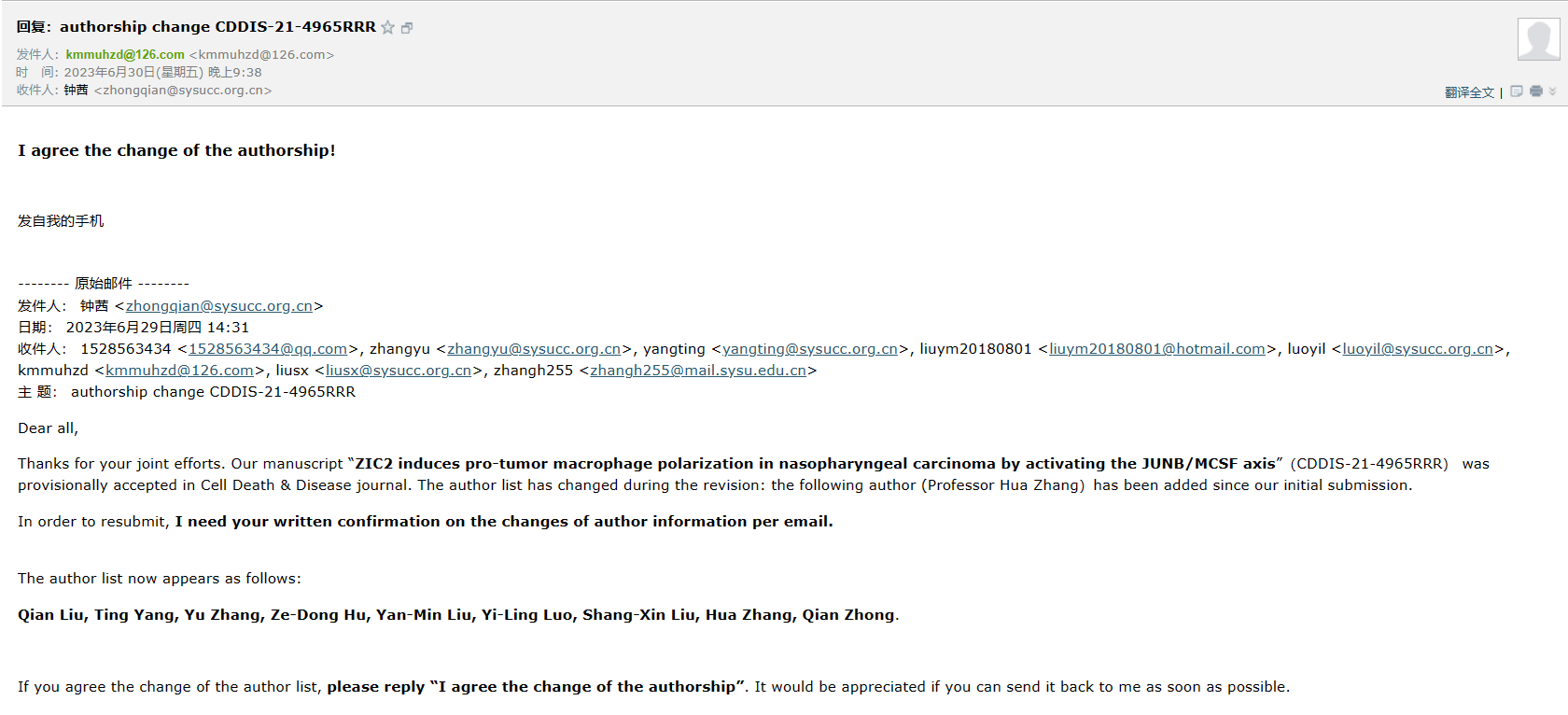


Dr. Yan-Min Liu's agreement:


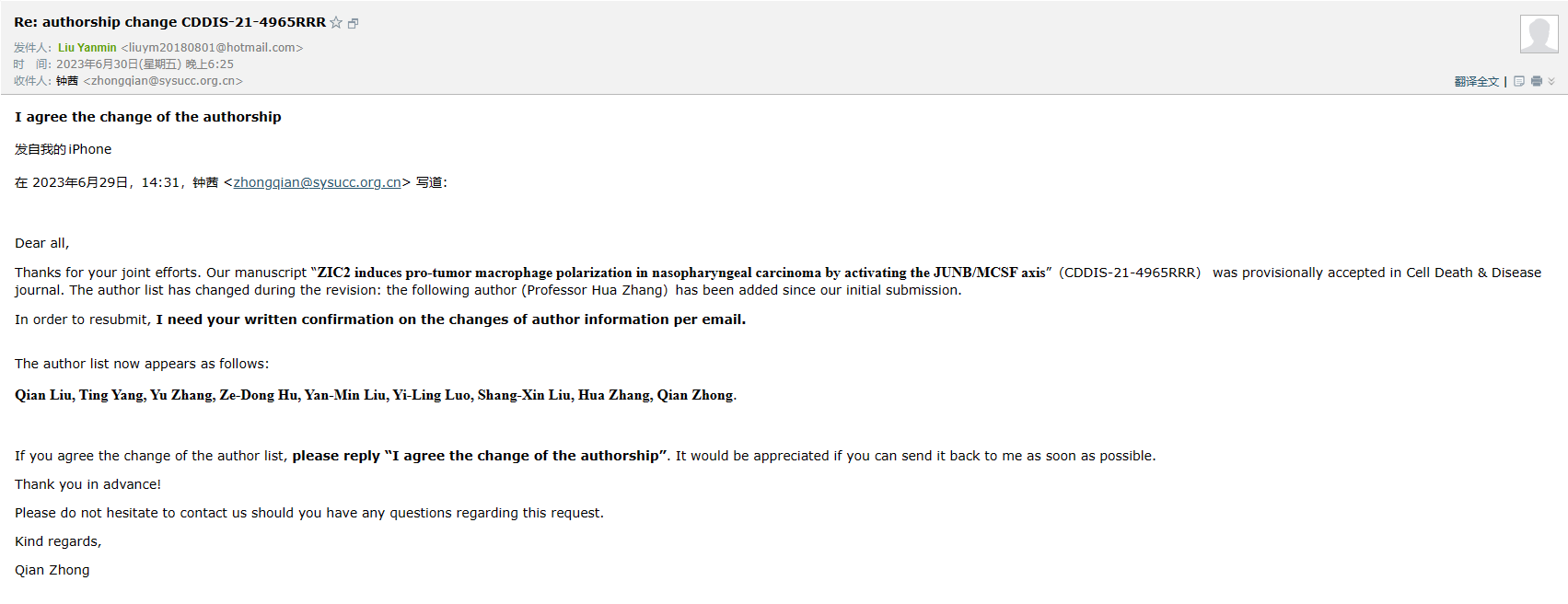


Dr. Yi-Ling Luo's agreement:


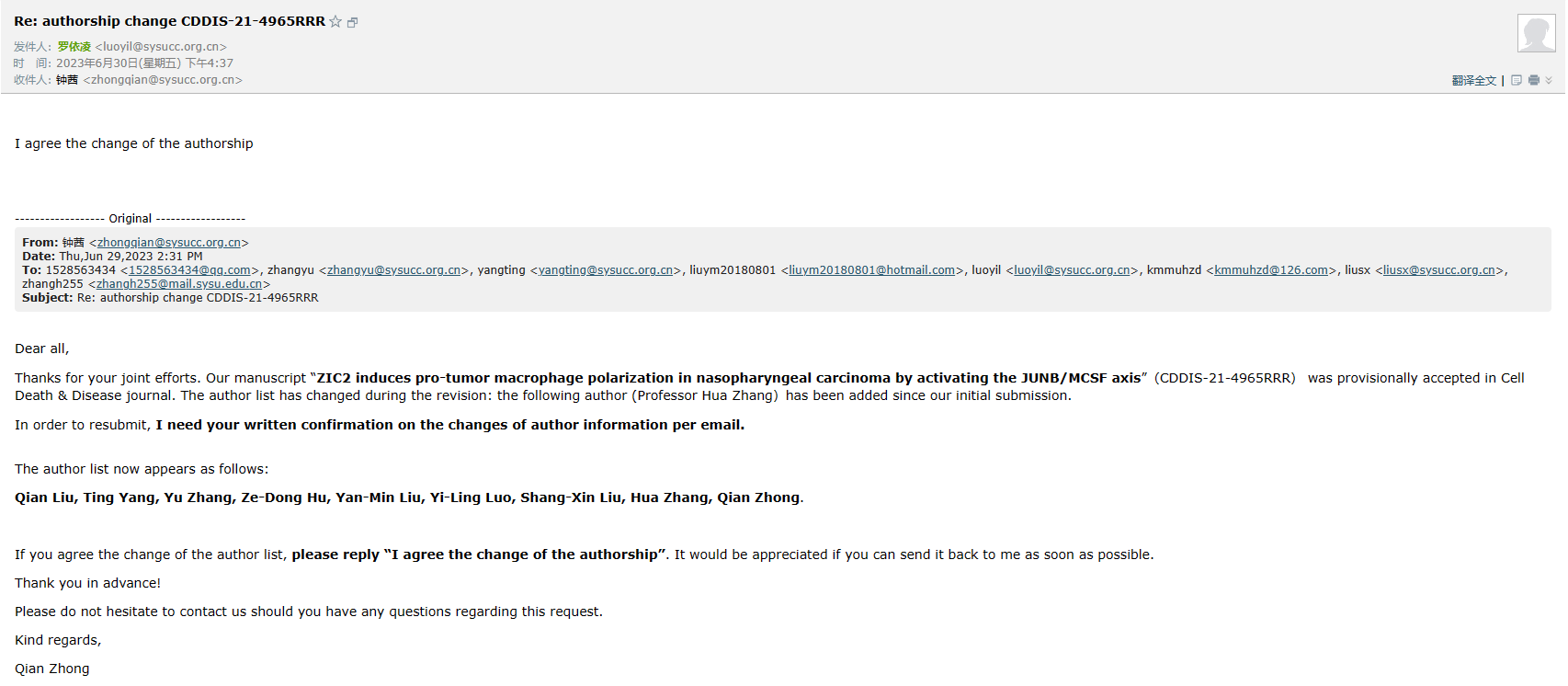


Dr. Shang-Xin Liu's agreement:


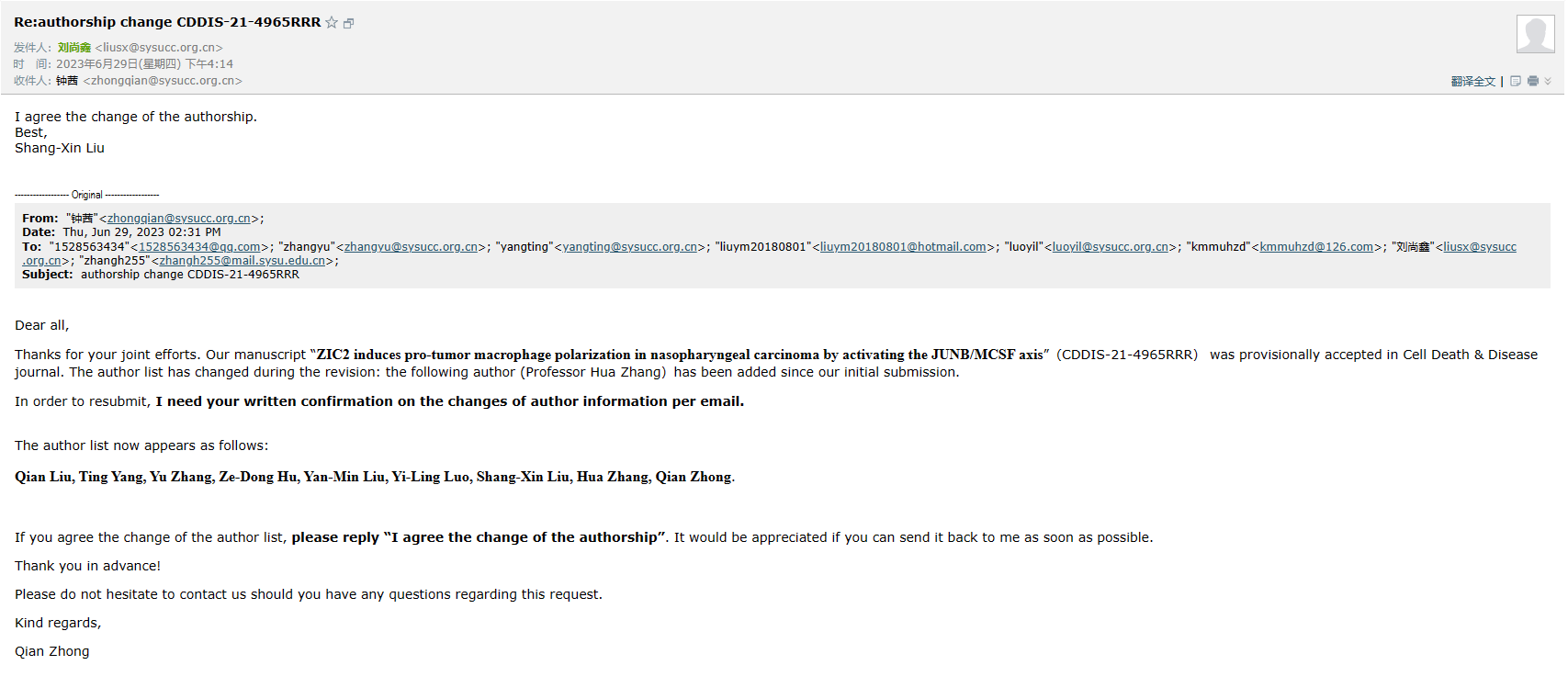


Dr. Hua Zhang's agreement:


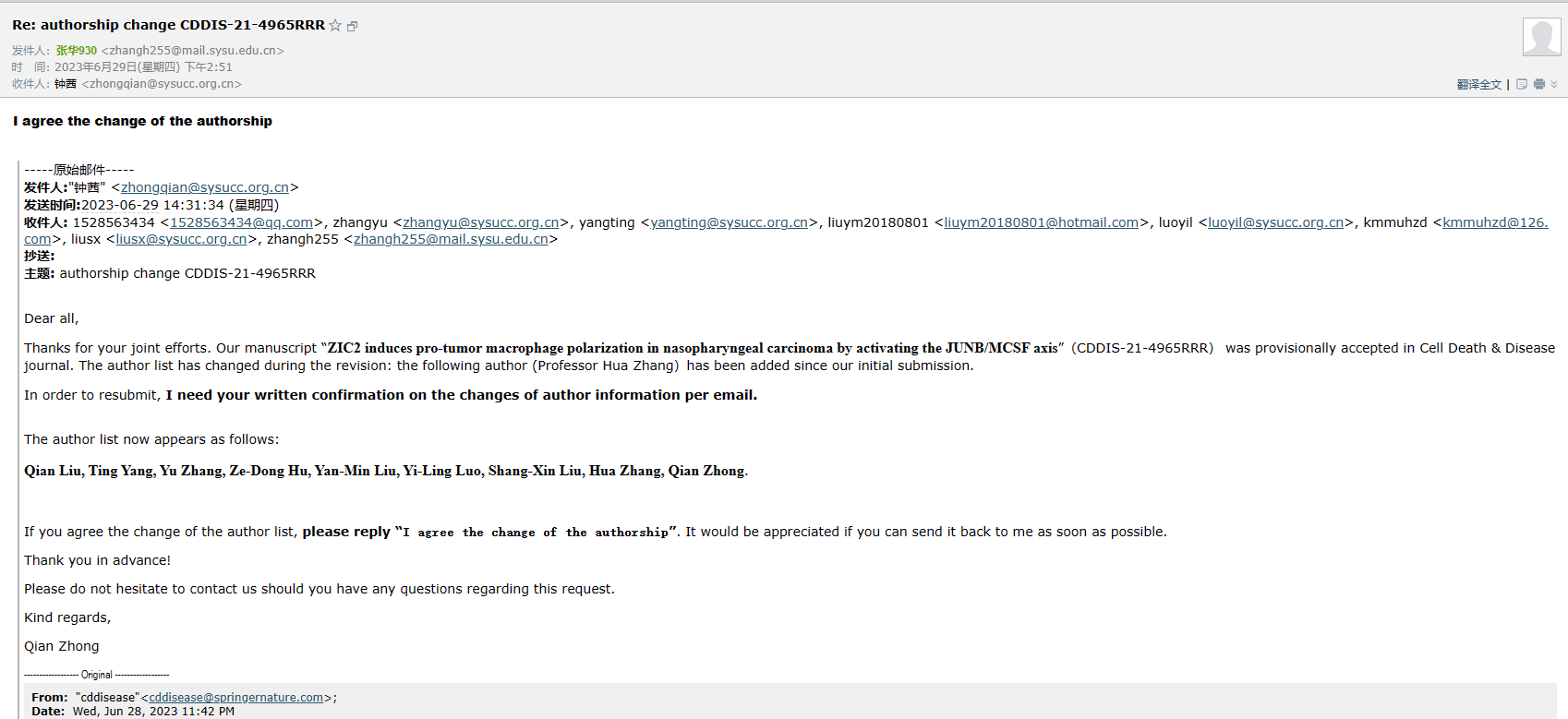

Supplement: Supplementary file 8 — authors' ageements [file 41419_2023_5983_MOESM8_ESM.docx]
