# Supplementary material for: ZIC2 induces pro-tumor macrophage polarization in nasopharyngeal carcinoma by activating the JUNB/MCSF axis
Source: Cell Death Dis. 2023 Jul 21;14(7):455. doi: 10.1038/s41419-023-05983-x (PMC10362010; doi:10.1038/s41419-023-05983-x)
Supplement: Supplementary file 10 — Original Data File [file 41419_2023_5983_MOESM10_ESM.pdf]

Figure 1 C

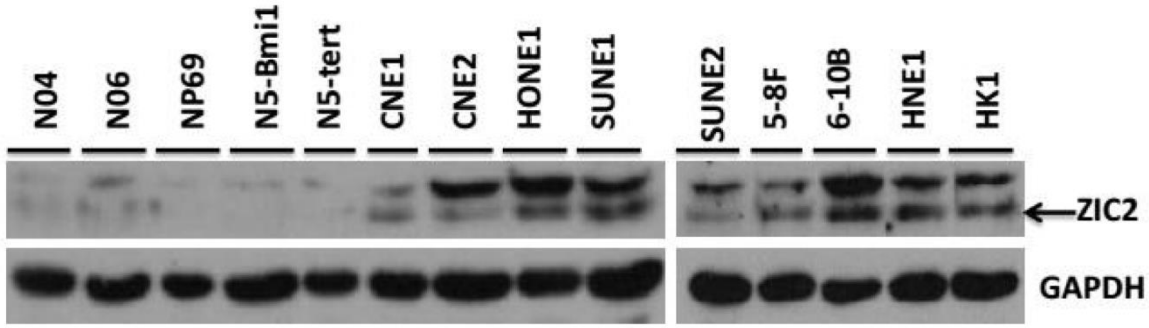
N04
N06
NP69
N5-Bmi1
N5-tert
CNE1
CNE2
HONE1
SUNE1
SUNE2
5-8F
6-10B
HNE1
HK1
ZIC2
GAPDH

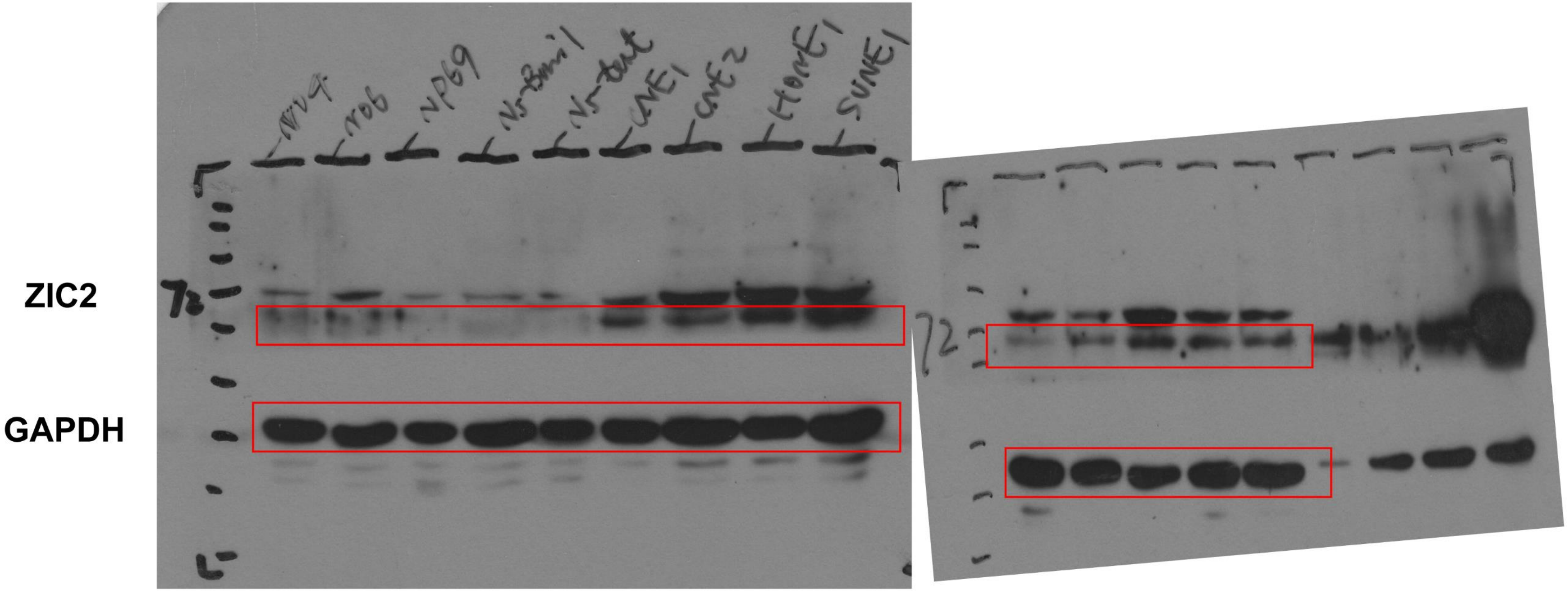
ZIC2
GAPDH

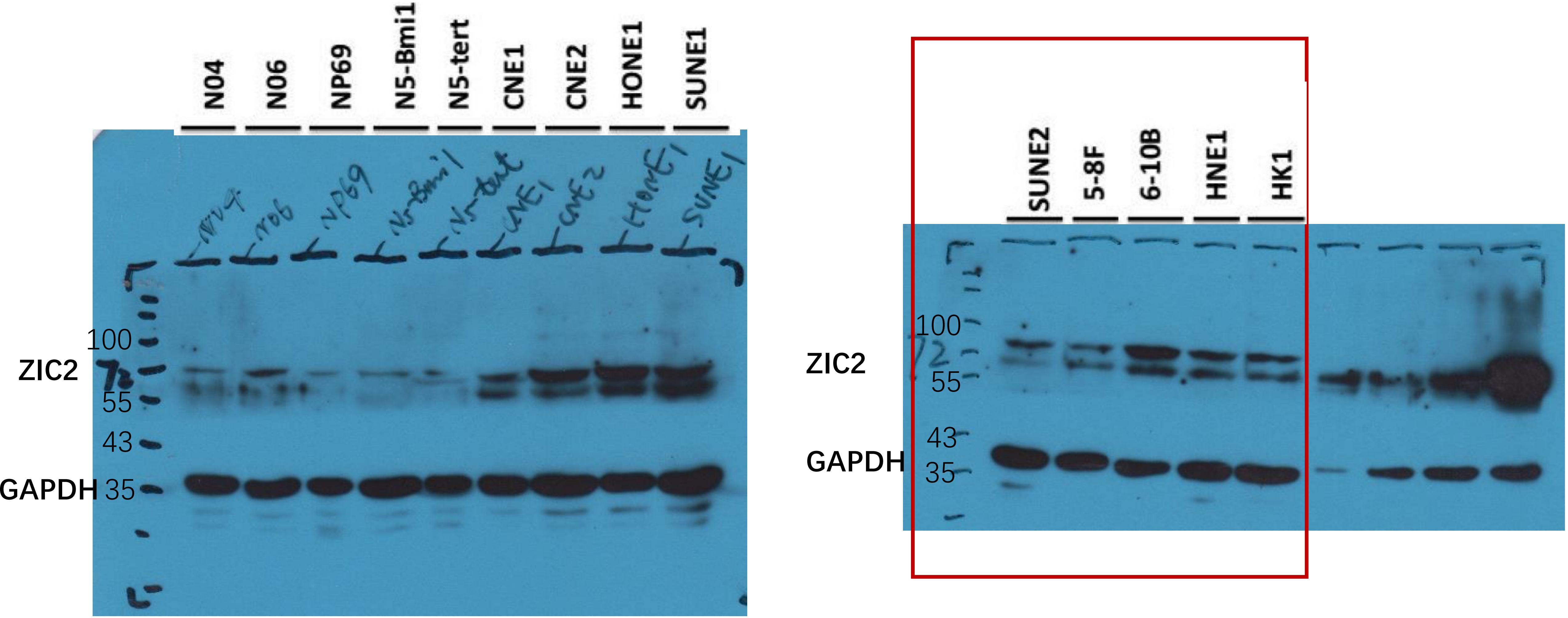
N04
N06
NP69
N5-Bmi1
N5-tert
CNE1
CNE2
HONE1
SUNE1
100
ZIC2
55
43
GAPDH
35
SUNE2
5-8F
6-10B
HNE1
HK1
100
ZIC2
55
43
GAPDH
35

Figure 1 D

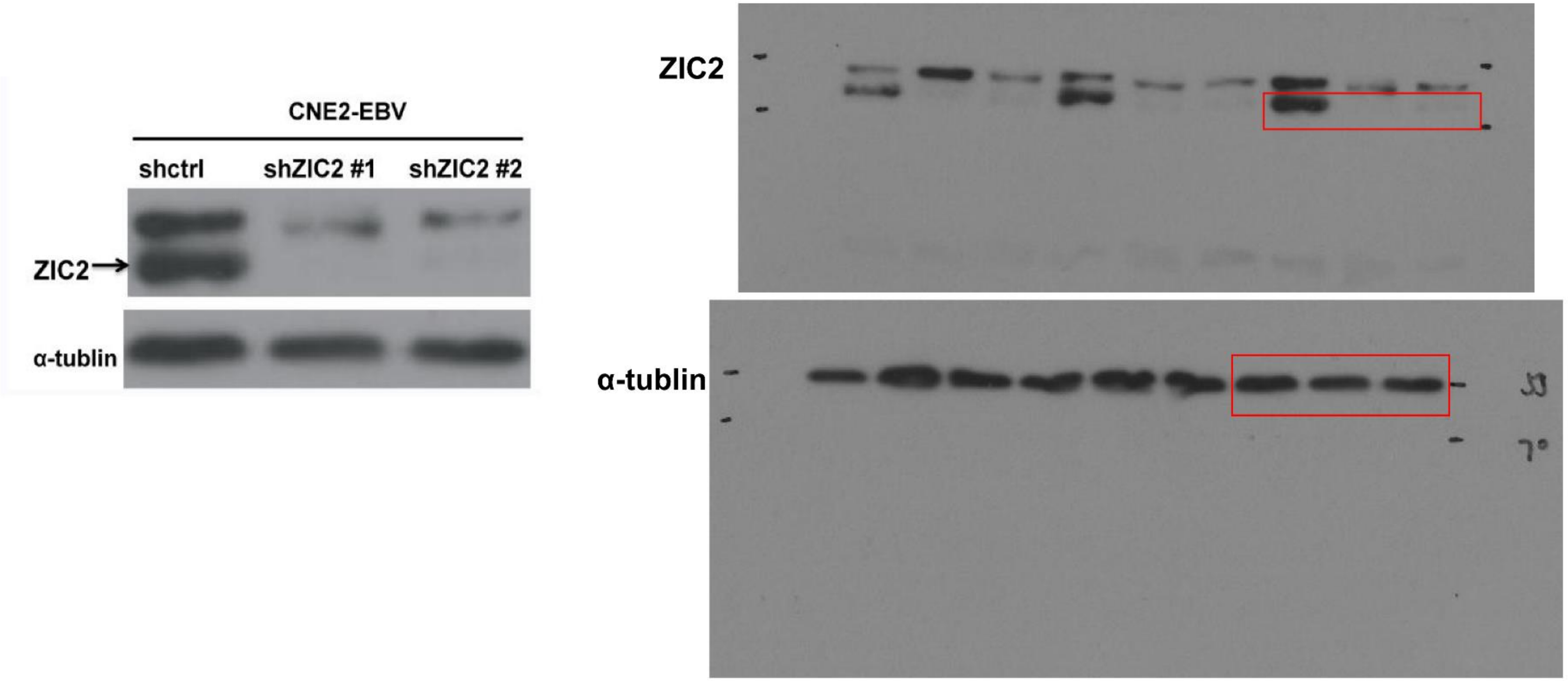

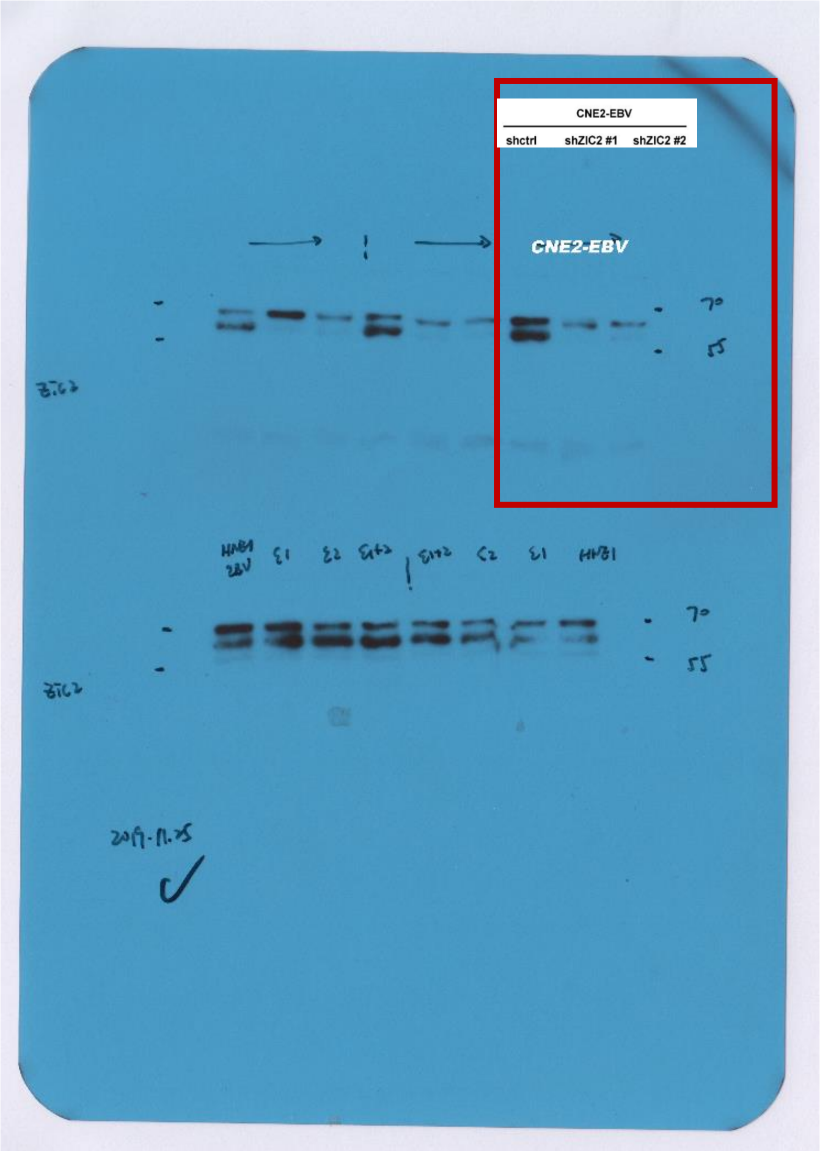
CNE2-EBV
shctrl shZIC2 #1 shZIC2 #2
CNE2-EBV

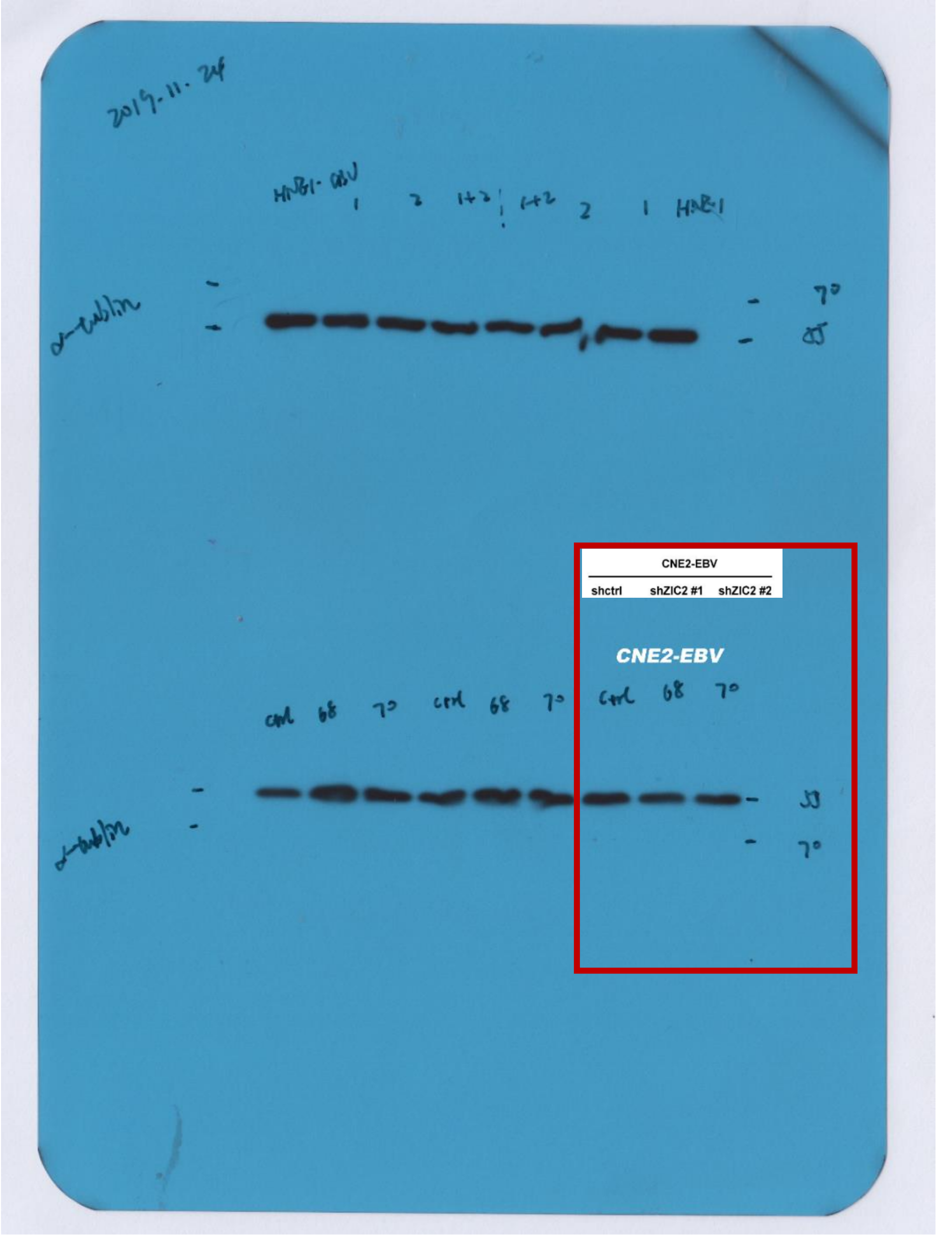
CNE2-EBV
shctrl shZIC2 #1 shZIC2 #2
CNE2-EBV

Figure 1 D

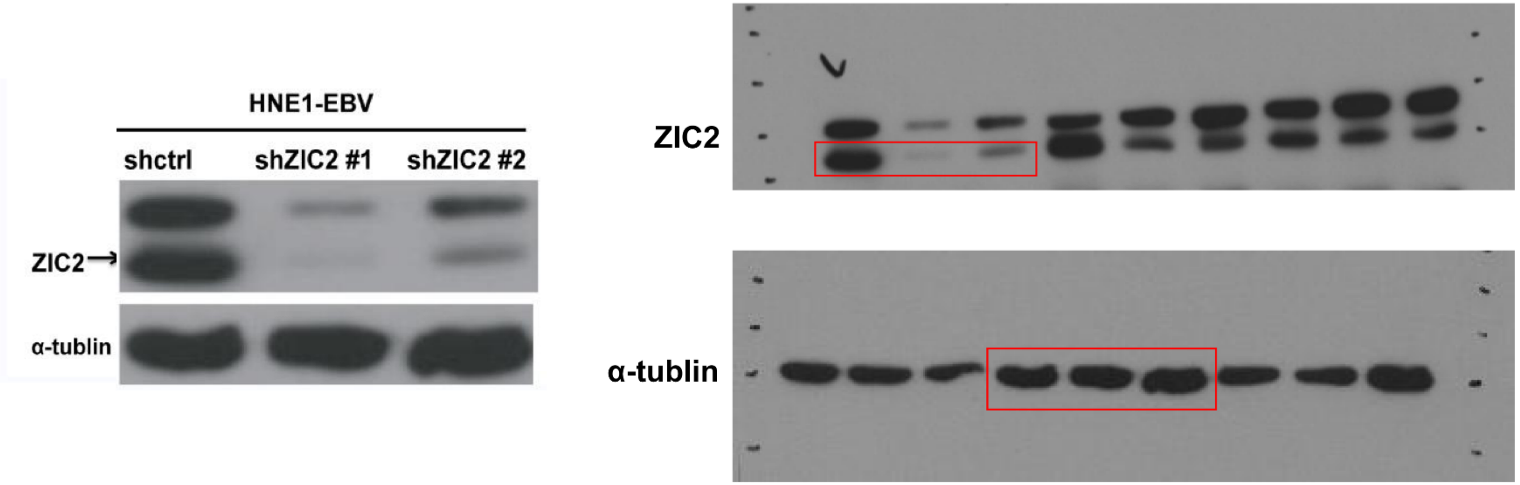

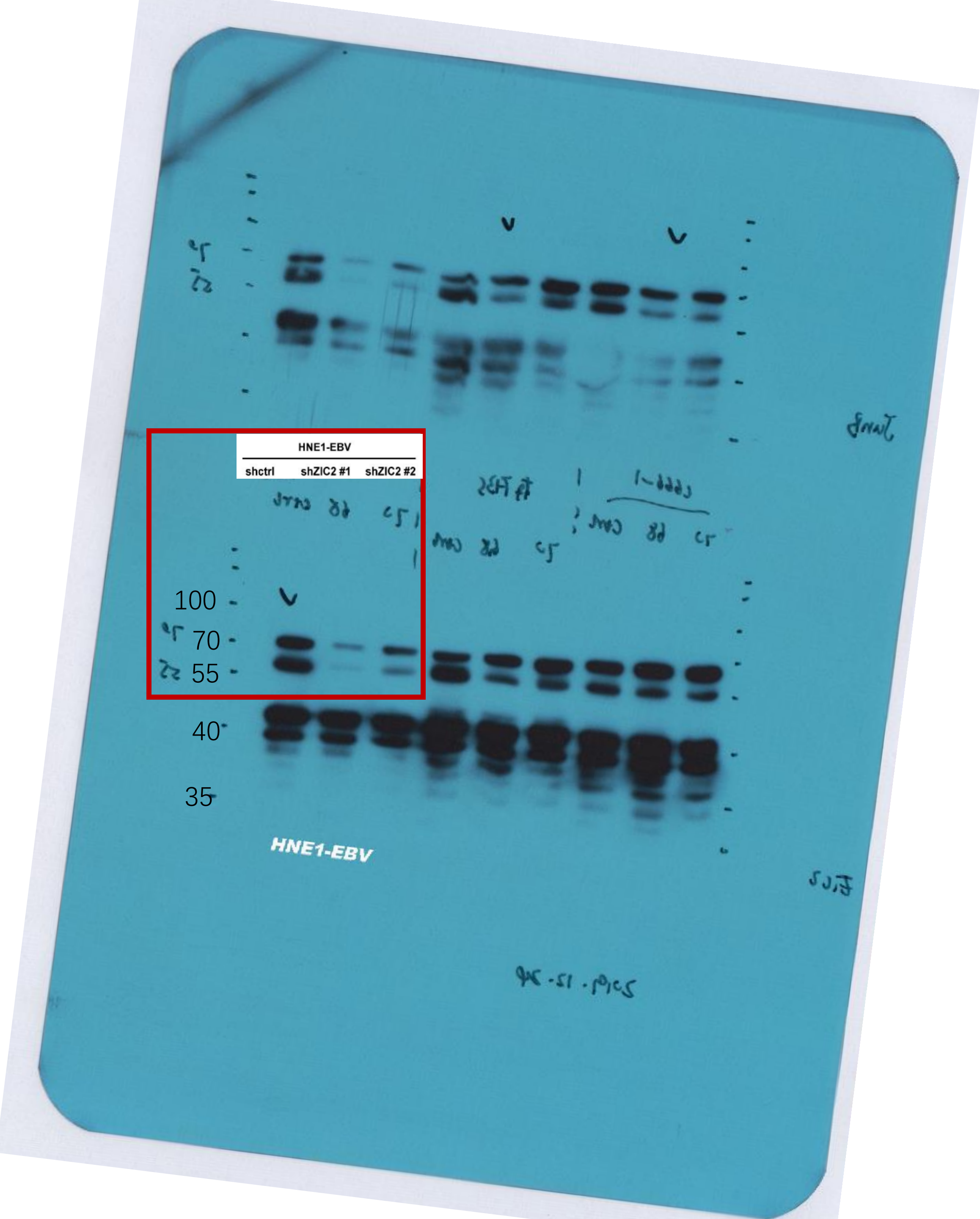
HNE1-EBV
shctrl
shZIC2 #1
shZIC2 #2
100
70
55
40
35
HNE1-EBV

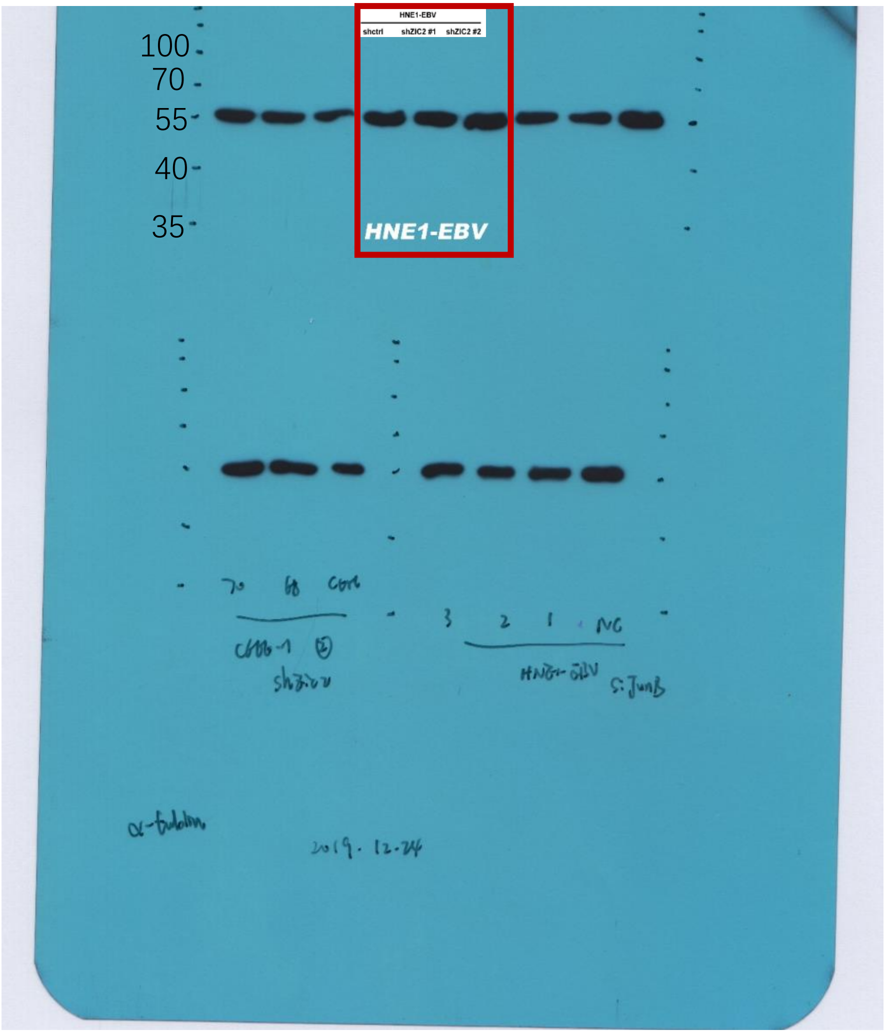
HNE1-EBV
shctrl
shZIC2 #1
shZIC2 #2
100
70
55
40
35
HNE1-EBV

Figure 3 C

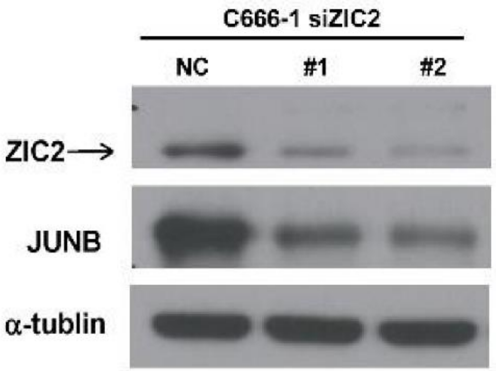

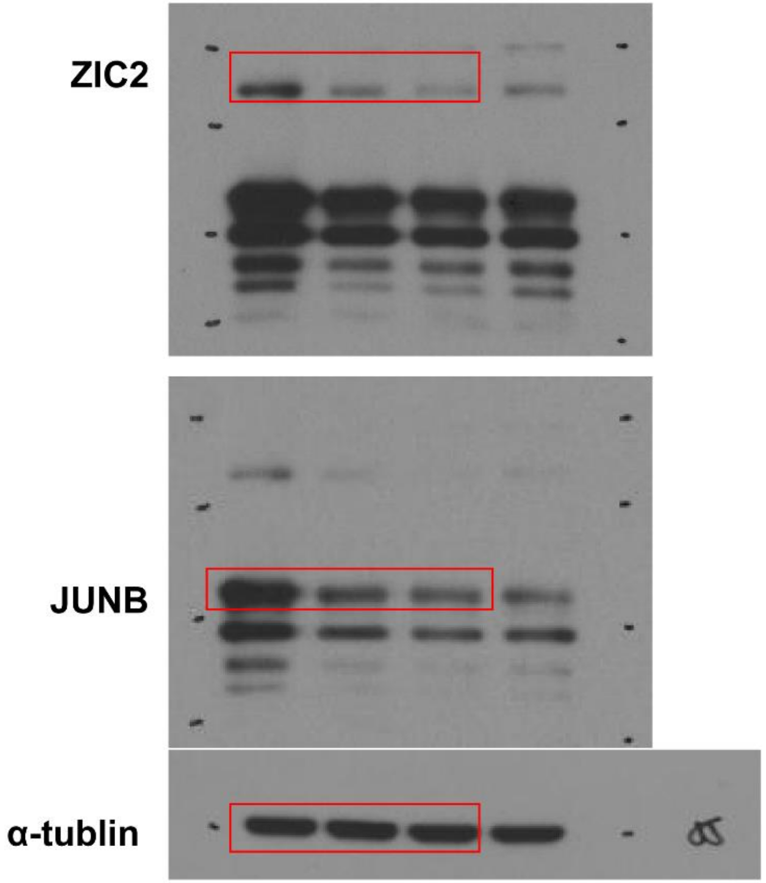

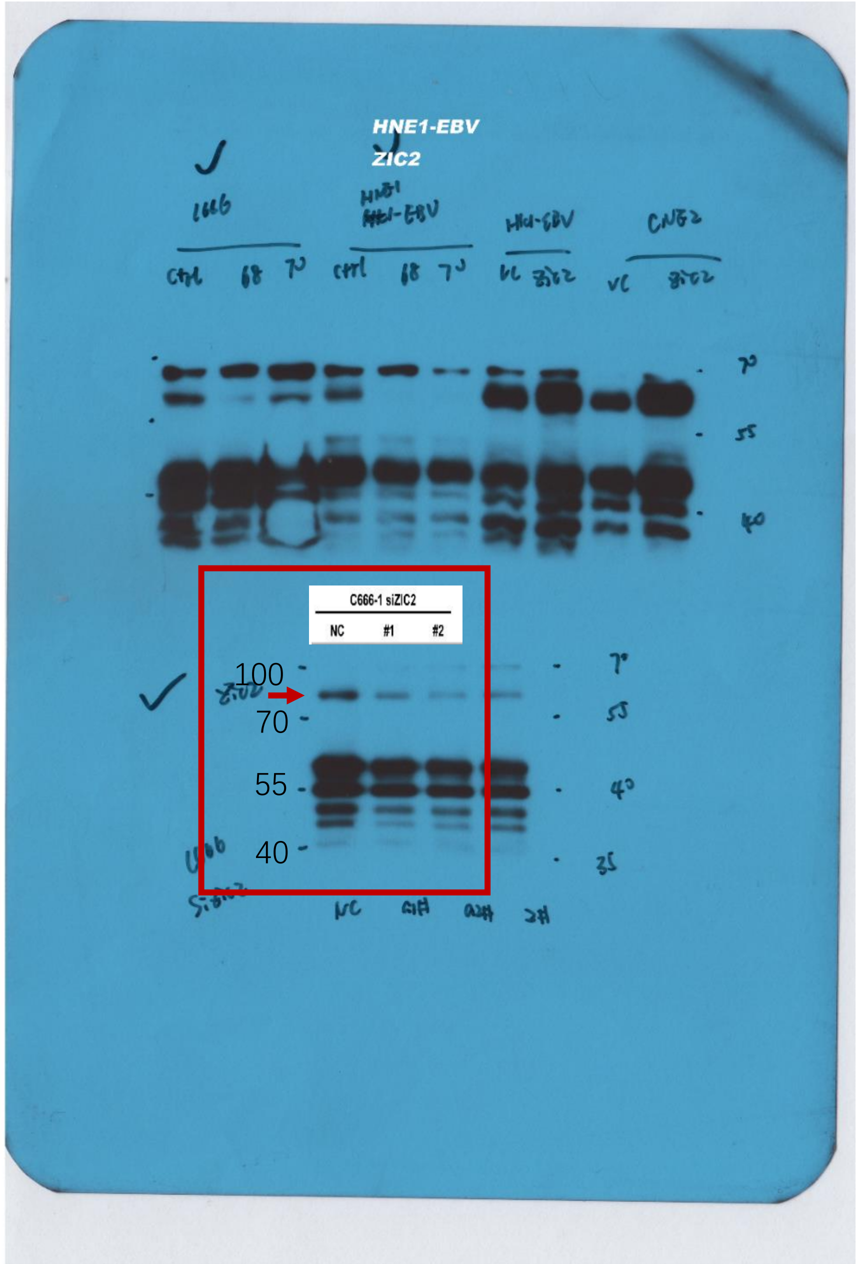
HNE1-EBV
ZIC2
C666-1 siZIC2
NC
#1
#2
100
70
55
40

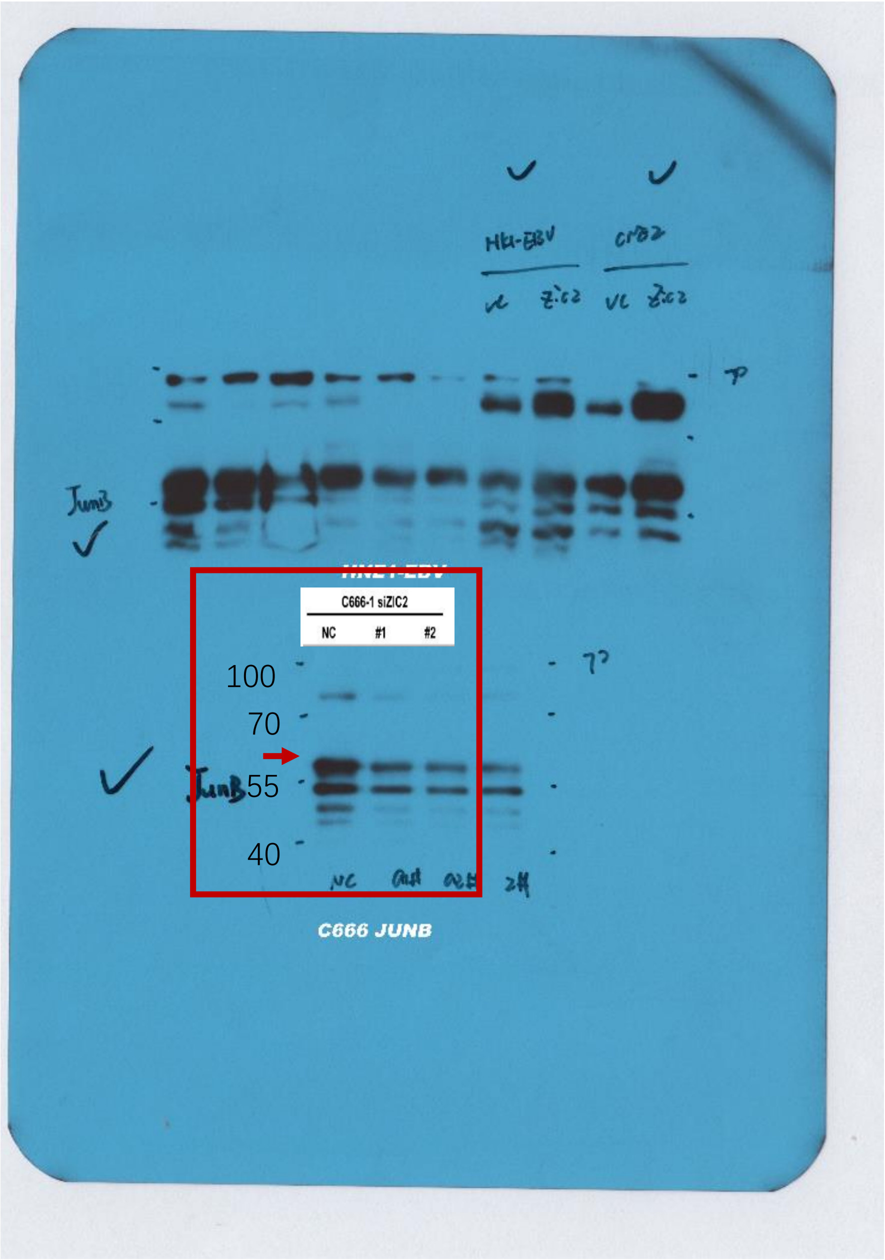
C666-1 siZIC2
NC
#1
#2
100
70
55
40
C666 JUNB

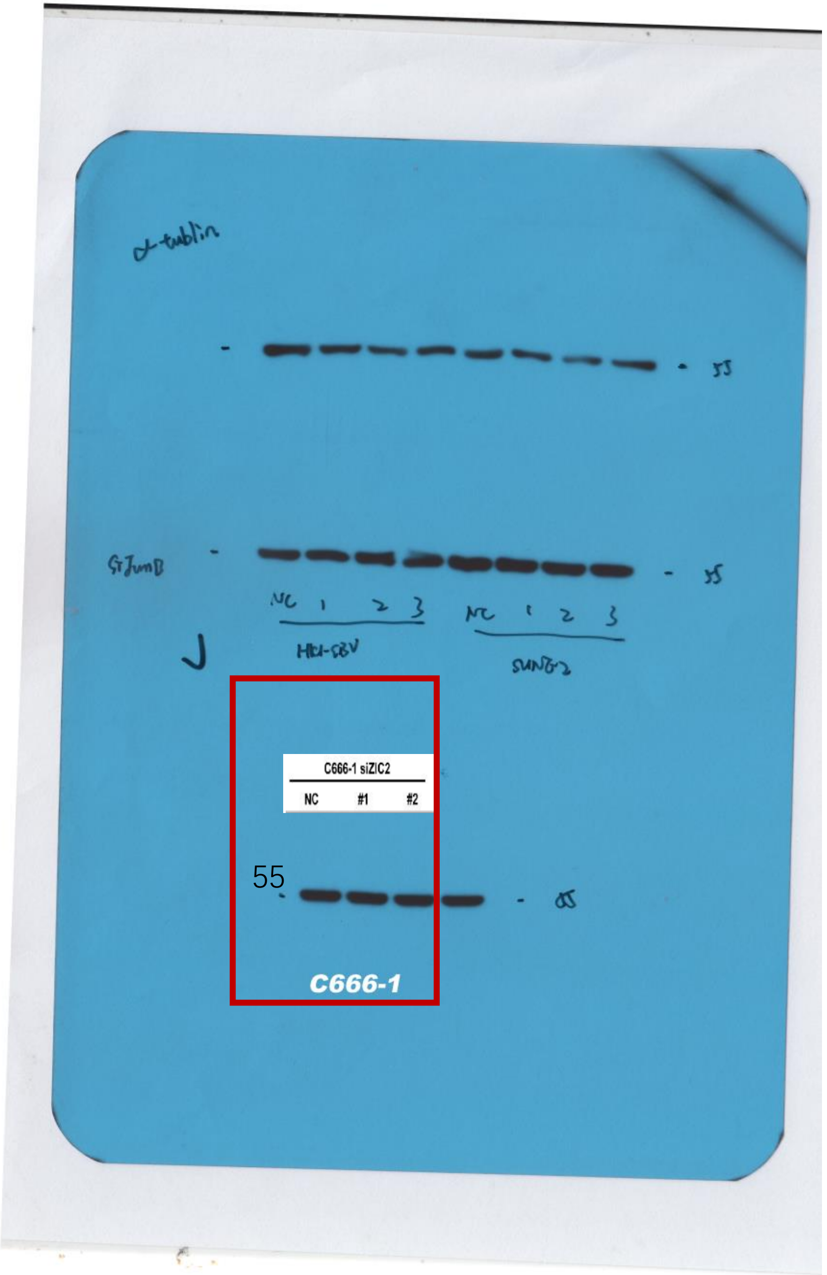

α-tublin
55
GTJunB
55
NC 1 2 3
HK1-EBV
NC 1 2 3
SUNE2
C666-1 siZIC2
NC
#1
#2
55
55
C666-1

Figure 3 C

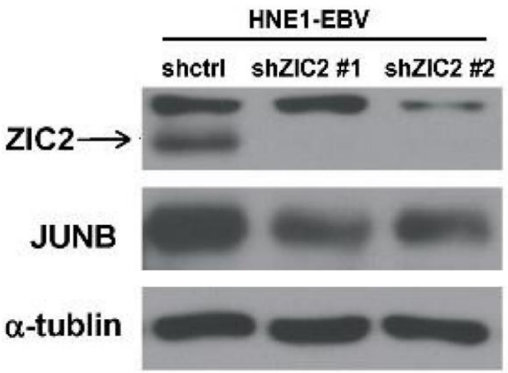
HNE1-EBV
shctrl
shZIC2 #1
shZIC2 #2
ZIC2
JUNB
α-tublin

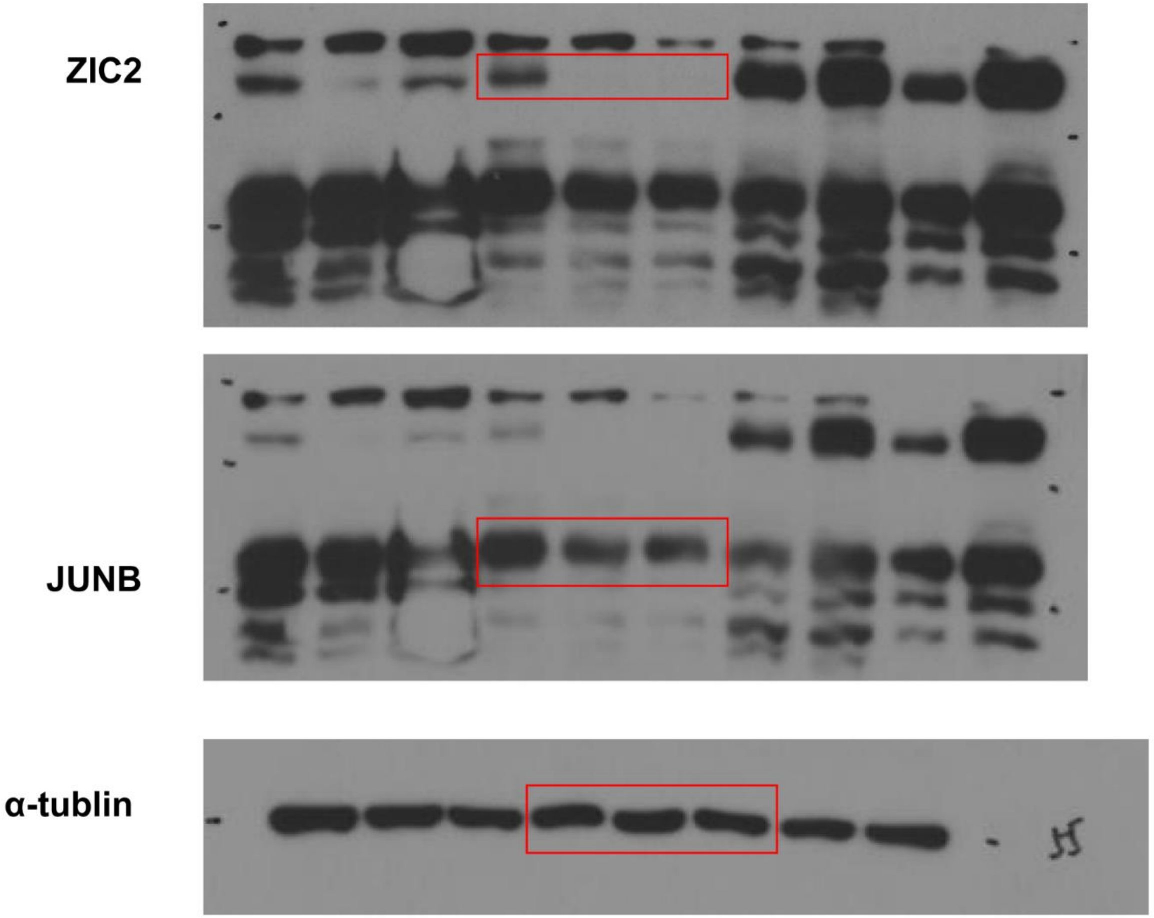
ZIC2
JUNB
α-tublin

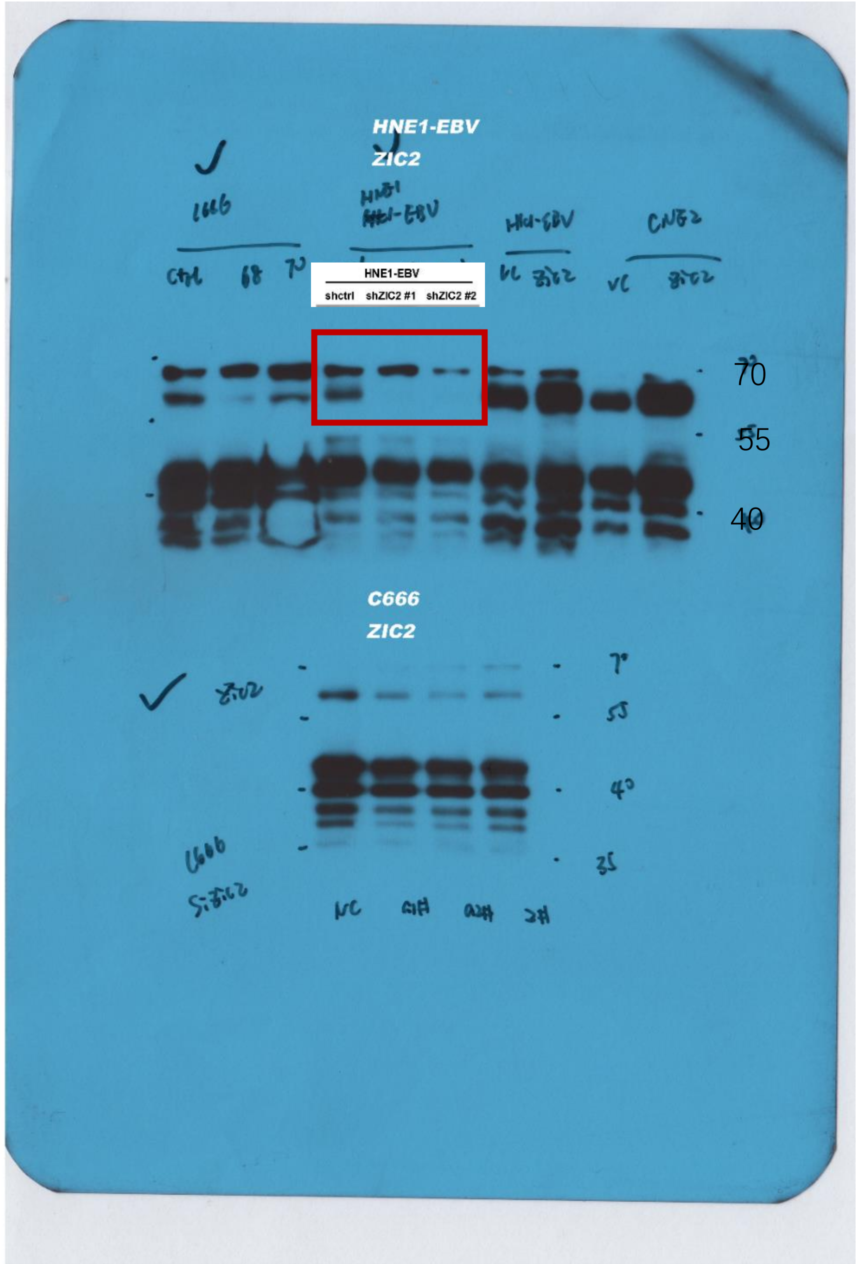
HNE1-EBV
ZIC2
HNE1-EBV
shctrl
shZIC2 #1
shZIC2 #2
70
55
40
C666
ZIC2

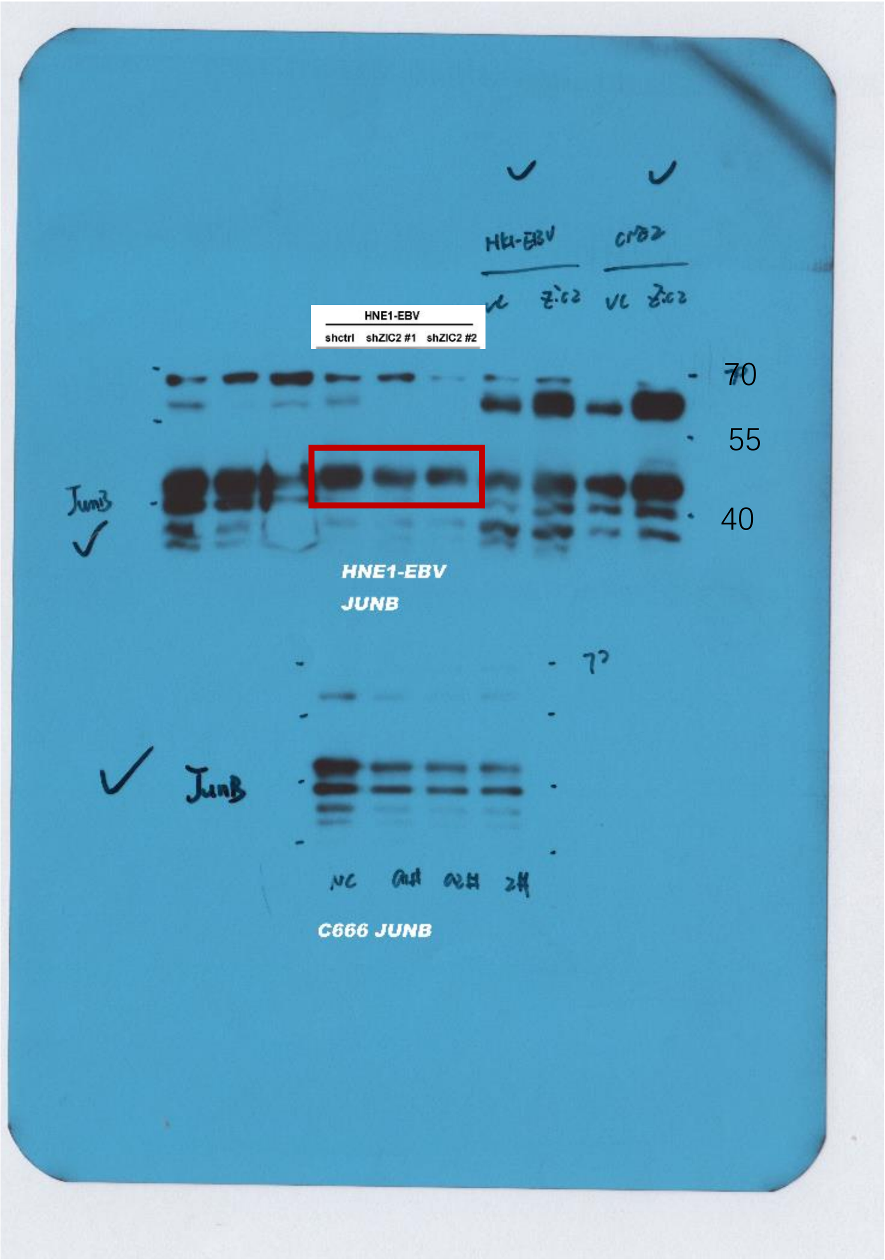
HNE1-EBV
shctrl
shZIC2 #1
shZIC2 #2
70
55
40
HNE1-EBV
JUNB
C666 JUNB

55

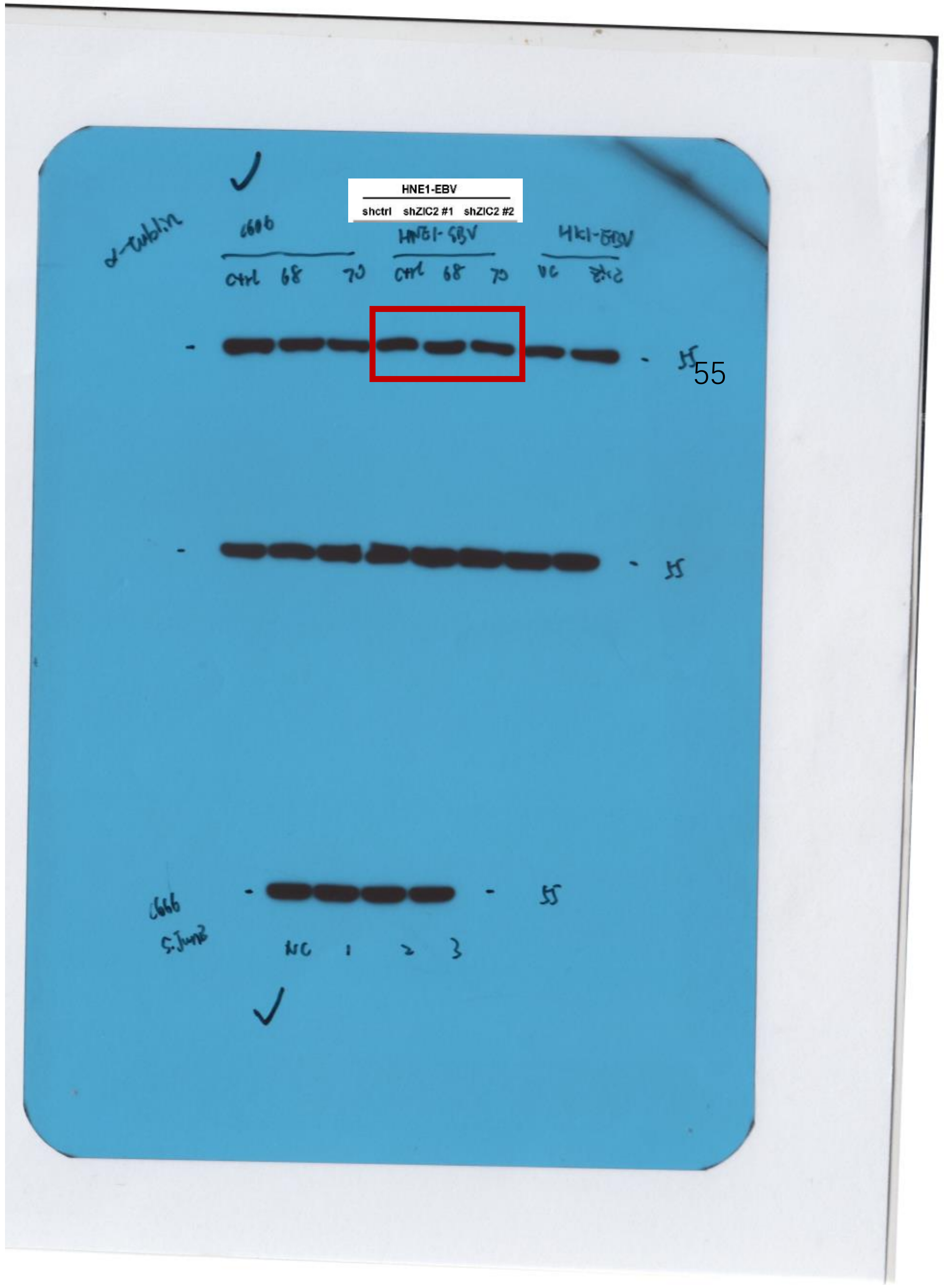

Figure 3 C

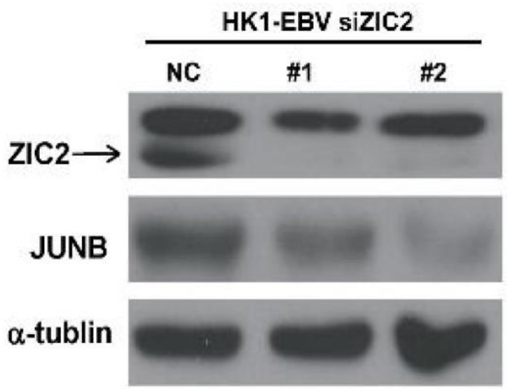

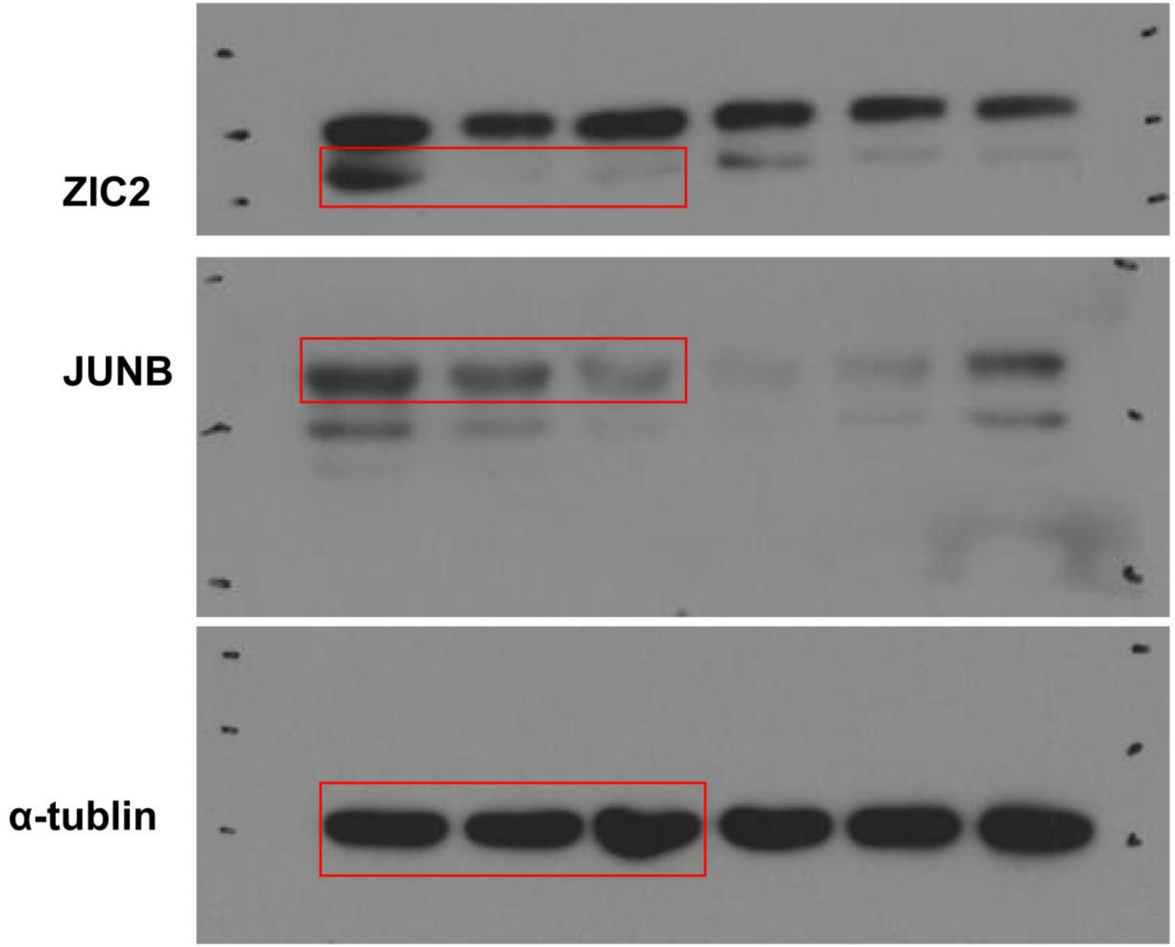

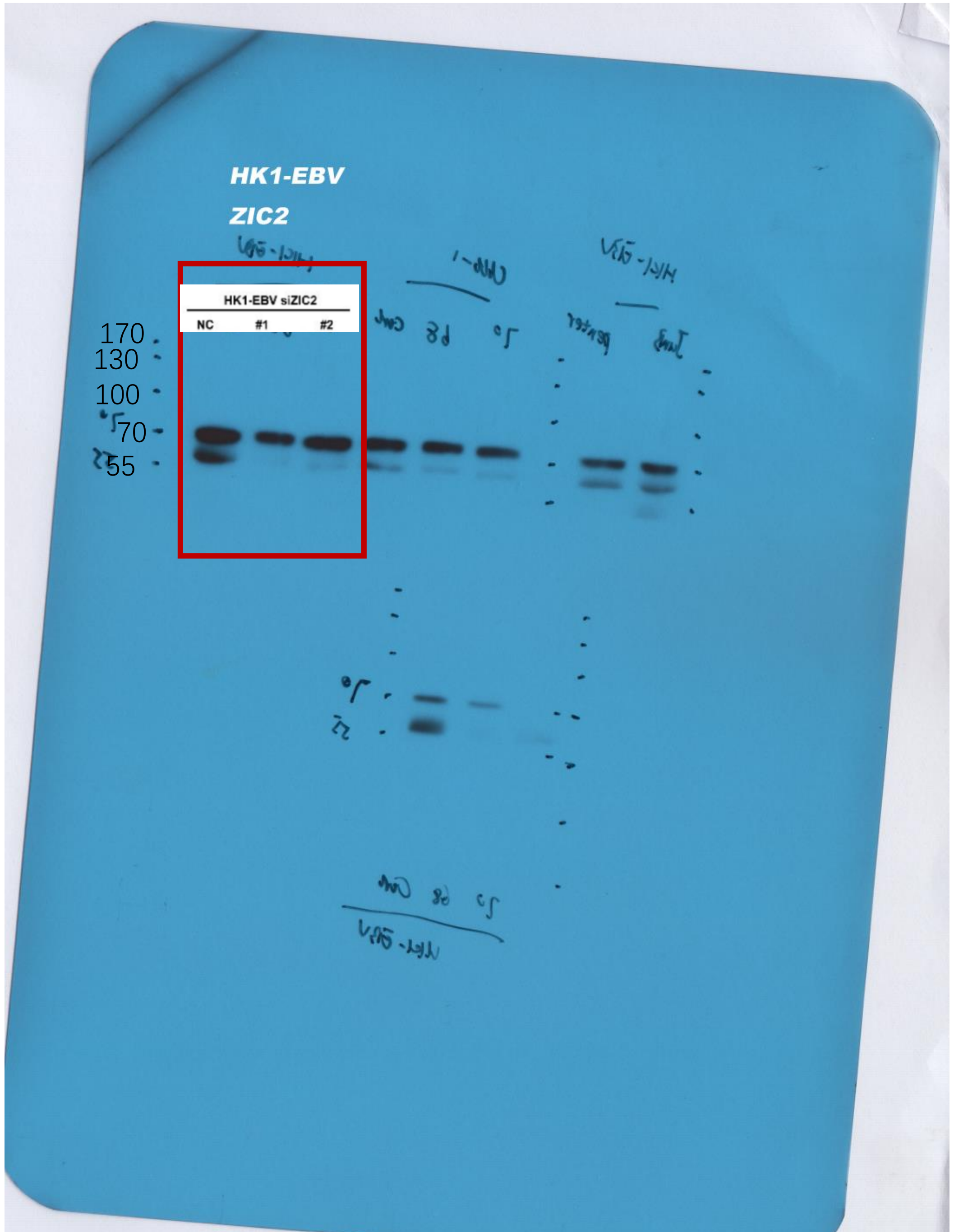
HK1-EBV
ZIC2
HK1-EBV siZIC2
NC
#1
#2
170
130
100
70
55

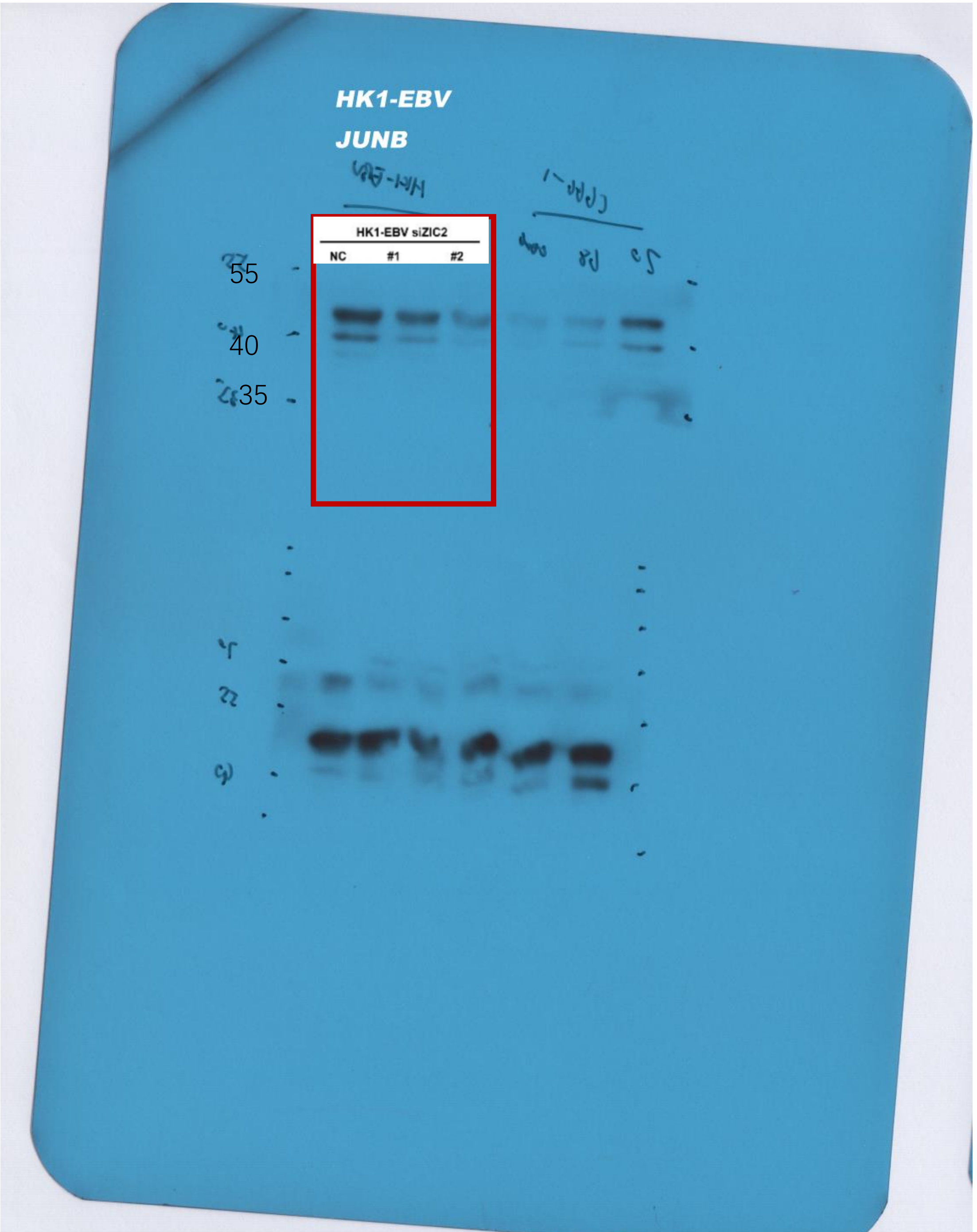
HK1-EBV
JUNB
HK1-EBV siZIC2
NC
#1
#2
55
40
35

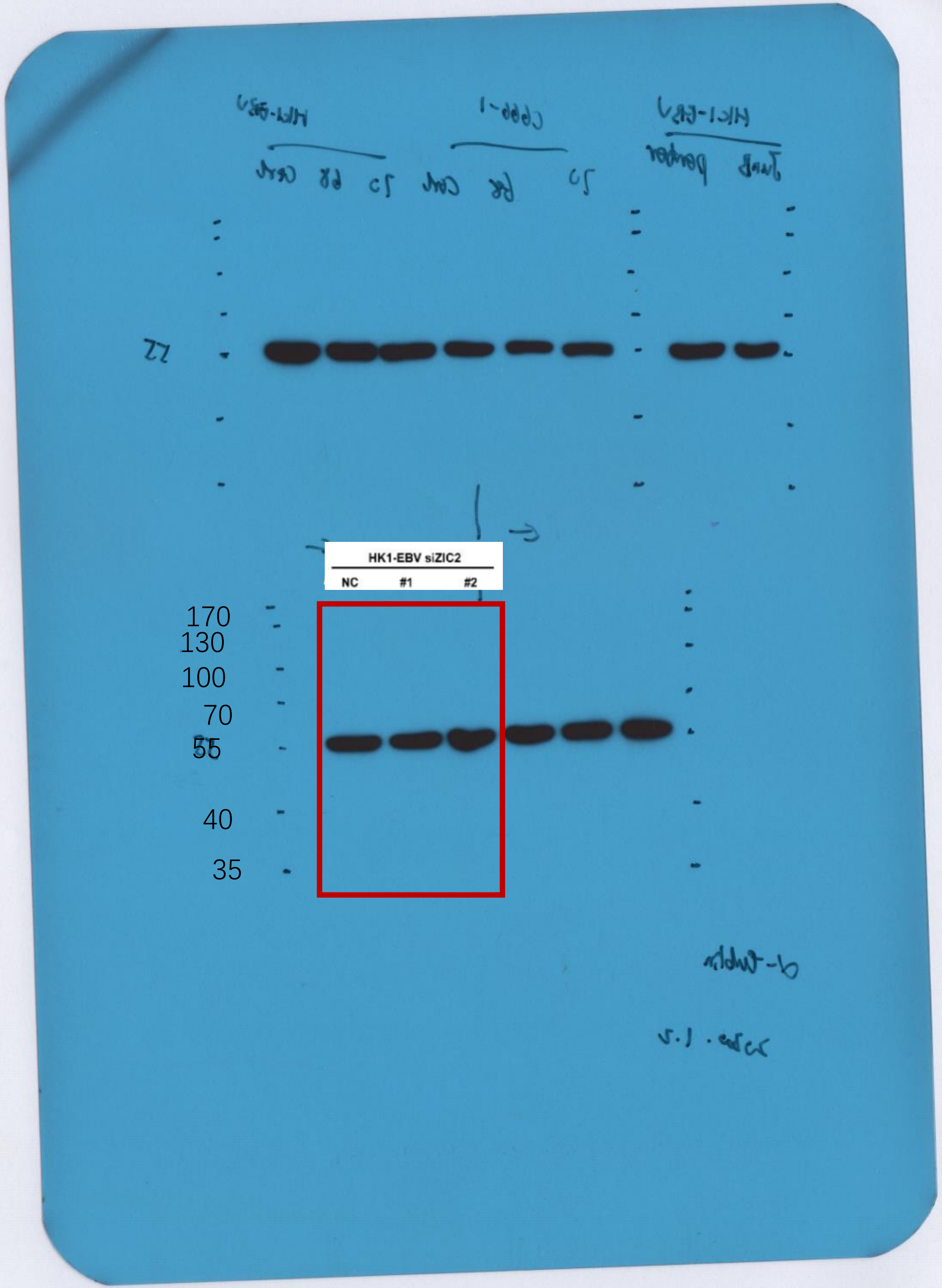
HK1-EBV siZIC2
NC
#1
#2
170
130
100
70
55
40
35

Figure 3 D

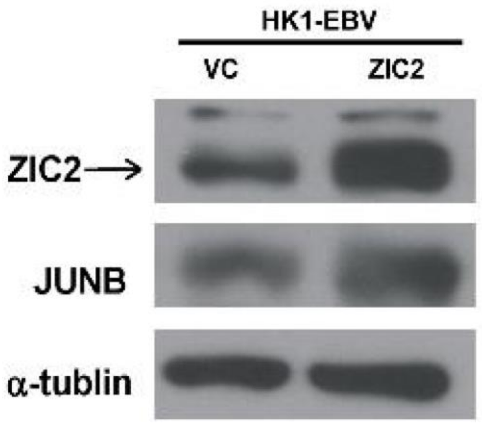

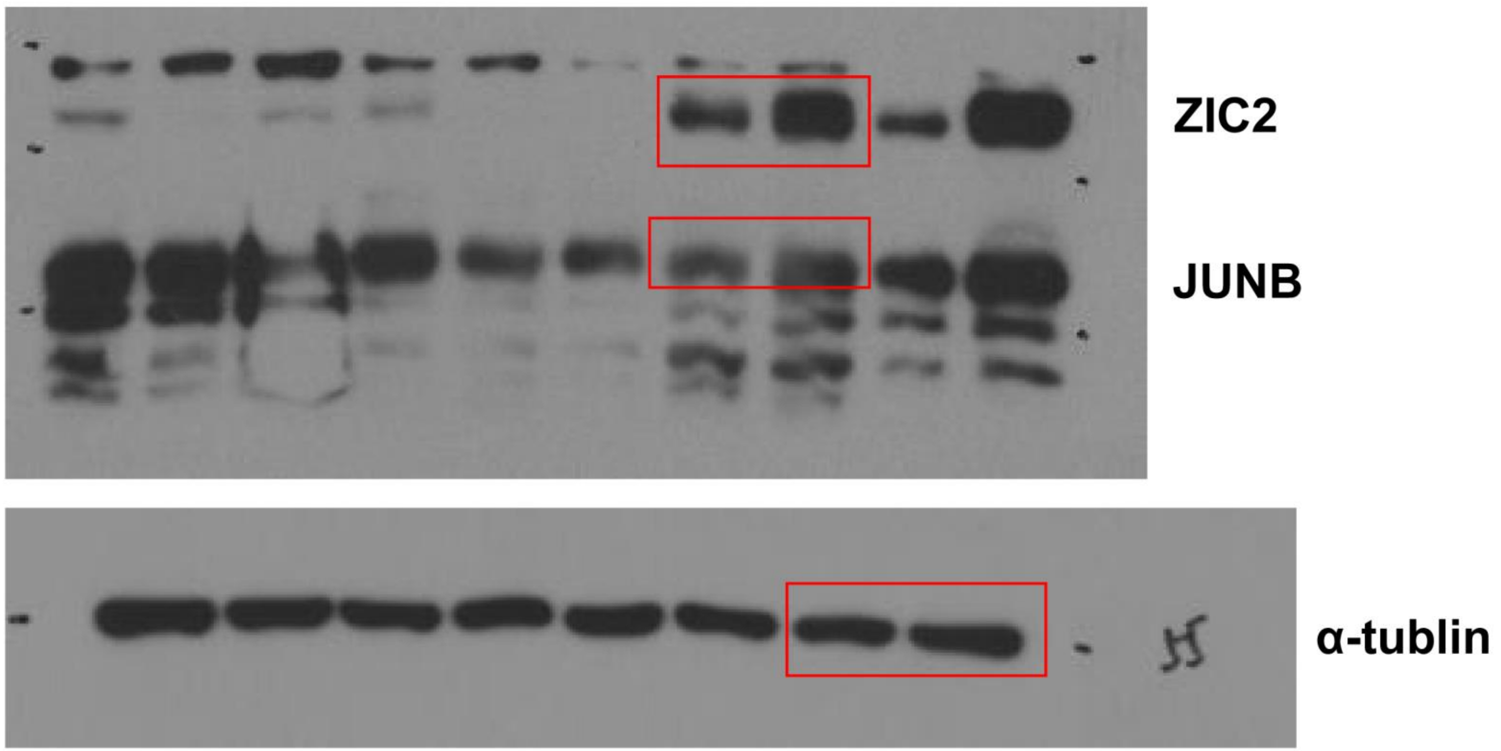

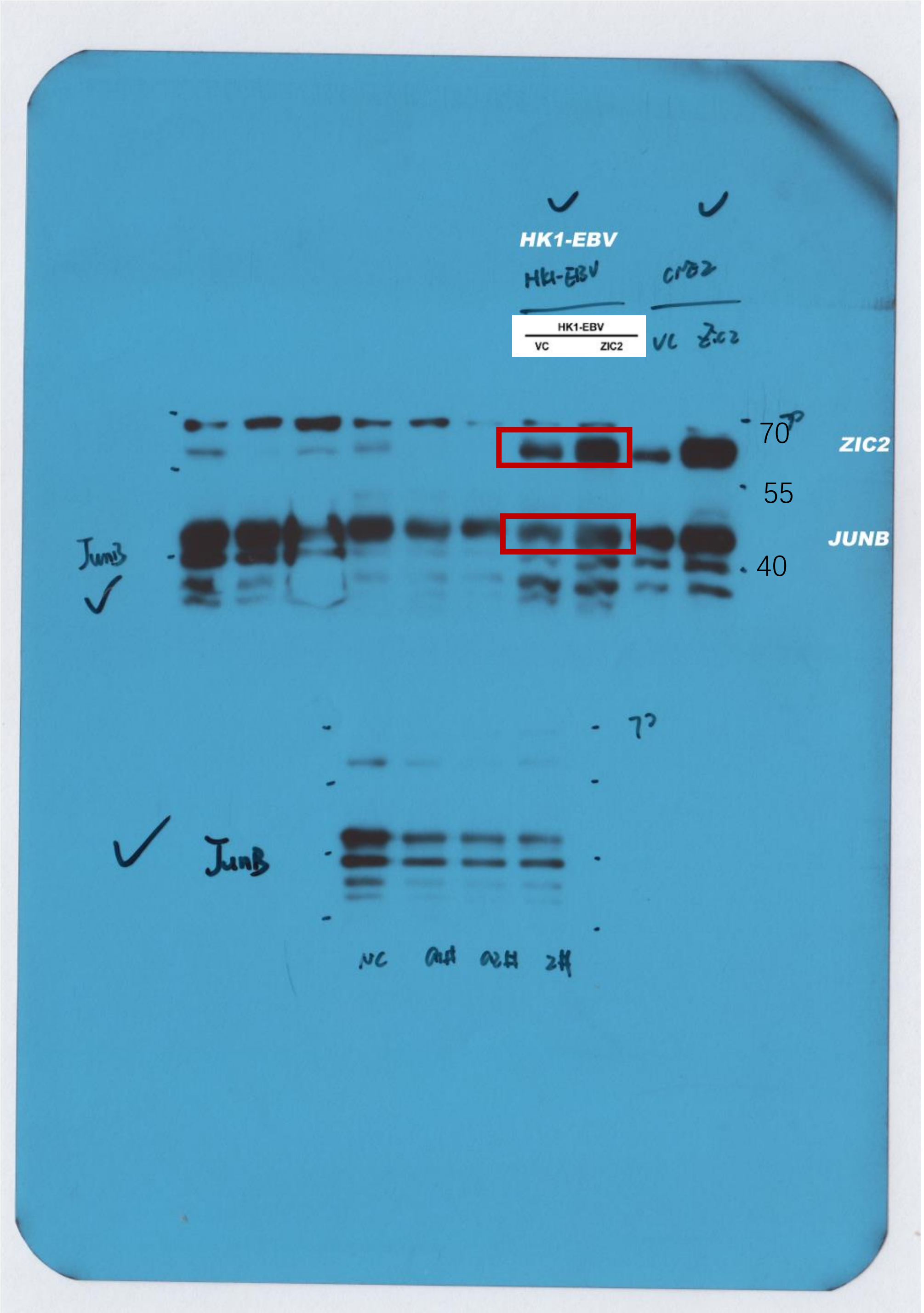
HK1-EBV
HK1-EBV
VC
ZIC2
70
55
40
ZIC2
JUNB
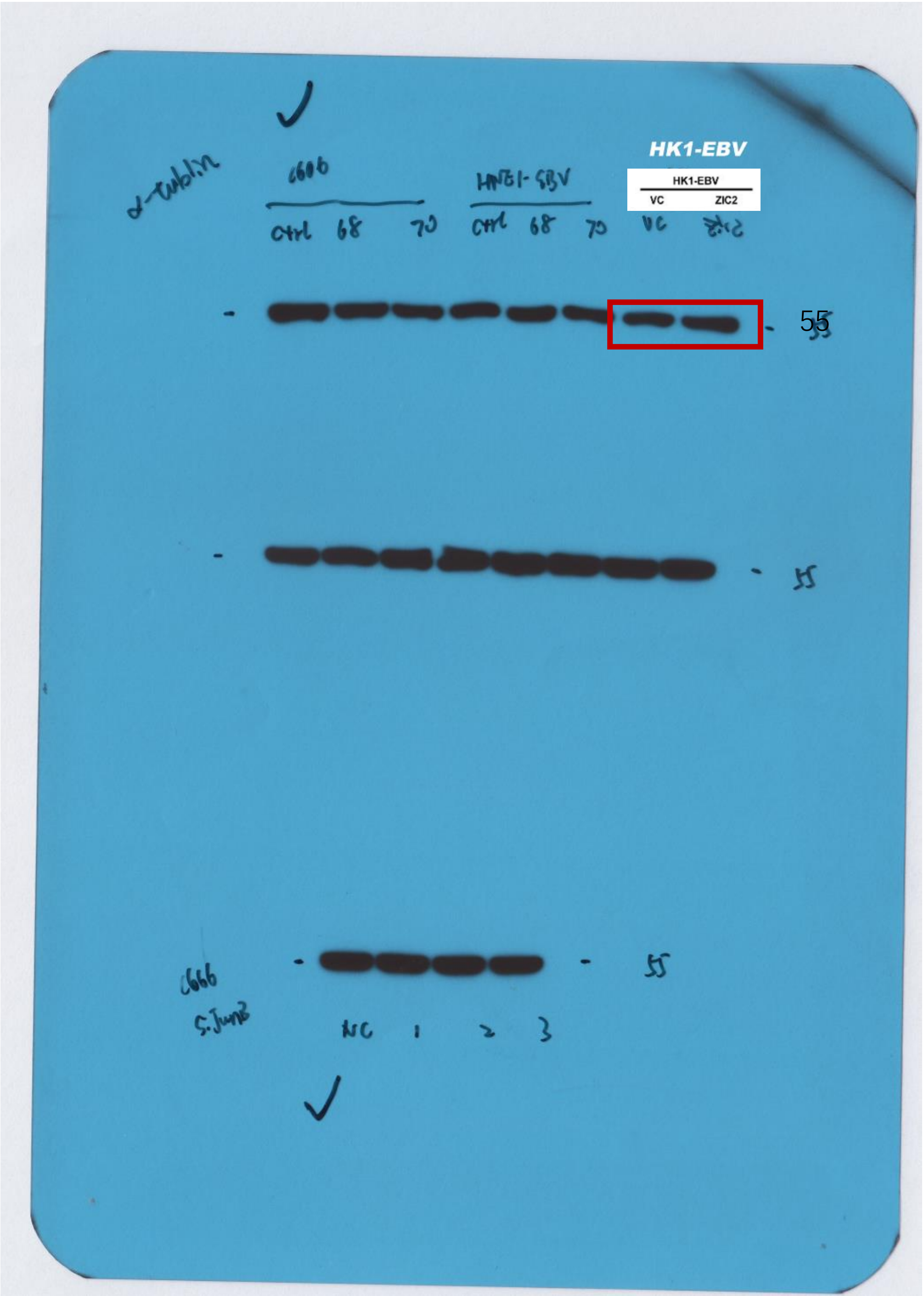
HK1-EBV
HK1-EBV
VC
ZIC2
55

Figure 3 D

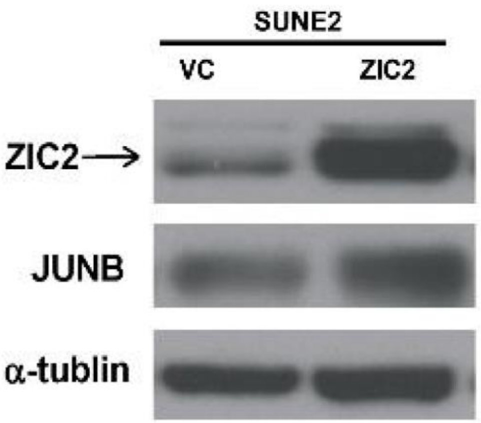
SUNE2
VC
ZIC2
ZIC2
JUNB
α-tublin

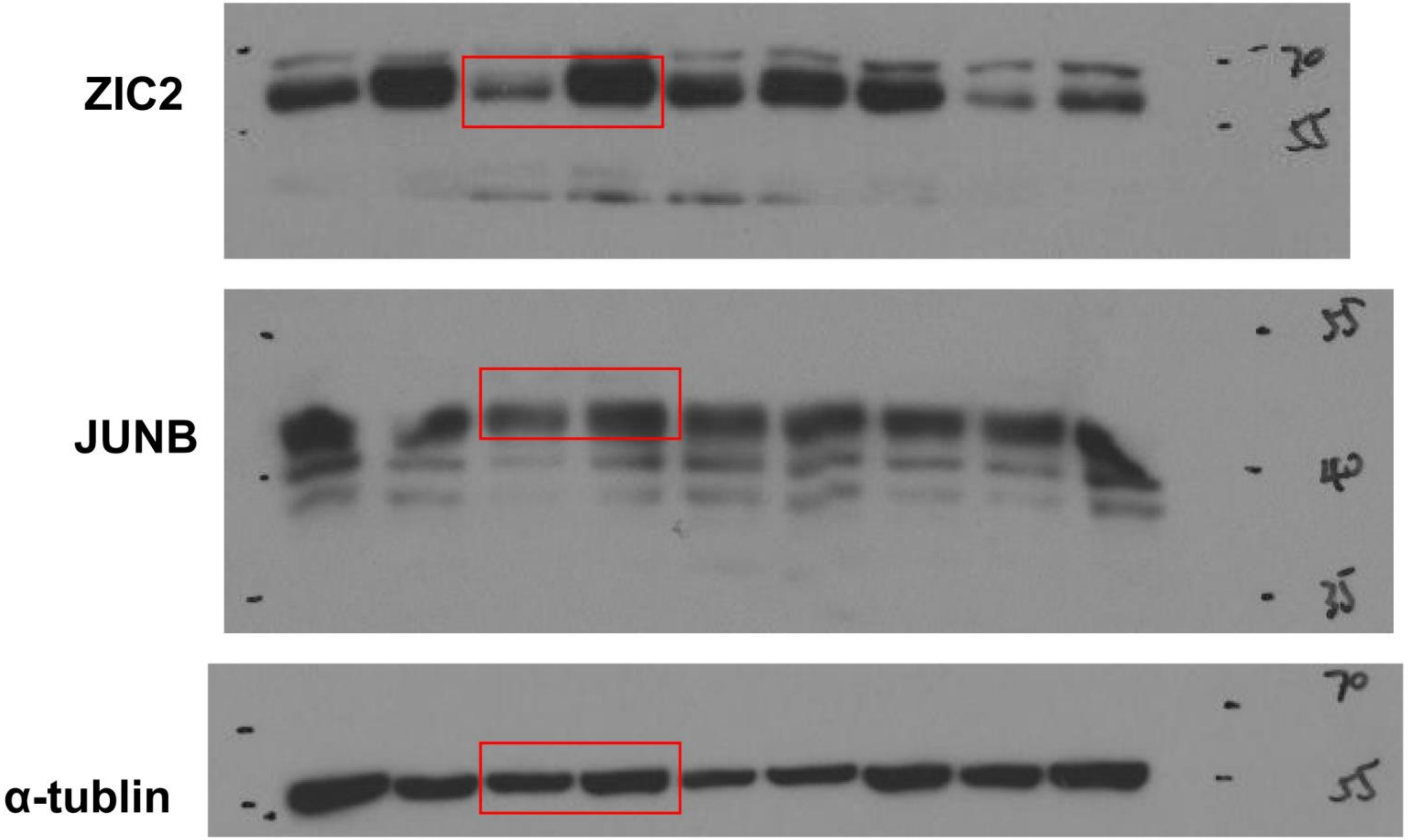
ZIC2
JUNB
α-tublin
70
55
55
40
35
70
55

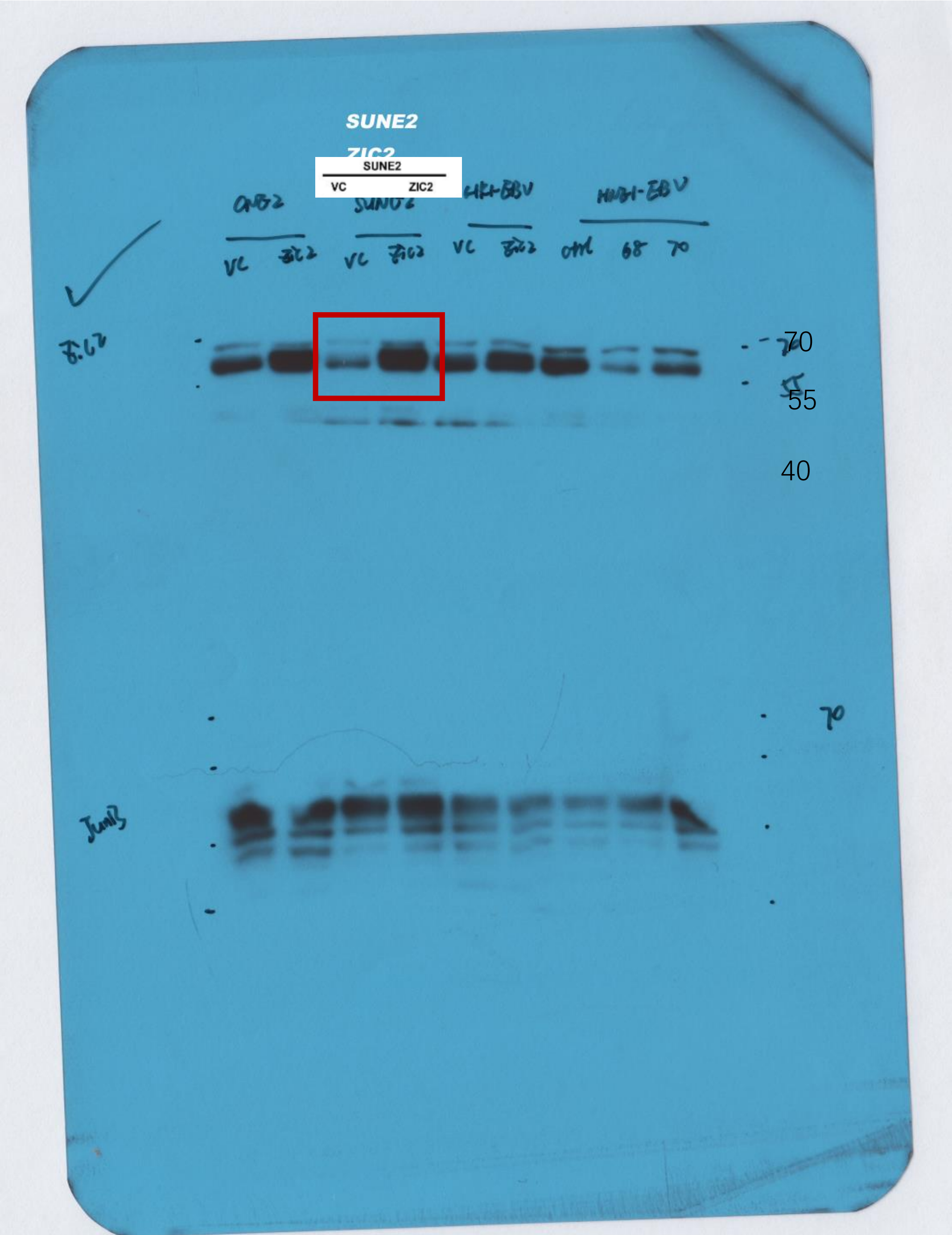
SUNE2
SUNE2
VC
ZIC2
70
55
40

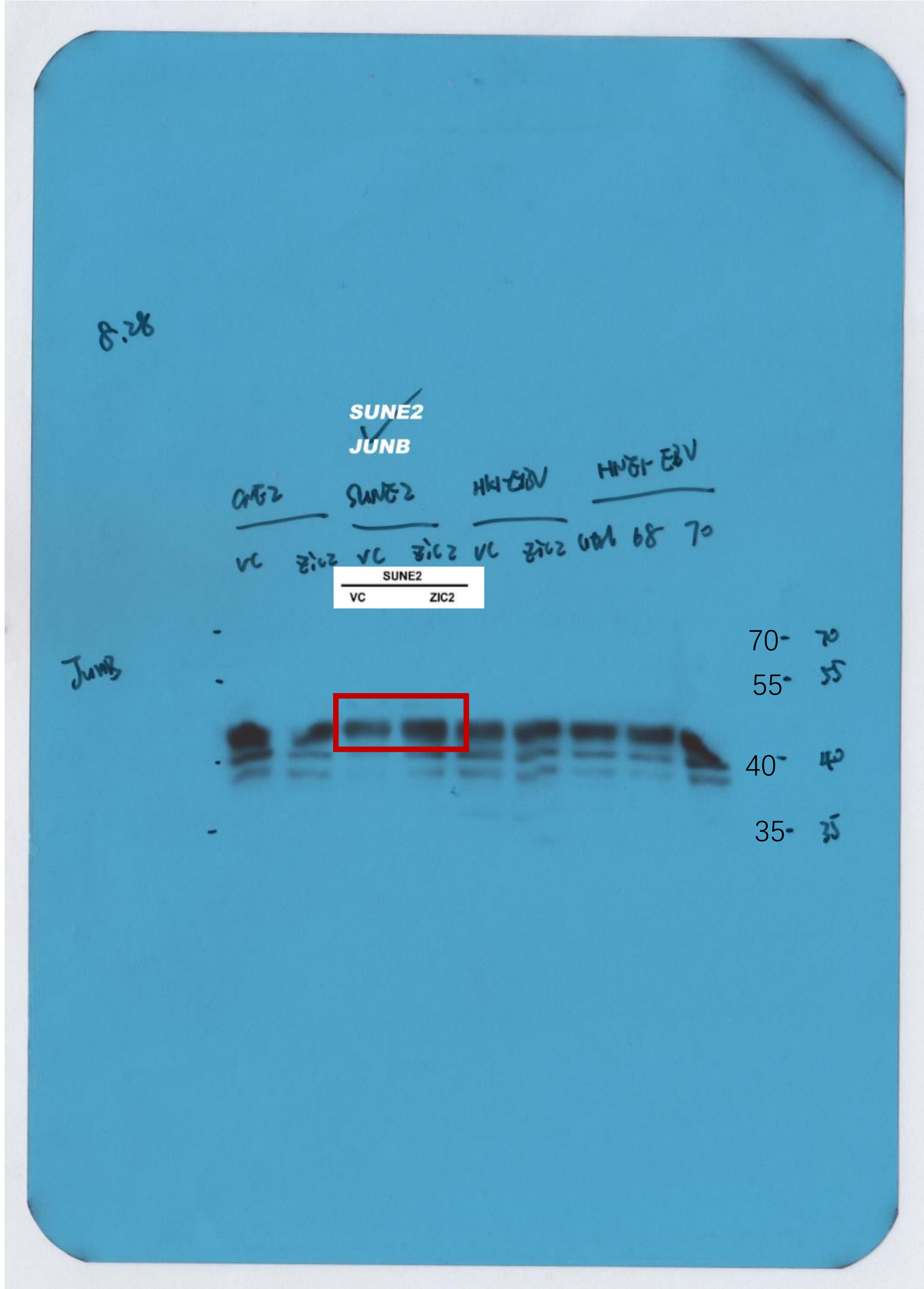
SUNE2
JUNB
SUNE2
VC
ZIC2
70
55
40
35

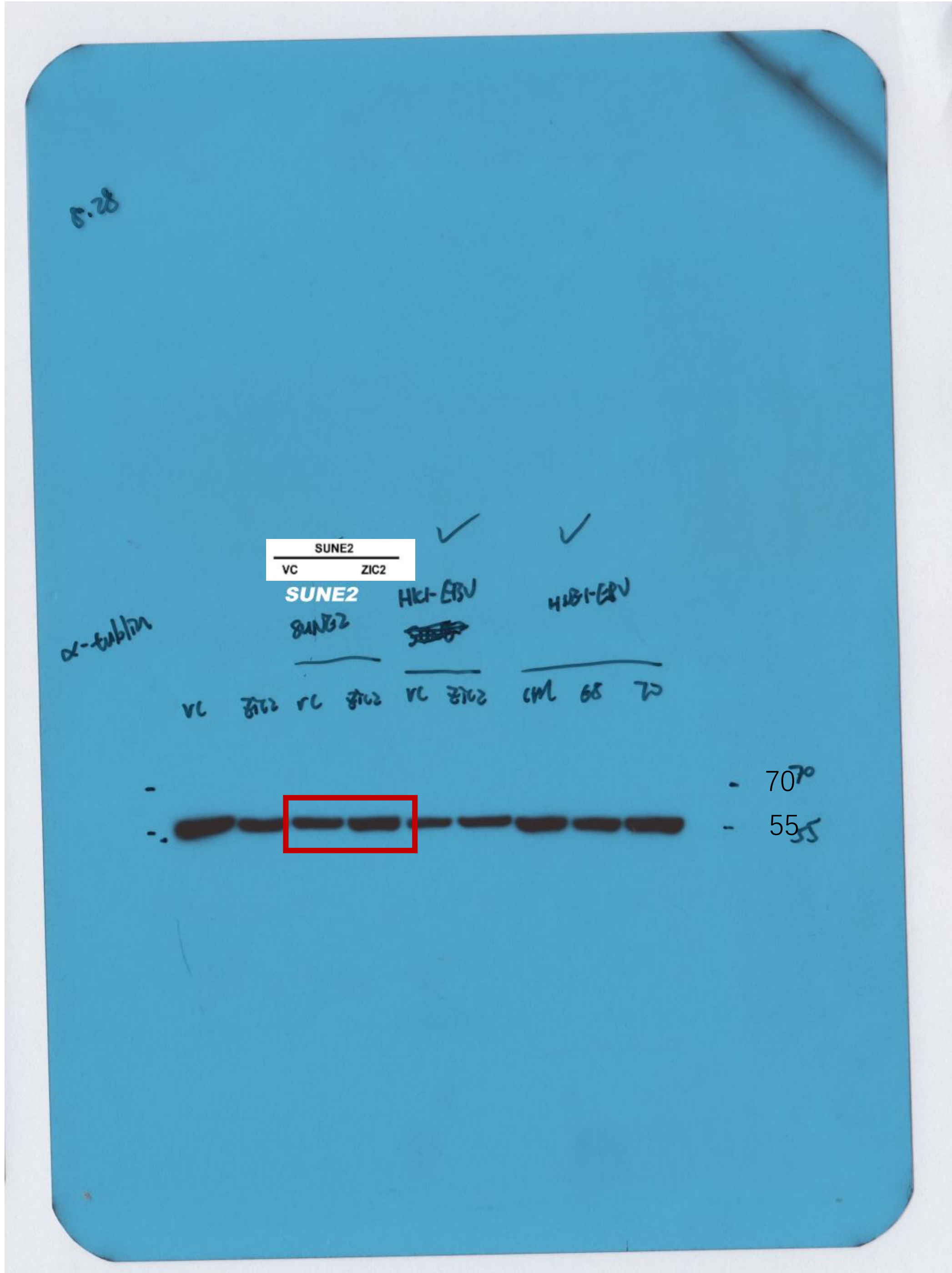

SUNE2
VC
ZIC2
SUNE2
70
55

Figure 7B

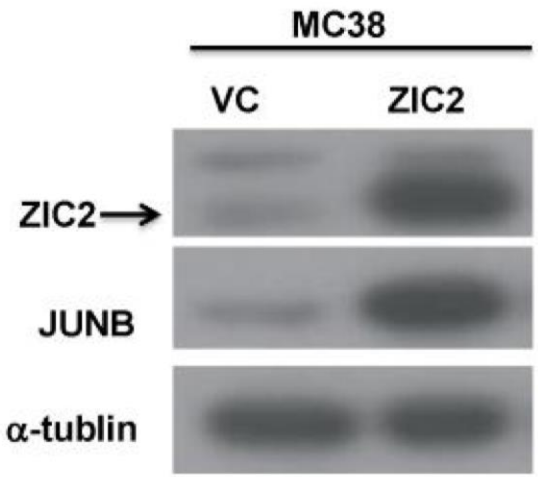

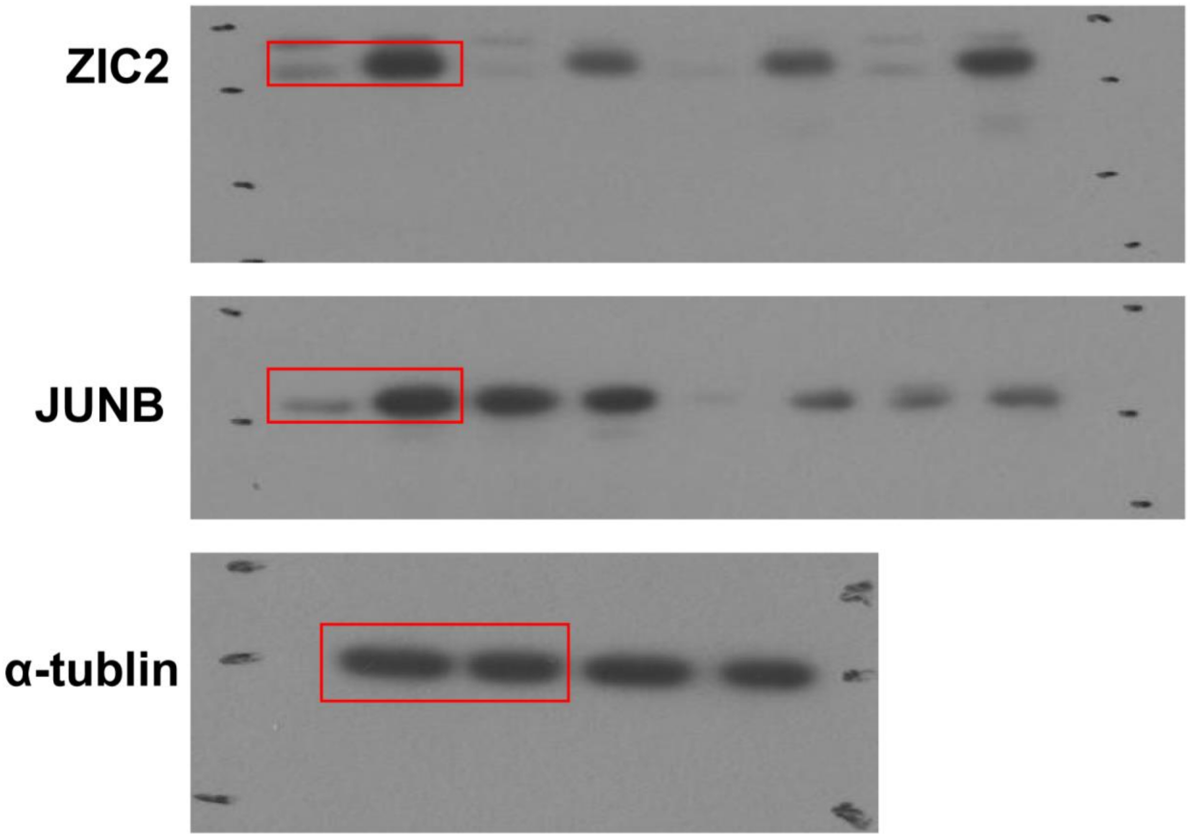

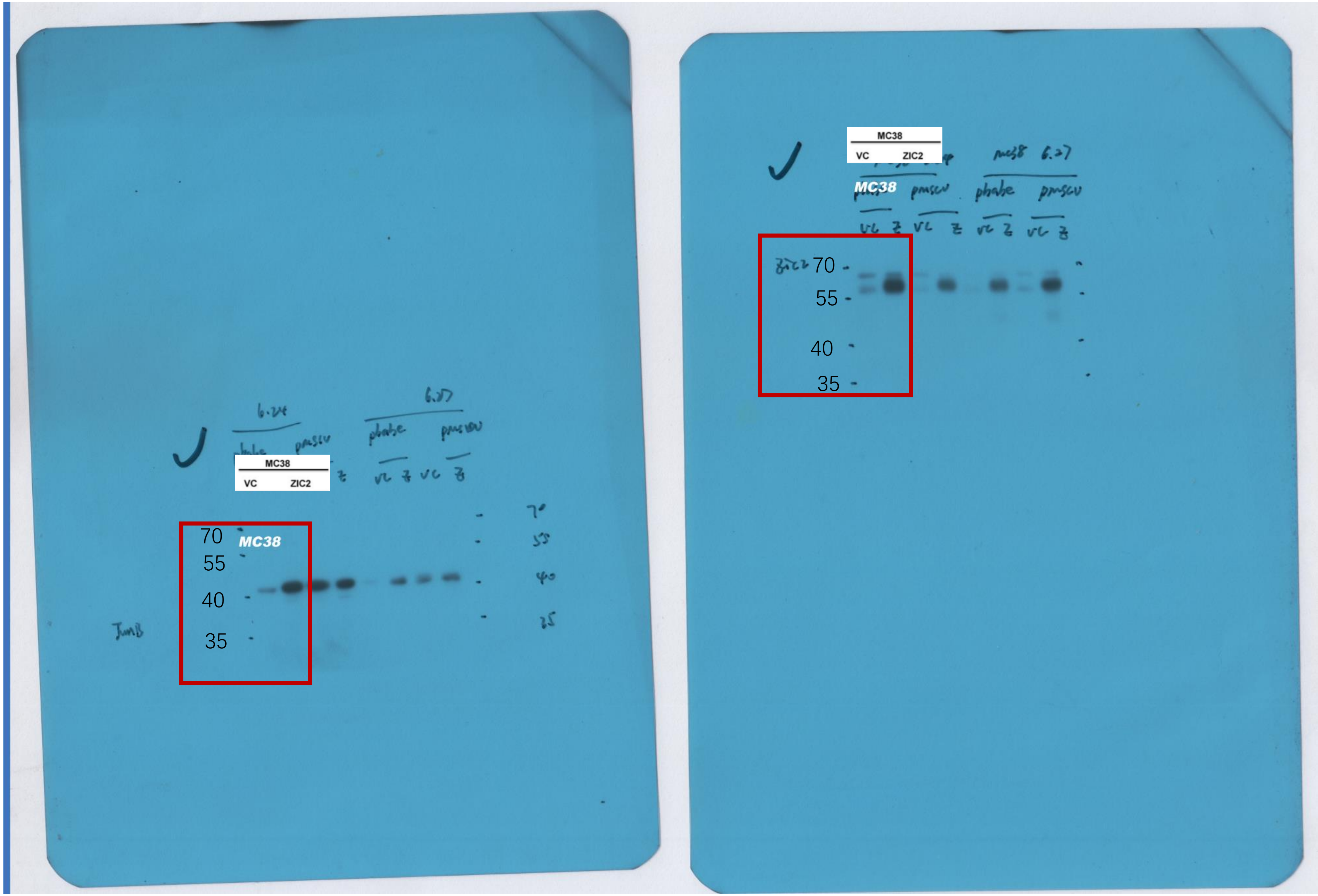
MC38
VC
ZIC2
MC38
70
55
40
35
MC38
VC
ZIC2
MC38
70
55
40
35

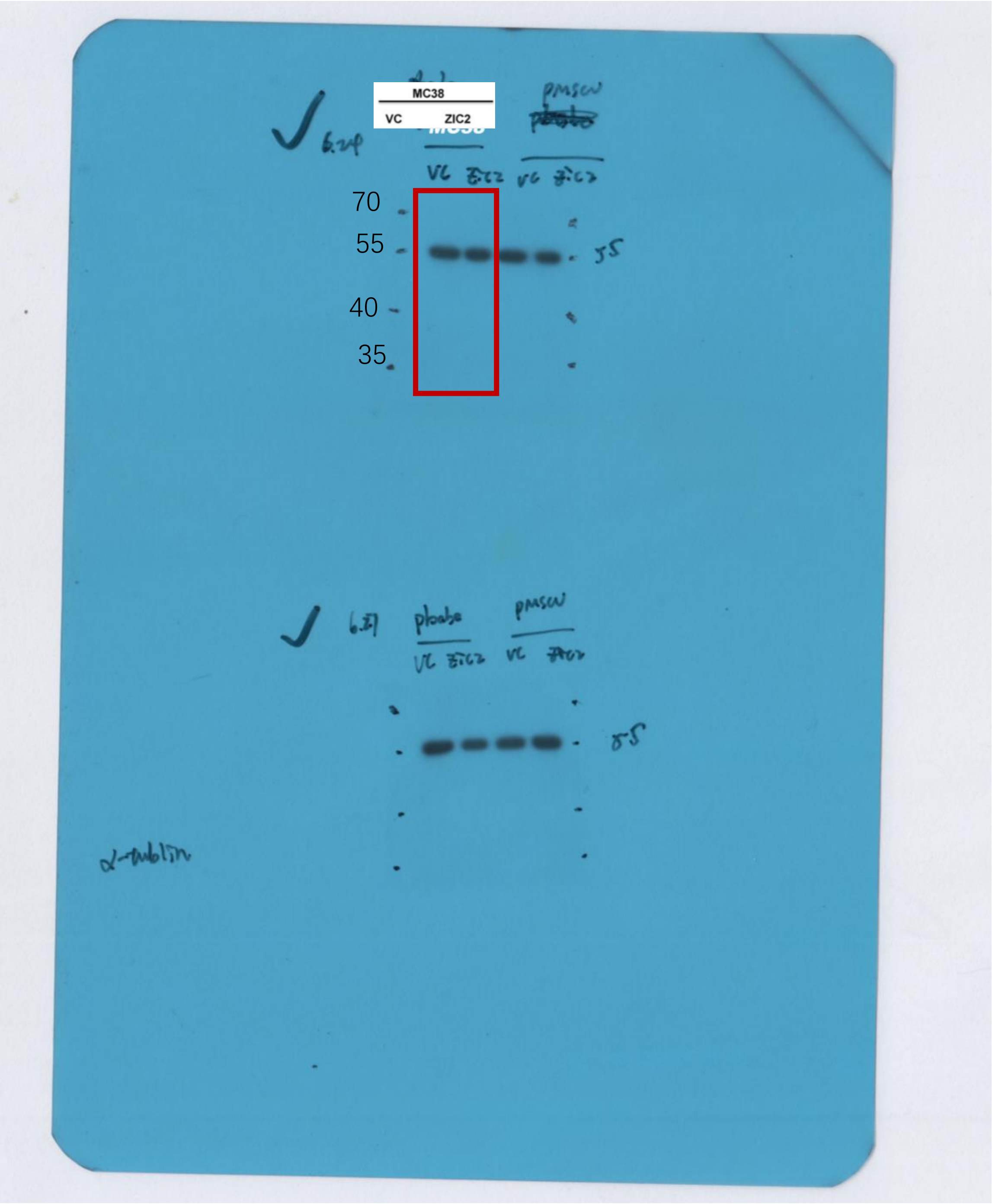
MC38
VC
ZIC2
70
55
40
35
pMSCV
VC
55
pbabe
pMSCV
VC
55
α-tubulin

Figure 7D

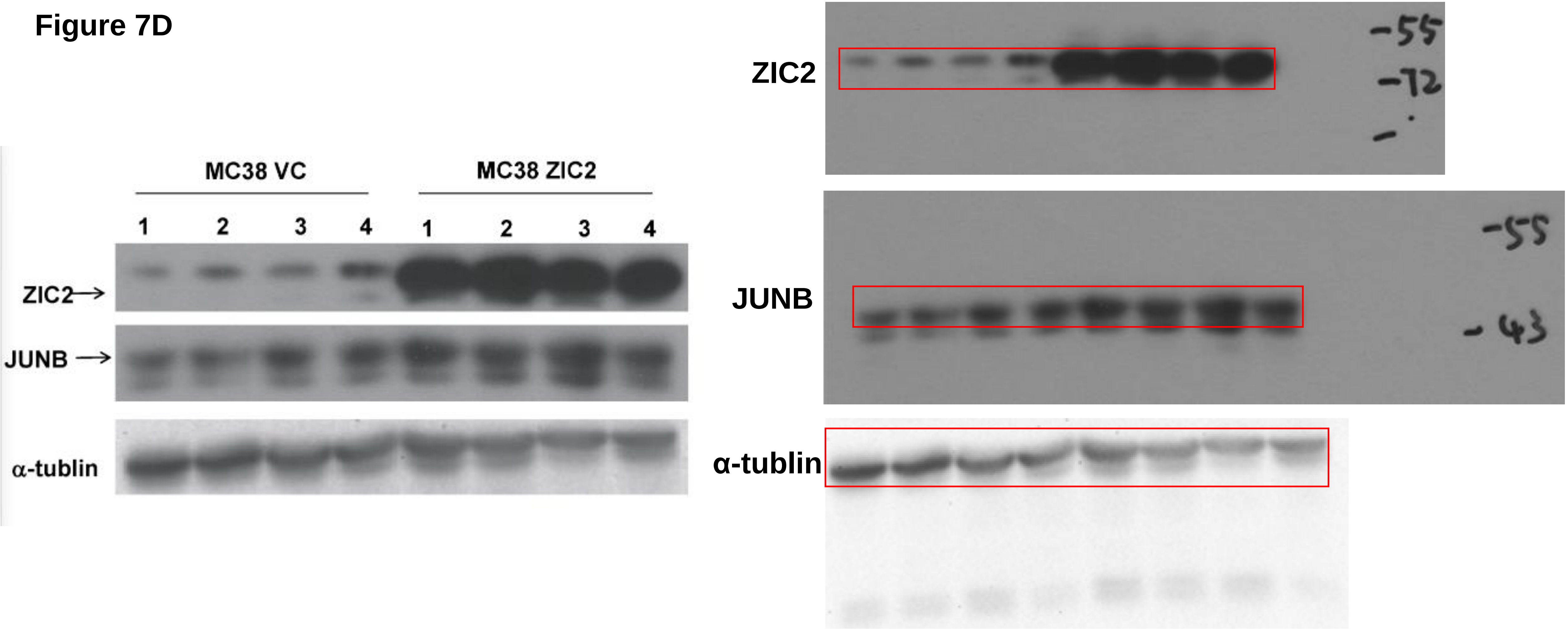
MC38 VC
MC38 ZIC2
1
2
3
4
1
2
3
4
ZIC2
JUNB
α-tublin
ZIC2
-55
-72
JUNB
-55
-43
α-tublin

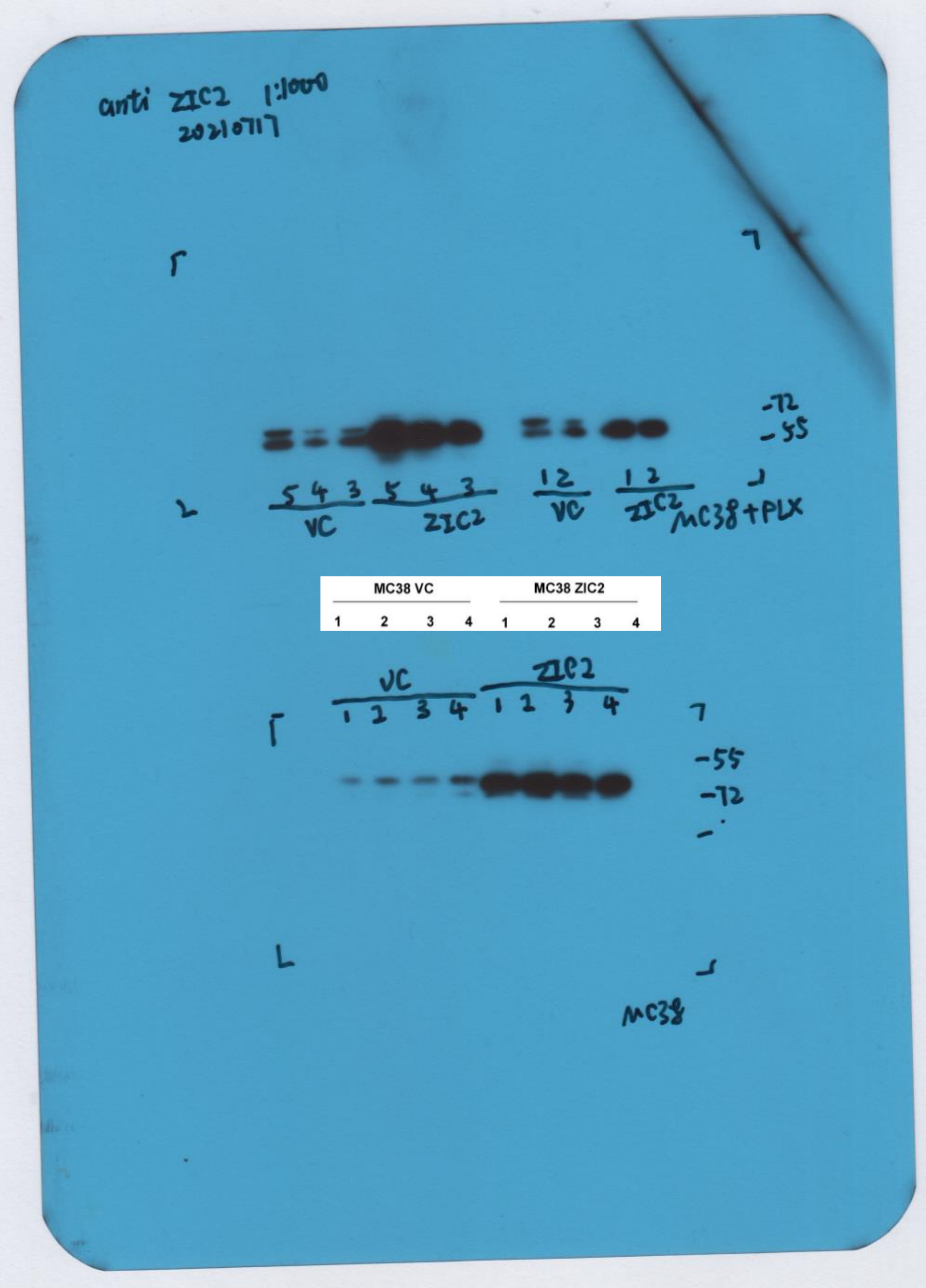
anti ZIC2 1:1000
20210717
-72
-55
5 4 3
VC
5 4 3
ZIC2
1 2
VC
1 2
ZIC2
MC38+PLX
MC38 VC
MC38 ZIC2
1 2 3 4 1 2 3 4
VC
ZIC2
1 2 3 4 1 2 3 4
-55
-72
MC38

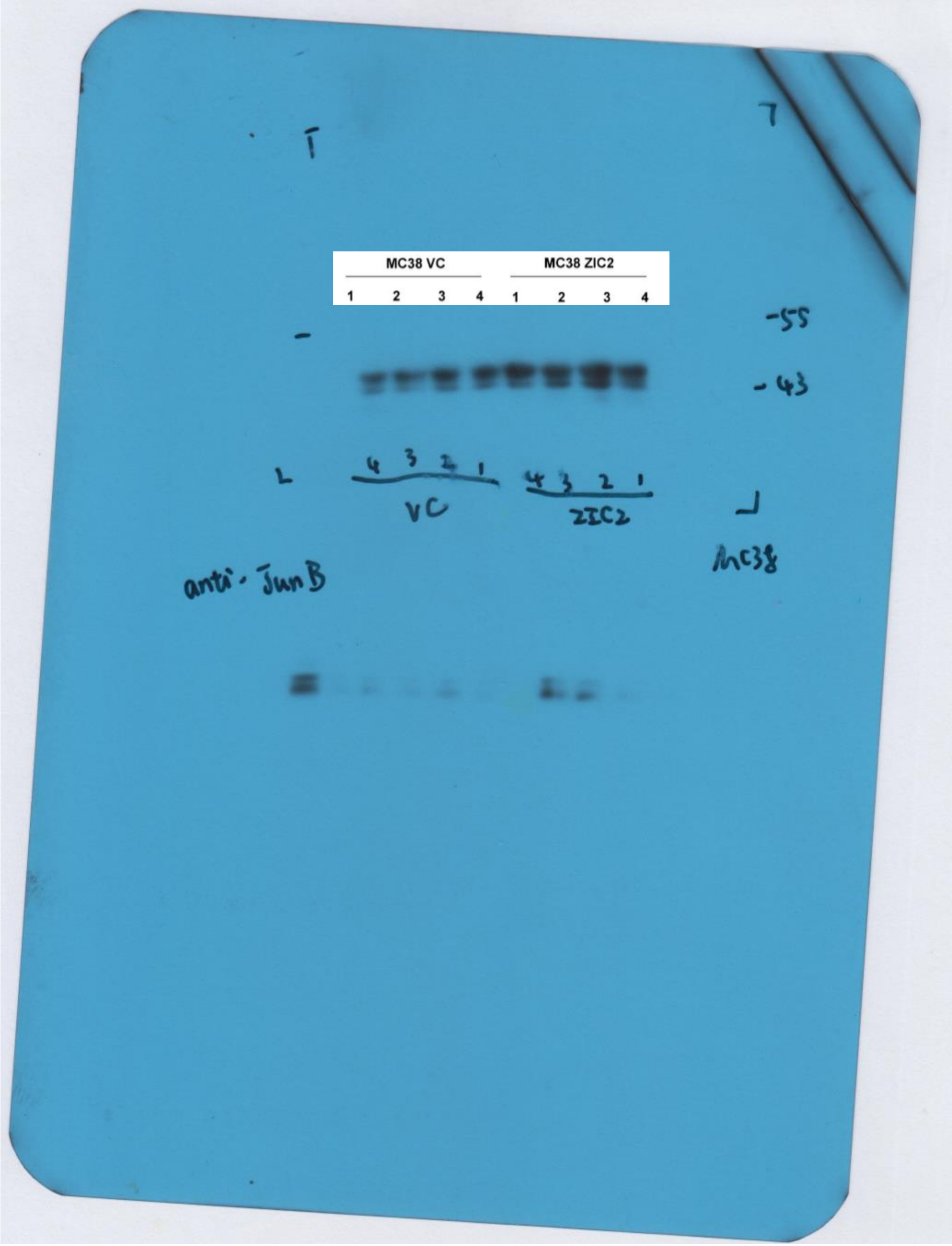
MC38 VC
MC38 ZIC2
1 2 3 4 1 2 3 4
-55
-43
4 3 2 1
VC
4 3 2 1
ZIC2
MC38
anti-JunB

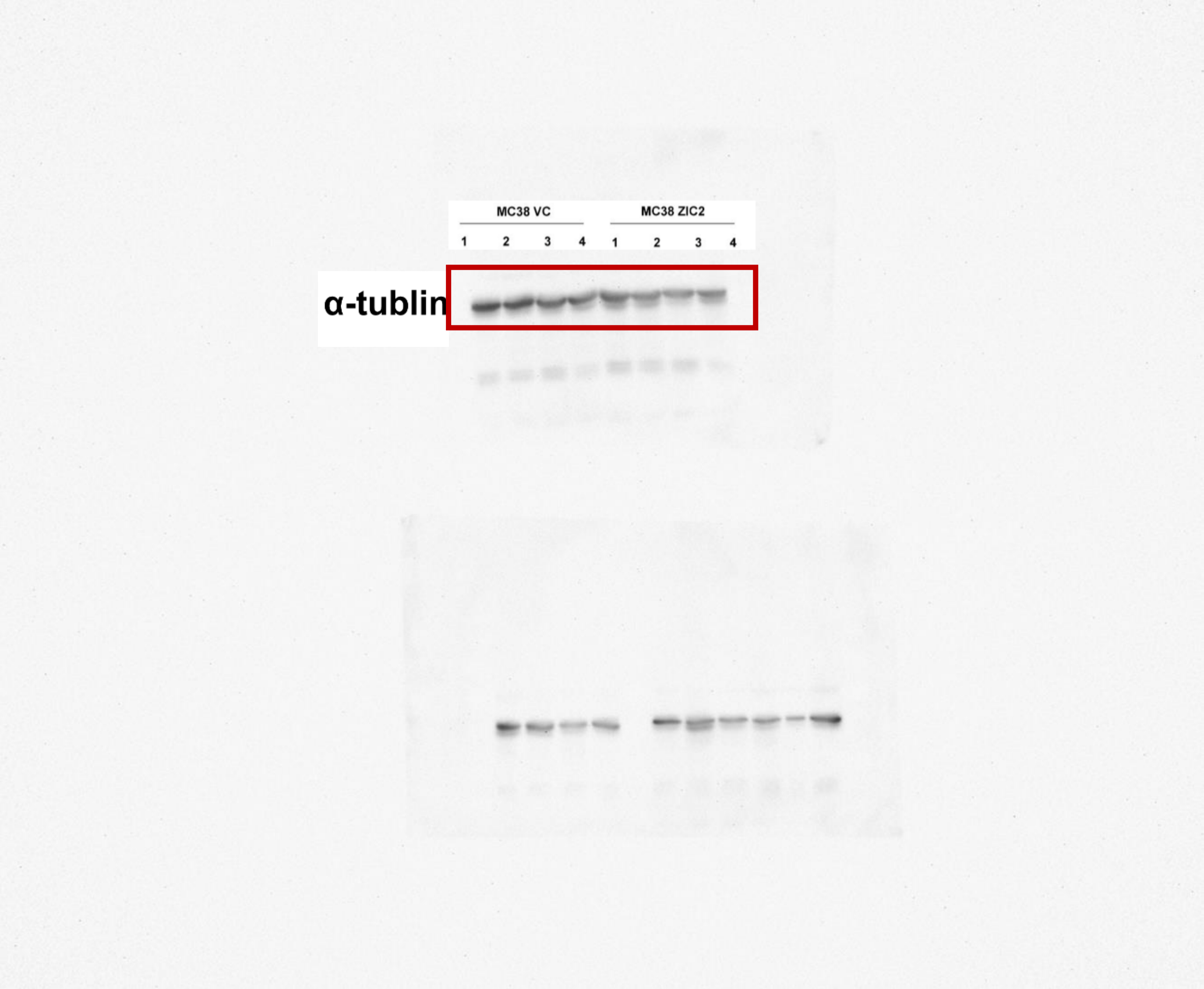
MC38 VC
MC38 ZIC2
1 2 3 4 1 2 3 4
α-tublin

Supplement Figure 2 B

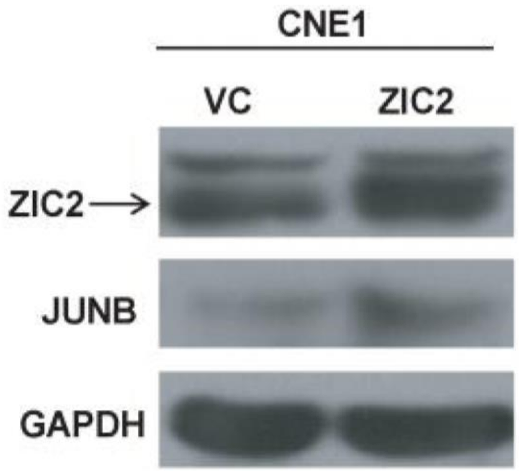

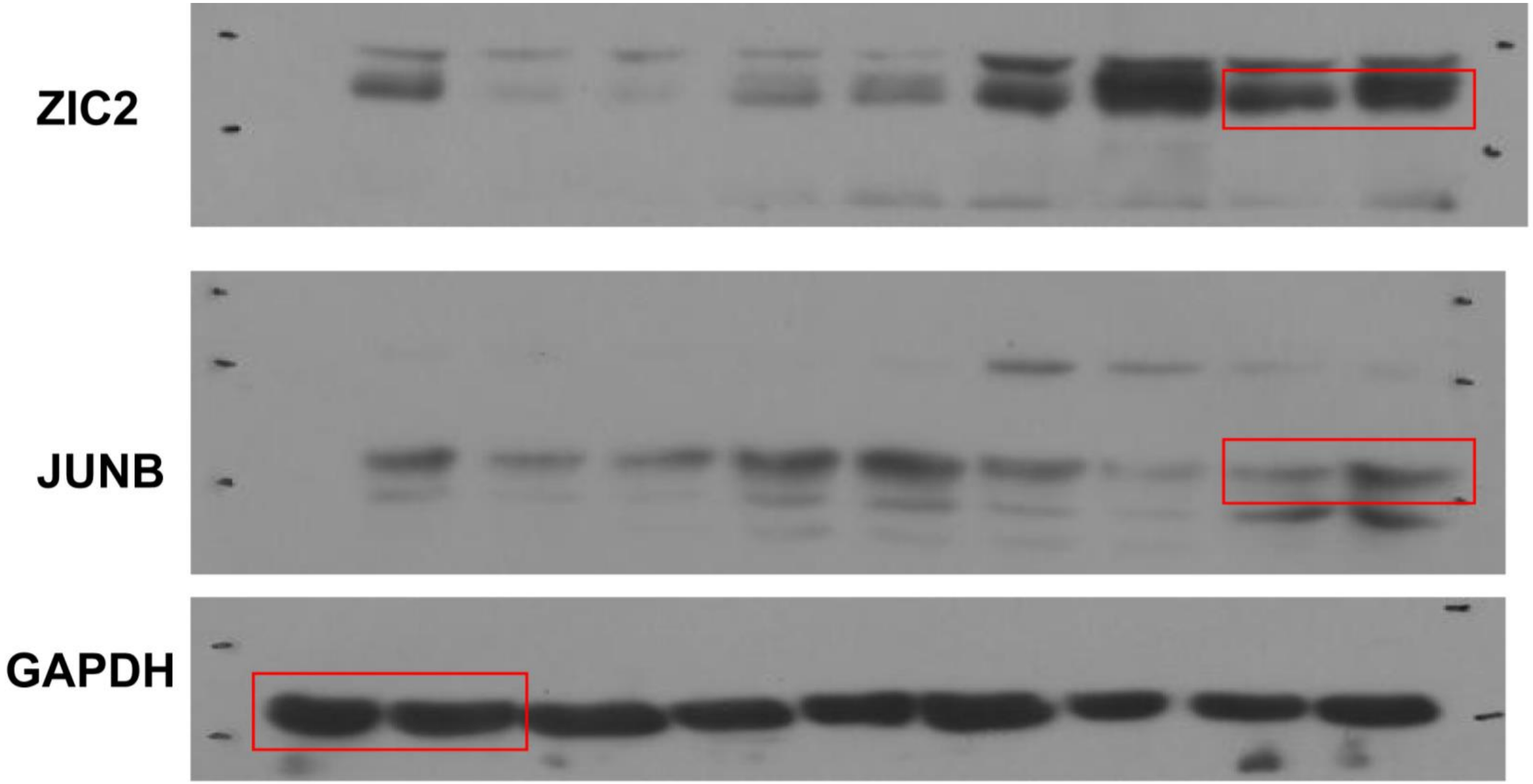

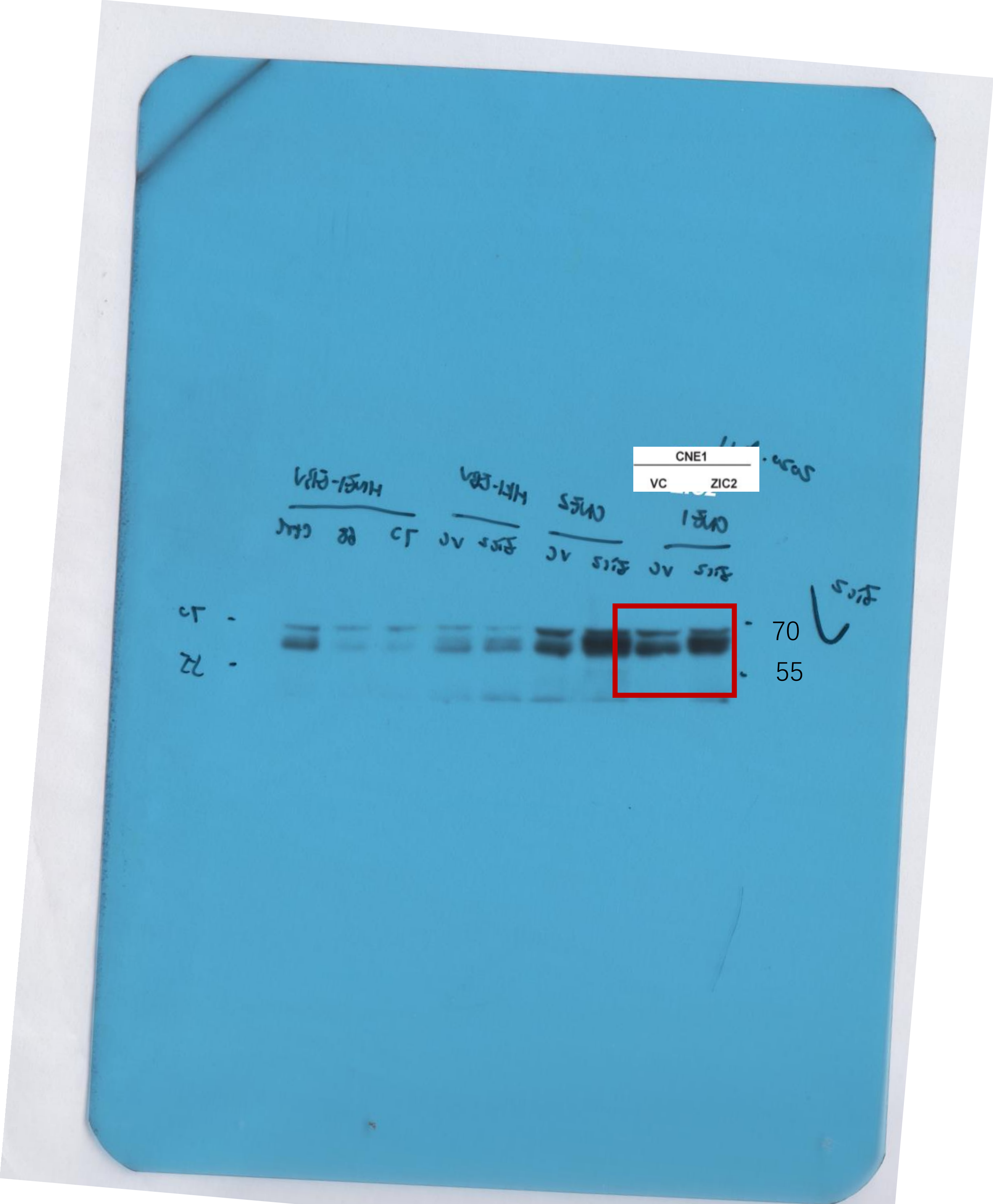
CNE1
VC
ZIC2
70
55

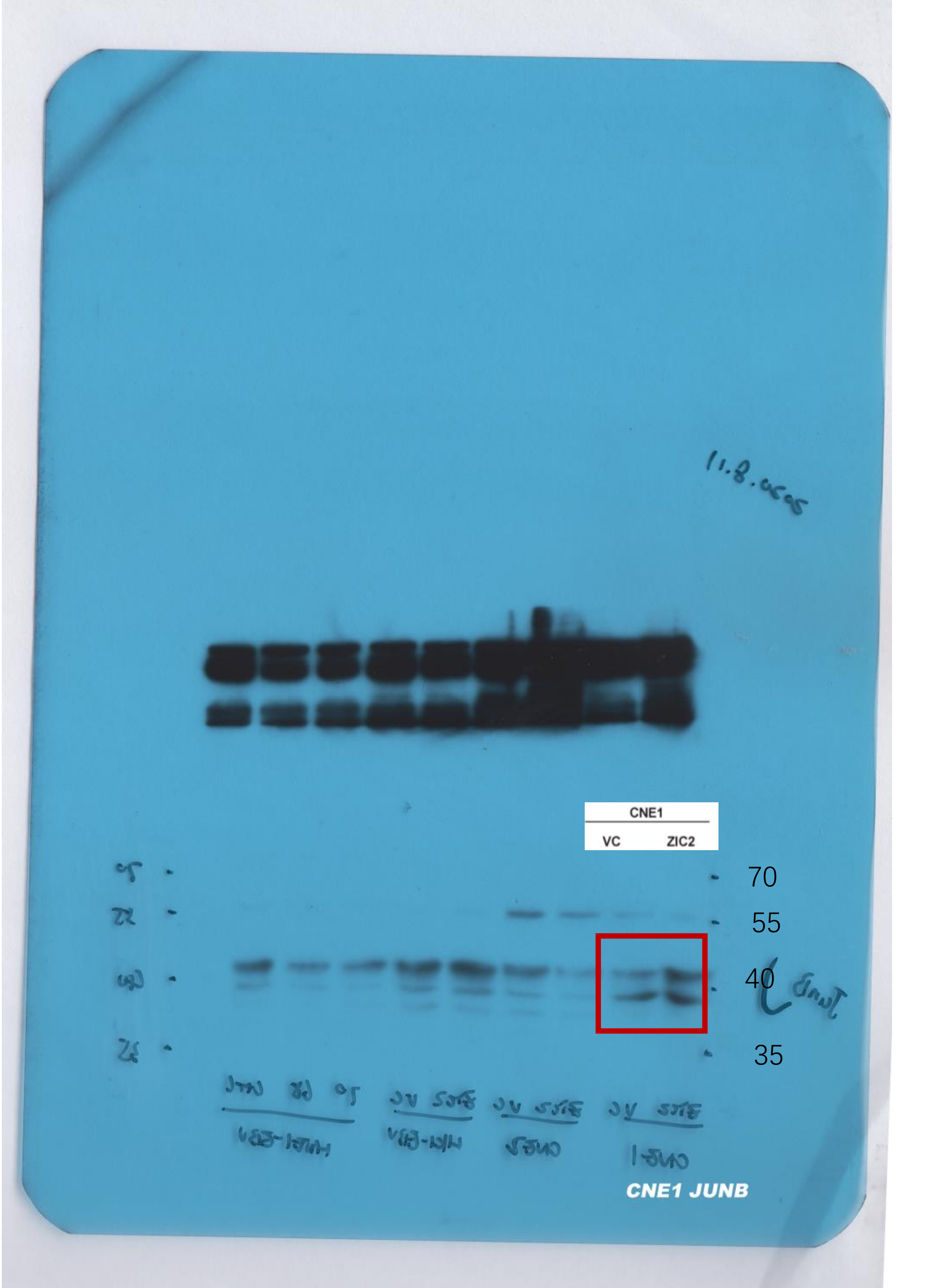
CNE1
VC
ZIC2
70
55
40
35
CNE1 JUNB

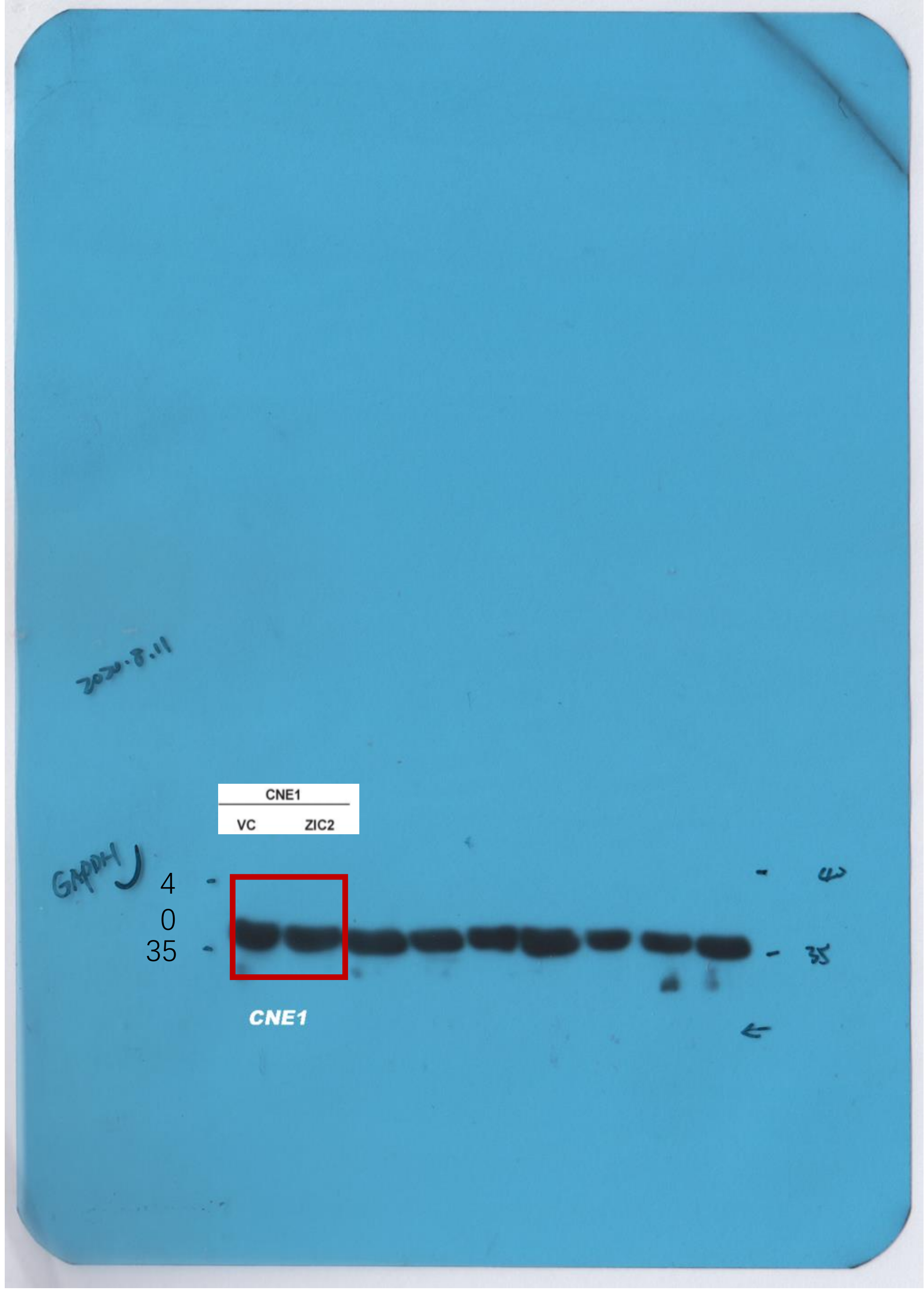
CNE1
VC
ZIC2
4
0
35
CNE1

Supplement Figure 2 B

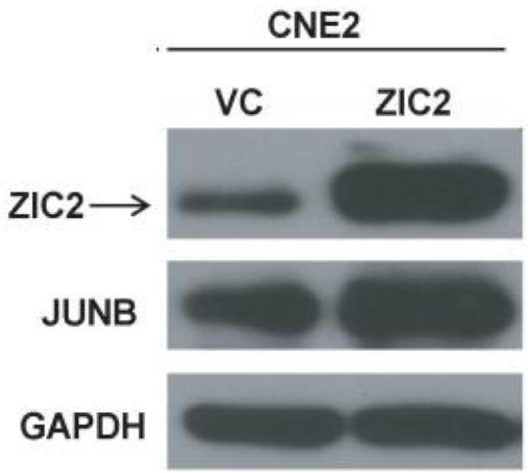

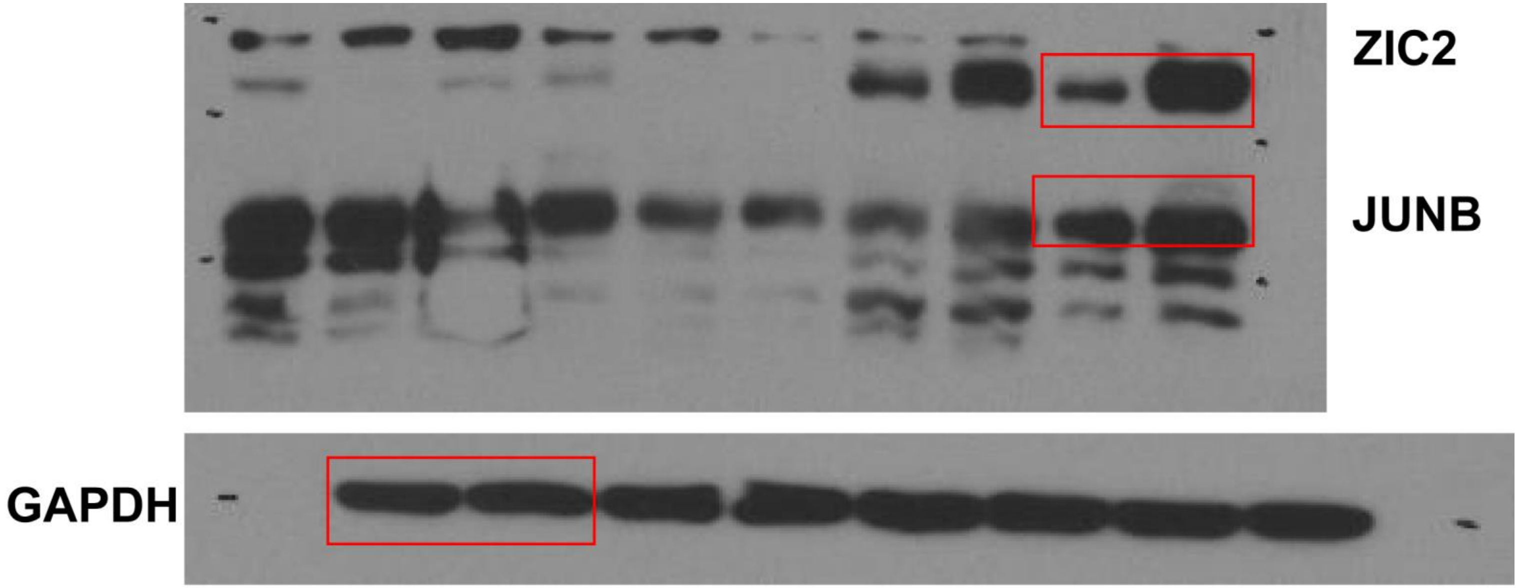

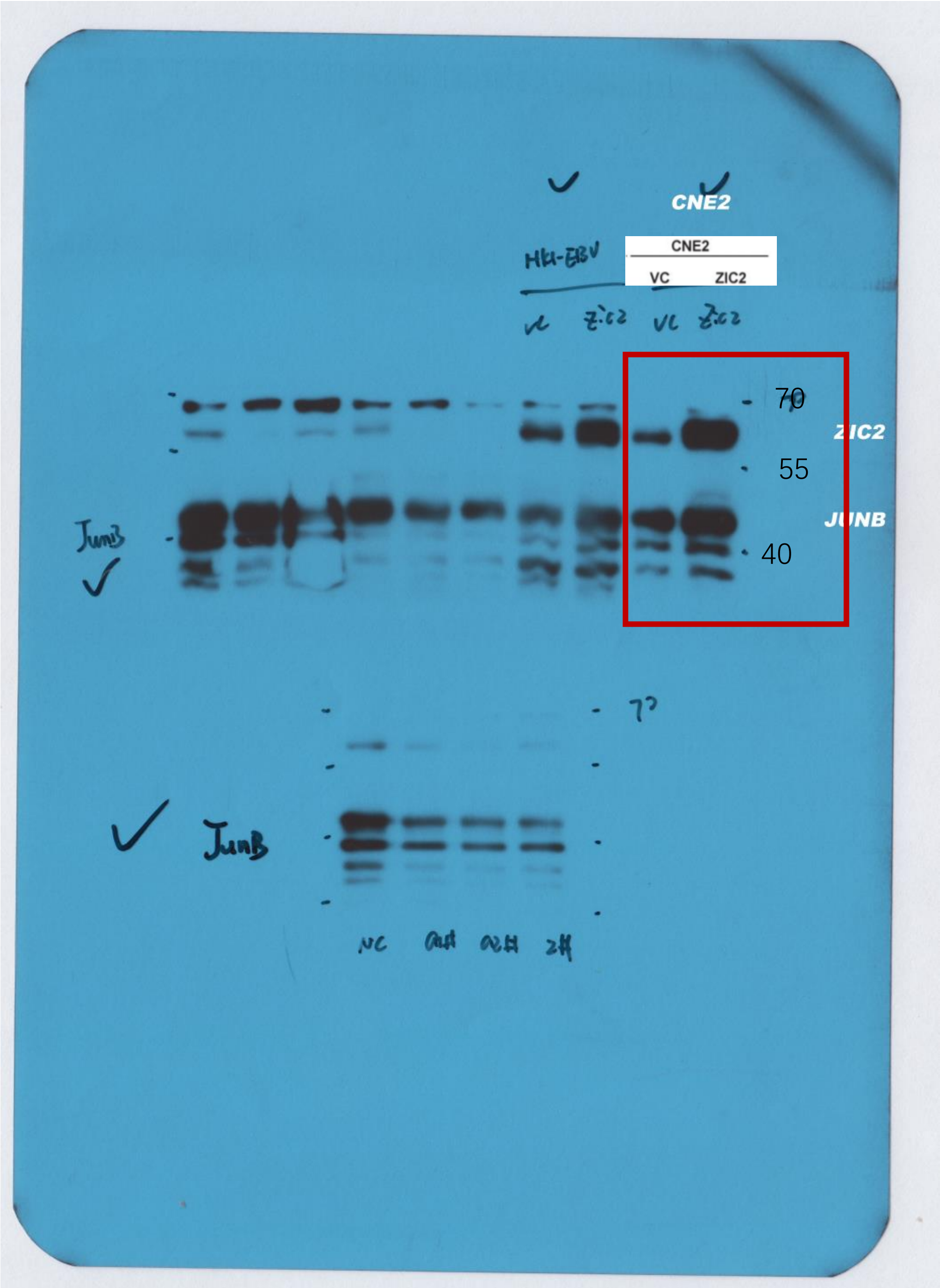
CNE2
CNE2
VC
ZIC2
HK1-EBV
70
55
40
ZIC2
JUNB
JunB
JunB

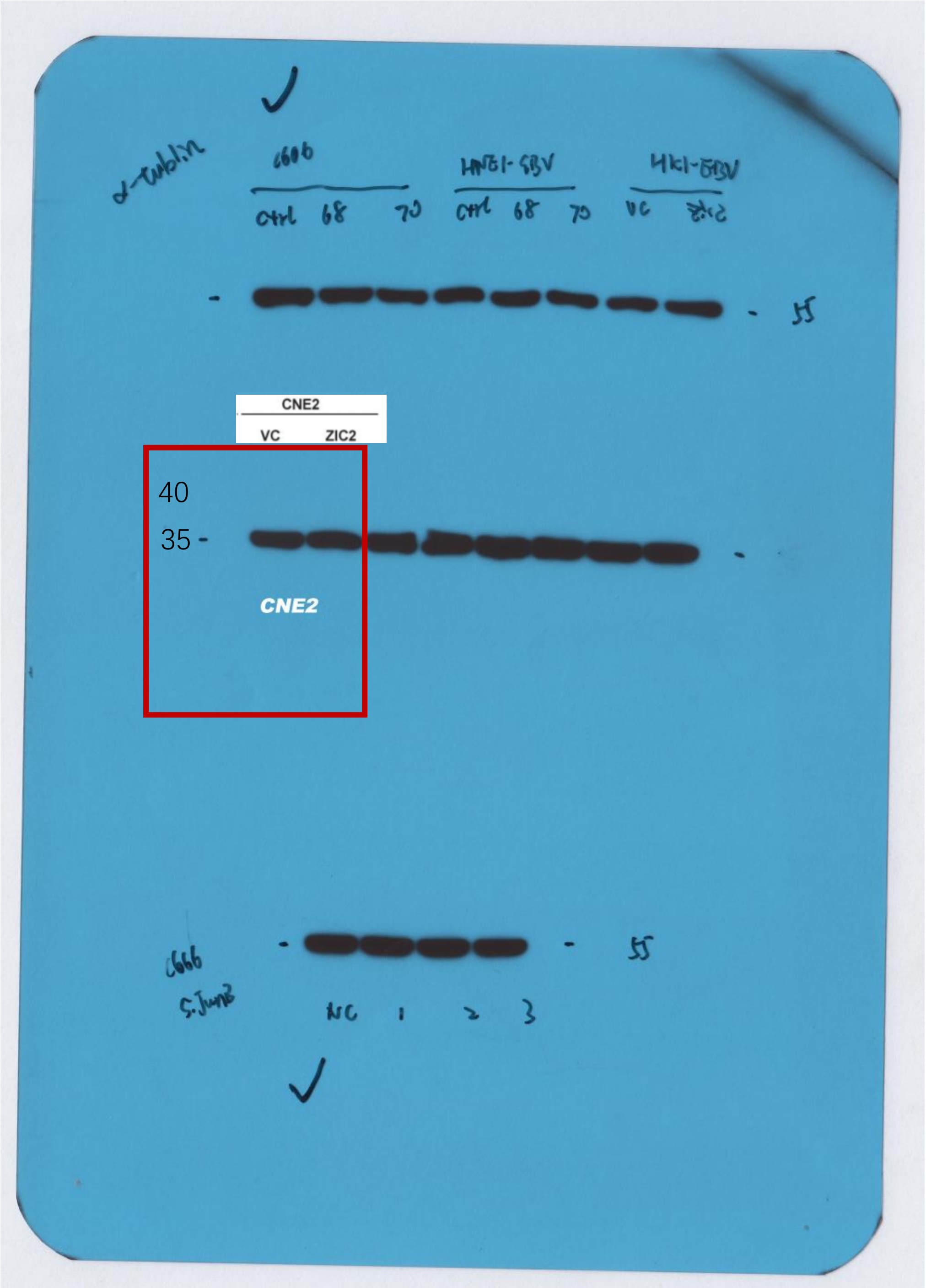
C666
HNE1-EBV
HK1-EBV
Ctrl
68
70
VC
55
CNE2
VC
ZIC2
40
35
CNE2
55
NC
1
2
3
